# Hypothalamus proteomics from mouse models with obesity and anorexia reveals therapeutic targets of appetite regulation

**DOI:** 10.1038/nutd.2016.10

**Published:** 2016-04-25

**Authors:** A Manousopoulou, Y Koutmani, S Karaliota, C H Woelk, E S Manolakos, K Karalis, S D Garbis

**Affiliations:** 1 Clinical and Experimental Sciences Unit, University of Southampton, Southampton, UK; 2 Centre of Basic Research, Biomedical Research Foundation of the Academy of Athens, Greece; 3 Information Technologies in Medicine and Biology, Department of Informatics and Telecommunications, University of Athens, Athens, Greece; 4 Wyss Institute of Biological Inspired Engineering, Boston, MA, USA; 5 Institute for Life Sciences, University of Southampton, Southampton, UK; 6 Cancer Sciences and CES Unit, University of Southampton, Southampton, UK

## Abstract

**Objective:**

This study examined the proteomic profile of the hypothalamus in mice exposed to a high-fat diet (HFD) or with the anorexia of acute illness. This comparison could provide insight on the effects of these two opposite states of energy balance on appetite regulation.

**Methods:**

Four to six-week-old male C56BL/6J mice were fed a normal (control 1 group; *n*=7) or a HFD (HFD group; *n*=10) for 8 weeks. The control 2 (*n*=7) and lipopolysaccharide (LPS) groups (*n*=10) were fed a normal diet for 8 weeks before receiving an injection of saline and LPS, respectively. Hypothalamic regions were analysed using a quantitative proteomics method based on a combination of techniques including iTRAQ stable isotope labeling, orthogonal two-dimensional liquid chromatography hyphenated with nanospray ionization and high-resolution mass spectrometry. Key proteins were validated with quantitative PCR.

**Results:**

Quantitative proteomics of the hypothalamous regions profiled a total of 9249 protein groups (*q*<0.05). Of these, 7718 protein groups were profiled with a minimum of two unique peptides for each. Hierachical clustering of the differentiated proteome revealed distinct proteomic signatures for the hypothalamus under the HFD and LPS nutritional conditions. Literature research with in silico bioinformatics interpretation of the differentiated proteome identified key biological relevant proteins and implicated pathways. Furthermore, the study identified potential pharmacologic targets. In the LPS groups, the anorexigen pro-opiomelanocortin was downregulated. In mice with obesity, nuclear factor-κB, glycine receptor subunit alpha-4 (GlyR) and neuropeptide Y levels were elevated, whereas serotonin receptor 1B levels decreased.

**Conclusions:**

High-precision quantitative proteomics revealed that under acute systemic inflammation in the hypothalamus as a response to LPS, homeostatic mechanisms mediating loss of appetite take effect. Conversely, under chronic inflammation in the hypothalamus as a response to HFD, mechanisms mediating a sustained ‘perpetual cycle' of appetite enhancement were observed. The GlyR protein may constitute a novel treatment target for the reduction of central orexigenic signals in obesity.

## Introduction

Overweight and obesity as a result of positive energy balance constitute major public health burdens with significant economic and social implications.^1^ The hypothalamus is the key brain site for the regulation of food intake and energy balance in mammals. Under physiological conditions, a variety of peripheral signals regulate appetite and adjust energy intake to match energy consumption requirements.^2^ Systemic acute inflammatory signals can cause profound anorexia by disrupting the physiological regulation of appetite in the hypothalamus.^3^ Conversely, emerging evidence suggests that hypothalamic ‘inflammatory' activation as a result of a high-fat diet (HFD) and obesity can disturb anorexigenic and thermogenic signals and promote abnormal body weight control.^4^ Deciphering the molecular events of these two contradictory observations in a global and directly comparative way allows for a more causal understanding on the relations between diet, inflammatory signals and appetite regulation. Provided that nutritional intervention protocols are likely to interfere with multiple pathways inside the cell, a system-wide interrogation of the host response using non-targeted quantitative proteomics was warranted.^5^

The aim of this study was to compare the global proteomic profile of the hypothalamus in mice exposed to a HFD or with anorexia of acute illness. We hypothesise that such a comparison can shed new insight on the effects of these two opposite states of energy balance on appetite regulation.

## Materials and methods

### Animal model

Studies were conducted in male mice on C56BL/6J background. Mice were bred and maintained at regular housing temperatures (23 °C) and 12-h light/dark cycle starting at 0700 hours. Animals had *ad libitum* access to water and food and were weighed at weekly intervals. Four to six-week-old mice (*n*=34) were randomly divided into four groups: the control 1 (C1) group (*n*=7), the HFD group (*n*=10), the control 2 (C2) group (*n*=7) and the lipopolysaccharide (LPS) group (*n*=10). Mice in the C1 group were fed a control diet (4.5% fat, 34% starch, 5.0% sugar and 22.0% protein), whereas the HFD group was fed a HFD (24% fat, 41% carbohydrate, and 24% protein; Research diets D12451 formula) for 8 weeks after which they were anaesthetised and perfused intracardially with phosphate-buffered saline. A diet containing 24% fat (45% kcal fat) was selected because it efficiently induces obesity and better simulates a human HFD as opposed to a more extreme 60% kcal fat diet.^6, 7^ Mice in the C2 and LPS groups were fed a control diet for 8 weeks. After this period, mice in the C2 group received an intraperitoneal injection of 0.3 ml saline, whereas in the LPS group received an intraperitoneal injection of LPS (*Escherichia coli* O111:B4 (Sigma No. L-2630)) at a dose of 120 μg per mouse dissolved in 0.3 ml saline. Mice in the C2 and LPS groups were killed 18 h after the injection as described above. As protein expression in the hypothalamus is known to be under circadian regulation,^8^ mice were killed at 0700 hours. Experimental procedures were approved by the Institutional Animal Care and Use Committee at the Centre of Basic Research, Biomedical Research Foundation of the Academy of Athens.

### Determination of insulin resistance by HOMA-IR

HOMA-IR analysis was used to assess insulin resistance in HFD-fed mice. After overnight fasting, values for homeostasis model assessment of insulin resistance (HOMA-IR) were calculated from the values of fasting serum glucose (mg dl^−1^) and fasting serum insulin (μU ml^−1^) by using the following formula: HOMA-IR=fasting glucose value (mg dl^−1^) × fasting insulin value (μU ml^−1^)/405. Low HOMA-IR values indicate high insulin sensitivity, whereas high HOMA-IR values indicate low insulin sensitivity (insulin resistance). Fasting glucose concentrations were measured using a hand-held glucometer (Biorad, Hercules, CA, USA), whereas serum insulin levels were quantified by ELISA (Mercodia, Uppsala, Sweden).

### Quantitative proteomics sample processing

**Figure 1:**
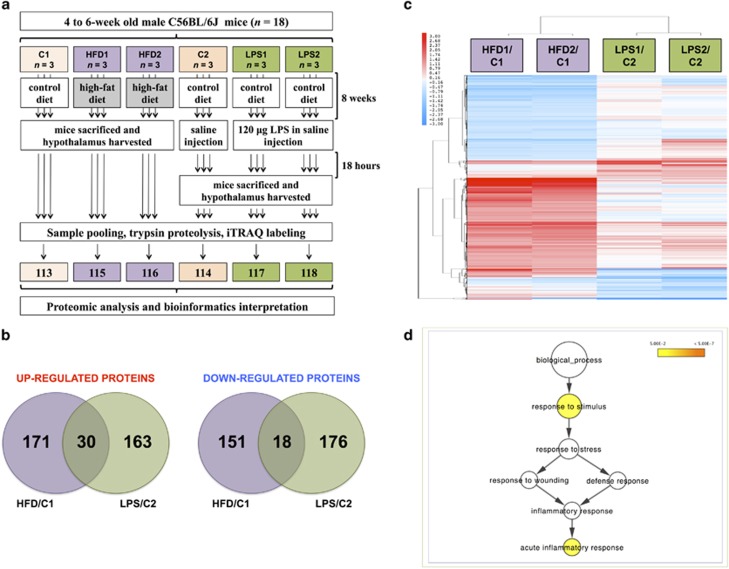
(**a**) Overview of the proteomics pipeline (**b**) Venn diagram description of the differentiated proteins between the HFD and LPS groups relative to their respective controls. (**c**) Hierarchical clustering analysis of modulated proteins: the HFD and LPS groups have a distinct endophenotypic portrait in the hypothalamus. (**d**) Gene ontology analysis using BiNGO of the commonly modulated proteins in the HFD and LPS conditions compared with their respective control showed a significant enrichment for acute inflammatory response. Coloured nodes representing GO terms correspond to those that were significant according to the *P*-value colour scale, whereas white nodes were not significant. The size of the node reflects the number of proteins that mapped to the corresponding GO term.

Hypothalamic regions were removed and snap frozen at −80 °C. Specimens were dissolved in 0.5 m triethylammonium bicarbonate, 0.05% sodium dodecyl sulphate, homogenised using the Fast Prep system (Savant Bio, Cedex, France) followed by pulsed probe sonication (Misonix, Farmingdale, NY, USA). Lysates were centrifuged (16 000 *g*, 10 min, 4 °C) and supernatants were measured for protein content using infra-red spectroscopy (Merck Millipore, Darmstadt, Germany). For the quantitative proteomic analysis, the hypothalamic regions from three mice were used for the C1 and C2 groups and from six mice for the HFD and LPS groups (*n*=18 in total). Three individual protein extracts were pooled (33.3 μg from each lysate giving 100 μg final protein content) to form one sample for the C1 and C2 groups and two biological replicates for the HFD and LPS groups. Lysates were then reduced, alkylated and subjected to trypsin proteolysis. Peptides were labelled using six of the eight-plex iTRAQ reagent kit (113=C1, 114=C2, 115=HFD1, 116=HFD2, 117=LPS1, 118=LPS2) and analysed using high-precision two-dimensional liquid chromatography with nanospray ionization tandem mass spectrometry as reported previously by the authors^9, 10, 11, 12^ (Figure 1a) (Supplementary Methods 1).

### Database searching

Unprocessed raw files were submitted to Proteome Discoverer 1.4 for target decoy searching against the UniProtKB/TrEMBL mus musculus database comprised of 57 475 entries (release date 03 September 2014), allowing for up to two missed cleavages, a precursor mass tolerance of 10 ppm, a minimum peptide length of six and a maximum of two variable (one equal) modifications of; iTRAQ 8-plex (Y), oxidation (M), deamidation (N, Q) or phosphorylation (S, T, Y). Methylthio (C) and iTRAQ (K, N terminus) were set as fixed modifications. Reporter ion ratios from unique peptides only were taken into consideration for the quantitation of the respective protein. Quantification ratios were median-normalised and log2 transformed. Proteins were grouped using the protein grouping inference algorithm in the Proteome Discoverer 1.4 application (Supplementary Methods 2). A protein was considered modulated in the HFD or LPS group relative to control when its log2 ratio was above or below±1 s.d. across both biological replicates.^13^ In adherence to the Paris Publication Guidelines for the analysis and documentation of peptide and protein identifications (http://www.mcponline.org/site/misc/ParisReport_Final.xhtml) and the recently published guidelines for high-confidence protein identification by Omenn *et al.*,^14^ only proteins identified with at least two unique peptides were further subjected to bioinformatics analysis. Proteins of biological relevance, two of which were identified with one unique peptide, were validated using targeted quantitative PCR. Proteomics data were deposited to the ProteomeXchange Consortium via the PRIDE partner repository with the dataset identifier PXD003475.

### Bioinformatics analysis

Heatmap construction of differentially expressed proteins in the HFD and LPS groups compared with their respective control was generated using Cluster 3.0 (http://bonsai.hgc.jp/~mdehoon/software/cluster/software.htm) and Java Treeview (http://jtreeview.sourceforge.net). MetaCore (GeneGo, St Joseph, MI, USA) and BiNGO were applied to differentially expressed proteins analysed with at least two unique peptides to identify over-represented processes in the modulated proteome of each group compared with their respective control. FDR-corrected *P-*values <0.05 were considered significant.

### Total RNA isolation and cDNA synthesis

For the quantitative PCR analysis the hypothalamic regions from four mice were utilised per group (C1, C2, HFD, LPS) (*n*=16 in total). Total RNA from mouse hypothalamus was extracted using an RNA Isolation Kit (Qiagen, Hilden, Germany) according to the manufacturer's instructions. RNA concentration and purity were determined by NanoDrop 2000c Spectrophotometer (Thermo Scientific, Waltham, MA, USA). Ribosomal RNA band integrity was evaluated by conventional 1% agarose gel electrophoresis and the Agilent Bioanalyzer with the RNA 6000 Nano Kit (Agilent, Santa Clara, CA, USA).

For complementary DNA (cDNA) synthesis, 300 ng total RNA from each sample was reverse transcribed into cDNA using M-MLV Reverse Transcriptase (Thermo Scientific) according to the manufacturer's instructions. All the cDNA samples were stored at −20 °C until quantitative PCR analyses.

### Quantification of mRNA

Quantification of mRNA was performed for the following proteins: NFκ-B, neuropeptide Y (NPY), 5-hydroxytryptamine (serotonin) receptor 1B, glycine receptor alpha-4 subunit, pro-opiomelanocortin. The PCR mixture contained 1 μl diluted cDNA, 10 μM gene-specific primer (forward and reverse mixed together) and 10 μl of 2 × Fast SYBR Green Master Mix (Roche Diagnostics, Rotkreuz, Switzerland) in a total volume of 20 μl. Amplification was performed in 96-well optical reaction plates (Roche Diagnostics) on LightCycler 480 (Roche Diagnostics) using the following programme: 94 °C for 3 min to activate polymerase, 40 cycles at 94 °C for 20 s, 60 °C for 20 s and 72 °C for 20 s; melting curve analysis was performed after every run by heating up to 95 °C to monitor presence of unspecific products. Two negative controls were included in each assay run, with water instead of template. Three replicate measurements for each sample were performed. Primers were designed and checked with Primer Quest Tool (IDT) and NCBI primer BLAST tool and synthesised by Macrogen (Seoul, South Korea). Primer sequences are listed in Supplementary Table 1.

### Quantitative PCR data analysis

The mRNA expression of genes in the hypothalamus of LPS-treated and HFD-fed mice was calculated relative to the expression in their respective control mice, according to the delta–delta Ct method (2−ΔΔCt) using the most and least stable reference genes found, as well as the most commonly used glyceraldehyde-3-phosphate dehydrogenase and beta-actin.

## Results

Mice fed a HFD weighed significantly more than those fed a control diet (Supplementary Figure 1A). Furthermore, they were insulin resistant with significantly higher HOMA-IR values (Supplementary Figure 1B). The proteomic analysis of the hypothalamic regions resulted in the profiling of 9249 protein groups (*q*<0.05) (Supplementary Tables 2 and 3), of which 7718 were identified with at least two unique peptides. Among the proteins identified with at least two unique peptides, 201 were upregulated in the HFD and 193 in the LPS groups compared with their respective control, of which 30 were common between the two conditions. Furthermore, 169 proteins were downregulated in the HFD and 194 in the LPS groups, of which 18 were common between the two conditions (Figure 1b, Supplementary Tables 4–6). The *R*^2^ value between biological replicates was 0.69 and 0.89 for the differentially expressed proteins of the HFD and LPS groups, respectively. Hierarchical clustering of the differentiated proteome revealed a distinct proteomic signatures of the hypothalamus under the HFD and LPS (Figure 1c) nutritional conditions.

**Figure 2:**
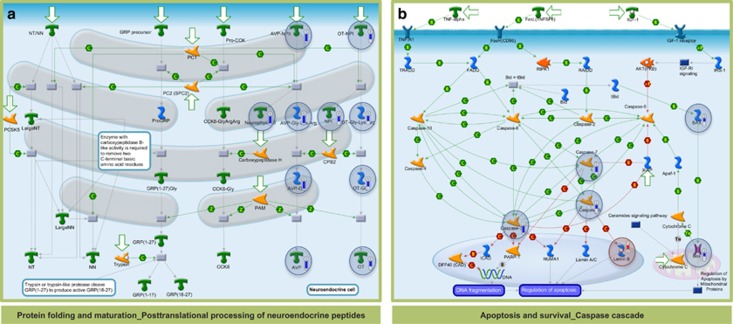
*In silico* analysis using MetaCore showed that (**a**) protein folding and maturation_posttranslational processing of neuroendocrine peptides (FDR-corrected *P*-value=1.2E-6) and (**b**) apoptosis and survival_caspase cascade (FDR-corrected *P*-value=1.8E-2) were significantly enriched in the differentially expressed proteins of the LPS groups compared with control. Analysed proteins are denoted with a circle (red=upregulation, blue=downregulation).

Gene ontology analysis using BiNGO of the 48 commonly modulated proteins showed a significant enrichment for acute inflammatory response (Figure 1d). In the hypothalamus of the LPS-exposed mice *protein folding and maturation_posttranslational processing of neuroendocrine peptides* (FDR-corrected *P*-value=1.2E-6) (Figure 2a) and *apoptosis and survival_caspase cascade* (FDR-corrected *P*-value=1.8E-2) (Figure 2b) were significantly enriched.

**Figure 3:**
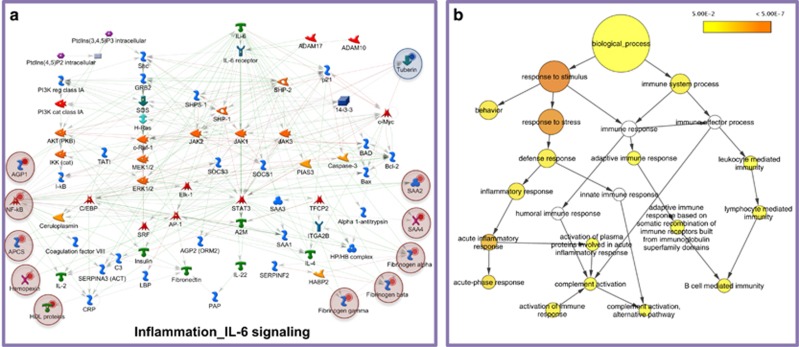
(**a**) *In silico* analysis using MetaCore showed that inflammation_IL-6 signalling was significantly enriched in the differentially expressed proteins of the HFD groups compared with control (FDR-corrected *P*-value=6.5E-6). Analysed proteins are denoted with a circle (red=upregulation, blue=downregulation). (**b**) Gene ontology analysis using BiNGO confirmed that acute and chronic inflammatory responses are significantly enriched in the differentially expressed hypothalamic proteins of HFD-fed mice compared with control. Coloured nodes representing GO terms correspond to those that were significant according to the *P*-value colour scale, whereas white nodes were not significant. The size of the node reflects the number of proteins that mapped to the corresponding GO term.

In the differentially expressed proteins of the HFD groups, *inflammation_IL-6 signalling* (FDR-corrected *P-*value=6.5E-6) (Figure 3a), *inflammation_kallikrein-kinin system* (FDR-corrected *P-*value=9.3E-6), and *inflammation_protein C signalling* (FDR-corrected *P-value*=9.2E-3) were significantly over-represented. A nodal protein in all three inflammatory pathways was NF-κB, analysed to increase in the HFD groups compared with control (Figure 1c). Gene ontology analysis using BiNGO confirmed that both acute and chronic inflammatory responses were significantly over-represented in the modulated proteome of the hypothalamus in HFD-fed mice compared with control (Figure 3b).

**Figure 4:**
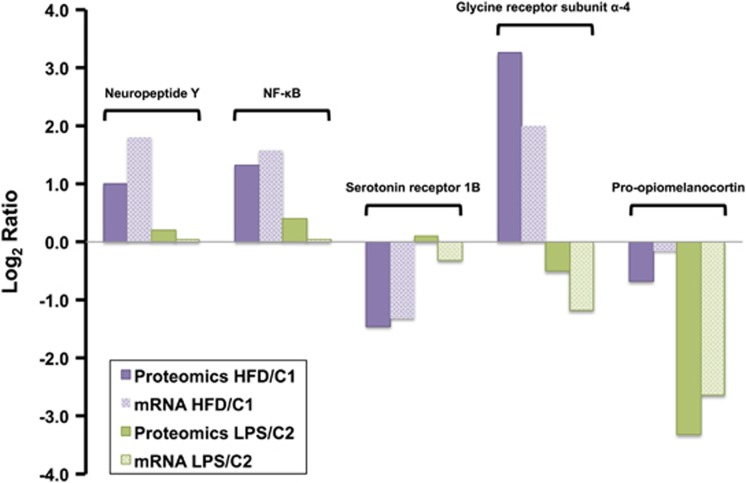
Validation of key proteomic findings using qPCR: NF-κB, pro-neuropeptide Y and glycine receptor alpha-4 subunit levels increased, whereas serotonin receptor 1B levels were reduced in the HFD groups relative to control. In the LPS groups compared with control, pro-opiomelanocortin was profiled to be downregulated.

Of potential biomedical interest to be discussed, nuclear factor-κB (NF-κB), pro-NPY and glycine receptor alpha-4 subunit levels increased, whereas serotonin receptor 1B levels were reduced in the HFD groups relative to control. In the LPS groups compared with control, pro-opiomelanocortin was profiled to be downregulated. The relative quantitation of these proteins at the mRNA level was validated using quantitative PCR (Figure 4).

## Discussion

Molecular perturbations in the hypothalamus as a result of obesity have been studied primarily at the transcriptome but not the proteome level.^15, 16^ This study compared the global proteomic profile of the mouse hypothalamus between two experimental groups in opposite states of energy balance, that is, mice with increased caloric intake through a HFD, versus mice with severely compromised food intake due to acute systemic inflammation. Acute inflammatory response was significantly enriched in the commonly modulated proteins of both groups (Figure 1d), in agreement with a number of studies indicating the altered ‘inflammatory' state in these experimental models.^3, 17^

It is well established that LPS induces anorexia in mouse models.^18^ Our results showed that the acute systemic inflammatory response caused by LPS treatment specifically decreased pro-opiomelanocortin (Figure 4) and the related ACTH and alpha-MSH peptides with well-shown anorexigenic effects (Figure 2a). This paradoxical downregulation of anorexigenic signals possibly highlights the presence of a negative feedback loop, aiming to re-establish homoeostasis. Furthermore, hypothalamic sampling was done 18 h after LPS administration, as the mice were recovering from the endotoxin effects, as the administered LPS dose was at a medium range. Along this theme, reduced expression levels of caspase isoforms 3, 6 and 7 in the hypothalamus of LPS-treated mice was observed (Figure 2b). This finding suggests control of apoptotic processes and protection of neurons during re-establishment of homoeostasis, supporting the theory that inflammatory and homoeostatic control mechanisms may antagonise in a time- and immune activation phase-dependent manner.^19^

In the modulated proteome of the HFD groups, a number of inflammatory pathways, including the IL-6 pathway (Figure 3a), kallikrein-kinin system and protein C signalling, were significantly enriched, reflecting a more pronounced, chronic inflammatory response, also evident in the gene ontology analysis (Figure 3b). Upregulation of pro-inflammatory mediators in the hypothalamus is a hallmark of obesity and constitutes a major component of insulin resistance pathogenesis.^20^ The exact mechanisms mediating this phenotype remain a subject of intense investigation with endoplasmic reticulum stress, oxidative stress or direct activation of inflammatory-related transcription factors among the implicated processes.

A central node in the hypothalamic inflammatory processes was NF-κB, found to be upregulated as a result of HFD (Figure 4). NF-κB, an important mediator of metabolic inflammation, is enriched in hypothalamic neurons but normally remains inactive.^21^ Over-nutrition alone, even before the onset of obesity, activates hypothalamic NF-κB partly through increased endoplasmic reticulum stress.^21^ Furthermore, activation of hypothalamic NF-κB interrupts central insulin and leptin signalling and actions, whereas local suppression in the mediobasal hypothalamus, or more specifically in hypothalamic Agouti-related peptide neurons, significantly protects against obesity and glucose intolerance.^21^

NPY was profiled to increase in the hypothalamus of HFD-fed mice compared with control (Figure 4). Central administration of NPY, a well-established orexigenic peptide in the hypothalamus,^22^ has been found to increase food intake and body weight, with chronic administration leading to development of obesity.^23, 24^ Furthermore, NPY overexpression in the dorsomedial hypothalamus induces hyperphagia and obesity,^25, 26^ whereas knockdown of NPY improves these changes.^27, 28^ A recent study showed that dorsomedial hypothalamus NPY knockdown rats on HFD had normal food intake, body weight and glucose tolerance/insulin sensitivity indexes as observed in lean control rats.^28^ These authors suggest that dorsomedial hypothalamus NPY could be a potential therapeutic target for obesity and diabetes.

In the hypothalamus of HFD-fed mice compared with control, levels of serotonin receptor 1B (5-hydroxytryptamin receptor 1B; 5-HT1BR), a key modulator of energy homeostasis and a therapeutic target for obesity, were analysed to decrease (Figure 4). Serotonin reduces the activity of the orexigenic AgRP/NPY neurons via the 5-HT1BR in mice (or 5-HT1DBR in humans).^29^ Studies have shown that mice lacking the 5-HT1BR develop hyperphagia, resulting in body weight increase.^30^ In addition, administration of CP-94,253, a highly selective 5-HT1BR agonist, significantly decreases food intake^31^ and potentiates the anorectic effects of pro-opiomelanocortin.

GlyR was analysed to be upregulated in the hypothalamus of mice with obesity compared with control (Figure 4). Although glycine is a well-established inhibitory neurotransmitter in the spinal cord and brain stem,^32^ studies examining the function of glycine receptors in higher brain structures including the hypothalamus are limited. A study by Karnani *et al.*^33^ showed that glycine receptor agonists exert a hyperpolarizing action on mature hypocretin/orexin (hcrt/orx) neurons in the hypothalamus, important regulators of arousal and reward seeking behaviour. To the best of our knowledge, our study is the first to identify an increase of GlyR levels in the hypothalamus of mice exposed to HFD. GlyR receptor antagonists, could be tested for their efficacy to reduce central orexigenic signals in obesity.

## Conclusion

High-precision quantitative proteomics revealed that under acute systemic inflammation in the hypothalamus as a response to LPS, homeostatic mechanisms mediating loss of appetite take effect. Conversely, under chronic inflammation in the hypothalamus as a response to HFD, mechanisms mediating a sustained ‘perpetual cycle' of appetite enhancement were observed. The combinatorial protein level modulation of the NF-κB, 5-HT1BR, GlyR and pro-NPY provides strong evidence for the enhancement of orexigenic signals as a response to HFD. Our study is the first to identify an increase of GlyR levels in the hypothalamus of mice exposed to HFD. Antagonists for this receptor could be tested for their efficacy to reduce central orexigenic signals in obesity.

## References

[bib1] Agborsangaya CB, Ngwakongnwi E, Lahtinen M, Cooke T, Johnson JA. Multimorbidity prevalence in the general population: the role of obesity in chronic disease clustering. BMC Public Health 2013; 13: 1161. 24325303 10.1186/1471-2458-13-1161 PMC4029057

[bib2] Schwartz MW, Woods SC, Porte D Jr, Seeley RJ, Baskin DG. Central nervous system control of food intake. Nature 2000; 404: 661–671. 10766253 10.1038/35007534

[bib3] Hart BL. Biological basis of the behavior of sick animals. Neurosci Biobehav Rev 1988; 12: 123–137. 3050629 10.1016/s0149-7634(88)80004-6

[bib4] Thaler JP, Schwartz MW. Minireview: Inflammation and obesity pathogenesis: the hypothalamus heats up. Endocrinology 2010; 151: 4109–4115. 20573720 10.1210/en.2010-0336 PMC2940486

[bib5] Moresco JJ, Dong MQ, Yates JR 3rd. Quantitative mass spectrometry as a tool for nutritional proteomics. Am J Clin Nutr 2008; 88: 597–604. 18779273 10.1093/ajcn/88.3.597

[bib6] Ghibaudi L, Cook J, Farley C, van Heek M, Hwa JJ. Fat intake affects adiposity, comorbidity factors, and energy metabolism of sprague-dawley rats. Obes Res 2002; 10: 956–963. 12226145 10.1038/oby.2002.130

[bib7] Johnston SL, Souter DM, Tolkamp BJ, Gordon IJ, Illius AW, Kyriazakis I et al. Intake compensates for resting metabolic rate variation in female C57BL/6J mice fed high-fat diets. Obesity (Silver Spring) 2007; 15: 600–606. 17372309 10.1038/oby.2007.550

[bib8] Nicolaides NC, Charmandari E, Chrousos GP, Kino T. Circadian endocrine rhythms: the hypothalamic-pituitary-adrenal axis and its actions. Ann N Y Acad Sci 2014; 1318: 71–80. 24890877 10.1111/nyas.12464 PMC4104011

[bib9] White CH, Johnston HE, Moesker B, Manousopoulou A, Margolis DM, Richman DD et al. Mixed effects of suberoylanilide hydroxamic acid (SAHA) on the host transcriptome and proteome and their implications for HIV reactivation from latency. Antiviral Res 2015; 123: 78–85. 26343910 10.1016/j.antiviral.2015.09.002 PMC5606336

[bib10] Delehouzé C, Godl K, Loaëc N, Bruyère C, Desban N, Oumata N et al. CDK/CK1 inhibitors roscovitine and CR8 downregulate amplified MYCN in neuroblastoma cells. Oncogene 2014; 33: 5675–5687. 24317512 10.1038/onc.2013.513 PMC4087096

[bib11] Papachristou EK, Roumeliotis TI, Chrysagi A, Trigoni C, Charvalos E, Townsend PA et al. The shotgun proteomic study of the human ThinPrep cervical smear using iTRAQ mass-tagging and 2D LC-FT-Orbitrap-MS: the detection of the human papillomavirus at the protein level. J Proteome Res 2013; 12: 2078–2089. 23510160 10.1021/pr301067r

[bib12] Hanley CJ, Noble F, Ward M, Bullock M, Drifka C, Mellone M et al. A subset of myofibroblastic cancer-associated fibroblasts regulate collagen fiber elongation, which is prognostic in multiple cancers. Oncotarget 2016; 7: 6159–6174. 26716418 10.18632/oncotarget.6740 PMC4868747

[bib13] Manousopoulou A, Woo J, Woelk CH, Johnston HE, Singhania A, Hawkes C et al. Are you also what your mother eats? Distinct proteomic portrait as a result of maternal high-fat diet in the cerebral cortex of the adult mouse. Int J Obes (Lond) 2015; 39: 1325–1328. 25797609 10.1038/ijo.2015.35 PMC5399160

[bib14] Omenn GS, Lane L, Lundberg EK, Beavis RC, Nesvizhskii AI, Deutsch EW. Metrics for the Human Proteome Project 2015: progress on the human proteome and guidelines for high-confidence proteins identification. J Proteome Res 2015; 14: 3452–3460. 26155816 10.1021/acs.jproteome.5b00499 PMC4755311

[bib15] Paternain L, Batlle MA, De la Garza AL, Milagro FI, Martínez JA, Campión J. Transcriptomic and epigenetic changes in the hypothalamus are involved in an increased susceptibility to a high-fat-sucrose diet in prenatally stressed female rats. Neuroendocrinology 2012; 96: 249–260. 22986707 10.1159/000341684

[bib16] Jovanovic Z, Tung YC, Lam BY, O'Rahilly S, Yeo GS. Identification of the global transcriptomic response of the hypothalamic arcuate nucleus to fasting and leptin. J Neuroendocrinol 2010; 22: 915–925. 20553370 10.1111/j.1365-2826.2010.02026.x PMC7617485

[bib17] Kälin S, Heppner FL, Bechmann I, Prinz M, Tschöp MH, Yi CX. Hypothalamic innate immune reaction in obesity. Nat Rev Endocrinol 2015; 11: 339–351. 25824676 10.1038/nrendo.2015.48

[bib18] Larson SJ, Collins SM, Weingarten HP. Dissociation of temperature changes and anorexia after experimental colitis and LPS administration in rats. Am J Physiol 1996; 271: R967–R972. 8897989 10.1152/ajpregu.1996.271.4.R967

[bib19] Okin D, Medzhitov R. Evolution of inflammatory diseases. Curr Biol 2012; 22: R733–R740. 22975004 10.1016/j.cub.2012.07.029 PMC3601794

[bib20] de Git KC, Adan RA. Leptin resistance in diet-induced obesity: the role of hypothalamic inflammation. Obes Rev 2015; 16: 207–224. 25589226 10.1111/obr.12243

[bib21] Zhang X, Zhang G, Zhang H, Karin M, Bai H, Cai D. Hypothalamic IKKβ/NF-κB and ER stress link overnutrition to energy imbalance and obesity. Cell 2008; 135: 61–73. 18854155 10.1016/j.cell.2008.07.043 PMC2586330

[bib22] Bi S, Kim YJ, Zheng F. Dorsomedial hypothalamic NPY and energy balance control. Neuropeptides 2012; 46: 309–314. 23083763 10.1016/j.npep.2012.09.002 PMC3508095

[bib23] Clark JT, Kalra PS, Crowley WR, Kalra SP. Neuropeptide Y and human pancreatic polypeptide stimulate feeding behavior in rats. Endocrinology 1984; 115: 427–429. 6547387 10.1210/endo-115-1-427

[bib24] Zarjevski N, Cusin I, Vettor R, Rohner-Jeanrenaud F, Jeanrenaud B. Chronic intracerebroventricular neuropeptide-Y administration to normal rats mimics hormonal and metabolic changes of obesity. Endocrinology 1993; 133: 1753–1758. 8404618 10.1210/endo.133.4.8404618

[bib25] Bi S, Ladenheim EE, Schwartz GJ, Moran TH. A role for NPY overexpression in the dorsomedial hypothalamus in hyperphagia and obesity of OLETF rats. Am J Physiol Regul Integr Comp Physiol 2001; 281: R254–R260. 11404301 10.1152/ajpregu.2001.281.1.R254

[bib26] Schroeder M, Zagoory-Sharon O, Shbiro L, Marco A, Hyun J, Moran TH et al. Development of obesity in the Otsuka Long-Evans Tokushima Fatty rat. Am J Physiol Regul Integr Comp Physiol 2009; 297: R1749–R1760. 19793959 10.1152/ajpregu.00461.2009 PMC2803618

[bib27] Yang L, Scott KA, Hyun J, Tamashiro KL, Tray N, Moran TH et al. Role of dorsomedial hypothalamic neuropeptide Y in modulating food intake and energy balance. J Neurosci 2009; 29: 179–190. 19129396 10.1523/JNEUROSCI.4379-08.2009 PMC2742174

[bib28] Kim YJ, Bi S. Knockdown of neuropeptide Y in the dorsomedial hypothalamus reverses high-fat diet-induced obesity and impaired glucose tolerance in rats. Am J Physiol Regul Integr Comp Physiol 2016; 310: R134–R142. 26561644 10.1152/ajpregu.00174.2015 PMC4796644

[bib29] Kennett GA, Dourish CT, Curzon G. 5-HT1B agonists induce anorexia at a postsynaptic site. Eur J Pharmacol 1987; 141: 429–435. 3666036 10.1016/0014-2999(87)90561-9

[bib30] Bouwknecht JA, van der Gugten J, Hijzen TH, Maes RA, Hen R, Olivier B. Male and female 5-HT(1B) receptor knockout mice have higher body weights than wildtypes. Physiol Behav 2001; 74: 507–516. 11790410 10.1016/s0031-9384(01)00589-3

[bib31] Lee MD, Kennett GA, Dourish CT, Clifton PG. 5-HT1B receptors modulate components of satiety in the rat: behavioural and pharmacological analyses of the selective serotonin1B agonist CP- 94,253. Psychopharmacology 2002; 164: 49–60. 12373419 10.1007/s00213-002-1162-7

[bib32] Gold MR, Martin AR. Characteristics of inhibitory post-synaptic currents in brain-stem neurons of the lamprey. J Physiol 1983; 342: 85–98. 6138429 10.1113/jphysiol.1983.sp014841 PMC1193949

[bib33] Karnani MM, Venner A, Jensen LT, Fugger L, Burdakov D. Direct and indirect control of orexin/hypocretin neurons by glycine receptors. J Physiol 2011; 589: 639–651. 21135047 10.1113/jphysiol.2010.198457 PMC3055548

