# Supplementary figures and images for: Hypothalamus proteomics from mouse models with obesity and anorexia reveals therapeutic targets of appetite regulation
Source: Nutr Diabetes. 2016 Apr 25;6(4):e204–. doi: 10.1038/nutd.2016.10 (PMC4855256; doi:10.1038/nutd.2016.10)

**A** Weight curve

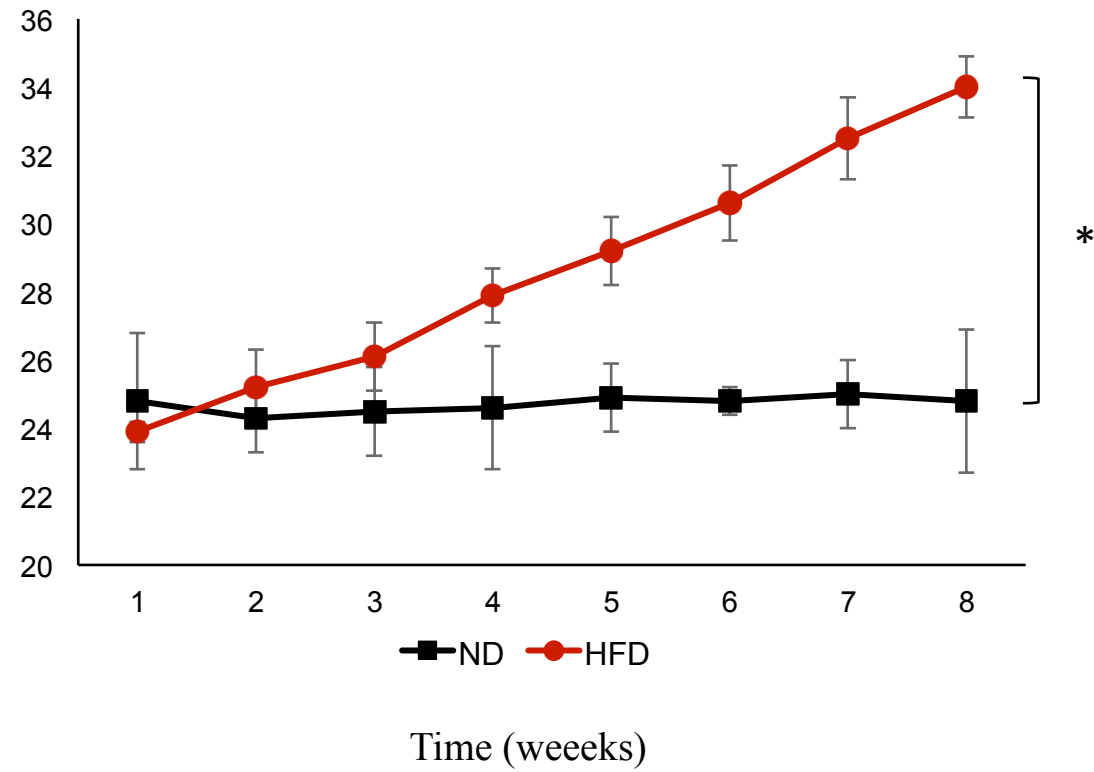

**B** Homeostatic model assessment (HOMA)

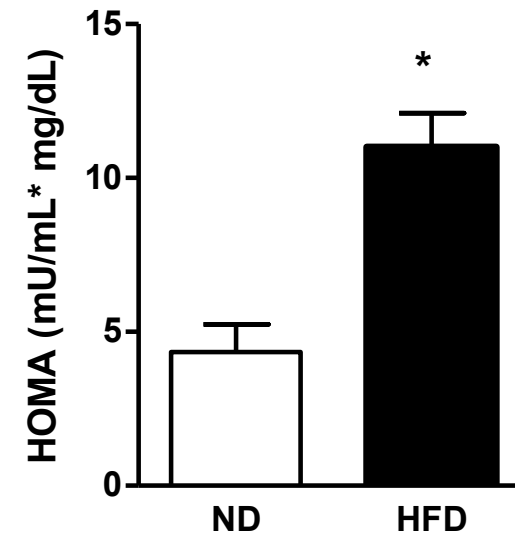

Supplement: Supplementary Figure 1 [file nutd201610x1.pdf]
