# Supplementary material for: Hypothalamus proteomics from mouse models with obesity and anorexia reveals therapeutic targets of appetite regulation
Source: Nutr Diabetes. 2016 Apr 25;6(4):e204–. doi: 10.1038/nutd.2016.10 (PMC4855256; doi:10.1038/nutd.2016.10)
Supplement: Supplementary Methods 1 [file nutd201610x2.pdf]

Supplementary Methods

High-pH Reverse Phase (RP) Peptide Fractionation

High-PH RP C_8 fractionation of the iTRAQ labelled peptides was performed on the Shimadzu LC-20AD HPLC system using the Waters, XBridge C8 column (150 × 3 mm, 3.5 μm particle). The composition of mobile phase (A) was 2% acetonitrile, 0.1% ammonium hydroxide, whereas the composition of mobile phase (B) was 100% acetonitrile, 0.1% ammonium hydroxide. The dried-up peptide pellet was dissolved in 100 μL of 98% mobile phase (A) and 2% mobile phase (B) with extensive vortex mixing. The sample was centrifuged at 13,000 rpm for 10 min, and the supernatant was injected in a 100 μL sample loop. The gradient used was the following: for 10 min isocratic 2% (B), for 10 min isocratic 5% (B), for 70 min gradient up to 20% (B), for 20 min gradient up to 85% (B), for 10 min isocratic 85% (B), for 10 min down at 2% (B) at a flow rate 0.2 mL/min. Signal response was monitored at 215 and the column temperature was set at 30 °C. Fraction collection was performed on a peak-dependent manner and a total of 65 fractions were collected. The peptide fractions were dried-up using a speedvac concentrator for 4−5 h at 30°C and stored at −20 °C until the LC−MS analysis.

LC-nESI- FT- MS Analysis

The LC-MS experiments were performed on the Dionex Ultimate 3000 UHPLC system hyphenated with the high-resolution nano-ESI LTQ-Velos Pro Orbitrap Elite mass spectrometer (Thermo Scientific, Waltham, MA, US). Individual peptide fractions were reconstituted in 2% acetonitrile, 0.1% formic acid and approximately 500ng of peptide material was loaded onto an Acclaim PepMap 100 (100 μm x 2 cm C_{18}, 5 μm particle) trapping column at 5 μL/min flow rate for 8 min. Peptides were eluted at 300 nl x min^{-1}

over a gradient of 3-22 % (140 mins), and 22-85 % (30 mins) organic phase (95% ACN, 5% DMSO, 0.1% FA) in aqueous phase (2% ACN, 5% DMSO, 0.1% FA) and were resolved on an Acclaim PepMap 100 column (C18 75 μm × 50 cm, 2 μm particle) retrofitted to a PicoTip nESI emitter (New Objective, Woburn, MA). Electrospray ionisation was conducted at 2.4 kV and precursor ions were characterised with an Obitrap Elite – Velos pro mass spectrometer (Thermo Scientific, Waltham, MA, US) at 60,000 mass resolution. The top 8 precursor ions per MS scan (minimum intensity; 500, minimum charge state; +2) were characterised by HCD (15,000 mass resolution, 1.2 Da isolation window, 40 keV collision energy) and CID (ion trap MS, 2 Da isolation window, 35 keV) with a dynamic exclusion (±10 ppm) of 120 seconds.
