# Supplementary material for: Hypothalamus proteomics from mouse models with obesity and anorexia reveals therapeutic targets of appetite regulation
Source: Nutr Diabetes. 2016 Apr 25;6(4):e204–. doi: 10.1038/nutd.2016.10 (PMC4855256; doi:10.1038/nutd.2016.10)
Supplement: Supplementary Methods 2 [file nutd201610x3.pdf]

Supplementary Methods 2

Protein grouping inference process in the Proteome Discoverer application

1. In the first step, the application collects all peptide spectrum matches (PSMs) that meet the selection criteria that you specified through the settings of the parameters in the Protein Grouping (Enabled) area on the Result Filters page. The Help explains these parameters. You can use these settings to specify which PSMs to consider for the inference of the protein groups. For example, if you set the Consider Only PSMs with Confidence at Least parameter to Medium, the Proteome Discoverer application considers only PSMs with a medium- or high-identification confidence when it creates the protein groups and ignores PSMs with a low-identification confidence. You can further use the Consider Only PSMs with Delta Cn Better Than parameter to filter out PSMs over a normalized score and consider the remaining PSMs for inclusion in the protein group inference process if their confidence levels fit.

   This first step prevents protein groups from including low-scoring, low-confidence PSMs. Even if the Proteome Discoverer application loads all PSMs initially identified by the search engines without applying further result filters, it considers only those PSMs meeting the specified criteria when inferring protein groups. If the set result filters filter out PSMs, the application does not consider them for the protein grouping process, even if they would otherwise fit the set grouping criteria.

2. In the second step, the application creates preliminary protein groups from the PSMs collected in the first step. It combines all proteins into one protein group that contains the same subset of peptides.

The Proteome Discoverer application takes the next steps in the protein grouping process if you select the Apply Strict Maximum Parsimony Principle parameter in the Result Filters page.

3. In the third step, the application removes all protein groups that have no unique peptides among the peptides that it considers for the protein grouping process. If a protein group does not contain at least one unique peptide, all of its peptides are also included by other protein groups, so there is no supporting evidence for the existence of this protein group. At this point, the application explicitly retains all protein groups that form circular rings of overlapping shared peptides. For example, suppose a circular ring is composed of the protein groups:
    - ABCD (identified by peptides a, b, c, and d)
    - CDEF (identified by peptides c, d, e, and f)
    - EFAB (identified by peptides e, f, a, and b)

    To explain all identified peptides, only two of the three protein groups are needed, but at this point it is not clear which to take and which to reject. The application postpones the resolution of this issue until step 5.

4. In the fourth step, the application first collects all spectra with more than one peptide match to consider for the protein grouping process. It then resolves these ambiguous cases and selects one of the PSMs to use for the protein grouping process while rejecting the remaining peptide matches of a spectrum. In cases where more than one PSM is considered for a spectrum, it resolves this ambiguity by selecting the PSM that is connected to the "best" protein group and rejecting the other PSMs. The "best" protein group is the group with the highest number of unambiguous and unique peptides and the highest protein score.
5. In the fifth step, the application resolves the cases where protein groups form circular rings of overlapping identified peptides. This step is the last step of the protein group inference

process, resulting in the final list of protein groups that are reported in the Proteins page of the MSF file.
