# Supplementary material for: Hypothalamus proteomics from mouse models with obesity and anorexia reveals therapeutic targets of appetite regulation
Source: Nutr Diabetes. 2016 Apr 25;6(4):e204–. doi: 10.1038/nutd.2016.10 (PMC4855256; doi:10.1038/nutd.2016.10)
Supplement: Supplementary Table 1 [file nutd201610x4.pdf]

Supplementary Table 1. Name, primer sequences, product size of reference genes and two target genes.

Symbol	Gene name	Primer sequence
**Htr1b**	5-hydroxytryptamine (serotonin) receptor 1B	F: GCGGTGGAGTATTCTGCTAAA<br>R:GAAGGGTGGCAACGAAATAGA
**Npy**	neuropeptide Y	F: CAGCCCTGAGACACTGATTT<br>R:GAGATGAGATGAGGGTGGAAAC
**Pro-Pomc**	pro-opiomelanocortin-alpha (transcript variant 5)	F:CTAAGAGAGGCCACTGAACATC<br>R: GTAGCAGAATCTCGGCATCTT
**Glra4**	glycine receptor alpha4 subunit	F: GCCTAAGGTGTCCTACGTAAAG<br>R: CTCAAGCAAGGCAGCAAATAC
**NFκ-B**	Nuclear factor of kappa light polypeptide gene enhancer in B-cells 2	F: CCGCCCTATCACAAGATGAA<br>R: GTCTTCCACCAGAGGGTAATATG
**β-actin**	Actin, beta	F: AGAAAATCTGGCACCACACC<br>R: TAGCACAGCCTGGATAGCAA
**GAPDH**	Glyceraldehyde-3-phosphate dehydrogenase	F: GACAGTCAGCCGCATCTTCT<br>R: TTAAAAGCAGCCCTGGTGAC
