# Supplementary material for: Hypothalamus proteomics from mouse models with obesity and anorexia reveals therapeutic targets of appetite regulation
Source: Nutr Diabetes. 2016 Apr 25;6(4):e204–. doi: 10.1038/nutd.2016.10 (PMC4855256; doi:10.1038/nutd.2016.10)
Supplement: Supplementary Table 2 [file nutd201610x5.pdf]

Supplementary Table 2. Total proteome

Accession	Description	ΣCoverage	Σ# Proteins	Unique Pepti	Σ# Peptides	Σ# PSMs	HFD1/C1	HFD2/C1	LPS1/C2	LPS2/C2	Score A(3,6)	overage A(3,	Peptides A(3	# PSM A(3,6)	# AAs	MW [kDa]	calc. pI
Q9D902	General transcription factor IIE subunit 2 OS=Mus musculus GN=Gtf2e2 PE=2 SV=2 - [T2EB_MOUSE]	15.41	1	4	5	8	0.695	0.148	0.018	0.013	19.03	15.41	6	8	292	33.0	9.64
Q62056	MCG130054 OS=Mus musculus GN=Psg17 PE=2 SV=1 - [Q62056_MOUSE]	1.68	1	1	1	1	2.849	0.107	0.034	0.019	0.00	1.68	1	1	475	52.9	7.44
D6MZJ6	MCG22096 OS=Mus musculus GN=Smpd5 PE=2 SV=1 - [D6MZJ6_MOUSE]	3.73	1	1	1	1	4.156	0.292	0.016	0.019	1.91	3.73	1	1	483	53.8	8.65
P49772-4	Isoform 4 of Fms-related tyrosine kinase 3 ligand OS=Mus musculus GN=Flt3lg -	3.95	1	1	1	1	3.788	0.040	0.019	0.021	0.00	3.95	1	1	253	28.2	8.63
G3X9G6	Luteinizing hormone beta OS=Mus musculus GN=Lhb PE=3 SV=1 - [G3X9G6_MOUSE]	21.99	2	2	2	9	0.203	0.133	0.036	0.033	21.98	21.99	4	9	141	15.1	8.10
K4DI78	SH3 domain-binding glutamic acid-rich protein OS=Mus musculus GN=Sh3bgr PE=4 SV=1 - [K4DI78_MOUSE]	19.23	5	2	2	3	2.869	0.250	0.054	0.048	6.69	19.23	3	3	130	14.5	4.56
Q3UKG2	Protein Prob1 (Fragment) OS=Mus musculus GN=Prob1 PE=2 SV=1 - [Q3UKG2_MOUSE]	1.59	1	1	1	2	36.455	1.251	0.068	0.056	2.51	1.59	1	2	1006	106.4	9.55
Q810Y9	PRAMEl6 OS=Mus musculus GN=Pramel6 PE=2 SV=1 - [Q810Y9_MOUSE]	5.98	1	2	2	2	3.163	0.352	0.085	0.062	4.96	5.98	2	2	485	55.8	5.88
A2CG22	Butyrophilin-like 6 OS=Mus musculus GN=Btnl6 PE=4 SV=1 - [A2CG22_MOUSE]	2.23	1	1	1	1	3.535	0.252	0.071	0.063	2.51	2.23	1	1	539	61.0	8.62
E9Q2X6	Structural maintenance of chromosomes protein OS=Mus musculus GN=Smc4 PE=2 SV=1 - [E9Q2X6_MOUSE]	2.46	2	2	4	5	27.198	0.913	0.055	0.066	15.31	2.46	4	5	1261	144.1	7.58
E9Q4F3	Thyrotropin subunit beta OS=Mus musculus GN=Tshb PE=3 SV=1 - [E9Q4F3_MOUSE]	7.97	2	1	1	4	0.260	0.164	0.058	0.071	15.37	7.97	2	4	138	15.4	7.44
Q8CDP0-4	Isoform 4 of Cytosolic carboxypeptidase 3 OS=Mus musculus GN=Agbl3 - [CBPC3_MOUSE]	3.13	4	1	1	1	5.319	0.273	0.067	0.072	0.00	3.13	1	1	511	59.3	7.27
A2RTF1	Cation channel sperm-associated protein subunit beta OS=Mus musculus GN=Catsperb PE=1 SV=1 - [CTSRB_MOUSE]	3.97	1	3	3	6	0.896	0.403	0.120	0.091	2.58	3.97	3	6	1109	126.0	8.15
Q3TML0	Protein disulfide-isomerase A6 OS=Mus musculus GN=Pdia6 PE=2 SV=1 - [Q3TML0_MOUSE]	40.22	1	1	15	66	0.748	0.040	0.620	0.096	201.23	40.22	24	66	445	48.7	5.19
Q64152	Transcription factor BTF3 OS=Mus musculus GN=Btf3 PE=2 SV=3 - [BTF3_MOUSE]	56.86	1	1	8	36	3.235	0.797	0.103	0.102	104.53	56.86	14	36	204	22.0	9.52
Q9CPQ0	Prolactin OS=Mus musculus GN=Prl PE=2 SV=2 - [Q9CPQ0_MOUSE]	24.00	3	6	6	34	0.569	0.448	0.118	0.104	83.55	24.00	10	34	225	25.4	5.43
P01193	Pro-opiomelanocortin OS=Mus musculus GN=Pomc PE=2 SV=1 - [COLI_MOUSE]	53.62	1	13	13	150	0.701	0.552	0.093	0.107	330.88	53.62	20	150	235	26.7	8.06
A2AVN2	Glycoprotein hormones alpha chain (Fragment) OS=Mus musculus GN=Cga PE=2 SV=1 - [A2AVN2_MOUSE]	12.82	2	3	3	16	0.503	0.402	0.108	0.112	35.91	12.82	4	16	117	13.2	8.47
O88904-2	Isoform 2 of Homeodomain-interacting protein kinase 1 OS=Mus musculus GN=Hipk1 - [HIPK1_MOUSE]	4.55	4	3	3	3	1.784	1.575	0.067	0.117	10.51	4.55	3	3	1165	126.1	8.28
P35455	Vasopressin-neurophysin 2-copeptin OS=Mus musculus GN=Avp PE=2 SV=1 - [NEU2_MOUSE]	72.02	1	7	9	109	1.218	1.200	0.080	0.118	257.14	72.02	17	109	168	17.9	7.58
P10637-4	Isoform Tau-C of Microtubule-associated protein tau OS=Mus musculus GN=Mapt - [TAU_MOUSE]	78.30	5	1	28	443	0.757	0.165	0.138	0.127	1210.77	78.30	50	443	341	35.7	9.44
Q7TQE7-2	Isoform 2 of Uncharacterized protein KIAA0895 OS=Mus musculus GN=Kiaa0895 - [K0895_MOUSE]	10.55	2	2	2	2	18.512	5.102	0.375	0.158	2.93	10.55	2	2	275	32.1	7.61
F8VQD3	Protein Vmn2r37 OS=Mus musculus GN=Vmn2r37 PE=3 SV=1 - [F8VQD3_MOUSE]	4.24	2	2	2	3	0.095	0.208	0.133	0.168	2.72	4.24	2	3	802	92.1	7.96
Q9DBZ1-2	Isoform 2 of Inhibitor of nuclear factor kappa-B kinase-interacting protein OS=Mus musculus GN=Ikbip - [IKIP_MOUSE]	5.51	1	2	2	5	0.841	0.491	0.165	0.182	13.56	5.51	3	5	345	38.5	8.66

P28667	MARCKS-related protein OS=Mus musculus GN=Marcksl1 PE=1 SV=2 - [MRP_MOUSE]	6.50	1	2	2	8	0.669	0.172	0.238	0.182	17.43	6.50	3	8	200	20.2	4.61
F8VPK5	Rho-associated protein kinase 2 OS=Mus musculus GN=Rock2 PE=2 SV=1 - [F8VPK5_MOUSE]	50.36	2	1	62	301	0.812	0.223	1.276	0.184	830.29	50.36	107	301	1388	160.5	5.99
Q14BE7	Family with sequence similarity 47, member A OS=Mus musculus GN=Fam47c PE=2 SV=1 - [Q14BE7_MOUSE]	6.98	1	3	3	3	6.603	0.990	0.215	0.185	5.17	6.98	3	3	430	50.6	8.65
P35454	Oxytocin-neurophysin 1 OS=Mus musculus GN=Oxt PE=2 SV=1 - [NEU1_MOUSE]	50.40	1	2	4	57	1.090	1.511	0.160	0.186	157.06	50.40	7	57	125	12.8	5.44
P29594	Caspase-2 OS=Mus musculus GN=Casp2 PE=1 SV=5 - [CASP2_MOUSE]	1.33	1	1	1	2	0.254	0.270	0.175	0.196	4.39	1.33	1	2	452	50.6	6.52
Q8BML2	Protein Oacyl OS=Mus musculus GN=Oacyl PE=2 SV=1 - [Q8BML2_MOUSE]	1.75	1	1	1	2	0.786	0.982	0.286	0.207	6.30	1.75	2	2	685	76.5	8.02
Q8CGN4-4	Isoform 4 of BCL-6 corepressor OS=Mus musculus GN=Bcor - [BCOR_MOUSE]	0.76	4	1	1	2	1.523	0.113	0.397	0.208	3.19	0.76	1	2	1707	186.6	6.87
Q8BVR6	RING finger and SPRY domain-containing protein 1 OS=Mus musculus GN=Rspry1 PE=2 SV=1 - [RSPRY_MOUSE]	8.16	3	4	4	4	1.624	0.680	0.370	0.210	11.42	8.16	4	4	576	64.3	5.50
H3BLA9	Sentrin-specific protease 6 (Fragment) OS=Mus musculus GN=Senp6 PE=2 SV=1 - [H3BLA9_MOUSE]	3.43	1	1	1	3	0.303	0.204	0.197	0.232	6.09	3.43	2	3	204	23.5	5.85
Q8BP71-5	Isoform 5 of RNA binding protein fox-1 homolog 2 OS=Mus musculus GN=Rbfox2 -	25.06	9	3	9	25	5.205	1.271	0.322	0.252	63.05	25.06	14	25	435	46.2	5.68
D3Z3A9	Reticulocalbin-3 (Fragment) OS=Mus musculus GN=Rcn3 PE=2 SV=1 - [D3Z3A9_MOUSE]	22.26	4	5	5	9	1.186	1.664	0.217	0.262	22.59	22.26	7	9	274	31.8	4.89
Q7TSI0-2	Isoform 2 of Zinc finger protein 12 OS=Mus musculus GN=Znf12 - [ZNF12_MOUSE]	1.99	2	1	1	1	4.081	0.911	0.287	0.267	0.00	1.99	1	1	654	75.0	7.91
Q04888	Transcription factor SOX-10 OS=Mus musculus GN=Sox10 PE=2 SV=2 - [SOX10_MOUSE]	5.58	1	1	1	2	1.126	0.302	0.454	0.269	7.72	5.58	2	2	466	49.9	6.60
Q3UHJ1	TNF receptor-associated factor 3 OS=Mus musculus GN=Traf3 PE=2 SV=1 - [Q3UHJ1_MOUSE]	40.04	4	1	23	87	1.205	1.962	0.451	0.279	261.56	40.04	36	87	542	61.5	8.03
Q61941	NAD(P) transhydrogenase, mitochondrial OS=Mus musculus GN=Nnt PE=1 SV=2 - [NNTM_MOUSE]	25.41	4	28	28	107	0.593	1.088	0.327	0.282	286.58	25.41	51	107	1086	113.8	7.64
P06880	Somatotropin OS=Mus musculus GN=Gh1 PE=2 SV=1 - [SOMA_MOUSE]	65.74	1	16	17	303	0.524	0.440	0.275	0.282	862.23	65.74	30	303	216	24.7	6.34
P09813	Apolipoprotein A-II OS=Mus musculus GN=Apoa2 PE=1 SV=2 - [APOA2_MOUSE]	18.63	1	2	2	10	3.448	0.451	0.317	0.291	26.46	18.63	4	10	102	11.3	7.18
P51880	Fatty acid-binding protein, brain OS=Mus musculus GN=Fabp7 PE=1 SV=2 - [FABP7_MOUSE]	69.70	2	6	7	237	1.814	0.406	0.267	0.293	712.63	69.70	12	237	132	14.9	5.63
B2RRL2	Jerky protein homolog-like OS=Mus musculus GN=Jrkl PE=2 SV=1 - [JERKL_MOUSE]	2.29	1	1	1	1	14.228	0.999	0.487	0.294	2.10	2.29	1	1	523	59.7	7.91
Q6GT24	Peroxiredoxin 6 OS=Mus musculus GN=Prdx6 PE=2 SV=1 - [Q6GT24_MOUSE]	66.07	2	1	15	164	1.361	0.957	0.896	0.294	449.52	66.07	29	164	224	24.8	6.37
Q8BGD6	Putative sodium-coupled neutral amino acid transporter 9 OS=Mus musculus GN=Slc38a9 PE=1 SV=1 - [S38A9_MOUSE]	4.46	1	2	2	5	4.947	0.814	0.398	0.298	19.43	4.46	3	5	560	63.4	7.50
Q80VP5	Probable peptide chain release factor C12orf65 homolog, mitochondrial OS=Mus musculus PE=1 SV=1 - [CL065_MOUSE]	13.59	3	2	2	2	4.469	0.424	0.357	0.311	2.41	13.59	2	2	184	20.8	9.92
Q80YN3	Breast carcinoma-amplified sequence 1 homolog OS=Mus musculus GN=Bcas1 PE=1 SV=3 - [BCAS1_MOUSE]	45.02	3	4	26	241	1.089	0.080	0.612	0.313	669.91	45.02	44	241	633	67.3	6.21
P84228	Histone H3.2 OS=Mus musculus GN=Hist1h3b PE=1 SV=2 - [H32_MOUSE]	59.56	2	3	11	54	0.469	0.271	0.297	0.314	120.02	59.56	17	54	136	15.4	11.27
P05622	Platelet-derived growth factor receptor beta OS=Mus musculus GN=Pdgfrb PE=1 SV=1 - [PGFRB_MOUSE]	9.93	9	9	10	28	2.084	0.922	0.316	0.314	74.60	9.93	15	28	1098	122.7	5.12

P97792	Coxsackievirus and adenovirus receptor homolog OS=Mus musculus GN=Cxadr PE=1 SV=1 - [CXAR_MOUSE]	26.03	3	9	9	30	0.616	0.687	0.403	0.317	75.82	26.03	17	30	365	39.9	6.96
G3XA11	Cornichon homolog 3 (Drosophila), isoform CRA_a OS=Mus musculus GN=Cnih3 PE=4 SV=1 - [G3XA11_MOUSE]	10.71	3	1	1	4	2.215	1.351	0.368	0.321	17.10	10.71	2	4	112	13.2	7.31
Q4V9V3	Elongation of very long chain fatty acids (FEN1/Elo2, SUR4/Elo3, yeast)-like 1 OS=Mus musculus GN=Elovl1 PE=2 SV=1 - [Q4V9V3_MOUSE]	5.94	2	1	1	8	0.580	0.140	0.231	0.324	13.66	5.94	2	8	202	23.7	9.76
Q9D6E4	Tetratricopeptide repeat protein 9B OS=Mus musculus GN=Ttc9b PE=2 SV=1 - [TTC9B_MOUSE]	39.75	1	5	7	16	0.873	1.493	0.748	0.326	43.84	39.75	12	16	239	25.9	9.48
Q19LI2	Alpha-1B-glycoprotein OS=Mus musculus GN=A1bg PE=1 SV=1 - [A1BG_MOUSE]	8.79	1	4	4	5	0.873	0.334	0.300	0.334	12.95	8.79	5	5	512	56.5	6.79
D3Z7S5	Transmembrane protein 208 OS=Mus musculus GN=Tmem208 PE=2 SV=1 - [D3Z7S5_MOUSE]	6.36	3	1	1	1	0.739	1.119	0.401	0.340	2.46	6.36	1	1	110	12.1	7.12
A2AMG5	Nibrin (Fragment) OS=Mus musculus GN=Nbn PE=2 SV=1 - [A2AMG5_MOUSE]	2.92	2	1	1	1	0.695	0.415	0.749	0.341	4.31	2.92	1	1	548	60.5	7.28
Q8VIG1	RE1-silencing transcription factor OS=Mus musculus GN=Rest PE=2 SV=2 - [REST_MOUSE]	1.76	1	1	2	2	70.853	4.067	1.022	0.351	5.07	1.76	2	2	1082	117.7	6.65
E9PWG6	Protein Ncapg OS=Mus musculus GN=Ncapg PE=4 SV=1 - [E9PWG6_MOUSE]	1.49	1	1	1	1	4.024	4.058	1.584	0.351	2.19	1.49	1	1	1004	112.8	5.55
F6TQW2	Protein Ighg2c OS=Mus musculus GN=Ighg2c PE=4 SV=1 - [F6TQW2_MOUSE]	10.42	2	2	4	8	1.935	0.760	0.770	0.353	24.77	10.42	5	8	403	44.1	6.90
Q3ULB5	Serine/threonine-protein kinase PAK 6 OS=Mus musculus GN=Pak6 PE=2 SV=1 - [PAK6_MOUSE]	7.62	1	2	3	8	1.700	1.612	1.013	0.353	13.17	7.62	5	8	682	74.8	9.44
P16014	Secretogranin-1 OS=Mus musculus GN=Chgb PE=1 SV=2 - [SCG1_MOUSE]	31.31	1	20	20	99	1.144	0.805	0.519	0.356	302.09	31.31	38	99	677	77.9	5.07
G3UZJ2	Microtubule-associated protein (Fragment) OS=Mus musculus GN=Map2 PE=2 SV=1 - [G3UZJ2_MOUSE]	71.78	1	2	22	183	0.991	0.542	0.415	0.363	549.97	71.78	38	183	241	25.7	11.09
Q8CGT2	Baculoviral IAP repeat-containing protein 1e OS=Mus musculus GN=Naip5 PE=2 SV=1 - [Q8CGT2_MOUSE]	2.21	2	2	2	2	18.228	2.803	0.824	0.368	2.96	2.21	2	2	1403	159.7	5.87
Q9Z0F1-2	Isoform Nesp55-2 of Neuroendocrine secretory protein 55 OS=Mus musculus GN=Gnas - [GNAS3_MOUSE]	7.91	2	2	2	7	1.175	0.446	0.301	0.372	14.20	7.91	4	7	253	28.9	5.05
Q9ER41	Torsin-1B OS=Mus musculus GN=Tor1b PE=2 SV=2 - [TOR1B_MOUSE]	10.12	4	2	2	3	2.055	0.464	0.600	0.372	5.66	10.12	3	3	336	37.8	8.02
P26339	Chromogranin-A OS=Mus musculus GN=Chga PE=1 SV=1 - [CMGA_MOUSE]	13.61	1	5	5	28	1.522	0.627	0.465	0.379	77.66	13.61	8	28	463	51.8	4.72
Q64676	2-hydroxyacylsphingosine 1-beta-galactosyltransferase OS=Mus musculus GN=Ugt8 PE=2 SV=2 - [illegible]	24.77	1	15	15	37	0.883	0.131	0.291	0.380	94.12	24.77	24	37	541	61.2	9.42
Q6NS86	Protein Zfp366 OS=Mus musculus GN=Zfp366 PE=2 SV=1 - [Q6NS86_MOUSE]	1.47	1	1	1	1	0.770	0.579	0.516	0.386	0.00	1.47	1	1	746	84.8	7.97
Q497J1	Lysoplasmalogenase OS=Mus musculus GN=Tmem86b PE=2 SV=1 - [TM86B_MOUSE]	8.85	1	1	1	1	5.906	1.152	0.358	0.390	2.70	8.85	1	1	226	25.0	6.73
P30681	High mobility group protein B2 OS=Mus musculus GN=Hmgb2 PE=1 SV=3 -	24.76	1	5	6	16	0.720	0.485	0.502	0.390	39.28	24.76	10	16	210	24.1	7.31
G5E8I7	Ubiquitin carboxyl-terminal hydrolase OS=Mus musculus GN=Usp17lc PE=3 SV=1 - [G5E8I7_MOUSE]	2.75	1	1	1	2	0.485	0.264	0.343	0.392	2.21	2.75	1	2	545	61.4	7.53
Q8VI24-2	Isoform 2 of DNA-binding protein SATB2 OS=Mus musculus GN=Satb2 - [SATB2_MOUSE]	2.97	3	2	2	2	1.063	1.887	0.401	0.394	5.31	2.97	2	2	674	76.0	7.50
H3BJH0	Low-density lipoprotein receptor-related protein 5 OS=Mus musculus GN=Lrp5 PE=2 SV=1 - [H3BJH0_MOUSE]	3.94	3	1	1	1	4.623	1.342	0.327	0.396	2.18	3.94	1	1	279	30.9	6.92

Q8BX10-2	Isoform 2 of Serine/threonine-protein phosphatase PGAM5, mitochondrial OS=Mus musculus GN=Pgam5 - [illegible]	47.74	3	1	14	45	1.220	0.686	0.534	0.397	103.38	47.74	24	45	287	31.8	9.04
Q9JJF0	Nucleosome assembly protein 1-like 5 OS=Mus musculus GN=Nap1l5 PE=2 SV=1 -	21.79	1	5	5	28	0.592	0.292	0.377	0.398	61.56	21.79	10	28	156	17.0	4.32
Q04886	Transcription factor SOX-8 OS=Mus musculus GN=Sox8 PE=2 SV=2 - [SOX8_MOUSE]	15.52	2	3	3	6	1.249	0.527	0.970	0.401	16.60	15.52	4	6	464	49.8	7.15
F7C9E8	Uncharacterized protein OS=Mus musculus GN=Gm5501 PE=4 SV=1 - [F7C9E8_MOUSE]	4.53	1	1	1	1	2.298	0.733	0.349	0.401	2.57	4.53	1	1	287	31.7	8.19
Q9CXL6	Doublecortin, isoform CRA_a OS=Mus musculus GN=Dcx PE=2 SV=1 - [Q9CXL6_MOUSE]	30.56	3	7	11	44	0.594	0.542	0.609	0.401	115.12	30.56	18	44	360	40.0	9.39
Q6NS65	Uracil nucleotide/cysteinyl leukotriene receptor OS=Mus musculus GN=Gpr17 PE=2 SV=1 - [GPR17_MOUSE]	5.31	1	2	2	5	0.868	0.597	0.479	0.401	14.46	5.31	4	5	339	37.8	8.72
Q4QRL3	Coiled-coil domain-containing protein 88B OS=Mus musculus GN=Ccdc88b PE=1 SV=2 - [CC88B_MOUSE]	3.04	2	2	3	3	1.330	0.288	0.279	0.412	8.00	3.04	3	3	1481	166.5	5.33
E9PYP8	Beta-crystallin B1 (Fragment) OS=Mus musculus GN=Crybb1 PE=2 SV=1 -	16.95	2	3	3	9	0.856	0.574	0.580	0.415	28.76	16.95	5	9	236	26.5	7.88
Q7TS75	APC membrane recruitment protein 1 OS=Mus musculus GN=Amer1 PE=2 SV=3 -	1.68	1	1	1	1	24.277	0.948	0.491	0.416	3.65	1.68	1	1	1132	124.1	4.68
Q8CGC4	Protein LSM14 homolog B OS=Mus musculus GN=Lsm14b PE=2 SV=3 - [LS14B_MOUSE]	18.18	1	1	6	15	1.718	0.833	0.579	0.419	38.25	18.18	9	15	385	42.3	9.63
Q9CY02	Alpha-hemoglobin-stabilizing protein OS=Mus musculus GN=Ahsp PE=2 SV=1 - [AHSP_MOUSE]	7.84	1	1	1	2	4.220	1.422	0.366	0.425	5.62	7.84	2	2	102	11.8	4.74
E9Q0M2	Membrane-associated guanylate kinase, WW and PDZ domain-containing protein 2 OS=Mus musculus GN=Magi2 PE=4 SV=1 - [E9Q0M2_MOUSE]	14.73	1	1	1	2	2.371	2.907	0.678	0.429	0.00	14.73	1	2	129	14.5	7.05
Q61603	Glycine receptor subunit alpha-4 OS=Mus musculus GN=Glra4 PE=2 SV=3 - [GLRA4_MOUSE]	4.39	1	1	2	3	14.349	6.330	1.152	0.429	8.27	4.39	3	3	456	52.5	8.44
Q9D9X8-2	Isoform 2 of Sperm acrosome membrane-associated protein 3 OS=Mus musculus GN=Spaca3 - [illegible]	32.52	2	2	2	4	1.222	0.440	0.478	0.430	1.79	32.52	2	4	163	18.4	6.86
F7B6A4	Protein Kctd17 (Fragment) OS=Mus musculus GN=Kctd17 PE=2 SV=1 - [F7B6A4_MOUSE]	10.19	5	1	1	1	0.840	0.585	0.916	0.430	3.03	10.19	1	1	206	23.6	4.63
D3YUR6	Transmembrane protein 169 (Fragment) OS=Mus musculus GN=Tmem169 PE=2 SV=1 - [D3YUR6_MOUSE]	21.67	2	2	2	3	0.614	0.403	0.682	0.442	10.52	21.67	3	3	120	13.4	5.02
A2AWR2	GPR155 variant 5 OS=Mus musculus GN=Gpr155 PE=2 SV=1 - [A2AWR2_MOUSE]	1.55	2	1	1	3	2.574	0.817	0.528	0.444	5.45	1.55	2	3	840	93.1	6.52
Q61500	Integral membrane protein 2A OS=Mus musculus GN=Itm2a PE=2 SV=2 - [ITM2A_MOUSE]	4.18	1	1	1	5	1.000	0.938	0.466	0.446	13.24	4.18	2	5	263	29.7	5.72
H3BK32	Sentrin-specific protease 6 (Fragment) OS=Mus musculus GN=Senp6 PE=2 SV=1 - [H3BK32_MOUSE]	2.62	5	2	2	3	4.459	1.281	0.815	0.446	5.88	2.62	2	3	917	103.6	6.37
E9Q4D5	GTP-binding protein REM 2 OS=Mus musculus GN=Rem2 PE=2 SV=1 - [E9Q4D5_MOUSE]	12.90	5	3	3	5	0.844	0.304	2.572	0.447	19.82	12.90	5	5	341	37.4	7.39
Q8K4K3	Tribbles homolog 2 OS=Mus musculus GN=Trib2 PE=2 SV=2 - [TRIB2_MOUSE]	3.50	1	1	1	2	4.391	1.447	0.910	0.448	2.39	3.50	1	2	343	38.7	6.15
E9Q8Z7	Mirror-image polydactyly gene 1 protein homolog OS=Mus musculus GN=Mipol1 PE=2 SV=1 - [E9Q8Z7_MOUSE]	10.03	5	2	4	5	1.356	0.968	1.035	0.454	11.28	10.03	5	5	379	43.5	5.43
Q80VW5-2	Isoform 2 of Whirlin OS=Mus musculus GN=Dfnb31 - [WHRN_MOUSE]	5.38	8	4	4	5	3.034	0.680	0.452	0.455	17.79	5.38	5	5	911	97.0	8.70
Q9QWK5	Baculoviral IAP repeat-containing protein 1a OS=Mus musculus GN=Naip1 PE=2 SV=3 - [BIR1A_MOUSE]	1.85	3	2	2	4	2.012	1.380	0.523	0.459	2.89	1.85	2	4	1403	158.6	6.30

Q8K094	Poliovirus receptor OS=Mus musculus GN=Pvr PE=2 SV=1 - [Q8K094_MOUSE]	9.80	1	2	2	4	1.524	0.845	0.546	0.460	10.73	9.80	3	4	408	44.6	8.06
P28651	Carbonic anhydrase-related protein OS=Mus musculus GN=Ca8 PE=1 SV=5 - [CAH8_MOUSE]	20.96	1	5	5	18	1.135	0.783	1.425	0.460	42.57	20.96	8	18	291	33.1	4.78
Q9CX53	Gem-associated protein 6 OS=Mus musculus GN=Gemin6 PE=2 SV=2 - [GEMI6_MOUSE]	7.83	1	1	1	6	3.834	0.903	0.449	0.460	10.93	7.83	2	6	166	18.7	5.25
O35887	Calumenin OS=Mus musculus GN=Calu PE=1 SV=1 - [CALU_MOUSE]	64.13	7	3	16	78	1.229	0.652	1.000	0.462	242.01	64.13	26	78	315	37.0	4.67
Q3TTY5	Keratin, type II cytoskeletal 2 epidermal OS=Mus musculus GN=Krt2 PE=1 SV=1 - [K22E_MOUSE]	10.47	1	4	6	26	5.182	0.804	0.674	0.468	54.43	10.47	9	26	707	70.9	8.06
P97378	Interleukin-12 receptor subunit beta-2 OS=Mus musculus GN=Il12rb2 PE=1 SV=1 - [I12R2_MOUSE]	3.20	2	2	2	3	2.999	1.099	0.379	0.470	3.31	3.20	3	3	874	98.1	7.81
P19324	Serpin H1 OS=Mus musculus GN=Serpinh1 PE=1 SV=3 - [SERPH_MOUSE]	37.65	1	15	15	45	0.690	0.792	0.360	0.470	171.60	37.65	29	45	417	46.5	8.82
Q9D6G9-2	Isoform 2 of CKLF-like MARVEL transmembrane domain-containing protein 5 OS=Mus musculus GN=Cmtm5 - [illegible]	17.59	2	1	1	2	1.106	0.405	1.193	0.471	7.10	17.59	1	2	108	11.8	4.77
Q8CGR5	Kallikrein-14 OS=Mus musculus GN=Klk14 PE=2 SV=1 - [KLK14_MOUSE]	8.00	1	1	1	1	2.015	1.532	0.471	0.473	3.21	8.00	1	1	250	27.0	9.09
Q6S5C2	N-acetylglucosamine-1-phosphotransferase subunit gamma OS=Mus musculus GN=Gnptg PE=2 SV=1 - [GNPTG_MOUSE]	10.42	2	2	2	4	1.511	0.925	0.403	0.473	11.32	10.42	4	4	307	34.1	6.92
D3Z6X8	Protein orai-3 OS=Mus musculus GN=Orai3 PE=2 SV=1 - [D3Z6X8_MOUSE]	19.12	1	1	1	2	3.800	1.796	1.209	0.474	5.18	19.12	1	2	136	14.5	8.21
F8VQ01	Protein Olfr66 OS=Mus musculus GN=Olfr66 PE=3 SV=1 - [F8VQ01_MOUSE]	10.29	1	1	1	2	2.026	1.073	0.347	0.475	0.00	10.29	1	2	311	35.3	8.53
G3X8T9	Serine (Or cysteine) peptidase inhibitor, clade A, member 3N, isoform CRA_a OS=Mus musculus GN=Serpina3n PE=3 SV=1 - [G3X8T9_MOUSE]	37.32	1	1	14	59	8.671	3.473	7.027	0.475	162.42	37.32	24	59	418	46.7	5.82
P43275	Histone H1.1 OS=Mus musculus GN=Hist1h1a PE=1 SV=2 - [H11_MOUSE]	28.64	1	4	7	36	0.612	0.777	0.575	0.475	99.19	28.64	13	36	213	21.8	10.93
Q9CQP0	39S ribosomal protein L33, mitochondrial OS=Mus musculus GN=Mrpl33 PE=2 SV=1 - [RM33_MOUSE]	18.46	1	1	1	2	1.439	0.458	0.628	0.480	3.56	18.46	2	2	65	7.4	10.89
Q60605-2	Isoform Smooth muscle of Myosin light polypeptide 6 OS=Mus musculus GN=Myl6 - [MYL6_MOUSE]	80.13	1	1	10	150	1.809	1.244	1.236	0.480	400.13	80.13	18	150	151	17.0	4.55
Q9CWF2	Tubulin beta-2B chain OS=Mus musculus GN=Tubb2b PE=1 SV=1 - [TBB2B_MOUSE]	83.82	2	2	33	4745	0.606	0.493	0.417	0.485	12689.53	83.82	61	4745	445	49.9	4.89
Q9QY93	dCTP pyrophosphatase 1 OS=Mus musculus GN=Dctpp1 PE=1 SV=1 - [DCTP1_MOUSE]	17.65	1	2	2	4	1.578	0.748	1.018	0.486	9.80	17.65	4	4	170	18.8	5.03
Q8R4E6	Purine-rich element-binding protein gamma OS=Mus musculus GN=Purg PE=1 SV=1 - [PURG_MOUSE]	27.71	2	9	13	33	0.442	0.342	0.586	0.486	94.85	27.71	19	33	350	39.9	9.51
P97363	Serine palmitoyltransferase 2 OS=Mus musculus GN=Sptlc2 PE=2 SV=2 - [SPTC2_MOUSE]	11.07	1	5	5	7	0.694	0.486	0.471	0.486	18.27	11.07	7	7	560	62.9	8.18
Q9R210	Transcription factor EB OS=Mus musculus GN=Tfeb PE=1 SV=2 - [TFEB_MOUSE]	7.37	13	2	5	6	2.079	0.431	0.739	0.487	15.67	7.37	6	6	475	52.6	6.29
Q3V188	Poly(U)-specific endoribonuclease OS=Mus musculus GN=Endou PE=2 SV=1 - [ENDOU_MOUSE]	5.83	2	2	2	6	0.692	0.508	0.722	0.487	18.75	5.83	3	6	412	47.0	5.30
Q8R0K2	E3 ubiquitin-protein ligase TRIM31 OS=Mus musculus GN=Trim31 PE=1 SV=1 - [TRI31_MOUSE]	7.10	1	2	3	4	1.611	0.778	0.457	0.491	5.64	7.10	3	4	507	57.1	7.85
Q8CEG5	Coiled-coil domain-containing protein 28B OS=Mus musculus GN=Ccdc28b PE=2 SV=3 - [CC28B_MOUSE]	20.50	4	3	3	7	0.532	0.321	0.907	0.492	23.30	20.50	5	7	200	22.0	5.36
Q91XZ5	Protein Pcdhb5 OS=Mus musculus GN=Pcdhb5 PE=2 SV=1 - [Q91XZ5_MOUSE]	2.15	1	1	1	1	2.690	0.985	0.533	0.493	2.65	2.15	1	1	792	87.2	4.94

Q61166	Microtubule-associated protein RP/EB family member 1 OS=Mus musculus GN=Mapre1 PE=1 SV=3 - [illegible]	42.54	1	7	11	88	0.932	0.678	0.612	0.498	260.07	42.54		21	88	268	30.0	5.22
Q9EPR2-2	Isoform 2 of Group XIIA secretory phospholipase A2 OS=Mus musculus GN=Pla2g12a - [PG12A_MOUSE]	8.50	2	1	1	1	3.423	1.769	0.589	0.500	3.51	8.50		1	1	153	17.2	6.49
Q9JIP4	Pannexin-1 OS=Mus musculus GN=Panx1 PE=1 SV=3 - [PANX1_MOUSE]	5.87	1	1	1	3	1.054	0.820	0.464	0.500	13.53	5.87		2	3	426	48.1	7.03
Q8BPB5	EGF-containing fibulin-like extracellular matrix protein 1 OS=Mus musculus GN=Efemp1 PE=2 SV=1 - [FBLN3_MOUSE]	12.98	1	4	4	16	0.856	1.861	0.431	0.500	33.41	12.98		6	16	493	54.9	5.14
Q80YX1	Tenascin OS=Mus musculus GN=Tnc PE=1 SV=1 - [TENA_MOUSE]	26.73	6	45	45	127	0.772	0.809	0.700	0.505	360.99	26.73		78	127	2110	231.7	4.89
B1AU75	Nuclear autoantigenic sperm protein OS=Mus musculus GN=Nasp PE=4 SV=1 - [B1AU75_MOUSE]	16.56	4	9	10	21	1.945	0.705	0.707	0.506	60.38	16.56		14	21	773	84.0	4.37
P02469	Laminin subunit beta-1 OS=Mus musculus GN=Lamb1 PE=1 SV=3 - [LAMB1_MOUSE]	13.10	3	19	21	46	1.091	0.606	0.483	0.508	128.21	13.10		33	46	1786	197.0	4.94
D3YZ69	Microtubule-associated tumor suppressor candidate 2 homolog OS=Mus musculus GN=Mtus2 PE=2 SV=1 - [illegible]	33.65	4	1	10	22	1.114	1.481	0.874	0.510	42.22	33.65		15	22	315	36.9	5.44
Q8BHT6	Beta-1,3-glucosyltransferase OS=Mus musculus GN=B3galtl PE=2 SV=3 -	13.09	2	3	5	15	0.762	1.038	0.871	0.512	27.99	13.09		8	15	489	55.3	6.92
O08664	B-cell CLL/lymphoma 7 protein family member C OS=Mus musculus GN=Bcl7c PE=1 SV=1 - [BCL7C_MOUSE]	5.53	1	1	1	2	1.254	1.023	0.371	0.513	3.45	5.53		2	2	217	23.4	5.20
D3Z6B9	Mitochondrial 10-formyltetrahydrofolate dehydrogenase OS=Mus musculus GN=Aldh1l2 PE=2 SV=1 - [D3Z6B9_MOUSE]	32.35	2	15	23	73	0.880	0.436	0.382	0.516	203.53	32.35		40	73	810	88.8	5.58
Q9D115	Zinc finger protein 706 OS=Mus musculus GN=Znf706 PE=2 SV=1 - [ZN706_MOUSE]	25.00	1	2	2	3	1.288	0.550	0.851	0.520	4.88	25.00		2	3	76	8.5	10.01
P70677	Caspase-3 OS=Mus musculus GN=Casp3 PE=1 SV=1 - [CASP3_MOUSE]	25.63	1	7	7	18	1.453	0.888	0.571	0.521	42.71	25.63		12	18	277	31.5	6.92
P47955	60S acidic ribosomal protein P1 OS=Mus musculus GN=Rplp1 PE=1 SV=1 - [RLA1_MOUSE]	57.02	3	3	3	9	1.153	0.877	0.755	0.521	19.27	57.02		4	9	114	11.5	4.32
Q8CHK4-3	Isoform 3 of Histone acetyltransferase KAT5 OS=Mus musculus GN=Kat5 - [KAT5_MOUSE]	5.49	4	2	2	2	3.496	0.228	0.961	0.524	6.07	5.49		2	2	546	61.8	8.57
O88566	Axin-2 OS=Mus musculus GN=Axin2 PE=1 SV=2 - [AXIN2_MOUSE]	2.14	2	2	2	2	0.857	0.671	0.624	0.525	4.56	2.14		2	2	840	92.8	7.75
Q9D7E4	UPF0449 protein C19orf25 homolog OS=Mus musculus PE=1 SV=1 - [CS025_MOUSE]	51.38	1	3	3	8	2.166	1.037	1.578	0.532	18.11	51.38		5	8	109	12.1	5.25
P43025	Tetranectin OS=Mus musculus GN=Clec3b PE=1 SV=2 - [TETN_MOUSE]	26.73	2	3	3	3	2.198	0.996	0.994	0.534	12.40	26.73		3	3	202	22.2	5.66
Q99PL5	Ribosome-binding protein 1 OS=Mus musculus GN=Rrbp1 PE=2 SV=2 - [RRBP1_MOUSE]	54.39	12	57	69	298	1.273	0.480	0.669	0.537	755.89	54.39		118	298	1605	172.8	9.33
P20917	Myelin-associated glycoprotein OS=Mus musculus GN=Mag PE=1 SV=2 - [MAG_MOUSE]	31.31	1	3	17	196	1.588	0.224	0.646	0.537	630.89	31.31		30	196	626	69.2	5.10
F6ZMJ4	Trinucleotide repeat-containing gene 6A protein OS=Mus musculus GN=Tnrc6a PE=2 SV=2 - [F6ZMJ4_MOUSE]	3.06	2	3	3	9	1.580	0.430	0.922	0.537	13.09	3.06		4	9	1896	203.0	6.99
A2AFS3	UPF0577 protein KIAA1324 OS=Mus musculus GN=Kiaa1324 PE=2 SV=1 -	9.91	3	8	8	19	0.923	0.933	0.521	0.537	57.15	9.91		13	19	1009	110.6	6.38
E0CY96	tRNA-splicing endonuclease subunit Sen15 OS=Mus musculus GN=Tsen15 PE=2 SV=1 - [E0CY96_MOUSE]	14.06	2	1	1	2	1.008	0.515	0.667	0.537	5.89	14.06		2	2	128	14.0	4.41
Q9QYI6	DnaJ homolog subfamily B member 9 OS=Mus musculus GN=Dnajb9 PE=2 SV=2 -	6.76	1	1	1	4	1.147	0.467	0.430	0.544	14.21	6.76		2	4	222	25.6	8.28

Q5MPP0-2	Isoform 2 of Fatty acid 2-hydroxylase OS=Mus musculus GN=Fa2h - [FA2H_MOUSE]	8.63	2	2	2	6	2.114	0.304	0.481	0.544	22.68	8.63	4	6	313	36.4	7.09
B8JJD1	Protein Fance (Fragment) OS=Mus musculus GN=Fance PE=2 SV=1 - [B8JJD1_MOUSE]	11.63	4	1	1	1	0.596	0.747	0.928	0.545	0.00	11.63	1	1	129	14.6	5.15
E9Q9D6	R3H domain-containing protein 2 OS=Mus musculus GN=R3hdm2 PE=2 SV=1 -	9.30	7	5	7	16	0.649	0.619	0.830	0.546	37.20	9.30	10	16	978	106.8	8.91
Q9JMG1	Endothelial differentiation-related factor 1 OS=Mus musculus GN=Edf1 PE=1 SV=1 - [EDF1_MOUSE]	37.84	1	7	7	28	1.121	0.505	0.853	0.546	72.94	37.84	12	28	148	16.4	9.99
Q9QZI8	Serine incorporator 1 OS=Mus musculus GN=Serinc1 PE=1 SV=1 - [SERC1_MOUSE]	5.74	1	2	2	2	0.350	0.638	0.731	0.546	7.37	5.74	2	2	453	50.5	6.28
Q91WD8	Protein Slc24a1 OS=Mus musculus GN=Slc24a1 PE=2 SV=1 - [Q91WD8_MOUSE]	5.31	1	3	3	4	3.640	2.786	0.738	0.548	2.23	5.31	3	4	1130	124.6	4.65
Q9WVS8-5	Isoform 5 of Mitogen-activated protein kinase 7 OS=Mus musculus GN=Mapk7 -	7.67	6	4	4	11	0.676	0.466	0.944	0.548	22.04	7.67	6	11	756	82.3	6.46
G3UYZ1	Immunoglobulin superfamily member 8 OS=Mus musculus GN=Igsf8 PE=2 SV=1 - [G3UYZ1_MOUSE]	39.78	2	1	16	185	0.956	0.386	0.989	0.551	681.36	39.78	31	185	548	58.1	7.91
Q569L8	Centromere protein J OS=Mus musculus GN=Cenpj PE=2 SV=2 - [CENPJ_MOUSE]	2.38	1	2	4	9	0.638	1.714	1.930	0.551	23.35	2.38	4	9	1344	153.0	6.55
Q9ERB0	Synaptosomal-associated protein 29 OS=Mus musculus GN=Snap29 PE=1 SV=1 -	50.77	1	12	12	87	1.485	0.923	0.962	0.554	169.81	50.77	21	87	260	29.6	5.38
Q6A078	Centrosomal protein of 290 kDa OS=Mus musculus GN=Cep290 PE=1 SV=2 -	2.79	3	4	8	18	0.824	0.697	0.471	0.555	44.11	2.79	10	18	2472	288.9	6.10
Q3UK98	Chromobox protein homolog 6 OS=Mus musculus GN=Cbx6 PE=2 SV=1 - [Q3UK98_MOUSE]	10.10	4	4	4	4	0.741	0.720	0.576	0.555	12.14	10.10	4	4	396	42.5	9.95
Q9R0B9	Procollagen-lysine,2-oxoglutarate 5-dioxygenase 2 OS=Mus musculus GN=Plod2 PE=2 SV=2 - [PLOD2_MOUSE]	1.63	2	1	1	1	2.399	0.735	0.622	0.555	2.69	1.63	1	1	737	84.4	6.89
O08538	Angiopoietin-1 OS=Mus musculus GN=Angpt1 PE=2 SV=2 - [ANGP1_MOUSE]	2.81	1	1	1	1	2.360	1.037	0.631	0.556	4.04	2.81	1	1	498	57.5	6.76
Q8K4L2	Archvillin OS=Mus musculus GN=Svil PE=2 SV=1 - [Q8K4L2_MOUSE]	4.58	5	7	8	10	1.072	0.859	0.493	0.558	25.10	4.58	9	10	2031	226.7	7.94
Q6NXL1	Protein Sec24d OS=Mus musculus GN=Sec24d PE=2 SV=1 - [Q6NXL1_MOUSE]	3.00	1	2	3	5	0.638	1.122	0.501	0.558	12.92	3.00	5	5	1032	112.6	7.18
D3YTQ9	40S ribosomal protein S15 OS=Mus musculus GN=Rps15 PE=2 SV=1 - [D3YTQ9_MOUSE]	33.05	3	4	4	9	0.652	0.282	0.830	0.559	12.73	33.05	5	9	118	13.7	10.59
Q9D735	Uncharacterized protein C19orf43 homolog OS=Mus musculus PE=2 SV=1 - [CS043_MOUSE]	21.97	1	3	3	16	0.748	0.320	0.979	0.560	33.45	21.97	6	16	173	18.4	9.67
O88508	DNA (cytosine-5)-methyltransferase 3A OS=Mus musculus GN=Dnmt3a PE=1 SV=2 -	5.51	6	3	3	6	2.335	1.734	1.305	0.561	9.31	5.51	3	6	908	101.6	6.65
P50543	Protein S100-A11 OS=Mus musculus GN=S100a11 PE=2 SV=1 - [S10AB_MOUSE]	43.88	1	4	4	19	1.350	1.993	0.451	0.563	51.60	43.88	7	19	98	11.1	5.45
P29699	Alpha-2-HS-glycoprotein OS=Mus musculus GN=Ahsg PE=1 SV=1 - [FETUA_MOUSE]	44.06	1	10	10	63	2.666	1.391	0.380	0.564	244.73	44.06	19	63	345	37.3	6.51
Q9NWG9	Melanoma-associated antigen H1 OS=Mus musculus GN=Mageh1 PE=2 SV=1 -	9.17	1	1	1	4	0.459	0.607	0.586	0.565	17.86	9.17	2	4	218	24.3	9.48
P36423	Thromboxane-A synthase OS=Mus musculus GN=Tbxas1 PE=2 SV=2 - [THAS_MOUSE]	2.63	1	1	1	2	1.617	1.251	0.913	0.565	2.24	2.63	1	2	533	60.4	7.15
Q768S4	Rab effector Noc2 OS=Mus musculus GN=Rph3al PE=1 SV=1 - [RPH3L_MOUSE]	1.99	1	1	1	1	0.582	1.145	0.744	0.567	2.04	1.99	1	1	302	33.2	8.82
G5E8Q8	MCG115189 OS=Mus musculus GN=Gpr116 PE=4 SV=1 - [G5E8Q8_MOUSE]	3.41	1	2	2	4	0.966	1.069	0.649	0.567	11.06	3.41	3	4	1348	149.3	7.02
Q80W00-2	Isoform 2 of Serine/threonine-protein phosphatase 1 regulatory subunit 10 OS=Mus musculus GN=Ppp1r10 - [illegible]	4.35	2	3	3	4	3.152	0.566	0.860	0.567	11.53	4.35	4	4	874	93.0	9.16

Q8C2K1-2	Isoform 2 of Differentially expressed in FDCP 6 OS=Mus musculus GN=Def6 - [DEFI6_MOUSE]	7.00	2	2	2	4	2.939	0.537	0.771	0.567	11.46	7.00	4	4	257	29.8	8.29
Q9WTL4	Insulin receptor-related protein OS=Mus musculus GN=Insrr PE=1 SV=2 - [INSRR_MOUSE]	2.54	1	2	3	4	5.023	12.078	0.754	0.567	4.22	2.54	3	4	1300	144.8	6.64
P43276	Histone H1.5 OS=Mus musculus GN=Hist1h1b PE=1 SV=2 - [H15_MOUSE]	38.57	1	7	13	68	0.533	0.528	0.656	0.568	162.52	38.57	24	68	223	22.6	10.92
Q6ZWQ9	MCG5400 OS=Mus musculus GN=Myl12a PE=2 SV=1 - [Q6ZWQ9_MOUSE]	49.42	3	1	7	117	1.432	0.490	0.464	0.568	350.96	49.42	13	117	172	19.9	4.81
Q8R0A6	V-set and transmembrane domain-containing protein 2A OS=Mus musculus GN=Vstm2a PE=2 SV=2 - [VTM2A_MOUSE]	5.51	2	1	1	3	1.217	0.909	0.761	0.570	7.53	5.51	2	3	236	25.9	8.27
E9PWE8	Dihydropyrimidinase-related protein 3 OS=Mus musculus GN=Dpysl3 PE=2 SV=1 -	61.49	5	27	33	852	1.045	0.565	0.539	0.570	2338.20	61.49	61	852	683	73.8	6.46
K4DID3	Developmental pluripotency-associated protein 3 (Fragment) OS=Mus musculus GN=Dppa3 PE=4 SV=1 - [illegible]	9.93	1	1	1	1	12.292	4.121	1.280	0.571	2.36	9.93	1	1	141	16.6	10.80
E9Q7T7	Protein Chadl OS=Mus musculus GN=Chadl PE=2 SV=1 - [E9Q7T7_MOUSE]	10.43	3	8	8	14	0.721	0.578	0.552	0.573	37.39	10.43	13	14	748	81.3	8.87
Q8VE98	CD276 antigen OS=Mus musculus GN=Cd276 PE=1 SV=1 - [CD276_MOUSE]	17.72	1	4	4	6	0.679	1.181	0.552	0.574	18.40	17.72	6	6	316	34.0	4.82
P97452	Ribosome biogenesis protein BOP1 OS=Mus musculus GN=Bop1 PE=1 SV=1 - [BOP1_MOUSE]	3.14	1	3	3	6	1.331	0.897	0.814	0.576	17.10	3.14	4	6	732	82.5	6.28
P97864	Caspase-7 OS=Mus musculus GN=Casp7 PE=1 SV=2 - [CASP7_MOUSE]	7.92	1	2	2	3	1.435	0.549	0.657	0.578	8.78	7.92	3	3	303	34.0	6.33
P12660	Purkinje cell protein 2 OS=Mus musculus GN=Pcp2 PE=2 SV=2 - [PCP2_MOUSE]	40.83	2	4	4	19	3.253	1.190	2.159	0.579	65.20	40.83	6	19	120	13.0	5.14
Q8CG48	Structural maintenance of chromosomes protein 2 OS=Mus musculus GN=Smc2 PE=1 SV=2 - [SMC2_MOUSE]	2.69	1	3	3	9	1.089	0.776	0.628	0.580	21.01	2.69	4	9	1191	134.2	8.41
Q921K2	Poly (ADP-ribose) polymerase family, member 1 OS=Mus musculus GN=Parp1 PE=2 SV=1 - [Q921K2_MOUSE]	17.16	3	15	15	34	0.871	0.526	0.787	0.582	86.51	17.16	23	34	1014	112.7	8.95
Q9R0X2	ADAM DEC1 OS=Mus musculus GN=Adamdec1 PE=2 SV=1 - [ADEC1_MOUSE]	3.21	1	1	1	1	3.247	1.467	0.441	0.582	0.00	3.21	1	1	467	52.9	8.48
P70213	Friend virus susceptibility protein 1 OS=Mus musculus GN=Fv1 PE=2 SV=1 - [FV1_MOUSE]	7.41	1	2	2	3	0.669	0.782	0.325	0.583	12.47	7.41	3	3	459	52.0	5.26
Q8VDP2	UPF0428 protein CXorf56 homolog OS=Mus musculus PE=2 SV=1 - [CX056_MOUSE]	22.52	2	5	5	14	1.310	0.625	0.807	0.583	34.46	22.52	7	14	222	25.6	8.73
Q9Z0F8	Disintegrin and metalloproteinase domain-containing protein 17 OS=Mus musculus GN=Adam17 PE=1 SV=3 - [illegible]	11.97	5	8	8	19	0.975	0.881	0.775	0.583	41.72	11.97	14	19	827	93.0	5.94
Q8R344	Coiled-coil domain-containing protein 12 OS=Mus musculus GN=Ccdc12 PE=1 SV=2 - [CCD12_MOUSE]	18.07	1	2	2	8	0.929	0.361	0.962	0.584	14.09	18.07	4	8	166	18.9	7.21
Q69ZA1-2	Isoform 2 of Cyclin-dependent kinase 13 OS=Mus musculus GN=Cdk13 -	4.62	16	3	5	31	1.370	1.228	0.840	0.585	56.43	4.62	8	31	1451	158.0	9.70
O88874	Cyclin-K OS=Mus musculus GN=Ccnk PE=1 SV=3 - [CCNK_MOUSE]	9.39	2	4	5	10	48.442	1.402	0.950	0.588	34.36	9.39	7	10	554	61.3	8.41
K7N6K8	Protein Sult2a7 OS=Mus musculus GN=Sult2a7 PE=4 SV=1 - [K7N6K8_MOUSE]	2.68	1	1	1	2	2.388	1.136	0.727	0.589	1.73	2.68	1	2	298	34.7	6.70
Q80W32	IQ domain-containing protein G OS=Mus musculus GN=Iqcg PE=2 SV=1 - [IQCG_MOUSE]	3.10	1	1	1	1	2.111	1.439	1.490	0.590	2.24	3.10	1	1	419	49.1	5.73
Q61555	Fibrillin-2 OS=Mus musculus GN=Fbn2 PE=1 SV=2 - [FBN2_MOUSE]	1.79	1	4	6	9	0.889	2.638	0.633	0.590	18.37	1.79	7	9	2907	313.6	4.84
Q3TIV5	Zinc finger CCCH domain-containing protein 15 OS=Mus musculus GN=Zc3h15 PE=1 SV=2 - [ZC3HF_MOUSE]	14.79	3	6	6	23	1.004	0.585	1.056	0.591	56.33	14.79	12	23	426	48.3	5.30

Q8BMK4	Cytoskeleton-associated protein 4 OS=Mus musculus GN=Ckap4 PE=2 SV=2 - [CKAP4_MOUSE]	55.30	6	28	29	164	1.048	0.932	0.673	0.593	447.66	55.30	53	164	575	63.7	5.64
Q8R1N0	Zinc finger protein 830 OS=Mus musculus GN=Znf830 PE=1 SV=1 - [ZN830_MOUSE]	10.74	1	4	4	9	1.500	0.479	0.972	0.594	28.05	10.74	5	9	363	40.6	5.40
Q9CQ94	DnaJ homolog subfamily C member 5B OS=Mus musculus GN=Dnajc5b PE=1 SV=1 -	11.56	1	1	2	3	7.609	0.931	0.744	0.595	7.78	11.56	3	3	199	22.6	5.62
Q9D1C2	Protein chibby homolog 1 OS=Mus musculus GN=Cby1 PE=2 SV=1 - [CBY1_MOUSE]	9.45	1	1	1	1	1.284	1.151	1.091	0.595	0.00	9.45	1	1	127	14.5	9.35
F8VQH0	Abelson tyrosine-protein kinase 2 OS=Mus musculus GN=Abl2 PE=2 SV=1 - [F8VQH0_MOUSE]	26.14	3	1	25	79	0.596	1.023	1.025	0.595	184.08	26.14	41	79	1182	128.1	7.84
E9Q4X2	Protein Uggt2 OS=Mus musculus GN=Uggt2 PE=2 SV=1 - [E9Q4X2_MOUSE]	3.72	3	3	8	9	0.880	0.841	0.549	0.596	20.05	3.72	9	9	1504	172.7	6.20
Q9DBW3	Protein GTLF3B OS=Mus musculus GN=Gtlf3b PE=2 SV=1 - [GTL3B_MOUSE]	21.82	1	2	2	3	3.162	0.768	0.775	0.596	5.47	21.82	3	3	110	12.7	8.47
Q6PGA0-2	Isoform 2 of REST corepressor 3 OS=Mus musculus GN=Rcor3 - [RCOR3_MOUSE]	11.06	5	4	4	8	0.997	0.787	0.652	0.598	20.56	11.06	5	8	434	47.4	6.80
Q3UX62	Coiled-coil domain-containing protein 114 OS=Mus musculus GN=Ccdc114 PE=2 SV=1 - [CC114_MOUSE]	6.99	1	1	2	2	1.834	1.067	0.930	0.598	1.93	6.99	2	2	658	74.1	7.25
O35930	Platelet glycoprotein Ib alpha chain OS=Mus musculus GN=Gp1ba PE=2 SV=2 -	3.95	1	1	1	3	1.722	0.454	0.814	0.600	0.00	3.95	1	3	734	80.0	5.87
Q3UNY1	Protein Gm16833 (Fragment) OS=Mus musculus GN=Gm16833 PE=2 SV=1 -	10.45	1	1	1	1	1.130	0.458	0.596	0.600	0.00	10.45	1	1	67	7.7	8.41
Q60972	Histone-binding protein RBBP4 OS=Mus musculus GN=Rbbp4 PE=1 SV=5 - [RBBP4_MOUSE]	36.47	2	6	12	55	1.330	0.575	0.817	0.600	146.88	36.47	22	55	425	47.6	4.89
Q9CUU3	Synaptonemal complex protein 2 OS=Mus musculus GN=Sycp2 PE=1 SV=2 - [SYCP2_MOUSE]	2.93	1	3	3	6	3.494	1.493	0.349	0.601	10.82	2.93	3	6	1500	172.0	7.93
Q9EQ08	Heparan N-sulfatase OS=Mus musculus GN=Sgsh PE=2 SV=1 - [Q9EQ08_MOUSE]	6.57	1	2	2	3	5.458	0.480	0.590	0.602	10.46	6.57	3	3	502	56.7	6.37
Q8R0J4-2	Isoform 2 of Transmembrane protein 134 OS=Mus musculus GN=Tmem134 -	4.44	3	1	1	2	1.187	0.858	0.731	0.603	6.01	4.44	2	2	180	20.2	6.54
Q8R409	Protein HEXIM1 OS=Mus musculus GN=Hexim1 PE=1 SV=1 - [HEXI1_MOUSE]	21.07	1	5	5	15	1.014	0.871	0.874	0.603	39.79	21.07	9	15	356	40.2	5.38
E0CX20	Protein BUD31 homolog OS=Mus musculus GN=Bud31 PE=4 SV=1 - [E0CX20_MOUSE]	32.64	2	5	5	9	0.986	0.556	0.602	0.603	30.98	32.64	9	9	144	17.0	8.82
Q61098	Interleukin-18 receptor 1 OS=Mus musculus GN=Il18r1 PE=2 SV=1 - [IL18R_MOUSE]	5.77	1	2	2	2	0.898	1.205	1.160	0.604	2.36	5.77	2	2	537	61.6	7.77
Q9QYS9-4	Isoform 4 of Protein quaking OS=Mus musculus GN=Qki - [QKI_MOUSE]	32.92	8	9	10	52	1.305	0.420	0.635	0.605	137.84	32.92	19	52	319	35.1	7.43
Q8CH77	Neuron navigator 1 OS=Mus musculus GN=Nav1 PE=1 SV=2 - [NAV1_MOUSE]	23.20	4	35	36	87	0.772	0.614	1.014	0.605	261.87	23.20	61	87	1875	202.2	8.06
G3X9I4	Aly/REF export factor 2 OS=Mus musculus GN=Alyref2 PE=4 SV=1 - [G3X9I4_MOUSE]	18.35	2	1	3	23	1.534	0.341	0.702	0.606	60.33	18.35	6	23	218	23.8	10.14
Q9JLG8-2	Isoform 2 of Calpain-15 OS=Mus musculus GN=Solh - [CAN15_MOUSE]	1.40	2	1	1	2	0.767	1.415	1.115	0.607	7.27	1.40	1	2	1072	115.3	6.52
P83917	Chromobox protein homolog 1 OS=Mus musculus GN=Cbx1 PE=1 SV=1 - [CBX1_MOUSE]	33.51	3	4	5	21	1.569	0.805	0.913	0.607	69.40	33.51	9	21	185	21.4	4.93
Q8JZK9	Hydroxymethylglutaryl-CoA synthase, cytoplasmic OS=Mus musculus GN=Hmgcs1 PE=1 SV=1 - [HMCS1_MOUSE]	28.46	1	14	15	60	0.946	0.377	0.517	0.607	181.84	28.46	27	60	520	57.5	5.99
Q8BXJ2	Transcriptional-regulating factor 1 OS=Mus musculus GN=Trerf1 PE=1 SV=1 - [TREF1_MOUSE]	1.83	3	1	1	3	0.790	1.153	1.641	0.607	3.67	1.83	2	3	1205	132.3	6.77
Q8R5A3	Amyloid beta A4 precursor protein-binding family B member 1-interacting protein OS=Mus musculus GN=Apbb1ip PE=1 SV=2 - [AB1IP_MOUSE]	7.01	1	3	4	9	1.305	1.006	0.579	0.608	20.41	7.01	7	9	670	74.3	5.35
P04370-9	Isoform 9 of Myelin basic protein OS=Mus musculus GN=Mbp - [MBP_MOUSE]	77.72	6	1	22	2309	1.373	0.072	0.754	0.609	6552.04	77.72	40	2309	184	20.2	11.02

Q9D0K2	Succinyl-CoA:3-ketoacid coenzyme A transferase 1, mitochondrial OS=Mus musculus GN=Oxct1 PE=1 SV=1 - [SCOT1_MOUSE]	54.23	2	20	20	259	0.800	0.672	0.671	0.610	771.07	54.23	37	259	520	56.0	8.53
Q8BGC0	HIV Tat-specific factor 1 homolog OS=Mus musculus GN=Htatsf1 PE=1 SV=1 -	23.65	2	11	12	42	1.339	0.828	0.879	0.611	119.68	23.65	20	42	757	86.2	4.40
O54990-6	Isoform 6 of Prominin-1 OS=Mus musculus GN=Prom1 - [PROM1_MOUSE]	23.69	6	1	14	37	2.024	0.959	0.693	0.611	91.55	23.69	22	37	823	92.1	6.55
Q8BVF4-2	Isoform 2 of Coiled-coil domain-containing protein 30 OS=Mus musculus GN=Ccdc30 - [CCD30_MOUSE]	5.08	4	3	4	7	1.986	1.001	0.760	0.612	14.67	5.08	6	7	649	75.5	6.48
Q91WE2	Protein FAM192A OS=Mus musculus GN=Fam192a PE=2 SV=1 - [F192A_MOUSE]	9.45	1	2	3	4	1.135	0.824	1.016	0.612	12.32	9.45	4	4	254	28.7	5.14
Q07646-2	Isoform 2 of Mesoderm-specific transcript protein OS=Mus musculus GN=Mest - [MEST_MOUSE]	22.39	5	4	4	10	0.672	0.688	0.421	0.613	34.76	22.39	8	10	326	37.6	9.44
H3BLH4	TSC22 domain family protein 1 (Fragment) OS=Mus musculus GN=Tsc22d1 PE=2 SV=1 - [H3BLH4_MOUSE]	11.36	1	1	1	1	11.331	1.767	1.614	0.613	2.53	11.36	1	1	132	13.5	4.59
Q8K083	Zinc finger protein 536 OS=Mus musculus GN=Znf536 PE=2 SV=1 - [ZN536_MOUSE]	4.30	146	2	5	9	0.796	0.650	1.418	0.613	16.62	4.30	7	9	1302	141.5	7.33
D3Z4D1	Protein JTB OS=Mus musculus GN=Jtb PE=2 SV=1 - [D3Z4D1_MOUSE]	21.05	2	2	2	4	1.143	0.760	0.321	0.613	9.08	21.05	2	4	95	10.5	7.55
E9Q507	GON-4-like protein OS=Mus musculus GN=Gon4l PE=4 SV=1 - [E9Q507_MOUSE]	2.32	3	3	3	6	1.172	0.907	1.098	0.614	12.32	2.32	4	6	2242	246.6	4.86
E9PZT5	Tumor necrosis factor receptor superfamily member 12A OS=Mus musculus GN=Tnfrsf12a PE=2 SV=1 - [illegible]	19.15	1	1	1	3	1.238	0.355	0.503	0.614	3.83	19.15	2	3	94	10.0	10.52
P62960	Nuclease-sensitive element binding protein 1 OS=Mus musculus GN=Ybx1 PE=1 SV=3 - [YBOX1_MOUSE]	31.68	4	4	7	14	0.955	0.343	0.632	0.615	35.04	31.68	10	14	322	35.7	9.88
Q811B5	Proline-rich protein 3 OS=Mus musculus GN=Prr3 PE=2 SV=1 - [PRR3_MOUSE]	13.68	6	3	3	14	0.901	0.441	0.809	0.615	36.45	13.68	5	14	190	21.2	9.85
Q80ZD3-2	Isoform 2 of Sodium-independent sulfate anion transporter OS=Mus musculus GN=Slc26a11 - [S2611_MOUSE]	1.90	2	1	1	1	1.311	0.576	0.956	0.615	0.00	1.90	1	1	421	45.2	7.14
Q9D6V8	Polyadenylate-binding protein-interacting protein 2 OS=Mus musculus GN=Paip2 PE=2 SV=1 - [PAIP2_MOUSE]	45.97	1	3	3	16	1.685	0.767	0.902	0.616	41.16	45.97	5	16	124	14.7	4.12
Q9CY58	Plasminogen activator inhibitor 1 RNA-binding protein OS=Mus musculus GN=Serbp1 PE=1 SV=2 - [PAIRB_MOUSE]	33.42	4	14	14	133	0.802	0.465	1.035	0.616	363.99	33.42	26	133	407	44.7	8.54
O89103	Complement component C1q receptor OS=Mus musculus GN=Cd93 PE=1 SV=1 - [C1QR1_MOUSE]	10.25	1	5	5	12	0.911	0.595	0.656	0.617	39.22	10.25	8	12	644	69.3	5.07
E9Q9A3	Clathrin coat assembly protein AP180 OS=Mus musculus GN=Snap91 PE=4 SV=2 - [E9Q9A3_MOUSE]	37.11	5	1	29	390	2.333	0.687	0.851	0.618	1155.75	37.11	51	390	838	86.0	5.07
E9QMD2	Ubiquitin-conjugating enzyme E2Q-like protein 1 OS=Mus musculus GN=Ube2ql1 PE=2 SV=1 - [E9QMD2_MOUSE]	13.16	1	2	2	7	0.555	0.497	0.910	0.618	22.00	13.16	3	7	304	32.6	10.10
Q7TT18	Activating transcription factor 7-interacting protein 1 OS=Mus musculus GN=Atf7ip PE=1 SV=1 - [MCAF1_MOUSE]	7.96	1	5	5	11	1.130	0.959	0.955	0.621	38.92	7.96	9	11	1306	138.5	4.77
Q8VCH6	Delta(24)-sterol reductase OS=Mus musculus GN=Dhcr24 PE=2 SV=1 - [DHC24_MOUSE]	6.20	1	4	4	9	0.834	0.442	0.579	0.623	20.41	6.20	8	9	516	60.1	8.16
Q80XN0	D-beta-hydroxybutyrate dehydrogenase, mitochondrial OS=Mus musculus GN=Bdh1 PE=1 SV=2 - [BDH_MOUSE]	55.10	2	15	17	189	0.605	0.531	0.639	0.623	606.91	55.10	31	189	343	38.3	9.01
P47867-2	Isoform 2 of Secretogranin 3 OS=Mus musculus GN=Scg3 - [SCG3_MOUSE]	35.62	2	12	12	57	1.711	0.828	0.703	0.624	155.28	35.62	21	57	466	52.8	4.91

Q9CQ40	39S ribosomal protein L49, mitochondrial OS=Mus musculus GN=Mrpl49 PE=2 SV=1 - [RM49_MOUSE]	33.73	1	5	5	12	2.730	0.998	0.733	0.624	34.13	33.73	7	12	166	19.1	9.50
F6XR20	Formin-like protein 2 (Fragment) OS=Mus musculus GN=Fmnl2 PE=4 SV=1 - [F6XR20_MOUSE]	31.96	2	1	15	38	1.191	0.836	0.541	0.625	111.48	31.96	27	38	510	59.1	9.35
D3Z5Q8	Synaptoporin (Fragment) OS=Mus musculus GN=Synpr PE=2 SV=1 - [D3Z5Q8_MOUSE]	26.40	1	1	2	6	2.343	1.255	0.716	0.625	18.89	26.40	3	6	125	13.7	5.33
Q80ZP8	Armet protein OS=Mus musculus GN=Manf PE=2 SV=1 - [Q80ZP8_MOUSE]	55.76	7	12	12	52	1.162	0.771	0.785	0.626	154.92	55.76	21	52	165	19.0	8.13
Q9EQF6	Dihydropyrimidinase-related protein 5 OS=Mus musculus GN=Dpysl5 PE=1 SV=1 -	71.10	2	36	36	334	0.683	0.435	0.509	0.626	983.16	71.10	63	334	564	61.5	7.09
E9PZM4	Protein Chd2 OS=Mus musculus GN=Chd2 PE=2 SV=1 - [E9PZM4_MOUSE]	2.96	3	3	4	5	1.419	1.284	0.855	0.626	9.13	2.96	5	5	1827	210.7	8.06
Q62386	Interleukin-17A OS=Mus musculus GN=Il17a PE=2 SV=1 - [IL17_MOUSE]	15.19	1	1	2	2	1.743	0.412	0.583	0.627	2.34	15.19	2	2	158	17.5	8.92
P21460	Cystatin-C OS=Mus musculus GN=Cst3 PE=2 SV=2 - [CYTC_MOUSE]	46.43	2	5	6	27	1.384	0.910	0.760	0.628	91.41	46.43	11	27	140	15.5	9.00
Q8R0L9-3	Isoform 3 of Transcriptional adapter 3 OS=Mus musculus GN=Tada3 -	23.72	3	3	3	5	1.595	0.900	1.300	0.628	12.71	23.72	4	5	156	17.7	8.00
Q8K0X8	Fasciculation and elongation protein zeta-1 OS=Mus musculus GN=Fez1 PE=1 SV=2 -	26.79	5	9	9	28	1.448	0.745	0.892	0.628	88.66	26.79	14	28	392	45.2	4.36
Q91X43	SH3 domain-containing protein 19 OS=Mus musculus GN=Sh3d19 PE=1 SV=2 -	15.08	2	8	9	18	1.650	0.799	0.878	0.629	44.33	15.08	13	18	789	86.0	8.53
Q9CQE1	Protein NipSnap homolog 3B OS=Mus musculus GN=Nipsnap3b PE=1 SV=1 - [NPS3B_MOUSE]	18.22	4	5	5	24	1.770	1.012	0.854	0.630	68.22	18.22	9	24	247	28.3	9.48
Q3U0J8	TBC1 domain family member 2B OS=Mus musculus GN=Tbc1d2b PE=1 SV=2 -	4.15	2	3	4	5	0.918	1.120	0.634	0.630	14.16	4.15	5	5	965	109.9	6.02
Q80Y14	Glutaredoxin-related protein 5, mitochondrial OS=Mus musculus GN=Glrx5 PE=2 SV=2 - [GLRX5_MOUSE]	31.58	1	4	5	51	2.300	0.622	0.913	0.631	143.17	31.58	9	51	152	16.3	6.55
F6UK66	Coiled-coil domain-containing protein 50 (Fragment) OS=Mus musculus GN=Ccdc50 PE=4 SV=1 - [F6UK66_MOUSE]	18.36	4	4	5	12	1.285	0.479	0.919	0.631	31.79	18.36	8	12	256	29.8	6.67
Q9DBK0	Acyl-coenzyme A thioesterase 12 OS=Mus musculus GN=Acot12 PE=2 SV=1 -	1.80	1	1	1	2	12.316	1.836	0.853	0.631	3.34	1.80	2	2	556	61.7	7.18
D3Z6H8	S-adenosylmethionine decarboxylase proenzyme OS=Mus musculus GN=Amd2 PE=3 SV=1 - [D3Z6H8_MOUSE]	2.40	2	1	1	2	1.700	1.031	1.018	0.632	5.57	2.40	2	2	334	38.3	6.42
P81117	Nucleobindin-2 OS=Mus musculus GN=Nucb2 PE=1 SV=2 - [NUCB2_MOUSE]	36.67	1	11	12	46	1.379	1.404	0.835	0.632	135.81	36.67	21	46	420	50.3	5.15
Q6PGH2	Hematological and neurological expressed 1-like protein OS=Mus musculus GN=Hn1l PE=2 SV=1 - [HN1L_MOUSE]	41.05	2	6	6	30	3.235	0.521	0.609	0.632	86.45	41.05	12	30	190	20.0	8.62
Q9JHP7-3	Isoform 3 of KDEL motif-containing protein 1 OS=Mus musculus GN=Kdelc1 -	9.93	4	2	2	3	2.088	0.816	0.434	0.633	6.57	9.93	2	3	282	32.1	7.77
Q8BXA1	Golgi integral membrane protein 4 OS=Mus musculus GN=Golim4 PE=1 SV=1 -	5.95	2	3	3	21	1.837	0.992	0.743	0.633	43.77	5.95	6	21	655	76.7	4.83
Q8BG75	Transmembrane protein 198 OS=Mus musculus GN=Tmem198 PE=2 SV=1 - [TM198_MOUSE]	6.94	1	2	2	3	0.778	0.625	0.837	0.634	6.28	6.94	3	3	360	39.7	9.92
Q9D9Q0	Leucine-rich repeat-containing protein 69 OS=Mus musculus GN=Lrrc69 PE=2 SV=1 - [LRC69_MOUSE]	6.05	1	2	2	4	2.071	1.137	0.691	0.634	2.75	6.05	2	4	347	40.0	8.65
Q61414	Keratin, type I cytoskeletal 15 OS=Mus musculus GN=Krt15 PE=1 SV=2 - [K1C15_MOUSE]	21.02	9	4	8	24	1.720	0.346	1.171	0.635	49.30	21.02	11	24	452	49.1	4.86
F6THH9	E3 ubiquitin-protein ligase MARCH8 (Fragment) OS=Mus musculus GN=March8 PE=2 SV=1 - [F6THH9_MOUSE]	6.83	2	1	1	2	1.161	0.772	0.716	0.635	7.44	6.83	2	2	249	28.2	8.22
Q8CHT3	Integrator complex subunit 5 OS=Mus musculus GN=Ints5 PE=2 SV=1 - [INT5_MOUSE]	8.74	1	5	5	9	1.485	0.984	0.602	0.635	24.41	8.74	7	9	1018	108.3	7.06

F2Z469	Matrilin-4 OS=Mus musculus GN=Matn4 PE=2 SV=1 - [F2Z469_MOUSE]	20.11	5	10	10	22	0.424	0.987	0.473	0.636	53.55	20.11	16	22	542	60.1	5.39
Q68FE8-2	Isoform 2 of Zinc finger protein 280D OS=Mus musculus GN=Znf280d - [Z280D_MOUSE]	4.07	2	3	3	5	0.649	0.740	0.925	0.636	9.36	4.07	5	5	787	88.4	8.53
Q8VE42	Ankyrin repeat domain-containing protein 49 OS=Mus musculus GN=Ankrd49 PE=1 SV=1 - [ANR49_MOUSE]	4.62	1	1	1	2	1.658	1.157	0.965	0.636	7.44	4.62	2	2	238	27.1	5.16
P15261	Interferon gamma receptor 1 OS=Mus musculus GN=Ifngr1 PE=1 SV=3 - [INGR1_MOUSE]	9.85	1	3	3	6	3.179	1.224	0.737	0.636	6.63	9.85	4	6	477	52.3	5.53
Q8BGM5	Bestrophin-2 OS=Mus musculus GN=Best2 PE=2 SV=1 - [BEST2_MOUSE]	1.97	1	1	1	1	1.438	1.470	0.560	0.637	2.15	1.97	1	1	508	57.0	5.39
F6SLR4	Testican-2 OS=Mus musculus GN=Spock2 PE=4 SV=1 - [F6SLR4_MOUSE]	33.10	2	13	13	30	0.929	0.538	0.833	0.637	79.89	33.10	19	30	423	46.8	4.98
P50429	Arylsulfatase B OS=Mus musculus GN=Arsb PE=2 SV=3 - [ARSB_MOUSE]	18.91	3	10	10	55	1.229	0.965	0.718	0.638	112.86	18.91	18	55	534	59.6	7.24
Q9QYL0	Spermatid-specific linker histone H1-like protein OS=Mus musculus GN=Hils1 PE=1 SV=1 - [HILS1_MOUSE]	5.88	1	1	1	1	2.278	1.298	0.304	0.641	0.00	5.88	1	1	170	19.2	9.94
Q8BHK1	Magnesium transporter NIPA1 OS=Mus musculus GN=Nipa1 PE=1 SV=1 - [NIPA1_MOUSE]	1.86	1	1	1	1	0.486	1.291	0.841	0.642	2.24	1.86	1	1	323	34.1	8.37
Q9WVB4	Slit homolog 3 protein OS=Mus musculus GN=Slit3 PE=2 SV=2 - [SLIT3_MOUSE]	0.98	1	1	2	2	0.701	0.791	0.936	0.643	5.39	0.98	2	2	1523	167.6	7.62
Q80UK0	SEC14 domain and spectrin repeat-containing protein 1 OS=Mus musculus GN=Sestd1 PE=2 SV=1 - [SESD1_MOUSE]	26.44	1	14	15	44	0.646	1.059	0.820	0.643	143.11	26.44	26	44	696	79.3	5.10
Q8QZR8	Cyclin-related protein FAM58B OS=Mus musculus GN=Fam58b PE=2 SV=2 -	4.00	1	1	1	1	3.229	0.917	0.453	0.644	2.71	4.00	1	1	250	28.9	6.28
P07091	Protein S100-A4 OS=Mus musculus GN=S100a4 PE=1 SV=1 - [S10A4_MOUSE]	7.92	1	1	1	2	1.375	1.393	0.667	0.644	3.80	7.92	2	2	101	11.7	5.31
Q8BHC4	Dephospho-CoA kinase domain-containing protein OS=Mus musculus GN=Dcakd PE=2 SV=1 - [DCAKD_MOUSE]	42.86	2	8	8	31	0.765	0.681	0.559	0.645	106.20	42.86	15	31	231	26.5	9.58
Q8C3R1	BRCA1-associated ATM activator 1 OS=Mus musculus GN=Brat1 PE=2 SV=1 - [BRAT1_MOUSE]	7.54	4	4	4	7	3.653	0.919	1.033	0.645	21.13	7.54	6	7	822	89.0	5.31
P28867	Protein kinase C delta type OS=Mus musculus GN=Prkcd PE=1 SV=3 - [KPCD_MOUSE]	39.17	6	21	24	93	0.636	0.329	0.602	0.646	215.41	39.17	43	93	674	77.5	7.39
Q3TVI8	Pre-B-cell leukemia transcription factor-interacting protein 1 OS=Mus musculus GN=Pbxip1 PE=1 SV=2 - [PBIP1_MOUSE]	11.69	2	6	6	11	1.023	1.054	0.698	0.646	27.32	11.69	9	11	727	81.1	5.36
O35153	BET1-like protein OS=Mus musculus GN=Bet1l PE=3 SV=1 - [BET1L_MOUSE]	11.71	1	1	1	7	0.743	0.829	0.771	0.649	22.39	11.71	2	7	111	12.4	8.82
Q9CQ01	Ribonuclease T2 OS=Mus musculus GN=Rnaset2 PE=2 SV=1 - [RNT2_MOUSE]	20.85	1	5	5	10	1.211	0.867	0.811	0.649	23.15	20.85	7	10	259	29.6	6.37
Q8VC57	BTB/POZ domain-containing protein KCTD5 OS=Mus musculus GN=Kctd5 PE=2 SV=1 - [KCTD5_MOUSE]	20.09	1	3	3	5	1.448	1.035	0.830	0.649	15.68	20.09	4	5	234	26.1	5.95
Q0P5V2	Sine oculis-binding protein homolog OS=Mus musculus GN=Sobp PE=2 SV=1 - [SOBP_MOUSE]	0.81	1	1	1	2	0.612	1.154	1.149	0.650	2.52	0.81	2	2	864	91.7	7.97
P16254	Signal recognition particle 14 kDa protein OS=Mus musculus GN=Srp14 PE=1 SV=1 - [SRP14_MOUSE]	27.27	2	3	3	7	1.433	0.553	0.682	0.651	14.41	27.27	5	7	110	12.5	10.17
D3YVV9	Synaptopodin-2 OS=Mus musculus GN=Synpo2 PE=2 SV=1 - [D3YVV9_MOUSE]	26.21	5	21	21	62	0.609	0.742	0.820	0.651	170.35	26.21	34	62	1198	129.5	7.81
P52479-2	Isoform 2 of Ubiquitin carboxyl-terminal hydrolase 10 OS=Mus musculus GN=Usp10 - [UBP10_MOUSE]	34.68	3	1	21	76	1.957	1.506	0.957	0.651	207.72	34.68	37	76	793	87.0	5.17
Q99JP6-2	Isoform 2 of Homer protein homolog 3 OS=Mus musculus GN=Homer3 -	22.56	3	3	9	21	1.496	0.580	1.945	0.651	51.05	22.56	16	21	359	40.0	5.29

Q6PAM1	Alpha-taxilin OS=Mus musculus GN=Txlna PE=2 SV=1 - [TXLNA_MOUSE]	25.81	9	13	14	38	1.895	0.546	0.951	0.651	115.62	25.81	25	38	554	62.3	6.74
Q5S006	Leucine-rich repeat serine/threonine-protein kinase 2 OS=Mus musculus GN=Lrrk2 PE=1 SV=2 - [LRRK2_MOUSE]	3.17	1	4	6	9	1.950	2.235	0.941	0.651	17.38	3.17	7	9	2527	284.5	6.83
F7BJ98	Protein Nbeal1 (Fragment) OS=Mus musculus GN=Nbeal1 PE=2 SV=1 - [F7BJ98_MOUSE]	3.81	2	3	3	4	14.626	2.464	1.006	0.651	5.00	3.81	3	4	734	85.2	5.25
P09803	Cadherin-1 OS=Mus musculus GN=Cdh1 PE=1 SV=1 - [CADH1_MOUSE]	9.73	1	4	4	15	6.268	3.331	0.780	0.652	34.75	9.73	7	15	884	98.2	4.83
Q9QY40	Plexin-B3 OS=Mus musculus GN=Plxnb3 PE=1 SV=2 - [PLXB3_MOUSE]	2.73	1	2	6	23	1.005	0.341	0.622	0.652	56.51	2.73	10	23	1902	208.2	5.95
Q9CR56	NF-kappa-B inhibitor-interacting Ras-like protein 2 OS=Mus musculus GN=Nkiras2 PE=2 SV=1 - [KBRS2_MOUSE]	19.37	2	4	4	6	0.872	0.770	0.754	0.652	17.35	19.37	5	6	191	21.5	8.05
D3Z7F1	Cytochrome P450 2S1 OS=Mus musculus GN=Cyp2s1 PE=2 SV=1 - [D3Z7F1_MOUSE]	20.98	3	8	8	17	1.107	0.640	1.139	0.653	57.54	20.98	14	17	448	49.8	9.19
P49962	Signal recognition particle 9 kDa protein OS=Mus musculus GN=Srp9 PE=1 SV=2 - [SRP09_MOUSE]	40.70	2	4	4	8	1.977	0.801	0.773	0.654	21.14	40.70	6	8	86	10.2	7.99
A6PWD2	Forkhead-associated domain-containing protein 1 OS=Mus musculus GN=Fhad1 PE=2 SV=1 - [FHAD1_MOUSE]	3.59	3	4	5	6	7.151	1.845	1.450	0.654	8.78	3.59	5	6	1420	163.6	6.64
Q9Z1M8	Protein Red OS=Mus musculus GN=Ik PE=2 SV=2 - [RED_MOUSE]	7.18	1	4	4	14	0.691	0.568	0.954	0.655	38.91	7.18	7	14	557	65.6	6.64
Q3UX37	Protein Plekhg1 OS=Mus musculus GN=Plekhg1 PE=2 SV=1 - [Q3UX37_MOUSE]	18.27	4	22	23	52	0.576	0.370	0.624	0.655	143.72	18.27	34	52	1390	155.8	6.40
Q9DBY8	Nuclear valosin-containing protein-like OS=Mus musculus GN=Nvl PE=1 SV=1 - [NVL_MOUSE]	10.53	1	7	7	12	0.985	0.761	0.700	0.655	27.16	10.53	10	12	855	94.4	6.35
O89017	Legumain OS=Mus musculus GN=Lgmn PE=1 SV=1 - [LGMN_MOUSE]	8.51	1	4	4	14	1.715	0.605	0.808	0.655	43.76	8.51	6	14	435	49.3	6.39
Q80W41	Condensin-2 complex subunit D3 OS=Mus musculus GN=Ncapd3 PE=2 SV=1 -	1.55	3	2	2	2	1.365	1.633	1.364	0.655	2.09	1.55	2	2	1223	139.0	7.11
P09581	Macrophage colony-stimulating factor 1 receptor OS=Mus musculus GN=Csf1r PE=1 SV=3 - [CSF1R_MOUSE]	0.82	1	1	1	1	1.350	1.058	1.126	0.657	2.92	0.82	1	1	977	109.1	6.21
Q9DCD6	Gamma-aminobutyric acid receptor-associated protein OS=Mus musculus GN=Gabarap PE=1 SV=2 - [GBRAP_MOUSE]	39.32	2	2	6	14	1.334	0.610	0.678	0.657	48.01	39.32	8	14	117	13.9	8.79
Q62393-3	Isoform 3 of Tumor protein D52 OS=Mus musculus GN=Tpd52 - [TPD52_MOUSE]	52.76	9	12	12	57	1.333	0.739	0.849	0.657	180.78	52.76	20	57	199	21.6	5.03
Q8CJ27	Abnormal spindle-like microcephaly-associated protein homolog OS=Mus musculus GN=Aspm PE=2 SV=2 - [ASPM_MOUSE]	1.28	2	4	5	22	1.054	1.130	0.924	0.658	38.38	1.28	8	22	3122	364.0	10.62
Q8BP99	UPF0500 protein C1orf216 homolog OS=Mus musculus PE=2 SV=2 - [CA216_MOUSE]	10.43	1	1	2	2	1.860	0.932	0.680	0.658	8.21	10.43	2	2	230	25.2	5.30
Q60865	Caprin-1 OS=Mus musculus GN=Caprin1 PE=1 SV=2 - [CAPR1_MOUSE]	26.73	4	14	14	91	1.383	0.757	0.880	0.658	230.32	26.73	21	91	707	78.1	5.25
Q059P4	Filamin A interacting protein 1 OS=Mus musculus GN=Filip1 PE=2 SV=1 - [Q059P4_MOUSE]	5.77	3	4	5	11	2.207	2.204	1.320	0.658	26.76	5.77	7	11	1214	137.8	8.19
Q792Y8	MCG15081 OS=Mus musculus GN=Gm10334 PE=3 SV=1 - [Q792Y8_MOUSE]	12.20	3	1	2	23	0.584	0.990	1.201	0.659	44.33	12.20	3	23	246	26.1	4.92
Q9R257	Heme-binding protein 1 OS=Mus musculus GN=Hebp1 PE=1 SV=2 - [HEBP1_MOUSE]	69.47	1	9	9	50	1.113	2.150	1.412	0.659	128.58	69.47	17	50	190	21.1	5.26
Q8R4P9-2	Isoform 2 of Multidrug resistance-associated protein 7 OS=Mus musculus GN=Abcc10 - [MRP7_MOUSE]	1.99	4	2	2	3	1.104	0.758	0.677	0.659	7.58	1.99	3	3	1460	159.5	7.37
Q8CJ40-2	Isoform 2 of Rootletin OS=Mus musculus GN=Crocc - [CROCC_MOUSE]	34.74	3	1	58	162	2.320	1.463	0.727	0.660	502.72	34.74	99	162	1845	208.1	5.53
B9EKI3	TATA element modulatory factor OS=Mus musculus GN=Tmf1 PE=1 SV=2 - [TMF1_MOUSE]	18.15	2	18	18	33	1.767	0.792	1.058	0.660	85.01	18.15	28	33	1091	121.7	4.87

P63300	Selenoprotein W OS=Mus musculus GN=Sepw1 PE=1 SV=3 - [SELW_MOUSE]	7.95	1	1	1	3	0.728	0.675	0.622	0.661	8.85	7.95	2	3	88	9.7	8.72
B1AVH0	Matrix metalloproteinase-16 (Fragment) OS=Mus musculus GN=Mmp16 PE=2 SV=1 - [B1AVH0_MOUSE]	8.05	1	1	1	1	45.234	3.265	1.562	0.661	2.98	8.05	1	1	174	19.7	9.60
Q8K284-2	Isoform 2 of General transcription factor 3C polypeptide 1 OS=Mus musculus GN=Gtf3c1 - [TF3C1_MOUSE]	5.84	3	10	10	15	0.707	0.735	0.743	0.661	41.37	5.84	12	15	2005	226.6	7.59
P62071	Ras-related protein R-Ras2 OS=Mus musculus GN=Rras2 PE=1 SV=1 - [RRAS2_MOUSE]	60.78	1	9	12	74	0.893	0.440	0.669	0.661	202.83	60.78	23	74	204	23.4	6.01
Q8R105	Vacuolar protein sorting-associated protein 37C OS=Mus musculus GN=Vps37c PE=2 SV=1 - [VP37C_MOUSE]	28.69	2	9	9	35	1.734	0.902	0.874	0.662	116.26	28.69	15	35	352	38.4	5.31
Q8VHI3	GDP-fucose protein O-fucosyltransferase 2 OS=Mus musculus GN=Pofut2 PE=1 SV=1 - [OFUT2_MOUSE]	8.86	1	4	4	7	1.862	1.023	0.740	0.662	16.26	8.86	6	7	429	49.4	6.48
Q64519	Syndecan-3 OS=Mus musculus GN=Sdc3 PE=2 SV=2 - [SDC3_MOUSE]	7.92	1	2	2	12	0.416	0.920	0.628	0.662	17.57	7.92	3	12	442	46.0	4.59
G5E887	Lebercilin OS=Mus musculus GN=Lca5 PE=4 SV=1 - [G5E887_MOUSE]	5.49	5	1	3	7	0.792	0.849	0.970	0.662	12.54	5.49	4	7	419	48.4	9.06
Q6P549	Phosphatidylinositol 3,4,5-trisphosphate 5-phosphatase 2 OS=Mus musculus GN=Inppl1 PE=1 SV=1 - [SHIP2_MOUSE]	8.19	1	6	7	20	0.983	0.650	0.626	0.663	53.95	8.19	12	20	1257	138.9	6.54
E9PZ67	Calsequestrin (Fragment) OS=Mus musculus GN=Casq2 PE=2 SV=1 - [E9PZ67_MOUSE]	8.65	3	2	2	3	5.438	2.156	1.416	0.663	6.45	8.65	3	3	312	36.4	4.58
P18608	Non-histone chromosomal protein HMG-14 OS=Mus musculus GN=Hmgn1 PE=1 SV=2 - [HMGN1_MOUSE]	18.75	2	3	3	13	0.853	0.621	0.852	0.663	28.57	18.75	5	13	96	10.1	9.76
D3Z598	Latent-transforming growth factor beta-binding protein 4 OS=Mus musculus GN=Ltbp4 PE=2 SV=1 - [D3Z598_MOUSE]	7.45	7	9	9	19	0.813	1.317	0.707	0.663	62.52	7.45	17	19	1558	166.7	5.11
D3Z1W6	Protein polybromo-1 OS=Mus musculus GN=Pbrm1 PE=2 SV=1 - [D3Z1W6_MOUSE]	4.17	13	6	6	7	0.585	0.764	0.917	0.664	25.48	4.17	7	7	1582	181.0	6.95
P36552	Coproporphyrinogen-III oxidase, mitochondrial OS=Mus musculus GN=Cpox PE=1 SV=2 - [HEM6_MOUSE]	39.73	1	14	14	66	2.236	0.623	0.575	0.664	207.14	39.73	28	66	443	49.7	8.53
Q5RJY2	G2/M phase-specific E3 ubiquitin-protein ligase OS=Mus musculus GN=G2e3 PE=2 SV=2 - [G2E3_MOUSE]	1.40	2	1	1	1	0.682	0.843	1.019	0.664	0.00	1.40	1	1	716	81.7	7.37
Q9JJI8	60S ribosomal protein L38 OS=Mus musculus GN=Rpl38 PE=2 SV=3 - [RL38_MOUSE]	50.00	1	4	5	17	0.963	0.602	0.770	0.664	42.91	50.00	7	17	70	8.2	10.10
P51655	Glypican-4 OS=Mus musculus GN=Gpc4 PE=2 SV=2 - [GPC4_MOUSE]	34.65	1	14	16	43	0.571	2.821	0.705	0.664	112.03	34.65	28	43	557	62.5	6.33
Q8CAY6	Acetyl-CoA acetyltransferase, cytosolic OS=Mus musculus GN=Acat2 PE=1 SV=2 - [THIC_MOUSE]	58.69	2	3	17	204	1.628	0.788	0.643	0.664	649.38	58.69	29	204	397	41.3	7.50
A2AQ53	Fibrillin-1 OS=Mus musculus GN=Fbn1 PE=4 SV=1 - [A2AQ53_MOUSE]	5.01	2	13	16	27	0.803	3.896	0.516	0.665	66.65	5.01	21	27	2873	312.1	4.92
E9Q8L9	Rab11 family-interacting protein 1 OS=Mus musculus GN=Rab11fip1 PE=2 SV=1 -	10.12	2	6	7	8	3.244	1.366	0.998	0.665	22.38	10.12	8	8	1166	124.9	5.80
O35295	Transcriptional activator protein Pur-beta OS=Mus musculus GN=Purb PE=1 SV=3 - [PURB_MOUSE]	62.65	1	11	18	67	1.096	0.803	0.745	0.665	223.27	62.65	27	67	324	33.9	5.43
F7AA26	Protein Gm20459 (Fragment) OS=Mus musculus GN=Gm20459 PE=4 SV=1 -	26.47	6	1	26	130	0.717	0.352	0.795	0.666	373.54	26.47	45	130	1137	126.0	5.27
A2BE93	Protein SET (Fragment) OS=Mus musculus GN=Set PE=2 SV=1 - [A2BE93_MOUSE]	32.56	5	8	8	66	1.356	0.779	0.958	0.666	181.82	32.56	14	66	215	24.9	5.55
Q5U5I3	28S ribosomal protein S18a, mitochondrial OS=Mus musculus GN=Mrps18a PE=2 SV=1 - [Q5U5I3_MOUSE]	21.94	3	4	4	5	0.443	0.537	0.773	0.667	7.49	21.94	4	5	196	22.2	10.11

P97376	Protein FRG1 OS=Mus musculus GN=Frg1 PE=1 SV=2 - [FRG1_MOUSE]	4.26	1	1	1	1	1.684	0.742	1.201	0.667	3.77	4.26	1	1	258	29.1	8.92
O88811	Signal transducing adapter molecule 2 OS=Mus musculus GN=Stam2 PE=1 SV=1 - [STAM2_MOUSE]	29.06	3	12	14	28	1.638	1.107	0.936	0.667	74.29	29.06	19	28	523	57.4	5.07
P62500-2	Isoform 2 of TSC22 domain family protein 1 OS=Mus musculus GN=Tsc22d1 -	34.27	4	1	5	42	0.792	1.517	0.879	0.668	93.67	34.27	10	42	143	15.6	5.20
E9QK04	Neogenin OS=Mus musculus GN=Neo1 PE=2 SV=1 - [E9QK04_MOUSE]	26.54	5	2	31	147	2.364	1.178	1.157	0.668	464.15	26.54	52	147	1492	162.8	6.49
P41317	Mannose-binding protein C OS=Mus musculus GN=Mbl2 PE=2 SV=2 - [MBL2_MOUSE]	12.30	1	2	2	4	2.261	1.309	0.386	0.669	14.70	12.30	4	4	244	25.9	5.06
Q99PG2-2	Isoform 2 of Opioid growth factor receptor OS=Mus musculus GN=Ogfr - [OGFR_MOUSE]	21.78	2	10	10	37	1.003	1.064	0.818	0.669	109.23	21.78	19	37	597	66.7	4.84
Q8C156	Condensin complex subunit 2 OS=Mus musculus GN=Ncaph PE=2 SV=1 - [CND2_MOUSE]	0.96	1	1	1	1	0.492	0.559	0.530	0.669	1.75	0.96	1	1	731	82.3	4.96
O35309	N-myc-interactor OS=Mus musculus GN=Nmi PE=2 SV=1 - [NMI_MOUSE]	5.10	1	1	1	2	1.206	0.938	0.561	0.669	4.63	5.10	2	2	314	35.2	5.05
Q8R034	Anaphase-promoting complex subunit 13 OS=Mus musculus GN=Anapc13 PE=2 SV=1 - [APC13_MOUSE]	13.51	1	1	1	2	2.517	0.943	1.060	0.670	6.57	13.51	2	2	74	8.3	4.18
A2ALK8	Tyrosine-protein phosphatase non-receptor type 3 OS=Mus musculus GN=Ptpn3 PE=2 SV=1 - [PTN3_MOUSE]	1.64	1	2	2	3	0.459	0.953	0.853	0.671	5.00	1.64	3	3	913	103.8	7.20
P23953	Carboxylesterase 1C OS=Mus musculus GN=Ces1c PE=1 SV=4 - [EST1C_MOUSE]	40.79	2	10	16	65	2.877	1.172	0.341	0.671	201.19	40.79	25	65	554	61.0	5.06
H7BWX9	Small ubiquitin-related modifier 2 OS=Mus musculus GN=Sumo2 PE=2 SV=1 -	49.06	4	2	3	14	1.413	1.066	1.263	0.671	47.63	49.06	4	14	53	6.0	8.91
E9Q2W9	Alpha-actinin-4 (Fragment) OS=Mus musculus GN=Actn4 PE=2 SV=1 - [E9Q2W9_MOUSE]	70.27	2	1	32	224	0.688	2.578	1.151	0.671	581.39	70.27	57	224	518	59.9	6.01
D3Z5X2	RAC-beta serine/threonine-protein kinase (Fragment) OS=Mus musculus GN=Akt2 PE=2 SV=1 - [D3Z5X2_MOUSE]	9.38	1	1	1	3	2.226	2.443	1.602	0.671	5.78	9.38	1	3	64	7.8	7.30
P26645	Myristoylated alanine-rich C-kinase substrate OS=Mus musculus GN=Marcks PE=1 SV=2 - [MARCS_MOUSE]	23.95	1	4	4	55	0.760	0.400	0.696	0.671	160.29	23.95	6	55	309	29.6	4.34
J3KMU3	Uncharacterized protein OS=Mus musculus GN=Gm2058 PE=3 SV=1 - [J3KMU3_MOUSE]	22.95	4	5	5	13	1.034	0.978	0.722	0.672	40.00	22.95	8	13	183	20.7	4.68
D3Z564	TGF-beta-activated kinase 1 and MAP3K7-binding protein 2 (Fragment) OS=Mus musculus GN=Tab2 PE=2 SV=1 - [D3Z564_MOUSE]	11.43	3	3	3	8	1.345	0.921	1.005	0.672	25.71	11.43	4	8	385	41.3	10.39
G5E920	MCG114640 OS=Mus musculus GN=Gm6104 PE=4 SV=1 - [G5E920_MOUSE]	29.50	3	7	7	18	0.570	0.416	0.889	0.672	51.48	29.50	9	18	200	23.0	8.37
E9PWI6	GRIP and coiled-coil domain-containing protein 2 OS=Mus musculus GN=Gcc2 PE=2 SV=1 - [E9PWI6_MOUSE]	32.91	6	48	48	120	1.692	0.979	1.045	0.672	365.89	32.91	80	120	1644	190.6	5.12
P62858	40S ribosomal protein S28 OS=Mus musculus GN=Rps28 PE=2 SV=1 - [RS28_MOUSE]	44.93	3	3	3	88	2.090	0.653	0.908	0.673	244.29	44.93	5	88	69	7.8	10.70
P63158	High mobility group protein B1 OS=Mus musculus GN=Hmgb1 PE=1 SV=2 -	43.72	3	15	16	101	0.493	0.494	0.986	0.673	265.51	43.72	26	101	215	24.9	5.74
Q9ET43	Claudin-12 OS=Mus musculus GN=Cldn12 PE=1 SV=2 - [CLD12_MOUSE]	6.15	1	1	1	2	1.639	2.290	1.069	0.673	6.04	6.15	2	2	244	27.0	8.47
Q9ERG0	LIM domain and actin-binding protein 1 OS=Mus musculus GN=Lima1 PE=1 SV=3 - [LIMA1_MOUSE]	18.46	2	13	13	36	1.713	0.766	0.800	0.673	92.58	18.46	21	36	753	84.0	6.60
P60761	Neurogranin OS=Mus musculus GN=Nrgn PE=1 SV=1 - [NEUG_MOUSE]	46.15	1	3	4	70	0.675	1.438	2.343	0.673	173.62	46.15	7	70	78	7.5	7.05
Q8K3C3	Protein LZIC OS=Mus musculus GN=Lzic PE=1 SV=1 - [LZIC_MOUSE]	45.26	2	6	8	24	1.700	0.728	0.865	0.673	76.65	45.26	12	24	190	21.5	4.96

E9Q585	Protein Slc4a1ap OS=Mus musculus GN=Slc4a1ap PE=2 SV=1 - [E9Q585_MOUSE]	10.07	3	6	6	12	1.275	0.879	0.823	0.674	25.95	10.07	9	12	715	79.6	5.10
Q3UCQ1	Forkhead box protein K2 OS=Mus musculus GN=Foxk2 PE=2 SV=3 - [FOXK2_MOUSE]	14.90	2	6	6	14	1.856	1.108	0.966	0.674	43.59	14.90	9	14	651	68.4	9.51
Q8K341-4	Isoform 4 of Alpha-tubulin N-acetyltransferase OS=Mus musculus GN=Atat1 - [ATAT_MOUSE]	43.24	9	13	13	57	0.787	0.683	0.736	0.674	176.08	43.24	26	57	333	37.5	10.10
Q9ERR7	15 kDa selenoprotein OS=Mus musculus GN=Sep15 PE=1 SV=3 - [SEP15_MOUSE]	30.86	2	4	4	18	1.179	1.043	0.697	0.675	55.09	30.86	8	18	162	17.8	5.35
Q8R1U2	Cell growth regulator with EF hand domain protein 1 OS=Mus musculus GN=Cgref1 PE=2 SV=1 - [CGRE1_MOUSE]	10.32	2	2	2	7	1.492	0.824	0.813	0.675	25.44	10.32	4	7	281	30.8	4.31
Q99JP4	Anaphase-promoting complex subunit CDC26 OS=Mus musculus GN=Cdc26 PE=2 SV=1 - [CDC26_MOUSE]	9.41	1	1	1	1	1.046	0.830	1.022	0.676	2.32	9.41	1	1	85	9.8	6.81
D3Z660	Protein Gm46 OS=Mus musculus GN=Gm46 PE=3 SV=1 - [D3Z660_MOUSE]	6.46	1	1	2	2	1.004	1.482	0.858	0.676	3.15	6.46	2	2	418	46.7	9.94
Q5XJV5	Nuclear receptor coactivator 6 OS=Mus musculus GN=Ncoa6 PE=2 SV=1 - [Q5XJV5_MOUSE]	5.61	6	7	7	10	1.368	1.253	0.942	0.676	15.58	5.61	9	10	2069	219.8	9.35
Q9D132	Uroplakin-1a OS=Mus musculus GN=Upk1a PE=1 SV=1 - [UPK1A_MOUSE]	12.06	1	1	1	1	1.555	1.148	0.782	0.676	2.25	12.06	1	1	257	28.8	5.33
E9Q9Q5	Protein 1700113H08Rik OS=Mus musculus GN=1700113H08Rik PE=4 SV=1 - [E9Q9Q5_MOUSE]	3.67	1	1	1	1	1.483	1.751	0.961	0.677	2.51	3.67	1	1	327	35.5	9.14
Q9CQ79	Thioredoxin domain-containing protein 9 OS=Mus musculus GN=Txndc9 PE=1 SV=1 - [TXND9_MOUSE]	19.91	1	4	5	12	1.447	0.939	0.958	0.677	31.34	19.91	8	12	226	26.2	5.95
P98064	Mannan-binding lectin serine protease 1 OS=Mus musculus GN=Masp1 PE=1 SV=2 - [MASP1_MOUSE]	1.99	2	1	1	2	3.707	0.858	0.858	0.677	6.00	1.99	2	2	704	79.9	5.55
Q8HW98	IgLON family member 5 OS=Mus musculus GN=Iglon5 PE=2 SV=2 - [IGLO5_MOUSE]	29.46	1	8	9	49	1.442	0.376	0.542	0.678	156.77	29.46	15	49	336	36.7	7.69
Q5F2E7	Nuclear fragile X mental retardation-interacting protein 2 OS=Mus musculus GN=Nufip2 PE=1 SV=1 - [illegible]	34.39	2	16	16	54	1.306	0.539	0.910	0.678	164.60	34.39	26	54	692	75.6	8.70
Q60872	Eukaryotic translation initiation factor 1A OS=Mus musculus GN=Eif1a PE=2 SV=3 - [IF1A_MOUSE]	26.39	16	5	5	9	0.869	0.837	0.990	0.678	20.24	26.39	7	9	144	16.5	5.24
Q80VN0	Coiled-coil domain-containing protein 37 OS=Mus musculus GN=Ccdc37 PE=2 SV=1 - [CCD37_MOUSE]	5.66	2	3	3	3	3.394	0.760	1.136	0.679	6.76	5.66	3	3	459	53.8	8.29
Q05816	Fatty acid-binding protein, epidermal OS=Mus musculus GN=Fabp5 PE=1 SV=3 - [FABP5_MOUSE]	65.93	1	7	7	169	1.770	0.484	0.821	0.679	474.08	65.93	14	169	135	15.1	6.54
P63089	Pleiotrophin OS=Mus musculus GN=Ptn PE=2 SV=1 - [PTN_MOUSE]	21.43	1	4	4	23	0.468	1.300	1.015	0.680	58.82	21.43	8	23	168	18.9	9.60
Q62469	Integrin alpha-2 OS=Mus musculus GN=Itga2 PE=1 SV=2 - [ITA2_MOUSE]	1.87	1	1	2	3	0.591	0.849	0.404	0.680	7.07	1.87	3	3	1178	128.9	5.21
Q9QZC8	Abhydrolase domain-containing protein 1 OS=Mus musculus GN=Abhd1 PE=2 SV=2 - [ABHD1_MOUSE]	1.94	1	1	1	3	0.595	0.673	0.675	0.680	6.90	1.94	2	3	412	45.7	7.24
H3BLK2	Dual-specificity protein phosphatase 3 OS=Mus musculus GN=Dusp3 PE=2 SV=1 - [H3BLK2_MOUSE]	13.41	1	1	1	2	1.069	1.098	0.969	0.681	4.98	13.41	2	2	82	8.8	4.35
Q32M26	Uncharacterized protein C11orf87 homolog OS=Mus musculus PE=2 SV=1 - [CK087_MOUSE]	5.03	1	1	1	2	0.672	0.695	1.162	0.682	8.55	5.03	2	2	199	20.7	9.29
Q3UJ00	Homeobox protein Meis1 OS=Mus musculus GN=Meis1 PE=2 SV=1 - [Q3UJ00_MOUSE]	10.95	14	2	2	3	1.765	1.122	1.389	0.682	4.31	10.95	3	3	210	23.0	5.95
Q4VBE7	BTB/POZ domain-containing protein KCTD2 OS=Mus musculus GN=Kctd2 PE=2 SV=1 - [Q4VBE7_MOUSE]	14.83	3	2	2	2	1.567	2.020	0.741	0.682	5.52	14.83	2	2	263	28.6	5.29

Q8BI84	Melanoma inhibitory activity protein 3 OS=Mus musculus GN=Mia3 PE=1 SV=2 - [MIA3_MOUSE]	23.11	3	19	37	119	1.522	0.996	0.937	0.684	324.62	23.11	62	119	1930	213.5	4.75
Q80TJ7	Histone lysine demethylase PHF8 OS=Mus musculus GN=Phf8 PE=1 SV=2 -	3.52	4	2	3	5	0.592	0.659	0.787	0.684	11.26	3.52	5	5	1023	113.5	8.44
Q99N91	39S ribosomal protein L34, mitochondrial OS=Mus musculus GN=Mrpl34 PE=2 SV=1 - [RM34_MOUSE]	23.91	1	2	2	11	0.713	0.471	0.872	0.684	25.96	23.91	4	11	92	10.5	11.81
A2A4H9	Peptidyl-prolyl cis-trans isomerase FKBP10 OS=Mus musculus GN=Fkbp10 PE=2 SV=1 - [A2A4H9_MOUSE]	13.86	2	5	5	12	1.348	1.372	0.609	0.684	23.96	13.86	7	12	469	52.3	5.53
Q9D7M8	DNA-directed RNA polymerase II subunit RPB4 OS=Mus musculus GN=Polr2d PE=2 SV=2 - [RPB4_MOUSE]	10.56	1	1	1	4	1.800	0.913	0.805	0.684	14.17	10.56	1	4	142	16.3	4.79
Q921I1	Serotransferrin OS=Mus musculus GN=Tf PE=1 SV=1 - [TRFE_MOUSE]	57.96	6	3	44	479	3.484	1.210	0.530	0.685	1364.79	57.96	81	479	697	76.7	7.18
Q60764	Probable E3 ubiquitin-protein ligase makorin-3 OS=Mus musculus GN=Mkrn3 PE=2 SV=2 - [MKRN3_MOUSE]	10.29	1	4	5	8	1.069	0.626	0.627	0.685	27.74	10.29	8	8	544	59.4	6.64
Q3TGF2	Protein FAM107B OS=Mus musculus GN=Fam107b PE=2 SV=2 - [F107B_MOUSE]	26.72	5	3	4	11	1.621	0.604	1.147	0.685	25.82	26.72	7	11	131	15.6	8.31
G3UW85	ATP-dependent RNA helicase DDX3X OS=Mus musculus GN=Erh PE=4 SV=1 - [G3UW85_MOUSE]	47.97	3	5	5	32	1.349	0.828	0.785	0.685	80.78	47.97	8	32	123	14.3	5.54
Q9CPV9	P2Y purinoceptor 12 OS=Mus musculus GN=P2ry12 PE=2 SV=1 - [P2Y12_MOUSE]	8.07	1	3	3	10	0.732	1.133	0.943	0.685	26.15	8.07	4	10	347	39.4	9.58
Q8BP27-2	Isoform 2 of Swi5-dependent recombination DNA repair protein 1 homolog OS=Mus musculus GN=Sfr1 - [illegible]	46.86	3	10	10	34	1.281	0.885	0.965	0.686	89.90	46.86	17	34	303	33.5	5.14
Q8R0A0	General transcription factor IIF subunit 2 OS=Mus musculus GN=Gtf2f2 PE=1 SV=1 - [T2FB_MOUSE]	21.29	1	5	6	11	1.769	0.813	0.944	0.686	23.27	21.29	10	11	249	28.4	9.22
G3UYF9	Prefoldin subunit 6 OS=Mus musculus GN=H2-Ke2 PE=2 SV=1 - [G3UYF9_MOUSE]	57.43	1	1	7	44	1.539	1.242	1.021	0.686	130.09	57.43	14	44	101	11.4	8.44
Q3UH53	Protein sidekick-1 OS=Mus musculus GN=Sdk1 PE=2 SV=1 - [SDK1_MOUSE]	2.64	2	4	5	8	1.384	1.041	0.791	0.686	11.10	2.64	6	8	2193	240.2	6.77
P63276	40S ribosomal protein S17 OS=Mus musculus GN=Rps17 PE=1 SV=2 - [RS17_MOUSE]	47.41	1	5	5	29	0.357	0.634	0.668	0.686	86.97	47.41	7	29	135	15.5	9.85
Q8BP78	Protein FRA10AC1 homolog OS=Mus musculus GN=Fra10ac1 PE=1 SV=3 -	9.21	1	2	2	3	0.609	0.494	0.794	0.686	10.23	9.21	2	3	315	37.2	7.28
P13595	Neural cell adhesion molecule 1 OS=Mus musculus GN=Ncam1 PE=1 SV=3 -	42.42	4	10	42	842	0.763	0.531	0.673	0.687	2584.70	42.42	79	842	1115	119.4	4.83
Q6PDM1-4	Isoform 4 of Male-specific lethal 1 homolog OS=Mus musculus GN=Msl1 - [MSL1_MOUSE]	13.61	6	4	4	9	1.448	0.722	1.190	0.687	28.94	13.61	8	9	463	49.2	8.94
Q99KV1	DnaJ homolog subfamily B member 11 OS=Mus musculus GN=Dnajb11 PE=1 SV=1 -	32.40	1	9	9	31	1.046	0.663	0.801	0.687	85.15	32.40	14	31	358	40.5	6.32
Q9JIS8	Solute carrier family 12 member 4 OS=Mus musculus GN=Slc12a4 PE=1 SV=2 -	13.09	3	6	12	65	0.761	0.560	0.595	0.688	171.99	13.09	21	65	1085	120.5	6.67
Q9DCC5	Cbx3 protein OS=Mus musculus GN=Cbx3 PE=2 SV=1 - [Q9DCC5_MOUSE]	50.82	4	4	6	32	1.495	0.696	0.929	0.688	115.46	50.82	9	32	183	20.8	5.33
Q5DU31-2	Isoform 2 of Interactor protein for cytohesin exchange factors 1 OS=Mus musculus GN=Ipcef1 - [illegible]	14.39	6	5	5	11	0.704	1.134	1.427	0.689	26.18	14.39	8	11	410	46.3	6.73
Q99K30	Epidermal growth factor receptor kinase substrate 8-like protein 2 OS=Mus musculus GN=Eps8l2 PE=1 SV=1 - [illegible]	13.85	2	6	6	9	1.597	0.923	1.268	0.690	27.26	13.85	7	9	729	82.2	7.18
F8WGL3	Cofilin-1 OS=Mus musculus GN=Cfl1 PE=2 SV=1 - [F8WGL3_MOUSE]	57.71	1	1	23	542	0.898	0.669	1.299	0.690	1400.79	57.71	42	542	227	24.6	8.03
J3QNH8	Protein Gm7694 OS=Mus musculus GN=Gm7694 PE=4 SV=1 - [J3QNH8_MOUSE]	6.30	1	1	1	2	3.091	1.355	0.716	0.691	6.70	6.30	1	2	270	28.5	5.50

Q9CPW7	Zinc finger matrin-type protein 2 OS=Mus musculus GN=Zmat2 PE=2 SV=1 - [ZMAT2_MOUSE]	15.58	1	4	4	5	0.633	0.492	1.177	0.691	13.90	15.58	5	5	199	23.6	9.01
Q99NH0	Ankyrin repeat domain-containing protein 17 OS=Mus musculus GN=Ankrd17 PE=1 SV=2 - [ANR17_MOUSE]	10.83	6	13	20	53	1.110	0.817	0.931	0.691	132.88	10.83	35	53	2603	274.0	6.52
Q8BJF9	Charged multivesicular body protein 2b OS=Mus musculus GN=Chmp2b PE=2 SV=1 -	30.52	1	7	8	22	1.312	0.538	1.269	0.691	69.59	30.52	15	22	213	23.9	8.78
Q9Z304	Protein S-Myc OS=Mus musculus GN=Mycs PE=2 SV=2 - [MYCS_MOUSE]	2.09	1	1	1	1	1.225	1.095	0.976	0.691	3.31	2.09	1	1	431	47.6	6.09
Q9D173	Mitochondrial import receptor subunit TOM7 homolog OS=Mus musculus GN=Tomm7 PE=3 SV=1 - [TOM7_MOUSE]	41.82	1	2	2	2	1.649	0.766	0.680	0.691	5.86	41.82	2	2	55	6.2	10.17
Q9R226	KH domain-containing, RNA-binding, signal transduction-associated protein 3 OS=Mus musculus GN=Khdrbs3 PE=1 SV=1 - [KHDR3_MOUSE]	28.32	2	7	12	57	1.061	1.118	1.240	0.691	129.61	28.32	21	57	346	38.8	8.10
P09242	Alkaline phosphatase, tissue-nonspecific isozyme OS=Mus musculus GN=Alpl PE=1 SV=2 - [PPBT_MOUSE]	34.92	4	15	15	43	0.933	1.065	0.499	0.691	128.66	34.92	23	43	524	57.5	7.01
Q3UH85	Protein 8430427H17Rik OS=Mus musculus GN=8430427H17Rik PE=2 SV=1 - [Q3UH85_MOUSE]	14.87	3	5	7	13	1.616	1.033	0.820	0.692	33.14	14.87	10	13	679	74.2	5.77
Q9DCT5	Stromal cell-derived factor 2 OS=Mus musculus GN=Sdf2 PE=2 SV=1 - [SDF2_MOUSE]	43.13	1	6	6	30	1.517	0.860	0.931	0.692	86.82	43.13	11	30	211	23.1	7.33
Q8BQ30	Phostensin OS=Mus musculus GN=Ppp1r18 PE=1 SV=1 - [PPR18_MOUSE]	28.45	2	10	10	15	1.933	0.796	1.152	0.692	39.05	28.45	13	15	594	65.6	5.39
B1AS04	Protein LZIC (Fragment) OS=Mus musculus GN=Lzic PE=2 SV=1 - [B1AS04_MOUSE]	40.00	2	1	3	12	1.439	0.827	1.007	0.692	37.53	40.00	5	12	65	7.7	4.51
Q3TXT3	SOSS complex subunit C OS=Mus musculus GN=Inip PE=2 SV=1 - [SOSSC_MOUSE]	10.58	1	1	1	2	1.290	1.379	1.254	0.692	5.80	10.58	2	2	104	11.4	9.25
E9PYH0	Versican core protein OS=Mus musculus GN=Vcan PE=2 SV=1 - [E9PYH0_MOUSE]	10.17	10	4	30	212	1.799	0.618	0.875	0.692	593.78	10.17	52	212	3354	366.7	4.63
Q91ZU6-5	Isoform 5 of Dystonin OS=Mus musculus GN=Dst - [DYST_MOUSE]	10.99	1	2	28	62	1.246	1.038	1.254	0.692	184.58	10.99	42	62	2611	301.5	6.55
Q3UPH1	Protein PRRC1 OS=Mus musculus GN=Prrc1 PE=2 SV=1 - [PRRC1_MOUSE]	13.54	1	5	5	10	0.939	1.727	0.782	0.693	26.47	13.54	8	10	443	46.3	5.95
Q8R3P8	Protein Rnf113a1 OS=Mus musculus GN=Rnf113a1 PE=2 SV=1 - [Q8R3P8_MOUSE]	22.29	1	7	7	17	1.406	0.707	1.072	0.693	47.58	22.29	11	17	341	38.4	7.06
F8WGD4	Serine/threonine-protein kinase Nek3 OS=Mus musculus GN=Nek3 PE=2 SV=1 - [F8WGD4_MOUSE]	6.88	2	3	3	8	1.277	0.814	0.756	0.693	20.62	6.88	5	8	509	57.2	7.01
E9QB02	Methionine--tRNA ligase, cytoplasmic OS=Mus musculus GN=Mars PE=2 SV=1 - [E9QB02_MOUSE]	37.69	2	1	32	104	0.711	0.630	0.795	0.693	279.57	37.69	57	104	910	102.3	6.92
Q99L45	Eukaryotic translation initiation factor 2 subunit 2 OS=Mus musculus GN=Eif2s2 PE=1 SV=1 - [IF2B_MOUSE]	32.63	3	12	12	44	0.622	0.669	0.802	0.694	130.31	32.63	19	44	331	38.1	5.80
Q62446	Peptidyl-prolyl cis-trans isomerase FKBP3 OS=Mus musculus GN=Fkbp3 PE=1 SV=2 - [FKBP3_MOUSE]	44.64	1	12	12	38	1.318	0.466	0.960	0.694	95.05	44.64	18	38	224	25.1	9.28
Q91W67	Ubiquitin-like protein 7 OS=Mus musculus GN=Ubl7 PE=1 SV=2 - [UBL7_MOUSE]	15.79	2	5	5	14	1.202	0.981	0.787	0.694	34.34	15.79	9	14	380	40.4	5.01
Q8BVF7	Gamma-secretase subunit APH-1A OS=Mus musculus GN=Aph1a PE=2 SV=2 - [APH1A_MOUSE]	8.30	2	2	2	5	1.078	1.029	0.807	0.694	13.06	8.30	4	5	265	29.0	7.90
A2AMY5	Ubiquitin-associated protein 2 OS=Mus musculus GN=Ubap2 PE=2 SV=1 -	18.83	5	17	17	60	1.442	0.776	0.905	0.695	155.82	18.83	26	60	1131	117.8	7.72
Q6P5U8	Coiled-coil domain-containing protein 148 OS=Mus musculus GN=Ccdc148 PE=2 SV=1 - [CC148_MOUSE]	2.66	2	1	2	2	3.853	1.707	1.025	0.695	5.55	2.66	2	2	527	63.4	6.62
Q99KK1	Receptor expression-enhancing protein 3 OS=Mus musculus GN=Reep3 PE=1 SV=1 - [REEP3_MOUSE]	19.29	2	5	5	12	1.190	0.515	0.616	0.695	25.09	19.29	9	12	254	29.2	9.58

E9QP46	Nesprin-2 OS=Mus musculus GN=Syne2 PE=2 SV=1 - [E9QP46_MOUSE]	3.70	4	11	23	34	2.166	1.151	1.171	0.695	64.08	3.70	29	34	6870	781.7	5.31
Q9WVM6	Tolloid-like protein 2 OS=Mus musculus GN=Tll2 PE=1 SV=1 - [TLL2_MOUSE]	0.99	1	1	1	1	4.476	1.242	0.716	0.695	1.88	0.99	1	1	1012	113.2	5.96
P01867-2	Isoform 2 of Ig gamma-2B chain C region OS=Mus musculus GN=Igh-3 - [IGG2B_MOUSE]	25.97	2	5	5	9	1.827	3.055	0.844	0.695	27.74	25.97	8	9	335	36.6	7.36
Q9Z131-2	Isoform 2 of SH3 domain-binding protein 5 OS=Mus musculus GN=Sh3bp5 - [3BP5_MOUSE]	7.42	3	3	4	11	1.330	0.780	1.088	0.696	23.59	7.42	7	11	458	51.0	5.11
E9Q1N0	Protein 4932431P20Rik OS=Mus musculus GN=4932431P20Rik PE=2 SV=1 - [E9Q1N0_MOUSE]	3.55	1	5	6	8	3.481	3.027	0.945	0.696	14.90	3.55	6	8	3095	357.3	5.49
Q6XLQ8	Calumenin OS=Mus musculus GN=Calu PE=2 SV=1 - [Q6XLQ8_MOUSE]	58.73	5	2	15	77	1.554	0.957	1.183	0.697	237.38	58.73	25	77	315	37.1	4.59
Q9D727	Uncharacterized protein C6orf226 homolog OS=Mus musculus PE=4 SV=1 - [CF226_MOUSE]	53.51	1	4	4	9	1.927	1.135	0.841	0.697	29.06	53.51	7	9	114	12.0	6.29
Q9CQY5-2	Isoform 2 of Magnesium transporter protein 1 OS=Mus musculus GN=Magt1 - [MAGT1_MOUSE]	11.76	6	3	4	7	1.039	0.843	0.644	0.698	14.37	11.76	5	7	306	34.5	9.72
F6XWQ4	Protein Ciz1 (Fragment) OS=Mus musculus GN=Ciz1 PE=4 SV=1 - [F6XWQ4_MOUSE]	17.71	6	4	4	9	1.534	2.125	1.890	0.698	11.54	17.71	5	9	288	31.3	5.11
Q9CRC3	UPF0235 protein C15orf40 homolog OS=Mus musculus PE=2 SV=1 - [CO040_MOUSE]	10.32	1	1	1	3	1.291	1.002	0.852	0.698	5.16	10.32	2	3	126	13.2	9.16
E9PWH4	Protein Abca15 OS=Mus musculus GN=Abca15 PE=2 SV=1 - [E9PWH4_MOUSE]	2.64	2	3	3	4	1.697	0.531	0.899	0.698	4.39	2.64	4	4	1668	190.4	8.53
Q8BTT6	Digestive organ expansion factor homolog OS=Mus musculus GN=Diexf PE=2 SV=2 - [DIEXF_MOUSE]	5.18	1	2	4	5	1.487	0.711	0.895	0.699	4.49	5.18	4	5	772	88.8	5.74
Q9DCI9	39S ribosomal protein L32, mitochondrial OS=Mus musculus GN=Mrpl32 PE=2 SV=1 - [RM32_MOUSE]	16.04	1	2	2	12	0.990	0.565	1.037	0.699	35.00	16.04	4	12	187	21.7	9.70
E9PZK7	ADP-ribosylation factor-related protein 1 OS=Mus musculus GN=Arfrp1 PE=2 SV=1 - [E9PZK7_MOUSE]	11.69	2	1	1	1	1.630	2.198	0.711	0.699	3.32	11.69	1	1	154	17.1	5.39
E9PZH0	Protein Tmem29 OS=Mus musculus GN=Tmem29 PE=4 SV=1 - [E9PZH0_MOUSE]	8.13	1	1	1	1	1.575	0.883	0.613	0.700	3.12	8.13	1	1	123	14.3	4.92
O88736	3-keto-steroid reductase OS=Mus musculus GN=Hsd17b7 PE=2 SV=1 - [DHB7_MOUSE]	20.06	2	5	5	16	0.684	0.348	0.655	0.700	44.12	20.06	9	16	334	37.3	6.73
Q9ERB5	Solute carrier organic anion transporter family member 1C1 OS=Mus musculus GN=Slco1c1 PE=1 SV=1 - [SO1C1_MOUSE]	6.29	2	2	2	4	0.641	0.881	1.266	0.701	6.84	6.29	3	4	715	78.3	8.81
Q61792	LIM and SH3 domain protein 1 OS=Mus musculus GN=Lasp1 PE=1 SV=1 - [LASP1_MOUSE]	46.01	12	9	16	200	1.431	0.964	1.062	0.701	535.76	46.01	27	200	263	30.0	7.05
Q8VCI5	Peroxisomal biogenesis factor 19 OS=Mus musculus GN=Pex19 PE=1 SV=1 - [PEX19_MOUSE]	55.18	2	11	11	46	1.668	0.653	1.036	0.701	150.99	55.18	18	46	299	32.7	4.34
Q9CYA0	Cysteine-rich with EGF-like domain protein 2 OS=Mus musculus GN=Creld2 PE=2 SV=1 - [CREL2_MOUSE]	24.86	1	10	10	25	2.033	0.780	0.817	0.701	67.56	24.86	17	25	350	38.2	4.58
Q8K2J0	1-phosphatidylinositol 4,5-bisphosphate phosphodiesterase delta-3 OS=Mus musculus GN=Plcd3 PE=1 SV=2 - [PLCD3_MOUSE]	8.92	2	6	6	10	0.422	0.944	0.788	0.702	27.66	8.92	10	10	785	88.6	6.83
A2A9I0	Golgi SNAP receptor complex member 2 OS=Mus musculus GN=Gosr2 PE=4 SV=1 - [A2A9I0_MOUSE]	14.55	2	3	3	8	0.713	1.119	0.645	0.702	16.82	14.55	6	8	165	19.6	9.50
P30558	Proteinase-activated receptor 1 OS=Mus musculus GN=F2r PE=1 SV=2 - [PAR1_MOUSE]	2.33	1	1	1	1	1.246	0.765	0.553	0.702	0.00	2.33	1	1	430	47.8	7.43
Q6PB93	Polypeptide N-acetylgalactosaminyltransferase 2 OS=Mus musculus GN=Galnt2 PE=2 SV=1 - [GALT2_MOUSE]	23.51	2	10	10	24	0.762	0.798	0.611	0.703	59.20	23.51	14	24	570	64.5	8.53
P07724	Serum albumin OS=Mus musculus GN=Alb PE=1 SV=3 - [ALBU_MOUSE]	75.49	4	49	49	2294	3.914	1.644	0.408	0.703	6501.08	75.49	93	2294	608	68.6	6.07

D3YX57	Fanconi anemia group I protein homolog OS=Mus musculus GN=Fanci PE=2 SV=1 - [D3YX57_MOUSE]	5.51	7	3	5	6	0.284	0.763	0.855	0.703	9.49	5.51	6	6	1325	148.7	6.16
Q8R2Y9	SOSS complex subunit B1 OS=Mus musculus GN=Nabp2 PE=2 SV=1 - [SOSB1_MOUSE]	14.62	4	1	2	9	0.934	0.780	0.949	0.703	22.36	14.62	4	9	212	22.6	9.06
Q8BUV8	Protein GPR107 OS=Mus musculus GN=Gpr107 PE=2 SV=2 - [GP107_MOUSE]	10.89	1	5	6	10	0.809	0.813	0.679	0.703	26.41	10.89	9	10	551	62.0	7.59
Q8VHC3	Selenoprotein M OS=Mus musculus GN=Selm PE=1 SV=3 - [SELM_MOUSE]	55.17	1	5	5	43	1.578	0.665	0.891	0.704	141.88	55.17	8	43	145	16.4	5.54
Q8C4C4	Repulsive guidance molecule A OS=Mus musculus GN=Rgma PE=2 SV=1 - [Q8C4C4_MOUSE]	35.17	3	10	10	19	0.629	0.536	0.592	0.704	52.29	35.17	15	19	344	37.8	6.60
P99027	60S acidic ribosomal protein P2 OS=Mus musculus GN=Rplp2 PE=1 SV=3 - [RLA2_MOUSE]	48.70	1	5	5	53	1.552	0.880	0.989	0.704	159.12	48.70	9	53	115	11.6	4.54
Q9CUB6	OTU domain-containing protein 1 OS=Mus musculus GN=Otud1 PE=2 SV=2 - [OTUD1_MOUSE]	6.39	1	1	1	1	1.682	2.365	1.020	0.704	4.01	6.39	1	1	454	48.8	6.28
Q5XKN4	Protein jagunal homolog 1 OS=Mus musculus GN=Jagn1 PE=2 SV=2 - [JAGN1_MOUSE]	10.38	1	2	2	4	0.826	1.159	0.730	0.704	2.44	10.38	3	4	183	21.1	9.80
Q8BNJ2	A disintegrin and metalloproteinase with thrombospondin motifs 4 OS=Mus musculus GN=Adamts4 PE=2 SV=2 - [ATS4_MOUSE]	2.04	3	2	2	3	0.988	1.144	0.961	0.704	7.06	2.04	3	3	833	90.0	8.28
D3YWT0	Signal peptidase complex catalytic subunit SEC11A OS=Mus musculus GN=Sec11a PE=2 SV=1 - [D3YWT0_MOUSE]	25.29	4	5	5	21	0.564	1.040	0.583	0.705	54.74	25.29	10	21	170	19.6	9.48
Q810D6	Glutamate-rich WD repeat-containing protein 1 OS=Mus musculus GN=Grwd1 PE=2 SV=2 - [GRWD1_MOUSE]	15.02	3	3	3	4	2.055	1.605	1.366	0.705	15.49	15.02	4	4	446	49.2	4.81
P62301	40S ribosomal protein S13 OS=Mus musculus GN=Rps13 PE=1 SV=2 - [RS13_MOUSE]	47.68	1	9	9	43	0.528	0.700	0.723	0.705	115.04	47.68	16	43	151	17.2	10.54
A2ADR8	Nuclear inhibitor of protein phosphatase 1 OS=Mus musculus GN=Ppp1r8 PE=2 SV=1 - [A2ADR8_MOUSE]	30.29	2	7	7	27	0.874	0.421	0.843	0.705	68.98	30.29	12	27	350	38.4	7.15
Q6P1J1	Crmp1 protein OS=Mus musculus GN=Crmp1 PE=2 SV=1 - [Q6P1J1_MOUSE]	72.16	1	4	36	961	0.914	0.726	0.900	0.705	2628.42	72.16	67	961	686	74.2	6.81
P25911-2	Isoform 2 of Tyrosine-protein kinase Lyn OS=Mus musculus GN=Lyn - [LYN_MOUSE]	20.77	16	5	9	38	0.608	1.463	0.850	0.706	90.92	20.77	16	38	491	56.2	6.57
Q99PF4-2	Isoform 2 of Cadherin-23 OS=Mus musculus GN=Cdh23 - [CAD23_MOUSE]	3.77	5	9	10	19	1.143	1.152	1.094	0.706	22.83	3.77	12	19	3319	365.4	4.63
P14211	Calreticulin OS=Mus musculus GN=Calr PE=1 SV=1 - [CALR_MOUSE]	57.21	1	23	23	234	1.287	0.976	0.866	0.706	658.96	57.21	41	234	416	48.0	4.49
Q689Z5	Protein strawberry notch homolog 1 OS=Mus musculus GN=Sbno1 PE=1 SV=2 - [SBNO1_MOUSE]	3.24	3	3	4	9	0.697	1.011	0.892	0.706	33.94	3.24	6	9	1390	153.6	8.07
Q6P2L6-4	Isoform 4 of Histone-lysine N-methyltransferase NSD3 OS=Mus musculus GN=Whsc1l1 - [NSD3_MOUSE]	6.76	5	3	3	5	0.994	0.967	0.961	0.706	15.49	6.76	4	5	621	70.0	9.17
Q9CR25	Diphthamide biosynthesis protein 2 OS=Mus musculus GN=Dph2 PE=1 SV=1 - [DPH2_MOUSE]	12.68	2	5	5	12	3.084	1.081	0.845	0.706	38.88	12.68	9	12	489	52.3	5.53
Q921K9	B-cell CLL/lymphoma 7 protein family member B OS=Mus musculus GN=Bcl7b PE=1 SV=1 - [BCL7B_MOUSE]	23.76	2	2	2	2	2.333	0.882	0.983	0.706	7.00	23.76	2	2	202	22.2	4.75
Q69Z38	Pseudopodium-enriched atypical kinase 1 OS=Mus musculus GN=Peak1 PE=1 SV=4 - [PEAK1_MOUSE]	14.41	1	17	17	32	0.745	1.144	1.187	0.707	96.88	14.41	24	32	1735	191.0	6.87
P62254	Ubiquitin-conjugating enzyme E2 G1 OS=Mus musculus GN=Ube2g1 PE=2 SV=3 -	37.06	2	6	6	24	2.047	0.839	0.972	0.707	71.61	37.06	11	24	170	19.5	5.30
D3Z3M7	CAP-Gly domain-containing linker protein 1 OS=Mus musculus GN=Clip1 PE=2 SV=1 - [D3Z3M7_MOUSE]	50.91	5	1	66	268	1.146	1.533	1.199	0.707	762.55	50.91	117	268	1320	147.9	5.31
P62830	60S ribosomal protein L23 OS=Mus musculus GN=Rpl23 PE=1 SV=1 - [RL23_MOUSE]	71.43	2	10	10	36	0.474	0.580	0.648	0.707	109.80	71.43	18	36	140	14.9	10.51

P48437	Prospero homeobox protein 1 OS=Mus musculus GN=Prox1 PE=1 SV=2 - [PROX1_MOUSE]	6.65	1	3	3	6	0.831	0.530	0.857	0.707	13.69	6.65	4	6	737	83.1	7.08
E0CYH6	Zinc finger protein 346 OS=Mus musculus GN=Zfp346 PE=2 SV=1 - [E0CYH6_MOUSE]	8.91	9	1	1	2	1.244	0.894	1.206	0.708	3.40	8.91	2	2	101	11.2	5.10
Q9Z2W0	Aspartyl aminopeptidase OS=Mus musculus GN=Dnpep PE=2 SV=2 - [DNPEP_MOUSE]	46.72	1	15	15	78	2.072	0.803	0.751	0.708	275.41	46.72	24	78	473	52.2	7.25
Q9D0B0	Serine/arginine-rich splicing factor 9 OS=Mus musculus GN=Srsf9 PE=1 SV=1 - [SRSF9_MOUSE]	18.92	1	2	4	12	1.814	0.861	0.762	0.708	31.31	18.92	7	12	222	25.6	8.65
Q8BM41	Protein LSM14 homolog B OS=Mus musculus GN=Lsm14b PE=2 SV=1 - [Q8BM41_MOUSE]	21.31	1	1	5	11	0.832	0.683	1.060	0.708	33.73	21.31	7	11	305	33.2	9.74
Q9ER65	Calsyntenin-2 OS=Mus musculus GN=Clstn2 PE=1 SV=2 - [CSTN2_MOUSE]	7.87	2	5	5	14	1.148	0.871	0.685	0.709	20.95	7.87	6	14	966	107.8	5.68
Q52KR6	Acin1 protein OS=Mus musculus GN=Acin1 PE=2 SV=1 - [Q52KR6_MOUSE]	18.48	6	1	11	36	0.869	0.803	0.877	0.709	100.50	18.48	19	36	552	64.0	8.63
A2A791-2	Isoform 2 of Zinc finger MYM-type protein 4 OS=Mus musculus GN=Zmym4 -	2.60	3	3	3	4	1.182	1.250	0.791	0.709	13.40	2.60	4	4	1460	162.7	6.54
Q8R138	Transmembrane protein 119 OS=Mus musculus GN=Tmem119 PE=2 SV=1 - [TM119_MOUSE]	15.36	1	3	3	7	0.983	1.251	0.925	0.709	26.71	15.36	6	7	280	29.4	4.51
P09103	Protein disulfide-isomerase OS=Mus musculus GN=P4hb PE=1 SV=2 - [PDIA1_MOUSE]	60.51	2	30	33	260	1.788	0.856	0.884	0.709	604.83	60.51	58	260	509	57.0	4.88
Q9Z2D8	Methyl-CpG-binding domain protein 3 OS=Mus musculus GN=Mbd3 PE=1 SV=1 - [MBD3_MOUSE]	28.07	5	8	8	21	0.930	0.652	0.955	0.709	57.69	28.07	12	21	285	32.1	5.82
Q8BY05	Poly(A) polymerase gamma OS=Mus musculus GN=Papolg PE=2 SV=1 - [Q8BY05_MOUSE]	1.86	2	1	1	1	2.799	0.709	0.689	0.710	2.15	1.86	1	1	429	49.0	7.62
Q9CZP0	Ufm1-specific protease 1 OS=Mus musculus GN=Ufsp1 PE=1 SV=1 - [UFSP1_MOUSE]	17.51	1	2	2	3	1.288	0.873	0.716	0.710	5.59	17.51	2	3	217	23.4	6.70
D3YYA0	Set1/Ash2 histone methyltransferase complex subunit ASH2 OS=Mus musculus GN=Ash2l PE=2 SV=1 - [D3YYA0_MOUSE]	7.58	3	5	5	9	0.671	0.848	0.803	0.710	24.40	7.58	6	9	501	56.4	6.83
Q68FG0-2	Isoform 2 of Zinc finger C4H2 domain-containing protein OS=Mus musculus GN=Zc4h2 - [ZC4H2_MOUSE]	27.19	3	7	7	9	1.378	0.424	1.110	0.710	18.38	27.19	9	9	217	25.4	7.14
Q8CIM8	Integrator complex subunit 4 OS=Mus musculus GN=Ints4 PE=2 SV=1 - [INT4_MOUSE]	14.52	3	9	9	21	0.948	1.180	0.659	0.711	28.25	14.52	14	21	964	108.1	6.42
Q9ESP1	Stromal cell-derived factor 2-like protein 1 OS=Mus musculus GN=Sdf2l1 PE=2 SV=2 - [SDF2L_MOUSE]	42.53	1	6	6	18	1.424	0.651	0.928	0.711	62.24	42.53	9	18	221	23.6	7.42
Q9CQQ8	U6 snRNA-associated Sm-like protein LSm7 OS=Mus musculus GN=Lsm7 PE=3 SV=1 - [LSM7_MOUSE]	64.08	1	4	5	15	1.570	0.813	0.942	0.711	29.77	64.08	9	15	103	11.6	5.27
Q9CPW2	Adrenodoxin-like protein, mitochondrial OS=Mus musculus GN=Fdx1l PE=2 SV=1 - [ADXL_MOUSE]	14.94	1	2	2	7	2.342	1.531	2.125	0.711	24.51	14.94	3	7	174	18.8	6.04
E9PUQ5	Golgin subfamily A member 2 OS=Mus musculus GN=Golga2 PE=2 SV=1 -	30.21	11	24	26	76	1.417	0.782	0.935	0.712	207.17	30.21	42	76	1026	116.2	5.05
Q8JZU0	Nucleoside diphosphate-linked moiety X motif 13 OS=Mus musculus GN=Nudt13 PE=2 SV=2 - [NUD13_MOUSE]	6.25	1	1	1	2	3.487	1.796	0.931	0.712	12.61	6.25	2	2	352	39.1	8.02
Q6NSR8	Probable aminopeptidase NPEPL1 OS=Mus musculus GN=Npepl1 PE=2 SV=1 - [PEPL1_MOUSE]	15.46	2	8	8	28	2.288	0.646	0.751	0.712	91.10	15.46	15	28	524	55.9	6.84
E9Q8R5	TraB domain-containing protein OS=Mus musculus GN=Trabd PE=2 SV=1 - [E9Q8R5_MOUSE]	8.13	3	2	2	5	0.812	0.835	0.659	0.712	12.65	8.13	3	5	320	36.0	8.31
Q9CWU4	UPF0690 protein C1orf52 homolog OS=Mus musculus PE=1 SV=1 - [CA052_MOUSE]	20.00	3	4	4	11	1.745	0.779	1.125	0.712	24.22	20.00	6	11	180	20.1	5.11
F7C957	PDZ and LIM domain protein 2 (Fragment) OS=Mus musculus GN=Pdlim2 PE=4 SV=1 - [F7C957_MOUSE]	35.10	2	4	4	14	2.937	0.509	0.825	0.712	45.90	35.10	8	14	151	16.7	8.60

Q9D6F4	Gamma-aminobutyric acid receptor subunit alpha-4 OS=Mus musculus GN=Gabra4 PE=2 SV=1 - [GBRA4_MOUSE]	10.69	1	5	5	19	0.650	0.792	1.110	0.712	57.62	10.69	10	19	552	60.8	9.32
P62313	U6 snRNA-associated Sm-like protein LSm6 OS=Mus musculus GN=Lsm6 PE=2 SV=1 - [LSM6_MOUSE]	55.00	1	5	5	34	1.466	0.838	0.895	0.712	88.93	55.00	10	34	80	9.1	9.58
Q99MX7	Cat eye syndrome critical region protein 6 homolog OS=Mus musculus GN=Cecr6 PE=1 SV=1 - [CECR6_MOUSE]	19.23	1	6	6	28	0.921	1.485	1.387	0.713	101.64	19.23	9	28	572	58.1	9.01
Q3TGW2	Endonuclease/exonuclease /phosphatase family domain-containing protein 1 OS=Mus musculus GN=Eepd1 PE=1 SV=1 - [EEPD1_MOUSE]	2.28	1	1	1	5	0.441	2.193	1.436	0.713	14.08	2.28	2	5	569	62.9	8.35
Q99LQ4	Coiled-coil domain-containing protein 23 OS=Mus musculus GN=Ccdc23 PE=2 SV=1 - [CCD23_MOUSE]	33.33	3	2	2	4	1.096	0.600	0.872	0.713	10.66	33.33	3	4	66	7.8	9.16
Q8R3Q0	Store-operated calcium entry-associated regulatory factor OS=Mus musculus GN=Tmem66 PE=2 SV=2 - [illegible]	17.37	1	5	5	9	0.653	0.887	0.599	0.713	28.42	17.37	9	9	334	35.8	8.19
Q9Z255	Ubiquitin-conjugating enzyme E2 A OS=Mus musculus GN=Ube2a PE=2 SV=1 -	11.18	1	1	1	8	1.525	0.989	0.746	0.713	33.91	11.18	2	8	152	17.3	5.15
P14733	Lamin-B1 OS=Mus musculus GN=Lmnb1 PE=1 SV=3 - [LMNB1_MOUSE]	56.12	1	31	36	244	1.165	0.649	0.745	0.714	670.42	56.12	63	244	588	66.7	5.16
Q3U962	Collagen alpha-2(V) chain OS=Mus musculus GN=Col5a2 PE=1 SV=1 - [CO5A2_MOUSE]	3.94	1	3	3	8	0.989	2.336	0.738	0.714	27.97	3.94	5	8	1497	144.9	6.70
Q505F4	Protein Zfp280b OS=Mus musculus GN=Zfp280b PE=2 SV=1 - [Q505F4_MOUSE]	8.80	1	2	2	5	1.425	0.639	0.837	0.714	19.73	8.80	3	5	534	59.5	6.70
O70251	Elongation factor 1-beta OS=Mus musculus GN=Eef1b PE=1 SV=5 - [EF1B_MOUSE]	58.67	5	10	12	183	1.679	0.776	1.024	0.714	546.46	58.67	22	183	225	24.7	4.69
Q62351	Transferrin receptor protein 1 OS=Mus musculus GN=Tfrc PE=1 SV=1 - [TFR1_MOUSE]	27.52	2	21	21	55	0.857	0.611	0.659	0.714	148.18	27.52	37	55	763	85.7	6.57
F6YA33	TFIIH basal transcription factor complex helicase XPD subunit (Fragment) OS=Mus musculus GN=Ercc2 PE=4 SV=1 - [F6YA33_MOUSE]	11.70	2	1	4	6	0.327	0.565	1.597	0.715	9.65	11.70	4	6	684	77.9	6.74
Q91XE0-2	Isoform 2 of Glycine N-acyltransferase OS=Mus musculus GN=Glyat - [GLYAT_MOUSE]	5.34	2	1	1	1	0.601	1.178	1.023	0.715	2.11	5.34	1	1	262	30.1	7.80
P23475	X-ray repair cross-complementing protein 6 OS=Mus musculus GN=Xrcc6 PE=1 SV=5 - [XRCC6_MOUSE]	11.51	3	4	8	13	0.795	0.734	0.632	0.715	32.60	11.51	11	13	608	69.4	6.79
Q148R4	Protein Spink5 OS=Mus musculus GN=Spink5 PE=2 SV=1 - [Q148R4_MOUSE]	2.26	1	2	2	2	1.026	0.637	0.885	0.715	4.79	2.26	2	2	1017	114.8	8.29
P08003	Protein disulfide-isomerase A4 OS=Mus musculus GN=Pdia4 PE=1 SV=3 - [PDIA4_MOUSE]	50.47	3	33	34	157	1.178	0.681	0.787	0.715	436.40	50.47	59	157	638	71.9	5.31
Q9CR89-2	Isoform 2 of Endoplasmic reticulum-Golgi intermediate compartment protein 2 OS=Mus musculus GN=Ergic2 - [illegible]	15.56	2	3	3	4	0.384	0.817	0.726	0.716	8.80	15.56	4	4	302	34.2	6.89
Q99LJ6	Glutathione peroxidase 7 OS=Mus musculus GN=Gpx7 PE=2 SV=1 - [GPX7_MOUSE]	22.58	1	4	5	15	0.771	0.786	0.536	0.716	38.56	22.58	8	15	186	21.0	8.27
Q8BZX4-2	Isoform 2 of Splicing regulatory glutamine/lysine-rich protein 1 OS=Mus musculus GN=Srek1 - [illegible]	4.43	2	2	2	8	0.298	0.781	0.670	0.716	33.95	4.43	4	8	610	69.1	10.27
Q8BTQ0	Polycomb group RING finger protein 3 OS=Mus musculus GN=Pcgf3 PE=2 SV=1 - [PCGF3_MOUSE]	8.30	1	1	1	2	1.778	1.219	1.098	0.716	0.00	8.30	1	2	241	28.0	8.22
Q923Q2	StAR-related lipid transfer protein 13 OS=Mus musculus GN=Stard13 PE=1 SV=5 -	2.34	2	2	2	3	2.224	0.832	0.687	0.716	8.70	2.34	3	3	1113	125.0	7.53
Q3UZ39	Leucine-rich repeat flightless-interacting protein 1 OS=Mus musculus GN=Lrrfip1 PE=1 SV=2 - [illegible]	16.19	2	2	8	31	0.571	0.934	1.108	0.716	81.36	16.19	13	31	729	79.2	4.82

Q5SUF2	Luc7-like protein 3 OS=Mus musculus GN=Luc7l3 PE=1 SV=1 - [LC7L3_MOUSE]	13.89	3	5	6	20	0.850	0.815	0.809	0.716	44.59	13.89	10	20	432	51.4	9.77
Q3V0Y1	Putative SMEK homolog 3 OS=Mus musculus GN=Smek3p PE=5 SV=2 - [SMEK3_MOUSE]	6.27	1	2	2	4	0.515	1.451	2.281	0.716	2.27	6.27	2	4	813	95.4	5.85
Q8BLU0	Fibronectin leucine rich transmembrane protein 2 OS=Mus musculus GN=Flrt2 PE=2 SV=1 - [Q8BLU0_MOUSE]	6.36	1	2	2	19	1.400	1.188	0.865	0.717	12.80	6.36	4	19	660	73.9	7.91
Q921H9	Sel1 repeat-containing protein 1 OS=Mus musculus GN=Selrc1 PE=1 SV=1 - [SELR1_MOUSE]	44.16	1	8	8	33	1.360	0.672	0.897	0.717	95.58	44.16	15	33	231	25.6	6.29
Q8BUR9	Mitotic-spindle organizing protein 1 OS=Mus musculus GN=Mzt1 PE=1 SV=1 - [MZT1_MOUSE]	55.13	1	3	3	6	0.947	0.711	0.960	0.717	25.65	55.13	5	6	78	8.1	4.78
Q5I012-4	Isoform 4 of Putative sodium-coupled neutral amino acid transporter 10 OS=Mus musculus GN=Slc38a10 - [S38AA_MOUSE]	8.14	5	7	8	10	1.352	1.024	0.635	0.717	27.56	8.14	9	10	1081	116.2	5.90
Q62010	Oviduct-specific glycoprotein OS=Mus musculus GN=Ovgp1 PE=2 SV=1 -	1.94	1	2	2	2	1.194	0.488	0.603	0.717	4.47	1.94	2	2	721	78.8	9.19
P11688	Integrin alpha-5 OS=Mus musculus GN=Itga5 PE=1 SV=3 - [ITA5_MOUSE]	6.08	1	6	6	9	0.977	1.096	1.131	0.717	18.48	6.08	8	9	1053	115.0	5.95
Q6PCS8	Protein Zbtb40 OS=Mus musculus GN=Zbtb40 PE=2 SV=1 - [Q6PCS8_MOUSE]	1.43	1	1	1	1	2.492	0.587	0.641	0.717	2.69	1.43	1	1	1258	138.1	6.15
P06537-3	Isoform 1-B of Glucocorticoid receptor OS=Mus musculus GN=Nr3c1 -	23.81	8	13	13	32	1.196	0.950	0.811	0.717	97.66	23.81	21	32	756	83.2	6.34
Q8R4G6	Alpha-1,6-mannosylglycoprotein 6-beta-N-acetylglucosaminyltransferase A OS=Mus musculus GN=Mgat5 PE=2 SV=1 - [MGT5A_MOUSE]	10.00	1	4	5	14	0.741	0.653	0.658	0.718	27.47	10.00	8	14	740	84.5	8.19
Q91XD7	Cysteine-rich with EGF-like domain protein 1 OS=Mus musculus GN=Creld1 PE=2 SV=1 - [CREL1_MOUSE]	20.95	1	9	9	30	0.755	0.779	0.686	0.718	86.88	20.95	16	30	420	45.7	5.02
E9Q0B8	X-ray repair cross-complementing protein 6 (Fragment) OS=Mus musculus GN=Xrcc6 PE=2 SV=1 - [E9Q0B8_MOUSE]	14.75	2	1	3	4	3.387	1.363	0.437	0.718	13.89	14.75	4	4	217	24.7	5.11
Q3UMG5-2	Isoform 2 of Leucine-rich repeat and calponin homology domain-containing protein 2 OS=Mus musculus GN=Lrch2 - [LRCH2_MOUSE]	6.92	6	2	2	3	0.854	1.435	0.518	0.718	5.72	6.92	3	3	751	82.3	6.33
P0C7T6	Ataxin-1-like OS=Mus musculus GN=Atxn1l PE=1 SV=1 - [ATX1L_MOUSE]	5.82	1	4	4	15	1.729	1.092	1.204	0.718	42.22	5.82	6	15	687	73.3	6.49
A2A5R8	Double-stranded RNA-binding protein Staufen homolog 1 OS=Mus musculus GN=Stau1 PE=2 SV=1 - [A2A5R8_MOUSE]	34.43	4	14	15	31	0.640	0.377	0.889	0.718	90.23	34.43	26	31	485	53.7	9.55
Q922G2	Protein FAM76A OS=Mus musculus GN=Fam76a PE=2 SV=1 - [FA76A_MOUSE]	23.78	3	4	6	10	0.805	0.603	1.167	0.718	23.62	23.78	7	10	307	35.1	9.19
E1U8D0	Protein SOGA1 OS=Mus musculus GN=Soga1 PE=1 SV=3 - [SOGA1_MOUSE]	12.48	2	12	14	39	0.980	0.521	0.858	0.719	118.71	12.48	23	39	1418	159.1	6.46
Q64444	Carbonic anhydrase 4 OS=Mus musculus GN=Ca4 PE=1 SV=1 - [CAH4_MOUSE]	26.56	2	6	6	29	1.008	1.443	1.000	0.719	88.65	26.56	9	29	305	34.3	8.21
Q8R3N6	THO complex subunit 1 OS=Mus musculus GN=Thoc1 PE=1 SV=1 - [THOC1_MOUSE]	9.59	1	5	5	13	1.477	0.806	1.031	0.719	27.39	9.59	8	13	657	75.4	4.97
Q9ESZ8-5	Isoform 5 of General transcription factor II-I OS=Mus musculus GN=Gtf2i -	41.96	13	31	32	75	0.742	0.924	0.682	0.719	224.80	41.96	49	75	939	106.0	8.47
Q6P539	Protein FAM222B OS=Mus musculus GN=Fam222b PE=2 SV=2 - [F222B_MOUSE]	7.30	2	3	3	6	1.539	1.123	1.174	0.719	15.66	7.30	5	6	562	59.6	9.23
O09106	Histone deacetylase 1 OS=Mus musculus GN=Hdac1 PE=1 SV=1 - [HDAC1_MOUSE]	25.10	2	4	10	33	0.558	0.864	0.537	0.719	82.71	25.10	17	33	482	55.0	5.48
Q8BGJ5	MCG13402, isoform CRA_a OS=Mus musculus GN=Ptbp1 PE=2 SV=1 - [Q8BGJ5_MOUSE]	27.22	9	8	10	43	0.961	0.908	0.540	0.719	119.02	27.22	18	43	529	56.9	9.17
P60764	Ras-related C3 botulinum toxin substrate 3 OS=Mus musculus GN=Rac3 PE=1 SV=1 - [RAC3_MOUSE]	42.71	8	4	10	79	0.606	0.679	0.511	0.719	226.30	42.71	18	79	192	21.4	8.15

Q3URU2	Paternally-expressed gene 3 protein OS=Mus musculus GN=Peg3 PE=1 SV=1 - [PEG3_MOUSE]	8.40	1	3	10	18	1.459	0.811	0.656	0.720	42.28	8.40	14	18	1571	178.8	5.45
A2AM05-4	Isoform 4 of Centlein OS=Mus musculus GN=Cntln - [CNTLN_MOUSE]	1.50	3	1	3	3	0.958	1.392	0.605	0.720	5.32	1.50	3	3	1396	160.6	7.99
Q3TQI7	Uncharacterized protein C9orf78 homolog OS=Mus musculus PE=1 SV=2 - [CI078_MOUSE]	11.76	1	3	3	11	1.607	0.692	0.993	0.720	35.25	11.76	6	11	289	33.5	6.34
Q9CY50	Translocon-associated protein subunit alpha OS=Mus musculus GN=Ssr1 PE=1 SV=1 - [SSRA_MOUSE]	23.43	1	4	4	31	0.596	0.791	0.644	0.720	93.37	23.43	8	31	286	32.0	4.45
Q61183-4	Isoform 4 of Poly(A) polymerase alpha OS=Mus musculus GN=Papola - [PAPOA_MOUSE]	7.46	13	3	4	5	0.685	0.702	0.673	0.720	7.23	7.46	5	5	657	72.8	8.07
Q9R0S3	Matrix metalloproteinase-17 OS=Mus musculus GN=Mmp17 PE=1 SV=3 - [MMP17_MOUSE]	3.81	1	1	1	2	2.515	1.088	0.826	0.720	9.39	3.81	1	2	578	64.3	6.38
P35922-9	Isoform ISO9 of Fragile X mental retardation protein 1 homolog OS=Mus musculus GN=Fmr1 - [FMR1_MOUSE]	25.53	18	10	14	70	0.954	0.877	0.934	0.721	178.87	25.53	24	70	568	64.1	8.53
Q9CQU5	ZW10 interactor OS=Mus musculus GN=Zwint PE=2 SV=1 - [ZWINT_MOUSE]	46.03	1	12	12	47	0.810	0.782	0.757	0.721	118.29	46.03	21	47	252	28.7	8.43
A2AJY2	Collagen alpha-1(XV) chain OS=Mus musculus GN=Col15a1 PE=2 SV=1 - [A2AJY2_MOUSE]	3.05	4	4	4	7	1.565	0.886	0.771	0.721	17.41	3.05	6	7	1345	138.2	4.88
Q9JJN6	Beta-catenin-interacting protein 1 OS=Mus musculus GN=Ctnnbip1 PE=1 SV=1 -	69.14	1	4	4	20	1.878	0.693	0.922	0.721	61.55	69.14	8	20	81	9.2	5.41
Q8K2C7-2	Isoform 2 of Protein OS-9 OS=Mus musculus GN=Os9 - [OS9_MOUSE]	21.72	3	11	12	34	1.707	0.949	0.949	0.722	93.69	21.72	18	34	617	69.7	4.83
D3Z2Z1	CAP-Gly domain-containing linker protein 1 OS=Mus musculus GN=Clip1 PE=2 SV=1 - [D3Z2Z1_MOUSE]	47.62	6	2	67	268	1.737	1.123	2.006	0.722	751.17	47.62	118	268	1426	159.9	5.26
P63325	40S ribosomal protein S10 OS=Mus musculus GN=Rps10 PE=1 SV=1 - [RS10_MOUSE]	60.00	3	13	13	78	0.869	0.709	0.690	0.722	207.82	60.00	23	78	165	18.9	10.15
A2A484	Protein Zmynd8 OS=Mus musculus GN=Zmynd8 PE=2 SV=1 - [A2A484_MOUSE]	10.45	11	11	12	31	1.106	0.832	0.799	0.722	70.38	10.45	19	31	1235	136.9	7.52
F6V8M6	Ataxin-2 (Fragment) OS=Mus musculus GN=Atxn2 PE=2 SV=1 - [F6V8M6_MOUSE]	20.05	1	1	8	28	1.183	0.606	0.836	0.722	94.53	20.05	13	28	399	42.8	8.95
Q9QXA5	U6 snRNA-associated Sm-like protein LSm4 OS=Mus musculus GN=Lsm4 PE=2 SV=1 - [LSM4_MOUSE]	27.01	3	4	4	9	1.827	0.754	0.858	0.723	16.35	27.01	6	9	137	15.1	10.05
Q6NZB0	DnaJ homolog subfamily C member 8 OS=Mus musculus GN=Dnajc8 PE=2 SV=2 -	24.90	8	8	8	22	0.984	0.384	1.026	0.723	52.88	24.90	14	22	253	29.8	9.06
Q8C7E9	Cleavage stimulation factor subunit 2 tau variant OS=Mus musculus GN=Cstf2t PE=1 SV=2 - [CSTFT_MOUSE]	34.81	1	9	14	38	1.820	1.097	1.021	0.723	122.97	34.81	21	38	632	65.8	7.25
P0CW02	Lymphocyte antigen 6C1 OS=Mus musculus GN=Ly6c1 PE=2 SV=1 - [LY6C1_MOUSE]	14.50	2	1	1	1	1.152	0.594	0.877	0.723	5.07	14.50	1	1	131	14.2	6.01
Q60848-2	Isoform 2 of Lymphocyte-specific helicase OS=Mus musculus GN=Hells - [HELLS_MOUSE]	1.98	2	1	2	3	0.309	0.187	0.809	0.723	7.16	1.98	2	3	808	93.7	8.19
Q8CFN5-3	Isoform 3 of Myocyte-specific enhancer factor 2C OS=Mus musculus GN=Mef2c - [MEF2C_MOUSE]	6.33	6	1	3	4	0.478	2.213	1.872	0.723	10.10	6.33	3	4	442	47.9	7.84
Q8C407	Protein YIPF4 OS=Mus musculus GN=Yipf4 PE=2 SV=1 - [YIPF4_MOUSE]	8.13	2	2	2	6	0.722	0.853	0.651	0.724	14.28	8.13	4	6	246	27.3	4.65
P83877	Thioredoxin-like protein 4A OS=Mus musculus GN=Txnl4a PE=2 SV=1 - [TXN4A_MOUSE]	38.03	2	5	5	13	1.483	1.267	0.794	0.724	25.45	38.03	8	13	142	16.8	5.85
Q8CCK0	Core histone macro-H2A.2 OS=Mus musculus GN=H2afy2 PE=1 SV=3 - [H2AW_MOUSE]	48.92	1	14	16	92	0.539	0.731	0.630	0.724	308.51	48.92	28	92	372	40.1	9.69
Q61686	Chromobox protein homolog 5 OS=Mus musculus GN=Cbx5 PE=1 SV=1 - [CBX5_MOUSE]	45.55	2	7	8	21	0.711	0.515	0.745	0.724	58.85	45.55	15	21	191	22.2	5.86
G3UXI2	Transmembrane protein 127 (Fragment) OS=Mus musculus GN=Tmem127 PE=2 SV=1 - [G3UXI2_MOUSE]	6.85	2	1	1	3	0.490	0.624	0.735	0.724	5.53	6.85	2	3	146	15.4	9.22

P13020-2	Isoform 2 of Gelsolin OS=Mus musculus GN=Gsn - [GELS_MOUSE]	49.25	3	27	28	146	1.723	0.467	0.689	0.725	437.24	49.25	49	146	731	80.7	5.76
Q9D8T4	Golgi apparatus membrane protein TVP23 homolog B OS=Mus musculus GN=Tvp23b PE=1 SV=1 - [TV23B_MOUSE]	11.71	2	3	3	4	0.531	0.746	0.713	0.725	7.86	11.71	4	4	205	23.3	8.34
Q00420-3	Isoform 3 of GA-binding protein subunit beta-1 OS=Mus musculus GN=Gabpb1 -	4.19	9	1	2	2	1.046	1.151	0.700	0.725	2.11	4.19	2	2	382	41.2	4.84
G3V018	Heterogeneous nuclear ribonucleoprotein Q OS=Mus musculus GN=Syncrip PE=2 SV=1 - [G3V018_MOUSE]	46.09	5	19	24	166	1.003	0.801	0.973	0.725	467.07	46.09	45	166	588	65.7	8.60
Q8K3D3-2	Isoform 2 of DNA repair protein SWI5 homolog OS=Mus musculus GN=Swi5 -	41.32	3	3	4	18	1.614	1.102	1.008	0.725	36.33	41.32	5	18	121	13.8	5.17
J3QNA8	Protein Rnpepl1 (Fragment) OS=Mus musculus GN=Rnpepl1 PE=4 SV=1 -	10.53	3	2	2	3	17.921	1.669	1.059	0.725	6.12	10.53	2	3	133	14.8	4.82
E0CX30	Zinc finger protein ZFAT OS=Mus musculus GN=Zfat PE=2 SV=1 - [E0CX30_MOUSE]	1.13	3	1	2	2	2.379	1.711	1.285	0.725	5.76	1.13	2	2	1152	128.9	7.62
Q8K352	SAM and SH3 domain-containing protein 3 OS=Mus musculus GN=Sash3 PE=2 SV=2 - [SASH3_MOUSE]	2.11	1	1	1	2	1.012	0.580	1.161	0.725	6.53	2.11	2	2	380	41.6	5.22
O35544	Excitatory amino acid transporter 4 OS=Mus musculus GN=Slc1a6 PE=2 SV=1 -	14.97	1	4	6	73	0.639	0.877	1.372	0.726	201.96	14.97	10	73	561	60.7	8.75
Q6ZWM4	N-alpha-acetyltransferase 38, NatC auxiliary subunit OS=Mus musculus GN=Naa38 PE=3 SV=3 - [NAA38_MOUSE]	81.25	1	5	5	35	2.292	0.899	1.063	0.726	142.72	81.25	9	35	96	10.4	4.48
D3YUJ3	Protein Ccnyl1 OS=Mus musculus GN=Ccnyl1 PE=2 SV=1 - [D3YUJ3_MOUSE]	20.98	2	5	7	16	0.909	0.799	0.907	0.726	39.54	20.98	12	16	367	41.6	6.34
P12961	Neuroendocrine protein 7B2 OS=Mus musculus GN=Scg5 PE=1 SV=1 - [7B2_MOUSE]	25.47	1	3	4	28	1.442	0.929	0.942	0.726	87.51	25.47	6	28	212	23.9	5.81
F6WPW1	Polyprenol reductase OS=Mus musculus GN=Srd5a3 PE=4 SV=1 - [F6WPW1_MOUSE]	3.40	2	1	1	1	1.414	1.129	0.985	0.726	1.99	3.40	1	1	206	24.1	9.54
Q9CQK7	RWD domain-containing protein 1 OS=Mus musculus GN=Rwdd1 PE=1 SV=1 -	9.88	1	3	3	12	1.707	1.138	1.007	0.726	27.32	9.88	5	12	243	27.8	4.26
Q99M08	Uncharacterized protein C4orf3 homolog OS=Mus musculus PE=1 SV=1 - [CD003_MOUSE]	24.62	1	1	1	4	0.841	0.686	0.778	0.727	12.61	24.62	2	4	65	7.4	5.24
Q99KF1	Transmembrane emp24 domain-containing protein 9 OS=Mus musculus GN=Tmed9 PE=2 SV=2 - [TMED9_MOUSE]	27.66	1	6	7	41	0.652	0.883	0.666	0.727	102.35	27.66	12	41	235	27.1	8.41
H3BKC5	U6 snRNA-associated Sm-like protein LSm5 (Fragment) OS=Mus musculus GN=Lsm5 PE=2 SV=1 - [H3BKC5_MOUSE]	52.38	2	2	2	6	1.433	0.920	1.021	0.727	8.79	52.38	4	6	42	4.6	5.10
Q8K2T8	RNA polymerase II-associated factor 1 homolog OS=Mus musculus GN=Paf1 PE=2 SV=1 - [PAF1_MOUSE]	18.50	1	8	8	18	2.424	0.885	0.888	0.727	45.91	18.50	11	18	535	60.5	4.65
Q3ULZ2	FH2 domain-containing protein 1 OS=Mus musculus GN=Fhdc1 PE=2 SV=3 - [FHDC1_MOUSE]	3.48	1	3	3	4	2.137	0.872	1.917	0.727	5.33	3.48	3	4	1149	125.3	8.82
Q9D3P8	Plasminogen receptor (KT) OS=Mus musculus GN=Plgrkt PE=1 SV=1 - [PLRKT_MOUSE]	19.05	4	3	3	13	0.470	0.890	0.672	0.727	34.95	19.05	5	13	147	17.2	9.50
Q8N7N5	DDB1- and CUL4-associated factor 8 OS=Mus musculus GN=Dcaf8 PE=1 SV=1 -	8.63	2	4	4	7	0.827	0.687	0.795	0.728	16.61	8.63	7	7	591	66.0	5.87
E9Q4M8	Delta(14)-sterol reductase OS=Mus musculus GN=Tm7sf2 PE=2 SV=1 - [E9Q4M8_MOUSE]	8.77	7	3	3	4	0.992	0.568	0.703	0.728	10.42	8.77	4	4	399	43.4	8.65
Q61586	Glycerol-3-phosphate acyltransferase 1, mitochondrial OS=Mus musculus GN=Gpam PE=1 SV=2 - [GPAT1_MOUSE]	6.05	1	3	3	5	0.671	1.607	1.560	0.728	18.51	6.05	4	5	827	93.6	7.84
P08030	Adenine phosphoribosyltransferase OS=Mus musculus GN=Aprt PE=2 SV=2 -	43.89	1	8	8	21	1.383	1.435	0.562	0.728	54.39	43.89	14	21	180	19.7	6.79
Q7TPR4	Alpha-actinin-1 OS=Mus musculus GN=Actn1 PE=1 SV=1 - [ACTN1_MOUSE]	66.37	2	1	56	556	0.632	2.249	1.737	0.728	1486.37	66.37	103	556	892	103.0	5.38

Q61624	Zinc finger protein 148 OS=Mus musculus GN=Znf148 PE=1 SV=2 - [ZN148_MOUSE]	13.73	1	7	7	11	2.423	0.986	1.149	0.729	30.58	13.73	9	11	794	88.7	6.48
Q8R035	Peptidyl-tRNA hydrolase ICT1, mitochondrial OS=Mus musculus GN=Ict1 PE=1 SV=1 - [ICT1_MOUSE]	49.03	4	9	10	22	0.920	0.505	0.898	0.729	68.01	49.03	17	22	206	23.5	10.18
P33215-2	Isoform 2 of Protein NEDD1 OS=Mus musculus GN=Nedd1 - [NEDD1_MOUSE]	3.54	2	1	1	2	1.851	1.029	0.859	0.729	3.37	3.54	1	2	508	54.3	8.53
P46978	Dolichyl-diphosphooligosaccharide--protein glycosyltransferase subunit STT3A OS=Mus musculus GN=Stt3a PE=1 SV=1 - [STT3A_MOUSE]	14.04	2	10	12	32	0.685	0.855	0.642	0.729	83.99	14.04	20	32	705	80.5	8.10
D3YVF0	A-kinase anchor protein 5 OS=Mus musculus GN=Akap5 PE=2 SV=2 - [AKAP5_MOUSE]	40.00	2	20	20	101	1.258	2.705	2.945	0.729	282.11	40.00	34	101	745	79.3	4.75
F8WJJ9	Myelin regulatory factor OS=Mus musculus GN=Myrf PE=4 SV=1 - [F8WJJ9_MOUSE]	0.90	2	1	1	1	1.654	0.498	0.632	0.729	2.29	0.90	1	1	1112	120.6	7.33
Q7TNR6	Immunoglobulin superfamily member 21 OS=Mus musculus GN=Igsf21 PE=2 SV=1 - [IGS21_MOUSE]	31.62	1	12	13	40	0.771	0.813	0.886	0.729	126.36	31.62	23	40	468	51.9	6.95
Q60598	Src substrate cortactin OS=Mus musculus GN=Cttn PE=1 SV=2 - [SRC8_MOUSE]	54.03	2	28	28	215	1.547	1.080	1.235	0.729	566.82	54.03	47	215	546	61.2	5.40
Q69ZL1	FYVE, RhoGEF and PH domain-containing protein 6 OS=Mus musculus GN=Fgd6 PE=1 SV=2 - [FGD6_MOUSE]	3.50	2	5	5	5	5.658	1.570	1.704	0.729	12.96	3.50	5	5	1399	155.1	7.87
Q8BSI6	R3H and coiled-coil domain-containing protein 1 OS=Mus musculus GN=R3hcc1 PE=2 SV=2 - [R3HC1_MOUSE]	8.05	2	3	3	4	2.598	1.548	0.924	0.730	10.92	8.05	4	4	596	65.7	5.44
Q62086	Serum paraoxonase/arylesterase 2 OS=Mus musculus GN=Pon2 PE=1 SV=2 -	17.23	1	4	4	14	0.704	0.622	0.608	0.730	32.87	17.23	8	14	354	39.6	5.83
Q07813	Apoptosis regulator BAX OS=Mus musculus GN=Bax PE=1 SV=1 - [BAX_MOUSE]	33.33	1	5	5	21	0.874	1.015	0.724	0.730	69.75	33.33	8	21	192	21.4	4.98
O08738	Caspase-6 OS=Mus musculus GN=Casp6 PE=2 SV=1 - [CASP6_MOUSE]	7.25	1	2	2	3	1.464	1.567	0.683	0.731	10.35	7.25	3	3	276	31.6	6.89
Q7TQA1-5	Isoform 5 of Immunoglobulin superfamily member 1 OS=Mus musculus GN=Igsf1 - [illegible]	12.52	4	11	11	50	0.869	0.500	0.440	0.731	137.14	12.52	18	50	775	85.1	5.00
O09164	Extracellular superoxide dismutase [Cu-Zn] OS=Mus musculus GN=Sod3 PE=1 SV=1 - [SODE_MOUSE]	19.12	1	4	4	9	1.918	0.812	0.833	0.731	25.38	19.12	7	9	251	27.4	6.84
O89020	Afamin OS=Mus musculus GN=Afm PE=1 SV=2 - [illegible]	16.78	3	9	9	18	3.142	1.544	0.291	0.731	46.29	16.78	14	18	608	69.3	5.78
Q497V6-2	Isoform 2 of Bromo adjacent homology domain containing 1 protein OS=Mus musculus GN=Bahd1 - [illegible]	2.08	2	1	1	1	2.846	2.373	0.592	0.731	1.89	2.08	1	1	769	83.5	9.01
Q8CCT4	Transcription elongation factor A protein-like 5 OS=Mus musculus GN=Tceal5 PE=1 SV=1 - [TCAL5_MOUSE]	41.50	5	3	8	79	1.152	0.565	1.167	0.731	204.95	41.50	13	79	200	22.0	6.20
P35951	Low-density lipoprotein receptor OS=Mus musculus GN=Ldlr PE=1 SV=2 - [LDLR_MOUSE]	11.25	1	8	8	19	1.263	0.536	0.641	0.732	54.08	11.25	14	19	862	94.9	5.02
Q9Z2X1-2	Isoform 2 of Heterogeneous nuclear ribonucleoprotein F OS=Mus musculus GN=Hnrnpf - [illegible]	39.24	5	10	12	140	1.386	0.885	0.805	0.732	466.95	39.24	21	140	395	43.7	5.48
Q5SUH7	Clathrin interactor 1 OS=Mus musculus GN=Clint1 PE=2 SV=1 - [Q5SUH7_MOUSE]	26.32	3	1	15	43	1.314	1.042	1.156	0.732	124.15	26.32	27	43	623	67.7	6.42
Q8BGZ2-2	Isoform 2 of Protein FAM168A OS=Mus musculus GN=Fam168a - [F168A_MOUSE]	7.66	2	2	2	11	1.044	0.651	0.882	0.732	28.77	7.66	4	11	235	25.0	9.16
P56677	Suppressor of tumorigenicity 14 protein homolog OS=Mus musculus GN=St14 PE=1 SV=2 - [ST14_MOUSE]	1.05	1	1	1	1	4.471	2.687	1.031	0.733	2.19	1.05	1	1	855	94.6	6.81
Q9DBP5	UMP-CMP kinase OS=Mus musculus GN=Cmpk1 PE=1 SV=1 - [KCY_MOUSE]	62.24	1	11	12	129	1.378	0.736	0.923	0.733	326.62	62.24	21	129	196	22.2	5.83

H3BJC4	Potassium voltage-gated channel subfamily H member 2 OS=Mus musculus GN=Erg PE=2 SV=1 - [H3BJC4_MOUSE]	3.03	6	1	1	1	1.246	0.795	0.779	0.733	2.64	3.03	1	1	363	41.4	7.49
Q8CI95	Oxysterol-binding protein-related protein 11 OS=Mus musculus GN=Osbpl11 PE=1 SV=2 - [OSB11_MOUSE]	24.37	2	8	10	31	1.076	1.031	0.935	0.733	87.68	24.37	18	31	751	83.6	7.01
O08583	THO complex subunit 4 OS=Mus musculus GN=Alyref PE=1 SV=3 - [THOC4_MOUSE]	40.78	8	5	8	68	1.289	0.436	1.151	0.734	200.11	40.78	14	68	255	26.9	11.15
A6H6E2	Multimerin-2 OS=Mus musculus GN=Mmrn2 PE=2 SV=1 - [MMRN2_MOUSE]	4.24	1	3	4	11	1.269	1.161	0.624	0.734	17.72	4.24	7	11	943	105.1	5.57
O08644	Ephrin type-B receptor 6 OS=Mus musculus GN=Ephb6 PE=2 SV=4 - [EPHB6_MOUSE]	7.59	2	5	5	14	0.699	0.950	0.989	0.734	35.72	7.59	8	14	1014	110.0	6.83
Q8BUN5	Mothers against decapentaplegic homolog 3 OS=Mus musculus GN=Smad3 PE=1 SV=2 - [SMAD3_MOUSE]	12.24	4	2	6	13	0.507	2.303	1.287	0.734	36.64	12.24	9	13	425	48.0	7.15
Q8BH24	Transmembrane 9 superfamily member 4 OS=Mus musculus GN=Tm9sf4 PE=2 SV=1 - [TM9S4_MOUSE]	15.86	1	9	9	27	0.699	0.713	0.600	0.734	74.55	15.86	17	27	643	74.6	7.23
Q6PGG6	Guanine nucleotide-binding protein-like 3-like protein OS=Mus musculus GN=Gnl3l PE=1 SV=1 - [GNL3L_MOUSE]	7.45	1	4	4	6	1.211	0.714	0.751	0.734	12.69	7.45	4	6	577	65.2	8.60
Q9D2R8	28S ribosomal protein S33, mitochondrial OS=Mus musculus GN=Mrps33 PE=2 SV=1 - [RT33_MOUSE]	11.32	1	1	1	2	0.800	0.594	0.784	0.735	6.38	11.32	2	2	106	12.5	10.26
Q63850	Nuclear pore glycoprotein p62 OS=Mus musculus GN=Nup62 PE=1 SV=2 - [NUP62_MOUSE]	10.46	1	3	5	12	1.553	1.145	1.177	0.735	28.91	10.46	8	12	526	53.2	5.31
Q3TLH4	Protein PRRC2C OS=Mus musculus GN=Prrc2c PE=1 SV=3 - [PRC2C_MOUSE]	19.96	2	45	49	190	1.333	0.761	1.055	0.735	484.90	19.96	81	190	2846	310.7	9.10
O88271	Craniofacial development protein 1 OS=Mus musculus GN=Cfdp1 PE=1 SV=1 - [CFDP1_MOUSE]	29.15	1	7	7	23	1.424	0.667	1.011	0.735	74.88	29.15	12	23	295	32.9	4.86
Q921L3	Transmembrane and coiled coil domain-containing protein 1 OS=Mus musculus GN=Tmco1 PE=2 SV=1 - [TMCO1_MOUSE]	12.77	1	2	2	6	1.049	0.676	0.810	0.735	19.02	12.77	3	6	188	21.2	9.74
Q9CZ49	Kelch-like protein 35 OS=Mus musculus GN=Klhl35 PE=2 SV=2 - [KLH35_MOUSE]	2.79	1	1	1	1	1.208	0.884	0.472	0.735	2.50	2.79	1	1	574	62.3	7.42
Q62167	ATP-dependent RNA helicase DDX3X OS=Mus musculus GN=Ddx3x PE=1 SV=3 - [DDX3X_MOUSE]	56.19	2	8	35	181	0.704	0.552	0.773	0.735	539.92	56.19	59	181	662	73.1	7.18
Q9Z0J0	Epididymal secretory protein E1 OS=Mus musculus GN=Npc2 PE=2 SV=1 - [NPC2_MOUSE]	51.68	1	9	9	26	2.192	0.567	0.765	0.735	71.57	51.68	14	26	149	16.4	7.68
A2A9C8	Mediator of RNA polymerase II transcription subunit 8 OS=Mus musculus GN=Med8 PE=2 SV=1 - [A2A9C8_MOUSE]	8.53	4	1	1	2	2.413	1.285	1.012	0.736	0.00	8.53	2	2	129	14.2	9.09
Q02819	Nucleobindin-1 OS=Mus musculus GN=Nucb1 PE=1 SV=2 - [NUCB1_MOUSE]	51.42	4	20	21	175	2.460	0.923	0.891	0.736	570.56	51.42	39	175	459	53.4	5.07
Q9D8L5	Coiled-coil domain-containing protein 91 OS=Mus musculus GN=Ccdc91 PE=2 SV=2 - [CCD91_MOUSE]	30.32	2	17	17	52	1.487	1.016	0.994	0.736	136.66	30.32	29	52	442	50.0	5.07
E9Q6B2	Coiled-coil domain-containing protein 85C OS=Mus musculus GN=Ccdc85c PE=2 SV=1 - [CC85C_MOUSE]	27.14	1	10	10	26	1.562	0.910	1.381	0.737	99.48	27.14	16	26	420	45.3	6.96
Q78HU3	Multivesicular body subunit 12A OS=Mus musculus GN=Mvb12a PE=1 SV=1 - [MB12A_MOUSE]	21.03	1	3	3	5	1.043	1.065	1.028	0.737	8.05	21.03	3	5	271	28.7	9.11
Q08EC4-3	Isoform 3 of Cas scaffolding protein family member 4 OS=Mus musculus GN=Cass4 - [CASS4_MOUSE]	2.19	3	1	1	1	0.687	0.614	0.687	0.737	2.87	2.19	1	1	685	75.5	6.71
Q9D1J3	SAP domain-containing ribonucleoprotein OS=Mus musculus GN=Sarnp PE=1 SV=3 - [SARNP_MOUSE]	40.95	1	8	8	30	0.822	0.545	1.039	0.737	85.58	40.95	14	30	210	23.5	6.65
Q8BLN5	Lanosterol synthase OS=Mus musculus GN=Lss PE=2 SV=2 - [ERG7_MOUSE]	20.87	1	13	13	27	0.926	0.334	0.591	0.737	77.24	20.87	21	27	733	83.1	6.40

Q80X32	UPF0461 protein C5orf24 homolog OS=Mus musculus PE=1 SV=1 - [CE024_MOUSE]	19.15	1	3	3	7	0.711	0.588	0.944	0.737	7.91	19.15	3	7	188	20.1	9.92
E9Q6I3	Protein Shroom3 OS=Mus musculus GN=Shroom3 PE=2 SV=1 - [E9Q6I3_MOUSE]	4.49	6	3	6	20	1.423	1.030	1.032	0.737	49.36	4.49	8	20	1849	199.8	7.49
Q99PI8	Reticulon-4 receptor OS=Mus musculus GN=Rtn4r PE=2 SV=1 - [RTN4R_MOUSE]	23.04	1	7	7	28	0.532	1.561	0.922	0.738	68.34	23.04	13	28	473	51.0	8.69
Q80XP8	Protein FAM76B OS=Mus musculus GN=Fam76b PE=2 SV=1 - [FA76B_MOUSE]	16.22	3	4	5	8	1.678	0.757	0.869	0.738	15.02	16.22	7	8	339	38.5	9.29
A2A8T9	Uncharacterized protein OS=Mus musculus GN=Efcab14 PE=2 SV=1 - [A2A8T9_MOUSE]	9.29	4	3	3	4	1.219	0.699	0.723	0.738	11.34	9.29	4	4	420	46.5	6.47
Q80U04-2	Isoform 2 of E3 ubiquitin-protein ligase Praja-2 OS=Mus musculus GN=Pja2 - [PJA2_MOUSE]	22.33	2	8	8	40	1.685	0.892	1.177	0.738	115.16	22.33	14	40	645	71.2	4.53
Q0VGU4	MCG18019 OS=Mus musculus GN=Vgf PE=2 SV=1 - [Q0VGU4_MOUSE]	41.49	1	19	19	200	1.452	0.736	0.878	0.738	568.07	41.49	35	200	617	68.2	4.70
E9PV69	CDK2-associated and cullin domain-containing protein 1 OS=Mus musculus GN=Cacul1 PE=2 SV=1 - [E9PV69_MOUSE]	14.70	3	3	3	4	0.942	0.861	0.843	0.738	9.20	14.70	4	4	347	38.8	5.25
B7ZDD9	Carnitine O-acetyltransferase OS=Mus musculus GN=Crat PE=2 SV=1 - [B7ZDD9_MOUSE]	10.34	1	1	1	2	1.881	2.263	0.923	0.738	3.74	10.34	2	2	58	6.2	5.78
Q8C4J7	Transducin beta-like protein 3 OS=Mus musculus GN=Tbl3 PE=2 SV=1 - [TBL3_MOUSE]	8.24	2	4	6	8	0.923	0.957	0.921	0.738	21.74	8.24	8	8	801	88.2	6.81
Q3URQ0	Testis-expressed sequence 10 protein OS=Mus musculus GN=Tex10 PE=1 SV=1 - [TEX10_MOUSE]	8.73	2	5	5	10	0.747	0.817	0.640	0.738	18.19	8.73	8	10	928	105.1	9.16
D3Z1R2	Methenyltetrahydrofolate synthase domain-containing protein (Fragment) OS=Mus musculus GN=Mthfsd PE=2 SV=1 - [D3Z1R2_MOUSE]	7.17	3	2	2	3	1.152	1.049	1.211	0.738	5.94	7.17	3	3	279	29.9	8.65
Q9EQC8	Papillary renal cell carcinoma (Translocation-associated) OS=Mus musculus GN=Prcc PE=2 SV=1 - [Q9EQC8_MOUSE]	15.68	1	6	6	14	0.955	0.857	1.106	0.739	40.34	15.68	9	14	491	52.3	4.94
Q99JF8	PC4 and SFRS1-interacting protein OS=Mus musculus GN=Psip1 PE=1 SV=1 - [PSIP1_MOUSE]	39.02	4	18	20	90	0.616	0.611	1.009	0.739	237.82	39.02	32	90	528	59.7	9.13
Q9JII5-2	Isoform 2 of DAZ-associated protein 1 OS=Mus musculus GN=Dazap1 -	21.48	4	7	7	39	1.984	0.754	0.953	0.739	113.30	21.48	11	39	405	43.1	8.56
P61219	DNA-directed RNA polymerases I, II, and III subunit RPABC2 OS=Mus musculus GN=Polr2f PE=2 SV=1 - [RPAB2_MOUSE]	11.81	1	2	2	7	1.259	0.970	0.881	0.739	17.54	11.81	4	7	127	14.5	4.22
P62274	40S ribosomal protein S29 OS=Mus musculus GN=Rps29 PE=2 SV=2 - [RS29_MOUSE]	25.00	2	3	3	5	0.485	0.506	0.730	0.739	9.53	25.00	4	5	56	6.7	10.13
Q3UXU7	Vang-like protein 1 OS=Mus musculus GN=Vangl1 PE=2 SV=1 - [Q3UXU7_MOUSE]	10.29	5	3	3	6	0.695	0.558	0.597	0.739	28.25	10.29	4	6	476	54.3	8.57
D3Z3E6	Transmembrane protein 56 (Fragment) OS=Mus musculus GN=Tmem56 PE=2 SV=1 - [D3Z3E6_MOUSE]	8.70	3	1	1	2	0.788	0.781	0.780	0.739	7.01	8.70	2	2	138	15.3	6.51
Q9CQ20	Mid1-interacting protein 1 OS=Mus musculus GN=Mid1ip1 PE=1 SV=1 - [M1IP1_MOUSE]	35.71	2	5	5	19	1.640	0.621	0.613	0.739	53.58	35.71	8	19	182	20.3	5.50
Q4VBE8	WD repeat-containing protein 18 OS=Mus musculus GN=Wdr18 PE=1 SV=1 -	15.31	1	6	6	11	0.938	0.992	0.770	0.739	33.09	15.31	11	11	431	47.2	6.89
Q9Z204	Heterogeneous nuclear ribonucleoproteins C1/C2 OS=Mus musculus GN=Hnrnpc PE=1 SV=1 - [HNRPC_MOUSE]	42.49	1	1	13	61	0.986	0.903	0.980	0.739	161.46	42.49	22	61	313	34.4	5.05
Q9R0Q1	Synaptotagmin-like protein 4 OS=Mus musculus GN=Sytl4 PE=1 SV=1 - [SYTL4_MOUSE]	11.14	4	5	7	17	0.880	0.931	0.493	0.739	34.37	11.14	12	17	673	76.0	8.84
Q7TPB0	Lipid phosphate phosphatase-related protein type 3 OS=Mus musculus GN=Lppr3 PE=2 SV=1 - [LPPR3_MOUSE]	33.24	7	18	18	76	0.708	0.692	0.711	0.740	234.31	33.24	28	76	716	76.6	6.01
F8VQ29	Protein Iqgap3 OS=Mus musculus GN=Iqgap3 PE=2 SV=1 - [F8VQ29_MOUSE]	1.41	1	1	2	2	1.416	2.676	0.997	0.740	4.50	1.41	2	2	1632	185.2	7.52

Q91W98	Solute carrier family 15 member 4 OS=Mus musculus GN=Slc15a4 PE=1 SV=1 -	2.26	1	1	1	2	0.828	0.818	0.785	0.740	8.98	2.26	2	2	574	62.2	9.16
Q9JI44	DNA methyltransferase 1-associated protein 1 OS=Mus musculus GN=Dmap1 PE=1 SV=1 - [DMAP1_MOUSE]	11.97	2	4	4	6	0.436	0.666	0.787	0.740	12.18	11.97	6	6	468	53.1	9.50
Q543V3	Insulin receptor substrate 1 OS=Mus musculus GN=Irs1 PE=2 SV=1 - [Q543V3_MOUSE]	6.74	2	6	6	24	0.884	0.676	0.904	0.740	50.11	6.74	10	24	1231	130.5	8.78
Q62419	Endophilin-A2 OS=Mus musculus GN=Sh3gl1 PE=1 SV=1 - [SH3G1_MOUSE]	62.77	1	16	21	123	0.957	0.963	1.265	0.740	344.47	62.77	38	123	368	41.5	5.72
F6V0H0	Protein Pagr1b (Fragment) OS=Mus musculus GN=Pagr1b PE=4 SV=1 - [F6V0H0_MOUSE]	32.41	2	3	3	6	1.661	0.877	1.047	0.741	17.63	32.41	5	6	108	12.5	5.66
Q9CPZ8	COX assembly mitochondrial protein homolog OS=Mus musculus GN=Cmc1 PE=3 SV=1 - [COXM1_MOUSE]	40.57	1	4	4	19	1.266	0.615	0.980	0.741	50.33	40.57	6	19	106	12.5	8.24
Q9CQI7	U2 small nuclear ribonucleoprotein B'' OS=Mus musculus GN=Snrpb2 PE=2 SV=1 -	30.22	2	5	7	20	1.411	0.927	1.074	0.741	52.27	30.22	10	20	225	25.3	9.72
Q60709	Amyloid-like protein 2 OS=Mus musculus GN=Aplp2 PE=2 SV=1 - [Q60709_MOUSE]	33.56	2	3	22	72	0.630	0.844	1.120	0.741	211.90	33.56	39	72	751	85.2	4.73
O70551	SRSF protein kinase 1 OS=Mus musculus GN=Srpk1 PE=1 SV=2 - [SRPK1_MOUSE]	10.96	3	6	9	14	3.149	0.927	0.865	0.741	31.68	10.96	12	14	648	73.0	6.19
Q6PGF2	MORN repeat-containing protein 4 OS=Mus musculus GN=Morn4 PE=2 SV=1 - [MORN4_MOUSE]	28.08	1	4	4	6	1.631	1.235	1.113	0.741	16.33	28.08	6	6	146	16.2	8.15
P20029	78 kDa glucose-regulated protein OS=Mus musculus GN=Hspa5 PE=1 SV=3 - [GRP78_MOUSE]	58.17	1	42	45	688	1.199	0.880	0.898	0.741	1937.18	58.17	83	688	655	72.4	5.16
Q4JK59-3	Isoform 3 of Methylcytosine dioxygenase TET2 OS=Mus musculus GN=Tet2 - [TET2_MOUSE]	2.07	3	1	1	3	2.632	1.267	1.106	0.742	7.37	2.07	1	3	580	64.5	8.84
D3Z6M7	MCG8002 OS=Mus musculus GN=Sult2a4 PE=4 SV=2 - [D3Z6M7_MOUSE]	3.17	3	1	1	1	2.080	1.903	0.971	0.742	0.00	3.17	1	1	284	33.2	8.18
B0R106	Proline-rich protein 5-like (Fragment) OS=Mus musculus GN=Prr5l PE=2 SV=1 - [B0R106_MOUSE]	6.25	2	1	1	2	2.521	1.395	0.854	0.742	5.12	6.25	1	2	128	14.4	9.54
Q5SXY1	Cytospin-B OS=Mus musculus GN=Specc1 PE=1 SV=2 - [CYTSB_MOUSE]	39.46	2	7	39	147	0.787	0.568	1.354	0.742	405.66	39.46	69	147	1067	118.0	6.64
P49182	Heparin cofactor 2 OS=Mus musculus GN=Serpind1 PE=1 SV=1 - [HEP2_MOUSE]	4.18	1	2	2	5	2.222	1.933	0.533	0.742	16.77	4.18	3	5	478	54.5	7.34
Q9DC33	High mobility group protein 20A OS=Mus musculus GN=Hmg20a PE=2 SV=1 -	26.88	3	8	8	25	0.894	0.730	0.996	0.742	76.96	26.88	13	25	346	39.9	6.40
Q9Z307	Inward rectifier potassium channel 16 OS=Mus musculus GN=Kcnj16 PE=2 SV=2 -	15.75	3	7	7	20	0.880	0.544	0.593	0.743	47.98	15.75	13	20	419	48.0	7.83
Q60715	Prolyl 4-hydroxylase subunit alpha-1 OS=Mus musculus GN=P4ha1 PE=2 SV=2 - [P4HA1_MOUSE]	12.73	3	6	6	10	0.918	1.063	0.675	0.743	31.45	12.73	8	10	534	60.9	5.90
Q9R099	Transducin beta-like protein 2 OS=Mus musculus GN=Tbl2 PE=2 SV=2 - [TBL2_MOUSE]	18.10	3	7	7	9	0.759	0.781	0.753	0.743	24.51	18.10	8	9	442	49.6	9.04
A2AHX8	Bcl-2-like protein 1 (Fragment) OS=Mus musculus GN=Bcl2l1 PE=2 SV=1 - [A2AHX8_MOUSE]	34.38	8	3	3	9	0.957	1.018	0.653	0.743	21.54	34.38	5	9	96	10.5	4.56
Q9Z0H3	SWI/SNF-related matrix-associated actin-dependent regulator of chromatin subfamily B member 1 OS=Mus musculus GN=Smarcb1 PE=1 SV=1 -	11.69	4	5	5	15	1.109	0.736	0.810	0.743	44.89	11.69	9	15	385	44.1	6.23
Q8BU40	NACHT, LRR and PYD domains-containing protein 4A OS=Mus musculus GN=Nlrp4a PE=2 SV=1 - [illegible]	4.48	1	2	3	4	1.028	0.925	1.011	0.744	13.38	4.48	3	4	982	112.5	6.27
O09159	Lysosomal alpha-mannosidase OS=Mus musculus GN=Man2b1 PE=2 SV=4 -	12.64	1	10	10	25	1.797	0.761	0.676	0.744	73.96	12.64	17	25	1013	114.6	8.13
Q3ZAQ4	Autophagy 9-like 1 protein OS=Mus musculus GN=Atg9a PE=2 SV=1 - [Q3ZAQ4_MOUSE]	12.99	2	9	9	28	1.080	0.937	0.864	0.744	74.06	12.99	17	28	839	94.4	6.64
Q9CXX9	CUE domain-containing protein 2 OS=Mus musculus GN=Cuedc2 PE=2 SV=1 -	21.83	2	4	5	24	1.284	0.763	1.396	0.744	73.81	21.83	9	24	284	31.8	4.98

Q9WV03	Protein FAM50A OS=Mus musculus GN=Fam50a PE=2 SV=1 - [FA50A_MOUSE]	7.37	1	2	2	6	0.425	0.529	0.852	0.744	13.08	7.37	4	6	339	40.2	6.83
O35459	Delta(3,5)-Delta(2,4)-dienoyl-CoA isomerase, mitochondrial OS=Mus musculus GN=Ech1 PE=2 SV=1 - [ECH1_MOUSE]	48.32	2	14	14	82	2.166	1.197	0.738	0.744	232.35	48.32	25	82	327	36.1	7.71
A2AEY2	Four and a half LIM domains 1, isoform CRA_c OS=Mus musculus GN=Fhl1 PE=4 SV=1 - [A2AEY2_MOUSE]	35.29	6	1	11	51	1.463	0.866	0.824	0.745	121.63	35.29	18	51	323	36.2	9.01
Q99LD8	N(G),N(G)-dimethylarginine dimethylaminohydrolase 2 OS=Mus musculus GN=Ddah2 PE=1 SV=1 - [illegible]	64.91	3	12	13	81	1.624	0.819	0.647	0.745	230.87	64.91	26	81	285	29.6	6.01
Q9DBX1	Regulator of cell cycle RGCC OS=Mus musculus GN=Rgcc PE=1 SV=1 - [RGCC_MOUSE]	21.90	1	2	2	10	2.511	1.609	1.364	0.745	29.44	21.90	4	10	137	14.7	4.84
Q923D4	Splicing factor 3B subunit 5 OS=Mus musculus GN=Sf3b5 PE=2 SV=1 - [SF3B5_MOUSE]	62.79	1	5	5	14	1.094	0.903	0.786	0.745	42.25	62.79	9	14	86	10.1	6.35
Q80X81	Acetyl-Coenzyme A acetyltransferase 3 OS=Mus musculus GN=Acat3 PE=2 SV=1 - [Q80X81_MOUSE]	53.15	2	3	17	199	2.543	0.705	0.597	0.746	619.36	53.15	28	199	397	41.4	7.94
Q9QXT0	Protein canopy homolog 2 OS=Mus musculus GN=Cnpy2 PE=2 SV=1 - [CNPY2_MOUSE]	61.54	1	11	11	50	1.923	0.992	0.956	0.746	138.35	61.54	22	50	182	20.8	5.07
P57787	Monocarboxylate transporter 4 OS=Mus musculus GN=Slc16a3 PE=1 SV=1 -	1.49	1	1	1	4	0.479	0.436	0.974	0.746	5.79	1.49	2	4	470	50.3	7.96
F8WIC0	Adenylate kinase 7 OS=Mus musculus GN=Ak7 PE=2 SV=1 - [F8WIC0_MOUSE]	2.21	1	1	1	1	2.202	1.000	0.681	0.746	0.00	2.21	1	1	723	82.5	4.88
E9Q4A0	cAMP-regulated phosphoprotein 21 OS=Mus musculus GN=Arpp21 PE=2 SV=2 -	12.65	17	7	7	23	0.932	0.957	1.374	0.746	68.23	12.65	11	23	601	65.2	6.87
Q5FW52-2	Isoform 2 of Muscular LMNA-interacting protein OS=Mus musculus GN=Mlip - [MLIP_MOUSE]	30.84	2	5	5	12	1.713	2.086	2.884	0.746	44.89	30.84	7	12	227	25.0	6.81
Q80Y50-5	Isoform 5 of Calmodulin-binding transcription activator 2 OS=Mus musculus GN=Camta2 - [CMTA2_MOUSE]	7.09	2	1	4	4	1.887	1.335	1.402	0.746	4.02	7.09	4	4	1001	107.8	6.42
B1AQG7	Protein Zfp207 (Fragment) OS=Mus musculus GN=Zfp207 PE=2 SV=1 - [B1AQG7_MOUSE]	18.06	5	3	3	16	1.411	0.718	1.002	0.746	50.81	18.06	6	16	227	25.0	6.52
O09105	Neurogenic differentiation factor 4 OS=Mus musculus GN=Neurod4 PE=1 SV=1 - [NDF4_MOUSE]	2.73	1	1	1	1	1.126	0.435	0.808	0.746	1.97	2.73	1	1	330	37.1	7.17
Q3V341	Protein kinase C zeta type OS=Mus musculus GN=Prkcz PE=2 SV=1 - [Q3V341_MOUSE]	8.31	2	4	4	10	0.531	0.910	0.942	0.746	9.05	8.31	5	10	409	46.6	4.84
Q05DT2	Hmg20b protein OS=Mus musculus GN=Hmg20b PE=2 SV=1 - [Q05DT2_MOUSE]	11.16	4	2	2	2	3.008	0.898	0.827	0.747	5.73	11.16	2	2	215	24.7	6.15
Q8R507	Fukutin OS=Mus musculus GN=Fktn PE=2 SV=1 - [FKTN_MOUSE]	1.95	1	1	1	4	0.717	0.753	0.704	0.747	10.12	1.95	2	4	461	53.5	8.51
Q9Z1Q5	Chloride intracellular channel protein 1 OS=Mus musculus GN=Clic1 PE=1 SV=3 - [CLIC1_MOUSE]	49.79	2	10	11	27	0.624	1.538	0.727	0.747	80.91	49.79	18	27	241	27.0	5.17
F6QKD2	Probable ATP-dependent RNA helicase DDX47 (Fragment) OS=Mus musculus GN=Ddx47 PE=3 SV=1 - [illegible]	8.33	2	2	2	4	3.367	1.322	0.719	0.747	8.71	8.33	3	4	360	40.0	8.85
Q3TCT4-2	Isoform 2 of Ectonucleoside triphosphate diphosphohydrolase 7 OS=Mus musculus [illegible]	7.91	2	1	1	1	1.139	0.918	0.768	0.747	0.00	7.91	1	1	354	40.0	6.80
P56212-2	Isoform ARPP-16 of cAMP-regulated phosphoprotein 19 OS=Mus musculus GN=Arpp19 - [ARP19_MOUSE]	60.42	5	4	5	37	1.188	0.624	2.100	0.748	110.53	60.42	9	37	96	10.6	9.70
B2LVG5	Cleavage and polyadenylation specific factor 4 isoform 1 OS=Mus musculus GN=Cpsf4 PE=2 SV=1 - [B2LVG5_MOUSE]	16.05	7	3	3	7	0.947	0.900	0.842	0.748	23.49	16.05	5	7	243	27.4	8.22
Q61548-3	Isoform 3 of Clathrin coat assembly protein AP180 OS=Mus musculus GN=Snap91 -	36.64	6	1	29	438	0.717	1.181	1.027	0.748	1236.96	36.64	51	438	868	88.8	4.91

Q9D281	Protein Noxp20 OS=Mus musculus GN=Fam114a1 PE=2 SV=1 - [NXP20_MOUSE]	11.25	1	5	5	8	1.484	1.392	0.692	0.748	23.81	11.25	8	8	569	61.0	4.56
Q3THK3	General transcription factor IIF subunit 1 OS=Mus musculus GN=Gtf2f1 PE=1 SV=2 - [T2FA_MOUSE]	6.69	1	4	4	6	0.484	0.681	0.808	0.748	11.55	6.69	5	6	508	57.2	7.01
P27773	Protein disulfide-isomerase A3 OS=Mus musculus GN=Pdia3 PE=1 SV=2 - [PDIA3_MOUSE]	71.49	2	38	38	428	1.247	0.858	0.791	0.748	1175.77	71.49	69	428	505	56.6	6.21
Q6ZPR1	UPF0501 protein KIAA1430 OS=Mus musculus GN=Kiaa1430 PE=1 SV=2 -	1.48	2	1	1	1	0.637	0.677	0.886	0.748	0.00	1.48	1	1	541	60.8	9.51
P53702	Cytochrome c-type heme lyase OS=Mus musculus GN=Hccs PE=2 SV=2 - [CCHL_MOUSE]	31.99	1	5	7	41	0.769	0.925	0.752	0.748	98.41	31.99	13	41	272	31.0	7.12
Q8BUB6	Uronyl 2-sulfotransferase OS=Mus musculus GN=Ust PE=2 SV=3 - [UST_MOUSE]	3.19	1	1	1	2	0.897	0.911	0.561	0.748	6.35	3.19	2	2	407	47.7	8.57
E9PYK7	Contactin-5 OS=Mus musculus GN=Cntn5 PE=4 SV=1 - [E9PYK7_MOUSE]	6.49	2	4	5	10	1.025	1.308	0.861	0.749	26.56	6.49	8	10	893	98.3	6.68
F8WHT3	Protein PRRC2B OS=Mus musculus GN=Prrc2b PE=2 SV=1 - [F8WHT3_MOUSE]	19.91	4	11	36	91	1.072	0.880	1.110	0.749	245.07	19.91	59	91	2230	243.0	8.47
Q9DCC1	RNA guanylyltransferase and 5'-phosphatase, isoform CRA_a OS=Mus musculus GN=Rngtt PE=2 SV=1 - [Q9DCC1_MOUSE]	9.47	2	4	4	6	1.209	0.760	0.833	0.749	21.51	9.47	6	6	581	66.8	8.22
Q00724	Retinol-binding protein 4 OS=Mus musculus GN=Rbp4 PE=2 SV=2 - [RET4_MOUSE]	8.96	2	1	1	3	5.787	2.541	0.473	0.749	4.95	8.96	2	3	201	23.2	5.99
Q5BKQ4	Inactive pancreatic lipase-related protein 1 OS=Mus musculus GN=Pnliprp1 PE=2 SV=2 - [LIPR1_MOUSE]	3.59	1	1	1	1	52.288	5.390	3.385	0.749	2.06	3.59	1	1	473	52.7	6.34
Q9Z2E1	Methyl-CpG-binding domain protein 2 OS=Mus musculus GN=Mbd2 PE=2 SV=2 - [MBD2_MOUSE]	22.22	3	6	6	9	0.901	0.713	1.087	0.749	26.54	22.22	8	9	414	43.5	10.04
Q99JF5	Diphosphomevalonate decarboxylase OS=Mus musculus GN=Mvd PE=1 SV=2 - [MVD1_MOUSE]	32.17	1	7	7	13	2.247	0.558	0.724	0.750	37.67	32.17	10	13	401	44.0	6.30
Q7TPW1	Nexilin OS=Mus musculus GN=Nexn PE=1 SV=3 - [NEXN_MOUSE]	13.18	1	7	8	17	1.696	1.030	1.523	0.750	49.19	13.18	12	17	607	72.1	5.01
P29319	Ephrin type-A receptor 3 OS=Mus musculus GN=Epha3 PE=1 SV=1 - [EPHA3_MOUSE]	5.19	5	2	4	9	1.508	2.332	0.959	0.750	25.39	5.19	5	9	983	109.9	7.20
Q3U957	Derlin-2 OS=Mus musculus GN=Derl2 PE=2 SV=1 - [Q3U957_MOUSE]	18.18	2	1	1	2	0.457	0.686	0.660	0.750	3.68	18.18	2	2	165	18.9	6.06
Q8C3W1	Uncharacterized protein C1orf198 homolog OS=Mus musculus PE=1 SV=1 - [CA198_MOUSE]	71.12	1	15	15	141	1.320	0.760	0.908	0.750	345.49	71.12	23	141	322	35.3	5.24
Q8BIQ6	MCG23335 OS=Mus musculus GN=Zfp947 PE=2 SV=1 - [Q8BIQ6_MOUSE]	8.70	145	1	2	3	1.430	0.925	0.820	0.750	7.03	8.70	3	3	437	51.4	8.87
Q9CYP7	Sestrin-3 OS=Mus musculus GN=Sesn3 PE=2 SV=1 - [SESN3_MOUSE]	2.85	1	1	1	3	1.941	2.087	0.810	0.750	6.25	2.85	2	3	492	57.0	6.25
Q3U0V2	Tumor necrosis factor receptor type 1-associated DEATH domain protein OS=Mus musculus GN=Tradd PE=1 SV=1 - [TRADD_MOUSE]	6.77	1	1	2	3	15.653	2.026	1.655	0.750	6.03	6.77	3	3	310	34.6	5.20
O54851	Gap junction delta-2 protein OS=Mus musculus GN=Gjd2 PE=2 SV=2 - [CXD2_MOUSE]	2.49	1	1	1	1	1.001	0.688	0.818	0.750	2.48	2.49	1	1	321	36.1	8.68
O70230-3	Isoform 3 of Zinc finger protein 143 OS=Mus musculus GN=Znf143 - [ZN143_MOUSE]	5.41	3	2	2	4	1.662	1.065	0.844	0.750	10.34	5.41	4	4	610	66.0	6.32
Q8BIF9	Zinc finger protein 787 OS=Mus musculus GN=Znf787 PE=2 SV=3 - [ZN787_MOUSE]	5.25	1	2	2	5	0.992	0.764	1.155	0.751	12.82	5.25	4	5	381	40.5	8.24
F8WJ93	Echinoderm microtubule-associated protein-like 4 OS=Mus musculus GN=Eml4 PE=2 SV=1 - [F8WJ93_MOUSE]	29.55	5	21	23	76	0.981	0.763	0.844	0.751	192.42	29.55	40	76	988	109.9	6.49
Q6ZQ29-2	Isoform 2 of Serine/threonine-protein kinase TAO2 OS=Mus musculus GN=Taok2 - [TAOK2_MOUSE]	15.55	2	3	16	55	0.847	0.656	0.988	0.751	171.60	15.55	26	55	1055	119.9	7.28

Q91Z67	SLIT-ROBO Rho GTPase-activating protein 2 OS=Mus musculus GN=Srgap2 PE=1 SV=2 - [SRGP2_MOUSE]	40.06	1	30	33	124	0.757	0.809	0.932	0.751	371.14	40.06	58	124	1071	120.7	6.64
Q8C131	Hydroxycarboxylic acid receptor 1 OS=Mus musculus GN=Hcar1 PE=1 SV=1 - [HCAR1_MOUSE]	3.50	2	1	1	1	1.397	1.562	1.581	0.751	2.90	3.50	1	1	343	38.9	8.98
Q8BUY9	Geranylgeranyl transferase type-1 subunit beta OS=Mus musculus GN=Pggt1b PE=2 SV=1 - [PGTB1_MOUSE]	19.89	1	6	6	6	1.706	0.820	0.696	0.752	19.77	19.89	6	6	377	42.3	6.83
O35098	Dihydropyrimidinase-related protein 4 OS=Mus musculus GN=Dpysl4 PE=1 SV=1 -	76.22	3	25	34	428	0.857	0.703	0.743	0.752	1406.47	76.22	62	428	572	61.9	6.98
Q9CQG9	Transmembrane protein 100 OS=Mus musculus GN=Tmem100 PE=1 SV=1 - [TM100_MOUSE]	21.64	1	5	5	13	1.001	0.879	0.648	0.752	27.81	21.64	9	13	134	14.5	9.50
Q8BJH1	Zinc finger C2HC domain-containing protein 1A OS=Mus musculus GN=Zc2hc1a PE=2 SV=1 - [ZC21A_MOUSE]	48.77	4	17	17	112	0.972	0.519	1.139	0.752	272.00	48.77	32	112	324	35.1	9.88
A2A9X5	5'(3')-deoxyribonucleotidase, cytosolic type OS=Mus musculus GN=Nt5c PE=2 SV=1 - [A2A9X5_MOUSE]	57.22	1	1	8	52	1.998	1.243	0.723	0.753	176.57	57.22	15	52	194	21.9	5.33
O70404	Vesicle-associated membrane protein 8 OS=Mus musculus GN=Vamp8 PE=1 SV=1 - [VAMP8_MOUSE]	16.83	1	2	2	16	0.852	1.086	0.661	0.753	32.70	16.83	4	16	101	11.4	8.19
A2AJG1	Acyl-CoA-binding domain-containing protein 7 OS=Mus musculus GN=Acbd7 PE=4 SV=1 - [A2AJG1_MOUSE]	11.36	2	1	1	2	1.782	2.134	0.703	0.753	6.56	11.36	1	2	88	10.0	5.55
Q91VH1	Adiponectin receptor protein 1 OS=Mus musculus GN=Adipor1 PE=1 SV=1 -	16.27	1	3	3	6	0.941	0.863	1.312	0.753	16.48	16.27	4	6	375	42.3	7.02
Q8K4G5-2	Isoform 2 of Actin-binding LIM protein 1 OS=Mus musculus GN=Ablim1 - [ABLM1_MOUSE]	42.65	12	1	25	140	1.440	0.968	1.154	0.753	358.49	42.65	48	140	701	78.9	8.25
Q91VS8	FERM, RhoGEF and pleckstrin domain-containing protein 2 OS=Mus musculus GN=Farp2 PE=1 SV=2 - [illegible]	3.00	2	3	4	5	0.485	1.007	0.704	0.753	11.87	3.00	5	5	1065	121.2	8.44
Q99N11	Dual specificity protein phosphatase 22 OS=Mus musculus GN=Dusp22 PE=1 SV=1 -	14.67	2	2	2	8	0.730	0.629	0.604	0.753	28.85	14.67	4	8	184	21.0	7.83
Q3UEB3-2	Isoform 2 of Poly(U)-binding-splicing factor PUF60 OS=Mus musculus GN=Puf60 - [PUF60_MOUSE]	40.77	2	1	20	66	0.928	0.991	0.731	0.753	215.86	40.77	34	66	547	58.5	5.35
D3Z7C0	39S ribosomal protein L40, mitochondrial OS=Mus musculus GN=Mrpl40 PE=2 SV=1 - [D3Z7C0_MOUSE]	36.42	4	5	5	21	1.134	0.629	0.948	0.753	52.92	36.42	10	21	162	19.4	9.19
Q9R0X5	X-linked retinitis pigmentosa GTPase regulator OS=Mus musculus GN=Rpgr PE=1 SV=2 - [RPGR_MOUSE]	7.49	9	2	6	9	0.902	1.049	1.216	0.753	19.22	7.49	9	9	1001	111.7	4.61
O88968	Transcobalamin-2 OS=Mus musculus GN=Tcn2 PE=2 SV=1 - [TCO2_MOUSE]	10.23	1	3	3	6	2.898	1.534	0.838	0.754	17.51	10.23	4	6	430	47.6	6.33
Q9JHL1	Na(+)/H(+) exchange regulatory cofactor NHE-RF2 OS=Mus musculus GN=Slc9a3r2 PE=1 SV=2 - [NHRF2_MOUSE]	45.40	2	13	13	48	1.447	0.654	0.882	0.754	131.01	45.40	22	48	337	37.4	7.59
Q9JIX0	Enhancer of yellow 2 transcription factor homolog OS=Mus musculus GN=Eny2 PE=2 SV=1 - [ENY2_MOUSE]	53.47	1	6	6	13	1.349	0.990	0.943	0.754	33.26	53.47	7	13	101	11.5	9.33
E9Q7G1	Protein Tmed7 OS=Mus musculus GN=Tmed7 PE=2 SV=1 - [E9Q7G1_MOUSE]	28.19	2	4	4	15	0.930	0.913	0.714	0.754	57.45	28.19	8	15	188	21.3	6.20
D3YWX2	YLP motif-containing protein 1 OS=Mus musculus GN=Ylpm1 PE=2 SV=2 - [D3YWX2_MOUSE]	20.48	4	33	34	132	1.062	0.758	1.137	0.754	368.36	20.48	59	132	2139	240.9	6.68
Q3U8Y1	28S ribosomal protein S11, mitochondrial OS=Mus musculus GN=Mrps11 PE=2 SV=1 - [Q3U8Y1_MOUSE]	28.80	2	4	4	11	0.751	0.498	1.048	0.755	46.11	28.80	7	11	191	20.2	10.95
Q9CQI3	Glia maturation factor beta OS=Mus musculus GN=Gmfb PE=1 SV=3 - [GMFB_MOUSE]	65.49	2	6	8	117	2.006	1.212	0.965	0.755	355.73	65.49	16	117	142	16.7	5.16
A2AIR4	Protein Grin3a OS=Mus musculus GN=Grin3a PE=2 SV=1 - [A2AIR4_MOUSE]	9.24	4	8	9	18	1.308	1.416	0.865	0.755	42.00	9.24	14	18	1115	125.0	7.09

A2TJV2	Paralemmin-3 OS=Mus musculus GN=Palm3 PE=2 SV=1 - [PALM3_MOUSE]	11.58	1	6	6	14	1.545	0.656	0.973	0.755	41.53	11.58	10	14	734	78.7	4.50
P14869	60S acidic ribosomal protein P0 OS=Mus musculus GN=Rplp0 PE=1 SV=3 - [RLA0_MOUSE]	40.06	2	1	13	65	0.468	0.846	0.696	0.755	160.23	40.06	24	65	317	34.2	6.25
Q3UGC7	Eukaryotic translation initiation factor 3 subunit J-A OS=Mus musculus GN=Eif3j1 PE=2 SV=1 - [EI3JA_MOUSE]	34.10	2	11	11	72	1.165	0.493	1.033	0.755	206.68	34.10	20	72	261	29.3	4.81
Q8R048	C-Myc-binding protein OS=Mus musculus GN=Mycbp PE=4 SV=1 - [Q8R048_MOUSE]	53.40	2	4	4	8	1.125	0.766	0.716	0.755	21.66	53.40	6	8	103	12.0	5.91
Q00899	Transcriptional repressor protein YY1 OS=Mus musculus GN=Yy1 PE=1 SV=1 - [TYY1_MOUSE]	21.01	4	6	6	10	0.694	0.746	0.995	0.755	23.01	21.01	8	10	414	44.7	6.29
A2AUM2	Eukaryotic translation initiation factor 2-alpha kinase 4 OS=Mus musculus GN=Eif2ak4 PE=2 SV=1 - [illegible]	6.35	10	8	8	13	1.103	0.791	0.759	0.755	25.58	6.35	10	13	1527	173.0	6.46
Q9D668	Arrestin domain-containing protein 2 OS=Mus musculus GN=Arrdc2 PE=2 SV=1 -	6.14	1	2	2	2	1.055	0.879	0.528	0.755	4.73	6.14	2	2	407	44.2	9.14
Q91VM5	RNA binding motif protein, X-linked-like-1 OS=Mus musculus GN=Rbmxl1 PE=1 SV=1 - [RMXL1_MOUSE]	34.02	1	2	13	97	0.934	0.412	1.252	0.755	260.19	34.02	25	97	388	42.1	9.99
A2AFR3-2	Isoform 2 of FERM and PDZ domain-containing protein 4 OS=Mus musculus GN=Frmpd4 - [FRPD4_MOUSE]	5.23	3	5	5	9	0.940	1.101	1.160	0.755	30.94	5.23	7	9	1280	140.4	5.20
Q80WJ7	Protein LYRIC OS=Mus musculus GN=Mtdh PE=1 SV=1 - [LYRIC_MOUSE]	34.72	5	12	17	76	0.716	0.649	0.980	0.755	195.26	34.72	30	76	579	63.8	9.33
Q8BYU6	Torsin-1A-interacting protein 2 OS=Mus musculus GN=Tor1aip2 PE=1 SV=1 -	7.77	1	3	3	4	1.250	1.162	0.905	0.755	6.71	7.77	4	4	502	54.5	4.86
Q921C3-2	Isoform B of Bromodomain and WD repeat-containing protein 1 OS=Mus musculus GN=Brwd1 - [BRWD1_MOUSE]	4.12	6	5	5	8	1.040	1.729	0.991	0.756	5.03	4.12	6	8	2259	253.8	8.19
G3X9V7	MCG19133, isoform CRA_b OS=Mus musculus GN=A830010M20Rik PE=4 SV=1 - [G3X9V7_MOUSE]	37.50	3	1	31	128	0.748	0.939	1.921	0.756	389.66	37.50	50	128	1088	117.2	7.80
Q8BQ47	Protein canopy homolog 4 OS=Mus musculus GN=Cnpy4 PE=1 SV=1 - [CNPY4_MOUSE]	26.94	2	6	6	21	1.815	1.024	0.903	0.756	63.15	26.94	11	21	245	28.1	4.77
Q3UMQ8	H/ACA ribonucleoprotein complex non-core subunit NAF1 OS=Mus musculus GN=Naf1 PE=1 SV=2 - [NAF1_MOUSE]	3.48	2	1	1	4	2.254	0.953	0.929	0.756	8.64	3.48	2	4	489	53.2	5.16
P60879-2	Isoform 2 of Synaptosomal associated protein 25 OS=Mus musculus GN=Snap25 - [SNP25_MOUSE]	66.99	1	3	17	790	1.047	2.093	1.557	0.756	2151.59	66.99	34	790	206	23.3	4.86
Q62000	Mimecan OS=Mus musculus GN=Ogn PE=2 SV=1 - [MIME_MOUSE]	20.47	1	4	4	7	0.841	6.356	0.728	0.756	17.98	20.47	6	7	298	34.0	5.74
Q9D2R0	Acetoacetyl-CoA synthetase OS=Mus musculus GN=Aacs PE=1 SV=1 - [AACS_MOUSE]	20.98	1	16	16	59	0.971	0.549	0.627	0.756	132.67	20.98	29	59	672	75.2	6.71
G8JL54	COMM domain-containing protein 1 (Fragment) OS=Mus musculus GN=Commd1 PE=2 SV=1 - [G8JL54_MOUSE]	19.35	1	1	2	6	5.632	0.824	0.884	0.756	14.90	19.35	3	6	93	10.3	5.19
E9Q774	Protein Akap11 OS=Mus musculus GN=Akap11 PE=2 SV=1 - [E9Q774_MOUSE]	4.07	2	7	7	14	0.781	0.735	0.705	0.757	32.15	4.07	10	14	1894	208.6	5.36
Q8K190	SAYSvFN domain-containing protein 1 OS=Mus musculus GN=Saysd1 PE=2 SV=1 -	32.98	1	3	3	7	1.004	0.983	0.761	0.757	13.45	32.98	3	7	188	20.7	8.60
Q9D5V6	Synapse-associated protein 1 OS=Mus musculus GN=Syap1 PE=1 SV=1 - [SYAP1_MOUSE]	34.25	1	14	14	44	1.318	1.132	1.112	0.757	96.79	34.25	23	44	365	41.3	4.54
Q9JLM8	Serine/threonine-protein kinase DCLK1 OS=Mus musculus GN=Dclk1 PE=1 SV=1 - [DCLK1_MOUSE]	41.01	2	10	29	159	1.097	1.073	1.196	0.757	506.93	41.01	49	159	756	84.1	8.87
O88455	7-dehydrocholesterol reductase OS=Mus musculus GN=Dhcr7 PE=2 SV=1 - [DHCR7_MOUSE]	9.13	2	3	3	6	0.777	0.537	0.749	0.757	13.71	9.13	4	6	471	53.9	8.35
Q922P8	Transmembrane protein 132A OS=Mus musculus GN=Tmem132a PE=2 SV=2 - [T132A_MOUSE]	20.04	2	13	13	58	0.732	0.993	0.782	0.758	140.39	20.04	22	58	1018	110.2	5.63

P62270	40S ribosomal protein S18 OS=Mus musculus GN=Rps18 PE=1 SV=3 - [RS18_MOUSE]	49.34	4	11	11	47	0.448	0.572	0.652	0.758	130.10	49.34	16	47	152	17.7	10.99
P58044	Isopentenyl-diphosphate Delta-isomerase 1 OS=Mus musculus GN=Idi1 PE=2 SV=1 - [IDI1_MOUSE]	51.54	5	14	14	37	2.651	0.704	0.831	0.758	104.21	51.54	22	37	227	26.3	6.16
Q9CY57-3	Isoform 3 of Chromatin target of PRMT1 protein OS=Mus musculus GN=Chtop -	40.59	8	7	7	49	0.698	0.571	0.921	0.758	148.02	40.59	11	49	202	22.0	11.97
P63147	Ubiquitin-conjugating enzyme E2 B OS=Mus musculus GN=Ube2b PE=1 SV=1 -	19.08	1	2	2	9	1.793	1.271	1.134	0.758	16.03	19.08	3	9	152	17.3	5.01
Q5SXJ3	Fanconi anemia group J protein homolog OS=Mus musculus GN=Brip1 PE=2 SV=1 - [FANCJ_MOUSE]	1.53	1	1	1	1	0.820	1.164	0.914	0.758	0.00	1.53	1	1	1174	131.3	7.50
Q9D1N9	39S ribosomal protein L21, mitochondrial OS=Mus musculus GN=Mrpl21 PE=2 SV=1 - [RM21_MOUSE]	18.66	2	3	3	6	0.567	0.668	0.812	0.759	18.57	18.66	5	6	209	23.4	9.99
E9PV60	Protein Wdfy4 OS=Mus musculus GN=Wdfy4 PE=2 SV=1 - [E9PV60_MOUSE]	1.36	2	2	4	4	1.898	1.869	1.637	0.759	3.97	1.36	4	4	3024	337.1	6.35
P97473	RISC-loading complex subunit TARBP2 OS=Mus musculus GN=Tarbp2 PE=1 SV=2 - [TRBP2_MOUSE]	4.93	6	2	2	6	1.781	1.064	0.831	0.759	13.27	4.93	4	6	365	38.8	6.01
O09127	Ephrin type-A receptor 8 OS=Mus musculus GN=Epha8 PE=1 SV=2 - [EPHA8_MOUSE]	3.09	3	2	3	6	1.142	0.521	0.927	0.759	11.60	3.09	4	6	1004	110.6	7.94
Q99KG3-3	Isoform 3 of RNA-binding protein 10 OS=Mus musculus GN=Rbm10 - [RBM10_MOUSE]	28.20	3	19	22	50	1.178	0.626	0.881	0.759	139.68	28.20	33	50	929	103.3	5.92
Q8VDU0	G-protein-signaling modulator 2 OS=Mus musculus GN=Gpsm2 PE=1 SV=2 -	10.16	2	3	6	9	1.580	0.393	0.948	0.759	30.09	10.16	9	9	679	75.5	6.95
Q99J23	GH3 domain-containing protein OS=Mus musculus GN=Ghdc PE=2 SV=2 - [GHDC_MOUSE]	1.88	1	1	1	2	0.949	0.767	0.814	0.759	6.94	1.88	2	2	532	58.5	7.56
Q9QUR6	Prolyl endopeptidase OS=Mus musculus GN=Prep PE=2 SV=1 - [PPCE_MOUSE]	51.69	1	29	29	133	0.608	0.749	0.798	0.759	399.60	51.69	52	133	710	80.7	5.73
Q9QWI6	SRC kinase signaling inhibitor 1 OS=Mus musculus GN=Srcin1 PE=1 SV=2 - [SRCN1_MOUSE]	61.76	4	2	67	453	0.739	0.670	1.337	0.760	1420.49	61.76	125	453	1250	134.8	9.32
Q3ULB0	Protein Rbm6 OS=Mus musculus GN=Rbm6 PE=2 SV=1 - [Q3ULB0_MOUSE]	12.68	1	10	12	23	1.386	0.763	0.973	0.760	54.77	12.68	17	23	986	113.6	6.20
Q8BQ46	Protein Taf15 OS=Mus musculus GN=Taf15 PE=2 SV=1 - [Q8BQ46_MOUSE]	21.54	2	4	8	48	1.258	0.622	0.948	0.760	129.69	21.54	12	48	557	58.6	8.40
Q3URQ4	Protein C78339 OS=Mus musculus GN=C78339 PE=2 SV=1 - [Q3URQ4_MOUSE]	7.77	1	2	2	6	0.849	0.892	0.896	0.760	26.45	7.77	3	6	399	42.3	6.71
P51807	Dynein light chain Tctex-type 1 OS=Mus musculus GN=Dynlt1 PE=1 SV=1 - [DYLT1_MOUSE]	14.16	1	1	1	4	1.022	0.626	0.680	0.760	14.01	14.16	2	4	113	12.5	5.08
Q61074	Protein phosphatase 1G OS=Mus musculus GN=Ppm1g PE=2 SV=3 - [PPM1G_MOUSE]	23.80	1	12	12	39	1.144	0.875	0.989	0.760	104.43	23.80	21	39	542	58.7	4.39
Q8VDY9	Caspase activity and apoptosis inhibitor 1 OS=Mus musculus GN=Caap1 PE=1 SV=2 - [CAAP1_MOUSE]	29.78	1	5	5	12	1.347	0.738	0.934	0.760	28.86	29.78	7	12	356	37.8	4.72
Q5EBP8	Heterogeneous nuclear ribonucleoprotein A1 OS=Mus musculus GN=Hnrnpa1 PE=2 SV=1 - [Q5EBP8_MOUSE]	56.30	4	14	17	272	1.671	0.711	0.959	0.760	874.56	56.30	30	272	373	38.8	9.13
Q99020	Heterogeneous nuclear ribonucleoprotein A/B OS=Mus musculus GN=Hnrnpab PE=1 SV=1 - [ROAA_MOUSE]	42.81	3	12	13	102	1.138	0.540	1.074	0.760	279.81	42.81	22	102	285	30.8	7.91
E9QAH1	Protein Golgb1 OS=Mus musculus GN=Golgb1 PE=2 SV=1 - [E9QAH1_MOUSE]	34.59	5	94	98	255	1.716	0.970	0.937	0.761	733.77	34.59	151	255	3197	365.1	5.06
P70663	SPARC-like protein 1 OS=Mus musculus GN=Sparcl1 PE=1 SV=3 - [SPRL1_MOUSE]	34.77	1	20	20	122	1.783	0.716	0.820	0.761	389.47	34.77	36	122	650	72.2	4.60
Q9CWM2	Cell division cycle-associated protein 4 OS=Mus musculus GN=Cdca4 PE=2 SV=2 - [CDCA4_MOUSE]	4.22	1	1	1	4	3.437	1.531	1.122	0.761	13.44	4.22	1	4	237	26.1	4.56

Q9D0E3	LysM and putative peptidoglycan-binding domain-containing protein 1 OS=Mus musculus GN=Lysmd1 PE=1 SV=1 - [illegible]	53.98	1	9	11	51	1.358	0.609	1.039	0.761	167.08	53.98	20	51	226	24.8	8.92
Q60750	Ephrin type-A receptor 1 OS=Mus musculus GN=Epha1 PE=1 SV=2 - [EPHA1_MOUSE]	1.74	1	2	2	3	3.595	1.652	1.366	0.762	4.53	1.74	3	3	977	108.5	6.33
Q8CCH2	NHL repeat-containing protein 3 OS=Mus musculus GN=Nhlrc3 PE=2 SV=1 -	23.34	2	5	5	7	2.515	1.164	0.643	0.762	15.59	23.34	7	7	347	38.2	6.23
O08912	Polypeptide N-acetylgalactosaminyltransferase 1 OS=Mus musculus GN=Galnt1 PE=1 SV=1 - [GALT1_MOUSE]	10.02	1	4	5	15	0.789	0.744	0.630	0.762	32.34	10.02	8	15	559	64.2	7.71
Q60648	Ganglioside GM2 activator OS=Mus musculus GN=Gm2a PE=1 SV=2 - [SAP3_MOUSE]	16.06	1	4	4	11	2.223	0.833	0.935	0.762	22.35	16.06	6	11	193	20.8	5.90
P58404-2	Isoform 2 of Striatin-4 OS=Mus musculus GN=Strn4 - [STRN4_MOUSE]	35.99	2	13	19	61	0.894	1.495	1.216	0.762	204.70	35.99	32	61	753	80.9	5.38
D3Z3Z8	DNA-directed RNA polymerase II subunit RPB9 (Fragment) OS=Mus musculus GN=Polr2i PE=2 SV=1 - [D3Z3Z8_MOUSE]	43.37	3	3	3	13	1.632	1.105	1.174	0.762	35.08	43.37	5	13	83	9.6	5.17
P32233	Developmentally-regulated GTP-binding protein 1 OS=Mus musculus GN=Drg1 PE=1 SV=1 - [DRG1_MOUSE]	40.60	2	13	14	34	0.741	0.965	0.826	0.762	97.27	40.60	21	34	367	40.5	8.90
Q09143	High affinity cationic amino acid transporter 1 OS=Mus musculus GN=Slc7a1 PE=2 SV=1 - [CTR1_MOUSE]	9.32	2	2	4	9	0.699	0.607	0.667	0.762	28.85	9.32	5	9	622	67.0	7.06
Q5SNZ0-2	Isoform 2 of Girdin OS=Mus musculus GN=Ccdc88a - [GRDN_MOUSE]	35.72	4	57	59	165	1.272	0.860	1.195	0.762	445.52	35.72	94	165	1845	212.4	5.99
Q8BG81	Polymerase delta-interacting protein 3 OS=Mus musculus GN=Poldip3 PE=2 SV=1 -	40.95	3	13	13	43	1.142	0.830	1.002	0.762	134.08	40.95	23	43	420	46.1	10.05
Q9WU60	Attractin OS=Mus musculus GN=Atrn PE=2 SV=3 - [ATRN_MOUSE]	5.67	1	8	8	17	1.188	0.914	1.059	0.762	37.52	5.67	14	17	1428	158.0	7.27
Q6A0A2	La-related protein 4B OS=Mus musculus GN=Larp4b PE=1 SV=2 - [LAR4B_MOUSE]	28.48	3	17	17	42	1.111	0.715	0.899	0.762	111.71	28.48	33	42	741	81.6	7.49
P29387	Guanine nucleotide-binding protein subunit beta-4 OS=Mus musculus GN=Gnb4 PE=2 SV=4 - [GBB4_MOUSE]	38.24	1	7	10	71	0.990	0.765	0.893	0.763	234.17	38.24	18	71	340	37.4	6.16
E9Q4Q2	Splicing factor 1 OS=Mus musculus GN=Sf1 PE=2 SV=1 - [E9Q4Q2_MOUSE]	25.91	9	9	11	31	1.146	0.580	1.029	0.763	79.50	25.91	19	31	548	59.7	9.50
F6ZL86	DnaJ homolog subfamily C member 1 (Fragment) OS=Mus musculus GN=Dnajc1 PE=4 SV=1 - [F6ZL86_MOUSE]	10.08	2	3	3	5	1.317	0.581	0.764	0.763	15.27	10.08	5	5	357	41.1	5.99
Q8K2Q9-2	Isoform 2 of Shootin-1 OS=Mus musculus GN=Kiaa1598 - [SHOT1_MOUSE]	44.30	2	14	15	66	1.445	0.906	1.092	0.763	157.59	44.30	26	66	456	52.6	5.55
Q8R4S0	Protein phosphatase 1 regulatory subunit 14C OS=Mus musculus GN=Ppp1r14c PE=1 SV=1 [PP14C_MOUSE]	28.66	1	3	3	13	1.510	0.494	0.885	0.763	28.08	28.66	4	13	164	17.7	5.27
Q9CSV6	Vesicle transport protein SFT2C OS=Mus musculus GN=Sft2d3 PE=2 SV=2 - [SFT2C_MOUSE]	6.70	2	1	1	3	1.029	0.695	0.635	0.764	7.56	6.70	2	3	209	21.6	10.08
B1AU22	Fibroblast growth factor 13 (Fragment) OS=Mus musculus GN=Fgf13 PE=2 SV=1 - [B1AU22_MOUSE]	37.21	3	1	1	4	0.995	0.897	0.664	0.764	14.68	37.21	2	4	43	4.8	6.65
Q9WVH9	Fibulin-5 OS=Mus musculus GN=Fbln5 PE=2 SV=1 - [FBLN5_MOUSE]	18.08	1	7	7	33	0.369	1.345	0.585	0.764	88.95	18.08	14	33	448	50.2	4.70
O54941	SWI/SNF-related matrix-associated actin-dependent regulator of chromatin subfamily E member 1 OS=Mus musculus GN=Smarce1 PE=1 SV=1 -	23.60	1	10	10	28	1.216	1.021	0.975	0.764	88.09	23.60	16	28	411	46.6	4.88
Q9DBG6	Dolichyl-diphosphooligosaccharide--protein glycosyltransferase subunit 2 OS=Mus musculus GN=Rpn2 PE=2 [illegible]	51.19	2	18	18	66	0.597	0.818	0.652	0.764	214.65	51.19	30	66	631	69.0	5.81
O88573	AF4/FMR2 family member 1 OS=Mus musculus GN=Aff1 PE=2 SV=2 - [AFF1_MOUSE]	3.78	4	3	3	6	1.291	0.776	0.744	0.764	2.00	3.78	4	6	1216	131.5	9.33

P08207	Protein S100-A10 OS=Mus musculus GN=S100a10 PE=2 SV=2 - [S10AA_MOUSE]	17.53	1	3	3	19	1.487	5.506	0.691	0.764	52.06	17.53	5	19	97	11.2	6.77
D3Z6C3	40S ribosomal protein S3a OS=Mus musculus GN=Rps3a2 PE=3 SV=1 - [D3Z6C3_MOUSE]	51.89	3	14	14	63	0.496	0.631	0.702	0.764	187.11	51.89	24	63	264	29.8	9.73
Q8BKI2	Trinucleotide repeat-containing gene 6B protein OS=Mus musculus GN=Tnrc6b PE=2 SV=2 - [TNR6B_MOUSE]	20.72	2	27	27	99	1.422	0.896	1.023	0.765	284.93	20.72	42	99	1810	191.8	6.30
Q9D0K1	Peroxisomal membrane protein PEX13 OS=Mus musculus GN=Pex13 PE=1 SV=1 - [PEX13_MOUSE]	12.59	1	3	3	5	0.915	0.770	0.699	0.765	15.40	12.59	5	5	405	44.6	7.53
Q9Z0H8	CAP-Gly domain-containing linker protein 2 OS=Mus musculus GN=Clip2 PE=1 SV=2 - [CLIP2_MOUSE]	56.06	6	1	58	282	1.481	0.879	1.202	0.765	806.64	56.06	101	282	1047	115.8	6.48
F8VPK8	Protein Pcdh9 OS=Mus musculus GN=Pcdh9 PE=2 SV=1 - [F8VPK8_MOUSE]	27.24	1	24	25	76	0.923	0.732	0.971	0.765	179.48	27.24	43	76	1237	136.1	5.50
O08997	Copper transport protein ATOX1 OS=Mus musculus GN=Atox1 PE=2 SV=1 - [ATOX1_MOUSE]	85.29	1	5	5	36	1.684	0.792	1.109	0.765	111.62	85.29	10	36	68	7.3	6.51
Q9QZF2	Glypican-1 OS=Mus musculus GN=Gpc1 PE=1 SV=1 - [GPC1_MOUSE]	40.75	1	20	20	70	0.787	1.063	0.848	0.765	213.88	40.75	39	70	557	61.3	7.05
H3BKQ3	Protein Evi5l OS=Mus musculus GN=Evi5l PE=2 SV=1 - [H3BKQ3_MOUSE]	30.26	11	21	24	70	0.978	0.790	0.856	0.765	200.92	30.26	43	70	813	93.4	5.25
Q9CQZ1	Heat shock factor-binding protein 1 OS=Mus musculus GN=Hsbp1 PE=2 SV=1 - [HSBP1_MOUSE]	72.37	1	4	4	52	1.236	1.445	0.970	0.765	164.20	72.37	8	52	76	8.6	4.31
Q8CIC2-2	Isoform 2 of Nucleoporin-like protein 2 OS=Mus musculus GN=Nupl2 - [NUPL2_MOUSE]	20.00	3	2	2	13	1.410	1.169	0.959	0.765	19.94	20.00	4	13	160	17.6	9.25
Q9CRD0	OCIA domain-containing protein 1 OS=Mus musculus GN=Ociad1 PE=1 SV=1 -	62.35	1	5	11	56	0.983	0.637	0.934	0.765	162.76	62.35	18	56	247	27.6	7.81
Q99N13-3	Isoform 3 of Histone deacetylase 9 OS=Mus musculus GN=Hdac9 - [HDAC9_MOUSE]	11.43	3	3	5	10	0.848	1.008	1.092	0.766	26.62	11.43	10	10	586	65.4	8.73
Q9ERN0	Secretory carrier-associated membrane protein 2 OS=Mus musculus GN=Scamp2 PE=2 SV=1 - [illegible]	9.73	1	2	2	20	0.594	0.923	0.755	0.766	47.63	9.73	4	20	329	36.4	6.32
Q8BWG9	Calcium release-activated calcium channel protein 1 OS=Mus musculus GN=Orai1 PE=2 SV=1 - [CRCM1_MOUSE]	2.63	1	1	1	6	0.836	0.786	0.723	0.766	15.16	2.63	2	6	304	33.0	7.06
A2AFG8	Neural cell adhesion molecule L1 OS=Mus musculus GN=L1cam PE=2 SV=1 -	34.42	6	5	36	315	0.987	0.983	0.931	0.766	936.34	34.42	63	315	1255	140.3	5.96
Q8BGZ1	Hippocalcin-like protein 4 OS=Mus musculus GN=Hpcal4 PE=2 SV=3 - [HPCL4_MOUSE]	80.63	2	9	14	205	1.098	2.897	1.103	0.766	522.53	80.63	26	205	191	22.2	4.89
Q9CQC6	Basic leucine zipper and W2 domain-containing protein 1 OS=Mus musculus GN=Bzw1 PE=1 SV=1 - [BZW1_MOUSE]	37.95	1	14	17	58	0.896	0.862	0.801	0.766	135.39	37.95	27	58	419	48.0	5.92
Q8R3E3-2	Isoform 2 of WD repeat domain phosphoinositide-interacting protein 1 OS=Mus musculus GN=Wipi1 - [illegible]	16.48	4	4	4	13	1.236	1.081	0.942	0.766	38.10	16.48	7	13	437	47.8	6.06
Q64152-2	Isoform 2 of Transcription factor BTF3 OS=Mus musculus GN=Btf3 - [BTF3_MOUSE]	60.49	1	1	8	38	1.149	0.634	0.889	0.766	107.65	60.49	15	38	162	17.7	7.50
F8VQ46	Trichohyalin-like protein 1 OS=Mus musculus GN=Tchhl1 PE=4 SV=1 - [F8VQ46_MOUSE]	2.35	2	1	1	1	0.676	1.589	0.949	0.766	2.67	2.35	1	1	638	70.7	4.73
Q9JHS9	Spliceosome-associated protein CWC15 homolog OS=Mus musculus GN=Cwc15 PE=1 SV=1 - [CWC15_MOUSE]	16.59	1	5	5	12	1.092	0.561	1.101	0.766	25.56	16.59	8	12	229	26.6	5.71
Q8K0P3	TLD domain-containing protein 1 OS=Mus musculus GN=Tldc1 PE=2 SV=1 - [TLDC1_MOUSE]	7.25	1	2	2	5	0.622	0.661	0.747	0.767	18.17	7.25	4	5	455	50.8	6.52
P58021	Transmembrane 9 superfamily member 2 OS=Mus musculus GN=Tm9sf2 PE=2 SV=1 - [TM9S2_MOUSE]	13.14	2	8	8	24	0.698	0.826	0.721	0.767	90.13	13.14	12	24	662	75.3	7.43
E9PVY8	Microtubule-actin cross-linking factor 1 OS=Mus musculus GN=Macf1 PE=2 SV=2 - [E9PVY8_MOUSE]	35.39	11	7	225	924	1.370	0.887	1.166	0.767	2661.50	35.39	395	924	7355	831.4	5.41

Q9Z2U1	Proteasome subunit alpha type-5 OS=Mus musculus GN=Psma5 PE=1 SV=1 - [PSA5_MOUSE]	51.04	1	2	9	108	1.104	0.984	0.645	0.767	390.46	51.04	17	108	241	26.4	4.79
Q8BQU6	Gap junction gamma-2 protein OS=Mus musculus GN=Gjc2 PE=1 SV=2 - [CXG2_MOUSE]	9.09	1	3	3	11	1.597	0.651	0.634	0.767	39.72	9.09	6	11	440	47.0	7.12
P27546-2	Isoform 2 of Microtubule-associated protein 4 OS=Mus musculus GN=Map4 -	73.13	5	28	65	532	1.979	0.562	1.032	0.767	1413.89	73.13	118	532	1124	117.3	4.98
F6RUI8	Protein Maskbp3 (Fragment) OS=Mus musculus GN=Ankhd1 PE=4 SV=1 -	5.97	2	1	3	8	1.394	0.653	0.698	0.767	22.50	5.97	5	8	637	66.3	7.53
Q9QYY0	GRB2-associated-binding protein 1 OS=Mus musculus GN=Gab1 PE=1 SV=2 - [GAB1_MOUSE]	26.62	1	13	13	51	1.940	0.394	0.982	0.767	170.82	26.62	24	51	695	76.8	5.67
Q9QXS6	Drebrin OS=Mus musculus GN=Dbn1 PE=1 SV=4 - [DREB_MOUSE]	40.08	4	22	22	248	1.174	1.429	1.427	0.767	735.35	40.08	38	248	706	77.2	4.49
Q80X50	Ubiquitin-associated protein 2-like OS=Mus musculus GN=Ubap2l PE=1 SV=1 -	22.13	5	19	19	199	1.526	0.938	1.177	0.768	489.47	22.13	34	199	1107	116.7	7.11
P70211-3	Isoform B of Netrin receptor DCC OS=Mus musculus GN=Dcc - [DCC_MOUSE]	13.57	3	13	13	31	0.864	0.895	0.765	0.768	97.70	13.57	22	31	1363	149.3	6.62
A2AG36	GPI ethanolamine phosphate transferase 3 OS=Mus musculus GN=Pigo PE=2 SV=1 - [A2AG36_MOUSE]	5.33	4	4	4	9	0.640	1.154	0.693	0.768	28.07	5.33	8	9	1069	116.4	8.84
E9QAS7	Protein Inpp5a OS=Mus musculus GN=Inpp5a PE=2 SV=1 - [E9QAS7_MOUSE]	32.46	1	1	16	43	1.024	0.960	0.788	0.768	111.21	32.46	26	43	422	48.8	7.09
Q8BSL7	ADP-ribosylation factor 2 OS=Mus musculus GN=Arf2 PE=1 SV=2 - [ARF2_MOUSE]	57.46	5	1	10	171	0.681	0.753	0.603	0.768	519.08	57.46	18	171	181	20.7	6.58
P23204	Peroxisome proliferator-activated receptor alpha OS=Mus musculus GN=Ppara PE=1 SV=2 - [PPARA_MOUSE]	2.14	1	1	1	1	1.232	1.213	1.710	0.768	0.00	2.14	1	1	468	52.3	6.15
B1B034	Protein Gm15155 OS=Mus musculus GN=Gm15155 PE=2 SV=1 - [B1B034_MOUSE]	9.42	1	1	1	2	3.102	1.527	0.515	0.768	0.00	9.42	1	2	138	15.6	9.25
F6TQP9	Solute carrier organic anion transporter family member 2B1 (Fragment) OS=Mus musculus GN=Slco2b1 PE=4 SV=1 - [F6TQP9_MOUSE]	8.87	3	2	2	3	1.136	0.460	1.004	0.768	7.46	8.87	3	3	203	22.1	7.09
Q5XJF6	Ribosomal protein OS=Mus musculus GN=Rpl10a PE=2 SV=1 - [Q5XJF6_MOUSE]	41.94	4	10	10	58	0.386	0.481	0.694	0.768	205.06	41.94	16	58	217	24.8	9.94
Q9JJ43-2	Isoform 2 of RNA binding protein fox-1 homolog 1 OS=Mus musculus GN=Rbfox1 -	30.32	7	3	9	33	1.204	0.852	1.184	0.769	86.80	30.32	15	33	376	40.5	7.33
Q6P542	ATP-binding cassette sub-family F member 1 OS=Mus musculus GN=Abcf1 PE=1 SV=1 - [ABCF1_MOUSE]	18.28	2	12	13	36	0.723	0.622	0.795	0.769	99.49	18.28	21	36	837	94.9	6.51
P17095	High mobility group protein HMG-I/HMG-Y OS=Mus musculus GN=Hmga1 PE=1 SV=4 - [HMGA1_MOUSE]	38.32	1	2	5	31	0.483	0.819	0.977	0.769	85.54	38.32	9	31	107	11.6	10.32
J3QN27	Calcium-activated potassium channel subunit alpha-1 OS=Mus musculus GN=Kcnma1 PE=4 SV=1 - [J3QN27_MOUSE]	13.69	11	2	12	66	0.728	0.799	1.592	0.769	201.71	13.69	22	66	1132	127.1	6.62
P97783	Protein AF1q OS=Mus musculus GN=Mllt11 PE=2 SV=1 - [AF1Q_MOUSE]	42.22	1	3	3	4	2.130	1.211	0.957	0.769	11.11	42.22	4	4	90	10.0	4.41
Q64191	N(4)-(beta-N-acetylglucosaminyl)-L-asparaginase OS=Mus musculus GN=Aga PE=2 SV=1 - [ASPG_MOUSE]	10.12	1	3	3	17	1.939	0.729	0.586	0.769	46.32	10.12	6	17	346	37.0	6.44
Q5SVR5	E3 ubiquitin-protein ligase RNF130 OS=Mus musculus GN=Rnf130 PE=2 SV=1 - [Q5SVR5_MOUSE]	4.17	2	1	1	2	1.469	0.786	0.987	0.770	6.95	4.17	2	2	384	42.4	9.19
Q9D850	Transmembrane protein 68 OS=Mus musculus GN=Tmem68 PE=2 SV=1 - [TMM68_MOUSE]	3.65	1	1	1	2	1.279	1.237	1.094	0.770	4.51	3.65	2	2	329	37.8	8.46
E9Q3G8	Protein Nup153 OS=Mus musculus GN=Nup153 PE=2 SV=1 - [E9Q3G8_MOUSE]	17.31	1	19	19	49	1.081	0.801	1.060	0.770	127.13	17.31	30	49	1462	151.9	8.84
Q9CQN7	39S ribosomal protein L41, mitochondrial OS=Mus musculus GN=Mrpl41 PE=2 SV=1 - [RM41_MOUSE]	45.19	2	6	6	18	1.248	0.635	1.103	0.770	56.20	45.19	10	18	135	15.3	9.82

Q9WU01	KH domain-containing, RNA-binding, signal transduction-associated protein 2 OS=Mus musculus GN=Khdrbs2 PE=1 SV=1 - [KHDR2_MOUSE]	29.23	3	8	13	46	0.959	0.771	0.819	0.770	117.07	29.23	24	46	349	38.8	6.48
Q9D394	Protein RUFY3 OS=Mus musculus GN=Rufy3 PE=1 SV=1 - [RUFY3_MOUSE]	57.78	2	1	26	239	1.055	1.075	0.703	0.770	654.62	57.78	48	239	469	53.0	5.49
E9Q7Z8	Protein Ythdc1 OS=Mus musculus GN=Ythdc1 PE=2 SV=1 - [E9Q7Z8_MOUSE]	8.73	4	5	6	18	0.583	0.730	1.011	0.770	40.94	8.73	10	18	710	82.7	5.91
E9Q6K1	TFIIH basal transcription factor complex helicase XPD subunit OS=Mus musculus GN=Ercc2 PE=2 SV=1 - [E9Q6K1_MOUSE]	8.80	3	1	4	7	0.481	0.762	0.773	0.770	13.96	8.80	5	7	739	84.4	6.92
Q8CI96-3	Isoform 3 of CAP-Gly domain-containing linker protein 4 OS=Mus musculus GN=Clip4 - [CLIP4_MOUSE]	13.24	1	1	3	5	1.122	0.813	1.114	0.770	9.65	13.24	5	5	219	24.0	4.96
G3UXK7	Cytochrome c-type heme lyase OS=Mus musculus GN=Hccs PE=2 SV=1 - [G3UXK7_MOUSE]	45.16	1	1	3	11	3.803	1.722	0.952	0.770	40.71	45.16	4	11	93	9.5	6.27
J3QP80	MCG15559 OS=Mus musculus GN=Gm5814 PE=4 SV=1 - [J3QP80_MOUSE]	32.80	2	3	3	11	1.833	0.987	0.943	0.770	41.84	32.80	6	11	125	14.2	4.86
B9EJX2	Nance-Horan syndrome (Human) OS=Mus musculus GN=Nhs PE=2 SV=1 - [B9EJX2_MOUSE]	9.78	2	8	8	11	1.350	1.113	0.852	0.771	30.43	9.78	10	11	1626	176.5	6.93
Q9R194	Cryptochrome-2 OS=Mus musculus GN=Cry2 PE=1 SV=1 - [CRY2_MOUSE]	11.49	1	4	5	9	1.230	0.836	0.787	0.771	24.53	11.49	8	9	592	66.8	8.35
B2RU80	Receptor-type tyrosine-protein phosphatase beta OS=Mus musculus GN=Ptprb PE=1 SV=1 - [PTPRB_MOUSE]	0.65	1	1	2	3	2.412	0.702	0.989	0.771	7.99	0.65	2	3	1998	224.4	7.18
O35450	FK506-binding protein-like OS=Mus musculus GN=Fkbpl PE=2 SV=1 - [FKBPL_MOUSE]	11.53	1	3	3	6	0.898	1.215	1.136	0.771	22.11	11.53	6	6	347	38.3	7.74
P27545	Ceramide synthase 1 OS=Mus musculus GN=Cers1 PE=1 SV=1 - [CERS1_MOUSE]	11.71	1	3	3	12	0.652	0.908	0.868	0.771	32.53	11.71	5	12	350	40.1	8.38
Q91YW3	DnaJ homolog subfamily C member 3 OS=Mus musculus GN=Dnajc3 PE=1 SV=1 -	18.06	1	9	10	26	1.101	0.789	0.773	0.771	58.18	18.06	16	26	504	57.4	5.85
Q921C5	Protein bicaudal D homolog 2 OS=Mus musculus GN=Bicd2 PE=1 SV=1 - [BICD2_MOUSE]	30.85	5	19	23	65	1.974	0.852	0.938	0.771	174.18	30.85	37	65	820	93.3	5.44
Q3UM18-2	Isoform 2 of Large subunit GTPase 1 homolog OS=Mus musculus GN=Lsg1 - [LSG1_MOUSE]	4.55	2	2	2	7	2.114	1.018	0.809	0.771	10.13	4.55	4	7	615	69.4	6.58
Q7M732	Retrotransposon-like protein 1 OS=Mus musculus GN=Rtl1 PE=2 SV=1 - [RTL1_MOUSE]	5.05	1	1	1	4	3.147	0.636	0.439	0.771	12.62	5.05	2	4	1744	198.9	4.26
Q6GQT9	Nodal modulator 1 OS=Mus musculus GN=Nomo1 PE=1 SV=1 - [NOMO1_MOUSE]	34.93	1	31	31	105	1.086	0.732	0.791	0.771	363.90	34.93	54	105	1214	133.3	6.09
Q9D0F3	Protein ERGIC-53 OS=Mus musculus GN=Lman1 PE=2 SV=1 - [LMAN1_MOUSE]	28.63	2	14	15	48	0.668	0.965	0.651	0.771	127.54	28.63	24	48	517	57.8	6.34
Q9ERI2	Ras-related protein Rab-27A OS=Mus musculus GN=Rab27a PE=1 SV=1 - [RB27A_MOUSE]	20.81	1	3	4	9	0.899	1.490	0.724	0.771	24.09	20.81	7	9	221	25.0	5.36
Q6NXY1	WD repeat-containing protein 67 OS=Mus musculus GN=Wdr67 PE=2 SV=1 -	2.11	3	2	2	2	36.551	4.119	3.770	0.772	0.00	2.11	2	2	996	115.8	8.12
Q6R0H7-3	Isoform XLas-3 of Guanine nucleotide-binding protein G(s) subunit alpha isoforms XLas OS=Mus musculus GN=Gnas - [GNAS1_MOUSE]	9.55	2	1	6	20	1.408	0.586	0.708	0.772	64.34	9.55	9	20	827	85.9	4.72
Q3UNV4	Cytochrome P450, family 2, subfamily j, polypeptide 13, isoform CRA_a OS=Mus musculus GN=Cyp2j13 PE=2 SV=1 - [Q3UNV4_MOUSE]	9.34	5	1	4	6	11.021	0.858	2.357	0.772	17.51	9.34	5	6	503	57.5	8.44
Q8K1I7	WAS/WASL-interacting protein family member 1 OS=Mus musculus GN=Wipf1 PE=1 SV=1 - [WIPF1_MOUSE]	21.30	2	8	8	26	2.316	0.727	1.002	0.772	78.90	21.30	14	26	493	50.0	11.41
Q8BHA1	Leucine-rich repeat-containing protein 24 OS=Mus musculus GN=Lrrc24 PE=2 SV=1 - [LRC24_MOUSE]	3.84	1	2	2	5	0.664	0.556	0.747	0.772	14.18	3.84	3	5	521	56.3	8.21

Q8BXG3	Intraflagellar transport protein 57 homolog OS=Mus musculus GN=Ift57 PE=1 SV=1 - [IFT57_MOUSE]	8.62	3	3	3	7	1.162	1.495	0.885	0.772	10.37	8.62	5	7	429	48.7	4.98
P62915	Transcription initiation factor IIB OS=Mus musculus GN=Gtf2b PE=1 SV=1 - [TF2B_MOUSE]	22.78	1	7	7	15	1.317	0.922	0.848	0.773	48.29	22.78	12	15	316	34.8	8.35
G5E8C4	MCG142017, isoform CRA_a OS=Mus musculus GN=Tmtc3 PE=4 SV=1 - [G5E8C4_MOUSE]	10.33	3	7	9	25	0.753	0.837	0.753	0.773	53.23	10.33	14	25	920	104.1	8.53
P62281	40S ribosomal protein S11 OS=Mus musculus GN=Rps11 PE=2 SV=3 - [RS11_MOUSE]	49.37	1	11	11	48	0.479	0.432	0.698	0.773	128.40	49.37	20	48	158	18.4	10.30
Q91V04	Translocating chain-associated membrane protein 1 OS=Mus musculus GN=Tram1 PE=1 SV=3 - [TRAM1_MOUSE]	8.02	1	3	3	5	1.003	1.176	0.645	0.773	14.22	8.02	5	5	374	43.0	9.69
I7HLV2	60S ribosomal protein L10 (Fragment) OS=Mus musculus GN=Rpl10 PE=4 SV=1 - [I7HLV2_MOUSE]	50.75	4	10	10	69	0.449	0.587	0.676	0.773	219.84	50.75	16	69	201	23.1	10.01
Q8K4R4	Cytoplasmic phosphatidylinositol transfer protein 1 OS=Mus musculus GN=Pitpnc1 PE=1 SV=1 - [illegible]	41.57	3	4	13	39	0.474	0.569	0.681	0.773	92.40	41.57	21	39	332	38.4	6.32
E9QNR5	Thrombospondin type-1 domain-containing protein 7A OS=Mus musculus GN=Thsd7a PE=2 SV=1 - [E9QNR5_MOUSE]	15.02	6	19	19	57	0.765	0.844	1.074	0.773	151.74	15.02	31	57	1644	183.3	7.20
Q91VJ5	Polyglutamine-binding protein 1 OS=Mus musculus GN=Pqbp1 PE=2 SV=1 - [PQBP1_MOUSE]	15.97	2	3	3	9	0.912	0.415	1.068	0.773	25.01	15.97	5	9	263	30.6	6.23
F8VQ95	Transforming acidic coiled-coil-containing protein 1 OS=Mus musculus GN=Tacc1 PE=2 SV=1 - [F8VQ95_MOUSE]	34.15	3	19	23	84	1.365	0.824	1.179	0.773	225.05	34.15	39	84	776	84.2	5.03
B2RUG9	Adenomatosis polyposis coli OS=Mus musculus GN=Apc PE=2 SV=1 - [B2RUG9_MOUSE]	28.85	8	59	63	176	0.955	0.646	1.204	0.773	513.51	28.85	104	176	2842	310.7	7.58
Q78JW9	Ubiquitin domain-containing protein UBFD1 OS=Mus musculus GN=Ubfd1 PE=1 SV=2 - [UBFD1_MOUSE]	25.82	1	9	9	49	1.678	0.769	0.771	0.773	139.75	25.82	15	49	368	40.1	8.85
Q9CQH7	Transcription factor BTF3 homolog 4 OS=Mus musculus GN=Btf3l4 PE=2 SV=1 - [BT3L4_MOUSE]	34.81	1	6	8	25	1.116	0.778	1.083	0.773	55.39	34.81	11	25	158	17.3	6.35
Q9CQS8	Protein transport protein Sec61 subunit beta OS=Mus musculus GN=Sec61b PE=1 SV=3 - [SC61B_MOUSE]	11.46	2	1	1	11	0.685	0.703	0.705	0.774	28.68	11.46	2	11	96	10.0	11.56
Q3UNH4	G protein-regulated inducer of neurite outgrowth 1 OS=Mus musculus GN=Gprin1 PE=1 SV=2 - [illegible]	64.91	1	57	57	392	0.922	1.867	1.398	0.774	1068.53	64.91	102	392	932	95.4	7.93
Q91YD3	mRNA-decapping enzyme 1A OS=Mus musculus GN=Dcp1a PE=1 SV=1 - [DCP1A_MOUSE]	17.94	1	9	9	22	1.266	1.296	0.872	0.774	66.36	17.94	16	22	602	65.2	6.99
F2Z470	3-hydroxy-3-methylglutaryl coenzyme A reductase OS=Mus musculus GN=Hmgcr PE=2 SV=1 - [F2Z470_MOUSE]	3.05	2	2	2	5	1.277	0.487	0.611	0.774	10.10	3.05	3	5	819	89.8	6.06
P24638	Lysosomal acid phosphatase OS=Mus musculus GN=Acp2 PE=2 SV=2 - [PPAL_MOUSE]	7.57	3	3	3	9	0.998	0.887	0.635	0.774	24.40	7.57	6	9	423	48.5	7.02
P09055	Integrin beta-1 OS=Mus musculus GN=Itgb1 PE=1 SV=1 - [ITB1_MOUSE]	24.06	1	17	17	66	0.745	1.272	0.605	0.774	218.04	24.06	30	66	798	88.2	5.94
J3QNY1	Uncharacterized protein OS=Mus musculus GN=Gm9242 PE=4 SV=1 - [J3QNY1_MOUSE]	45.38	7	13	15	244	1.099	0.683	1.112	0.774	797.43	45.38	28	244	357	37.1	8.31
Q9CPP0	Nucleoplasmin-3 OS=Mus musculus GN=Npm3 PE=2 SV=3 - [NPM3_MOUSE]	8.57	1	1	1	3	2.175	0.988	0.942	0.774	5.76	8.57	2	3	175	19.0	4.82
P63168	Dynein light chain 1, cytoplasmic OS=Mus musculus GN=Dynll1 PE=1 SV=1 - [DYL1_MOUSE]	61.80	2	3	5	72	1.136	1.193	1.015	0.774	287.01	61.80	8	72	89	10.4	7.40
Q9JIW9	Ras-related protein Ral-B OS=Mus musculus GN=Ralb PE=2 SV=1 - [RALB_MOUSE]	52.91	2	3	8	27	1.106	0.960	0.801	0.774	66.40	52.91	10	27	206	23.3	6.62
Q8K0Z5	Tropomyosin 3, gamma OS=Mus musculus GN=Tpm3 PE=2 SV=1 - [Q8K0Z5_MOUSE]	37.32	3	5	15	264	1.676	1.181	1.531	0.774	678.01	37.32	27	264	284	33.1	4.77

Q6NZF1	Zinc finger CCCH domain-containing protein 11A OS=Mus musculus GN=Zc3h11a PE=1 SV=1 - [ZC11A_MOUSE]	26.89	1	16	16	41	1.012	0.624	1.008	0.774	104.24	26.89	26	41	792	86.4	8.13
P60867	40S ribosomal protein S20 OS=Mus musculus GN=Rps20 PE=1 SV=1 - [RS20_MOUSE]	41.18	2	5	6	35	0.601	0.510	0.731	0.774	81.43	41.18	11	35	119	13.4	9.94
B9EJA2-5	Isoform 5 of Cortactin-binding protein 2 OS=Mus musculus GN=Cttnbp2 - [CTTB2_MOUSE]	20.22	7	3	29	145	0.846	0.870	1.129	0.774	440.26	20.22	49	145	1607	175.1	8.29
Q9JL35	High mobility group nucleosome-binding domain-containing protein 5 OS=Mus musculus GN=Hmgn5 PE=1 SV=2 - [illegible]	5.67	1	2	2	3	0.904	0.720	0.863	0.775	5.10	5.67	3	3	406	45.3	4.37
P51885	Lumican OS=Mus musculus GN=Lum PE=1 SV=2 - [LUM_MOUSE]	12.13	1	4	4	10	1.764	1.485	0.619	0.775	21.83	12.13	6	10	338	38.2	6.43
Q5NCR9	Nuclear speckle splicing regulatory protein 1 OS=Mus musculus GN=Nsrp1 PE=1 SV=1 - [NSRP1_MOUSE]	7.38	1	3	3	8	0.782	0.706	1.079	0.775	13.95	7.38	6	8	542	63.8	8.76
P97855	Ras GTPase-activating protein-binding protein 1 OS=Mus musculus GN=G3bp1 PE=1 SV=1 - [G3BP1_MOUSE]	33.98	1	11	12	45	1.147	0.750	0.814	0.775	154.66	33.98	19	45	465	51.8	5.59
P62889	60S ribosomal protein L30 OS=Mus musculus GN=Rpl30 PE=2 SV=2 - [RL30_MOUSE]	36.52	1	5	5	20	0.640	0.594	0.672	0.775	56.71	36.52	8	20	115	12.8	9.63
P62774	Myotrophin OS=Mus musculus GN=Mtpn PE=1 SV=2 - [MTPN_MOUSE]	52.54	1	5	5	63	1.438	1.429	1.291	0.775	159.72	52.54	9	63	118	12.9	5.52
D3Z0V7	TSC22 domain family protein 1 OS=Mus musculus GN=Tsc22d1 PE=2 SV=1 -	11.56	4	1	10	64	1.548	0.860	1.421	0.775	142.18	11.56	19	64	995	102.0	5.77
Q8BXN9	Transmembrane protein 87A OS=Mus musculus GN=Tmem87a PE=1 SV=1 - [TM87A_MOUSE]	15.14	6	8	8	27	0.855	1.056	0.797	0.775	71.50	15.14	14	27	555	63.3	6.67
Q4VA53-3	Isoform 3 of Sister chromatid cohesion protein PDS5 homolog B OS=Mus musculus GN=Pds5b - [PDS5B_MOUSE]	10.57	4	12	15	34	0.549	0.724	0.786	0.775	77.72	10.57	22	34	1448	164.6	8.54
Q8BJA2	Solute carrier family 41 member 1 OS=Mus musculus GN=Slc41a1 PE=2 SV=1 -	4.30	1	1	1	1	1.675	1.265	0.650	0.775	5.40	4.30	1	1	512	54.9	5.31
B2RQG2	PHD finger protein 3 OS=Mus musculus GN=Phf3 PE=2 SV=1 - [B2RQG2_MOUSE]	4.79	1	8	8	21	0.679	0.766	0.975	0.775	51.07	4.79	14	21	2025	225.4	6.70
Q62347	Cyclic AMP-responsive element-binding protein 1 OS=Mus musculus GN=Creb1 PE=2 SV=1 - [Q62347_MOUSE]	25.09	36	7	7	18	1.334	0.860	1.123	0.775	58.03	25.09	10	18	287	30.9	6.37
Q9Z2I2	Peptidyl-prolyl cis-trans isomerase FKBP1B OS=Mus musculus GN=Fkbp1b PE=1 SV=3 - [FKB1B_MOUSE]	19.44	1	1	2	28	2.246	0.799	1.572	0.776	97.45	19.44	3	28	108	11.8	8.47
Q99LD9	Translation initiation factor eIF-2B subunit beta OS=Mus musculus GN=Eif2b2 PE=2 SV=1 - [EI2BB_MOUSE]	23.93	3	7	7	13	1.760	1.311	0.793	0.776	33.39	23.93	10	13	351	38.9	6.24
O89016	ATP-binding cassette sub-family D member 4 OS=Mus musculus GN=Abcd4 PE=2 SV=2 - [ABCD4_MOUSE]	9.08	1	4	4	6	2.250	1.071	0.792	0.776	16.30	9.08	5	6	606	68.5	6.42
Q9CYI4	Putative RNA-binding protein Luc7-like 1 OS=Mus musculus GN=Luc7l PE=2 SV=2 -	15.36	6	2	6	27	0.732	1.021	0.810	0.776	73.46	15.36	10	27	371	43.9	9.88
Q8BH60	Golgi-associated PDZ and coiled-coil motif-containing protein OS=Mus musculus GN=Gopc PE=1 SV=1 - [GOPC_MOUSE]	39.09	3	15	15	43	1.217	0.900	1.032	0.776	116.29	39.09	24	43	463	50.6	6.25
F7BGR7	RNA-binding protein 4 OS=Mus musculus GN=Rbm4 PE=4 SV=1 - [F7BGR7_MOUSE]	29.35	10	1	12	42	1.490	1.120	1.333	0.776	133.97	29.35	21	42	477	52.8	7.30
O35126	Atrophin-1 OS=Mus musculus GN=Atn1 PE=1 SV=1 - [ATN1_MOUSE]	11.32	2	5	6	11	1.352	0.637	1.186	0.776	32.44	11.32	10	11	1175	123.6	8.95
Q76HL1	Protein Prss43 OS=Mus musculus GN=Prss43 PE=2 SV=1 - [Q76HL1_MOUSE]	7.33	1	2	2	2	0.883	0.958	0.946	0.776	5.63	7.33	2	2	382	42.2	5.80
Q9JIH2	Nuclear pore complex protein Nup50 OS=Mus musculus GN=Nup50 PE=1 SV=3 - [NUP50_MOUSE]	21.67	1	8	8	19	1.217	0.681	0.917	0.776	54.52	21.67	14	19	466	49.5	6.24

A2AQ19	RNA polymerase-associated protein RTF1 homolog OS=Mus musculus GN=Rtf1 PE=2 SV=1 - [RTF1_MOUSE]	19.58	1	14	14	26	0.995	0.864	0.969	0.776	58.92	19.58	21	26	715	80.7	8.16
Q62422	Osteoclast-stimulating factor 1 OS=Mus musculus GN=Ostf1 PE=1 SV=2 - [OSTF1_MOUSE]	27.44	1	5	5	14	1.764	0.831	0.906	0.776	37.35	27.44	8	14	215	23.8	5.68
O09131	Glutathione S-transferase omega-1 OS=Mus musculus GN=Gsto1 PE=2 SV=2 - [GSTO1_MOUSE]	50.00	3	12	12	57	1.376	0.741	0.583	0.776	142.30	50.00	22	57	240	27.5	7.36
Q5SUT0	RNA-binding protein EWS OS=Mus musculus GN=Ewsr1 PE=2 SV=1 - [Q5SUT0_MOUSE]	11.81	4	7	7	82	1.425	0.936	1.168	0.776	282.68	11.81	13	82	618	64.9	9.38
Q3TRR0-2	Isoform 2 of Microtubule-associated protein 9 OS=Mus musculus GN=Map9 -	17.05	3	7	8	13	0.924	0.976	1.408	0.776	27.35	17.05	10	13	616	69.6	7.59
A2AL85	Aspartyl/asparaginyl beta-hydroxylase OS=Mus musculus GN=Asph PE=2 SV=1 - [A2AL85_MOUSE]	17.10	9	8	9	14	1.000	1.069	0.827	0.776	34.08	17.10	13	14	725	81.4	5.11
Q9CQT2	RNA-binding protein 7 OS=Mus musculus GN=Rbm7 PE=1 SV=1 - [RBM7_MOUSE]	12.45	1	3	3	12	1.321	0.891	1.039	0.776	32.19	12.45	5	12	265	30.1	9.38
Q8R1V4	Transmembrane emp24 domain-containing protein 4 OS=Mus musculus GN=Tmed4 PE=2 SV=1 - [TMED4_MOUSE]	27.31	2	4	5	24	0.849	0.925	0.685	0.777	62.04	27.31	8	24	227	26.0	8.18
Q80UW0-2	Isoform 2 of Heparan-sulfate 6-O-sulfotransferase 2 OS=Mus musculus GN=Hs6st2 - [H6ST2_MOUSE]	6.22	3	2	2	4	1.666	0.842	0.913	0.777	1.99	6.22	2	4	466	53.7	8.98
Q8C7K6	Prenylcysteine oxidase-like OS=Mus musculus GN=Pcyox1l PE=2 SV=1 - [PCYXL_MOUSE]	17.58	1	10	10	19	1.140	0.849	0.796	0.777	65.10	17.58	14	19	495	54.8	7.65
Q61739	Integrin alpha-6 OS=Mus musculus GN=Itga6 PE=1 SV=3 - [ITA6_MOUSE]	20.07	3	18	18	40	0.938	0.729	0.739	0.777	110.55	20.07	31	40	1091	122.1	7.03
Q69ZQ2	Pre-mRNA-splicing factor ISY1 homolog OS=Mus musculus GN=Isy1 PE=1 SV=2 - [ISY1_MOUSE]	25.96	1	6	7	16	1.324	0.972	1.004	0.777	41.44	25.96	9	16	285	33.0	5.17
A2AWI9	Endophilin-B2 OS=Mus musculus GN=Sh3glb2 PE=4 SV=1 - [A2AWI9_MOUSE]	51.07	2	2	19	117	1.833	1.217	1.074	0.777	338.51	51.07	33	117	374	41.8	5.97
G3UW90	Trichorhinophalangeal syndrome I (Human), isoform CRA_b OS=Mus musculus GN=Trps1 PE=4 SV=1 - [G3UW90_MOUSE]	2.34	2	2	2	3	1.789	0.743	0.858	0.777	10.41	2.34	3	3	1281	140.9	7.64
Q9QYY8	Spastin OS=Mus musculus GN=Spast PE=2 SV=3 - [SPAST_MOUSE]	42.67	1	19	19	52	0.962	0.661	0.804	0.777	148.48	42.67	29	52	614	66.4	9.69
Q9R1P1	Proteasome subunit beta type-3 OS=Mus musculus GN=Psmb3 PE=1 SV=1 - [PSB3_MOUSE]	41.46	3	7	7	28	1.553	0.952	0.736	0.778	96.82	41.46	12	28	205	22.9	6.55
E9PWQ6	Serine/threonine-protein kinase 31 OS=Mus musculus GN=Stk31 PE=2 SV=1 - [E9PWQ6_MOUSE]	3.80	4	3	3	4	0.454	0.345	0.997	0.778	9.50	3.80	3	4	973	109.8	5.43
Q8BH50-2	Isoform 2 of Uncharacterized protein C18orf25 homolog OS=Mus musculus -	7.41	3	1	1	1	0.911	1.081	1.042	0.778	2.59	7.41	1	1	162	17.3	4.67
B1AQX6	SRC kinase-signaling inhibitor 1 OS=Mus musculus GN=Srcin1 PE=2 SV=1 - [B1AQX6_MOUSE]	71.81	4	10	75	479	0.559	0.958	0.984	0.778	1495.43	71.81	139	479	1174	126.9	9.36
Q9WVC3	Caveolin-2 OS=Mus musculus GN=Cav2 PE=1 SV=1 - [CAV2_MOUSE]	7.41	1	1	1	1	4.486	1.814	0.885	0.778	3.55	7.41	1	1	162	18.2	5.63
Q9WUK2	Eukaryotic translation initiation factor 4H OS=Mus musculus GN=Eif4h PE=1 SV=3 - [IF4H_MOUSE]	52.42	2	10	10	84	1.739	0.741	1.307	0.778	202.80	52.42	15	84	248	27.3	7.23
Q8BGH7	CDC42 small effector protein 2 OS=Mus musculus GN=Cdc42se2 PE=1 SV=1 -	22.62	1	1	1	5	0.712	0.780	1.097	0.778	20.59	22.62	2	5	84	9.2	8.35
Q9CR10	Oxidoreductase-like domain-containing protein 1 OS=Mus musculus GN=Oxld1 PE=2 SV=1 - [OXLD1_MOUSE]	7.46	1	1	1	8	1.689	0.980	1.278	0.779	25.76	7.46	2	8	201	22.2	8.05
Q3V118	Protein 4922505E12Rik OS=Mus musculus GN=4922505E12Rik PE=2 SV=1 - [Q3V118_MOUSE]	2.74	1	1	1	1	0.817	0.791	0.943	0.779	0.00	2.74	1	1	621	71.3	9.60
Q80UF4	Serologically defined colon cancer antigen 8 homolog OS=Mus musculus GN=Sdccag8 PE=1 SV=1 - [SDCG8_MOUSE]	5.58	4	3	4	13	1.573	0.670	1.233	0.779	29.54	5.58	6	13	717	82.9	6.52

P62311	U6 snRNA-associated Sm-like protein LSm3 OS=Mus musculus GN=Lsm3 PE=3 SV=2 - [LSM3_MOUSE]	11.76	1	1	1	8	2.126	0.869	1.025	0.779	30.11	11.76	2	8	102	11.8	4.70
P61600	N-alpha-acetyltransferase 20 OS=Mus musculus GN=Naa20 PE=2 SV=1 - [NAA20_MOUSE]	19.66	6	3	3	8	0.756	1.191	0.643	0.779	25.52	19.66	6	8	178	20.4	5.03
Q9CQU0	Thioredoxin domain-containing protein 12 OS=Mus musculus GN=Txndc12 PE=2 SV=1 - [TXD12_MOUSE]	40.59	1	6	6	44	1.635	0.906	0.893	0.779	147.75	40.59	12	44	170	19.0	5.26
Q8C4Y1	Max protein OS=Mus musculus GN=Max PE=2 SV=1 - [Q8C4Y1_MOUSE]	10.60	3	2	2	5	1.283	0.826	0.876	0.779	14.39	10.60	3	5	151	17.2	6.55
P50580	Proliferation-associated protein 2G4 OS=Mus musculus GN=Pa2g4 PE=1 SV=3 - [PA2G4_MOUSE]	45.94	2	16	16	92	0.710	0.563	0.698	0.779	257.44	45.94	29	92	394	43.7	6.86
Q06335	Amyloid-like protein 2 OS=Mus musculus GN=Aplp2 PE=1 SV=4 - [APLP2_MOUSE]	32.96	2	2	21	65	0.683	0.705	0.898	0.779	191.81	32.96	36	65	707	80.4	4.70
P10711	Transcription elongation factor A protein 1 OS=Mus musculus GN=Tcea1 PE=1 SV=2 - [TCEA1_MOUSE]	39.87	6	12	12	72	1.510	0.739	1.057	0.779	209.15	39.87	23	72	301	33.9	8.38
P57759	Endoplasmic reticulum resident protein 29 OS=Mus musculus GN=Erp29 PE=1 SV=2 - [ERP29_MOUSE]	51.91	4	12	12	79	1.881	1.040	0.858	0.779	262.03	51.91	19	79	262	28.8	6.15
Q3UGS4	Protein FAM195B OS=Mus musculus GN=Fam195b PE=1 SV=1 - [F195B_MOUSE]	64.95	1	7	7	21	1.376	0.799	1.150	0.780	56.09	64.95	12	21	97	11.1	9.14
G3X937	Solute carrier family 41 member 3 OS=Mus musculus GN=Slc41a3 PE=4 SV=1 -	6.56	3	3	3	5	0.905	0.552	0.733	0.780	14.96	6.56	4	5	488	53.3	7.65
Q8BJ05-2	Isoform 2 of Zinc finger CCCH domain-containing protein 14 OS=Mus musculus GN=Zc3h14 - [ZC3HE_MOUSE]	32.30	3	13	14	47	1.387	0.847	1.112	0.780	138.76	32.30	25	47	579	64.9	8.15
Q61103	Zinc finger protein ubi-d4 OS=Mus musculus GN=Dpf2 PE=1 SV=1 - [REQU_MOUSE]	14.58	3	4	4	8	0.683	0.840	0.862	0.781	27.25	14.58	7	8	391	44.2	6.47
P49135	TFIIH basal transcription factor complex helicase XPB subunit OS=Mus musculus GN=Ercc3 PE=2 SV=1 - [ERCC3_MOUSE]	9.45	1	5	5	8	1.016	0.906	0.743	0.781	32.26	9.45	6	8	783	89.1	7.18
A6PWB3	Protein C87977 (Fragment) OS=Mus musculus GN=C87977 PE=2 SV=1 -	14.81	15	2	2	3	2.302	2.699	1.443	0.781	8.15	14.81	2	3	189	21.4	4.79
Q6PHZ8	Kv channel-interacting protein 4 OS=Mus musculus GN=Kcnip4 PE=1 SV=1 -	55.20	7	10	12	30	0.549	0.687	0.876	0.781	89.68	55.20	19	30	250	28.7	5.21
B1AXI9	5-azacytidine induced gene 1 OS=Mus musculus GN=Azi1 PE=2 SV=1 - [B1AXI9_MOUSE]	14.64	3	13	13	28	0.888	0.895	0.911	0.781	86.41	14.64	22	28	1059	120.2	8.65
Q8C3X8	Lipase maturation factor 2 OS=Mus musculus GN=Lmf2 PE=2 SV=1 - [LMF2_MOUSE]	8.40	1	5	5	10	0.670	0.634	0.620	0.781	12.26	8.40	6	10	702	79.9	9.99
E9Q1M6	Protein Maskbp3 OS=Mus musculus GN=Ankhd1 PE=4 SV=1 - [E9Q1M6_MOUSE]	8.21	7	7	16	36	1.006	0.809	1.064	0.781	93.99	8.21	28	36	2485	263.2	5.92
P15864	Histone H1.2 OS=Mus musculus GN=Hist1h1c PE=1 SV=2 - [H12_MOUSE]	49.53	3	9	18	114	0.415	1.226	0.883	0.781	304.94	49.53	32	114	212	21.3	11.00
Q922H9-2	Isoform 2 of Zinc finger protein 330 OS=Mus musculus GN=Znf330 - [ZN330_MOUSE]	2.57	2	1	1	3	1.138	0.497	0.915	0.781	8.99	2.57	2	3	311	34.9	6.44
O35678	Monoglyceride lipase OS=Mus musculus GN=Mgll PE=1 SV=1 - [MGLL_MOUSE]	42.90	5	11	11	42	0.611	0.863	1.078	0.781	122.32	42.90	20	42	303	33.4	7.15
Q4LDD4-3	Isoform 3 of Arf-GAP with Rho-GAP domain, ANK repeat and PH domain-containing protein 1 OS=Mus musculus GN=Arap1 - [ARAP1_MOUSE]	3.77	5	4	5	7	1.446	1.085	0.765	0.781	17.01	3.77	7	7	1193	134.5	6.24
P19253	60S ribosomal protein L13a OS=Mus musculus GN=Rpl13a PE=1 SV=4 - [RL13A_MOUSE]	37.44	3	10	10	58	0.544	0.649	0.730	0.781	148.00	37.44	17	58	203	23.4	11.02
Q61193	Ral guanine nucleotide dissociation stimulator-like 2 OS=Mus musculus GN=Rgl2 PE=1 SV=2 - [RGL2_MOUSE]	7.33	2	4	4	7	0.705	0.572	0.805	0.781	25.09	7.33	7	7	778	83.8	6.73
F6V6T4	Transmembrane emp24 domain-containing protein 2 (Fragment) OS=Mus musculus GN=Tmed2 PE=3 SV=1 - [F6V6T4_MOUSE]	34.09	3	5	5	13	0.684	0.829	0.721	0.781	35.03	34.09	9	13	176	20.0	9.07

Q62165	Dystroglycan OS=Mus musculus GN=Dag1 PE=1 SV=4 - [DAG1_MOUSE]	19.37	1	12	13	40	1.191	0.919	1.046	0.782	104.56	19.37	21	40	893	96.8	8.44
P15209-2	Isoform GP95-TRKB of BDNF/NT-3 growth factors receptor OS=Mus musculus GN=Ntrk2 - [NTRK2_MOUSE]	21.64	1	1	10	66	0.246	1.352	0.637	0.782	201.97	21.64	17	66	476	53.2	6.47
Q8R1B5	Complexin-3 OS=Mus musculus GN=Cplx3 PE=1 SV=1 - [CPLX3_MOUSE]	35.44	1	5	5	11	0.471	1.147	2.206	0.782	28.26	35.44	7	11	158	17.6	4.89
Q9DAU1	Protein canopy homolog 3 OS=Mus musculus GN=Cnpy3 PE=1 SV=1 - [CNPY3_MOUSE]	31.16	3	8	8	19	1.414	0.818	1.042	0.782	53.21	31.16	15	19	276	30.5	5.62
E9PVK4	Tetratricopeptide repeat protein 21B OS=Mus musculus GN=Ttc21b PE=2 SV=1 -	3.23	2	3	4	6	1.382	2.168	0.969	0.782	15.57	3.23	4	6	1269	145.3	6.86
D3YWP3	MCG1034428 OS=Mus musculus GN=Rpl23a-ps3 PE=3 SV=1 - [D3YWP3_MOUSE]	46.79	7	8	8	27	0.878	0.900	0.882	0.782	69.89	46.79	13	27	156	17.7	10.42
P61965	WD repeat-containing protein 5 OS=Mus musculus GN=Wdr5 PE=1 SV=1 - [WDR5_MOUSE]	19.46	2	5	5	11	1.499	0.995	0.717	0.782	25.33	19.46	9	11	334	36.6	8.27
P49710	Hematopoietic lineage cell-specific protein OS=Mus musculus GN=Hcls1 PE=1 SV=2 - [HCLS1_MOUSE]	26.75	2	11	11	40	1.760	1.060	1.065	0.782	103.44	26.75	20	40	486	54.2	4.84
Q8BM75-2	Isoform 2 of AT-rich interactive domain-containing protein 5B OS=Mus musculus GN=Arid5b - [ARI5B_MOUSE]	1.38	2	1	1	3	1.362	1.241	0.934	0.783	10.10	1.38	2	3	945	104.0	9.23
D3YU17	Nicalin OS=Mus musculus GN=Ncln PE=2 SV=1 - [D3YU17_MOUSE]	29.18	3	16	16	54	0.624	0.728	0.708	0.783	167.70	29.18	26	54	562	62.7	6.49
Q99JR8-2	Isoform 2 of SWI/SNF-related matrix-associated actin-dependent regulator of chromatin subfamily D member 2 OS=Mus musculus GN=Smarcd2 - [SMRD2_MOUSE]	16.53	3	6	7	10	0.841	0.805	0.822	0.783	28.52	16.53	9	10	484	55.3	9.48
P59326	YTH domain family protein 1 OS=Mus musculus GN=Ythdf1 PE=2 SV=1 - [YTHD1_MOUSE]	27.37	3	8	12	40	1.011	0.839	1.023	0.783	71.53	27.37	18	40	559	60.8	8.95
A2BE28-2	Isoform 2 of Ribosomal biogenesis protein LAS1L OS=Mus musculus GN=Las1l -	13.97	2	8	8	23	0.831	0.991	0.766	0.783	64.52	13.97	13	23	759	87.5	4.42
Q8R311	cTAGE family member 5 OS=Mus musculus GN=Ctage5 PE=1 SV=1 - [CTGE5_MOUSE]	37.23	15	25	25	71	1.149	0.898	0.955	0.784	205.68	37.23	41	71	779	87.7	5.11
P09405	Nucleolin OS=Mus musculus GN=Ncl PE=1 SV=2 - [NUCL_MOUSE]	44.41	1	41	42	284	1.012	0.471	1.125	0.784	796.88	44.41	72	284	707	76.7	4.75
A0T1G3	C-type lectin domain family 1 member B OS=Mus musculus GN=Clec1b PE=4 SV=1 - [A0T1G3_MOUSE]	13.25	2	1	1	1	0.365	2.820	2.423	0.784	1.76	13.25	1	1	83	9.6	8.46
Q9Z247	Peptidyl-prolyl cis-trans isomerase FKBP9 OS=Mus musculus GN=Fkbp9 PE=1 SV=1 - [FKBP9_MOUSE]	10.70	1	7	7	13	1.295	1.090	0.860	0.784	33.89	10.70	10	13	570	63.0	5.21
Q925G2	Cytochrome b reductase 1 OS=Mus musculus GN=Cybrd1 PE=1 SV=2 - [CYBR1_MOUSE]	19.66	2	3	3	15	0.871	1.025	0.503	0.784	27.41	19.66	5	15	290	31.8	9.06
Q8K2J7-2	Isoform 2 of RELT-like protein 1 OS=Mus musculus GN=Rell1 - [RELL1_MOUSE]	10.40	2	3	3	6	0.886	0.653	0.866	0.784	14.01	10.40	6	6	202	22.2	8.34
Q3U1Z5	G-protein-signaling modulator 3 OS=Mus musculus GN=Gpsm3 PE=1 SV=2 -	13.84	1	1	1	2	1.629	1.204	1.232	0.784	5.16	13.84	1	2	159	17.6	5.20
Q91VK1	Basic leucine zipper and W2 domain-containing protein 2 OS=Mus musculus GN=Bzw2 PE=1 SV=1 - [BZW2_MOUSE]	29.59	1	9	13	24	0.993	1.212	1.056	0.784	61.02	29.59	20	24	419	48.0	6.68
Q4VAA7	Sorting nexin-33 OS=Mus musculus GN=Snx33 PE=2 SV=1 - [SNX33_MOUSE]	1.39	1	1	1	3	4.101	1.157	0.928	0.784	7.96	1.39	2	3	574	65.3	6.79
F6VUN6	MICAL C-terminal-like protein (Fragment) OS=Mus musculus GN=Micalcl PE=4 SV=1 - [F6VUN6_MOUSE]	4.20	3	2	2	2	1.461	0.883	0.914	0.784	3.16	4.20	2	2	572	63.7	8.94
Q9CR37	Pancreatic progenitor cell differentiation and proliferation factor OS=Mus musculus GN=Ppdpf PE=2 SV=1 - [PPDPF_MOUSE]	24.35	1	2	2	10	1.242	0.746	1.089	0.785	21.69	24.35	4	10	115	12.3	7.44
Q8C779-2	Isoform 2 of Uncharacterized protein CXorf57 homolog OS=Mus musculus -	2.59	2	1	1	3	0.956	1.543	0.686	0.785	0.00	2.59	1	3	849	96.7	8.73

Q9D824	Pre-mRNA 3'-end-processing factor FIP1 OS=Mus musculus GN=Fip1l1 PE=1 SV=1 -	17.38	7	9	9	25	1.107	0.800	0.917	0.785	64.49	17.38	17	25	581	64.9	5.77
A3KGK3-2	Isoform 2 of Fer-1-like protein 4 OS=Mus musculus GN=Fer1l4 - [FR1L4_MOUSE]	4.52	2	4	4	6	0.765	0.751	1.028	0.785	6.96	4.52	4	6	1901	213.0	5.96
O88532	Zinc finger RNA-binding protein OS=Mus musculus GN=Zfr PE=1 SV=2 - [ZFR_MOUSE]	25.51	5	20	20	93	0.697	0.577	0.912	0.785	307.55	25.51	36	93	1074	116.8	9.04
P62315	Small nuclear ribonucleoprotein Sm D1 OS=Mus musculus GN=Snrpd1 PE=1 SV=1 -	36.97	1	4	4	15	0.732	0.586	0.697	0.785	43.74	36.97	8	15	119	13.3	11.56
Q9CQR2	40S ribosomal protein S21 OS=Mus musculus GN=Rps21 PE=2 SV=1 - [RS21_MOUSE]	36.14	1	3	3	38	1.835	0.853	1.065	0.785	116.72	36.14	6	38	83	9.1	8.51
Q80YR4-2	Isoform 2 of Zinc finger protein 598 OS=Mus musculus GN=Znf598 - [ZN598_MOUSE]	17.50	3	12	12	23	0.728	0.685	0.873	0.786	76.77	17.50	17	23	880	96.3	8.29
P08113	Endoplasmin OS=Mus musculus GN=Hsp90b1 PE=1 SV=2 - [ENPL_MOUSE]	47.63	2	38	40	277	0.885	1.109	0.764	0.786	762.12	47.63	67	277	802	92.4	4.82
Q9CXU9	Eukaryotic translation initiation factor 1b OS=Mus musculus GN=Eif1b PE=2 SV=2 - [EIF1B_MOUSE]	60.18	5	1	5	32	1.557	0.525	0.916	0.786	128.18	60.18	9	32	113	12.8	7.37
B7ZCE0	N-acetyltransferase 6 (Fragment) OS=Mus musculus GN=Nat6 PE=4 SV=1 - [B7ZCE0_MOUSE]	5.39	2	1	1	1	2.668	0.558	0.935	0.786	2.81	5.39	1	1	204	22.5	8.24
Q8C0C7	Phenylalanine--tRNA ligase alpha subunit OS=Mus musculus GN=Farsa PE=2 SV=1 - [SYFA_MOUSE]	43.31	3	20	20	63	0.681	0.734	0.702	0.786	195.76	43.31	32	63	508	57.6	8.28
F6QRE9	Protein BC007180 (Fragment) OS=Mus musculus GN=BC007180 PE=4 SV=2 -	6.30	1	3	3	4	2.360	1.251	1.005	0.786	9.55	6.30	4	4	889	95.3	4.37
Q61595-3	Isoform 3 of Kinectin OS=Mus musculus GN=Ktn1 - [KTN1_MOUSE]	49.27	4	4	57	181	1.715	1.212	1.057	0.786	518.38	49.27	96	181	1228	140.9	6.13
A2AWT6	Nucleolar transcription factor 1 OS=Mus musculus GN=Ubtf PE=4 SV=1 - [A2AWT6_MOUSE]	19.53	7	14	15	24	0.483	0.814	0.918	0.786	62.57	19.53	21	24	727	85.0	5.55
Q8BRG8-2	Isoform 2 of Transmembrane protein 209 OS=Mus musculus GN=Tmem209 -	11.96	5	5	5	6	0.872	0.998	0.818	0.787	8.12	11.96	6	6	560	62.8	8.73
Q6GU68	Immunoglobulin superfamily containing leucine-rich repeat protein OS=Mus musculus GN=Islr PE=1 SV=1 - [illegible]	8.18	1	2	2	2	1.122	2.220	0.813	0.787	2.04	8.18	2	2	428	45.6	5.38
E9Q7E2	Protein Arid2 OS=Mus musculus GN=Arid2 PE=2 SV=1 - [E9Q7E2_MOUSE]	4.21	2	6	8	10	0.662	0.969	0.716	0.787	25.81	4.21	10	10	1828	195.9	7.47
P24369	Peptidyl-prolyl cis-trans isomerase B OS=Mus musculus GN=Ppib PE=2 SV=2 - [PPIB_MOUSE]	51.85	1	11	12	146	1.383	0.845	0.734	0.787	358.72	51.85	24	146	216	23.7	9.55
Q9D6T0	Nitric oxide synthase-interacting protein OS=Mus musculus GN=Nosip PE=2 SV=1 - [NOSIP_MOUSE]	24.58	3	7	7	11	0.971	0.835	0.979	0.787	35.26	24.58	11	11	301	33.2	8.62
R4GML6	Centriolin (Fragment) OS=Mus musculus GN=Cep110 PE=4 SV=1 - [R4GML6_MOUSE]	1.47	15	2	5	6	2.306	2.099	1.138	0.787	14.60	1.47	5	6	2316	266.6	5.52
E9QM77	Ataxin-2 OS=Mus musculus GN=Atxn2 PE=2 SV=1 - [E9QM77_MOUSE]	29.24	6	21	29	119	1.396	0.658	0.964	0.787	374.86	29.24	46	119	1286	136.4	9.54
Q3UN90	LYR motif-containing protein 9 OS=Mus musculus GN=Lyrm9 PE=2 SV=1 - [LYRM9_MOUSE]	21.79	5	1	2	5	1.001	1.408	1.373	0.787	11.93	21.79	2	5	78	9.3	8.91
Q9CU65	Zinc finger MYM-type protein 2 OS=Mus musculus GN=Zmym2 PE=2 SV=3 -	4.80	2	6	6	17	1.600	0.833	1.088	0.787	37.86	4.80	10	17	1376	154.5	6.37
Q8K2Y7	39S ribosomal protein L47, mitochondrial OS=Mus musculus GN=Mrpl47 PE=2 SV=2 - [RM47_MOUSE]	21.83	1	5	5	18	0.728	0.494	0.770	0.787	46.30	21.83	8	18	252	29.7	10.21
E9Q9S5	Protein Gm9964 OS=Mus musculus GN=Gm9964 PE=4 SV=1 - [E9Q9S5_MOUSE]	16.67	1	1	1	1	2.552	0.934	1.094	0.787	0.00	16.67	1	1	144	16.6	10.01
Q8CFB8	NAD+ ADP-ribosyltransferase 3 PARP-3 OS=Mus musculus GN=Parp3 PE=2 SV=1 - [Q8CFB8_MOUSE]	4.17	4	2	2	5	2.285	1.553	0.891	0.787	9.52	4.17	3	5	528	59.4	7.12
D3Z2K2	28S ribosomal protein S14, mitochondrial OS=Mus musculus GN=Mrps14 PE=2 SV=1 - [D3Z2K2_MOUSE]	18.03	2	2	2	11	0.765	0.461	1.015	0.787	31.40	18.03	4	11	122	14.3	10.86

Q9D614	Syntaxin 17, isoform CRA_b OS=Mus musculus GN=Stx17 PE=2 SV=1 - [Q9D614_MOUSE]	14.86	2	1	5	11	8.017	1.877	0.519	0.787	26.26	14.86	7	11	276	31.5	6.47
Q9R0P3	S-formylglutathione hydrolase OS=Mus musculus GN=Esd PE=2 SV=1 - [ESTD_MOUSE]	57.80	8	10	10	54	0.892	0.715	0.823	0.788	192.70	57.80	18	54	282	31.3	7.12
Q3USH5	Splicing factor, suppressor of white-apricot homolog OS=Mus musculus GN=Sfswap PE=1 SV=2 - [SFSWA_MOUSE]	3.28	1	3	3	6	0.795	0.686	0.856	0.788	11.48	3.28	4	6	945	104.1	8.05
Q8R2S9	Actin-related protein 8 OS=Mus musculus GN=Actr8 PE=2 SV=1 - [ARP8_MOUSE]	3.37	1	2	2	3	0.761	0.732	0.702	0.788	6.18	3.37	3	3	624	70.5	7.77
F6V035	Protein Ccdc149 OS=Mus musculus GN=Ccdc149 PE=4 SV=1 - [F6V035_MOUSE]	11.26	1	5	6	18	1.425	1.188	1.070	0.788	52.07	11.26	10	18	524	58.8	5.57
Q9CR41	Huntingtin-interacting protein K OS=Mus musculus GN=Hypk PE=2 SV=2 - [HYPK_MOUSE]	42.64	2	5	5	34	2.150	0.922	1.212	0.788	105.45	42.64	9	34	129	14.7	4.93
Q9QZQ1-2	Isoform 1 of Afadin OS=Mus musculus GN=Mllt4 - [AFAD_MOUSE]	36.40	4	1	53	173	1.038	1.176	0.766	0.788	501.17	36.40	88	173	1805	204.4	6.24
P28798	Granulins OS=Mus musculus GN=Grn PE=1 SV=2 - [GRN_MOUSE]	11.04	5	5	5	16	1.396	0.758	1.042	0.788	45.63	11.04	10	16	589	63.4	6.80
Q9CPT4	UPF0556 protein C19orf10 homolog OS=Mus musculus GN=D17Wsu104e PE=2 SV=1 - [CS010_MOUSE]	28.31	1	4	4	18	1.443	0.895	0.757	0.788	45.43	28.31	8	18	166	18.0	6.79
Q91ZD4	Vang-like protein 2 OS=Mus musculus GN=Vangl2 PE=1 SV=3 - [VANG2_MOUSE]	13.44	4	5	5	14	0.632	0.540	0.560	0.788	46.39	13.44	8	14	521	59.7	9.22
P61804	Dolichyl-diphosphooligosaccharide--protein glycosyltransferase subunit DAD1 OS=Mus musculus GN=Dad1 PE=2 [illegible]	19.47	1	2	2	9	0.593	1.075	0.609	0.789	29.41	19.47	4	9	113	12.5	7.08
E9QML5	Zinc finger protein 638 OS=Mus musculus GN=Zfml PE=2 SV=1 - [E9QML5_MOUSE]	17.76	10	26	26	65	0.940	0.643	1.131	0.789	177.67	17.76	44	65	1926	214.3	6.76
P97427	Dihydropyrimidinase-related protein 1 OS=Mus musculus GN=Crmp1 PE=1 SV=1 -	75.87	1	2	34	951	1.050	0.887	0.874	0.789	2555.72	75.87	64	951	572	62.1	7.12
Q8BHG9	CGG triplet repeat-binding protein 1 OS=Mus musculus GN=Cggbp1 PE=2 SV=1 -	22.16	1	3	3	5	0.795	0.637	0.883	0.789	18.54	22.16	5	5	167	18.7	8.95
Q3UQN2	FCH domain only protein 2 OS=Mus musculus GN=Fcho2 PE=1 SV=1 - [FCHO2_MOUSE]	17.43	5	12	13	35	0.921	1.201	0.800	0.789	110.97	17.43	23	35	809	88.7	6.89
P70195	Proteasome subunit beta type-7 OS=Mus musculus GN=Psmb7 PE=1 SV=1 - [PSB7_MOUSE]	14.80	1	4	4	37	1.545	0.871	0.758	0.789	96.06	14.80	8	37	277	29.9	7.99
O09012	Peroxisomal targeting signal 1 receptor OS=Mus musculus GN=Pex5 PE=2 SV=2 - [PEX5_MOUSE]	33.49	3	15	15	57	1.093	1.079	0.746	0.789	192.39	33.49	25	57	639	70.7	4.55
P70208	Plexin-A3 OS=Mus musculus GN=Plxna3 PE=1 SV=2 - [PLXA3_MOUSE]	14.85	2	14	28	68	0.736	0.965	0.649	0.789	189.57	14.85	49	68	1872	207.8	7.09
Q9EQC4	Elongation of very long chain fatty acids protein 4 OS=Mus musculus GN=Elovl4 PE=1 SV=2 - [ELOV4_MOUSE]	2.88	1	1	1	1	0.725	0.725	0.528	0.789	0.00	2.88	1	1	312	36.5	9.23
P27792	Protein lyl-1 OS=Mus musculus GN=Lyl1 PE=2 SV=2 - [LYL1_MOUSE]	6.12	1	1	1	1	21.216	1.527	0.773	0.789	3.23	6.12	1	1	278	30.1	10.29
F8WHP5	Phospholipase DDHD1 OS=Mus musculus GN=Ddhd1 PE=2 SV=1 - [F8WHP5_MOUSE]	23.25	8	15	15	42	1.128	0.792	0.897	0.789	118.67	23.25	24	42	856	94.7	5.69
Q8BIE6	FERM domain-containing protein 4A OS=Mus musculus GN=Frmd4a PE=1 SV=2 -	25.88	10	21	22	78	0.839	0.590	0.927	0.789	274.32	25.88	37	78	1020	113.8	8.92
B9EKR1	Protein Ptprz1 OS=Mus musculus GN=Ptprz1 PE=2 SV=1 - [B9EKR1_MOUSE]	15.44	1	32	33	244	1.314	1.058	1.170	0.790	671.43	15.44	62	244	2312	254.2	4.88
Q8BXA0	Leucine-rich repeat and fibronectin type-III domain containing protein 5 OS=Mus musculus GN=Lrfn5 PE=1 SV=1 - [illegible]	17.94	2	9	10	24	0.752	0.839	0.801	0.790	79.70	17.94	17	24	719	79.3	7.24
O35381	Acidic leucine-rich nuclear phosphoprotein 32 family member A OS=Mus musculus GN=Anp32a PE=1 SV=1 - [illegible]	46.96	4	7	14	205	1.229	1.008	1.202	0.790	506.43	46.96	23	205	247	28.5	4.07

P51949	CDK-activating kinase assembly factor MAT1 OS=Mus musculus GN=Mnat1 PE=2 SV=2 - [MAT1_MOUSE]	18.45	1	5	5	13	1.234	0.999	0.993	0.790	29.00	18.45	10	13	309	35.8	5.82
O35900	U6 snRNA-associated Sm-like protein LSm2 OS=Mus musculus GN=Lsm2 PE=2 SV=1 - [LSM2_MOUSE]	74.74	3	7	7	24	1.774	0.802	1.115	0.790	43.16	74.74	11	24	95	10.8	6.52
Q60929-2	Isoform 2 of Myocyte-specific enhancer factor 2A OS=Mus musculus GN=Mef2a - [MEF2A_MOUSE]	13.27	6	3	5	13	1.091	1.233	1.885	0.790	28.86	13.27	7	13	490	52.6	8.69
Q6P8I4	PEST proteolytic signal-containing nuclear protein OS=Mus musculus GN=Pcnp PE=1 SV=1 - [PCNP_MOUSE]	26.97	6	6	6	37	1.746	0.817	1.209	0.790	92.11	26.97	10	37	178	19.0	7.49
Q80ZI1	TMF-regulated nuclear protein 1 OS=Mus musculus GN=Trnp1 PE=1 SV=1 - [TRNP1_MOUSE]	14.80	1	2	2	3	0.588	0.422	1.100	0.790	11.86	14.80	3	3	223	23.1	11.43
Q80U16-7	Isoform 7 of Protein FAM65B OS=Mus musculus GN=Fam65b - [FA65B_MOUSE]	13.06	8	6	6	11	1.069	1.427	1.213	0.791	25.75	13.06	11	11	605	67.1	5.54
Q99KI3	ER membrane protein complex subunit 3 OS=Mus musculus GN=Emc3 PE=2 SV=3 - [EMC3_MOUSE]	19.16	1	7	7	33	0.586	0.658	0.707	0.791	87.87	19.16	12	33	261	30.0	6.81
Q7TQH0-3	Isoform 3 of Ataxin-2-like protein OS=Mus musculus GN=Atxn2l - [ATX2L_MOUSE]	28.19	6	22	24	127	1.471	0.867	1.073	0.791	381.18	28.19	43	127	1043	109.9	8.85
Q810A5	Uncharacterized protein KIAA0895-like OS=Mus musculus GN=Kiaa0895l PE=2 SV=1 - [K895L_MOUSE]	5.14	1	1	1	3	1.762	1.000	0.892	0.791	0.00	5.14	1	3	467	53.1	8.38
Q8VDC1	FYVE and coiled-coil domain-containing protein 1 OS=Mus musculus GN=Fyco1 PE=1 SV=1 - [FYCO1_MOUSE]	11.00	5	10	11	29	1.612	0.916	1.031	0.791	104.48	11.00	18	29	1437	162.2	4.98
Q6WVG3	BTB/POZ domain-containing protein KCTD12 OS=Mus musculus GN=Kctd12 PE=1 SV=1 - [KCD12_MOUSE]	39.45	1	9	12	90	1.152	1.115	0.798	0.791	311.37	39.45	22	90	327	35.9	5.81
Q9CPT5	Nucleolar protein 16 OS=Mus musculus GN=Nop16 PE=2 SV=1 - [NOP16_MOUSE]	20.22	3	4	4	7	0.791	0.901	0.861	0.791	11.83	20.22	6	7	178	21.1	9.91
Q5NC05	Transcription termination factor 2 OS=Mus musculus GN=Ttf2 PE=1 SV=2 - [TTF2_MOUSE]	4.31	1	3	5	9	1.613	0.734	1.061	0.791	6.86	4.31	5	9	1138	125.5	8.90
Q8R370	Usher syndrome type-1C protein-binding protein 1 OS=Mus musculus GN=Ushbp1 PE=1 SV=2 - [USBP1_MOUSE]	3.38	1	2	2	2	1.889	0.732	1.314	0.791	4.75	3.38	2	2	680	74.9	5.54
D3YY06	tRNA-splicing endonuclease subunit Sen34 (Fragment) OS=Mus musculus GN=Tsen34 PE=2 SV=1 - [illegible]	32.45	7	4	4	5	5.023	0.831	0.255	0.791	15.10	32.45	5	5	151	16.1	5.66
O08575-2	Isoform 2 of Eyes absent homolog 2 OS=Mus musculus GN=Eya2 - [EYA2_MOUSE]	3.38	2	1	1	1	7.307	1.207	1.087	0.791	2.37	3.38	1	1	503	55.3	6.05
Q8CH02	SURP and G-patch domain-containing protein 1 OS=Mus musculus GN=Sugp1 PE=1 SV=1 - [SUGP1_MOUSE]	24.11	1	11	11	25	1.468	0.835	0.984	0.791	73.18	24.11	20	25	643	72.6	7.64
Q19A41	Krueppel-like factor 14 OS=Mus musculus GN=Klf14 PE=2 SV=1 - [KLF14_MOUSE]	12.00	1	1	1	1	0.941	0.454	1.447	0.791	0.00	12.00	1	1	325	35.1	6.23
Q8C025	Cholinephosphotransferase 1 OS=Mus musculus GN=Chpt1 PE=2 SV=1 - [CHPT1_MOUSE]	6.03	6	2	2	4	0.818	0.493	0.675	0.792	10.01	6.03	4	4	398	44.6	7.08
Q9D1I6	39S ribosomal protein L14, mitochondrial OS=Mus musculus GN=Mrpl14 PE=2 SV=1 - [RM14_MOUSE]	41.38	1	5	5	13	1.211	0.558	0.746	0.792	35.12	41.38	8	13	145	15.9	10.99
Q9DCD5	Tight junction-associated protein 1 OS=Mus musculus GN=Tjap1 PE=1 SV=1 - [TJAP1_MOUSE]	4.27	1	1	2	6	1.544	0.998	0.848	0.792	19.61	4.27	4	6	539	59.4	6.07
Q61142	Spindlin-1 OS=Mus musculus GN=Spin1 PE=1 SV=2 - [SPIN1_MOUSE]	48.47	3	8	8	28	0.666	0.504	0.722	0.792	78.98	48.47	15	28	262	29.6	6.96
Q8BWP8-2	Isoform 2 of N-acetyllactosaminide beta-1,3-N-acetylglucosaminyltransferase OS=Mus musculus GN=B3gnt1 - [B3GN1_MOUSE]	14.25	2	4	4	10	1.120	0.938	0.679	0.792	32.01	14.25	6	10	379	43.0	7.71

E9Q705	BolA-like protein 3 OS=Mus musculus GN=Bola3 PE=2 SV=1 - [E9Q705_MOUSE]	42.11	3	3	3	16	1.811	0.568	1.069	0.793	56.00	42.11	6	16	95	10.8	8.31
Q91YA2	Serine/threonine-protein kinase H1 OS=Mus musculus GN=Pskh1 PE=2 SV=3 - [KPSH1_MOUSE]	5.90	1	2	2	6	1.762	0.809	0.944	0.793	4.29	5.90	4	6	424	48.1	9.83
Q9JIQ3	Diablo homolog, mitochondrial OS=Mus musculus GN=Diablo PE=1 SV=2 - [DBLOH_MOUSE]	13.92	3	4	4	5	1.939	0.570	1.029	0.793	11.86	13.92	5	5	237	26.8	6.37
Q04692	SWI/SNF-related matrix-associated actin-dependent regulator of chromatin subfamily A containing DEAD/H box 1 OS=Mus musculus GN=Smarcad1 PE=1 SV=2 - [SMRCD_MOUSE]	4.31	1	2	3	5	0.815	0.759	0.845	0.793	15.02	4.31	4	5	1021	116.4	5.68
Q3TJZ6	Protein FAM98A OS=Mus musculus GN=Fam98a PE=2 SV=1 - [FA98A_MOUSE]	19.81	1	5	8	51	1.447	0.673	0.899	0.793	214.25	19.81	15	51	515	55.0	8.95
G3X9J0	MCG122846 OS=Mus musculus GN=Sipa1l3 PE=4 SV=1 - [G3X9J0_MOUSE]	16.84	1	22	24	36	0.915	0.789	1.394	0.793	84.26	16.84	32	36	1776	194.9	8.32
Q8CB87	Ras-related protein Rab-44 OS=Mus musculus GN=Rab44 PE=2 SV=1 - [RAB44_MOUSE]	4.00	1	3	3	3	1.166	3.146	0.933	0.793	6.86	4.00	3	3	725	78.2	4.83
Q9D1M7	Peptidyl-prolyl cis-trans isomerase FKBP11 OS=Mus musculus GN=Fkbp11 PE=2 SV=1 - [FKB11_MOUSE]	19.40	1	3	3	5	0.936	0.628	0.554	0.793	12.35	19.40	5	5	201	22.1	9.36
Q91XD2	LIM and senescent cell antigen-like-containing domain protein 2 OS=Mus musculus GN=Lims2 PE=1 SV=1 - [LIMS2_MOUSE]	13.78	1	2	5	16	1.498	1.058	0.840	0.793	44.14	13.78	9	16	341	39.0	7.97
Q91UZ1	Phospholipase C beta 4 OS=Mus musculus GN=Plcb4 PE=2 SV=1 - [Q91UZ1_MOUSE]	37.45	2	32	43	132	0.581	0.355	0.595	0.793	402.79	37.45	72	132	1175	134.4	6.90
Q91XV3	Brain acid soluble protein 1 OS=Mus musculus GN=Basp1 PE=1 SV=3 - [BASP1_MOUSE]	81.86	1	13	13	173	0.793	0.928	1.045	0.793	408.92	81.86	20	173	226	22.1	4.51
Q9WV06	Ankyrin repeat domain-containing protein 2 OS=Mus musculus GN=Ankrd2 PE=2 SV=2 - [ANKR2_MOUSE]	6.70	1	1	1	1	4.817	1.120	1.436	0.793	3.44	6.70	1	1	358	39.8	6.67
O55142	60S ribosomal protein L35a OS=Mus musculus GN=Rpl35a PE=2 SV=2 - [RL35A_MOUSE]	49.09	1	7	7	17	0.442	0.638	0.764	0.793	36.66	49.09	10	17	110	12.5	10.89
Q9CRD2	ER membrane protein complex subunit 2 OS=Mus musculus GN=Emc2 PE=2 SV=1 - [EMC2_MOUSE]	34.34	1	9	9	37	1.334	0.624	0.846	0.793	103.86	34.34	14	37	297	34.9	6.81
Q9D024	Coiled-coil domain-containing protein 47 OS=Mus musculus GN=Ccdc47 PE=2 SV=2 - [CCD47_MOUSE]	32.71	4	13	13	36	0.760	0.671	0.809	0.794	98.57	32.71	20	36	483	55.8	4.84
A6PWC3	Nardilysin OS=Mus musculus GN=Nrd1 PE=2 SV=1 - [A6PWC3_MOUSE]	28.74	4	28	28	95	1.051	0.973	0.763	0.794	264.90	28.74	48	95	1117	127.7	4.87
Q8VIJ6	Splicing factor, proline- and glutamine-rich OS=Mus musculus GN=Sfpq PE=1 SV=1 - [SFPQ_MOUSE]	32.76	1	22	23	234	1.380	0.801	1.031	0.794	597.99	32.76	41	234	699	75.4	9.44
Q9EP82	tRNA (guanine-N(7)-)-methyltransferase subunit WDR4 OS=Mus musculus GN=Wdr4 PE=1 SV=2 - [WDR4_MOUSE]	1.45	3	1	1	2	0.891	1.011	0.793	0.794	0.00	1.45	1	2	413	45.7	6.44
Q8CGB3	Uveal autoantigen with coiled-coil domains and ankyrin repeats OS=Mus musculus GN=Uaca PE=1 SV=2 - [UACA_MOUSE]	7.58	3	9	11	19	1.659	1.984	0.806	0.794	49.19	7.58	16	19	1411	160.7	7.20
Q3UY34	Uncharacterized protein C12orf43 homolog OS=Mus musculus PE=2 SV=1 - [CL043_MOUSE]	19.53	1	4	4	8	1.727	1.254	0.881	0.794	20.43	19.53	6	8	256	27.6	8.70
Q99LJ0	CTTNBP2 N-terminal-like protein OS=Mus musculus GN=Cttnbp2nl PE=1 SV=1 - [CT2NL_MOUSE]	29.94	1	15	16	51	1.602	0.629	1.078	0.794	141.66	29.94	27	51	638	69.8	7.71
Q9JHQ5	Leucine zipper transcription factor-like protein 1 OS=Mus musculus GN=Lztfl1 PE=2 SV=1 - [LZTL1_MOUSE]	49.16	4	14	14	82	1.746	1.101	0.979	0.794	203.63	49.16	25	82	299	34.8	5.17
A2ACJ2	Fanconi anemia-associated protein of 100 kDa OS=Mus musculus GN=Faap100 PE=2 SV=1 - [FP100_MOUSE]	7.39	1	4	4	6	2.448	1.451	1.041	0.794	17.09	7.39	6	6	879	94.2	5.36
Q80WT0	Junctophilin-4 OS=Mus musculus GN=Jph4 PE=2 SV=1 - [JPH4_MOUSE]	6.53	1	3	3	8	1.058	1.966	1.202	0.794	16.11	6.53	6	8	628	66.0	6.71

Q8R2M2	Deoxynucleotidyltransferase terminal-interacting protein 2 OS=Mus musculus GN=Dnttip2 PE=1 SV=1 - [illegible]	8.84	2	3	4	8	0.645	0.993	0.626	0.794	18.94	8.84	6	8	758	84.2	6.44
Q3UNZ2	Gene model 853, (NCBI) OS=Mus musculus GN=Gm853 PE=2 SV=1 - [Q3UNZ2_MOUSE]	1.88	1	1	1	1	1.004	1.031	0.819	0.795	0.00	1.88	1	1	425	46.5	6.64
Q3UKU4	Protein FAM83F OS=Mus musculus GN=Fam83f PE=1 SV=1 - [FA83F_MOUSE]	6.26	1	3	3	3	1.012	2.857	2.417	0.795	3.03	6.26	3	3	495	55.0	8.78
Q9DB38	Zinc finger protein 580 OS=Mus musculus GN=Znf580 PE=2 SV=1 - [ZN580_MOUSE]	10.47	1	1	1	1	1.048	1.059	0.859	0.795	2.44	10.47	1	1	172	18.8	10.08
D3Z6G3	Microtubule-associated protein RP/EB family member 3 OS=Mus musculus GN=Mapre3 PE=2 SV=1 - [illegible]	39.47	2	1	13	100	1.132	0.673	0.642	0.795	273.62	39.47	24	100	266	30.3	5.30
P14576	Signal recognition particle 54 kDa protein OS=Mus musculus GN=Srp54 PE=1 SV=2 - [SRP54_MOUSE]	39.09	3	16	16	52	0.655	0.805	0.706	0.795	181.35	39.09	28	52	504	55.7	8.75
O54988-2	Isoform 2 of STE20-like serine/threonine-protein kinase OS=Mus musculus GN=Slk - [SLK_MOUSE]	31.86	3	30	32	134	0.905	0.879	1.285	0.795	342.07	31.86	57	134	1202	137.6	5.08
D3Z285	NFU1 iron-sulfur cluster scaffold homolog, mitochondrial (Fragment) OS=Mus musculus GN=Nfu1 PE=2 SV=1 - [illegible]	28.64	1	1	5	43	1.323	0.884	1.091	0.795	123.05	28.64	8	43	199	22.0	4.96
D6RDE5	DNA-binding protein Rfx5 OS=Mus musculus GN=Rfx5 PE=4 SV=1 - [D6RDE5_MOUSE]	48.78	8	1	1	3	3.283	1.123	0.902	0.795	13.03	48.78	2	3	41	4.4	9.99
B1ATC3	Trinucleotide repeat-containing gene 6C protein OS=Mus musculus GN=Tnrc6c PE=4 SV=1 - [B1ATC3_MOUSE]	6.74	3	7	7	17	1.518	1.029	0.883	0.795	43.89	6.74	11	17	1900	198.1	7.17
Q922H4	Mannose-1-phosphate guanyltransferase alpha OS=Mus musculus GN=Gmppa PE=2 SV=1 - [GMPPA_MOUSE]	28.33	8	9	9	21	0.550	0.891	0.793	0.795	53.87	28.33	14	21	420	46.2	7.62
P70670	Nascent polypeptide-associated complex subunit alpha, muscle-specific form OS=Mus musculus GN=Naca PE=1 SV=2 - [NACAM_MOUSE]	6.45	2	10	10	43	1.386	0.738	0.971	0.795	115.62	6.45	14	43	2187	220.4	9.35
Q9DCC8	Mitochondrial import receptor subunit TOM20 homolog OS=Mus musculus GN=Tomm20 PE=1 SV=1 - [illegible]	18.62	1	2	2	7	0.985	0.613	0.955	0.796	21.66	18.62	3	7	145	16.3	8.60
Q60900-2	Isoform HuC-S of ELAV-like protein 3 OS=Mus musculus GN=Elavl3 - [ELAV3_MOUSE]	44.72	2	9	15	74	0.867	0.663	0.828	0.796	209.00	44.72	27	74	360	38.8	9.28
Q80X82	Symplekin OS=Mus musculus GN=Sympk PE=1 SV=1 - [SYMPK_MOUSE]	15.73	4	15	15	33	0.654	0.946	0.795	0.796	96.83	15.73	22	33	1284	142.2	6.05
Q99KK7	Dipeptidyl peptidase 3 OS=Mus musculus GN=Dpp3 PE=2 SV=2 - [DPP3_MOUSE]	56.78	1	30	32	105	0.625	0.887	0.781	0.796	331.07	56.78	52	105	738	82.8	5.38
Q5HZK2	Neurensin-2 OS=Mus musculus GN=Nrsn2 PE=2 SV=1 - [NRSN2_MOUSE]	17.33	1	1	1	2	1.329	0.951	1.924	0.796	0.00	17.33	1	2	202	22.4	4.77
Q9CZH3	Proteasome assembly chaperone 3 OS=Mus musculus GN=Psmg3 PE=1 SV=1 -	22.95	1	2	2	4	1.405	1.083	0.696	0.796	15.26	22.95	4	4	122	13.3	8.66
Q91XU0	ATPase WRNIP1 OS=Mus musculus GN=Wrnip1 PE=1 SV=2 - [WRIP1_MOUSE]	23.48	2	14	14	26	0.613	0.808	0.736	0.796	64.22	23.48	21	26	660	71.7	6.18
D3YXP6	Phosphomevalonate kinase OS=Mus musculus GN=Pmvk PE=2 SV=1 - [D3YXP6_MOUSE]	15.82	4	3	3	7	0.663	0.618	0.635	0.796	13.14	15.82	5	7	158	17.9	9.86
Q9D1Q6	Endoplasmic reticulum resident protein 44 OS=Mus musculus GN=Erp44 PE=1 SV=1 - [ERP44_MOUSE]	50.00	1	15	17	86	1.413	0.820	0.710	0.796	250.08	50.00	29	86	406	46.8	5.27
Q921W0	Charged multivesicular body protein 1a OS=Mus musculus GN=Chmp1a PE=1 SV=1 -	23.47	1	6	6	25	1.275	0.748	1.244	0.797	68.24	23.47	11	25	196	21.6	8.06
F7DEU6	Inosine-5'-monophosphate dehydrogenase (Fragment) OS=Mus musculus GN=Impdh1 PE=2 SV=1 - [F7DEU6_MOUSE]	18.84	5	7	10	30	1.267	1.138	0.945	0.797	106.46	18.84	16	30	568	60.8	7.59
Q3UMR0-3	Isoform 3 of Ankyrin repeat domain-containing protein 27 OS=Mus musculus GN=Ankrd27 - [ANR27_MOUSE]	5.86	3	6	6	10	1.400	1.111	0.961	0.797	19.84	5.86	9	10	1007	112.3	6.65

Q91Z96	BMP-2-inducible protein kinase OS=Mus musculus GN=Bmp2k PE=1 SV=1 - [BMP2K_MOUSE]	5.89	2	4	6	10	1.130	0.724	0.890	0.797	24.18	5.89	9	10	1138	126.1	6.80
Q5RKR3	Immunoglobulin superfamily containing leucine-rich repeat protein 2 OS=Mus musculus GN=Islr2 PE=1 SV=1 - [ISLR2_MOUSE]	16.11	2	9	10	27	1.064	1.066	1.017	0.797	59.76	16.11	14	27	745	79.7	5.49
Q920A5	Retinoid-inducible serine carboxypeptidase OS=Mus musculus GN=Scpep1 PE=2 SV=2 - [RISC_MOUSE]	8.19	1	4	4	9	1.671	1.132	0.720	0.797	23.86	8.19	6	9	452	50.9	5.66
O55187-2	Isoform 2 of E3 SUMO-protein ligase CBX4 OS=Mus musculus GN=Cbx4 -	4.74	2	1	1	2	0.827	1.088	1.398	0.797	0.00	4.74	1	2	485	52.5	9.35
Q9JK48-2	Isoform 2 of Endophilin-B1 OS=Mus musculus GN=Sh3glb1 - [SHLB1_MOUSE]	30.57	2	10	11	54	1.188	1.235	1.063	0.797	142.68	30.57	20	54	386	43.2	5.68
Q3UU56	General transcription factor 3C polypeptide 2 OS=Mus musculus GN=Gtf3c2 PE=2 SV=1 - [Q3UU56_MOUSE]	4.96	5	3	3	4	0.694	0.522	1.312	0.797	10.97	4.96	4	4	907	100.2	6.76
Q5SS00	DBF4-type zinc finger-containing protein 2 homolog OS=Mus musculus GN=Zdbf2 PE=2 SV=1 - [ZDBF2_MOUSE]	7.94	2	16	16	35	1.272	1.029	0.639	0.797	85.00	7.94	25	35	2493	273.6	5.22
Q6PDM2-3	Isoform 3 of Serine/arginine-rich splicing factor 1 OS=Mus musculus GN=Srsf1 -	46.94	6	10	10	126	1.053	0.738	0.874	0.797	391.89	46.94	19	126	196	21.8	8.69
E0CXE3	Contactin-6 OS=Mus musculus GN=Cntn6 PE=2 SV=1 - [E0CXE3_MOUSE]	18.41	3	13	13	33	0.946	0.918	0.714	0.797	96.23	18.41	23	33	956	105.5	6.58
P45878	Peptidyl-prolyl cis-trans isomerase FKBP2 OS=Mus musculus GN=Fkbp2 PE=1 SV=1 - [FKBP2_MOUSE]	49.29	1	9	9	37	1.643	0.846	1.003	0.798	115.28	49.29	16	37	140	15.3	8.88
P25444	40S ribosomal protein S2 OS=Mus musculus GN=Rps2 PE=1 SV=3 - [RS2_MOUSE]	62.46	11	20	20	91	0.552	0.665	0.729	0.798	234.50	62.46	34	91	293	31.2	10.24
E9PV44	ATPase inhibitor, mitochondrial OS=Mus musculus GN=Atpif1 PE=2 SV=1 - [E9PV44_MOUSE]	48.65	2	8	8	60	1.816	0.786	1.158	0.798	125.05	48.65	13	60	74	8.8	9.31
B0F2B4	Neuroligin 4-like OS=Mus musculus GN=Nlgn4l PE=1 SV=1 - [NLGN4_MOUSE]	18.10	1	7	9	22	0.760	1.004	1.015	0.798	70.19	18.10	13	22	945	97.3	6.32
P62983	Ubiquitin-40S ribosomal protein S27a OS=Mus musculus GN=Rps27a PE=1 SV=2 - [RS27A_MOUSE]	66.67	7	2	13	387	0.843	1.036	0.790	0.798	945.85	66.67	25	387	156	17.9	9.64
Q9D1R9	60S ribosomal protein L34 OS=Mus musculus GN=Rpl34 PE=3 SV=2 - [RL34_MOUSE]	38.46	2	7	7	34	0.475	0.655	0.731	0.798	94.87	38.46	14	34	117	13.3	11.47
Q9EST5-2	Isoform 2 of Acidic leucine-rich nuclear phosphoprotein 32 family member B OS=Mus musculus GN=Anp32b - [illegible]	24.92	2	5	9	57	1.261	1.274	0.748	0.798	157.98	24.92	15	57	329	37.4	4.18
Q8K0V2	DCN1-like protein 3 OS=Mus musculus GN=Dcun1d3 PE=2 SV=1 - [DCNL3_MOUSE]	25.66	3	6	6	7	2.288	1.308	0.954	0.798	19.59	25.66	7	7	304	34.4	5.12
Q6PGH0	Ubiquitin domain-containing protein 2 OS=Mus musculus GN=Ubtd2 PE=2 SV=1 -	32.91	2	7	7	26	1.320	0.859	1.028	0.798	68.41	32.91	12	26	234	26.1	5.83
Q7JJ13-2	Isoform 2 of Bromodomain containing protein 2 OS=Mus musculus GN=Brd2 - [BRD2_MOUSE]	10.24	3	6	8	14	0.676	0.920	0.792	0.798	32.78	10.24	11	14	752	83.2	9.11
P61620	Protein transport protein Sec61 subunit alpha isoform 1 OS=Mus musculus GN=Sec61a1 PE=2 SV=2 - [illegible]	13.87	2	4	7	27	0.797	0.982	0.677	0.798	64.80	13.87	13	27	476	52.2	8.06
Q9EQW6	Oligodendrocyte transcription factor 2 OS=Mus musculus GN=Olig2 PE=1 SV=1 -	5.88	1	1	1	3	1.100	0.247	0.775	0.799	8.61	5.88	2	3	323	32.4	9.13
Q9CX66	Uncharacterized protein C12orf45 homolog OS=Mus musculus GN=D10Wsu102e PE=2 SV=1 - [CL045_MOUSE]	11.35	1	2	2	3	1.422	0.852	1.026	0.799	8.62	11.35	3	3	185	20.0	4.84
Q7TNC4-2	Isoform 2 of Putative RNA-binding protein Luc7-like 2 OS=Mus musculus GN=Luc7l2 - [LC7L2_MOUSE]	27.08	7	5	8	36	0.796	0.927	0.778	0.799	95.39	27.08	15	36	325	38.6	9.91
Q7TNB5	Glutamate receptor 1 OS=Mus musculus GN=Gria1 PE=2 SV=1 - [Q7TNB5_MOUSE]	34.29	1	1	28	122	0.526	1.775	0.942	0.799	337.23	34.29	53	122	907	101.5	7.53

Q9CZ00-2	Isoform 2 of Dysbindin domain-containing protein 1 OS=Mus musculus GN=Dbndd1 - [DBND1_MOUSE]	65.49	8	4	4	14	2.139	0.737	0.872	0.799	49.00	65.49	5	14	113	12.5	4.91
Q925F2	Endothelial cell-selective adhesion molecule OS=Mus musculus GN=Esam PE=1 SV=1 - [ESAM_MOUSE]	16.50	2	4	4	8	1.050	1.057	0.854	0.799	23.31	16.50	8	8	394	41.8	9.26
Q8BV13	COP9 signalosome complex subunit 7b OS=Mus musculus GN=Cops7b PE=1 SV=1 -	23.11	1	4	4	14	1.096	0.949	0.957	0.799	52.85	23.11	5	14	264	29.7	6.32
P26043	Radixin OS=Mus musculus GN=Rdx PE=1 SV=3 - [RADI_MOUSE]	42.54	2	19	29	149	1.214	0.377	0.773	0.799	392.54	42.54	53	149	583	68.5	6.20
Q3V038	Tetratricopeptide repeat protein 9A OS=Mus musculus GN=Ttc9 PE=2 SV=1 - [TTC9A_MOUSE]	51.14	1	9	10	37	1.664	0.548	0.845	0.799	102.59	51.14	19	37	219	24.3	8.85
Q62018	RNA polymerase-associated protein CTR9 homolog OS=Mus musculus GN=Ctr9 PE=1 SV=2 - [CTR9_MOUSE]	2.39	9	2	3	9	0.733	1.270	0.935	0.799	14.35	2.39	5	9	1173	133.3	6.49
P29351	Tyrosine-protein phosphatase non-receptor type 6 OS=Mus musculus GN=Ptpn6 PE=1 SV=2 - [PTN6_MOUSE]	13.95	3	7	7	15	1.214	0.979	0.716	0.800	35.18	13.95	11	15	595	67.5	7.81
F6W8T3	E3 ubiquitin-protein ligase MARCH5 (Fragment) OS=Mus musculus GN=March5 PE=4 SV=1 - [F6W8T3_MOUSE]	12.30	1	1	1	1	2.100	1.487	1.193	0.800	2.89	12.30	1	1	187	20.9	9.35
A2API3	Integrator complex subunit 8 OS=Mus musculus GN=Ints8 PE=2 SV=1 - [A2API3_MOUSE]	2.04	2	1	1	2	1.715	1.229	0.947	0.800	2.58	2.04	1	2	978	111.3	6.71
O55047-2	Isoform 2 of Serine/threonine-protein kinase tousled-like 2 OS=Mus musculus GN=Tlk2 - [TLK2_MOUSE]	11.91	12	4	8	17	1.089	0.748	0.889	0.800	42.95	11.91	14	17	697	79.6	8.57
Q9D1Z3	Protein FAM173B OS=Mus musculus GN=Fam173b PE=2 SV=1 - [F173B_MOUSE]	9.31	1	1	2	4	1.061	0.482	0.716	0.800	8.36	9.31	3	4	247	27.4	8.75
Q8K4Z5	Splicing factor 3A subunit 1 OS=Mus musculus GN=Sf3a1 PE=1 SV=1 - [SF3A1_MOUSE]	36.41	1	24	25	100	1.257	0.997	0.907	0.800	282.79	36.41	44	100	791	88.5	5.22
P09925	Surfeit locus protein 1 OS=Mus musculus GN=Surf1 PE=2 SV=3 - [SURF1_MOUSE]	17.32	2	4	4	16	0.863	0.896	0.760	0.800	38.47	17.32	7	16	306	34.8	9.72
Q91YP2	Neurolysin, mitochondrial OS=Mus musculus GN=Nln PE=2 SV=1 - [NEUL_MOUSE]	39.63	1	26	26	71	1.179	0.964	0.849	0.800	208.12	39.63	41	71	704	80.4	6.44
Q5DTM8	E3 ubiquitin-protein ligase BRE1A OS=Mus musculus GN=Rnf20 PE=1 SV=2 - [BRE1A_MOUSE]	14.39	7	12	12	35	1.202	0.708	1.021	0.800	91.33	14.39	20	35	973	113.5	5.96
Q8C8U0	Liprin-beta-1 OS=Mus musculus GN=Ppfibp1 PE=1 SV=3 - [LIPB1_MOUSE]	8.67	4	6	7	14	1.207	1.015	0.815	0.801	39.83	8.67	12	14	969	108.5	5.49
Q91ZV7	Plexin domain-containing protein 1 OS=Mus musculus GN=Plxdc1 PE=1 SV=2 -	4.00	3	2	2	4	0.555	0.749	0.761	0.801	11.64	4.00	3	4	500	55.6	6.62
Q8BU14	Translocation protein SEC62 OS=Mus musculus GN=Sec62 PE=1 SV=1 - [SEC62_MOUSE]	11.06	1	4	4	14	1.300	0.583	0.765	0.801	26.44	11.06	8	14	398	45.6	7.31
Q91XX4	MCG133388, isoform CRA_n OS=Mus musculus GN=Pcdhgb6 PE=2 SV=1 - [Q91XX4_MOUSE]	5.27	1	2	4	22	1.387	0.985	0.817	0.801	56.75	5.27	7	22	930	100.6	5.34
P08414	Calcium/calmodulin-dependent protein kinase type IV OS=Mus musculus GN=Camk4 PE=1 SV=2 - [KCC4_MOUSE]	35.39	2	14	15	51	0.565	1.121	1.570	0.801	161.54	35.39	24	51	469	52.6	4.93
O08600	Endonuclease G, mitochondrial OS=Mus musculus GN=Endog PE=2 SV=1 - [NUCG_MOUSE]	26.19	1	7	7	16	1.149	0.441	0.787	0.801	41.39	26.19	11	16	294	32.2	9.54
Q6V4S5	Protein sidekick-2 OS=Mus musculus GN=Sdk2 PE=2 SV=1 - [SDK2_MOUSE]	16.59	3	18	29	64	0.727	0.590	0.692	0.801	179.34	16.59	46	64	2176	239.8	7.36
D3Z4U5	Janus kinase and microtubule-interacting protein 1 OS=Mus musculus GN=Jakmip1 PE=2 SV=1 - [D3Z4U5_MOUSE]	27.32	6	2	21	76	0.462	0.814	0.885	0.801	209.56	27.32	36	76	831	97.2	5.80
Q69ZS0-2	Isoform 2 of E3 ubiquitin-protein ligase PDZRN3 OS=Mus musculus GN=Pdzrn3 -	4.75	4	3	4	7	0.854	2.243	1.524	0.801	15.02	4.75	6	7	1011	113.5	5.83
Q60902-4	Isoform 4 of Epidermal growth factor receptor substrate 15-like 1 OS=Mus musculus GN=Eps15l1 - [EP15R_MOUSE]	63.70	1	1	43	251	1.345	1.288	1.087	0.802	721.92	63.70	74	251	763	84.7	5.02

Q6NZN0-5	Isoform 5 of RNA-binding protein 26 OS=Mus musculus GN=Rbm26 - [RBM26_MOUSE]	21.33	8	18	19	39	1.296	0.748	0.916	0.802	108.35	21.33	27	39	989	111.2	9.44
Q9D8X2	Coiled-coil domain-containing protein 124 OS=Mus musculus GN=Ccdc124 PE=2 SV=1 - [CC124_MOUSE]	34.56	1	6	8	34	1.809	0.662	1.202	0.802	89.56	34.56	13	34	217	25.3	9.64
Q8K2H2	OTU domain-containing protein 6B OS=Mus musculus GN=Otud6b PE=2 SV=1 -	35.37	3	6	7	20	1.072	0.475	1.011	0.802	64.88	35.37	12	20	294	33.7	5.53
Q02496	Mucin-1 OS=Mus musculus GN=Muc1 PE=1 SV=2 - [MUC1_MOUSE]	2.38	1	1	1	1	1.766	0.912	0.870	0.802	2.88	2.38	1	1	630	64.5	5.67
O70250	Phosphoglycerate mutase 2 OS=Mus musculus GN=Pgam2 PE=1 SV=3 - [PGAM2_MOUSE]	50.59	1	7	12	204	2.247	2.290	0.896	0.802	662.26	50.59	23	204	253	28.8	8.50
P02802	Metallothionein-1 OS=Mus musculus GN=Mt1 PE=1 SV=1 - [MT1_MOUSE]	13.11	1	1	1	2	1.471	0.996	1.053	0.802	7.64	13.11	1	2	61	6.0	7.96
Q9DB34	Charged multivesicular body protein 2a OS=Mus musculus GN=Chmp2a PE=1 SV=1 -	25.23	5	7	8	28	0.754	0.868	0.835	0.802	70.78	25.23	14	28	222	25.1	5.97
O08796	Eukaryotic elongation factor 2 kinase OS=Mus musculus GN=Eef2k PE=1 SV=1 - [EF2K_MOUSE]	4.70	2	3	4	7	0.947	1.164	0.729	0.802	15.90	4.70	6	7	724	81.7	5.38
P52483	Ubiquitin-conjugating enzyme E2 E3 OS=Mus musculus GN=Ube2e3 PE=1 SV=2 -	30.92	6	3	4	11	0.624	0.785	0.814	0.803	26.86	30.92	7	11	207	22.9	7.18
D3YUE3	Lysine-specific demethylase 2B (Fragment) OS=Mus musculus GN=Kdm2b PE=2 SV=1 - [illegible]	1.17	6	1	1	1	0.744	1.026	0.876	0.803	2.06	1.17	1	1	514	59.7	5.06
P63242	Eukaryotic translation initiation factor 5A-1 OS=Mus musculus GN=Eif5a PE=1 SV=2 - [IF5A1_MOUSE]	68.83	2	5	11	173	1.487	0.844	1.170	0.803	539.74	68.83	21	173	154	16.8	5.24
Q6ZWZ6	40S ribosomal protein S12 OS=Mus musculus GN=Rps12 PE=2 SV=1 - [Q6ZWZ6_MOUSE]	53.79	2	6	6	65	1.224	0.777	0.971	0.803	191.51	53.79	11	65	132	14.5	7.21
Q9D0Y8-2	Isoform 2 of 39S ribosomal protein L52, mitochondrial OS=Mus musculus GN=Mrpl52 - [RM52_MOUSE]	11.11	2	1	1	1	1.079	0.837	0.960	0.803	0.00	11.11	1	1	63	7.5	11.03
Q3U6Q4-3	Isoform 3 of Phosphoinositide 3-kinase regulatory subunit 6 OS=Mus musculus GN=Pik3r6 - [illegible]	6.56	3	1	1	12	1.421	1.629	0.853	0.803	0.00	6.56	1	12	305	34.3	7.44
O88693	Ceramide glucosyltransferase OS=Mus musculus GN=Ugcg PE=2 SV=1 -	4.31	1	2	2	3	1.742	0.589	1.517	0.803	2.33	4.31	2	3	394	44.8	7.80
Q8VE97	Serine/arginine-rich splicing factor 4 OS=Mus musculus GN=Srsf4 PE=2 SV=1 - [SRSF4_MOUSE]	13.91	3	6	8	31	0.920	0.795	0.897	0.803	77.20	13.91	13	31	489	55.9	11.40
E9QNK8	Kv channel-interacting protein 2 OS=Mus musculus GN=Kcnip2 PE=2 SV=1 -	27.78	11	2	5	13	1.909	2.093	1.538	0.803	38.29	27.78	8	13	252	29.0	4.91
A2AKA9	Bromodomain-containing protein 3 (Fragment) OS=Mus musculus GN=Brd3 PE=4 SV=1 - [A2AKA9_MOUSE]	9.68	3	4	6	17	0.582	0.785	0.718	0.803	39.54	9.68	10	17	682	75.3	9.29
Q05DI3	Stathmin OS=Mus musculus GN=Stmn4 PE=2 SV=1 - [Q05DI3_MOUSE]	11.36	4	2	2	10	1.463	0.843	0.794	0.803	16.22	11.36	4	10	176	20.4	5.73
Q80YP0	Cyclin-dependent kinase 3 OS=Mus musculus GN=Cdk3 PE=1 SV=2 - [CDK3_MOUSE]	6.93	15	1	2	22	1.445	0.956	0.953	0.803	40.43	6.93	3	22	303	33.9	9.00
Q9DBG9	Tax1-binding protein 3 OS=Mus musculus GN=Tax1bp3 PE=1 SV=1 - [TX1B3_MOUSE]	28.23	1	2	2	4	2.243	1.105	0.858	0.804	13.32	28.23	3	4	124	13.7	8.48
E9QKY7	Transcription factor 20 OS=Mus musculus GN=Tcf20 PE=2 SV=1 - [E9QKY7_MOUSE]	16.13	4	19	20	61	0.997	0.891	1.321	0.804	172.00	16.13	31	61	1965	213.4	9.03
Q80WW9	DDRGK domain-containing protein 1 OS=Mus musculus GN=Ddrgk1 PE=1 SV=2 -	9.84	1	2	2	4	0.632	0.662	0.740	0.804	14.12	9.84	4	4	315	36.0	5.35
F8WI14	Extracellular matrix protein 1 OS=Mus musculus GN=Ecm1 PE=2 SV=1 - [F8WI14_MOUSE]	7.89	8	4	4	7	1.820	1.267	1.103	0.804	19.85	7.89	7	7	558	62.7	6.89
Q8K114-2	Isoform 2 of Integrator complex subunit 9 OS=Mus musculus GN=Ints9 -	2.08	2	1	1	2	0.485	0.781	0.885	0.804	7.98	2.08	2	2	576	64.7	6.84

Q9DAW6	U4/U6 small nuclear ribonucleoprotein Prp4 OS=Mus musculus GN=Prpf4 PE=2 SV=1 - [PRP4_MOUSE]	18.81	1	7	7	21	0.952	0.818	0.720	0.804	80.68	18.81	12	21	521	58.3	7.28
Q7M6Y3-5	Isoform 5 of Phosphatidylinositol-binding clathrin assembly protein OS=Mus musculus GN=Picalm - [illegible]	33.44	3	1	17	122	0.765	1.007	1.131	0.804	311.81	33.44	29	122	655	70.9	8.37
Q9QXK7	Cleavage and polyadenylation specificity factor subunit 3 OS=Mus musculus GN=Cpsf3 PE=1 SV=2 - [CPSF3_MOUSE]	11.70	1	7	8	15	0.579	0.916	0.691	0.804	40.98	11.70	13	15	684	77.5	5.60
P10639	Thioredoxin OS=Mus musculus GN=Txn PE=1 SV=3 - [THIO_MOUSE]	65.71	1	8	8	106	1.750	0.882	0.984	0.804	245.63	65.71	14	106	105	11.7	4.92
Q9Z2L6	Multiple inositol polyphosphate phosphatase 1 OS=Mus musculus GN=Minpp1 PE=1 SV=3 - [illegible]	18.50	1	8	8	14	1.529	0.895	0.734	0.805	37.91	18.50	12	14	481	54.5	7.49
Q80VM8	PNMA-like protein 1 OS=Mus musculus GN=Pnmal1 PE=2 SV=1 - [PNML1_MOUSE]	8.60	1	2	2	2	2.584	1.067	1.369	0.805	4.60	8.60	2	2	430	48.0	9.45
P01820	Ig heavy chain V region PJ14 OS=Mus musculus PE=1 SV=1 - [HVM44_MOUSE]	5.22	2	1	1	1	0.982	1.033	0.750	0.805	1.97	5.22	1	1	115	12.4	8.16
Q6PIX5	Inactive rhomboid protein 1 OS=Mus musculus GN=Rhbdf1 PE=1 SV=2 - [RHDF1_MOUSE]	6.89	5	4	4	8	1.437	0.694	1.006	0.805	23.55	6.89	7	8	856	97.2	8.59
O08795-2	Isoform 2 of Glucosidase 2 subunit beta OS=Mus musculus GN=Prkcsh - [GLU2B_MOUSE]	28.03	2	14	14	132	1.854	0.965	1.113	0.805	380.92	28.03	26	132	528	59.5	4.48
Q6PIU9	Uncharacterized protein FLJ45252 homolog OS=Mus musculus PE=1 SV=2 - [YJ005_MOUSE]	37.29	1	10	10	39	1.932	0.876	0.964	0.805	129.43	37.29	18	39	354	37.7	5.07
Q9D958	Signal peptidase complex subunit 1 OS=Mus musculus GN=Spcs1 PE=2 SV=3 - [SPCS1_MOUSE]	8.07	1	2	2	10	0.658	0.752	0.747	0.805	27.37	8.07	3	10	161	18.2	10.01
Q8R1F0	Leydig cell tumor 10 kDa protein homolog OS=Mus musculus GN=D8Ertd738e PE=2 SV=1 - [L10K_MOUSE]	25.53	1	3	3	5	0.787	0.612	0.967	0.805	14.48	25.53	4	5	94	10.2	11.63
Q8BKH7-2	Isoform 2 of Target of rapamycin complex 2 subunit MAPKAP1 OS=Mus musculus GN=Mapkap1 - [SIN1_MOUSE]	1.65	3	1	1	2	0.492	0.737	0.995	0.805	0.00	1.65	2	2	486	54.8	8.22
G3UYQ3	Phosphatidylserine synthase 2 OS=Mus musculus GN=Ptdss2 PE=4 SV=1 -	16.25	3	1	1	1	1.413	0.842	0.847	0.805	2.77	16.25	1	1	80	8.9	6.58
Q8CCS2	Transmembrane protein FAM155A OS=Mus musculus GN=Fam155a PE=2 SV=1 -	4.28	2	2	2	3	0.961	1.271	0.768	0.805	6.05	4.28	3	3	467	52.7	6.86
Q6P5C5	Single-strand selective monofunctional uracil DNA glycosylase OS=Mus musculus GN=Smug1 PE=2 SV=1 - [SMUG1_MOUSE]	21.15	1	4	4	9	1.131	2.292	1.054	0.805	36.64	21.15	7	9	279	30.6	6.77
H3BJU7	Rho guanine nucleotide exchange factor 2 OS=Mus musculus GN=Arhgef2 PE=2 SV=1 - [H3BJU7_MOUSE]	47.59	17	36	36	152	0.640	1.259	1.493	0.805	459.17	47.59	66	152	956	108.5	6.87
A6X8Z3	Insulin-like growth factor 2 mRNA-binding protein 2 OS=Mus musculus GN=Igf2bp2 PE=2 SV=1 - [A6X8Z3_MOUSE]	4.77	3	2	2	6	1.279	0.671	0.784	0.805	1.65	4.77	2	6	524	58.0	7.78
D3YZ05	Translation initiation factor eIF-2B subunit alpha (Fragment) OS=Mus musculus GN=Eif2b1 PE=2 SV=1 - [D3YZ05_MOUSE]	37.59	6	1	5	7	0.685	0.784	0.915	0.805	11.71	37.59	7	7	133	14.9	9.51
Q99J21	Mucolipin-1 OS=Mus musculus GN=Mcoln1 PE=1 SV=1 - [MCLN1_MOUSE]	9.31	3	3	3	18	0.896	1.185	0.939	0.806	25.30	9.31	4	18	580	65.5	7.65
Q6NVF9	Cleavage and polyadenylation specificity factor subunit 6 OS=Mus musculus GN=Cpsf6 PE=1 SV=1 - [CPSF6_MOUSE]	31.03	5	12	12	40	0.783	0.793	0.766	0.806	107.28	31.03	20	40	551	59.1	7.15
Q9WTR5	Cadherin-13 OS=Mus musculus GN=Cdh13 PE=1 SV=2 - [CAD13_MOUSE]	30.67	1	18	18	150	0.939	1.142	0.873	0.806	442.65	30.67	30	150	714	78.1	5.12
Q923S6-3	Isoform 3 of Neuralized-like protein 1A OS=Mus musculus GN=Neurl - [NEU1A_MOUSE]	7.34	3	1	1	4	2.207	1.570	0.792	0.806	13.13	7.34	1	4	218	24.1	9.82
Q2KN98	Cytospin-A OS=Mus musculus GN=Specc1l PE=1 SV=1 - [CYTSA_MOUSE]	15.92	1	16	16	47	1.269	0.767	1.044	0.806	156.67	15.92	28	47	1118	124.4	5.76

P30412	Peptidyl-prolyl cis-trans isomerase C OS=Mus musculus GN=Ppic PE=1 SV=1 - [PPIC_MOUSE]	27.83	1	5	6	80	1.508	1.217	0.947	0.806	166.57	27.83	8	80	212	22.8	7.50
Q8BMN4	Leishmanolysin-like peptidase OS=Mus musculus GN=Lmln PE=2 SV=1 - [LMLN_MOUSE]	6.31	1	4	4	6	2.615	1.039	0.661	0.806	12.05	6.31	5	6	681	76.5	7.28
O09167	60S ribosomal protein L21 OS=Mus musculus GN=Rpl21 PE=2 SV=3 - [RL21_MOUSE]	45.63	3	4	9	19	0.473	0.553	0.758	0.806	46.20	45.63	12	19	160	18.6	10.49
G8JL40	Lysine-specific histone demethylase 1A (Fragment) OS=Mus musculus GN=Kdm1a PE=4 SV=1 - [illegible]	8.35	5	6	6	11	0.509	0.784	0.732	0.806	25.25	8.35	9	11	683	75.5	6.80
Q80U78-2	Isoform 2 of Pumilio homolog 1 OS=Mus musculus GN=Pum1 - [PUM1_MOUSE]	15.18	7	11	15	66	1.062	1.055	0.986	0.807	175.28	15.18	25	66	1186	126.3	6.86
Q61559	IgG receptor FcRn large subunit p51 OS=Mus musculus GN=Fcgrt PE=1 SV=1 - [FCGRN_MOUSE]	7.95	1	3	3	7	1.039	1.567	0.936	0.807	21.15	7.95	6	7	365	40.1	5.29
Q3UGF1-3	Isoform 3 of WD repeat-containing protein 19 OS=Mus musculus GN=Wdr19 -	2.84	3	3	3	8	0.796	0.924	1.307	0.807	18.12	2.84	4	8	1021	115.1	6.29
Q8BTZ5	Ankyrin repeat domain-containing protein 46 OS=Mus musculus GN=Ankrd46 PE=2 SV=1 - [ANR46_MOUSE]	20.61	1	4	4	14	0.717	1.023	0.882	0.807	41.13	20.61	8	14	228	25.2	5.73
Q9D1C8	Vacuolar protein sorting-associated protein 28 homolog OS=Mus musculus GN=Vps28 PE=2 SV=1 - [VPS28_MOUSE]	71.04	1	13	13	52	1.443	1.187	0.837	0.807	136.53	71.04	20	52	221	25.4	5.54
O70433	Four and a half LIM domains protein 2 OS=Mus musculus GN=Fhl2 PE=1 SV=1 - [FHL2_MOUSE]	29.03	1	8	8	15	0.953	2.740	2.873	0.807	41.55	29.03	13	15	279	32.1	7.30
Q8CAK3	UPF0515 protein C19orf66 homolog OS=Mus musculus PE=2 SV=1 - [CS066_MOUSE]	35.86	2	10	10	42	1.425	0.613	1.015	0.807	111.27	35.86	18	42	290	33.0	7.28
P41731	CD63 antigen OS=Mus musculus GN=Cd63 PE=1 SV=2 - [CD63_MOUSE]	4.62	1	1	1	4	1.286	1.211	0.745	0.807	15.06	4.62	2	4	238	25.7	6.98
Q6ZPT1	Kelch-like protein 9 OS=Mus musculus GN=Klhl9 PE=2 SV=2 - [KLHL9_MOUSE]	5.67	6	3	3	5	1.249	0.912	0.898	0.807	9.64	5.67	5	5	617	69.4	6.44
Q61070	Etoposide-induced protein 2.4 OS=Mus musculus GN=Ei24 PE=1 SV=3 - [EI24_MOUSE]	6.47	1	3	3	7	0.551	0.630	0.826	0.807	16.57	6.47	4	7	340	38.9	9.72
Q3TDN0	Protein dispatched homolog 1 OS=Mus musculus GN=Disp1 PE=1 SV=2 - [DISP1_MOUSE]	4.93	1	4	4	6	1.111	0.161	0.983	0.808	3.40	4.93	4	6	1521	170.0	6.84
Q9WVH4	Forkhead box protein O3 OS=Mus musculus GN=Foxo3 PE=1 SV=1 - [FOXO3_MOUSE]	8.63	2	3	3	7	1.090	1.106	1.407	0.808	22.40	8.63	4	7	672	71.0	5.12
P70158	Acid sphingomyelinase-like phosphodiesterase 3a OS=Mus musculus GN=Smpdl3a PE=2 SV=2 - [ASM3A_MOUSE]	3.15	1	3	3	5	1.054	0.693	0.788	0.808	12.29	3.15	4	5	445	49.8	6.46
Q61749	Translation initiation factor eIF-2B subunit delta OS=Mus musculus GN=Eif2b4 PE=2 SV=2 - [EI2BD_MOUSE]	25.00	4	9	9	39	0.901	0.743	0.877	0.808	109.06	25.00	15	39	524	57.6	9.25
G3X9G4	Dynamin-2 OS=Mus musculus GN=Dnm2 PE=2 SV=1 - [G3X9G4_MOUSE]	46.90	6	1	42	367	3.468	1.026	0.881	0.808	921.31	46.90	75	367	870	98.0	7.44
O88745	Scrapie-responsive protein 1 OS=Mus musculus GN=Scrg1 PE=2 SV=1 - [SCRG1_MOUSE]	40.82	1	4	4	15	1.787	0.975	1.081	0.808	42.41	40.82	7	15	98	11.2	7.91
Q920L1	Fatty acid desaturase 1 OS=Mus musculus GN=Fads1 PE=2 SV=1 - [FADS1_MOUSE]	10.96	1	4	4	8	0.636	0.698	0.683	0.808	27.40	10.96	7	8	447	52.3	9.29
Q8CC21	Tetratricopeptide repeat protein 19, mitochondrial OS=Mus musculus GN=Ttc19 PE=1 SV=1 - [TTC19_MOUSE]	13.15	3	4	4	17	1.172	1.028	0.733	0.808	52.62	13.15	8	17	365	41.2	6.21
Q501J6	Probable ATP-dependent RNA helicase DDX17 OS=Mus musculus GN=Ddx17 PE=2 SV=1 - [DDX17_MOUSE]	42.92	3	20	29	203	0.751	0.606	0.745	0.808	605.36	42.92	48	203	650	72.4	8.59
Q01341	Adenylate cyclase type 6 OS=Mus musculus GN=Adcy6 PE=1 SV=1 - [ADCY6_MOUSE]	8.93	2	6	10	36	0.769	1.287	0.784	0.809	93.97	8.93	19	36	1165	130.2	7.87
P49935	Pro-cathepsin H OS=Mus musculus GN=Ctsh PE=2 SV=2 - [CATH_MOUSE]	9.31	2	2	2	6	1.349	1.352	0.872	0.809	24.48	9.31	4	6	333	37.1	8.40

O70200	Allograft inflammatory factor 1 OS=Mus musculus GN=Aif1 PE=1 SV=1 - [AIF1_MOUSE]	49.66	4	7	8	20	1.731	1.148	1.112	0.809	59.10	49.66	13	20	147	16.9	8.76
Q8R480	Nuclear pore complex protein Nup85 OS=Mus musculus GN=Nup85 PE=1 SV=1 - [NUP85_MOUSE]	4.57	1	2	2	4	0.710	1.173	0.816	0.809	9.00	4.57	4	4	656	74.7	5.57
Q8QZY9	Splicing factor 3B subunit 4 OS=Mus musculus GN=Sf3b4 PE=2 SV=1 - [SF3B4_MOUSE]	10.61	1	3	3	17	1.567	0.921	1.025	0.809	42.17	10.61	5	17	424	44.3	8.56
J3QJZ3	MCG116065 OS=Mus musculus GN=Gm6472 PE=4 SV=1 - [J3QJZ3_MOUSE]	39.69	3	7	7	34	0.596	0.725	0.717	0.809	95.52	39.69	12	34	194	22.1	10.10
Q9Z1Z2	Serine-threonine kinase receptor-associated protein OS=Mus musculus GN=Strap PE=1 SV=2 - [STRAP_MOUSE]	56.29	1	16	16	84	1.639	0.914	0.790	0.809	271.78	56.29	29	84	350	38.4	5.12
P70318	Nucleolysin TIAR OS=Mus musculus GN=Tial1 PE=2 SV=1 - [TIAR_MOUSE]	27.30	4	6	10	40	1.340	1.016	0.762	0.809	120.48	27.30	17	40	392	43.4	7.99
P62204	Calmodulin OS=Mus musculus GN=Calm1 PE=1 SV=2 - [CALM_MOUSE]	82.55	6	12	13	1097	1.140	0.993	1.429	0.809	2702.47	82.55	21	1097	149	16.8	4.22
Q9ESD7-3	Isoform 3 of Dysferlin OS=Mus musculus GN=Dysf - [DYSF_MOUSE]	3.53	6	4	5	10	1.100	0.873	0.773	0.809	25.37	3.53	7	10	2069	235.8	5.78
G3UX23	Bifunctional UDP-N-acetylglucosamine 2-epimerase/N-acetylmannosamine kinase OS=Mus musculus GN=Gne PE=2 SV=1 - [G3UX23_MOUSE]	6.02	7	3	3	4	0.856	1.014	0.673	0.809	5.27	6.02	4	4	648	71.2	6.90
Q6ZPZ3-2	Isoform 2 of Zinc finger CCCH domain-containing protein 4 OS=Mus musculus GN=Zc3h4 - [ZC3H4_MOUSE]	8.35	3	6	6	22	1.544	1.057	1.103	0.809	58.64	8.35	12	22	1221	131.5	6.52
Q64327	Male-enhanced antigen 1 OS=Mus musculus GN=Mea1 PE=2 SV=1 - [MEA1_MOUSE]	11.49	1	2	2	5	1.628	0.968	1.022	0.809	16.02	11.49	3	5	174	18.6	4.08
Q63871	DNA-directed RNA polymerases I, II, and III subunit RPABC4 OS=Mus musculus GN=Polr2k PE=2 SV=2 - [RPAB4_MOUSE]	12.07	2	1	1	7	1.502	0.735	1.046	0.809	15.41	12.07	2	7	58	7.0	9.06
Q9CRD4	Dysbindin domain-containing protein 2 OS=Mus musculus GN=Dbndd2 PE=1 SV=1 - [DBND2_MOUSE]	7.59	1	1	1	7	2.717	1.008	1.331	0.809	15.87	7.59	2	7	158	17.2	4.22
Q6EDY6	Leucine-rich repeat-containing protein 16A OS=Mus musculus GN=Lrrc16a PE=1 SV=2 - [LR16A_MOUSE]	21.83	5	24	27	79	0.817	0.532	0.677	0.809	236.55	21.83	48	79	1374	151.8	7.80
Q8R554	OTU domain-containing protein 7A OS=Mus musculus GN=Otud7a PE=2 SV=1 -	15.44	2	7	12	39	1.127	0.971	1.122	0.810	99.60	15.44	20	39	926	100.7	8.22
Q8CD10	Calcium uptake protein 2, mitochondrial OS=Mus musculus GN=Micu2 PE=1 SV=2 - [MICU2_MOUSE]	5.32	1	2	2	3	1.263	1.083	0.799	0.810	8.84	5.32	3	3	432	49.4	9.45
Q6DID7	Protein wntless homolog OS=Mus musculus GN=Wls PE=1 SV=1 - [WLS_MOUSE]	7.02	2	4	4	11	0.859	1.054	0.904	0.810	30.02	7.02	6	11	541	62.1	7.65
Q7TME0	Lipid phosphate phosphatase-related protein type 4 OS=Mus musculus GN=Lppr4 PE=1 SV=2 - [LPPR4_MOUSE]	35.77	1	20	20	89	0.634	1.762	1.644	0.810	255.83	35.77	38	89	766	83.2	8.84
E9Q944	Protein Kank1 (Fragment) OS=Mus musculus GN=Kank1 PE=2 SV=1 - [E9Q944_MOUSE]	6.45	2	5	6	14	1.436	0.771	0.710	0.810	32.50	6.45	9	14	1147	126.0	5.63
E9Q9C7	Actin-binding LIM protein 1 OS=Mus musculus GN=Ablim1 PE=2 SV=1 - [E9Q9C7_MOUSE]	45.21	6	1	25	139	1.754	0.950	1.335	0.810	353.52	45.21	47	139	668	75.2	8.09
E9Q842	Protein Nav2 OS=Mus musculus GN=Nav2 PE=2 SV=1 - [E9Q842_MOUSE]	3.74	2	5	7	20	0.845	0.592	0.934	0.810	53.55	3.74	11	20	2432	261.5	8.95
Q63ZW7-2	Isoform 2 of InaD-like protein OS=Mus musculus GN=Inadl - [INADL_MOUSE]	12.69	7	9	11	25	1.562	0.305	0.656	0.810	67.14	12.69	15	25	1261	136.5	4.83
Q3UHX0	Nucleolar protein 8 OS=Mus musculus GN=Nol8 PE=1 SV=2 - [NOL8_MOUSE]	4.36	2	4	5	10	0.875	0.726	1.020	0.810	24.76	4.36	8	10	1147	128.6	6.55
Q8BLA8	Transient receptor potential cation channel subfamily A member 1 OS=Mus musculus GN=Trpa1 PE=1 SV=1 - [TRPA1_MOUSE]	1.42	1	1	2	4	0.547	0.709	0.698	0.810	2.35	1.42	2	4	1125	128.4	7.31

Q9CQB2	Protein FAM195A OS=Mus musculus GN=Fam195a PE=1 SV=1 - [F195A_MOUSE]	34.38	1	2	2	5	0.958	0.558	1.226	0.811	8.49	34.38	3	5	160	17.9	7.30
Q8C0Q5	Protein Zfp317 OS=Mus musculus GN=Zfp317 PE=2 SV=1 - [Q8C0Q5_MOUSE]	2.47	1	1	1	1	0.726	1.278	0.867	0.811	0.00	2.47	1	1	607	69.5	8.78
Q923D5	WW domain-binding protein 11 OS=Mus musculus GN=Wbp11 PE=1 SV=2 -	17.00	1	10	10	24	0.713	0.829	1.089	0.811	67.95	17.00	15	24	641	69.8	8.40
G5E8V9	MCG18094 (Fragment) OS=Mus musculus GN=Arfip1 PE=4 SV=1 - [G5E8V9_MOUSE]	24.13	4	7	8	24	0.777	1.451	1.032	0.811	64.65	24.13	13	24	373	41.5	6.76
P55821	Stathmin-2 OS=Mus musculus GN=Stmn2 PE=1 SV=1 - [STMN2_MOUSE]	43.58	1	5	8	103	1.171	0.968	0.840	0.811	222.60	43.58	14	103	179	20.8	8.32
Q8VHY0	Chondroitin sulfate proteoglycan 4 OS=Mus musculus GN=Cspg4 PE=1 SV=3 - [CSPG4_MOUSE]	29.65	3	46	46	173	1.026	0.922	0.791	0.811	503.65	29.65	85	173	2327	252.2	5.44
Q80T85	DDB1- and CUL4-associated factor 5 OS=Mus musculus GN=Dcaf5 PE=1 SV=2 -	8.88	1	7	7	15	1.369	0.840	0.811	0.811	37.03	8.88	11	15	946	103.6	5.80
Q62418-3	Isoform 3 of Drebrin-like protein OS=Mus musculus GN=Dbnl - [DBNL_MOUSE]	45.37	3	15	15	214	1.640	1.224	1.279	0.811	669.71	45.37	29	214	432	48.3	4.92
Q05D44	Eukaryotic translation initiation factor 5B OS=Mus musculus GN=Eif5b PE=1 SV=2 - [IF2P_MOUSE]	19.08	1	19	19	48	0.968	0.864	0.840	0.811	142.54	19.08	32	48	1216	137.5	5.59
Q6ZWN5	40S ribosomal protein S9 OS=Mus musculus GN=Rps9 PE=2 SV=3 - [RS9_MOUSE]	41.24	6	11	12	74	0.396	0.448	0.657	0.811	165.99	41.24	23	74	194	22.6	10.65
Q9Z0L8-2	Isoform II of Gamma-glutamyl hydrolase OS=Mus musculus GN=Ggh - [GGH_MOUSE]	5.71	2	1	1	5	1.532	1.237	0.679	0.811	18.70	5.71	2	5	315	35.4	8.29
Q9D8N1	Uncharacterized protein C11orf24 homolog OS=Mus musculus PE=2 SV=1 - [CK024_MOUSE]	12.50	1	2	3	4	2.640	1.235	0.965	0.811	4.51	12.50	3	4	392	41.1	8.15
P0C192	Leucine-rich repeat-containing protein 4B OS=Mus musculus GN=Lrrc4b PE=1 SV=1 - [LRC4B_MOUSE]	31.03	3	12	14	37	0.906	1.691	0.982	0.811	109.26	31.03	25	37	709	76.1	7.24
E9Q5M4	Protein Zfr2 OS=Mus musculus GN=Zfr2 PE=2 SV=1 - [E9Q5M4_MOUSE]	11.33	2	8	8	21	0.763	0.476	0.665	0.811	48.60	11.33	11	21	874	94.9	9.51
B1ARW8	Uncharacterized protein C1orf122 homolog OS=Mus musculus PE=2 SV=1 - [CA122_MOUSE]	33.64	2	3	3	11	1.601	0.991	1.009	0.812	34.11	33.64	5	11	110	11.3	8.21
Q91WK2	Eukaryotic translation initiation factor 3 subunit H OS=Mus musculus GN=Eif3h PE=1 SV=1 - [EIF3H_MOUSE]	36.36	1	12	12	29	0.877	0.928	0.748	0.812	70.43	36.36	20	29	352	39.8	6.67
Q8BI72	CDKN2A-interacting protein OS=Mus musculus GN=Cdkn2aip PE=2 SV=1 [CARF_MOUSE]	20.07	1	9	10	20	0.842	0.942	0.940	0.812	62.55	20.07	17	20	563	59.7	9.16
Q8R0J2	Mannose-P-dolichol utilization defect 1 OS=Mus musculus GN=Mpdu1 PE=2 SV=1 - [Q8R0J2_MOUSE]	8.10	5	2	2	4	0.443	0.870	0.644	0.812	11.19	8.10	4	4	247	26.4	8.54
Q9CR76	Transmembrane protein 186 OS=Mus musculus GN=Tmem186 PE=2 SV=2 - [TM186_MOUSE]	22.22	1	3	3	3	1.222	0.992	0.755	0.812	7.98	22.22	3	3	216	24.6	10.11
Q8K377	Leucine-rich repeat transmembrane neuronal protein 1 OS=Mus musculus GN=Lrrtm1 PE=2 SV=1 - [illegible]	9.58	2	7	7	30	0.538	1.261	0.821	0.812	75.39	9.58	13	30	522	58.7	7.11
B2RSI6	Leucine rich repeat containing 8 family, member B OS=Mus musculus GN=Lrrc8b PE=2 SV=1 - [B2RSI6_MOUSE]	8.84	2	6	6	16	4.271	1.880	1.353	0.812	46.75	8.84	8	16	803	92.1	6.70
P52194	Calmegin OS=Mus musculus GN=Clgn PE=2 SV=2 - [CLGN_MOUSE]	11.29	1	4	5	11	1.198	0.906	1.254	0.812	23.32	11.29	7	11	611	69.4	4.68
P70362	Ubiquitin fusion degradation protein 1 homolog OS=Mus musculus GN=Ufd1l PE=1 SV=2 - [UFD1_MOUSE]	26.38	2	7	7	30	0.920	0.761	0.808	0.812	77.07	26.38	13	30	307	34.5	6.70
Q3UFS4	G patch domain-containing protein 11 OS=Mus musculus GN=Gpatch11 PE=2 SV=2 - [GPT11_MOUSE]	9.92	1	2	3	4	1.226	0.626	0.919	0.812	6.34	9.92	4	4	262	30.6	5.08
Q9CXG3	Peptidyl-prolyl cis-trans isomerase-like 4 OS=Mus musculus GN=Ppil4 PE=2 SV=2 - [PPIL4_MOUSE]	6.30	2	2	2	5	0.951	1.080	1.010	0.812	12.87	6.30	3	5	492	57.2	6.11

F6YIY0	Protein YIPF3 (Fragment) OS=Mus musculus GN=Yipf3 PE=4 SV=1 - [F6YIY0_MOUSE]	7.96	3	2	2	5	0.771	1.045	0.601	0.812	9.10	7.96	3	5	201	22.4	8.47
Q7TQE6	Macoilin OS=Mus musculus GN=Tmem57 PE=2 SV=1 - [MACOI_MOUSE]	17.77	1	12	12	30	1.278	0.808	1.133	0.812	68.19	17.77	19	30	664	76.0	9.07
E9Q5X9	Protein Zfp819 OS=Mus musculus GN=Zfp819 PE=2 SV=1 - [E9Q5X9_MOUSE]	8.10	2	2	2	2	3.844	3.713	1.425	0.812	2.07	8.10	2	2	531	59.1	8.66
G3X9B5	Disintegrin and metalloproteinase domain-containing protein 29 OS=Mus musculus GN=Adam29 PE=4 SV=1 - [illegible]	1.97	2	1	1	1	2.193	0.641	1.022	0.812	2.80	1.97	1	1	763	86.4	8.12
G3X8T6	Kelch-like protein 29 OS=Mus musculus GN=Klhl29 PE=4 SV=1 - [G3X8T6_MOUSE]	3.89	2	3	3	4	1.087	0.794	1.302	0.812	7.70	3.89	4	4	875	94.3	7.17
Q9DCT6-4	Isoform 4 of Chromatin complexes subunit BAP18 OS=Mus musculus GN=Bap18 -	42.62	11	3	3	4	1.466	1.133	0.870	0.812	16.59	42.62	4	4	122	12.4	9.04
Q62470-2	Isoform 2 of Integrin alpha 3 OS=Mus musculus GN=Itga3 - [ITA3_MOUSE]	12.64	4	10	11	18	0.681	1.111	0.658	0.813	39.86	12.64	17	18	1068	118.8	6.81
Q80TC5	Pogo transposable element with KRAB domain OS=Mus musculus GN=Pogk PE=2 SV=2 - [POGK_MOUSE]	3.13	2	2	2	2	0.598	0.476	0.782	0.813	4.86	3.13	2	2	607	69.6	5.29
Q91WG2	Rab GTPase-binding effector protein 2 OS=Mus musculus GN=Rabep2 PE=2 SV=3 - [RABE2_MOUSE]	34.84	5	15	15	55	2.160	1.098	1.099	0.813	176.85	34.84	28	55	554	62.1	4.89
Q5SWP3	NAC-alpha domain-containing protein 1 OS=Mus musculus GN=Nacad PE=1 SV=1 - [NACAD_MOUSE]	23.87	2	24	24	85	1.985	0.494	1.034	0.813	261.91	23.87	41	85	1504	156.7	4.56
P61211	ADP-ribosylation factor-like protein 1 OS=Mus musculus GN=Arl1 PE=2 SV=1 - [ARL1_MOUSE]	34.25	3	6	6	22	0.571	0.689	0.752	0.813	69.79	34.25	11	22	181	20.4	5.72
Q9R087	Glypican-6 OS=Mus musculus GN=Gpc6 PE=1 SV=1 - [GPC6_MOUSE]	17.84	2	7	9	25	1.027	1.164	0.767	0.813	84.25	17.84	14	25	555	63.0	5.43
Q99J09	Methylosome protein 50 OS=Mus musculus GN=Wdr77 PE=1 SV=1 - [MEP50_MOUSE]	30.12	3	9	9	45	1.273	0.902	0.819	0.813	162.46	30.12	17	45	342	36.9	5.27
Q80VJ2	Steroid receptor RNA activator 1 OS=Mus musculus GN=Sra1 PE=1 SV=3 - [SRA1_MOUSE]	30.17	2	6	6	27	1.583	0.846	1.337	0.813	53.63	30.17	9	27	232	25.5	6.43
Q8BRT1-5	Isoform 2 of CLIP-associating protein 2 OS=Mus musculus GN=Clasp2 -	30.00	2	1	15	54	2.193	1.304	0.690	0.813	133.46	30.00	24	54	600	66.3	6.67
Q8VDM6	Heterogeneous nuclear ribonucleoprotein U-like protein 1 OS=Mus musculus GN=Hnrnpul1 PE=1 SV=1 - [illegible]	26.19	3	20	20	73	0.752	0.798	0.777	0.813	197.92	26.19	34	73	859	95.9	6.58
P62717	60S ribosomal protein L18a OS=Mus musculus GN=Rpl18a PE=1 SV=1 - [RL18A_MOUSE]	39.20	2	8	8	43	0.419	0.606	0.648	0.813	111.71	39.20	14	43	176	20.7	10.71
F6TBA6	Kv channel-interacting protein 2 (Fragment) OS=Mus musculus GN=Kcnip2 PE=2 SV=1 - [F6TBA6_MOUSE]	26.74	2	1	4	11	10.601	1.845	1.447	0.814	30.03	26.74	6	11	172	19.9	6.05
Q9R1C7	Pre-mRNA-processing factor 40 homolog A OS=Mus musculus GN=Prpf40a PE=1 SV=1 - [PR40A_MOUSE]	17.94	3	14	15	40	1.048	0.954	0.997	0.814	104.24	17.94	26	40	953	108.4	7.69
Q91V55	40S ribosomal protein S5 OS=Mus musculus GN=Rps5 PE=2 SV=1 - [Q91V55_MOUSE]	39.22	3	8	8	116	0.612	0.722	0.768	0.814	313.25	39.22	15	116	204	22.9	9.72
Q8BMA6	Signal recognition particle subunit SRP68 OS=Mus musculus GN=Srp68 PE=2 SV=2 - [SRP68_MOUSE]	37.12	2	19	19	58	0.624	0.733	0.759	0.814	157.48	37.12	31	58	625	70.5	8.57
O70435	Proteasome subunit alpha type-3 OS=Mus musculus GN=Psma3 PE=1 SV=3 - [PSA3_MOUSE]	36.86	5	10	10	44	1.809	0.843	0.770	0.814	142.52	36.86	18	44	255	28.4	5.44
Q6ZWY8	Thymosin beta-10 OS=Mus musculus GN=Tmsb10 PE=2 SV=3 - [TYB10_MOUSE]	45.45	2	4	5	28	1.516	0.847	1.069	0.814	36.70	45.45	9	28	44	5.0	5.36
Q8VHE0	Translocation protein SEC63 homolog OS=Mus musculus GN=Sec63 PE=1 SV=4 - [SEC63_MOUSE]	21.32	1	13	13	36	0.767	0.770	0.884	0.814	118.63	21.32	22	36	760	87.8	5.38
Q9DC23	DnaJ homolog subfamily C member 10 OS=Mus musculus GN=Dnajc10 PE=1 SV=2 -	11.48	1	8	8	17	0.730	1.019	0.843	0.814	52.16	11.48	12	17	793	90.5	6.96

Q99LC8	Translation initiation factor eIF-2B subunit alpha OS=Mus musculus GN=Eif2b1 PE=2 SV=1 - [EI2BA_MOUSE]	33.44	7	5	9	19	0.684	0.906	0.808	0.814	44.47	33.44	15	19	305	33.8	8.32
Q91XW9	MCG133388, isoform CRA_f OS=Mus musculus GN=Pcdhgc5 PE=2 SV=1 - [Q91XW9_MOUSE]	12.82	1	6	8	35	0.512	2.046	1.949	0.814	98.50	12.82	15	35	944	101.8	5.27
O54962	Barrier-to-autointegration factor OS=Mus musculus GN=Banf1 PE=1 SV=1 - [BAF_MOUSE]	43.82	1	3	3	18	0.750	0.961	0.959	0.814	52.24	43.82	5	18	89	10.1	6.09
P16297	Interleukin-2 receptor subunit beta OS=Mus musculus GN=Il2rb PE=2 SV=1 - [IL2RB_MOUSE]	2.97	1	1	1	1	1.585	1.831	0.855	0.814	3.28	2.97	1	1	539	60.5	5.64
O54734	Dolichyl-diphosphooligosaccharide--protein glycosyltransferase 48 kDa subunit OS=Mus musculus GN=Ddost PE=1 SV=2 - [OST48_MOUSE]	24.49	1	9	9	33	0.584	0.731	0.686	0.814	100.51	24.49	14	33	441	49.0	5.83
A2AT93	Protein Plcb4 (Fragment) OS=Mus musculus GN=Plcb4 PE=2 SV=1 - [A2AT93_MOUSE]	46.27	1	2	13	44	0.804	0.336	0.836	0.814	133.67	46.27	22	44	255	29.4	9.14
A6H644	MCG130490 OS=Mus musculus GN=Ppp1r12b PE=2 SV=1 - [A6H644_MOUSE]	16.73	2	13	14	35	1.196	0.997	1.174	0.814	79.69	16.73	21	35	992	110.9	5.74
A2AJW4	Protein phosphatase 1 regulatory subunit 3 OS=Mus musculus GN=Ppp1r3d PE=4 SV=1 - [A2AJW4_MOUSE]	19.71	1	4	4	10	1.106	0.767	0.778	0.815	28.02	19.71	8	10	279	30.5	8.25
P61750	ADP-ribosylation factor 4 OS=Mus musculus GN=Arf4 PE=2 SV=2 - [ARF4_MOUSE]	67.78	6	4	10	112	0.787	0.944	0.729	0.815	356.16	67.78	18	112	180	20.4	7.14
Q8R2T8-2	Isoform 2 of General transcription factor 3C polypeptide 5 OS=Mus musculus GN=Gtf3c5 - [TF3C5_MOUSE]	1.75	2	1	1	2	1.191	0.745	0.887	0.815	4.41	1.75	2	2	514	59.8	6.73
Q01339	Beta-2-glycoprotein 1 OS=Mus musculus GN=Apoh PE=1 SV=1 - [APOH_MOUSE]	35.65	3	11	11	39	3.454	2.397	0.626	0.815	100.20	35.65	19	39	345	38.6	8.22
Q0VGB7	Serine/threonine-protein phosphatase 4 regulatory subunit 2 OS=Mus musculus GN=Ppp4r2 PE=1 SV=1 - [illegible]	25.18	1	7	8	9	1.574	1.302	1.014	0.815	23.88	25.18	9	9	417	46.4	4.56
E9QQ99	E3 ubiquitin-protein ligase synoviolin OS=Mus musculus GN=Syvn1 PE=2 SV=1 - [E9QQ99_MOUSE]	4.10	4	3	3	15	0.875	0.825	0.872	0.815	36.64	4.10	5	15	561	61.4	6.84
Q9Z0N1	Eukaryotic translation initiation factor 2 subunit 3, X-linked OS=Mus musculus GN=Eif2s3x PE=1 SV=2 - [illegible]	32.20	3	13	13	56	0.709	0.658	0.750	0.815	151.80	32.20	23	56	472	51.0	8.40
Q80TN7	Neuron navigator 3 OS=Mus musculus GN=Nav3 PE=1 SV=2 - [NAV3_MOUSE]	9.11	4	15	16	32	0.935	0.873	1.006	0.815	81.26	9.11	23	32	2359	252.1	8.76
Q5XJY5	Coatomer subunit delta OS=Mus musculus GN=Arcn1 PE=2 SV=2 - [COPD_MOUSE]	34.25	1	19	19	69	0.821	0.815	0.787	0.815	166.22	34.25	35	69	511	57.2	6.21
P51859	Hepatoma-derived growth factor OS=Mus musculus GN=Hdgf PE=1 SV=2 - [HDGF_MOUSE]	41.77	3	10	11	67	0.999	0.559	1.303	0.815	202.95	41.77	19	67	237	26.3	4.83
Q7TNS2	Mitochondrial inner membrane organizing system protein 1 OS=Mus musculus GN=Minos1 PE=2 SV=1 - [illegible]	10.53	1	1	1	4	2.349	0.911	0.632	0.815	9.63	10.53	2	4	76	8.6	8.94
P62908	40S ribosomal protein S3 OS=Mus musculus GN=Rps3 PE=1 SV=1 - [RS3_MOUSE]	59.67	2	15	15	105	0.624	0.761	0.751	0.815	306.90	59.67	30	105	243	26.7	9.66
Q8K2F8	Protein LSM14 homolog A OS=Mus musculus GN=Lsm14a PE=1 SV=1 - [LS14A_MOUSE]	23.16	3	10	11	42	1.176	0.636	0.990	0.815	95.01	23.16	19	42	462	50.5	9.52
Q8VDM1	Zinc finger CCCH-type with G patch domain-containing protein OS=Mus musculus GN=Zgpat PE=2 SV=1 - [ZGPAT_MOUSE]	10.18	2	4	4	10	0.797	0.743	0.801	0.815	29.91	10.18	7	10	511	56.4	5.33
P62702	40S ribosomal protein S4, X isoform OS=Mus musculus GN=Rps4x PE=2 SV=2 - [RS4X_MOUSE]	55.51	2	13	15	68	0.518	0.548	0.717	0.815	177.67	55.51	27	68	263	29.6	10.15
Q8BHP3	Thrombospondin type-1 domain-containing protein 7B OS=Mus musculus GN=Thsd7b PE=2 SV=1 - [Q8BHP3_MOUSE]	1.90	3	2	2	3	0.997	0.713	0.748	0.815	7.32	1.90	3	3	1107	123.6	7.62
Q91WR3-2	Isoform 2 of Activating signal cointegrator 1 complex subunit 2 OS=Mus musculus GN=Ascc2 - [illegible]	1.41	3	1	1	1	1.171	0.944	1.050	0.816	1.88	1.41	1	1	709	80.9	4.94

E0CYV4	Desumoylating isopeptidase 2 (Fragment) OS=Mus musculus GN=Desi2 PE=2 SV=1 - [E0CYV4_MOUSE]	12.50	3	1	1	2	0.633	0.496	1.012	0.816	5.27	12.50	2	2	136	15.3	5.25
Q810U4-2	Isoform 2 of Neuronal cell adhesion molecule OS=Mus musculus GN=Nrcam -	38.53	2	1	35	248	1.679	1.293	1.052	0.816	765.45	38.53	65	248	1186	131.2	6.06
P97784	Cryptochrome-1 OS=Mus musculus GN=Cry1 PE=1 SV=1 - [CRY1_MOUSE]	2.48	1	1	2	3	3.354	1.256	0.498	0.816	7.65	2.48	3	3	606	68.0	7.97
Q9CZX8	40S ribosomal protein S19 OS=Mus musculus GN=Rps19 PE=1 SV=3 - [RS19_MOUSE]	53.79	4	1	10	67	0.797	0.724	0.846	0.816	189.71	53.79	19	67	145	16.1	10.40
Q91VW5	Golgin subfamily A member 4 OS=Mus musculus GN=Golga4 PE=1 SV=2 -	28.19	1	51	55	156	1.699	1.018	1.072	0.816	433.64	28.19	86	156	2238	257.4	5.36
A0AUP1	Coiled-coil domain-containing protein 112 OS=Mus musculus GN=Ccdc112 PE=2 SV=2 - [CC112_MOUSE]	8.60	2	1	5	8	0.846	0.975	0.867	0.816	11.50	8.60	5	8	442	52.7	9.60
Q9D031	Ras suppressor protein 1 OS=Mus musculus GN=Rsu1 PE=2 SV=1 - [Q9D031_MOUSE]	50.90	6	9	9	31	0.846	1.234	0.672	0.816	86.64	50.90	17	31	277	31.4	8.63
Q9CSN1	SNW domain-containing protein 1 OS=Mus musculus GN=Snw1 PE=1 SV=3 - [SNW1_MOUSE]	36.19	1	16	17	60	1.074	0.473	1.090	0.816	175.76	36.19	28	60	536	61.4	9.48
Q8BW86-2	Isoform 2 of Rho guanine nucleotide exchange factor 33 OS=Mus musculus GN=Arhgef33 - [ARG33_MOUSE]	6.41	2	5	5	6	1.172	0.756	2.177	0.816	16.17	6.41	6	6	764	84.8	6.81
Q64012-2	Isoform 1 of RNA-binding protein Raly OS=Mus musculus GN=Raly - [RALY_MOUSE]	45.95	5	12	12	49	0.417	0.550	0.785	0.816	163.93	45.95	22	49	296	31.2	9.38
Q8BM88	Cathepsin O OS=Mus musculus GN=Ctso PE=2 SV=1 - [CATO_MOUSE]	8.65	1	3	3	5	1.374	0.865	0.731	0.816	12.04	8.65	5	5	312	34.7	7.80
Q8BZB3	Transmembrane protein C15orf27 homolog OS=Mus musculus PE=2 SV=3 - [CO027_MOUSE]	18.03	2	5	5	14	1.383	0.653	1.001	0.816	38.51	18.03	8	14	538	58.9	4.87
Q9D0B6	Protein PBDC1 OS=Mus musculus GN=Pbdc1 PE=2 SV=1 - [PBDC1_MOUSE]	33.84	2	7	7	20	1.232	1.008	0.789	0.816	49.08	33.84	13	20	198	22.2	4.55
O55029	Coatomer subunit beta' OS=Mus musculus GN=Copb2 PE=2 SV=2 - [COPB2_MOUSE]	36.24	1	26	26	103	1.080	0.901	0.758	0.816	312.11	36.24	49	103	905	102.4	5.30
Q8BGA8-2	Isoform 2 of Acyl-coenzyme A synthetase ACSM5, mitochondrial OS=Mus musculus GN=Acsm5 - [ACSM5_MOUSE]	5.79	2	1	1	1	1.567	1.104	0.686	0.816	2.78	5.79	1	1	311	34.6	9.06
Q8VEB4	Group XV phospholipase A2 OS=Mus musculus GN=Pla2g15 PE=1 SV=1 - [PAG15_MOUSE]	10.92	1	5	5	13	1.552	1.189	0.898	0.816	32.33	10.92	8	13	412	47.3	6.47
E9Q0G1	Density-regulated protein (Fragment) OS=Mus musculus GN=Denr PE=2 SV=1 - [E9Q0G1_MOUSE]	19.67	2	4	4	15	0.949	0.476	1.010	0.816	36.97	19.67	7	15	183	20.5	7.53
A2AAY5	SH3 and PX domain-containing protein 2B OS=Mus musculus GN=Sh3pxd2b PE=1 SV=1 - [SPD2B_MOUSE]	16.85	1	12	12	23	1.220	0.636	0.854	0.817	63.86	16.85	17	23	908	101.5	8.66
Q9CQ48	NudC domain-containing protein 2 OS=Mus musculus GN=Nudcd2 PE=1 SV=1 -	41.40	2	5	5	20	1.431	0.949	0.804	0.817	59.44	41.40	8	20	157	17.6	5.07
D6RI51	Trehalase OS=Mus musculus GN=Treh PE=2 SV=1 - [D6RI51_MOUSE]	18.00	2	1	1	2	0.403	0.827	1.062	0.817	0.00	18.00	1	2	150	17.2	5.69
Q6P4S8	Integrator complex subunit 1 OS=Mus musculus GN=Ints1 PE=1 SV=2 - [INT1_MOUSE]	4.15	2	7	7	12	0.558	0.805	0.703	0.817	42.95	4.15	11	12	2195	245.0	6.28
Q3TJ22	Angio-associated migratory protein OS=Mus musculus GN=Aamp PE=2 SV=1 - [Q3TJ22_MOUSE]	17.24	2	7	7	22	1.551	1.070	0.817	0.817	79.50	17.24	13	22	435	46.9	4.41
Q8CFX3	Protein Pcdh1 OS=Mus musculus GN=Pcdh1 PE=2 SV=1 - [Q8CFX3_MOUSE]	37.86	1	3	26	169	0.701	2.278	2.013	0.817	511.43	37.86	45	169	1038	112.3	5.02
Q9CW46	Ribonucleoprotein PTB-binding 1 OS=Mus musculus GN=Raver1 PE=1 SV=2 -	16.04	2	8	8	28	1.429	1.070	0.844	0.817	59.59	16.04	14	28	748	79.3	8.72
E0CZ61	Protein Gm16039 (Fragment) OS=Mus musculus GN=Gm16039 PE=2 SV=1 -	27.88	6	2	2	7	1.785	1.368	1.141	0.817	27.15	27.88	3	7	104	11.0	4.59
Q4VAA2	Protein CDV3 OS=Mus musculus GN=Cdv3 PE=1 SV=2 - [CDV3_MOUSE]	53.02	3	10	10	68	1.677	0.717	1.230	0.817	173.19	53.02	17	68	281	29.7	6.10

P58252	Elongation factor 2 OS=Mus musculus GN=Eef2 PE=1 SV=2 - [EF2_MOUSE]	61.77	1	48	50	323	0.798	0.896	0.815	0.817	890.13	61.77	92	323	858	95.3	6.83
E9Q0G0	Protein 4932415D10Rik OS=Mus musculus GN=4932415D10Rik PE=4 SV=1 - [E9Q0G0_MOUSE]	1.42	1	1	1	1	1.155	0.580	1.021	0.818	1.86	1.42	1	1	563	63.7	10.02
A2AL79	Aspartyl/asparaginyl beta-hydroxylase OS=Mus musculus GN=Asph PE=2 SV=1 - [A2AL79_MOUSE]	25.89	4	2	3	9	0.843	0.909	0.864	0.818	18.47	25.89	5	9	197	20.7	4.39
Q7TN98-5	Isoform 5 of Cytoplasmic polyadenylation element-binding protein 4 OS=Mus musculus GN=Cpeb4 - [CPEB4_MOUSE]	26.14	7	6	11	31	1.048	1.075	1.037	0.818	91.22	26.14	19	31	704	77.2	6.90
Q9CZS1	Aldehyde dehydrogenase X, mitochondrial OS=Mus musculus GN=Aldh1b1 PE=2 SV=1 - [AL1B1_MOUSE]	49.52	3	20	22	127	0.577	0.692	0.774	0.818	403.50	49.52	39	127	519	57.5	7.02
P97465	Docking protein 1 OS=Mus musculus GN=Dok1 PE=1 SV=2 - [DOK1_MOUSE]	6.02	1	1	1	1	1.741	1.206	0.886	0.818	2.17	6.02	1	1	482	52.4	6.57
Q07079	Insulin-like growth factor-binding protein 5 OS=Mus musculus GN=Igfbp5 PE=1 SV=1 - [IBP5_MOUSE]	19.93	1	4	5	13	1.424	1.104	0.785	0.818	39.75	19.93	9	13	271	30.4	8.12
Q0V8T8-2	Isoform 2 of Contactin-associated protein like 5-2 OS=Mus musculus GN=Cntnap5b -	3.24	2	1	3	11	0.398	0.875	0.882	0.818	30.58	3.24	6	11	1235	137.7	6.30
P11881-8	Isoform 8 of Inositol 1,4,5-trisphosphate receptor type 1 OS=Mus musculus GN=Itpr1 - [ITPR1_MOUSE]	26.99	8	56	66	165	0.964	1.729	1.914	0.818	463.80	26.99	112	165	2694	306.7	6.29
P62900	60S ribosomal protein L31 OS=Mus musculus GN=Rpl31 PE=2 SV=1 - [RL31_MOUSE]	30.40	1	4	4	22	0.770	0.782	0.792	0.818	62.71	30.40	8	22	125	14.5	10.54
E9Q8P5	PDZ and LIM domain protein 5 OS=Mus musculus GN=Pdlim5 PE=2 SV=2 -	35.98	3	1	5	16	0.994	1.592	1.114	0.818	55.62	35.98	9	16	239	26.3	9.67
G3X9N3	MCG16539 OS=Mus musculus GN=Pnmal2 PE=4 SV=1 - [G3X9N3_MOUSE]	25.11	1	13	13	54	0.697	1.410	0.851	0.818	140.04	25.11	22	54	661	71.7	5.17
Q6PGB6-3	Isoform 3 of N-alpha-acetyltransferase 50 OS=Mus musculus GN=Naa50 - [NAA50_MOUSE]	61.24	5	9	9	24	0.667	0.793	0.782	0.818	49.17	61.24	17	24	129	14.9	8.56
Q8BND5-2	Isoform 2 of Sulfhydryl oxidase 1 OS=Mus musculus GN=Qsox1 - [QSOX1_MOUSE]	11.50	4	6	6	10	1.508	1.039	0.720	0.818	37.13	11.50	9	10	661	73.3	6.89
Q9Z277	Tyrosine-protein kinase BAZ1B OS=Mus musculus GN=Baz1b PE=1 SV=2 - [BAZ1B_MOUSE]	7.10	2	9	10	16	0.501	0.760	0.824	0.818	38.20	7.10	14	16	1479	170.5	8.60
Q8BRV5	Uncharacterized protein KIAA1671 OS=Mus musculus GN=Kiaa1671 PE=2 SV=1 -	18.18	1	4	4	20	0.819	1.095	1.304	0.818	52.31	18.18	8	20	308	34.6	6.09
E9PYQ0	Protein Rsph10b OS=Mus musculus GN=Rsph10b PE=2 SV=1 - [E9PYQ0_MOUSE]	4.68	1	2	2	2	1.367	1.089	1.372	0.818	0.00	4.68	2	2	876	101.5	6.84
Q811F1	Zinc finger and BTB domain-containing protein 41 OS=Mus musculus GN=Zbtb41 PE=2 SV=4 - [ZBT41_MOUSE]	1.54	1	1	2	2	1.941	1.553	0.983	0.819	4.73	1.54	2	2	908	104.6	7.91
Q91X97	Neurocalcin-delta OS=Mus musculus GN=Ncald PE=1 SV=4 - [NCALD_MOUSE]	75.13	4	8	13	207	0.949	1.481	1.008	0.819	587.88	75.13	25	207	193	22.2	5.35
E9Q9U8	Protein Ccdc74a OS=Mus musculus GN=Ccdc74a PE=4 SV=1 - [E9Q9U8_MOUSE]	17.86	2	3	4	7	0.701	1.433	0.757	0.819	11.28	17.86	5	7	364	39.7	10.24
Q9CYZ2	Tumor protein D54 OS=Mus musculus GN=Tpd52l2 PE=1 SV=1 - [TPD54_MOUSE]	71.36	2	2	16	74	1.212	1.359	1.012	0.819	232.11	71.36	26	74	220	24.0	6.15
Q9DAS5	Ceacam12 protein OS=Mus musculus GN=Ceacam12 PE=2 SV=1 - [Q9DAS5_MOUSE]	4.58	3	1	1	1	2.613	1.816	1.244	0.819	1.92	4.58	1	1	262	29.5	7.09
Q09XV5	Chromodomain-helicase-DNA-binding protein 8 OS=Mus musculus GN=Chd8 PE=1 SV=1 - [CHD8_MOUSE]	3.21	3	4	7	10	1.132	0.679	0.978	0.820	23.54	3.21	10	10	2582	290.7	6.42
Q9ERE7	LDLR chaperone MESD OS=Mus musculus GN=Mesdc2 PE=1 SV=1 - [MESD_MOUSE]	25.45	2	6	6	21	1.456	0.647	0.942	0.820	58.31	25.45	9	21	224	25.2	6.34
Q6R891	Neurabin-2 OS=Mus musculus GN=Ppp1r9b PE=1 SV=1 - [NEB2_MOUSE]	43.45	2	24	27	176	1.194	1.132	1.369	0.820	556.73	43.45	49	176	817	89.5	4.92

Q8BNU0	Armadillo repeat-containing protein 6 OS=Mus musculus GN=Armc6 PE=2 SV=1 -	36.32	3	14	14	76	0.626	1.016	0.768	0.820	231.57	36.32	25	76	468	50.7	6.00
Q9CXU0	Mediator of RNA polymerase II transcription subunit 10 OS=Mus musculus GN=Med10 PE=1 SV=1 - [MED10_MOUSE]	5.19	1	1	1	1	1.265	1.098	0.823	0.820	2.60	5.19	1	1	135	15.7	6.19
Q9CQ10	Charged multivesicular body protein 3 OS=Mus musculus GN=Chmp3 PE=1 SV=3 -	25.89	1	7	7	18	0.942	0.802	1.050	0.820	31.58	25.89	12	18	224	25.2	5.06
Q924A2	Protein capicua homolog OS=Mus musculus GN=Cic PE=1 SV=2 - [CIC_MOUSE]	7.33	4	11	11	19	1.160	0.962	1.476	0.820	54.69	7.33	16	19	2510	258.0	8.02
B7ZNG0-2	Isoform 2 of Kinesin-like protein KIF7 OS=Mus musculus GN=Kif7 - [KIF7_MOUSE]	3.79	4	2	4	10	1.704	0.747	0.752	0.820	28.99	3.79	5	10	1347	151.4	6.44
P62137	Serine/threonine-protein phosphatase PP1-alpha catalytic subunit OS=Mus musculus GN=Ppp1ca PE=1 SV=1 - [PP1A_MOUSE]	56.06	1	3	16	138	1.437	1.268	1.026	0.820	428.95	56.06	30	138	330	37.5	6.33
B0QZM1	Ubiquitin-conjugating enzyme E2 J2 OS=Mus musculus GN=Ube2j2 PE=2 SV=1 -	9.48	4	2	2	8	1.447	0.819	0.839	0.820	22.62	9.48	3	8	232	25.8	8.78
Q8CH25-2	Isoform 2 of SAFB-like transcription modulator OS=Mus musculus GN=Sltm - [SLTM_MOUSE]	12.64	2	12	12	41	1.108	0.811	0.987	0.820	117.20	12.64	21	41	1013	115.1	8.05
Q9ESJ0	Exportin-4 OS=Mus musculus GN=Xpo4 PE=1 SV=2 - [XPO4_MOUSE]	5.65	1	5	5	9	0.931	0.951	0.887	0.820	24.42	5.65	7	9	1151	129.9	5.12
Q922W5	Pyrroline-5-carboxylate reductase 1, mitochondrial OS=Mus musculus GN=Pycr1 PE=1 SV=1 - [P5CR1_MOUSE]	28.16	4	3	6	19	1.089	0.752	0.690	0.820	69.47	28.16	11	19	309	32.4	6.86
Q8VCG1	Deoxyuridine triphosphatase, isoform CRA_b OS=Mus musculus GN=Dut PE=2 SV=1 - [Q8VCG1_MOUSE]	39.50	2	7	7	19	1.599	0.906	0.897	0.820	60.95	39.50	13	19	200	21.2	9.58
A2CG49	Kalirin OS=Mus musculus GN=Kalrn PE=1 SV=1 - [KALRN_MOUSE]	18.52	14	25	45	129	0.620	1.351	1.195	0.821	350.54	18.52	77	129	2964	336.8	6.07
A2CG63-2	Isoform 2 of AT-rich interactive domain-containing protein 4B OS=Mus musculus GN=Arid4b - [ARI4B_MOUSE]	2.61	3	3	3	5	0.883	0.993	0.869	0.821	12.95	2.61	3	5	1227	137.2	5.43
A2AU72-2	Isoform 2 of Armadillo repeat-containing protein 3 OS=Mus musculus GN=Armc3 - [ARMC3_MOUSE]	2.19	3	1	1	1	1.111	1.080	1.109	0.821	2.51	2.19	1	1	684	74.8	6.05
A2AJ72	MCG130458 OS=Mus musculus GN=Fubp3 PE=4 SV=1 - [A2AJ72_MOUSE]	51.67	6	18	24	119	1.159	0.769	0.855	0.821	319.17	51.67	41	119	569	61.4	8.28
Q9QWW1-2	Isoform 2 of Homer protein homolog 2 OS=Mus musculus GN=Homer2 -	37.32	3	12	12	46	0.793	1.404	1.049	0.821	144.69	37.32	20	46	343	39.4	6.38
Q9DC11	Plexin domain-containing protein 2 OS=Mus musculus GN=Plxdc2 PE=1 SV=1 -	15.66	3	8	8	16	0.856	0.934	0.718	0.821	44.29	15.66	13	16	530	59.6	6.60
E9QQ02	VPS10 domain-containing receptor SorCS1 OS=Mus musculus GN=Sorcs1 PE=4 SV=2 - [E9QQ02_MOUSE]	9.06	8	9	9	21	1.093	1.046	1.052	0.821	69.49	9.06	16	21	1192	132.6	7.27
Q9CW79-2	Isoform 2 of Golgin subfamily A member 1 OS=Mus musculus GN=Golga1 -	30.42	3	18	19	60	1.795	0.887	0.991	0.821	153.97	30.42	31	60	733	84.2	5.50
Q9CQ60	6-phosphogluconolactonase OS=Mus musculus GN=Pgls PE=2 SV=1 -	65.76	3	5	12	69	1.422	0.845	0.646	0.821	224.10	65.76	22	69	257	27.2	5.85
Q9Z148-2	Isoform 2 of Histone-lysine N-methyltransferase EHMT2 OS=Mus musculus GN=Ehmt2 - [EHMT2_MOUSE]	3.24	5	2	2	3	1.126	1.102	1.050	0.821	12.04	3.24	3	3	1172	128.3	5.40
P63239	Neuroendocrine convertase 1 OS=Mus musculus GN=Pcsk1 PE=1 SV=1 - [NEC1_MOUSE]	21.25	1	14	14	33	1.051	1.006	0.734	0.821	82.15	21.25	23	33	753	84.1	6.43
Q8CEE7	Retinol dehydrogenase 13 OS=Mus musculus GN=Rdh13 PE=2 SV=1 - [RDH13_MOUSE]	16.47	2	4	4	11	0.903	0.623	0.649	0.821	41.75	16.47	6	11	334	36.4	8.85
Q3TYD6	Serine/threonine-protein kinase LMTK2 OS=Mus musculus GN=Lmtk2 PE=1 SV=3 - [LMTK2_MOUSE]	21.21	1	22	23	67	1.224	0.969	1.318	0.821	209.14	21.21	38	67	1471	160.4	4.51

Q9D0B5	Thiosulfate sulfurtransferase/rhodanese-like domain-containing protein 3 OS=Mus musculus GN=Tstd3 PE=2 SV=1 - [TSTD3_MOUSE]	37.58	1	5	5	20	1.873	1.056	1.274	0.822	54.10	37.58	8	20	157	17.3	8.13
Q8BW10	RNA-binding protein NOB1 OS=Mus musculus GN=Nob1 PE=1 SV=1 - [NOB1_MOUSE]	4.22	2	2	2	2	0.824	0.607	0.750	0.822	3.95	4.22	2	2	403	45.4	7.31
Q60749	KH domain-containing, RNA-binding, signal transduction-associated protein 1 OS=Mus musculus GN=Khdrbs1 PE=1 SV=2 - [KHDR1_MOUSE]	26.64	1	9	13	51	1.081	0.838	1.011	0.822	129.41	26.64	21	51	443	48.3	8.72
Q8VHG2	Angiomotin OS=Mus musculus GN=Amot PE=1 SV=3 - [AMOT_MOUSE]	28.86	7	21	27	81	1.175	1.099	1.441	0.822	247.52	28.86	44	81	1126	120.8	7.50
P54728	UV excision repair protein RAD23 homolog B OS=Mus musculus GN=Rad23b PE=1 SV=2 - [RD23B_MOUSE]	32.69	1	9	12	117	1.845	1.000	1.113	0.822	302.16	32.69	22	117	416	43.5	4.83
E9Q616	Protein Ahnak OS=Mus musculus GN=Ahnak PE=2 SV=1 - [E9Q616_MOUSE]	58.59	2	141	146	454	1.451	1.892	0.930	0.822	1195.27	58.59	237	454	5656	603.9	6.30
A2AKB4	FERM and PDZ domain-containing protein 1 OS=Mus musculus GN=Frmpd1 PE=1 SV=1 - [FRPD1_MOUSE]	5.94	2	6	6	8	0.907	0.790	0.882	0.822	14.85	5.94	7	8	1549	169.1	5.16
Q9D7N3	28S ribosomal protein S9, mitochondrial OS=Mus musculus GN=Mrps9 PE=1 SV=3 - [RT09_MOUSE]	36.41	1	12	12	25	0.999	0.679	0.925	0.822	65.40	36.41	18	25	390	44.9	8.81
P14131	40S ribosomal protein S16 OS=Mus musculus GN=Rps16 PE=2 SV=4 - [RS16_MOUSE]	67.12	1	12	12	65	0.632	0.523	0.731	0.822	175.43	67.12	22	65	146	16.4	10.21
Q61247	Alpha-2-antiplasmin OS=Mus musculus GN=Serpinf2 PE=1 SV=1 - [A2AP_MOUSE]	14.26	3	7	7	22	2.771	1.299	0.354	0.822	43.53	14.26	11	22	491	54.9	6.30
B7ZP47	Wapal protein OS=Mus musculus GN=Wapal PE=2 SV=1 - [B7ZP47_MOUSE]	8.46	3	9	9	15	1.032	0.682	1.038	0.822	42.99	8.46	12	15	1194	133.3	5.47
A2A6P4	Protein Fam104a OS=Mus musculus GN=Fam104a PE=2 SV=1 - [A2A6P4_MOUSE]	15.68	1	2	2	5	0.480	0.480	0.811	0.823	7.95	15.68	3	5	185	19.6	11.18
P62192	26S protease regulatory subunit 4 OS=Mus musculus GN=Psmc1 PE=1 SV=1 -	59.77	1	19	21	101	0.915	0.793	0.776	0.823	247.07	59.77	34	101	440	49.2	6.21
Q60739-2	Isoform 2 of BAG family molecular chaperone regulator 1 OS=Mus musculus GN=Bag1 - [BAG1_MOUSE]	54.34	3	7	7	26	1.892	0.779	0.902	0.823	62.70	54.34	11	26	219	24.9	4.97
Q99PM3	Transcription initiation factor IIA subunit 1 OS=Mus musculus GN=Gtf2a1 PE=2 SV=2 - [TF2AA_MOUSE]	7.41	2	2	2	18	1.811	0.986	0.999	0.823	52.53	7.41	4	18	378	41.6	4.55
P62342	Selenoprotein T OS=Mus musculus GN=Selt PE=2 SV=2 - [SELT_MOUSE]	22.05	1	5	5	15	0.783	0.931	0.756	0.823	46.77	22.05	9	15	195	22.3	8.60
G3X9K8	TP53RK-binding protein OS=Mus musculus GN=Tprkb PE=3 SV=1 - [G3X9K8_MOUSE]	16.46	2	1	1	9	1.786	0.952	0.859	0.823	25.67	16.46	2	9	158	17.7	6.93
Q9CWD8	Iron-sulfur protein NUBPL OS=Mus musculus GN=Nubpl PE=2 SV=2 - [NUBPL_MOUSE]	12.85	2	3	3	10	2.018	1.360	0.874	0.823	37.91	12.85	4	10	319	34.1	9.07
Q9D164-2	Isoform 2 of FXYD domain-containing ion transport regulator 6 OS=Mus musculus GN=Fxyd6 - [FXYD6_MOUSE]	46.24	2	3	3	34	0.601	1.129	0.669	0.823	113.11	46.24	3	34	93	10.3	5.11
G5E843	Roundabout homolog 1 OS=Mus musculus GN=Robo1 PE=4 SV=1 - [G5E843_MOUSE]	19.85	2	25	26	63	1.103	0.701	0.878	0.824	186.34	19.85	42	63	1612	176.3	6.19
Q9JJR9	Nuclear receptor-interacting protein 3 OS=Mus musculus GN=Nrip3 PE=2 SV=1 -	6.67	2	2	2	4	1.177	0.828	1.005	0.824	8.98	6.67	4	4	240	26.9	8.50
Q6IQX8	Protein Zfp219 OS=Mus musculus GN=Zfp219 PE=2 SV=1 - [Q6IQX8_MOUSE]	12.40	1	4	4	8	1.065	0.854	0.882	0.824	16.73	12.40	5	8	726	77.7	9.17
F6ZQL0	Protein LYRIC (Fragment) OS=Mus musculus GN=Mtdh PE=4 SV=1 - [F6ZQL0_MOUSE]	34.09	1	1	6	32	0.791	0.803	0.912	0.824	87.39	34.09	11	32	220	24.4	9.31
Q921I6	SH3 domain-binding protein 4 OS=Mus musculus GN=Sh3bp4 PE=2 SV=1 -	12.89	1	10	10	20	1.053	1.388	0.831	0.824	45.70	12.89	16	20	962	107.5	7.34

Q9DBN5	Lon protease homolog 2, peroxisomal OS=Mus musculus GN=Lonp2 PE=2 SV=1 - [LONP2_MOUSE]	8.22	4	6	7	10	0.874	0.823	0.692	0.824	19.42	8.22	10	10	852	94.5	7.23
E9Q5E0	Myocyte-specific enhancer factor 2D OS=Mus musculus GN=Mef2d PE=2 SV=1 - [E9Q5E0_MOUSE]	17.79	5	7	7	30	0.983	1.157	1.286	0.824	84.67	17.79	13	30	506	54.1	8.38
E9QNH6	Unconventional myosin-Ib OS=Mus musculus GN=Myo1b PE=2 SV=1 - [E9QNH6_MOUSE]	26.90	6	19	24	60	0.620	0.981	0.998	0.824	179.91	26.90	39	60	1078	125.0	9.14
Q924Z4	Ceramide synthase 2 OS=Mus musculus GN=Cers2 PE=1 SV=1 - [CERS2_MOUSE]	11.32	4	3	3	13	0.700	0.483	0.783	0.824	42.46	11.32	5	13	380	45.0	8.75
Q9QWV9	Cyclin-T1 OS=Mus musculus GN=Ccnt1 PE=1 SV=3 - [CCNT1_MOUSE]	3.87	2	1	2	4	1.315	0.514	1.108	0.824	12.86	3.87	4	4	724	80.5	8.68
P20065-2	Isoform Short of Thymosin beta-4 OS=Mus musculus GN=Tmsb4x - [TYB4_MOUSE]	45.45	3	3	4	21	1.500	1.506	1.622	0.824	28.73	45.45	6	21	44	5.0	5.06
Q61553	Fascin OS=Mus musculus GN=Fscn1 PE=1 SV=4 - [FSCN1_MOUSE]	49.70	5	24	24	226	1.025	0.856	0.910	0.824	660.53	49.70	45	226	493	54.5	6.89
B1AU74	Motile sperm domain-containing protein 2 OS=Mus musculus GN=Mospd2 PE=2 SV=1 - [B1AU74_MOUSE]	11.58	5	6	6	14	0.889	0.720	0.746	0.824	45.97	11.58	10	14	518	59.8	5.76
Q99JT9	1,2-dihydroxy-3-keto-5-methylthiopentene dioxygenase OS=Mus musculus GN=Adi1 PE=1 SV=1 - [MTND_MOUSE]	24.58	2	3	4	21	2.069	1.013	0.761	0.824	68.91	24.58	6	21	179	21.5	5.50
Q99MI1	ELKS/Rab6-interacting/CAST family member 1 OS=Mus musculus GN=Erc1 PE=1 SV=1 - [RB6I2_MOUSE]	42.86	5	39	50	202	1.259	0.895	1.188	0.824	510.19	42.86	81	202	1120	128.3	5.87
Q99L59	X-linked lymphocyte-regulated protein 3A OS=Mus musculus GN=Xlr3a PE=2 SV=1 - [Q99L59_MOUSE]	7.69	5	2	2	2	0.999	1.074	0.784	0.825	2.50	7.69	2	2	221	25.7	7.85
Q9D8S3	ADP-ribosylation factor GTPase-activating protein 3 OS=Mus musculus GN=Arfgap3 PE=2 SV=2 - [ARFG3_MOUSE]	30.59	1	14	15	37	1.172	0.855	0.847	0.825	106.00	30.59	26	37	523	57.4	8.47
G5E8A7	Protein Smaug homolog 2 OS=Mus musculus GN=Samd4b PE=4 SV=1 - [G5E8A7_MOUSE]	19.21	2	8	8	31	0.915	0.809	0.836	0.825	95.40	19.21	15	31	687	74.9	6.83
E9QP99	Golgin subfamily A member 3 OS=Mus musculus GN=Golga3 PE=2 SV=1 -	41.43	4	48	49	150	1.656	0.812	1.133	0.825	470.23	41.43	82	150	1487	167.2	5.38
O54781	SRSF protein kinase 2 OS=Mus musculus GN=Srpk2 PE=1 SV=2 - [SRPK2_MOUSE]	24.38	2	10	14	56	0.663	0.866	0.905	0.825	153.65	24.38	23	56	681	76.7	4.91
Q9D338	39S ribosomal protein L19, mitochondrial OS=Mus musculus GN=Mrpl19 PE=2 SV=1 - [RM19_MOUSE]	33.22	1	7	7	21	0.756	0.827	0.836	0.825	60.04	33.22	12	21	292	33.6	9.44
Q8BFQ4	WD repeat-containing protein 82 OS=Mus musculus GN=Wdr82 PE=1 SV=1 -	18.21	1	7	7	15	0.965	1.058	0.752	0.825	37.07	18.21	10	15	313	35.1	7.69
Q8K4E0	Alstrom syndrome protein 1 homolog OS=Mus musculus GN=Alms1 PE=1 SV=2 - [ALMS1_MOUSE]	2.06	1	4	4	5	0.711	1.160	0.975	0.825	6.42	2.06	4	5	3251	360.0	6.39
E9QP09	Cullin-9 OS=Mus musculus GN=Cul9 PE=3 SV=1 - [E9QP09_MOUSE]	12.10	2	6	21	51	0.564	1.136	0.969	0.825	140.09	12.10	34	51	2520	281.1	5.59
Q5IR70-2	Isoform 2 of Cancer-associated gene 1 protein homolog OS=Mus musculus GN=Cage1 - [CAGE1_MOUSE]	3.69	3	2	3	3	0.596	1.157	0.683	0.825	7.90	3.69	3	3	515	58.7	5.26
E9PV16	Adenosylhomocysteinase OS=Mus musculus GN=Ahcyl2 PE=2 SV=1 - [E9PV16_MOUSE]	43.86	10	1	32	258	1.810	0.845	1.092	0.825	688.62	43.86	58	258	643	69.9	6.79
Q07113	Cation-independent mannose-6-phosphate receptor OS=Mus musculus GN=Igf2r PE=1 SV=1 - [MPRI_MOUSE]	10.11	1	20	20	37	0.853	0.965	0.859	0.825	97.95	10.11	30	37	2483	273.6	5.71
Q9CRA5	Golgi phosphoprotein 3 OS=Mus musculus GN=Golph3 PE=2 SV=1 - [GOLP3_MOUSE]	27.52	3	5	6	18	0.660	1.023	0.766	0.825	52.05	27.52	11	18	298	33.7	6.44
Q8R205	Zinc finger CCCH domain-containing protein 10 OS=Mus musculus GN=Zc3h10 PE=2 SV=1 - [ZC3HA_MOUSE]	12.18	1	2	2	6	1.546	1.117	1.151	0.825	5.18	12.18	3	6	435	46.1	7.62

P14148	60S ribosomal protein L7 OS=Mus musculus GN=Rpl7 PE=2 SV=2 - [RL7_MOUSE]	61.48	2	20	22	98	0.558	0.790	0.688	0.825	265.96	61.48	42	98	270	31.4	10.89
Q9QY31	Zinc finger protein SNAI3 OS=Mus musculus GN=Snai3 PE=2 SV=1 - [SNAI3_MOUSE]	6.62	1	1	1	3	2.257	1.548	1.111	0.825	0.00	6.62	1	3	287	31.6	8.65
Q9D0A3	UPF0552 protein C15orf38 homolog OS=Mus musculus PE=2 SV=1 - [CO038_MOUSE]	38.50	1	8	8	38	1.925	0.821	1.176	0.825	99.89	38.50	15	38	226	25.2	5.19
Q9DCJ9	N-acetylneuraminate lyase OS=Mus musculus GN=Npl PE=1 SV=1 - [NPL_MOUSE]	50.94	1	12	12	41	1.351	0.947	0.742	0.826	125.33	50.94	21	41	320	35.1	8.03
D3YTQ4	High affinity cGMP-specific 3',5'-cyclic phosphodiesterase 9A OS=Mus musculus GN=Pde9a PE=2 SV=1 - [illegible]	10.24	4	4	4	8	1.026	0.706	0.843	0.826	24.65	10.24	6	8	508	58.8	6.24
P83870	PHD finger-like domain-containing protein 5A OS=Mus musculus GN=Phf5a PE=1 SV=1 - [PHF5A_MOUSE]	48.18	1	7	7	21	1.932	0.790	1.134	0.826	46.90	48.18	13	21	110	12.4	8.41
Q9WVA3	Mitotic checkpoint protein BUB3 OS=Mus musculus GN=Bub3 PE=2 SV=2 - [BUB3_MOUSE]	29.75	1	8	8	17	0.749	0.840	0.734	0.826	43.36	29.75	12	17	326	36.9	6.84
F6ZKC7	Cleavage stimulation factor subunit 2 (Fragment) OS=Mus musculus GN=Cstf2 PE=4 SV=1 - [F6ZKC7_MOUSE]	16.60	1	1	3	15	2.260	1.061	0.953	0.826	44.31	16.60	6	15	259	26.6	8.32
P11859	Angiotensinogen OS=Mus musculus GN=Agt PE=1 SV=1 - [ANGT_MOUSE]	26.00	1	1	9	28	1.442	1.480	1.209	0.826	75.63	26.00	16	28	477	52.0	5.44
Q99LI5	Zinc finger protein 281 OS=Mus musculus GN=Znf281 PE=1 SV=1 - [ZN281_MOUSE]	2.58	1	2	2	3	1.687	0.817	0.980	0.826	9.33	2.58	2	3	893	96.6	8.56
P32067	Lupus La protein homolog OS=Mus musculus GN=Ssb PE=2 SV=1 - [LA_MOUSE]	50.84	4	21	21	66	0.746	0.884	0.856	0.826	182.81	50.84	35	66	415	47.7	9.77
Q8C9B9	Death-inducer obliterator 1 OS=Mus musculus GN=Dido1 PE=1 SV=4 - [DIDO1_MOUSE]	6.91	3	10	10	30	0.978	1.080	0.958	0.826	82.38	6.91	17	30	2256	247.0	7.91
P35276	Ras-related protein Rab-3D OS=Mus musculus GN=Rab3d PE=1 SV=1 - [RAB3D_MOUSE]	47.03	4	2	10	125	2.740	1.908	1.081	0.826	381.57	47.03	17	125	219	24.4	4.93
Q9QUG9	RAS guanyl-releasing protein 2 OS=Mus musculus GN=Rasgrp2 PE=1 SV=2 -	29.61	11	17	17	61	0.458	0.521	1.112	0.826	162.04	29.61	28	61	608	69.4	7.66
Q04750	DNA topoisomerase 1 OS=Mus musculus GN=Top1 PE=1 SV=2 - [TOP1_MOUSE]	21.25	2	16	16	38	0.596	0.789	0.817	0.827	92.47	21.25	28	38	767	90.8	9.33
E9QKY4	FYVE, RhoGEF and PH domain-containing protein 5 OS=Mus musculus GN=Fgd5 PE=2 SV=1 - [E9QKY4_MOUSE]	4.69	3	4	5	10	1.295	1.008	0.858	0.827	21.83	4.69	7	10	1514	166.8	4.91
Q6DFY2	Opioid binding protein/cell adhesion molecule-like OS=Mus musculus GN=Opcml PE=2 SV=1 - [Q6DFY2_MOUSE]	42.73	1	2	16	123	0.839	1.090	1.002	0.827	377.08	42.73	30	123	337	37.1	6.68
Q8BQ33	Treslin OS=Mus musculus GN=Ticrr PE=2 SV=2 - [illegible]	1.22	1	2	3	3	1.630	1.406	0.579	0.827	6.90	1.22	3	3	1889	208.2	7.94
O88792	Junctional adhesion molecule A OS=Mus musculus GN=F11r PE=1 SV=2 - [JAM1_MOUSE]	12.00	1	4	4	7	0.917	0.974	0.821	0.827	18.88	12.00	5	7	300	32.4	6.77
Q8CDM1	ATPase family AAA domain-containing protein 2 OS=Mus musculus GN=Atad2 PE=1 SV=1 - [ATAD2_MOUSE]	1.06	2	1	1	1	10.527	1.441	1.052	0.827	3.14	1.06	1	1	1040	117.9	6.89
Q6PDX6	E3 ubiquitin-protein ligase Rnf220 OS=Mus musculus GN=Rnf220 PE=1 SV=1 - [RN220_MOUSE]	13.07	6	7	7	23	1.444	0.802	0.937	0.827	48.36	13.07	12	23	566	62.7	6.04
F8VQL9	Nuclear receptor corepressor 2 OS=Mus musculus GN=Ncor2 PE=2 SV=1 - [F8VQL9_MOUSE]	15.52	12	23	31	59	1.076	0.766	0.983	0.827	182.56	15.52	49	59	2507	273.2	7.44
Q5SQP1-2	Isoform 2 of HORMA domain-containing protein 2 OS=Mus musculus GN=Hormad2 - [HORM2_MOUSE]	5.67	2	1	1	3	1.411	0.895	1.644	0.827	0.00	5.67	1	3	247	28.2	6.93
Q8BHN0	Protein phosphatase 1L OS=Mus musculus GN=Ppm1l PE=1 SV=1 - [PPM1L_MOUSE]	13.89	3	5	5	12	0.770	0.728	0.748	0.827	31.66	13.89	9	12	360	41.0	5.99
Q9WVI9-2	Isoform JIP-1a of C-Jun-amino-terminal kinase-interacting protein 1 OS=Mus musculus GN=Mapk8ip1 - [illegible]	27.58	4	3	11	34	1.536	0.782	0.906	0.828	101.07	27.58	17	34	660	71.9	4.84

Q3UHI0	Serine-rich coiled-coil domain-containing protein 2 OS=Mus musculus GN=Ccser2 PE=1 SV=1 - [CCSE2_MOUSE]	6.84	2	4	5	7	1.051	0.700	1.219	0.828	16.65	6.84	7	7	833	92.9	6.46
Q3UJB0	Protein Sf3b2 OS=Mus musculus GN=Sf3b2 PE=2 SV=1 - [Q3UJB0_MOUSE]	23.92	1	19	19	60	0.714	0.799	0.983	0.828	157.72	23.92	33	60	878	98.1	5.62
P54130	Fibroblast growth factor 9 OS=Mus musculus GN=Fgf9 PE=2 SV=2 - [FGF9_MOUSE]	6.25	1	1	1	1	0.334	2.653	0.714	0.828	0.00	6.25	1	1	208	23.4	7.58
P35979	60S ribosomal protein L12 OS=Mus musculus GN=Rpl12 PE=1 SV=2 - [RL12_MOUSE]	67.27	3	9	9	76	0.906	0.788	0.760	0.828	238.53	67.27	16	76	165	17.8	9.42
E9Q1V2	Copine-5 (Fragment) OS=Mus musculus GN=Cpne5 PE=2 SV=1 - [E9Q1V2_MOUSE]	35.98	2	1	6	25	1.023	1.740	2.234	0.828	68.40	35.98	10	25	214	23.7	5.11
Q6NVG1	Lysophospholipid acyltransferase LPCAT4 OS=Mus musculus GN=Lpcat4 PE=1 SV=1 - [LPCT4_MOUSE]	19.85	1	7	7	15	0.595	0.838	1.104	0.828	48.71	19.85	12	15	524	57.1	8.75
Q8R0W6	NEDD4 family-interacting protein 1 OS=Mus musculus GN=Ndfip1 PE=1 SV=1 -	24.43	1	3	3	5	0.775	0.738	0.772	0.828	11.77	24.43	3	5	221	24.9	4.61
Q9D0C1	E3 ubiquitin-protein ligase RNF115 OS=Mus musculus GN=Rnf115 PE=1 SV=1 - [RN115_MOUSE]	4.59	1	2	2	4	1.534	0.933	1.194	0.828	8.49	4.59	2	4	305	33.8	5.83
Q61743	ATP-sensitive inward rectifier potassium channel 11 OS=Mus musculus GN=Kcnj11 PE=2 SV=1 - [IRK11_MOUSE]	11.54	1	4	4	5	1.521	1.080	0.976	0.828	11.65	11.54	5	5	390	43.5	8.19
B1AVU6	Protein Gm436 OS=Mus musculus GN=Gm436 PE=4 SV=1 - [B1AVU6_MOUSE]	4.42	1	1	1	1	0.529	0.448	0.838	0.828	2.64	4.42	1	1	407	45.8	8.44
Q9QYC1-2	Isoform 2 of Pecanex-like protein 1 OS=Mus musculus GN=Pcnx - [PCX1_MOUSE]	3.37	2	6	7	13	0.696	0.644	0.910	0.828	28.78	3.37	10	13	2252	248.0	7.20
Q8BYZ1	ABI gene family member 3 OS=Mus musculus GN=Abi3 PE=2 SV=3 - [ABI3_MOUSE]	11.72	3	3	4	20	1.447	1.077	1.116	0.828	51.15	11.72	7	20	367	39.1	5.54
A2T4N6	Programmed cell death 2 alternative transcript OS=Mus musculus GN=Pdcd2 PE=2 SV=1 - [A2T4N6_MOUSE]	17.05	2	4	4	4	1.197	1.110	0.872	0.828	14.08	17.05	4	4	258	28.7	5.36
Q6P5D3-2	Isoform 2 of Putative ATP-dependent RNA helicase DHX57 OS=Mus musculus GN=Dhx57 - [DHX57_MOUSE]	8.54	5	8	11	27	1.213	0.898	0.965	0.828	83.79	8.54	18	27	1335	149.8	7.75
Q8BZL4	Probable G-protein coupled receptor 22 OS=Mus musculus GN=Gpr22 PE=2 SV=1 - [GPR22_MOUSE]	2.31	2	1	1	2	0.682	1.472	1.269	0.828	4.07	2.31	2	2	432	49.0	9.28
Q91WV0	Protein Dr1 OS=Mus musculus GN=Dr1 PE=2 SV=1 - [NC2B_MOUSE]	15.34	1	3	3	9	1.462	0.933	0.924	0.828	23.29	15.34	6	9	176	19.4	4.75
Q8VIF2	Serine protease 42 OS=Mus musculus GN=Prss42 PE=2 SV=1 - [PRS42_MOUSE]	3.88	1	1	2	3	1.072	1.240	0.794	0.828	6.21	3.88	3	3	335	36.7	8.48
Q8BJU0-2	Isoform 2 of Small glutamine-rich tetratricopeptide repeat-containing protein alpha OS=Mus musculus GN=Sgta - [SGTA_MOUSE]	31.53	3	9	9	43	1.199	0.935	0.758	0.828	129.12	31.53	16	43	314	34.2	5.06
E9Q1L5	Sentrin-specific protease 7 OS=Mus musculus GN=Senp7 PE=2 SV=1 - [E9Q1L5_MOUSE]	12.67	3	9	9	17	1.069	0.907	0.924	0.829	45.41	12.67	14	17	1010	113.3	6.47
Q3UL36	Arginine and glutamate-rich protein 1 OS=Mus musculus GN=Arglu1 PE=1 SV=2 -	16.61	2	6	7	37	0.915	0.927	1.050	0.829	80.80	16.61	12	37	271	32.9	10.36
Q9Z315	U4/U6.U5 tri-snRNP-associated protein 1 OS=Mus musculus GN=Sart1 PE=2 SV=1 - [SNUT1_MOUSE]	18.86	1	11	11	25	0.772	0.712	0.949	0.829	77.39	18.86	18	25	806	90.8	5.82
Q8BM72	Heat shock 70 kDa protein 13 OS=Mus musculus GN=Hspa13 PE=2 SV=1 - [HSP13_MOUSE]	29.30	3	13	13	45	0.804	0.999	0.616	0.829	116.85	29.30	25	45	471	51.7	5.63
Q8VI59	Pecanex-like protein 3 OS=Mus musculus GN=Pcnxl3 PE=1 SV=2 - [PCX3_MOUSE]	6.90	4	10	10	12	1.355	0.837	1.048	0.829	36.05	6.90	10	12	2028	221.4	6.62
F8VQE2	Kinesin-like protein KIF21B OS=Mus musculus GN=Kif21b PE=2 SV=1 - [F8VQE2_MOUSE]	11.76	4	11	16	45	0.603	0.711	0.814	0.829	118.74	11.76	25	45	1624	181.3	6.96
P57780	Alpha-actinin-4 OS=Mus musculus GN=Actn4 PE=1 SV=1 - [ACTN4_MOUSE]	74.56	6	20	59	522	1.088	1.851	1.243	0.829	1497.25	74.56	105	522	912	104.9	5.41

A3KG01	Eph receptor B2 OS=Mus musculus GN=Ephb2 PE=2 SV=2 - [A3KG01_MOUSE]	19.98	8	13	17	39	0.546	0.560	0.719	0.829	107.56	19.98	28	39	986	109.8	5.71
Q7TNV0	Protein DEK OS=Mus musculus GN=Dek PE=1 SV=1 - [DEK_MOUSE]	24.47	3	9	9	33	0.641	0.953	0.908	0.829	86.90	24.47	13	33	380	43.1	6.86
G3UWD4	Periphilin 1, isoform CRA_b OS=Mus musculus GN=Pphln1 PE=4 SV=1 - [G3UWD4_MOUSE]	6.14	5	2	2	3	1.333	0.791	1.059	0.829	7.82	6.14	3	3	293	33.8	6.32
Q60596	DNA repair protein XRCC1 OS=Mus musculus GN=Xrcc1 PE=1 SV=2 - [XRCC1_MOUSE]	8.24	1	5	5	10	0.670	0.648	0.906	0.829	25.89	8.24	8	10	631	68.9	6.33
D3YXK2	Scaffold attachment factor B1 OS=Mus musculus GN=Safb PE=1 SV=2 - [SAFB1_MOUSE]	25.40	4	11	23	63	0.929	0.883	1.056	0.829	148.22	25.40	33	63	937	105.0	5.35
Q9QYE6	Golgin subfamily A member 5 OS=Mus musculus GN=Golga5 PE=1 SV=2 -	33.33	1	18	19	51	1.554	0.930	0.920	0.830	176.65	33.33	30	51	729	82.3	6.23
Q673H1	Tumor suppressor candidate gene 1 protein homolog OS=Mus musculus GN=Tusc1 PE=2 SV=1 - [TUSC1_MOUSE]	13.17	1	2	2	5	0.908	0.732	1.245	0.830	23.37	13.17	3	5	205	22.7	11.09
Q9CS74	Protein SGT1 homolog OS=Mus musculus GN=Ecd PE=2 SV=2 - [SGT1_MOUSE]	3.28	1	2	2	4	1.744	1.159	1.105	0.830	9.34	3.28	2	4	641	71.7	4.94
Q9D303	Uncharacterized protein OS=Mus musculus GN=Gm8989 PE=2 SV=1 - [Q9D303_MOUSE]	6.23	1	1	1	1	1.646	1.925	0.703	0.830	0.00	6.23	1	1	353	41.0	7.78
Q9D7M1	Glucose-induced degradation protein 8 homolog OS=Mus musculus GN=Gid8 PE=2 SV=1 - [GID8_MOUSE]	50.00	1	7	7	34	1.446	1.078	0.943	0.830	159.59	50.00	12	34	228	26.8	4.97
P62317	Small nuclear ribonucleoprotein Sm D2 OS=Mus musculus GN=Snrpd2 PE=1 SV=1 -	46.61	2	6	6	29	1.369	0.901	0.844	0.830	96.47	46.61	11	29	118	13.5	9.91
O08784	Treacle protein OS=Mus musculus GN=Tcof1 PE=1 SV=1 - [TCOF_MOUSE]	21.44	5	23	23	65	1.013	0.738	0.967	0.830	209.19	21.44	37	65	1320	134.9	9.35
Q9D8N0	Elongation factor 1-gamma OS=Mus musculus GN=Eef1g PE=1 SV=3 - [EF1G_MOUSE]	43.71	1	17	17	146	0.563	0.728	0.699	0.830	351.68	43.71	31	146	437	50.0	6.74
Q9D8E6	60S ribosomal protein L4 OS=Mus musculus GN=Rpl4 PE=1 SV=3 - [RL4_MOUSE]	44.63	1	21	21	121	0.490	0.572	0.691	0.830	313.49	44.63	35	121	419	47.1	11.00
Q00PI9	Heterogeneous nuclear ribonucleoprotein U-like protein 2 OS=Mus musculus GN=Hnrnpul2 PE=1 SV=2 - [illegible]	36.64	1	24	24	118	0.723	0.859	0.893	0.830	346.77	36.64	38	118	745	84.9	4.89
D9J2V6	Pre-B-cell leukemia transcription factor 1 OS=Mus musculus GN=Pbx1 PE=2 SV=1 - [D9J2V6_MOUSE]	27.14	11	4	6	9	0.590	1.654	1.095	0.830	33.43	27.14	8	9	339	37.0	6.60
P97822-2	Isoform 2 of Acidic leucine-rich nuclear phosphoprotein 32 family member E OS=Mus musculus GN=Anp32e - [illegible]	37.10	8	6	8	41	1.257	0.988	1.092	0.830	127.40	37.10	14	41	248	28.2	3.87
E9PXX7	Thioredoxin domain-containing protein 5 OS=Mus musculus GN=Txndc5 PE=2 SV=1 - [E9PXX7_MOUSE]	62.50	2	15	17	60	1.259	1.184	0.922	0.830	158.85	62.50	29	60	344	38.5	5.39
Q7M6Y3-6	Isoform 6 of Phosphatidylinositol-binding clathrin assembly protein OS=Mus musculus GN=Picalm - [illegible]	34.36	3	1	17	98	0.856	0.630	1.109	0.830	277.36	34.36	29	98	652	70.5	7.90
Q6P2L7	Protein CASC4 OS=Mus musculus GN=Casc4 PE=1 SV=1 - [CASC4_MOUSE]	29.66	5	10	10	31	1.313	1.115	0.866	0.831	94.65	29.66	17	31	435	49.4	5.45
Q8CIR4	Transient receptor potential cation channel subfamily M member 6 OS=Mus musculus GN=Trpm6 PE=2 SV=1 - [illegible]	0.99	1	1	2	2	0.839	1.017	0.781	0.831	5.55	0.99	2	2	2028	232.7	8.27
Q91X96	Guanine nucleotide exchange factor MSS4 OS=Mus musculus GN=Rabif PE=2 SV=1 -	13.01	1	3	3	8	2.292	0.928	1.074	0.831	22.20	13.01	4	8	123	13.9	5.52
B1B1D8	39S ribosomal protein L2, mitochondrial OS=Mus musculus GN=Mrpl2 PE=2 SV=1 - [B1B1D8_MOUSE]	21.71	2	3	4	11	1.141	0.632	0.830	0.831	38.48	21.71	8	11	304	33.1	11.06
G3UWF7	Polypeptide N-acetylgalactosaminyltransferase 15 OS=Mus musculus GN=Galnt15 PE=4 SV=1 - [illegible]	1.56	2	1	1	1	0.673	0.656	1.031	0.831	2.16	1.56	1	1	514	58.5	7.21
Q5SXY1-3	Isoform 4 of Cytospin-B OS=Mus musculus GN=Specc1 - [CYTSB_MOUSE]	36.17	1	1	33	125	1.083	0.363	0.927	0.831	334.43	36.17	57	125	987	109.8	5.86

Q9CXY6	Interleukin enhancer-binding factor 2 OS=Mus musculus GN=Ilf2 PE=1 SV=1 - [ILF2_MOUSE]	38.97	1	13	13	49	0.702	1.002	0.777	0.831	124.76	38.97	25	49	390	43.0	5.26
Q3U0V1	Far upstream element-binding protein 2 OS=Mus musculus GN=Khsrp PE=1 SV=2 - [FUBP2_MOUSE]	52.01	1	28	35	268	1.090	0.886	1.129	0.831	761.98	52.01	62	268	748	76.7	7.33
Q8BK67	Protein RCC2 OS=Mus musculus GN=Rcc2 PE=2 SV=1 - [RCC2_MOUSE]	20.58	2	10	10	26	0.811	0.742	0.906	0.831	86.08	20.58	16	26	520	55.9	8.72
Q6ZQA6	Immunoglobulin superfamily member 3 OS=Mus musculus GN=Igsf3 PE=1 SV=2 - [IGSF3_MOUSE]	8.96	1	9	9	21	0.973	0.838	0.730	0.831	63.16	8.96	16	21	1194	134.6	6.05
P62911	60S ribosomal protein L32 OS=Mus musculus GN=Rpl32 PE=2 SV=2 - [RL32_MOUSE]	44.44	4	7	7	53	0.512	0.702	0.720	0.831	147.30	44.44	13	53	135	15.8	11.33
O88974-3	Isoform 3 of Histone-lysine N-methyltransferase SETDB1 OS=Mus musculus GN=Setdb1 - [SETB1_MOUSE]	5.00	5	3	3	6	0.949	0.653	0.914	0.831	8.33	5.00	4	6	500	54.4	5.11
F2Z4B3	Cyclin-dependent kinase 2-associated protein 1 OS=Mus musculus GN=Cdk2ap1 PE=2 SV=1 - [F2Z4B3_MOUSE]	26.44	4	2	2	4	0.869	1.204	0.966	0.831	12.76	26.44	3	4	87	9.6	9.19
Q9Z0H4-11	Isoform 11 of CUGBP Elav-like family member 2 OS=Mus musculus GN=Celf2 - [CELF2_MOUSE]	24.14	13	9	11	50	0.719	1.763	1.472	0.831	128.59	24.14	19	50	464	49.2	9.09
E9QKA4	Serine/arginine repetitive matrix protein 1 OS=Mus musculus GN=Srrm1 PE=2 SV=1 - [E9QKA4_MOUSE]	7.58	9	3	7	19	0.744	0.794	0.759	0.831	38.20	7.58	12	19	897	101.1	11.94
P01756	Ig heavy chain V region MOPC 104E OS=Mus musculus PE=1 SV=1 - [HVM12_MOUSE]	16.24	3	1	1	4	2.646	2.898	0.828	0.831	15.21	16.24	2	4	117	13.0	7.11
Q8CIE6	Coatomer subunit alpha OS=Mus musculus GN=Copa PE=1 SV=2 - [COPA_MOUSE]	42.48	4	44	44	129	0.705	0.884	0.713	0.832	372.98	42.48	67	129	1224	138.3	7.65
Q6DFZ1	Golgi-specific brefeldin A-resistance factor 1 OS=Mus musculus GN=Gbf1 PE=1 SV=1 - [Q6DFZ1_MOUSE]	15.05	4	21	24	73	0.683	0.704	0.712	0.832	202.09	15.05	39	73	1861	206.7	5.80
P29341	Polyadenylate-binding protein 1 OS=Mus musculus GN=Pabpc1 PE=1 SV=2 -	37.11	3	15	24	126	1.311	0.820	0.898	0.832	393.20	37.11	41	126	636	70.6	9.50
Q9DBH5	Vesicular integral-membrane protein VIP36 OS=Mus musculus GN=Lman2 PE=2 SV=2 - [LMAN2_MOUSE]	36.59	1	10	10	38	0.797	0.881	0.727	0.832	116.90	36.59	18	38	358	40.4	6.95
D3Z069	Pleckstrin homology-like domain family B member 2 (Fragment) OS=Mus musculus GN=Phldb2 PE=2 SV=1 - [D3Z069_MOUSE]	1.95	4	2	2	3	1.430	1.205	1.290	0.832	7.30	1.95	3	3	1131	127.1	6.27
Q9D1M0	Protein SEC13 homolog OS=Mus musculus GN=Sec13 PE=2 SV=3 - [SEC13_MOUSE]	23.91	1	6	6	40	1.315	1.003	0.802	0.832	112.72	23.91	11	40	322	35.5	5.38
Q3THE2	Myosin regulatory light chain 12B OS=Mus musculus GN=Myl12b PE=1 SV=2 -	59.88	3	1	9	135	0.961	0.361	1.283	0.832	407.22	59.88	17	135	172	19.8	4.84
Q80W85	Nucleoplasmin-2 OS=Mus musculus GN=Npm2 PE=2 SV=1 - [NPM2_MOUSE]	7.25	1	1	1	2	1.190	1.032	1.207	0.832	2.83	7.25	1	2	207	23.3	5.10
E9QMV2	Protein Gm6314 OS=Mus musculus GN=Abracl PE=4 SV=1 - [E9QMV2_MOUSE]	40.74	2	2	2	7	2.648	0.923	0.771	0.832	19.21	40.74	4	7	81	9.1	5.80
Q91WB2	Probable lipid phosphate phosphatase PPAPDC3 OS=Mus musculus GN=Ppapdc3 PE=1 SV=1 - [PPAC3_MOUSE]	6.27	1	2	2	16	0.442	0.597	1.280	0.833	54.98	6.27	3	16	271	29.7	9.99
E9QPI5	Sister chromatid cohesion protein PDS5 homolog A OS=Mus musculus GN=Pds5a PE=4 SV=1 - [E9QPI5_MOUSE]	11.19	4	9	12	19	0.816	0.807	0.861	0.833	40.63	11.19	14	19	1332	150.1	7.85
Q9Z1L5	Voltage-dependent calcium channel subunit alpha-2/delta-3 OS=Mus musculus GN=Cacna2d3 PE=1 SV=1 - [CA2D3_MOUSE]	20.62	1	18	18	58	0.558	0.778	1.102	0.833	160.20	20.62	31	58	1091	122.7	5.73
F2Z4A3	Protein Fat1 OS=Mus musculus GN=Fat1 PE=2 SV=1 - [F2Z4A3_MOUSE]	4.20	1	13	15	29	1.259	0.994	1.067	0.833	62.60	4.20	24	29	4590	506.0	5.00
E9PVG7	Mitogen-activated protein kinase kinase kinase kinase 4 OS=Mus musculus GN=Map4k4 PE=4 SV=2 - [E9PVG7_MOUSE]	20.42	3	1	24	66	1.044	0.794	0.991	0.833	177.33	20.42	37	66	1288	146.6	7.58

Q8K2D6	Deoxycytidylate deaminase OS=Mus musculus GN=Dctd PE=2 SV=1 - [DCTD_MOUSE]	3.93	2	1	1	4	1.222	1.020	1.329	0.833	4.54	3.93	1	4	178	20.0	7.91
Q8BY02	NF-kappa-B-repressing factor OS=Mus musculus GN=Nkrf PE=2 SV=3 - [NKRF_MOUSE]	19.57	1	12	13	29	1.780	0.717	0.919	0.833	68.03	19.57	19	29	690	77.7	8.97
E9PWK7	Ephrin type-B receptor 4 OS=Mus musculus GN=Ephb4 PE=2 SV=1 - [E9PWK7_MOUSE]	9.52	5	3	6	10	1.612	1.277	0.645	0.833	25.16	9.52	7	10	935	103.1	7.08
Q80TE7	Leucine-rich repeat-containing protein 7 OS=Mus musculus GN=Lrrc7 PE=1 SV=2 - [LRRC7_MOUSE]	42.75	8	47	53	170	0.843	1.544	1.494	0.833	471.87	42.75	80	170	1490	166.8	6.61
Q2MKA5-2	Isoform 2 of Neuronal acetylcholine receptor subunit alpha-5 OS=Mus musculus GN=Chrna5 - [ACHA5_MOUSE]	3.20	2	1	1	1	1.754	0.710	0.840	0.834	0.00	3.20	1	1	438	50.3	6.01
Q8VDD5	Myosin-9 OS=Mus musculus GN=Myh9 PE=1 SV=4 - [MYH9_MOUSE]	47.91	2	68	95	521	1.366	1.043	1.122	0.834	1526.05	47.91	167	521	1960	226.2	5.66
A4FUP9	Glycosyltransferase 1 domain-containing protein 1 OS=Mus musculus GN=Glt1d1 PE=2 SV=2 - [GL1D1_MOUSE]	10.98	1	2	2	4	1.367	0.805	1.137	0.834	7.41	10.98	3	4	346	38.8	5.66
O55234	Proteasome subunit beta type-5 OS=Mus musculus GN=Psmb5 PE=1 SV=3 - [PSB5_MOUSE]	53.79	1	15	15	93	1.923	0.846	0.760	0.834	300.21	53.79	28	93	264	28.5	7.02
D3Z4U0	Zinc finger Ran-binding domain-containing protein 2 OS=Mus musculus GN=Zranb2 PE=2 SV=1 - [D3Z4U0_MOUSE]	23.03	5	7	7	25	0.846	0.741	0.958	0.834	56.77	23.03	12	25	330	37.3	9.63
Q9CQM9	Glutaredoxin-3 OS=Mus musculus GN=Glrx3 PE=1 SV=1 - [GLRX3_MOUSE]	55.79	1	15	15	84	1.764	0.799	0.873	0.834	258.76	55.79	28	84	337	37.8	5.59
P67984	60S ribosomal protein L22 OS=Mus musculus GN=Rpl22 PE=2 SV=2 - [RL22_MOUSE]	43.75	1	4	4	12	1.119	0.796	0.924	0.834	29.35	43.75	6	12	128	14.7	9.19
Q91YY0	Very low-density lipoprotein receptor OS=Mus musculus GN=Vldlr PE=2 SV=1 - [Q91YY0_MOUSE]	23.91	5	15	15	39	0.764	1.316	0.979	0.834	98.12	23.91	23	39	845	93.5	4.79
P62264	40S ribosomal protein S14 OS=Mus musculus GN=Rps14 PE=2 SV=3 - [RS14_MOUSE]	41.72	3	7	7	61	0.662	0.562	0.841	0.834	187.17	41.72	13	61	151	16.3	10.05
Q8VDT9	39S ribosomal protein L50, mitochondrial OS=Mus musculus GN=Mrpl50 PE=2 SV=2 - [RM50_MOUSE]	60.38	1	8	8	26	1.252	0.976	0.981	0.834	77.98	60.38	13	26	159	18.2	9.33
Q61048	WW domain-binding protein 4 OS=Mus musculus GN=Wbp4 PE=1 SV=4 - [WBP4_MOUSE]	2.93	1	1	1	5	0.957	0.514	1.074	0.834	10.95	2.93	2	5	376	42.1	7.36
Q6ZWZ7	60S ribosomal protein L17 OS=Mus musculus GN=Rpl17 PE=2 SV=1 - [Q6ZWZ7_MOUSE]	38.59	2	4	7	37	0.602	0.811	0.703	0.834	100.87	38.59	12	37	184	21.4	10.18
Q9DCT8	Cysteine-rich protein 2 OS=Mus musculus GN=Crip2 PE=1 SV=1 - [CRIP2_MOUSE]	46.15	1	7	8	83	1.156	1.255	1.257	0.835	258.79	46.15	13	83	208	22.7	8.63
F6T9K1	Dr1-associated corepressor OS=Mus musculus GN=Drap1 PE=2 SV=2 - [F6T9K1_MOUSE]	20.33	5	4	4	9	0.709	0.671	0.814	0.835	21.26	20.33	6	9	182	20.0	8.25
Q61809	Leucine-rich repeat neuronal protein 1 OS=Mus musculus GN=Lrrn1 PE=2 SV=1 - [LRRN1_MOUSE]	17.46	1	11	12	24	0.849	0.705	0.862	0.835	72.97	17.46	20	24	716	80.5	6.15
Q68FD9	UPF0606 protein KIAA1549 OS=Mus musculus GN=Kiaa1549 PE=1 SV=3 -	15.46	3	20	20	46	0.688	1.118	1.216	0.835	149.31	15.46	31	46	1940	209.1	6.10
Q08879-2	Isoform C of Fibulin-1 OS=Mus musculus GN=Fbln1 - [FBLN1_MOUSE]	7.30	2	5	5	7	1.305	1.279	0.672	0.835	16.72	7.30	7	7	685	75.2	5.27
Q9QZ82	Cholesterol side-chain cleavage enzyme, mitochondrial OS=Mus musculus GN=Cyp11a1 PE=2 SV=1 - [illegible]	3.99	1	2	2	2	3.119	1.655	1.057	0.835	6.21	3.99	2	2	526	60.3	9.39
H3BL26	Tetraspanin-6 (Fragment) OS=Mus musculus GN=Tspan6 PE=2 SV=1 - [H3BL26_MOUSE]	16.77	4	3	3	9	0.549	0.882	0.548	0.835	18.22	16.77	4	9	167	18.7	7.61
Q920A7	AFG3-like protein 1 OS=Mus musculus GN=Afg3l1 PE=2 SV=2 - [AFG31_MOUSE]	16.48	2	7	12	32	0.818	0.708	0.845	0.835	74.88	16.48	19	32	789	87.0	8.85
Q61189	Methylosome subunit pICln OS=Mus musculus GN=Clns1a PE=1 SV=1 - [ICLN_MOUSE]	41.95	2	6	6	43	1.639	1.089	1.026	0.835	128.85	41.95	11	43	236	26.0	4.12

Q9DBD5	Proline-, glutamic acid- and leucine-rich protein 1 OS=Mus musculus GN=Pelp1 PE=1 SV=2 - [PELP1_MOUSE]	5.34	1	4	4	8	0.727	1.037	0.628	0.835	22.81	5.34	6	8	1123	118.0	4.36
Q8CFZ0	SUMO-conjugating enzyme UBC9 OS=Mus musculus GN=Ube2i PE=4 SV=1 - [Q8CFZ0_MOUSE]	36.63	3	5	5	19	0.737	0.972	0.812	0.836	65.63	36.63	9	19	101	11.2	8.84
Q8C341	SUN domain-containing ossification factor OS=Mus musculus GN=Suco PE=1 SV=3 - [SUCO_MOUSE]	6.00	3	5	6	10	1.065	1.011	1.002	0.836	20.90	6.00	7	10	1250	139.1	5.02
Q8BP01	Vimentin-type intermediate filament-associated coiled-coil protein OS=Mus musculus GN=Vmac PE=2 SV=1 - [illegible]	13.79	1	2	3	9	1.429	1.167	0.838	0.836	22.53	13.79	5	9	174	19.0	5.39
Q8BH43	Wiskott-Aldrich syndrome protein family member 2 OS=Mus musculus GN=Wasf2 PE=1 SV=1 - [WASF2_MOUSE]	23.34	2	11	12	30	1.194	0.932	0.963	0.836	83.53	23.34	21	30	497	54.0	5.53
Q8C129	Leucyl-cystinyl aminopeptidase OS=Mus musculus GN=Lnpep PE=1 SV=1 - [LCAP_MOUSE]	17.46	1	16	16	40	0.615	1.140	0.626	0.836	131.24	17.46	28	40	1025	117.2	5.96
Q9DBR4-2	Isoform 2 of Amyloid beta A4 precursor protein-binding family B member 2 OS=Mus musculus GN=Apbb2 - [illegible]	8.66	11	5	6	15	1.732	0.694	1.616	0.836	40.33	8.66	10	15	739	80.8	6.05
E9PWC0	Microtubule-associated protein OS=Mus musculus GN=Map4 PE=2 SV=1 - [E9PWC0_MOUSE]	71.62	2	30	60	401	1.269	0.635	1.163	0.836	1055.17	71.62	110	401	902	94.5	8.97
Q920P5	Adenylate kinase isoenzyme 5 OS=Mus musculus GN=Ak5 PE=2 SV=2 - [KAD5_MOUSE]	35.94	2	17	18	82	0.827	2.701	1.332	0.836	218.08	35.94	31	82	562	63.3	5.29
A6H690	IQ and AAA domain-containing protein 1-like OS=Mus musculus GN=Iqca1p1 PE=2 SV=3 - [IQCAL_MOUSE]	4.36	4	2	3	9	0.426	1.286	1.638	0.836	14.96	4.36	4	9	825	95.9	9.51
O55060	Thiopurine S-methyltransferase OS=Mus musculus GN=Tpmt PE=1 SV=1 - [TPMT_MOUSE]	20.42	4	4	4	10	1.379	1.073	0.784	0.836	30.40	20.42	6	10	240	27.6	6.44
Q6PCM2-2	Isoform 2 of Integrator complex subunit 6 OS=Mus musculus GN=Ints6 -	8.92	5	6	6	12	0.518	1.019	0.807	0.836	26.81	8.92	10	12	874	98.6	8.66
Q9R0M8	UDP-galactose translocator OS=Mus musculus GN=Slc35a2 PE=2 SV=1 - [S35A2_MOUSE]	13.59	2	3	3	16	0.966	0.664	0.682	0.836	44.94	13.59	5	16	390	40.7	9.86
E9Q3B5	Uncharacterized protein OS=Mus musculus GN=Gm10774 PE=3 SV=1 - [E9Q3B5_MOUSE]	29.85	2	2	2	6	0.972	1.111	0.885	0.836	20.31	29.85	4	6	67	7.6	8.48
E9QKH8	Catenin delta-2 OS=Mus musculus GN=Ctnnd2 PE=2 SV=1 - [E9QKH8_MOUSE]	49.60	1	1	49	455	0.898	0.548	1.302	0.837	1407.33	49.60	85	455	1246	134.9	7.65
Q8CGF7-2	Isoform 2 of Transcription elongation regulator 1 OS=Mus musculus GN=Tcerg1 - [TCRG1_MOUSE]	17.24	3	21	22	60	1.014	0.633	1.042	0.837	155.47	17.24	33	60	1079	121.5	8.54
F7CDW2	Serine-rich coiled-coil domain-containing protein 1 (Fragment) OS=Mus musculus GN=Ccser1 PE=4 SV=1 - [illegible]	14.88	3	2	2	3	2.047	1.186	1.043	0.837	8.83	14.88	2	3	168	18.7	5.21
Q80ZM5	H1 histone family, member X OS=Mus musculus GN=H1fx PE=2 SV=1 - [Q80ZM5_MOUSE]	21.81	1	4	4	15	0.519	1.036	0.904	0.837	36.01	21.81	8	15	188	20.1	11.22
O55201-2	Isoform 2 of Transcription elongation factor SPT5 OS=Mus musculus GN=Supt5h - [SPT5H_MOUSE]	28.49	2	22	22	80	0.954	0.987	0.978	0.837	213.67	28.49	39	80	888	98.2	7.15
D3YTQ3	Heterogeneous nuclear ribonucleoprotein D-like OS=Mus musculus GN=Hnrpdl PE=2 SV=1 - [D3YTQ3_MOUSE]	35.00	5	12	15	88	1.207	0.790	1.404	0.837	269.21	35.00	25	88	420	46.2	9.57
O89079	Coatomer subunit epsilon OS=Mus musculus GN=Cope PE=2 SV=3 - [COPE_MOUSE]	39.94	5	10	11	27	1.135	0.884	0.797	0.837	81.83	39.94	19	27	308	34.5	5.06
D3Z667	Dynein heavy chain 2, axonemal OS=Mus musculus GN=Dnah2 PE=2 SV=1 -	2.58	2	8	9	19	1.835	1.201	1.432	0.837	23.02	2.58	10	19	4462	512.0	6.47
Q9QUR7	Peptidyl-prolyl cis-trans isomerase NIMA-interacting 1 OS=Mus musculus GN=Pin1 PE=1 SV=1 - [PIN1_MOUSE]	55.15	1	8	8	82	1.745	1.046	1.193	0.837	276.20	55.15	13	82	165	18.4	8.79
Q3TDQ1	Dolichyl-diphosphooligosaccharide--protein glycosyltransferase subunit STT3B OS=Mus musculus GN=Stt3b PE=1 SV=2 - [STT3B_MOUSE]	11.54	1	8	10	36	0.579	0.733	0.725	0.837	90.24	11.54	20	36	823	93.2	8.95

Q9CQ06	39S ribosomal protein L24, mitochondrial OS=Mus musculus GN=Mrpl24 PE=2 SV=1 - [RM24_MOUSE]	28.70	1	6	6	16	1.205	0.561	0.847	0.837	37.17	28.70	9	16	216	24.9	9.50
P63037	DnaJ homolog subfamily A member 1 OS=Mus musculus GN=Dnaja1 PE=1 SV=1 -	48.36	5	14	17	95	0.861	0.654	0.828	0.837	281.40	48.36	31	95	397	44.8	7.08
Q0VBV7	Uncharacterized protein KIAA1377 OS=Mus musculus GN=Kiaa1377 PE=2 SV=1 -	0.54	1	1	1	2	1.861	1.269	1.420	0.837	6.02	0.54	1	2	1103	123.6	9.07
E9QQ10	A-kinase anchor protein 9 OS=Mus musculus GN=Akap9 PE=2 SV=1 - [E9QQ10_MOUSE]	18.95	5	63	66	159	1.227	1.089	1.088	0.838	419.82	18.95	105	159	3779	433.9	5.03
P63271	Transcription elongation factor SPT4-A OS=Mus musculus GN=Supt4h1a PE=2 SV=1 - [SPT4A_MOUSE]	19.66	2	1	2	6	0.694	0.843	0.873	0.838	17.49	19.66	3	6	117	13.2	8.06
Q8BIF2-2	Isoform 2 of RNA binding protein fox-1 homolog 3 OS=Mus musculus GN=Rbfox3 -	35.00	7	5	10	35	1.096	0.946	1.361	0.838	124.78	35.00	17	35	360	39.0	7.23
Q8BUL6	Pleckstrin homology domain-containing family A member 1 OS=Mus musculus GN=Plekha1 PE=1 SV=1 - [illegible]	28.46	5	11	11	19	0.932	0.631	0.840	0.838	35.99	28.46	15	19	383	43.3	9.22
Q9D365	Signal peptidase complex subunit 3 OS=Mus musculus GN=Spcs3 PE=2 SV=1 - [SPCS3_MOUSE]	21.67	1	4	4	8	0.583	0.851	0.622	0.838	21.57	21.67	5	8	180	20.1	9.60
P08556	GTPase NRas OS=Mus musculus GN=Nras PE=2 SV=1 - [RASN_MOUSE]	61.90	5	2	9	90	0.658	0.901	0.856	0.838	204.56	61.90	15	90	189	21.2	5.17
H3BL28	Protein Ppp1r9a (Fragment) OS=Mus musculus GN=Ppp1r9a PE=2 SV=1 -	27.54	9	23	26	123	1.031	1.094	2.095	0.838	324.04	27.54	45	123	1042	116.4	4.96
Q8BSK8-2	Isoform Alpha II of Ribosomal protein S6 kinase beta-1 OS=Mus musculus GN=Rps6kb1 - [KS6B1_MOUSE]	12.95	4	5	7	16	0.856	0.992	0.895	0.838	46.65	12.95	12	16	502	56.1	6.44
F8WI56	Disco-interacting protein 2 homolog A OS=Mus musculus GN=Dip2a PE=2 SV=1 - [F8WI56_MOUSE]	22.86	3	2	29	71	0.910	0.607	1.148	0.838	191.30	22.86	48	71	1562	169.4	7.77
Q8VBT0	Thioredoxin-related transmembrane protein 1 OS=Mus musculus GN=Tmx1 PE=1 SV=1 - [TMX1_MOUSE]	20.50	2	7	7	32	0.576	0.786	0.627	0.838	77.89	20.50	12	32	278	31.4	5.29
B8JK56	Adenylate cyclase type 3 OS=Mus musculus GN=Adcy3 PE=3 SV=1 - [B8JK56_MOUSE]	14.86	2	15	15	36	0.634	0.734	0.804	0.838	101.72	14.86	25	36	1144	128.9	6.29
P23116	Eukaryotic translation initiation factor 3 subunit A OS=Mus musculus GN=Eif3a PE=1 SV=5 - [EIF3A_MOUSE]	29.02	1	40	40	176	0.780	0.727	0.940	0.838	451.53	29.02	70	176	1344	161.8	6.77
P01027	Complement C3 OS=Mus musculus GN=C3 PE=1 SV=3 - [CO3_MOUSE]	45.46	4	68	68	255	2.293	1.342	0.516	0.838	733.34	45.46	114	255	1663	186.4	6.73
D3Z742	MCG125361, isoform CRA_a OS=Mus musculus GN=Mb21d2 PE=4 SV=1 - [D3Z742_MOUSE]	17.92	2	8	8	18	0.767	0.886	0.812	0.838	45.87	17.92	12	18	491	55.6	7.50
Q8BFX3	BTB/POZ domain-containing protein KCTD3 OS=Mus musculus GN=Kctd3 PE=2 SV=1 - [KCTD3_MOUSE]	8.59	2	6	6	11	1.256	0.834	0.651	0.838	29.68	8.59	10	11	815	88.8	7.15
P35377-4	Isoform KOR3C of Nociceptin receptor OS=Mus musculus GN=Oprl1 -	11.58	1	1	1	1	1.142	1.324	0.857	0.838	0.00	11.58	1	1	95	10.3	7.94
P12265	Beta-glucuronidase OS=Mus musculus GN=Gusb PE=2 SV=2 - [BGLR_MOUSE]	12.50	2	7	7	20	2.100	0.774	0.754	0.838	66.68	12.50	13	20	648	74.1	6.70
P59017	Bcl-2-like protein 13 OS=Mus musculus GN=Bcl2l13 PE=1 SV=2 - [B2L13_MOUSE]	19.35	1	5	6	16	0.520	0.897	0.897	0.838	31.19	19.35	10	16	434	46.7	4.59
Q8CB96-2	Isoform 2 of Ras association domain-containing protein 4 OS=Mus musculus GN=Rassf4 - [illegible]	15.51	3	1	4	8	1.707	1.329	0.756	0.838	16.88	15.51	5	8	303	34.6	6.54
F6UPY1	PAP-associated domain-containing protein 5 (Fragment) OS=Mus musculus GN=Papd5 PE=2 SV=1 - [F6UPY1_MOUSE]	16.00	4	2	2	5	2.151	0.916	1.345	0.839	16.80	16.00	3	5	200	21.9	9.63
Q5NCU4	SPARC OS=Mus musculus GN=Sparc PE=2 SV=1 - [Q5NCU4_MOUSE]	27.91	3	8	8	51	1.501	0.526	0.621	0.839	144.99	27.91	15	51	301	34.3	4.86
Q9WTK2-2	Isoform 2 of Chromodomain Y-like protein OS=Mus musculus GN=Cdyl - [CDYL_MOUSE]	4.60	2	2	2	3	1.927	0.778	0.857	0.839	8.28	4.60	3	3	544	60.1	9.10

E9Q6C7	Latrophilin-2 OS=Mus musculus GN=Lphn2 PE=2 SV=1 - [E9Q6C7_MOUSE]	14.93	4	16	18	68	0.967	0.949	0.987	0.839	181.90	14.93	30	68	1487	166.5	6.71
Q03146	Epithelial discoidin domain-containing receptor 1 OS=Mus musculus GN=Ddr1 PE=2 SV=2 - [DDR1_MOUSE]	8.01	8	5	5	13	1.282	0.690	0.893	0.839	34.38	8.01	10	13	911	101.1	6.58
D3Z3S1	Prolactin regulatory element binding, isoform CRA_b OS=Mus musculus GN=Preb PE=4 SV=1 - [D3Z3S1_MOUSE]	45.43	3	12	12	33	0.868	0.800	0.834	0.839	85.75	45.43	20	33	350	37.9	8.56
Q91YQ5	Dolichyl-diphosphooligosaccharide--protein glycosyltransferase subunit 1 OS=Mus musculus GN=Rpn1 PE=2 SV=1 - [RPN1_MOUSE]	46.22	1	25	25	141	0.754	0.937	0.711	0.839	437.45	46.22	42	141	608	68.5	6.46
Q569Z6	Thyroid hormone receptor-associated protein 3 OS=Mus musculus GN=Thrap3 PE=1 SV=1 - [TR150_MOUSE]	15.46	5	15	15	52	0.678	0.732	0.922	0.839	151.65	15.46	25	52	951	108.1	10.17
Q6A068	Cell division cycle 5-like protein OS=Mus musculus GN=Cdc5l PE=1 SV=2 - [CDC5L_MOUSE]	34.16	1	22	22	72	1.056	0.888	0.997	0.839	183.67	34.16	36	72	802	92.1	8.02
Q6JHY2-3	Isoform 4 of Submandibular gland protein C OS=Mus musculus GN=Muc19 -	10.26	1	1	1	1	1.100	0.824	1.440	0.839	2.69	10.26	1	1	341	34.1	9.09
Q9Z0R9	Fatty acid desaturase 2 OS=Mus musculus GN=Fads2 PE=2 SV=1 - [FADS2_MOUSE]	22.75	1	9	9	23	0.913	0.755	0.530	0.839	62.52	22.75	18	23	444	52.4	8.82
Q9Z1A1	Protein Tfg OS=Mus musculus GN=Tfg PE=2 SV=1 - [Q9Z1A1_MOUSE]	41.81	7	13	13	85	1.670	0.852	0.937	0.839	280.34	41.81	23	85	397	43.0	5.10
Q9D0R2	Threonine--tRNA ligase, cytoplasmic OS=Mus musculus GN=Tars PE=1 SV=2 - [SYTC_MOUSE]	43.21	1	25	29	92	0.924	0.918	0.844	0.839	281.67	43.21	51	92	722	83.3	7.36
Q80XU3	Nuclear ubiquitous casein and cyclin-dependent kinase substrate 1 OS=Mus musculus GN=Nucks1 PE=1 SV=1 - [NUCKS_MOUSE]	7.26	1	2	2	11	0.625	0.754	1.122	0.839	27.61	7.26	4	11	234	26.3	5.14
E9Q0N0	Intersectin-1 OS=Mus musculus GN=Itsn1 PE=2 SV=1 - [E9Q0N0_MOUSE]	38.86	13	2	63	237	5.230	2.149	0.993	0.839	693.78	38.86	108	237	1719	194.8	7.91
Q9WUA2	Phenylalanine--tRNA ligase beta subunit OS=Mus musculus GN=Farsb PE=2 SV=2 - [SYFB_MOUSE]	48.56	1	29	29	122	0.652	0.849	0.738	0.839	363.71	48.56	50	122	589	65.7	7.12
Q9ERE8	Mesoderm development candidate 1 OS=Mus musculus GN=Mesdc1 PE=1 SV=1 -	8.84	1	2	2	8	3.491	1.254	0.821	0.840	24.47	8.84	4	8	362	37.8	8.15
Q5SS80	Dehydrogenase/reductase SDR family member 13 OS=Mus musculus GN=Dhrs13 PE=1 SV=1 - [DHR13_MOUSE]	16.22	4	5	5	18	0.780	0.481	0.749	0.840	62.63	16.22	10	18	376	40.7	7.93
P46938-2	Isoform 2 of Yorkie homolog OS=Mus musculus GN=Yap1 - [YAP1_MOUSE]	39.41	6	9	9	25	2.034	1.499	1.171	0.840	76.92	39.41	14	25	472	50.7	5.00
P59708	Pre-mRNA branch site protein p14 OS=Mus musculus GN=Sf3b14 PE=2 SV=1 -	39.20	1	4	4	13	0.641	0.926	0.937	0.840	44.79	39.20	7	13	125	14.6	9.38
Q0V8T9	Contactin-associated protein like 5-1 OS=Mus musculus GN=Cntnap5a PE=2 SV=1 -	10.43	2	8	11	40	1.066	1.353	0.940	0.840	113.80	10.43	21	40	1304	145.6	6.28
Q61112	45 kDa calcium-binding protein OS=Mus musculus GN=Sdf4 PE=2 SV=1 - [CAB45_MOUSE]	27.98	2	10	10	34	1.251	0.707	0.926	0.840	86.26	27.98	18	34	361	42.0	4.96
P70122	Ribosome maturation protein SBDS OS=Mus musculus GN=Sbds PE=1 SV=4 - [SBDS_MOUSE]	45.60	3	12	12	35	1.061	0.790	0.862	0.840	87.65	45.60	20	35	250	28.8	8.76
Q64264	5-hydroxytryptamine receptor 1A OS=Mus musculus GN=Htr1a PE=2 SV=2 - [5HT1A_MOUSE]	11.16	1	3	4	7	0.558	1.044	1.039	0.840	18.84	11.16	7	7	421	46.1	8.90
Q9ERD6-2	Isoform 2 of Ras-specific guanine nucleotide-releasing factor RalGPS2 OS=Mus musculus GN=Ralgps2 - [RGPS2_MOUSE]	2.88	4	1	1	2	3.684	1.940	1.295	0.840	7.79	2.88	1	2	555	61.7	8.94
Q6A025	Protein phosphatase 1 regulatory subunit 26 OS=Mus musculus GN=Ppp1r26 PE=2 SV=2 - [PPR26_MOUSE]	5.07	1	2	2	2	1.311	0.553	1.145	0.840	2.27	5.07	2	2	1163	124.9	8.76
Q91ZD1-2	Isoform 2 of Protein odd-skipped-related 2 OS=Mus musculus GN=Osr2 - [OSR2_MOUSE]	9.42	3	2	2	2	26.798	2.940	1.224	0.840	1.77	9.42	2	2	276	30.5	9.41
E9QAT4	Protein Sec16a OS=Mus musculus GN=Sec16a PE=2 SV=1 - [E9QAT4_MOUSE]	27.15	4	46	47	141	1.612	1.005	1.150	0.840	418.25	27.15	71	141	2357	254.0	5.81

E9Q6U4	Protein Pfdn4 OS=Mus musculus GN=Pfdn4 PE=2 SV=1 - [E9Q6U4_MOUSE]	41.54	5	5	6	18	1.929	0.985	1.152	0.841	38.11	41.54	8	18	130	14.7	4.50
B6ZND9	EGAM1C OS=Mus musculus GN=Crxos1 PE=2 SV=1 - [B6ZND9_MOUSE]	8.45	2	1	1	3	2.009	1.153	0.967	0.841	7.77	8.45	1	3	142	17.2	9.38
Q8R326	Paraspeckle component 1 OS=Mus musculus GN=Pspc1 PE=1 SV=1 - [PSPC1_MOUSE]	53.73	4	21	23	124	1.311	0.866	1.154	0.841	309.79	53.73	42	124	523	58.7	6.67
Q06770	Corticosteroid-binding globulin OS=Mus musculus GN=Serpina6 PE=1 SV=1 - [CBG_MOUSE]	9.32	1	4	4	8	2.134	1.199	0.468	0.841	11.83	9.32	6	8	397	44.7	5.24
E9PVA6	ARF GTPase-activating protein GIT2 OS=Mus musculus GN=Git2 PE=2 SV=1 - [E9PVA6_MOUSE]	27.14	13	14	17	60	0.971	0.944	0.722	0.841	134.16	27.14	26	60	759	84.4	7.59
Q8R050-2	Isoform 2 of Eukaryotic peptide chain release factor GTP-binding subunit ERF3A OS=Mus musculus GN=Gspt1 - [illegible]	37.95	3	10	22	94	1.485	0.946	0.968	0.841	283.65	37.95	41	94	635	68.5	5.21
Q8BWY3	Eukaryotic peptide chain release factor subunit 1 OS=Mus musculus GN=Etf1 PE=1 SV=4 - [ERF1_MOUSE]	37.76	1	14	14	30	0.692	0.872	0.762	0.841	99.17	37.76	22	30	437	49.0	5.71
Q6ZPR6-2	Isoform 2 of Inhibitor of Bruton tyrosine kinase OS=Mus musculus GN=Ibtk - [IBTK_MOUSE]	3.84	2	3	3	3	1.110	0.942	0.944	0.841	4.51	3.84	3	3	1251	138.5	7.74
M0QWQ7	Semaphorin-3B OS=Mus musculus GN=Sema3b PE=4 SV=1 - [M0QWQ7_MOUSE]	8.05	3	2	2	4	4.955	2.211	0.584	0.841	4.39	8.05	2	4	534	59.1	7.71
A2AR02	Peptidyl-prolyl cis-trans isomerase G OS=Mus musculus GN=Ppig PE=1 SV=1 - [PPIG_MOUSE]	11.17	2	7	7	16	0.522	0.768	0.861	0.841	46.73	11.17	11	16	752	88.3	10.27
G3X8Q6	Kinesin family member C2 OS=Mus musculus GN=Kifc2 PE=3 SV=1 - [G3X8Q6_MOUSE]	7.70	2	5	5	9	1.907	1.194	1.244	0.841	21.71	7.70	9	9	792	85.5	6.16
Q9D554	Splicing factor 3A subunit 3 OS=Mus musculus GN=Sf3a3 PE=2 SV=2 - [SF3A3_MOUSE]	26.95	1	13	13	35	1.029	0.935	0.919	0.841	104.65	26.95	22	35	501	58.8	5.34
P97333	Neuropilin-1 OS=Mus musculus GN=Nrp1 PE=1 SV=2 - [NRP1_MOUSE]	12.35	1	9	9	27	0.569	0.714	0.571	0.841	75.26	12.35	15	27	923	102.9	5.90
Q6DFY8	Protein FAM5B OS=Mus musculus GN=Fam5b PE=2 SV=1 - [FAM5B_MOUSE]	9.83	1	7	8	16	0.736	1.614	1.115	0.841	39.75	9.83	13	16	783	89.2	7.88
Q8R173	Palmitoyltransferase ZDHHC3 OS=Mus musculus GN=Zdhhc3 PE=1 SV=1 -	5.35	1	1	1	4	0.351	0.542	0.684	0.841	14.23	5.35	2	4	299	34.0	8.09
Q8CFZ4	Glypican-3 OS=Mus musculus GN=Gpc3 PE=2 SV=1 - [GPC3_MOUSE]	5.70	2	3	3	7	0.514	1.236	0.467	0.841	11.63	5.70	5	7	579	65.3	6.58
F8VQK6	Protein Fbxl18 OS=Mus musculus GN=Fbxl18 PE=2 SV=1 - [F8VQK6_MOUSE]	17.54	2	9	9	16	0.777	0.934	0.887	0.841	47.71	17.54	14	16	707	77.7	8.13
Q99LT0	Protein dpy-30 homolog OS=Mus musculus GN=Dpy30 PE=1 SV=1 - [DPY30_MOUSE]	47.47	1	4	4	14	1.337	0.844	0.941	0.841	39.40	47.47	8	14	99	11.2	4.88
Q6PAJ3	GRB2-associated and regulator of MAPK protein-like OS=Mus musculus GN=Gareml PE=2 SV=2 - [GAREL_MOUSE]	6.93	1	2	2	6	1.000	0.540	1.200	0.841	22.54	6.93	3	6	880	93.5	6.73
Q9DCG9	tRNA methyltransferase 112 homolog OS=Mus musculus GN=Trmt112 PE=2 SV=1 - [TR112_MOUSE]	27.20	2	3	3	13	1.420	1.065	1.141	0.842	33.65	27.20	5	13	125	14.1	5.27
Q8CGZ0	Calcium homeostasis endoplasmic reticulum protein OS=Mus musculus GN=Cherp PE=1 SV=1 - [CHERP_MOUSE]	8.12	2	8	8	19	1.071	0.876	1.003	0.842	46.96	8.12	15	19	936	106.1	9.14
Q8VDN4	Coiled-coil domain-containing protein 92 OS=Mus musculus GN=Ccdc92 PE=1 SV=1 - [CCD92_MOUSE]	58.28	3	16	16	61	2.029	0.582	1.083	0.842	169.82	58.28	27	61	314	35.2	9.38
A2AWF8	Receptor-type tyrosine-protein phosphatase eta OS=Mus musculus GN=Ptprj PE=2 SV=1 - [A2AWF8_MOUSE]	8.59	4	9	9	24	0.756	1.272	1.397	0.842	72.29	8.59	15	24	1164	128.6	5.39
P51855	Glutathione synthetase OS=Mus musculus GN=Gss PE=2 SV=1 - [GSHB_MOUSE]	49.58	5	22	22	93	2.364	0.625	0.708	0.842	243.88	49.58	40	93	474	52.2	5.80
Q8R5H6	Wiskott-Aldrich syndrome protein family member 1 OS=Mus musculus GN=Wasf1 PE=1 SV=2 - [WASF1_MOUSE]	35.42	1	16	19	115	0.918	1.685	1.536	0.842	369.53	35.42	35	115	559	61.5	6.37

Q9D2W5	GRAM domain-containing protein 3 OS=Mus musculus GN=Gramd3 PE=2 SV=1 -	5.32	2	1	1	2	0.743	0.886	1.024	0.842	9.18	5.32	1	2	432	47.9	7.75
Q62313	Trans-Golgi network integral membrane protein 1 OS=Mus musculus GN=Tgoln1 PE=1 SV=1 - [TGON1_MOUSE]	5.10	2	2	2	3	1.094	1.031	0.821	0.842	9.24	5.10	3	3	353	37.8	5.34
Q3TFP0	Serine/arginine-rich-splicing factor 10 OS=Mus musculus GN=Srsf10 PE=2 SV=1 - [Q3TFP0_MOUSE]	35.71	4	3	6	15	0.501	1.003	0.870	0.842	48.96	35.71	11	15	182	22.1	10.33
Q8CHY6	Transcriptional repressor p66 alpha OS=Mus musculus GN=Gatad2a PE=1 SV=2 -	18.28	2	7	9	30	1.127	0.635	0.938	0.842	89.80	18.28	18	30	629	67.3	9.89
Q8BYR2	Serine/threonine-protein kinase LATS1 OS=Mus musculus GN=Lats1 PE=1 SV=3 - [LATS1_MOUSE]	5.76	1	4	5	10	1.202	0.977	0.984	0.842	23.50	5.76	7	10	1129	126.2	8.73
Q02780-5	Isoform 5 of Nuclear factor 1 A-type OS=Mus musculus GN=Nfia - [NFIA_MOUSE]	16.09	13	3	5	11	1.013	0.884	0.982	0.842	25.02	16.09	9	11	466	51.5	7.77
Q99K48	Non-POU domain-containing octamer-binding protein OS=Mus musculus GN=Nono PE=1 SV=3 - [NONO_MOUSE]	45.88	3	21	23	228	1.405	0.858	0.952	0.842	668.31	45.88	43	228	473	54.5	8.95
Q9CR67	Transmembrane protein 33 OS=Mus musculus GN=Tmem33 PE=2 SV=1 - [TMM33_MOUSE]	22.27	3	4	5	11	0.903	0.827	0.815	0.842	29.84	22.27	8	11	247	28.0	9.66
Q61823	Programmed cell death protein 4 OS=Mus musculus GN=Pdcd4 PE=1 SV=1 - [PDCD4_MOUSE]	16.63	1	6	7	13	0.866	1.213	0.812	0.842	31.02	16.63	11	13	469	51.7	5.16
Q5F2E8	Serine/threonine-protein kinase TAO1 OS=Mus musculus GN=Taok1 PE=1 SV=1 - [TAOK1_MOUSE]	28.37	2	20	28	70	0.676	0.864	1.011	0.842	191.88	28.37	44	70	1001	116.0	7.55
Q8BGT7	Survival of motor neuron-related-splicing factor 30 OS=Mus musculus GN=Smndc1 PE=2 SV=1 - [SPF30_MOUSE]	35.29	1	6	6	9	1.336	0.965	0.920	0.842	23.73	35.29	7	9	238	26.7	7.24
Q8BGS2	BolA-like protein 2 OS=Mus musculus GN=Bola2 PE=1 SV=1 - [BOLA2_MOUSE]	58.14	2	5	5	19	2.467	0.789	1.047	0.843	44.93	58.14	8	19	86	10.2	6.16
G3X997	Metallo-beta-lactamase domain-containing protein 2 OS=Mus musculus GN=Mblac2 PE=4 SV=1 - [G3X997_MOUSE]	55.56	2	13	13	60	0.697	1.322	0.690	0.843	186.34	55.56	24	60	279	31.2	6.86
B2KG29	Mediator of RNA polymerase II transcription subunit 21 OS=Mus musculus GN=Med21 PE=4 SV=1 - [illegible]	22.22	2	1	1	3	1.336	1.115	1.272	0.843	12.15	22.22	2	3	90	9.7	4.35
Q80V26	Inositol monophosphatase 3 OS=Mus musculus GN=Impad1 PE=1 SV=1 - [IMPA3_MOUSE]	28.65	1	8	8	18	0.803	0.701	0.688	0.843	48.41	28.65	13	18	356	38.6	6.47
Q5SRI3	Dedicator of cytokinesis protein 2 OS=Mus musculus GN=Dock2 PE=2 SV=2 - [Q5SRI3_MOUSE]	2.30	3	2	3	4	0.751	0.906	0.835	0.843	5.64	2.30	4	4	1175	135.7	6.99
E9Q1P5	SH3 and multiple ankyrin repeat domains protein 3 (Fragment) OS=Mus musculus GN=Shank3 PE=2 SV=1 - [illegible]	41.91	1	1	5	11	1.314	1.335	2.158	0.843	42.05	41.91	8	11	136	15.3	9.82
Q8C8I3	Eukaryotic translation initiation factor 2D OS=Mus musculus GN=Eif2d PE=2 SV=1 - [Q8C8I3_MOUSE]	6.18	4	3	3	4	7.458	1.141	0.627	0.843	10.62	6.18	4	4	453	50.1	7.99
Q8VI36-2	Isoform Alpha of Paxillin OS=Mus musculus GN=Pxn - [PAXI_MOUSE]	29.44	3	10	10	37	1.509	1.153	1.026	0.843	115.71	29.44	19	37	557	60.8	6.27
Q9CPW4	Actin-related protein 2/3 complex subunit 5 OS=Mus musculus GN=Arpc5 PE=2 SV=3 - [ARPC5_MOUSE]	52.32	3	8	8	70	0.746	1.585	0.986	0.843	212.12	52.32	16	70	151	16.3	5.67
D3Z6S1	Transmembrane protein 214 OS=Mus musculus GN=Tmem214 PE=2 SV=1 - [D3Z6S1_MOUSE]	22.90	4	12	13	45	0.572	0.666	0.728	0.843	104.29	22.90	23	45	642	71.3	9.41
Q8BLQ7	Cationic amino acid transporter 4 OS=Mus musculus GN=Slc7a4 PE=1 SV=1 -	7.87	5	3	3	8	0.928	1.069	0.764	0.844	11.80	7.87	5	8	635	68.3	7.08
F2Z429	Tudor domain-containing protein 6 OS=Mus musculus GN=Tdrd6 PE=2 SV=1 - [F2Z429_MOUSE]	0.89	3	2	2	3	0.423	1.005	0.889	0.844	5.75	0.89	2	3	2134	237.9	5.35
Q8CGM2	Retinitis pigmentosa 1-like 1 protein OS=Mus musculus GN=Rp1l1 PE=1 SV=1 - [RP1L1_MOUSE]	3.01	1	2	3	3	1.917	0.881	1.264	0.844	7.72	3.01	3	3	1859	199.6	6.48
E9PY16	Protein Adap1 OS=Mus musculus GN=Adap1 PE=2 SV=1 - [E9PY16_MOUSE]	49.20	2	18	18	83	0.751	0.761	0.911	0.844	247.87	49.20	31	83	374	43.3	8.65
Q9ES18	Forkhead box protein J2 OS=Mus musculus GN=Foxj2 PE=2 SV=1 - [FOXJ2_MOUSE]	2.48	1	1	1	4	1.088	1.051	1.018	0.844	13.81	2.48	2	4	565	61.5	6.65

Q5U405	Transmembrane protease serine 13 OS=Mus musculus GN=Tmprss13 PE=2 SV=2 -	3.31	2	1	2	132	0.797	1.400	0.877	0.844	143.97	3.31	3	132	543	59.8	9.01
Q9Z1Q9	Valine--tRNA ligase OS=Mus musculus GN=Vars PE=2 SV=1 - [SYVC_MOUSE]	40.22	6	42	42	196	0.622	0.564	0.725	0.844	558.22	40.22	77	196	1263	140.1	7.77
Q9QXN3	Activating signal cointegrator 1 OS=Mus musculus GN=Trip4 PE=1 SV=2 - [TRIP4_MOUSE]	19.79	2	10	11	19	1.860	1.208	0.866	0.844	55.90	19.79	15	19	581	66.2	7.55
Q9EQH2	Endoplasmic reticulum aminopeptidase 1 OS=Mus musculus GN=Erap1 PE=2 SV=2 - [ERAP1_MOUSE]	7.74	1	7	7	11	0.902	1.232	0.682	0.844	32.84	7.74	11	11	930	106.5	6.20
O35618-2	Isoform Short of Protein Mdm4 OS=Mus musculus GN=Mdm4 - [MDM4_MOUSE]	9.45	2	1	1	4	1.065	1.275	1.214	0.844	6.55	9.45	2	4	127	14.1	8.35
E9Q6J5	Protein Bod1l OS=Mus musculus GN=Bod1l PE=2 SV=1 - [E9Q6J5_MOUSE]	6.56	1	11	14	27	1.392	0.786	1.147	0.844	77.79	6.56	21	27	3032	327.3	5.33
A0A4W9	Neuronal growth regulator 1 OS=Mus musculus GN=Negr1 PE=2 SV=1 - [A0A4W9_MOUSE]	49.54	4	14	14	143	1.045	1.487	0.984	0.844	449.79	49.54	25	143	329	36.1	6.86
Q6ZQI3	Malectin OS=Mus musculus GN=Mlec PE=2 SV=2 - [MLEC_MOUSE]	39.86	2	11	11	37	0.805	0.872	0.661	0.844	89.16	39.86	17	37	291	32.3	6.05
O54782	Epididymis-specific alpha-mannosidase OS=Mus musculus GN=Man2b2 PE=2 SV=2 - [MA2B2_MOUSE]	5.21	2	4	4	7	0.967	1.263	0.721	0.844	21.26	5.21	7	7	1018	115.5	7.39
Q9D706	RNA polymerase II-associated protein 3 OS=Mus musculus GN=Rpap3 PE=1 SV=1 - [RPAP3_MOUSE]	36.97	1	20	20	63	1.169	0.745	0.940	0.844	174.22	36.97	35	63	660	74.1	7.99
Q9QYI3	DnaJ homolog subfamily C member 7 OS=Mus musculus GN=Dnajc7 PE=1 SV=2 -	42.51	3	21	21	60	0.972	0.736	0.918	0.844	166.82	42.51	34	60	494	56.4	6.49
P14115	60S ribosomal protein L27a OS=Mus musculus GN=Rpl27a PE=2 SV=5 - [RL27A_MOUSE]	27.70	1	4	4	22	0.663	0.597	0.649	0.844	61.07	27.70	8	22	148	16.6	11.12
O70279	Protein DGCR14 OS=Mus musculus GN=Dgcr14 PE=2 SV=2 - [DGC14_MOUSE]	15.03	2	5	5	11	1.124	0.879	1.078	0.844	39.24	15.03	8	11	479	52.6	6.70
Q61733	28S ribosomal protein S31, mitochondrial OS=Mus musculus GN=Mrps31 PE=2 SV=1 - [RT31_MOUSE]	28.65	1	9	10	29	1.163	0.593	1.089	0.844	72.18	28.65	18	29	384	43.9	8.51
P62245	40S ribosomal protein S15a OS=Mus musculus GN=Rps15a PE=2 SV=2 - [RS15A_MOUSE]	66.92	4	9	9	50	0.566	0.666	0.714	0.845	150.49	66.92	18	50	130	14.8	10.13
Q99N94	39S ribosomal protein L9, mitochondrial OS=Mus musculus GN=Mrpl9 PE=2 SV=2 - [RM09_MOUSE]	21.13	2	5	5	11	0.585	0.572	0.895	0.845	34.22	21.13	9	11	265	30.2	10.08
A2ARV4	Low-density lipoprotein receptor-related protein 2 OS=Mus musculus GN=Lrp2 PE=1 SV=1 - [LRP2_MOUSE]	2.55	3	4	5	5	2.039	1.251	0.931	0.845	4.70	2.55	5	5	4660	518.9	5.15
Q149F1	RNA pseudouridylate synthase domain-containing protein 2 OS=Mus musculus GN=Rpusd2 PE=2 SV=2 - [RUSD2_MOUSE]	11.03	1	4	4	6	0.512	0.981	1.372	0.845	16.88	11.03	5	6	553	61.5	6.77
D3Z5F7	Protein Gm20521 OS=Mus musculus GN=Gm20521 PE=2 SV=1 - [D3Z5F7_MOUSE]	34.83	11	8	8	26	1.087	0.781	0.943	0.845	79.04	34.83	15	26	333	37.2	8.40
Q3UG37	Histone deacetylase 6 (Fragment) OS=Mus musculus GN=Hdac6 PE=2 SV=1 - [Q3UG37_MOUSE]	21.11	3	15	15	69	0.678	0.776	0.867	0.845	231.49	21.11	27	69	1009	110.2	5.83
Q9D3B1	Very-long-chain (3R)-3-hydroxyacyl-[acyl-carrier protein] dehydratase 2 OS=Mus musculus GN=Ptplb PE=2 SV=1 - [HACD2_MOUSE]	10.63	1	2	2	5	1.065	1.336	0.797	0.845	11.75	10.63	3	5	254	28.4	9.58
Q8VHR5	Transcriptional repressor p66-beta OS=Mus musculus GN=Gatad2b PE=1 SV=1 -	42.09	2	18	20	70	1.181	0.941	1.117	0.845	223.47	42.09	32	70	594	65.4	9.70
Q8BGT6	MICAL-like protein 1 OS=Mus musculus GN=Micall1 PE=1 SV=3 - [MILK1_MOUSE]	30.80	3	18	20	78	1.368	0.291	0.960	0.845	248.59	30.80	35	78	870	94.0	6.68
Q9Z2W9	Glutamate receptor 3 OS=Mus musculus GN=Gria3 PE=1 SV=2 - [GRIA3_MOUSE]	33.33	3	1	28	107	1.076	1.788	1.637	0.845	283.93	33.33	49	107	888	100.5	8.38
Q9DB15	39S ribosomal protein L12, mitochondrial OS=Mus musculus GN=Mrpl12 PE=1 SV=2 - [RM12_MOUSE]	46.77	1	8	8	42	1.723	0.892	1.073	0.845	116.63	46.77	14	42	201	21.7	9.29

Q9EQG7	Ectonucleotide pyrophosphatase/phosphodiesterase family member 5 OS=Mus musculus GN=Enpp5 PE=2 SV=3 - [ENPP5_MOUSE]	10.48	1	3	4	18	1.085	1.151	0.848	0.846	51.34	10.48	7	18	477	54.4	5.90
Q61937	Nucleophosmin OS=Mus musculus GN=Npm1 PE=1 SV=1 - [NPM_MOUSE]	57.19	5	18	18	100	1.010	0.676	1.118	0.846	276.36	57.19	31	100	292	32.5	4.77
Q8C0D7-4	Isoform 4 of Inhibitor of growth protein 4 OS=Mus musculus GN=Ing4 - [ING4_MOUSE]	4.82	5	1	1	3	1.236	0.691	1.043	0.846	9.19	4.82	2	3	166	19.2	8.47
P21841	Pulmonary surfactant-associated protein C OS=Mus musculus GN=Sftpc PE=2 SV=1 - [PSPC_MOUSE]	8.81	2	1	1	22	0.770	1.384	0.914	0.846	0.00	8.81	1	22	193	21.0	6.90
Q3UFY0	Ribosomal RNA processing protein 36 homolog OS=Mus musculus GN=Rrp36 PE=2 SV=1 - [RRP36_MOUSE]	2.87	1	1	1	4	1.701	3.145	0.720	0.846	12.93	2.87	1	4	244	28.6	10.56
Q01098	Glutamate receptor ionotropic, NMDA 2C OS=Mus musculus GN=Grin2c PE=2 SV=2 - [NMDE3_MOUSE]	1.78	1	1	2	2	1.739	1.259	0.666	0.846	4.43	1.78	2	2	1239	135.3	8.66
D3Z0A2	Protein arginine N-methyltransferase 1 OS=Mus musculus GN=Prmt1 PE=2 SV=1 - [D3Z0A2_MOUSE]	40.88	6	9	12	30	1.093	1.197	0.760	0.846	88.50	40.88	19	30	318	36.5	6.19
Q9CR09	Ubiquitin-fold modifier-conjugating enzyme 1 OS=Mus musculus GN=Ufc1 PE=2 SV=1 - [UFC1_MOUSE]	32.34	2	8	8	23	1.294	0.871	0.890	0.846	56.36	32.34	12	23	167	19.5	7.40
F8VQ54	Endoribonuclease Dicer OS=Mus musculus GN=Dicer1 PE=2 SV=1 - [F8VQ54_MOUSE]	2.73	6	4	5	6	0.654	0.847	0.667	0.846	15.15	2.73	6	6	1906	215.6	5.80
Q3TZ02	Cytohesin-1 OS=Mus musculus GN=Cyth1 PE=2 SV=1 - [Q3TZ02_MOUSE]	46.25	6	12	18	54	1.082	1.092	1.096	0.846	132.92	46.25	27	54	400	46.4	5.74
Q91W59-2	Isoform 2 of RNA-binding motif, single-stranded-interacting protein 1 OS=Mus musculus GN=Rbms1 - [illegible]	16.49	4	3	5	12	1.115	0.560	1.053	0.846	45.22	16.49	8	12	370	40.0	8.12
Q61543	Golgi apparatus protein 1 OS=Mus musculus GN=Glg1 PE=1 SV=1 - [GSLG1_MOUSE]	37.45	4	41	41	136	0.941	0.725	0.790	0.846	405.23	37.45	67	136	1175	133.6	6.84
Q8K2K6-3	Isoform 3 of Arf-GAP domain and FG repeat-containing protein 1 OS=Mus musculus GN=Agfg1 - [illegible]	26.04	4	10	12	62	1.502	1.028	1.126	0.846	199.94	26.04	23	62	530	55.0	8.47
Q60967	Bifunctional 3'-phosphoadenosine 5'-phosphosulfate synthase 1 OS=Mus musculus GN=Papss1 PE=2 SV=1 - [illegible]	36.86	1	16	19	63	0.870	0.691	0.762	0.846	181.68	36.86	33	63	624	70.7	6.77
P68040	Guanine nucleotide-binding protein subunit beta-2-like 1 OS=Mus musculus GN=Gnb2l1 PE=1 SV=3 - [illegible]	60.88	1	17	17	73	0.733	0.844	0.714	0.846	229.03	60.88	29	73	317	35.1	7.69
E9PX94	Coiled-coil and C2 domain-containing protein 1A OS=Mus musculus GN=Cc2d1a PE=2 SV=2 - [E9PX94_MOUSE]	36.23	1	2	30	86	0.957	0.933	1.005	0.846	239.17	36.23	54	86	897	98.6	7.36
Q99KN2	Probable cytosolic iron-sulfur protein assembly protein CIAO1 OS=Mus musculus GN=Ciao1 PE=2 SV=1 - [CIAO1_MOUSE]	15.34	4	4	4	16	1.979	1.164	0.968	0.846	49.76	15.34	6	16	339	37.6	4.88
Q62318	Transcription intermediary factor 1-beta OS=Mus musculus GN=Trim28 PE=1 SV=3 -	41.85	2	26	26	148	0.847	1.075	0.792	0.847	477.81	41.85	44	148	834	88.8	5.77
Q3TUE1	Far upstream element-binding protein 1 OS=Mus musculus GN=Fubp1 PE=2 SV=1 - [Q3TUE1_MOUSE]	49.84	1	2	28	172	1.649	1.245	1.200	0.847	444.63	49.84	49	172	642	67.4	7.61
B2RVL6	Zinc finger CCHC domain-containing protein 24 OS=Mus musculus GN=Zcchc24 PE=2 SV=1 - [ZCH24_MOUSE]	7.05	1	2	2	4	1.219	0.521	0.956	0.847	8.64	7.05	4	4	241	26.9	8.70
O88444	Adenylate cyclase type 1 OS=Mus musculus GN=Adcy1 PE=2 SV=2 - [ADCY1_MOUSE]	12.16	1	10	11	27	0.396	0.757	0.946	0.847	93.68	12.16	19	27	1118	123.3	8.47
Q3UHQ6-2	Isoform 2 of Protein dopey-2 OS=Mus musculus GN=Dopey2 - [DOP2_MOUSE]	8.82	6	14	15	23	0.645	0.934	0.834	0.847	78.40	8.82	23	23	2177	243.9	6.01
F8VQC7	Kinectin OS=Mus musculus GN=Ktn1 PE=2 SV=1 - [F8VQC7_MOUSE]	47.10	2	5	58	190	1.892	0.735	1.138	0.847	538.28	47.10	98	190	1327	152.4	5.90

Q8BL66	Early endosome antigen 1 OS=Mus musculus GN=Eea1 PE=1 SV=2 - [EEA1_MOUSE]	57.26	5	83	90	417	1.429	0.854	1.128	0.847	1130.16	57.26	162	417	1411	160.8	5.77
Q8BG15-5	Isoform 5 of CTD small phosphatase-like protein 2 OS=Mus musculus GN=Ctdspl2 - [CTSL2_MOUSE]	5.08	6	2	2	7	0.340	0.409	0.958	0.847	23.92	5.08	4	7	256	28.1	5.82
Q9R1Z8	Vinexin OS=Mus musculus GN=Sorbs3 PE=1 SV=1 - [VINEX_MOUSE]	11.73	1	8	8	26	1.812	0.998	0.897	0.847	80.38	11.73	14	26	733	82.3	9.20
Q8CCF0-3	Isoform 3 of U4/U6 small nuclear ribonucleoprotein Prp31 OS=Mus musculus GN=Prpf31 - [PRP31_MOUSE]	25.05	5	12	13	38	0.692	0.666	0.877	0.847	93.05	25.05	23	38	495	55.0	5.90
Q9Z1N5	Spliceosome RNA helicase Ddx39b OS=Mus musculus GN=Ddx39b PE=1 SV=1 - [DX39B_MOUSE]	45.56	2	9	19	133	0.504	0.962	0.784	0.847	402.62	45.56	36	133	428	49.0	5.67
F6ZV59	Heterogeneous nuclear ribonucleoprotein D0 (Fragment) OS=Mus musculus GN=Hnrnpd PE=4 SV=1 - [illegible]	52.78	11	11	13	132	1.050	0.601	1.074	0.847	374.25	52.78	23	132	216	24.7	9.52
P21661	Neuroendocrine convertase 2 OS=Mus musculus GN=Pcsk2 PE=2 SV=1 - [NEC2_MOUSE]	22.14	1	11	11	48	1.034	1.234	0.768	0.847	167.88	22.14	19	48	637	70.7	6.42
P48725-3	Isoform 3 of Pericentrin OS=Mus musculus GN=Pcnt - [PCNT_MOUSE]	7.24	4	14	17	30	0.801	0.991	1.132	0.847	72.38	7.24	21	30	2500	284.4	5.08
Q9CR39	WD repeat domain phosphoinositide-interacting protein 3 OS=Mus musculus GN=Wdr45b PE=2 SV=2 - [illegible]	20.64	3	5	6	21	1.625	0.976	0.733	0.847	72.58	20.64	10	21	344	38.0	7.56
Q99M87	DnaJ homolog subfamily A member 3, mitochondrial OS=Mus musculus GN=Dnaja3 PE=1 SV=1 - [DNJA3_MOUSE]	46.25	2	2	17	76	1.650	0.785	0.923	0.847	258.03	46.25	31	76	480	52.4	9.22
E9Q8T1	Transforming acidic coiled-coil-containing protein 2 OS=Mus musculus GN=Tacc2 PE=2 SV=1 - [E9Q8T1_MOUSE]	10.94	7	22	26	72	1.401	0.673	1.172	0.847	173.82	10.94	39	72	2879	305.0	4.79
Q9Z2A9-2	Isoform 2 of Gamma-glutamyltransferase 5 OS=Mus musculus GN=Ggt5 - [GGT5_MOUSE]	3.51	3	1	2	3	1.810	1.562	0.738	0.848	6.80	3.51	3	3	456	49.3	7.84
Q9ESU6	Bromodomain-containing protein 4 OS=Mus musculus GN=Brd4 PE=1 SV=2 - [BRD4_MOUSE]	7.79	5	7	11	27	0.720	0.827	0.886	0.848	66.99	7.79	17	27	1400	155.8	9.19
Q7TSV4	Phosphoglucomutase-2 OS=Mus musculus GN=Pgm2 PE=1 SV=1 - [PGM2_MOUSE]	27.90	1	15	15	45	1.022	0.878	0.714	0.848	106.99	27.90	26	45	620	68.7	6.14
Q3UHX2	28 kDa heat- and acid-stable phosphoprotein OS=Mus musculus GN=Pdap1 PE=1 SV=1 - [HAP28_MOUSE]	33.70	1	8	8	73	1.197	0.530	1.465	0.848	213.76	33.70	15	73	181	20.6	7.39
O88569	Heterogeneous nuclear ribonucleoproteins A2/B1 OS=Mus musculus GN=Hnrnpa2b1 PE=1 SV=2 - [ROA2_MOUSE]	68.27	3	24	27	646	1.636	0.873	1.153	0.848	2169.44	68.27	52	646	353	37.4	8.95
Q64310	Surfeit locus protein 4 OS=Mus musculus GN=Surf4 PE=2 SV=1 - [SURF4_MOUSE]	17.47	3	4	4	21	0.503	0.965	0.635	0.848	59.43	17.47	8	21	269	30.4	7.78
Q7M739	Nuclear pore complex-associated intranuclear coiled-coil protein TPR OS=Mus musculus GN=Tpr PE=2 SV=1 - [illegible]	34.92	8	74	75	350	1.580	0.977	1.165	0.848	986.49	34.92	133	350	2357	266.8	5.01
Q5U4C9	Dual specificity tyrosine-phosphorylation-regulated kinase 2 OS=Mus musculus GN=Dyrk2 PE=2 SV=1 - [DYRK2_MOUSE]	8.18	2	4	5	8	2.193	0.745	1.328	0.848	18.92	8.18	7	8	599	66.5	9.67
Q9DAC7	Tetratricopeptide repeat protein 32 OS=Mus musculus GN=Ttc32 PE=2 SV=1 - [TTC32_MOUSE]	10.14	1	1	1	1	2.617	0.958	1.092	0.848	4.62	10.14	1	1	148	16.8	5.40
Q8BKG3	Inactive tyrosine-protein kinase 7 OS=Mus musculus GN=Ptk7 PE=1 SV=1 - [PTK7_MOUSE]	13.94	1	12	13	30	0.860	1.019	0.743	0.849	85.18	13.94	22	30	1062	117.5	6.84
J3QSN2	Ubiquitin carboxyl-terminal hydrolase OS=Mus musculus GN=Usp13 PE=3 SV=1 - [J3QSN2_MOUSE]	15.40	3	9	9	18	0.964	1.182	0.892	0.849	43.95	15.40	15	18	857	96.5	5.49
P70365-4	Isoform 4 of Nuclear receptor coactivator 1 OS=Mus musculus GN=Ncoa1 -	9.32	4	8	8	20	1.361	1.381	1.235	0.849	58.13	9.32	15	20	1330	144.7	6.24
E0CZA5	Neurexin-1 OS=Mus musculus GN=Nrxn1 PE=2 SV=1 - [E0CZA5_MOUSE]	34.89	6	1	42	183	0.468	0.826	0.903	0.849	550.87	34.89	74	183	1476	162.1	5.99

Q9D9E0-2	Isoform 2 of Solute carrier family 22 member 17 OS=Mus musculus GN=Slc22a17 -	8.74	2	4	4	13	0.506	0.864	0.622	0.849	34.42	8.74	6	13	366	39.9	8.43
F2Z4B7	Protein Raph1 OS=Mus musculus GN=Raph1 PE=2 SV=1 - [F2Z4B7_MOUSE]	26.05	5	15	16	33	1.036	0.975	0.994	0.849	82.25	26.05	25	33	645	72.7	6.35
Q8VDI9	Alpha-1,2-mannosyltransferase ALG9 OS=Mus musculus GN=Alg9 PE=2 SV=1 -	6.06	2	3	3	4	0.476	0.763	0.733	0.849	6.52	6.06	4	4	611	69.5	8.94
Q9JKS5	Intracellular hyaluronan-binding protein 4 OS=Mus musculus GN=Habp4 PE=2 SV=2 - [HABP4_MOUSE]	30.17	2	10	10	51	1.428	0.525	1.060	0.849	139.34	30.17	18	51	411	45.9	6.84
P58283	E3 ubiquitin-protein ligase RNF216 OS=Mus musculus GN=Rnf216 PE=1 SV=3 - [RN216_MOUSE]	7.03	4	4	5	12	1.160	1.257	1.010	0.849	29.07	7.03	6	12	853	97.6	5.01
K4DI59	DNA ligase OS=Mus musculus GN=Lig3 PE=3 SV=1 - [K4DI59_MOUSE]	9.74	7	7	7	11	1.754	0.880	0.925	0.849	33.65	9.74	11	11	1016	113.1	8.98
F8VQK5	SAM and SH3 domain-containing protein 1 OS=Mus musculus GN=Sash1 PE=2 SV=1 - [F8VQK5_MOUSE]	25.45	2	27	27	65	0.829	0.578	0.971	0.849	175.28	25.45	41	65	1230	135.5	6.20
O88998	Noelin OS=Mus musculus GN=Olfm1 PE=1 SV=1 - [NOE1_MOUSE]	31.96	7	12	12	57	1.006	1.890	1.500	0.849	154.29	31.96	19	57	485	55.4	6.95
P56183-2	Isoform 2 of Ribosomal RNA processing protein 1 homolog A OS=Mus musculus GN=Rrp1 - [RRP1_MOUSE]	4.74	3	2	2	3	0.649	1.279	0.794	0.849	8.82	4.74	3	3	443	50.0	8.24
Q8VCE1	DnaJ homolog subfamily C member 28 OS=Mus musculus GN=Dnajc28 PE=2 SV=2 -	7.01	3	3	3	8	2.220	1.675	1.095	0.849	22.32	7.01	6	8	385	44.8	8.65
P63087-2	Isoform Gamma-2 of Serine/threonine-protein phosphatase PP1-gamma catalytic subunit OS=Mus musculus GN=Ppp1cc - [PP1G_MOUSE]	55.49	2	3	17	151	1.376	0.943	1.007	0.849	470.52	55.49	32	151	337	38.5	6.13
Q60973	Histone-binding protein RBBP7 OS=Mus musculus GN=Rbbp7 PE=1 SV=1 - [RBBP7_MOUSE]	32.00	4	1	12	61	1.821	2.153	0.815	0.849	166.39	32.00	19	61	425	47.8	5.05
Q3UTQ8	Cyclin-dependent kinase-like 5 OS=Mus musculus GN=Cdkl5 PE=2 SV=1 - [CDKL5_MOUSE]	25.91	3	19	20	72	0.925	1.002	1.600	0.849	176.08	25.91	36	72	938	105.4	9.58
P49586	Choline-phosphate cytidylyltransferase A OS=Mus musculus GN=Pcyt1a PE=1 SV=1 - [PCY1A_MOUSE]	37.60	2	8	12	30	1.042	0.706	0.844	0.849	80.43	37.60	21	30	367	41.6	7.03
O55074	A-kinase anchor protein 7 isoform alpha OS=Mus musculus GN=Akap7 PE=1 SV=4 - [AKA7A_MOUSE]	38.27	2	4	4	32	0.586	0.982	0.879	0.849	101.73	38.27	7	32	81	9.2	5.27
Q920Q6-3	Isoform 3 of RNA-binding protein Musashi homolog 2 OS=Mus musculus GN=Msi2 - [MSI2H_MOUSE]	36.88	5	7	8	29	1.098	0.740	0.819	0.849	78.66	36.88	16	29	282	30.9	9.10
D3YZE7	Protein 2700050L05Rik OS=Mus musculus GN=2700050L05Rik PE=4 SV=2 - [D3YZE7_MOUSE]	1.24	3	1	1	1	0.830	1.524	0.677	0.849	1.90	1.24	1	1	1206	135.3	6.24
P13011	Acyl-CoA desaturase 2 OS=Mus musculus GN=Scd2 PE=2 SV=2 - [ACOD2_MOUSE]	3.91	3	2	2	5	0.656	0.401	0.722	0.850	11.56	3.91	3	5	358	40.9	9.01
Q9EQI8	39S ribosomal protein L46, mitochondrial OS=Mus musculus GN=Mrpl46 PE=2 SV=1 - [RM46_MOUSE]	38.87	1	9	9	19	1.191	1.107	0.768	0.850	56.78	38.87	13	19	283	32.1	7.40
O08807	Peroxiredoxin-4 OS=Mus musculus GN=Prdx4 PE=1 SV=1 - [PRDX4_MOUSE]	38.69	2	6	8	145	1.825	0.902	0.751	0.850	335.12	38.69	15	145	274	31.0	7.15
Q64355	Embryonal Fyn-associated substrate OS=Mus musculus GN=Efs PE=1 SV=2 - [EFS_MOUSE]	6.61	1	2	2	5	1.402	0.837	0.930	0.850	9.68	6.61	3	5	560	58.9	5.12
O35841	Apoptosis inhibitor 5 OS=Mus musculus GN=Api5 PE=2 SV=2 - [API5_MOUSE]	46.63	1	20	21	69	0.702	1.028	0.807	0.850	214.94	46.63	35	69	504	56.7	5.92
Q6P1E7-2	Isoform 2 of Coiled-coil domain-containing protein 111 OS=Mus musculus GN=Ccdc111 - [CC111_MOUSE]	4.88	1	1	1	1	1.163	1.086	1.035	0.850	0.00	4.88	1	1	328	37.9	8.32
F8VQK0	Protein Nlrp4g OS=Mus musculus GN=Nlrp4g PE=2 SV=2 - [F8VQK0_MOUSE]	2.01	3	1	2	4	1.910	0.740	1.106	0.850	6.93	2.01	2	4	646	74.3	6.52
G3X8R5	Glutamine-rich protein 1 OS=Mus musculus GN=Qrich1 PE=4 SV=1 - [G3X8R5_MOUSE]	3.73	2	3	3	5	1.484	1.184	1.073	0.850	14.56	3.73	5	5	777	86.5	5.87

Q8WTY4	Anamorsin OS=Mus musculus GN=Ciapin1 PE=1 SV=1 - [CPIN1_MOUSE]	44.01	4	9	9	42	1.319	0.701	1.021	0.850	158.24	44.01	16	42	309	33.4	5.20
P61222	ATP-binding cassette sub-family E member 1 OS=Mus musculus GN=Abce1 PE=2 SV=1 - [ABCE1_MOUSE]	31.55	1	16	16	44	0.780	0.861	0.811	0.850	139.03	31.55	26	44	599	67.3	8.34
P62852	40S ribosomal protein S25 OS=Mus musculus GN=Rps25 PE=2 SV=1 - [RS25_MOUSE]	34.40	1	6	6	29	0.547	0.797	0.790	0.850	66.78	34.40	11	29	125	13.7	10.11
Q8CHR9-2	Isoform 2 of PIH1 domain-containing protein 2 OS=Mus musculus GN=Pih1d2 - [PIHD2_MOUSE]	13.16	5	1	1	1	0.914	1.003	1.041	0.850	0.00	13.16	1	1	114	13.1	9.22
Q9DBF7	Pre-mRNA-splicing factor CWC25 homolog OS=Mus musculus GN=Cwc25 PE=2 SV=2 - [CWC25_MOUSE]	13.46	4	4	4	5	1.108	1.033	0.974	0.851	6.87	13.46	5	5	416	48.8	10.23
Q8BMQ2	General transcription factor 3C polypeptide 4 OS=Mus musculus GN=Gtf3c4 PE=1 SV=2 - [TF3C4_MOUSE]	11.63	4	7	7	13	1.023	0.909	0.764	0.851	42.42	11.63	10	13	817	91.6	6.73
Q8BZJ8-3	Isoform 3 of Uncharacterized protein C8orf34 homolog OS=Mus musculus -	7.93	5	1	1	1	1.814	1.064	0.798	0.851	3.23	7.93	1	1	227	25.6	8.76
Q8BR75	Serine/Arginine-related protein 53 OS=Mus musculus GN=Rsrc1 PE=2 SV=1 - [Q8BR75_MOUSE]	8.45	4	2	2	6	0.925	0.668	0.920	0.851	12.86	8.45	3	6	213	24.8	11.31
E2QRQ3	Double-stranded RNA-binding protein Staufen homolog 2 OS=Mus musculus GN=Stau2 PE=2 SV=1 - [E2QRQ3_MOUSE]	30.02	8	10	13	34	0.819	0.540	0.929	0.851	96.25	30.02	23	34	473	52.0	9.67
P97379	Ras GTPase-activating protein-binding protein 2 OS=Mus musculus GN=G3bp2 PE=1 SV=2 - [G3BP2_MOUSE]	41.08	1	3	17	261	1.256	0.826	1.003	0.851	771.05	41.08	31	261	482	54.1	5.62
Q60809	CCR4-NOT transcription complex subunit 7 OS=Mus musculus GN=Cnot7 PE=1 SV=1 - [CNOT7_MOUSE]	11.93	1	2	2	11	0.660	1.413	0.864	0.851	42.15	11.93	3	11	285	32.7	4.84
Q61025-2	Isoform 2 of Intraflagellar transport protein 20 homolog OS=Mus musculus GN=Ift20 - [IFT20_MOUSE]	40.57	2	3	3	12	1.359	1.049	0.973	0.851	40.63	40.57	6	12	106	12.0	5.60
Q920P3	Deleted in bladder cancer protein 1 homolog OS=Mus musculus GN=Dbc1 PE=2 SV=1 - [DBC1_MOUSE]	12.50	2	8	9	28	0.409	1.193	1.072	0.851	91.53	12.50	15	28	760	88.6	8.97
O88909	Solute carrier family 22 member 8 OS=Mus musculus GN=Slc22a8 PE=2 SV=2 -	3.72	1	2	2	4	0.996	3.248	0.718	0.851	13.04	3.72	4	4	537	59.2	8.40
P97927	Laminin subunit alpha-4 OS=Mus musculus GN=Lama4 PE=1 SV=2 - [LAMA4_MOUSE]	11.56	7	19	20	53	1.077	1.006	0.859	0.851	157.70	11.56	31	53	1816	201.7	6.21
Q8BT14-2	Isoform 2 of CCR4-NOT transcription complex subunit 4 OS=Mus musculus GN=Cnot4 - [CNOT4_MOUSE]	16.08	4	6	6	35	1.251	0.885	0.961	0.851	72.30	16.08	11	35	572	63.0	7.03
P41105	60S ribosomal protein L28 OS=Mus musculus GN=Rpl28 PE=1 SV=2 - [RL28_MOUSE]	56.93	2	11	11	103	0.458	0.454	0.686	0.851	272.00	56.93	19	103	137	15.7	12.02
Q8BIQ5	Cleavage stimulation factor subunit 2 OS=Mus musculus GN=Cstf2 PE=1 SV=2 - [CSTF2_MOUSE]	28.62	4	7	14	48	1.937	0.896	0.915	0.851	126.01	28.62	24	48	580	61.3	6.83
P62309	Small nuclear ribonucleoprotein G OS=Mus musculus GN=Snrpg PE=1 SV=1 -	34.21	1	3	3	12	1.295	0.800	0.815	0.852	30.14	34.21	5	12	76	8.5	8.88
P31786	Acyl-CoA-binding protein OS=Mus musculus GN=Dbi PE=1 SV=2 - [ACBP_MOUSE]	65.52	5	9	9	379	1.908	0.776	0.947	0.852	1394.29	65.52	12	379	87	10.0	8.82
F6RND9	Myosin phosphatase Rho-interacting protein (Fragment) OS=Mus musculus GN=Mprip PE=2 SV=1 - [F6RND9_MOUSE]	44.46	8	3	36	83	0.942	1.060	1.064	0.852	238.68	44.46	59	83	1010	114.0	6.51
Q8CFQ9	Fusion, derived from t(1216) malignant liposarcoma (Human) OS=Mus musculus GN=Fus PE=2 SV=1 - [illegible]	29.01	5	10	14	134	1.245	0.737	1.196	0.852	362.60	29.01	24	134	517	52.6	9.36
P07742	Ribonucleoside-diphosphate reductase large subunit OS=Mus musculus GN=Rrm1 PE=1 SV=2 - [RIR1_MOUSE]	11.24	1	7	7	14	1.068	0.704	0.633	0.852	30.51	11.24	11	14	792	90.2	6.70
Q3V4B5	COMM domain-containing protein 6 OS=Mus musculus GN=Commd6 PE=2 SV=1 -	74.71	2	6	6	16	1.091	1.145	0.886	0.852	37.22	74.71	9	16	87	9.8	5.38

Q9CXW4	60S ribosomal protein L11 OS=Mus musculus GN=Rpl11 PE=1 SV=4 - [RL11_MOUSE]	47.75	6	10	10	33	0.625	0.669	0.757	0.852	103.94	47.75	18	33	178	20.2	9.60
P60670	Nuclear protein localization protein 4 homolog OS=Mus musculus GN=Nploc4 PE=1 SV=3 - [NPL4_MOUSE]	49.51	2	26	26	68	0.823	0.818	0.833	0.852	188.41	49.51	45	68	608	68.0	6.46
Q3UUQ7	GPI inositol-deacylase OS=Mus musculus GN=Pgap1 PE=1 SV=3 - [PGAP1_MOUSE]	9.22	1	10	10	29	0.513	0.619	0.606	0.852	70.02	9.22	18	29	922	104.5	8.91
Q8C9H6-3	Isoform 3 of Striatin-interacting proteins 2 OS=Mus musculus GN=Strip2 - [illegible]	10.78	5	7	9	19	0.716	0.955	1.585	0.852	49.31	10.78	12	19	816	93.0	5.45
F6RPJ9	Insulin-degrading enzyme (Fragment) OS=Mus musculus GN=Ide PE=3 SV=1 - [F6RPJ9_MOUSE]	33.94	2	35	35	113	1.544	0.785	0.801	0.852	296.63	33.94	62	113	987	114.2	6.27
E9Q5D5	WW domain-containing adapter protein with coiled coil OS=Mus musculus GN=Wac PE=2 SV=1 - [E9Q5D5_MOUSE]	15.75	12	3	5	12	1.648	1.044	0.931	0.852	31.18	15.75	9	12	597	65.0	9.35
Q9JJU8	SH3 domain-binding glutamic acid-rich-like protein OS=Mus musculus GN=Sh3bgrl PE=3 SV=1 - [SH3L1_MOUSE]	75.44	1	7	7	110	1.671	1.351	1.176	0.852	298.76	75.44	14	110	114	12.8	4.92
Q91ZS8-5	Isoform 5 of Double-stranded RNA-specific editase 1 OS=Mus musculus GN=Adarb1 - [RED1_MOUSE]	26.14	5	11	12	25	0.785	0.531	0.787	0.852	91.13	26.14	19	25	677	74.1	9.35
Q8CG46-2	Isoform 2 of Structural maintenance of chromosomes protein 5 OS=Mus musculus GN=Smc5 - [illegible]	4.32	2	3	3	5	1.491	1.276	0.862	0.853	10.33	4.32	4	5	1087	127.3	8.56
P16675	Lysosomal protective protein OS=Mus musculus GN=Ctsa PE=1 SV=1 - [PPGB_MOUSE]	13.71	2	5	5	14	1.024	0.907	0.800	0.853	30.81	13.71	8	14	474	53.8	5.86
Q8CIG8	Protein arginine N-methyltransferase 5 OS=Mus musculus GN=Prmt5 PE=1 SV=3 - [ANM5_MOUSE]	30.30	2	18	19	58	0.711	0.787	0.710	0.853	167.10	30.30	34	58	637	72.6	6.42
F6QL70	Protein Gm17669 OS=Mus musculus GN=Gm17669 PE=4 SV=1 - [F6QL70_MOUSE]	33.77	6	7	7	22	0.524	0.899	0.739	0.853	43.86	33.77	11	22	154	17.0	11.84
Q9D864	Actin-related protein 6 OS=Mus musculus GN=Actr6 PE=2 SV=2 - [ARP6_MOUSE]	14.65	1	3	3	3	2.458	0.873	0.993	0.853	5.40	14.65	3	3	396	45.8	5.03
A2ALB1	RNA exonuclease 4 (Fragment) OS=Mus musculus GN=Rexo4 PE=2 SV=1 - [A2ALB1_MOUSE]	5.23	3	2	2	7	1.502	1.104	1.024	0.853	18.00	5.23	3	7	363	40.1	9.80
Q80SY4	E3 ubiquitin-protein ligase MIB1 OS=Mus musculus GN=Mib1 PE=1 SV=1 - [MIB1_MOUSE]	9.74	3	7	8	18	0.841	0.883	0.987	0.853	45.77	9.74	13	18	1006	110.0	6.92
P70662-2	Isoform 2 of LIM domain-binding protein 1 OS=Mus musculus GN=Ldb1 - [LDB1_MOUSE]	7.52	4	1	2	3	0.878	0.719	0.944	0.853	9.36	7.52	3	3	319	36.6	8.63
Q6P5D8	Structural maintenance of chromosomes flexible hinge domain-containing protein 1 OS=Mus musculus GN=Smchd1 PE=1 SV=2 - [SMHD1_MOUSE]	15.60	1	28	28	46	0.853	0.990	0.844	0.853	121.20	15.60	41	46	2007	225.5	7.24
Q8VEA4	Mitochondrial intermembrane space import and assembly protein 40 OS=Mus musculus GN=Chchd4 PE=1 SV=1 [illegible]	16.55	1	3	3	19	1.844	0.988	1.209	0.854	53.35	16.55	5	19	139	15.5	4.32
P11438	Lysosome-associated membrane glycoprotein 1 OS=Mus musculus GN=Lamp1 PE=1 SV=2 - [LAMP1_MOUSE]	8.37	1	3	3	29	1.290	0.818	0.818	0.854	85.55	8.37	6	29	406	43.8	8.40
Q3UFQ8	Leucine-rich repeat-containing protein 16B OS=Mus musculus GN=Lrrc16b PE=2 SV=2 - [LR16B_MOUSE]	15.71	1	16	16	36	0.799	0.579	0.761	0.854	90.61	15.71	26	36	1375	150.3	7.72
Q9CR00	26S proteasome non-ATPase regulatory subunit 9 OS=Mus musculus GN=Psmd9 PE=1 SV=1 - [PSMD9_MOUSE]	27.48	1	5	5	58	2.127	0.855	1.124	0.854	202.37	27.48	10	58	222	24.7	6.43
Q3UHD1	Brain-specific angiogenesis inhibitor 1 OS=Mus musculus GN=Bai1 PE=1 SV=1 - [BAI1_MOUSE]	15.23	2	24	25	89	0.775	1.024	1.192	0.854	254.36	15.23	43	89	1582	173.2	7.56
Q61191	Host cell factor 1 OS=Mus musculus GN=Hcfc1 PE=1 SV=2 - [HCFC1_MOUSE]	24.21	5	35	35	133	1.126	0.968	0.965	0.854	343.27	24.21	60	133	2045	210.3	7.18

Q8BIZ1-2	Isoform 2 of Ankyrin repeat and sterile alpha motif domain-containing protein 1B OS=Mus musculus GN=Anks1b - [ANS1B_MOUSE]	38.50	4	1	15	53	0.988	3.568	1.910	0.854	149.99	38.50	28	53	426	48.3	7.97
Q8R555	Cartilage acidic protein 1 OS=Mus musculus GN=Crtac1 PE=2 SV=1 - [CRAC1_MOUSE]	31.11	1	15	15	53	0.844	1.355	0.935	0.854	163.89	31.11	25	53	646	70.3	5.14
P20060	Beta-hexosaminidase subunit beta OS=Mus musculus GN=Hexb PE=2 SV=2 - [HEXB_MOUSE]	23.88	1	14	14	55	0.630	1.041	0.960	0.854	137.60	23.88	27	55	536	61.1	8.12
Q99K28	ADP-ribosylation factor GTPase-activating protein 2 OS=Mus musculus GN=Arfgap2 PE=1 SV=1 - [ARFG2_MOUSE]	25.38	2	14	14	51	1.163	0.762	1.014	0.854	124.55	25.38	23	51	520	56.6	8.18
Q9DBG7	Signal recognition particle receptor subunit alpha OS=Mus musculus GN=Srpr PE=1 SV=1 - [SRPR_MOUSE]	27.36	2	17	17	44	0.632	0.755	0.724	0.854	104.79	27.36	31	44	636	69.6	8.95
P61924	Coatomer subunit zeta-1 OS=Mus musculus GN=Copz1 PE=2 SV=1 - [COPZ1_MOUSE]	41.24	1	4	4	24	0.901	1.059	0.856	0.854	84.36	41.24	8	24	177	20.2	4.81
Q9CR57	60S ribosomal protein L14 OS=Mus musculus GN=Rpl14 PE=2 SV=3 - [RL14_MOUSE]	42.40	1	9	10	73	0.543	0.737	0.739	0.854	196.26	42.40	16	73	217	23.5	11.02
Q9QZR9	Collagen alpha-4(IV) chain OS=Mus musculus GN=Col4a4 PE=2 SV=1 - [CO4A4_MOUSE]	2.73	1	2	2	2	1.446	0.685	1.022	0.854	2.99	2.73	2	2	1682	164.0	8.54
Q8R033	LYR motif-containing protein 2 OS=Mus musculus GN=Lyrm2 PE=2 SV=1 - [LYRM2_MOUSE]	7.95	1	1	1	1	3.953	1.077	0.988	0.854	1.85	7.95	1	1	88	10.4	10.40
Q69AB2	Thioredoxin domain-containing protein 8 OS=Mus musculus GN=Txndc8 PE=1 SV=1 - [TXND8_MOUSE]	18.90	2	2	2	2	5.843	1.097	1.036	0.854	5.51	18.90	2	2	127	14.5	8.98
Q9WU56-2	Isoform 2 of tRNA pseudouridine synthase A, mitochondrial OS=Mus musculus GN=Pus1 - [TRUA_MOUSE]	15.27	7	5	5	8	1.408	0.954	1.083	0.855	25.14	15.27	7	8	393	44.1	8.21
Q61187	Tumor susceptibility gene 101 protein OS=Mus musculus GN=Tsg101 PE=1 SV=2 - [TS101_MOUSE]	31.71	3	11	12	42	0.691	0.854	0.925	0.855	105.71	31.71	18	42	391	44.1	6.71
P11499	Heat shock protein HSP 90-beta OS=Mus musculus GN=Hsp90ab1 PE=1 SV=3 - [HS90B_MOUSE]	57.32	5	30	48	1131	0.732	0.934	0.778	0.855	3441.33	57.32	90	1131	724	83.2	5.03
G3X9V2	Catenin (Cadherin associated protein), delta 1, isoform CRA_a OS=Mus musculus GN=Ctnnd1 PE=4 SV=1 - [G3X9V2_MOUSE]	39.38	19	29	29	117	0.861	1.056	0.879	0.855	343.66	39.38	53	117	932	104.0	6.95
Q9WTL8-2	Isoform 2 of Aryl hydrocarbon receptor nuclear translocator-like protein 1 OS=Mus musculus GN=Arntl - [BMAL1_MOUSE]	7.68	5	3	3	7	0.743	1.051	0.973	0.855	25.55	7.68	5	7	625	68.6	6.86
Q8CH09	SURP and G-patch domain-containing protein 2 OS=Mus musculus GN=Sugp2 PE=2 SV=2 - [SUGP2_MOUSE]	22.21	4	20	20	87	0.536	0.629	0.804	0.855	250.00	22.21	36	87	1067	118.0	8.31
Q812E0	Cytoplasmic polyadenylation element-binding protein 2 OS=Mus musculus GN=Cpeb2 PE=1 SV=1 - [CPEB2_MOUSE]	31.86	3	1	10	55	0.959	0.828	0.754	0.855	141.12	31.86	18	55	521	58.4	7.50
Q8BKX1-3	Isoform 3 of Brain-specific angiogenesis inhibitor 1-associated protein 2 OS=Mus musculus GN=Baiap2 - [BAIP2_MOUSE]	70.18	4	2	31	244	0.763	1.911	2.071	0.855	766.03	70.18	53	244	513	56.8	8.97
P50586-2	Isoform Short of Tubby protein OS=Mus musculus GN=Tub - [TUB_MOUSE]	18.49	2	6	7	16	0.977	0.752	0.851	0.855	43.08	18.49	11	16	449	49.5	9.41
P06797	Cathepsin L1 OS=Mus musculus GN=Ctsl1 PE=1 SV=2 - [CATL1_MOUSE]	14.37	2	5	5	32	1.894	0.855	1.242	0.855	86.78	14.37	10	32	334	37.5	6.83
Q9R117	Non-receptor tyrosine-protein kinase TYK2 OS=Mus musculus GN=Tyk2 PE=1 SV=2 - [TYK2_MOUSE]	2.46	3	1	3	5	7.368	1.551	0.774	0.855	8.55	2.46	5	5	1180	132.8	7.12
Q99N96	39S ribosomal protein L1, mitochondrial OS=Mus musculus GN=Mrpl1 PE=1 SV=2 - [RM01_MOUSE]	26.49	3	6	8	18	0.749	0.801	0.794	0.855	47.06	26.49	11	18	336	37.6	8.72
Q6NZQ4	PAX-interacting protein 1 OS=Mus musculus GN=Paxip1 PE=1 SV=1 - [PAXI1_MOUSE]	3.41	1	2	2	2	1.497	1.563	0.937	0.855	2.97	3.41	2	2	1056	119.2	7.20

Q5RL20	39S ribosomal protein L43, mitochondrial OS=Mus musculus GN=Mrpl43 PE=2 SV=1 - [Q5RL20_MOUSE]	37.74	2	6	6	18	0.826	1.073	0.774	0.855	46.34	37.74	11	18	159	17.9	10.58
Q99MR6-3	Isoform C of Serrate RNA effector molecule homolog OS=Mus musculus GN=Srrt - [SRRT_MOUSE]	21.18	4	17	18	39	1.176	0.881	0.957	0.855	107.90	21.18	28	39	864	99.4	5.92
Q9JKF6	Poliovirus receptor-related protein 1 OS=Mus musculus GN=Pvrl1 PE=1 SV=3 - [PVRL1_MOUSE]	41.36	1	15	15	66	0.998	1.780	1.056	0.855	220.72	41.36	26	66	515	57.0	6.35
A2AU91	Tumor suppressor p53-binding protein 1 OS=Mus musculus GN=Trp53bp1 PE=2 SV=1 - [A2AU91_MOUSE]	35.70	9	39	39	183	1.233	0.771	0.839	0.855	587.60	35.70	65	183	1969	212.6	4.63
Q56A08	G patch domain and KOW motifs-containing protein OS=Mus musculus GN=Gpkow PE=2 SV=2 - [GPKOW_MOUSE]	19.26	2	9	9	27	1.076	0.795	0.905	0.855	63.05	19.26	15	27	488	53.8	8.07
Q8BPB0	MOB kinase activator 1B OS=Mus musculus GN=Mob1b PE=1 SV=3 - [MOB1B_MOUSE]	19.91	4	4	4	15	0.804	1.579	0.818	0.855	28.42	19.91	7	15	216	25.1	6.73
P60853	Leucine zipper putative tumor suppressor 1 OS=Mus musculus GN=Lzts1 PE=2 SV=3 - [LZTS1_MOUSE]	21.04	1	9	9	20	1.170	2.061	1.672	0.855	57.91	21.04	15	20	599	67.2	7.56
Q920B0-2	Isoform 2 of FERM domain-containing protein 4B OS=Mus musculus GN=Frmd4b - [FRM4B_MOUSE]	5.30	4	3	4	9	1.017	0.462	0.922	0.855	23.60	5.30	6	9	981	111.5	8.51
Q9D9I4	TBC1 domain family member 20 OS=Mus musculus GN=Tbc1d20 PE=2 SV=1 - [illegible]	10.20	2	3	3	11	0.867	0.879	0.886	0.856	35.95	10.20	5	11	402	45.8	6.96
G5E8E3	Protein 2310022A10Rik OS=Mus musculus GN=2310022A10Rik PE=4 SV=1 - [G5E8E3_MOUSE]	9.93	3	4	4	7	0.823	0.909	0.958	0.856	17.11	9.93	5	7	413	44.4	10.26
Q5DTX6	Junctional protein associated with coronary artery disease OS=Mus musculus GN=Jcad PE=1 SV=2 - [JCAD_MOUSE]	29.62	1	27	27	74	1.207	1.072	2.001	0.856	220.51	29.62	49	74	1320	144.7	6.52
O88522	NF-kappa-B essential modulator OS=Mus musculus GN=Ikbkg PE=1 SV=2 - [NEMO_MOUSE]	25.00	11	9	10	43	1.623	1.104	1.237	0.856	137.12	25.00	19	43	412	47.9	5.85
Q05C51	Ribonuclease OS=Mus musculus GN=Rnaseh2a PE=2 SV=1 - [Q05C51_MOUSE]	18.11	4	4	4	13	1.257	1.128	0.948	0.856	34.80	18.11	5	13	254	28.1	5.07
Q8K004	Protein Spata2 OS=Mus musculus GN=Spata2 PE=2 SV=1 - [Q8K004_MOUSE]	11.07	2	5	5	11	1.026	1.404	1.216	0.856	31.18	11.07	8	11	515	57.8	8.63
Q8VDR9	Dedicator of cytokinesis protein 6 OS=Mus musculus GN=Dock6 PE=1 SV=4 - [DOCK6_MOUSE]	4.04	4	5	9	17	0.807	0.914	0.731	0.856	38.87	4.04	14	17	2080	233.1	6.80
P43407	Syndecan-2 OS=Mus musculus GN=Sdc2 PE=1 SV=1 - [SDC2_MOUSE]	4.46	1	1	1	1	0.439	1.010	0.688	0.856	0.00	4.46	1	1	202	22.1	4.58
P31324	cAMP-dependent protein kinase type II-beta regulatory subunit OS=Mus musculus GN=Prkar2b PE=1 SV=3 - [illegible]	46.63	2	12	18	108	0.509	1.299	0.993	0.856	362.46	46.63	32	108	416	46.1	4.98
G5E829	MCG13663, isoform CRA_a OS=Mus musculus GN=Atp2b1 PE=3 SV=1 - [G5E829_MOUSE]	35.25	1	20	44	430	0.507	0.951	1.308	0.856	1234.61	35.25	80	430	1220	134.7	5.91
Q3UVK2	Protein Zfp940 OS=Mus musculus GN=Zfp940 PE=2 SV=1 - [Q3UVK2_MOUSE]	25.78	1	2	2	2	1.333	1.490	1.058	0.856	2.95	25.78	2	2	128	14.3	5.08
Q3TUA9	Probable inactive protein kinase-like protein SgK196 OS=Mus musculus GN=Sgk196 PE=2 SV=2 - [SG196_MOUSE]	9.46	1	3	3	5	1.094	1.052	0.906	0.856	13.55	9.46	5	5	349	39.9	6.62
Q80TI1	Pleckstrin homology domain-containing family H member 1 OS=Mus musculus GN=Plekhh1 PE=2 SV=2 - [illegible]	6.27	2	6	7	10	1.087	0.844	1.004	0.856	20.27	6.27	9	10	1356	150.8	7.59
Q00493	Carboxypeptidase E OS=Mus musculus GN=Cpe PE=1 SV=2 - [CBPE_MOUSE]	62.61	1	26	26	173	0.749	1.090	0.721	0.856	511.52	62.61	45	173	476	53.2	5.19
Q3TRP8	Cartilage acidic protein 1 OS=Mus musculus GN=Cep68 PE=2 SV=1 - [Q3TRP8_MOUSE]	12.01	3	3	4	10	1.325	1.778	0.861	0.856	23.86	12.01	7	10	633	67.7	5.94
O88708	Origin recognition complex subunit 4 OS=Mus musculus GN=Orc4 PE=1 SV=2 - [ORC4_MOUSE]	4.85	1	2	2	3	3.018	1.220	1.305	0.856	5.67	4.85	2	3	433	49.9	7.15
B2RRE7	OTU domain-containing protein 4 OS=Mus musculus GN=Otud4 PE=1 SV=1 - [OTUD4_MOUSE]	4.61	1	3	3	5	1.269	0.800	1.161	0.856	13.13	4.61	4	5	1107	123.0	6.76

Q9CWZ3-2	Isoform 2 of RNA-binding protein 8A OS=Mus musculus GN=Rbm8a - [RBM8A_MOUSE]	15.61	2	3	3	9	0.959	1.016	0.887	0.856	23.47	15.61	5	9	173	19.7	5.92
E9Q4V5	Protein Gm4301 OS=Mus musculus GN=Gm4301 PE=4 SV=1 - [E9Q4V5_MOUSE]	9.00	13	1	3	4	1.274	3.385	0.677	0.856	6.87	9.00	3	4	289	33.0	9.79
Q91WK1	SPRY domain-containing protein 4 OS=Mus musculus GN=Spryd4 PE=2 SV=1 -	43.00	1	7	7	19	0.859	0.638	0.816	0.856	61.30	43.00	11	19	207	23.3	9.45
D3Z723	Nuclear transcription factor Y subunit beta (Fragment) OS=Mus musculus GN=Nfyb PE=2 SV=1 - [D3Z723_MOUSE]	19.81	2	2	2	8	1.197	1.154	0.856	0.857	22.12	19.81	4	8	106	11.7	4.58
Q9D4H9-3	Isoform 3 of PHD finger protein 14 OS=Mus musculus GN=Phf14 - [PHF14_MOUSE]	3.76	5	3	4	4	0.872	0.810	1.018	0.857	4.99	3.76	4	4	878	98.7	5.34
Q6P9Q6	FK506-binding protein 15 OS=Mus musculus GN=Fkbp15 PE=1 SV=2 - [FKB15_MOUSE]	22.20	6	19	20	94	1.442	0.919	1.063	0.857	243.69	22.20	36	94	1216	132.9	5.07
A2AV25	Fibrinogen C domain-containing protein 1 OS=Mus musculus GN=Fibcd1 PE=2 SV=1 - [FBCD1_MOUSE]	2.61	1	1	1	2	0.545	1.013	0.635	0.857	7.54	2.61	2	2	459	50.6	6.67
O70472	Transmembrane protein 131 OS=Mus musculus GN=Tmem131 PE=1 SV=2 - [TM131_MOUSE]	2.45	1	3	3	3	0.904	0.673	0.843	0.857	7.23	2.45	3	3	1877	204.5	8.54
E9Q066	La-related protein 4 OS=Mus musculus GN=Larp4 PE=2 SV=1 - [E9Q066_MOUSE]	19.92	3	11	11	39	1.501	0.784	1.030	0.857	80.93	19.92	15	39	718	79.6	6.51
Q05A62-2	Isoform 2 of Dynein light chain 1, axonemal OS=Mus musculus GN=Dnal1 -	50.57	6	8	8	19	1.064	2.545	0.828	0.857	51.89	50.57	14	19	176	19.9	5.49
A2ALU4	Protein Shroom2 OS=Mus musculus GN=Shroom2 PE=1 SV=1 - [SHRM2_MOUSE]	29.24	5	33	35	111	1.638	0.939	1.254	0.857	318.02	29.24	53	111	1481	164.6	6.70
Q8BFW7	Lipoma-preferred partner homolog OS=Mus musculus GN=Lpp PE=1 SV=1 - [LPP_MOUSE]	32.63	5	14	14	94	1.377	1.991	0.969	0.857	272.59	32.63	25	94	613	65.8	7.37
P70698	CTP synthase 1 OS=Mus musculus GN=Ctps1 PE=1 SV=2 - [PYRG1_MOUSE]	25.04	1	12	14	36	0.693	0.739	0.717	0.857	97.42	25.04	25	36	591	66.6	6.58
Q9CQE9	FLYWCH family member 2 OS=Mus musculus GN=Flywch2 PE=2 SV=1 - [FWCH2_MOUSE]	53.24	3	4	4	9	1.265	0.409	0.824	0.857	22.68	53.24	6	9	139	14.4	8.13
Q1RLL3	Copine-9 OS=Mus musculus GN=Cpne9 PE=2 SV=1 - [CPNE9_MOUSE]	37.43	1	9	15	100	0.357	0.215	0.557	0.857	279.29	37.43	29	100	553	61.8	5.40
Q9JIF7	Coatomer subunit beta OS=Mus musculus GN=Copb1 PE=1 SV=1 - [COPB_MOUSE]	35.26	1	28	28	111	0.597	0.904	0.719	0.858	339.84	35.26	49	111	953	107.0	6.00
E9Q394	Protein Akap13 OS=Mus musculus GN=Akap13 PE=2 SV=1 - [E9Q394_MOUSE]	3.75	6	8	8	12	0.773	0.730	0.815	0.858	25.00	3.75	10	12	2776	303.8	5.38
Q9CQE8	UPF0568 protein C14orf166 homolog OS=Mus musculus PE=2 SV=1 - [CN166_MOUSE]	58.20	2	13	13	71	1.280	0.828	0.831	0.858	186.97	58.20	23	71	244	28.1	6.89
Q61033	Lamina-associated polypeptide 2, isoforms alpha/zeta OS=Mus musculus GN=Tmpo PE=1 SV=4 - [LAP2A_MOUSE]	23.67	2	5	13	71	1.145	0.725	0.830	0.858	227.38	23.67	23	71	693	75.1	8.05
Q8K248	4-hydroxyphenylpyruvate dioxygenase-like protein OS=Mus musculus GN=Hpdl PE=2 SV=1 - [HPDL_MOUSE]	3.23	1	1	1	2	1.090	0.811	1.137	0.858	2.73	3.23	2	2	371	39.9	6.84
Q9CZ62	Centrosomal protein of 97 kDa OS=Mus musculus GN=Cep97 PE=2 SV=1 - [CEP97_MOUSE]	10.86	5	6	6	9	1.341	1.048	0.851	0.858	24.95	10.86	7	9	856	94.6	5.00
Q3UZA1	CapZ-interacting protein OS=Mus musculus GN=Rcsd1 PE=2 SV=1 - [CPZIP_MOUSE]	18.20	2	4	4	14	1.590	1.143	1.239	0.858	45.25	18.20	6	14	412	44.1	5.16
Q9Z1D1	Eukaryotic translation initiation factor 3 subunit G OS=Mus musculus GN=Eif3g PE=1 SV=2 - [EIF3G_MOUSE]	31.87	1	9	9	43	1.119	0.697	0.920	0.858	102.20	31.87	15	43	320	35.6	5.90
Q8CFI2	Ubiquitin-conjugating enzyme E2 R1 OS=Mus musculus GN=Cdc34 PE=1 SV=1 - [UB2R1_MOUSE]	9.36	1	2	3	6	1.239	0.978	1.070	0.858	10.28	9.36	5	6	235	26.6	4.56
Q62481	Vacuolar protein sorting-associated protein 72 homolog OS=Mus musculus GN=Vps72 PE=2 SV=2 - [VPS72_MOUSE]	7.34	1	3	3	8	0.777	0.632	1.055	0.858	15.21	7.34	5	8	368	40.8	6.40
Q810A7	ATP-dependent RNA helicase DDX42 OS=Mus musculus GN=Ddx42 PE=1 SV=3 - [DDX42_MOUSE]	25.30	2	17	18	64	0.845	0.824	1.061	0.858	201.46	25.30	30	64	929	101.9	6.98

E9QAZ2	Ribosomal protein L15 OS=Mus musculus GN=Gm10020 PE=3 SV=1 - [E9QAZ2_MOUSE]	39.71	4	8	8	58	0.438	0.592	0.735	0.859	182.19	39.71	15	58	204	24.1	11.58
Q6P8I6	Cytochrome c oxidase assembly protein COX11, mitochondrial OS=Mus musculus GN=Cox11 PE=2 SV=1 - [COX11_MOUSE]	28.36	2	5	5	7	0.814	0.742	0.810	0.859	10.32	28.36	5	7	275	30.8	8.78
Q8BIX3	ARL14 effector protein OS=Mus musculus GN=Arl14ep PE=2 SV=1 - [AL14E_MOUSE]	3.26	1	1	1	4	0.928	0.656	0.913	0.859	8.23	3.26	2	4	276	31.0	8.24
Q9QXA1-2	Isoform 2 of Cysteine and histidine-rich protein 1 OS=Mus musculus GN=Cyhr1 -	9.33	4	2	2	11	1.259	1.287	0.666	0.859	35.14	9.33	4	11	375	39.7	6.28
O35227	Disintegrin and metalloproteinase domain-containing protein 7 OS=Mus musculus GN=Adam7 PE=2 SV=2 - [illegible]	1.90	1	1	1	6	1.493	1.277	1.780	0.859	0.00	1.90	1	6	789	89.1	6.38
Q3UJT0	Threonine aspartase subunit beta OS=Mus musculus GN=Tasp1 PE=2 SV=1 - [Q3UJT0_MOUSE]	4.96	2	1	1	2	1.109	0.803	0.930	0.859	5.34	4.96	2	2	383	40.2	7.88
P51954	Serine/threonine-protein kinase Nek1 OS=Mus musculus GN=Nek1 PE=1 SV=2 - [NEK1_MOUSE]	8.23	2	7	7	11	1.594	0.787	1.177	0.859	31.07	8.23	9	11	1203	136.6	5.45
Q8BVA5	UPF0554 protein C2orf43 homolog OS=Mus musculus PE=2 SV=1 - [CB043_MOUSE]	14.42	5	4	5	20	0.639	0.605	0.924	0.859	49.55	14.42	8	20	326	37.3	8.28
P61514	60S ribosomal protein L37a OS=Mus musculus GN=Rpl37a PE=2 SV=2 - [RL37A_MOUSE]	30.43	1	4	4	12	0.559	0.845	0.851	0.860	35.62	30.43	7	12	92	10.3	10.43
Q8BYB9	Protein O-glucosyltransferase 1 OS=Mus musculus GN=Poglut1 PE=2 SV=2 -	19.90	1	9	9	16	1.064	0.744	0.636	0.860	37.53	19.90	15	16	392	46.3	8.85
Q8CIB9	N-acetyltransferase ESCO2 OS=Mus musculus GN=Esco2 PE=2 SV=3 - [ESCO2_MOUSE]	1.69	1	1	1	1	1.356	1.214	0.683	0.860	3.18	1.69	1	1	592	67.2	9.48
P97352	Protein S100-A13 OS=Mus musculus GN=S100a13 PE=1 SV=1 - [S10AD_MOUSE]	35.71	1	5	5	21	1.624	0.883	1.171	0.860	69.73	35.71	8	21	98	11.2	6.13
Q91VA6	Polymerase delta-interacting protein 2 OS=Mus musculus GN=Poldip2 PE=2 SV=1 -	13.59	2	4	4	10	0.933	0.717	0.965	0.860	30.86	13.59	6	10	368	41.8	8.63
F6UNU0	Rho GTPase-activating protein 29 (Fragment) OS=Mus musculus GN=Arhgap29 PE=4 SV=1 - [F6UNU0_MOUSE]	0.70	2	1	1	2	1.268	0.835	1.120	0.860	6.85	0.70	1	2	859	95.8	6.77
Q5SUE8-2	Isoform 2 of Ankyrin repeat domain-containing protein 40 OS=Mus musculus GN=Ankrd40 - [ANR40_MOUSE]	23.29	4	6	7	19	1.681	0.725	0.767	0.860	40.52	23.29	11	19	322	35.7	5.16
Q8R3V5-1	Isoform 1 of Endophilin-B2 OS=Mus musculus GN=Sh3glb2 - [SHLB2_MOUSE]	53.67	2	1	19	119	1.176	0.923	0.847	0.860	351.14	53.67	35	119	395	44.1	5.99
Q9QZE5	Coatomer subunit gamma-1 OS=Mus musculus GN=Copg1 PE=2 SV=1 - [COPG1_MOUSE]	37.53	2	20	23	83	0.637	0.995	0.714	0.860	264.72	37.53	41	83	874	97.5	5.35
Q6PDI5	Proteasome-associated protein ECM29 homolog OS=Mus musculus GN=Ecm29 PE=1 SV=3 - [ECM29_MOUSE]	29.84	7	38	38	96	0.656	0.801	0.784	0.860	297.77	29.84	63	96	1840	203.6	7.06
E9PWM3	Protein Armcx4 OS=Mus musculus GN=Armcx4 PE=2 SV=1 - [E9PWM3_MOUSE]	10.06	1	14	14	37	1.255	0.743	1.055	0.860	83.88	10.06	21	37	2356	242.8	4.87
P53986	Monocarboxylate transporter 1 OS=Mus musculus GN=Slc16a1 PE=1 SV=1 -	11.76	1	4	4	16	0.824	0.603	0.647	0.860	46.34	11.76	7	16	493	53.2	7.47
E9Q447	Spectrin alpha chain, non-erythrocytic 1 OS=Mus musculus GN=Sptan1 PE=2 SV=1 - [E9Q447_MOUSE]	77.08	6	2	207	3707	1.174	0.674	0.725	0.860	10916.43	77.08	379	3707	2478	285.2	5.34
Q9D0J8	Parathymosin OS=Mus musculus GN=Ptms PE=2 SV=3 - [PTMS_MOUSE]	10.89	1	1	1	36	1.323	1.116	1.422	0.860	96.83	10.89	2	36	101	11.4	4.22
Q8R3D1	TBC1 domain family member 13 OS=Mus musculus GN=Tbc1d13 PE=2 SV=1 -	17.25	3	7	7	19	0.584	1.046	0.669	0.860	51.32	17.25	12	19	400	46.4	5.36
Q61908	Cx9C motif-containing protein 4 OS=Mus musculus GN=Cmc4 PE=2 SV=1 - [CMC4_MOUSE]	23.53	1	2	2	3	1.270	0.816	1.271	0.860	7.61	23.53	3	3	68	7.7	8.38
Q8VCC9	Spondin-1 OS=Mus musculus GN=Spon1 PE=2 SV=1 - [SPON1_MOUSE]	20.20	2	13	13	32	0.796	1.015	0.844	0.861	92.70	20.20	23	32	807	90.8	6.02
Q91VJ4	Serine/threonine-protein kinase 38 OS=Mus musculus GN=Stk38 PE=1 SV=1 - [STK38_MOUSE]	16.56	1	5	6	13	1.373	0.910	0.854	0.861	48.08	16.56	8	13	465	54.1	7.34

P47879	Insulin-like growth factor-binding protein 4 OS=Mus musculus GN=Igfbp4 PE=2 SV=2 - [IBP4_MOUSE]	14.17	3	3	3	8	1.869	0.957	1.176	0.861	28.87	14.17	4	8	254	27.8	7.17
Q9CZX9	ER membrane protein complex subunit 4 OS=Mus musculus GN=Emc4 PE=2 SV=1 - [EMC4_MOUSE]	14.21	1	2	2	7	1.393	1.082	1.028	0.861	18.20	14.21	3	7	183	20.1	8.62
Q8BZ32	Putative Polycomb group protein ASXL2 OS=Mus musculus GN=Asxl2 PE=2 SV=1 - [ASXL2_MOUSE]	4.74	4	3	3	3	0.987	0.502	0.831	0.861	5.09	4.74	3	3	1370	147.0	8.81
Q8K411-2	Isoform 2 of Presequence protease, mitochondrial OS=Mus musculus GN=Pitrm1 -	40.87	3	33	33	94	1.113	0.792	0.806	0.861	278.12	40.87	57	94	1035	117.1	7.12
Q9ERT9	Protein phosphatase 1 regulatory subunit 1A OS=Mus musculus GN=Ppp1r1a PE=2 SV=1 - [PPR1A_MOUSE]	83.04	1	11	11	74	1.403	1.504	1.602	0.861	217.41	83.04	17	74	171	18.7	5.25
E9QN31	Putative ribosomal RNA methyltransferase NOP2 OS=Mus musculus GN=Nop2 PE=4 SV=1 - [E9QN31_MOUSE]	4.28	2	3	3	7	0.858	0.974	1.063	0.861	16.46	4.28	4	7	794	86.9	9.19
P70445	Eukaryotic translation initiation factor 4E-binding protein 2 OS=Mus musculus GN=Eif4ebp2 PE=2 SV=1 - [illegible]	47.50	1	3	3	23	1.825	1.088	1.022	0.861	61.96	47.50	6	23	120	12.9	6.52
E9Q2S0	Protein Pcdh7 OS=Mus musculus GN=Pcdh7 PE=2 SV=1 - [E9Q2S0_MOUSE]	32.59	1	5	27	100	1.108	1.012	1.112	0.861	302.18	32.59	43	100	1255	136.7	5.17
E9Q634	Unconventional myosin-Ie OS=Mus musculus GN=Myo1e PE=1 SV=1 - [MYO1E_MOUSE]	6.23	1	6	6	17	0.999	0.611	0.794	0.861	46.66	6.23	9	17	1107	126.7	9.07
P61375	LIM/homeobox protein Lhx5 OS=Mus musculus GN=Lhx5 PE=2 SV=1 - [LHX5_MOUSE]	4.48	5	2	2	3	0.726	0.722	0.817	0.861	8.47	4.48	2	3	402	44.4	7.71
Q8BYW1-2	Isoform 2 of Rho GTPase-activating protein 25 OS=Mus musculus GN=Arhgap25 -	10.38	3	4	4	8	1.340	0.675	1.053	0.861	13.39	10.38	5	8	559	62.8	5.48
Q9R0P5	Destrin OS=Mus musculus GN=Dstn PE=1 SV=3 - [DEST_MOUSE]	75.15	1	13	15	142	1.336	0.847	1.002	0.861	343.08	75.15	26	142	165	18.5	7.97
Q8K327	Chromosome alignment-maintaining phosphoprotein 1 OS=Mus musculus GN=Champ1 PE=1 SV=1 - [illegible]	24.94	1	17	17	25	1.072	0.825	1.086	0.861	81.10	24.94	23	25	802	87.5	7.85
P62267	40S ribosomal protein S23 OS=Mus musculus GN=Rps23 PE=2 SV=3 - [RS23_MOUSE]	53.85	1	7	8	24	0.633	1.026	0.769	0.862	74.58	53.85	15	24	143	15.8	10.49
Q9D819	Inorganic pyrophosphatase OS=Mus musculus GN=Ppa1 PE=1 SV=1 - [IPYR_MOUSE]	78.55	1	21	21	136	1.088	0.822	0.933	0.862	378.20	78.55	42	136	289	32.6	5.60
G3X928	SEC23-interacting protein OS=Mus musculus GN=Sec23ip PE=4 SV=1 - [G3X928_MOUSE]	21.44	2	18	20	89	0.972	0.946	0.806	0.862	256.82	21.44	35	89	998	110.7	5.72
Q8VHV1	Brain and acute leukemia cytoplasmic protein OS=Mus musculus GN=Baalc PE=2 SV=3 - [BAALC_MOUSE]	19.31	3	3	3	12	1.174	0.798	0.935	0.862	20.97	19.31	5	12	145	15.5	7.06
Q8CHP5-2	Isoform 2 of Partner of Y14 and mago OS=Mus musculus GN=Wibg - [WIBG_MOUSE]	26.73	2	3	3	16	1.352	0.662	1.160	0.862	36.11	26.73	5	16	202	22.7	9.48
Q9CQZ0-2	Isoform 2 of ORM1-like protein 2 OS=Mus musculus GN=Ormdl2 - [ORML2_MOUSE]	11.70	3	1	1	2	0.523	0.880	0.750	0.862	6.23	11.70	2	2	94	10.8	9.52
Q8K1C0-3	Isoform 3 of Protein angel homolog 2 OS=Mus musculus GN=Angel2 - [ANGE2_MOUSE]	5.87	5	2	2	3	0.643	0.962	0.965	0.862	10.51	5.87	3	3	375	41.9	7.18
O55028	[3-methyl-2-oxobutanoate dehydrogenase [lipoamide]] kinase, mitochondrial OS=Mus musculus GN=Bckdk PE=1 SV=1 - [BCKD_MOUSE]	25.73	2	8	8	17	0.839	1.019	0.704	0.862	54.17	25.73	12	17	412	46.6	8.91
Q921I9	Exosome complex component RRP41 OS=Mus musculus GN=Exosc4 PE=2 SV=3 - [EXOS4_MOUSE]	53.88	1	8	8	14	1.185	1.018	0.763	0.862	32.26	53.88	12	14	245	26.2	6.15
Q61333	Tumor necrosis factor alpha-induced protein 2 OS=Mus musculus GN=Tnfaip2 PE=2 SV=2 - [TNAP2_MOUSE]	3.76	2	2	2	3	1.540	1.234	1.237	0.862	2.51	3.76	2	3	691	78.1	6.07
Q9DAM5	Mitochondrial thiamine pyrophosphate carrier OS=Mus musculus GN=Slc25a19 PE=2 SV=1 - [TPC_MOUSE]	13.21	3	5	5	9	0.757	0.805	0.842	0.862	22.63	13.21	9	9	318	35.6	9.20

Q8BYK6-2	Isoform 2 of YTH domain family protein 3 OS=Mus musculus GN=Ythdf3 - [YTHD3_MOUSE]	16.13	1	1	8	32	0.745	1.013	0.777	0.862	52.70	16.13	13	32	589	64.6	8.24
Q8BH69	Selenide, water dikinase 1 OS=Mus musculus GN=Sephs1 PE=2 SV=1 - [SPS1_MOUSE]	20.66	1	6	6	9	0.929	0.861	0.886	0.862	28.23	20.66	8	9	392	42.9	5.97
E9Q3Z4	Hexokinase-3 OS=Mus musculus GN=Hk3 PE=2 SV=1 - [E9Q3Z4_MOUSE]	4.38	3	2	3	15	0.746	0.889	0.741	0.862	48.82	4.38	5	15	867	94.2	5.80
Q8BP92	Reticulocalbin-2 OS=Mus musculus GN=Rcn2 PE=2 SV=1 - [RCN2_MOUSE]	48.13	1	11	13	140	2.096	0.879	1.017	0.863	454.32	48.13	23	140	320	37.2	4.42
F6Q537	Leucine-rich repeat-containing protein 48 OS=Mus musculus GN=Lrrc48 PE=4 SV=1 - [F6Q537_MOUSE]	6.31	2	2	2	2	1.517	1.199	0.946	0.863	2.29	6.31	2	2	523	60.8	4.78
G3X9K3	Brefeldin A-inhibited guanine nucleotide-exchange protein 1 OS=Mus musculus GN=Arfgef1 PE=2 SV=1 - [illegible]	28.22	2	31	45	128	0.712	0.838	0.847	0.863	353.07	28.22	72	128	1846	208.4	5.86
Q80UM7	Mannosyl-oligosaccharide glucosidase OS=Mus musculus GN=Mogs PE=2 SV=1 - [MOGS_MOUSE]	23.26	1	15	15	43	0.699	0.901	0.714	0.863	130.87	23.26	26	43	834	91.8	9.00
E9Q295	Protein Gm973 OS=Mus musculus GN=Gm973 PE=4 SV=1 - [E9Q295_MOUSE]	2.17	1	1	3	8	1.581	0.979	1.048	0.863	14.22	2.17	4	8	1059	120.9	9.42
Q3U3V8	X-ray radiation resistance-associated protein 1 OS=Mus musculus GN=Xrra1 PE=2 SV=1 - [XRRA1_MOUSE]	3.05	1	1	1	7	2.238	0.939	1.122	0.863	10.91	3.05	2	7	786	88.8	9.61
Q9Z329-3	Isoform 3 of Inositol 1,4,5-trisphosphate receptor type 2 OS=Mus musculus GN=Itpr2 - [ITPR2_MOUSE]	7.61	5	10	21	43	1.221	0.801	0.887	0.863	122.91	7.61	32	43	2668	303.8	6.47
Q9DAM7	UPF0444 transmembrane protein C12orf23 homolog OS=Mus musculus PE=2 SV=1 - [CL023_MOUSE]	70.43	1	5	5	23	1.201	0.889	1.366	0.863	86.79	70.43	10	23	115	11.5	9.32
Q9D1F4	Proline-rich AKT1 substrate 1 OS=Mus musculus GN=Akt1s1 PE=1 SV=1 -	18.29	8	4	4	8	1.063	1.166	1.014	0.863	18.88	18.29	7	8	257	27.5	4.72
P47856-2	Isoform 2 of Glutamine--fructose-6-phosphate aminotransferase [isomerizing] 1 OS=Mus musculus GN=Gfpt1 - [illegible]	42.29	5	23	23	70	0.648	0.867	0.646	0.863	234.56	42.29	42	70	681	76.7	6.77
Q9D1P4	Cysteine and histidine-rich domain-containing protein 1 OS=Mus musculus GN=Chordc1 PE=1 SV=1 - [CHRD1_MOUSE]	38.37	1	10	10	29	0.963	0.836	0.818	0.863	90.20	38.37	16	29	331	37.3	7.90
Q9WUU8-2	Isoform 2 of TNFAIP3-interacting protein 1 OS=Mus musculus GN=Tnip1 -	19.87	6	8	9	38	1.547	0.820	1.123	0.863	113.81	19.87	16	38	594	67.2	6.21
Q923T9-2	Isoform 2 of Calcium/calmodulin-dependent protein kinase type II subunit gamma OS=Mus musculus [illegible]	52.51	2	1	25	329	0.481	0.701	1.044	0.864	849.89	52.51	45	329	518	58.3	7.18
O09005	Sphingolipid delta(4)-desaturase DES1 OS=Mus musculus GN=Degs1 PE=2 SV=1 - [DEGS1_MOUSE]	14.86	1	3	3	10	0.941	0.647	1.175	0.864	44.12	14.86	5	10	323	38.2	7.77
E9PWD9	DNA-directed RNA polymerase OS=Mus musculus GN=Polrmt PE=2 SV=1 -	5.65	4	5	5	9	0.375	0.487	0.494	0.864	22.00	5.65	9	9	1133	128.3	9.03
Q9CZA6-2	Isoform 2 of Nuclear distribution protein nudE homolog 1 OS=Mus musculus GN=Nde1 - [NDE1_MOUSE]	52.37	6	13	16	35	2.274	0.681	1.065	0.864	103.59	52.37	28	35	317	35.9	5.19
G3UZZ3	Dynamin-2 (Fragment) OS=Mus musculus GN=Dnm2 PE=3 SV=1 - [G3UZZ3_MOUSE]	36.06	2	1	33	292	0.675	0.436	0.666	0.864	719.63	36.06	60	292	807	91.6	8.19
Q9DB90	Protein SMG9 OS=Mus musculus GN=Smg9 PE=2 SV=1 - [SMG9_MOUSE]	19.42	4	8	8	29	1.162	1.033	1.041	0.864	86.21	19.42	13	29	520	57.6	7.01
Q8R0F6	Integrin-linked kinase-associated serine/threonine phosphatase 2C OS=Mus musculus GN=Ilkap PE=2 [illegible]	24.74	2	10	10	20	0.781	0.886	0.777	0.864	58.41	24.74	17	20	392	42.7	7.36
Q91YY5	Solute carrier organic anion transporter family member 1A5 OS=Mus musculus GN=Slco1a5 PE=2 SV=1 - [illegible]	2.69	1	1	1	1	1.907	1.396	1.097	0.864	3.39	2.69	1	1	670	74.6	7.94
Q9CQ02	COMM domain-containing protein 4 OS=Mus musculus GN=Commd4 PE=2 SV=1 -	31.16	1	6	6	8	1.387	1.003	1.108	0.864	24.47	31.16	6	8	199	21.8	6.67

Q8K2I4	Beta-mannosidase OS=Mus musculus GN=Manba PE=2 SV=1 - [MANBA_MOUSE]	4.89	2	4	4	11	1.829	0.899	0.824	0.864	23.18	4.89	6	11	879	100.8	7.25
Q8VDI7	Ubiquitin-associated domain-containing protein 1 OS=Mus musculus GN=Ubac1 PE=2 SV=2 - [UBAC1_MOUSE]	28.61	2	3	11	29	1.795	1.376	1.170	0.864	88.32	28.61	17	29	409	45.5	4.97
G3UWH9	Conserved oligomeric Golgi complex subunit 4 (Fragment) OS=Mus musculus GN=Cog4 PE=2 SV=1 - [illegible]	15.86	6	10	10	21	0.654	0.837	0.700	0.865	60.98	15.86	17	21	763	86.2	5.16
Q8VDC0	Probable leucine--tRNA ligase, mitochondrial OS=Mus musculus GN=Lars2 PE=2 SV=1 - [SYLM_MOUSE]	14.41	3	11	11	22	1.034	0.938	0.980	0.865	52.28	14.41	18	22	902	101.4	8.19
Q6WQJ1	Sn1-specific diacylglycerol lipase alpha OS=Mus musculus GN=Dagla PE=1 SV=2 - [DGLA_MOUSE]	24.90	1	22	22	88	0.766	1.041	1.290	0.865	233.70	24.90	39	88	1044	115.3	6.42
D3YYY8	Protein Arhgef26 OS=Mus musculus GN=Arhgef26 PE=4 SV=1 - [D3YYY8_MOUSE]	10.24	1	6	6	9	1.130	0.618	0.969	0.865	27.46	10.24	9	9	869	97.3	8.72
Q8C5W0-4	Isoform 4 of Calmin OS=Mus musculus GN=Clmn - [CLMN_MOUSE]	28.31	4	21	21	70	0.804	0.923	0.956	0.865	200.25	28.31	34	70	1021	113.4	4.97
P11031	Activated RNA polymerase II transcriptional coactivator p15 OS=Mus musculus GN=Sub1 PE=1 SV=3 - [TCP4_MOUSE]	29.92	1	5	5	18	0.708	1.004	0.909	0.865	37.53	29.92	9	18	127	14.4	9.60
P39688	Tyrosine-protein kinase Fyn OS=Mus musculus GN=Fyn PE=1 SV=4 - [FYN_MOUSE]	47.30	20	17	24	100	0.749	0.666	0.756	0.865	260.80	47.30	41	100	537	60.6	6.67
Q8BFQ8	Parkinson disease 7 domain-containing protein 1 OS=Mus musculus GN=Pddc1 PE=1 SV=1 - [PDDC1_MOUSE]	45.91	1	7	7	28	0.955	0.978	0.918	0.865	97.22	45.91	12	28	220	23.3	7.05
B2RY56	RNA-binding protein 25 OS=Mus musculus GN=Rbm25 PE=1 SV=2 - [RBM25_MOUSE]	14.08	1	10	11	41	0.732	0.887	0.886	0.865	126.16	14.08	17	41	838	99.5	6.32
Q9Z2M6	Ubiquitin-like protein 3 OS=Mus musculus GN=Ubl3 PE=1 SV=1 - [UBL3_MOUSE]	52.99	1	5	5	11	1.448	0.595	0.865	0.865	33.39	52.99	9	11	117	13.2	6.92
Q7TSC1	Protein PRRC2A OS=Mus musculus GN=Prrc2a PE=1 SV=1 - [PRC2A_MOUSE]	21.73	2	34	38	167	1.194	0.787	1.232	0.865	412.10	21.73	66	167	2158	229.1	9.39
P07356	Annexin A2 OS=Mus musculus GN=Anxa2 PE=1 SV=2 - [ANXA2_MOUSE]	67.85	4	21	21	75	0.933	4.251	0.673	0.865	226.18	67.85	36	75	339	38.7	7.69
A2BGN7	Protein MANBAL OS=Mus musculus GN=Manbal PE=4 SV=1 - [A2BGN7_MOUSE]	9.41	1	1	1	2	0.539	0.771	0.755	0.866	4.75	9.41	2	2	85	9.4	9.38
Q63918	Serum deprivation-response protein OS=Mus musculus GN=Sdpr PE=1 SV=3 - [SDPR_MOUSE]	15.79	1	6	6	12	1.533	1.506	0.909	0.866	30.58	15.79	8	12	418	46.7	5.21
Q3TZX8-3	Isoform 3 of Polynucleotide 5'-hydroxyl-kinase NOL9 OS=Mus musculus GN=Nol9 - [NOL9_MOUSE]	3.03	3	3	3	5	0.570	0.706	0.755	0.866	10.60	3.03	4	5	627	70.9	9.44
A2AJ15	Endoplasmic reticulum mannosyl-oligosaccharide 1,2-alpha-mannosidase OS=Mus musculus GN=Man1b1 PE=2 SV=1 - [MA1B1_MOUSE]	10.79	1	7	7	11	1.033	0.825	0.742	0.866	20.90	10.79	8	11	658	75.1	8.48
Q61324-2	Isoform 2 of Aryl hydrocarbon receptor nuclear translocator 2 OS=Mus musculus GN=Arnt2 - [illegible]	20.40	2	8	8	31	0.865	0.714	1.005	0.866	94.70	20.40	14	31	701	76.8	6.80
Q6ZWX6	Eukaryotic translation initiation factor 2 subunit 1 OS=Mus musculus GN=Eif2s1 PE=1 SV=3 - [IF2A_MOUSE]	58.41	1	20	20	69	0.769	0.876	0.837	0.866	196.88	58.41	37	69	315	36.1	5.08
Q80YS6	Actin filament-associated protein 1 OS=Mus musculus GN=Afap1 PE=1 SV=1 - [AFAP1_MOUSE]	8.07	2	5	6	11	1.365	0.913	0.986	0.866	29.93	8.07	9	11	731	80.6	8.68
Q9WTS6-2	Isoform 2 of Teneurin-3 OS=Mus musculus GN=Tenm3 - [TEN3_MOUSE]	10.60	5	16	21	59	0.659	0.654	0.808	0.866	154.72	10.60	32	59	2699	300.9	6.42
F7AYW2	Protein PRRC2B (Fragment) OS=Mus musculus GN=Prrc2b PE=4 SV=1 - [illegible]	21.13	2	1	23	48	0.963	0.429	1.420	0.866	148.37	21.13	37	48	1472	158.7	7.93
G3XA10	Heterogeneous nuclear ribonucleoprotein U OS=Mus musculus GN=Hnrnpu PE=4 SV=1 - [G3XA10_MOUSE]	36.95	2	31	31	194	0.826	0.741	0.929	0.866	483.04	36.95	51	194	793	86.8	5.92

Q9CZ28	Vacuolar-sorting protein SNF8 OS=Mus musculus GN=Snf8 PE=2 SV=1 - [SNF8_MOUSE]	29.07	2	7	7	20	1.049	1.312	0.829	0.866	51.30	29.07	12	20	258	28.9	6.65
Q8JZP9	GAS2-like protein 1 OS=Mus musculus GN=Gas2l1 PE=2 SV=1 - [GA2L1_MOUSE]	13.42	3	6	6	18	1.048	1.047	1.206	0.866	66.40	13.42	11	18	678	72.4	10.01
Q8VED5	Keratin, type II cytoskeletal 79 OS=Mus musculus GN=Krt79 PE=1 SV=2 - [K2C79_MOUSE]	12.05	2	2	3	18	1.402	1.116	1.031	0.866	32.81	12.05	4	18	531	57.5	7.69
P35980	60S ribosomal protein L18 OS=Mus musculus GN=Rpl18 PE=2 SV=3 - [RL18_MOUSE]	32.45	5	6	6	36	0.464	0.519	0.696	0.867	107.29	32.45	11	36	188	21.6	11.78
E9QKB1	Dynein light chain 1, cytoplasmic OS=Mus musculus GN=Dlc1 PE=2 SV=1 - [E9QKB1_MOUSE]	2.47	4	2	2	4	0.782	0.814	0.782	0.867	7.08	2.47	4	4	1092	123.3	7.58
O55106	Striatin OS=Mus musculus GN=Strn PE=1 SV=2 - [STRN_MOUSE]	35.64	3	17	20	90	1.069	0.869	1.235	0.867	299.36	35.64	33	90	780	85.9	5.27
Q8BRK9	Alpha-mannosidase 2x OS=Mus musculus GN=Man2a2 PE=2 SV=2 - [MA2A2_MOUSE]	6.16	2	6	6	13	0.496	0.906	0.753	0.867	43.58	6.16	11	13	1152	130.6	6.87
E9Q1V5	Sphingomyelin phosphodiesterase 4 OS=Mus musculus GN=Smpd4 PE=2 SV=1 -	12.07	10	6	6	7	1.413	1.200	0.899	0.867	19.43	12.07	7	7	721	81.7	8.65
Q8BGD9	Eukaryotic translation initiation factor 4B OS=Mus musculus GN=Eif4b PE=1 SV=1 - [IF4B_MOUSE]	31.59	1	20	20	145	1.170	0.706	1.300	0.867	380.53	31.59	37	145	611	68.8	5.67
Q9QYC0	Alpha-adducin OS=Mus musculus GN=Add1 PE=1 SV=2 - [ADDA_MOUSE]	48.71	4	1	26	280	3.911	1.988	3.153	0.867	735.99	48.71	47	280	735	80.6	5.90
Q9R008	Mevalonate kinase OS=Mus musculus GN=Mvk PE=2 SV=1 - [KIME_MOUSE]	36.20	3	12	12	31	0.745	0.472	0.623	0.867	89.45	36.20	20	31	395	41.9	6.68
Q91WE4	UPF0729 protein C18orf32 homolog OS=Mus musculus PE=2 SV=1 - [CR032_MOUSE]	37.50	1	3	3	7	0.480	0.721	0.836	0.867	7.66	37.50	5	7	72	8.0	9.35
Q8BWZ3	N-alpha-acetyltransferase 25, NatB auxiliary subunit OS=Mus musculus GN=Naa25 PE=1 SV=1 - [NAA25_MOUSE]	14.51	3	13	14	41	0.775	0.811	0.821	0.867	104.82	14.51	24	41	972	111.6	6.52
P61215	Carbonic anhydrase-related protein 10 OS=Mus musculus GN=Ca10 PE=2 SV=1 - [CAH10_MOUSE]	30.49	4	6	6	17	1.007	0.993	0.814	0.867	40.00	30.49	9	17	328	37.5	8.09
D3YXJ0	Protein Dgkh OS=Mus musculus GN=Dgkh PE=4 SV=1 - [D3YXJ0_MOUSE]	21.89	1	15	19	44	0.763	1.251	1.514	0.867	134.15	21.89	29	44	1156	127.3	6.33
P20918	Plasminogen OS=Mus musculus GN=Plg PE=1 SV=3 - [PLMN_MOUSE]	26.60	2	17	18	70	3.526	1.758	0.485	0.867	198.22	26.60	32	70	812	90.7	6.60
Q80VL1	Tudor and KH domain-containing protein OS=Mus musculus GN=Tdrkh PE=1 SV=1 - [TDRKH_MOUSE]	28.57	1	12	12	56	1.044	0.951	0.791	0.867	206.04	28.57	20	56	560	62.1	4.94
P55096	ATP-binding cassette sub-family D member 3 OS=Mus musculus GN=Abcd3 PE=1 SV=2 - [ABCD3_MOUSE]	36.42	1	19	20	66	0.478	0.623	0.686	0.867	159.70	36.42	35	66	659	75.4	9.26
D3YZP9	Coiled-coil domain-containing protein 6 OS=Mus musculus GN=Ccdc6 PE=3 SV=1 - [CCDC6_MOUSE]	44.56	3	22	22	115	1.951	0.988	1.127	0.867	316.53	44.56	39	115	469	52.9	7.34
E9QQ25	Striated muscle-specific serine/threonine-protein kinase OS=Mus musculus GN=Speg PE=2 SV=1 - [E9QQ25_MOUSE]	6.53	6	13	16	42	0.512	1.160	1.763	0.868	118.80	6.53	26	42	3262	354.1	8.18
Q9Z0H8-2	Isoform 2 of CAP-Gly domain-containing linker protein 2 OS=Mus musculus GN=Clip2 - [CLIP2_MOUSE]	57.91	6	1	58	277	1.881	1.146	0.903	0.868	787.74	57.91	100	277	1012	111.8	6.55
Q9EP97	Sentrin-specific protease 3 OS=Mus musculus GN=Senp3 PE=1 SV=1 - [SENP3_MOUSE]	7.57	1	3	3	4	0.466	0.826	0.754	0.868	14.94	7.57	4	4	568	64.4	8.75
Q922Q8	Leucine-rich repeat-containing protein 59 OS=Mus musculus GN=Lrrc59 PE=2 SV=1 - [LRC59_MOUSE]	42.67	1	11	11	49	0.609	0.582	0.851	0.868	127.07	42.67	19	49	307	34.9	9.52
G3X9I1	DNA segment, Chr 6, Wayne State University 176, expressed, isoform CRA_a OS=Mus musculus GN=Fam3c PE=4 SV=1 - [G3X9I1_MOUSE]	43.65	6	7	7	13	0.614	1.017	0.959	0.868	40.95	43.65	11	13	197	21.5	7.71
Q3U5C7	Prickle-like protein 1 OS=Mus musculus GN=Prickle1 PE=1 SV=1 - [PRIC1_MOUSE]	9.01	1	5	5	9	1.166	1.009	1.123	0.868	12.90	9.01	6	9	832	94.1	6.28

Q9ERY9	Probable ergosterol biosynthetic protein 28 OS=Mus musculus GN=ORF11 PE=2 SV=1 - [ERG28_MOUSE]	17.14	2	2	2	8	0.438	0.761	0.698	0.868	21.95	17.14	4	8	140	15.8	9.91
Q3U1F9	Phosphoprotein associated with glycosphingolipid-enriched microdomains 1 OS=Mus musculus GN=Pag1 PE=1 SV=2 - [PHAG1_MOUSE]	37.53	1	11	11	39	1.226	0.990	1.006	0.868	126.66	37.53	17	39	429	46.5	4.81
D3Z3B2	V-type proton ATPase 16 kDa proteolipid subunit (Fragment) OS=Mus musculus GN=Atp6v0c PE=2 SV=1 - [illegible]	15.52	3	1	1	9	0.424	2.805	1.031	0.868	38.14	15.52	2	9	116	11.9	6.64
Q9CQ13	Coordinator of PRMT5 and differentiation stimulator OS=Mus musculus GN=Coprs PE=1 SV=1 - [COPRS_MOUSE]	28.90	1	2	2	5	2.039	0.915	0.771	0.868	20.75	28.90	3	5	173	18.7	4.08
Q9CQD4	Charged multivesicular body protein 1b-2 OS=Mus musculus GN=Chmp1b2 PE=2 SV=2 - [CH1B2_MOUSE]	30.15	1	3	9	38	1.218	0.679	0.926	0.868	90.66	30.15	16	38	199	22.1	8.10
O35400	Sulfotransferase family cytosolic 2B member 1 OS=Mus musculus GN=Sult2b1 PE=2 SV=2 - [ST2B1_MOUSE]	1.78	3	1	1	3	1.248	1.223	0.927	0.868	5.78	1.78	1	3	338	38.3	5.17
D3YXA6	Tripartite motif-containing protein 46 OS=Mus musculus GN=Trim46 PE=2 SV=1 -	30.84	5	19	20	42	0.830	1.059	1.239	0.868	117.07	30.84	35	42	736	80.9	7.87
P97863-3	Isoform 3 of Nuclear factor 1 B-type OS=Mus musculus GN=Nfib - [NFIB_MOUSE]	10.00	8	3	4	13	1.028	1.265	0.944	0.868	28.33	10.00	7	13	420	47.4	8.87
O89086	Putative RNA-binding protein 3 OS=Mus musculus GN=Rbm3 PE=1 SV=1 - [RBM3_MOUSE]	41.83	2	3	3	46	1.968	0.864	1.339	0.868	146.97	41.83	6	46	153	16.6	7.50
B2RRE2	Myo18a protein OS=Mus musculus GN=Myo18a PE=2 SV=1 - [B2RRE2_MOUSE]	36.30	14	71	74	291	0.958	0.659	1.065	0.868	886.22	36.30	124	291	2047	232.0	6.10
G5E8L7	Hematopoietic cell transcript 1, isoform CRA_a OS=Mus musculus GN=Hemt1 PE=4 SV=1 - [G5E8L7_MOUSE]	3.41	1	1	1	1	1.761	0.833	1.202	0.868	0.00	3.41	1	1	176	19.9	5.54
P23819-4	Isoform 4 of Glutamate receptor 2 OS=Mus musculus GN=Gria2 - [GRIA2_MOUSE]	48.32	4	2	42	168	1.464	1.744	1.304	0.869	450.82	48.32	70	168	921	102.8	7.72
B8JJI3	Uncharacterized protein (Fragment) OS=Mus musculus GN=Crebrf PE=2 SV=1 - [B8JJI3_MOUSE]	6.57	3	1	1	1	1.875	1.060	0.815	0.869	0.00	6.57	1	1	137	16.1	10.51
A2ACD2	V-set and transmembrane domain-containing protein 2-like protein OS=Mus musculus GN=Vstm2l PE=4 SV=1 - [illegible]	21.78	2	3	3	7	1.766	1.284	0.861	0.869	31.52	21.78	5	7	202	22.1	8.84
Q8R2Y0-2	Isoform 2 of Monoacylglycerol lipase ABHD6 OS=Mus musculus GN=Abhd6 -	37.02	2	9	9	24	0.759	1.194	1.118	0.869	65.14	37.02	17	24	289	32.8	8.34
E9Q6H8	Protein Plekha5 OS=Mus musculus GN=Plekha5 PE=2 SV=1 - [E9Q6H8_MOUSE]	24.35	1	25	26	58	0.928	0.944	1.108	0.869	165.11	24.35	43	58	1269	144.3	7.39
A2AGH8	Mediator of RNA polymerase II transcription subunit 12 OS=Mus musculus GN=Med12 PE=2 SV=1 - [illegible]	1.48	5	2	3	6	2.105	1.381	1.109	0.869	24.55	1.48	4	6	2157	240.7	7.17
Q9D0K0	TBC1 domain family member 7 OS=Mus musculus GN=Tbc1d7 PE=1 SV=1 -	26.62	5	7	8	16	1.107	0.897	0.781	0.869	33.92	26.62	12	16	293	33.8	7.20
B9EHJ3	Tight junction protein ZO-1 OS=Mus musculus GN=Tjp1 PE=2 SV=1 - [B9EHJ3_MOUSE]	32.17	1	1	42	185	1.285	0.971	1.083	0.869	528.74	32.17	72	185	1685	188.7	6.67
P10923	Osteopontin OS=Mus musculus GN=Spp1 PE=1 SV=1 - [OSTP_MOUSE]	10.54	3	2	2	2	3.668	1.386	0.754	0.869	8.28	10.54	2	2	294	32.4	4.53
Q9CQU3	Protein RER1 OS=Mus musculus GN=Rer1 PE=1 SV=1 - [RER1_MOUSE]	18.37	1	3	3	14	0.894	0.782	0.635	0.869	41.98	18.37	4	14	196	23.0	9.51
Q8K019-3	Isoform 3 of Bcl-2-associated transcription factor 1 OS=Mus musculus GN=Bclaf1 - [BCLF1_MOUSE]	11.23	4	8	9	28	0.875	0.881	0.950	0.869	58.52	11.23	15	28	748	86.0	10.08
Q9QX47-3	Isoform 3 of Protein SON OS=Mus musculus [illegible]	15.14	9	24	24	58	1.175	0.999	0.909	0.869	175.83	15.14	35	58	2404	261.3	5.68
Q91UZ5	Inositol monophosphatase 2 OS=Mus musculus GN=Impa2 PE=1 SV=1 - [IMPA2_MOUSE]	4.83	1	2	2	2	0.761	1.273	1.443	0.869	4.35	4.83	2	2	290	31.7	6.23
B9EKN8	TRAF2 and NCK interacting kinase OS=Mus musculus GN=Tnik PE=2 SV=1 - [B9EKN8_MOUSE]	28.48	10	27	35	118	0.947	0.908	1.257	0.869	346.22	28.48	61	118	1352	153.9	7.09

Q8CGQ2	A disintegrin and metalloproteinase domain 4 OS=Mus musculus GN=Adam4 PE=2 SV=1 - [Q8CGQ2_MOUSE]	1.83	1	1	1	1	0.966	0.644	0.775	0.869	0.00	1.83	1	1	763	84.4	7.77
Q9CQF3	Cleavage and polyadenylation specificity factor subunit 5 OS=Mus musculus GN=Nudt21 PE=2 SV=1 - [illegible]	49.34	1	10	10	27	0.737	0.891	0.757	0.869	73.74	49.34	15	27	227	26.2	8.82
E9PVG8	Protein 9530053A07Rik OS=Mus musculus GN=9530053A07Rik PE=4 SV=1 - [E9PVG8_MOUSE]	1.39	1	1	2	6	1.358	0.766	1.068	0.869	14.32	1.39	3	6	2581	280.0	6.18
Q6NZK8	Protein tyrosine phosphatase domain-containing protein 1 OS=Mus musculus GN=Ptpdc1 PE=2 SV=1 - [illegible]	21.29	3	11	11	20	1.070	0.994	0.888	0.869	41.54	21.29	15	20	747	83.9	6.34
P53996-2	Isoform 2 of Cellular nucleic acid-binding protein OS=Mus musculus GN=Cnbp -	47.06	5	7	7	43	2.101	1.035	1.291	0.869	137.50	47.06	14	43	170	18.7	7.71
Q9CU62	Structural maintenance of chromosomes protein 1A OS=Mus musculus GN=Smc1a PE=1 SV=4 - [SMC1A_MOUSE]	30.82	2	41	41	106	0.744	0.716	0.929	0.869	274.34	30.82	62	106	1233	143.1	7.64
E9Q614	Protein Chd3 OS=Mus musculus GN=Chd3 PE=2 SV=1 - [E9Q614_MOUSE]	7.97	4	4	15	34	0.865	1.108	0.878	0.869	92.60	7.97	23	34	2021	228.8	6.35
Q9JLI8	Squamous cell carcinoma antigen recognized by T-cells 3 OS=Mus musculus GN=Sart3 PE=2 SV=1 - [SART3_MOUSE]	32.54	2	23	24	68	0.596	0.779	0.821	0.870	172.21	32.54	39	68	962	109.5	5.24
Q6PH08	ERC protein 2 OS=Mus musculus GN=Erc2 PE=1 SV=2 - [ERC2_MOUSE]	51.93	3	28	56	262	1.187	1.465	1.886	0.870	630.40	51.93	96	262	957	110.6	7.03
Q9DBZ5	Eukaryotic translation initiation factor 3 subunit K OS=Mus musculus GN=Eif3k PE=1 SV=1 - [EIF3K_MOUSE]	31.65	2	5	5	28	1.279	1.319	0.880	0.870	80.54	31.65	10	28	218	25.1	4.93
D3YU00	StAR-related lipid transfer (START) domain containing 5, isoform CRA_a OS=Mus musculus GN=Stard5 PE=4 SV=1 - [D3YU00_MOUSE]	4.37	2	1	1	5	1.288	0.473	0.785	0.870	13.88	4.37	2	5	206	23.2	5.45
E9PYF7	AP1AR OS=Mus musculus GN=Ap1ar PE=4 SV=1 - [E9PYF7_MOUSE]	11.74	1	4	4	10	1.206	1.163	1.235	0.870	18.35	11.74	7	10	298	33.8	4.87
Q922J9	Fatty acyl-CoA reductase 1 OS=Mus musculus GN=Far1 PE=1 SV=1 - [FACR1_MOUSE]	13.79	6	6	6	12	1.071	0.806	0.694	0.870	29.63	13.79	9	12	515	59.4	9.19
F6TRJ8	Coiled-coil domain-containing protein 85A (Fragment) OS=Mus musculus GN=Ccdc85a PE=4 SV=1 - [illegible]	38.19	2	2	5	12	1.301	1.021	1.205	0.870	34.82	38.19	9	12	144	16.0	6.27
Q6IR42	Zinc finger CW-type PWWP domain protein 1 OS=Mus musculus GN=Zcwpw1 PE=2 SV=2 - [ZCPW1_MOUSE]	1.75	1	1	1	1	1.446	1.211	0.787	0.870	2.35	1.75	1	1	630	70.5	5.45
Q3U1T9-4	Isoform 4 of DENN domain containing protein 1B OS=Mus musculus GN=Dennd1b - [DEN1B_MOUSE]	5.41	3	2	2	5	1.659	0.755	1.068	0.870	20.45	5.41	3	5	499	55.1	6.61
P47962	60S ribosomal protein L5 OS=Mus musculus GN=Rpl5 PE=1 SV=3 - [RL5_MOUSE]	41.75	2	15	16	96	0.674	0.714	0.759	0.870	267.40	41.75	27	96	297	34.4	9.77
Q5SXC4	Protein Vezf1 OS=Mus musculus GN=Vezf1 PE=2 SV=1 - [Q5SXC4_MOUSE]	2.51	8	1	2	7	1.331	0.776	0.842	0.870	14.78	2.51	2	7	518	56.5	9.57
Q8BH15-2	Isoform 2 of CCR4-NOT transcription complex subunit 10 OS=Mus musculus GN=Cnot10 - [CNO10_MOUSE]	10.36	6	7	7	11	0.877	1.122	0.846	0.870	26.70	10.36	8	11	743	81.7	7.68
Q921M3	Splicing factor 3B subunit 3 OS=Mus musculus GN=Sf3b3 PE=2 SV=1 - [SF3B3_MOUSE]	24.73	2	27	27	107	1.244	0.981	0.855	0.870	312.87	24.73	44	107	1217	135.5	5.26
Q5SV64	Myosin-10 OS=Mus musculus GN=Myh10 PE=2 SV=1 - [Q5SV64_MOUSE]	47.93	5	72	102	696	1.297	1.065	1.386	0.870	2008.88	47.93	175	696	2007	232.3	5.54
Q8BL48-2	Isoform 2 of RING finger protein unkempt homolog OS=Mus musculus GN=Unk - [UNK_MOUSE]	10.16	2	6	6	9	0.867	0.714	1.279	0.871	20.59	10.16	9	9	797	86.8	6.92
Q8BGF7-4	Isoform 4 of PAB-dependent poly(A)-specific ribonuclease subunit 2 OS=Mus musculus GN=Pan2 - [illegible]	2.23	4	2	2	4	0.514	1.070	0.887	0.871	2.29	2.23	2	4	1166	131.1	5.96
Q3UHU5-3	Isoform 3 of Protein SOGA2 OS=Mus musculus GN=Soga2 - [SOGA2_MOUSE]	20.71	8	25	31	74	0.972	0.701	1.348	0.871	190.81	20.71	50	74	1893	208.0	6.18

Q8VE65	Transcription initiation factor TFIID subunit 12 OS=Mus musculus GN=Taf12 PE=1 SV=1 - [TAF12_MOUSE]	16.77	2	2	3	5	1.518	0.896	1.041	0.871	10.78	16.77	5	5	161	17.9	7.99
A2A473	Protein Tox2 OS=Mus musculus GN=Tox2 PE=2 SV=1 - [A2A473_MOUSE]	17.62	4	5	7	15	1.009	0.582	0.940	0.871	43.33	17.62	11	15	505	53.2	7.42
Q9R059	Four and a half LIM domains protein 3 OS=Mus musculus GN=Fhl3 PE=2 SV=2 - [FHL3_MOUSE]	13.84	2	3	3	14	1.632	1.725	0.890	0.871	57.71	13.84	6	14	289	31.8	6.20
Q8CA71	Protein shisa-4 OS=Mus musculus GN=Shisa4 PE=2 SV=1 - [SHSA4_MOUSE]	8.63	1	2	2	7	0.727	1.179	0.821	0.871	18.87	8.63	4	7	197	21.5	7.42
Q9CYA6	Zinc finger CCHC domain-containing protein 8 OS=Mus musculus GN=Zcchc8 PE=2 SV=3 - [ZCHC8_MOUSE]	7.05	1	4	4	13	1.746	1.119	1.165	0.871	15.28	7.05	6	13	709	78.0	4.73
D3Z2B4	Xenotropic and polytropic retrovirus receptor 1 OS=Mus musculus GN=Xpr1 PE=2 SV=1 - [D3Z2B4_MOUSE]	8.73	3	6	6	10	0.821	0.794	0.771	0.871	27.35	8.73	10	10	630	74.0	7.68
O35926	Cyclin-dependent kinase 5 activator 2 OS=Mus musculus GN=Cdk5r2 PE=3 SV=2 -	14.36	1	3	3	7	0.837	0.758	1.244	0.871	18.95	14.36	4	7	369	38.9	9.72
Q6P5E4	UDP-glucose:glycoprotein glucosyltransferase 1 OS=Mus musculus GN=Uggt1 PE=1 SV=4 - [UGGG1_MOUSE]	30.63	7	36	38	133	0.782	0.974	0.811	0.871	393.22	30.63	66	133	1551	176.3	5.62
D3YVV7	Protein Nova2 OS=Mus musculus GN=Nova2 PE=4 SV=2 - [D3YVV7_MOUSE]	40.45	2	9	16	81	1.160	1.293	0.998	0.871	257.40	40.45	31	81	492	49.0	8.16
O08688	Calpain-5 OS=Mus musculus GN=Capn5 PE=2 SV=1 - [CAN5_MOUSE]	41.25	3	23	23	63	0.700	0.702	0.766	0.871	195.91	41.25	34	63	640	72.9	7.36
A2AHC3	Calmodulin-regulated spectrin-associated protein 1 OS=Mus musculus GN=Camsap1 PE=1 SV=1 [CAMP1_MOUSE]	27.45	8	35	38	101	0.833	0.722	1.117	0.871	308.40	27.45	65	101	1581	175.8	6.95
Q8BH27	Multiple epidermal growth factor-like domains protein 9 OS=Mus musculus GN=Megf9 PE=2 SV=1 - [MEGF9_MOUSE]	8.00	1	5	5	16	0.852	1.397	1.206	0.871	40.62	8.00	10	16	600	62.8	5.47
Q8CES0	N-alpha-acetyltransferase 30 OS=Mus musculus GN=Naa30 PE=2 SV=2 - [NAA30_MOUSE]	15.93	3	4	4	12	1.173	0.973	0.882	0.871	37.03	15.93	7	12	364	39.4	5.64
Q3UVU3	Zinc transporter 10 OS=Mus musculus GN=Slc30a10 PE=1 SV=1 - [ZNT10_MOUSE]	21.06	2	6	6	19	0.398	0.644	0.729	0.871	74.92	21.06	10	19	470	50.9	7.02
P0CG14	Chromosome transmission fidelity protein 8 homolog isoform 2 OS=Mus musculus GN=Chtf8 PE=2 SV=1 - [CTF8A_MOUSE]	12.57	1	4	4	7	1.399	1.185	1.162	0.871	22.35	12.57	6	7	533	52.2	12.32
Q8VEG6-3	Isoform 3 of CCR4-NOT transcription complex subunit 6-like OS=Mus musculus GN=Cnot6l - [CNO6L_MOUSE]	4.83	4	3	3	3	1.335	0.780	0.780	0.871	7.31	4.83	3	3	435	49.4	6.30
Q8K339	DNA/RNA-binding protein KIN17 OS=Mus musculus GN=Kin PE=2 SV=1 - [KIN17_MOUSE]	8.95	1	3	4	7	1.185	0.823	0.773	0.871	17.32	8.95	7	7	391	44.7	9.04
Q8BIL5	Protein Hook homolog 1 OS=Mus musculus GN=Hook1 PE=1 SV=2 - [HOOK1_MOUSE]	21.43	2	15	15	44	1.298	1.431	1.089	0.871	113.05	21.43	25	44	728	84.4	5.22
Q8R5H1	Ubiquitin carboxyl-terminal hydrolase 15 OS=Mus musculus GN=Usp15 PE=2 SV=1 - [UBP15_MOUSE]	40.06	3	2	28	68	0.906	1.337	1.250	0.871	201.66	40.06	42	68	981	112.3	5.17
Q8BY71	Histone acetyltransferase type B catalytic subunit OS=Mus musculus GN=Hat1 PE=2 SV=1 - [HAT1_MOUSE]	2.16	2	1	1	3	0.690	0.755	0.668	0.871	7.94	2.16	2	3	416	49.2	6.33
Q62348	Translin OS=Mus musculus GN=Tsn PE=1 SV=1 - [TSN_MOUSE]	56.14	1	14	14	49	0.770	1.030	0.631	0.871	128.06	56.14	24	49	228	26.2	6.44
Q9DBR3-2	Isoform 2 of Armadillo repeat-containing protein 8 OS=Mus musculus GN=Armc8 - [ARMC8_MOUSE]	33.33	2	2	12	38	0.947	1.179	0.844	0.871	93.71	33.33	20	38	399	44.4	7.21
P28659-2	Isoform 2 of CUGBP Elav-like family member 1 OS=Mus musculus GN=Celf1 -	17.01	4	6	8	23	1.077	1.292	1.043	0.871	70.89	17.01	13	23	482	51.6	8.47
Q9D219	B-cell CLL/lymphoma 9 protein OS=Mus musculus GN=Bcl9 PE=1 SV=3 - [BCL9_MOUSE]	9.33	1	9	11	23	1.411	1.153	1.001	0.872	56.78	9.33	15	23	1425	148.9	8.91

Q91VE0	Long-chain fatty acid transport protein 4 OS=Mus musculus GN=Slc27a4 PE=1 SV=1 - [S27A4_MOUSE]	34.37	1	20	21	73	0.829	0.695	0.814	0.872	220.92	34.37	36	73	643	72.3	8.59
Q56A07	Sodium channel subunit beta-2 OS=Mus musculus GN=Scn2b PE=1 SV=1 - [SCN2B_MOUSE]	36.74	1	7	7	24	0.990	0.992	1.023	0.872	69.37	36.74	11	24	215	24.2	6.54
Q571B6-3	Isoform 3 of WASP homolog-associated protein with actin, membranes and microtubules OS=Mus musculus GN=Whamm - [WHAMM_MOUSE]	1.45	4	1	1	1	0.595	0.707	0.834	0.872	2.26	1.45	1	1	415	47.5	5.38
Q99LM2	CDK5 regulatory subunit-associated protein 3 OS=Mus musculus GN=Cdk5rap3 PE=2 SV=1 [CK5P3_MOUSE]	26.24	7	11	11	32	0.851	0.960	0.828	0.872	85.96	26.24	18	32	503	57.0	4.83
Q3U285-2	Isoform 2 of Ligand-dependent nuclear receptor corepressor-like protein OS=Mus musculus GN=Lcorl - [illegible]	2.90	2	1	1	1	1.189	1.416	0.966	0.872	2.13	2.90	1	1	517	57.3	7.93
Q5EBG8	Uncharacterized protein C1orf50 homolog OS=Mus musculus PE=2 SV=1 - [CA050_MOUSE]	38.19	1	4	4	10	2.550	0.878	0.960	0.872	24.81	38.19	7	10	199	21.8	5.53
Q8JZY2	COMM domain-containing protein 10 OS=Mus musculus GN=Commd10 PE=2 SV=1 - [COMDA_MOUSE]	25.74	1	5	5	10	1.157	1.462	0.881	0.872	29.84	25.74	9	10	202	22.8	6.65
Q8C1A5	Thimet oligopeptidase OS=Mus musculus GN=Thop1 PE=1 SV=1 - [THOP1_MOUSE]	44.25	1	32	32	131	1.574	1.115	0.778	0.872	345.73	44.25	56	131	687	78.0	6.06
K3W4Q8	Basigin OS=Mus musculus GN=Bsg PE=4 SV=1 - [K3W4Q8_MOUSE]	41.74	3	2	8	47	0.650	1.052	0.781	0.872	124.11	41.74	12	47	218	24.1	5.36
Q8R1B4	Eukaryotic translation initiation factor 3 subunit C OS=Mus musculus GN=Eif3c PE=1 SV=1 - [EIF3C_MOUSE]	24.37	1	25	25	118	0.779	0.853	0.846	0.872	291.03	24.37	44	118	911	105.5	5.78
P45591	Cofilin-2 OS=Mus musculus GN=Cfl2 PE=1 SV=1 - [COF2_MOUSE]	83.13	1	10	16	231	2.161	0.625	0.892	0.872	567.53	83.13	26	231	166	18.7	7.88
E0CZ16	Kelch-like protein 3 OS=Mus musculus GN=Klhl3 PE=1 SV=2 - [KLHL3_MOUSE]	10.90	3	3	5	8	0.785	1.492	1.013	0.872	28.02	10.90	8	8	587	64.9	5.60
Q8VHL1	Histone-lysine N-methyltransferase SETD7 OS=Mus musculus GN=Setd7 PE=2 SV=2 - [SETD7_MOUSE]	51.64	1	14	14	81	1.743	0.822	1.399	0.872	251.18	51.64	25	81	366	40.5	4.65
Q99N95	39S ribosomal protein L3, mitochondrial OS=Mus musculus GN=Mrpl3 PE=2 SV=1 - [RM03_MOUSE]	12.93	3	4	4	8	0.920	0.958	1.016	0.872	24.90	12.93	7	8	348	39.1	9.55
F6RJ39	Apoptotic chromatin condensation inducer in the nucleus (Fragment) OS=Mus musculus GN=Acin1 PE=4 SV=1 - [illegible]	18.71	12	12	23	72	1.331	0.700	1.053	0.873	174.75	18.71	38	72	1272	143.6	5.74
Q9JKR6	Hypoxia up-regulated protein 1 OS=Mus musculus GN=Hyou1 PE=1 SV=1 -	42.04	3	36	36	178	0.871	1.079	0.857	0.873	546.30	42.04	64	178	999	111.1	5.19
Q99LM9	Transcriptional adapter 1 OS=Mus musculus GN=Tada1 PE=2 SV=1 - [TADA1_MOUSE]	7.46	1	2	2	4	0.347	0.712	0.806	0.873	13.64	7.46	3	4	335	37.4	7.62
P57776-3	Isoform 3 of Elongation factor 1-delta OS=Mus musculus GN=Eef1d - [EF1D_MOUSE]	50.76	7	3	28	235	1.762	0.812	0.925	0.873	776.55	50.76	49	235	660	72.9	6.43
Q9WV91	Prostaglandin F2 receptor negative regulator OS=Mus musculus GN=Ptgfrn PE=1 SV=2 - [FPRP_MOUSE]	14.11	1	12	12	32	0.700	0.705	0.909	0.873	84.04	14.11	19	32	879	98.7	6.61
O35855	Branched-chain-amino-acid aminotransferase, mitochondrial OS=Mus musculus GN=Bcat2 PE=2 SV=2 - [BCAT2_MOUSE]	21.37	4	7	7	36	1.217	1.555	0.897	0.873	107.77	21.37	12	36	393	44.1	8.29
P21956-2	Isoform 2 of Lactadherin OS=Mus musculus GN=Mfge8 - [MFGM_MOUSE]	24.41	2	9	9	23	1.053	2.524	0.824	0.873	52.01	24.41	16	23	426	47.1	7.05
Q8CIE2-2	Isoform 2 of Zinc finger MIZ domain-containing protein 2 OS=Mus musculus GN=Zmiz2 - [ZMIZ2_MOUSE]	5.86	5	2	2	3	0.814	1.085	0.792	0.873	5.35	5.86	3	3	888	93.8	7.14
P27046	Alpha-mannosidase 2 OS=Mus musculus GN=Man2a1 PE=1 SV=2 - [MA2A1_MOUSE]	4.17	1	4	4	16	0.994	1.399	0.752	0.873	53.37	4.17	6	16	1150	131.5	8.03
Q61655	ATP-dependent RNA helicase DDX19A OS=Mus musculus GN=Ddx19a PE=2 SV=2 - [DD19A_MOUSE]	26.57	3	10	12	53	0.805	0.995	0.972	0.873	155.57	26.57	20	53	478	53.9	6.67

Q9WVL3-2	Isoform 2 of Solute carrier family 12 member 7 OS=Mus musculus GN=Slc12a7 -	6.74	2	6	8	17	2.245	1.844	0.768	0.873	28.13	6.74	10	17	1054	116.5	6.58
Q62241	U1 small nuclear ribonucleoprotein C OS=Mus musculus GN=Snrpc PE=2 SV=1 -	24.53	1	3	3	27	1.488	1.053	1.204	0.873	71.18	24.53	6	27	159	17.4	9.67
Q8R3V6	CUE domain-containing protein 1 OS=Mus musculus GN=Cuedc1 PE=1 SV=2 -	10.82	2	3	3	10	1.241	0.649	0.902	0.873	16.45	10.82	6	10	388	42.8	5.58
G3X8Q1	Calcineurin binding protein 1, isoform CRA_a OS=Mus musculus GN=Cabin1 PE=4 SV=1 - [G3X8Q1_MOUSE]	3.93	1	6	8	15	1.083	1.326	1.357	0.873	36.95	3.93	11	15	2187	243.0	6.01
Q9CPS8	Small membrane A-kinase anchor protein OS=Mus musculus PE=1 SV=1 - [SMAKA_MOUSE]	43.40	1	4	4	11	1.850	0.853	1.071	0.873	37.20	43.40	7	11	106	11.9	4.25
Q9CQV4	Protein FAM134C OS=Mus musculus GN=Fam134c PE=2 SV=1 - [F134C_MOUSE]	10.52	2	3	5	22	0.865	0.796	0.963	0.873	53.97	10.52	8	22	466	51.6	4.97
Q80UV9-2	Isoform 2 of Transcription initiation factor TFIID subunit 1 OS=Mus musculus GN=Taf1 - [TAF1_MOUSE]	0.97	4	1	1	1	0.725	0.926	0.951	0.873	0.00	0.97	1	1	929	105.7	5.07
Q8VED2	Biogenesis of lysosome-related organelles complex 1 subunit 4 OS=Mus musculus GN=Bloc1s4 PE=1 SV=1 - [illegible]	21.40	1	4	4	5	1.304	1.356	0.950	0.873	10.74	21.40	5	5	215	23.1	5.14
Q9Z1Z0	General vesicular transport factor p115 OS=Mus musculus GN=Uso1 PE=1 SV=2 - [USO1_MOUSE]	39.42	4	31	32	156	0.714	0.947	0.958	0.874	457.16	39.42	56	156	959	106.9	4.93
F6XPV7	Microtubule-associated protein (Fragment) OS=Mus musculus GN=Map4 PE=2 SV=1 - [F6XPV7_MOUSE]	32.00	2	1	2	9	0.521	0.348	0.966	0.874	22.07	32.00	4	9	75	8.0	10.58
P03975	IgE-binding protein OS=Mus musculus GN=Iap PE=2 SV=1 - [IGEB_MOUSE]	10.41	1	4	5	8	1.155	0.474	0.685	0.874	22.70	10.41	8	8	557	62.7	9.31
A2AC80	Metalloendopeptidase homolog PEX (Fragment) OS=Mus musculus GN=Phex PE=2 SV=1 - [A2AC80_MOUSE]	1.09	2	1	1	1	1.021	0.637	0.744	0.874	2.36	1.09	1	1	548	63.1	8.43
F8VQC1	Signal recognition particle subunit SRP72 OS=Mus musculus GN=Srp72 PE=2 SV=1 - [F8VQC1_MOUSE]	30.85	2	16	17	65	0.858	0.856	0.845	0.874	161.08	30.85	30	65	671	74.6	9.23
Q8BGT1	Fibronectin leucine rich transmembrane protein 3 OS=Mus musculus GN=Flrt3 PE=2 SV=1 - [Q8BGT1_MOUSE]	9.24	1	4	4	10	0.852	1.029	0.908	0.874	34.23	9.24	8	10	649	72.8	8.06
Q5XJE5	RNA polymerase-associated protein LEO1 OS=Mus musculus GN=Leo1 PE=1 SV=2 -	5.10	1	3	3	7	0.917	0.768	1.031	0.874	15.67	5.10	4	7	667	75.6	4.46
Q6NS60	F-box only protein 41 OS=Mus musculus GN=Fbxo41 PE=1 SV=3 - [FBX41_MOUSE]	29.32	1	21	21	63	0.655	1.035	1.132	0.874	186.38	29.32	34	63	873	94.3	8.24
P55066	Neurocan core protein OS=Mus musculus GN=Ncan PE=2 SV=1 - [NCAN_MOUSE]	25.32	1	28	29	197	1.648	1.273	1.502	0.874	657.77	25.32	57	197	1268	137.1	5.72
Q80XU8	Leucine-rich repeat and fibronectin type-III domain containing protein 4 OS=Mus musculus GN=Lrfn4 PE=1 SV=1 - [illegible]	17.14	1	8	8	21	1.044	1.040	0.887	0.874	77.86	17.14	13	21	636	67.2	6.81
P70372	ELAV-like protein 1 OS=Mus musculus GN=Elavl1 PE=1 SV=2 - [ELAV1_MOUSE]	30.98	1	9	10	46	0.897	0.947	0.826	0.874	123.83	30.98	17	46	326	36.1	9.04
P57080	Ubiquitin carboxyl-terminal hydrolase 25 OS=Mus musculus GN=Usp25 PE=1 SV=2 - [UBP25_MOUSE]	8.53	1	7	8	31	1.106	0.989	0.870	0.874	75.54	8.53	13	31	1055	121.3	5.31
B0V2N1-4	Isoform 4 of Receptor-type tyrosine-protein phosphatase S OS=Mus musculus GN=Ptprs - [PTPRS_MOUSE]	36.94	3	1	39	183	0.497	1.495	1.111	0.874	598.04	36.94	72	183	1497	167.8	6.81
Q3U5C8	Rho guanine nucleotide exchange factor 16 OS=Mus musculus GN=Arhgef16 PE=1 SV=3 - [ARHGG_MOUSE]	2.66	1	2	2	2	4.744	1.355	1.118	0.874	5.23	2.66	2	2	713	80.3	7.49
O35566	CD151 antigen OS=Mus musculus GN=Cd151 PE=2 SV=2 - [CD151_MOUSE]	9.09	1	2	2	4	0.897	0.797	0.813	0.874	11.38	9.09	3	4	253	28.2	7.47
Q80TL0	Protein phosphatase 1E OS=Mus musculus GN=Ppm1e PE=1 SV=2 - [PPM1E_MOUSE]	19.49	1	12	12	60	1.028	1.127	0.987	0.874	199.60	19.49	22	60	749	83.4	4.97

Q6PGA2	GTP-binding protein RAD OS=Mus musculus GN=Rrad PE=2 SV=1 - [Q6PGA2_MOUSE]	6.19	2	1	1	4	0.934	1.474	0.513	0.874	16.21	6.19	2	4	307	33.2	9.26
Q61129	Complement factor I OS=Mus musculus GN=Cfi PE=1 SV=3 - [CFAI_MOUSE]	7.79	1	4	4	6	3.033	1.523	0.860	0.874	20.82	7.79	6	6	603	67.2	7.46
Q91YR7	Pre-mRNA-processing factor 6 OS=Mus musculus GN=Prpf6 PE=2 SV=1 - [PRP6_MOUSE]	21.68	3	18	18	48	0.680	0.862	0.926	0.874	125.35	21.68	29	48	941	106.7	8.09
P70414	Sodium/calcium exchanger 1 OS=Mus musculus GN=Slc8a1 PE=1 SV=1 - [NAC1_MOUSE]	24.33	1	1	17	83	0.699	0.694	1.160	0.875	224.93	24.33	31	83	970	108.0	5.00
Q99KK2	N-acylneuraminate cytidylyltransferase OS=Mus musculus GN=Cmas PE=1 SV=2 - [NEUA_MOUSE]	44.91	2	16	17	72	0.520	0.571	0.678	0.875	212.32	44.91	33	72	432	48.0	8.10
Q8CBY0	Glutamyl-tRNA(Gln) amidotransferase subunit C, mitochondrial OS=Mus musculus GN=Gatc PE=2 SV=1 - [GATC_MOUSE]	42.58	1	5	5	16	1.970	1.075	1.338	0.875	45.84	42.58	10	16	155	16.7	5.16
Q3UPF5-2	Isoform 2 of Zinc finger CCCH-type antiviral protein 1 OS=Mus musculus GN=Zc3hav1 - [ZCCHV_MOUSE]	7.73	4	4	5	15	1.535	1.228	0.700	0.875	46.07	7.73	8	15	789	88.2	7.96
Q61550	Double-strand-break repair protein rad21 homolog OS=Mus musculus GN=Rad21 PE=1 SV=3 - [RAD21_MOUSE]	8.82	1	4	4	8	0.812	0.957	0.767	0.875	22.66	8.82	7	8	635	72.0	4.64
E9Q5E1	Protocadherin-19 OS=Mus musculus GN=Pcdh19 PE=2 SV=1 - [E9Q5E1_MOUSE]	12.85	5	13	14	43	0.965	1.351	0.822	0.875	120.20	12.85	25	43	1097	120.8	5.40
Q8CJG0	Protein argonaute-2 OS=Mus musculus GN=Ago2 PE=1 SV=3 - [AGO2_MOUSE]	32.67	1	17	25	65	0.986	1.027	0.730	0.875	189.83	32.67	40	65	860	97.2	9.19
Q8C6B2-2	Isoform 2 of Rhotekin OS=Mus musculus GN=Rtkn - [RTKN_MOUSE]	29.76	4	8	13	27	0.942	0.372	0.959	0.875	95.30	29.76	19	27	551	61.5	6.00
Q9EP71	Ankycorbin OS=Mus musculus GN=Rai14 PE=1 SV=1 - [RAI14_MOUSE]	15.12	1	13	13	20	1.744	1.172	1.096	0.875	48.25	15.12	19	20	979	108.8	6.27
Q8BUK6	Protein Hook homolog 3 OS=Mus musculus GN=Hook3 PE=1 SV=2 - [HOOK3_MOUSE]	39.69	2	27	29	165	1.582	0.983	1.047	0.875	454.65	39.69	53	165	718	83.2	5.19
H3BJ26	Homeobox protein cut-like 1 OS=Mus musculus GN=Cux1 PE=2 SV=1 - [H3BJ26_MOUSE]	22.84	7	1	9	35	1.007	0.650	0.727	0.875	94.14	22.84	13	35	543	61.6	5.17
B8QI36	Liprin-alpha 4 OS=Mus musculus GN=Ppfia4 PE=2 SV=1 - [B8QI36_MOUSE]	40.44	2	24	42	157	1.088	0.843	1.018	0.875	450.62	40.44	68	157	1187	133.6	6.27
Q08093	Calponin-2 OS=Mus musculus GN=Cnn2 PE=2 SV=1 - [CNN2_MOUSE]	23.61	2	5	8	16	1.050	2.056	0.650	0.875	43.65	23.61	13	16	305	33.1	7.62
Q3UX43	Ankyrin repeat domain-containing protein 13C OS=Mus musculus GN=Ankrd13c PE=2 SV=2 [AN13C_MOUSE]	2.40	1	1	1	3	1.679	1.048	0.896	0.875	6.01	2.40	2	3	541	60.1	6.90
A2ANP1	2310028H24Rik protein OS=Mus musculus GN=Fam219a PE=2 SV=1 [A2ANP1_MOUSE]	25.60	3	3	3	9	1.457	1.020	1.191	0.875	24.86	25.60	5	9	168	18.6	4.70
Q60692	Proteasome subunit beta type-6 OS=Mus musculus GN=Psmb6 PE=1 SV=3 - [PSB6_MOUSE]	43.70	1	7	7	47	1.805	0.758	0.714	0.876	159.35	43.70	13	47	238	25.4	5.11
Q3UM29	Conserved oligomeric Golgi complex subunit 7 OS=Mus musculus GN=Cog7 PE=2 SV=1 - [COG7_MOUSE]	18.05	1	14	14	36	0.562	0.933	0.761	0.876	89.60	18.05	23	36	770	86.0	5.38
F6UUZ3	Protein Dgkk (Fragment) OS=Mus musculus GN=Dgkk PE=4 SV=1 - [F6UUZ3_MOUSE]	2.82	2	2	3	3	1.192	0.949	0.785	0.876	7.61	2.82	3	3	1062	119.8	7.25
Q9EPE9	Probable cation-transporting ATPase 13A1 OS=Mus musculus GN=Atp13a1 PE=1 SV=2 - [AT131_MOUSE]	11.50	1	11	11	28	0.802	0.935	0.814	0.876	67.22	11.50	20	28	1200	132.3	8.03
P32507-2	Isoform Alpha of Poliovirus receptor-related protein 2 OS=Mus musculus GN=Pvrl2 -	14.35	2	4	4	17	1.131	1.110	0.820	0.876	51.14	14.35	8	17	467	50.7	5.48
Q9CQE3	28S ribosomal protein S17, mitochondrial OS=Mus musculus GN=Mrps17 PE=2 SV=1 - [RT17_MOUSE]	35.00	2	3	3	8	0.639	0.855	0.890	0.876	23.89	35.00	5	8	120	13.4	9.89
Q80TR0	MKIAA0827 protein (Fragment) OS=Mus musculus GN=Nfat5 PE=2 SV=1 - [Q80TR0_MOUSE]	4.45	1	1	3	7	2.282	1.171	1.049	0.876	18.18	4.45	5	7	1033	111.6	4.67

O35085	Homeobox protein ARX OS=Mus musculus GN=Arx PE=2 SV=3 - [ARX_MOUSE]	9.93	1	2	2	2	1.554	0.374	1.707	0.876	2.16	9.93	2	2	564	58.5	5.24
D3Z0Z6	Elongation of very long chain fatty acids protein 5 (Fragment) OS=Mus musculus GN=Elovl5 PE=2 SV=1 - [D3Z0Z6_MOUSE]	8.45	3	1	1	4	0.727	0.539	0.662	0.876	7.41	8.45	2	4	71	8.3	9.25
E9Q1U6	CBP80/20-dependent translation initiation factor OS=Mus musculus GN=Ctif PE=4 SV=1 - [E9Q1U6_MOUSE]	18.62	3	11	11	22	0.840	0.746	0.755	0.876	57.49	18.62	17	22	623	70.2	6.61
Q8K003	Translation machinery-associated protein 7 OS=Mus musculus GN=Tma7 PE=2 SV=1 - [TMA7_MOUSE]	23.44	1	1	2	4	0.817	0.491	1.096	0.876	6.35	23.44	3	4	64	7.1	9.99
F8WIA3	Modulator of apoptosis 1 OS=Mus musculus GN=Moap1 PE=2 SV=1 - [F8WIA3_MOUSE]	28.00	4	1	1	3	1.355	0.715	0.981	0.876	14.33	28.00	2	3	75	7.9	4.09
Q8CHW4	Translation initiation factor eIF-2B subunit epsilon OS=Mus musculus GN=Eif2b5 PE=1 SV=1 - [EI2BE_MOUSE]	13.95	1	8	8	25	0.876	0.824	0.836	0.876	80.63	13.95	13	25	717	80.0	5.07
F7BE84	Protein Ankrd50 (Fragment) OS=Mus musculus GN=Ankrd50 PE=2 SV=1 -	11.29	2	12	12	29	1.143	0.753	0.908	0.877	80.40	11.29	21	29	1390	150.8	6.47
G5E8K5-2	Isoform 2 of Ankyrin-3 OS=Mus musculus GN=Ank3 - [ANK3_MOUSE]	48.03	3	1	63	322	1.160	0.710	1.318	0.877	957.26	48.03	119	322	1726	188.1	7.75
P62320	Small nuclear ribonucleoprotein Sm D3 OS=Mus musculus GN=Snrpd3 PE=1 SV=1 -	31.75	1	3	3	26	1.233	0.782	0.835	0.877	79.70	31.75	6	26	126	13.9	10.32
Q8BJZ4	28S ribosomal protein S35, mitochondrial OS=Mus musculus GN=Mrps35 PE=2 SV=2 - [RT35_MOUSE]	20.63	1	5	5	12	0.986	0.676	0.932	0.877	34.95	20.63	8	12	320	36.0	8.59
Q8VDP4	DBIRD complex subunit KIAA1967 homolog OS=Mus musculus PE=1 SV=2 - [K1967_MOUSE]	32.97	1	22	22	74	0.859	0.850	0.809	0.877	213.65	32.97	38	74	922	102.9	5.25
Q810U4	Neuronal cell adhesion molecule OS=Mus musculus GN=Nrcam PE=1 SV=2 -	38.06	3	3	37	266	0.921	0.822	0.884	0.877	843.91	38.06	67	266	1256	138.4	5.91
D3YU96	Cytohesin-2 OS=Mus musculus GN=Cyth2 PE=2 SV=1 - [D3YU96_MOUSE]	42.30	9	10	16	55	0.811	1.292	1.208	0.877	142.61	42.30	26	55	383	44.6	6.38
Q99LI7	Cleavage stimulation factor subunit 3 OS=Mus musculus GN=Cstf3 PE=1 SV=1 - [CSTF3_MOUSE]	16.46	1	8	9	25	0.937	0.761	0.812	0.877	80.52	16.46	13	25	717	82.8	8.12
Q3UVX5	Metabotropic glutamate receptor 5 OS=Mus musculus GN=Grm5 PE=2 SV=2 - [GRM5_MOUSE]	28.35	3	2	32	117	0.476	1.975	1.758	0.877	320.89	28.35	50	117	1203	131.8	7.84
Q05BC3-2	Isoform 2 of Echinoderm microtubule-associated protein-like 1 OS=Mus musculus GN=Eml1 - [EMAL1_MOUSE]	31.67	5	22	24	124	1.159	0.382	0.798	0.877	312.84	31.67	44	124	783	86.4	7.46
Q9JHU9	Inositol-3-phosphate synthase 1 OS=Mus musculus GN=Isyna1 PE=2 SV=1 -	28.37	1	13	13	56	1.422	2.151	0.842	0.877	152.51	28.37	21	56	557	60.9	6.42
Q6A098	Selenocysteine insertion sequence-binding protein 2-like OS=Mus musculus GN=Secisbp2l PE=1 SV=2 [SBP2L_MOUSE]	4.51	2	5	5	5	1.542	0.789	0.623	0.877	9.91	4.51	5	5	1086	119.6	6.35
Q810C1	SLIT and NTRK-like protein 1 OS=Mus musculus GN=Slitrk1 PE=2 SV=1 - [SLIK1_MOUSE]	6.47	2	4	4	7	0.974	1.679	0.995	0.877	16.81	6.47	5	7	696	77.8	6.49
P57724	Poly(rC)-binding protein 4 OS=Mus musculus GN=Pcbp4 PE=2 SV=1 - [PCBP4_MOUSE]	18.86	1	4	5	17	0.838	0.388	0.767	0.877	59.84	18.86	9	17	403	41.4	8.34
Q922L6	Negative elongation factor D OS=Mus musculus GN=Nelfcd PE=2 SV=2 - [NELFD_MOUSE]	7.28	2	3	3	9	1.624	1.397	0.795	0.877	25.09	7.28	5	9	591	66.2	5.16
Q91WI7	Integrin-alpha FG-GAP repeat-containing protein 2 OS=Mus musculus GN=Itfg2 PE=2 SV=1 - [ITFG2_MOUSE]	6.09	2	2	3	7	1.511	1.333	0.751	0.877	19.86	6.09	5	7	443	48.9	5.11
E9Q7G0	Protein Numa1 OS=Mus musculus GN=Numa1 PE=2 SV=1 - [E9Q7G0_MOUSE]	35.39	1	41	65	207	1.266	1.096	0.942	0.877	552.62	35.39	113	207	2094	235.5	5.87
D6RGR0	Calpain-10 OS=Mus musculus GN=Capn10 PE=2 SV=1 - [D6RGR0_MOUSE]	9.66	3	3	3	4	1.247	1.071	0.859	0.877	10.12	9.66	3	4	507	57.5	7.94
P58158	Galactosylgalactosylxylosyl protein 3-beta-glucuronosyltransferase 3 OS=Mus musculus GN=B3gat3 PE=2 SV=1 - [B3GA3_MOUSE]	25.67	1	5	5	19	1.080	0.817	0.744	0.877	62.88	25.67	8	19	335	37.0	8.78

P55288	Cadherin-11 OS=Mus musculus GN=Cdh11 PE=1 SV=1 - [CAD11_MOUSE]	32.41	4	17	17	57	1.314	0.944	1.087	0.877	173.35	32.41	29	57	796	88.1	4.89
Q64449	C-type mannose receptor 2 OS=Mus musculus GN=Mrc2 PE=1 SV=3 - [MRC2_MOUSE]	3.72	2	5	5	9	0.871	3.318	0.577	0.877	29.23	3.72	7	9	1479	167.0	5.99
D3YW42	Coiled-coil domain-containing protein 9 OS=Mus musculus GN=Ccdc9 PE=2 SV=2 - [D3YW42_MOUSE]	11.21	6	7	7	18	1.440	0.841	1.262	0.878	52.93	11.21	13	18	589	65.9	4.94
E9PZF4	Nebulin-related-anchoring protein OS=Mus musculus GN=Nrap PE=2 SV=1 - [E9PZF4_MOUSE]	1.30	6	1	2	3	1.478	0.774	0.969	0.878	2.72	1.30	2	3	1692	191.8	9.28
Q6BCL1	PML-RARA-regulated adapter molecule 1 OS=Mus musculus GN=Pram1 PE=1 SV=2 - [PRAM_MOUSE]	1.33	1	1	1	1	0.782	0.645	1.360	0.878	2.17	1.33	1	1	675	75.5	9.63
Q99LW6-2	Isoform 2 of YY1-associated factor 2 OS=Mus musculus GN=Yaf2 - [YAF2_MOUSE]	17.65	1	1	1	2	1.880	0.933	1.365	0.878	7.89	17.65	2	2	68	7.5	9.36
Q91XD6	Vacuolar protein-sorting-associated protein 36 OS=Mus musculus GN=Vps36 PE=1 SV=1 - [VPS36_MOUSE]	24.61	1	10	10	33	0.800	1.048	0.983	0.878	105.92	24.61	18	33	386	43.7	7.15
Q8CI04	Conserved oligomeric Golgi complex subunit 3 OS=Mus musculus GN=Cog3 PE=1 SV=3 - [COG3_MOUSE]	11.71	2	9	9	18	0.652	0.840	0.691	0.878	55.18	11.71	16	18	820	93.2	5.78
Q9CZN7	Serine hydroxymethyltransferase OS=Mus musculus GN=Shmt2 PE=2 SV=1 -	33.73	1	15	16	51	0.817	0.738	0.644	0.878	142.57	33.73	28	51	504	55.7	8.47
G5E8L8	Mitogen activated protein kinase kinase kinase 2 OS=Mus musculus GN=Map3k2 PE=4 SV=1 - [G5E8L8_MOUSE]	2.58	2	2	2	3	0.978	1.231	1.030	0.878	6.78	2.58	3	3	619	69.6	8.32
P62627	Dynein light chain roadblock-type 1 OS=Mus musculus GN=Dynlrb1 PE=1 SV=3 -	73.96	2	4	4	59	1.776	0.842	1.220	0.878	157.24	73.96	7	59	96	11.0	7.25
P32848	Parvalbumin alpha OS=Mus musculus GN=Pvalb PE=1 SV=3 - [PRVA_MOUSE]	76.36	1	10	10	31	2.894	0.810	1.565	0.878	83.76	76.36	17	31	110	11.9	5.19
E9Q9K8	Protein Akap6 OS=Mus musculus GN=Akap6 PE=2 SV=1 - [E9Q9K8_MOUSE]	4.20	1	10	11	22	1.575	1.110	0.978	0.878	47.41	4.20	16	22	2307	254.2	5.06
Q8VD75	Huntingtin-interacting protein 1 OS=Mus musculus GN=Hip1 PE=1 SV=2 - [HIP1_MOUSE]	42.66	1	35	35	114	1.126	1.012	0.969	0.878	384.49	42.66	60	114	1029	115.1	5.43
G3X934	MCG115964 OS=Mus musculus GN=Wdr70 PE=4 SV=1 - [G3X934_MOUSE]	11.42	2	5	6	8	1.018	0.884	0.974	0.878	15.59	11.42	8	8	657	73.0	6.05
P62754	40S ribosomal protein S6 OS=Mus musculus GN=Rps6 PE=1 SV=1 - [RS6_MOUSE]	36.95	1	10	11	54	0.706	0.728	0.765	0.878	156.37	36.95	16	54	249	28.7	10.84
J3QNB1	La-related protein 1 OS=Mus musculus GN=Larp1 PE=4 SV=1 - [J3QNB1_MOUSE]	25.19	2	20	21	76	1.406	0.886	1.154	0.878	193.89	25.19	31	76	1072	121.0	8.79
Q61120	SHC-transforming protein 3 OS=Mus musculus GN=Shc3 PE=1 SV=2 - [SHC3_MOUSE]	40.72	1	14	14	63	0.763	1.005	1.117	0.878	211.23	40.72	25	63	474	52.1	7.30
Q80WC1-2	Isoform 2 of Ubinuclein-2 OS=Mus musculus GN=Ubn2 - [UBN2_MOUSE]	1.66	5	1	2	3	10.960	1.034	1.995	0.878	7.95	1.66	2	3	1203	130.7	9.26
Q8C9S4	Uncharacterized protein C10orf118 homolog OS=Mus musculus GN=Otg1 PE=2 SV=2 - [CJ118_MOUSE]	15.27	2	12	13	30	1.195	0.707	1.016	0.878	79.71	15.27	20	30	917	104.8	5.53
Q6PDS3	Sterile alpha and TIR motif containing protein 1 OS=Mus musculus GN=Sarm1 PE=1 SV=1 - [SARM1_MOUSE]	31.35	4	18	19	43	0.658	1.043	0.912	0.878	126.98	31.35	29	43	724	79.6	6.27
P53798	Squalene synthase OS=Mus musculus GN=Fdft1 PE=2 SV=2 - [FDFT_MOUSE]	11.30	1	4	4	12	0.790	0.450	0.614	0.878	44.14	11.30	7	12	416	48.1	6.32
O55042	Alpha-synuclein OS=Mus musculus GN=Snca PE=1 SV=2 - [SYUA_MOUSE]	91.43	2	9	12	329	1.135	1.350	1.440	0.879	979.09	91.43	21	329	140	14.5	4.77
Q8C7H1	Methylmalonic aciduria type A homolog, mitochondrial OS=Mus musculus GN=Mmaa PE=2 SV=1 - [MMAA_MOUSE]	24.58	1	10	10	23	0.821	1.016	0.853	0.879	41.28	24.58	15	23	415	45.9	9.32
Q9R1P3	Proteasome subunit beta type-2 OS=Mus musculus GN=Psmb2 PE=1 SV=1 - [PSB2_MOUSE]	56.72	1	12	13	62	1.604	0.980	0.717	0.879	209.59	56.72	21	62	201	22.9	7.02

Q5F2D9	Arrestin, beta 2 OS=Mus musculus GN=Arrb2 PE=4 SV=1 - [Q5F2D9_MOUSE]	7.88	3	2	3	11	1.500	1.053	0.815	0.879	30.49	7.88	5	11	406	45.7	7.83
P28740-2	Isoform 2 of Kinesin-like protein KIF2A OS=Mus musculus GN=Kif2a - [KIF2A_MOUSE]	49.17	7	1	29	178	0.770	0.564	1.079	0.879	479.98	49.17	49	178	659	74.8	6.46
P10126	Elongation factor 1-alpha 1 OS=Mus musculus GN=Eef1a1 PE=1 SV=3 - [EF1A1_MOUSE]	59.52	3	12	25	328	0.812	0.855	0.843	0.879	961.25	59.52	40	328	462	50.1	9.01
Q8R4G0-8	Isoform 1H of Netrin-G1 OS=Mus musculus GN=Ntng1 - [NTNG1_MOUSE]	15.07	9	1	6	20	0.546	1.373	1.089	0.879	59.14	15.07	12	20	438	50.1	6.46
E9Q9E1	Eukaryotic translation initiation factor 4 gamma 1 OS=Mus musculus GN=Eif4g1 PE=2 SV=1 - [E9Q9E1_MOUSE]	31.32	8	1	44	186	0.648	0.576	0.792	0.879	472.28	31.32	74	186	1593	175.2	5.38
Q8VHM5	Heterogeneous nuclear ribonucleoprotein R OS=Mus musculus GN=Hnrnpr PE=2 SV=1 - [Q8VHM5_MOUSE]	40.19	5	17	23	144	0.836	0.657	0.949	0.879	458.56	40.19	40	144	632	70.8	8.13
Q9D1P0	39S ribosomal protein L13, mitochondrial OS=Mus musculus GN=Mrpl13 PE=2 SV=1 - [RM13_MOUSE]	38.76	3	7	7	24	0.767	0.831	0.861	0.879	72.58	38.76	13	24	178	20.7	9.35
E9PXF0	Protein Pcdh17 OS=Mus musculus GN=Pcdh17 PE=2 SV=1 - [E9PXF0_MOUSE]	31.03	1	28	28	100	1.090	1.085	0.811	0.879	324.33	31.03	48	100	1157	126.1	5.21
Q9DC50	Peroxisomal carnitine O-octanoyltransferase OS=Mus musculus GN=Crot PE=1 SV=1 - [OCTC_MOUSE]	20.92	1	9	9	23	1.110	0.923	1.124	0.879	79.81	20.92	14	23	612	70.2	6.73
Q9CRD0-2	Isoform 2 of OCIA domain-containing protein 1 OS=Mus musculus GN=Ociad1 - [OCAD1_MOUSE]	58.67	1	1	7	38	0.966	1.114	1.414	0.879	113.37	58.67	13	38	196	21.7	6.90
Q91WM1	Spermatid perinuclear RNA binding protein OS=Mus musculus GN=Strbp PE=1 SV=1 - [STRBP_MOUSE]	31.55	4	13	18	45	0.651	0.693	0.816	0.879	96.86	31.55	28	45	672	73.7	8.72
Q9DBR7	Protein phosphatase 1 regulatory subunit 12A OS=Mus musculus GN=Ppp1r12a PE=1 SV=2 - [MYPT1_MOUSE]	34.11	2	32	33	117	1.216	0.907	1.357	0.879	308.07	34.11	55	117	1029	114.9	5.49
Q8VDJ3	Vigilin OS=Mus musculus GN=Hdlbp PE=1 SV=1 - [VIGLN_MOUSE]	44.40	1	49	50	163	1.150	0.793	0.871	0.879	443.03	44.40	81	163	1268	141.7	6.87
O35226-2	Isoform Rpn10B of 26S proteasome non-ATPase regulatory subunit 4 OS=Mus musculus GN=Psmd4 - [PSMD4_MOUSE]	32.72	4	3	12	89	1.538	0.917	1.321	0.879	274.85	32.72	22	89	379	41.0	4.79
F6TLV3	Fibronectin type-III domain-containing protein 3A (Fragment) OS=Mus musculus GN=Fndc3a PE=4 SV=1 - [F6TLV3_MOUSE]	20.28	3	16	16	33	0.917	0.991	0.755	0.880	94.36	20.28	22	33	1154	127.2	7.15
G3UVU2	Splicing factor 3A subunit 2 OS=Mus musculus GN=Sf3a2 PE=4 SV=1 - [G3UVU2_MOUSE]	8.66	3	4	4	12	0.822	0.586	0.817	0.880	36.48	8.66	7	12	485	51.2	9.64
F6QZ23	Intraflagellar transport protein 88 homolog (Fragment) OS=Mus musculus GN=Ift88 PE=4 SV=1 - [F6QZ23_MOUSE]	10.29	1	1	2	3	2.598	1.335	1.172	0.880	6.01	10.29	2	3	175	20.0	9.32
F8WJK8	Hsc70-interacting protein OS=Mus musculus GN=St13 PE=2 SV=1 - [F8WJK8_MOUSE]	37.29	5	16	16	135	1.062	0.557	1.048	0.880	366.86	37.29	30	135	362	40.5	5.30
Q9ERA6	Tuftelin-interacting protein 11 OS=Mus musculus GN=Tfip11 PE=1 SV=1 - [TFP11_MOUSE]	13.84	2	9	11	31	0.532	0.728	0.705	0.880	85.98	13.84	18	31	838	96.2	5.90
O88507	Ciliary neurotrophic factor receptor subunit alpha OS=Mus musculus GN=Cntfr PE=1 SV=2 - [CNTFR_MOUSE]	26.88	3	9	9	37	1.348	0.931	0.892	0.880	103.41	26.88	15	37	372	40.8	6.83
J3QNY8	Uncharacterized protein OS=Mus musculus GN=Gm19965 PE=4 SV=1 - [J3QNY8_MOUSE]	2.62	1	1	1	1	2.982	1.496	1.095	0.880	2.59	2.62	1	1	420	49.6	6.14
C3S7Q5	Ojoplano variant A OS=Mus musculus GN=Ofcc1 PE=2 SV=1 - [C3S7Q5_MOUSE]	5.08	1	2	2	4	1.336	0.988	1.092	0.880	7.10	5.08	3	4	926	105.0	6.54
Q3UH68	LIM and calponin homology domains-containing protein 1 OS=Mus musculus GN=Limch1 PE=1 SV=2 - [LIMC1_MOUSE]	37.75	9	29	29	145	1.608	0.738	1.301	0.880	406.44	37.75	52	145	1057	118.1	5.48
Q8BMP6	Golgi resident protein GCP60 OS=Mus musculus GN=Acbd3 PE=1 SV=3 - [GCP60_MOUSE]	28.57	1	9	9	59	0.957	0.742	0.786	0.880	155.70	28.57	16	59	525	60.1	5.11

O88844	Isocitrate dehydrogenase [NADP] cytoplasmic OS=Mus musculus GN=Idh1 PE=1 SV=2 - [IDHC_MOUSE]	72.22	2	29	31	216	0.833	0.854	0.763	0.880	640.84	72.22	55	216	414	46.6	7.17
Q810B7	SLIT and NTRK-like protein 5 OS=Mus musculus GN=Slitrk5 PE=2 SV=1 - [SLIK5_MOUSE]	12.54	3	10	10	24	0.809	1.539	1.084	0.880	66.86	12.54	16	24	957	107.1	6.93
Q9Z266	SNARE-associated protein Snapin OS=Mus musculus GN=Snapin PE=1 SV=1 - [SNAPN_MOUSE]	42.65	2	5	5	13	1.392	1.408	0.993	0.881	44.15	42.65	8	13	136	14.9	9.31
Q9D823	60S ribosomal protein L37 OS=Mus musculus GN=Rpl37 PE=2 SV=3 - [RL37_MOUSE]	28.87	1	5	5	11	0.615	1.046	0.748	0.881	25.11	28.87	7	11	97	11.1	11.74
Q62415	Apoptosis-stimulating of p53 protein 1 OS=Mus musculus GN=Ppp1r13b PE=1 SV=2 - [ASPP1_MOUSE]	31.74	2	26	28	96	1.102	0.974	1.162	0.881	290.30	31.74	44	96	1087	119.1	6.68
Q8CIN4	Serine/threonine-protein kinase PAK 2 OS=Mus musculus GN=Pak2 PE=1 SV=1 - [PAK2_MOUSE]	42.18	1	10	21	145	1.264	0.707	1.021	0.881	416.21	42.18	41	145	524	57.9	5.77
Q9DB27	Malignant T-cell-amplified sequence 1 OS=Mus musculus GN=Mcts1 PE=2 SV=1 - [MCTS1_MOUSE]	60.77	3	8	8	25	0.796	0.918	0.798	0.881	87.44	60.77	12	25	181	20.5	8.82
G3XA57	Protein Rab11fip2 OS=Mus musculus GN=Rab11fip2 PE=4 SV=1 - [G3XA57_MOUSE]	39.84	3	15	17	47	0.913	0.892	1.226	0.881	161.47	39.84	30	47	512	58.2	9.45
P19426-2	Isoform 2 of Negative elongation factor E OS=Mus musculus GN=Nelfe -	13.08	3	5	5	9	1.378	0.899	1.076	0.881	23.98	13.08	8	9	367	41.5	9.39
G3UXZ5	Proteasome activator complex subunit 1 (Fragment) OS=Mus musculus GN=Psme1 PE=2 SV=1 - [illegible]	54.20	6	12	12	56	1.046	1.848	0.785	0.881	153.84	54.20	20	56	238	27.4	5.60
Q9D1L9	Ragulator complex protein LAMTOR5 OS=Mus musculus GN=Lamtor5 PE=2 SV=1 - [LTOR5_MOUSE]	54.95	2	3	3	28	1.727	0.978	1.181	0.881	102.16	54.95	6	28	91	9.6	4.87
O08810	116 kDa U5 small nuclear ribonucleoprotein component OS=Mus musculus GN=Eftud2 PE=2 SV=1 - [illegible]	43.77	5	34	35	104	0.713	0.983	0.837	0.881	309.31	43.77	59	104	971	109.3	5.00
Q08509	Epidermal growth factor receptor kinase substrate 8 OS=Mus musculus GN=Eps8 PE=1 SV=2 - [EPS8_MOUSE]	41.05	4	26	26	67	1.111	0.975	0.950	0.881	167.35	41.05	43	67	821	91.7	7.65
Q9D1B9	39S ribosomal protein L28, mitochondrial OS=Mus musculus GN=Mrpl28 PE=2 SV=3 - [RM28_MOUSE]	23.74	2	6	6	12	0.507	0.707	0.796	0.881	33.05	23.74	10	12	257	30.2	9.29
Q9D0V7	Receptor-binding cancer antigen expressed on SiSo cells OS=Mus musculus GN=Ebag9 PE=1 SV=2 - [RCAS1_MOUSE]	21.13	1	3	3	5	1.420	1.091	0.872	0.881	15.92	21.13	4	5	213	24.3	6.29
Q6A051-3	Isoform 3 of Attractin-like protein 1 OS=Mus musculus GN=Atrnl1 - [ATRN1_MOUSE]	3.43	3	3	3	6	0.984	1.101	1.255	0.881	12.22	3.43	4	6	787	87.2	7.88
P0C027	Diphosphoinositol polyphosphate phosphohydrolase 3-alpha OS=Mus musculus GN=Nudt10 PE=1 SV=1 - [illegible]	36.59	1	3	5	33	1.214	1.267	0.778	0.881	90.35	36.59	9	33	164	18.6	5.69
P32020	Non-specific lipid-transfer protein OS=Mus musculus GN=Scp2 PE=1 SV=3 - [NLTP_MOUSE]	40.22	4	23	23	109	1.603	0.823	0.972	0.881	320.56	40.22	39	109	547	59.1	7.44
Q9WTP6-2	Isoform 2 of Adenylate kinase 2, mitochondrial OS=Mus musculus GN=Ak2 - [KAD2_MOUSE]	47.84	3	11	11	55	0.958	0.781	0.732	0.882	159.41	47.84	20	55	232	25.6	7.42
Q80TN5	Palmitoyltransferase ZDHHC17 OS=Mus musculus GN=Zdhhc17 PE=1 SV=2 -	8.23	3	5	6	15	0.976	0.640	0.844	0.882	36.17	8.23	10	15	632	72.6	7.39
P13864-2	Isoform 2 of DNA (cytosine-5)-methyltransferase 1 OS=Mus musculus GN=Dnmt1 - [illegible]	4.73	3	5	5	7	2.461	1.568	1.073	0.882	16.45	4.73	7	7	1502	169.9	7.69
P33766	fMet-Leu-Phe receptor OS=Mus musculus GN=Fpr1 PE=2 SV=1 - [FPR1_MOUSE]	3.02	1	1	1	10	1.553	0.761	1.122	0.882	31.52	3.02	1	10	364	40.3	9.31
P47939	Regulated endocrine-specific protein 18 OS=Mus musculus GN=Resp18 PE=2 SV=1 - [RES18_MOUSE]	10.86	1	2	2	4	1.177	0.814	0.535	0.882	6.88	10.86	3	4	175	19.5	8.91
A2AWS5	Histone deacetylase 5 OS=Mus musculus GN=Hdac5 PE=2 SV=1 - [A2AWS5_MOUSE]	19.81	7	9	16	49	1.415	1.185	1.178	0.882	98.86	19.81	27	49	1030	111.4	5.72

Q8VDM4	26S proteasome non-ATPase regulatory subunit 2 OS=Mus musculus GN=Psmd2 PE=1 SV=1 - [PSMD2_MOUSE]	48.68	3	36	36	180	0.778	0.968	0.782	0.882	530.85	48.68	64	180	908	100.1	5.17
Q9CX86	Heterogeneous nuclear ribonucleoprotein A0 OS=Mus musculus GN=Hnrnpa0 PE=1 SV=1 - [ROA0_MOUSE]	32.46	1	5	7	59	0.817	0.782	0.890	0.882	175.28	32.46	13	59	305	30.5	9.31
Q99P72-1	Isoform 3 of Reticulon-4 OS=Mus musculus GN=Rtn4 - [RTN4_MOUSE]	37.19	1	2	7	70	0.232	1.449	1.403	0.882	190.31	37.19	12	70	199	22.5	9.41
Q9Z0P4-2	Isoform 2 of Paralemmin-1 OS=Mus musculus GN=Palm - [PALM_MOUSE]	57.52	1	1	18	306	0.761	1.428	0.975	0.882	788.53	57.52	32	306	339	36.7	4.84
Q9DBR0	A-kinase anchor protein 8 OS=Mus musculus GN=Akap8 PE=1 SV=1 - [AKAP8_MOUSE]	10.33	1	5	6	12	0.872	0.738	0.953	0.882	33.87	10.33	9	12	687	76.2	5.14
E9Q452	Tropomyosin alpha-1 chain OS=Mus musculus GN=Tpm1 PE=2 SV=1 - [E9Q452_MOUSE]	47.69	5	3	18	375	2.258	1.321	1.730	0.882	1073.15	47.69	31	375	281	32.5	4.77
O08842	GDNF family receptor alpha-2 OS=Mus musculus GN=Gfra2 PE=2 SV=1 - [GFRA2_MOUSE]	31.10	3	13	14	44	0.580	0.454	0.628	0.882	118.59	31.10	22	44	463	51.6	7.75
Q0VBL3	Protein Rbm15 OS=Mus musculus GN=Rbm15 PE=2 SV=1 - [Q0VBL3_MOUSE]	18.71	1	12	15	37	0.686	0.702	0.884	0.882	114.37	18.71	21	37	962	105.7	10.08
E9QPQ8	39S ribosomal protein L48, mitochondrial OS=Mus musculus GN=Mrpl48 PE=2 SV=1 - [E9QPQ8_MOUSE]	13.74	2	3	3	7	1.065	0.632	0.885	0.882	12.56	13.74	5	7	211	24.0	9.52
Q8BFU3-2	Isoform 2 of RING finger protein 214 OS=Mus musculus GN=Rnf214 - [RN214_MOUSE]	34.49	3	1	18	70	1.297	0.665	0.708	0.882	200.68	34.49	32	70	548	61.2	8.15
A2AIG3	Exostosin-2 (Fragment) OS=Mus musculus GN=Ext2 PE=2 SV=1 - [A2AIG3_MOUSE]	18.78	3	2	2	4	0.762	0.921	0.920	0.883	11.00	18.78	3	4	213	24.0	8.53
Q9CPR5	39S ribosomal protein L15, mitochondrial OS=Mus musculus GN=Mrpl15 PE=1 SV=1 - [RM15_MOUSE]	45.76	3	13	13	23	0.925	0.921	0.813	0.883	66.20	45.76	21	23	295	33.5	10.07
D3Z5N2	Stathmin OS=Mus musculus GN=Stmn1 PE=2 SV=1 - [D3Z5N2_MOUSE]	43.54	3	7	10	321	1.770	1.123	1.474	0.883	875.30	43.54	18	321	147	17.0	9.09
Q9CY97	RNA polymerase II subunit A C-terminal domain phosphatase SSU72 OS=Mus musculus GN=Ssu72 PE=2 SV=1 - [SSU72_MOUSE]	39.69	2	7	7	25	1.845	1.049	1.100	0.883	78.30	39.69	12	25	194	22.5	5.21
Q8BZQ7	Anaphase-promoting complex subunit 2 OS=Mus musculus GN=Anapc2 PE=1 SV=2 - [ANC2_MOUSE]	11.83	1	9	11	33	0.937	1.029	0.767	0.883	97.52	11.83	16	33	837	95.2	5.31
E9QAQ7	AT-rich interactive domain-containing protein 1A OS=Mus musculus GN=Arid1a PE=2 SV=1 - [E9QAQ7_MOUSE]	18.85	5	25	27	127	1.201	0.973	1.121	0.883	374.53	18.85	44	127	2287	242.5	6.71
Q5UE59	Kinesin light chain 1 OS=Mus musculus GN=Klc1 PE=2 SV=1 - [Q5UE59_MOUSE]	60.70	6	22	30	190	1.237	0.906	0.946	0.883	551.94	60.70	54	190	542	61.6	5.76
P61963	DDB1- and CUL4-associated factor 7 OS=Mus musculus GN=Dcaf7 PE=2 SV=1 -	29.82	1	9	9	63	1.382	1.099	0.528	0.883	177.39	29.82	17	63	342	38.9	5.52
Q8BYP3	Rho-related GTP-binding protein RhoF OS=Mus musculus GN=Rhof PE=2 SV=1 - [RHOF_MOUSE]	14.22	1	2	2	6	1.004	1.178	0.811	0.883	18.96	14.22	4	6	211	23.6	8.43
Q9JIA7	Sphingosine kinase 2 OS=Mus musculus GN=Sphk2 PE=1 SV=2 - [SPHK2_MOUSE]	12.80	1	5	5	12	0.612	1.051	1.111	0.883	45.05	12.80	9	12	617	65.6	6.57
Q8K1J5	Protein SDE2 homolog OS=Mus musculus GN=Sde2 PE=1 SV=1 - [SDE2_MOUSE]	4.46	1	1	2	4	0.487	1.327	0.919	0.883	6.94	4.46	3	4	448	48.5	5.66
Q8BGU2	Cerebellin-2 OS=Mus musculus GN=Cbln2 PE=1 SV=1 - [CBLN2_MOUSE]	12.50	1	2	3	14	1.105	0.776	0.766	0.883	31.40	12.50	6	14	224	24.0	8.18
F7BX42	Neuronal pentraxin receptor OS=Mus musculus GN=Nptxr PE=2 SV=1 - [F7BX42_MOUSE]	43.61	2	15	16	92	1.116	4.374	1.498	0.883	247.51	43.61	28	92	493	52.3	6.13
P17047	Lysosome-associated membrane glycoprotein 2 OS=Mus musculus GN=Lamp2 PE=2 SV=2 - [LAMP2_MOUSE]	5.78	3	3	3	9	1.321	1.082	0.812	0.883	21.55	5.78	5	9	415	45.7	7.39
Q9D787	Peptidyl-prolyl cis-trans isomerase-like 2 OS=Mus musculus GN=Ppil2 PE=2 SV=2 - [PPIL2_MOUSE]	8.64	2	4	4	8	0.973	0.746	0.806	0.883	20.10	8.64	5	8	521	59.0	8.38

Q9CQE7	Endoplasmic reticulum-Golgi intermediate compartment protein 3 OS=Mus musculus GN=Ergic3 PE=2 SV=1 - [illegible]	20.37	4	8	8	25	0.788	0.814	0.727	0.883	66.32	20.37	15	25	383	43.2	6.47
Q9D1D4	Transmembrane emp24 domain-containing protein 10 OS=Mus musculus GN=Tmed10 PE=2 SV=1 - [TMEDA_MOUSE]	37.90	2	8	8	25	0.948	0.955	0.708	0.883	83.46	37.90	11	25	219	24.9	6.70
Q8CIV2	Membralin OS=Mus musculus GN=Tmem259 PE=1 SV=2 - [MBRL_MOUSE]	11.85	3	3	3	8	0.688	0.990	0.944	0.884	15.28	11.85	4	8	574	63.5	5.49
B1AQW2	Microtubule-associated protein tau OS=Mus musculus GN=Mapt PE=2 SV=1 - [B1AQW2_MOUSE]	77.95	4	2	33	430	1.121	0.421	1.499	0.884	1223.40	77.95	56	430	390	40.9	9.64
P57722-2	Isoform 2 of Poly(rC)-binding protein 3 OS=Mus musculus GN=Pcbp3 - [PCBP3_MOUSE]	47.84	4	6	13	124	0.700	0.885	0.774	0.884	394.73	47.84	25	124	370	39.1	7.52
O54965	E3 ubiquitin-protein ligase RNF13 OS=Mus musculus GN=Rnf13 PE=1 SV=2 - [RNF13_MOUSE]	12.34	2	4	4	5	1.820	0.892	0.727	0.884	15.56	12.34	5	5	381	42.7	5.06
Q8C078	Calcium/calmodulin-dependent protein kinase kinase 2 OS=Mus musculus GN=Camkk2 PE=1 SV=2 - [illegible]	38.78	5	18	18	57	0.773	1.362	1.464	0.884	183.86	38.78	28	57	588	64.6	5.90
Q9CQF8	Ribosomal protein 63, mitochondrial OS=Mus musculus GN=Mrp63 PE=2 SV=1 - [RT63_MOUSE]	20.59	1	2	2	4	1.092	0.656	0.968	0.884	12.77	20.59	4	4	102	11.9	10.15
Q6NXK2	Zinc finger protein 532 OS=Mus musculus GN=Znf532 PE=2 SV=1 - [ZN532_MOUSE]	5.69	2	4	5	9	0.717	0.798	1.033	0.884	13.26	5.69	7	9	1036	110.9	8.29
F8VPU2	FERM, RhoGEF and pleckstrin domain-containing protein 1 OS=Mus musculus GN=Farp1 PE=1 SV=1 - [illegible]	31.39	2	26	28	149	0.661	1.335	0.955	0.884	453.26	31.39	50	149	1048	118.8	7.88
Q921Z5	Tumor necrosis factor alpha-induced protein 8 OS=Mus musculus GN=Tnfaip8 PE=2 SV=1 - [TFIP8_MOUSE]	22.73	11	4	4	11	0.930	1.586	0.765	0.884	34.73	22.73	7	11	198	22.9	7.93
D3Z729	Eukaryotic translation initiation factor 4E type 2 OS=Mus musculus GN=Eif4e2 PE=2 SV=1 - [D3Z729_MOUSE]	24.15	8	5	5	19	0.870	1.280	0.937	0.884	45.27	24.15	8	19	236	27.1	7.18
Q99KW3-4	Isoform 4 of TRIO and F-actin-binding protein OS=Mus musculus GN=Triobp -	8.74	9	9	11	27	1.309	1.167	0.929	0.884	53.95	8.74	18	27	1968	218.1	7.75
Q80X41-2	Isoform 2 of Serine/threonine-protein kinase VRK1 OS=Mus musculus GN=Vrk1 - [VRK1_MOUSE]	9.60	4	3	3	5	0.621	0.666	1.038	0.884	11.22	9.60	5	5	396	45.0	8.98
Q9EPB4	Apoptosis-associated speck like protein containing a CARD OS=Mus musculus GN=Pycard PE=1 SV=1 - [ASC_MOUSE]	47.15	1	7	7	15	1.862	1.192	0.738	0.884	49.61	47.15	10	15	193	21.4	5.43
Q9Z2G6	Protein sel-1 homolog 1 OS=Mus musculus GN=Sel1l PE=2 SV=2 - [SE1L1_MOUSE]	13.54	3	8	8	31	0.928	1.033	0.766	0.884	126.99	13.54	14	31	790	88.3	5.57
Q9CPX7	28S ribosomal protein S16, mitochondrial OS=Mus musculus GN=Mrps16 PE=2 SV=1 - [RT16_MOUSE]	25.19	1	3	3	16	1.008	0.879	0.963	0.884	43.35	25.19	6	16	135	15.2	9.67
Q8C8N2	Protein SCAI OS=Mus musculus GN=Scai PE=1 SV=2 - [SCAI_MOUSE]	43.23	3	21	21	111	0.496	1.005	0.897	0.884	332.89	43.23	39	111	606	70.2	8.60
E9Q9B7	Protein Kidins220 OS=Mus musculus GN=Kidins220 PE=2 SV=1 - [E9Q9B7_MOUSE]	23.42	1	33	33	105	1.065	0.892	0.884	0.884	285.44	23.42	56	105	1793	199.1	6.67
G3UYC3	RAS guanyl-releasing protein 1 OS=Mus musculus GN=Rasgrp1 PE=2 SV=1 -	8.82	5	4	4	8	0.679	1.165	1.203	0.885	31.62	8.82	6	8	760	86.5	7.96
Q8BU11	TOX high mobility group box family member 4 OS=Mus musculus GN=Tox4 PE=1 SV=3 - [TOX4_MOUSE]	14.54	4	4	7	23	1.199	0.683	0.902	0.885	64.03	14.54	13	23	619	65.9	5.01
F7ACR9	Microtubule-actin cross-linking factor 1 (Fragment) OS=Mus musculus GN=Macf1 PE=2 SV=1 - [F7ACR9_MOUSE]	46.97	4	2	217	907	1.752	1.240	0.972	0.885	2618.47	46.97	384	907	5333	607.9	5.40
Q3KNZ4	Avpr2 protein OS=Mus musculus GN=Avpr2 PE=2 SV=1 - [Q3KNZ4_MOUSE]	11.88	1	1	1	3	1.792	1.013	0.576	0.885	5.35	11.88	1	3	160	17.4	8.31
Q3U9R5	Ficolin-2 OS=Mus musculus GN=Fcnb PE=2 SV=1 - [Q3U9R5_MOUSE]	3.56	2	1	1	1	0.610	1.076	0.913	0.885	1.89	3.56	1	1	253	26.9	5.34

P63028	Translationally-controlled tumor protein OS=Mus musculus GN=Tpt1 PE=1 SV=1 - [TCTP_MOUSE]	52.91	2	6	6	107	1.623	0.817	1.203	0.885	250.99	52.91	11	107	172	19.4	4.86
E9Q865	Serpin A9 (Fragment) OS=Mus musculus GN=Serpina9 PE=2 SV=1 - [E9Q865_MOUSE]	6.34	2	1	1	3	0.643	0.337	2.073	0.885	5.72	6.34	2	3	205	22.9	9.39
Q99JX3	Golgi reassembly-stacking protein 2 OS=Mus musculus GN=Gorasp2 PE=1 SV=3 -	30.60	5	11	11	45	1.131	1.136	0.968	0.885	124.77	30.60	18	45	451	47.0	4.79
F6YRX3	Protein kinase domain-containing protein, cytoplasmic (Fragment) OS=Mus musculus GN=Pkdcc PE=4 SV=1 - [illegible]	5.36	3	1	1	1	1.495	1.136	0.920	0.885	0.00	5.36	1	1	224	25.3	5.25
Q62523	Zyxin OS=Mus musculus GN=Zyx PE=1 SV=2 - [illegible]	30.67	1	14	14	165	2.191	1.307	1.212	0.885	260.27	30.67	25	165	564	60.5	6.40
Q60994	Adiponectin OS=Mus musculus GN=Adipoq PE=1 SV=2 - [ADIPO_MOUSE]	9.72	2	2	2	3	2.910	2.742	0.540	0.885	9.80	9.72	3	3	247	26.8	5.57
E9Q9U5	Protein Ildr2 OS=Mus musculus GN=Ildr2 PE=2 SV=1 - [E9Q9U5_MOUSE]	33.43	1	19	19	60	0.603	1.395	0.992	0.885	166.32	33.43	35	60	661	73.2	7.68
Q64514-2	Isoform Short of Tripeptidyl-peptidase 2 OS=Mus musculus GN=Tpp2 -	47.80	2	54	54	201	0.691	0.808	0.894	0.885	565.07	47.80	91	201	1249	138.4	6.38
Q8JZQ9	Eukaryotic translation initiation factor 3 subunit B OS=Mus musculus GN=Eif3b PE=1 SV=1 - [EIF3B_MOUSE]	32.38	1	22	22	110	0.905	0.968	0.861	0.885	346.95	32.38	40	110	803	91.3	5.02
Q9WV55	Vesicle-associated membrane protein-associated protein A OS=Mus musculus GN=Vapa PE=1 SV=2 - [illegible]	45.78	1	15	16	130	0.692	0.935	0.922	0.886	305.65	45.78	28	130	249	27.8	8.40
D3Z4S3	Putative peptidyl-tRNA hydrolase PTRHD1 OS=Mus musculus GN=Ptrhd1 PE=3 SV=1 - [PTRD1_MOUSE]	39.29	1	5	5	16	1.453	0.967	1.166	0.886	50.19	39.29	8	16	140	16.0	9.32
E9QLD2	Myelin transcription factor 1-like protein OS=Mus musculus GN=Myt1l PE=2 SV=1 - [E9QLD2_MOUSE]	4.81	13	4	4	9	0.943	1.532	1.224	0.886	21.54	4.81	6	9	1185	132.7	4.93
Q499E5	Storkhead-box protein 2 OS=Mus musculus GN=Stox2 PE=2 SV=2 - [STOX2_MOUSE]	9.40	3	6	6	13	1.754	1.275	1.468	0.886	26.08	9.40	8	13	926	102.7	8.46
Q9QXC1	Fetuin-B OS=Mus musculus GN=Fetub PE=1 SV=1 - [FETUB_MOUSE]	14.18	2	4	4	14	3.773	1.682	0.537	0.886	46.96	14.18	8	14	388	42.7	6.61
Q9CQR6	Serine/threonine-protein phosphatase 6 catalytic subunit OS=Mus musculus GN=Ppp6c PE=2 SV=1 - [PPP6_MOUSE]	27.21	1	9	9	20	0.918	0.940	0.876	0.886	53.52	27.21	13	20	305	35.1	5.69
P35413	G-protein coupled receptor 3 OS=Mus musculus GN=Gpr3 PE=2 SV=1 - [GPR3_MOUSE]	9.70	1	1	1	1	0.794	2.938	0.822	0.886	0.00	9.70	1	1	330	35.4	7.72
Q3TTL0	Uncharacterized protein C3orf38 homolog OS=Mus musculus PE=2 SV=1 - [CC038_MOUSE]	11.49	1	2	3	7	1.226	0.977	1.019	0.886	19.90	11.49	4	7	348	39.5	6.20
Q8BR92	Paralemmin-2 OS=Mus musculus GN=Palm2 PE=1 SV=1 - [PALM2_MOUSE]	52.66	2	8	16	105	1.100	0.878	0.988	0.886	305.85	52.66	28	105	376	42.1	5.15
E9Q8N5	CLIP-associating protein 2 OS=Mus musculus GN=Clasp2 PE=2 SV=1 - [E9Q8N5_MOUSE]	52.49	4	1	57	287	0.420	0.466	0.852	0.886	786.44	52.49	100	287	1286	140.6	8.63
Q62192	CD180 antigen OS=Mus musculus GN=Cd180 PE=1 SV=2 - [CD180_MOUSE]	10.14	1	5	5	9	0.560	0.776	0.412	0.886	23.68	10.14	8	9	661	74.3	5.88
F8WJI3	Coiled-coil domain-containing protein 58 OS=Mus musculus GN=Ccdc58 PE=2 SV=1 - [F8WJI3_MOUSE]	54.48	3	6	6	24	1.402	0.963	1.402	0.886	85.04	54.48	10	24	134	15.6	8.60
Q8BVF2	Phosducin-like protein 3 OS=Mus musculus GN=Pdcl3 PE=1 SV=1 - [PDCL3_MOUSE]	24.17	1	6	6	15	1.198	1.132	0.768	0.886	42.38	24.17	10	15	240	27.6	4.72
G3X9J1	Sodium/calcium exchanger 1 OS=Mus musculus GN=Slc8a1 PE=4 SV=1 - [G3X9J1_MOUSE]	24.33	3	1	17	88	0.228	0.677	0.827	0.887	242.80	24.33	32	88	970	108.0	4.98
A2BIE1	Protein Qser1 OS=Mus musculus GN=Qser1 PE=4 SV=2 - [A2BIE1_MOUSE]	1.59	1	2	2	5	2.965	1.260	1.087	0.887	9.95	1.59	2	5	1698	185.3	6.95
Q6P9J9	Anoctamin-6 OS=Mus musculus GN=Ano6 PE=1 SV=1 - [ANO6_MOUSE]	8.67	1	6	6	13	0.785	1.503	0.693	0.887	42.19	8.67	10	13	911	106.2	6.76

Q3TAA7	Serine/threonine-protein kinase 11-interacting protein OS=Mus musculus GN=Stk11ip PE=1 SV=1 - [S11IP_MOUSE]	6.62	1	5	5	7	0.837	0.986	0.882	0.887	17.59	6.62	7	7	1072	117.9	4.96
P10630	Eukaryotic initiation factor 4A-II OS=Mus musculus GN=Eif4a2 PE=2 SV=2 - [IF4A2_MOUSE]	69.78	6	13	28	193	0.574	0.904	0.829	0.887	568.86	69.78	49	193	407	46.4	5.48
D3Z0K8	Thioredoxin reductase 2, mitochondrial OS=Mus musculus GN=Txnrd2 PE=2 SV=1 - [D3Z0K8_MOUSE]	50.71	7	18	19	52	1.653	0.886	0.814	0.887	134.60	50.71	31	52	491	53.0	9.04
Q8K1H7	T-complex protein 11-like protein 2 OS=Mus musculus GN=Tcp11l2 PE=2 SV=1 -	2.71	1	1	1	2	1.490	1.618	0.776	0.887	4.32	2.71	2	2	517	57.9	5.08
Q9JHJ0	Tropomodulin-3 OS=Mus musculus GN=Tmod3 PE=1 SV=1 - [TMOD3_MOUSE]	28.41	1	8	9	27	1.944	1.840	1.074	0.887	73.67	28.41	15	27	352	39.5	5.14
Q9D1J1	Adaptin ear-binding coat-associated protein 2 OS=Mus musculus GN=Necap2 PE=1 SV=1 - [NECP2_MOUSE]	34.59	2	6	6	30	1.384	0.776	0.928	0.887	84.27	34.59	12	30	266	28.6	7.94
Q9JLQ0	CD2-associated protein OS=Mus musculus GN=Cd2ap PE=1 SV=3 - [CD2AP_MOUSE]	31.87	1	19	19	46	2.008	0.922	0.958	0.887	133.87	31.87	30	46	637	70.4	6.38
G3UW40	MCG4620, isoform CRA_b OS=Mus musculus GN=Mcc PE=4 SV=1 - [G3UW40_MOUSE]	15.70	2	9	10	18	1.025	0.956	1.368	0.887	57.82	15.70	14	18	828	92.8	5.48
Q08481-3	Isoform 3 of Platelet endothelial cell adhesion molecule OS=Mus musculus GN=Pecam1 - [PECA1_MOUSE]	13.55	6	7	7	12	1.143	1.223	0.892	0.887	31.22	13.55	9	12	716	80.0	6.89
Q9EP72	ER membrane protein complex subunit 7 OS=Mus musculus GN=Emc7 PE=2 SV=1 - [EMC7_MOUSE]	24.48	1	4	4	12	0.598	0.604	0.804	0.887	28.56	24.48	6	12	241	26.3	9.23
Q811Q9-2	Isoform 2 of Choline-phosphate cytidylyltransferase B OS=Mus musculus GN=Pcyt1b - [illegible]	28.91	2	5	9	22	1.494	1.094	0.965	0.887	58.97	28.91	13	22	339	38.7	7.55
Q99N93	39S ribosomal protein L16, mitochondrial OS=Mus musculus GN=Mrpl16 PE=2 SV=1 - [RM16_MOUSE]	28.29	1	6	6	9	0.901	0.666	0.848	0.887	25.92	28.29	8	9	251	28.8	10.27
E9PUC5	PH and SEC7 domain-containing protein 3 OS=Mus musculus GN=Psd3 PE=2 SV=1 - [E9PUC5_MOUSE]	44.41	2	1	17	117	0.519	0.984	1.678	0.887	297.01	44.41	31	117	376	42.3	9.60
Q9DB24	Protein Tceal6 OS=Mus musculus GN=Tceal6 PE=2 SV=1 - [Q9DB24_MOUSE]	27.00	1	1	6	68	1.040	0.473	1.887	0.887	183.44	27.00	11	68	200	22.6	5.55
O70194	Eukaryotic translation initiation factor 3 subunit D OS=Mus musculus GN=Eif3d PE=1 SV=2 - [EIF3D_MOUSE]	36.86	1	14	14	67	0.818	0.783	0.878	0.887	191.04	36.86	25	67	548	63.9	6.05
B1AXZ4	ELAV-like protein 2 OS=Mus musculus GN=Elavl2 PE=2 SV=1 - [B1AXZ4_MOUSE]	48.97	10	6	17	107	0.808	0.559	0.683	0.887	297.57	48.97	31	107	388	42.5	9.04
Q8BGI0	Lathosterol oxidase OS=Mus musculus GN=Sc5d PE=2 SV=1 - [Q8BGI0_MOUSE]	2.68	1	1	1	2	0.982	0.490	0.796	0.887	4.58	2.68	1	2	299	35.0	8.75
A2A7B5	Protein Prdm2 OS=Mus musculus GN=Prdm2 PE=2 SV=1 - [A2A7B5_MOUSE]	3.34	1	4	5	17	1.342	1.032	0.984	0.887	45.60	3.34	7	17	1709	187.1	8.00
Q91V01	Lysophospholipid acyltransferase 5 OS=Mus musculus GN=Lpcat3 PE=1 SV=1 -	4.52	2	2	2	3	0.944	1.158	0.800	0.887	8.38	4.52	3	3	487	56.1	8.56
Q7TSY6-4	Isoform 4 of CUGBP Elav-like family member 4 OS=Mus musculus GN=Celf4 -	22.81	9	4	9	23	0.775	1.176	0.834	0.887	55.37	22.81	15	23	456	48.5	8.78
Q5SFM8-2	Isoform 2 of RNA-binding protein 27 OS=Mus musculus GN=Rbm27 - [RBM27_MOUSE]	8.36	3	6	7	18	1.057	0.761	1.044	0.888	50.73	8.36	11	18	1005	112.6	9.13
Q8K4Q8	Collectin-12 OS=Mus musculus GN=Colec12 PE=1 SV=1 - [COL12_MOUSE]	8.89	1	6	7	9	2.025	2.674	0.832	0.888	26.31	8.89	8	9	742	81.3	5.49
E9PVR3	Acetylcholinesterase collagenic tail peptide OS=Mus musculus GN=Colq PE=2 SV=1 - [E9PVR3_MOUSE]	2.68	2	1	1	1	1.009	1.042	1.214	0.888	2.61	2.68	1	1	447	46.5	8.51
Q8K4M5	COMM domain-containing protein 1 OS=Mus musculus GN=Commd1 PE=2 SV=2 -	27.66	4	4	5	16	1.548	1.043	0.744	0.888	47.30	27.66	9	16	188	21.0	7.59
E9Q6R4	Protein Arid1b OS=Mus musculus GN=Arid1b PE=2 SV=1 - [E9Q6R4_MOUSE]	12.84	3	21	22	71	1.275	0.970	1.168	0.888	203.48	12.84	38	71	2243	236.9	6.80

O88630	Golgi SNAP receptor complex member 1 OS=Mus musculus GN=Gosr1 PE=1 SV=2 - [GOSR1_MOUSE]	29.60	3	8	8	30	1.174	0.846	0.752	0.888	82.10	29.60	14	30	250	28.5	9.29
H7BX26	Centrosomal protein of 170 kDa OS=Mus musculus GN=Cep170 PE=2 SV=1 -	40.56	6	52	55	246	1.093	0.648	1.133	0.888	696.30	40.56	97	246	1578	173.8	7.20
P58462-2	Isoform 2 of Forkhead box protein P1 OS=Mus musculus GN=Foxp1 - [FOXP1_MOUSE]	7.02	13	2	2	3	0.678	0.669	1.272	0.888	18.15	7.02	3	3	641	71.5	6.25
D6RH86	Ribosomal protein S6 kinase beta-2 OS=Mus musculus GN=Rps6kb2 PE=2 SV=1 -	14.74	5	2	3	6	2.541	1.704	1.341	0.888	18.17	14.74	4	6	285	31.4	5.36
Q3UIX4	Protein Srsf11 OS=Mus musculus GN=Srsf11 PE=2 SV=1 - [Q3UIX4_MOUSE]	18.91	5	3	5	11	0.623	0.860	0.957	0.888	30.63	18.91	8	11	476	53.1	10.58
Q6A0A9	Constitutive coactivator of PPAR-gamma-like protein 1 OS=Mus musculus GN=FAM120A PE=1 SV=2 [F120A_MOUSE]	30.67	1	27	29	106	0.896	1.207	1.049	0.888	309.74	30.67	51	106	1112	121.6	8.92
B2RXC2	Inositol 1,4,5-trisphosphate 3-kinase B OS=Mus musculus GN=Itpkb PE=2 SV=1 - [B2RXC2_MOUSE]	24.10	1	14	15	43	1.702	0.579	0.843	0.888	120.50	24.10	23	43	942	102.6	8.00
Q8VDS7	Centrosomal protein CEP57L1 OS=Mus musculus GN=Cep57l1 PE=2 SV=1 -	17.50	5	3	3	6	0.630	0.644	1.081	0.888	3.06	17.50	3	6	400	46.8	8.38
Q9Z0L0	Trophoblast glycoprotein OS=Mus musculus GN=Tpbg PE=1 SV=3 - [TPBG_MOUSE]	13.85	1	5	5	22	0.556	0.856	0.657	0.888	89.21	13.85	9	22	426	46.4	6.83
Q8BVG4	Dipeptidyl peptidase 9 OS=Mus musculus GN=Dpp9 PE=2 SV=2 - [DPP9_MOUSE]	23.32	2	14	16	46	0.656	0.995	1.012	0.888	137.29	23.32	28	46	862	97.9	6.65
F8VPP8	Protein Zc3h7b OS=Mus musculus GN=Zc3h7b PE=2 SV=1 - [F8VPP8_MOUSE]	12.73	1	12	12	23	0.768	0.925	0.821	0.888	61.54	12.73	18	23	982	110.2	7.42
D3Z396	Neurotrimin OS=Mus musculus GN=Ntm PE=2 SV=1 - [D3Z396_MOUSE]	45.25	5	10	14	203	0.842	1.003	1.042	0.888	642.49	45.25	26	203	316	34.9	6.96
Q9ET30	Transmembrane 9 superfamily member 3 OS=Mus musculus GN=Tm9sf3 PE=1 SV=1 - [TM9S3_MOUSE]	12.61	1	9	9	30	0.807	0.884	0.750	0.888	86.02	12.61	13	30	587	67.5	7.21
Q5SPL2	PHD finger protein 12 OS=Mus musculus GN=Phf12 PE=2 SV=1 - [PHF12_MOUSE]	1.40	1	1	1	1	9.223	4.165	1.090	0.888	2.11	1.40	1	1	1003	109.4	7.72
Q8C6Y6-2	Isoform 2 of Ankyrin repeat and SOCS box protein 14 OS=Mus musculus GN=Asb14 - [ASB14_MOUSE]	3.46	3	1	1	2	0.404	1.535	1.866	0.888	4.54	3.46	1	2	433	48.3	8.57
P99026	Proteasome subunit beta type-4 OS=Mus musculus GN=Psmb4 PE=1 SV=1 - [PSB4_MOUSE]	45.45	1	7	7	66	1.685	0.917	0.732	0.889	259.73	45.45	14	66	264	29.1	5.64
Q19AB2	ROBO2 isoform b OS=Mus musculus GN=Robo2 PE=2 SV=1 - [Q19AB2_MOUSE]	28.18	5	29	30	79	0.899	1.394	1.544	0.889	247.74	28.18	51	79	1508	165.3	6.28
E9Q3A0	E3 ubiquitin-protein ligase Trim36 OS=Mus musculus GN=Trim36 PE=2 SV=1 - [E9Q3A0_MOUSE]	9.07	2	6	6	14	1.472	0.444	0.735	0.889	35.68	9.07	10	14	717	81.3	6.25
E9Q9H2	DnaJ homolog subfamily C member 2 OS=Mus musculus GN=Dnajc2 PE=2 SV=1 -	17.00	4	8	8	23	1.184	0.884	1.079	0.889	62.84	17.00	12	23	547	63.4	9.17
Q9D2K6	Protein D930007J09Rik OS=Mus musculus GN=D930007J09Rik PE=2 SV=1 - [Q9D2K6_MOUSE]	5.34	1	1	1	2	1.104	1.172	0.677	0.889	4.52	5.34	1	2	131	13.6	12.13
Q8BVU5	ADP-ribose pyrophosphatase, mitochondrial OS=Mus musculus GN=Nudt9 PE=2 SV=1 - [NUDT9_MOUSE]	26.29	5	9	9	27	1.381	0.960	1.088	0.889	74.59	26.29	15	27	350	38.6	6.76
Q91VW3	SH3 domain-binding glutamic acid-rich-like protein 3 OS=Mus musculus GN=Sh3bgrl3 PE=1 SV=1 - [illegible]	51.61	2	6	6	137	1.925	1.136	1.498	0.889	398.77	51.61	12	137	93	10.5	5.14
Q78WH7	Calcium/calmodulin-dependent protein kinase II inhibitor 2 OS=Mus musculus GN=Camk2n2 PE=2 SV=1 - [illegible]	67.09	1	3	6	31	1.447	0.769	1.093	0.889	80.33	67.09	10	31	79	8.6	5.48
Q6P9S0	MTSS1-like protein OS=Mus musculus GN=Mtss1l PE=1 SV=1 - [MTSSL_MOUSE]	27.55	2	9	16	71	0.970	0.854	0.928	0.889	205.96	27.55	27	71	715	76.8	6.99
Q7TSK3-3	Isoform 3 of Protocadherin 8 OS=Mus musculus GN=Pcdh8 - [PCDH8_MOUSE]	13.87	3	8	9	32	0.975	1.846	0.959	0.889	100.17	13.87	15	32	966	101.8	5.44

Q3UVU8	Disks large-associated protein 1 OS=Mus musculus GN=Dlgap1 PE=2 SV=1 -	36.32	1	1	18	72	0.819	1.525	1.778	0.889	186.91	36.32	24	72	636	70.6	5.58
Q9BCZ4	Selenoprotein S OS=Mus musculus GN=Vimp PE=2 SV=3 - [SELS_MOUSE]	25.26	1	3	3	7	0.656	0.871	0.712	0.889	19.73	25.26	5	7	190	21.5	9.39
O70166	Stathmin-3 OS=Mus musculus GN=Stmn3 PE=1 SV=1 - [STMN3_MOUSE]	30.00	1	7	7	38	1.102	0.862	0.923	0.889	76.82	30.00	13	38	180	20.9	7.49
Q99PV8-3	Isoform 3 of B-cell lymphoma/leukemia 11B OS=Mus musculus GN=Bcl11b - [BC11B_MOUSE]	7.97	3	3	3	5	0.857	1.388	2.017	0.889	10.21	7.97	4	5	690	74.1	7.69
P70288	Histone deacetylase 2 OS=Mus musculus GN=Hdac2 PE=1 SV=1 - [HDAC2_MOUSE]	24.80	2	4	10	37	0.987	1.047	0.942	0.889	103.76	24.80	17	37	488	55.3	5.91
Q6P4T1	Protein Snx19 OS=Mus musculus GN=Snx19 PE=2 SV=1 - [Q6P4T1_MOUSE]	11.63	1	9	9	17	0.854	0.740	0.954	0.889	49.37	11.63	13	17	997	109.7	5.12
O35943	Frataxin, mitochondrial OS=Mus musculus GN=Fxn PE=1 SV=1 - [FRDA_MOUSE]	20.29	1	5	5	13	1.813	0.932	1.079	0.889	36.19	20.29	8	13	207	22.9	8.02
G3UW47	Kinesin family member 1A OS=Mus musculus GN=Kif1a PE=3 SV=1 - [G3UW47_MOUSE]	40.37	4	1	56	228	0.964	0.977	0.984	0.889	692.39	40.37	97	228	1697	191.7	6.20
Q6PE01	U5 small nuclear ribonucleoprotein 40 kDa protein OS=Mus musculus GN=Snrnp40 PE=2 SV=1 - [SNR40_MOUSE]	32.96	1	6	6	29	1.046	0.796	0.898	0.889	85.70	32.96	10	29	358	39.3	8.10
Q58A65-2	Isoform 2 of C-Jun-amino-terminal kinase-interacting protein 4 OS=Mus musculus GN=Spag9 - [JIP4_MOUSE]	29.46	9	2	30	110	1.543	0.995	1.047	0.889	342.83	29.46	55	110	1307	144.6	5.15
G3UZY2	Thioredoxin (Fragment) OS=Mus musculus GN=Txn2 PE=3 SV=1 - [G3UZY2_MOUSE]	52.31	4	3	3	12	1.545	1.099	1.371	0.890	39.33	52.31	4	12	130	14.2	5.81
G5E893	Ankyrin repeat domain 12, isoform CRA_a OS=Mus musculus GN=Ankrd12 PE=4 SV=1 - [G5E893_MOUSE]	2.94	3	5	5	6	1.214	1.011	1.006	0.890	12.40	2.94	6	6	2041	232.8	7.02
Q6KAU4	Multivesicular body subunit 12B OS=Mus musculus GN=Mvb12b PE=2 SV=2 - [MB12B_MOUSE]	38.17	2	9	9	37	1.071	0.829	0.884	0.890	123.92	38.17	16	37	317	35.4	7.93
Q6PAK3	Protein arginine N-methyltransferase 8 OS=Mus musculus GN=Prmt8 PE=2 SV=2 - [ANM8_MOUSE]	30.71	1	9	12	34	1.027	1.325	1.104	0.890	90.29	30.71	17	34	394	45.2	6.93
Q03157	Amyloid-like protein 1 OS=Mus musculus GN=Aplp1 PE=1 SV=1 - [APLP1_MOUSE]	28.94	1	18	18	76	1.490	0.969	1.222	0.890	225.91	28.94	31	76	653	72.7	5.67
Q8R3I3	Conserved oligomeric Golgi complex subunit 6 OS=Mus musculus GN=Cog6 PE=2 SV=2 - [COG6_MOUSE]	13.24	1	6	7	11	0.806	1.082	0.750	0.890	26.31	13.24	9	11	657	73.0	6.14
Q9D2V7	Coronin-7 OS=Mus musculus GN=Coro7 PE=2 SV=2 - [CORO7_MOUSE]	32.54	3	21	21	77	1.175	1.130	1.179	0.890	216.93	32.54	39	77	922	100.7	5.77
Q99JA0	Calcitonin gene-related peptide 1 OS=Mus musculus GN=Calca PE=2 SV=1 - [CALCA_MOUSE]	14.06	2	1	1	6	2.963	0.736	1.213	0.890	21.27	14.06	2	6	128	14.1	6.09
Q8VDV3-2	Isoform 2 of Guanine nucleotide exchange factor for Rab-3A OS=Mus musculus GN=Rab3il1 - [R3GEF_MOUSE]	16.56	5	3	3	8	1.774	1.413	1.019	0.890	17.41	16.56	4	8	314	35.4	7.09
Q6XUX1-3	Isoform 3 of Dual serine/threonine and tyrosine protein kinase OS=Mus musculus GN=Dstyk - [illegible]	4.47	4	4	4	7	0.384	0.451	0.840	0.890	22.37	4.47	5	7	918	103.8	6.54
Q5PR69	Uncharacterized protein KIAA1211 OS=Mus musculus GN=Kiaa1211 PE=2 SV=3 -	23.61	5	22	22	76	1.187	0.627	0.892	0.890	212.38	23.61	38	76	1207	132.2	5.40
Q3UHA3	Spatacsin OS=Mus musculus GN=Spg11 PE=2 SV=3 - [SPTCS_MOUSE]	0.95	2	2	2	3	1.632	1.248	0.841	0.890	6.41	0.95	3	3	2430	273.8	5.94
Q9D9K3	Cell death regulator Aven OS=Mus musculus GN=Aven PE=1 SV=2 - [AVEN_MOUSE]	21.05	3	6	6	16	1.561	0.803	1.114	0.890	44.79	21.05	11	16	342	37.2	4.98
G3UZP2	N6-adenosine-methyltransferase 70 kDa subunit OS=Mus musculus GN=Mettl3 PE=2 SV=1 - [G3UZP2_MOUSE]	25.31	9	3	4	6	1.284	0.906	0.963	0.890	15.63	25.31	6	6	245	26.4	7.21
E9PUI5	Formin-binding protein 1-like OS=Mus musculus GN=Fnbp1l PE=2 SV=1 - [E9PUI5_MOUSE]	35.14	4	16	18	72	1.089	1.007	1.065	0.890	197.91	35.14	30	72	609	70.4	6.73

O09110-2	Isoform 1 of Dual specificity mitogen-activated protein kinase kinase 3 OS=Mus musculus GN=Map2k3 - [illegible]	9.87	2	1	3	8	0.596	1.036	0.590	0.890	21.49	9.87	6	8	314	35.8	6.25
Q91YE7-2	Isoform 2 of RNA-binding protein 5 OS=Mus musculus GN=Rbm5 - [RBM5_MOUSE]	13.76	2	7	10	28	1.558	0.767	1.021	0.890	74.87	13.76	16	28	814	92.2	6.21
P24547	Inosine-5'-monophosphate dehydrogenase 2 OS=Mus musculus GN=Impdh2 PE=1 SV=2 - [IMDH2_MOUSE]	40.66	1	18	20	70	1.627	0.953	0.713	0.890	209.00	40.66	35	70	514	55.8	7.28
Q9WV19	Cytochrome P450, family 2, subfamily g, polypeptide 1 OS=Mus musculus GN=Cyp2g1 PE=2 SV=1 - [Q9WV19_MOUSE]	6.48	1	2	2	2	0.386	0.540	0.902	0.890	1.91	6.48	2	2	494	56.8	8.75
D3YTU7	Suppressor of IKBKE 1 OS=Mus musculus GN=Sike1 PE=2 SV=1 - [D3YTU7_MOUSE]	37.10	2	4	4	14	1.555	1.068	1.312	0.890	36.91	37.10	8	14	124	13.8	5.05
Q8CGC7	Bifunctional glutamate/proline--tRNA ligase OS=Mus musculus GN=Eprs PE=1 SV=4 - [SYEP_MOUSE]	43.19	2	52	55	209	0.700	0.796	0.893	0.891	591.34	43.19	94	209	1512	170.0	7.66
Q64704-5	Isoform 3D-1 of Syntaxin-3 OS=Mus musculus GN=Stx3 - [STX3_MOUSE]	13.84	6	3	4	8	1.799	2.148	1.121	0.891	21.88	13.84	6	8	289	33.2	5.35
D3YZ20	DNA-directed RNA polymerase II subunit RPB11 OS=Mus musculus GN=Polr2j PE=2 SV=1 - [D3YZ20_MOUSE]	22.52	4	3	3	6	0.788	1.171	0.964	0.891	19.05	22.52	4	6	111	12.6	6.77
P31230	Aminoacyl tRNA synthase complex-interacting multifunctional protein 1 OS=Mus musculus GN=Aimp1 PE=1 SV=2 - [illegible]	71.61	2	15	15	94	1.206	0.850	1.042	0.891	269.13	71.61	27	94	310	34.0	8.35
Q8K135	Dyslexia-associated protein KIAA0319-like protein OS=Mus musculus GN=Kiaa0319l PE=1 SV=1 - [K319L_MOUSE]	10.02	5	9	10	19	1.058	1.284	0.995	0.891	40.95	10.02	18	19	1048	115.2	6.16
F6Y0D0	Protein 1110008J03Rik (Fragment) OS=Mus musculus GN=1110008J03Rik PE=2 SV=1 - [F6Y0D0_MOUSE]	8.79	4	1	1	1	1.275	0.895	1.131	0.891	2.24	8.79	1	1	91	10.2	10.96
B2RT44	LON peptidase N-terminal domain and ring finger 2 OS=Mus musculus GN=Lonrf2 PE=2 SV=1 - [B2RT44_MOUSE]	11.97	1	5	5	10	0.760	1.026	0.846	0.891	28.97	11.97	8	10	518	58.4	5.31
D3YZZ4	TSC22 domain family protein 4 OS=Mus musculus GN=Tsc22d4 PE=2 SV=1 -	46.43	2	2	7	45	1.556	0.636	0.999	0.891	114.07	46.43	12	45	168	17.6	8.51
P52734	FYVE, RhoGEF and PH domain-containing protein 1 OS=Mus musculus GN=Fgd1 PE=1 SV=2 - [FGD1_MOUSE]	12.92	2	8	8	15	1.032	0.900	0.915	0.891	44.59	12.92	13	15	960	106.3	6.58
P47708	Rabphilin-3A OS=Mus musculus GN=Rph3a PE=1 SV=2 - [RP3A_MOUSE]	53.74	1	31	33	233	1.082	0.608	1.169	0.891	766.71	53.74	58	233	681	75.4	8.27
Q5QD14	Trace amine-associated receptor 5 OS=Mus musculus GN=Taar5 PE=2 SV=1 - [TAAR5_MOUSE]	3.26	1	1	1	1	0.484	0.815	0.676	0.891	2.30	3.26	1	1	337	38.2	8.35
Q3UHC1	Protein Rapgef1 OS=Mus musculus GN=Rapgef1 PE=2 SV=1 - [Q3UHC1_MOUSE]	11.76	5	13	13	29	0.623	0.989	0.978	0.891	79.11	11.76	22	29	1224	136.4	6.24
O88685	26S protease regulatory subunit 6A OS=Mus musculus GN=Psmc3 PE=1 SV=2 -	69.46	4	27	27	162	1.163	1.049	0.919	0.891	497.03	69.46	47	162	442	49.5	5.19
P97433-2	Isoform 2 of Rho guanine nucleotide exchange factor 28 OS=Mus musculus GN=Arhgef28 - [ARG28_MOUSE]	4.61	3	5	6	8	1.168	1.091	1.037	0.891	18.52	4.61	8	8	1324	147.7	5.76
B1AT92	Growth factor receptor-bound protein 2 OS=Mus musculus GN=Grb2 PE=2 SV=1 - [B1AT92_MOUSE]	55.67	4	12	12	104	1.790	1.315	1.082	0.891	299.22	55.67	24	104	203	23.6	6.80
Q99M51	Cytoplasmic protein NCK1 OS=Mus musculus GN=Nck1 PE=1 SV=1 - [NCK1_MOUSE]	39.79	2	11	13	56	1.672	0.820	1.117	0.892	147.09	39.79	23	56	377	42.9	6.47
P53783	Transcription factor SOX-1 OS=Mus musculus GN=Sox1 PE=2 SV=1 - [SOX1_MOUSE]	8.95	2	1	1	1	4.160	2.237	0.801	0.892	2.15	8.95	1	1	391	39.2	9.82
D3Z6Q9	Bridging integrator 2 OS=Mus musculus GN=Bin2 PE=2 SV=1 - [BIN2_MOUSE]	28.83	1	8	10	53	1.189	1.115	0.967	0.892	134.73	28.83	18	53	489	52.5	5.34
Q8CAF4-3	Isoform 3 of NHS-like protein 1 OS=Mus musculus GN=Nhsl1 - [NHSL1_MOUSE]	10.04	3	12	13	25	1.176	0.884	1.397	0.892	67.65	10.04	19	25	1583	169.3	8.07

Q9CR51	V-type proton ATPase subunit G 1 OS=Mus musculus GN=Atp6v1g1 PE=2 SV=3 -	36.44	1	3	3	21	1.477	0.589	1.285	0.892	73.12	36.44	5	21	118	13.7	7.97
Q9QY33	Tetraspanin-3 OS=Mus musculus GN=Tspan3 PE=1 SV=1 - [TSN3_MOUSE]	9.09	1	2	2	13	0.816	0.776	0.811	0.892	30.69	9.09	3	13	253	28.0	5.86
D3YZR1	Protein TSSC4 (Fragment) OS=Mus musculus GN=Tssc4 PE=2 SV=1 - [D3YZR1_MOUSE]	52.07	5	7	7	20	1.673	0.772	1.304	0.892	54.79	52.07	11	20	217	23.3	4.64
A2A4A1	DnaJ homolog subfamily C member 24 OS=Mus musculus GN=Dnajc24 PE=2 SV=1 -	29.73	3	4	4	11	1.871	0.959	1.073	0.892	34.05	29.73	7	11	148	16.9	4.92
Q3UID0	SWI/SNF complex subunit SMARCC2 OS=Mus musculus GN=Smarcc2 PE=2 SV=1 - [Q3UID0_MOUSE]	24.60	3	19	26	80	0.835	0.875	0.924	0.892	223.90	24.60	47	80	1130	124.6	5.47
Q6PFX9-2	Isoform 2 of Tankyrase-1 OS=Mus musculus GN=Tnks - [TNKS1_MOUSE]	6.56	4	6	6	10	1.173	0.713	0.959	0.892	24.84	6.56	9	10	976	106.6	7.14
Q8K409	DNA polymerase beta OS=Mus musculus GN=Polb PE=2 SV=3 - [DPOLB_MOUSE]	12.84	1	5	5	11	1.047	0.708	0.857	0.892	28.75	12.84	8	11	335	38.3	8.85
Q8BTH6	Transcriptional repressor scratch 2 OS=Mus musculus GN=Scrt2 PE=2 SV=2 - [SCRT2_MOUSE]	11.25	2	2	2	3	0.935	0.443	1.217	0.892	8.48	11.25	3	3	311	32.7	8.98
P50428	Arylsulfatase A OS=Mus musculus GN=Arsa PE=2 SV=2 - [ARSA_MOUSE]	31.42	2	10	11	40	1.704	1.166	0.803	0.892	108.96	31.42	17	40	506	53.7	5.87
Q9D8W5	26S proteasome non-ATPase regulatory subunit 12 OS=Mus musculus GN=Psmd12 PE=1 SV=4 - [PSD12_MOUSE]	49.34	3	21	22	85	0.714	1.069	0.882	0.892	247.53	49.34	38	85	456	52.9	7.06
Q8BGF6	ELMO domain-containing protein 2 OS=Mus musculus GN=Elmod2 PE=2 SV=1 -	9.90	1	3	3	11	0.846	0.771	0.865	0.892	33.04	9.90	6	11	293	34.7	8.57
Q80WT5-2	Isoform 2 of Aftiphilin OS=Mus musculus GN=Aftph - [AFTIN_MOUSE]	25.66	5	18	18	68	1.457	1.078	1.307	0.892	182.16	25.66	27	68	904	98.4	4.51
P52479	Ubiquitin carboxyl-terminal hydrolase 10 OS=Mus musculus GN=Usp10 PE=1 SV=3 - [UBP10_MOUSE]	34.60	3	1	21	82	2.230	1.103	1.249	0.892	223.63	34.60	37	82	792	87.0	5.17
J3QNX5	Protein AI848285 OS=Mus musculus GN=AI848285 PE=4 SV=1 - [J3QNX5_MOUSE]	8.52	1	2	2	2	1.001	1.038	1.220	0.892	2.96	8.52	2	2	399	42.4	9.96
B2KG46	Protein bicaudal D homolog 1 OS=Mus musculus GN=Bicd1 PE=2 SV=1 - [B2KG46_MOUSE]	18.67	7	11	15	35	1.402	0.842	0.915	0.892	95.21	18.67	24	35	975	110.9	5.91
O35551	Rab GTPase-binding effector protein 1 OS=Mus musculus GN=Rabep1 PE=1 SV=2 - [RABE1_MOUSE]	44.78	9	36	39	193	1.650	1.162	1.267	0.892	560.84	44.78	68	193	862	99.5	5.01
Q9CZJ9	DnaJ homolog subfamily C member 18 OS=Mus musculus GN=Dnajc18 PE=2 SV=1 -	4.20	1	1	1	2	1.910	0.692	0.951	0.892	8.51	4.20	1	2	357	41.4	8.78
Q8BX09-2	Isoform 2 of Retinoblastoma-binding protein 5 OS=Mus musculus GN=Rbbp5 - [RBBP5_MOUSE]	21.98	2	7	7	17	1.442	0.614	0.854	0.893	55.91	21.98	11	17	373	40.7	4.83
Q5M8N0	CB1 cannabinoid receptor-interacting protein 1 OS=Mus musculus GN=Cnrip1 PE=1 SV=1 - [CNRP1_MOUSE]	73.17	2	9	9	56	1.208	1.500	0.838	0.893	161.61	73.17	16	56	164	18.6	7.96
Q9WUD1	STIP1 homology and U box-containing protein 1 OS=Mus musculus GN=Stub1 PE=1 SV=1 - [CHIP_MOUSE]	69.74	1	20	21	68	1.305	0.963	1.103	0.893	179.37	69.74	35	68	304	34.9	6.01
Q3U4G3	Xyloside xylosyltransferase 1 OS=Mus musculus GN=Xxylt1 PE=2 SV=2 - [XXLT1_MOUSE]	11.22	1	3	3	8	1.064	0.693	0.916	0.893	26.55	11.22	6	8	392	43.8	7.78
Q6PAT0	Probable inactive tRNA-specific adenosine deaminase-like protein 3 OS=Mus musculus GN=Adat3 PE=2 SV=1 - [illegible]	9.46	1	2	2	5	1.144	0.834	0.825	0.893	18.49	9.46	4	5	349	37.5	7.23
D3YW48	Calpain small subunit 1 (Fragment) OS=Mus musculus GN=Capns1 PE=2 SV=1 -	48.13	3	9	9	37	1.430	1.290	0.780	0.893	114.55	48.13	16	37	241	25.3	5.60
Q0GGX2-2	Isoform 2 of Zinc finger protein 541 OS=Mus musculus GN=Znf541 - [ZN541_MOUSE]	3.69	2	3	3	6	3.686	4.499	2.314	0.893	8.56	3.69	3	6	1302	141.7	7.42
Q811P8	Rho GTPase-activating protein 32 OS=Mus musculus GN=Arhgap32 PE=1 SV=2 -	27.86	5	42	43	155	0.895	1.136	1.380	0.893	447.32	27.86	77	155	2089	229.6	6.86

Q91WE1	Sorting nexin-15 OS=Mus musculus GN=Snx15 PE=2 SV=1 - [SNX15_MOUSE]	50.15	3	12	12	33	1.493	0.944	1.191	0.893	85.32	50.15	20	33	337	37.7	5.24
Q6IRU5-2	Isoform 2 of Clathrin light chain B OS=Mus musculus GN=Cltb - [CLCB_MOUSE]	34.60	3	1	11	181	1.957	0.898	1.338	0.893	469.53	34.60	20	181	211	23.2	4.68
A2A9P6	Cyclin-dependent kinase 11B OS=Mus musculus GN=Cdk11b PE=2 SV=1 - [A2A9P6_MOUSE]	8.93	3	6	8	17	0.627	0.895	0.848	0.893	45.81	8.93	11	17	750	87.3	5.31
Q9CZT4-2	Isoform 2 of DNA-directed RNA polymerase III subunit RPC5 OS=Mus musculus GN=Polr3e - [RPC5_MOUSE]	9.94	2	5	5	17	0.597	0.761	0.916	0.893	55.43	9.94	9	17	684	76.8	6.04
Q69ZT9	TBC1 domain family member 30 OS=Mus musculus GN=Tbc1d30 PE=2 SV=2 -	1.57	1	1	1	1	14.581	3.667	2.524	0.893	2.48	1.57	1	1	766	84.8	8.53
F6QA74	DNA-(apurinic or apyrimidinic site) lyase (Fragment) OS=Mus musculus GN=Apex1 PE=2 SV=1 - [F6QA74_MOUSE]	41.18	3	10	10	47	0.668	0.859	0.882	0.893	129.86	41.18	17	47	289	32.3	8.57
O09061	Proteasome subunit beta type-1 OS=Mus musculus GN=Psmb1 PE=1 SV=1 - [PSB1_MOUSE]	64.58	1	12	13	89	1.873	0.996	0.721	0.893	284.56	64.58	24	89	240	26.4	7.81
Q91WM2	Cat eye syndrome critical region protein 5 homolog OS=Mus musculus GN=Cecr5 PE=2 SV=1 - [CECR5_MOUSE]	28.16	1	9	9	25	0.768	0.833	0.745	0.893	81.80	28.16	16	25	419	46.3	7.88
Q8CGI1	Protein FAM193A OS=Mus musculus GN=Fam193a PE=1 SV=2 - [F193A_MOUSE]	3.09	2	2	3	6	1.009	0.968	0.850	0.893	14.29	3.09	3	6	1231	136.6	6.47
Q9D2C2	Protein SAAL1 OS=Mus musculus GN=Saal1 PE=1 SV=1 - [SAAL1_MOUSE]	6.75	2	3	3	7	0.786	0.986	0.689	0.894	22.46	6.75	5	7	474	52.7	4.53
Q8C6E0	Coiled-coil domain-containing protein 104 OS=Mus musculus GN=Ccdc104 PE=2 SV=1 - [CC104_MOUSE]	52.48	2	13	13	75	1.592	0.903	1.234	0.894	212.01	52.48	23	75	343	39.6	4.93
Q3TXU5	Deoxyhypusine synthase OS=Mus musculus GN=Dhps PE=2 SV=2 - [DHYS_MOUSE]	31.44	2	9	9	31	1.711	0.937	0.726	0.894	92.31	31.44	14	31	369	40.6	5.67
Q3UPL0	Protein transport protein Sec31A OS=Mus musculus GN=Sec31a PE=1 SV=2 - [SC31A_MOUSE]	35.28	3	34	37	153	0.748	0.915	0.850	0.894	489.94	35.28	64	153	1230	133.5	6.76
E9PZY1	Hermansky-Pudlak syndrome 3 protein homolog OS=Mus musculus GN=Hps3 PE=2 SV=1 - [E9PZY1_MOUSE]	0.69	2	1	1	1	0.938	0.931	0.950	0.894	1.71	0.69	1	1	870	98.1	5.67
Q80XL6	Acyl-CoA dehydrogenase family member 11 OS=Mus musculus GN=Acad11 PE=1 SV=2 - [ACD11_MOUSE]	16.94	2	11	12	22	0.806	0.664	0.798	0.894	61.14	16.94	19	22	779	87.3	8.43
Q9QYE3-15	Isoform 7 of B-cell lymphoma/leukemia 11A OS=Mus musculus GN=Bcl11a - [BC11A_MOUSE]	26.15	6	2	2	2	0.990	1.061	1.460	0.894	3.68	26.15	2	2	130	14.3	5.54
P61961	Ubiquitin-fold modifier 1 OS=Mus musculus GN=Ufm1 PE=1 SV=1 - [UFM1_MOUSE]	68.24	4	4	4	15	1.770	1.036	1.059	0.894	45.94	68.24	7	15	85	9.1	9.31
P27612	Phospholipase A-2-activating protein OS=Mus musculus GN=Plaa PE=2 SV=4 - [PLAP_MOUSE]	38.79	2	25	25	110	0.996	0.959	0.876	0.894	336.13	38.79	47	110	794	87.2	6.14
P70206	Plexin-A1 OS=Mus musculus GN=Plxna1 PE=1 SV=1 - [PLXA1_MOUSE]	32.00	1	36	56	193	0.540	0.876	0.650	0.894	564.20	32.00	95	193	1894	211.0	6.90
D3Z3N4	MCG11326, isoform CRA_a OS=Mus musculus GN=Hnrnph3 PE=4 SV=1 - [D3Z3N4_MOUSE]	50.58	3	10	11	47	1.593	1.130	1.075	0.894	169.12	50.58	19	47	346	36.8	6.87
P70460	Vasodilator-stimulated phosphoprotein OS=Mus musculus GN=Vasp PE=1 SV=4 - [VASP_MOUSE]	18.40	1	8	8	23	1.466	1.075	0.903	0.894	57.05	18.40	13	23	375	39.6	8.53
Q91WC0	Histone-lysine N-methyltransferase setd3 OS=Mus musculus GN=Setd3 PE=1 SV=1 -	16.33	7	11	11	28	0.822	0.887	0.797	0.894	66.06	16.33	18	28	594	67.1	5.60
P61087	Ubiquitin-conjugating enzyme E2 K OS=Mus musculus GN=Ube2k PE=1 SV=3 - [UBE2K_MOUSE]	41.50	3	7	8	43	0.982	0.968	0.927	0.894	117.98	41.50	14	43	200	22.4	5.44
Q3TFD2	Lysophosphatidylcholine acyltransferase 1 OS=Mus musculus GN=Lpcat1 PE=1 SV=1 - [PCAT1_MOUSE]	19.29	5	8	8	13	0.877	1.026	0.826	0.894	29.08	19.29	12	13	534	59.7	6.34
Q63810-2	Isoform 2 of Calcineurin subunit B type 1 OS=Mus musculus GN=Ppp3r1 - [CANB1_MOUSE]	77.50	3	13	13	421	0.828	1.496	1.949	0.895	1491.11	77.50	26	421	160	18.2	4.92

P47911	60S ribosomal protein L6 OS=Mus musculus GN=Rpl6 PE=1 SV=3 - [RL6_MOUSE]	39.86	2	14	14	61	0.618	0.826	0.763	0.895	134.22	39.86	24	61	296	33.5	10.70
O35375-5	Isoform B0 of Neuropilin-2 OS=Mus musculus GN=Nrp2 - [NRP2_MOUSE]	27.75	6	19	19	69	0.969	2.452	0.800	0.895	192.67	27.75	31	69	901	101.4	5.81
Q99JX7	Nuclear RNA export factor 1 OS=Mus musculus GN=Nxf1 PE=1 SV=3 - [NXF1_MOUSE]	17.96	1	9	9	19	0.582	0.753	0.848	0.895	60.63	17.96	14	19	618	70.3	8.73
Q91ZM2	SH2B adapter protein 1 OS=Mus musculus GN=Sh2b1 PE=1 SV=2 - [SH2B1_MOUSE]	25.79	6	11	11	25	1.003	1.101	0.921	0.895	85.60	25.79	16	25	756	79.6	5.25
Q8C0I1	Alkyldihydroxyacetonephosphate synthase, peroxisomal OS=Mus musculus GN=Agps PE=1 SV=1 - [ADAS_MOUSE]	28.22	5	12	12	29	0.941	0.840	0.811	0.895	80.41	28.22	19	29	645	71.6	7.50
P54775	26S protease regulatory subunit 6B OS=Mus musculus GN=Psmc4 PE=1 SV=2 -	64.11	1	22	23	106	0.965	1.134	0.842	0.895	304.61	64.11	39	106	418	47.4	5.21
Q9EQQ2	Protein YIPF5 OS=Mus musculus GN=Yipf5 PE=2 SV=1 - [YIPF5_MOUSE]	4.67	1	1	1	10	0.679	0.594	0.826	0.895	23.73	4.67	2	10	257	27.9	4.36
Q8BFZ2-2	Isoform 2 of Lipid phosphate phosphatase-related protein type 1 OS=Mus musculus GN=Lppr1 - [LPPR1_MOUSE]	14.24	2	2	2	12	1.269	0.923	1.046	0.895	25.39	14.24	4	12	295	32.7	7.01
G3X948	DNA polymerase sigma OS=Mus musculus GN=Papd7 PE=4 SV=1 - [G3X948_MOUSE]	2.58	2	1	1	1	1.564	1.211	0.871	0.895	0.00	2.58	1	1	542	60.0	9.39
Q8C0J6	Ankyrin repeat domain-containing protein SOWAHC OS=Mus musculus GN=Sowahc PE=2 SV=2 - [SWAHC_MOUSE]	12.30	1	5	5	9	0.898	0.989	0.970	0.895	23.00	12.30	6	9	512	54.9	6.00
P63056-2	Isoform 2 of Noelin-3 OS=Mus musculus GN=Olfm3 - [NOE3_MOUSE]	9.17	3	3	3	8	0.870	0.309	0.792	0.895	22.03	9.17	5	8	458	52.8	8.15
Q8BYM5	Neuroligin-3 OS=Mus musculus GN=Nlgn3 PE=1 SV=2 - [NLGN3_MOUSE]	30.42	4	13	16	59	0.655	1.243	0.929	0.895	165.06	30.42	28	59	825	91.1	5.80
Q8CFT0	RELT-like 2 OS=Mus musculus GN=Rell2 PE=2 SV=1 - [Q8CFT0_MOUSE]	36.05	6	7	7	63	0.823	0.422	0.965	0.895	178.99	36.05	11	63	294	31.4	6.46
Q9JKV1	Proteasomal ubiquitin receptor ADRM1 OS=Mus musculus GN=Adrm1 PE=1 SV=2 - [ADRM1_MOUSE]	17.94	2	8	8	63	1.152	1.012	0.924	0.895	189.71	17.94	12	63	407	42.0	5.07
Q80Y83-13	Isoform 13 of Dixin OS=Mus musculus GN=Dixdc1 - [DIXC1_MOUSE]	22.25	15	8	9	30	1.182	0.794	1.007	0.895	83.17	22.25	14	30	391	44.7	6.27
O54825	Bystin OS=Mus musculus GN=Bysl PE=1 SV=3 - [BYST_MOUSE]	6.65	1	2	3	3	0.573	0.733	0.647	0.895	7.75	6.65	3	3	436	49.8	7.78
Q9DD03	Ras-related protein Rab-13 OS=Mus musculus GN=Rab13 PE=1 SV=1 - [RAB13_MOUSE]	27.72	3	2	6	50	0.609	0.756	0.755	0.895	132.57	27.72	9	50	202	22.8	9.50
D3YVE6	MCG18601 OS=Mus musculus GN=Rpl7a-ps10 PE=4 SV=1 - [D3YVE6_MOUSE]	47.37	11	15	16	66	0.567	0.786	0.764	0.895	179.97	47.37	28	66	266	30.0	10.45
A2AJ95	NMDA receptor synaptonuclear-signaling and neuronal migration factor OS=Mus musculus GN=Nsmf PE=2 SV=1 - [A2AJ95_MOUSE]	22.96	12	9	10	24	0.603	1.198	1.053	0.895	57.63	22.96	16	24	479	53.9	8.51
Q9QXX4	Calcium-binding mitochondrial carrier protein Aralar2 OS=Mus musculus GN=Slc25a13 PE=1 SV=1 - [CMC2_MOUSE]	25.00	1	9	15	76	1.393	1.377	0.550	0.895	205.75	25.00	23	76	676	74.4	8.60
P57784	U2 small nuclear ribonucleoprotein A' OS=Mus musculus GN=Snrpa1 PE=1 SV=2 -	49.80	2	12	12	56	0.756	0.694	0.851	0.895	159.65	49.80	19	56	255	28.3	8.62
P22935	Cellular retinoic acid-binding protein 2 OS=Mus musculus GN=Crabp2 PE=2 SV=2 -	18.12	1	2	3	7	1.267	4.666	0.619	0.895	16.17	18.12	5	7	138	15.7	5.71
Q9CQ80	Vacuolar protein-sorting-associated protein 25 OS=Mus musculus GN=Vps25 PE=2 SV=1 - [VPS25_MOUSE]	35.80	6	5	5	23	0.989	1.338	0.945	0.895	63.54	35.80	9	23	176	20.7	6.34
Q8K3W3	Protein CASC3 OS=Mus musculus GN=Casc3 PE=1 SV=3 - [CASC3_MOUSE]	8.17	1	3	3	10	1.261	1.009	0.982	0.895	24.54	8.17	5	10	698	75.7	6.44
E9Q4G7	Casein kinase I isoform alpha OS=Mus musculus GN=Csnk1a1 PE=2 SV=1 - [E9Q4G7_MOUSE]	37.20	8	12	12	43	0.883	0.824	0.867	0.895	106.82	37.20	20	43	336	38.7	9.57
Q64487-7	Isoform G of Receptor-type tyrosine-protein phosphatase delta OS=Mus musculus GN=Ptprd - [PTPRD_MOUSE]	34.86	23	42	48	220	1.260	1.065	1.118	0.896	650.21	34.86	90	220	1905	213.4	6.62

Q811U4	Mitofusin-1 OS=Mus musculus GN=Mfn1 PE=1 SV=3 - [MFN1_MOUSE]	19.84	4	11	12	22	0.743	0.699	0.857	0.896	63.38	19.84	18	22	741	83.7	6.51
Q62092	Neuron-specific protein family member 1 OS=Mus musculus GN=Nsg1 PE=2 SV=3 - [NSG1_MOUSE]	22.70	1	3	3	6	0.859	0.882	0.929	0.896	19.76	22.70	5	6	185	20.9	6.21
Q8K224	N-acetyltransferase 10 OS=Mus musculus GN=Nat10 PE=2 SV=1 - [NAT10_MOUSE]	15.72	4	13	14	24	1.311	0.976	0.789	0.896	62.54	15.72	22	24	1024	115.3	8.32
Q91YE9-2	Isoform 2 of Cytosolic 5'-nucleotidase 1B OS=Mus musculus GN=Nt5c1b - [5NT1B_MOUSE]	5.95	4	2	3	5	1.145	0.688	1.147	0.896	12.58	5.95	4	5	555	62.9	8.28
Q7TSH9	Zinc finger protein 184 OS=Mus musculus GN=Zfp184 PE=2 SV=1 - [ZN184_MOUSE]	6.78	145	2	3	3	1.439	0.872	1.279	0.896	4.58	6.78	3	3	737	84.0	8.37
Q9Z1X4	Interleukin enhancer-binding factor 3 OS=Mus musculus GN=Ilf3 PE=1 SV=2 - [ILF3_MOUSE]	50.00	4	25	30	163	0.929	0.680	0.837	0.896	393.01	50.00	52	163	898	96.0	8.76
Q6PAL7	AT-hook DNA-binding motif containing protein 1 OS=Mus musculus GN=Ahdc1 PE=1 SV=1 - [AHDC1_MOUSE]	3.51	1	5	5	11	0.592	0.851	0.961	0.896	35.71	3.51	9	11	1594	168.0	8.97
P26369	Splicing factor U2AF 65 kDa subunit OS=Mus musculus GN=U2af2 PE=1 SV=3 - [U2AF2_MOUSE]	29.47	2	12	12	42	0.886	0.982	0.855	0.896	109.46	29.47	22	42	475	53.5	9.09
O08739	AMP deaminase 3 OS=Mus musculus GN=Ampd3 PE=2 SV=2 - [AMPD3_MOUSE]	19.97	6	13	14	28	0.701	0.712	0.738	0.896	69.01	19.97	19	28	766	88.6	7.33
O35449	Proline-rich transmembrane protein 1 OS=Mus musculus GN=Prrt1 PE=1 SV=1 -	12.09	1	3	3	31	1.076	1.677	1.256	0.896	83.18	12.09	6	31	306	31.4	7.65
Q99J95	Cyclin-dependent kinase 9 OS=Mus musculus GN=Cdk9 PE=1 SV=1 - [CDK9_MOUSE]	26.34	20	7	9	49	0.671	0.981	0.877	0.896	111.26	26.34	15	49	372	42.7	8.79
Q8BGS1-3	Isoform 3 of Band 4.1-like protein 5 OS=Mus musculus GN=Epb41l5 - [E41L5_MOUSE]	10.01	3	5	6	8	0.925	0.675	0.776	0.896	26.05	10.01	8	8	739	82.5	6.47
Q9D8Z2	TP53-regulated inhibitor of apoptosis 1 OS=Mus musculus GN=Triap1 PE=2 SV=1 - [TRIA1_MOUSE]	55.26	1	4	4	19	1.725	1.002	1.305	0.896	72.84	55.26	6	19	76	8.8	5.48
Q9JHZ2	Progressive ankylosis protein OS=Mus musculus GN=Ankh PE=2 SV=1 - [ANKH_MOUSE]	9.76	1	3	4	9	0.669	0.807	0.814	0.896	25.48	9.76	7	9	492	54.3	7.88
Q9WVJ2	26S proteasome non-ATPase regulatory subunit 13 OS=Mus musculus GN=Psmd13 PE=1 SV=1 - [PSD13_MOUSE]	52.93	6	18	18	72	0.893	1.139	0.784	0.896	209.05	52.93	34	72	376	42.8	5.71
F6ZFU0	Elongation factor 1-delta (Fragment) OS=Mus musculus GN=Eef1d PE=2 SV=1 - [F6ZFU0_MOUSE]	58.88	4	1	27	201	1.042	1.080	0.732	0.896	613.30	58.88	46	201	552	61.4	7.39
Q8BZB2	Phosphopantothenoylcysteine decarboxylase OS=Mus musculus GN=Ppcdc PE=2 SV=1 - [COAC_MOUSE]	35.29	1	6	6	17	1.729	1.220	0.990	0.896	42.64	35.29	9	17	204	22.3	6.52
P70268	Serine/threonine-protein kinase N1 OS=Mus musculus GN=Pkn1 PE=1 SV=3 - [PKN1_MOUSE]	13.42	3	7	10	30	0.570	1.015	0.851	0.896	80.76	13.42	13	30	946	104.3	6.27
Q9QXK3	Coatomer subunit gamma-2 OS=Mus musculus GN=Copg2 PE=2 SV=1 - [COPG2_MOUSE]	31.00	5	13	22	56	0.619	0.828	0.873	0.896	157.90	31.00	38	56	871	97.6	5.80
P62073	Mitochondrial import inner membrane translocase subunit Tim10 OS=Mus musculus GN=Timm10 PE=1 SV=1 - [TIM10_MOUSE]	75.56	1	8	8	102	1.971	1.001	1.293	0.896	291.88	75.56	15	102	90	10.3	6.29
Q8C8R3-3	Isoform 3 of Ankyrin-2 OS=Mus musculus GN=Ank2 - [ANK2_MOUSE]	55.71	1	2	43	429	1.349	0.845	1.127	0.896	1325.79	55.71	73	429	1050	117.4	4.92
Q02614	SAP30-binding protein OS=Mus musculus GN=Sap30bp PE=2 SV=2 - [S30BP_MOUSE]	28.57	1	6	6	8	1.374	0.905	1.119	0.896	22.48	28.57	8	8	308	33.8	4.87
Q78PG9	Coiled-coil domain-containing protein 25 OS=Mus musculus GN=Ccdc25 PE=1 SV=1 - [CCD25_MOUSE]	38.46	1	9	9	25	1.317	0.808	1.004	0.896	77.35	38.46	15	25	208	24.5	6.95
Q91Y09	Protein Pcdha9 OS=Mus musculus GN=Pcdhac2 PE=2 SV=1 - [Q91Y09_MOUSE]	8.65	2	6	7	14	0.795	0.975	1.018	0.896	44.12	8.65	12	14	1006	109.4	5.47
G3X8R4	General transcription factor II H, polypeptide 1 OS=Mus musculus GN=Gtf2h1 PE=4 SV=1 - [G3X8R4_MOUSE]	7.86	4	4	4	6	1.562	2.050	1.168	0.896	15.03	7.86	6	6	547	61.8	8.76

O35972	39S ribosomal protein L23, mitochondrial OS=Mus musculus GN=Mrpl23 PE=2 SV=1 - [RM23_MOUSE]	33.56	1	3	3	10	0.555	0.588	0.790	0.896	38.98	33.56	5	10	146	17.1	9.76
Q9EST3	Eukaryotic translation initiation factor 4E transporter OS=Mus musculus GN=Eif4enif1 PE=1 SV=2 - [illegible]	11.29	5	8	8	27	1.232	0.853	0.988	0.896	77.50	11.29	12	27	983	107.9	7.74
Q9QZK2-2	Isoform 2 of Breast cancer anti-estrogen resistance protein 3 OS=Mus musculus GN=Bcar3 - [BCAR3_MOUSE]	4.57	2	3	3	6	0.569	0.701	0.904	0.896	14.02	4.57	4	6	700	78.7	7.14
Q61081	Hsp90 co-chaperone Cdc37 OS=Mus musculus GN=Cdc37 PE=2 SV=1 - [CDC37_MOUSE]	26.65	1	10	10	69	0.902	0.845	0.961	0.896	169.70	26.65	18	69	379	44.6	5.34
Q9QY81	Nuclear pore membrane glycoprotein 210 OS=Mus musculus GN=Nup210 PE=1 SV=2 - [PO210_MOUSE]	12.04	1	18	19	44	0.923	1.007	0.921	0.896	138.12	12.04	29	44	1886	204.0	6.65
Q9D6I0	Cxx1c protein OS=Mus musculus GN=Cxx1c PE=4 SV=1 - [Q9D6I0_MOUSE]	15.18	1	1	1	1	1.378	1.128	0.929	0.897	4.58	15.18	1	1	112	13.6	5.01
F6Z1Z9	Calcium uptake protein 1, mitochondrial (Fragment) OS=Mus musculus GN=Micu1 PE=2 SV=1 - [F6Z1Z9_MOUSE]	44.87	1	1	2	5	2.225	2.405	0.831	0.897	21.06	44.87	3	5	78	8.9	7.46
Q9Z0G0	PDZ domain-containing protein GIPC1 OS=Mus musculus GN=Gipc1 PE=1 SV=1 - [GIPC1_MOUSE]	45.05	3	12	12	68	0.914	1.058	0.963	0.897	206.85	45.05	21	68	333	36.1	5.91
Q8VCV1	Alpha/beta hydrolase domain-containing protein 17C OS=Mus musculus GN=Abhd17c PE=2 SV=2 - [AB17C_MOUSE]	12.81	1	3	3	7	0.735	0.756	0.821	0.897	21.12	12.81	5	7	320	35.1	5.64
Q3TBW2	39S ribosomal protein L10, mitochondrial OS=Mus musculus GN=Mrpl10 PE=2 SV=2 - [RM10_MOUSE]	29.01	2	5	5	13	0.641	0.614	0.974	0.897	27.10	29.01	9	13	262	29.4	9.69
P61290	Proteasome activator complex subunit 3 OS=Mus musculus GN=Psme3 PE=1 SV=1 - [PSME3_MOUSE]	29.53	3	8	8	30	1.059	1.819	0.710	0.897	96.22	29.53	13	30	254	29.5	5.95
Q8VFH8	MCG58953 OS=Mus musculus GN=Olfr786 PE=3 SV=1 - [Q8VFH8_MOUSE]	3.21	2	1	1	1	0.964	1.143	1.247	0.897	2.36	3.21	1	1	312	35.6	8.68
B9EJA2-2	Isoform 2 of Cortactin-binding protein 2 OS=Mus musculus GN=Cttnbp2 - [CTTB2_MOUSE]	51.43	4	1	27	144	1.043	1.479	2.558	0.897	438.40	51.43	47	144	630	67.5	8.22
Q9D0L8	mRNA cap guanine-N7 methyltransferase OS=Mus musculus GN=Rnmt PE=1 SV=1 - [MCES_MOUSE]	41.72	6	14	15	53	0.772	0.837	0.938	0.897	151.12	41.72	23	53	465	53.3	6.48
P16951-2	Isoform 2 of Cyclic AMP-dependent transcription factor ATF-2 OS=Mus musculus GN=Atf2 - [ATF2_MOUSE]	20.57	8	5	6	16	1.021	1.027	1.329	0.897	52.70	20.57	10	16	389	42.3	7.17
Q8R4X3	RNA-binding protein 12 OS=Mus musculus GN=Rbm12 PE=1 SV=3 - [RBM12_MOUSE]	16.94	5	14	14	33	1.442	1.164	0.965	0.897	92.50	16.94	20	33	992	102.7	8.32
Q3U9G9	Lamin-B receptor OS=Mus musculus GN=Lbr PE=1 SV=2 - [LBR_MOUSE]	10.70	1	7	7	21	0.508	0.823	0.787	0.898	61.50	10.70	11	21	626	71.4	9.36
P49722	Proteasome subunit alpha type-2 OS=Mus musculus GN=Psma2 PE=1 SV=3 - [PSA2_MOUSE]	67.09	1	14	14	93	1.535	0.909	0.706	0.898	259.54	67.09	25	93	234	25.9	7.43
Q3U332	Ras and Rab interactor 3 OS=Mus musculus GN=Rin3 PE=2 SV=1 - [Q3U332_MOUSE]	1.56	2	1	1	1	1.668	1.180	0.777	0.898	2.90	1.56	1	1	900	98.6	7.36
O88520	Leucine-rich repeat protein SHOC-2 OS=Mus musculus GN=Shoc2 PE=2 SV=2 - [SHOC2_MOUSE]	22.16	1	9	10	25	0.603	0.754	0.842	0.898	59.62	22.16	16	25	582	64.9	8.46
A2AQ25	Sickle tail protein OS=Mus musculus GN=Skt PE=1 SV=1 - [SKT_MOUSE]	26.72	18	6	44	156	1.239	1.346	1.185	0.898	410.92	26.72	75	156	1946	212.9	7.77
D3YVL0	Putative helicase MOV-10 OS=Mus musculus GN=Mov10 PE=4 SV=1 - [D3YVL0_MOUSE]	6.57	3	5	5	8	1.557	1.221	0.817	0.898	19.39	6.57	6	8	1004	113.5	8.98
Q9CQF0	39S ribosomal protein L11, mitochondrial OS=Mus musculus GN=Mrpl11 PE=2 SV=1 - [RM11_MOUSE]	31.77	1	7	7	15	1.228	1.045	0.911	0.898	43.13	31.77	11	15	192	20.7	9.73
Q9DB42	Zinc finger protein 593 OS=Mus musculus GN=Znf593 PE=2 SV=2 - [ZN593_MOUSE]	8.96	1	1	1	8	1.484	0.772	1.369	0.898	15.08	8.96	2	8	134	15.1	9.55

Q9JKC8	AP-3 complex subunit mu-1 OS=Mus musculus GN=Ap3m1 PE=1 SV=1 - [AP3M1_MOUSE]	36.84	4	9	11	31	0.644	0.718	0.898	0.898	84.54	36.84	17	31	418	46.9	6.93
P28271	Cytoplasmic aconitate hydratase OS=Mus musculus GN=Aco1 PE=1 SV=3 - [ACOC_MOUSE]	45.11	1	33	34	150	1.495	0.730	0.740	0.898	451.98	45.11	61	150	889	98.1	7.50
Q8VCQ3	Nuclear receptor-binding factor 2 OS=Mus musculus GN=Nrbf2 PE=1 SV=1 - [NRBF2_MOUSE]	42.51	2	10	10	21	1.528	1.133	1.246	0.898	53.32	42.51	14	21	287	32.5	5.76
P62878	E3 ubiquitin-protein ligase RBX1 OS=Mus musculus GN=Rbx1 PE=1 SV=1 - [RBX1_MOUSE]	23.15	1	2	2	8	5.942	2.945	1.483	0.898	15.55	23.15	2	8	108	12.3	6.96
E9Q9E9	Protein 1190002N15Rik OS=Mus musculus GN=1190002N15Rik PE=2 SV=1 - [E9Q9E9_MOUSE]	4.42	2	2	2	4	0.686	0.919	0.656	0.898	11.47	4.42	4	4	430	49.4	8.63
E9Q137	Protein Tex264 OS=Mus musculus GN=Tex264 PE=4 SV=1 - [E9Q137_MOUSE]	26.21	2	5	5	24	0.911	1.110	0.862	0.898	70.42	26.21	9	24	309	33.6	5.40
Q9WUA6-2	Isoform 2 of RAC-gamma serine/threonine-protein kinase OS=Mus musculus GN=Akt3 - [AKT3_MOUSE]	30.64	2	9	14	36	0.710	1.067	1.008	0.898	100.87	30.64	23	36	470	54.6	6.70
Q9DC22	DDB1- and CUL4-associated factor 6 OS=Mus musculus GN=Dcaf6 PE=1 SV=1 -	8.11	1	7	7	13	1.097	1.516	1.241	0.898	34.00	8.11	9	13	876	97.5	5.22
P48302	Endothelin B receptor OS=Mus musculus GN=Ednrb PE=2 SV=1 - [EDNRB_MOUSE]	4.30	1	2	2	7	0.794	0.736	0.730	0.899	12.14	4.30	2	7	442	49.5	9.28
Q9WVD4	H(+)/Cl(-) exchange transporter 5 OS=Mus musculus GN=Clcn5 PE=2 SV=1 - [CLCN5_MOUSE]	8.18	2	4	6	12	1.194	0.615	0.648	0.899	28.73	8.18	10	12	746	83.0	7.01
Q9JKB1	Ubiquitin carboxyl-terminal hydrolase isozyme L3 OS=Mus musculus GN=Uchl3 PE=1 SV=2 - [UCHL3_MOUSE]	50.87	2	10	10	99	1.524	1.025	1.135	0.899	301.48	50.87	20	99	230	26.1	5.05
P60840-2	Isoform 2 of Alpha-endosulfine OS=Mus musculus GN=Ensa - [ENSA_MOUSE]	62.39	3	6	7	57	1.131	1.117	1.655	0.899	158.56	62.39	13	57	117	12.9	8.27
Q3U1V8	Mitogen-activated protein kinase kinase kinase 9 OS=Mus musculus GN=Map3k9 PE=2 SV=2 - [M3K9_MOUSE]	3.90	2	3	4	10	1.116	0.684	1.128	0.899	25.04	3.90	8	10	1077	118.7	5.86
Q5XG71	Small subunit processome component 20 homolog OS=Mus musculus GN=Utp20 PE=2 SV=2 - [UTP20_MOUSE]	1.61	2	2	3	4	1.719	0.568	1.011	0.899	7.85	1.61	3	4	2788	317.5	8.09
P38585	Tubulin--tyrosine ligase OS=Mus musculus GN=Ttl PE=2 SV=2 - [TTL_MOUSE]	16.45	2	4	5	17	0.780	0.797	1.027	0.899	47.93	16.45	10	17	377	43.1	6.46
P70429-2	Isoform 1 of Ena/VASP-like protein OS=Mus musculus GN=Evl - [EVL_MOUSE]	44.78	4	16	16	63	0.999	0.938	0.997	0.899	174.44	44.78	25	63	393	42.1	8.35
F6ZGR6	Protein D430041D05Rik (Fragment) OS=Mus musculus GN=D430041D05Rik PE=4 SV=1 - [F6ZGR6_MOUSE]	13.29	4	16	16	56	0.845	1.187	1.585	0.899	178.48	13.29	28	56	1678	181.0	9.38
Q9QXT8	Calsenilin OS=Mus musculus GN=Kcnip3 PE=1 SV=2 - [CSEN_MOUSE]	30.86	5	6	6	13	1.578	0.829	0.814	0.899	35.27	30.86	9	13	256	29.4	5.69
Q9D8M7-2	Isoform 2 of PHD finger protein 10 OS=Mus musculus GN=Phf10 - [PHF10_MOUSE]	7.68	4	3	3	10	1.687	0.980	0.936	0.899	17.89	7.68	4	10	495	55.6	6.73
Q9QZM0	Ubiquilin-2 OS=Mus musculus GN=Ubqln2 PE=1 SV=2 - [UBQL2_MOUSE]	23.82	1	7	11	189	1.915	1.216	0.967	0.899	574.41	23.82	21	189	638	67.3	5.22
Q68EF0	Rab-3A-interacting protein OS=Mus musculus GN=Rab3ip PE=1 SV=1 - [RAB3I_MOUSE]	27.57	1	8	8	13	1.010	0.809	1.192	0.899	42.90	27.57	11	13	428	47.1	6.71
P54276	DNA mismatch repair protein Msh6 OS=Mus musculus GN=Msh6 PE=1 SV=3 - [MSH6_MOUSE]	8.39	1	10	10	12	0.945	0.775	0.896	0.899	37.12	8.39	12	12	1358	151.0	6.73
Q8CFV4	Neuritin OS=Mus musculus GN=Nrn1 PE=1 SV=1 - [NRN1_MOUSE]	19.01	2	3	3	17	0.674	2.177	2.344	0.899	61.36	19.01	6	17	142	15.3	6.99
Q8C7X2-2	Isoform 2 of ER membrane protein complex subunit 1 OS=Mus musculus GN=Emc1 - [EMC1_MOUSE]	29.48	3	24	24	84	0.845	0.747	0.817	0.899	276.70	29.48	43	84	994	111.2	7.43
G3UYG6	PERQ amino acid-rich with GYF domain-containing protein 2 (Fragment) OS=Mus musculus GN=Gigyf2 PE=2 SV=1 - [G3UYG6_MOUSE]	16.58	11	17	19	99	1.420	0.846	1.166	0.899	233.31	16.58	32	99	1285	148.5	5.57

A2AA71	Protein transport protein Sec24A OS=Mus musculus GN=Sec24a PE=2 SV=1 - [A2AA71_MOUSE]	21.49	4	14	14	33	0.724	0.613	0.886	0.899	70.99	21.49	23	33	1089	118.6	7.83
E9PX30	Mitogen-activated protein kinase kinase kinase kinase OS=Mus musculus GN=Map4k5 PE=2 SV=1 - [E9PX30_MOUSE]	10.15	4	7	7	18	1.153	0.940	0.905	0.899	58.92	10.15	11	18	847	94.9	7.91
Q5SVQ0-4	Isoform 4 of Histone acetyltransferase KAT7 OS=Mus musculus GN=Kat7 - [KAT7_MOUSE]	7.40	5	3	3	9	0.774	0.619	0.954	0.899	25.42	7.40	6	9	581	66.9	8.72
Q61160	Protein FADD OS=Mus musculus GN=Fadd PE=1 SV=1 - [FADD_MOUSE]	7.80	1	2	2	3	1.540	1.227	0.960	0.899	6.65	7.80	3	3	205	22.9	6.04
P51829	Adenylate cyclase type 7 OS=Mus musculus GN=Adcy7 PE=2 SV=2 - [ADCY7_MOUSE]	7.92	1	3	6	10	1.388	0.794	0.655	0.899	26.80	7.92	9	10	1099	122.6	7.87
Q9Z2Y3	Homer protein homolog 1 OS=Mus musculus GN=Homer1 PE=1 SV=2 - [HOME1_MOUSE]	60.11	11	19	19	106	1.150	1.958	2.211	0.899	273.72	60.11	33	106	366	41.4	5.53
Q8BGH4	Receptor expression-enhancing protein 1 OS=Mus musculus GN=Reep1 PE=1 SV=1 - [REEP1_MOUSE]	40.30	1	8	8	30	0.845	0.554	1.025	0.899	84.31	40.30	13	30	201	22.3	9.50
F7CD11	Synaptojanin-1 (Fragment) OS=Mus musculus GN=Synj1 PE=4 SV=1 - [F7CD11_MOUSE]	46.03	3	3	6	65	1.061	0.307	0.894	0.899	172.89	46.03	10	65	189	19.3	11.14
Q8VDS4	Regulation of nuclear pre-mRNA domain-containing protein 1A OS=Mus musculus GN=Rprd1a PE=2 SV=1 - [illegible]	18.91	1	5	6	16	0.677	1.048	0.787	0.900	47.15	18.91	10	16	312	35.7	7.58
B1AUN2	MCG14442 OS=Mus musculus GN=Eif2b3 PE=4 SV=1 - [B1AUN2_MOUSE]	33.85	3	14	14	43	0.757	0.887	0.859	0.900	97.98	33.85	23	43	452	50.5	6.74
G3X973	Stabilin 1, isoform CRA_a OS=Mus musculus GN=Stab1 PE=4 SV=1 - [G3X973_MOUSE]	2.88	3	3	4	5	0.994	1.416	0.961	0.900	12.04	2.88	5	5	2571	276.3	6.61
Q6PFE3	DNA repair and recombination protein RAD54B OS=Mus musculus GN=Rad54b PE=2 SV=1 - [illegible]	4.74	1	2	2	2	1.183	1.037	1.006	0.900	2.85	4.74	2	2	886	99.3	8.22
Q64127-2	Isoform Short of Transcription intermediary factor 1-alpha OS=Mus musculus GN=Trim24 - [TIF1A_MOUSE]	3.74	3	4	4	5	0.849	0.721	0.575	0.900	11.35	3.74	5	5	1017	112.8	6.80
P57716	Nicastrin OS=Mus musculus GN=Ncstn PE=1 SV=3 - [NICA_MOUSE]	12.99	1	8	8	27	0.953	0.878	0.746	0.900	77.13	12.99	14	27	708	78.4	6.09
Q3TWI9	Transmembrane protein 63B OS=Mus musculus GN=Tmem63b PE=1 SV=1 - [TM63B_MOUSE]	10.70	6	9	9	19	0.593	0.908	0.869	0.900	49.56	10.70	12	19	832	94.7	7.49
Q80TN4	DnaJ homolog subfamily C member 16 OS=Mus musculus GN=Dnajc16 PE=1 SV=2 - [illegible]	14.51	2	9	10	21	0.793	0.820	0.854	0.900	55.86	14.51	15	21	772	89.1	7.55
Q6PAR0-2	Isoform 2 of Kelch domain-containing protein 10 OS=Mus musculus GN=Klhdc10 - [KLD10_MOUSE]	9.02	3	3	3	5	1.082	1.941	0.759	0.900	21.41	9.02	5	5	410	45.4	8.60
Q9D8B3	Charged multivesicular body protein 4b OS=Mus musculus GN=Chmp4b PE=2 SV=2 - [illegible]	53.13	2	11	11	68	0.931	0.466	1.251	0.900	197.85	53.13	21	68	224	24.9	4.82
Q03137	Ephrin type-A receptor 4 OS=Mus musculus GN=Epha4 PE=1 SV=2 - [EPHA4_MOUSE]	23.12	2	13	17	54	0.610	1.020	1.289	0.900	184.37	23.12	30	54	986	109.7	6.51
Q64518	Sarcoplasmic/endoplasmic reticulum calcium ATPase 3 OS=Mus musculus GN=Atp2a3 PE=2 SV=3 - [AT2A3_MOUSE]	12.14	6	5	14	56	0.851	1.553	1.063	0.900	161.27	12.14	24	56	1038	113.6	5.85
Q922M7	Ashwin OS=Mus musculus PE=1 SV=1 - [illegible]	26.72	1	5	5	16	1.581	0.804	1.119	0.900	61.30	26.72	10	16	232	26.0	9.55
Q5SUF5	Protein Cacna1g OS=Mus musculus GN=Cacna1g PE=2 SV=1 - [Q5SUF5_MOUSE]	4.54	11	6	6	26	0.750	0.378	0.815	0.900	65.20	4.54	11	26	2247	248.8	7.27
Q8R2K3	Single-stranded DNA-binding protein OS=Mus musculus GN=Ssbp1 PE=2 SV=1 - [Q8R2K3_MOUSE]	60.14	3	9	9	38	2.351	1.150	1.226	0.900	107.64	60.14	13	38	148	17.1	9.79
Q8R3R8	Gamma-aminobutyric acid receptor-associated protein-like 1 OS=Mus musculus GN=Gabarapl1 PE=1 SV=2 - [illegible]	41.03	1	2	6	16	1.390	0.852	1.014	0.900	49.46	41.03	8	16	117	14.0	8.73
P60843	Eukaryotic initiation factor 4A-I OS=Mus musculus GN=Eif4a1 PE=2 SV=1 - [IF4A1_MOUSE]	57.14	1	12	27	202	0.770	0.912	0.898	0.900	594.90	57.14	48	202	406	46.1	5.48

Q6P5B5	Fragile X mental retardation syndrome-related protein 2 OS=Mus musculus GN=Fxr2 PE=2 SV=1 - [Q6P5B5_MOUSE]	34.72	2	14	19	60	0.899	0.802	0.878	0.900	197.95	34.72	32	60	674	74.2	6.23
O55241	Orexin OS=Mus musculus GN=Hcrt PE=2 SV=1 - [illegible]	13.08	1	1	1	5	1.189	0.753	0.882	0.901	8.76	13.08	2	5	130	13.5	9.70
Q91Y86	Mitogen-activated protein kinase 8 OS=Mus musculus GN=Mapk8 PE=1 SV=1 -	40.10	12	4	13	64	1.670	1.549	0.893	0.901	174.65	40.10	24	64	384	44.2	7.69
Q922P9	Putative oxidoreductase GLYR1 OS=Mus musculus GN=Glyr1 PE=2 SV=1 - [GLYR1_MOUSE]	13.74	2	6	6	26	0.587	0.781	0.878	0.901	85.40	13.74	11	26	546	59.7	9.22
Q7TSI3	Serine/threonine-protein phosphatase 6 regulatory subunit 1 OS=Mus musculus GN=Ppp6r1 PE=1 SV=1 - [illegible]	22.90	1	12	13	42	1.521	1.193	1.309	0.901	111.77	22.90	21	42	856	94.5	4.64
Q9WTX2	Interferon-inducible double stranded RNA-dependent protein kinase activator A OS=Mus musculus GN=Prkra PE=1 SV=1 - [PRKRA_MOUSE]	31.95	1	9	9	38	0.925	0.605	0.707	0.901	99.61	31.95	18	38	313	34.3	8.43
P97449	Aminopeptidase N OS=Mus musculus GN=Anpep PE=1 SV=4 - [AMPN_MOUSE]	23.19	1	19	19	88	0.829	2.295	0.810	0.901	267.38	23.19	34	88	966	109.6	5.90
Q8VD29	Sodium/potassium/calcium exchanger 3 OS=Mus musculus GN=Slc24a3 PE=2 SV=1 - [Q8VD29_MOUSE]	2.69	2	2	2	3	0.525	0.819	0.693	0.901	6.77	2.69	2	3	595	66.0	4.88
Q9CZG9	PDZ domain-containing protein 11 OS=Mus musculus GN=Pdzd11 PE=1 SV=1 -	79.29	1	7	8	33	1.457	1.207	0.968	0.901	97.98	79.29	15	33	140	16.2	7.14
D3YUS5	Protein Rasal2 OS=Mus musculus GN=Rasal2 PE=2 SV=1 - [D3YUS5_MOUSE]	29.42	6	25	30	82	1.099	1.026	1.172	0.901	218.25	29.42	46	82	1261	141.9	7.80
Q9R0N3	Synaptotagmin-11 OS=Mus musculus GN=Syt11 PE=2 SV=2 - [SYT11_MOUSE]	30.00	2	10	10	39	0.916	0.523	0.850	0.901	114.07	30.00	18	39	430	48.3	9.11
Q9JJA2	Conserved oligomeric Golgi complex subunit 8 OS=Mus musculus GN=Cog8 PE=2 SV=3 - [COG8_MOUSE]	22.19	2	11	11	19	0.688	0.890	0.719	0.901	50.56	22.19	17	19	640	71.6	5.19
Q9WTK3	Glycosylphosphatidylinositol anchor attachment 1 protein OS=Mus musculus GN=Gpaa1 PE=1 SV=3 - [GPAA1_MOUSE]	9.18	4	4	4	9	0.251	0.519	0.785	0.901	27.37	9.18	6	9	621	67.9	8.40
Q8BUB4	WD repeat and FYVE domain-containing protein 2 OS=Mus musculus GN=Wdfy2 PE=2 SV=2 - [WDFY2_MOUSE]	5.25	1	2	2	3	0.929	0.821	0.743	0.901	10.09	5.25	2	3	400	45.1	6.81
Q6PDH0	Pleckstrin homology-like domain family B member 1 OS=Mus musculus GN=Phldb1 PE=1 SV=1 - [PHLB1_MOUSE]	29.03	14	31	31	88	1.056	0.616	1.017	0.901	241.98	29.03	53	88	1371	150.0	8.87
Q68FE6	Protein FAM65A OS=Mus musculus GN=Fam65a PE=1 SV=2 - [FA65A_MOUSE]	14.80	1	13	13	28	0.854	0.968	0.904	0.901	91.79	14.80	18	28	1223	132.3	5.88
Q99J77	N-acetylneuraminic acid synthase (Sialic acid synthase) OS=Mus musculus GN=Nans PE=2 SV=1 - [Q99J77_MOUSE]	44.01	1	15	15	56	1.029	0.904	0.838	0.901	155.26	44.01	25	56	359	40.0	7.06
Q9D517	1-acyl-sn-glycerol-3-phosphate acyltransferase gamma OS=Mus musculus GN=Agpat3 PE=1 SV=2 - [PLCC_MOUSE]	32.18	1	14	14	48	0.659	0.761	0.821	0.902	133.34	32.18	24	48	376	43.3	8.51
D3YVR9	MCG7316, isoform CRA_b OS=Mus musculus GN=1110004F10Rik PE=4 SV=1 - [D3YVR9_MOUSE]	17.52	3	3	3	28	1.376	0.983	1.097	0.902	114.74	17.52	6	28	137	15.2	4.65
A2A547	60S ribosomal protein L19 OS=Mus musculus GN=Rpl19 PE=2 SV=1 - [A2A547_MOUSE]	36.60	2	8	10	82	0.652	0.647	0.773	0.902	166.36	36.60	18	82	194	23.2	11.47
Q6P5F6	Zinc transporter ZIP10 OS=Mus musculus GN=Slc39a10 PE=1 SV=1 - [S39AA_MOUSE]	15.61	2	11	11	45	0.713	1.112	1.098	0.902	119.25	15.61	18	45	833	94.3	6.71
Q61249	Immunoglobulin-binding protein 1 OS=Mus musculus GN=Igbp1 PE=1 SV=1 - [IGBP1_MOUSE]	27.06	1	10	10	41	1.309	0.999	1.040	0.902	102.40	27.06	18	41	340	38.9	6.18
O35593	26S proteasome non-ATPase regulatory subunit 14 OS=Mus musculus GN=Psmd14 PE=1 SV=2 - [PSDE_MOUSE]	56.45	1	13	13	59	0.692	0.926	0.816	0.902	170.88	56.45	22	59	310	34.6	6.52
A2A6Q8	Myosin light chain 4 (Fragment) OS=Mus musculus GN=Myl4 PE=2 SV=1 - [A2A6Q8_MOUSE]	36.46	3	6	6	14	0.966	1.042	1.732	0.902	29.65	36.46	9	14	192	21.1	5.03

Q8BTI7	Serine/threonine-protein phosphatase 6 regulatory ankyrin repeat subunit C OS=Mus musculus GN=Ankrd52 PE=2 SV=1 - [ANR52_MOUSE]	2.70	2	2	3	5	1.354	0.810	0.707	0.902	15.40	2.70	5	5	1076	115.0	6.54
D3YXK0	MCG4624, isoform CRA_c OS=Mus musculus GN=Jakmip2 PE=4 SV=2 - [D3YXK0_MOUSE]	23.78	1	14	16	43	1.031	0.881	1.236	0.902	118.86	23.78	26	43	820	96.3	5.97
B2RY58	Hyperpolarization-activated, cyclic nucleotide-gated K+ 4 OS=Mus musculus GN=Hcn4 PE=2 SV=1 - [B2RY58_MOUSE]	20.90	2	10	18	78	1.237	0.786	0.926	0.902	233.19	20.90	33	78	1201	129.0	9.04
Q8CI08	SLAIN motif-containing protein 2 OS=Mus musculus GN=Slain2 PE=1 SV=2 - [SLAI2_MOUSE]	16.70	2	7	7	17	1.658	0.962	1.262	0.902	49.74	16.70	10	17	581	62.3	9.47
P42337	Phosphatidylinositol 4,5-bisphosphate 3-kinase catalytic subunit alpha isoform OS=Mus musculus GN=Pik3ca PE=1 SV=2 - [PK3CA_MOUSE]	8.33	4	8	8	16	0.773	0.769	0.849	0.902	48.76	8.33	13	16	1068	124.3	7.15
Q8BU88	39S ribosomal protein L22, mitochondrial OS=Mus musculus GN=Mrpl22 PE=2 SV=1 - [RM22_MOUSE]	24.76	1	5	5	14	0.724	0.694	0.794	0.902	38.90	24.76	8	14	206	23.8	9.86
Q922V4	Pleiotropic regulator 1 OS=Mus musculus GN=Plrg1 PE=2 SV=1 - [PLRG1_MOUSE]	28.27	3	9	9	49	1.304	0.942	0.935	0.902	151.80	28.27	17	49	513	56.9	9.17
G5E8S8	MAGUK p55 subfamily member 7 OS=Mus musculus GN=Mpp7 PE=4 SV=1 - [G5E8S8_MOUSE]	11.98	4	7	8	11	1.212	0.803	1.091	0.902	23.87	11.98	10	11	576	65.5	7.30
O70591	Prefoldin subunit 2 OS=Mus musculus GN=Pfdn2 PE=2 SV=2 - [PFD2_MOUSE]	57.14	2	9	9	57	1.526	0.946	1.154	0.902	157.13	57.14	16	57	154	16.5	6.58
Q9JLV2-2	Isoform 2 of Short transient receptor potential channel 4-associated protein OS=Mus musculus GN=Trpc4ap - [TP4AP_MOUSE]	1.77	2	1	1	2	0.815	0.824	0.775	0.902	5.04	1.77	2	2	789	89.9	7.59
Q8CDU6	Probable E3 ubiquitin-protein ligase HECTD2 OS=Mus musculus GN=Hectd2 PE=2 SV=2 - [HECD2_MOUSE]	3.49	3	2	2	3	1.715	1.265	1.121	0.902	8.99	3.49	3	3	774	87.7	7.88
Q99ME2	WD repeat-containing protein 6 OS=Mus musculus GN=Wdr6 PE=2 SV=1 - [WDR6_MOUSE]	10.40	1	10	10	29	1.196	1.433	0.698	0.902	78.99	10.40	16	29	1125	121.8	6.98
Q9CX97	WD repeat-containing protein 55 OS=Mus musculus GN=Wdr55 PE=2 SV=2 -	4.64	1	2	2	3	1.714	0.928	0.794	0.902	7.53	4.64	3	3	388	42.6	4.89
Q3UFK1	Coiled-coil domain containing 28A OS=Mus musculus GN=Ccdc28a PE=2 SV=1 -	69.57	5	8	8	24	0.681	0.568	1.606	0.902	87.18	69.57	13	24	184	20.3	8.12
Q9CQT5	Proteasome maturation protein OS=Mus musculus GN=Pomp PE=2 SV=1 - [POMP_MOUSE]	25.53	1	3	3	4	1.617	1.273	0.954	0.902	15.88	25.53	4	4	141	15.8	5.49
Q9D6I9	Leucine rich adaptor protein 1 OS=Mus musculus GN=Lurap1 PE=2 SV=1 -	20.92	1	4	4	7	1.186	1.052	1.162	0.902	19.23	20.92	6	7	239	25.8	4.72
O35969	Guanidinoacetate N-methyltransferase OS=Mus musculus GN=Gamt PE=1 SV=1 - [GAMT_MOUSE]	22.88	2	4	4	12	0.904	0.411	0.650	0.902	49.62	22.88	8	12	236	26.3	5.71
Q8K4R4-2	Isoform 2 of Cytoplasmic phosphatidylinositol transfer protein 1 OS=Mus musculus GN=Pitpnc1 - [PITC1_MOUSE]	30.22	3	1	10	30	0.625	0.610	0.790	0.902	80.73	30.22	17	30	268	31.8	5.64
Q91WN1	DnaJ homolog subfamily C member 9 OS=Mus musculus GN=Dnajc9 PE=2 SV=2 -	28.96	1	6	7	16	1.266	1.045	1.278	0.903	39.44	28.96	10	16	259	30.0	5.94
P56818	Beta-secretase 1 OS=Mus musculus GN=Bace1 PE=1 SV=2 - [BACE1_MOUSE]	10.78	3	4	4	10	0.564	0.972	0.820	0.903	25.21	10.78	8	10	501	55.7	5.60
B0V2M3	Zinc finger protein 318 OS=Mus musculus GN=Zfp318 PE=2 SV=1 - [B0V2M3_MOUSE]	1.79	3	2	4	8	0.854	0.759	1.179	0.903	22.34	1.79	4	8	2237	246.2	7.01
Q8K0H5	Transcription initiation factor TFIID subunit 10 OS=Mus musculus GN=Taf10 PE=1 SV=1 - [TAF10_MOUSE]	11.47	1	1	1	6	1.958	1.111	1.218	0.903	33.03	11.47	2	6	218	21.8	6.55
O35668	Huntingtin-associated protein 1 OS=Mus musculus GN=Hap1 PE=1 SV=1 - [HAP1_MOUSE]	50.64	5	27	27	136	1.104	1.120	0.619	0.903	395.72	50.64	46	136	628	70.1	4.74
P56376	Acylphosphatase-1 OS=Mus musculus GN=Acyp1 PE=2 SV=2 - [ACYP1_MOUSE]	73.74	2	8	9	49	2.431	0.879	1.200	0.903	139.70	73.74	18	49	99	11.2	9.04

G5E924	Heterogeneous nuclear ribonucleoprotein L (Fragment) OS=Mus musculus GN=Hnrnpl PE=4 SV=1 - [illegible]	56.75	5	23	23	282	0.925	0.950	0.941	0.903	809.60	56.75	42	282	615	66.9	8.18
Q0VBL6	Hypoxia-inducible factor 3-alpha OS=Mus musculus GN=Hif3a PE=1 SV=2 - [HIF3A_MOUSE]	1.81	2	1	2	2	0.439	0.805	0.752	0.903	4.37	1.81	2	2	662	73.0	8.34
Q80XH2-2	Isoform 2 of Interphotoreceptor matrix proteoglycan 2 OS=Mus musculus GN=Impg2 - [IMPG2_MOUSE]	1.23	2	1	1	1	2.055	1.112	1.177	0.903	2.37	1.23	1	1	1134	126.6	4.78
Q99PI5	Phosphatidate phosphatase LPIN2 OS=Mus musculus GN=Lpin2 PE=1 SV=2 -	6.38	4	4	6	11	1.110	1.269	1.167	0.903	27.51	6.38	10	11	893	99.6	5.47
Q9ES52-2	Isoform 2 of Phosphatidylinositol 3,4,5-trisphosphate 5-phosphatase 1 OS=Mus musculus GN=Inpp5d - [illegible]	3.03	8	3	4	11	1.067	1.127	0.964	0.903	24.26	3.03	7	11	1190	133.4	7.80
Q9D9Z5	DET1- and DDB1-associated protein 1 OS=Mus musculus GN=Dda1 PE=2 SV=1 -	26.47	2	3	3	8	1.418	1.010	1.115	0.903	21.36	26.47	5	8	102	11.7	8.68
P63005	Platelet-activating factor acetylhydrolase IB subunit alpha OS=Mus musculus GN=Pafah1b1 PE=1 SV=2 [LIS1_MOUSE]	62.68	3	23	24	138	1.678	0.861	0.857	0.903	450.16	62.68	40	138	410	46.6	7.37
Q8VHK9	Probable ATP-dependent RNA helicase DHX36 OS=Mus musculus GN=Dhx36 PE=2 SV=2 - [DHX36_MOUSE]	19.58	1	14	16	39	1.191	0.792	0.821	0.903	111.39	19.58	25	39	1001	113.8	8.29
P55302	Alpha-2-macroglobulin receptor-associated protein OS=Mus musculus GN=Lrpap1 PE=1 SV=1 - [AMRP_MOUSE]	47.78	3	20	21	103	1.818	1.156	1.264	0.903	305.49	47.78	35	103	360	42.2	7.87
E9PVA8	Protein Gcn1l1 OS=Mus musculus GN=Gcn1l1 PE=2 SV=1 - [E9PVA8_MOUSE]	33.99	2	78	80	216	0.681	0.825	0.690	0.903	623.43	33.99	133	216	2671	292.8	7.36
Q9CZR2	N-acetylated-alpha-linked acidic dipeptidase 2 OS=Mus musculus GN=Naalad2 PE=1 SV=2 - [NALD2_MOUSE]	4.46	2	3	3	11	1.034	0.895	0.738	0.903	20.69	4.46	6	11	740	82.7	8.37
F7BI35	Synaptojanin-2 (Fragment) OS=Mus musculus GN=Synj2 PE=2 SV=1 - [F7BI35_MOUSE]	10.07	15	5	6	17	1.069	0.453	0.920	0.903	46.18	10.07	8	17	725	80.7	8.62
Q05921	2-5A-dependent ribonuclease OS=Mus musculus GN=Rnasel PE=2 SV=2 -	3.81	1	3	3	4	1.996	1.337	0.986	0.903	6.41	3.81	4	4	735	83.2	6.55
Q8BGV7	BTB/POZ domain-containing adapter for CUL3-mediated RhoA degradation protein 1 OS=Mus musculus GN=Kctd13 PE=2 SV=1 - [BACD1_MOUSE]	13.07	2	3	4	9	1.030	1.098	1.033	0.904	27.41	13.07	7	9	329	36.4	6.99
Q3TCN2	Putative phospholipase B-like 2 OS=Mus musculus GN=Plbd2 PE=1 SV=2 - [PLBL2_MOUSE]	11.95	2	5	5	24	2.513	0.833	0.873	0.904	87.00	11.95	9	24	594	66.2	6.13
Q9JM96	Cdc42 effector protein 4 OS=Mus musculus GN=Cdc42ep4 PE=1 SV=1 - [BORG4_MOUSE]	41.26	3	10	10	45	1.520	0.867	1.376	0.904	164.09	41.26	18	45	349	37.8	5.36
J3QP68	Uncharacterized protein OS=Mus musculus GN=Gm4204 PE=3 SV=1 - [J3QP68_MOUSE]	33.62	3	9	9	53	1.158	1.024	1.361	0.904	169.08	33.62	16	53	354	41.1	4.55
G5E8G3	MCG9827 OS=Mus musculus GN=Opcml PE=4 SV=1 - [G5E8G3_MOUSE]	42.03	1	1	15	116	0.501	0.691	1.067	0.904	349.66	42.03	28	116	345	38.0	6.87
Q3TN34	JRAB OS=Mus musculus GN=Micall2 PE=1 SV=1 - [Q3TN34_MOUSE]	13.28	2	8	10	24	1.561	0.640	0.925	0.904	69.75	13.28	18	24	1009	108.2	9.55
Q80U93	Nuclear pore complex protein Nup214 OS=Mus musculus GN=Nup214 PE=1 SV=2 - [NU214_MOUSE]	16.12	2	25	25	62	1.263	1.014	1.017	0.904	177.35	16.12	40	62	2085	212.8	7.08
Q924S7	Sprouty-related, EVH1 domain-containing protein 2 OS=Mus musculus GN=Spred2 PE=1 SV=1 - [SPRE2_MOUSE]	16.59	3	4	5	11	1.603	1.176	1.061	0.904	36.95	16.59	8	11	410	46.8	6.81
Q8C0C0	Zinc fingers and homeoboxes protein 2 OS=Mus musculus GN=Zhx2 PE=1 SV=1 - [ZHX2_MOUSE]	5.14	1	4	4	7	1.281	0.868	0.888	0.904	18.73	5.14	6	7	836	92.2	7.46
Q8BKX1-4	Isoform 4 of Brain-specific angiogenesis inhibitor 1-associated protein 2 OS=Mus musculus GN=Baiap2 - [illegible]	73.24	1	1	30	223	0.729	2.384	2.967	0.904	713.04	73.24	51	223	482	53.2	8.98
Q3UYK3	TBC1 domain family member 9 OS=Mus musculus GN=Tbc1d9 PE=2 SV=2 -	3.09	2	2	4	9	0.676	0.766	0.769	0.904	22.22	3.09	6	9	1264	142.9	5.34

P70257-1	Isoform NFIX1 of Nuclear factor 1 X-type OS=Mus musculus GN=Nfix - [NFIX_MOUSE]	15.87	6	3	4	10	1.023	1.381	0.853	0.904	35.91	15.87	7	10	441	48.8	8.47
O70342	Neuropeptide Y receptor type 5 OS=Mus musculus GN=Npy5r PE=2 SV=2 - [NPY5R_MOUSE]	2.15	1	1	1	1	0.412	1.257	1.647	0.904	0.00	2.15	1	1	466	52.8	8.94
Q6P1H4	Exostoses (Multiple)-like 3 OS=Mus musculus GN=Extl3 PE=2 SV=1 - [Q6P1H4_MOUSE]	6.31	2	5	6	10	0.553	0.943	0.893	0.904	25.44	6.31	10	10	919	104.5	6.57
Q6PD21	SH2 domain-containing adapter protein B OS=Mus musculus GN=Shb PE=1 SV=2 - [SHB_MOUSE]	13.72	4	5	5	5	1.229	0.853	1.257	0.905	14.09	13.72	5	5	503	54.7	8.78
Q7M6Z0	Reticulon-4 receptor-like 2 OS=Mus musculus GN=Rtn4rl2 PE=2 SV=1 - [R4RL2_MOUSE]	17.14	2	7	7	32	0.955	2.127	1.059	0.905	73.72	17.14	14	32	420	46.0	7.65
Q8BWM0	Prostaglandin E synthase 2 OS=Mus musculus GN=Ptges2 PE=1 SV=3 - [PGES2_MOUSE]	57.81	1	15	15	51	0.748	0.704	0.777	0.905	144.04	57.81	27	51	384	43.3	9.00
Q8VD63	Testis-specific Y-encoded-like protein 4 OS=Mus musculus GN=Tspyl4 PE=1 SV=1 - [TSYL4_MOUSE]	23.89	1	6	7	25	1.372	0.889	0.901	0.905	73.41	23.89	12	25	406	44.8	6.99
Q8C985-2	Isoform 2b of Neurexin-3-beta OS=Mus musculus GN=Nrxn3 - [NRX3B_MOUSE]	29.26	3	2	14	40	1.271	1.518	0.981	0.905	120.91	29.26	26	40	564	62.0	9.31
Q8R5K2-2	Isoform 2 of Ubiquitin carboxyl-terminal hydrolase 33 OS=Mus musculus GN=Usp33 - [UBP33_MOUSE]	6.88	3	4	5	7	1.342	0.913	0.934	0.905	15.18	6.88	6	7	901	101.8	5.68
P23591	GDP-L-fucose synthase OS=Mus musculus GN=Tsta3 PE=2 SV=3 - [FCL_MOUSE]	21.18	1	5	5	17	1.114	0.853	0.891	0.905	59.66	21.18	9	17	321	35.9	6.74
Q9CQP3	Coiled-coil-helix-coiled-coil-helix domain-containing protein 5 OS=Mus musculus GN=Chchd5 PE=2 SV=1 - [illegible]	31.82	1	3	3	7	1.227	0.835	0.893	0.905	21.43	31.82	5	7	110	12.3	7.31
Q640M6	Glycerophosphodiester phosphodiesterase domain-containing protein 5 OS=Mus musculus GN=Gdpd5 PE=2 SV=1 - [GDPD5_MOUSE]	10.38	1	5	5	11	0.329	0.814	1.244	0.905	29.77	10.38	10	11	607	68.8	8.00
Q68ED7	CREB-regulated transcription coactivator 1 OS=Mus musculus GN=Crtc1 PE=2 SV=1 - [CRTC1_MOUSE]	20.63	1	9	9	48	1.366	1.378	1.701	0.905	158.28	20.63	17	48	630	66.9	6.05
Q8JZX4	Splicing factor 45 OS=Mus musculus GN=Rbm17 PE=1 SV=1 - [SPF45_MOUSE]	42.22	3	15	16	36	1.345	0.799	1.020	0.905	93.85	42.22	24	36	405	45.3	5.82
Q99J62	Replication factor C subunit 4 OS=Mus musculus GN=Rfc4 PE=1 SV=1 - [RFC4_MOUSE]	15.66	4	6	6	9	1.145	1.326	0.892	0.905	20.42	15.66	9	9	364	39.8	6.70
E9PXC2	Upstream stimulatory factor 2 OS=Mus musculus GN=Usf2 PE=2 SV=1 - [E9PXC2_MOUSE]	13.95	7	2	3	11	1.728	1.660	1.194	0.905	30.69	13.95	5	11	215	24.0	5.86
Q61687	Transcriptional regulator ATRX OS=Mus musculus GN=Atrx PE=1 SV=3 - [ATRX_MOUSE]	6.34	8	12	12	25	0.748	1.285	1.325	0.905	74.31	6.34	16	25	2476	278.4	6.68
Q9D842-2	Isoform 2 of Aprataxin and PNK-like factor OS=Mus musculus GN=Aplf - [APLF_MOUSE]	7.42	3	2	2	4	16.210	1.553	1.156	0.905	12.39	7.42	2	4	391	43.0	5.07
E0CYF6	Protein AW554918 OS=Mus musculus GN=AW554918 PE=4 SV=1 - [E0CYF6_MOUSE]	13.75	1	1	1	2	0.674	6.100	0.785	0.905	5.16	13.75	1	2	80	8.4	6.70
Q9Z1F9	SUMO-activating enzyme subunit 2 OS=Mus musculus GN=Uba2 PE=2 SV=1 - [SAE2_MOUSE]	47.18	5	23	23	79	0.702	0.970	0.807	0.905	198.76	47.18	37	79	638	70.5	5.24
E9Q0F0	Protein Krt78 OS=Mus musculus GN=Krt78 PE=2 SV=1 - [E9Q0F0_MOUSE]	3.37	2	2	4	19	2.818	1.625	1.500	0.905	44.23	3.37	5	19	1068	112.2	7.97
Q7TN29	Stromal membrane-associated protein 2 OS=Mus musculus GN=Smap2 PE=1 SV=1 - [SMAP2_MOUSE]	32.48	2	11	12	42	1.299	0.883	1.182	0.905	143.61	32.48	20	42	428	46.5	8.87
E9Q9Q6	Protein Cdh18 OS=Mus musculus GN=Cdh18 PE=2 SV=1 - [E9Q9Q6_MOUSE]	16.58	4	9	9	24	0.939	0.857	0.883	0.905	52.29	16.58	15	24	790	87.9	5.16
P02104	Hemoglobin subunit epsilon-Y2 OS=Mus musculus GN=Hbb-y PE=1 SV=2 - [HBE_MOUSE]	17.01	4	1	3	30	2.475	2.348	0.433	0.905	73.97	17.01	6	30	147	16.1	8.18
Q9EPV8	Ubiquitin-like protein 5 OS=Mus musculus GN=Ubl5 PE=1 SV=1 - [UBL5_MOUSE]	79.45	7	6	6	15	1.574	0.705	0.844	0.905	41.00	79.45	9	15	73	8.5	8.44

Q8C2Q3	RNA-binding protein 14 OS=Mus musculus GN=Rbm14 PE=1 SV=1 - [RBM14_MOUSE]	37.52	6	18	22	121	1.227	0.719	1.009	0.905	368.20	37.52	40	121	669	69.4	9.67
E0CZD9	Zinc finger protein-like 1 (Fragment) OS=Mus musculus GN=Zfpl1 PE=2 SV=1 - [E0CZD9_MOUSE]	9.92	7	2	2	8	0.659	0.958	0.843	0.905	25.75	9.92	4	8	242	26.8	7.11
Q8JZL3	Thiamine-triphosphatase OS=Mus musculus GN=Thtpa PE=1 SV=3 - [THTPA_MOUSE]	25.89	1	5	6	15	1.738	0.878	0.912	0.905	52.79	25.89	8	15	224	24.2	4.72
P50544	Very long-chain specific acyl-CoA dehydrogenase, mitochondrial OS=Mus musculus GN=Acadvl PE=1 SV=3 - [illegible]	57.77	2	31	31	133	1.106	0.900	0.716	0.906	395.68	57.77	57	133	656	70.8	8.75
Q9D0R8	Protein LSM12 homolog OS=Mus musculus GN=Lsm12 PE=1 SV=1 - [LSM12_MOUSE]	22.56	1	4	4	17	1.375	0.901	1.060	0.906	50.62	22.56	7	17	195	21.7	7.74
Q9D287	Pre-mRNA-splicing factor SPF27 OS=Mus musculus GN=Bcas2 PE=2 SV=1 - [SPF27_MOUSE]	29.33	1	6	6	17	1.235	0.984	0.934	0.906	52.22	29.33	10	17	225	26.1	5.66
O70340	Neuronal pentraxin-2 OS=Mus musculus GN=Nptx2 PE=2 SV=1 - [NPTX2_MOUSE]	29.14	1	10	11	39	0.921	1.270	0.888	0.906	105.19	29.14	17	39	429	47.1	5.81
Q8VE80	THO complex subunit 3 OS=Mus musculus GN=Thoc3 PE=2 SV=1 - [THOC3_MOUSE]	21.94	1	7	7	13	1.447	1.065	1.053	0.906	37.53	21.94	11	13	351	38.7	6.09
P60762-2	Isoform 2 of Mortality factor 4-like protein 1 OS=Mus musculus GN=Morf4l1 -	11.15	2	4	4	7	0.725	0.607	0.826	0.906	16.56	11.15	6	7	323	37.2	9.19
Q8BWS5	G protein-regulated inducer of neurite outgrowth 3 OS=Mus musculus GN=Gprin3 PE=1 SV=1 - [illegible]	48.10	1	25	26	91	1.026	0.659	1.628	0.906	259.30	48.10	40	91	763	80.4	7.02
Q0PMG2	MAM domain-containing glycosylphosphatidylinositol anchor protein 1 OS=Mus musculus GN=Mdga1 PE=2 SV=2 - [MDGA1_MOUSE]	15.74	6	11	11	31	0.738	0.814	0.723	0.906	81.68	15.74	20	31	940	104.1	8.31
Q8BJW6	Eukaryotic translation initiation factor 2A OS=Mus musculus GN=Eif2a PE=2 SV=2 - [EIF2A_MOUSE]	31.84	5	14	14	36	0.980	0.574	0.878	0.906	83.57	31.84	23	36	581	64.4	8.91
Q9CQV6	Microtubule-associated proteins 1A/1B light chain 3B OS=Mus musculus GN=Map1lc3b PE=1 SV=3 - [MLP3B_MOUSE]	22.40	3	1	3	37	2.889	1.125	1.132	0.906	83.38	22.40	6	37	125	14.6	8.43
Q8BU30	Isoleucine--tRNA ligase, cytoplasmic OS=Mus musculus GN=Iars PE=2 SV=2 - [SYIC_MOUSE]	29.64	3	32	32	82	0.614	0.785	0.776	0.906	215.37	29.64	51	82	1262	144.2	6.55
Q99LX5	Multiple myeloma tumor-associated protein 2 homolog OS=Mus musculus GN=Mmtag2 PE=2 SV=1 - [illegible]	5.38	1	1	1	3	0.865	0.841	0.759	0.906	8.99	5.38	2	3	260	29.3	9.86
P58501	PAX3- and PAX7-binding protein 1 OS=Mus musculus GN=Paxbp1 PE=1 SV=3 -	2.29	3	3	3	11	0.560	0.598	0.864	0.906	25.96	2.29	5	11	919	104.8	5.67
D3Z131	THO complex subunit 6 homolog OS=Mus musculus GN=Thoc6 PE=2 SV=1 - [D3Z131_MOUSE]	7.09	4	2	2	6	1.128	1.111	0.772	0.906	18.32	7.09	4	6	296	32.7	7.11
F6YRI7	Calcium/calmodulin-dependent protein kinase type 1G (Fragment) OS=Mus musculus GN=Camk1g PE=4 SV=1 - [illegible]	6.75	3	1	1	2	1.606	1.000	0.995	0.906	2.86	6.75	2	2	252	29.0	5.54
Q9DCR2	AP-3 complex subunit sigma-1 OS=Mus musculus GN=Ap3s1 PE=1 SV=2 - [AP3S1_MOUSE]	26.42	1	4	5	24	0.639	1.063	0.847	0.906	63.02	26.42	9	24	193	21.7	5.39
E0CYB8	Probable palmitoyltransferase ZDHHC24 OS=Mus musculus GN=Zdhhc24 PE=2 SV=1 - [illegible]	6.67	2	1	1	1	0.736	1.015	1.054	0.906	0.00	6.67	1	1	105	10.8	10.24
Q9DAJ5-2	Isoform 2 of Dynein light chain roadblock-type 2 OS=Mus musculus GN=Dynlrb2 -	18.82	2	1	1	2	0.668	0.599	0.466	0.906	5.92	18.82	2	2	85	9.6	8.56
Q8BWC6	Nebulette (Fragment) OS=Mus musculus GN=Nebl PE=2 SV=2 - [Q8BWC6_MOUSE]	3.35	3	1	1	1	1.082	1.556	1.683	0.906	0.00	3.35	1	1	448	51.7	6.86
P49025-3	Isoform 3 of Citron Rho-interacting kinase OS=Mus musculus GN=Cit - [CTRO_MOUSE]	44.08	10	73	74	258	1.064	0.677	1.115	0.906	741.11	44.08	128	258	1597	183.3	6.61
Q8VI75	Importin-4 OS=Mus musculus GN=Ipo4 PE=1 SV=1 - [IPO4_MOUSE]	22.92	3	19	19	64	0.531	0.822	0.629	0.906	172.10	22.92	36	64	1082	119.2	5.03

Q5U4C3	Splicing factor, arginine/serine-rich 19 OS=Mus musculus GN=Scaf1 PE=1 SV=1 -	9.08	1	7	7	17	1.078	1.197	0.928	0.906	41.50	9.08	10	17	1256	133.8	9.45
J3QQ00	Homer protein homolog 3 (Fragment) OS=Mus musculus GN=Homer3 PE=4 SV=1 - [J3QQ00_MOUSE]	60.22	1	1	7	18	1.062	0.643	0.914	0.906	39.79	60.22	13	18	93	10.7	4.98
Q5RL57	A kinase (PRKA) anchor protein 8-like OS=Mus musculus GN=Akap8l PE=2 SV=1 -	6.55	2	4	4	8	0.583	1.022	1.114	0.907	29.51	6.55	6	8	641	71.3	5.06
Q61137-2	Isoform 2 of Astrotactin-1 OS=Mus musculus GN=Astn1 - [ASTN1_MOUSE]	24.81	3	23	23	89	0.765	1.220	1.139	0.907	307.13	24.81	39	89	1294	143.9	5.15
P58871	182 kDa tankyrase-1-binding protein OS=Mus musculus GN=Tnks1bp1 PE=1 SV=2 - [TB182_MOUSE]	39.19	1	49	49	184	1.356	1.340	1.538	0.907	536.53	39.19	85	184	1720	181.7	4.88
G3UZX2	Ubiquitin-conjugating enzyme E2 G2 (Fragment) OS=Mus musculus GN=Ube2g2 PE=2 SV=1 - [G3UZX2_MOUSE]	52.11	2	3	3	6	0.747	1.274	0.832	0.907	19.49	52.11	5	6	71	7.8	4.50
Q8BQY8	Checkpoint protein HUS1 OS=Mus musculus GN=Hus1 PE=2 SV=1 - [HUS1_MOUSE]	4.64	3	1	1	1	1.519	1.489	0.953	0.907	2.43	4.64	1	1	280	31.7	7.37
Q8K2A4	MCG140700, isoform CRA_c OS=Mus musculus GN=Zfp87 PE=2 SV=1 - [Q8K2A4_MOUSE]	4.81	145	1	2	2	0.649	0.799	0.779	0.907	4.27	4.81	2	2	540	63.0	8.53
Q9Z275	Retinaldehyde-binding protein 1 OS=Mus musculus GN=Rlbp1 PE=2 SV=3 - [RLBP1_MOUSE]	35.02	1	9	9	24	1.165	1.616	1.459	0.907	76.83	35.02	17	24	317	36.4	5.06
Q8R0N6	Hydroxyacid-oxoacid transhydrogenase, mitochondrial OS=Mus musculus GN=Adhfe1 PE=2 SV=2 - [illegible]	25.81	2	8	8	29	1.812	0.984	0.638	0.907	93.10	25.81	14	29	465	49.9	7.58
Q64010	Adapter molecule crk OS=Mus musculus GN=Crk PE=1 SV=1 - [CRK_MOUSE]	63.16	6	17	17	93	2.082	1.256	1.037	0.907	282.39	63.16	32	93	304	33.8	5.55
D3Z6X7	Transmembrane 9 superfamily member 1 OS=Mus musculus GN=Tm9sf1 PE=2 SV=1 - [D3Z6X7_MOUSE]	2.72	6	2	2	3	1.170	0.721	1.060	0.907	6.29	2.72	3	3	589	66.9	6.98
O70378	ER membrane protein complex subunit 8 OS=Mus musculus GN=Emc8 PE=1 SV=1 - [EMC8_MOUSE]	64.25	7	11	11	55	0.983	0.855	0.792	0.907	160.09	64.25	21	55	207	23.3	6.15
B2RXR6	Serine/threonine-protein phosphatase 6 regulatory ankyrin repeat subunit B OS=Mus musculus GN=Ankrd44 PE=2 SV=1 - [ANR44_MOUSE]	4.53	4	3	4	8	1.072	0.802	0.713	0.907	27.82	4.53	6	8	993	107.3	6.42
P43247	DNA mismatch repair protein Msh2 OS=Mus musculus GN=Msh2 PE=2 SV=1 - [MSH2_MOUSE]	13.37	3	11	12	26	0.597	0.842	0.734	0.907	75.16	13.37	19	26	935	104.1	5.96
Q9R1P0	Proteasome subunit alpha type-4 OS=Mus musculus GN=Psma4 PE=1 SV=1 - [PSA4_MOUSE]	41.76	3	10	11	56	1.535	0.913	0.860	0.907	167.79	41.76	20	56	261	29.5	7.72
Q9D0Q7	39S ribosomal protein L45, mitochondrial OS=Mus musculus GN=Mrpl45 PE=2 SV=1 - [RM45_MOUSE]	26.47	2	11	11	25	0.711	0.670	0.832	0.907	67.53	26.47	18	25	306	35.4	9.23
Q9WVQ5	Methylthioribulose-1-phosphate dehydratase OS=Mus musculus GN=Apip PE=1 SV=1 - [MTNB_MOUSE]	58.09	1	9	9	31	1.489	0.781	0.868	0.907	97.61	58.09	14	31	241	26.9	6.90
E9PVU9	Bis(5'-adenosyl)-triphosphatase (Fragment) OS=Mus musculus GN=Fhit PE=2 SV=1 - [E9PVU9_MOUSE]	39.52	5	6	6	27	1.894	1.092	0.910	0.907	90.78	39.52	9	27	124	14.2	7.68
P51141	Segment polarity protein dishevelled homolog DVL-1 OS=Mus musculus GN=Dvl1 PE=1 SV=2 - [DVL1_MOUSE]	17.41	1	6	8	15	0.972	0.761	0.952	0.907	39.83	17.41	13	15	695	75.3	7.02
Q9R1E6	Ectonucleotide pyrophosphatase/phosphodiesterase family member 2 OS=Mus musculus GN=Enpp2 PE=1 SV=3 - [ENPP2_MOUSE]	17.05	4	11	12	26	2.248	0.490	0.714	0.907	87.65	17.05	19	26	862	98.8	7.27
Q9QZ26	Potassium voltage-gated channel subfamily E member 1-like protein OS=Mus musculus GN=Kcne1l PE=2 SV=1 - [illegible]	5.59	1	1	1	4	1.193	0.717	0.649	0.907	15.56	5.59	2	4	143	15.0	5.41
F6UHR6	Oxysterol-binding protein OS=Mus musculus GN=Osbpl9 PE=2 SV=2 - [F6UHR6_MOUSE]	16.69	6	9	9	15	0.875	0.940	0.914	0.907	46.33	16.69	12	15	719	81.0	5.72

E9Q0M7	Protein Atp11b OS=Mus musculus GN=Atp11b PE=2 SV=1 - [E9Q0M7_MOUSE]	6.78	3	5	6	21	1.893	1.323	0.867	0.907	49.59	6.78	8	21	841	95.3	6.24
Q9Z160	Conserved oligomeric Golgi complex subunit 1 OS=Mus musculus GN=Cog1 PE=2 SV=3 - [COG1_MOUSE]	17.55	5	9	11	26	0.762	0.787	0.844	0.907	77.79	17.55	20	26	980	109.0	7.18
Q61771	Kinesin-like protein KIF3B OS=Mus musculus GN=Kif3b PE=1 SV=1 - [KIF3B_MOUSE]	26.91	2	13	19	77	0.747	0.753	1.380	0.907	232.78	26.91	33	77	747	85.2	7.69
Q8C0M9	Isoaspartyl peptidase/L-asparaginase OS=Mus musculus GN=Asrgl1 PE=1 SV=1 - [ASGL1_MOUSE]	62.27	1	16	16	176	1.743	0.828	0.915	0.907	512.18	62.27	31	176	326	33.9	7.65
Q80TA9	Ectopic P granules protein 5 homolog OS=Mus musculus GN=Epg5 PE=2 SV=2 - [EPG5_MOUSE]	6.88	1	13	13	29	1.457	1.096	0.963	0.908	65.30	6.88	20	29	2572	290.6	6.37
P61982	14-3-3 protein gamma OS=Mus musculus GN=Ywhag PE=1 SV=2 - [1433G_MOUSE]	72.87	2	15	22	764	1.515	1.120	0.882	0.908	2122.95	72.87	36	764	247	28.3	4.89
Q9CT10	Ran-binding protein 3 OS=Mus musculus GN=Ranbp3 PE=1 SV=2 - [RANB3_MOUSE]	27.29	1	11	11	41	1.257	0.814	1.012	0.908	114.43	27.29	18	41	491	52.5	5.12
P54754	Ephrin type-B receptor 3 OS=Mus musculus GN=Ephb3 PE=1 SV=2 - [EPHB3_MOUSE]	19.94	3	9	16	35	1.205	0.852	1.076	0.908	97.02	19.94	24	35	993	109.6	6.28
E9QAS4	Chromodomain-helicase-DNA-binding protein 4 OS=Mus musculus GN=Chd4 PE=2 SV=1 - [E9QAS4_MOUSE]	15.09	5	15	26	60	0.856	0.949	0.917	0.908	162.84	15.09	38	60	1902	216.2	5.92
Q5SW19	Clustered mitochondria protein homolog OS=Mus musculus GN=Cluh PE=2 SV=2 - [CLU_MOUSE]	20.38	3	22	23	78	0.840	0.726	0.804	0.908	204.29	20.38	38	78	1315	148.0	6.02
Q9CQG2	Methyltransferase-like protein 16 OS=Mus musculus GN=Mettl16 PE=1 SV=1 -	10.31	3	4	4	10	0.866	0.905	0.636	0.908	29.15	10.31	6	10	553	62.3	7.36
P49769	Presenilin-1 OS=Mus musculus GN=Psen1 PE=1 SV=1 - [PSN1_MOUSE]	13.92	2	4	5	12	0.908	0.934	0.893	0.908	37.73	13.92	8	12	467	52.6	5.38
Q9R0C8	Guanine nucleotide exchange factor VAV3 OS=Mus musculus GN=Vav3 PE=1 SV=2 -	8.15	5	4	5	10	0.672	0.524	0.823	0.908	18.03	8.15	9	10	847	97.9	7.06
Q3UBX0	Transmembrane protein 109 OS=Mus musculus GN=Tmem109 PE=1 SV=2 - [TM109_MOUSE]	13.99	8	5	5	16	0.592	1.450	0.929	0.908	42.63	13.99	8	16	243	26.3	9.89
Q922B2	Aspartate--tRNA ligase, cytoplasmic OS=Mus musculus GN=Dars PE=2 SV=2 - [SYDC_MOUSE]	55.49	2	26	26	135	0.697	0.753	0.785	0.908	321.46	55.49	46	135	501	57.1	6.49
Q9DC40	Telomere length regulation protein TEL2 homolog OS=Mus musculus GN=Telo2 PE=1 SV=2 - [TELO2_MOUSE]	3.33	1	3	3	5	0.795	1.078	0.850	0.908	11.11	3.33	5	5	840	93.3	5.25
O55057	Retinal rod rhodopsin-sensitive cGMP 3',5'-cyclic phosphodiesterase subunit delta OS=Mus musculus GN=Pde6d PE=1 SV=1 - [PDE6D_MOUSE]	28.67	2	3	3	22	1.715	0.984	0.722	0.908	84.62	28.67	5	22	150	17.3	5.67
Q9Z2A7	Diacylglycerol O-acyltransferase 1 OS=Mus musculus GN=Dgat1 PE=1 SV=1 - [DGAT1_MOUSE]	13.25	3	5	5	19	0.829	0.727	0.829	0.908	80.81	13.25	9	19	498	56.8	9.39
Q8BHJ5	F-box-like/WD repeat-containing protein TBL1XR1 OS=Mus musculus GN=Tbl1xr1 PE=2 SV=1 - [illegible]	16.73	1	3	6	13	2.002	1.211	0.955	0.908	41.38	16.73	9	13	514	55.6	5.63
Q0V8T7	Contactin-associated protein like 5-3 OS=Mus musculus GN=Cntnap5c PE=2 SV=1 -	4.29	1	2	5	14	1.051	1.451	1.015	0.908	39.74	4.29	9	14	1305	146.1	5.97
Q8BTE5	Protein 1110001A16Rik OS=Mus musculus GN=1110001A16Rik PE=4 SV=2 - [Q8BTE5_MOUSE]	36.25	1	3	3	7	1.308	1.507	1.054	0.908	15.48	36.25	6	7	80	9.3	9.45
Q9WTS2	Alpha-(1,6)-fucosyltransferase OS=Mus musculus GN=Fut8 PE=2 SV=2 -	18.96	1	11	11	25	0.926	0.850	0.773	0.908	55.37	18.96	16	25	575	66.5	7.52
Q8K3I4	Rab effector MyRIP OS=Mus musculus GN=Myrip PE=1 SV=1 - [MYRIP_MOUSE]	12.62	2	8	8	23	0.960	0.689	1.157	0.908	57.82	12.62	13	23	856	94.9	6.07
Q8K0C9	GDP-mannose 4,6 dehydratase OS=Mus musculus GN=Gmds PE=2 SV=1 - [GMDS_MOUSE]	38.17	2	14	14	36	1.360	1.037	0.812	0.908	93.89	38.17	22	36	372	42.0	7.03
Q9D0E1-2	Isoform 2 of Heterogeneous nuclear ribonucleoprotein M OS=Mus musculus GN=Hnrnpm - [illegible]	58.84	6	38	38	227	0.909	0.978	0.893	0.908	694.02	58.84	69	227	690	73.7	8.75

O55033	Cytoplasmic protein NCK2 OS=Mus musculus GN=Nck2 PE=1 SV=1 - [NCK2_MOUSE]	43.16	1	12	13	37	1.204	0.949	1.304	0.908	110.34	43.16	22	37	380	42.9	6.95
Q9EQ61	Pescadillo homolog OS=Mus musculus GN=Pes1 PE=1 SV=1 - [PESC_MOUSE]	3.60	2	2	2	3	0.488	0.614	0.622	0.909	5.43	3.60	3	3	584	67.8	6.84
Q6P5D4	Centrosomal protein of 135 kDa OS=Mus musculus GN=Cep135 PE=1 SV=1 -	10.00	1	8	12	32	1.328	0.934	1.633	0.909	69.57	10.00	16	32	1140	133.3	6.48
Q78ZA7	Nucleosome assembly protein 1-like 4 OS=Mus musculus GN=Nap1l4 PE=1 SV=1 -	32.27	1	11	11	60	1.428	0.764	0.984	0.909	208.12	32.27	19	60	375	42.7	4.67
P41241	Tyrosine-protein kinase CSK OS=Mus musculus GN=Csk PE=1 SV=2 - [CSK_MOUSE]	37.56	2	13	15	37	1.117	0.819	0.689	0.909	105.55	37.56	26	37	450	50.7	7.06
P06837	Neuromodulin OS=Mus musculus GN=Gap43 PE=1 SV=1 - [NEUM_MOUSE]	51.98	1	10	11	148	1.258	0.960	1.037	0.909	400.28	51.98	16	148	227	23.6	4.73
Q8R4U7	Leucine zipper protein 1 OS=Mus musculus GN=Luzp1 PE=1 SV=2 - [LUZP1_MOUSE]	47.19	2	43	43	128	1.551	0.426	1.185	0.909	400.71	47.19	68	128	1068	119.2	7.99
P70459	ETS domain-containing transcription factor ERF OS=Mus musculus GN=Erf PE=2 SV=1 - [ERF_MOUSE]	6.90	1	1	2	4	1.002	1.215	1.181	0.909	9.72	6.90	4	4	551	59.0	7.28
Q9D2D7-3	Isoform 3 of Zinc finger protein 687 OS=Mus musculus GN=Znf687 - [ZN687_MOUSE]	7.23	3	2	2	3	0.574	0.844	0.800	0.909	9.16	7.23	3	3	775	80.9	6.38
Q7TPM6	Fibronectin type III and SPRY domain-containing protein 1 OS=Mus musculus GN=Fsd1 PE=2 SV=1 - [FSD1_MOUSE]	42.74	2	19	19	86	0.847	0.678	0.743	0.909	274.27	42.74	34	86	496	55.5	6.67
Q8R1S4-2	Isoform 2 of Metastasis suppressor protein 1 OS=Mus musculus GN=Mtss1 -	15.91	7	9	11	35	2.295	0.764	1.000	0.909	74.57	15.91	16	35	723	78.8	7.15
Q5Y5T5	Probable palmitoyltransferase ZDHHC8 OS=Mus musculus GN=Zdhhc8 PE=1 SV=1 - [ZDHC8_MOUSE]	13.65	1	7	8	21	1.073	1.292	1.105	0.909	70.36	13.65	14	21	762	82.0	9.38
Q4LDF6	Protein Cfhr2 (Precursor) OS=Mus musculus GN=Cfhr2 PE=2 SV=1 - [Q4LDF6_MOUSE]	19.28	1	2	5	18	2.637	2.021	0.715	0.909	58.49	19.28	9	18	332	37.9	7.69
Q9DC71	28S ribosomal protein S15, mitochondrial OS=Mus musculus GN=Mrps15 PE=2 SV=2 - [RT15_MOUSE]	21.32	1	6	6	12	0.775	0.906	0.921	0.909	27.97	21.32	10	12	258	29.4	10.13
Q8BYC6	Serine/threonine-protein kinase TAO3 OS=Mus musculus GN=Taok3 PE=1 SV=2 - [TAOK3_MOUSE]	12.14	3	6	12	23	0.999	0.962	0.970	0.909	49.79	12.14	18	23	898	105.3	7.36
Q78PY7	Staphylococcal nuclease domain-containing protein 1 OS=Mus musculus GN=Snd1 PE=1 SV=1 - [SND1_MOUSE]	42.97	3	35	35	168	0.801	0.899	0.770	0.909	482.04	42.97	66	168	910	102.0	7.43
Q61592	Growth arrest-specific protein 6 OS=Mus musculus GN=Gas6 PE=2 SV=2 - [GAS6_MOUSE]	1.93	1	1	1	2	2.639	1.274	0.587	0.909	4.63	1.93	2	2	674	74.6	5.52
E9Q6K3	Protein Pibf1 OS=Mus musculus GN=Pibf1 PE=2 SV=1 - [E9Q6K3_MOUSE]	2.65	1	2	3	7	0.638	0.625	0.872	0.909	15.99	2.65	4	7	756	89.5	6.16
Q8K449	ATP-binding cassette sub-family A member 9 OS=Mus musculus GN=Abca9 PE=1 SV=2 - [ABCA9_MOUSE]	2.65	4	4	4	6	1.422	1.346	1.007	0.909	17.45	2.65	6	6	1623	183.0	6.93
A2AW15	Tau-tubulin kinase 2 OS=Mus musculus GN=Ttbk2 PE=2 SV=1 - [A2AW15_MOUSE]	17.76	4	6	15	37	1.550	0.611	0.952	0.909	104.56	17.76	25	37	1312	144.4	7.65
Q3TC46	Protein PAT1 homolog 1 OS=Mus musculus GN=Patl1 PE=1 SV=2 - [PATL1_MOUSE]	7.53	1	5	5	7	1.386	0.692	0.843	0.909	17.84	7.53	6	7	770	86.7	6.37
Q80U72	Protein scribble homolog OS=Mus musculus GN=Scrib PE=1 SV=2 - [SCRIB_MOUSE]	20.97	6	21	24	49	1.109	0.774	0.853	0.910	135.47	20.97	37	49	1612	174.0	5.12
Q8BKY8	mTERF domain-containing protein 3, mitochondrial OS=Mus musculus GN=Mterfd3 PE=1 SV=1 - [MTER3_MOUSE]	15.58	2	5	6	13	1.072	1.026	0.760	0.910	30.84	15.58	11	13	385	43.4	8.94
D3Z2J4	AKT-interacting protein (Fragment) OS=Mus musculus GN=Aktip PE=2 SV=1 - [D3Z2J4_MOUSE]	15.75	3	2	2	9	0.794	0.984	0.902	0.910	37.21	15.75	4	9	273	30.8	9.28
Q8VDK1-2	Isoform 2 of Nitrilase homolog 1 OS=Mus musculus GN=Nit1 - [NIT1_MOUSE]	52.76	5	12	12	33	1.913	1.003	0.778	0.910	88.63	52.76	21	33	290	31.9	7.05

Q91ZW3	SWI/SNF-related matrix-associated actin-dependent regulator of chromatin subfamily A member 5 OS=Mus musculus GN=Smarca5 PE=1 SV=1 -	18.46	1	15	22	51	0.727	0.791	0.753	0.910	115.05	18.46	34	51	1051	121.6	8.15
Q6ZPF3-3	Isoform 3 of T-lymphoma invasion and metastasis-inducing protein 2 OS=Mus musculus GN=Tiam2 - [illegible]	26.01	5	10	12	38	0.765	1.751	1.914	0.910	100.75	26.01	19	38	619	70.1	6.33
P58334	Krueppel-like factor 16 OS=Mus musculus GN=Klf16 PE=2 SV=2 - [KLF16_MOUSE]	6.77	1	1	1	2	1.324	1.317	1.018	0.910	4.87	6.77	2	2	251	25.6	10.13
P46061	Ran GTPase-activating protein 1 OS=Mus musculus GN=Rangap1 PE=1 SV=2 -	41.60	1	21	21	70	0.656	1.165	0.776	0.910	201.01	41.60	36	70	589	63.5	4.68
Q91VN6	Probable ATP-dependent RNA helicase DDX41 OS=Mus musculus GN=Ddx41 PE=1 SV=2 - [DDX41_MOUSE]	3.86	1	3	3	5	0.867	0.723	0.859	0.910	10.14	3.86	4	5	622	69.8	6.70
P63330	Serine/threonine-protein phosphatase 2A catalytic subunit alpha isoform OS=Mus musculus GN=Ppp2ca PE=1 SV=1 - [PP2AA_MOUSE]	66.02	1	2	15	302	1.277	0.867	0.982	0.910	948.91	66.02	30	302	309	35.6	5.54
Q5GH67	XK-related protein 4 OS=Mus musculus GN=Xkr4 PE=1 SV=1 - [XKR4_MOUSE]	12.83	1	7	7	30	0.556	1.156	1.058	0.910	91.37	12.83	12	30	647	71.5	8.03
E9PZJ7	Protein Vmn2r107 OS=Mus musculus GN=Vmn2r107 PE=2 SV=1 - [E9PZJ7_MOUSE]	0.70	1	1	1	1	0.958	0.790	0.871	0.910	1.86	0.70	1	1	861	99.2	7.24
P39098-2	Isoform 2 of Mannosyl-oligosaccharide 1,2-alpha-mannosidase IB OS=Mus musculus GN=Man1a2 - [MA1A2_MOUSE]	5.86	3	3	3	4	0.736	1.229	0.908	0.910	8.41	5.86	4	4	614	69.9	8.68
Q9WVQ0	Polyamine-modulated factor 1-binding protein 1 OS=Mus musculus GN=Pmfbp1 PE=2 SV=1 - [PMFBP_MOUSE]	3.52	3	2	3	3	1.172	1.059	1.021	0.910	5.81	3.52	3	3	1022	119.3	6.58
Q9DC04-1	Isoform 5 of Regulator of G-protein signaling 3 OS=Mus musculus GN=Rgs3 -	11.27	7	6	6	10	1.689	0.969	1.104	0.910	29.32	11.27	10	10	568	61.5	4.94
Q9JI11	Serine/threonine-protein kinase 4 OS=Mus musculus GN=Stk4 PE=1 SV=1 - [STK4_MOUSE]	24.23	5	6	10	25	1.428	1.068	0.912	0.910	65.76	24.23	16	25	487	55.5	5.19
G5E870	E3 ubiquitin-protein ligase TRIP12 OS=Mus musculus GN=Trip12 PE=1 SV=1 - [TRIPC_MOUSE]	13.93	1	21	21	47	0.620	1.008	0.873	0.910	120.39	13.93	36	47	2025	224.0	8.35
F6QXA3	BTB/POZ domain-containing protein KCTD7 (Fragment) OS=Mus musculus GN=Kctd7 PE=2 SV=1 - [F6QXA3_MOUSE]	15.09	2	2	2	3	1.618	0.822	0.886	0.910	12.09	15.09	3	3	232	25.6	7.17
Q7TQK1	Integrator complex subunit 7 OS=Mus musculus GN=Ints7 PE=1 SV=1 - [INT7_MOUSE]	5.07	1	4	4	7	0.811	1.016	1.017	0.911	15.57	5.07	6	7	966	106.8	8.22
Q9CQU1	Microfibrillar-associated protein 1 OS=Mus musculus GN=Mfap1 PE=1 SV=1 - [MFAP1_MOUSE]	13.21	1	5	5	8	1.546	0.834	1.146	0.911	18.67	13.21	6	8	439	51.9	4.98
Q8VCX5	Calcium uptake protein 1, mitochondrial OS=Mus musculus GN=Micu1 PE=1 SV=1 - [MICU1_MOUSE]	26.83	4	10	11	31	0.700	1.007	1.139	0.911	94.49	26.83	18	31	477	54.3	8.59
P98192	Dihydroxyacetone phosphate acyltransferase OS=Mus musculus GN=Gnpat PE=2 SV=1 - [GNPAT_MOUSE]	3.69	2	3	3	5	0.948	0.959	0.705	0.911	11.66	3.69	5	5	678	76.8	8.13
Q920D3	Mediator of RNA polymerase II transcription subunit 28 OS=Mus musculus GN=Med28 PE=2 SV=2 - [illegible]	29.78	2	4	4	6	1.001	0.803	0.871	0.911	16.97	29.78	5	6	178	19.5	5.58
O35627-2	Isoform CAR2 of Nuclear receptor subfamily 1 group I member 3 OS=Mus musculus GN=Nr1i3 - [NR1I3_MOUSE]	3.50	3	1	1	1	2.704	0.405	1.067	0.911	0.00	3.50	1	1	286	32.5	8.53
P07310	Creatine kinase M-type OS=Mus musculus GN=Ckm PE=1 SV=1 - [KCRM_MOUSE]	44.88	1	13	16	123	7.322	12.065	0.657	0.911	348.69	44.88	30	123	381	43.0	7.06
B9EKS2	Jumonji domain containing 1B OS=Mus musculus GN=Kdm3b PE=2 SV=1 - [B9EKS2_MOUSE]	6.53	3	7	8	19	1.080	0.923	1.015	0.911	48.90	6.53	12	19	1762	191.3	7.23
Q925H0	Acid-sensing ion channel 2 OS=Mus musculus GN=Asic2 PE=1 SV=1 - [ASIC2_MOUSE]	7.42	1	1	4	6	0.838	1.062	0.870	0.911	18.90	7.42	6	6	512	57.7	5.19

Q3UV20	MAM domain-containing glycosylphosphatidylinositol anchor protein 2 OS=Mus musculus GN=Mdga2 PE=2 SV=1 - [Q3UV20_MOUSE]	27.12	4	22	22	51	0.794	0.739	0.795	0.911	137.53	27.12	34	51	1025	114.9	7.93
Q9CYK1	Tryptophan--tRNA ligase, mitochondrial OS=Mus musculus GN=Wars2 PE=2 SV=2 - [SYWM_MOUSE]	25.28	1	10	10	34	0.689	1.417	0.928	0.911	99.78	25.28	18	34	360	40.1	8.82
Q02257	Junction plakoglobin OS=Mus musculus GN=Jup PE=1 SV=3 - [PLAK_MOUSE]	36.64	1	17	22	60	0.881	0.915	0.973	0.911	200.09	36.64	36	60	745	81.7	6.14
P14873	Microtubule-associated protein 1B OS=Mus musculus GN=Map1b PE=1 SV=2 -	49.19	1	108	110	1242	1.643	0.860	1.263	0.911	3516.47	49.19	191	1242	2464	270.1	4.83
Q8BJ71	Nuclear pore complex protein Nup93 OS=Mus musculus GN=Nup93 PE=2 SV=1 - [NUP93_MOUSE]	28.57	2	24	24	81	0.872	1.072	0.908	0.911	217.76	28.57	41	81	819	93.2	5.72
Q9ESB3	Histidine-rich glycoprotein OS=Mus musculus GN=Hrg PE=1 SV=2 - [HRG_MOUSE]	14.29	1	7	7	25	3.501	2.827	0.508	0.911	69.98	14.29	13	25	525	59.1	7.66
Q8R317	Ubiquilin-1 OS=Mus musculus GN=Ubqln1 PE=1 SV=1 - [UBQL1_MOUSE]	34.02	2	9	13	161	1.655	1.015	0.855	0.911	485.56	34.02	22	161	582	61.9	4.94
Q8JZM7	Parafibromin OS=Mus musculus GN=Cdc73 PE=2 SV=1 - [CDC73_MOUSE]	12.05	1	4	5	16	1.054	0.971	1.010	0.911	43.22	12.05	8	16	531	60.5	9.61
G5E8Q0	Lysosomal trafficking regulator, isoform CRA_a OS=Mus musculus GN=Lyst PE=4 SV=1 - [G5E8Q0_MOUSE]	3.56	3	9	10	26	0.757	1.144	1.060	0.911	61.02	3.56	15	26	3787	425.1	6.60
Q6NV83-3	Isoform 3 of U2 snRNP-associated SURP motif-containing protein OS=Mus musculus GN=U2surp - [SR140_MOUSE]	19.49	3	15	15	35	0.631	0.870	0.919	0.912	93.52	19.49	23	35	985	113.2	8.35
P46662	Merlin OS=Mus musculus GN=Nf2 PE=1 SV=2 - [MERL_MOUSE]	18.46	6	9	10	19	1.083	0.758	1.071	0.912	36.43	18.46	13	19	596	69.7	6.35
E0CYV9	Protein 1110002E22Rik OS=Mus musculus GN=1110002E22Rik PE=4 SV=1 - [E0CYV9_MOUSE]	0.67	1	1	1	1	1.408	0.772	0.869	0.912	2.06	0.67	1	1	1786	189.6	8.57
Q925I8	OL-protocadherin isoform OS=Mus musculus GN=Pcdh10 PE=2 SV=1 - [Q925I8_MOUSE]	25.58	4	17	17	65	1.238	0.956	1.056	0.912	170.50	25.58	29	65	1040	113.1	4.92
Q61072	Disintegrin and metalloproteinase domain-containing protein 9 OS=Mus musculus GN=Adam9 PE=1 SV=2 - [ADAM9_MOUSE]	5.68	2	4	4	9	0.662	0.786	0.919	0.912	24.43	5.68	5	9	845	92.0	7.47
Q91W18-2	Isoform 2 of Tudor domain containing protein 3 OS=Mus musculus GN=Tdrd3 - [TDRD3_MOUSE]	21.79	5	14	14	29	1.489	0.790	1.178	0.912	85.69	21.79	22	29	716	79.0	9.13
E9Q945	Protein Kctd18 OS=Mus musculus GN=Kctd18 PE=2 SV=1 - [E9Q945_MOUSE]	12.98	3	3	3	5	0.958	0.873	0.871	0.912	13.92	12.98	5	5	262	28.1	9.55
Q6DFZ2	Nesprin-2 (Fragment) OS=Mus musculus GN=Syne2 PE=2 SV=1 - [Q6DFZ2_MOUSE]	4.28	2	1	11	19	0.493	1.121	0.962	0.912	38.27	4.28	15	19	2152	247.7	5.34
Q9CQW0	ER membrane protein complex subunit 6 OS=Mus musculus GN=Emc6 PE=2 SV=1 - [EMC6_MOUSE]	20.00	1	2	2	6	0.736	0.724	0.763	0.912	15.52	20.00	4	6	110	12.0	10.07
Q8BR10	Interleukin-1 receptor-associated kinase 1 OS=Mus musculus GN=Irak1 PE=2 SV=1 - [Q8BR10_MOUSE]	7.45	11	3	3	7	0.656	1.006	0.908	0.912	16.01	7.45	5	7	711	77.3	6.27
Q3U5F4	YrdC domain-containing protein, mitochondrial OS=Mus musculus GN=Yrdc PE=1 SV=1 - [YRDC_MOUSE]	21.43	2	3	3	5	2.017	1.106	1.111	0.912	9.10	21.43	4	5	280	29.4	6.07
Q62377	U2 small nuclear ribonucleoprotein auxiliary factor 35 kDa subunit-related protein 2 OS=Mus musculus GN=Zrsr2 PE=2 SV=1 - [U2AFM_MOUSE]	4.55	4	2	2	2	0.975	0.922	1.105	0.912	6.75	4.55	2	2	462	55.3	8.43
Q91WU0	Expressed sequence AU018778 OS=Mus musculus GN=Ces1f PE=2 SV=1 - [Q91WU0_MOUSE]	5.88	1	2	3	5	1.776	1.018	0.961	0.912	8.47	5.88	3	5	561	61.6	6.48
Q5U452	MCG14616 OS=Mus musculus GN=2810408M09Rik PE=2 SV=1 - [Q5U452_MOUSE]	13.52	4	3	3	5	0.557	0.537	0.763	0.912	12.06	13.52	4	5	244	27.4	9.07
Q8CCJ3	E3 UFM1-protein ligase 1 OS=Mus musculus GN=Ufl1 PE=1 SV=2 - [UFL1_MOUSE]	25.22	3	18	18	53	0.713	0.780	0.770	0.912	169.40	25.22	33	53	793	89.5	6.67

Q07279	Transcription factor NF-E2 45 kDa subunit OS=Mus musculus GN=Nfe2 PE=1 SV=1 - [NFE2_MOUSE]	2.95	2	1	1	1	8.229	1.484	1.402	0.912	2.40	2.95	1	1	373	41.5	4.94
P63054	Purkinje cell protein 4 OS=Mus musculus GN=Pcp4 PE=2 SV=2 - [PCP4_MOUSE]	62.90	2	4	4	74	1.292	0.720	2.123	0.912	217.47	62.90	7	74	62	6.8	6.71
Q9D2U9	Histone H2B type 3-A OS=Mus musculus GN=Hist3h2ba PE=1 SV=3 - [H2B3A_MOUSE]	77.78	2	3	14	216	0.637	0.655	0.773	0.912	633.42	77.78	25	216	126	14.0	10.37
Q60989	E3 ubiquitin-protein ligase XIAP OS=Mus musculus GN=Xiap PE=1 SV=2 - [XIAP_MOUSE]	12.30	2	4	4	10	1.758	0.962	0.959	0.912	35.32	12.30	8	10	496	56.0	6.23
P08775	DNA-directed RNA polymerase II subunit RPB1 OS=Mus musculus GN=Polr2a PE=1 SV=3 - [RPB1_MOUSE]	14.47	1	23	24	47	0.693	0.845	0.996	0.912	121.78	14.47	35	47	1970	217.0	7.37
P51410	60S ribosomal protein L9 OS=Mus musculus GN=Rpl9 PE=2 SV=2 - [RL9_MOUSE]	53.65	4	10	10	36	0.529	0.688	0.792	0.913	102.55	53.65	17	36	192	21.9	9.95
Q3U3T8-2	Isoform 2 of WD repeat-containing protein 62 OS=Mus musculus GN=Wdr62 -	1.12	5	1	1	1	0.955	1.039	0.953	0.913	2.97	1.12	1	1	1075	119.1	5.71
P70407	Cadherin-9 OS=Mus musculus GN=Cdh9 PE=2 SV=2 - [CADH9_MOUSE]	9.92	2	5	8	15	1.038	1.516	1.011	0.913	44.12	9.92	11	15	786	88.2	4.96
Q9D666-3	Isoform 3 of SUN domain-containing protein 1 OS=Mus musculus GN=Sun1 - [SUN1_MOUSE]	13.70	10	2	9	23	0.569	0.986	0.944	0.913	63.96	13.70	15	23	876	97.9	6.64
Q9Z2U0	Proteasome subunit alpha type-7 OS=Mus musculus GN=Psma7 PE=1 SV=1 - [PSA7_MOUSE]	58.47	2	13	13	80	1.840	1.023	0.832	0.913	214.27	58.47	24	80	248	27.8	8.46
Q6DFV5	Probable helicase with zinc finger domain OS=Mus musculus GN=Helz PE=1 SV=2 - [HELZ_MOUSE]	2.04	5	3	3	7	1.340	1.140	1.395	0.913	25.52	2.04	5	7	1964	219.7	7.40
G3X939	Sodium/hydrogen exchanger OS=Mus musculus GN=Slc9a3 PE=3 SV=1 -	3.98	1	2	2	3	0.654	0.950	1.001	0.913	0.00	3.98	2	3	829	93.0	7.71
E0CZ78	Serine/threonine-protein phosphatase OS=Mus musculus GN=Ppp3cb PE=2 SV=1 - [E0CZ78_MOUSE]	37.60	5	9	16	182	0.758	1.639	1.147	0.913	477.20	37.60	29	182	524	59.0	5.91
B1AUH1-2	Isoform 2 of Receptor-type tyrosine-protein phosphatase U OS=Mus musculus GN=Ptpru - [PTPRU_MOUSE]	7.17	3	7	8	15	1.160	1.353	0.823	0.913	42.83	7.17	13	15	1436	161.1	6.81
Q91WX5	Bak1 protein OS=Mus musculus GN=Bak1 PE=2 SV=1 - [Q91WX5_MOUSE]	16.56	4	2	2	5	0.999	0.773	0.636	0.913	16.26	16.56	3	5	151	16.4	4.46
P97318-8	Isoform DAB553 of Disabled homolog 1 OS=Mus musculus GN=Dab1 -	9.76	9	4	4	14	1.068	1.036	1.183	0.913	46.95	9.76	7	14	553	59.4	5.36
P04925	Major prion protein OS=Mus musculus GN=Prnp PE=1 SV=2 - [PRIO_MOUSE]	28.74	1	4	4	42	0.850	0.840	1.027	0.913	115.18	28.74	6	42	254	28.0	9.33
H9KUX8	Baculoviral IAP repeat-containing protein 6 OS=Mus musculus GN=Birc6 PE=2 SV=1 - [H9KUX8_MOUSE]	13.62	6	48	49	107	0.875	0.811	0.885	0.913	288.57	13.62	76	107	4854	529.1	6.06
F8VPR5	CREB-binding protein OS=Mus musculus GN=Crebbp PE=2 SV=1 - [F8VPR5_MOUSE]	7.54	2	8	12	42	0.875	0.911	0.918	0.913	74.64	7.54	19	42	2441	265.4	8.50
E9Q1Z2	Protein Enthd1 OS=Mus musculus GN=Enthd1 PE=4 SV=1 - [E9Q1Z2_MOUSE]	2.91	1	1	2	5	0.405	0.801	0.666	0.913	10.68	2.91	3	5	618	68.4	6.84
Q9CX56	26S proteasome non-ATPase regulatory subunit 8 OS=Mus musculus GN=Psmd8 PE=1 SV=2 - [PSMD8_MOUSE]	41.93	1	15	15	76	0.684	1.105	0.812	0.913	164.53	41.93	29	76	353	39.9	9.58
P14685	26S proteasome non-ATPase regulatory subunit 3 OS=Mus musculus GN=Psmd3 PE=1 SV=3 - [PSMD3_MOUSE]	51.70	2	26	26	130	0.732	0.907	0.839	0.913	329.15	51.70	45	130	530	60.7	8.44
F6YIW9	Leucine-rich repeat serine/threonine-protein kinase 1 (Fragment) OS=Mus musculus GN=Lrrk1 PE=2 SV=1 - [F6YIW9_MOUSE]	9.06	2	3	3	3	0.429	1.028	1.275	0.913	6.93	9.06	3	3	508	57.6	6.25
E9Q6C6	Tubulin polyglutamylase TTLL7 (Fragment) OS=Mus musculus GN=Ttll7 PE=2 SV=1 - [E9Q6C6_MOUSE]	5.41	4	3	4	12	0.815	0.626	0.997	0.913	25.54	5.41	6	12	887	102.6	9.23

P97379-2	Isoform B of Ras GTPase-activating protein-binding protein 2 OS=Mus musculus GN=G3bp2 - [G3BP2_MOUSE]	38.31	1	1	15	184	2.266	1.047	0.884	0.913	466.83	38.31	27	184	449	50.8	5.43
D3Z0K6	MCG120108, isoform CRA_a OS=Mus musculus GN=Rsbn1l PE=4 SV=1 - [D3Z0K6_MOUSE]	2.92	1	1	1	1	0.995	1.112	0.768	0.913	2.08	2.92	1	1	821	91.7	8.95
E9Q7G4	BAH and coiled-coil domain-containing protein 1 OS=Mus musculus GN=Bahcc1 PE=2 SV=1 - [E9Q7G4_MOUSE]	2.38	2	4	4	4	2.071	1.083	0.971	0.913	10.52	2.38	4	4	2643	282.4	9.03
Q3UUI3	Acyl-coenzyme A thioesterase THEM4 OS=Mus musculus GN=Them4 PE=1 SV=1 - [THEM4_MOUSE]	32.61	2	7	7	15	0.455	0.972	1.006	0.914	39.44	32.61	10	15	230	26.0	9.64
E0CX32	Coiled-coil domain-containing protein 85A OS=Mus musculus GN=Ccdc85a PE=2 SV=1 - [E0CX32_MOUSE]	21.65	4	8	11	41	0.729	0.907	1.001	0.914	115.65	21.65	21	41	545	59.6	8.68
Q9CZT6	Protein CMSS1 OS=Mus musculus GN=Cmss1 PE=2 SV=1 - [CMS1_MOUSE]	8.70	1	2	2	3	1.083	1.045	0.984	0.914	5.03	8.70	3	3	276	31.6	8.46
Q6DID3	Protein SCAF8 OS=Mus musculus GN=Scaf8 PE=1 SV=1 - [SCAF8_MOUSE]	4.97	1	5	6	11	0.958	1.085	0.990	0.914	27.45	4.97	8	11	1268	139.5	8.24
P62849-2	Isoform 2 of 40S ribosomal protein S24 OS=Mus musculus GN=Rps24 -	40.00	3	6	6	25	0.688	0.920	0.831	0.914	68.42	40.00	11	25	130	15.1	10.89
Q8C0L9	Glycerophosphocholine phosphodiesterase GPCPD1 OS=Mus musculus GN=Gpcpd1 PE=1 SV=1 - [illegible]	20.59	9	11	11	29	0.859	0.730	1.084	0.914	72.32	20.59	17	29	675	76.5	5.58
G3X9D3	Podocalyxin-like 2 OS=Mus musculus GN=Podxl2 PE=4 SV=1 - [G3X9D3_MOUSE]	7.98	3	3	3	6	0.854	0.921	0.805	0.914	18.68	7.98	6	6	539	58.4	4.39
Q9CQE5	Regulator of G-protein signaling 10 OS=Mus musculus GN=Rgs10 PE=2 SV=1 - [RGS10_MOUSE]	49.72	1	6	7	24	1.420	0.913	1.082	0.914	78.30	49.72	11	24	181	21.1	6.81
Q80YR9	RNA-binding protein 12B-A OS=Mus musculus GN=Rbm12b1 PE=1 SV=2 - [R12BA_MOUSE]	7.54	1	2	6	8	0.868	0.815	0.916	0.914	10.14	7.54	7	8	836	96.5	8.07
Q8K2C9	Very-long-chain (3R)-3-hydroxyacyl-[acyl-carrier protein] dehydratase 3 OS=Mus musculus GN=ptplad1 PE=1 SV=2 - [illegible]	20.72	1	7	7	32	0.655	0.849	0.736	0.914	97.15	20.72	11	32	362	43.1	9.13
Q3TEL6-2	Isoform 2 of RING finger protein 157 OS=Mus musculus GN=Rnf157 - [RN157_MOUSE]	17.62	5	5	7	20	1.344	0.968	1.262	0.914	55.37	17.62	11	20	681	74.5	4.75
Q03141-3	Isoform 3 of MAP/microtubule affinity-regulating kinase 3 OS=Mus musculus GN=Mark3 - [illegible]	39.38	3	16	29	127	0.796	0.851	1.092	0.914	398.22	39.38	49	127	744	83.2	9.42
Q7TSK2-2	Isoform 2 of Seizure protein 6 OS=Mus musculus GN=Sez6 - [SEZ6_MOUSE]	7.06	3	1	5	9	1.223	1.876	2.170	0.914	26.82	7.06	8	9	977	106.0	5.36
F6Q475	Protein Ftsj1 (Fragment) OS=Mus musculus GN=Ftsj1 PE=4 SV=1 - [F6Q475_MOUSE]	16.22	3	1	1	4	0.618	0.833	0.796	0.914	13.40	16.22	2	4	111	12.4	4.87
Q6GQU6	Leucine-rich repeat and immunoglobulin-like domain-containing nogo receptor-interacting protein 3 OS=Mus musculus GN=Lingo3 PE=2 SV=2 - [LIGO3_MOUSE]	7.98	1	4	4	13	0.923	0.924	0.959	0.914	32.27	7.98	8	13	589	64.6	8.31
Q9DBX2	Phosducin-like protein OS=Mus musculus GN=Pdcl PE=1 SV=1 - [PHLP_MOUSE]	26.91	2	7	7	31	1.383	1.067	1.158	0.914	84.69	26.91	12	31	301	34.4	4.87
Q8C761	WD repeat-containing protein 60 OS=Mus musculus GN=Wdr60 PE=2 SV=1 -	3.00	1	3	3	4	2.150	0.964	0.785	0.914	4.60	3.00	4	4	999	115.3	7.24
Q8BFU3	RING finger protein 214 OS=Mus musculus GN=Rnf214 PE=2 SV=1 - [RN214_MOUSE]	38.32	6	6	23	99	1.273	0.783	1.106	0.914	301.68	38.32	40	99	668	73.6	6.23
Q9JHU2	Palmdelphin OS=Mus musculus GN=Palmd PE=1 SV=1 - [PALMD_MOUSE]	30.49	3	14	14	43	1.813	1.934	1.429	0.914	132.76	30.49	22	43	551	62.7	5.66
Q8CFP6	DnaJ homolog subfamily C member 27 OS=Mus musculus GN=Dnajc27 PE=2 SV=1 -	11.72	1	3	3	8	0.655	0.630	0.860	0.914	24.88	11.72	6	8	273	30.8	8.47
Q5SRY7-4	Isoform 4 of F-box/WD repeat-containing protein 11 OS=Mus musculus GN=Fbxw11 - [FBW1B_MOUSE]	17.72	4	4	6	13	1.664	0.874	1.043	0.914	46.72	17.72	11	13	508	58.4	7.02

Q8CGN5-4	Isoform 4 of Perilipin-1 OS=Mus musculus GN=Plin1 - [PLIN1_MOUSE]	2.46	4	1	1	1	0.796	0.658	0.857	0.914	1.93	2.46	1	1	244	25.9	6.34
Q8BSE0	Regulator of microtubule dynamics protein 2 OS=Mus musculus GN=Rmdn2 PE=2 SV=1 - [RMD2_MOUSE]	6.34	1	2	3	5	1.062	1.295	0.862	0.914	18.25	6.34	4	5	410	47.0	7.56
O88307	Sortilin-related receptor OS=Mus musculus GN=Sorl1 PE=2 SV=3 - [SORL_MOUSE]	8.31	1	17	17	39	1.187	0.897	0.926	0.915	110.03	8.31	28	39	2215	246.9	5.54
O35704	Serine palmitoyltransferase 1 OS=Mus musculus GN=Sptlc1 PE=2 SV=2 - [SPTC1_MOUSE]	10.36	1	4	4	6	1.262	1.096	0.759	0.915	17.60	10.36	6	6	473	52.5	6.40
Q8R1F6-2	Isoform 2 of Protein HID1 OS=Mus musculus GN=Hid1 - [HID1_MOUSE]	16.52	3	13	13	38	0.565	0.850	0.798	0.915	96.75	16.52	17	38	787	88.6	5.94
Q9QYI4	DnaJ homolog subfamily B member 12 OS=Mus musculus GN=Dnajb12 PE=2 SV=2 -	16.49	3	6	6	18	0.847	0.946	0.980	0.915	63.98	16.49	11	18	376	42.0	8.51
F8WHY8	Metastasis-associated protein MTA1 OS=Mus musculus GN=Mta1 PE=2 SV=1 - [F8WHY8_MOUSE]	27.79	3	12	16	62	0.708	0.804	0.886	0.915	150.51	27.79	25	62	698	79.1	9.42
Q9WV98	Mitochondrial import inner membrane translocase subunit Tim9 OS=Mus musculus GN=Timm9 PE=1 SV=1 - [TIM9_MOUSE]	65.17	1	5	5	52	1.540	1.078	1.025	0.915	133.91	65.17	10	52	89	10.3	7.21
Q8R010	Aminoacyl tRNA synthase complex-interacting multifunctional protein 2 OS=Mus musculus GN=Aimp2 PE=1 SV=2 - [illegible]	58.75	2	11	11	38	0.766	0.942	0.844	0.915	119.63	58.75	20	38	320	35.4	7.83
Q3TQP0	Protein Gm10767 OS=Mus musculus GN=Gm10767 PE=4 SV=1 - [Q3TQP0_MOUSE]	30.10	2	3	3	9	1.649	0.964	1.064	0.915	32.96	30.10	5	9	103	12.5	7.25
Q99JI4	26S proteasome non-ATPase regulatory subunit 6 OS=Mus musculus GN=Psmd6 PE=1 SV=1 - [PSMD6_MOUSE]	38.05	1	13	13	64	0.900	0.970	0.847	0.915	196.19	38.05	25	64	389	45.5	5.52
E9Q1S3	Protein transport protein Sec23A OS=Mus musculus GN=Sec23a PE=2 SV=1 - [E9Q1S3_MOUSE]	30.84	2	16	20	136	0.690	0.909	0.847	0.915	426.89	30.84	38	136	736	82.9	7.46
Q8VE22	28S ribosomal protein S23, mitochondrial OS=Mus musculus GN=Mrps23 PE=2 SV=1 - [RT23_MOUSE]	40.68	5	7	7	14	0.638	0.704	0.977	0.915	40.74	40.68	12	14	177	20.3	8.59
Q61301-2	Isoform 2 of Catenin alpha-2 OS=Mus musculus GN=Ctnna2 - [CTNA2_MOUSE]	59.12	8	35	48	236	0.592	0.930	0.946	0.915	673.36	59.12	86	236	905	100.4	5.96
P84104-2	Isoform Short of Serine/arginine-rich splicing factor 3 OS=Mus musculus GN=Srsf3 - [SRSF3_MOUSE]	45.16	3	5	6	38	0.548	0.879	0.921	0.915	84.08	45.16	11	38	124	14.2	10.08
E9Q3V9	Latrophilin-1 OS=Mus musculus GN=Lphn1 PE=2 SV=1 - [E9Q3V9_MOUSE]	33.05	7	31	33	203	0.928	1.088	1.008	0.915	636.01	33.05	58	203	1516	166.9	6.70
Q3TAS6-2	Isoform 2 of ER membrane protein complex subunit 10 OS=Mus musculus GN=Emc10 - [EMC10_MOUSE]	13.78	3	3	3	11	1.030	0.825	0.780	0.915	38.55	13.78	6	11	254	26.7	6.13
Q6IR34-3	Isoform 3 of G-protein-signaling modulator 1 OS=Mus musculus GN=Gpsm1 -	51.54	9	22	25	78	0.975	0.866	1.124	0.915	243.38	51.54	43	78	650	71.9	6.15
Q8R4C2	RUN and FYVE domain-containing protein 2 OS=Mus musculus GN=Rufy2 PE=1 SV=2 - [RUFY2_MOUSE]	28.88	3	4	17	53	1.508	1.152	0.863	0.915	150.24	28.88	30	53	606	70.0	5.83
P12023-2	Isoform APP695 of Amyloid beta A4 protein OS=Mus musculus GN=App - [A4_MOUSE]	34.96	1	1	21	144	0.984	0.789	1.143	0.915	455.90	34.96	40	144	695	78.4	4.83
Q8BFZ9	Erlin-2 OS=Mus musculus GN=Erlin2 PE=1 SV=1 - [ERLN2_MOUSE]	50.88	2	14	17	78	0.757	1.249	0.832	0.916	234.81	50.88	29	78	340	37.8	5.50
P63101	14-3-3 protein zeta/delta OS=Mus musculus GN=Ywhaz PE=1 SV=1 - [1433Z_MOUSE]	84.90	4	16	26	1458	1.140	1.427	1.075	0.916	3906.44	84.90	48	1458	245	27.8	4.79
Q91VR5	ATP-dependent RNA helicase DDX1 OS=Mus musculus GN=Ddx1 PE=1 SV=1 - [DDX1_MOUSE]	57.43	1	37	37	163	0.925	0.848	0.893	0.916	484.34	57.43	68	163	740	82.4	7.21
Q6PGL7	WASH complex subunit FAM21 OS=Mus musculus GN=Fam21 PE=1 SV=1 - [FAM21_MOUSE]	43.78	2	38	38	152	1.370	0.870	1.138	0.916	497.40	43.78	61	152	1334	145.2	4.77
Q8CC96	Uncharacterized protein C6orf222 homolog OS=Mus musculus PE=2 SV=1 - [CF222_MOUSE]	1.49	1	1	1	1	1.543	1.073	1.466	0.916	2.01	1.49	1	1	669	72.9	9.01

Q07409	Contactin-3 OS=Mus musculus GN=Cntn3 PE=2 SV=2 - [CNTN3_MOUSE]	16.54	1	12	14	28	1.035	1.122	0.895	0.916	74.91	16.54	22	28	1028	113.2	6.23
Q9D3L0	Membrane protein FAM174A OS=Mus musculus GN=Fam174a PE=2 SV=2 -	38.95	1	3	3	13	0.927	0.973	1.011	0.916	41.75	38.95	5	13	190	20.0	6.79
G5E8R8	MCG129950, isoform CRA_a OS=Mus musculus GN=Ubxn7 PE=4 SV=1 - [G5E8R8_MOUSE]	34.56	6	14	15	40	1.259	1.088	1.161	0.916	110.09	34.56	25	40	489	54.8	5.16
Q61466-2	Isoform 1 of SWI/SNF-related matrix-associated actin-dependent regulator of chromatin subfamily D member 1 OS=Mus musculus GN=Smarcd1 - [SMRD1_MOUSE]	23.54	2	8	10	20	1.099	0.876	1.128	0.916	64.33	23.54	16	20	514	58.0	9.17
Q8BUR3-2	Isoform 2 of Forkhead box protein J3 OS=Mus musculus GN=Foxj3 - [FOXJ3_MOUSE]	5.77	4	2	2	5	1.458	1.131	0.958	0.916	11.63	5.77	4	5	589	65.5	7.17
Q7TS73	Protein FAM101A OS=Mus musculus GN=Fam101a PE=2 SV=1 - [F101A_MOUSE]	12.25	1	1	1	2	1.740	0.911	0.636	0.916	0.00	12.25	1	2	204	22.6	7.40
P70232	Neural cell adhesion molecule L1-like protein OS=Mus musculus GN=Chl1 PE=1 SV=2 - [CHL1_MOUSE]	42.10	1	6	40	159	0.952	1.048	0.827	0.916	464.40	42.10	67	159	1209	135.0	5.57
Q9WTI7-3	Isoform 3 of Unconventional myosin-Ic OS=Mus musculus GN=Myo1c -	27.30	5	24	24	64	0.713	1.217	0.791	0.916	195.99	27.30	40	64	1044	119.8	9.39
Q99J83	Autophagy protein 5 OS=Mus musculus GN=Atg5 PE=1 SV=1 - [ATG5_MOUSE]	32.36	1	8	8	21	0.748	0.968	0.881	0.916	52.37	32.36	13	21	275	32.4	5.91
Q99KJ8	Dynactin subunit 2 OS=Mus musculus GN=Dctn2 PE=1 SV=3 - [DCTN2_MOUSE]	69.40	1	24	25	188	1.579	1.069	1.042	0.916	505.06	69.40	45	188	402	44.1	5.26
Q8CE50	Sorting nexin-30 OS=Mus musculus GN=Snx30 PE=2 SV=1 - [SNX30_MOUSE]	47.14	1	18	18	54	0.892	0.969	0.699	0.916	144.96	47.14	31	54	437	49.5	5.35
Q01853	Transitional endoplasmic reticulum ATPase OS=Mus musculus GN=Vcp PE=1 SV=4 - [TERA_MOUSE]	60.30	8	42	43	429	0.766	0.685	0.870	0.916	1159.02	60.30	80	429	806	89.3	5.26
Q9DCC4	Pyrroline-5-carboxylate reductase 3 OS=Mus musculus GN=Pycrl PE=2 SV=2 - [P5CR3_MOUSE]	42.34	1	10	10	59	1.191	1.062	0.926	0.916	162.21	42.34	20	59	274	28.7	7.27
Q6PE13	Proline-rich transmembrane protein 3 OS=Mus musculus GN=Prrt3 PE=1 SV=1 -	25.85	1	16	16	85	1.062	0.936	1.036	0.916	231.57	25.85	27	85	971	101.2	7.52
B2RXW8	Ppfia1 protein OS=Mus musculus GN=Ppfia1 PE=2 SV=1 - [B2RXW8_MOUSE]	35.70	5	22	40	144	1.198	0.916	0.965	0.916	430.53	35.70	64	144	1241	140.0	6.14
Q9CYC6	m7GpppN-mRNA hydrolase OS=Mus musculus GN=Dcp2 PE=1 SV=2 - [DCP2_MOUSE]	10.90	1	4	4	7	0.754	0.805	1.014	0.916	14.94	10.90	5	7	422	48.3	7.94
Q8BGA3	Leucine-rich repeat transmembrane neuronal protein 2 OS=Mus musculus GN=Lrrtm2 PE=2 SV=1 - [illegible]	8.35	1	3	5	10	0.502	1.333	0.736	0.916	26.67	8.35	8	10	515	58.8	8.00
Q9D7P6	Iron-sulfur cluster assembly enzyme ISCU, mitochondrial OS=Mus musculus GN=Iscu PE=1 SV=1 - [ISCU_MOUSE]	50.60	4	11	11	47	1.683	0.843	1.147	0.916	133.15	50.60	20	47	168	18.1	9.29
Q3TTY0	Phospholipase B1, membrane-associated OS=Mus musculus GN=Plb1 PE=2 SV=2 - [PLB1_MOUSE]	2.77	3	2	2	2	0.889	1.069	0.798	0.916	5.19	2.77	2	2	1478	164.4	6.86
Q3TKT4	Transcription activator BRG1 OS=Mus musculus GN=Smarca4 PE=1 SV=1 - [SMCA4_MOUSE]	11.28	3	3	17	37	0.959	0.832	1.209	0.916	104.83	11.28	25	37	1613	181.3	8.00
B2RUJ8	Rho GTPase activating protein 12 OS=Mus musculus GN=Arhgap12 PE=2 SV=1 -	18.33	2	11	11	31	0.873	0.885	0.971	0.916	74.71	18.33	20	31	813	92.6	7.96
Q9WVL1	AP-4 complex subunit sigma-1 OS=Mus musculus GN=Ap4s1 PE=2 SV=1 - [AP4S1_MOUSE]	13.19	1	2	2	4	0.859	0.769	0.790	0.916	9.80	13.19	4	4	144	16.8	5.34
Q9CQ73	Plakophilin 2 OS=Mus musculus GN=Pkp2 PE=2 SV=1 - [Q9CQ73_MOUSE]	2.64	3	2	3	7	0.397	1.604	1.036	0.916	16.98	2.64	4	7	795	88.0	9.33
Q69ZW3-2	Isoform 2 of EH domain-binding protein 1 OS=Mus musculus GN=Ehbp1 - [EHBP1_MOUSE]	34.74	3	36	37	106	0.961	1.074	1.120	0.916	312.84	34.74	60	106	1206	136.2	5.39
P47968	Ribose-5-phosphate isomerase OS=Mus musculus GN=Rpia PE=2 SV=2 - [RPIA_MOUSE]	30.03	1	7	7	18	1.878	1.063	0.904	0.916	47.80	30.03	12	18	303	32.4	8.02
Q99J36	THUMP domain-containing protein 1 OS=Mus musculus GN=Thumpd1 PE=1 SV=1 -	16.29	1	7	7	16	0.561	0.737	0.995	0.916	56.25	16.29	14	16	350	38.9	6.07

Q60612	Adenosine receptor A1 OS=Mus musculus GN=Adora1 PE=2 SV=4 - [AA1R_MOUSE]	3.37	1	2	2	3	0.517	0.870	1.316	0.916	8.75	3.37	2	3	326	36.6	8.48
Q9CXW2	28S ribosomal protein S22, mitochondrial OS=Mus musculus GN=Mrps22 PE=2 SV=1 - [RT22_MOUSE]	34.82	1	11	11	33	1.317	1.018	1.083	0.917	79.74	34.82	20	33	359	41.2	8.56
Q61768	Kinesin-1 heavy chain OS=Mus musculus GN=Kif5b PE=1 SV=3 - [KINH_MOUSE]	51.61	2	34	46	262	1.287	0.756	1.016	0.917	785.92	51.61	82	262	963	109.5	6.44
Q8JZU2	Protein Slc25a1 OS=Mus musculus GN=Slc25a1 PE=2 SV=1 - [Q8JZU2_MOUSE]	42.77	2	13	13	76	0.871	0.712	0.658	0.917	224.99	42.77	23	76	311	33.9	9.89
E9Q9A5	Bifunctional polynucleotide phosphatase/kinase OS=Mus musculus GN=Pnkp PE=2 SV=1 - [E9Q9A5_MOUSE]	11.73	4	4	4	11	1.236	0.891	0.760	0.917	27.69	11.73	6	11	486	53.3	7.39
Q921F2	TAR DNA-binding protein 43 OS=Mus musculus GN=Tardbp PE=1 SV=1 - [TADBP_MOUSE]	39.61	5	11	11	82	0.684	0.804	0.799	0.917	228.38	39.61	19	82	414	44.5	6.70
Q6P9R2	Serine/threonine-protein kinase OSR1 OS=Mus musculus GN=Oxsr1 PE=1 SV=1 - [OXSR1_MOUSE]	19.73	3	7	10	45	0.953	0.921	0.838	0.917	122.70	19.73	17	45	527	58.2	6.43
Q8CI51	PDZ and LIM domain protein 5 OS=Mus musculus GN=Pdlim5 PE=1 SV=4 -	39.09	3	13	18	55	1.547	1.260	1.148	0.917	181.85	39.09	30	55	591	63.3	8.25
Q9Z2Z6	Mitochondrial carnitine/acylcarnitine carrier protein OS=Mus musculus GN=Slc25a20 PE=1 SV=1 - [MCAT_MOUSE]	43.52	1	14	14	40	0.756	0.926	0.784	0.917	95.49	43.52	25	40	301	33.0	9.11
Q8BXV2	BRI3-binding protein OS=Mus musculus GN=Bri3bp PE=2 SV=1 - [BRI3B_MOUSE]	19.76	1	5	5	27	0.622	1.112	0.826	0.917	97.62	19.76	10	27	253	28.2	9.52
Q99LH9	SH3 domain-binding protein 5-like OS=Mus musculus GN=Sh3bp5l PE=1 SV=1 -	17.60	1	6	6	13	1.529	0.851	0.833	0.917	30.60	17.60	11	13	392	43.3	6.00
Q8K4P0	pre-mRNA 3' end processing protein WDR33 OS=Mus musculus GN=Wdr33 PE=2 SV=1 - [WDR33_MOUSE]	11.73	2	11	12	34	1.050	0.784	0.957	0.917	91.98	11.73	19	34	1330	145.2	9.13
P47963	60S ribosomal protein L13 OS=Mus musculus GN=Rpl13 PE=2 SV=3 - [RL13_MOUSE]	19.43	2	5	5	25	0.645	0.595	0.768	0.917	59.97	19.43	8	25	211	24.3	11.55
Q923D3	Prostate androgen-regulated mucin-like protein 1 homolog OS=Mus musculus GN=Parm1 PE=2 SV=1 - [PARM1_MOUSE]	5.74	1	1	1	1	1.918	2.144	1.505	0.917	3.24	5.74	1	1	296	30.6	5.49
Q8K3X4	Interferon regulatory factor 2-binding protein-like OS=Mus musculus GN=Irf2bpl PE=1 SV=1 - [I2BPL_MOUSE]	22.84	1	13	14	35	0.998	1.259	1.064	0.917	114.03	22.84	24	35	775	80.5	8.24
Q7M729	Sodium channel subunit beta-4 OS=Mus musculus GN=Scn4b PE=2 SV=1 - [SCN4B_MOUSE]	19.74	1	5	5	19	0.769	0.347	1.788	0.917	46.94	19.74	8	19	228	25.2	8.82
D3YYW8	RNA-binding protein 42 OS=Mus musculus GN=Rbm42 PE=2 SV=1 - [D3YYW8_MOUSE]	5.35	3	3	3	5	1.087	0.908	0.844	0.917	12.48	5.35	4	5	449	47.2	9.50
P0C7M9	C-type lectin domain family 2 member L OS=Mus musculus GN=Clec2l PE=2 SV=1 - [CLC2L_MOUSE]	28.44	1	7	7	25	0.715	0.446	0.785	0.917	73.28	28.44	12	25	211	23.6	7.20
Q8VDD8	WAS protein family homolog 1 OS=Mus musculus GN=Wash1 PE=2 SV=1 -	26.53	1	9	10	27	0.873	0.868	1.060	0.917	87.52	26.53	16	27	475	51.6	5.44
Q9DB25	Dolichyl-phosphate beta-glucosyltransferase OS=Mus musculus GN=Alg5 PE=2 SV=1 - [ALG5_MOUSE]	7.10	2	2	2	5	0.976	0.868	0.783	0.917	14.66	7.10	3	5	324	36.8	8.60
Q7TSJ6	Serine/threonine-protein kinase LATS2 OS=Mus musculus GN=Lats2 PE=1 SV=1 - [LATS2_MOUSE]	4.03	4	2	3	4	2.199	1.289	1.072	0.917	8.92	4.03	3	4	1042	115.4	8.22
Q920N7	Synaptotagmin-12 OS=Mus musculus GN=Syt12 PE=2 SV=1 - [SYT12_MOUSE]	45.37	1	15	15	69	0.711	0.602	1.111	0.917	199.62	45.37	26	69	421	46.7	5.64
B1AXS8	Zinc finger MYM-type protein 3 OS=Mus musculus GN=Zmym3 PE=2 SV=1 -	6.71	7	8	8	13	0.739	0.745	1.289	0.917	33.27	6.71	12	13	1356	151.3	6.39
A2AJ26	ATP-binding cassette sub-family A member 2 OS=Mus musculus GN=Abca2 PE=3 SV=1 - [A2AJ26_MOUSE]	12.66	2	23	24	60	0.936	0.673	0.798	0.917	185.36	12.66	40	60	2433	270.4	6.47
E9QAN8	Protein Pik3c2b OS=Mus musculus GN=Pik3c2b PE=2 SV=1 - [E9QAN8_MOUSE]	3.19	2	2	4	12	2.721	1.476	1.289	0.918	35.58	3.19	6	12	1632	184.1	7.05

Q7M6U3-2	Isoform 2 of Inactive serine/threonine-protein kinase TEX14 OS=Mus musculus GN=Tex14 - [TEX14_MOUSE]	2.03	3	3	3	4	1.463	1.267	1.054	0.918	7.13	2.03	4	4	1232	138.8	5.19
P27659	60S ribosomal protein L3 OS=Mus musculus GN=Rpl3 PE=2 SV=3 - [RL3_MOUSE]	38.21	3	18	18	102	0.616	0.801	0.740	0.918	231.69	38.21	34	102	403	46.1	10.21
Q9WVF7	DNA polymerase epsilon catalytic subunit A OS=Mus musculus GN=Pole PE=2 SV=3 - [DPOE1_MOUSE]	0.70	1	1	1	1	0.595	0.783	0.965	0.918	0.00	0.70	1	1	2283	261.9	6.44
Q6P1D5	Seizure 6-like protein OS=Mus musculus GN=Sez6l PE=2 SV=1 - [SE6L1_MOUSE]	18.48	2	14	15	47	1.008	1.324	1.171	0.918	138.86	18.48	22	47	963	104.8	4.69
Q99M07	Cytochrome c oxidase assembly factor 5 OS=Mus musculus GN=Coa5 PE=2 SV=1 - [COA5_MOUSE]	33.78	1	2	2	6	0.743	0.589	1.385	0.918	16.53	33.78	4	6	74	8.4	8.70
Q9DCP2	Sodium-coupled neutral amino acid transporter 3 OS=Mus musculus GN=Slc38a3 PE=1 SV=1 - [S38A3_MOUSE]	11.68	1	6	6	26	0.842	1.029	0.945	0.918	74.74	11.68	9	26	505	55.6	7.09
P48678	Prelamin-A/C OS=Mus musculus GN=Lmna PE=1 SV=2 - [LMNA_MOUSE]	61.80	4	39	40	312	1.597	1.207	1.002	0.918	873.89	61.80	73	312	665	74.2	6.98
Q8VCD6	Receptor expression-enhancing protein 2 OS=Mus musculus GN=Reep2 PE=2 SV=2 - [REEP2_MOUSE]	44.88	1	11	11	40	0.669	0.647	1.010	0.918	108.33	44.88	20	40	254	28.4	9.41
Q810C0	SLIT and NTRK-like protein 2 OS=Mus musculus GN=Slitrk2 PE=1 SV=1 - [SLIK2_MOUSE]	8.27	1	6	6	10	0.644	0.907	0.832	0.918	26.44	8.27	10	10	846	95.4	7.65
Q60829	Protein phosphatase 1 regulatory subunit 1B OS=Mus musculus GN=Ppp1r1b PE=1 SV=2 - [PPR1B_MOUSE]	61.86	4	10	10	161	0.950	0.739	3.717	0.918	457.72	61.86	17	161	194	21.8	4.65
Q9QZH3	Peptidyl-prolyl cis-trans isomerase E OS=Mus musculus GN=Ppie PE=2 SV=2 - [PPIE_MOUSE]	36.21	1	8	9	30	1.576	1.012	1.011	0.918	71.14	36.21	14	30	301	33.4	5.60
F8VQB2	Neuronal PAS domain-containing protein 3 OS=Mus musculus GN=Npas3 PE=4 SV=1 - [F8VQB2_MOUSE]	5.73	2	3	3	3	1.084	0.557	1.044	0.918	4.44	5.73	3	3	925	100.3	6.67
Q6ZQ29	Serine/threonine-protein kinase TAO2 OS=Mus musculus GN=Taok2 PE=1 SV=3 - [TAOK2_MOUSE]	12.66	2	2	15	56	1.791	1.304	1.084	0.918	127.70	12.66	24	56	1240	139.2	7.06
Q5RJG7-5	Isoform 5 of Isoprenoid synthase domain-containing protein OS=Mus musculus GN=Ispd - [ISPD_MOUSE]	20.76	5	7	7	18	1.202	1.245	0.885	0.918	52.48	20.76	12	18	419	46.3	6.52
B1APX2	Protein 5031439G07Rik OS=Mus musculus GN=5031439G07Rik PE=2 SV=1 - [B1APX2_MOUSE]	14.99	2	6	6	17	0.974	0.454	0.922	0.918	57.29	14.99	11	17	447	50.2	8.09
P12658	Calbindin OS=Mus musculus GN=Calb1 PE=1 SV=2 - [CALB1_MOUSE]	79.69	1	17	18	347	1.863	2.087	1.321	0.919	863.73	79.69	32	347	261	30.0	4.83
O55137	Acyl-coenzyme A thioesterase 1 OS=Mus musculus GN=Acot1 PE=1 SV=1 - [ACOT1_MOUSE]	44.63	1	5	14	48	1.708	0.999	0.822	0.919	152.83	44.63	27	48	419	46.1	6.58
Q9CWE6	Oocyte-expressed protein homolog OS=Mus musculus GN=Ooep PE=1 SV=1 - [OOEP_MOUSE]	6.71	1	1	1	1	0.785	0.841	1.030	0.919	0.00	6.71	1	1	164	18.4	6.01
Q6A028	Switch-associated protein 70 OS=Mus musculus GN=Swap70 PE=1 SV=2 - [SWP70_MOUSE]	22.91	1	11	11	24	1.296	1.174	0.974	0.919	64.12	22.91	20	24	585	69.0	6.05
P26516	26S proteasome non-ATPase regulatory subunit 7 OS=Mus musculus GN=Psmd7 PE=1 SV=2 - [PSMD7_MOUSE]	53.27	1	12	12	43	0.688	0.948	0.785	0.919	139.56	53.27	21	43	321	36.5	6.77
Q8QZR0	Translocating chain-associated membrane protein 1-like 1 OS=Mus musculus GN=Tram1l1 PE=2 SV=1 - [illegible]	3.31	1	1	1	1	0.944	0.857	0.464	0.919	2.95	3.31	1	1	363	41.0	9.45
Q9JKY5	Huntingtin-interacting protein 1-related protein OS=Mus musculus GN=Hip1r PE=1 SV=2 - [HIP1R_MOUSE]	45.41	3	42	44	165	0.837	0.779	0.952	0.919	468.73	45.41	77	165	1068	119.4	6.52
Q80ZF8	Brain-specific angiogenesis inhibitor 3 OS=Mus musculus GN=Bai3 PE=1 SV=2 - [BAI3_MOUSE]	15.44	4	16	20	50	0.897	1.752	1.630	0.919	127.90	15.44	30	50	1522	171.2	7.02
O09000	Nuclear receptor coactivator 3 OS=Mus musculus GN=Ncoa3 PE=1 SV=2 - [NCOA3_MOUSE]	2.07	3	2	3	4	1.623	0.919	0.839	0.919	9.15	2.07	3	4	1398	151.5	7.77

O88983	Syntaxin-8 OS=Mus musculus GN=Stx8 PE=1 SV=1 - [STX8_MOUSE]	26.69	2	6	6	18	0.614	0.786	0.892	0.919	60.50	26.69	11	18	236	26.9	5.01
Q05186	Reticulocalbin-1 OS=Mus musculus GN=Rcn1 PE=1 SV=1 - [RCN1_MOUSE]	32.92	1	11	11	26	1.578	1.647	1.074	0.919	65.73	32.92	16	26	325	38.1	4.84
Q3UQ44	Ras GTPase-activating-like protein IQGAP2 OS=Mus musculus GN=Iqgap2 PE=1 SV=2 - [IQGA2_MOUSE]	13.71	1	14	17	31	0.780	2.025	1.103	0.919	92.16	13.71	26	31	1575	180.4	5.64
Q60625	Intercellular adhesion molecule 5 OS=Mus musculus GN=Icam5 PE=1 SV=2 - [ICAM5_MOUSE]	29.77	1	21	22	117	0.777	4.992	2.903	0.919	334.63	29.77	39	117	917	96.9	6.32
Q9CR40	Kelch-like protein 28 OS=Mus musculus GN=Klhl28 PE=2 SV=1 - [KLH28_MOUSE]	3.85	2	2	2	2	0.781	0.779	0.946	0.919	2.89	3.85	2	2	571	64.1	5.96
G3UW60	MCG14773 OS=Mus musculus GN=Susd5 PE=4 SV=1 - [G3UW60_MOUSE]	5.01	1	4	4	6	0.895	1.003	0.968	0.919	16.17	5.01	6	6	619	66.8	4.93
Q571I4	Tyrosine-protein kinase SgK223 OS=Mus musculus GN=Sgk223 PE=2 SV=2 - [SG223_MOUSE]	3.39	2	3	3	10	0.810	1.322	1.207	0.919	40.96	3.39	6	10	1179	126.6	7.21
Q91W39	Nuclear receptor coactivator 5 OS=Mus musculus GN=Ncoa5 PE=1 SV=1 - [NCOA5_MOUSE]	25.73	4	10	10	24	0.869	0.818	0.981	0.919	67.52	25.73	17	24	579	65.3	9.82
E0CX81	Glucose 1,6-bisphosphate synthase OS=Mus musculus GN=Pgm2l1 PE=2 SV=1 -	47.43	1	1	15	82	0.305	0.898	1.453	0.919	236.49	47.43	28	82	253	28.9	7.15
H7BX02	Probable E3 ubiquitin-protein ligase Roquin OS=Mus musculus GN=Rc3h1 PE=2 SV=1 - [H7BX02_MOUSE]	17.66	2	11	12	30	1.208	1.009	1.098	0.919	77.29	17.66	18	30	1121	124.3	7.18
Q91W50	Cold shock domain-containing protein E1 OS=Mus musculus GN=Csde1 PE=2 SV=1 - [CSDE1_MOUSE]	51.25	1	35	35	130	1.275	0.882	1.031	0.919	347.02	51.25	60	130	798	88.7	6.37
Q7TPE5	Probable RNA polymerase II nuclear localization protein SLC7A6OS OS=Mus musculus GN=Slc7a6os PE=1 SV=1 - [S7A6O_MOUSE]	3.59	1	1	1	2	1.384	0.892	1.100	0.919	5.12	3.59	2	2	306	35.0	4.58
Q8VC98-2	Isoform 2 of Pleckstrin homology domain-containing family A member 4 OS=Mus musculus GN=Plekha4 - [PKHA4_MOUSE]	5.07	6	2	2	2	0.844	0.609	0.935	0.919	1.93	5.07	2	2	473	52.0	7.96
E9Q0V6	NHS-like protein 2 OS=Mus musculus GN=Nhsl2 PE=2 SV=1 - [E9Q0V6_MOUSE]	16.16	2	12	12	31	0.797	1.264	1.739	0.919	80.90	16.16	19	31	1219	132.3	8.48
P26040	Ezrin OS=Mus musculus GN=Ezr PE=1 SV=3 - [EZRI_MOUSE]	36.69	1	13	24	103	0.960	1.226	1.516	0.920	269.52	36.69	42	103	586	69.4	6.10
Q9WV31	Activity-regulated cytoskeleton-associated protein OS=Mus musculus GN=Arc PE=1 SV=1 - [ARC_MOUSE]	8.84	1	3	3	10	0.826	2.111	1.918	0.920	30.49	8.84	6	10	396	45.3	5.39
O35737	Heterogeneous nuclear ribonucleoprotein H OS=Mus musculus GN=Hnrnph1 PE=1 SV=3 - [HNRH1_MOUSE]	53.23	3	9	17	278	1.199	0.955	0.932	0.920	901.62	53.23	30	278	449	49.2	6.30
P51150	Ras-related protein Rab-7a OS=Mus musculus GN=Rab7a PE=1 SV=2 - [RAB7A_MOUSE]	77.78	1	17	18	138	0.729	0.881	0.855	0.920	430.37	77.78	32	138	207	23.5	6.70
Q2HXL6	ER degradation-enhancing alpha-mannosidase-like protein 3 OS=Mus musculus GN=Edem3 PE=1 SV=2 - [EDEM3_MOUSE]	3.22	1	2	3	8	1.276	1.163	1.156	0.920	15.07	3.22	6	8	931	104.1	5.06
F7C259	WD repeat domain-containing protein 83 (Fragment) OS=Mus musculus GN=Wdr83 PE=4 SV=1 - [F7C259_MOUSE]	27.27	3	1	1	1	1.253	1.606	0.897	0.920	0.00	27.27	1	1	55	6.5	5.71
Q9CPR8	Melanoma-associated antigen G1 OS=Mus musculus GN=Ndnl2 PE=1 SV=1 - [MAGG1_MOUSE]	3.94	1	1	1	2	1.304	1.398	0.915	0.920	4.48	3.94	2	2	279	31.4	9.07
Q9CWY6	RNA-binding protein with multiple-splicing 2 OS=Mus musculus GN=Rbpms2 PE=2 SV=1 - [Q9CWY6_MOUSE]	11.31	1	1	1	1	1.787	1.882	1.173	0.920	3.34	11.31	1	1	168	18.3	9.29
H3BIX7	Calcium-transporting ATPase type 2C member 1 OS=Mus musculus GN=Atp2c1 PE=2 SV=1 - [H3BIX7_MOUSE]	22.89	12	16	18	64	0.734	1.027	0.920	0.920	156.67	22.89	31	64	926	101.2	7.44
F6UI76	F-box only protein 44 (Fragment) OS=Mus musculus GN=Fbxo44 PE=4 SV=1 -	9.72	10	1	2	3	1.071	1.114	0.887	0.920	4.58	9.72	2	3	144	16.9	5.21

Q05860-5	Isoform 5 of Formin-1 OS=Mus musculus GN=Fmn1 - [FMN1_MOUSE]	1.66	2	1	2	3	1.672	1.246	1.618	0.920	0.00	1.66	2	3	1204	133.1	5.71
Q9R1Z7	6-pyruvoyl tetrahydrobiopterin synthase OS=Mus musculus GN=Pts PE=2 SV=2 - [PTPS_MOUSE]	39.58	1	6	6	34	1.584	1.192	0.907	0.920	102.96	39.58	12	34	144	16.2	6.52
P21126	Ubiquitin-like protein 4A OS=Mus musculus GN=Ubl4a PE=2 SV=1 - [UBL4A_MOUSE]	59.87	2	9	9	41	1.324	0.979	1.002	0.920	117.48	59.87	16	41	157	17.8	8.44
Q8VE70	Programmed cell death protein 10 OS=Mus musculus GN=Pdcd10 PE=1 SV=1 -	38.68	3	8	8	30	0.982	1.381	0.889	0.920	79.94	38.68	14	30	212	24.7	8.19
Q8K093	Thyrotropin-releasing hormone-degrading ectoenzyme OS=Mus musculus GN=Trhde PE=2 SV=1 - [TRHDE_MOUSE]	11.41	1	8	8	13	1.004	0.973	0.940	0.920	21.13	11.41	11	13	1025	117.4	7.06
Q9JHW4	Selenocysteine-specific elongation factor OS=Mus musculus GN=Eefsec PE=2 SV=2 - [SELB_MOUSE]	16.98	1	8	8	18	1.008	1.043	0.906	0.920	44.07	16.98	12	18	583	63.5	8.29
Q91X84	CREB-regulated transcription coactivator 3 OS=Mus musculus GN=Crtc3 PE=2 SV=2 - [CRTC3_MOUSE]	5.98	2	3	3	8	1.799	1.110	1.029	0.920	23.09	5.98	6	8	619	67.0	6.83
P59240	Nephrocystin-4 OS=Mus musculus GN=Nphp4 PE=1 SV=2 - [NPHP4_MOUSE]	4.14	1	4	4	13	0.615	1.171	0.991	0.920	37.76	4.14	8	13	1425	157.2	7.77
P32043	Homeobox protein Hox-C5 OS=Mus musculus GN=Hoxc5 PE=2 SV=3 - [HXC5_MOUSE]	11.71	1	2	2	3	1.637	1.090	0.882	0.921	6.00	11.71	2	3	222	25.0	9.52
Q8CCJ4	APC membrane recruitment protein 2 OS=Mus musculus GN=Amer2 PE=1 SV=2 -	23.51	2	15	15	47	1.244	0.891	1.339	0.921	121.61	23.51	26	47	672	69.9	6.64
Q921L5	Conserved oligomeric Golgi complex subunit 2 OS=Mus musculus GN=Cog2 PE=2 SV=2 - [COG2_MOUSE]	23.12	2	13	14	29	0.872	0.993	0.671	0.921	90.33	23.12	22	29	731	82.0	6.21
O09010	Beta-1,3-N-acetylglucosaminyltransferase lunatic fringe OS=Mus musculus GN=Lfng PE=1 SV=1 - [LFNG_MOUSE]	3.97	1	1	1	2	0.958	0.405	0.825	0.921	3.76	3.97	2	2	378	41.9	8.81
Q91W52	Transmembrane protein 19 OS=Mus musculus GN=Tmem19 PE=2 SV=1 - [TMM19_MOUSE]	3.87	2	1	1	4	0.562	0.713	0.782	0.921	13.50	3.87	2	4	336	36.3	7.72
Q06890	Clusterin OS=Mus musculus GN=Clu PE=1 SV=1 - [CLUS_MOUSE]	35.04	6	15	15	128	1.137	1.353	1.055	0.921	373.14	35.04	29	128	448	51.6	5.67
E9PUB7	Protein misato homolog 1 OS=Mus musculus GN=Msto1 PE=2 SV=1 - [E9PUB7_MOUSE]	15.47	4	8	8	25	0.763	0.734	0.722	0.921	74.06	15.47	13	25	556	61.2	6.46
Q8CI96	CAP-Gly domain-containing linker protein 4 OS=Mus musculus GN=Clip4 PE=1 SV=1 - [CLIP4_MOUSE]	8.38	2	3	5	10	0.993	1.152	0.975	0.921	24.04	8.38	10	10	704	75.7	8.62
Q9D753	Exosome complex component RRP43 OS=Mus musculus GN=Exosc8 PE=2 SV=1 - [EXOS8_MOUSE]	9.78	3	3	3	5	1.354	1.183	0.722	0.921	7.99	9.78	4	5	276	29.9	5.20
P42208	Septin-2 OS=Mus musculus GN=Sept2 PE=1 SV=2 - [SEPT2_MOUSE]	66.76	10	16	17	144	1.355	0.712	0.932	0.921	432.37	66.76	33	144	361	41.5	6.55
B1ASU0	Interleukin-1 receptor accessory protein-like 1 OS=Mus musculus GN=Il1rapl1 PE=4 SV=1 - [B1ASU0_MOUSE]	11.21	3	6	7	14	0.788	2.329	1.251	0.921	41.74	11.21	10	14	696	79.7	6.32
Q64511	DNA topoisomerase 2-beta OS=Mus musculus GN=Top2b PE=1 SV=2 - [TOP2B_MOUSE]	26.49	7	38	40	91	0.677	0.924	0.939	0.921	246.69	26.49	62	91	1612	181.8	8.29
Q8BTZ4-2	Isoform 2 of Anaphase-promoting complex subunit 5 OS=Mus musculus GN=Anapc5 - [APC5_MOUSE]	9.08	2	6	7	15	1.236	1.042	0.804	0.921	34.93	9.08	10	15	727	81.6	6.71
Q9R1P4	Proteasome subunit alpha type-1 OS=Mus musculus GN=Psma1 PE=1 SV=1 - [PSA1_MOUSE]	67.30	1	16	16	114	1.869	0.930	0.793	0.921	322.52	67.30	29	114	263	29.5	6.46
Q6ZQK5	Arf-GAP with coiled-coil, ANK repeat and PH domain-containing protein 2 OS=Mus musculus GN=Acap2 PE=1 SV=2 - [ACAP2_MOUSE]	25.58	4	15	17	55	0.812	1.256	1.205	0.921	183.57	25.58	27	55	770	87.2	6.68
Q91VL8	Telomeric repeat-binding factor 2-interacting protein 1 OS=Mus musculus GN=Terf2ip PE=1 SV=1 - [TE2IP_MOUSE]	32.32	1	9	9	25	1.432	0.816	0.996	0.921	68.17	32.32	16	25	393	43.3	4.81

Q9JJC6	RILP-like protein 1 OS=Mus musculus GN=Rilpl1 PE=1 SV=1 - [RIPL1_MOUSE]	39.66	2	13	14	40	1.249	2.212	1.223	0.921	125.52	39.66	25	40	406	47.3	5.16
Q8K382	DENN domain-containing protein 1A OS=Mus musculus GN=Dennd1a PE=1 SV=2 - [DEN1A_MOUSE]	11.91	3	9	10	23	1.302	1.546	1.272	0.922	72.31	11.91	17	23	1016	111.5	6.79
A2AWA9	Rab GTPase-activating protein 1 OS=Mus musculus GN=Rabgap1 PE=2 SV=1 -	28.38	5	23	26	80	1.013	1.128	0.951	0.922	211.04	28.38	43	80	1064	120.7	5.25
P19221	Prothrombin OS=Mus musculus GN=F2 PE=1 SV=1 - [THRB_MOUSE]	27.67	3	15	16	52	4.055	2.377	0.498	0.922	137.66	27.67	30	52	618	70.2	6.43
D6RJ18	CD209 antigen-like protein C OS=Mus musculus GN=Cd209c PE=2 SV=1 - [D6RJ18_MOUSE]	12.73	2	1	1	1	5.826	0.659	1.669	0.922	2.98	12.73	1	1	110	12.3	9.06
Q9Z2N8	Actin-like protein 6A OS=Mus musculus GN=Actl6a PE=1 SV=2 - [ACL6A_MOUSE]	27.04	2	7	9	17	1.013	0.836	0.732	0.922	44.27	27.04	14	17	429	47.4	5.60
Q5U430-3	Isoform 3 of E3 ubiquitin-protein ligase UBR3 OS=Mus musculus GN=Ubr3 -	11.48	5	18	19	53	0.711	0.911	1.023	0.922	136.16	11.48	32	53	1769	198.5	5.67
Q8CI59	Metalloreductase STEAP3 OS=Mus musculus GN=Steap3 PE=1 SV=1 - [STEA3_MOUSE]	14.75	4	6	6	13	1.012	0.960	0.884	0.922	24.67	14.75	11	13	488	54.7	9.22
Q3U3J1	2-oxoisovalerate dehydrogenase subunit alpha, mitochondrial OS=Mus musculus GN=Bckdha PE=2 SV=1 - [Q3U3J1_MOUSE]	51.12	2	17	17	88	0.919	0.929	0.804	0.922	262.13	51.12	29	88	446	50.7	8.06
O88487	Cytoplasmic dynein 1 intermediate chain 2 OS=Mus musculus GN=Dync1i2 PE=2 SV=1 - [DC1I2_MOUSE]	25.49	2	1	10	76	3.142	0.381	0.748	0.922	178.34	25.49	18	76	612	68.4	5.29
Q6P9Q4	FH1/FH2 domain-containing protein 1 OS=Mus musculus GN=Fhod1 PE=2 SV=3 -	4.34	1	3	3	4	0.931	1.270	1.048	0.922	8.69	4.34	3	4	1197	129.5	6.28
Q80Y56	Rabenosyn-5 OS=Mus musculus GN=Zfyve20 PE=2 SV=1 - [RBNS5_MOUSE]	17.37	1	11	11	39	1.385	1.023	1.129	0.923	132.26	17.37	20	39	783	88.4	5.30
Q8BZR9	Uncharacterized protein C17orf85 homolog OS=Mus musculus PE=1 SV=1 - [CQ085_MOUSE]	14.15	1	7	8	14	0.818	0.720	1.020	0.923	32.80	14.15	12	14	615	70.0	5.80
E9PZ19	Protein Igsf9b OS=Mus musculus GN=Igsf9b PE=2 SV=1 - [E9PZ19_MOUSE]	17.39	2	20	20	36	0.937	0.704	1.002	0.923	106.20	17.39	31	36	1328	144.9	6.70
Q6PAN7	Proline-rich protein 18 OS=Mus musculus GN=Prr18 PE=2 SV=2 - [PRR18_MOUSE]	20.85	2	3	3	8	1.343	0.407	0.849	0.923	34.85	20.85	6	8	307	32.2	11.11
O35089	Protein cornichon homolog 2 OS=Mus musculus GN=Cnih2 PE=1 SV=2 - [CNIH2_MOUSE]	8.75	1	1	1	1	2.550	2.092	1.167	0.923	3.41	8.75	1	1	160	18.9	7.25
Q91VD8	MCG141290 OS=Mus musculus GN=Pcdhb17 PE=2 SV=1 - [Q91VD8_MOUSE]	0.88	1	1	1	1	0.953	1.212	1.052	0.923	1.73	0.88	1	1	799	87.7	4.86
B1AXN9	Ribosomal protein S6 kinase alpha-3 OS=Mus musculus GN=Rps6ka3 PE=2 SV=1 -	28.09	14	9	19	53	0.874	1.089	1.003	0.923	135.58	28.09	32	53	712	80.6	7.15
Q61043-2	Isoform 2 of Ninein OS=Mus musculus GN=Nin - [NIN_MOUSE]	1.48	3	2	3	3	1.488	0.933	0.952	0.923	3.31	1.48	3	3	2026	233.5	4.97
Q91ZU6	Dystonin OS=Mus musculus GN=Dst PE=1 SV=1 - [DYST_MOUSE]	24.24	5	2	160	508	0.496	0.413	0.854	0.923	1474.41	24.24	266	508	7389	833.1	5.31
E9PYH6	Protein Setd1a OS=Mus musculus GN=Setd1a PE=4 SV=1 - [E9PYH6_MOUSE]	4.25	2	5	5	11	0.724	0.847	0.945	0.923	35.50	4.25	9	11	1716	185.9	5.06
F7B2J7	Mucolipin-2 (Fragment) OS=Mus musculus GN=Mcoln2 PE=2 SV=1 - [F7B2J7_MOUSE]	8.85	1	1	1	1	0.906	1.102	1.416	0.923	1.66	8.85	1	1	113	12.9	9.22
A2A8R0	MCG17975 OS=Mus musculus GN=Zfyve9 PE=4 SV=1 - [A2A8R0_MOUSE]	11.02	3	9	10	25	1.216	0.765	1.097	0.923	79.50	11.02	18	25	1397	152.3	5.00
A8Y5Q0	[Pyruvate dehydrogenase [acetyl-transferring]]-phosphatase 1, mitochondrial OS=Mus musculus GN=Pdp1 PE=2 SV=1 - [A8Y5Q0_MOUSE]	44.81	4	17	17	66	0.828	0.886	0.936	0.923	210.38	44.81	31	66	578	65.4	6.90
Q9D4H2	GRIP and coiled-coil domain-containing protein 1 OS=Mus musculus GN=Gcc1 PE=1 SV=2 - [GCC1_MOUSE]	18.12	2	11	14	48	1.310	0.970	1.073	0.923	107.12	18.12	22	48	778	87.6	5.54
Q9CWW6	Peptidyl-prolyl cis-trans isomerase NIMA-interacting 4 OS=Mus musculus GN=Pin4 PE=2 SV=1 - [PIN4_MOUSE]	30.53	1	4	4	18	1.479	0.473	1.089	0.923	52.10	30.53	7	18	131	13.8	9.77

Q0KL01	UBX domain-containing protein 2B OS=Mus musculus GN=Ubxn2b PE=1 SV=2 -	42.90	1	10	10	31	1.572	1.175	1.200	0.923	89.55	42.90	17	31	331	37.4	5.94
Q91ZX7	Prolow-density lipoprotein receptor-related protein 1 OS=Mus musculus GN=Lrp1 PE=1 SV=1 - [LRP1_MOUSE]	27.52	3	100	105	484	1.100	1.085	0.924	0.923	1504.58	27.52	188	484	4545	504.4	5.36
Q8CJG1	Protein argonaute-1 OS=Mus musculus GN=Ago1 PE=1 SV=2 - [AGO1_MOUSE]	26.14	4	7	18	53	1.288	0.910	0.773	0.923	166.33	26.14	32	53	857	97.2	9.16
Q9EQS9	Immunoglobulin superfamily DCC subclass member 4 OS=Mus musculus GN=Igdcc4 PE=2 SV=1 - [illegible]	7.11	2	4	4	7	0.750	1.923	1.070	0.923	18.24	7.11	6	7	1252	134.7	6.18
Q01815-2	Isoform 2 of Voltage-dependent L-type calcium channel subunit alpha-1C OS=Mus musculus GN=Cacna1c - [illegible]	9.59	15	12	14	35	0.754	1.517	1.186	0.924	98.27	9.59	22	35	2128	238.8	6.93
E9Q132	60S ribosomal protein L24 OS=Mus musculus GN=Rpl24 PE=2 SV=1 - [E9Q132_MOUSE]	48.87	3	8	8	31	0.667	0.735	0.785	0.924	96.20	48.87	14	31	133	15.3	10.87
Q7TSG2	RNA polymerase II subunit A C-terminal domain phosphatase OS=Mus musculus GN=Ctdp1 PE=1 SV=1 - [CTDP1_MOUSE]	17.81	3	12	12	38	1.553	0.882	1.117	0.924	123.75	17.81	18	38	960	104.5	5.39
P52800	Ephrin-B2 OS=Mus musculus GN=Efnb2 PE=1 SV=1 - [EFNB2_MOUSE]	27.38	2	6	7	50	1.050	1.871	1.294	0.924	117.33	27.38	12	50	336	37.2	8.97
E9Q5F9	Protein Setd2 OS=Mus musculus GN=Setd2 PE=2 SV=1 - [E9Q5F9_MOUSE]	2.52	3	4	6	12	0.857	0.700	1.006	0.924	30.59	2.52	8	12	2537	285.5	6.30
Q0PHV7	Dapper homolog 3 OS=Mus musculus GN=Dact3 PE=1 SV=1 - [DACT3_MOUSE]	10.98	1	5	5	11	0.747	1.378	1.364	0.924	35.50	10.98	9	11	610	63.2	10.42
A8Y5H7	Protein Sec14l1 OS=Mus musculus GN=Sec14l1 PE=2 SV=1 - [A8Y5H7_MOUSE]	5.87	3	4	4	7	1.576	1.483	1.260	0.924	18.68	5.87	7	7	715	81.2	6.34
G3UVV3	MCG1556, isoform CRA_b OS=Mus musculus GN=Zfp407 PE=4 SV=1 - [G3UVV3_MOUSE]	2.54	1	2	2	2	2.762	0.519	1.127	0.924	2.39	2.54	2	2	2246	245.8	6.47
A2AQA7	Intron-binding protein aquarius OS=Mus musculus GN=Aqr PE=2 SV=1 - [A2AQA7_MOUSE]	9.21	2	9	10	22	0.720	0.868	0.761	0.924	76.50	9.21	16	22	1400	161.8	7.17
A2BDX0	Activity-dependent neuroprotector homeobox protein OS=Mus musculus GN=Adnp PE=3 SV=1 - [A2BDX0_MOUSE]	14.89	2	14	14	43	0.897	1.066	0.925	0.924	114.56	14.89	23	43	1108	124.2	6.86
E9QKT1	Ubiquitin-protein ligase E3A OS=Mus musculus GN=Ube3a PE=2 SV=1 - [E9QKT1_MOUSE]	35.63	2	28	29	127	0.876	1.029	0.863	0.924	383.37	35.63	48	127	870	99.8	5.08
Q9QZD9	Eukaryotic translation initiation factor 3 subunit I OS=Mus musculus GN=Eif3i PE=1 SV=1 - [EIF3I_MOUSE]	53.85	2	13	14	54	1.549	1.072	0.889	0.924	154.24	53.85	25	54	325	36.4	5.64
E9PVM1	Type II inositol 3,4-bisphosphate 4-phosphatase OS=Mus musculus GN=Inpp4b PE=2 SV=1 - [illegible]	13.81	6	11	12	25	0.707	1.012	0.999	0.924	58.95	13.81	21	25	927	105.1	6.25
Q99LF4	tRNA-splicing ligase RtcB homolog OS=Mus musculus GN=D10Wsu52e PE=2 SV=1 -	58.61	1	26	26	154	0.819	0.762	0.842	0.924	410.49	58.61	48	154	505	55.2	7.23
Q8BTF8	RNA-binding Raly-like protein OS=Mus musculus GN=Ralyl PE=2 SV=1 - [RALYL_MOUSE]	19.11	4	5	7	27	0.554	0.826	0.780	0.924	66.10	19.11	13	27	293	32.4	7.93
G3X957	Liprin-beta-2 OS=Mus musculus GN=Ppfibp2 PE=4 SV=1 - [G3X957_MOUSE]	13.49	5	5	7	19	1.444	1.710	1.107	0.924	27.21	13.49	10	19	882	98.6	6.27
Q8CFV9	Riboflavin kinase OS=Mus musculus GN=Rfk PE=1 SV=2 - [RIFK_MOUSE]	33.55	1	4	4	14	0.763	0.868	0.776	0.924	37.42	33.55	8	14	155	17.4	7.55
Q9EPJ9	ADP-ribosylation factor GTPase-activating protein 1 OS=Mus musculus GN=Arfgap1 PE=1 SV=2 - [ARFG1_MOUSE]	58.94	3	24	24	184	1.075	1.289	1.300	0.925	501.80	58.94	39	184	414	45.3	5.57
Q5NCI0-2	Isoform 2 of Up-regulator of cell proliferation OS=Mus musculus GN=Urgcp -	3.40	3	3	3	5	0.536	0.865	0.742	0.925	16.72	3.40	5	5	883	100.0	6.70
Q792Z1	MCG140784 OS=Mus musculus GN=Try10 PE=2 SV=1 - [Q792Z1_MOUSE]	4.07	1	1	1	25	1.407	1.303	1.002	0.925	63.17	4.07	2	25	246	26.2	5.83
P35564	Calnexin OS=Mus musculus GN=Canx PE=1 SV=1 - [CALX_MOUSE]	34.69	1	21	22	242	0.876	1.034	0.862	0.925	666.43	34.69	40	242	591	67.2	4.64

Q8CG73	Protein fantom OS=Mus musculus GN=Rpgrip1l PE=1 SV=2 - [FTM_MOUSE]	4.67	4	2	6	10	3.045	1.501	1.035	0.925	26.41	4.67	7	10	1264	144.9	5.43
Q9DBE0	Cysteine sulfinic acid decarboxylase OS=Mus musculus GN=Csad PE=2 SV=1 - [CSAD_MOUSE]	19.88	2	8	10	25	1.777	0.872	0.711	0.925	61.51	19.88	17	25	493	55.1	6.61
Q8BMA5	Protein NPAT OS=Mus musculus GN=Npat PE=2 SV=2 - [NPAT_MOUSE]	2.25	1	2	2	4	0.949	1.237	0.930	0.925	10.21	2.25	2	4	1420	152.1	5.54
P00520-2	Isoform II of Tyrosine-protein kinase ABL1 OS=Mus musculus GN=Abl1 - [ABL1_MOUSE]	14.59	4	8	12	24	0.809	0.633	1.185	0.925	58.84	14.59	18	24	1117	122.1	8.48
P12023	Amyloid beta A4 protein OS=Mus musculus GN=App PE=1 SV=3 - [A4_MOUSE]	32.60	2	2	22	144	1.647	0.936	0.928	0.925	454.01	32.60	41	144	770	86.7	4.79
B1AZF9	Apoptosis-associated tyrosine kinase OS=Mus musculus GN=Aatk PE=3 SV=1 - [B1AZF9_MOUSE]	20.96	6	17	17	51	1.548	0.885	1.151	0.925	178.46	20.96	29	51	1317	139.4	4.55
Q80UM5	NEL-like 2 (Chicken) OS=Mus musculus GN=Nell2 PE=2 SV=1 - [Q80UM5_MOUSE]	12.70	2	11	11	24	1.106	1.192	1.099	0.925	63.09	12.70	17	24	819	91.4	5.82
Q4VC33	Macrophage erythroblast attacher OS=Mus musculus GN=Maea PE=1 SV=1 - [MAEA_MOUSE]	20.71	2	7	7	17	0.986	1.416	0.807	0.925	47.79	20.71	10	17	396	45.3	8.69
P06909	Complement factor H OS=Mus musculus GN=Cfh PE=1 SV=2 - [CFAH_MOUSE]	20.75	4	17	20	59	3.029	1.508	0.756	0.925	174.87	20.75	35	59	1234	139.0	6.99
Q3TWW8	Protein Srsf6 OS=Mus musculus GN=Srsf6 PE=2 SV=1 - [Q3TWW8_MOUSE]	18.58	1	7	8	24	0.831	0.857	0.856	0.925	57.23	18.58	11	24	339	39.0	11.46
Q8K0V4	CCR4-NOT transcription complex subunit 3 OS=Mus musculus GN=Cnot3 PE=1 SV=1 - [CNOT3_MOUSE]	10.65	2	9	9	27	1.360	0.840	1.128	0.925	66.27	10.65	14	27	751	81.9	6.20
Q6NXJ0	Protein WWC2 OS=Mus musculus GN=Wwc2 PE=2 SV=1 - [WWC2_MOUSE]	8.59	1	9	9	16	1.841	0.907	0.738	0.925	39.57	8.59	13	16	1187	132.5	5.71
Q9CY66-2	Isoform 2 of H/ACA ribonucleoprotein complex subunit 1 OS=Mus musculus GN=Gar1 - [GAR1_MOUSE]	25.33	4	5	5	10	1.051	0.799	1.038	0.925	37.01	25.33	8	10	229	23.3	11.02
Q9R0E1	Procollagen-lysine,2-oxoglutarate 5-dioxygenase 3 OS=Mus musculus GN=Plod3 PE=1 SV=1 - [PLOD3_MOUSE]	11.61	1	7	7	15	1.219	1.020	0.800	0.925	42.74	11.61	10	15	741	84.9	6.23
Q9CRC0	Vitamin K epoxide reductase complex subunit 1 OS=Mus musculus GN=Vkorc1 PE=2 SV=1 - [VKOR1_MOUSE]	8.07	1	1	1	2	0.501	0.769	0.621	0.926	7.06	8.07	2	2	161	17.8	9.14
F2Z497	Protein D130043K22Rik OS=Mus musculus GN=D130043K22Rik PE=2 SV=1 - [F2Z497_MOUSE]	5.37	2	3	3	6	1.445	1.307	0.951	0.926	21.32	5.37	5	6	782	84.4	4.77
Q3UMU9-2	Isoform 2 of Hepatoma-derived growth factor-related protein 2 OS=Mus musculus GN=Hdgfrp2 - [HDGR2_MOUSE]	13.34	4	8	11	27	0.689	0.916	0.914	0.926	65.59	13.34	19	27	667	74.0	8.54
Q9Z0M5	Lysosomal acid lipase/cholesteryl ester hydrolase OS=Mus musculus GN=Lipa PE=2 SV=2 - [LICH_MOUSE]	4.03	1	1	1	1	1.355	0.951	1.256	0.926	0.00	4.03	1	1	397	45.3	8.07
P97793	ALK tyrosine kinase receptor OS=Mus musculus GN=Alk PE=1 SV=2 - [ALK_MOUSE]	13.82	1	15	16	33	0.865	0.874	0.904	0.926	107.78	13.82	25	33	1621	174.8	6.93
Q9JKN1	Zinc transporter 7 OS=Mus musculus GN=Slc30a7 PE=1 SV=1 - [ZNT7_MOUSE]	3.97	1	1	1	8	0.914	0.868	0.830	0.926	17.02	3.97	2	8	378	41.8	6.84
Q9D4H4-2	Isoform 2 of Angiomotin-like protein 1 OS=Mus musculus GN=Amotl1 - [AMOL1_MOUSE]	10.43	2	7	10	29	1.067	0.671	1.018	0.926	72.45	10.43	18	29	882	98.4	7.37
Q6P5H2	Nestin OS=Mus musculus GN=Nes PE=1 SV=1 - [NEST_MOUSE]	16.85	2	22	23	46	1.614	1.106	0.887	0.926	125.04	16.85	34	46	1864	207.0	4.34
Q9D662	Protein transport protein Sec23B OS=Mus musculus GN=Sec23b PE=2 SV=1 - [SC23B_MOUSE]	16.04	2	7	11	43	0.800	0.777	0.841	0.926	148.56	16.04	20	43	767	86.4	6.96
B1AVP0	Protein Phactr2 OS=Mus musculus GN=Phactr2 PE=2 SV=1 - [B1AVP0_MOUSE]	10.76	5	6	8	18	0.859	1.134	1.587	0.926	42.24	10.76	12	18	632	69.2	7.18
Q8BRE0	Protein RD3 OS=Mus musculus GN=Rd3 PE=2 SV=1 - [RD3_MOUSE]	12.82	3	1	2	3	1.025	1.110	1.077	0.926	4.58	12.82	2	3	195	22.7	8.66
Q8CH18-3	Isoform 3 of Cell division cycle and apoptosis regulator protein 1 OS=Mus musculus GN=Ccar1 - [CCAR1_MOUSE]	16.24	4	15	16	60	0.971	0.926	0.925	0.926	131.91	16.24	27	60	1084	124.7	6.02

Q9QYH6	Melanoma-associated antigen D1 OS=Mus musculus GN=Maged1 PE=1 SV=1 -	19.23	1	8	10	59	1.467	0.821	0.984	0.926	164.76	19.23	15	59	775	85.6	7.50
E9Q084	TATA-box-binding protein (Fragment) OS=Mus musculus GN=Tbp PE=2 SV=1 - [E9Q084_MOUSE]	3.89	5	1	1	2	0.779	0.924	0.793	0.926	2.22	3.89	1	2	180	19.4	8.19
Q3UH93	Plexin-D1 OS=Mus musculus GN=Plxnd1 PE=1 SV=1 - [PLXD1_MOUSE]	15.01	2	19	21	57	1.008	2.672	1.558	0.926	186.80	15.01	36	57	1925	211.5	7.11
Q9D6S7	Ribosome-recycling factor, mitochondrial OS=Mus musculus GN=Mrrf PE=1 SV=1 - [RRFM_MOUSE]	35.11	1	6	6	40	1.111	0.493	1.271	0.926	141.38	35.11	12	40	262	29.0	9.85
Q6PFR5	Transformer-2 protein homolog alpha OS=Mus musculus GN=Tra2a PE=1 SV=1 - [TRA2A_MOUSE]	34.88	2	6	7	20	0.650	0.977	0.867	0.926	62.21	34.88	11	20	281	32.3	11.28
Q60605	Myosin light polypeptide 6 OS=Mus musculus GN=Myl6 PE=1 SV=3 - [MYL6_MOUSE]	80.13	1	1	10	146	2.194	1.095	1.012	0.926	396.80	80.13	18	146	151	16.9	4.65
P21550	Beta-enolase OS=Mus musculus GN=Eno3 PE=1 SV=3 - [ENOB_MOUSE]	54.61	9	9	21	615	1.626	2.215	0.906	0.926	2158.84	54.61	37	615	434	47.0	7.18
Q5SQY2	Biorientation of chromosomes in cell division protein 1 OS=Mus musculus GN=Bod1 PE=2 SV=1 - [BOD1_MOUSE]	44.51	1	5	7	16	1.414	1.419	1.018	0.926	47.92	44.51	12	16	173	18.4	5.90
P62862	40S ribosomal protein S30 OS=Mus musculus GN=Fau PE=2 SV=1 - [RS30_MOUSE]	16.95	2	1	1	4	1.233	1.201	0.965	0.927	9.17	16.95	2	4	59	6.6	12.15
F7D291	MTSS1-like protein (Fragment) OS=Mus musculus GN=Mtss1l PE=4 SV=1 - [F7D291_MOUSE]	34.34	2	4	10	35	0.689	0.818	0.942	0.927	121.57	34.34	18	35	364	39.1	8.00
G3X952	MCG20985, isoform CRA_a OS=Mus musculus GN=Zkscan2 PE=4 SV=1 - [G3X952_MOUSE]	2.08	1	1	1	2	2.036	1.395	0.802	0.927	3.77	2.08	1	2	960	110.4	7.85
Q8K1A6	Coiled-coil and C2 domain-containing protein 1A OS=Mus musculus GN=Cc2d1a PE=1 SV=2 - [C2D1A_MOUSE]	36.90	1	4	32	95	1.413	1.048	1.100	0.927	269.56	36.90	56	95	943	103.6	7.84
E9Q7P2	Protein Cacna1i OS=Mus musculus GN=Cacna1i PE=2 SV=1 - [E9Q7P2_MOUSE]	3.59	3	3	5	7	4.596	1.613	1.254	0.927	10.81	3.59	6	7	2199	242.7	6.47
Q9D1X9	Transmembrane protein 50B OS=Mus musculus GN=Tmem50b PE=2 SV=1 - [TM50B_MOUSE]	14.56	3	2	2	4	1.174	0.960	0.937	0.927	10.78	14.56	3	4	158	17.9	5.39
D3Z3N8	G1/S-specific cyclin-E1 (Fragment) OS=Mus musculus GN=Ccne1 PE=2 SV=1 - [D3Z3N8_MOUSE]	9.58	2	1	1	1	15.167	3.563	3.876	0.927	0.00	9.58	1	1	167	19.7	6.23
Q9EPU5	Tumor necrosis factor receptor superfamily member 21 OS=Mus musculus GN=Tnfrsf21 PE=1 SV=2 - [illegible]	16.34	1	9	9	20	0.994	0.793	0.903	0.927	55.23	16.34	17	20	655	71.9	7.46
Q9CQF7	Prefoldin 1 OS=Mus musculus GN=Pfdn1 PE=2 SV=1 - [Q9CQF7_MOUSE]	60.66	2	8	8	61	1.608	1.024	1.154	0.927	131.75	60.66	14	61	122	14.2	6.81
Q6IMB2	ADP-ribosylation factor-like 9 OS=Mus musculus GN=Arl9 PE=2 SV=1 - [Q6IMB2_MOUSE]	10.53	2	1	1	1	1.174	1.249	0.820	0.927	2.33	10.53	1	1	133	15.1	8.19
P15066	Transcription factor jun-D OS=Mus musculus GN=Jund PE=1 SV=1 - [JUND_MOUSE]	16.42	2	4	5	12	1.136	1.180	1.177	0.927	40.46	16.42	7	12	341	34.9	7.44
F6TKE8	Ly6/PLAUR domain-containing protein 1 (Fragment) OS=Mus musculus GN=Lypd1 PE=4 SV=1 - [F6TKE8_MOUSE]	14.63	4	1	1	15	1.188	2.475	0.993	0.927	51.10	14.63	2	15	82	9.1	4.88
Q9JM52-3	Isoform 3 of Misshapen-like kinase 1 OS=Mus musculus GN=Mink1 - [MINK1_MOUSE]	35.24	7	1	37	123	1.848	2.079	0.800	0.927	353.12	35.24	60	123	1345	151.2	7.96
P59325	Eukaryotic translation initiation factor 5 OS=Mus musculus GN=Eif5 PE=1 SV=1 - [IF5_MOUSE]	35.90	2	15	16	80	0.930	0.648	0.908	0.927	243.03	35.90	30	80	429	48.9	5.52
K4DI65	Cold inducible RNA binding protein, isoform CRA_a OS=Mus musculus GN=Cirbp PE=4 SV=1 - [K4DI65_MOUSE]	45.24	3	6	6	48	1.496	0.763	1.281	0.927	140.75	45.24	11	48	168	18.1	9.73
Q8R349	Cell division cycle protein 16 homolog OS=Mus musculus GN=Cdc16 PE=2 SV=1 - [CDC16_MOUSE]	15.16	1	8	8	30	0.661	0.986	0.739	0.927	86.49	15.16	13	30	620	71.4	5.76
Q9EPU0-2	Isoform 2 of Regulator of nonsense transcripts 1 OS=Mus musculus GN=Upf1 -	40.88	2	35	35	104	0.788	0.965	0.866	0.927	296.53	40.88	58	104	1113	122.6	6.68

Q69ZK0	Phosphatidylinositol 3,4,5-trisphosphate-dependent Rac exchanger 1 protein OS=Mus musculus GN=Prex1 PE=1 SV=2 - [PREX1_MOUSE]	20.79	5	32	33	73	0.626	0.921	0.891	0.927	202.15	20.79	54	73	1650	184.8	6.29
Q8BMK5-2	Isoform 2 of Uncharacterized protein C12orf68 homolog OS=Mus musculus -	32.89	2	3	4	16	1.483	0.837	1.003	0.927	52.65	32.89	7	16	152	16.2	4.27
Q3U2G2	Heat shock 70 kDa protein 4 OS=Mus musculus GN=Hspa4 PE=2 SV=1 - [Q3U2G2_MOUSE]	59.14	1	3	51	417	0.881	1.037	1.132	0.927	1224.79	59.14	95	417	842	94.1	5.21
B1ATZ0	Hepatocyte growth factor-regulated tyrosine kinase substrate OS=Mus musculus GN=Hgs PE=2 SV=1 - [B1ATZ0_MOUSE]	26.50	6	18	19	88	1.123	1.033	1.027	0.927	216.85	26.50	36	88	766	85.7	7.47
Q8C0W0	Protein Tmsb15b2 OS=Mus musculus GN=Tmsb15l PE=2 SV=1 - [Q8C0W0_MOUSE]	75.00	2	1	4	23	1.762	1.007	1.480	0.928	39.34	75.00	8	23	80	9.2	6.13
Q9EPK6	Nucleotide exchange factor SIL1 OS=Mus musculus GN=Sil1 PE=1 SV=2 - [SIL1_MOUSE]	11.61	1	4	5	13	1.352	1.188	0.973	0.928	28.89	11.61	9	13	465	52.4	5.27
Q6KAS7-2	Isoform 2 of Zinc finger protein 521 OS=Mus musculus GN=Znf521 - [ZN521_MOUSE]	4.05	2	4	4	4	1.105	1.416	0.837	0.928	7.38	4.05	4	4	1310	147.4	7.01
Q9DB52	Protein FAM122A OS=Mus musculus GN=Fam122a PE=1 SV=1 - [F122A_MOUSE]	38.38	1	6	6	19	2.029	0.917	1.117	0.928	79.60	38.38	9	19	284	30.3	6.79
Q8BM92	Cadherin-7 OS=Mus musculus GN=Cdh7 PE=2 SV=1 - [CADH7_MOUSE]	15.03	2	9	9	17	1.127	0.854	0.916	0.928	45.71	15.03	11	17	785	87.1	4.82
P63073	Eukaryotic translation initiation factor 4E OS=Mus musculus GN=Eif4e PE=1 SV=1 - [IF4E_MOUSE]	31.34	3	6	7	30	1.026	1.040	0.895	0.928	98.33	31.34	12	30	217	25.0	6.15
Q91Z69	SLIT-ROBO Rho GTPase-activating protein 1 OS=Mus musculus GN=Srgap1 PE=1 SV=2 - [SRGP1_MOUSE]	25.42	3	15	24	59	1.095	0.692	0.802	0.928	161.41	25.42	39	59	1062	121.4	6.74
Q9CQ45	Neudesin OS=Mus musculus GN=Nenf PE=1 SV=1 - [NENF_MOUSE]	63.16	1	8	8	49	1.988	1.184	1.146	0.928	145.60	63.16	14	49	171	18.9	5.27
Q14B46-2	Isoform 2 of Rhotekin-2 OS=Mus musculus GN=Rtkn2 - [RTKN2_MOUSE]	2.00	3	1	1	1	0.902	1.036	0.905	0.928	2.13	2.00	1	1	601	66.6	8.07
P62484	Abl interactor 2 OS=Mus musculus GN=Abi2 PE=1 SV=1 - [ABI2_MOUSE]	35.20	1	9	12	72	0.985	1.339	1.283	0.928	173.88	35.20	22	72	446	49.4	6.01
Q8QZY1	Eukaryotic translation initiation factor 3 subunit L OS=Mus musculus GN=Eif3l PE=1 SV=1 - [EIF3L_MOUSE]	35.28	1	18	18	77	0.676	0.986	0.773	0.928	221.03	35.28	31	77	564	66.6	6.44
Q03958	Prefoldin subunit 6 OS=Mus musculus GN=Pfdn6 PE=2 SV=1 - [PFD6_MOUSE]	55.91	1	2	9	55	1.290	1.045	1.201	0.928	145.38	55.91	18	55	127	14.4	8.88
Q9ERV1	Probable E3 ubiquitin-protein ligase makorin-2 OS=Mus musculus GN=Mkrn2 PE=2 SV=2 - [MKRN2_MOUSE]	29.57	1	11	11	19	1.469	0.726	1.053	0.928	44.27	29.57	16	19	416	46.6	7.34
E9Q7N5	Ribosome-releasing factor 2, mitochondrial OS=Mus musculus GN=Gfm2 PE=2 SV=1 - [E9Q7N5_MOUSE]	20.78	6	15	15	34	0.620	0.750	0.738	0.928	88.55	20.78	25	34	741	82.0	6.32
H7BX22	Ran-specific GTPase-activating protein OS=Mus musculus GN=Ranbp1 PE=2 SV=1 - [H7BX22_MOUSE]	31.37	2	4	4	61	1.839	0.943	1.280	0.928	157.01	31.37	7	61	153	17.9	8.24
P39087	Glutamate receptor ionotropic, kainate 2 OS=Mus musculus GN=Grik2 PE=1 SV=4 - [GRIK2_MOUSE]	19.27	4	14	16	34	0.716	1.185	0.978	0.928	106.75	19.27	23	34	908	102.4	7.77
Q9CWL8	Beta-catenin-like protein 1 OS=Mus musculus GN=Ctnnbl1 PE=1 SV=1 - [CTBL1_MOUSE]	13.85	1	8	8	10	0.688	0.990	0.757	0.928	23.46	13.85	9	10	563	64.9	5.07
A2AMW0	Capping protein (Actin filament) muscle Z-line, beta, isoform CRA_a OS=Mus musculus GN=Capzb PE=4 SV=1 - [A2AMW0_MOUSE]	62.69	6	3	20	292	0.842	1.382	1.131	0.928	722.71	62.69	34	292	260	29.3	6.92
Q4KL52	Zfyve19 protein OS=Mus musculus GN=Zfyve19 PE=2 SV=1 - [Q4KL52_MOUSE]	32.73	2	8	8	29	1.833	0.964	1.305	0.929	83.73	32.73	14	29	330	36.3	7.81
Q00897	Alpha-1-antitrypsin 1-4 OS=Mus musculus GN=Serpina1d PE=2 SV=1 - [A1AT4_MOUSE]	49.64	1	5	18	128	2.649	1.546	1.023	0.929	346.72	49.64	33	128	413	46.0	5.44

Q62120	Tyrosine-protein kinase JAK2 OS=Mus musculus GN=Jak2 PE=1 SV=2 - [JAK2_MOUSE]	4.52	3	3	5	11	0.501	1.048	0.793	0.929	27.50	4.52	7	11	1129	130.2	7.52
Q8C5W3	Tubulin-specific chaperone cofactor E-like protein OS=Mus musculus GN=Tbcel PE=1 SV=1 - [TBCEL_MOUSE]	54.48	3	18	18	53	1.386	0.955	0.873	0.929	137.46	54.48	34	53	424	48.0	5.53
P62869	Transcription elongation factor B polypeptide 2 OS=Mus musculus GN=Tceb2 PE=1 SV=1 - [ELOB_MOUSE]	84.75	1	8	8	25	1.164	0.986	1.028	0.929	67.27	84.75	12	25	118	13.2	5.01
Q9QYX7	Protein piccolo OS=Mus musculus GN=Pclo PE=1 SV=4 - [PCLO_MOUSE]	41.20	3	157	161	886	0.924	1.013	1.691	0.929	2412.24	41.20	276	886	5068	550.5	6.51
Q02248	Catenin beta-1 OS=Mus musculus GN=Ctnnb1 PE=1 SV=1 - [CTNB1_MOUSE]	54.29	8	30	35	210	0.695	1.141	0.973	0.929	605.06	54.29	64	210	781	85.4	5.86
Q3U319	E3 ubiquitin-protein ligase BRE1B OS=Mus musculus GN=Rnf40 PE=2 SV=2 - [BRE1B_MOUSE]	24.58	4	18	19	48	1.201	0.792	0.943	0.929	145.29	24.58	30	48	1001	113.9	6.48
E9Q813	Ras-GEF domain-containing family member 1B OS=Mus musculus GN=Rasgef1b PE=2 SV=1 [E9Q813_MOUSE]	1.86	3	1	1	1	0.355	0.592	0.841	0.929	0.00	1.86	1	1	431	50.3	8.06
Q6TXD4	Dynamin-binding protein OS=Mus musculus GN=Dnmbp PE=1 SV=2 - [DNMBP_MOUSE]	3.10	2	3	3	3	0.441	0.899	1.023	0.929	5.66	3.10	3	3	1580	177.2	5.41
Q9DCJ1	Target of rapamycin complex subunit LST8 OS=Mus musculus GN=Mlst8 PE=1 SV=1 - [LST8_MOUSE]	27.61	1	7	7	17	1.768	1.006	0.956	0.929	58.80	27.61	12	17	326	35.8	5.86
Q921S7	39S ribosomal protein L37, mitochondrial OS=Mus musculus GN=Mrpl37 PE=2 SV=1 - [RM37_MOUSE]	32.62	1	13	13	46	0.997	0.787	0.866	0.929	152.79	32.62	23	46	423	48.3	8.84
Q9R062	Glycogenin-1 OS=Mus musculus GN=Gyg1 PE=2 SV=3 - [GLYG_MOUSE]	16.82	3	5	5	24	1.349	0.670	0.740	0.929	83.47	16.82	7	24	333	37.4	5.29
Q80W54	CAAX prenyl protease 1 homolog OS=Mus musculus GN=Zmpste24 PE=1 SV=2 -	15.79	2	6	6	18	0.776	0.910	0.765	0.929	45.89	15.79	10	18	475	54.7	6.95
Q9Z2W1	Serine/threonine-protein kinase 25 OS=Mus musculus GN=Stk25 PE=1 SV=2 - [STK25_MOUSE]	21.60	2	5	9	18	0.419	1.115	0.902	0.929	50.53	21.60	13	18	426	48.1	6.81
E9PVC5	Eukaryotic translation initiation factor 4 gamma 1 OS=Mus musculus GN=Eif4g1 PE=2 SV=1 - [E9PVC5_MOUSE]	31.37	6	1	44	188	0.695	0.556	0.754	0.929	476.27	31.37	74	188	1594	175.4	5.38
Q8C5X1	Protein 4931406P16Rik OS=Mus musculus GN=4931406P16Rik PE=2 SV=1 - [Q8C5X1_MOUSE]	4.72	1	2	2	3	1.546	0.356	0.774	0.929	8.07	4.72	3	3	1059	115.5	7.24
Q99ME6-2	Isoform B of Protein ATP1B4 OS=Mus musculus GN=Atp1b4 - [AT1B4_MOUSE]	1.99	2	1	1	1	2.037	1.658	1.440	0.929	0.00	1.99	1	1	352	40.9	4.78
Q6P5F7	Protein tweety homolog 3 OS=Mus musculus GN=Ttyh3 PE=1 SV=1 - [TTYH3_MOUSE]	14.89	2	6	6	32	0.612	1.252	1.078	0.929	91.15	14.89	10	32	524	57.7	5.85
Q9ER72	Cysteine--tRNA ligase, cytoplasmic OS=Mus musculus GN=Cars PE=1 SV=2 - [SYCC_MOUSE]	39.59	2	32	32	106	0.765	0.670	0.737	0.929	295.31	39.59	56	106	831	94.8	6.76
A9C437	Chloride channel protein 2 OS=Mus musculus GN=Clcn2 PE=2 SV=1 - [A9C437_MOUSE]	6.40	3	4	4	8	14.813	2.293	0.526	0.930	25.14	6.40	6	8	891	97.6	8.56
Q8C0D5	Elongation factor Tu GTP-binding domain-containing protein 1 OS=Mus musculus GN=Eftud1 PE=2 SV=1 - [ETUD1_MOUSE]	21.03	2	21	22	41	0.755	0.866	0.871	0.930	112.92	21.03	33	41	1127	125.7	6.16
P59759	MKL/myocardin-like protein 2 OS=Mus musculus GN=Mkl2 PE=1 SV=1 - [MKL2_MOUSE]	32.96	2	1	25	102	1.087	1.479	1.696	0.930	267.66	32.96	41	102	1080	117.5	6.16
Q62426	Cystatin-B OS=Mus musculus GN=Cstb PE=1 SV=1 - [CYTB_MOUSE]	47.96	1	3	4	21	1.363	0.792	0.960	0.930	54.17	47.96	7	21	98	11.0	7.39
O35286	Putative pre-mRNA-splicing factor ATP-dependent RNA helicase DHX15 OS=Mus musculus GN=Dhx15 PE=2 SV=2 - [DHX15_MOUSE]	32.33	1	21	22	88	0.552	0.675	0.824	0.930	245.89	32.33	35	88	795	90.9	7.46
P14069	Protein S100-A6 OS=Mus musculus GN=S100a6 PE=1 SV=3 - [S10A6_MOUSE]	47.19	1	4	4	12	2.621	1.704	0.517	0.930	28.16	47.19	6	12	89	10.0	5.48
Q0P5V9-3	Isoform 3 of Solute carrier family 45 member 4 OS=Mus musculus GN=Slc45a4 -	5.92	4	2	2	2	0.516	0.491	0.979	0.930	3.56	5.92	2	2	591	64.6	6.25

Q5SWZ5	Myosin phosphatase Rho-interacting protein OS=Mus musculus GN=Mprip PE=2 SV=1 - [Q5SWZ5_MOUSE]	35.79	3	26	61	168	0.993	0.956	1.332	0.930	488.46	35.79	102	168	2269	257.1	5.59
Q9QYM9	Tomoregulin-2 OS=Mus musculus GN=Tmeff2 PE=2 SV=1 - [TEFF2_MOUSE]	12.03	2	5	5	13	0.907	1.278	1.029	0.930	23.48	12.03	8	13	374	41.4	5.15
H3BKM2	Nuclear-interacting partner of ALK OS=Mus musculus GN=Zc3hc1 PE=2 SV=1 - [H3BKM2_MOUSE]	13.29	6	4	4	11	0.968	1.165	1.007	0.930	37.70	13.29	7	11	459	50.5	5.50
Q61584	Fragile X mental retardation syndrome-related protein 1 OS=Mus musculus GN=Fxr1 PE=1 SV=2 - [FXR1_MOUSE]	33.97	7	14	18	83	0.866	0.589	0.779	0.930	277.50	33.97	33	83	677	76.2	6.98
Q91WE6-3	Isoform 3 of Threonylcarbamoyladenosine tRNA methylthiotransferase OS=Mus musculus GN=Cdkal1 - [CDKAL_MOUSE]	7.41	4	2	2	4	0.789	0.859	0.737	0.930	11.07	7.41	4	4	351	39.7	7.40
D3YVV6	Protein Hs3st4 OS=Mus musculus GN=Hs3st4 PE=4 SV=1 - [D3YVV6_MOUSE]	1.78	1	1	1	1	0.975	1.401	1.416	0.930	0.00	1.78	1	1	449	49.1	9.23
Q61475	Complement decay-accelerating factor, GPI-anchored OS=Mus musculus GN=Cd55 PE=2 SV=2 - [DAF1_MOUSE]	5.13	1	2	2	3	0.989	0.946	0.820	0.930	6.12	5.13	3	3	390	42.6	8.40
D3Z4L8	Protein Ifi27l1 (Fragment) OS=Mus musculus GN=Ifi27l1 PE=2 SV=1 - [D3Z4L8_MOUSE]	13.33	6	1	1	1	1.082	1.062	1.231	0.930	3.24	13.33	1	1	105	10.3	11.19
D3Z3R1	60S ribosomal protein L36 OS=Mus musculus GN=Gm5745 PE=3 SV=1 - [D3Z3R1_MOUSE]	35.58	5	4	5	43	0.702	0.824	0.802	0.930	120.34	35.58	10	43	104	12.1	11.36
E9PUQ8	Protein Dgkd OS=Mus musculus GN=Dgkd PE=2 SV=1 - [E9PUQ8_MOUSE]	12.95	6	11	15	47	0.595	1.087	1.360	0.931	155.45	12.95	23	47	1220	135.1	7.81
Q69ZX8	Actin-binding LIM protein 3 OS=Mus musculus GN=Ablim3 PE=1 SV=2 - [ABLM3_MOUSE]	22.14	1	11	12	50	1.182	0.892	1.219	0.931	114.12	22.14	23	50	682	77.6	8.54
Q8C0V7	Sperm motility kinase W OS=Mus musculus GN=Gm4776 PE=2 SV=2 - [SMKW_MOUSE]	2.40	1	1	1	1	2.001	1.706	0.816	0.931	3.67	2.40	1	1	499	55.4	8.68
A2AHG0	Leucine zipper putative tumor suppressor 3 OS=Mus musculus GN=Lzts3 PE=2 SV=1 - [LZTS3_MOUSE]	42.14	4	20	21	81	1.439	1.395	2.180	0.931	253.64	42.14	33	81	700	74.9	6.99
Q91YN9	BAG family molecular chaperone regulator 2 OS=Mus musculus GN=Bag2 PE=1 SV=1 - [BAG2_MOUSE]	32.86	1	7	8	22	1.187	1.089	0.807	0.931	56.71	32.86	14	22	210	23.5	6.42
Q9CR20	Immediate early response 3-interacting protein 1 OS=Mus musculus GN=Ier3ip1 PE=2 SV=1 - [IR3IP_MOUSE]	24.39	2	1	1	6	1.113	1.133	0.905	0.931	22.07	24.39	2	6	82	9.0	8.22
P34152-3	Isoform 3 of Focal adhesion kinase 1 OS=Mus musculus GN=Ptk2 - [FAK1_MOUSE]	33.65	9	29	31	90	0.871	1.267	1.129	0.931	246.11	33.65	46	90	1052	119.2	6.62
Q8VH51-2	Isoform 2 of RNA-binding protein 39 OS=Mus musculus GN=Rbm39 - [RBM39_MOUSE]	44.66	7	17	17	42	0.723	0.886	0.885	0.931	120.58	44.66	28	42	524	58.6	10.13
P46471	26S protease regulatory subunit 7 OS=Mus musculus GN=Psmc2 PE=1 SV=5 -	69.05	2	28	28	137	0.716	1.008	0.896	0.931	407.06	69.05	51	137	433	48.6	5.95
Q9CRA8	Exosome complex component RRP46 OS=Mus musculus GN=Exosc5 PE=1 SV=1 - [EXOS5_MOUSE]	14.04	1	4	4	4	1.547	1.166	1.033	0.931	3.78	14.04	4	4	235	25.2	7.56
F6RGI3	Protein Gm340 OS=Mus musculus GN=Gm340 PE=4 SV=1 - [F6RGI3_MOUSE]	2.90	1	1	2	3	0.209	0.669	0.696	0.931	3.15	2.90	2	3	1242	136.1	7.52
A2RS76	Vomeronasal 1 receptor, B3 OS=Mus musculus GN=Vmn1r53 PE=2 SV=1 - [A2RS76_MOUSE]	3.55	2	1	1	1	1.983	1.328	1.312	0.931	0.00	3.55	1	1	310	35.4	9.66
Q69Z99	Zinc finger protein 512 OS=Mus musculus GN=Znf512 PE=2 SV=2 - [ZN512_MOUSE]	19.04	1	10	10	22	0.659	0.751	0.871	0.931	57.87	19.04	16	22	562	63.9	9.51
O88291	DBIRD complex subunit ZNF326 OS=Mus musculus GN=Znf326 PE=1 SV=1 - [ZN326_MOUSE]	21.03	3	10	10	41	1.000	0.775	0.956	0.931	115.19	21.03	16	41	580	65.2	5.19
Q6DFW4	Nucleolar protein 58 OS=Mus musculus GN=Nop58 PE=1 SV=1 - [NOP58_MOUSE]	27.99	1	11	11	34	0.619	0.740	0.730	0.932	116.92	27.99	18	34	536	60.3	8.34
D6RI31	Disrupted in renal carcinoma protein 2 homolog OS=Mus musculus GN=Dirc2 PE=2 SV=1 - [D6RI31_MOUSE]	13.91	2	1	1	4	0.974	0.650	0.847	0.932	13.57	13.91	2	4	115	12.5	9.72

G5E8E7	Tyrosine-protein phosphatase non-receptor type OS=Mus musculus GN=Ptpn4 PE=3 SV=1 - [G5E8E7_MOUSE]	13.39	4	8	8	25	0.727	0.839	0.888	0.932	53.29	13.39		14	25	926	105.8	7.25
Q3U4S0	Pantothenate kinase 2 (Hallervorden-Spatz syndrome) OS=Mus musculus GN=Pank2 PE=2 SV=1 - [Q3U4S0_MOUSE]	15.12	4	5	7	14	0.993	0.983	0.925	0.932	41.78	15.12		11	14	443	48.6	8.44
Q99K41	EMILIN-1 OS=Mus musculus GN=Emilin1 PE=1 SV=1 - [EMIL1_MOUSE]	9.34	1	6	6	15	1.030	3.125	0.679	0.932	40.44	9.34		11	15	1017	107.5	5.30
Q543K9	Purine nucleoside phosphorylase OS=Mus musculus GN=Pnp PE=2 SV=1 - [Q543K9_MOUSE]	51.56	3	11	11	56	1.937	1.031	0.763	0.932	168.34	51.56		22	56	289	32.2	6.16
Q91VX9	Transmembrane protein 168 OS=Mus musculus GN=Tmem168 PE=2 SV=1 - [TM168_MOUSE]	2.44	1	1	1	2	1.056	1.625	0.721	0.932	7.59	2.44		1	2	697	79.6	7.96
G5E8E1	Leucine rich repeat (In FLII) interacting protein 1, isoform CRA_e OS=Mus musculus GN=Lrrfip1 PE=4 SV=1 - [G5E8E1_MOUSE]	33.64	2	1	13	49	1.472	0.497	1.325	0.932	154.52	33.64		21	49	428	48.9	5.57
Q9CRA4	Methylsterol monooxygenase 1 OS=Mus musculus GN=Msmo1 PE=2 SV=1 -	8.53	2	2	2	7	0.852	0.554	0.893	0.932	28.69	8.53		3	7	293	34.7	7.66
Q8BYU9	Protein Secisbp2 OS=Mus musculus GN=Secisbp2 PE=2 SV=1 - [Q8BYU9_MOUSE]	3.14	2	1	1	3	1.374	0.714	1.116	0.932	4.99	3.14		1	3	414	45.1	8.76
E9QAE3	Protein Btaf1 OS=Mus musculus GN=Btaf1 PE=2 SV=1 - [E9QAE3_MOUSE]	4.92	1	6	6	15	0.970	1.336	0.940	0.932	26.08	4.92		10	15	1848	206.9	6.43
Q8K2P7	Sodium-coupled neutral amino acid transporter 1 OS=Mus musculus GN=Slc38a1 PE=1 SV=1 - [S38A1_MOUSE]	5.36	2	3	3	6	0.546	0.516	0.668	0.932	12.95	5.36		4	6	485	53.8	7.08
Q8VGL0	MCG60008 OS=Mus musculus GN=Olfr340 PE=2 SV=1 - [Q8VGL0_MOUSE]	1.92	1	1	1	1	1.161	1.190	0.988	0.932	1.72	1.92		1	1	312	34.9	7.47
Q9QXD8	LIM domain-containing protein 1 OS=Mus musculus GN=Limd1 PE=1 SV=2 - [LIMD1_MOUSE]	17.66	1	6	7	13	1.721	1.419	0.987	0.932	35.83	17.66		10	13	668	71.4	6.32
Q6PCN7	Helicase-like transcription factor OS=Mus musculus GN=Hltf PE=1 SV=1 - [HLTF_MOUSE]	3.99	3	2	5	14	1.559	1.099	1.006	0.932	34.92	3.99		7	14	1003	113.2	8.09
Q8CGK3	Lon protease homolog, mitochondrial OS=Mus musculus GN=Lonp1 PE=1 SV=2 - [LONM_MOUSE]	43.52	2	34	37	174	0.597	0.747	0.837	0.932	521.71	43.52		66	174	949	105.8	6.57
Q9R0H0	Peroxisomal acyl-coenzyme A oxidase 1 OS=Mus musculus GN=Acox1 PE=1 SV=5 - [ACOX1_MOUSE]	45.08	3	21	21	81	1.052	0.712	0.705	0.932	284.09	45.08		37	81	661	74.6	8.48
P28740-1	Isoform 1 of Kinesin-like protein KIF2A OS=Mus musculus GN=Kif2a - [KIF2A_MOUSE]	51.82	11	2	30	176	0.484	0.614	1.062	0.932	451.42	51.82		51	176	716	80.8	6.16
P10518	Delta-aminolevulinic acid dehydratase OS=Mus musculus GN=Alad PE=1 SV=1 - [HEM2_MOUSE]	32.42	1	8	8	111	2.579	1.452	1.689	0.932	300.82	32.42		16	111	330	36.0	6.79
F6VQ81	Tumor protein D54 (Fragment) OS=Mus musculus GN=Tpd52l2 PE=4 SV=1 -	78.88	1	1	12	79	1.176	0.997	1.155	0.932	253.57	78.88		22	79	161	17.7	8.19
Q91V76	Ester hydrolase C11orf54 homolog OS=Mus musculus PE=2 SV=1 - [CK054_MOUSE]	35.87	1	9	9	27	1.609	0.945	0.833	0.933	65.66	35.87		17	27	315	35.0	6.29
Q3TY65	Islet cell autoantigen 1-like protein OS=Mus musculus GN=Ica1l PE=2 SV=1 - [ICA1L_MOUSE]	15.78	1	4	5	12	0.806	1.425	0.827	0.933	32.70	15.78		8	12	431	48.1	4.91
Q80U38	Protein KHNYN OS=Mus musculus GN=Khnyn PE=2 SV=2 - [KHNYN_MOUSE]	18.93	1	9	9	16	1.296	1.055	0.782	0.933	50.97	18.93		13	16	671	74.5	7.87
E9QA61	MCG113054, isoform CRA_a OS=Mus musculus GN=Cep85l PE=4 SV=2 - [E9QA61_MOUSE]	2.23	3	1	3	16	1.110	1.287	0.909	0.933	37.03	2.23		5	16	806	91.8	6.30
Q91WE9-2	Isoform 2 of Protein FAM19A5 OS=Mus musculus GN=Fam19a5 - [F19A5_MOUSE]	23.20	2	3	3	7	1.452	0.975	1.304	0.933	13.55	23.20		5	7	125	13.6	8.82
E9PYI7	Protein Lmo7 OS=Mus musculus GN=Lmo7 PE=2 SV=1 - [E9PYI7_MOUSE]	23.95	6	25	25	67	1.577	1.789	2.663	0.933	213.41	23.95		40	67	1453	164.6	5.73
E9QNN1	ATP-dependent RNA helicase A OS=Mus musculus GN=Dhx9 PE=2 SV=1 - [E9QNN1_MOUSE]	34.90	6	40	41	191	0.758	0.900	0.811	0.933	640.53	34.90		68	191	1384	149.6	6.83

Q99PT1	Rho GDP-dissociation inhibitor 1 OS=Mus musculus GN=Arhgdia PE=1 SV=3 -	74.02	1	19	19	187	1.312	0.890	0.803	0.933	509.85	74.02	31	187	204	23.4	5.20
H7BWZ3	Actin-related protein 2/3 complex subunit 3 OS=Mus musculus GN=Arpc3 PE=2 SV=1 - [H7BWZ3_MOUSE]	38.24	4	8	8	36	0.615	1.581	1.076	0.933	84.40	38.24	14	36	170	19.6	8.60
P84084	ADP-ribosylation factor 5 OS=Mus musculus GN=Arf5 PE=2 SV=2 - [ARF5_MOUSE]	57.78	4	2	9	215	0.769	1.207	1.010	0.933	567.74	57.78	17	215	180	20.5	6.79
E9Q0P8	Serine/threonine-protein kinase A-Raf OS=Mus musculus GN=Araf PE=2 SV=1 - [E9Q0P8_MOUSE]	34.37	2	3	11	45	0.748	0.948	0.953	0.933	112.72	34.37	21	45	419	46.2	9.28
Q8BNU1	Ras-related protein Rab-26 OS=Mus musculus GN=Rab26 PE=2 SV=1 - [Q8BNU1_MOUSE]	16.24	3	1	2	51	0.764	0.951	1.088	0.933	146.47	16.24	4	51	197	20.7	9.58
Q9QYR6	Microtubule-associated protein 1A OS=Mus musculus GN=Map1a PE=1 SV=2 -	51.08	3	106	108	1446	1.937	1.045	1.553	0.934	4501.76	51.08	181	1446	2776	300.0	5.00
E9PUU4	Gem-associated protein 5 OS=Mus musculus GN=Gemin5 PE=2 SV=1 - [E9PUU4_MOUSE]	11.71	4	16	16	36	1.191	1.042	0.938	0.934	94.36	11.71	26	36	1503	166.6	6.74
G3UWG1	MCG115977 OS=Mus musculus GN=Gm10108 PE=3 SV=1 - [G3UWG1_MOUSE]	69.52	2	8	12	455	1.750	0.731	1.337	0.934	1363.73	69.52	22	455	105	11.7	9.64
D3Z3A0	MCG126099, isoform CRA_b OS=Mus musculus GN=Ppp1r2 PE=4 SV=1 - [D3Z3A0_MOUSE]	52.82	6	8	8	39	1.607	1.216	1.538	0.934	105.05	52.82	15	39	195	21.9	4.73
Q6P8X1	Sorting nexin-6 OS=Mus musculus GN=Snx6 PE=1 SV=2 - [SNX6_MOUSE]	42.61	1	14	17	84	1.018	1.076	0.872	0.934	232.16	42.61	32	84	406	46.6	6.16
Q8VCQ8	Caldesmon 1 OS=Mus musculus GN=Cald1 PE=2 SV=1 - [Q8VCQ8_MOUSE]	35.85	4	11	19	94	1.010	1.144	1.022	0.934	239.82	35.85	32	94	530	60.4	7.37
Q9DAP0	Leucine-rich repeat-containing protein 46 OS=Mus musculus GN=Lrrc46 PE=2 SV=2 - [LRC46_MOUSE]	4.02	1	1	1	8	1.148	1.063	1.407	0.934	22.17	4.02	1	8	323	36.0	5.39
F6ZFT1	Acyl carrier protein (Fragment) OS=Mus musculus GN=Ndufab1 PE=2 SV=1 -	55.12	3	6	6	51	1.789	1.161	1.356	0.934	148.94	55.12	11	51	127	14.3	4.77
Q8R5H1-5	Isoform 5 of Ubiquitin carboxyl-terminal hydrolase 15 OS=Mus musculus GN=Usp15 - [UBP15_MOUSE]	39.18	4	1	27	68	0.940	0.832	0.887	0.934	198.88	39.18	41	68	952	109.2	5.11
Q30D77	Collagen alpha-1(XXIV) chain OS=Mus musculus GN=Col24a1 PE=2 SV=2 - [COOA1_MOUSE]	5.25	1	5	5	8	0.836	3.625	1.010	0.934	17.49	5.25	6	8	1733	175.6	7.34
Q9EP75	Leukotriene-B4 omega-hydroxylase 3 OS=Mus musculus GN=Cyp4f14 PE=2 SV=1 -	4.58	4	2	2	3	0.531	1.069	0.963	0.934	11.12	4.58	3	3	524	59.8	6.87
Q9DBL2	Ganglioside-induced differentiation-associated protein 2 OS=Mus musculus GN=Gdap2 PE=2 SV=1 - [GDAP2_MOUSE]	19.48	2	8	8	14	1.139	1.251	0.897	0.934	39.88	19.48	11	14	498	56.2	5.39
E9PY46	Protein Ift140 OS=Mus musculus GN=Ift140 PE=2 SV=1 - [E9PY46_MOUSE]	5.19	2	7	7	12	0.604	0.907	0.707	0.934	33.04	5.19	12	12	1464	165.8	5.91
Q69ZU8-2	Isoform 2 of Ankyrin repeat domain-containing protein 6 OS=Mus musculus GN=Ankrd6 - [ANKR6_MOUSE]	3.55	2	1	1	4	1.270	0.768	1.207	0.934	0.00	3.55	1	4	677	73.7	8.92
Q922H1-2	Isoform 2 of Protein arginine N-methyltransferase 3 OS=Mus musculus GN=Prmt3 - [ANM3_MOUSE]	17.05	4	7	8	15	0.764	0.808	0.857	0.934	42.05	17.05	13	15	528	59.4	5.30
Q8VE92	RNA-binding protein 4B OS=Mus musculus GN=Rbm4b PE=1 SV=1 - [RBM4B_MOUSE]	26.33	4	1	8	35	1.555	0.809	0.887	0.934	104.55	26.33	16	35	357	40.0	6.74
Q921F4	Heterogeneous nuclear ribonucleoprotein L-like OS=Mus musculus GN=Hnrnpll PE=1 SV=3 - [HNRLL_MOUSE]	43.82	3	20	20	71	0.865	0.961	0.865	0.934	228.44	43.82	35	71	591	64.1	5.85
G5E884	Serine/threonine-protein kinase PAK 1 OS=Mus musculus GN=Pak1 PE=4 SV=1 - [G5E884_MOUSE]	54.23	2	13	27	297	1.200	1.116	1.581	0.934	808.50	54.23	51	297	544	60.6	5.86
Q8VDP6	CDP-diacylglycerol--inositol 3-phosphatidyltransferase OS=Mus musculus GN=Cdipt PE=1 SV=1 - [CDIPT_MOUSE]	9.86	1	2	2	26	0.909	1.281	0.912	0.934	69.69	9.86	4	26	213	23.6	8.27
G3XA30	MCG1618, isoform CRA_c OS=Mus musculus GN=Nsmce4a PE=4 SV=1 - [G3XA30_MOUSE]	7.09	1	2	2	7	1.374	0.890	0.850	0.935	16.03	7.09	3	7	381	43.7	5.30

Q9CQB5	CDGSH iron-sulfur domain-containing protein 2 OS=Mus musculus GN=Cisd2 PE=1 SV=1 - [CISD2_MOUSE]	50.37	2	6	6	20	0.684	0.923	0.741	0.935	55.51	50.37	11	20	135	15.2	9.51
Q3TDD9	Protein phosphatase 1 regulatory subunit 21 OS=Mus musculus GN=Ppp1r21 PE=2 SV=2 - [PPR21_MOUSE]	39.10	3	31	32	101	1.060	0.980	0.956	0.935	312.89	39.10	49	101	780	88.3	6.90
Q8R5L3-2	Isoform 2 of Vam6/Vps39-like protein OS=Mus musculus GN=Vps39 - [VPS39_MOUSE]	14.97	2	10	11	29	0.803	0.993	0.764	0.935	73.54	14.97	19	29	875	100.6	7.12
Q505D9	Tripartite motif-containing protein 67 OS=Mus musculus GN=Trim67 PE=2 SV=1 -	9.77	1	4	6	8	0.948	1.124	1.433	0.935	19.26	9.77	7	8	768	82.7	7.08
Q3TA40	Protein 6430548M08Rik OS=Mus musculus GN=6430548M08Rik PE=2 SV=1 - [Q3TA40_MOUSE]	40.81	4	16	16	85	0.761	1.667	1.287	0.935	225.43	40.81	29	85	397	45.2	5.07
Q8BMJ2	Leucine--tRNA ligase, cytoplasmic OS=Mus musculus GN=Lars PE=2 SV=2 - [SYLC_MOUSE]	35.57	1	36	36	139	0.875	0.790	0.875	0.935	405.20	35.57	62	139	1178	134.1	7.05
Q8BNV1	tRNA (uracil-5-)-methyltransferase homolog A OS=Mus musculus GN=Trmt2a PE=2 SV=1 - [TRM2A_MOUSE]	11.32	5	4	4	6	0.954	0.976	0.883	0.935	14.37	11.32	5	6	574	63.3	7.25
D3Z5G2	Solute carrier family 25 member 45 (Fragment) OS=Mus musculus GN=Slc25a45 PE=2 SV=1 - [D3Z5G2_MOUSE]	21.03	2	2	2	2	1.918	1.217	0.941	0.935	2.85	21.03	2	2	195	21.3	8.82
Q6GQX2	Nck-associated protein 5-like OS=Mus musculus GN=Nckap5l PE=2 SV=1 - [NCK5L_MOUSE]	3.78	2	3	3	5	0.889	0.553	1.111	0.935	11.74	3.78	5	5	1323	138.1	8.37
Q9CR13	UPF0562 protein C7orf55 homolog OS=Mus musculus PE=3 SV=1 - [CG055_MOUSE]	13.27	1	1	1	2	1.202	0.835	0.626	0.935	10.42	13.27	2	2	113	12.7	10.08
Q9CZ30	Obg-like ATPase 1 OS=Mus musculus GN=Ola1 PE=1 SV=1 - [OLA1_MOUSE]	51.77	3	19	20	93	0.677	1.018	0.908	0.935	263.52	51.77	35	93	396	44.7	7.81
Q08943	FACT complex subunit SSRP1 OS=Mus musculus GN=Ssrp1 PE=1 SV=2 - [SSRP1_MOUSE]	17.23	3	11	11	31	0.929	1.027	0.790	0.935	88.87	17.23	20	31	708	80.8	6.76
Q3LAC4	Phosphatidylinositol 3,4,5-trisphosphate-dependent Rac exchanger 2 protein OS=Mus musculus GN=Prex2 PE=2 SV=2 - [PREX2_MOUSE]	7.51	4	10	11	17	0.762	1.038	1.026	0.935	48.77	7.51	15	17	1598	181.6	7.34
Q6PFD9	Nuclear pore complex protein Nup98-Nup96 OS=Mus musculus GN=Nup98 PE=1 SV=2 - [NUP98_MOUSE]	19.55	1	28	29	89	1.232	1.044	1.133	0.935	212.84	19.55	46	89	1816	197.1	6.18
E9Q035	Protein Gm20425 OS=Mus musculus GN=Gm20425 PE=4 SV=1 - [E9Q035_MOUSE]	51.43	6	10	52	497	1.048	1.056	0.705	0.935	1415.80	51.43	95	497	978	107.7	7.77
Q5FWI3	Transmembrane protein 2 OS=Mus musculus GN=Tmem2 PE=1 SV=1 - [TMEM2_MOUSE]	5.06	1	6	7	11	0.957	1.244	0.974	0.935	27.87	5.06	9	11	1383	153.7	7.78
Q9Z1R9	MCG124046 OS=Mus musculus GN=Prss1 PE=2 SV=1 - [Q9Z1R9_MOUSE]	12.20	2	1	2	776	1.092	1.360	0.944	0.935	1720.88	12.20	4	776	246	26.1	4.94
F6QMQ3	Protein CBFA2T2 (Fragment) OS=Mus musculus GN=Cbfa2t2 PE=4 SV=1 -	8.85	1	1	1	1	2.022	1.122	0.981	0.935	2.72	8.85	1	1	192	20.3	9.74
Q60520	Paired amphipathic helix protein Sin3a OS=Mus musculus GN=Sin3a PE=1 SV=3 - [SIN3A_MOUSE]	11.38	4	11	11	25	0.674	1.082	0.807	0.935	70.89	11.38	17	25	1274	145.0	7.25
Q6PHQ8	N-alpha-acetyltransferase 35, NatC auxiliary subunit OS=Mus musculus GN=Naa35 PE=1 SV=1 - [NAA35_MOUSE]	26.76	6	15	17	42	0.701	0.846	0.914	0.935	128.05	26.76	28	42	725	83.3	7.30
Q8BP22	Protein FAM92A1 OS=Mus musculus GN=Fam92a1 PE=1 SV=1 - [F92A1_MOUSE]	16.06	4	6	6	14	1.163	0.945	0.903	0.936	30.86	16.06	12	14	355	39.9	7.36
P58459-3	Isoform 3 of A disintegrin and metalloproteinase with thrombospondin motifs 10 OS=Mus musculus GN=Adamts10 - [ATS10_MOUSE]	9.86	1	1	1	1	0.714	0.663	1.390	0.936	3.02	9.86	1	1	213	24.0	9.41
Q8CGF6	WD repeat-containing protein 47 OS=Mus musculus GN=Wdr47 PE=1 SV=2 -	40.00	2	28	29	121	1.016	1.053	0.960	0.936	412.16	40.00	51	121	920	102.2	5.97
Q9JME9	V-set and transmembrane domain-containing protein 2B OS=Mus musculus GN=Vstm2b PE=2 SV=1 - [VTM2B_MOUSE]	25.96	1	5	5	20	1.263	1.555	1.145	0.936	68.09	25.96	9	20	285	30.1	7.15

G5E8J3	Bromodomain and WD repeat domain containing 2, isoform CRA_a OS=Mus musculus GN=Wdr11 PE=4 SV=1 - [G5E8J3_MOUSE]	18.23	2	17	18	44	0.717	1.038	0.902	0.936	127.63	18.23	28	44	1223	135.9	7.11
Q6PD03	Serine/threonine-protein phosphatase 2A 56 kDa regulatory subunit alpha isoform OS=Mus musculus GN=Ppp2r5a PE=1 SV=1 - [2A5A_MOUSE]	26.54	1	10	12	24	1.966	1.391	1.161	0.936	73.18	26.54	19	24	486	56.3	7.03
Q8C5L3-2	Isoform 2 of CCR4-NOT transcription complex subunit 2 OS=Mus musculus GN=Cnot2 - [CNOT2_MOUSE]	25.42	5	10	10	41	1.124	1.011	1.107	0.936	119.52	25.42	16	41	531	58.6	7.37
Q8K4P8	E3 ubiquitin-protein ligase HECW1 OS=Mus musculus GN=Hecw1 PE=2 SV=3 - [HECW1_MOUSE]	11.91	6	13	16	37	0.658	0.654	1.113	0.936	98.10	11.91	27	37	1604	179.4	5.39
Q925B0-2	Isoform 2 of PRKC apoptosis WT1 regulator protein OS=Mus musculus GN=Pawr -	22.84	2	6	6	9	0.926	1.355	0.908	0.936	26.71	22.84	7	9	289	30.9	8.66
E9Q150	Segment polarity protein dishevelled homolog DVL-3 OS=Mus musculus GN=Dvl3 PE=2 SV=1 - [E9Q150_MOUSE]	10.59	4	6	8	18	1.055	0.767	1.060	0.936	46.02	10.59	12	18	699	76.3	6.70
Q91VR8	Protein BRICK1 OS=Mus musculus GN=Brk1 PE=2 SV=1 - [BRK1_MOUSE]	73.33	1	8	8	61	0.877	1.589	1.216	0.936	171.09	73.33	16	61	75	8.8	5.45
Q9DB54	Protein FAM216A OS=Mus musculus GN=Fam216a PE=2 SV=1 - [F216A_MOUSE]	6.77	1	1	2	5	1.236	1.170	1.418	0.936	10.49	6.77	3	5	251	28.3	9.70
Q9QYK7	RING finger protein 11 OS=Mus musculus GN=Rnf11 PE=1 SV=1 - [RNF11_MOUSE]	46.75	1	4	4	12	1.001	1.258	1.093	0.936	33.66	46.75	7	12	154	17.4	4.78
Q8CG79	Apoptosis-stimulating of p53 protein 2 OS=Mus musculus GN=Tp53bp2 PE=1 SV=3 - [ASPP2_MOUSE]	18.44	2	13	14	39	1.435	0.709	1.107	0.936	118.68	18.44	22	39	1128	125.2	6.34
O35601-2	Isoform FYB-120 of FYN-binding protein OS=Mus musculus GN=Fyb - [FYB_MOUSE]	2.59	2	2	2	2	1.473	1.017	1.201	0.936	4.27	2.59	2	2	773	84.8	6.71
F8VQJ3	Laminin subunit gamma-1 OS=Mus musculus GN=Lamc1 PE=2 SV=1 - [F8VQJ3_MOUSE]	29.50	3	2	38	150	1.212	1.498	0.757	0.936	424.42	29.50	67	150	1607	177.1	5.19
A2RSY6	TRMT1-like protein OS=Mus musculus GN=Trmt1l PE=2 SV=1 - [TRM1L_MOUSE]	5.49	4	4	4	7	2.081	1.152	0.760	0.936	16.46	5.49	5	7	728	80.8	7.50
G3X8W7	CDC23 (Cell division cycle 23, yeast, homolog), isoform CRA_a OS=Mus musculus GN=Cdc23 PE=4 SV=1 - [G3X8W7_MOUSE]	19.61	3	9	10	21	0.696	1.005	0.854	0.936	75.26	19.61	15	21	571	65.8	6.83
F8WIU7	Putative protein arginine N-methyltransferase 10 OS=Mus musculus GN=Prmt10 PE=2 SV=1 - [F8WIU7_MOUSE]	2.36	3	2	2	3	0.883	1.065	0.942	0.936	5.47	2.36	3	3	846	94.1	5.36
Q9CQG1	Cation transport regulator-like protein 2 OS=Mus musculus GN=Chac2 PE=2 SV=1 - [CHAC2_MOUSE]	30.34	2	4	4	12	1.010	0.769	0.846	0.936	36.61	30.34	7	12	178	20.1	5.68
Q8CI11-2	Isoform 2 of Guanine nucleotide-binding protein-like 3 OS=Mus musculus GN=Gnl3 - [GNL3_MOUSE]	9.90	5	2	4	12	1.087	0.956	0.817	0.936	27.09	9.90	6	12	505	57.2	9.44
P70671	Interferon regulatory factor 3 OS=Mus musculus GN=Irf3 PE=1 SV=1 - [IRF3_MOUSE]	10.98	1	5	5	14	0.893	0.973	0.850	0.936	30.38	10.98	9	14	419	46.8	5.43
Q00560	Interleukin-6 receptor subunit beta OS=Mus musculus GN=Il6st PE=1 SV=2 - [IL6RB_MOUSE]	14.18	1	9	9	28	1.335	0.995	0.982	0.936	77.94	14.18	17	28	917	102.4	5.52
Q8K2L8	Trafficking protein particle complex subunit 12 OS=Mus musculus GN=Trappc12 PE=1 SV=2 [TPC12_MOUSE]	29.86	2	20	20	60	1.051	1.109	1.008	0.937	162.21	29.86	32	60	797	87.6	4.81
Q07235	Glia-derived nexin OS=Mus musculus GN=Serpine2 PE=2 SV=2 - [GDN_MOUSE]	37.78	1	13	14	38	0.574	0.583	0.746	0.937	98.14	37.78	25	38	397	44.2	9.85
Q9CZP7-2	Isoform 2 of Hsp90 co-chaperone Cdc37-like 1 OS=Mus musculus GN=Cdc37l1 -	9.77	4	4	4	9	1.437	0.703	1.006	0.937	25.44	9.77	7	9	307	35.4	5.92
Q9CR59	Growth arrest and DNA damage-inducible proteins-interacting protein 1 OS=Mus musculus GN=Gadd45gip1 PE=2 SV=1 - [G45IP_MOUSE]	27.48	1	5	5	14	1.114	0.684	1.008	0.937	33.63	27.48	8	14	222	25.8	10.27

Q62383	Transcription elongation factor SPT6 OS=Mus musculus GN=Supt6h PE=1 SV=2 - [SPT6H_MOUSE]	11.53	1	17	17	32	0.762	0.937	0.843	0.937	101.75	11.53	27	32	1726	199.0	4.93
Q9Z1K5	E3 ubiquitin-protein ligase ARIH1 OS=Mus musculus GN=Arih1 PE=2 SV=3 - [ARI1_MOUSE]	20.90	3	10	10	25	0.949	1.196	0.969	0.937	80.21	20.90	16	25	555	64.0	5.08
P22724	Protein Wnt-4 OS=Mus musculus GN=Wnt4 PE=1 SV=1 - [WNT4_MOUSE]	3.99	1	1	1	1	1.327	1.261	0.696	0.937	2.63	3.99	1	1	351	39.0	8.51
Q9QYL7	Activator of basal transcription 1 OS=Mus musculus GN=Abt1 PE=2 SV=1 - [ABT1_MOUSE]	9.29	1	2	2	2	1.508	1.129	0.933	0.937	5.51	9.29	2	2	269	30.6	10.01
Q9WUL7	ADP-ribosylation factor-like protein 3 OS=Mus musculus GN=Arl3 PE=1 SV=1 - [ARL3_MOUSE]	48.35	1	6	6	27	1.676	1.027	0.896	0.937	112.10	48.35	12	27	182	20.5	7.24
F8WHV4	Collagen alpha-1(XIX) chain OS=Mus musculus GN=Col19a1 PE=2 SV=1 - [F8WHV4_MOUSE]	4.96	2	2	3	7	0.898	0.905	0.749	0.937	17.89	4.96	3	7	1069	108.2	8.25
Q9JMG7	Hepatoma-derived growth factor-related protein 3 OS=Mus musculus GN=Hdgfrp3 PE=1 SV=2 - [HDGR3_MOUSE]	30.69	2	6	7	27	0.798	0.632	1.157	0.937	64.11	30.69	12	27	202	22.4	8.40
E9Q816	Protein Cyp2w1 OS=Mus musculus GN=Cyp2w1 PE=3 SV=1 - [E9Q816_MOUSE]	1.22	1	1	1	1	3.545	1.275	0.970	0.937	2.19	1.22	1	1	493	54.4	9.14
Q8BUY8	G-protein coupled receptor associated sorting protein 2 OS=Mus musculus GN=Gprasp2 PE=2 SV=2 - [GASP2_MOUSE]	20.22	1	11	11	21	0.972	0.629	0.662	0.937	53.20	20.22	17	21	826	92.7	5.11
Q8BIZ1-3	Isoform 3 of Ankyrin repeat and sterile alpha motif domain-containing protein 1B OS=Mus musculus GN=Anks1b - [ANS1B_MOUSE]	36.88	2	1	15	63	0.403	2.111	2.921	0.937	167.30	36.88	28	63	404	45.5	8.38
P26231	Catenin alpha-1 OS=Mus musculus GN=Ctnna1 PE=1 SV=1 - [CTNA1_MOUSE]	53.42	1	23	39	153	0.785	1.145	0.817	0.937	442.60	53.42	69	153	906	100.0	6.23
Q9CQ22	Ragulator complex protein LAMTOR1 OS=Mus musculus GN=Lamtor1 PE=1 SV=1 - [LTOR1_MOUSE]	47.83	1	6	6	36	0.775	1.124	0.796	0.937	95.02	47.83	10	36	161	17.7	5.15
Q8CB77	Transcription elongation factor B polypeptide 3 OS=Mus musculus GN=Tceb3 PE=1 SV=3 - [ELOA1_MOUSE]	6.34	1	4	5	8	1.591	0.925	0.984	0.937	15.77	6.34	6	8	773	87.1	9.61
Q6P9K8	Caskin-1 OS=Mus musculus GN=Caskin1 PE=1 SV=2 - [CSKI1_MOUSE]	48.57	5	45	50	352	0.953	0.628	1.119	0.937	1084.81	48.57	92	352	1431	150.4	9.17
Q5SV85-2	Isoform 2 of Synergin gamma OS=Mus musculus GN=Synrg - [SYNRG_MOUSE]	42.53	6	36	37	150	1.336	1.113	1.336	0.938	446.25	42.53	61	150	1138	122.0	5.00
D3Z7A9	Protein Lilra5 OS=Mus musculus GN=Lilra5 PE=4 SV=1 - [D3Z7A9_MOUSE]	5.76	1	1	1	2	0.786	1.136	0.966	0.938	6.15	5.76	1	2	295	32.7	7.14
Q80UP3	Diacylglycerol kinase zeta OS=Mus musculus GN=Dgkz PE=1 SV=2 - [DGKZ_MOUSE]	14.53	3	9	11	46	0.644	1.539	1.731	0.938	129.36	14.53	20	46	929	104.0	8.09
Q69ZK7	Protein FAM214A OS=Mus musculus GN=Fam214a PE=2 SV=3 - [F214A_MOUSE]	2.33	2	2	2	4	1.323	0.892	1.124	0.938	3.92	2.33	2	4	1075	119.2	7.74
Q9WUM4	Coronin-1C OS=Mus musculus GN=Coro1c PE=1 SV=2 - [COR1C_MOUSE]	36.29	4	18	22	135	0.590	0.741	0.960	0.938	353.91	36.29	41	135	474	53.1	7.08
E9Q9M5	Ubiquitin carboxyl-terminal hydrolase OS=Mus musculus GN=Usp19 PE=2 SV=1 - [E9Q9M5_MOUSE]	18.01	4	16	16	56	0.921	0.924	0.919	0.938	146.99	18.01	26	56	1299	143.4	6.23
Q9CQB0	snRNA-activating protein complex subunit 3 OS=Mus musculus GN=Snapc3 PE=2 SV=1 - [Q9CQB0_MOUSE]	10.41	2	1	1	1	0.966	1.072	1.189	0.938	0.00	10.41	1	1	269	29.9	4.70
Q9DC29	ATP-binding cassette sub-family B member 6, mitochondrial OS=Mus musculus GN=Abcb6 PE=1 SV=1 - [ABCB6_MOUSE]	15.56	1	6	8	24	1.143	1.192	0.797	0.938	86.80	15.56	12	24	842	93.7	8.05
F8VQ05	Protein Fryl OS=Mus musculus GN=Fryl PE=4 SV=1 - [F8VQ05_MOUSE]	6.98	1	14	16	29	1.249	0.855	0.879	0.938	64.92	6.98	26	29	3007	337.6	5.63
D3Z5P5	DNA-binding protein SMUBP-2 OS=Mus musculus GN=Ighmbp2 PE=2 SV=1 -	3.05	2	2	2	4	0.718	1.144	1.021	0.938	7.44	3.05	3	4	885	97.4	8.05

Q9DA69-3	Isoform 3 of Intraflagellar transport protein 43 homolog OS=Mus musculus GN=Ift43 - [IFT43_MOUSE]	7.77	3	1	1	2	1.884	1.075	1.276	0.938	5.14	7.77	2	2	193	21.9	4.70
P70333	Heterogeneous nuclear ribonucleoprotein H2 OS=Mus musculus GN=Hnrnph2 PE=1 SV=1 - [HNRH2_MOUSE]	42.98	3	8	15	190	1.064	1.030	1.157	0.938	605.84	42.98	25	190	449	49.2	6.30
Q8CDV6-2	Isoform 2 of Coiled-coil domain-containing protein 63 OS=Mus musculus GN=Ccdc63 - [CCD63_MOUSE]	3.08	2	1	2	4	1.136	1.393	0.907	0.938	8.17	3.08	3	4	487	56.9	7.59
Q8C115-3	Isoform 3 of Pleckstrin homology domain-containing family H member 2 OS=Mus musculus GN=Plekhh2 - [illegible]	1.28	3	2	3	9	0.948	1.029	1.124	0.938	23.79	1.28	4	9	1481	166.5	7.40
Q8C119	Protein NDNF OS=Mus musculus GN=Ndnf PE=1 SV=2 - [NDNF_MOUSE]	1.94	1	1	1	1	1.142	0.910	0.992	0.938	1.87	1.94	1	1	568	65.0	8.97
Q99P72	Reticulon-4 OS=Mus musculus GN=Rtn4 PE=1 SV=2 - [RTN4_MOUSE]	63.68	4	45	51	606	1.744	0.915	1.362	0.938	1820.17	63.68	87	606	1162	126.5	4.54
Q6P2K6	Serine/threonine-protein phosphatase 4 regulatory subunit 3A OS=Mus musculus GN=Smek1 PE=1 SV=1 - [illegible]	11.95	4	4	7	15	0.967	0.903	0.950	0.938	41.52	11.95	10	15	820	93.8	4.89
Q68FE7	Transmembrane protein 151B OS=Mus musculus GN=Tmem151b PE=2 SV=1 - [T151B_MOUSE]	8.73	1	4	4	6	1.013	0.812	1.002	0.938	16.59	8.73	6	6	561	61.7	7.03
Q80ZJ6-2	Isoform 2 of Protein zer-1 homolog OS=Mus musculus GN=Zer1 - [ZER1_MOUSE]	17.75	3	13	14	25	0.667	0.897	0.839	0.938	66.34	17.75	22	25	766	87.7	5.80
Q5RJH6-3	Isoform 3 of Protein SMG7 OS=Mus musculus GN=Smg7 - [SMG7_MOUSE]	5.73	3	7	7	13	0.992	0.819	1.029	0.939	28.86	5.73	12	13	1134	126.5	8.69
Q9CXW3	Calcyclin-binding protein OS=Mus musculus GN=Cacybp PE=1 SV=1 - [CYBP_MOUSE]	53.71	1	10	11	41	1.278	0.939	0.791	0.939	106.03	53.71	18	41	229	26.5	7.87
Q5DTZ6-3	Isoform 3 of RING finger protein 150 OS=Mus musculus GN=Rnf150 - [RN150_MOUSE]	6.99	3	2	2	4	0.892	1.406	1.169	0.939	12.40	6.99	3	4	286	31.7	8.46
Q8K0C4	Lanosterol 14-alpha demethylase OS=Mus musculus GN=Cyp51a1 PE=2 SV=1 -	32.21	1	14	14	68	0.735	0.396	0.709	0.939	213.97	32.21	25	68	503	56.7	8.41
Q3B807	Transcription elongation regulator 1-like protein OS=Mus musculus GN=Tcerg1l PE=2 SV=3 - [TCRGL_MOUSE]	3.90	3	2	3	10	1.427	1.197	1.210	0.939	16.31	3.90	4	10	590	66.3	9.58
P47753	F-actin-capping protein subunit alpha-1 OS=Mus musculus GN=Capza1 PE=1 SV=4 - [CAZA1_MOUSE]	54.20	1	1	11	111	1.904	1.548	1.132	0.939	324.25	54.20	19	111	286	32.9	5.55
Q99MP8-2	Isoform 2 of BRCA1-associated protein OS=Mus musculus GN=Brap -	32.62	4	12	13	29	1.261	0.970	1.116	0.939	75.12	32.62	21	29	561	63.9	5.71
F7CK47	Microtubule-associated protein (Fragment) OS=Mus musculus GN=Map4 PE=4 SV=1 - [F7CK47_MOUSE]	21.65	1	1	34	262	2.451	1.363	1.012	0.939	670.78	21.65	63	262	1441	152.7	9.28
Q8BR30	RUN and FYVE domain-containing protein 2 OS=Mus musculus GN=Rufy2 PE=2 SV=1 - [Q8BR30_MOUSE]	35.70	4	1	14	49	1.666	1.152	0.977	0.939	146.02	35.70	26	49	437	50.2	5.47
Q8CF96	Synaptotagmin VIIbeta OS=Mus musculus GN=Syt7 PE=2 SV=1 - [Q8CF96_MOUSE]	58.17	2	5	25	87	0.814	1.294	1.303	0.939	249.05	58.17	43	87	447	50.6	9.11
Q04735	Cyclin-dependent kinase 16 OS=Mus musculus GN=Cdk16 PE=1 SV=1 - [CDK16_MOUSE]	38.31	18	9	17	65	0.611	0.808	1.027	0.939	161.43	38.31	28	65	496	55.9	7.87
Q3TDT0	Tripartite motif-containing protein 3 OS=Mus musculus GN=Trim3 PE=2 SV=1 - [Q3TDT0_MOUSE]	47.00	5	23	25	126	0.639	0.784	0.954	0.939	348.48	47.00	43	126	717	78.2	8.16
G5E8B6	Kin of IRRE like 3 (Drosophila), isoform CRA_b OS=Mus musculus GN=Kirrel3 PE=4 SV=1 - [G5E8B6_MOUSE]	20.60	5	11	11	21	0.991	1.164	1.108	0.939	58.83	20.60	16	21	733	80.4	6.93
Q8VEE4	Replication protein A 70 kDa DNA-binding subunit OS=Mus musculus GN=Rpa1 PE=2 SV=1 - [RFA1_MOUSE]	12.52	2	7	7	15	0.881	0.843	0.816	0.939	36.00	12.52	11	15	623	69.0	7.91
Q62472	Vomeronasal secretory protein 2 OS=Mus musculus GN=Lcn4 PE=2 SV=1 - [VNS2_MOUSE]	5.41	1	1	1	1	1.088	1.031	1.500	0.939	0.00	5.41	1	1	185	21.4	5.73

Q80ZS3	28S ribosomal protein S26, mitochondrial OS=Mus musculus GN=Mrps26 PE=2 SV=1 - [RT26_MOUSE]	25.50	1	4	4	11	0.917	0.815	1.022	0.939	28.86	25.50	8	11	200	23.4	9.96
Q9DCB8	Iron-sulfur cluster assembly 2 homolog, mitochondrial OS=Mus musculus GN=Isca2 PE=2 SV=2 - [ISCA2_MOUSE]	50.65	1	7	7	39	2.038	0.884	1.164	0.939	126.38	50.65	13	39	154	16.7	5.60
Q61136	Serine/threonine-protein kinase PRP4 homolog OS=Mus musculus GN=Prpf4b PE=1 SV=3 - [PRP4B_MOUSE]	10.82	1	8	9	26	0.870	1.059	0.888	0.939	53.56	10.82	13	26	1007	116.9	10.23
B2KF50	Protein Uhrf1bp1 OS=Mus musculus GN=Uhrf1bp1 PE=2 SV=1 - [B2KF50_MOUSE]	19.94	1	17	21	56	1.065	0.810	0.829	0.940	153.55	19.94	35	56	1429	156.9	6.21
Q8BTV1	Tumor suppressor candidate 3 OS=Mus musculus GN=Tusc3 PE=2 SV=1 - [TUSC3_MOUSE]	13.54	2	5	6	16	1.087	0.884	0.821	0.940	45.94	13.54	11	16	347	39.5	10.11
G3XA26	Carbonic anhydrase 7 OS=Mus musculus GN=Car7 PE=4 SV=1 - [G3XA26_MOUSE]	6.25	2	1	1	2	1.323	1.196	1.408	0.940	4.47	6.25	2	2	208	23.6	7.64
Q8CE20	Protein 9030612E09Rik OS=Mus musculus GN=9030612E09Rik PE=2 SV=1 - [Q8CE20_MOUSE]	15.00	1	2	2	2	0.832	0.819	0.863	0.940	2.24	15.00	2	2	140	14.4	10.11
Q8BG18	N-terminal EF-hand calcium-binding protein 1 OS=Mus musculus GN=Necab1 PE=1 SV=1 - [NECA1_MOUSE]	32.10	1	9	9	39	0.560	1.554	1.532	0.940	110.87	32.10	16	39	352	40.9	4.89
P20444	Protein kinase C alpha type OS=Mus musculus GN=Prkca PE=1 SV=3 - [KPCA_MOUSE]	29.02	1	1	19	103	1.049	0.975	1.331	0.940	249.01	29.02	30	103	672	76.8	7.14
D6RHR4	Protein Snhg11 OS=Mus musculus GN=Snhg11 PE=2 SV=1 - [D6RHR4_MOUSE]	23.88	3	1	2	2	0.343	1.229	1.084	0.940	1.71	23.88	2	2	67	7.4	10.17
Q8K2Y3	Protein eva-1 homolog B OS=Mus musculus GN=Eva1b PE=2 SV=1 - [EVA1B_MOUSE]	13.41	1	1	1	4	0.872	1.307	0.760	0.940	20.16	13.41	2	4	164	18.3	4.68
Q8C788	Sorting nexin 18 OS=Mus musculus GN=Snx18 PE=2 SV=1 - [Q8C788_MOUSE]	17.56	2	9	9	39	1.461	1.002	0.901	0.940	113.65	17.56	16	39	615	67.7	6.46
Q3TXX3-2	Isoform 2 of Protrudin OS=Mus musculus GN=Zfyve27 - [ZFY27_MOUSE]	7.11	3	2	2	8	0.642	0.596	0.923	0.940	26.13	7.11	4	8	408	45.4	5.35
A2AKQ8	Tetratricopeptide repeat protein 30A2 OS=Mus musculus GN=Ttc30a2 PE=2 SV=1 - [T30A2_MOUSE]	9.49	1	5	6	7	0.494	0.909	0.716	0.940	15.14	9.49	7	7	664	76.1	5.16
Q8BR90	UPF0600 protein C5orf51 homolog OS=Mus musculus PE=2 SV=1 - [CE051_MOUSE]	28.91	2	8	8	27	0.637	1.163	0.868	0.940	81.92	28.91	14	27	294	33.4	5.19
Q91VD1	Galectin-12 OS=Mus musculus GN=Lgals12 PE=1 SV=2 - [LEG12_MOUSE]	8.60	7	2	2	2	1.520	0.854	0.975	0.940	5.73	8.60	2	2	314	35.4	8.60
D3Z601	Calsyntenin-3 OS=Mus musculus GN=Clstn3 PE=2 SV=1 - [D3Z601_MOUSE]	11.10	2	10	10	34	1.016	0.857	0.854	0.940	84.31	11.10	16	34	919	101.7	5.27
O88325	Alpha-N-acetylglucosaminidase OS=Mus musculus GN=Naglu PE=2 SV=1 -	15.29	1	8	8	13	1.984	1.104	1.035	0.940	47.23	15.29	12	13	739	82.5	6.61
Q9WV32	Actin-related protein 2/3 complex subunit 1B OS=Mus musculus GN=Arpc1b PE=1 SV=4 - [ARC1B_MOUSE]	27.42	4	8	8	19	1.721	0.969	1.124	0.940	64.04	27.42	13	19	372	41.0	8.35
P67871	Casein kinase II subunit beta OS=Mus musculus GN=Csnk2b PE=1 SV=1 - [CSK2B_MOUSE]	44.65	7	8	8	52	1.331	1.122	0.967	0.940	161.05	44.65	14	52	215	24.9	5.55
Q9JJL8	Serine--tRNA ligase, mitochondrial OS=Mus musculus GN=Sars2 PE=2 SV=2 - [SYSM_MOUSE]	37.07	1	14	14	54	0.843	0.940	0.889	0.940	158.59	37.07	24	54	518	58.3	7.90
Q9CQA5-2	Isoform 2 of Mediator of RNA polymerase II transcription subunit 4 OS=Mus musculus GN=Med4 - [illegible]	25.99	2	4	4	7	1.479	1.077	0.932	0.940	22.76	25.99	7	7	177	19.9	6.57
Q3UCV8	Ubiquitin thioesterase otulin OS=Mus musculus GN=Fam105b PE=1 SV=1 [OTUL_MOUSE]	14.77	1	6	6	10	0.356	1.595	0.828	0.940	15.18	14.77	9	10	352	40.3	5.68
Q9CQT0	MCG22296, isoform CRA_b OS=Mus musculus GN=Thg1l PE=2 SV=1 - [Q9CQT0_MOUSE]	7.56	2	2	2	3	0.646	1.105	0.876	0.940	5.44	7.56	3	3	238	28.0	7.11
Q8VCY8-2	Isoform 2 of Lipid phosphate phosphatase-related protein type 2 OS=Mus musculus GN=Lppr2 - [illegible]	12.83	2	4	4	21	0.382	1.650	1.350	0.941	85.04	12.83	7	21	452	48.4	9.45

Q69ZS7	HBS1-like protein OS=Mus musculus GN=Hbs1l PE=1 SV=2 - [HBS1L_MOUSE]	32.26	3	11	17	38	1.152	0.740	0.862	0.941	115.55	32.26	29	38	682	75.1	6.46
Q7TSF4	Leucine-rich repeat-containing protein FAM211A OS=Mus musculus GN=Fam211a PE=1 SV=1 - [F211A_MOUSE]	15.04	2	3	3	5	0.721	0.882	1.141	0.941	14.23	15.04	4	5	339	37.6	8.76
Q80Y61	Brain-specific angiogenesis inhibitor 1-associated protein 2-like protein 2 OS=Mus musculus GN=Baiap2l2 PE=1 SV=1 - [BI2L2_MOUSE]	4.21	1	2	2	3	0.926	1.640	1.768	0.941	4.86	4.21	3	3	522	58.4	9.50
Q6NZM9	Histone deacetylase 4 OS=Mus musculus GN=Hdac4 PE=1 SV=1 - [HDAC4_MOUSE]	18.31	2	16	17	50	1.375	0.993	1.029	0.941	140.90	18.31	29	50	1076	118.5	6.92
Q8VEH8	Endoplasmic reticulum lectin 1 OS=Mus musculus GN=Erlec1 PE=2 SV=1 - [ERLEC_MOUSE]	31.47	3	12	12	25	1.014	0.945	0.851	0.941	69.34	31.47	18	25	483	54.9	6.25
Q91VC3	Eukaryotic initiation factor 4A-III OS=Mus musculus GN=Eif4a3 PE=2 SV=3 - [IF4A3_MOUSE]	54.50	3	19	22	84	0.617	0.850	0.832	0.941	258.05	54.50	39	84	411	46.8	6.73
Q3TJ55	Syntaxin-2 OS=Mus musculus GN=Stx2 PE=2 SV=1 - [Q3TJ55_MOUSE]	20.49	4	5	7	42	0.944	0.836	0.837	0.941	82.69	20.49	10	42	288	33.2	6.21
Q6P6J9	Thioredoxin domain-containing protein 15 OS=Mus musculus GN=Txndc15 PE=1 SV=1 - [TXD15_MOUSE]	8.72	1	2	2	6	1.511	1.508	0.957	0.941	22.80	8.72	4	6	344	38.1	4.77
P83882	60S ribosomal protein L36a OS=Mus musculus GN=Rpl36a PE=2 SV=2 - [RL36A_MOUSE]	23.58	1	3	3	14	0.964	1.219	0.903	0.941	31.96	23.58	6	14	106	12.4	10.58
P69566	Ran-binding protein 9 OS=Mus musculus GN=Ranbp9 PE=1 SV=1 - [RANB9_MOUSE]	22.05	3	12	12	35	0.926	1.280	0.942	0.941	105.71	22.05	20	35	653	71.0	6.84
Q3ZK22	Vezatin OS=Mus musculus GN=Vezt PE=1 SV=2 - [VEZA_MOUSE]	13.33	8	8	9	27	0.834	1.413	1.242	0.941	83.71	13.33	16	27	780	87.9	5.20
Q6VN19	Ran-binding protein 10 OS=Mus musculus GN=Ranbp10 PE=1 SV=2 - [RBP10_MOUSE]	14.68	1	6	6	13	0.800	1.086	0.847	0.941	36.67	14.68	10	13	620	67.1	6.58
Q8C0Q2	Zinc fingers and homeoboxes protein 3 OS=Mus musculus GN=Zhx3 PE=2 SV=2 - [ZHX3_MOUSE]	9.46	1	6	6	14	1.555	0.980	1.124	0.941	46.23	9.46	10	14	951	104.3	6.57
O70480	Vesicle-associated membrane protein 4 OS=Mus musculus GN=Vamp4 PE=1 SV=1 - [VAMP4_MOUSE]	28.37	2	3	3	20	1.064	1.091	0.757	0.941	75.70	28.37	4	20	141	16.3	7.36
Q9EQT6	Synaptotagmin-13 OS=Mus musculus GN=Syt13 PE=1 SV=1 - [SYT13_MOUSE]	10.56	1	4	4	13	0.622	1.441	0.963	0.941	33.87	10.56	7	13	426	46.8	7.53
Q7M6Z4	Kinesin-like protein KIF27 OS=Mus musculus GN=Kif27 PE=1 SV=1 - [KIF27_MOUSE]	2.87	2	2	5	14	1.170	0.665	0.985	0.941	39.42	2.87	7	14	1394	158.9	7.14
Q80WQ2	Protein VAC14 homolog OS=Mus musculus GN=Vac14 PE=1 SV=1 - [VAC14_MOUSE]	27.37	1	15	16	55	0.647	1.079	0.892	0.941	161.93	27.37	24	55	782	88.0	6.13
Q9QUM9	Proteasome subunit alpha type-6 OS=Mus musculus GN=Psma6 PE=1 SV=1 - [PSA6_MOUSE]	51.22	3	12	12	118	1.789	1.024	0.811	0.941	355.52	51.22	22	118	246	27.4	6.76
Q8BJD1	Inter-alpha-trypsin inhibitor heavy chain H5 OS=Mus musculus GN=Itih5 PE=2 SV=1 - [ITIH5_MOUSE]	7.04	1	6	6	14	1.254	1.307	0.867	0.941	33.93	7.04	10	14	952	106.7	8.16
Q6A0D4	Raftlin OS=Mus musculus GN=Rftn1 PE=1 SV=4 - [RFTN1_MOUSE]	29.06	2	12	12	39	0.757	0.408	0.593	0.941	132.73	29.06	20	39	554	61.5	7.68
Q3UVG3	Protein FAM91A1 OS=Mus musculus GN=Fam91a1 PE=1 SV=1 - [F91A1_MOUSE]	19.35	1	13	14	35	0.527	0.941	0.771	0.942	97.25	19.35	23	35	837	93.4	6.35
Q9CZ83	39S ribosomal protein L55, mitochondrial OS=Mus musculus GN=Mrpl55 PE=2 SV=1 - [RM55_MOUSE]	11.81	2	2	2	3	1.508	1.417	0.894	0.942	8.70	11.81	2	3	127	15.1	10.51
F7BJK1	Protein Pcdh1 (Fragment) OS=Mus musculus GN=Pcdh1 PE=2 SV=1 - [F7BJK1_MOUSE]	39.09	1	4	27	179	1.053	1.829	1.583	0.942	559.98	39.09	47	179	1077	116.4	5.08
Q60864	Stress-induced-phosphoprotein 1 OS=Mus musculus GN=Stip1 PE=1 SV=1 - [STIP1_MOUSE]	61.51	1	41	41	524	1.684	0.815	1.282	0.942	1336.43	61.51	73	524	543	62.5	6.80
Q01721	Growth arrest-specific protein 1 OS=Mus musculus GN=Gas1 PE=2 SV=2 - [GAS1_MOUSE]	8.16	1	2	2	4	0.897	0.688	0.743	0.942	10.39	8.16	3	4	343	35.7	6.04
Q149L6	DnaJ homolog subfamily B member 14 OS=Mus musculus GN=Dnajb14 PE=2 SV=1 -	21.90	3	8	8	28	0.895	0.834	0.848	0.942	69.52	21.90	16	28	379	42.3	8.59

Q61609	Sodium-dependent phosphate transporter 1 OS=Mus musculus GN=Slc20a1 PE=1 SV=1 - [S20A1_MOUSE]	6.61	1	3	5	12	0.898	0.805	0.952	0.942	26.88	6.61	8	12	681	74.1	6.89
Q9CQA9	Cancer-related nucleoside-triphosphatase homolog OS=Mus musculus GN=Ntpcr PE=2 SV=1 - [NTPCR_MOUSE]	30.00	4	5	5	11	0.725	0.905	0.800	0.942	29.15	30.00	8	11	190	20.7	9.72
Q9ER64	Oxysterol-binding protein-related protein 5 OS=Mus musculus GN=Osbpl5 PE=2 SV=3 - [OSBL5_MOUSE]	2.29	2	1	2	6	1.160	0.607	0.857	0.942	19.50	2.29	4	6	874	98.9	7.44
Q9WV04	Kinesin-like protein KIF9 OS=Mus musculus GN=Kif9 PE=2 SV=2 - [KIF9_MOUSE]	3.92	5	1	3	5	0.167	1.493	1.092	0.942	11.71	3.92	4	5	790	89.8	6.86
Q8R3L2	Transcription factor 25 OS=Mus musculus GN=Tcf25 PE=1 SV=2 - [TCF25_MOUSE]	15.38	4	2	7	20	0.969	1.030	0.781	0.942	46.15	15.38	13	20	676	76.6	6.51
A2A7Z8	Arylacetamide deacetylase-like 3 OS=Mus musculus GN=Aadacl3 PE=3 SV=1 - [ADCL3_MOUSE]	3.92	1	1	1	1	1.002	1.160	1.250	0.942	0.00	3.92	1	1	408	46.3	8.28
Q3UZ39-2	Isoform 2 of Leucine-rich repeat flightless-interacting protein 1 OS=Mus musculus GN=Lrrfip1 - [illegible]	28.82	3	6	18	54	1.693	0.867	1.611	0.942	164.00	28.82	27	54	628	71.3	6.04
O88559-2	Isoform 2 of Menin OS=Mus musculus GN=Men1 - [MEN1_MOUSE]	3.96	5	2	2	4	0.504	0.825	0.861	0.942	5.78	3.96	3	4	556	61.4	6.62
Q505H4-3	Isoform 3 of von Willebrand factor C domain containing protein 2-like OS=Mus musculus GN=Vwc2l - [illegible]	8.73	3	1	1	1	1.352	0.672	1.403	0.942	3.90	8.73	1	1	126	14.3	6.84
Q3UH45	Protein Tecpr2 OS=Mus musculus GN=Tecpr2 PE=2 SV=1 - [Q3UH45_MOUSE]	2.95	1	4	4	7	1.289	0.916	1.376	0.942	24.09	2.95	5	7	1423	154.8	5.38
Q8BGA9	Mitochondrial inner membrane protein OXA1L OS=Mus musculus GN=Oxa1l PE=2 SV=1 - [OXA1L_MOUSE]	8.31	1	4	4	12	0.743	0.815	0.883	0.942	23.42	8.31	6	12	433	48.2	9.61
Q9QYA2	Mitochondrial import receptor subunit TOM40 homolog OS=Mus musculus GN=Tomm40 PE=1 SV=3 - [illegible]	45.43	2	10	10	98	0.793	0.850	0.873	0.942	324.44	45.43	18	98	361	37.9	7.74
Q5PPR2	Exocyst complex component 1 OS=Mus musculus GN=Exoc1 PE=2 SV=1 - [Q5PPR2_MOUSE]	33.33	2	1	25	82	0.540	1.305	1.627	0.942	220.56	33.33	44	82	879	100.1	6.52
Q8C5U9	Ubiquilin-3 OS=Mus musculus GN=Ubqln3 PE=2 SV=1 - [UBQL3_MOUSE]	3.04	1	1	2	13	1.642	0.972	0.902	0.942	15.35	3.04	4	13	658	70.7	6.43
Q8VBZ3	Cleft lip and palate transmembrane protein 1 homolog OS=Mus musculus GN=Clptm1 PE=1 SV=1 - [illegible]	25.60	1	12	12	52	0.806	0.737	0.883	0.942	170.78	25.60	20	52	664	75.2	6.30
Q9ERE9	C-Jun-amino-terminal kinase-interacting protein 2 OS=Mus musculus GN=Mapk8ip2 PE=1 SV=1 - [JIP2_MOUSE]	7.47	1	4	4	23	1.616	0.947	0.913	0.942	65.55	7.47	8	23	830	89.8	4.42
Q8R007	Poliovirus receptor-related protein 4 OS=Mus musculus GN=Pvrl4 PE=1 SV=1 - [PVRL4_MOUSE]	1.77	2	1	1	1	1.349	1.360	1.586	0.942	0.00	1.77	1	1	508	55.6	5.34
Q9D0G0	28S ribosomal protein S30, mitochondrial OS=Mus musculus GN=Mrps30 PE=2 SV=1 - [RT30_MOUSE]	24.21	1	7	7	21	0.854	0.711	0.899	0.942	51.87	24.21	11	21	442	49.9	9.38
Q9JKN6	RNA-binding protein Nova-1 OS=Mus musculus GN=Nova1 PE=1 SV=2 - [NOVA1_MOUSE]	50.89	1	15	22	120	1.313	0.417	0.715	0.942	348.75	50.89	43	120	507	51.7	8.72
Q9D7S9	Charged multivesicular body protein 5 OS=Mus musculus GN=Chmp5 PE=2 SV=1 -	32.88	1	7	7	31	1.143	0.666	1.229	0.943	85.00	32.88	11	31	219	24.6	4.79
Q6PHP6	Leucine rich repeat protein 2, neuronal OS=Mus musculus GN=Lrrn2 PE=2 SV=1 - [Q6PHP6_MOUSE]	2.05	1	1	1	1	2.006	1.142	1.180	0.943	0.00	2.05	1	1	730	81.1	7.97
A2AEW8	GRIP1-associated protein 1 OS=Mus musculus GN=Gripap1 PE=2 SV=1 - [A2AEW8_MOUSE]	65.35	7	52	53	235	1.751	1.097	1.458	0.943	678.65	65.35	89	235	837	95.9	5.17
Q9CW03	Structural maintenance of chromosomes protein 3 OS=Mus musculus GN=Smc3 PE=1 SV=2 - [SMC3_MOUSE]	35.99	2	38	41	117	0.862	1.004	0.815	0.943	330.10	35.99	66	117	1217	141.5	7.18
Q6DIC0	Probable global transcription activator SNF2L2 OS=Mus musculus GN=Smarca2 PE=1 SV=1 - [SMCA2_MOUSE]	12.37	12	10	18	46	0.708	1.082	0.999	0.943	140.89	12.37	30	46	1577	180.1	7.20

Q78T81	Protein FAM102A OS=Mus musculus GN=Fam102a PE=1 SV=1 - [F102A_MOUSE]	23.47	1	7	7	31	0.734	0.824	1.119	0.943	111.51	23.47	11	31	392	42.8	8.56
Q91YE3	Egl nine homolog 1 OS=Mus musculus GN=Egln1 PE=2 SV=2 - [EGLN1_MOUSE]	8.50	1	3	3	8	1.696	0.860	1.232	0.943	16.15	8.50	5	8	400	43.1	8.29
Q8K4D7	1-phosphatidylinositol 4,5-bisphosphate phosphodiesterase zeta-1 OS=Mus musculus GN=Plcz1 PE=1 SV=1 - [PLCZ1_MOUSE]	1.85	1	1	1	2	37.377	1.229	2.071	0.943	2.37	1.85	1	2	647	74.6	5.63
H3BJP5	POU domain, class 2, transcription factor 1 (Fragment) OS=Mus musculus GN=Pou2f1 PE=2 SV=1 - [H3BJP5_MOUSE]	39.13	17	1	1	1	1.047	1.038	1.194	0.943	1.66	39.13	1	1	23	2.5	4.91
O54946-2	Isoform B of DnaJ homolog subfamily B member 6 OS=Mus musculus GN=Dnajb6 - [DNJB6_MOUSE]	40.50	3	2	10	35	0.839	1.607	1.101	0.943	90.40	40.50	16	35	242	27.0	7.61
E9Q412	Protein Brd1 OS=Mus musculus GN=Brd1 PE=2 SV=1 - [E9Q412_MOUSE]	1.04	3	1	1	1	1.190	0.923	1.034	0.943	1.86	1.04	1	1	867	98.9	8.68
P62196	26S protease regulatory subunit 8 OS=Mus musculus GN=Psmc5 PE=1 SV=1 -	67.73	2	27	29	142	0.946	1.142	0.887	0.943	396.66	67.73	49	142	406	45.6	7.55
Q8BG32	26S proteasome non-ATPase regulatory subunit 11 OS=Mus musculus GN=Psmd11 PE=1 SV=3 - [PSD11_MOUSE]	66.82	8	8	26	116	0.732	1.047	0.855	0.943	371.49	66.82	45	116	422	47.4	6.48
Q9QXV8	Protein sprouty homolog 2 OS=Mus musculus GN=Spry2 PE=2 SV=1 - [SPY2_MOUSE]	22.86	1	5	5	20	1.073	0.944	1.094	0.943	54.73	22.86	10	20	315	34.6	8.29
P26041	Moesin OS=Mus musculus GN=Msn PE=1 SV=3 - [MOES_MOUSE]	42.46	1	19	29	126	1.446	0.864	0.944	0.943	337.49	42.46	50	126	577	67.7	6.60
Q9JLL3	Tumor necrosis factor receptor superfamily member 19 OS=Mus musculus GN=Tnfrsf19 PE=2 SV=2 - [TNR19_MOUSE]	7.45	2	2	2	12	1.646	0.960	0.974	0.943	0.00	7.45	2	12	416	45.2	5.82
Q3UFB2	Box C/D snoRNA protein 1 OS=Mus musculus GN=Znhit6 PE=2 SV=2 - [BCD1_MOUSE]	12.61	2	3	3	5	0.990	1.617	0.915	0.943	9.16	12.61	3	5	460	52.2	5.57
P56812	Programmed cell death protein 5 OS=Mus musculus GN=Pdcd5 PE=1 SV=3 - [PDCD5_MOUSE]	53.97	2	7	7	64	1.659	1.003	1.264	0.943	174.49	53.97	13	64	126	14.3	5.68
Q32NY4	Metal transporter CNNM3 OS=Mus musculus GN=Cnnm3 PE=1 SV=2 - [CNNM3_MOUSE]	19.21	2	11	14	39	1.204	1.172	0.975	0.944	109.72	19.21	25	39	713	76.2	5.58
O54818	Tumor protein D53 OS=Mus musculus GN=Tpd52l1 PE=2 SV=1 - [TPD53_MOUSE]	40.69	1	8	8	17	0.739	1.368	1.102	0.944	49.60	40.69	11	17	204	22.5	6.21
Q9CZB0	Succinate dehydrogenase cytochrome b560 subunit, mitochondrial OS=Mus musculus GN=Sdhc PE=2 SV=1 - [C560_MOUSE]	14.20	3	2	2	8	0.768	0.986	0.929	0.944	16.33	14.20	3	8	169	18.4	9.94
G3UYG5	Potassium voltage-gated channel subfamily KQT member 5 OS=Mus musculus GN=Kcnq5 PE=2 SV=1 - [G3UYG5_MOUSE]	5.30	5	3	4	10	1.409	0.953	1.663	0.944	22.39	5.30	7	10	924	101.3	9.50
P53995	Anaphase-promoting complex subunit 1 OS=Mus musculus GN=Anapc1 PE=1 SV=2 - [APC1_MOUSE]	15.07	3	21	21	54	0.756	0.962	0.898	0.944	156.58	15.07	35	54	1944	215.9	6.35
O88796	Ribonuclease P protein subunit p30 OS=Mus musculus GN=Rpp30 PE=2 SV=1 -	3.36	1	1	1	1	1.232	0.651	0.894	0.944	2.93	3.36	1	1	268	29.5	9.06
Q9R060	Cytosolic Fe-S cluster assembly factor NUBP1 OS=Mus musculus GN=Nubp1 PE=1 SV=1 - [NUBP1_MOUSE]	14.69	1	3	3	9	1.205	0.972	1.051	0.944	39.15	14.69	6	9	320	34.1	5.90
P68254-2	Isoform 2 of 14-3-3 protein theta OS=Mus musculus GN=Ywhaq - [1433T_MOUSE]	66.67	5	15	21	775	0.925	1.018	0.904	0.944	2169.88	66.67	36	775	243	27.7	4.93
Q8K3K8	Optineurin OS=Mus musculus GN=Optn PE=1 SV=1 - [OPTN_MOUSE]	24.49	1	13	13	36	2.256	0.907	1.038	0.944	93.48	24.49	25	36	584	67.0	5.26
Q8C0J2-2	Isoform 2 of Autophagy-related protein 16-1 OS=Mus musculus GN=Atg16l1 -	30.61	8	13	14	38	1.221	0.984	1.030	0.944	85.24	30.61	24	38	588	66.2	6.29
P35278	Ras-related protein Rab-5C OS=Mus musculus GN=Rab5c PE=1 SV=2 - [RAB5C_MOUSE]	65.74	4	7	11	62	0.797	0.984	0.854	0.944	176.20	65.74	19	62	216	23.4	8.41
P62242	40S ribosomal protein S8 OS=Mus musculus GN=Rps8 PE=1 SV=2 - [RS8_MOUSE]	39.90	1	8	8	35	0.623	0.807	0.772	0.944	100.24	39.90	13	35	208	24.2	10.32

Q61672	Equilibrative nucleoside transporter 2 OS=Mus musculus GN=Slc29a2 PE=1 SV=2 -	2.63	1	1	1	5	0.723	0.555	0.859	0.944	11.72	2.63	2	5	456	50.2	6.51
Q3UHY7	Coiled-coil domain containing 32 OS=Mus musculus GN=Ccdc32 PE=2 SV=1 -	37.99	3	4	4	11	1.289	0.757	1.326	0.944	32.43	37.99	7	11	179	19.8	4.78
H3BL05	Zinc finger protein 428 (Fragment) OS=Mus musculus GN=Zfp428 PE=2 SV=1 -	31.37	3	3	3	15	1.514	0.924	1.210	0.944	55.18	31.37	6	15	153	16.3	4.21
O35405	Phospholipase D3 OS=Mus musculus GN=Pld3 PE=2 SV=1 - [PLD3_MOUSE]	25.82	4	10	11	36	0.709	1.381	1.117	0.944	111.62	25.82	19	36	488	54.4	6.52
Q99M31	Heat shock 70 kDa protein 14 OS=Mus musculus GN=Hspa14 PE=2 SV=2 - [HSP7E_MOUSE]	17.49	5	7	8	18	1.240	1.085	0.885	0.944	53.20	17.49	12	18	509	54.6	5.92
E0CYJ0	MOB-like protein phocein (Fragment) OS=Mus musculus GN=Mob4 PE=2 SV=1 - [E0CYJ0_MOUSE]	41.94	4	7	7	24	1.209	1.114	1.009	0.944	76.63	41.94	11	24	186	21.6	6.51
Q9DC16	Endoplasmic reticulum-Golgi intermediate compartment protein 1 OS=Mus musculus GN=Ergic1 PE=1 SV=1 - [illegible]	33.45	3	8	8	33	0.706	1.035	0.765	0.944	110.47	33.45	15	33	290	32.5	7.06
Q6PB51	Coiled-coil domain-containing protein 117 OS=Mus musculus GN=Ccdc117 PE=2 SV=1 - [CC117_MOUSE]	3.97	1	1	1	2	1.215	0.971	1.039	0.944	3.45	3.97	2	2	277	30.4	5.88
Q8CDM8	Protein FAM160B1 OS=Mus musculus GN=Fam160b1 PE=2 SV=2 - [F16B1_MOUSE]	10.47	2	6	6	15	0.876	0.984	0.869	0.944	42.67	10.47	10	15	764	86.0	5.25
P22907-2	Isoform 2 of Porphobilinogen deaminase OS=Mus musculus GN=Hmbs -	22.09	2	5	6	13	0.992	1.134	0.870	0.944	22.04	22.09	11	13	344	37.8	7.15
Q91YT7	Protein Ythdf2 OS=Mus musculus GN=Ythdf2 PE=2 SV=1 - [Q91YT7_MOUSE]	12.44	1	4	7	23	0.880	0.820	0.985	0.944	37.66	12.44	11	23	579	62.2	8.79
Q3UI43	BRISC and BRCA1-A complex member 1 OS=Mus musculus GN=Babam1 PE=2 SV=1 - [BABA1_MOUSE]	42.64	1	9	9	41	1.461	1.224	1.195	0.944	127.47	42.64	14	41	333	36.8	4.55
D3YZC7	Bromodomain-containing protein 8 OS=Mus musculus GN=Brd8 PE=2 SV=1 - [D3YZC7_MOUSE]	9.19	4	7	7	16	2.094	0.969	1.043	0.945	47.33	9.19	13	16	881	95.6	4.72
Q9R171	Cerebellin-1 OS=Mus musculus GN=Cbln1 PE=1 SV=1 - [CBLN1_MOUSE]	37.82	1	5	7	47	1.211	0.553	0.748	0.945	122.29	37.82	14	47	193	21.1	7.28
Q6PDL0	Cytoplasmic dynein 1 light intermediate chain 2 OS=Mus musculus GN=Dync1li2 PE=1 SV=2 - [DC1L2_MOUSE]	42.48	2	18	19	114	1.235	0.728	1.116	0.945	372.00	42.48	33	114	492	54.2	6.28
Q9CXU4	MCG11298 OS=Mus musculus GN=Timm23 PE=2 SV=1 - [Q9CXU4_MOUSE]	12.92	2	2	2	3	1.063	1.118	0.841	0.945	10.26	12.92	3	3	209	21.9	8.60
Q61990	Poly(rC)-binding protein 2 OS=Mus musculus GN=Pcbp2 PE=1 SV=1 - [PCBP2_MOUSE]	59.12	4	7	15	172	0.857	0.909	0.871	0.945	478.48	59.12	27	172	362	38.2	6.79
P08074	Carbonyl reductase [NADPH] 2 OS=Mus musculus GN=Cbr2 PE=1 SV=1 - [CBR2_MOUSE]	25.41	2	5	6	20	1.148	2.731	0.659	0.945	58.70	25.41	12	20	244	25.9	9.01
P62334	26S protease regulatory subunit 10B OS=Mus musculus GN=Psmc6 PE=1 SV=1 -	62.98	1	19	19	116	0.985	1.101	0.893	0.945	339.09	62.98	36	116	389	44.1	7.49
Q9ES28-3	Isoform C of Rho guanine nucleotide exchange factor 7 OS=Mus musculus GN=Arhgef7 - [ARHG7_MOUSE]	43.33	9	24	28	91	0.872	0.998	1.051	0.945	235.79	43.33	46	91	787	88.9	6.28
Q3UHK6	Teneurin-4 OS=Mus musculus GN=Tenm4 PE=1 SV=2 - [TEN4_MOUSE]	17.94	6	34	40	107	0.854	1.177	1.018	0.945	289.63	17.94	68	107	2771	308.2	6.57
Q9R0L6	Pericentriolar material 1 protein OS=Mus musculus GN=Pcm1 PE=1 SV=2 - [PCM1_MOUSE]	15.75	2	27	28	71	1.119	0.792	1.014	0.945	202.94	15.75	46	71	2025	228.7	5.01
Q3TVA9	Coiled-coil domain-containing protein 136 OS=Mus musculus GN=Ccdc136 PE=2 SV=2 - [CC136_MOUSE]	23.86	7	18	21	90	1.163	0.661	0.887	0.946	320.71	23.86	36	90	1136	131.8	4.84
A3KGR9	Minor histocompatibility antigen H13 (Fragment) OS=Mus musculus GN=H13 PE=2 SV=1 - [A3KGR9_MOUSE]	11.53	6	3	3	18	0.775	0.957	0.820	0.946	52.16	11.53	5	18	347	38.1	8.40
Q06138	Calcium-binding protein 39 OS=Mus musculus GN=Cab39 PE=1 SV=2 - [CAB39_MOUSE]	41.06	3	13	16	76	0.864	1.189	0.935	0.946	224.74	41.06	30	76	341	39.8	6.89

O89114	DnaJ homolog subfamily B member 5 OS=Mus musculus GN=Dnajb5 PE=2 SV=1 -	27.01	2	9	9	23	1.461	1.228	1.279	0.946	67.35	27.01	12	23	348	39.1	9.04
Q923W1	Trimethylguanosine synthase OS=Mus musculus GN=Tgs1 PE=1 SV=2 - [TGS1_MOUSE]	5.98	2	4	4	6	0.781	0.856	0.998	0.946	15.32	5.98	5	6	853	96.7	4.97
O08663	Methionine aminopeptidase 2 OS=Mus musculus GN=Metap2 PE=1 SV=1 -	28.87	7	13	13	47	0.947	0.755	0.832	0.946	136.06	28.87	25	47	478	52.9	5.82
P27048	Small nuclear ribonucleoprotein-associated protein B OS=Mus musculus GN=Snrpb PE=1 SV=1 - [illegible]	30.30	2	7	7	37	0.699	0.856	0.806	0.946	119.95	30.30	12	37	231	23.6	10.90
Q8K0W9	DPH3 homolog OS=Mus musculus GN=Dph3 PE=1 SV=1 - [DPH3_MOUSE]	25.61	1	1	1	3	2.928	1.202	1.228	0.946	10.48	25.61	2	3	82	9.3	4.09
Q8BFY9-2	Isoform 2 of Transportin-1 OS=Mus musculus GN=Tnpo1 - [TNPO1_MOUSE]	15.96	4	7	12	24	0.760	1.133	0.808	0.946	64.80	15.96	20	24	890	101.2	4.96
Q99KL7	Ras-related protein Rab-28 OS=Mus musculus GN=Rab28 PE=2 SV=1 - [RAB28_MOUSE]	17.19	1	3	3	7	1.251	1.232	1.083	0.946	22.24	17.19	5	7	221	24.7	5.67
D3YVM2	Transmembrane protein 59 OS=Mus musculus GN=Tmem59 PE=2 SV=1 - [D3YVM2_MOUSE]	3.91	2	1	1	2	0.996	0.841	0.783	0.946	4.07	3.91	1	2	256	28.6	5.39
Q62141-1	Isoform 1 of Paired amphipathic helix protein Sin3b OS=Mus musculus GN=Sin3b - [SIN3B_MOUSE]	5.56	6	3	4	5	1.133	1.219	1.459	0.946	10.68	5.56	5	5	954	109.3	6.55
Q9D187	Mitotic spindle-associated MMXD complex subunit MIP18 OS=Mus musculus GN=Fam96b PE=2 SV=1 - [MIP18_MOUSE]	30.06	2	3	3	8	1.046	1.078	0.906	0.946	21.67	30.06	6	8	163	17.7	4.98
E9QAJ9	Rho GTPase-activating protein 17 OS=Mus musculus GN=Arhgap17 PE=2 SV=1 -	14.67	5	7	9	33	0.782	0.806	0.728	0.946	92.03	14.67	15	33	818	88.9	8.12
Q80UY2	E3 ubiquitin-protein ligase KCMF1 OS=Mus musculus GN=Kcmf1 PE=2 SV=1 - [KCMF1_MOUSE]	34.91	2	7	7	40	1.586	1.038	1.289	0.946	133.68	34.91	11	40	381	41.8	5.76
O54983	Thiomorpholine-carboxylate dehydrogenase OS=Mus musculus GN=Crym PE=1 SV=1 - [CRYM_MOUSE]	39.94	1	12	12	128	1.041	2.399	1.151	0.946	343.74	39.94	22	128	313	33.5	5.67
E9QAI5	DNA fragmentation factor subunit beta OS=Mus musculus GN=Cad PE=2 SV=1 - [E9QAI5_MOUSE]	18.83	6	29	34	94	0.719	0.966	0.780	0.946	246.30	18.83	56	94	2162	236.1	6.49
Q60902	Epidermal growth factor receptor substrate 15-like 1 OS=Mus musculus GN=Eps15l1 PE=1 SV=3 - [EP15R_MOUSE]	59.65	3	7	49	278	1.864	1.067	1.218	0.946	765.11	59.65	83	278	907	99.2	5.02
Q9CQV1	Mitochondrial import inner membrane translocase subunit TIM16 OS=Mus musculus GN=Pam16 PE=2 SV=1 - [TIM16_MOUSE]	47.20	1	4	4	19	1.185	0.956	0.963	0.946	56.38	47.20	8	19	125	13.8	9.64
E9QLT0	Hydroxypyruvate isomerase OS=Mus musculus GN=Hyi PE=2 SV=1 - [E9QLT0_MOUSE]	2.62	2	1	1	1	1.854	1.695	12.561	0.946	0.00	2.62	1	1	267	29.4	5.62
Q9JL15	Galectin-8 OS=Mus musculus GN=Lgals8 PE=2 SV=1 - [LEG8_MOUSE]	11.71	3	3	3	6	1.105	0.822	0.624	0.946	24.76	11.71	5	6	316	36.1	8.95
Q9JIZ5	Activator protein 4 OS=Mus musculus GN=Tfap4 PE=2 SV=1 - [Q9JIZ5_MOUSE]	3.25	1	1	1	1	2.073	1.464	1.397	0.946	1.89	3.25	1	1	338	38.7	6.09
Q91Z49	UAP56-interacting factor OS=Mus musculus GN=Fyttd1 PE=1 SV=1 - [UIF_MOUSE]	31.23	4	8	8	13	1.004	0.859	0.987	0.946	36.52	31.23	11	13	317	35.9	11.84
Q8C3F2	Constitutive coactivator of PPAR-gamma-like protein 2 OS=Mus musculus GN=Fam120c PE=2 SV=3 - [F120C_MOUSE]	24.11	3	18	20	81	0.627	0.690	0.783	0.947	236.78	24.11	35	81	1091	119.6	8.46
Q8R3Y8-2	Isoform 2 of Interferon regulatory factor 2-binding protein 1 OS=Mus musculus GN=Irf2bp1 - [I2BP1_MOUSE]	23.54	2	11	11	33	1.005	0.972	0.803	0.947	101.21	23.54	18	33	565	59.5	8.02
Q810U4-5	Isoform 5 of Neuronal cell adhesion molecule OS=Mus musculus GN=Nrcam -	35.77	2	1	7	94	1.273	1.127	1.121	0.947	274.92	35.77	14	94	260	28.9	8.43
P97447	Four and a half LIM domains protein 1 OS=Mus musculus GN=Fhl1 PE=2 SV=3 - [FHL1_MOUSE]	47.86	7	4	14	60	1.848	1.481	1.178	0.947	151.87	47.86	24	60	280	31.9	8.37

D3YXN0	AP-1 complex subunit sigma-1A (Fragment) OS=Mus musculus GN=Ap1s1 PE=2 SV=1 - [D3YXN0_MOUSE]	21.69	3	1	3	13	1.318	0.975	1.356	0.947	36.99	21.69	5	13	189	22.0	5.52
D3YWN7	Ensconsin OS=Mus musculus GN=Map7 PE=4 SV=2 - [D3YWN7_MOUSE]	17.07	4	12	13	50	0.942	0.960	1.400	0.947	154.60	17.07	22	50	738	82.8	9.38
Q8R4R6	Nucleoporin NUP53 OS=Mus musculus GN=Nup35 PE=1 SV=2 - [NUP53_MOUSE]	23.08	2	5	5	20	1.230	0.970	0.958	0.947	72.70	23.08	9	20	325	34.8	9.25
Q80UW8	DNA-directed RNA polymerases I, II, and III subunit RPABC1 OS=Mus musculus GN=Polr2e PE=2 SV=1 - [RPAB1_MOUSE]	28.10	1	5	5	18	1.634	1.185	0.800	0.947	56.31	28.10	9	18	210	24.6	5.95
Q91Y08	Protein Pcdhb1 OS=Mus musculus GN=Pcdhb1 PE=2 SV=1 - [Q91Y08_MOUSE]	1.34	1	1	1	1	1.749	1.246	1.128	0.947	2.47	1.34	1	1	818	90.4	5.33
Q9D6K8	FUN14 domain-containing protein 2 OS=Mus musculus GN=Fundc2 PE=2 SV=1 -	26.49	1	4	4	11	0.618	0.849	0.863	0.947	31.40	26.49	6	11	151	16.6	9.70
O08914	Fatty-acid amide hydrolase 1 OS=Mus musculus GN=Faah PE=2 SV=1 - [FAAH1_MOUSE]	36.79	1	15	15	40	0.737	1.237	1.143	0.947	131.08	36.79	25	40	579	63.2	7.87
E9PYJ8	Histone acetyltransferase p300 OS=Mus musculus GN=Ep300 PE=4 SV=1 - [E9PYJ8_MOUSE]	8.50	2	7	12	28	0.673	0.909	0.830	0.947	48.12	8.50	16	28	2412	263.1	8.54
A2AF33	Protein Tmsb15b1 OS=Mus musculus GN=Tmsb15b1 PE=4 SV=1 - [A2AF33_MOUSE]	77.78	1	1	3	16	1.542	0.973	1.425	0.947	25.98	77.78	6	16	45	5.2	5.34
P63250	G protein-activated inward rectifier potassium channel 1 OS=Mus musculus GN=Kcnj3 PE=1 SV=1 - [IRK3_MOUSE]	14.37	2	6	6	12	0.976	1.160	1.420	0.947	34.23	14.37	10	12	501	56.5	8.37
Q9D1T0	Leucine-rich repeat and immunoglobulin-like domain-containing nogo receptor-interacting protein 1 OS=Mus musculus GN=Lingo1 PE=1 SV=1 - [LIGO1_MOUSE]	43.00	2	19	22	72	0.567	1.684	1.367	0.947	217.37	43.00	38	72	614	69.1	8.31
P98084	Amyloid beta A4 precursor protein-binding family A member 2 OS=Mus musculus GN=Apba2 PE=1 SV=2 - [APBA2_MOUSE]	22.13	5	8	12	47	1.485	0.958	1.017	0.947	134.76	22.13	21	47	750	82.7	4.88
Q922M3	BTB/POZ domain-containing adapter for CUL3-mediated RhoA degradation protein 3 OS=Mus musculus GN=Kctd10 PE=1 SV=1 - [BACD3_MOUSE]	13.33	3	4	5	8	1.236	1.144	0.943	0.947	16.91	13.33	8	8	315	35.7	6.34
Q3KNM2	E3 ubiquitin-protein ligase MARCH5 OS=Mus musculus GN=March5 PE=2 SV=1 -	8.99	1	2	2	5	1.015	0.785	0.757	0.947	13.27	8.99	3	5	278	31.2	8.70
Q9QYB5	Gamma-adducin OS=Mus musculus GN=Add3 PE=1 SV=2 - [ADDG_MOUSE]	39.52	1	2	21	144	1.214	1.258	1.493	0.947	347.32	39.52	35	144	706	78.7	5.95
Q99J10	Cytoplasmic tRNA 2-thiolation protein 1 OS=Mus musculus GN=Ctu1 PE=2 SV=1 - [CTU1_MOUSE]	8.57	1	3	3	9	0.917	0.709	0.934	0.947	26.22	8.57	6	9	420	43.8	8.10
Q91YR1	Twinfilin-1 OS=Mus musculus GN=Twf1 PE=1 SV=2 - [TWF1_MOUSE]	30.86	2	8	11	46	0.934	0.912	0.916	0.947	134.19	30.86	20	46	350	40.1	6.67
P15626	Glutathione S-transferase Mu 2 OS=Mus musculus GN=Gstm2 PE=1 SV=2 - [GSTM2_MOUSE]	72.02	4	9	17	163	0.691	4.770	0.645	0.947	565.58	72.02	28	163	218	25.7	7.39
Q61625	Glutamate receptor ionotropic, delta-2 OS=Mus musculus GN=Grid2 PE=1 SV=1 - [GRID2_MOUSE]	12.81	2	10	11	26	0.979	0.792	1.284	0.947	81.11	12.81	17	26	1007	113.0	6.10
B1AXJ6	Low-density lipoprotein receptor-related protein 8 OS=Mus musculus GN=Lrp8 PE=2 SV=1 - [B1AXJ6_MOUSE]	13.83	7	5	5	7	1.059	1.017	1.075	0.947	5.83	13.83	6	7	694	77.2	5.00
Q6NXK8	Acid-sensing ion channel 1 OS=Mus musculus GN=Asic1 PE=1 SV=1 - [ASIC1_MOUSE]	21.10	2	9	11	23	0.826	1.181	0.963	0.948	57.83	21.10	16	23	526	59.6	5.58
A2AUK8	Band 4.1-like protein 1 OS=Mus musculus GN=Epb4.1l1 PE=4 SV=1 - [A2AUK8_MOUSE]	53.75	5	19	41	346	1.243	1.326	1.381	0.948	1000.42	53.75	73	346	867	96.8	5.66
Q8BV23	Phospholipase A2, group III, isoform CRA_b OS=Mus musculus GN=Pla2g3 PE=2 SV=1 - [Q8BV23_MOUSE]	3.81	2	1	1	4	1.868	1.069	0.890	0.948	0.00	3.81	1	4	446	49.8	8.29
F8WJG3	Transformer-2 protein homolog beta OS=Mus musculus GN=Tra2b PE=2 SV=1 - [F8WJG3_MOUSE]	29.79	2	4	5	28	0.648	1.133	0.863	0.948	89.56	29.79	10	28	188	21.9	10.15

E9PX95	Protein Abca17 OS=Mus musculus GN=Abca17 PE=3 SV=1 - [E9PX95_MOUSE]	2.31	1	2	3	4	2.200	1.676	1.175	0.948	6.04	2.31	3	4	1733	195.9	7.83
Q0KK55	Protein very KIND OS=Mus musculus GN=Kndc1 PE=1 SV=2 - [VKIND_MOUSE]	13.95	6	19	20	53	0.861	0.614	0.752	0.948	114.62	13.95	32	53	1742	191.2	6.30
Q9CYN9	Renin receptor OS=Mus musculus GN=Atp6ap2 PE=2 SV=2 - [RENR_MOUSE]	26.00	1	8	8	26	0.688	1.053	0.730	0.948	64.36	26.00	13	26	350	39.1	5.54
P41242-3	Isoform 3 of Megakaryocyte-associated tyrosine-protein kinase OS=Mus musculus GN=Matk - [illegible]	21.72	6	7	8	20	0.732	1.057	1.037	0.948	67.52	21.72	12	20	465	51.5	8.40
Q8VE19	WD repeat-containing protein mio OS=Mus musculus GN=Mios PE=2 SV=2 - [MIO_MOUSE]	14.17	1	11	11	22	0.879	1.340	0.937	0.948	58.61	14.17	17	22	875	98.3	6.71
P97291	Cadherin-8 OS=Mus musculus GN=Cdh8 PE=1 SV=2 - [CADH8_MOUSE]	21.28	6	10	10	22	1.227	0.833	1.098	0.948	63.91	21.28	15	22	799	88.2	4.74
E9QPX3	NADH dehydrogenase [ubiquinone] iron-sulfur protein 4, mitochondrial OS=Mus musculus GN=Ndufs4 PE=4 SV=1 - [illegible]	62.29	2	11	11	202	1.346	0.541	1.469	0.948	502.96	62.29	19	202	175	19.8	9.99
E0CZ60	Protein E330034G19Rik (Fragment) OS=Mus musculus GN=E330034G19Rik PE=2 SV=1 - [E0CZ60_MOUSE]	2.91	5	1	1	1	0.881	1.383	0.903	0.948	1.66	2.91	1	1	206	23.8	6.95
B4XVP9	Phf11-1 OS=Mus musculus GN=Phf11c PE=2 SV=1 - [B4XVP9_MOUSE]	7.37	1	1	1	2	6.035	1.071	1.137	0.948	0.00	7.37	1	2	339	38.1	7.93
Q9CX34	Suppressor of G2 allele of SKP1 homolog OS=Mus musculus GN=Sugt1 PE=2 SV=3 - [SUGT1_MOUSE]	60.12	1	16	16	75	1.096	0.989	1.050	0.948	220.32	60.12	26	75	336	38.1	5.45
Q3TIV9	AP-1 complex subunit sigma-2 OS=Mus musculus GN=Ap1s2 PE=2 SV=1 - [Q3TIV9_MOUSE]	23.57	4	3	3	11	1.220	0.738	0.974	0.948	28.31	23.57	4	11	157	18.6	5.47
Q9WTX8	Mitotic spindle assembly checkpoint protein MAD1 OS=Mus musculus GN=Mad1l1 PE=2 SV=1 - [MD1L1_MOUSE]	17.43	2	11	11	24	1.489	0.991	0.877	0.948	61.27	17.43	18	24	717	83.5	5.66
Q9CY62	E3 ubiquitin-protein ligase RNF181 OS=Mus musculus GN=Rnf181 PE=2 SV=1 - [RN181_MOUSE]	10.91	3	2	2	9	1.468	0.915	0.951	0.948	21.75	10.91	4	9	165	19.1	6.04
P70408	Cadherin-10 OS=Mus musculus GN=Cdh10 PE=2 SV=3 - [CAD10_MOUSE]	17.26	2	12	14	37	1.088	1.191	1.255	0.949	102.00	17.26	24	37	788	88.3	4.94
Q9DAU5	Protein delta homolog 1 OS=Mus musculus GN=Dlk1 PE=2 SV=1 - [Q9DAU5_MOUSE]	4.48	14	2	2	5	1.055	0.610	0.598	0.949	8.18	4.48	3	5	290	31.1	5.06
Q9D125	28S ribosomal protein S25, mitochondrial OS=Mus musculus GN=Mrps25 PE=1 SV=1 - [RT25_MOUSE]	35.67	1	5	6	18	0.750	0.925	0.942	0.949	48.42	35.67	10	18	171	19.9	9.23
Q8BTV2-2	Isoform 2 of Cleavage and polyadenylation specificity factor subunit 7 OS=Mus musculus GN=Cpsf7 - [CPSF7_MOUSE]	22.94	3	9	9	30	0.926	0.774	0.938	0.949	75.32	22.94	16	30	462	51.0	7.78
Q3UH60	Disco-interacting protein 2 homolog B OS=Mus musculus GN=Dip2b PE=1 SV=1 - [DIP2B_MOUSE]	35.90	3	12	43	162	1.233	0.568	1.104	0.949	476.93	35.90	76	162	1574	171.0	8.09
Q9CQ25	Mitotic-spindle organizing protein 2 OS=Mus musculus GN=Mzt2 PE=1 SV=1 - [MZT2_MOUSE]	22.01	1	2	2	8	1.116	1.001	1.026	0.949	32.53	22.01	4	8	159	16.5	10.18
E0CYH0	MCG16685, isoform CRA_d OS=Mus musculus GN=Wtap PE=4 SV=1 - [E0CYH0_MOUSE]	15.40	5	4	4	12	1.455	1.320	1.022	0.949	28.20	15.40	6	12	396	44.2	5.25
G5E832	Adenomatosis polyposis coli 2 OS=Mus musculus GN=Apc2 PE=4 SV=1 - [G5E832_MOUSE]	11.43	6	17	21	47	0.718	0.776	0.947	0.949	115.12	11.43	32	47	2274	243.0	8.88
A2A4U9	Solute carrier family 13 member 3 OS=Mus musculus GN=Slc13a3 PE=2 SV=1 - [illegible]	4.84	2	2	2	5	1.199	1.364	0.748	0.949	15.25	4.84	4	5	558	61.1	8.02
Q8R3H7	Heparan sulfate 2-O-sulfotransferase 1 OS=Mus musculus GN=Hs2st1 PE=1 SV=2 - [HS2ST_MOUSE]	8.15	1	2	2	3	1.092	0.961	0.816	0.949	7.29	8.15	3	3	356	41.8	8.57
E9Q3Q6	CD166 antigen OS=Mus musculus GN=Alcam PE=2 SV=1 - [E9Q3Q6_MOUSE]	47.54	5	25	25	129	0.967	1.998	1.210	0.949	381.81	47.54	40	129	570	63.6	7.27
Q9R1K9	Centrin-2 OS=Mus musculus GN=Cetn2 PE=1 SV=1 - [CETN2_MOUSE]	41.28	2	7	8	34	1.165	1.092	1.088	0.949	75.71	41.28	13	34	172	19.8	5.00

Q9QYF9	Protein NDRG3 OS=Mus musculus GN=Ndrg3 PE=1 SV=1 - [NDRG3_MOUSE]	31.20	3	8	9	73	0.850	1.094	0.893	0.949	186.08	31.20	17	73	375	41.5	5.25
Q9DCU6	39S ribosomal protein L4, mitochondrial OS=Mus musculus GN=Mrpl4 PE=2 SV=1 - [RM04_MOUSE]	22.11	1	6	6	13	0.533	0.662	0.853	0.949	42.60	22.11	11	13	294	33.1	9.82
Q99JX4	Eukaryotic translation initiation factor 3 subunit M OS=Mus musculus GN=Eif3m PE=2 SV=1 - [EIF3M_MOUSE]	9.89	3	2	3	5	0.294	1.217	0.687	0.949	12.12	9.89	5	5	374	42.5	5.74
Q8BK72	28S ribosomal protein S27, mitochondrial OS=Mus musculus GN=Mrps27 PE=2 SV=2 - [RT27_MOUSE]	36.63	1	13	14	40	1.103	0.956	0.908	0.949	105.82	36.63	22	40	415	47.7	5.50
O70174	Neuronal acetylcholine receptor subunit alpha-4 OS=Mus musculus GN=Chrna4 PE=2 SV=2 - [ACHA4_MOUSE]	11.92	2	6	6	20	0.587	0.469	0.890	0.949	73.64	11.92	10	20	629	70.3	7.25
Q7TMR0	Lysosomal Pro-X carboxypeptidase OS=Mus musculus GN=Prcp PE=2 SV=2 - [PCP_MOUSE]	14.66	2	7	7	14	1.461	1.442	1.038	0.949	41.42	14.66	11	14	491	55.0	6.79
Q8BXQ2	GPI transamidase component PIG-T OS=Mus musculus GN=Pigt PE=1 SV=2 - [PIGT_MOUSE]	14.43	2	6	8	18	0.936	0.778	0.823	0.949	61.30	14.43	12	18	582	65.7	8.40
B2RXS4	Plexin-B2 OS=Mus musculus GN=Plxnb2 PE=1 SV=1 - [PLXB2_MOUSE]	21.44	1	31	33	85	0.855	1.038	0.818	0.949	257.77	21.44	56	85	1842	206.1	5.87
Q8VEJ9	Vacuolar protein sorting-associated protein 4A OS=Mus musculus GN=Vps4a PE=1 SV=1 - [VPS4A_MOUSE]	38.90	2	9	16	64	0.950	1.048	0.951	0.949	163.32	38.90	30	64	437	48.9	7.80
B9EJ80	PDZ domain containing 8 OS=Mus musculus GN=Pdzd8 PE=2 SV=1 - [B9EJ80_MOUSE]	16.74	1	16	16	41	1.004	0.734	0.993	0.949	106.03	16.74	24	41	1147	127.7	6.04
A2AG50	MAP7 domain-containing protein 2 OS=Mus musculus GN=Map7d2 PE=1 SV=1 -	40.85	5	29	32	180	0.987	0.352	1.164	0.950	538.44	40.85	59	180	781	86.0	8.81
G5E8T9	Hydroxyacyl glutathione hydrolase OS=Mus musculus GN=Hagh PE=3 SV=1 - [G5E8T9_MOUSE]	62.14	8	14	14	102	2.043	1.030	1.024	0.950	281.63	62.14	24	102	309	34.1	7.94
B2RPU8	MCG130675 OS=Mus musculus GN=2410018M08Rik PE=2 SV=1 - [B2RPU8_MOUSE]	7.41	3	5	5	29	1.810	0.847	1.257	0.950	99.63	7.41	9	29	756	84.2	8.44
Q9JHC9-2	Isoform 1 of ETS-related transcription factor Elf-2 OS=Mus musculus GN=Elf2 - [ELF2_MOUSE]	10.15	4	2	2	5	1.042	0.807	0.680	0.950	7.84	10.15	3	5	581	61.9	6.55
O35685	Nuclear migration protein nudC OS=Mus musculus GN=Nudc PE=1 SV=1 - [NUDC_MOUSE]	51.51	1	18	19	90	1.372	1.158	0.906	0.950	257.78	51.51	35	90	332	38.3	5.26
Q14C51	Pentatricopeptide repeat domain-containing protein 3, mitochondrial OS=Mus musculus GN=Ptcd3 PE=2 SV=2 - [PTCD3_MOUSE]	27.15	1	14	17	38	0.865	0.918	1.011	0.950	110.22	27.15	26	38	685	77.7	5.88
Q61010-3	Isoform 3 of E3 ubiquitin-protein ligase DTX1 OS=Mus musculus GN=Dtx1 -	2.04	3	1	1	2	0.812	0.874	0.942	0.950	5.42	2.04	2	2	539	58.1	9.67
Q6W8Q3	Purkinje cell protein 4-like protein 1 OS=Mus musculus GN=Pcp4l1 PE=2 SV=1 -	11.76	1	1	1	9	1.839	0.540	2.586	0.950	21.24	11.76	2	9	68	7.5	5.52
Q9QZE7	Translin-associated protein X OS=Mus musculus GN=Tsnax PE=1 SV=1 - [TSNAX_MOUSE]	60.69	1	14	14	58	0.947	1.094	0.791	0.950	211.33	60.69	24	58	290	32.9	6.55
Q64674	Spermidine synthase OS=Mus musculus GN=Srm PE=2 SV=1 - [SPEE_MOUSE]	47.35	1	13	13	56	0.924	0.892	0.955	0.950	194.09	47.35	23	56	302	34.0	5.50
P22682	E3 ubiquitin-protein ligase CBL OS=Mus musculus GN=Cbl PE=1 SV=3 - [CBL_MOUSE]	31.11	1	19	21	66	1.544	1.425	1.182	0.950	206.50	31.11	37	66	913	100.5	6.67
Q6ZPU9	KIF1-binding protein OS=Mus musculus GN=Kbp PE=2 SV=2 - [KBP_MOUSE]	31.60	6	14	14	59	0.748	0.944	0.907	0.950	188.20	31.60	25	59	617	71.0	5.48
Q8VDG3	Poly(A)-specific ribonuclease PARN OS=Mus musculus GN=Parn PE=1 SV=1 -	5.13	1	3	3	6	0.808	0.681	0.860	0.950	14.41	5.13	4	6	624	71.5	5.97
Q9CYI0	Protein Njmu-R1 OS=Mus musculus PE=2 SV=2 - [NJMU_MOUSE]	19.34	3	7	8	17	0.585	1.209	0.753	0.950	47.13	19.34	12	17	393	44.4	5.05
Q8C170	Unconventional myosin-IXa OS=Mus musculus GN=Myo9a PE=1 SV=2 - [MYO9A_MOUSE]	9.72	3	20	22	41	0.827	0.802	1.065	0.950	110.05	9.72	32	41	2542	291.9	8.97
Q9D4I9	RAB23, member RAS oncogene family, isoform CRA_a OS=Mus musculus GN=Rab23 PE=2 SV=1 - [Q9D4I9_MOUSE]	60.76	3	11	11	37	1.026	1.039	0.993	0.951	108.84	60.76	19	37	237	26.8	7.30

Q5DTT2	PH and SEC7 domain-containing protein 1 OS=Mus musculus GN=Psd PE=1 SV=2 - [PSD1_MOUSE]	17.09	3	13	13	38	0.942	1.049	1.159	0.951	92.32	17.09	19	38	1024	109.6	7.05
Q91VR7	Microtubule-associated proteins 1A/1B light chain 3A OS=Mus musculus GN=Map1lc3a PE=1 SV=1 - [MLP3A_MOUSE]	23.14	2	1	3	34	2.061	1.278	1.094	0.951	78.04	23.14	6	34	121	14.3	8.68
Q6ZWZ2	Ubiquitin-conjugating enzyme E2 R2 OS=Mus musculus GN=Ube2r2 PE=2 SV=1 -	21.01	1	4	5	27	1.827	1.214	0.838	0.951	70.80	21.01	10	27	238	27.1	4.42
P62761	Visinin-like protein 1 OS=Mus musculus GN=Vsnl1 PE=1 SV=2 - [VISL1_MOUSE]	78.01	1	11	16	441	1.238	0.986	0.974	0.951	1086.95	78.01	29	441	191	22.1	5.15
E0CYC4	Cyclin-dependent kinase 8 (Fragment) OS=Mus musculus GN=Cdk8 PE=2 SV=1 - [E0CYC4_MOUSE]	26.12	8	6	6	10	0.961	1.099	1.175	0.951	30.31	26.12	8	10	245	28.5	8.98
Q6PGC1	ATP-dependent RNA helicase Dhx29 OS=Mus musculus GN=Dhx29 PE=2 SV=1 - [DHX29_MOUSE]	12.75	1	14	14	28	0.820	0.825	0.926	0.951	74.66	12.75	23	28	1365	153.9	7.94
Q91YU6	Leucine zipper putative tumor suppressor 2 OS=Mus musculus GN=Lzts2 PE=2 SV=3 - [LZTS2_MOUSE]	11.18	1	5	5	14	0.911	1.330	1.054	0.951	33.98	11.18	7	14	671	72.5	7.11
Q9D4Q1	RIB43A-like with coiled-coils protein 2 OS=Mus musculus GN=Ribc2 PE=2 SV=2 - [RIBC2_MOUSE]	2.91	2	1	1	1	1.038	1.151	0.648	0.951	2.07	2.91	1	1	309	37.3	9.38
Q9CQH8	Ribonuclease P protein subunit p14 OS=Mus musculus GN=Rpp14 PE=2 SV=1 -	12.30	1	1	1	1	1.593	0.902	0.880	0.951	2.47	12.30	1	1	122	13.6	8.28
Q8K3A6	Protein ARMCX6 OS=Mus musculus GN=Armcx6 PE=2 SV=1 - [ARMX6_MOUSE]	2.66	1	1	1	13	1.035	0.592	1.287	0.951	13.54	2.66	2	13	301	33.3	5.17
G3X8R0	Receptor accessory protein 5, isoform CRA_a OS=Mus musculus GN=Reep5 PE=4 SV=1 - [G3X8R0_MOUSE]	25.40	2	6	6	44	0.836	0.881	0.918	0.951	98.80	25.40	10	44	189	21.4	8.12
P61809	Cyclin-dependent kinase 5 activator 1 OS=Mus musculus GN=Cdk5r1 PE=1 SV=1 -	13.03	2	3	3	4	0.792	1.869	1.647	0.951	8.17	13.03	4	4	307	34.0	9.35
Q9ES97-2	Isoform 2 of Reticulon-3 OS=Mus musculus GN=Rtn3 - [RTN3_MOUSE]	55.13	4	37	42	290	1.183	1.126	1.491	0.952	851.76	55.13	73	290	945	101.9	4.93
Q2M2N2	Speckle-type POZ protein-like OS=Mus musculus GN=Spopl PE=2 SV=3 - [SPOPL_MOUSE]	5.87	5	2	2	2	1.222	1.310	1.084	0.952	6.06	5.87	2	2	392	44.7	7.01
G3X9X1	MCG13111, isoform CRA_a OS=Mus musculus GN=Kbtbd2 PE=4 SV=1 - [G3X9X1_MOUSE]	9.95	1	6	6	11	0.876	0.923	0.879	0.952	27.94	9.95	9	11	623	71.2	5.62
Q9Z2X2	26S proteasome non-ATPase regulatory subunit 10 OS=Mus musculus GN=Psmd10 PE=1 SV=3 - [PSD10_MOUSE]	19.05	1	3	3	11	1.258	1.075	0.982	0.952	34.50	19.05	5	11	231	25.1	6.06
Q8VE91-3	Isoform 3 of Protein FAM134B OS=Mus musculus GN=Fam134b - [F134B_MOUSE]	13.58	4	3	3	8	0.923	0.627	1.161	0.952	16.81	13.58	5	8	346	38.1	4.67
Q1HFZ0-2	Isoform 2 of tRNA (cytosine(34)-C(5))-methyltransferase OS=Mus musculus GN=Nsun2 - [NSUN2_MOUSE]	29.38	4	17	18	42	0.945	1.041	0.856	0.952	121.24	29.38	28	42	691	77.7	6.71
Q9CQ86	Migration and invasion enhancer 1 OS=Mus musculus GN=Mien1 PE=1 SV=1 - [MIEN1_MOUSE]	14.78	1	2	2	21	1.986	1.108	1.218	0.952	53.90	14.78	4	21	115	12.3	4.51
F8VQD7	Receptor-type tyrosine-protein phosphatase gamma OS=Mus musculus GN=Ptprg PE=2 SV=1 - [F8VQD7_MOUSE]	8.95	5	10	11	34	0.995	1.288	0.913	0.952	87.10	8.95	18	34	1442	161.2	6.37
P11930	Nucleoside diphosphate-linked moiety X motif 19, mitochondrial OS=Mus musculus GN=Nudt19 PE=1 SV=2 - [NUD19_MOUSE]	16.81	1	3	4	12	1.244	0.695	0.784	0.952	40.49	16.81	6	12	357	40.3	6.68
E9Q7M2	Protein Tsc22d2 OS=Mus musculus GN=Tsc22d2 PE=2 SV=1 - [E9Q7M2_MOUSE]	8.19	1	3	6	42	2.250	0.859	1.267	0.952	119.93	8.19	11	42	769	78.1	4.89
B2RWC4	Gm88 protein OS=Mus musculus GN=Lrrc73 PE=2 SV=1 - [B2RWC4_MOUSE]	2.22	1	1	1	1	0.624	1.767	1.321	0.952	1.86	2.22	1	1	316	33.4	4.84
D3YWB9	Contactin-associated protein-like 4 OS=Mus musculus GN=Cntnap4 PE=2 SV=1 -	18.40	2	20	20	49	0.806	0.765	0.854	0.952	147.35	18.40	34	49	1310	144.7	7.02
Q5PR73	GTP-binding protein Di-Ras2 OS=Mus musculus GN=Diras2 PE=2 SV=1 - [DIRA2_MOUSE]	58.79	1	7	10	28	0.672	0.746	1.198	0.952	90.04	58.79	17	28	199	22.5	8.76

Q6P4T2	U5 small nuclear ribonucleoprotein 200 kDa helicase OS=Mus musculus GN=Snrnp200 PE=2 SV=1 - [U520_MOUSE]	33.94	1	58	59	179	0.782	0.832	0.871	0.952	588.27	33.94	100	179	2136	244.4	6.06
Q9DAW9	Calponin-3 OS=Mus musculus GN=Cnn3 PE=2 SV=1 - [CNN3_MOUSE]	41.52	1	8	13	78	1.651	1.097	0.993	0.952	218.04	41.52	23	78	330	36.4	5.72
Q6ZPS2	Carnosine synthase 1 OS=Mus musculus GN=Carns1 PE=1 SV=2 - [CRNS1_MOUSE]	5.20	4	3	3	11	1.269	0.398	0.935	0.952	35.97	5.20	6	11	827	89.2	6.04
Q3U155	Coiled-coil domain-containing protein 174 OS=Mus musculus GN=Ccdc174 PE=2 SV=1 - [CC174_MOUSE]	3.85	1	1	1	4	1.154	1.269	1.256	0.952	11.84	3.85	2	4	467	53.9	6.21
Q9EPU4	Cleavage and polyadenylation specificity factor subunit 1 OS=Mus musculus GN=Cpsf1 PE=1 SV=1 - [CPSF1_MOUSE]	11.31	1	13	14	35	0.666	0.899	0.814	0.952	86.54	11.31	21	35	1441	160.7	6.39
D3Z6X3	Peroxisomal biogenesis factor 3 OS=Mus musculus GN=Pex3 PE=2 SV=1 - [D3Z6X3_MOUSE]	3.75	4	1	1	1	2.130	1.125	0.971	0.952	3.98	3.75	1	1	293	32.7	6.64
O08547	Vesicle-trafficking protein SEC22b OS=Mus musculus GN=Sec22b PE=1 SV=3 - [SC22B_MOUSE]	47.44	2	8	8	76	0.658	1.030	0.924	0.952	241.29	47.44	15	76	215	24.7	8.51
G5E866	Splicing factor 3B subunit 1 OS=Mus musculus GN=Sf3b1 PE=4 SV=1 - [G5E866_MOUSE]	38.27	2	39	39	123	0.963	0.889	1.045	0.952	393.00	38.27	66	123	1304	145.7	7.09
Q9Z204-3	Isoform 3 of Heterogeneous nuclear ribonucleoproteins C1/C2 OS=Mus musculus GN=Hnrnpc - [HNRPC_MOUSE]	38.57	2	1	13	64	1.340	0.793	0.905	0.952	175.54	38.57	21	64	293	32.3	5.08
Q9CQM5	Thioredoxin domain-containing protein 17 OS=Mus musculus GN=Txndc17 PE=1 SV=1 - [TXD17_MOUSE]	47.15	1	5	5	17	1.762	1.325	1.180	0.952	41.87	47.15	9	17	123	14.0	4.77
Q9QYS2	Metabotropic glutamate receptor 3 OS=Mus musculus GN=Grm3 PE=2 SV=1 - [GRM3_MOUSE]	33.67	1	25	29	106	0.451	0.997	1.350	0.953	281.51	33.67	48	106	879	99.0	7.75
Q62186	Translocon-associated protein subunit delta OS=Mus musculus GN=Ssr4 PE=2 SV=1 - [SSRD_MOUSE]	17.44	2	2	2	15	1.153	1.050	0.784	0.953	56.56	17.44	4	15	172	18.9	5.78
Q8BMS9	Ras association domain-containing protein 2 OS=Mus musculus GN=Rassf2 PE=2 SV=1 - [RASF2_MOUSE]	28.22	3	5	7	16	0.865	0.863	0.970	0.953	44.02	28.22	12	16	326	37.9	7.94
E0CXQ6	Tandem C2 domains nuclear protein OS=Mus musculus GN=Tc2n PE=2 SV=1 - [E0CXQ6_MOUSE]	2.35	2	1	1	1	1.335	1.583	1.230	0.953	0.00	2.35	1	1	425	47.7	8.84
Q8VDU5	SNF-related serine/threonine-protein kinase OS=Mus musculus GN=Snrk PE=1 SV=1 - [SNRK_MOUSE]	29.28	5	18	18	51	1.066	0.946	1.156	0.953	169.00	29.28	27	51	748	81.9	7.49
J3QNH3	Uncharacterized protein OS=Mus musculus GN=Gm21788 PE=4 SV=1 - [J3QNH3_MOUSE]	7.22	1	1	1	2	1.767	1.234	1.132	0.953	3.11	7.22	1	2	180	20.4	9.04
Q3TXS7	26S proteasome non-ATPase regulatory subunit 1 OS=Mus musculus GN=Psmd1 PE=1 SV=1 - [PSMD1_MOUSE]	42.60	3	33	33	138	0.711	0.987	0.847	0.953	396.95	42.60	59	138	953	105.7	5.39
Q91YJ3	Thymocyte nuclear protein 1 OS=Mus musculus GN=Thyn1 PE=1 SV=1 - [THYN1_MOUSE]	41.15	1	10	10	21	0.846	0.759	0.886	0.953	47.13	41.15	17	21	226	26.2	9.11
P60521	Gamma-aminobutyric acid receptor-associated protein-like 2 OS=Mus musculus GN=Gabarapl2 PE=1 SV=1 - [GBRL2_MOUSE]	65.81	1	7	10	50	1.890	0.969	0.822	0.953	148.73	65.81	19	50	117	13.7	8.10
Q9DCH4	Eukaryotic translation initiation factor 3 subunit F OS=Mus musculus GN=Eif3f PE=1 SV=2 - [EIF3F_MOUSE]	33.24	1	10	10	45	0.741	0.933	0.773	0.953	154.89	33.24	17	45	361	38.0	5.58
Q8VHQ3	Protein phosphatase 1 regulatory inhibitor subunit 16B OS=Mus musculus GN=Ppp1r16b PE=2 SV=1 - [PP16B_MOUSE]	9.33	2	5	5	10	0.965	0.526	1.405	0.953	28.84	9.33	8	10	568	63.5	6.58
O35607	Bone morphogenetic protein receptor type-2 OS=Mus musculus GN=Bmpr2 PE=2 SV=1 - [BMPR2_MOUSE]	22.06	1	17	17	44	1.001	1.240	1.171	0.953	119.80	22.06	30	44	1038	114.9	6.23
P33622	Apolipoprotein C-III OS=Mus musculus GN=Apoc3 PE=2 SV=2 - [APOC3_MOUSE]	27.27	2	2	2	16	3.865	4.664	0.482	0.953	61.32	27.27	4	16	99	11.0	4.75

G3UWG2	T-cell lymphoma invasion and metastasis 1 OS=Mus musculus GN=Tiam1 PE=4 SV=1 - [G3UWG2_MOUSE]	19.99	6	22	23	62	0.933	2.370	1.875	0.953	183.34	19.99	41	62	1591	177.3	6.64
P47941	Crk-like protein OS=Mus musculus GN=Crkl PE=1 SV=2 - [CRKL_MOUSE]	56.44	1	13	13	95	1.844	0.898	0.919	0.953	277.25	56.44	24	95	303	33.8	6.74
Q6PFF0	Protein Scaf4 OS=Mus musculus GN=Scaf4 PE=2 SV=1 - [Q6PFF0_MOUSE]	3.56	2	2	3	7	1.195	1.195	1.155	0.953	26.07	3.56	4	7	1209	131.6	9.60
Q5RJH3	Cadherin-12 OS=Mus musculus GN=Cdh12 PE=2 SV=1 - [CAD12_MOUSE]	8.19	1	4	5	9	1.178	1.100	1.189	0.953	27.73	8.19	9	9	794	88.4	4.83
F6WKY8	Phospholipase D1 (Fragment) OS=Mus musculus GN=Pld1 PE=4 SV=1 - [F6WKY8_MOUSE]	8.61	1	1	5	7	0.766	1.029	0.940	0.953	16.16	8.61	7	7	871	100.1	8.78
G3X972	Protein Sec24c OS=Mus musculus GN=Sec24c PE=4 SV=1 - [G3X972_MOUSE]	30.57	1	28	30	112	0.791	0.985	0.922	0.954	305.24	30.57	50	112	1096	118.5	6.84
Q91ZV0	Melanoma inhibitory activity protein 2 OS=Mus musculus GN=Mia2 PE=2 SV=2 - [MIA2_MOUSE]	1.55	1	1	1	1	0.639	1.037	0.756	0.954	1.64	1.55	1	1	517	57.7	4.12
Q8BL99-5	Isoform 5 of Protein dopey-1 OS=Mus musculus GN=Dopey1 - [DOP1_MOUSE]	3.85	7	7	8	12	0.752	1.324	0.816	0.954	33.84	3.85	12	12	2391	268.1	6.21
P52430	Serum paraoxonase/arylesterase 1 OS=Mus musculus GN=Pon1 PE=1 SV=2 -	11.55	3	4	4	6	2.170	1.487	0.744	0.954	8.93	11.55	5	6	355	39.5	5.22
P47757	F-actin-capping protein subunit beta OS=Mus musculus GN=Capzb PE=1 SV=3 - [CAPZB_MOUSE]	58.12	4	2	19	269	0.892	0.657	0.740	0.954	682.18	58.12	32	269	277	31.3	5.74
Q76LL6-3	Isoform 3 of FH1/FH2 domain-containing protein 3 OS=Mus musculus GN=Fhod3 - [FHOD3_MOUSE]	4.32	4	5	5	13	0.609	0.500	1.003	0.954	37.68	4.32	9	13	1413	158.2	5.83
D3Z497	Uncharacterized protein OS=Mus musculus GN=Gm7075 PE=4 SV=1 - [D3Z497_MOUSE]	8.04	2	1	1	8	1.867	1.060	1.434	0.954	18.69	8.04	2	8	112	12.6	6.70
Q6P8N8	ATP-dependent Clp protease ATP-binding subunit clpX-like, mitochondrial OS=Mus musculus GN=Clpx PE=2 SV=1 - [Q6P8N8_MOUSE]	32.42	3	17	17	31	0.944	0.859	0.880	0.954	90.04	32.42	27	31	620	67.3	7.55
Q8BKE6	Cytochrome P450 20A1 OS=Mus musculus GN=Cyp20a1 PE=2 SV=1 - [CP20A_MOUSE]	11.04	2	3	4	6	1.742	1.184	0.929	0.954	18.10	11.04	5	6	462	52.1	6.95
Q9D0T2	Dual specificity protein phosphatase 12 OS=Mus musculus GN=Dusp12 PE=2 SV=1 -	4.72	4	2	2	6	0.717	1.337	0.904	0.954	13.26	4.72	4	6	339	37.1	7.99
E9PZF0	Nucleoside diphosphate kinase OS=Mus musculus GN=Gm20390 PE=2 SV=1 - [E9PZF0_MOUSE]	80.15	3	6	16	168	0.950	1.189	0.811	0.954	504.45	80.15	29	168	267	30.2	8.72
Q6P1B3	PILR alpha-associated neural protein OS=Mus musculus GN=Pianp PE=1 SV=1 - [PIANP_MOUSE]	20.14	1	3	3	45	0.547	2.497	1.234	0.954	102.16	20.14	6	45	278	29.7	8.72
E9QKQ5	Phosphatidate phosphatase LPIN1 OS=Mus musculus GN=Lpin1 PE=2 SV=1 -	6.96	3	3	4	7	1.055	0.755	0.799	0.954	19.15	6.96	6	7	891	98.5	6.79
Q60960	Importin subunit alpha-5 OS=Mus musculus GN=Kpna1 PE=1 SV=2 - [IMA5_MOUSE]	28.44	6	6	15	72	0.883	0.901	0.921	0.954	189.76	28.44	25	72	538	60.1	5.01
Q8C878	NEDD8-activating enzyme E1 catalytic subunit OS=Mus musculus GN=Uba3 PE=1 SV=2 - [UBA3_MOUSE]	45.02	3	17	17	52	0.768	0.953	0.953	0.954	141.93	45.02	29	52	462	51.7	5.45
A2AMC3	GDP-fucose protein O-fucosyltransferase 1 OS=Mus musculus GN=Pofut1 PE=2 SV=1 - [A2AMC3_MOUSE]	22.44	3	3	6	14	0.928	0.984	0.791	0.954	46.38	22.44	12	14	352	40.0	8.70
Q6KCD5-2	Isoform 2 of Nipped-B-like protein OS=Mus musculus GN=Nipbl - [NIPBL_MOUSE]	3.31	3	8	8	16	1.222	1.007	0.985	0.954	27.94	3.31	15	16	2691	303.6	7.81
Q8BWU5	Probable tRNA threonylcarbamoyladenosine biosynthesis protein Osgep OS=Mus musculus GN=Osgep PE=2 SV=2 - [OSGEP_MOUSE]	33.43	3	6	9	27	1.074	1.243	0.644	0.954	74.80	33.43	18	27	335	36.3	6.24
E9QNG1	Intersectin-2 OS=Mus musculus GN=Itsn2 PE=2 SV=1 - [E9QNG1_MOUSE]	18.40	4	18	27	78	1.480	0.931	1.352	0.954	236.79	18.40	45	78	1658	188.7	8.25
G3X8R8	MCG123888 OS=Mus musculus GN=Mkl2 PE=4 SV=1 - [G3X8R8_MOUSE]	32.45	1	1	25	102	0.850	0.988	1.614	0.954	267.90	32.45	41	102	1091	118.5	6.09

Q6P1B1	Xaa-Pro aminopeptidase 1 OS=Mus musculus GN=Xpnpep1 PE=2 SV=1 - [XPP1_MOUSE]	34.83	1	18	18	66	0.921	1.261	1.035	0.954	199.49	34.83	31	66	623	69.5	5.54
Q8C635	Glycerol kinase-like 1 OS=Mus musculus GN=Gykl1 PE=2 SV=1 - [Q8C635_MOUSE]	15.85	1	2	5	28	1.104	0.904	1.058	0.955	87.46	15.85	8	28	549	59.9	5.27
K3W4S5	Semaphorin-6A OS=Mus musculus GN=Sema6a PE=4 SV=1 - [K3W4S5_MOUSE]	9.55	7	6	7	13	2.126	0.828	1.277	0.955	30.78	9.55	11	13	1005	111.7	8.46
Q99MR0	Actin-like protein 6B OS=Mus musculus GN=Actl6b PE=1 SV=1 - [ACL6B_MOUSE]	27.70	4	9	10	24	0.718	1.189	0.991	0.955	62.12	27.70	17	24	426	46.9	5.71
Q62170	P-selectin glycoprotein ligand 1 OS=Mus musculus GN=Selplg PE=1 SV=2 - [SELPL_MOUSE]	12.59	2	2	2	6	1.408	1.160	0.931	0.955	8.76	12.59	2	6	397	41.8	4.44
Q9CXI3	DBH-like monooxygenase protein 1 OS=Mus musculus GN=Moxd1 PE=1 SV=1 -	10.11	1	5	6	9	1.046	1.136	0.957	0.955	23.84	10.11	8	9	613	69.6	6.67
P70196	TNF receptor-associated factor 6 OS=Mus musculus GN=Traf6 PE=1 SV=2 - [TRAF6_MOUSE]	2.64	1	1	1	3	1.106	0.961	1.018	0.955	5.63	2.64	2	3	530	60.0	6.55
Q9CQL5	39S ribosomal protein L18, mitochondrial OS=Mus musculus GN=Mrpl18 PE=2 SV=1 - [RM18_MOUSE]	22.78	1	4	4	15	0.956	0.883	0.854	0.955	38.68	22.78	8	15	180	20.7	9.28
Q8K273	Membrane magnesium transporter 1 OS=Mus musculus GN=Mmgt1 PE=2 SV=1 -	26.72	1	2	2	6	0.941	1.040	0.845	0.955	19.36	26.72	3	6	131	14.7	9.16
P22366	Myeloid differentiation primary response protein MyD88 OS=Mus musculus GN=Myd88 PE=1 SV=3 - [MYD88_MOUSE]	10.81	3	3	3	4	0.966	1.084	0.961	0.955	6.85	10.81	4	4	296	33.7	5.52
Q2M3X8-4	Isoform 4 of Phosphatase and actin regulator 1 OS=Mus musculus GN=Phactr1 -	32.97	3	1	23	83	0.958	0.493	0.879	0.955	225.62	32.97	37	83	649	73.5	7.05
G3XA20	MCG130431, isoform CRA_a OS=Mus musculus GN=Grip2 PE=4 SV=1 - [G3XA20_MOUSE]	18.91	4	12	14	34	0.686	0.878	1.054	0.955	86.29	18.91	24	34	1042	112.2	6.86
Q8R1Q8	Cytoplasmic dynein 1 light intermediate chain 1 OS=Mus musculus GN=Dync1li1 PE=1 SV=1 - [DC1L1_MOUSE]	63.86	1	28	29	189	1.045	1.145	1.057	0.955	669.67	63.86	49	189	523	56.6	6.42
Q8BN57	Uncharacterized protein C3orf33 homolog OS=Mus musculus PE=2 SV=1 - [CC033_MOUSE]	9.52	1	2	2	4	0.734	1.079	0.987	0.955	11.70	9.52	4	4	294	33.6	9.89
Q8K0Z7	Translational activator of cytochrome c oxidase 1 OS=Mus musculus GN=Taco1 PE=2 SV=1 - [TACO1_MOUSE]	48.30	1	10	11	34	1.322	0.820	0.895	0.955	117.44	48.30	19	34	294	32.3	8.12
D3YTT7	40S ribosomal protein SA OS=Mus musculus GN=Rpsa-ps10 PE=3 SV=1 - [D3YTT7_MOUSE]	48.81	2	11	11	55	0.931	0.986	0.758	0.955	178.22	48.81	22	55	295	32.8	4.93
P40124	Adenylyl cyclase-associated protein 1 OS=Mus musculus GN=Cap1 PE=1 SV=4 -	63.29	2	23	25	221	1.008	1.247	1.186	0.956	705.46	63.29	46	221	474	51.5	7.52
Q6QNU9	Toll-like receptor 12 OS=Mus musculus GN=Tlr12 PE=2 SV=1 - [TLR12_MOUSE]	1.55	1	1	1	1	0.742	0.332	0.933	0.956	0.00	1.55	1	1	906	99.9	8.40
Q61200	Neurexophilin-1 OS=Mus musculus GN=Nxph1 PE=2 SV=2 - [NXPH1_MOUSE]	8.49	2	2	2	4	1.422	1.641	1.128	0.956	11.77	8.49	3	4	271	31.0	8.63
Q8CIP4	MAP/microtubule affinity-regulating kinase 4 OS=Mus musculus GN=Mark4 PE=1 SV=1 - [MARK4_MOUSE]	16.36	1	9	11	58	0.880	0.838	1.040	0.956	149.08	16.36	21	58	752	82.6	9.67
Q91V09	WD repeat-containing protein 13 OS=Mus musculus GN=Wdr13 PE=1 SV=1 -	37.94	2	14	14	70	1.079	1.032	0.982	0.956	218.92	37.94	24	70	485	53.6	9.14
Q9EQ09	Oxidized low-density lipoprotein receptor 1 OS=Mus musculus GN=Olr1 PE=2 SV=2 - [OLR1_MOUSE]	3.58	1	1	2	2	0.507	0.840	1.075	0.956	4.16	3.58	2	2	363	41.6	7.64
E9Q3M9	Protein 2010300C02Rik OS=Mus musculus GN=2010300C02Rik PE=2 SV=1 - [E9Q3M9_MOUSE]	53.87	4	15	46	214	1.060	1.812	2.555	0.956	541.74	53.87	74	214	1175	125.8	7.37
Q3UHD3-2	Isoform 2 of Microtubule-associated tumor suppressor candidate 2 homolog OS=Mus musculus GN=Mtus2 - [MTUS2_MOUSE]	24.62	6	14	25	70	0.957	0.873	1.442	0.956	175.09	24.62	39	70	1316	143.1	8.75
P97300-3	Isoform 3 of Neuroplastin OS=Mus musculus GN=Nptn - [NPTN_MOUSE]	33.57	5	1	11	174	0.734	1.334	1.457	0.956	431.32	33.57	19	174	277	30.8	7.49

A2AJH3	Glycylpeptide N-tetradecanoyltransferase 2 OS=Mus musculus GN=Nmt2 PE=2 SV=1 - [A2AJH3_MOUSE]	27.11	3	11	13	27	0.592	0.718	1.223	0.956	62.11	27.11	21	27	498	56.9	8.16
Q7TNC8	Glycine receptor subunit alpha-2 OS=Mus musculus GN=Glra2 PE=2 SV=1 - [GLRA2_MOUSE]	8.19	1	3	4	17	0.553	0.786	0.816	0.956	41.56	8.19	8	17	452	52.0	8.90
Q9Z0W3	Nuclear pore complex protein Nup160 OS=Mus musculus GN=Nup160 PE=1 SV=2 - [NU160_MOUSE]	8.49	2	10	10	25	0.656	0.991	0.988	0.956	65.63	8.49	15	25	1402	158.1	5.52
P26883	Peptidyl-prolyl cis-trans isomerase FKBP1A OS=Mus musculus GN=Fkbp1a PE=1 SV=2 - [FKB1A_MOUSE]	44.44	2	4	5	145	2.012	1.484	1.436	0.956	506.41	44.44	8	145	108	11.9	8.16
P05132	cAMP-dependent protein kinase catalytic subunit alpha OS=Mus musculus GN=Prkaca PE=1 SV=3 - [KAPCA_MOUSE]	51.85	2	9	20	86	0.476	0.875	0.942	0.956	252.01	51.85	31	86	351	40.5	8.79
Q8K124	Pleckstrin homology domain-containing family O member 2 OS=Mus musculus GN=Plekho2 PE=1 SV=1 - [illegible]	26.46	1	10	10	31	1.250	0.804	1.106	0.956	81.14	26.46	18	31	495	53.8	5.50
D6RIQ7	Transcription initiation factor IIA subunit 2 OS=Mus musculus GN=Gtf2a2 PE=2 SV=1 - [D6RIQ7_MOUSE]	14.52	6	1	1	4	0.930	0.949	0.875	0.956	10.04	14.52	2	4	62	7.2	9.04
Q9CX60	Protein LBH OS=Mus musculus GN=Lbh PE=1 SV=1 - [LBH_MOUSE]	36.19	1	4	4	39	2.720	0.531	0.953	0.956	150.18	36.19	8	39	105	12.1	4.31
Q8C9X6-2	Isoform 2 of Enhancer of polycomb homolog 1 OS=Mus musculus GN=Epc1 -	3.93	2	2	2	4	1.316	0.975	1.120	0.956	6.28	3.93	3	4	763	84.7	8.57
Q9JMK2	Casein kinase I isoform epsilon OS=Mus musculus GN=Csnk1e PE=1 SV=2 - [KC1E_MOUSE]	26.92	3	4	10	59	0.922	0.690	1.092	0.956	168.93	26.92	18	59	416	47.3	9.66
Q3UHD9	Arf-GAP with GTPase, ANK repeat and PH domain-containing protein 2 OS=Mus musculus GN=Agap2 PE=1 SV=1 - [illegible]	44.94	2	38	39	168	0.578	1.261	1.445	0.956	532.97	44.94	69	168	1186	124.4	9.89
Q80VI1	E3 ubiquitin-protein ligase TRIM56 OS=Mus musculus GN=Trim56 PE=1 SV=1 - [TRI56_MOUSE]	2.86	2	3	3	5	0.986	1.015	0.803	0.957	9.95	2.86	5	5	734	79.5	7.96
Q8VE11	Myotubularin-related protein 6 OS=Mus musculus GN=Mtmr6 PE=1 SV=1 -	23.34	1	12	13	28	1.020	1.035	0.872	0.957	88.63	23.34	20	28	617	70.9	7.83
O35188	Fractalkine OS=Mus musculus GN=Cx3cl1 PE=2 SV=3 - [X3CL1_MOUSE]	12.15	1	4	4	16	1.605	3.236	1.751	0.957	50.46	12.15	6	16	395	42.1	5.54
Q99LC9	Peroxisome assembly factor 2 OS=Mus musculus GN=Pex6 PE=2 SV=1 - [PEX6_MOUSE]	8.87	1	6	6	11	0.648	0.706	0.806	0.957	39.82	8.87	11	11	981	104.5	7.34
Q91V64	Isochorismatase domain-containing protein 1 OS=Mus musculus GN=Isoc1 PE=2 SV=1 - [ISOC1_MOUSE]	49.83	2	12	13	40	0.571	0.675	0.564	0.957	126.94	49.83	24	40	297	32.0	7.39
Q5SWU9	Acetyl-CoA carboxylase 1 OS=Mus musculus GN=Acaca PE=1 SV=1 - [ACACA_MOUSE]	33.52	3	56	64	183	0.826	0.698	0.819	0.957	532.92	33.52	104	183	2345	265.1	6.39
Q3U1J4	DNA damage-binding protein 1 OS=Mus musculus GN=Ddb1 PE=1 SV=2 - [DDB1_MOUSE]	42.28	1	45	45	195	0.958	1.028	0.921	0.957	580.30	42.28	84	195	1140	126.8	5.26
Q0P678	Zinc finger CCCH domain-containing protein 18 OS=Mus musculus GN=Zc3h18 PE=1 SV=1 - [ZCH18_MOUSE]	14.66	5	11	12	22	0.660	0.765	0.841	0.957	67.85	14.66	19	22	948	105.6	7.80
Q6PDJ6	F-box only protein 42 OS=Mus musculus GN=Fbxo42 PE=1 SV=1 - [FBX42_MOUSE]	5.44	1	4	4	8	0.720	1.047	0.835	0.957	23.29	5.44	6	8	717	77.7	7.58
P11404	Fatty acid-binding protein, heart OS=Mus musculus GN=Fabp3 PE=1 SV=5 - [FABPH_MOUSE]	75.94	2	12	13	129	2.105	1.180	1.208	0.957	319.42	75.94	23	129	133	14.8	6.57
Q5SV54	Ortholog of human amyotrophic lateral sclerosis 2 (Juvenile) chromosome region, candidate 19 (ALS2CR19) OS=Mus musculus GN=Pard3b PE=4 SV=1 - [Q5SV54_MOUSE]	1.54	4	2	2	5	1.413	1.253	1.095	0.957	9.67	1.54	2	5	1104	121.7	8.69
Q9QZC2	Plexin-C1 OS=Mus musculus GN=Plxnc1 PE=1 SV=1 - [PLXC1_MOUSE]	22.24	1	30	32	91	0.947	1.971	0.766	0.957	258.17	22.24	55	91	1574	176.4	7.75
O88273	Gremlin-2 OS=Mus musculus GN=Grem2 PE=1 SV=1 - [GREM2_MOUSE]	8.93	1	1	1	1	1.134	0.976	0.947	0.957	2.43	8.93	1	1	168	19.3	9.17

E9QMD3	Zinc finger homeobox protein 3 OS=Mus musculus GN=Zfhx3 PE=3 SV=1 - [E9QMD3_MOUSE]	3.20	2	8	9	13	2.350	0.621	1.340	0.957	23.39	3.20	9	13	3723	405.9	6.24
P14824	Annexin A6 OS=Mus musculus GN=Anxa6 PE=1 SV=3 - [ANXA6_MOUSE]	62.11	2	41	42	289	0.970	1.108	0.909	0.957	857.19	62.11	76	289	673	75.8	5.50
E9Q6A7	Protein Bptf OS=Mus musculus GN=Bptf PE=2 SV=1 - [E9Q6A7_MOUSE]	6.03	3	13	13	17	1.490	1.101	0.873	0.958	44.04	6.03	16	17	2921	321.4	7.59
D3Z5K8	SH3 and multiple ankyrin repeat domains protein 2 OS=Mus musculus GN=Shank2 PE=2 SV=1 - [D3Z5K8_MOUSE]	33.19	1	1	45	192	0.444	1.062	1.732	0.958	595.40	33.19	79	192	1841	200.0	6.68
Q62093	Serine/arginine-rich splicing factor 2 OS=Mus musculus GN=Srsf2 PE=1 SV=4 - [SRSF2_MOUSE]	9.50	1	3	3	27	0.830	0.964	0.946	0.958	58.49	9.50	5	27	221	25.5	11.85
Q811D0-2	Isoform 2 of Disks large homolog 1 OS=Mus musculus GN=Dlg1 - [DLG1_MOUSE]	44.68	5	3	32	207	0.707	0.727	0.837	0.958	585.74	44.68	58	207	893	99.6	6.07
Q8CFJ9	WD repeat-containing protein 24 OS=Mus musculus GN=Wdr24 PE=2 SV=1 -	4.43	1	3	3	6	1.059	1.307	1.160	0.958	20.34	4.43	4	6	790	88.1	6.46
Q5NBX1-2	Isoform 2 of Protein cordon-bleu OS=Mus musculus GN=Cobl - [COBL_MOUSE]	11.00	11	11	11	26	0.991	1.092	1.620	0.958	74.35	11.00	19	26	1255	135.4	8.21
Q6PB66	Leucine-rich PPR motif-containing protein, mitochondrial OS=Mus musculus GN=Lrpprc PE=1 SV=2 - [LPPRC_MOUSE]	53.23	3	67	68	242	0.650	1.008	0.858	0.958	729.33	53.23	121	242	1392	156.5	6.83
Q8BVA9	ELAV (Embryonic lethal, abnormal vision, Drosophila)-like 4 (Hu antigen D) OS=Mus musculus GN=Elavl4 PE=2 SV=1 - [Q8BVA9_MOUSE]	52.63	11	6	17	85	0.822	0.657	0.745	0.958	254.47	52.63	31	85	380	41.7	9.39
Q93092	Transaldolase OS=Mus musculus GN=Taldo1 PE=1 SV=2 - [TALDO_MOUSE]	54.30	1	24	24	127	1.137	0.790	0.902	0.958	315.71	54.30	42	127	337	37.4	7.03
G5E895	MCG142264, isoform CRA_b OS=Mus musculus GN=Akr1b10 PE=4 SV=1 - [G5E895_MOUSE]	54.43	4	7	14	59	1.510	0.998	0.904	0.958	212.25	54.43	21	59	316	35.8	7.28
B0QZL3	Proton-associated sugar transporter A OS=Mus musculus GN=Slc45a1 PE=2 SV=1 -	12.30	2	6	6	8	1.410	0.761	0.793	0.958	26.00	12.30	8	8	675	73.3	7.40
Q6P6L5	Protein Mybpc1 OS=Mus musculus GN=Mybpc1 PE=2 SV=1 - [Q6P6L5_MOUSE]	6.23	4	3	4	4	2.115	1.133	0.720	0.958	8.41	6.23	4	4	1124	126.1	5.95
Q61627	Glutamate receptor ionotropic, delta-1 OS=Mus musculus GN=Grid1 PE=1 SV=2 - [GRID1_MOUSE]	20.32	1	16	17	32	1.030	1.233	0.944	0.958	94.80	20.32	28	32	1009	112.1	6.70
Q923X1	EGF, latrophilin seven transmembrane domain-containing protein 1 OS=Mus musculus GN=Eltd1 PE=2 SV=3 - [ELTD1_MOUSE]	2.44	1	1	1	1	1.641	1.250	1.068	0.958	1.69	2.44	1	1	739	82.2	6.86
Q8BFX1	E3 ubiquitin-protein ligase RNF187 OS=Mus musculus GN=Rnf187 PE=1 SV=2 - [RN187_MOUSE]	10.17	1	2	2	3	1.301	0.716	1.068	0.958	8.87	10.17	2	3	236	26.3	5.59
O09044	Synaptosomal-associated protein 23 OS=Mus musculus GN=Snap23 PE=1 SV=1 -	60.00	5	8	9	114	1.544	0.834	0.907	0.958	308.35	60.00	15	114	210	23.2	4.98
Q66X22	NACHT, LRR and PYD domains-containing protein 9B OS=Mus musculus GN=Nlrp9b PE=2 SV=1 - [illegible]	1.50	7	1	2	2	3.369	0.868	0.702	0.958	4.28	1.50	2	2	1003	114.7	7.59
Q8C419	Probable G-protein coupled receptor 158 OS=Mus musculus GN=Gpr158 PE=1 SV=2 - [GP158_MOUSE]	35.00	1	33	33	238	0.779	0.868	1.461	0.958	669.28	35.00	60	238	1200	134.3	8.09
J3KMN0	Protein Kmt2d OS=Mus musculus GN=Kmt2d PE=4 SV=1 - [J3KMN0_MOUSE]	2.36	2	9	10	20	1.185	1.037	1.120	0.958	52.31	2.36	14	20	5588	599.9	5.78
O35639	Annexin A3 OS=Mus musculus GN=Anxa3 PE=1 SV=4 - [ANXA3_MOUSE]	55.11	1	16	18	59	1.277	2.529	0.834	0.958	155.53	55.11	33	59	323	36.4	5.76
Q8CI71	Coiled-coil domain-containing protein 132 OS=Mus musculus GN=Ccdc132 PE=1 SV=2 - [CC132_MOUSE]	28.94	5	25	25	69	0.569	1.263	1.021	0.958	209.72	28.94	42	69	964	111.1	6.07
F6ZZL0	Alpha-protein kinase 2 (Fragment) OS=Mus musculus GN=Alpk2 PE=2 SV=1 - [F6ZZL0_MOUSE]	3.05	3	4	4	4	2.285	1.273	1.398	0.958	11.49	3.05	4	4	1608	174.1	4.93
O35526	Syntaxin-1A OS=Mus musculus GN=Stx1a PE=1 SV=3 - [STX1A_MOUSE]	72.92	2	15	21	235	0.557	2.208	1.538	0.959	696.85	72.92	32	235	288	33.0	5.24

P68510	14-3-3 protein eta OS=Mus musculus GN=Ywhah PE=1 SV=2 - [1433F_MOUSE]	59.35	2	11	17	677	0.963	1.194	1.027	0.959	1826.75	59.35	30	677	246	28.2	4.89
Q9WUM3	Coronin-1B OS=Mus musculus GN=Coro1b PE=1 SV=1 - [COR1B_MOUSE]	38.84	2	16	16	68	1.034	0.987	1.101	0.959	192.20	38.84	27	68	484	53.9	5.78
Q5Y5T2	Palmitoyltransferase ZDHHC18 OS=Mus musculus GN=Zdhhc18 PE=1 SV=4 -	3.95	1	1	1	3	1.018	0.942	1.031	0.959	5.28	3.95	1	3	380	41.1	8.95
A6X8Z5	Rho GTPase-activating protein 31 OS=Mus musculus GN=Arhgap31 PE=1 SV=1 -	2.11	1	2	2	5	0.553	0.970	1.064	0.959	12.96	2.11	3	5	1425	155.2	5.77
Q61037-4	Isoform C of Tuberin OS=Mus musculus GN=Tsc2 - [TSC2_MOUSE]	20.45	5	2	24	80	0.413	0.637	1.032	0.959	206.36	20.45	40	80	1770	197.0	6.80
Q9R0B6	Laminin subunit gamma-3 OS=Mus musculus GN=Lamc3 PE=2 SV=2 - [LAMC3_MOUSE]	2.59	2	3	3	3	1.206	1.184	1.325	0.959	9.64	2.59	3	3	1581	172.2	6.96
P41230-2	Isoform 2 of Lysine-specific demethylase 5C OS=Mus musculus GN=Kdm5c -	4.38	3	4	6	12	1.147	0.880	1.041	0.959	22.73	4.38	8	12	1551	174.9	5.40
A2BI14	Coiled-coil domain-containing protein 171 (Fragment) OS=Mus musculus GN=Ccdc171 PE=2 SV=1 - [illegible]	7.03	6	6	8	12	1.972	1.345	1.254	0.959	22.56	7.03	8	12	1166	135.0	6.76
Q8K310	Matrin-3 OS=Mus musculus GN=Matr3 PE=1 SV=1 - [MATR3_MOUSE]	43.97	2	32	32	297	0.705	0.818	0.989	0.959	857.72	43.97	57	297	846	94.6	6.25
Q8BZR4	Acyl-coenzyme A thioesterase 8 OS=Mus musculus GN=Acot8 PE=2 SV=1 - [Q8BZR4_MOUSE]	17.54	3	4	4	7	0.842	1.164	0.627	0.959	20.73	17.54	7	7	268	29.5	7.12
H3BJR6	Sodium channel subunit beta-3 OS=Mus musculus GN=Scn3b PE=2 SV=1 - [H3BJR6_MOUSE]	22.86	4	4	4	10	0.743	1.538	0.940	0.959	28.51	22.86	7	10	175	20.3	4.75
Q6ZPY2	Protein SMG5 OS=Mus musculus GN=Smg5 PE=2 SV=2 - [SMG5_MOUSE]	5.31	2	5	5	8	1.242	0.847	0.949	0.959	12.36	5.31	7	8	1017	114.0	6.00
Q8R016	Bleomycin hydrolase OS=Mus musculus GN=Blmh PE=2 SV=1 - [BLMH_MOUSE]	45.93	4	5	18	115	0.812	1.179	0.838	0.959	318.70	45.93	33	115	455	52.5	6.48
E0CYH7	Filamin A-interacting protein 1-like OS=Mus musculus GN=Filip1l PE=2 SV=1 - [E0CYH7_MOUSE]	4.95	5	3	4	4	0.779	0.913	0.928	0.959	6.92	4.95	4	4	1131	129.8	6.33
Q3UKC1	Tax1-binding protein 1 homolog OS=Mus musculus GN=Tax1bp1 PE=1 SV=2 -	29.36	3	21	22	67	1.480	1.035	1.245	0.959	180.65	29.36	39	67	814	93.6	5.33
Q99KQ4	Nicotinamide phosphoribosyltransferase OS=Mus musculus GN=Nampt PE=1 SV=1 - [NAMPT_MOUSE]	62.12	1	25	25	93	0.896	0.950	0.859	0.959	291.48	62.12	45	93	491	55.4	7.15
E9Q2S9	Coiled-coil domain-containing protein 43 OS=Mus musculus GN=Ccdc43 PE=2 SV=1 - [E9Q2S9_MOUSE]	18.92	3	5	5	23	1.233	0.800	1.313	0.959	54.98	18.92	10	23	222	25.0	5.02
B0R0Y8	SH3 domain-containing kinase-binding protein 1 (Fragment) OS=Mus musculus GN=Sh3kbp1 PE=2 SV=1 - [illegible]	66.51	1	1	9	52	1.763	1.018	1.354	0.959	161.80	66.51	17	52	209	22.4	9.48
P47759	Neuron-specific protein family member 2 OS=Mus musculus GN=Nsg2 PE=2 SV=1 - [NSG2_MOUSE]	15.20	3	2	2	4	0.897	1.232	1.023	0.959	12.28	15.20	4	4	171	19.0	9.39
Q7TNK1	Transcription factor RFX4 OS=Mus musculus GN=Rfx4 PE=1 SV=1 - [RFX4_MOUSE]	11.16	4	3	3	4	0.346	0.568	1.035	0.959	2.90	11.16	3	4	735	83.3	6.80
O35174	Potassium voltage-gated channel subfamily S member 2 OS=Mus musculus GN=Kcns2 PE=2 SV=1 - [KCNS2_MOUSE]	3.56	2	2	2	3	1.103	0.930	1.036	0.959	6.24	3.56	2	3	477	54.3	5.72
Q8BGE6	Cysteine protease ATG4B OS=Mus musculus GN=Atg4b PE=1 SV=2 - [ATG4B_MOUSE]	28.75	2	8	8	24	0.692	1.009	0.849	0.959	87.36	28.75	14	24	393	44.3	5.07
P63321	Ras-related protein Ral-A OS=Mus musculus GN=Rala PE=1 SV=1 - [RALA_MOUSE]	51.94	1	3	8	60	0.782	0.699	0.878	0.959	163.58	51.94	13	60	206	23.5	7.11
O55192	Sodium-dependent noradrenaline transporter OS=Mus musculus GN=Slc6a2 PE=2 SV=2 - [SC6A2_MOUSE]	5.02	1	1	2	8	1.420	0.671	0.848	0.959	24.21	5.02	4	8	617	69.2	7.44
Q501J7-3	Isoform 3 of Phosphatase and actin regulator 4 OS=Mus musculus GN=Phactr4 -	29.40	4	10	13	44	1.611	0.679	1.083	0.959	127.47	29.40	22	44	704	77.6	6.58

Q99M71	Mammalian ependymin-related protein 1 OS=Mus musculus GN=Epdr1 PE=2 SV=1 - [EPDR1_MOUSE]	30.36	2	6	6	131	1.697	0.929	1.272	0.960	416.46	30.36	12	131	224	25.5	7.58
Q9WTU6	Mitogen-activated protein kinase 9 OS=Mus musculus GN=Mapk9 PE=1 SV=2 -	44.92	11	7	14	52	0.929	0.865	0.828	0.960	137.30	44.92	24	52	423	48.2	5.97
Q9DAA6-2	Isoform 2 of Exosome complex component CSL4 OS=Mus musculus GN=Exosc1 - [EXOS1_MOUSE]	9.09	2	1	1	2	1.565	1.115	1.086	0.960	7.02	9.09	2	2	154	16.8	6.29
Q9CR16	Peptidyl-prolyl cis-trans isomerase D OS=Mus musculus GN=Ppid PE=1 SV=3 - [PPID_MOUSE]	63.24	1	21	22	122	0.918	1.043	0.933	0.960	272.38	63.24	39	122	370	40.7	7.43
Q60850-3	Isoform 3 of Solute carrier family 23 member 3 OS=Mus musculus GN=Slc23a3 -	13.93	3	1	1	2	0.843	0.826	0.938	0.960	0.00	13.93	1	2	122	13.2	6.05
Q99M04	Lipoyl synthase, mitochondrial OS=Mus musculus GN=Lias PE=2 SV=1 - [LIAS_MOUSE]	23.86	2	7	7	14	1.113	0.629	0.884	0.960	48.69	23.86	10	14	373	41.9	8.88
Q9Z0V2	Potassium voltage-gated channel subfamily D member 2 OS=Mus musculus GN=Kcnd2 PE=1 SV=1 - [KCND2_MOUSE]	23.97	1	11	12	70	0.702	0.640	1.023	0.960	193.42	23.97	20	70	630	70.5	8.07
Q60575-2	Isoform 2 of Kinesin-like protein KIF1B OS=Mus musculus GN=Kif1b - [KIF1B_MOUSE]	20.73	8	15	32	97	0.857	0.785	0.941	0.960	244.20	20.73	49	97	1770	198.8	5.59
O54786-2	Isoform ICAD-S of DNA fragmentation factor subunit alpha OS=Mus musculus GN=Dffa - [DFFA_MOUSE]	36.60	2	9	9	20	2.659	0.788	1.371	0.960	56.33	36.60	14	20	265	29.2	4.59
E9Q6W4	Protein Zfp296 OS=Mus musculus GN=Zfp296 PE=2 SV=1 - [E9Q6W4_MOUSE]	19.10	1	3	3	3	1.380	0.856	1.101	0.960	4.23	19.10	3	3	445	47.8	9.16
Q91YJ5	Translation initiation factor IF-2, mitochondrial OS=Mus musculus GN=Mtif2 PE=1 SV=2 - [IF2M_MOUSE]	26.55	5	17	17	33	1.045	0.670	1.043	0.960	86.12	26.55	25	33	727	81.2	7.24
Q9QUH0	Glutaredoxin-1 OS=Mus musculus GN=Glrx PE=1 SV=3 - [GLRX1_MOUSE]	37.38	1	4	4	19	1.333	0.749	0.989	0.960	61.87	37.38	7	19	107	11.9	8.37
Q8C854	Myelin expression factor 2 OS=Mus musculus GN=Myef2 PE=1 SV=1 - [MYEF2_MOUSE]	42.13	8	21	22	92	0.772	0.879	0.868	0.960	229.33	42.13	38	92	591	63.3	8.87
Q8BIH0-2	Isoform 2 of Histone deacetylase complex subunit SAP130 OS=Mus musculus GN=Sap130 - [SP130_MOUSE]	5.35	4	4	4	10	1.629	0.878	1.242	0.960	26.45	5.35	7	10	879	92.8	9.98
Q8C015	Serine/threonine-protein kinase PAK 7 OS=Mus musculus GN=Pak7 PE=1 SV=1 - [PAK7_MOUSE]	27.82	1	14	15	40	0.936	0.941	1.264	0.960	119.40	27.82	27	40	719	80.9	8.21
Q9QXW9	Large neutral amino acids transporter small subunit 2 OS=Mus musculus GN=Slc7a8 PE=1 SV=1 - [LAT2_MOUSE]	6.03	1	2	2	5	0.468	1.180	1.280	0.960	13.54	6.03	3	5	531	57.8	6.67
P62046	Leucine-rich repeat and calponin homology domain containing protein 1 OS=Mus musculus GN=Lrch1 PE=1 SV=2 - [LRCH1_MOUSE]	19.89	1	9	10	21	1.385	0.859	0.778	0.960	55.11	19.89	16	21	709	79.0	6.13
D3Z6I8	Tropomyosin alpha-3 chain OS=Mus musculus GN=Tpm3 PE=2 SV=1 - [D3Z6I8_MOUSE]	38.06	2	4	15	319	1.726	1.227	1.407	0.960	851.77	38.06	26	319	247	28.7	4.79
Q3V3R4	Integrin alpha-1 OS=Mus musculus GN=Itga1 PE=2 SV=2 - [ITA1_MOUSE]	10.60	2	13	13	23	1.083	1.301	1.054	0.960	60.26	10.60	17	23	1179	130.7	6.20
E9PWJ4	Protein Auts2 OS=Mus musculus GN=Auts2 PE=2 SV=1 - [E9PWJ4_MOUSE]	3.93	3	2	2	2	1.691	1.682	1.358	0.960	4.58	3.93	2	2	789	87.4	9.23
O55242-2	Isoform 2 of Sigma non-opioid intracellular receptor 1 OS=Mus musculus GN=Sigmar1 - [SGMR1_MOUSE]	14.06	2	2	3	8	0.835	0.957	0.720	0.960	22.93	14.06	5	8	192	21.6	5.94
O35136	Neural cell adhesion molecule 2 OS=Mus musculus GN=Ncam2 PE=1 SV=1 -	42.05	2	30	32	222	0.922	1.191	0.957	0.961	641.86	42.05	60	222	837	93.1	6.07
Q80Y55	BSD domain-containing protein 1 OS=Mus musculus GN=Bsdc1 PE=2 SV=1 - [BSDC1_MOUSE]	28.34	2	8	8	27	1.816	1.341	1.379	0.961	92.46	28.34	16	27	427	46.9	4.40
Q9JJQ0	GPI mannosyltransferase 3 OS=Mus musculus GN=Pigb PE=1 SV=2 - [PIGB_MOUSE]	3.87	1	2	2	5	0.756	0.943	0.929	0.961	19.97	3.87	3	5	542	63.1	9.23

O35857	Mitochondrial import inner membrane translocase subunit TIM44 OS=Mus musculus GN=Timm44 PE=2 SV=2 - [TIM44_MOUSE]	40.04	2	20	20	77	0.824	0.799	0.944	0.961	239.73	40.04	38	77	452	51.1	8.13
Q8R550	SH3 domain-containing kinase-binding protein 1 OS=Mus musculus GN=Sh3kbp1 PE=1 SV=1 - [SH3K1_MOUSE]	48.80	9	18	26	147	1.645	0.825	1.435	0.961	437.94	48.80	48	147	709	78.1	7.55
Q9CZX7	Transmembrane protein 55A OS=Mus musculus GN=Tmem55a PE=1 SV=1 - [TM55A_MOUSE]	32.30	1	6	6	21	1.090	1.073	0.953	0.961	69.11	32.30	11	21	257	28.0	8.68
Q99N57	RAF proto-oncogene serine/threonine-protein kinase OS=Mus musculus GN=Raf1 PE=1 SV=2 - [RAF1_MOUSE]	17.44	3	4	11	34	0.776	1.000	0.897	0.961	107.14	17.44	21	34	648	72.9	9.16
Q6NXI6-2	Isoform 2 of Regulation of nuclear pre-mRNA domain-containing protein 2 OS=Mus musculus GN=Rprd2 - [illegible]	18.11	2	16	16	45	1.447	1.169	1.234	0.961	125.51	18.11	25	45	1425	151.3	6.93
Q8BLR9-2	Isoform 2 of Hypoxia-inducible factor 1-alpha inhibitor OS=Mus musculus GN=Hif1an - [HIF1N_MOUSE]	5.32	2	2	2	7	1.016	0.956	0.989	0.961	14.95	5.32	4	7	263	30.4	6.55
Q3V3Q7	Phosphofurin acidic cluster sorting protein 2 OS=Mus musculus GN=Pacs2 PE=1 SV=2 - [PACS2_MOUSE]	17.52	2	9	11	35	0.811	0.375	0.821	0.961	111.54	17.52	17	35	862	94.9	6.51
Q8R0W0	Epiplakin OS=Mus musculus GN=Eppk1 PE=1 SV=2 - [EPIPL_MOUSE]	2.46	1	7	10	31	1.883	1.018	0.922	0.961	62.05	2.46	14	31	6548	724.2	6.07
A2RTL5	Arginine/serine-rich coiled-coil protein 2 OS=Mus musculus GN=Rsrc2 PE=2 SV=1 - [RSRC2_MOUSE]	6.12	3	2	2	5	1.518	0.868	0.804	0.961	13.17	6.12	4	5	376	43.8	11.46
F7DCH5	CLIP-associating protein 2 OS=Mus musculus GN=Clasp2 PE=4 SV=1 - [F7DCH5_MOUSE]	52.95	2	2	58	285	0.664	0.296	0.782	0.961	768.05	52.95	102	285	1307	142.8	8.66
E0CXZ8	Dynamin-3 OS=Mus musculus GN=Dnm3 PE=2 SV=1 - [E0CXZ8_MOUSE]	46.08	3	1	15	56	1.345	0.651	0.883	0.961	167.11	46.08	23	56	319	35.7	5.62
Q9WV96	Mitochondrial import inner membrane translocase subunit Tim10 B OS=Mus musculus GN=Timm10b PE=2 SV=1 - [T10B_MOUSE]	51.00	5	4	4	13	1.218	1.017	1.027	0.961	46.42	51.00	8	13	100	11.3	7.08
Q8K2Q7	BRO1 domain-containing protein BROX OS=Mus musculus GN=Brox PE=2 SV=1 - [BROX_MOUSE]	33.82	3	10	11	26	0.574	0.611	0.722	0.961	77.04	33.82	17	26	411	46.2	7.69
Q9QZ88-2	Isoform 2 of Vacuolar protein sorting-associated protein 29 OS=Mus musculus GN=Vps29 - [VPS29_MOUSE]	39.78	5	8	8	47	1.065	0.992	0.866	0.961	148.48	39.78	14	47	186	20.9	7.05
Q8BGW5-2	Isoform 2 of SOSS complex subunit B2 OS=Mus musculus GN=Nabp1 -	12.71	4	1	2	3	1.086	0.958	1.050	0.961	4.32	12.71	3	3	118	13.0	9.45
O08648-2	Isoform B of Mitogen-activated protein kinase kinase kinase 4 OS=Mus musculus GN=Map3k4 - [M3K4_MOUSE]	13.14	2	19	19	36	0.848	0.869	0.870	0.961	100.55	13.14	28	36	1545	175.0	6.37
Q8VEE0	Ribulose-phosphate 3-epimerase OS=Mus musculus GN=Rpe PE=2 SV=1 - [RPE_MOUSE]	15.79	4	3	3	9	1.549	0.950	0.966	0.961	27.00	15.79	6	9	228	24.9	5.41
Q9D513	Meiosis-specific with OB domain-containing protein OS=Mus musculus GN=Meiob PE=2 SV=3 - [MEIOB_MOUSE]	1.91	1	1	1	1	1.006	2.696	0.917	0.961	2.31	1.91	1	1	470	52.9	6.96
O70293-2	Isoform GRK6B of G protein-coupled receptor kinase 6 OS=Mus musculus GN=Grk6 - [GRK6_MOUSE]	25.47	3	12	13	32	0.776	0.824	0.967	0.962	95.43	25.47	20	32	589	67.1	8.66
F8VQA4	Peptidyl-glycine alpha-amidating monooxygenase OS=Mus musculus GN=Pam PE=2 SV=1 - [F8VQA4_MOUSE]	37.12	6	32	32	95	1.143	0.928	0.735	0.962	276.50	37.12	52	95	978	108.8	6.60
P12960	Contactin-1 OS=Mus musculus GN=Cntn1 PE=1 SV=1 - [CNTN1_MOUSE]	58.33	1	53	54	735	0.935	1.002	0.951	0.962	2116.83	58.33	98	735	1020	113.3	6.16
Q62384	Zinc finger protein ZPR1 OS=Mus musculus GN=Znf259 PE=1 SV=1 - [ZPR1_MOUSE]	44.88	3	15	15	49	1.642	1.160	1.057	0.962	125.63	44.88	25	49	459	50.7	4.78
Q8QZT1	Acetyl-CoA acetyltransferase, mitochondrial OS=Mus musculus GN=Acat1 PE=1 SV=1 - [THIL_MOUSE]	74.76	1	24	24	286	1.018	0.823	0.868	0.962	938.88	74.76	40	286	424	44.8	8.51

O70572	Sphingomyelin phosphodiesterase 2 OS=Mus musculus GN=Smpd2 PE=1 SV=1 -	12.89	1	5	5	18	0.710	0.834	0.818	0.962	53.26	12.89	10	18	419	47.4	6.44
D3Z5H8	Nucleoside diphosphate-linked moiety X motif 8, mitochondrial OS=Mus musculus GN=Nudt8 PE=4 SV=1 - [D3Z5H8_MOUSE]	18.56	2	2	2	8	3.733	0.829	0.771	0.962	9.36	18.56	3	8	167	18.7	5.03
P97772	Metabotropic glutamate receptor 1 OS=Mus musculus GN=Grm1 PE=2 SV=2 - [GRM1_MOUSE]	19.18	3	17	19	88	0.856	1.032	0.951	0.962	191.59	19.18	32	88	1199	133.1	6.86
Q7TPD2	Protein FAM185A OS=Mus musculus GN=Fam185a PE=2 SV=1 - [F185A_MOUSE]	13.76	2	4	4	9	0.872	0.862	0.963	0.962	29.94	13.76	6	9	378	40.0	6.38
P53811	Phosphatidylinositol transfer protein beta isoform OS=Mus musculus GN=Pitpnb PE=1 SV=2 - [PIPNB_MOUSE]	53.51	1	12	15	38	0.729	1.115	0.921	0.962	98.71	53.51	24	38	271	31.5	6.95
G3UYI5	Protein mago nashi homolog 2 (Fragment) OS=Mus musculus GN=Magohb PE=2 SV=1 - [G3UYI5_MOUSE]	40.00	4	4	4	25	0.661	0.822	0.821	0.962	51.49	40.00	6	25	115	13.4	5.54
F7B2D1	Synaptophysin-like protein 2 (Fragment) OS=Mus musculus GN=Sypl2 PE=4 SV=1 - [F7B2D1_MOUSE]	2.34	1	1	1	1	0.762	0.995	0.911	0.962	2.27	2.34	1	1	256	28.6	10.21
Q3U284	Transmembrane protein 231 OS=Mus musculus GN=Tmem231 PE=1 SV=1 - [TM231_MOUSE]	6.35	1	3	3	3	0.749	0.740	0.900	0.962	5.89	6.35	3	3	315	36.2	8.16
Q9DBN4	Putative monooxygenase p33MONOX OS=Mus musculus GN=P33monox PE=1 SV=1 - [P33MX_MOUSE]	51.49	6	12	12	31	1.447	0.794	1.314	0.962	95.27	51.49	19	31	303	32.7	9.42
Q8BFQ9	Kelch-like protein 42 OS=Mus musculus GN=Klhl42 PE=2 SV=1 - [KLH42_MOUSE]	1.83	1	1	1	2	1.222	0.859	0.906	0.962	4.42	1.83	2	2	493	55.6	6.32
G3X9J4	Protein 2310035C23Rik OS=Mus musculus GN=2310035C23Rik PE=4 SV=1 - [G3X9J4_MOUSE]	33.14	5	33	33	134	0.900	1.146	1.030	0.962	366.77	33.14	58	134	1216	134.5	5.34
B8QI34	Liprin-alpha 2 OS=Mus musculus GN=Ppfia2 PE=2 SV=1 - [B8QI34_MOUSE]	37.47	3	20	41	182	1.011	1.345	1.398	0.962	540.20	37.47	70	182	1257	143.2	6.11
E9Q8S5	Microtubule-associated serine/threonine-protein kinase 3 OS=Mus musculus GN=Mast3 PE=2 SV=1 - [E9Q8S5_MOUSE]	16.93	4	12	15	23	1.453	1.200	1.463	0.962	62.81	16.93	20	23	1305	142.5	8.65
P20357	Microtubule-associated protein 2 OS=Mus musculus GN=Map2 PE=1 SV=2 - [MTAP2_MOUSE]	65.32	10	86	111	1529	1.453	1.574	1.540	0.963	4451.42	65.32	192	1529	1828	199.0	4.91
Q9CQC9	GTP-binding protein SAR1b OS=Mus musculus GN=Sar1b PE=1 SV=1 - [SAR1B_MOUSE]	55.05	1	6	9	32	0.525	0.600	0.622	0.963	103.24	55.05	14	32	198	22.4	6.11
Q9CY34	NEDD8-conjugating enzyme UBE2F OS=Mus musculus GN=Ube2f PE=1 SV=1 - [UB2FA_MOUSE]	52.43	2	7	7	14	1.154	0.880	1.215	0.963	35.78	52.43	11	14	185	21.1	6.54
Q8CEC2	Aryl hydrocarbon receptor nuclear translocator OS=Mus musculus GN=Arnt PE=2 SV=1 - [Q8CEC2_MOUSE]	1.84	5	1	1	1	0.981	0.777	0.926	0.963	2.43	1.84	1	1	760	83.5	6.73
Q3TNH5-3	Isoform 3 of Protein FAM172A OS=Mus musculus GN=Fam172a - [F172A_MOUSE]	12.70	4	4	4	11	0.813	0.617	0.809	0.963	30.08	12.70	7	11	307	35.8	6.61
Q8BVP5	Casein kinase I isoform gamma-2 OS=Mus musculus GN=Csnk1g2 PE=1 SV=1 -	25.54	2	2	9	18	0.492	0.740	0.756	0.963	65.91	25.54	13	18	415	47.6	9.06
P06151	L-lactate dehydrogenase A chain OS=Mus musculus GN=Ldha PE=1 SV=3 - [LDHA_MOUSE]	63.86	7	22	25	263	0.876	1.453	1.031	0.963	707.65	63.86	43	263	332	36.5	7.74
Q69Z98	Serine/threonine-protein kinase BRSK2 OS=Mus musculus GN=Brsk2 PE=1 SV=2 - [BRSK2_MOUSE]	42.99	7	4	26	124	1.168	1.000	0.908	0.963	385.30	42.99	47	124	735	81.7	8.79
Q8K4X7	1-acyl-sn-glycerol-3-phosphate acyltransferase delta OS=Mus musculus GN=Agpat4 PE=2 SV=1 - [PLCD_MOUSE]	21.69	2	9	10	42	0.863	0.778	0.855	0.963	105.70	21.69	19	42	378	43.8	8.38
Q99MY8	Histone-lysine N-methyltransferase ASH1L OS=Mus musculus GN=Ash1l PE=1 SV=3 - [ASH1L_MOUSE]	2.37	1	5	5	5	1.775	1.036	0.917	0.963	6.44	2.37	5	5	2958	331.1	9.35
Q9DC51	Guanine nucleotide-binding protein G(k) subunit alpha OS=Mus musculus GN=Gnai3 PE=1 SV=3 - [GNAI3_MOUSE]	48.31	6	6	15	272	0.780	0.954	1.041	0.963	759.78	48.31	28	272	354	40.5	5.69

P70188-2	Isoform KAP3B of Kinesin-associated protein 3 OS=Mus musculus GN=Kifap3 - [KIFA3_MOUSE]	27.72	2	16	19	49	0.798	1.216	1.049	0.963	134.83	27.72	32	49	772	89.0	5.20
Q9Z1J2-2	Isoform 2 of Serine/threonine-protein kinase Nek4 OS=Mus musculus GN=Nek4 - [NEK4_MOUSE]	13.04	3	6	7	13	1.023	0.888	0.760	0.963	28.36	13.04	9	13	744	83.5	6.95
Q8CAG6	Pleckstrin OS=Mus musculus GN=Plek PE=2 SV=1 - [Q8CAG6_MOUSE]	6.42	2	2	2	4	1.662	1.732	1.001	0.963	8.14	6.42	4	4	327	37.1	7.43
P51660	Peroxisomal multifunctional enzyme type 2 OS=Mus musculus GN=Hsd17b4 PE=1 SV=3 - [DHB4_MOUSE]	33.06	1	20	20	103	0.718	0.608	0.751	0.963	328.46	33.06	36	103	735	79.4	8.57
P98203-4	Isoform 4 of Armadillo repeat protein deleted in velo-cardio-facial syndrome homolog OS=Mus musculus GN=Arvcf - [ARVC_MOUSE]	32.74	5	22	22	69	0.686	1.010	0.905	0.963	180.51	32.74	37	69	892	97.1	6.79
E9QK16	Protocadherin Fat 3 OS=Mus musculus GN=Fat3 PE=2 SV=1 - [E9QK16_MOUSE]	4.00	3	13	15	27	1.217	0.968	0.997	0.963	65.50	4.00	20	27	4551	501.3	4.87
Q4VAC9-2	Isoform 2 of Pleckstrin homology domain-containing family G member 3 OS=Mus musculus GN=Plekhg3 - [illegible]	4.63	6	3	4	11	0.987	0.563	1.015	0.963	28.28	4.63	6	11	1340	148.3	5.62
Q9D3E6	Cohesin subunit SA-1 OS=Mus musculus GN=Stag1 PE=1 SV=3 - [STAG1_MOUSE]	7.87	9	5	11	18	0.874	0.861	0.887	0.963	42.98	7.87	15	18	1258	144.3	5.59
Q9R1L5	Microtubule-associated serine/threonine-protein kinase 1 OS=Mus musculus GN=Mast1 PE=1 SV=3 - [MAST1_MOUSE]	26.69	4	28	33	109	1.006	0.813	1.025	0.963	331.29	26.69	57	109	1570	170.9	8.44
Q9WV92-2	Isoform 2 of Band 4.1-like protein 3 OS=Mus musculus GN=Epb41l3 - [E41L3_MOUSE]	50.69	4	4	47	449	1.090	0.996	1.327	0.963	1371.09	50.69	84	449	939	104.5	5.50
Q8CI61	BAG family molecular chaperone regulator 4 OS=Mus musculus GN=Bag4 PE=1 SV=2 - [BAG4_MOUSE]	20.35	1	7	7	28	1.688	1.440	1.504	0.964	67.73	20.35	13	28	457	49.1	5.34
F6QBN8	Protein Gm6772 OS=Mus musculus GN=Gm6772 PE=4 SV=2 - [F6QBN8_MOUSE]	3.08	1	1	1	1	1.518	1.220	1.685	0.964	1.64	3.08	1	1	487	54.1	4.96
Q9CQL6	39S ribosomal protein L35, mitochondrial OS=Mus musculus GN=Mrpl35 PE=2 SV=1 - [RM35_MOUSE]	7.45	1	1	1	2	1.231	1.062	0.910	0.964	9.15	7.45	2	2	188	21.5	11.47
Q9CR23	Transmembrane protein 9 OS=Mus musculus GN=Tmem9 PE=2 SV=1 - [TMEM9_MOUSE]	14.75	1	2	3	18	0.842	0.734	0.695	0.964	47.97	14.75	5	18	183	20.6	6.95
Q5FWH7	Zinc transporter ZIP12 OS=Mus musculus GN=Slc39a12 PE=2 SV=1 - [S39AC_MOUSE]	13.35	4	9	9	30	0.745	0.532	0.720	0.964	82.61	13.35	16	30	689	76.2	5.77
Q8CFG9	Complement C1r-B subcomponent OS=Mus musculus GN=C1rb PE=2 SV=1 - [C1RB_MOUSE]	3.68	2	1	1	1	2.462	1.897	0.763	0.964	2.40	3.68	1	1	706	79.9	5.81
A2ARI4-2	Isoform 2 of Leucine-rich repeat-containing G-protein coupled receptor 4 OS=Mus musculus GN=Lgr4 - [LGR4_MOUSE]	2.48	2	2	3	4	1.085	1.176	1.184	0.964	3.80	2.48	3	4	927	101.3	6.35
Q91Y82	Kallikrein 6, isoform CRA_a OS=Mus musculus GN=Klk6 PE=2 SV=1 - [Q91Y82_MOUSE]	15.42	1	2	2	9	4.816	0.604	1.074	0.964	37.15	15.42	4	9	253	28.3	7.93
Q6IQX7	Chondroitin sulfate synthase 2 OS=Mus musculus GN=Chpf PE=2 SV=1 - [CHSS2_MOUSE]	6.59	4	4	4	7	1.135	0.901	0.936	0.964	24.80	6.59	7	7	774	85.5	6.84
Q9D0I9	Arginine--tRNA ligase, cytoplasmic OS=Mus musculus GN=Rars PE=2 SV=2 - [SYRC_MOUSE]	43.48	1	27	27	111	0.723	0.960	0.774	0.964	309.73	43.48	46	111	660	75.6	7.55
Q8R1R3	StAR-related lipid transfer protein 7, mitochondrial OS=Mus musculus GN=Stard7 PE=2 SV=2 - [STAR7_MOUSE]	5.90	2	2	2	3	1.115	1.293	0.815	0.964	5.74	5.90	3	3	373	43.1	8.95
Q3V3W4	Protein Zbtb2 OS=Mus musculus GN=Zbtb2 PE=2 SV=1 - [Q3V3W4_MOUSE]	1.17	1	1	1	2	1.742	1.340	0.968	0.964	4.88	1.17	1	2	514	57.2	6.01
P52189	Inward rectifier potassium channel 4 OS=Mus musculus GN=Kcnj4 PE=1 SV=1 - [IRK4_MOUSE]	9.21	2	3	3	11	1.444	2.862	3.290	0.964	27.16	9.21	5	11	445	49.9	6.21
Q9D4J1	EF-hand domain-containing protein D1 OS=Mus musculus GN=Efhd1 PE=2 SV=1 -	28.75	1	3	6	60	2.277	0.823	0.981	0.964	147.16	28.75	10	60	240	27.0	6.02

E9QLP0	Tubulin polyglutamylase complex subunit 2 OS=Mus musculus GN=Tpgs2 PE=2 SV=1 - [E9QLP0_MOUSE]	10.81	2	3	3	19	0.562	0.823	0.860	0.964	49.62	10.81	6	19	296	33.2	7.24
A2AWP8-4	Isoform 4 of Rho guanine nucleotide exchange factor 10-like protein OS=Mus musculus GN=Arhgef10l - [ARGAL_MOUSE]	6.12	10	7	8	16	0.981	0.790	0.836	0.964	46.86	6.12	13	16	1275	139.3	5.74
Q3UHC7-2	Isoform 2 of Disabled homolog 2-interacting protein OS=Mus musculus GN=Dab2ip - [DAB2P_MOUSE]	24.69	9	19	25	86	0.886	0.851	1.136	0.964	233.70	24.69	43	86	1065	117.6	6.79
P59470	DNA-directed RNA polymerase III subunit RPC2 OS=Mus musculus GN=Polr3b PE=2 SV=2 - [RPC2_MOUSE]	4.06	1	5	5	8	1.079	0.866	1.085	0.964	14.13	4.06	6	8	1133	127.6	8.50
Q9QXJ1-2	Isoform 2 of Amyloid beta A4 precursor protein-binding family B member 1 OS=Mus musculus GN=Apbb1 - [APBB1_MOUSE]	31.21	3	13	15	44	0.914	1.055	1.044	0.964	143.15	31.21	27	44	708	77.1	4.97
Q62443	Neuronal pentraxin-1 OS=Mus musculus GN=Nptx1 PE=2 SV=1 - [NPTX1_MOUSE]	33.56	1	13	15	54	1.205	1.984	1.127	0.964	169.15	33.56	25	54	432	47.1	6.44
Q9WUK4	Replication factor C subunit 2 OS=Mus musculus GN=Rfc2 PE=2 SV=1 - [RFC2_MOUSE]	5.44	1	2	2	3	0.889	1.357	0.939	0.964	7.99	5.44	3	3	349	38.7	6.44
Q3TXX4	Vesicular glutamate transporter 1 OS=Mus musculus GN=Slc17a7 PE=2 SV=2 -	15.54	3	7	7	60	0.414	2.438	1.840	0.964	132.64	15.54	13	60	560	61.6	7.34
Q9R0A0	Peroxisomal membrane protein PEX14 OS=Mus musculus GN=Pex14 PE=1 SV=1 - [PEX14_MOUSE]	17.55	2	6	6	16	0.717	0.907	0.773	0.964	43.71	17.55	10	16	376	41.2	5.11
Q3KNY0	Immunoglobulin-like and fibronectin type III domain containing protein 1 OS=Mus musculus GN=Igfn1 PE=1 SV=3 - [IGFN1_MOUSE]	1.68	7	3	3	4	1.081	1.008	0.969	0.965	5.02	1.68	3	4	2849	303.5	6.58
Q3TKX1	ATPase, H+ transporting, lysosomal accessory protein OS=Mus musculus GN=Atp6ap1 PE=2 SV=1 - [Q3TKX1_MOUSE]	23.61	6	8	8	24	0.943	1.208	0.949	0.965	69.90	23.61	15	24	415	45.5	5.63
O55143	Sarcoplasmic/endoplasmic reticulum calcium ATPase 2 OS=Mus musculus GN=Atp2a2 PE=1 SV=2 - [AT2A2_MOUSE]	51.25	4	39	48	285	0.535	0.869	1.156	0.965	890.06	51.25	84	285	1044	114.8	5.34
Q80W71-2	Isoform 2 of Pleckstrin homology domain-containing family A member 8 OS=Mus musculus GN=Plekha8 - [PKHA8_MOUSE]	2.53	2	1	1	3	1.007	1.741	0.889	0.965	2.93	2.53	2	3	474	52.7	4.87
A2AVP4	RING finger and CCCH-type zinc finger domain-containing protein 2 OS=Mus musculus GN=Rc3h2 PE=2 SV=1 - [A2AVP4_MOUSE]	4.62	2	3	4	6	1.671	0.990	1.060	0.965	16.28	4.62	5	6	1125	124.6	7.20
Q69Z26	Contactin-4 OS=Mus musculus GN=Cntn4 PE=2 SV=2 - [CNTN4_MOUSE]	12.96	4	11	12	26	0.762	1.133	1.093	0.965	66.88	12.96	17	26	1026	113.4	7.36
B1ARA3	60S ribosomal protein L26 (Fragment) OS=Mus musculus GN=Rpl26 PE=2 SV=1 - [B1ARA3_MOUSE]	47.57	4	6	6	31	0.624	0.854	0.856	0.965	75.53	47.57	11	31	103	12.2	10.92
Q03173	Protein enabled homolog OS=Mus musculus GN=Enah PE=1 SV=2 - [ENAH_MOUSE]	28.80	6	1	19	117	1.872	0.848	0.950	0.965	288.29	28.80	29	117	802	85.8	7.72
A2A9W1	MIF4G domain-containing protein (Fragment) OS=Mus musculus GN=Mif4gd PE=2 SV=1 - [A2A9W1_MOUSE]	39.51	4	5	5	7	1.236	0.951	0.948	0.965	14.73	39.51	7	7	162	18.6	5.14
Q99JW1-2	Isoform 2 of Alpha/beta hydrolase domain-containing protein 17A OS=Mus musculus GN=Abhd17a - [AB17A_MOUSE]	16.67	2	2	3	13	0.769	0.631	1.221	0.965	38.55	16.67	6	13	246	26.9	6.95
P70451-2	Isoform 2 of Tyrosine-protein kinase Fer OS=Mus musculus GN=Fer - [FER_MOUSE]	10.98	5	7	9	22	0.910	0.900	0.806	0.965	58.22	10.98	14	22	765	87.7	6.77
Q8BU04	Putative E3 ubiquitin-protein ligase UBR7 OS=Mus musculus GN=Ubr7 PE=2 SV=1 -	7.06	2	3	3	10	1.383	1.188	1.157	0.965	23.92	7.06	6	10	425	48.0	4.75
Q8C1W1	Vasohibin-1 OS=Mus musculus GN=Vash1 PE=2 SV=4 - [VASH1_MOUSE]	10.40	1	3	4	5	0.813	0.768	0.964	0.965	6.64	10.40	5	5	375	41.8	9.39
Q5SSI6	U3 small nucleolar RNA-associated protein 18 homolog OS=Mus musculus GN=Utp18 PE=1 SV=1 - [UTP18_MOUSE]	11.05	1	3	3	6	0.898	0.806	1.281	0.965	16.45	11.05	4	6	552	61.2	8.78

A2RTT5	Protein Vmn1r232 OS=Mus musculus GN=Vmn1r232 PE=2 SV=1 - [A2RTT5_MOUSE]	5.98	1	1	1	1	2.813	1.167	1.640	0.965	3.74	5.98	1	1	351	40.3	9.16
D3Z2P2	Chitinase-3-like protein 1 OS=Mus musculus GN=Chi3l1 PE=2 SV=1 - [D3Z2P2_MOUSE]	7.92	4	2	2	6	1.395	1.230	0.947	0.965	11.32	7.92	4	6	379	42.8	8.41
P15209	BDNF/NT-3 growth factors receptor OS=Mus musculus GN=Ntrk2 PE=1 SV=1 - [NTRK2_MOUSE]	25.58	3	5	19	102	0.641	1.676	1.063	0.966	297.36	25.58	33	102	821	92.1	6.55
O89001	Carboxypeptidase D OS=Mus musculus GN=Cpd PE=1 SV=2 - [CBPD_MOUSE]	16.19	2	20	21	58	1.355	1.286	0.992	0.966	144.13	16.19	33	58	1377	152.3	6.18
Q6I6G8	E3 ubiquitin-protein ligase HECW2 OS=Mus musculus GN=Hecw2 PE=2 SV=1 - [HECW2_MOUSE]	15.46	4	20	23	54	0.669	0.844	1.107	0.966	152.73	15.46	40	54	1578	176.1	5.41
J3QM95	FSD1-like protein OS=Mus musculus GN=Fsd1l PE=4 SV=1 - [J3QM95_MOUSE]	34.61	6	15	15	51	1.084	1.076	0.970	0.966	146.21	34.61	26	51	497	55.8	6.40
Q78RX3	Small integral membrane protein 12 OS=Mus musculus GN=Smim12 PE=3 SV=1 -	18.48	1	2	2	6	0.635	0.708	1.062	0.966	16.22	18.48	4	6	92	10.8	9.04
E9QQ33	E3 ubiquitin-protein ligase SH3RF1 OS=Mus musculus GN=Sh3rf1 PE=4 SV=1 - [E9QQ33_MOUSE]	14.14	6	9	12	26	1.110	1.156	1.225	0.966	61.42	14.14	21	26	891	93.3	8.48
A2AN08-3	Isoform 3 of E3 ubiquitin-protein ligase UBR4 OS=Mus musculus GN=Ubr4 -	18.25	6	74	76	255	0.679	0.903	0.893	0.966	706.74	18.25	131	255	5156	569.9	6.06
Q80WQ8	Mis18-binding protein 1 OS=Mus musculus GN=Mis18bp1 PE=1 SV=1 - [M18BP_MOUSE]	3.91	3	3	3	6	0.854	1.428	0.830	0.966	6.17	3.91	3	6	998	113.9	9.09
P16092-4	Isoform 4 of Fibroblast growth factor receptor 1 OS=Mus musculus GN=Fgfr1 -	4.24	21	1	3	23	1.004	1.110	1.185	0.966	49.01	4.24	5	23	731	81.8	6.57
G3XA17	Eukaryotic translation initiation factor 4 gamma 2 OS=Mus musculus GN=Eif4g2 PE=4 SV=1 - [G3XA17_MOUSE]	31.13	7	24	24	116	0.824	0.998	0.963	0.966	312.81	31.13	41	116	906	102.0	7.14
Q03517	Secretogranin-2 OS=Mus musculus GN=Scg2 PE=1 SV=1 - [SCG2_MOUSE]	52.84	1	26	27	496	1.577	0.942	0.800	0.966	1208.53	52.84	47	496	617	70.6	4.75
D3Z2Z6	Probable phospholipid-transporting ATPase IB (Fragment) OS=Mus musculus GN=Atp8a2 PE=2 SV=1 - [D3Z2Z6_MOUSE]	45.21	1	1	3	10	1.034	0.744	0.863	0.966	31.10	45.21	6	10	73	8.0	9.72
Q8VCY6	U3 small nucleolar RNA-associated protein 6 homolog OS=Mus musculus GN=Utp6 PE=2 SV=1 - [UTP6_MOUSE]	2.18	1	1	1	2	0.877	0.820	0.742	0.966	5.36	2.18	2	2	597	70.4	7.71
Q9JKC7	AP-4 complex subunit mu-1 OS=Mus musculus GN=Ap4m1 PE=2 SV=1 - [AP4M1_MOUSE]	7.57	3	3	3	5	0.846	0.768	0.788	0.966	14.96	7.57	5	5	449	49.5	6.64
Q9JLV1	BAG family molecular chaperone regulator 3 OS=Mus musculus GN=Bag3 PE=1 SV=2 - [BAG3_MOUSE]	35.18	1	15	15	56	2.540	0.893	1.049	0.966	162.80	35.18	27	56	577	61.8	7.27
J3QMM7	ATP-dependent (S)-NAD(P)H-hydrate dehydratase OS=Mus musculus GN=Carkd PE=3 SV=1 - [J3QMM7_MOUSE]	52.91	7	12	12	131	1.594	1.031	0.825	0.966	392.10	52.91	23	131	327	35.1	8.13
Q9DBT3	Coiled-coil domain-containing protein 97 OS=Mus musculus GN=Ccdc97 PE=1 SV=1 - [CCD97_MOUSE]	19.41	1	5	5	11	0.826	0.877	1.074	0.967	37.58	19.41	8	11	340	38.7	4.60
Q8JZW4	Copine-5 OS=Mus musculus GN=Cpne5 PE=2 SV=1 - [CPNE5_MOUSE]	33.05	2	3	17	124	1.313	1.605	1.421	0.967	340.37	33.05	30	124	593	65.6	5.77
P83940	Transcription elongation factor B polypeptide 1 OS=Mus musculus GN=Tceb1 PE=1 SV=1 - [ELOC_MOUSE]	66.07	1	7	7	51	0.997	0.939	1.018	0.967	199.60	66.07	12	51	112	12.5	4.78
H3BK39	Rap guanine nucleotide exchange factor 3 OS=Mus musculus GN=Rapgef3 PE=2 SV=1 - [H3BK39_MOUSE]	6.11	5	3	6	17	0.996	0.841	0.814	0.967	46.33	6.11	11	17	884	100.1	8.07
O88384	Vesicle transport through interaction with t-SNAREs homolog 1B OS=Mus musculus GN=Vti1b PE=1 SV=1 - [VTI1B_MOUSE]	40.95	5	8	8	45	0.911	1.009	0.975	0.967	145.06	40.95	13	45	232	26.7	8.79
Q6ZWQ5	Sorting nexin 12, isoform CRA_c OS=Mus musculus GN=Snx12 PE=2 SV=1 - [Q6ZWQ5_MOUSE]	61.11	4	8	10	44	0.956	1.015	1.064	0.967	113.11	61.11	19	44	162	18.9	8.44
Q9WVK4	EH domain-containing protein 1 OS=Mus musculus GN=Ehd1 PE=1 SV=1 - [EHD1_MOUSE]	61.42	1	19	31	184	0.813	1.041	0.918	0.967	554.12	61.42	58	184	534	60.6	6.83

Q8BH48-2	Isoform 2 of Ubiquitin-associated protein 1 OS=Mus musculus GN=Ubap1 -	22.90	3	6	7	30	1.890	1.029	1.343	0.967	76.84	22.90	12	30	441	48.2	5.07
Q99P88	Nuclear pore complex protein Nup155 OS=Mus musculus GN=Nup155 PE=2 SV=1 - [NU155_MOUSE]	17.54	7	19	20	56	0.787	1.061	0.890	0.967	154.90	17.54	33	56	1391	155.0	6.15
Q3UMR5	Calcium uniporter protein, mitochondrial OS=Mus musculus GN=Mcu PE=1 SV=2 - [MCU_MOUSE]	31.71	2	10	10	49	0.848	1.438	1.150	0.967	153.05	31.71	19	49	350	39.7	8.56
B1AZP2-2	Isoform 2 of Disks large-associated protein 4 OS=Mus musculus GN=Dlgap4 -	30.84	9	16	25	133	0.581	1.101	1.522	0.967	366.67	30.84	45	133	989	107.5	7.30
Q9QZ23	NFU1 iron-sulfur cluster scaffold homolog, mitochondrial OS=Mus musculus GN=Nfu1 PE=1 SV=2 - [NFU1_MOUSE]	28.63	1	2	6	42	2.028	1.096	1.247	0.967	121.85	28.63	10	42	255	28.5	5.03
P30416	Peptidyl-prolyl cis-trans isomerase FKBP4 OS=Mus musculus GN=Fkbp4 PE=1 SV=5 - [FKBP4_MOUSE]	44.32	4	20	20	114	1.253	0.830	0.864	0.967	300.89	44.32	35	114	458	51.5	5.72
Q61285	ATP-binding cassette sub-family D member 2 OS=Mus musculus GN=Abcd2 PE=1 SV=1 - [ABCD2_MOUSE]	11.07	2	6	6	10	1.276	0.680	0.821	0.967	31.61	11.07	8	10	741	83.4	9.09
F7BPE8	Protein Zkscan2 (Fragment) OS=Mus musculus GN=Zkscan2 PE=4 SV=1 -	14.29	1	1	1	1	0.353	1.304	0.698	0.967	2.34	14.29	1	1	42	4.8	8.75
Q5NCP6	Peripheral-type benzodiazepine receptor-associated protein 1 OS=Mus musculus GN=Bzrap1 PE=2 SV=1 - [Q5NCP6_MOUSE]	2.07	7	2	4	11	0.899	0.611	1.130	0.967	24.89	2.07	5	11	1786	193.3	5.19
Q91V14	Solute carrier family 12 member 5 OS=Mus musculus GN=Slc12a5 PE=1 SV=2 -	28.21	3	28	34	334	0.629	0.903	1.062	0.967	1051.56	28.21	59	334	1138	126.2	6.74
E9Q3Y4	Lipopolysaccharide-responsive and beige-like anchor protein OS=Mus musculus GN=Lrba PE=2 SV=1 - [E9Q3Y4_MOUSE]	11.91	4	22	29	78	0.714	1.019	1.090	0.967	218.11	11.91	48	78	2854	316.6	5.54
P28481-4	Isoform 4 of Collagen alpha-1(II) chain OS=Mus musculus GN=Col2a1 - [CO2A1_MOUSE]	17.35	7	11	11	14	0.785	3.578	1.288	0.967	33.17	17.35	13	14	1458	138.9	7.09
E9QMY1	OX-2 membrane glycoprotein OS=Mus musculus GN=Cd200 PE=2 SV=1 -	23.05	5	7	7	47	0.664	1.234	0.866	0.967	124.63	23.05	14	47	269	30.3	8.51
Q99J27	Acetyl-coenzyme A transporter 1 OS=Mus musculus GN=Slc33a1 PE=1 SV=1 -	12.36	1	6	6	12	0.770	0.787	1.068	0.967	32.84	12.36	9	12	550	61.0	8.16
Q9EQQ9-3	Isoform 3 of Bifunctional protein NCOAT OS=Mus musculus GN=Mgea5 - [NCOAT_MOUSE]	32.49	3	26	27	78	0.942	1.225	1.007	0.967	203.59	32.49	42	78	954	107.3	5.12
Q91XY5	Protein Pcdhgb6 OS=Mus musculus GN=Pcdhga3 PE=2 SV=1 - [Q91XY5_MOUSE]	11.64	1	4	7	24	1.502	1.115	1.345	0.967	69.03	11.64	11	24	928	100.1	4.98
O35691	Pinin OS=Mus musculus GN=Pnn PE=1 SV=4 - [PININ_MOUSE]	15.31	2	12	12	26	0.737	0.845	0.963	0.967	76.56	15.31	19	26	725	82.4	7.01
P35585	AP-1 complex subunit mu-1 OS=Mus musculus GN=Ap1m1 PE=1 SV=3 - [AP1M1_MOUSE]	48.46	2	15	18	91	0.738	0.879	0.946	0.968	224.59	48.46	32	91	423	48.5	7.30
P45700	Mannosyl-oligosaccharide 1,2-alpha-mannosidase IA OS=Mus musculus GN=Man1a1 PE=1 SV=1 - [MA1A1_MOUSE]	2.75	1	2	2	4	1.282	0.762	0.820	0.968	7.10	2.75	4	4	655	73.2	6.81
Q9R0Q7	Prostaglandin E synthase 3 OS=Mus musculus GN=Ptges3 PE=1 SV=1 - [TEBP_MOUSE]	51.25	2	9	9	96	1.266	0.825	1.102	0.968	285.03	51.25	18	96	160	18.7	4.55
Q80Y17	Lethal(2) giant larvae protein homolog 1 OS=Mus musculus GN=Llgl1 PE=1 SV=1 - [L2GL1_MOUSE]	36.39	1	27	28	117	0.897	0.464	0.819	0.968	404.29	36.39	49	117	1036	112.5	6.48
E9PUF7	Rho guanine nucleotide exchange factor 1 OS=Mus musculus GN=Arhgef1 PE=2 SV=1 - [E9PUF7_MOUSE]	18.65	7	14	14	36	0.915	0.995	0.855	0.968	110.52	18.65	24	36	976	108.6	6.09
Q3UXZ6	Protein FAM81A OS=Mus musculus GN=Fam81a PE=2 SV=2 - [FA81A_MOUSE]	44.23	5	14	15	34	0.794	5.093	1.719	0.968	88.54	44.23	24	34	364	41.7	8.75
P32921	Tryptophan--tRNA ligase, cytoplasmic OS=Mus musculus GN=Wars PE=1 SV=2 - [SYWC_MOUSE]	48.65	1	1	22	181	0.457	0.973	0.921	0.968	519.96	48.65	38	181	481	54.3	6.89
E9PUM9	Protein Nrxn2 OS=Mus musculus GN=Nrxn2 PE=2 SV=1 - [E9PUM9_MOUSE]	26.48	8	31	38	158	0.752	0.993	0.845	0.968	498.14	26.48	70	158	1703	183.9	5.82

Q80ZX0	Protein Sec24b OS=Mus musculus GN=Sec24b PE=2 SV=1 - [Q80ZX0_MOUSE]	19.58	3	18	18	93	1.011	0.950	1.014	0.968	277.43	19.58	32	93	1251	135.5	6.96
Q8BGD8	Cytochrome c oxidase assembly factor 6 homolog OS=Mus musculus GN=Coa6 PE=1 SV=1 - [COA6_MOUSE]	46.84	1	4	4	21	1.759	1.119	1.240	0.968	70.08	46.84	6	21	79	9.3	8.16
Q91YT2	E3 ubiquitin-protein ligase RNF185 OS=Mus musculus GN=Rnf185 PE=2 SV=1 - [RN185_MOUSE]	3.65	2	1	1	2	0.607	1.042	0.890	0.968	4.35	3.65	1	2	192	20.5	6.52
Q91XQ0-2	Isoform 2 of Dynein heavy chain 8, axonemal OS=Mus musculus GN=Dnahc8 -	2.38	3	7	8	9	0.827	0.530	0.936	0.968	21.03	2.38	8	9	4202	479.8	6.13
Q9EPL8	Importin-7 OS=Mus musculus GN=Ipo7 PE=1 SV=2 - [IPO7_MOUSE]	22.25	1	19	19	72	0.600	1.113	0.809	0.968	215.41	22.25	35	72	1038	119.4	4.82
G3UW89	Zinc finger protein 423 OS=Mus musculus GN=Zfp423 PE=4 SV=1 - [G3UW89_MOUSE]	0.86	3	1	1	1	0.701	1.229	0.741	0.968	2.05	0.86	1	1	1167	131.5	7.42
E9Q9Q2	Protein R3hdm1 OS=Mus musculus GN=R3hdm1 PE=2 SV=1 - [E9Q9Q2_MOUSE]	11.37	1	7	9	21	1.073	1.054	0.981	0.968	65.82	11.37	13	21	1135	124.3	8.70
G3UYK7	Protein Gm20537 (Fragment) OS=Mus musculus GN=Gm20537 PE=4 SV=1 -	24.74	2	2	2	11	1.317	1.054	1.093	0.968	22.43	24.74	3	11	97	10.3	10.05
E9PUM4	Talin-2 OS=Mus musculus GN=Tln2 PE=4 SV=1 - [E9PUM4_MOUSE]	52.83	2	88	109	513	0.674	1.005	0.930	0.968	1587.14	52.83	189	513	2542	271.5	5.55
Q9D074	E3 ubiquitin-protein ligase MGRN1 OS=Mus musculus GN=Mgrn1 PE=1 SV=2 - [MGRN1_MOUSE]	16.92	5	5	7	17	1.000	1.006	0.859	0.968	49.34	16.92	11	17	532	58.4	4.88
B1AZI6	THO complex subunit 2 OS=Mus musculus GN=Thoc2 PE=2 SV=1 - [THOC2_MOUSE]	6.90	3	8	10	17	1.950	0.925	1.098	0.968	31.00	6.90	14	17	1594	182.7	8.44
Q3UMT1	Protein phosphatase 1 regulatory subunit 12C OS=Mus musculus GN=Ppp1r12c PE=1 SV=1 [PP12C_MOUSE]	34.65	2	19	19	81	1.201	0.711	1.177	0.968	241.26	34.65	35	81	782	84.6	6.00
Q8VEJ4	Notchless protein homolog 1 OS=Mus musculus GN=Nle1 PE=2 SV=4 - [NLE1_MOUSE]	12.37	1	4	4	7	2.018	1.024	0.986	0.968	17.56	12.37	5	7	485	53.1	7.17
O35594	Intraflagellar transport protein 81 homolog OS=Mus musculus GN=Ift81 PE=2 SV=4 - [IFT81_MOUSE]	15.24	3	9	10	25	1.062	0.971	0.914	0.968	76.41	15.24	16	25	676	79.2	8.75
Q9QXG4	Acetyl-coenzyme A synthetase, cytoplasmic OS=Mus musculus GN=Acss2 PE=1 SV=2 - [ACSA_MOUSE]	19.26	5	12	12	48	0.919	0.558	0.813	0.968	121.68	19.26	21	48	701	78.8	6.64
Q8BGT8	Phytanoyl-CoA hydroxylase interacting protein-like OS=Mus musculus GN=Phyhipl PE=2 SV=1 - [PHIPL_MOUSE]	49.07	5	17	19	215	0.954	0.875	0.892	0.968	569.64	49.07	36	215	375	42.3	6.35
Q9JM13	Rab5 GDP/GTP exchange factor OS=Mus musculus GN=Rabgef1 PE=1 SV=1 - [RABX5_MOUSE]	38.49	3	18	18	49	1.032	1.045	1.000	0.968	135.11	38.49	29	49	491	56.8	6.79
O35852	Preprodynorphin (Fragment) OS=Mus musculus GN=Pdyn PE=2 SV=1 - [O35852_MOUSE]	22.58	2	4	4	11	1.246	0.780	1.154	0.968	37.20	22.58	7	11	248	28.0	5.63
Q45VK7	Cytoplasmic dynein 2 heavy chain 1 OS=Mus musculus GN=Dync2h1 PE=1 SV=1 -	5.16	5	20	20	38	0.713	1.000	0.777	0.968	86.25	5.16	29	38	4306	492.0	6.60
P40936	Indolethylamine N-methyltransferase OS=Mus musculus GN=Inmt PE=1 SV=1 - [INMT_MOUSE]	14.39	1	3	3	6	3.539	1.139	1.128	0.968	16.21	14.39	5	6	264	29.4	6.39
E9PXE2	Guanine nucleotide exchange factor DBS OS=Mus musculus GN=Mcf2l PE=2 SV=1 -	19.23	16	16	17	53	1.002	0.910	0.932	0.968	157.06	19.23	28	53	1175	132.3	6.27
Q99LR1	Monoacylglycerol lipase ABHD12 OS=Mus musculus GN=Abhd12 PE=1 SV=2 -	43.47	4	14	14	64	0.650	0.809	0.942	0.968	192.52	43.47	27	64	398	45.2	8.72
E9Q9J4	Inositol hexakisphosphate and diphosphoinositol-pentakisphosphate kinase 2 OS=Mus musculus GN=Ppip5k2 PE=2 SV=1 - [E9Q9J4_MOUSE]	21.01	5	14	20	53	1.059	0.443	0.834	0.968	152.74	21.01	29	53	1242	140.3	8.22
Q8VI33	Transcription initiation factor TFIID subunit 9 OS=Mus musculus GN=Taf9 PE=2 SV=1 - [TAF9_MOUSE]	18.94	2	2	4	9	1.048	0.836	1.010	0.969	20.86	18.94	7	9	264	29.0	7.91
O35075	Down syndrome critical region protein 3 homolog OS=Mus musculus GN=Dscr3 PE=2 SV=1 - [DSCR3_MOUSE]	16.16	1	3	3	5	2.295	1.139	0.851	0.969	12.98	16.16	4	5	297	32.9	7.68

Q9CZN4-3	Isoform 3 of Protein shisa-9 OS=Mus musculus GN=Shisa9 - [SHSA9_MOUSE]	19.86	4	8	8	23	0.770	0.937	1.040	0.969	63.07	19.86	16	23	423	46.7	8.62
Q9DCD2	Pre-mRNA-splicing factor SYF1 OS=Mus musculus GN=Xab2 PE=2 SV=1 - [SYF1_MOUSE]	10.29	3	9	9	19	0.895	0.910	0.911	0.969	48.62	10.29	13	19	855	99.9	6.23
Q3TQ29	Pumilio homolog 2 OS=Mus musculus GN=Pum2 PE=2 SV=1 - [Q3TQ29_MOUSE]	11.02	10	6	10	36	1.039	1.043	0.891	0.969	117.38	11.02	16	36	980	105.6	6.86
G3X915	LysM and putative peptidoglycan-binding domain-containing protein 2 OS=Mus musculus GN=Lysmd2 PE=4 SV=1 - [illegible]	35.35	3	5	7	28	1.354	1.080	1.520	0.969	71.14	35.35	13	28	215	23.6	5.52
Q3UP87	Neutrophil elastase OS=Mus musculus GN=Elane PE=2 SV=1 - [ELNE_MOUSE]	10.19	1	1	1	1	7.921	1.187	0.983	0.969	2.44	10.19	1	1	265	28.6	10.01
A2AI21	Glutamate receptor ionotropic, NMDA 1 OS=Mus musculus GN=Grin1 PE=2 SV=1 - [A2AI21_MOUSE]	16.37	10	17	17	56	0.663	1.952	1.297	0.969	158.13	16.37	29	56	959	107.9	8.97
Q6P3A8	2-oxoisovalerate dehydrogenase subunit beta, mitochondrial OS=Mus musculus GN=Bckdhb PE=2 SV=2 - [illegible]	36.67	2	9	9	22	0.655	0.687	0.700	0.969	70.48	36.67	15	22	390	42.9	6.70
Q8C5P7-2	Isoform 2 of Testis development-related protein OS=Mus musculus GN=Tdrp -	8.97	1	1	1	2	1.707	1.470	1.263	0.969	5.69	8.97	2	2	156	16.7	9.31
Q8BYN2	DNA segment, Chr 2, ERATO Doi 435, expressed, isoform CRA_e OS=Mus musculus GN=Tubgcp4 PE=2 SV=1 - [illegible]	9.16	3	3	5	14	2.143	2.847	1.059	0.969	38.40	9.16	7	14	666	76.0	6.65
Q4VA55	PWWP domain-containing protein MUM1L1 OS=Mus musculus GN=Mum1l1 PE=1 SV=2 - [MUML1_MOUSE]	4.26	3	2	2	2	1.012	1.228	2.047	0.969	0.00	4.26	2	2	681	76.9	4.93
P58059	28S ribosomal protein S21, mitochondrial OS=Mus musculus GN=Mrps21 PE=2 SV=1 - [RT21_MOUSE]	48.28	3	4	4	8	0.589	0.587	0.894	0.970	23.80	48.28	6	8	87	10.6	10.32
Q9Z1B8	PHD finger protein 1 OS=Mus musculus GN=Phf1 PE=1 SV=2 - [PHF1_MOUSE]	9.12	1	2	2	5	0.580	0.669	0.821	0.970	7.38	9.12	3	5	559	61.1	8.84
E9Q3H6	Protein Coq10a OS=Mus musculus GN=Coq10a PE=2 SV=1 - [E9Q3H6_MOUSE]	22.39	4	6	6	16	0.551	0.906	0.871	0.970	40.30	22.39	10	16	259	28.9	9.82
Q99N84	28S ribosomal protein S18b, mitochondrial OS=Mus musculus GN=Mrps18b PE=2 SV=1 - [RT18B_MOUSE]	20.08	5	4	4	13	1.189	0.778	0.732	0.970	43.10	20.08	6	13	254	28.7	8.35
P55772	Ectonucleoside triphosphate diphosphohydrolase 1 OS=Mus musculus GN=Entpd1 PE=2 SV=1 - [illegible]	22.55	5	8	8	24	1.176	1.453	1.072	0.970	64.75	22.55	14	24	510	57.2	5.86
Q80XJ3	Tetratricopeptide repeat protein 28 OS=Mus musculus GN=Ttc28 PE=2 SV=3 - [TTC28_MOUSE]	2.49	1	5	5	12	1.576	1.075	1.164	0.970	29.61	2.49	8	12	2450	267.3	7.15
P58686	UPF0184 protein C9orf16 homolog OS=Mus musculus PE=2 SV=1 - [CI016_MOUSE]	30.12	1	2	2	5	2.184	1.486	0.901	0.970	13.71	30.12	2	5	83	9.0	4.26
Q9CYH6	Ribosome biogenesis regulatory protein homolog OS=Mus musculus GN=Rrs1 PE=2 SV=1 - [RRS1_MOUSE]	18.08	1	6	7	11	0.907	0.974	0.940	0.970	23.62	18.08	9	11	365	41.5	10.77
P61166	Transmembrane protein 258 OS=Mus musculus GN=Tmem258 PE=2 SV=1 - [TM258_MOUSE]	10.13	1	1	1	2	1.040	1.060	0.931	0.970	4.51	10.13	2	2	79	9.1	5.83
Q01149	Collagen alpha-2(I) chain OS=Mus musculus GN=Col1a2 PE=2 SV=2 - [CO1A2_MOUSE]	27.26	2	20	20	75	0.995	4.268	1.008	0.970	222.82	27.26	29	75	1372	129.5	9.19
P50295	Arylamine N-acetyltransferase 2 OS=Mus musculus GN=Nat2 PE=1 SV=1 -	16.55	3	4	4	8	1.894	1.338	0.902	0.970	15.85	16.55	5	8	290	33.7	5.88
Q8BXC6	COMM domain-containing protein 2 OS=Mus musculus GN=Commd2 PE=1 SV=1 -	13.07	2	3	3	11	0.880	1.556	0.848	0.970	25.28	13.07	5	11	199	22.8	6.62
Q8BL65-3	Isoform 3 of Actin-binding LIM protein 2 OS=Mus musculus GN=Ablim2 - [ABLM2_MOUSE]	49.01	7	1	23	165	4.585	1.409	1.403	0.970	530.91	49.01	42	165	606	67.5	8.13
Q3TCX3-2	Isoform 2 of UPF0469 protein KIAA0907 OS=Mus musculus GN=Kiaa0907 - [K0907_MOUSE]	7.39	2	2	2	4	1.546	1.531	1.581	0.970	16.46	7.39	2	4	487	51.2	9.23
Q9D023	Mitochondrial pyruvate carrier 2 OS=Mus musculus GN=Mpc2 PE=1 SV=1 - [MPC2_MOUSE]	21.26	3	3	4	25	0.398	0.662	0.849	0.970	46.86	21.26	7	25	127	14.3	10.61

J3QP81	CLIP-associating protein 1 OS=Mus musculus GN=Clasp1 PE=4 SV=1 - [J3QP81_MOUSE]	41.74	3	1	52	213	0.379	0.364	0.893	0.970	641.97	41.74	92	213	1459	160.8	8.68
Q9JK83	Partitioning defective 6 homolog beta OS=Mus musculus GN=Pard6b PE=1 SV=2 -	9.97	2	3	3	5	1.335	1.197	1.256	0.970	12.10	9.97	5	5	371	41.0	5.59
Q9CZU3	Superkiller viralicidic activity 2-like 2 OS=Mus musculus GN=Skiv2l2 PE=2 SV=1 -	10.58	1	10	10	20	1.130	1.076	0.962	0.970	55.90	10.58	17	20	1040	117.6	6.40
Q8VC85	U6 snRNA-associated Sm-like protein LSm1 OS=Mus musculus GN=Lsm1 PE=2 SV=1 - [LSM1_MOUSE]	24.81	1	2	2	4	2.817	1.321	1.053	0.970	9.25	24.81	3	4	133	15.2	5.38
Q8K209	G-protein coupled receptor 56 OS=Mus musculus GN=Gpr56 PE=1 SV=1 - [GPR56_MOUSE]	14.99	1	10	10	44	1.242	0.737	0.650	0.970	122.71	14.99	17	44	687	77.2	8.59
Q62283	Tetraspanin-7 OS=Mus musculus GN=Tspan7 PE=2 SV=2 - [TSN7_MOUSE]	14.46	2	3	3	9	0.674	1.010	1.250	0.970	18.99	14.46	3	9	249	27.5	7.20
L7N1Y6	Protein Olfr889 OS=Mus musculus GN=Olfr889 PE=3 SV=1 - [L7N1Y6_MOUSE]	3.18	1	1	1	1	20.429	1.882	1.875	0.970	2.31	3.18	1	1	314	35.1	7.81
Q9QYC0-2	Isoform 2 of Alpha-adducin OS=Mus musculus GN=Add1 - [ADDA_MOUSE]	56.33	6	1	26	267	0.878	0.794	1.005	0.970	721.91	56.33	46	267	632	69.8	6.51
Q8BFV3	Dual specificity protein phosphatase 4 OS=Mus musculus GN=Dusp4 PE=2 SV=1 - [DUS4_MOUSE]	3.27	1	1	1	1	2.081	1.523	1.455	0.970	3.04	3.27	1	1	398	43.3	7.55
Q61830	Macrophage mannose receptor 1 OS=Mus musculus GN=Mrc1 PE=1 SV=2 - [MRC1_MOUSE]	3.98	1	5	5	11	0.819	1.379	0.951	0.970	31.53	3.98	9	11	1456	164.9	6.83
Q9JI90	E3 ubiquitin-protein ligase RNF14 OS=Mus musculus GN=Rnf14 PE=2 SV=2 - [RNF14_MOUSE]	15.05	3	7	7	25	0.984	1.024	0.999	0.971	71.71	15.05	12	25	485	54.9	4.70
P39447	Tight junction protein ZO-1 OS=Mus musculus GN=Tjp1 PE=1 SV=2 - [ZO1_MOUSE]	31.75	1	2	43	186	2.598	1.638	1.146	0.971	532.23	31.75	73	186	1745	194.6	6.64
Q923U0	TOM1-like protein 1 OS=Mus musculus GN=Tom1l1 PE=1 SV=1 - [TM1L1_MOUSE]	16.88	5	5	6	19	1.121	1.113	0.892	0.971	52.14	16.88	11	19	474	52.7	5.36
Q8CIH9	Amidophosphoribosyltransferase OS=Mus musculus GN=Ppat PE=2 SV=1 - [Q8CIH9_MOUSE]	26.69	1	13	13	26	1.290	1.113	0.880	0.971	58.99	26.69	21	26	517	57.4	6.96
E9QP59	Inner nuclear membrane protein Man1 OS=Mus musculus GN=Lemd3 PE=2 SV=1 - [E9QP59_MOUSE]	24.84	4	16	16	32	0.787	0.765	1.024	0.971	87.75	24.84	25	32	918	100.1	7.55
Q91YN5-2	Isoform AGX1 of UDP-N-acetylhexosamine pyrophosphorylase OS=Mus musculus GN=Uap1 - [UAP1_MOUSE]	23.56	4	10	10	27	0.940	0.978	1.027	0.971	81.66	23.56	16	27	505	56.9	6.54
P59913	Protein-L-isoaspartate O-methyltransferase domain-containing protein 1 OS=Mus musculus GN=Pcmtd1 PE=2 SV=1 - [PCMD1_MOUSE]	10.64	1	4	5	13	0.926	0.663	1.075	0.971	26.70	10.64	9	13	357	40.7	5.66
Q9DCF9-2	Isoform 2 of Translocon-associated protein subunit gamma OS=Mus musculus GN=Ssr3 - [SSRG_MOUSE]	12.29	2	2	2	3	1.091	0.813	0.800	0.971	5.41	12.29	2	3	179	20.7	9.35
Q8CFC7-2	Isoform 2 of CLK4-associating serine/arginine rich protein OS=Mus musculus GN=Clasrp - [CLASR_MOUSE]	1.36	3	1	1	1	0.410	0.966	1.032	0.971	0.00	1.36	1	1	588	65.9	9.98
P63080	Gamma-aminobutyric acid receptor subunit beta-3 OS=Mus musculus GN=Gabrb3 PE=2 SV=1 - [GBRB3_MOUSE]	14.16	3	5	7	34	0.690	1.124	1.124	0.971	113.65	14.16	12	34	473	54.1	9.10
E9Q1P0	Protein Olfr739 OS=Mus musculus GN=Olfr739 PE=3 SV=1 - [E9Q1P0_MOUSE]	6.15	2	1	1	1	1.494	1.007	0.868	0.971	0.00	6.15	1	1	309	34.7	8.22
Q5NCX5-2	Isoform 2 of Neuralized-like protein 4 OS=Mus musculus GN=Neurl4 - [NEUL4_MOUSE]	6.81	7	7	8	11	1.660	1.139	0.860	0.971	31.49	6.81	11	11	1541	165.1	5.96
E9QKZ2	Importin-9 OS=Mus musculus GN=Ipo9 PE=2 SV=1 - [E9QKZ2_MOUSE]	17.60	6	16	16	43	0.689	1.061	0.795	0.971	127.39	17.60	28	43	1040	115.9	4.82
E9Q1P8	Interferon regulatory factor 2-binding protein 2 OS=Mus musculus GN=Irf2bp2 PE=1 SV=1 - [I2BP2_MOUSE]	33.16	1	11	12	33	1.457	0.959	1.164	0.971	120.41	33.16	20	33	570	59.3	8.69

P97450	ATP synthase-coupling factor 6, mitochondrial OS=Mus musculus GN=Atp5j PE=1 SV=1 - [ATP5J_MOUSE]	46.30	2	6	6	83	1.930	1.002	1.347	0.971	219.90	46.30	12	83	108	12.5	9.36
Q6PGE7	Sodium-dependent proline transporter OS=Mus musculus GN=Slc6a7 PE=2 SV=1 - [SC6A7_MOUSE]	7.85	1	3	3	22	0.563	1.385	0.986	0.972	60.43	7.85	6	22	637	71.0	6.61
Q8VEG4	Exonuclease 3'-5' domain-containing protein 2 OS=Mus musculus GN=Exd2 PE=2 SV=1 - [EXD2_MOUSE]	14.52	2	6	6	19	0.664	0.722	1.037	0.972	50.79	14.52	9	19	496	56.7	8.16
Q8CBC4	Consortin OS=Mus musculus GN=Cnst PE=1 SV=1 - [CNST_MOUSE]	24.75	3	10	10	24	1.327	0.881	1.080	0.972	76.38	24.75	14	24	711	76.8	4.59
Q8JZN7	Mitochondrial Rho GTPase 2 OS=Mus musculus GN=Rhot2 PE=2 SV=1 - [MIRO2_MOUSE]	21.94	4	9	11	40	1.007	0.930	0.973	0.972	121.14	21.94	20	40	620	69.0	6.00
Q5DTY9	BTB/POZ domain-containing protein KCTD16 OS=Mus musculus GN=Kctd16 PE=1 SV=2 - [KCD16_MOUSE]	28.57	1	12	14	77	0.575	1.254	1.270	0.972	224.94	28.57	26	77	427	48.9	8.19
Q80SY5	Pre-mRNA-splicing factor 38B OS=Mus musculus GN=Prpf38b PE=1 SV=1 - [PR38B_MOUSE]	6.09	1	3	3	6	0.540	0.581	0.871	0.972	12.17	6.09	6	6	542	63.7	10.54
P62812	Gamma-aminobutyric acid receptor subunit alpha-1 OS=Mus musculus GN=Gabra1 PE=1 SV=1 - [GBRA1_MOUSE]	31.87	2	12	18	104	0.484	0.687	1.115	0.972	277.74	31.87	34	104	455	51.7	9.31
Q8VEB3	UNC119-binding protein C5orf30 homolog OS=Mus musculus GN=D1Ertd622e PE=2 SV=1 - [CE030_MOUSE]	9.66	2	2	2	3	1.408	1.000	0.905	0.972	7.07	9.66	3	3	207	23.1	9.45
Q8R332-4	Isoform 4 of Nucleoporin p58/p45 OS=Mus musculus GN=Nupl1 - [NUPL1_MOUSE]	14.66	4	7	7	15	1.193	1.024	0.993	0.972	36.05	14.66	11	15	532	54.0	8.31
E0CYX7	FXYD domain-containing ion transport regulator 5 (Fragment) OS=Mus musculus GN=Fxyd5 PE=2 SV=1 - [E0CYX7_MOUSE]	14.41	5	1	1	2	0.998	2.207	0.998	0.972	8.57	14.41	2	2	111	11.7	8.19
Q80ZE5	Membrane progestin receptor beta OS=Mus musculus GN=Paqr8 PE=2 SV=2 - [MPRB_MOUSE]	5.65	1	2	2	3	1.181	1.297	1.645	0.972	4.17	5.65	3	3	354	40.4	8.15
Q8R4I7	Neuropilin and tolloid-like protein 1 OS=Mus musculus GN=Neto1 PE=1 SV=2 - [NETO1_MOUSE]	16.70	1	6	6	12	1.125	1.296	1.453	0.972	43.42	16.70	9	12	533	60.2	6.99
Q9DC53	Copine-8 OS=Mus musculus GN=Cpne8 PE=2 SV=2 - [CPNE8_MOUSE]	23.94	3	3	12	89	1.792	1.305	1.048	0.972	245.23	23.94	21	89	564	63.1	5.96
F7CTH4	Protein Zfp811 OS=Mus musculus GN=Zfp811 PE=4 SV=1 - [F7CTH4_MOUSE]	7.80	1	3	3	3	1.238	1.145	1.253	0.972	2.69	7.80	3	3	551	62.8	9.31
Q78ZM0	Sorting nexin 3 OS=Mus musculus GN=Snx3 PE=2 SV=1 - [Q78ZM0_MOUSE]	55.56	4	8	10	43	1.204	0.916	0.985	0.972	109.78	55.56	18	43	162	18.8	8.66
A2AKI5	Integrin alpha-V light chain OS=Mus musculus GN=Itgav PE=2 SV=1 - [A2AKI5_MOUSE]	36.41	4	33	33	128	0.897	1.614	0.973	0.972	353.18	36.41	61	128	1008	111.4	5.67
Q8BHN5	RNA-binding protein 45 OS=Mus musculus GN=Rbm45 PE=2 SV=1 - [RBM45_MOUSE]	3.36	1	2	2	2	1.334	0.924	0.971	0.972	2.46	3.36	2	2	476	53.3	7.69
Q8R0X7	Sphingosine-1-phosphate lyase 1 OS=Mus musculus GN=Sgpl1 PE=2 SV=1 - [SGPL1_MOUSE]	16.37	5	9	10	17	0.704	0.852	0.920	0.972	40.24	16.37	14	17	568	63.6	9.10
Q8C996	Transmembrane protein 163 OS=Mus musculus GN=Tmem163 PE=1 SV=1 - [TM163_MOUSE]	20.14	1	4	4	54	1.190	1.141	0.959	0.972	193.00	20.14	6	54	288	31.2	7.93
F6ZGI7	Molybdopterin synthase catalytic subunit OS=Mus musculus GN=Mocs2 PE=4 SV=1 - [F6ZGI7_MOUSE]	45.45	2	4	4	13	2.120	0.893	1.040	0.972	27.36	45.45	8	13	88	9.7	4.61
Q9JHG2	Calcipressin-2 OS=Mus musculus GN=Rcan2 PE=2 SV=1 - [RCAN2_MOUSE]	13.20	2	3	3	9	1.899	0.859	0.987	0.972	20.37	13.20	5	9	197	22.0	6.68
D3Z5N5	1-phosphatidylinositol 3-phosphate 5-kinase OS=Mus musculus GN=Pikfyve PE=2 SV=2 - [D3Z5N5_MOUSE]	15.01	4	25	25	51	0.897	1.122	1.098	0.972	139.00	15.01	36	51	2052	231.9	6.64
A8C756	Thyroid adenoma-associated protein homolog OS=Mus musculus GN=Thada PE=2 SV=1 - [THADA_MOUSE]	1.34	3	2	2	3	0.839	0.834	0.956	0.972	5.67	1.34	3	3	1938	217.1	6.25
Q8BMF8	Gliomedin OS=Mus musculus GN=Gldn PE=1 SV=1 - [GLDN_MOUSE]	2.55	1	1	1	1	7.704	1.324	1.457	0.973	2.97	2.55	1	1	549	59.1	8.51

Q8K013	GTP-binding protein 10 OS=Mus musculus GN=Gtpbp10 PE=2 SV=1 - [GTPBA_MOUSE]	14.21	10	4	6	14	0.951	1.013	1.023	0.973	28.42	14.21	10	14	366	40.1	9.14
P51174	Long-chain specific acyl-CoA dehydrogenase, mitochondrial OS=Mus musculus GN=Acadl PE=2 SV=2 - [ACADL_MOUSE]	46.98	1	20	20	145	1.889	0.989	0.764	0.973	452.41	46.98	38	145	430	47.9	8.31
E9QAQ5	Glycogen synthase kinase-3 beta OS=Mus musculus GN=Gsk3b PE=2 SV=1 - [E9QAQ5_MOUSE]	64.43	2	14	21	169	0.614	0.837	0.969	0.973	482.36	64.43	39	169	433	48.0	8.78
Q03173-4	Isoform 3 of Protein enabled homolog OS=Mus musculus GN=Enah - [ENAH_MOUSE]	31.67	8	1	19	122	2.848	1.846	0.684	0.973	296.94	31.67	29	122	783	83.7	7.53
Q9CTY5	Calcium uptake protein 3, mitochondrial OS=Mus musculus GN=Micu3 PE=2 SV=2 - [MICU3_MOUSE]	34.61	2	15	15	56	0.599	1.029	1.027	0.973	196.83	34.61	30	56	523	59.8	7.15
Q9JLR1	Protein transport protein Sec61 subunit alpha isoform 2 OS=Mus musculus GN=Sec61a2 PE=2 SV=3 - [illegible]	14.92	2	4	7	22	0.732	0.562	0.911	0.973	63.33	14.92	13	22	476	52.2	8.06
P52503	NADH dehydrogenase [ubiquinone] iron-sulfur protein 6, mitochondrial OS=Mus musculus GN=Ndufs6 PE=1 SV=2 - [illegible]	62.07	2	7	7	231	1.915	1.000	1.438	0.973	677.55	62.07	14	231	116	13.0	8.65
Q61221-2	Isoform 2 of Hypoxia-inducible factor 1-alpha OS=Mus musculus GN=Hif1a -	3.04	2	2	2	2	0.400	0.672	0.724	0.973	6.18	3.04	2	2	822	91.8	5.30
Q5SW75	Protein phosphatase Slingshot homolog 2 OS=Mus musculus GN=Ssh2 PE=1 SV=2 - [SSH2_MOUSE]	11.88	3	11	11	24	0.959	1.043	1.227	0.973	73.49	11.88	16	24	1423	158.1	5.67
Q7TSQ8	Pyruvate dehydrogenase phosphatase regulatory subunit, mitochondrial OS=Mus musculus GN=Pdpr PE=2 SV=1 - [PDPR_MOUSE]	24.72	3	22	22	60	1.026	0.920	0.875	0.973	178.66	24.72	38	60	878	99.2	6.35
Q6NXH2	Glycoprotein endo-alpha-1,2-mannosidase OS=Mus musculus GN=Manea PE=2 SV=1 - [MANEA_MOUSE]	5.84	1	1	1	4	1.263	0.843	1.012	0.973	15.38	5.84	2	4	462	53.1	8.62
Q8C0E2	Vacuolar protein sorting-associated protein 26B OS=Mus musculus GN=Vps26b PE=1 SV=1 - [VP26B_MOUSE]	49.11	2	17	18	121	0.853	0.966	0.998	0.973	329.48	49.11	33	121	336	39.1	7.37
Q6NZA9	Transcription initiation factor TFIID subunit 9B OS=Mus musculus GN=Taf9b PE=1 SV=2 - [TAF9B_MOUSE]	12.85	3	1	3	13	1.598	1.363	1.123	0.973	33.18	12.85	6	13	249	27.2	9.58
E9PX52	Arf-GAP with SH3 domain, ANK repeat and PH domain-containing protein 2 OS=Mus musculus GN=Asap2 PE=2 SV=1 - [E9PX52_MOUSE]	33.90	6	23	27	134	1.191	0.704	0.995	0.973	366.69	33.90	44	134	1000	111.1	6.80
Q6ZPS6	Ankyrin repeat and IBR domain-containing protein 1 OS=Mus musculus GN=Ankib1 PE=1 SV=2 - [AKIB1_MOUSE]	20.18	1	13	13	33	1.134	1.003	0.964	0.973	123.85	20.18	19	33	1085	121.8	5.16
D3YY36	Protein 1300017J02Rik OS=Mus musculus GN=1300017J02Rik PE=2 SV=1 - [D3YY36_MOUSE]	22.67	3	12	13	40	4.561	1.817	0.577	0.973	100.42	22.67	23	40	622	68.6	7.88
Q8C0L8	Conserved oligomeric Golgi complex subunit 5 OS=Mus musculus GN=Cog5 PE=2 SV=3 - [COG5_MOUSE]	11.94	1	7	7	24	1.016	0.821	0.783	0.974	58.87	11.94	12	24	829	91.3	6.27
Q9QXW4	Fascin-3 OS=Mus musculus GN=Fscn3 PE=2 SV=2 - [FSCN3_MOUSE]	2.61	1	1	1	1	1.441	1.502	1.368	0.974	2.65	2.61	1	1	498	56.4	7.53
Q9D486	C-Maf-inducing protein OS=Mus musculus GN=Cmip PE=2 SV=3 - [CMIP_MOUSE]	13.71	3	9	9	22	0.579	1.278	0.897	0.974	61.21	13.71	16	22	773	86.2	6.81
Q68FL6	Methionine--tRNA ligase, cytoplasmic OS=Mus musculus GN=Mars PE=2 SV=1 - [SYMC_MOUSE]	37.36	1	1	32	107	0.643	0.944	0.947	0.974	285.22	37.36	58	107	902	101.4	7.14
B2RXE2	Sodium/hydrogen exchanger OS=Mus musculus GN=Slc9a5 PE=2 SV=1 -	6.35	1	4	4	12	1.475	1.113	0.873	0.974	33.90	6.35	7	12	898	98.9	7.31
E9PVY0	Serine/threonine-protein kinase MRCK alpha OS=Mus musculus GN=Cdc42bpa PE=2 SV=1 - [E9PVY0_MOUSE]	34.24	12	42	52	156	0.962	0.806	1.031	0.974	418.46	34.24	90	156	1732	196.8	6.62
Q7TMK6	Protein Hook homolog 2 OS=Mus musculus GN=Hook2 PE=1 SV=3 - [HOOK2_MOUSE]	10.47	1	6	6	9	1.214	1.047	1.057	0.974	28.02	10.47	8	9	716	83.3	5.43

P61807	Stannin OS=Mus musculus GN=Snn PE=1 SV=1 - [SNN_MOUSE]	67.05	1	3	3	6	1.458	0.931	1.026	0.974	11.41	67.05	5	6	88	9.5	5.31
Q9CWK3	CD2 antigen cytoplasmic tail-binding protein 2 OS=Mus musculus GN=Cd2bp2 PE=1 SV=1 - [CD2B2_MOUSE]	38.60	1	7	7	17	1.397	1.341	1.228	0.974	51.27	38.60	11	17	342	37.7	4.60
Q6PIP5	NudC domain-containing protein 1 OS=Mus musculus GN=Nudcd1 PE=2 SV=2 -	20.45	1	10	11	32	1.115	0.991	0.899	0.974	71.20	20.45	18	32	582	66.7	5.30
Q3UZV7-2	Isoform 2 of UPF0577 protein KIAA1324-like homolog OS=Mus musculus -	16.77	8	13	13	27	0.941	0.791	0.927	0.974	76.77	16.77	18	27	1014	112.0	6.16
A2AMI2	Eukaryotic translation initiation factor 4 gamma 3 OS=Mus musculus GN=Eif4g3 PE=2 SV=1 - [A2AMI2_MOUSE]	29.70	6	1	39	155	0.983	0.784	0.965	0.974	437.09	29.70	70	155	1579	174.7	5.55
Q9QXZ0-3	Isoform 3 of Microtubule-actin cross-linking factor 1 OS=Mus musculus GN=Macf1 - [MACF1_MOUSE]	46.26	7	1	213	900	0.341	0.754	0.733	0.974	2601.48	46.26	378	900	5270	601.4	5.45
P49813	Tropomodulin-1 OS=Mus musculus GN=Tmod1 PE=2 SV=2 - [TMOD1_MOUSE]	44.29	1	13	15	49	1.597	2.504	2.381	0.974	126.68	44.29	25	49	359	40.4	5.10
Q8BXN7	Protein phosphatase 1K, mitochondrial OS=Mus musculus GN=Ppm1k PE=1 SV=1 - [PPM1K_MOUSE]	9.14	1	3	3	8	0.954	0.837	0.925	0.974	20.50	9.14	6	8	372	40.9	6.39
Q62132-2	Isoform Beta of Receptor-type tyrosine-protein phosphatase R OS=Mus musculus GN=Ptprr - [PTPRR_MOUSE]	12.75	3	7	7	13	0.817	1.517	1.214	0.975	36.65	12.75	10	13	549	61.9	8.12
P15116	Cadherin-2 OS=Mus musculus GN=Cdh2 PE=1 SV=2 - [CADH2_MOUSE]	34.77	2	20	20	97	0.841	1.265	1.193	0.975	255.51	34.77	37	97	906	99.7	4.78
Q8BFS6	Calcineurin-like phosphoesterase domain-containing protein 1 OS=Mus musculus GN=Cpped1 PE=2 SV=1 - [CPPED_MOUSE]	25.32	6	6	6	12	1.645	1.135	1.158	0.975	36.98	25.32	10	12	312	35.2	5.34
Q91V89	Protein Ppp2r5d OS=Mus musculus GN=Ppp2r5d PE=2 SV=1 - [Q91V89_MOUSE]	48.48	1	20	26	113	0.585	0.843	1.006	0.975	344.85	48.48	46	113	594	69.0	7.96
F6S7U1	Protein C030046E11Rik (Fragment) OS=Mus musculus GN=C030046E11Rik PE=4 SV=1 - [F6S7U1_MOUSE]	2.89	4	1	2	3	1.805	1.227	0.912	0.975	8.96	2.89	3	3	1314	146.7	6.00
Q9CYV5	Transmembrane protein 135 OS=Mus musculus GN=Tmem135 PE=2 SV=1 - [TM135_MOUSE]	5.46	3	2	3	4	0.764	0.771	0.894	0.975	11.16	5.46	4	4	458	52.3	9.50
O88597	Beclin-1 OS=Mus musculus GN=Becn1 PE=1 SV=3 - [BECN1_MOUSE]	24.78	5	7	8	17	0.736	1.163	1.167	0.975	49.52	24.78	11	17	448	51.6	4.93
B1AVF4	Midline 2 OS=Mus musculus GN=Mid2 PE=4 SV=1 - [B1AVF4_MOUSE]	10.95	4	6	6	12	1.102	0.915	0.847	0.975	30.18	10.95	8	12	685	77.8	7.03
Q6NVE8	WD repeat-containing protein 44 OS=Mus musculus GN=Wdr44 PE=1 SV=1 -	34.21	2	28	28	74	1.169	1.173	1.195	0.975	189.46	34.21	46	74	915	101.5	5.24
Q8BKR5	Protein phosphatase 1 regulatory subunit 37 OS=Mus musculus GN=Ppp1r37 PE=2 SV=1 - [PPR37_MOUSE]	16.85	1	11	11	29	0.939	1.061	1.121	0.975	68.69	16.85	18	29	712	77.5	5.06
Q8CHY3	Dymeclin OS=Mus musculus GN=Dym PE=2 SV=1 - [DYM_MOUSE]	6.13	4	5	5	12	1.150	1.252	0.817	0.975	26.52	6.13	8	12	669	75.8	5.77
Q9JIA6	Galactokinase OS=Mus musculus GN=Galk1 PE=2 SV=1 - [Q9JIA6_MOUSE]	27.55	2	8	8	36	0.701	1.050	0.817	0.975	124.23	27.55	14	36	392	42.3	5.26
Q8R4V1-3	Isoform 3 of NFAT activation molecule 1 OS=Mus musculus GN=Nfam1 -	12.21	3	2	2	3	0.614	0.934	0.881	0.975	6.29	12.21	3	3	213	24.2	7.77
Q91YK2	Ribosomal RNA processing protein 1 homolog B OS=Mus musculus GN=Rrp1b PE=2 SV=2 - [RRP1B_MOUSE]	5.39	1	2	4	6	0.826	0.527	1.009	0.975	15.39	5.39	5	6	724	80.5	9.72
Q9CWR1	WD repeat-containing protein 73 OS=Mus musculus GN=Wdr73 PE=2 SV=1 -	11.05	4	1	4	10	1.838	1.020	0.936	0.975	27.19	11.05	6	10	371	40.8	5.54
Q99PV0	Pre-mRNA-processing-splicing factor 8 OS=Mus musculus GN=Prpf8 PE=1 SV=2 - [PRP8_MOUSE]	29.42	2	60	60	171	0.724	0.877	0.838	0.975	480.26	29.42	97	171	2335	273.4	8.84
P97770-2	Isoform 2 of THUMP domain-containing protein 3 OS=Mus musculus GN=Thumpd3 - [THUM3_MOUSE]	18.85	4	8	8	15	0.632	0.762	0.805	0.975	46.67	18.85	13	15	504	56.3	7.44

Q8BTY2	Sodium bicarbonate cotransporter 3 OS=Mus musculus GN=Slc4a7 PE=1 SV=2 -	11.41	3	8	11	34	0.678	0.968	1.153	0.975	94.70	11.41	19	34	1034	116.4	6.24
Q7TSG1-2	Isoform 2 of Centrosomal protein of 120 kDa OS=Mus musculus GN=Cep120 -	6.24	3	5	5	13	1.665	0.949	1.053	0.976	26.84	6.24	7	13	929	105.8	6.24
O35127	Protein C10 OS=Mus musculus GN=Grcc10 PE=2 SV=1 - [C10_MOUSE]	76.19	1	6	6	26	1.815	1.190	1.118	0.976	80.21	76.19	12	26	126	13.2	5.14
Q924L1	LETM1 domain-containing protein 1 OS=Mus musculus GN=Letmd1 PE=2 SV=1 -	13.89	3	5	5	15	1.192	1.215	0.943	0.976	53.53	13.89	9	15	360	41.7	10.54
Q99LP6	GrpE protein homolog 1, mitochondrial OS=Mus musculus GN=Grpel1 PE=1 SV=1 - [GRPE1_MOUSE]	44.24	1	10	10	46	1.636	1.111	1.231	0.976	111.51	44.24	17	46	217	24.3	8.38
P42567	Epidermal growth factor receptor substrate 15 OS=Mus musculus GN=Eps15 PE=1 SV=1 - [EPS15_MOUSE]	41.58	6	25	28	123	1.408	0.731	1.039	0.976	346.83	41.58	48	123	897	98.4	4.60
Q9D071-3	Isoform 3 of MMS19 nucleotide excision repair protein homolog OS=Mus musculus GN=Mms19 - [MMS19_MOUSE]	19.74	8	12	12	27	0.668	0.921	0.821	0.976	79.37	19.74	20	27	988	108.2	6.28
O54946	DnaJ homolog subfamily B member 6 OS=Mus musculus GN=Dnajb6 PE=1 SV=4 -	33.42	4	2	10	38	0.997	0.845	0.896	0.976	107.52	33.42	16	38	365	39.8	9.36
Q6P1J0	Glycoprotein endo-alpha-1,2-mannosidase-like protein OS=Mus musculus GN=Maneal PE=2 SV=1 - [MANEL_MOUSE]	4.65	1	2	2	3	1.082	0.965	0.859	0.976	7.79	4.65	3	3	452	51.1	7.21
Q9R0Q6	Actin-related protein 2/3 complex subunit 1A OS=Mus musculus GN=Arpc1a PE=1 SV=1 - [ARC1A_MOUSE]	44.59	2	16	16	107	0.564	1.323	1.165	0.976	260.17	44.59	30	107	370	41.6	8.18
O70582	Arachidonate 12-lipoxygenase, 12R-type OS=Mus musculus GN=Alox12b PE=2 SV=1 - [LX12B_MOUSE]	2.43	1	1	2	2	2.252	0.565	1.884	0.976	2.92	2.43	2	2	701	80.5	6.71
M0QWF3	Protein 0610010F05Rik OS=Mus musculus GN=0610010F05Rik PE=4 SV=1 -	9.40	3	5	6	8	1.771	0.861	1.112	0.976	25.67	9.40	7	8	713	81.5	6.46
F6SMY7	Probable E3 ubiquitin-protein ligase MYCBP2 (Fragment) OS=Mus musculus GN=Mycbp2 PE=2 SV=1 - [illegible]	11.24	6	42	43	81	1.077	0.936	0.942	0.976	178.06	11.24	66	81	4636	509.0	7.09
Q8BNY6	Neuronal calcium sensor 1 OS=Mus musculus GN=Ncs1 PE=2 SV=3 - [NCS1_MOUSE]	64.74	1	12	12	68	0.847	1.283	1.120	0.976	183.99	64.74	22	68	190	21.9	4.83
E9Q6L4	TBC1 domain family member 1 OS=Mus musculus GN=Tbc1d1 PE=2 SV=1 -	7.84	4	5	5	12	1.042	0.999	1.096	0.976	39.23	7.84	7	12	1033	117.2	6.80
E9PZA8	Synaptotagmin-7 OS=Mus musculus GN=Syt7 PE=2 SV=1 - [E9PZA8_MOUSE]	51.50	2	5	25	155	0.477	1.491	1.713	0.976	452.23	51.50	43	155	567	63.1	9.44
Q9CX00	IST1 homolog OS=Mus musculus GN=Ist1 PE=2 SV=1 - [IST1_MOUSE]	26.52	1	9	9	38	0.852	1.394	0.931	0.976	101.31	26.52	17	38	362	39.4	5.44
Q9DBQ7-3	Isoform 3 of Protein-associating with the carboxyl-terminal domain of ezrin OS=Mus musculus GN=Scyl3 - [illegible]	1.66	2	1	1	2	1.172	1.323	0.770	0.976	2.93	1.66	1	2	722	80.0	5.15
Q9EPS2	Peptide YY OS=Mus musculus GN=Pyy PE=2 SV=2 - [PYY_MOUSE]	6.12	2	1	1	1	1.134	1.023	0.732	0.976	1.90	6.12	1	1	98	11.1	5.45
Q7TSS2	Ubiquitin-conjugating enzyme E2 Q1 OS=Mus musculus GN=Ube2q1 PE=2 SV=2 -	26.30	3	8	10	22	0.867	1.054	0.898	0.976	61.26	26.30	17	22	422	46.1	5.10
D3Z108	Transmembrane and ubiquitin-like domain-containing protein 1 (Fragment) OS=Mus musculus GN=Tmub1 PE=2 SV=1 - [D3Z108_MOUSE]	19.61	4	3	3	10	0.928	0.883	0.887	0.977	31.09	19.61	5	10	204	21.6	4.82
P70425	GTP-binding protein Rit2 OS=Mus musculus GN=Rit2 PE=1 SV=1 - [RIT2_MOUSE]	25.81	3	5	5	11	0.634	0.719	0.762	0.977	33.93	25.81	9	11	217	24.8	6.92
Q9D0M5	Dynein light chain 2, cytoplasmic OS=Mus musculus GN=Dynll2 PE=1 SV=1 - [DYL2_MOUSE]	67.42	2	4	6	215	1.420	0.889	0.846	0.977	682.30	67.42	10	215	89	10.3	7.37
E9QAD4	Coiled-coil domain-containing protein 93 OS=Mus musculus GN=Ccdc93 PE=2 SV=1 - [E9QAD4_MOUSE]	32.64	2	19	19	47	1.109	0.850	1.004	0.977	135.73	32.64	31	47	628	72.4	8.29

P98063	Bone morphogenetic protein 1 OS=Mus musculus GN=Bmp1 PE=1 SV=2 - [BMP1_MOUSE]	1.61	1	1	1	4	1.050	0.603	1.624	0.977	10.15	1.61	2	4	991	111.6	6.95
Q6ZQ08	CCR4-NOT transcription complex subunit 1 OS=Mus musculus GN=Cnot1 PE=1 SV=2 - [CNOT1_MOUSE]	17.64	5	35	35	114	0.747	1.022	0.907	0.977	306.46	17.64	61	114	2375	266.6	7.11
Q8CGQ8-2	Isoform 2 of Sodium/potassium/calcium exchanger 4 OS=Mus musculus GN=Slc24a4 - [NCKX4_MOUSE]	12.27	7	7	7	28	0.652	1.725	1.265	0.977	61.46	12.27	12	28	554	61.1	7.93
Q9ES00	Ubiquitin conjugation factor E4 B OS=Mus musculus GN=Ube4b PE=1 SV=3 -	23.70	4	18	20	67	0.738	0.955	0.869	0.977	184.76	23.70	33	67	1173	133.2	6.07
P02463	Collagen alpha-1(IV) chain OS=Mus musculus GN=Col4a1 PE=2 SV=4 - [CO4A1_MOUSE]	9.11	1	7	9	53	1.679	1.198	0.999	0.977	126.86	9.11	14	53	1669	160.6	8.24
Q8BFW4	Tripartite motif-containing protein 65 OS=Mus musculus GN=Trim65 PE=2 SV=1 -	5.75	2	2	3	5	1.731	0.969	1.171	0.977	0.00	5.75	3	5	522	58.4	6.93
Q14BI2	Metabotropic glutamate receptor 2 OS=Mus musculus GN=Grm2 PE=2 SV=2 - [GRM2_MOUSE]	23.28	1	15	21	69	0.499	0.964	1.177	0.977	199.11	23.28	30	69	872	95.8	8.06
Q6ZQ06	Centrosomal protein of 162 kDa OS=Mus musculus GN=Cep162 PE=1 SV=2 -	3.56	2	3	5	13	3.956	1.478	0.606	0.977	29.13	3.56	6	13	1403	160.8	5.53
Q3UHE1-2	Isoform 2 of Membrane-associated phosphatidylinositol transfer protein 3 OS=Mus musculus GN=Pitpnm3 - [PITM3_MOUSE]	16.08	2	11	11	37	0.946	0.974	1.081	0.977	112.54	16.08	18	37	958	104.7	7.12
Q922U1	U4/U6 small nuclear ribonucleoprotein Prp3 OS=Mus musculus GN=Prpf3 PE=1 SV=1 - [PRPF3_MOUSE]	10.98	2	6	7	17	0.923	0.875	1.025	0.977	33.01	10.98	12	17	683	77.4	9.50
Q3U422	NADH dehydrogenase [ubiquinone] flavoprotein 3, mitochondrial OS=Mus musculus GN=Ndufv3 PE=2 SV=1 - [Q3U422_MOUSE]	64.32	1	16	21	94	1.680	0.817	1.335	0.977	201.63	64.32	37	94	468	50.5	8.97
Q91YJ2	Sorting nexin-4 OS=Mus musculus GN=Snx4 PE=2 SV=1 - [SNX4_MOUSE]	52.00	1	21	21	59	0.815	0.995	0.847	0.978	169.27	52.00	37	59	450	51.7	5.80
P84086	Complexin-2 OS=Mus musculus GN=Cplx2 PE=1 SV=1 - [CPLX2_MOUSE]	47.76	2	3	6	89	1.024	1.736	1.749	0.978	225.21	47.76	10	89	134	15.4	5.08
P54923	[Protein ADP-ribosylarginine] hydrolase OS=Mus musculus GN=Adprh PE=2 SV=1 - [ADPRH_MOUSE]	44.48	1	13	13	93	0.936	1.167	0.874	0.978	273.63	44.48	25	93	362	40.0	5.76
Q9DB29	Isoamyl acetate-hydrolyzing esterase 1 homolog OS=Mus musculus GN=Iah1 PE=2 SV=1 - [IAH1_MOUSE]	42.97	1	8	8	26	1.309	0.807	0.749	0.978	67.87	42.97	14	26	249	28.0	5.52
Q8BGR2	Leucine-rich repeat-containing protein 8D OS=Mus musculus GN=Lrrc8d PE=2 SV=1 - [LRC8D_MOUSE]	15.37	1	9	10	18	0.823	1.186	1.052	0.978	60.24	15.37	14	18	859	98.1	7.44
P70279	Surfeit locus protein 6 OS=Mus musculus GN=Surf6 PE=2 SV=1 - [SURF6_MOUSE]	10.14	2	2	4	4	0.560	0.658	1.024	0.978	8.02	10.14	4	4	355	41.2	10.52
E9PV26	Protein BC068157 OS=Mus musculus GN=BC068157 PE=2 SV=1 - [E9PV26_MOUSE]	23.41	3	18	18	74	1.095	1.045	1.376	0.978	245.63	23.41	34	74	1098	110.6	9.98
Q8R3H9	Tetratricopeptide repeat protein 4 OS=Mus musculus GN=Ttc4 PE=2 SV=1 - [TTC4_MOUSE]	8.29	3	4	4	8	1.156	0.890	0.893	0.978	21.04	8.29	7	8	386	44.3	5.22
O55176	E3 ubiquitin-protein ligase Praja-1 OS=Mus musculus GN=Pja1 PE=1 SV=3 - [PJA1_MOUSE]	15.40	6	6	6	16	1.192	0.588	1.305	0.978	41.67	15.40	11	16	578	63.9	4.97
E9QMC2	Metabotropic glutamate receptor 5 OS=Mus musculus GN=Grm5 PE=2 SV=1 - [E9QMC2_MOUSE]	28.69	3	1	31	111	0.738	0.530	1.175	0.978	308.13	28.69	48	111	1171	128.2	7.58
P05480	Neuronal proto-oncogene tyrosine-protein kinase Src OS=Mus musculus GN=Src PE=1 SV=4 - [SRC_MOUSE]	40.48	4	14	20	63	0.777	1.263	0.964	0.978	160.21	40.48	34	63	541	60.6	7.84
P59481	VIP36-like protein OS=Mus musculus GN=Lman2l PE=2 SV=1 - [LMA2L_MOUSE]	28.53	4	8	8	19	0.621	0.762	0.716	0.978	54.49	28.53	14	19	347	39.9	9.41
Q8CBW3-2	Isoform 2 of Abl interactor 1 OS=Mus musculus GN=Abi1 - [ABI1_MOUSE]	45.74	11	12	15	105	0.991	1.031	1.380	0.978	287.27	45.74	28	105	446	48.5	7.06
Q8C180	Fibroblast growth factor receptor substrate 2 OS=Mus musculus GN=Frs2 PE=1 SV=3 - [FRS2_MOUSE]	22.24	1	7	8	25	1.072	1.099	1.231	0.979	76.04	22.24	13	25	508	56.8	6.16

Q61029-3	Isoform Epsilon of Lamina-associated polypeptide 2, isoforms beta/delta/epsilon/gamma OS=Mus musculus GN=Tmpo - [LAP2B_MOUSE]	48.54	5	7	15	101	0.864	0.772	0.883	0.979	310.55	48.54	29	101	412	46.0	9.22
Q99K95	Protein RTF2 homolog OS=Mus musculus GN=Rtfdc1 PE=2 SV=1 - [RTF2_MOUSE]	6.51	1	2	2	3	0.942	0.646	1.269	0.979	6.13	6.51	3	3	307	33.9	8.81
P32261	Antithrombin-III OS=Mus musculus GN=Serpinc1 PE=1 SV=1 - [ANT3_MOUSE]	35.05	1	14	14	49	2.787	2.336	0.543	0.979	158.97	35.05	24	49	465	52.0	6.46
Q6PGN3-3	Isoform 3 of Serine/threonine-protein kinase DCLK2 OS=Mus musculus GN=Dclk2 - [DCLK2_MOUSE]	45.01	6	24	25	92	0.603	0.800	1.033	0.979	301.90	45.01	40	92	771	84.4	8.62
Q80TL7-2	Isoform 2 of Protein MON2 homolog OS=Mus musculus GN=Mon2 - [MON2_MOUSE]	15.93	3	24	25	57	0.760	1.084	0.850	0.979	154.10	15.93	39	57	1708	188.2	6.09
Q9EQN3	TSC22 domain family protein 4 OS=Mus musculus GN=Tsc22d4 PE=2 SV=2 -	43.15	3	7	12	58	1.453	0.984	1.097	0.979	154.05	43.15	21	58	387	40.0	8.13
P60229	Eukaryotic translation initiation factor 3 subunit E OS=Mus musculus GN=Eif3e PE=1 SV=1 - [EIF3E_MOUSE]	35.28	1	14	14	63	0.774	1.099	0.865	0.979	161.54	35.28	24	63	445	52.2	6.04
Q8BGX3	Leucine-rich repeat and transmembrane domain-containing protein 2 OS=Mus musculus GN=Lrtm2 PE=2 SV=1 - [LRTM2_MOUSE]	20.54	1	6	6	18	0.567	1.138	1.065	0.979	58.75	20.54	9	18	370	41.0	6.40
Q3U308	Cytoplasmic tRNA 2-thiolation protein 2 OS=Mus musculus GN=Ctu2 PE=2 SV=1 - [CTU2_MOUSE]	14.59	4	5	5	26	0.720	0.899	0.761	0.979	76.22	14.59	10	26	514	56.1	7.08
P56481	Gastrin/cholecystokinin type B receptor OS=Mus musculus GN=Cckbr PE=2 SV=1 - [GASR_MOUSE]	5.08	1	1	1	2	0.341	2.015	1.990	0.979	3.24	5.08	2	2	453	49.1	9.79
Q8R3Z5-4	Isoform 4 of Voltage-dependent L-type calcium channel subunit beta-1 OS=Mus musculus GN=Cacnb1 - [CACB1_MOUSE]	38.36	3	1	15	73	1.944	1.805	1.471	0.979	246.64	38.36	27	73	524	57.7	6.81
Q8R5A6	TBC1 domain family member 22A OS=Mus musculus GN=Tbc1d22a PE=2 SV=3 -	9.88	2	3	5	10	1.142	1.094	1.267	0.979	24.75	9.88	7	10	516	59.3	6.46
Q570Y9-2	Isoform 2 of DEP domain-containing mTOR-interacting protein OS=Mus musculus GN=Deptor - [DPTOR_MOUSE]	18.14	4	5	5	10	1.013	0.769	1.248	0.979	19.47	18.14	9	10	397	45.2	8.65
Q9D9M5	Pyridoxal phosphate phosphatase PHOSPHO2 OS=Mus musculus GN=Phospho2 PE=2 SV=1 - [PHOP2_MOUSE]	12.03	2	3	3	4	0.905	0.903	0.884	0.979	10.67	12.03	4	4	241	27.5	6.54
Q3UH99	Protein shisa-6 homolog OS=Mus musculus GN=Shisa6 PE=2 SV=1 - [SHSA6_MOUSE]	18.67	1	1	9	31	0.936	0.871	1.148	0.979	70.07	18.67	16	31	525	58.4	9.48
Q9CZW6	E3 ubiquitin-protein ligase RNF146 OS=Mus musculus GN=Rnf146 PE=1 SV=2 - [RN146_MOUSE]	17.55	2	6	6	12	2.172	1.065	1.327	0.979	29.77	17.55	9	12	359	38.9	5.20
Q9ES46	Beta-parvin OS=Mus musculus GN=Parvb PE=1 SV=1 - [PARVB_MOUSE]	26.03	3	5	7	30	0.659	1.050	0.716	0.979	94.41	26.03	13	30	365	41.6	6.33
O88878	AN1-type zinc finger protein 5 OS=Mus musculus GN=Zfand5 PE=1 SV=1 -	37.09	3	6	6	10	2.249	0.979	1.254	0.979	32.32	37.09	7	10	213	23.0	8.51
Q8CCB4	Vacuolar protein sorting-associated protein 53 homolog OS=Mus musculus GN=Vps53 PE=2 SV=1 - [VPS53_MOUSE]	33.53	8	22	22	52	0.535	1.091	0.961	0.979	146.16	33.53	34	52	832	94.4	6.61
Q99PL8	Thiamine transporter 2 OS=Mus musculus GN=Slc19a3 PE=2 SV=2 - [S19A3_MOUSE]	3.28	1	1	1	2	0.806	0.816	1.187	0.979	3.07	3.28	2	2	488	55.0	6.96
Q62376	U1 small nuclear ribonucleoprotein 70 kDa OS=Mus musculus GN=Snrnp70 PE=1 SV=2 - [RU17_MOUSE]	18.97	2	9	9	34	0.715	0.984	0.916	0.979	89.79	18.97	15	34	448	52.0	9.94
D3YWN5	Leucine-rich repeat-containing protein 49 OS=Mus musculus GN=Lrrc49 PE=2 SV=1 - [D3YWN5_MOUSE]	10.00	9	7	8	12	1.165	0.873	0.892	0.979	27.89	10.00	10	12	680	78.3	8.00
O35344	Importin subunit alpha-4 OS=Mus musculus GN=Kpna3 PE=1 SV=1 - [IMA4_MOUSE]	18.62	1	4	8	35	0.772	0.921	0.963	0.979	84.16	18.62	14	35	521	57.7	4.94

E9QP62	LIM and senescent cell antigen-like-containing domain protein 1 OS=Mus musculus GN=Lims1 PE=2 SV=1 - [E9QP62_MOUSE]	17.83	3	4	7	14	1.734	1.433	1.040	0.979	36.98	17.83	12	14	387	44.3	7.88
O55003	BCL2/adenovirus E1B 19 kDa protein-interacting protein 3 OS=Mus musculus GN=Bnip3 PE=1 SV=1 - [BNIP3_MOUSE]	22.99	1	6	6	18	0.920	0.814	1.110	0.979	50.93	22.99	10	18	187	21.0	7.14
Q9CQT1	Methylthioribose-1-phosphate isomerase OS=Mus musculus GN=Mri1 PE=2 SV=1 - [MTNA_MOUSE]	44.44	3	11	11	39	1.425	0.975	0.904	0.980	82.56	44.44	17	39	369	39.4	5.91
P17918	Proliferating cell nuclear antigen OS=Mus musculus GN=Pcna PE=1 SV=2 - [PCNA_MOUSE]	21.07	2	4	4	15	1.922	0.961	1.202	0.980	49.38	21.07	5	15	261	28.8	4.77
A2AI08	Taperin OS=Mus musculus GN=Tprn PE=1 SV=1 - [TPRN_MOUSE]	20.43	1	7	8	19	1.538	0.621	0.959	0.980	54.13	20.43	13	19	749	80.0	7.09
Q3TCW6	Macrophage metalloelastase OS=Mus musculus GN=Mmp12 PE=2 SV=1 -	2.48	2	1	1	1	1.044	0.638	1.339	0.980	2.05	2.48	1	1	403	46.8	9.03
O08989	Ras-related protein M-Ras OS=Mus musculus GN=Mras PE=1 SV=1 - [RASM_MOUSE]	45.19	2	9	9	33	0.711	1.013	0.967	0.980	89.43	45.19	12	33	208	23.9	8.78
Q8R1F1	Niban-like protein 1 OS=Mus musculus GN=Fam129b PE=1 SV=2 [NIBL1_MOUSE]	17.36	2	13	14	29	0.918	1.377	0.819	0.980	62.53	17.36	24	29	749	84.8	5.94
Q8BW22	Calcium-responsive transactivator OS=Mus musculus GN=Ss18l1 PE=1 SV=1 -	6.22	1	3	3	11	1.072	1.001	1.088	0.980	44.01	6.22	6	11	402	43.7	6.58
P26450	Phosphatidylinositol 3-kinase regulatory subunit alpha OS=Mus musculus GN=Pik3r1 PE=1 SV=2 - [P85A_MOUSE]	19.20	7	11	14	35	1.081	1.056	0.996	0.980	71.63	19.20	23	35	724	83.5	6.28
Q8BGY4-3	Isoform 3 of Kelch-like protein 26 OS=Mus musculus GN=Klhl26 - [KLH26_MOUSE]	2.10	4	1	1	1	2.039	1.229	0.916	0.980	2.39	2.10	1	1	525	58.7	6.93
P97298	Pigment epithelium-derived factor OS=Mus musculus GN=Serpinf1 PE=1 SV=2 -	5.28	1	1	1	2	1.594	1.653	1.161	0.980	8.47	5.28	2	2	417	46.2	6.98
Q8BTU1	UPF0468 protein C16orf80 homolog OS=Mus musculus GN=Gtl3 PE=2 SV=1 - [CP080_MOUSE]	10.36	1	2	2	2	0.706	0.846	0.630	0.980	6.74	10.36	2	2	193	22.7	9.76
Q9CQJ2	PIH1 domain-containing protein 1 OS=Mus musculus GN=Pih1d1 PE=2 SV=1 -	16.90	4	4	4	12	1.109	0.899	0.880	0.980	28.41	16.90	8	12	290	32.2	5.21
Q3TYE5	Limbic system-associated membrane protein OS=Mus musculus GN=Lsamp PE=2 SV=1 - [Q3TYE5_MOUSE]	52.66	3	17	18	322	0.988	1.052	1.017	0.980	977.02	52.66	33	322	338	37.3	6.68
Q8CHE4	PH domain leucine-rich repeat-containing protein phosphatase 1 OS=Mus musculus GN=Phlpp1 PE=2 SV=2 - [illegible]	15.59	1	19	20	47	1.214	0.575	0.944	0.980	132.02	15.59	30	47	1687	182.3	6.19
B9EJ86	Oxysterol-binding protein OS=Mus musculus GN=Osbpl8 PE=2 SV=1 - [B9EJ86_MOUSE]	28.23	2	19	21	55	1.094	0.846	1.230	0.980	161.62	28.23	33	55	889	101.2	6.96
Q8JZM4	Delta and Notch-like epidermal growth factor-related receptor OS=Mus musculus GN=Dner PE=1 SV=1 - [DNER_MOUSE]	8.28	1	5	5	18	0.758	1.282	1.027	0.980	52.41	8.28	9	18	737	78.7	5.10
D3YU40	Insulin-like growth factor-binding protein 2 OS=Mus musculus GN=Igfbp2 PE=2 SV=1 - [D3YU40_MOUSE]	27.22	2	2	3	9	3.544	0.838	0.832	0.980	33.75	27.22	5	9	158	17.7	8.85
F8WJA7	AF4/FMR2 family member 3 OS=Mus musculus GN=Aff3 PE=2 SV=1 - [F8WJA7_MOUSE]	1.63	6	2	2	2	1.848	1.070	1.179	0.980	2.26	1.63	2	2	1228	133.1	8.22
Q61335	B-cell receptor-associated protein 31 OS=Mus musculus GN=Bcap31 PE=1 SV=4 -	32.65	1	8	8	27	0.744	0.835	0.783	0.980	74.84	32.65	13	27	245	27.9	8.70
Q8BHE5	Bone morphogenetic protein 3 OS=Mus musculus GN=Bmp3 PE=1 SV=1 - [BMP3_MOUSE]	2.78	1	1	1	2	0.890	1.183	1.067	0.980	5.06	2.78	2	2	468	52.7	9.38
P61089	Ubiquitin-conjugating enzyme E2 N OS=Mus musculus GN=Ube2n PE=1 SV=1 -	50.66	2	8	8	46	0.832	0.979	0.874	0.980	150.47	50.66	14	46	152	17.1	6.57
P62881-2	Isoform 2 of Guanine nucleotide-binding protein subunit beta-5 OS=Mus musculus GN=Gnb5 - [GBB5_MOUSE]	46.46	2	16	16	89	0.996	1.133	1.143	0.981	303.33	46.46	29	89	353	38.7	6.06
A2RT62	F-box/LRR-repeat protein 16 OS=Mus musculus GN=Fbxl16 PE=2 SV=1 - [FXL16_MOUSE]	32.78	1	11	11	60	0.672	2.042	1.638	0.981	170.59	32.78	22	60	479	51.8	6.60

A2ANY6	Midasin OS=Mus musculus GN=Mdn1 PE=2 SV=1 - [A2ANY6_MOUSE]	0.54	2	3	3	4	1.733	0.979	0.795	0.981	7.35	0.54	3	4	5589	629.9	5.72
Q9QYE9-4	Isoform 4 of Pleckstrin homology domain-containing family B member 1 OS=Mus musculus GN=Plekhb1 - [illegible]	48.15	7	4	7	51	1.922	0.596	0.894	0.981	165.42	48.15	13	51	189	21.3	6.10
E0CYK4	Repressor of RNA polymerase III transcription MAF1 homolog (Fragment) OS=Mus musculus GN=Maf1 PE=2 SV=1 - [E0CYK4_MOUSE]	25.74	3	2	2	8	1.253	1.268	1.010	0.981	30.40	25.74	4	8	202	22.2	5.39
E9PUL5	Proline-rich transmembrane protein 2 OS=Mus musculus GN=Prrt2 PE=1 SV=1 -	37.86	1	6	6	102	0.875	1.203	1.425	0.981	186.25	37.86	10	102	346	35.9	4.63
E9QJS0	28S ribosomal protein S10, mitochondrial OS=Mus musculus GN=Mrps10 PE=3 SV=1 - [E9QJS0_MOUSE]	19.38	5	3	3	8	0.982	0.784	0.985	0.981	17.03	19.38	6	8	160	18.7	8.13
D3YZR8	Bcl2 antagonist of cell death OS=Mus musculus GN=Bad PE=2 SV=1 - [D3YZR8_MOUSE]	50.56	5	5	5	34	0.984	0.792	1.079	0.981	129.85	50.56	10	34	178	19.3	6.11
B2KF67	Diphosphoinositol polyphosphate phosphohydrolase 1 OS=Mus musculus GN=Nudt3 PE=2 SV=1 - [illegible]	61.87	5	5	6	60	1.779	1.033	1.060	0.981	151.38	61.87	12	60	139	15.7	6.79
Q149M0	C-type lectin domain family 12 member B OS=Mus musculus GN=Clec12b PE=2 SV=1 - [CL12B_MOUSE]	6.18	1	1	1	1	1.619	1.201	1.030	0.981	0.00	6.18	1	1	275	31.2	5.17
F6YWH6	Disintegrin and metalloproteinase domain-containing protein 12 (Fragment) OS=Mus musculus GN=Adam12 PE=2 SV=1 - [F6YWH6_MOUSE]	1.03	2	1	1	1	1.603	0.850	0.857	0.981	1.96	1.03	1	1	581	62.6	8.31
Q4VBD2	Transmembrane anterior posterior transformation protein 1 OS=Mus musculus GN=Tapt1 PE=2 SV=2 - [TAPT1_MOUSE]	23.23	1	11	11	26	0.809	0.793	0.919	0.981	67.72	23.23	18	26	564	63.9	8.22
Q99MK8	Beta-adrenergic receptor kinase 1 OS=Mus musculus GN=Adrbk1 PE=2 SV=2 -	28.01	7	14	19	73	0.730	1.124	0.928	0.981	207.53	28.01	34	73	689	79.6	7.28
Q99KP6	Pre-mRNA-processing factor 19 OS=Mus musculus GN=Prpf19 PE=2 SV=1 -	35.12	4	11	11	41	0.909	0.916	0.810	0.981	106.92	35.12	18	41	504	55.2	6.61
Q99N23	Carbonic anhydrase 15 OS=Mus musculus GN=Ca15 PE=2 SV=1 - [CAH15_MOUSE]	4.01	1	1	1	1	0.362	1.521	0.861	0.981	2.67	4.01	1	1	324	35.5	6.79
Q8BUU7	N-acetylgalactosamine kinase OS=Mus musculus GN=Galk2 PE=2 SV=1 - [Q8BUU7_MOUSE]	23.94	2	7	7	19	0.668	1.565	0.838	0.981	59.96	23.94	12	19	447	49.3	6.73
Q9CQL4	39S ribosomal protein L20, mitochondrial OS=Mus musculus GN=Mrpl20 PE=1 SV=1 - [RM20_MOUSE]	21.48	1	3	3	5	1.634	1.278	0.914	0.981	15.06	21.48	5	5	149	17.6	11.11
Q6VNS1	NT-3 growth factor receptor OS=Mus musculus GN=Ntrk3 PE=1 SV=1 - [NTRK3_MOUSE]	20.73	3	11	16	46	0.981	1.752	1.095	0.981	127.31	20.73	26	46	825	92.7	6.67
O35954	Membrane-associated phosphatidylinositol transfer protein 1 OS=Mus musculus GN=Pitpnm1 PE=1 SV=1 - [illegible]	38.37	2	30	31	140	0.596	0.930	0.935	0.981	444.16	38.37	56	140	1243	134.9	6.06
A2AQ45	Formin-binding protein 1 OS=Mus musculus GN=Fnbp1 PE=2 SV=1 - [A2AQ45_MOUSE]	42.30	13	22	22	82	1.470	0.577	0.966	0.981	228.10	42.30	35	82	591	68.9	5.68
Q9WVP1	AP-1 complex subunit mu-2 OS=Mus musculus GN=Ap1m2 PE=1 SV=3 - [AP1M2_MOUSE]	11.35	2	1	4	12	1.191	1.193	1.216	0.981	30.87	11.35	6	12	423	48.1	7.55
Q6ZPJ3	Ubiquitin-conjugating enzyme E2 O OS=Mus musculus GN=Ube2o PE=1 SV=3 -	38.28	1	39	40	202	0.792	0.844	0.948	0.981	564.73	38.28	72	202	1288	140.7	5.06
Q9JJV2	Profilin-2 OS=Mus musculus GN=Pfn2 PE=1 SV=3 - [PROF2_MOUSE]	67.86	4	10	10	181	1.558	1.051	0.994	0.981	557.71	67.86	19	181	140	15.0	6.99
Q8R3C0	Mini-chromosome maintenance complex-binding protein OS=Mus musculus GN=Mcmbp PE=2 SV=1 - [illegible]	11.37	2	5	5	8	0.779	1.047	0.865	0.981	22.15	11.37	7	8	642	72.8	5.66
P49443	Protein phosphatase 1A OS=Mus musculus GN=Ppm1a PE=1 SV=1 - [PPM1A_MOUSE]	47.64	1	12	16	131	1.014	0.916	0.985	0.981	374.82	47.64	31	131	382	42.4	5.36
Q8BH79	Anoctamin-10 OS=Mus musculus GN=Ano10 PE=2 SV=1 - [ANO10_MOUSE]	14.26	4	8	9	16	0.815	0.898	0.790	0.981	43.81	14.26	12	16	659	76.1	6.70

Q6ZQH8	Nucleoporin NUP188 homolog OS=Mus musculus GN=Nup188 PE=1 SV=2 -	8.13	5	9	9	16	0.508	0.916	0.920	0.981	52.59	8.13	16	16	1759	196.6	7.01
Q9D8T7-2	Isoform 2 of SRA stem-loop-interacting RNA-binding protein, mitochondrial OS=Mus musculus GN=Slirp - [illegible]	50.51	4	3	3	21	0.988	0.964	1.013	0.982	50.63	50.51	6	21	99	10.9	8.59
Q8BTI8	Serine/arginine repetitive matrix protein 2 OS=Mus musculus GN=Srrm2 PE=1 SV=3 - [SRRM2_MOUSE]	24.23	3	46	46	187	0.994	0.906	1.047	0.982	519.54	24.23	73	187	2703	294.7	12.03
Q3UI47	Kinesin family member 3A, isoform CRA_b OS=Mus musculus GN=Kif3a PE=2 SV=1 - [Q3UI47_MOUSE]	26.79	6	17	18	51	0.810	0.931	1.064	0.982	149.92	26.79	28	51	698	79.8	6.64
Q8VD12	Zinc finger protein 385A OS=Mus musculus GN=Znf385a PE=1 SV=2 - [Z385A_MOUSE]	19.95	2	6	6	9	1.023	0.480	0.952	0.982	20.82	19.95	7	9	386	40.4	9.86
Q8VC69	Solute carrier family 22 member 6 OS=Mus musculus GN=Slc22a6 PE=1 SV=1 -	9.72	1	5	5	17	0.758	2.653	0.885	0.982	30.82	9.72	7	17	545	60.0	9.10
P35821	Tyrosine-protein phosphatase non-receptor type 1 OS=Mus musculus GN=Ptpn1 PE=1 SV=2 - [PTN1_MOUSE]	20.37	1	7	7	20	1.015	1.112	1.001	0.982	49.02	20.37	11	20	432	49.6	6.16
P05532-2	Isoform 2 of Mast/stem cell growth factor receptor Kit OS=Mus musculus GN=Kit - [KIT_MOUSE]	10.05	10	9	10	36	0.886	1.582	1.015	0.982	86.05	10.05	18	36	975	108.9	7.11
Q9D0B1	Zinc finger protein 524 OS=Mus musculus GN=Znf524 PE=2 SV=1 - [ZN524_MOUSE]	4.05	1	1	1	1	1.475	1.119	1.013	0.982	2.61	4.05	1	1	321	34.8	8.62
Q91XX0	MCG133388, isoform CRA_r OS=Mus musculus GN=Pcdhgc4 PE=2 SV=1 - [Q91XX0_MOUSE]	7.44	1	3	5	21	0.903	0.818	0.814	0.982	57.11	7.44	8	21	941	101.2	5.27
Q9ERU9	E3 SUMO-protein ligase RanBP2 OS=Mus musculus GN=Ranbp2 PE=1 SV=2 - [RBP2_MOUSE]	23.29	1	60	62	158	0.819	0.983	1.017	0.982	476.32	23.29	95	158	3053	340.9	6.18
B9EKI5	Casitas B-lineage lymphoma b OS=Mus musculus GN=Cblb PE=1 SV=1 - [B9EKI5_MOUSE]	9.59	3	4	6	10	1.543	1.294	1.188	0.982	33.28	9.59	10	10	938	104.5	8.02
Q9WUD8	Fas apoptotic inhibitory molecule 1 OS=Mus musculus GN=Faim PE=1 SV=1 - [FAIM1_MOUSE]	21.79	3	5	5	9	1.627	0.917	1.064	0.982	22.28	21.79	8	9	179	20.2	5.64
Q9JMH6-2	Isoform 2 of Thioredoxin reductase 1, cytoplasmic OS=Mus musculus GN=Txnrd1 -	43.69	2	15	16	71	1.570	0.833	0.761	0.982	218.06	43.69	29	71	499	54.5	6.33
Q9R0K7	Plasma membrane calcium-transporting ATPase 2 OS=Mus musculus GN=Atp2b2 PE=1 SV=2 - [AT2B2_MOUSE]	43.49	2	30	50	494	0.713	0.735	1.264	0.982	1391.82	43.49	89	494	1198	132.5	5.96
Q8C845	EF-hand domain-containing protein D2 OS=Mus musculus GN=Efhd2 PE=2 SV=1 -	51.67	2	13	16	206	1.627	0.994	1.375	0.982	592.65	51.67	29	206	240	26.8	5.14
Q6TCG2	Progestin and adipoQ receptor family member 9 OS=Mus musculus GN=Paqr9 PE=2 SV=2 - [PAQR9_MOUSE]	2.40	1	1	1	5	0.956	0.852	1.101	0.982	10.72	2.40	2	5	375	42.7	8.76
D3Z375	Astrocytic phosphoprotein PEA-15 (Fragment) OS=Mus musculus GN=Pea15a PE=2 SV=1 - [D3Z375_MOUSE]	64.13	2	3	5	96	1.933	1.586	1.407	0.982	311.89	64.13	10	96	92	10.7	4.64
O88967	ATP-dependent zinc metalloprotease YME1L1 OS=Mus musculus GN=Yme1l1 PE=2 SV=1 - [YMEL1_MOUSE]	22.10	1	12	13	28	0.943	1.197	0.853	0.982	74.40	22.10	22	28	715	80.0	8.97
P97470	Serine/threonine-protein phosphatase 4 catalytic subunit OS=Mus musculus GN=Ppp4c PE=1 SV=2 - [PP4C_MOUSE]	19.87	1	3	5	32	1.670	1.045	0.836	0.982	105.99	19.87	8	32	307	35.1	5.06
Q11136	Xaa-Pro dipeptidase OS=Mus musculus GN=Pepd PE=2 SV=3 - [PEPD_MOUSE]	43.00	2	19	19	84	2.089	0.825	0.698	0.982	253.62	43.00	36	84	493	55.0	5.78
Q7TMQ7	WD repeat-containing protein 91 OS=Mus musculus GN=Wdr91 PE=1 SV=1 -	16.84	3	12	12	27	0.779	0.961	0.947	0.982	67.79	16.84	19	27	748	83.4	6.64
F8WIE5	E3 ubiquitin-protein ligase HECTD1 OS=Mus musculus GN=Hectd1 PE=2 SV=1 -	18.47	4	42	43	117	0.723	0.871	0.812	0.982	323.09	18.47	68	117	2610	289.0	5.36
Q6ZWR4	Serine/threonine-protein phosphatase 2A 55 kDa regulatory subunit B beta isoform OS=Mus musculus GN=Ppp2r2b PE=1 SV=1 - [2ABB_MOUSE]	21.44	4	3	10	34	0.488	1.387	1.126	0.983	79.64	21.44	16	34	443	51.7	6.44

A2ANU3	Synapse differentiation-inducing gene protein 1 OS=Mus musculus GN=Syndig1 PE=1 SV=1 - [SYNG1_MOUSE]	12.40	5	3	3	5	0.773	0.587	1.028	0.983	13.04	12.40	5	5	258	28.4	4.88
Q8BT60	Copine-3 OS=Mus musculus GN=Cpne3 PE=1 SV=2 - [CPNE3_MOUSE]	25.52	1	11	13	68	1.045	1.007	0.864	0.983	163.46	25.52	20	68	533	59.5	5.78
Q810K9	Glucoside xylosyltransferase 2 OS=Mus musculus GN=Gxylt2 PE=2 SV=1 -	3.38	1	1	1	1	0.354	1.193	0.922	0.983	2.64	3.38	1	1	444	51.4	9.80
Q62074	Protein kinase C iota type OS=Mus musculus GN=Prkci PE=1 SV=3 - [KPCI_MOUSE]	6.39	2	3	3	5	0.921	0.771	1.117	0.983	14.73	6.39	4	5	595	68.2	5.85
Q91VU7	Protein Pus7 OS=Mus musculus GN=Pus7 PE=2 SV=2 - [Q91VU7_MOUSE]	15.45	4	8	8	20	1.591	1.284	0.948	0.983	42.42	15.45	11	20	660	74.7	5.87
Q6NS59-2	Isoform 2 of Protein FAM135A OS=Mus musculus GN=Fam135a - [F135A_MOUSE]	2.13	2	2	2	3	1.214	1.184	1.264	0.983	5.74	2.13	2	3	1081	118.3	5.45
Q3UH13	Tubby-related protein 4 OS=Mus musculus GN=Tulp4 PE=2 SV=1 - [Q3UH13_MOUSE]	6.20	3	6	6	11	0.752	0.754	1.011	0.983	28.97	6.20	9	11	1354	147.8	7.66
Q9CPQ9	Fructose-bisphosphate aldolase OS=Mus musculus GN=Aldoart1 PE=2 SV=1 -	52.75	2	1	22	753	1.527	3.523	1.093	0.983	2226.89	52.75	39	753	364	39.3	7.37
E9Q446	E3 ubiquitin-protein ligase RNF25 OS=Mus musculus GN=Rnf25 PE=2 SV=1 - [E9Q446_MOUSE]	23.52	6	7	7	24	1.165	0.761	1.104	0.984	69.24	23.52	11	24	455	51.1	6.19
P38060	Hydroxymethylglutaryl-CoA lyase, mitochondrial OS=Mus musculus GN=Hmgcl PE=1 SV=2 - [HMGCL_MOUSE]	37.23	1	9	10	30	0.857	1.131	0.910	0.984	88.12	37.23	19	30	325	34.2	8.41
Q7TQK4	Exosome complex component RRP40 OS=Mus musculus GN=Exosc3 PE=1 SV=3 - [EXOS3_MOUSE]	27.37	2	4	4	5	1.990	1.243	0.991	0.984	20.19	27.37	5	5	274	29.5	8.06
Q8BTZ7	Mannose-1-phosphate guanyltransferase beta OS=Mus musculus GN=Gmppb PE=2 SV=1 - [GMPPB_MOUSE]	20.00	1	5	5	18	0.624	0.867	0.859	0.984	56.63	20.00	10	18	360	39.9	6.74
Q80Z10-2	Isoform 2 of Astrotactin-2 OS=Mus musculus GN=Astn2 - [ASTN2_MOUSE]	17.36	3	15	15	49	0.801	0.787	0.890	0.984	138.82	17.36	24	49	1348	148.8	6.02
D6RFQ2	Peroxisomal membrane protein 11B OS=Mus musculus GN=Pex11b PE=2 SV=1 -	16.67	1	1	5	11	1.354	1.082	0.968	0.984	23.05	16.67	8	11	366	39.8	9.22
P70255-5	Isoform 5 of Nuclear factor 1 C-type OS=Mus musculus GN=Nfic - [NFIC_MOUSE]	9.02	7	3	3	7	1.231	0.801	0.924	0.984	18.78	9.02	6	7	377	42.1	8.38
Q8BH74	Nuclear pore complex protein Nup107 OS=Mus musculus GN=Nup107 PE=2 SV=1 - [NU107_MOUSE]	17.93	2	13	14	28	0.946	1.078	0.929	0.984	76.08	17.93	21	28	926	106.6	5.43
Q9ESX5	H/ACA ribonucleoprotein complex subunit 4 OS=Mus musculus GN=Dkc1 PE=1 SV=4 - [DKC1_MOUSE]	10.02	4	5	5	11	0.591	0.708	0.802	0.984	34.28	10.02	8	11	509	57.4	9.28
E9Q855	Secretory carrier-associated membrane protein 3 OS=Mus musculus GN=Scamp3 PE=2 SV=1 - [illegible]	34.60	3	7	7	44	0.714	1.054	0.983	0.984	157.70	34.60	11	44	315	34.5	8.16
Q9JHW2	Omega-amidase NIT2 OS=Mus musculus GN=Nit2 PE=1 SV=1 - [NIT2_MOUSE]	62.68	1	15	15	58	1.524	1.039	0.889	0.984	167.87	62.68	27	58	276	30.5	6.90
Q8R0J7	Vacuolar protein sorting-associated protein 37B OS=Mus musculus GN=Vps37b PE=2 SV=1 - [VP37B_MOUSE]	30.18	1	5	5	16	2.177	1.453	1.206	0.984	36.21	30.18	9	16	285	31.0	7.05
Q99K01	Pyridoxal-dependent decarboxylase domain-containing protein 1 OS=Mus musculus GN=Pdxdc1 PE=1 SV=2 - [illegible]	28.46	6	17	17	65	0.739	0.800	0.761	0.984	218.88	28.46	30	65	787	87.3	5.48
Q9QZB9	Dynactin subunit 5 OS=Mus musculus GN=Dctn5 PE=1 SV=1 - [DCTN5_MOUSE]	24.18	1	4	4	12	1.057	0.941	0.946	0.984	31.87	24.18	6	12	182	20.1	8.02
P29416	Beta-hexosaminidase subunit alpha OS=Mus musculus GN=Hexa PE=2 SV=2 - [HEXA_MOUSE]	23.48	1	9	9	27	0.834	1.233	0.905	0.984	86.09	23.48	16	27	528	60.6	6.54
P46935	E3 ubiquitin-protein ligase NEDD4 OS=Mus musculus GN=Nedd4 PE=1 SV=3 - [NEDD4_MOUSE]	37.43	3	25	29	118	0.734	1.037	0.881	0.984	363.17	37.43	52	118	887	102.6	5.26
Q6DIC6	Protein Tro OS=Mus musculus GN=Tro PE=2 SV=1 - [Q6DIC6_MOUSE]	6.37	6	8	9	25	0.687	0.543	0.922	0.984	62.18	6.37	14	25	2087	205.9	9.31

Q9JKY0	Cell differentiation protein RCD1 homolog OS=Mus musculus GN=Rqcd1 PE=1 SV=1 - [RCD1_MOUSE]	9.70	1	3	3	8	0.970	1.213	0.936	0.984	21.64	9.70	6	8	299	33.6	8.03
Q5XJV6	Serine/threonine-protein kinase LMTK3 OS=Mus musculus GN=Lmtk3 PE=1 SV=1 - [LMTK3_MOUSE]	21.35	4	23	23	86	1.391	1.280	1.338	0.984	220.03	21.35	43	86	1424	150.8	4.88
Q8K199	COX assembly mitochondrial protein 2 homolog OS=Mus musculus GN=Cmc2 PE=2 SV=1 - [COXM2_MOUSE]	12.66	1	1	1	4	0.999	0.529	1.019	0.984	7.35	12.66	2	4	79	9.4	8.24
B7ZNY3	Pard3 protein OS=Mus musculus GN=Pard3 PE=2 SV=1 - [B7ZNY3_MOUSE]	13.34	20	2	13	33	1.023	0.552	0.907	0.984	99.26	13.34	21	33	1319	147.4	8.15
O89032-3	Isoform 3 of SH3 and PX domain-containing protein 2A OS=Mus musculus GN=Sh3pxd2a - [SPD2A_MOUSE]	10.18	3	8	8	18	0.877	1.042	1.988	0.984	56.41	10.18	13	18	1081	119.0	8.18
Q9CPU4	Microsomal glutathione S-transferase 3 OS=Mus musculus GN=Mgst3 PE=1 SV=1 - [MGST3_MOUSE]	24.84	1	3	3	53	1.070	0.894	1.103	0.985	228.47	24.84	6	53	153	16.9	9.50
Q6NSW3-4	Isoform 4 of A-kinase anchor protein SPHKAP OS=Mus musculus GN=Sphkap -	15.57	5	17	18	42	0.929	1.378	1.145	0.985	100.40	15.57	30	42	1586	173.2	5.08
P47811-3	Isoform 3 of Mitogen-activated protein kinase 14 OS=Mus musculus GN=Mapk14 - [MK14_MOUSE]	16.67	13	4	5	28	0.838	1.092	1.089	0.985	54.40	16.67	8	28	360	41.5	5.97
Q8BHS8-3	Isoform 3 of Syntabulin OS=Mus musculus GN=Sybu - [SYBU_MOUSE]	7.08	6	2	4	13	0.920	1.093	1.011	0.985	42.63	7.08	8	13	593	64.6	5.91
Q9CPQ3	Mitochondrial import receptor subunit TOM22 homolog OS=Mus musculus GN=Tomm22 PE=2 SV=3 - [illegible]	33.10	1	3	3	46	0.789	0.963	0.985	0.985	132.65	33.10	6	46	142	15.5	4.34
Q3UVD5	Leucine-rich repeat-containing G-protein coupled receptor 6 OS=Mus musculus GN=Lgr6 PE=2 SV=1 - [illegible]	3.21	1	2	2	2	20.074	2.481	2.235	0.985	2.44	3.21	2	2	967	104.2	6.07
Q8BZ81	Leucine-rich repeat transmembrane neuronal protein 3 OS=Mus musculus GN=Lrrtm3 PE=2 SV=2 - [illegible]	16.49	3	4	5	9	1.172	1.425	1.158	0.985	27.17	16.49	7	9	582	66.0	9.19
Q91W53	Golgin subfamily A member 7 OS=Mus musculus GN=Golga7 PE=2 SV=1 -	35.04	2	5	5	18	1.000	0.601	0.901	0.985	35.90	35.04	9	18	137	15.8	7.05
P22892	AP-1 complex subunit gamma-1 OS=Mus musculus GN=Ap1g1 PE=1 SV=3 - [AP1G1_MOUSE]	33.09	3	23	23	62	0.643	0.970	0.815	0.985	185.91	33.09	36	62	822	91.3	6.80
Q2VPA6-3	Isoform 3 of Helicase POLQ-like OS=Mus musculus GN=Helq - [HELQ_MOUSE]	6.07	5	3	4	4	1.005	0.884	0.841	0.985	9.83	6.07	4	4	824	92.0	6.20
Q9CQW2	ADP-ribosylation factor-like protein 8B OS=Mus musculus GN=Arl8b PE=2 SV=1 - [ARL8B_MOUSE]	47.85	1	4	9	42	0.838	1.015	0.866	0.985	123.48	47.85	15	42	186	21.5	8.43
Q80X71	Transmembrane protein 106B OS=Mus musculus GN=Tmem106b PE=2 SV=1 - [T106B_MOUSE]	12.00	4	3	3	8	0.896	1.008	1.000	0.985	27.31	12.00	5	8	275	31.2	6.68
Q8C0M2	Abhydrolase domain-containing protein 4 OS=Mus musculus GN=Abhd4 PE=2 SV=1 - [Q8C0M2_MOUSE]	22.96	3	8	8	29	0.642	0.576	0.741	0.985	82.48	22.96	14	29	318	36.0	8.27
H3BL68	PAB-dependent poly(A)-specific ribonuclease subunit 3 (Fragment) OS=Mus musculus GN=Pan3 PE=4 SV=1 - [illegible]	10.93	4	1	1	2	0.828	0.982	1.005	0.985	7.04	10.93	2	2	183	19.0	6.77
Q62361	Pro-thyrotropin-releasing hormone OS=Mus musculus GN=Trh PE=2 SV=2 - [TRH_MOUSE]	10.94	1	3	3	4	2.538	1.762	0.608	0.985	6.92	10.94	4	4	256	29.2	5.88
E9Q8I9	Protein furry homolog OS=Mus musculus GN=Fry PE=1 SV=1 - [FRY_MOUSE]	14.04	3	34	38	95	0.785	1.010	1.071	0.985	272.58	14.04	64	95	3020	338.9	6.01
D3Z795	Proteasome assembly chaperone 1 OS=Mus musculus GN=Psmg1 PE=2 SV=1 -	24.25	2	5	5	13	0.805	0.935	0.834	0.985	44.56	24.25	10	13	268	30.5	7.12
E9PYL2	Protein Prr12 OS=Mus musculus GN=Prr12 PE=2 SV=1 - [E9PYL2_MOUSE]	9.09	1	9	9	21	1.175	1.177	1.014	0.985	56.63	9.09	13	21	2035	211.7	7.65
D3YUI2	MKL/myocardin-like protein 1 OS=Mus musculus GN=Mkl1 PE=2 SV=1 - [D3YUI2_MOUSE]	8.99	4	6	6	14	1.121	0.925	1.587	0.985	41.55	8.99	10	14	879	93.3	5.54

Q9CRD0-3	Isoform 3 of OCIA domain-containing protein 1 OS=Mus musculus GN=Ociad1 - [OCAD1_MOUSE]	60.85	1	1	7	40	1.008	0.923	1.016	0.985	123.11	60.85	13	40	189	20.8	7.81
D3Z3D9	Beta-chimaerin OS=Mus musculus GN=Chn2 PE=2 SV=1 - [D3Z3D9_MOUSE]	21.43	2	3	3	8	1.268	0.779	1.213	0.985	24.73	21.43	5	8	196	22.4	8.37
Q9ERI6	Retinol dehydrogenase 14 OS=Mus musculus GN=Rdh14 PE=1 SV=1 - [RDH14_MOUSE]	41.32	1	11	11	42	0.656	1.069	0.914	0.985	127.59	41.32	18	42	334	36.3	8.18
Q9WV30	Nuclear factor of activated T-cells 5 OS=Mus musculus GN=Nfat5 PE=2 SV=1 - [NFAT5_MOUSE]	10.45	5	8	10	22	1.155	0.751	1.286	0.986	62.76	10.45	16	22	1225	132.1	5.35
P00755	Kallikrein 1-related peptidase b1 OS=Mus musculus GN=Klk1b1 PE=2 SV=1 -	4.98	8	2	2	2	0.337	2.019	1.082	0.986	2.21	4.98	2	2	261	29.0	8.10
Q3TXR6	Peroxisomal N(1)-acetyl-spermine/spermidine oxidase OS=Mus musculus GN=Paox PE=2 SV=1 - [Q3TXR6_MOUSE]	18.61	3	6	6	18	1.495	1.059	0.955	0.986	42.31	18.61	11	18	274	30.3	6.01
Q3UPH7-3	Isoform 3 of Rho guanine nucleotide exchange factor 40 OS=Mus musculus GN=Arhgef40 - [ARH40_MOUSE]	5.72	4	6	7	13	0.782	0.900	0.873	0.986	32.83	5.72	11	13	1469	160.1	5.58
Q8N9S3-3	Isoform 3 of Activator of 90 kDa heat shock protein ATPase homolog 2 OS=Mus musculus GN=Ahsa2 - [illegible]	18.18	6	4	5	14	1.031	0.900	0.880	0.986	36.66	18.18	9	14	286	32.4	8.10
Q91Z78	BCL2/adenovirus E1B 19 kDa protein-interacting protein 3-like OS=Mus musculus GN=Bnip3l PE=2 SV=1 - [Q91Z78_MOUSE]	11.76	2	1	2	6	0.848	1.159	1.038	0.986	16.43	11.76	4	6	204	22.3	5.52
Q99JN2	Kelch-like protein 22 OS=Mus musculus GN=Klhl22 PE=1 SV=1 - [KLH22_MOUSE]	16.40	7	9	10	26	0.586	1.049	0.951	0.986	64.23	16.40	15	26	634	71.6	5.71
E9Q4K7	Protein Kif13b OS=Mus musculus GN=Kif13b PE=2 SV=1 - [E9Q4K7_MOUSE]	11.45	1	14	16	29	0.995	0.897	0.864	0.986	81.21	11.45	26	29	1843	204.4	5.80
P37172	Activin receptor type-1 OS=Mus musculus GN=Acvr1 PE=2 SV=2 - [ACVR1_MOUSE]	8.45	1	2	2	5	1.849	0.748	0.851	0.986	2.52	8.45	3	5	509	57.2	7.33
P62259	14-3-3 protein epsilon OS=Mus musculus GN=Ywhae PE=1 SV=1 - [1433E_MOUSE]	80.00	3	21	24	1129	1.669	1.231	0.992	0.986	3385.42	80.00	42	1129	255	29.2	4.74
Q9DCM7	Nucleus accumbens-associated protein 2 OS=Mus musculus GN=Nacc2 PE=2 SV=1 - [NACC2_MOUSE]	10.75	1	4	5	12	1.358	1.180	0.830	0.986	37.85	10.75	8	12	586	63.2	5.90
P62631	Elongation factor 1-alpha 2 OS=Mus musculus GN=Eef1a2 PE=1 SV=1 - [EF1A2_MOUSE]	64.79	1	13	25	320	0.639	0.559	0.924	0.986	874.41	64.79	46	320	463	50.4	9.03
A3KFU5	Protein Pabpc4 OS=Mus musculus GN=Pabpc4 PE=2 SV=1 - [A3KFU5_MOUSE]	33.44	6	12	20	76	1.017	0.986	0.917	0.986	227.63	33.44	36	76	631	69.4	9.57
Q80X73	Protein pelota homolog OS=Mus musculus GN=Pelo PE=2 SV=3 - [PELO_MOUSE]	20.52	1	7	7	18	0.623	0.951	0.966	0.986	41.46	20.52	10	18	385	43.3	5.99
Q61024	Asparagine synthetase [glutamine-hydrolyzing] OS=Mus musculus GN=Asns PE=2 SV=3 - [ASNS_MOUSE]	39.93	4	19	19	70	0.636	0.991	0.835	0.986	170.86	39.93	34	70	561	64.2	6.58
Q9DBL7	Bifunctional coenzyme A synthase OS=Mus musculus GN=Coasy PE=1 SV=2 - [COASY_MOUSE]	30.37	1	10	10	34	0.693	0.659	0.790	0.986	118.92	30.37	16	34	563	62.0	7.11
P59235	Nucleoporin Nup43 OS=Mus musculus GN=Nup43 PE=2 SV=2 - [NUP43_MOUSE]	22.37	1	7	7	18	1.374	1.085	0.830	0.986	41.61	22.37	13	18	380	42.0	5.39
Q8BLF1	Neutral cholesterol ester hydrolase 1 OS=Mus musculus GN=Nceh1 PE=1 SV=1 - [NCEH1_MOUSE]	29.17	3	12	12	45	0.609	0.924	1.004	0.986	117.06	29.17	20	45	408	45.7	7.05
A2AMS3	Galactose-1-phosphate uridylyltransferase OS=Mus musculus GN=Galt PE=3 SV=1 - [A2AMS3_MOUSE]	17.22	3	5	5	19	0.800	0.829	0.941	0.986	55.92	17.22	9	19	360	41.2	7.03
Q8BGC1	UPF0489 protein C5orf22 homolog OS=Mus musculus PE=2 SV=1 - [CE022_MOUSE]	16.06	3	4	4	5	0.902	1.405	1.167	0.986	13.78	16.06	5	5	442	49.4	4.81
Q3UHI4	Protein TMED8 OS=Mus musculus GN=Tmed8 PE=1 SV=1 - [TMED8_MOUSE]	31.60	1	7	7	22	1.306	1.147	0.903	0.986	68.62	31.60	12	22	326	35.8	4.74
Q3UBG2-2	Isoform 2 of PTB-containing, cubilin and LRP1-interacting protein OS=Mus musculus GN=Pid1 - [illegible]	6.90	2	1	1	2	1.017	1.358	0.983	0.986	8.45	6.90	2	2	203	23.0	6.95

D3Z7P3	Glutaminase kidney isoform, mitochondrial OS=Mus musculus GN=Gls PE=1 SV=1 - [GLSK_MOUSE]	48.66	6	6	30	169	1.126	1.194	1.184	0.986	562.79	48.66	51	169	674	73.9	7.99
Q99LC2	Cleavage stimulation factor subunit 1 OS=Mus musculus GN=Cstf1 PE=2 SV=1 - [CSTF1_MOUSE]	16.01	2	5	5	14	0.972	0.948	0.955	0.987	35.00	16.01	9	14	431	48.4	6.58
Q9EPN1-4	Isoform 4 of Neurobeachin OS=Mus musculus GN=Nbea - [NBEA_MOUSE]	28.66	4	62	69	256	0.667	1.291	1.110	0.987	781.18	28.66	114	256	2931	325.9	6.20
Q91XL9	Oxysterol-binding protein-related protein 1 OS=Mus musculus GN=Osbpl1a PE=1 SV=2 - [OSBL1_MOUSE]	14.63	7	12	14	57	0.760	0.725	1.041	0.987	156.74	14.63	23	57	950	107.7	6.44
Q9R190	Metastasis-associated protein MTA2 OS=Mus musculus GN=Mta2 PE=1 SV=1 - [MTA2_MOUSE]	22.90	1	10	15	46	1.068	0.955	1.025	0.987	116.62	22.90	24	46	668	75.0	9.67
Q3UFT3	GRB2-associated and regulator of MAPK protein OS=Mus musculus GN=Garem PE=1 SV=2 - [GAREM_MOUSE]	9.02	2	7	7	16	0.996	1.077	1.056	0.987	41.72	9.02	12	16	876	97.2	6.60
Q6NZR5	Protein Skiv2l OS=Mus musculus GN=Skiv2l PE=2 SV=1 - [Q6NZR5_MOUSE]	15.11	2	15	16	33	0.889	1.014	0.850	0.987	86.40	15.11	25	33	1244	137.4	6.28
P61148	Fibroblast growth factor 1 OS=Mus musculus GN=Fgf1 PE=2 SV=1 - [FGF1_MOUSE]	45.81	5	6	6	29	2.454	0.708	0.985	0.987	95.53	45.81	10	29	155	17.4	7.02
Q3V0J1-3	Isoform 3 of Transmembrane protein 237 OS=Mus musculus GN=Tmem237 -	13.15	3	4	4	6	0.668	0.640	0.933	0.987	14.38	13.15	6	6	403	44.6	6.48
Q61527-3	Isoform JM-A CYT-2 of Receptor tyrosine-protein kinase erbB-4 OS=Mus musculus GN=Erbb4 - [ERBB4_MOUSE]	9.37	6	8	9	29	1.309	1.248	1.241	0.987	71.03	9.37	14	29	1292	145.2	6.39
Q99LG4	Tetratricopeptide repeat protein 5 OS=Mus musculus GN=Ttc5 PE=1 SV=2 - [TTC5_MOUSE]	9.32	1	4	4	12	0.821	1.034	0.862	0.987	38.87	9.32	6	12	440	48.8	6.29
Q9JKX6	ADP-sugar pyrophosphatase OS=Mus musculus GN=Nudt5 PE=1 SV=1 - [NUDT5_MOUSE]	29.82	2	6	6	19	1.652	1.050	1.012	0.987	48.00	29.82	10	19	218	24.0	5.48
P82198	Transforming growth factor-beta-induced protein ig-h3 OS=Mus musculus GN=Tgfbi PE=2 SV=1 - [BGH3_MOUSE]	4.83	1	3	3	5	1.691	3.119	0.808	0.987	11.95	4.83	5	5	683	74.5	7.06
Q5SW88	Ras-related protein Rab-1A OS=Mus musculus GN=Rab1 PE=2 SV=1 - [Q5SW88_MOUSE]	87.13	4	9	18	197	0.688	1.022	0.939	0.987	538.18	87.13	33	197	202	22.4	6.21
Q9CY73	39S ribosomal protein L44, mitochondrial OS=Mus musculus GN=Mrpl44 PE=1 SV=3 - [RM44_MOUSE]	15.62	2	4	4	19	1.170	0.892	0.863	0.987	56.29	15.62	7	19	333	37.5	8.51
P13439	Uridine 5'-monophosphate synthase OS=Mus musculus GN=Umps PE=2 SV=3 - [UMPS_MOUSE]	33.06	2	14	14	33	0.887	1.030	0.896	0.987	92.72	33.06	25	33	481	52.3	6.61
Q400C8	Archaemetzincin-2 OS=Mus musculus GN=Amz2 PE=2 SV=1 - [AMZ2_MOUSE]	9.47	4	3	3	7	0.978	1.343	1.022	0.987	19.60	9.47	5	7	359	41.3	7.66
Q6IRU5	Clathrin light chain B OS=Mus musculus GN=Cltb PE=2 SV=1 - [CLCB_MOUSE]	37.99	3	1	11	223	2.066	2.687	1.135	0.987	573.62	37.99	19	223	229	25.2	4.63
Q5SSZ5	Tensin-3 OS=Mus musculus GN=Tns3 PE=1 SV=1 - [TENS3_MOUSE]	11.32	2	10	13	42	1.372	0.909	0.979	0.987	133.85	11.32	22	42	1440	155.5	6.65
E9Q1F5	Protein Myo5c OS=Mus musculus GN=Myo5c PE=2 SV=1 - [E9Q1F5_MOUSE]	6.54	2	1	11	39	2.104	1.498	1.433	0.988	101.46	6.54	17	39	1742	202.6	7.59
Q9DBJ1	Phosphoglycerate mutase 1 OS=Mus musculus GN=Pgam1 PE=1 SV=3 - [PGAM1_MOUSE]	80.31	1	13	17	944	1.632	1.127	0.848	0.988	2899.82	80.31	33	944	254	28.8	7.18
Q8BRH4	Histone-lysine N-methyltransferase 2C OS=Mus musculus GN=Kmt2c PE=2 SV=2 -	1.16	7	3	5	5	1.179	0.957	1.086	0.988	10.78	1.16	5	5	4903	539.8	6.55
Q7TT37	Elongator complex protein 1 OS=Mus musculus GN=Ikbkap PE=2 SV=2 - [ELP1_MOUSE]	18.60	1	20	20	75	0.591	0.893	0.819	0.988	215.98	18.60	38	75	1333	149.5	6.00
E9Q2E4	Protein Gm15800 OS=Mus musculus GN=Gm15800 PE=2 SV=2 - [E9Q2E4_MOUSE]	17.75	6	62	66	171	0.645	0.779	0.975	0.988	480.80	17.75	108	171	4418	483.8	6.06
B8QI35	Liprin-alpha 3 OS=Mus musculus GN=Ppfia3 PE=2 SV=1 - [B8QI35_MOUSE]	50.17	5	43	53	253	0.816	1.296	1.355	0.988	793.52	50.17	92	253	1194	133.3	5.64
F6R5X6	Bicaudal D-related protein 1 (Fragment) OS=Mus musculus GN=Ccdc64 PE=2 SV=1 - [F6R5X6_MOUSE]	3.64	1	1	1	2	4.666	1.316	2.138	0.988	2.73	3.64	1	2	302	34.6	4.75

Q7M6W1	RTN1-C OS=Mus musculus GN=Rtn1 PE=2 SV=1 - [Q7M6W1_MOUSE]	47.60	2	2	9	51	0.501	1.146	0.736	0.988	153.67	47.60	17	51	208	23.5	8.91
Q9CVB6	Actin-related protein 2/3 complex subunit 2 OS=Mus musculus GN=Arpc2 PE=1 SV=3 - [ARPC2_MOUSE]	56.67	2	19	19	121	0.573	1.308	1.065	0.988	338.82	56.67	36	121	300	34.3	7.36
Q8BKC8-2	Isoform 2 of Phosphatidylinositol 4-kinase beta OS=Mus musculus GN=Pi4kb -	11.36	5	7	7	14	0.722	0.888	0.851	0.988	41.70	11.36	12	14	801	89.9	6.51
Q8VE52	Opioid growth factor receptor-like protein 1 OS=Mus musculus GN=Ogfrl1 PE=2 SV=2 - [OGRL1_MOUSE]	12.07	1	5	5	12	0.847	1.328	1.126	0.988	26.93	12.07	9	12	464	52.2	6.57
Q8BVA2	Transmembrane protein 222 OS=Mus musculus GN=Tmem222 PE=2 SV=1 - [TM222_MOUSE]	28.85	3	3	3	18	0.534	0.997	0.949	0.988	55.06	28.85	6	18	208	23.2	6.61
Q9JKK0	Calcipressin-3 OS=Mus musculus GN=Rcan3 PE=1 SV=1 - [RCAN3_MOUSE]	20.08	4	3	4	8	0.954	0.852	0.897	0.988	27.54	20.08	7	8	239	27.1	4.74
P97471	Mothers against decapentaplegic homolog 4 OS=Mus musculus GN=Smad4 PE=1 SV=2 - [SMAD4_MOUSE]	15.61	1	8	8	12	0.983	1.336	0.937	0.988	34.52	15.61	11	12	551	60.3	6.99
Q8BHS6	Armadillo repeat-containing X-linked protein 3 OS=Mus musculus GN=Armcx3 PE=1 SV=1 - [ARMX3_MOUSE]	21.90	1	6	6	19	0.842	1.100	0.963	0.988	35.02	21.90	10	19	379	42.6	8.68
E9Q1W7	TBC domain-containing protein kinase-like protein OS=Mus musculus GN=Tbck PE=2 SV=1 - [E9Q1W7_MOUSE]	18.03	3	12	13	35	0.599	0.935	0.894	0.988	104.68	18.03	23	35	893	100.6	6.43
D3YZU1	SH3 and multiple ankyrin repeat domains protein 1 OS=Mus musculus GN=Shank1 PE=2 SV=1 - [SHAN1_MOUSE]	30.32	3	44	46	198	0.896	1.256	1.548	0.988	606.66	30.32	79	198	2167	226.2	8.34
Q9CWK8	Sorting nexin-2 OS=Mus musculus GN=Snx2 PE=1 SV=2 - [SNX2_MOUSE]	43.55	1	20	22	91	0.882	1.145	0.888	0.988	285.82	43.55	41	91	519	58.4	5.12
Q5NCM9	B9 domain-containing protein 1 (Fragment) OS=Mus musculus GN=B9d1 PE=2 SV=1 - [Q5NCM9_MOUSE]	31.36	2	2	2	2	1.845	1.126	1.015	0.989	0.00	31.36	2	2	118	13.2	9.64
Q924B0	Inositol (Myo)-1(Or 4)-monophosphatase 1 OS=Mus musculus GN=Impa1 PE=2 SV=1 - [Q924B0_MOUSE]	56.32	4	14	14	98	1.009	0.871	0.961	0.989	240.86	56.32	27	98	277	30.4	5.19
Q9ERR1-2	Isoform 2 of Nuclear distribution protein nudE-like 1 OS=Mus musculus GN=Ndel1 - [NDEL1_MOUSE]	50.48	2	13	16	54	1.141	1.018	1.243	0.989	169.52	50.48	29	54	315	35.5	5.22
Q8BHR8	UPF0705 protein C11orf49 homolog OS=Mus musculus PE=2 SV=1 - [CK049_MOUSE]	18.13	3	4	4	11	0.920	1.056	0.927	0.989	37.75	18.13	8	11	331	37.5	5.41
Q7TPV4	Myb-binding protein 1A OS=Mus musculus GN=Mybbp1a PE=1 SV=2 [MBB1A_MOUSE]	22.77	1	23	23	69	0.586	0.722	0.766	0.989	197.27	22.77	37	69	1344	151.9	8.95
Q8C569-2	Isoform 2 of Protein FAM118B OS=Mus musculus GN=Fam118b - [F118B_MOUSE]	5.82	2	1	1	1	0.727	1.291	1.108	0.989	4.45	5.82	1	1	275	30.8	8.57
A2AR50-2	Isoform 2 of Ras-specific guanine nucleotide-releasing factor RalGPS1 OS=Mus musculus GN=Ralgps1 - [illegible]	6.04	4	3	3	4	0.851	0.971	0.866	0.989	11.02	6.04	3	4	497	55.9	9.64
Q5SSW2	Proteasome activator complex subunit 4 OS=Mus musculus GN=Psme4 PE=1 SV=1 - [PSME4_MOUSE]	5.43	2	6	8	12	0.833	0.857	0.895	0.989	31.93	5.43	9	12	1843	211.1	7.01
Q8VE38	Oxidoreductase NAD-binding domain-containing protein 1 OS=Mus musculus GN=Oxnad1 PE=2 SV=2 - [illegible]	14.47	6	4	5	8	1.191	0.677	0.900	0.989	20.10	14.47	8	8	311	34.7	8.13
P42227-2	Isoform Stat3B of Signal transducer and activator of transcription 3 OS=Mus musculus GN=Stat3 - [STAT3_MOUSE]	21.05	4	11	12	30	0.789	1.169	0.707	0.989	98.16	21.05	19	30	722	83.1	7.12
Q3TQB2	FAD-dependent oxidoreductase domain-containing protein 1 OS=Mus musculus GN=Foxred1 PE=2 SV=1 - [illegible]	8.42	4	4	4	8	0.811	0.848	0.879	0.989	25.93	8.42	6	8	487	54.1	7.53
Q8BYY4	Tetratricopeptide repeat protein 39B OS=Mus musculus GN=Ttc39b PE=1 SV=1 -	12.48	5	7	7	14	0.693	0.827	0.899	0.989	41.82	12.48	12	14	617	70.2	6.64

A2AA86	SUZ domain-containing protein 1 OS=Mus musculus GN=Szrd1 PE=2 SV=1 - [A2AA86_MOUSE]	17.42	4	2	2	14	2.288	0.691	1.065	0.989	50.31	17.42	4	14	132	14.6	11.46
Q924D1	Cytochrome P450 CYP2J9 OS=Mus musculus GN=Cyp2j9 PE=2 SV=1 - [Q924D1_MOUSE]	20.52	3	5	8	32	0.906	0.688	0.794	0.989	101.57	20.52	14	32	502	57.9	7.75
P51906	Excitatory amino acid transporter 3 OS=Mus musculus GN=Slc1a1 PE=1 SV=2 -	7.07	1	3	3	10	21.994	1.216	1.082	0.990	19.83	7.07	5	10	523	56.7	6.11
Q9D1H7	Golgi to ER traffic protein 4 homolog OS=Mus musculus GN=Get4 PE=2 SV=2 - [GET4_MOUSE]	18.65	4	6	7	17	0.794	0.952	1.018	0.990	36.17	18.65	11	17	327	36.5	5.41
P59108	Copine-2 OS=Mus musculus GN=Cpne2 PE=2 SV=1 - [CPNE2_MOUSE]	19.89	1	8	9	64	0.843	1.438	0.865	0.990	183.01	19.89	17	64	548	61.0	5.96
P19536	Cytochrome c oxidase subunit 5B, mitochondrial OS=Mus musculus GN=Cox5b PE=1 SV=1 - [COX5B_MOUSE]	58.59	2	8	8	180	1.600	1.047	1.297	0.990	466.17	58.59	16	180	128	13.8	8.38
Q5SUW3	Growth factor receptor-bound protein 10 OS=Mus musculus GN=Grb10 PE=2 SV=1 - [Q5SUW3_MOUSE]	12.73	5	6	6	17	0.757	0.649	0.658	0.990	49.68	12.73	9	17	550	62.2	8.47
Q924M7	Mannose-6-phosphate isomerase OS=Mus musculus GN=Mpi PE=2 SV=1 - [MPI_MOUSE]	39.24	2	13	13	44	0.858	1.083	1.032	0.990	148.99	39.24	19	44	423	46.5	5.95
P98191	Phosphatidate cytidylyltransferase 1 OS=Mus musculus GN=Cds1 PE=1 SV=2 -	8.03	1	4	4	12	0.687	0.584	0.929	0.990	34.94	8.03	7	12	461	52.8	7.72
Q922D4-2	Isoform 2 of Serine/threonine-protein phosphatase 6 regulatory subunit 3 OS=Mus musculus GN=Ppp6r3 - [illegible]	30.11	5	20	21	68	1.120	1.257	1.014	0.990	204.92	30.11	34	68	827	92.6	4.59
O55098	Serine/threonine-protein kinase 10 OS=Mus musculus GN=Stk10 PE=1 SV=2 - [STK10_MOUSE]	3.52	1	2	3	4	0.750	1.396	0.857	0.990	5.08	3.52	3	4	966	111.8	7.15
Q8BWG8-2	Isoform 1B of Beta-arrestin-1 OS=Mus musculus GN=Arrb1 - [ARRB1_MOUSE]	28.78	7	11	12	49	0.691	1.083	1.009	0.990	136.02	28.78	21	49	410	46.2	6.42
Q9CXT7-2	Isoform 2 of Transmembrane protein 192 OS=Mus musculus GN=Tmem192 -	14.93	2	3	3	6	1.392	1.146	0.912	0.990	11.26	14.93	6	6	221	25.4	9.14
Q3THJ3	Probable RNA-binding protein EIF1AD OS=Mus musculus GN=Eif1ad PE=2 SV=2 - [EIF1A_MOUSE]	21.76	1	3	3	4	1.779	1.316	1.478	0.990	11.61	21.76	4	4	170	19.5	4.87
Q7TQ95	Protein lunapark OS=Mus musculus GN=Lnp PE=1 SV=1 - [LNP_MOUSE]	41.18	5	14	14	71	0.984	1.006	0.956	0.990	220.50	41.18	25	71	425	47.5	5.27
Q91VC7	Protein phosphatase 1 regulatory subunit 14A OS=Mus musculus GN=Ppp1r14a PE=2 SV=1 - [PP14A_MOUSE]	43.54	1	6	6	36	1.501	0.369	1.236	0.990	96.89	43.54	11	36	147	16.6	7.34
P27038	Activin receptor type-2A OS=Mus musculus GN=Acvr2a PE=1 SV=1 - [AVR2A_MOUSE]	2.14	1	1	1	1	1.426	0.894	0.989	0.990	0.00	2.14	1	1	513	57.9	5.92
D3Z118	Islet cell autoantigen 1 OS=Mus musculus GN=Ica1 PE=2 SV=1 - [D3Z118_MOUSE]	20.65	8	9	10	33	0.680	1.205	0.957	0.990	93.65	20.65	18	33	465	52.9	6.20
Q6PDY0	Coiled-coil domain-containing protein 85B OS=Mus musculus GN=Ccdc85b PE=1 SV=1 - [CC85B_MOUSE]	32.67	1	4	6	13	0.950	1.025	1.056	0.990	23.36	32.67	8	13	202	22.1	5.08
Q62261	Spectrin beta chain, non-erythrocytic 1 OS=Mus musculus GN=Sptbn1 PE=1 SV=2 - [SPTB2_MOUSE]	73.55	1	13	163	2161	0.956	0.721	1.438	0.990	6100.33	73.55	298	2161	2363	274.1	5.58
A2ADB1	Msx2-interacting protein OS=Mus musculus GN=Spen PE=2 SV=1 - [A2ADB1_MOUSE]	3.48	5	8	8	13	1.174	1.218	1.018	0.990	26.20	3.48	11	13	3620	395.7	8.56
P17439	Glucosylceramidase OS=Mus musculus GN=Gba PE=1 SV=1 - [GLCM_MOUSE]	7.38	1	4	4	11	0.827	0.740	0.805	0.990	32.27	7.38	7	11	515	57.6	7.75
P61759	Prefoldin subunit 3 OS=Mus musculus GN=Vbp1 PE=2 SV=2 - [PFD3_MOUSE]	67.35	2	14	15	65	1.578	0.792	1.196	0.990	187.29	67.35	23	65	196	22.4	6.28
G3X918	RAS p21 protein activator 2 OS=Mus musculus GN=Rasa2 PE=4 SV=1 - [G3X918_MOUSE]	6.14	3	3	6	12	0.868	1.106	0.966	0.990	28.53	6.14	11	12	847	96.3	7.59
Q9D1I5	Methylmalonyl-CoA epimerase, mitochondrial OS=Mus musculus GN=Mcee PE=2 SV=1 - [MCEE_MOUSE]	44.38	1	4	5	11	1.319	1.094	1.440	0.990	42.62	44.38	7	11	178	19.0	9.09

Q9JI18	Low-density lipoprotein receptor-related protein 1B OS=Mus musculus GN=Lrp1b PE=2 SV=1 - [LRP1B_MOUSE]	6.00	4	23	27	57	1.378	1.273	1.144	0.991	150.46	6.00	41	57	4599	513.3	5.39
Q52KF7	Disks large homolog 3 OS=Mus musculus GN=Dlg3 PE=2 SV=1 - [Q52KF7_MOUSE]	53.98	3	32	34	217	0.866	1.503	1.354	0.991	670.04	53.98	62	217	817	90.3	7.03
Q7TQF7	Amphiphysin OS=Mus musculus GN=Amph PE=1 SV=1 - [AMPH_MOUSE]	43.44	1	26	28	397	1.256	1.694	1.493	0.991	1121.55	43.44	49	397	686	75.0	4.63
O55128	Histone deacetylase complex subunit SAP18 OS=Mus musculus GN=Sap18 PE=1 SV=1 - [SAP18_MOUSE]	38.56	5	6	6	38	1.016	0.854	0.853	0.991	85.40	38.56	11	38	153	17.6	9.35
A2AJ88-2	Isoform 2 of Patatin-like phospholipase domain-containing protein 7 OS=Mus musculus GN=Pnpla7 - [illegible]	4.15	4	4	5	8	0.438	0.918	0.805	0.991	10.37	4.15	7	8	1326	147.5	7.44
P25785	Metalloproteinase inhibitor 2 OS=Mus musculus GN=Timp2 PE=1 SV=2 - [TIMP2_MOUSE]	48.64	3	11	11	28	2.320	1.260	0.824	0.991	81.08	48.64	14	28	220	24.3	7.49
Q810L4	Transmembrane protein 39B OS=Mus musculus GN=Tmem39b PE=1 SV=1 - [TM39B_MOUSE]	2.03	1	1	1	2	1.245	1.006	1.022	0.991	5.01	2.03	2	2	492	56.3	9.45
Q01338	Alpha-2A adrenergic receptor OS=Mus musculus GN=Adra2a PE=1 SV=1 -	17.78	2	7	7	27	1.450	0.999	0.846	0.991	75.25	17.78	11	27	450	48.8	9.69
Q9WTQ5	A-kinase anchor protein 12 OS=Mus musculus GN=Akap12 PE=1 SV=1 - [AKA12_MOUSE]	30.82	2	35	35	154	1.639	0.626	0.990	0.991	483.15	30.82	59	154	1684	180.6	4.44
Q99L85	Elongator complex protein 5 OS=Mus musculus GN=Elp5 PE=2 SV=1 - [ELP5_MOUSE]	25.33	5	6	6	18	0.733	1.138	0.871	0.991	59.22	25.33	10	18	300	33.5	5.49
Q9CXD6	Mitochondrial calcium uniporter regulator 1 OS=Mus musculus GN=Mcur1 PE=2 SV=1 - [MCUR1_MOUSE]	10.88	3	3	5	7	1.171	1.571	0.822	0.991	12.42	10.88	6	7	340	37.8	10.20
P55284	Cadherin-5 OS=Mus musculus GN=Cdh5 PE=1 SV=2 - [CADH5_MOUSE]	20.03	1	13	13	36	1.457	1.991	0.973	0.991	111.96	20.03	20	36	784	87.8	5.30
Q6P1H6-2	Isoform 2 of Ankyrin repeat and LEM domain-containing protein 2 OS=Mus musculus GN=Ankle2 - [illegible]	18.38	4	17	17	28	1.198	1.203	1.042	0.991	72.59	18.38	23	28	963	106.1	7.37
Q6PD26	GPI transamidase component PIG-S OS=Mus musculus GN=Pigs PE=1 SV=3 - [PIGS_MOUSE]	27.93	1	10	10	24	0.624	0.835	0.759	0.992	73.34	27.93	17	24	555	61.7	6.93
Q8K2P6	Rieske domain-containing protein OS=Mus musculus GN=Rfesd PE=1 SV=1 - [RFESD_MOUSE]	41.40	2	6	6	17	2.444	1.185	0.997	0.992	34.90	41.40	9	17	157	18.0	6.86
Q91YY2	Beta-1,4-galactosyltransferase 3 OS=Mus musculus GN=B4galt3 PE=1 SV=1 -	4.30	1	2	2	3	1.121	0.983	0.973	0.992	3.93	4.30	2	3	395	44.1	9.25
Q9Z2A5	Arginyl-tRNA--protein transferase 1 OS=Mus musculus GN=Ate1 PE=1 SV=2 - [ATE1_MOUSE]	28.29	4	10	11	30	1.155	1.054	0.834	0.992	80.28	28.29	16	30	516	59.1	8.19
P35550	rRNA 2'-O-methyltransferase fibrillarin OS=Mus musculus GN=Fbl PE=1 SV=2 - [FBRL_MOUSE]	33.64	1	7	9	55	0.658	0.643	0.781	0.992	122.08	33.64	18	55	327	34.3	10.24
Q9D7H3	RNA 3'-terminal phosphate cyclase OS=Mus musculus GN=RtcA PE=2 SV=2 - [RTCA_MOUSE]	43.72	4	12	12	43	0.749	0.903	0.829	0.992	107.91	43.72	20	43	366	39.2	7.90
P70335-2	Isoform 2 of Rho-associated protein kinase 1 OS=Mus musculus GN=Rock1 -	23.73	2	23	32	92	1.252	0.802	1.060	0.992	215.30	23.73	54	92	1353	157.9	5.86
O88895	Histone deacetylase 3 OS=Mus musculus GN=Hdac3 PE=1 SV=1 - [HDAC3_MOUSE]	8.96	3	3	4	11	0.558	0.794	0.964	0.992	29.35	8.96	7	11	424	48.3	5.26
Q9WVE8	Protein kinase C and casein kinase substrate in neurons protein 2 OS=Mus musculus GN=Pacsin2 PE=1 SV=1 - [illegible]	24.07	1	11	12	61	1.511	1.159	1.260	0.992	168.72	24.07	23	61	486	55.8	5.20
O70372	Telomerase reverse transcriptase OS=Mus musculus GN=Tert PE=1 SV=1 - [TERT_MOUSE]	1.78	1	1	2	6	1.895	1.307	1.233	0.992	14.12	1.78	3	6	1122	127.9	9.91
O35316	Sodium- and chloride-dependent taurine transporter OS=Mus musculus GN=Slc6a6 PE=1 SV=2 - [illegible]	4.03	1	1	3	27	0.646	1.148	1.003	0.992	72.31	4.03	6	27	621	69.8	7.23
Q8K4T3	STE20-related kinase adapter protein beta OS=Mus musculus GN=Stradb PE=2 SV=1 - [STRAB_MOUSE]	11.24	1	3	3	9	0.983	1.134	0.986	0.992	29.28	11.24	5	9	418	46.8	6.90

A2A6Q5	Cell division cycle protein 27 homolog OS=Mus musculus GN=Cdc27 PE=1 SV=1 - [CDC27_MOUSE]	6.55	4	5	5	12	0.834	0.810	1.027	0.992	25.90	6.55	10	12	825	91.8	7.02
Q61526	Receptor tyrosine-protein kinase erbB-3 OS=Mus musculus GN=Erbb3 PE=1 SV=2 - [ERBB3_MOUSE]	1.34	1	1	1	4	1.694	0.975	1.146	0.992	15.46	1.34	2	4	1339	147.5	6.55
Q9D0W5	Peptidyl-prolyl cis-trans isomerase-like 1 OS=Mus musculus GN=Ppil1 PE=2 SV=1 - [PPIL1_MOUSE]	33.73	1	6	6	28	0.807	0.925	0.947	0.992	68.18	33.73	10	28	166	18.2	7.99
Q8BML9	Glutaminyl-tRNA synthetase OS=Mus musculus GN=Qars PE=2 SV=1 - [Q8BML9_MOUSE]	36.90	4	24	25	85	0.915	1.031	0.897	0.992	269.26	36.90	44	85	775	87.6	7.31
Q9CYN2	Signal peptidase complex subunit 2 OS=Mus musculus GN=Spcs2 PE=2 SV=1 - [SPCS2_MOUSE]	38.50	1	7	7	22	1.099	1.199	0.802	0.992	86.14	38.50	10	22	226	25.0	8.57
D3YY42	Protein YIF1B OS=Mus musculus GN=Yif1b PE=2 SV=1 - [D3YY42_MOUSE]	19.92	4	3	3	5	0.380	1.054	0.894	0.992	17.74	19.92	4	5	251	27.5	9.47
Q8K2D3	Enhancer of mRNA-decapping protein 3 OS=Mus musculus GN=Edc3 PE=2 SV=1 - [EDC3_MOUSE]	36.02	3	15	15	44	0.993	0.892	0.991	0.992	109.33	36.02	27	44	508	55.9	7.09
D3Z7Q2	Uncharacterized protein OS=Mus musculus GN=Smim20 PE=2 SV=1 - [D3Z7Q2_MOUSE]	40.58	1	3	3	11	0.971	0.749	1.005	0.992	29.62	40.58	5	11	69	7.8	9.70
Q3UUG6-2	Isoform 2 of TBC1 domain family member 24 OS=Mus musculus GN=Tbc1d24 -	30.99	2	14	14	49	0.685	1.024	0.956	0.992	130.27	30.99	24	49	555	62.6	7.11
Q8C0V0	Serine/threonine-protein kinase tousled-like 1 OS=Mus musculus GN=Tlk1 PE=2 SV=2 - [TLK1_MOUSE]	8.88	6	2	6	12	1.532	1.013	1.169	0.992	32.41	8.88	10	12	766	86.6	8.79
G3UWD8	RING finger and CHY zinc finger domain-containing protein 1 OS=Mus musculus GN=Rchy1 PE=4 SV=1 - [illegible]	11.76	2	3	3	6	1.743	0.894	1.306	0.992	14.34	11.76	5	6	221	25.2	7.30
F6XC25	Coiled-coil and C2 domain-containing protein 1B (Fragment) OS=Mus musculus GN=Cc2d1b PE=4 SV=1 - [illegible]	20.00	3	15	15	34	1.738	0.675	1.008	0.992	98.25	20.00	25	34	770	85.0	5.87
Q9D6Z1	Nucleolar protein 56 OS=Mus musculus GN=Nop56 PE=1 SV=2 - [NOP56_MOUSE]	33.62	7	8	16	45	0.813	0.788	0.793	0.992	123.73	33.62	28	45	580	64.4	9.14
Q3U0S6	Ras-interacting protein 1 OS=Mus musculus GN=Rasip1 PE=1 SV=3 - [RAIN_MOUSE]	7.49	1	4	4	7	0.780	0.729	1.046	0.992	30.19	7.49	5	7	961	103.5	7.83
G5E896	Enhancer of mRNA decapping 4, isoform CRA_b OS=Mus musculus GN=Edc4 PE=4 SV=1 - [G5E896_MOUSE]	27.10	5	3	27	76	1.095	1.239	0.824	0.992	232.25	27.10	45	76	1406	152.4	5.82
Q80U49	Centrosomal protein of 170 kDa protein B OS=Mus musculus GN=Cep170b PE=1 SV=2 - [C170B_MOUSE]	47.46	2	57	60	230	1.020	0.726	1.274	0.992	635.66	47.46	103	230	1574	170.7	6.87
Q6NXH3	Vasculin OS=Mus musculus GN=Gpbp1 PE=1 SV=1 - [GPBP1_MOUSE]	10.15	4	3	4	11	2.298	1.708	1.352	0.992	49.38	10.15	5	11	473	53.4	6.90
Q9CZU4	GTPase Era, mitochondrial OS=Mus musculus GN=Eral1 PE=2 SV=1 - [ERAL1_MOUSE]	9.38	1	2	2	2	0.815	1.133	0.814	0.993	6.17	9.38	2	2	437	48.2	8.78
B1AXP6	Mitochondrial import receptor subunit TOM5 homolog OS=Mus musculus GN=Tomm5 PE=3 SV=1 - [illegible]	58.82	4	4	4	10	0.858	1.087	1.032	0.993	22.27	58.82	6	10	51	6.0	9.60
Q9JL56	Glycerophosphodiester phosphodiesterase 1 OS=Mus musculus GN=Gde1 PE=2 SV=1 - [GDE1_MOUSE]	24.77	2	8	8	31	0.645	0.809	0.961	0.993	80.14	24.77	15	31	331	37.6	6.90
Q64433	10 kDa heat shock protein, mitochondrial OS=Mus musculus GN=Hspe1 PE=1 SV=2 - [CH10_MOUSE]	81.37	1	11	11	292	1.930	0.870	1.390	0.993	685.74	81.37	21	292	102	11.0	8.35
Q8R2U0-2	Isoform 2 of Nucleoporin SEH1 OS=Mus musculus GN=Seh1l - [SEH1_MOUSE]	40.06	2	9	9	20	0.991	1.037	0.859	0.993	67.59	40.06	14	20	357	39.4	7.88
P15920	V-type proton ATPase 116 kDa subunit a isoform 2 OS=Mus musculus GN=Atp6v0a2 PE=1 SV=2 [VPP2_MOUSE]	9.35	2	8	8	16	1.141	0.909	0.854	0.993	42.07	9.35	11	16	856	98.1	6.64
G3X9R0	MCG68164 OS=Mus musculus GN=Cylc1 PE=4 SV=1 - [G3X9R0_MOUSE]	5.47	1	3	3	3	3.456	2.648	1.236	0.993	6.92	5.47	3	3	640	71.4	9.74

Q921T2-2	Isoform 2 of Torsin-1A-interacting protein 1 OS=Mus musculus GN=Tor1aip1 -	29.86	8	13	13	48	0.764	0.972	0.869	0.993	143.16	29.86	22	48	576	64.8	7.14
Q8BLR7	Rho-related GTP-binding protein Rho6 OS=Mus musculus GN=Rnd1 PE=2 SV=1 - [RND1_MOUSE]	3.88	1	1	1	1	1.391	1.001	0.829	0.993	2.64	3.88	1	1	232	26.0	7.75
Q8VBW5-2	Isoform 2 of HMG box transcription factor BBX OS=Mus musculus GN=Bbx - [BBX_MOUSE]	3.98	5	2	3	3	1.390	1.145	1.045	0.993	2.87	3.98	3	3	829	91.8	9.06
Q8CAM5	Ras-related protein Rab-36 OS=Mus musculus GN=Rab36 PE=2 SV=1 - [RAB36_MOUSE]	6.37	1	1	1	6	2.016	0.898	0.996	0.993	19.14	6.37	2	6	267	29.8	6.23
Q91Z50	Flap endonuclease 1 OS=Mus musculus GN=Fen1 PE=2 SV=1 - [Q91Z50_MOUSE]	11.05	2	4	4	7	1.010	0.723	1.015	0.993	18.13	11.05	7	7	380	42.6	8.34
O08677	Kininogen-1 OS=Mus musculus GN=Kng1 PE=1 SV=1 - [KNG1_MOUSE]	26.78	4	16	17	82	3.103	1.731	0.727	0.993	262.09	26.78	31	82	661	73.1	6.54
Q8BYA0	Tubulin-specific chaperone D OS=Mus musculus GN=Tbcd PE=2 SV=1 - [TBCD_MOUSE]	30.43	3	28	29	74	0.733	0.791	0.797	0.993	216.83	30.43	47	74	1196	133.2	6.51
Q8BHN3	Neutral alpha-glucosidase AB OS=Mus musculus GN=Ganab PE=1 SV=1 - [GANAB_MOUSE]	29.13	3	24	24	116	0.797	1.008	0.876	0.993	349.20	29.13	45	116	944	106.8	6.06
Q921W4	Quinone oxidoreductase-like protein 1 OS=Mus musculus GN=Cryzl1 PE=2 SV=1 - [QORL1_MOUSE]	29.60	6	10	10	28	1.114	1.141	0.912	0.993	73.96	29.60	18	28	348	38.7	5.97
E9PV57	Peroxisome proliferator-activated receptor delta (Fragment) OS=Mus musculus GN=Ppard PE=2 SV=1 - [E9PV57_MOUSE]	7.54	2	1	1	1	0.742	0.547	0.987	0.993	0.00	7.54	1	1	252	28.2	8.18
P39038	Cadherin-4 OS=Mus musculus GN=Cdh4 PE=2 SV=1 - [CADH4_MOUSE]	17.74	2	12	12	36	0.891	1.987	1.120	0.993	70.72	17.74	21	36	913	100.0	4.81
G3UWD5	MCG66639, isoform CRA_b OS=Mus musculus GN=A330050F15Rik PE=4 SV=1 - [G3UWD5_MOUSE]	11.59	1	1	1	2	2.301	0.428	0.857	0.993	4.36	11.59	2	2	69	7.7	9.55
O54692	Centromere/kinetochore protein zw10 homolog OS=Mus musculus GN=Zw10 PE=2 SV=3 - [ZW10_MOUSE]	20.92	1	13	13	27	0.755	0.954	0.827	0.993	78.52	20.92	20	27	779	88.0	5.92
Q68EF6	Brain-enriched guanylate kinase-associated protein OS=Mus musculus GN=Begain PE=1 SV=2 - [BEGIN_MOUSE]	46.83	2	21	21	74	1.109	1.350	1.504	0.993	202.41	46.83	35	74	600	65.3	5.87
P06728	Apolipoprotein A-IV OS=Mus musculus GN=Apoa4 PE=2 SV=3 - [APOA4_MOUSE]	71.14	1	24	24	119	3.120	3.715	0.515	0.993	334.86	71.14	42	119	395	45.0	5.47
P42669	Transcriptional activator protein Pur-alpha OS=Mus musculus GN=Pura PE=1 SV=1 - [PURA_MOUSE]	66.36	1	21	25	175	1.117	0.823	0.883	0.993	453.93	66.36	43	175	321	34.9	6.44
Q9D3D0	Alpha-tocopherol transfer protein-like OS=Mus musculus GN=Ttpal PE=2 SV=3 - [TTPAL_MOUSE]	14.58	3	4	4	17	0.972	1.883	1.313	0.993	47.26	14.58	8	17	343	38.8	6.44
F8VPK0	Protein Ttc37 OS=Mus musculus GN=Ttc37 PE=2 SV=1 - [F8VPK0_MOUSE]	15.04	1	19	20	53	0.706	0.858	0.852	0.994	163.44	15.04	35	53	1563	173.8	7.12
A2ACM0	Regulatory-associated protein of mTOR OS=Mus musculus GN=Rptor PE=4 SV=1 - [A2ACM0_MOUSE]	18.13	10	20	21	63	0.626	0.923	0.884	0.994	182.25	18.13	36	63	1335	149.4	6.87
E9Q390	Myoferlin OS=Mus musculus GN=Myof PE=2 SV=2 - [E9Q390_MOUSE]	6.15	5	8	10	15	1.286	2.269	0.980	0.994	29.89	6.15	13	15	2048	233.2	6.23
Q91YM2	Rho GTPase-activating protein 35 OS=Mus musculus GN=Arhgap35 PE=1 SV=3 -	42.23	1	50	50	162	0.799	0.974	0.916	0.994	450.32	42.23	88	162	1499	170.3	6.61
Q9CYG7	Mitochondrial import receptor subunit TOM34 OS=Mus musculus GN=Tomm34 PE=2 SV=1 - [TOM34_MOUSE]	46.93	2	12	12	48	1.018	1.103	0.863	0.994	106.23	46.93	23	48	309	34.3	9.14
Q9Z2Y8	Proline synthase co-transcribed bacterial homolog protein OS=Mus musculus GN=Prosc PE=1 SV=1 - [PROSC_MOUSE]	46.72	2	12	13	58	1.766	1.077	0.977	0.994	158.71	46.72	22	58	274	30.0	8.27
E9Q3G7	Probable phospholipid-transporting ATPase IH OS=Mus musculus GN=Atp11a PE=2 SV=1 - [E9Q3G7_MOUSE]	7.97	4	6	9	17	1.303	0.845	0.893	0.994	39.92	7.97	13	17	1142	130.6	6.60
P15327	Bisphosphoglycerate mutase OS=Mus musculus GN=Bpgm PE=2 SV=2 - [PMGE_MOUSE]	57.14	4	11	11	56	1.931	1.191	0.605	0.994	175.67	57.14	20	56	259	30.0	7.06

B2RXC6	DNA-directed RNA polymerase OS=Mus musculus GN=Polr3a PE=2 SV=1 - [B2RXC6_MOUSE]	7.55	1	7	8	14	0.987	0.894	0.857	0.994	30.89	7.55	12	14	1390	155.6	8.50
Q8BHL8	Proteasome inhibitor PI31 subunit OS=Mus musculus GN=Psmf1 PE=1 SV=1 - [PSMF1_MOUSE]	21.77	1	7	7	26	1.658	1.066	1.054	0.994	55.41	21.77	12	26	271	29.6	5.25
Q9JLN9	Serine/threonine-protein kinase mTOR OS=Mus musculus GN=Mtor PE=1 SV=2 - [MTOR_MOUSE]	25.54	3	53	56	175	0.654	0.932	0.876	0.994	536.75	25.54	95	175	2549	288.6	7.17
G3X920	Armadillo repeat containing 8, isoform CRA_b OS=Mus musculus GN=Armc8 PE=4 SV=1 - [G3X920_MOUSE]	23.48	2	4	14	44	0.776	1.511	0.999	0.994	116.83	23.48	24	44	673	75.4	6.73
O55222	Integrin-linked protein kinase OS=Mus musculus GN=Ilk PE=1 SV=2 - [ILK_MOUSE]	24.56	3	11	12	37	0.775	1.289	0.918	0.994	92.90	24.56	20	37	452	51.3	8.07
Q9WV34	MAGUK p55 subfamily member 2 OS=Mus musculus GN=Mpp2 PE=1 SV=1 - [MPP2_MOUSE]	62.32	2	30	30	147	0.750	1.555	1.387	0.994	396.46	62.32	54	147	552	61.5	6.44
Q8BXR5	Sodium leak channel non-selective protein OS=Mus musculus GN=Nalcn PE=1 SV=2 - [NALCN_MOUSE]	2.88	3	4	5	5	5.278	1.787	1.307	0.994	8.86	2.88	5	5	1738	200.3	8.68
Q8VDS3	Chromobox protein homolog 7 OS=Mus musculus GN=Cbx7 PE=1 SV=1 - [CBX7_MOUSE]	6.33	3	1	1	2	1.273	1.105	1.525	0.994	4.37	6.33	2	2	158	18.1	5.20
Q6PEV3	WAS/WASL-interacting protein family member 2 OS=Mus musculus GN=Wipf2 PE=2 SV=1 - [WIPF2_MOUSE]	69.09	2	23	23	142	1.501	0.773	1.307	0.994	416.05	69.09	41	142	440	46.3	10.99
P62855	40S ribosomal protein S26 OS=Mus musculus GN=Rps26 PE=2 SV=3 - [RS26_MOUSE]	26.09	1	3	3	5	0.637	1.024	0.789	0.994	12.54	26.09	4	5	115	13.0	11.00
Q8CB62	Centrobin OS=Mus musculus GN=Cntrob PE=2 SV=2 - [CNTRB_MOUSE]	3.16	2	2	2	3	0.909	0.972	1.201	0.994	2.90	3.16	3	3	887	99.3	5.55
P06801	NADP-dependent malic enzyme OS=Mus musculus GN=Me1 PE=1 SV=2 - [MAOX_MOUSE]	50.70	1	22	22	109	0.767	0.657	0.756	0.994	304.97	50.70	40	109	572	63.9	7.44
A2AEW0	Transcription factor E3 OS=Mus musculus GN=Tfe3 PE=2 SV=1 - [A2AEW0_MOUSE]	24.77	16	3	7	8	1.258	1.478	1.140	0.995	20.64	24.77	8	8	537	57.7	6.37
D6RJ69	Bardet-Biedl syndrome 5 protein homolog OS=Mus musculus GN=Bbs5 PE=2 SV=1 - [D6RJ69_MOUSE]	20.45	3	1	1	2	0.754	1.640	0.744	0.995	2.88	20.45	2	2	88	9.9	6.01
Q3UKJ7	WD40 repeat-containing protein SMU1 OS=Mus musculus GN=Smu1 PE=2 SV=2 - [SMU1_MOUSE]	28.46	2	11	11	24	1.170	1.251	0.852	0.995	57.44	28.46	19	24	513	57.5	7.18
Q922Q9	Chitinase domain-containing protein 1 OS=Mus musculus GN=Chid1 PE=2 SV=1 - [illegible]	22.90	4	8	8	17	0.816	0.986	0.846	0.995	46.50	22.90	13	17	393	44.9	7.97
Q8R310	Transmembrane and coiled coil domains protein 3 OS=Mus musculus GN=Tmcc3 PE=1 SV=2 - [TMCC3_MOUSE]	29.98	5	10	11	31	1.300	0.982	0.864	0.995	75.78	29.98	20	31	477	53.7	8.60
A2AGS6	Tumor protein p53-inducible protein 11 (Fragment) OS=Mus musculus GN=Trp53i11 PE=2 SV=1 - [illegible]	19.54	2	2	2	4	0.752	2.302	0.881	0.995	9.81	19.54	3	4	87	9.6	9.60
E9Q9X1	Dystonin OS=Mus musculus GN=Dst PE=4 SV=2 - [E9Q9X1_MOUSE]	24.48	4	5	162	514	1.294	0.619	0.903	0.995	1497.42	24.48	268	514	7406	835.2	5.31
Q60963	Platelet-activating factor acetylhydrolase OS=Mus musculus GN=Pla2g7 PE=2 SV=2 - [PAFA_MOUSE]	29.55	5	12	12	28	1.374	1.035	0.946	0.995	83.00	29.55	19	28	440	49.2	7.12
Q91WQ5	TAF5-like RNA polymerase II p300/CBP-associated factor-associated factor 65 kDa subunit 5L OS=Mus musculus GN=Taf5l PE=2 SV=1 - [TAF5L_MOUSE]	1.87	1	1	1	1	0.894	0.935	1.039	0.995	2.86	1.87	1	1	589	65.9	5.92
Q8CJ19	Protein-methionine sulfoxide oxidase MICAL3 OS=Mus musculus GN=Mical3 PE=1 SV=2 - [MICA3_MOUSE]	29.35	9	51	56	186	0.826	0.938	1.191	0.995	489.29	29.35	96	186	1993	223.6	5.47
E9Q6X0	Microtubule-associated protein RP/EB family member 2 OS=Mus musculus GN=Mapre2 PE=2 SV=1 - [illegible]	63.73	5	13	16	89	0.737	0.960	1.041	0.995	249.65	63.73	28	89	284	32.2	5.26
Q9WVQ1	Membrane-associated guanylate kinase, WW and PDZ domain-containing protein 2 OS=Mus musculus GN=Magi2 PE=1 SV=2 - [MAGI2_MOUSE]	34.90	4	35	38	132	1.110	0.984	1.085	0.995	353.98	34.90	64	132	1275	140.8	6.19

Q922B9-3	Isoform 3 of Sperm-specific antigen 2 homolog OS=Mus musculus GN=Ssfa2 -	1.36	4	2	2	2	2.401	1.429	0.956	0.995	3.50	1.36	2	2	1101	120.1	5.38
Q9D415	Disks large-associated protein 1 OS=Mus musculus GN=Dlgap1 PE=1 SV=3 -	34.48	10	12	29	114	0.659	1.429	1.483	0.995	311.29	34.48	41	114	992	110.3	7.09
Q9DAK9	14 kDa phosphohistidine phosphatase OS=Mus musculus GN=Phpt1 PE=1 SV=1 - [PHP14_MOUSE]	51.61	1	7	7	74	1.487	0.897	0.996	0.996	186.08	51.61	13	74	124	14.0	5.53
Q91XY0	MCG133388, isoform CRA_m OS=Mus musculus GN=Pcdhga8 PE=2 SV=1 - [Q91XY0_MOUSE]	8.58	1	2	7	28	1.128	1.421	1.239	0.996	76.19	8.58	14	28	932	101.4	5.11
P61294	Ras-related protein Rab-6B OS=Mus musculus GN=Rab6b PE=1 SV=1 - [RAB6B_MOUSE]	60.58	1	6	12	134	0.714	0.904	1.030	0.996	361.46	60.58	22	134	208	23.4	5.53
O55091	Protein IMPACT OS=Mus musculus GN=Impact PE=1 SV=2 - [IMPCT_MOUSE]	39.94	1	13	14	88	0.991	0.716	0.709	0.996	266.54	39.94	23	88	318	36.3	5.05
Q99NB8	Ubiquilin-4 OS=Mus musculus GN=Ubqln4 PE=1 SV=1 - [UBQL4_MOUSE]	19.13	1	5	8	119	1.696	1.105	1.135	0.996	322.21	19.13	15	119	596	63.5	5.03
G3X956	FACT complex subunit SPT16 OS=Mus musculus GN=Supt16 PE=4 SV=1 - [G3X956_MOUSE]	20.34	2	22	22	57	0.805	0.717	0.877	0.996	161.61	20.34	36	57	1047	119.8	5.66
Q9Z1T1	AP-3 complex subunit beta 1 OS=Mus musculus GN=Ap3b1 PE=1 SV=2 - [AP3B1_MOUSE]	17.92	1	15	23	55	0.853	1.295	0.948	0.996	116.18	17.92	39	55	1105	122.7	5.66
E9PYE2	tRNA wybutosine-synthesizing protein 3 homolog OS=Mus musculus GN=Tyw3 PE=2 SV=1 - [E9PYE2_MOUSE]	18.06	3	2	2	5	1.412	0.935	1.186	0.996	14.94	18.06	3	5	144	15.7	7.34
Q9QUR8	Semaphorin-7A OS=Mus musculus GN=Sema7a PE=1 SV=1 - [SEM7A_MOUSE]	27.71	1	15	15	42	1.243	0.559	1.126	0.996	120.04	27.71	24	42	664	74.9	7.74
A2A699	Protein FAM171A2 OS=Mus musculus GN=Fam171a2 PE=2 SV=1 - [F1712_MOUSE]	18.25	2	12	12	48	0.601	1.211	1.097	0.996	165.69	18.25	22	48	822	87.4	8.05
Q05CL8	La-related protein 7 OS=Mus musculus GN=Larp7 PE=1 SV=2 - [LARP7_MOUSE]	21.58	3	11	11	22	0.871	0.979	0.937	0.996	53.21	21.58	16	22	570	64.8	9.54
O08747	Netrin receptor UNC5C OS=Mus musculus GN=Unc5c PE=1 SV=1 - [UNC5C_MOUSE]	8.16	3	7	7	14	0.665	1.528	1.018	0.996	36.06	8.16	11	14	931	103.0	6.01
Q8BPG6	Sulfatase-modifying factor 2 OS=Mus musculus GN=Sumf2 PE=2 SV=2 - [SUMF2_MOUSE]	21.43	2	4	5	11	1.383	1.566	0.990	0.996	35.44	21.43	9	11	308	34.7	7.11
Q3UJP5	Protein C8orf37 homolog OS=Mus musculus PE=1 SV=1 - [CH037_MOUSE]	27.75	2	6	6	27	1.547	1.177	1.140	0.996	58.53	27.75	11	27	209	23.8	6.86
Q69ZZ6	Transmembrane and coiled coil domains protein 1 OS=Mus musculus GN=Tmcc1 PE=1 SV=2 - [TMCC1_MOUSE]	28.35	8	13	15	37	1.232	0.979	1.232	0.996	87.06	28.35	24	37	649	71.6	6.55
E9PXG1	Transporter OS=Mus musculus GN=Slc6a12 PE=2 SV=1 - [E9PXG1_MOUSE]	8.26	4	3	4	22	0.517	3.304	0.553	0.996	64.83	8.26	6	22	557	63.0	6.11
A2RSJ4	UHRF1-binding protein 1-like OS=Mus musculus GN=Uhrf1bp1l PE=2 SV=2 - [UH1BL_MOUSE]	29.24	1	31	35	110	0.862	1.101	1.158	0.996	310.69	29.24	55	110	1457	161.8	6.61
Q5S003	Sperm-associated antigen 17 OS=Mus musculus GN=Spag17 PE=1 SV=1 - [SPG17_MOUSE]	2.85	2	4	5	10	2.071	1.286	1.611	0.997	12.92	2.85	5	10	2175	246.2	6.01
Q9CXE7	Transmembrane emp24 domain-containing protein 5 OS=Mus musculus GN=Tmed5 PE=2 SV=1 - [TMED5_MOUSE]	17.03	6	4	4	15	1.580	1.048	0.750	0.997	37.21	17.03	8	15	229	26.2	4.93
Q99LI2	Chloride channel CLIC-like protein 1 OS=Mus musculus GN=Clcc1 PE=1 SV=1 - [CLCC1_MOUSE]	17.25	2	8	8	14	1.025	1.138	1.026	0.997	34.02	17.25	13	14	539	60.6	5.68
Q3TZZ7	Extended synaptotagmin-2 OS=Mus musculus GN=Esyt2 PE=1 SV=1 - [ESYT2_MOUSE]	23.79	2	15	16	41	0.649	1.121	1.025	0.997	139.08	23.79	29	41	845	94.1	7.75
Q8BHS3	Pre-mRNA-splicing factor RBM22 OS=Mus musculus GN=Rbm22 PE=2 SV=1 - [RBM22_MOUSE]	19.05	1	7	8	16	1.081	0.782	1.152	0.997	41.03	19.05	11	16	420	46.9	8.54
Q9ES34	Ubiquitin-protein ligase E3B OS=Mus musculus GN=Ube3b PE=2 SV=3 - [UBE3B_MOUSE]	7.85	1	6	6	14	1.168	0.762	1.334	0.997	18.80	7.85	8	14	1070	122.7	8.32
Q9DAV9	Trimeric intracellular cation channel type B OS=Mus musculus GN=Tmem38b PE=2 SV=1 - [TM38B_MOUSE]	3.77	1	2	2	10	0.476	0.783	0.907	0.997	17.18	3.77	2	10	292	32.6	9.11

Q9CVD2	Ataxin-3 OS=Mus musculus GN=Atxn3 PE=1 SV=2 - [ATX3_MOUSE]	17.75	5	6	6	21	1.377	1.216	1.314	0.997	51.44	17.75	9	21	355	40.5	4.83
Q9CWI3	BRCA2 and CDKN1A-interacting protein OS=Mus musculus GN=Bccip PE=2 SV=1 - [BCCIP_MOUSE]	17.72	1	5	5	8	1.326	0.946	0.921	0.997	22.07	17.72	7	8	316	35.9	4.45
Q9QZ73	DCN1-like protein 1 OS=Mus musculus GN=Dcun1d1 PE=2 SV=1 - [DCNL1_MOUSE]	32.43	5	6	7	24	1.126	0.912	0.974	0.997	70.06	32.43	12	24	259	30.1	5.34
G3X8P5	Sodium/iodide cotransporter OS=Mus musculus GN=Slc5a5 PE=3 SV=1 -	1.62	2	1	1	2	1.264	3.523	0.767	0.997	1.64	1.62	2	2	618	65.5	7.49
Q9ERF3	WD repeat-containing protein 61 OS=Mus musculus GN=Wdr61 PE=2 SV=1 -	35.08	4	7	7	26	0.898	1.035	0.914	0.997	80.72	35.08	13	26	305	33.8	5.36
P15307	Proto-oncogene c-Rel OS=Mus musculus GN=Rel PE=1 SV=2 - [REL_MOUSE]	2.39	2	1	1	2	1.516	1.046	1.238	0.997	6.80	2.39	2	2	587	64.9	6.54
O55186	CD59A glycoprotein OS=Mus musculus GN=Cd59a PE=2 SV=1 - [CD59A_MOUSE]	13.82	1	1	1	2	3.014	0.839	0.930	0.997	9.17	13.82	1	2	123	13.6	7.47
P56391	Cytochrome c oxidase subunit 6B1 OS=Mus musculus GN=Cox6b1 PE=1 SV=2 -	70.93	1	8	8	202	1.834	0.705	1.360	0.997	518.77	70.93	16	202	86	10.1	8.72
E9Q6Q2	Serine/threonine-protein kinase WNK2 OS=Mus musculus GN=Wnk2 PE=2 SV=1 - [E9Q6Q2_MOUSE]	26.17	18	32	39	154	0.798	0.865	1.295	0.997	450.12	26.17	69	154	1991	211.0	5.78
Q9JIY5	Serine protease HTRA2, mitochondrial OS=Mus musculus GN=Htra2 PE=1 SV=2 - [HTRA2_MOUSE]	26.42	4	2	10	40	1.073	0.716	0.762	0.997	123.71	26.42	19	40	458	49.3	9.60
Q80YA7	Dipeptidyl peptidase 8 OS=Mus musculus GN=Dpp8 PE=1 SV=1 - [DPP8_MOUSE]	24.44	1	14	15	61	0.951	1.002	0.940	0.997	172.95	24.44	26	61	892	102.1	5.81
Q8BFU4	GAIP/RGS19 short isoform OS=Mus musculus GN=Rgs19 PE=2 SV=1 - [Q8BFU4_MOUSE]	10.31	5	1	2	8	0.521	1.424	0.997	0.997	22.85	10.31	4	8	194	22.2	6.38
Q9JIG7	Coiled-coil domain-containing protein 22 OS=Mus musculus GN=Ccdc22 PE=1 SV=1 - [CCD22_MOUSE]	40.83	1	20	20	71	0.849	1.259	0.908	0.997	224.02	40.83	36	71	627	70.8	6.01
O54774	AP-3 complex subunit delta-1 OS=Mus musculus GN=Ap3d1 PE=1 SV=1 - [AP3D1_MOUSE]	36.78	1	33	33	102	0.534	0.930	1.027	0.997	297.98	36.78	53	102	1199	135.0	7.37
Q9WV89-4	Isoform 4 of Syntaxin-binding protein 4 OS=Mus musculus GN=Stxbp4 - [STXB4_MOUSE]	5.77	4	2	3	5	1.197	1.108	1.208	0.997	11.56	5.77	4	5	520	57.5	5.44
Q8BZI0	Actin filament-associated protein 1-like 1 OS=Mus musculus GN=Afap1l1 PE=1 SV=1 - [AF1L1_MOUSE]	11.20	2	5	5	8	1.374	0.499	1.302	0.997	19.52	11.20	7	8	768	86.6	6.77
Q9QWR8	Alpha-N-acetylgalactosaminidase OS=Mus musculus GN=Naga PE=2 SV=2 -	19.76	1	7	8	14	1.611	1.224	1.069	0.997	39.21	19.76	11	14	415	47.2	6.44
Q80XC6	Protein NRDE2 homolog OS=Mus musculus GN=Nrde2 PE=2 SV=3 - [NRDE2_MOUSE]	3.16	1	3	3	4	1.334	0.775	1.388	0.997	5.00	3.16	3	4	1172	133.4	7.93
O35144-2	Isoform 2 of Telomeric repeat-binding factor 2 OS=Mus musculus GN=Terf2 -	15.32	5	5	5	10	1.308	1.063	1.011	0.997	29.63	15.32	8	10	470	51.8	9.55
O08919	Numb-like protein OS=Mus musculus GN=Numbl PE=1 SV=3 - [NUMBL_MOUSE]	26.16	1	11	14	60	0.886	1.367	1.272	0.997	184.33	26.16	27	60	604	64.1	8.82
P01101	Proto-oncogene c-Fos OS=Mus musculus GN=Fos PE=1 SV=1 - [FOS_MOUSE]	4.47	1	1	1	1	1.810	2.214	0.818	0.997	3.17	4.47	1	1	380	40.8	4.87
H3BLE4	Epidermal growth factor receptor substrate 15 (Fragment) OS=Mus musculus GN=Eps15 PE=4 SV=1 - [H3BLE4_MOUSE]	45.33	1	1	4	8	1.751	0.993	1.186	0.997	22.09	45.33	6	8	150	15.5	4.41
Q6QD59	Vesicle transport protein SEC20 OS=Mus musculus GN=Bnip1 PE=2 SV=1 - [SEC20_MOUSE]	44.74	4	9	9	35	0.945	1.081	0.932	0.998	102.66	44.74	16	35	228	26.2	8.75
G3X9V4	Glutamate receptor ionotropic, NMDA 2B OS=Mus musculus GN=Grin2b PE=4 SV=1 - [G3X9V4_MOUSE]	23.41	2	28	30	105	0.611	1.672	1.644	0.998	278.55	23.41	52	105	1482	165.9	6.87
P61460	DEP domain-containing protein 5 OS=Mus musculus GN=Depdc5 PE=1 SV=2 -	14.14	8	1	16	36	0.827	1.216	1.212	0.998	90.13	14.14	24	36	1591	180.3	6.74

Q5SWD9-3	Isoform 3 of Pre-rRNA-processing protein TSR1 homolog OS=Mus musculus GN=Tsr1 - [TSR1_MOUSE]	9.42	3	3	3	4	1.459	1.084	0.830	0.998	13.10	9.42	4	4	382	43.0	8.63
Q3UHH2	Solute carrier family 22 member 23 OS=Mus musculus GN=Slc22a23 PE=2 SV=1 -	16.40	4	8	8	25	0.980	0.696	1.018	0.998	88.77	16.40	15	25	689	74.3	7.83
Q8K3J5-2	Isoform 2 of Zinc finger protein 131 OS=Mus musculus GN=Znf131 - [ZN131_MOUSE]	10.43	3	3	3	4	2.149	1.269	0.819	0.998	2.67	10.43	3	4	585	66.9	5.16
Q9D4F2	Presqualene diphosphate phosphatase OS=Mus musculus GN=Ppapdc2 PE=1 SV=1 - [PPAC2_MOUSE]	8.22	1	2	2	8	0.288	0.865	1.117	0.998	35.22	8.22	4	8	292	31.7	10.29
Q9WV69	Dematin OS=Mus musculus GN=Dmtn PE=1 SV=1 - [DEMA_MOUSE]	62.22	4	19	20	206	1.495	1.047	1.701	0.998	575.62	62.22	38	206	405	45.4	8.41
D3Z3V2	CMP-N-acetylneuraminate-beta-galactosamide-alpha-2,3-sialyltransferase 2 OS=Mus musculus GN=St3gal2 PE=2 SV=1 - [D3Z3V2_MOUSE]	18.15	2	4	4	7	0.777	0.898	0.796	0.998	12.42	18.15	5	7	303	34.6	8.53
F7BAB2	Protein Tmem132b OS=Mus musculus GN=Tmem132b PE=4 SV=1 - [F7BAB2_MOUSE]	16.60	1	15	15	41	1.073	2.539	1.714	0.998	124.72	16.60	24	41	1078	119.3	4.87
P60122	RuvB-like 1 OS=Mus musculus GN=Ruvbl1 PE=1 SV=1 - [RUVB1_MOUSE]	50.44	3	17	17	90	0.949	1.169	0.845	0.998	290.42	50.44	29	90	456	50.2	6.42
Q684R7-2	Isoform 2 of FRAS1-related extracellular matrix protein 1 OS=Mus musculus GN=Frem1 - [FREM1_MOUSE]	4.19	4	3	3	7	0.641	1.101	0.909	0.998	6.46	4.19	3	7	2172	242.4	6.38
B1ATI9	Growth arrest-specific protein 7 OS=Mus musculus GN=Gas7 PE=4 SV=1 - [B1ATI9_MOUSE]	47.57	4	18	19	99	1.236	1.464	1.894	0.998	277.02	47.57	33	99	412	47.2	7.59
Q921J2	GTP-binding protein Rheb OS=Mus musculus GN=Rheb PE=1 SV=1 - [RHEB_MOUSE]	41.85	1	9	9	41	0.655	1.071	0.897	0.998	117.75	41.85	16	41	184	20.4	5.92
F6WKE4	Epsilon-sarcoglycan (Fragment) OS=Mus musculus GN=Sgce PE=2 SV=1 - [F6WKE4_MOUSE]	9.49	8	3	3	11	1.149	1.208	0.991	0.998	21.55	9.49	6	11	411	46.4	6.24
Q9EPC2	Fibroblast growth factor 23 OS=Mus musculus GN=Fgf23 PE=1 SV=1 - [FGF23_MOUSE]	11.95	1	2	2	4	2.546	0.851	1.006	0.998	6.41	11.95	2	4	251	27.7	9.45
Q8BJ63-3	Isoform 3 of AP-5 complex subunit mu-1 OS=Mus musculus GN=Ap5m1 - [AP5M1_MOUSE]	7.49	3	2	2	3	1.580	1.099	1.690	0.999	6.23	7.49	3	3	307	34.3	8.34
Q99KH8	Serine/threonine-protein kinase 24 OS=Mus musculus GN=Stk24 PE=1 SV=1 - [STK24_MOUSE]	38.52	1	7	13	64	0.937	0.964	0.938	0.999	168.88	38.52	25	64	431	47.9	5.43
Q8CDG3	Deubiquitinating protein VCIP135 OS=Mus musculus GN=Vcpip1 PE=2 SV=1 -	34.26	2	35	35	121	0.836	0.979	1.187	0.999	349.12	34.26	62	121	1220	134.4	7.17
P46414	Cyclin-dependent kinase inhibitor 1B OS=Mus musculus GN=Cdkn1b PE=1 SV=2 -	45.69	1	6	6	46	1.994	1.015	1.293	0.999	139.63	45.69	12	46	197	22.2	7.02
E0CYG3	Sperm flagellar protein 2 OS=Mus musculus GN=Spef2 PE=2 SV=1 - [E0CYG3_MOUSE]	4.29	7	5	6	12	1.237	1.472	1.007	0.999	28.21	4.29	7	12	1724	197.8	5.86
Q91W43	Glycine dehydrogenase [decarboxylating], mitochondrial OS=Mus musculus GN=Gldc PE=1 SV=1 - [GCSP_MOUSE]	13.95	1	11	11	20	0.760	1.842	1.626	0.999	49.20	13.95	16	20	1025	113.2	7.56
Q9D067-4	Isoform 4 of Nuclear protein MDM1 OS=Mus musculus GN=Mdm1 - [MDM1_MOUSE]	10.26	6	6	6	10	1.091	0.870	1.195	0.999	22.40	10.26	8	10	663	74.5	9.33
Q8VEH5	EPM2A-interacting protein 1 OS=Mus musculus GN=Epm2aip1 PE=2 SV=1 - [EPMIP_MOUSE]	33.83	1	19	19	64	0.695	1.282	1.008	0.999	183.14	33.83	32	64	606	70.1	5.87
B1AY10	Transcriptional repressor NF-X1 OS=Mus musculus GN=Nfx1 PE=2 SV=1 - [NFX1_MOUSE]	7.09	3	8	8	19	1.057	0.697	1.103	0.999	45.49	7.09	12	19	1114	123.7	8.31
Q924C1	Exportin-5 OS=Mus musculus GN=Xpo5 PE=2 SV=1 - [XPO5_MOUSE]	16.28	3	18	18	79	0.736	1.023	0.810	0.999	202.41	16.28	31	79	1204	136.9	5.82
Q8BTW3	Exosome complex component MTR3 OS=Mus musculus GN=Exosc6 PE=1 SV=1 - [EXOS6_MOUSE]	12.82	1	3	3	6	0.807	0.932	0.914	0.999	14.83	12.82	5	6	273	28.4	6.11
Q8BTI9	Phosphatidylinositol 4,5-bisphosphate 3-kinase catalytic subunit beta isoform OS=Mus musculus GN=Pik3cb PE=1 SV=2 - [PK3CB_MOUSE]	9.40	1	8	9	17	1.306	1.059	1.045	0.999	52.96	9.40	13	17	1064	121.6	7.09

Q62356	Follistatin-related protein 1 OS=Mus musculus GN=Fstl1 PE=1 SV=2 - [FSTL1_MOUSE]	7.84	1	2	2	4	3.324	0.494	0.907	0.999	14.08	7.84	4	4	306	34.5	5.85
Q8C6M1	Ubiquitin carboxyl-terminal hydrolase 20 OS=Mus musculus GN=Usp20 PE=1 SV=1 - [UBP20_MOUSE]	13.43	3	11	11	20	0.826	0.990	0.947	0.999	51.25	13.43	17	20	916	102.1	6.16
E0CYH4	WD repeat-containing protein 26 OS=Mus musculus GN=Wdr26 PE=2 SV=1 -	24.48	5	13	13	38	1.074	1.186	1.010	0.999	121.90	24.48	24	38	625	68.8	6.15
D3YX13	Inhibitor of growth protein 3 (Fragment) OS=Mus musculus GN=Ing3 PE=2 SV=1 - [D3YX13_MOUSE]	6.06	3	2	2	3	1.607	1.074	1.200	0.999	4.94	6.06	3	3	264	29.5	6.35
F6RJV6	LanC-like protein 2 (Fragment) OS=Mus musculus GN=Lancl2 PE=2 SV=1 -	51.25	2	16	16	69	0.674	1.382	0.970	0.999	211.11	51.25	29	69	441	49.7	7.14
H3BL51	Retrotransposon-derived protein PEG10 (Fragment) OS=Mus musculus GN=Peg10 PE=2 SV=1 - [H3BL51_MOUSE]	8.40	4	2	2	3	1.218	1.308	0.897	0.999	6.72	8.40	3	3	369	41.7	4.67
Q6PD28	Protein Ppp2r5b OS=Mus musculus GN=Ppp2r5b PE=2 SV=1 - [Q6PD28_MOUSE]	38.23	1	13	15	63	0.551	0.747	0.964	0.999	201.77	38.23	27	63	497	57.3	6.84
Q8K0T0	Reticulon-1 OS=Mus musculus GN=Rtn1 PE=1 SV=1 - [RTN1_MOUSE]	52.56	1	19	26	223	1.194	1.467	1.750	0.999	572.07	52.56	49	223	780	83.5	4.58
P39053-4	Isoform 4 of Dynamin-1 OS=Mus musculus GN=Dnm1 - [DYN1_MOUSE]	78.13	9	6	62	960	0.539	1.793	1.074	0.999	2507.64	78.13	113	960	864	97.4	7.17
F8VQ70	Protein Scaper OS=Mus musculus GN=Scaper PE=2 SV=1 - [F8VQ70_MOUSE]	4.58	1	6	6	8	0.889	0.766	1.050	0.999	17.49	4.58	7	8	1398	157.7	7.31
P50427	Steryl-sulfatase OS=Mus musculus GN=Sts PE=2 SV=1 - [STS_MOUSE]	4.65	1	2	3	4	0.875	0.649	0.980	0.999	8.00	4.65	4	4	624	66.5	8.50
Q99MN1	Lysine--tRNA ligase OS=Mus musculus GN=Kars PE=1 SV=1 - [SYK_MOUSE]	40.50	2	23	24	139	0.820	0.884	0.914	1.000	344.22	40.50	45	139	595	67.8	5.94
Q8VFC3	Olfactory receptor 266 OS=Mus musculus GN=Olfr266 PE=2 SV=1 - [Q8VFC3_MOUSE]	10.13	1	1	1	5	1.520	2.178	1.573	1.000	0.00	10.13	1	5	316	35.6	8.07
Q9WTS4	Teneurin-1 OS=Mus musculus GN=Tenm1 PE=1 SV=1 - [TEN1_MOUSE]	9.45	3	20	22	46	0.912	1.242	0.987	1.000	139.44	9.45	37	46	2731	305.6	6.37
E9Q4T0	Holliday junction recognition protein OS=Mus musculus GN=Hjurp PE=2 SV=1 -	4.57	6	2	2	6	1.480	0.643	0.880	1.000	21.24	4.57	3	6	591	65.7	9.63
P63318	Protein kinase C gamma type OS=Mus musculus GN=Prkcg PE=1 SV=1 - [KPCG_MOUSE]	46.48	2	25	28	210	0.661	1.553	1.511	1.000	581.33	46.48	49	210	697	78.3	7.46
Q7TQG1	Pleckstrin homology domain-containing family A member 6 OS=Mus musculus GN=Plekha6 PE=1 SV=1 - [illegible]	24.21	2	20	21	73	1.147	0.594	1.123	1.000	206.46	24.21	34	73	1173	131.3	8.97
Q8R180	ERO1-like protein alpha OS=Mus musculus GN=Ero1l PE=1 SV=2 - [ERO1A_MOUSE]	17.89	2	8	8	32	0.685	1.689	0.733	1.000	99.30	17.89	15	32	464	54.1	6.54
Q9CWW7	CpG-binding protein OS=Mus musculus GN=Cxxc1 PE=2 SV=1 - [CXXC1_MOUSE]	6.97	1	4	4	11	0.625	0.809	0.956	1.000	24.52	6.97	8	11	660	76.1	8.27
Q8BRK8	5'-AMP-activated protein kinase catalytic subunit alpha-2 OS=Mus musculus GN=Prkaa2 PE=1 SV=3 - [AAPK2_MOUSE]	20.29	1	5	9	34	0.786	0.926	1.350	1.000	119.56	20.29	16	34	552	62.0	7.87
Q9R1J0	Sterol-4-alpha-carboxylate 3-dehydrogenase, decarboxylating OS=Mus musculus GN=Nsdhl PE=2 SV=1 - [NSDHL_MOUSE]	29.01	1	9	9	27	1.133	0.673	0.784	1.000	94.99	29.01	15	27	362	40.7	7.85
Q91XM9-7	Isoform 7 of Disks large homolog 2 OS=Mus musculus GN=Dlg2 - [DLG2_MOUSE]	49.39	9	31	39	240	0.975	1.633	1.620	1.000	726.31	49.39	70	240	976	109.2	6.28
Q9Z140	Copine-6 OS=Mus musculus GN=Cpne6 PE=1 SV=1 - [CPNE6_MOUSE]	55.30	3	23	24	190	0.877	2.513	1.212	1.000	558.26	55.30	42	190	557	61.7	5.59
Q8C008	Double zinc ribbon and ankyrin repeat-containing protein 1 OS=Mus musculus GN=Dzank1 PE=1 SV=2 - [illegible]	31.11	2	20	20	53	0.898	0.744	0.974	1.000	172.96	31.11	33	53	778	85.0	8.18
Q8CIB6	Transmembrane protein 230 OS=Mus musculus GN=Tmem230 PE=1 SV=1 - [TM230_MOUSE]	20.83	1	2	2	10	0.953	0.937	0.953	1.000	30.15	20.83	4	10	120	13.2	9.31
Q9D832	DnaJ homolog subfamily B member 4 OS=Mus musculus GN=Dnajb4 PE=2 SV=1 -	49.55	1	12	13	48	1.019	1.020	1.047	1.000	173.56	49.55	20	48	337	37.8	8.59

Q9ER67	Maged2 protein OS=Mus musculus GN=Maged2 PE=2 SV=1 - [Q9ER67_MOUSE]	14.94	5	7	9	30	1.085	0.827	0.705	1.000	95.82	14.94	14	30	616	65.4	9.14
G3X9Y5	Protein Ube4a OS=Mus musculus GN=Ube4a PE=4 SV=1 - [G3X9Y5_MOUSE]	15.58	3	15	16	39	0.744	0.834	0.888	1.000	110.05	15.58	27	39	1085	124.4	5.19
Q9CQS2	H/ACA ribonucleoprotein complex subunit 3 OS=Mus musculus GN=Nop10 PE=3 SV=1 - [NOP10_MOUSE]	20.31	1	1	1	3	0.817	0.724	0.782	1.001	10.21	20.31	2	3	64	7.7	9.99
Q9ESL4	Mitogen-activated protein kinase kinase kinase MLT OS=Mus musculus GN=Mltk PE=1 SV=1 - [MLTK_MOUSE]	7.23	3	1	4	5	1.739	1.460	1.107	1.001	10.81	7.23	5	5	802	91.7	7.74
D3YUW7	Cingulin OS=Mus musculus GN=Cgn PE=2 SV=1 - [D3YUW7_MOUSE]	14.10	4	14	15	26	1.382	0.859	0.849	1.001	58.13	14.10	21	26	1184	135.7	5.86
Q7TPQ3-2	Isoform 2 of E3 ubiquitin-protein ligase SHPRH OS=Mus musculus GN=Shprh -	2.85	4	4	4	4	0.614	1.192	0.962	1.001	5.04	2.85	4	4	1616	185.0	7.53
Q9DC28	Casein kinase I isoform delta OS=Mus musculus GN=Csnk1d PE=1 SV=2 - [KC1D_MOUSE]	30.36	5	6	12	53	1.204	0.775	0.958	1.001	135.45	30.36	21	53	415	47.3	9.74
Q9CWT6	Probable ATP-dependent RNA helicase DDX28 OS=Mus musculus GN=Ddx28 PE=2 SV=2 - [DDX28_MOUSE]	1.85	1	1	1	1	1.172	0.792	1.099	1.001	1.84	1.85	1	1	540	59.5	10.04
B1AVZ0	Uracil phosphoribosyltransferase homolog OS=Mus musculus GN=Uprt PE=2 SV=1 - [UPP_MOUSE]	38.06	1	9	9	30	1.498	1.097	1.033	1.001	77.50	38.06	18	30	310	34.3	6.23
Q8BHG2	UPF0587 protein C1orf123 homolog OS=Mus musculus PE=2 SV=1 - [CA123_MOUSE]	68.13	9	8	8	29	1.744	0.969	1.215	1.001	85.96	68.13	13	29	160	18.0	5.12
Q9R1T2	SUMO-activating enzyme subunit 1 OS=Mus musculus GN=Sae1 PE=2 SV=1 - [SAE1_MOUSE]	48.00	2	13	13	38	0.818	1.027	0.914	1.001	94.05	48.00	24	38	350	38.6	5.36
A2ABW9	CDK5 and ABL1 enzyme substrate 2 OS=Mus musculus GN=Cables2 PE=2 SV=1 -	6.51	2	2	2	3	1.020	1.193	1.020	1.001	4.38	6.51	2	3	476	52.0	9.86
F8WI64	Vacuolar protein sorting-associated protein 8 homolog OS=Mus musculus GN=Vps8 PE=4 SV=2 - [F8WI64_MOUSE]	12.65	9	14	14	25	0.934	1.003	0.998	1.001	56.96	12.65	22	25	1399	157.9	5.60
Q9DC61	Mitochondrial-processing peptidase subunit alpha OS=Mus musculus GN=Pmpca PE=1 SV=1 - [MPPA_MOUSE]	28.44	2	15	15	46	0.882	1.096	0.934	1.001	142.03	28.44	27	46	524	58.2	6.83
Q6IEE6	Transmembrane protein 132E OS=Mus musculus GN=Tmem132e PE=2 SV=1 - [T132E_MOUSE]	13.14	1	10	10	18	1.125	1.117	1.049	1.001	53.31	13.14	16	18	982	106.9	6.04
Q9EP69	Phosphatidylinositide phosphatase SAC1 OS=Mus musculus GN=Sacm1l PE=2 SV=1 - [SAC1_MOUSE]	33.73	1	18	18	88	0.694	0.679	0.872	1.001	266.94	33.73	34	88	587	66.9	7.30
H3BJZ7	Protein unc-13 homolog B OS=Mus musculus GN=Unc13a PE=2 SV=1 - [H3BJZ7_MOUSE]	27.09	4	19	39	124	0.610	1.476	1.234	1.001	348.37	27.09	66	124	1731	195.7	5.26
Q3UYV9	Nuclear cap-binding protein subunit 1 OS=Mus musculus GN=Ncbp1 PE=1 SV=2 - [NCBP1_MOUSE]	13.42	1	8	8	20	0.921	0.991	0.918	1.001	55.61	13.42	12	20	790	91.9	6.64
Q62276-2	Isoform 2 of Mediator of RNA polymerase II transcription subunit 22 OS=Mus musculus GN=Med22 - [illegible]	19.29	3	2	2	3	1.604	1.561	0.848	1.001	12.06	19.29	3	3	140	16.4	5.02
P04117	Fatty acid-binding protein, adipocyte OS=Mus musculus GN=Fabp4 PE=1 SV=3 - [FABP4_MOUSE]	22.73	3	3	3	11	7.715	2.713	0.556	1.001	22.34	22.73	6	11	132	14.6	8.40
Q6PHZ5	Putative RNA-binding protein 15B OS=Mus musculus GN=Rbm15b PE=1 SV=2 -	5.64	1	5	7	7	0.776	0.811	1.119	1.001	12.84	5.64	7	7	887	97.0	9.91
Q3B7Z2	Oxysterol-binding protein 1 OS=Mus musculus GN=Osbp PE=1 SV=3 - [OSBP1_MOUSE]	32.92	2	18	21	84	0.939	0.943	1.052	1.001	246.34	32.92	37	84	805	88.7	7.20
Q5SUQ9	CST complex subunit CTC1 OS=Mus musculus GN=Ctc1 PE=1 SV=2 - [CTC1_MOUSE]	9.57	2	1	9	21	0.501	0.989	0.630	1.001	77.65	9.57	15	21	1212	133.9	8.12
Q8BL97-3	Isoform 3 of Serine/arginine-rich splicing factor 7 OS=Mus musculus GN=Srsf7 -	37.58	4	5	6	28	0.970	0.935	0.889	1.001	64.07	37.58	11	28	157	17.9	9.47
Q9JME7	Trafficking protein particle complex subunit 2-like protein OS=Mus musculus GN=Trappc2l PE=2 SV=1 - [TPC2L_MOUSE]	41.73	1	5	5	16	0.785	1.006	1.021	1.002	50.66	41.73	9	16	139	16.0	6.77

A2AJW5	Uncharacterized protein OS=Mus musculus GN=Fam217b PE=4 SV=1 - [A2AJW5_MOUSE]	13.95	1	4	4	18	1.188	1.197	1.406	1.002	44.05	13.95	7	18	387	42.1	9.45
Q5SX19	N-acetylglucosaminyl-phosphatidylinositol de-N-acetylase OS=Mus musculus GN=Pigl PE=2 SV=1 - [PIGL_MOUSE]	5.16	1	1	1	3	0.992	1.083	0.951	1.002	4.89	5.16	2	3	252	28.2	8.34
Q80TE4-2	Isoform 2 of Signal-induced proliferation-associated 1-like protein 2 OS=Mus musculus GN=Sipa1l2 - [SI1L2_MOUSE]	10.22	2	14	17	37	1.081	0.608	0.963	1.002	84.75	10.22	28	37	1703	187.2	6.95
Q80XE1	Synembryn-B OS=Mus musculus GN=Ric8b PE=1 SV=2 - [RIC8B_MOUSE]	11.92	5	3	6	16	1.116	1.238	0.881	1.002	43.42	11.92	9	16	520	58.6	5.72
Q3T9A5	Glomulin OS=Mus musculus GN=Glmn PE=2 SV=1 - [Q3T9A5_MOUSE]	5.08	2	3	3	7	0.798	0.822	0.625	1.002	14.11	5.08	5	7	532	60.6	5.05
Q9CZ44	NSFL1 cofactor p47 OS=Mus musculus GN=Nsfl1c PE=1 SV=1 - [NSF1C_MOUSE]	41.08	4	15	15	182	2.072	1.235	1.163	1.002	553.24	41.08	28	182	370	40.7	5.15
Q9D1M4	Eukaryotic translation elongation factor 1 epsilon-1 OS=Mus musculus GN=Eef1e1 PE=2 SV=1 - [MCA3_MOUSE]	38.51	1	6	6	17	1.589	1.345	0.816	1.002	35.58	38.51	10	17	174	19.8	8.59
Q80U35	Rho guanine nucleotide exchange factor 17 OS=Mus musculus GN=Arhgef17 PE=1 SV=2 - [ARHGH_MOUSE]	14.88	2	15	17	34	0.916	0.916	0.852	1.002	80.80	14.88	24	34	2057	221.5	6.30
P62700	Protein yippee-like 5 OS=Mus musculus GN=Ypel5 PE=2 SV=1 - [YPEL5_MOUSE]	23.97	1	3	3	6	2.494	1.332	0.992	1.002	12.46	23.97	6	6	121	13.8	7.31
Q9WTN0	Geranylgeranyl pyrophosphate synthase OS=Mus musculus GN=Ggps1 PE=2 SV=1 - [GGPPS_MOUSE]	32.00	1	9	9	23	0.671	1.015	0.812	1.002	63.90	32.00	15	23	300	34.7	6.46
Q9D1C9	Ribosomal RNA-processing protein 7 homolog A OS=Mus musculus GN=Rrp7a PE=2 SV=1 - [RRP7A_MOUSE]	9.29	4	2	2	2	0.920	1.283	0.589	1.002	2.88	9.29	2	2	280	32.4	9.48
Q9DBG1	Sterol 26-hydroxylase, mitochondrial OS=Mus musculus GN=Cyp27a1 PE=1 SV=1 - [CP27A_MOUSE]	10.13	1	3	3	8	1.036	1.100	0.983	1.002	16.99	10.13	5	8	533	60.7	9.14
Q9WTU0	Lysine-specific demethylase PHF2 OS=Mus musculus GN=Phf2 PE=1 SV=2 -	5.75	4	4	5	6	0.893	0.962	0.963	1.002	15.24	5.75	6	6	1096	120.7	9.17
Q9D1E6	Tubulin-folding cofactor B OS=Mus musculus GN=Tbcb PE=1 SV=2 - [TBCB_MOUSE]	33.61	1	7	7	41	1.747	0.710	0.860	1.002	133.63	33.61	14	41	244	27.4	5.24
Q61140	Breast cancer anti-estrogen resistance protein 1 OS=Mus musculus GN=Bcar1 PE=1 SV=2 - [BCAR1_MOUSE]	33.64	2	19	19	71	1.654	1.119	1.051	1.002	211.89	33.64	31	71	874	94.2	5.68
E9PWJ0	CUB and sushi domain-containing protein 3 OS=Mus musculus GN=Csmd3 PE=2 SV=1 - [E9PWJ0_MOUSE]	3.84	4	10	11	21	1.044	1.354	1.143	1.002	55.52	3.84	17	21	3538	386.9	5.91
Q9QWK4	CD5 antigen-like OS=Mus musculus GN=Cd5l PE=1 SV=3 - [CD5L_MOUSE]	3.13	1	1	1	4	2.154	1.607	0.796	1.003	11.40	3.13	2	4	352	38.8	5.16
Q9QYJ0	DnaJ homolog subfamily A member 2 OS=Mus musculus GN=Dnaja2 PE=1 SV=1 -	66.02	1	23	23	122	0.973	0.950	1.076	1.003	294.97	66.02	41	122	412	45.7	6.48
Q6ZPK7	Lateral signaling target protein 2 homolog OS=Mus musculus GN=Zfyve28 PE=1 SV=2 - [LST2_MOUSE]	3.98	3	3	3	7	0.960	0.979	1.290	1.003	19.20	3.98	5	7	905	99.7	5.10
A2A763	Nuclear transcription factor Y subunit gamma (Fragment) OS=Mus musculus GN=Nfyc PE=2 SV=1 - [A2A763_MOUSE]	27.55	4	3	3	4	1.137	1.398	0.530	1.003	10.76	27.55	4	4	98	11.4	5.60
P68037	Ubiquitin-conjugating enzyme E2 L3 OS=Mus musculus GN=Ube2l3 PE=2 SV=1 -	46.10	2	5	5	31	0.823	0.727	0.955	1.003	106.39	46.10	10	31	154	17.9	8.51
Q8BJ42	Disks large-associated protein 2 OS=Mus musculus GN=Dlgap2 PE=1 SV=2 -	27.95	3	20	27	113	0.877	1.967	2.618	1.003	319.34	27.95	46	113	1059	119.0	6.81
Q99JY9	Actin-related protein 3 OS=Mus musculus GN=Actr3 PE=1 SV=3 - [ARP3_MOUSE]	82.30	1	20	24	280	0.653	1.328	1.021	1.003	773.73	82.30	42	280	418	47.3	5.88
D3Z5J3	Sorbin and SH3 domain-containing protein 1 OS=Mus musculus GN=Sorbs1 PE=2 SV=1 - [D3Z5J3_MOUSE]	34.75	2	5	27	128	2.125	0.545	1.332	1.003	381.49	34.75	49	128	938	103.9	5.94

P45952	Medium-chain specific acyl-CoA dehydrogenase, mitochondrial OS=Mus musculus GN=Acadm PE=1 SV=1 - [ACADM_MOUSE]	42.52	2	15	15	66	0.922	1.103	0.829	1.003	191.35	42.52	28	66	421	46.5	8.37
A2ARZ3	Fibrous sheath-interacting protein 2 OS=Mus musculus GN=Fsip2 PE=1 SV=3 - [FSIP2_MOUSE]	1.54	2	8	12	36	1.581	1.760	1.979	1.003	64.92	1.54	16	36	6995	784.4	6.51
Q9CQW1	Synaptobrevin homolog YKT6 OS=Mus musculus GN=Ykt6 PE=2 SV=1 - [YKT6_MOUSE]	53.03	1	9	9	48	0.734	0.947	0.961	1.003	141.27	53.03	18	48	198	22.3	6.35
Q60607	BRK-1T OS=Mus musculus GN=Bmpr1a PE=2 SV=1 - [Q60607_MOUSE]	12.60	2	3	4	10	0.665	0.752	0.738	1.003	31.63	12.60	7	10	500	56.4	6.71
Q9CQV5	28S ribosomal protein S24, mitochondrial OS=Mus musculus GN=Mrps24 PE=2 SV=1 - [RT24_MOUSE]	13.77	1	3	3	5	0.632	0.665	1.003	1.003	13.83	13.77	5	5	167	18.9	9.76
P61161	Actin-related protein 2 OS=Mus musculus GN=Actr2 PE=1 SV=1 - [ARP2_MOUSE]	50.51	1	16	16	144	0.698	1.441	1.133	1.003	501.57	50.51	27	144	394	44.7	6.74
A2A8U2-2	Isoform Samp1 of Transmembrane protein 201 OS=Mus musculus GN=Tmem201 -	15.82	3	3	3	8	1.044	1.716	0.848	1.003	11.68	15.82	4	8	392	43.5	8.56
A2AHB7	Potassium channel subfamily T member 1 OS=Mus musculus GN=Kcnt1 PE=2 SV=1 - [A2AHB7_MOUSE]	7.89	6	7	7	21	1.067	0.895	0.906	1.003	63.18	7.89	13	21	1217	137.3	7.46
P43024	Cytochrome c oxidase subunit 6A1, mitochondrial OS=Mus musculus GN=Cox6a1 PE=1 SV=2 - [CX6A1_MOUSE]	40.54	2	2	2	14	0.600	0.780	1.049	1.003	46.08	40.54	3	14	111	12.3	9.98
Q9QYS1	Protein Wnt-16 OS=Mus musculus GN=Wnt16 PE=2 SV=2 - [WNT16_MOUSE]	3.30	1	1	2	2	0.618	1.054	1.091	1.003	4.97	3.30	2	2	364	40.7	8.63
E9PUA2	Terminal uridylyltransferase 7 OS=Mus musculus GN=Zcchc6 PE=4 SV=1 -	6.85	2	5	8	10	1.365	0.931	1.249	1.004	22.22	6.85	10	10	1474	167.0	6.52
O88351	Inhibitor of nuclear factor kappa-B kinase subunit beta OS=Mus musculus GN=Ikbkb PE=1 SV=1 - [IKKB_MOUSE]	10.04	4	6	6	11	0.773	1.488	1.000	1.004	33.05	10.04	8	11	757	86.6	6.40
Q4U2R1-2	Isoform 2 of E3 ubiquitin-protein ligase HERC2 OS=Mus musculus GN=Herc2 -	7.71	6	26	31	50	0.869	0.802	0.878	1.004	129.55	7.71	47	50	4800	523.1	6.27
Q8C1B1	Calmodulin-regulated spectrin-associated protein 2 OS=Mus musculus GN=Camsap2 PE=1 SV=3 [CAMP2_MOUSE]	24.09	9	27	29	93	1.085	1.004	1.303	1.004	248.97	24.09	46	93	1461	164.2	6.86
Q61699-2	Isoform HSP105-beta of Heat shock protein 105 kDa OS=Mus musculus GN=Hsph1 -	55.65	5	38	43	348	1.017	1.073	1.089	1.004	966.52	55.65	78	348	814	91.6	5.74
F7CGG2	Transformation/transcription domain-associated protein (Fragment) OS=Mus musculus GN=Trrap PE=4 SV=1 - [F7CGG2_MOUSE]	6.19	5	17	18	29	0.682	1.081	0.875	1.004	67.31	6.19	25	29	3587	405.3	8.19
Q8C050-2	Isoform 1 of Ribosomal protein S6 kinase alpha-5 OS=Mus musculus GN=Rps6ka5 -	20.55	2	13	15	47	0.674	0.856	1.117	1.004	132.21	20.55	27	47	798	89.5	7.25
Q9DCZ1	GMP reductase 1 OS=Mus musculus GN=Gmpr PE=2 SV=1 - [GMPR1_MOUSE]	37.39	2	9	11	43	1.664	0.724	0.994	1.004	133.47	37.39	20	43	345	37.5	7.09
P97929	Breast cancer type 2 susceptibility protein homolog OS=Mus musculus GN=Brca2 PE=1 SV=2 - [BRCA2_MOUSE]	2.40	1	6	6	6	0.718	0.877	0.908	1.004	9.71	2.40	6	6	3329	370.4	6.67
Q9QZD4	DNA repair endonuclease XPF OS=Mus musculus GN=Ercc4 PE=2 SV=3 - [XPF_MOUSE]	11.34	3	8	8	18	1.139	0.737	0.956	1.004	51.73	11.34	15	18	917	103.6	7.71
Q504M2	MCG53395 OS=Mus musculus GN=Pdp2 PE=2 SV=1 - [Q504M2_MOUSE]	12.41	1	4	4	10	1.009	1.110	0.892	1.004	14.32	12.41	5	10	532	59.9	6.32
Q91W27	Tuberoinfundibular peptide of 39 residues OS=Mus musculus GN=Pth2 PE=1 SV=1 - [TIP39_MOUSE]	18.00	1	2	2	6	1.198	1.179	0.758	1.004	15.59	18.00	3	6	100	11.0	11.28
P70261	Paladin OS=Mus musculus GN=Pald1 PE=1 SV=1 - [PALD_MOUSE]	13.85	1	9	9	18	0.778	0.707	0.836	1.004	60.36	13.85	14	18	859	96.7	6.58
Q9ESW4	Acylglycerol kinase, mitochondrial OS=Mus musculus GN=Agk PE=1 SV=1 - [AGK_MOUSE]	55.34	2	19	19	93	0.705	0.936	0.943	1.004	254.59	55.34	34	93	421	46.9	8.40

F6Y3V0	Receptor-type tyrosine-protein phosphatase F (Fragment) OS=Mus musculus GN=Ptprf PE=4 SV=1 - [F6Y3V0_MOUSE]	32.69	1	3	31	104	1.084	0.959	0.896	1.004	305.21	32.69	54	104	1291	145.4	6.73
E9Q0E9	Ankyrin repeat domain-containing protein 55 OS=Mus musculus GN=Ankrd55 PE=2 SV=1 - [E9Q0E9_MOUSE]	2.68	2	1	1	5	0.914	0.592	0.709	1.004	10.98	2.68	2	5	598	65.4	7.44
Q9ESC8	AF4/FMR2 family member 4 OS=Mus musculus GN=Aff4 PE=1 SV=1 - [AFF4_MOUSE]	1.72	1	1	2	2	2.734	1.100	0.994	1.004	3.68	1.72	2	2	1160	126.6	9.36
O08586	Phosphatidylinositol 3,4,5-trisphosphate 3-phosphatase and dual-specificity protein phosphatase PTEN OS=Mus musculus GN=Pten PE=1 SV=1 -	23.08	1	8	8	37	0.623	0.857	0.951	1.004	109.96	23.08	16	37	403	47.1	6.37
Q61656	Probable ATP-dependent RNA helicase DDX5 OS=Mus musculus GN=Ddx5 PE=1 SV=2 - [DDX5_MOUSE]	45.93	5	24	32	157	0.625	0.711	0.947	1.004	448.89	45.93	58	157	614	69.2	8.92
O08715-2	Isoform 1 of A-kinase anchor protein 1, mitochondrial OS=Mus musculus GN=Akap1 - [AKAP1_MOUSE]	35.85	6	13	13	38	1.302	0.951	1.220	1.004	115.59	35.85	22	38	544	57.6	4.94
Q4V9Z5-3	Isoform 3 of Seizure 6-like protein 2 OS=Mus musculus GN=Sez6l2 - [SE6L2_MOUSE]	27.00	3	14	15	67	1.423	1.131	1.109	1.004	195.79	27.00	28	67	863	93.3	4.84
F8VQ93	PH-interacting protein OS=Mus musculus GN=Phip PE=4 SV=1 - [F8VQ93_MOUSE]	1.70	3	2	3	6	0.603	1.114	0.913	1.004	15.19	1.70	4	6	1821	206.6	8.85
A2A9M5	Dedicator of cytokinesis protein 7 OS=Mus musculus GN=Dock7 PE=2 SV=1 - [A2A9M5_MOUSE]	17.67	7	25	28	70	0.708	1.039	0.887	1.004	207.36	17.67	51	70	2128	241.0	6.79
A2AS98	Nck-associated protein 1 OS=Mus musculus GN=Nckap1 PE=2 SV=1 - [A2AS98_MOUSE]	41.27	3	42	44	258	0.512	1.356	1.123	1.004	662.90	41.27	81	258	1134	129.4	6.68
E9QK48	Echinoderm microtubule-associated protein-like 2 OS=Mus musculus GN=Eml2 PE=2 SV=1 - [E9QK48_MOUSE]	39.00	5	26	27	104	1.019	0.642	0.785	1.004	305.53	39.00	48	104	841	90.7	6.98
Q9JIG8	PRA1 family protein 2 OS=Mus musculus GN=Praf2 PE=2 SV=1 - [PRAF2_MOUSE]	26.97	1	4	4	11	0.779	0.842	1.005	1.004	28.64	26.97	7	11	178	19.5	9.60
P68181	cAMP-dependent protein kinase catalytic subunit beta OS=Mus musculus GN=Prkacb PE=1 SV=2 - [KAPCB_MOUSE]	55.56	4	8	18	91	0.726	1.025	0.997	1.004	273.46	55.56	28	91	351	40.7	8.56
Q8BTH8	Casein kinase I isoform gamma-1 OS=Mus musculus GN=Csnk1g1 PE=1 SV=2 -	23.31	2	5	10	18	0.811	1.334	0.933	1.004	48.43	23.31	15	18	459	52.7	9.01
Q9CQX8	28S ribosomal protein S36, mitochondrial OS=Mus musculus GN=Mrps36 PE=1 SV=1 - [RT36_MOUSE]	70.59	4	5	5	97	1.753	0.868	1.568	1.004	251.39	70.59	10	97	102	11.1	9.99
Q7TPV2-2	Isoform 2 of E3 ubiquitin-protein ligase DZIP3 OS=Mus musculus GN=Dzip3 -	18.54	5	15	15	29	0.884	0.967	0.874	1.005	83.43	18.54	23	29	998	113.8	5.21
Q923G2	DNA-directed RNA polymerases I, II, and III subunit RPABC3 OS=Mus musculus GN=Polr2h PE=2 SV=3 - [RPAB3_MOUSE]	32.67	1	3	3	9	1.258	1.134	0.899	1.005	28.15	32.67	5	9	150	17.1	4.68
Q99NE5-7	Isoform 7 of Regulating synaptic membrane exocytosis protein 1 OS=Mus musculus GN=Rims1 - [RIMS1_MOUSE]	30.66	3	1	30	106	0.774	1.010	1.486	1.005	354.77	30.66	53	106	1184	132.9	9.33
Q8CJ26	Death domain-containing membrane protein NRADD OS=Mus musculus GN=Nradd PE=1 SV=1 - [NRADD_MOUSE]	19.30	1	3	3	3	1.799	0.845	1.049	1.005	11.70	19.30	3	3	228	24.7	5.01
Q9D1F0	CAAX box 1 homolog A (Human) OS=Mus musculus GN=Cxx1b PE=4 SV=1 - [Q9D1F0_MOUSE]	7.08	1	1	1	1	1.571	0.888	0.604	1.005	2.81	7.08	1	1	113	13.6	8.40
Q7TQF2	F-box only protein 10 OS=Mus musculus GN=Fbxo10 PE=1 SV=2 - [FBX10_MOUSE]	7.05	2	6	6	11	1.278	1.054	0.934	1.005	26.17	7.05	9	11	950	104.4	7.85
P35822	Receptor-type tyrosine-protein phosphatase kappa OS=Mus musculus GN=Ptprk PE=1 SV=1 - [PTPRK_MOUSE]	6.52	1	6	7	18	1.257	1.950	1.138	1.005	73.00	6.52	12	18	1457	164.1	5.95
P19096	Fatty acid synthase OS=Mus musculus GN=Fasn PE=1 SV=2 - [FAS_MOUSE]	54.59	4	102	103	614	0.720	0.601	0.817	1.005	1807.47	54.59	182	614	2504	272.3	6.58

Q91YD9	Neural Wiskott-Aldrich syndrome protein OS=Mus musculus GN=Wasl PE=1 SV=1 - [WASL_MOUSE]	27.54	2	13	13	99	1.067	0.951	1.287	1.005	375.44	27.54	23	99	501	54.2	7.93
E9Q912	Protein Rap1gds1 OS=Mus musculus GN=Rap1gds1 PE=2 SV=1 - [E9Q912_MOUSE]	50.08	2	25	25	222	0.887	0.930	1.013	1.005	693.72	50.08	46	222	607	66.0	5.35
G3X8Y3	N-alpha-acetyltransferase 15, NatA auxiliary subunit OS=Mus musculus GN=Naa15 PE=4 SV=1 - [G3X8Y3_MOUSE]	27.28	3	20	24	74	0.687	0.722	0.896	1.005	198.51	27.28	42	74	865	101.0	7.52
Q8CG64	Fukutin-related protein OS=Mus musculus GN=Fkrp PE=1 SV=1 - [FKRP_MOUSE]	4.66	1	2	2	6	0.944	0.952	0.803	1.005	18.36	4.66	4	6	494	54.8	7.17
Q52KG3	Melanoma antigen, family E, 2 OS=Mus musculus GN=Magee2 PE=2 SV=1 - [Q52KG3_MOUSE]	18.16	1	8	8	17	0.845	1.252	0.771	1.005	46.60	18.16	12	17	523	59.7	4.93
J3QPB5	Nucleoporin NDC1 OS=Mus musculus GN=Tmem48 PE=4 SV=1 - [J3QPB5_MOUSE]	5.55	2	2	2	4	1.167	0.856	0.958	1.005	14.15	5.55	4	4	541	60.3	8.70
Q8BW96	Calcium/calmodulin-dependent protein kinase type 1D OS=Mus musculus GN=Camk1d PE=1 SV=2 - [KCC1D_MOUSE]	55.58	3	12	16	100	0.993	1.915	1.504	1.005	340.18	55.58	28	100	385	42.9	7.17
A3KG57	Serine/arginine-rich-splicing factor 12 (Fragment) OS=Mus musculus GN=Srsf12 PE=2 SV=1 - [A3KG57_MOUSE]	17.24	1	2	4	11	1.200	1.084	0.925	1.005	34.33	17.24	7	11	261	30.3	11.58
Q8BGF0	Protein AW551984 OS=Mus musculus GN=AW551984 PE=2 SV=1 - [Q8BGF0_MOUSE]	35.70	3	23	26	82	0.798	0.794	0.515	1.005	237.13	35.70	43	82	804	89.4	6.92
Q9D168	Integrator complex subunit 12 OS=Mus musculus GN=Ints12 PE=1 SV=1 - [INT12_MOUSE]	9.76	1	4	4	5	1.538	0.685	1.224	1.005	13.21	9.76	4	5	461	48.5	9.69
Q3TIR3	Synembryn-A OS=Mus musculus GN=Ric8a PE=1 SV=2 - [RIC8A_MOUSE]	33.58	1	14	16	62	0.671	1.051	0.925	1.005	174.58	33.58	29	62	530	59.8	5.68
Q9JKF1	Ras GTPase-activating-like protein IQGAP1 OS=Mus musculus GN=Iqgap1 PE=1 SV=2 - [IQGA1_MOUSE]	24.98	1	29	32	80	0.958	1.334	0.789	1.005	220.65	24.98	53	80	1657	188.6	6.48
Q32NY6	COUP transcription factor 1 OS=Mus musculus GN=Nr2f1 PE=2 SV=1 - [Q32NY6_MOUSE]	18.33	4	1	5	10	0.556	0.619	0.916	1.005	16.01	18.33	7	10	420	45.9	8.25
Q3UIL6-2	Isoform 2 of Pleckstrin homology domain-containing family A member 7 OS=Mus musculus GN=Plekha7 - [illegible]	14.77	6	13	13	21	0.740	0.719	0.764	1.005	59.04	14.77	19	21	1266	143.7	9.01
D3Z580	Protein Celf5 OS=Mus musculus GN=Celf5 PE=2 SV=1 - [D3Z580_MOUSE]	14.72	5	2	6	15	0.460	1.994	1.428	1.006	37.45	14.72	11	15	394	42.5	7.90
Q9CQV8-2	Isoform Short of 14-3-3 protein beta/alpha OS=Mus musculus GN=Ywhab -	78.69	4	12	23	864	0.929	1.182	0.986	1.006	2357.11	78.69	40	864	244	27.8	4.83
Q9D2M8	Ubiquitin-conjugating enzyme E2 variant 2 OS=Mus musculus GN=Ube2v2 PE=2 SV=4 - [UB2V2_MOUSE]	70.34	5	5	10	101	0.773	0.998	0.889	1.006	264.42	70.34	16	101	145	16.4	8.09
Q9Z2S7-3	Isoform 3 of TSC22 domain family protein 3 OS=Mus musculus GN=Tsc22d3 -	23.38	4	4	5	28	1.925	0.775	0.958	1.006	72.02	23.38	8	28	201	22.6	4.77
Q5SVR0-2	Isoform 2 of TBC1 domain family member 9B OS=Mus musculus GN=Tbc1d9b -	19.58	2	19	21	57	1.060	0.964	0.880	1.006	159.03	19.58	35	57	1246	139.8	5.35
Q3TF37	Myosin-binding protein C, cardiac-type OS=Mus musculus GN=Mybpc3 PE=2 SV=1 - [Q3TF37_MOUSE]	5.84	4	3	4	4	0.556	1.014	1.244	1.006	7.32	5.84	4	4	1113	124.1	7.01
O70152	Dolichol-phosphate mannosyltransferase OS=Mus musculus GN=Dpm1 PE=2 SV=1 - [DPM1_MOUSE]	25.77	3	7	7	26	0.626	0.573	0.745	1.006	67.82	25.77	13	26	260	29.2	9.51
F6T4M4	Serine/arginine repetitive matrix protein 1 (Fragment) OS=Mus musculus GN=Srrm1 PE=2 SV=1 - [F6T4M4_MOUSE]	24.29	2	1	4	127	1.275	1.361	0.999	1.006	183.86	24.29	7	127	140	16.2	5.38
Q3TRM4-2	Isoform 2 of Neuropathy target esterase OS=Mus musculus GN=Pnpla6 - [PLPL6_MOUSE]	11.04	4	12	14	32	0.998	0.679	0.704	1.006	83.13	11.04	22	32	1323	146.0	7.84
Q9JI78	Peptide-N(4)-(N-acetyl-beta-glucosaminyl)asparagine amidase OS=Mus musculus GN=Ngly1 PE=1 [illegible]	26.42	1	16	16	38	0.915	0.922	0.886	1.006	95.19	26.42	24	38	651	74.2	6.89

Q80YR5	Scaffold attachment factor B2 OS=Mus musculus GN=Safb2 PE=1 SV=2 - [SAFB2_MOUSE]	22.00	7	7	18	43	1.047	0.665	1.180	1.006	125.25	22.00	26	43	991	111.8	6.38
Q922E6	FAST kinase domain-containing protein 2 OS=Mus musculus GN=Fastkd2 PE=2 SV=2 - [FAKD2_MOUSE]	15.09	1	9	9	25	0.962	0.856	0.969	1.006	65.33	15.09	16	25	689	78.9	8.90
Q9QXE7	F-box-like/WD repeat-containing protein TBL1X OS=Mus musculus GN=Tbl1x PE=2 SV=2 - [TBL1X_MOUSE]	13.09	1	2	5	11	1.999	1.125	0.925	1.006	28.90	13.09	8	11	527	56.8	5.72
Q3UJK4	GTP-binding protein 2 OS=Mus musculus GN=Gtpbp2 PE=2 SV=1 - [GTPB2_MOUSE]	3.16	2	1	1	1	2.039	1.437	0.664	1.006	3.61	3.16	1	1	602	65.7	8.05
Q8JZN5	Acyl-CoA dehydrogenase family member 9, mitochondrial OS=Mus musculus GN=Acad9 PE=2 SV=2 - [ACAD9_MOUSE]	45.28	3	23	23	97	0.932	0.790	0.832	1.006	277.50	45.28	40	97	625	68.7	7.46
Q9CZC8	Secernin-1 OS=Mus musculus GN=Scrn1 PE=1 SV=1 - [SCRN1_MOUSE]	50.00	2	19	20	220	1.515	0.940	1.120	1.006	624.47	50.00	35	220	414	46.3	4.79
F8VPQ1	Dedicator of cytokinesis protein 3 OS=Mus musculus GN=Dock3 PE=2 SV=1 - [F8VPQ1_MOUSE]	24.73	4	43	45	146	0.768	1.300	1.199	1.006	386.50	24.73	76	146	2030	233.1	6.99
Q8R086	Sulfite oxidase, mitochondrial OS=Mus musculus GN=Suox PE=1 SV=2 - [SUOX_MOUSE]	19.41	1	7	7	30	1.925	1.118	0.827	1.006	90.69	19.41	14	30	546	60.7	6.54
Q8VBT9	Tether containing UBX domain for GLUT4 OS=Mus musculus GN=Aspscr1 PE=1 SV=1 - [ASPC1_MOUSE]	40.91	14	14	14	53	1.020	0.813	0.888	1.006	163.87	40.91	23	53	550	59.8	6.96
Q9R0Y5	Adenylate kinase isoenzyme 1 OS=Mus musculus GN=Ak1 PE=1 SV=1 - [KAD1_MOUSE]	69.59	2	13	14	253	1.589	1.104	1.313	1.006	806.19	69.59	27	253	194	21.5	5.81
Q9D2Z4	Sentrin-specific protease 8 OS=Mus musculus GN=Senp8 PE=1 SV=2 - [SENP8_MOUSE]	19.91	1	3	3	7	1.221	1.029	0.874	1.006	28.67	19.91	5	7	221	25.0	5.95
Q5F4S8	Protein Trpm3 OS=Mus musculus GN=Trpm3 PE=2 SV=1 - [Q5F4S8_MOUSE]	17.36	3	1	25	70	0.821	1.464	0.933	1.006	191.49	17.36	45	70	1711	195.1	7.50
F6S6C3	Sentrin-specific protease 5 (Fragment) OS=Mus musculus GN=Senp5 PE=2 SV=1 - [F6S6C3_MOUSE]	11.20	3	4	5	5	1.134	0.878	0.992	1.006	7.21	11.20	5	5	607	69.4	9.09
Q9JKK8	Serine/threonine-protein kinase ATR OS=Mus musculus GN=Atr PE=1 SV=2 - [ATR_MOUSE]	1.21	2	3	3	3	0.531	0.518	0.924	1.007	6.30	1.21	3	3	2635	300.0	7.53
Q7TT15	Putative polypeptide N-acetylgalactosaminyltransferase-like protein 3 OS=Mus musculus GN=Wbscr17 PE=1 SV=1 - [GLTL3_MOUSE]	8.03	3	4	4	6	0.829	0.816	0.809	1.007	15.68	8.03	6	6	598	67.6	8.90
F7AGB5	Methyltransferase-like protein 17, mitochondrial (Fragment) OS=Mus musculus GN=Mettl17 PE=2 SV=1 - [illegible]	6.07	4	2	2	2	7.948	1.895	0.734	1.007	1.81	6.07	2	2	280	31.6	8.25
Q9ERG2	Striatin-3 OS=Mus musculus GN=Strn3 PE=1 SV=1 - [STRN3_MOUSE]	30.53	2	17	22	73	1.223	1.381	1.057	1.007	220.04	30.53	36	73	796	87.1	5.29
Q641P0	Actin-related protein 3B OS=Mus musculus GN=Actr3b PE=2 SV=1 - [ARP3B_MOUSE]	42.34	2	12	16	146	0.505	1.789	1.567	1.007	411.51	42.34	31	146	418	47.5	6.02
P48428	Tubulin-specific chaperone A OS=Mus musculus GN=Tbca PE=2 SV=3 - [TBCA_MOUSE]	58.33	1	8	8	43	1.775	0.766	1.139	1.007	101.76	58.33	15	43	108	12.7	5.27
P52482	Ubiquitin-conjugating enzyme E2 E1 OS=Mus musculus GN=Ube2e1 PE=2 SV=1 -	32.64	4	3	4	10	1.434	1.136	1.394	1.007	21.11	32.64	6	10	193	21.3	8.53
P62141	Serine/threonine-protein phosphatase PP1-beta catalytic subunit OS=Mus musculus GN=Ppp1cb PE=1 SV=3 - [PP1B_MOUSE]	62.08	1	7	19	134	1.417	1.202	1.051	1.007	411.03	62.08	34	134	327	37.2	6.19
P28474	Alcohol dehydrogenase class-3 OS=Mus musculus GN=Adh5 PE=1 SV=3 - [ADHX_MOUSE]	45.19	2	13	13	85	1.421	0.982	0.841	1.007	242.86	45.19	23	85	374	39.5	7.25
Q9CY27	Very-long-chain enoyl-CoA reductase OS=Mus musculus GN=Tecr PE=1 SV=1 - [TECR_MOUSE]	31.49	3	10	12	49	0.689	0.742	0.789	1.007	113.89	31.49	20	49	308	36.1	9.55
P42580	NK1 transcription factor-related protein 2 OS=Mus musculus GN=Nkx1-2 PE=1 SV=1 - [NKX12_MOUSE]	4.59	2	1	1	1	21.155	3.958	2.311	1.007	2.83	4.59	1	1	305	32.0	6.70

E9PUD2	Dynamin-1-like protein OS=Mus musculus GN=Dnm1l PE=2 SV=1 - [E9PUD2_MOUSE]	74.02	3	4	47	387	0.734	0.654	0.808	1.007	1093.79	74.02	85	387	712	79.5	7.08
Q8BPM0	Disheveled-associated activator of morphogenesis 1 OS=Mus musculus GN=Daam1 PE=1 SV=4 - [DAAM1_MOUSE]	30.08	5	27	32	118	0.669	0.834	1.037	1.007	301.61	30.08	52	118	1077	123.3	7.46
Q8BXQ0	Ethanolamine kinase 1 OS=Mus musculus GN=Etnk1 PE=2 SV=1 - [Q8BXQ0_MOUSE]	10.47	2	4	4	6	0.710	0.999	0.887	1.007	14.61	10.47	6	6	363	42.0	5.44
Q9CSU0-2	Isoform 2 of Regulation of nuclear pre-mRNA domain-containing protein 1B OS=Mus musculus GN=Rprd1b - [RPR1B_MOUSE]	30.95	5	6	7	18	1.180	1.014	0.756	1.007	68.85	30.95	11	18	294	33.5	5.64
Q6VH22	Intraflagellar transport protein 172 homolog OS=Mus musculus GN=Ift172 PE=1 SV=1 - [IF172_MOUSE]	5.09	3	5	7	12	1.445	1.266	0.771	1.007	36.06	5.09	8	12	1749	197.4	6.02
Q9CZD5	Translation initiation factor IF-3, mitochondrial OS=Mus musculus GN=Mtif3 PE=1 SV=1 - [IF3M_MOUSE]	13.41	1	2	2	6	1.965	1.258	1.057	1.007	23.73	13.41	4	6	276	31.7	9.42
Q922Q4	Pyrroline-5-carboxylate reductase 2 OS=Mus musculus GN=Pycr2 PE=2 SV=1 - [P5CR2_MOUSE]	49.38	1	10	13	58	0.818	0.907	0.858	1.008	186.25	49.38	25	58	320	33.6	7.77
Q9CZV8	F-box/LRR-repeat protein 20 OS=Mus musculus GN=Fbxl20 PE=1 SV=3 - [FXL20_MOUSE]	27.52	6	7	10	25	0.444	0.861	0.816	1.008	79.13	27.52	16	25	436	48.4	7.49
P25233	Necdin OS=Mus musculus GN=Ndn PE=1 SV=1 - [NECD_MOUSE]	8.00	1	2	2	6	1.407	0.857	0.620	1.008	20.69	8.00	4	6	325	36.8	8.51
Q8R395	COMM domain-containing protein 5 OS=Mus musculus GN=Commd5 PE=2 SV=1 -	33.48	1	6	6	15	1.095	1.246	0.885	1.008	48.09	33.48	10	15	224	24.5	8.06
Q3UII8	Metastasis-associated protein MTA3 OS=Mus musculus GN=Mta3 PE=2 SV=1 - [Q3UII8_MOUSE]	26.79	7	8	14	49	0.611	0.675	0.757	1.008	131.78	26.79	23	49	586	66.3	8.82
Q9JMH7	Sialidase-3 OS=Mus musculus GN=Neu3 PE=2 SV=1 - [NEUR3_MOUSE]	2.63	1	1	1	2	1.057	0.694	0.869	1.008	7.45	2.63	2	2	418	46.8	6.71
Q9CZX0	Elongator complex protein 3 OS=Mus musculus GN=Elp3 PE=2 SV=1 - [ELP3_MOUSE]	41.68	2	20	20	51	0.660	0.795	0.734	1.008	148.77	41.68	34	51	547	62.3	8.90
D3Z7G4	Protein BC024978 OS=Mus musculus GN=BC024978 PE=4 SV=1 - [D3Z7G4_MOUSE]	8.63	4	2	2	4	1.356	1.225	1.028	1.008	17.52	8.63	4	4	336	36.5	5.99
Q9D0F1	Kinetochore protein NDC80 homolog OS=Mus musculus GN=Ndc80 PE=2 SV=1 - [NDC80_MOUSE]	1.87	1	1	1	1	3.379	1.539	1.477	1.008	4.20	1.87	1	1	642	73.9	5.95
Q9CQI9	Mediator of RNA polymerase II transcription subunit 30 OS=Mus musculus GN=Med30 PE=1 SV=1 - [MED30_MOUSE]	21.91	1	4	4	7	1.086	1.175	1.451	1.008	17.72	21.91	5	7	178	20.3	8.28
E9Q5C9	Protein Nolc1 OS=Mus musculus GN=Nolc1 PE=2 SV=1 - [E9Q5C9_MOUSE]	24.36	1	17	17	42	0.681	0.847	0.951	1.008	98.74	24.36	29	42	702	73.7	9.36
Q71FD5	E3 ubiquitin-protein ligase ZNRF2 OS=Mus musculus GN=Znrf2 PE=1 SV=1 - [ZNRF2_MOUSE]	34.03	4	3	4	21	1.864	0.795	1.198	1.008	48.73	34.03	7	21	238	23.7	6.76
P70302	Stromal interaction molecule 1 OS=Mus musculus GN=Stim1 PE=1 SV=2 - [STIM1_MOUSE]	23.07	1	15	15	39	0.913	1.104	1.105	1.008	104.22	23.07	27	39	685	77.5	6.54
Q9D3V5	Fibrous sheath-interacting protein 1 OS=Mus musculus GN=Fsip1 PE=1 SV=1 - [FSIP1_MOUSE]	2.53	1	1	1	1	0.821	1.013	1.043	1.008	1.74	2.53	1	1	435	48.9	4.96
E9PX73	T-lymphocyte surface antigen Ly-9 OS=Mus musculus GN=Ly9 PE=2 SV=1 - [E9PX73_MOUSE]	5.33	3	2	2	4	1.302	0.882	0.923	1.008	0.00	5.33	2	4	544	61.1	7.11
Q91VK4	Integral membrane protein 2C OS=Mus musculus GN=Itm2c PE=1 SV=2 - [ITM2C_MOUSE]	32.71	1	5	5	18	1.387	1.025	0.993	1.008	79.92	32.71	9	18	269	30.5	8.59
G5E8J9	SCY1-like protein 2 OS=Mus musculus GN=Scyl2 PE=2 SV=1 - [G5E8J9_MOUSE]	26.70	3	1	22	84	0.731	1.124	1.240	1.008	249.38	26.70	38	84	929	103.1	8.02
Q99PH1	Leucine-rich repeat-containing protein 4 OS=Mus musculus GN=Lrrc4 PE=1 SV=2 - [LRRC4_MOUSE]	7.67	1	3	4	9	0.921	1.504	1.226	1.008	24.96	7.67	6	9	652	72.6	7.01
Q80U62-3	Isoform 3 of Run domain Beclin-1 interacting and cysteine-rich containing protein OS=Mus musculus GN=Kiaa0226 - [RUBIC_MOUSE]	4.31	6	4	4	8	1.095	0.774	1.089	1.008	13.31	4.31	5	8	927	103.9	6.01

P49194	Retinol-binding protein 3 OS=Mus musculus GN=Rbp3 PE=2 SV=3 - [RET3_MOUSE]	4.13	1	2	2	2	1.161	0.768	1.032	1.008	2.29	4.13	2	2	1234	134.4	5.11
Q6PAR5-2	Isoform 2 of GTPase-activating protein and VPS9 domain-containing protein 1 OS=Mus musculus GN=Gapvd1 - [GAPD1_MOUSE]	21.29	14	24	25	93	0.851	1.011	1.003	1.009	236.63	21.29	44	93	1437	160.0	5.20
Q02384	Son of sevenless homolog 2 OS=Mus musculus GN=Sos2 PE=1 SV=2 - [SOS2_MOUSE]	7.50	1	6	11	25	1.046	0.886	0.909	1.009	63.15	7.50	19	25	1333	153.0	6.76
Q8VE37	Regulator of chromosome condensation OS=Mus musculus GN=Rcc1 PE=1 SV=1 - [RCC1_MOUSE]	26.13	3	8	8	22	0.924	0.623	0.966	1.009	54.55	26.13	13	22	421	44.9	8.10
P54823	Probable ATP-dependent RNA helicase DDX6 OS=Mus musculus GN=Ddx6 PE=2 SV=1 - [DDX6_MOUSE]	39.13	3	14	15	115	0.632	0.839	0.831	1.009	301.61	39.13	28	115	483	54.2	8.66
Q9D0D3	Poly(A) RNA polymerase, mitochondrial OS=Mus musculus GN=Mtpap PE=2 SV=1 - [PAPD1_MOUSE]	19.32	1	9	9	25	0.784	0.787	0.994	1.009	63.17	19.32	14	25	585	65.2	8.88
F6SDD7	DNA topoisomerase (Fragment) OS=Mus musculus GN=Top3b PE=3 SV=1 - [F6SDD7_MOUSE]	20.49	2	9	9	22	1.535	0.923	1.086	1.009	57.33	20.49	16	22	527	60.0	7.40
Q8BT51	Cytochrome c oxidase assembly factor 4 homolog, mitochondrial OS=Mus musculus GN=COA4 PE=3 SV=1 - [illegible]	21.84	1	2	2	16	1.751	1.203	1.031	1.009	42.02	21.84	4	16	87	10.1	6.90
Q8BXQ8	Protein FAM53C OS=Mus musculus GN=Fam53c PE=1 SV=1 - [FA53C_MOUSE]	3.31	1	1	1	2	1.161	1.097	1.463	1.009	7.65	3.31	2	2	393	43.2	7.72
Q6P3D0	U8 snoRNA-decapping enzyme OS=Mus musculus GN=Nudt16 PE=1 SV=1 - [NUD16_MOUSE]	65.13	2	10	11	30	0.628	1.183	0.902	1.009	96.39	65.13	19	30	195	21.8	7.12
P40142	Transketolase OS=Mus musculus GN=Tkt PE=1 SV=1 - [TKT_MOUSE]	64.69	1	36	36	540	1.970	0.793	0.779	1.009	1436.55	64.69	66	540	623	67.6	7.50
E9QMI6	Pre-mRNA-processing factor 40 homolog B (Fragment) OS=Mus musculus GN=Prpf40b PE=2 SV=1 - [illegible]	6.46	7	4	5	19	1.038	0.986	0.961	1.009	37.85	6.46	10	19	852	97.0	7.03
Q9QZB7	Actin-related protein 10 OS=Mus musculus GN=Actr10 PE=1 SV=2 - [ARP10_MOUSE]	53.24	1	18	19	73	0.687	0.924	0.961	1.009	162.30	53.24	30	73	417	46.2	7.61
Q9WTS5	Teneurin-2 OS=Mus musculus GN=Tenm2 PE=2 SV=1 - [TEN2_MOUSE]	10.17	1	17	21	70	0.727	0.846	0.772	1.009	182.77	10.17	33	70	2764	306.3	6.68
P61979-3	Isoform 3 of Heterogeneous nuclear ribonucleoprotein K OS=Mus musculus GN=Hnrnpk - [illegible]	54.44	9	4	20	383	1.141	1.122	1.110	1.009	1033.81	54.44	39	383	439	48.5	5.92
Q8CHT1-2	Isoform 2 of Ephexin-1 OS=Mus musculus GN=Ngef - [NGEF_MOUSE]	31.61	4	19	19	66	0.566	1.384	1.859	1.009	198.56	31.61	35	66	620	71.4	5.24
I7HIP9	Cadherin-22 OS=Mus musculus GN=Cdh22 PE=3 SV=1 - [I7HIP9_MOUSE]	8.12	3	5	5	12	1.801	1.704	1.099	1.009	19.93	8.12	6	12	813	87.9	4.78
Q8BUV6	U7 snRNA-associated Sm-like protein LSm11 OS=Mus musculus GN=Lsm11 PE=1 SV=1 - [LSM11_MOUSE]	9.42	1	2	2	3	0.776	0.753	0.888	1.009	12.18	9.42	2	3	361	39.9	10.73
E9Q9X4	Nitric oxide synthase OS=Mus musculus GN=Nos3 PE=2 SV=1 - [E9Q9X4_MOUSE]	3.80	2	3	4	10	0.684	1.087	0.988	1.009	17.01	3.80	7	10	1106	122.0	7.52
O08601	Microsomal triglyceride transfer protein large subunit OS=Mus musculus GN=Mttp PE=2 SV=2 - [MTP_MOUSE]	7.27	2	6	6	7	0.881	1.037	0.812	1.010	18.71	7.27	7	7	894	99.0	7.62
Q9CPN9	Protein 2210010C04Rik OS=Mus musculus GN=2210010C04Rik PE=2 SV=1 - [Q9CPN9_MOUSE]	3.24	1	1	1	1	1.654	1.283	1.038	1.010	1.77	3.24	1	1	247	26.4	7.93
Q9Z280-2	Isoform PLD1B of Phospholipase D1 OS=Mus musculus GN=Pld1 - [PLD1_MOUSE]	6.56	4	2	6	14	1.119	0.501	0.825	1.010	45.61	6.56	9	14	1036	119.5	8.70
Q8CAV6	Protein kinase C theta type OS=Mus musculus GN=Prkcq PE=2 SV=1 - [Q8CAV6_MOUSE]	18.39	3	8	11	37	0.936	0.504	0.890	1.010	92.91	18.39	17	37	571	64.9	8.06
Q8BGF3	WD repeat-containing protein 92 OS=Mus musculus GN=Wdr92 PE=2 SV=1 -	3.36	1	1	1	2	1.835	0.910	0.763	1.010	6.11	3.36	2	2	357	39.8	8.15

Q8BFW3-2	Isoform 2 of Protein phosphatase 1 regulatory subunit 15B OS=Mus musculus GN=Ppp1r15b - [PR15B_MOUSE]	1.33	2	1	1	1	1.864	0.991	0.927	1.010	0.00	1.33	1	1	679	75.8	5.01
Q8JZR6	Electroneutral sodium bicarbonate exchanger 1 OS=Mus musculus GN=Slc4a8 PE=2 SV=1 - [S4A8_MOUSE]	20.48	2	13	18	59	0.681	0.822	0.960	1.010	165.14	20.48	31	59	1089	122.3	6.60
A8Y5P1	High affinity copper uptake protein 1 OS=Mus musculus GN=Slc31a1 PE=2 SV=1 - [illegible]	14.62	2	1	1	7	1.041	1.167	1.151	1.010	25.06	14.62	2	7	130	14.5	7.17
A2AR78	TRAF family member-associated NF-kappa-B activator OS=Mus musculus GN=Tank PE=2 SV=1 - [A2AR78_MOUSE]	15.00	7	4	4	5	1.164	0.971	1.517	1.010	21.41	15.00	4	5	360	40.9	5.66
Q8CAA7	Glucose 1,6-bisphosphate synthase OS=Mus musculus GN=Pgm2l1 PE=1 SV=1 - [illegible]	49.11	1	19	33	177	0.579	1.133	1.186	1.010	478.36	49.11	57	177	621	70.2	6.49
Q9ES63	Ubiquitin carboxyl-terminal hydrolase 29 OS=Mus musculus GN=Usp29 PE=2 SV=2 - [UBP29_MOUSE]	4.26	1	3	3	8	0.344	0.661	0.611	1.010	20.59	4.26	5	8	869	97.8	5.60
Q925J9-4	Isoform 4 of Mediator of RNA polymerase II transcription subunit 1 OS=Mus musculus GN=Med1 - [illegible]	3.91	3	3	4	5	2.440	1.112	1.277	1.010	14.55	3.91	5	5	1560	165.3	8.78
Q8BX80	Cytosolic endo-beta-N-acetylglucosaminidase OS=Mus musculus GN=Engase PE=2 SV=1 - [ENASE_MOUSE]	11.44	3	5	5	8	0.907	0.915	0.937	1.010	24.16	11.44	6	8	734	82.9	6.65
G5E861	Sodium channel and clathrin linker 1 OS=Mus musculus GN=Sclt1 PE=2 SV=1 - [SCLT1_MOUSE]	5.81	3	2	4	9	1.635	1.031	0.987	1.010	21.87	5.81	6	9	688	80.4	6.05
Q61216	Double-strand break repair protein MRE11A OS=Mus musculus GN=Mre11a PE=2 SV=1 - [MRE11_MOUSE]	9.92	3	7	7	11	0.726	1.011	0.973	1.010	28.40	9.92	9	11	706	80.2	6.01
P56375	Acylphosphatase-2 OS=Mus musculus GN=Acyp2 PE=2 SV=2 - [ACYP2_MOUSE]	47.17	1	6	6	51	2.210	0.743	1.210	1.011	187.24	47.17	10	51	106	11.9	9.26
E9Q0J5	Kinesin-like protein KIF21A OS=Mus musculus GN=Kif21a PE=3 SV=2 - [E9Q0J5_MOUSE]	32.36	4	1	45	201	0.842	0.558	1.115	1.011	629.25	32.36	75	201	1672	186.5	6.47
P97819-2	Isoform Short of 85/88 kDa calcium-independent phospholipase A2 OS=Mus musculus GN=Pla2g6 - [PLPL9_MOUSE]	9.97	4	5	5	13	0.701	1.202	0.950	1.011	45.93	9.97	8	13	752	83.6	7.18
F6W8I0	YjeF N-terminal domain-containing protein 3 OS=Mus musculus GN=Yjefn3 PE=2 SV=2 - [YJEN3_MOUSE]	29.48	1	7	7	11	0.760	4.431	4.653	1.011	37.45	29.48	11	11	251	27.0	7.40
Q3TDX8-5	Isoform 2 of Cytochrome b5 reductase 4 OS=Mus musculus GN=Cyb5r4 - [NB5R4_MOUSE]	22.64	3	6	8	37	1.159	1.024	0.978	1.011	111.50	22.64	14	37	477	54.0	6.44
B1AUY8	N-alpha-acetyltransferase 10 OS=Mus musculus GN=Naa10 PE=2 SV=1 - [B1AUY8_MOUSE]	27.27	6	3	7	28	1.009	1.028	1.019	1.011	86.11	27.27	13	28	220	24.8	5.52
O70309	Integrin beta-5 OS=Mus musculus GN=Itgb5 PE=1 SV=2 - [ITB5_MOUSE]	2.76	4	3	3	5	0.568	1.067	0.766	1.011	6.52	2.76	4	5	798	87.9	6.16
Q9D6H2	Heat shock protein beta-11 OS=Mus musculus GN=Hspb11 PE=1 SV=2 - [HSB11_MOUSE]	19.58	1	2	2	6	1.382	0.933	1.026	1.011	21.63	19.58	3	6	143	16.3	5.16
Q7TMJ8-2	Isoform 2 of Phosphoinositide-3-kinase-interacting protein 1 OS=Mus musculus GN=Pik3ip1 - [illegible]	13.15	3	2	2	6	0.946	1.325	0.977	1.011	22.51	13.15	4	6	213	23.0	5.59
M0QWA1	Protein Csmd2 (Fragment) OS=Mus musculus GN=Csmd2 PE=4 SV=1 - [M0QWA1_MOUSE]	3.10	1	9	9	21	0.841	1.115	1.084	1.011	60.00	3.10	15	21	3423	372.4	6.01
Q9CWV0	Mitochondrial assembly of ribosomal large subunit protein 1 OS=Mus musculus GN=Malsu1 PE=2 SV=1 - [illegible]	7.46	6	1	2	4	1.249	1.069	0.781	1.011	9.74	7.46	4	4	228	26.0	6.73
O54749	Cytochrome P450 2J5 OS=Mus musculus GN=Cyp2j5 PE=2 SV=1 - [CP2J5_MOUSE]	5.39	3	1	2	4	1.058	1.659	0.922	1.011	11.27	5.39	3	4	501	57.7	8.73
D3Z0M9	MCG18410, isoform CRA_a OS=Mus musculus GN=Ddx23 PE=3 SV=1 - [D3Z0M9_MOUSE]	18.19	1	15	15	34	0.849	1.005	0.889	1.011	110.11	18.19	25	34	819	95.4	9.58
F8WHJ1	FTS and Hook-interacting protein OS=Mus musculus GN=Fam160a2 PE=2 SV=1 - [F8WHJ1_MOUSE]	24.31	4	1	16	40	1.593	1.262	1.224	1.011	121.67	24.31	26	40	909	99.1	6.44

Q91Z61	GTP-binding protein Di-Ras1 OS=Mus musculus GN=Diras1 PE=2 SV=1 - [DIRA1_MOUSE]	67.68	2	9	13	51	0.704	0.802	1.024	1.011	162.00	67.68	21	51	198	22.3	8.90
Q63829	COMM domain-containing protein 3 OS=Mus musculus GN=Commd3 PE=2 SV=1 -	37.95	1	6	6	21	1.000	1.291	0.863	1.011	58.20	37.95	11	21	195	22.0	5.59
Q505D1	Serine/threonine-protein phosphatase 6 regulatory ankyrin repeat subunit A OS=Mus musculus GN=Ankrd28 PE=1 SV=1 - [ANR28_MOUSE]	9.78	1	7	8	13	1.051	1.347	1.325	1.011	28.38	9.78	12	13	1053	112.8	6.27
Q3UGY8	Brefeldin A-inhibited guanine nucleotide-exchange protein 3 OS=Mus musculus GN=Arfgef3 PE=1 SV=1 - [illegible]	21.66	1	39	39	122	0.720	0.902	0.895	1.011	328.11	21.66	68	122	2170	239.9	5.88
F6QM56	Acid trehalase-like protein 1 (Fragment) OS=Mus musculus GN=Athl1 PE=4 SV=1 - [F6QM56_MOUSE]	7.70	5	2	3	4	1.271	1.051	0.904	1.011	7.98	7.70	4	4	623	69.3	5.25
Q5SXA9	Protein KIBRA OS=Mus musculus GN=Wwc1 PE=1 SV=1 - [KIBRA_MOUSE]	12.59	1	10	10	24	0.721	0.980	1.133	1.011	66.33	12.59	18	24	1104	124.0	5.97
Q3USH1	Protein FAM196A OS=Mus musculus GN=Fam196a PE=2 SV=1 - [F196A_MOUSE]	24.17	2	7	7	14	1.105	0.812	1.063	1.012	45.94	24.17	10	14	422	46.2	7.14
P31649	Sodium- and chloride-dependent GABA transporter 2 OS=Mus musculus GN=Slc6a13 PE=1 SV=1 - [illegible]	10.47	2	4	5	23	0.417	3.825	0.752	1.012	65.59	10.47	9	23	602	68.2	6.25
P46467	Vacuolar protein sorting-associated protein 4B OS=Mus musculus GN=Vps4b PE=1 SV=2 - [VPS4B_MOUSE]	39.41	2	10	15	44	0.944	1.006	0.944	1.012	121.93	39.41	28	44	444	49.4	7.11
A2A6T1	Cerebellar degeneration-related protein 2-like OS=Mus musculus GN=Cdr2l PE=2 SV=1 - [CDR2L_MOUSE]	16.13	1	7	8	16	1.886	0.581	0.910	1.012	39.05	16.13	13	16	465	53.2	5.76
Q571K4	TGF-beta-activated kinase 1 and MAP3K7-binding protein 3 OS=Mus musculus GN=Tab3 PE=1 SV=2 - [TAB3_MOUSE]	13.13	1	5	5	13	2.028	1.000	1.197	1.012	35.79	13.13	9	13	716	79.0	8.50
Q9WTX5	S-phase kinase-associated protein 1 OS=Mus musculus GN=Skp1 PE=1 SV=3 - [SKP1_MOUSE]	63.80	4	12	12	107	1.459	1.031	1.291	1.012	336.53	63.80	22	107	163	18.7	4.54
D3Z2J3	Putative ATP-dependent RNA helicase DHX30 OS=Mus musculus GN=Dhx30 PE=2 SV=1 - [D3Z2J3_MOUSE]	27.15	5	25	25	71	0.760	0.763	0.942	1.012	212.99	27.15	40	71	1186	132.9	8.59
E9PZ00	Sulfated glycoprotein 1 OS=Mus musculus GN=Psap PE=2 SV=1 - [E9PZ00_MOUSE]	51.00	5	26	26	182	1.585	1.147	1.224	1.012	470.59	51.00	48	182	551	60.6	5.24
Q9CZT8	Ras-related protein Rab-3B OS=Mus musculus GN=Rab3b PE=1 SV=1 - [RAB3B_MOUSE]	53.88	3	5	10	157	0.545	2.018	1.102	1.012	475.81	53.88	17	157	219	24.7	5.11
Q80TI0-2	Isoform 2 of GRAM domain containing protein 1B OS=Mus musculus GN=Gramd1b - [GRM1B_MOUSE]	16.62	6	10	10	32	0.576	1.184	1.610	1.012	61.91	16.62	17	32	698	81.0	5.78
Q7TSH4	Centriolar coiled-coil protein of 110 kDa OS=Mus musculus GN=Ccp110 PE=1 SV=1 -	15.94	1	12	13	28	1.117	0.815	1.047	1.012	92.03	15.94	21	28	1004	111.1	8.85
P97287	Induced myeloid leukemia cell differentiation protein Mcl-1 homolog OS=Mus musculus GN=Mcl1 PE=1 SV=3 - [MCL1_MOUSE]	6.34	1	1	1	2	3.435	1.224	1.076	1.012	6.13	6.34	2	2	331	35.2	6.20
P29533	Vascular cell adhesion protein 1 OS=Mus musculus GN=Vcam1 PE=1 SV=1 -	30.99	3	21	21	79	1.183	1.159	1.230	1.012	237.33	30.99	35	79	739	81.3	5.30
Q91VH6	Protein MEMO1 OS=Mus musculus GN=Memo1 PE=1 SV=1 - [MEMO1_MOUSE]	18.86	1	5	5	12	0.942	1.198	0.793	1.012	27.79	18.86	7	12	297	33.7	7.14
Q99J56	Derlin-1 OS=Mus musculus GN=Derl1 PE=2 SV=1 - [DERL1_MOUSE]	8.76	1	2	2	20	0.815	1.328	0.854	1.012	63.90	8.76	3	20	251	28.8	9.51
Q68FM6	Protein phosphatase 1 regulatory subunit 29 OS=Mus musculus GN=Elfn2 PE=1 SV=1 - [PPR29_MOUSE]	28.80	1	16	19	77	0.881	1.303	1.228	1.012	234.01	28.80	33	77	823	90.0	7.52
O70252	Heme oxygenase 2 OS=Mus musculus GN=Hmox2 PE=2 SV=1 - [HMOX2_MOUSE]	58.41	4	14	15	57	0.640	1.075	0.902	1.012	181.38	58.41	27	57	315	35.7	5.87

F6QKK2	ADP-ribosylation factor-like protein 8A (Fragment) OS=Mus musculus GN=Arl8a PE=4 SV=1 - [F6QKK2_MOUSE]	56.36	2	3	8	31	0.704	0.724	0.869	1.012	81.18	56.36	14	31	165	18.9	8.16
Q99NE5-5	Isoform 5 of Regulating synaptic membrane exocytosis protein 1 OS=Mus musculus GN=Rims1 - [illegible]	41.33	13	15	44	138	0.687	1.199	1.296	1.012	446.11	41.33	74	138	1350	150.6	9.13
R4GMK9	MCG7133 OS=Mus musculus GN=Zfp618 PE=4 SV=1 - [R4GMK9_MOUSE]	2.94	3	1	1	2	1.316	1.056	1.052	1.012	1.71	2.94	1	2	374	41.1	6.81
B1ARW4	NADH dehydrogenase [ubiquinone] iron-sulfur protein 5 (Fragment) OS=Mus musculus GN=Ndufs5 PE=2 SV=1 - [illegible]	60.44	2	7	7	109	1.597	0.478	1.314	1.012	345.03	60.44	14	109	91	10.9	8.94
Q60660	Killer cell lectin-like receptor 2 OS=Mus musculus GN=Klra2 PE=2 SV=1 - [KLRA2_MOUSE]	3.13	3	1	1	1	2.158	1.508	0.970	1.012	2.41	3.13	1	1	288	33.6	8.09
O35730	E3 ubiquitin-protein ligase RING1 OS=Mus musculus GN=Ring1 PE=1 SV=2 - [RING1_MOUSE]	13.30	3	2	3	6	0.583	0.840	0.862	1.012	18.01	13.30	5	6	406	42.6	5.74
A2A8L5	Receptor-type tyrosine-protein phosphatase F OS=Mus musculus GN=Ptprf PE=1 SV=1 - [PTPRF_MOUSE]	29.61	1	10	41	145	1.108	1.136	0.863	1.012	412.87	29.61	73	145	1898	211.4	6.65
Q6GQT6	Sterol regulatory element-binding protein cleavage-activating protein OS=Mus musculus GN=Scap PE=1 SV=1 - [SCAP_MOUSE]	2.51	1	2	2	5	0.491	0.927	1.353	1.012	22.82	2.51	3	5	1276	139.5	7.08
P83887	Tubulin gamma-1 chain OS=Mus musculus GN=Tubg1 PE=1 SV=1 - [TBG1_MOUSE]	51.44	1	4	16	57	1.423	1.014	1.010	1.012	188.57	51.44	28	57	451	51.1	6.02
Q6P5B0	RRP12-like protein OS=Mus musculus GN=Rrp12 PE=1 SV=1 - [RRP12_MOUSE]	9.96	1	11	11	22	1.789	1.151	1.024	1.012	53.29	9.96	16	22	1295	143.0	8.91
Q99JI6	Ras-related protein Rap-1b OS=Mus musculus GN=Rap1b PE=2 SV=2 - [RAP1B_MOUSE]	75.00	1	6	12	129	0.882	1.027	0.937	1.012	380.79	75.00	21	129	184	20.8	5.78
Q80VQ1	Leucine-rich repeat-containing protein 1 OS=Mus musculus GN=Lrrc1 PE=2 SV=2 - [LRRC1_MOUSE]	15.65	4	6	7	14	1.259	0.897	0.815	1.012	33.21	15.65	10	14	524	59.4	5.16
A2AQ87	SH2 domain-containing adapter protein F (Fragment) OS=Mus musculus GN=Shf PE=2 SV=1 - [A2AQ87_MOUSE]	27.80	7	7	7	22	0.995	1.057	1.461	1.013	72.88	27.80	11	22	446	47.1	5.64
E9Q6A6	Collagen alpha-6(VI) chain OS=Mus musculus GN=Col6a6 PE=2 SV=2 - [E9Q6A6_MOUSE]	2.38	3	4	4	4	0.688	0.670	0.863	1.013	4.37	2.38	4	4	2265	246.3	6.90
Q5DTL9	Sodium-driven chloride bicarbonate exchanger OS=Mus musculus GN=Slc4a10 PE=1 SV=2 - [S4A10_MOUSE]	26.57	1	18	25	104	0.572	1.106	1.186	1.013	298.24	26.57	44	104	1118	125.7	6.51
Q8BJU9	Peptide chain release factor 1-like, mitochondrial OS=Mus musculus GN=Mtrf1l PE=2 SV=1 - [RF1ML_MOUSE]	22.79	1	6	7	14	1.740	0.997	0.902	1.013	40.97	22.79	11	14	373	42.2	8.16
P28738	Kinesin heavy chain isoform 5C OS=Mus musculus GN=Kif5c PE=1 SV=3 - [KIF5C_MOUSE]	48.74	3	32	48	312	1.239	0.969	1.279	1.013	960.79	48.74	87	312	956	109.2	6.19
O35465	Peptidyl-prolyl cis-trans isomerase FKBP8 OS=Mus musculus GN=Fkbp8 PE=1 SV=2 - [FKBP8_MOUSE]	28.86	2	1	11	57	0.788	0.651	0.940	1.013	182.29	28.86	19	57	402	43.5	5.16
Q8BFR4	N-acetylglucosamine-6-sulfatase OS=Mus musculus GN=Gns PE=2 SV=1 - [GNS_MOUSE]	17.10	1	9	9	26	1.300	1.180	1.060	1.013	68.42	17.10	16	26	544	61.1	8.24
Q91VJ2	Protein kinase C delta-binding protein OS=Mus musculus GN=Prkcdbp PE=1 SV=1 - [PRDBP_MOUSE]	15.77	1	4	4	9	0.840	1.850	0.876	1.013	26.33	15.77	8	9	260	27.8	5.57
Q62189	U1 small nuclear ribonucleoprotein A OS=Mus musculus GN=Snrpa PE=2 SV=3 -	39.72	4	8	10	66	0.949	0.853	1.048	1.013	177.28	39.72	17	66	287	31.8	9.80
Q8BLR2	Copine-4 OS=Mus musculus GN=Cpne4 PE=1 SV=1 - [CPNE4_MOUSE]	31.96	4	15	16	77	1.443	1.640	1.198	1.013	196.26	31.96	24	77	557	62.4	6.33
P18760	Cofilin-1 OS=Mus musculus GN=Cfl1 PE=1 SV=3 - [COF1_MOUSE]	87.35	1	1	23	591	1.394	1.051	1.112	1.013	1516.11	87.35	42	591	166	18.5	8.09
Q80YE7-2	Isoform 2 of Death-associated protein kinase 1 OS=Mus musculus GN=Dapk1 -	8.74	4	10	11	18	1.152	1.400	1.416	1.013	48.06	8.74	16	18	1430	159.9	6.92

Q8BRM2	RAB6-interacting golgin OS=Mus musculus GN=Gorab PE=1 SV=1 - [GORAB_MOUSE]	28.53	2	8	8	12	1.328	0.705	1.346	1.013	25.80	28.53	12	12	368	41.5	7.36
Q9QYJ3	DnaJ homolog subfamily B member 1 OS=Mus musculus GN=Dnajb1 PE=2 SV=3 -	28.82	1	9	10	43	1.155	0.962	1.121	1.013	128.02	28.82	17	43	340	38.1	8.63
Q8BK03	Protein FAM73B OS=Mus musculus GN=Fam73b PE=1 SV=1 - [FA73B_MOUSE]	28.50	6	11	12	37	0.731	0.771	0.764	1.013	97.93	28.50	20	37	593	65.5	5.41
A3KGK9	Dehydrodolichyl diphosphate synthase (Fragment) OS=Mus musculus GN=Dhdds PE=2 SV=1 - [illegible]	6.55	5	1	1	2	1.200	0.874	0.921	1.013	0.00	6.55	2	2	168	19.4	9.10
C3VPR6	Protein NLRC5 OS=Mus musculus GN=Nlrc5 PE=1 SV=2 - [NLRC5_MOUSE]	3.71	2	4	4	4	1.741	1.316	1.008	1.013	8.37	3.71	4	4	1915	211.6	6.38
Q99M28	RNA-binding protein with serine-rich domain 1 OS=Mus musculus GN=Rnps1 PE=2 SV=1 - [RNPS1_MOUSE]	10.82	3	2	3	6	1.343	0.719	0.972	1.013	17.38	10.82	3	6	305	34.2	11.84
Q8BIZ1	Ankyrin repeat and sterile alpha motif domain-containing protein 1B OS=Mus musculus GN=Anks1b PE=1 SV=3 - [illegible]	23.35	6	10	28	87	0.668	0.780	1.181	1.013	235.55	23.35	45	87	1259	139.0	6.29
O35066	Kinesin-like protein KIF3C OS=Mus musculus GN=Kif3c PE=2 SV=3 - [KIF3C_MOUSE]	28.39	2	15	20	47	1.434	0.915	1.134	1.013	124.07	28.39	31	47	796	89.9	8.06
Q924T2	28S ribosomal protein S2, mitochondrial OS=Mus musculus GN=Mrps2 PE=2 SV=1 - [RT02_MOUSE]	20.27	1	4	5	19	0.829	0.730	0.945	1.013	57.73	20.27	9	19	291	32.3	9.14
P57774	Pro-neuropeptide Y OS=Mus musculus GN=Npy PE=2 SV=2 - [NPY_MOUSE]	28.87	1	3	3	23	2.218	1.816	1.311	1.013	66.32	28.87	5	23	97	10.9	7.12
Q3TVF4	Geranylgeranyl transferase type-2 subunit beta OS=Mus musculus GN=Rabggtb PE=2 SV=1 - [Q3TVF4_MOUSE]	14.50	2	4	4	13	1.090	1.133	0.959	1.014	33.23	14.50	6	13	331	36.9	5.08
Q8BFZ3	Beta-actin-like protein 2 OS=Mus musculus GN=Actbl2 PE=1 SV=1 - [ACTBL_MOUSE]	49.47	1	4	18	1798	1.096	1.252	1.002	1.014	5511.01	49.47	29	1798	376	42.0	5.49
Q68ED2	Metabotropic glutamate receptor 7 OS=Mus musculus GN=Grm7 PE=1 SV=1 - [GRM7_MOUSE]	24.04	6	16	18	51	0.737	1.068	1.107	1.014	171.23	24.04	29	51	915	102.2	7.88
Q80W04	Transmembrane and coiled coil domains protein 2 OS=Mus musculus GN=Tmcc2 PE=1 SV=1 - [TMCC2_MOUSE]	32.72	3	15	17	65	0.878	0.960	1.033	1.014	169.49	32.72	30	65	706	77.0	6.84
Q8C7I4	Protein Apol9a OS=Mus musculus GN=Apol9b PE=2 SV=1 - [Q8C7I4_MOUSE]	3.87	2	1	1	1	1.522	0.050	1.281	1.014	1.87	3.87	1	1	310	33.4	6.98
P32648	VIP peptides OS=Mus musculus GN=Vip PE=1 SV=1 - [VIP_MOUSE]	41.18	1	5	5	14	1.475	1.638	1.616	1.014	40.80	41.18	7	14	170	19.0	6.60
Q3UFK8	FERM domain-containing protein 8 OS=Mus musculus GN=Frmd8 PE=1 SV=2 -	16.95	2	7	7	21	0.904	0.493	0.808	1.014	61.68	16.95	13	21	466	51.8	6.44
G3X922	MCG115602 OS=Mus musculus GN=Dnajc13 PE=4 SV=1 - [G3X922_MOUSE]	18.77	1	39	40	94	0.618	1.149	0.918	1.014	268.38	18.77	64	94	2243	254.3	6.77
Q3U182	CREB-regulated transcription coactivator 2 OS=Mus musculus GN=Crtc2 PE=1 SV=2 - [CRTC2_MOUSE]	9.10	1	5	5	10	1.289	1.277	1.224	1.014	22.80	9.10	7	10	692	73.2	7.01
Q8C079-3	Isoform 3 of Striatin-interacting protein 1 OS=Mus musculus GN=Strip1 -	23.49	4	12	14	55	0.915	1.251	0.952	1.014	155.99	23.49	24	55	762	86.8	5.66
P63058-3	Isoform Alpha-3 of Thyroid hormone receptor alpha OS=Mus musculus GN=Thra - [THA_MOUSE]	4.42	2	2	2	11	1.078	1.262	1.103	1.014	25.66	4.42	4	11	453	50.9	7.36
P0DJF2	Protein PET117 homolog, mitochondrial OS=Mus musculus GN=Pet117 PE=3 SV=1 - [PT117_MOUSE]	12.50	1	1	1	2	0.803	0.862	1.151	1.014	6.27	12.50	2	2	80	9.1	10.35
Q8VE88-2	Isoform 2 of Protein FAM114A2 OS=Mus musculus GN=Fam114a2 - [F1142_MOUSE]	24.29	2	10	10	28	0.847	1.555	1.057	1.014	93.05	24.29	16	28	490	53.3	4.89
P16045	Galectin-1 OS=Mus musculus GN=Lgals1 PE=1 SV=3 - [LEG1_MOUSE]	43.70	1	5	5	53	1.795	1.767	1.019	1.014	181.13	43.70	10	53	135	14.9	5.49
P24668	Cation-dependent mannose-6-phosphate receptor OS=Mus musculus GN=M6pr PE=1 SV=1 - [MPRD_MOUSE]	25.18	1	8	8	30	0.811	1.139	0.885	1.014	113.56	25.18	12	30	278	31.2	5.39

Q8VE99	Coiled-coil domain-containing protein 115 OS=Mus musculus GN=Ccdc115 PE=2 SV=1 - [CC115_MOUSE]	18.33	1	3	3	4	1.504	0.958	0.754	1.014	10.29	18.33	4	4	180	19.7	8.81
E9Q3M3	Dynactin subunit 1 OS=Mus musculus GN=Dctn1 PE=2 SV=1 - [E9Q3M3_MOUSE]	51.19	9	51	53	316	0.788	0.913	1.046	1.014	1034.59	51.19	99	316	1264	139.7	5.71
Q3USJ8	FCH and double SH3 domains protein 2 OS=Mus musculus GN=Fchsd2 PE=1 SV=2 - [FCSD2_MOUSE]	22.70	3	13	13	51	1.485	1.165	0.953	1.014	137.34	22.70	20	51	740	84.2	5.66
Q91V12-2	Isoform A of Cytosolic acyl coenzyme A thioester hydrolase OS=Mus musculus GN=Acot7 - [BACH_MOUSE]	58.28	5	15	15	136	0.858	0.734	0.840	1.014	401.47	58.28	27	136	338	37.5	7.52
E9PYJ6	Focadhesin OS=Mus musculus GN=Focad PE=2 SV=1 - [E9PYJ6_MOUSE]	4.21	4	6	7	13	0.685	0.785	0.760	1.014	30.85	4.21	12	13	1712	189.4	6.90
P26049	Gamma-aminobutyric acid receptor subunit alpha-3 OS=Mus musculus GN=Gabra3 PE=1 SV=1 - [GBRA3_MOUSE]	29.67	2	13	15	56	0.855	1.106	1.180	1.014	142.82	29.67	26	56	492	55.4	8.94
Q9DBS5	Kinesin light chain 4 OS=Mus musculus GN=Klc4 PE=1 SV=1 - [KLC4_MOUSE]	31.99	1	11	17	75	0.969	0.862	1.048	1.014	200.33	31.99	31	75	619	68.6	6.09
Q9CY16	28S ribosomal protein S28, mitochondrial OS=Mus musculus GN=Mrps28 PE=2 SV=1 - [RT28_MOUSE]	32.26	1	5	5	11	0.381	0.912	0.960	1.015	33.44	32.26	8	11	186	20.5	8.92
Q3V0G7-2	Isoform 2 of GTPase-activating Rap/Ran-GAP domain-like protein 3 OS=Mus musculus GN=Garnl3 - [illegible]	19.54	5	12	13	29	0.820	0.929	1.060	1.015	85.57	19.54	19	29	993	110.5	7.25
Q9WV85	Nucleoside diphosphate kinase 3 OS=Mus musculus GN=Nme3 PE=2 SV=3 - [NDK3_MOUSE]	63.31	1	10	10	24	0.687	1.012	0.698	1.015	42.87	63.31	16	24	169	19.1	6.68
Q6DVA0	LEM domain-containing protein 2 OS=Mus musculus GN=Lemd2 PE=1 SV=1 -	16.63	1	8	8	15	1.021	1.305	0.981	1.015	43.65	16.63	12	15	511	57.5	9.01
Q91WK5	Glycine cleavage system H protein, mitochondrial OS=Mus musculus GN=Gcsh PE=1 SV=2 - [GCSH_MOUSE]	39.41	1	3	3	13	1.380	0.806	1.013	1.015	50.56	39.41	4	13	170	18.6	4.75
Q3UTR7	Angiotensinogen OS=Mus musculus GN=Agt PE=2 SV=1 - [Q3UTR7_MOUSE]	23.03	1	1	9	39	1.380	0.486	0.604	1.015	107.61	23.03	17	39	482	52.6	5.54
P10493	Nidogen-1 OS=Mus musculus GN=Nid1 PE=1 SV=2 - [NID1_MOUSE]	22.81	1	24	24	76	1.408	1.276	0.818	1.015	227.33	22.81	44	76	1245	136.5	5.44
G5E897	KDEL (Lys-Asp-Glu-Leu) containing 2, isoform CRA_b OS=Mus musculus GN=Kdelc2 PE=4 SV=1 - [G5E897_MOUSE]	8.75	1	3	4	6	0.737	1.100	0.711	1.015	13.88	8.75	5	6	503	57.6	7.74
Q80TS3-5	Isoform 5 of Latrophilin-3 OS=Mus musculus GN=Lphn3 - [LPHN3_MOUSE]	29.91	23	39	40	159	0.901	1.436	1.139	1.015	494.30	29.91	71	159	1528	169.8	6.74
Q8R1N4	NudC domain-containing protein 3 OS=Mus musculus GN=Nudcd3 PE=2 SV=3 -	30.30	5	9	9	39	1.272	0.982	0.885	1.015	108.19	30.30	14	39	363	40.9	5.26
P61027	Ras-related protein Rab-10 OS=Mus musculus GN=Rab10 PE=1 SV=1 - [RAB10_MOUSE]	76.00	1	11	16	183	0.778	0.876	0.894	1.015	525.81	76.00	28	183	200	22.5	8.38
Q7TT23	Uncharacterized protein C20orf194 homolog OS=Mus musculus PE=2 SV=2 - [CT194_MOUSE]	5.77	3	4	4	6	1.075	0.901	0.824	1.015	15.98	5.77	5	6	1179	132.1	6.43
Q8K1J6	CCA tRNA nucleotidyltransferase 1, mitochondrial OS=Mus musculus GN=Trnt1 PE=2 SV=1 - [TRNT1_MOUSE]	37.10	2	16	16	44	0.738	0.878	0.912	1.015	114.60	37.10	28	44	434	49.9	8.56
G3UW55	MCG14937 OS=Mus musculus GN=A230065H16Rik PE=4 SV=1 - [G3UW55_MOUSE]	75.44	1	5	5	32	1.261	1.980	1.074	1.015	66.22	75.44	8	32	114	11.4	4.75
P63017	Heat shock cognate 71 kDa protein OS=Mus musculus GN=Hspa8 PE=1 SV=1 - [HSP7C_MOUSE]	80.80	4	35	54	2120	1.121	1.028	0.962	1.015	6346.71	80.80	102	2120	646	70.8	5.52
P59999	Actin-related protein 2/3 complex subunit 4 OS=Mus musculus GN=Arpc4 PE=1 SV=3 - [ARPC4_MOUSE]	55.95	2	8	8	44	0.560	1.314	1.101	1.015	122.91	55.95	13	44	168	19.7	8.43
Q8BTG7-3	Isoform 3 of Protein NDRG4 OS=Mus musculus GN=Ndrg4 - [NDRG4_MOUSE]	48.02	5	13	14	88	1.006	0.906	0.933	1.015	279.19	48.02	23	88	404	44.4	6.23

Q5XG69	Soluble lamin-associated protein of 75 kDa OS=Mus musculus GN=Fam169a PE=1 SV=3 - [F169A_MOUSE]	8.57	1	5	5	20	0.649	0.806	0.945	1.015	53.76	8.57	10	20	665	73.2	4.68
Q9Z1S8	GRB2-associated-binding protein 2 OS=Mus musculus GN=Gab2 PE=1 SV=2 - [GAB2_MOUSE]	18.95	2	10	10	23	1.402	0.871	1.166	1.016	61.38	18.95	15	23	665	73.2	8.31
P17742	Peptidyl-prolyl cis-trans isomerase A OS=Mus musculus GN=Ppia PE=1 SV=2 - [PPIA_MOUSE]	73.78	2	15	16	1072	1.518	1.013	1.295	1.016	2735.23	73.78	29	1072	164	18.0	7.90
B2RQ71	Dip2c protein OS=Mus musculus GN=Dip2c PE=2 SV=1 - [B2RQ71_MOUSE]	23.52	2	1	30	90	1.866	0.567	1.126	1.016	257.96	23.52	51	90	1556	170.8	7.39
Q9D8P4	39S ribosomal protein L17, mitochondrial OS=Mus musculus GN=Mrpl17 PE=1 SV=1 - [RM17_MOUSE]	34.09	4	7	7	10	0.945	1.148	0.922	1.016	23.53	34.09	10	10	176	20.2	9.82
Q8VFF4	MCG58903 OS=Mus musculus GN=Olfr847 PE=3 SV=1 - [Q8VFF4_MOUSE]	5.77	1	1	1	2	4.603	1.448	1.217	1.016	2.66	5.77	1	2	312	34.7	8.21
Q8BTY8	Sec1 family domain-containing protein 2 OS=Mus musculus GN=Scfd2 PE=2 SV=1 - [SCFD2_MOUSE]	17.25	5	11	11	26	0.672	0.843	0.807	1.016	68.32	17.25	20	26	684	74.7	6.81
P35290	Ras-related protein Rab-24 OS=Mus musculus GN=Rab24 PE=1 SV=2 - [RAB24_MOUSE]	36.45	1	7	7	19	0.836	1.019	0.894	1.016	56.54	36.45	13	19	203	23.1	6.23
E9QAG7	E3 ubiquitin-protein ligase makorin-1 OS=Mus musculus GN=Mkrn1 PE=2 SV=1 - [E9QAG7_MOUSE]	6.71	6	2	3	3	1.294	1.218	1.060	1.016	5.15	6.71	3	3	417	47.3	5.12
M0QWG7	Probable UDP-sugar transporter protein SLC35A5 (Fragment) OS=Mus musculus GN=Slc35a5 PE=4 SV=1 - [M0QWG7_MOUSE]	10.73	3	2	2	3	1.284	0.873	1.480	1.016	6.95	10.73	3	3	233	27.1	9.28
A2AGL3-2	Isoform 2 of Ryanodine receptor 3 OS=Mus musculus GN=Ryr3 - [RYR3_MOUSE]	3.79	3	7	14	26	1.090	1.087	1.428	1.016	56.97	3.79	20	26	4834	547.4	5.74
E0CXJ2	Seipin OS=Mus musculus GN=Bscl2 PE=2 SV=1 - [E0CXJ2_MOUSE]	7.51	2	2	2	6	0.731	0.987	0.755	1.016	10.25	7.51	3	6	213	23.6	8.88
Q99LL3	Carbohydrate sulfotransferase 12 OS=Mus musculus GN=Chst12 PE=2 SV=2 -	7.40	2	2	2	5	0.651	0.883	0.562	1.016	15.78	7.40	4	5	419	49.4	7.88
Q3TSR0	Exostosin-like 2 OS=Mus musculus GN=Extl2 PE=2 SV=1 - [Q3TSR0_MOUSE]	32.59	2	8	8	15	1.377	1.279	1.043	1.016	44.69	32.59	11	15	316	35.8	8.82
Q8R2Y2	Cell surface glycoprotein MUC18 OS=Mus musculus GN=Mcam PE=1 SV=1 - [MUC18_MOUSE]	24.85	3	12	12	30	1.375	0.832	0.992	1.016	87.66	24.85	17	30	648	71.5	5.83
Q9R061	Cytosolic Fe-S cluster assembly factor NUBP2 OS=Mus musculus GN=Nubp2 PE=1 SV=1 - [NUBP2_MOUSE]	18.18	1	4	4	9	0.915	1.087	0.973	1.016	30.35	18.18	7	9	275	29.5	6.52
Q925H0-2	Isoform 2 of Acid-sensing ion channel 2 OS=Mus musculus GN=Asic2 - [ASIC2_MOUSE]	7.10	1	1	4	5	0.888	1.151	1.108	1.016	14.30	7.10	5	5	563	63.2	7.11
Q9JKK7	Tropomodulin-2 OS=Mus musculus GN=Tmod2 PE=1 SV=2 - [TMOD2_MOUSE]	66.10	3	18	19	218	1.590	1.286	1.418	1.016	659.55	66.10	34	218	351	39.5	5.35
P28571-1	Isoform GlyT-1A of Sodium and chloride-dependent glycine transporter 1 OS=Mus musculus GN=Slc6a9 - [SC6A9_MOUSE]	16.43	7	1	9	67	0.734	0.470	0.728	1.016	178.01	16.43	15	67	633	70.5	7.91
Q99KY4	Cyclin-G-associated kinase OS=Mus musculus GN=Gak PE=1 SV=2 - [GAK_MOUSE]	24.98	5	25	28	119	0.749	1.192	1.048	1.016	316.40	24.98	48	119	1305	143.6	5.73
Q80VD1	Protein FAM98B OS=Mus musculus GN=Fam98b PE=2 SV=1 - [FA98B_MOUSE]	35.43	1	8	10	56	0.997	1.206	0.980	1.016	177.97	35.43	19	56	429	45.3	8.50
O54967-2	Isoform 2 of Activated CDC42 kinase 1 OS=Mus musculus GN=Tnk2 - [ACK1_MOUSE]	3.87	7	3	3	5	1.059	0.842	1.232	1.017	14.77	3.87	5	5	1008	111.6	7.31
Q8BU25	Inactive serine protease PAMR1 OS=Mus musculus GN=Pamr1 PE=2 SV=3 - [PAMR1_MOUSE]	3.61	1	2	2	3	2.384	1.218	1.366	1.017	6.68	3.61	2	3	720	80.3	7.30
Q91W86	Vacuolar protein sorting-associated protein 11 homolog OS=Mus musculus GN=Vps11 PE=1 SV=3 - [VPS11_MOUSE]	34.01	1	25	27	64	0.840	0.926	0.917	1.017	180.99	34.01	42	64	941	107.7	7.01
Q8C080	Sorting nexin-16 OS=Mus musculus GN=Snx16 PE=2 SV=2 - [SNX16_MOUSE]	36.92	2	9	9	25	1.083	0.996	1.151	1.017	73.90	36.92	14	25	344	38.8	5.21
Q64237	Dopamine beta-hydroxylase OS=Mus musculus GN=Dbh PE=2 SV=1 - [DOPO_MOUSE]	12.56	2	7	7	17	1.947	1.111	0.668	1.017	55.73	12.56	13	17	621	70.1	6.10

Q8BP48	Methionine aminopeptidase 1 OS=Mus musculus GN=Metap1 PE=2 SV=1 -	32.38	1	11	11	26	1.093	1.146	0.957	1.017	73.15	32.38	17	26	386	43.2	7.17
Q80ZV0	Ribonuclease H2 subunit B OS=Mus musculus GN=Rnaseh2b PE=1 SV=2 - [RNH2B_MOUSE]	8.44	2	3	3	3	1.082	1.300	0.743	1.017	1.72	8.44	3	3	308	34.7	9.45
Q4KMM3	Oxidation resistance protein 1 OS=Mus musculus GN=Oxr1 PE=1 SV=3 - [OXR1_MOUSE]	52.31	2	8	38	243	1.144	1.285	1.975	1.017	753.42	52.31	70	243	866	95.9	5.33
Q9CZD3	Glycine--tRNA ligase OS=Mus musculus GN=Gars PE=1 SV=1 - [SYG_MOUSE]	44.17	1	27	27	111	0.903	0.838	0.882	1.017	323.32	44.17	46	111	729	81.8	6.65
Q5SSL4	Active breakpoint cluster region-related protein OS=Mus musculus GN=Abr PE=2 SV=1 - [ABR_MOUSE]	50.41	9	32	36	175	0.790	1.175	0.949	1.017	526.02	50.41	63	175	859	97.6	6.58
F7AR15	Protein Nxf7 (Fragment) OS=Mus musculus GN=Nxf7 PE=4 SV=1 - [F7AR15_MOUSE]	1.26	2	1	1	2	0.992	1.090	1.006	1.017	4.33	1.26	1	2	475	55.9	8.15
Q68EF8	Rap guanine nucleotide exchange factor-like 1 OS=Mus musculus GN=Rapgefl1 PE=2 SV=2 - [RPGFL_MOUSE]	9.21	1	4	4	9	0.865	1.736	1.748	1.017	18.87	9.21	6	9	662	73.6	6.21
P48543	G protein-activated inward rectifier potassium channel 3 OS=Mus musculus GN=Kcnj9 PE=2 SV=2 - [IRK9_MOUSE]	2.29	1	1	1	4	0.655	0.692	1.080	1.018	8.82	2.29	2	4	393	43.9	5.00
Q8VCB2-2	Isoform 2 of Mediator of RNA polymerase II transcription subunit 25 OS=Mus musculus GN=Med25 - [illegible]	0.97	3	1	1	2	0.635	0.983	1.027	1.018	2.24	0.97	2	2	618	64.5	9.54
Q3V0I2	Proline-rich protein 7 OS=Mus musculus GN=Prr7 PE=2 SV=1 - [PRR7_MOUSE]	13.75	1	2	2	7	1.158	1.596	1.527	1.018	16.64	13.75	4	7	269	30.3	8.60
Q9R0H5	Keratin, type II cytoskeletal 71 OS=Mus musculus GN=Krt71 PE=1 SV=1 - [K2C71_MOUSE]	4.58	1	1	2	12	0.877	0.929	0.917	1.018	26.12	4.58	3	12	524	57.3	6.99
Q8QZS3	Folliculin OS=Mus musculus GN=Flcn PE=1 SV=1 - [FLCN_MOUSE]	4.49	1	2	2	5	0.710	0.962	0.882	1.018	15.33	4.49	3	5	579	64.3	6.38
Q91WJ0	Fibroblast growth factor receptor substrate 3 OS=Mus musculus GN=Frs3 PE=1 SV=3 - [FRS3_MOUSE]	26.02	1	8	9	32	1.131	0.907	0.898	1.018	95.09	26.02	16	32	492	53.9	6.95
Q3TPE9	Ankyrin repeat and MYND domain-containing protein 2 OS=Mus musculus GN=Ankmy2 PE=1 SV=1 - [ANKY2_MOUSE]	23.64	2	10	10	32	0.644	1.153	0.889	1.018	88.26	23.64	18	32	440	48.7	6.67
O35595	Protein patched homolog 2 OS=Mus musculus GN=Ptch2 PE=2 SV=2 - [PTC2_MOUSE]	3.21	2	2	2	3	1.457	1.343	1.162	1.018	2.53	3.21	2	3	1182	128.5	6.57
P35123	Ubiquitin carboxyl-terminal hydrolase 4 OS=Mus musculus GN=Usp4 PE=1 SV=3 - [UBP4_MOUSE]	32.54	1	21	22	70	0.845	1.041	0.967	1.018	222.19	32.54	38	70	962	108.3	5.64
Q8R2R9	AP-3 complex subunit mu-2 OS=Mus musculus GN=Ap3m2 PE=2 SV=1 - [AP3M2_MOUSE]	47.61	3	12	14	75	0.475	0.991	1.052	1.018	236.96	47.61	25	75	418	46.9	7.56
E9QL31	Disabled homolog 2 OS=Mus musculus GN=Dab2 PE=2 SV=1 - [E9QL31_MOUSE]	30.07	10	15	15	50	1.211	1.235	0.967	1.018	140.92	30.07	25	50	745	79.9	6.51
Q8C483	Serine--tRNA ligase, cytoplasmic OS=Mus musculus GN=Sars PE=2 SV=1 - [Q8C483_MOUSE]	55.60	4	25	25	91	0.858	0.906	0.951	1.018	272.65	55.60	39	91	536	61.1	7.43
Q6P5F9	Exportin-1 OS=Mus musculus GN=Xpo1 PE=1 SV=1 - [XPO1_MOUSE]	30.81	3	28	28	112	0.641	1.134	0.883	1.018	325.98	30.81	49	112	1071	123.0	6.07
Q3U9D6	Exocyst complex component 6 OS=Mus musculus GN=Exoc6 PE=2 SV=1 - [Q3U9D6_MOUSE]	14.18	2	11	13	29	0.951	1.386	1.167	1.018	64.89	14.18	22	29	804	93.2	6.15
Q3USC7	Ganglioside-induced differentiation-associated protein 1-like 1 OS=Mus musculus GN=Gdap1l1 PE=2 SV=1 - [illegible]	42.78	3	13	14	71	0.879	1.059	1.005	1.018	214.72	42.78	26	71	367	41.9	6.48
Q8K4S1	1-phosphatidylinositol 4,5-bisphosphate phosphodiesterase epsilon-1 OS=Mus musculus GN=Plce1 PE=1 SV=3 - [PLCE1_MOUSE]	0.92	1	1	1	1	1.782	1.532	1.513	1.018	0.00	0.92	1	1	2282	254.9	6.15
Q9ERS6	X-linked interleukin-1 receptor accessory protein-like 2 OS=Mus musculus GN=Il1rapl2 PE=2 SV=1 - [IRPL2_MOUSE]	1.46	1	1	1	1	1.432	1.688	0.849	1.018	2.41	1.46	1	1	686	78.7	6.18

O35409	Glutamate carboxypeptidase 2 OS=Mus musculus GN=Folh1 PE=2 SV=2 -	29.12	2	18	18	85	1.222	0.905	0.875	1.018	216.04	29.12	32	85	752	84.5	8.10
O70566	Protein diaphanous homolog 2 OS=Mus musculus GN=Diaph2 PE=1 SV=2 -	13.48	1	15	16	32	0.964	1.575	1.230	1.018	75.03	13.48	22	32	1098	124.8	6.92
Q9JHU4	Cytoplasmic dynein 1 heavy chain 1 OS=Mus musculus GN=Dync1h1 PE=1 SV=2 -	53.75	2	227	229	1502	0.628	0.801	0.932	1.018	4329.16	53.75	416	1502	4644	531.7	6.42
F6R9G0	E1A-binding protein p400 (Fragment) OS=Mus musculus GN=Ep400 PE=4 SV=1 - [F6R9G0_MOUSE]	22.15	1	2	5	17	0.904	1.268	1.073	1.018	37.65	22.15	9	17	325	34.6	10.81
Q9D1G3	Protein-cysteine N-palmitoyltransferase HHAT-like protein OS=Mus musculus GN=Hhatl PE=1 SV=2 - [HHATL_MOUSE]	2.39	1	1	1	2	1.167	1.056	1.031	1.018	5.77	2.39	2	2	503	56.4	6.65
Q5KU39	Vacuolar protein sorting-associated protein 41 homolog OS=Mus musculus GN=Vps41 PE=2 SV=1 - [VPS41_MOUSE]	27.08	1	20	21	46	0.707	0.985	0.864	1.018	120.93	27.08	35	46	853	98.5	5.81
Q811T9-2	Isoform 2 of Disrupted in schizophrenia 1 homolog OS=Mus musculus GN=Disc1 - [DISC1_MOUSE]	6.24	5	2	2	3	1.500	1.048	1.292	1.019	0.00	6.24	2	3	850	92.6	6.15
Q80TR8-4	Isoform 4 of Protein VPRBP OS=Mus musculus GN=Vprbp - [VPRBP_MOUSE]	6.13	4	7	7	10	0.926	1.259	0.973	1.019	16.91	6.13	9	10	1419	159.2	6.54
O89051	Integral membrane protein 2B OS=Mus musculus GN=Itm2b PE=2 SV=1 - [ITM2B_MOUSE]	45.49	1	8	8	28	0.691	0.704	0.928	1.019	77.88	45.49	11	28	266	30.2	5.30
A2AGR0	MAP kinase-activating death domain protein OS=Mus musculus GN=Madd PE=2 SV=1 - [A2AGR0_MOUSE]	41.22	28	52	52	215	0.588	1.040	1.052	1.019	628.88	41.22	88	215	1577	175.0	6.07
Q4G5Y1	Kelch domain-containing protein 2 OS=Mus musculus GN=Klhdc2 PE=2 SV=3 -	6.16	1	2	2	5	1.111	1.511	0.948	1.019	14.58	6.16	3	5	406	45.9	6.61
Q68FM7	Protein Arhgef11 OS=Mus musculus GN=Arhgef11 PE=2 SV=1 - [Q68FM7_MOUSE]	27.71	3	28	29	80	0.783	0.784	1.041	1.019	248.93	27.71	45	80	1552	171.7	5.58
Q8CJF7	Protein ELYS OS=Mus musculus GN=Ahctf1 PE=1 SV=1 - [ELYS_MOUSE]	4.59	3	7	8	17	1.009	1.048	1.131	1.019	55.20	4.59	13	17	2243	247.5	6.55
F8WGX0	FYVE, RhoGEF and PH domain-containing protein 4 (Fragment) OS=Mus musculus GN=Fgd4 PE=2 SV=1 - [F8WGX0_MOUSE]	11.27	6	6	7	12	1.339	1.425	0.957	1.019	40.77	11.27	11	12	754	85.2	5.82
Q8CA44	ATP synthase subunit s-like protein OS=Mus musculus GN=Atp5sl PE=2 SV=1 - [Q8CA44_MOUSE]	8.56	2	1	2	5	1.134	1.093	1.086	1.019	13.62	8.56	2	5	257	29.2	7.80
Q8BK30	NADH dehydrogenase [ubiquinone] flavoprotein 3, mitochondrial OS=Mus musculus GN=Ndufv3 PE=2 SV=1 - [illegible]	54.81	1	1	6	36	1.580	1.009	1.502	1.019	74.70	54.81	10	36	104	11.8	9.35
Q8BIJ7	RUN and FYVE domain-containing protein 1 OS=Mus musculus GN=Rufy1 PE=1 SV=1 - [RUFY1_MOUSE]	22.75	1	16	17	52	1.087	1.023	1.044	1.019	148.10	22.75	29	52	712	80.3	5.68
Q8BGU5	Cyclin-Y OS=Mus musculus GN=Ccny PE=1 SV=1 - [CCNY_MOUSE]	31.09	2	7	9	51	0.590	1.321	1.165	1.019	142.94	31.09	15	51	341	39.4	7.20
Q8BGG7	Ubiquitin-associated and SH3 domain-containing protein B OS=Mus musculus GN=Ubash3b PE=1 SV=1 - [illegible]	10.34	3	6	6	9	0.771	0.977	1.032	1.019	22.38	10.34	9	9	638	71.4	6.81
Q91VY9	Zinc finger protein 622 OS=Mus musculus GN=Znf622 PE=2 SV=1 - [ZN622_MOUSE]	4.62	1	2	2	4	1.522	0.894	0.951	1.019	12.70	4.62	3	4	476	53.4	6.10
O08742	Platelet glycoprotein V OS=Mus musculus GN=Gp5 PE=2 SV=1 - [GPV_MOUSE]	5.11	2	1	2	2	1.424	1.467	1.016	1.019	2.06	5.11	2	2	567	63.4	8.97
Q61233	Plastin-2 OS=Mus musculus GN=Lcp1 PE=1 SV=4 - [PLSL_MOUSE]	52.15	5	19	23	96	2.012	0.962	0.842	1.019	315.49	52.15	36	96	627	70.1	5.33
Q9JM63	ATP-sensitive inward rectifier potassium channel 10 OS=Mus musculus GN=Kcnj10 PE=1 SV=1 - [IRK10_MOUSE]	37.99	1	11	11	51	0.754	0.225	0.538	1.020	112.29	37.99	18	51	379	42.4	8.29
Q3TZI6-2	Isoform 2 of Cyclin-J OS=Mus musculus GN=Ccnj - [CCNJ_MOUSE]	3.27	2	1	1	1	1.771	1.054	1.098	1.020	1.68	3.27	1	1	367	42.1	7.34

O55135	Eukaryotic translation initiation factor 6 OS=Mus musculus GN=Eif6 PE=1 SV=2 - [IF6_MOUSE]	34.69	2	6	6	19	1.505	0.926	0.968	1.020	59.05	34.69	9	19	245	26.5	4.74
Q5DW34-2	Isoform 2 of Histone-lysine N-methyltransferase EHMT1 OS=Mus musculus GN=Ehmt1 - [EHMT1_MOUSE]	5.71	6	4	4	4	1.021	1.422	1.010	1.020	10.53	5.71	4	4	1243	136.4	5.97
Q9QY15	ATP-dependent RNA helicase DDX25 OS=Mus musculus GN=Ddx25 PE=1 SV=2 - [DDX25_MOUSE]	13.64	4	6	8	18	0.742	0.954	0.917	1.020	36.64	13.64	12	18	484	54.8	5.99
Q6DFV7	Nuclear receptor coactivator 7 OS=Mus musculus GN=Ncoa7 PE=2 SV=2 - [NCOA7_MOUSE]	21.53	2	17	19	43	1.053	0.840	1.331	1.020	115.79	21.53	28	43	943	106.3	5.43
P61082	NEDD8-conjugating enzyme Ubc12 OS=Mus musculus GN=Ube2m PE=2 SV=1 -	49.73	5	11	11	47	1.098	0.930	1.003	1.020	127.76	49.73	18	47	183	20.9	7.69
E9Q4I1	Receptor-type tyrosine-protein phosphatase O OS=Mus musculus GN=Ptpro PE=2 SV=1 - [E9Q4I1_MOUSE]	1.75	5	1	2	9	1.262	1.598	0.811	1.020	20.90	1.75	4	9	1198	135.1	5.92
Q80YQ8	Protein RMD5 homolog A OS=Mus musculus GN=Rmnd5a PE=2 SV=2 - [RMD5A_MOUSE]	30.95	3	10	11	30	0.758	1.124	1.025	1.020	97.62	30.95	17	30	391	44.0	6.06
Q9JJY3	Sphingomyelin phosphodiesterase 3 OS=Mus musculus GN=Smpd3 PE=1 SV=1 -	16.03	1	9	9	21	0.407	0.940	1.418	1.020	70.85	16.03	12	21	655	71.2	5.88
Q61086	Frizzled-3 OS=Mus musculus GN=Fzd3 PE=1 SV=1 - [FZD3_MOUSE]	5.56	1	2	2	2	1.097	1.241	0.878	1.020	7.58	5.56	2	2	666	76.2	7.64
Q9WVA4	Transgelin-2 OS=Mus musculus GN=Tagln2 PE=1 SV=4 - [TAGL2_MOUSE]	55.28	1	9	11	137	1.427	1.607	0.790	1.020	417.05	55.28	21	137	199	22.4	8.24
Q8VBU5	Phosphodiesterase 4B OS=Mus musculus GN=Pde4b PE=2 SV=1 - [Q8VBU5_MOUSE]	39.94	13	21	26	113	0.852	0.883	1.123	1.020	316.65	39.94	50	113	721	82.0	5.63
Q8CG03	cGMP-specific 3',5'-cyclic phosphodiesterase OS=Mus musculus GN=Pde5a PE=1 SV=2 - [PDE5A_MOUSE]	2.20	1	2	2	3	0.819	1.537	0.981	1.020	4.93	2.20	3	3	865	98.3	6.05
Q9D883	Splicing factor U2AF 35 kDa subunit OS=Mus musculus GN=U2af1 PE=1 SV=4 - [U2AF1_MOUSE]	30.13	2	4	5	39	0.629	1.114	0.879	1.020	111.54	30.13	9	39	239	27.8	8.81
Q9DAR7	m7GpppX diphosphatase OS=Mus musculus GN=Dcps PE=1 SV=1 - [DCPS_MOUSE]	30.47	3	9	9	23	1.652	1.067	0.863	1.020	64.07	30.47	16	23	338	39.0	6.48
Q9D0R4	Probable ATP-dependent RNA helicase DDX56 OS=Mus musculus GN=Ddx56 PE=2 SV=1 - [DDX56_MOUSE]	5.68	2	2	3	5	0.778	0.822	1.005	1.020	15.06	5.68	4	5	546	61.2	9.17
I3ITR1	MCG50313 OS=Mus musculus GN=AK157302 PE=4 SV=1 - [I3ITR1_MOUSE]	50.39	2	5	5	20	1.193	0.900	0.974	1.020	59.10	50.39	9	20	129	14.2	8.85
Q3TIX9	U4/U6.U5 tri-snRNP-associated protein 2 OS=Mus musculus GN=Usp39 PE=2 SV=2 - [SNUT2_MOUSE]	16.49	1	7	7	21	0.963	0.943	0.891	1.020	54.62	16.49	11	21	564	65.1	8.90
P46656	Adrenodoxin, mitochondrial OS=Mus musculus GN=Fdx1 PE=2 SV=1 - [ADX_MOUSE]	28.72	1	5	5	11	2.735	0.955	1.297	1.020	29.00	28.72	6	11	188	20.1	5.62
Q9D8S9	BolA-like protein 1 OS=Mus musculus GN=Bola1 PE=1 SV=1 - [BOLA1_MOUSE]	74.45	1	8	8	42	2.189	0.982	1.364	1.020	157.37	74.45	15	42	137	14.4	8.76
B1AS06	Disks large-associated protein 3 OS=Mus musculus GN=Dlgap3 PE=2 SV=1 -	33.44	2	23	24	137	0.714	1.341	1.679	1.021	406.24	33.44	42	137	966	104.6	8.81
P28658	Ataxin-10 OS=Mus musculus GN=Atxn10 PE=1 SV=2 - [ATX10_MOUSE]	46.74	1	20	21	99	0.715	0.929	0.688	1.021	261.45	46.74	38	99	475	53.7	5.25
Q9WUQ5	C-X-C motif chemokine 14 OS=Mus musculus GN=Cxcl14 PE=2 SV=2 - [CXL14_MOUSE]	7.07	1	1	1	2	0.635	0.836	0.984	1.021	4.56	7.07	2	2	99	11.7	9.88
E9Q3P4	Protein Cenpf OS=Mus musculus GN=Cenpf PE=2 SV=1 - [E9Q3P4_MOUSE]	2.40	3	3	9	16	1.396	1.390	1.373	1.021	39.90	2.40	10	16	2997	342.3	5.16
P56873	Sjoegren syndrome/scleroderma autoantigen 1 homolog OS=Mus musculus GN=Sssca1 PE=2 SV=1 - [illegible]	9.05	2	2	2	6	1.025	1.348	1.174	1.021	14.38	9.05	4	6	199	21.3	5.12
Q8K268	ATP-binding cassette sub-family F member 3 OS=Mus musculus GN=Abcf3 PE=1 SV=1 - [ABCF3_MOUSE]	28.63	1	15	16	33	0.917	0.713	0.871	1.021	86.91	28.63	23	33	709	79.8	6.16

Q60841-3	Isoform 3 of Reelin OS=Mus musculus GN=Reln - [RELN_MOUSE]	8.81	3	16	16	35	0.865	2.054	1.302	1.021	94.21	8.81	25	35	3428	383.0	5.55
D3Z373	Protein 2310045N01Rik OS=Mus musculus GN=2310045N01Rik PE=2 SV=1 - [D3Z373_MOUSE]	14.16	2	2	2	3	1.426	1.417	0.977	1.021	7.97	14.16	3	3	113	12.7	6.93
Q5RJG1	Nucleolar protein 10 OS=Mus musculus GN=Nol10 PE=2 SV=1 - [NOL10_MOUSE]	1.31	1	1	1	2	0.414	0.839	0.890	1.021	3.67	1.31	1	2	687	80.0	8.19
E9PZM7	Protein Scaf11 OS=Mus musculus GN=Scaf11 PE=2 SV=1 - [E9PZM7_MOUSE]	2.06	3	2	3	5	1.627	0.622	1.117	1.021	14.57	2.06	5	5	1456	162.0	6.77
E9PXY8	Ubiquitin carboxyl-terminal hydrolase 7 OS=Mus musculus GN=Usp7 PE=2 SV=1 - [E9PXY8_MOUSE]	41.73	7	41	41	116	1.021	1.158	1.037	1.021	326.46	41.73	66	116	1143	132.8	5.66
Q3TIR1-3	Isoform 3 of Trafficking protein particle complex subunit 13 OS=Mus musculus GN=Trappc13 - [TPC13_MOUSE]	8.25	4	3	3	12	0.728	0.866	0.864	1.021	32.47	8.25	5	12	412	45.9	5.60
Q6PB44-2	Isoform 2 of Tyrosine-protein phosphatase non-receptor type 23 OS=Mus musculus GN=Ptpn23 - [PTN23_MOUSE]	27.34	2	39	40	124	0.916	1.125	0.934	1.021	372.80	27.34	67	124	1690	184.8	6.76
P97326	Cadherin-6 OS=Mus musculus GN=Cdh6 PE=1 SV=2 - [CADH6_MOUSE]	13.54	1	9	11	21	0.918	0.630	1.117	1.021	56.20	13.54	17	21	790	88.3	5.00
Q6PFX2-2	Isoform 2 of BEN domain-containing protein 6 OS=Mus musculus GN=Bend6 - [BEND6_MOUSE]	11.16	3	2	2	11	1.120	0.520	0.859	1.021	33.95	11.16	4	11	251	28.2	8.22
Q9Z2D1	Myotubularin-related protein 2 OS=Mus musculus GN=Mtmr2 PE=1 SV=3 -	26.59	5	14	15	52	1.066	0.838	1.026	1.021	164.90	26.59	28	52	643	73.2	7.25
Q6RHR9-3	Isoform 3 of Membrane-associated guanylate kinase, WW and PDZ domain-containing protein 1 OS=Mus musculus GN=Magi1 - [MAGI1_MOUSE]	28.05	4	25	27	132	1.609	1.135	1.084	1.021	416.23	28.05	51	132	1237	134.3	5.78
P33242-2	Isoform 2 of Steroidogenic factor 1 OS=Mus musculus GN=Nr5a1 - [STF1_MOUSE]	6.70	2	1	1	3	2.663	1.502	1.153	1.021	0.00	6.70	1	3	388	43.4	8.09
J3QP71	Basigin (Fragment) OS=Mus musculus GN=Bsg PE=4 SV=1 - [J3QP71_MOUSE]	44.67	1	1	7	40	0.441	1.471	1.602	1.021	108.08	44.67	10	40	197	21.7	6.20
Q80X90	Filamin-B OS=Mus musculus GN=Flnb PE=1 SV=3 - [FLNB_MOUSE]	35.13	1	60	66	198	1.272	1.733	1.109	1.022	589.65	35.13	112	198	2602	277.7	5.71
H7BX88	Carnitine O-acetyltransferase OS=Mus musculus GN=Crat PE=2 SV=1 - [H7BX88_MOUSE]	31.07	5	19	19	64	0.835	0.949	0.904	1.022	163.31	31.07	33	64	605	68.6	8.07
Q80XG9	Leucine-rich repeat transmembrane neuronal protein 4 OS=Mus musculus GN=Lrrtm4 PE=1 SV=2 - [illegible]	17.12	3	8	10	26	0.860	1.600	1.358	1.022	74.72	17.12	17	26	590	67.1	9.00
Q7TT50	Serine/threonine-protein kinase MRCK beta OS=Mus musculus GN=Cdc42bpb PE=1 SV=2 - [MRCKB_MOUSE]	35.90	3	45	55	223	0.856	1.135	1.152	1.022	663.46	35.90	98	223	1713	194.6	6.46
Q62420	Endophilin-A1 OS=Mus musculus GN=Sh3gl2 PE=1 SV=2 - [SH3G2_MOUSE]	59.38	5	11	17	286	1.208	1.292	1.412	1.022	902.51	59.38	31	286	352	39.9	5.39
G3X9U9	Fission 1 (Mitochondrial outer membrane) homolog (Yeast), isoform CRA_c OS=Mus musculus GN=Fis1 PE=4 SV=1 - [G3X9U9_MOUSE]	46.90	3	6	6	35	1.343	0.939	1.081	1.022	83.05	46.90	9	35	145	16.3	9.25
O88327	Alpha-catulin OS=Mus musculus GN=Ctnnal1 PE=2 SV=1 - [CTNL1_MOUSE]	2.46	1	2	2	2	2.462	1.809	1.128	1.022	5.24	2.46	2	2	731	81.4	6.55
Q3V1I0	Lysozyme g-like protein 2 OS=Mus musculus GN=Lyg2 PE=2 SV=1 - [LYG2_MOUSE]	6.10	1	1	1	1	1.232	0.711	0.932	1.022	0.00	6.10	1	1	213	23.7	8.44
Q9D289	Trafficking protein particle complex subunit 6B OS=Mus musculus GN=Trappc6b PE=2 SV=1 - [TPC6B_MOUSE]	38.61	1	7	7	17	0.697	1.207	0.827	1.022	41.78	38.61	11	17	158	17.9	8.68
Q8VCL2	Protein SCO2 homolog, mitochondrial OS=Mus musculus GN=Sco2 PE=2 SV=1 - [SCO2_MOUSE]	15.29	1	3	3	13	1.037	0.923	0.876	1.022	42.92	15.29	6	13	255	28.9	8.29
Q505F5	Leucine-rich repeat-containing protein 47 OS=Mus musculus GN=Lrrc47 PE=1 SV=1 - [LRC47_MOUSE]	40.28	4	19	19	67	0.786	0.882	0.934	1.022	174.10	40.28	34	67	581	63.6	8.10

E9PUD6	Protein Zscan18 OS=Mus musculus GN=Zscan18 PE=4 SV=1 - [E9PUD6_MOUSE]	4.33	1	3	3	6	1.778	1.384	1.151	1.022	14.71	4.33	6	6	809	91.7	4.35
Q60738	Zinc transporter 1 OS=Mus musculus GN=Slc30a1 PE=2 SV=1 - [ZNT1_MOUSE]	27.24	2	10	10	40	1.053	1.057	1.145	1.022	125.83	27.24	16	40	503	54.7	6.62
P53810	Phosphatidylinositol transfer protein alpha isoform OS=Mus musculus GN=Pitpna PE=1 SV=2 - [PIPNA_MOUSE]	63.10	4	15	18	166	0.805	1.035	0.946	1.022	453.11	63.10	30	166	271	31.9	6.37
Q3TMX0	MCG4375, isoform CRA_b OS=Mus musculus GN=Sdcbp PE=2 SV=1 - [Q3TMX0_MOUSE]	30.20	5	6	7	14	1.320	1.329	1.152	1.022	34.64	30.20	9	14	298	32.2	7.15
Q9JIK9	28S ribosomal protein S34, mitochondrial OS=Mus musculus GN=Mrps34 PE=2 SV=1 - [RT34_MOUSE]	35.32	1	8	8	31	0.874	0.764	0.945	1.022	75.12	35.32	15	31	218	25.8	10.43
Q8R570	Synaptosomal-associated protein 47 OS=Mus musculus GN=Snap47 PE=1 SV=1 -	58.60	8	17	18	91	0.766	0.907	0.869	1.022	297.75	58.60	32	91	413	46.5	5.76
E9PYB0	Protein Ahnak2 (Fragment) OS=Mus musculus GN=Ahnak2 PE=2 SV=1 -	41.12	1	4	14	53	3.386	0.722	1.275	1.022	153.80	41.12	23	53	1739	182.6	7.08
Q99JP7	Gamma-glutamyltransferase 7 OS=Mus musculus GN=Ggt7 PE=1 SV=2 -	28.85	3	13	13	49	0.974	0.974	1.052	1.022	136.14	28.85	22	49	662	70.2	5.06
Q8CFA2	Aminomethyltransferase, mitochondrial OS=Mus musculus GN=Amt PE=2 SV=1 - [GCST_MOUSE]	38.46	1	11	11	26	0.813	0.646	0.785	1.022	58.18	38.46	16	26	403	44.0	8.70
Q5H8C4-2	Isoform 2 of Vacuolar protein sorting-associated protein 13A OS=Mus musculus GN=Vps13a - [VP13A_MOUSE]	12.48	2	30	32	74	0.678	1.131	1.097	1.022	192.22	12.48	50	74	3061	346.5	6.02
Q62417	Sorbin and SH3 domain-containing protein 1 OS=Mus musculus GN=Sorbs1 PE=1 SV=2 - [SRBS1_MOUSE]	32.64	1	9	35	141	0.600	1.274	1.603	1.022	410.35	32.64	64	141	1290	143.0	8.25
Q6NZL0	Protein SOGA3 OS=Mus musculus GN=Soga3 PE=2 SV=2 - [SOGA3_MOUSE]	35.98	1	29	33	160	0.839	0.772	1.148	1.022	441.83	35.98	59	160	945	103.4	6.14
P10853	Histone H2B type 1-F/J/L OS=Mus musculus GN=Hist1h2bf PE=1 SV=2 - [H2B1F_MOUSE]	73.02	11	2	13	252	0.338	0.952	0.891	1.022	721.67	73.02	24	252	126	13.9	10.32
Q6PHU5	Sortilin OS=Mus musculus GN=Sort1 PE=1 SV=1 - [SORT_MOUSE]	18.06	3	16	16	48	0.829	1.044	1.208	1.023	126.23	18.06	29	48	825	91.1	5.88
Q9CWN7	CCR4-NOT transcription complex subunit 11 OS=Mus musculus GN=Cnot11 PE=2 SV=1 - [CNO11_MOUSE]	15.64	1	4	4	21	0.616	1.231	1.002	1.023	54.43	15.64	7	21	505	54.9	6.55
Q6PGF3	Mediator of RNA polymerase II transcription subunit 16 OS=Mus musculus GN=Med16 PE=2 SV=2 - [illegible]	5.56	3	4	4	7	0.713	0.911	0.771	1.023	19.87	5.56	5	7	828	91.7	7.75
E9Q8N0	Ral guanine nucleotide dissociation stimulator-like 1 OS=Mus musculus GN=Rgl1 PE=2 SV=1 - [E9Q8N0_MOUSE]	4.70	7	3	4	7	1.323	1.233	1.258	1.023	15.24	4.70	5	7	766	85.9	5.83
Q04207-2	Isoform p65 delta of Transcription factor p65 OS=Mus musculus GN=Rela - [TF65_MOUSE]	9.83	2	4	4	6	1.128	1.043	1.029	1.023	8.95	9.83	5	6	539	59.0	6.10
P48771	Cytochrome c oxidase subunit 7A2, mitochondrial OS=Mus musculus GN=Cox7a2 PE=1 SV=2 - [CX7A2_MOUSE]	34.94	1	2	3	140	0.876	0.891	0.962	1.023	322.00	34.94	6	140	83	9.3	10.27
Q9JKK1	Syntaxin-6 OS=Mus musculus GN=Stx6 PE=1 SV=1 - [STX6_MOUSE]	58.82	2	12	12	41	0.976	1.144	1.043	1.023	135.00	58.82	20	41	255	29.0	4.92
E0CY63	Claudin domain-containing protein 1 (Fragment) OS=Mus musculus GN=Cldn25 PE=2 SV=1 - [E0CY63_MOUSE]	15.45	3	2	2	8	0.990	0.928	0.926	1.023	18.51	15.45	4	8	123	14.3	4.70
Q9WVM3	Anaphase-promoting complex subunit 7 OS=Mus musculus GN=Anapc7 PE=2 SV=3 - [APC7_MOUSE]	12.92	2	7	7	15	0.576	0.744	0.900	1.023	42.63	12.92	11	15	565	63.0	5.72
Q80TL1	Adenylate cyclase type 2 OS=Mus musculus GN=Adcy2 PE=2 SV=2 - [ADCY2_MOUSE]	10.46	2	9	10	26	0.837	0.931	1.014	1.023	96.00	10.46	16	26	1090	123.2	8.31
Q80TQ5-3	Isoform 3 of Pleckstrin homology domain-containing family M member 2 OS=Mus musculus GN=Plekhm2 - [illegible]	4.15	4	3	3	5	1.174	0.839	1.016	1.023	18.61	4.15	4	5	867	95.0	4.79

Q80YC5	Coagulation factor XII OS=Mus musculus GN=F12 PE=2 SV=2 - [FA12_MOUSE]	6.87	1	3	3	8	4.626	1.669	0.524	1.023	11.97	6.87	5	8	597	65.7	6.84
Q9D1K2	V-type proton ATPase subunit F OS=Mus musculus GN=Atp6v1f PE=1 SV=2 -	77.31	2	9	9	192	1.734	1.227	1.324	1.023	647.36	77.31	17	192	119	13.4	5.82
Q923Z0	G-protein coupled receptor family C group 5 member B OS=Mus musculus GN=Gprc5b PE=2 SV=1 - [GPC5B_MOUSE]	14.39	1	4	4	41	1.092	0.812	1.121	1.023	186.29	14.39	7	41	410	45.9	8.38
Q9JKW0	ADP-ribosylation factor-like protein 6-interacting protein 1 OS=Mus musculus GN=Arl6ip1 PE=2 SV=1 - [AR6P1_MOUSE]	11.82	1	3	3	14	0.445	0.824	0.748	1.023	33.22	11.82	4	14	203	23.4	9.32
Q8BGQ7	Alanine--tRNA ligase, cytoplasmic OS=Mus musculus GN=Aars PE=1 SV=1 - [SYAC_MOUSE]	45.97	3	33	34	150	0.741	0.816	0.958	1.023	450.80	45.97	60	150	968	106.8	5.67
A3KGQ6	Actin-related protein 2/3 complex subunit 5-like protein OS=Mus musculus GN=Arpc5l PE=2 SV=1 - [A3KGQ6_MOUSE]	61.44	2	10	10	43	0.685	2.074	1.198	1.023	127.76	61.44	15	43	153	17.1	6.80
Q8VCF0	Mitochondrial antiviral-signaling protein OS=Mus musculus GN=Mavs PE=1 SV=1 - [MAVS_MOUSE]	36.78	1	10	10	60	1.139	1.015	0.685	1.023	167.37	36.78	17	60	503	53.4	6.37
Q8CFI7	DNA-directed RNA polymerase II subunit RPB2 OS=Mus musculus GN=Polr2b PE=2 SV=2 - [RPB2_MOUSE]	13.71	1	15	15	37	0.676	0.930	0.801	1.024	108.22	13.71	27	37	1174	133.8	6.87
Q8BNJ6	Neuropilin and tolloid-like protein 2 OS=Mus musculus GN=Neto2 PE=1 SV=1 - [NETO2_MOUSE]	14.48	2	7	7	18	0.675	1.025	1.168	1.024	45.84	14.48	13	18	525	59.3	6.77
Q9QVP9	Protein-tyrosine kinase 2-beta OS=Mus musculus GN=Ptk2b PE=1 SV=2 - [FAK2_MOUSE]	46.58	4	41	42	167	0.795	3.696	2.376	1.024	462.63	46.58	74	167	1009	115.7	6.35
Q6A085	Zinc finger protein 629 OS=Mus musculus GN=Znf629 PE=2 SV=2 - [ZN629_MOUSE]	13.84	147	6	7	12	1.747	1.053	0.877	1.024	22.96	13.84	8	12	867	96.0	7.78
G3UWX1	Replication factor C subunit 1 OS=Mus musculus GN=Rfc1 PE=2 SV=1 - [G3UWX1_MOUSE]	2.39	6	2	2	2	15.144	3.880	2.531	1.024	5.67	2.39	2	2	1130	125.8	9.35
O35218	Cleavage and polyadenylation specificity factor subunit 2 OS=Mus musculus GN=Cpsf2 PE=1 SV=1 - [CPSF2_MOUSE]	10.23	1	6	6	14	1.141	1.089	0.944	1.024	39.68	10.23	11	14	782	88.3	5.11
Q8K2M0	39S ribosomal protein L38, mitochondrial OS=Mus musculus GN=Mrpl38 PE=2 SV=2 - [RM38_MOUSE]	18.95	2	7	7	15	0.630	0.690	0.826	1.024	42.32	18.95	11	15	380	45.0	8.10
O88413	Tubby-related protein 3 OS=Mus musculus GN=Tulp3 PE=1 SV=1 - [TULP3_MOUSE]	5.65	1	2	2	4	1.286	0.850	1.107	1.024	13.01	5.65	4	4	460	51.2	6.24
Q9CX80	Cytoglobin OS=Mus musculus GN=Cygb PE=2 SV=1 - [CYGB_MOUSE]	24.21	1	5	5	19	1.627	0.807	0.708	1.024	49.97	24.21	9	19	190	21.5	6.80
Q69ZP3	Probable hydrolase PNKD OS=Mus musculus GN=Pnkd PE=2 SV=2 - [PNKD_MOUSE]	19.22	3	7	9	27	0.904	0.623	1.005	1.024	71.35	19.22	14	27	385	43.0	8.94
E9Q5W5	Zinc finger ZZ-type and EF-hand domain-containing protein 1 OS=Mus musculus GN=Zzef1 PE=2 SV=1 - [E9Q5W5_MOUSE]	15.53	6	32	33	84	0.838	0.936	1.006	1.024	245.07	15.53	57	84	2924	328.1	6.10
Q9CQV7	Mitochondrial import inner membrane translocase subunit TIM14 OS=Mus musculus GN=Dnajc19 PE=2 SV=3 - [TIM14_MOUSE]	71.55	5	9	9	24	0.877	0.922	1.073	1.024	61.47	71.55	16	24	116	12.4	10.10
Q9D8N2	Protein FAM45A OS=Mus musculus GN=Fam45a PE=2 SV=2 - [FA45A_MOUSE]	30.25	4	8	8	26	0.832	0.939	1.016	1.024	84.42	30.25	13	26	357	40.4	6.47
P26039	Talin-1 OS=Mus musculus GN=Tln1 PE=1 SV=2 - [TLN1_MOUSE]	51.48	4	84	107	478	0.879	1.474	0.819	1.024	1473.57	51.48	190	478	2541	269.7	6.18
O70503	Estradiol 17-beta-dehydrogenase 12 OS=Mus musculus GN=Hsd17b12 PE=2 SV=1 - [DHB12_MOUSE]	44.55	2	15	15	58	0.548	0.603	0.775	1.024	163.09	44.55	28	58	312	34.7	9.52
Q80T41	Gamma-aminobutyric acid type B receptor subunit 2 OS=Mus musculus GN=Gabbr2 PE=2 SV=2 - [GABR2_MOUSE]	23.40	1	20	20	120	0.487	1.074	0.955	1.024	348.42	23.40	36	120	940	105.6	8.72
P60710	Actin, cytoplasmic 1 OS=Mus musculus GN=Actb PE=1 SV=1 - [ACTB_MOUSE]	81.07	10	5	29	3254	0.987	1.388	1.022	1.024	10463.95	81.07	51	3254	375	41.7	5.48

Q9CRB6	Tubulin polymerization-promoting protein family member 3 OS=Mus musculus GN=Tppp3 PE=1 SV=1 - [TPPP3_MOUSE]	57.39	2	13	14	88	2.288	0.492	1.001	1.024	269.85	57.39	25	88	176	19.0	9.11
D3Z2Y6	Protein S100a16 (Fragment) OS=Mus musculus GN=S100a16 PE=2 SV=1 -	47.95	2	3	3	24	1.093	1.017	1.083	1.024	75.48	47.95	5	24	73	8.5	9.35
A2A6A1	G patch domain-containing protein 8 OS=Mus musculus GN=Gpatch8 PE=2 SV=1 -	1.53	1	2	2	2	1.455	1.111	1.219	1.024	2.22	1.53	2	2	1505	164.9	7.64
Q8K3G5	Inactive serine/threonine-protein kinase VRK3 OS=Mus musculus GN=Vrk3 PE=1 SV=2 - [VRK3_MOUSE]	8.83	5	4	4	5	1.081	0.967	1.020	1.024	10.69	8.83	4	5	453	50.8	8.57
Q8C0Z1	Protein ITFG3 OS=Mus musculus GN=Itfg3 PE=1 SV=1 - [ITFG3_MOUSE]	21.26	3	8	8	15	1.444	0.925	1.243	1.024	46.11	21.26	13	15	555	60.5	5.68
P45376	Aldose reductase OS=Mus musculus GN=Akr1b1 PE=1 SV=3 - [ALDR_MOUSE]	62.03	3	17	18	96	0.966	1.141	0.862	1.024	295.41	62.03	31	96	316	35.7	7.18
Q9CWG9	Biogenesis of lysosome-related organelles complex 1 subunit 2 OS=Mus musculus GN=Bloc1s2 PE=1 SV=1 - [illegible]	26.57	1	4	4	14	1.777	1.101	1.053	1.025	36.80	26.57	7	14	143	16.3	4.88
A5A9W3	GLI-Kruppel family member (Fragment) OS=Mus musculus GN=Gli2 PE=2 SV=1 - [A5A9W3_MOUSE]	6.53	4	1	1	1	0.733	0.771	0.873	1.025	2.89	6.53	1	1	245	25.9	8.87
P60335	Poly(rC)-binding protein 1 OS=Mus musculus GN=Pcbp1 PE=1 SV=1 - [PCBP1_MOUSE]	67.70	1	11	17	196	0.935	0.896	0.975	1.025	535.69	67.70	33	196	356	37.5	7.09
Q8BV79	TPR and ankyrin repeat-containing protein 1 OS=Mus musculus GN=Trank1 PE=2 SV=3 - [TRNK1_MOUSE]	2.37	1	2	4	5	0.537	1.034	0.864	1.025	10.90	2.37	4	5	2999	343.1	7.81
Q8BP00-2	Isoform 2 of IQ calmodulin binding motif-containing protein 1 OS=Mus musculus GN=Iqcb1 - [IQCB1_MOUSE]	19.10	2	10	10	30	0.846	0.956	0.903	1.025	88.83	19.10	16	30	597	69.0	9.51
P47754	F-actin-capping protein subunit alpha-2 OS=Mus musculus GN=Capza2 PE=1 SV=3 - [CAZA2_MOUSE]	62.94	2	10	13	283	1.057	1.490	1.174	1.025	813.48	62.94	25	283	286	32.9	5.85
O08576-2	Isoform 2 of RUN domain-containing protein 3A OS=Mus musculus GN=Rundc3a - [RUN3A_MOUSE]	31.00	4	10	10	45	0.883	0.980	0.947	1.025	147.67	31.00	17	45	400	45.5	5.48
E9Q1M1	Protein Pdzd2 OS=Mus musculus GN=Pdzd2 PE=2 SV=1 - [E9Q1M1_MOUSE]	4.33	1	9	9	15	1.378	1.174	1.479	1.025	32.24	4.33	13	15	2796	296.5	7.65
Q8VI78	Phospholipase A1 member A OS=Mus musculus GN=Pla1a PE=2 SV=3 - [PLA1A_MOUSE]	10.96	1	2	2	2	1.246	0.978	0.756	1.025	2.96	10.96	2	2	456	50.0	7.40
Q8BHJ7	Gamma-aminobutyric acid receptor subunit alpha-5 OS=Mus musculus GN=Gabra5 PE=2 SV=1 - [GBRA5_MOUSE]	16.20	1	5	7	25	0.837	1.013	1.093	1.025	66.91	16.20	13	25	463	52.2	9.01
Q8BUE4	Apoptosis-inducing factor 2 OS=Mus musculus GN=Aifm2 PE=2 SV=1 - [AIFM2_MOUSE]	7.77	2	2	2	2	1.202	1.109	0.990	1.025	5.71	7.77	2	2	373	40.6	8.98
E9Q7D5	Protein Arhgef5 OS=Mus musculus GN=Arhgef5 PE=2 SV=1 - [E9Q7D5_MOUSE]	1.14	1	1	1	2	2.628	1.012	1.189	1.025	4.52	1.14	2	2	1581	176.6	5.83
A2ARS0	Ankyrin repeat domain-containing protein 63 OS=Mus musculus GN=Ankrd63 PE=4 SV=1 - [ANR63_MOUSE]	17.44	1	5	5	10	1.078	1.898	4.483	1.025	29.27	17.44	8	10	390	41.0	10.83
Q91W29	Cytochrome c oxidase subunit 4 isoform 2, mitochondrial OS=Mus musculus GN=Cox4i2 PE=2 SV=1 - [illegible]	23.26	1	3	3	9	1.402	1.754	1.195	1.025	25.67	23.26	4	9	172	20.2	9.47
P58391	Small conductance calcium activated potassium channel protein 3 OS=Mus musculus GN=Kcnn3 PE=2 SV=1 - [KCNN3_MOUSE]	6.02	2	3	5	7	0.519	0.967	0.806	1.025	20.54	6.02	7	7	731	81.2	8.95
Q9WV18	Gamma-aminobutyric acid type B receptor subunit 1 OS=Mus musculus GN=Gabbr1 PE=1 SV=1 - [GABR1_MOUSE]	19.48	5	19	19	96	0.601	1.066	0.999	1.025	277.10	19.48	36	96	960	108.1	8.21
Q8R307	Vacuolar protein sorting-associated protein 18 homolog OS=Mus musculus GN=Vps18 PE=1 SV=2 - [VPS18_MOUSE]	24.56	1	20	20	65	0.835	0.951	0.887	1.026	188.16	24.56	32	65	973	110.1	6.09

Q8BTN3	DNA segment, Chr 16, human D22S680E, expressed, isoform CRA_c OS=Mus musculus GN=Tango2 PE=2 SV=1 - [Q8BTN3_MOUSE]	39.13	4	9	9	20	1.492	1.013	1.031	1.026	59.00	39.13	14	20	276	30.9	5.10
Q78T54-2	Isoform 2 of Vacuolar ATPase assembly integral membrane protein VMA21 OS=Mus musculus GN=Vma21 - [illegible]	14.46	2	1	1	12	0.500	1.053	0.881	1.026	43.80	14.46	2	12	83	9.3	9.99
A2AQY8	Kynurenine--oxoglutarate transaminase 1 OS=Mus musculus GN=Ccbl1 PE=2 SV=1 - [A2AQY8_MOUSE]	30.21	3	7	8	35	1.808	0.905	0.993	1.026	107.80	30.21	14	35	374	42.2	7.53
Q9CWB7	Glutaredoxin-like protein C5orf63 homolog OS=Mus musculus PE=1 SV=1 - [YD286_MOUSE]	16.52	1	2	2	3	1.616	1.122	1.256	1.026	2.50	16.52	2	3	115	13.4	9.13
B9EJ54	MCG21756, isoform CRA_b OS=Mus musculus GN=Nup205 PE=2 SV=1 - [B9EJ54_MOUSE]	17.83	2	24	26	51	0.828	1.128	0.931	1.026	144.13	17.83	40	51	2008	227.3	6.34
Q3UF95	Large proline-rich protein BAG6 OS=Mus musculus GN=Bag6 PE=2 SV=1 - [Q3UF95_MOUSE]	32.31	8	27	27	124	0.971	1.023	1.059	1.026	407.04	32.31	47	124	1136	119.1	5.82
Q99LE6	ATP-binding cassette sub-family F member 2 OS=Mus musculus GN=Abcf2 PE=2 SV=1 - [ABCF2_MOUSE]	12.74	1	8	8	32	0.680	0.512	0.881	1.026	82.36	12.74	13	32	628	71.7	7.05
Q61418	H(+)/Cl(-) exchange transporter 4 OS=Mus musculus GN=Clcn4 PE=2 SV=2 - [CLCN4_MOUSE]	12.45	1	5	6	16	1.133	0.891	0.967	1.026	38.19	12.45	10	16	747	83.7	6.86
Q61735-2	Isoform 2 of Leukocyte surface antigen CD47 OS=Mus musculus GN=Cd47 -	22.53	3	7	7	77	0.752	1.153	1.109	1.026	251.40	22.53	12	77	324	35.3	8.25
Q8VEE1	LIM and cysteine-rich domains protein 1 OS=Mus musculus GN=Lmcd1 PE=1 SV=1 - [LMCD1_MOUSE]	30.68	1	9	9	20	1.778	1.425	1.539	1.026	59.33	30.68	14	20	365	41.0	7.93
D3YXQ9	Tubulin polyglutamylase TTLL13 OS=Mus musculus GN=Ttll13 PE=2 SV=1 - [D3YXQ9_MOUSE]	1.81	2	1	1	1	2.170	1.154	0.763	1.026	0.00	1.81	1	1	663	77.9	8.10
Q9D020	Cytosolic 5'-nucleotidase 3A OS=Mus musculus GN=Nt5c3a PE=1 SV=4 - [5NT3A_MOUSE]	53.17	2	17	17	59	1.007	1.313	0.935	1.026	151.32	53.17	29	59	331	37.2	6.65
Q99MU3-5	Isoform 5 of Double-stranded RNA-specific adenosine deaminase OS=Mus musculus GN=Adar - [illegible]	29.78	5	20	20	57	0.771	0.652	0.872	1.026	197.02	29.78	34	57	930	102.5	8.78
Q3U5D0	Spermatogenesis-associated protein 7 homolog OS=Mus musculus GN=Spata7 PE=2 SV=1 - [illegible]	3.86	4	1	2	18	1.061	1.438	1.055	1.026	34.84	3.86	2	18	415	47.0	6.33
Q9QYP6	5-azacytidine-induced protein 2 OS=Mus musculus GN=Azi2 PE=1 SV=1 - [AZI2_MOUSE]	23.21	5	9	9	22	1.550	0.972	1.344	1.026	70.67	23.21	15	22	405	46.1	6.70
G3UZJ3	Dystrobrevin beta OS=Mus musculus GN=Dtnb PE=2 SV=1 - [G3UZJ3_MOUSE]	15.56	16	4	8	36	0.851	1.599	1.254	1.026	87.51	15.56	13	36	559	63.4	8.38
Q8R5A0	N-lysine methyltransferase SMYD2 OS=Mus musculus GN=Smyd2 PE=1 SV=1 - [SMYD2_MOUSE]	12.01	1	4	4	8	0.927	0.987	0.877	1.026	15.81	12.01	7	8	433	49.5	6.95
Q8CFE6	Sodium-coupled neutral amino acid transporter 2 OS=Mus musculus GN=Slc38a2 PE=1 SV=1 - [S38A2_MOUSE]	4.17	1	1	1	3	2.255	1.070	0.998	1.026	13.80	4.17	1	3	504	55.5	7.94
B7ZNX6	Erbb2ip protein OS=Mus musculus GN=Erbb2ip PE=2 SV=1 - [B7ZNX6_MOUSE]	27.57	4	28	33	78	1.696	0.938	1.293	1.026	219.28	27.57	51	78	1411	157.8	5.87
Q3V1V0	NEDD4 family-interacting protein 2 OS=Mus musculus GN=Ndfip2 PE=2 SV=1 -	5.81	5	1	1	4	1.239	0.840	1.117	1.026	13.87	5.81	2	4	241	26.8	4.70
Q14AK4	Probable palmitoyltransferase ZDHHC11 OS=Mus musculus GN=Zdhhc11 PE=2 SV=2 - [illegible]	2.31	1	1	1	1	1.708	1.064	2.006	1.027	2.05	2.31	1	1	347	39.6	8.97
A2AI89	Phosphorylase b kinase regulatory subunit alpha, skeletal muscle isoform OS=Mus musculus GN=Phka1 PE=2 SV=1 - [A2AI89_MOUSE]	13.71	6	11	14	43	0.888	0.868	0.803	1.027	121.28	13.71	21	43	1211	135.5	5.86
Q4PJX1	Protein odr-4 homolog OS=Mus musculus GN=Odr4 PE=2 SV=2 - [ODR4_MOUSE]	16.33	3	8	8	31	0.780	0.764	0.825	1.027	79.35	16.33	15	31	447	50.0	6.14
A2ARZ0	Protein Olfr1049 OS=Mus musculus GN=Olfr1049 PE=4 SV=1 - [A2ARZ0_MOUSE]	10.06	20	1	2	2	1.292	1.010	0.828	1.027	0.00	10.06	2	2	308	34.9	8.48

Q923T9	Calcium/calmodulin-dependent protein kinase type II subunit gamma OS=Mus musculus GN=Camk2g PE=1 SV=1 - [KCC2G_MOUSE]	51.80	2	1	25	344	0.920	1.209	1.170	1.027	891.05	51.80	45	344	529	59.6	7.58
Q06180-2	Isoform PTPA of Tyrosine-protein phosphatase non-receptor type 2 OS=Mus musculus GN=Ptpn2 - [PTN2_MOUSE]	16.75	3	4	4	9	0.943	1.023	1.021	1.027	31.57	16.75	7	9	382	44.5	8.24
Q7TSZ8	Nucleus accumbens-associated protein 1 OS=Mus musculus GN=Nacc1 PE=1 SV=1 - [NACC1_MOUSE]	15.37	1	5	6	14	1.188	1.058	1.135	1.027	39.98	15.37	10	14	514	56.5	5.73
A2AKH7	Leucine-rich repeat-containing protein 57 (Fragment) OS=Mus musculus GN=Lrrc57 PE=2 SV=1 - [A2AKH7_MOUSE]	43.38	4	10	10	23	0.605	1.596	1.307	1.027	63.36	43.38	17	23	219	24.3	8.18
Q9D4V7	Rab-like protein 3 OS=Mus musculus GN=Rabl3 PE=2 SV=1 - [RABL3_MOUSE]	11.44	1	2	2	5	1.161	1.227	0.931	1.027	17.94	11.44	2	5	236	26.3	7.15
H7BX92	Brain-specific angiogenesis inhibitor 2 OS=Mus musculus GN=Bai2 PE=2 SV=1 - [H7BX92_MOUSE]	10.54	7	10	11	39	0.932	1.391	1.370	1.027	116.22	10.54	19	39	1489	162.5	7.50
Q9CRC9	Glucosamine-6-phosphate isomerase 2 OS=Mus musculus GN=Gnpda2 PE=2 SV=1 - [GNPI2_MOUSE]	53.26	5	8	11	46	1.554	1.020	0.806	1.027	146.26	53.26	18	46	276	31.1	6.90
Q769J6	A disintegrin and metalloproteinase with thrombospondin motifs 13 OS=Mus musculus GN=Adamts13 PE=2 SV=1 - [ATS13_MOUSE]	1.12	1	1	1	1	0.709	1.073	1.399	1.027	0.00	1.12	1	1	1426	155.3	6.57
Q61286-2	Isoform ALF1A of Transcription factor 12 OS=Mus musculus GN=Tcf12 -	5.72	2	1	2	3	1.297	0.822	1.489	1.027	7.97	5.72	3	3	682	72.9	7.02
Q3UN04	Ubiquitin carboxyl-terminal hydrolase 30 OS=Mus musculus GN=Usp30 PE=2 SV=1 - [UBP30_MOUSE]	12.96	1	3	3	8	0.603	0.741	0.788	1.027	14.36	12.96	4	8	517	58.2	8.46
Q9D7E3	Ovarian cancer-associated gene 2 protein homolog OS=Mus musculus GN=Ovca2 PE=2 SV=1 - [OVCA2_MOUSE]	5.78	1	1	1	8	0.696	1.128	0.919	1.027	14.64	5.78	2	8	225	24.2	5.88
E9QAF9	Protein TANC1 OS=Mus musculus GN=Tanc1 PE=2 SV=1 - [E9QAF9_MOUSE]	15.52	3	19	20	66	1.145	0.860	1.130	1.027	189.90	15.52	32	66	1849	199.9	8.32
Q9CQD1	Ras-related protein Rab-5A OS=Mus musculus GN=Rab5a PE=1 SV=1 - [RAB5A_MOUSE]	46.05	1	5	8	58	0.857	0.984	0.928	1.027	184.34	46.05	15	58	215	23.6	8.15
P60954-2	Isoform 2 of Nucleolar protein 4 OS=Mus musculus GN=Nol4 - [NOL4_MOUSE]	13.30	7	3	5	19	1.091	1.759	1.276	1.027	32.24	13.30	7	19	564	62.5	5.17
Q8C6I2	Succinate dehydrogenase assembly factor 2, mitochondrial OS=Mus musculus GN=Sdhaf2 PE=2 SV=1 - [SDHF2_MOUSE]	23.78	3	3	4	19	1.888	0.934	1.479	1.028	53.88	23.78	8	19	164	19.4	6.35
P07901	Heat shock protein HSP 90-alpha OS=Mus musculus GN=Hsp90aa1 PE=1 SV=4 - [HS90A_MOUSE]	59.89	7	37	53	1143	0.779	0.910	0.912	1.028	3532.93	59.89	98	1143	733	84.7	5.01
Q5BL07-2	Isoform 2 of Peroxisome biogenesis factor 1 OS=Mus musculus GN=Pex1 -	8.76	4	10	11	23	0.849	0.695	0.915	1.028	56.21	8.76	18	23	1244	136.7	6.21
G5E8T2	E3 ubiquitin-protein ligase TTC3 OS=Mus musculus GN=Ttc3 PE=4 SV=1 - [G5E8T2_MOUSE]	14.25	22	22	26	73	0.735	0.681	0.855	1.028	227.32	14.25	40	73	1979	223.8	6.25
P63166	Small ubiquitin-related modifier 1 OS=Mus musculus GN=Sumo1 PE=1 SV=1 -	35.64	1	4	4	20	1.594	1.002	1.210	1.028	34.57	35.64	7	20	101	11.5	5.52
P62746	Rho-related GTP-binding protein RhoB OS=Mus musculus GN=Rhob PE=1 SV=1 - [RHOB_MOUSE]	64.29	1	7	9	115	0.891	0.880	0.971	1.028	366.85	64.29	16	115	196	22.1	5.24
Q3TYH2	Ras-related protein Rab-15 OS=Mus musculus GN=Rab15 PE=2 SV=1 - [Q3TYH2_MOUSE]	34.43	7	5	8	90	0.455	1.503	1.159	1.028	261.53	34.43	14	90	212	24.3	5.71
E9QK34	Neuroligin-1 OS=Mus musculus GN=Nlgn1 PE=2 SV=1 - [E9QK34_MOUSE]	13.88	3	6	9	37	1.063	1.419	1.089	1.028	102.86	13.88	15	37	814	91.0	6.10
Q8BH17	A230074B11Rik protein OS=Mus musculus GN=Vwa3b PE=2 SV=1 - [Q8BH17_MOUSE]	9.04	1	1	2	2	1.116	0.707	0.960	1.028	2.84	9.04	2	2	343	39.1	5.92
P59281-2	Isoform 2 of Rho GTPase-activating protein 39 OS=Mus musculus GN=Arhgap39 -	21.93	5	22	22	54	0.723	1.028	1.145	1.028	150.66	21.93	39	54	1076	121.4	7.52
O08585	Clathrin light chain A OS=Mus musculus GN=Clta PE=1 SV=2 - [CLCA_MOUSE]	25.96	6	7	8	188	1.975	1.346	1.842	1.028	451.46	25.96	16	188	235	25.6	4.58

Q91XI1	tRNA-dihydrouridine(47) synthase [NAD(P)(+)]-like OS=Mus musculus GN=Dus3l PE=2 SV=1 - [DUS3L_MOUSE]	14.76	2	9	9	23	0.812	0.822	0.827	1.028	61.03	14.76	16	23	637	71.0	7.97
Q8C052	Microtubule-associated protein 1S OS=Mus musculus GN=Map1s PE=1 SV=2 -	24.36	1	19	19	86	0.691	0.945	0.954	1.028	256.35	24.36	36	86	973	102.9	7.02
Q8BUN9	Protein Slc24a2 OS=Mus musculus GN=Slc24a2 PE=2 SV=1 - [Q8BUN9_MOUSE]	18.62	3	2	8	68	0.533	0.952	1.498	1.028	170.37	18.62	14	68	666	74.2	6.54
Q9DBL9-2	Isoform 2 of 1-acylglycerol-3-phosphate O-acyltransferase ABHD5 OS=Mus musculus GN=Abhd5 - [ABHD5_MOUSE]	17.17	2	2	2	7	0.907	0.802	1.024	1.028	22.76	17.17	4	7	198	21.8	6.39
Q3UYG8	O-acetyl-ADP-ribose deacetylase MACROD2 OS=Mus musculus GN=Macrod2 PE=2 SV=1 - [MACD2_MOUSE]	20.63	2	6	6	13	1.168	1.173	1.155	1.028	40.14	20.63	11	13	475	52.1	4.75
Q9WTM5	RuvB-like 2 OS=Mus musculus GN=Ruvbl2 PE=2 SV=3 - [RUVB2_MOUSE]	52.92	2	22	22	82	0.876	1.119	0.846	1.028	235.30	52.92	40	82	463	51.1	5.64
E9Q6H7	Uncharacterized protein OS=Mus musculus GN=Tmem181b-ps PE=4 SV=1 - [E9Q6H7_MOUSE]	8.42	2	1	1	2	0.992	1.246	0.895	1.028	7.29	8.42	2	2	190	21.8	8.69
Q3UHD6-2	Isoform 2 of Sorting nexin-27 OS=Mus musculus GN=Snx27 - [SNX27_MOUSE]	42.59	5	17	17	77	0.869	1.051	0.929	1.028	278.82	42.59	32	77	526	59.5	6.24
Q8K448	ATP-binding cassette sub-family A member 5 OS=Mus musculus GN=Abca5 PE=1 SV=2 - [ABCA5_MOUSE]	5.97	3	10	11	28	0.869	1.032	0.924	1.029	87.13	5.97	15	28	1642	185.8	7.20
P68404-2	Isoform Beta-II of Protein kinase C beta type OS=Mus musculus GN=Prkcb -	43.98	1	2	28	225	0.567	0.842	1.771	1.029	601.76	43.98	50	225	673	76.8	7.02
F8VQE9	Arf-GAP with GTPase, ANK repeat and PH domain-containing protein 3 OS=Mus musculus GN=Agap3 PE=2 SV=1 - [F8VQE9_MOUSE]	23.74	4	12	17	85	0.691	1.119	1.044	1.029	267.02	23.74	31	85	910	97.6	7.77
Q9JM14	5'(3')-deoxyribonucleotidase, cytosolic type OS=Mus musculus GN=Nt5c PE=1 SV=1 - [NT5C_MOUSE]	62.50	1	2	9	46	1.157	1.681	0.848	1.029	150.90	62.50	17	46	200	23.1	5.49
E9Q7C0	Protein 1700030K09Rik OS=Mus musculus GN=1700030K09Rik PE=2 SV=1 - [E9Q7C0_MOUSE]	8.07	2	3	3	4	1.045	0.907	1.134	1.029	6.40	8.07	4	4	607	65.4	5.94
Q6NXL5	ArfGAP with coiled-coil, ankyrin repeat and PH domains 3 OS=Mus musculus GN=Acap3 PE=2 SV=1 - [Q6NXL5_MOUSE]	16.33	4	8	10	20	1.264	1.159	1.284	1.029	50.79	16.33	16	20	833	92.7	6.02
Q9D880	Mitochondrial import inner membrane translocase subunit TIM50 OS=Mus musculus GN=Timm50 PE=1 SV=1 - [TIM50_MOUSE]	36.26	1	12	12	65	0.695	0.918	0.896	1.029	163.36	36.26	22	65	353	39.8	8.13
Q8R361	Rab11 family-interacting protein 5 OS=Mus musculus GN=Rab11fip5 PE=1 SV=2 -	33.80	1	14	14	91	1.130	0.741	1.018	1.029	369.23	33.80	26	91	645	69.5	9.07
D3Z444	RIKEN cDNA 1810048P08, isoform CRA_b OS=Mus musculus GN=Rab43 PE=3 SV=1 - [D3Z444_MOUSE]	16.67	3	1	2	45	2.166	1.751	1.203	1.029	125.99	16.67	3	45	150	16.6	8.78
E9Q512	Protein Trip11 OS=Mus musculus GN=Trip11 PE=2 SV=1 - [E9Q512_MOUSE]	22.82	4	8	40	84	1.958	1.142	0.984	1.029	214.55	22.82	58	84	1976	226.3	5.26
A2A543	Voltage-dependent L-type calcium channel subunit beta-1 OS=Mus musculus GN=Cacnb1 PE=2 SV=1 - [A2A543_MOUSE]	35.68	6	3	17	82	1.194	1.205	1.185	1.029	275.46	35.68	32	82	597	65.4	6.62
P97393	Rho GTPase-activating protein 5 OS=Mus musculus GN=Arhgap5 PE=2 SV=2 -	22.19	2	26	27	67	0.835	0.922	0.964	1.029	198.81	22.19	42	67	1501	172.0	6.34
Q3TZM9-2	Isoform 2 of GDP-Man:Man(3)GlcNAc(2)-PP-Dol alpha-1,2-mannosyltransferase OS=Mus musculus GN=Alg11 - [ALG11_MOUSE]	7.56	4	2	2	8	0.520	0.597	0.732	1.029	36.18	7.56	4	8	450	50.4	8.19
Q3TEA8-2	Isoform 2 of Heterochromatin protein 1-binding protein 3 OS=Mus musculus GN=Hp1bp3 - [HP1B3_MOUSE]	34.75	8	19	19	79	0.467	0.902	0.928	1.029	213.92	34.75	35	79	541	59.5	9.79
Q8BL03	Mitochondrial carnitine/acylcarnitine carrier protein CACL OS=Mus musculus GN=Slc25a29 PE=2 SV=1 - [MCATL_MOUSE]	19.28	1	4	4	6	0.893	1.062	0.964	1.029	14.98	19.28	6	6	306	32.7	8.62

Q9D3W4	GPN-loop GTPase 3 OS=Mus musculus GN=Gpn3 PE=2 SV=1 - [GPN3_MOUSE]	14.44	2	2	2	4	0.878	1.324	0.735	1.029	18.05	14.44	4	4	284	32.8	4.53
A8Y5G7	DNA polymerase delta subunit 2 (Fragment) OS=Mus musculus GN=Pold2 PE=2 SV=1 - [A8Y5G7_MOUSE]	17.01	2	3	3	5	1.037	1.024	0.906	1.029	12.63	17.01	5	5	241	26.3	6.64
Q8VCF1	Soluble calcium-activated nucleotidase 1 OS=Mus musculus GN=Cant1 PE=2 SV=1 - [CANT1_MOUSE]	18.86	3	5	5	9	0.725	1.011	0.955	1.029	17.22	18.86	8	9	403	45.6	6.77
Q504P2	C-type lectin domain family 12 member A OS=Mus musculus GN=Clec12a PE=2 SV=1 - [CL12A_MOUSE]	11.24	2	2	3	4	1.110	0.836	1.216	1.029	7.78	11.24	4	4	267	30.7	7.77
Q7TMY4	THO complex subunit 7 homolog OS=Mus musculus GN=Thoc7 PE=1 SV=2 - [THOC7_MOUSE]	15.20	3	4	4	7	1.518	1.141	1.056	1.030	14.65	15.20	6	7	204	23.7	5.67
Q3U3E2	Protein FAM117B OS=Mus musculus GN=Fam117b PE=1 SV=1 - [F117B_MOUSE]	29.28	1	11	11	31	1.203	0.962	1.240	1.030	96.88	29.28	16	31	584	61.3	9.95
Q9D853	Methyltransferase-like protein 10 OS=Mus musculus GN=Mettl10 PE=2 SV=1 -	23.36	2	4	4	7	1.000	1.314	0.931	1.030	22.01	23.36	7	7	244	26.8	5.15
O08579	Emerin OS=Mus musculus GN=Emd PE=1 SV=1 - [EMD_MOUSE]	39.38	3	3	8	39	0.722	1.340	0.938	1.030	133.84	39.38	13	39	259	29.4	5.01
Q3TPJ8	Cytoplasmic dynein 1 intermediate chain 2 OS=Mus musculus GN=Dync1i2 PE=2 SV=1 - [Q3TPJ8_MOUSE]	24.53	3	1	10	78	1.333	1.251	1.028	1.030	196.17	24.53	18	78	632	70.6	5.24
Q8C172	Ceramide synthase 6 OS=Mus musculus GN=Cers6 PE=1 SV=1 - [CERS6_MOUSE]	7.81	2	3	3	3	0.663	1.016	0.786	1.030	6.23	7.81	3	3	384	44.8	7.64
Q80W65	Proprotein convertase subtilisin/kexin type 9 OS=Mus musculus GN=Pcsk9 PE=1 SV=2 - [PCSK9_MOUSE]	0.86	1	1	1	1	0.733	1.288	1.531	1.030	1.87	0.86	1	1	694	74.8	6.96
A2ASS6	Titin OS=Mus musculus GN=Ttn PE=1 SV=1 - [TITIN_MOUSE]	1.84	13	45	53	91	1.188	1.050	1.030	1.030	163.31	1.84	58	91	35213	3904.1	6.20
Q99LJ1	Tissue alpha-L-fucosidase OS=Mus musculus GN=Fuca1 PE=2 SV=1 - [FUCO_MOUSE]	1.55	1	1	1	1	0.917	1.317	0.844	1.030	2.59	1.55	1	1	452	52.2	6.95
D6RG73	Bifunctional apoptosis regulator OS=Mus musculus GN=Bfar PE=2 SV=1 - [D6RG73_MOUSE]	7.95	2	1	1	7	0.861	0.716	0.935	1.030	23.10	7.95	2	7	176	20.1	6.65
A2AMV1	RIKEN cDNA 2610012O22, isoform CRA_a OS=Mus musculus GN=Mrto4 PE=3 SV=1 - [A2AMV1_MOUSE]	20.59	3	5	5	7	0.677	0.905	0.906	1.030	9.52	20.59	7	7	238	27.4	8.28
Q9R1S3-2	Isoform 2 of GPI ethanolamine phosphate transferase 1 OS=Mus musculus GN=Pign - [PIGN_MOUSE]	2.51	4	2	2	3	0.992	0.993	0.778	1.030	7.43	2.51	3	3	798	90.4	7.59
P09671	Superoxide dismutase [Mn], mitochondrial OS=Mus musculus GN=Sod2 PE=1 SV=3 - [SODM_MOUSE]	67.12	1	12	12	193	1.668	0.976	1.040	1.030	511.46	67.12	23	193	222	24.6	8.62
Q8VDV7	Protein Tbc1d19 OS=Mus musculus GN=Tbc1d19 PE=2 SV=1 - [Q8VDV7_MOUSE]	12.36	1	5	5	17	0.938	1.140	0.963	1.030	43.15	12.36	9	17	526	60.4	6.10
Q8BG02	Serine/threonine-protein phosphatase 2A 55 kDa regulatory subunit B gamma isoform OS=Mus musculus GN=Ppp2r2c PE=2 SV=1 - [2ABG_MOUSE]	16.78	1	6	10	22	1.140	1.088	1.298	1.030	61.96	16.78	13	22	447	51.4	6.37
E9PU87	Serine/threonine-protein kinase SIK3 OS=Mus musculus GN=Sik3 PE=2 SV=1 - [E9PU87_MOUSE]	31.85	7	31	34	130	0.944	0.693	0.927	1.030	354.74	31.85	57	130	1369	150.6	7.02
Q5XPI3	E3 ubiquitin-protein ligase RNF123 OS=Mus musculus GN=Rnf123 PE=2 SV=1 - [RN123_MOUSE]	8.22	7	9	11	32	0.892	0.929	1.007	1.030	76.24	8.22	19	32	1314	148.6	6.70
E9PY61	Protein Gpr179 OS=Mus musculus GN=Gpr179 PE=4 SV=1 - [E9PY61_MOUSE]	0.78	1	1	1	1	1.473	0.715	0.998	1.030	2.81	0.78	1	1	2293	248.6	5.55
Q8BUY5	Translocase of inner mitochondrial membrane domain-containing protein 1 OS=Mus musculus GN=Timmdc1 PE=2 SV=1 [TIDC1_MOUSE]	11.58	1	3	3	6	1.009	0.749	0.859	1.030	17.51	11.58	5	6	285	31.8	9.29
Q9D6Z0-2	Isoform 2 of Alpha-ketoglutarate-dependent dioxygenase alkB homolog 7 OS=Mus musculus GN=Alkbh7 - [ALKB7_MOUSE]	5.52	2	1	1	2	1.514	1.326	0.935	1.031	4.72	5.52	2	2	163	18.5	6.11

Q2TPA8	Hydroxysteroid dehydrogenase-like protein 2 OS=Mus musculus GN=Hsdl2 PE=2 SV=1 - [HSDL2_MOUSE]	28.57	3	11	11	49	1.157	0.774	0.873	1.031	144.90	28.57	20	49	490	54.2	6.74
E9PUU5	Protein Zfp839 OS=Mus musculus GN=Zfp839 PE=4 SV=1 - [E9PUU5_MOUSE]	1.52	1	1	1	1	1.416	0.897	0.868	1.031	2.76	1.52	1	1	921	98.9	5.94
Q99PU5	Long-chain-fatty-acid--CoA ligase ACSBG1 OS=Mus musculus GN=Acsbg1 PE=1 SV=1 - [ACBG1_MOUSE]	55.76	3	35	35	168	0.727	0.560	0.803	1.031	521.69	55.76	61	168	721	80.4	5.94
Q8CBC8	Branched-chain-amino-acid aminotransferase OS=Mus musculus GN=Bcat1 PE=2 SV=1 - [Q8CBC8_MOUSE]	30.68	8	12	12	77	2.158	0.680	0.751	1.031	208.95	30.68	20	77	453	49.9	6.77
P58242	Acid sphingomyelinase-like phosphodiesterase 3b OS=Mus musculus GN=Smpdl3b PE=1 SV=1 - [ASM3B_MOUSE]	12.94	2	4	5	17	1.313	3.424	1.216	1.031	56.70	12.94	9	17	456	51.6	6.43
Q4VBD9	GDNF-inducible zinc finger protein 1 OS=Mus musculus GN=Gzf1 PE=1 SV=2 - [GZF1_MOUSE]	2.27	1	1	1	2	1.652	0.990	1.263	1.031	5.32	2.27	1	2	706	79.5	7.97
A2AKQ3	Protein Slc35d1 OS=Mus musculus GN=Slc35d1 PE=2 SV=1 - [A2AKQ3_MOUSE]	6.76	3	1	1	1	2.654	2.276	1.166	1.031	1.79	6.76	1	1	148	16.4	10.67
Q9D5V2	Kelch-like protein 10 OS=Mus musculus GN=Klhl10 PE=1 SV=1 - [KLH10_MOUSE]	0.99	1	1	1	1	5.553	1.822	0.885	1.031	2.51	0.99	1	1	608	68.9	5.77
F8WJ31	Zinc finger and SCAN domain-containing protein 26 OS=Mus musculus GN=Zscan26 PE=2 SV=1 - [F8WJ31_MOUSE]	7.35	3	3	3	5	0.833	0.785	1.064	1.031	11.09	7.35	5	5	340	38.8	8.78
E9PWW9	Protein Rsf1 OS=Mus musculus GN=Rsf1 PE=2 SV=1 - [E9PWW9_MOUSE]	3.37	2	4	4	7	1.345	0.865	0.951	1.031	13.35	3.37	5	7	1426	160.5	5.02
Q9DB60	Prostamide/prostaglandin F synthase OS=Mus musculus GN=Fam213b PE=1 SV=1 - [PGFS_MOUSE]	51.74	2	7	7	36	0.551	1.055	0.840	1.031	109.91	51.74	13	36	201	21.7	6.74
B7ZDF4	Histone deacetylase 5 (Fragment) OS=Mus musculus GN=Hdac5 PE=2 SV=1 - [B7ZDF4_MOUSE]	44.57	1	1	7	18	1.318	0.908	0.808	1.031	43.58	44.57	12	18	175	20.0	9.03
Q8C0T5	Signal-induced proliferation-associated 1-like protein 1 OS=Mus musculus GN=Sipa1l1 PE=1 SV=2 - [illegible]	37.43	2	43	47	129	1.136	1.622	1.885	1.031	412.99	37.43	76	129	1782	196.9	8.13
E9Q605	Inhibitor of nuclear factor kappa-B kinase subunit alpha OS=Mus musculus GN=Chuk PE=2 SV=1 - [E9Q605_MOUSE]	3.06	4	2	2	2	2.260	1.567	1.056	1.031	3.74	3.06	2	2	719	82.2	7.71
Q9Z0U1	Tight junction protein ZO-2 OS=Mus musculus GN=Tjp2 PE=1 SV=2 - [ZO2_MOUSE]	31.88	2	31	32	138	1.160	0.636	1.096	1.031	382.66	31.88	55	138	1167	131.2	6.79
E0CY18	AN1-type zinc finger protein 2B (Fragment) OS=Mus musculus GN=Zfand2b PE=2 SV=1 - [E0CY18_MOUSE]	33.94	2	7	7	42	1.803	1.067	1.548	1.031	100.03	33.94	12	42	218	23.7	7.72
B2RR83	Probable ATP-dependent RNA helicase YTHDC2 OS=Mus musculus GN=Ythdc2 PE=2 SV=1 - [YTDC2_MOUSE]	10.10	1	13	13	29	0.703	0.932	0.968	1.031	83.92	10.10	22	29	1445	161.0	8.51
J3QMK2	MCG22959, isoform CRA_a OS=Mus musculus GN=Bbip1 PE=4 SV=1 - [J3QMK2_MOUSE]	14.93	1	1	1	4	1.799	1.016	1.272	1.031	14.01	14.93	2	4	67	7.6	8.76
Q9R0N8-2	Isoform 2 of Synaptotagmin-6 OS=Mus musculus GN=Syt6 - [SYT6_MOUSE]	38.03	4	10	12	41	0.886	0.532	0.836	1.031	108.81	38.03	21	41	426	48.3	8.43
P03953-2	Isoform 2 of Complement factor D OS=Mus musculus GN=Cfd - [CFAD_MOUSE]	13.18	2	3	3	6	1.871	1.088	0.937	1.031	15.35	13.18	5	6	258	28.0	6.67
P82347	Delta-sarcoglycan OS=Mus musculus GN=Sgcd PE=1 SV=1 - [SGCD_MOUSE]	13.84	2	4	4	7	6.909	1.666	1.119	1.031	20.18	13.84	6	7	289	32.1	9.10
P57746	V-type proton ATPase subunit D OS=Mus musculus GN=Atp6v1d PE=1 SV=1 -	72.87	1	18	19	135	0.786	1.318	1.052	1.032	403.13	72.87	31	135	247	28.4	9.45
Q80UU9	Membrane-associated progesterone receptor component 2 OS=Mus musculus GN=Pgrmc2 PE=1 SV=2 - [illegible]	52.07	1	9	11	53	0.676	1.137	0.886	1.032	169.31	52.07	20	53	217	23.3	5.15
Q9R1R8	Retinol dehydrogenase 11 OS=Mus musculus GN=Rdh11 PE=2 SV=1 - [Q9R1R8_MOUSE]	19.67	4	5	5	18	0.738	0.736	0.969	1.032	58.97	19.67	8	18	300	33.2	8.79

A2ATK9	Family with sequence similarity 171, member A1 OS=Mus musculus GN=Fam171a1 PE=2 SV=1 - [A2ATK9_MOUSE]	22.09	4	14	15	41	0.838	1.305	1.293	1.032	89.25	22.09	27	41	892	97.9	6.40
Q6P6L6	Gem (Nuclear organelle) associated protein 4 OS=Mus musculus GN=Gemin4 PE=2 SV=1 - [Q6P6L6_MOUSE]	2.46	1	2	2	4	0.446	0.940	0.957	1.032	10.56	2.46	4	4	1058	120.1	5.15
Q9Z1B3-3	Isoform C of 1-phosphatidylinositol 4,5-bisphosphate phosphodiesterase beta-1 OS=Mus musculus GN=Plcb1 - [PLCB1_MOUSE]	52.09	4	49	53	239	0.670	1.380	1.894	1.032	653.56	52.09	93	239	1198	136.2	6.34
Q3UQ28	Peroxidasin homolog OS=Mus musculus GN=Pxdn PE=2 SV=2 - [PXDN_MOUSE]	3.19	1	4	4	7	1.526	1.679	1.560	1.032	5.96	3.19	4	7	1475	165.0	7.14
J3QQ22	Serine/threonine-protein kinase SMG1 OS=Mus musculus GN=Smg1 PE=4 SV=1 - [J3QQ22_MOUSE]	5.03	5	15	17	34	0.977	1.026	0.954	1.032	104.03	5.03	25	34	3582	401.4	6.30
P30677	Guanine nucleotide-binding protein subunit alpha-14 OS=Mus musculus GN=Gna14 PE=1 SV=2 - [illegible]	18.31	1	1	6	23	0.541	1.381	0.864	1.032	58.96	18.31	8	23	355	41.5	6.30
Q8R104	NAD-dependent protein deacetylase sirtuin-3 OS=Mus musculus GN=Sirt3 PE=2 SV=1 - [SIR3_MOUSE]	17.51	3	1	4	14	0.574	0.955	0.878	1.032	42.48	17.51	8	14	257	28.8	6.23
G3X9Y1	Synaptotagmin III, isoform CRA_a OS=Mus musculus GN=Syt3 PE=4 SV=1 - [G3X9Y1_MOUSE]	28.96	4	12	16	59	0.638	0.631	1.003	1.032	159.85	28.96	30	59	587	63.2	6.62
B2RXC1	Trafficking protein particle complex subunit 11 OS=Mus musculus GN=Trappc11 PE=2 SV=1 [TPC11_MOUSE]	15.53	2	15	16	34	0.571	0.917	0.946	1.032	82.92	15.53	27	34	1133	128.3	7.58
Q80U63	Mitofusin-2 OS=Mus musculus GN=Mfn2 PE=1 SV=3 - [MFN2_MOUSE]	32.23	4	19	20	66	0.660	0.995	0.987	1.032	203.16	32.23	30	66	757	86.1	6.77
D3YYH1	PEX5-related protein OS=Mus musculus GN=Pex5l PE=2 SV=1 - [D3YYH1_MOUSE]	48.79	1	2	13	70	2.005	0.805	1.358	1.032	167.33	48.79	22	70	373	41.4	5.08
Q8BG30	Negative elongation factor A OS=Mus musculus GN=Nelfa PE=1 SV=1 - [NELFA_MOUSE]	20.94	1	9	9	28	1.004	0.709	1.029	1.032	64.41	20.94	14	28	530	57.5	9.11
P49070	Calcium signal-modulating cyclophilin ligand OS=Mus musculus GN=Camlg PE=1 SV=2 - [CAMLG_MOUSE]	28.57	1	8	8	21	1.205	0.934	0.955	1.032	69.58	28.57	13	21	294	32.5	7.74
Q6NZD2	Sorting nexin 1 OS=Mus musculus GN=Snx1 PE=2 SV=1 - [Q6NZD2_MOUSE]	44.15	3	20	22	99	1.092	1.128	0.857	1.032	322.04	44.15	37	99	521	58.8	5.26
Q9QXB9	Developmentally-regulated GTP-binding protein 2 OS=Mus musculus GN=Drg2 PE=1 SV=1 - [DRG2_MOUSE]	36.54	1	10	12	41	0.508	0.843	0.849	1.032	140.28	36.54	20	41	364	40.7	8.88
Q6IRU2	Tropomyosin alpha-4 chain OS=Mus musculus GN=Tpm4 PE=2 SV=3 - [TPM4_MOUSE]	45.97	1	9	12	91	1.891	0.979	1.145	1.032	209.11	45.97	20	91	248	28.5	4.68
Q8CFE4	SCY1-like protein 2 OS=Mus musculus GN=Scyl2 PE=1 SV=1 - [SCYL2_MOUSE]	26.77	2	1	22	84	0.671	0.960	1.109	1.032	249.48	26.77	38	84	930	103.3	8.02
F6SPK0	Ubiquitin-conjugating enzyme E2 J1 (Fragment) OS=Mus musculus GN=Ube2j1 PE=4 SV=1 - [F6SPK0_MOUSE]	19.08	2	3	3	31	1.191	0.801	1.052	1.032	100.91	19.08	6	31	262	28.9	7.25
Q0KL02	Triple functional domain protein OS=Mus musculus GN=Trio PE=1 SV=3 - [TRIO_MOUSE]	28.14	3	1	74	226	1.476	2.221	1.018	1.032	640.96	28.14	125	226	3102	347.6	6.35
Q8R516-2	Isoform 2 of E3 ubiquitin-protein ligase MIB2 OS=Mus musculus GN=Mib2 -	8.90	6	9	9	16	0.909	1.021	0.957	1.032	35.91	8.90	13	16	921	100.2	7.97
Q3UPZ0	Sema domain, seven thrombospondin repeats (Type 1 and type 1-like), transmembrane domain (TM) and short cytoplasmic domain, (Semaphorin) 5A, isoform CRA_a OS=Mus musculus GN=Sema5a PE=2 SV=1 - [Q3UPZ0_MOUSE]	2.70	2	3	3	3	1.328	0.781	1.009	1.033	3.97	2.70	3	3	1074	120.2	7.06
Q99J39-2	Isoform Cytoplasmic+peroxisomal of Malonyl-CoA decarboxylase, mitochondrial OS=Mus [illegible]	36.56	2	11	12	45	0.781	0.933	0.924	1.033	142.98	36.56	21	45	454	50.7	7.88
P08226	Apolipoprotein E OS=Mus musculus GN=Apoe PE=1 SV=2 - [APOE_MOUSE]	36.01	8	14	14	128	1.325	2.078	0.956	1.033	380.28	36.01	25	128	311	35.8	5.68

F7A5I8	Prostate tumor-overexpressed gene 1 protein homolog (Fragment) OS=Mus musculus GN=Ptov1 PE=4 SV=1 - [F7A5I8_MOUSE]	6.96	2	1	1	4	0.831	0.675	0.698	1.033	13.38	6.96	2	4	158	18.6	9.73
Q3UYI5	Ral guanine nucleotide dissociation stimulator-like 3 OS=Mus musculus GN=Rgl3 PE=1 SV=2 - [RGL3_MOUSE]	2.68	1	2	2	5	1.469	1.055	0.927	1.033	14.40	2.68	4	5	709	77.9	8.60
P10605	Cathepsin B OS=Mus musculus GN=Ctsb PE=1 SV=2 - [CATB_MOUSE]	45.72	1	13	13	64	1.494	0.992	1.016	1.033	223.27	45.72	23	64	339	37.3	5.91
E9PWV3	Ribosomal protein S6 kinase OS=Mus musculus GN=Rps6ka1 PE=2 SV=1 - [E9PWV3_MOUSE]	27.26	15	6	16	52	0.921	0.800	0.858	1.033	136.63	27.26	28	52	719	81.3	8.54
Q9ER00	Syntaxin-12 OS=Mus musculus GN=Stx12 PE=1 SV=1 - [STX12_MOUSE]	43.43	1	10	10	56	0.898	1.198	1.023	1.033	173.74	43.43	19	56	274	31.2	5.44
Q811L6	Microtubule-associated serine/threonine-protein kinase 4 OS=Mus musculus GN=Mast4 PE=1 SV=3 - [MAST4_MOUSE]	11.96	13	18	24	66	1.380	0.716	1.455	1.033	177.20	11.96	39	66	2618	283.8	8.62
Q810B6	Ankyrin repeat and FYVE domain-containing protein 1 OS=Mus musculus GN=Ankfy1 PE=2 SV=2 - [ANFY1_MOUSE]	15.83	2	15	17	50	0.673	1.059	0.886	1.033	155.35	15.83	25	50	1169	128.6	5.91
J3QPB6	G-protein-coupled receptor 124 (Fragment) OS=Mus musculus GN=Gpr124 PE=4 SV=1 - [J3QPB6_MOUSE]	10.95	1	1	1	1	3.201	1.094	0.950	1.033	0.00	10.95	1	1	210	23.2	6.51
Q8BLY2	Probable threonine--tRNA ligase 2, cytoplasmic OS=Mus musculus GN=Tarsl2 PE=2 SV=1 - [SYTC2_MOUSE]	34.94	1	21	24	62	1.116	0.951	1.124	1.033	168.09	34.94	38	62	790	91.3	7.53
Q61239	Protein farnesyltransferase/geranylgeranyltransferase type-1 subunit alpha OS=Mus musculus GN=Fnta PE=1 SV=1 - [FNTA_MOUSE]	35.28	2	10	11	36	1.354	0.814	0.881	1.033	102.27	35.28	18	36	377	44.0	4.93
D3YX28	Serine protease HTRA2, mitochondrial OS=Mus musculus GN=Htra2 PE=2 SV=1 - [D3YX28_MOUSE]	34.35	1	1	9	31	1.199	0.910	1.182	1.033	97.78	34.35	16	31	361	38.9	10.13
D3Z442	Uncharacterized protein OS=Mus musculus GN=Aamdc PE=2 SV=1 - [D3Z442_MOUSE]	62.59	9	9	9	26	2.076	0.789	1.171	1.033	72.10	62.59	15	26	139	15.2	9.10
P14901	Heme oxygenase 1 OS=Mus musculus GN=Hmox1 PE=1 SV=1 - [HMOX1_MOUSE]	15.22	2	3	3	10	1.146	1.056	1.097	1.033	37.55	15.22	5	10	289	32.9	6.54
Q9QXM1-3	Isoform 3 of Junction-mediating and -regulatory protein OS=Mus musculus GN=Jmy - [JMY_MOUSE]	13.17	5	10	11	20	0.909	0.584	0.972	1.033	59.59	13.17	15	20	964	108.6	6.13
Q5ND28	Scavenger receptor class F member 1 OS=Mus musculus GN=Scarf1 PE=1 SV=1 - [SREC_MOUSE]	1.22	1	1	1	1	1.719	1.202	1.211	1.033	1.83	1.22	1	1	820	87.4	6.04
F7CC56	RB1-inducible coiled-coil protein 1 (Fragment) OS=Mus musculus GN=Rb1cc1 PE=4 SV=1 - [F7CC56_MOUSE]	25.75	6	36	37	94	0.885	0.871	1.118	1.033	274.90	25.75	61	94	1468	168.9	5.52
F8WIZ7	Oxysterol-binding protein OS=Mus musculus GN=Osbpl6 PE=2 SV=1 - [F8WIZ7_MOUSE]	20.99	1	2	16	48	1.092	1.010	1.286	1.033	128.97	20.99	27	48	967	109.7	7.27
Q8BYI8	Uncharacterized protein KIAA1467 OS=Mus musculus GN=Kiaa1467 PE=2 SV=1 -	11.38	2	7	7	22	0.671	0.924	0.917	1.033	60.67	11.38	10	22	624	67.0	5.03
P63213	Guanine nucleotide-binding protein G(I)/G(S)/G(O) subunit gamma-2 OS=Mus musculus GN=Gng2 PE=1 SV=2 - [GBG2_MOUSE]	61.97	1	4	5	182	0.782	1.189	1.053	1.033	534.64	61.97	10	182	71	7.8	7.99
G5E819	Serine/threonine-protein phosphatase OS=Mus musculus GN=Ppp5c PE=3 SV=1 - [G5E819_MOUSE]	51.10	13	25	25	132	1.060	1.030	1.040	1.033	394.09	51.10	44	132	499	56.8	6.20
Q3U2A8	Valine--tRNA ligase, mitochondrial OS=Mus musculus GN=Vars2 PE=2 SV=2 - [SYVM_MOUSE]	18.58	6	12	15	33	0.801	0.888	0.949	1.033	82.17	18.58	27	33	1060	118.4	7.14
Q8BPU7	Engulfment and cell motility protein 1 OS=Mus musculus GN=Elmo1 PE=1 SV=2 - [ELMO1_MOUSE]	34.39	4	18	24	74	0.803	0.871	1.027	1.033	197.64	34.39	36	74	727	83.9	6.28
Q8VDL4-3	Isoform 3 of ADP-dependent glucokinase OS=Mus musculus GN=Adpgk -	30.91	3	10	10	25	0.883	0.815	0.763	1.033	72.80	30.91	18	25	495	53.7	5.62

Q8R5L1	Complement component 1 Q subcomponent-binding protein, mitochondrial OS=Mus musculus GN=C1qbp PE=2 SV=1 - [Q8R5L1_MOUSE]	51.97	2	8	8	181	2.234	1.047	1.397	1.034	582.48	51.97	16	181	279	31.0	4.87
P63002	Amino-terminal enhancer of split OS=Mus musculus GN=Aes PE=1 SV=1 - [AES_MOUSE]	16.75	2	3	3	11	0.776	0.786	0.958	1.034	26.79	16.75	6	11	197	22.0	6.37
Q9CZN8	Glutamyl-tRNA(Gln) amidotransferase subunit A, mitochondrial OS=Mus musculus GN=Qrsl1 PE=2 SV=1 - [GATA_MOUSE]	29.90	1	12	12	35	0.934	0.966	1.035	1.034	98.35	29.90	23	35	525	56.7	5.95
Q61599	Rho GDP-dissociation inhibitor 2 OS=Mus musculus GN=Arhgdib PE=1 SV=3 -	28.50	2	4	4	16	1.454	1.235	1.029	1.034	51.62	28.50	8	16	200	22.8	5.11
Q60823	RAC-beta serine/threonine-protein kinase OS=Mus musculus GN=Akt2 PE=1 SV=1 - [AKT2_MOUSE]	27.23	7	7	12	35	1.437	1.422	1.215	1.034	86.57	27.23	23	35	481	55.7	6.37
Q9CQW3	Vitamin K-dependent protein Z OS=Mus musculus GN=Proz PE=1 SV=1 - [PROZ_MOUSE]	5.01	1	2	2	5	5.350	2.554	0.612	1.034	14.94	5.01	3	5	399	44.3	5.90
P56371	Ras-related protein Rab-4A OS=Mus musculus GN=Rab4a PE=1 SV=2 - [RAB4A_MOUSE]	34.40	1	4	7	60	1.023	0.851	0.743	1.034	170.41	34.40	12	60	218	24.4	6.07
Q9ESN9-4	Isoform 1d of C-Jun-amino-terminal kinase-interacting protein 3 OS=Mus musculus GN=Mapk8ip3 - [JIP3_MOUSE]	28.37	10	3	31	128	0.575	0.717	0.829	1.034	391.37	28.37	53	128	1336	147.4	5.45
Q3UDP0	WD repeat-containing protein 41 OS=Mus musculus GN=Wdr41 PE=2 SV=1 -	26.96	4	8	9	18	1.217	1.107	0.923	1.034	46.26	26.96	13	18	460	51.5	5.15
Q3TSG4	RNA demethylase ALKBH5 OS=Mus musculus GN=Alkbh5 PE=1 SV=2 - [ALKB5_MOUSE]	4.56	1	2	2	4	1.527	0.789	0.957	1.034	5.13	4.56	3	4	395	44.4	9.09
E9Q1G8	Septin-7 OS=Mus musculus GN=Sept7 PE=3 SV=2 - [E9Q1G8_MOUSE]	63.16	3	1	27	547	0.619	0.884	1.006	1.034	1661.00	63.16	50	547	437	50.6	8.57
Q80T11	Usher syndrome type-1G protein homolog OS=Mus musculus GN=Ush1g PE=1 SV=1 - [USH1G_MOUSE]	4.77	1	2	2	2	1.251	1.381	1.376	1.034	0.00	4.77	2	2	461	51.5	6.99
P35700	Peroxiredoxin-1 OS=Mus musculus GN=Prdx1 PE=1 SV=1 - [PRDX1_MOUSE]	66.33	4	13	15	349	1.664	0.907	1.074	1.034	856.65	66.33	30	349	199	22.2	8.12
E9QMP6	Serine/threonine-protein kinase MARK2 OS=Mus musculus GN=Mark2 PE=4 SV=2 - [E9QMP6_MOUSE]	48.84	19	26	35	165	0.750	1.243	1.405	1.034	506.48	48.84	61	165	776	86.3	9.67
Q8R420	ATP-binding cassette sub-family A member 3 OS=Mus musculus GN=Abca3 PE=2 SV=3 - [ABCA3_MOUSE]	4.58	2	7	8	18	1.260	0.891	1.007	1.034	47.57	4.58	13	18	1704	191.8	7.17
Q9DBR1-2	Isoform 2 of 5'-3' exoribonuclease 2 OS=Mus musculus GN=Xrn2 -	15.52	2	14	14	28	0.770	0.781	0.973	1.035	70.18	15.52	22	28	947	108.0	7.25
F7BSN3	Ubiquitin carboxyl-terminal hydrolase OS=Mus musculus GN=Usp34 PE=2 SV=1 - [F7BSN3_MOUSE]	4.15	7	12	12	30	0.809	0.987	0.776	1.035	84.13	4.15	19	30	3255	370.4	5.72
E9PZ15	GDP-fucose protein O-fucosyltransferase 1 OS=Mus musculus GN=Pofut1 PE=2 SV=1 - [E9PZ15_MOUSE]	19.19	2	1	4	8	1.058	1.374	0.937	1.035	23.50	19.19	7	8	297	33.6	8.46
Q9WUI1	Mitogen-activated protein kinase 11 OS=Mus musculus GN=Mapk11 PE=1 SV=2 -	9.34	8	1	2	15	0.974	1.326	1.137	1.035	35.05	9.34	4	15	364	41.4	5.78
Q69ZP3-2	Isoform 2 of Probable hydrolase PNKD OS=Mus musculus GN=Pnkd - [PNKD_MOUSE]	19.01	1	1	2	4	1.437	1.124	1.390	1.035	13.35	19.01	3	4	142	15.6	10.18
Q8CE33-2	Isoform 2 of Kelch-like protein 11 OS=Mus musculus GN=Klhl11 - [KLH11_MOUSE]	4.92	2	2	2	6	1.177	1.252	0.909	1.035	12.92	4.92	3	6	569	66.0	6.58
P22005	Proenkephalin-A OS=Mus musculus GN=Penk PE=2 SV=2 - [PENK_MOUSE]	32.84	2	7	7	30	3.321	1.103	2.512	1.035	81.57	32.84	10	30	268	31.0	5.68
Q61488	Desert hedgehog protein OS=Mus musculus GN=Dhh PE=1 SV=1 - [DHH_MOUSE]	4.29	1	1	1	3	1.152	0.827	1.284	1.035	7.31	4.29	1	3	396	43.5	9.39
H3BK66	WD repeat-containing protein 37 (Fragment) OS=Mus musculus GN=Wdr37 PE=2 SV=1 - [H3BK66_MOUSE]	51.13	5	1	6	35	0.720	0.608	0.852	1.035	105.57	51.13	11	35	133	14.9	9.31

Q64689	Sia-alpha-2,3-Gal-beta-1,4-GlcNAc-R:alpha 2,8-sialyltransferase OS=Mus musculus GN=St8sia3 PE=2 SV=2 - [SIA8C_MOUSE]	19.47	2	7	7	15	0.829	0.926	0.894	1.035	43.82	19.47	12	15	380	43.9	9.52
Q6PAL8	DENN domain-containing protein 5A OS=Mus musculus GN=Dennd5a PE=1 SV=1 - [DEN5A_MOUSE]	15.23	1	12	16	47	0.804	0.728	0.865	1.035	118.33	15.23	28	47	1287	146.6	6.70
Q8CCX5	Keratin-like protein KRT222 OS=Mus musculus GN=Krt222 PE=2 SV=1 - [KT222_MOUSE]	33.67	2	8	9	28	1.743	0.431	1.080	1.035	63.19	33.67	16	28	294	34.2	5.78
P59764	Dedicator of cytokinesis protein 4 OS=Mus musculus GN=Dock4 PE=1 SV=1 - [DOCK4_MOUSE]	16.43	2	27	29	103	0.864	1.340	1.171	1.035	257.07	16.43	50	103	1978	226.4	7.65
Q9JKD3	Secretory carrier-associated membrane protein 5 OS=Mus musculus GN=Scamp5 PE=2 SV=1 - [SCAM5_MOUSE]	28.51	1	4	4	37	0.531	1.055	1.006	1.036	76.42	28.51	7	37	235	26.1	8.54
Q9D8C6	Mediator of RNA polymerase II transcription subunit 11 OS=Mus musculus GN=Med11 PE=2 SV=1 - [MED11_MOUSE]	31.62	2	2	2	4	1.100	1.288	1.065	1.036	12.49	31.62	4	4	117	13.1	5.96
Q9CRC6	UPF0693 protein C10orf32 homolog OS=Mus musculus PE=1 SV=1 - [CJ032_MOUSE]	58.10	1	4	4	21	1.711	1.144	1.398	1.036	94.38	58.10	8	21	105	11.5	6.79
E9QLB2	Lysophospholipase-like protein 1 OS=Mus musculus GN=Lyplal1 PE=4 SV=1 -	20.50	2	4	4	11	0.838	0.775	0.763	1.036	35.98	20.50	6	11	239	26.3	7.88
Q64314-2	Isoform Short of Hematopoietic progenitor cell antigen CD34 OS=Mus musculus GN=Cd34 - [CD34_MOUSE]	11.08	2	5	5	14	0.844	0.861	1.185	1.036	40.97	11.08	9	14	325	35.2	5.16
O88485	Cytoplasmic dynein 1 intermediate chain 1 OS=Mus musculus GN=Dync1i1 PE=1 SV=2 - [DC1I1_MOUSE]	31.37	5	12	13	102	1.629	0.926	1.061	1.036	293.28	31.37	24	102	628	70.7	5.12
Q9ER88	28S ribosomal protein S29, mitochondrial OS=Mus musculus GN=Dap3 PE=2 SV=1 - [RT29_MOUSE]	19.69	9	6	6	21	0.885	0.918	0.986	1.036	69.51	19.69	11	21	391	44.7	8.94
Q9DAI2	Rab-like protein 5 OS=Mus musculus GN=Rabl5 PE=2 SV=1 - [RABL5_MOUSE]	36.22	1	5	5	29	1.472	1.400	0.931	1.036	82.82	36.22	10	29	185	20.8	5.25
E9QAC9	Calcium-independent phospholipase A2-gamma OS=Mus musculus GN=Pnpla8 PE=2 SV=1 - [E9QAC9_MOUSE]	27.71	4	17	17	43	0.825	1.023	0.990	1.036	129.02	27.71	28	43	711	80.2	9.19
P21447	Multidrug resistance protein 1A OS=Mus musculus GN=Abcb1a PE=1 SV=3 -	25.78	3	26	29	109	0.933	1.505	0.962	1.036	298.30	25.78	51	109	1276	140.6	8.85
O70589	Peripheral plasma membrane protein CASK OS=Mus musculus GN=Cask PE=1 SV=2 - [CSKP_MOUSE]	50.32	9	36	40	159	0.722	1.055	0.996	1.036	458.35	50.32	65	159	926	105.0	6.43
Q8K2Q5	Coiled-coil-helix-coiled-coil-helix domain-containing protein 7 OS=Mus musculus GN=Chchd7 PE=2 SV=1 - [CHCH7_MOUSE]	49.41	5	4	4	27	1.621	1.155	1.416	1.036	64.63	49.41	8	27	85	10.1	8.51
P62715	Serine/threonine-protein phosphatase 2A catalytic subunit beta isoform OS=Mus musculus GN=Ppp2cb PE=1 SV=1 - [PP2AB_MOUSE]	66.02	1	2	15	297	1.003	0.988	1.022	1.036	938.81	66.02	30	297	309	35.6	5.43
Q9D142	Uridine diphosphate glucose pyrophosphatase OS=Mus musculus GN=Nudt14 PE=2 SV=1 - [NUD14_MOUSE]	16.22	1	3	3	10	1.499	0.870	0.753	1.036	28.53	16.22	6	10	222	24.4	5.14
A2A4A6	1-phosphatidylinositol 4,5-bisphosphate phosphodiesterase gamma-1 OS=Mus musculus GN=Plcg1 PE=4 SV=1 - [A2A4A6_MOUSE]	25.19	5	28	28	97	0.624	0.870	0.934	1.036	267.34	25.19	53	97	1290	148.4	5.99
P18242	Cathepsin D OS=Mus musculus GN=Ctsd PE=1 SV=1 - [CATD_MOUSE]	43.90	3	15	15	65	1.374	0.989	0.962	1.036	183.81	43.90	27	65	410	44.9	7.15
O88952	Protein lin-7 homolog C OS=Mus musculus GN=Lin7c PE=1 SV=2 - [LIN7C_MOUSE]	56.35	5	5	11	145	1.386	1.062	1.128	1.036	331.63	56.35	19	145	197	21.8	8.43
Q8C754	Vacuolar protein sorting-associated protein 52 homolog OS=Mus musculus GN=Vps52 PE=2 SV=1 - [VPS52_MOUSE]	40.11	3	22	22	87	0.728	1.108	0.982	1.036	265.56	40.11	37	87	723	82.0	5.90

Q64322	Neural proliferation differentiation and control protein 1 OS=Mus musculus GN=Npdc1 PE=2 SV=2 - [NPDC1_MOUSE]	40.06	5	9	10	28	1.039	1.085	0.879	1.036	83.06	40.06	17	28	332	35.7	7.12
D3YUW8	Pogo transposable element with ZNF domain OS=Mus musculus GN=Pogz PE=2 SV=1 - [D3YUW8_MOUSE]	9.74	5	10	11	20	0.760	0.946	1.056	1.036	47.56	9.74	17	20	1314	144.8	7.28
O35245-5	Isoform 5 of Polycystin-2 OS=Mus musculus GN=Pkd2 - [PKD2_MOUSE]	6.19	5	4	4	6	0.940	1.500	0.801	1.037	21.40	6.19	5	6	872	98.0	6.68
Q3UHF7	Transcription factor HIVEP2 OS=Mus musculus GN=Hivep2 PE=1 SV=1 - [ZEP2_MOUSE]	6.71	1	12	12	15	0.979	1.264	1.561	1.037	36.62	6.71	15	15	2430	266.5	6.96
Q80Y81-2	Isoform 2 of Zinc phosphodiesterase ELAC protein 2 OS=Mus musculus GN=Elac2 - [RNZ2_MOUSE]	14.08	4	10	10	19	1.106	0.763	1.009	1.037	60.20	14.08	15	19	824	92.0	7.43
D3YUE4	Protein Fam151b OS=Mus musculus GN=Fam151b PE=4 SV=1 - [D3YUE4_MOUSE]	13.98	1	3	3	6	1.095	1.206	1.263	1.037	11.80	13.98	3	6	279	31.4	6.11
Q8BTJ4-2	Isoform 2 of Bis(5'-adenosyl)-triphosphatase enpp4 OS=Mus musculus GN=Enpp4 - [ENPP4_MOUSE]	24.82	4	7	7	10	1.249	0.974	0.742	1.037	20.15	24.82	10	10	423	47.9	6.73
Q8BRF7-3	Isoform 3 of Sec1 family domain-containing protein 1 OS=Mus musculus GN=Scfd1 - [SCFD1_MOUSE]	45.39	3	20	20	72	0.668	0.803	0.820	1.037	233.15	45.39	33	72	597	67.4	5.74
Q9CR95	Adaptin ear-binding coat-associated protein 1 OS=Mus musculus GN=Necap1 PE=1 SV=2 - [NECP1_MOUSE]	44.00	1	9	9	102	1.350	1.354	1.191	1.037	300.60	44.00	17	102	275	29.6	6.38
P35441	Thrombospondin-1 OS=Mus musculus GN=Thbs1 PE=1 SV=1 - [TSP1_MOUSE]	7.52	2	9	9	22	1.575	2.566	0.714	1.037	62.61	7.52	13	22	1170	129.6	4.96
P70697	Uroporphyrinogen decarboxylase OS=Mus musculus GN=Urod PE=1 SV=2 - [DCUP_MOUSE]	46.05	1	11	11	38	1.234	0.829	0.866	1.037	127.26	46.05	18	38	367	40.7	6.65
Q9Z0P5-2	Isoform 2 of Twinfilin-2 OS=Mus musculus GN=Twf2 - [TWF2_MOUSE]	53.03	4	10	15	57	1.029	1.215	1.073	1.037	155.98	53.03	27	57	347	39.3	6.70
O88622-2	Isoform 2 of Poly(ADP-ribose) glycohydrolase OS=Mus musculus GN=Parg -	11.85	6	9	10	19	0.766	0.872	1.055	1.037	48.93	11.85	12	19	920	103.7	6.80
Q52KH6	Predicted gene, OTTMUSG00000017677 OS=Mus musculus GN=DXBay18 PE=2 SV=1 - [Q52KH6_MOUSE]	3.31	1	1	2	3	3.358	1.344	1.192	1.037	5.87	3.31	2	3	695	77.0	10.13
F8VPT3	Protein Lct OS=Mus musculus GN=Lct PE=3 SV=1 - [F8VPT3_MOUSE]	1.97	1	2	2	2	1.461	1.713	1.343	1.037	2.80	1.97	2	2	1931	217.7	6.74
Q3UN10	Wolframin OS=Mus musculus GN=Wfs1 PE=2 SV=1 - [Q3UN10_MOUSE]	14.99	3	12	12	43	0.706	1.758	1.653	1.037	95.27	14.99	18	43	814	91.8	7.99
Q9CWS0	N(G),N(G)-dimethylarginine dimethylaminohydrolase 1 OS=Mus musculus GN=Ddah1 PE=1 SV=3 - [DDAH1_MOUSE]	77.19	2	18	19	188	1.186	1.039	1.311	1.037	567.14	77.19	37	188	285	31.4	5.97
Q8BNE1-3	Isoform 3 of Protein FAM115A OS=Mus musculus GN=Fam115a - [F115A_MOUSE]	17.67	3	7	7	12	0.820	0.832	0.655	1.037	21.63	17.67	9	12	498	56.6	8.75
Q99J31	Oligophrenin-1 OS=Mus musculus GN=Ophn1 PE=2 SV=1 - [OPHN1_MOUSE]	13.97	3	12	12	29	1.080	1.292	0.963	1.037	68.18	13.97	21	29	802	91.9	7.96
Q8BHZ0	Protein FAM49A OS=Mus musculus GN=Fam49a PE=2 SV=1 - [FA49A_MOUSE]	49.85	1	8	12	84	0.923	1.545	1.330	1.037	254.96	49.85	21	84	323	37.3	6.01
Q8C2E7	WASH complex subunit strumpellin OS=Mus musculus GN=Kiaa0196 PE=2 SV=2 -	29.34	2	29	30	78	0.730	0.992	0.858	1.038	251.56	29.34	53	78	1159	134.0	7.12
Q99LU0	Charged multivesicular body protein 1b-1 OS=Mus musculus GN=Chmp1b1 PE=1 SV=1 - [CH1B1_MOUSE]	30.15	1	3	9	35	1.811	0.742	1.072	1.038	85.15	30.15	15	35	199	22.1	8.10
E9Q2H1	E3 ubiquitin-protein ligase UBR5 OS=Mus musculus GN=Ubr5 PE=2 SV=1 - [E9Q2H1_MOUSE]	4.07	2	8	8	12	1.114	0.955	1.197	1.038	39.91	4.07	10	12	2798	308.8	5.83
O89053	Coronin-1A OS=Mus musculus GN=Coro1a PE=1 SV=5 - [COR1A_MOUSE]	41.87	4	6	19	94	0.882	1.575	1.533	1.038	255.85	41.87	34	94	461	51.0	6.48
Q60737	Casein kinase II subunit alpha OS=Mus musculus GN=Csnk2a1 PE=1 SV=2 - [CSK21_MOUSE]	53.71	3	15	18	140	1.013	0.836	1.096	1.038	388.01	53.71	35	140	391	45.1	7.74

Q00558	Factor VIII intron 22 protein OS=Mus musculus GN=F8a1 PE=2 SV=1 - [F8I2_MOUSE]	24.47	2	5	5	18	0.687	1.175	1.372	1.038	72.38	24.47	10	18	380	40.5	6.89
Q9EQM3	Homeobox protein Nkx-2.4 OS=Mus musculus GN=Nkx2-4 PE=2 SV=2 - [NKX24_MOUSE]	6.21	1	1	1	1	1.137	0.645	1.115	1.038	2.47	6.21	1	1	354	36.2	9.58
Q9QXZ0-4	Isoform 4 of Microtubule-actin cross-linking factor 1 OS=Mus musculus GN=Macf1 - [MACF1_MOUSE]	44.32	7	2	212	896	0.919	0.794	0.944	1.038	2586.69	44.32	377	896	5478	621.8	5.40
E9Q7U2	Calcium-binding and coiled coil domain-containing protein 1 OS=Mus musculus GN=Calcoco1 PE=2 SV=1 - [illegible]	32.70	3	21	21	82	1.412	1.157	1.814	1.038	238.17	32.70	34	82	633	70.9	4.86
A2AIX0	72 kDa inositol polyphosphate 5-phosphatase OS=Mus musculus GN=Inpp5e PE=4 SV=1 - [illegible]	12.73	3	4	7	17	1.451	0.845	1.362	1.038	41.01	12.73	11	17	605	67.2	9.07
Q9EQH3	Vacuolar protein sorting-associated protein 35 OS=Mus musculus GN=Vps35 PE=1 SV=1 - [VPS35_MOUSE]	44.85	1	29	30	159	0.580	1.141	0.879	1.038	468.47	44.85	49	159	796	91.7	5.44
Q9Z2G0	Protein fem-1 homolog B OS=Mus musculus GN=Fem1b PE=1 SV=1 - [FEM1B_MOUSE]	8.29	1	3	4	7	1.543	1.078	0.798	1.038	7.15	8.29	5	7	627	70.2	6.61
Q3TYD4	Arylsulfatase G OS=Mus musculus GN=Arsg PE=2 SV=1 - [ARSG_MOUSE]	12.57	2	6	6	18	3.592	0.440	1.356	1.038	53.70	12.57	12	18	525	57.4	6.39
Q8BGN3	Ectonucleotide pyrophosphatase/phosphodiesterase family member 6 OS=Mus musculus GN=Enpp6 PE=2 SV=1 - [ENPP6_MOUSE]	43.41	3	15	15	79	1.964	0.487	0.900	1.038	235.05	43.41	28	79	440	50.6	7.31
Q9DC07	LIM zinc-binding domain-containing Nebulette OS=Mus musculus GN=Nebl PE=2 SV=1 - [LNEBL_MOUSE]	54.81	1	8	16	127	1.743	1.292	1.551	1.038	362.41	54.81	29	127	270	31.1	8.31
D3YXV8	Leukocyte receptor cluster member 8 homolog (Fragment) OS=Mus musculus GN=Leng8 PE=2 SV=1 - [D3YXV8_MOUSE]	6.17	7	3	3	7	0.964	1.011	0.928	1.039	17.40	6.17	5	7	729	80.7	9.36
Q8BN59	La-related protein 6 OS=Mus musculus GN=Larp6 PE=1 SV=1 - [LARP6_MOUSE]	26.83	1	11	11	31	0.996	0.770	1.236	1.039	81.97	26.83	18	31	492	54.8	7.90
Q5DQR4	Syntaxin-binding protein 5-like OS=Mus musculus GN=Stxbp5l PE=1 SV=1 - [STB5L_MOUSE]	32.41	4	3	29	116	0.686	1.099	1.617	1.039	374.73	32.41	51	116	1185	131.8	6.86
A2AQH1	Ceramide kinase-like isoform 1 OS=Mus musculus GN=Cerkl PE=2 SV=1 - [A2AQH1_MOUSE]	5.33	1	3	3	3	1.660	1.524	0.825	1.039	5.11	5.33	3	3	525	58.7	8.16
E9PV86	Protein Mctp1 OS=Mus musculus GN=Mctp1 PE=2 SV=1 - [E9PV86_MOUSE]	11.67	4	7	7	9	0.638	1.719	2.061	1.039	31.58	11.67	8	9	951	106.7	8.37
Q8C3X4-2	Isoform 2 of Translation factor Guf1, mitochondrial OS=Mus musculus GN=Guf1 - [GUF1_MOUSE]	3.37	4	2	2	4	1.359	1.326	1.127	1.039	11.29	3.37	3	4	563	62.9	8.94
P61971	Nuclear transport factor 2 OS=Mus musculus GN=Nutf2 PE=2 SV=1 - [NTF2_MOUSE]	55.91	1	4	5	41	0.894	0.990	0.831	1.039	134.18	55.91	9	41	127	14.5	5.38
P61358	60S ribosomal protein L27 OS=Mus musculus GN=Rpl27 PE=2 SV=2 - [RL27_MOUSE]	47.06	3	5	6	28	0.533	1.006	0.892	1.039	70.53	47.06	12	28	136	15.8	10.56
O88834	SH2 domain-containing adapter protein D OS=Mus musculus GN=Shd PE=1 SV=1 - [SHD_MOUSE]	34.99	2	8	9	32	1.770	0.659	1.238	1.039	88.07	34.99	16	32	343	38.5	5.05
Q5RJI5	Serine/threonine-protein kinase BRSK1 OS=Mus musculus GN=Brsk1 PE=1 SV=1 - [BRSK1_MOUSE]	43.19	5	22	27	122	0.945	0.793	1.116	1.039	361.63	43.19	46	122	778	85.1	9.32
Q9QXY7	Membrane transport protein XK OS=Mus musculus GN=Xk PE=1 SV=1 - [XK_MOUSE]	4.26	1	1	2	6	1.298	0.885	1.133	1.039	14.69	4.26	4	6	446	51.1	8.19
Q62230	Sialoadhesin OS=Mus musculus GN=Siglec1 PE=1 SV=2 - [SN_MOUSE]	5.55	6	5	5	8	1.496	1.902	1.283	1.039	16.73	5.55	5	8	1695	182.9	6.67
Q5F4S9	Protein Trpm3 OS=Mus musculus GN=Trpm3 PE=2 SV=1 - [Q5F4S9_MOUSE]	17.49	4	1	25	77	0.502	0.793	0.752	1.039	211.45	17.49	46	77	1721	196.2	7.56
A2AJK8	Tetratricopeptide repeat protein 1 OS=Mus musculus GN=Ttc1 PE=2 SV=1 - [A2AJK8_MOUSE]	27.60	2	9	9	37	1.291	0.934	1.110	1.039	92.69	27.60	15	37	250	28.5	4.93

Q61598	Rab GDP dissociation inhibitor beta OS=Mus musculus GN=Gdi2 PE=1 SV=1 - [GDIB_MOUSE]	81.12	2	30	36	495	0.817	0.866	0.901	1.039	1393.38	81.12	66	495	445	50.5	6.25
Q9D011	M-phase-specific PLK1-interacting protein OS=Mus musculus GN=Mplkip PE=2 SV=1 - [MPLKI_MOUSE]	22.47	2	2	2	16	1.329	1.168	1.329	1.039	17.95	22.47	3	16	178	19.0	10.23
P70441	Na(+)/H(+) exchange regulatory cofactor NHE-RF1 OS=Mus musculus GN=Slc9a3r1 PE=1 SV=3 - [NHRF1_MOUSE]	51.83	2	19	19	119	1.381	1.226	1.722	1.039	270.26	51.83	36	119	355	38.6	5.90
Q8BUX1	Protein Klhl4 OS=Mus musculus GN=Klhl4 PE=2 SV=1 - [Q8BUX1_MOUSE]	1.87	4	1	1	3	0.895	0.500	0.790	1.039	10.13	1.87	2	3	589	65.4	6.37
Q9EQX4	Allograft inflammatory factor 1-like OS=Mus musculus GN=Aif1l PE=2 SV=1 - [AIF1L_MOUSE]	29.33	3	3	4	13	2.360	0.646	1.356	1.039	31.08	29.33	6	13	150	17.0	7.18
A2AS93	Caspase OS=Mus musculus GN=Casp9 PE=3 SV=1 - [A2AS93_MOUSE]	12.59	3	3	3	5	1.024	1.023	1.032	1.039	7.40	12.59	5	5	405	44.3	5.83
Q9ET22	Dipeptidyl peptidase 2 OS=Mus musculus GN=Dpp7 PE=2 SV=2 - [DPP2_MOUSE]	10.67	1	5	5	9	0.847	1.019	0.802	1.039	26.99	10.67	9	9	506	56.2	5.39
Q91WD0-2	Isoform 2 of Protein GPR108 OS=Mus musculus GN=Gpr108 - [GP108_MOUSE]	4.27	2	1	1	1	1.555	1.145	0.986	1.040	0.00	4.27	1	1	562	63.2	7.55
P51125-3	Isoform 3 of Calpastatin OS=Mus musculus GN=Cast - [ICAL_MOUSE]	37.30	8	18	18	72	2.382	0.968	1.224	1.040	216.91	37.30	32	72	740	79.6	5.54
Q920R0	Alsin OS=Mus musculus GN=Als2 PE=1 SV=3 - [ALS2_MOUSE]	15.20	2	16	18	46	0.784	0.940	1.093	1.040	133.12	15.20	32	46	1651	182.5	6.30
Q9D2D9	Kelch domain-containing protein 8B OS=Mus musculus GN=Klhdc8b PE=2 SV=1 -	14.41	2	3	4	5	0.939	1.490	0.916	1.040	8.88	14.41	5	5	354	37.6	8.09
P50518	V-type proton ATPase subunit E 1 OS=Mus musculus GN=Atp6v1e1 PE=1 SV=2 -	64.60	2	16	16	537	1.009	1.565	1.088	1.040	1247.01	64.60	29	537	226	26.1	8.43
Q60603	Potassium voltage-gated channel subfamily H member 1 OS=Mus musculus GN=Kcnh1 PE=1 SV=1 - [KCNH1_MOUSE]	3.94	2	3	4	12	1.717	1.221	1.311	1.040	25.86	3.94	6	12	989	111.2	7.47
Q9JLB0-2	Isoform Alpha of MAGUK p55 subfamily member 6 OS=Mus musculus GN=Mpp6 - [MPP6_MOUSE]	56.03	2	1	30	138	1.553	1.462	2.689	1.040	392.18	56.03	55	138	539	60.9	6.18
Q6ZQ89-3	Isoform 3 of E3 ubiquitin-protein ligase MARCH6 OS=Mus musculus GN=March6 -	6.00	3	3	3	5	0.958	1.005	0.908	1.040	9.86	6.00	4	5	883	99.2	6.07
P62075	Mitochondrial import inner membrane translocase subunit Tim13 OS=Mus musculus GN=Timm13 PE=1 SV=1 - [TIM13_MOUSE]	61.05	1	5	5	54	1.914	0.720	1.331	1.040	119.85	61.05	10	54	95	10.5	8.18
Q9Z0Y1	Dynactin subunit 3 OS=Mus musculus GN=Dctn3 PE=2 SV=2 - [DCTN3_MOUSE]	55.38	4	12	12	43	0.924	1.229	0.932	1.040	94.16	55.38	20	43	186	21.0	6.06
P62918	60S ribosomal protein L8 OS=Mus musculus GN=Rpl8 PE=2 SV=2 - [RL8_MOUSE]	50.97	1	13	13	78	0.669	0.879	0.863	1.040	188.65	50.97	21	78	257	28.0	11.03
Q8BJN4	Fibronectin type III domain-containing protein 9 OS=Mus musculus GN=Fndc9 PE=2 SV=1 - [FNDC9_MOUSE]	25.22	1	4	4	8	1.607	1.021	0.778	1.040	22.14	25.22	6	8	226	25.6	5.90
Q8VHI6	Wiskott-Aldrich syndrome protein family member 3 OS=Mus musculus GN=Wasf3 PE=2 SV=1 - [WASF3_MOUSE]	37.72	1	10	13	83	1.411	1.016	1.267	1.040	247.63	37.72	22	83	501	55.2	6.37
H3BK68	tRNA (guanine(26)-N(2))-dimethyltransferase (Fragment) OS=Mus musculus GN=Trmt1 PE=2 SV=1 - [H3BK68_MOUSE]	25.34	10	3	4	4	1.204	0.917	0.908	1.041	11.83	25.34	4	4	221	24.1	5.66
Q8BY87-2	Isoform 2 of Ubiquitin carboxyl-terminal hydrolase 47 OS=Mus musculus GN=Usp47 - [UBP47_MOUSE]	20.94	2	24	24	61	0.919	1.090	0.954	1.041	178.63	20.94	41	61	1356	154.7	5.08
Q3TDN2-2	Isoform 2 of FAS-associated factor 2 OS=Mus musculus GN=Faf2 - [FAF2_MOUSE]	33.33	5	10	10	44	0.769	0.690	0.872	1.041	133.88	33.33	18	44	426	50.2	5.27
E9Q421	GPI-anchor transamidase OS=Mus musculus GN=Pigk PE=2 SV=1 - [E9Q421_MOUSE]	9.12	4	4	4	9	0.713	0.915	0.859	1.041	21.20	9.12	6	9	362	40.8	5.80
Q6ZQ73	Cullin-associated NEDD8-dissociated protein 2 OS=Mus musculus GN=Cand2 PE=1 SV=2 - [CAND2_MOUSE]	5.10	1	4	6	13	0.863	1.113	0.817	1.041	37.50	5.10	10	13	1235	135.5	5.58

Q8JZV7	Putative N-acetylglucosamine-6-phosphate deacetylase OS=Mus musculus GN=Amdhd2 PE=2 SV=1 - [illegible]	38.39	2	10	10	32	1.614	1.141	0.960	1.041	98.77	38.39	16	32	409	43.5	6.23
Q9R1S8	Calpain-7 OS=Mus musculus GN=Capn7 PE=2 SV=1 - [CAN7_MOUSE]	4.43	2	3	3	12	2.462	1.049	1.009	1.041	20.95	4.43	6	12	813	92.5	7.97
Q99MJ9	ATP-dependent RNA helicase DDX50 OS=Mus musculus GN=Ddx50 PE=2 SV=1 - [DDX50_MOUSE]	7.49	1	3	5	7	0.537	0.793	0.965	1.041	13.33	7.49	7	7	734	82.1	9.25
P97445	Voltage-dependent P/Q-type calcium channel subunit alpha-1A OS=Mus musculus GN=Cacna1a PE=1 SV=2 - [illegible]	18.54	2	28	33	103	0.608	0.863	1.276	1.041	303.72	18.54	51	103	2368	267.5	8.85
F8WIA8	MAGE-like protein 2 OS=Mus musculus GN=Magel2 PE=2 SV=1 - [F8WIA8_MOUSE]	1.95	2	2	2	2	1.056	0.861	0.613	1.041	6.45	1.95	2	2	1284	137.9	7.47
P30873	Somatostatin receptor type 1 OS=Mus musculus GN=Sstr1 PE=2 SV=1 - [SSR1_MOUSE]	4.86	1	1	1	1	0.606	1.252	1.357	1.041	4.54	4.86	1	1	391	42.7	8.28
P50153	Guanine nucleotide-binding protein G(I)/G(S)/G(O) subunit gamma-4 OS=Mus musculus GN=Gng4 PE=2 [illegible]	88.00	1	7	8	187	0.996	1.222	1.069	1.041	540.26	88.00	14	187	75	8.4	7.08
Q8BXZ1	Protein disulfide-isomerase TMX3 OS=Mus musculus GN=Tmx3 PE=1 SV=2 - [TMX3_MOUSE]	31.14	1	13	14	41	0.779	1.024	0.846	1.041	121.25	31.14	23	41	456	51.8	5.16
Q9WVK8	Cholesterol 24-hydroxylase OS=Mus musculus GN=Cyp46a1 PE=2 SV=1 - [CP46A_MOUSE]	43.20	1	22	22	75	0.612	1.497	1.180	1.041	198.00	43.20	36	75	500	56.8	8.82
Q9CQU8	Mitochondrial inner membrane protease subunit 1 OS=Mus musculus GN=Immp1l PE=2 SV=1 - [illegible]	11.45	1	1	2	4	0.460	0.633	0.993	1.041	9.75	11.45	3	4	166	18.5	8.16
Q9D9H8	UPF0565 protein C2orf69 homolog OS=Mus musculus PE=2 SV=3 - [CB069_MOUSE]	9.32	1	3	3	9	0.890	1.063	1.023	1.041	33.51	9.32	6	9	365	41.7	7.87
E9QNX9	Tyrosine-protein kinase receptor OS=Mus musculus GN=Igf1r PE=2 SV=1 - [E9QNX9_MOUSE]	9.13	2	8	9	19	1.138	1.434	1.133	1.041	34.64	9.13	12	19	1369	155.2	5.83
O08908	Phosphatidylinositol 3-kinase regulatory subunit beta OS=Mus musculus GN=Pik3r2 PE=1 SV=2 - [P85B_MOUSE]	22.71	3	10	13	32	0.916	1.067	1.228	1.041	82.54	22.71	20	32	722	81.2	6.07
Q91VV4-2	Isoform 2 of DENN domain containing protein 2D OS=Mus musculus GN=Dennd2d - [DEN2D_MOUSE]	8.80	2	2	2	2	1.607	1.659	1.292	1.041	0.00	8.80	2	2	466	52.9	7.97
Q80XP9-2	Isoform 2 of Serine/threonine-protein kinase WNK3 OS=Mus musculus GN=Wnk3 - [WNK3_MOUSE]	20.18	3	18	25	96	1.051	0.867	1.047	1.042	272.05	20.18	41	96	1710	188.3	5.62
B2RUJ5	Amyloid beta A4 precursor protein-binding family A member 1 OS=Mus musculus GN=Apba1 PE=2 SV=2 - [APBA1_MOUSE]	26.37	2	13	16	117	1.494	0.948	1.190	1.042	307.80	26.37	30	117	842	92.9	4.88
Q9CQA3	Succinate dehydrogenase [ubiquinone] iron-sulfur subunit, mitochondrial OS=Mus musculus GN=Sdhb PE=1 SV=1 - [DHSB_MOUSE]	50.35	2	17	17	102	1.254	0.876	0.869	1.042	269.90	50.35	28	102	282	31.8	8.68
Q9QUK9	MCG15083 OS=Mus musculus GN=Try5 PE=2 SV=1 - [Q9QUK9_MOUSE]	12.20	3	1	2	114	2.005	1.064	1.072	1.042	248.17	12.20	4	114	246	26.3	5.30
Q9JJF3	Bifunctional lysine-specific demethylase and histidyl-hydroxylase NO66 OS=Mus musculus GN=No66 PE=1 SV=2 - [NO66_MOUSE]	10.28	1	6	8	11	0.908	1.489	1.120	1.042	20.20	10.28	10	11	603	67.5	8.66
P59528	Taste receptor type 2 member 123 OS=Mus musculus GN=Tas2r123 PE=2 SV=2 -	3.30	1	1	1	1	1.296	2.155	1.431	1.042	1.92	3.30	1	1	333	38.0	9.64
Q7TPD1-2	Isoform 2 of F-box only protein 11 OS=Mus musculus GN=Fbxo11 - [FBX11_MOUSE]	2.61	4	2	2	4	1.446	0.825	1.188	1.042	9.12	2.61	3	4	842	94.0	6.90
Q9D2N9	Vacuolar protein sorting-associated protein 33A OS=Mus musculus GN=Vps33a PE=1 SV=2 - [VP33A_MOUSE]	39.30	1	19	19	59	0.613	0.857	0.843	1.042	170.90	39.30	35	59	598	67.5	7.08
P59672	Ankyrin repeat and SAM domain-containing protein 1A OS=Mus musculus GN=Anks1a PE=1 SV=3 - [ANS1A_MOUSE]	10.52	3	10	12	25	1.181	0.943	0.983	1.042	68.26	10.52	18	25	1150	125.2	6.05

A2AJK6-3	Isoform 3 of Chromodomain-helicase-DNA-binding protein 7 OS=Mus musculus GN=Chd7 - [illegible]	1.96	3	2	6	10	0.798	1.281	1.006	1.042	25.44	1.96	9	10	2960	331.0	6.57
Q14AI0	Sister chromatid cohesion protein DCC1 OS=Mus musculus GN=DSCC1 PE=2 SV=1 - [DCC1_MOUSE]	6.02	2	1	1	1	0.704	1.136	1.033	1.042	0.00	6.02	1	1	399	45.5	4.91
E9PYQ9	Solute carrier family 15 member 2 OS=Mus musculus GN=Slc15a2 PE=2 SV=1 -	8.04	6	4	4	10	1.173	1.108	1.037	1.042	27.74	8.04	7	10	709	79.6	6.79
D3Z074	Protein diaphanous homolog 1 OS=Mus musculus GN=Diap1 PE=2 SV=2 - [D3Z074_MOUSE]	22.95	5	22	22	47	0.778	1.260	1.026	1.042	132.05	22.95	33	47	1220	135.7	5.34
Q9CRB0	Sorting nexin-24 OS=Mus musculus GN=Snx24 PE=2 SV=1 - [SNX24_MOUSE]	9.47	1	2	2	3	0.547	0.696	0.910	1.042	9.09	9.47	3	3	169	19.6	7.01
Q921H8	3-ketoacyl-CoA thiolase A, peroxisomal OS=Mus musculus GN=Acaa1a PE=2 SV=1 - [THIKA_MOUSE]	58.25	6	10	19	111	0.958	0.892	0.817	1.042	355.23	58.25	30	111	424	43.9	8.44
B7ZC21	cAMP-dependent protein kinase inhibitor gamma (Fragment) OS=Mus musculus GN=Pkig PE=2 SV=1 - [B7ZC21_MOUSE]	30.36	3	2	2	6	1.946	1.634	2.010	1.042	20.71	30.36	4	6	56	5.9	4.28
P48377	MHC class II regulatory factor RFX1 OS=Mus musculus GN=Rfx1 PE=2 SV=2 - [RFX1_MOUSE]	6.54	1	4	4	11	2.407	1.705	1.188	1.042	32.31	6.54	6	11	963	103.6	6.34
P59158	Solute carrier family 12 member 3 OS=Mus musculus GN=Slc12a3 PE=1 SV=1 -	1.40	1	1	1	1	1.523	1.325	0.900	1.042	2.55	1.40	1	1	1002	110.6	7.56
Q9WUH2-2	Isoform 2 of LIM/homeobox protein Lhx9 OS=Mus musculus GN=Lhx9 -	4.98	2	1	1	1	0.555	1.100	0.880	1.042	0.00	4.98	1	1	321	36.1	9.01
Q6QI06	Rapamycin-insensitive companion of mTOR OS=Mus musculus GN=Rictor PE=1 SV=2 - [RICTR_MOUSE]	10.54	2	12	12	23	0.959	1.045	0.920	1.042	60.26	10.54	20	23	1708	191.4	7.23
Q8K0D0-2	Isoform 2 of Cyclin-dependent kinase 17 OS=Mus musculus GN=Cdk17 -	37.44	18	7	15	65	1.329	1.298	1.224	1.042	162.42	37.44	25	65	430	48.9	8.06
F6ZBR8	Protein Wiz (Fragment) OS=Mus musculus GN=Wiz PE=4 SV=1 - [F6ZBR8_MOUSE]	3.80	4	4	4	6	1.424	0.954	1.019	1.043	9.57	3.80	6	6	973	104.5	9.41
P00375	Dihydrofolate reductase OS=Mus musculus GN=Dhfr PE=1 SV=3 - [DYR_MOUSE]	12.30	1	1	2	6	0.765	1.261	0.899	1.043	11.16	12.30	4	6	187	21.6	8.53
Q3UUF8	Ankyrin repeat domain-containing protein 34B OS=Mus musculus GN=Ankrd34b PE=1 SV=1 - [AN34B_MOUSE]	28.74	1	12	12	33	0.847	0.544	1.035	1.043	92.60	28.74	19	33	508	55.4	8.02
Q62311	Transcription initiation factor TFIID subunit 6 OS=Mus musculus GN=Taf6 PE=2 SV=1 - [TAF6_MOUSE]	5.75	3	4	4	11	1.675	1.290	1.558	1.043	23.75	5.75	6	11	678	72.6	8.60
Q3TPX4	Exocyst complex component 5 OS=Mus musculus GN=Exoc5 PE=1 SV=2 - [EXOC5_MOUSE]	37.29	3	25	25	73	0.697	1.404	1.073	1.043	220.72	37.29	42	73	708	81.7	6.71
Q78Y63-2	Isoform 2 of Phosducin-like protein 2 OS=Mus musculus GN=Pdcl2 - [PDCL2_MOUSE]	16.67	2	2	2	3	1.247	1.341	1.350	1.043	5.66	16.67	2	3	192	22.0	5.24
Q8C605	6-phosphofructokinase OS=Mus musculus GN=Pfkp PE=2 SV=1 - [Q8C605_MOUSE]	54.46	5	32	36	289	1.560	1.110	0.984	1.043	927.18	54.46	65	289	784	85.5	6.89
A2BDX3	Adenylyltransferase and sulfurtransferase MOCS3 OS=Mus musculus GN=Mocs3 PE=2 SV=1 - [MOCS3_MOUSE]	27.61	1	10	11	28	0.871	0.970	0.949	1.043	98.56	27.61	17	28	460	49.3	7.49
Q8K304-2	Isoform 2 of Transmembrane protein 129 OS=Mus musculus GN=Tmem129 -	15.46	2	3	3	5	0.793	0.907	0.889	1.043	14.97	15.46	5	5	291	32.7	7.66
G5E850	Cytochrome b-5, isoform CRA_a OS=Mus musculus GN=Cyb5 PE=3 SV=1 - [G5E850_MOUSE]	40.82	3	4	4	42	1.151	0.995	0.871	1.043	132.62	40.82	7	42	98	11.1	5.30
Q2WF71	Leucine-rich repeat and fibronectin type III domain containing protein 1 OS=Mus musculus GN=Lrfn1 PE=1 SV=1 - [illegible]	15.67	3	7	9	19	0.562	0.944	0.933	1.043	71.34	15.67	15	19	766	81.9	7.59
Q80U12	Small G protein signaling modulator 2 OS=Mus musculus GN=Sgsm2 PE=2 SV=2 -	11.84	3	6	8	26	2.622	1.345	0.868	1.043	61.25	11.84	13	26	1005	113.0	6.60
B1AXV0	DOMON domain-containing protein FRRS1L OS=Mus musculus GN=Frrs1l PE=1 SV=1 - [FRS1L_MOUSE]	23.21	2	5	5	15	0.420	1.320	1.237	1.043	41.90	23.21	9	15	293	32.5	5.43

Q8CJ96	Ras association domain-containing protein 8 OS=Mus musculus GN=Rassf8 PE=1 SV=1 - [RASF8_MOUSE]	10.02	1	4	4	6	2.128	0.994	1.021	1.043	14.71	10.02	6	6	419	48.1	5.95
Q9CZR8	Elongation factor Ts, mitochondrial OS=Mus musculus GN=Tsfm PE=2 SV=1 - [EFTS_MOUSE]	43.52	3	10	10	48	1.111	1.203	0.958	1.043	167.82	43.52	19	48	324	35.3	7.06
P40630-2	Isoform Nuclear of Transcription factor A, mitochondrial OS=Mus musculus GN=Tfam - [TFAM_MOUSE]	33.02	3	8	8	25	1.091	0.819	1.252	1.044	59.04	33.02	13	25	215	25.0	9.66
Q9JID9	SH2B adapter protein 2 OS=Mus musculus GN=Sh2b2 PE=1 SV=2 - [SH2B2_MOUSE]	2.58	1	1	1	1	4.784	1.707	1.038	1.044	4.01	2.58	1	1	621	66.5	6.35
A2A864	Integrin beta OS=Mus musculus GN=Itgb4 PE=3 SV=1 - [A2A864_MOUSE]	1.66	4	3	3	4	3.661	0.751	0.729	1.044	7.28	1.66	4	4	1802	199.7	6.02
P21836	Acetylcholinesterase OS=Mus musculus GN=Ache PE=1 SV=1 - [ACES_MOUSE]	18.73	1	8	8	26	1.443	0.715	1.276	1.044	80.87	18.73	14	26	614	68.1	6.33
Q2PFD7-5	Isoform 5 of PH and SEC7 domain-containing protein 3 OS=Mus musculus GN=Psd3 - [PSD3_MOUSE]	37.55	6	15	31	165	1.063	0.945	1.326	1.044	437.55	37.55	54	165	1004	111.1	6.25
O88737	Protein bassoon OS=Mus musculus GN=Bsn PE=1 SV=4 - [BSN_MOUSE]	45.38	2	120	122	887	0.941	1.104	2.137	1.044	2704.82	45.38	221	887	3942	418.6	7.71
Q8VHJ5	Serine/threonine-protein kinase MARK1 OS=Mus musculus GN=Mark1 PE=1 SV=2 - [MARK1_MOUSE]	43.40	1	25	36	152	0.782	0.815	1.124	1.044	427.05	43.40	59	152	795	88.3	9.39
F8VQF9	Trafficking protein particle complex subunit 10 OS=Mus musculus GN=Trappc10 PE=4 SV=1 [F8VQF9_MOUSE]	19.79	2	20	20	42	0.802	1.095	0.998	1.044	118.21	19.79	31	42	1258	141.4	5.92
H3BLL3	Leucine-rich repeat and calponin homology domain containing protein 4 OS=Mus musculus GN=Lrch4 PE=2 SV=1 - [H3BLL3_MOUSE]	16.80	6	6	6	9	1.413	1.478	0.798	1.044	19.24	16.80	8	9	649	69.9	6.89
O88958	Glucosamine-6-phosphate isomerase 1 OS=Mus musculus GN=Gnpda1 PE=2 SV=3 - [GNPI1_MOUSE]	39.45	2	7	10	54	1.492	1.069	0.800	1.044	163.36	39.45	19	54	289	32.5	6.60
P51569	Alpha-galactosidase A OS=Mus musculus GN=Gla PE=1 SV=1 - [AGAL_MOUSE]	4.30	2	2	2	4	0.747	1.036	0.969	1.044	12.81	4.30	3	4	419	47.6	5.72
P0C0A3	Charged multivesicular body protein 6 OS=Mus musculus GN=Chmp6 PE=2 SV=2 -	19.50	2	4	6	26	0.872	0.733	1.133	1.044	69.80	19.50	11	26	200	23.4	5.44
Q7TRG4	Olfactory receptor 843 OS=Mus musculus GN=Olfr843 PE=2 SV=1 - [Q7TRG4_MOUSE]	2.88	1	1	1	1	1.937	1.601	1.312	1.044	2.79	2.88	1	1	313	35.1	7.84
Q5DU28	Pecanex-like protein 2 OS=Mus musculus GN=Pcnxl2 PE=2 SV=2 - [PCX2_MOUSE]	2.36	6	3	3	3	3.374	1.112	1.202	1.045	6.50	2.36	3	3	2122	234.0	6.95
Q91Z31	Polypyrimidine tract-binding protein 2 OS=Mus musculus GN=Ptbp2 PE=1 SV=2 - [PTBP2_MOUSE]	46.33	3	13	15	54	0.684	1.032	0.924	1.045	152.18	46.33	21	54	531	57.5	8.66
Q5SYL3	UPF0378 protein KIAA0100 OS=Mus musculus GN=Kiaa0100 PE=2 SV=1 -	4.97	1	9	9	20	0.794	0.712	0.984	1.045	54.50	4.97	17	20	2234	254.3	7.34
Q8VCS3	Glycosaminoglycan xylosylkinase OS=Mus musculus GN=Fam20b PE=2 SV=1 -	14.67	1	5	5	13	1.212	1.027	0.740	1.045	38.67	14.67	9	13	409	46.6	6.95
P61793-2	Isoform 2 of Lysophosphatidic acid receptor 1 OS=Mus musculus GN=Lpar1 -	8.67	2	2	2	5	1.353	0.661	1.350	1.045	14.31	8.67	3	5	346	39.3	8.53
Q8BGI5-2	Isoform 2 of Peroxisome assembly protein 26 OS=Mus musculus GN=Pex26 -	11.51	4	4	4	10	0.621	0.946	0.868	1.045	23.60	11.51	7	10	304	33.9	6.80
E9PVP1	Aurora kinase B OS=Mus musculus GN=Aim1 PE=2 SV=1 - [E9PVP1_MOUSE]	1.24	1	2	3	3	1.506	0.810	1.003	1.045	6.14	1.24	3	3	1691	184.6	6.01
Q76LS9-2	Isoform 2 of Protein FAM63A OS=Mus musculus GN=Fam63a - [FA63A_MOUSE]	17.86	3	5	6	14	0.826	0.680	0.978	1.045	40.68	17.86	11	14	459	50.2	4.74
Q6ZPQ6-2	Isoform 2 of Membrane-associated phosphatidylinositol transfer protein 2 OS=Mus musculus GN=Pitpnm2 - [illegible]	18.11	4	23	24	64	0.581	1.913	1.687	1.045	146.61	18.11	40	64	1281	142.2	7.71
Q99PA7	MCG117379 OS=Mus musculus GN=4930550L24Rik PE=2 SV=1 - [Q99PA7_MOUSE]	7.50	1	1	1	1	0.835	0.842	1.713	1.045	3.09	7.50	1	1	320	35.0	5.14

P63046	Sulfotransferase 4A1 OS=Mus musculus GN=Sult4a1 PE=2 SV=1 - [ST4A1_MOUSE]	34.86	2	10	10	44	0.806	0.754	0.893	1.045	99.08	34.86	19	44	284	33.0	5.53
Q8R323	Replication factor C subunit 3 OS=Mus musculus GN=Rfc3 PE=2 SV=1 - [RFC3_MOUSE]	3.93	1	1	1	2	0.584	1.025	0.960	1.045	10.49	3.93	2	2	356	40.5	8.38
P97868-2	Isoform 2 of E3 ubiquitin-protein ligase RBBP6 OS=Mus musculus GN=Rbbp6 -	6.15	4	11	11	18	2.799	1.201	1.238	1.045	39.41	6.15	16	18	1756	195.2	9.61
Q3THK7	GMP synthase [glutamine-hydrolyzing] OS=Mus musculus GN=Gmps PE=1 SV=2 - [GUAA_MOUSE]	56.28	1	33	33	131	0.714	0.988	0.924	1.045	424.85	56.28	58	131	693	76.7	6.73
Q9D1Q4	Dolichol-phosphate mannosyltransferase subunit 3 OS=Mus musculus GN=Dpm3 PE=3 SV=1 - [DPM3_MOUSE]	23.91	1	2	2	8	1.063	0.805	0.863	1.046	21.21	23.91	4	8	92	10.1	7.08
Q8K2V6	Importin-11 OS=Mus musculus GN=Ipo11 PE=1 SV=1 - [IPO11_MOUSE]	8.31	2	6	6	11	0.900	1.168	0.976	1.046	32.26	8.31	9	11	975	112.3	5.26
A2A9C3-2	Isoform 2 of Protein SZT2 OS=Mus musculus GN=Szt2 - [SZT2_MOUSE]	3.15	2	7	7	11	1.955	1.181	1.133	1.046	23.71	3.15	10	11	3430	377.3	6.19
P80316	T-complex protein 1 subunit epsilon OS=Mus musculus GN=Cct5 PE=1 SV=1 - [TCPE_MOUSE]	61.92	2	33	34	230	0.827	1.114	0.941	1.046	682.63	61.92	64	230	541	59.6	6.02
Q9D5T0	ATPase family AAA domain-containing protein 1 OS=Mus musculus GN=Atad1 PE=1 SV=1 - [ATAD1_MOUSE]	34.90	1	12	12	37	0.813	0.826	0.962	1.046	107.37	34.90	20	37	361	40.7	6.90
O55022	Membrane-associated progesterone receptor component 1 OS=Mus musculus GN=Pgrmc1 PE=1 SV=4 - [illegible]	44.62	1	8	10	115	0.750	1.696	0.832	1.046	346.91	44.62	17	115	195	21.7	4.70
Q6PR54-2	Isoform 2 of Telomere-associated protein RIF1 OS=Mus musculus GN=Rif1 - [RIF1_MOUSE]	1.67	3	3	3	3	5.713	1.432	1.326	1.046	7.97	1.67	3	3	2393	263.3	5.48
F7AX87	Low-density lipoprotein receptor-related protein 11 (Fragment) OS=Mus musculus GN=Lrp11 PE=2 SV=1 - [F7AX87_MOUSE]	25.31	4	4	5	15	0.911	0.953	0.902	1.046	35.83	25.31	7	15	245	26.3	8.98
Q7TMW6	Cytosolic Fe-S cluster assembly factor NARFL OS=Mus musculus GN=Narfl PE=2 SV=2 - [NARFL_MOUSE]	30.25	4	9	9	22	0.812	0.980	0.992	1.046	71.19	30.25	14	22	476	53.1	6.48
Q9JJY4	Probable ATP-dependent RNA helicase DDX20 OS=Mus musculus GN=Ddx20 PE=1 SV=2 - [DDX20_MOUSE]	10.79	1	6	6	13	0.684	0.850	1.212	1.046	26.75	10.79	11	13	825	91.7	6.74
A2RSQ0	DENN domain-containing protein 5B OS=Mus musculus GN=Dennd5b PE=1 SV=2 -	8.32	1	6	10	34	0.907	0.839	0.910	1.047	84.30	8.32	19	34	1274	144.5	6.68
A2A9T0-2	Isoform 2 of TANK-binding kinase 1-binding protein 1 OS=Mus musculus GN=Tbkbp1 - [TBKB1_MOUSE]	7.55	7	4	5	12	0.726	1.425	0.829	1.047	29.55	7.55	7	12	596	65.4	5.62
Q8BJ03-2	Isoform 2 of Cytochrome c oxidase assembly protein COX15 homolog OS=Mus musculus GN=Cox15 - [COX15_MOUSE]	15.40	2	5	5	10	1.207	1.222	0.973	1.047	17.62	15.40	8	10	396	44.0	9.70
Q80VE5	Protein Tbc1d22b OS=Mus musculus GN=Tbc1d22b PE=2 SV=1 - [Q80VE5_MOUSE]	24.75	1	9	10	22	0.769	0.585	0.862	1.047	65.44	24.75	16	22	505	59.1	7.43
E9Q9A9	2'-5'-oligoadenylate synthase 2 OS=Mus musculus GN=Oas2 PE=2 SV=1 - [OAS2_MOUSE]	2.29	2	1	1	1	1.463	0.726	0.690	1.047	0.00	2.29	1	1	742	85.0	6.79
F6VFS9	Guanylate cyclase OS=Mus musculus GN=Gucy2d PE=2 SV=1 - [F6VFS9_MOUSE]	1.70	1	1	1	1	4.017	1.398	1.155	1.047	2.98	1.70	1	1	1116	122.3	6.60
P70266-2	Isoform 2 of 6-phosphofructo-2-kinase/fructose-2,6-bisphosphatase 1 OS=Mus musculus GN=Pfkfb1 - [illegible]	4.69	2	1	2	6	1.103	0.397	0.671	1.047	11.56	4.69	3	6	448	52.1	6.38
P09528	Ferritin heavy chain OS=Mus musculus GN=Fth1 PE=1 SV=2 - [FRIH_MOUSE]	50.00	1	9	9	56	1.796	1.039	1.293	1.047	173.36	50.00	14	56	182	21.1	5.88
Q8C650-2	Isoform 2 of Septin-10 OS=Mus musculus GN=Sept10 - [SEP10_MOUSE]	18.27	3	3	7	56	1.959	0.938	0.791	1.047	103.23	18.27	12	56	427	49.8	6.71
Q3UWE6	MCG14935, isoform CRA_a OS=Mus musculus GN=Wdr20a PE=2 SV=1 - [Q3UWE6_MOUSE]	23.73	2	10	11	36	1.464	0.968	1.075	1.047	86.31	23.73	16	36	569	62.8	8.00
Q8VEB1	G protein-coupled receptor kinase 5 OS=Mus musculus GN=Grk5 PE=1 SV=2 - [GRK5_MOUSE]	3.90	1	1	2	7	1.625	1.223	2.095	1.047	13.77	3.90	3	7	590	67.7	8.19

P97429	Annexin A4 OS=Mus musculus GN=Anxa4 PE=2 SV=4 - [ANXA4_MOUSE]	39.81	3	10	11	37	1.322	2.500	0.870	1.047	112.39	39.81	18	37	319	35.9	5.57
Q9WTP2	Protein sprouty homolog 4 OS=Mus musculus GN=Spry4 PE=2 SV=1 - [SPY4_MOUSE]	12.67	1	2	2	3	1.908	0.994	1.071	1.047	10.85	12.67	3	3	300	32.5	7.88
Q9JMA1	Ubiquitin carboxyl-terminal hydrolase 14 OS=Mus musculus GN=Usp14 PE=1 SV=3 - [UBP14_MOUSE]	45.03	3	21	21	118	0.982	1.233	0.918	1.047	341.61	45.03	37	118	493	56.0	5.24
E9Q3A5	Protein Vmn2r105 OS=Mus musculus GN=Vmn2r105 PE=3 SV=1 - [E9Q3A5_MOUSE]	2.44	1	2	2	2	1.086	0.685	1.201	1.047	5.75	2.44	2	2	860	98.7	7.59
O88587-2	Isoform Soluble of Catechol O-methyltransferase OS=Mus musculus GN=Comt - [COMT_MOUSE]	56.31	3	11	11	60	0.799	1.111	1.032	1.047	139.39	56.31	19	60	222	24.7	5.47
Q811S7	Upstream-binding protein 1 OS=Mus musculus GN=Ubp1 PE=1 SV=1 - [UBIP1_MOUSE]	19.63	2	7	8	21	1.008	1.109	1.094	1.047	64.45	19.63	14	21	540	60.2	6.27
B1ATD5	MCG9626, isoform CRA_b OS=Mus musculus GN=Mtmr3 PE=4 SV=1 - [B1ATD5_MOUSE]	12.17	8	11	12	28	1.299	1.327	1.071	1.047	77.23	12.17	17	28	1159	129.6	6.07
D3Z2J6	Thioredoxin-related transmembrane protein 2 OS=Mus musculus GN=Tmx2 PE=2 SV=1 - [D3Z2J6_MOUSE]	28.02	3	7	7	24	0.686	0.636	0.849	1.047	71.56	28.02	12	24	257	29.6	8.72
Q8BK64	Activator of 90 kDa heat shock protein ATPase homolog 1 OS=Mus musculus GN=Ahsa1 PE=2 SV=2 - [AHSA1_MOUSE]	65.98	1	18	19	179	0.972	0.900	0.902	1.047	551.68	65.98	32	179	338	38.1	5.53
Q9CQA1	Trafficking protein particle complex subunit 5 OS=Mus musculus GN=Trappc5 PE=1 SV=1 - [TPPC5_MOUSE]	28.19	1	6	6	24	0.554	0.972	0.856	1.047	66.95	28.19	12	24	188	20.8	9.66
Q7TPG7	Protein FAM19A2 OS=Mus musculus GN=Fam19a2 PE=2 SV=1 - [F19A2_MOUSE]	10.69	1	1	1	2	0.815	1.731	2.176	1.047	5.85	10.69	1	2	131	14.6	9.17
Q76I24	Protein Mettl7a2 OS=Mus musculus GN=Mettl7a2 PE=2 SV=1 - [Q76I24_MOUSE]	23.77	5	1	6	7	2.522	1.549	0.171	1.047	18.97	23.77	7	7	244	28.2	8.82
A3KMP2	Tetratricopeptide repeat protein 38 OS=Mus musculus GN=Ttc38 PE=2 SV=2 - [TTC38_MOUSE]	19.57	3	7	7	22	1.328	1.195	0.661	1.048	73.85	19.57	13	22	465	52.2	6.30
B2RW11	Ankyrin repeat domain 34A OS=Mus musculus GN=Ankrd34a PE=2 SV=1 [B2RW11_MOUSE]	38.99	1	14	14	65	0.966	0.986	1.500	1.048	231.52	38.99	26	65	495	52.4	9.45
Q80U95	Ubiquitin-protein ligase E3C OS=Mus musculus GN=Ube3c PE=2 SV=2 - [UBE3C_MOUSE]	24.19	1	19	21	57	0.662	0.865	0.918	1.048	167.91	24.19	35	57	1083	123.9	6.39
Q6QWF9	Calcium/calmodulin-dependent protein kinase II inhibitor 1 OS=Mus musculus GN=Camk2n1 PE=1 SV=1 - [illegible]	57.69	1	2	5	31	1.191	1.674	2.218	1.048	79.42	57.69	9	31	78	8.5	5.45
P54729	NEDD8 ultimate buster 1 OS=Mus musculus GN=Nub1 PE=1 SV=2 - [NUB1_MOUSE]	15.31	1	11	12	34	0.665	0.969	0.884	1.048	95.87	15.31	19	34	614	70.3	5.88
Q3UDW8-2	Isoform 2 of Heparan-alpha-glucosaminide N-acetyltransferase OS=Mus musculus GN=Hgsnat - [HGNAT_MOUSE]	1.76	2	1	1	3	0.865	0.684	0.925	1.048	9.25	1.76	2	3	624	69.1	7.85
Q8CBW4	DDB1- and CUL4-associated factor 12-like protein 1 OS=Mus musculus GN=Dcaf12l1 PE=2 SV=1 - [illegible]	9.78	2	4	4	7	0.804	1.110	0.819	1.048	12.34	9.78	6	7	501	54.3	8.13
Q6PFY1	FCH and double SH3 domains protein 1 OS=Mus musculus GN=Fchsd1 PE=2 SV=1 - [FCSD1_MOUSE]	1.89	1	1	1	6	0.905	1.642	0.925	1.048	15.74	1.89	2	6	688	76.2	5.55
Q59J78	Mimitin, mitochondrial OS=Mus musculus GN=Ndufaf2 PE=2 SV=1 - [MIMIT_MOUSE]	66.07	1	12	12	47	1.602	0.626	0.823	1.048	118.42	66.07	22	47	168	19.6	8.25
Q8BGY3	Leucine zipper protein 2 OS=Mus musculus GN=Luzp2 PE=2 SV=1 - [LUZP2_MOUSE]	28.12	1	10	11	35	1.323	0.931	1.046	1.048	97.44	28.12	19	35	345	39.0	8.50
Q9Z2C9	Myotubularin-related protein 7 OS=Mus musculus GN=Mtmr7 PE=2 SV=2 -	26.36	5	13	14	45	0.817	0.865	0.931	1.048	142.38	26.36	23	45	660	75.6	6.43
Q91YL2	E3 ubiquitin-protein ligase RNF126 OS=Mus musculus GN=Rnf126 PE=1 SV=1 - [RN126_MOUSE]	10.22	1	2	2	4	1.229	1.351	1.387	1.048	10.57	10.22	3	4	313	34.1	5.17
Q8CBY8-2	Isoform 2 of Dynactin subunit 4 OS=Mus musculus GN=Dctn4 - [DCTN4_MOUSE]	25.87	2	11	11	39	0.862	0.932	1.154	1.048	120.81	25.87	19	39	460	52.2	7.69

Q91V41	Ras-related protein Rab-14 OS=Mus musculus GN=Rab14 PE=1 SV=3 - [RAB14_MOUSE]	72.09	2	10	12	154	0.756	0.897	0.918	1.048	450.18	72.09	24	154	215	23.9	6.21
Q60649	Caseinolytic peptidase B protein homolog OS=Mus musculus GN=Clpb PE=1 SV=1 - [CLPB_MOUSE]	30.58	3	13	17	45	1.115	1.134	1.056	1.048	146.10	30.58	31	45	677	76.0	8.51
Q3V1Z0	Iporin OS=Mus musculus GN=Rusc2 PE=2 SV=1 - [Q3V1Z0_MOUSE]	9.46	4	7	7	16	0.813	0.780	0.950	1.048	50.22	9.46	11	16	1523	162.2	6.58
P40336	Vacuolar protein sorting-associated protein 26A OS=Mus musculus GN=Vps26a PE=2 SV=1 - [VP26A_MOUSE]	32.11	2	10	11	48	0.940	1.062	1.020	1.048	122.90	32.11	21	48	327	38.1	6.57
Q8C145	Zinc transporter ZIP6 OS=Mus musculus GN=Slc39a6 PE=1 SV=1 - [S39A6_MOUSE]	11.37	2	8	8	27	0.925	1.251	1.162	1.048	107.50	11.37	13	27	765	86.3	6.84
G3UWY5	E3 ubiquitin-protein ligase ZNRF1 (Fragment) OS=Mus musculus GN=Znrf1 PE=4 SV=1 - [G3UWY5_MOUSE]	26.14	7	1	2	6	1.231	2.441	1.241	1.048	23.33	26.14	4	6	88	9.8	5.63
Q9WU28	Prefoldin subunit 5 OS=Mus musculus GN=Pfdn5 PE=1 SV=1 - [PFD5_MOUSE]	64.94	6	9	9	39	1.349	1.102	1.115	1.048	109.57	64.94	14	39	154	17.3	6.33
P98200	Probable phospholipid-transporting ATPase IB OS=Mus musculus GN=Atp8a2 PE=1 SV=1 - [AT8A2_MOUSE]	12.20	3	9	14	31	0.770	0.780	0.725	1.048	89.58	12.20	23	31	1148	129.3	7.33
Q76MZ3	Serine/threonine-protein phosphatase 2A 65 kDa regulatory subunit A alpha isoform OS=Mus musculus GN=Ppp2r1a PE=1 SV=3 - [2AAA_MOUSE]	55.52	4	21	28	292	0.663	1.239	1.026	1.049	835.43	55.52	50	292	589	65.3	5.11
D3YZI9	Protein Pgbd5 OS=Mus musculus GN=Pgbd5 PE=2 SV=1 - [D3YZI9_MOUSE]	20.65	3	8	8	26	0.938	1.409	1.255	1.049	75.46	20.65	12	26	523	58.3	8.85
Q08331	Calretinin OS=Mus musculus GN=Calb2 PE=1 SV=3 - [CALB2_MOUSE]	78.23	1	23	24	613	1.642	0.933	0.888	1.049	1655.95	78.23	45	613	271	31.4	5.02
F8VPR1	Leiomodin-1 OS=Mus musculus GN=Lmod1 PE=2 SV=1 - [F8VPR1_MOUSE]	4.03	2	2	2	2	0.610	1.543	0.865	1.049	0.00	4.03	2	2	595	66.2	9.19
Q3UHJ0-2	Isoform 2 of AP2-associated protein kinase 1 OS=Mus musculus GN=Aak1 -	56.83	2	31	34	309	1.120	1.252	1.382	1.049	1007.66	56.83	64	309	878	94.6	6.61
Q9CZG3	COMM domain-containing protein 8 OS=Mus musculus GN=Commd8 PE=2 SV=1 -	46.99	1	7	7	25	1.344	1.159	1.023	1.049	56.76	46.99	11	25	183	20.8	5.59
P70168	Importin subunit beta-1 OS=Mus musculus GN=Kpnb1 PE=1 SV=2 - [IMB1_MOUSE]	46.35	1	32	32	225	0.662	1.382	0.892	1.049	683.70	46.35	57	225	876	97.1	4.78
Q80ZI6	E3 ubiquitin-protein ligase LRSAM1 OS=Mus musculus GN=Lrsam1 PE=2 SV=1 -	24.21	4	14	15	43	1.164	1.476	0.978	1.049	125.06	24.21	26	43	727	83.9	6.14
P58873	Rhomboid-related protein 3 OS=Mus musculus GN=Rhbdl3 PE=2 SV=1 - [RHBL3_MOUSE]	5.69	1	2	3	8	0.852	1.094	1.032	1.049	15.31	5.69	5	8	404	45.2	7.58
D3YUH8	Rho GTPase-activating protein 20 OS=Mus musculus GN=Arhgap20 PE=2 SV=1 -	2.97	2	2	3	5	1.094	1.018	1.437	1.049	14.26	2.97	4	5	1146	127.7	6.99
Q8K1B8	Fermitin family homolog 3 OS=Mus musculus GN=Fermt3 PE=1 SV=1 - [URP2_MOUSE]	14.59	1	7	7	11	0.954	1.478	0.986	1.049	37.08	14.59	10	11	665	75.6	7.05
Q8BH61	Coagulation factor XIII A chain OS=Mus musculus GN=F13a1 PE=2 SV=3 - [F13A_MOUSE]	20.08	1	10	10	27	1.046	2.157	0.831	1.049	53.70	20.08	15	27	732	83.2	5.92
P35969	Vascular endothelial growth factor receptor 1 OS=Mus musculus GN=Flt1 PE=1 SV=1 - [VGFR1_MOUSE]	3.45	9	3	4	16	1.207	1.061	1.111	1.049	36.95	3.45	6	16	1333	149.8	8.31
B2RUR8	OTU domain-containing protein 7B OS=Mus musculus GN=Otud7b PE=1 SV=1 -	16.55	3	6	11	36	1.853	0.599	1.021	1.049	84.35	16.55	17	36	840	91.9	6.86
Q6P6J0	tRNA-specific adenosine deaminase 2 OS=Mus musculus GN=Adat2 PE=2 SV=1 - [ADAT2_MOUSE]	27.23	1	3	3	5	1.048	1.347	1.182	1.049	11.91	27.23	4	5	191	21.3	5.88
Q6P1I6	PH and SEC7 domain-containing protein 2 OS=Mus musculus GN=Psd2 PE=2 SV=1 - [PSD2_MOUSE]	17.92	4	14	14	39	0.998	0.685	0.815	1.049	112.02	17.92	26	39	770	84.2	5.21
G3UWV4	Serine/threonine-protein kinase BRSK2 OS=Mus musculus GN=Brsk2 PE=2 SV=1 - [G3UWV4_MOUSE]	42.32	4	3	25	123	1.280	0.866	1.121	1.050	383.17	42.32	44	123	697	77.7	8.82

Q6PA06	Atlastin-2 OS=Mus musculus GN=Atl2 PE=1 SV=1 - [ATLA2_MOUSE]	29.16	3	11	13	47	0.610	0.914	1.005	1.050	147.27	29.16	24	47	583	66.2	5.43
B2RPU2	Pleckstrin homology domain-containing family D member 1 OS=Mus musculus GN=Plekhd1 PE=2 SV=1 - [illegible]	10.30	1	4	4	6	1.130	0.555	1.170	1.050	15.64	10.30	5	6	505	59.1	6.44
Q80YA9	Connector enhancer of kinase suppressor of ras 2 OS=Mus musculus GN=Cnksr2 PE=1 SV=1 - [CNKR2_MOUSE]	32.66	3	28	29	75	0.641	1.847	2.050	1.050	216.46	32.66	48	75	1032	117.3	6.79
Q8BPN8-2	Isoform 2 of DmX-like protein 2 OS=Mus musculus GN=Dmxl2 - [DMXL2_MOUSE]	35.90	7	85	92	433	0.639	1.129	1.093	1.050	1343.23	35.90	164	433	3022	336.8	6.46
Q921P9	Transcription elongation factor A protein-like 1 OS=Mus musculus GN=Tceal1 PE=2 SV=1 - [TCAL1_MOUSE]	15.76	1	3	3	6	1.349	1.225	1.105	1.050	20.13	15.76	6	6	165	19.3	4.54
Q6URW6-3	Isoform 3 of Myosin-14 OS=Mus musculus GN=Myh14 - [MYH14_MOUSE]	37.73	6	2	72	236	0.738	0.983	0.994	1.050	673.67	37.73	124	236	2033	231.5	5.55
P97480-2	Isoform 2 of Eyes absent homolog 3 OS=Mus musculus GN=Eya3 - [EYA3_MOUSE]	2.64	3	1	1	2	0.891	1.433	0.917	1.050	5.82	2.64	2	2	416	45.5	5.85
Q99KW9	T-cell immunomodulatory protein OS=Mus musculus GN=Itfg1 PE=2 SV=2 - [TIP_MOUSE]	16.23	1	7	7	19	0.891	1.068	0.856	1.050	53.18	16.23	10	19	610	67.4	5.73
D3Z2Q7	Protein Hmcn1 OS=Mus musculus GN=Hmcn1 PE=2 SV=1 - [D3Z2Q7_MOUSE]	2.45	2	7	9	14	1.873	1.009	1.067	1.050	16.58	2.45	9	14	5517	597.9	6.58
Q9CWG8	NADH dehydrogenase [ubiquinone] complex I, assembly factor 7 OS=Mus musculus GN=Ndufaf7 PE=2 SV=4 - [illegible]	31.19	2	11	11	37	0.787	1.110	0.932	1.050	94.91	31.19	20	37	436	48.4	6.95
G3UXG8	All-trans retinoic acid-induced differentiation factor (Fragment) OS=Mus musculus GN=Atraid PE=2 SV=1 - [G3UXG8_MOUSE]	11.89	4	2	2	8	1.013	0.947	1.069	1.050	16.45	11.89	4	8	143	15.1	5.44
Q6ZQF7	Protein Jade-2 OS=Mus musculus GN=Phf15 PE=2 SV=2 - [JADE2_MOUSE]	6.88	3	3	3	5	1.636	1.192	1.235	1.050	6.99	6.88	5	5	829	92.1	5.77
A2AJ76	Hemicentin-2 OS=Mus musculus GN=Hmcn2 PE=1 SV=1 - [HMCN2_MOUSE]	1.16	2	6	6	10	2.746	0.976	0.720	1.050	13.92	1.16	6	10	5100	546.9	5.71
A2AFS9	MCG1031886 OS=Mus musculus GN=Tex13a PE=4 SV=1 - [A2AFS9_MOUSE]	2.92	1	1	1	1	0.762	1.485	1.888	1.050	2.73	2.92	1	1	377	42.7	8.12
Q3TT38	Uncharacterized protein C6orf106 homolog OS=Mus musculus GN=D17Wsu92e PE=2 SV=2 - [CF106_MOUSE]	12.03	3	2	2	3	1.373	1.061	1.166	1.050	8.81	12.03	3	3	291	32.1	4.48
Q8C4B4	Protein unc-119 homolog B OS=Mus musculus GN=Unc119b PE=2 SV=1 - [U119B_MOUSE]	25.50	2	4	5	15	1.044	0.953	1.019	1.050	60.11	25.50	8	15	251	28.3	5.72
P11087-2	Isoform 2 of Collagen alpha-1(I) chain OS=Mus musculus GN=Col1a1 - [CO1A1_MOUSE]	25.31	2	15	15	92	0.836	4.705	1.071	1.050	307.88	25.31	22	92	1225	117.7	5.72
F6TZU3	Gigaxonin (Fragment) OS=Mus musculus GN=Gan PE=4 SV=1 - [F6TZU3_MOUSE]	12.40	2	5	5	11	0.990	0.919	1.160	1.050	29.34	12.40	7	11	597	67.6	5.92
Q3TCJ1	BRISC complex subunit Abro1 OS=Mus musculus GN=Fam175b PE=2 SV=1 [F175B_MOUSE]	37.59	3	11	11	32	1.010	1.085	1.023	1.050	84.90	37.59	18	32	415	46.9	6.18
B1AX58	Plastin-3 OS=Mus musculus GN=Pls3 PE=2 SV=1 - [B1AX58_MOUSE]	51.33	2	24	30	162	0.920	0.811	0.956	1.050	501.01	51.33	49	162	639	71.7	5.62
Q3UN02	Lysocardiolipin acyltransferase 1 OS=Mus musculus GN=Lclat1 PE=2 SV=2 - [LCLT1_MOUSE]	21.01	2	7	7	16	0.667	1.284	0.965	1.050	49.28	21.01	11	16	376	44.4	8.53
Q8CAD1	Ras-binding protein DA-Raf OS=Mus musculus GN=Araf PE=2 SV=1 - [Q8CAD1_MOUSE]	50.54	2	1	8	42	0.721	0.872	0.810	1.050	117.90	50.54	16	42	186	20.9	8.47
F6SEU4	Ras/Rap GTPase-activating protein SynGAP OS=Mus musculus GN=Syngap1 PE=3 SV=2 - [SYGP1_MOUSE]	42.46	1	3	52	389	0.752	2.985	2.887	1.050	1139.12	42.46	93	389	1340	148.1	8.98
Q8BH80	Vesicle-associated membrane protein, associated protein B and C OS=Mus musculus GN=Vapb PE=2 SV=1 - [illegible]	58.02	2	13	14	84	0.901	0.737	0.915	1.051	220.50	58.02	25	84	243	26.9	7.78
P97372	Proteasome activator complex subunit 2 OS=Mus musculus GN=Psme2 PE=2 SV=4 - [PSME2_MOUSE]	53.56	3	12	12	36	0.797	1.817	0.685	1.051	109.38	53.56	20	36	239	27.0	5.76

Q60571	Corticotropin-releasing factor-binding protein OS=Mus musculus GN=Crhbp PE=2 SV=1 - [CRHBP_MOUSE]	15.22	1	3	3	3	1.833	2.194	1.009	1.051	8.87	15.22	3	3	322	36.0	6.60
Q8CE96	tRNA (adenine(58)-N(1))-methyltransferase non-catalytic subunit TRM6 OS=Mus musculus GN=Trmt6 PE=1 SV=1 - [TRM6_MOUSE]	7.24	3	3	3	6	0.872	0.656	0.997	1.051	23.74	7.24	4	6	497	55.5	6.95
P48455	Serine/threonine-protein phosphatase 2B catalytic subunit gamma isoform OS=Mus musculus GN=Ppp3cc PE=2 SV=1 - [PP2BC_MOUSE]	13.84	1	3	7	47	0.888	1.275	1.165	1.051	129.14	13.84	13	47	513	58.7	7.25
Q9D7X8	Gamma-glutamylcyclotransferase OS=Mus musculus GN=Ggct PE=2 SV=1 -	22.34	1	5	5	17	1.117	0.791	0.978	1.051	47.41	22.34	8	17	188	21.2	5.67
Q80WS3	rRNA/tRNA 2'-O-methyltransferase fibrillarin-like protein 1 OS=Mus musculus GN=Fbll1 PE=2 SV=1 - [illegible]	35.67	1	10	12	28	0.911	0.613	0.818	1.051	74.49	35.67	20	28	314	33.3	10.15
P61202	COP9 signalosome complex subunit 2 OS=Mus musculus GN=Cops2 PE=1 SV=1 -	48.76	3	21	21	101	0.703	1.155	0.875	1.051	298.64	48.76	36	101	443	51.6	5.53
A2AHJ4	Bromodomain and WD repeat-containing protein 3 OS=Mus musculus GN=Brwd3 PE=2 SV=1 - [BRWD3_MOUSE]	3.11	1	3	3	3	1.426	0.935	1.043	1.051	4.93	3.11	3	3	1799	202.8	7.42
F7ABZ6	Protein Dnah10 (Fragment) OS=Mus musculus GN=Dnah10 PE=4 SV=1 -	1.49	2	3	7	13	2.350	1.071	1.230	1.051	27.97	1.49	8	13	4365	502.5	5.94
Q91YN1-2	Isoform 2 of Protein FAM118A OS=Mus musculus GN=Fam118a - [F118A_MOUSE]	4.40	2	1	1	2	0.616	1.378	1.083	1.051	7.91	4.40	2	2	250	27.9	7.06
P17665	Cytochrome c oxidase subunit 7C, mitochondrial OS=Mus musculus GN=Cox7c PE=1 SV=1 - [COX7C_MOUSE]	38.10	4	3	3	8	2.116	0.976	1.062	1.051	23.55	38.10	4	8	63	7.3	11.00
Q8K0T4	Katanin p60 ATPase-containing subunit A-like 1 OS=Mus musculus GN=Katnal1 PE=1 SV=1 - [KATL1_MOUSE]	36.27	7	17	17	58	1.016	1.030	1.049	1.051	156.02	36.27	30	58	488	55.1	7.09
Q3URE9	Leucine-rich repeat and immunoglobulin-like domain-containing nogo receptor-interacting protein 2 OS=Mus musculus GN=Lingo2 PE=2 SV=1 - [LIGO2_MOUSE]	7.10	1	2	4	11	0.895	1.156	0.953	1.051	37.08	7.10	7	11	606	68.0	8.13
O88572	Low-density lipoprotein receptor-related protein 6 OS=Mus musculus GN=Lrp6 PE=1 SV=1 - [LRP6_MOUSE]	0.68	1	1	1	4	4.625	0.776	1.253	1.052	7.42	0.68	2	4	1613	180.1	5.33
Q8BLK9-3	Isoform 3 of Ribosomal protein S6 kinase delta-1 OS=Mus musculus GN=Rps6kc1 -	23.91	5	16	16	41	1.279	1.262	1.139	1.052	114.84	23.91	24	41	916	100.3	4.92
P60879	Synaptosomal-associated protein 25 OS=Mus musculus GN=Snap25 PE=1 SV=1 -	72.33	1	5	20	1019	1.203	0.899	1.352	1.052	2931.08	72.33	40	1019	206	23.3	4.77
E9Q6Q8	TBC1 domain family member 4 OS=Mus musculus GN=Tbc1d4 PE=2 SV=1 -	7.88	9	8	8	15	1.405	0.822	1.128	1.052	44.63	7.88	12	15	1243	140.2	7.31
E9Q4P1	Protein Wdfy1 OS=Mus musculus GN=Wdfy1 PE=4 SV=1 - [E9Q4P1_MOUSE]	25.61	4	9	9	37	0.963	0.972	0.853	1.052	107.89	25.61	16	37	410	46.2	7.34
Q8BPI1-2	Isoform 2 of Protein kintoun OS=Mus musculus GN=Dnaaf2 - [KTU_MOUSE]	3.48	2	2	2	2	1.243	1.038	0.997	1.052	7.03	3.48	2	2	776	84.6	5.05
Q62087	Serum paraoxonase/lactonase 3 OS=Mus musculus GN=Pon3 PE=1 SV=2 -	17.80	2	3	3	9	1.516	1.628	0.791	1.052	34.98	17.80	6	9	354	39.3	5.74
Q0VBD0	Integrin beta OS=Mus musculus GN=Itgb8 PE=2 SV=1 - [Q0VBD0_MOUSE]	23.86	1	15	16	41	0.836	1.561	1.134	1.052	120.07	23.86	29	41	767	84.5	7.09
Q14CH7	Alanine--tRNA ligase, mitochondrial OS=Mus musculus GN=Aars2 PE=2 SV=1 - [SYAM_MOUSE]	12.35	1	11	11	38	0.977	0.768	0.902	1.052	103.05	12.35	19	38	980	106.7	6.18
Q8K1X4	NCK associated protein 1 like OS=Mus musculus GN=Nckap1l PE=2 SV=1 - [Q8K1X4_MOUSE]	2.82	1	1	3	32	2.900	1.248	1.595	1.052	80.52	2.82	5	32	1134	128.8	6.64
Q91YP3	Putative deoxyribose-phosphate aldolase OS=Mus musculus GN=Dera PE=2 SV=1 - [DEOC_MOUSE]	8.18	1	2	2	3	1.501	1.091	0.812	1.052	6.52	8.18	3	3	318	35.0	8.72

Q9WUN2	Serine/threonine-protein kinase TBK1 OS=Mus musculus GN=Tbk1 PE=1 SV=1 - [TBK1_MOUSE]	16.74	1	10	10	28	0.851	0.945	0.926	1.052	79.93	16.74	18	28	729	83.4	6.87
Q8VHN8	Protein syndesmos OS=Mus musculus GN=Nudt16l1 PE=1 SV=2 - [SDOS_MOUSE]	17.54	4	3	4	13	2.027	0.989	0.979	1.052	43.64	17.54	7	13	211	23.4	9.26
E9Q7X6-2	Isoform 2 of Protein HEG homolog 1 OS=Mus musculus GN=Heg1 - [HEG1_MOUSE]	3.95	5	3	3	4	1.921	1.078	1.074	1.052	13.52	3.95	4	4	1240	131.5	6.67
B8JJ53	Tripartite motif-containing protein 26 (Fragment) OS=Mus musculus GN=Trim26 PE=2 SV=1 - [B8JJ53_MOUSE]	9.74	6	1	1	3	2.886	1.131	1.166	1.052	8.43	9.74	2	3	195	22.7	9.74
Q8CIP5	Protein dispatched homolog 2 OS=Mus musculus GN=Disp2 PE=2 SV=1 - [DISP2_MOUSE]	11.08	4	9	9	20	1.000	0.672	0.690	1.052	59.05	11.08	13	20	1345	147.8	6.76
Q9R013	Cathepsin F OS=Mus musculus GN=Ctsf PE=2 SV=1 - [CATF_MOUSE]	12.55	1	5	5	11	0.730	1.223	1.061	1.052	41.91	12.55	8	11	462	51.6	6.55
O88207	Collagen alpha-1(V) chain OS=Mus musculus GN=Col5a1 PE=2 SV=2 - [CO5A1_MOUSE]	1.41	1	1	2	3	0.470	1.296	1.012	1.052	6.98	1.41	3	3	1838	183.6	4.98
P63216	Guanine nucleotide-binding protein G(I)/G(S)/G(O) subunit gamma-3 OS=Mus musculus GN=Gng3 PE=1 SV=1 - [GBG3_MOUSE]	81.33	1	6	6	143	0.748	1.498	1.398	1.053	443.82	81.33	12	143	75	8.3	7.78
Q63ZW6	Col4a5 protein OS=Mus musculus GN=Col4a5 PE=2 SV=1 - [Q63ZW6_MOUSE]	6.74	2	4	6	18	1.406	1.612	0.892	1.053	59.52	6.74	9	18	1691	161.7	8.10
Q8VDZ4	Palmitoyltransferase ZDHHC5 OS=Mus musculus GN=Zdhhc5 PE=1 SV=1 -	38.32	6	18	20	64	1.070	0.886	1.262	1.053	179.28	38.32	31	64	715	77.5	9.01
P99024	Tubulin beta-5 chain OS=Mus musculus GN=Tubb5 PE=1 SV=1 - [TBB5_MOUSE]	83.11	2	6	32	4204	0.840	1.291	1.075	1.053	11113.59	83.11	60	4204	444	49.6	4.89
P70202	Latexin OS=Mus musculus GN=Lxn PE=1 SV=2 - [LXN_MOUSE]	25.23	1	4	4	32	1.189	1.059	0.865	1.053	129.46	25.23	7	32	222	25.5	5.74
Q5DTN8	Janus kinase and microtubule-interacting protein 3 OS=Mus musculus GN=Jakmip3 PE=2 SV=2 - [JKIP3_MOUSE]	18.13	5	12	16	56	1.738	0.768	1.273	1.053	163.06	18.13	27	56	844	98.7	5.74
Q9D8Z6	Autophagy-related protein 101 OS=Mus musculus GN=Atg101 PE=2 SV=1 - [ATGA1_MOUSE]	19.72	1	4	4	11	1.251	1.268	1.012	1.053	31.39	19.72	7	11	218	25.0	6.15
E9Q467	Protein Abcc4 OS=Mus musculus GN=Abcc4 PE=2 SV=1 - [E9Q467_MOUSE]	7.12	2	7	7	16	1.171	1.584	1.041	1.053	43.99	7.12	12	16	1250	140.1	8.29
Q8BFT9	Synaptic vesicle 2-related protein OS=Mus musculus GN=Svop PE=1 SV=1 - [SVOP_MOUSE]	17.34	2	6	6	22	0.815	1.101	0.937	1.053	56.21	17.34	9	22	548	60.7	5.85
G5E8T6	Membrane associated guanylate kinase, WW and PDZ domain containing 3, isoform CRA_a OS=Mus musculus GN=Magi3 PE=4 SV=1 - [G5E8T6_MOUSE]	18.70	3	22	25	61	1.035	0.996	1.199	1.053	143.92	18.70	39	61	1476	161.5	8.05
D3Z5R4	WAS/WASL-interacting protein family member 3 OS=Mus musculus GN=Wipf3 PE=4 SV=1 - [D3Z5R4_MOUSE]	36.30	4	12	12	49	1.108	2.095	2.558	1.053	128.13	36.30	19	49	449	45.1	10.84
Q8BH57-2	Isoform 2 of WD repeat-containing protein 48 OS=Mus musculus GN=Wdr48 -	38.37	4	24	24	109	1.265	1.161	0.996	1.053	291.31	38.37	39	109	662	74.4	7.17
Q9DC63	F-box only protein 3 OS=Mus musculus GN=Fbxo3 PE=2 SV=1 - [FBX3_MOUSE]	23.54	4	9	10	29	0.860	0.945	0.844	1.053	91.92	23.54	17	29	480	55.2	5.02
Q3T995	E3 ubiquitin-protein ligase UHRF2 OS=Mus musculus GN=Uhrf2 PE=2 SV=1 - [Q3T995_MOUSE]	3.96	4	1	1	2	2.162	1.051	0.996	1.053	4.57	3.96	2	2	202	22.4	6.30
P08122	Collagen alpha-2(IV) chain OS=Mus musculus GN=Col4a2 PE=2 SV=4 - [CO4A2_MOUSE]	9.20	2	10	11	36	1.657	1.283	1.197	1.053	115.72	9.20	19	36	1707	167.2	8.48
P97481	Endothelial PAS domain-containing protein 1 OS=Mus musculus GN=Epas1 PE=1 SV=2 - [EPAS1_MOUSE]	3.43	1	1	2	3	0.902	1.415	2.049	1.053	2.09	3.43	2	3	874	96.6	5.73
Q641K1	Cytosolic carboxypeptidase 1 OS=Mus musculus GN=Agtpbp1 PE=1 SV=2 - [CBPC1_MOUSE]	11.74	6	10	10	41	0.883	0.954	1.232	1.054	111.40	11.74	18	41	1218	137.1	6.52
Q8CDN9-2	Isoform 2 of Leucine-rich repeat-containing protein 9 OS=Mus musculus GN=Lrrc9 - [LRRC9_MOUSE]	1.04	3	1	1	1	1.177	0.996	1.151	1.054	0.00	1.04	1	1	1154	133.0	7.88

Q6PDJ1-2	Isoform 2 of VWFA and cache domain-containing protein 1 OS=Mus musculus GN=Cachd1 - [CAHD1_MOUSE]	5.54	2	7	7	11	0.684	0.539	0.758	1.054	29.74	5.54	9	11	1282	143.1	6.52
Q91V36	Nuclear receptor-binding protein 2 OS=Mus musculus GN=Nrbp2 PE=1 SV=2 - [NRBP2_MOUSE]	22.85	1	10	11	34	0.924	0.974	0.915	1.054	88.04	22.85	19	34	499	57.3	6.35
Q08761	Vitamin K-dependent protein S OS=Mus musculus GN=Pros1 PE=2 SV=1 - [PROS_MOUSE]	2.22	1	2	2	5	1.364	0.959	1.145	1.054	10.69	2.22	3	5	675	74.9	5.82
D3YXA8	Solute carrier organic anion transporter family member 3A1 OS=Mus musculus GN=Slco3a1 PE=2 SV=1 - [illegible]	2.66	3	1	1	2	2.413	0.976	0.865	1.054	6.49	2.66	2	2	602	64.3	7.43
Q99NG0	Helicase ARIP4 OS=Mus musculus GN=Rad54l2 PE=1 SV=1 - [ARIP4_MOUSE]	4.09	2	4	5	11	0.422	0.350	0.637	1.054	13.21	4.09	5	11	1466	162.4	6.13
B1AR76	Breast carcinoma-amplified sequence 3 homolog (Fragment) OS=Mus musculus GN=Bcas3 PE=2 SV=1 - [B1AR76_MOUSE]	43.01	2	1	15	92	0.927	0.926	1.083	1.054	275.08	43.01	30	92	458	49.4	6.20
Q8VCU2	Glycosylphosphatidylinositol specific phospholipase D1 OS=Mus musculus GN=Gpld1 PE=2 SV=1 - [Q8VCU2_MOUSE]	28.74	3	20	20	54	1.335	1.101	0.676	1.054	158.76	28.74	30	54	842	93.6	6.80
P46638	Ras-related protein Rab-11B OS=Mus musculus GN=Rab11b PE=1 SV=3 - [RB11B_MOUSE]	58.26	6	7	13	64	0.738	0.939	0.954	1.054	179.16	58.26	24	64	218	24.5	5.94
D3Z0B9	Aldehyde dehydrogenase family 16 member A1 OS=Mus musculus GN=Aldh16a1 PE=2 SV=1 [D3Z0B9_MOUSE]	9.95	2	6	6	19	1.182	0.956	0.945	1.054	52.75	9.95	11	19	754	79.4	6.25
Q8CAQ8-3	Isoform 3 of Mitochondrial inner membrane protein OS=Mus musculus GN=Immt - [IMMT_MOUSE]	69.25	2	2	52	300	1.197	1.233	1.101	1.054	907.55	69.25	92	300	709	78.7	7.25
D3Z0A5	Protein lifeguard 3 (Fragment) OS=Mus musculus GN=Tmbim1 PE=2 SV=1 -	15.05	5	1	1	6	0.926	0.500	0.764	1.054	11.59	15.05	2	6	93	10.2	5.26
F7ANC5	Leucine-rich repeat and coiled-coil domain-containing protein 1 (Fragment) OS=Mus musculus GN=Lrrcc1 PE=2 SV=1 - [F7ANC5_MOUSE]	3.61	9	1	3	7	2.168	1.003	0.838	1.054	13.37	3.61	4	7	581	68.6	6.20
Q9D4C9	Clavesin-1 OS=Mus musculus GN=Clvs1 PE=2 SV=1 - [CLVS1_MOUSE]	28.25	3	7	9	30	0.627	1.023	0.943	1.054	97.86	28.25	15	30	354	40.6	6.73
Q9JI10	Serine/threonine-protein kinase 3 OS=Mus musculus GN=Stk3 PE=1 SV=1 - [STK3_MOUSE]	21.73	3	5	9	26	1.233	1.134	0.910	1.054	69.07	21.73	15	26	497	56.8	4.98
Q8K363	ATP-dependent RNA helicase DDX18 OS=Mus musculus GN=Ddx18 PE=2 SV=1 - [DDX18_MOUSE]	3.94	1	2	2	2	0.441	0.874	0.753	1.054	2.70	3.94	2	2	660	74.1	9.52
P97789-3	Isoform 3 of 5'-3' exoribonuclease 1 OS=Mus musculus GN=Xrn1 -	7.53	4	10	10	23	0.741	1.064	1.121	1.055	69.40	7.53	17	23	1687	191.3	7.40
D3Z2W1	Protein Dgki OS=Mus musculus GN=Dgki PE=2 SV=1 - [D3Z2W1_MOUSE]	23.24	3	18	19	43	0.752	1.085	1.534	1.055	135.11	23.24	30	43	1050	115.9	7.85
Q91WA6-2	Isoform 2 of Sharpin OS=Mus musculus GN=Sharpin - [SHRPN_MOUSE]	7.54	2	2	2	7	0.957	1.607	0.999	1.055	12.51	7.54	3	7	305	31.8	5.41
E9PUE6	Transmembrane protein 145 OS=Mus musculus GN=Tmem145 PE=2 SV=1 - [E9PUE6_MOUSE]	7.28	3	3	3	8	0.564	0.812	0.693	1.055	19.19	7.28	6	8	563	62.8	8.75
Q8K1R7	Serine/threonine-protein kinase Nek9 OS=Mus musculus GN=Nek9 PE=1 SV=2 - [NEK9_MOUSE]	12.70	1	9	9	13	1.423	1.264	1.112	1.055	36.86	12.70	13	13	984	107.1	5.63
Q8R189	Monocyte to macrophage differentiation factor 2 OS=Mus musculus GN=Mmd2 PE=2 SV=1 - [PAQRA_MOUSE]	2.43	1	1	1	1	0.451	0.507	0.899	1.055	2.02	2.43	1	1	247	28.9	8.53
E9Q8G5	Protein A430033K04Rik OS=Mus musculus GN=A430033K04Rik PE=2 SV=1 - [E9Q8G5_MOUSE]	2.50	1	1	1	1	0.694	1.115	0.877	1.055	0.00	2.50	1	1	680	79.4	8.03
Q3UMB9	WASH complex subunit 7 OS=Mus musculus GN=Kiaa1033 PE=2 SV=2 [WASH7_MOUSE]	17.05	1	20	21	53	0.599	0.920	0.864	1.055	149.88	17.05	37	53	1173	136.3	7.37
Q8BME9	Cerebellin-4 OS=Mus musculus GN=Cbln4 PE=1 SV=1 - [CBLN4_MOUSE]	17.68	1	3	3	10	1.548	0.663	0.892	1.055	27.56	17.68	6	10	198	21.6	8.73
Q6NS82-3	Isoform 3 of Protein FAM134A OS=Mus musculus GN=Fam134a - [F134A_MOUSE]	13.89	3	4	4	11	1.001	1.202	1.030	1.055	25.46	13.89	5	11	504	53.8	4.49

P99025	GTP cyclohydrolase 1 feedback regulatory protein OS=Mus musculus GN=Gchfr PE=1 SV=3 - [GFRP_MOUSE]	20.24	1	1	1	7	2.303	0.693	0.862	1.055	16.03	20.24	2	7	84	9.6	6.27
Q80UG2	Plexin-A4 OS=Mus musculus GN=Plxna4 PE=1 SV=3 - [PLXA4_MOUSE]	33.97	1	38	56	211	0.709	0.874	0.957	1.055	615.38	33.97	97	211	1893	212.4	6.83
Q5ICG5	Long chain acyl-CoA synthetase 6 isoform 3 OS=Mus musculus GN=Acsl6 PE=2 SV=1 - [Q5ICG5_MOUSE]	47.06	9	1	31	162	1.097	1.011	0.989	1.055	477.94	47.06	57	162	697	77.9	7.24
B0V2N1-6	Isoform 6 of Receptor-type tyrosine-protein phosphatase S OS=Mus musculus GN=Ptprs - [PTPRS_MOUSE]	34.12	4	5	44	193	0.880	0.649	1.192	1.055	622.23	34.12	80	193	1861	206.7	7.28
Q8C547	HEAT repeat-containing protein 5B OS=Mus musculus GN=Heatr5b PE=2 SV=3 -	14.20	5	23	24	58	0.866	1.039	0.995	1.055	206.09	14.20	37	58	2070	224.2	7.14
Q8VC88	Grancalcin OS=Mus musculus GN=Gca PE=2 SV=1 - [GRAN_MOUSE]	3.18	1	1	1	1	1.048	1.316	0.839	1.055	1.94	3.18	1	1	220	24.6	5.07
B9EI21	Zinc finger matrin type 3 OS=Mus musculus GN=Zmat3 PE=2 SV=1 - [B9EI21_MOUSE]	12.41	2	2	2	8	1.538	0.826	1.109	1.055	22.27	12.41	3	8	290	32.0	9.11
F6W8F5	Otoconin-90 (Fragment) OS=Mus musculus GN=Oc90 PE=3 SV=1 - [F6W8F5_MOUSE]	7.27	9	1	1	1	1.402	1.048	0.997	1.055	2.44	7.27	1	1	220	23.5	5.26
Q3U1U4	Integrin alpha-M OS=Mus musculus GN=Itgam PE=2 SV=1 - [Q3U1U4_MOUSE]	13.39	10	11	13	31	1.057	1.202	1.129	1.056	98.72	13.39	19	31	1232	135.9	8.21
Q62507	Cochlin OS=Mus musculus GN=Coch PE=2 SV=2 - [COCH_MOUSE]	9.42	1	5	5	9	2.012	2.077	0.904	1.056	24.11	9.42	9	9	552	59.9	8.44
Q60803	TNF receptor-associated factor 3 OS=Mus musculus GN=Traf3 PE=1 SV=2 - [TRAF3_MOUSE]	42.50	4	3	25	97	1.085	0.686	0.732	1.056	294.18	42.50	40	97	567	64.3	8.05
Q80WC3	Trinucleotide repeat-containing gene 18 protein OS=Mus musculus GN=Tnrc18 PE=1 SV=2 - [TNC18_MOUSE]	1.22	4	1	3	3	1.660	1.815	0.865	1.056	4.85	1.22	3	3	2878	307.4	8.40
Q99J47	Dehydrogenase/reductase SDR family member 7B OS=Mus musculus GN=Dhrs7b PE=2 SV=1 - [DRS7B_MOUSE]	43.96	2	12	12	35	0.892	1.055	1.023	1.056	112.79	43.96	22	35	323	35.0	9.63
D6RH67	Transmembrane protein 62 OS=Mus musculus GN=Tmem62 PE=2 SV=1 - [D6RH67_MOUSE]	22.82	3	5	5	9	1.233	1.023	1.049	1.056	26.97	22.82	8	9	333	37.3	8.95
A2AJL3-2	Isoform 2 of FGGY carbohydrate kinase domain-containing protein OS=Mus musculus GN=Fggy - [illegible]	6.20	6	2	2	4	1.719	1.119	0.930	1.056	13.55	6.20	4	4	387	42.3	6.57
Q9JME5	AP-3 complex subunit beta 2 OS=Mus musculus GN=Ap3b2 PE=1 SV=2 - [AP3B2_MOUSE]	47.69	2	38	47	204	0.576	1.135	1.024	1.056	567.04	47.69	82	204	1082	119.1	5.63
Q8CGY8	UDP-N-acetylglucosamine--peptide N-acetylglucosaminyltransferase 110 kDa subunit OS=Mus musculus GN=Ogt PE=1 SV=2 - [OGT1_MOUSE]	34.51	2	30	30	125	0.680	1.386	1.176	1.056	382.35	34.51	56	125	1046	116.9	6.70
A2A891-2	Isoform 2 of Calmodulin-binding transcription activator 1 OS=Mus musculus GN=Camta1 - [CMTA1_MOUSE]	4.84	5	5	6	13	0.631	0.543	0.843	1.056	38.89	4.84	11	13	1675	183.4	7.71
M0QW59	Kinase suppressor of Ras 2 OS=Mus musculus GN=Ksr2 PE=4 SV=1 - [M0QW59_MOUSE]	18.40	2	13	13	35	0.824	1.197	1.124	1.056	110.46	18.40	21	35	951	107.6	8.69
Q6GV12	3-ketodihydrosphingosine reductase OS=Mus musculus GN=Kdsr PE=2 SV=1 - [KDSR_MOUSE]	10.54	1	3	3	12	1.022	0.960	0.910	1.056	26.75	10.54	5	12	332	35.9	7.90
F6Y616	Polyadenylate-binding protein-interacting protein 1 OS=Mus musculus GN=Paip1 PE=2 SV=2 - [F6Y616_MOUSE]	35.33	7	13	13	56	1.241	0.903	1.038	1.056	138.81	35.33	23	56	484	53.9	4.78
Q9DCS3	Trans-2-enoyl-CoA reductase, mitochondrial OS=Mus musculus GN=Mecr PE=2 SV=2 - [MECR_MOUSE]	54.42	2	12	13	52	0.765	0.919	1.115	1.056	143.06	54.42	21	52	373	40.3	9.07
Q8BTG3	T-complex protein 11-like protein 1 OS=Mus musculus GN=Tcp11l1 PE=2 SV=1 -	29.67	1	12	12	32	0.839	1.244	0.839	1.056	99.82	29.67	20	32	509	56.3	5.50
E9Q6W2	Cytochrome c oxidase assembly protein 3 homolog, mitochondrial OS=Mus musculus GN=Coa3 PE=2 SV=1 - [illegible]	33.33	2	4	4	14	0.698	0.940	0.924	1.056	43.31	33.33	7	14	90	9.8	11.44

O08582	GTP-binding protein 1 OS=Mus musculus GN=Gtpbp1 PE=1 SV=2 - [GTPB1_MOUSE]	32.34	1	18	18	58	0.715	0.931	1.198	1.056	171.36	32.34	33	58	668	72.3	8.29
Q8C3S2-2	Isoform 2 of Transport and Golgi organization protein 6 homolog OS=Mus musculus GN=Tango6 - [TNG6_MOUSE]	1.31	3	1	1	3	0.799	1.087	0.971	1.057	9.72	1.31	2	3	915	100.3	6.43
Q9CXF4	TBC1 domain family member 15 OS=Mus musculus GN=Tbc1d15 PE=1 SV=1 -	17.88	1	10	11	35	1.094	1.042	1.030	1.057	94.65	17.88	19	35	671	76.5	5.30
Q8R0S4	Voltage-dependent L-type calcium channel subunit beta-4 OS=Mus musculus GN=Cacnb4 PE=1 SV=2 - [CACB4_MOUSE]	41.43	4	12	17	76	0.689	0.747	1.448	1.057	229.10	41.43	30	76	519	57.9	9.28
G3UXB6	Sorting nexin-14 OS=Mus musculus GN=Snx14 PE=2 SV=1 - [G3UXB6_MOUSE]	8.91	5	8	8	23	0.698	0.902	0.946	1.057	51.06	8.91	12	23	920	106.4	6.99
Q99K23	Ufm1-specific protease 2 OS=Mus musculus GN=Ufsp2 PE=1 SV=1 - [UFSP2_MOUSE]	23.64	1	10	10	30	1.025	0.847	0.887	1.057	92.52	23.64	17	30	461	52.5	6.76
Q8R3F5	Malonyl-CoA-acyl carrier protein transacylase, mitochondrial OS=Mus musculus GN=Mcat PE=2 SV=3 - [FABD_MOUSE]	44.88	1	12	12	46	0.934	1.087	0.960	1.057	152.17	44.88	22	46	381	41.9	8.10
Q9CPR0	MCG3437 OS=Mus musculus GN=Tpbpa PE=2 SV=1 - [Q9CPR0_MOUSE]	26.61	1	1	1	1	0.370	0.503	1.933	1.057	3.17	26.61	1	1	124	13.9	4.23
Q7TMB8	Cytoplasmic FMR1-interacting protein 1 OS=Mus musculus GN=Cyfip1 PE=1 SV=1 - [CYFP1_MOUSE]	28.97	4	15	35	187	0.775	1.203	0.976	1.057	538.28	28.97	65	187	1253	145.1	6.90
Q571E4	N-acetylgalactosamine-6-sulfatase OS=Mus musculus GN=Galns PE=2 SV=2 - [GALNS_MOUSE]	24.23	1	10	11	24	2.015	1.123	0.903	1.057	48.25	24.23	19	24	520	57.6	6.52
D3Z041	Long-chain-fatty-acid--CoA ligase 1 OS=Mus musculus GN=Acsl1 PE=2 SV=1 - [D3Z041_MOUSE]	42.49	5	24	27	115	0.631	1.174	0.984	1.057	316.26	42.49	48	115	699	78.0	7.47
Q8R071	Inositol-trisphosphate 3-kinase A OS=Mus musculus GN=Itpka PE=2 SV=1 - [IP3KA_MOUSE]	43.36	1	14	15	51	0.767	3.654	2.823	1.057	152.11	43.36	26	51	459	50.9	7.72
Q5DTK1	Chondroitin sulfate synthase 3 OS=Mus musculus GN=Chsy3 PE=1 SV=3 - [CHSS3_MOUSE]	2.26	2	2	3	6	1.529	0.742	0.772	1.057	10.77	2.26	4	6	884	100.0	8.38
Q9D1H6	NADH dehydrogenase [ubiquinone] 1 alpha subcomplex assembly factor 4 OS=Mus musculus GN=Ndufaf4 PE=2 SV=1 - [NDUF4_MOUSE]	36.99	3	7	8	22	0.987	0.823	1.089	1.057	50.34	36.99	12	22	173	20.1	9.39
Q9D7X3	Dual specificity protein phosphatase 3 OS=Mus musculus GN=Dusp3 PE=1 SV=1 - [DUS3_MOUSE]	60.00	5	8	8	43	1.202	0.952	1.131	1.057	159.80	60.00	15	43	185	20.5	6.54
P35279-2	Isoform 2 of Ras-related protein Rab-6A OS=Mus musculus GN=Rab6a - [RAB6A_MOUSE]	56.25	2	6	12	121	0.830	0.910	0.946	1.057	330.13	56.25	20	121	208	23.5	5.54
F7C3I9	Afadin (Fragment) OS=Mus musculus GN=Mllt4 PE=4 SV=1 - [F7C3I9_MOUSE]	39.44	1	1	10	48	1.513	1.542	1.734	1.057	141.25	39.44	17	48	355	41.4	5.06
Q8VID5	Protein Recql5 OS=Mus musculus GN=Recql5 PE=2 SV=1 - [Q8VID5_MOUSE]	4.38	1	3	4	4	2.892	0.910	0.938	1.057	8.19	4.38	4	4	982	108.2	8.68
P29595	NEDD8 OS=Mus musculus GN=Nedd8 PE=1 SV=2 - [NEDD8_MOUSE]	43.21	1	3	4	146	2.033	1.103	1.275	1.057	300.27	43.21	7	146	81	9.0	7.25
D3Z286	Protein Tmppe OS=Mus musculus GN=Tmppe PE=4 SV=2 - [D3Z286_MOUSE]	13.87	1	4	4	14	0.821	0.938	1.007	1.057	40.11	13.87	8	14	447	49.4	6.89
Q3UH06-2	Isoform 2 of Ras-responsive element-binding protein 1 OS=Mus musculus GN=Rreb1 - [RREB1_MOUSE]	3.83	5	3	3	3	3.569	0.838	0.568	1.057	8.12	3.83	3	3	1618	175.3	6.68
Q8R4T1	Cystin-1 OS=Mus musculus GN=Cys1 PE=1 SV=1 - [CYS1_MOUSE]	22.07	2	2	2	7	2.080	1.513	1.149	1.058	29.19	22.07	4	7	145	15.5	5.25
E9PUA3	IQ motif and SEC7 domain-containing protein 1 OS=Mus musculus GN=Iqsec1 PE=2 SV=1 - [E9PUA3_MOUSE]	37.22	3	31	39	207	0.636	0.754	1.099	1.058	561.63	37.22	71	207	1099	122.6	8.47
Q5EBH1	Ras association domain-containing protein 5 OS=Mus musculus GN=Rassf5 PE=1 SV=1 - [RASF5_MOUSE]	8.23	3	3	4	20	0.989	0.633	0.725	1.058	43.96	8.23	7	20	413	46.7	9.28
Q9WTP9	Ventral anterior homeobox 2 OS=Mus musculus GN=Vax2 PE=1 SV=1 - [VAX2_MOUSE]	17.81	4	3	3	3	0.854	1.013	0.932	1.058	5.25	17.81	3	3	292	31.7	9.74

Q9EPR5	VPS10 domain-containing receptor SorCS2 OS=Mus musculus GN=Sorcs2 PE=1 SV=2 - [SORC2_MOUSE]	29.16	3	30	31	91	0.687	1.216	0.965	1.058	233.10	29.16	54	91	1159	128.8	7.34
Q8K2Y9	Malcavernin OS=Mus musculus GN=Ccm2 PE=1 SV=1 - [CCM2_MOUSE]	20.09	6	7	7	23	1.198	1.088	1.433	1.058	79.93	20.09	14	23	453	49.9	5.62
Q99N87	28S ribosomal protein S5, mitochondrial OS=Mus musculus GN=Mrps5 PE=2 SV=1 - [RT05_MOUSE]	20.14	2	9	9	21	0.793	0.772	1.036	1.058	57.70	20.14	15	21	432	48.2	10.14
Q9Z0Z4-2	Isoform 2 of Hephaestin OS=Mus musculus GN=Heph - [HEPH_MOUSE]	15.48	3	14	14	34	0.934	0.570	0.798	1.058	113.55	15.48	21	34	1156	129.5	6.13
Q3UPL5	Uncharacterized protein C11orf96 homolog OS=Mus musculus GN=Ag2 PE=1 SV=2 - [CK096_MOUSE]	3.61	1	1	1	4	1.554	0.700	1.381	1.058	11.81	3.61	2	4	249	27.1	10.89
P47809	Dual specificity mitogen-activated protein kinase kinase 4 OS=Mus musculus GN=Map2k4 PE=1 SV=2 - [MP2K4_MOUSE]	59.45	5	17	17	61	0.720	1.051	0.980	1.058	180.17	59.45	28	61	397	44.1	8.07
Q8CCP0	Nuclear export mediator factor Nemf OS=Mus musculus GN=Nemf PE=1 SV=2 - [NEMF_MOUSE]	7.89	7	7	9	17	0.800	0.846	0.907	1.058	44.28	7.89	12	17	1064	121.1	6.80
Q9CRY7	Glycerophosphodiester phosphodiesterase domain-containing protein 1 OS=Mus musculus GN=Gdpd1 PE=2 SV=1 - [GDPD1_MOUSE]	37.90	1	10	10	29	0.476	0.783	0.948	1.058	88.25	37.90	17	29	314	35.8	8.31
P00493	Hypoxanthine-guanine phosphoribosyltransferase OS=Mus musculus GN=Hprt1 PE=1 SV=3 - [HPRT_MOUSE]	72.94	1	12	12	105	1.608	1.043	0.998	1.058	292.18	72.94	23	105	218	24.6	6.68
Q2TBE6	Phosphatidylinositol 4-kinase type 2-alpha OS=Mus musculus GN=Pi4k2a PE=1 SV=1 - [P4K2A_MOUSE]	36.74	3	14	15	50	0.930	0.979	0.935	1.058	139.59	36.74	28	50	479	54.2	8.05
Q99PP9-3	Isoform 3 of Tripartite motif-containing protein 16 OS=Mus musculus GN=Trim16 - [TRI16_MOUSE]	2.56	5	1	1	2	2.242	1.123	1.516	1.058	2.56	2.56	1	2	469	54.0	6.57
Q8R2T7	Oxysterol-binding protein OS=Mus musculus GN=Osbpl10 PE=2 SV=1 - [Q8R2T7_MOUSE]	17.77	1	6	8	17	1.176	1.126	1.068	1.058	53.06	17.77	13	17	591	65.8	7.39
B2RQE8	Rho GTPase-activating protein 42 OS=Mus musculus GN=Arhgap42 PE=2 SV=1 -	2.14	1	1	2	3	0.814	0.811	1.012	1.058	6.70	2.14	3	3	841	94.6	8.00
E9Q247	SID1 transmembrane family member 1 OS=Mus musculus GN=Sidt1 PE=2 SV=1 - [E9Q247_MOUSE]	7.81	3	5	5	7	0.808	1.861	0.992	1.058	15.40	7.81	7	7	832	94.4	6.71
Q6PNC0	DmX-like protein 1 OS=Mus musculus GN=Dmxl1 PE=1 SV=1 - [DMXL1_MOUSE]	10.85	2	25	30	69	0.800	0.892	1.002	1.058	170.72	10.85	46	69	3013	335.8	6.42
P58742	Aladin OS=Mus musculus GN=Aaas PE=1 SV=1 - [AAAS_MOUSE]	14.29	1	5	5	16	0.876	1.109	1.059	1.058	34.93	14.29	7	16	546	59.4	6.87
Q8CI32	BAG family molecular chaperone regulator 5 OS=Mus musculus GN=Bag5 PE=1 SV=1 - [BAG5_MOUSE]	50.78	3	16	19	51	1.030	1.147	1.088	1.058	152.01	50.78	28	51	447	50.9	6.05
P70207	Plexin-A2 OS=Mus musculus GN=Plxna2 PE=1 SV=2 - [PLXA2_MOUSE]	18.37	1	17	32	100	0.819	1.636	1.475	1.058	282.25	18.37	54	100	1894	211.4	6.54
P63038	60 kDa heat shock protein, mitochondrial OS=Mus musculus GN=Hspd1 PE=1 SV=1 - [CH60_MOUSE]	80.10	4	48	48	1124	1.233	1.008	1.035	1.059	3433.96	80.10	88	1124	573	60.9	6.18
P97742	Carnitine O-palmitoyltransferase 1, liver isoform OS=Mus musculus GN=Cpt1a PE=1 SV=4 - [CPT1A_MOUSE]	24.58	1	14	14	32	0.862	0.870	0.795	1.059	87.58	24.58	23	32	773	88.2	8.62
Q9CQ26	STAM-binding protein OS=Mus musculus GN=Stambp PE=2 SV=1 - [STABP_MOUSE]	20.28	1	7	7	26	1.405	1.272	0.935	1.059	78.18	20.28	13	26	424	48.5	6.64
Q5SVL6	Rap1 GTPase-activating protein 2 OS=Mus musculus GN=Rap1gap2 PE=1 SV=1 -	50.28	3	24	26	99	0.834	1.254	1.306	1.059	300.45	50.28	45	99	712	78.2	6.43
Q9CQN1	Heat shock protein 75 kDa, mitochondrial OS=Mus musculus GN=Trap1 PE=1 SV=1 - [TRAP1_MOUSE]	50.99	1	31	32	240	0.699	0.958	0.951	1.059	658.60	50.99	54	240	706	80.2	6.68
F8VQC3	Protein Slfn10-ps OS=Mus musculus GN=Slfn10-ps PE=4 SV=1 - [F8VQC3_MOUSE]	1.18	1	1	1	2	0.978	2.026	1.598	1.059	0.00	1.18	1	2	847	96.8	6.77

P15532	Nucleoside diphosphate kinase A OS=Mus musculus GN=Nme1 PE=1 SV=1 - [NDKA_MOUSE]	83.55	3	3	13	172	1.452	1.236	0.928	1.059	495.30	83.55	23	172	152	17.2	7.37
P01029	Complement C4-B OS=Mus musculus GN=C4b PE=1 SV=3 - [CO4B_MOUSE]	10.70	2	16	16	34	3.037	1.554	0.703	1.059	78.79	10.70	27	34	1738	192.8	7.53
Q8BTS4	Nuclear pore complex protein Nup54 OS=Mus musculus GN=Nup54 PE=1 SV=1 - [NUP54_MOUSE]	22.75	1	9	9	19	1.216	1.170	0.989	1.059	45.86	22.75	13	19	510	55.7	7.02
Q91YS8	Calcium/calmodulin-dependent protein kinase type 1 OS=Mus musculus GN=Camk1 PE=1 SV=1 - [KCC1A_MOUSE]	53.21	2	11	15	88	1.065	1.271	0.923	1.059	295.48	53.21	28	88	374	41.6	5.35
D3YV00	Probable phospholipid-transporting ATPase IIB OS=Mus musculus GN=Atp9b PE=4 SV=1 - [D3YV00_MOUSE]	8.19	3	6	8	22	1.492	0.967	0.837	1.059	72.54	8.19	15	22	1135	127.9	7.64
P59648	FXYD domain-containing ion transport regulator 7 OS=Mus musculus GN=Fxyd7 PE=1 SV=1 - [FXYD7_MOUSE]	25.00	1	2	2	6	0.981	0.861	1.176	1.059	11.81	25.00	2	6	80	8.5	7.84
Q6XQH0	Galactose-3-O-sulfotransferase 2 OS=Mus musculus GN=Gal3st2 PE=2 SV=1 -	3.30	4	1	1	2	0.537	1.237	1.101	1.060	0.00	3.30	1	2	394	46.9	8.18
Q8BIW1	Protein prune homolog OS=Mus musculus GN=Prune PE=2 SV=1 - [PRUNE_MOUSE]	45.37	1	17	17	116	0.794	1.028	0.964	1.060	320.24	45.37	29	116	454	50.2	5.11
Q3UHL1	CaM kinase-like vesicle-associated protein OS=Mus musculus GN=Camkv PE=1 SV=2 - [CAMKV_MOUSE]	40.43	1	18	19	117	0.425	1.612	1.554	1.060	343.85	40.43	37	117	512	54.8	5.54
Q8BX94	Oxysterol-binding protein-related protein 2 OS=Mus musculus GN=Osbpl2 PE=2 SV=1 - [OSBL2_MOUSE]	17.36	1	6	9	27	0.936	0.936	0.902	1.060	66.08	17.36	15	27	484	55.3	6.20
Q9Z0H7	B-cell lymphoma/leukemia 10 OS=Mus musculus GN=Bcl10 PE=1 SV=1 - [BCL10_MOUSE]	4.29	1	1	1	3	1.650	1.121	1.165	1.060	7.84	4.29	2	3	233	25.9	6.55
E9PWG2	Protein Trappc8 OS=Mus musculus GN=Trappc8 PE=2 SV=1 - [E9PWG2_MOUSE]	26.93	2	31	31	117	1.100	0.899	0.960	1.060	308.24	26.93	54	117	1437	160.8	7.01
P59598	Putative Polycomb group protein ASXL1 OS=Mus musculus GN=Asxl1 PE=1 SV=1 - [ASXL1_MOUSE]	2.64	1	2	2	3	2.259	1.445	1.041	1.060	5.24	2.64	2	3	1514	162.6	5.99
O88851	Putative hydrolase RBBP9 OS=Mus musculus GN=Rbbp9 PE=1 SV=2 - [RBBP9_MOUSE]	58.60	1	8	8	25	0.802	0.869	0.952	1.060	73.42	58.60	13	25	186	20.9	5.97
Q9DC48	Pre-mRNA-processing factor 17 OS=Mus musculus GN=Cdc40 PE=1 SV=1 - [PRP17_MOUSE]	1.38	1	1	1	1	0.841	0.708	0.933	1.060	2.83	1.38	1	1	579	65.4	7.06
P63154	Crooked neck-like protein 1 OS=Mus musculus GN=Crnkl1 PE=2 SV=1 - [CRNL1_MOUSE]	9.28	2	6	6	12	0.305	0.960	0.987	1.060	34.35	9.28	11	12	690	83.4	6.93
E9Q9X9	Uncharacterized protein OS=Mus musculus GN=Gm3033 PE=4 SV=1 - [E9Q9X9_MOUSE]	10.34	1	1	1	1	0.611	1.128	1.079	1.060	0.00	10.34	1	1	145	17.0	5.10
Q8BH58	TIP41-like protein OS=Mus musculus GN=Tiprl PE=2 SV=1 - [TIPRL_MOUSE]	51.66	1	11	11	45	0.636	1.064	0.938	1.060	156.74	51.66	19	45	271	31.2	5.63
Q3UGN9	Signal transducing adapter molecule 1 OS=Mus musculus GN=Stam PE=2 SV=1 - [Q3UGN9_MOUSE]	50.00	3	15	17	84	1.378	1.118	1.182	1.060	264.12	50.00	30	84	462	51.1	4.87
Q8BG92	Clavesin-2 OS=Mus musculus GN=Clvs2 PE=2 SV=1 - [CLVS2_MOUSE]	31.19	2	5	8	40	0.744	1.115	0.892	1.060	134.46	31.19	14	40	327	37.9	6.20
Q05915	GTP cyclohydrolase 1 OS=Mus musculus GN=Gch1 PE=2 SV=1 - [GCH1_MOUSE]	20.33	1	5	5	7	1.518	1.354	1.280	1.060	12.00	20.33	7	7	241	27.0	8.27
Q80TS7	Dendrin OS=Mus musculus GN=Ddn PE=1 SV=3 - [DEND_MOUSE]	7.04	1	4	4	6	0.680	2.622	2.421	1.060	15.47	7.04	6	6	710	76.4	9.98
Q9CR98	Protein FAM136A OS=Mus musculus GN=Fam136a PE=1 SV=1 - [F136A_MOUSE]	76.81	2	10	10	43	1.538	1.317	1.336	1.061	132.11	76.81	16	43	138	15.7	7.61
Q9R0X4	Acyl-coenzyme A thioesterase 9, mitochondrial OS=Mus musculus GN=Acot9 PE=1 SV=1 - [ACOT9_MOUSE]	45.33	2	19	19	96	0.650	0.788	0.911	1.061	290.99	45.33	36	96	439	50.5	8.59
Q9WTX6	Cullin-1 OS=Mus musculus GN=Cul1 PE=1 SV=1 - [CUL1_MOUSE]	38.02	3	28	28	96	0.756	0.886	0.975	1.061	249.17	38.02	47	96	776	89.6	8.00

Q8C460-2	Isoform 2 of ERI1 exoribonuclease 3 OS=Mus musculus GN=Eri3 - [ERI3_MOUSE]	11.15	2	3	3	12	0.352	1.233	0.970	1.061	37.33	11.15	5	12	296	32.9	7.99
Q60597	2-oxoglutarate dehydrogenase, mitochondrial OS=Mus musculus GN=Ogdh PE=1 SV=3 - [ODO1_MOUSE]	55.52	6	41	49	429	0.754	0.790	0.960	1.061	1344.12	55.52	90	429	1023	116.4	6.83
Q91YQ3	Cold shock domain-containing protein C2 OS=Mus musculus GN=Csdc2 PE=2 SV=2 - [CSDC2_MOUSE]	33.12	1	4	4	10	1.296	0.986	1.267	1.061	22.57	33.12	7	10	154	16.8	7.55
A2ALS5	Protein Rap1gap OS=Mus musculus GN=Rap1gap PE=2 SV=1 - [A2ALS5_MOUSE]	60.37	4	1	30	164	1.373	0.679	1.267	1.061	433.82	60.37	53	164	694	76.7	6.07
Q8BM13	Noelin-2 OS=Mus musculus GN=Olfm2 PE=1 SV=2 - [NOE2_MOUSE]	15.85	1	7	7	15	1.202	0.920	0.897	1.061	45.64	15.85	12	15	448	50.7	8.51
A2AEE7	Cadherin EGF LAG seven-pass G-type receptor 2 OS=Mus musculus GN=Celsr2 PE=4 SV=1 - [A2AEE7_MOUSE]	13.46	3	6	29	66	0.866	1.135	1.085	1.061	169.61	13.46	47	66	2919	316.8	5.47
Q3UQ84	Threonine--tRNA ligase, mitochondrial OS=Mus musculus GN=Tars2 PE=2 SV=1 - [SYTM_MOUSE]	24.07	4	16	17	38	0.748	1.026	0.953	1.061	105.75	24.07	29	38	723	81.6	7.87
Q8CIB5	Fermitin family homolog 2 OS=Mus musculus GN=Fermt2 PE=1 SV=1 - [FERM2_MOUSE]	42.79	2	19	25	85	0.773	0.910	0.932	1.061	257.61	42.79	43	85	680	77.8	6.70
Q91YL3	Uridine-cytidine kinase-like 1 OS=Mus musculus GN=Uckl1 PE=1 SV=1 - [UCKL1_MOUSE]	24.45	5	12	12	27	0.928	0.759	0.856	1.061	62.59	24.45	20	27	548	60.8	7.15
P60766-1	Isoform 1 of Cell division control protein 42 homolog OS=Mus musculus GN=Cdc42 - [CDC42_MOUSE]	61.26	7	3	9	134	1.463	1.139	0.971	1.061	316.69	61.26	16	134	191	21.3	6.04
Q8C804	Spindle and centriole-associated protein 1 OS=Mus musculus GN=Spice1 PE=1 SV=2 - [SPICE_MOUSE]	5.35	1	3	3	4	2.623	1.623	1.319	1.061	5.97	5.35	3	4	860	95.6	7.05
Q91WT4	DnaJ homolog subfamily C member 17 OS=Mus musculus GN=Dnajc17 PE=2 SV=2 -	16.50	1	3	4	6	0.825	1.069	1.062	1.061	14.21	16.50	5	6	303	34.4	8.57
Q91Y17	Protein Pcdha9 OS=Mus musculus GN=Pcdha2 PE=2 SV=1 - [Q91Y17_MOUSE]	5.59	4	1	4	8	8.445	2.267	1.494	1.061	21.09	5.59	6	8	948	102.2	5.05
Q9DBC7	cAMP-dependent protein kinase type I-alpha regulatory subunit OS=Mus musculus GN=Prkar1a PE=1 SV=3 - [illegible]	49.34	3	15	18	98	0.969	0.911	1.080	1.062	302.88	49.34	32	98	381	43.2	5.35
Q9R0M4	Podocalyxin OS=Mus musculus GN=Podxl PE=1 SV=2 - [PODXL_MOUSE]	5.57	3	3	4	9	1.258	1.649	1.074	1.062	27.64	5.57	5	9	503	53.4	4.97
Q99KR7	Peptidyl-prolyl cis-trans isomerase F, mitochondrial OS=Mus musculus GN=Ppif PE=1 SV=1 - [PPIF_MOUSE]	52.43	1	9	11	60	1.765	0.744	1.253	1.062	178.86	52.43	19	60	206	21.7	9.16
Q8BKC5	Importin-5 OS=Mus musculus GN=Ipo5 PE=1 SV=3 - [IPO5_MOUSE]	37.01	2	31	32	133	0.606	1.153	0.887	1.062	363.62	37.01	57	133	1097	123.5	4.93
Q8C1B7-3	Isoform 3 of Septin-11 OS=Mus musculus GN=Sept11 - [SEP11_MOUSE]	49.18	4	10	22	248	0.926	1.365	1.340	1.062	643.49	49.18	38	248	425	48.9	6.81
Q8C7R4	Ubiquitin-like modifier-activating enzyme 6 OS=Mus musculus GN=Uba6 PE=1 SV=1 - [UBA6_MOUSE]	27.92	2	24	24	86	0.743	1.025	0.946	1.062	287.84	27.92	41	86	1053	117.9	6.11
Q9WUC3	Lymphocyte antigen 6H OS=Mus musculus GN=Ly6h PE=2 SV=2 - [LY6H_MOUSE]	56.83	2	7	7	69	0.754	1.438	0.923	1.062	187.36	56.83	12	69	139	14.7	7.53
E9Q8V6	Protein Dennd4a OS=Mus musculus GN=Dennd4a PE=4 SV=1 - [E9Q8V6_MOUSE]	9.42	1	13	14	26	0.797	0.754	1.011	1.062	62.48	9.42	19	26	1869	209.2	7.56
D3Z1L0	Transcription factor 7-like 2 (Fragment) OS=Mus musculus GN=Tcf7l2 PE=2 SV=1 - [D3Z1L0_MOUSE]	8.30	18	2	2	3	1.341	0.865	0.884	1.062	9.45	8.30	2	3	265	29.6	9.31
Q6P6M7	O-phosphoseryl-tRNA(Sec) selenium transferase OS=Mus musculus GN=Sepsecs PE=1 SV=2 - [SPCS_MOUSE]	17.26	4	7	7	20	1.846	1.205	0.949	1.062	65.87	17.26	12	20	504	55.3	8.06
P16406	Glutamyl aminopeptidase OS=Mus musculus GN=Enpep PE=1 SV=1 - [AMPE_MOUSE]	10.37	3	9	9	17	0.939	1.242	1.007	1.062	49.35	10.37	14	17	945	107.9	5.44

Q8BZ64	Tectonic-1 OS=Mus musculus GN=Tctn1 PE=1 SV=1 - [TECT1_MOUSE]	6.58	2	2	2	3	0.747	1.152	0.876	1.062	7.99	6.58	3	3	593	63.4	7.28
Q9Z268	RasGAP-activating-like protein 1 OS=Mus musculus GN=Rasal1 PE=2 SV=2 -	42.55	5	32	32	115	0.510	2.394	1.169	1.062	355.76	42.55	57	115	799	89.3	6.37
A2APT9	Kelch domain-containing protein 7A OS=Mus musculus GN=Klhdc7a PE=2 SV=3 -	15.91	1	6	7	13	1.451	0.905	1.428	1.062	27.89	15.91	10	13	773	83.5	6.33
Q5EBG6	Heat shock protein beta-6 OS=Mus musculus GN=Hspb6 PE=1 SV=1 - [HSPB6_MOUSE]	8.02	1	1	1	2	2.582	1.631	1.056	1.062	6.02	8.02	2	2	162	17.5	6.00
Q9DBX3	Sushi domain-containing protein 2 OS=Mus musculus GN=Susd2 PE=1 SV=1 - [SUSD2_MOUSE]	14.51	3	11	11	38	1.012	1.378	0.858	1.062	104.05	14.51	19	38	820	90.6	6.62
Q61194-2	Isoform 2 of Phosphatidylinositol 4-phosphate 3-kinase C2 domain-containing subunit alpha OS=Mus musculus GN=Pik3c2a - [P3C2A_MOUSE]	7.66	3	10	10	17	1.750	0.784	1.016	1.062	53.56	7.66	17	17	1658	187.4	8.24
Q80W68	Kin of IRRE-like protein 1 OS=Mus musculus GN=Kirrel PE=1 SV=1 - [KIRR1_MOUSE]	7.86	2	3	3	4	1.888	1.085	1.254	1.062	13.45	7.86	4	4	789	87.1	5.92
D3Z5I9	RNA-binding protein 33 OS=Mus musculus GN=Rbm33 PE=2 SV=1 - [D3Z5I9_MOUSE]	2.10	2	2	2	6	1.768	1.117	1.003	1.063	20.99	2.10	4	6	1191	132.7	6.87
Q9CZW5	Mitochondrial import receptor subunit TOM70 OS=Mus musculus GN=Tomm70a PE=1 SV=2 - [TOM70_MOUSE]	49.26	3	30	30	167	0.674	1.052	1.085	1.063	469.64	49.26	51	167	611	67.5	7.53
Q91XX9	Protein Pcdhgb6 OS=Mus musculus GN=Pcdhga9 PE=2 SV=1 - [Q91XX9_MOUSE]	6.65	1	1	5	27	1.521	1.943	1.301	1.063	74.53	6.65	10	27	932	101.1	5.12
F6TU77	Protein Fam184a (Fragment) OS=Mus musculus GN=Fam184a PE=2 SV=1 -	19.04	4	15	18	39	1.656	1.173	1.129	1.063	105.02	19.04	29	39	1003	117.0	5.74
Q6DIB5	Multiple epidermal growth factor-like domains protein 10 OS=Mus musculus GN=Megf10 PE=1 SV=1 - [MEG10_MOUSE]	4.45	1	5	5	11	1.992	1.070	1.266	1.063	31.83	4.45	8	11	1147	122.9	7.05
Q8BKE9	Intraflagellar transport protein 74 homolog OS=Mus musculus GN=Ift74 PE=1 SV=2 - [IFT74_MOUSE]	33.33	2	16	17	45	1.576	1.127	0.994	1.063	139.52	33.33	27	45	600	69.3	6.00
Q8K2B3	Succinate dehydrogenase [ubiquinone] flavoprotein subunit, mitochondrial OS=Mus musculus GN=Sdha PE=1 SV=1 - [DHSA_MOUSE]	71.54	1	35	35	504	1.488	0.859	0.971	1.063	1761.39	71.54	66	504	664	72.5	7.37
Q6P9N8	Protein Trak2 OS=Mus musculus GN=Trak2 PE=2 SV=1 - [Q6P9N8_MOUSE]	6.46	2	4	4	6	1.053	1.100	1.538	1.063	14.67	6.46	5	6	913	101.2	5.16
Q9CQ65	S-methyl-5'-thioadenosine phosphorylase OS=Mus musculus GN=Mtap PE=2 SV=1 - [MTAP_MOUSE]	55.12	1	12	12	62	3.020	0.731	0.785	1.063	200.01	55.12	23	62	283	31.0	7.14
D3YWB7	Protein Gm4297 OS=Mus musculus GN=Gm4297 PE=4 SV=1 - [D3YWB7_MOUSE]	2.84	1	1	1	2	1.526	1.728	1.067	1.063	2.11	2.84	1	2	211	24.4	4.50
Q99KR6	E3 ubiquitin-protein ligase RNF34 OS=Mus musculus GN=Rnf34 PE=1 SV=1 - [RNF34_MOUSE]	14.63	1	4	4	21	1.154	1.368	1.364	1.063	65.16	14.63	7	21	376	42.0	4.84
Q8C190	VPS9 domain-containing protein 1 OS=Mus musculus GN=Vps9d1 PE=2 SV=1 -	18.18	3	3	8	17	1.251	1.461	1.045	1.063	47.41	18.18	13	17	649	71.2	6.87
Q8R322	Nucleoporin GLE1 OS=Mus musculus GN=Gle1 PE=2 SV=2 - [GLE1_MOUSE]	10.73	1	3	3	4	0.642	0.961	0.954	1.063	11.06	10.73	4	4	699	79.5	7.61
Q0VE82	Copine-7 OS=Mus musculus GN=Cpne7 PE=2 SV=1 - [CPNE7_MOUSE]	29.44	1	12	13	70	0.693	1.729	0.974	1.063	174.64	29.44	21	70	557	61.9	5.40
Q3UVL4	Vacuolar protein sorting-associated protein 51 homolog OS=Mus musculus GN=Vps51 PE=2 SV=2 - [VPS51_MOUSE]	48.08	10	28	28	87	0.550	1.207	1.047	1.063	276.62	48.08	48	87	782	86.1	6.24
Q7TMM9	Tubulin beta-2A chain OS=Mus musculus GN=Tubb2a PE=1 SV=1 - [TBB2A_MOUSE]	83.82	2	3	33	4988	0.731	1.309	1.106	1.063	13478.21	83.82	61	4988	445	49.9	4.89
Q6PCM1	Lysine-specific demethylase 3A OS=Mus musculus GN=Kdm3a PE=1 SV=1 -	1.13	1	1	1	1	0.742	0.962	1.044	1.063	2.46	1.13	1	1	1323	147.8	7.74

Q03963	Interferon-induced, double stranded RNA-activated protein kinase OS=Mus musculus GN=Eif2ak2 PE=1 SV=2 - [E2AK2_MOUSE]	11.84	1	4	5	9	0.983	1.168	0.793	1.063	34.25	11.84	8	9	515	58.2	8.57
Q8K4K6	Pantothenate kinase 1 OS=Mus musculus GN=Pank1 PE=1 SV=1 - [PANK1_MOUSE]	11.50	2	3	5	10	1.275	1.127	1.200	1.063	21.93	11.50	8	10	548	60.1	8.03
Q8CF89	TGF-beta-activated kinase 1 and MAP3K7-binding protein 1 OS=Mus musculus GN=Tab1 PE=1 SV=2 - [TAB1_MOUSE]	32.07	1	11	11	42	1.163	1.008	1.030	1.063	119.96	32.07	18	42	502	54.6	5.52
Q8JZS0	Protein lin-7 homolog A OS=Mus musculus GN=Lin7a PE=1 SV=2 - [LIN7A_MOUSE]	52.79	3	6	12	98	0.663	1.653	1.417	1.063	231.29	52.79	22	98	233	26.0	8.72
Q80TG9	Leucine-rich repeat and fibronectin type-III domain containing protein 2 OS=Mus musculus GN=Lrfn2 PE=1 SV=2 - [illegible]	9.52	1	5	5	8	1.066	1.397	0.872	1.064	26.16	9.52	7	8	788	84.9	6.74
Q64151	Semaphorin-4C OS=Mus musculus GN=Sema4c PE=1 SV=1 - [SEM4C_MOUSE]	4.44	1	3	3	4	2.431	0.345	1.012	1.064	10.69	4.44	3	4	834	92.5	7.24
Q9D1E8	1-acyl-sn-glycerol-3-phosphate acyltransferase epsilon OS=Mus musculus GN=Agpat5 PE=2 SV=2 - [PLCE_MOUSE]	23.56	2	9	9	30	0.576	0.650	0.748	1.064	86.87	23.56	15	30	365	42.2	9.38
Q3TE40	DNA-directed RNA polymerase I subunit RPA2 OS=Mus musculus GN=Rpa2 PE=2 SV=1 - [Q3TE40_MOUSE]	33.70	3	6	6	17	1.067	0.989	0.986	1.064	55.03	33.70	9	17	270	29.4	5.76
Q9CPX6	Ubiquitin-like-conjugating enzyme ATG3 OS=Mus musculus GN=Atg3 PE=1 SV=1 - [ATG3_MOUSE]	27.71	1	8	8	19	0.873	1.064	1.052	1.064	51.36	27.71	14	19	314	35.8	4.72
O88888	Amyloid beta A4 precursor protein-binding family A member 3 OS=Mus musculus GN=Apba3 PE=1 SV=1 - [APBA3_MOUSE]	3.68	1	2	2	4	1.516	0.854	0.946	1.064	11.86	3.68	3	4	571	60.7	5.08
Q9CW07	Protein phosphatase 1 regulatory subunit 3G OS=Mus musculus GN=Ppp1r3g PE=2 SV=2 - [PP13G_MOUSE]	2.31	1	1	1	3	0.849	1.458	1.971	1.064	5.53	2.31	2	3	347	37.8	5.00
E9Q1T1	Calcium/calmodulin-dependent protein kinase type II subunit beta OS=Mus musculus GN=Camk2d PE=2 SV=1 - [illegible]	49.72	13	3	26	382	0.728	1.540	1.128	1.064	1087.34	49.72	46	382	533	60.0	7.27
P61021	Ras-related protein Rab-5B OS=Mus musculus GN=Rab5b PE=1 SV=1 - [RAB5B_MOUSE]	64.65	1	8	11	74	0.751	0.999	0.953	1.064	219.58	64.65	19	74	215	23.7	8.13
Q4ACU6	SH3 and multiple ankyrin repeat domains protein 3 OS=Mus musculus GN=Shank3 PE=1 SV=2 - [SHAN3_MOUSE]	33.80	2	41	48	174	0.672	1.441	2.389	1.064	530.77	33.80	82	174	1805	192.1	8.90
Q9EPL2	Calsyntenin-1 OS=Mus musculus GN=Clstn1 PE=1 SV=1 - [CSTN1_MOUSE]	25.23	1	1	23	77	1.038	0.720	1.778	1.064	242.25	25.23	39	77	979	108.8	4.92
Q05BC8	Nbr1 protein OS=Mus musculus GN=Nbr1 PE=2 SV=1 - [Q05BC8_MOUSE]	3.27	7	3	3	4	1.202	1.242	1.111	1.064	8.31	3.27	4	4	888	98.8	4.88
Q9DCU2	Plasmolipin OS=Mus musculus GN=Pllp PE=2 SV=1 - [PLLP_MOUSE]	21.43	1	2	2	24	1.776	1.082	1.144	1.064	82.16	21.43	3	24	182	19.8	9.41
P62838	Ubiquitin-conjugating enzyme E2 D2 OS=Mus musculus GN=Ube2d2 PE=1 SV=1 -	28.57	2	3	4	32	0.495	1.044	0.976	1.064	100.29	28.57	7	32	147	16.7	7.83
Q811I0	ATP synthase mitochondrial F1 complex assembly factor 1 OS=Mus musculus GN=Atpaf1 PE=2 SV=1 - [illegible]	24.38	6	6	6	13	2.372	1.322	1.064	1.064	43.27	24.38	9	13	324	36.3	8.19
Q9CPR1	RWD domain-containing protein 4 OS=Mus musculus GN=Rwdd4 PE=2 SV=1 -	18.62	3	4	4	12	1.332	0.844	1.121	1.064	29.77	18.62	7	12	188	21.1	5.63
A2A690	Protein TANC2 OS=Mus musculus GN=Tanc2 PE=1 SV=1 - [TANC2_MOUSE]	23.37	4	33	34	131	0.908	1.002	1.275	1.064	373.01	23.37	60	131	1994	220.1	7.97
Q8VD69	Arrestin domain containing 4 OS=Mus musculus GN=Arrdc4 PE=2 SV=1 - [Q8VD69_MOUSE]	12.84	1	2	2	2	0.980	1.112	1.159	1.064	6.81	12.84	2	2	296	31.6	8.10
Q8BS95	Golgi pH regulator OS=Mus musculus GN=Gpr89a PE=2 SV=2 - [GPHR_MOUSE]	8.57	1	2	2	6	0.770	1.182	1.189	1.065	19.38	8.57	4	6	455	52.7	9.22
E9QAQ3	Rho GTPase-activating protein 26 OS=Mus musculus GN=Arhgap26 PE=2 SV=1 -	31.23	7	23	23	69	0.658	1.078	1.030	1.065	181.19	31.23	40	69	759	85.9	6.74

B2RXA1	Plcxd2 protein OS=Mus musculus GN=Plcxd2 PE=2 SV=1 - [B2RXA1_MOUSE]	6.18	1	2	2	4	0.798	0.571	0.980	1.065	10.42	6.18	4	4	340	38.6	8.88
Q91VZ6	Stromal membrane-associated protein 1 OS=Mus musculus GN=Smap1 PE=1 SV=1 - [SMAP1_MOUSE]	13.64	2	5	6	48	1.122	0.930	1.168	1.065	158.10	13.64	12	48	440	47.6	8.51
Q80W47	WD repeat domain phosphoinositide-interacting protein 2 OS=Mus musculus GN=Wipi2 PE=1 SV=1 - [WIPI2_MOUSE]	34.61	2	10	11	55	1.251	1.109	1.127	1.065	168.92	34.61	18	55	445	48.4	5.86
Q6PGF7	Exocyst complex component 8 OS=Mus musculus GN=Exoc8 PE=1 SV=1 - [EXOC8_MOUSE]	45.53	1	30	30	90	0.844	1.366	1.062	1.065	243.06	45.53	49	90	716	81.0	5.40
P19091	Androgen receptor OS=Mus musculus GN=Ar PE=1 SV=1 - [ANDR_MOUSE]	6.12	1	3	3	3	1.175	1.446	1.183	1.065	6.62	6.12	3	3	899	98.1	6.79
Q8C561-2	Isoform 2 of LMBR1 domain-containing protein 2 OS=Mus musculus GN=Lmbrd2 - [LMBD2_MOUSE]	9.40	3	4	4	5	0.555	1.065	1.099	1.065	16.68	9.40	5	5	649	75.7	7.50
E9Q3T3	Protein Kif14 OS=Mus musculus GN=Kif14 PE=2 SV=1 - [E9Q3T3_MOUSE]	1.66	2	2	3	4	1.938	0.987	0.878	1.065	10.58	1.66	4	4	1624	180.8	7.58
Q9ET26	RING finger protein 114 OS=Mus musculus GN=Rnf114 PE=2 SV=2 - [RN114_MOUSE]	26.64	1	7	7	22	1.729	0.766	1.320	1.065	63.17	26.64	12	22	229	25.7	7.03
Q923M0	Protein phosphatase 1 regulatory subunit 16A OS=Mus musculus GN=Ppp1r16a PE=1 SV=1 - [PP16A_MOUSE]	10.31	2	6	6	15	1.156	0.808	1.190	1.065	38.68	10.31	11	15	524	57.5	5.81
Q6P5U7	Leucine-rich repeat and WD repeat-containing protein KIAA1239 OS=Mus musculus GN=Kiaa1239 PE=2 SV=2 - [K1239_MOUSE]	14.87	1	24	25	67	0.649	2.023	1.572	1.065	184.13	14.87	39	67	1742	197.3	6.15
Q9CWZ7	Gamma-soluble NSF attachment protein OS=Mus musculus GN=Napg PE=1 SV=1 - [SNAG_MOUSE]	62.18	2	17	19	203	1.148	1.384	1.253	1.065	595.36	62.18	34	203	312	34.7	5.41
O89084	cAMP-specific 3',5'-cyclic phosphodiesterase 4A OS=Mus musculus GN=Pde4a PE=2 SV=2 - [PDE4A_MOUSE]	24.76	6	10	16	81	0.813	0.989	1.326	1.065	200.75	24.76	28	81	844	93.5	5.26
Q3UDE2	Tubulin--tyrosine ligase-like protein 12 OS=Mus musculus GN=Ttll12 PE=1 SV=1 - [TTL12_MOUSE]	28.48	2	13	13	56	0.940	0.971	0.934	1.065	177.01	28.48	25	56	639	74.0	5.63
Q8K1E0	Syntaxin-5 OS=Mus musculus GN=Stx5 PE=1 SV=3 - [STX5_MOUSE]	41.97	11	10	10	18	0.741	0.803	0.850	1.065	55.28	41.97	17	18	355	39.7	8.92
A2A4B4	EMILIN-3 OS=Mus musculus GN=Emilin3 PE=2 SV=1 - [A2A4B4_MOUSE]	3.23	2	2	2	3	2.638	3.487	0.507	1.065	4.98	3.23	2	3	711	77.3	6.74
P42932	T-complex protein 1 subunit theta OS=Mus musculus GN=Cct8 PE=1 SV=3 - [TCPQ_MOUSE]	80.84	6	37	37	249	0.702	1.012	0.866	1.065	757.93	80.84	69	249	548	59.5	5.62
F2Z3X6	3-phosphoinositide-dependent protein kinase 1 OS=Mus musculus GN=Pdpk1 PE=2 SV=1 - [F2Z3X6_MOUSE]	42.67	8	19	19	61	0.726	0.921	0.974	1.065	183.38	42.67	31	61	532	60.9	7.39
G3UYR1	Protein Rgs22 (Fragment) OS=Mus musculus GN=Rgs22 PE=2 SV=1 - [G3UYR1_MOUSE]	1.23	3	1	2	2	0.794	1.073	1.321	1.066	5.15	1.23	2	2	1134	131.2	8.51
Q9CZY3	Ubiquitin-conjugating enzyme E2 variant 1 OS=Mus musculus GN=Ube2v1 PE=1 SV=1 - [UB2V1_MOUSE]	69.39	6	5	10	59	0.892	1.058	1.060	1.066	155.75	69.39	15	59	147	16.3	7.96
Q64727	Vinculin OS=Mus musculus GN=Vcl PE=1 SV=4 - [VINC_MOUSE]	60.13	1	59	60	333	1.048	1.682	0.956	1.066	919.14	60.13	101	333	1066	116.6	6.00
Q69ZQ1	Uncharacterized family 31 glucosidase KIAA1161 OS=Mus musculus GN=Kiaa1161 PE=1 SV=2 [K1161_MOUSE]	8.24	1	5	5	8	0.890	0.754	1.015	1.066	20.44	8.24	8	8	716	81.3	8.29
P55065	Phospholipid transfer protein OS=Mus musculus GN=Pltp PE=1 SV=1 - [PLTP_MOUSE]	8.11	4	3	3	4	1.313	1.312	0.870	1.066	5.84	8.11	3	4	493	54.4	6.62
Q8K203	Endonuclease 8-like 3 OS=Mus musculus GN=Neil3 PE=1 SV=1 - [NEIL3_MOUSE]	8.42	4	4	4	8	1.633	1.976	0.787	1.066	11.02	8.42	5	8	606	67.4	9.01
E9Q8I7	Protein Nfxl1 OS=Mus musculus GN=Nfxl1 PE=2 SV=1 - [E9Q8I7_MOUSE]	6.43	3	5	6	12	1.609	1.243	1.065	1.066	32.55	6.43	8	12	918	101.4	8.40
G5E8E0	Regulator of G-protein signaling 17, isoform CRA_b OS=Mus musculus GN=Rgs17 PE=4 SV=1 - [G5E8E0_MOUSE]	33.04	3	6	7	22	1.871	1.395	1.060	1.066	59.95	33.04	11	22	230	26.6	6.01

Q8R5J9	PRA1 family protein 3 OS=Mus musculus GN=Arl6ip5 PE=1 SV=2 - [PRAF3_MOUSE]	27.66	1	6	6	40	0.692	1.102	1.067	1.066	123.35	27.66	10	40	188	21.5	9.61
P22723	Gamma-aminobutyric acid receptor subunit gamma-2 OS=Mus musculus GN=Gabrg2 PE=1 SV=3 - [GBRG2_MOUSE]	19.62	4	7	9	23	1.094	0.822	1.061	1.066	59.31	19.62	14	23	474	55.1	8.66
E9Q0A7	Oxidation resistance protein 1 OS=Mus musculus GN=Oxr1 PE=2 SV=1 - [E9Q0A7_MOUSE]	48.20	2	2	32	193	1.376	0.708	1.288	1.066	581.62	48.20	57	193	832	91.7	5.07
Q9WUU7	Cathepsin Z OS=Mus musculus GN=Ctsz PE=2 SV=1 - [CATZ_MOUSE]	15.03	1	4	4	31	1.778	1.165	1.014	1.066	72.02	15.03	8	31	306	34.0	6.60
A2AJI0	MAP7 domain-containing protein 1 OS=Mus musculus GN=Map7d1 PE=1 SV=1 -	28.61	3	1	22	78	1.363	0.788	1.014	1.066	167.13	28.61	35	78	846	93.2	10.15
Q8BGR8	GSK3-beta interaction protein OS=Mus musculus GN=Gskip PE=2 SV=1 - [GSKIP_MOUSE]	37.41	2	3	3	12	1.802	1.024	1.212	1.066	37.49	37.41	5	12	139	15.6	4.41
Q3UFS0	Protein zyg-11 homolog B OS=Mus musculus GN=Zyg11b PE=2 SV=2 - [ZY11B_MOUSE]	26.48	3	14	14	32	0.743	1.016	0.925	1.066	96.62	26.48	22	32	744	83.9	6.87
P33175	Kinesin heavy chain isoform 5A OS=Mus musculus GN=Kif5a PE=1 SV=3 - [KIF5A_MOUSE]	35.93	1	24	35	161	1.227	0.594	1.049	1.066	427.88	35.93	63	161	1027	116.9	5.94
Q8C8N3	Brorin OS=Mus musculus GN=Vwc2 PE=1 SV=1 - [VWC2_MOUSE]	4.63	1	1	1	1	1.464	1.236	1.341	1.066	4.74	4.63	1	1	324	35.4	5.38
Q4PNJ2	Sodium/potassium-transporting ATPase subunit beta-1-interacting protein 2 OS=Mus musculus GN=Nkain2 PE=1 SV=2 - [NKAI2_MOUSE]	6.25	1	1	1	3	0.800	1.168	1.139	1.066	6.76	6.25	2	3	208	23.9	5.07
Q9CPP6	NADH dehydrogenase [ubiquinone] 1 alpha subcomplex subunit 5 OS=Mus musculus GN=Ndufa5 PE=1 SV=3 - [NDUA5_MOUSE]	75.00	2	10	10	72	1.126	0.992	1.037	1.067	206.35	75.00	17	72	116	13.4	8.10
Q7TSH2	Phosphorylase b kinase regulatory subunit beta OS=Mus musculus GN=Phkb PE=1 SV=1 - [KPBB_MOUSE]	19.08	5	17	17	47	0.711	0.937	0.993	1.067	137.17	19.08	29	47	1085	123.8	6.83
Q99LG2	Transportin-2 OS=Mus musculus GN=Tnpo2 PE=2 SV=1 - [TNPO2_MOUSE]	17.47	2	7	13	32	0.519	1.130	1.001	1.067	88.56	17.47	22	32	887	100.4	4.98
A2BGI8	Peptidyl-prolyl cis-trans isomerase H (Fragment) OS=Mus musculus GN=Ppih PE=2 SV=1 - [A2BGI8_MOUSE]	62.03	4	9	10	93	1.172	1.406	0.989	1.067	214.21	62.03	16	93	158	17.2	8.34
P61028	Ras-related protein Rab-8B OS=Mus musculus GN=Rab8b PE=1 SV=1 - [RAB8B_MOUSE]	59.90	1	6	16	137	0.964	1.164	0.889	1.067	379.21	59.90	27	137	207	23.6	9.07
P70170-3	Isoform SUR2C of ATP-binding cassette sub-family C member 9 OS=Mus musculus GN=Abcc9 - [ABCC9_MOUSE]	3.51	8	2	4	6	0.510	1.170	1.258	1.067	7.29	3.51	5	6	1511	170.0	7.24
Q99MR3	Solute carrier family 12 member 9 OS=Mus musculus GN=Slc12a9 PE=1 SV=2 -	16.08	1	11	11	32	0.791	0.883	1.001	1.067	100.53	16.08	18	32	914	96.3	7.02
Q9D8Y1	Transmembrane protein 126A OS=Mus musculus GN=Tmem126a PE=2 SV=1 - [T126A_MOUSE]	19.90	2	4	4	21	0.735	0.987	0.891	1.067	53.85	19.90	8	21	196	21.5	9.41
A6H6A9-3	Isoform 3 of Rab GTPase-activating protein 1-like OS=Mus musculus GN=Rabgap1l -	37.84	2	11	15	50	1.157	1.135	1.100	1.067	123.95	37.84	25	50	370	42.7	7.33
Q80W82	JNK3 alpha1 protein kinase OS=Mus musculus GN=Mapk10 PE=2 SV=1 - [Q80W82_MOUSE]	48.10	15	7	17	77	0.930	1.155	1.210	1.067	222.07	48.10	30	77	422	48.5	7.69
Q9JKP5	Muscleblind-like protein 1 OS=Mus musculus GN=Mbnl1 PE=1 SV=1 - [MBNL1_MOUSE]	14.37	5	2	6	22	0.946	0.553	0.829	1.067	49.09	14.37	10	22	341	37.0	8.60
P83741-2	Isoform 2 of Serine/threonine-protein kinase WNK1 OS=Mus musculus GN=Wnk1 - [WNK1_MOUSE]	16.07	12	30	37	132	1.390	0.670	1.275	1.067	370.66	16.07	65	132	2626	279.0	6.61
Q3TYA4	Syntaxin-binding protein 6 OS=Mus musculus GN=Stxbp6 PE=2 SV=1 - [Q3TYA4_MOUSE]	50.00	4	10	10	35	0.765	0.783	1.073	1.067	95.26	50.00	19	35	210	23.7	9.19
Q03147	Cyclin-dependent kinase 7 OS=Mus musculus GN=Cdk7 PE=1 SV=2 - [CDK7_MOUSE]	5.49	1	2	2	4	0.862	0.969	1.089	1.067	9.63	5.49	3	4	346	38.9	8.47
P62806	Histone H4 OS=Mus musculus GN=Hist1h4a PE=1 SV=2 - [H4_MOUSE]	61.17	1	10	10	337	0.504	0.790	0.787	1.067	886.68	61.17	18	337	103	11.4	11.36

Q8BG22	Calcium-activated chloride channel regulator 2 OS=Mus musculus GN=Clca2 PE=2 SV=1 - [CLCA2_MOUSE]	1.27	2	2	2	2	0.544	0.932	0.773	1.067	3.76	1.27	2	2	942	103.6	6.19
O54916	RalBP1-associated Eps domain-containing protein 1 OS=Mus musculus GN=Reps1 PE=1 SV=2 - [REPS1_MOUSE]	30.57	10	19	21	64	1.408	1.109	1.183	1.067	190.81	30.57	36	64	795	86.5	5.58
Q9WTR1	Transient receptor potential cation channel subfamily V member 2 OS=Mus musculus GN=Trpv2 PE=1 SV=2 - [illegible]	19.18	1	13	13	36	1.001	1.097	1.008	1.068	105.94	19.18	21	36	756	85.9	6.99
D3YY16	Glia maturation factor gamma OS=Mus musculus GN=Gmfg PE=2 SV=1 - [D3YY16_MOUSE]	48.51	3	2	4	14	1.691	1.744	0.992	1.068	44.56	48.51	6	14	101	11.9	5.24
P63011	Ras-related protein Rab-3A OS=Mus musculus GN=Rab3a PE=1 SV=1 - [RAB3A_MOUSE]	57.27	2	4	12	298	0.702	1.286	1.199	1.068	877.52	57.27	23	298	220	25.0	5.03
O88704	Potassium/sodium hyperpolarization-activated cyclic nucleotide-gated channel 1 OS=Mus musculus GN=Hcn1 PE=1 SV=1 - [HCN1_MOUSE]	23.30	1	12	20	155	1.263	0.900	1.224	1.068	370.33	23.30	37	155	910	102.4	8.37
Q3UHD2	Glucose-fructose oxidoreductase domain-containing protein 1 OS=Mus musculus GN=Gfod1 PE=2 SV=1 - [illegible]	22.82	1	7	8	19	0.832	1.198	1.112	1.068	53.68	22.82	13	19	390	43.3	5.92
P11983	T-complex protein 1 subunit alpha OS=Mus musculus GN=Tcp1 PE=1 SV=3 - [TCPA_MOUSE]	63.67	3	28	28	161	0.673	0.948	0.913	1.068	505.30	63.67	52	161	556	60.4	6.16
Q6PDY2	2-aminoethanethiol dioxygenase OS=Mus musculus GN=Ado PE=1 SV=2 - [AEDO_MOUSE]	64.45	1	15	15	69	0.989	0.699	0.989	1.068	186.27	64.45	26	69	256	28.4	5.97
Q91WG5	5'-AMP-activated protein kinase subunit gamma-2 OS=Mus musculus GN=Prkag2 PE=1 SV=2 - [AAKG2_MOUSE]	23.14	4	9	11	25	0.653	1.351	1.170	1.068	69.39	23.14	20	25	566	62.9	9.36
Q6P6I6	DNA-directed RNA polymerase II subunit GRINL1A OS=Mus musculus GN=Polr2m PE=2 SV=2 - [illegible]	10.66	4	3	3	10	1.597	1.428	1.201	1.068	27.39	10.66	6	10	366	41.2	6.55
Q8BG58	Transmembrane prolyl 4-hydroxylase OS=Mus musculus GN=P4htm PE=2 SV=1 - [P4HTM_MOUSE]	11.93	2	4	4	9	0.934	1.163	0.979	1.068	21.95	11.93	5	9	503	57.0	6.09
P21614	Vitamin D-binding protein OS=Mus musculus GN=Gc PE=1 SV=2 - [VTDB_MOUSE]	49.79	1	18	19	63	2.963	1.981	0.719	1.068	218.58	49.79	31	63	476	53.6	5.50
P35831	Tyrosine-protein phosphatase non-receptor type 12 OS=Mus musculus GN=Ptpn12 PE=1 SV=3 - [PTN12_MOUSE]	18.32	1	10	10	20	1.126	1.273	1.222	1.068	62.59	18.32	16	20	775	86.5	5.99
P80314	T-complex protein 1 subunit beta OS=Mus musculus GN=Cct2 PE=1 SV=4 - [TCPB_MOUSE]	74.39	1	33	33	310	0.873	1.102	0.902	1.068	997.30	74.39	63	310	535	57.4	6.40
Q62178	Semaphorin-4A OS=Mus musculus GN=Sema4a PE=1 SV=2 - [SEM4A_MOUSE]	23.68	6	16	17	46	0.736	0.874	0.865	1.068	112.57	23.68	30	46	760	83.4	7.72
P16546	Spectrin alpha chain, non-erythrocytic 1 OS=Mus musculus GN=Sptan1 PE=1 SV=4 - [SPTN1_MOUSE]	77.18	5	2	206	3711	1.300	1.170	1.236	1.068	10872.33	77.18	377	3711	2472	284.4	5.33
Q0VF22	Coiled-coil domain-containing protein 138 OS=Mus musculus GN=Ccdc138 PE=2 SV=1 - [CC138_MOUSE]	1.47	1	1	1	1	2.639	0.980	1.141	1.068	3.24	1.47	1	1	680	77.9	8.16
Q9JHI5	Isovaleryl-CoA dehydrogenase, mitochondrial OS=Mus musculus GN=Ivd PE=1 SV=1 - [IVD_MOUSE]	54.95	1	19	19	109	1.507	1.043	0.759	1.068	325.62	54.95	34	109	424	46.3	8.29
Q3U108-3	Isoform 3 of AT-rich interactive domain-containing protein 5A OS=Mus musculus GN=Arid5a - [illegible]	2.05	1	1	1	1	2.008	1.159	1.200	1.068	3.73	2.05	1	1	684	73.3	8.76
P61080	Ubiquitin-conjugating enzyme E2 D1 OS=Mus musculus GN=Ube2d1 PE=2 SV=1 -	24.49	2	2	3	24	0.924	0.906	1.084	1.068	82.46	24.49	6	24	147	16.6	7.42
Q8BKQ3	Testican-1 OS=Mus musculus GN=Spock1 PE=2 SV=1 - [Q8BKQ3_MOUSE]	18.45	2	6	8	15	1.548	0.919	1.553	1.068	44.80	18.45	13	15	439	49.2	6.01
Q8BXZ8	Putative methyltransferase NSUN6 OS=Mus musculus GN=Nsun6 PE=4 SV=1 - [Q8BXZ8_MOUSE]	21.74	2	2	2	5	1.506	1.192	1.084	1.068	15.12	21.74	2	5	115	13.3	6.93

Q9JLB2	MAGUK p55 subfamily member 5 OS=Mus musculus GN=Mpp5 PE=1 SV=1 - [MPP5_MOUSE]	29.04	1	14	15	30	0.853	0.816	0.999	1.068	78.69	29.04	25	30	675	77.2	6.09
Q8VIM9	Immunity-related GTPase family Q protein OS=Mus musculus GN=Irgq PE=2 SV=1 - [IRGQ_MOUSE]	26.93	1	13	13	53	1.020	0.956	1.023	1.068	144.45	26.93	21	53	583	59.3	4.83
Q69ZF3	Non-lysosomal glucosylceramidase OS=Mus musculus GN=Gba2 PE=1 SV=2 -	16.67	2	9	9	48	0.727	1.112	0.996	1.068	156.26	16.67	16	48	918	103.2	5.59
Q9JL26-2	Isoform 2 of Formin-like protein 1 OS=Mus musculus GN=Fmnl1 - [FMNL_MOUSE]	23.97	3	16	24	66	0.646	1.192	1.107	1.068	166.91	23.97	42	66	1064	119.0	5.66
F7DBB3	Protein Ahnak2 (Fragment) OS=Mus musculus GN=Ahnak2 PE=2 SV=1 -	28.43	2	10	20	58	3.109	0.876	1.068	1.069	167.74	28.43	34	58	1576	166.4	5.78
E9PYG6	Protein Rasa1 OS=Mus musculus GN=Rasa1 PE=2 SV=1 - [E9PYG6_MOUSE]	11.75	1	11	11	38	0.900	1.191	0.889	1.069	101.85	11.75	19	38	1038	115.4	6.60
D3YW20	Protein 9030624J02Rik (Fragment) OS=Mus musculus GN=9030624J02Rik PE=2 SV=2 - [D3YW20_MOUSE]	17.73	8	15	15	34	0.915	1.057	0.905	1.069	88.61	17.73	25	34	874	98.9	7.08
P61164	Alpha-centractin OS=Mus musculus GN=Actr1a PE=2 SV=1 - [ACTZ_MOUSE]	55.59	1	9	17	110	0.654	0.835	0.970	1.069	368.43	55.59	31	110	376	42.6	6.64
P01831	Thy-1 membrane glycoprotein OS=Mus musculus GN=Thy1 PE=1 SV=1 - [THY1_MOUSE]	50.00	1	8	8	237	1.222	1.111	1.310	1.069	711.32	50.00	13	237	162	18.1	8.97
O54950	5'-AMP-activated protein kinase subunit gamma-1 OS=Mus musculus GN=Prkag1 PE=1 SV=2 - [AAKG1_MOUSE]	23.94	1	5	7	25	0.808	1.146	1.127	1.069	67.03	23.94	13	25	330	37.5	7.14
P16627	Heat shock 70 kDa protein 1-like OS=Mus musculus GN=Hspa1l PE=2 SV=4 - [HS71L_MOUSE]	35.10	1	4	24	428	1.241	1.156	0.980	1.069	1092.58	35.10	42	428	641	70.6	6.24
P97300	Neuroplastin OS=Mus musculus GN=Nptn PE=1 SV=3 - [NPTN_MOUSE]	36.27	5	7	17	252	0.390	2.680	2.476	1.069	691.42	36.27	31	252	397	44.3	7.74
Q5SVJ0	Calcium/calmodulin-dependent protein kinase II, beta, isoform CRA_b OS=Mus musculus GN=Camk2b PE=4 SV=1 - [Q5SVJ0_MOUSE]	58.11	4	19	31	623	0.631	0.965	1.277	1.069	1862.52	58.11	55	623	666	72.9	7.27
D3YVS7	Uncharacterized protein (Fragment) OS=Mus musculus GN=Cystm1 PE=2 SV=1 -	11.90	2	1	1	11	1.553	0.869	1.150	1.069	34.36	11.90	2	11	84	9.2	4.44
Q99N15	17beta-hydroxysteroid dehydrogenase type 10/short chain L-3-hydroxyacyl-CoA dehydrogenase OS=Mus musculus GN=Hsd17b10 PE=2 SV=1 - [Q99N15_MOUSE]	76.25	3	13	13	73	0.851	0.954	0.977	1.069	234.50	76.25	24	73	261	27.3	8.76
Q8C5P7	Testis development-related protein OS=Mus musculus GN=Tdrp PE=2 SV=1 - [TDRP_MOUSE]	34.07	2	4	4	16	2.093	0.747	1.488	1.069	53.56	34.07	7	16	182	20.2	5.73
Q8VHK1	Caskin-2 OS=Mus musculus GN=Caskin2 PE=1 SV=3 - [CSKI2_MOUSE]	18.82	2	10	15	48	1.604	0.963	1.099	1.069	125.92	18.82	26	48	1201	126.7	7.39
Q7TMS5	ATP-binding cassette sub-family G member 2 OS=Mus musculus GN=Abcg2 PE=2 SV=1 - [ABCG2_MOUSE]	8.37	3	4	5	24	0.643	1.106	0.998	1.069	79.91	8.37	9	24	657	72.9	8.54
E9Q179	G-rich sequence factor 1 OS=Mus musculus GN=Grsf1 PE=2 SV=1 - [E9Q179_MOUSE]	21.55	4	7	7	29	1.364	0.704	1.027	1.069	89.29	21.55	14	29	362	41.6	5.33
Q9JKC6	Cell cycle exit and neuronal differentiation protein 1 OS=Mus musculus GN=Cend1 PE=1 SV=1 - [CEND_MOUSE]	71.14	1	14	14	164	0.784	0.707	1.031	1.069	286.85	71.14	24	164	149	15.0	8.97
Q4FE56	Ubiquitin carboxyl-terminal hydrolase OS=Mus musculus GN=Usp9x PE=2 SV=1 - [Q4FE56_MOUSE]	27.17	8	44	63	254	0.625	1.069	0.883	1.069	697.58	27.17	115	254	2554	290.0	5.83
F8WGW3	S1 RNA-binding domain-containing protein 1 OS=Mus musculus GN=Srbd1 PE=2 SV=1 - [F8WGW3_MOUSE]	4.49	3	3	4	5	1.353	0.872	0.944	1.069	11.27	4.49	5	5	981	110.1	8.78
Q3U0P5	Ectonucleoside triphosphate diphosphohydrolase 6, isoform CRA_a OS=Mus musculus GN=Entpd6 PE=2 SV=1 - [Q3U0P5_MOUSE]	11.21	1	4	4	19	0.718	1.135	0.928	1.069	49.52	11.21	8	19	455	49.8	8.62
Q3TY60-2	Isoform 2 of Protein FAM131B OS=Mus musculus GN=Fam131b - [F131B_MOUSE]	22.70	3	8	8	39	0.778	1.229	1.523	1.069	105.91	22.70	14	39	348	37.5	4.48

D3YVU0	Ubiquitin carboxyl-terminal hydrolase 46 OS=Mus musculus GN=Usp46 PE=2 SV=1 - [D3YVU0_MOUSE]	22.12	5	7	7	20	0.966	1.292	1.125	1.070	66.14	22.12	11	20	339	39.5	7.36
Q3TQF0	F-box only protein 31 OS=Mus musculus GN=Fbxo31 PE=1 SV=1 - [FBX31_MOUSE]	7.89	1	2	2	3	0.712	1.598	0.985	1.070	5.77	7.89	2	3	507	57.2	6.98
Q80TJ1-4	Isoform 4 of Calcium-dependent secretion activator 1 OS=Mus musculus GN=Cadps - [CAPS1_MOUSE]	51.67	4	1	58	411	0.633	0.696	0.990	1.070	1231.70	51.67	101	411	1351	152.6	5.74
B9EHT4	CAP-Gly domain-containing linker protein 3 OS=Mus musculus GN=Clip3 PE=1 SV=1 - [CLIP3_MOUSE]	36.01	2	17	17	42	0.784	0.680	1.096	1.070	115.74	36.01	27	42	547	59.5	7.94
Q8K215	LYR motif-containing protein 4 OS=Mus musculus GN=Lyrm4 PE=2 SV=1 - [LYRM4_MOUSE]	56.04	1	5	6	20	1.101	0.973	0.953	1.070	46.27	56.04	9	20	91	10.8	10.13
Q9Z211	Peroxisomal membrane protein 11A OS=Mus musculus GN=Pex11a PE=2 SV=1 -	7.32	1	2	3	4	1.339	1.049	1.344	1.070	8.73	7.32	4	4	246	28.1	9.83
Q8R2Y8	Peptidyl-tRNA hydrolase 2, mitochondrial OS=Mus musculus GN=Ptrh2 PE=2 SV=1 - [PTH2_MOUSE]	38.12	1	5	5	19	0.938	1.032	0.941	1.070	59.31	38.12	8	19	181	19.5	7.42
Q6P1F6	Serine/threonine-protein phosphatase 2A 55 kDa regulatory subunit B alpha isoform OS=Mus musculus GN=Ppp2r2a PE=1 SV=1 - [2ABA_MOUSE]	48.32	2	12	20	93	0.842	0.954	1.230	1.070	264.19	48.32	36	93	447	51.7	6.20
P05627	Transcription factor AP-1 OS=Mus musculus GN=Jun PE=1 SV=3 - [JUN_MOUSE]	10.48	2	2	3	4	0.763	1.065	0.917	1.070	11.49	10.48	4	4	334	35.9	8.75
O88990	Alpha-actinin-3 OS=Mus musculus GN=Actn3 PE=2 SV=1 - [ACTN3_MOUSE]	18.89	3	3	17	76	1.468	0.639	1.329	1.070	186.59	18.89	29	76	900	103.0	5.45
O89116	Vesicle transport through interaction with t-SNAREs homolog 1A OS=Mus musculus GN=Vti1a PE=1 SV=1 - [VTI1A_MOUSE]	19.82	3	6	6	19	1.024	1.432	1.324	1.070	56.71	19.82	11	19	217	25.0	6.40
P37040	NADPH--cytochrome P450 reductase OS=Mus musculus GN=Por PE=1 SV=2 - [NCPR_MOUSE]	45.72	5	24	24	101	0.732	1.136	0.901	1.070	325.56	45.72	41	101	678	77.0	5.53
Q9ES56	Trafficking protein particle complex subunit 4 OS=Mus musculus GN=Trappc4 PE=1 SV=1 - [TPPC4_MOUSE]	30.14	1	6	6	15	1.060	1.119	0.953	1.070	40.01	30.14	10	15	219	24.4	6.21
Q7TMY8-3	Isoform 3 of E3 ubiquitin-protein ligase HUWE1 OS=Mus musculus GN=Huwe1 -	23.27	7	74	74	249	0.794	0.963	1.055	1.070	724.90	23.27	123	249	4362	480.6	5.20
Q9Z1W8	Potassium-transporting ATPase alpha chain 2 OS=Mus musculus GN=Atp12a PE=1 SV=3 - [AT12A_MOUSE]	8.99	1	2	11	575	0.969	0.679	1.455	1.070	1331.11	8.99	19	575	1035	114.7	6.33
A2A9F7	GPN-loop GTPase 2 OS=Mus musculus GN=Gpn2 PE=2 SV=1 - [A2A9F7_MOUSE]	14.63	2	2	2	4	0.540	1.382	0.933	1.070	17.93	14.63	3	4	287	31.8	4.93
Q0VBM2	Protein FAM83B OS=Mus musculus GN=Fam83b PE=1 SV=1 - [FA83B_MOUSE]	2.67	1	2	2	4	1.361	0.978	1.125	1.070	5.86	2.67	2	4	1012	114.6	9.11
Q8BGR6	ADP-ribosylation factor-like protein 15 OS=Mus musculus GN=Arl15 PE=2 SV=1 - [ARL15_MOUSE]	22.06	1	4	4	13	0.615	1.180	1.138	1.070	39.39	22.06	7	13	204	22.9	5.29
Q9R0D8	WD repeat-containing protein 54 OS=Mus musculus GN=Wdr54 PE=2 SV=1 -	47.90	2	9	9	37	1.999	1.220	1.096	1.070	154.90	47.90	16	37	334	35.6	6.21
Q6P9Z1	SWI/SNF-related matrix-associated actin-dependent regulator of chromatin subfamily D member 3 OS=Mus musculus GN=Smarcd3 PE=1 SV=2 -	18.84	1	5	7	20	0.646	0.863	0.757	1.070	40.55	18.84	10	20	483	55.0	9.35
Q5NCM5	Epsin-2 OS=Mus musculus GN=Epn2 PE=2 SV=1 - [Q5NCM5_MOUSE]	33.12	5	1	16	182	1.234	1.380	1.865	1.070	512.09	33.12	31	182	634	67.7	7.01
Q9EPW0	Type I inositol 3,4-bisphosphate 4-phosphatase OS=Mus musculus GN=Inpp4a PE=2 SV=1 - [illegible]	35.78	1	11	25	94	0.596	1.026	1.045	1.070	261.37	35.78	44	94	939	105.5	7.05
O35379	Multidrug resistance-associated protein 1 OS=Mus musculus GN=Abcc1 PE=1 SV=1 - [MRP1_MOUSE]	4.12	2	5	5	12	0.997	0.911	1.220	1.070	38.21	4.12	8	12	1528	171.1	7.36

Q9DCV4	Regulator of microtubule dynamics protein 1 OS=Mus musculus GN=Rmdn1 PE=2 SV=2 - [RMD1_MOUSE]	30.16	2	7	8	17	1.976	0.910	0.818	1.070	55.12	30.16	11	17	305	35.0	8.70
P12849	cAMP-dependent protein kinase type I-beta regulatory subunit OS=Mus musculus GN=Prkar1b PE=2 SV=2 - [illegible]	38.32	8	9	12	67	0.854	1.004	1.183	1.070	231.69	38.32	23	67	381	43.2	5.96
O35250	Exocyst complex component 7 OS=Mus musculus GN=Exoc7 PE=1 SV=2 - [EXOC7_MOUSE]	49.78	5	31	31	112	0.732	1.302	1.092	1.071	336.02	49.78	54	112	697	79.9	6.98
O08749	Dihydrolipoyl dehydrogenase, mitochondrial OS=Mus musculus GN=Dld PE=1 SV=2 - [DLDH_MOUSE]	67.98	1	26	26	546	1.714	1.175	1.269	1.071	1705.02	67.98	45	546	509	54.2	7.90
Q9CZ86	Protein Zfp248 OS=Mus musculus GN=Zfp248 PE=2 SV=1 - [Q9CZ86_MOUSE]	2.32	2	1	1	1	9.386	1.586	1.209	1.071	2.31	2.32	1	1	475	54.5	8.88
Q8R059	UDP-glucose 4-epimerase OS=Mus musculus GN=Gale PE=2 SV=1 - [GALE_MOUSE]	25.65	3	8	8	24	0.887	0.964	0.935	1.071	72.04	25.65	14	24	347	38.2	6.74
Q9D358	Low molecular weight phosphotyrosine protein phosphatase OS=Mus musculus GN=Acp1 PE=1 SV=3 - [PPAC_MOUSE]	50.63	1	3	8	43	1.940	1.212	0.931	1.071	141.13	50.63	12	43	158	18.2	6.74
Q9D061	Acyl-CoA-binding domain-containing protein 6 OS=Mus musculus GN=Acbd6 PE=1 SV=2 - [ACBD6_MOUSE]	51.06	3	13	13	54	1.161	1.202	1.249	1.071	164.22	51.06	23	54	282	30.9	5.11
D3YVE8	Solute carrier family 35 member G2 OS=Mus musculus GN=Slc35g2 PE=3 SV=1 -	9.71	1	4	4	17	0.660	0.610	0.669	1.071	43.06	9.71	6	17	412	46.4	7.87
F7D6J5	Sodium channel protein type 8 subunit alpha OS=Mus musculus GN=Scn8a PE=2 SV=1 - [F7D6J5_MOUSE]	12.87	13	16	20	40	0.701	0.660	1.236	1.071	125.89	12.87	27	40	1849	210.9	6.11
O35083	1-acyl-sn-glycerol-3-phosphate acyltransferase alpha OS=Mus musculus GN=Agpat1 PE=2 SV=1 - [PLCA_MOUSE]	31.23	1	2	7	18	0.746	0.963	0.967	1.071	55.79	31.23	12	18	285	31.7	9.14
P48168	Glycine receptor subunit beta OS=Mus musculus GN=Glrb PE=1 SV=2 - [GLRB_MOUSE]	15.32	2	6	6	23	1.211	0.415	0.748	1.071	68.97	15.32	12	23	496	55.9	8.81
Q8R502	Leucine-rich repeat-containing protein 8C OS=Mus musculus GN=Lrrc8c PE=1 SV=1 - [LRC8C_MOUSE]	14.82	3	8	9	27	0.829	0.961	1.069	1.071	102.26	14.82	16	27	803	92.3	7.81
P35235	Tyrosine-protein phosphatase non-receptor type 11 OS=Mus musculus GN=Ptpn11 PE=1 SV=2 - [PTN11_MOUSE]	50.75	2	28	28	145	0.942	0.795	1.016	1.071	432.32	50.75	52	145	597	68.4	7.30
E9Q6P4	Protein Tubgcp6 OS=Mus musculus GN=Tubgcp6 PE=2 SV=1 - [E9Q6P4_MOUSE]	4.20	2	3	4	12	0.724	0.878	1.109	1.071	33.76	4.20	8	12	1761	196.1	5.92
Q9CRA9	FGFR1 oncogene partner 2 homolog OS=Mus musculus GN=Fgfr1op2 PE=2 SV=1 - [FGOP2_MOUSE]	32.81	2	1	8	34	1.972	1.216	1.218	1.071	108.91	32.81	11	34	253	29.4	5.83
Q9DB32	Hydroxyacylglutathione hydrolase-like protein OS=Mus musculus GN=Haghl PE=2 SV=1 - [HAGHL_MOUSE]	46.64	4	9	9	26	1.700	0.851	0.731	1.071	89.32	46.64	16	26	283	31.5	5.55
Q5DU56-5	Isoform 5 of Protein NLRC3 OS=Mus musculus GN=Nlrc3 - [NLRC3_MOUSE]	7.11	5	1	2	2	2.541	0.897	1.045	1.071	3.89	7.11	2	2	239	25.5	9.17
Q9ESM6	Glycerophosphoinositol inositolphosphodiesterase GDPD2 OS=Mus musculus GN=Gdpd2 PE=1 SV=1 - [GDPD2_MOUSE]	7.61	1	3	3	7	0.724	0.699	0.660	1.072	18.63	7.61	5	7	539	61.1	8.16
Q8R1C3-2	Isoform 2 of Vesicular, overexpressed in cancer, prosurvival protein 1 OS=Mus musculus GN=Vopp1 - [illegible]	7.89	2	1	1	2	0.740	0.995	0.957	1.072	3.69	7.89	2	2	114	13.3	8.25
Q9QX60-2	Isoform 2 of Deoxyguanosine kinase, mitochondrial OS=Mus musculus GN=Dguok - [DGUOK_MOUSE]	15.45	3	2	2	5	1.441	0.776	0.945	1.072	13.31	15.45	3	5	246	28.8	6.28
D3Z641	Tetraspanin-5 OS=Mus musculus GN=Tspan5 PE=4 SV=1 - [D3Z641_MOUSE]	15.23	2	2	2	10	0.668	0.851	0.895	1.072	35.01	15.23	3	10	197	22.4	4.60
Q03717	Potassium voltage-gated channel subfamily B member 1 OS=Mus musculus GN=Kcnb1 PE=1 SV=2 - [KCNB1_MOUSE]	11.90	1	8	8	27	0.864	1.288	1.596	1.072	74.74	11.90	15	27	857	95.5	8.16

P26618	Platelet-derived growth factor receptor alpha OS=Mus musculus GN=Pdgfra PE=1 SV=3 - [PGFRA_MOUSE]	7.99	8	7	8	26	1.339	1.770	1.028	1.072	62.62	7.99	12	26	1089	122.6	5.15
Q00898	Alpha-1-antitrypsin 1-5 OS=Mus musculus GN=Serpina1e PE=1 SV=1 - [A1AT5_MOUSE]	35.35	2	4	13	133	10.152	5.515	3.309	1.072	419.13	35.35	24	133	413	45.9	5.73
Q6PD10	Inositol hexakisphosphate kinase 1 OS=Mus musculus GN=Ip6k1 PE=2 SV=1 - [IP6K1_MOUSE]	17.09	4	7	7	19	0.709	0.770	0.919	1.072	55.72	17.09	11	19	433	49.3	7.23
Q564G0	Guanylate kinase OS=Mus musculus GN=Guk1 PE=2 SV=1 - [Q564G0_MOUSE]	59.82	3	10	10	35	1.350	1.257	1.127	1.072	95.53	59.82	18	35	219	24.0	7.44
Q8C4Y3	Negative elongation factor B OS=Mus musculus GN=Nelfb PE=2 SV=2 - [NELFB_MOUSE]	17.07	2	10	11	21	1.472	1.153	0.843	1.072	54.20	17.07	14	21	580	65.6	6.02
Q8CF93	Polypeptide N-acetylgalactosaminyltransferase 13 OS=Mus musculus GN=Galnt13 PE=2 SV=1 - [illegible]	4.86	3	2	3	8	1.610	0.812	1.020	1.072	21.95	4.86	4	8	556	63.9	6.83
P35546-2	Isoform 2 of Proto-oncogene tyrosine-protein kinase receptor Ret OS=Mus musculus GN=Ret - [RET_MOUSE]	5.78	2	5	6	7	1.090	0.727	1.090	1.072	13.18	5.78	7	7	1073	119.4	7.06
Q9D906	Ubiquitin-like modifier-activating enzyme ATG7 OS=Mus musculus GN=Atg7 PE=1 SV=1 - [ATG7_MOUSE]	32.38	1	17	17	58	0.879	0.891	1.061	1.072	166.81	32.38	29	58	698	77.5	6.40
Q80VM7	Ankyrin repeat domain-containing protein 24 OS=Mus musculus GN=Ankrd24 PE=2 SV=4 - [ANR24_MOUSE]	5.18	2	4	5	12	0.844	1.347	1.204	1.072	22.62	5.18	7	12	985	106.2	4.89
Q8K337	Type II inositol 1,4,5-trisphosphate 5-phosphatase OS=Mus musculus GN=Inpp5b PE=1 SV=1 - [illegible]	7.15	3	6	7	11	1.178	1.229	1.118	1.073	26.63	7.15	9	11	993	112.7	5.64
Q8C0K5	Graves disease carrier protein homolog OS=Mus musculus GN=Slc25a16 PE=2 SV=1 - [GDC_MOUSE]	16.27	3	3	4	11	0.807	1.203	1.037	1.073	30.17	16.27	7	11	332	36.2	9.88
O88951	Protein lin-7 homolog B OS=Mus musculus GN=Lin7b PE=1 SV=2 - [LIN7B_MOUSE]	43.00	4	5	10	67	1.212	1.141	1.080	1.073	175.24	43.00	17	67	207	22.9	8.68
Q3UMF0-4	Isoform 4 of Cordon-bleu protein-like 1 OS=Mus musculus GN=Cobll1 - [COBL1_MOUSE]	22.86	6	1	20	76	1.266	0.659	1.042	1.073	231.18	22.86	34	76	1203	129.5	8.00
P58750	Serine/threonine-protein kinase pim-3 OS=Mus musculus GN=Pim3 PE=1 SV=1 - [PIM3_MOUSE]	6.44	1	1	1	1	1.419	0.759	1.059	1.073	2.58	6.44	1	1	326	35.9	5.83
Q8VC16	Leucine-rich repeat-containing protein 14 OS=Mus musculus GN=Lrrc14 PE=2 SV=1 - [LRC14_MOUSE]	12.58	3	4	5	9	1.067	0.901	0.939	1.073	26.11	12.58	7	9	493	54.9	6.67
Q9DBS1	Transmembrane protein 43 OS=Mus musculus GN=Tmem43 PE=1 SV=1 - [TMM43_MOUSE]	39.75	1	11	11	29	0.636	1.293	0.849	1.073	95.67	39.75	16	29	400	44.8	7.36
A6H8H5	Potassium voltage-gated channel subfamily B member 2 OS=Mus musculus GN=Kcnb2 PE=2 SV=2 - [KCNB2_MOUSE]	8.27	1	5	5	9	1.368	0.996	1.116	1.073	28.42	8.27	7	9	907	102.3	6.10
Q99MR1	PERQ amino acid-rich with GYF domain-containing protein 1 OS=Mus musculus GN=Gigyf1 PE=1 SV=2 - [illegible]	7.47	1	6	6	10	0.808	0.761	1.182	1.073	26.04	7.47	9	10	1044	116.2	5.39
Q60700	Mitogen-activated protein kinase kinase kinase 12 OS=Mus musculus GN=Map3k12 PE=1 SV=1 [illegible] [M3K12_MOUSE]	10.92	3	7	8	18	0.902	0.701	1.021	1.073	49.18	10.92	13	18	888	96.0	6.24
Q9CPV2	Anaphase-promoting complex subunit 16 OS=Mus musculus GN=Anapc16 PE=2 SV=1 - [APC16_MOUSE]	6.36	1	1	1	2	1.075	0.956	1.052	1.073	4.40	6.36	2	2	110	11.7	4.97
Q8BGT5	Alanine aminotransferase 2 OS=Mus musculus GN=Gpt2 PE=2 SV=1 - [ALAT2_MOUSE]	26.44	2	9	11	38	0.832	0.909	0.894	1.073	107.60	26.44	20	38	522	57.9	8.00
Q8K1H1	Tudor domain-containing protein 7 OS=Mus musculus GN=Tdrd7 PE=1 SV=1 - [TDRD7_MOUSE]	13.54	1	13	13	21	1.109	1.102	1.040	1.073	47.46	13.54	18	21	1086	122.1	6.70
P54254	Ataxin-1 OS=Mus musculus GN=Atxn1 PE=1 SV=2 - [ATX1_MOUSE]	14.66	2	8	8	21	1.522	1.414	1.860	1.073	42.87	14.66	15	21	791	83.7	8.16
D3Z495	Protein spire homolog 1 OS=Mus musculus GN=Spire1 PE=2 SV=1 - [D3Z495_MOUSE]	16.12	6	7	8	36	0.713	0.835	1.118	1.073	102.82	16.12	14	36	515	59.4	9.52

Q8C4U8	EGF-like repeat and discoidin I-like domain-containing protein 3 OS=Mus musculus GN=Edil3 PE=2 SV=1 - [illegible]	25.32	4	11	11	21	1.054	0.639	0.804	1.073	65.20	25.32	17	21	470	52.7	7.80
Q61171	Peroxiredoxin-2 OS=Mus musculus GN=Prdx2 PE=1 SV=3 - [PRDX2_MOUSE]	53.54	2	12	12	343	1.528	1.115	0.964	1.074	1001.15	53.54	24	343	198	21.8	5.41
P70660	Neurogenin-1 OS=Mus musculus GN=Neurog1 PE=1 SV=1 - [NGN1_MOUSE]	8.61	1	1	1	2	1.836	1.581	1.101	1.074	5.92	8.61	1	2	244	26.3	6.54
Q8VD65	Phosphoinositide 3-kinase regulatory subunit 4 OS=Mus musculus GN=Pik3r4 PE=1 SV=3 - [PI3R4_MOUSE]	21.87	1	24	24	66	0.690	0.998	1.025	1.074	172.71	21.87	40	66	1358	152.5	7.12
Q64448	Gap junction alpha-3 protein OS=Mus musculus GN=Gja3 PE=2 SV=4 - [CXA3_MOUSE]	1.68	1	1	1	2	0.953	1.406	0.914	1.074	4.22	1.68	2	2	417	46.3	7.53
Q8CHG7	Rap guanine nucleotide exchange factor 2 OS=Mus musculus GN=Rapgef2 PE=1 SV=2 - [RPGF2_MOUSE]	35.56	3	32	38	154	1.003	1.146	1.316	1.074	504.85	35.56	61	154	1496	166.3	6.61
A2AIL4	NADH dehydrogenase (ubiquinone) complex I, assembly factor 6 OS=Mus musculus GN=Ndufaf6 PE=1 SV=1 - [illegible]	5.71	1	2	2	3	1.091	1.048	0.890	1.074	8.79	5.71	3	3	333	38.3	9.44
E9PZP8	Protein Herc1 OS=Mus musculus GN=Herc1 PE=2 SV=1 - [E9PZP8_MOUSE]	12.02	2	41	41	97	0.738	0.802	1.000	1.074	281.45	12.02	63	97	4859	532.3	6.04
D3Z451	MCG54087 OS=Mus musculus GN=Serpina3j PE=3 SV=1 - [D3Z451_MOUSE]	11.19	1	1	3	7	1.669	1.075	1.038	1.074	19.94	11.19	4	7	420	47.3	7.50
P28063	Proteasome subunit beta type-8 OS=Mus musculus GN=Psmb8 PE=1 SV=2 - [PSB8_MOUSE]	12.32	2	3	3	9	1.560	1.516	0.772	1.074	16.18	12.32	6	9	276	30.2	6.68
Q9ER73	Elongator complex protein 4 OS=Mus musculus GN=Elp4 PE=2 SV=2 - [ELP4_MOUSE]	30.09	2	11	11	41	0.699	0.963	1.007	1.074	119.50	30.09	20	41	422	46.3	8.78
Q80Y24	Prickle-like protein 2 OS=Mus musculus GN=Prickle2 PE=1 SV=3 - [PRIC2_MOUSE]	24.14	2	15	15	47	0.821	1.704	1.608	1.074	143.81	24.14	27	47	845	95.7	7.27
Q7TN33	CUGBP Elav-like family member 6 OS=Mus musculus GN=Celf6 PE=2 SV=1 - [CELF6_MOUSE]	16.96	6	3	7	34	0.713	1.061	0.528	1.074	90.30	16.96	13	34	460	48.2	8.65
P70390-2	Isoform 2 of Short stature homeobox protein 2 OS=Mus musculus GN=Shox2 - [SHOX2_MOUSE]	15.05	2	4	4	8	2.109	0.944	1.167	1.074	14.26	15.05	4	8	319	33.6	9.11
Q7TQI7	Ankyrin repeat and BTB/POZ domain-containing protein 2 OS=Mus musculus GN=Abtb2 PE=2 SV=1 - [illegible]	13.77	1	10	10	14	2.517	1.123	1.242	1.074	33.76	13.77	14	14	1024	113.4	6.28
O70281	Protein-tyrosine sulfotransferase 1 OS=Mus musculus GN=Tpst1 PE=2 SV=1 - [TPST1_MOUSE]	11.89	2	3	3	8	1.206	1.019	0.896	1.074	13.69	11.89	5	8	370	42.1	9.09
O55026	Ectonucleoside triphosphate diphosphohydrolase 2 OS=Mus musculus GN=Entpd2 PE=1 SV=2 - [illegible]	12.53	2	6	6	21	0.745	1.189	0.658	1.074	57.03	12.53	10	21	495	54.3	8.37
F7B0R9	Protein 2010300C02Rik (Fragment) OS=Mus musculus GN=2010300C02Rik PE=4 SV=1 - [F7B0R9_MOUSE]	64.88	1	1	31	118	3.729	1.282	2.470	1.074	337.17	64.88	51	118	652	69.5	9.39
P80313	T-complex protein 1 subunit eta OS=Mus musculus GN=Cct7 PE=1 SV=1 - [TCPH_MOUSE]	58.82	1	28	28	146	0.631	1.027	0.859	1.074	462.79	58.82	49	146	544	59.6	7.84
D3YZG8	Probable bifunctional methylenetetrahydrofolate dehydrogenase/cyclohydrolase 2 OS=Mus musculus GN=Mthfd2l PE=3 SV=1 - [MTD2L_MOUSE]	35.50	1	8	9	16	2.501	0.559	0.531	1.074	41.08	35.50	14	16	338	36.4	9.39
Q8R0G9	Nuclear pore complex protein Nup133 OS=Mus musculus GN=Nup133 PE=1 SV=2 - [NU133_MOUSE]	16.71	1	12	13	42	0.947	0.975	0.919	1.074	127.02	16.71	21	42	1155	128.5	5.20
Q9CQS4	Solute carrier family 25 member 46 OS=Mus musculus GN=Slc25a46 PE=1 SV=1 -	44.02	1	12	12	67	0.717	0.981	0.946	1.074	161.70	44.02	21	67	418	46.2	7.64
Q9CXR1	Dehydrogenase/reductase SDR family member 7 OS=Mus musculus GN=Dhrs7 PE=2 SV=2 - [DHRS7_MOUSE]	44.97	1	11	11	27	1.264	0.957	1.261	1.074	77.26	44.97	16	27	338	38.1	8.32
Q9CXT8	Mitochondrial-processing peptidase subunit beta OS=Mus musculus GN=Pmpcb PE=2 SV=1 - [MPPB_MOUSE]	43.15	3	15	16	58	0.995	1.055	0.858	1.074	179.60	43.15	28	58	489	54.6	6.99

Q0VF62	Bcas3 protein OS=Mus musculus GN=Bcas3 PE=2 SV=1 - [Q0VF62_MOUSE]	32.75	5	9	23	121	0.741	1.165	1.115	1.074	368.90	32.75	46	121	913	99.4	6.64
Q02053	Ubiquitin-like modifier-activating enzyme 1 OS=Mus musculus GN=Uba1 PE=1 SV=1 - [UBA1_MOUSE]	59.26	2	46	46	597	0.702	1.105	1.003	1.074	1972.15	59.26	84	597	1058	117.7	5.66
Q91X51	Golgi reassembly-stacking protein 1 OS=Mus musculus GN=Gorasp1 PE=2 SV=3 -	12.56	1	3	3	20	1.885	1.050	0.981	1.074	64.44	12.56	6	20	446	46.9	4.65
Q6A037	NEDD4-binding protein 1 OS=Mus musculus GN=N4bp1 PE=1 SV=2 - [N4BP1_MOUSE]	13.44	1	9	10	37	0.902	1.094	0.995	1.074	96.05	13.44	12	37	893	99.1	5.74
Q80WJ1-2	Isoform 2 of Gametogenetin OS=Mus musculus GN=Ggn - [GGN_MOUSE]	10.04	3	2	2	2	1.260	1.084	1.197	1.075	4.63	10.04	2	2	269	28.6	10.59
Q3TDK6	Protein rogdi homolog OS=Mus musculus GN=Rogdi PE=2 SV=2 - [ROGDI_MOUSE]	40.07	3	10	10	47	0.530	1.397	1.018	1.075	141.10	40.07	18	47	287	32.1	8.18
Q3ULL6	Protein Upf3b OS=Mus musculus GN=Upf3b PE=2 SV=1 - [Q3ULL6_MOUSE]	15.68	2	6	6	12	1.474	1.162	1.073	1.075	32.16	15.68	9	12	472	57.0	9.44
P63137-2	Isoform 2 of Gamma-aminobutyric acid receptor subunit beta-2 OS=Mus musculus GN=Gabrb2 - [GBRB2_MOUSE]	19.62	3	5	8	30	0.456	0.808	1.145	1.075	93.37	19.62	13	30	474	54.6	9.32
O88697	Serine/threonine-protein kinase 16 OS=Mus musculus GN=Stk16 PE=1 SV=3 - [STK16_MOUSE]	18.03	2	3	3	12	1.049	1.495	1.212	1.075	35.18	18.03	5	12	305	34.4	6.46
P01863	Ig gamma-2A chain C region, A allele OS=Mus musculus GN=Ighg PE=1 SV=1 - [GCAA_MOUSE]	15.45	3	3	4	14	3.594	9.571	0.177	1.075	35.93	15.45	8	14	330	36.4	7.40
P22599	Alpha-1-antitrypsin 1-2 OS=Mus musculus GN=Serpina1b PE=1 SV=2 - [A1AT2_MOUSE]	47.46	2	3	15	166	2.929	2.390	0.614	1.075	467.41	47.46	27	166	413	45.9	5.54
P61406	Telomerase-binding protein EST1A OS=Mus musculus GN=Smg6 PE=2 SV=1 - [EST1A_MOUSE]	5.57	1	6	7	12	0.764	0.883	0.858	1.075	36.08	5.57	9	12	1418	160.4	6.77
Q91XX1	Protein Pcdhgb6 OS=Mus musculus GN=Pcdhgc3 PE=2 SV=1 - [Q91XX1_MOUSE]	19.70	1	10	12	43	0.774	0.870	1.024	1.075	125.52	19.70	23	43	934	101.0	5.07
Q8C570	mRNA export factor OS=Mus musculus GN=Rae1 PE=1 SV=1 - [RAE1L_MOUSE]	40.22	2	14	14	44	1.213	1.100	1.023	1.075	131.43	40.22	22	44	368	40.9	7.83
Q8K3Z9	Nuclear envelope pore membrane protein POM 121 OS=Mus musculus GN=Pom121 PE=1 SV=2 - [PO121_MOUSE]	9.75	2	8	8	12	1.156	0.767	1.250	1.075	38.12	9.75	12	12	1200	120.9	10.20
Q91ZZ3	Beta-synuclein OS=Mus musculus GN=Sncb PE=1 SV=1 - [SYUB_MOUSE]	50.38	1	5	8	297	1.329	1.233	1.549	1.076	948.47	50.38	15	297	133	14.0	4.37
P28663	Beta-soluble NSF attachment protein OS=Mus musculus GN=Napb PE=1 SV=2 - [SNAB_MOUSE]	78.86	2	18	23	278	0.804	1.621	1.226	1.076	798.89	78.86	41	278	298	33.5	5.47
P52480	Pyruvate kinase PKM OS=Mus musculus GN=Pkm PE=1 SV=4 - [KPYM_MOUSE]	80.41	1	2	45	1748	1.300	0.999	0.777	1.076	5260.02	80.41	83	1748	531	57.8	7.47
O88705	Potassium/sodium hyperpolarization-activated cyclic nucleotide-gated channel 3 OS=Mus musculus GN=Hcn3 PE=1 SV=1 - [HCN3_MOUSE]	15.66	1	10	11	39	0.895	0.886	1.061	1.076	111.88	15.66	17	39	779	86.6	9.86
Q8BLS7	Ankyrin repeat domain-containing protein SOWAHA OS=Mus musculus GN=Sowaha PE=2 SV=2 - [illegible]	10.40	1	4	4	7	1.595	1.624	1.504	1.076	20.18	10.40	6	7	548	57.7	9.82
E9Q9C3	Afadin OS=Mus musculus GN=Mllt4 PE=2 SV=1 - [illegible]	36.73	4	2	54	177	0.636	1.437	1.242	1.076	512.92	36.73	88	177	1827	207.2	6.23
P35436	Glutamate receptor ionotropic, NMDA 2A OS=Mus musculus GN=Grin2a PE=1 SV=2 - [NMDE1_MOUSE]	21.38	1	24	24	72	0.783	1.321	2.259	1.076	209.77	21.38	39	72	1464	165.3	7.01
Q9CRA9-2	Isoform 2 of FGFR1 oncogene partner 2 homolog OS=Mus musculus GN=Fgfr1op2 - [FGOP2_MOUSE]	36.28	2	1	8	37	1.496	1.084	1.335	1.076	123.01	36.28	11	37	215	24.9	5.57
Q6PDN3	Myosin light chain kinase, smooth muscle OS=Mus musculus GN=Mylk PE=1 SV=3 - [MYLK_MOUSE]	14.17	12	24	25	77	0.950	2.448	0.905	1.076	198.83	14.17	43	77	1941	212.8	6.25
Q8C4G3	Probable phospholipid-transporting ATPase IIA OS=Mus musculus GN=Atp9a PE=2 SV=1 - [Q8C4G3_MOUSE]	22.25	8	21	23	50	0.739	0.900	0.909	1.076	136.45	22.25	30	50	1029	116.3	7.27

Q9D1G1	Ras-related protein Rab-1B OS=Mus musculus GN=Rab1b PE=1 SV=1 - [RAB1B_MOUSE]	85.57	1	7	16	165	1.081	1.225	1.046	1.076	459.71	85.57	30	165	201	22.2	5.73
O09117-2	Isoform 2 of Synaptophysin-like protein 1 OS=Mus musculus GN=Sypl1 -	17.28	3	4	4	20	1.411	0.810	1.081	1.076	59.78	17.28	7	20	243	26.7	7.72
Q6PAM0	5'-AMP-activated protein kinase subunit beta-2 OS=Mus musculus GN=Prkab2 PE=1 SV=1 - [AAKB2_MOUSE]	27.68	3	5	5	25	0.843	1.117	1.197	1.077	47.08	27.68	8	25	271	30.2	6.46
Q80UP5	Ankyrin repeat domain-containing protein 13A OS=Mus musculus GN=Ankrd13a PE=1 SV=2 - [AN13A_MOUSE]	14.80	2	6	6	12	1.122	0.741	0.891	1.077	39.98	14.80	10	12	588	67.1	5.08
Q9ERK4	Exportin-2 OS=Mus musculus GN=Cse1l PE=2 SV=1 - [XPO2_MOUSE]	34.09	4	2	30	113	0.613	1.183	0.831	1.077	324.10	34.09	50	113	971	110.4	5.77
G3X9S1	Protein FAM13C OS=Mus musculus GN=Fam13c PE=4 SV=1 - [G3X9S1_MOUSE]	9.83	3	4	5	16	1.140	1.221	1.571	1.077	48.86	9.83	9	16	519	57.9	6.73
Q99NF8	Ran-binding protein 17 OS=Mus musculus GN=Ranbp17 PE=2 SV=1 - [RBP17_MOUSE]	0.83	1	1	1	1	0.805	0.807	1.065	1.077	2.19	0.83	1	1	1088	124.0	6.38
Q5SVG5	AP-1 complex subunit beta 1 OS=Mus musculus GN=Ap1b1 PE=2 SV=1 - [Q5SVG5_MOUSE]	61.46	7	26	53	396	0.736	1.026	0.992	1.077	1158.92	61.46	93	396	916	101.1	5.22
Q8C167	Prolyl endopeptidase-like OS=Mus musculus GN=Prepl PE=2 SV=1 - [PPCEL_MOUSE]	38.48	4	25	25	127	0.843	0.819	0.676	1.077	381.37	38.48	40	127	725	83.1	6.65
Q4FJZ2	Importin subunit alpha OS=Mus musculus GN=Kpna6 PE=2 SV=1 - [Q4FJZ2_MOUSE]	40.71	3	12	19	76	0.802	0.866	0.983	1.077	201.61	40.71	32	76	533	59.6	4.98
O54828	Regulator of G-protein signaling 9 OS=Mus musculus GN=Rgs9 PE=1 SV=3 - [RGS9_MOUSE]	10.07	4	5	6	14	1.490	1.029	5.186	1.077	30.54	10.07	10	14	675	76.9	9.33
P16301	Phosphatidylcholine-sterol acyltransferase OS=Mus musculus GN=Lcat PE=1 SV=2 - [LCAT_MOUSE]	5.48	1	2	2	3	2.471	1.518	0.827	1.077	10.11	5.48	3	3	438	49.7	6.43
F8WI30	Sorting nexin-7 OS=Mus musculus GN=Snx7 PE=2 SV=1 - [F8WI30_MOUSE]	22.25	4	10	10	22	1.048	2.418	1.093	1.077	68.76	22.25	17	22	445	50.8	4.82
Q8K2Q0	COMM domain-containing protein 9 OS=Mus musculus GN=Commd9 PE=2 SV=3 -	36.87	1	6	6	23	1.084	1.252	0.936	1.077	70.06	36.87	11	23	198	21.8	5.82
A2BFQ5	Histone H2A OS=Mus musculus GN=Gm14474 PE=3 SV=1 - [A2BFQ5_MOUSE]	31.43	3	1	1	2	0.500	1.399	0.607	1.077	2.84	31.43	1	2	105	12.1	10.23
Q8BMI0	F-box only protein 38 OS=Mus musculus GN=Fbxo38 PE=1 SV=1 - [FBX38_MOUSE]	7.37	1	7	7	10	0.894	0.809	1.067	1.077	24.67	7.37	10	10	1194	133.8	6.09
Q6P0X2	Zinc finger protein 511 OS=Mus musculus GN=Znf511 PE=2 SV=2 - [ZN511_MOUSE]	11.01	2	2	2	3	1.479	1.102	1.198	1.077	5.67	11.01	3	3	227	25.7	6.25
E9Q5E2	Protein BC005561 OS=Mus musculus GN=BC005561 PE=2 SV=1 - [E9Q5E2_MOUSE]	1.83	1	1	3	6	1.741	1.272	1.480	1.077	6.75	1.83	4	6	1589	182.6	8.56
A2AFI8	RalBP1-associated Eps domain-containing protein 2 OS=Mus musculus GN=Reps2 PE=2 SV=1 - [A2AFI8_MOUSE]	42.97	4	20	22	76	1.114	1.173	1.460	1.077	238.32	42.97	38	76	647	70.4	7.25
Q9D4H1	Exocyst complex component 2 OS=Mus musculus GN=Exoc2 PE=1 SV=1 - [EXOC2_MOUSE]	22.40	1	22	22	58	0.561	1.300	1.078	1.077	172.68	22.40	40	58	924	103.9	7.18
B0QZL5	Phosphatidylinositol 4,5-bisphosphate 3-kinase catalytic subunit delta isoform OS=Mus musculus GN=Pik3cd PE=2 SV=1 - [B0QZL5_MOUSE]	2.31	10	1	3	9	0.889	1.307	0.980	1.078	12.40	2.31	5	9	1040	119.3	7.34
E9Q6Y8	Ubiquitin carboxyl-terminal hydrolase OS=Mus musculus GN=Usp31 PE=2 SV=1 - [E9Q6Y8_MOUSE]	21.50	1	21	21	80	0.914	0.804	1.369	1.078	199.93	21.50	35	80	1344	146.2	9.17
O35874	Neutral amino acid transporter A OS=Mus musculus GN=Slc1a4 PE=1 SV=1 -	23.50	2	9	11	61	0.596	0.908	0.865	1.078	156.82	23.50	19	61	532	56.0	5.87
E9Q2X2	Neurexin-3 OS=Mus musculus GN=Nrxn3 PE=2 SV=1 - [E9Q2X2_MOUSE]	33.50	1	3	40	159	0.861	0.975	0.863	1.078	509.10	33.50	72	159	1391	153.5	5.77
P61205	ADP-ribosylation factor 3 OS=Mus musculus GN=Arf3 PE=2 SV=2 - [ARF3_MOUSE]	67.40	5	2	12	275	1.039	1.322	1.020	1.078	749.67	67.40	20	275	181	20.6	7.43
Q68FF6	ARF GTPase-activating protein GIT1 OS=Mus musculus GN=Git1 PE=1 SV=1 - [GIT1_MOUSE]	57.01	5	33	36	150	0.585	1.130	1.207	1.078	448.32	57.01	60	150	770	85.2	6.93

G3UY87	PCTP-like protein (Fragment) OS=Mus musculus GN=Stard10 PE=4 SV=1 -	13.43	8	4	4	8	0.858	0.835	0.810	1.078	24.90	13.43	5	8	283	31.6	8.70
P17156	Heat shock-related 70 kDa protein 2 OS=Mus musculus GN=Hspa2 PE=1 SV=2 - [HSP72_MOUSE]	59.40	1	20	40	852	0.917	2.344	1.224	1.078	2313.73	59.40	74	852	633	69.6	5.67
Q8BYZ7-2	Isoform 2 of Engulfment and cell motility protein 3 OS=Mus musculus GN=Elmo3 - [ELMO3_MOUSE]	7.25	2	3	4	5	1.051	1.066	1.089	1.078	8.25	7.25	4	5	607	69.2	6.30
Q9D4H8	Cullin-2 OS=Mus musculus GN=Cul2 PE=1 SV=2 - [CUL2_MOUSE]	38.52	4	28	29	97	0.697	0.933	0.947	1.078	289.01	38.52	51	97	745	86.8	7.01
Q920I9	WD repeat-containing protein 7 OS=Mus musculus GN=Wdr7 PE=1 SV=3 - [WDR7_MOUSE]	40.36	2	47	47	238	0.553	1.083	1.050	1.078	728.94	40.36	83	238	1489	163.3	6.90
Q8BG94	COMM domain-containing protein 7 OS=Mus musculus GN=Commd7 PE=2 SV=1 -	24.00	2	3	3	10	0.745	0.838	1.021	1.078	28.38	24.00	6	10	200	22.6	5.94
Q60769	Tumor necrosis factor alpha-induced protein 3 OS=Mus musculus GN=Tnfaip3 PE=1 SV=2 - [TNAP3_MOUSE]	1.55	1	1	1	2	1.070	1.572	1.417	1.078	2.66	1.55	2	2	775	87.6	8.16
Q9R111	Guanine deaminase OS=Mus musculus GN=Gda PE=1 SV=1 - [GUAD_MOUSE]	68.06	2	26	26	237	0.948	2.831	1.109	1.078	662.56	68.06	47	237	454	51.0	5.53
Q99K74-2	Isoform 2 of Mediator of RNA polymerase II transcription subunit 24 OS=Mus musculus GN=Med24 - [MED24_MOUSE]	4.18	4	3	3	5	0.512	0.881	0.869	1.078	17.81	4.18	5	5	956	105.9	6.79
P02089	Hemoglobin subunit beta-2 OS=Mus musculus GN=Hbb-b2 PE=1 SV=2 - [HBB2_MOUSE]	85.03	2	5	14	904	1.579	7.987	1.146	1.079	2507.89	85.03	28	904	147	15.9	8.05
Q8R5C5	Beta-centractin OS=Mus musculus GN=Actr1b PE=1 SV=1 - [ACTY_MOUSE]	56.12	3	9	17	101	0.720	0.983	1.071	1.079	297.29	56.12	30	101	376	42.3	6.40
O88322	Nidogen-2 OS=Mus musculus GN=Nid2 PE=1 SV=2 - [NID2_MOUSE]	11.76	1	11	11	35	1.537	1.447	1.136	1.079	124.02	11.76	20	35	1403	153.8	5.38
O70496	H(+)/Cl(-) exchange transporter 7 OS=Mus musculus GN=Clcn7 PE=1 SV=1 - [CLCN7_MOUSE]	13.20	3	8	8	27	0.915	0.839	0.874	1.079	61.49	13.20	12	27	803	88.7	7.27
Q9D8Z1	Activating signal cointegrator 1 complex subunit 1 OS=Mus musculus GN=Ascc1 PE=2 SV=1 - [ASCC1_MOUSE]	8.71	1	3	3	5	0.846	1.004	0.893	1.079	13.92	8.71	5	5	356	41.3	6.54
E9PYX5	Probable cation-transporting ATPase 13A2 (Fragment) OS=Mus musculus GN=Atp13a2 PE=2 SV=1 - [E9PYX5_MOUSE]	2.16	3	1	2	2	0.591	0.799	1.042	1.079	5.43	2.16	2	2	970	104.9	7.91
Q3SXD3	HD domain-containing protein 2 OS=Mus musculus GN=Hddc2 PE=2 SV=1 - [HDDC2_MOUSE]	31.16	2	4	4	20	1.044	1.098	1.040	1.079	69.59	31.16	8	20	199	22.7	5.02
Q9WUR2-2	Isoform 2 of Enoyl-CoA delta isomerase 2, mitochondrial OS=Mus musculus GN=Eci2 - [ECI2_MOUSE]	37.15	11	11	11	57	0.856	1.208	0.903	1.079	154.16	37.15	19	57	358	39.5	8.43
A6H5Z3	Exocyst complex component 6B OS=Mus musculus GN=Exoc6b PE=2 SV=1 -	35.80	4	23	25	56	0.601	1.475	1.053	1.079	170.38	35.80	41	56	810	94.1	6.39
Q8K3J1	NADH dehydrogenase [ubiquinone] iron-sulfur protein 8, mitochondrial OS=Mus musculus GN=Ndufs8 PE=1 SV=1 - [NDUS8_MOUSE]	32.08	1	6	6	57	1.003	1.133	1.015	1.079	130.50	32.08	11	57	212	24.0	6.21
Q925I1	ATPase family AAA domain-containing protein 3 OS=Mus musculus GN=Atad3 PE=1 SV=1 - [ATAD3_MOUSE]	47.88	5	25	26	106	0.760	0.908	0.917	1.079	324.21	47.88	44	106	591	66.7	9.29
D6RIQ3	Phytanoyl-CoA dioxygenase domain-containing protein 1 OS=Mus musculus GN=Phyhd1 PE=2 SV=1 - [D6RIQ3_MOUSE]	21.38	6	3	3	11	1.328	0.991	0.958	1.079	32.42	21.38	6	11	159	18.0	6.68
P56974-2	Isoform DON-1M of Pro-neuregulin-2, membrane-bound isoform OS=Mus musculus GN=Nrg2 - [NRG2_MOUSE]	2.52	4	1	1	3	1.064	0.903	1.053	1.079	13.02	2.52	2	3	754	82.0	9.19
P97329	Kinesin-like protein KIF20A OS=Mus musculus GN=Kif20a PE=2 SV=1 - [KI20A_MOUSE]	5.19	1	2	4	9	1.721	0.819	1.072	1.079	22.15	5.19	4	9	887	99.8	6.99
Q09200	Beta-1,4 N-acetylgalactosaminyltransferase 1 OS=Mus musculus GN=B4galnt1 PE=2 SV=1 - [B4GN1_MOUSE]	12.01	1	6	7	12	0.911	1.039	1.132	1.079	31.26	12.01	10	12	533	59.2	8.59

Q80X95	Ras-related GTP-binding protein A OS=Mus musculus GN=Rraga PE=2 SV=1 - [RRAGA_MOUSE]	32.59	3	3	11	31	0.464	0.935	0.905	1.079	108.10	32.59	19	31	313	36.5	7.72
P47739	Aldehyde dehydrogenase, dimeric NADP-preferring OS=Mus musculus GN=Aldh3a1 PE=2 SV=2 - [AL3A1_MOUSE]	9.71	1	3	5	9	1.074	1.179	1.079	1.080	21.25	9.71	7	9	453	50.4	6.95
P08249	Malate dehydrogenase, mitochondrial OS=Mus musculus GN=Mdh2 PE=1 SV=3 - [MDHM_MOUSE]	75.44	1	29	29	1483	1.951	1.067	0.947	1.080	4515.14	75.44	57	1483	338	35.6	8.68
Q99P31	Hsp70-binding protein 1 OS=Mus musculus GN=Hspbp1 PE=2 SV=1 - [HPBP1_MOUSE]	30.81	1	8	8	22	0.890	0.938	0.861	1.080	70.26	30.81	13	22	357	39.1	5.36
Q9EP89	Serine beta-lactamase-like protein LACTB, mitochondrial OS=Mus musculus GN=Lactb PE=1 SV=1 - [LACTB_MOUSE]	21.42	1	12	12	31	0.784	1.285	1.395	1.080	90.14	21.42	17	31	551	60.7	8.90
Q6IMP4-2	Isoform 2 of Pannexin-2 OS=Mus musculus GN=Panx2 - [PANX2_MOUSE]	12.90	2	5	6	12	1.039	0.617	0.910	1.080	40.44	12.90	9	12	651	71.6	8.69
Q3U2J5	Calmodulin-lysine N-methyltransferase OS=Mus musculus GN=Camkmt PE=1 SV=1 - [CMKMT_MOUSE]	19.50	2	6	6	13	0.912	1.051	0.963	1.080	41.30	19.50	9	13	323	35.9	7.47
G3X8X1	RIKEN cDNA 2810439F02 OS=Mus musculus GN=Ttc39c PE=4 SV=1 - [G3X8X1_MOUSE]	9.83	3	6	6	16	0.821	1.240	1.026	1.080	46.02	9.83	12	16	580	65.4	6.87
Q8K330	Protein phosphatase Slingshot homolog 3 OS=Mus musculus GN=Ssh3 PE=1 SV=1 - [SSH3_MOUSE]	7.70	6	4	5	11	1.162	0.973	1.032	1.080	30.77	7.70	8	11	649	72.2	5.57
Q3KNK3	NADH-cytochrome b5 reductase 2 OS=Mus musculus GN=Cyb5r2 PE=2 SV=2 -	10.87	2	2	2	2	13.157	1.199	1.334	1.080	4.29	10.87	2	2	276	31.3	6.76
P51830	Adenylate cyclase type 9 OS=Mus musculus GN=Adcy9 PE=1 SV=1 - [ADCY9_MOUSE]	22.62	2	29	29	87	0.626	0.908	1.220	1.080	219.94	22.62	46	87	1353	150.9	7.21
P49615	Cyclin-dependent kinase 5 OS=Mus musculus GN=Cdk5 PE=1 SV=1 - [CDK5_MOUSE]	54.79	15	13	15	86	0.547	0.917	1.001	1.080	229.19	54.79	27	86	292	33.3	7.66
P54320	Elastin OS=Mus musculus GN=Eln PE=2 SV=2 - [ELN_MOUSE]	12.33	1	4	4	9	0.448	0.827	0.928	1.080	21.38	12.33	5	9	860	71.9	10.43
E9QP54	Jouberin OS=Mus musculus GN=Ahi1 PE=2 SV=1 - [E9QP54_MOUSE]	55.01	4	49	50	242	0.859	1.013	0.661	1.080	677.90	55.01	82	242	1047	119.6	7.20
P61329-2	Isoform 2 of Fibroblast growth factor 12 OS=Mus musculus GN=Fgf12 - [FGF12_MOUSE]	45.30	5	8	8	36	1.187	0.826	1.150	1.081	117.51	45.30	15	36	181	20.4	8.87
Q80WG5	Leucine-rich repeat-containing protein 8A OS=Mus musculus GN=Lrrc8a PE=1 SV=1 - [LRC8A_MOUSE]	30.12	1	17	19	46	0.770	0.815	0.838	1.081	136.80	30.12	32	46	810	94.1	7.94
Q78JE5	F-box only protein 22 OS=Mus musculus GN=Fbxo22 PE=2 SV=2 - [FBX22_MOUSE]	24.13	4	7	8	15	0.696	1.059	0.907	1.081	50.73	24.13	12	15	402	44.2	7.61
Q91X78	Erlin-1 OS=Mus musculus GN=Erlin1 PE=1 SV=1 - [ERLN1_MOUSE]	26.88	2	6	10	46	0.887	1.630	1.269	1.081	122.66	26.88	18	46	346	38.9	7.21
Q9QZQ8-2	Isoform 1 of Core histone macro-H2A.1 OS=Mus musculus GN=H2afy - [H2AY_MOUSE]	44.99	2	13	15	85	0.377	0.828	0.772	1.081	283.94	44.99	26	85	369	39.3	9.83
H7BX79	Coiled-coil domain-containing protein 162 OS=Mus musculus GN=Ccdc162 PE=2 SV=2 - [H7BX79_MOUSE]	7.76	7	5	5	9	7.951	3.140	2.087	1.081	13.83	7.76	5	9	722	83.4	8.10
Q03719	Potassium voltage-gated channel subfamily D member 1 OS=Mus musculus GN=Kcnd1 PE=1 SV=1 - [KCND1_MOUSE]	8.14	1	4	5	6	0.985	0.783	1.168	1.081	9.85	8.14	6	6	651	71.7	8.21
Q76KF0-3	Isoform 2 of Semaphorin-6D OS=Mus musculus GN=Sema6d - [SEM6D_MOUSE]	7.21	7	7	7	13	1.056	1.071	1.007	1.081	39.42	7.21	9	13	998	111.7	8.66
Q9QZX7-2	Isoform 2 of Serine racemase OS=Mus musculus GN=Srr - [SRR_MOUSE]	46.82	4	10	10	60	0.453	2.289	1.729	1.081	225.86	46.82	19	60	314	34.0	6.01
E0CZ01	von Willebrand factor A domain-containing protein 5B2 OS=Mus musculus GN=Vwa5b2 PE=2 SV=1 - [E0CZ01_MOUSE]	3.21	4	3	4	14	0.949	0.965	0.936	1.081	38.42	3.21	6	14	1248	133.3	6.25
Q7TSJ2	Microtubule-associated protein 6 OS=Mus musculus GN=Map6 PE=1 SV=2 - [MAP6_MOUSE]	80.46	3	56	74	903	1.354	0.850	1.397	1.081	2442.24	80.46	140	903	906	96.4	9.50

Q61206	Platelet-activating factor acetylhydrolase IB subunit beta OS=Mus musculus GN=Pafah1b2 PE=1 SV=2 [PA1B2_MOUSE]	45.85	1	6	7	86	1.265	1.083	1.038	1.081	287.70	45.85	11	86	229	25.6	5.92
Q9Z1G3	V-type proton ATPase subunit C 1 OS=Mus musculus GN=Atp6v1c1 PE=1 SV=4 -	77.75	5	33	33	234	0.615	1.287	1.032	1.081	620.23	77.75	58	234	382	43.9	7.46
Q99LB6-2	Isoform 2 of Methionine adenosyltransferase 2 subunit beta OS=Mus musculus GN=Mat2b - [MAT2B_MOUSE]	44.89	3	13	13	41	1.069	1.240	1.229	1.081	128.78	44.89	23	41	323	36.2	6.60
P47791	Glutathione reductase, mitochondrial OS=Mus musculus GN=Gsr PE=2 SV=3 - [GSHR_MOUSE]	43.40	2	18	18	65	1.850	1.192	1.250	1.081	230.60	43.40	31	65	500	53.6	7.99
Q9D7N9	Adipocyte plasma membrane-associated protein OS=Mus musculus GN=Apmap PE=1 SV=1 - [APMAP_MOUSE]	53.98	1	20	20	62	0.807	1.021	0.943	1.082	192.54	53.98	33	62	415	46.4	6.32
P62835	Ras-related protein Rap-1A OS=Mus musculus GN=Rap1a PE=2 SV=1 - [RAP1A_MOUSE]	64.13	1	3	9	109	0.875	0.489	0.957	1.082	306.44	64.13	17	109	184	21.0	6.67
P06800-3	Isoform 3 of Receptor-type tyrosine-protein phosphatase C OS=Mus musculus GN=Ptprc - [PTPRC_MOUSE]	0.95	3	1	1	3	0.936	1.510	1.092	1.082	9.72	0.95	2	3	1152	130.5	6.33
Q99JT1	Glutamyl-tRNA(Gln) amidotransferase subunit B, mitochondrial OS=Mus musculus GN=Pet112 PE=2 SV=1 - [GATB_MOUSE]	31.06	3	13	13	38	1.039	0.950	1.036	1.082	106.25	31.06	19	38	557	62.1	8.54
P27671	Ras-specific guanine nucleotide-releasing factor 1 OS=Mus musculus GN=Rasgrf1 PE=1 SV=2 - [RGRF1_MOUSE]	27.97	2	27	33	81	0.641	0.919	0.877	1.082	249.54	27.97	47	81	1262	144.0	7.17
P24161	T-cell surface glycoprotein CD3 zeta chain OS=Mus musculus GN=Cd247 PE=1 SV=1 - [CD3Z_MOUSE]	6.71	5	1	1	1	1.205	0.804	0.837	1.082	0.00	6.71	1	1	164	18.6	9.28
Q6PD31	Trafficking kinesin-binding protein 1 OS=Mus musculus GN=Trak1 PE=1 SV=1 - [TRAK1_MOUSE]	10.22	2	7	7	14	2.175	1.442	1.145	1.082	33.77	10.22	10	14	939	104.4	5.78
Q9Z0S9	Prenylated Rab acceptor protein 1 OS=Mus musculus GN=Rabac1 PE=1 SV=1 -	24.32	1	4	4	14	0.610	0.815	0.761	1.082	38.34	24.32	7	14	185	20.6	7.90
Q7TPD0	Integrator complex subunit 3 OS=Mus musculus GN=Ints3 PE=1 SV=2 - [INT3_MOUSE]	5.76	2	4	5	9	0.703	1.133	1.325	1.082	34.08	5.76	8	9	1041	117.9	5.80
Q5RKZ7	Molybdenum cofactor biosynthesis protein 1 OS=Mus musculus GN=Mocs1 PE=2 SV=2 - [MOCS1_MOUSE]	10.38	6	4	5	9	1.298	1.318	1.081	1.082	13.08	10.38	7	9	636	69.8	9.14
P70388-2	Isoform 2 of DNA repair protein RAD50 OS=Mus musculus GN=Rad50 - [RAD50_MOUSE]	6.16	7	6	8	16	1.490	1.142	1.505	1.082	35.93	6.16	14	16	1251	146.2	7.05
Q3V460	Gene model 561, (NCBI) OS=Mus musculus GN=Gm561 PE=4 SV=1 - [Q3V460_MOUSE]	15.89	1	2	2	3	0.846	0.905	1.054	1.082	7.76	15.89	3	3	107	12.0	5.19
Q7TNI2	Transmembrane protein 59 like OS=Mus musculus GN=Tmem59l PE=2 SV=1 [TM59L_MOUSE]	9.79	1	3	3	5	0.616	1.046	0.775	1.082	17.61	9.79	4	5	337	37.6	7.69
Q99L27	GMP reductase 2 OS=Mus musculus GN=Gmpr2 PE=2 SV=2 - [GMPR2_MOUSE]	24.43	1	5	7	30	1.667	0.824	0.832	1.082	89.07	24.43	13	30	348	38.0	7.44
A2AKS7	Cadherin 17, isoform CRA_a OS=Mus musculus GN=Cdh17 PE=4 SV=1 - [A2AKS7_MOUSE]	2.88	2	1	1	1	3.985	1.240	0.702	1.082	3.46	2.88	1	1	728	80.6	4.70
Q0PD20	Rab34 OS=Mus musculus GN=Rab34 PE=2 SV=1 - [Q0PD20_MOUSE]	22.01	5	5	6	17	0.654	0.647	0.752	1.082	51.73	22.01	9	17	259	29.1	8.27
Q8QZT2	Centriole, cilia and spindle-associated protein OS=Mus musculus GN=Ccsap PE=2 SV=1 - [CCSAP_MOUSE]	27.38	1	6	6	20	0.975	1.610	3.031	1.082	53.46	27.38	11	20	252	28.4	9.22
P55144-3	Isoform 1 of Tyrosine-protein kinase receptor TYRO3 OS=Mus musculus GN=Tyro3 - [TYRO3_MOUSE]	13.13	3	10	10	23	1.284	0.872	1.802	1.082	66.82	13.13	17	23	876	96.0	5.60
Q8BQR7	MCG61680 OS=Mus musculus GN=Mageb18 PE=2 SV=1 - [Q8BQR7_MOUSE]	3.06	1	1	1	1	1.397	1.484	1.233	1.083	2.78	3.06	1	1	327	37.2	6.90
Q8BJW5-2	Isoform 2 of Nucleolar protein 11 OS=Mus musculus GN=Nol11 - [NOL11_MOUSE]	3.27	2	2	2	2	0.667	0.620	0.935	1.083	4.17	3.27	2	2	703	78.5	6.21

M0QWN7	Ubiquitin carboxyl-terminal hydrolase OS=Mus musculus GN=Usp35 PE=3 SV=1 - [M0QWN7_MOUSE]	12.39	2	9	10	28	0.713	0.879	0.955	1.083	73.16	12.39	17	28	1009	112.4	6.16
Q8BU85	Methionine-R-sulfoxide reductase B3, mitochondrial OS=Mus musculus GN=Msrb3 PE=1 SV=2 - [MSRB3_MOUSE]	3.56	1	1	1	2	1.837	1.438	1.033	1.083	5.26	3.56	2	2	253	26.8	7.99
Q3V3A3	MCG22200, isoform CRA_b OS=Mus musculus GN=Gpr165 PE=2 SV=1 - [Q3V3A3_MOUSE]	6.15	1	2	2	8	1.237	1.370	0.950	1.083	24.86	6.15	3	8	455	51.0	7.33
Q91YM4-2	Isoform 2 of Protein TBRG4 OS=Mus musculus GN=Tbrg4 - [TBRG4_MOUSE]	20.00	5	8	8	29	0.698	0.867	0.996	1.083	95.15	20.00	14	29	600	68.1	8.32
Q99NF7	Ppm1b protein OS=Mus musculus GN=Ppm1b PE=2 SV=1 - [Q99NF7_MOUSE]	34.80	5	1	14	102	0.964	1.064	1.036	1.083	281.58	34.80	26	102	477	52.1	5.11
O35902-2	Isoform 2 of Desmoglein-3 OS=Mus musculus GN=Dsg3 - [DSG3_MOUSE]	1.72	3	1	2	2	2.112	1.368	0.948	1.083	5.14	1.72	2	2	929	101.3	4.98
Q8R278	Protein Vmn1r213 OS=Mus musculus GN=Vmn1r213 PE=4 SV=1 - [Q8R278_MOUSE]	1.82	1	1	1	1	1.532	0.779	0.968	1.083	2.22	1.82	1	1	384	44.2	9.19
Q56A10-2	Isoform 2 of Zinc finger protein 608 OS=Mus musculus GN=Znf608 - [ZN608_MOUSE]	0.62	2	1	1	2	1.368	1.076	1.095	1.083	2.41	0.62	1	2	1455	155.9	8.79
Q8BMD5-3	Isoform 3 of Cytidine and dCMP deaminase domain-containing protein 1 OS=Mus musculus GN=Cdadc1 - [CDAC1_MOUSE]	4.83	6	2	2	6	0.921	0.643	0.941	1.083	20.27	4.83	3	6	497	56.1	6.79
E9PXL4	Gamma-aminobutyric acid receptor subunit alpha-1 (Fragment) OS=Mus musculus GN=Gabra1 PE=2 SV=1 - [E9PXL4_MOUSE]	8.33	1	1	1	1	1.644	1.186	1.295	1.083	2.54	8.33	1	1	132	14.5	11.19
Q791V5	Mitochondrial carrier homolog 2 OS=Mus musculus GN=Mtch2 PE=1 SV=1 - [MTCH2_MOUSE]	26.40	3	7	7	37	0.599	1.117	0.985	1.083	107.18	26.40	12	37	303	33.5	8.25
Q8BX70-3	Isoform 3 of Vacuolar protein sorting-associated protein 13C OS=Mus musculus GN=Vps13c - [VP13C_MOUSE]	24.46	4	75	76	185	0.952	0.891	0.971	1.083	512.84	24.46	116	185	3708	415.0	6.77
P49117	Nuclear receptor subfamily 2 group C member 2 OS=Mus musculus GN=Nr2c2 PE=1 SV=1 - [NR2C2_MOUSE]	9.90	2	4	4	6	1.392	2.066	1.134	1.083	16.90	9.90	5	6	596	65.2	6.28
Q9ERK7	Neuronal acetylcholine receptor subunit beta-2 OS=Mus musculus GN=Chrnb2 PE=2 SV=1 - [ACHB2_MOUSE]	15.57	2	5	5	9	1.085	1.040	0.877	1.084	22.01	15.57	7	9	501	57.1	7.03
Q9QX66-3	Isoform 3 of Zinc finger protein neuro-d4 OS=Mus musculus GN=Dpf1 - [DPF1_MOUSE]	8.73	6	2	2	2	2.849	1.707	0.543	1.084	4.73	8.73	2	2	332	37.9	7.23
O88543	COP9 signalosome complex subunit 3 OS=Mus musculus GN=Cops3 PE=1 SV=3 -	43.50	3	14	14	83	0.856	1.047	0.958	1.084	250.12	43.50	26	83	423	47.8	6.65
Q80YF9	Rho GTPase-activating protein 33 OS=Mus musculus GN=Arhgap33 PE=1 SV=1 -	14.10	1	13	14	32	0.906	1.541	1.484	1.084	101.82	14.10	20	32	1305	139.7	9.19
D3Z001	Protein 2310057J18Rik OS=Mus musculus GN=2310057J18Rik PE=4 SV=1 - [D3Z001_MOUSE]	10.16	2	1	1	1	0.794	0.838	1.018	1.084	2.19	10.16	1	1	246	28.1	4.27
Q91V48	MCG1033322 OS=Mus musculus GN=Pcdhb21 PE=2 SV=1 - [Q91V48_MOUSE]	5.30	1	2	2	3	2.104	1.119	1.203	1.084	2.83	5.30	2	3	774	85.2	4.83
Q8BVQ5	Protein phosphatase methylesterase 1 OS=Mus musculus GN=Ppme1 PE=1 SV=5 - [PPME1_MOUSE]	46.63	1	15	15	89	0.613	1.087	0.946	1.084	207.80	46.63	28	89	386	42.2	5.97
Q8R4V2	Dual specificity protein phosphatase 15 OS=Mus musculus GN=Dusp15 PE=2 SV=3 -	37.02	3	7	7	23	1.293	0.306	0.920	1.084	83.45	37.02	11	23	235	26.2	9.26
P53994	Ras-related protein Rab-2A OS=Mus musculus GN=Rab2a PE=1 SV=1 - [RAB2A_MOUSE]	67.45	3	6	13	141	1.311	1.071	1.004	1.084	410.24	67.45	26	141	212	23.5	6.54
P08508	Low affinity immunoglobulin gamma Fc region receptor III OS=Mus musculus GN=Fcgr3 PE=1 SV=1 - [FCGR3_MOUSE]	3.83	5	1	1	2	0.734	1.919	1.076	1.084	7.24	3.83	2	2	261	30.0	9.29
Q91YN0	Uncharacterized protein C12orf4 homolog OS=Mus musculus GN=D6Wsu163e PE=1 SV=1 - [CL004_MOUSE]	20.65	1	9	9	26	0.476	0.890	0.922	1.084	57.26	20.65	15	26	552	63.6	6.18
J3QPX8	Uncharacterized protein OS=Mus musculus GN=Olfr1187-ps1 PE=4 SV=1 - [J3QPX8_MOUSE]	23.91	1	1	1	4	1.398	1.298	0.796	1.084	0.00	23.91	1	4	92	10.4	7.18

A3KGB4	TBC1 domain family member 8B OS=Mus musculus GN=Tbc1d8b PE=2 SV=1 -	3.32	1	2	3	17	0.545	1.640	0.829	1.084	27.65	3.32	4	17	1114	127.8	5.49
D3Z7E5	Glycogen synthase kinase-3 alpha OS=Mus musculus GN=Gsk3a PE=2 SV=1 - [D3Z7E5_MOUSE]	44.03	2	9	16	101	0.881	1.084	1.124	1.084	294.72	44.03	29	101	486	51.2	8.91
Q7TNS5	Protein B630019K06Rik OS=Mus musculus GN=B630019K06Rik PE=2 SV=1 - [Q7TNS5_MOUSE]	27.03	2	7	7	31	0.760	0.763	0.576	1.084	97.25	27.03	11	31	333	34.8	6.65
P53690	Matrix metalloproteinase-14 OS=Mus musculus GN=Mmp14 PE=2 SV=3 - [MMP14_MOUSE]	5.15	1	2	2	3	0.914	1.029	0.941	1.084	5.85	5.15	2	3	582	65.9	8.03
P53395	Lipoamide acyltransferase component of branched-chain alpha-keto acid dehydrogenase complex, mitochondrial OS=Mus musculus GN=Dbt PE=2 SV=2 - [ODB2_MOUSE]	34.02	1	17	17	65	1.032	1.134	1.096	1.084	156.22	34.02	30	65	482	53.2	8.60
D3Z6H3	Dynactin 6, isoform CRA_b OS=Mus musculus GN=Dctn6 PE=4 SV=1 - [D3Z6H3_MOUSE]	31.89	4	3	5	19	0.873	0.911	1.096	1.084	57.53	31.89	9	19	185	20.0	6.38
O88746	Target of Myb protein 1 OS=Mus musculus GN=Tom1 PE=1 SV=1 - [TOM1_MOUSE]	56.10	3	18	18	92	2.000	1.074	1.220	1.084	259.19	56.10	30	92	492	54.3	4.94
O88602	Voltage-dependent calcium channel gamma-2 subunit OS=Mus musculus GN=Cacng2 PE=1 SV=1 - [CCG2_MOUSE]	29.72	1	6	7	51	0.632	0.648	1.024	1.084	176.57	29.72	13	51	323	35.9	8.98
Q99KC8	von Willebrand factor A domain-containing protein 5A OS=Mus musculus GN=Vwa5a PE=1 SV=2 - [VMA5A_MOUSE]	35.69	2	20	23	74	1.027	0.931	0.836	1.084	217.98	35.69	39	74	793	87.1	6.58
Q8K1M6	Dynamin-1-like protein OS=Mus musculus GN=Dnm1l PE=1 SV=2 - [DNM1L_MOUSE]	72.24	4	2	45	396	0.992	1.333	1.065	1.085	1122.31	72.24	81	396	742	82.6	7.05
D3YUR7	Lipoxygenase homology domain-containing protein 1 (Fragment) OS=Mus musculus GN=Loxhd1 PE=2 SV=1 - [illegible]	1.76	4	2	2	2	3.753	1.578	1.230	1.085	2.48	1.76	2	2	1478	168.6	5.14
P46737	Lys-63-specific deubiquitinase BRCC36 OS=Mus musculus GN=Brcc3 PE=2 SV=1 - [BRCC3_MOUSE]	29.55	5	8	8	21	0.870	1.086	0.972	1.085	66.58	29.55	14	21	291	33.3	5.83
D3YY50	Copper homeostasis protein cutC homolog (Fragment) OS=Mus musculus GN=Cutc PE=2 SV=1 - [D3YY50_MOUSE]	26.77	3	5	5	8	1.470	1.508	1.115	1.085	27.18	26.77	7	8	254	27.1	6.68
P28828	Receptor-type tyrosine-protein phosphatase mu OS=Mus musculus GN=Ptprm PE=2 SV=2 - [PTPRM_MOUSE]	8.82	1	8	12	31	1.563	0.993	1.080	1.085	74.80	8.82	20	31	1452	163.5	6.68
Q61730	Interleukin-1 receptor accessory protein OS=Mus musculus GN=Il1rap PE=1 SV=1 - [IL1AP_MOUSE]	17.54	3	1	11	40	0.772	0.259	0.957	1.085	110.80	17.54	19	40	570	65.7	7.77
Q9CRA7	ATP synthase subunit s, mitochondrial OS=Mus musculus GN=Atp5s PE=2 SV=1 - [ATP5S_MOUSE]	26.50	1	5	5	16	0.958	1.589	0.883	1.085	47.17	26.50	10	16	200	23.3	8.06
D6RIK0	Prestin OS=Mus musculus GN=Slc26a5 PE=2 SV=1 - [D6RIK0_MOUSE]	3.36	4	1	1	2	1.182	0.832	1.552	1.085	5.15	3.36	1	2	447	48.6	8.59
P55258	Ras-related protein Rab-8A OS=Mus musculus GN=Rab8a PE=1 SV=2 - [RAB8A_MOUSE]	56.04	1	4	13	110	1.051	0.833	1.026	1.085	307.70	56.04	23	110	207	23.7	9.07
Q9D964	Glycine amidinotransferase, mitochondrial OS=Mus musculus GN=Gatm PE=1 SV=1 - [GATM_MOUSE]	29.31	1	10	10	26	0.931	0.556	0.866	1.085	73.02	29.31	19	26	423	48.3	7.88
Q91WT9-2	Isoform 2 of Cystathionine beta-synthase OS=Mus musculus GN=Cbs - [CBS_MOUSE]	24.68	5	10	11	26	0.891	0.699	0.867	1.085	71.90	24.68	18	26	547	60.2	6.61
P01635	Ig kappa chain V-V region K2 (Fragment) OS=Mus musculus PE=1 SV=1 - [KV5A3_MOUSE]	8.70	1	1	1	2	2.883	4.349	0.766	1.085	5.70	8.70	2	2	115	12.6	8.31
Q5XG73-2	Isoform 2 of Acyl-CoA-binding domain-containing protein 5 OS=Mus musculus GN=Acbd5 - [ACBD5_MOUSE]	16.95	4	7	7	18	1.045	0.908	0.939	1.085	61.00	16.95	12	18	472	52.3	5.29
Q8K3B1	F-box/SPRY domain-containing protein 1 OS=Mus musculus GN=Fbxo45 PE=1 SV=2 - [FBSP1_MOUSE]	29.37	1	6	6	10	1.057	1.259	1.072	1.085	17.39	29.37	8	10	286	30.6	7.85

G3X8X7	Vacuolar protein sorting 16 (Yeast) OS=Mus musculus GN=Vps16 PE=4 SV=1 - [G3X8X7_MOUSE]	24.79	4	16	16	36	0.639	0.825	0.976	1.085	141.14	24.79	24	36	839	94.8	6.92
Q3TFQ1	SPRY domain-containing protein 7 OS=Mus musculus GN=Spryd7 PE=2 SV=2 -	36.22	2	6	6	33	0.856	0.895	0.874	1.085	93.95	36.22	11	33	196	21.7	6.70
Q99NF3	Centrosomal protein of 41 kDa OS=Mus musculus GN=Cep41 PE=1 SV=1 - [CEP41_MOUSE]	35.39	2	9	9	30	1.153	1.157	1.083	1.085	94.72	35.39	14	30	373	41.4	8.12
P68134	Actin, alpha skeletal muscle OS=Mus musculus GN=Acta1 PE=1 SV=1 - [ACTS_MOUSE]	67.37	3	2	27	2327	0.772	1.243	1.197	1.085	6680.19	67.37	45	2327	377	42.0	5.39
Q6A009	E3 ubiquitin-protein ligase listerin OS=Mus musculus GN=Ltn1 PE=1 SV=3 - [LTN1_MOUSE]	6.90	1	12	12	27	0.864	0.920	0.806	1.086	69.46	6.90	20	27	1767	198.8	6.55
P47937	Neuromedin-K receptor OS=Mus musculus GN=Tacr3 PE=2 SV=2 - [NK3R_MOUSE]	1.99	1	1	1	1	1.229	0.877	0.908	1.086	1.69	1.99	1	1	452	51.0	9.31
Q99KD5	Protein unc-45 homolog A OS=Mus musculus GN=Unc45a PE=1 SV=2 - [UN45A_MOUSE]	17.37	4	14	14	27	0.949	1.377	0.844	1.086	84.80	17.37	20	27	944	103.4	6.34
Q3UMB5	Smith-Magenis syndrome chromosomal region candidate gene 8 protein homolog OS=Mus musculus GN=Smcr8 PE=1 SV=2 - [SMCR8_MOUSE]	29.84	2	20	20	53	1.043	0.954	0.988	1.086	160.36	29.84	32	53	935	104.9	5.40
Q8BYK5-2	Isoform 2 of Phosphatase and actin regulator 3 OS=Mus musculus GN=Phactr3 -	19.68	5	9	10	21	0.975	1.035	1.401	1.086	55.05	19.68	16	21	559	63.1	9.45
E9Q3C1	Protein C2cd2 OS=Mus musculus GN=C2cd2 PE=2 SV=1 - [E9Q3C1_MOUSE]	5.60	1	4	4	7	1.028	1.217	1.177	1.086	8.09	5.60	6	7	696	76.5	7.72
Q9JI74	Kelch-like protein 1 OS=Mus musculus GN=Klhl1 PE=1 SV=2 - [KLHL1_MOUSE]	6.92	1	3	3	9	1.334	0.582	0.909	1.086	15.63	6.92	4	9	751	82.8	6.42
Q8CHX7	Raftlin-2 OS=Mus musculus GN=Rftn2 PE=2 SV=3 - [RFTN2_MOUSE]	28.00	2	10	10	61	1.015	1.017	0.946	1.086	209.07	28.00	19	61	500	54.9	5.50
Q8VCH8	UBX domain-containing protein 4 OS=Mus musculus GN=Ubxn4 PE=1 SV=1 -	27.67	1	8	8	20	0.813	0.663	0.963	1.086	59.36	27.67	13	20	506	56.4	6.61
P62331	ADP-ribosylation factor 6 OS=Mus musculus GN=Arf6 PE=1 SV=2 - [ARF6_MOUSE]	45.71	5	6	7	17	0.861	1.014	1.000	1.086	51.48	45.71	10	17	175	20.1	8.95
O35495	Cyclin-dependent kinase 14 OS=Mus musculus GN=Cdk14 PE=2 SV=2 - [CDK14_MOUSE]	29.00	20	8	11	67	1.223	1.213	1.107	1.086	204.82	29.00	19	67	469	53.0	8.92
Q3UHK1	Proton myo-inositol cotransporter OS=Mus musculus GN=Slc2a13 PE=2 SV=2 -	13.50	1	8	8	54	0.797	1.074	0.900	1.086	163.18	13.50	16	54	637	69.0	6.86
P56393	Cytochrome c oxidase subunit 7B, mitochondrial OS=Mus musculus GN=Cox7b PE=2 SV=1 - [COX7B_MOUSE]	10.00	1	2	2	5	0.975	0.919	1.088	1.086	15.44	10.00	4	5	80	9.0	10.27
Q9EQG9	Collagen type IV alpha-3-binding protein OS=Mus musculus GN=Col4a3bp PE=1 SV=1 - [C43BP_MOUSE]	17.15	3	12	12	23	1.314	0.963	0.722	1.086	58.08	17.15	17	23	624	71.1	5.44
Q9DBJ6	Josephin-1 OS=Mus musculus GN=Josd1 PE=2 SV=1 - [JOS1_MOUSE]	5.45	1	1	1	2	1.527	1.455	1.232	1.086	7.28	5.45	2	2	202	23.1	8.53
A2AV19	Exosome complex component RRP4 (Fragment) OS=Mus musculus GN=Exosc2 PE=2 SV=1 - [A2AV19_MOUSE]	23.89	2	3	3	6	1.360	1.074	1.086	1.086	20.29	23.89	6	6	247	27.3	8.16
Q9ERA0	Alpha-globin transcription factor CP2 OS=Mus musculus GN=Tfcp2 PE=1 SV=1 - [TFCP2_MOUSE]	14.34	3	4	5	12	0.600	1.029	0.911	1.086	36.73	14.34	8	12	502	57.0	5.54
Q3UNT6	B-lymphocyte antigen CD20 OS=Mus musculus GN=Ms4a2 PE=2 SV=1 - [Q3UNT6_MOUSE]	9.09	2	1	1	1	2.366	0.986	0.863	1.086	0.00	9.09	1	1	198	22.2	4.44
P31001	Desmin OS=Mus musculus GN=Des PE=1 SV=3 - [DESM_MOUSE]	41.36	1	12	18	194	1.264	2.792	1.073	1.086	475.64	41.36	31	194	469	53.5	5.27
Q64133	Amine oxidase [flavin-containing] A OS=Mus musculus GN=Maoa PE=1 SV=3 - [AOFA_MOUSE]	45.63	1	18	23	109	0.846	0.845	0.784	1.086	296.11	45.63	43	109	526	59.6	7.81
Q6F5F2	Protein Sirpb1a OS=Mus musculus GN=Sirpb1a PE=2 SV=1 - [Q6F5F2_MOUSE]	3.84	4	1	3	26	0.800	0.881	1.152	1.086	53.23	3.84	5	26	391	43.5	7.99
Q9Z2X8	Kelch-like ECH-associated protein 1 OS=Mus musculus GN=Keap1 PE=1 SV=1 - [KEAP1_MOUSE]	14.90	1	9	9	18	1.080	1.004	0.892	1.086	39.07	14.90	14	18	624	69.5	6.44

Q62073	Mitogen-activated protein kinase kinase kinase 7 OS=Mus musculus GN=Map3k7 PE=1 SV=1 - [M3K7_MOUSE]	18.48	4	9	9	25	1.588	0.969	1.354	1.086	80.75	18.48	15	25	579	64.2	6.67
P61315	Galactose-3-O-sulfotransferase 3 OS=Mus musculus GN=Gal3st3 PE=2 SV=1 -	5.57	1	2	2	4	0.848	1.534	1.121	1.087	9.59	5.57	4	4	431	49.2	9.76
Q9D3X9-2	Isoform 2 of Microfibrillar-associated protein 3-like OS=Mus musculus GN=Mfap3l - [MFA3L_MOUSE]	7.52	3	3	3	5	0.728	1.248	1.012	1.087	13.13	7.52	5	5	306	34.2	4.89
G3UYK8	Coronin OS=Mus musculus GN=Coro1a PE=2 SV=1 - [G3UYK8_MOUSE]	33.68	4	1	14	68	0.452	1.596	1.470	1.087	195.76	33.68	25	68	389	43.2	6.74
P59041	DnaJ homolog subfamily C member 30 OS=Mus musculus GN=Dnajc30 PE=2 SV=1 -	11.42	1	2	2	2	0.557	0.901	0.967	1.087	4.99	11.42	2	2	219	24.7	10.21
Q9D6Y9	1,4-alpha-glucan-branching enzyme OS=Mus musculus GN=Gbe1 PE=2 SV=1 -	31.62	3	19	19	50	0.833	0.713	0.907	1.087	133.65	31.62	35	50	702	80.3	6.43
Q5U4C1	G-protein coupled receptor associated sorting protein 1 OS=Mus musculus GN=Gprasp1 PE=2 SV=1 - [GASP1_MOUSE]	23.61	2	30	31	90	1.043	1.094	0.755	1.087	241.32	23.61	51	90	1347	151.6	5.08
Q8BX37	Iron/zinc purple acid phosphatase-like protein OS=Mus musculus GN=Papl PE=2 SV=2 - [PAPL_MOUSE]	2.51	2	1	1	2	0.551	1.245	1.348	1.087	2.01	2.51	1	2	438	50.6	9.04
O54984	ATPase Asna1 OS=Mus musculus GN=Asna1 PE=1 SV=2 - [ASNA_MOUSE]	37.36	1	9	9	45	0.756	1.323	0.984	1.087	132.16	37.36	18	45	348	38.8	4.91
Q8R146-2	Isoform 2 of Acylamino-acid-releasing enzyme OS=Mus musculus GN=Apeh - [APEH_MOUSE]	50.63	2	26	26	81	1.204	0.936	0.801	1.087	272.54	50.63	43	81	717	79.9	5.54
Q62009-5	Isoform 5 of Periostin OS=Mus musculus GN=Postn - [POSTN_MOUSE]	3.83	5	2	3	5	1.204	1.294	0.853	1.087	11.48	3.83	4	5	783	87.0	7.66
Q9DBE8	Alpha-1,3/1,6-mannosyltransferase ALG2 OS=Mus musculus GN=Alg2 PE=2 SV=2 -	41.69	2	16	16	82	0.626	0.823	0.834	1.087	244.02	41.69	29	82	415	47.4	7.97
Q922Y1	UBX domain-containing protein 1 OS=Mus musculus GN=Ubxn1 PE=1 SV=1 -	40.40	1	10	10	54	1.686	0.898	1.114	1.087	127.48	40.40	17	54	297	33.6	5.26
Q6PCX9	E3 ubiquitin-protein ligase TRIM37 OS=Mus musculus GN=Trim37 PE=2 SV=1 - [TRI37_MOUSE]	1.14	1	1	1	2	1.442	1.037	1.663	1.087	7.23	1.14	1	2	961	107.6	5.21
Q8VI51	VPS10 domain-containing receptor SorCS3 OS=Mus musculus GN=Sorcs3 PE=1 SV=1 - [SORC3_MOUSE]	6.56	1	8	8	15	0.920	1.298	1.296	1.088	32.62	6.56	12	15	1219	135.9	6.49
P52840	Sulfotransferase 1A1 OS=Mus musculus GN=Sult1a1 PE=2 SV=1 - [ST1A1_MOUSE]	13.40	5	2	3	4	1.452	1.318	0.820	1.088	5.95	13.40	3	4	291	34.0	8.10
O70310	Glycylpeptide N-tetradecanoyltransferase 1 OS=Mus musculus GN=Nmt1 PE=1 SV=1 - [NMT1_MOUSE]	21.77	1	7	10	31	0.760	0.800	1.122	1.088	94.29	21.77	17	31	496	56.9	8.00
D3YZ62	Unconventional myosin-Va OS=Mus musculus GN=Myo5a PE=2 SV=1 - [D3YZ62_MOUSE]	50.00	8	81	92	550	0.679	0.912	1.092	1.088	1526.21	50.00	158	550	1828	212.2	8.62
Q9D7S7-2	Isoform 2 of 60S ribosomal protein L22-like 1 OS=Mus musculus GN=Rpl22l1 -	25.62	2	3	3	5	0.694	0.840	0.811	1.088	12.25	25.62	4	5	121	14.3	9.45
G3X8Y7	Long-chain fatty acid transport protein 3 OS=Mus musculus GN=Slc27a3 PE=4 SV=1 - [G3X8Y7_MOUSE]	3.90	3	2	2	3	0.564	0.731	0.942	1.088	12.99	3.90	3	3	667	73.0	7.65
Q8C0I4	Enhancer of polycomb homolog 2 OS=Mus musculus GN=Epc2 PE=2 SV=2 - [EPC2_MOUSE]	5.69	1	4	4	8	0.619	1.080	1.116	1.088	16.02	5.69	6	8	808	90.9	8.73
Q8R2K1-2	Isoform 2 of Fucose mutarotase OS=Mus musculus GN=Fuom - [FUCM_MOUSE]	24.81	8	3	3	9	1.139	1.217	0.852	1.088	29.18	24.81	5	9	133	14.7	5.31
E9Q722	Upstream stimulatory factor 1 OS=Mus musculus GN=Usf1 PE=2 SV=1 - [E9Q722_MOUSE]	6.42	2	1	1	7	0.779	1.511	0.918	1.088	18.67	6.42	2	7	296	32.0	5.50
Q9D0T1	NHP2-like protein 1 OS=Mus musculus GN=Nhp2l1 PE=2 SV=4 - [NH2L1_MOUSE]	40.63	2	6	6	25	0.930	1.054	0.793	1.088	69.85	40.63	11	25	128	14.2	8.46
Q9D5V5	Cullin-5 OS=Mus musculus GN=Cul5 PE=1 SV=3 - [CUL5_MOUSE]	37.31	4	24	26	98	0.757	1.043	1.082	1.089	266.13	37.31	44	98	780	90.9	7.81

E9QAD8	IQ motif and SEC7 domain-containing protein 2 OS=Mus musculus GN=Iqsec2 PE=2 SV=1 - [E9QAD8_MOUSE]	38.24	5	36	42	186	1.559	1.611	1.802	1.089	580.92	38.24	72	186	1488	162.7	8.56
Q8CDN6	Thioredoxin-like protein 1 OS=Mus musculus GN=Txnl1 PE=1 SV=3 - [TXNL1_MOUSE]	48.10	1	9	9	82	1.346	1.140	0.966	1.089	228.02	48.10	17	82	289	32.2	4.96
Q91YZ2	C-terminal binding protein 2, isoform CRA_b OS=Mus musculus GN=Ctbp2 PE=2 SV=1 - [Q91YZ2_MOUSE]	30.24	5	6	9	37	0.700	1.054	0.731	1.089	102.85	30.24	16	37	420	45.9	6.48
D3Z6S7	Immunoglobulin superfamily member 11 OS=Mus musculus GN=Igsf11 PE=4 SV=1 - [D3Z6S7_MOUSE]	16.80	2	4	4	38	0.905	1.142	1.327	1.089	88.62	16.80	7	38	369	39.9	7.17
E9Q6P9	Voltage-dependent T-type calcium channel subunit alpha-1H OS=Mus musculus GN=Cacna1h PE=4 SV=1 - [illegible]	3.22	7	5	7	11	1.254	1.277	1.262	1.089	23.49	3.22	11	11	2359	261.1	6.92
Q8K0G5	Protein TSSC1 OS=Mus musculus GN=Tssc1 PE=1 SV=2 - [TSSC1_MOUSE]	24.61	1	7	7	35	1.008	1.342	1.085	1.089	119.70	24.61	12	35	386	43.1	5.14
Q920E5	Farnesyl pyrophosphate synthase OS=Mus musculus GN=Fdps PE=2 SV=1 - [FPPS_MOUSE]	33.71	1	11	11	38	0.906	0.914	0.849	1.089	123.43	33.71	19	38	353	40.6	5.66
Q8K0D5	Elongation factor G, mitochondrial OS=Mus musculus GN=Gfm1 PE=2 SV=1 - [EFGM_MOUSE]	31.29	2	23	23	81	0.797	0.801	1.006	1.089	219.65	31.29	43	81	751	83.5	6.92
Q8BQP9	Regulator of G-protein signaling 7-binding protein OS=Mus musculus GN=Rgs7bp PE=1 SV=1 - [R7BP_MOUSE]	38.52	1	6	6	29	0.653	1.191	1.414	1.089	84.13	38.52	10	29	257	29.0	8.46
P62962	Profilin-1 OS=Mus musculus GN=Pfn1 PE=1 SV=2 - [PROF1_MOUSE]	73.57	3	10	10	175	0.907	0.940	0.980	1.089	547.76	73.57	20	175	140	14.9	8.28
Q6GQW0	Ankyrin repeat and BTB/POZ domain-containing protein BTBD11 OS=Mus musculus GN=Btbd11 PE=2 SV=2 - [illegible]	12.17	6	10	10	26	0.687	0.850	0.753	1.089	60.89	12.17	19	26	1109	121.5	6.81
A2AQ17	Complex I intermediate-associated protein 30, mitochondrial OS=Mus musculus GN=Ndufaf1 PE=4 SV=1 - [illegible]	33.23	2	10	10	28	1.110	1.095	1.053	1.089	88.87	33.23	17	28	328	37.8	8.60
Q9Z2B2-2	Isoform 2 of Brain mitochondrial carrier protein 1 OS=Mus musculus GN=Slc25a14 -	11.49	2	2	4	11	1.082	1.152	1.017	1.089	11.76	11.49	7	11	322	36.0	9.69
Q9JK81	UPF0160 protein MYG1, mitochondrial OS=Mus musculus GN=Myg1 PE=2 SV=1 - [MYG1_MOUSE]	41.84	3	13	13	48	0.634	0.970	0.873	1.089	134.52	41.84	22	48	380	42.7	7.02
Q9D8S4	Oligoribonuclease, mitochondrial OS=Mus musculus GN=Rexo2 PE=1 SV=2 - [ORN_MOUSE]	39.66	1	8	9	25	1.312	0.972	1.161	1.089	76.52	39.66	17	25	237	26.7	7.15
O54824	Pro-interleukin-16 OS=Mus musculus GN=Il16 PE=1 SV=3 - [IL16_MOUSE]	7.87	2	6	7	8	1.489	0.861	1.213	1.090	18.87	7.87	8	8	1322	141.3	7.78
O70439	Syntaxin-7 OS=Mus musculus GN=Stx7 PE=1 SV=3 - [STX7_MOUSE]	54.41	1	10	11	67	1.008	1.195	1.080	1.090	204.87	54.41	19	67	261	29.8	5.78
Q8BJI1	Sodium-dependent neutral amino acid transporter SLC6A17 OS=Mus musculus GN=Slc6a17 PE=1 SV=1 - [illegible]	18.84	2	11	11	78	0.681	1.237	1.130	1.090	190.12	18.84	19	78	727	81.0	6.23
E9Q3K3	Arf-GAP with GTPase, ANK repeat and PH domain-containing protein 1 OS=Mus musculus GN=Agap1 PE=2 SV=1 - [illegible]	21.77	3	10	16	62	0.624	0.710	0.886	1.090	197.11	21.77	27	62	804	89.0	8.03
Q8CEC0-3	Isoform 3 of Nuclear pore complex protein Nup88 OS=Mus musculus GN=Nup88 -	14.56	3	9	10	15	1.324	1.149	1.085	1.090	41.17	14.56	13	15	742	83.6	6.10
Q8CHK3	Lysophospholipid acyltransferase 7 OS=Mus musculus GN=Mboat7 PE=2 SV=1 -	9.73	2	4	4	28	0.356	1.054	0.920	1.090	86.45	9.73	6	28	473	53.4	8.69
Q6PCP5	Mitochondrial fission factor OS=Mus musculus GN=Mff PE=1 SV=1 - [MFF_MOUSE]	45.36	7	3	10	83	1.176	0.972	1.090	1.090	269.07	45.36	17	83	291	32.9	6.83
Q3V2Q8	NEDD4-binding protein 2-like 1 OS=Mus musculus GN=N4bp2l1 PE=2 SV=1 - [N42L1_MOUSE]	7.56	2	1	2	14	1.781	1.456	1.048	1.090	25.04	7.56	4	14	238	28.0	9.94
Q06185	ATP synthase subunit e, mitochondrial OS=Mus musculus GN=Atp5i PE=1 SV=2 - [ATP5I_MOUSE]	53.52	2	3	4	16	0.713	1.644	1.115	1.090	34.63	53.52	7	16	71	8.2	9.35

Q8BFR5	Elongation factor Tu, mitochondrial OS=Mus musculus GN=Tufm PE=1 SV=1 - [EFTU_MOUSE]	69.25	3	27	27	282	0.969	1.045	1.086	1.090	891.65	69.25	52	282	452	49.5	7.56
B1AZA5	Transmembrane protein 245 OS=Mus musculus GN=Tmem245 PE=1 SV=1 - [TM245_MOUSE]	7.76	3	6	6	18	0.850	1.415	1.104	1.090	41.16	7.76	12	18	876	97.3	8.75
Q8VCG3	WD repeat-containing protein 74 OS=Mus musculus GN=Wdr74 PE=2 SV=1 -	3.13	1	1	1	3	0.872	0.890	1.092	1.090	6.14	3.13	2	3	384	42.6	7.78
Q8C6I5	Cysteine desulfurase, mitochondrial OS=Mus musculus GN=Nfs1 PE=2 SV=1 - [Q8C6I5_MOUSE]	53.38	6	19	19	72	1.062	0.899	0.978	1.090	248.52	53.38	37	72	459	50.5	8.16
A2A6J4	Lymphocyte-specific protein 1 OS=Mus musculus GN=Lsp1 PE=2 SV=1 - [A2A6J4_MOUSE]	13.98	4	4	4	5	3.207	1.490	1.519	1.090	16.13	13.98	5	5	322	35.7	5.12
Q91YX0	Protein THEMIS2 OS=Mus musculus GN=Themis2 PE=1 SV=2 - [THMS2_MOUSE]	1.51	1	1	1	1	0.873	0.774	0.931	1.090	2.64	1.51	1	1	663	74.3	6.19
P97384	Annexin A11 OS=Mus musculus GN=Anxa11 PE=1 SV=2 - [ANX11_MOUSE]	26.24	2	11	12	34	0.962	2.226	1.169	1.091	108.43	26.24	21	34	503	54.0	7.66
Q91YS7	Dual-specificity mitogen-activated protein kinase kinase 2 OS=Mus musculus GN=Map2k2 PE=2 SV=1 - [illegible]	41.00	4	10	17	75	0.567	1.075	0.904	1.091	194.46	41.00	29	75	400	44.3	7.05
Q9D6J6	NADH dehydrogenase [ubiquinone] flavoprotein 2, mitochondrial OS=Mus musculus GN=Ndufv2 PE=1 SV=2 - [illegible]	55.24	4	13	14	312	1.851	0.914	1.254	1.091	960.98	55.24	23	312	248	27.3	7.40
O54724	Polymerase I and transcript release factor OS=Mus musculus GN=Ptrf PE=1 SV=1 -	26.79	1	9	12	33	1.016	1.980	1.142	1.091	98.31	26.79	19	33	392	43.9	5.52
Q80TB8	Synaptic vesicle membrane protein VAT-1 homolog-like OS=Mus musculus GN=Vat1l PE=2 SV=2 - [VAT1L_MOUSE]	43.88	1	12	16	93	0.905	0.841	0.755	1.091	264.90	43.88	28	93	417	45.8	5.06
Q8K221	Arfaptin-2 OS=Mus musculus GN=Arfip2 PE=2 SV=2 - [ARFP2_MOUSE]	45.16	3	14	14	51	0.831	1.653	0.982	1.091	150.71	45.16	22	51	341	37.7	5.87
Q8C0K8	Protein Uvrag OS=Mus musculus GN=Uvrag PE=2 SV=1 - [Q8C0K8_MOUSE]	17.19	1	10	11	18	0.676	0.951	1.119	1.091	49.92	17.19	17	18	698	77.5	7.97
O35114	Lysosome membrane protein 2 OS=Mus musculus GN=Scarb2 PE=1 SV=3 -	16.53	1	9	9	31	0.868	1.027	0.789	1.091	79.34	16.53	16	31	478	54.0	5.10
Q91W34	UPF0420 protein C16orf58 homolog OS=Mus musculus PE=2 SV=1 - [CP058_MOUSE]	16.31	3	7	7	22	0.591	0.846	0.775	1.091	58.57	16.31	12	22	466	50.4	7.15
Q80Y39	Uncharacterized protein C10orf62 homolog OS=Mus musculus PE=2 SV=1 - [CJ062_MOUSE]	8.22	1	1	2	2	2.228	1.519	1.896	1.091	5.00	8.22	2	2	304	34.0	9.51
Q80TL4	Protein KIAA1045 OS=Mus musculus GN=Kiaa1045 PE=1 SV=2 - [K1045_MOUSE]	35.25	3	15	15	61	0.750	1.402	1.526	1.091	160.17	35.25	28	61	400	45.2	5.77
A2AD03	Rab proteins geranylgeranyltransferase component A 1 OS=Mus musculus GN=Chm PE=2 SV=1 - [A2AD03_MOUSE]	9.67	3	5	5	12	0.516	1.160	1.195	1.091	39.09	9.67	8	12	662	73.6	4.69
F6UDR6	E3 ubiquitin-protein ligase RNF170 (Fragment) OS=Mus musculus GN=Rnf170 PE=4 SV=1 - [F6UDR6_MOUSE]	12.39	8	1	1	5	0.804	1.062	1.135	1.091	19.36	12.39	2	5	113	12.9	4.70
Q7TPM9	Myotubularin-related protein 10 OS=Mus musculus GN=Mtmr10 PE=2 SV=2 -	2.46	1	2	2	3	1.101	1.015	0.870	1.091	10.43	2.46	3	3	771	88.1	8.68
Q9CQM0	Nicolin-1 OS=Mus musculus GN=Nicn1 PE=1 SV=1 - [NICN1_MOUSE]	9.39	1	2	2	5	0.872	0.874	0.935	1.091	12.30	9.39	4	5	213	24.3	7.08
Q8BP40	Lysophosphatidic acid phosphatase type 6 OS=Mus musculus GN=Acp6 PE=2 SV=1 -	37.32	1	11	11	38	1.913	1.336	1.059	1.091	113.45	37.32	18	38	418	47.6	7.72
Q8K0S5	Reticulon-4 receptor-like 1 OS=Mus musculus GN=Rtn4rl1 PE=2 SV=1 - [R4RL1_MOUSE]	8.09	1	3	3	5	0.837	1.252	1.090	1.091	14.67	8.09	5	5	445	49.8	8.65
B1AR09	Protein Mllt6 OS=Mus musculus GN=Mllt6 PE=2 SV=1 - [B1AR09_MOUSE]	9.27	2	6	6	8	1.919	1.047	1.529	1.092	27.14	9.27	6	8	1079	110.5	8.47
E9QPH0	cGMP-dependent protein kinase OS=Mus musculus GN=Prkg2 PE=2 SV=1 - [E9QPH0_MOUSE]	18.42	3	10	13	25	1.020	1.874	1.258	1.092	57.71	18.42	18	25	733	84.2	8.40
Q8K2T4	UPF0723 protein C11orf83 homolog OS=Mus musculus PE=2 SV=1 - [CK083_MOUSE]	11.24	1	1	1	3	1.374	1.250	1.277	1.092	5.72	11.24	2	3	89	9.6	9.52

Q80VP0-2	Isoform 2 of Tectonin beta-propeller repeat-containing protein 1 OS=Mus musculus GN=Tecpr1 - [TCPR1_MOUSE]	20.49	3	18	19	63	0.709	1.031	1.118	1.092	202.40	20.49	32	63	1025	114.0	6.96
P58854	Gamma-tubulin complex component 3 OS=Mus musculus GN=Tubgcp3 PE=2 SV=2 - [GCP3_MOUSE]	15.58	3	13	13	33	0.706	1.018	0.890	1.092	93.63	15.58	23	33	905	103.4	8.32
E9Q242	Adenylosuccinate lyase OS=Mus musculus GN=Adsl PE=2 SV=1 - [E9Q242_MOUSE]	35.39	5	13	13	54	0.633	0.886	0.879	1.092	161.02	35.39	20	54	469	53.1	7.27
O08545	Ephrin-A3 OS=Mus musculus GN=Efna3 PE=1 SV=4 - [EFNA3_MOUSE]	6.96	1	1	1	3	1.934	1.053	0.978	1.092	7.84	6.96	1	3	230	25.6	8.44
Q8K4T5	Dual specificity protein phosphatase 19 OS=Mus musculus GN=Dusp19 PE=2 SV=1 -	5.91	2	2	2	4	0.787	0.865	1.009	1.092	5.87	5.91	2	4	220	24.2	5.99
Q8JZP2	Synapsin-3 OS=Mus musculus GN=Syn3 PE=1 SV=2 - [SYN3_MOUSE]	48.70	2	19	21	136	1.157	1.021	1.183	1.092	369.52	48.70	35	136	579	63.3	9.38
E9Q411	Protein Nbas OS=Mus musculus GN=Nbas PE=2 SV=1 - [E9Q411_MOUSE]	14.90	1	29	30	68	1.029	0.885	0.879	1.092	184.31	14.90	52	68	2356	265.6	5.95
Q9Z2D3	Non-syndromic hearing impairment protein 5 homolog OS=Mus musculus GN=Dfna5 PE=2 SV=1 - [DFNA5_MOUSE]	22.66	4	10	10	24	0.869	1.052	0.989	1.092	68.35	22.66	17	24	512	56.6	5.39
Q8C3Q5-2	Isoform 2 of Protein shisa-7 OS=Mus musculus GN=Shisa7 - [SHSA7_MOUSE]	29.39	3	11	11	60	0.853	3.109	2.820	1.092	183.31	29.39	21	60	541	56.4	9.86
P35285	Ras-related protein Rab-22A OS=Mus musculus GN=Rab22a PE=1 SV=2 - [RB22A_MOUSE]	48.45	3	6	8	28	1.163	1.054	1.049	1.092	94.90	48.45	13	28	194	21.8	8.15
E9PUW6	Cardiolipin synthase OS=Mus musculus GN=Crls1 PE=2 SV=1 - [E9PUW6_MOUSE]	12.99	3	1	1	2	0.735	0.943	0.951	1.092	7.58	12.99	2	2	154	15.9	10.98
Q8R2Z5	von Willebrand factor A domain-containing protein 1 OS=Mus musculus GN=Vwa1 PE=1 SV=1 - [VWA1_MOUSE]	16.87	1	4	4	14	1.114	1.079	0.979	1.092	63.14	16.87	7	14	415	44.7	6.54
Q9Z0V7	Mitochondrial import inner membrane translocase subunit Tim17-B OS=Mus musculus GN=Timm17b PE=2 SV=1 - [TI17B_MOUSE]	30.23	2	3	3	7	0.840	0.854	0.884	1.092	26.60	30.23	5	7	172	18.3	9.03
Q9D7B6	Isobutyryl-CoA dehydrogenase, mitochondrial OS=Mus musculus GN=Acad8 PE=2 SV=2 - [ACAD8_MOUSE]	52.06	2	21	21	57	0.973	1.130	0.989	1.092	163.11	52.06	33	57	413	45.0	8.13
B7FAU9	Filamin, alpha OS=Mus musculus GN=Flna PE=4 SV=1 - [B7FAU9_MOUSE]	47.82	8	91	97	370	0.835	3.002	0.895	1.092	1182.14	47.82	170	370	2639	280.3	6.01
H7BX09	Nuclear receptor coactivator 2 OS=Mus musculus GN=Grip1 PE=2 SV=1 - [H7BX09_MOUSE]	16.68	13	15	17	44	1.344	1.222	1.117	1.093	127.24	16.68	27	44	1061	114.3	5.78
D3Z7V3	Sarcolemmal membrane-associated protein OS=Mus musculus GN=Slmap PE=2 SV=1 - [D3Z7V3_MOUSE]	43.04	13	31	32	105	0.964	1.317	1.311	1.093	270.68	43.04	57	105	790	90.7	5.25
Q3UFY8	Mitochondrial ribonuclease P protein 1 OS=Mus musculus GN=Trmt10c PE=2 SV=2 - [MRRP1_MOUSE]	9.18	1	3	3	7	0.875	0.838	1.123	1.093	16.44	9.18	5	7	414	48.4	9.38
G3X9K4	SAPS domain family, member 2, isoform CRA_a OS=Mus musculus GN=Ppp6r2 PE=4 SV=1 - [G3X9K4_MOUSE]	34.24	2	23	24	85	0.987	1.135	1.207	1.093	304.53	34.24	40	85	923	100.4	4.82
P46096	Synaptotagmin-1 OS=Mus musculus GN=Syt1 PE=1 SV=1 - [SYT1_MOUSE]	54.39	2	16	26	427	0.551	1.167	1.334	1.093	1178.65	54.39	45	427	421	47.4	8.53
Q91VU6	DDB1- and CUL4-associated factor 11 OS=Mus musculus GN=Dcaf11 PE=1 SV=1 -	18.03	10	7	7	24	0.882	0.952	1.184	1.093	74.84	18.03	13	24	549	62.0	6.44
Q8R0V5	Indoleamine 2,3-dioxygenase 2 OS=Mus musculus GN=Ido2 PE=1 SV=2 - [I23O2_MOUSE]	3.77	2	1	1	2	2.731	1.585	0.863	1.093	0.00	3.77	1	2	398	44.4	6.80
P70296	Phosphatidylethanolamine-binding protein 1 OS=Mus musculus GN=Pebp1 PE=1 SV=3 - [PEBP1_MOUSE]	63.64	6	9	9	492	1.646	1.299	1.310	1.093	1925.55	63.64	17	492	187	20.8	5.40
Q5MJS3	Extracellular serine/threonine protein kinase FAM20C OS=Mus musculus GN=Fam20c PE=2 SV=1 - [DMP4_MOUSE]	7.25	2	3	3	8	1.517	0.965	1.035	1.093	23.34	7.25	5	8	579	65.7	7.47

A2AE98	WD and tetratricopeptide repeats protein 1 OS=Mus musculus GN=Wdtc1 PE=2 SV=1 - [A2AE98_MOUSE]	3.25	2	2	2	3	1.131	0.896	0.996	1.093	3.40	3.25	3	3	615	69.0	8.40
Q8VBX6	Multiple PDZ domain protein OS=Mus musculus GN=Mpdz PE=1 SV=2 - [MPDZ_MOUSE]	14.89	12	22	24	63	1.130	1.148	0.856	1.093	187.75	14.89	38	63	2055	218.6	5.02
E9Q6R7	Protein Utrn OS=Mus musculus GN=Utrn PE=2 SV=1 - [E9Q6R7_MOUSE]	21.20	3	58	63	152	0.942	1.571	1.011	1.093	446.57	21.20	101	152	3430	392.5	5.33
Q68FH0	Plakophilin-4 OS=Mus musculus GN=Pkp4 PE=1 SV=1 - [PKP4_MOUSE]	58.07	6	49	54	242	1.123	1.242	1.153	1.093	662.51	58.07	93	242	1190	131.5	8.94
Q3TC93	HCLS1-binding protein 3 OS=Mus musculus GN=Hs1bp3 PE=1 SV=2 - [H1BP3_MOUSE]	12.15	1	4	4	9	1.491	1.033	1.087	1.093	18.08	12.15	8	9	395	43.7	4.98
Q05AA6	Dystrophin-related protein 2 OS=Mus musculus GN=Drp2 PE=1 SV=1 - [DRP2_MOUSE]	7.52	2	7	9	17	1.389	1.121	1.164	1.093	30.62	7.52	12	17	957	108.0	6.25
Q80UP8	Sodium-dependent phosphate transporter 2 OS=Mus musculus GN=Slc20a2 PE=1 SV=2 - [S20A2_MOUSE]	9.60	1	6	8	20	0.970	1.154	0.930	1.093	42.30	9.60	13	20	656	70.8	6.48
K4DI77	WD repeat-containing protein 81 (Fragment) OS=Mus musculus GN=Wdr81 PE=4 SV=1 - [K4DI77_MOUSE]	11.58	5	16	16	36	0.709	0.875	0.968	1.093	75.47	11.58	28	36	1934	211.8	5.77
O54829	Regulator of G-protein signaling 7 OS=Mus musculus GN=Rgs7 PE=1 SV=2 - [RGS7_MOUSE]	28.57	1	11	14	71	0.782	1.399	1.256	1.094	191.58	28.57	24	71	469	54.8	8.13
F8WH29	Fibrocystin-L OS=Mus musculus GN=Pkhd1l1 PE=2 SV=1 - [F8WH29_MOUSE]	1.37	4	5	5	5	1.935	0.542	1.502	1.094	4.14	1.37	5	5	4247	464.2	6.42
P63024	Vesicle-associated membrane protein 3 OS=Mus musculus GN=Vamp3 PE=1 SV=1 - [VAMP3_MOUSE]	48.54	1	2	5	179	1.519	0.636	0.882	1.094	522.22	48.54	9	179	103	11.5	8.50
Q9WVS4	MAPK/MAK/MRK overlapping kinase OS=Mus musculus GN=Mok PE=1 SV=1 -	3.10	3	1	2	13	1.050	0.965	1.079	1.094	37.98	3.10	2	13	420	48.0	9.48
Q8BIJ6	Isoleucine--tRNA ligase, mitochondrial OS=Mus musculus GN=Iars2 PE=2 SV=1 - [SYIM_MOUSE]	40.12	3	31	31	137	0.852	1.074	0.996	1.094	433.38	40.12	52	137	1012	112.7	6.81
P47857	6-phosphofructokinase, muscle type OS=Mus musculus GN=Pfkm PE=1 SV=3 - [K6PF_MOUSE]	51.92	1	31	36	551	1.709	0.979	0.986	1.094	1900.35	51.92	66	551	780	85.2	8.00
Q91WG4	Elongator complex protein 2 OS=Mus musculus GN=Elp2 PE=1 SV=1 - [ELP2_MOUSE]	15.64	2	12	12	22	1.270	1.100	0.835	1.094	63.48	15.64	17	22	831	93.0	5.82
Q9JLK3	Calcium-binding protein 5 OS=Mus musculus GN=Cabp5 PE=1 SV=1 - [CABP5_MOUSE]	8.67	2	1	1	1	1.250	1.332	1.448	1.094	2.82	8.67	1	1	173	19.7	4.45
Q5HZI2	C2 calcium-dependent domain-containing protein 4C OS=Mus musculus GN=C2cd4cC2CD4 family PE=2 SV=1 - [illegible]	40.10	1	10	11	37	1.066	1.448	1.060	1.094	121.14	40.10	17	37	419	44.6	9.73
Q8K394	Inactive phospholipase C-like protein 2 OS=Mus musculus GN=Plcl2 PE=1 SV=2 - [PLCL2_MOUSE]	23.05	1	21	22	78	0.525	0.880	1.330	1.094	220.81	23.05	39	78	1128	125.7	6.92
O35343	Importin subunit alpha-3 OS=Mus musculus GN=Kpna4 PE=2 SV=1 - [IMA3_MOUSE]	18.62	2	4	8	30	1.280	1.369	1.080	1.094	93.72	18.62	14	30	521	57.9	4.94
D3YWS5	Glutamate decarboxylase-like protein 1 OS=Mus musculus GN=Gadl1 PE=2 SV=1 - [D3YWS5_MOUSE]	3.35	4	1	2	4	1.050	1.007	1.054	1.094	11.78	3.35	3	4	478	54.4	7.39
Q99N32	Beta-klotho OS=Mus musculus GN=Klb PE=1 SV=1 - [KLOTB_MOUSE]	2.01	2	2	2	2	0.521	0.659	1.251	1.094	4.18	2.01	2	2	1043	120.1	9.04
P42859	Huntingtin OS=Mus musculus GN=Htt PE=1 SV=2 - [HD_MOUSE]	24.43	3	58	58	173	0.649	0.989	1.056	1.095	486.65	24.43	95	173	3119	344.5	6.29
Q9ES74	Serine/threonine-protein kinase Nek7 OS=Mus musculus GN=Nek7 PE=2 SV=1 - [NEK7_MOUSE]	35.10	1	8	9	25	0.792	1.113	1.024	1.095	82.73	35.10	17	25	302	34.5	8.25
Q9D6W8	Uncharacterized protein C17orf59 homolog OS=Mus musculus PE=1 SV=1 - [CQ059_MOUSE]	26.94	1	6	6	24	1.962	1.182	1.361	1.095	74.62	26.94	11	24	360	38.0	5.34
D3Z7G3	Protein 2010107G23Rik (Fragment) OS=Mus musculus GN=2010107G23Rik PE=2 SV=1 - [D3Z7G3_MOUSE]	24.24	5	2	2	19	0.567	1.399	0.980	1.095	64.32	24.24	4	19	99	10.8	11.15

Q8C669	E3 ubiquitin-protein ligase pellino homolog 1 OS=Mus musculus GN=Peli1 PE=1 SV=2 - [PELI1_MOUSE]	4.55	4	2	2	3	1.220	1.439	1.237	1.095	5.13	4.55	3	3	418	46.2	8.03
Q6KAR6	Exocyst complex component 3 OS=Mus musculus GN=Exoc3 PE=1 SV=2 - [EXOC3_MOUSE]	27.28	1	19	19	58	0.685	1.469	1.066	1.095	158.62	27.28	35	58	755	86.4	6.20
Q8CAK1	Putative transferase CAF17 homolog, mitochondrial OS=Mus musculus GN=Iba57 PE=2 SV=1 - [CAF17_MOUSE]	24.02	2	6	6	13	1.017	1.362	1.263	1.095	38.64	24.02	9	13	358	38.4	9.01
P80560	Receptor-type tyrosine-protein phosphatase N2 OS=Mus musculus GN=Ptprn2 PE=1 SV=2 - [PTPR2_MOUSE]	37.56	1	30	31	132	0.977	1.176	0.881	1.095	417.47	37.56	53	132	1001	111.4	5.95
Q8BZA9	Fructose-2,6-bisphosphatase TIGAR OS=Mus musculus GN=Tigar PE=2 SV=1 -	36.06	1	5	5	19	1.019	1.081	0.901	1.095	61.86	36.06	9	19	269	29.2	8.18
P49446-2	Isoform 2 of Receptor-type tyrosine-protein phosphatase epsilon OS=Mus musculus GN=Ptpre - [illegible]	30.06	3	14	16	58	0.947	1.222	1.051	1.095	159.47	30.06	28	58	642	74.7	7.80
A2A4N9	ATP-dependent RNA helicase DHX8 (Fragment) OS=Mus musculus GN=Dhx8 PE=2 SV=1 - [A2A4N9_MOUSE]	3.15	2	2	3	7	0.458	0.858	1.058	1.095	14.19	3.15	3	7	1175	134.4	7.40
Q8K212	Phosphofurin acidic cluster sorting protein 1 OS=Mus musculus GN=Pacs1 PE=1 SV=2 - [PACS1_MOUSE]	37.98	1	27	29	117	0.716	1.056	1.097	1.095	314.77	37.98	51	117	961	104.8	7.74
P21237	Brain-derived neurotrophic factor OS=Mus musculus GN=Bdnf PE=1 SV=1 - [BDNF_MOUSE]	11.24	3	2	2	3	1.614	0.720	0.860	1.095	7.40	11.24	3	3	249	28.1	8.66
P17427	AP-2 complex subunit alpha-2 OS=Mus musculus GN=Ap2a2 PE=1 SV=2 - [AP2A2_MOUSE]	54.80	2	34	50	355	0.527	1.486	1.147	1.095	995.62	54.80	92	355	938	104.0	6.93
Q921G8	Gamma-tubulin complex component 2 OS=Mus musculus GN=Tubgcp2 PE=2 SV=2 - [GCP2_MOUSE]	25.30	1	19	19	57	0.736	1.210	0.907	1.096	176.19	25.30	32	57	905	103.2	6.77
Q8VCN9	Tubulin-specific chaperone C OS=Mus musculus GN=Tbcc PE=2 SV=1 - [TBCC_MOUSE]	31.38	1	10	10	32	1.158	1.033	1.001	1.096	118.60	31.38	17	32	341	38.1	5.30
P49442	Inositol polyphosphate 1-phosphatase OS=Mus musculus GN=Inpp1 PE=1 SV=2 - [INPP_MOUSE]	38.64	5	14	14	35	0.567	1.054	0.901	1.096	105.46	38.64	22	35	396	43.3	5.01
P58281-2	Isoform 2 of Dynamin-like 120 kDa protein, mitochondrial OS=Mus musculus GN=Opa1 - [OPA1_MOUSE]	52.26	5	50	51	255	0.791	1.126	1.136	1.096	810.68	52.26	87	255	997	115.5	7.55
Q80UG1	Fatty acid desaturase 6 OS=Mus musculus GN=Fads6 PE=2 SV=1 - [FADS6_MOUSE]	2.05	1	1	1	1	0.372	0.506	0.959	1.096	2.27	2.05	1	1	342	38.8	8.46
Q8VHX6-2	Isoform 2 of Filamin-C OS=Mus musculus GN=Flnc - [FLNC_MOUSE]	17.23	4	27	35	90	1.821	2.037	0.798	1.096	274.95	17.23	58	90	2693	287.2	5.95
Q9CPU0	Lactoylglutathione lyase OS=Mus musculus GN=Glo1 PE=1 SV=3 - [LGUL_MOUSE]	57.07	1	13	13	90	2.050	0.890	0.870	1.096	262.52	57.07	23	90	184	20.8	5.47
P32037	Solute carrier family 2, facilitated glucose transporter member 3 OS=Mus musculus GN=Slc2a3 PE=1 SV=1 - [illegible]	20.69	2	9	9	65	0.492	1.156	1.202	1.096	205.08	20.69	15	65	493	53.4	4.98
Q8VBV7	COP9 signalosome complex subunit 8 OS=Mus musculus GN=Cops8 PE=1 SV=1 -	38.28	1	6	6	48	0.884	1.271	1.055	1.096	141.03	38.28	11	48	209	23.2	5.20
A2ARL5	Protein Gm14419 OS=Mus musculus GN=Gm14419 PE=2 SV=1 - [A2ARL5_MOUSE]	1.37	1	1	1	1	0.782	1.214	1.233	1.097	0.00	1.37	1	1	582	67.1	9.23
Q9DB20	ATP synthase subunit O, mitochondrial OS=Mus musculus GN=Atp5o PE=1 SV=1 - [ATPO_MOUSE]	62.44	2	10	15	223	0.747	0.883	1.035	1.097	573.73	62.44	28	223	213	23.3	9.99
P21995	Embigin OS=Mus musculus GN=Emb PE=1 SV=2 - [EMB_MOUSE]	22.42	1	7	8	44	0.987	1.073	0.876	1.097	99.16	22.42	14	44	330	37.0	6.02
Q4QQM5	Protein FAM73A OS=Mus musculus GN=Fam73a PE=2 SV=1 - [FA73A_MOUSE]	23.00	3	9	9	21	0.654	0.838	0.922	1.097	72.25	23.00	14	21	600	67.5	5.48
Q3UM45	Protein phosphatase 1 regulatory subunit 7 OS=Mus musculus GN=Ppp1r7 PE=1 SV=2 - [PP1R7_MOUSE]	54.57	2	17	17	104	1.143	2.092	1.342	1.097	294.84	54.57	32	104	361	41.3	4.92

A2AST1	Coiled-coil protein associated with myosin II and DISC1 OS=Mus musculus GN=Ccdc141 PE=2 SV=1 - [A2AST1_MOUSE]	8.48	4	12	12	29	1.132	0.964	0.941	1.097	78.86	8.48	20	29	1451	164.8	5.63
Q80WC7-2	Isoform 2 of Arf-GAP domain and FG repeat-containing protein 2 OS=Mus musculus GN=Agfg2 - [AGFG2_MOUSE]	46.02	4	10	12	36	1.030	1.336	1.208	1.097	102.99	46.02	22	36	289	30.7	9.44
Q8BYI6-2	Isoform 2 of Lysophosphatidylcholine acyltransferase 2 OS=Mus musculus GN=Lpcat2 - [PCAT2_MOUSE]	6.94	3	4	4	8	0.773	0.669	0.857	1.097	11.74	6.94	7	8	504	55.8	5.48
P80317	T-complex protein 1 subunit zeta OS=Mus musculus GN=Cct6a PE=1 SV=3 - [TCPZ_MOUSE]	60.83	4	31	31	164	0.949	1.114	0.930	1.097	514.79	60.83	51	164	531	58.0	7.08
Q9DBG3	AP-2 complex subunit beta OS=Mus musculus GN=Ap2b1 PE=1 SV=1 - [AP2B1_MOUSE]	66.60	10	32	59	552	0.881	1.371	1.126	1.097	1521.41	66.60	103	552	937	104.5	5.38
D3YXV0	Otoferlin (Fragment) OS=Mus musculus GN=Otof PE=2 SV=1 - [D3YXV0_MOUSE]	4.92	5	7	8	10	1.335	1.001	1.451	1.097	23.41	4.92	10	10	1932	219.0	5.53
Q9DB40-2	Isoform 2 of Mediator of RNA polymerase II transcription subunit 27 OS=Mus musculus GN=Med27 - [MED27_MOUSE]	15.87	2	2	2	12	0.959	1.072	0.968	1.097	28.84	15.87	4	12	208	23.1	9.26
Q80XC3	USP6 N-terminal-like protein OS=Mus musculus GN=Usp6nl PE=1 SV=2 - [US6NL_MOUSE]	8.67	2	6	6	9	1.038	1.002	1.246	1.097	16.71	8.67	7	9	819	93.5	9.13
Q8K3G9	DCC-interacting protein 13-beta OS=Mus musculus GN=Appl2 PE=2 SV=1 - [DP13B_MOUSE]	38.82	5	19	20	57	0.710	0.905	0.948	1.097	166.24	38.82	32	57	662	73.8	5.03
Q8BGW1	Alpha-ketoglutarate-dependent dioxygenase FTO OS=Mus musculus GN=Fto PE=1 SV=1 - [FTO_MOUSE]	33.07	6	16	16	35	0.855	0.863	0.878	1.097	93.23	33.07	27	35	502	58.0	5.12
Q9D6L8	Peptidyl-prolyl cis-trans isomerase-like 3 OS=Mus musculus GN=Ppil3 PE=2 SV=1 - [PPIL3_MOUSE]	27.33	2	4	4	13	1.393	1.340	1.127	1.098	31.38	27.33	7	13	161	18.1	6.79
O89050	Muskelin OS=Mus musculus GN=Mkln1 PE=2 SV=1 - [MKLN1_MOUSE]	10.88	1	6	6	15	0.902	1.328	0.945	1.098	38.35	10.88	10	15	735	84.8	6.34
P63040	Complexin-1 OS=Mus musculus GN=Cplx1 PE=1 SV=1 - [CPLX1_MOUSE]	51.49	2	3	6	123	2.037	0.789	1.623	1.098	353.31	51.49	10	123	134	15.1	4.97
A2AJA9	Uncharacterized protein C9orf172 homolog OS=Mus musculus GN=Gm996 PE=1 SV=1 - [CI172_MOUSE]	36.65	2	26	26	84	0.704	1.191	1.379	1.098	279.33	36.65	44	84	974	107.1	9.11
Q9D114	Guanosine-3',5'-bis(diphosphate) 3'-pyrophosphohydrolase MESH1 OS=Mus musculus GN=Hddc3 PE=2 SV=1 - [MESH1_MOUSE]	15.64	1	2	2	4	1.418	1.289	1.250	1.098	12.33	15.64	4	4	179	20.2	5.96
G3X909	Slit homolog 2 protein OS=Mus musculus GN=Slit2 PE=2 SV=1 - [G3X909_MOUSE]	2.00	6	1	1	3	1.453	0.776	2.419	1.098	0.00	2.00	1	3	851	95.4	8.54
Q8R4G0-4	Isoform 1D of Netrin-G1 OS=Mus musculus GN=Ntng1 - [NTNG1_MOUSE]	18.96	9	2	7	22	0.627	1.402	1.199	1.098	65.97	18.96	13	22	480	53.9	6.92
G3UXW9	COP9 signalosome complex subunit 1 OS=Mus musculus GN=Gps1 PE=4 SV=1 -	42.40	5	19	20	71	0.881	1.257	0.890	1.098	228.71	42.40	33	71	526	58.8	6.74
E9QAN9	Interferon-activable protein 203 OS=Mus musculus GN=Ifi203 PE=2 SV=1 - [E9QAN9_MOUSE]	3.33	4	2	2	4	1.482	1.231	1.589	1.098	2.64	3.33	2	4	871	95.2	9.77
Q8VHK5	Membrane protein MLC1 OS=Mus musculus GN=Mlc1 PE=2 SV=1 - [MLC1_MOUSE]	9.95	2	4	4	15	0.567	0.728	1.049	1.099	48.15	9.95	7	15	382	41.6	7.90
Q91VE6-2	Isoform 2 of MKI67 FHA domain-interacting nucleolar phosphoprotein OS=Mus musculus GN=Mki67ip - [MK67I_MOUSE]	12.64	2	3	3	6	0.709	0.821	0.850	1.099	23.55	12.64	4	6	269	30.6	10.20
P80315	T-complex protein 1 subunit delta OS=Mus musculus GN=Cct4 PE=1 SV=3 - [TCPD_MOUSE]	66.42	5	32	33	233	0.629	0.904	0.851	1.099	681.62	66.42	61	233	539	58.0	8.02
Q8BGC4	Zinc-binding alcohol dehydrogenase domain-containing protein 2 OS=Mus musculus GN=Zadh2 PE=2 SV=1 - [ZADH2_MOUSE]	41.11	1	11	11	36	0.922	1.157	1.048	1.099	103.07	41.11	21	36	377	40.5	7.42
Q6WKZ8	E3 ubiquitin-protein ligase UBR2 OS=Mus musculus GN=Ubr2 PE=1 SV=2 - [UBR2_MOUSE]	4.22	3	8	8	21	0.777	0.873	1.048	1.099	57.70	4.22	11	21	1755	199.0	6.33
D3YXZ3	Kinesin light chain 2 OS=Mus musculus GN=Klc2 PE=4 SV=1 - [D3YXZ3_MOUSE]	56.40	4	25	31	171	1.201	0.830	1.145	1.099	485.72	56.40	58	171	617	68.1	7.06

E9PX19	Microcephalin OS=Mus musculus GN=Mcph1 PE=2 SV=1 - [E9PX19_MOUSE]	3.50	4	2	2	2	2.674	1.115	1.043	1.099	3.04	3.50		2	2	600	65.6	7.11
Q8BGZ3	DDB1- and CUL4-associated factor 12 OS=Mus musculus GN=Dcaf12 PE=2 SV=1 -	1.55	1	1	1	1	1.701	0.980	1.842	1.099	2.47	1.55		1	1	453	50.4	9.03
O09172	Glutamate--cysteine ligase regulatory subunit OS=Mus musculus GN=Gclm PE=2 SV=1 - [GSH0_MOUSE]	32.85	3	9	9	37	1.156	0.845	0.810	1.099	127.46	32.85		16	37	274	30.5	5.52
Q9Z2L7	Cytokine receptor-like factor 3 OS=Mus musculus GN=Crlf3 PE=2 SV=1 - [CRLF3_MOUSE]	5.43	4	2	2	4	1.208	1.131	0.908	1.099	14.07	5.43		2	4	442	49.5	5.03
Q99M80-4	Isoform 4 of Receptor-type tyrosine-protein phosphatase T OS=Mus musculus GN=Ptprt - [PTPRT_MOUSE]	22.72	6	23	24	67	1.049	1.363	1.049	1.099	199.61	22.72		43	67	1435	161.1	6.84
Q78J03	Methionine-R-sulfoxide reductase B2, mitochondrial OS=Mus musculus GN=Msrb2 PE=1 SV=1 - [MSRB2_MOUSE]	34.86	1	8	8	28	1.607	1.025	1.529	1.099	83.40	34.86		14	28	175	19.1	9.06
E9QKE4	Rab3 GTPase-activating protein non-catalytic subunit OS=Mus musculus GN=Rab3gap2 PE=2 SV=1 - [E9QKE4_MOUSE]	33.74	4	42	42	152	0.583	1.182	0.978	1.099	498.00	33.74		69	152	1387	154.5	6.07
O08532-5	Isoform 2E of Voltage-dependent calcium channel subunit alpha-2/delta-1 OS=Mus musculus GN=Cacna2d1 - [illegible]	44.64	6	41	42	268	0.780	1.913	1.013	1.099	737.72	44.64		73	268	1091	123.2	5.27
E9QL80	Acyl-CoA:lysophosphatidylglycerol acyltransferase 1 OS=Mus musculus GN=Lpgat1 PE=2 SV=1 - [illegible]	19.80	4	9	9	24	0.521	0.894	0.968	1.099	66.31	19.80		16	24	409	47.0	9.03
P70280	Vesicle-associated membrane protein 7 OS=Mus musculus GN=Vamp7 PE=1 SV=1 - [VAMP7_MOUSE]	31.36	1	8	8	26	0.548	0.861	0.891	1.099	70.74	31.36		13	26	220	25.0	8.60
P62077	Mitochondrial import inner membrane translocase subunit Tim8 B OS=Mus musculus GN=Timm8b PE=3 SV=1 - [TIM8B_MOUSE]	54.22	1	5	5	19	1.717	0.776	1.521	1.099	54.75	54.22		8	19	83	9.3	5.12
Q14CH0-2	Isoform 2 of Protein FAM171B OS=Mus musculus GN=Fam171b - [F171B_MOUSE]	33.13	3	20	21	87	1.351	1.228	1.412	1.099	236.76	33.13		37	87	821	91.5	8.37
P54818	Galactocerebrosidase OS=Mus musculus GN=Galc PE=1 SV=2 - [GALC_MOUSE]	6.43	1	4	4	15	1.049	0.877	0.933	1.100	37.76	6.43		7	15	684	77.2	6.74
Q8BG26	RUN and SH3 domain-containing protein 1 OS=Mus musculus GN=Rusc1 PE=1 SV=2 - [RUSC1_MOUSE]	13.21	3	9	9	16	1.051	0.912	1.091	1.100	50.47	13.21		13	16	893	95.1	6.02
Q0GA42	Metal transporter CNNM1 OS=Mus musculus GN=Cnnm1 PE=1 SV=5 - [CNNM1_MOUSE]	19.24	1	12	16	53	0.960	0.919	1.055	1.100	136.71	19.24		25	53	951	103.9	6.51
Q9DCL9	Multifunctional protein ADE2 OS=Mus musculus GN=Paics PE=1 SV=4 - [PUR6_MOUSE]	44.47	3	20	21	87	0.926	0.926	1.025	1.100	261.20	44.47		36	87	425	47.0	7.23
Q3UE37	Ubiquitin-conjugating enzyme E2 Z OS=Mus musculus GN=Ube2z PE=1 SV=2 - [UBE2Z_MOUSE]	19.66	1	7	7	34	0.633	0.940	0.916	1.100	86.77	19.66		13	34	356	38.3	5.62
A2AED5	RIKEN cDNA 4921515J06 OS=Mus musculus GN=Henmt1 PE=4 SV=1 - [A2AED5_MOUSE]	6.87	2	1	1	1	0.864	1.333	0.884	1.100	2.31	6.87		1	1	262	29.5	6.61
Q64337	Sequestosome-1 OS=Mus musculus GN=Sqstm1 PE=1 SV=1 - [SQSTM_MOUSE]	26.92	4	7	7	25	1.283	0.938	1.088	1.100	52.92	26.92		10	25	442	48.1	5.21
Q6PHS9-4	Isoform 4 of Voltage-dependent calcium channel subunit alpha-2/delta-2 OS=Mus musculus GN=Cacna2d2 - [illegible]	26.42	7	26	27	134	0.620	0.465	0.792	1.100	403.56	26.42		47	134	1147	129.6	5.60
Q8K021	Secretory carrier-associated membrane protein 1 OS=Mus musculus GN=Scamp1 PE=1 SV=1 - [illegible]	36.69	3	9	9	127	0.928	1.138	1.095	1.100	376.74	36.69		17	127	338	38.0	7.71
Q923X4-2	Isoform 2 of Glutaredoxin-2, mitochondrial OS=Mus musculus GN=Glrx2 - [GLRX2_MOUSE]	23.58	3	2	2	3	1.762	1.174	1.159	1.100	10.04	23.58		3	3	123	14.0	8.94
Q9CYW4	Haloacid dehalogenase-like hydrolase domain-containing protein 3 OS=Mus musculus GN=Hdhd3 PE=2 SV=1 - [HDHD3_MOUSE]	39.44	1	8	8	27	1.268	1.307	1.322	1.100	76.46	39.44		16	27	251	28.0	6.80

Q9D2G2	Dihydrolipoyllysine-residue succinyltransferase component of 2-oxoglutarate dehydrogenase complex, mitochondrial OS=Mus musculus GN=Dlst PE=1 SV=1 - [ODO2_MOUSE]	38.99	2	15	15	191	1.706	0.839	1.007	1.100	534.17	38.99	26	191	454	49.0	8.95
Q8VCE2	GPN-loop GTPase 1 OS=Mus musculus GN=Gpn1 PE=1 SV=1 - [GPN1_MOUSE]	15.32	1	4	4	14	0.799	1.160	0.954	1.100	44.36	15.32	7	14	372	41.6	4.88
P21761	Thyrotropin-releasing hormone receptor OS=Mus musculus GN=Trhr PE=2 SV=1 - [TRFR_MOUSE]	10.18	1	3	3	6	0.873	2.010	1.176	1.100	17.45	10.18	4	6	393	44.5	8.37
P80318	T-complex protein 1 subunit gamma OS=Mus musculus GN=Cct3 PE=1 SV=1 - [TCPG_MOUSE]	61.65	6	33	33	242	0.630	0.926	0.855	1.101	726.46	61.65	60	242	545	60.6	6.70
A2ARA8-2	Isoform 2 of Integrin alpha 8 OS=Mus musculus GN=Itga8 - [ITA8_MOUSE]	3.50	3	1	1	2	1.374	2.237	0.788	1.101	5.41	3.50	2	2	286	31.0	7.27
E9Q5Y2	Splicing factor U2AF 26 kDa subunit OS=Mus musculus GN=U2af1l4 PE=2 SV=1 -	9.39	8	1	2	6	1.344	0.749	1.226	1.101	8.72	9.39	3	6	181	21.3	8.98
Q8K0W3	RING finger protein 208 OS=Mus musculus GN=Rnf208 PE=2 SV=2 - [RN208_MOUSE]	8.30	1	1	1	1	6.261	5.943	1.168	1.101	3.54	8.30	1	1	265	28.4	7.49
Q99JP0	Mitogen-activated protein kinase kinase kinase kinase 3 OS=Mus musculus GN=Map4k3 PE=1 SV=4 - [illegible]	16.89	2	12	14	33	0.910	1.025	1.081	1.101	91.27	16.89	25	33	894	101.1	7.64
Q8BUW1	Potassium channel, subfamily K, member 10 OS=Mus musculus GN=Kcnk10 PE=2 SV=1 - [Q8BUW1_MOUSE]	5.23	1	2	2	6	1.301	0.907	1.159	1.101	9.41	5.23	3	6	535	59.4	8.82
O54833	Casein kinase II subunit alpha' OS=Mus musculus GN=Csnk2a2 PE=2 SV=1 - [CSK22_MOUSE]	49.71	1	13	16	71	0.685	0.973	1.093	1.101	215.14	49.71	28	71	350	41.2	8.56
O88428	Bifunctional 3'-phosphoadenosine 5'-phosphosulfate synthase 2 OS=Mus musculus GN=Papss2 PE=1 SV=2 - [illegible]	15.46	1	5	8	22	1.309	0.999	1.179	1.101	63.48	15.46	13	22	621	70.3	7.58
D3Z7P3-2	Isoform 2 of Glutaminase kidney isoform, mitochondrial OS=Mus musculus GN=Gls -	43.95	4	1	24	118	1.496	1.343	1.720	1.101	398.40	43.95	40	118	603	66.0	8.12
P60882	Multiple epidermal growth factor-like domains protein 8 OS=Mus musculus GN=Megf8 PE=2 SV=2 - [MEGF8_MOUSE]	1.69	1	4	4	8	0.808	0.851	0.898	1.101	17.39	1.69	7	8	2789	297.3	6.80
Q8R2U4	N-terminal Xaa-Pro-Lys N-methyltransferase 1 OS=Mus musculus GN=Ntmt1 PE=2 SV=3 - [NTM1A_MOUSE]	30.04	2	5	5	15	1.274	1.227	1.165	1.101	40.53	30.04	7	15	223	25.4	6.90
Q9JII6	Alcohol dehydrogenase [NADP(+)] OS=Mus musculus GN=Akr1a1 PE=1 SV=3 -	53.54	2	16	17	142	0.819	1.225	1.028	1.101	382.57	53.54	28	142	325	36.6	7.39
Q8BNN1	Spermatogenesis-associated protein 2-like protein OS=Mus musculus GN=Spata2l PE=2 SV=1 - [SPA2L_MOUSE]	25.59	1	7	7	24	1.506	2.083	1.742	1.101	58.97	25.59	12	24	426	46.7	5.20
Q9CRB9	Coiled-coil-helix-coiled-coil-helix domain-containing protein 3, mitochondrial OS=Mus musculus GN=Chchd3 PE=1 SV=1 - [CHCH3_MOUSE]	51.98	4	17	17	187	1.451	0.941	1.220	1.101	501.57	51.98	29	187	227	26.3	8.37
Q8C7D2-2	Isoform 2 of Protein cereblon OS=Mus musculus GN=Crbn - [CRBN_MOUSE]	12.76	3	4	4	16	1.048	0.880	1.141	1.101	47.35	12.76	7	16	431	49.5	5.45
P63248	cAMP-dependent protein kinase inhibitor alpha OS=Mus musculus GN=Pkia PE=1 SV=2 - [IPKA_MOUSE]	51.32	1	3	3	33	1.632	1.451	1.488	1.101	90.21	51.32	6	33	76	8.0	4.54
Q99KK9	Probable histidine--tRNA ligase, mitochondrial OS=Mus musculus GN=Hars2 PE=2 SV=1 - [SYHM_MOUSE]	27.92	2	11	14	38	0.837	0.892	0.979	1.101	91.73	27.92	21	38	505	56.9	8.32
O88441	Metaxin-2 OS=Mus musculus GN=Mtx2 PE=1 SV=1 - [MTX2_MOUSE]	53.23	1	10	10	78	0.910	1.259	1.038	1.101	255.41	53.23	18	78	263	29.7	5.63
Q8CG72	Poly(ADP-ribose) glycohydrolase ARH3 OS=Mus musculus GN=Adprhl2 PE=1 SV=1 - [ARHL2_MOUSE]	31.62	4	8	9	35	1.069	0.908	0.964	1.101	118.15	31.62	15	35	370	39.4	4.96
A2AF47	Dedicator of cytokinesis protein 11 OS=Mus musculus GN=Dock11 PE=1 SV=1 -	7.81	2	8	14	27	0.876	0.996	0.814	1.101	70.65	7.81	22	27	2073	237.6	7.78

Q0VF55	ATPase, Ca++ transporting, plasma membrane 3 OS=Mus musculus GN=Atp2b3 PE=2 SV=1 - [Q0VF55_MOUSE]	37.87	2	1	46	320	0.495	0.667	1.313	1.102	875.61	37.87	77	320	1220	134.3	5.69
Q9QZ59-2	Isoform 2 of Doublesex- and mab-3-related transcription factor 1 OS=Mus musculus GN=Dmrt1 - [DMRT1_MOUSE]	15.57	3	1	1	18	1.444	1.481	1.028	1.102	42.15	15.57	1	18	212	21.9	8.90
Q8BVI5	Syntaxin-16 OS=Mus musculus GN=Stx16 PE=1 SV=3 - [STX16_MOUSE]	28.22	6	8	8	36	0.514	1.084	0.883	1.102	112.10	28.22	15	36	326	37.1	5.86
Q8CFE2	UPF0609 protein C4orf27 homolog OS=Mus musculus PE=2 SV=1 - [CD027_MOUSE]	8.96	2	3	3	6	1.390	1.061	0.711	1.102	13.47	8.96	6	6	346	39.3	7.69
P97813	Phospholipase D2 OS=Mus musculus GN=Pld2 PE=1 SV=2 - [PLD2_MOUSE]	6.11	4	5	5	12	0.799	1.061	0.926	1.102	40.58	6.11	8	12	933	106.1	7.39
B1AS29	Glutamate receptor ionotropic, kainate 3 OS=Mus musculus GN=Grik3 PE=2 SV=1 - [GRIK3_MOUSE]	12.19	1	7	8	14	0.762	1.021	1.037	1.102	35.21	12.19	13	14	919	104.0	7.52
B1ASQ7	Protein Gm13212 OS=Mus musculus GN=Gm13212 PE=2 SV=1 - [B1ASQ7_MOUSE]	14.64	151	1	2	2	1.968	1.484	1.461	1.102	4.90	14.64	2	2	799	94.6	8.79
P35293	Ras-related protein Rab-18 OS=Mus musculus GN=Rab18 PE=2 SV=2 - [RAB18_MOUSE]	62.14	1	11	12	50	0.746	1.000	1.037	1.102	151.60	62.14	22	50	206	23.0	5.36
Q80U30	Protein CLEC16A OS=Mus musculus GN=Clec16a PE=2 SV=2 - [CL16A_MOUSE]	8.88	6	8	8	15	0.804	1.031	1.135	1.102	31.84	8.88	11	15	1036	116.2	6.05
Q9R0P9	Ubiquitin carboxyl-terminal hydrolase isozyme L1 OS=Mus musculus GN=Uchl1 PE=1 SV=1 - [UCHL1_MOUSE]	87.44	1	18	19	771	1.750	1.207	1.340	1.102	2122.45	87.44	38	771	223	24.8	5.24
E9PX66	STAGA complex 65 subunit gamma OS=Mus musculus GN=Supt7l PE=2 SV=1 - [E9PX66_MOUSE]	7.85	3	2	2	2	1.682	1.292	1.243	1.102	6.36	7.85	2	2	344	38.5	5.35
Q3V3N7	Protein Bbs1 OS=Mus musculus GN=Bbs1 PE=2 SV=1 - [Q3V3N7_MOUSE]	14.33	1	5	6	21	1.004	1.053	0.971	1.102	54.94	14.33	9	21	593	65.1	8.54
Q922D8	C-1-tetrahydrofolate synthase, cytoplasmic OS=Mus musculus GN=Mthfd1 PE=1 SV=4 - [C1TC_MOUSE]	43.42	1	36	37	154	0.622	0.808	0.827	1.102	442.52	43.42	63	154	935	101.1	7.14
Q921M7	Protein FAM49B OS=Mus musculus GN=Fam49b PE=2 SV=1 - [FA49B_MOUSE]	62.96	1	15	18	151	0.595	1.116	1.041	1.102	467.32	62.96	30	151	324	36.8	6.06
P84244	Histone H3.3 OS=Mus musculus GN=H3f3a PE=1 SV=2 - [H33_MOUSE]	59.56	6	3	11	60	0.414	1.073	0.964	1.102	167.46	59.56	18	60	136	15.3	11.27
Q9CZW4	Long-chain-fatty-acid--CoA ligase 3 OS=Mus musculus GN=Acsl3 PE=2 SV=2 - [ACSL3_MOUSE]	35.14	3	18	20	54	0.579	0.671	0.839	1.102	169.65	35.14	32	54	720	80.4	8.54
Q6NXM2	RCC1 and BTB domain-containing protein 1 OS=Mus musculus GN=Rcbtb1 PE=2 SV=1 - [RCBT1_MOUSE]	7.16	3	3	3	6	1.411	0.830	0.857	1.102	19.56	7.16	6	6	531	58.3	6.43
P15208	Insulin receptor OS=Mus musculus GN=Insr PE=1 SV=2 - [INSR_MOUSE]	12.17	1	14	15	29	0.912	1.093	0.985	1.102	76.61	12.17	25	29	1372	155.5	5.95
O35454	Chloride transport protein 6 OS=Mus musculus GN=Clcn6 PE=1 SV=1 - [CLCN6_MOUSE]	15.17	3	9	9	24	0.924	0.812	0.962	1.103	72.98	15.17	14	24	870	96.9	7.02
Q9QYR7-2	Isoform 2 of Acyl-coenzyme A thioesterase 3 OS=Mus musculus GN=Acot3 -	20.90	4	2	5	12	0.962	0.940	0.879	1.103	37.20	20.90	9	12	421	46.2	7.24
Q91WF7	Polyphosphoinositide phosphatase OS=Mus musculus GN=Fig4 PE=1 SV=1 - [FIG4_MOUSE]	9.59	1	8	8	17	0.760	1.152	0.921	1.103	42.31	9.59	13	17	907	103.4	6.98
Q8K0S2	Uncharacterized protein C8orf47 homolog OS=Mus musculus PE=2 SV=1 - [CH047_MOUSE]	16.02	1	5	5	14	1.634	1.959	1.620	1.103	37.94	16.02	7	14	362	38.5	4.82
Q9D0C4-2	Isoform 2 of tRNA (guanine(37)-N1)-methyltransferase OS=Mus musculus GN=Trmt5 - [TRM5_MOUSE]	7.39	2	3	3	4	1.062	1.243	0.950	1.103	9.36	7.39	4	4	487	55.4	8.32
P24527	Leukotriene A-4 hydrolase OS=Mus musculus GN=Lta4h PE=1 SV=4 - [LKHA4_MOUSE]	42.39	1	24	24	105	0.873	1.449	1.022	1.103	320.59	42.39	44	105	611	69.0	6.42
Q8BIV3	Ran-binding protein 6 OS=Mus musculus GN=Ranbp6 PE=2 SV=3 - [RNBP6_MOUSE]	18.73	2	14	16	37	0.664	0.850	0.860	1.103	89.59	18.73	25	37	1105	124.5	5.03
Q3U487	E3 ubiquitin-protein ligase HECTD3 OS=Mus musculus GN=Hectd3 PE=1 SV=2 -	20.79	2	14	15	35	0.875	0.832	0.890	1.103	100.37	20.79	26	35	861	97.3	5.47

Q9Z2M7	Phosphomannomutase 2 OS=Mus musculus GN=Pmm2 PE=2 SV=1 - [PMM2_MOUSE]	50.41	1	9	11	41	0.923	1.137	1.101	1.103	111.35	50.41	17	41	242	27.6	6.42
O35382	Exocyst complex component 4 OS=Mus musculus GN=Exoc4 PE=1 SV=2 - [EXOC4_MOUSE]	31.28	3	25	28	105	0.621	1.227	1.066	1.103	331.21	31.28	46	105	975	110.5	6.49
Q91XE9	Cyclic AMP-responsive element-binding protein 3-like protein 3 OS=Mus musculus GN=Creb3l3 PE=2 SV=1 - [CR3L3_MOUSE]	2.09	1	1	1	1	1.601	1.477	1.187	1.103	3.03	2.09	1	1	479	52.1	5.07
A1L314	Macrophage-expressed gene 1 protein OS=Mus musculus GN=Mpeg1 PE=2 SV=1 - [MPEG1_MOUSE]	3.51	2	1	1	2	2.144	1.508	1.080	1.103	0.00	3.51	1	2	713	78.3	5.92
P70295	Ancient ubiquitous protein 1 OS=Mus musculus GN=Aup1 PE=2 SV=1 - [AUP1_MOUSE]	17.80	2	4	4	14	1.145	1.037	0.967	1.103	46.90	17.80	8	14	410	46.1	8.40
Q8R0H9	ADP-ribosylation factor-binding protein GGA1 OS=Mus musculus GN=Gga1 PE=1 SV=1 - [GGA1_MOUSE]	21.10	1	10	11	63	0.899	1.002	1.051	1.103	158.75	21.10	19	63	635	69.9	5.27
Q04736	Tyrosine-protein kinase Yes OS=Mus musculus GN=Yes1 PE=1 SV=3 - [YES_MOUSE]	36.78	15	9	19	96	0.659	0.626	0.831	1.104	233.22	36.78	34	96	541	60.6	6.64
Q61474	RNA-binding protein Musashi homolog 1 OS=Mus musculus GN=Msi1 PE=1 SV=1 - [MSI1H_MOUSE]	32.32	3	8	9	21	0.854	0.683	0.828	1.104	66.11	32.32	15	21	362	39.1	7.87
Q91XU3	Phosphatidylinositol 5-phosphate 4-kinase type-2 gamma OS=Mus musculus GN=Pip4k2c PE=2 SV=1 - [PI42C_MOUSE]	38.24	1	13	13	30	0.938	1.581	1.584	1.104	78.30	38.24	22	30	421	47.3	6.89
Q03734	Serine protease inhibitor A3M OS=Mus musculus GN=Serpina3m PE=1 SV=2 - [SPA3M_MOUSE]	25.12	1	1	8	40	2.810	1.005	1.311	1.104	132.32	25.12	11	40	418	47.0	6.10
Q9D892	Inosine triphosphate pyrophosphatase OS=Mus musculus GN=Itpa PE=1 SV=2 - [ITPA_MOUSE]	36.87	1	6	6	55	0.754	1.365	1.044	1.104	179.11	36.87	12	55	198	21.9	5.87
Q8K366	Exosc10 protein OS=Mus musculus GN=Exosc10 PE=2 SV=1 - [Q8K366_MOUSE]	12.99	3	10	10	15	0.700	0.948	1.021	1.104	26.89	12.99	13	15	862	98.1	8.29
Q6P069-2	Isoform 2 of Sorcin OS=Mus musculus GN=Sri - [SORCN_MOUSE]	46.45	2	10	10	53	1.424	1.401	0.984	1.104	150.21	46.45	17	53	183	20.3	5.34
Q05144	Ras-related C3 botulinum toxin substrate 2 OS=Mus musculus GN=Rac2 PE=2 SV=1 - [RAC2_MOUSE]	28.65	7	2	6	40	0.820	1.074	0.934	1.104	103.88	28.65	10	40	192	21.4	7.61
Q80V91	Probable E3 ubiquitin-protein ligase DTX3 OS=Mus musculus GN=Dtx3 PE=2 SV=2 -	15.85	3	3	3	35	0.759	0.836	1.052	1.104	72.47	15.85	6	35	347	38.0	8.73
P63085	Mitogen-activated protein kinase 1 OS=Mus musculus GN=Mapk1 PE=1 SV=3 -	56.98	10	13	22	249	0.753	2.513	1.211	1.104	637.23	56.98	44	249	358	41.2	6.98
O08797	Protein Serpinb9 OS=Mus musculus GN=Serpinb9 PE=2 SV=1 - [O08797_MOUSE]	39.57	2	11	14	49	0.769	1.541	0.799	1.104	139.07	39.57	27	49	374	42.2	5.40
P60904	DnaJ homolog subfamily C member 5 OS=Mus musculus GN=Dnajc5 PE=1 SV=1 -	44.44	3	5	6	209	1.238	1.624	1.281	1.104	334.27	44.44	11	209	198	22.1	5.07
Q921G7	Electron transfer flavoprotein-ubiquinone oxidoreductase, mitochondrial OS=Mus musculus GN=Etfdh PE=1 SV=1 - [ETFD_MOUSE]	42.53	2	22	22	119	1.029	1.172	1.007	1.104	368.77	42.53	39	119	616	68.0	7.58
Q91ZU6-6	Isoform 6 of Dystonin OS=Mus musculus GN=Dst - [DYST_MOUSE]	14.52	2	4	7	17	1.177	0.762	0.986	1.105	48.20	14.52	13	17	482	55.0	9.38
O08967	Cytohesin-3 OS=Mus musculus GN=Cyth3 PE=1 SV=1 - [CYH3_MOUSE]	30.33	4	7	12	31	0.978	0.996	1.280	1.105	81.20	30.33	19	31	399	46.3	5.54
Q61068	Ubiquitin carboxyl-terminal hydrolase DUB-1 OS=Mus musculus GN=Dub1 PE=1 SV=1 - [UBPW_MOUSE]	2.28	1	1	1	1	1.640	1.017	0.989	1.105	2.48	2.28	1	1	526	59.0	7.74
O35393	Ephrin-B3 OS=Mus musculus GN=Efnb3 PE=1 SV=1 - [EFNB3_MOUSE]	46.18	1	10	11	47	1.123	0.863	0.968	1.105	142.11	46.18	20	47	340	35.9	8.25
Q9CXJ1	Probable glutamate--tRNA ligase, mitochondrial OS=Mus musculus GN=Ears2 PE=2 SV=1 - [SYEM_MOUSE]	16.44	1	7	7	18	0.627	1.862	1.439	1.105	53.91	16.44	12	18	523	58.3	8.75
Q9JJ00	Phospholipid scramblase 1 OS=Mus musculus GN=Plscr1 PE=2 SV=1 - [PLS1_MOUSE]	4.27	1	2	2	3	1.321	1.162	0.992	1.105	6.26	4.27	3	3	328	35.9	5.06

Q8R1Q9	Ribokinase OS=Mus musculus GN=Rbks PE=1 SV=1 - [RBSK_MOUSE]	7.74	1	2	2	5	3.147	0.742	1.189	1.105	15.03	7.74	3	5	323	34.1	5.47
P35283	Ras-related protein Rab-12 OS=Mus musculus GN=Rab12 PE=1 SV=3 - [RAB12_MOUSE]	49.79	2	13	14	86	0.753	1.084	0.992	1.105	239.35	49.79	22	86	243	27.3	8.41
Q91Z40	Gbp6 protein OS=Mus musculus GN=Gbp7 PE=2 SV=2 - [Q91Z40_MOUSE]	10.19	3	3	7	13	1.279	1.330	0.920	1.105	35.31	10.19	11	13	638	72.7	6.33
Q99LF7	Protein Dcaf4 OS=Mus musculus GN=Dcaf4 PE=2 SV=1 - [Q99LF7_MOUSE]	2.12	1	1	1	2	1.515	1.503	0.789	1.105	0.00	2.12	1	2	519	58.1	8.37
G3XA00	Glutamate receptor, metabotropic 4 OS=Mus musculus GN=Grm4 PE=3 SV=1 - [G3XA00_MOUSE]	16.78	5	11	13	40	0.594	0.508	1.193	1.105	123.42	16.78	23	40	912	101.7	8.85
Q9WV56	Growth/differentiation factor 2 OS=Mus musculus GN=Gdf2 PE=2 SV=2 - [GDF2_MOUSE]	3.04	1	1	1	1	1.732	1.349	1.151	1.105	2.25	3.04	1	1	428	47.7	5.44
Q8K0B2-3	Isoform 3 of Probable lysosomal cobalamin transporter OS=Mus musculus GN=Lmbrd1 - [LMBD1_MOUSE]	3.85	3	1	1	11	0.892	0.558	1.090	1.105	49.25	3.85	2	11	467	53.4	7.80
Q921Q3-2	Isoform 2 of Chitobiosyldiphosphodolichol beta-mannosyltransferase OS=Mus musculus GN=Alg1 - [ALG1_MOUSE]	17.54	2	6	6	10	0.566	0.945	0.921	1.106	28.38	17.54	10	10	456	51.4	8.21
Q9WTK5	Nuclear factor NF-kappa-B p100 subunit OS=Mus musculus GN=Nfkb2 PE=1 SV=1 - [NFKB2_MOUSE]	3.56	1	2	2	4	3.757	1.674	1.592	1.106	3.69	3.56	2	4	899	96.8	6.37
Q8C3X2	Coiled-coil domain-containing protein 90B, mitochondrial OS=Mus musculus GN=Ccdc90b PE=2 SV=1 - [illegible]	30.86	2	7	9	26	0.904	1.829	0.946	1.106	83.27	30.86	14	26	256	29.6	8.53
Q6RI63	Constitutive coactivator of peroxisome proliferator-activated receptor gamma OS=Mus musculus GN=Fam120b PE=1 SV=1 [F120B_MOUSE]	12.34	2	8	8	27	0.737	1.051	0.919	1.106	52.02	12.34	12	27	786	89.3	6.52
Q9Z127	Large neutral amino acids transporter small subunit 1 OS=Mus musculus GN=Slc7a5 PE=1 SV=2 - [LAT1_MOUSE]	2.54	1	1	1	2	1.524	1.212	1.441	1.106	8.22	2.54	1	2	512	55.8	7.90
O88845	A-kinase anchor protein 10, mitochondrial OS=Mus musculus GN=Akap10 PE=1 SV=3 - [AKA10_MOUSE]	35.20	3	16	16	47	0.763	0.918	1.144	1.106	128.37	35.20	26	47	662	73.6	6.79
P97346	Nucleoredoxin OS=Mus musculus GN=Nxn PE=1 SV=1 - [NXN_MOUSE]	20.00	2	6	6	13	1.081	1.284	0.821	1.106	39.13	20.00	12	13	435	48.3	4.93
Q8BHN1	Gamma-taxilin OS=Mus musculus GN=Txlng PE=1 SV=1 - [TXLNG_MOUSE]	12.79	6	2	6	9	1.417	1.728	1.252	1.106	19.61	12.79	7	9	524	60.3	8.12
Q5SUA5	Unconventional myosin-Ig OS=Mus musculus GN=Myo1g PE=2 SV=1 - [MYO1G_MOUSE]	6.05	1	4	5	6	1.774	1.099	1.004	1.106	15.69	6.05	6	6	1024	117.2	8.59
Q3TES0	IQ motif and SEC7 domain-containing protein 3 OS=Mus musculus GN=Iqsec3 PE=1 SV=1 - [IQEC3_MOUSE]	25.27	2	24	29	115	1.109	0.814	0.978	1.106	328.80	25.27	50	115	1195	129.0	6.19
Q9CYH2	Redox-regulatory protein FAM213A OS=Mus musculus GN=Fam213a PE=1 SV=2 -	42.66	8	12	12	51	0.765	1.004	0.884	1.106	122.96	42.66	20	51	218	24.4	9.17
Q0MW30	E3 ubiquitin-protein ligase NEURL1B OS=Mus musculus GN=Neurl1b PE=1 SV=1 - [NEU1B_MOUSE]	5.68	1	2	2	3	0.860	0.782	0.948	1.106	2.56	5.68	2	3	546	58.5	8.37
Q9JJ61	Polypeptide N-acetylgalactosaminyltransferase 16 OS=Mus musculus GN=Galnt16 PE=2 SV=2 - [illegible]	22.58	1	9	10	25	0.799	1.022	0.840	1.106	78.29	22.58	15	25	558	62.8	8.92
G3UWF6	MCG1027453 OS=Mus musculus GN=2010015L04Rik PE=4 SV=1 - [G3UWF6_MOUSE]	25.58	3	3	4	6	2.802	1.705	1.114	1.106	13.56	25.58	6	6	129	14.9	4.86
Q8BU33	Acetolactate synthase-like protein OS=Mus musculus GN=Ilvbl PE=2 SV=1 - [ILVBL_MOUSE]	16.93	3	9	9	21	1.023	0.689	0.873	1.106	54.29	16.93	16	21	632	68.1	8.69
O55013	Trafficking protein particle complex subunit 3 OS=Mus musculus GN=Trappc3 PE=1 SV=1 - [TPPC3_MOUSE]	39.44	1	6	6	25	0.805	1.052	0.941	1.106	60.90	39.44	10	25	180	20.3	4.96

Q5Y5T1-2	Isoform 2 of Probable palmitoyltransferase ZDHHC20 OS=Mus musculus GN=Zdhhc20 - [ZDH20_MOUSE]	13.86	2	2	4	15	0.668	0.629	0.759	1.106	36.91	13.86	7	15	368	42.5	8.00
Q9D7A8	Armadillo repeat-containing protein 1 OS=Mus musculus GN=Armc1 PE=2 SV=1 -	49.29	1	13	13	44	0.763	1.020	1.093	1.106	113.07	49.29	23	44	282	31.2	5.57
Q8R0F8	Acylpyruvase FAHD1, mitochondrial OS=Mus musculus GN=Fahd1 PE=1 SV=2 - [FAHD1_MOUSE]	70.48	1	13	13	35	1.889	0.972	1.188	1.107	109.12	70.48	20	35	227	25.2	7.69
F6UIK0	Protein Dusp16 (Fragment) OS=Mus musculus GN=Dusp16 PE=2 SV=1 -	2.58	1	1	1	3	0.774	1.168	1.053	1.107	7.48	2.58	1	3	271	30.3	7.28
D3Z0F5	COP9 signalosome complex subunit 6 OS=Mus musculus GN=Cops6 PE=2 SV=1 -	70.71	3	14	14	58	0.581	0.956	0.943	1.107	178.19	70.71	25	58	297	33.6	6.00
Q8BHC1	Ras-related protein Rab-39B OS=Mus musculus GN=Rab39b PE=2 SV=1 - [RB39B_MOUSE]	69.95	2	15	17	98	0.773	1.022	0.998	1.107	269.04	69.95	30	98	213	24.6	7.83
A2AMH5	Choline transporter-like protein 1 OS=Mus musculus GN=Slc44a1 PE=2 SV=1 -	17.07	4	1	11	41	0.639	0.442	1.111	1.107	128.11	17.07	19	41	656	73.0	8.60
Q924T7-2	Isoform 2 of E3 ubiquitin-protein ligase RNF31 OS=Mus musculus GN=Rnf31 -	17.41	4	15	15	44	1.082	0.983	1.051	1.107	137.18	17.41	27	44	1057	118.1	6.49
Q8BGV8	Mitochondrial dynamic protein MID51 OS=Mus musculus GN=Smcr7l PE=1 SV=1 - [MID51_MOUSE]	14.69	3	7	7	18	1.105	1.026	1.070	1.107	38.30	14.69	12	18	463	51.2	7.88
Q9CQQ4	Gem-associated protein 2 OS=Mus musculus GN=Gemin2 PE=2 SV=1 - [GEMI2_MOUSE]	7.06	1	2	2	8	0.549	1.496	1.013	1.107	16.09	7.06	3	8	269	30.4	5.53
Q8R1Y2	Uncharacterized protein C16orf45 homolog OS=Mus musculus PE=2 SV=1 - [CP045_MOUSE]	23.15	3	6	6	18	0.891	1.272	1.075	1.107	38.89	23.15	11	18	203	23.5	5.63
Q9CR26	Vacuolar protein sorting-associated protein VTA1 homolog OS=Mus musculus GN=Vta1 PE=1 SV=1 - [VTA1_MOUSE]	34.30	2	10	10	37	0.935	1.048	1.044	1.107	104.43	34.30	17	37	309	33.9	6.13
Q6NXK7	Inactive dipeptidyl peptidase 10 OS=Mus musculus GN=Dpp10 PE=2 SV=1 -	48.31	3	34	36	133	0.625	1.386	1.140	1.107	363.34	48.31	60	133	797	90.8	6.48
M0QWJ9	Protein Tmem178b OS=Mus musculus GN=Tmem178b PE=4 SV=1 - [M0QWJ9_MOUSE]	18.60	1	3	3	6	0.522	1.905	1.113	1.107	13.79	18.60	6	6	172	19.4	9.77
Q9QY01	Serine/threonine-protein kinase ULK2 OS=Mus musculus GN=Ulk2 PE=1 SV=1 - [ULK2_MOUSE]	11.96	1	6	6	12	0.704	0.782	1.119	1.107	34.74	11.96	9	12	1037	112.8	8.53
A2AEV2	WD repeat domain phosphoinositide-interacting protein 4 OS=Mus musculus GN=Wdr45 PE=2 SV=1 - [illegible]	25.78	7	6	6	15	1.465	0.981	1.018	1.107	45.60	25.78	11	15	256	28.5	7.65
Q9D428	Golgin subfamily A member 7B OS=Mus musculus GN=GOLGA7B PE=2 SV=1 -	22.16	1	4	4	15	1.161	1.155	0.927	1.107	34.66	22.16	6	15	167	18.3	6.01
Q8K297	Procollagen galactosyltransferase 1 OS=Mus musculus GN=Colgalt1 PE=1 SV=2 -	3.24	1	2	2	3	0.827	1.520	0.829	1.107	3.16	3.24	3	3	617	71.0	7.28
Q99JY0	Trifunctional enzyme subunit beta, mitochondrial OS=Mus musculus GN=Hadhb PE=1 SV=1 - [illegible]	47.79	2	18	19	121	0.830	0.874	0.856	1.107	301.07	47.79	36	121	475	51.4	9.38
Q61035	Histidine--tRNA ligase, cytoplasmic OS=Mus musculus GN=Hars PE=2 SV=2 - [SYHC_MOUSE]	45.78	1	19	22	88	1.046	1.014	0.871	1.107	240.65	45.78	37	88	509	57.4	6.00
Q0QWG9-2	Isoform 2 of Delphilin OS=Mus musculus GN=Grid2ip - [GRD2I_MOUSE]	8.01	3	6	6	13	1.109	0.630	1.113	1.107	16.12	8.01	8	13	1024	112.5	6.89
Q8VDP3-2	Isoform 2 of Protein-methionine sulfoxide oxidase MICAL1 OS=Mus musculus GN=Mical1 - [MICA1_MOUSE]	17.95	4	15	15	44	1.248	1.256	1.128	1.108	128.33	17.95	26	44	975	108.6	5.57
E9QPY6	DNA mismatch repair protein Msh3 OS=Mus musculus GN=Msh3 PE=2 SV=1 - [E9QPY6_MOUSE]	5.84	2	3	3	4	1.404	1.136	1.140	1.108	9.32	5.84	4	4	1095	123.3	7.83
Q62179	Semaphorin-4B OS=Mus musculus GN=Sema4b PE=1 SV=2 - [SEM4B_MOUSE]	10.57	2	8	9	21	0.831	1.181	0.988	1.108	45.58	10.57	15	21	823	91.3	8.15
F6WKU5	Multidrug resistance-associated protein 9 (Fragment) OS=Mus musculus GN=Abcc12 PE=4 SV=1 - [illegible]	5.34	2	2	2	6	1.930	1.206	0.982	1.108	2.47	5.34	2	6	524	58.7	6.79

Q11011	Puromycin-sensitive aminopeptidase OS=Mus musculus GN=Npepps PE=1 SV=2 - [PSA_MOUSE]	57.39	7	44	44	301	0.642	1.160	1.004	1.108	826.73	57.39	81	301	920	103.3	5.90
Q91V24	ATP-binding cassette sub-family A member 7 OS=Mus musculus GN=Abca7 PE=1 SV=1 - [ABCA7_MOUSE]	8.99	2	13	13	32	0.714	0.786	1.045	1.108	97.15	8.99	20	32	2159	236.7	7.28
Q9JLC8-3	Isoform 3 of Sacsin OS=Mus musculus GN=Sacs - [SACS_MOUSE]	13.10	6	51	52	96	0.999	0.735	1.037	1.108	263.98	13.10	77	96	4579	519.9	7.09
Q05860-3	Isoform 3 of Formin-1 OS=Mus musculus GN=Fmn1 - [FMN1_MOUSE]	1.61	6	1	3	4	1.271	1.321	1.142	1.108	4.04	1.61	4	4	1368	152.5	8.82
Q8K2I1	Protein farnesyltransferase subunit beta OS=Mus musculus GN=Fntb PE=1 SV=1 - [FNTB_MOUSE]	17.62	2	5	6	15	1.132	0.866	0.896	1.108	36.96	17.62	10	15	437	48.8	5.80
E9Q5C3	Fibroblast growth factor receptor (Fragment) OS=Mus musculus GN=Fgfr2 PE=2 SV=1 - [E9Q5C3_MOUSE]	8.48	34	2	5	27	2.120	1.458	1.233	1.108	54.73	8.48	7	27	696	78.3	8.10
Q61334	B-cell receptor-associated protein 29 OS=Mus musculus GN=Bcap29 PE=1 SV=1 -	10.42	1	4	4	19	0.490	0.895	0.915	1.108	42.75	10.42	8	19	240	27.9	9.72
P52795	Ephrin-B1 OS=Mus musculus GN=Efnb1 PE=1 SV=1 - [EFNB1_MOUSE]	34.20	1	7	9	27	0.696	2.685	1.130	1.108	75.65	34.20	16	27	345	37.8	9.03
Q8K070	Sterile alpha motif domain-containing protein 14 OS=Mus musculus GN=Samd14 PE=1 SV=1 - [SAM14_MOUSE]	26.38	2	8	8	14	0.907	0.716	1.101	1.108	39.55	26.38	12	14	417	45.1	9.47
F8WH72	Schlafen family member 5 (Fragment) OS=Mus musculus GN=Slfn5 PE=2 SV=1 - [F8WH72_MOUSE]	1.85	2	1	1	1	0.830	0.356	0.941	1.109	1.91	1.85	1	1	378	43.0	6.04
Q8C6B0	MCG20149, isoform CRA_a OS=Mus musculus GN=Mettl7a1 PE=2 SV=1 - [Q8C6B0_MOUSE]	26.23	5	1	6	10	0.913	1.539	1.085	1.109	22.48	26.23	8	10	244	28.1	8.31
Q61578	NADPH:adrenodoxin oxidoreductase, mitochondrial OS=Mus musculus GN=Fdxr PE=2 SV=1 - [ADRO_MOUSE]	44.94	1	15	15	50	0.707	0.874	0.839	1.109	146.64	44.94	24	50	494	54.2	8.66
P20108	Thioredoxin-dependent peroxide reductase, mitochondrial OS=Mus musculus GN=Prdx3 PE=1 SV=1 - [PRDX3_MOUSE]	49.42	1	10	10	225	2.044	1.172	0.916	1.109	700.26	49.42	19	225	257	28.1	7.58
A2ADY9	Protein DDI1 homolog 2 OS=Mus musculus GN=Ddi2 PE=1 SV=1 - [DDI2_MOUSE]	44.11	2	13	13	58	1.423	1.488	1.110	1.109	178.33	44.11	24	58	399	44.6	5.05
O09126	Semaphorin-4D OS=Mus musculus GN=Sema4d PE=1 SV=2 - [SEM4D_MOUSE]	20.56	1	15	15	35	0.867	1.093	0.948	1.109	102.32	20.56	23	35	861	95.6	7.81
E9Q175	Unconventional myosin-VI OS=Mus musculus GN=Myo6 PE=2 SV=1 - [E9Q175_MOUSE]	44.77	2	12	55	172	0.890	0.851	1.092	1.109	487.44	44.77	92	172	1253	144.7	8.51
Q5NCF2	Trafficking protein particle complex subunit 1 OS=Mus musculus GN=Trappc1 PE=1 SV=1 - [TPPC1_MOUSE]	11.03	3	2	2	7	0.641	0.838	0.830	1.109	16.64	11.03	3	7	145	16.9	9.16
Q8C0L0	Thioredoxin-related transmembrane protein 4 OS=Mus musculus GN=Tmx4 PE=1 SV=2 - [TMX4_MOUSE]	17.31	3	6	6	35	0.715	1.141	1.002	1.109	108.66	17.31	11	35	335	37.1	4.37
Q922R1	UPF0183 protein C16orf70 homolog OS=Mus musculus PE=2 SV=2 - [CP070_MOUSE]	16.59	4	6	6	12	0.536	1.112	1.080	1.109	46.64	16.59	8	12	422	47.4	7.74
Q9QUI0	Transforming protein RhoA OS=Mus musculus GN=Rhoa PE=1 SV=1 - [RHOA_MOUSE]	65.28	3	2	9	142	0.692	0.804	0.854	1.109	424.66	65.28	18	142	193	21.8	6.10
Q3TZX3	Solute carrier family 25 member 33 OS=Mus musculus GN=Slc25a33 PE=2 SV=1 -	5.00	1	2	2	4	0.848	0.799	0.687	1.109	5.52	5.00	3	4	320	35.0	9.67
Q99LB4	Capping protein (Actin filament), gelsolin-like OS=Mus musculus GN=Capg PE=1 SV=1 - [Q99LB4_MOUSE]	24.93	8	8	8	24	1.211	1.259	0.693	1.110	75.83	24.93	12	24	349	38.7	6.95
D3YU06	Abhydrolase domain-containing protein 3 OS=Mus musculus GN=Abhd3 PE=2 SV=1 - [D3YU06_MOUSE]	14.43	5	5	5	14	1.102	0.932	0.924	1.110	32.12	14.43	7	14	402	45.2	7.30
Q8BHH2	Ras-related protein Rab-9B OS=Mus musculus GN=Rab9b PE=2 SV=1 - [RAB9B_MOUSE]	46.77	1	6	8	36	0.899	0.822	0.626	1.110	125.70	46.77	13	36	201	22.7	4.93

Q60805	Tyrosine-protein kinase Mer OS=Mus musculus GN=Mertk PE=1 SV=1 - [MERTK_MOUSE]	11.37	1	10	11	27	0.965	1.506	1.390	1.110	65.60	11.37	18	27	994	110.1	5.50
P59644	Phosphatidylinositol 4,5-bisphosphate 5-phosphatase A OS=Mus musculus GN=Inpp5j PE=1 SV=2 - [PI5PA_MOUSE]	27.62	2	18	18	56	1.065	0.819	1.310	1.110	141.55	27.62	28	56	1003	107.5	9.36
Q921E2	Ras-related protein Rab-31 OS=Mus musculus GN=Rab31 PE=1 SV=1 - [RAB31_MOUSE]	69.59	3	9	11	47	0.905	0.646	0.962	1.110	159.17	69.59	19	47	194	21.3	7.40
Q8BH66	Atlastin-1 OS=Mus musculus GN=Atl1 PE=1 SV=1 - [ATLA1_MOUSE]	42.47	1	21	23	152	0.601	1.118	1.096	1.110	446.61	42.47	45	152	558	63.3	6.49
Q9WVJ3-2	Isoform 2 of Carboxypeptidase Q OS=Mus musculus GN=Cpq - [CBPQ_MOUSE]	13.97	2	5	5	14	1.988	1.463	1.049	1.110	40.67	13.97	8	14	458	50.5	6.40
Q9QWT9-3	Isoform 3 of Kinesin-like protein KIFC1 OS=Mus musculus GN=Kifc1 - [KIFC1_MOUSE]	1.15	4	1	1	3	0.955	1.117	1.050	1.110	8.24	1.15	1	3	611	67.1	8.59
E9PZY0	Dual-specificity protein phosphatase isoform MDSP OS=Mus musculus GN=Dusp13 PE=2 SV=1 - [E9PZY0_MOUSE]	7.38	2	1	1	1	1.118	1.011	1.100	1.110	0.00	7.38	1	1	149	16.3	9.13
A2ACC6	Ral GTPase-activating protein subunit beta OS=Mus musculus GN=Ralgapb PE=2 SV=1 - [A2ACC6_MOUSE]	23.55	6	22	22	95	0.951	1.109	1.043	1.110	289.25	23.55	40	95	1495	166.5	6.74
Q8BG40	Katanin p80 WD40 repeat-containing subunit B1 OS=Mus musculus GN=Katnb1 PE=1 SV=1 - [KTNB1_MOUSE]	29.03	1	14	14	51	0.926	1.029	1.076	1.110	156.21	29.03	21	51	658	72.6	7.27
Q9D1H8	39S ribosomal protein L53, mitochondrial OS=Mus musculus GN=Mrpl53 PE=2 SV=1 - [RM53_MOUSE]	35.59	1	3	3	11	0.883	0.814	0.951	1.110	38.19	35.59	6	11	118	12.7	9.52
P09225	Lymphotoxin-alpha OS=Mus musculus GN=Lta PE=2 SV=1 - [TNFB_MOUSE]	12.87	1	2	3	8	1.148	1.109	0.909	1.110	13.66	12.87	4	8	202	22.0	10.14
Q5SUC9	Protein SCO1 homolog, mitochondrial OS=Mus musculus GN=Sco1 PE=2 SV=1 - [SCO1_MOUSE]	17.25	2	6	6	12	0.881	1.096	1.076	1.111	32.85	17.25	11	12	284	31.6	8.47
Q8CIT0	Corticoliberin OS=Mus musculus GN=Crh PE=2 SV=1 - [CRF_MOUSE]	24.60	1	4	4	14	1.234	1.209	0.968	1.111	27.16	24.60	7	14	187	20.8	10.52
G3X9B1	HEAT repeat containing 1 OS=Mus musculus GN=Heatr1 PE=4 SV=1 - [G3X9B1_MOUSE]	2.24	1	3	5	11	1.020	0.904	0.831	1.111	18.04	2.24	7	11	2143	241.9	6.98
D3Z4X7	ADP-ribosylation factor-like protein 10 (Fragment) OS=Mus musculus GN=Arl10 PE=2 SV=1 - [D3Z4X7_MOUSE]	32.67	3	4	4	12	0.791	0.868	0.661	1.111	39.19	32.67	8	12	202	22.8	4.77
Q920M7	Synaptotagmin-17 OS=Mus musculus GN=Syt17 PE=2 SV=1 - [SYT17_MOUSE]	17.66	1	8	8	12	0.509	2.479	1.446	1.111	29.99	17.66	9	12	470	53.3	7.15
P49718	DNA replication licensing factor MCM5 OS=Mus musculus GN=Mcm5 PE=2 SV=1 - [MCM5_MOUSE]	8.05	2	2	2	2	1.759	1.666	1.655	1.111	5.71	8.05	2	2	733	82.3	8.43
Q8BLI4	Dermatan-sulfate epimerase OS=Mus musculus GN=Dse PE=2 SV=1 - [DSE_MOUSE]	1.46	1	2	2	2	3.585	1.742	0.876	1.111	5.06	1.46	2	2	958	109.7	7.25
Q91WQ3	Tyrosine--tRNA ligase, cytoplasmic OS=Mus musculus GN=Yars PE=2 SV=3 - [SYYC_MOUSE]	70.45	3	39	40	171	1.012	0.945	0.983	1.111	474.14	70.45	70	171	528	59.1	7.01
B1ASP2	Tyrosine-protein kinase OS=Mus musculus GN=Jak1 PE=3 SV=1 - [B1ASP2_MOUSE]	15.26	3	13	15	29	0.845	0.925	0.939	1.111	81.65	15.26	28	29	1153	133.3	7.53
E9Q2V5	Protein BC037034 OS=Mus musculus GN=BC037034 PE=4 SV=1 - [E9Q2V5_MOUSE]	14.31	2	6	6	19	0.669	1.007	0.985	1.111	47.47	14.31	11	19	580	62.7	8.68
Q9QUJ7-2	Isoform Short of Long-chain-fatty-acid--CoA ligase 4 OS=Mus musculus GN=Acsl4 - [ACSL4_MOUSE]	23.73	2	12	14	33	0.889	1.048	1.002	1.111	86.05	23.73	22	33	670	74.3	8.00
Q3UYV8	Dynein assembly factor 3, axonemal OS=Mus musculus GN=Dnaaf3 PE=2 SV=1 - [DAAF3_MOUSE]	1.88	1	1	1	2	0.886	1.055	1.297	1.111	5.85	1.88	1	2	586	64.6	6.11
Q8BSB7	Tissue factor pathway inhibitor OS=Mus musculus GN=Tfpi PE=2 SV=1 - [Q8BSB7_MOUSE]	3.40	6	1	1	1	0.725	1.673	1.350	1.111	2.04	3.40	1	1	235	26.8	5.71
Q99KJ0	UBX domain-containing protein 2A OS=Mus musculus GN=Ubxn2a PE=2 SV=1 -	26.74	2	5	5	8	1.567	1.188	1.066	1.111	24.18	26.74	8	8	258	29.2	5.80

Q8BHD8	Protein-L-isoaspartate O-methyltransferase domain-containing protein 2 OS=Mus musculus GN=Pcmtd2 PE=2 SV=1 - [PCMD2_MOUSE]	15.04	3	5	6	8	1.381	1.003	1.125	1.111	17.57	15.04	7	8	359	40.7	6.46
Q8CBF3	Ephrin type-B receptor 1 OS=Mus musculus GN=Ephb1 PE=1 SV=1 - [EPHB1_MOUSE]	18.29	4	7	14	32	0.672	0.382	0.902	1.111	82.39	18.29	24	32	984	109.8	6.40
Q9CQX6	MCG141091, isoform CRA_a OS=Mus musculus GN=Gm16286 PE=2 SV=1 - [Q9CQX6_MOUSE]	36.82	5	4	8	22	1.025	0.673	0.963	1.111	60.09	36.82	13	22	220	24.8	6.23
Q9CPY7	Cytosol aminopeptidase OS=Mus musculus GN=Lap3 PE=1 SV=3 - [AMPL_MOUSE]	71.87	2	29	29	188	1.744	0.888	1.017	1.111	558.50	71.87	53	188	519	56.1	7.72
Q9WTP7	GTP:AMP phosphotransferase AK3, mitochondrial OS=Mus musculus GN=Ak3 PE=1 SV=3 - [KAD3_MOUSE]	81.94	2	18	18	96	0.812	1.234	1.030	1.112	298.50	81.94	33	96	227	25.4	8.84
Q8R2H9	Phosphoethanolamine/phosphocholine phosphatase OS=Mus musculus GN=Phospho1 PE=2 SV=1 - [PHOP1_MOUSE]	26.97	3	5	5	10	1.030	0.763	0.977	1.112	32.11	26.97	9	10	267	29.9	7.94
Q80TQ2	Ubiquitin carboxyl-terminal hydrolase CYLD OS=Mus musculus GN=Cyld PE=1 SV=2 - [CYLD_MOUSE]	28.26	3	22	22	54	1.037	1.494	1.625	1.112	177.09	28.26	36	54	952	106.5	5.63
Q8R404	Protein QIL1 OS=Mus musculus GN=Qil1 PE=2 SV=1 - [QIL1_MOUSE]	41.18	1	3	3	12	0.984	1.330	1.034	1.112	53.71	41.18	4	12	119	13.4	8.63
B1AWN6	Protein Scn2a1 OS=Mus musculus GN=Scn2a1 PE=3 SV=1 - [B1AWN6_MOUSE]	19.79	11	19	36	130	0.622	1.292	1.428	1.112	390.20	19.79	61	130	2006	227.8	5.67
Q8VI63	MOB kinase activator 2 OS=Mus musculus GN=Mob2 PE=2 SV=1 - [MOB2_MOUSE]	11.49	2	3	3	12	0.750	1.007	0.953	1.112	31.85	11.49	6	12	235	26.8	6.52
Q7M759	Alpha/beta hydrolase domain-containing protein 17B OS=Mus musculus GN=Abhd17b PE=2 SV=1 - [AB17B_MOUSE]	39.58	1	7	8	19	0.628	0.571	1.141	1.112	47.67	39.58	14	19	288	32.2	6.29
Q61411	GTPase HRas OS=Mus musculus GN=Hras1 PE=1 SV=2 - [RASH_MOUSE]	77.78	5	5	12	109	0.581	0.925	1.112	1.112	266.41	77.78	22	109	189	21.3	5.31
Q8K2Z8-2	Isoform 2 of Ubiquitin-conjugating enzyme E2 Q2 OS=Mus musculus GN=Ube2q2 -	12.94	4	1	3	6	1.180	0.939	1.064	1.112	18.17	12.94	5	6	255	28.9	4.83
H3BKS9	Atrial natriuretic peptide-converting enzyme OS=Mus musculus GN=Corin PE=2 SV=1 - [H3BKS9_MOUSE]	5.00	5	2	3	4	0.804	1.160	1.243	1.112	4.50	5.00	3	4	980	109.3	5.22
P42128	Forkhead box protein K1 OS=Mus musculus GN=Foxk1 PE=1 SV=2 - [FOXK1_MOUSE]	19.33	1	8	8	15	0.820	1.233	1.222	1.113	42.21	19.33	11	15	719	74.9	9.17
O35604	Niemann-Pick C1 protein OS=Mus musculus GN=Npc1 PE=1 SV=2 - [NPC1_MOUSE]	6.50	1	6	6	10	0.908	0.785	1.011	1.113	24.12	6.50	8	10	1277	142.8	5.71
Q18PE0-2	Isoform 2 of Protein Dok-7 OS=Mus musculus GN=Dok7 - [DOK7_MOUSE]	10.00	3	2	2	3	1.296	0.907	0.880	1.113	10.28	10.00	3	3	360	37.1	7.14
Q8R2D7	Protein Vmn1r26 OS=Mus musculus GN=Vmn1r26 PE=4 SV=1 - [Q8R2D7_MOUSE]	7.96	1	1	1	1	1.568	3.943	1.094	1.113	0.00	7.96	1	1	339	38.8	9.45
Q8BLE7	Vesicular glutamate transporter 2 OS=Mus musculus GN=Slc17a6 PE=1 SV=1 -	16.84	1	10	10	85	0.613	0.483	0.744	1.113	237.62	16.84	17	85	582	64.5	6.68
A6H8H2	DENN domain-containing protein 4C OS=Mus musculus GN=Dennd4c PE=1 SV=1 -	7.87	4	12	12	24	0.672	1.061	1.037	1.113	61.96	7.87	19	24	1906	211.3	6.40
Q3TYX3	SET and MYND domain-containing protein 5 OS=Mus musculus GN=Smyd5 PE=2 SV=2 - [SMYD5_MOUSE]	20.91	1	8	8	19	0.671	1.339	1.064	1.113	35.50	20.91	12	19	416	47.1	5.21
Q14BJ1	Protein FAM89A OS=Mus musculus GN=Fam89a PE=2 SV=2 - [FA89A_MOUSE]	12.00	1	1	1	1	8.778	2.671	1.043	1.113	2.60	12.00	1	1	175	18.7	5.80
Q8BU31	Ras-related protein Rap-2c OS=Mus musculus GN=Rap2c PE=1 SV=1 - [RAP2C_MOUSE]	73.22	2	4	10	66	0.643	0.837	0.940	1.113	223.95	73.22	20	66	183	20.7	4.94
Q3UX10	Tubulin alpha chain-like 3 OS=Mus musculus GN=Tubal3 PE=2 SV=2 - [TBAL3_MOUSE]	16.59	1	3	6	58	1.076	1.195	0.981	1.113	124.11	16.59	10	58	446	50.0	5.58
Q91YH5	Atlastin-3 OS=Mus musculus GN=Atl3 PE=2 SV=1 - [ATLA3_MOUSE]	40.11	3	14	16	45	0.995	1.148	1.031	1.113	142.30	40.11	30	45	541	60.5	6.10

Q8K2A1	PTB domain-containing engulfment adapter protein 1 OS=Mus musculus GN=Gulp1 PE=1 SV=1 - [GULP1_MOUSE]	9.54	3	3	3	3	1.539	1.147	1.051	1.113	8.13	9.54	3	3	304	34.4	7.90
Q9ESN6	Tripartite motif-containing protein 2 OS=Mus musculus GN=Trim2 PE=1 SV=1 - [TRIM2_MOUSE]	43.55	8	23	25	172	0.968	0.846	1.200	1.113	508.73	43.55	44	172	744	81.4	6.96
Q99JY8	Lipid phosphate phosphohydrolase 3 OS=Mus musculus GN=Ppap2b PE=1 SV=1 -	24.36	1	10	10	62	0.508	1.077	1.057	1.113	178.96	24.36	17	62	312	35.2	9.07
Q9JI91	Alpha-actinin-2 OS=Mus musculus GN=Actn2 PE=1 SV=2 - [ACTN2_MOUSE]	63.09	4	34	49	223	0.975	3.948	3.777	1.113	634.19	63.09	89	223	894	103.8	5.45
Q8BGN5	NIPA-like protein 3 OS=Mus musculus GN=Nipal3 PE=2 SV=1 - [NPAL3_MOUSE]	12.20	3	3	3	7	0.853	0.882	1.393	1.113	34.71	12.20	4	7	410	44.8	7.75
Q810J8	Zinc finger FYVE domain-containing protein 1 OS=Mus musculus GN=Zfyve1 PE=2 SV=2 - [ZFYV1_MOUSE]	25.23	1	15	15	39	0.724	0.973	1.030	1.113	118.45	25.23	26	39	777	86.9	7.34
Q9Z1P6	NADH dehydrogenase [ubiquinone] 1 alpha subcomplex subunit 7 OS=Mus musculus GN=Ndufa7 PE=1 SV=3 - [illegible]	73.45	1	13	13	104	1.063	1.012	1.373	1.113	296.03	73.45	24	104	113	12.6	10.17
Q8BKZ9	Pyruvate dehydrogenase protein X component, mitochondrial OS=Mus musculus GN=Pdhx PE=2 SV=1 - [ODPX_MOUSE]	45.71	3	18	18	140	1.069	1.114	1.248	1.113	358.44	45.71	31	140	501	54.0	7.75
O35864	COP9 signalosome complex subunit 5 OS=Mus musculus GN=Cops5 PE=1 SV=3 -	56.29	1	15	15	80	0.912	1.090	0.969	1.113	233.02	56.29	25	80	334	37.5	6.54
Q61644	Protein kinase C and casein kinase substrate in neurons protein 1 OS=Mus musculus GN=Pacsin1 PE=1 SV=1 - [illegible]	61.90	1	28	30	439	1.420	1.313	1.660	1.113	1358.09	61.90	54	439	441	50.5	5.24
I7HJR1	Collagen alpha-2(IX) chain OS=Mus musculus GN=Col9a2 PE=4 SV=1 - [I7HJR1_MOUSE]	2.03	2	1	1	1	0.875	0.724	1.270	1.113	2.94	2.03	1	1	688	65.3	9.52
D3YV40	Protein kinase C-binding protein NELL1 (Fragment) OS=Mus musculus GN=Nell1 PE=2 SV=1 - [D3YV40_MOUSE]	7.92	3	3	3	5	0.676	0.811	0.873	1.113	11.39	7.92	5	5	379	42.3	8.65
F6TY66	Claudin-10A (Fragment) OS=Mus musculus GN=Cldn10 PE=4 SV=1 - [F6TY66_MOUSE]	21.31	5	2	2	9	0.752	0.667	1.125	1.113	28.09	21.31	3	9	122	13.1	8.76
Q8BHC9	Alpha-(1,3)-fucosyltransferase 11 OS=Mus musculus GN=Fut11 PE=2 SV=1 -	7.16	1	2	2	4	1.033	1.053	0.979	1.113	10.80	7.16	4	4	489	55.5	6.23
Q9JL62	Glycolipid transfer protein OS=Mus musculus GN=Gltp PE=2 SV=3 - [GLTP_MOUSE]	53.11	3	9	9	46	0.892	0.338	0.899	1.113	136.48	53.11	16	46	209	23.7	7.39
P62823	Ras-related protein Rab-3C OS=Mus musculus GN=Rab3c PE=1 SV=1 - [RAB3C_MOUSE]	60.79	1	6	13	255	0.820	1.149	1.148	1.114	733.06	60.79	25	255	227	25.9	5.24
A2AIV2	Protein virilizer homolog OS=Mus musculus GN=Kiaa1429 PE=1 SV=1 [VIR_MOUSE]	3.09	3	3	5	11	2.460	1.002	0.895	1.114	34.37	3.09	7	11	1811	201.3	4.96
P16388	Potassium voltage-gated channel subfamily A member 1 OS=Mus musculus GN=Kcna1 PE=2 SV=1 - [KCNA1_MOUSE]	26.26	3	5	10	50	1.062	0.479	1.425	1.114	156.26	26.26	17	50	495	56.4	5.14
A2AG06-2	Isoform 2 of Uncharacterized protein C17orf104 homolog OS=Mus musculus GN=Gm1564 - [illegible]	2.53	2	2	2	4	0.990	1.175	0.904	1.114	8.24	2.53	2	4	909	102.8	6.80
Q8CAL5	Glypican-5 OS=Mus musculus GN=Gpc5 PE=2 SV=1 - [GPC5_MOUSE]	12.94	3	7	7	15	0.916	0.975	0.878	1.114	38.85	12.94	11	15	572	63.8	7.30
Q9EQZ6-3	Isoform 3 of Rap guanine nucleotide exchange factor 4 OS=Mus musculus GN=Rapgef4 - [RPGF4_MOUSE]	31.92	4	27	30	107	0.614	1.228	1.084	1.114	283.23	31.92	52	107	993	113.4	6.83
A2ASQ1	Agrin OS=Mus musculus GN=Agrn PE=1 SV=1 - [illegible]	21.23	1	2	33	117	0.858	1.354	1.096	1.114	341.40	21.23	59	117	1950	207.4	6.32
Q9R049-2	Isoform 2 of E3 ubiquitin-protein ligase AMFR OS=Mus musculus GN=Amfr -	12.52	2	5	5	6	0.830	0.970	1.115	1.114	18.20	12.52	6	6	639	72.7	6.46
P97785	GDNF family receptor alpha-1 OS=Mus musculus GN=Gfra1 PE=2 SV=2 - [GFRA1_MOUSE]	24.36	5	9	10	21	0.679	0.446	0.735	1.114	61.92	24.36	16	21	468	51.7	7.84
Q6PF93	Phosphatidylinositol 3-kinase catalytic subunit type 3 OS=Mus musculus GN=Pik3c3 PE=1 SV=1 - [PK3C3_MOUSE]	25.48	5	19	20	44	0.832	1.027	1.068	1.114	127.17	25.48	32	44	887	101.4	6.73

Q8BXA7	PH domain leucine-rich repeat-containing protein phosphatase 2 OS=Mus musculus GN=Phlpp2 PE=2 SV=3 - [PHLP2_MOUSE]	2.35	2	3	3	4	0.889	0.922	0.933	1.114	13.23	2.35	4	4	1320	145.9	5.95
Q9D8U8	Sorting nexin-5 OS=Mus musculus GN=Snx5 PE=1 SV=1 - [SNX5_MOUSE]	40.35	1	15	16	63	1.421	1.264	0.926	1.114	185.06	40.35	28	63	404	46.8	6.62
P0C913	Overexpressed in colon carcinoma 1 protein homolog OS=Mus musculus PE=3 SV=1 -	46.03	1	2	2	3	1.244	1.254	0.894	1.114	14.17	46.03	2	3	63	6.4	6.57
Q9CQF9	Prenylcysteine oxidase OS=Mus musculus GN=Pcyox1 PE=1 SV=1 - [PCYOX_MOUSE]	38.22	3	14	14	70	0.968	1.364	0.940	1.114	199.26	38.22	25	70	505	56.5	6.92
E9QKV6	Unconventional myosin-IXb OS=Mus musculus GN=Myo9b PE=2 SV=1 - [E9QKV6_MOUSE]	4.08	6	5	5	13	1.363	0.782	1.009	1.114	14.96	4.08	7	13	1961	222.3	8.40
Q9D3D9	ATP synthase subunit delta, mitochondrial OS=Mus musculus GN=Atp5d PE=1 SV=1 - [ATPD_MOUSE]	44.05	2	4	5	49	1.836	1.257	1.557	1.115	121.61	44.05	8	49	168	17.6	5.08
P62827	GTP-binding nuclear protein Ran OS=Mus musculus GN=Ran PE=1 SV=3 - [RAN_MOUSE]	48.61	2	4	11	92	0.425	0.914	0.857	1.115	257.50	48.61	21	92	216	24.4	7.49
Q8VC03	Echinoderm microtubule-associated protein-like 3 OS=Mus musculus GN=Eml3 PE=2 SV=1 - [EMAL3_MOUSE]	5.80	1	4	4	8	1.736	0.803	1.070	1.115	23.16	5.80	8	8	897	95.6	7.05
P12032	Metalloproteinase inhibitor 1 OS=Mus musculus GN=Timp1 PE=2 SV=2 - [TIMP1_MOUSE]	4.39	1	1	1	2	1.322	1.192	0.939	1.115	2.24	4.39	1	2	205	22.6	8.82
Q80UJ7	Rab3 GTPase-activating protein catalytic subunit OS=Mus musculus GN=Rab3gap1 PE=2 SV=4 - [RB3GP_MOUSE]	39.76	1	34	34	158	0.695	1.172	1.009	1.115	470.67	39.76	64	158	981	110.1	5.73
Q9DB05	Alpha-soluble NSF attachment protein OS=Mus musculus GN=Napa PE=1 SV=1 - [SNAA_MOUSE]	70.17	1	13	18	154	0.842	1.172	1.006	1.115	472.47	70.17	32	154	295	33.2	5.45
Q3UHB1	5'-nucleotidase domain-containing protein 3 OS=Mus musculus GN=Nt5dc3 PE=2 SV=1 - [NT5D3_MOUSE]	46.34	1	20	20	73	0.635	0.993	1.034	1.115	220.22	46.34	36	73	546	63.1	8.56
Q8CC35	Synaptopodin OS=Mus musculus GN=Synpo PE=1 SV=2 - [SYNPO_MOUSE]	37.24	4	23	23	132	1.086	2.870	4.190	1.115	435.04	37.24	39	132	929	99.5	9.42
Q9DAC9	POU domain, class 5, transcription factor 2 OS=Mus musculus GN=Pou5f2 PE=2 SV=1 - [PO5F2_MOUSE]	27.36	1	4	5	8	2.426	0.150	1.658	1.115	13.45	27.36	5	8	329	36.4	8.91
Q3UDK1-2	Isoform 2 of TRAF-type zinc finger domain-containing protein 1 OS=Mus musculus GN=Trafd1 - [TRAD1_MOUSE]	12.85	3	6	6	12	1.589	1.251	1.458	1.115	28.48	12.85	9	12	576	63.8	5.41
D3Z5R9	Cytoplasmic polyadenylation element-binding protein 3 OS=Mus musculus GN=Cpeb3 PE=2 SV=1 - [D3Z5R9_MOUSE]	27.61	1	2	14	52	1.747	0.959	1.247	1.115	140.57	27.61	24	52	699	76.2	7.03
J3QPN5	Uncharacterized protein OS=Mus musculus GN=Flg PE=4 SV=1 - [J3QPN5_MOUSE]	5.16	8	1	1	1	0.498	7.025	0.808	1.115	1.86	5.16	1	1	252	26.7	10.90
D3Z399	F-box/LRR-repeat protein 13 OS=Mus musculus GN=Fbxl13 PE=2 SV=1 - [D3Z399_MOUSE]	2.85	2	1	1	1	0.951	0.816	1.270	1.115	0.00	2.85	1	1	632	72.5	9.03
Q8R5I6	Glutathione S-transferase mu 4 OS=Mus musculus GN=Gstm4 PE=2 SV=1 - [Q8R5I6_MOUSE]	30.28	3	2	7	130	0.830	1.889	1.032	1.115	479.25	30.28	12	130	218	25.5	6.93
Q8VE18	Protein SMG8 OS=Mus musculus GN=Smg8 PE=2 SV=1 - [SMG8_MOUSE]	6.16	2	5	5	12	1.099	1.106	1.097	1.115	26.08	6.16	9	12	991	109.6	7.77
P12787	Cytochrome c oxidase subunit 5A, mitochondrial OS=Mus musculus GN=Cox5a PE=1 SV=2 - [COX5A_MOUSE]	59.59	1	9	10	429	1.472	0.974	1.374	1.115	1124.29	59.59	18	429	146	16.1	6.54
Q8BGC9	Protein CREG2 OS=Mus musculus GN=Creg2 PE=1 SV=1 - [CREG2_MOUSE]	9.03	1	2	2	5	1.902	1.137	0.890	1.116	17.14	9.03	3	5	288	31.7	7.77
Q3UU35	Ovostatin homolog OS=Mus musculus GN=Ovos PE=2 SV=2 - [OVOS_MOUSE]	4.40	1	3	3	3	3.032	1.767	0.814	1.116	6.24	4.40	3	3	1456	162.2	5.26
Q9D1X0	Nucleolar protein 3 OS=Mus musculus GN=Nol3 PE=1 SV=1 - [NOL3_MOUSE]	47.73	1	7	7	48	1.531	0.855	1.250	1.116	184.78	47.73	14	48	220	24.6	4.07

Q8R1K1	Ubiquitin-associated domain-containing protein 2 OS=Mus musculus GN=Ubac2 PE=2 SV=1 - [UBAC2_MOUSE]	12.17	1	4	4	19	0.838	0.893	0.897	1.116	51.96	12.17	7	19	345	39.0	9.44
Q78YZ6-3	Isoform 3 of Short coiled-coil protein OS=Mus musculus GN=Scoc - [SCOC_MOUSE]	22.22	3	1	1	13	2.691	0.826	1.208	1.116	35.92	22.22	2	13	81	9.3	4.75
Q8C739	Protein FAM110B OS=Mus musculus GN=Fam110b PE=2 SV=1 - [F110B_MOUSE]	34.70	2	9	9	24	1.339	0.964	1.378	1.116	59.44	34.70	15	24	366	40.3	9.23
O88342	WD repeat-containing protein 1 OS=Mus musculus GN=Wdr1 PE=1 SV=3 - [WDR1_MOUSE]	62.21	1	31	31	243	1.165	1.256	0.947	1.116	830.65	62.21	55	243	606	66.4	6.60
P31750	RAC-alpha serine/threonine-protein kinase OS=Mus musculus GN=Akt1 PE=1 SV=2 -	34.79	4	7	14	32	0.738	0.914	1.016	1.116	82.53	34.79	24	32	480	55.7	5.90
D3YXS8	Mitochondrial import receptor subunit TOM40B OS=Mus musculus GN=Tomm40l PE=2 SV=1 [D3YXS8_MOUSE]	22.99	5	5	5	18	0.878	1.345	1.284	1.116	58.88	22.99	8	18	274	30.4	7.27
O08539	Myc box-dependent-interacting protein 1 OS=Mus musculus GN=Bin1 PE=1 SV=1 - [BIN1_MOUSE]	57.31	3	29	31	471	1.535	1.018	1.508	1.116	1304.70	57.31	59	471	588	64.4	5.03
Q5U3K5	Rab-like protein 6 OS=Mus musculus GN=Rabl6 PE=1 SV=2 - [RABL6_MOUSE]	29.93	1	19	19	50	0.858	1.053	1.161	1.116	162.72	29.93	29	50	725	79.8	5.53
Q9CY45	N(6)-adenine-specific DNA methyltransferase 2 OS=Mus musculus GN=N6amt2 PE=2 SV=1 - [N6MT2_MOUSE]	22.90	1	4	4	7	2.235	1.028	1.044	1.117	20.47	22.90	5	7	214	24.5	4.67
Q61391	Neprilysin OS=Mus musculus GN=Mme PE=1 SV=3 - [NEP_MOUSE]	11.20	1	9	9	17	0.987	0.871	1.262	1.117	30.34	11.20	14	17	750	85.6	5.81
E9PUZ5	PRKCA-binding protein OS=Mus musculus GN=Pick1 PE=4 SV=1 - [E9PUZ5_MOUSE]	33.65	2	2	13	48	0.751	1.553	0.920	1.117	143.95	33.65	22	48	416	46.5	5.30
Q6P8M1	Putative deoxyribonuclease TATDN1 OS=Mus musculus GN=Tatdn1 PE=2 SV=1 -	30.51	2	7	7	12	1.244	1.144	1.096	1.117	34.79	30.51	10	12	295	33.3	6.18
B1AYL1	Protein Scn7a OS=Mus musculus GN=Scn7a PE=2 SV=1 - [B1AYL1_MOUSE]	7.61	1	8	8	14	1.777	1.699	0.858	1.117	33.64	7.61	10	14	1681	192.1	7.42
B1AYM9	Protein CK137956 OS=Mus musculus GN=CK137956 PE=2 SV=1 - [B1AYM9_MOUSE]	10.07	1	3	3	6	0.611	1.599	0.870	1.117	2.80	10.07	3	6	596	65.4	8.92
Q8K482	EMILIN-2 OS=Mus musculus GN=Emilin2 PE=1 SV=1 - [EMIL2_MOUSE]	3.54	1	3	3	8	1.435	1.188	1.130	1.117	20.64	3.54	6	8	1074	117.2	5.62
P08553	Neurofilament medium polypeptide OS=Mus musculus GN=Nefm PE=1 SV=4 - [NFM_MOUSE]	57.08	2	47	52	1382	3.004	0.348	1.579	1.117	3833.99	57.08	93	1382	848	95.9	4.77
Q9DCJ5	NADH dehydrogenase [ubiquinone] 1 alpha subcomplex subunit 8 OS=Mus musculus GN=Ndufa8 PE=1 SV=3 - [illegible]	47.09	1	7	7	61	1.017	0.905	1.099	1.117	147.79	47.09	14	61	172	20.0	8.46
Q9DBC3	Cap-specific mRNA (nucleoside-2'-O-)-methyltransferase 1 OS=Mus musculus GN=Ftsjd2 PE=1 SV=1 - [illegible]	23.30	4	15	17	37	0.775	1.022	0.961	1.118	86.76	23.30	26	37	837	95.6	7.27
O70176	Pituitary adenylate cyclase-activating polypeptide OS=Mus musculus GN=Adcyap1 PE=2 SV=1 - [PACA_MOUSE]	40.57	1	7	7	21	1.551	0.902	0.669	1.118	57.62	40.57	13	21	175	19.4	9.23
A2AL18	Rho GTPase-activating protein 11A OS=Mus musculus GN=Arhgap11a PE=2 SV=1 -	4.60	3	1	1	1	0.938	1.072	1.319	1.118	3.49	4.60	1	1	457	50.4	9.86
Q3U4B1	Protein Ssbp4 OS=Mus musculus GN=Ssbp4 PE=2 SV=1 - [Q3U4B1_MOUSE]	8.26	1	2	2	4	1.426	1.256	1.718	1.118	10.90	8.26	4	4	363	37.4	6.64
K3W4R5	Cytoskeleton-associated protein 5 OS=Mus musculus GN=Ckap5 PE=4 SV=1 - [K3W4R5_MOUSE]	42.62	4	78	79	278	0.627	0.883	0.997	1.118	733.67	42.62	140	278	2032	225.5	7.85
P24270	Catalase OS=Mus musculus GN=Cat PE=1 SV=4 - [CATA_MOUSE]	61.48	2	25	25	161	1.592	0.757	0.717	1.118	468.13	61.48	45	161	527	59.8	7.88
Q9JKF7	39S ribosomal protein L39, mitochondrial OS=Mus musculus GN=Mrpl39 PE=2 SV=4 - [RM39_MOUSE]	25.00	1	7	7	9	1.151	1.058	1.123	1.118	25.83	25.00	8	9	336	38.5	7.94
Q66JW3	Thymocyte selection-associated high mobility group box protein TOX OS=Mus musculus GN=Tox PE=1 SV=2 - [illegible]	6.65	1	2	4	9	6.312	0.563	1.584	1.118	20.98	6.65	7	9	526	57.2	7.27

J3QP43	Protein 6330403A02Rik OS=Mus musculus GN=6330403A02Rik PE=4 SV=1 - [J3QP43_MOUSE]	21.28	2	2	2	74	0.836	3.066	1.855	1.118	256.14	21.28	4	74	141	15.0	6.51
Q9D975	Sulfiredoxin-1 OS=Mus musculus GN=Srxn1 PE=2 SV=1 - [SRXN1_MOUSE]	23.53	2	3	3	6	1.784	0.941	0.935	1.118	17.81	23.53	5	6	136	14.1	8.02
Q9D328	Transmembrane protein 35 OS=Mus musculus GN=Tmem35 PE=2 SV=1 - [TMM35_MOUSE]	13.17	2	3	3	13	1.510	1.513	1.195	1.118	42.37	13.17	4	13	167	18.5	9.99
Q9CPQ8	ATP synthase subunit g, mitochondrial OS=Mus musculus GN=Atp5l PE=1 SV=1 - [ATP5L_MOUSE]	63.11	3	7	8	116	0.633	1.142	1.069	1.118	328.27	63.11	14	116	103	11.4	9.74
Q9CRT8	Exportin-T OS=Mus musculus GN=Xpot PE=2 SV=3 - [XPOT_MOUSE]	9.14	1	8	8	30	0.995	1.289	0.902	1.118	76.41	9.14	14	30	963	109.7	5.25
O54791	Transcription factor MafF OS=Mus musculus GN=Maff PE=2 SV=1 - [MAFF_MOUSE]	4.49	3	1	1	2	0.908	0.467	1.066	1.118	4.99	4.49	2	2	156	16.9	9.80
Q8R015-2	Isoform 2 of Biogenesis of lysosome-related organelles complex 1 subunit 5 OS=Mus musculus GN=Bloc1s5 - [BL1S5_MOUSE]	13.61	2	2	2	2	1.293	1.246	1.000	1.118	4.77	13.61	2	2	169	18.8	6.87
Q8K3H0	DCC-interacting protein 13-alpha OS=Mus musculus GN=Appl1 PE=1 SV=1 - [DP13A_MOUSE]	47.95	1	25	27	131	0.834	1.264	1.141	1.118	430.41	47.95	46	131	707	79.3	5.41
Q8C4X2	Casein kinase I isoform gamma-3 OS=Mus musculus GN=Csnk1g3 PE=1 SV=2 -	36.32	1	5	12	26	0.708	0.578	0.979	1.118	102.31	36.32	18	26	424	48.9	9.11
O70370	Cathepsin S OS=Mus musculus GN=Ctss PE=2 SV=2 - [CATS_MOUSE]	22.35	2	5	5	11	1.318	1.340	1.631	1.119	31.16	22.35	8	11	340	38.4	6.96
Q5EBJ4	Ermin OS=Mus musculus GN=Ermn PE=1 SV=1 - [ERMIN_MOUSE]	53.02	1	12	13	243	2.826	0.441	1.432	1.119	679.78	53.02	22	243	281	32.1	4.59
Q8C7U7	Polypeptide N-acetylgalactosaminyltransferase 6 OS=Mus musculus GN=Galnt6 PE=2 SV=1 - [GALT6_MOUSE]	0.96	1	1	1	1	0.717	1.227	1.327	1.119	1.86	0.96	1	1	622	71.5	8.53
Q8BLQ9-3	Isoform 3 of Cell adhesion molecule 2 OS=Mus musculus GN=Cadm2 - [CADM2_MOUSE]	42.03	5	14	14	118	0.638	1.227	1.707	1.119	307.82	42.03	24	118	395	43.5	5.33
E9Q9S2	Centrosomal protein of 89 kDa OS=Mus musculus GN=Cep89 PE=2 SV=1 - [E9Q9S2_MOUSE]	2.19	2	1	1	1	1.141	0.897	1.252	1.119	3.00	2.19	1	1	639	73.0	8.19
Q3TLS3	GDP-D-glucose phosphorylase 1 OS=Mus musculus GN=Gdpgp1 PE=2 SV=2 -	25.65	1	8	8	32	0.710	1.091	1.157	1.119	92.47	25.65	16	32	386	42.5	6.19
A2ABY3	Ethanolamine-phosphate cytidylyltransferase OS=Mus musculus GN=Pcyt2 PE=4 SV=1 - [A2ABY3_MOUSE]	43.52	2	17	17	75	1.394	0.822	1.067	1.119	210.51	43.52	30	75	386	43.4	6.73
Q80US4-3	Isoform 3 of Actin-related protein 5 OS=Mus musculus GN=Actr5 - [ARP5_MOUSE]	12.03	2	2	2	8	0.689	1.000	0.854	1.119	19.23	12.03	3	8	266	28.9	6.86
F8WJ74	GRAM domain-containing protein 1A OS=Mus musculus GN=Gramd1a PE=2 SV=1 - [F8WJ74_MOUSE]	10.47	4	5	5	19	1.135	1.006	1.006	1.120	70.43	10.47	10	19	688	76.5	6.80
Q99K70	Ras-related GTP-binding protein C OS=Mus musculus GN=Rragc PE=2 SV=1 - [RRAGC_MOUSE]	26.63	2	6	8	25	1.014	0.985	1.057	1.120	81.92	26.63	15	25	398	44.1	5.10
E9QP44	Arf-GAP with Rho-GAP domain, ANK repeat and PH domain-containing protein 2 OS=Mus musculus GN=Arap2 PE=2 SV=1 - [E9QP44_MOUSE]	3.17	4	5	5	5	0.895	0.768	1.116	1.120	10.22	3.17	5	5	1703	193.3	7.17
Q3U7U3	F-box only protein 7 OS=Mus musculus GN=Fbxo7 PE=2 SV=2 - [FBX7_MOUSE]	12.24	5	4	4	14	1.285	1.184	1.186	1.120	35.33	12.24	8	14	523	57.6	5.83
Q9D967	Magnesium-dependent phosphatase 1 OS=Mus musculus GN=Mdp1 PE=1 SV=1 - [MGDP1_MOUSE]	47.56	1	7	7	24	1.109	1.200	0.989	1.120	90.82	47.56	13	24	164	18.6	6.80
Q9CQZ5	NADH dehydrogenase [ubiquinone] 1 alpha subcomplex subunit 6 OS=Mus musculus GN=Ndufa6 PE=1 SV=1 - [NDUA6_MOUSE]	44.27	1	6	6	37	0.467	0.978	1.037	1.120	101.45	44.27	10	37	131	15.3	10.11
F7BTZ2	Protein PML (Fragment) OS=Mus musculus GN=Pml PE=4 SV=1 - [F7BTZ2_MOUSE]	7.79	6	3	3	5	0.957	1.524	0.777	1.120	14.99	7.79	5	5	578	64.6	5.07
Q8C799	Uncharacterized protein (Fragment) OS=Mus musculus GN=D330020A13Rik PE=2 SV=1 - [Q8C799_MOUSE]	9.95	1	1	1	3	1.208	0.955	1.707	1.120	3.88	9.95	1	3	211	22.6	11.43

E9Q0D4	Protein Sbf2 OS=Mus musculus GN=Sbf2 PE=2 SV=1 - [E9Q0D4_MOUSE]	11.39	6	13	16	35	0.905	0.941	1.096	1.120	74.27	11.39	26	35	1826	204.9	7.37
Q6PDI6	Protein FAM63B OS=Mus musculus GN=Fam63b PE=1 SV=1 - [FA63B_MOUSE]	21.13	3	8	9	30	1.272	1.215	1.339	1.120	85.14	21.13	16	30	601	65.6	4.63
Q61703	Inter-alpha-trypsin inhibitor heavy chain H2 OS=Mus musculus GN=Itih2 PE=1 SV=1 - [ITIH2_MOUSE]	13.64	3	11	11	26	1.777	1.979	0.605	1.120	68.54	13.64	19	26	946	105.9	7.27
Q3UGR5	Haloacid dehalogenase-like hydrolase domain-containing protein 2 OS=Mus musculus GN=Hdhd2 PE=1 SV=2 - [HDHD2_MOUSE]	84.56	4	14	15	78	2.077	1.011	0.905	1.121	221.72	84.56	28	78	259	28.7	6.05
F6UII7	Poliovirus receptor-related protein 3 (Fragment) OS=Mus musculus GN=Pvrl3 PE=4 SV=1 - [F6UII7_MOUSE]	14.75	2	2	2	2	0.771	1.611	1.076	1.121	2.54	14.75	2	2	122	13.2	9.76
Q9EQ32-2	Isoform 2 of Phosphoinositide 3-kinase adapter protein 1 OS=Mus musculus GN=Pik3ap1 - [BCAP_MOUSE]	4.11	2	1	1	2	0.927	0.979	0.911	1.121	6.96	4.11	2	2	632	70.8	5.48
P02088	Hemoglobin subunit beta-1 OS=Mus musculus GN=Hbb-b1 PE=1 SV=2 - [HBB1_MOUSE]	85.03	1	3	14	2055	1.455	11.761	1.046	1.121	6554.71	85.03	28	2055	147	15.8	7.65
J3QPU9	Protein Zfp853 OS=Mus musculus GN=Zfp853 PE=4 SV=1 - [J3QPU9_MOUSE]	1.69	1	1	1	1	0.726	1.070	1.052	1.121	2.31	1.69	1	1	652	75.0	6.18
F6V3Y9	Autophagy-related protein 2 homolog A (Fragment) OS=Mus musculus GN=Atg2a PE=4 SV=1 - [F6V3Y9_MOUSE]	8.09	2	10	11	24	0.617	0.859	1.172	1.121	61.34	8.09	15	24	1718	188.6	6.20
Q99K46	Ubiquitin carboxyl-terminal hydrolase 11 OS=Mus musculus GN=Usp11 PE=2 SV=4 - [UBP11_MOUSE]	25.30	1	17	18	57	0.804	1.689	1.052	1.121	159.57	25.30	31	57	921	105.3	4.96
Q64332-2	Isoform IIb of Synapsin-2 OS=Mus musculus GN=Syn2 - [SYN2_MOUSE]	66.39	1	1	24	643	0.915	1.029	1.064	1.121	1990.22	66.39	45	643	479	52.4	7.72
G3X8U3	MCG6895 OS=Mus musculus GN=2210016F16Rik PE=4 SV=1 - [G3X8U3_MOUSE]	18.05	1	6	6	13	0.823	1.116	0.777	1.121	34.28	18.05	9	13	338	38.6	5.64
Q9WVS7-2	Isoform 2 of Dual specificity mitogen-activated protein kinase kinase 5 OS=Mus musculus GN=Map2k5 - [MP2K5_MOUSE]	20.05	4	6	6	20	0.883	1.031	1.057	1.121	57.41	20.05	12	20	439	49.1	6.20
Q6PB70	Anoctamin-8 OS=Mus musculus GN=Ano8 PE=2 SV=3 - [ANO8_MOUSE]	11.51	2	10	10	21	0.722	0.753	0.797	1.121	57.16	11.51	15	21	1060	119.0	5.74
Q80XQ2	TBC1 domain family member 5 OS=Mus musculus GN=Tbc1d5 PE=1 SV=2 -	24.66	1	14	14	42	1.006	0.797	1.222	1.121	123.85	24.66	24	42	815	91.8	6.79
Q9D720	Non-structural maintenance of chromosomes element 1 homolog OS=Mus musculus GN=Nsmce1 PE=2 SV=1	7.52	3	2	2	3	0.951	1.093	0.837	1.121	5.51	7.52	3	3	266	30.7	6.89
Q8R1X6	Spartin OS=Mus musculus GN=Spg20 PE=2 SV=1 - [SPG20_MOUSE]	29.81	5	18	19	56	1.205	0.863	0.975	1.121	154.05	29.81	32	56	671	72.6	5.86
E9Q6C8	XK-related protein 6 OS=Mus musculus GN=Xkr6 PE=2 SV=1 - [XKR6_MOUSE]	9.40	5	4	4	16	0.954	0.767	0.758	1.121	61.38	9.40	7	16	638	70.9	7.97
O55131	Septin-7 OS=Mus musculus GN=Sept7 PE=1 SV=1 - [SEPT7_MOUSE]	64.68	3	1	28	542	0.712	0.777	1.119	1.121	1637.52	64.68	51	542	436	50.5	8.57
P27600	Guanine nucleotide-binding protein subunit alpha-12 OS=Mus musculus GN=Gna12 PE=1 SV=3 - [GNA12_MOUSE]	26.12	3	9	11	99	0.596	0.438	0.779	1.121	246.86	26.12	20	99	379	44.1	9.83
Q8C6G1	Protein C21orf2 homolog OS=Mus musculus PE=2 SV=1 - [CU002_MOUSE]	18.07	2	4	4	13	1.058	1.156	1.026	1.121	35.50	18.07	8	13	249	28.2	6.93
Q8BGX2	Uncharacterized protein C19orf52 homolog OS=Mus musculus PE=2 SV=1 - [CS052_MOUSE]	23.31	1	4	4	19	0.878	1.258	0.971	1.122	48.30	23.31	8	19	266	29.4	6.73
Q9DCS9	NADH dehydrogenase [ubiquinone] 1 beta subcomplex subunit 10 OS=Mus musculus GN=Ndufb10 PE=1 SV=3 - [NDUBA_MOUSE]	49.43	2	9	9	75	1.401	1.362	1.136	1.122	227.14	49.43	18	75	176	21.0	8.03
Q80YD1	ATP-dependent RNA helicase SUPV3L1, mitochondrial OS=Mus musculus GN=Supv3l1 PE=2 SV=1 - [SUV3_MOUSE]	22.08	2	16	16	37	0.770	0.912	1.057	1.122	115.44	22.08	26	37	779	87.0	7.84

Q66JZ4	T-cell activation inhibitor, mitochondrial OS=Mus musculus GN=TCAIM PE=2 SV=1 - [TCAIM_MOUSE]	8.42	3	3	3	5	0.632	0.623	0.995	1.122	10.85	8.42	4	5	499	57.4	9.25
Q3UED7	Interferon-gamma-inducible GTPase Ifgga2 protein OS=Mus musculus GN=Gm4951 PE=2 SV=1 - [Q3UED7_MOUSE]	5.42	1	1	2	4	0.630	0.819	1.106	1.122	11.81	5.42	2	4	406	47.2	5.71
Q9CZ52	Anthrax toxin receptor 1 OS=Mus musculus GN=Antxr1 PE=2 SV=2 - [ANTR1_MOUSE]	6.94	2	3	3	8	1.117	0.752	0.661	1.122	20.51	6.94	5	8	562	62.3	7.61
Q8BLN6	Protein unc-80 homolog OS=Mus musculus GN=Unc80 PE=1 SV=2 - [UNC80_MOUSE]	8.31	5	17	18	37	0.767	0.923	0.895	1.122	99.56	8.31	29	37	3261	363.3	6.89
E9PXM6	Equilibrative nucleoside transporter 1 OS=Mus musculus GN=Slc29a1 PE=2 SV=1 -	7.82	11	3	3	12	0.698	0.802	0.905	1.122	36.40	7.82	6	12	358	38.9	7.15
P17751	Triosephosphate isomerase OS=Mus musculus GN=Tpi1 PE=1 SV=4 - [TPIS_MOUSE]	60.54	2	16	16	812	1.490	1.143	1.346	1.122	2513.86	60.54	31	812	299	32.2	5.74
P17012	Zinc finger X-chromosomal protein OS=Mus musculus GN=Zfx PE=1 SV=2 - [ZFX_MOUSE]	1.25	1	1	1	1	1.895	1.238	1.408	1.122	1.98	1.25	1	1	799	90.0	6.19
Q5DTZ0	Protein NYNRIN OS=Mus musculus GN=Nynrin PE=1 SV=2 - [NYNRI_MOUSE]	5.38	1	5	5	7	0.906	0.865	0.697	1.122	8.02	5.38	6	7	1840	202.9	6.99
Q3U0M1	Trafficking protein particle complex subunit 9 OS=Mus musculus GN=Trappc9 PE=1 SV=2 - [TPPC9_MOUSE]	21.95	7	26	26	72	0.599	1.068	0.948	1.122	214.75	21.95	46	72	1148	128.2	6.47
Q3U186	Probable arginine--tRNA ligase, mitochondrial OS=Mus musculus GN=Rars2 PE=2 SV=1 - [SYRM_MOUSE]	28.37	2	15	15	31	1.092	1.174	1.044	1.122	77.92	28.37	24	31	578	65.3	8.02
Q9EQJ0	Two pore calcium channel protein 1 OS=Mus musculus GN=Tpcn1 PE=2 SV=1 - [TPC1_MOUSE]	3.67	1	3	3	8	0.702	1.210	1.049	1.122	25.78	3.67	4	8	817	94.4	8.53
E9Q296	Acyl-coenzyme A oxidase OS=Mus musculus GN=Acox3 PE=2 SV=1 - [E9Q296_MOUSE]	17.31	1	1	10	24	0.949	0.589	0.660	1.122	54.19	17.31	16	24	728	81.2	6.86
Q9JIY0	Pleckstrin homology domain-containing family O member 1 OS=Mus musculus GN=Plekho1 PE=1 SV=1 - [PKHO1_MOUSE]	10.05	3	3	4	8	0.982	0.645	1.123	1.122	6.86	10.05	6	8	408	46.0	8.97
Q8C4G9	MCG21623, isoform CRA_b OS=Mus musculus GN=Gpr123 PE=2 SV=1 - [Q8C4G9_MOUSE]	7.61	1	4	4	16	0.797	0.948	1.024	1.123	35.10	7.61	7	16	578	63.5	7.43
Q8VE85	Protein slowmo homolog 1 OS=Mus musculus GN=Slmo1 PE=2 SV=1 - [SLMO1_MOUSE]	33.14	1	5	5	12	0.726	0.975	1.116	1.123	35.49	33.14	9	12	172	19.0	7.91
Q9JJA9	General receptor for phosphoinositides 1-associated scaffold protein OS=Mus musculus GN=Grasp PE=1 SV=2 - [GRASP_MOUSE]	20.41	1	4	5	8	1.128	3.013	2.699	1.123	26.31	20.41	6	8	392	42.3	9.10
Q8BHL5-2	Isoform 2 of Engulfment and cell motility protein 2 OS=Mus musculus GN=Elmo2 - [ELMO2_MOUSE]	31.67	6	15	21	78	0.900	1.666	1.257	1.123	222.51	31.67	36	78	720	82.5	5.96
E9QN52	Leucine-rich repeat flightless-interacting protein 2 OS=Mus musculus GN=Lrrfip2 PE=2 SV=1 - [E9QN52_MOUSE]	38.07	3	13	14	79	2.036	0.654	1.467	1.123	214.55	38.07	24	79	415	47.1	5.68
Q9JLV5	Cullin-3 OS=Mus musculus GN=Cul3 PE=1 SV=1 - [CUL3_MOUSE]	42.45	5	29	29	147	0.664	1.055	0.951	1.123	389.60	42.45	54	147	768	88.9	8.46
Q9JI59	Junctional adhesion molecule B OS=Mus musculus GN=Jam2 PE=1 SV=1 - [JAM2_MOUSE]	23.15	2	7	7	19	1.167	1.239	1.068	1.123	44.11	23.15	12	19	298	33.0	8.40
Q8CI75	DIS3-like exonuclease 2 OS=Mus musculus GN=Dis3l2 PE=1 SV=1 - [DI3L2_MOUSE]	18.97	2	16	17	48	0.892	0.908	1.166	1.123	155.83	18.97	31	48	870	97.7	5.90
Q8R1H0	Homeodomain-only protein OS=Mus musculus GN=Hopx PE=1 SV=1 - [HOP_MOUSE]	12.33	1	1	1	8	2.555	1.325	1.601	1.123	20.97	12.33	2	8	73	8.3	4.88
D3YUC0	DNA ligase (Fragment) OS=Mus musculus GN=Lig1 PE=3 SV=1 - [D3YUC0_MOUSE]	2.47	3	1	2	5	1.222	0.967	0.981	1.123	12.69	2.47	3	5	687	76.3	8.54
Q9QX96	Sal-like protein 2 OS=Mus musculus GN=Sall2 PE=2 SV=2 - [SALL2_MOUSE]	2.79	2	1	1	4	0.933	0.744	1.183	1.123	10.35	2.79	2	4	1004	104.9	6.28
Q8CCM6-2	Isoform 2 of Mitochondrial import inner membrane translocase subunit Tim21 OS=Mus musculus GN=Timm21 - [TIM21_MOUSE]	18.03	3	3	3	8	1.265	1.173	0.994	1.123	22.75	18.03	6	8	244	27.9	9.95

P0C605-2	Isoform Beta of cGMP-dependent protein kinase 1 OS=Mus musculus GN=Prkg1 -	13.56	1	3	7	22	0.689	2.371	0.919	1.123	59.87	13.56	12	22	686	77.7	5.44
Q62425	NADH dehydrogenase [ubiquinone] 1 alpha subcomplex subunit 4 OS=Mus musculus GN=Ndufa4 PE=1 SV=2 - [NDUA4_MOUSE]	41.46	1	5	5	93	0.801	0.979	1.058	1.123	217.88	41.46	9	93	82	9.3	9.52
Q9D273	Cob(I)yrinic acid a,c-diamide adenosyltransferase, mitochondrial OS=Mus musculus GN=Mmab PE=2 SV=1 - [MMAB_MOUSE]	59.07	3	11	11	35	1.428	1.315	1.026	1.123	103.96	59.07	21	35	237	26.3	9.20
Q8CFR5	Dystrobrevin alpha OS=Mus musculus GN=Dtna PE=2 SV=1 - [Q8CFR5_MOUSE]	32.40	9	12	16	96	0.870	1.168	1.142	1.123	289.95	32.40	30	96	682	76.9	6.89
E9QNP0	KxDL motif-containing protein 1 OS=Mus musculus GN=Kxd1 PE=2 SV=1 - [E9QNP0_MOUSE]	45.69	9	4	14	386	1.002	1.345	0.968	1.123	939.67	45.69	26	386	232	26.8	9.83
Q7TQI3	Ubiquitin thioesterase OTUB1 OS=Mus musculus GN=Otub1 PE=1 SV=2 - [OTUB1_MOUSE]	62.73	3	13	13	121	0.924	1.257	1.084	1.123	388.81	62.73	22	121	271	31.3	4.94
P22437	Prostaglandin G/H synthase 1 OS=Mus musculus GN=Ptgs1 PE=2 SV=1 - [PGH1_MOUSE]	14.45	1	6	7	15	1.256	1.570	1.101	1.124	33.83	14.45	11	15	602	69.0	6.83
F8WGM8	Actin, cytoplasmic 2 (Fragment) OS=Mus musculus GN=Actg1 PE=2 SV=1 - [F8WGM8_MOUSE]	87.62	1	1	8	650	2.620	2.775	1.096	1.124	2589.54	87.62	15	650	105	11.9	7.21
Q8R242	Di-N-acetylchitobiase OS=Mus musculus GN=Ctbs PE=2 SV=2 - [DIAC_MOUSE]	11.75	2	4	4	13	1.556	1.374	1.287	1.124	32.24	11.75	6	13	366	41.3	5.60
Q5SSM3	Rho GTPase-activating protein 44 OS=Mus musculus GN=Arhgap44 PE=2 SV=1 -	38.33	5	24	25	130	1.012	1.212	1.202	1.124	324.51	38.33	42	130	814	88.9	6.60
Q8VD24	Zinc finger CCHC domain-containing protein 18 OS=Mus musculus GN=Zcchc18 PE=2 SV=1 - [ZCC18_MOUSE]	10.18	1	3	3	5	0.963	1.085	0.805	1.124	19.74	10.18	5	5	393	43.9	5.38
Q04690-2	Isoform 1 of Neurofibromin OS=Mus musculus GN=Nf1 - [NF1_MOUSE]	28.55	5	66	67	207	0.601	0.840	0.896	1.124	576.68	28.55	113	207	2820	317.0	7.27
P60766	Cell division control protein 42 homolog OS=Mus musculus GN=Cdc42 PE=1 SV=2 - [CDC42_MOUSE]	53.40	7	1	7	121	1.970	1.012	1.145	1.124	286.53	53.40	13	121	191	21.2	6.55
P19788	Matrix Gla protein OS=Mus musculus GN=Mgp PE=2 SV=1 - [MGP_MOUSE]	10.58	1	1	1	3	1.056	1.056	1.192	1.124	7.06	10.58	2	3	104	12.4	9.55
Q9ERD7	Tubulin beta-3 chain OS=Mus musculus GN=Tubb3 PE=1 SV=1 - [TBB3_MOUSE]	77.33	1	12	28	3849	0.830	0.965	1.025	1.124	10640.96	77.33	52	3849	450	50.4	4.93
Q3UPE3-2	Isoform 2 of RecQ-mediated genome instability protein 2 OS=Mus musculus GN=Rmi2 - [RMI2_MOUSE]	4.83	2	1	1	3	1.023	0.868	1.339	1.124	6.16	4.83	2	3	145	15.4	10.76
Q8CB27	Ubiquitin thioesterase OTU1 OS=Mus musculus GN=Yod1 PE=1 SV=1 - [OTU1_MOUSE]	5.25	1	1	1	2	0.911	2.282	1.021	1.125	9.33	5.25	2	2	343	37.5	5.76
O09118	Netrin-1 OS=Mus musculus GN=Ntn1 PE=1 SV=3 - [NET1_MOUSE]	2.48	1	1	1	4	1.519	1.099	0.901	1.125	12.77	2.48	2	4	604	67.8	8.78
Q8BHA3	Probable D-tyrosyl-tRNA(Tyr) deacylase 2 OS=Mus musculus GN=Dtd2 PE=2 SV=1 - [DTD2_MOUSE]	28.57	2	4	4	10	2.116	1.165	1.123	1.125	20.28	28.57	6	10	168	18.2	7.91
Q9D6U8	Protein FAM162A OS=Mus musculus GN=Fam162a PE=2 SV=1 - [F162A_MOUSE]	32.90	1	6	6	19	1.035	1.318	1.105	1.125	71.23	32.90	10	19	155	17.7	9.88
E9QKX5	Vacuolar protein sorting-associated protein 13B OS=Mus musculus GN=Vps13b PE=4 SV=1 - [E9QKX5_MOUSE]	2.10	2	5	5	6	1.026	1.113	1.221	1.125	11.49	2.10	6	6	3993	443.7	6.46
Q6PDG8-2	Isoform 2 of Vacuolar fusion protein MON1 homolog A OS=Mus musculus GN=Mon1a - [MON1A_MOUSE]	19.31	2	6	6	23	0.990	1.156	1.146	1.125	74.84	19.31	11	23	461	51.1	6.06
P11531	Dystrophin OS=Mus musculus GN=Dmd PE=1 SV=3 - [DMD_MOUSE]	11.61	3	32	38	133	1.171	1.092	1.173	1.125	361.77	11.61	65	133	3678	425.6	5.94
Q66GT5	Phosphatidylglycerophosphatase and protein-tyrosine phosphatase 1 OS=Mus musculus GN=Ptpmt1 PE=1 SV=1 - [PTPM1_MOUSE]	25.39	2	4	4	12	0.812	0.937	1.119	1.125	38.21	25.39	7	12	193	21.9	9.72

Q8K3R3-2	Isoform 2 of 1-phosphatidylinositol 4,5-bisphosphate phosphodiesterase delta-4 OS=Mus musculus GN=Plcd4 - [PLCD4_MOUSE]	6.97	5	5	5	8	1.718	0.982	0.838	1.125	17.90	6.97	7	8	775	89.2	5.07
Q3UMF0-3	Isoform 3 of Cordon-bleu protein-like 1 OS=Mus musculus GN=Cobll1 - [COBL1_MOUSE]	22.88	4	1	20	74	1.607	0.628	1.215	1.125	226.96	22.88	34	74	1202	129.4	7.88
E9Q2I4	Uncharacterized protein OS=Mus musculus GN=Elmsan1 PE=4 SV=1 - [E9Q2I4_MOUSE]	2.66	1	2	2	5	1.167	1.113	1.227	1.125	16.12	2.66	3	5	1089	119.6	9.19
D3Z1B6	Regulator of G-protein-signaling 10 (Fragment) OS=Mus musculus GN=Rgs10 PE=2 SV=1 - [D3Z1B6_MOUSE]	38.82	1	1	2	7	1.873	1.411	1.555	1.125	27.96	38.82	4	7	85	9.7	4.91
Q5PR72	Phosphodiesterase 2A, cGMP-stimulated OS=Mus musculus GN=Pde2a PE=2 SV=1 - [Q5PR72_MOUSE]	47.91	3	38	38	174	0.492	1.866	1.448	1.125	489.83	47.91	64	174	935	105.2	5.41
Q8BHN7-2	Isoform 2 of Uncharacterized protein C12orf29 homolog OS=Mus musculus -	11.72	2	3	3	3	1.184	1.019	0.974	1.125	7.45	11.72	3	3	273	31.0	7.49
B7ZCR6	Protein Znf512b OS=Mus musculus GN=Znf512b PE=2 SV=1 - [B7ZCR6_MOUSE]	4.03	3	2	3	6	2.568	1.037	1.478	1.125	5.54	4.03	4	6	869	95.3	9.77
G3XA50	MCG51226 OS=Mus musculus GN=Lrrc10b PE=4 SV=1 - [G3XA50_MOUSE]	6.51	1	1	2	2	1.488	1.133	2.395	1.126	4.80	6.51	2	2	292	33.0	7.36
Q3UP23	Transmembrane protein 26 OS=Mus musculus GN=Tmem26 PE=2 SV=2 - [TMM26_MOUSE]	3.55	1	1	1	1	0.822	1.522	1.016	1.126	0.00	3.55	1	1	366	41.6	7.58
Q61738-4	Isoform Alpha-7X2A of Integrin alpha-7 OS=Mus musculus GN=Itga7 - [ITA7_MOUSE]	9.95	7	11	11	27	1.006	1.651	0.952	1.126	54.76	9.95	18	27	1116	122.1	6.00
Q6PHS6	Sorting nexin-13 OS=Mus musculus GN=Snx13 PE=2 SV=1 - [SNX13_MOUSE]	7.94	3	7	7	14	0.623	0.933	0.883	1.126	46.40	7.94	12	14	957	110.7	6.62
E0CYK3	Protein RRNAD1 OS=Mus musculus GN=Rrnad1 PE=2 SV=1 - [E0CYK3_MOUSE]	27.80	1	2	3	3	0.723	0.643	0.921	1.126	2.53	27.80	3	3	277	30.1	9.16
Q8VC30	Bifunctional ATP-dependent dihydroxyacetone kinase/FAD-AMP lyase (cyclizing) OS=Mus musculus GN=Dak PE=2 SV=1 - [DHAK_MOUSE]	36.33	1	15	15	52	0.697	0.848	0.963	1.126	141.12	36.33	26	52	578	59.7	6.92
Q3TZU5	Protein kinase inhibitor beta, cAMP dependent, testis specific, isoform CRA_c OS=Mus musculus GN=Pkib PE=4 SV=1 - [illegible]	53.85	4	2	2	5	1.646	1.548	1.550	1.126	19.42	53.85	3	5	78	8.3	5.15
Q80X85	28S ribosomal protein S7, mitochondrial OS=Mus musculus GN=Mrps7 PE=2 SV=1 - [RT07_MOUSE]	14.88	1	3	3	9	0.585	0.904	0.936	1.126	33.73	14.88	6	9	242	28.0	9.94
Q8BP47	Asparagine--tRNA ligase, cytoplasmic OS=Mus musculus GN=Nars PE=1 SV=2 - [SYNC_MOUSE]	39.00	1	19	19	105	0.694	0.691	0.954	1.126	257.96	39.00	35	105	559	64.2	5.86
Q8BFV2	PCI domain-containing protein 2 OS=Mus musculus GN=Pcid2 PE=2 SV=1 - [PCID2_MOUSE]	20.30	3	7	7	13	1.143	1.012	1.153	1.126	28.36	20.30	11	13	399	46.1	8.53
P59279	Ras-related protein Rab-2B OS=Mus musculus GN=Rab2b PE=2 SV=1 - [RAB2B_MOUSE]	62.50	4	4	11	81	1.373	1.141	1.046	1.127	269.31	62.50	21	81	216	24.2	6.68
Q9DCT1	1,5-anhydro-D-fructose reductase OS=Mus musculus GN=Akr1e2 PE=1 SV=1 -	42.19	2	12	13	46	0.849	1.088	1.081	1.127	110.84	42.19	23	46	301	34.4	7.33
Q6P9N1	Hyccin OS=Mus musculus GN=Fam126a PE=1 SV=2 [HYCCI_MOUSE]	13.05	3	5	5	38	1.028	1.111	1.084	1.127	19.01	13.05	8	38	521	57.3	7.88
Q6DFW0	Protein C9orf72 homolog OS=Mus musculus PE=2 SV=1 - [CI072_MOUSE]	7.38	3	4	4	10	0.572	1.111	0.892	1.127	24.00	7.38	6	10	420	47.3	5.67
Q8K3V7	UPF0258 protein KIAA1024 OS=Mus musculus GN=Kiaa1024 PE=2 SV=1 -	4.47	2	3	4	14	1.512	0.778	0.887	1.127	27.59	4.47	7	14	917	102.7	7.24
Q9D0P0	Emopamil-binding protein-like OS=Mus musculus GN=Ebpl PE=2 SV=1 - [EBPL_MOUSE]	4.37	1	1	1	1	4.146	1.108	1.399	1.127	2.71	4.37	1	1	206	23.3	6.89
Q8R0K4	Coiled-coil domain-containing protein 137 OS=Mus musculus GN=Ccdc137 PE=2 SV=1 - [CC137_MOUSE]	7.59	1	1	2	2	9.261	0.560	1.570	1.127	5.62	7.59	2	2	290	32.9	10.68
P63254	Cysteine-rich protein 1 OS=Mus musculus GN=Crip1 PE=2 SV=2 - [CRIP1_MOUSE]	32.47	1	3	4	20	1.266	2.989	0.987	1.127	47.92	32.47	7	20	77	8.5	8.57

Q9D0I4	Syntaxin-17 OS=Mus musculus GN=Stx17 PE=1 SV=1 - [STX17_MOUSE]	15.28	2	1	5	14	1.341	0.905	0.915	1.127	38.76	15.28	8	14	301	33.2	6.74
Q9CQ62	2,4-dienoyl-CoA reductase, mitochondrial OS=Mus musculus GN=Decr1 PE=1 SV=1 - [DECR_MOUSE]	29.25	1	10	10	55	1.050	1.081	0.903	1.127	175.97	29.25	18	55	335	36.2	8.95
Q9Z1W9	STE20/SPS1-related proline-alanine-rich protein kinase OS=Mus musculus GN=Stk39 PE=1 SV=1 - [STK39_MOUSE]	42.81	2	16	18	67	1.000	0.600	0.894	1.127	188.12	42.81	28	67	556	60.3	6.29
E9PWX7	TBC1 domain family member 25 OS=Mus musculus GN=Tbc1d25 PE=2 SV=1 -	1.52	2	1	1	2	1.135	1.265	0.940	1.127	7.24	1.52	2	2	723	80.2	6.37
Q9EQC5	N-terminal kinase-like protein OS=Mus musculus GN=Scyl1 PE=1 SV=1 - [NTKL_MOUSE]	20.47	1	13	13	35	0.779	0.841	1.003	1.128	104.58	20.47	21	35	806	89.1	6.44
P07934	Phosphorylase b kinase gamma catalytic chain, skeletal muscle/heart isoform OS=Mus musculus GN=Phkg1 PE=2 SV=3 - [PHKG1_MOUSE]	27.58	3	9	10	17	1.182	1.190	0.927	1.128	46.75	27.58	14	17	388	44.9	6.55
P56473	Agouti-related protein OS=Mus musculus GN=Agrp PE=1 SV=1 - [AGRP_MOUSE]	38.93	1	4	4	21	2.000	1.030	0.440	1.128	69.02	38.93	6	21	131	14.4	8.29
A2AAE1-6	Isoform 6 of Uncharacterized protein KIAA1109 OS=Mus musculus GN=Kiaa1109 - [K1109_MOUSE]	7.26	10	31	32	64	1.016	1.026	1.144	1.128	162.83	7.26	47	64	4970	551.5	6.60
P83093	Stromal interaction molecule 2 OS=Mus musculus GN=Stim2 PE=1 SV=2 - [STIM2_MOUSE]	21.98	3	13	13	41	0.839	1.147	1.467	1.128	133.11	21.98	21	41	746	83.9	6.79
O70325-2	Isoform Cytoplasmic of Phospholipid hydroperoxide glutathione peroxidase, mitochondrial OS=Mus musculus GN=Gpx4 - [GPX41_MOUSE]	55.29	4	10	10	53	0.996	0.818	1.091	1.128	140.90	55.29	18	53	170	19.5	8.05
Q9R1V4	Disintegrin and metalloproteinase domain-containing protein 11 OS=Mus musculus GN=Adam11 PE=1 SV=2 - [ADA11_MOUSE]	23.16	2	12	12	47	0.579	0.997	1.186	1.128	169.12	23.16	23	47	773	84.1	7.50
Q9ERZ4	Muscarinic acetylcholine receptor M2 OS=Mus musculus GN=Chrm2 PE=1 SV=2 -	12.66	1	3	3	26	1.142	0.403	1.070	1.128	81.95	12.66	5	26	466	51.5	8.92
Q9D920	Loss of heterozygosity 12 chromosomal region 1 protein homolog OS=Mus musculus GN=Loh12cr1 PE=2 SV=1 - [L12R1_MOUSE]	51.28	2	9	9	36	1.294	1.225	1.003	1.128	108.88	51.28	16	36	195	22.1	6.55
E9PXB7	E3 ubiquitin-protein ligase NEDD4-like OS=Mus musculus GN=Nedd4l PE=2 SV=1 -	28.18	5	18	23	62	0.642	1.179	1.224	1.128	196.86	28.18	37	62	976	112.1	5.87
Q8BL06	Inactive ubiquitin carboxyl-terminal hydrolase 54 OS=Mus musculus GN=Usp54 PE=1 SV=2 - [UBP54_MOUSE]	4.85	3	5	5	8	0.945	0.693	1.099	1.128	18.11	4.85	7	8	1588	176.6	7.62
P70303	CTP synthase 2 OS=Mus musculus GN=Ctps2 PE=1 SV=1 - [PYRG2_MOUSE]	32.94	4	13	16	56	0.746	1.030	0.781	1.128	149.11	32.94	27	56	586	65.5	6.49
P84075	Neuron-specific calcium-binding protein hippocalcin OS=Mus musculus GN=Hpca PE=1 SV=2 - [HPCA_MOUSE]	66.84	4	5	14	241	0.799	0.978	2.211	1.129	637.82	66.84	26	241	193	22.4	4.97
Q8CAQ8	Mitochondrial inner membrane protein OS=Mus musculus GN=Immt PE=1 SV=1 - [IMMT_MOUSE]	69.48	2	2	53	307	1.047	1.174	1.274	1.129	933.41	69.48	95	307	757	83.8	6.61
O54834-2	Isoform 2 of Rho GTPase-activating protein 6 OS=Mus musculus GN=Arhgap6 -	15.76	7	7	7	16	0.867	2.165	1.060	1.129	45.36	15.76	12	16	660	72.7	8.88
Q6ZPF4-2	Isoform 2 of Formin-like protein 3 OS=Mus musculus GN=Fmnl3 - [FMNL3_MOUSE]	6.45	3	2	7	19	1.199	2.393	1.253	1.129	49.07	6.45	11	19	976	111.2	6.92
Q2PZL6	Protocadherin Fat 4 OS=Mus musculus GN=Fat4 PE=1 SV=2 - [FAT4_MOUSE]	1.53	2	4	4	4	14.817	1.138	1.402	1.129	6.38	1.53	4	4	4981	540.0	4.91
Q8C8R3-7	Isoform 7 of Ankyrin-2 OS=Mus musculus GN=Ank2 - [ANK2_MOUSE]	61.89	2	2	23	246	0.512	0.957	1.267	1.129	600.48	61.89	41	246	454	49.5	9.48
Q9DCN1	Peroxisomal NADH pyrophosphatase NUDT12 OS=Mus musculus GN=Nudt12 PE=2 SV=1 - [NUD12_MOUSE]	13.42	2	5	5	8	0.750	0.805	0.807	1.129	24.39	13.42	8	8	462	51.5	7.12

Q9D0J4	ADP-ribosylation factor-like protein 2 OS=Mus musculus GN=Arl2 PE=1 SV=1 - [ARL2_MOUSE]	26.63	1	4	4	12	1.046	0.712	0.740	1.129	39.16	26.63	7	12	184	20.9	5.96
Q8VGW0	MCG53396 OS=Mus musculus GN=Olfr584 PE=2 SV=1 - [Q8VGW0_MOUSE]	11.60	1	2	2	3	0.993	0.755	1.935	1.129	2.75	11.60	2	3	319	36.0	9.29
G5E8F4	MCG140999 OS=Mus musculus GN=Fpgt PE=4 SV=1 - [G5E8F4_MOUSE]	5.08	1	1	3	6	1.352	1.012	0.854	1.129	14.70	5.08	4	6	590	65.3	6.48
O08529	Calpain-2 catalytic subunit OS=Mus musculus GN=Capn2 PE=2 SV=4 - [CAN2_MOUSE]	38.86	2	19	19	93	1.028	0.905	0.924	1.129	274.41	38.86	32	93	700	79.8	4.96
Q8VDQ1	Prostaglandin reductase 2 OS=Mus musculus GN=Ptgr2 PE=1 SV=2 - [PTGR2_MOUSE]	37.04	4	12	12	56	1.375	0.896	0.979	1.129	166.78	37.04	18	56	351	38.0	5.41
O88544	COP9 signalosome complex subunit 4 OS=Mus musculus GN=Cops4 PE=1 SV=1 -	72.91	5	24	25	172	1.132	1.272	0.955	1.129	537.32	72.91	44	172	406	46.3	5.83
Q8R1I1	Cytochrome b-c1 complex subunit 9 OS=Mus musculus GN=Uqcr10 PE=1 SV=1 -	26.56	1	1	1	20	0.974	0.929	1.100	1.129	72.84	26.56	2	20	64	7.4	9.19
E9PVD3	Protein Dchs1 OS=Mus musculus GN=Dchs1 PE=2 SV=1 - [E9PVD3_MOUSE]	6.62	3	13	13	31	0.983	0.857	0.849	1.129	96.68	6.62	21	31	3291	346.2	5.00
E9QKK8	Probable phospholipid-transporting ATPase 11C OS=Mus musculus GN=Atp11c PE=2 SV=1 - [E9QKK8_MOUSE]	9.50	4	6	7	13	1.316	1.060	1.003	1.130	46.87	9.50	10	13	1116	127.7	7.05
Q91VK2	Eef1d protein OS=Mus musculus GN=Eef1d PE=2 SV=1 - [Q91VK2_MOUSE]	79.71	2	1	18	171	0.976	1.054	1.084	1.130	529.31	79.71	32	171	276	30.6	4.98
Q8JZW8	Paraneoplastic antigen Ma3 homolog OS=Mus musculus GN=Pnma3 PE=2 SV=1 - [PNMA3_MOUSE]	6.87	1	3	3	8	0.934	0.754	0.805	1.130	18.71	6.87	6	8	466	54.0	9.25
Q6ZWV7	60S ribosomal protein L35 OS=Mus musculus GN=Rpl35 PE=2 SV=1 - [RL35_MOUSE]	40.65	4	5	7	14	0.816	0.736	0.872	1.130	35.98	40.65	9	14	123	14.5	11.05
P51863	V-type proton ATPase subunit d 1 OS=Mus musculus GN=Atp6v0d1 PE=1 SV=2 -	49.00	1	15	15	81	0.531	1.422	1.150	1.130	211.88	49.00	25	81	351	40.3	5.00
Q501P1	Fibulin-7 OS=Mus musculus GN=Fbln7 PE=1 SV=1 - [FBLN7_MOUSE]	2.95	1	1	2	2	1.210	1.462	1.343	1.130	4.33	2.95	2	2	440	47.9	7.83
F7CZ64	Voltage-dependent calcium channel gamma-8 subunit OS=Mus musculus GN=Cacng8 PE=4 SV=2 - [F7CZ64_MOUSE]	30.02	2	6	6	62	0.620	2.242	1.530	1.130	232.65	30.02	11	62	423	43.4	9.20
Q9JIG4	Protein phosphatase 1 regulatory subunit 3F OS=Mus musculus GN=Ppp1r3f PE=1 SV=3 - [PPR3F_MOUSE]	33.17	5	15	15	40	0.717	0.918	0.943	1.130	93.41	33.17	25	40	799	84.1	4.70
A2AH25	Rho GTPase-activating protein 1 OS=Mus musculus GN=Arhgap1 PE=2 SV=1 -	56.16	2	19	20	95	0.663	1.366	1.029	1.130	291.69	56.16	36	95	479	54.4	5.87
Q8C353	Transmembrane protein 252 OS=Mus musculus GN=Tmem252 PE=2 SV=1 - [TM252_MOUSE]	8.74	1	1	1	1	0.946	0.985	1.444	1.130	0.00	8.74	1	1	183	20.4	4.82
Q8QZV4	Serine/threonine-protein kinase 32C OS=Mus musculus GN=Stk32c PE=2 SV=1 -	16.39	2	7	8	74	0.860	1.359	1.527	1.130	185.63	16.39	14	74	488	55.2	6.16
E9Q3A7	Protein Cdh19 OS=Mus musculus GN=Cdh19 PE=3 SV=1 - [E9Q3A7_MOUSE]	10.26	1	5	6	11	1.481	0.833	1.254	1.130	23.82	10.26	7	11	770	87.2	4.70
P58802	TBC1 domain family member 10A OS=Mus musculus GN=Tbc1d10a PE=1 SV=1 -	24.80	2	10	10	31	0.800	0.969	1.011	1.130	77.59	24.80	17	31	500	56.2	7.85
Q8BG39	Synaptic vesicle glycoprotein 2B OS=Mus musculus GN=Sv2b PE=1 SV=1 - [SV2B_MOUSE]	15.52	1	10	11	119	1.297	2.071	1.809	1.131	374.32	15.52	19	119	683	77.4	5.57
Q9WV02	RNA-binding motif protein, X chromosome OS=Mus musculus GN=Rbmx PE=1 SV=1 - [RBMX_MOUSE]	35.55	4	2	14	91	1.605	0.934	1.383	1.131	235.41	35.55	26	91	391	42.3	10.05
Q8BX02	KN motif and ankyrin repeat domain-containing protein 2 OS=Mus musculus GN=Kank2 PE=1 SV=1 - [KANK2_MOUSE]	11.15	2	6	6	22	0.633	2.329	0.935	1.131	42.42	11.15	9	22	843	90.2	5.55
Q8BH55	Threonine synthase-like 1 OS=Mus musculus GN=Thnsl1 PE=2 SV=1 - [THNS1_MOUSE]	35.74	6	21	22	83	1.012	1.146	0.950	1.131	254.46	35.74	37	83	747	83.0	7.20
Q9DCI3	MLN64 N-terminal domain homolog OS=Mus musculus GN=Stard3nl PE=1 SV=2 -	9.79	1	2	2	8	1.339	1.058	0.921	1.131	23.42	9.79	4	8	235	26.8	5.02

Q9QZD5	Rab proteins geranylgeranyltransferase component A 2 OS=Mus musculus GN=Chml PE=2 SV=2 - [RAE2_MOUSE]	2.09	1	1	1	2	1.312	0.435	0.870	1.131	5.64	2.09	2	2	621	70.0	5.19
P62814	V-type proton ATPase subunit B, brain isoform OS=Mus musculus GN=Atp6v1b2 PE=1 SV=1 [VATB2_MOUSE]	86.89	2	34	34	784	0.873	1.049	1.130	1.131	2445.46	86.89	62	784	511	56.5	5.81
E9Q5J6	Protein A730017C20Rik OS=Mus musculus GN=A730017C20Rik PE=2 SV=1 - [E9Q5J6_MOUSE]	36.27	3	4	4	10	1.433	1.065	0.849	1.131	30.83	36.27	4	10	193	21.8	9.36
A2AIH8	Pirin (Fragment) OS=Mus musculus GN=Pir PE=2 SV=1 - [A2AIH8_MOUSE]	22.13	2	5	5	8	1.085	1.258	1.333	1.131	17.67	22.13	7	8	253	27.9	6.92
A6X919	Probable C-mannosyltransferase DPY19L1 OS=Mus musculus GN=Dpy19l1 PE=2 SV=1 - [illegible]	11.26	3	5	5	12	0.727	0.540	0.846	1.132	38.37	11.26	7	12	746	84.1	9.16
Q80VP9	Aspartate beta-hydroxylase domain-containing protein 2 OS=Mus musculus GN=Asphd2 PE=2 SV=1 - [illegible]	14.29	3	4	5	12	0.732	1.181	1.309	1.132	33.07	14.29	7	12	343	38.7	7.05
Q9DCS2	UPF0585 protein C16orf13 homolog OS=Mus musculus PE=1 SV=1 - [CP013_MOUSE]	48.04	5	9	9	28	0.738	0.989	0.785	1.132	68.27	48.04	15	28	204	22.7	6.52
F6WVB0	Ephrin type-A receptor 10 (Fragment) OS=Mus musculus GN=Epha10 PE=4 SV=1 - [F6WVB0_MOUSE]	17.52	3	7	8	12	0.936	0.931	1.289	1.132	31.51	17.52	9	12	862	93.1	7.08
P35330	Intercellular adhesion molecule 2 OS=Mus musculus GN=Icam2 PE=1 SV=1 - [ICAM2_MOUSE]	19.86	4	4	4	9	1.144	1.252	0.922	1.132	21.18	19.86	7	9	277	31.4	7.91
Q61151	Serine/threonine-protein phosphatase 2A 56 kDa regulatory subunit epsilon isoform OS=Mus musculus GN=Ppp2r5e PE=2 SV=3 - [2A5E_MOUSE]	34.26	1	10	16	54	0.598	0.849	1.026	1.132	178.28	34.26	27	54	467	54.7	6.95
Q8BUM6	Protein FAM163B OS=Mus musculus GN=Fam163b PE=1 SV=1 - [F163B_MOUSE]	41.32	1	3	3	17	2.670	1.475	1.952	1.132	57.81	41.32	5	17	167	18.3	5.15
Q8BFP9	[Pyruvate dehydrogenase [lipoamide]] kinase isozyme 1, mitochondrial OS=Mus musculus GN=Pdk1 PE=2 SV=2 - [PDK1_MOUSE]	33.18	1	9	12	38	0.726	1.386	1.012	1.132	101.82	33.18	19	38	434	49.0	8.19
A2A5R2	Brefeldin A-inhibited guanine nucleotide-exchange protein 2 OS=Mus musculus GN=Arfgef2 PE=1 SV=1 - [illegible]	29.69	2	30	44	122	0.812	1.010	1.101	1.132	361.38	29.69	72	122	1792	202.1	6.55
Q8VCT3	Aminopeptidase B OS=Mus musculus GN=Rnpep PE=2 SV=2 - [AMPB_MOUSE]	36.15	2	21	21	103	0.734	1.260	1.128	1.133	294.77	36.15	40	103	650	72.4	5.35
Q80U87	Ubiquitin carboxyl-terminal hydrolase 8 OS=Mus musculus GN=Usp8 PE=1 SV=2 - [UBP8_MOUSE]	20.93	2	19	20	68	1.448	0.747	1.076	1.133	192.65	20.93	33	68	1080	122.5	8.47
Q9CQF4	Uncharacterized protein C6orf203 homolog OS=Mus musculus PE=1 SV=1 - [CF203_MOUSE]	27.92	1	3	9	19	2.973	1.131	1.189	1.133	39.04	27.92	12	19	240	27.8	9.36
Q8BGY2	Eukaryotic translation initiation factor 5A-2 OS=Mus musculus GN=Eif5a2 PE=2 SV=3 - [IF5A2_MOUSE]	50.98	1	3	9	94	3.291	1.402	1.087	1.133	276.96	50.98	15	94	153	16.8	5.58
Q91WK7	Ankyrin repeat domain-containing protein 54 OS=Mus musculus GN=Ankrd54 PE=1 SV=1 - [ANR54_MOUSE]	26.42	5	6	6	9	0.839	0.794	0.910	1.133	18.50	26.42	9	9	299	32.5	6.68
P11798	Calcium/calmodulin-dependent protein kinase type II subunit alpha OS=Mus musculus GN=Camk2a PE=1 SV=2 - [illegible]	53.35	6	16	25	621	0.625	1.978	1.675	1.133	1668.22	53.35	46	621	478	54.1	7.08
Q9D2V5	Protein AAR2 homolog OS=Mus musculus GN=Aar2 PE=2 SV=3 - [AAR2_MOUSE]	8.59	1	2	2	4	1.037	0.807	0.929	1.133	14.16	8.59	4	4	384	43.4	5.48
Q5DU14-3	Isoform 3 of Unconventional myosin-XVI OS=Mus musculus GN=Myo16 -	3.38	4	4	6	10	2.639	1.855	1.516	1.133	22.69	3.38	8	10	1863	205.0	7.21
A2ARP1-3	Isoform 3 of Inositol hexakisphosphate and diphosphoinositol-pentakisphosphate kinase 1 OS=Mus musculus GN=Ppip5k1 - [VIP1_MOUSE]	21.98	11	19	26	55	0.687	0.896	0.959	1.133	144.73	21.98	42	55	1415	157.5	5.33

Q9CZU6	Citrate synthase, mitochondrial OS=Mus musculus GN=Cs PE=1 SV=1 - [CISY_MOUSE]	51.08	1	13	21	406	0.672	1.074	1.070	1.133	1048.36	51.08	39	406	464	51.7	8.57
O35710-2	Isoform 2 of Nocturnin OS=Mus musculus GN=Ccrn4l - [NOCT_MOUSE]	9.86	2	3	3	8	0.520	1.130	1.055	1.133	21.30	9.86	4	8	365	41.5	5.87
P97950	Ras-related protein Rab-33A OS=Mus musculus GN=Rab33a PE=2 SV=1 - [RB33A_MOUSE]	32.07	1	5	7	17	1.152	1.525	0.969	1.133	52.94	32.07	13	17	237	26.5	7.88
P20152	Vimentin OS=Mus musculus GN=Vim PE=1 SV=3 - [VIME_MOUSE]	61.59	3	23	30	404	1.015	2.285	0.721	1.133	1063.94	61.59	57	404	466	53.7	5.12
O54890	Integrin beta-3 OS=Mus musculus GN=Itgb3 PE=1 SV=2 - [ITB3_MOUSE]	8.51	1	6	6	14	1.265	1.647	0.841	1.134	43.46	8.51	10	14	787	86.7	5.24
Q3UHF3-2	Isoform 2 of Mesoderm induction early response protein 3 OS=Mus musculus GN=Mier3 - [MIER3_MOUSE]	13.58	3	3	3	8	0.996	2.021	1.247	1.134	2.23	13.58	3	8	523	58.6	4.64
Q8CF60	Protein Zfp263 OS=Mus musculus GN=Zfp263 PE=2 SV=1 - [Q8CF60_MOUSE]	5.74	2	2	3	3	1.770	1.063	0.999	1.134	5.49	5.74	3	3	680	77.5	6.87
Q5RKN9	Capping protein (Actin filament) muscle Z-line, alpha 1 OS=Mus musculus GN=Capza1 PE=2 SV=1 - [Q5RKN9_MOUSE]	54.20	1	1	11	117	1.631	1.161	1.412	1.134	339.85	54.20	20	117	286	32.9	5.55
Q8R179	Kelch repeat and BTB domain-containing protein 4 OS=Mus musculus GN=Kbtbd4 PE=2 SV=1 - [KBTB4_MOUSE]	8.80	2	3	4	5	1.130	1.143	0.887	1.134	12.50	8.80	5	5	534	59.8	5.74
O88962	7-alpha-hydroxycholest-4-en-3-one 12-alpha-hydroxylase OS=Mus musculus GN=Cyp8b1 PE=2 SV=1 - [illegible]	1.80	1	1	1	2	1.240	1.953	1.785	1.134	6.73	1.80	1	2	500	57.7	8.84
Q99NF1	Beta,beta-carotene 9',10'-oxygenase OS=Mus musculus GN=Bco2 PE=1 SV=1 - [BCDO2_MOUSE]	8.27	1	2	2	2	1.940	2.441	0.760	1.134	0.00	8.27	2	2	532	60.1	6.54
Q7TNE3	Sperm-associated antigen 7 OS=Mus musculus GN=Spag7 PE=1 SV=1 - [SPAG7_MOUSE]	28.19	2	6	6	20	1.983	1.038	1.293	1.134	55.93	28.19	11	20	227	25.9	7.43
Q6PCN3	Tau-tubulin kinase 1 OS=Mus musculus GN=Ttbk1 PE=2 SV=3 - [TTBK1_MOUSE]	20.41	1	18	20	63	1.538	1.030	1.503	1.134	153.16	20.41	26	63	1308	141.5	5.62
A2AKD7	Alpha-1-syntrophin OS=Mus musculus GN=Snta1 PE=2 SV=1 - [A2AKD7_MOUSE]	41.88	2	16	17	54	0.716	1.025	1.022	1.134	156.63	41.88	29	54	499	53.2	6.80
Q9ESL4-2	Isoform 2 of Mitogen-activated protein kinase kinase kinase MLT OS=Mus musculus GN=Mltk - [MLTK_MOUSE]	10.35	3	1	4	10	1.196	1.182	0.897	1.134	39.13	10.35	6	10	454	51.3	5.35
Q60710	Deoxynucleoside triphosphate triphosphohydrolase SAMHD1 OS=Mus musculus GN=Samhd1 [illegible]	30.94	6	16	16	29	0.798	1.099	0.937	1.134	71.38	30.94	25	29	627	72.6	7.96
Q307W7	Kinocilin OS=Mus musculus GN=Kncn PE=1 SV=1 - [KNCN_MOUSE]	6.45	1	1	1	2	0.913	1.045	1.031	1.134	4.56	6.45	1	2	124	12.8	9.41
E9Q9I2	Protein Dlg5 OS=Mus musculus GN=Dlg5 PE=2 SV=1 - [E9Q9I2_MOUSE]	7.90	4	11	11	16	1.151	0.925	1.294	1.135	39.38	7.90	16	16	1898	212.0	7.52
P36916	Guanine nucleotide-binding protein-like 1 OS=Mus musculus GN=Gnl1 PE=1 SV=4 - [illegible]	22.57	2	13	13	46	0.633	0.921	1.055	1.135	147.50	22.57	23	46	607	68.7	5.68
Q99JR1	Sideroflexin-1 OS=Mus musculus GN=Sfxn1 PE=1 SV=3 - [SFXN1_MOUSE]	46.58	1	11	13	120	0.699	1.126	0.861	1.135	319.54	46.58	23	120	322	35.6	9.23
Q8BVY0	Protein Rsl1d1 OS=Mus musculus GN=Rsl1d1 PE=2 SV=1 - [Q8BVY0_MOUSE]	16.59	1	6	6	9	0.769	1.010	0.862	1.135	17.26	16.59	8	9	452	50.4	9.98
P56942	Pro-MCH OS=Mus musculus GN=Pmch PE=1 SV=2 - [MCH_MOUSE]	36.97	1	6	6	42	1.700	0.330	1.063	1.135	139.04	36.97	10	42	165	18.5	7.14
Q8CD92-2	Isoform 2 of Tetratricopeptide repeat protein 27 OS=Mus musculus GN=Ttc27 - [TTC27_MOUSE]	6.38	2	5	5	10	0.687	1.113	1.006	1.135	22.54	6.38	9	10	846	96.2	5.77
Q8CBX0	Transmembrane protein 63C OS=Mus musculus GN=Tmem63c PE=2 SV=1 - [TM63C_MOUSE]	7.48	1	5	5	11	0.885	1.300	0.936	1.135	22.98	7.48	8	11	802	93.0	7.77
P48036	Annexin A5 OS=Mus musculus GN=Anxa5 PE=1 SV=1 - [ANXA5_MOUSE]	64.58	1	20	20	210	1.894	1.517	0.795	1.135	634.17	64.58	35	210	319	35.7	4.96

P70349	Histidine triad nucleotide-binding protein 1 OS=Mus musculus GN=Hint1 PE=1 SV=3 - [HINT1_MOUSE]	78.57	2	7	7	61	1.317	0.989	1.255	1.135	222.41	78.57	14	61	126	13.8	6.87
P27808	Alpha-1,3-mannosyl-glycoprotein 2-beta-N-acetylglucosaminyltransferase OS=Mus musculus GN=Mgat1 PE=2 SV=1 - [MGAT1_MOUSE]	2.68	1	1	1	2	1.518	0.455	1.476	1.135	5.65	2.68	1	2	447	51.7	8.90
Q922X9	Protein arginine N-methyltransferase 7 OS=Mus musculus GN=Prmt7 PE=1 SV=1 - [ANM7_MOUSE]	3.61	2	2	2	8	1.845	0.808	1.013	1.135	17.71	3.61	4	8	692	78.3	5.74
Q8C166	Copine-1 OS=Mus musculus GN=Cpne1 PE=1 SV=1 - [CPNE1_MOUSE]	31.72	8	10	16	46	0.607	0.990	0.851	1.135	131.88	31.72	28	46	536	58.8	5.66
Q9D9V3-2	Isoform 2 of Ethylmalonyl-CoA decarboxylase OS=Mus musculus GN=Echdc1 -	45.48	3	12	12	50	1.275	0.744	0.826	1.135	152.42	45.48	22	50	299	32.7	7.02
P09470	Angiotensin-converting enzyme OS=Mus musculus GN=Ace PE=1 SV=3 - [ACE_MOUSE]	13.19	5	14	16	42	0.779	1.614	0.812	1.135	102.43	13.19	29	42	1312	150.8	6.55
Q149S1	Tektin-4 OS=Mus musculus GN=Tekt4 PE=2 SV=1 - [TEKT4_MOUSE]	5.37	1	2	2	2	1.690	1.212	1.287	1.135	4.51	5.37	2	2	447	52.0	6.16
F8VQ75	Kinesin-like protein KIF13A OS=Mus musculus GN=Kif13a PE=2 SV=1 - [F8VQ75_MOUSE]	5.55	2	6	9	12	1.051	0.893	1.365	1.135	25.85	5.55	10	12	1749	195.6	5.38
Q8BVE3	V-type proton ATPase subunit H OS=Mus musculus GN=Atp6v1h PE=1 SV=1 -	50.93	1	21	21	305	0.808	1.313	1.110	1.135	1144.71	50.93	37	305	483	55.8	6.61
Q61161	Mitogen-activated protein kinase kinase kinase kinase 2 OS=Mus musculus GN=Map4k2 PE=1 SV=1 - [illegible]	23.51	5	13	14	32	1.065	0.805	1.046	1.136	110.16	23.51	23	32	821	91.2	6.46
Q9CQ19	Myosin regulatory light polypeptide 9 OS=Mus musculus GN=Myl9 PE=1 SV=3 - [MYL9_MOUSE]	48.26	3	1	8	118	0.852	1.826	0.971	1.136	350.20	48.26	15	118	172	19.8	4.92
Q9CR86	Calcium-regulated heat stable protein 1 OS=Mus musculus GN=Carhsp1 PE=1 SV=1 - [CHSP1_MOUSE]	66.89	2	8	8	36	1.990	0.428	1.418	1.136	90.80	66.89	12	36	148	16.1	8.21
Q8BG51	Mitochondrial Rho GTPase 1 OS=Mus musculus GN=Rhot1 PE=2 SV=1 - [MIRO1_MOUSE]	29.79	5	15	17	77	0.770	1.062	0.990	1.136	239.06	29.79	26	77	631	72.2	6.49
Q61702	Inter-alpha-trypsin inhibitor heavy chain H1 OS=Mus musculus GN=Itih1 PE=1 SV=2 - [ITIH1_MOUSE]	8.71	2	5	5	13	1.635	1.912	0.477	1.136	41.76	8.71	9	13	907	101.0	6.96
P51791-2	Isoform 2 of H(+)/Cl(-) exchange transporter 3 OS=Mus musculus GN=Clcn3 -	17.24	5	10	12	36	0.759	0.967	1.104	1.136	81.43	17.24	20	36	760	84.4	7.25
Q9CR61	NADH dehydrogenase [ubiquinone] 1 beta subcomplex subunit 7 OS=Mus musculus GN=Ndufb7 PE=1 SV=3 - [illegible]	59.85	1	8	8	67	0.622	0.962	1.027	1.136	197.09	59.85	16	67	137	16.3	8.18
P27661	Histone H2A.x OS=Mus musculus GN=H2afx PE=1 SV=2 - [H2AX_MOUSE]	37.76	1	2	5	130	0.766	1.054	1.156	1.136	308.81	37.76	10	130	143	15.1	10.74
Q8C8C1	Receptor-transporting protein 1 OS=Mus musculus GN=Rtp1 PE=1 SV=1 - [RTP1_MOUSE]	3.80	1	1	1	1	3.115	1.767	1.802	1.136	1.77	3.80	1	1	263	30.7	7.61
P08228	Superoxide dismutase [Cu-Zn] OS=Mus musculus GN=Sod1 PE=1 SV=2 - [SODC_MOUSE]	47.40	1	8	8	359	1.637	1.239	1.416	1.136	1176.09	47.40	15	359	154	15.9	6.51
P50396	Rab GDP dissociation inhibitor alpha OS=Mus musculus GN=Gdi1 PE=1 SV=3 - [GDIA_MOUSE]	73.38	3	25	31	561	0.978	1.117	1.043	1.136	1547.78	73.38	59	561	447	50.5	5.08
P17426-2	Isoform B of AP-2 complex subunit alpha-1 OS=Mus musculus GN=Ap2a1 - [AP2A1_MOUSE]	56.13	5	33	49	445	0.617	1.081	1.104	1.136	1205.03	56.13	91	445	955	105.4	7.66
Q8BZ36-2	Isoform 2 of RAD50-interacting protein 1 OS=Mus musculus GN=Rint1 -	7.08	2	3	3	3	0.465	0.864	1.084	1.136	5.51	7.08	3	3	734	83.4	5.07
Q9D5U0	Lysophosphatidylcholine acyltransferase 2B OS=Mus musculus GN=Lpcat2b PE=2 SV=1 - [PCT2B_MOUSE]	3.29	1	1	1	1	2.085	1.549	1.438	1.136	3.12	3.29	1	1	516	58.3	8.91
P97302	Transcription regulator protein BACH1 OS=Mus musculus GN=Bach1 PE=1 SV=1 - [BACH1_MOUSE]	4.60	1	3	3	4	1.319	1.073	0.843	1.137	10.80	4.60	4	4	739	81.3	5.01
P05784	Keratin, type I cytoskeletal 18 OS=Mus musculus GN=Krt18 PE=1 SV=5 - [K1C18_MOUSE]	35.93	13	10	13	36	1.201	1.326	0.883	1.137	104.54	35.93	22	36	423	47.5	5.33

Q6ZWR6-4	Isoform 4 of Nesprin-1 OS=Mus musculus GN=Syne1 - [SYNE1_MOUSE]	24.92	10	154	192	470	0.855	1.050	1.111	1.137	1327.83	24.92	310	470	8797	1009.0	5.59
P63001	Ras-related C3 botulinum toxin substrate 1 OS=Mus musculus GN=Rac1 PE=1 SV=1 - [RAC1_MOUSE]	43.23	8	6	12	102	0.546	1.020	1.071	1.137	318.38	43.23	22	102	192	21.4	8.50
Q9DAI6	Protein FAM135B OS=Mus musculus GN=Fam135b PE=2 SV=3 - [F135B_MOUSE]	3.92	1	5	5	7	0.442	0.859	0.938	1.137	12.83	3.92	7	7	1403	155.4	6.02
F8VPY2	Transcription initiation factor TFIID subunit 5 OS=Mus musculus GN=Taf5 PE=4 SV=1 - [F8VPY2_MOUSE]	4.49	2	1	1	2	1.278	0.764	0.795	1.137	3.28	4.49	1	2	801	86.9	5.64
Q9D279	Mitotic interactor and substrate of PLK1 OS=Mus musculus GN=Misp PE=2 SV=1 - [MISP_MOUSE]	1.08	1	1	1	2	1.508	1.628	1.128	1.137	3.84	1.08	1	2	648	72.2	6.18
Q91V92	ATP-citrate synthase OS=Mus musculus GN=Acly PE=1 SV=1 - [ACLY_MOUSE]	50.41	1	1	49	323	0.623	0.643	0.978	1.137	991.93	50.41	93	323	1091	119.7	7.44
Q8R3U1	HRAS-like suppressor 3 OS=Mus musculus GN=Pla2g16 PE=1 SV=2 - [HRSL3_MOUSE]	24.69	3	4	4	11	0.943	0.569	0.909	1.137	37.66	24.69	7	11	162	17.9	8.72
Q62148	Retinal dehydrogenase 2 OS=Mus musculus GN=Aldh1a2 PE=1 SV=2 - [AL1A2_MOUSE]	27.61	3	10	13	56	0.820	3.044	0.886	1.137	175.27	27.61	24	56	518	56.6	5.74
P34884	Macrophage migration inhibitory factor OS=Mus musculus GN=Mif PE=1 SV=2 - [MIF_MOUSE]	23.48	1	4	4	106	1.749	0.875	0.931	1.137	227.99	23.48	8	106	115	12.5	7.34
Q6NS45-3	Isoform 3 of Coiled-coil domain-containing protein 66 OS=Mus musculus GN=Ccdc66 - [CCD66_MOUSE]	2.11	3	2	2	2	1.834	1.534	0.926	1.138	0.00	2.11	2	2	902	103.7	7.71
Q9D8V7	Signal peptidase complex catalytic subunit SEC11C OS=Mus musculus GN=Sec11c PE=2 SV=3 - [SC11C_MOUSE]	23.96	3	4	4	15	0.773	0.841	0.692	1.138	54.79	23.96	8	15	192	21.6	9.23
Q8BXR1	Probable cationic amino acid transporter OS=Mus musculus GN=Slc7a14 PE=2 SV=1 - [S7A14_MOUSE]	15.43	2	8	8	59	0.572	1.420	1.074	1.138	195.84	15.43	12	59	771	83.9	5.35
Q8BR76	Meckelin OS=Mus musculus GN=Tmem67 PE=1 SV=2 - [MKS3_MOUSE]	1.92	3	2	2	3	1.486	0.909	0.817	1.138	5.62	1.92	3	3	992	111.7	7.74
Q62277	Synaptophysin OS=Mus musculus GN=Syp PE=1 SV=2 - [SYPH_MOUSE]	33.76	1	6	11	165	1.175	1.413	1.236	1.138	489.34	33.76	19	165	314	34.0	4.94
Q8BXR9-2	Isoform 2 of Oxysterol-binding protein-related protein 6 OS=Mus musculus GN=Osbpl6 - [OSBL6_MOUSE]	21.01	2	1	15	42	0.835	0.419	0.718	1.138	125.80	21.01	25	42	928	105.2	6.79
Q8CGE9	Regulator of G-protein signaling 12 OS=Mus musculus GN=Rgs12 PE=1 SV=2 - [RGS12_MOUSE]	5.00	11	5	5	12	1.037	1.944	1.853	1.138	36.64	5.00	9	12	1381	149.5	7.59
Q61425	Hydroxyacyl-coenzyme A dehydrogenase, mitochondrial OS=Mus musculus GN=Hadh PE=1 SV=2 - [HCDH_MOUSE]	62.42	1	17	18	77	0.817	0.981	0.892	1.138	201.01	62.42	27	77	314	34.4	8.65
E9PXX4	High affinity cAMP-specific and IBMX-insensitive 3',5'-cyclic phosphodiesterase 8B OS=Mus musculus GN=Pde8b PE=2 SV=1 - [E9PXX4_MOUSE]	9.04	9	6	7	13	0.932	1.261	1.129	1.138	32.37	9.04	10	13	730	82.6	6.35
Q9QXL8	Nucleoside diphosphate kinase 7 OS=Mus musculus GN=Nme7 PE=2 SV=1 - [NDK7_MOUSE]	21.27	2	7	8	15	0.855	1.367	1.024	1.139	44.88	21.27	13	15	395	44.4	7.12
Q8BUE5	Alpha-1D adrenergic receptor OS=Mus musculus GN=Adra1a PE=2 SV=1 -	4.22	2	2	2	2	0.972	0.906	0.885	1.139	3.80	4.22	2	2	427	47.5	9.36
Q62101	Serine/threonine-protein kinase D1 OS=Mus musculus GN=Prkd1 PE=1 SV=2 - [KPCD1_MOUSE]	4.68	4	3	3	6	1.563	1.148	0.949	1.139	18.44	4.68	4	6	918	102.0	6.58
P35492	Histidine ammonia-lyase OS=Mus musculus GN=Hal PE=1 SV=1 - [HUTH_MOUSE]	2.59	1	1	2	2	1.034	0.654	1.215	1.139	4.76	2.59	2	2	657	72.2	6.34
Q6P5E6	ADP-ribosylation factor-binding protein GGA2 OS=Mus musculus GN=Gga2 PE=1 SV=1 - [GGA2_MOUSE]	11.61	2	4	5	18	1.055	1.120	1.045	1.139	51.85	11.61	9	18	603	66.0	7.30
Q9D1F9	Protein Slc37a4 OS=Mus musculus GN=Slc37a4 PE=2 SV=1 - [Q9D1F9_MOUSE]	3.03	1	1	1	2	0.737	0.927	1.052	1.139	4.07	3.03	2	2	429	46.0	8.88
Q8CBE3	WD repeat-containing protein 37 OS=Mus musculus GN=Wdr37 PE=2 SV=1 -	30.24	9	10	15	68	0.651	0.981	1.106	1.139	188.79	30.24	28	68	496	55.0	7.23

P46664	Adenylosuccinate synthetase isozyme 2 OS=Mus musculus GN=Adss PE=1 SV=2 - [PURA2_MOUSE]	47.59	1	16	18	92	0.884	0.820	0.988	1.139	268.03	47.59	33	92	456	50.0	6.38
G5E8M7	Glutathione S-transferase Mu 6 OS=Mus musculus GN=Gstm6 PE=3 SV=1 - [G5E8M7_MOUSE]	21.12	3	1	6	38	1.195	1.185	1.001	1.139	86.59	21.12	11	38	232	27.3	5.91
Q9JL04	Formin-2 OS=Mus musculus GN=Fmn2 PE=1 SV=2 - [FMN2_MOUSE]	18.06	1	21	23	100	0.741	0.977	1.526	1.139	282.98	18.06	41	100	1578	167.3	5.48
P55264-2	Isoform Short of Adenosine kinase OS=Mus musculus GN=Adk - [ADK_MOUSE]	41.74	2	14	14	99	0.778	0.853	1.039	1.139	235.15	41.74	23	99	345	38.4	6.04
Q8CH72	E3 ubiquitin-protein ligase TRIM32 OS=Mus musculus GN=Trim32 PE=1 SV=2 - [TRI32_MOUSE]	31.15	3	16	16	54	1.088	1.077	1.055	1.139	153.27	31.15	28	54	655	72.0	6.90
D3Z2Q2	Syntaxin-binding protein 5 OS=Mus musculus GN=Stxbp5 PE=2 SV=1 - [D3Z2Q2_MOUSE]	35.49	5	29	30	121	0.715	1.144	1.045	1.139	375.57	35.49	49	121	1099	121.6	6.96
Q9D6D0	Protein Slc25a27 OS=Mus musculus GN=Slc25a27 PE=2 SV=1 - [Q9D6D0_MOUSE]	44.72	1	13	13	28	1.022	1.547	1.134	1.139	80.90	44.72	22	28	322	35.8	9.39
Q9CQN3	Mitochondrial import receptor subunit TOM6 homolog OS=Mus musculus GN=Tomm6 PE=3 SV=1 - [illegible]	18.92	1	1	1	1	1.224	0.833	1.198	1.140	2.43	18.92	1	1	74	7.9	4.89
Q8BUR4	Dedicator of cytokinesis protein 1 OS=Mus musculus GN=Dock1 PE=1 SV=3 - [DOCK1_MOUSE]	14.64	2	21	24	58	0.960	0.672	0.996	1.140	152.93	14.64	42	58	1865	214.9	7.62
P59016	Vacuolar protein sorting-associated protein 33B OS=Mus musculus GN=Vps33b PE=1 SV=1 - [VP33B_MOUSE]	35.49	1	18	18	38	0.836	1.112	0.996	1.140	126.99	35.49	29	38	617	70.5	6.86
Q8C033	Rho guanine nucleotide exchange factor 10 OS=Mus musculus GN=Arhgef10 PE=1 SV=2 - [ARHGA_MOUSE]	13.38	4	13	13	31	0.861	0.353	0.893	1.140	87.18	13.38	21	31	1345	147.9	5.73
Q8C181	Muscleblind-like protein 2 OS=Mus musculus GN=Mbnl2 PE=2 SV=2 - [MBNL2_MOUSE]	15.82	5	1	6	22	0.861	1.172	1.158	1.140	58.33	15.82	10	22	373	40.1	9.00
Q61012	Guanine nucleotide-binding protein G(T) subunit gamma-T1 OS=Mus musculus GN=Gngt1 PE=1 SV=3 - [illegible]	40.54	2	2	3	3	0.797	0.471	0.916	1.140	9.42	40.54	3	3	74	8.5	4.82
Q9Z1G4-3	Isoform A1-III of V-type proton ATPase 116 kDa subunit a isoform 1 OS=Mus musculus GN=Atp6v0a1 - [illegible]	40.75	7	32	33	528	0.671	1.261	1.206	1.141	1695.39	40.75	59	528	832	95.6	6.77
Q9R078	5'-AMP-activated protein kinase subunit beta-1 OS=Mus musculus GN=Prkab1 PE=1 SV=2 - [AAKB1_MOUSE]	19.63	1	3	3	13	1.039	0.900	0.931	1.141	52.11	19.63	5	13	270	30.3	6.23
Q9D9D5-2	Isoform 2 of Transmembrane and coiled coil domain-containing protein 5A OS=Mus musculus GN=Tmco5a - [illegible]	3.53	2	1	1	1	0.888	1.007	1.223	1.141	2.58	3.53	1	1	283	33.5	4.91
Q80TA6	Myotubularin-related protein 12 OS=Mus musculus GN=Mtmr12 PE=2 SV=2 -	7.50	4	4	4	10	0.906	1.633	1.740	1.141	24.88	7.50	7	10	747	85.5	6.84
O35638	Cohesin subunit SA-2 OS=Mus musculus GN=Stag2 PE=1 SV=3 - [STAG2_MOUSE]	7.55	3	5	9	15	1.037	1.135	0.850	1.141	43.86	7.55	13	15	1231	141.2	5.43
E9PUB6	Protein Fbrsl1 OS=Mus musculus GN=Fbrsl1 PE=2 SV=1 - [E9PUB6_MOUSE]	6.41	2	2	3	6	0.751	0.964	1.283	1.141	12.86	6.41	5	6	593	63.8	7.58
P97825	Hematological and neurological expressed 1 protein OS=Mus musculus GN=Hn1 PE=1 SV=3 - [HN1_MOUSE]	41.56	1	4	4	45	1.824	1.654	1.456	1.142	178.83	41.56	7	45	154	16.1	5.31
P15379-2	Isoform 13 of CD44 antigen OS=Mus musculus GN=Cd44 - [CD44_MOUSE]	18.73	21	5	5	7	1.302	1.398	0.951	1.142	18.45	18.73	7	7	363	40.0	5.92
Q63870	Collagen alpha-1(VII) chain OS=Mus musculus GN=Col7a1 PE=2 SV=3 - [CO7A1_MOUSE]	0.78	1	2	2	3	5.681	1.891	1.452	1.142	7.42	0.78	2	3	2944	295.1	6.34
Q9DBK7	MCG18845, isoform CRA_d OS=Mus musculus GN=Uba7 PE=2 SV=1 - [Q9DBK7_MOUSE]	4.20	2	3	3	4	0.882	1.426	0.975	1.142	4.78	4.20	3	4	977	108.6	5.97
F6VEG4	Lysosomal alpha-glucosidase (Fragment) OS=Mus musculus GN=Gaa PE=4 SV=1 - [F6VEG4_MOUSE]	48.11	1	1	4	39	1.355	1.103	1.294	1.142	180.52	48.11	7	39	106	12.4	6.28

G5E8J4	Protein tyrosine phosphatase, non-receptor type 21, isoform CRA_a OS=Mus musculus GN=Ptpn21 PE=4 SV=1 - [illegible]	1.28	2	2	2	2	11.499	1.437	0.480	1.142	4.97	1.28	2	2	1176	133.4	7.85
Q3TYW3	Protein BC023829 OS=Mus musculus GN=BC023829 PE=2 SV=1 - [Q3TYW3_MOUSE]	2.80	3	1	1	2	0.574	0.819	1.122	1.142	5.50	2.80	2	2	322	37.2	7.20
B2RY04	Dedicator of cytokinesis protein 5 OS=Mus musculus GN=Dock5 PE=1 SV=2 - [DOCK5_MOUSE]	7.76	1	11	13	26	1.464	0.947	0.947	1.142	51.96	7.76	22	26	1868	214.3	7.55
A2ALK6	Band 4.1-like protein 4B OS=Mus musculus GN=Epb4.1l4b PE=2 SV=1 - [A2ALK6_MOUSE]	12.01	2	7	9	18	0.905	1.238	1.089	1.142	60.49	12.01	15	18	899	99.7	9.17
Q78IK2	Up-regulated during skeletal muscle growth protein 5 OS=Mus musculus GN=Usmg5 PE=1 SV=1 - [illegible]	29.31	1	3	3	7	0.934	1.482	1.052	1.143	22.75	29.31	5	7	58	6.4	9.83
Q07417	Short-chain specific acyl-CoA dehydrogenase, mitochondrial OS=Mus musculus GN=Acads PE=2 SV=2 - [ACADS_MOUSE]	43.69	2	14	14	34	0.988	1.030	0.990	1.143	101.26	43.69	22	34	412	44.9	8.47
Q8BND3-2	Isoform 2 of WD repeat-containing protein 35 OS=Mus musculus GN=Wdr35 -	3.59	2	3	5	7	0.977	1.083	0.619	1.143	15.82	3.59	7	7	1170	132.7	6.58
Q99MS8	Tubulin polyglutamylase complex subunit 1 OS=Mus musculus GN=Tpgs1 PE=1 SV=1 - [TPGS1_MOUSE]	35.97	1	8	8	20	1.036	1.220	0.906	1.143	69.46	35.97	15	20	303	32.6	8.94
Q62432-2	Isoform Short of Mothers against decapentaplegic homolog 2 OS=Mus musculus GN=Smad2 - [SMAD2_MOUSE]	21.97	8	4	8	21	1.462	1.164	0.996	1.143	57.70	21.97	13	21	437	48.9	7.09
Q9DCD0	6-phosphogluconate dehydrogenase, decarboxylating OS=Mus musculus GN=Pgd PE=2 SV=3 - [6PGD_MOUSE]	52.38	1	20	22	125	0.698	1.151	0.917	1.143	361.29	52.38	39	125	483	53.2	7.23
O35326	Serine/arginine-rich splicing factor 5 OS=Mus musculus GN=Srsf5 PE=1 SV=2 - [SRSF5_MOUSE]	30.11	2	6	8	45	1.046	0.896	1.067	1.143	122.85	30.11	12	45	269	30.9	11.56
O35129	Prohibitin-2 OS=Mus musculus GN=Phb2 PE=1 SV=1 - [PHB2_MOUSE]	69.90	3	24	24	158	0.758	0.924	0.982	1.143	463.26	69.90	43	158	299	33.3	9.83
F6TFF2	Pituitary tumor-transforming gene 1 protein-interacting protein (Fragment) OS=Mus musculus GN=Pttg1ip PE=4 SV=1 - [F6TFF2_MOUSE]	18.02	3	3	3	6	1.028	1.099	1.067	1.143	12.55	18.02	4	6	172	19.8	8.68
Q6GTL7	6-phosphofructo-2-kinase/fructose-2, 6-biphosphatase 2 OS=Mus musculus GN=Pfkfb2 PE=2 SV=1 - [Q6GTL7_MOUSE]	44.40	4	18	21	49	0.903	0.955	0.977	1.143	137.18	44.40	37	49	518	59.8	8.12
Q6PJN8	DALR anticodon-binding domain-containing protein 3 OS=Mus musculus GN=Dalrd3 PE=2 SV=1 - [DALD3_MOUSE]	6.51	1	3	3	6	0.743	0.933	0.871	1.143	16.25	6.51	6	6	538	58.7	8.15
Q66X19	NACHT, LRR and PYD domains-containing protein 4E OS=Mus musculus GN=Nlrp4e PE=2 SV=2 - [illegible]	1.64	1	1	2	2	1.062	1.161	1.236	1.143	4.82	1.64	2	2	978	112.4	6.52
Q149F3	Eukaryotic peptide chain release factor GTP-binding subunit ERF3B OS=Mus musculus GN=Gspt2 PE=1 SV=1 - [ERF3B_MOUSE]	30.38	2	7	19	61	1.051	1.163	0.958	1.143	164.33	30.38	32	61	632	69.1	5.20
Q3TWV4	AP-2 complex subunit mu OS=Mus musculus GN=Ap2m1 PE=2 SV=1 - [Q3TWV4_MOUSE]	66.28	2	31	31	289	0.493	1.051	1.174	1.143	832.99	66.28	55	289	433	49.4	9.54
O08599	Syntaxin-binding protein 1 OS=Mus musculus GN=Stxbp1 PE=1 SV=2 - [STXB1_MOUSE]	76.94	1	2	50	835	0.284	1.348	1.261	1.144	2453.51	76.94	91	835	594	67.5	6.96
P54731	FAS-associated factor 1 OS=Mus musculus GN=Faf1 PE=1 SV=2 - [FAF1_MOUSE]	29.12	1	16	16	36	1.206	0.981	0.976	1.144	90.71	29.12	27	36	649	73.8	4.86
Q8BLV3	Sodium/hydrogen exchanger 7 OS=Mus musculus GN=Slc9a7 PE=2 SV=1 -	10.19	2	6	6	31	0.726	0.750	0.907	1.144	71.02	10.19	11	31	726	80.2	6.40
Q3V1L4	Cytosolic purine 5'-nucleotidase OS=Mus musculus GN=Nt5c2 PE=1 SV=2 - [5NTC_MOUSE]	34.29	3	15	15	37	0.935	1.018	0.945	1.144	100.41	34.29	24	37	560	64.8	6.21
Q6GYP7	Ral GTPase-activating protein subunit alpha-1 OS=Mus musculus GN=Ralgapa1 PE=1 SV=1 [RGPA1_MOUSE]	15.82	6	26	28	84	0.749	0.945	1.067	1.144	230.30	15.82	46	84	2035	229.2	6.10
P63030	Mitochondrial pyruvate carrier 1 OS=Mus musculus GN=Mpc1 PE=1 SV=1 - [MPC1_MOUSE]	39.45	5	4	4	20	0.805	0.963	1.146	1.144	54.24	39.45	8	20	109	12.4	9.61

Q64378	Peptidyl-prolyl cis-trans isomerase FKBP5 OS=Mus musculus GN=Fkbp5 PE=1 SV=1 - [FKBP5_MOUSE]	26.10	2	10	10	30	0.842	1.433	1.193	1.144	82.73	26.10	15	30	456	50.9	7.80
P58064	28S ribosomal protein S6, mitochondrial OS=Mus musculus GN=Mrps6 PE=2 SV=3 - [RT06_MOUSE]	64.00	1	7	7	19	0.889	0.906	1.068	1.144	45.45	64.00	14	19	125	14.3	9.50
E9Q070	Uncharacterized protein OS=Mus musculus GN=Gm8730 PE=2 SV=1 - [E9Q070_MOUSE]	41.32	2	1	13	65	0.330	2.604	0.475	1.144	160.75	41.32	24	65	317	34.2	6.25
H3BKG0	Caveolin-1 OS=Mus musculus GN=Cav1 PE=2 SV=1 - [H3BKG0_MOUSE]	54.84	6	4	6	28	0.917	1.874	1.097	1.144	59.37	54.84	10	28	93	10.4	6.05
P51432	1-phosphatidylinositol 4,5-bisphosphate phosphodiesterase beta-3 OS=Mus musculus GN=Plcb3 PE=2 SV=2 - [PLCB3_MOUSE]	16.61	1	17	18	49	1.024	1.056	0.961	1.144	149.29	16.61	30	49	1234	139.4	5.94
Q9D7I5-2	Isoform 2 of Phospholysine phosphohistidine inorganic pyrophosphate phosphatase OS=Mus musculus GN=Lhpp - [LHPP_MOUSE]	33.47	2	5	5	14	1.589	0.917	1.088	1.144	35.35	33.47	10	14	242	26.0	5.69
Q9Z320	Keratin, type I cytoskeletal 27 OS=Mus musculus GN=Krt27 PE=1 SV=1 - [K1C27_MOUSE]	5.58	1	1	2	7	0.536	0.810	2.558	1.145	12.38	5.58	3	7	448	49.1	5.05
F8VPV8	Protein BC030307 OS=Mus musculus GN=BC030307 PE=2 SV=1 - [F8VPV8_MOUSE]	5.54	5	4	5	12	1.111	1.282	1.372	1.145	21.50	5.54	6	12	1318	150.7	8.62
Q9WV68	Peroxisomal 2,4-dienoyl-CoA reductase OS=Mus musculus GN=Decr2 PE=1 SV=1 - [DECR2_MOUSE]	8.22	1	2	2	5	0.887	1.120	0.824	1.145	15.98	8.22	4	5	292	31.3	8.81
P09411	Phosphoglycerate kinase 1 OS=Mus musculus GN=Pgk1 PE=1 SV=4 - [PGK1_MOUSE]	87.53	5	36	36	584	0.864	1.179	1.087	1.145	1702.60	87.53	66	584	417	44.5	7.90
Q9DA03	Complex III assembly factor LYRM7 OS=Mus musculus GN=Lyrm7 PE=2 SV=2 - [LYRM7_MOUSE]	37.50	2	4	4	12	1.611	1.077	1.571	1.145	35.66	37.50	6	12	104	12.0	9.45
Q6RKD8	Fibronectin leucine rich transmembrane protein 1 OS=Mus musculus GN=Flrt1 PE=2 SV=1 - [Q6RKD8_MOUSE]	8.75	1	5	5	10	0.869	1.147	1.028	1.145	28.03	8.75	10	10	674	74.1	6.60
Q8VCA8	Secernin-2 OS=Mus musculus GN=Scrn2 PE=2 SV=1 - [SCRN2_MOUSE]	20.47	2	5	5	10	1.684	1.241	1.120	1.145	34.61	20.47	9	10	425	46.6	5.71
Q3TJI8	Corticosteroid 11-beta-dehydrogenase isozyme 1 OS=Mus musculus GN=Hsd11b1 PE=2 SV=1 - [Q3TJI8_MOUSE]	13.74	4	5	5	13	0.913	0.451	1.113	1.145	31.22	13.74	9	13	262	28.7	8.72
E9PYI4	Uncharacterized protein OS=Mus musculus GN=Mroh8 PE=2 SV=1 - [E9PYI4_MOUSE]	6.10	1	3	3	3	1.096	1.347	1.120	1.145	6.49	6.10	3	3	1049	119.4	8.21
Q6NXM6	Gene model 1008, (NCBI) OS=Mus musculus GN=Zfp663 PE=2 SV=1 - [Q6NXM6_MOUSE]	1.98	1	1	1	1	2.167	0.935	1.116	1.145	3.45	1.98	1	1	656	74.2	8.97
F8VPM0	Bromodomain adjacent to zinc finger domain protein 2A OS=Mus musculus GN=Baz2a PE=2 SV=1 - [F8VPM0_MOUSE]	2.91	5	3	4	5	0.370	0.838	0.983	1.145	14.11	2.91	4	5	1887	209.3	6.51
P48193	Protein 4.1 OS=Mus musculus GN=Epb41 PE=1 SV=2 - [41_MOUSE]	39.04	9	16	26	92	0.856	1.064	0.863	1.146	251.05	39.04	41	92	858	95.9	5.60
Q61563	Fibroblast growth factor receptor OS=Mus musculus GN=Fgfr3 PE=2 SV=2 - [Q61563_MOUSE]	14.71	21	7	10	39	1.491	1.120	1.064	1.146	83.41	14.71	16	39	782	85.8	6.92
Q9DCN2-2	Isoform 2 of NADH-cytochrome b5 reductase 3 OS=Mus musculus GN=Cyb5r3 -	61.15	4	14	14	94	0.817	1.379	0.897	1.146	233.29	61.15	26	94	278	31.5	8.38
P70339	Proto-oncogene FRAT1 OS=Mus musculus GN=Frat1 PE=1 SV=2 - [FRAT1_MOUSE]	7.30	1	1	1	4	1.254	0.843	1.145	1.146	17.47	7.30	2	4	274	28.9	6.54
Q91ZR1	Ras-related protein Rab-4B OS=Mus musculus GN=Rab4b PE=2 SV=2 - [RAB4B_MOUSE]	52.11	1	7	10	72	0.735	0.736	0.969	1.146	203.16	52.11	19	72	213	23.6	6.06
P97390	Vacuolar protein sorting-associated protein 45 OS=Mus musculus GN=Vps45 PE=1 SV=1 - [VPS45_MOUSE]	43.16	1	24	24	76	0.614	0.994	0.910	1.146	192.20	43.16	43	76	570	65.0	8.25
Q8BFY6	Peflin OS=Mus musculus GN=Pef1 PE=2 SV=1 - [PEF1_MOUSE]	15.64	1	5	5	22	0.963	1.669	1.126	1.146	51.27	15.64	10	22	275	29.2	6.30
B1ART1	Protein Vps13d OS=Mus musculus GN=Vps13d PE=2 SV=1 - [B1ART1_MOUSE]	10.19	3	32	32	55	0.684	0.882	0.955	1.147	137.48	10.19	48	55	4359	487.0	6.49

Q9QYK9	Calcium/calmodulin-dependent protein kinase type 1B OS=Mus musculus GN=Pnck PE=2 SV=1 - [KCC1B_MOUSE]	44.02	2	12	12	34	0.964	1.683	0.855	1.147	95.08	44.02	21	34	343	38.5	6.47
Q80VP1	Epsin-1 OS=Mus musculus GN=Epn1 PE=1 SV=3 - [EPN1_MOUSE]	34.78	3	12	14	74	1.180	1.636	1.620	1.147	197.82	34.78	24	74	575	60.2	4.81
Q9QYB1	Chloride intracellular channel protein 4 OS=Mus musculus GN=Clic4 PE=1 SV=3 - [CLIC4_MOUSE]	76.28	2	16	17	96	1.160	0.500	0.926	1.147	262.52	76.28	26	96	253	28.7	5.59
Q3U3Q1-2	Isoform 2 of Serine/threonine-protein kinase ULK3 OS=Mus musculus GN=Ulk3 - [ULK3_MOUSE]	11.89	2	4	4	7	1.365	0.772	0.993	1.147	24.94	11.89	5	7	471	53.3	7.36
Q9Z1K6	E3 ubiquitin-protein ligase ARIH2 OS=Mus musculus GN=Arih2 PE=2 SV=1 - [ARI2_MOUSE]	22.56	1	10	10	43	0.981	1.107	1.106	1.147	98.33	22.56	17	43	492	57.7	5.69
Q8CHS8	Vacuolar protein sorting-associated protein 37A OS=Mus musculus GN=Vps37a PE=2 SV=1 - [VP37A_MOUSE]	18.14	3	6	7	15	1.632	1.112	1.250	1.147	44.78	18.14	10	15	397	44.4	5.67
Q7TS58	Antigen presenting cell lectin-like receptor A2 OS=Mus musculus GN=Clec4b1 PE=2 SV=1 - [Q7TS58_MOUSE]	9.57	5	1	2	2	5.222	1.067	1.504	1.147	1.75	9.57	2	2	209	24.2	7.08
D3YTZ7	Josephin-2 OS=Mus musculus GN=Josd2 PE=2 SV=1 - [D3YTZ7_MOUSE]	31.51	4	3	3	14	0.836	0.307	0.872	1.147	21.15	31.51	5	14	146	16.1	5.49
Q9DB75-2	Isoform 2 of Cell death-inducing p53-target protein 1 OS=Mus musculus GN=Cdip1 - [CDIP1_MOUSE]	8.38	3	2	2	10	0.824	0.895	0.911	1.147	24.51	8.38	4	10	191	20.0	5.34
Q91VN4	Coiled-coil-helix-coiled-coil-helix domain-containing protein 6, mitochondrial OS=Mus musculus GN=Chchd6 PE=2 SV=2 - [CHCH6_MOUSE]	54.21	1	3	16	107	1.396	0.952	1.192	1.147	349.42	54.21	25	107	273	29.8	8.41
Q62465	Synaptic vesicle membrane protein VAT-1 homolog OS=Mus musculus GN=Vat1 PE=1 SV=3 - [VAT1_MOUSE]	42.12	1	14	14	79	0.942	1.005	0.589	1.147	229.16	42.12	25	79	406	43.1	6.37
Q9Z2D0	Myotubularin-related protein 9 OS=Mus musculus GN=Mtmr9 PE=2 SV=2 -	15.05	1	8	8	29	0.747	0.912	0.986	1.147	92.00	15.05	14	29	545	62.9	6.62
F8VQ43	Laminin subunit alpha-2 OS=Mus musculus GN=Lama2 PE=2 SV=1 - [F8VQ43_MOUSE]	13.31	2	36	37	85	1.457	1.031	1.087	1.147	227.45	13.31	59	85	3118	343.6	6.09
Q8K0S0	Phytanoyl-CoA hydroxylase interacting protein OS=Mus musculus GN=Phyhip PE=1 SV=1 - [PHYIP_MOUSE]	33.94	2	10	12	103	0.727	2.061	1.682	1.147	312.08	33.94	21	103	330	37.5	7.01
Q8BI22-2	Isoform 2 of Centrosomal protein of 128 kDa OS=Mus musculus GN=Cep128 -	6.34	4	5	9	21	1.401	1.085	1.097	1.147	32.38	6.34	12	21	1026	120.1	6.39
D3Z3B8	Disks large homolog 1 OS=Mus musculus GN=Dlg1 PE=2 SV=1 - [D3Z3B8_MOUSE]	47.84	2	2	31	224	0.514	1.317	1.886	1.147	608.03	47.84	56	224	834	91.6	6.34
A2RTH5	Leucine carboxyl methyltransferase 1 OS=Mus musculus GN=Lcmt1 PE=2 SV=1 -	35.84	1	11	11	36	0.563	1.289	1.056	1.147	102.68	35.84	18	36	332	38.2	5.62
Q8BVW0	Neutral alpha-glucosidase C OS=Mus musculus GN=Ganc PE=2 SV=2 - [GANC_MOUSE]	5.79	3	4	4	11	1.196	1.573	0.942	1.147	36.57	5.79	7	11	898	101.9	6.32
Q08024-4	Isoform 4 of Core-binding factor subunit beta OS=Mus musculus GN=Cbfb - [PEBB_MOUSE]	20.95	4	2	2	2	1.464	0.920	1.041	1.147	6.97	20.95	2	2	148	17.6	6.01
Q78IK4	Apolipoprotein O-like OS=Mus musculus GN=Apool PE=2 SV=1 - [APOOL_MOUSE]	38.49	2	9	9	32	0.881	0.819	1.074	1.148	90.86	38.49	18	32	265	29.2	9.31
G5E876	Hect domain and RLD 3, isoform CRA_a OS=Mus musculus GN=Herc3 PE=4 SV=1 - [G5E876_MOUSE]	6.02	3	4	4	5	0.765	0.829	1.144	1.148	3.70	6.02	5	5	913	101.4	6.79
Q8VG21	Olfactory receptor 1195 OS=Mus musculus GN=Olfr1195 PE=3 SV=1 - [Q8VG21_MOUSE]	2.60	2	1	1	1	3.428	1.241	1.675	1.148	2.31	2.60	1	1	308	34.8	8.05
Q9D180	WD repeat-containing protein 65 OS=Mus musculus GN=Wdr65 PE=2 SV=3 -	4.96	1	5	7	10	1.680	0.976	1.266	1.148	20.02	4.96	7	10	1249	144.8	5.74
Q9JLB9	Poliovirus receptor-related protein 3 OS=Mus musculus GN=Pvrl3 PE=1 SV=1 - [PVRL3_MOUSE]	15.12	2	7	7	14	1.360	1.636	1.035	1.148	39.79	15.12	11	14	549	60.5	6.54

Q8CDJ3	Beclin 1-associated autophagy-related key regulator OS=Mus musculus GN=Atg14 PE=1 SV=1 - [BAKOR_MOUSE]	1.42	1	1	1	1	1.083	1.150	0.992	1.148	1.70	1.42	1	1	492	55.4	7.81
Q91WA9	ATP-binding cassette transporter ABCG4 OS=Mus musculus GN=Abcg4 PE=2 SV=2 - [Q91WA9_MOUSE]	5.11	2	2	3	5	0.841	0.751	1.056	1.148	9.10	5.11	4	5	646	72.1	8.19
Q924N4	Solute carrier family 12 member 6 OS=Mus musculus GN=Slc12a6 PE=1 SV=2 -	15.30	5	9	15	79	0.758	0.849	1.060	1.148	212.42	15.30	26	79	1150	127.4	7.03
P56392	Cytochrome c oxidase subunit 7A1, mitochondrial OS=Mus musculus GN=Cox7a1 PE=2 SV=1 - [CX7A1_MOUSE]	47.50	1	2	2	3	1.148	1.560	1.054	1.148	4.13	47.50	2	3	80	9.0	9.79
Q8BJL0-2	Isoform 2 of SWI/SNF-related matrix-associated actin-dependent regulator of chromatin subfamily A-like protein 1 OS=Mus musculus GN=Smarcal1 - [SMAL1_MOUSE]	10.41	6	5	5	19	0.829	0.746	1.059	1.148	52.05	10.41	9	19	788	87.1	9.74
Q9WVA2	Mitochondrial import inner membrane translocase subunit Tim8 A OS=Mus musculus GN=Timm8a1 PE=1 SV=1 - [TIM8A_MOUSE]	42.27	2	4	4	32	2.129	0.767	1.551	1.148	89.84	42.27	6	32	97	11.0	5.16
G3UZ36	Interferon-inducible protein AIM2 (Fragment) OS=Mus musculus GN=Aim2 PE=2 SV=1 - [G3UZ36_MOUSE]	31.91	3	2	2	2	0.832	1.226	1.231	1.149	2.30	31.91	2	2	94	10.8	5.85
P46686	Tubby-related protein 2 OS=Mus musculus GN=Tulp2 PE=2 SV=3 - [TULP2_MOUSE]	2.67	2	1	1	2	1.025	0.755	1.219	1.149	2.81	2.67	1	2	562	62.7	6.65
Q8CHQ0	F-box only protein 4 OS=Mus musculus GN=Fbxo4 PE=1 SV=2 - [FBX4_MOUSE]	19.22	1	6	6	9	1.053	1.272	1.010	1.149	24.28	19.22	9	9	385	43.7	6.11
Q8BYN3	Inositol-tetrakisphosphate 1-kinase OS=Mus musculus GN=Itpk1 PE=2 SV=1 - [ITPK1_MOUSE]	23.15	2	7	7	20	1.070	1.003	0.844	1.149	48.19	23.15	10	20	419	46.1	6.29
P35802	Neuronal membrane glycoprotein M6-a OS=Mus musculus GN=Gpm6a PE=1 SV=1 - [GPM6A_MOUSE]	29.50	1	7	8	109	0.501	1.670	1.210	1.149	258.17	29.50	13	109	278	31.1	5.27
Q8CHC4	Synaptojanin-1 OS=Mus musculus GN=Synj1 PE=1 SV=3 - [SYNJ1_MOUSE]	45.49	6	52	56	375	0.711	1.094	1.139	1.149	1067.24	45.49	97	375	1574	172.5	6.89
P97315	Cysteine and glycine-rich protein 1 OS=Mus musculus GN=Csrp1 PE=1 SV=3 - [CSRP1_MOUSE]	66.84	2	15	15	212	1.702	0.946	1.588	1.149	753.56	66.84	26	212	193	20.6	8.57
Q91V35	Protein tyrosin phosphatase receptor type alpha OS=Mus musculus GN=Ptpra PE=2 SV=1 - [Q91V35_MOUSE]	27.62	3	18	21	78	0.689	1.442	1.038	1.149	217.42	27.62	37	78	793	89.8	6.64
A3KG59-2	Isoform 2 of Peptidase M20 domain-containing protein 2 OS=Mus musculus GN=Pm20d2 - [P20D2_MOUSE]	6.89	2	1	2	7	0.609	0.744	0.814	1.149	19.00	6.89	3	7	305	32.8	5.63
Q8BZW8	NHL repeat-containing protein 2 OS=Mus musculus GN=Nhlrc2 PE=2 SV=1 -	20.97	1	12	12	31	1.419	1.034	1.149	1.149	90.10	20.97	20	31	725	78.4	5.54
Q8CI33-2	Isoform 2 of CWF19-like protein 1 OS=Mus musculus GN=Cwf19l1 - [C19L1_MOUSE]	18.30	2	4	5	8	1.386	1.404	1.338	1.149	18.26	18.30	7	8	399	45.1	7.68
B2RWE3	CDGSH iron sulfur domain 3 OS=Mus musculus GN=Cisd3 PE=2 SV=1 - [B2RWE3_MOUSE]	38.06	2	5	5	15	0.555	0.853	0.818	1.149	52.85	38.06	7	15	134	15.0	9.95
Q8K126	Peptide chain release factor 1, mitochondrial OS=Mus musculus GN=Mtrf1 PE=2 SV=1 - [RF1M_MOUSE]	2.24	1	1	1	2	1.529	1.089	1.331	1.150	5.79	2.24	2	2	446	52.4	8.35
P70236	Dual specificity mitogen-activated protein kinase kinase 6 OS=Mus musculus GN=Map2k6 PE=1 SV=1 - [MP2K6_MOUSE]	38.32	2	9	11	56	0.754	1.264	1.266	1.150	171.43	38.32	20	56	334	37.4	7.39
Q80ZJ7	Sorting nexin-32 OS=Mus musculus GN=Snx32 PE=2 SV=1 - [SNX32_MOUSE]	28.47	1	11	12	31	1.005	1.153	0.933	1.150	86.96	28.47	20	31	404	46.6	6.96
Q8BJY1	26S proteasome non-ATPase regulatory subunit 5 OS=Mus musculus GN=Psmd5 PE=1 SV=4 - [PSMD5_MOUSE]	47.22	2	19	20	77	0.600	1.019	0.796	1.150	257.97	47.22	37	77	504	55.9	5.21
A2AF65	Dedicator of cytokinesis protein 11 OS=Mus musculus GN=Dock11 PE=2 SV=1 -	10.63	1	1	6	11	1.422	0.903	0.898	1.150	23.63	10.63	8	11	527	61.0	7.85

P97807-2	Isoform Cytoplasmic of Fumarate hydratase, mitochondrial OS=Mus musculus GN=Fh -	76.45	3	28	29	413	1.535	1.022	0.989	1.150	1279.32	76.45	54	413	467	50.0	7.94
Q8BMS1	Trifunctional enzyme subunit alpha, mitochondrial OS=Mus musculus GN=Hadha PE=1 SV=1 - [illegible]	61.73	1	38	39	182	0.813	0.883	0.875	1.150	528.47	61.73	68	182	763	82.6	9.14
Q7TSE6	Serine/threonine-protein kinase 38-like OS=Mus musculus GN=Stk38l PE=1 SV=2 - [ST38L_MOUSE]	23.92	2	8	9	23	1.025	0.954	1.252	1.150	60.69	23.92	15	23	464	53.7	6.96
Q6P3E7	Histone deacetylase 10 OS=Mus musculus GN=Hdac10 PE=2 SV=2 - [HDA10_MOUSE]	1.65	1	1	1	2	0.730	0.828	0.907	1.150	5.28	1.65	2	2	666	72.1	5.39
F6YUL1	Cbp/p300-interacting transactivator 1 (Fragment) OS=Mus musculus GN=Cited1 PE=2 SV=1 - [illegible]	29.66	4	2	2	2	1.990	1.400	1.224	1.150	6.45	29.66	2	2	118	12.0	9.64
Q99K85	Phosphoserine aminotransferase OS=Mus musculus GN=Psat1 PE=1 SV=1 - [SERC_MOUSE]	63.78	3	21	21	134	2.211	0.576	1.059	1.150	407.70	63.78	37	134	370	40.4	8.03
Q9QYB5-2	Isoform 1 of Gamma-adducin OS=Mus musculus GN=Add3 - [ADDG_MOUSE]	36.65	1	2	21	132	0.981	1.018	1.283	1.150	325.10	36.65	36	132	674	75.4	6.34
B1AY13	Ubiquitin carboxyl-terminal hydrolase 24 OS=Mus musculus GN=Usp24 PE=1 SV=1 - [UBP24_MOUSE]	21.09	6	36	40	99	0.752	1.158	1.011	1.150	292.06	21.09	63	99	2617	293.8	6.19
Q3TMV7	Pyridine nucleotide-disulfide oxidoreductase domain-containing protein 1 OS=Mus musculus GN=Pyroxd1 PE=2 SV=1 - [PYRD1_MOUSE]	4.02	1	2	2	3	0.925	0.938	0.962	1.150	5.02	4.02	3	3	498	55.6	5.33
Q924Z6-2	Isoform 2 of Exportin-6 OS=Mus musculus GN=Xpo6 - [XPO6_MOUSE]	2.31	3	2	2	4	0.582	0.747	1.169	1.150	13.69	2.31	4	4	1124	128.5	6.37
P84309	Adenylate cyclase type 5 OS=Mus musculus GN=Adcy5 PE=1 SV=2 - [ADCY5_MOUSE]	23.30	2	15	21	106	0.742	0.943	2.231	1.150	331.05	23.30	35	106	1262	139.0	7.06
Q99M01	Phenylalanine--tRNA ligase, mitochondrial OS=Mus musculus GN=Fars2 PE=2 SV=1 - [SYFM_MOUSE]	19.29	2	7	7	17	1.324	1.167	1.005	1.150	41.99	19.29	12	17	451	52.3	7.17
Q9Z0V1-2	Isoform 2 of Potassium voltage-gated channel subfamily D member 3 OS=Mus musculus GN=Kcnd3 - [illegible]	18.24	3	8	10	35	0.850	1.091	1.168	1.150	114.26	18.24	14	35	636	71.4	8.25
E9Q401	Ryanodine receptor 2 OS=Mus musculus GN=Ryr2 PE=1 SV=1 - [RYR2_MOUSE]	20.66	3	76	84	242	0.676	1.799	1.561	1.151	719.85	20.66	142	242	4966	564.5	6.09
D3Z0Q6	Glycogen [starch] synthase, muscle OS=Mus musculus GN=Gys1 PE=2 SV=1 - [D3Z0Q6_MOUSE]	13.80	2	8	8	22	0.573	1.404	1.170	1.151	62.66	13.80	12	22	674	76.6	6.47
Q921Q7	Ras and Rab interactor 1 OS=Mus musculus GN=Rin1 PE=1 SV=1 - [RIN1_MOUSE]	18.87	2	12	13	40	0.613	2.605	1.894	1.151	95.56	18.87	22	40	763	83.0	8.72
Q3TCH7	Cullin-4A OS=Mus musculus GN=Cul4a PE=1 SV=1 - [CUL4A_MOUSE]	36.63	3	17	27	71	0.727	1.146	1.006	1.151	186.82	36.63	44	71	759	87.7	8.35
Q99KR3	Beta-lactamase-like protein 2 OS=Mus musculus GN=Lactb2 PE=1 SV=1 -	20.83	1	7	7	18	2.319	1.233	1.219	1.151	52.41	20.83	13	18	288	32.7	6.33
Q61290	Voltage-dependent R-type calcium channel subunit alpha-1E OS=Mus musculus GN=Cacna1e PE=1 SV=1 - [illegible]	14.17	2	20	25	81	0.730	1.494	1.791	1.151	279.01	14.17	39	81	2272	257.1	8.18
Q9Z120	tRNA (guanine-N(7)-)-methyltransferase OS=Mus musculus GN=Mettl1 PE=2 SV=1 - [TRMB_MOUSE]	13.81	2	2	2	4	0.653	0.909	0.960	1.151	9.86	13.81	3	4	268	30.6	7.34
Q8C8T7	Protein ELFN1 OS=Mus musculus GN=Elfn1 PE=2 SV=1 - [ELFN1_MOUSE]	24.88	1	13	16	39	0.677	1.300	1.547	1.151	131.51	24.88	26	39	828	90.8	8.18
Q91V51	Probable tubulin polyglutamylase TTLL1 OS=Mus musculus GN=Ttll1 PE=1 SV=1 -	7.80	2	2	2	2	0.665	1.063	1.048	1.151	7.94	7.80	2	2	423	49.1	8.72
Q8K285	FCH domain only protein 1 OS=Mus musculus GN=Fcho1 PE=1 SV=2 - [FCHO1_MOUSE]	2.75	4	2	2	3	0.805	1.271	1.253	1.152	8.56	2.75	3	3	873	95.1	7.11
Q9Z2U2-2	Isoform 2 of Zinc finger protein 292 OS=Mus musculus GN=Zfp292 - [ZN292_MOUSE]	2.67	2	5	7	16	1.690	1.036	0.817	1.152	35.01	2.67	8	16	2693	300.3	7.56
Q8R3S6	Exocyst complex component 1 OS=Mus musculus GN=Exoc1 PE=2 SV=4 - [EXOC1_MOUSE]	32.44	3	1	26	82	0.638	0.531	0.729	1.152	221.58	32.44	44	82	894	101.8	6.52

Q9D051	Pyruvate dehydrogenase E1 component subunit beta, mitochondrial OS=Mus musculus GN=Pdhb PE=1 SV=1 - [ODPB_MOUSE]	61.84	1	16	16	301	0.896	1.116	1.130	1.152	912.37	61.84	28	301	359	38.9	6.87
P50247	Adenosylhomocysteinase OS=Mus musculus GN=Ahcy PE=1 SV=3 - [SAHH_MOUSE]	68.29	2	25	27	169	1.231	0.956	0.847	1.152	458.32	68.29	50	169	432	47.7	6.54
P30548	Substance-P receptor OS=Mus musculus GN=Tacr1 PE=2 SV=2 - [NK1R_MOUSE]	3.69	1	1	1	7	1.591	1.486	1.378	1.152	18.10	3.69	2	7	407	46.3	7.64
Q5QNQ6	Oxysterol-binding protein 2 OS=Mus musculus GN=Osbp2 PE=2 SV=1 - [OSBP2_MOUSE]	10.35	4	4	8	17	0.709	1.079	1.121	1.152	42.21	10.35	12	17	908	101.3	7.14
P43274	Histone H1.4 OS=Mus musculus GN=Hist1h1e PE=1 SV=2 - [H14_MOUSE]	49.77	3	6	20	128	0.366	1.080	1.045	1.152	304.10	49.77	37	128	219	22.0	11.11
Q80U56	Late secretory pathway protein AVL9 homolog OS=Mus musculus GN=Avl9 PE=2 SV=2 - [AVL9_MOUSE]	33.28	1	15	15	74	0.606	1.183	1.057	1.152	166.60	33.28	27	74	649	72.1	6.14
Q8CGP6	Histone H2A type 1-H OS=Mus musculus GN=Hist1h2ah PE=1 SV=3 - [H2A1H_MOUSE]	58.59	7	2	5	184	1.159	1.130	1.012	1.152	387.73	58.59	10	184	128	13.9	11.03
Q6P5E8	Diacylglycerol kinase theta OS=Mus musculus GN=Dgkq PE=1 SV=1 - [DGKQ_MOUSE]	26.34	4	19	19	63	0.956	0.859	0.953	1.152	171.63	26.34	34	63	934	102.2	7.52
Q9CWE0	Mitochondrial fission regulator 1-like OS=Mus musculus GN=Mtfr1l PE=1 SV=1 - [MFR1L_MOUSE]	41.87	5	9	9	52	1.326	0.898	1.476	1.153	158.97	41.87	16	52	289	31.7	6.10
Q8R092-2	Isoform 3 of Uncharacterized protein C1orf43 homolog OS=Mus musculus -	23.28	5	4	5	9	0.913	1.167	1.063	1.153	22.72	23.28	6	9	189	21.4	8.72
E0CZE0	NEDD8-activating enzyme E1 regulatory subunit OS=Mus musculus GN=Nae1 PE=2 SV=1 - [E0CZE0_MOUSE]	25.29	2	12	12	45	0.891	0.961	0.970	1.153	118.45	25.29	19	45	510	57.6	5.85
Q9EPL4	Methyltransferase-like protein 9 OS=Mus musculus GN=Mettl9 PE=2 SV=1 - [METL9_MOUSE]	11.01	1	3	3	7	2.135	1.162	1.378	1.153	15.87	11.01	5	7	318	36.4	6.68
Q05BH3	Dhx16 protein OS=Mus musculus GN=Dhx16 PE=2 SV=1 - [Q05BH3_MOUSE]	7.15	2	3	4	11	1.543	1.302	1.255	1.153	24.94	7.15	5	11	741	84.0	6.55
Q8BHW6	Spermatogenesis-associated protein 21 OS=Mus musculus GN=Spata21 PE=2 SV=1 -	9.10	1	4	4	4	1.051	0.623	1.095	1.153	4.47	9.10	4	4	681	75.5	8.48
P48760-2	Isoform 2 of Folylpolyglutamate synthase, mitochondrial OS=Mus musculus GN=Fpgs - [illegible]	10.28	5	2	2	6	1.200	1.385	1.053	1.153	2.41	10.28	2	6	545	60.6	7.84
Q9JMC3	DnaJ homolog subfamily A member 4 OS=Mus musculus GN=Dnaja4 PE=2 SV=1 -	43.83	2	13	15	31	1.259	1.396	1.415	1.153	75.05	43.83	22	31	397	44.9	7.58
P50285	Dimethylaniline monooxygenase [N-oxide-forming] 1 OS=Mus musculus GN=Fmo1 PE=1 SV=1 - [FMO1_MOUSE]	17.48	3	6	6	11	0.864	1.657	0.814	1.153	29.53	17.48	10	11	532	59.9	8.47
Q6P2B1	Transportin-3 OS=Mus musculus GN=Tnpo3 PE=1 SV=1 - [TNPO3_MOUSE]	16.36	4	11	12	36	0.670	1.586	1.039	1.153	111.49	16.36	23	36	923	104.1	5.57
Q8CDA1	Phosphatidylinositide phosphatase SAC2 OS=Mus musculus GN=Inpp5f PE=1 SV=1 - [SAC2_MOUSE]	24.56	4	25	25	68	1.157	0.605	1.053	1.153	175.75	24.56	42	68	1132	127.5	7.11
Q9JIS5	Synaptic vesicle glycoprotein 2A OS=Mus musculus GN=Sv2a PE=1 SV=1 - [SV2A_MOUSE]	21.29	1	17	17	171	0.783	1.087	1.120	1.154	489.60	21.29	30	171	742	82.6	5.57
Q8R373	CXADR-like membrane protein OS=Mus musculus GN=Clmp PE=1 SV=1 - [CLMP_MOUSE]	6.70	1	2	2	3	0.545	1.949	1.827	1.154	7.26	6.70	2	3	373	41.2	8.38
P10833	Ras-related protein R-Ras OS=Mus musculus GN=Rras PE=2 SV=1 - [RRAS_MOUSE]	34.86	1	4	7	49	1.157	1.114	1.093	1.154	132.07	34.86	13	49	218	23.7	6.79
P62932	F-box only protein 40 OS=Mus musculus GN=Fbxo40 PE=2 SV=1 - [FBX40_MOUSE]	1.55	1	1	1	4	0.731	1.304	0.989	1.154	3.32	1.55	1	4	710	79.9	6.92
D3Z781	Sickle tail protein OS=Mus musculus GN=Etl4 PE=2 SV=2 - [D3Z781_MOUSE]	32.74	16	1	39	149	1.091	1.121	1.972	1.154	395.98	32.74	68	149	1347	146.5	9.25
Q50H33	BTB/POZ domain-containing protein KCTD8 OS=Mus musculus GN=Kctd8 PE=1 SV=1 - [KCTD8_MOUSE]	23.53	2	9	11	35	0.410	0.400	0.861	1.154	102.91	23.53	19	35	476	52.7	8.38

Q9WVC2-2	Isoform 2 of Ly-6/neurotoxin-like protein 1 OS=Mus musculus GN=Lynx1 -	19.83	1	2	2	7	0.892	0.376	0.952	1.154	17.21	19.83	4	7	116	12.8	8.19
Q8K596	Protein Slc8a2 OS=Mus musculus GN=Slc8a2 PE=2 SV=1 - [Q8K596_MOUSE]	29.97	1	19	20	134	0.741	1.815	1.619	1.155	414.27	29.97	39	134	921	100.6	5.12
Q9QZ06	Toll-interacting protein OS=Mus musculus GN=Tollip PE=1 SV=1 - [TOLIP_MOUSE]	45.99	4	10	10	103	1.300	1.022	1.029	1.155	314.01	45.99	18	103	274	30.3	5.17
Q9CZJ2	Heat shock 70 kDa protein 12B OS=Mus musculus GN=Hspa12b PE=1 SV=1 - [HS12B_MOUSE]	24.38	2	12	15	48	0.941	1.235	1.028	1.155	122.57	24.38	26	48	685	76.1	8.47
P52927	High mobility group protein HMGI-C OS=Mus musculus GN=Hmga2 PE=1 SV=1 -	32.41	2	2	2	3	2.788	4.293	3.987	1.155	5.42	32.41	2	3	108	11.8	10.62
Q61626	Glutamate receptor ionotropic, kainate 5 OS=Mus musculus GN=Grik5 PE=2 SV=2 - [GRIK5_MOUSE]	5.52	1	4	5	6	0.784	2.078	1.186	1.155	18.53	5.52	6	6	979	109.2	8.21
Q9D0S9	Histidine triad nucleotide-binding protein 2, mitochondrial OS=Mus musculus GN=Hint2 PE=2 SV=1 - [HINT2_MOUSE]	49.08	1	6	6	32	0.979	1.089	0.980	1.155	107.52	49.08	12	32	163	17.3	9.82
K3W4N2	Interleukin-18 OS=Mus musculus GN=Il18 PE=3 SV=1 - [K3W4N2_MOUSE]	20.31	2	3	3	3	1.439	0.961	1.261	1.155	8.01	20.31	3	3	192	22.1	4.91
O08638	Myosin-11 OS=Mus musculus GN=Myh11 PE=1 SV=1 - [MYH11_MOUSE]	45.03	4	3	96	488	0.888	2.275	1.205	1.155	1386.19	45.03	166	488	1972	226.9	5.45
Q8BH70	F-box/LRR-repeat protein 4 OS=Mus musculus GN=Fbxl4 PE=2 SV=1 - [FBXL4_MOUSE]	13.69	1	6	6	13	1.215	0.875	0.934	1.155	36.88	13.69	11	13	621	70.2	6.37
E5CYJ9	Protein unc-79 homolog OS=Mus musculus GN=Unc79 PE=2 SV=1 - [E5CYJ9_MOUSE]	1.21	4	3	3	5	1.275	1.014	1.158	1.155	10.13	1.21	5	5	2654	297.1	6.39
A6ZI44	Fructose-bisphosphate aldolase OS=Mus musculus GN=Aldoa PE=2 SV=1 - [A6ZI44_MOUSE]	79.67	5	6	35	1834	1.242	1.151	1.066	1.156	5613.81	79.67	63	1834	418	45.1	7.91
Q6NWV3	Intraflagellar transport protein 122 homolog OS=Mus musculus GN=Ift122 PE=2 SV=1 - [IF122_MOUSE]	5.33	3	5	5	8	0.650	1.104	0.951	1.156	27.83	5.33	8	8	1182	134.7	6.98
Q3U2V3	8-oxo-dGDP phosphatase NUDT18 OS=Mus musculus GN=Nudt18 PE=2 SV=1 -	17.03	2	4	4	7	0.527	1.100	1.006	1.156	18.35	17.03	7	7	323	35.7	6.62
A6H6A9	Rab GTPase-activating protein 1-like OS=Mus musculus GN=Rabgap1l PE=1 SV=1 - [RBG1L_MOUSE]	15.58	3	8	14	41	1.021	1.320	1.209	1.156	104.79	15.58	23	41	815	92.3	5.31
Q9WUE4	Nitrogen permease regulator 2-like protein OS=Mus musculus GN=Nprl2 PE=2 SV=1 - [NPRL2_MOUSE]	10.00	1	2	2	2	1.184	0.964	0.967	1.156	6.32	10.00	2	2	380	43.6	6.67
Q8CHT0	Delta-1-pyrroline-5-carboxylate dehydrogenase, mitochondrial OS=Mus musculus GN=Aldh4a1 [illegible]	43.59	2	23	23	107	0.676	1.533	0.880	1.156	332.94	43.59	40	107	562	61.8	8.24
Q3URS9-2	Isoform 2 of Coiled-coil domain-containing protein 51 OS=Mus musculus GN=Ccdc51 - [CCD51_MOUSE]	27.20	2	8	8	35	0.987	1.212	1.268	1.156	132.81	27.20	13	35	375	41.7	7.56
Q3UVK0	Endoplasmic reticulum metallopeptidase 1 OS=Mus musculus GN=Ermp1 PE=1 SV=2 - [ERMP1_MOUSE]	14.59	4	9	9	24	0.703	0.855	0.884	1.156	75.53	14.59	15	24	898	100.1	7.49
P38647	Stress-70 protein, mitochondrial OS=Mus musculus GN=Hspa9 PE=1 SV=3 - [GRP75_MOUSE]	61.71	1	38	39	501	1.290	1.010	1.202	1.156	1538.68	61.71	72	501	679	73.4	6.07
E9PWY6	2-amino-3-ketobutyrate coenzyme A ligase, mitochondrial OS=Mus musculus GN=Gcat PE=2 SV=1 - [E9PWY6_MOUSE]	38.74	2	9	9	21	1.245	1.204	0.867	1.156	79.37	38.74	15	21	382	41.3	7.91
O70481	E3 ubiquitin-protein ligase UBR1 OS=Mus musculus GN=Ubr1 PE=1 SV=2 - [UBR1_MOUSE]	12.58	1	17	17	33	0.889	0.985	1.057	1.156	105.62	12.58	27	33	1757	200.1	6.05
Q9D8W7	OCIA domain-containing protein 2 OS=Mus musculus GN=Ociad2 PE=2 SV=1 -	17.53	1	3	3	14	0.987	1.680	1.305	1.156	42.59	17.53	5	14	154	16.9	9.41
Q60613	Adenosine receptor A2a OS=Mus musculus GN=Adora2a PE=2 SV=3 - [AA2AR_MOUSE]	2.68	1	1	1	2	1.676	0.943	6.648	1.156	5.88	2.68	2	2	410	44.9	8.43
Q9Z110-2	Isoform Short of Delta-1-pyrroline-5-carboxylate synthase OS=Mus musculus GN=Aldh18a1 - [P5CS_MOUSE]	30.64	5	20	20	63	0.728	0.979	0.955	1.156	188.64	30.64	34	63	793	87.0	7.55

D3Z4N1	Rhotekin (Fragment) OS=Mus musculus GN=Rtkn PE=2 SV=1 - [D3Z4N1_MOUSE]	31.34	2	1	6	11	1.378	0.688	0.815	1.156	30.70	31.34	9	11	217	24.0	6.35
Q04519	Sphingomyelin phosphodiesterase OS=Mus musculus GN=Smpd1 PE=2 SV=2 -	9.41	1	6	6	11	0.964	1.433	1.128	1.157	33.18	9.41	9	11	627	69.9	7.20
F8VPN4	Protein Agl OS=Mus musculus GN=Agl PE=4 SV=1 - [F8VPN4_MOUSE]	38.90	5	45	47	153	0.766	0.950	0.976	1.157	493.20	38.90	83	153	1532	174.2	6.74
P08032	Spectrin alpha chain, erythrocytic 1 OS=Mus musculus GN=Spta1 PE=2 SV=3 - [SPTA1_MOUSE]	43.35	3	82	84	302	2.768	1.785	0.696	1.157	913.90	43.35	138	302	2415	279.7	5.03
Q6PAV2-2	Isoform 2 of Probable E3 ubiquitin-protein ligase HERC4 OS=Mus musculus GN=Herc4 - [HERC4_MOUSE]	17.06	3	16	16	36	1.022	1.300	1.110	1.157	99.47	17.06	26	36	1049	117.5	6.37
Q99JB2	Stomatin-like protein 2, mitochondrial OS=Mus musculus GN=Stoml2 PE=1 SV=1 - [STML2_MOUSE]	55.24	4	15	15	79	1.057	1.221	1.070	1.157	194.47	55.24	25	79	353	38.4	8.87
Q8BUM3	Tyrosine-protein phosphatase non-receptor type 7 OS=Mus musculus GN=Ptpn7 PE=1 SV=1 - [PTN7_MOUSE]	2.23	1	1	1	3	2.851	0.950	0.980	1.157	8.66	2.23	1	3	359	40.3	6.70
F7A6H4	E3 ubiquitin-protein ligase RNF213 OS=Mus musculus GN=Rnf213 PE=4 SV=2 - [F7A6H4_MOUSE]	1.67	3	7	7	9	1.193	1.187	1.161	1.157	12.24	1.67	8	9	5152	584.4	6.80
E9PX29	Protein Sptbn4 OS=Mus musculus GN=Sptbn4 PE=2 SV=1 - [E9PX29_MOUSE]	39.48	13	75	82	305	0.857	0.829	1.428	1.157	912.26	39.48	140	305	2561	288.6	6.00
D3Z1D7	Protein Gprin2 OS=Mus musculus GN=Gprin2 PE=4 SV=1 - [D3Z1D7_MOUSE]	17.36	1	4	4	13	1.032	0.913	0.870	1.157	25.51	17.36	7	13	455	47.0	6.40
P52633	Signal transducer and transcription activator 6 OS=Mus musculus GN=Stat6 PE=2 SV=2 - [STAT6_MOUSE]	1.67	1	1	2	2	1.246	1.233	0.970	1.157	4.32	1.67	2	2	837	93.4	6.23
Q8CC88	von Willebrand factor A domain-containing protein 8 OS=Mus musculus GN=Vwa8 PE=2 SV=2 - [VWA8_MOUSE]	30.76	3	49	49	128	0.840	0.787	0.881	1.157	374.38	30.76	83	128	1905	213.3	6.60
Q8VHQ9	Acyl-coenzyme A thioesterase 11 OS=Mus musculus GN=Acot11 PE=2 SV=1 -	31.99	6	16	16	56	0.747	0.630	0.899	1.157	171.33	31.99	28	56	594	67.3	6.80
Q9R1C6	Diacylglycerol kinase epsilon OS=Mus musculus GN=Dgke PE=2 SV=1 - [DGKE_MOUSE]	16.31	2	9	9	25	0.718	1.137	1.202	1.158	69.35	16.31	15	25	564	63.6	7.44
Q9JJ28	Protein flightless-1 homolog OS=Mus musculus GN=Flii PE=1 SV=1 - [FLII_MOUSE]	20.69	1	24	24	55	0.772	0.916	1.077	1.158	175.52	20.69	38	55	1271	144.7	6.06
P59267	Palmitoyltransferase ZDHHC2 OS=Mus musculus GN=Zdhhc2 PE=2 SV=1 -	11.75	1	3	5	10	1.633	0.765	0.986	1.158	20.18	11.75	7	10	366	42.0	8.48
Q6XPS7	L-threonine aldolase OS=Mus musculus GN=Tha1 PE=2 SV=1 - [Q6XPS7_MOUSE]	5.50	1	2	2	4	0.693	1.572	0.940	1.158	12.67	5.50	4	4	400	43.5	7.20
E9Q8Y7	Sarcospan OS=Mus musculus GN=Sspn PE=4 SV=1 - [E9Q8Y7_MOUSE]	6.43	3	1	1	2	1.184	0.892	1.044	1.158	3.57	6.43	1	2	140	15.7	7.81
Q9JLI6	Selenocysteine lyase OS=Mus musculus GN=Scly PE=1 SV=1 - [SCLY_MOUSE]	25.93	8	9	9	34	1.864	0.986	1.008	1.158	97.85	25.93	15	34	432	47.1	6.80
Q99P58	Ras-related protein Rab-27B OS=Mus musculus GN=Rab27b PE=1 SV=3 - [RB27B_MOUSE]	36.70	1	5	6	33	1.899	0.988	0.984	1.158	105.46	36.70	12	33	218	24.5	5.54
E9Q0S6	Protein Tns1 OS=Mus musculus GN=Tns1 PE=2 SV=1 - [E9Q0S6_MOUSE]	33.74	1	35	38	169	1.508	1.513	1.126	1.158	505.21	33.74	67	169	1888	201.3	7.75
Q6NT99	Dual specificity protein phosphatase 23 OS=Mus musculus GN=Dusp23 PE=2 SV=1 -	31.33	1	4	4	9	1.223	1.207	1.198	1.158	29.18	31.33	7	9	150	16.6	8.21
D3YXN7	Tetraspanin-9 (Fragment) OS=Mus musculus GN=Tspan9 PE=2 SV=1 - [D3YXN7_MOUSE]	15.12	2	2	2	10	1.357	1.132	0.984	1.158	18.78	15.12	4	10	172	19.1	4.93
A1L341	Dual-specificity tyrosine-phosphorylation-regulated kinase 1A OS=Mus musculus GN=Dyrk1a PE=2 SV=1 - [A1L341_MOUSE]	20.03	4	9	11	27	1.342	1.136	1.227	1.158	80.02	20.03	17	27	754	84.4	8.82
P05201	Aspartate aminotransferase, cytoplasmic OS=Mus musculus GN=Got1 PE=1 SV=3 - [AATC_MOUSE]	75.54	2	30	30	815	1.997	1.038	0.907	1.158	2203.85	75.54	54	815	413	46.2	7.14

O54865	Guanylate cyclase soluble subunit beta-1 OS=Mus musculus GN=Gucy1b3 PE=2 SV=1 - [GCYB1_MOUSE]	38.87	3	27	27	97	0.593	1.242	1.258	1.158	269.74	38.87	50	97	620	70.6	5.31
Q8CHI8-5	Isoform 5 of E1A-binding protein p400 OS=Mus musculus GN=Ep400 - [EP400_MOUSE]	6.29	6	10	13	40	0.841	0.888	1.173	1.158	79.15	6.29	19	40	2909	318.0	9.11
Q5SRX1-2	Isoform 2 of TOM1-like protein 2 OS=Mus musculus GN=Tom1l2 - [TM1L2_MOUSE]	48.67	9	15	15	158	1.661	1.225	1.247	1.159	556.19	48.67	27	158	487	53.3	4.87
Q3V3V9-2	Isoform 2 of Leucine-rich repeat-containing protein 16C OS=Mus musculus GN=Rltpr - [LR16C_MOUSE]	26.29	2	12	12	71	1.225	0.938	1.531	1.159	220.35	26.29	22	71	738	79.7	6.77
Q8VD72-2	Isoform 2 of Tetratricopeptide repeat protein 8 OS=Mus musculus GN=Ttc8 -	2.57	2	1	2	6	0.979	1.086	1.049	1.159	10.04	2.57	4	6	505	57.4	7.20
Q8K0T7	Protein unc-13 homolog C OS=Mus musculus GN=Unc13c PE=1 SV=3 - [UN13C_MOUSE]	12.04	1	18	25	57	0.811	0.697	1.177	1.159	148.64	12.04	38	57	2210	249.7	6.07
Q9D8B4	NADH dehydrogenase [ubiquinone] 1 alpha subcomplex subunit 11 OS=Mus musculus GN=Ndufa11 PE=2 SV=2 - [illegible]	32.62	2	2	2	3	1.618	1.134	1.008	1.159	11.60	32.62	3	3	141	15.0	8.35
P50516	V-type proton ATPase catalytic subunit A OS=Mus musculus GN=Atp6v1a PE=1 SV=2 - [VATA_MOUSE]	84.93	5	47	47	949	0.928	1.228	1.122	1.159	2847.02	84.93	83	949	617	68.3	5.58
Q8BZN6	Dedicator of cytokinesis protein 10 OS=Mus musculus GN=Dock10 PE=1 SV=3 -	15.91	4	29	29	58	0.924	0.868	1.072	1.159	169.10	15.91	44	58	2150	245.6	7.05
G3UZJ7	Protein Obox3 (Fragment) OS=Mus musculus GN=Obox3 PE=2 SV=1 - [G3UZJ7_MOUSE]	11.92	4	1	2	2	0.841	0.648	0.961	1.159	5.38	11.92	2	2	151	17.5	10.10
Q9DBC0	Selenoprotein O OS=Mus musculus GN=Selo PE=1 SV=4 - [SELO_MOUSE]	22.34	1	11	11	22	0.868	0.888	0.941	1.159	59.40	22.34	17	22	667	74.2	5.83
Q9JJX7	Tyrosyl-DNA phosphodiesterase 2 OS=Mus musculus GN=Tdp2 PE=1 SV=1 -	7.03	1	2	2	2	1.052	1.120	1.009	1.159	4.78	7.03	2	2	370	41.0	5.50
Q7TT21	Tuberin OS=Mus musculus GN=Tsc2 PE=2 SV=1 - [Q7TT21_MOUSE]	19.29	3	3	25	80	0.728	1.460	1.090	1.160	220.89	19.29	44	80	1742	194.0	6.89
Q8BTE0	UPF0369 protein C6orf57 homolog OS=Mus musculus PE=2 SV=2 - [CF057_MOUSE]	9.62	1	1	1	1	0.920	1.196	1.094	1.160	3.07	9.62	1	1	104	11.9	9.50
P21279	Guanine nucleotide-binding protein G(q) subunit alpha OS=Mus musculus GN=Gnaq PE=1 SV=4 - [GNAQ_MOUSE]	54.04	1	10	18	132	0.719	1.007	0.980	1.160	357.88	54.04	31	132	359	42.1	5.68
P97499	Telomerase protein component 1 OS=Mus musculus GN=Tep1 PE=1 SV=1 - [TEP1_MOUSE]	2.32	1	3	4	9	1.095	0.940	1.162	1.160	16.41	2.32	4	9	2629	291.3	7.24
Q9DBS2	Tumor protein p63-regulated gene 1-like protein OS=Mus musculus GN=Tprg1l PE=1 SV=1 - [TPRGL_MOUSE]	50.38	2	9	9	74	0.774	1.365	1.084	1.160	163.87	50.38	17	74	266	29.8	7.37
Q4PZA2-3	Isoform C of Endothelin-converting enzyme 1 OS=Mus musculus GN=Ece1 -	7.30	4	5	5	11	1.058	1.269	0.858	1.160	39.75	7.30	9	11	753	85.4	5.82
G3UVU9	Bifunctional arginine demethylase and lysyl-hydroxylase JMJD6 OS=Mus musculus GN=Jmjd6 PE=4 SV=1 - [illegible]	11.11	5	2	2	4	2.075	0.978	0.984	1.160	10.62	11.11	3	4	270	32.0	9.28
D3Z1K9	Protein Ttc18 OS=Mus musculus GN=Ttc18 PE=2 SV=2 - [D3Z1K9_MOUSE]	1.91	2	1	1	1	2.501	1.227	1.126	1.160	3.80	1.91	1	1	1097	123.0	5.52
Q99KF0	Caspase recruitment domain-containing protein 14 OS=Mus musculus GN=Card14 PE=2 SV=2 - [CAR14_MOUSE]	3.50	1	2	2	6	1.003	0.689	1.412	1.160	2.64	3.50	2	6	999	113.4	6.10
D6RET7	GRAM domain-containing protein 4 OS=Mus musculus GN=Gramd4 PE=2 SV=1 -	12.69	3	2	3	10	0.880	1.177	1.092	1.160	24.30	12.69	5	10	323	37.6	8.13
A2ASZ8-5	Isoform 5 of Calcium-binding mitochondrial carrier protein SCaMC-2 OS=Mus musculus GN=Slc25a25 - [illegible]	48.50	8	18	21	80	0.936	0.700	0.867	1.160	229.16	48.50	36	80	501	56.0	8.87
Q810B8	SLIT and NTRK-like protein 4 OS=Mus musculus GN=Slitrk4 PE=2 SV=2 - [SLIK4_MOUSE]	6.81	1	3	4	7	0.853	0.897	1.012	1.160	19.47	6.81	6	7	837	94.5	7.80
Q5SYH2	Transmembrane protein 199 OS=Mus musculus GN=Tmem199 PE=2 SV=1 - [TM199_MOUSE]	7.69	1	2	2	3	0.805	0.957	0.855	1.160	6.77	7.69	3	3	208	23.1	9.10

Q14BB9	MAP6 domain-containing protein 1 OS=Mus musculus GN=Map6d1 PE=1 SV=1 -	56.54	1	6	6	33	1.636	1.051	1.617	1.160	112.37	56.54	10	33	191	20.4	9.88
Q80Y98	Phospholipase DDHD2 OS=Mus musculus GN=Ddhd2 PE=1 SV=3 - [DDHD2_MOUSE]	22.03	3	16	16	58	0.785	1.040	0.989	1.160	142.61	22.03	28	58	699	79.5	5.31
Q64737	Trifunctional purine biosynthetic protein adenosine-3 OS=Mus musculus GN=Gart PE=2 SV=3 - [PUR2_MOUSE]	45.25	3	34	34	108	0.944	0.982	0.998	1.160	361.35	45.25	59	108	1010	107.4	6.68
Q3UR44	Protein MRVI1 (Fragment) OS=Mus musculus GN=Mrvi1 PE=2 SV=1 - [Q3UR44_MOUSE]	5.87	4	2	3	5	2.433	2.455	1.136	1.160	11.16	5.87	3	5	715	78.4	5.66
Q9ER47	Potassium voltage-gated channel subfamily H member 7 OS=Mus musculus GN=Kcnh7 PE=2 SV=2 - [KCNH7_MOUSE]	3.10	1	3	3	8	1.478	1.082	0.868	1.160	18.57	3.10	5	8	1195	135.0	8.00
Q99LS3	Phosphoserine phosphatase OS=Mus musculus GN=Psph PE=1 SV=1 - [SERB_MOUSE]	24.89	3	4	4	13	0.675	1.453	1.116	1.160	40.51	24.89	6	13	225	25.1	6.14
P21278	Guanine nucleotide-binding protein subunit alpha-11 OS=Mus musculus GN=Gna11 PE=1 SV=1 - [illegible]	55.43	1	13	20	109	0.693	1.255	0.986	1.161	310.51	55.43	36	109	359	42.0	5.97
Q8CBY1-4	Isoform 4 of Protein Smaug homolog 1 OS=Mus musculus GN=Samd4a -	11.59	4	2	2	4	0.565	0.288	0.681	1.161	8.80	11.59	3	4	302	32.8	9.35
Q9Z2C4	Myotubularin-related protein 1 OS=Mus musculus GN=Mtmr1 PE=1 SV=1 -	25.26	7	13	15	50	0.593	1.265	1.237	1.161	129.86	25.26	26	50	669	75.3	6.80
Q9D1A2	Cytosolic non-specific dipeptidase OS=Mus musculus GN=Cndp2 PE=1 SV=1 -	60.42	1	21	21	103	1.016	1.178	1.098	1.161	323.42	60.42	39	103	475	52.7	5.66
D3YVV3	Protein Ankrd29 OS=Mus musculus GN=Ankrd29 PE=2 SV=1 - [D3YVV3_MOUSE]	35.60	3	9	9	29	1.098	0.632	1.096	1.161	74.44	35.60	17	29	309	33.2	8.43
Q9WU78	Programmed cell death 6-interacting protein OS=Mus musculus GN=Pdcd6ip PE=1 SV=3 - [PDC6I_MOUSE]	57.08	3	40	40	179	0.851	1.020	1.012	1.161	612.41	57.08	66	179	869	96.0	6.52
Q569Z5	Probable ATP-dependent RNA helicase DDX46 OS=Mus musculus GN=Ddx46 PE=1 SV=2 - [DDX46_MOUSE]	20.54	1	3	21	45	1.277	1.002	1.167	1.161	139.09	20.54	35	45	1032	117.4	9.26
P97355	Spermine synthase OS=Mus musculus GN=Sms PE=1 SV=1 - [SPSY_MOUSE]	52.73	3	15	15	48	0.952	1.055	1.094	1.161	125.33	52.73	27	48	366	41.3	5.06
G3UZ30	Protein phosphatase 1 regulatory subunit 11 (Fragment) OS=Mus musculus GN=Ppp1r11 PE=2 SV=2 - [illegible]	36.67	3	3	3	24	1.403	0.698	2.015	1.162	75.38	36.67	6	24	120	13.8	8.59
Q9Z0J4	Nitric oxide synthase, brain OS=Mus musculus GN=Nos1 PE=1 SV=1 - [NOS1_MOUSE]	40.80	6	51	53	191	0.694	1.752	0.887	1.162	530.63	40.80	92	191	1429	160.4	7.15
Q99KR8	Plasma alpha-L-fucosidase OS=Mus musculus GN=Fuca2 PE=2 SV=1 - [FUCO2_MOUSE]	4.12	1	2	2	8	1.083	1.159	0.818	1.162	17.49	4.12	3	8	461	53.6	6.61
P35282	Ras-related protein Rab-21 OS=Mus musculus GN=Rab21 PE=1 SV=4 - [RAB21_MOUSE]	46.40	1	9	10	33	0.929	0.776	0.961	1.162	105.94	46.40	17	33	222	24.1	7.94
P36993	Protein phosphatase 1B OS=Mus musculus GN=Ppm1b PE=2 SV=1 - [PPM1B_MOUSE]	43.59	5	1	13	91	0.861	0.856	1.610	1.162	254.22	43.59	24	91	390	42.8	5.19
E9QN14	SLIT-ROBO Rho GTPase-activating protein 3 OS=Mus musculus GN=Srgap3 PE=2 SV=1 - [E9QN14_MOUSE]	38.60	3	27	33	158	1.015	1.109	1.161	1.162	471.83	38.60	59	158	1075	121.6	6.57
E9QB08	Protein shisa-5 (Fragment) OS=Mus musculus GN=Shisa5 PE=2 SV=1 - [E9QB08_MOUSE]	9.59	1	1	1	6	1.578	1.280	1.257	1.162	0.00	9.59	1	6	146	15.8	7.66
A2AB59	Rho GTPase-activating protein 27 OS=Mus musculus GN=Arhgap27 PE=1 SV=1 -	5.29	3	3	3	11	1.047	0.708	1.102	1.162	24.56	5.29	6	11	869	97.0	5.94
Q6P6I8	Signal-regulatory protein alpha OS=Mus musculus GN=Sirpa PE=2 SV=1 - [Q6P6I8_MOUSE]	43.61	8	14	16	230	0.909	1.989	1.553	1.162	664.42	43.61	28	230	509	56.0	8.28
Q9R0N5	Synaptotagmin-5 OS=Mus musculus GN=Syt5 PE=1 SV=1 - [SYT5_MOUSE]	34.97	1	8	13	69	0.766	1.510	0.889	1.162	143.72	34.97	22	69	386	43.1	9.60
Q9CZL5	Pterin-4-alpha-carbinolamine dehydratase 2 OS=Mus musculus GN=Pcbd2 PE=1 SV=2 - [PHS2_MOUSE]	34.56	1	3	5	11	1.183	1.264	1.967	1.162	26.82	34.56	8	11	136	14.8	9.16

Q9CQI6	Coactosin-like protein OS=Mus musculus GN=Cotl1 PE=1 SV=3 - [COTL1_MOUSE]	66.90	1	8	8	65	1.060	1.090	0.899	1.162	183.26	66.90	15	65	142	15.9	5.40
A2AGR6	DNA damage-binding protein 2 OS=Mus musculus GN=Ddb2 PE=2 SV=1 - [A2AGR6_MOUSE]	6.42	2	2	2	2	1.167	0.810	0.951	1.162	4.79	6.42	2	2	296	33.4	8.35
Q3UTH8-2	Isoform 2 of Rho guanine nucleotide exchange factor 9 OS=Mus musculus GN=Arhgef9 - [ARHG9_MOUSE]	27.07	10	13	15	40	0.897	1.366	1.065	1.162	105.70	27.07	23	40	495	58.6	5.92
Q791T5-2	Isoform 2 of Mitochondrial carrier homolog 1 OS=Mus musculus GN=Mtch1 - [MTCH1_MOUSE]	44.89	3	14	14	72	0.808	1.048	0.938	1.162	202.58	44.89	23	72	372	39.9	9.48
P23780	Beta-galactosidase OS=Mus musculus GN=Glb1 PE=2 SV=1 - [BGAL_MOUSE]	13.91	2	8	8	16	0.646	1.175	1.006	1.162	48.76	13.91	12	16	647	73.1	7.47
Q6NS52-2	Isoform 2 of Diacylglycerol kinase beta OS=Mus musculus GN=Dgkb - [DGKB_MOUSE]	33.96	2	21	22	67	0.817	2.232	1.735	1.162	191.98	33.96	34	67	795	89.4	7.93
Q8BW41-2	Isoform 2 of Glycosyltransferase-like domain-containing protein 2 OS=Mus musculus GN=Gtdc2 - [illegible]	25.69	2	9	9	26	0.957	1.003	0.816	1.163	82.62	25.69	17	26	580	66.6	8.47
G3X9L6	MCG55033 OS=Mus musculus GN=Gm10250 PE=4 SV=1 - [G3X9L6_MOUSE]	88.75	3	13	15	170	1.052	1.061	1.337	1.163	453.80	88.75	24	170	160	18.6	5.44
Q9D404	3-oxoacyl-[acyl-carrier-protein] synthase, mitochondrial OS=Mus musculus GN=Oxsm PE=2 SV=1 - [OXSM_MOUSE]	23.31	2	6	6	24	1.589	1.001	0.888	1.163	70.43	23.31	11	24	459	48.6	7.06
P36898	Bone morphogenetic protein receptor type-1B OS=Mus musculus GN=Bmpr1b PE=1 SV=1 - [BMR1B_MOUSE]	8.57	1	2	3	3	1.943	1.274	1.570	1.163	5.92	8.57	3	3	502	56.9	7.53
Q8K3I9	Glucocorticoid-induced transcript 1 protein OS=Mus musculus GN=Glcci1 PE=1 SV=1 - [GLCI1_MOUSE]	16.01	5	5	5	8	1.137	0.823	1.930	1.163	18.88	16.01	8	8	537	57.4	9.47
P97313	DNA-dependent protein kinase catalytic subunit OS=Mus musculus GN=Prkdc PE=1 SV=3 - [PRKDC_MOUSE]	2.18	3	5	7	14	0.812	0.934	0.901	1.163	28.98	2.18	8	14	4128	471.2	7.12
Q80U44	Zinc finger FYVE domain-containing protein 16 OS=Mus musculus GN=Zfyve16 PE=1 SV=2 - [ZFY16_MOUSE]	1.83	1	3	3	4	1.325	1.311	1.176	1.163	8.53	1.83	4	4	1528	166.6	4.78
Q6ZPJ0	Testis-expressed sequence 2 protein OS=Mus musculus GN=Tex2 PE=1 SV=2 - [TEX2_MOUSE]	24.65	3	21	21	49	0.847	0.910	1.116	1.163	134.87	24.65	33	49	1128	125.1	5.80
Q3UJU9	Regulator of microtubule dynamics protein 3 OS=Mus musculus GN=Rmdn3 PE=1 SV=2 - [RMD3_MOUSE]	35.74	4	12	12	73	0.895	1.356	1.025	1.163	202.02	35.74	22	73	470	52.0	5.21
P70271	PDZ and LIM domain protein 4 OS=Mus musculus GN=Pdlim4 PE=1 SV=3 -	36.67	3	8	8	28	1.353	1.268	1.131	1.164	58.32	36.67	14	28	330	35.5	8.05
D3Z2R5	Protein Sepn1 OS=Mus musculus GN=Sepn1 PE=4 SV=1 - [D3Z2R5_MOUSE]	5.75	1	3	3	5	1.568	1.104	0.637	1.164	16.29	5.75	5	5	557	62.4	5.55
Q99NH8	Triggering receptor expressed on myeloid cells 2 OS=Mus musculus GN=Trem2 PE=1 SV=1 - [TREM2_MOUSE]	12.78	2	2	2	2	1.606	1.251	1.307	1.164	5.53	12.78	2	2	227	24.5	6.67
Q9DC70	NADH dehydrogenase [ubiquinone] iron-sulfur protein 7, mitochondrial OS=Mus musculus GN=Ndufs7 PE=1 SV=1 - [illegible]	14.73	1	4	4	24	0.555	0.804	0.985	1.164	67.36	14.73	7	24	224	24.7	9.92
Q920N2	Biotin--protein ligase OS=Mus musculus GN=Hlcs PE=1 SV=1 - [BPL1_MOUSE]	15.65	1	6	6	15	1.708	1.025	1.251	1.164	34.27	15.65	11	15	722	78.5	5.34
Q91WD5	NADH dehydrogenase [ubiquinone] iron-sulfur protein 2, mitochondrial OS=Mus musculus GN=Ndufs2 PE=1 SV=1 - [illegible]	61.99	3	22	22	241	0.747	1.114	1.046	1.164	749.89	61.99	41	241	463	52.6	6.99
Q8BGR9	Ubiquitin-like domain-containing CTD phosphatase 1 OS=Mus musculus GN=Ublcp1 PE=1 SV=1 - [illegible]	27.36	2	8	8	25	0.971	0.548	0.799	1.164	63.93	27.36	13	25	318	36.8	6.46
Q8CI43	Myosin light chain 6B OS=Mus musculus GN=Myl6b PE=2 SV=1 - [MYL6B_MOUSE]	56.04	1	10	12	63	1.900	0.702	1.398	1.165	172.64	56.04	19	63	207	22.7	5.53
Q9ER74	Sal-like protein 1 OS=Mus musculus GN=Sall1 PE=1 SV=1 - [SALL1_MOUSE]	3.86	2	2	2	4	0.593	0.825	0.855	1.165	10.38	3.86	2	4	1322	140.1	7.08

Q9D2E2	Target of EGR1 protein 1 OS=Mus musculus GN=Toe1 PE=2 SV=1 - [TOE1_MOUSE]	3.52	2	2	2	5	1.525	1.394	1.202	1.165	5.86	3.52	3	5	511	56.8	6.64
Q80ZD8	Amphoterin-induced protein 1 OS=Mus musculus GN=Amigo1 PE=1 SV=1 -	15.45	3	6	6	8	0.774	1.312	1.068	1.165	27.35	15.45	8	8	492	55.3	6.86
E9QMR2	Dedicator of cytokinesis protein 9 OS=Mus musculus GN=Dock9 PE=2 SV=1 - [E9QMR2_MOUSE]	20.36	2	1	37	98	0.848	1.055	1.007	1.165	257.11	20.36	60	98	2058	234.8	7.56
D3YZ99	Protein N-terminal asparagine amidohydrolase OS=Mus musculus GN=Ntan1 PE=2 SV=1 - [D3YZ99_MOUSE]	7.93	2	1	1	3	1.673	0.750	1.060	1.165	17.11	7.93	2	3	227	24.9	5.88
Q4VA93	Protein kinase C OS=Mus musculus GN=Prkca PE=2 SV=1 - [Q4VA93_MOUSE]	38.54	2	4	23	130	0.732	1.944	1.597	1.165	330.59	38.54	37	130	672	76.8	7.05
Q91XP5	Glycine receptor subunit alpha-3 OS=Mus musculus GN=Glra3 PE=2 SV=2 - [GLRA3_MOUSE]	8.19	2	4	4	9	0.759	0.883	0.954	1.166	28.19	8.19	5	9	464	53.7	8.50
Q9Z2D6	Methyl-CpG-binding protein 2 OS=Mus musculus GN=Mecp2 PE=1 SV=1 -	50.21	4	19	19	122	0.744	1.187	1.204	1.166	369.74	50.21	36	122	484	52.3	9.96
O35621	Phosphomannomutase 1 OS=Mus musculus GN=Pmm1 PE=1 SV=1 - [PMM1_MOUSE]	51.15	5	11	13	68	1.188	1.470	1.235	1.166	177.47	51.15	22	68	262	29.8	5.47
P70178	Homeobox protein SIX5 OS=Mus musculus GN=Six5 PE=1 SV=2 - [SIX5_MOUSE]	6.54	1	2	2	2	2.113	0.584	0.876	1.166	4.44	6.54	2	2	719	73.7	5.14
O70146	Dual specificity testis-specific protein kinase 1 OS=Mus musculus GN=Tesk1 PE=2 SV=3 - [TESK1_MOUSE]	1.91	1	1	1	2	0.598	1.152	0.991	1.166	4.43	1.91	1	2	627	68.0	8.06
E9QKU6	Serine/threonine-protein phosphatase 4 regulatory subunit 4 OS=Mus musculus GN=Ppp4r4 PE=4 SV=1 - [illegible]	21.83	4	16	16	46	0.737	0.954	1.151	1.166	114.85	21.83	27	46	875	99.4	7.71
Q8C8T8	Pre-rRNA-processing protein TSR2 homolog OS=Mus musculus GN=Tsr2 PE=2 SV=1 - [TSR2_MOUSE]	13.61	2	2	2	26	1.543	1.431	1.349	1.166	81.51	13.61	4	26	191	20.9	4.23
P16054	Protein kinase C epsilon type OS=Mus musculus GN=Prkce PE=1 SV=1 - [KPCE_MOUSE]	51.70	3	32	34	212	0.663	1.605	1.552	1.166	581.66	51.70	58	212	737	83.5	7.03
A2AWI7	Endophilin-B2 OS=Mus musculus GN=Sh3glb2 PE=2 SV=1 - [A2AWI7_MOUSE]	53.47	1	1	19	125	0.841	1.197	1.329	1.166	373.90	53.47	35	125	404	44.9	5.58
P67778	Prohibitin OS=Mus musculus GN=Phb PE=1 SV=1 - [PHB_MOUSE]	53.31	2	14	14	110	0.749	1.112	1.035	1.166	410.01	53.31	27	110	272	29.8	5.76
E9Q6Y0	Transmembrane channel-like protein 3 OS=Mus musculus GN=Tmc3 PE=2 SV=1 - [E9Q6Y0_MOUSE]	1.18	3	1	1	1	2.675	4.078	1.062	1.166	2.48	1.18	1	1	1101	125.6	9.11
Q62240	Lysine-specific demethylase 5D OS=Mus musculus GN=Kdm5d PE=2 SV=2 -	1.94	4	2	4	5	1.302	0.999	1.345	1.166	5.81	1.94	4	5	1548	176.9	6.16
B7ZCJ1	Rho GTPase-activating protein 21 OS=Mus musculus GN=Arhgap21 PE=4 SV=1 -	28.08	6	42	43	121	1.009	1.184	1.414	1.166	352.94	28.08	71	121	1955	216.8	7.64
Q99KI0	Aconitate hydratase, mitochondrial OS=Mus musculus GN=Aco2 PE=1 SV=1 - [ACON_MOUSE]	58.33	1	47	47	1028	0.926	0.854	1.113	1.167	2947.45	58.33	86	1028	780	85.4	7.93
Q6S5J6-2	Isoform 2 of Krev interaction trapped protein 1 OS=Mus musculus GN=Krit1 - [KRIT1_MOUSE]	7.03	4	4	4	9	1.329	1.014	1.427	1.167	20.07	7.03	7	9	697	79.5	8.41
Q80VM5	Dipeptidyl aminopeptidase-like protein 6 OS=Mus musculus GN=Dpp6 PE=2 SV=1 - [Q80VM5_MOUSE]	33.25	3	1	25	174	1.479	1.087	2.022	1.167	480.88	33.25	49	174	803	91.2	6.48
Q8CC86	Nicotinate phosphoribosyltransferase OS=Mus musculus GN=Naprt1 PE=2 SV=1 - [PNCB_MOUSE]	22.86	1	9	9	25	1.221	1.172	0.892	1.167	80.52	22.86	16	25	538	58.2	6.46
Q62108-3	Isoform 3 of Disks large homolog 4 OS=Mus musculus GN=Dlg4 - [DLG4_MOUSE]	48.27	9	25	31	252	0.844	1.633	1.447	1.167	830.65	48.27	58	252	721	80.1	5.92
Q8K3F6	Potassium voltage-gated channel subfamily KQT member 3 OS=Mus musculus GN=Kcnq3 PE=1 SV=2 - [KCNQ3_MOUSE]	13.40	1	6	7	15	0.711	1.139	1.595	1.167	48.68	13.40	11	15	873	96.8	8.79
F6SMJ7	GTP-binding protein 8 (Fragment) OS=Mus musculus GN=Gtpbp8 PE=4 SV=1 -	27.89	4	3	3	10	0.870	0.840	1.041	1.168	33.34	27.89	6	10	147	16.3	8.85

A2AT37	Protein Upf2 OS=Mus musculus GN=Upf2 PE=2 SV=1 - [A2AT37_MOUSE]	11.74	2	11	11	25	1.301	1.125	1.026	1.168	73.83	11.74	19	25	1269	147.5	5.64
O35633-2	Isoform 2 of Vesicular inhibitory amino acid transporter OS=Mus musculus GN=Slc32a1 - [VIAAT_MOUSE]	30.52	2	13	14	96	0.573	0.631	0.956	1.168	257.70	30.52	26	96	521	56.8	6.90
Q91V57	N-chimaerin OS=Mus musculus GN=Chn1 PE=1 SV=2 - [CHIN_MOUSE]	28.54	8	12	12	36	0.895	0.819	1.021	1.168	116.75	28.54	18	36	459	53.2	6.76
O08530	Sphingosine 1-phosphate receptor 1 OS=Mus musculus GN=S1pr1 PE=2 SV=3 - [S1PR1_MOUSE]	8.38	1	3	3	12	0.492	0.981	1.117	1.168	37.91	8.38	6	12	382	42.6	9.42
Q7TQ33	RGM domain family member B OS=Mus musculus GN=Rgmb PE=1 SV=1 - [RGMB_MOUSE]	6.19	1	2	2	2	1.389	1.011	1.424	1.168	7.28	6.19	2	2	436	47.2	6.34
Q99LC5	Electron transfer flavoprotein subunit alpha, mitochondrial OS=Mus musculus GN=Etfa PE=1 SV=2 - [ETFA_MOUSE]	73.87	1	20	20	88	1.160	1.061	0.994	1.168	305.15	73.87	36	88	333	35.0	8.38
P12657	Muscarinic acetylcholine receptor M1 OS=Mus musculus GN=Chrm1 PE=2 SV=2 -	5.65	1	2	2	8	0.596	2.753	2.864	1.168	17.43	5.65	4	8	460	51.3	9.32
B2RUS7	ATP-binding cassette, sub-family C (CFTR/MRP), member 8 OS=Mus musculus GN=Abcc8 PE=2 SV=1 - [B2RUS7_MOUSE]	8.56	5	9	11	17	0.869	1.011	0.974	1.168	31.81	8.56	14	17	1588	178.0	8.19
P56379	6.8 kDa mitochondrial proteolipid OS=Mus musculus GN=Mp68 PE=2 SV=1 - [68MP_MOUSE]	29.31	1	2	2	15	0.945	1.012	1.284	1.169	32.20	29.31	3	15	58	6.7	9.99
Q91XY8	Protein Pcdhgb6 OS=Mus musculus GN=Pcdhga11 PE=2 SV=1 - [Q91XY8_MOUSE]	10.40	1	3	8	30	1.370	1.568	1.558	1.169	83.09	10.40	16	30	933	101.1	4.88
Q8BZ60	Stonin-2 OS=Mus musculus GN=Ston2 PE=2 SV=1 - [STON2_MOUSE]	16.54	2	11	11	25	1.056	1.129	1.025	1.169	73.44	16.54	17	25	895	99.5	5.26
D9HP81	Regulating synaptic membrane exocytosis 2 OS=Mus musculus GN=Rims2 PE=2 SV=1 - [D9HP81_MOUSE]	18.51	4	21	24	73	0.946	0.888	1.283	1.169	209.33	18.51	40	73	1572	177.5	9.36
P04104	Keratin, type II cytoskeletal 1 OS=Mus musculus GN=Krt1 PE=1 SV=4 - [K2C1_MOUSE]	4.87	1	2	3	16	2.738	1.212	1.178	1.169	35.67	4.87	5	16	637	65.6	8.15
H3BIW6	1-phosphatidylinositol 4,5-bisphosphate phosphodiesterase eta-2 OS=Mus musculus GN=Plch2 PE=2 SV=1 - [illegible]	19.70	15	17	21	79	0.505	1.223	1.309	1.169	222.52	19.70	36	79	1137	125.7	6.79
Q9WVG6-2	Isoform 2 of Histone-arginine methyltransferase CARM1 OS=Mus musculus GN=Carm1 - [CARM1_MOUSE]	17.44	4	7	7	18	0.952	1.113	1.063	1.169	47.34	17.44	12	18	585	63.4	6.61
Q8BGQ1	Spermatogenesis-defective protein 39 homolog OS=Mus musculus GN=Vipas39 PE=1 SV=1 - [SPE39_MOUSE]	33.81	2	15	15	55	0.992	1.156	1.001	1.169	169.08	33.81	26	55	491	56.6	7.17
Q3UGX2	Spectrin beta 1 OS=Mus musculus GN=Sptb PE=2 SV=1 - [Q3UGX2_MOUSE]	54.27	1	7	109	433	0.901	1.149	1.112	1.169	1366.96	54.27	193	433	2329	267.9	5.49
D3YY34	Polyhomeotic-like protein 3 OS=Mus musculus GN=Phc3 PE=4 SV=1 - [D3YY34_MOUSE]	7.28	7	4	4	13	1.989	1.757	1.610	1.169	27.37	7.28	6	13	948	102.1	6.65
Q6ZQ38	Cullin-associated NEDD8-dissociated protein 1 OS=Mus musculus GN=Cand1 PE=2 SV=2 - [CAND1_MOUSE]	38.05	2	42	44	231	0.637	1.225	0.956	1.170	753.86	38.05	79	231	1230	136.2	5.78
P97454	Mothers against decapentaplegic homolog 5 OS=Mus musculus GN=Smad5 PE=1 SV=2 - [SMAD5_MOUSE]	7.31	4	2	4	7	0.739	0.968	0.889	1.170	21.71	7.31	7	7	465	52.1	7.71
Q9QZH6	Evolutionarily conserved signaling intermediate in Toll pathway, mitochondrial OS=Mus musculus GN=Ecsit PE=1 SV=2 - [ECSIT_MOUSE]	32.64	2	12	12	26	0.828	0.879	0.987	1.170	66.71	32.64	21	26	435	49.8	6.60
Q8K0U4	Heat shock 70 kDa protein 12A OS=Mus musculus GN=Hspa12a PE=1 SV=1 - [HS12A_MOUSE]	62.37	1	34	37	259	0.935	0.976	1.094	1.170	734.25	62.37	66	259	675	74.8	6.77
Q3TIU4	2',5'-phosphodiesterase 12 OS=Mus musculus GN=Pde12 PE=2 SV=2 - [PDE12_MOUSE]	22.70	1	9	9	26	0.954	0.887	1.024	1.170	70.09	22.70	16	26	608	67.5	6.92
Q9Z0X1	Apoptosis-inducing factor 1, mitochondrial OS=Mus musculus GN=Aifm1 PE=1 SV=1 - [AIFM1_MOUSE]	33.82	2	17	17	94	1.020	1.018	1.090	1.170	286.59	33.82	32	94	612	66.7	9.17

Q60759	Glutaryl-CoA dehydrogenase, mitochondrial OS=Mus musculus GN=Gcdh PE=2 SV=2 - [GCDH_MOUSE]	29.91	2	8	11	52	0.586	0.989	0.942	1.170	124.56	29.91	16	52	438	48.6	8.73
Q6ZPK0-5	Isoform 5 of PHD finger protein 21A OS=Mus musculus GN=Phf21a - [PF21A_MOUSE]	5.31	18	3	3	4	0.713	0.551	1.168	1.170	8.86	5.31	4	4	527	59.1	9.54
Q8VDG5	Phosphopantothenate--cysteine ligase OS=Mus musculus GN=Ppcs PE=2 SV=1 - [PPCS_MOUSE]	23.15	2	7	7	12	1.316	0.998	0.967	1.170	26.44	23.15	8	12	311	33.8	6.55
P60041	Somatostatin OS=Mus musculus GN=Sst PE=2 SV=1 - [SMS_MOUSE]	44.83	1	3	3	15	1.273	1.506	1.278	1.170	31.46	44.83	5	15	116	12.7	5.52
Q3V209-2	Isoform 2 of Transmembrane and ubiquitin-like domain-containing protein 2 OS=Mus musculus [illegible]	10.37	3	1	1	5	0.707	0.560	0.858	1.170	17.70	10.37	2	5	299	31.6	4.92
Q60996	Serine/threonine-protein phosphatase 2A 56 kDa regulatory subunit gamma isoform OS=Mus musculus GN=Ppp2r5c PE=1 SV=2 - [2A5G_MOUSE]	33.78	4	8	15	35	0.586	1.031	1.049	1.170	119.38	33.78	24	35	524	60.8	6.64
Q8BMK1	Methyltransferase-like protein 2 OS=Mus musculus GN=Mettl2 PE=2 SV=2 - [METL2_MOUSE]	7.46	1	2	2	4	1.447	1.200	1.281	1.170	6.64	7.46	4	4	389	43.9	6.20
Q5SQM0	Echinoderm microtubule-associated protein-like 6 OS=Mus musculus GN=Eml6 PE=2 SV=1 - [EMAL6_MOUSE]	3.27	1	4	6	9	1.275	1.411	1.135	1.170	19.18	3.27	9	9	1958	217.8	7.42
O35071	Kinesin-like protein KIF1C OS=Mus musculus GN=Kif1c PE=1 SV=2 - [KIF1C_MOUSE]	12.09	3	5	13	51	1.075	0.610	1.414	1.170	116.75	12.09	20	51	1100	122.4	7.03
Q3UPC7	Uncharacterized protein KIAA0825 homolog OS=Mus musculus PE=2 SV=3 - [K0825_MOUSE]	0.86	1	1	1	1	0.815	0.798	1.085	1.170	0.00	0.86	1	1	1272	145.5	6.23
Q99LY2	Glycerophosphodiester phosphodiesterase domain-containing protein 3 OS=Mus musculus GN=Gdpd3 PE=2 SV=2 - [GDPD3_MOUSE]	3.03	1	1	1	1	0.433	0.989	1.139	1.170	0.00	3.03	1	1	330	38.5	7.25
Q9D820-2	Isoform 2 of Prolyl-tRNA synthetase associated domain-containing protein 1 OS=Mus musculus GN=Prorsd1 - [illegible]	26.67	2	3	3	6	1.374	0.831	0.996	1.170	15.66	26.67	4	6	135	15.0	7.18
P28843	Dipeptidyl peptidase 4 OS=Mus musculus GN=Dpp4 PE=1 SV=3 - [DPP4_MOUSE]	6.97	1	3	4	10	0.828	4.083	0.948	1.170	16.80	6.97	6	10	760	87.4	6.42
Q8C1D8	Protein IWS1 homolog OS=Mus musculus GN=Iws1 PE=1 SV=1 - [IWS1_MOUSE]	8.49	2	6	6	8	1.195	1.018	1.139	1.170	18.20	8.49	8	8	766	85.2	4.67
O08852	Polycystin-1 OS=Mus musculus GN=Pkd1 PE=1 SV=2 - [PKD1_MOUSE]	0.82	1	2	3	4	1.078	0.938	1.221	1.171	9.18	0.82	4	4	4293	466.3	5.87
P32921-2	Isoform 2 of Tryptophan--tRNA ligase, cytoplasmic OS=Mus musculus GN=Wars - [SYWC_MOUSE]	50.53	1	1	22	184	1.001	1.019	1.159	1.171	522.95	50.53	38	184	475	53.6	7.08
Q9JHI7	Exosome complex component RRP45 OS=Mus musculus GN=Exosc9 PE=2 SV=1 - [EXOS9_MOUSE]	4.79	1	2	2	3	1.049	0.795	0.919	1.171	3.25	4.79	3	3	438	48.9	5.11
Q499X9	Methionine--tRNA ligase, mitochondrial OS=Mus musculus GN=Mars2 PE=2 SV=2 - [SYMM_MOUSE]	13.48	1	8	8	17	0.810	0.856	1.060	1.171	51.28	13.48	15	17	586	65.8	8.09
Q9CZ57	5-methylcytosine rRNA methyltransferase NSUN4 OS=Mus musculus GN=Nsun4 PE=2 SV=1 - [NSUN4_MOUSE]	14.17	2	4	4	6	0.706	1.117	1.003	1.171	18.47	14.17	6	6	381	42.8	8.25
Q8K358	Phosphatidylinositol glycan anchor biosynthesis class U protein OS=Mus musculus GN=Pigu PE=2 SV=4 - [PIGU_MOUSE]	3.92	2	1	1	2	0.663	0.975	0.945	1.171	8.47	3.92	2	2	434	49.8	7.72
P42703	Leukemia inhibitory factor receptor OS=Mus musculus GN=Lifr PE=1 SV=1 - [LIFR_MOUSE]	13.83	1	3	13	28	1.644	0.911	1.131	1.171	83.68	13.83	20	28	1092	122.5	6.04
A2AQP0	Myosin-7B OS=Mus musculus GN=Myh7b PE=3 SV=1 - [MYH7B_MOUSE]	3.76	1	4	8	23	1.784	0.877	1.348	1.171	51.16	3.76	11	23	1941	221.4	5.88
B1AWL4	Protein Zfp462 OS=Mus musculus GN=Zfp462 PE=2 SV=1 - [B1AWL4_MOUSE]	1.49	3	2	2	2	0.855	0.782	0.835	1.171	3.31	1.49	2	2	1407	160.6	7.69
E9PWK1	Epoxide hydrolase 1 OS=Mus musculus GN=Ephx1 PE=2 SV=1 - [E9PWK1_MOUSE]	29.93	4	13	13	48	0.907	1.953	1.070	1.171	143.79	29.93	24	48	441	50.9	8.15

Q8BVG8	N-acetyltransferase 14 OS=Mus musculus GN=Nat14 PE=2 SV=1 - [NAT14_MOUSE]	14.56	1	3	3	6	0.468	1.707	1.301	1.171	11.96	14.56	5	6	206	21.8	10.74
A2A716	Protein Osbpl7 OS=Mus musculus GN=Osbpl7 PE=2 SV=1 - [A2A716_MOUSE]	14.02	2	10	12	41	1.125	0.855	1.152	1.171	109.20	14.02	20	41	963	108.1	7.69
P60487	Pyridoxal phosphate phosphatase OS=Mus musculus GN=Pdxp PE=1 SV=1 - [PLPP_MOUSE]	75.68	1	18	18	125	1.542	1.466	1.052	1.171	365.02	75.68	33	125	292	31.5	5.74
E9Q1I1	Protein Gm3893 OS=Mus musculus GN=Gm3893 PE=4 SV=1 - [E9Q1I1_MOUSE]	3.64	3	1	1	1	0.872	0.332	0.745	1.171	0.00	3.64	1	1	302	33.2	5.57
Q6PHN9	Ras-related protein Rab-35 OS=Mus musculus GN=Rab35 PE=1 SV=1 - [RAB35_MOUSE]	78.61	1	11	16	118	0.708	0.993	0.972	1.172	356.20	78.61	26	118	201	23.0	8.29
E9Q368	Protein Gm17296 OS=Mus musculus GN=Gm17296 PE=4 SV=1 - [E9Q368_MOUSE]	0.82	1	1	1	2	1.165	5.336	1.079	1.172	0.00	0.82	1	2	1579	175.6	6.62
Q9EPC1	Alpha-parvin OS=Mus musculus GN=Parva PE=1 SV=1 - [PARVA_MOUSE]	24.73	4	6	9	29	0.930	1.598	0.931	1.172	75.97	24.73	16	29	372	42.3	5.95
Q9D6K7	Tetratricopeptide repeat protein 33 OS=Mus musculus GN=Ttc33 PE=2 SV=1 - [TTC33_MOUSE]	21.76	4	5	5	5	1.132	0.960	1.052	1.172	10.78	21.76	5	5	262	29.4	5.35
D3YTR2	Receptor expression-enhancing protein 6 OS=Mus musculus GN=Reep6 PE=2 SV=1 - [D3YTR2_MOUSE]	8.92	2	2	2	3	0.913	0.676	1.277	1.172	5.35	8.92	2	3	572	60.5	11.69
A2AC60	Protein D630003M21Rik OS=Mus musculus GN=D630003M21Rik PE=4 SV=1 - [A2AC60_MOUSE]	3.44	2	2	2	2	0.530	0.908	0.983	1.172	5.44	3.44	2	2	1106	120.3	6.44
H7BX05	Obscurin OS=Mus musculus GN=Obscn PE=2 SV=2 - [H7BX05_MOUSE]	1.56	9	9	10	16	1.418	1.545	1.152	1.172	23.44	1.56	12	16	8032	874.0	5.96
Q5U4D8	Sodium-dependent multivitamin transporter OS=Mus musculus GN=Slc5a6 PE=2 SV=2 - [SC5A6_MOUSE]	2.05	1	1	1	2	0.757	1.558	0.961	1.172	6.97	2.05	2	2	634	68.5	7.85
Q91ZJ5	UTP--glucose-1-phosphate uridylyltransferase OS=Mus musculus GN=Ugp2 PE=2 SV=3 - [UGPA_MOUSE]	54.92	2	27	27	104	0.695	1.037	1.110	1.172	313.57	54.92	49	104	508	56.9	7.61
O55100-2	Isoform 1B of Synaptogyrin-1 OS=Mus musculus GN=Syngr1 - [SNG1_MOUSE]	16.23	2	3	3	18	0.631	1.447	1.262	1.172	46.61	16.23	5	18	191	21.3	5.26
F7BWT7	Tetraspanin-15 OS=Mus musculus GN=Tspan15 PE=2 SV=2 - [TSN15_MOUSE]	10.20	2	3	3	5	0.881	0.528	1.008	1.173	15.55	10.20	3	5	294	33.0	5.97
D3Z4H6	Nucleolysin TIA-1 OS=Mus musculus GN=Tia1 PE=2 SV=1 - [D3Z4H6_MOUSE]	24.94	6	5	9	31	2.167	1.595	1.039	1.173	94.72	24.94	14	31	385	42.7	7.99
Q9DAT5	Mitochondrial tRNA-specific 2-thiouridylase 1 OS=Mus musculus GN=Trmu PE=2 SV=1 - [MTU1_MOUSE]	15.59	2	6	6	11	0.674	0.775	0.871	1.173	36.79	15.59	9	11	417	47.2	8.40
P16390	Potassium voltage-gated channel subfamily A member 3 OS=Mus musculus GN=Kcna3 PE=2 SV=3 - [KCNA3_MOUSE]	23.11	2	5	9	67	0.868	1.010	1.158	1.173	235.00	23.11	14	67	528	58.5	5.33
Q8CFX0	Protein Wdr17 OS=Mus musculus GN=Wdr17 PE=2 SV=1 - [Q8CFX0_MOUSE]	3.61	5	4	4	5	1.193	1.526	1.447	1.173	9.19	3.61	5	5	1273	141.5	6.81
F6Z5N5	Enoyl-CoA hydratase domain-containing protein 2, mitochondrial (Fragment) OS=Mus musculus GN=Echdc2 PE=4 SV=1 - [F6Z5N5_MOUSE]	47.06	5	4	4	12	0.784	1.475	0.802	1.173	25.94	47.06	7	12	119	12.7	10.24
Q7TS72	Inositol-trisphosphate 3-kinase C OS=Mus musculus GN=Itpkc PE=1 SV=1 - [IP3KC_MOUSE]	4.57	1	2	2	3	1.704	1.141	1.044	1.173	12.37	4.57	2	3	678	74.4	5.36
Q04857	Collagen alpha-1(VI) chain OS=Mus musculus GN=Col6a1 PE=2 SV=1 - [CO6A1_MOUSE]	14.44	1	12	12	58	0.632	4.928	0.781	1.173	172.58	14.44	21	58	1025	108.4	5.36
Q6NVE9	Protein phosphatase PTC7 homolog OS=Mus musculus GN=Pptc7 PE=2 SV=1 - [PPTC7_MOUSE]	33.87	2	7	7	28	1.719	0.755	1.204	1.173	94.97	33.87	14	28	310	33.0	5.27
Q8R3I2-2	Isoform 2 of Lysophospholipid acyltransferase 2 OS=Mus musculus GN=Mboat2 - [MBOA2_MOUSE]	10.06	3	3	3	10	0.696	0.539	1.041	1.173	12.89	10.06	5	10	487	55.3	8.60
Q62245	Son of sevenless homolog 1 OS=Mus musculus GN=Sos1 PE=1 SV=2 - [SOS1_MOUSE]	18.04	1	20	25	58	0.833	0.841	0.920	1.173	148.41	18.04	42	58	1319	150.8	6.89

Q4VAE3	Transmembrane protein 65 OS=Mus musculus GN=Tmem65 PE=2 SV=1 - [TMM65_MOUSE]	26.50	1	6	6	49	0.796	1.147	1.156	1.173	130.36	26.50	12	49	234	24.9	7.78
Q00623	Apolipoprotein A-I OS=Mus musculus GN=Apoa1 PE=1 SV=2 - [APOA1_MOUSE]	49.62	1	17	17	157	3.132	3.819	0.617	1.174	428.53	49.62	31	157	264	30.6	5.73
P70699	Lysosomal alpha-glucosidase OS=Mus musculus GN=Gaa PE=1 SV=2 - [LYAG_MOUSE]	26.44	4	16	19	139	1.394	1.067	0.842	1.174	451.90	26.44	38	139	953	106.2	5.83
Q8R4H2	Rho guanine nucleotide exchange factor 12 OS=Mus musculus GN=Arhgef12 PE=1 SV=2 - [ARHGC_MOUSE]	31.37	1	1	36	107	0.664	0.749	1.150	1.174	305.49	31.37	61	107	1543	172.2	5.74
Q8CHP8	Phosphoglycolate phosphatase OS=Mus musculus GN=Pgp PE=2 SV=1 - [PGP_MOUSE]	56.70	1	15	15	98	1.364	0.901	0.993	1.174	274.77	56.70	29	98	321	34.5	5.35
Q9R0S0	GADD45G OS=Mus musculus GN=Gadd45g PE=2 SV=1 - [Q9R0S0_MOUSE]	3.77	2	1	1	1	1.378	1.470	1.731	1.174	2.90	3.77	1	1	159	17.3	4.30
Q9D483	DNA-directed RNA polymerase III subunit RPC3 OS=Mus musculus GN=Polr3c PE=2 SV=1 - [RPC3_MOUSE]	4.69	3	3	3	4	0.581	1.041	1.172	1.174	4.15	4.69	4	4	533	60.7	7.58
Q6PFX7-2	Isoform 2 of Neuronal tyrosine-phosphorylated phosphoinositide-3-kinase adapter 1 OS=Mus musculus GN=Nyap1 - [NYAP1_MOUSE]	15.14	4	10	10	28	0.891	0.785	1.046	1.174	61.30	15.14	18	28	832	87.5	9.55
Q80XI4	Phosphatidylinositol 5-phosphate 4-kinase type-2 beta OS=Mus musculus GN=Pip4k2b PE=1 SV=1 - [PI42B_MOUSE]	49.28	1	17	23	81	0.708	1.184	1.205	1.174	230.56	49.28	35	81	416	47.3	7.33
Q8C163	Nuclease EXOG, mitochondrial OS=Mus musculus GN=Exog PE=2 SV=1 - [EXOG_MOUSE]	41.58	2	12	13	49	0.910	1.232	1.058	1.174	148.49	41.58	22	49	368	41.4	8.12
Q922G0	Solute carrier family 25 member 36 OS=Mus musculus GN=Slc25a36 PE=2 SV=1 -	10.61	4	3	3	7	0.676	0.957	0.854	1.174	22.11	10.61	5	7	311	34.1	8.65
P20033-2	Isoform Short of Platelet-derived growth factor subunit A OS=Mus musculus GN=Pdgfa - [PDGFA_MOUSE]	9.18	4	1	1	4	2.080	1.386	0.902	1.175	8.48	9.18	2	4	196	22.3	8.21
Q8C863	E3 ubiquitin-protein ligase Itchy OS=Mus musculus GN=Itch PE=1 SV=2 - [ITCH_MOUSE]	21.06	2	12	16	42	0.711	0.904	1.131	1.175	126.60	21.06	25	42	864	98.9	6.28
Q9D0I6	WD repeat, SAM and U-box domain-containing protein 1 OS=Mus musculus GN=Wdsub1 PE=2 SV=1 - [WSDU1_MOUSE]	14.14	3	5	6	14	1.325	0.843	0.899	1.175	36.54	14.14	10	14	474	51.7	6.34
E9Q5B5	Hexokinase-2 OS=Mus musculus GN=Hk2 PE=2 SV=1 - [E9Q5B5_MOUSE]	9.34	3	2	9	71	0.974	1.343	1.004	1.175	201.95	9.34	14	71	889	99.2	6.09
P46097	Synaptotagmin-2 OS=Mus musculus GN=Syt2 PE=1 SV=1 - [SYT2_MOUSE]	44.31	1	11	20	154	0.898	0.211	0.915	1.175	448.76	44.31	38	154	422	47.2	7.99
Q8C5J7	Regulator of G-protein-signaling 20 OS=Mus musculus GN=Rgs20 PE=2 SV=1 - [Q8C5J7_MOUSE]	23.08	4	3	4	17	0.599	1.403	1.768	1.175	54.23	23.08	8	17	208	23.9	5.11
Q561M1	Acp1 protein OS=Mus musculus GN=Acp1 PE=2 SV=1 - [Q561M1_MOUSE]	47.47	2	2	7	32	1.381	1.027	0.996	1.175	89.69	47.47	10	32	158	17.9	6.54
Q9QZS3-4	Isoform 4 of Protein numb homolog OS=Mus musculus GN=Numb - [NUMB_MOUSE]	28.16	10	11	14	46	1.115	1.198	1.702	1.175	109.45	28.16	26	46	593	64.4	8.47
Q8K3W0	BRCA1-A complex subunit BRE OS=Mus musculus GN=Bre PE=1 SV=2 - [BRE_MOUSE]	24.80	8	9	9	28	0.847	1.122	1.065	1.175	75.99	24.80	16	28	383	43.5	5.94
P99028	Cytochrome b-c1 complex subunit 6, mitochondrial OS=Mus musculus GN=Uqcrh PE=1 SV=2 - [QCR6_MOUSE]	48.31	2	5	5	198	2.259	1.103	1.707	1.175	751.85	48.31	9	198	89	10.4	4.87
Q9JI38	tRNA pseudouridine(38/39) synthase OS=Mus musculus GN=Pus3 PE=1 SV=2 - [PUS3_MOUSE]	3.74	2	2	2	2	0.720	1.071	1.010	1.175	2.30	3.74	2	2	481	55.5	8.12
G3UWA6	4F2 cell-surface antigen heavy chain OS=Mus musculus GN=Slc3a2 PE=4 SV=1 - [G3UWA6_MOUSE]	47.08	2	22	22	145	0.705	1.011	0.960	1.175	387.72	47.08	38	145	565	62.2	5.50
Q8K1Z0	Ubiquinone biosynthesis protein COQ9, mitochondrial OS=Mus musculus GN=Coq9 PE=1 SV=1 - [COQ9_MOUSE]	21.73	2	6	6	38	1.126	1.641	1.121	1.175	118.58	21.73	12	38	313	35.1	5.92
Q9CWQ0	Diphthine synthase OS=Mus musculus GN=Dph5 PE=2 SV=2 - [DPH5_MOUSE]	16.01	1	3	3	5	1.702	0.979	0.907	1.175	21.55	16.01	5	5	281	31.2	5.29

B1APT1	Metallophosphoesterase MPPED2 (Fragment) OS=Mus musculus GN=Mpped2 PE=4 SV=1 - [B1APT1_MOUSE]	12.37	3	2	2	9	1.427	0.967	1.077	1.176	39.88	12.37	4	9	283	32.2	6.46
Q9Z1Q2	Abhydrolase domain-containing protein 16A OS=Mus musculus GN=Abhd16a PE=1 SV=3 - [ABHGA_MOUSE]	36.56	6	16	16	52	0.827	1.095	1.031	1.176	177.82	36.56	29	52	558	63.0	8.25
P56565	Protein S100-A1 OS=Mus musculus GN=S100a1 PE=1 SV=2 - [S10A1_MOUSE]	19.15	2	2	2	16	1.309	0.917	1.232	1.176	45.67	19.15	3	16	94	10.5	4.50
P05202	Aspartate aminotransferase, mitochondrial OS=Mus musculus GN=Got2 PE=1 SV=1 - [AATM_MOUSE]	77.44	1	35	36	698	1.873	1.098	0.961	1.176	1849.68	77.44	71	698	430	47.4	9.00
Q8BVG5-2	Isoform 2 of Polypeptide N acetylgalactosaminyltransferase 14 OS=Mus musculus GN=Galnt14 - [GLT14_MOUSE]	4.43	3	1	2	6	0.510	0.958	0.859	1.176	16.10	4.43	3	6	429	50.0	8.41
Q8C1Y8	Vacuolar fusion protein CCZ1 homolog OS=Mus musculus GN=Ccz1 PE=1 SV=1 - [CCZ1_MOUSE]	16.04	1	7	7	13	1.031	1.124	1.187	1.176	30.37	16.04	11	13	480	55.5	6.02
B1H3M0	OXR1 short isoform C7C OS=Mus musculus GN=Oxr1 PE=2 SV=1 - [B1H3M0_MOUSE]	47.22	1	1	8	37	1.078	0.849	1.034	1.177	102.13	47.22	15	37	216	24.4	6.43
Q9ET77	Junctophilin-3 OS=Mus musculus GN=Jph3 PE=1 SV=1 - [JPH3_MOUSE]	20.56	2	14	14	33	1.023	1.104	1.526	1.177	80.14	20.56	22	33	744	81.2	9.22
P52196	Thiosulfate sulfurtransferase OS=Mus musculus GN=Tst PE=1 SV=3 - [THTR_MOUSE]	45.45	1	14	14	64	0.877	0.776	0.820	1.177	209.66	45.45	25	64	297	33.4	7.85
Q9EPK7	Exportin-7 OS=Mus musculus GN=Xpo7 PE=2 SV=3 - [XPO7_MOUSE]	31.37	3	30	30	107	0.855	1.405	0.994	1.177	305.02	31.37	51	107	1087	123.7	6.38
B8ZXI1-2	Isoform 2 of Queuine tRNA ribosyltransferase subunit QTRTD1 OS=Mus musculus GN=Qtrtd1 - [QTRD1_MOUSE]	15.99	2	3	3	3	1.542	1.402	1.008	1.177	12.76	15.99	3	3	344	38.3	6.52
Q8BGW6-2	Isoform 2 of Serine/threonine-protein kinase 32A OS=Mus musculus GN=Stk32a - [ST32A_MOUSE]	9.78	2	2	2	3	1.269	1.294	1.280	1.177	4.10	9.78	3	3	368	43.2	6.40
O88188	Lymphocyte antigen 86 OS=Mus musculus GN=Ly86 PE=1 SV=1 - [LY86_MOUSE]	5.56	1	1	1	2	1.508	0.976	1.037	1.177	3.91	5.56	2	2	162	17.8	5.22
G3X9A7	MCG49978 OS=Mus musculus GN=Plcxd3 PE=4 SV=1 - [G3X9A7_MOUSE]	43.30	1	12	12	40	0.863	1.509	1.109	1.177	126.83	43.30	18	40	321	36.3	6.46
P97794	ATP-sensitive inward rectifier potassium channel 8 OS=Mus musculus GN=Kcnj8 PE=2 SV=1 - [IRK8_MOUSE]	11.56	1	2	2	3	0.852	0.612	1.190	1.177	2.89	11.56	2	3	424	47.9	9.20
B2RUF0	Y box protein 2 OS=Mus musculus GN=Ybx2 PE=2 SV=1 - [B2RUF0_MOUSE]	9.75	3	1	3	5	0.770	1.292	1.304	1.177	9.48	9.75	4	5	359	38.1	10.76
Q3THG9	Alanyl-tRNA editing protein Aarsd1 OS=Mus musculus GN=Aarsd1 PE=1 SV=2 - [AASD1_MOUSE]	37.38	3	12	12	36	1.010	0.907	0.873	1.177	112.90	37.38	22	36	412	44.9	6.42
Q8BR63	Protein FAM177A1 OS=Mus musculus GN=Fam177a1 PE=2 SV=1 - [F177A_MOUSE]	31.88	1	5	5	29	1.321	0.587	1.095	1.177	107.69	31.88	10	29	207	23.6	4.59
Q5NCJ0	Protein Rapgef6 OS=Mus musculus GN=Rapgef6 PE=2 SV=1 - [Q5NCJ0_MOUSE]	9.56	6	8	14	25	1.274	0.995	1.127	1.178	67.82	9.56	20	25	1601	179.1	6.35
E9Q4K0	Actin-binding LIM protein 2 OS=Mus musculus GN=Ablim2 PE=2 SV=1 - [E9Q4K0_MOUSE]	49.70	4	4	25	161	2.183	0.758	1.670	1.178	516.75	49.70	46	161	664	73.6	8.07
Q7TNF0	Double C2-like domain-containing protein alpha OS=Mus musculus GN=Doc2a PE=2 SV=1 - [DOC2A_MOUSE]	28.15	6	8	10	38	0.743	1.171	1.172	1.178	109.65	28.15	17	38	405	44.6	7.12
O08915	AH receptor-interacting protein OS=Mus musculus GN=Aip PE=1 SV=1 - [AIP_MOUSE]	40.00	2	11	11	33	1.011	0.945	1.097	1.178	88.25	40.00	19	33	330	37.6	6.40
Q9DD18-2	Isoform 2 of D-tyrosyl-tRNA(Tyr) deacylase 1 OS=Mus musculus GN=Dtd1 -	30.85	2	4	4	21	0.690	0.886	1.043	1.178	63.03	30.85	6	21	201	22.5	8.76
Q8BWR2	PITH domain-containing protein 1 OS=Mus musculus GN=Pithd1 PE=2 SV=1 - [PITH1_MOUSE]	55.45	4	11	11	73	2.065	1.124	0.940	1.178	198.99	55.45	21	73	211	24.2	5.74
P70704	Probable phospholipid-transporting ATPase IA OS=Mus musculus GN=Atp8a1 PE=1 SV=1 - [AT8A1_MOUSE]	32.11	5	1	34	183	0.849	0.989	0.926	1.178	527.99	32.11	61	183	1149	129.7	7.27

Q8BK75-2	Isoform 2 of Elongator complex protein 6 OS=Mus musculus GN=Elp6 - [ELP6_MOUSE]	21.15	2	4	4	6	0.711	1.163	1.081	1.178	16.92	21.15	5	6	260	28.3	6.79
F7D8Z3	Uncharacterized protein OS=Mus musculus GN=Gm9894 PE=4 SV=2 - [F7D8Z3_MOUSE]	4.31	145	1	2	2	23.986	1.356	1.252	1.178	4.25	4.31	2	2	371	43.6	8.87
F8VPZ3	Ubiquitin carboxyl-terminal hydrolase OS=Mus musculus GN=Usp32 PE=2 SV=1 - [F8VPZ3_MOUSE]	17.08	4	19	19	44	0.798	0.995	1.053	1.179	113.42	17.08	31	44	1604	181.6	6.51
F2Z3Y1	DNA-3-methyladenine glycosylase OS=Mus musculus GN=Mpg PE=4 SV=1 - [F2Z3Y1_MOUSE]	7.53	2	1	1	2	0.881	1.510	1.407	1.179	3.13	7.53	1	2	146	15.8	10.83
Q9EQ80	NIF3-like protein 1 OS=Mus musculus GN=Nif3l1 PE=1 SV=4 - [NIF3L_MOUSE]	26.33	3	9	9	40	0.980	0.831	0.926	1.179	120.64	26.33	17	40	376	41.7	6.76
B0R091	Protein Gm20056 OS=Mus musculus GN=Chp1 PE=2 SV=1 - [B0R091_MOUSE]	79.58	3	14	14	68	1.134	0.967	0.981	1.179	190.54	79.58	25	68	191	22.0	4.93
Q7TNY3	Calcipressin 1 large isoform OS=Mus musculus GN=Rcan1 PE=2 SV=1 - [Q7TNY3_MOUSE]	15.94	4	3	4	18	1.078	1.643	1.397	1.179	54.07	15.94	8	18	251	28.1	5.41
Q8K1M3	Protein kinase, cAMP dependent regulatory, type II alpha OS=Mus musculus GN=Prkar2a PE=2 SV=1 - [Q8K1M3_MOUSE]	55.97	2	16	22	110	0.895	1.591	0.977	1.180	334.89	55.97	38	110	402	45.6	4.93
A2AHC7	Protein Gm6592 OS=Mus musculus GN=Gm6592 PE=4 SV=1 - [A2AHC7_MOUSE]	5.36	1	1	1	1	0.432	0.881	1.209	1.180	2.29	5.36	1	1	168	19.4	5.08
Q60932-2	Isoform Mt-VDAC1 of Voltage-dependent anion-selective channel protein 1 OS=Mus musculus GN=Vdac1 - [illegible]	78.09	3	20	22	974	0.961	1.126	1.192	1.180	2989.33	78.09	40	974	283	30.7	8.54
D3Z3P2	Occludin/ELL domain-containing protein 1 OS=Mus musculus GN=Ocel1 PE=2 SV=1 - [D3Z3P2_MOUSE]	8.89	1	1	1	1	2.423	1.322	1.043	1.180	2.17	8.89	1	1	135	15.1	11.75
A2AEG6	Glycoprotein m6b, isoform CRA_g OS=Mus musculus GN=Gpm6b PE=4 SV=1 - [A2AEG6_MOUSE]	18.90	6	2	6	36	0.926	1.460	1.951	1.180	85.59	18.90	12	36	328	36.2	6.14
Q69ZK9	Neuroligin-2 OS=Mus musculus GN=Nlgn2 PE=1 SV=2 - [NLGN2_MOUSE]	27.99	2	14	17	91	0.610	1.105	1.055	1.180	253.69	27.99	32	91	836	90.9	6.18
P42125	Enoyl-CoA delta isomerase 1, mitochondrial OS=Mus musculus GN=Eci1 PE=2 SV=2 - [ECI1_MOUSE]	50.17	1	12	12	67	1.955	1.181	0.864	1.180	223.56	50.17	22	67	289	32.2	8.98
Q91W45	Polyadenylate-binding protein-interacting protein 2B OS=Mus musculus GN=Paip2b PE=2 SV=2 - [PAI2B_MOUSE]	19.12	1	1	2	3	2.860	1.563	1.600	1.181	10.56	19.12	3	3	136	15.5	4.32
Q80SW1	Putative adenosylhomocysteinase 2 OS=Mus musculus GN=Ahcyl1 PE=1 SV=1 -	58.87	5	13	35	342	0.793	0.809	0.974	1.181	995.59	58.87	65	342	530	58.9	6.89
Q8K157	Aldose 1-epimerase OS=Mus musculus GN=Galm PE=2 SV=1 - [GALM_MOUSE]	7.31	1	2	2	8	4.180	2.413	1.250	1.181	26.56	7.31	3	8	342	37.8	6.74
E0CYM0	Protein 1700019G17Rik OS=Mus musculus GN=1700019G17Rik PE=2 SV=1 - [E0CYM0_MOUSE]	11.76	2	1	1	1	2.302	1.432	1.192	1.181	2.47	11.76	1	1	136	15.3	8.06
Q6P1G0	HEAT repeat-containing protein 6 OS=Mus musculus GN=Heatr6 PE=2 SV=1 -	6.59	1	4	5	11	1.289	1.095	1.011	1.181	14.26	6.59	6	11	1184	128.8	7.24
Q6ZPE2-2	Isoform 2 of Myotubularin-related protein 5 OS=Mus musculus GN=Sbf1 - [MTMR5_MOUSE]	32.33	6	48	52	196	0.638	0.987	1.180	1.181	597.87	32.33	95	196	1893	211.3	7.33
Q9JHK4	Geranylgeranyl transferase type-2 subunit alpha OS=Mus musculus GN=Rabggta PE=1 SV=1 - [PGTA_MOUSE]	44.62	2	21	21	75	0.849	1.049	1.010	1.181	233.78	44.62	38	75	567	64.9	5.80
Q8BHL3	TBC1 domain family member 10B OS=Mus musculus GN=Tbc1d10b PE=1 SV=2 -	36.97	1	17	17	91	0.707	0.882	1.092	1.181	307.13	36.97	31	91	798	87.2	8.90
J3KMK0	Uncharacterized protein OS=Mus musculus GN=Gm9923 PE=4 SV=1 - [J3KMK0_MOUSE]	16.50	3	3	8	18	1.592	1.387	1.378	1.181	50.45	16.50	14	18	612	67.4	5.71
Q9WTR6	Cystine/glutamate transporter OS=Mus musculus GN=Slc7a11 PE=1 SV=1 -	6.77	1	3	3	37	0.495	1.466	1.092	1.181	105.57	6.77	5	37	502	55.4	9.23
Q8VDN2	Sodium/potassium-transporting ATPase subunit alpha-1 OS=Mus musculus GN=Atp1a1 PE=1 SV=1 - [illegible]	54.45	1	31	61	2252	0.608	1.398	1.488	1.182	6617.10	54.45	115	2252	1023	112.9	5.45

A2ALW2	Protein Zkscan16 OS=Mus musculus GN=Zkscan16 PE=4 SV=1 - [A2ALW2_MOUSE]	7.28	145	4	6	7	0.591	0.986	0.829	1.182	15.33	7.28	7	7	728	83.8	8.91
Q9D4S6	MCG10597 OS=Mus musculus GN=4930564B18Rik PE=2 SV=1 - [Q9D4S6_MOUSE]	7.73	1	1	1	1	2.727	1.090	0.884	1.182	0.00	7.73	1	1	220	24.7	4.65
Q80XK6	Autophagy-related protein 2 homolog B OS=Mus musculus GN=Atg2b PE=1 SV=3 - [ATG2B_MOUSE]	12.67	3	24	24	56	0.770	0.914	1.048	1.182	161.94	12.67	39	56	2075	231.3	5.88
P48722	Heat shock 70 kDa protein 4L OS=Mus musculus GN=Hspa4l PE=1 SV=2 - [HS74L_MOUSE]	62.41	5	47	51	346	0.923	0.738	1.091	1.182	996.62	62.41	90	346	838	94.3	5.74
D3Z0R2	Protein phosphatase 1 regulatory subunit 36 OS=Mus musculus GN=Ppp1r36 PE=3 SV=1 - [PPR36_MOUSE]	4.87	1	2	2	4	0.639	1.087	1.471	1.182	9.45	4.87	2	4	411	47.3	9.36
Q8BQU3	Protein ACN9 homolog, mitochondrial OS=Mus musculus GN=Acn9 PE=2 SV=1 - [ACN9_MOUSE]	50.40	1	5	5	12	1.244	0.761	0.940	1.182	33.90	50.40	9	12	125	14.5	9.07
Q8BMG8	Mitochondrial folate transporter/carrier OS=Mus musculus GN=Slc25a32 PE=2 SV=1 - [MFTC_MOUSE]	6.96	1	2	2	5	0.962	1.289	1.138	1.182	11.33	6.96	4	5	316	35.0	9.47
Q9D211	CDKN2AIP N-terminal-like protein OS=Mus musculus GN=Cdkn2aipnl PE=2 SV=1 - [C2AIL_MOUSE]	20.69	1	1	2	3	2.083	1.963	1.217	1.183	6.19	20.69	3	3	116	13.2	5.29
Q8BGB7	Enolase-phosphatase E1 OS=Mus musculus GN=Enoph1 PE=2 SV=1 - [ENOPH_MOUSE]	35.02	2	7	7	38	0.754	1.265	1.049	1.183	112.68	35.02	13	38	257	28.6	4.92
Q8R0Y6	Cytosolic 10-formyltetrahydrofolate dehydrogenase OS=Mus musculus GN=Aldh1l1 PE=2 SV=1 - [illegible]	65.30	1	43	51	274	0.724	0.842	0.971	1.183	791.26	65.30	91	274	902	98.6	5.91
Q8K1S2	Netrin receptor UNC5D OS=Mus musculus GN=Unc5d PE=2 SV=1 - [UNC5D_MOUSE]	6.90	1	5	5	12	0.680	1.827	0.916	1.183	33.52	6.90	8	12	956	106.3	6.14
E9QJR0	Protein 4833403I15Rik OS=Mus musculus GN=4833403I15Rik PE=4 SV=1 - [E9QJR0_MOUSE]	0.71	1	1	1	3	1.637	0.760	1.537	1.183	8.73	0.71	1	3	991	113.2	5.47
Q9CQ75	NADH dehydrogenase [ubiquinone] 1 alpha subcomplex subunit 2 OS=Mus musculus GN=Ndufa2 PE=1 SV=3 - [illegible]	66.67	1	9	10	105	1.171	1.122	1.127	1.183	304.30	66.67	17	105	99	10.9	9.99
Q80ZW2	Protein THEM6 OS=Mus musculus GN=Them6 PE=2 SV=1 - [THEM6_MOUSE]	38.65	1	8	8	30	0.983	0.492	1.237	1.183	92.93	38.65	16	30	207	23.8	9.11
E9Q933	Transmembrane protein 11, mitochondrial OS=Mus musculus GN=Tmem11 PE=2 SV=1 - [E9Q933_MOUSE]	15.34	2	3	3	8	0.619	1.277	1.085	1.183	19.48	15.34	6	8	176	19.8	6.25
Q8BRC6	Protein MAATS1 OS=Mus musculus GN=Maats1 PE=2 SV=3 - [MAAT1_MOUSE]	1.92	1	1	1	4	1.453	1.433	0.864	1.183	2.70	1.92	2	4	783	91.6	6.83
Q9Z188	Dual specificity tyrosine-phosphorylation-regulated kinase 1B OS=Mus musculus GN=Dyrk1b PE=1 SV=3 - [illegible]	12.40	3	4	6	14	1.314	1.402	1.119	1.183	33.53	12.40	8	14	629	69.1	9.09
E9Q350	BAI1-associated protein 3 OS=Mus musculus GN=Baiap3 PE=4 SV=1 - [E9Q350_MOUSE]	22.17	3	21	21	71	0.690	0.755	0.594	1.184	218.09	22.17	38	71	1150	129.0	6.25
P03958	Adenosine deaminase OS=Mus musculus GN=Ada PE=1 SV=3 - [ADA_MOUSE]	8.52	1	2	2	3	0.862	1.101	0.654	1.184	10.37	8.52	3	3	352	40.0	5.72
Q5EG47	5'-AMP-activated protein kinase catalytic subunit alpha-1 OS=Mus musculus GN=Prkaa1 PE=1 SV=2 - [AAPK1_MOUSE]	24.69	1	7	11	40	0.751	0.981	1.055	1.184	130.60	24.69	18	40	559	63.9	8.12
P50114	Protein S100-B OS=Mus musculus GN=S100b PE=2 SV=2 - [S100B_MOUSE]	85.87	1	4	5	167	2.239	0.816	1.384	1.184	545.41	85.87	10	167	92	10.7	4.55
Q14BN8	RWD domain containing 2, isoform CRA_a OS=Mus musculus GN=Rwdd2a PE=2 SV=1 - [Q14BN8_MOUSE]	8.90	2	2	2	5	0.576	0.933	0.695	1.184	14.24	8.90	3	5	292	33.8	5.87
F8VQN6	Rho guanine nucleotide exchange factor 12 OS=Mus musculus GN=Arhgef12 PE=2 SV=1 - [F8VQN6_MOUSE]	31.41	1	1	36	105	5.536	1.148	2.450	1.184	292.54	31.41	60	105	1544	172.4	5.74
P23506	Protein-L-isoaspartate(D-aspartate) O-methyltransferase OS=Mus musculus GN=Pcmt1 PE=1 SV=3 - [PIMT_MOUSE]	75.33	7	15	16	124	1.119	1.280	1.165	1.184	315.26	75.33	28	124	227	24.6	7.65

Q0GNC1-3	Isoform 2 of Inverted formin-2 OS=Mus musculus GN=Inf2 - [INF2_MOUSE]	15.98	5	14	16	61	0.921	0.410	1.797	1.184	173.48	15.98	26	61	1264	137.4	5.31
Q9D1D6	Collagen triple helix repeat containing protein 1 OS=Mus musculus GN=Cthrc1 PE=2 SV=2 - [CTHR1_MOUSE]	4.90	1	1	1	6	1.562	0.544	0.752	1.184	11.50	4.90	2	6	245	26.4	7.50
Q6GQS1	Calcium-binding mitochondrial carrier protein SCaMC-3 OS=Mus musculus GN=Slc25a23 PE=2 SV=1 - [illegible]	33.19	6	13	16	68	0.692	1.749	1.320	1.184	187.78	33.19	28	68	467	52.5	7.59
F6W6X9	RNA pseudouridylate synthase domain-containing protein 4 (Fragment) OS=Mus musculus GN=Rpusd4 PE=4 SV=1 - [F6W6X9_MOUSE]	34.29	2	1	1	2	0.595	0.562	1.091	1.184	2.79	34.29	2	2	35	4.1	11.43
E9Q641	Nebulin-related-anchoring protein OS=Mus musculus GN=Nrap PE=2 SV=1 - [E9Q641_MOUSE]	1.21	4	1	2	3	5.294	2.124	1.090	1.184	2.57	1.21	2	3	1485	167.4	9.20
Q8VCE6	5'(3')-deoxyribonucleotidase, mitochondrial OS=Mus musculus GN=Nt5m PE=2 SV=1 - [NT5M_MOUSE]	40.00	1	5	6	14	0.884	1.082	1.014	1.184	56.34	40.00	8	14	220	25.6	8.35
Q9D387	Lysosome-associated membrane glycoprotein 5 OS=Mus musculus GN=Lamp5 PE=1 SV=2 - [LAMP5_MOUSE]	10.71	1	3	3	9	1.483	0.680	1.666	1.184	24.90	10.71	6	9	280	31.7	6.14
Q9D0L7	Armadillo repeat-containing protein 10 OS=Mus musculus GN=Armc10 PE=1 SV=1 - [illegible]	60.13	3	11	11	53	0.711	1.474	1.017	1.184	156.64	60.13	18	53	306	33.3	7.99
Q64735-2	Isoform 2 of Complement component receptor 1-like protein OS=Mus musculus GN=Cr1l - [CR1L_MOUSE]	13.86	2	6	6	22	0.700	1.192	0.871	1.185	68.29	13.86	11	22	440	49.0	5.73
Q8CIV8	Tubulin-specific chaperone E OS=Mus musculus GN=Tbce PE=1 SV=1 - [TBCE_MOUSE]	21.56	3	9	10	29	1.029	1.422	1.047	1.185	80.81	21.56	17	29	524	59.0	6.29
Q8BMQ8	Vacuolar fusion protein MON1 homolog B OS=Mus musculus GN=Mon1b PE=2 SV=1 - [MON1B_MOUSE]	13.92	1	6	6	11	1.312	1.274	0.999	1.185	29.92	13.92	9	11	553	60.0	5.57
Q8VDQ8-2	Isoform 2 of NAD-dependent protein deacetylase sirtuin-2 OS=Mus musculus GN=Sirt2 - [SIR2_MOUSE]	68.18	3	22	22	236	1.095	0.259	0.971	1.185	690.26	68.18	38	236	352	39.5	7.02
Q8R2U6	Diphosphoinositol polyphosphate phosphohydrolase 2 OS=Mus musculus GN=Nudt4 PE=1 SV=1 - [illegible]	31.28	1	3	4	12	1.101	0.633	1.516	1.185	31.83	31.28	6	12	179	20.1	6.35
Q60714	Long-chain fatty acid transport protein 1 OS=Mus musculus GN=Slc27a1 PE=1 SV=1 - [S27A1_MOUSE]	34.37	1	14	15	66	0.948	1.121	1.079	1.185	184.13	34.37	26	66	646	71.2	8.38
Q6SKR2	HemK methyltransferase family member 2, isoform CRA_c OS=Mus musculus GN=N6amt1 PE=2 SV=1 - [Q6SKR2_MOUSE]	19.63	2	3	3	11	0.759	1.130	0.950	1.185	28.25	19.63	6	11	214	23.0	5.62
P22315	Ferrochelatase, mitochondrial OS=Mus musculus GN=Fech PE=1 SV=2 - [HEMH_MOUSE]	49.05	2	18	18	60	0.891	1.144	1.095	1.185	158.21	49.05	29	60	420	47.1	8.91
Q3TUF7-2	Isoform 2 of YEATS domain-containing protein 2 OS=Mus musculus GN=Yeats2 - [YETS2_MOUSE]	0.92	3	1	1	1	0.919	1.144	1.168	1.185	2.80	0.92	1	1	1305	137.2	9.14
Q9R0M6	Ras-related protein Rab-9A OS=Mus musculus GN=Rab9a PE=1 SV=1 - [RAB9A_MOUSE]	55.22	3	7	9	32	0.881	1.040	0.941	1.185	94.37	55.22	15	32	201	22.9	5.66
F7C846	Metaxin-1 OS=Mus musculus GN=Mtx1 PE=2 SV=1 - [F7C846_MOUSE]	19.09	9	9	9	47	0.594	1.191	1.096	1.185	147.94	19.09	17	47	461	51.3	9.60
Q8BTX9	Inactive hydroxysteroid dehydrogenase-like protein 1 OS=Mus musculus GN=Hsdl1 PE=2 SV=1 - [HSDL1_MOUSE]	42.42	1	12	13	40	0.847	1.129	0.905	1.186	121.39	42.42	22	40	330	36.8	8.48
Q8BGD5	Carnitine O-palmitoyltransferase 1, brain isoform OS=Mus musculus GN=Cpt1c PE=1 SV=1 - [CPT1C_MOUSE]	19.80	1	12	12	29	0.545	1.248	1.128	1.186	79.21	19.80	22	29	798	90.0	7.49
Q7TSY8	Shugoshin-like 2 OS=Mus musculus GN=Sgol2 PE=1 SV=1 - [SGOL2_MOUSE]	2.58	1	2	3	5	0.814	0.917	1.764	1.186	8.82	2.58	3	5	1164	130.2	8.76
Q8C0T7	Major facilitator superfamily domain-containing protein 9 OS=Mus musculus GN=Mfsd9 PE=2 SV=1 - [illegible]	3.22	1	1	1	1	1.259	1.060	0.984	1.186	4.83	3.22	1	1	466	49.6	8.28

Q9JLM9	Growth factor receptor-bound protein 14 OS=Mus musculus GN=Grb14 PE=1 SV=1 - [GRB14_MOUSE]	15.61	3	6	6	11	1.107	0.763	1.151	1.186	34.75	15.61		10	11	538	60.5	8.44
D3YXQ4	Beta-chimaerin (Fragment) OS=Mus musculus GN=Chn2 PE=2 SV=1 - [D3YXQ4_MOUSE]	11.65	1	1	1	1	8.135	1.301	1.571	1.186	1.94	11.65		1	1	103	11.8	8.91
E9Q8N8	Electrogenic sodium bicarbonate cotransporter 1 OS=Mus musculus GN=Slc4a4 PE=2 SV=1 - [E9Q8N8_MOUSE]	28.52	6	4	28	170	0.833	0.512	0.860	1.186	453.50	28.52		49	170	1094	123.0	6.89
Q8R527	Rho-related GTP-binding protein RhoQ OS=Mus musculus GN=Rhoq PE=1 SV=2 - [RHOQ_MOUSE]	12.20	7	1	2	8	0.799	0.642	0.602	1.186	26.34	12.20		4	8	205	22.6	6.32
P34928	Apolipoprotein C-I OS=Mus musculus GN=Apoc1 PE=2 SV=1 - [APOC1_MOUSE]	10.23	1	1	1	2	2.160	3.386	0.492	1.186	7.64	10.23		2	2	88	9.7	9.09
Q924D0	Reticulon-4-interacting protein 1, mitochondrial OS=Mus musculus GN=Rtn4ip1 PE=1 SV=2 - [RT4I1_MOUSE]	36.36	2	12	12	27	0.912	1.034	0.909	1.186	78.23	36.36		21	27	396	43.3	9.20
Q80TM9	Nischarin OS=Mus musculus GN=Nisch PE=1 SV=2 - [NISCH_MOUSE]	17.51	14	22	22	90	0.779	1.387	0.991	1.187	280.10	17.51		36	90	1593	174.9	5.16
E9QP85	Protein Dos OS=Mus musculus GN=Dos PE=4 SV=1 - [E9QP85_MOUSE]	25.93	3	12	12	62	1.068	1.276	1.191	1.187	180.31	25.93		22	62	698	74.1	6.33
G5E889	Mitochondrial translation optimization 1 homolog (S. cerevisiae), isoform CRA_b OS=Mus musculus GN=Mto1 PE=3 SV=1 - [G5E889_MOUSE]	12.11	4	7	7	10	1.250	1.033	1.266	1.187	28.23	12.11		10	10	669	74.3	8.92
E9Q968	Pituitary adenylate cyclase-activating polypeptide type I receptor OS=Mus musculus GN=Adcyap1r1 PE=2 SV=1 - [E9Q968_MOUSE]	9.29	7	5	5	22	0.942	1.063	1.025	1.187	64.54	9.29		8	22	495	56.5	6.52
P56399	Ubiquitin carboxyl-terminal hydrolase 5 OS=Mus musculus GN=Usp5 PE=1 SV=1 - [UBP5_MOUSE]	44.64	4	31	32	268	0.884	1.256	1.108	1.187	764.48	44.64		57	268	858	95.8	5.01
Q80X80	C2 domain-containing protein 2-like OS=Mus musculus GN=C2cd2l PE=1 SV=3 -	44.05	1	19	19	133	0.881	1.495	1.687	1.187	427.86	44.05		34	133	706	76.3	7.21
P97490	Adenylate cyclase type 8 OS=Mus musculus GN=Adcy8 PE=2 SV=2 - [ADCY8_MOUSE]	12.01	1	13	13	32	0.653	1.042	0.949	1.187	76.51	12.01		23	32	1249	140.0	6.93
P56388	Cocaine- and amphetamine-regulated transcript protein OS=Mus musculus GN=Cartpt PE=2 SV=2 - [CART_MOUSE]	58.14	1	2	7	20	1.281	0.917	0.820	1.187	59.96	58.14		13	20	129	14.3	8.87
Q8VDH1-2	Isoform 2 of F-box only protein 21 OS=Mus musculus GN=Fbxo21 - [FBX21_MOUSE]	12.90	2	5	5	19	1.660	1.220	1.094	1.187	69.37	12.90		8	19	620	71.3	5.99
Q3UHG7	Protein LCHN OS=Mus musculus GN=Lchn PE=2 SV=1 - [LCHN_MOUSE]	14.29	2	6	6	16	0.973	0.789	1.140	1.187	37.62	14.29		9	16	455	51.4	5.25
O35985	Submaxillary gland androgen-regulated protein 2, isoform gamma OS=Mus musculus GN=Smr2 PE=2 SV=1 - [SMR2C_MOUSE]	15.38	1	1	1	3	1.319	1.828	1.173	1.188	0.00	15.38		1	3	143	16.1	7.40
Q9ESN4	Complement C1q-like protein 3 OS=Mus musculus GN=C1ql3 PE=1 SV=1 - [C1QL3_MOUSE]	28.24	2	3	4	11	1.238	1.583	1.848	1.188	26.08	28.24		8	11	255	26.7	6.79
P24472	Glutathione S-transferase A4 OS=Mus musculus GN=Gsta4 PE=1 SV=3 - [GSTA4_MOUSE]	59.91	3	10	13	85	1.488	1.493	0.851	1.188	211.74	59.91		23	85	222	25.5	7.39
D3YUM1	NADH dehydrogenase [ubiquinone] flavoprotein 1, mitochondrial OS=Mus musculus GN=Ndufv1 PE=2 SV=1 - [illegible]	67.25	6	26	26	166	0.772	0.879	1.070	1.188	430.86	67.25		47	166	455	49.9	8.07
F8WIV2	Serpin B6 OS=Mus musculus GN=Serpinb6a PE=2 SV=1 - [F8WIV2_MOUSE]	50.38	11	16	17	71	0.759	1.574	1.097	1.188	206.56	50.38		27	71	399	44.7	6.38
Q8VBY2	Calcium/calmodulin-dependent protein kinase kinase 1 OS=Mus musculus GN=Camkk1 PE=1 SV=1 - [illegible]	32.08	2	15	15	34	1.009	1.027	1.254	1.188	105.77	32.08		25	34	505	55.8	5.99
Q6P9J5	KN motif and ankyrin repeat domain-containing protein 4 OS=Mus musculus GN=Kank4 PE=2 SV=1 - [KANK4_MOUSE]	13.48	1	9	9	36	2.119	0.410	1.281	1.188	72.41	13.48		13	36	1016	110.3	4.88

Q9Z1A9	TBC1 domain family member 8 OS=Mus musculus GN=Tbc1d8 PE=2 SV=2 -	3.97	1	4	4	8	0.991	1.557	1.503	1.188	24.44	3.97	6	8	1134	130.0	5.48
Q8BHF7	CDP-diacylglycerol--glycerol-3-phosphate 3-phosphatidyltransferase, mitochondrial OS=Mus musculus GN=Pgs1 PE=2 SV=1 - [PGPS1_MOUSE]	27.12	3	11	11	35	0.815	1.055	0.964	1.188	128.69	27.12	20	35	553	62.4	8.90
O88842	FYVE, RhoGEF and PH domain-containing protein 3 OS=Mus musculus GN=Fgd3 PE=2 SV=1 - [FGD3_MOUSE]	6.28	3	2	2	4	1.641	1.096	1.024	1.188	16.92	6.28	3	4	733	80.6	5.76
Q8VEK0	Cell cycle control protein 50A OS=Mus musculus GN=Tmem30a PE=1 SV=1 - [CC50A_MOUSE]	26.37	3	11	11	78	1.393	1.282	1.159	1.188	183.63	26.37	18	78	364	41.0	8.37
Q8C726	BTB/POZ domain-containing protein 9 OS=Mus musculus GN=Btbd9 PE=2 SV=1 -	4.08	1	1	2	3	1.933	1.312	1.235	1.189	11.70	4.08	3	3	612	69.1	5.68
Q922F4	Tubulin beta-6 chain OS=Mus musculus GN=Tubb6 PE=1 SV=1 - [TBB6_MOUSE]	60.40	1	9	23	2054	0.978	1.333	0.909	1.189	5436.67	60.40	40	2054	447	50.1	4.89
Q9D0L4	Uncharacterized aarF domain-containing protein kinase 1 OS=Mus musculus GN=Adck1 PE=2 SV=1 - [ADCK1_MOUSE]	26.29	2	12	12	40	0.694	1.052	0.964	1.189	120.29	26.29	20	40	525	59.7	8.05
Q9DCM2	Glutathione S-transferase kappa 1 OS=Mus musculus GN=Gstk1 PE=1 SV=3 - [GSTK1_MOUSE]	64.60	1	12	13	43	1.003	1.335	1.014	1.189	133.89	64.60	20	43	226	25.7	8.88
B7ZMP1	Probable Xaa-Pro aminopeptidase 3 OS=Mus musculus GN=Xpnpep3 PE=2 SV=1 -	25.49	2	3	8	23	1.022	1.282	0.955	1.189	78.38	25.49	15	23	506	56.6	7.68
Q3UGP9	Leucine-rich repeat-containing protein 58 OS=Mus musculus GN=Lrrc58 PE=2 SV=1 - [LRC58_MOUSE]	4.10	1	1	1	4	0.655	1.271	1.002	1.189	9.65	4.10	2	4	366	40.1	6.62
O88653	Ragulator complex protein LAMTOR3 OS=Mus musculus GN=Lamtor3 PE=1 SV=1 - [LTOR3_MOUSE]	21.77	1	3	3	8	1.574	1.238	0.965	1.189	24.29	21.77	6	8	124	13.5	7.34
P11214	Tissue-type plasminogen activator OS=Mus musculus GN=Plat PE=1 SV=3 - [TPA_MOUSE]	9.30	1	5	5	12	1.101	1.042	0.830	1.189	38.22	9.30	10	12	559	63.1	8.15
B7ZBW1	Potassium voltage-gated channel subfamily KQT member 2 OS=Mus musculus GN=Kcnq2 PE=2 SV=1 - [B7ZBW1_MOUSE]	32.86	28	25	25	121	0.788	1.080	1.249	1.189	364.23	32.86	42	121	852	93.9	9.28
Q69ZB3	Testis-specific Y-encoded-like protein 5 OS=Mus musculus GN=Tspyl5 PE=2 SV=2 - [TSYL5_MOUSE]	7.88	1	2	2	3	3.182	2.822	1.441	1.189	1.81	7.88	2	3	406	44.3	9.77
O09111	NADH dehydrogenase [ubiquinone] 1 beta subcomplex subunit 11, mitochondrial OS=Mus musculus GN=Ndufb11 PE=1 SV=2 - [NDUBB_MOUSE]	30.46	1	3	3	55	0.937	1.037	1.108	1.189	172.51	30.46	6	55	151	17.4	5.22
Q91W96	Anaphase-promoting complex subunit 4 OS=Mus musculus GN=Anapc4 PE=2 SV=1 - [APC4_MOUSE]	10.90	2	7	7	14	0.903	1.122	0.963	1.190	51.16	10.90	11	14	807	91.6	5.40
D3YTU2	2-oxoglutarate and iron-dependent oxygenase domain-containing protein 1 OS=Mus musculus GN=Ogfod1 PE=2 SV=1 - [D3YTU2_MOUSE]	38.49	4	19	19	38	1.046	1.004	0.836	1.190	116.62	38.49	27	38	530	61.1	4.87
P43432	Interleukin-12 subunit beta OS=Mus musculus GN=Il12b PE=1 SV=1 - [IL12B_MOUSE]	3.28	1	1	1	1	0.606	0.519	1.133	1.190	2.79	3.28	1	1	335	38.2	6.54
Q8BGF9	Solute carrier family 25 member 44 OS=Mus musculus GN=Slc25a44 PE=2 SV=1 -	6.37	3	2	2	4	0.767	1.270	1.179	1.190	10.00	6.37	3	4	314	35.3	9.66
P04247	Myoglobin OS=Mus musculus GN=Mb PE=1 SV=3 - [MYG_MOUSE]	36.36	1	4	4	19	6.019	11.650	0.537	1.190	64.96	36.36	7	19	154	17.1	7.62
P28028	Serine/threonine-protein kinase B-raf OS=Mus musculus GN=Braf PE=1 SV=3 - [BRAF_MOUSE]	37.94	2	5	26	80	1.157	0.861	1.099	1.190	231.21	37.94	46	80	804	88.7	7.65
Q9CQB4	Cytochrome b-c1 complex subunit 7 OS=Mus musculus GN=Uqcrb PE=3 SV=1 - [Q9CQB4_MOUSE]	54.95	2	10	10	140	1.304	1.050	1.448	1.190	354.75	54.95	19	140	111	13.6	9.11
Q925N0	Sideroflexin-5 OS=Mus musculus GN=Sfxn5 PE=1 SV=2 - [SFXN5_MOUSE]	35.38	3	11	11	71	0.578	0.682	0.880	1.191	176.98	35.38	19	71	342	37.3	9.44

O70443	Guanine nucleotide-binding protein G(z) subunit alpha OS=Mus musculus GN=Gnaz PE=2 SV=4 - [GNAZ_MOUSE]	48.17	2	13	16	147	0.615	1.248	1.178	1.191	451.91	48.17	28	147	355	40.8	7.61
E9QLB8	Uncharacterized aarF domain-containing protein kinase 4 OS=Mus musculus GN=Adck4 PE=4 SV=1 - [E9QLB8_MOUSE]	11.07	2	4	4	11	1.361	0.881	0.915	1.191	43.17	11.07	7	11	533	59.1	7.75
P28184	Metallothionein-3 OS=Mus musculus GN=Mt3 PE=1 SV=1 - [MT3_MOUSE]	33.82	1	2	2	4	0.941	0.960	2.292	1.191	8.73	33.82	4	4	68	7.0	7.47
Q8BRR9	Calcium/calmodulin-dependent 3',5'-cyclic nucleotide phosphodiesterase 1A OS=Mus musculus GN=Pde1a PE=2 SV=1	35.05	7	12	13	65	0.881	2.185	1.407	1.191	236.94	35.05	23	65	545	62.3	5.91
Q80ZJ1	Ras-related protein Rap-2a OS=Mus musculus GN=Rap2a PE=1 SV=2 - [RAP2A_MOUSE]	67.76	3	2	9	61	0.644	1.100	0.929	1.191	214.38	67.76	18	61	183	20.6	4.82
Q3URE1	Acyl-CoA synthetase family member 3, mitochondrial OS=Mus musculus GN=Acsf3 PE=2 SV=2 - [ACSF3_MOUSE]	32.76	2	16	16	54	1.003	0.927	0.986	1.191	144.45	32.76	28	54	583	65.0	8.02
Q80UW5	Serine/threonine-protein kinase MRCK gamma OS=Mus musculus GN=Cdc42bpg PE=2 SV=2 - [MRCKG_MOUSE]	4.26	1	3	6	11	1.372	0.904	1.232	1.191	33.51	4.26	8	11	1551	172.0	6.05
A2AIG8-2	Isoform 2 of 1-aminocyclopropane-1-carboxylate synthase-like protein 1 OS=Mus musculus GN=Accs - [1A1L1_MOUSE]	9.39	3	3	3	12	0.890	0.977	0.792	1.191	18.08	9.39	6	12	479	54.5	6.33
P62743	AP-2 complex subunit sigma OS=Mus musculus GN=Ap2s1 PE=1 SV=1 - [AP2S1_MOUSE]	42.96	1	6	6	86	0.699	1.404	1.131	1.191	256.95	42.96	10	86	142	17.0	6.18
Q9R0X0	Mediator of RNA polymerase II transcription subunit 20 OS=Mus musculus GN=Med20 PE=1 SV=1 - [MED20_MOUSE]	10.38	4	2	2	2	1.160	1.009	0.966	1.191	6.53	10.38	2	2	212	23.2	6.87
Q9CR68	Cytochrome b-c1 complex subunit Rieske, mitochondrial OS=Mus musculus GN=Uqcrfs1 PE=1 SV=1 - [UCRI_MOUSE]	48.91	1	12	12	149	0.967	0.897	1.278	1.191	419.85	48.91	20	149	274	29.3	8.70
Q9ES97-3	Isoform 3 of Reticulon-3 OS=Mus musculus GN=Rtn3 - [RTN3_MOUSE]	21.10	2	1	6	41	0.716	0.443	0.966	1.191	124.47	21.10	11	41	237	25.4	8.50
Q91VN1	Zinc finger protein 24 OS=Mus musculus GN=Znf24 PE=1 SV=1 - [ZNF24_MOUSE]	9.51	1	2	2	5	0.481	0.720	1.216	1.191	5.69	9.51	3	5	368	41.9	6.04
Q7TMC8	L-fucose kinase OS=Mus musculus GN=Fuk PE=2 SV=1 - [Q7TMC8_MOUSE]	14.95	1	14	14	37	0.894	1.311	1.211	1.191	105.59	14.95	24	37	1090	119.2	6.60
Q8K1R3	Polyribonucleotide nucleotidyltransferase 1, mitochondrial OS=Mus musculus GN=Pnpt1 PE=1 SV=1 - [PNPT1_MOUSE]	20.05	2	13	15	37	1.032	1.067	1.042	1.192	96.34	20.05	22	37	783	85.6	8.03
Q91WA3	Histone deacetylase 11 OS=Mus musculus GN=Hdac11 PE=2 SV=1 - [HDA11_MOUSE]	36.89	3	11	12	60	0.854	0.883	0.965	1.192	187.85	36.89	22	60	347	39.1	7.14
Q9CYK2	Glutaminyl-peptide cyclotransferase OS=Mus musculus GN=Qpct PE=1 SV=2 - [QPCT_MOUSE]	9.39	2	3	3	9	0.890	0.899	0.517	1.192	28.96	9.39	5	9	362	41.1	6.92
Q9EQM6	Microprocessor complex subunit DGCR8 OS=Mus musculus GN=Dgcr8 PE=2 SV=2 - [DGCR8_MOUSE]	1.81	1	1	1	1	1.218	0.743	0.774	1.192	0.00	1.81	1	1	773	86.3	6.06
Q6PER3	Microtubule-associated protein RP/EB family member 3 OS=Mus musculus GN=Mapre3 PE=1 SV=1 - [MARE3_MOUSE]	41.99	2	3	15	128	1.191	0.884	1.165	1.192	328.14	41.99	28	128	281	31.9	5.54
Q69ZS6	Synaptic vesicle glycoprotein 2C OS=Mus musculus GN=Sv2c PE=1 SV=2 - [SV2C_MOUSE]	12.65	1	8	9	42	1.460	0.686	1.521	1.192	118.94	12.65	14	42	727	82.2	5.11
F7B8J4	Probable carboxypeptidase PM20D1 (Fragment) OS=Mus musculus GN=Pm20d1 PE=4 SV=1 - [F7B8J4_MOUSE]	31.31	2	2	2	5	0.801	1.462	0.957	1.192	15.87	31.31	3	5	99	11.4	5.67
Q1JQR8	Glucagon-like peptide 1 receptor OS=Mus musculus GN=Glp1r PE=2 SV=1 - [Q1JQR8_MOUSE]	1.73	2	1	1	2	0.703	0.898	0.863	1.192	6.68	1.73	2	2	463	53.0	8.44
Q3U1D9	UPF0602 protein C4orf47 homolog OS=Mus musculus PE=2 SV=1 - [CD047_MOUSE]	5.70	1	1	1	2	2.041	1.266	0.979	1.192	0.00	5.70	1	2	316	35.2	9.73

Q6VNB8	WD repeat and FYVE domain-containing protein 3 OS=Mus musculus GN=Wdfy3 PE=1 SV=1 - [WDFY3_MOUSE]	12.29	6	37	39	125	0.863	1.012	1.092	1.192	359.70	12.29	69	125	3508	392.1	6.80
F6W8W7	Protein Taf4a (Fragment) OS=Mus musculus GN=Taf4a PE=4 SV=1 - [F6W8W7_MOUSE]	5.00	2	3	3	9	2.069	1.291	1.365	1.192	17.76	5.00	4	9	680	73.2	9.94
F8VQK3	Protein Gucy1a2 OS=Mus musculus GN=Gucy1a2 PE=2 SV=1 - [F8VQK3_MOUSE]	40.41	1	22	22	65	0.547	0.935	1.239	1.193	208.49	40.41	37	65	730	81.8	6.87
P81122	Insulin receptor substrate 2 OS=Mus musculus GN=Irs2 PE=1 SV=2 - [IRS2_MOUSE]	26.12	1	22	22	63	1.229	1.140	1.176	1.193	175.64	26.12	32	63	1321	136.7	8.69
Q9CZ04	COP9 signalosome complex subunit 7a OS=Mus musculus GN=Cops7a PE=1 SV=2 -	40.73	5	10	10	24	1.012	0.735	1.142	1.193	65.51	40.73	18	24	275	30.2	7.87
P97930	Thymidylate kinase OS=Mus musculus GN=Dtymk PE=2 SV=2 - [KTHY_MOUSE]	33.96	3	8	9	20	0.986	1.349	1.038	1.193	51.98	33.96	17	20	212	23.9	7.90
Q99LC3	NADH dehydrogenase [ubiquinone] 1 alpha subcomplex subunit 10, mitochondrial OS=Mus musculus GN=Ndufa10 PE=1 SV=1 - [NDUAA_MOUSE]	66.48	1	20	20	127	0.717	0.903	1.054	1.193	412.39	66.48	32	127	355	40.6	7.78
Q9CPW9-2	Isoform 2 of Methionine aminopeptidase 1D, mitochondrial OS=Mus musculus GN=Metap1d - [MAP12_MOUSE]	10.14	2	2	2	3	1.867	1.806	0.555	1.193	8.69	10.14	3	3	217	24.0	5.26
Q3ULJ0-2	Isoform 2 of Glycerol-3-phosphate dehydrogenase 1-like protein OS=Mus musculus GN=Gpd1l - [GPD1L_MOUSE]	64.38	3	20	22	134	0.497	1.671	1.198	1.193	361.87	64.38	39	134	320	34.6	6.87
Q8BZG5-2	Isoform 2 of Protein RRNAD1 OS=Mus musculus GN=Rrnad1 - [RRNAD_MOUSE]	11.29	4	1	2	2	2.078	0.931	1.165	1.193	0.00	11.29	2	2	310	34.3	8.65
P31938	Dual specificity mitogen-activated protein kinase kinase 1 OS=Mus musculus GN=Map2k1 PE=1 SV=2 - [illegible]	67.43	1	13	21	127	0.551	2.034	1.249	1.193	333.66	67.43	36	127	393	43.4	6.70
P05063	Fructose-bisphosphate aldolase C OS=Mus musculus GN=Aldoc PE=1 SV=4 - [ALDOC_MOUSE]	81.54	1	24	32	1090	1.408	0.731	1.011	1.193	2812.67	81.54	59	1090	363	39.4	7.12
Q6NVF0	Inositol polyphosphate 5-phosphatase OCRL-1 OS=Mus musculus GN=Ocrl PE=2 SV=1 - [OCRL_MOUSE]	29.44	4	24	24	82	0.740	1.168	1.016	1.193	210.66	29.44	36	82	900	104.2	6.27
Q80TC6	Zinc finger SWIM domain-containing protein 5 OS=Mus musculus GN=Zswim5 PE=2 SV=2 - [ZSWM5_MOUSE]	1.60	1	1	2	2	1.287	1.853	1.362	1.193	5.52	1.60	2	2	1188	130.8	7.39
Q8BH82	N-acyl-phosphatidylethanolamine-hydrolyzing phospholipase D OS=Mus musculus GN=Napepld PE=1 SV=1 - [illegible]	19.95	1	7	7	12	1.491	1.206	1.060	1.194	36.86	19.95	11	12	396	45.8	5.94
Q9JHR9-2	Isoform 2 of Nuclear receptor-interacting protein 2 OS=Mus musculus GN=Nrip2 - [NRIP2_MOUSE]	34.93	4	7	7	32	1.340	0.696	0.948	1.194	91.21	34.93	14	32	229	25.3	7.34
G3X925	Pyruvate kinase OS=Mus musculus GN=Pklr PE=3 SV=1 - [G3X925_MOUSE]	23.17	4	6	9	69	1.441	1.522	1.372	1.194	158.76	23.17	16	69	574	62.2	7.09
Q1HKZ5	Mitogen-activated protein kinase kinase kinase 13 OS=Mus musculus GN=Map3k13 PE=2 SV=1 - [M3K13_MOUSE]	4.90	1	2	4	13	0.891	0.726	1.588	1.194	40.13	4.90	7	13	959	106.9	6.74
Q9DCM0	Persulfide dioxygenase ETHE1, mitochondrial OS=Mus musculus GN=Ethe1 PE=1 SV=2 - [ETHE1_MOUSE]	63.78	1	11	11	62	1.218	0.969	0.902	1.194	188.11	63.78	20	62	254	27.7	7.23
Q921I0	ORM1-like protein 1 OS=Mus musculus GN=Ormdl1 PE=2 SV=1 - [ORML1_MOUSE]	7.19	1	1	1	1	1.948	1.169	0.987	1.194	0.00	7.19	1	1	153	17.3	9.54
Q9WUB0	RanBP-type and C3HC4-type zinc finger-containing protein 1 OS=Mus musculus GN=Rbck1 PE=1 SV=2 - [HOIL1_MOUSE]	32.87	2	16	16	47	1.590	1.276	1.058	1.195	149.40	32.87	27	47	508	57.5	6.19
Q8BG67	Protein EFR3 homolog A OS=Mus musculus GN=Efr3a PE=1 SV=1 - [EFR3A_MOUSE]	23.93	3	15	17	55	0.680	1.091	0.987	1.195	166.96	23.93	29	55	819	92.6	6.83
B1ASN7	EF-hand calcium-binding domain-containing protein 3 (Fragment) OS=Mus musculus GN=Efcab3 PE=2 SV=2 - [illegible]	3.06	1	1	1	1	4.224	1.943	1.378	1.195	2.27	3.06	1	1	359	40.8	9.00

J3QP99	FTS and Hook-interacting protein OS=Mus musculus GN=Fam160a2 PE=4 SV=1 - [J3QP99_MOUSE]	22.75	3	1	16	40	2.118	1.369	1.087	1.195	119.01	22.75	25	40	989	108.0	6.76
Q9CPV4	Glyoxalase domain-containing protein 4 OS=Mus musculus GN=Glod4 PE=2 SV=1 - [GLOD4_MOUSE]	80.87	8	19	19	127	1.081	1.116	1.039	1.195	389.11	80.87	37	127	298	33.3	5.47
Q9DBL1	Short/branched chain specific acyl-CoA dehydrogenase, mitochondrial OS=Mus musculus GN=Acadsb [illegible]	41.90	2	15	15	65	0.796	1.135	0.871	1.195	207.49	41.90	27	65	432	47.8	7.87
Q91VC9	Growth hormone-inducible transmembrane protein OS=Mus musculus GN=Ghitm PE=2 SV=1 - [GHITM_MOUSE]	6.36	1	2	2	8	1.307	1.537	1.633	1.195	20.34	6.36	4	8	346	37.3	9.80
A2ARM1	Protein PAT1 homolog 2 OS=Mus musculus GN=Patl2 PE=2 SV=1 - [PATL2_MOUSE]	3.02	1	1	1	1	2.055	0.992	0.898	1.195	0.00	3.02	1	1	529	59.6	7.42
Q91ZP9	N-terminal EF-hand calcium-binding protein 2 OS=Mus musculus GN=Necab2 PE=1 SV=1 - [NECA2_MOUSE]	60.67	3	19	19	95	0.807	1.686	1.390	1.196	297.97	60.67	35	95	389	43.4	5.30
G3UZG5	Kelch domain-containing protein 4 OS=Mus musculus GN=Klhdc4 PE=2 SV=1 -	2.09	3	1	1	1	1.172	0.951	1.045	1.196	2.06	2.09	1	1	527	58.5	5.81
B1AWN4	Protein Atp10b OS=Mus musculus GN=Atp10b PE=4 SV=1 - [B1AWN4_MOUSE]	0.81	1	1	1	2	1.219	1.682	1.094	1.196	4.63	0.81	1	2	1474	166.3	6.60
Q2TA57	Aspartate beta-hydroxylase domain-containing protein 1 OS=Mus musculus GN=Asphd1 PE=2 SV=2 - [illegible]	10.56	1	3	3	3	1.603	1.260	1.450	1.196	7.51	10.56	3	3	360	38.2	8.25
D3Z1M4	Calcium-binding protein 1 OS=Mus musculus GN=Cabp1 PE=2 SV=1 - [D3Z1M4_MOUSE]	15.71	4	5	6	20	2.035	0.846	1.915	1.196	50.89	15.71	11	20	350	37.9	8.28
G3X9A8	Ankyrin repeat domain 13 family, member D, isoform CRA_a OS=Mus musculus GN=Ankrd13d PE=4 SV=1 - [G3X9A8_MOUSE]	10.08	5	5	5	11	0.742	1.010	0.931	1.196	33.87	10.08	8	11	605	68.0	5.47
P10107	Annexin A1 OS=Mus musculus GN=Anxa1 PE=1 SV=2 - [ANXA1_MOUSE]	52.89	1	13	15	51	0.888	3.290	0.566	1.196	149.03	52.89	26	51	346	38.7	7.37
Q8CA95-3	Isoform 3 of cAMP and cAMP-inhibited cGMP 3',5'-cyclic phosphodiesterase 10A OS=Mus musculus GN=Pde10a - [PDE10_MOUSE]	30.53	7	21	21	81	0.415	0.432	2.673	1.196	270.72	30.53	37	81	796	90.3	6.65
P18872-2	Isoform Alpha-2 of Guanine nucleotide-binding protein G(o) subunit alpha OS=Mus musculus GN=Gnao1 - [illegible]	64.97	5	3	23	492	0.397	0.548	1.070	1.196	1295.84	64.97	44	492	354	40.0	6.01
Q8BVL3	Sorting nexin-17 OS=Mus musculus GN=Snx17 PE=1 SV=2 - [SNX17_MOUSE]	31.49	4	10	10	37	0.774	1.145	0.910	1.196	107.53	31.49	17	37	470	52.8	7.49
Q9D8B6	Protein FAM210B OS=Mus musculus GN=Fam210b PE=2 SV=3 - [F210B_MOUSE]	13.16	1	2	2	6	0.480	0.608	1.304	1.196	11.73	13.16	4	6	190	20.3	10.51
O54785-3	Isoform 3 of LIM domain kinase 2 OS=Mus musculus GN=Limk2 - [LIMK2_MOUSE]	8.87	4	3	3	5	0.624	1.100	1.320	1.197	13.00	8.87	4	5	451	51.1	8.28
Q8CJC5-2	Isoform 2 of E3 ubiquitin-protein ligase NEURL3 OS=Mus musculus GN=Neurl3 -	10.31	2	1	1	1	1.707	1.078	2.032	1.197	0.00	10.31	1	1	194	21.4	6.89
Q811G0-2	Isoform 2 of Protein PTHB1 OS=Mus musculus GN=Bbs9 - [PTHB1_MOUSE]	10.11	6	7	7	15	1.333	1.924	0.992	1.197	47.20	10.11	11	15	880	98.4	6.19
P28653	Biglycan OS=Mus musculus GN=Bgn PE=2 SV=1 - [PGS1_MOUSE]	22.76	1	7	7	27	0.908	4.387	0.985	1.197	59.37	22.76	11	27	369	41.6	7.27
P58774-2	Isoform 2 of Tropomyosin beta chain OS=Mus musculus GN=Tpm2 - [TPM2_MOUSE]	48.94	2	2	16	208	1.911	2.715	1.241	1.197	537.98	48.94	30	208	284	32.9	4.67
Q63ZV0	Insulinoma-associated protein 1 OS=Mus musculus GN=Insm1 PE=2 SV=1 - [INSM1_MOUSE]	3.07	1	1	1	1	0.298	1.056	0.931	1.197	3.33	3.07	1	1	521	54.0	9.01
Q9ESJ1	CDK5 and ABL1 enzyme substrate 1 OS=Mus musculus GN=Cables1 PE=1 SV=2 -	14.44	2	5	5	12	1.736	1.330	1.391	1.197	39.15	14.44	7	12	568	61.4	9.23
Q9QXL2-4	Isoform 4 of Kinesin-like protein KIF21A OS=Mus musculus GN=Kif21a - [KI21A_MOUSE]	33.29	2	1	45	203	0.650	0.837	1.028	1.197	630.82	33.29	75	203	1616	180.4	6.34
Q80U40-2	Isoform 2 of RIMS-binding protein 2 OS=Mus musculus GN=Rimbp2 - [RIMB2_MOUSE]	23.29	3	16	17	57	1.292	1.306	1.758	1.198	161.25	23.29	30	57	1069	117.9	5.27

A6H5Y3	Methionine synthase OS=Mus musculus GN=Mtr PE=2 SV=1 - [METH_MOUSE]	7.66	1	6	6	14	1.031	1.611	0.971	1.198	38.70	7.66	10	14	1253	139.0	5.57
P31648	Sodium- and chloride-dependent GABA transporter 1 OS=Mus musculus GN=Slc6a1 PE=1 SV=2 - [illegible]	16.69	1	7	7	121	0.663	0.792	1.036	1.198	203.76	16.69	12	121	599	67.0	7.96
Q9WU84	Copper chaperone for superoxide dismutase OS=Mus musculus GN=Ccs PE=1 SV=1 - [CCS_MOUSE]	9.49	1	4	4	8	1.402	1.305	1.102	1.198	22.13	9.49	6	8	274	28.9	6.10
D3Z093	Lipoyltransferase 1, mitochondrial (Fragment) OS=Mus musculus GN=Lipt1 PE=2 SV=1 - [D3Z093_MOUSE]	9.13	4	2	2	6	1.246	0.944	0.944	1.198	15.90	9.13	4	6	230	25.7	9.25
Q80U57	Regulating synaptic membrane exocytosis protein 3 OS=Mus musculus GN=Rims3 PE=1 SV=2 - [RIMS3_MOUSE]	57.98	1	11	12	41	0.940	0.780	0.998	1.198	113.82	57.98	21	41	307	32.6	9.29
P14152	Malate dehydrogenase, cytoplasmic OS=Mus musculus GN=Mdh1 PE=1 SV=3 - [MDHC_MOUSE]	60.78	2	26	26	797	0.900	1.160	1.132	1.198	2166.75	60.78	47	797	334	36.5	6.58
O70405	Serine/threonine-protein kinase ULK1 OS=Mus musculus GN=Ulk1 PE=1 SV=1 - [ULK1_MOUSE]	12.18	1	10	10	24	0.962	0.975	1.058	1.199	51.50	12.18	14	24	1051	112.4	8.66
Q9QZV9	NTF2-related export protein 1 OS=Mus musculus GN=Nxt1 PE=1 SV=2 - [NXT1_MOUSE]	16.43	3	2	2	3	1.378	1.246	1.086	1.199	7.87	16.43	3	3	140	15.8	5.03
O88839	Disintegrin and metalloproteinase domain-containing protein 15 OS=Mus musculus GN=Adam15 PE=1 SV=2 - [illegible]	15.63	4	9	9	28	0.922	0.924	1.098	1.199	55.93	15.63	15	28	864	92.6	6.54
Q8R4N0	Citrate lyase subunit beta-like protein, mitochondrial OS=Mus musculus GN=Clybl PE=2 SV=2 - [CLYBL_MOUSE]	58.28	1	16	16	76	1.056	1.184	1.007	1.199	228.37	58.28	28	76	338	37.5	8.54
Q6PCZ4	Melanoma-associated antigen E1 OS=Mus musculus GN=Magee1 PE=1 SV=1 -	20.59	1	14	14	42	1.211	0.770	1.143	1.200	131.39	20.59	25	42	918	101.6	6.67
P48962	ADP/ATP translocase 1 OS=Mus musculus GN=Slc25a4 PE=1 SV=4 - [ADT1_MOUSE]	72.82	1	16	30	830	0.654	0.923	1.017	1.200	2216.36	72.82	56	830	298	32.9	9.72
O35683	NADH dehydrogenase [ubiquinone] 1 alpha subcomplex subunit 1 OS=Mus musculus GN=Ndufa1 PE=2 SV=1 - [illegible]	22.86	1	2	2	11	0.899	1.071	1.102	1.200	28.66	22.86	4	11	70	8.1	9.48
Q9D394-4	Isoform 4 of Protein RUFY3 OS=Mus musculus GN=Rufy3 - [RUFY3_MOUSE]	53.56	2	2	27	250	2.155	0.739	1.363	1.200	676.08	53.56	50	250	519	57.4	5.85
Q69ZH9	Rho GTPase-activating protein 23 OS=Mus musculus GN=Arhgap23 PE=1 SV=2 -	21.44	3	11	26	66	0.812	0.773	1.050	1.201	201.50	21.44	40	66	1483	161.7	8.98
Q8K0D7	Tail-anchored protein insertion receptor WRB OS=Mus musculus GN=Wrb PE=2 SV=1 - [WRB_MOUSE]	16.09	1	3	3	19	0.467	0.927	0.906	1.201	50.36	16.09	6	19	174	19.9	9.79
Q9Z1B7	Mitogen-activated protein kinase 13 OS=Mus musculus GN=Mapk13 PE=1 SV=2 -	5.19	8	1	2	13	1.124	0.888	0.958	1.201	30.60	5.19	3	13	366	42.0	8.57
P33146	Cadherin-15 OS=Mus musculus GN=Cdh15 PE=1 SV=3 - [CAD15_MOUSE]	1.66	1	1	1	1	0.660	1.853	0.978	1.201	2.03	1.66	1	1	784	85.6	4.86
G5E8B1	Protein tyrosine phosphatase, non-receptor type 13 OS=Mus musculus GN=Ptpn13 PE=4 SV=1 - [G5E8B1_MOUSE]	4.00	2	7	8	13	0.691	1.320	1.164	1.201	27.64	4.00	10	13	2451	270.1	6.35
Q9CXP8	Guanine nucleotide-binding protein G(I)/G(S)/G(O) subunit gamma-10 OS=Mus musculus GN=Gng10 [illegible]	61.76	2	3	4	12	0.889	5.644	1.872	1.201	42.32	61.76	8	12	68	7.2	7.85
Q8R164	Valacyclovir hydrolase OS=Mus musculus GN=Bphl PE=2 SV=1 - [BPHL_MOUSE]	55.33	1	14	14	55	1.885	1.189	1.060	1.201	158.82	55.33	26	55	291	32.8	8.94
Q8BFW6	Ectonucleoside triphosphate diphosphohydrolase 3 OS=Mus musculus GN=Entpd3 PE=2 SV=1 - [illegible]	13.61	1	7	7	17	1.654	1.081	1.068	1.202	40.80	13.61	11	17	529	58.9	6.15
Q6ZQ03	Formin-binding protein 4 OS=Mus musculus GN=Fnbp4 PE=1 SV=2 - [FNBP4_MOUSE]	2.62	3	3	3	4	1.881	1.529	1.315	1.202	5.04	2.62	4	4	1031	111.2	4.73
Q9CQ91	NADH dehydrogenase [ubiquinone] 1 alpha subcomplex subunit 3 OS=Mus musculus GN=Ndufa3 PE=1 SV=1 - [illegible]	57.14	2	5	5	82	0.870	0.954	1.072	1.202	204.87	57.14	9	82	84	9.3	8.47

G3UXN4	KN motif and ankyrin repeat domain-containing protein 3 OS=Mus musculus GN=Kank3 PE=2 SV=1 - [G3UXN4_MOUSE]	3.81	4	2	3	4	1.107	1.381	0.960	1.202	8.50	3.81	4	4	603	64.3	4.84
P32883-2	Isoform 2B of GTPase KRas OS=Mus musculus GN=Kras - [RASK_MOUSE]	66.49	5	4	9	87	1.543	1.086	1.030	1.203	193.75	66.49	15	87	188	21.5	8.12
Q5SSE9	ATP-binding cassette sub-family A member 13 OS=Mus musculus GN=Abca13 PE=2 SV=1 - [ABCAD_MOUSE]	1.75	2	4	9	19	2.853	1.266	1.151	1.203	35.66	1.75	11	19	5034	568.5	6.52
Q9WTL7	Acyl-protein thioesterase 2 OS=Mus musculus GN=Lypla2 PE=1 SV=1 - [LYPA2_MOUSE]	61.47	1	11	11	62	0.705	1.133	1.061	1.203	218.60	61.47	19	62	231	24.8	7.23
Q8K183	Pyridoxal kinase OS=Mus musculus GN=Pdxk PE=1 SV=1 - [PDXK_MOUSE]	60.26	2	16	16	311	1.409	1.041	0.906	1.203	869.71	60.26	29	311	312	35.0	6.29
Q5U458	DnaJ homolog subfamily C member 11 OS=Mus musculus GN=Dnajc11 PE=2 SV=2 -	42.04	3	21	21	71	1.004	1.071	1.130	1.203	220.30	42.04	37	71	559	63.2	8.32
P97821	Dipeptidyl peptidase 1 OS=Mus musculus GN=Ctsc PE=2 SV=1 - [CATC_MOUSE]	5.41	2	3	3	10	1.136	1.448	1.044	1.204	21.72	5.41	5	10	462	52.3	6.89
H3BJL3	Protein unc-13 homolog B (Fragment) OS=Mus musculus GN=Unc13a PE=4 SV=1 - [H3BJL3_MOUSE]	43.31	2	1	7	15	2.240	1.702	1.444	1.204	45.76	43.31	10	15	254	27.9	5.44
Q29SA9	Peripheral clock protein 1 OS=Mus musculus GN=Nckap5 PE=2 SV=1 - [Q29SA9_MOUSE]	1.74	6	2	2	2	1.826	1.704	1.140	1.204	5.97	1.74	2	2	1777	194.1	7.09
D6RHQ6	Ubiquitin carboxyl-terminal hydrolase 16 OS=Mus musculus GN=Usp16 PE=2 SV=1 - [D6RHQ6_MOUSE]	8.38	5	2	2	3	1.698	0.528	0.941	1.204	4.26	8.38	2	3	334	38.1	8.34
Q9QXZ7	Photoreceptor-specific nuclear receptor OS=Mus musculus GN=Nr2e3 PE=1 SV=1 - [NR2E3_MOUSE]	6.84	1	1	2	2	1.140	0.873	1.289	1.204	5.06	6.84	2	2	395	43.1	7.74
Q9CWY3	N-lysine methyltransferase SETD6 OS=Mus musculus GN=Setd6 PE=2 SV=1 - [SETD6_MOUSE]	6.98	1	3	3	4	1.586	2.467	1.093	1.204	7.52	6.98	4	4	473	52.9	4.86
O55229	Choline/ethanolamine kinase OS=Mus musculus GN=Chkb PE=1 SV=3 - [CHKB_MOUSE]	19.80	3	6	6	18	0.881	1.242	1.032	1.204	66.63	19.80	9	18	394	45.1	5.41
Q8BYM8	Probable cysteine--tRNA ligase, mitochondrial OS=Mus musculus GN=Cars2 PE=2 SV=2 - [SYCM_MOUSE]	20.15	2	8	8	24	0.852	1.382	1.036	1.205	65.13	20.15	11	24	551	61.2	8.18
P51175	Protoporphyrinogen oxidase OS=Mus musculus GN=Ppox PE=2 SV=1 - [PPOX_MOUSE]	26.00	1	8	9	22	0.913	0.945	1.008	1.205	72.72	26.00	17	22	477	50.8	8.84
Q3UFY7-4	Isoform 4 of 7-methylguanosine phosphate-specific 5'-nucleotidase OS=Mus musculus GN=Nt5c3b - [illegible]	41.78	4	11	11	38	0.966	1.172	0.912	1.205	84.44	41.78	19	38	292	33.6	6.54
E9PWX1	Dipeptidyl aminopeptidase-like protein 6 OS=Mus musculus GN=Dpp6 PE=2 SV=1 - [E9PWX1_MOUSE]	33.29	3	2	26	184	0.734	1.115	1.133	1.205	507.51	33.29	51	184	859	97.2	6.48
A2AKU9	ATP synthase subunit gamma OS=Mus musculus GN=Atp5c1 PE=2 SV=1 - [A2AKU9_MOUSE]	51.52	7	14	15	177	0.647	0.797	1.069	1.205	486.81	51.52	27	177	297	32.8	9.13
Q9ES82	Popeye domain-containing protein 2 OS=Mus musculus GN=Popdc2 PE=2 SV=1 -	7.90	2	1	1	2	1.857	0.894	1.161	1.205	6.03	7.90	1	2	367	41.2	5.83
Q8BHE3	Caytaxin OS=Mus musculus GN=Atcay PE=1 SV=1 - [ATCAY_MOUSE]	22.85	1	6	6	60	0.745	1.347	1.171	1.205	165.89	22.85	11	60	372	42.2	4.68
Q8BYR5	Calcium-dependent secretion activator 2 OS=Mus musculus GN=Cadps2 PE=1 SV=2 - [CAPS2_MOUSE]	31.77	9	3	34	122	0.606	1.527	1.075	1.205	330.41	31.77	62	122	1297	147.7	6.14
Q9JLZ8-2	Isoform 2 of Single Ig IL-1-related receptor OS=Mus musculus GN=Sigirr - [SIGIR_MOUSE]	2.07	2	1	1	1	1.076	1.233	1.111	1.205	1.76	2.07	1	1	387	43.7	6.92
P26262	Plasma kallikrein OS=Mus musculus GN=Klkb1 PE=1 SV=2 - [KLKB1_MOUSE]	12.07	1	7	7	18	2.208	1.847	0.600	1.206	41.35	12.07	11	18	638	71.3	8.02
O70318	Band 4.1-like protein 2 OS=Mus musculus GN=Epb41l2 PE=1 SV=2 - [E41L2_MOUSE]	38.46	1	30	36	185	1.155	0.831	1.281	1.206	511.01	38.46	64	185	988	109.9	5.43
O55126	Protein NipSnap homolog 2 OS=Mus musculus GN=Gbas PE=2 SV=1 - [NIPS2_MOUSE]	53.02	2	14	15	68	0.845	1.099	1.116	1.206	204.51	53.02	26	68	281	32.9	9.26

Q9ER35	Fructosamine-3-kinase OS=Mus musculus GN=Fn3k PE=2 SV=1 - [FN3K_MOUSE]	38.51	3	11	11	46	1.101	0.812	1.046	1.206	132.18	38.51	20	46	309	35.0	8.40
Q9QXY6	EH domain-containing protein 3 OS=Mus musculus GN=Ehd3 PE=1 SV=2 - [EHD3_MOUSE]	56.64	1	18	31	193	0.859	1.505	1.307	1.206	556.85	56.64	57	193	535	60.8	6.46
B7ZCV4	Ubiquinone biosynthesis protein COQ4 homolog, mitochondrial (Fragment) OS=Mus musculus GN=Coq4 PE=2 SV=1 - [B7ZCV4_MOUSE]	17.50	2	1	3	8	0.854	1.515	0.969	1.206	23.86	17.50	6	8	200	22.4	8.84
Q9ES70	Serine/threonine-protein kinase Nek6 OS=Mus musculus GN=Nek6 PE=1 SV=1 - [NEK6_MOUSE]	7.67	4	2	3	4	0.900	0.848	1.023	1.206	7.39	7.67	4	4	313	35.7	7.90
Q91VT4	Carbonyl reductase family member 4 OS=Mus musculus GN=Cbr4 PE=2 SV=2 - [CBR4_MOUSE]	39.83	2	8	8	38	0.618	1.043	1.091	1.207	117.30	39.83	15	38	236	25.4	9.69
Q91VI7	Ribonuclease inhibitor OS=Mus musculus GN=Rnh1 PE=1 SV=1 - [RINI_MOUSE]	56.14	1	19	19	106	1.384	1.220	0.978	1.207	331.87	56.14	32	106	456	49.8	4.78
E9Q1N6	Protein Rasgef1a OS=Mus musculus GN=Rasgef1a PE=2 SV=1 - [E9Q1N6_MOUSE]	16.77	1	7	7	12	1.040	3.785	1.645	1.207	43.44	16.77	11	12	489	55.8	8.02
Q9CPT3	N-acylneuraminate-9-phosphatase OS=Mus musculus GN=Nanp PE=1 SV=1 - [NANP_MOUSE]	23.39	1	5	5	11	0.981	1.074	0.862	1.207	28.63	23.39	9	11	248	27.8	6.07
P56480	ATP synthase subunit beta, mitochondrial OS=Mus musculus GN=Atp5b PE=1 SV=2 - [ATPB_MOUSE]	84.31	1	35	35	3211	1.313	1.144	1.069	1.208	9996.32	84.31	65	3211	529	56.3	5.34
Q5U4F6	WD repeat-containing protein 34 OS=Mus musculus GN=Wdr34 PE=2 SV=2 -	17.38	2	5	6	20	1.039	1.045	1.031	1.208	60.52	17.38	11	20	535	57.9	6.52
Q9WTZ8	Protein BEX2 OS=Mus musculus GN=Bex2 PE=1 SV=1 - [BEX2_MOUSE]	24.03	1	3	3	13	0.910	1.429	1.012	1.208	24.81	24.03	5	13	129	15.4	6.96
Q91V61-2	Isoform 2 of Sideroflexin-3 OS=Mus musculus GN=Sfxn3 - [SFXN3_MOUSE]	51.39	3	12	13	86	0.646	1.289	1.450	1.208	265.06	51.39	24	86	288	31.6	9.45
F6VIG0	Protein RMD5 homolog B (Fragment) OS=Mus musculus GN=Rmnd5b PE=4 SV=1 - [F6VIG0_MOUSE]	5.95	2	1	1	5	2.165	1.149	1.111	1.208	11.10	5.95	1	5	185	20.4	7.25
A2AH22-7	Isoform 7 of Activating molecule in BECN1-regulated autophagy protein 1 OS=Mus musculus GN=Ambra1 - [illegible]	4.49	7	5	5	10	1.242	1.098	1.158	1.208	24.36	4.49	8	10	1180	129.8	6.81
Q8CDI6	Coiled-coil domain-containing protein 158 OS=Mus musculus GN=Ccdc158 PE=2 SV=1 - [CD158_MOUSE]	4.42	2	3	6	9	1.043	2.192	1.982	1.208	14.37	4.42	7	9	1109	126.7	6.40
Q6PEE3	Ribonucleoside-diphosphate reductase subunit M2 B OS=Mus musculus GN=Rrm2b PE=1 SV=1 - [illegible]	13.11	8	4	5	8	0.790	1.450	0.936	1.208	21.86	13.11	8	8	351	40.8	5.01
O88329	Unconventional myosin-Ia OS=Mus musculus GN=Myo1a PE=2 SV=2 - [MYO1A_MOUSE]	3.93	1	1	4	13	0.921	1.320	1.023	1.209	25.46	3.93	5	13	1043	118.6	9.44
Q8BGN8-2	Isoform 2 of Synaptoporin OS=Mus musculus GN=Synpr - [SYNPR_MOUSE]	16.84	2	3	5	21	1.398	2.135	1.629	1.209	55.91	16.84	9	21	285	31.3	6.33
Q8VE47	Ubiquitin-like modifier-activating enzyme 5 OS=Mus musculus GN=Uba5 PE=2 SV=2 - [UBA5_MOUSE]	24.07	3	6	6	37	0.774	1.213	0.992	1.209	112.71	24.07	11	37	403	44.8	4.96
E9QN99	Alpha/beta hydrolase domain-containing protein 14B OS=Mus musculus GN=Abhd14b PE=4 SV=1 - [E9QN99_MOUSE]	25.71	2	4	4	15	1.748	1.072	1.079	1.209	47.19	25.71	7	15	210	22.4	6.52
Q9CXS4	Centromere protein V OS=Mus musculus GN=Cenpv PE=2 SV=2 - [CENPV_MOUSE]	55.56	3	11	11	33	0.717	0.861	1.196	1.209	109.56	55.56	18	33	252	27.5	9.79
Q91W69	Epsin-3 OS=Mus musculus GN=Epn3 PE=2 SV=1 - [EPN3_MOUSE]	15.88	1	4	8	25	2.056	0.443	1.233	1.209	62.90	15.88	13	25	636	68.2	5.88
Q9JI75	Ribosyldihydronicotinamide dehydrogenase [quinone] OS=Mus musculus GN=Nqo2 PE=2 SV=3 - [NQO2_MOUSE]	47.19	1	7	7	30	1.637	1.273	0.966	1.209	94.05	47.19	13	30	231	26.2	7.01
Q9DCT2	NADH dehydrogenase [ubiquinone] iron-sulfur protein 3, mitochondrial OS=Mus musculus GN=Ndufs3 PE=1 SV=2 - [illegible]	61.22	1	15	15	169	1.031	1.088	1.076	1.209	506.04	61.22	28	169	263	30.1	7.17

E9Q5G3	Protein Kif23 OS=Mus musculus GN=Kif23 PE=2 SV=1 - [E9Q5G3_MOUSE]	2.20	1	1	2	4	1.622	1.711	1.367	1.209	9.44	2.20	3	4	953	108.7	8.66
Q8R1T1	Charged multivesicular body protein 7 OS=Mus musculus GN=Chmp7 PE=1 SV=1 -	30.60	2	9	9	24	0.726	1.291	1.124	1.210	46.26	30.60	13	24	451	50.6	5.22
Q8CGV2	Tryptophan 5-hydroxylase 2 OS=Mus musculus GN=Tph2 PE=2 SV=2 - [TPH2_MOUSE]	28.89	4	10	11	35	1.142	0.710	0.973	1.210	100.14	28.89	18	35	488	55.8	6.24
Q9CY64	Biliverdin reductase A OS=Mus musculus GN=Blvra PE=2 SV=1 - [BIEA_MOUSE]	53.22	4	15	15	69	0.769	0.983	1.038	1.210	195.75	53.22	29	69	295	33.5	7.02
Q9CRC8	Leucine-rich repeat-containing protein 40 OS=Mus musculus GN=Lrrc40 PE=2 SV=2 - [LRC40_MOUSE]	52.33	5	29	29	73	0.737	1.039	0.950	1.210	201.39	52.33	50	73	602	68.0	6.89
Q8R066	Complement C1q tumor necrosis factor-related protein 4 OS=Mus musculus GN=C1qtnf4 PE=2 SV=1 - [illegible]	43.56	1	9	9	38	0.819	1.339	1.244	1.210	145.47	43.56	14	38	326	35.0	8.98
Q64522	Histone H2A type 2-B OS=Mus musculus GN=Hist2h2ab PE=1 SV=3 - [H2A2B_MOUSE]	34.62	1	2	3	92	0.520	0.637	0.845	1.210	219.64	34.62	6	92	130	14.0	10.89
Q3UV17	Keratin, type II cytoskeletal 2 oral OS=Mus musculus GN=Krt76 PE=2 SV=1 -	5.72	3	1	5	34	8.604	1.172	1.400	1.211	72.10	5.72	9	34	594	62.8	8.43
Q8K4I3	Rho guanine nucleotide exchange factor 6 OS=Mus musculus GN=Arhgef6 PE=1 SV=1 - [ARHG6_MOUSE]	12.58	5	6	10	33	0.863	0.863	0.984	1.211	85.60	12.58	17	33	771	87.0	5.87
Q99L88	Beta-1-syntrophin OS=Mus musculus GN=Sntb1 PE=1 SV=4 - [SNTB1_MOUSE]	31.10	2	11	13	37	1.013	1.117	1.007	1.211	111.19	31.10	20	37	537	58.0	8.31
P70444	BH3-interacting domain death agonist OS=Mus musculus GN=Bid PE=1 SV=2 - [BID_MOUSE]	40.00	2	7	7	25	1.373	1.142	1.266	1.211	81.71	40.00	12	25	195	21.9	4.81
Q8BXA5	Cleft lip and palate transmembrane protein 1-like protein OS=Mus musculus GN=Clptm1l PE=2 SV=1 - [illegible]	7.24	1	3	3	7	1.319	1.151	0.861	1.211	24.90	7.24	5	7	539	62.1	8.84
P63044	Vesicle-associated membrane protein 2 OS=Mus musculus GN=Vamp2 PE=1 SV=2 - [VAMP2_MOUSE]	43.10	2	2	5	365	0.776	1.979	1.353	1.211	1233.43	43.10	9	365	116	12.7	8.13
P33609	DNA polymerase alpha catalytic subunit OS=Mus musculus GN=Pola1 PE=1 SV=2 - [DPOLA_MOUSE]	1.02	3	1	2	3	3.154	1.275	1.441	1.211	6.75	1.02	3	3	1465	167.2	5.74
G3UZL4	Ras-related protein Rab-11B (Fragment) OS=Mus musculus GN=Rab11b PE=3 SV=1 - [G3UZL4_MOUSE]	72.00	2	1	7	39	0.772	1.860	1.084	1.211	105.59	72.00	12	39	125	14.3	6.18
Q91WU5	Arsenite methyltransferase OS=Mus musculus GN=As3mt PE=2 SV=2 - [AS3MT_MOUSE]	38.83	1	13	13	32	1.282	1.140	0.782	1.211	92.96	38.83	20	32	376	41.8	5.86
B9EJI9	Transmembrane protein 229A OS=Mus musculus GN=Tmem229a PE=2 SV=1 - [T229A_MOUSE]	11.05	1	3	3	6	1.007	0.615	1.055	1.211	25.03	11.05	5	6	371	41.5	9.35
Q91VM9	Inorganic pyrophosphatase 2, mitochondrial OS=Mus musculus GN=Ppa2 PE=2 SV=1 - [IPYR2_MOUSE]	57.58	5	17	17	86	1.159	1.019	1.182	1.211	318.54	57.58	29	86	330	38.1	6.98
Q91VD9	NADH-ubiquinone oxidoreductase 75 kDa subunit, mitochondrial OS=Mus musculus GN=Ndufs1 PE=1 SV=2 - [illegible]	63.14	1	39	39	519	1.021	1.091	1.118	1.211	1695.98	63.14	75	519	727	79.7	5.72
Q8CDI7	Coiled-coil domain-containing protein 150 OS=Mus musculus GN=Ccdc150 PE=2 SV=2 - [CC150_MOUSE]	1.17	4	1	2	9	2.933	1.523	1.562	1.211	23.83	1.17	2	9	1110	128.4	6.93
Q62130	Tyrosine-protein phosphatase non-receptor type 14 OS=Mus musculus GN=Ptpn14 PE=1 SV=2 - [PTN14_MOUSE]	2.19	1	1	2	3	2.506	1.026	1.509	1.211	7.30	2.19	2	3	1189	135.0	8.34
P54071	Isocitrate dehydrogenase [NADP], mitochondrial OS=Mus musculus GN=Idh2 PE=1 SV=3 - [IDHP_MOUSE]	48.01	2	22	25	106	0.669	1.683	0.864	1.212	288.29	48.01	43	106	452	50.9	8.69
Q9JKY7	Cytochrome P450 CYP2D22 OS=Mus musculus GN=Cyp2d22 PE=2 SV=1 -	22.40	2	8	9	34	0.799	0.998	0.820	1.212	104.33	22.40	16	34	500	56.4	6.98
Q4VBE4-2	Isoform 2 of Pikachurin OS=Mus musculus GN=Egflam - [EGFLA_MOUSE]	2.78	2	2	2	2	2.980	2.231	0.785	1.212	0.00	2.78	2	2	1009	109.8	7.18

P33174	Chromosome-associated kinesin KIF4 OS=Mus musculus GN=Kif4 PE=1 SV=3 - [KIF4_MOUSE]	6.09	1	3	7	11	1.591	1.818	1.056	1.212	19.77	6.09	8	11	1231	139.4	6.68
Q6PAJ1	Breakpoint cluster region protein OS=Mus musculus GN=Bcr PE=1 SV=3 - [BCR_MOUSE]	22.52	1	21	26	100	0.838	1.095	1.475	1.212	286.81	22.52	43	100	1270	143.0	6.92
A2A8K5	Probable G-protein-coupled receptor 153 OS=Mus musculus GN=Gpr153 PE=2 SV=1 - [A2A8K5_MOUSE]	2.47	2	1	1	4	0.765	0.811	0.789	1.212	17.31	2.47	2	4	608	66.2	8.06
Q9EQP2	EH domain-containing protein 4 OS=Mus musculus GN=Ehd4 PE=1 SV=1 - [EHD4_MOUSE]	47.50	1	20	26	98	0.765	1.482	1.058	1.213	285.55	47.50	43	98	541	61.4	6.76
O08848	60 kDa SS-A/Ro ribonucleoprotein OS=Mus musculus GN=Trove2 PE=1 SV=1 -	17.29	1	7	7	20	0.763	0.824	0.927	1.213	58.25	17.29	12	20	538	60.1	7.90
Q8K1S3-2	Isoform 2 of Netrin receptor UNC5B OS=Mus musculus GN=Unc5b - [UNC5B_MOUSE]	6.53	2	4	5	8	1.470	1.058	1.174	1.213	14.98	6.53	7	8	934	102.4	6.05
Q80T62	Probable G-protein coupled receptor 101 OS=Mus musculus GN=Gpr101 PE=2 SV=2 - [GP101_MOUSE]	3.13	1	2	2	2	1.242	1.366	0.961	1.213	4.11	3.13	2	2	511	56.2	5.77
Q8BIQ3	Zinc finger protein 2 OS=Mus musculus GN=Znf2 PE=2 SV=1 - [ZNF2_MOUSE]	6.32	1	2	3	4	1.492	1.970	1.347	1.213	11.46	6.32	4	4	427	48.6	8.79
Q8CHZ9	Transcription termination factor, mitochondrial OS=Mus musculus GN=Mterf PE=2 SV=1 - [MTERF_MOUSE]	5.80	2	2	2	3	2.153	1.035	1.208	1.213	6.53	5.80	2	3	379	43.8	9.06
Q9D075	tRNA methyltransferase 10 homolog B OS=Mus musculus GN=Trmt10b PE=2 SV=2 -	1.89	3	1	1	1	0.743	0.784	0.899	1.214	2.26	1.89	1	1	318	36.4	7.71
Q9JKZ2	Sodium/myo-inositol cotransporter OS=Mus musculus GN=Slc5a3 PE=1 SV=2 -	11.84	1	5	5	33	1.362	1.010	0.979	1.214	47.00	11.84	8	33	718	79.5	7.30
Q7TPS5	C2 domain-containing protein 5 OS=Mus musculus GN=C2cd5 PE=1 SV=2 - [C2CD5_MOUSE]	15.45	4	14	14	28	1.331	1.283	1.189	1.214	63.91	15.45	20	28	1016	111.6	5.78
O70423	Membrane primary amine oxidase OS=Mus musculus GN=Aoc3 PE=2 SV=3 - [AOC3_MOUSE]	15.82	3	11	11	43	0.723	2.710	0.899	1.214	162.58	15.82	20	43	765	84.5	6.42
M0QWU0	CXXC-type zinc finger protein 4 OS=Mus musculus GN=Cxxc4 PE=4 SV=1 -	11.20	1	1	1	4	1.100	1.222	1.048	1.214	2.02	11.20	1	4	366	36.4	9.07
Q3UN16	Probable G-protein coupled receptor 162 OS=Mus musculus GN=Gpr162 PE=2 SV=2 - [GP162_MOUSE]	22.28	1	10	10	26	0.742	1.066	1.421	1.214	81.73	22.28	16	26	588	64.1	8.79
Q8BIK4	Dedicator of cytokinesis protein 9 OS=Mus musculus GN=Dock9 PE=1 SV=2 - [DOCK9_MOUSE]	20.00	3	1	36	96	0.832	0.686	1.181	1.214	253.76	20.00	58	96	2055	235.2	7.25
P01887	Beta-2-microglobulin OS=Mus musculus GN=B2m PE=1 SV=2 - [B2MG_MOUSE]	40.34	1	4	4	13	2.400	1.435	1.095	1.214	36.41	40.34	6	13	119	13.8	8.44
Q8BZF8	Phosphoglucomutase-like protein 5 OS=Mus musculus GN=Pgm5 PE=1 SV=2 - [PGM5_MOUSE]	21.52	1	9	11	30	1.244	2.382	0.962	1.214	71.94	21.52	17	30	567	62.2	7.18
Q0VGM9-4	Isoform 4 of Regulator of telomere elongation helicase 1 OS=Mus musculus GN=Rtel1 - [RTEL1_MOUSE]	2.13	5	2	2	2	2.476	1.915	1.522	1.214	4.33	2.13	2	2	1128	125.8	8.15
P97765	WW domain-binding protein 2 OS=Mus musculus GN=Wbp2 PE=1 SV=1 - [WBP2_MOUSE]	49.04	2	11	12	68	1.834	1.196	1.147	1.214	203.73	49.04	20	68	261	28.0	6.33
Q6P5G0	Mitogen-activated protein kinase 4 OS=Mus musculus GN=Mapk4 PE=1 SV=1 -	5.66	3	2	3	5	1.285	1.548	1.592	1.214	14.82	5.66	5	5	583	65.5	5.40
P61264	Syntaxin-1B OS=Mus musculus GN=Stx1b PE=1 SV=1 - [STX1B_MOUSE]	72.92	1	18	24	496	1.081	1.043	1.115	1.215	1605.03	72.92	38	496	288	33.2	5.38
E9Q0C6	Protein Gm14569 OS=Mus musculus GN=Gm14569 PE=4 SV=1 - [E9Q0C6_MOUSE]	1.89	6	3	3	4	0.875	0.848	0.983	1.215	6.69	1.89	3	4	1637	180.9	8.70
Q8VCW8	Acyl-CoA synthetase family member 2, mitochondrial OS=Mus musculus GN=Acsf2 PE=2 SV=1 - [ACSF2_MOUSE]	37.40	1	20	20	101	0.828	1.145	0.873	1.215	322.08	37.40	37	101	615	67.9	8.18
Q8R127	Saccharopine dehydrogenase-like oxidoreductase OS=Mus musculus GN=Sccpdh PE=2 SV=1 - [illegible]	43.59	4	14	14	67	0.775	0.574	0.895	1.215	202.33	43.59	24	67	429	47.1	8.60

E9PWG9	Mitogen-activated protein kinase kinase kinase 5 OS=Mus musculus GN=Map3k5 PE=2 SV=1 - [E9PWG9_MOUSE]	12.83	4	14	16	42	0.616	1.779	1.369	1.215	131.04	12.83	26	42	1372	153.5	5.78
Q3UHH1-2	Isoform 2 of Zinc finger SWIM domain-containing protein 8 OS=Mus musculus GN=Zswim8 - [ZSWM8_MOUSE]	8.40	5	11	11	32	0.994	0.817	1.310	1.215	90.49	8.40	19	32	1798	193.4	6.99
P47199	Quinone oxidoreductase OS=Mus musculus GN=Cryz PE=2 SV=1 - [QOR_MOUSE]	45.92	5	12	12	48	0.912	1.335	1.172	1.216	148.44	45.92	21	48	331	35.2	8.07
Q9WV27	Sodium/potassium-transporting ATPase subunit alpha-4 OS=Mus musculus GN=Atp1a4 PE=1 SV=3 - [illegible]	17.83	1	4	19	1179	1.217	0.994	0.945	1.216	3429.14	17.83	34	1179	1032	114.8	5.71
Q9D0P8	Intraflagellar transport protein 27 homolog OS=Mus musculus GN=Ift27 PE=1 SV=1 - [IFT27_MOUSE]	19.89	1	3	3	8	0.945	1.276	0.930	1.216	25.52	19.89	6	8	186	20.8	5.20
P47746	Cannabinoid receptor 1 OS=Mus musculus GN=Cnr1 PE=2 SV=1 - [CNR1_MOUSE]	12.47	1	5	5	24	0.573	1.206	1.261	1.216	79.28	12.47	8	24	473	52.8	8.27
Q8R3B1	1-phosphatidylinositol 4,5-bisphosphate phosphodiesterase delta-1 OS=Mus musculus GN=Plcd1 PE=2 SV=2 - [PLCD1_MOUSE]	17.33	1	10	11	40	0.850	1.479	1.304	1.216	105.32	17.33	19	40	756	85.8	6.21
J3QQ16	Protein Col6a3 OS=Mus musculus GN=Col6a3 PE=4 SV=1 - [J3QQ16_MOUSE]	19.09	3	42	43	99	0.817	3.622	0.828	1.216	261.73	19.09	74	99	2677	288.5	8.53
H7BX60	Protein Pot1b OS=Mus musculus GN=Pot1b PE=4 SV=1 - [H7BX60_MOUSE]	2.03	1	1	1	1	1.988	1.666	2.152	1.217	0.00	2.03	1	1	640	71.6	6.10
Q9JJV5	Voltage-dependent calcium channel gamma-3 subunit OS=Mus musculus GN=Cacng3 PE=1 SV=2 - [CCG3_MOUSE]	24.44	1	6	7	13	0.501	2.782	2.286	1.217	46.73	24.44	13	13	315	35.5	9.48
Q8BIP0	Aspartate--tRNA ligase, mitochondrial OS=Mus musculus GN=Dars2 PE=2 SV=1 - [SYDM_MOUSE]	18.68	1	10	10	19	0.889	1.143	1.114	1.217	49.02	18.68	15	19	653	74.1	6.98
Q99JB8	Protein kinase C and casein kinase II substrate protein 3 OS=Mus musculus GN=Pacsin3 PE=1 SV=1 - [illegible]	30.19	8	10	10	42	1.253	0.930	1.356	1.217	144.25	30.19	19	42	424	48.6	6.10
Q62442-2	Isoform 2 of Vesicle-associated membrane protein 1 OS=Mus musculus GN=Vamp1 - [VAMP1_MOUSE]	70.09	3	5	6	65	1.141	0.759	0.978	1.217	172.83	70.09	9	65	117	12.7	8.47
D3Z0Z9	Glyceraldehyde-3-phosphate dehydrogenase OS=Mus musculus GN=Gm5069 PE=3 SV=1 - [D3Z0Z9_MOUSE]	62.31	1	4	21	976	0.646	1.098	1.349	1.217	2532.43	62.31	40	976	337	36.5	7.74
E9Q0K9	ELMO domain-containing protein 1 OS=Mus musculus GN=Elmod1 PE=2 SV=1 - [illegible]	28.53	2	7	8	25	0.701	1.016	1.325	1.217	59.26	28.53	11	25	326	37.9	8.28
P62748	Hippocalcin-like protein 1 OS=Mus musculus GN=Hpcal1 PE=2 SV=2 - [HPCL1_MOUSE]	66.84	1	5	13	200	1.408	2.491	1.038	1.217	560.23	66.84	24	200	193	22.3	5.50
D3YZV2	Potassium voltage-gated channel subfamily C member 3 OS=Mus musculus GN=Kcnc3 PE=2 SV=1 - [D3YZV2_MOUSE]	9.23	6	5	7	16	1.663	0.381	1.013	1.217	39.47	9.23	12	16	769	81.9	6.98
A2AIR9	Voltage-dependent N-type calcium channel subunit alpha-1B OS=Mus musculus GN=Cacna1b PE=2 SV=1 - [illegible]	11.27	6	15	20	51	0.917	1.457	1.439	1.217	160.71	11.27	32	51	2325	261.1	8.56
Q60790	Ras GTPase-activating protein 3 OS=Mus musculus GN=Rasa3 PE=1 SV=2 - [RASA3_MOUSE]	29.86	2	22	24	61	0.788	0.791	1.208	1.218	166.68	29.86	44	61	834	95.9	7.34
B7ZCA7	Kazrin (Fragment) OS=Mus musculus GN=Kazn PE=2 SV=1 - [B7ZCA7_MOUSE]	48.28	1	1	11	55	1.237	0.688	1.399	1.218	148.03	48.28	17	55	261	29.6	8.54
Q6P8J2	Diamine acetyltransferase 2 OS=Mus musculus GN=Sat2 PE=2 SV=1 - [SAT2_MOUSE]	8.24	1	2	2	7	1.249	1.044	0.894	1.218	16.48	8.24	4	7	170	19.3	6.04
O35427	DNA-directed RNA polymerase III subunit RPC9 OS=Mus musculus GN=Crcp PE=2 SV=1 - [RPC9_MOUSE]	25.00	1	2	2	5	1.408	1.421	1.222	1.218	20.52	25.00	3	5	148	16.7	5.00
P30275	Creatine kinase U-type, mitochondrial OS=Mus musculus GN=Ckmt1 PE=1 SV=1 - [KCRU_MOUSE]	64.35	5	19	25	565	1.047	1.097	1.123	1.218	1667.11	64.35	46	565	418	47.0	8.16

P62482	Voltage-gated potassium channel subunit beta-2 OS=Mus musculus GN=Kcnab2 PE=1 SV=1 - [KCAB2_MOUSE]	56.13	5	17	20	125	0.942	0.685	1.151	1.218	325.50	56.13	34	125	367	41.0	9.00
Q8BQR4-4	Isoform 3 of KAT8 regulatory NSL complex subunit 2 OS=Mus musculus GN=Kansl2 - [KANL2_MOUSE]	7.64	4	1	1	2	0.899	1.002	0.955	1.218	5.48	7.64	1	2	157	17.6	9.63
E9Q3H7	Protein Dnah6 OS=Mus musculus GN=Dnah6 PE=2 SV=1 - [E9Q3H7_MOUSE]	1.64	3	5	6	6	0.946	1.361	1.340	1.218	10.82	1.64	6	6	4092	468.3	5.66
Q7TSF1	Desmoglein-1-beta OS=Mus musculus GN=Dsg1b PE=1 SV=1 - [DSG1B_MOUSE]	8.11	4	4	5	5	2.511	1.062	1.380	1.218	13.30	8.11	5	5	1060	114.4	4.84
Q9CQH3	NADH dehydrogenase [ubiquinone] 1 beta subcomplex subunit 5, mitochondrial OS=Mus musculus GN=Ndufb5 PE=1 SV=1 - [NDUB5_MOUSE]	31.75	5	6	6	34	0.911	1.070	1.160	1.218	105.54	31.75	11	34	189	21.7	9.41
Q8C7J6	Potassium channel tetramerisation domain containing 12b OS=Mus musculus GN=Kctd12b PE=2 SV=1 - [illegible]	11.99	1	1	4	12	1.422	0.598	0.460	1.218	29.72	11.99	7	12	292	33.3	5.95
P47740	Fatty aldehyde dehydrogenase OS=Mus musculus GN=Aldh3a2 PE=2 SV=2 -	28.72	3	9	13	52	0.838	1.078	1.043	1.219	148.75	28.72	22	52	484	53.9	8.35
B1AXP3	MCG1697, isoform CRA_a OS=Mus musculus GN=Fam154a PE=4 SV=1 - [B1AXP3_MOUSE]	3.57	1	1	1	2	1.595	1.364	1.169	1.219	0.00	3.57	1	2	476	54.7	8.82
Q7M760	Ubiquitin thioesterase Zranb1 OS=Mus musculus GN=Zranb1 PE=2 SV=1 - [ZRAN1_MOUSE]	3.25	1	2	2	4	1.792	1.165	1.400	1.219	8.20	3.25	3	4	708	80.9	5.68
Q52KR2	Leucine-rich repeats and immunoglobulin-like domains protein 2 OS=Mus musculus GN=Lrig2 PE=2 SV=1 - [LRIG2_MOUSE]	3.70	2	3	3	9	1.040	1.088	0.944	1.219	32.13	3.70	5	9	1054	117.8	5.49
P16125	L-lactate dehydrogenase B chain OS=Mus musculus GN=Ldhb PE=1 SV=2 - [LDHB_MOUSE]	64.67	5	21	24	426	0.868	0.975	1.207	1.219	1142.27	64.67	44	426	334	36.5	6.05
Q8BGY6	Lactosylceramide 1,3-N-acetyl-beta-D-glucosaminyltransferase OS=Mus musculus GN=B3gnt5 PE=2 SV=1 - [illegible]	11.17	1	2	2	2	0.850	0.425	1.819	1.220	2.94	11.17	2	2	376	43.9	9.10
Q8C3V4	Signal transducer and activator of transcription 1 OS=Mus musculus GN=Stat1 PE=2 SV=1 - [Q8C3V4_MOUSE]	25.77	2	15	15	29	1.112	1.190	1.057	1.220	82.99	25.77	23	29	749	87.3	5.71
G5E881	Mitochondrial translocator assembly and maintenance protein 41 homolog OS=Mus musculus GN=Tamm41 PE=4 SV=1 - [G5E881_MOUSE]	34.12	6	9	9	29	0.687	0.755	1.144	1.220	70.11	34.12	15	29	337	37.9	9.11
Q3U7R1	Extended synaptotagmin-1 OS=Mus musculus GN=Esyt1 PE=2 SV=2 - [ESYT1_MOUSE]	24.45	6	19	21	58	1.075	0.992	0.883	1.220	195.62	24.45	38	58	1092	121.5	5.95
Q99LB2	Dehydrogenase/reductase SDR family member 4 OS=Mus musculus GN=Dhrs4 PE=2 SV=3 - [DHRS4_MOUSE]	45.16	1	12	12	30	1.492	1.162	0.990	1.220	87.11	45.16	22	30	279	29.9	9.38
Q9CPQ1	Cytochrome c oxidase subunit 6C OS=Mus musculus GN=Cox6c PE=1 SV=3 - [COX6C_MOUSE]	47.37	2	8	8	72	0.895	0.973	1.122	1.220	157.35	47.37	14	72	76	8.5	10.14
P70423	Cationic amino acid transporter 3 OS=Mus musculus GN=Slc7a3 PE=2 SV=1 -	4.21	1	2	2	5	0.848	1.025	0.943	1.220	21.02	4.21	3	5	618	67.4	7.37
Q04447	Creatine kinase B-type OS=Mus musculus GN=Ckb PE=1 SV=1 - [KCRB_MOUSE]	89.76	1	27	30	2525	1.509	0.997	1.264	1.221	7554.81	89.76	55	2525	381	42.7	5.67
Q8BZZ3	NEDD4-like E3 ubiquitin-protein ligase WWP1 OS=Mus musculus GN=Wwp1 PE=1 SV=2 - [WWP1_MOUSE]	17.76	1	9	14	40	1.062	0.900	1.170	1.221	110.60	17.76	22	40	918	104.6	6.38
Q3ULD5	Methylcrotonoyl-CoA carboxylase beta chain, mitochondrial OS=Mus musculus GN=Mccc2 PE=2 SV=1 - [MCCB_MOUSE]	46.18	1	18	18	90	0.792	1.084	0.958	1.221	277.02	46.18	33	90	563	61.3	8.00
Q8BG73	SH3 domain-binding glutamic acid-rich-like protein 2 OS=Mus musculus GN=Sh3bgrl2 PE=1 SV=1 - [illegible]	66.36	1	5	5	55	1.491	1.294	1.327	1.221	156.02	66.36	9	55	107	12.2	5.55
Q6A058	Armadillo repeat-containing X-linked protein 2 OS=Mus musculus GN=Armcx2 PE=2 SV=2 - [ARMX2_MOUSE]	24.36	1	12	12	44	1.501	0.848	1.097	1.221	168.43	24.36	20	44	784	81.0	9.33

Q5DTT3	Protein FAM208B OS=Mus musculus GN=Fam208b PE=2 SV=2 - [F208B_MOUSE]	0.71	1	1	2	4	0.601	0.187	1.080	1.221	5.86	0.71	2	4	2382	264.1	6.44
B0R0S4	Rab9 effector protein with kelch motifs OS=Mus musculus GN=Rabepk PE=2 SV=1 - [B0R0S4_MOUSE]	20.43	5	6	6	18	1.508	1.105	1.044	1.221	56.97	20.43	11	18	372	40.2	5.74
Q8VCN5	Cystathionine gamma-lyase OS=Mus musculus GN=Cth PE=1 SV=1 - [CGL_MOUSE]	20.60	1	6	6	18	1.582	0.770	0.953	1.221	57.96	20.60	11	18	398	43.5	7.65
Q5NCB9	Tripartite motif-containing protein 7 (Fragment) OS=Mus musculus GN=Trim7 PE=2 SV=2 - [Q5NCB9_MOUSE]	25.93	1	1	1	3	0.596	6.035	1.218	1.221	7.72	25.93	1	3	54	6.1	4.78
P61458	Pterin-4-alpha-carbinolamine dehydratase OS=Mus musculus GN=Pcbd1 PE=1 SV=2 - [PHS_MOUSE]	51.92	1	4	5	14	1.996	0.833	0.968	1.221	36.95	51.92	8	14	104	12.0	6.80
Q8C413	Diacylglycerol kinase gamma OS=Mus musculus GN=Dgkg PE=2 SV=1 - [Q8C413_MOUSE]	17.36	3	10	11	25	0.912	1.983	1.198	1.222	68.21	17.36	18	25	749	83.9	6.39
Q91VA7	Isocitrate dehydrogenase 3 (NAD+) beta OS=Mus musculus GN=Idh3b PE=2 SV=1 - [Q91VA7_MOUSE]	54.69	1	19	21	257	0.788	0.979	1.127	1.222	798.59	54.69	38	257	384	42.2	8.60
Q542I8	Integrin beta OS=Mus musculus GN=Itgb2 PE=2 SV=1 - [Q542I8_MOUSE]	16.36	5	11	11	20	1.015	1.172	1.142	1.222	60.37	16.36	18	20	770	84.8	7.05
P18872	Guanine nucleotide-binding protein G(o) subunit alpha OS=Mus musculus GN=Gnao1 PE=1 SV=3 - [illegible]	64.97	7	7	24	547	0.571	1.459	1.302	1.222	1450.52	64.97	46	547	354	40.1	5.53
P01642	Ig kappa chain V-V region L7 (Fragment) OS=Mus musculus GN=Gm10881 PE=1 SV=1 - [KV5A9_MOUSE]	10.43	1	1	1	4	2.325	1.933	1.418	1.222	10.25	10.43	2	4	115	12.6	5.94
Q9CYZ8-2	Isoform 2 of Single-stranded DNA-binding protein 2 OS=Mus musculus GN=Ssbp2 - [SSBP2_MOUSE]	8.76	3	2	2	7	1.527	1.045	1.422	1.222	18.60	8.76	3	7	331	35.0	6.87
D3YTK5	THAP domain-containing protein 1 (Fragment) OS=Mus musculus GN=Thap1 PE=2 SV=1 - [D3YTK5_MOUSE]	3.89	2	1	1	1	1.221	2.183	1.277	1.222	2.39	3.89	1	1	180	21.2	8.43
Q810A3	Tetratricopeptide repeat protein 9C OS=Mus musculus GN=Ttc9c PE=2 SV=1 - [TTC9C_MOUSE]	24.56	2	3	4	10	0.716	1.233	1.061	1.222	26.40	24.56	7	10	171	20.0	9.29
P52825	Carnitine O-palmitoyltransferase 2, mitochondrial OS=Mus musculus GN=Cpt2 PE=1 SV=2 - [CPT2_MOUSE]	32.22	4	16	16	51	0.906	1.093	0.765	1.222	156.88	32.22	27	51	658	73.9	8.37
G3X9S2	Ectonucleotide pyrophosphatase/phosphodiesterase 1, isoform CRA_d OS=Mus musculus GN=Enpp1 PE=1 SV=1 - [G3X9S2_MOUSE]	15.91	4	12	12	26	1.273	1.755	1.096	1.223	75.51	15.91	20	26	905	103.0	6.57
A7E1Z5	Electrogenic Na-bicarbonate cotransporter splice variant NBCe1-E OS=Mus musculus GN=Slc4a4 PE=2 SV=1 - [illegible]	26.92	7	2	26	160	0.877	0.694	1.144	1.223	425.87	26.92	46	160	1070	120.4	6.84
Q99JZ4	GTP-binding protein SAR1a OS=Mus musculus GN=Sar1a PE=2 SV=1 - [Q99JZ4_MOUSE]	44.44	2	3	7	31	1.398	1.090	1.071	1.223	104.39	44.44	12	31	198	22.4	6.68
Q61503	5'-nucleotidase OS=Mus musculus GN=Nt5e PE=1 SV=2 - [5NTD_MOUSE]	11.63	1	6	6	21	1.441	1.415	1.733	1.223	58.33	11.63	11	21	576	63.8	6.64
Q64669	NAD(P)H dehydrogenase [quinone] 1 OS=Mus musculus GN=Nqo1 PE=1 SV=3 - [NQO1_MOUSE]	25.18	1	7	7	24	0.661	1.425	0.840	1.223	76.28	25.18	13	24	274	30.9	8.72
P56528	ADP-ribosyl cyclase 1 OS=Mus musculus GN=Cd38 PE=1 SV=2 - [CD38_MOUSE]	15.13	1	5	5	14	1.046	0.436	0.639	1.223	47.40	15.13	8	14	304	34.4	8.29
Q80VR3	Sulfotransferase 1C1 OS=Mus musculus GN=Sult1c1 PE=1 SV=1 - [ST1C1_MOUSE]	5.26	1	1	1	1	0.763	0.251	1.714	1.223	2.56	5.26	1	1	304	35.8	6.96
Q6P9S1	ATM interactor OS=Mus musculus GN=Atmin PE=2 SV=2 - [ATMIN_MOUSE]	3.30	1	2	2	5	1.737	0.830	1.407	1.223	6.71	3.30	4	5	818	87.4	5.64
Q9D186	Apolipoprotein O OS=Mus musculus GN=Apoo PE=2 SV=1 - [Q9D186_MOUSE]	12.80	3	2	2	11	0.753	1.124	1.191	1.223	35.60	12.80	3	11	164	18.8	8.37
E9Q1Y3	Apolipoprotein B-100 (Fragment) OS=Mus musculus GN=Apob PE=2 SV=1 - [E9Q1Y3_MOUSE]	2.36	4	7	8	18	1.382	1.222	0.874	1.223	36.80	2.36	13	18	4456	503.6	6.83

D3YZN4	Paraplegin OS=Mus musculus GN=Spg7 PE=2 SV=1 - [D3YZN4_MOUSE]	35.06	8	17	17	37	0.755	1.081	1.019	1.223	109.06	35.06	24	37	676	75.0	7.40
Q9CQX0	Ubiquitin thioesterase OTUB2 OS=Mus musculus GN=Otub2 PE=2 SV=1 - [OTUB2_MOUSE]	14.10	3	2	3	6	2.005	1.332	1.085	1.223	10.78	14.10	5	6	234	27.3	6.28
Q4FZD7	Inactive serine/threonine-protein kinase PLK5 OS=Mus musculus GN=Plk5 PE=1 SV=1 - [PLK5_MOUSE]	2.18	2	1	1	1	1.239	0.724	1.572	1.223	2.34	2.18	1	1	595	66.0	9.50
A6H611	Mitochondrial intermediate peptidase OS=Mus musculus GN=Mipep PE=2 SV=1 - [MIPEP_MOUSE]	19.69	1	12	12	29	0.714	1.078	1.068	1.224	75.77	19.69	19	29	711	80.8	7.15
Q61581	Insulin-like growth factor-binding protein 7 OS=Mus musculus GN=Igfbp7 PE=2 SV=3 - [IBP7_MOUSE]	13.52	3	4	4	14	1.505	0.814	1.170	1.224	35.79	13.52	8	14	281	29.0	8.31
Q9D3A9	Protein tweety homolog 1 OS=Mus musculus GN=Ttyh1 PE=1 SV=1 - [TTYH1_MOUSE]	15.56	6	7	7	56	0.582	1.307	1.125	1.224	166.56	15.56	11	56	450	49.0	5.03
Q8JZX9	Cdc42 effector protein 2 OS=Mus musculus GN=Cdc42ep2 PE=1 SV=1 - [BORG1_MOUSE]	27.10	1	4	4	14	1.996	1.191	1.335	1.224	49.04	27.10	6	14	214	23.0	5.54
O88712-2	Isoform 2 of C-terminal-binding protein 1 OS=Mus musculus GN=Ctbp1 - [CTBP1_MOUSE]	43.02	3	9	13	89	0.688	1.352	1.306	1.224	292.59	43.02	25	89	430	46.6	6.65
P99029-2	Isoform Cytoplasmic+peroxisomal of Peroxiredoxin-5, mitochondrial OS=Mus musculus GN=Prdx5 - [illegible]	74.69	4	13	13	350	1.402	1.158	1.193	1.224	1155.88	74.69	25	350	162	17.0	7.87
P70248	Unconventional myosin-If OS=Mus musculus GN=Myo1f PE=1 SV=1 - [MYO1F_MOUSE]	4.19	3	3	3	3	1.203	1.003	0.836	1.224	6.68	4.19	3	3	1099	125.9	8.92
P12815	Programmed cell death protein 6 OS=Mus musculus GN=Pdcd6 PE=1 SV=2 - [PDCD6_MOUSE]	47.12	1	7	7	41	0.760	1.339	1.105	1.225	133.38	47.12	13	41	191	21.9	5.40
P46460	Vesicle-fusing ATPase OS=Mus musculus GN=Nsf PE=1 SV=2 - [NSF_MOUSE]	70.56	3	48	48	771	0.677	1.285	1.252	1.225	2074.57	70.56	82	771	744	82.6	6.95
D3YWM6	Uncharacterized protein OS=Mus musculus GN=Ces2d-ps PE=4 SV=1 [D3YWM6_MOUSE]	3.58	3	2	2	2	1.302	0.733	1.022	1.225	4.08	3.58	2	2	558	62.2	5.88
F8VQ40	Laminin subunit alpha-1 OS=Mus musculus GN=Lama1 PE=4 SV=1 - [F8VQ40_MOUSE]	10.22	2	27	27	59	1.168	1.423	1.051	1.225	160.67	10.22	43	59	3083	337.9	6.71
Q99JW2	Aminoacylase-1 OS=Mus musculus GN=Acy1 PE=1 SV=1 - [ACY1_MOUSE]	27.70	1	10	10	23	1.934	1.161	1.102	1.226	76.35	27.70	17	23	408	45.8	6.32
J3QK48	Acyl-protein thioesterase 1 (Fragment) OS=Mus musculus GN=Lypla1 PE=4 SV=1 - [J3QK48_MOUSE]	32.73	7	6	6	36	0.906	1.131	1.074	1.226	102.34	32.73	10	36	220	23.2	6.77
O70400	PDZ and LIM domain protein 1 OS=Mus musculus GN=Pdlim1 PE=2 SV=4 -	44.04	1	11	11	49	1.454	2.219	1.548	1.226	135.10	44.04	21	49	327	35.8	6.84
F8VPX2	Protein Fbxw9 OS=Mus musculus GN=Fbxw9 PE=2 SV=1 - [F8VPX2_MOUSE]	4.15	1	2	2	3	0.790	1.004	1.070	1.226	10.31	4.15	3	3	458	50.5	6.90
P0C1Q2	Dual 3',5'-cyclic-AMP and -GMP phosphodiesterase 11A OS=Mus musculus GN=Pde11a PE=1 SV=1 - [PDE11_MOUSE]	7.40	1	4	4	8	0.688	1.444	1.218	1.226	23.13	7.40	6	8	933	104.5	6.43
Q61493	DNA polymerase zeta catalytic subunit OS=Mus musculus GN=Rev3l PE=1 SV=3 - [DPOLZ_MOUSE]	0.38	1	1	1	1	1.539	1.156	0.958	1.226	2.33	0.38	1	1	3122	350.5	8.32
P15508	Spectrin beta chain, erythrocytic OS=Mus musculus GN=Sptb PE=1 SV=4 - [SPTB1_MOUSE]	55.69	1	2	103	407	2.050	1.853	1.067	1.227	1298.93	55.69	182	407	2128	245.1	5.33
Q9QZN4	F-box only protein 6 OS=Mus musculus GN=Fbxo6 PE=1 SV=1 - [FBX6_MOUSE]	29.49	3	7	7	19	1.102	1.196	1.014	1.227	56.39	29.49	13	19	295	34.5	8.18
D3Z553	Protein Churchill OS=Mus musculus GN=Churc1 PE=2 SV=1 - [D3Z553_MOUSE]	37.14	2	1	1	1	7.813	3.158	1.399	1.228	3.23	37.14	1	1	35	4.1	4.08
Q3TG86	Cytochrome P450, family 11, subfamily b, polypeptide 1 OS=Mus musculus GN=Cyp11b1 PE=2 SV=1 - [illegible]	9.38	1	2	2	2	12.085	2.162	1.613	1.228	4.59	9.38	2	2	501	57.7	9.38
Q8VCT4	Carboxylesterase 1D OS=Mus musculus GN=Ces1d PE=1 SV=1 - [CES1D_MOUSE]	10.80	1	3	5	19	1.319	0.846	0.692	1.228	49.78	10.80	9	19	565	61.7	6.61

Q8BPX9	Solute carrier family 15 member 3 OS=Mus musculus GN=Slc15a3 PE=2 SV=1 -	1.90	1	1	1	1	0.819	1.246	1.061	1.228	2.01	1.90	1	1	578	64.0	8.81
F8VPN2	Protein Tex15 OS=Mus musculus GN=Tex15 PE=2 SV=1 - [F8VPN2_MOUSE]	0.86	1	2	3	4	2.042	1.422	0.962	1.228	9.85	0.86	3	4	2785	311.1	6.39
F6Q8C0	Apoptotic chromatin condensation inducer in the nucleus (Fragment) OS=Mus musculus GN=Acin1 PE=4 SV=1 - [illegible]	21.28	1	1	2	5	1.179	1.091	1.455	1.228	12.79	21.28	3	5	141	16.7	10.55
Q03391	Glutamate receptor ionotropic, NMDA 2D OS=Mus musculus GN=Grin2d PE=1 SV=3 - [NMDE4_MOUSE]	13.45	2	11	12	33	1.181	0.639	0.843	1.228	95.91	13.45	20	33	1323	142.9	8.37
Q5PPQ4	Protein Zfp658 OS=Mus musculus GN=Zfp658 PE=2 SV=1 - [Q5PPQ4_MOUSE]	4.62	145	1	2	6	2.306	1.631	0.915	1.228	2.26	4.62	2	6	887	101.4	9.19
P12382	6-phosphofructokinase, liver type OS=Mus musculus GN=Pfkl PE=1 SV=4 - [K6PL_MOUSE]	41.15	2	21	29	223	1.050	1.422	1.138	1.228	683.67	41.15	57	223	780	85.3	7.17
Q8BKT7	THO complex subunit 5 homolog OS=Mus musculus GN=Thoc5 PE=1 SV=2 - [THOC5_MOUSE]	7.03	3	4	5	9	0.805	1.056	0.831	1.228	17.61	7.03	7	9	683	78.6	7.12
Q9Z1R4	Uncharacterized protein C6orf47 homolog OS=Mus musculus GN=D17h6s53e PE=2 SV=1 - [CF047_MOUSE]	12.29	1	4	4	11	0.792	0.754	1.084	1.228	35.28	12.29	8	11	293	32.0	6.89
Q8CEC6	Peptidylprolyl isomerase domain and WD repeat-containing protein 1 OS=Mus musculus GN=Ppwd1 PE=2 SV=2 - [illegible]	3.10	1	2	2	3	0.880	0.770	1.202	1.228	8.36	3.10	3	3	646	73.3	7.15
P97441	Zinc transporter 3 OS=Mus musculus GN=Slc30a3 PE=1 SV=1 - [ZNT3_MOUSE]	16.75	1	7	7	45	0.768	5.787	3.971	1.229	125.14	16.75	14	45	388	41.8	6.38
Q64018	Glycine receptor subunit alpha-1 OS=Mus musculus GN=Glra1 PE=1 SV=2 - [GLRA1_MOUSE]	15.10	3	4	5	15	1.424	0.642	1.061	1.229	27.68	15.10	8	15	457	52.6	8.90
Q9D1X8	Tetraspanin 2, isoform CRA_b OS=Mus musculus GN=Tspan2 PE=2 SV=1 - [Q9D1X8_MOUSE]	15.21	2	2	3	32	1.297	0.791	1.253	1.229	91.53	15.21	6	32	217	23.4	8.00
Q9CYT6	Adenylyl cyclase-associated protein 2 OS=Mus musculus GN=Cap2 PE=1 SV=1 -	52.73	2	21	23	146	0.870	1.755	1.550	1.229	397.66	52.73	41	146	476	52.8	6.43
A2APY7	NADH dehydrogenase [ubiquinone] 1 alpha subcomplex assembly factor 5 OS=Mus musculus GN=Ndufaf5 PE=2 SV=1 - [NDUF5_MOUSE]	21.87	2	7	7	16	1.143	1.249	1.254	1.229	50.60	21.87	12	16	343	38.4	6.10
Q03311	Cholinesterase OS=Mus musculus GN=Bche PE=2 SV=2 - [CHLE_MOUSE]	6.63	1	3	3	5	1.135	0.663	1.070	1.229	9.34	6.63	4	5	603	68.4	7.25
Q9Z1M0	P2X purinoceptor 7 OS=Mus musculus GN=P2rx7 PE=1 SV=2 - [P2RX7_MOUSE]	15.63	3	9	9	27	0.877	0.740	1.035	1.229	72.69	15.63	15	27	595	68.3	8.22
O54827	Probable phospholipid-transporting ATPase VA OS=Mus musculus GN=Atp10a PE=1 SV=4 - [AT10A_MOUSE]	2.06	1	2	2	2	1.277	1.025	1.140	1.229	6.55	2.06	2	2	1508	168.7	6.67
E9PYK8	Rho guanine nucleotide exchange factor 4 OS=Mus musculus GN=Arhgef4 PE=2 SV=2 - [E9PYK8_MOUSE]	8.46	8	8	11	23	1.960	0.808	1.247	1.229	69.15	8.46	15	23	1855	206.2	6.52
P68373	Tubulin alpha-1C chain OS=Mus musculus GN=Tuba1c PE=1 SV=1 - [TBA1C_MOUSE]	61.25	1	7	28	3586	1.108	1.370	1.100	1.230	11123.93	61.25	54	3586	449	49.9	5.10
Q8BKK4-3	Isoform 3 of Protein phosphatase 1 regulatory subunit 1C OS=Mus musculus GN=Ppp1r1c - [PPR1C_MOUSE]	15.38	2	2	2	3	2.612	0.823	2.499	1.230	6.13	15.38	2	3	91	10.4	9.63
Q14B80	Potassium voltage-gated channel subfamily C member 2 OS=Mus musculus GN=Kcnc2 PE=1 SV=1 - [KCNC2_MOUSE]	13.55	2	5	7	12	0.660	0.463	1.339	1.230	44.74	13.55	11	12	642	70.5	7.69
Q3UY51	Leucine-rich repeat-containing protein 55 OS=Mus musculus GN=Lrrc55 PE=2 SV=1 - [LRC55_MOUSE]	3.22	1	1	1	2	0.335	0.464	1.006	1.230	5.17	3.22	2	2	311	34.4	6.39
Q68FD5	Clathrin heavy chain 1 OS=Mus musculus GN=Cltc PE=1 SV=3 - [CLH1_MOUSE]	76.36	2	96	107	2314	0.695	1.286	1.202	1.231	6669.61	76.36	194	2314	1675	191.4	5.69
Q9JJA4	Ribosome biogenesis protein WDR12 OS=Mus musculus GN=Wdr12 PE=2 SV=1 - [WDR12_MOUSE]	16.78	1	5	5	10	1.620	0.974	1.173	1.231	28.59	16.78	7	10	423	47.3	5.60

Q9D6R2	Isocitrate dehydrogenase [NAD] subunit alpha, mitochondrial OS=Mus musculus GN=Idh3a PE=1 SV=1 - [IDH3A_MOUSE]	54.37	2	23	23	294	0.990	1.209	1.091	1.231	777.01	54.37	44	294	366	39.6	6.73
E9Q9D5	Protein Rabl2 OS=Mus musculus GN=Rabl2 PE=2 SV=1 - [E9Q9D5_MOUSE]	21.97	7	3	4	4	1.019	0.789	1.221	1.231	11.11	21.97	4	4	223	25.6	5.66
Q7TT28	RNA exonuclease 1 homolog OS=Mus musculus GN=Rexo1 PE=1 SV=1 - [REXO1_MOUSE]	0.74	1	1	1	1	1.401	1.599	1.390	1.231	1.79	0.74	1	1	1213	130.7	8.97
O35988	Syndecan-4 OS=Mus musculus GN=Sdc4 PE=1 SV=1 - [SDC4_MOUSE]	14.14	1	2	2	11	1.311	1.251	1.514	1.231	18.74	14.14	3	11	198	21.5	4.41
Q6PD19	UPF0668 protein C10orf76 homolog OS=Mus musculus PE=2 SV=2 - [CJ076_MOUSE]	14.22	3	7	7	12	0.907	1.166	0.936	1.231	39.41	14.22	12	12	689	78.6	6.60
Q3UYC0	Protein phosphatase 1H OS=Mus musculus GN=Ppm1h PE=1 SV=1 - [PPM1H_MOUSE]	38.21	3	15	17	81	0.755	1.401	1.382	1.231	245.75	38.21	32	81	513	56.3	6.73
P62874	Guanine nucleotide-binding protein G(I)/G(S)/G(T) subunit beta-1 OS=Mus musculus GN=Gnb1 PE=1 SV=3 - [illegible]	59.41	3	8	15	387	0.630	1.250	1.173	1.231	1221.49	59.41	28	387	340	37.4	6.00
Q9Z0E0	Neurochondrin OS=Mus musculus GN=Ncdn PE=1 SV=1 - [NCDN_MOUSE]	47.87	1	1	28	204	0.787	0.915	1.349	1.231	605.01	47.87	50	204	729	78.8	5.54
Q09LZ8-2	Isoform 2 of Cytosolic carboxypeptidase 6 OS=Mus musculus GN=Agbl4 - [CBPC6_MOUSE]	9.07	7	4	4	11	0.808	1.111	1.117	1.232	27.56	9.07	7	11	463	53.9	8.43
Q9JMI0	Endothelin-converting enzyme-like 1 OS=Mus musculus GN=Ecel1 PE=2 SV=2 - [ECEL1_MOUSE]	12.39	1	10	10	20	1.488	1.299	0.769	1.232	60.91	12.39	17	20	775	87.9	7.80
J3QMF0	Protein Gm5709 OS=Mus musculus GN=Gm5709 PE=4 SV=1 - [J3QMF0_MOUSE]	6.48	3	2	2	2	0.653	1.195	0.916	1.232	2.63	6.48	2	2	401	46.1	8.69
Q69ZB8-2	Isoform 2 of Zinc finger CCHC domain-containing protein 2 OS=Mus musculus GN=Zcchc2 - [ZCHC2_MOUSE]	2.68	2	1	1	1	2.437	0.984	1.018	1.232	2.20	2.68	1	1	1155	123.2	6.95
Q9D1H9	Microfibril-associated glycoprotein 4 OS=Mus musculus GN=Mfap4 PE=1 SV=1 - [MFAP4_MOUSE]	7.78	2	1	1	1	1.424	1.110	1.240	1.232	2.48	7.78	1	1	257	28.9	5.44
E9Q828	Protein Atp2b4 OS=Mus musculus GN=Atp2b4 PE=2 SV=1 - [E9Q828_MOUSE]	39.93	3	6	45	331	0.651	2.765	1.487	1.232	921.11	39.93	74	331	1172	129.2	6.73
Q8BZ20	Poly [ADP-ribose] polymerase 12 OS=Mus musculus GN=Parp12 PE=2 SV=3 -	4.36	1	2	2	4	1.283	1.446	0.851	1.232	13.51	4.36	2	4	711	79.9	8.54
E9Q8M8	Mineralocorticoid receptor OS=Mus musculus GN=Nr3c2 PE=2 SV=1 - [E9Q8M8_MOUSE]	5.28	7	2	3	3	1.160	1.124	1.346	1.232	6.90	5.28	3	3	984	107.0	7.87
Q8CFK6	DENN domain-containing protein 1C OS=Mus musculus GN=Dennd1c PE=2 SV=1 -	2.80	1	2	2	2	1.336	1.021	1.585	1.232	5.22	2.80	2	2	786	86.6	5.96
F2Z461	E3 ISG15--protein ligase Herc6 OS=Mus musculus GN=Herc6 PE=2 SV=1 - [HERC6_MOUSE]	1.79	1	2	2	2	1.051	1.156	1.165	1.232	3.65	1.79	2	2	1003	112.9	7.52
Q6NV72-2	Isoform 2 of WD repeat-containing protein C2orf44 homolog OS=Mus musculus -	5.04	2	3	3	6	1.066	0.834	1.241	1.233	6.15	5.04	5	6	635	69.2	7.65
Q8BSM7	Large neutral amino acids transporter small subunit 3 OS=Mus musculus GN=Slc43a1 PE=1 SV=1 - [LAT3_MOUSE]	7.62	3	3	3	4	1.711	1.306	0.752	1.233	14.73	7.62	4	4	564	62.6	8.12
Q9DBH0	NEDD4-like E3 ubiquitin-protein ligase WWP2 OS=Mus musculus GN=Wwp2 PE=1 SV=1 - [WWP2_MOUSE]	12.07	1	6	11	18	1.071	0.912	1.066	1.233	49.83	12.07	15	18	870	98.7	7.11
D3YXX0	Transmembrane gamma-carboxyglutamic acid protein 2 (Fragment) OS=Mus musculus GN=Prrg2 PE=2 SV=1 - [D3YXX0_MOUSE]	14.71	3	1	1	1	1.430	1.148	1.623	1.233	2.05	14.71	1	1	68	7.4	8.56
Q14BV6-2	Isoform 2 of UPF0249 protein ydjC homolog OS=Mus musculus GN=Ydjc - [YDJC_MOUSE]	11.07	3	3	3	5	0.877	1.525	1.010	1.233	15.56	11.07	5	5	271	28.9	6.77
Q8BWF0	Succinate-semialdehyde dehydrogenase, mitochondrial OS=Mus musculus GN=Aldh5a1 PE=1 SV=1 - [illegible]	60.42	1	27	27	263	0.780	0.837	1.007	1.234	812.18	60.42	47	263	523	55.9	8.25
Q99PL6	UBX domain-containing protein 6 OS=Mus musculus GN=Ubxn6 PE=1 SV=1 -	43.21	3	16	16	76	1.035	1.197	1.329	1.234	240.27	43.21	24	76	442	49.8	8.54

F8VPU6	Ubiquitin carboxyl-terminal hydrolase OS=Mus musculus GN=Usp9y PE=3 SV=1 - [F8VPU6_MOUSE]	7.16	5	1	20	75	0.994	1.430	1.009	1.234	192.41	7.16	35	75	2556	292.1	6.07
O35682	Myeloid-associated differentiation marker OS=Mus musculus GN=Myadm PE=2 SV=2 - [MYADM_MOUSE]	12.50	1	3	3	65	0.535	0.676	1.087	1.234	241.16	12.50	6	65	320	35.3	8.31
Q91YT8	Transmembrane protein 63A OS=Mus musculus GN=Tmem63a PE=2 SV=1 - [TM63A_MOUSE]	6.97	3	5	5	21	1.300	0.246	0.940	1.234	52.51	6.97	9	21	804	91.8	7.93
F6WDS8	Regulator of G-protein-signaling 6 OS=Mus musculus GN=Rgs6 PE=4 SV=1 - [F6WDS8_MOUSE]	34.21	3	12	15	78	0.482	0.575	1.107	1.234	238.52	34.21	27	78	456	52.9	6.67
Q8R191	Synaptogyrin-3 OS=Mus musculus GN=Syngr3 PE=1 SV=1 - [SNG3_MOUSE]	35.81	1	6	6	59	0.613	1.592	1.198	1.234	165.19	35.81	10	59	229	24.5	8.18
Q8K354	Carbonyl reductase [NADPH] 3 OS=Mus musculus GN=Cbr3 PE=2 SV=1 - [CBR3_MOUSE]	65.34	1	13	16	77	1.444	1.519	1.793	1.234	228.35	65.34	29	77	277	30.9	6.57
Q9CWF6	Bardet-Biedl syndrome 2 protein homolog OS=Mus musculus GN=Bbs2 PE=2 SV=1 - [BBS2_MOUSE]	9.71	1	7	7	16	0.802	1.079	0.962	1.234	44.56	9.71	11	16	721	79.9	5.86
P14094	Sodium/potassium-transporting ATPase subunit beta-1 OS=Mus musculus GN=Atp1b1 PE=1 SV=1 - [AT1B1_MOUSE]	42.43	1	17	17	731	0.602	0.839	1.125	1.234	1957.45	42.43	32	731	304	35.2	8.65
B2RQX9	Transporter OS=Mus musculus GN=Slc6a5 PE=2 SV=1 - [B2RQX9_MOUSE]	18.20	3	9	10	43	3.975	0.298	0.840	1.234	146.19	18.20	18	43	791	87.0	7.40
Q3THS6	S-adenosylmethionine synthase isoform type-2 OS=Mus musculus GN=Mat2a PE=2 SV=2 - [METK2_MOUSE]	46.33	1	14	16	103	1.077	0.911	1.122	1.234	299.30	46.33	30	103	395	43.7	6.48
P19783	Cytochrome c oxidase subunit 4 isoform 1, mitochondrial OS=Mus musculus GN=Cox4i1 PE=1 SV=2 - [COX41_MOUSE]	57.99	4	11	11	342	1.013	1.123	1.145	1.235	1012.36	57.99	22	342	169	19.5	9.23
P97364	Selenide, water dikinase 2 OS=Mus musculus GN=Sephs2 PE=1 SV=3 - [SPS2_MOUSE]	7.96	3	3	4	7	0.670	1.039	1.155	1.235	13.15	7.96	5	7	452	47.8	6.06
Q61036-2	Isoform 2 of Serine/threonine-protein kinase PAK 3 OS=Mus musculus GN=Pak3 - [PAK3_MOUSE]	45.40	5	9	24	287	0.993	1.068	1.162	1.235	868.05	45.40	45	287	544	60.7	5.45
Q5PSV9	Mediator of DNA damage checkpoint protein 1 OS=Mus musculus GN=Mdc1 PE=1 SV=1 - [MDC1_MOUSE]	5.21	3	6	6	9	1.258	0.921	1.043	1.235	22.82	5.21	9	9	1707	184.6	5.19
F6XGJ4	Protein Gm17093 (Fragment) OS=Mus musculus GN=Gm17093 PE=4 SV=1 -	9.81	1	1	1	2	1.313	0.937	1.240	1.235	5.25	9.81	1	2	214	25.0	8.34
Q9QYI5	DnaJ homolog subfamily B member 2 OS=Mus musculus GN=Dnajb2 PE=2 SV=2 -	45.13	2	10	10	49	1.377	0.967	1.060	1.236	136.86	45.13	18	49	277	30.6	4.98
Q80UK7	Spindle assembly abnormal protein 6 homolog OS=Mus musculus GN=Sass6 PE=2 SV=2 - [SAS6_MOUSE]	5.96	2	2	4	11	0.903	0.910	0.889	1.236	23.00	5.96	7	11	654	74.0	7.37
Q14DN9-2	Isoform 2 of Ankyrin repeat and death domain-containing protein 1B OS=Mus musculus GN=Ankdd1b - [AKD1B_MOUSE]	1.72	3	1	1	2	0.628	0.844	0.716	1.236	4.72	1.72	2	2	465	52.1	7.31
O88829-2	Isoform 2 of Lactosylceramide alpha-2,3-sialyltransferase OS=Mus musculus GN=St3gal5 - [SIAT9_MOUSE]	2.73	2	1	1	2	0.636	1.236	1.179	1.236	9.99	2.73	2	2	403	46.1	8.28
Q9Z2I0	LETM1 and EF-hand domain-containing protein 1, mitochondrial OS=Mus musculus GN=Letm1 PE=2 SV=1 - [LETM1_MOUSE]	47.43	1	35	35	143	0.728	1.277	1.250	1.236	414.57	47.43	57	143	738	82.9	6.52
A2RSX7	tRNA wybutosine-synthesizing protein 5 OS=Mus musculus GN=Tyw5 PE=2 SV=2 - [TYW5_MOUSE]	8.25	1	1	2	3	0.613	0.992	1.109	1.237	10.34	8.25	2	3	315	36.6	6.34
P70181	Phosphatidylinositol 4-phosphate 5-kinase type-1 beta OS=Mus musculus GN=Pip5k1b PE=1 SV=1 - [PI51B_MOUSE]	16.33	2	4	6	18	1.364	1.687	1.022	1.237	52.02	16.33	10	18	539	60.8	6.86
Q61327	Sodium-dependent dopamine transporter OS=Mus musculus GN=Slc6a3 PE=1 SV=2 - [SC6A3_MOUSE]	6.14	1	3	4	16	0.836	0.438	3.812	1.237	37.04	6.14	8	16	619	68.8	7.21

Q3TWN3-2	Isoform 2 of Metal transporter CNNM2 OS=Mus musculus GN=Cnnm2 -	30.83	2	13	20	49	0.623	0.739	0.888	1.237	133.71	30.83	30	49	853	94.4	6.14
Q8R5M8-4	Isoform 4 of Cell adhesion molecule 1 OS=Mus musculus GN=Cadm1 - [CADM1_MOUSE]	42.45	9	13	13	77	0.724	2.020	1.409	1.237	268.21	42.45	23	77	417	45.8	5.12
Q8VCK3	Tubulin gamma-2 chain OS=Mus musculus GN=Tubg2 PE=1 SV=1 - [TBG2_MOUSE]	36.81	1	1	14	52	0.588	1.360	1.013	1.237	167.04	36.81	24	52	451	51.1	6.21
P56382	ATP synthase subunit epsilon, mitochondrial OS=Mus musculus GN=Atp5e PE=2 SV=2 - [ATP5E_MOUSE]	42.31	1	3	3	18	1.296	0.714	1.136	1.238	38.96	42.31	5	18	52	5.8	10.01
Q62172	RalA-binding protein 1 OS=Mus musculus GN=Ralbp1 PE=1 SV=4 - [RBP1_MOUSE]	19.14	3	14	14	43	0.896	1.135	1.199	1.238	116.50	19.14	22	43	648	75.0	5.92
Q70IV5-2	Isoform 2 of Synemin OS=Mus musculus GN=Synm - [SYNEM_MOUSE]	22.00	3	19	21	64	1.172	1.621	0.809	1.238	188.40	22.00	34	64	1259	140.8	5.15
P97952	Sodium channel subunit beta-1 OS=Mus musculus GN=Scn1b PE=2 SV=1 - [SCN1B_MOUSE]	30.73	1	5	5	22	1.225	1.976	1.510	1.238	71.92	30.73	9	22	218	24.6	4.83
M0QWP1	Agrin OS=Mus musculus GN=Agrn PE=4 SV=1 - [M0QWP1_MOUSE]	22.71	2	4	35	121	1.733	1.066	1.265	1.238	361.81	22.71	62	121	2034	216.5	6.15
Q9CQC7	NADH dehydrogenase [ubiquinone] 1 beta subcomplex subunit 4 OS=Mus musculus GN=Ndufb4 PE=1 SV=3 - [NDUB4_MOUSE]	51.16	2	5	6	56	0.602	0.866	1.095	1.238	159.44	51.16	11	56	129	15.1	9.89
P63328	Serine/threonine-protein phosphatase 2B catalytic subunit alpha isoform OS=Mus musculus GN=Ppp3ca PE=1 SV=1 - [PP2BA_MOUSE]	44.34	1	1	20	365	1.417	1.695	1.829	1.239	982.08	44.34	37	365	521	58.6	5.86
Q9JJF9	Signal peptide peptidase-like 2A OS=Mus musculus GN=Sppl2a PE=1 SV=2 - [SPP2A_MOUSE]	1.91	1	1	1	2	1.631	1.196	0.967	1.239	5.52	1.91	1	2	523	58.1	7.06
Q9D6K5	Synaptojanin-2-binding protein OS=Mus musculus GN=Synj2bp PE=1 SV=1 - [SYJ2B_MOUSE]	57.93	6	1	6	47	1.091	0.987	1.042	1.239	127.58	57.93	11	47	145	15.8	6.30
Q3V132	ADP/ATP translocase 4 OS=Mus musculus GN=Slc25a31 PE=2 SV=1 - [ADT4_MOUSE]	24.38	1	5	10	256	0.590	1.023	0.959	1.239	679.56	24.38	17	256	320	35.2	9.58
P97817	Cerebellar degeneration-related protein 2 OS=Mus musculus GN=Cdr2 PE=1 SV=2 - [CDR2_MOUSE]	9.45	1	3	3	5	2.137	1.470	1.392	1.240	6.48	9.45	3	5	455	52.1	5.00
Q61361	Brevican core protein OS=Mus musculus GN=Bcan PE=1 SV=2 - [PGCB_MOUSE]	35.90	1	30	31	284	1.669	1.050	1.131	1.240	811.76	35.90	55	284	883	95.8	4.93
H3BK50	Serine/threonine-protein phosphatase 2A 65 kDa regulatory subunit A beta isoform (Fragment) OS=Mus musculus GN=Ppp2r1b PE=2 SV=1 - [H3BK50_MOUSE]	35.48	1	1	2	4	0.737	1.298	1.178	1.240	14.22	35.48	4	4	93	10.2	8.51
Q8CCB5	Methyltransferase-like protein 24 OS=Mus musculus GN=Mettl24 PE=2 SV=1 -	6.34	1	2	2	2	1.187	1.779	0.885	1.240	0.00	6.34	2	2	363	40.6	9.33
P13707	Glycerol-3-phosphate dehydrogenase [NAD(+)], cytoplasmic OS=Mus musculus GN=Gpd1 PE=1 SV=3 - [GPDA_MOUSE]	74.50	2	23	25	106	0.978	0.627	1.294	1.240	291.28	74.50	47	106	349	37.5	7.17
E9QNR6	Protein AI837181 OS=Mus musculus GN=AI837181 PE=2 SV=1 - [E9QNR6_MOUSE]	43.62	2	11	11	45	1.036	0.996	1.127	1.240	131.73	43.62	20	45	298	31.8	6.10
J3QM67	Protein LOC100040083 OS=Mus musculus GN=Gm20825 PE=4 SV=1 - [J3QM67_MOUSE]	7.05	11	1	1	1	1.037	0.828	1.123	1.241	2.65	7.05	1	1	227	25.7	8.44
G3X9P9	MCG1048453 OS=Mus musculus GN=AF366264 PE=4 SV=1 - [G3X9P9_MOUSE]	7.37	2	2	2	2	2.161	1.263	1.056	1.241	4.86	7.37	2	2	502	57.0	7.64
Q9EPK2-3	Isoform 3 of Protein XRP2 OS=Mus musculus GN=Rp2 - [XRP2_MOUSE]	21.43	4	7	7	22	0.642	1.087	1.099	1.241	62.11	21.43	13	22	322	36.5	5.48
Q9Z123	Semaphorin-4F OS=Mus musculus GN=Sema4f PE=2 SV=2 - [SEM4F_MOUSE]	6.18	2	4	4	12	1.127	0.884	0.966	1.241	30.56	6.18	7	12	777	84.4	6.93
P62737	Actin, aortic smooth muscle OS=Mus musculus GN=Acta2 PE=1 SV=1 - [ACTA_MOUSE]	73.47	6	3	28	2426	0.699	1.682	1.066	1.241	6932.08	73.47	47	2426	377	42.0	5.39
Q91VF2	Histamine N-methyltransferase OS=Mus musculus GN=Hnmt PE=1 SV=1 - [HNMT_MOUSE]	22.03	1	7	7	20	0.647	1.218	0.969	1.241	56.63	22.03	12	20	295	33.6	5.06

Q8BHW2	Protein OSCP1 OS=Mus musculus GN=Oscp1 PE=2 SV=1 - [OSCP1_MOUSE]	38.26	1	14	14	50	0.773	1.481	1.130	1.242	127.92	38.26	25	50	379	43.2	7.20
Q8K207	Uncharacterized protein C1orf21 homolog OS=Mus musculus PE=1 SV=1 - [CA021_MOUSE]	19.83	1	2	2	4	2.335	1.214	1.369	1.242	13.43	19.83	3	4	121	13.9	5.22
B1AS81	CMP-N-acetylneuraminate-beta-1,4-galactoside alpha-2,3-sialyltransferase (Fragment) OS=Mus musculus GN=St3gal3 PE=2 SV=1 - [B1AS81_MOUSE]	10.06	4	1	1	1	1.842	0.893	1.450	1.242	0.00	10.06	1	1	159	18.3	9.57
Q9R1T4-2	Isoform I of Septin-6 OS=Mus musculus GN=Sept6 - [SEPT6_MOUSE]	50.12	4	10	21	209	0.784	0.966	1.352	1.242	553.62	50.12	38	209	427	48.7	6.55
Q9D032-2	Isoform 2 of Single-stranded DNA-binding protein 3 OS=Mus musculus GN=Ssbp3 - [SSBP3_MOUSE]	8.31	4	2	2	3	1.701	1.495	1.372	1.242	10.52	8.31	3	3	361	37.7	6.73
P06745	Glucose-6-phosphate isomerase OS=Mus musculus GN=Gpi PE=1 SV=4 - [G6PI_MOUSE]	57.17	1	16	34	739	1.353	1.283	1.145	1.242	1912.52	57.17	65	739	558	62.7	8.13
Q60936	Chaperone activity of bc1 complex-like, mitochondrial OS=Mus musculus GN=Adck3 PE=2 SV=2 - [ADCK3_MOUSE]	14.26	4	6	7	22	1.137	1.367	1.126	1.242	76.20	14.26	13	22	645	71.7	6.54
A2A6U3	Septin-9 OS=Mus musculus GN=Sept9 PE=2 SV=1 - [A2A6U3_MOUSE]	55.22	6	28	28	118	1.061	1.342	1.437	1.243	335.05	55.22	49	118	565	63.7	8.32
Q8K4Z0-2	Isoform 2 of Leucine-rich repeat LGI family member 2 OS=Mus musculus GN=Lgi2 - [LGI2_MOUSE]	24.91	2	13	13	38	0.840	0.604	1.088	1.243	111.52	24.91	23	38	542	62.0	6.54
Q8JZZ0	UDP-glucuronosyltransferase 3A2 OS=Mus musculus GN=Ugt3a2 PE=2 SV=2 -	7.07	1	3	3	3	3.441	1.061	1.163	1.243	5.36	7.07	3	3	523	59.6	7.53
Q9R1Q8	Transgelin-3 OS=Mus musculus GN=Tagln3 PE=1 SV=1 - [TAGL3_MOUSE]	80.90	1	14	17	266	1.363	0.896	1.206	1.243	767.64	80.90	32	266	199	22.5	7.33
A2A8L1	Protein Chd5 OS=Mus musculus GN=Chd5 PE=2 SV=1 - [A2A8L1_MOUSE]	9.04	5	4	16	35	0.890	0.904	1.234	1.243	92.63	9.04	24	35	1946	222.4	6.24
E9PX70	Collagen alpha-1(XII) chain OS=Mus musculus GN=Col12a1 PE=2 SV=1 - [E9PX70_MOUSE]	12.60	8	24	24	69	1.097	4.647	1.189	1.243	222.10	12.60	40	69	3064	333.5	5.80
Q99KP3	Lambda-crystallin homolog OS=Mus musculus GN=Cryl1 PE=2 SV=3 - [CRYL1_MOUSE]	54.86	1	14	14	33	0.667	1.720	1.286	1.243	104.32	54.86	22	33	319	35.2	5.86
Q7TQD2	Tubulin polymerization-promoting protein OS=Mus musculus GN=Tppp PE=1 SV=1 - [TPPP_MOUSE]	68.35	2	16	17	356	1.290	0.728	1.827	1.243	1017.44	68.35	27	356	218	23.6	9.42
Q6VVW5	Atrial natriuretic peptide receptor 2 OS=Mus musculus GN=Npr2 PE=2 SV=2 - [ANPRB_MOUSE]	11.37	7	9	10	14	0.884	1.099	0.830	1.243	31.95	11.37	12	14	1047	117.0	6.87
P43006-2	Isoform Glt-1A of Excitatory amino acid transporter 2 OS=Mus musculus GN=Slc1a2 - [EAA2_MOUSE]	33.04	5	3	20	704	0.689	1.203	1.442	1.243	2203.04	33.04	33	704	569	61.7	6.84
Q9R053	Sodium channel protein type 11 subunit alpha OS=Mus musculus GN=Scn11a PE=1 SV=2 - [SCNBA_MOUSE]	1.76	1	1	3	11	1.563	1.378	1.050	1.244	23.06	1.76	3	11	1765	201.3	8.27
Q9WU79	Proline dehydrogenase 1, mitochondrial OS=Mus musculus GN=Prodh PE=2 SV=2 - [PROD_MOUSE]	30.05	5	14	14	58	0.844	1.508	1.212	1.244	193.93	30.05	26	58	599	68.0	8.24
Q61753	D-3-phosphoglycerate dehydrogenase OS=Mus musculus GN=Phgdh PE=1 SV=3 - [SERA_MOUSE]	40.34	2	21	21	146	0.741	0.953	1.097	1.244	457.57	40.34	40	146	533	56.5	6.54
P97494	Glutamate--cysteine ligase catalytic subunit OS=Mus musculus GN=Gclc PE=2 SV=4 - [GSH1_MOUSE]	45.37	1	20	20	76	0.871	1.146	1.113	1.244	226.87	45.37	32	76	637	72.5	5.90
Q9D0M1	Phosphoribosyl pyrophosphate synthase-associated protein 1 OS=Mus musculus GN=Prpsap1 PE=1 SV=1 - [illegible]	58.71	4	11	15	62	0.860	1.141	1.009	1.244	212.95	58.71	28	62	356	39.4	7.20
Q9WTK7	Serine/threonine-protein kinase STK11 OS=Mus musculus GN=Stk11 PE=1 SV=1 - [STK11_MOUSE]	27.29	4	11	12	25	0.909	1.035	1.117	1.244	68.89	27.29	20	25	436	49.2	6.84

Q8CGK7	Guanine nucleotide-binding protein G(olf) subunit alpha OS=Mus musculus GN=Gnal PE=1 SV=1 - [GNAL_MOUSE]	42.26	4	10	13	109	1.076	1.563	3.245	1.245	298.89	42.26	22	109	381	44.3	6.65
Q6PBC0	Cds2 protein OS=Mus musculus GN=Cds2 PE=2 SV=1 - [Q6PBC0_MOUSE]	20.93	3	2	2	29	0.572	1.188	0.966	1.245	94.76	20.93	4	29	129	14.9	4.94
F6SZ47	Serine/threonine-protein kinase B-raf (Fragment) OS=Mus musculus GN=Braf PE=2 SV=1 - [F6SZ47_MOUSE]	38.40	3	3	24	82	1.078	1.186	1.245	1.245	245.26	38.40	44	82	750	83.1	7.68
Q8BWT1	3-ketoacyl-CoA thiolase, mitochondrial OS=Mus musculus GN=Acaa2 PE=1 SV=3 - [THIM_MOUSE]	75.82	1	22	23	150	1.247	1.510	0.989	1.245	521.46	75.82	37	150	397	41.8	8.09
P27681	Gamma-aminobutyric acid receptor subunit gamma-3 OS=Mus musculus GN=Gabrg3 PE=2 SV=2 - [GBRG3_MOUSE]	4.28	2	2	2	3	1.660	1.509	1.021	1.245	4.09	4.28	3	3	467	54.3	8.05
Q02357-5	Isoform 5 of Ankyrin-1 OS=Mus musculus GN=Ank1 - [ANK1_MOUSE]	38.47	17	55	59	223	1.054	0.728	0.841	1.245	682.99	38.47	103	223	1744	191.9	7.87
O88741	Ganglioside-induced differentiation-associated protein 1 OS=Mus musculus GN=Gdap1 PE=1 SV=1 - [GDAP1_MOUSE]	33.80	2	11	12	119	0.739	0.737	1.006	1.245	330.82	33.80	24	119	358	41.3	8.37
E9Q2P0	Slit homolog 1 protein OS=Mus musculus GN=Slit1 PE=2 SV=1 - [E9Q2P0_MOUSE]	7.82	4	9	9	19	1.672	0.767	0.814	1.245	59.00	7.82	14	19	1458	159.0	6.68
Q9D3P9	Neurotensin/neuromedin N OS=Mus musculus GN=Nts PE=2 SV=1 - [NEUT_MOUSE]	12.43	1	2	2	5	1.074	1.073	0.979	1.245	15.97	12.43	3	5	169	19.6	7.30
Q8K1G2	Limbin OS=Mus musculus GN=Evc2 PE=1 SV=1 - [LBN_MOUSE]	5.90	1	3	6	7	0.820	1.244	1.358	1.246	19.79	5.90	6	7	1220	137.6	6.20
Q7TMF3	NADH dehydrogenase [ubiquinone] 1 alpha subcomplex subunit 12 OS=Mus musculus GN=Ndufa12 PE=1 SV=2 - [NDUAC_MOUSE]	87.59	2	12	12	99	0.938	1.133	1.157	1.246	351.54	87.59	22	99	145	17.1	9.36
Q8R123	FAD synthase OS=Mus musculus GN=Flad1 PE=1 SV=1 - [FAD1_MOUSE]	35.57	4	13	13	33	0.686	1.155	1.138	1.246	88.99	35.57	20	33	492	54.7	6.64
Q9JKL5	Calcineurin B homologous protein 3 OS=Mus musculus GN=Tesc PE=1 SV=2 - [CHP3_MOUSE]	35.98	1	6	6	36	1.349	1.656	2.098	1.246	107.64	35.98	11	36	214	24.6	4.98
Q6P3B9	Putative ribosome-binding factor A, mitochondrial OS=Mus musculus GN=Rbfa PE=2 SV=1 - [RBFA_MOUSE]	6.29	1	2	2	4	1.227	0.721	1.131	1.247	7.74	6.29	3	4	350	39.3	7.33
A9Z1V5	von Willebrand factor A domain-containing protein 5B1 OS=Mus musculus GN=Vwa5b1 PE=2 SV=1 - [VW5B1_MOUSE]	12.35	2	11	11	35	0.931	1.159	0.743	1.247	82.79	12.35	20	35	1215	133.9	7.93
P02798	Metallothionein-2 OS=Mus musculus GN=Mt2 PE=1 SV=2 - [MT2_MOUSE]	32.79	1	2	2	3	1.127	0.783	1.630	1.247	8.44	32.79	3	3	61	6.1	7.83
Q9DBB8	Trans-1,2-dihydrobenzene-1,2-diol dehydrogenase OS=Mus musculus GN=Dhdh PE=2 SV=1 - [DHDH_MOUSE]	40.54	1	11	11	45	1.339	1.599	1.119	1.247	158.40	40.54	20	45	333	36.3	6.44
J3QQ18	Ras GTPase-activating protein SynGAP OS=Mus musculus GN=Syngap1 PE=4 SV=1 - [J3QQ18_MOUSE]	40.67	1	1	49	371	0.992	2.477	2.599	1.248	1090.43	40.67	87	371	1308	144.8	9.01
Q8K097-2	Isoform 2 of Protein lifeguard 2 OS=Mus musculus GN=Faim2 - [LFG2_MOUSE]	15.08	2	3	3	24	1.530	1.286	1.126	1.248	69.71	15.08	6	24	305	34.2	7.46
Q9DAC2	Complement component 8, gamma subunit, isoform CRA_b OS=Mus musculus GN=C8g PE=2 SV=1 - [Q9DAC2_MOUSE]	19.05	2	2	2	9	2.035	1.405	1.021	1.248	35.96	19.05	4	9	168	18.9	8.32
Q02788	Collagen alpha-2(VI) chain OS=Mus musculus GN=Col6a2 PE=2 SV=3 - [CO6A2_MOUSE]	14.80	3	12	13	37	1.032	4.204	0.887	1.248	80.63	14.80	21	37	1034	110.3	6.42
Q8K274	Ketosamine-3-kinase OS=Mus musculus GN=Fn3krp PE=2 SV=2 - [KT3K_MOUSE]	33.01	1	10	10	48	0.760	1.036	1.133	1.248	133.95	33.01	17	48	309	34.4	7.87
P17183	Gamma-enolase OS=Mus musculus GN=Eno2 PE=1 SV=2 - [ENOG_MOUSE]	79.95	8	20	30	1753	1.785	1.147	1.123	1.248	5705.41	79.95	55	1753	434	47.3	5.11
O35350	Calpain-1 catalytic subunit OS=Mus musculus GN=Capn1 PE=2 SV=1 - [CAN1_MOUSE]	31.14	1	18	18	42	0.803	1.160	1.031	1.248	128.06	31.14	29	42	713	82.1	5.87
Q9ET54-3	Isoform 3 of Palladin OS=Mus musculus GN=Palld - [PALLD_MOUSE]	21.72	7	11	11	27	1.002	3.141	0.936	1.249	70.89	21.72	17	27	663	72.3	7.68

Q5DQR4-3	Isoform 3 of Syntaxin-binding protein 5-like OS=Mus musculus GN=Stxbp5l -	31.27	5	1	27	109	0.760	0.656	1.197	1.249	350.16	31.27	49	109	1161	128.8	6.76
Q99ML4	Protein FAM69B OS=Mus musculus GN=Fam69b PE=1 SV=1 - [FA69B_MOUSE]	12.06	3	5	5	9	0.916	0.742	1.291	1.250	23.04	12.06	9	9	431	48.8	8.15
Q924S8	Sprouty-related, EVH1 domain-containing protein 1 OS=Mus musculus GN=Spred1 PE=1 SV=1 - [SPRE1_MOUSE]	22.52	2	8	9	26	0.900	0.818	1.214	1.250	67.82	22.52	17	26	444	50.6	6.47
Q9JIK5	Nucleolar RNA helicase 2 OS=Mus musculus GN=Ddx21 PE=1 SV=3 - [DDX21_MOUSE]	8.93	1	7	7	13	0.954	0.852	0.913	1.250	34.02	8.93	11	13	851	93.5	9.11
Q9QXL2-3	Isoform 3 of Kinesin-like protein KIF21A OS=Mus musculus GN=Kif21a - [KI21A_MOUSE]	33.44	2	1	43	198	0.968	0.708	0.892	1.250	620.49	33.44	72	198	1567	174.7	6.06
Q61146	Occludin OS=Mus musculus GN=Ocln PE=1 SV=1 - [OCLN_MOUSE]	9.02	2	3	3	7	1.606	0.984	0.885	1.250	12.35	9.02	5	7	521	59.0	6.48
Q8BGP6	Solute carrier family 25 member 40 OS=Mus musculus GN=Slc25a40 PE=2 SV=1 -	5.34	2	2	2	8	0.835	1.030	0.935	1.250	9.54	5.34	3	8	337	37.9	8.95
Q80WB5	Protein N-terminal glutamine amidohydrolase OS=Mus musculus GN=Wdyhv1 PE=1 SV=1 - [NTAQ1_MOUSE]	20.57	1	3	3	8	3.148	1.244	0.685	1.250	23.24	20.57	4	8	209	24.3	5.43
Q6NY15-2	Isoform 2 of Testis-specific gene 10 protein OS=Mus musculus GN=Tsga10 - [TSG10_MOUSE]	3.33	4	2	3	5	0.931	0.908	1.061	1.250	9.74	3.33	5	5	690	80.3	5.80
Q9EQ06	Estradiol 17-beta-dehydrogenase 11 OS=Mus musculus GN=Hsd17b11 PE=2 SV=1 - [DHB11_MOUSE]	51.01	5	13	13	36	0.664	1.182	0.964	1.250	119.66	51.01	25	36	298	32.9	8.66
A2APV2-3	Isoform 3 of Formin-like protein 2 OS=Mus musculus GN=Fmnl2 - [FMNL2_MOUSE]	24.56	7	9	28	85	0.969	0.773	1.229	1.250	256.92	24.56	52	85	1091	123.9	7.65
P52293	Importin subunit alpha-1 OS=Mus musculus GN=Kpna2 PE=1 SV=2 - [IMA1_MOUSE]	7.37	4	2	3	8	0.864	0.583	0.696	1.250	31.05	7.37	5	8	529	57.9	5.68
A7M7C7	SKI family transcriptional corepressor 2 OS=Mus musculus GN=Skor2 PE=1 SV=1 - [SKOR2_MOUSE]	3.27	1	2	2	3	1.180	1.253	1.281	1.250	0.00	3.27	2	3	1008	105.6	6.67
E9Q6E9	Uncharacterized protein OS=Mus musculus GN=Simc1 PE=2 SV=1 - [E9Q6E9_MOUSE]	3.69	2	3	3	7	1.176	2.086	1.874	1.251	16.97	3.69	5	7	1354	146.5	6.39
P16283	Anion exchange protein 3 OS=Mus musculus GN=Slc4a3 PE=1 SV=2 - [B3A3_MOUSE]	18.09	9	14	17	59	0.729	0.702	1.229	1.251	167.43	18.09	28	59	1227	135.3	6.51
Q9ESJ4	NCK-interacting protein with SH3 domain OS=Mus musculus GN=Nckipsd PE=2 SV=2 - [SPN90_MOUSE]	35.85	1	24	24	167	0.781	1.197	1.229	1.251	479.31	35.85	45	167	714	78.5	6.05
P97314	Cysteine and glycine-rich protein 2 OS=Mus musculus GN=Csrp2 PE=1 SV=3 - [CSRP2_MOUSE]	45.08	1	7	7	39	1.257	3.909	1.068	1.252	118.35	45.08	14	39	193	20.9	8.62
P41969	ETS domain-containing protein Elk-1 OS=Mus musculus GN=Elk1 PE=2 SV=3 - [ELK1_MOUSE]	15.62	1	3	3	4	2.609	1.166	1.238	1.252	8.26	15.62	4	4	429	45.2	6.80
Q3U1V6	Ubiquitin-conjugating enzyme E2 variant 3 OS=Mus musculus GN=Uevld PE=2 SV=1 - [UEVLD_MOUSE]	19.11	3	8	9	38	0.691	1.034	0.924	1.252	105.91	19.11	16	38	471	51.6	7.28
B2RY83	Inactive heparanase-2 OS=Mus musculus GN=Hpse2 PE=2 SV=1 - [HPSE2_MOUSE]	1.69	1	1	1	1	0.872	1.082	2.082	1.252	1.73	1.69	1	1	592	66.6	9.96
Q9CWD3	Nucleoside diphosphate-linked moiety X motif 17 OS=Mus musculus GN=Nudt17 PE=2 SV=1 - [NUD17_MOUSE]	5.41	2	1	1	3	0.742	1.115	0.895	1.252	15.26	5.41	2	3	296	32.7	5.63
Q9D1R2	Protein KTI12 homolog OS=Mus musculus GN=Kti12 PE=1 SV=1 - [KTI12_MOUSE]	4.56	1	2	2	2	1.347	1.095	1.174	1.252	3.71	4.56	2	2	351	38.4	6.92
Q8CDJ6	Protein 4932411E22Rik OS=Mus musculus GN=4932411E22Rik PE=2 SV=1 - [Q8CDJ6_MOUSE]	9.68	1	3	3	4	1.818	1.541	1.350	1.253	8.08	9.68	4	4	403	44.0	6.11
Q8K4Z3	NAD(P)H-hydrate epimerase OS=Mus musculus GN=Apoa1bp PE=1 SV=1 -	55.67	1	10	10	74	0.837	1.080	1.012	1.253	212.98	55.67	17	74	282	31.0	7.69
Q91WE3	Ribonuclease P protein subunit p25 OS=Mus musculus GN=Rpp25 PE=2 SV=1 -	41.71	1	5	5	25	1.289	0.781	1.937	1.253	34.27	41.71	5	25	199	21.0	9.16

B1AU41	Alpha-1B adrenergic receptor (Fragment) OS=Mus musculus GN=Adra1b PE=2 SV=1 - [B1AU41_MOUSE]	5.84	3	2	2	3	1.358	0.603	1.171	1.253	6.56	5.84	3	3	462	51.1	9.54
Q8VIG0-2	Isoform 2 of Zinc finger CCHC domain-containing protein 14 OS=Mus musculus GN=Zcchc14 - [ZCH14_MOUSE]	3.71	3	1	1	1	4.880	4.567	0.647	1.253	2.24	3.71	1	1	944	98.6	8.25
H3BLS1	Probable tRNA pseudouridine synthase 2 OS=Mus musculus GN=Trub2 PE=4 SV=1 - [H3BLS1_MOUSE]	10.74	4	1	1	4	3.247	3.221	0.495	1.253	7.04	10.74	1	4	121	13.2	10.67
F6UQD0	Protein Zfp273 OS=Mus musculus GN=Zfp273 PE=4 SV=1 - [F6UQD0_MOUSE]	16.24	146	1	2	3	1.928	1.218	1.016	1.253	5.05	16.24	2	3	468	54.3	9.06
F8VQ72	Mitogen-activated protein kinase kinase kinase 1 OS=Mus musculus GN=Map3k1 PE=2 SV=1 - [F8VQ72_MOUSE]	5.29	2	3	3	6	1.428	1.090	1.162	1.253	14.86	5.29	3	6	1493	161.3	7.88
Q9JKL4	NADH dehydrogenase [ubiquinone] 1 alpha subcomplex assembly factor 3 OS=Mus musculus GN=Ndufaf3 PE=2 SV=1 - [NDUF3_MOUSE]	29.19	1	3	3	17	1.229	1.137	1.179	1.254	52.76	29.19	5	17	185	20.7	8.05
Q8C5L6	Inositol polyphosphate 5-phosphatase K OS=Mus musculus GN=Inpp5k PE=2 SV=2 - [INP5K_MOUSE]	9.62	2	4	4	7	0.718	1.146	1.012	1.254	14.54	9.62	6	7	468	54.1	5.88
Q9QYB8	Beta-adducin OS=Mus musculus GN=Add2 PE=1 SV=4 - [ADDB_MOUSE]	44.41	3	29	30	228	0.909	1.154	1.701	1.255	662.42	44.41	52	228	725	80.6	6.21
Q8BGV9	Cysteine protease ATG4D OS=Mus musculus GN=Atg4d PE=1 SV=1 - [ATG4D_MOUSE]	2.11	1	1	1	2	0.594	0.603	0.923	1.255	7.51	2.11	2	2	474	52.9	8.62
O89112	LanC-like protein 1 OS=Mus musculus GN=Lancl1 PE=1 SV=1 - [LANC1_MOUSE]	29.07	2	10	10	59	0.727	1.445	1.413	1.255	166.90	29.07	15	59	399	45.3	7.77
Q64471	Glutathione S-transferase theta-1 OS=Mus musculus GN=Gstt1 PE=1 SV=4 - [GSTT1_MOUSE]	32.08	3	9	9	29	0.900	1.553	0.887	1.256	83.06	32.08	17	29	240	27.4	7.27
Q9D2I5-7	Isoform 7 of LisH domain-containing protein ARMC9 OS=Mus musculus GN=Armc9 - [ARMC9_MOUSE]	3.02	7	2	2	3	0.899	0.622	0.975	1.256	4.02	3.02	3	3	496	55.3	5.77
E9PZ16	Basement membrane-specific heparan sulfate proteoglycan core protein OS=Mus musculus GN=Hspg2 PE=2 SV=1 - [E9PZ16_MOUSE]	22.34	3	73	73	300	1.266	1.610	1.064	1.256	883.47	22.34	132	300	4383	469.5	6.47
Q8R464	Cell adhesion molecule 4 OS=Mus musculus GN=Cadm4 PE=1 SV=1 - [CADM4_MOUSE]	39.95	1	12	12	122	1.358	0.683	1.056	1.256	408.91	39.95	24	122	388	42.7	6.30
A6ZI47	Fructose-bisphosphate aldolase OS=Mus musculus GN=Aldoart2 PE=2 SV=1 -	28.02	1	2	15	317	0.889	1.010	1.061	1.256	790.46	28.02	27	317	364	39.3	7.44
Q80UW3	Ras and Rab interactor-like protein OS=Mus musculus GN=Rinl PE=1 SV=1 - [RINL_MOUSE]	1.07	1	1	1	2	1.550	1.710	1.102	1.257	0.00	1.07	1	2	563	62.3	6.20
Q9Z1S5-2	Isoform 2 of Neuronal-specific septin-3 OS=Mus musculus GN=Sept3 - [SEPT3_MOUSE]	56.38	2	15	15	151	1.007	1.394	1.274	1.257	383.42	56.38	26	151	337	38.7	6.81
P97370	Sodium/potassium-transporting ATPase subunit beta-3 OS=Mus musculus GN=Atp1b3 PE=1 SV=1 - [AT1B3_MOUSE]	48.20	1	11	11	35	0.653	1.150	0.936	1.257	93.11	48.20	19	35	278	31.8	8.51
Q812A5-2	Isoform 2 of Proline-rich protein 5 OS=Mus musculus GN=Prr5 - [PRR5_MOUSE]	11.11	2	2	2	6	1.420	1.236	1.009	1.257	20.37	11.11	4	6	378	41.3	6.86
D3Z0U3	Transmembrane protein 120A (Fragment) OS=Mus musculus GN=Tmem120a PE=2 SV=1 - [D3Z0U3_MOUSE]	10.75	2	2	2	3	0.919	1.052	1.182	1.257	6.00	10.75	3	3	93	10.7	5.15
Q8CJH3	Plexin-B1 OS=Mus musculus GN=Plxnb1 PE=1 SV=2 - [PLXB1_MOUSE]	12.65	1	21	24	116	0.965	1.135	1.091	1.257	354.79	12.65	42	116	2119	231.2	5.55
Q3UZD7	Protein FAM71F1 OS=Mus musculus GN=Fam71f1 PE=2 SV=1 - [F71F1_MOUSE]	3.52	3	1	1	1	0.599	2.710	2.274	1.258	2.59	3.52	1	1	341	38.7	9.23
A7XUY5-2	Isoform 2 of Selection and upkeep of intraepithelial T-cells protein 5 OS=Mus musculus GN=Skint5 - [SKIT5_MOUSE]	5.21	6	4	5	6	1.598	2.784	1.855	1.258	9.52	5.21	5	6	1440	163.4	8.16
Q8K1S6-2	Isoform 2 of Protein spire homolog 2 OS=Mus musculus GN=Spire2 - [SPIR2_MOUSE]	4.69	3	2	3	9	3.187	1.687	1.578	1.258	16.45	4.69	4	9	618	69.6	7.75

O70622-2	Isoform 2 of Reticulon-2 OS=Mus musculus GN=Rtn2 - [RTN2_MOUSE]	8.33	2	1	1	2	0.356	0.817	0.982	1.258	5.52	8.33	2	2	204	22.2	9.44
Q9ES03-2	Isoform 2 of T-box transcription factor TBX20 OS=Mus musculus GN=Tbx20 -	5.39	2	1	1	1	1.075	1.030	1.291	1.258	2.87	5.39	1	1	297	33.2	7.58
Q99NB1	Acetyl-coenzyme A synthetase 2-like, mitochondrial OS=Mus musculus GN=Acss1 PE=1 SV=1 - [ACS2L_MOUSE]	30.06	1	17	17	54	1.007	1.062	0.948	1.258	154.26	30.06	29	54	682	74.6	6.98
Q6IMK7	Diamine oxidase-like protein 2 OS=Mus musculus GN=Doxl2 PE=3 SV=1 - [Q6IMK7_MOUSE]	1.52	1	1	1	1	0.934	1.546	0.996	1.259	2.88	1.52	1	1	723	81.8	7.06
F8WHW6	Phosphatidylinositol 4-phosphate 5-kinase type-1 gamma OS=Mus musculus GN=Pip5k1c PE=2 SV=1 - [F8WHW6_MOUSE]	32.75	7	13	16	89	0.604	1.351	1.568	1.259	349.57	32.75	29	89	687	75.5	6.14
Q3V117	ATP-citrate synthase OS=Mus musculus GN=Acly PE=2 SV=1 - [Q3V117_MOUSE]	50.41	2	1	49	326	0.825	1.024	0.995	1.259	1011.73	50.41	93	326	1101	120.7	7.44
E9QME5	E3 ubiquitin-protein ligase TRIM33 OS=Mus musculus GN=Trim33 PE=2 SV=1 - [E9QME5_MOUSE]	4.99	4	5	5	7	1.562	0.997	1.385	1.259	20.13	4.99	7	7	1123	121.6	6.73
E9Q4T9	Transmembrane protease serine 4 OS=Mus musculus GN=Tmprss4 PE=2 SV=1 - [E9Q4T9_MOUSE]	8.06	2	1	1	1	1.345	0.709	0.859	1.259	0.00	8.06	1	1	335	36.7	7.52
Q7TN22-2	Isoform 2 of Thioredoxin domain-containing protein 16 OS=Mus musculus GN=Txndc16 - [TXD16_MOUSE]	3.66	2	2	2	2	1.873	1.016	1.532	1.259	4.63	3.66	2	2	819	92.0	5.31
E9Q2C0	Protein 9330101J02Rik (Fragment) OS=Mus musculus GN=9330101J02Rik PE=2 SV=1 - [E9Q2C0_MOUSE]	1.76	3	2	2	2	1.325	0.476	1.180	1.259	5.09	1.76	2	2	1423	163.6	7.61
M0QWY6	Cell surface glycoprotein CD200 receptor 1 OS=Mus musculus GN=Cd200r1 PE=4 SV=1 - [M0QWY6_MOUSE]	7.35	2	1	1	1	1.539	3.264	1.040	1.259	2.71	7.35	1	1	245	26.7	6.64
Q60992	Guanine nucleotide exchange factor VAV2 OS=Mus musculus GN=Vav2 PE=1 SV=1 -	4.95	1	3	3	5	1.746	0.971	1.048	1.259	6.33	4.95	3	5	868	99.9	6.84
Q9CWR0-2	Isoform 2 of Rho guanine nucleotide exchange factor 25 OS=Mus musculus GN=Arhgef25 - [ARHGP_MOUSE]	8.05	3	2	5	16	0.575	1.545	1.212	1.260	51.78	8.05	9	16	609	67.4	5.19
Q497Y9	C130050O18Rik protein OS=Mus musculus GN=C130050O18Rik PE=2 SV=1 - [Q497Y9_MOUSE]	1.97	1	1	1	2	0.577	1.868	1.402	1.260	4.89	1.97	2	2	356	38.7	8.34
Q3UW66	Sulfurtransferase OS=Mus musculus GN=Mpst PE=2 SV=1 - [Q3UW66_MOUSE]	44.44	2	11	11	62	0.752	1.748	1.664	1.260	191.70	44.44	20	62	297	33.1	6.47
A1L3P4	Sodium/hydrogen exchanger OS=Mus musculus GN=Slc9a6 PE=2 SV=1 -	10.40	3	7	7	55	0.951	1.012	1.104	1.260	187.57	10.40	13	55	702	77.9	6.37
Q01065	Calcium/calmodulin-dependent 3',5'-cyclic nucleotide phosphodiesterase 1B OS=Mus musculus [illegible]	50.28	1	20	21	128	0.668	1.057	2.300	1.261	389.00	50.28	40	128	535	61.2	5.72
Q80TJ1-2	Isoform 2 of Calcium-dependent secretion activator 1 OS=Mus musculus GN=Cadps - [CAPS1_MOUSE]	53.49	3	3	60	419	0.433	1.400	1.280	1.261	1262.61	53.49	103	419	1361	153.9	5.86
Q99KE8-3	Isoform 3 of Zinc finger protein 64 OS=Mus musculus GN=Zfp64 - [ZFP64_MOUSE]	1.69	2	1	1	1	1.646	1.310	1.168	1.261	2.38	1.69	1	1	473	53.3	8.78
E9PVB6	LIM domain kinase 1 OS=Mus musculus GN=Limk1 PE=2 SV=1 - [E9PVB6_MOUSE]	12.05	3	6	6	23	1.169	0.396	0.856	1.261	92.43	12.05	11	23	639	71.8	7.77
Q8C4Q6	Axin interactor, dorsalization-associated protein OS=Mus musculus GN=Aida PE=1 SV=1 - [AIDA_MOUSE]	38.03	2	11	11	39	1.032	0.880	0.904	1.261	103.30	38.03	18	39	305	34.9	6.74
P04919-2	Isoform Kidney of Band 3 anion transport protein OS=Mus musculus GN=Slc4a1 - [B3AT_MOUSE]	25.06	2	13	13	67	1.213	1.497	0.608	1.261	185.80	25.06	24	67	850	94.2	6.68
Q3V3R1	Monofunctional C1-tetrahydrofolate synthase, mitochondrial OS=Mus musculus GN=Mthfd1l PE=1 SV=2 - [illegible]	38.28	1	32	33	110	0.803	1.150	0.959	1.261	310.20	38.28	60	110	977	105.7	7.02
A2AWL7-3	Isoform 3 of MAX gene-associated protein OS=Mus musculus GN=Mga -	2.46	6	3	4	10	1.395	1.080	0.909	1.261	28.34	2.46	5	10	2924	320.1	7.23

D2EAC2-2	Isoform 2 of Zinc finger BED domain-containing protein 6 OS=Mus musculus GN=Zbed6 - [ZBED6_MOUSE]	1.07	2	1	1	1	1.889	2.279	1.250	1.261	0.00	1.07	1	1	934	104.4	6.89
Q8VI56-2	Isoform 2 of Low-density lipoprotein receptor-related protein 4 OS=Mus musculus GN=Lrp4 - [LRP4_MOUSE]	3.03	2	5	5	10	0.976	1.080	1.316	1.262	24.06	3.03	8	10	1848	205.2	5.36
Q99KE1	NAD-dependent malic enzyme, mitochondrial OS=Mus musculus GN=Me2 PE=2 SV=1 - [MAOM_MOUSE]	52.63	1	23	24	130	1.481	1.202	1.006	1.262	359.12	52.63	43	130	589	65.8	7.61
Q9CQ54	NADH dehydrogenase [ubiquinone] 1 subunit C2 OS=Mus musculus GN=Ndufc2 PE=1 SV=1 - [NDUC2_MOUSE]	59.17	1	8	8	68	0.806	1.001	1.086	1.262	186.08	59.17	14	68	120	14.2	9.20
Q924W6-2	Isoform 2 of Tripartite motif-containing protein 66 OS=Mus musculus GN=Trim66 - [TRI66_MOUSE]	5.54	4	5	5	10	0.842	0.591	0.979	1.262	23.92	5.54	7	10	1209	133.2	7.06
Q8K1C9	Leucine-rich repeat-containing protein 41 OS=Mus musculus GN=Lrrc41 PE=1 SV=3 - [LRC41_MOUSE]	9.29	1	4	5	25	1.175	0.912	1.545	1.262	13.57	9.29	6	25	807	88.1	8.31
F8WIE1	Alpha-mannosidase 2C1 OS=Mus musculus GN=Man2c1 PE=2 SV=1 - [F8WIE1_MOUSE]	21.89	5	14	14	37	0.619	1.049	0.985	1.262	133.02	21.89	24	37	1037	115.4	6.46
Q6PDC8	Major facilitator superfamily domain-containing protein 4 OS=Mus musculus GN=Mfsd4 PE=2 SV=2 - [illegible]	8.43	9	4	4	9	0.673	1.652	1.498	1.263	24.89	8.43	7	9	510	56.1	8.00
Q8VEB6	Zinc phosphodiesterase ELAC protein 1 OS=Mus musculus GN=Elac1 PE=2 SV=1 - [RNZ1_MOUSE]	11.88	2	4	4	13	1.660	1.113	0.912	1.263	31.83	11.88	7	13	362	39.7	6.60
Q9R112	Sulfide:quinone oxidoreductase, mitochondrial OS=Mus musculus GN=Sqrdl PE=2 SV=3 - [SQRD_MOUSE]	24.67	4	12	12	34	0.772	1.365	0.875	1.263	97.78	24.67	21	34	450	50.2	9.09
P17182	Alpha-enolase OS=Mus musculus GN=Eno1 PE=1 SV=3 - [ENOA_MOUSE]	86.41	4	17	39	1795	0.955	1.259	1.226	1.263	5858.03	86.41	71	1795	434	47.1	6.80
Q9R244-2	Isoform 2 of Short transient receptor potential channel 2 OS=Mus musculus GN=Trpc2 - [illegible]	1.12	9	1	2	4	1.236	1.407	1.512	1.263	7.35	1.12	2	4	1072	119.4	8.13
Q64692	CMP-N-acetylneuraminate-poly-alpha-2,8-sialyltransferase OS=Mus musculus GN=St8sia4 PE=2 SV=1 - [illegible]	5.57	1	1	1	1	2.057	1.354	1.339	1.263	3.12	5.57	1	1	359	41.2	9.80
Q3UYH7	Beta-adrenergic receptor kinase 2 OS=Mus musculus GN=Adrbk2 PE=2 SV=2 -	15.70	1	7	12	26	1.123	1.569	1.064	1.264	70.03	15.70	17	26	688	79.6	7.69
Q9R1V7	Disintegrin and metalloproteinase domain-containing protein 23 OS=Mus musculus GN=Adam23 PE=1 SV=1 - [illegible]	21.71	7	15	15	96	0.837	0.751	1.162	1.264	294.76	21.71	28	96	829	91.5	7.68
O08553	Dihydropyrimidinase-related protein 2 OS=Mus musculus GN=Dpysl2 PE=1 SV=2 -	80.07	1	37	43	2645	1.413	1.275	1.172	1.264	8139.76	80.07	81	2645	572	62.2	6.38
D3YWN6	Protein downstream neighbor of Son OS=Mus musculus GN=Donson PE=2 SV=1 -	7.36	7	2	2	3	0.699	2.372	1.944	1.264	6.04	7.36	2	3	489	54.2	7.78
P10922	Histone H1.0 OS=Mus musculus GN=H1f0 PE=2 SV=4 - [H10_MOUSE]	43.81	1	11	11	46	0.640	1.365	1.161	1.264	109.34	43.81	16	46	194	20.8	10.90
Q3U1Y4	DENN domain-containing protein 4B OS=Mus musculus GN=Dennd4b PE=2 SV=2 -	12.61	6	13	13	38	1.195	0.851	1.100	1.264	124.54	12.61	22	38	1499	164.6	7.71
P97290	Plasma protease C1 inhibitor OS=Mus musculus GN=Serping1 PE=1 SV=3 -	12.10	2	5	5	13	1.493	1.608	0.618	1.264	49.19	12.10	9	13	504	55.5	6.29
Q5SX40	Myosin-1 OS=Mus musculus GN=Myh1 PE=1 SV=1 - [MYH1_MOUSE]	4.53	2	2	7	24	1.512	1.295	1.322	1.264	48.89	4.53	9	24	1942	223.2	5.76
Q8VCM3	Zinc finger FYVE domain-containing protein 21 OS=Mus musculus GN=Zfyve21 PE=2 SV=2 - [ZFY21_MOUSE]	17.95	1	4	5	9	1.441	0.798	0.970	1.265	24.23	17.95	7	9	234	26.0	8.34
Q8CGB6-2	Isoform 2 of Tensin-like C1 domain-containing phosphatase OS=Mus musculus GN=Tenc1 - [TENC1_MOUSE]	12.27	4	9	11	21	0.929	2.198	1.226	1.265	66.92	12.27	18	21	1377	149.4	8.34

P17879	Heat shock 70 kDa protein 1B OS=Mus musculus GN=Hspa1b PE=1 SV=3 - [HS71B_MOUSE]	55.30	2	13	32	380	0.771	2.374	1.214	1.265	985.95	55.30	55	380	642	70.1	5.72
Q8CIA5-2	Isoform 2 of UDP-xylose and UDP-N-acetylglucosamine transporter OS=Mus musculus GN=Slc35b4 - [illegible]	18.87	2	1	1	1	1.085	1.004	1.289	1.265	0.00	18.87	1	1	159	18.6	8.41
Q8VC42	Uncharacterized protein C18orf8 homolog OS=Mus musculus GN=Mic1 PE=2 SV=1 - [MIC1_MOUSE]	17.20	3	8	8	14	0.744	1.242	1.153	1.265	38.34	17.20	12	14	657	74.9	7.83
Q8K010	5-oxoprolinase OS=Mus musculus GN=Oplah PE=2 SV=1 - [OPLA_MOUSE]	32.45	5	32	34	111	0.937	1.465	1.143	1.265	361.03	32.45	60	111	1288	137.5	6.28
A2AQA9	Protein Neb OS=Mus musculus GN=Neb PE=2 SV=1 - [A2AQA9_MOUSE]	1.51	5	7	8	8	2.650	1.480	1.360	1.265	13.79	1.51	8	8	6909	800.1	9.09
Q14BI1	Protein Slc24a2 OS=Mus musculus GN=Slc24a2 PE=2 SV=1 - [Q14BI1_MOUSE]	16.17	1	2	8	98	0.870	0.685	1.255	1.266	240.07	16.17	14	98	711	78.9	6.20
Q8BWW3	Phosphoacetylglucosamine mutase OS=Mus musculus GN=Pgm3 PE=2 SV=1 - [Q8BWW3_MOUSE]	43.51	2	17	17	54	0.702	1.154	1.005	1.266	150.53	43.51	26	54	501	55.0	6.29
Q8CFI5	Probable proline--tRNA ligase, mitochondrial OS=Mus musculus GN=Pars2 PE=2 SV=2 - [SYPM_MOUSE]	22.74	3	8	8	17	1.280	1.369	1.503	1.266	57.85	22.74	12	17	475	53.5	7.84
F8WGT1	Adenosylhomocysteinase OS=Mus musculus GN=Ahcyl2 PE=2 SV=1 - [F8WGT1_MOUSE]	45.42	11	2	33	258	0.535	0.844	1.074	1.266	684.04	45.42	60	258	612	66.7	7.36
A2A9Y7	Cytochrome P450 4X1 OS=Mus musculus GN=Cyp4x1 PE=2 SV=1 - [A2A9Y7_MOUSE]	8.52	2	4	4	5	0.957	1.406	1.223	1.266	13.03	8.52	5	5	481	55.5	7.21
Q07797	Galectin-3-binding protein OS=Mus musculus GN=Lgals3bp PE=1 SV=1 - [LG3BP_MOUSE]	15.25	2	7	7	17	1.761	1.378	0.995	1.266	50.71	15.25	13	17	577	64.4	5.14
P97477	Aurora kinase A OS=Mus musculus GN=Aurka PE=1 SV=1 - [AURKA_MOUSE]	11.14	2	2	2	2	0.392	0.722	0.984	1.266	2.30	11.14	2	2	395	44.7	9.38
Q00915	Retinol-binding protein 1 OS=Mus musculus GN=Rbp1 PE=2 SV=2 - [RET1_MOUSE]	68.15	1	8	9	36	0.939	3.198	1.140	1.267	122.14	68.15	16	36	135	15.8	5.25
Q9CQZ6	NADH dehydrogenase [ubiquinone] 1 beta subcomplex subunit 3 OS=Mus musculus GN=Ndufb3 PE=1 SV=1 - [illegible]	17.31	1	2	2	16	0.818	1.051	1.166	1.267	31.81	17.31	4	16	104	11.7	9.04
E9QP49	EH domain-binding protein 1-like protein 1 OS=Mus musculus GN=Ehbp1l1 PE=2 SV=1 - [E9QP49_MOUSE]	8.09	7	8	9	14	1.501	1.558	0.963	1.267	33.08	8.09	12	14	1718	184.7	4.74
Q9D7J4	Cytochrome c oxidase protein 20 homolog OS=Mus musculus GN=Cox20 PE=2 SV=1 - [COX20_MOUSE]	22.22	1	2	2	5	0.829	1.044	1.047	1.267	11.22	22.22	4	5	117	13.2	9.01
O54830-2	Isoform 2 of Prolactin-7A1 OS=Mus musculus GN=Prl7a1 - [PR7A1_MOUSE]	7.17	2	1	1	3	1.449	1.102	1.067	1.267	0.00	7.17	1	3	265	29.9	4.56
Q91Y57	Sialic acid-binding Ig-like lectin 12 OS=Mus musculus GN=Siglec12 PE=1 SV=3 - [illegible]	1.71	1	1	1	1	1.132	1.259	1.006	1.267	2.51	1.71	1	1	467	51.9	7.84
Q99PP6-3	Isoform Gamma of Tripartite motif-containing protein 34A OS=Mus musculus GN=Trim34a - [TR34A_MOUSE]	4.91	6	1	1	1	0.624	0.229	1.721	1.267	3.58	4.91	1	1	326	37.6	8.44
Q9CR53	Neuromedin-B OS=Mus musculus GN=Nmb PE=2 SV=1 - [NMB_MOUSE]	7.44	2	1	1	2	1.969	7.014	1.925	1.267	7.91	7.44	2	2	121	13.6	11.72
O35219	Potassium voltage-gated channel subfamily H member 2 OS=Mus musculus GN=Kcnh2 PE=2 SV=2 - [KCNH2_MOUSE]	11.02	4	10	10	20	1.177	0.963	0.992	1.267	61.14	11.02	14	20	1162	126.8	7.78
Q8C8R3-2	Isoform 2 of Ankyrin-2 OS=Mus musculus GN=Ank2 - [ANK2_MOUSE]	54.02	2	6	171	1396	0.800	1.078	1.206	1.268	4047.97	54.02	301	1396	3926	428.5	5.16
Q9JHJ8	ICOS ligand OS=Mus musculus GN=Icoslg PE=1 SV=1 - [ICOSL_MOUSE]	10.25	2	3	3	3	1.054	1.357	1.319	1.268	4.32	10.25	3	3	322	35.9	7.91
F6TQG5	Adenosine deaminase domain-containing protein 1 (Fragment) OS=Mus musculus GN=Adad1 PE=4 SV=1 - [F6TQG5_MOUSE]	10.95	4	1	1	1	1.552	1.457	2.029	1.268	0.00	10.95	1	1	201	22.5	8.63
Q04899	Cyclin-dependent kinase 18 OS=Mus musculus GN=Cdk18 PE=1 SV=1 - [CDK18_MOUSE]	36.14	17	10	16	65	0.869	1.319	1.096	1.268	166.85	36.14	27	65	451	51.8	8.51

Q60857	Sodium-dependent serotonin transporter OS=Mus musculus GN=Slc6a4 PE=1 SV=4 - [SC6A4_MOUSE]	3.97	1	2	2	10	0.529	0.699	1.026	1.268	34.85	3.97	4	10	630	70.0	6.18
G5E8R3	Pyruvate carboxylase OS=Mus musculus GN=Pcx PE=3 SV=1 - [G5E8R3_MOUSE]	52.80	3	50	51	306	0.820	0.849	1.021	1.268	928.20	52.80	91	306	1178	129.6	6.71
Q8BGE4	Protein 4933426M11Rik OS=Mus musculus GN=4933426M11Rik PE=2 SV=1 - [Q8BGE4_MOUSE]	4.64	1	1	1	4	1.238	1.042	1.151	1.269	12.84	4.64	2	4	302	32.0	5.00
Q61205	Platelet-activating factor acetylhydrolase IB subunit gamma OS=Mus musculus GN=Pafah1b3 PE=1 SV=1 [PA1B3_MOUSE]	18.97	4	6	6	27	0.918	0.941	0.689	1.269	62.33	18.97	12	27	232	25.8	6.93
Q810Y8	PRAMEl7 OS=Mus musculus GN=Pramel7 PE=2 SV=1 - [Q810Y8_MOUSE]	1.67	1	1	1	1	1.478	1.001	1.237	1.269	2.19	1.67	1	1	479	55.8	7.24
E9Q8G3	Protein Srcap OS=Mus musculus GN=Srcap PE=2 SV=1 - [E9Q8G3_MOUSE]	5.34	1	2	2	5	1.385	0.981	2.485	1.269	13.64	5.34	2	5	431	45.3	9.83
Q9CZ13	Cytochrome b-c1 complex subunit 1, mitochondrial OS=Mus musculus GN=Uqcrc1 PE=1 SV=2 - [QCR1_MOUSE]	52.29	1	18	19	504	0.995	1.135	1.168	1.269	1366.00	52.29	34	504	480	52.8	6.21
Q3U1N2-2	Isoform 2 of Sterol regulatory element-binding protein 2 OS=Mus musculus GN=Srebf2 - [SRBP2_MOUSE]	2.01	2	2	3	4	1.205	1.325	1.437	1.269	6.83	2.01	4	4	1096	119.8	8.32
Q6R0H7-4	Isoform XLas-4 of Guanine nucleotide-binding protein G(s) subunit alpha isoforms XLas OS=Mus musculus GN=Gnas - [GNAS1_MOUSE]	22.79	7	13	21	165	0.545	1.246	0.951	1.269	462.21	22.79	35	165	1119	120.0	4.83
Q91XE8	Transmembrane protein 205 OS=Mus musculus GN=Tmem205 PE=1 SV=1 - [TM205_MOUSE]	17.46	1	3	3	13	0.796	0.809	0.806	1.269	41.54	17.46	5	13	189	21.2	9.42
P16332	Methylmalonyl-CoA mutase, mitochondrial OS=Mus musculus GN=Mut PE=2 SV=2 - [MUTA_MOUSE]	44.52	1	24	24	86	0.990	1.481	1.194	1.269	266.26	44.52	40	86	748	82.8	6.89
Q3TC72	Fumarylacetoacetate hydrolase domain-containing protein 2A OS=Mus musculus GN=Fahd2 PE=1 SV=1 - [illegible]	64.22	1	14	14	67	1.724	1.433	1.097	1.269	248.21	64.22	21	67	313	34.7	8.16
O55226	Chondroadherin OS=Mus musculus GN=Chad PE=2 SV=1 - [CHAD_MOUSE]	3.91	1	2	2	7	0.789	0.556	0.950	1.269	14.07	3.91	2	7	358	40.3	9.39
A3KFX0	Cytosolic 5'-nucleotidase 1A OS=Mus musculus GN=Nt5c1a PE=2 SV=1 - [5NT1A_MOUSE]	33.42	1	9	10	25	1.091	1.166	0.924	1.269	78.81	33.42	18	25	365	40.7	7.06
P01786	Ig heavy chain V region MOPC 47A OS=Mus musculus PE=1 SV=1 - [HVM17_MOUSE]	13.68	9	1	1	2	1.334	1.703	0.664	1.269	7.02	13.68	2	2	117	13.0	8.84
P29758	Ornithine aminotransferase, mitochondrial OS=Mus musculus GN=Oat PE=1 SV=1 - [OAT_MOUSE]	51.03	3	20	21	82	1.545	1.466	1.012	1.270	256.96	51.03	35	82	439	48.3	6.62
Q8BVZ5	Interleukin-33 OS=Mus musculus GN=Il33 PE=1 SV=1 - [IL33_MOUSE]	10.53	3	2	2	5	1.671	1.504	1.270	1.270	16.46	10.53	3	5	266	30.0	8.60
Q6PIC6	Sodium/potassium-transporting ATPase subunit alpha-3 OS=Mus musculus GN=Atp1a3 PE=1 SV=1 - [illegible]	61.30	2	41	71	4449	0.703	0.681	1.122	1.270	13378.25	61.30	136	4449	1013	111.6	5.41
Q6P6N5	Sprouty-related, EVH1 domain-containing protein 3 OS=Mus musculus GN=Spred3 PE=2 SV=1 - [SPRE3_MOUSE]	3.92	1	1	1	3	0.860	0.975	1.497	1.270	12.79	3.92	2	3	408	42.8	8.34
A2ASS6-3	Isoform 3 of Titin OS=Mus musculus GN=Ttn - [TITIN_MOUSE]	1.53	1	4	5	5	3.021	1.305	0.949	1.270	11.36	1.53	5	5	5508	618.9	5.68
Q922H2	[Pyruvate dehydrogenase [lipoamide]] kinase isozyme 3, mitochondrial OS=Mus musculus GN=Pdk3 PE=2 SV=1 - [PDK3_MOUSE]	32.53	1	11	12	51	0.728	1.224	1.259	1.270	141.84	32.53	21	51	415	47.9	8.82
Q921U8-2	Isoform L2 of Smoothelin OS=Mus musculus GN=Smtn - [SMTN_MOUSE]	15.31	7	12	12	24	0.734	2.466	0.880	1.271	62.20	15.31	17	24	921	100.1	8.81
Q9ET80	Junctophilin-1 OS=Mus musculus GN=Jph1 PE=1 SV=1 - [JPH1_MOUSE]	13.94	1	7	8	14	1.167	1.293	3.205	1.271	36.74	13.94	9	14	660	71.9	9.22

O35598	Disintegrin and metalloproteinase domain-containing protein 10 OS=Mus musculus GN=Adam10 PE=1 SV=2 - [ADA10_MOUSE]	20.69	3	13	13	49	1.151	0.890	1.086	1.271	137.81	20.69	22	49	749	83.9	7.94
Q80ZJ8	EF-hand calcium-binding domain-containing protein 4A OS=Mus musculus GN=Efcab4a PE=2 SV=2 - [EFC4A_MOUSE]	8.63	3	2	3	5	2.018	3.306	1.550	1.271	13.02	8.63	5	5	394	44.4	5.20
Q810F8-2	Isoform 2 of T-box transcription factor TBX10 OS=Mus musculus GN=Tbx10 -	5.81	2	2	2	2	1.511	0.845	1.083	1.271	5.39	5.81	2	2	344	38.1	9.39
Q923S9	Ras-related protein Rab-30 OS=Mus musculus GN=Rab30 PE=2 SV=1 - [RAB30_MOUSE]	36.95	2	4	7	64	0.720	1.013	1.037	1.271	193.59	36.95	11	64	203	23.0	4.97
Q6GQV0-2	Isoform 2 of Uncharacterized protein C16orf59 homolog OS=Mus musculus -	4.78	2	1	1	1	1.711	1.198	1.245	1.272	3.08	4.78	1	1	251	27.7	6.52
Q80U23	Syntaphilin OS=Mus musculus GN=Snph PE=1 SV=3 - [SNPH_MOUSE]	35.96	3	10	12	43	0.711	0.844	1.298	1.272	135.91	35.96	21	43	495	53.7	5.90
P28654	Decorin OS=Mus musculus GN=Dcn PE=2 SV=1 - [PGS2_MOUSE]	25.99	1	7	8	20	1.636	5.846	0.963	1.272	53.92	25.99	14	20	354	39.8	8.68
Q8BLY3	Leucine-rich repeat and fibronectin type-III domain containing protein 3 OS=Mus musculus GN=Lrfn3 PE=1 SV=1 - [LRFN3_MOUSE]	8.95	1	3	4	10	1.170	1.411	1.174	1.272	30.98	8.95	6	10	626	66.0	7.08
Q9WU39	Amiloride-sensitive sodium channel subunit gamma OS=Mus musculus GN=Scnn1g PE=1 SV=1 - [SCNNG_MOUSE]	2.75	1	1	1	1	0.735	1.233	1.349	1.272	3.50	2.75	1	1	655	74.6	7.47
Q80XI3-2	Isoform 2 of Eukaryotic translation initiation factor 4 gamma 3 OS=Mus musculus GN=Eif4g3 - [IF4G3_MOUSE]	29.87	6	1	39	155	0.944	1.039	1.130	1.272	437.82	29.87	70	155	1560	172.8	5.50
P70193	Leucine-rich repeats and immunoglobulin-like domains protein 1 OS=Mus musculus GN=Lrig1 PE=2 SV=2 - [LRIG1_MOUSE]	10.72	1	10	10	16	0.822	0.651	0.740	1.272	51.29	10.72	14	16	1091	119.1	6.86
M0QWX4	Sentrin-specific protease 1 OS=Mus musculus GN=Senp1 PE=4 SV=1 - [M0QWX4_MOUSE]	6.31	3	2	3	13	1.107	1.192	1.156	1.272	24.83	6.31	4	13	666	75.5	8.59
P35486	Pyruvate dehydrogenase E1 component subunit alpha, somatic form, mitochondrial OS=Mus musculus GN=Pdha1 PE=1 SV=1 - [ODPA_MOUSE]	75.13	1	29	30	332	0.789	1.075	1.193	1.272	832.83	75.13	54	332	390	43.2	8.19
F6XCE3	Myosin regulatory light chain 2, ventricular/cardiac muscle isoform (Fragment) OS=Mus musculus GN=Myl2 PE=4 SV=1 - [F6XCE3_MOUSE]	17.74	5	1	1	1	1.686	1.251	1.009	1.273	0.00	17.74	1	1	62	7.2	5.99
F6SBP3	Serine/threonine-protein kinase SIK3 (Fragment) OS=Mus musculus GN=Sik3 PE=4 SV=1 - [F6SBP3_MOUSE]	49.30	1	1	2	2	1.404	1.477	1.078	1.273	1.76	49.30	2	2	71	7.7	4.28
P51450-2	Isoform 2 of Nuclear receptor ROR-gamma OS=Mus musculus GN=Rorc -	5.25	5	2	3	3	1.092	1.258	1.499	1.273	2.51	5.25	3	3	495	55.7	7.72
O35600	Retinal-specific ATP-binding cassette transporter OS=Mus musculus GN=Abca4 PE=2 SV=1 - [ABCA4_MOUSE]	1.17	1	1	3	6	1.033	1.201	1.522	1.273	10.41	1.17	5	6	2310	260.0	6.20
Q9CPU2	NADH dehydrogenase [ubiquinone] 1 beta subcomplex subunit 2, mitochondrial OS=Mus musculus GN=Ndufb2 PE=3 SV=1 - [NDUB2_MOUSE]	8.57	1	2	2	3	1.493	1.078	1.240	1.273	5.04	8.57	3	3	105	12.0	6.52
Q8R3P0	Aspartoacylase OS=Mus musculus GN=Aspa PE=1 SV=2 - [ACY2_MOUSE]	37.82	3	10	10	54	2.704	0.360	1.005	1.273	171.79	37.82	17	54	312	35.3	6.58
D3Z1B2	MCG50540 OS=Mus musculus GN=Gm4953 PE=4 SV=1 - [D3Z1B2_MOUSE]	16.15	1	1	3	19	0.988	0.835	1.475	1.273	48.26	16.15	5	19	161	18.5	5.44
Q3TMH2	Secernin-3 OS=Mus musculus GN=Scrn3 PE=1 SV=1 - [SCRN3_MOUSE]	32.30	2	14	15	58	1.234	1.084	1.240	1.273	160.47	32.30	25	58	418	47.6	5.66
Q8BGY9	High affinity choline transporter 1 OS=Mus musculus GN=Slc5a7 PE=1 SV=1 -	5.00	1	3	3	6	0.701	0.660	1.885	1.274	17.53	5.00	6	6	580	63.3	5.33
Q9JI19	Acidic fibroblast growth factor intracellular-binding protein OS=Mus musculus GN=Fibp PE=2 SV=1 - [FIBP_MOUSE]	32.49	2	10	10	34	0.740	1.283	1.099	1.274	115.14	32.49	18	34	357	41.2	6.76

E9QMK2	Versican core protein OS=Mus musculus GN=Vcan PE=2 SV=1 - [E9QMK2_MOUSE]	18.33	8	1	27	209	1.619	0.753	1.774	1.274	591.22	18.33	48	209	1615	178.4	4.96
Q63844	Mitogen-activated protein kinase 3 OS=Mus musculus GN=Mapk3 PE=1 SV=5 -	55.00	11	14	22	237	1.010	2.237	1.176	1.274	599.67	55.00	44	237	380	43.0	6.61
D6RH74	UPF0462 protein C4orf33 homolog OS=Mus musculus GN=D3Ertd751e PE=2 SV=1 -	32.93	4	3	3	8	1.843	0.954	1.148	1.274	18.27	32.93	6	8	82	9.4	4.41
E9PVV3	Potassium voltage-gated channel subfamily C member 1 OS=Mus musculus GN=Kcnc1 PE=2 SV=1 - [E9PVV3_MOUSE]	16.58	2	6	8	28	0.727	0.435	1.013	1.275	80.51	16.58	12	28	585	65.8	6.18
Q9CQJ8	NADH dehydrogenase [ubiquinone] 1 beta subcomplex subunit 9 OS=Mus musculus GN=Ndufb9 PE=1 SV=3 - [NDUB9_MOUSE]	56.98	1	7	7	137	0.842	0.960	1.122	1.275	356.37	56.98	13	137	179	22.0	7.80
P52875	Transmembrane protein 165 OS=Mus musculus GN=Tmem165 PE=2 SV=2 - [TM165_MOUSE]	7.74	1	1	1	4	3.073	1.435	1.398	1.275	7.75	7.74	1	4	323	34.8	7.46
Q6PE15	Mycophenolic acid acyl-glucuronide esterase, mitochondrial OS=Mus musculus GN=Abhd10 PE=2 SV=1 - [ABHDA_MOUSE]	34.01	3	10	10	39	1.160	0.813	0.955	1.275	116.54	34.01	17	39	297	33.0	8.79
Q5SQX6	Cytoplasmic FMR1-interacting protein 2 OS=Mus musculus GN=Cyfip2 PE=1 SV=2 - [CYFP2_MOUSE]	37.51	1	1	48	254	1.411	1.721	1.133	1.275	725.08	37.51	88	254	1253	145.6	7.05
P50571	Gamma-aminobutyric acid receptor subunit beta-1 OS=Mus musculus GN=Gabrb1 PE=1 SV=1 - [GBRB1_MOUSE]	22.57	2	5	8	34	0.844	1.459	1.032	1.276	113.02	22.57	13	34	474	54.1	8.78
Q9Z2Q6	Septin-5 OS=Mus musculus GN=Sept5 PE=1 SV=2 - [SEPT5_MOUSE]	69.65	5	22	25	245	0.786	1.504	1.550	1.276	704.98	69.65	45	245	369	42.7	6.67
Q9DC42	CD97 antigen OS=Mus musculus GN=Cd97 PE=2 SV=1 - [Q9DC42_MOUSE]	4.29	6	2	2	2	0.773	1.039	1.014	1.276	4.60	4.29	2	2	722	80.1	7.27
Q76N33	AMSH-like protease OS=Mus musculus GN=Stambpl1 PE=2 SV=1 [STALP_MOUSE]	16.74	4	5	6	18	1.367	1.197	0.892	1.276	43.32	16.74	9	18	436	49.6	7.53
Q61235	Beta-2-syntrophin OS=Mus musculus GN=Sntb2 PE=1 SV=2 - [SNTB2_MOUSE]	22.31	1	9	11	25	0.827	2.186	1.252	1.276	68.26	22.31	17	25	520	56.3	8.69
Q8BYI9	Tenascin-R OS=Mus musculus GN=Tnr PE=1 SV=2 - [TENR_MOUSE]	41.61	4	45	46	492	1.525	1.365	1.444	1.276	1455.53	41.61	82	492	1358	149.5	4.94
Q3UUJ4	STE20-related kinase adapter protein alpha OS=Mus musculus GN=Strada PE=2 SV=1 - [STRAA_MOUSE]	22.74	4	8	8	18	1.305	1.317	1.240	1.276	59.73	22.74	12	18	431	48.2	6.43
Q60673	Receptor-type tyrosine-protein phosphatase-like N OS=Mus musculus GN=Ptprn PE=2 SV=2 - [PTPRN_MOUSE]	20.33	2	13	14	62	1.187	1.012	0.848	1.276	219.94	20.33	25	62	979	106.0	7.09
Q80TZ3	Putative tyrosine-protein phosphatase auxilin OS=Mus musculus GN=Dnajc6 PE=2 SV=2 - [AUXI_MOUSE]	40.94	4	2	30	174	0.956	0.933	1.045	1.276	546.22	40.94	56	174	938	102.2	7.23
P58389	Serine/threonine-protein phosphatase 2A activator OS=Mus musculus GN=Ppp2r4 PE=1 SV=1 - [PTPA_MOUSE]	58.51	6	15	15	120	0.736	1.285	0.947	1.277	325.33	58.51	26	120	323	36.7	6.39
Q3UNZ8	Quinone oxidoreductase-like protein 2 OS=Mus musculus PE=2 SV=1 - [QORL2_MOUSE]	41.43	2	9	9	30	1.098	1.101	0.993	1.277	112.11	41.43	14	30	350	37.8	8.90
D3Z482	Ankyrin repeat domain-containing protein 26 OS=Mus musculus GN=Ankrd26 PE=4 SV=1 - [D3Z482_MOUSE]	1.84	2	3	3	5	1.763	1.321	0.891	1.277	7.63	1.84	4	5	1681	192.1	5.95
P11276	Fibronectin OS=Mus musculus GN=Fn1 PE=1 SV=4 - [FINC_MOUSE]	26.60	1	49	49	153	1.803	2.211	0.806	1.277	447.76	26.60	87	153	2477	272.4	5.59
Q8CDV2	Calcium/calmodulin-dependent 3',5'-cyclic nucleotide phosphodiesterase 1C OS=Mus musculus GN=Pde1c PE=2 SV=1	6.97	6	2	3	16	1.328	0.775	1.485	1.277	59.01	6.97	5	16	603	68.3	6.89
Q9Z2B9	Ribosomal protein S6 kinase alpha-4 OS=Mus musculus GN=Rps6ka4 PE=1 SV=2 -	10.61	1	5	8	15	1.000	1.026	1.066	1.277	33.06	10.61	10	15	773	85.6	8.27
P18581-2	Isoform 2 of Low affinity cationic amino acid transporter 2 OS=Mus musculus GN=Slc7a2 - [CTR2_MOUSE]	11.25	3	5	6	13	0.947	0.993	1.178	1.278	47.87	11.25	9	13	658	71.7	6.28

Q8K1S4-2	Isoform 2 of Netrin receptor UNC5A OS=Mus musculus GN=Unc5a - [UNC5A_MOUSE]	12.95	3	7	7	24	1.514	1.701	1.266	1.278	76.41	12.95	13	24	842	92.9	6.71
Q9D6M3	Mitochondrial glutamate carrier 1 OS=Mus musculus GN=Slc25a22 PE=1 SV=1 -	57.28	7	12	15	154	0.608	1.701	1.343	1.278	387.88	57.28	29	154	323	34.6	9.09
P13808-2	Isoform B1 of Anion exchange protein 2 OS=Mus musculus GN=Slc4a2 -	7.11	5	4	5	8	1.458	0.663	0.891	1.278	19.83	7.11	7	8	1223	135.4	6.07
Q8BGF8	Solute carrier family 35 member D3 OS=Mus musculus GN=Slc35d3 PE=2 SV=1 -	5.69	1	2	2	5	0.568	0.654	1.424	1.279	7.06	5.69	4	5	422	44.9	7.33
P09240	Cholecystokinin OS=Mus musculus GN=Cck PE=1 SV=3 - [CCKN_MOUSE]	13.91	1	1	2	8	1.165	2.431	1.869	1.279	22.20	13.91	4	8	115	12.8	9.35
Q99LX0	Protein DJ-1 OS=Mus musculus GN=Park7 PE=1 SV=1 - [PARK7_MOUSE]	77.78	5	16	16	238	1.482	1.361	1.244	1.279	674.21	77.78	30	238	189	20.0	6.77
Q505B7-2	Isoform 2 of Protein archease OS=Mus musculus GN=Zbtb8os - [ARCH_MOUSE]	20.55	5	3	3	7	2.017	1.106	1.237	1.279	21.00	20.55	4	7	146	17.2	4.68
A2AU34	Protein Tmem74b OS=Mus musculus GN=Tmem74b PE=4 SV=1 - [A2AU34_MOUSE]	17.24	1	2	2	6	1.027	0.791	1.013	1.279	22.09	17.24	4	6	232	25.0	6.79
D3YTZ9	Protein RIC-3 OS=Mus musculus GN=Ric3 PE=2 SV=1 - [D3YTZ9_MOUSE]	12.57	4	3	3	8	1.955	1.476	1.122	1.280	25.64	12.57	4	8	366	40.2	5.05
Q8JZQ2	AFG3-like protein 2 OS=Mus musculus GN=Afg3l2 PE=1 SV=1 - [AFG32_MOUSE]	47.26	1	32	40	157	0.825	1.164	1.127	1.280	445.68	47.26	69	157	802	89.5	8.60
Q5SUR0	Phosphoribosylformylglycinamidine synthase OS=Mus musculus GN=Pfas PE=2 SV=1 - [PUR4_MOUSE]	30.22	3	28	28	109	0.869	0.938	0.905	1.280	325.81	30.22	50	109	1337	144.5	5.67
E9Q7L0	Protein Ogdhl OS=Mus musculus GN=Ogdhl PE=2 SV=1 - [E9Q7L0_MOUSE]	49.27	1	32	41	308	0.721	0.846	1.104	1.280	994.43	49.27	77	308	1029	116.5	6.84
P16858	Glyceraldehyde-3-phosphate dehydrogenase OS=Mus musculus GN=Gapdh PE=1 SV=2 - [G3P_MOUSE]	93.69	5	7	31	2111	0.683	1.066	1.208	1.280	6394.99	93.69	58	2111	333	35.8	8.25
Q9WUH1	Transmembrane protein 115 OS=Mus musculus GN=Tmem115 PE=1 SV=1 - [TM115_MOUSE]	11.43	1	2	2	4	1.521	0.817	1.272	1.280	15.56	11.43	3	4	350	38.1	7.50
Q8BH44	Coronin-2B OS=Mus musculus GN=Coro2b PE=2 SV=2 - [COR2B_MOUSE]	38.96	4	15	15	62	0.895	1.271	1.345	1.280	171.36	38.96	26	62	480	54.9	8.27
Q9QUK3	Protein CLN8 OS=Mus musculus GN=Cln8 PE=2 SV=1 - [CLN8_MOUSE]	6.25	1	1	1	4	0.925	0.827	0.957	1.280	13.13	6.25	2	4	288	33.1	8.82
Q9DBM2	Peroxisomal bifunctional enzyme OS=Mus musculus GN=Ehhadh PE=1 SV=4 - [ECHP_MOUSE]	17.97	1	10	10	24	1.240	0.817	0.917	1.281	67.89	17.97	16	24	718	78.3	9.13
P59511	A disintegrin and metalloproteinase with thrombospondin motifs 20 OS=Mus musculus GN=Adamts20 PE=2 SV=2 [illegible]	1.15	2	2	2	2	0.614	0.763	1.184	1.281	5.31	1.15	2	2	1906	211.9	7.94
Q3TE80	Protein 2410002F23Rik OS=Mus musculus GN=2410002F23Rik PE=2 SV=1 - [Q3TE80_MOUSE]	27.82	3	4	4	27	0.653	1.427	1.203	1.281	61.00	27.82	8	27	284	32.4	6.32
Q8CJ53-4	Isoform 4 of Cdc42-interacting protein 4 OS=Mus musculus GN=Trip10 -	11.72	4	5	6	9	1.278	1.218	1.283	1.281	18.47	11.72	8	9	546	62.5	5.41
P03899	NADH-ubiquinone oxidoreductase chain 3 OS=Mus musculus GN=Mtnd3 PE=3 SV=3 - [NU3M_MOUSE]	13.04	1	1	1	4	0.919	1.371	1.280	1.281	13.59	13.04	2	4	115	13.2	4.64
Q9DCW4	Electron transfer flavoprotein subunit beta OS=Mus musculus GN=Etfb PE=1 SV=3 - [ETFB_MOUSE]	60.39	1	16	16	102	1.527	1.036	1.029	1.281	308.13	60.39	30	102	255	27.6	8.10
O35435	Dihydroorotate dehydrogenase (quinone), mitochondrial OS=Mus musculus GN=Dhodh PE=2 SV=2 - [illegible]	42.28	5	11	12	29	1.116	1.196	0.981	1.281	65.71	42.28	20	29	395	42.7	9.55
P56380	Bis(5'-nucleosyl)-tetraphosphatase [asymmetrical] OS=Mus musculus GN=Nudt2 PE=1 SV=3 - [AP4A_MOUSE]	39.46	1	6	6	19	1.099	1.234	1.050	1.282	48.84	39.46	10	19	147	17.0	6.28
Q8BH64	EH domain-containing protein 2 OS=Mus musculus GN=Ehd2 PE=1 SV=1 - [EHD2_MOUSE]	29.83	2	14	16	41	0.788	1.876	0.886	1.282	112.78	29.83	26	41	543	61.1	6.51
Q9Z0E8	Solute carrier family 22 member 5 OS=Mus musculus GN=Slc22a5 PE=1 SV=1 -	5.03	4	2	2	5	0.647	1.037	0.847	1.282	11.21	5.03	3	5	557	62.7	6.79

Q9D9Y8	RIKEN cDNA 1700025E21, isoform CRA_b OS=Mus musculus GN=Ssmem1 PE=2 SV=1 - [Q9D9Y8_MOUSE]	3.69	1	1	1	1	0.986	1.036	0.933	1.282	2.63	3.69	1	1	244	27.9	7.74
P11260	Putative transposase element L1Md-A101/L1Md-A102/L1Md-A2 OS=Mus musculus PE=1 SV=2 - [TR22_MOUSE]	5.60	3	1	2	4	0.685	1.074	2.146	1.283	9.24	5.60	3	4	357	41.2	9.48
Q8CIZ8	von Willebrand factor OS=Mus musculus GN=Vwf PE=1 SV=2 - [VWF_MOUSE]	2.99	2	6	7	11	1.175	1.864	1.302	1.283	35.13	2.99	11	11	2813	309.1	5.50
Q99KX1	Myeloid leukemia factor 2 OS=Mus musculus GN=Mlf2 PE=1 SV=1 - [MLF2_MOUSE]	12.15	1	2	2	27	1.216	1.606	1.762	1.283	79.89	12.15	4	27	247	28.0	6.98
Q9D8U3	Endoplasmic reticulum resident protein 27 OS=Mus musculus GN=Erp27 PE=2 SV=1 - [ERP27_MOUSE]	5.51	1	1	1	1	1.342	0.955	0.755	1.283	2.59	5.51	1	1	272	30.7	5.00
Q5F284	Phospholipid scramblase 3 (Fragment) OS=Mus musculus GN=Plscr3 PE=2 SV=1 - [Q5F284_MOUSE]	12.05	2	2	2	7	0.702	1.207	0.944	1.283	19.75	12.05	4	7	224	24.0	6.35
P97492	Regulator of G-protein signaling 14 OS=Mus musculus GN=Rgs14 PE=1 SV=2 - [RGS14_MOUSE]	31.99	1	12	13	33	1.189	2.286	2.040	1.284	109.09	31.99	19	33	547	59.8	7.43
E9Q8A5	Protein Pms1 (Fragment) OS=Mus musculus GN=Pms1 PE=2 SV=1 - [E9Q8A5_MOUSE]	4.19	2	1	1	1	0.749	1.284	1.001	1.284	2.38	4.19	1	1	669	74.8	6.89
Q8CJ61	CKLF-like MARVEL transmembrane domain-containing protein 4 OS=Mus musculus GN=Cmtm4 PE=2 SV=1 - [CKLF4_MOUSE]	29.33	1	4	4	16	0.920	1.943	1.209	1.284	29.96	29.33	5	16	208	22.9	5.59
Q62210	Baculoviral IAP repeat-containing protein 2 OS=Mus musculus GN=Birc2 PE=1 SV=1 - [BIRC2_MOUSE]	15.03	4	8	8	13	1.949	1.402	1.154	1.285	26.17	15.03	13	13	612	69.6	6.54
Q5M8N4-2	Isoform 2 of Epimerase family protein SDR39U1 OS=Mus musculus GN=Sdr39u1 - [D39U1_MOUSE]	33.11	2	9	9	30	0.713	0.915	1.076	1.285	85.96	33.11	14	30	293	31.4	9.31
Q8CG76	Aflatoxin B1 aldehyde reductase member 2 OS=Mus musculus GN=Akr7a2 PE=1 SV=3 - [ARK72_MOUSE]	48.23	1	14	14	68	1.041	1.277	1.116	1.285	204.98	48.23	26	68	367	40.6	8.12
Q5SX79-2	Isoform 2 of Protein Shroom1 OS=Mus musculus GN=Shroom1 - [SHRM1_MOUSE]	7.63	5	4	7	8	1.771	0.845	2.074	1.285	15.84	7.63	8	8	760	83.3	7.28
Q80Z38-3	Isoform 3 of SH3 and multiple ankyrin repeat domains protein 2 OS=Mus musculus GN=Shank2 - [SHAN2_MOUSE]	44.06	1	1	40	178	1.888	1.278	1.921	1.286	560.07	44.06	70	178	1262	135.3	6.04
B7ZCI2	Nebulette OS=Mus musculus GN=Nebl PE=2 SV=1 - [B7ZCI2_MOUSE]	8.68	1	2	8	62	1.056	0.967	1.098	1.286	180.40	8.68	14	62	1014	115.9	8.51
Q61164	Transcriptional repressor CTCF OS=Mus musculus GN=Ctcf PE=1 SV=2 - [CTCF_MOUSE]	5.57	1	5	5	7	1.123	0.934	0.970	1.286	19.93	5.57	7	7	736	83.7	6.90
E9Q7T5	Histamine H3 receptor OS=Mus musculus GN=Hrh3 PE=2 SV=1 - [E9Q7T5_MOUSE]	5.41	5	2	2	11	0.456	0.826	1.519	1.286	21.84	5.41	4	11	407	45.3	9.25
E9PYH3	Ethanolamine-phosphate phospho-lyase OS=Mus musculus GN=Agxt2l1 PE=2 SV=1 - [E9PYH3_MOUSE]	33.67	2	13	14	37	1.010	0.518	0.876	1.287	104.01	33.67	26	37	493	54.8	6.87
G5E846	Peripherin OS=Mus musculus GN=Prph PE=3 SV=1 - [G5E846_MOUSE]	42.32	5	15	18	75	3.764	0.497	1.067	1.287	197.84	42.32	33	75	475	54.3	5.52
Q80UW2	F-box only protein 2 OS=Mus musculus GN=Fbxo2 PE=1 SV=1 - [FBX2_MOUSE]	39.39	1	11	11	36	2.291	0.844	1.096	1.287	119.20	39.39	19	36	297	33.7	4.28
P61922	4-aminobutyrate aminotransferase, mitochondrial OS=Mus musculus GN=Abat PE=1 SV=1 - [GABT_MOUSE]	77.00	5	28	28	942	1.617	0.700	0.856	1.287	2698.39	77.00	53	942	500	56.4	8.09
G3X8R1	Kaptin OS=Mus musculus GN=Kptn PE=4 SV=1 - [G3X8R1_MOUSE]	8.84	2	3	3	6	0.712	1.227	1.031	1.287	22.04	8.84	6	6	430	47.5	5.00
R4GML5	Dynein intermediate chain 2, axonemal (Fragment) OS=Mus musculus GN=Dnaic2 PE=4 SV=1 - [R4GML5_MOUSE]	5.16	2	1	1	1	1.755	1.427	1.830	1.288	2.48	5.16	1	1	562	64.2	5.20
Q8BYL4	Tyrosine--tRNA ligase, mitochondrial OS=Mus musculus GN=Yars2 PE=2 SV=2 - [SYYM_MOUSE]	24.15	3	10	10	32	0.750	0.784	0.978	1.288	93.38	24.15	18	32	472	52.6	9.16

Q64387	Prepronociceptin OS=Mus musculus GN=Pnoc PE=2 SV=1 - [PNOC_MOUSE]	40.64	2	7	7	23	0.989	0.958	0.841	1.288	67.54	40.64	10	23	187	20.9	8.56
Q8BUV3	Gephyrin OS=Mus musculus GN=Gphn PE=1 SV=2 - [GEPH_MOUSE]	56.31	1	1	30	159	0.920	0.472	0.898	1.288	489.76	56.31	56	159	769	83.2	5.60
Q9DB77	Cytochrome b-c1 complex subunit 2, mitochondrial OS=Mus musculus GN=Uqcrc2 PE=1 SV=1 - [QCR2_MOUSE]	58.50	1	25	26	625	0.890	1.102	1.158	1.288	1840.08	58.50	50	625	453	48.2	9.25
Q5U5V2	Hydroxylysine kinase OS=Mus musculus GN=Agphd1 PE=2 SV=2 - [AGPD1_MOUSE]	14.10	2	5	5	8	0.931	1.477	0.927	1.288	10.56	14.10	6	8	376	42.3	6.02
Q5I1X5	RelA-associated inhibitor OS=Mus musculus GN=Ppp1r13l PE=1 SV=1 - [IASPP_MOUSE]	4.98	4	3	3	4	1.398	1.270	1.015	1.289	11.85	4.98	4	4	824	88.9	6.86
Q8BN58-2	Isoform 2 of Rho GTPase-activating protein 28 OS=Mus musculus GN=Arhgap28 -	2.80	8	1	2	2	2.483	1.233	1.749	1.289	5.08	2.80	2	2	678	76.1	6.93
B1AVK5	Protein Col4a6 OS=Mus musculus GN=Col4a6 PE=2 SV=1 - [B1AVK5_MOUSE]	3.19	1	2	3	5	1.283	4.533	1.841	1.289	9.82	3.19	4	5	1691	163.9	9.06
Q9JJR8	Transmembrane protein 9B OS=Mus musculus GN=Tmem9b PE=1 SV=1 - [TMM9B_MOUSE]	30.15	3	5	7	27	0.587	1.084	1.130	1.289	63.82	30.15	12	27	199	22.6	8.18
Q3UMF9	Failed axon connections homolog OS=Mus musculus GN=Faxc PE=2 SV=1 - [FAXC_MOUSE]	8.56	1	3	3	5	0.543	1.237	1.215	1.289	12.70	8.56	5	5	409	46.8	5.49
Q9DBF1-2	Isoform 2 of Alpha-aminoadipic semialdehyde dehydrogenase OS=Mus musculus GN=Aldh7a1 - [AL7A1_MOUSE]	42.07	6	17	17	102	0.629	1.282	1.063	1.289	318.81	42.07	31	102	511	55.6	6.40
Q69Z89-2	Isoform 2 of Ras-associating and dilute domain-containing protein OS=Mus musculus GN=Radil - [illegible]	5.71	7	4	4	7	0.851	1.146	1.105	1.289	27.05	5.71	7	7	1050	114.9	6.06
E0CYX9	Coiled-coil domain-containing protein 127 (Fragment) OS=Mus musculus GN=Ccdc127 PE=2 SV=1 - [illegible]	34.45	5	6	7	25	1.028	1.432	1.238	1.289	66.60	34.45	11	25	238	27.7	8.76
Q64726	Zinc-alpha-2-glycoprotein OS=Mus musculus GN=Azgp1 PE=1 SV=2 - [ZA2G_MOUSE]	11.73	1	3	3	3	1.947	2.363	0.769	1.289	8.79	11.73	3	3	307	35.3	6.18
Q14AA6	MCG49183 OS=Mus musculus GN=1700009N14Rik PE=2 SV=1 - [Q14AA6_MOUSE]	37.04	2	1	8	81	0.686	1.047	0.967	1.290	212.06	37.04	15	81	216	24.3	7.97
Q923D2	Flavin reductase (NADPH) OS=Mus musculus GN=Blvrb PE=2 SV=3 - [BLVRB_MOUSE]	77.67	3	10	10	84	2.472	1.700	0.790	1.290	306.90	77.67	19	84	206	22.2	7.01
Q9DBB9	Carboxypeptidase N subunit 2 OS=Mus musculus GN=Cpn2 PE=1 SV=2 - [CPN2_MOUSE]	11.15	1	7	7	10	1.123	1.329	1.000	1.290	22.80	11.15	10	10	547	60.4	5.88
Q99J08	SEC14-like protein 2 OS=Mus musculus GN=Sec14l2 PE=2 SV=1 - [S14L2_MOUSE]	59.80	1	15	18	69	0.874	1.124	1.163	1.290	203.02	59.80	28	69	403	46.3	7.11
Q9WUM5	Succinyl-CoA ligase [ADP/GDP-forming] subunit alpha, mitochondrial OS=Mus musculus GN=Suclg1 PE=1 SV=4 - [SUCA_MOUSE]	45.38	1	10	10	124	1.146	0.870	1.331	1.290	416.52	45.38	18	124	346	36.1	9.39
P70392	Ras-specific guanine nucleotide-releasing factor 2 OS=Mus musculus GN=Rasgrf2 PE=1 SV=2 - [RGRF2_MOUSE]	31.54	6	33	39	98	0.622	1.449	0.969	1.291	289.14	31.54	64	98	1189	135.6	7.85
Q9WUB3	Glycogen phosphorylase, muscle form OS=Mus musculus GN=Pygm PE=1 SV=3 - [PYGM_MOUSE]	57.48	2	37	50	414	0.817	0.832	0.848	1.291	1114.84	57.48	94	414	842	97.2	7.11
Q60837	Interleukin-12 receptor subunit beta-1 OS=Mus musculus GN=Il12rb1 PE=1 SV=2 - [I12R1_MOUSE]	2.03	1	1	1	1	0.604	1.469	1.273	1.291	3.17	2.03	1	1	738	81.7	8.18
Q9D7B1	tRNA-dihydrouridine(20) synthase [NAD(P)+]-like OS=Mus musculus GN=Dus2l PE=1 SV=1 - [DUS2L_MOUSE]	14.20	2	3	4	11	1.178	1.065	1.224	1.291	10.85	14.20	4	11	493	55.3	6.34
Q8BSZ2	AP-3 complex subunit sigma-2 OS=Mus musculus GN=Ap3s2 PE=1 SV=1 - [AP3S2_MOUSE]	22.80	1	4	5	19	1.069	1.449	1.192	1.292	52.42	22.80	9	19	193	22.0	5.22
O35074	Prostacyclin synthase OS=Mus musculus GN=Ptgis PE=2 SV=1 - [PTGIS_MOUSE]	16.97	2	7	7	22	0.850	4.274	0.780	1.292	59.41	16.97	11	22	501	57.0	6.73
Q80YV4-2	Isoform 2 of Pantothenate kinase 4 OS=Mus musculus GN=Pank4 - [PANK4_MOUSE]	25.10	4	16	17	49	0.789	0.865	1.108	1.292	136.27	25.10	28	49	773	86.3	6.39

E9Q784	Protein Zc3h13 OS=Mus musculus GN=Zc3h13 PE=2 SV=1 - [E9Q784_MOUSE]	2.95	1	4	6	7	3.212	2.148	0.581	1.293	13.77	2.95	7	7	1729	203.6	9.38
P41233	ATP-binding cassette sub-family A member 1 OS=Mus musculus GN=Abca1 PE=1 SV=4 - [ABCA1_MOUSE]	5.26	1	9	11	24	1.097	1.099	1.030	1.293	76.92	5.26	17	24	2261	253.7	6.81
O88398	Advillin OS=Mus musculus GN=Avil PE=1 SV=2 - [AVIL_MOUSE]	6.47	1	3	4	5	1.259	0.955	1.304	1.293	12.13	6.47	4	5	819	92.0	5.54
O88696	Putative ATP-dependent Clp protease proteolytic subunit, mitochondrial OS=Mus musculus GN=Clpp PE=2 SV=1 - [CLPP_MOUSE]	31.99	1	8	8	73	1.060	1.249	1.041	1.293	218.10	31.99	16	73	272	29.8	7.47
Q99M15	Proline-serine-threonine phosphatase-interacting protein 2 OS=Mus musculus GN=Pstpip2 PE=1 SV=4 - [PPIP2_MOUSE]	5.39	1	1	1	2	1.244	2.750	1.295	1.293	4.56	5.39	2	2	334	38.9	8.37
B1AZR7	Protein Pcdh11x OS=Mus musculus GN=Pcdh11x PE=2 SV=1 - [B1AZR7_MOUSE]	2.80	3	3	4	5	1.571	1.524	1.250	1.293	9.64	2.80	5	5	1320	145.3	5.30
P40338	Von Hippel-Lindau disease tumor suppressor OS=Mus musculus GN=Vhl PE=2 SV=1 - [VHL_MOUSE]	7.18	1	1	1	6	1.169	1.323	1.259	1.293	18.85	7.18	2	6	181	20.8	6.11
Q62441	Transducin-like enhancer protein 4 OS=Mus musculus GN=Tle4 PE=1 SV=4 - [TLE4_MOUSE]	6.34	7	2	4	9	1.878	1.072	1.207	1.293	28.73	6.34	5	9	773	83.7	7.50
Q68FG3	Protein SPT2 homolog OS=Mus musculus GN=Spty2d1 PE=2 SV=1 - [SPT2_MOUSE]	10.26	1	6	6	12	0.599	1.027	1.644	1.294	26.37	10.26	7	12	682	74.8	9.76
P53808	Phosphatidylcholine transfer protein OS=Mus musculus GN=Pctp PE=1 SV=2 - [PPCT_MOUSE]	6.07	2	1	1	2	1.154	0.954	1.844	1.294	7.22	6.07	1	2	214	24.8	7.03
Q148W0	Probable phospholipid-transporting ATPase IC OS=Mus musculus GN=Atp8b1 PE=2 SV=2 - [AT8B1_MOUSE]	2.96	1	1	3	10	2.169	0.953	1.224	1.294	22.94	2.96	4	10	1251	143.7	7.36
Q9JJ59	ATP-binding cassette sub-family B member 9 OS=Mus musculus GN=Abcb9 PE=2 SV=1 - [ABCB9_MOUSE]	22.83	3	11	13	49	1.172	0.622	1.134	1.294	138.99	22.83	23	49	762	83.9	7.62
P08905	Lysozyme C-2 OS=Mus musculus GN=Lyz2 PE=1 SV=2 - [LYZ2_MOUSE]	14.86	2	2	2	3	2.809	2.376	0.906	1.294	11.86	14.86	3	3	148	16.7	8.81
Q8CI03	FLYWCH-type zinc finger-containing protein 1 OS=Mus musculus GN=Flywch1 PE=2 SV=2 - [FWCH1_MOUSE]	11.59	3	6	6	14	0.638	0.784	0.923	1.295	38.54	11.59	10	14	673	77.0	9.91
G3X926	MCG1407, isoform CRA_a OS=Mus musculus GN=Rpf2 PE=4 SV=1 - [G3X926_MOUSE]	13.19	2	3	3	7	1.409	1.157	0.934	1.295	20.43	13.19	6	7	273	31.5	9.98
Q00612	Glucose-6-phosphate 1-dehydrogenase X OS=Mus musculus GN=G6pdx PE=1 SV=3 - [G6PD1_MOUSE]	59.42	4	27	27	143	0.749	0.693	1.047	1.295	441.60	59.42	48	143	515	59.2	6.49
F6R4N2	Type I inositol 3,4-bisphosphate 4-phosphatase OS=Mus musculus GN=Inpp4a PE=4 SV=1 - [F6R4N2_MOUSE]	31.96	2	3	17	60	0.518	1.135	1.051	1.295	185.17	31.96	31	60	679	76.4	7.28
Q9JK92	Heat shock protein beta-8 OS=Mus musculus GN=Hspb8 PE=1 SV=1 - [HSPB8_MOUSE]	13.78	1	3	3	7	3.056	0.946	0.918	1.295	19.24	13.78	5	7	196	21.5	5.02
O35608	Angiopoietin-2 OS=Mus musculus GN=Angpt2 PE=2 SV=2 - [ANGP2_MOUSE]	2.82	1	1	1	1	1.182	0.981	1.405	1.295	0.00	2.82	1	1	496	56.5	5.57
P40240	CD9 antigen OS=Mus musculus GN=Cd9 PE=1 SV=2 - [CD9_MOUSE]	14.16	1	2	2	13	0.872	0.684	1.205	1.295	38.69	14.16	3	13	226	25.2	7.23
Q9CQQ7	ATP synthase subunit b, mitochondrial OS=Mus musculus GN=Atp5f1 PE=1 SV=1 - [AT5F1_MOUSE]	44.53	1	15	16	153	0.826	1.117	1.153	1.295	372.91	44.53	29	153	256	28.9	9.06
Q7TRE0	Olfactory receptor Olfr885 OS=Mus musculus GN=Olfr885 PE=3 SV=1 - [Q7TRE0_MOUSE]	6.80	1	1	1	1	0.557	0.732	0.954	1.296	3.19	6.80	1	1	309	34.4	8.02
Q68FG2	Protein Sptbn2 OS=Mus musculus GN=Sptbn2 PE=2 SV=1 - [Q68FG2_MOUSE]	62.65	1	119	136	925	0.740	2.006	2.243	1.296	2826.03	62.65	244	925	2388	270.8	5.86
Q91WL8	WW domain-containing oxidoreductase OS=Mus musculus GN=Wwox PE=1 SV=1 - [WWOX_MOUSE]	28.50	4	10	10	39	0.689	0.792	1.055	1.296	94.59	28.50	18	39	414	46.5	7.02

G3X8V5	RIKEN cDNA 2610206B13, isoform CRA_b OS=Mus musculus GN=Rnf219 PE=4 SV=1 - [G3X8V5_MOUSE]	8.31	4	5	5	12	1.624	0.879	1.562	1.297	31.19	8.31	7	12	722	79.9	6.33
D3Z3Z3	Voltage-dependent L-type calcium channel subunit beta-3 OS=Mus musculus GN=Cacnb3 PE=2 SV=1 - [D3Z3Z3_MOUSE]	39.96	4	12	16	63	0.746	2.325	1.659	1.297	199.84	39.96	28	63	483	54.4	6.54
Q8VCZ6	Small G protein signaling modulator 3 OS=Mus musculus GN=Sgsm3 PE=1 SV=1 -	6.40	3	4	4	9	0.619	0.755	0.753	1.297	25.90	6.40	7	9	750	85.4	5.87
P28334	5-hydroxytryptamine receptor 1B OS=Mus musculus GN=Htr1b PE=2 SV=1 - [5HT1B_MOUSE]	5.18	1	1	1	7	0.408	0.325	0.879	1.298	32.32	5.18	2	7	386	43.1	8.69
B2RQR5	Small G protein signaling modulator 1 OS=Mus musculus GN=Sgsm1 PE=2 SV=1 -	25.80	5	20	23	113	0.774	1.247	0.935	1.298	345.97	25.80	41	113	1093	123.2	5.66
Q61147	Ceruloplasmin OS=Mus musculus GN=Cp PE=1 SV=2 - [CERU_MOUSE]	32.99	5	1	26	94	1.202	2.390	0.862	1.298	275.32	32.99	46	94	1061	121.1	5.85
Q80X98	DEAH (Asp-Glu-Ala-His) box polypeptide 38 OS=Mus musculus GN=Dhx38 PE=2 SV=1 - [Q80X98_MOUSE]	2.85	1	2	3	5	1.468	1.224	1.267	1.298	16.47	2.85	5	5	1228	140.5	6.49
Q9ESN5-2	Isoform 2 of Centromere protein K OS=Mus musculus GN=Cenpk - [CENPK_MOUSE]	11.46	3	1	2	3	1.009	1.029	1.290	1.298	0.00	11.46	2	3	253	29.0	4.63
P48774	Glutathione S-transferase Mu 5 OS=Mus musculus GN=Gstm5 PE=1 SV=1 - [GSTM5_MOUSE]	60.27	6	13	15	113	0.752	1.170	1.240	1.298	309.22	60.27	28	113	224	26.6	7.21
Q8R574	Phosphoribosyl pyrophosphate synthase-associated protein 2 OS=Mus musculus GN=Prpsap2 PE=1 SV=1 - [illegible]	60.43	1	13	16	73	0.731	1.408	1.046	1.299	238.51	60.43	29	73	369	40.9	7.17
Q8BY79	Solute carrier family 35 member G1 OS=Mus musculus GN=Slc35g1 PE=2 SV=1 -	4.89	1	1	1	1	3.088	2.115	2.087	1.299	2.38	4.89	1	1	368	40.2	7.84
Q91VB8	Alpha globin 1 OS=Mus musculus GN=Hba-a1 PE=2 SV=1 - [Q91VB8_MOUSE]	69.72	2	9	10	1680	3.520	2.768	0.540	1.299	5450.00	69.72	18	1680	142	15.1	8.22
Q9R0N9	Synaptotagmin-9 OS=Mus musculus GN=Syt9 PE=1 SV=2 - [SYT9_MOUSE]	11.00	2	3	7	22	0.861	0.807	1.264	1.299	51.19	11.00	12	22	491	56.2	7.24
E9Q2M1	Protein Gm5799 OS=Mus musculus GN=Gm5799 PE=4 SV=1 - [E9Q2M1_MOUSE]	2.99	1	1	1	1	1.018	0.745	1.295	1.300	0.00	2.99	1	1	201	24.2	8.76
P70182	Phosphatidylinositol 4-phosphate 5-kinase type-1 alpha OS=Mus musculus GN=Pip5k1a PE=1 SV=2 - [PI51A_MOUSE]	21.25	5	6	9	36	0.895	1.622	1.316	1.300	98.10	21.25	16	36	546	60.4	8.59
Q7TNU7	LETM1 domain-containing protein LETM2, mitochondrial OS=Mus musculus GN=Letm2 PE=2 SV=1 - [LETM2_MOUSE]	2.92	1	2	2	3	1.914	1.261	1.637	1.300	4.28	2.92	3	3	480	54.4	9.63
Q91WS0	CDGSH iron-sulfur domain-containing protein 1 OS=Mus musculus GN=Cisd1 PE=1 SV=1 - [CISD1_MOUSE]	44.44	1	5	5	123	0.653	1.151	1.172	1.300	383.04	44.44	10	123	108	12.1	9.06
G3UX26	Voltage-dependent anion-selective channel protein 2 (Fragment) OS=Mus musculus GN=Vdac2 PE=4 SV=1 - [G3UX26_MOUSE]	70.32	4	15	16	197	0.766	1.128	1.106	1.301	592.08	70.32	27	197	283	30.4	7.58
A2ALL9	Protein Atp2b3 OS=Mus musculus GN=Atp2b3 PE=2 SV=1 - [A2ALL9_MOUSE]	40.07	2	2	47	331	0.985	0.999	1.096	1.301	903.26	40.07	79	331	1173	128.7	6.04
A2A5Z6-2	Isoform 2 of E3 ubiquitin-protein ligase SMURF2 OS=Mus musculus GN=Smurf2 -	7.21	5	4	5	15	1.144	1.203	1.047	1.301	31.96	7.21	9	15	735	84.5	7.68
Q9Z223-1	Isoform 2 of Molybdopterin synthase catalytic subunit OS=Mus musculus GN=Mocs2 - [MOC2B_MOUSE]	18.24	5	3	3	4	1.327	1.336	1.914	1.301	9.85	18.24	4	4	170	18.6	6.79
Q60687	Follitropin subunit beta OS=Mus musculus GN=Fshb PE=2 SV=1 - [FSHB_MOUSE]	6.92	1	1	1	1	0.575	0.572	1.038	1.301	1.65	6.92	1	1	130	14.9	6.67
J3QPE8	MCG16555 OS=Mus musculus GN=Vdac3-ps1 PE=4 SV=1 - [J3QPE8_MOUSE]	65.37	3	14	16	296	0.877	1.107	1.105	1.301	736.87	65.37	30	296	283	30.7	8.66
A2AHL1	Anoctamin-3 OS=Mus musculus GN=Ano3 PE=2 SV=1 - [ANO3_MOUSE]	3.77	2	2	2	7	0.879	1.065	2.099	1.301	29.59	3.77	4	7	981	114.5	8.87

Q9DC69	NADH dehydrogenase [ubiquinone] 1 alpha subcomplex subunit 9, mitochondrial OS=Mus musculus GN=Ndufa9 PE=1 SV=2 - [NDUA9_MOUSE]	54.11	2	19	19	204	0.681	0.834	1.103	1.301	577.86	54.11	37	204	377	42.5	9.74
P14106	Complement C1q subcomponent subunit B OS=Mus musculus GN=C1qb PE=1 SV=2 - [C1QB_MOUSE]	7.51	1	1	2	20	1.369	2.148	1.391	1.301	52.86	7.51	4	20	253	26.7	8.15
Q3UV95	Erythrocyte membrane protein band 4.2 OS=Mus musculus GN=Epb4.2 PE=2 SV=1 - [Q3UV95_MOUSE]	18.57	2	11	11	30	1.405	1.767	0.578	1.301	74.61	18.57	20	30	673	74.8	7.84
Q91X52	L-xylulose reductase OS=Mus musculus GN=Dcxr PE=2 SV=2 - [DCXR_MOUSE]	31.97	2	5	6	21	1.041	1.269	1.260	1.302	66.67	31.97	11	21	244	25.7	7.31
A2APX7	Protein Scn1a OS=Mus musculus GN=Scn1a PE=2 SV=1 - [A2APX7_MOUSE]	12.57	13	12	24	106	0.661	0.543	1.176	1.302	304.62	12.57	38	106	1981	225.9	5.99
Q8K0F1	TBC1 domain family member 23 OS=Mus musculus GN=Tbc1d23 PE=2 SV=1 -	17.25	2	10	10	24	0.983	1.493	1.140	1.302	71.56	17.25	14	24	684	76.4	5.36
Q91ZA3	Propionyl-CoA carboxylase alpha chain, mitochondrial OS=Mus musculus GN=Pcca PE=2 SV=2 - [PCCA_MOUSE]	51.80	6	30	33	150	0.949	1.380	1.203	1.302	463.77	51.80	58	150	724	79.9	7.25
Q9CZD0	Methylmalonic aciduria and homocystinuria type C protein homolog OS=Mus musculus GN=Mmachc PE=2 SV=2 - [MMAC_MOUSE]	3.58	1	1	1	1	1.402	1.266	1.305	1.303	0.00	3.58	1	1	279	31.6	7.55
E9PXB9	Mitochondrial import inner membrane translocase subunit Tim22 OS=Mus musculus GN=Timm22 PE=2 SV=1 - [E9PXB9_MOUSE]	16.67	2	1	1	6	0.644	1.017	1.015	1.303	19.73	16.67	2	6	90	9.2	5.36
P60191	Regulating synaptic membrane exocytosis protein 4 OS=Mus musculus GN=Rims4 PE=2 SV=1 - [RIMS4_MOUSE]	18.96	1	4	4	11	1.272	0.851	1.272	1.303	29.63	18.96	7	11	269	29.3	5.80
A2AEV7	Coiled-coil domain containing 120 OS=Mus musculus GN=Ccdc120 PE=2 SV=1 -	5.41	2	2	2	4	0.885	0.756	0.936	1.303	10.83	5.41	3	4	629	67.4	9.55
P33267	Cytochrome P450 2F2 OS=Mus musculus GN=Cyp2f2 PE=2 SV=1 - [CP2F2_MOUSE]	5.30	1	3	3	4	0.292	1.460	0.999	1.303	9.55	5.30	4	4	491	55.9	7.90
P11627	Neural cell adhesion molecule L1 OS=Mus musculus GN=L1cam PE=1 SV=1 -	28.97	3	1	32	239	0.965	1.380	0.944	1.303	682.78	28.97	56	239	1260	140.9	5.97
F6W0I8	Carbonic anhydrase 12 (Fragment) OS=Mus musculus GN=Car12 PE=2 SV=1 - [F6W0I8_MOUSE]	10.29	1	1	1	2	0.517	1.806	1.257	1.303	0.00	10.29	1	2	204	22.8	6.68
Q8BMF4	Dihydrolipoyllysine-residue acetyltransferase component of pyruvate dehydrogenase complex, mitochondrial OS=Mus musculus GN=Dlat PE=1 SV=2 - [ODP2_MOUSE]	49.53	1	24	24	325	0.906	1.164	1.270	1.303	897.49	49.53	43	325	642	67.9	8.57
Q9D771	Transmembrane protein 206 OS=Mus musculus GN=Tmem206 PE=2 SV=1 - [TM206_MOUSE]	16.57	1	3	3	10	1.113	0.742	1.524	1.303	20.02	16.57	4	10	350	40.2	7.77
Q8BHE8	Uncharacterized protein C2orf47 homolog, mitochondrial OS=Mus musculus PE=2 SV=1 - [CB047_MOUSE]	14.09	1	3	4	16	0.904	1.422	1.269	1.304	40.66	14.09	8	16	291	33.0	9.14
D3Z5M7	Peroxidasin homolog OS=Mus musculus GN=Pxdn PE=2 SV=1 - [D3Z5M7_MOUSE]	4.23	1	1	1	1	1.014	1.134	1.366	1.304	3.22	4.23	1	1	331	37.6	7.75
Q60866	Phosphotriesterase-related protein OS=Mus musculus GN=Pter PE=2 SV=1 - [PTER_MOUSE]	17.77	4	5	5	8	2.390	0.910	0.917	1.304	19.47	17.77	8	8	349	39.2	6.67
Q60952	Centrosome-associated protein CEP250 OS=Mus musculus GN=Cep250 PE=1 SV=4 - [CP250_MOUSE]	5.26	6	6	10	12	1.325	1.379	1.306	1.304	29.35	5.26	10	12	2414	276.6	5.07
Q9DB30	Phosphorylase b kinase gamma catalytic chain, liver/testis isoform OS=Mus musculus GN=Phkg2 PE=2 SV=2 - [illegible]	11.58	3	4	4	10	1.321	1.129	0.884	1.305	37.71	11.58	6	10	406	46.5	6.48
Q7TT36	Probable G-protein coupled receptor 125 OS=Mus musculus GN=Gpr125 PE=2 SV=3 - [GP125_MOUSE]	3.89	1	2	3	3	1.427	0.993	1.686	1.305	4.07	3.89	3	3	1310	144.6	8.40

P52623	Uridine-cytidine kinase 1 OS=Mus musculus GN=Uck1 PE=2 SV=2 - [UCK1_MOUSE]	37.18	3	8	10	36	1.278	1.288	0.952	1.305	87.00	37.18	17	36	277	31.0	7.88
P06684	Complement C5 OS=Mus musculus GN=C5 PE=1 SV=2 - [CO5_MOUSE]	1.37	1	3	3	4	1.639	2.474	0.821	1.306	6.37	1.37	4	4	1680	188.8	6.81
Q8BIF0-2	Isoform 2 of CD99 antigen-like protein 2 OS=Mus musculus GN=Cd99l2 - [C99L2_MOUSE]	35.51	4	5	5	29	1.196	0.964	1.577	1.306	106.56	35.51	7	29	214	22.8	4.65
E9Q7Q0	Mucin-4 OS=Mus musculus GN=Muc4 PE=2 SV=1 - [E9Q7Q0_MOUSE]	1.70	2	2	2	5	1.049	1.491	1.406	1.306	2.14	1.70	2	5	3470	368.2	7.33
Q99MR8	Methylcrotonoyl-CoA carboxylase subunit alpha, mitochondrial OS=Mus musculus GN=Mccc1 PE=2 SV=2 - [MCCA_MOUSE]	51.46	3	26	28	116	1.031	1.162	1.045	1.306	358.24	51.46	47	116	717	79.3	7.83
E9QKH0	CLIP-associating protein 1 OS=Mus musculus GN=Clasp1 PE=2 SV=1 - [E9QKH0_MOUSE]	42.12	3	2	54	223	0.982	1.110	1.085	1.307	671.87	42.12	95	223	1536	169.2	9.03
E0CXZ0	Nucleolar protein 56 OS=Mus musculus GN=Nop56 PE=2 SV=1 - [E0CXZ0_MOUSE]	39.08	2	1	9	25	0.206	0.310	0.840	1.307	60.76	39.08	16	25	261	29.1	7.18
E9Q2Y0	Protein Eid2b OS=Mus musculus GN=Eid2b PE=4 SV=1 - [E9Q2Y0_MOUSE]	15.89	1	1	2	6	1.953	1.294	1.172	1.308	10.07	15.89	4	6	151	16.2	5.24
P56389	Cytidine deaminase OS=Mus musculus GN=Cda PE=1 SV=2 - [CDD_MOUSE]	56.85	1	5	6	32	1.923	0.499	0.902	1.308	92.46	56.85	12	32	146	16.1	5.58
Q01279	Epidermal growth factor receptor OS=Mus musculus GN=Egfr PE=1 SV=1 - [EGFR_MOUSE]	12.15	2	12	13	33	1.425	1.444	0.927	1.308	93.05	12.15	20	33	1210	134.8	6.86
Q8BML1-2	Isoform 2 of Protein-methionine sulfoxide oxidase MICAL2 OS=Mus musculus GN=Mical2 - [MICA2_MOUSE]	8.17	4	3	8	14	3.312	2.763	0.448	1.308	24.99	8.17	9	14	1102	124.2	8.54
D3Z0D9	DnaJ (Hsp40) homolog, subfamily C, member 12, isoform CRA_a (Fragment) OS=Mus musculus GN=Dnajc12 PE=4 SV=1 - [D3Z0D9_MOUSE]	21.55	2	1	1	10	2.063	1.112	1.147	1.308	32.32	21.55	2	10	116	13.3	6.57
Q8CE90-5	Isoform 5 of Dual specificity mitogen-activated protein kinase kinase 7 OS=Mus musculus GN=Map2k7 - [illegible]	41.43	8	12	14	48	0.670	0.695	1.050	1.308	127.87	41.43	25	48	391	44.2	8.73
Q9DAS9	Guanine nucleotide-binding protein G(I)/G(S)/G(O) subunit gamma-12 OS=Mus musculus GN=Gng12 [illegible]	69.44	1	5	5	94	1.357	0.788	1.215	1.309	289.70	69.44	10	94	72	8.0	8.97
Q9D6J5	NADH dehydrogenase [ubiquinone] 1 beta subcomplex subunit 8, mitochondrial OS=Mus musculus GN=Ndufb8 PE=1 SV=1 - [NDUB8_MOUSE]	29.57	4	4	4	130	0.991	1.109	1.194	1.309	424.44	29.57	7	130	186	21.9	6.64
F8SLP9	PEX5-related protein OS=Mus musculus GN=Pex5l PE=2 SV=1 - [F8SLP9_MOUSE]	56.61	5	11	22	95	0.911	0.811	1.133	1.309	240.05	56.61	37	95	567	63.1	5.33
Q8BJS4-3	Isoform 3 of SUN domain-containing protein 2 OS=Mus musculus GN=Sun2 - [SUN2_MOUSE]	13.16	5	7	7	19	1.064	1.517	1.101	1.309	56.55	13.16	12	19	699	78.1	6.90
H9KUZ7	Splicing regulator RBM11 OS=Mus musculus GN=Rbm11 PE=2 SV=1 - [H9KUZ7_MOUSE]	4.62	2	1	1	1	3.122	1.286	1.232	1.309	2.03	4.62	1	1	238	27.0	8.81
F6UCX4	Protein Sptbn5 (Fragment) OS=Mus musculus GN=Sptbn5 PE=4 SV=1 - [F6UCX4_MOUSE]	1.24	1	1	2	10	0.835	0.846	1.229	1.310	21.92	1.24	3	10	1209	138.7	5.92
Q8R0Y8	Mitochondrial coenzyme A transporter SLC25A42 OS=Mus musculus GN=Slc25a42 PE=2 SV=1 - [S2542_MOUSE]	20.13	1	6	6	8	1.273	1.295	1.187	1.310	25.99	20.13	8	8	318	35.2	10.05
E9Q448	Tropomyosin alpha-1 chain OS=Mus musculus GN=Tpm1 PE=2 SV=1 - [E9Q448_MOUSE]	41.20	2	1	15	264	1.123	4.430	0.956	1.310	677.90	41.20	29	264	284	32.8	4.75
Q8C178	Acyl-coenzyme A oxidase OS=Mus musculus GN=Acox3 PE=2 SV=1 - [Q8C178_MOUSE]	16.93	3	1	10	24	0.740	0.663	0.953	1.310	52.23	16.93	16	24	697	77.8	7.11
Q8VGU2	Olfactory receptor 283 OS=Mus musculus GN=Olfr283 PE=2 SV=1 - [Q8VGU2_MOUSE]	4.53	1	1	1	1	3.221	2.264	1.343	1.310	0.00	4.53	1	1	309	34.3	8.18
Q8BH04	Phosphoenolpyruvate carboxykinase [GTP], mitochondrial OS=Mus musculus GN=Pck2 PE=2 SV=1 - [PCKGM_MOUSE]	38.13	1	20	20	81	0.758	1.231	0.917	1.311	219.92	38.13	33	81	640	70.5	7.28

P03930	ATP synthase protein 8 OS=Mus musculus GN=Mtatp8 PE=1 SV=1 - [ATP8_MOUSE]	43.28	1	3	3	19	0.757	1.010	1.234	1.311	48.69	43.28	5	19	67	7.8	9.88
P59114	Phosphorylated CTD-interacting factor 1 OS=Mus musculus GN=Pcif1 PE=1 SV=1 - [PCIF1_MOUSE]	10.91	1	5	5	13	0.609	0.943	1.115	1.311	41.94	10.91	8	13	706	80.5	7.64
P11679	Keratin, type II cytoskeletal 8 OS=Mus musculus GN=Krt8 PE=1 SV=4 - [K2C8_MOUSE]	37.14	3	12	15	64	0.843	1.083	0.965	1.311	151.48	37.14	25	64	490	54.5	5.82
Q8VIF5	Low affinity neurotensin receptor OS=Mus musculus GN=Ntsr2 PE=2 SV=1 - [Q8VIF5_MOUSE]	6.25	2	2	2	9	0.482	0.558	0.735	1.312	34.04	6.25	3	9	416	46.3	9.19
E9Q1K0	Sorting nexin-25 OS=Mus musculus GN=Snx25 PE=2 SV=1 - [E9Q1K0_MOUSE]	9.33	2	8	9	17	0.889	0.898	1.150	1.312	47.30	9.33	15	17	986	112.1	7.01
Q571H0	Nucleolar pre-ribosomal-associated protein 1 OS=Mus musculus GN=Urb1 PE=2 SV=2 - [NPA1P_MOUSE]	2.24	5	4	5	7	1.713	0.520	1.169	1.312	16.67	2.24	5	7	2274	254.5	6.96
Q9ERL9	Guanylate cyclase soluble subunit alpha-3 OS=Mus musculus GN=Gucy1a3 PE=2 SV=2 - [GCYA3_MOUSE]	31.84	1	19	19	50	0.589	1.396	1.425	1.312	146.58	31.84	30	50	691	77.5	7.17
A2AP32	NADH dehydrogenase [ubiquinone] 1 beta subcomplex subunit 6 OS=Mus musculus GN=Ndufb6 PE=2 SV=1 - [illegible]	39.18	2	4	4	74	1.147	0.950	1.191	1.313	172.57	39.18	6	74	97	11.7	9.60
Q64516	Glycerol kinase OS=Mus musculus GN=Gk PE=2 SV=2 - [GLPK_MOUSE]	34.53	5	14	18	80	0.999	1.178	1.192	1.313	249.69	34.53	31	80	559	61.2	5.87
F6TUA2	CAS1 domain-containing protein 1 (Fragment) OS=Mus musculus GN=Casd1 PE=4 SV=1 - [F6TUA2_MOUSE]	4.95	3	1	1	1	1.481	1.229	1.212	1.313	2.10	4.95	1	1	182	20.7	8.98
Q8VD33	Small glutamine-rich tetratricopeptide repeat-containing protein beta OS=Mus musculus GN=Sgtb PE=2 SV=1 - [SGTB_MOUSE]	26.97	2	1	6	30	1.652	1.523	1.884	1.313	73.05	26.97	11	30	304	33.4	4.92
Q8K2C6	NAD-dependent protein deacylase sirtuin-5, mitochondrial OS=Mus musculus GN=Sirt5 PE=1 SV=1 - [SIR5_MOUSE]	30.97	1	9	9	24	1.335	0.973	1.025	1.313	81.92	30.97	18	24	310	34.1	8.53
D3Z3F7	UDP-glucose 6-dehydrogenase OS=Mus musculus GN=Ugdh PE=2 SV=1 - [D3Z3F7_MOUSE]	10.09	3	3	4	5	0.942	0.983	0.840	1.313	13.04	10.09	5	5	426	47.4	6.23
O35955	Proteasome subunit beta type-10 OS=Mus musculus GN=Psmb10 PE=1 SV=1 - [PSB10_MOUSE]	17.95	1	4	4	8	1.278	1.271	0.892	1.314	25.83	17.95	6	8	273	29.0	6.87
Q8VEM8	Phosphate carrier protein, mitochondrial OS=Mus musculus GN=Slc25a3 PE=1 SV=1 - [MPCP_MOUSE]	35.57	2	16	16	390	0.797	1.089	1.127	1.314	1024.41	35.57	28	390	357	39.6	9.26
P42230	Signal transducer and activator of transcription 5A OS=Mus musculus GN=Stat5a PE=1 SV=1 - [STA5A_MOUSE]	20.81	3	1	15	30	1.071	0.880	0.707	1.314	92.93	20.81	21	30	793	90.8	6.39
Q64521	Glycerol-3-phosphate dehydrogenase, mitochondrial OS=Mus musculus GN=Gpd2 PE=1 SV=2 - [GPDM_MOUSE]	68.64	2	42	42	264	0.864	1.525	1.138	1.314	859.55	68.64	76	264	727	80.9	6.61
Q91W61	F-box/LRR-repeat protein 15 OS=Mus musculus GN=Fbxl15 PE=1 SV=2 - [FXL15_MOUSE]	41.00	1	9	9	25	0.692	0.941	0.875	1.314	72.49	41.00	17	25	300	33.1	7.43
Q922Q1	MOSC domain-containing protein 2, mitochondrial OS=Mus musculus GN=Marc2 PE=1 SV=1 - [MOSC2_MOUSE]	42.31	4	12	14	54	0.683	1.776	1.221	1.315	140.50	42.31	24	54	338	38.2	8.68
Q8VHE6	Dynein heavy chain 5, axonemal OS=Mus musculus GN=Dnah5 PE=1 SV=2 -	1.47	1	4	5	7	1.431	0.967	1.133	1.315	10.11	1.47	5	7	4621	527.2	6.15
E9Q6P5	Protein Ttc7b OS=Mus musculus GN=Ttc7b PE=2 SV=1 - [E9Q6P5_MOUSE]	23.25	1	19	19	67	0.743	1.230	1.199	1.315	187.74	23.25	30	67	843	94.1	6.90
P48542-2	Isoform GIRK2-1 of G protein-activated inward rectifier potassium channel 2 OS=Mus musculus GN=Kcnj6 - [illegible]	8.70	5	2	2	12	1.094	0.808	1.314	1.315	26.76	8.70	4	12	414	47.4	5.52
F6T4Z4	Decaprenyl-diphosphate synthase subunit 2 (Fragment) OS=Mus musculus GN=Pdss2 PE=4 SV=1 - [F6T4Z4_MOUSE]	7.97	1	1	1	1	1.465	1.725	1.848	1.315	0.00	7.97	1	1	138	15.2	7.93

Q61001	Laminin subunit alpha-5 OS=Mus musculus GN=Lama5 PE=1 SV=4 - [LAMA5_MOUSE]	13.13	3	35	36	119	1.210	1.907	1.058	1.316	353.47	13.13	62	119	3718	403.8	6.73
A1BN54	Alpha actinin 1a OS=Mus musculus GN=Actn1 PE=2 SV=1 - [A1BN54_MOUSE]	68.55	2	3	58	583	1.114	3.514	2.380	1.316	1544.13	68.55	107	583	887	102.7	5.48
D6RGB6	Uncharacterized protein OS=Mus musculus GN=Vps9d1 PE=2 SV=1 - [D6RGB6_MOUSE]	27.47	1	1	6	8	0.369	0.705	1.119	1.316	17.56	27.47	8	8	233	25.6	9.29
Q8BZ98-2	Isoform 2 of Dynamin-3 OS=Mus musculus GN=Dnm3 - [DYN3_MOUSE]	56.23	4	23	53	476	0.580	0.810	1.076	1.317	1210.33	56.23	93	476	859	96.7	8.24
Q9CWQ8	GATS-like protein 3 OS=Mus musculus GN=Gatsl3 PE=2 SV=1 - [GATL3_MOUSE]	5.74	1	1	1	1	1.516	1.674	1.429	1.317	0.00	5.74	1	1	331	36.6	4.98
P56213	FAD-linked sulfhydryl oxidase ALR OS=Mus musculus GN=Gfer PE=2 SV=2 - [ALR_MOUSE]	27.78	1	5	5	12	1.124	1.157	1.450	1.318	27.66	27.78	8	12	198	22.9	7.93
P34968	5-hydroxytryptamine receptor 2C OS=Mus musculus GN=Htr2c PE=2 SV=2 - [5HT2C_MOUSE]	1.96	1	1	1	1	1.072	1.350	1.456	1.318	2.40	1.96	1	1	459	51.9	9.31
Q3TY86	Apoptosis-inducing factor 3 OS=Mus musculus GN=Aifm3 PE=2 SV=1 - [AIFM3_MOUSE]	27.44	3	16	16	36	0.632	1.062	1.132	1.318	102.01	27.44	27	36	605	66.7	9.04
P54116	Erythrocyte band 7 integral membrane protein OS=Mus musculus GN=Stom PE=1 SV=3 - [STOM_MOUSE]	23.24	2	6	6	21	1.243	1.858	0.693	1.318	70.92	23.24	10	21	284	31.4	6.93
Q9D799	Methionyl-tRNA formyltransferase, mitochondrial OS=Mus musculus GN=Mtfmt PE=2 SV=3 - [FMT_MOUSE]	6.99	1	2	2	3	0.805	0.865	1.229	1.319	3.25	6.99	2	3	386	43.1	9.42
Q9Z0S1	3'(2'),5'-bisphosphate nucleotidase 1 OS=Mus musculus GN=Bpnt1 PE=1 SV=2 - [BPNT1_MOUSE]	56.82	3	13	13	72	0.724	0.973	1.213	1.319	218.19	56.82	25	72	308	33.2	5.82
E9Q8T7	Protein Dnah1 OS=Mus musculus GN=Dnah1 PE=4 SV=1 - [E9Q8T7_MOUSE]	1.13	1	3	5	6	0.667	0.913	1.012	1.319	15.53	1.13	5	6	4250	486.8	5.57
Q9D0F9	Phosphoglucomutase-1 OS=Mus musculus GN=Pgm1 PE=1 SV=4 - [PGM1_MOUSE]	65.48	2	34	35	318	0.717	0.858	0.807	1.319	903.61	65.48	66	318	562	61.4	6.57
Q8CGR6	Epidermal growth factor-binding protein type B OS=Mus musculus GN=Klk13 PE=3 SV=1 - [Q8CGR6_MOUSE]	5.80	1	1	1	1	1.664	1.284	0.993	1.320	0.00	5.80	1	1	276	30.3	8.31
F7ANP4	Protein Gm16506 (Fragment) OS=Mus musculus GN=Gm16506 PE=4 SV=1 -	9.39	1	1	1	1	2.438	0.738	1.125	1.320	0.00	9.39	1	1	213	24.7	9.09
A2A4U6	Protein Zfp334 OS=Mus musculus GN=Zfp334 PE=4 SV=1 - [A2A4U6_MOUSE]	4.42	1	2	2	5	1.028	0.718	1.048	1.320	24.56	4.42	3	5	679	78.2	9.45
Q8CI94	Glycogen phosphorylase, brain form OS=Mus musculus GN=Pygb PE=1 SV=3 - [PYGB_MOUSE]	62.75	1	37	50	483	0.665	1.262	1.053	1.320	1448.15	62.75	89	483	843	96.7	6.73
Q8C0M0-2	Isoform 2 of WD repeat-containing protein 59 OS=Mus musculus GN=Wdr59 -	5.54	4	4	4	9	1.095	1.028	0.970	1.320	15.74	5.54	6	9	974	109.7	7.80
Q08274	Dystrophia myotonica WD repeat-containing protein OS=Mus musculus GN=Dmwd PE=2 SV=3 - [DMWD_MOUSE]	35.64	1	2	17	55	1.310	1.925	1.464	1.321	181.69	35.64	27	55	665	69.8	7.24
F8WHT2	Protein phosphatase Slingshot homolog 1 OS=Mus musculus GN=Ssh1 PE=2 SV=1 - [F8WHT2_MOUSE]	6.71	3	4	4	16	1.114	1.021	1.086	1.321	47.15	6.71	7	16	999	110.7	6.83
Q8R1B0	SH3 and cysteine-rich domain-containing protein 2 OS=Mus musculus GN=Stac2 PE=2 SV=1 - [STAC2_MOUSE]	7.84	1	3	3	3	1.543	0.344	1.346	1.321	8.62	7.84	3	3	408	44.8	7.75
E9QQ30	Eukaryotic translation initiation factor 2-alpha kinase 3 OS=Mus musculus GN=Eif2ak3 PE=2 SV=1 - [illegible]	1.53	2	2	2	3	0.876	1.626	0.985	1.321	6.26	1.53	3	3	1114	124.6	5.25
Q03265	ATP synthase subunit alpha, mitochondrial OS=Mus musculus GN=Atp5a1 PE=1 SV=1 - [ATPA_MOUSE]	69.44	4	46	46	1988	0.941	1.005	1.197	1.322	5853.59	69.44	79	1988	553	59.7	9.19
Q04592	Proprotein convertase subtilisin/kexin type 5 OS=Mus musculus GN=Pcsk5 PE=1 SV=3 - [PCSK5_MOUSE]	0.59	1	1	1	2	1.029	0.825	0.897	1.322	2.75	0.59	1	2	1877	209.1	6.09
Q9Z2I8-2	Isoform 2 of Succinyl-CoA ligase [GDP-forming] subunit beta, mitochondrial OS=Mus musculus GN=Suclg2 - [illegible]	46.09	2	15	16	60	0.983	1.289	0.957	1.322	155.23	46.09	29	60	384	41.3	5.44

P26443	Glutamate dehydrogenase 1, mitochondrial OS=Mus musculus GN=Glud1 PE=1 SV=1 - [DHE3_MOUSE]	65.77	3	36	36	460	0.858	0.905	0.917	1.322	1438.33	65.77	63	460	558	61.3	8.00
Q9JK53	Prolargin OS=Mus musculus GN=Prelp PE=2 SV=2 - [PRELP_MOUSE]	19.05	1	7	7	23	0.760	7.465	0.925	1.322	66.41	19.05	12	23	378	43.3	9.54
Q8BS40	Glycolipid transfer protein domain-containing protein 1 OS=Mus musculus GN=Gltpd1 PE=2 SV=1 - [GLTD1_MOUSE]	18.52	1	3	3	11	1.083	1.223	0.984	1.323	29.13	18.52	5	11	216	24.6	6.74
Q3U269	Thiosulfate sulfurtransferase/rhodanese-like domain-containing protein 2 OS=Mus musculus GN=Tstd2 PE=2 SV=3 - [TSTD2_MOUSE]	2.22	1	1	1	1	1.775	1.204	1.254	1.323	2.28	2.22	1	1	495	55.4	7.28
B8JJ66	MCG141136, isoform CRA_a OS=Mus musculus GN=2310061I04Rik PE=4 SV=1 - [B8JJ66_MOUSE]	16.81	5	3	3	14	0.890	1.034	1.085	1.323	49.42	16.81	6	14	232	26.7	8.35
Q7M750	Opalin OS=Mus musculus GN=Opalin PE=1 SV=1 - [OPALI_MOUSE]	32.87	1	3	3	12	1.050	0.391	1.381	1.324	36.32	32.87	4	12	143	15.8	4.88
J3QPG9	Uncharacterized protein OS=Mus musculus GN=Gm21718 PE=4 SV=1 - [J3QPG9_MOUSE]	10.53	1	1	2	5	1.280	1.113	1.309	1.324	12.26	10.53	4	5	171	18.8	6.77
E0CXR8	Partitioning defective 3 homolog (Fragment) OS=Mus musculus GN=Pard3 PE=2 SV=1 - [E0CXR8_MOUSE]	16.67	10	1	12	29	0.961	0.610	0.821	1.324	88.49	16.67	19	29	990	108.8	6.51
Q9CWJ9	Bifunctional purine biosynthesis protein PURH OS=Mus musculus GN=Atic PE=1 SV=2 - [PUR9_MOUSE]	63.51	1	27	27	111	0.820	1.193	0.946	1.324	379.68	63.51	51	111	592	64.2	6.76
P08551	Neurofilament light polypeptide OS=Mus musculus GN=Nefl PE=1 SV=5 - [NFL_MOUSE]	64.46	1	31	34	945	2.474	0.263	1.364	1.325	2646.58	64.46	62	945	543	61.5	4.64
Q6PCQ0	IQ domain-containing protein E OS=Mus musculus GN=Iqce PE=2 SV=1 - [IQCE_MOUSE]	8.23	4	2	3	7	2.017	0.647	1.408	1.325	13.40	8.23	4	7	778	86.3	7.18
Q8R1G2	Carboxymethylenebutenolidase homolog OS=Mus musculus GN=Cmbl PE=2 SV=1 - [CMBL_MOUSE]	43.27	3	7	8	33	1.034	2.085	0.767	1.325	95.14	43.27	13	33	245	27.9	7.18
P11352	Glutathione peroxidase 1 OS=Mus musculus GN=Gpx1 PE=1 SV=2 - [GPX1_MOUSE]	41.79	1	6	6	29	1.633	1.261	0.855	1.325	85.75	41.79	11	29	201	22.3	7.21
O35684	Neuroserpin OS=Mus musculus GN=Serpini1 PE=1 SV=1 - [NEUS_MOUSE]	20.00	2	6	6	9	0.704	1.067	1.716	1.326	21.19	20.00	8	9	410	46.3	4.72
O89023	Tripeptidyl-peptidase 1 OS=Mus musculus GN=Tpp1 PE=1 SV=2 - [TPP1_MOUSE]	17.79	1	5	5	18	0.716	2.679	1.275	1.326	57.07	17.79	9	18	562	61.3	6.57
Q9CQC8-2	Isoform 2 of Maspardin OS=Mus musculus GN=Spg21 - [SPG21_MOUSE]	17.53	2	4	4	9	1.120	0.945	1.073	1.327	31.91	17.53	7	9	291	33.1	6.54
G5E8Q5	DCN1-like protein 2 OS=Mus musculus GN=Dcun1d2 PE=4 SV=1 - [G5E8Q5_MOUSE]	34.52	5	5	7	20	1.243	1.373	1.210	1.327	51.75	34.52	11	20	197	22.8	7.97
Q61423	Potassium voltage-gated channel subfamily A member 4 OS=Mus musculus GN=Kcna4 PE=1 SV=2 - [KCNA4_MOUSE]	11.62	2	5	7	32	0.609	1.013	1.621	1.327	104.63	11.62	12	32	654	73.4	5.29
Q9QZ08	N-acetyl-D-glucosamine kinase OS=Mus musculus GN=Nagk PE=2 SV=3 - [NAGK_MOUSE]	51.31	3	13	13	54	1.064	1.812	1.129	1.327	165.60	51.31	23	54	343	37.2	5.73
O08791-2	Isoform Short of Transcription factor COE3 OS=Mus musculus GN=Ebf3 -	2.36	8	1	1	2	0.685	0.539	0.869	1.328	6.25	2.36	2	2	551	60.4	8.81
P51163	Uroporphyrinogen-III synthase OS=Mus musculus GN=Uros PE=2 SV=1 - [HEM4_MOUSE]	26.79	2	5	5	19	1.222	1.280	1.152	1.328	72.20	26.79	8	19	265	28.5	6.54
Q3UND0-2	Isoform 2 of Src kinase-associated phosphoprotein 2 OS=Mus musculus GN=Skap2 - [SKAP2_MOUSE]	8.83	2	2	2	3	0.698	2.011	1.123	1.328	4.64	8.83	2	3	351	39.9	4.74
Q8BS45	Tetratricopeptide repeat protein 26 OS=Mus musculus GN=Ttc26 PE=2 SV=1 - [TTC26_MOUSE]	4.51	1	2	2	5	0.538	0.907	0.947	1.328	11.69	4.51	3	5	554	64.1	6.80
Q62084	Protein phosphatase 1 regulatory subunit 14B OS=Mus musculus GN=Ppp1r14b PE=1 SV=2 - [PP14B_MOUSE]	30.61	1	3	3	9	1.812	0.802	0.947	1.328	39.99	30.61	5	9	147	15.9	4.86

F6UT49	Glyceraldehyde-3-phosphate dehydrogenase OS=Mus musculus GN=Gm7251 PE=3 SV=1 - [F6UT49_MOUSE]	28.66	1	3	11	197	1.826	1.439	1.267	1.329	424.32	28.66	19	197	335	36.2	8.05
Q9ERS2	NADH dehydrogenase [ubiquinone] 1 alpha subcomplex subunit 13 OS=Mus musculus GN=Ndufa13 PE=1 SV=3 - [illegible]	63.19	1	11	12	131	0.855	1.083	1.087	1.329	365.84	63.19	21	131	144	16.8	9.48
Q9D7X1	BTB/POZ domain-containing protein KCTD4 OS=Mus musculus GN=Kctd4 PE=2 SV=1 - [KCTD4_MOUSE]	23.55	1	5	5	13	0.961	1.762	1.273	1.329	48.97	23.55	9	13	259	30.0	7.06
Q61133	Glutathione S-transferase theta-2 OS=Mus musculus GN=Gstt2 PE=2 SV=4 - [GSTT2_MOUSE]	13.11	1	2	3	10	2.146	1.636	1.015	1.329	26.45	13.11	6	10	244	27.6	7.55
A2AQ07	Tubulin beta-1 chain OS=Mus musculus GN=Tubb1 PE=1 SV=1 - [TBB1_MOUSE]	31.93	1	3	11	485	2.244	2.123	1.194	1.329	1036.46	31.93	18	485	451	50.4	5.07
Q9QUG3	Prion-like protein doppel OS=Mus musculus GN=Prnd PE=1 SV=1 - [PRND_MOUSE]	6.15	1	1	1	1	0.628	1.115	1.612	1.330	0.00	6.15	1	1	179	20.4	9.41
P21107	Tropomyosin alpha-3 chain OS=Mus musculus GN=Tpm3 PE=1 SV=3 - [TPM3_MOUSE]	32.28	2	1	14	247	2.450	1.393	2.583	1.330	640.89	32.28	24	247	285	33.0	4.72
Q3TL44	NLR family member X1 OS=Mus musculus GN=Nlrx1 PE=2 SV=1 - [NLRX1_MOUSE]	13.44	1	12	12	31	0.656	1.235	1.149	1.330	94.06	13.44	21	31	975	107.8	7.37
F7CVL0	Protein Dot1l (Fragment) OS=Mus musculus GN=Dot1l PE=2 SV=1 - [F7CVL0_MOUSE]	4.08	2	4	4	5	0.772	1.240	1.640	1.330	13.25	4.08	4	5	1202	128.6	9.72
Q9QZD8	Mitochondrial dicarboxylate carrier OS=Mus musculus GN=Slc25a10 PE=2 SV=2 -	27.87	1	9	9	26	1.272	1.256	1.061	1.330	58.88	27.87	15	26	287	31.7	9.32
F6QP10	Sn1-specific diacylglycerol lipase beta (Fragment) OS=Mus musculus GN=Daglb PE=4 SV=2 - [F6QP10_MOUSE]	4.09	2	2	2	3	0.277	0.624	1.179	1.330	7.89	4.09	3	3	587	64.6	6.44
Q8BGH2	Sorting and assembly machinery component 50 homolog OS=Mus musculus GN=Samm50 PE=1 SV=1 - [illegible]	44.56	1	20	20	94	0.765	1.071	1.134	1.331	297.88	44.56	35	94	469	51.8	6.80
Q9D8N3	Lysoplasmalogenase-like protein TMEM86A OS=Mus musculus GN=Tmem86a PE=2 SV=1 - [TM86A_MOUSE]	15.35	1	2	3	5	0.417	0.752	1.437	1.331	2.56	15.35	3	5	241	26.3	8.56
P39053-3	Isoform 3 of Dynamin-1 OS=Mus musculus GN=Dnm1 - [DYN1_MOUSE]	75.44	5	2	59	946	1.398	1.023	1.593	1.331	2459.25	75.44	110	946	851	95.9	6.76
Q9D7V9	N-acylethanolamine-hydrolyzing acid amidase OS=Mus musculus GN=Naaa PE=2 SV=2 - [NAAA_MOUSE]	9.67	3	3	3	6	1.210	2.361	1.708	1.331	13.04	9.67	4	6	362	40.0	6.46
Q9D6Y7-2	Isoform 2 of Mitochondrial peptide methionine sulfoxide reductase OS=Mus musculus GN=Msra - [illegible]	48.36	4	8	8	53	1.000	0.847	1.145	1.331	150.93	48.36	16	53	213	23.8	7.20
D3Z511	Protein eva-1 homolog A (Fragment) OS=Mus musculus GN=Eva1a PE=2 SV=1 - [D3Z511_MOUSE]	16.13	2	2	2	10	1.008	0.858	1.329	1.331	30.96	16.13	3	10	155	17.6	6.55
Q9EPX2	Papilin OS=Mus musculus GN=Papln PE=2 SV=2 - [PPN_MOUSE]	5.47	2	5	5	7	2.073	1.020	1.218	1.331	20.71	5.47	7	7	1280	138.8	7.75
A2AM67	SH2 domain-containing protein 5 OS=Mus musculus GN=Sh2d5 PE=2 SV=1 - [A2AM67_MOUSE]	19.81	3	7	7	23	0.570	1.221	1.471	1.332	81.06	19.81	13	23	429	47.4	8.47
H7BXB9	Protein 1700037H04Rik (Fragment) OS=Mus musculus GN=1700037H04Rik PE=2 SV=1 - [H7BXB9_MOUSE]	46.10	3	5	5	15	1.317	1.167	0.965	1.332	52.28	46.10	9	15	141	15.8	8.32
D3Z2L5	Beta-crystallin B3 (Fragment) OS=Mus musculus GN=Crybb3 PE=2 SV=1 -	18.46	4	1	1	1	1.534	1.415	1.116	1.332	0.00	18.46	1	1	65	6.8	4.94
Q9WV92-8	Isoform 8 of Band 4.1-like protein 3 OS=Mus musculus GN=Epb41l3 - [E41L3_MOUSE]	41.25	2	3	44	403	0.897	0.981	1.427	1.332	1243.53	41.25	80	403	1052	116.7	5.22
J3QQ15	Protein Wdr49 OS=Mus musculus GN=Wdr49 PE=4 SV=1 - [J3QQ15_MOUSE]	2.90	1	1	1	3	1.669	0.472	1.496	1.332	2.12	2.90	1	3	448	51.0	7.80
Q9CQ69	Cytochrome b-c1 complex subunit 8 OS=Mus musculus GN=Uqcrq PE=1 SV=3 - [QCR8_MOUSE]	82.93	2	6	6	101	0.690	0.940	1.143	1.333	273.63	82.93	11	101	82	9.8	10.26
Q9D0M3-2	Isoform 2 of Cytochrome c1, heme protein, mitochondrial OS=Mus musculus GN=Cyc1 -	47.37	2	10	10	307	0.885	1.054	1.179	1.333	939.47	47.37	18	307	266	29.5	7.05

Q8BHJ6	Serine incorporator 5 OS=Mus musculus GN=Serinc5 PE=2 SV=1 - [SERC5_MOUSE]	7.59	1	3	3	4	0.645	0.360	1.127	1.334	9.54	7.59	4	4	461	51.8	8.07
B0V2H4	V-type proton ATPase subunit G 2 OS=Mus musculus GN=Atp6v1g2 PE=2 SV=1 -	48.39	2	1	3	101	1.195	0.938	1.837	1.334	283.36	48.39	5	101	93	10.9	10.24
E9QAS3	Tyrosine-protein phosphatase non-receptor type 22 OS=Mus musculus GN=Ptpn22 PE=2 SV=1 - [E9QAS3_MOUSE]	5.45	2	2	2	4	1.606	1.339	1.605	1.334	0.00	5.45	2	4	715	80.1	6.16
L7N2D1	Uncharacterized protein OS=Mus musculus GN=Gm9312 PE=4 SV=1 - [L7N2D1_MOUSE]	15.32	4	1	2	5	3.148	1.293	2.095	1.334	9.34	15.32	4	5	111	11.7	7.25
Q8BIG7	Catechol O-methyltransferase domain-containing protein 1 OS=Mus musculus GN=Comtd1 PE=2 SV=1 - [CMTD1_MOUSE]	41.98	2	7	7	28	0.812	0.992	0.875	1.334	79.14	41.98	11	28	262	28.9	8.32
E9Q7N9	Protein Dnah11 OS=Mus musculus GN=Dnah11 PE=4 SV=2 - [E9Q7N9_MOUSE]	1.00	2	2	5	9	0.755	1.005	1.592	1.334	15.48	1.00	8	9	4488	516.0	6.14
Q924W5	Structural maintenance of chromosomes protein 6 OS=Mus musculus GN=Smc6 PE=2 SV=1 - [SMC6_MOUSE]	1.46	1	2	2	2	1.833	0.570	2.048	1.334	4.04	1.46	2	2	1097	127.1	7.17
Q60991	25-hydroxycholesterol 7-alpha-hydroxylase OS=Mus musculus GN=Cyp7b1 PE=2 SV=2 - [CP7B1_MOUSE]	8.28	1	3	4	17	0.847	0.912	1.108	1.335	39.30	8.28	7	17	507	58.4	9.09
Q91XF0	Pyridoxine-5'-phosphate oxidase OS=Mus musculus GN=Pnpo PE=1 SV=1 - [PNPO_MOUSE]	40.23	2	9	9	48	1.669	0.825	1.122	1.335	127.11	40.23	15	48	261	30.1	8.22
P08071	Lactotransferrin OS=Mus musculus GN=Ltf PE=2 SV=4 - [TRFL_MOUSE]	8.06	1	3	4	8	3.545	1.537	1.112	1.335	9.07	8.06	4	8	707	77.8	8.53
P62880	Guanine nucleotide-binding protein G(I)/G(S)/G(T) subunit beta-2 OS=Mus musculus GN=Gnb2 PE=1 SV=3 - [GBB2_MOUSE]	60.29	6	8	16	263	1.136	1.330	1.248	1.336	962.14	60.29	28	263	340	37.3	6.00
E5FYH1	Testis- and ovary-specific PAZ domain-containing protein 1 OS=Mus musculus GN=Topaz1 PE=2 SV=1 - [TOPZ1_MOUSE]	1.57	1	2	2	2	5.173	1.929	1.963	1.336	2.10	1.57	2	2	1653	185.4	7.72
D6RI23	Major facilitator superfamily domain-containing protein 10 OS=Mus musculus GN=Mfsd10 PE=2 SV=1 - [D6RI23_MOUSE]	10.89	4	1	1	4	1.211	1.549	1.354	1.336	9.84	10.89	2	4	101	11.3	9.74
Q9DBG5	Perilipin-3 OS=Mus musculus GN=Plin3 PE=1 SV=1 - [PLIN3_MOUSE]	46.68	1	14	14	78	1.698	0.838	1.338	1.336	241.16	46.68	23	78	437	47.2	5.62
Q8BHJ9	Pre-mRNA-splicing factor SLU7 OS=Mus musculus GN=Slu7 PE=1 SV=1 - [SLU7_MOUSE]	12.48	1	5	5	10	19.127	1.349	1.989	1.337	23.51	12.48	6	10	585	68.0	7.08
H3BJY2	Arf-GAP with SH3 domain, ANK repeat and PH domain-containing protein 1 OS=Mus musculus GN=Asap1 PE=2 SV=1 - [H3BJY2_MOUSE]	42.48	9	4	37	151	0.976	1.177	0.993	1.337	439.30	42.48	61	151	1144	126.9	7.43
E9Q3L2	Protein Pi4ka OS=Mus musculus GN=Pi4ka PE=4 SV=1 - [E9Q3L2_MOUSE]	33.61	6	62	62	211	0.535	1.112	1.247	1.338	602.84	33.61	107	211	2044	231.2	6.87
P47738	Aldehyde dehydrogenase, mitochondrial OS=Mus musculus GN=Aldh2 PE=1 SV=1 - [ALDH2_MOUSE]	57.42	3	22	25	190	0.700	1.387	0.908	1.338	634.46	57.42	43	190	519	56.5	7.62
Q8CC27-4	Isoform 4 of Voltage-dependent L-type calcium channel subunit beta-2 OS=Mus musculus GN=Cacnb2 - [CACB2_MOUSE]	26.53	6	6	12	51	0.845	1.106	1.362	1.339	129.63	26.53	22	51	573	64.7	9.07
P31650	Sodium- and chloride-dependent GABA transporter 3 OS=Mus musculus GN=Slc6a11 PE=1 SV=2 - [S6A11_MOUSE]	15.31	1	9	11	168	0.718	0.429	0.755	1.339	489.93	15.31	21	168	627	69.9	6.98
B1AUE5	Peroxisome biogenesis factor 10 OS=Mus musculus GN=Pex10 PE=2 SV=1 - [PEX10_MOUSE]	8.33	1	2	2	7	0.785	1.148	1.398	1.339	8.20	8.33	3	7	324	37.1	9.94
Q9JKS4-3	Isoform 3 of LIM domain-binding protein 3 OS=Mus musculus GN=Ldb3 - [LDB3_MOUSE]	15.89	7	8	9	19	1.031	3.030	1.416	1.340	54.15	15.89	13	19	661	70.7	7.96
P20352	Tissue factor OS=Mus musculus GN=F3 PE=1 SV=2 - [TF_MOUSE]	30.61	1	7	7	18	1.148	1.290	1.287	1.340	50.25	30.61	12	18	294	32.9	9.32
Q9CXI0	2-methoxy-6-polyprenyl-1,4-benzoquinol methylase, mitochondrial OS=Mus musculus GN=Coq5 PE=2 SV=2 - [COQ5_MOUSE]	40.98	2	10	10	32	1.265	1.643	1.229	1.340	96.39	40.98	18	32	327	37.3	7.49

Q62219-2	Isoform 2 of Transforming growth factor beta-1-induced transcript 1 protein OS=Mus musculus GN=Tgfb1i1 - [illegible]	26.80	11	7	7	37	1.335	2.482	1.276	1.341	112.21	26.80	12	37	444	48.2	7.23
P56564	Excitatory amino acid transporter 1 OS=Mus musculus GN=Slc1a3 PE=1 SV=2 -	18.23	2	5	9	229	0.443	1.518	1.095	1.341	797.15	18.23	14	229	543	59.6	8.40
Q9QUT0	Ammonium transporter Rh type A OS=Mus musculus GN=Rhag PE=2 SV=1 - [RHAG_MOUSE]	9.82	1	2	2	9	1.243	0.470	1.679	1.341	11.28	9.82	2	9	438	47.8	6.47
Q9CZP5	Mitochondrial chaperone BCS1 OS=Mus musculus GN=Bcs1l PE=1 SV=1 - [BCS1_MOUSE]	44.98	1	16	16	48	0.618	1.155	1.172	1.341	148.91	44.98	28	48	418	47.4	7.93
Q9CYS6	Uncharacterized protein C2orf72 homolog OS=Mus musculus PE=2 SV=2 - [CB072_MOUSE]	31.82	2	4	4	14	0.437	1.010	1.143	1.342	33.92	31.82	6	14	286	30.3	6.05
P56388-2	Isoform Short of Cocaine- and amphetamine-regulated transcript protein OS=Mus musculus GN=Cartpt - [illegible]	43.97	1	1	6	16	0.880	0.653	1.196	1.343	49.50	43.97	11	16	116	12.8	8.25
O88935	Synapsin-1 OS=Mus musculus GN=Syn1 PE=1 SV=2 - [SYN1_MOUSE]	73.65	3	38	41	1507	1.009	1.588	1.627	1.343	4429.29	73.65	78	1507	706	74.1	9.80
Q9JJG7	Calcium-binding protein 8 OS=Mus musculus GN=Caln1 PE=2 SV=1 - [CABP8_MOUSE]	7.76	2	1	1	1	1.242	1.069	1.611	1.343	2.86	7.76	1	1	219	24.8	4.84
P56135	ATP synthase subunit f, mitochondrial OS=Mus musculus GN=Atp5j2 PE=1 SV=3 - [ATPK_MOUSE]	32.95	2	3	3	125	0.746	0.854	1.161	1.343	332.36	32.95	5	125	88	10.3	9.95
A2AE27	AMP deaminase 2 OS=Mus musculus GN=Ampd2 PE=4 SV=1 - [A2AE27_MOUSE]	33.13	7	28	28	109	1.017	0.890	1.026	1.343	304.33	33.13	45	109	824	94.6	6.51
Q60976	Jerky protein OS=Mus musculus GN=Jrk PE=2 SV=2 - [JERKY_MOUSE]	2.15	1	1	1	1	1.896	1.066	1.227	1.344	2.07	2.15	1	1	557	62.4	8.95
P51881	ADP/ATP translocase 2 OS=Mus musculus GN=Slc25a5 PE=1 SV=3 - [ADT2_MOUSE]	66.11	1	10	24	594	0.722	0.783	1.231	1.344	1594.31	66.11	46	594	298	32.9	9.73
Q91W92	Cdc42 effector protein 1 OS=Mus musculus GN=Cdc42ep1 PE=1 SV=1 - [BORG5_MOUSE]	19.07	1	4	4	17	2.024	0.609	1.342	1.344	65.16	19.07	8	17	409	43.1	7.21
Q8BH95	Enoyl-CoA hydratase, mitochondrial OS=Mus musculus GN=Echs1 PE=1 SV=1 - [ECHM_MOUSE]	51.03	2	17	17	118	1.311	1.234	1.118	1.344	360.43	51.03	33	118	290	31.5	8.48
Q66L42	Mitogen-activated protein kinase kinase kinase 10 OS=Mus musculus GN=Map3k10 PE=2 SV=2 [M3K10_MOUSE]	5.43	3	3	4	9	0.805	1.275	1.190	1.345	20.22	5.43	7	9	940	103.1	6.87
Q3U3R4-2	Isoform 2 of Lipase maturation factor 1 OS=Mus musculus GN=Lmf1 -	13.58	7	7	7	17	1.035	1.297	1.185	1.345	39.08	13.58	11	17	567	65.0	9.29
E9PV80	Nuclear receptor coactivator 2 OS=Mus musculus GN=Ncoa2 PE=4 SV=1 - [E9PV80_MOUSE]	7.75	3	8	8	19	2.688	1.338	1.172	1.345	43.92	7.75	10	19	1393	151.3	6.62
P20491	High affinity immunoglobulin epsilon receptor subunit gamma OS=Mus musculus GN=Fcer1g PE=1 SV=1 - [illegible]	25.58	1	3	3	7	0.977	1.700	1.233	1.345	19.42	25.58	6	7	86	9.6	7.97
J3QP51	Endophilin-A3 (Fragment) OS=Mus musculus GN=Sh3gl3 PE=4 SV=1 - [J3QP51_MOUSE]	68.12	1	1	15	51	1.445	1.238	1.146	1.345	159.94	68.12	25	51	229	24.9	5.81
Q8CGA0	Protein phosphatase 1F OS=Mus musculus GN=Ppm1f PE=2 SV=1 - [PPM1F_MOUSE]	40.71	1	12	12	46	0.799	1.107	0.974	1.346	141.57	40.71	21	46	452	49.6	5.30
Q5F226	Protocadherin Fat 2 OS=Mus musculus GN=Fat2 PE=2 SV=1 - [FAT2_MOUSE]	3.75	1	8	8	9	1.361	1.258	0.904	1.346	10.27	3.75	8	9	4351	479.8	5.12
Q99MN9	Propionyl-CoA carboxylase beta chain, mitochondrial OS=Mus musculus GN=Pccb PE=1 SV=2 - [PCCB_MOUSE]	48.80	3	20	20	96	0.700	1.184	1.112	1.346	299.69	48.80	37	96	541	58.4	7.66
Q8C522	Endonuclease domain-containing 1 protein OS=Mus musculus GN=Endod1 PE=1 SV=2 - [ENDD1_MOUSE]	33.33	1	12	12	135	0.893	0.548	0.909	1.346	394.35	33.33	22	135	501	55.2	6.16
P70404	Isocitrate dehydrogenase [NAD] subunit gamma 1, mitochondrial OS=Mus musculus GN=Idh3g PE=1 SV=1 - [IDHG1_MOUSE]	51.91	1	16	17	182	0.722	0.895	1.160	1.346	567.50	51.91	31	182	393	42.8	9.01
E9PX91	Phosphatidylserine decarboxylase proenzyme OS=Mus musculus GN=Pisd PE=2 SV=1 - [E9PX91_MOUSE]	14.40	6	5	5	12	0.643	0.900	1.037	1.347	24.35	14.40	10	12	375	42.7	9.60

G3UVV4	Hexokinase 1, isoform CRA_f OS=Mus musculus GN=Hk1 PE=3 SV=1 - [G3UVV4_MOUSE]	52.45	10	44	51	620	0.574	1.314	1.153	1.347	1826.72	52.45	93	620	917	101.8	6.67
Q6P1E8	EF-hand calcium-binding domain-containing protein 6 OS=Mus musculus GN=Efcab6 PE=1 SV=2 - [EFCB6_MOUSE]	1.19	1	1	1	2	11.654	2.339	2.810	1.347	2.95	1.19	1	2	1516	175.7	8.87
Q000W5	Guanylate-binding protein 10 OS=Mus musculus GN=Gbp10 PE=2 SV=1 - [Q000W5_MOUSE]	9.33	2	2	4	6	0.754	1.043	0.895	1.348	15.02	9.33	6	6	611	70.4	6.46
P63115	Asc-type amino acid transporter 1 OS=Mus musculus GN=Slc7a10 PE=1 SV=1 -	8.30	4	3	3	12	1.119	0.428	0.920	1.349	38.54	8.30	4	12	530	57.5	8.44
F7BNZ5	Breast carcinoma-amplified sequence 1 homolog (Fragment) OS=Mus musculus GN=Bcas1 PE=4 SV=1 - [F7BNZ5_MOUSE]	58.31	1	1	23	236	1.133	0.167	2.169	1.349	658.13	58.31	39	236	379	40.5	9.03
O88502	High affinity cAMP-specific and IBMX-insensitive 3',5'-cyclic phosphodiesterase 8A OS=Mus musculus GN=Pde8a PE=1 SV=1 - [PDE8A_MOUSE]	12.88	2	6	8	13	0.907	0.529	1.191	1.349	31.29	12.88	11	13	823	93.1	5.85
B1AQZ0	Septin-8 OS=Mus musculus GN=Sept8 PE=2 SV=1 - [B1AQZ0_MOUSE]	52.89	7	15	20	249	1.252	1.068	1.545	1.350	701.64	52.89	38	249	484	55.8	6.10
P41539	Protachykinin-1 OS=Mus musculus GN=Tac1 PE=2 SV=1 - [TKN1_MOUSE]	21.54	2	3	3	19	1.605	0.914	1.262	1.350	60.12	21.54	5	19	130	15.0	9.23
Q8BMF3	NADP-dependent malic enzyme, mitochondrial OS=Mus musculus GN=Me3 PE=1 SV=2 - [MAON_MOUSE]	38.41	2	19	20	119	1.043	3.164	1.308	1.350	344.06	38.41	35	119	604	67.1	7.83
E9PVS5	Mitochondrial inner membrane protein (Fragment) OS=Mus musculus GN=Immt PE=2 SV=1 - [E9PVS5_MOUSE]	67.55	1	2	13	63	0.818	1.154	1.306	1.351	166.58	67.55	23	63	188	19.7	9.09
Q80T23-2	Isoform 2 of Synaptotagmin-like protein 5 OS=Mus musculus GN=Sytl5 -	10.12	2	4	6	11	1.463	1.168	1.503	1.352	32.14	10.12	9	11	731	81.7	8.78
Q9EPR4	Solute carrier family 23 member 2 OS=Mus musculus GN=Slc23a2 PE=1 SV=2 -	3.70	2	2	2	2	0.970	1.206	0.990	1.352	5.99	3.70	2	2	648	70.0	7.50
P09026	Homeobox protein Hox-B3 OS=Mus musculus GN=Hoxb3 PE=2 SV=4 - [HXB3_MOUSE]	4.16	2	2	2	2	5.290	2.139	1.654	1.353	5.23	4.16	2	2	433	44.3	9.16
Q91YI0	Argininosuccinate lyase OS=Mus musculus GN=Asl PE=2 SV=1 - [ARLY_MOUSE]	31.90	5	14	15	45	0.993	1.325	1.035	1.353	129.12	31.90	25	45	464	51.7	6.96
A8Y5N8	Pleckstrin homology domain-containing family F member 2 (Fragment) OS=Mus musculus GN=Plekhf2 PE=2 SV=1 - [A8Y5N8_MOUSE]	15.74	2	1	2	3	0.605	0.430	1.118	1.353	10.00	15.74	2	3	197	22.1	9.45
Q924X2	Carnitine O-palmitoyltransferase 1, muscle isoform OS=Mus musculus GN=Cpt1b PE=2 SV=1 - [CPT1B_MOUSE]	3.11	1	1	1	1	0.970	2.037	1.755	1.353	0.00	3.11	1	1	772	88.2	8.38
Q91XY7	Protein Pcdhgb6 OS=Mus musculus GN=Pcdhga12 PE=2 SV=1 - [Q91XY7_MOUSE]	15.67	1	5	10	33	2.177	1.356	1.072	1.354	84.62	15.67	15	33	932	100.8	4.97
Q8BMD8	Calcium-binding mitochondrial carrier protein SCaMC-1 OS=Mus musculus GN=Slc25a24 PE=2 SV=1 - [illegible]	17.26	1	8	8	15	1.765	2.533	1.156	1.354	36.40	17.26	14	15	475	52.9	7.43
G3UX44	Estradiol 17-beta-dehydrogenase 8 (Fragment) OS=Mus musculus GN=H2-Ke6 PE=2 SV=1 - [illegible]	27.78	3	5	5	31	0.851	1.137	1.089	1.354	99.22	27.78	9	31	234	24.0	8.79
F6X767	Inositol polyphosphate multikinase (Fragment) OS=Mus musculus GN=Ipmk PE=4 SV=1 - [F6X767_MOUSE]	47.83	1	1	1	1	1.279	1.139	1.028	1.355	0.00	47.83	1	1	23	2.6	5.72
Q9ERE3	Serine/threonine-protein kinase Sgk3 OS=Mus musculus GN=Sgk3 PE=1 SV=1 - [SGK3_MOUSE]	1.81	1	1	1	2	2.235	1.079	1.455	1.355	4.26	1.81	2	2	496	57.1	7.15
O35452	Protein Tnxb OS=Mus musculus GN=Tnxb PE=4 SV=1 - [O35452_MOUSE]	4.07	2	5	5	12	1.308	1.299	1.372	1.356	12.86	4.07	7	12	4006	435.2	5.22
Q9D8B7	Junctional adhesion molecule C OS=Mus musculus GN=Jam3 PE=1 SV=2 - [JAM3_MOUSE]	30.32	2	9	9	40	1.009	0.372	1.218	1.356	108.92	30.32	16	40	310	34.8	7.03
Q04573	Neuropeptide Y receptor type 1 OS=Mus musculus GN=Npy1r PE=2 SV=1 - [NPY1R_MOUSE]	2.09	1	1	1	2	1.690	3.874	1.826	1.356	5.77	2.09	2	2	382	44.0	8.21

Q61292	Laminin subunit beta-2 OS=Mus musculus GN=Lamb2 PE=2 SV=2 - [LAMB2_MOUSE]	26.51	1	39	41	177	1.201	1.686	1.257	1.356	532.28	26.51	75	177	1799	196.5	6.67
Q8VCL5	Solute carrier family 17 member 9 OS=Mus musculus GN=Slc17a9 PE=1 SV=3 -	4.03	1	1	1	1	2.348	1.958	1.476	1.356	3.52	4.03	1	1	447	48.4	8.50
Q9WV54	Acid ceramidase OS=Mus musculus GN=Asah1 PE=1 SV=1 - [ASAH1_MOUSE]	31.47	3	12	12	57	1.319	1.352	1.245	1.357	154.89	31.47	24	57	394	44.6	8.46
Q3UGX3	N-acetylaspartate synthetase OS=Mus musculus GN=Nat8l PE=1 SV=1 - [NAT8L_MOUSE]	9.70	1	3	3	4	1.121	0.686	1.146	1.357	9.03	9.70	4	4	299	32.8	8.53
Q923K4	tRNA modification GTPase GTPBP3, mitochondrial OS=Mus musculus GN=Gtpbp3 PE=2 SV=1 - [GTPB3_MOUSE]	18.50	4	4	4	9	0.637	1.378	0.931	1.357	26.22	18.50	7	9	492	52.1	6.05
Q9Z2I9	Succinyl-CoA ligase [ADP-forming] subunit beta, mitochondrial OS=Mus musculus GN=Sucla2 PE=1 SV=2 - [illegible]	56.37	1	25	25	313	0.744	1.039	1.177	1.358	893.20	56.37	49	313	463	50.1	7.01
Q6PFE7-3	Isoform 3 of Tomoregulin-1 OS=Mus musculus GN=Tmeff1 - [TEFF1_MOUSE]	15.36	4	6	6	9	0.922	0.906	1.029	1.358	21.79	15.36	8	9	371	40.0	6.95
B7ZCL8	55 kDa erythrocyte membrane protein OS=Mus musculus GN=Mpp1 PE=2 SV=1 - [B7ZCL8_MOUSE]	42.38	7	14	14	41	1.269	0.931	1.068	1.358	131.50	42.38	23	41	446	49.8	7.20
P08121	Collagen alpha-1(III) chain OS=Mus musculus GN=Col3a1 PE=1 SV=4 - [CO3A1_MOUSE]	14.21	1	8	8	10	0.711	10.535	0.977	1.358	26.35	14.21	9	10	1464	138.9	6.52
Q91ZB5	Mas-related G-protein coupled receptor member G OS=Mus musculus GN=Mrgprg PE=2 SV=1 - [MRGRG_MOUSE]	4.50	1	1	1	1	1.610	1.806	1.529	1.359	1.60	4.50	1	1	289	32.0	8.81
Q3UGH2	Integrin beta-1-binding protein 1 OS=Mus musculus GN=Itgb1bp1 PE=2 SV=1 -	10.11	3	1	2	2	1.078	1.240	2.207	1.359	6.40	10.11	2	2	188	20.5	7.40
E9Q4A5	Protein Gm1818 OS=Mus musculus GN=Gm1818 PE=4 SV=1 - [E9Q4A5_MOUSE]	3.54	1	1	1	1	0.851	0.273	1.542	1.359	0.00	3.54	1	1	396	45.8	9.09
O35387	HCLS1-associated protein X-1 OS=Mus musculus GN=Hax1 PE=1 SV=1 - [HAX1_MOUSE]	21.79	1	4	4	13	0.934	1.487	1.101	1.360	57.86	21.79	7	13	280	31.6	4.91
Q8JZR0	Long-chain-fatty-acid--CoA ligase 5 OS=Mus musculus GN=Acsl5 PE=2 SV=1 - [ACSL5_MOUSE]	12.59	1	7	9	21	1.039	1.360	1.419	1.360	48.03	12.59	15	21	683	76.2	7.09
Q3TJD7	PDZ and LIM domain protein 7 OS=Mus musculus GN=Pdlim7 PE=2 SV=1 -	23.85	7	9	10	24	0.866	3.413	1.022	1.360	72.35	23.85	15	24	457	50.1	8.47
Q9QYG0-2	Isoform 2 of Protein NDRG2 OS=Mus musculus GN=Ndrg2 - [NDRG2_MOUSE]	69.75	1	1	17	292	0.268	0.823	0.855	1.360	875.55	69.75	31	292	357	39.3	5.63
O88703	Potassium/sodium hyperpolarization-activated cyclic nucleotide-gated channel 2 OS=Mus musculus GN=Hcn2 PE=1 SV=1 - [HCN2_MOUSE]	32.56	1	15	24	132	1.192	0.513	1.179	1.360	354.74	32.56	46	132	863	94.7	8.73
Q9EQR6	Fanconi anemia group G protein homolog OS=Mus musculus GN=Fancg PE=2 SV=1 - [FANCG_MOUSE]	13.16	1	3	3	4	4.872	3.148	1.117	1.361	2.05	13.16	3	4	623	68.5	5.38
A3KGV1-2	Isoform 2 of Outer dense fiber protein 2 OS=Mus musculus GN=Odf2 - [ODFP2_MOUSE]	5.33	10	3	3	4	1.453	1.191	1.318	1.361	11.12	5.33	3	4	806	93.2	7.06
J3QK54	Protein Fignl2 OS=Mus musculus GN=Fignl2 PE=4 SV=1 - [J3QK54_MOUSE]	1.09	1	1	1	1	0.772	0.988	1.086	1.362	2.01	1.09	1	1	644	65.9	6.62
P48758	Carbonyl reductase [NADPH] 1 OS=Mus musculus GN=Cbr1 PE=1 SV=3 - [CBR1_MOUSE]	79.42	1	19	21	141	0.759	1.534	1.078	1.362	392.76	79.42	39	141	277	30.6	8.31
Q9D5I4	Tctex1 domain-containing protein 1 OS=Mus musculus GN=Tctex1d1 PE=2 SV=1 -	5.20	1	1	1	1	2.383	1.547	1.058	1.362	2.84	5.20	1	1	173	19.7	6.93
O88848	ADP-ribosylation factor-like protein 6 OS=Mus musculus GN=Arl6 PE=2 SV=1 - [ARL6_MOUSE]	55.38	3	7	8	21	0.878	1.146	1.073	1.363	59.98	55.38	12	21	186	20.9	8.25
G5E869	MCG133876, isoform CRA_a OS=Mus musculus GN=Zfp142 PE=4 SV=1 - [G5E869_MOUSE]	0.92	3	1	2	5	0.774	2.069	1.759	1.363	11.98	0.92	3	5	1843	206.1	7.88
Q640R3	Hepatocyte cell adhesion molecule OS=Mus musculus GN=Hepacam PE=1 SV=2 -	26.79	1	9	9	71	0.842	0.600	1.220	1.364	245.34	26.79	16	71	418	46.3	9.42
P29391	Ferritin light chain 1 OS=Mus musculus GN=Ftl1 PE=1 SV=2 - [FRIL1_MOUSE]	52.46	4	7	7	39	1.892	1.833	1.348	1.364	115.82	52.46	14	39	183	20.8	6.00

K7N759	Protein Gm8356 OS=Mus musculus GN=Gm8356 PE=4 SV=1 - [K7N759_MOUSE]	5.39	3	1	1	1	1.639	0.574	1.928	1.364	2.84	5.39	1	1	204	24.2	8.21
P70452	Syntaxin-4 OS=Mus musculus GN=Stx4 PE=1 SV=1 - [STX4_MOUSE]	35.57	2	10	10	41	1.377	0.946	1.226	1.364	104.70	35.57	19	41	298	34.1	6.14
D3YTP3	Protein Mtx3 OS=Mus musculus GN=Mtx3 PE=4 SV=2 - [D3YTP3_MOUSE]	32.05	1	8	8	39	0.840	0.706	1.011	1.364	122.38	32.05	14	39	312	34.9	7.99
Q9DB41-2	Isoform 2 of Mitochondrial glutamate carrier 2 OS=Mus musculus GN=Slc25a18 - [GHC2_MOUSE]	32.05	2	8	11	74	0.721	0.900	0.815	1.365	192.47	32.05	21	74	312	33.3	9.29
Q9CWU6	Ubiquinol-cytochrome c reductase complex chaperone CBP3 homolog OS=Mus musculus GN=Uqcc PE=2 SV=1 - [UQCC_MOUSE]	29.15	8	7	7	34	0.837	0.993	1.104	1.366	89.32	29.15	12	34	295	34.3	8.48
E9PV14	Band 4.1-like protein 1 (Fragment) OS=Mus musculus GN=Epb4.1l1 PE=2 SV=1 -	52.07	1	22	44	254	2.270	0.575	1.549	1.367	794.72	52.07	77	254	1064	117.1	5.92
E9Q7W7	Protein Gm5155 OS=Mus musculus GN=Gm5155 PE=4 SV=2 - [E9Q7W7_MOUSE]	4.01	3	2	2	4	1.488	1.200	1.090	1.367	0.00	4.01	2	4	947	106.3	6.52
P01837	Ig kappa chain C region OS=Mus musculus PE=1 SV=1 - [IGKC_MOUSE]	49.06	1	5	5	25	2.529	4.941	0.980	1.367	61.67	49.06	10	25	106	11.8	5.41
Q7TNG8	Probable D-lactate dehydrogenase, mitochondrial OS=Mus musculus GN=Ldhd PE=1 SV=1 - [LDHD_MOUSE]	13.64	1	6	6	20	1.014	1.078	1.117	1.367	54.78	13.64	11	20	484	51.8	6.62
Q3UXL4	Centrosomal protein kizuna OS=Mus musculus GN=Plk1s1 PE=1 SV=1 - [KIZ_MOUSE]	2.88	1	2	2	4	1.909	1.479	1.096	1.367	10.13	2.88	4	4	695	76.5	5.62
Q61923	Potassium voltage-gated channel subfamily A member 6 OS=Mus musculus GN=Kcna6 PE=1 SV=1 - [KCNA6_MOUSE]	20.42	2	6	8	46	0.689	0.885	1.145	1.367	138.85	20.42	16	46	529	58.6	5.03
Q8BN51	Thymidine kinase 2, mitochondrial OS=Mus musculus GN=Tk2 PE=2 SV=1 - [Q8BN51_MOUSE]	6.67	2	2	2	10	0.869	1.363	1.062	1.368	22.38	6.67	4	10	270	31.2	7.21
P48318	Glutamate decarboxylase 1 OS=Mus musculus GN=Gad1 PE=2 SV=2 - [DCE1_MOUSE]	35.75	5	10	19	119	0.626	0.932	1.065	1.369	342.54	35.75	31	119	593	66.6	7.17
Q9QYR9	Acyl-coenzyme A thioesterase 2, mitochondrial OS=Mus musculus GN=Acot2 PE=1 SV=2 - [ACOT2_MOUSE]	44.59	1	7	14	61	0.721	1.309	1.000	1.369	207.50	44.59	25	61	453	49.6	7.36
Q5DTW7-3	Isoform 3 of Uncharacterized protein KIAA1551 OS=Mus musculus GN=Kiaa1551 - [K1551_MOUSE]	2.76	5	3	3	5	4.686	2.113	1.018	1.369	4.56	2.76	3	5	1519	168.7	7.44
P28230	Gap junction beta-1 protein OS=Mus musculus GN=Gjb1 PE=1 SV=3 - [CXB1_MOUSE]	8.83	1	2	2	2	1.896	0.714	1.198	1.369	4.51	8.83	2	2	283	32.0	8.95
Q3TW96	UDP-N-acetylhexosamine pyrophosphorylase-like protein 1 OS=Mus musculus GN=Uap1l1 PE=2 SV=1 - [UAP1L_MOUSE]	31.56	2	14	15	54	0.928	1.435	1.049	1.369	159.27	31.56	28	54	507	56.6	5.43
D3YZ54	2-hydroxyacyl-CoA lyase 1 OS=Mus musculus GN=Hacl1 PE=2 SV=2 - [D3YZ54_MOUSE]	21.84	7	9	9	19	1.484	0.926	0.984	1.370	54.05	21.84	14	19	554	61.0	6.18
Q9QYC7	Vitamin K-dependent gamma-carboxylase OS=Mus musculus GN=Ggcx PE=2 SV=1 - [VKGC_MOUSE]	3.70	1	2	3	4	1.985	1.192	1.417	1.370	4.24	3.70	3	4	757	87.1	8.02
Q8CI78	Required for meiotic nuclear division protein 1 homolog OS=Mus musculus GN=Rmnd1 PE=2 SV=1 - [RMND1_MOUSE]	13.11	2	6	6	20	1.058	1.259	1.101	1.370	49.96	13.11	10	20	450	51.8	7.99
Q9QXV0	ProSAAS OS=Mus musculus GN=Pcsk1n PE=1 SV=2 - [PCSK1_MOUSE]	43.02	1	9	9	122	1.734	0.819	1.007	1.370	364.12	43.02	16	122	258	27.3	5.85
Q9CQX2	Cytochrome b5 type B OS=Mus musculus GN=Cyb5b PE=1 SV=1 - [CYB5B_MOUSE]	32.19	1	5	5	27	1.873	2.102	1.833	1.370	81.04	32.19	9	27	146	16.3	4.89
Q9EQF5	Dihydropyrimidinase OS=Mus musculus GN=Dpys PE=2 SV=2 - [DPYS_MOUSE]	10.60	1	4	4	15	0.738	1.700	0.893	1.370	35.05	10.60	6	15	519	56.7	7.20
F7BA29	Glutamate receptor ionotropic, NMDA 3B (Fragment) OS=Mus musculus GN=Grin3b PE=4 SV=1 - [F7BA29_MOUSE]	2.77	2	1	1	1	1.134	1.190	1.365	1.370	1.65	2.77	1	1	542	60.2	8.44
Q9DB72	BTB/POZ domain-containing protein 17 OS=Mus musculus GN=Btbd17 PE=2 SV=1 -	25.52	2	11	12	52	0.542	0.803	1.054	1.370	147.49	25.52	23	52	478	52.6	9.42

Q9JK42	[Pyruvate dehydrogenase [lipoamide]] kinase isozyme 2, mitochondrial OS=Mus musculus GN=Pdk2 PE=1 SV=2 - [PDK2_MOUSE]	44.47	1	11	15	64	0.696	1.421	1.293	1.370	172.08	44.47	24	64	407	46.0	6.61
D3YWC7	MCG15626, isoform CRA_b OS=Mus musculus GN=Fam47e PE=4 SV=2 - [D3YWC7_MOUSE]	2.99	1	1	1	1	1.511	1.216	1.119	1.370	1.82	2.99	1	1	402	46.4	9.38
Q9JLJ0	Lipopolysaccharide-induced tumor necrosis factor-alpha factor homolog OS=Mus musculus GN=Litaf PE=1 SV=1 - [LITAF_MOUSE]	4.35	1	1	1	4	0.757	0.287	1.203	1.370	8.87	4.35	2	4	161	16.9	5.88
D3YX47	MCG20932 OS=Mus musculus GN=Rpl21-ps6 PE=4 SV=1 - [D3YX47_MOUSE]	38.06	1	1	6	13	0.906	1.861	1.132	1.371	30.44	38.06	8	13	155	18.0	10.43
P42232	Signal transducer and activator of transcription 5B OS=Mus musculus GN=Stat5b PE=1 SV=1 - [STA5B_MOUSE]	24.05	1	3	16	35	1.204	0.586	0.670	1.371	103.48	24.05	23	35	786	89.9	6.05
Q63912	Oligodendrocyte-myelin glycoprotein OS=Mus musculus GN=Omg PE=1 SV=1 - [OMGP_MOUSE]	32.73	2	17	17	225	1.315	1.326	1.513	1.372	474.47	32.73	31	225	440	49.3	8.41
Q3TKY6	Peptidyl-prolyl cis-trans isomerase CWC27 homolog OS=Mus musculus GN=Cwc27 PE=1 SV=1 - [illegible]	6.40	1	3	3	3	0.669	1.308	1.351	1.372	8.63	6.40	3	3	469	53.5	5.53
Q8BGV0	Probable asparagine--tRNA ligase, mitochondrial OS=Mus musculus GN=Nars2 PE=2 SV=1 - [SYNM_MOUSE]	6.92	2	4	4	11	0.564	1.010	1.229	1.373	22.41	6.92	7	11	477	53.9	7.36
B1AVY7	Kinesin-like protein KIF16B OS=Mus musculus GN=Kif16b PE=1 SV=1 - [KI16B_MOUSE]	4.34	1	3	6	13	1.735	0.999	1.430	1.373	32.52	4.34	8	13	1312	150.0	6.44
Q9D172	ES1 protein homolog, mitochondrial OS=Mus musculus GN=D10Jhu81e PE=1 SV=1 -	58.27	1	11	11	87	1.483	1.212	1.233	1.373	259.56	58.27	20	87	266	28.1	8.78
Q69ZF7	Metal transporter CNNM4 OS=Mus musculus GN=Cnnm4 PE=1 SV=2 - [CNNM4_MOUSE]	8.43	1	2	7	19	0.735	0.704	0.987	1.373	42.75	8.43	11	19	771	86.6	6.07
D3Z7J7	Deoxyhypusine hydroxylase (Fragment) OS=Mus musculus GN=Dohh PE=2 SV=1 - [D3Z7J7_MOUSE]	10.64	4	1	1	2	2.472	1.456	1.447	1.373	4.47	10.64	2	2	188	20.5	4.94
Q8BI08	Protein MAL2 OS=Mus musculus GN=Mal2 PE=1 SV=1 - [MAL2_MOUSE]	6.29	1	1	1	7	0.316	1.844	1.484	1.373	20.13	6.29	2	7	175	19.1	6.49
E9Q9M2	Cyclin-dependent kinase 14 (Fragment) OS=Mus musculus GN=Cdk14 PE=2 SV=1 - [E9Q9M2_MOUSE]	26.19	3	1	3	4	2.264	1.370	1.739	1.373	10.22	26.19	4	4	168	18.6	9.50
O08573-2	Isoform Short of Galectin-9 OS=Mus musculus GN=Lgals9 - [LEG9_MOUSE]	13.98	4	4	4	10	1.061	0.855	0.824	1.375	33.84	13.98	6	10	322	36.5	8.69
Q8CBH5-2	Isoform 2 of Major facilitator superfamily domain-containing protein 6 OS=Mus musculus GN=Mfsd6 - [illegible]	5.81	3	3	6	31	0.722	0.902	1.146	1.375	86.43	5.81	10	31	774	85.7	5.72
A6PWA4	Protein Gm13023 (Fragment) OS=Mus musculus GN=Gm13023 PE=2 SV=1 -	6.62	2	1	1	1	0.824	0.638	1.497	1.375	0.00	6.62	1	1	151	17.6	7.74
Q70KF4	Cardiomyopathy-associated protein 5 OS=Mus musculus GN=Cmya5 PE=1 SV=2 -	0.78	2	2	2	2	1.975	1.149	1.178	1.376	6.02	0.78	2	2	3739	412.8	4.75
P27601	Guanine nucleotide-binding protein subunit alpha-13 OS=Mus musculus GN=Gna13 PE=1 SV=1 - [illegible]	45.89	4	14	16	168	0.643	0.907	0.940	1.377	439.52	45.89	32	168	377	44.0	8.21
Q8K3P5	CCR4-NOT transcription complex subunit 6 OS=Mus musculus GN=Cnot6 PE=1 SV=2 - [CNOT6_MOUSE]	3.59	3	2	2	2	1.491	1.067	1.084	1.377	1.73	3.59	2	2	557	63.3	7.01
G3UXL2	Ribose-phosphate pyrophosphokinase OS=Mus musculus GN=Prps1l3 PE=2 SV=1 - [G3UXL2_MOUSE]	51.26	2	4	16	83	0.623	1.612	1.162	1.378	266.06	51.26	29	83	318	34.8	6.98
P19246	Neurofilament heavy polypeptide OS=Mus musculus GN=Nefh PE=1 SV=3 - [NFH_MOUSE]	41.10	1	43	47	635	3.026	0.390	1.414	1.379	1870.73	41.10	79	635	1090	116.9	5.81
Q9JIA1	Leucine-rich glioma-inactivated protein 1 OS=Mus musculus GN=Lgi1 PE=1 SV=1 - [LGI1_MOUSE]	40.04	2	23	23	136	0.616	1.344	1.421	1.379	385.52	40.04	41	136	557	63.6	8.02

Q02105	Complement C1q subcomponent subunit C OS=Mus musculus GN=C1qc PE=2 SV=2 - [C1QC_MOUSE]	25.20	1	5	5	52	1.410	1.841	1.500	1.379	191.09	25.20	9	52	246	26.0	8.54
P16330	2',3'-cyclic-nucleotide 3'-phosphodiesterase OS=Mus musculus GN=Cnp PE=1 SV=3 - [CN37_MOUSE]	84.29	4	45	47	1328	0.945	0.295	1.214	1.379	3614.91	84.29	82	1328	420	47.1	8.97
P03921	NADH-ubiquinone oxidoreductase chain 5 OS=Mus musculus GN=Mtnd5 PE=3 SV=2 - [NU5M_MOUSE]	7.41	3	4	4	12	0.648	1.117	1.262	1.381	30.84	7.41	8	12	607	68.4	9.03
Q8BY89-2	Isoform 2 of Choline transporter-like protein 2 OS=Mus musculus GN=Slc44a2 -	18.18	2	12	13	58	0.580	1.293	1.228	1.381	142.91	18.18	23	58	704	79.9	8.66
E0CZ04	Gamma-glutamylaminecyclotransferase (Fragment) OS=Mus musculus GN=Ggact PE=2 SV=1 - [E0CZ04_MOUSE]	54.29	5	3	3	7	1.685	1.194	0.857	1.381	24.66	54.29	5	7	105	11.9	5.39
P54830	Tyrosine-protein phosphatase non-receptor type 5 OS=Mus musculus GN=Ptpn5 PE=2 SV=2 - [PTN5_MOUSE]	32.90	4	14	14	47	0.788	1.249	1.909	1.382	118.72	32.90	23	47	541	60.8	5.20
Q9QZS8-2	Isoform 2 of SH2 domain-containing protein 3C OS=Mus musculus GN=Sh2d3c - [SH2D3_MOUSE]	11.82	3	7	7	20	1.042	1.083	1.340	1.382	65.74	11.82	12	20	702	77.4	8.15
G3XA42	MCG148135 OS=Mus musculus GN=Gm17227 PE=4 SV=1 - [G3XA42_MOUSE]	17.65	1	1	1	1	1.311	0.818	1.544	1.384	0.00	17.65	1	1	51	5.6	10.71
Q8BTW9	Serine/threonine-protein kinase PAK 4 OS=Mus musculus GN=Pak4 PE=1 SV=1 - [PAK4_MOUSE]	11.47	4	3	5	17	1.261	1.269	1.251	1.384	52.88	11.47	9	17	593	64.6	9.85
O35239	Tyrosine-protein phosphatase non-receptor type 9 OS=Mus musculus GN=Ptpn9 PE=2 SV=2 - [PTN9_MOUSE]	21.59	1	11	12	43	0.560	1.351	1.222	1.385	135.38	21.59	22	43	593	67.9	8.10
A2ALI5	Adherens junction-associated protein 1 OS=Mus musculus GN=Ajap1 PE=2 SV=1 - [AJAP1_MOUSE]	11.65	1	3	3	6	1.612	1.528	1.781	1.385	25.36	11.65	5	6	412	44.7	9.42
B1AWT2	Ras-related GTP binding D, isoform CRA_a OS=Mus musculus GN=Rragd PE=4 SV=1 - [B1AWT2_MOUSE]	13.53	4	3	5	14	0.686	2.228	1.929	1.386	43.32	13.53	10	14	399	45.1	4.92
P54310	Hormone-sensitive lipase OS=Mus musculus GN=Lipe PE=1 SV=2 - [LIPS_MOUSE]	14.76	3	9	9	17	0.708	0.868	1.072	1.386	40.53	14.76	13	17	759	83.3	6.95
Q8BTR5	Dual specificity phosphatase 28 OS=Mus musculus GN=Dusp28 PE=1 SV=1 -	24.54	1	2	2	14	1.333	1.019	1.146	1.387	45.99	24.54	4	14	163	17.5	7.75
Q91W97	Putative hexokinase HKDC1 OS=Mus musculus GN=Hkdc1 PE=2 SV=1 - [HKDC1_MOUSE]	10.05	1	4	8	25	1.305	1.320	1.097	1.387	53.25	10.05	13	25	915	102.2	7.09
Q9Z0P4	Paralemmin-1 OS=Mus musculus GN=Palm PE=1 SV=1 - [PALM_MOUSE]	58.49	1	4	21	275	1.817	1.188	1.153	1.387	726.36	58.49	36	275	383	41.6	4.84
O08917	Flotillin-1 OS=Mus musculus GN=Flot1 PE=1 SV=1 - [FLOT1_MOUSE]	62.62	5	23	23	143	0.987	1.707	1.318	1.387	422.34	62.62	44	143	428	47.5	7.15
P21619	Lamin-B2 OS=Mus musculus GN=Lmnb2 PE=1 SV=2 - [LMNB2_MOUSE]	55.20	2	26	31	185	1.302	1.296	1.482	1.387	611.86	55.20	56	185	596	67.3	5.50
Q9WVD5	Mitochondrial ornithine transporter 1 OS=Mus musculus GN=Slc25a15 PE=2 SV=1 -	15.28	1	4	4	10	0.810	0.862	0.923	1.388	28.43	15.28	8	10	301	32.8	8.85
Q8K0C1	Importin-13 OS=Mus musculus GN=Ipo13 PE=2 SV=1 - [IPO13_MOUSE]	6.02	2	5	5	11	0.694	0.983	1.135	1.388	28.86	6.02	7	11	963	108.2	5.30
Q80ZX8-2	Isoform 2 of Sperm-associated antigen 1 OS=Mus musculus GN=Spag1 -	26.84	3	12	12	24	1.881	0.852	1.260	1.389	65.11	26.84	21	24	529	58.5	5.78
Q80SX0	StAR-related lipid transfer protein 4 OS=Mus musculus GN=Stard4 PE=2 SV=1 -	21.43	4	3	3	4	1.957	1.350	1.213	1.390	9.82	21.43	4	4	224	25.5	5.34
Q9WUR9	GTP:AMP phosphotransferase AK4, mitochondrial OS=Mus musculus GN=Ak4 PE=2 SV=1 - [KAD4_MOUSE]	57.40	4	10	10	77	1.005	2.145	1.162	1.390	199.85	57.40	19	77	223	25.0	7.53
J3QPY0	Protein 1600014C10Rik OS=Mus musculus GN=1600014C10Rik PE=4 SV=1 - [J3QPY0_MOUSE]	61.82	1	1	2	6	0.699	1.006	1.186	1.391	15.11	61.82	3	6	55	5.6	8.98

P00405	Cytochrome c oxidase subunit 2 OS=Mus musculus GN=Mtco2 PE=1 SV=1 - [COX2_MOUSE]	30.84	1	7	7	204	0.862	0.958	1.165	1.391	508.27	30.84	12	204	227	26.0	4.73
Q9JLZ3	Methylglutaconyl-CoA hydratase, mitochondrial OS=Mus musculus GN=Auh PE=2 SV=1 - [AUHM_MOUSE]	38.54	7	12	13	94	0.891	0.884	1.167	1.393	300.47	38.54	25	94	314	33.4	9.51
Q8K298	Actin-binding protein anillin OS=Mus musculus GN=Anln PE=1 SV=2 - [ANLN_MOUSE]	14.72	1	16	16	52	1.708	0.379	1.819	1.393	146.11	14.72	29	52	1121	122.7	6.98
Q6V595	Kelch-like protein 6 OS=Mus musculus GN=Klhl6 PE=2 SV=2 - [KLHL6_MOUSE]	3.72	1	1	2	2	1.459	1.241	1.433	1.393	6.22	3.72	2	2	619	70.1	6.34
Q9R187	Tumor necrosis factor receptor superfamily member EDAR OS=Mus musculus GN=Edar PE=1 SV=1 - [EDAR_MOUSE]	1.34	1	1	1	1	0.896	1.661	2.742	1.394	2.57	1.34	1	1	448	48.4	5.43
Q921N6	Probable ATP-dependent RNA helicase DDX27 OS=Mus musculus GN=Ddx27 PE=1 SV=3 - [DDX27_MOUSE]	2.63	1	2	2	3	2.986	1.357	1.207	1.394	3.99	2.63	3	3	760	85.9	9.25
Q80VA0	N-acetylgalactosaminyltransferase 7 OS=Mus musculus GN=Galnt7 PE=2 SV=2 -	3.81	3	2	2	2	0.656	0.214	1.699	1.394	0.00	3.81	2	2	657	75.4	7.93
A8DUK4	Beta-globin OS=Mus musculus GN=Hbb-b1 PE=2 SV=1 - [A8DUK4_MOUSE]	85.03	2	5	14	2661	3.594	0.374	0.447	1.395	8645.53	85.03	28	2661	147	15.7	7.69
Q4KMS1	Tripartite motif-containing 44 OS=Mus musculus GN=Trim44 PE=2 SV=1 - [Q4KMS1_MOUSE]	12.75	3	2	2	4	1.986	1.663	1.631	1.395	12.89	12.75	3	4	345	38.1	4.18
P01648	Ig kappa chain V-V region HP 91A3 OS=Mus musculus PE=1 SV=1 - [KV5AF_MOUSE]	23.15	7	1	2	4	3.555	2.872	0.762	1.395	10.55	23.15	4	4	108	12.0	8.47
Q6TAC4	Vomeronasal type-2 receptor 26 OS=Mus musculus GN=Vmn2r26 PE=2 SV=2 -	2.34	1	2	2	2	1.755	2.363	1.134	1.395	2.30	2.34	2	2	855	96.1	7.68
Q91ZB7	Mas-related G-protein coupled receptor member E OS=Mus musculus GN=Mrgpre PE=2 SV=1 - [MRGRE_MOUSE]	3.87	1	1	1	1	4.354	1.219	1.016	1.396	2.67	3.87	1	1	310	34.6	6.68
G5E8L5	MCG140820 OS=Mus musculus GN=Zfp777 PE=4 SV=1 - [G5E8L5_MOUSE]	1.07	2	1	1	1	0.854	1.024	1.327	1.396	3.17	1.07	1	1	841	94.9	6.67
D3YY46	Calcium and integrin-binding protein 1 (Fragment) OS=Mus musculus GN=Cib1 PE=2 SV=1 - [D3YY46_MOUSE]	26.36	3	2	2	7	1.219	0.887	1.158	1.396	26.76	26.36	3	7	110	12.4	4.23
Q61496	Probable ATP-dependent RNA helicase DDX4 OS=Mus musculus GN=Ddx4 PE=1 SV=2 - [DDX4_MOUSE]	7.98	2	2	5	20	1.746	2.130	1.426	1.396	52.95	7.98	7	20	702	76.4	6.09
A2AMZ4	Uncharacterized protein C17orf89 homolog OS=Mus musculus PE=4 SV=1 - [CQ089_MOUSE]	33.78	1	3	3	8	1.157	1.176	1.195	1.397	22.67	33.78	4	8	74	7.8	9.55
P37804	Transgelin OS=Mus musculus GN=Tagln PE=1 SV=3 - [TAGL_MOUSE]	76.62	1	14	15	115	0.853	3.715	0.926	1.397	329.45	76.62	28	115	201	22.6	8.81
Q7TNV1-2	Isoform 2 of Protein FAM57B OS=Mus musculus GN=Fam57b - [FA57B_MOUSE]	4.44	3	1	1	2	1.052	0.708	1.020	1.398	1.61	4.44	1	2	225	25.4	8.72
Q8CEC5	NF-kappa-B inhibitor-interacting Ras-like protein 1 OS=Mus musculus GN=Nkiras1 PE=2 SV=1 - [KBRS1_MOUSE]	16.15	2	3	3	6	0.778	1.033	1.262	1.398	12.74	16.15	6	6	192	21.6	7.33
Q8C729-2	Isoform 2 of Protein FAM126B OS=Mus musculus GN=Fam126b - [F126B_MOUSE]	27.13	3	13	13	48	1.037	1.312	1.417	1.399	144.99	27.13	25	48	586	64.4	6.67
O89019-2	Isoform 2 of Inversin OS=Mus musculus GN=Invs - [INVS_MOUSE]	3.46	8	2	3	6	2.572	1.227	1.067	1.399	8.67	3.46	3	6	897	99.6	9.07
Q71RI9-2	Isoform 2 of Kynurenine--oxoglutarate transaminase 3 OS=Mus musculus GN=Ccbl2 - [KAT3_MOUSE]	30.24	3	10	11	33	1.268	1.209	1.158	1.399	97.92	30.24	16	33	420	47.3	7.20
Q3U0B3	Dehydrogenase/reductase SDR family member 11 OS=Mus musculus GN=Dhrs11 PE=2 SV=1 - [DHR11_MOUSE]	16.92	2	4	4	10	1.329	1.619	0.997	1.399	31.08	16.92	8	10	260	28.3	6.34
Q922R8	Protein disulfide-isomerase A6 OS=Mus musculus GN=Pdia6 PE=1 SV=3 - [PDIA6_MOUSE]	40.68	1	1	15	68	0.739	2.555	0.340	1.399	203.03	40.68	24	68	440	48.1	5.14
P52963	Band 4.1-like protein 4A OS=Mus musculus GN=Epb41l4a PE=2 SV=2 [E41LA_MOUSE]	5.83	1	3	3	4	1.303	0.663	1.444	1.400	9.08	5.83	4	4	686	78.5	9.44

Q60634	Flotillin-2 OS=Mus musculus GN=Flot2 PE=1 SV=2 - [FLOT2_MOUSE]	68.93	4	30	30	128	0.920	1.720	1.251	1.400	364.38	68.93	53	128	428	47.0	5.20
Q80T14	Extracellular matrix protein FRAS1 OS=Mus musculus GN=Fras1 PE=2 SV=2 - [FRAS1_MOUSE]	1.25	1	3	5	13	1.417	1.323	1.012	1.400	30.32	1.25	6	13	4010	442.1	5.82
Q99JG2	Endothelin B receptor-like protein 2 OS=Mus musculus GN=Gpr37l1 PE=1 SV=2 -	8.94	1	4	4	22	0.574	0.822	1.192	1.401	53.70	8.94	7	22	481	52.7	7.36
Q8K1N4	Spermatogenesis-associated serine-rich protein 2 OS=Mus musculus GN=Spats2 PE=1 SV=1 - [illegible]	4.04	2	2	2	2	2.797	1.581	1.104	1.401	6.28	4.04	2	2	545	58.9	9.00
Q3UQA7	Selenoprotein H OS=Mus musculus GN=Selh PE=2 SV=2 - [SELH_MOUSE]	17.24	1	2	2	4	1.657	1.503	1.025	1.402	12.84	17.24	3	4	116	13.0	9.80
Q9ESU7	Neutral amino acid transporter ASCT2 OS=Mus musculus GN=Slc1a5 PE=2 SV=1 - [Q9ESU7_MOUSE]	11.35	3	3	3	7	0.768	0.932	0.945	1.402	19.38	11.35	5	7	555	58.4	7.12
A7M7S2	Nitrogen permease regulator 3-like protein OS=Mus musculus GN=Nprl3 PE=2 SV=1 - [A7M7S2_MOUSE]	6.43	2	3	3	5	1.657	0.947	1.123	1.402	11.34	6.43	5	5	544	60.8	6.83
Q99JX1	Transcription initiation factor TFIID subunit 11 OS=Mus musculus GN=Taf11 PE=2 SV=1 - [TAF11_MOUSE]	5.21	1	1	1	2	1.742	0.876	1.317	1.403	3.74	5.21	2	2	211	23.3	5.24
P35505	Fumarylacetoacetase OS=Mus musculus GN=Fah PE=1 SV=2 - [FAAA_MOUSE]	42.24	2	12	12	57	2.842	1.169	1.154	1.403	193.57	42.24	20	57	419	46.1	7.18
F6QDU1	Uncharacterized protein OS=Mus musculus GN=Rpl28-ps4 PE=4 SV=1 - [F6QDU1_MOUSE]	8.40	1	1	1	1	0.958	1.230	1.614	1.404	2.35	8.40	1	1	131	15.2	11.05
Q8BG16	Sodium-dependent neutral amino acid transporter B(0)AT2 OS=Mus musculus GN=Slc6a15 PE=1 SV=1 - [illegible]	7.82	1	4	4	4	1.720	1.383	1.816	1.404	12.53	7.82	4	4	729	81.7	5.19
P52480-2	Isoform M1 of Pyruvate kinase PKM OS=Mus musculus GN=Pkm - [KPYM_MOUSE]	80.60	1	3	46	1956	1.654	1.098	1.168	1.404	5864.96	80.60	85	1956	531	57.9	7.14
O35226	26S proteasome non-ATPase regulatory subunit 4 OS=Mus musculus GN=Psmd4 PE=1 SV=1 - [PSMD4_MOUSE]	31.91	2	1	10	85	0.707	1.046	1.469	1.406	268.63	31.91	19	85	376	40.7	4.79
Q9DB16	Calcium-binding protein 39 like OS=Mus musculus GN=Cab39l PE=1 SV=3 - [CB39L_MOUSE]	14.24	2	2	5	20	0.701	1.421	0.944	1.406	46.35	14.24	9	20	337	39.1	8.31
J3QNE4	Protein E230008N13Rik OS=Mus musculus GN=E230008N13Rik PE=4 SV=1 - [J3QNE4_MOUSE]	3.43	3	2	6	13	0.889	1.372	1.352	1.407	25.98	3.43	7	13	1664	195.7	5.31
Q9CS42	Ribose-phosphate pyrophosphokinase 2 OS=Mus musculus GN=Prps2 PE=1 SV=4 - [PRPS2_MOUSE]	43.08	1	6	14	50	0.869	1.081	1.119	1.407	164.31	43.08	24	50	318	34.8	6.61
Q9JJZ2	Tubulin alpha-8 chain OS=Mus musculus GN=Tuba8 PE=1 SV=1 - [TBA8_MOUSE]	50.33	1	6	22	2388	1.147	1.546	1.892	1.407	7485.50	50.33	42	2388	449	50.0	5.10
E9Q152	Protein Als2cr11 OS=Mus musculus GN=Als2cr11 PE=2 SV=1 - [E9Q152_MOUSE]	2.15	1	1	1	2	2.431	0.931	0.569	1.407	4.58	2.15	1	2	557	63.1	8.87
Q3USB7	Inactive phospholipase C-like protein 1 OS=Mus musculus GN=Plcl1 PE=1 SV=3 - [PLCL1_MOUSE]	31.57	1	29	30	115	0.993	0.645	1.217	1.407	366.03	31.57	51	115	1096	122.6	5.64
Q9D500	Protein 4930533L02Rik OS=Mus musculus GN=4930533L02Rik PE=2 SV=1 - [Q9D500_MOUSE]	12.90	1	1	1	5	2.323	1.678	1.561	1.407	10.20	12.90	1	5	124	14.0	9.66
D3Z090	Macrophage colony-stimulating factor 1 (Fragment) OS=Mus musculus GN=Csf1 PE=2 SV=1 - [D3Z090_MOUSE]	7.69	3	1	1	2	1.891	0.742	1.614	1.408	6.47	7.69	2	2	182	21.3	7.25
P68404	Protein kinase C beta type OS=Mus musculus GN=Prkcb PE=1 SV=4 - [KPCB_MOUSE]	41.73	1	2	28	140	0.543	0.252	0.969	1.409	378.82	41.73	49	140	671	76.7	7.01
Q8K1K4	Centromere protein I OS=Mus musculus GN=Cenpi PE=2 SV=1 - [CENPI_MOUSE]	3.35	1	1	1	6	0.857	0.928	0.854	1.409	0.00	3.35	1	6	746	85.3	8.05
Q9CPS6	Histidine triad nucleotide-binding protein 3 OS=Mus musculus GN=Hint3 PE=2 SV=1 - [HINT3_MOUSE]	24.24	3	4	4	17	0.912	1.078	1.103	1.409	42.62	24.24	7	17	165	18.8	6.33
Q7TMA2	Zinc finger protein 503 OS=Mus musculus GN=Znf503 PE=2 SV=1 - [ZN503_MOUSE]	5.67	2	2	3	5	3.969	2.607	1.154	1.410	18.48	5.67	4	5	652	63.0	8.66

A2AHQ7	Phosphorylase b kinase regulatory subunit alpha, liver isoform OS=Mus musculus GN=Phka2 PE=2 SV=1 - [A2AHQ7_MOUSE]	3.44	3	3	4	16	1.000	1.109	1.165	1.411	42.47	3.44	6	16	1191	133.7	6.40
E9Q166	Protein Atad2b OS=Mus musculus GN=Atad2b PE=2 SV=1 - [E9Q166_MOUSE]	2.40	1	2	3	3	0.899	0.936	1.430	1.412	4.70	2.40	3	3	1460	164.6	6.96
Q91VH2	Sorting nexin-9 OS=Mus musculus GN=Snx9 PE=1 SV=1 - [SNX9_MOUSE]	7.73	1	5	5	7	0.698	1.354	1.163	1.412	18.53	7.73	7	7	595	66.5	5.52
P20934	Protein EVI2A OS=Mus musculus GN=Evi2a PE=1 SV=2 - [EVI2A_MOUSE]	11.66	1	2	2	9	0.954	0.274	1.357	1.414	25.11	11.66	4	9	223	24.1	9.52
Q3UHN9	Bifunctional heparan sulfate N-deacetylase/N-sulfotransferase 1 OS=Mus musculus GN=Ndst1 PE=1 SV=2 - [NDST1_MOUSE]	5.56	2	3	4	6	1.051	0.700	1.871	1.414	13.00	5.56	5	6	882	100.7	7.77
Q8CDI2	F-box only protein 43 OS=Mus musculus GN=Fbxo43 PE=1 SV=2 - [FBX43_MOUSE]	4.06	2	2	2	3	1.073	0.898	1.330	1.415	6.05	4.06	2	3	640	71.1	8.16
Q8QZS1	3-hydroxyisobutyryl-CoA hydrolase, mitochondrial OS=Mus musculus GN=Hibch PE=1 SV=1 - [HIBCH_MOUSE]	49.09	2	16	16	77	0.841	1.127	1.026	1.415	226.43	49.09	31	77	385	43.0	8.06
Q52KR3	Protein prune homolog 2 OS=Mus musculus GN=Prune2 PE=2 SV=2 - [PRUN2_MOUSE]	6.65	4	2	14	47	1.300	1.642	1.445	1.415	139.60	6.65	21	47	3084	339.3	4.46
Q60928	Gamma-glutamyltranspeptidase 1 OS=Mus musculus GN=Ggt1 PE=1 SV=1 -	4.23	8	2	2	4	1.354	1.117	1.252	1.415	14.49	4.23	3	4	568	61.5	7.15
Q8BL80	Rho GTPase-activating protein 22 OS=Mus musculus GN=Arhgap22 PE=1 SV=2 -	8.12	4	4	5	13	0.575	1.386	0.973	1.416	30.92	8.12	7	13	702	77.7	7.88
Q9QUP5	Hyaluronan and proteoglycan link protein 1 OS=Mus musculus GN=Hapln1 PE=2 SV=1 - [HPLN1_MOUSE]	47.47	1	13	14	82	1.543	1.872	2.289	1.417	217.91	47.47	24	82	356	40.5	7.80
Q3UR70	Transforming growth factor-beta receptor-associated protein 1 OS=Mus musculus GN=Tgfbrap1 PE=2 SV=1 - [TGFA1_MOUSE]	11.40	1	4	7	19	0.851	0.596	1.134	1.417	53.47	11.40	14	19	860	97.2	6.70
P70174	Histamine H1 receptor OS=Mus musculus GN=Hrh1 PE=2 SV=2 - [HRH1_MOUSE]	14.14	1	6	6	12	1.125	1.217	1.106	1.417	35.29	14.14	9	12	488	55.6	9.32
Q3URU2-2	Isoform 2 of Paternally-expressed gene 3 protein OS=Mus musculus GN=Peg3 -	8.12	1	1	8	15	1.164	1.185	1.253	1.418	32.12	8.12	11	15	1194	136.9	7.17
G3X8P9	Aldehyde oxidase OS=Mus musculus GN=Aox1 PE=4 SV=1 - [G3X8P9_MOUSE]	3.90	2	5	6	10	2.146	1.918	1.082	1.418	26.20	3.90	10	10	1333	146.6	6.93
P48320	Glutamate decarboxylase 2 OS=Mus musculus GN=Gad2 PE=2 SV=1 - [DCE2_MOUSE]	43.25	1	19	21	181	0.569	0.884	1.038	1.419	580.86	43.25	38	181	585	65.2	6.90
F6TS96	Transducin-like enhancer protein 3 (Fragment) OS=Mus musculus GN=Tle3 PE=2 SV=1 - [F6TS96_MOUSE]	9.69	14	5	5	8	1.552	0.956	1.263	1.420	17.93	9.69	8	8	702	75.3	7.23
Q9DD02-2	Isoform 2 of Protein Hikeshi OS=Mus musculus GN=L7rn6 - [HIKES_MOUSE]	15.82	3	2	2	9	0.938	1.131	1.305	1.420	25.22	15.82	4	9	158	17.2	7.24
Q8CJF9	Protein argonaute-3 OS=Mus musculus GN=Ago3 PE=2 SV=2 - [AGO3_MOUSE]	20.23	1	5	15	39	2.587	0.509	1.428	1.421	106.94	20.23	24	39	860	97.2	9.10
P14602	Heat shock protein beta-1 OS=Mus musculus GN=Hspb1 PE=1 SV=3 - [HSPB1_MOUSE]	61.72	4	9	9	35	2.000	2.039	1.144	1.421	108.80	61.72	16	35	209	23.0	6.55
Q8VE95	UPF0598 protein C8orf82 homolog OS=Mus musculus PE=2 SV=1 - [CH082_MOUSE]	35.78	1	6	6	18	1.270	0.995	1.027	1.421	40.18	35.78	11	18	218	24.3	8.70
B2KGD7	Importin-8 (Fragment) OS=Mus musculus GN=Ipo8 PE=2 SV=1 - [B2KGD7_MOUSE]	9.97	3	2	3	6	0.932	0.977	0.919	1.421	11.30	9.97	5	6	361	42.5	5.91
P97478	Ubiquinone biosynthesis protein COQ7 homolog OS=Mus musculus GN=Coq7 PE=2 SV=3 - [COQ7_MOUSE]	27.19	2	5	5	16	0.546	1.930	1.289	1.422	54.83	27.19	10	16	217	24.0	7.17
Q9ERE2	Keratin, type II cuticular Hb1 (Fragment) OS=Mus musculus GN=Krt81 PE=2 SV=1 - [KRT81_MOUSE]	4.10	4	1	2	17	6.802	0.598	1.832	1.422	37.01	4.10	4	17	390	43.7	5.20
A2AIT6	Alpha-tocopherol transfer protein OS=Mus musculus GN=Ttpa PE=2 SV=2 - [A2AIT6_MOUSE]	7.63	2	1	1	4	1.311	1.216	1.195	1.423	20.53	7.63	2	4	262	29.8	9.67

A6H630	UPF0364 protein C6orf211 homolog OS=Mus musculus PE=2 SV=1 - [CF211_MOUSE]	29.38	5	12	12	30	0.880	1.342	1.579	1.423	82.53	29.38	19	30	439	50.5	5.92
Q9Z2R6	Protein unc-119 homolog A OS=Mus musculus GN=Unc119 PE=1 SV=1 - [U119A_MOUSE]	39.17	2	1	7	26	1.746	1.248	1.589	1.423	77.42	39.17	11	26	240	27.0	6.15
Q8BX57-3	Isoform 3 of PX domain-containing protein kinase-like protein OS=Mus musculus GN=Pxk -	4.91	3	2	2	3	0.750	0.897	0.940	1.424	10.02	4.91	3	3	448	50.9	8.79
Q3TH73-2	Isoform 2 of Protein tweety homolog 2 OS=Mus musculus GN=Ttyh2 -	5.32	2	3	3	12	0.835	0.565	0.937	1.424	39.14	5.32	6	12	470	52.1	6.51
P39061-2	Isoform 3 of Collagen alpha-1(XVIII) chain OS=Mus musculus GN=Col18a1 -	10.11	4	11	11	30	0.880	2.573	0.918	1.424	83.69	10.11	20	30	1315	134.1	6.00
Q91V93	Rho-related BTB domain-containing protein 2 OS=Mus musculus GN=Rhobtb2 PE=2 SV=2 - [RHBT2_MOUSE]	7.69	1	4	4	7	1.332	1.614	1.167	1.424	16.49	7.69	4	7	728	82.6	6.95
Q8C0S1	DIS3-like exonuclease 1 OS=Mus musculus GN=Dis3l PE=2 SV=2 - [DI3L1_MOUSE]	5.41	2	5	5	8	3.095	1.467	1.200	1.425	17.82	5.41	5	8	1053	120.2	6.81
E9Q354	Scm-like with four MBT domains protein 1 (Fragment) OS=Mus musculus GN=Sfmbt1 PE=2 SV=1 - [E9Q354_MOUSE]	3.46	2	1	1	1	0.978	1.211	1.147	1.426	0.00	3.46	1	1	636	72.1	6.65
O70172	Phosphatidylinositol 5-phosphate 4-kinase type-2 alpha OS=Mus musculus GN=Pip4k2a PE=1 SV=1 - [PI42A_MOUSE]	36.79	3	9	15	112	1.027	0.783	1.386	1.426	317.13	36.79	28	112	405	46.1	6.99
Q8BQV2	Choline acetyltransferase OS=Mus musculus GN=Chat PE=2 SV=1 - [Q8BQV2_MOUSE]	18.45	2	8	9	16	0.992	1.077	2.084	1.427	47.67	18.45	14	16	645	72.3	7.62
Q8BMV3	Acylphosphatase-1 OS=Mus musculus GN=Acyp1 PE=4 SV=1 - [Q8BMV3_MOUSE]	16.67	1	1	2	3	0.946	1.123	1.042	1.427	4.85	16.67	3	3	108	11.9	6.90
Q99N28	Cell adhesion molecule 3 OS=Mus musculus GN=Cadm3 PE=1 SV=1 - [CADM3_MOUSE]	42.17	2	9	9	87	0.652	1.458	1.266	1.428	237.08	42.17	17	87	396	42.9	5.80
B2RSH2	Guanine nucleotide-binding protein G(i) subunit alpha-1 OS=Mus musculus GN=Gnai1 PE=2 SV=1 - [GNAI1_MOUSE]	66.38	6	10	20	385	0.751	1.462	1.329	1.429	1115.70	66.38	37	385	354	40.3	5.97
Q7TSH7	Potassium voltage-gated channel subfamily F member 1 OS=Mus musculus GN=Kcnf1 PE=2 SV=1 - [KCNF1_MOUSE]	3.25	2	1	1	1	1.301	1.165	1.378	1.429	0.00	3.25	1	1	493	55.6	6.06
D3YVB6	Ketohexokinase (Fragment) OS=Mus musculus GN=Khk PE=2 SV=1 - [D3YVB6_MOUSE]	11.07	1	2	3	8	0.852	1.080	1.089	1.429	22.91	11.07	6	8	244	27.2	6.23
P63078	Guanine nucleotide-binding protein G(I)/G(S)/G(O) subunit gamma-8 OS=Mus musculus GN=Gng8 PE=2 SV=1 - [GBG8_MOUSE]	47.14	1	2	2	13	0.745	0.466	0.723	1.429	53.66	47.14	4	13	70	7.8	7.14
O35914	Zinc finger protein basonuclin-1 OS=Mus musculus GN=Bnc1 PE=2 SV=1 - [BNC1_MOUSE]	1.56	2	1	1	1	35.658	1.265	3.003	1.430	3.21	1.56	1	1	961	106.6	6.42
Q9D871	Carcinoembryonic antigen-related cell adhesion molecule 18 OS=Mus musculus GN=Ceacam18 PE=2 SV=1 - [CEA18_MOUSE]	2.13	1	1	1	1	1.606	1.241	1.871	1.430	2.69	2.13	1	1	376	42.2	6.76
P00416	Cytochrome c oxidase subunit 3 OS=Mus musculus GN=Mtco3 PE=3 SV=1 - [COX3_MOUSE]	5.36	2	1	1	3	2.035	1.472	1.380	1.430	8.05	5.36	2	3	261	29.9	7.30
Q8BVL9	Janus kinase and microtubule-interacting protein 1 OS=Mus musculus GN=Jakmip1 PE=1 SV=2 - [JKIP1_MOUSE]	34.82	5	2	21	77	2.268	1.867	2.029	1.432	206.85	34.82	36	77	626	73.1	6.09
O55125	Protein NipSnap homolog 1 OS=Mus musculus GN=Nipsnap1 PE=1 SV=1 [NIPS1_MOUSE]	49.65	2	15	16	93	0.757	1.200	1.205	1.432	236.42	49.65	27	93	284	33.3	9.44
Q9JKV5	Secretory carrier-associated membrane protein 4 OS=Mus musculus GN=Scamp4 PE=2 SV=1 - [SCAM4_MOUSE]	4.78	1	1	1	2	0.800	1.477	1.229	1.432	6.59	4.78	2	2	230	25.3	8.66
P04186	Complement factor B OS=Mus musculus GN=Cfb PE=1 SV=2 - [CFAB_MOUSE]	15.64	9	13	13	33	2.516	1.983	0.616	1.433	85.06	15.64	23	33	761	85.0	7.37
P35762	CD81 antigen OS=Mus musculus GN=Cd81 PE=1 SV=2 - [CD81_MOUSE]	30.93	1	4	4	240	1.525	1.166	1.110	1.433	714.62	30.93	6	240	236	25.8	5.83
P04370-4	Isoform 4 of Myelin basic protein OS=Mus musculus GN=Mbp - [MBP_MOUSE]	75.90	7	1	24	2328	1.059	0.395	1.899	1.433	6593.36	75.90	42	2328	195	21.5	11.24

Q8C5H8	NAD kinase 2, mitochondrial OS=Mus musculus GN=Nadk2 PE=1 SV=2 - [NAKD2_MOUSE]	48.89	4	17	18	57	1.144	1.182	1.095	1.434	159.15	48.89	33	57	452	50.8	8.07
P14231	Sodium/potassium-transporting ATPase subunit beta-2 OS=Mus musculus GN=Atp1b2 PE=1 SV=2 - [AT1B2_MOUSE]	47.59	2	12	12	227	0.708	0.670	1.013	1.434	533.54	47.59	22	227	290	33.3	8.31
P28650	Adenylosuccinate synthetase isozyme 1 OS=Mus musculus GN=Adssl1 PE=1 SV=2 - [PURA1_MOUSE]	45.95	3	16	18	63	1.206	0.384	1.228	1.434	193.27	45.95	32	63	457	50.2	8.43
P08752	Guanine nucleotide-binding protein G(i) subunit alpha-2 OS=Mus musculus GN=Gnai2 PE=1 SV=5 - [GNAI2_MOUSE]	68.17	6	12	22	361	0.627	1.095	0.954	1.434	1072.42	68.17	41	361	355	40.5	5.45
E9PZS8	Cytochrome c oxidase subunit 7A-related protein, mitochondrial OS=Mus musculus GN=Cox7a2l PE=2 SV=1 - [E9PZS8_MOUSE]	47.01	3	6	6	11	0.822	1.042	1.220	1.435	37.34	47.01	9	11	134	14.9	9.57
Q9Z2G9	Oxidoreductase HTATIP2 OS=Mus musculus GN=Htatip2 PE=1 SV=3 - [HTAI2_MOUSE]	15.70	2	4	4	4	1.015	1.420	1.068	1.435	10.87	15.70	4	4	242	26.9	8.59
O09174	Alpha-methylacyl-CoA racemase OS=Mus musculus GN=Amacr PE=1 SV=4 - [AMACR_MOUSE]	24.67	1	7	7	13	2.338	1.124	1.184	1.436	39.38	24.67	11	13	381	41.7	7.40
Q8BPE4	Transmembrane protein 177 OS=Mus musculus GN=Tmem177 PE=2 SV=1 - [TM177_MOUSE]	5.79	1	2	2	8	1.031	1.245	1.266	1.436	22.61	5.79	4	8	311	34.0	9.77
Q6P9P6	Kinesin-like protein KIF11 OS=Mus musculus GN=Kif11 PE=2 SV=1 - [KIF11_MOUSE]	1.24	1	1	1	1	1.603	2.402	1.706	1.436	2.95	1.24	1	1	1052	118.0	6.00
Q6PIE5	Sodium/potassium-transporting ATPase subunit alpha-2 OS=Mus musculus GN=Atp1a2 PE=1 SV=1 - [AT1A2_MOUSE]	61.57	3	43	72	2881	0.803	0.730	1.055	1.436	8239.21	61.57	135	2881	1020	112.1	5.55
Q61532	Mitogen-activated protein kinase 6 OS=Mus musculus GN=Mapk6 PE=1 SV=3 -	3.47	3	1	2	3	2.172	0.811	1.679	1.436	7.83	3.47	3	3	720	82.1	5.20
Q8K4F5	Alpha/beta hydrolase domain-containing protein 11 OS=Mus musculus GN=Abhd11 PE=2 SV=1 - [ABHDB_MOUSE]	25.08	2	7	7	21	1.040	0.979	1.040	1.437	60.87	25.08	13	21	307	33.5	9.61
A2BDQ4	Eph receptor A7, isoform CRA_b OS=Mus musculus GN=Epha7 PE=4 SV=1 - [A2BDQ4_MOUSE]	11.77	10	4	9	20	1.885	1.283	1.696	1.437	57.37	11.77	14	20	994	111.3	5.80
P23242	Gap junction alpha-1 protein OS=Mus musculus GN=Gja1 PE=1 SV=2 - [CXA1_MOUSE]	43.72	1	11	11	164	1.043	0.881	1.322	1.439	490.10	43.72	19	164	382	43.0	8.76
Q67BT3	Solute carrier family 13 member 5 OS=Mus musculus GN=Slc13a5 PE=2 SV=1 -	3.32	1	2	2	11	0.685	1.311	1.114	1.439	35.82	3.32	4	11	572	63.8	7.64
Q6XE40	MAGUK p55 subfamily member 3 OS=Mus musculus GN=Mpp3 PE=2 SV=1 - [Q6XE40_MOUSE]	46.15	3	22	23	84	0.797	1.423	1.712	1.439	257.96	46.15	42	84	585	66.4	6.47
E9Q5P6	Protein Pdzrn4 OS=Mus musculus GN=Pdzrn4 PE=2 SV=1 - [E9Q5P6_MOUSE]	1.55	2	1	1	1	4.921	2.310	1.946	1.440	2.39	1.55	1	1	775	88.6	5.30
Q9EQK5	Major vault protein OS=Mus musculus GN=Mvp PE=1 SV=4 - [MVP_MOUSE]	20.33	3	14	14	40	1.017	1.081	1.113	1.440	118.82	20.33	22	40	861	95.9	5.59
Q61165	Sodium/hydrogen exchanger 1 OS=Mus musculus GN=Slc9a1 PE=1 SV=1 -	18.90	1	12	12	46	0.792	0.776	1.397	1.440	142.00	18.90	21	46	820	91.4	7.12
Q14DI5	Protein Zbtb4 OS=Mus musculus GN=Zbtb4 PE=2 SV=1 - [Q14DI5_MOUSE]	8.24	2	1	1	2	2.258	1.721	0.951	1.440	6.56	8.24	1	2	364	38.1	8.60
Q9EQ20	Methylmalonate-semialdehyde dehydrogenase [acylating], mitochondrial OS=Mus musculus GN=Aldh6a1 PE=1 SV=1 - [MMSA_MOUSE]	58.88	2	27	27	212	0.767	1.363	1.088	1.441	641.31	58.88	52	212	535	57.9	8.07
B0R0D5	FERM domain-containing protein 5 (Fragment) OS=Mus musculus GN=Frmd5 PE=2 SV=1 - [B0R0D5_MOUSE]	15.40	10	5	5	11	0.613	0.598	1.156	1.442	34.02	15.40	8	11	500	56.3	8.91
E9Q800	Mitochondrial inner membrane protein OS=Mus musculus GN=Immt PE=2 SV=1 - [E9Q800_MOUSE]	68.63	2	2	51	290	1.335	1.593	1.862	1.442	881.80	68.63	91	290	679	75.6	7.80
Q9D267	Epididymal-specific lipocalin-9 OS=Mus musculus GN=Lcn9 PE=2 SV=1 - [LCN9_MOUSE]	11.24	1	1	1	2	1.300	1.205	1.579	1.442	0.00	11.24	1	2	178	20.5	6.77

Q6PFX8	N-acetylaspartyl-glutamate synthetase A OS=Mus musculus GN=Rimkla PE=1 SV=1 - [RIMKA_MOUSE]	8.42	1	3	3	6	3.616	0.649	1.366	1.443	15.47	8.42	4	6	380	41.5	6.65
Q99PE8	ATP-binding cassette sub-family G member 5 OS=Mus musculus GN=Abcg5 PE=1 SV=1 - [ABCG5_MOUSE]	5.98	2	1	2	14	1.651	0.883	1.335	1.443	33.56	5.98	3	14	652	73.2	8.06
Q80V42	Carboxypeptidase M OS=Mus musculus GN=Cpm PE=2 SV=2 - [CBPM_MOUSE]	24.83	2	9	9	33	1.901	0.461	1.295	1.443	102.76	24.83	17	33	443	50.5	7.78
P31809-3	Isoform 3 of Carcinoembryonic antigen-related cell adhesion molecule 1 OS=Mus musculus GN=Ceacam1 - [illegible]	9.38	5	2	2	3	1.565	1.837	1.255	1.444	8.82	9.38	3	3	341	37.2	6.23
Q9CR62	Mitochondrial 2-oxoglutarate/malate carrier protein OS=Mus musculus GN=Slc25a11 PE=1 SV=3 - [illegible]	58.92	3	15	15	80	0.609	1.164	1.194	1.444	245.44	58.92	28	80	314	34.1	9.94
P63143	Voltage-gated potassium channel subunit beta-1 OS=Mus musculus GN=Kcnab1 PE=2 SV=2 - [KCAB1_MOUSE]	28.18	5	8	10	18	1.017	0.758	1.301	1.444	37.39	28.18	15	18	401	44.7	9.32
E9Q951	Zinc finger protein 426 OS=Mus musculus GN=Zfp426 PE=2 SV=1 - [E9Q951_MOUSE]	4.55	8	1	3	9	1.578	2.088	1.051	1.445	16.41	4.55	5	9	483	55.5	8.73
Q91VM4	Protein spinster homolog 2 OS=Mus musculus GN=Spns2 PE=2 SV=2 - [SPNS2_MOUSE]	2.73	1	1	1	2	1.016	0.747	1.203	1.447	3.91	2.73	2	2	549	58.2	9.17
A2AR81	T-box brain protein 1 OS=Mus musculus GN=Tbr1 PE=2 SV=1 - [A2AR81_MOUSE]	24.40	2	7	7	9	1.016	2.179	2.269	1.447	28.88	24.40	9	9	418	44.8	7.17
O35324	SH2 domain-containing protein 1B OS=Mus musculus GN=Sh2d1b PE=1 SV=1 -	13.64	2	1	1	1	0.551	0.819	1.912	1.448	0.00	13.64	1	1	132	15.2	7.33
Q5NCE8	Magnesium transporter MRS2 homolog, mitochondrial OS=Mus musculus GN=Mrs2 PE=2 SV=2 - [MRS2_MOUSE]	21.89	1	8	8	15	1.157	1.052	1.303	1.448	38.96	21.89	11	15	434	49.3	7.53
P03995	Glial fibrillary acidic protein OS=Mus musculus GN=Gfap PE=1 SV=4 - [GFAP_MOUSE]	77.67	4	3	37	510	1.505	1.699	0.886	1.449	1433.72	77.67	68	510	430	49.9	5.34
A2ASI5	Protein Scn3a OS=Mus musculus GN=Scn3a PE=3 SV=1 - [A2ASI5_MOUSE]	10.79	11	6	20	70	1.278	1.494	1.087	1.451	212.47	10.79	34	70	1947	220.7	5.72
Q8BKV0-2	Isoform 2 of Testican-3 OS=Mus musculus GN=Spock3 - [TICN3_MOUSE]	13.16	2	3	5	9	3.848	1.004	1.411	1.451	19.76	13.16	9	9	433	48.7	5.16
P61967	AP-1 complex subunit sigma-1A OS=Mus musculus GN=Ap1s1 PE=1 SV=1 - [AP1S1_MOUSE]	23.42	3	1	3	13	0.893	1.336	1.404	1.452	36.84	23.42	5	13	158	18.7	5.73
Q91YP0	L-2-hydroxyglutarate dehydrogenase, mitochondrial OS=Mus musculus GN=L2hgdh PE=2 SV=1 - [illegible]	40.30	1	16	16	56	0.770	1.672	1.173	1.453	146.66	40.30	25	56	464	50.9	8.29
D6RCZ6	Presenilins-associated rhomboid-like protein, mitochondrial OS=Mus musculus GN=Parl PE=2 SV=1 - [D6RCZ6_MOUSE]	16.81	3	1	1	4	1.193	2.017	2.360	1.453	12.39	16.81	2	4	119	13.3	10.68
D3YWD9	Growth arrest-specific protein 2 OS=Mus musculus GN=Gas2 PE=2 SV=1 - [D3YWD9_MOUSE]	4.15	2	1	1	1	1.498	1.087	1.190	1.454	2.59	4.15	1	1	265	29.3	7.56
O35242	Protein FAN OS=Mus musculus GN=Nsmaf PE=2 SV=2 - [FAN_MOUSE]	3.80	1	3	3	4	0.879	0.488	1.301	1.454	8.00	3.80	4	4	920	104.5	5.91
P21981	Protein-glutamine gamma-glutamyltransferase 2 OS=Mus musculus GN=Tgm2 PE=1 SV=4 - [TGM2_MOUSE]	38.63	2	20	20	72	0.922	2.581	1.161	1.454	249.70	38.63	34	72	686	77.0	5.10
G3UXZ6	Polypyrimidine tract-binding protein 3 OS=Mus musculus GN=Ptbp3 PE=2 SV=1 - [G3UXZ6_MOUSE]	60.00	1	1	1	1	0.569	0.698	1.644	1.454	3.08	60.00	1	1	45	4.6	4.91
Q80X19-2	Isoform 2 of Collagen alpha-1(XIV) chain OS=Mus musculus GN=Col14a1 -	2.98	4	4	4	5	1.990	1.298	1.047	1.455	6.47	2.98	5	5	1781	191.2	5.15
Q9CZ69	CKLF-like MARVEL transmembrane domain-containing protein 6 OS=Mus musculus GN=Cmtm6 PE=2 SV=1 - [illegible]	5.46	1	1	1	1	6.708	3.154	1.162	1.456	2.17	5.46	1	1	183	19.8	6.29
Q78TU8	Family with sequence similarity 107, member A OS=Mus musculus GN=Fam107a PE=2 SV=1 - [Q78TU8_MOUSE]	24.31	4	3	4	14	0.901	0.793	2.261	1.456	33.10	24.31	7	14	144	17.5	9.74

Q8BNW9	Kelch repeat and BTB domain-containing protein 11 OS=Mus musculus GN=Kbtbd11 PE=1 SV=3 - [KBTBB_MOUSE]	45.66	1	19	19	99	0.750	1.571	1.463	1.456	306.02	45.66	35	99	633	67.9	5.39
A2A5V4	SH3 domain-binding protein 1 OS=Mus musculus GN=Sh3bp1 PE=2 SV=1 -	12.21	4	6	6	20	0.940	1.353	1.442	1.456	78.32	12.21	10	20	680	74.1	6.60
Q8VHP7	Leukocyte elastase inhibitor B OS=Mus musculus GN=Serpinb1b PE=1 SV=1 -	23.30	4	5	10	19	1.198	0.801	0.858	1.457	47.75	23.30	15	19	382	42.9	6.74
Q6ZQ18-2	Isoform 2 of Protein EFR3 homolog B OS=Mus musculus GN=Efr3b - [EFR3B_MOUSE]	28.59	2	15	17	55	0.671	1.218	1.419	1.459	194.13	28.59	30	55	773	87.5	6.68
Q91WT7	3-alpha-hydroxysteroid dehydrogenase type 1 OS=Mus musculus GN=Akr1c14 PE=2 SV=1 - [Q91WT7_MOUSE]	5.88	1	1	1	2	1.325	1.202	1.158	1.460	4.36	5.88	2	2	323	37.2	7.20
Q9WUD0	Cytochrome P450 2B10 OS=Mus musculus GN=Cyp2b10 PE=2 SV=1 - [Q9WUD0_MOUSE]	3.05	3	1	1	2	3.937	3.056	0.442	1.460	2.43	3.05	2	2	491	55.8	7.83
Q8BHX3-2	Isoform 2 of Borealin OS=Mus musculus GN=Cdca8 - [BOREA_MOUSE]	12.27	4	3	3	5	1.391	1.215	1.325	1.461	3.97	12.27	3	5	326	36.6	10.24
Q9WUT3	Ribosomal protein S6 kinase alpha-2 OS=Mus musculus GN=Rps6ka2 PE=2 SV=1 -	45.43	9	20	29	95	1.030	1.472	1.141	1.461	263.54	45.43	48	95	733	83.1	8.50
P56400	Platelet glycoprotein Ib beta chain OS=Mus musculus GN=Gp1bb PE=1 SV=1 -	13.11	2	2	2	4	1.973	3.141	0.958	1.462	9.56	13.11	3	4	206	21.7	10.43
O54991	Contactin-associated protein 1 OS=Mus musculus GN=Cntnap1 PE=1 SV=2 -	31.19	1	36	36	177	0.798	0.736	1.288	1.462	529.97	31.19	64	177	1385	156.2	7.06
Q9JI39	ATP-binding cassette sub-family B member 10, mitochondrial OS=Mus musculus GN=Abcb10 PE=2 SV=1 - [illegible]	20.28	1	8	9	20	0.731	1.049	1.028	1.462	76.03	20.28	14	20	715	77.1	9.60
Q8VEH6	COBW domain-containing protein 1 OS=Mus musculus GN=Cbwd1 PE=2 SV=1 -	7.38	2	2	2	6	1.654	1.459	1.555	1.463	15.95	7.38	3	6	393	43.7	4.89
Q99L13	3-hydroxyisobutyrate dehydrogenase, mitochondrial OS=Mus musculus GN=Hibadh PE=2 SV=1 - [illegible]	36.72	1	10	10	62	1.264	0.739	1.065	1.463	193.08	36.72	19	62	335	35.4	8.13
Q80SZ7	Guanine nucleotide-binding protein G(I)/G(S)/G(O) subunit gamma-5 OS=Mus musculus GN=Gng5 PE=2 [illegible]	33.82	2	3	3	12	1.982	1.605	1.455	1.463	29.19	33.82	5	12	68	7.3	9.85
Q8BGT9	Polypeptide N-acetylgalactosaminyltransferase 12 OS=Mus musculus GN=Galnt12 PE=2 SV=1 - [illegible]	1.74	1	1	1	1	1.191	1.101	1.334	1.465	1.97	1.74	1	1	576	66.5	7.24
Q80YT7	Myomegalin OS=Mus musculus GN=Pde4dip PE=2 SV=2 - [MYOME_MOUSE]	7.64	5	5	16	38	2.068	0.990	1.172	1.466	104.97	7.64	26	38	2224	250.5	5.53
P68368	Tubulin alpha-4A chain OS=Mus musculus GN=Tuba4a PE=1 SV=1 - [TBA4A_MOUSE]	68.75	1	12	31	3452	0.999	1.608	1.427	1.467	10425.83	68.75	58	3452	448	49.9	5.06
Q8CGC6	RNA-binding protein 28 OS=Mus musculus GN=Rbm28 PE=1 SV=4 - [RBM28_MOUSE]	0.93	1	1	1	2	1.499	1.188	1.096	1.467	4.47	0.93	1	2	750	84.2	9.54
Q64446	Copper-transporting ATPase 2 OS=Mus musculus GN=Atp7b PE=1 SV=2 - [ATP7B_MOUSE]	3.76	2	4	4	4	1.275	1.269	1.540	1.468	9.33	3.76	4	4	1462	157.1	6.65
Q8CIH5	1-phosphatidylinositol 4,5-bisphosphate phosphodiesterase gamma-2 OS=Mus musculus GN=Plcg2 PE=1 SV=1 - [PLCG2_MOUSE]	5.38	4	3	5	9	0.676	0.978	1.251	1.468	19.47	5.38	7	9	1265	147.5	6.77
Q5NCB5	Immunity-related GTPase family M protein 1 (Fragment) OS=Mus musculus GN=Irgm1 PE=2 SV=1 - [Q5NCB5_MOUSE]	29.51	3	8	8	14	0.701	1.228	1.022	1.468	45.55	29.51	12	14	288	32.3	6.42
J3QP58	Synphilin-1 OS=Mus musculus GN=Sncaip PE=4 SV=1 - [J3QP58_MOUSE]	7.16	6	2	2	11	1.143	1.466	1.305	1.469	1.86	7.16	2	11	503	54.9	7.50
Q80YR2-2	Isoform 2 of Protein FAM160B2 OS=Mus musculus GN=Fam160b2 - [F16B2_MOUSE]	5.15	2	2	2	2	1.409	0.707	0.592	1.469	7.60	5.15	2	2	544	59.8	5.35
D3YU12	NmrA-like family domain-containing protein 1 OS=Mus musculus GN=Nmral1 PE=2 SV=1 - [D3YU12_MOUSE]	39.80	5	12	12	45	0.897	0.743	1.234	1.470	120.70	39.80	22	45	299	33.1	6.80

P26048	Gamma-aminobutyric acid receptor subunit alpha-2 OS=Mus musculus GN=Gabra2 PE=1 SV=1 - [GBRA2_MOUSE]	15.52	1	3	8	47	1.398	2.330	1.066	1.470	115.82	15.52	15	47	451	51.1	9.06
Q7TSH8	Uncharacterized protein KIAA0195 OS=Mus musculus GN=Kiaa0195 PE=1 SV=1 -	6.69	5	9	10	17	0.570	1.059	1.224	1.470	41.31	6.69	15	17	1360	151.7	6.46
Q8CD19	LanC-like protein 3 OS=Mus musculus GN=Lancl3 PE=2 SV=2 - [LANC3_MOUSE]	15.24	2	4	4	8	0.884	1.024	0.888	1.470	14.61	15.24	5	8	420	46.4	7.05
F6VUT6	Protein Gm8251 OS=Mus musculus GN=Gm8251 PE=4 SV=1 - [F6VUT6_MOUSE]	2.16	1	2	3	5	1.598	1.273	1.436	1.471	9.71	2.16	3	5	1994	225.8	9.74
O35963	Ras-related protein Rab-33B OS=Mus musculus GN=Rab33b PE=1 SV=1 - [RB33B_MOUSE]	38.86	1	4	9	68	0.763	1.037	1.352	1.472	193.19	38.86	15	68	229	25.7	7.69
Q5HZI9	Solute carrier family 25 member 51 OS=Mus musculus GN=Slc25a51 PE=2 SV=1 -	29.53	4	8	8	24	0.968	1.278	1.046	1.472	67.65	29.53	16	24	298	33.7	9.66
E9PW06	Neurofascin OS=Mus musculus GN=Nfasc PE=2 SV=1 - [E9PW06_MOUSE]	46.59	2	9	51	339	1.726	0.271	1.161	1.473	1046.21	46.59	86	339	1174	132.1	6.79
Q69ZN6-2	Isoform 2 of N-acetylglucosamine-1-phosphotransferase subunits alpha/beta OS=Mus musculus	1.01	2	1	1	2	0.791	2.869	1.535	1.473	0.00	1.01	1	2	1189	135.2	7.59
Q8R238	Serine dehydratase-like OS=Mus musculus GN=Sdsl PE=2 SV=1 - [SDSL_MOUSE]	16.72	2	5	5	8	0.996	1.033	0.960	1.473	19.11	16.72	6	8	329	34.7	6.43
Q8K440-2	Isoform 2 of ATP-binding cassette sub-family A member 8-B OS=Mus musculus GN=Abca8b - [ABC8B_MOUSE]	3.27	2	3	3	5	1.061	1.630	0.840	1.473	10.19	3.27	5	5	1558	176.1	7.03
Q5H8B9	FRAS1-related extracellular matrix protein 3 OS=Mus musculus GN=Frem3 PE=2 SV=2 - [FREM3_MOUSE]	1.22	2	2	3	4	0.472	0.943	1.126	1.474	8.75	1.22	3	4	2123	234.9	5.17
Q8VFJ3	MCG141852 OS=Mus musculus GN=Olfr836 PE=2 SV=1 - [Q8VFJ3_MOUSE]	3.47	1	1	1	3	1.005	1.006	1.021	1.477	9.47	3.47	1	3	317	35.7	7.31
A7UAK4	6-phosphofructo-2-kinase/fructose-2, 6-biphosphatase 3 splice variant 1 OS=Mus musculus GN=Pfkfb3 PE=2	9.09	8	2	4	9	1.264	0.814	1.185	1.477	23.77	9.09	6	9	462	53.4	7.91
Q9DA75	Major facilitator superfamily domain-containing protein 2A OS=Mus musculus GN=Mfsd2a PE=2 SV=1 -	3.37	1	1	1	1	2.220	2.164	1.150	1.477	0.00	3.37	1	1	534	58.9	7.02
P61226	Ras-related protein Rap-2b OS=Mus musculus GN=Rap2b PE=1 SV=1 - [RAP2B_MOUSE]	50.82	1	2	7	51	0.673	1.751	1.534	1.478	167.68	50.82	14	51	183	20.5	4.81
A6PWR8	Ubiquitin carboxyl-terminal hydrolase 43 OS=Mus musculus GN=Usp43 PE=2 SV=1 - [A6PWR8_MOUSE]	2.31	3	2	3	3	1.805	2.467	1.630	1.478	4.67	2.31	3	3	1127	123.3	9.39
Q8K0L9-2	Isoform 2 of Zinc finger and BTB domain-containing protein 20 OS=Mus musculus GN=Zbtb20 -	15.72	3	6	7	12	0.749	0.864	1.727	1.478	25.19	15.72	10	12	668	73.3	6.49
Q8R151	NFX1-type zinc finger-containing protein 1 OS=Mus musculus GN=Znfx1 PE=2 SV=3 - [ZNFX1_MOUSE]	11.05	4	19	21	40	0.986	1.319	1.478	1.480	97.47	11.05	31	40	1909	218.7	7.47
Q14BE9	Protein Tekt5 OS=Mus musculus GN=Tekt5 PE=2 SV=1 - [Q14BE9_MOUSE]	7.23	2	1	3	4	2.940	0.879	1.582	1.480	10.31	7.23	3	4	415	48.1	7.11
P98086	Complement C1q subcomponent subunit A OS=Mus musculus GN=C1qa PE=1 SV=2 - [C1QA_MOUSE]	10.20	1	2	2	13	0.953	1.782	1.631	1.481	38.04	10.20	4	13	245	26.0	9.11
Q8BH02	Torsin-4A OS=Mus musculus GN=Tor4a PE=2 SV=1 - [TOR4A_MOUSE]	9.62	1	2	2	2	1.495	0.241	1.626	1.481	2.41	9.62	2	2	426	47.6	8.62
Q9CXJ4	ATP-binding cassette sub-family B member 8, mitochondrial OS=Mus musculus GN=Abcb8 PE=2 SV=1 - [ABCB8_MOUSE]	26.22	3	15	16	57	0.924	1.240	1.240	1.482	158.63	26.22	30	57	717	78.0	9.07
Q8K3A0	Iron-sulfur cluster co-chaperone protein HscB, mitochondrial OS=Mus musculus GN=Hscb PE=2 SV=2 - [HSC20_MOUSE]	45.30	3	10	10	34	1.741	1.413	1.997	1.482	113.64	45.30	15	34	234	26.6	7.37
O88533	Aromatic-L-amino-acid decarboxylase OS=Mus musculus GN=Ddc PE=2 SV=1 - [DDC_MOUSE]	31.25	2	12	12	50	1.370	0.580	1.069	1.482	161.88	31.25	22	50	480	53.8	6.60

P07309	Transthyretin OS=Mus musculus GN=Ttr PE=1 SV=1 - [TTHY_MOUSE]	15.65	4	3	3	6	4.412	3.025	1.004	1.483	10.68	15.65	5	6	147	15.8	6.16
Q62181	Semaphorin-3C OS=Mus musculus GN=Sema3c PE=1 SV=2 - [SEM3C_MOUSE]	1.46	1	1	1	1	1.569	1.546	1.408	1.484	2.19	1.46	1	1	751	85.2	8.65
H3BLR1	G/T mismatch-specific thymine DNA glycosylase (Fragment) OS=Mus musculus GN=Tdg PE=4 SV=1 - [H3BLR1_MOUSE]	11.71	5	1	1	1	1.060	0.594	1.629	1.484	1.90	11.71	1	1	111	12.4	8.18
M0QWK0	Zinc finger protein 451 OS=Mus musculus GN=Zfp451 PE=4 SV=1 - [M0QWK0_MOUSE]	9.48	1	1	1	1	2.567	0.591	0.678	1.484	2.02	9.48	1	1	116	12.3	4.46
Q80W21	Glutathione S-transferase Mu 7 OS=Mus musculus GN=Gstm7 PE=1 SV=1 - [GSTM7_MOUSE]	75.23	6	8	16	217	1.011	1.393	0.976	1.484	739.72	75.23	29	217	218	25.7	6.80
Q8VD37	SH3-containing GRB2-like protein 3-interacting protein 1 OS=Mus musculus GN=Sgip1 PE=1 SV=1 - [SGIP1_MOUSE]	52.73	5	1	33	202	1.257	1.104	1.710	1.485	556.81	52.73	59	202	806	86.0	7.87
Q9CPN8	Insulin-like growth factor 2 mRNA-binding protein 3 OS=Mus musculus GN=Igf2bp3 PE=1 SV=1 - [IF2B3_MOUSE]	2.42	1	1	1	1	1.980	3.958	2.174	1.485	2.73	2.42	1	1	579	63.5	8.87
Q9CQY6	Mitochondrial nucleoid factor 1 OS=Mus musculus GN=Mnf1 PE=1 SV=1 - [MNF1_MOUSE]	62.50	3	7	7	28	0.822	1.024	1.138	1.485	100.84	62.50	12	28	136	16.3	9.19
Q62417-2	Isoform 2 of Sorbin and SH3 domain-containing protein 1 OS=Mus musculus GN=Sorbs1 - [SRBS1_MOUSE]	45.14	5	1	26	126	1.111	1.264	1.847	1.488	364.15	45.14	48	126	740	82.8	7.85
Q8BG93	Probable 8-oxo-dGTP diphosphatase NUDT15 OS=Mus musculus GN=Nudt15 PE=1 SV=1 - [NUD15_MOUSE]	6.47	1	1	1	4	1.441	1.506	1.206	1.489	10.50	6.47	2	4	170	19.6	5.27
P55200-2	Isoform 2 of Histone-lysine N-methyltransferase 2A OS=Mus musculus GN=Kmt2a - [KMT2A_MOUSE]	2.65	2	6	6	7	1.374	1.115	1.133	1.489	23.31	2.65	7	7	3963	429.1	9.16
Q8R100	Protein FAM26E OS=Mus musculus GN=Fam26e PE=2 SV=1 - [FA26E_MOUSE]	10.68	1	2	2	3	0.889	2.114	1.524	1.490	4.03	10.68	3	3	309	35.3	8.18
Q8BLB8	Ankyrin repeat domain-containing protein 34C OS=Mus musculus GN=Ankrd34c PE=2 SV=1 [AN34C_MOUSE]	4.68	1	2	2	2	1.738	0.774	0.814	1.490	2.55	4.68	2	2	534	58.1	9.17
Q9JMF3	Guanine nucleotide-binding protein G(I)/G(S)/G(O) subunit gamma-13 OS=Mus musculus GN=Gng13 PE=2 SV=1	46.27	1	4	4	23	0.811	2.646	2.117	1.490	56.12	46.27	6	23	67	8.0	5.45
Q9D952	Envoplakin OS=Mus musculus GN=Evpl PE=2 SV=3 - [EVPL_MOUSE]	1.43	1	1	4	7	2.117	0.947	1.817	1.491	10.68	1.43	4	7	2035	231.9	6.60
P0C7A0	KH homology domain-containing protein 1B OS=Mus musculus GN=Khdc1b PE=2 SV=1 - [KHD1B_MOUSE]	6.35	3	1	1	2	1.444	1.636	0.968	1.492	0.00	6.35	1	2	126	14.8	6.07
E9QJV4	Pre-mRNA-processing factor 39 OS=Mus musculus GN=Prpf39 PE=2 SV=1 -	5.56	3	3	3	6	2.332	1.319	1.438	1.492	14.22	5.56	5	6	665	77.9	5.44
Q64430	Copper-transporting ATPase 1 OS=Mus musculus GN=Atp7a PE=1 SV=3 - [ATP7A_MOUSE]	6.17	2	5	7	18	0.962	1.159	0.990	1.493	49.04	6.17	10	18	1491	161.9	6.62
Q9WUF3	CASP8-associated protein 2 OS=Mus musculus GN=Casp8ap2 PE=1 SV=2 - [C8AP2_MOUSE]	0.71	2	2	2	2	1.784	1.699	1.025	1.494	4.12	0.71	2	2	1962	219.0	6.67
Q8BHW4	Protein 4933430I17Rik OS=Mus musculus GN=4933430I17Rik PE=2 SV=1 -	5.25	1	2	2	2	1.304	1.197	1.985	1.494	2.18	5.25	2	2	495	56.2	8.46
Q6XAS3	MCG116991, isoform CRA_b OS=Mus musculus GN=Ssx9 PE=2 SV=1 - [Q6XAS3_MOUSE]	14.12	1	1	1	1	1.223	1.111	1.471	1.495	4.04	14.12	1	1	170	20.0	5.10
P15105	Glutamine synthetase OS=Mus musculus GN=Glul PE=1 SV=6 - [GLNA_MOUSE]	58.98	3	20	20	646	0.924	1.049	1.328	1.495	2018.90	58.98	37	646	373	42.1	7.08
Q2MV57-2	Isoform 2 of Tectonic-2 OS=Mus musculus GN=Tctn2 - [TECT2_MOUSE]	5.35	3	3	3	4	2.618	1.207	1.228	1.497	11.21	5.35	4	4	579	63.9	5.91
Q61330	Contactin-2 OS=Mus musculus GN=Cntn2 PE=2 SV=2 - [CNTN2_MOUSE]	30.58	1	25	25	114	1.183	0.681	1.257	1.497	379.68	30.58	42	114	1040	113.1	7.62
P28661-2	Isoform 2 of Septin-4 OS=Mus musculus GN=Sept4 - [SEPT4_MOUSE]	54.68	10	18	22	145	1.493	0.500	1.369	1.498	436.82	54.68	39	145	459	53.0	6.18

Q7TS17	MCG1027552 OS=Mus musculus GN=Olfr285 PE=3 SV=1 - [Q7TS17_MOUSE]	3.13	1	1	1	3	0.828	1.660	0.958	1.498	8.14	3.13	1	3	319	35.6	7.91
Q8BMS4	Hexaprenyldihydroxybenzoate methyltransferase, mitochondrial OS=Mus musculus GN=Coq3 PE=2 SV=1 - [COQ3_MOUSE]	20.81	1	6	6	23	1.221	1.295	1.202	1.498	66.29	20.81	11	23	370	40.9	7.96
Q505D7	Optic atrophy 3 protein homolog OS=Mus musculus GN=Opa3 PE=1 SV=1 - [OPA3_MOUSE]	33.52	1	5	5	20	0.593	1.308	1.285	1.499	49.97	33.52	8	20	179	20.1	8.34
Q60770	Syntaxin-binding protein 3 OS=Mus musculus GN=Stxbp3 PE=1 SV=1 - [STXB3_MOUSE]	33.95	4	18	18	38	1.001	0.784	1.246	1.499	118.07	33.95	29	38	592	67.9	8.02
Q9CRB8	Mitochondrial fission process protein 1 OS=Mus musculus GN=Mtfp1 PE=1 SV=1 - [MTFP1_MOUSE]	28.92	1	5	5	11	0.749	1.735	1.267	1.500	29.82	28.92	9	11	166	18.3	8.68
P57016	Ladinin-1 OS=Mus musculus GN=Lad1 PE=1 SV=1 - [LAD1_MOUSE]	5.68	1	2	2	3	0.697	0.923	1.592	1.500	8.17	5.68	2	3	528	58.8	9.73
Q6ZPV2-2	Isoform 2 of DNA helicase INO80 OS=Mus musculus GN=Ino80 - [INO80_MOUSE]	1.29	2	2	2	2	0.673	1.281	1.172	1.500	2.61	1.29	2	2	1552	175.4	9.32
A2AFI9	Histone-binding protein RBBP7 OS=Mus musculus GN=Rbbp7 PE=2 SV=1 - [A2AFI9_MOUSE]	31.87	2	1	12	61	1.845	0.943	1.532	1.501	149.80	31.87	19	61	386	43.6	5.45
Q9Z239	Phospholemman OS=Mus musculus GN=Fxyd1 PE=1 SV=1 - [PLM_MOUSE]	8.70	1	1	1	1	0.698	0.587	1.067	1.502	2.85	8.70	1	1	92	10.3	8.54
Q9CWL2	Zinc finger protein castor homolog 1 OS=Mus musculus GN=Casz1 PE=2 SV=3 - [CASZ1_MOUSE]	0.57	2	1	1	1	1.227	1.599	1.597	1.503	2.66	0.57	1	1	1761	191.1	7.14
Q5F285	Transmembrane protein 256 OS=Mus musculus GN=Tmem256 PE=2 SV=1 - [TM256_MOUSE]	24.78	1	1	1	4	0.450	1.303	1.252	1.503	27.32	24.78	2	4	113	11.6	8.94
Q8CII2	Cell division cycle protein 123 homolog OS=Mus musculus GN=Cdc123 PE=2 SV=2 - [CD123_MOUSE]	11.31	2	3	4	16	1.848	2.211	1.878	1.505	39.08	11.31	7	16	336	38.8	4.67
P59055-2	Isoform 2 of Cysteine/serine-rich nuclear protein 3 OS=Mus musculus GN=Csrnp3 - [CSRN3_MOUSE]	2.56	2	1	1	1	2.478	1.115	1.149	1.505	3.01	2.56	1	1	585	64.9	4.73
Q9JJN2	Zinc finger homeobox protein 4 OS=Mus musculus GN=Zfhx4 PE=1 SV=1 - [ZFHX4_MOUSE]	2.08	4	4	5	6	1.424	1.407	1.560	1.505	8.33	2.08	5	6	3550	392.1	6.44
J3KMT3	Protein Gm6882 OS=Mus musculus GN=Gm6882 PE=4 SV=1 - [J3KMT3_MOUSE]	10.86	1	2	2	3	0.844	1.791	1.832	1.505	5.18	10.86	3	3	313	35.4	6.83
Q9D6F9	Tubulin beta-4A chain OS=Mus musculus GN=Tubb4a PE=1 SV=3 - [TBB4A_MOUSE]	84.01	1	9	31	3928	0.962	1.155	1.502	1.506	10069.98	84.01	59	3928	444	49.6	4.88
Q80WM4	Hyaluronan and proteoglycan link protein 4 OS=Mus musculus GN=Hapln4 PE=2 SV=2 - [HPLN4_MOUSE]	39.00	1	10	11	62	1.137	0.510	1.007	1.507	230.59	39.00	19	62	400	42.8	8.85
Q9R069	Basal cell adhesion molecule OS=Mus musculus GN=Bcam PE=2 SV=1 - [BCAM_MOUSE]	30.71	2	15	15	42	1.165	1.976	0.991	1.507	116.92	30.71	22	42	622	67.6	6.25
P63141	Potassium voltage-gated channel subfamily A member 2 OS=Mus musculus GN=Kcna2 PE=1 SV=1 - [KCNA2_MOUSE]	23.85	2	5	9	58	0.733	0.550	1.335	1.507	209.19	23.85	16	58	499	56.7	4.86
Q6DFV7-6	Isoform 6 of Nuclear receptor coactivator 7 OS=Mus musculus GN=Ncoa7 -	21.38	4	1	3	4	1.668	1.492	1.589	1.508	12.50	21.38	4	4	159	18.4	6.64
E9Q2N0	Protein Cpsf4l OS=Mus musculus GN=Cpsf4l PE=2 SV=1 - [E9Q2N0_MOUSE]	4.29	1	1	1	2	1.903	1.544	1.661	1.510	6.12	4.29	1	2	280	31.8	8.54
Q8R1M0	UPF0361 protein C3orf37 homolog OS=Mus musculus PE=2 SV=1 - [CC037_MOUSE]	6.52	1	2	2	5	4.807	1.118	1.558	1.510	14.49	6.52	3	5	353	40.1	8.57
E9PVX6	Protein Mki67 OS=Mus musculus GN=Mki67 PE=2 SV=1 - [E9PVX6_MOUSE]	1.07	1	3	3	3	3.173	2.041	3.846	1.510	5.00	1.07	3	3	3177	350.7	9.72
Q7M6Y6	Maestro heat-like repeat-containing protein family member 2B OS=Mus musculus GN=Mroh2b PE=1 SV=2 - [MRO2B_MOUSE]	1.14	1	1	1	1	1.666	1.038	1.690	1.511	0.00	1.14	1	1	1581	180.4	6.61
Q8K2C8	Glycerol-3-phosphate acyltransferase 4 OS=Mus musculus GN=Agpat6 PE=2 SV=1 - [GPAT4_MOUSE]	7.02	1	2	2	3	1.677	1.328	1.358	1.512	7.62	7.02	3	3	456	52.1	9.35

Q62264	Thyroid hormone-inducible hepatic protein OS=Mus musculus GN=Thrsp PE=1 SV=1 - [THRSP_MOUSE]	8.67	1	1	1	2	1.563	2.095	1.190	1.512	8.47	8.67	2	2	150	17.1	4.98
E9QAZ9	Protein Vmn2r100 OS=Mus musculus GN=Vmn2r100 PE=3 SV=1 - [E9QAZ9_MOUSE]	2.47	1	1	3	6	0.911	0.854	1.331	1.513	14.03	2.47	3	6	850	97.2	7.61
Q9JJ94	Sjoegren syndrome nuclear autoantigen 1 homolog OS=Mus musculus GN=Ssna1 PE=1 SV=1 - [SSNA1_MOUSE]	23.53	1	2	2	6	9.189	2.851	2.584	1.515	4.08	23.53	2	6	119	13.5	5.68
P54869	Hydroxymethylglutaryl-CoA synthase, mitochondrial OS=Mus musculus GN=Hmgcs2 PE=1 SV=2 - [illegible]	17.32	1	8	9	29	0.663	2.153	1.052	1.515	81.66	17.32	14	29	508	56.8	8.41
Q8BH59	Calcium-binding mitochondrial carrier protein Aralar1 OS=Mus musculus GN=Slc25a12 PE=1 SV=1 - [illegible]	70.90	1	33	39	443	0.743	1.245	1.200	1.516	1384.72	70.90	75	443	677	74.5	8.25
Q8BH00	Aldehyde dehydrogenase family 8 member A1 OS=Mus musculus GN=Aldh8a1 PE=1 SV=1 - [AL8A1_MOUSE]	7.80	2	2	3	3	2.729	1.370	1.081	1.516	7.81	7.80	3	3	487	53.6	7.55
P55099	Tachykinin-3 OS=Mus musculus GN=Tac3 PE=2 SV=2 - [TKNK_MOUSE]	11.21	1	1	1	17	1.325	1.001	1.119	1.516	51.07	11.21	2	17	116	12.7	8.15
A8E0Y8	Immunoglobulin superfamily member 2 OS=Mus musculus GN=Cd101 PE=2 SV=1 - [IGSF2_MOUSE]	0.77	1	1	1	3	2.287	0.352	1.779	1.517	6.26	0.77	1	3	1033	114.1	8.02
Q8BJG4	MOB kinase activator 3C OS=Mus musculus GN=Mob3c PE=2 SV=1 - [MOB3C_MOUSE]	6.48	1	1	2	2	1.861	1.428	1.087	1.518	3.54	6.48	2	2	216	25.5	8.44
Q9D5R3	Centrosomal protein of 83 kDa OS=Mus musculus GN=Ccdc41 PE=2 SV=2 - [CEP83_MOUSE]	3.03	6	1	2	5	0.882	1.027	1.686	1.519	9.08	3.03	3	5	692	81.9	6.61
Q99JZ7	ERBB receptor feedback inhibitor 1 OS=Mus musculus GN=Errfi1 PE=1 SV=1 - [ERRFI_MOUSE]	3.69	1	1	1	4	1.631	0.826	1.579	1.520	13.21	3.69	2	4	461	50.0	7.72
P02772	Alpha-fetoprotein OS=Mus musculus GN=Afp PE=2 SV=1 - [FETA_MOUSE]	1.65	1	1	1	1	0.564	1.104	1.606	1.521	2.04	1.65	1	1	605	67.3	5.92
Q91YI1-2	Isoform 2 of Autophagy-related protein 13 OS=Mus musculus GN=Atg13 - [ATG13_MOUSE]	9.19	2	4	4	10	1.200	1.471	1.334	1.522	24.51	9.19	6	10	479	52.6	5.16
Q8BJ56-3	Isoform 3 of Patatin-like phospholipase domain-containing protein 2 OS=Mus musculus GN=Pnpla2 - [illegible]	10.00	5	3	3	6	2.099	0.863	1.492	1.523	18.39	10.00	4	6	430	47.8	6.09
P03995-2	Isoform 2 of Glial fibrillary acidic protein OS=Mus musculus GN=Gfap - [GFAP_MOUSE]	78.04	4	1	35	506	1.885	1.595	0.971	1.523	1418.92	78.04	66	506	428	49.3	5.50
P17809	Solute carrier family 2, facilitated glucose transporter member 1 OS=Mus musculus GN=Slc2a1 PE=1 SV=4 - [illegible]	11.18	1	7	7	40	1.060	0.892	1.224	1.524	110.52	11.18	11	40	492	53.9	8.87
Q02013	Aquaporin-1 OS=Mus musculus GN=Aqp1 PE=1 SV=3 - [AQP1_MOUSE]	3.35	1	1	1	4	1.782	2.721	0.492	1.524	10.71	3.35	2	4	269	28.8	7.43
Q8VED9	Galectin-related protein OS=Mus musculus GN=Lgalsl PE=1 SV=1 - [LEGL_MOUSE]	62.79	1	6	9	75	0.828	1.699	1.642	1.525	219.87	62.79	18	75	172	18.9	5.35
Q8VCB3	Glycogen [starch] synthase, liver OS=Mus musculus GN=Gys2 PE=1 SV=2 - [GYS2_MOUSE]	2.56	1	1	1	1	1.524	2.682	1.032	1.525	3.41	2.56	1	1	704	80.8	6.89
Q00519	Xanthine dehydrogenase/oxidase OS=Mus musculus GN=Xdh PE=1 SV=5 -	2.70	2	2	4	10	2.019	1.868	1.570	1.527	20.65	2.70	7	10	1335	146.5	7.56
Q99LH1	Nucleolar GTP-binding protein 2 OS=Mus musculus GN=Gnl2 PE=2 SV=2 - [NOG2_MOUSE]	4.26	2	2	2	3	0.781	1.165	1.159	1.528	9.63	4.26	3	3	728	83.3	9.20
Q5GAM8	Probable inactive ribonuclease-like protein 12 OS=Mus musculus GN=Rnase12 PE=2 SV=1 - [RNS12_MOUSE]	4.83	1	1	1	1	1.470	1.170	1.034	1.529	0.00	4.83	1	1	145	16.9	6.51
Q8CHK2	Relaxin-3 OS=Mus musculus GN=Rln3 PE=2 SV=1 - [REL3_MOUSE]	14.18	1	2	2	6	1.088	0.416	1.270	1.530	13.45	14.18	3	6	141	14.9	5.91
E9QNK5	Corticotropin-releasing factor receptor 2 OS=Mus musculus GN=Crhr2 PE=2 SV=1 - [E9QNK5_MOUSE]	2.44	4	1	1	3	0.866	1.489	2.191	1.531	3.89	2.44	1	3	410	47.5	7.40

P43006-3	Isoform Glt-1B of Excitatory amino acid transporter 2 OS=Mus musculus GN=Slc1a2 - [EAA2_MOUSE]	32.08	5	1	18	685	1.426	1.299	1.312	1.532	2146.04	32.08	29	685	558	60.6	6.68
Q62433	Protein NDRG1 OS=Mus musculus GN=Ndrg1 PE=1 SV=1 - [NDRG1_MOUSE]	45.18	9	8	12	130	1.071	0.757	1.145	1.534	396.50	45.18	23	130	394	43.0	6.10
Q8K0M7	Protein D630039A03Rik OS=Mus musculus GN=D630039A03Rik PE=2 SV=2 - [Q8K0M7_MOUSE]	6.78	1	1	1	3	1.360	0.906	1.091	1.534	0.00	6.78	1	3	236	26.3	8.02
Q3V384	Lactation elevated protein 1 OS=Mus musculus GN=Lace1 PE=2 SV=1 - [LACE1_MOUSE]	21.88	3	8	8	21	0.840	0.916	1.264	1.536	65.86	21.88	15	21	480	54.3	8.03
Q9JLJ2	4-trimethylaminobutyraldehyde dehydrogenase OS=Mus musculus GN=Aldh9a1 PE=1 SV=1 - [AL9A1_MOUSE]	48.79	2	22	23	110	0.982	1.138	1.025	1.537	341.10	48.79	39	110	494	53.5	6.98
Q8CBX9	Basic immunoglobulin-like variable motif-containing protein OS=Mus musculus GN=Bivm PE=2 SV=1 - [BIVM_MOUSE]	4.98	1	2	2	2	1.320	2.009	1.450	1.539	5.22	4.98	2	2	502	56.6	8.81
Q3USL1	Kelch domain-containing protein 9 OS=Mus musculus GN=Klhdc9 PE=2 SV=1 -	4.57	1	1	1	9	0.850	1.100	0.856	1.539	17.91	4.57	2	9	350	38.2	8.50
Q07076	Annexin A7 OS=Mus musculus GN=Anxa7 PE=2 SV=2 - [ANXA7_MOUSE]	32.83	1	15	15	104	0.864	2.479	1.474	1.543	320.34	32.83	27	104	463	49.9	6.18
A2A880	Sorting nexin-11 (Fragment) OS=Mus musculus GN=Snx11 PE=2 SV=1 - [A2A880_MOUSE]	6.60	2	1	1	2	1.638	1.283	1.927	1.545	2.61	6.60	2	2	197	22.4	8.09
Q9JMA2	Queuine tRNA-ribosyltransferase OS=Mus musculus GN=Qtrt1 PE=1 SV=2 - [TGT_MOUSE]	19.35	1	5	6	18	1.223	1.419	1.719	1.546	49.08	19.35	9	18	403	44.1	7.68
O08966	Solute carrier family 22 member 1 OS=Mus musculus GN=Slc22a1 PE=2 SV=2 -	6.83	3	3	3	4	1.696	1.185	1.268	1.547	5.22	6.83	3	4	556	61.5	6.67
Q8VD73	Potassium voltage-gated channel, shaker-related subfamily, beta member 3 OS=Mus musculus GN=Kcnab3 PE=2 SV=1 - [Q8VD73_MOUSE]	19.80	2	4	6	14	0.807	0.461	1.106	1.551	43.52	19.80	10	14	404	43.8	8.54
Q61016	Guanine nucleotide-binding protein G(I)/G(S)/G(O) subunit gamma-7 OS=Mus musculus GN=Gng7 PE=2 SV=2 - [GBG7_MOUSE]	67.65	3	5	6	29	0.938	1.988	3.340	1.552	75.26	67.65	10	29	68	7.5	8.51
Q6NV92	Protein Zfp941 OS=Mus musculus GN=Zfp941 PE=2 SV=1 - [Q6NV92_MOUSE]	2.09	2	1	1	4	0.884	1.180	1.002	1.552	11.32	2.09	2	4	671	75.9	9.32
F6V5V4	Protein Gm11397 OS=Mus musculus GN=Gm11397 PE=3 SV=1 - [F6V5V4_MOUSE]	10.61	4	1	2	8	1.990	0.703	2.475	1.552	2.44	10.61	3	8	377	43.0	5.87
D3Z2D7	Phenazine biosynthesis-like domain-containing protein 2 (Fragment) OS=Mus musculus GN=Pbld2 PE=2 SV=1 - [D3Z2D7_MOUSE]	7.43	2	1	1	1	2.157	1.209	1.374	1.552	2.67	7.43	1	1	175	19.9	10.48
Q64339	Ubiquitin-like protein ISG15 OS=Mus musculus GN=Isg15 PE=1 SV=4 - [ISG15_MOUSE]	9.32	1	2	2	6	1.751	1.900	0.809	1.552	12.19	9.32	3	6	161	17.9	7.90
Q99LB7	Sarcosine dehydrogenase, mitochondrial OS=Mus musculus GN=Sardh PE=1 SV=1 - [SARDH_MOUSE]	17.52	3	12	12	36	1.083	0.725	1.083	1.552	103.26	17.52	22	36	919	101.6	6.74
O70209	PDZ and LIM domain protein 3 OS=Mus musculus GN=Pdlim3 PE=1 SV=1 -	28.48	1	5	5	15	1.046	3.816	1.469	1.553	55.87	28.48	9	15	316	34.3	7.91
P46660	Alpha-internexin OS=Mus musculus GN=Ina PE=1 SV=2 - [AINX_MOUSE]	67.06	1	31	35	1067	1.678	0.377	1.787	1.554	2981.82	67.06	68	1067	504	55.7	5.27
Q99PW8	Kinesin-like protein KIF17 OS=Mus musculus GN=Kif17 PE=1 SV=1 - [KIF17_MOUSE]	4.43	4	2	3	4	1.521	0.927	1.750	1.556	5.85	4.43	4	4	1038	116.3	5.26
Q8BH86	UPF0317 protein C14orf159 homolog, mitochondrial OS=Mus musculus PE=2 SV=1 - [CN159_MOUSE]	25.77	4	14	14	40	1.362	1.317	1.311	1.557	124.96	25.77	26	40	617	66.3	7.09
O55123	Stromelysin-2 OS=Mus musculus GN=Mmp10 PE=2 SV=1 - [MMP10_MOUSE]	2.73	3	1	1	4	1.553	1.195	1.470	1.558	1.90	2.73	1	4	476	53.9	6.37
Q7TN89	GTPase ERas OS=Mus musculus GN=Eras PE=1 SV=1 - [RASE_MOUSE]	3.08	1	1	1	1	0.349	2.188	1.431	1.562	2.56	3.08	1	1	227	24.3	6.83
Q6ZQM0-4	Isoform 4 of E3 ubiquitin-protein ligase rififylin OS=Mus musculus GN=Rffl - [RFFL_MOUSE]	6.48	5	1	1	3	2.659	0.944	1.230	1.562	6.12	6.48	2	3	293	33.0	5.83

Q80WM5	Hyaluronan and proteoglycan link protein 3 OS=Mus musculus GN=Hapln3 PE=2 SV=3 - [HPLN3_MOUSE]	16.99	1	5	5	13	1.189	1.337	1.328	1.564	26.80	16.99	8	13	359	40.7	6.52
P28666	Murinoglobulin-2 OS=Mus musculus GN=Mug2 PE=2 SV=2 - [MUG2_MOUSE]	13.58	1	1	17	55	0.657	1.494	1.063	1.566	157.69	13.58	24	55	1451	162.3	6.74
P34914	Bifunctional epoxide hydrolase 2 OS=Mus musculus GN=Ephx2 PE=1 SV=2 - [HYES_MOUSE]	38.27	2	15	15	47	0.975	1.729	1.702	1.567	148.33	38.27	27	47	554	62.5	6.19
E9PYP1	Protein Ces1a OS=Mus musculus GN=Ces1a PE=4 SV=1 - [E9PYP1_MOUSE]	4.26	1	1	2	6	6.271	5.555	1.579	1.567	12.88	4.26	3	6	563	61.7	5.26
Q9CZT5	Vasorin OS=Mus musculus GN=Vasn PE=2 SV=2 - [VASN_MOUSE]	6.09	1	2	2	3	1.510	0.752	1.605	1.569	7.60	6.09	3	3	673	72.2	7.08
P61372	Insulin gene enhancer protein ISL-1 OS=Mus musculus GN=Isl1 PE=1 SV=1 - [ISL1_MOUSE]	5.16	1	1	1	4	19.943	5.821	2.550	1.570	6.49	5.16	1	4	349	39.0	8.28
P85094	Isochorismatase domain-containing protein 2A, mitochondrial OS=Mus musculus GN=Isoc2a PE=2 SV=1 - [illegible]	58.74	1	7	7	20	1.160	1.579	1.143	1.571	71.82	58.74	13	20	206	22.4	8.02
Q9Z2E4	Protein phosphatase 1 regulatory subunit 17 OS=Mus musculus GN=Ppp1r17 PE=1 SV=1 - [PPR17_MOUSE]	11.32	1	1	1	2	1.763	1.324	1.545	1.573	5.37	11.32	2	2	159	17.8	6.23
Q6IQY5-2	Isoform 2 of Centrosomal protein of 70 kDa OS=Mus musculus GN=Cep70 - [CEP70_MOUSE]	3.98	2	2	2	2	1.925	2.304	0.513	1.573	4.37	3.98	2	2	578	66.8	6.51
F8VPN7	Dedicator of cytokinesis protein 9 OS=Mus musculus GN=Dock9 PE=2 SV=1 - [F8VPN7_MOUSE]	19.92	2	1	37	96	0.889	1.124	1.257	1.573	254.89	19.92	60	96	2113	241.2	7.43
Q9D3A8	Carboxyl-terminal PDZ ligand of neuronal nitric oxide synthase protein OS=Mus musculus GN=Nos1ap PE=1 SV=3 - [CAPON_MOUSE]	6.76	4	4	4	6	0.856	2.033	1.268	1.574	17.69	6.76	5	6	503	55.8	6.09
F6RVG2	Suppressor of cytokine-signaling 2 (Fragment) OS=Mus musculus GN=Socs2 PE=4 SV=1 - [F6RVG2_MOUSE]	2.99	1	1	1	1	2.217	2.363	2.129	1.575	2.51	2.99	1	1	201	22.9	8.92
B1AS67	Pleckstrin homology domain-containing family G member 5 OS=Mus musculus GN=Plekhg5 PE=2 SV=1 - [illegible]	4.62	5	3	4	9	0.947	1.064	1.719	1.578	18.48	4.62	5	9	1060	117.8	6.83
Q8C753-4	Isoform 4 of Uncharacterized protein KIAA0556 OS=Mus musculus GN=Kiaa0556 - [K0556_MOUSE]	2.61	6	3	3	3	1.762	1.357	1.360	1.579	5.86	2.61	3	3	1612	179.2	5.27
Q9ER56-2	Isoform 2 of Calpain-12 OS=Mus musculus GN=Capn12 - [CAN12_MOUSE]	2.45	5	1	1	6	4.653	0.193	2.137	1.579	0.00	2.45	1	6	449	50.4	7.62
Q61102	ATP-binding cassette sub-family B member 7, mitochondrial OS=Mus musculus GN=Abcb7 PE=1 SV=3 - [ABCB7_MOUSE]	37.63	1	23	24	75	0.812	1.206	1.455	1.580	223.96	37.63	41	75	752	82.5	9.32
Q3U213-2	Isoform 2 of Protein SERAC1 OS=Mus musculus GN=Serac1 - [SRAC1_MOUSE]	6.57	3	4	4	5	0.878	0.761	1.470	1.581	10.04	6.57	5	5	624	70.6	7.53
Q6X7S9	EP300-interacting inhibitor of differentiation 2 OS=Mus musculus GN=Eid2 PE=1 SV=1 - [EID2_MOUSE]	9.75	1	2	2	4	1.759	0.641	2.318	1.581	11.77	9.75	4	4	236	25.3	10.37
F6XLV1	Protein E030010N08Rik (Fragment) OS=Mus musculus GN=E030010N08Rik PE=4 SV=1 - [F6XLV1_MOUSE]	15.36	1	3	4	7	0.540	0.973	1.115	1.583	17.91	15.36	7	7	306	34.7	9.95
P57725	SAM domain-containing protein SAMSN-1 OS=Mus musculus GN=Samsn1 PE=1 SV=2 - [SAMN1_MOUSE]	8.33	2	2	2	2	3.672	4.490	1.636	1.583	2.38	8.33	2	2	372	41.6	5.25
O88531	Palmitoyl-protein thioesterase 1 OS=Mus musculus GN=Ppt1 PE=2 SV=2 - [PPT1_MOUSE]	34.31	3	8	9	51	0.877	1.920	1.300	1.584	179.93	34.31	16	51	306	34.5	8.00
D3YWJ0	Protein Nuggc OS=Mus musculus GN=Nuggc PE=4 SV=1 - [D3YWJ0_MOUSE]	8.17	2	3	3	4	4.478	2.128	0.896	1.586	2.71	8.17	3	4	796	91.2	8.95
P97799	Neurensin-1 OS=Mus musculus GN=Nrsn1 PE=2 SV=1 - [NRSN1_MOUSE]	11.73	1	3	3	5	0.714	1.044	0.838	1.587	11.88	11.73	5	5	196	21.6	7.99
P58468	Protein FAM207A OS=Mus musculus GN=Fam207a PE=1 SV=1 - [F207A_MOUSE]	23.74	1	4	4	7	1.549	1.698	1.192	1.589	22.38	23.74	7	7	219	24.8	11.00

Q9Z0F7	Gamma-synuclein OS=Mus musculus GN=Sncg PE=1 SV=1 - [SYUG_MOUSE]	64.23	1	9	9	112	2.427	0.599	1.259	1.591	364.21	64.23	16	112	123	13.2	4.65
Q3U5Q7	UMP-CMP kinase 2, mitochondrial OS=Mus musculus GN=Cmpk2 PE=2 SV=2 -	41.61	4	15	16	45	0.870	1.091	1.026	1.598	135.44	41.61	27	45	447	50.0	7.27
P97459	Neuronal PAS domain-containing protein 1 OS=Mus musculus GN=Npas1 PE=1 SV=1 - [NPAS1_MOUSE]	2.19	1	1	1	1	4.220	1.336	2.692	1.598	2.23	2.19	1	1	594	63.7	8.63
Q3UTJ2	Sorbin and SH3 domain-containing protein 2 OS=Mus musculus GN=Sorbs2 PE=1 SV=2 - [SRBS2_MOUSE]	42.03	12	1	40	150	0.722	2.105	1.549	1.599	445.58	42.03	71	150	1180	132.3	8.53
Q8BYH7	TBC1 domain family member 17 OS=Mus musculus GN=Tbc1d17 PE=2 SV=2 -	11.78	2	6	7	17	0.940	1.213	1.207	1.600	48.18	11.78	11	17	645	72.8	5.25
Q924Y0	Gamma-butyrobetaine dioxygenase OS=Mus musculus GN=Bbox1 PE=2 SV=1 - [BODG_MOUSE]	12.66	1	4	4	12	1.762	0.719	0.866	1.603	28.75	12.66	7	12	387	44.7	6.92
Q9D4K7	Coiled-coil domain-containing protein 105 OS=Mus musculus GN=Ccdc105 PE=2 SV=3 - [CC105_MOUSE]	6.21	1	3	3	3	1.758	2.363	1.043	1.605	2.40	6.21	3	3	499	57.3	9.82
Q9CQR4	Acyl-coenzyme A thioesterase 13 OS=Mus musculus GN=Acot13 PE=1 SV=1 -	53.57	1	7	7	30	0.589	1.208	1.464	1.607	99.05	53.57	11	30	140	15.2	8.82
Q8K2P2	Protein FAM83A OS=Mus musculus GN=Fam83a PE=2 SV=2 - [FA83A_MOUSE]	3.21	1	1	1	1	0.789	0.796	1.136	1.608	2.00	3.21	1	1	436	48.2	8.63
P82343-2	Isoform 2 of N-acylglucosamine 2-epimerase OS=Mus musculus GN=Renbp -	5.05	3	3	3	9	0.856	1.848	0.946	1.608	15.96	5.05	4	9	416	48.0	5.87
Q3UW53	Protein Niban OS=Mus musculus GN=Fam129a PE=1 SV=2 - [NIBAN_MOUSE]	13.71	3	7	7	10	1.449	1.394	1.188	1.608	25.86	13.71	9	10	926	102.6	4.78
Q99L20	Glutathione S-transferase theta 3 OS=Mus musculus GN=Gstt3 PE=2 SV=1 - [Q99L20_MOUSE]	36.10	4	6	8	33	0.490	0.884	0.727	1.609	114.68	36.10	14	33	241	27.4	7.74
Q80W22	Threonine synthase-like 2 OS=Mus musculus GN=Thnsl2 PE=2 SV=1 - [THNS2_MOUSE]	15.94	2	8	8	18	1.206	1.460	1.218	1.609	55.97	15.94	15	18	483	54.2	6.39
Q07456	Protein AMBP OS=Mus musculus GN=Ambp PE=1 SV=2 - [AMBP_MOUSE]	14.90	1	4	4	12	3.342	2.340	0.878	1.612	38.50	14.90	8	12	349	39.0	6.32
Q71B07	Probable C-mannosyltransferase DPY19L3 OS=Mus musculus GN=Dpy19l3 PE=2 SV=2 - [illegible]	3.63	1	3	3	7	0.642	1.085	1.206	1.614	21.96	3.63	6	7	716	82.9	8.75
B2RPV6	Multimerin-1 OS=Mus musculus GN=Mmrn1 PE=2 SV=2 - [MMRN1_MOUSE]	4.63	3	2	4	17	0.769	0.892	1.580	1.616	18.79	4.63	5	17	1210	136.0	7.71
D3YW52	Alpha-2-macroglobulin OS=Mus musculus GN=Pzp PE=2 SV=1 - [D3YW52_MOUSE]	36.70	3	42	45	220	3.034	3.079	0.802	1.617	618.30	36.70	80	220	1507	167.2	6.83
Q5IRJ6	Zinc transporter 9 OS=Mus musculus GN=Slc30a9 PE=1 SV=2 - [ZNT9_MOUSE]	26.46	3	14	14	38	0.892	1.116	1.289	1.617	97.17	26.46	23	38	567	62.8	8.69
G3X919	T-box transcription factor TBX18 OS=Mus musculus GN=Tbx18 PE=4 SV=1 - [G3X919_MOUSE]	5.22	2	2	2	2	1.487	2.062	1.608	1.618	2.48	5.22	2	2	613	65.4	8.43
Q8R3P6	von Willebrand factor A domain-containing protein 9 OS=Mus musculus GN=Vwa9 PE=2 SV=1 - [VWA9_MOUSE]	11.65	1	3	4	5	0.837	1.439	1.301	1.619	6.40	11.65	5	5	515	57.2	5.17
Q8BH35	Complement component C8 beta chain OS=Mus musculus GN=C8b PE=1 SV=1 - [CO8B_MOUSE]	10.19	2	4	4	11	1.858	1.956	1.136	1.619	10.42	10.19	6	11	589	66.2	7.77
Q3UHB8	Coiled-coil domain-containing protein 177 OS=Mus musculus GN=Ccdc177 PE=1 SV=1 - [CC177_MOUSE]	23.09	1	12	12	27	0.954	1.330	1.940	1.623	70.26	23.09	20	27	706	79.8	10.80
P40201	Chromodomain-helicase-DNA-binding protein 1 OS=Mus musculus GN=Chd1 PE=1 SV=3 - [CHD1_MOUSE]	1.81	3	2	3	5	1.248	0.795	1.354	1.623	6.97	1.81	4	5	1711	196.3	7.37
Q64442	Sorbitol dehydrogenase OS=Mus musculus GN=Sord PE=1 SV=3 - [DHSO_MOUSE]	49.30	1	15	15	49	1.228	1.130	1.254	1.625	156.21	49.30	26	49	357	38.2	7.02
P62965	Cellular retinoic acid-binding protein 1 OS=Mus musculus GN=Crabp1 PE=1 SV=2 -	57.66	1	6	7	15	1.614	1.246	1.123	1.625	47.83	57.66	11	15	137	15.6	5.38
O09116	Small proline-rich protein 3 OS=Mus musculus GN=Sprr3 PE=2 SV=1 - [SPRR3_MOUSE]	5.88	1	1	1	3	1.244	0.390	1.873	1.626	2.27	5.88	1	3	238	25.2	5.07

Q91XH5	Sepiapterin reductase OS=Mus musculus GN=Spr PE=2 SV=1 - [Q91XH5_MOUSE]	66.03	5	15	15	67	0.967	1.427	1.242	1.626	217.71	66.03	23	67	262	27.9	6.21
P10284	Homeobox protein Hox-B4 OS=Mus musculus GN=Hoxb4 PE=2 SV=1 - [HXB4_MOUSE]	6.80	1	1	1	1	0.924	0.658	0.977	1.627	3.44	6.80	1	1	250	27.5	9.77
Q8VDS8-3	Isoform 3 of Syntaxin-18 OS=Mus musculus GN=Stx18 - [STX18_MOUSE]	13.96	4	4	4	10	1.289	1.242	0.902	1.628	29.24	13.96	6	10	308	35.2	5.87
A2AGB2	Uncharacterized protein C1orf87 homolog OS=Mus musculus GN=Gm12695 PE=4 SV=1 - [CA087_MOUSE]	1.28	1	1	1	7	0.832	1.150	1.228	1.629	13.99	1.28	2	7	545	62.4	9.31
Q9CS00	Cactin OS=Mus musculus GN=Cactin PE=1 SV=2 - [CATIN_MOUSE]	1.17	1	1	1	1	1.418	0.832	1.172	1.633	2.82	1.17	1	1	772	90.6	9.79
P03911	NADH-ubiquinone oxidoreductase chain 4 OS=Mus musculus GN=Mtnd4 PE=1 SV=1 - [NU4M_MOUSE]	4.79	1	2	2	13	0.859	0.964	1.493	1.633	43.29	4.79	4	13	459	51.8	9.38
Q91WD9	Secretagogin OS=Mus musculus GN=Scgn PE=2 SV=1 - [SEGN_MOUSE]	11.96	1	3	3	8	0.824	0.670	0.958	1.634	16.45	11.96	6	8	276	32.1	5.19
Q6PDC0	RUN domain-containing protein 3B OS=Mus musculus GN=Rundc3b PE=2 SV=1 - [illegible]	23.04	1	1	7	17	3.332	1.393	1.225	1.635	48.81	23.04	12	17	408	45.1	5.17
P28665	Murinoglobulin-1 OS=Mus musculus GN=Mug1 PE=1 SV=3 - [MUG1_MOUSE]	35.70	1	24	40	155	3.585	3.763	1.047	1.635	478.66	35.70	63	155	1476	165.2	6.42
O88939-2	Isoform 2 of Zinc finger and BTB domain-containing protein 7A OS=Mus musculus GN=Zbtb7a - [illegible]	12.92	4	4	4	5	0.666	0.761	0.808	1.641	18.09	12.92	5	5	565	59.7	5.26
Q61282	Aggrecan core protein OS=Mus musculus GN=Acan PE=1 SV=2 - [PGCA_MOUSE]	13.56	1	21	22	73	3.715	1.590	2.171	1.642	210.31	13.56	37	73	2132	221.8	4.30
D3Z683	Alpha-1,3-mannosyl-glycoprotein 4-beta-N-acetylglucosaminyltransferase C (Fragment) OS=Mus musculus GN=Mgat4c PE=2 SV=1 - [D3Z683_MOUSE]	19.48	3	1	1	1	0.877	1.292	1.251	1.647	3.06	19.48	1	1	77	9.3	9.11
Q8BLR5	PH and SEC7 domain-containing protein 4 OS=Mus musculus GN=Psd4 PE=2 SV=1 - [PSD4_MOUSE]	4.88	1	2	3	17	0.712	6.919	1.615	1.648	30.42	4.88	4	17	1005	112.7	5.24
Q8BGY7	Protein FAM210A OS=Mus musculus GN=Fam210a PE=2 SV=1 - [F210A_MOUSE]	10.26	1	3	3	10	0.860	1.399	1.314	1.653	24.89	10.26	5	10	273	31.6	9.82
Q91X91	Nicotinate-nucleotide pyrophosphorylase [carboxylating] OS=Mus musculus GN=Qprt PE=2 SV=1 - [NADC_MOUSE]	17.39	1	5	5	11	1.716	1.810	1.225	1.653	28.41	17.39	10	11	299	31.5	6.71
Q99L04	Dehydrogenase/reductase SDR family member 1 OS=Mus musculus GN=Dhrs1 PE=2 SV=1 - [DHRS1_MOUSE]	41.21	1	11	11	48	0.821	1.092	1.572	1.655	153.43	41.21	17	48	313	34.0	8.35
Q3TVW5	Trichoplein keratin filament binding protein OS=Mus musculus GN=Tchp PE=1 SV=2 - [TCHP_MOUSE]	8.45	3	4	4	20	0.964	0.850	1.269	1.655	38.74	8.45	5	20	497	60.6	6.80
D3YU05	Glyceraldehyde-3-phosphate dehydrogenase OS=Mus musculus GN=Gm6316 PE=3 SV=1 - [D3YU05_MOUSE]	44.25	1	1	17	1318	3.427	2.848	1.880	1.656	3801.54	44.25	34	1318	339	36.5	8.46
Q8VD26-2	Isoform 2 of Transmembrane protein 143 OS=Mus musculus GN=Tmem143 -	8.61	4	3	3	11	0.526	1.177	1.496	1.658	31.04	8.61	6	11	453	51.0	9.14
Q5UAK0-5	Isoform 5 of Mesoderm induction early response protein 1 OS=Mus musculus GN=Mier1 - [MIER1_MOUSE]	10.00	5	2	3	9	4.702	2.203	1.298	1.664	18.89	10.00	6	9	330	37.3	5.31
P55088	Aquaporin-4 OS=Mus musculus GN=Aqp4 PE=2 SV=2 - [AQP4_MOUSE]	30.96	3	7	7	26	1.051	1.049	1.301	1.666	86.88	30.96	11	26	323	34.4	7.42
D3Z3B0	Nicotinamide mononucleotide adenylyltransferase 3 OS=Mus musculus GN=Nmnat3 PE=2 SV=1 - [illegible]	9.63	3	1	1	2	1.343	2.625	2.363	1.668	6.64	9.63	2	2	135	14.9	7.44
Q8R1S0	Ubiquinone biosynthesis monooxygenase COQ6 OS=Mus musculus GN=Coq6 PE=2 SV=3 - [COQ6_MOUSE]	14.29	4	7	7	19	1.045	1.647	1.332	1.669	56.26	14.29	11	19	476	51.4	7.17

Q8BHB9	Chloride intracellular channel protein 6 OS=Mus musculus GN=Clic6 PE=2 SV=1 - [CLIC6_MOUSE]	17.62	3	8	8	13	1.632	2.393	1.006	1.670	28.94	17.62	12	13	596	62.8	4.32
Q9CR27	WASH complex subunit CCDC53 OS=Mus musculus GN=Ccdc53 PE=2 SV=1 -	23.71	2	3	3	12	3.257	1.689	1.177	1.670	28.45	23.71	6	12	194	21.1	4.46
Q61818-2	Isoform 2 of Retinoic acid-induced protein 1 OS=Mus musculus GN=Rai1 - [RAI1_MOUSE]	5.43	2	6	6	12	0.831	0.899	1.505	1.673	28.96	5.43	7	12	1840	196.0	8.87
Q8K370	Acyl-CoA dehydrogenase family member 10 OS=Mus musculus GN=Acad10 PE=2 SV=1 - [ACD10_MOUSE]	10.57	3	2	9	17	1.995	1.318	0.815	1.675	57.23	10.57	13	17	1069	118.9	8.24
Q8C0P5	Coronin-2A OS=Mus musculus GN=Coro2a PE=2 SV=1 - [COR2A_MOUSE]	20.80	2	11	13	39	0.870	0.927	1.321	1.675	99.20	20.80	20	39	524	59.5	7.71
Q7TT41	DBH-like monooxygenase protein 2 OS=Mus musculus GN=Moxd2 PE=2 SV=1 -	4.68	1	1	1	5	0.719	0.363	1.531	1.676	0.00	4.68	1	5	619	69.4	4.92
Q62421	Endophilin-A3 OS=Mus musculus GN=Sh3gl3 PE=2 SV=1 - [SH3G3_MOUSE]	61.38	4	1	20	75	0.853	2.923	2.126	1.682	252.62	61.38	34	75	347	38.9	5.12
Q6URW6	Myosin-14 OS=Mus musculus GN=Myh14 PE=1 SV=1 - [MYH14_MOUSE]	37.95	7	2	72	242	1.001	0.705	1.290	1.683	695.15	37.95	126	242	2000	228.4	5.55
J3KMP5	Uncharacterized protein OS=Mus musculus GN=Gm21897 PE=4 SV=1 - [J3KMP5_MOUSE]	6.00	6	2	3	3	1.389	1.090	1.268	1.690	7.66	6.00	3	3	500	58.1	9.94
Q9QYG0	Protein NDRG2 OS=Mus musculus GN=Ndrg2 PE=1 SV=1 - [NDRG2_MOUSE]	69.00	1	1	17	292	0.196	0.518	0.970	1.690	865.35	69.00	31	292	371	40.8	5.40
O35488	Very long-chain acyl-CoA synthetase OS=Mus musculus GN=Slc27a2 PE=1 SV=2 - [S27A2_MOUSE]	14.03	2	9	9	17	0.823	0.659	0.956	1.690	38.06	14.03	13	17	620	70.4	8.90
J3QQ44	Endophilin-A3 OS=Mus musculus GN=Sh3gl3 PE=4 SV=1 - [J3QQ44_MOUSE]	64.42	3	1	17	62	0.930	1.878	1.700	1.692	206.85	64.42	29	62	312	35.1	5.03
Q9CQ56	Vesicle transport protein USE1 OS=Mus musculus GN=Use1 PE=2 SV=1 - [USE1_MOUSE]	23.70	4	6	6	17	0.796	1.173	1.089	1.694	47.37	23.70	11	17	270	30.6	9.11
P56501	Mitochondrial uncoupling protein 3 OS=Mus musculus GN=Ucp3 PE=2 SV=1 - [UCP3_MOUSE]	5.19	1	1	1	2	1.047	1.359	1.228	1.698	8.63	5.19	2	2	308	33.9	9.55
G3UYF8	Homeobox protein unc-4 homolog OS=Mus musculus GN=Uncx PE=2 SV=1 - [G3UYF8_MOUSE]	11.56	1	1	1	1	0.443	0.704	0.892	1.700	2.13	11.56	1	1	294	32.7	12.19
F6VJ09	Breast carcinoma-amplified sequence 3 homolog (Fragment) OS=Mus musculus GN=Bcas3 PE=4 SV=1 - [F6VJ09_MOUSE]	32.56	1	2	3	16	0.860	0.560	1.273	1.700	41.93	32.56	6	16	86	9.4	9.57
Q3V0K9	Plastin-1 OS=Mus musculus GN=Pls1 PE=2 SV=1 - [PLSI_MOUSE]	34.13	2	14	17	53	1.384	0.545	1.543	1.704	153.43	34.13	30	53	630	70.4	5.38
Q9JM62-2	Isoform 2 of Receptor expression-enhancing protein 6 OS=Mus musculus GN=Reep6 - [REEP6_MOUSE]	5.75	2	1	1	2	0.789	1.040	1.402	1.707	4.73	5.75	2	2	174	19.4	7.87
Q91Z53	Glyoxylate reductase/hydroxypyruvate reductase OS=Mus musculus GN=Grhpr PE=1 SV=1 - [GRHPR_MOUSE]	64.02	3	17	17	69	0.741	0.873	1.196	1.710	210.49	64.02	28	69	328	35.3	7.65
Q9QUG2-2	Isoform 2 of DNA polymerase kappa OS=Mus musculus GN=Polk - [POLK_MOUSE]	3.92	3	2	2	3	1.422	1.068	0.910	1.710	5.91	3.92	2	3	739	82.6	5.49
Q60773	Cyclin-dependent kinase 4 inhibitor D OS=Mus musculus GN=Cdkn2d PE=1 SV=2 -	18.67	1	2	2	3	2.089	1.086	2.173	1.712	12.38	18.67	3	3	166	17.8	6.25
G3UZ26	Serine hydroxymethyltransferase (Fragment) OS=Mus musculus GN=Shmt1 PE=3 SV=1 - [illegible]	15.49	4	6	7	12	1.252	0.995	1.448	1.712	30.60	15.49	11	12	439	48.4	7.01
P10649	Glutathione S-transferase Mu 1 OS=Mus musculus GN=Gstm1 PE=1 SV=2 - [GSTM1_MOUSE]	75.23	4	11	20	477	0.901	2.359	1.398	1.715	1568.50	75.23	38	477	218	26.0	7.94
P52760	Ribonuclease UK114 OS=Mus musculus GN=Hrsp12 PE=1 SV=3 - [UK114_MOUSE]	92.59	1	9	11	87	2.287	1.622	2.138	1.715	237.64	92.59	21	87	135	14.2	8.68
P16460	Argininosuccinate synthase OS=Mus musculus GN=Ass1 PE=1 SV=1 - [ASSY_MOUSE]	47.33	3	21	21	99	0.899	1.263	1.141	1.716	262.00	47.33	37	99	412	46.6	8.22

Q3UZP4	Small VCP/p97-interacting protein OS=Mus musculus GN=Svip PE=2 SV=1 - [SVIP_MOUSE]	32.47	1	2	2	27	1.591	0.952	1.729	1.717	73.92	32.47	4	27	77	8.4	8.91
Q9JHH6	Carboxypeptidase B2 OS=Mus musculus GN=Cpb2 PE=1 SV=1 - [CBPB2_MOUSE]	5.45	1	2	2	2	1.699	1.169	0.936	1.719	4.47	5.45	2	2	422	48.8	7.97
D3YZR0	Protein Gm15737 OS=Mus musculus GN=Gm15737 PE=4 SV=1 - [D3YZR0_MOUSE]	16.04	1	1	1	2	1.501	1.251	1.438	1.724	6.60	16.04	1	2	106	11.8	9.72
P01868	Ig gamma-1 chain C region secreted form OS=Mus musculus GN=Ighg1 PE=1 SV=1 - [IGHG1_MOUSE]	22.53	2	5	5	22	2.697	5.125	0.897	1.726	51.88	22.53	10	22	324	35.7	7.40
Q9JLB0	MAGUK p55 subfamily member 6 OS=Mus musculus GN=Mpp6 PE=1 SV=1 - [MPP6_MOUSE]	55.15	3	1	30	142	0.679	0.670	0.992	1.728	407.23	55.15	56	142	553	62.6	6.40
P50608	Fibromodulin OS=Mus musculus GN=Fmod PE=2 SV=1 - [FMOD_MOUSE]	16.22	1	5	5	18	0.526	10.188	1.173	1.729	45.91	16.22	10	18	376	43.0	6.04
F7D143	Uncharacterized protein KIAA0232 (Fragment) OS=Mus musculus GN=D5Ertd579e PE=2 SV=1 - [F7D143_MOUSE]	4.00	3	3	3	3	1.179	1.341	2.070	1.729	4.40	4.00	3	3	1274	141.0	4.89
Q9CQ71	Replication protein A 14 kDa subunit OS=Mus musculus GN=Rpa3 PE=2 SV=1 - [RFA3_MOUSE]	38.84	1	3	3	7	2.347	1.107	1.154	1.730	21.59	38.84	5	7	121	13.6	4.84
Q921C1	Gap junction gamma-3 protein OS=Mus musculus GN=Gjc3 PE=2 SV=2 - [CXG3_MOUSE]	8.92	1	2	2	4	0.820	0.290	1.478	1.733	11.84	8.92	3	4	269	30.3	8.37
P13597-2	Isoform 2 of Intercellular adhesion molecule 1 OS=Mus musculus GN=Icam1 -	7.53	2	4	4	8	1.524	2.365	1.691	1.734	20.43	7.53	6	8	531	58.7	6.21
Q9WVL0	Maleylacetoacetate isomerase OS=Mus musculus GN=Gstz1 PE=1 SV=1 - [MAAI_MOUSE]	73.15	1	13	13	67	0.593	1.207	1.008	1.737	206.90	73.15	24	67	216	24.3	7.85
Q80TE0-2	Isoform 2 of RNA polymerase II-associated protein 1 OS=Mus musculus GN=Rpap1 - [RPAP1_MOUSE]	5.19	1	1	5	6	0.640	2.851	2.060	1.737	15.90	5.19	6	6	1290	141.5	6.60
J3QNR5	Uncharacterized protein OS=Mus musculus GN=Tubb4b-ps1 PE=3 SV=1 - [J3QNR5_MOUSE]	70.72	2	5	28	3377	0.923	1.419	1.568	1.737	8456.17	70.72	51	3377	444	49.8	4.83
Q8K406	Leucine-rich repeat LGI family member 3 OS=Mus musculus GN=Lgi3 PE=1 SV=1 - [LGI3_MOUSE]	32.85	1	16	16	58	1.041	0.399	1.207	1.738	138.51	32.85	28	58	548	61.8	8.16
A2ARX0	Protein Gm14443 OS=Mus musculus GN=Gm14443 PE=4 SV=1 - [A2ARX0_MOUSE]	11.48	2	1	2	2	1.600	1.140	1.887	1.741	2.93	11.48	2	2	610	70.5	9.06
Q8CDK8	Prominin-1 OS=Mus musculus GN=Prom1 PE=2 SV=1 - [Q8CDK8_MOUSE]	23.58	4	1	14	39	0.977	0.529	0.944	1.743	93.39	23.58	23	39	827	92.7	6.55
D3Z2N8	Coiled-coil domain-containing protein 67 (Fragment) OS=Mus musculus GN=Ccdc67 PE=2 SV=1 - [illegible]	6.05	3	2	4	11	1.772	1.063	1.500	1.745	16.25	6.05	5	11	397	46.3	6.25
P84096	Rho-related GTP-binding protein RhoG OS=Mus musculus GN=Rhog PE=2 SV=1 - [RHOG_MOUSE]	54.45	7	10	11	92	0.936	0.369	1.453	1.747	273.37	54.45	20	92	191	21.3	8.12
Q6ZWR6-5	Isoform 5 of Nesprin-1 OS=Mus musculus GN=Syne1 - [SYNE1_MOUSE]	26.42	1	1	30	66	0.723	0.982	2.704	1.749	200.44	26.42	47	66	1431	165.1	6.42
Q8K1S1	Leucine-rich repeat LGI family member 4 OS=Mus musculus GN=Lgi4 PE=1 SV=1 - [LGI4_MOUSE]	6.70	1	4	4	9	0.969	1.439	1.356	1.749	21.17	6.70	7	9	537	59.3	7.78
P07759	Serine protease inhibitor A3K OS=Mus musculus GN=Serpina3k PE=1 SV=2 - [SPA3K_MOUSE]	46.89	1	11	16	126	3.569	2.223	0.810	1.752	395.72	46.89	28	126	418	46.8	5.16
D6RI42	Protein Zc3h7a OS=Mus musculus GN=Zc3h7a PE=2 SV=1 - [D6RI42_MOUSE]	6.67	7	2	2	4	1.826	1.143	1.219	1.754	6.67	6.67	2	4	390	43.1	4.87
Q810U3	Neurofascin OS=Mus musculus GN=Nfasc PE=1 SV=1 - [NFASC_MOUSE]	41.69	1	5	47	346	0.864	1.778	1.862	1.761	1044.89	41.69	80	346	1240	137.9	6.19
P19157	Glutathione S-transferase P 1 OS=Mus musculus GN=Gstp1 PE=1 SV=2 - [GSTP1_MOUSE]	59.05	3	11	11	197	0.802	1.469	1.305	1.764	587.08	59.05	18	197	210	23.6	7.87
Q99PJ1-24	Isoform 24 of Protocadherin-15 OS=Mus musculus GN=Pcdh15 - [PCD15_MOUSE]	3.45	21	4	4	7	0.359	0.745	1.328	1.767	9.77	3.45	4	7	1363	149.8	5.71
Q91XA9	Acidic mammalian chitinase OS=Mus musculus GN=Chia PE=1 SV=2 - [CHIA_MOUSE]	4.23	2	1	2	3	1.092	1.112	0.880	1.767	2.08	4.23	2	3	473	52.0	5.06

P24549	Retinal dehydrogenase 1 OS=Mus musculus GN=Aldh1a1 PE=1 SV=5 - [AL1A1_MOUSE]	42.71	2	17	18	91	0.891	0.689	1.078	1.769	284.86	42.71	33	91	501	54.4	7.80
Q6A044-2	Isoform 2 of Protein FAM189A1 OS=Mus musculus GN=Fam189a1 - [F1891_MOUSE]	20.86	2	2	2	3	2.274	2.044	2.971	1.776	10.31	20.86	3	3	139	14.7	6.25
Q14BP6-2	Isoform 2 of Leucine-rich repeat-containing protein LOC400891 homolog OS=Mus musculus - [YV012_MOUSE]	2.51	1	1	1	1	0.900	0.981	1.474	1.778	0.00	2.51	1	1	359	38.3	5.68
Q65CL1	Catenin alpha-3 OS=Mus musculus GN=Ctnna3 PE=1 SV=2 - [CTNA3_MOUSE]	8.27	1	5	6	12	0.912	0.455	1.209	1.778	35.43	8.27	10	12	895	99.7	6.52
Q8VIM5	Myocardin OS=Mus musculus GN=Myocd PE=1 SV=2 - [MYCD_MOUSE]	2.78	2	1	2	2	3.799	2.731	2.922	1.784	4.87	2.78	2	2	935	101.3	6.29
P97760	DNA-directed RNA polymerase II subunit RPB3 OS=Mus musculus GN=Polr2c PE=2 SV=1 - [RPB3_MOUSE]	17.09	2	4	4	9	1.313	0.667	1.761	1.791	30.08	17.09	6	9	275	31.3	4.97
Q8C5D8-3	Isoform 3 of E3 SUMO-protein ligase PIAS2 OS=Mus musculus GN=Pias2 -	3.73	9	1	2	2	1.056	0.971	0.854	1.791	5.46	3.73	2	2	563	62.3	8.22
A2ADE6	Filamin-binding LIM protein 1 (Fragment) OS=Mus musculus GN=Fblim1 PE=4 SV=1 - [A2ADE6_MOUSE]	39.43	9	3	3	6	1.690	3.821	1.356	1.792	12.65	39.43	5	6	175	18.3	5.14
O88700	Bloom syndrome protein homolog OS=Mus musculus GN=Blm PE=1 SV=1 - [BLM_MOUSE]	3.25	2	3	4	4	1.471	1.221	1.428	1.793	10.55	3.25	4	4	1416	158.3	7.27
Q80U19	Disheveled-associated activator of morphogenesis 2 OS=Mus musculus GN=Daam2 PE=2 SV=4 - [illegible]	24.48	4	22	26	69	0.991	0.737	1.873	1.801	187.69	24.48	43	69	1115	128.3	6.92
P01872	Ig mu chain C region secreted form OS=Mus musculus GN=Igh-6 PE=1 SV=2 - [IGHM_MOUSE]	32.38	2	11	12	35	2.533	3.177	1.278	1.801	110.10	32.38	20	35	454	49.9	7.01
E9Q5M6	Protein Wdr52 OS=Mus musculus GN=Wdr52 PE=4 SV=1 - [E9Q5M6_MOUSE]	2.22	2	4	4	5	0.675	0.938	1.048	1.803	7.25	2.22	4	5	1843	211.4	5.53
Q9JJG9	Nitric oxide-associated protein 1 OS=Mus musculus GN=Noa1 PE=1 SV=1 - [NOA1_MOUSE]	7.07	1	3	3	6	0.499	0.552	0.944	1.807	16.43	7.07	4	6	693	77.3	8.12
P70665-3	Isoform 3 of Sialate O-acetylesterase OS=Mus musculus GN=Siae - [SIAE_MOUSE]	13.59	3	4	4	7	3.045	0.834	0.957	1.809	18.48	13.59	7	7	390	43.9	5.86
O35231	Kinesin-like protein KIFC3 OS=Mus musculus GN=Kifc3 PE=1 SV=4 - [KIFC3_MOUSE]	9.34	4	6	7	10	2.135	1.607	1.201	1.816	21.70	9.34	8	10	824	92.5	7.71
Q9DCE9	Protein Igtp OS=Mus musculus GN=Igtp PE=2 SV=1 - [Q9DCE9_MOUSE]	5.44	1	2	2	4	1.371	1.442	1.172	1.818	13.66	5.44	4	4	423	48.5	7.77
Q9WVF8	Tumor suppressor candidate 2 OS=Mus musculus GN=Tusc2 PE=1 SV=3 - [TUSC2_MOUSE]	41.82	1	3	3	8	1.211	0.827	1.925	1.820	26.55	41.82	6	8	110	12.1	9.69
E9Q1Q9	Ketohexokinase OS=Mus musculus GN=Khk PE=2 SV=1 - [E9Q1Q9_MOUSE]	8.45	2	2	3	6	0.866	0.900	1.247	1.844	17.78	8.45	6	6	343	37.7	6.06
P51910	Apolipoprotein D OS=Mus musculus GN=Apod PE=2 SV=1 - [APOD_MOUSE]	34.92	1	6	6	34	1.819	0.624	1.477	1.851	89.85	34.92	9	34	189	21.5	4.91
Q5SYD0	Unconventional myosin-Id OS=Mus musculus GN=Myo1d PE=1 SV=1 - [MYO1D_MOUSE]	41.35	2	36	39	117	0.896	0.424	1.531	1.851	347.91	41.35	63	117	1006	116.0	9.41
P24529	Tyrosine 3-monooxygenase OS=Mus musculus GN=Th PE=1 SV=3 - [TY3H_MOUSE]	39.96	5	15	15	55	0.479	0.385	2.388	1.855	150.03	39.96	28	55	498	56.0	6.15
E9Q672	B-cell lymphoma/leukemia 11B OS=Mus musculus GN=Rit1 PE=2 SV=1 - [E9Q672_MOUSE]	6.56	2	1	1	1	1.057	0.917	1.497	1.855	0.00	6.56	1	1	183	21.6	9.09
E9QPE7	Myosin-11 OS=Mus musculus GN=Myh11 PE=4 SV=1 - [E9QPE7_MOUSE]	46.49	3	3	95	480	0.229	3.992	0.696	1.861	1356.36	46.49	164	480	1938	223.2	5.50
Q61704	Inter-alpha-trypsin inhibitor heavy chain H3 OS=Mus musculus GN=Itih3 PE=1 SV=3 - [ITIH3_MOUSE]	31.50	2	19	19	107	1.413	1.058	0.968	1.864	292.48	31.50	33	107	889	99.3	6.05
Q640L5	Coiled-coil domain-containing protein 18 OS=Mus musculus GN=Ccdc18 PE=2 SV=1 - [CCD18_MOUSE]	1.10	4	1	2	2	1.609	0.408	1.738	1.865	4.18	1.10	2	2	1455	169.6	5.49
Q8BW75	Amine oxidase [flavin-containing] B OS=Mus musculus GN=Maob PE=1 SV=4 - [AOFB_MOUSE]	56.73	3	21	25	132	0.773	1.397	1.417	1.865	343.15	56.73	45	132	520	58.5	8.29

E9PZQ0	Ryanodine receptor 1 OS=Mus musculus GN=Ryr1 PE=1 SV=1 - [RYR1_MOUSE]	1.37	2	4	8	11	2.178	1.914	1.979	1.868	24.43	1.37	9	11	5035	564.7	5.27
P29788	Vitronectin OS=Mus musculus GN=Vtn PE=1 SV=2 - [VTNC_MOUSE]	11.09	1	4	5	13	2.326	1.809	1.437	1.874	46.00	11.09	7	13	478	54.8	5.88
P01878	Ig alpha chain C region OS=Mus musculus PE=1 SV=1 - [IGHA_MOUSE]	9.01	1	2	2	3	1.600	2.605	0.819	1.874	9.13	9.01	3	3	344	36.9	5.06
P58660	Caspase recruitment domain-containing protein 10 OS=Mus musculus GN=Card10 PE=2 SV=1 - [CAR10_MOUSE]	4.31	2	2	4	6	26.984	1.766	1.988	1.875	12.13	4.31	4	6	1021	114.3	6.14
Q8QZR5	Alanine aminotransferase 1 OS=Mus musculus GN=Gpt PE=2 SV=3 - [ALAT1_MOUSE]	24.19	1	9	10	23	2.414	1.133	1.604	1.878	61.58	24.19	16	23	496	55.1	6.64
E9PX64	Kinesin-like protein KIF26B OS=Mus musculus GN=Kif26b PE=2 SV=1 - [E9PX64_MOUSE]	1.26	2	1	2	2	1.062	0.154	3.845	1.884	4.67	1.26	2	2	1665	179.1	8.15
P97496-2	Isoform 2 of SWI/SNF complex subunit SMARCC1 OS=Mus musculus GN=Smarcc1 - [SMRC1_MOUSE]	9.58	5	3	10	30	2.648	1.458	1.156	1.889	81.33	9.58	17	30	1075	120.0	5.81
Q9QY42	Probable G-protein coupled receptor 37 OS=Mus musculus GN=Gpr37 PE=2 SV=1 - [GPR37_MOUSE]	13.00	3	4	5	13	1.436	1.121	1.822	1.896	43.88	13.00	7	13	600	66.7	8.12
Q63886	UDP-glucuronosyltransferase 1-1 OS=Mus musculus GN=Ugt1a1 PE=2 SV=2 -	5.05	12	1	2	3	3.004	0.347	1.320	1.897	11.53	5.05	2	3	535	60.0	8.62
F6ZAW1	Protein Cald1 (Fragment) OS=Mus musculus GN=Cald1 PE=4 SV=1 - [F6ZAW1_MOUSE]	35.07	2	2	9	36	0.457	2.730	1.797	1.904	83.00	35.07	12	36	211	24.7	5.52
Q9ER97	Neuroglobin OS=Mus musculus GN=Ngb PE=1 SV=1 - [NGB_MOUSE]	42.38	3	4	4	19	1.287	2.110	0.787	1.908	43.25	42.38	8	19	151	17.0	5.88
Q6PGB8	Probable global transcription activator SNF2L1 OS=Mus musculus GN=Smarca1 PE=1 SV=1 - [SMCA1_MOUSE]	8.03	4	3	10	18	0.814	0.821	3.332	1.908	47.02	8.03	14	18	1046	121.6	8.27
Q9QWV4	Myeloid leukemia factor 1 OS=Mus musculus GN=Mlf1 PE=1 SV=1 - [MLF1_MOUSE]	3.37	2	1	1	3	1.440	0.870	0.789	1.908	7.27	3.37	2	3	267	30.4	9.32
P10810	Monocyte differentiation antigen CD14 OS=Mus musculus GN=Cd14 PE=1 SV=1 - [CD14_MOUSE]	4.37	1	1	1	2	0.792	1.213	1.325	1.911	7.43	4.37	2	2	366	39.2	5.21
Q00896	Alpha-1-antitrypsin 1-3 OS=Mus musculus GN=Serpina1c PE=1 SV=2 - [A1AT3_MOUSE]	52.91	2	4	17	175	3.895	4.353	0.707	1.913	503.99	52.91	31	175	412	45.8	5.44
P47212	Galanin peptides OS=Mus musculus GN=Gal PE=2 SV=1 - [GALA_MOUSE]	40.32	1	3	3	14	1.328	1.148	0.970	1.913	47.42	40.32	5	14	124	13.5	6.54
P43136	Nuclear receptor subfamily 2 group F member 6 OS=Mus musculus GN=Nr2f6 PE=1 SV=2 - [NR2F6_MOUSE]	11.03	3	2	2	6	0.703	0.523	2.328	1.919	5.71	11.03	3	6	390	41.9	7.56
O88492	Perilipin-4 OS=Mus musculus GN=Plin4 PE=1 SV=2 - [PLIN4_MOUSE]	32.15	2	15	15	36	1.102	1.529	2.327	1.924	85.91	32.15	21	36	1403	139.3	8.59
Q99J93	Interferon-induced transmembrane protein 2 OS=Mus musculus GN=Ifitm2 PE=1 SV=1 - [IFM2_MOUSE]	18.06	3	2	2	5	0.826	2.222	1.139	1.925	12.26	18.06	3	5	144	15.7	7.30
Q9WVT6	Carbonic anhydrase 14 OS=Mus musculus GN=Ca14 PE=1 SV=1 - [CAH14_MOUSE]	20.18	1	6	6	37	1.442	0.449	1.736	1.933	105.38	20.18	10	37	337	37.5	6.35
Q8CEE6	PAS domain-containing serine/threonine-protein kinase OS=Mus musculus GN=Pask PE=2 SV=3 - [PASK_MOUSE]	4.48	1	3	3	4	2.654	1.506	1.140	1.934	8.63	4.48	3	4	1383	151.2	5.07
Q8BGQ4-2	Isoform 2 of Protein O-mannosyl-transferase 2 OS=Mus musculus GN=Pomt2 -	4.48	3	2	2	2	1.334	1.322	1.325	1.936	6.39	4.48	2	2	603	67.8	9.47
P17563	Selenium-binding protein 1 OS=Mus musculus GN=Selenbp1 PE=1 SV=2 [SBP1_MOUSE]	54.66	6	21	21	101	1.743	1.576	1.367	1.943	359.28	54.66	37	101	472	52.5	6.29
Q922B1	O-acetyl-ADP-ribose deacetylase MACROD1 OS=Mus musculus GN=Macrod1 PE=2 SV=2 - [MACD1_MOUSE]	26.63	1	8	8	36	1.157	1.373	1.496	1.943	115.25	26.63	14	36	323	35.3	8.85
O35215	D-dopachrome decarboxylase OS=Mus musculus GN=Ddt PE=1 SV=3 - [DOPD_MOUSE]	55.93	2	5	5	13	1.661	1.087	1.605	1.960	36.00	55.93	7	13	118	13.1	6.54

Q8C838	Tumor suppressor candidate 5 homolog OS=Mus musculus GN=Tusc5 PE=2 SV=1 - [TUSC5_MOUSE]	17.34	1	2	2	3	1.267	0.788	1.943	1.961	5.51	17.34	2	3	173	18.7	8.00
F6TYB7	Myelin basic protein (Fragment) OS=Mus musculus GN=Mbp PE=4 SV=1 - [F6TYB7_MOUSE]	84.82	9	4	25	2287	1.599	0.143	2.407	1.963	6437.37	84.82	42	2287	191	21.2	10.73
P0C8K7	Small integral membrane protein 1 OS=Mus musculus GN=Smim1 PE=3 SV=1 -	23.08	1	1	1	8	0.735	0.302	1.568	1.964	29.89	23.08	2	8	78	8.8	8.66
P60202	Myelin proteolipid protein OS=Mus musculus GN=Plp1 PE=1 SV=2 - [MYPR_MOUSE]	28.16	2	12	12	709	0.995	0.251	1.631	1.966	2131.39	28.16	24	709	277	30.1	8.35
P31725	Protein S100-A9 OS=Mus musculus GN=S100a9 PE=1 SV=3 - [S10A9_MOUSE]	38.94	1	4	4	12	2.783	2.428	1.444	1.973	42.89	38.94	8	12	113	13.0	7.17
D3Z7Y0	Glutathione peroxidase (Fragment) OS=Mus musculus GN=Gpx3 PE=3 SV=1 - [D3Z7Y0_MOUSE]	35.94	2	1	5	16	8.522	2.701	3.840	1.984	38.69	35.94	8	16	217	24.0	5.66
E9PVD2	Inter alpha-trypsin inhibitor, heavy chain 4 OS=Mus musculus GN=Itih4 PE=2 SV=1 - [E9PVD2_MOUSE]	31.35	2	1	24	73	1.764	1.120	1.439	1.985	186.93	31.35	39	73	941	104.5	6.40
Q9CYL5	Golgi-associated plant pathogenesis-related protein 1 OS=Mus musculus GN=Glipr2 PE=2 SV=3 - [GAPR1_MOUSE]	33.12	2	4	4	14	1.096	1.533	1.099	1.995	45.47	33.12	8	14	154	17.1	9.51
P42703-2	Isoform 2 of Leukemia inhibitory factor receptor OS=Mus musculus GN=Lifr - [LIFR_MOUSE]	15.30	1	1	11	20	2.130	1.457	2.757	1.998	52.73	15.30	16	20	719	81.2	7.05
Q8BVI4	Dihydropteridine reductase OS=Mus musculus GN=Qdpr PE=1 SV=2 - [DHPR_MOUSE]	61.83	4	11	11	106	0.910	0.756	1.436	2.003	402.25	61.83	20	106	241	25.6	7.81
K3W4L8	Doublecortin domain-containing protein 2C OS=Mus musculus GN=Dcdc2c PE=4 SV=1 - [K3W4L8_MOUSE]	4.61	2	1	1	1	0.964	0.638	1.345	2.004	2.78	4.61	1	1	347	39.3	9.39
J3QNP7	Protein 1700014D04Rik (Fragment) OS=Mus musculus GN=1700014D04Rik PE=4 SV=1 - [J3QNP7_MOUSE]	21.21	2	1	1	2	3.446	1.176	1.771	2.019	3.43	21.21	1	2	99	11.2	9.63
D6RDB5	Rho GTPase-activating protein 23 OS=Mus musculus GN=Arhgap23 PE=2 SV=1 -	29.34	1	1	15	40	2.140	1.261	1.111	2.020	110.75	29.34	21	40	634	70.2	7.85
Q60771	Claudin-11 OS=Mus musculus GN=Cldn11 PE=1 SV=1 - [CLD11_MOUSE]	15.94	1	5	5	245	0.681	0.342	1.698	2.036	514.07	15.94	9	245	207	22.1	7.91
Q61885	Myelin-oligodendrocyte glycoprotein OS=Mus musculus GN=Mog PE=1 SV=1 - [MOG_MOUSE]	39.43	3	11	12	176	1.283	0.278	1.851	2.044	490.68	39.43	21	176	246	28.3	7.96
Q8CIM3	D-2-hydroxyglutarate dehydrogenase, mitochondrial OS=Mus musculus GN=D2hgdh PE=2 SV=3 - [illegible]	17.94	2	6	6	18	0.923	2.906	1.700	2.047	56.45	17.94	11	18	535	58.5	7.37
P07146	Anionic trypsin-2 OS=Mus musculus GN=Prss2 PE=2 SV=1 - [TRY2_MOUSE]	8.13	1	1	1	17	5.040	0.990	1.316	2.048	28.58	8.13	1	17	246	26.2	4.63
Q9DBB4	N-alpha-acetyltransferase 16, NatA auxiliary subunit OS=Mus musculus GN=Naa16 PE=2 SV=1 - [NAA16_MOUSE]	6.83	2	1	6	15	0.671	1.154	1.727	2.052	34.10	6.83	9	15	864	101.2	8.35
Q08642	Protein-arginine deiminase type-2 OS=Mus musculus GN=Padi2 PE=1 SV=2 - [PADI2_MOUSE]	31.20	1	18	18	64	1.029	0.445	1.694	2.053	215.51	31.20	32	64	673	76.2	5.53
P03987-2	Isoform 2 of Ig gamma-3 chain C region OS=Mus musculus - [IGHG3_MOUSE]	17.02	2	5	5	20	2.042	5.040	1.235	2.054	67.75	17.02	8	20	329	36.2	8.27
Q3U2C5-2	Isoform 2 of E3 ubiquitin-protein ligase RNF149 OS=Mus musculus GN=Rnf149 -	6.02	2	1	1	2	1.133	1.389	0.847	2.055	4.51	6.02	1	2	249	27.6	6.84
Q91X72	Hemopexin OS=Mus musculus GN=Hpx PE=1 SV=2 - [HEMO_MOUSE]	50.22	1	23	23	133	3.431	2.089	1.276	2.068	359.09	50.22	43	133	460	51.3	7.80
Q61635	GTP-binding protein OS=Mus musculus GN=Ifi47 PE=2 SV=1 - [Q61635_MOUSE]	14.52	6	4	7	25	2.034	2.091	1.352	2.072	59.46	14.52	10	25	420	46.8	6.74
P00920	Carbonic anhydrase 2 OS=Mus musculus GN=Ca2 PE=1 SV=4 - [CAH2_MOUSE]	65.38	1	14	14	421	1.284	1.229	1.543	2.076	931.03	65.38	28	421	260	29.0	7.01
Q91X56	Sphingosine 1-phosphate receptor 5 OS=Mus musculus GN=S1pr5 PE=2 SV=1 - [S1PR5_MOUSE]	13.00	1	3	3	6	1.437	0.846	2.229	2.077	18.18	13.00	5	6	400	42.3	9.83

D3YX11	GTPase IMAP family member 1 (Fragment) OS=Mus musculus GN=Gimap1 PE=2 SV=1 - [D3YX11_MOUSE]	14.86	3	1	1	1	1.647	2.631	2.017	2.094	2.24	14.86	1	1	74	7.9	9.99
Q9Z306	Solute carrier family 22 member 4 OS=Mus musculus GN=Slc22a4 PE=1 SV=1 -	7.41	1	4	4	9	0.793	0.635	1.142	2.094	23.59	7.41	7	9	553	62.2	6.40
P13562	Progonadoliberin-1 OS=Mus musculus GN=Gnrh1 PE=1 SV=1 - [GON1_MOUSE]	15.56	1	1	1	4	1.164	1.063	1.078	2.106	16.83	15.56	1	4	90	10.3	6.52
Q99K67	Alpha-aminoadipic semialdehyde synthase, mitochondrial OS=Mus musculus GN=Aass PE=2 SV=1 - [AASS_MOUSE]	17.93	2	13	13	24	1.004	1.804	1.817	2.107	53.99	17.93	21	24	926	102.9	6.87
Q08091	Calponin-1 OS=Mus musculus GN=Cnn1 PE=2 SV=1 - [CNN1_MOUSE]	51.52	2	12	16	65	0.716	8.691	1.112	2.111	181.65	51.52	26	65	297	33.3	8.97
O89110	Caspase-8 OS=Mus musculus GN=Casp8 PE=1 SV=1 - [CASP8_MOUSE]	4.79	1	1	2	2	1.371	1.383	1.848	2.119	4.96	4.79	2	2	480	55.3	5.25
Q9DB73	NADH-cytochrome b5 reductase 1 OS=Mus musculus GN=Cyb5r1 PE=2 SV=1 -	34.43	5	11	11	37	0.829	1.942	1.631	2.121	95.32	34.43	17	37	305	34.1	8.87
G3UYT0	Ubiquitin-conjugating enzyme E2 G2 OS=Mus musculus GN=Ube2g2 PE=2 SV=1 -	68.18	1	1	1	1	0.665	0.130	1.782	2.131	2.47	68.18	1	1	44	4.9	4.44
Q91WH7	ATPase, H+/K+ exchanging, gastric, alpha polypeptide OS=Mus musculus GN=Atp4a PE=2 SV=1 - [Q91WH7_MOUSE]	11.41	3	4	12	339	1.869	1.417	2.441	2.144	802.92	11.41	22	339	1025	113.0	5.81
Q8CFB4	Guanylate-binding protein 5 OS=Mus musculus GN=Gbp5 PE=1 SV=2 - [GBP5_MOUSE]	6.27	1	3	3	4	1.783	1.421	1.745	2.157	12.34	6.27	4	4	590	66.9	6.09
Q6NS46	Protein RRP5 homolog OS=Mus musculus GN=Pdcd11 PE=2 SV=2 - [RRP5_MOUSE]	4.46	1	4	5	7	1.402	0.688	1.141	2.161	21.43	4.46	6	7	1862	207.6	8.75
P0DM40	Fer-1-like protein 5 OS=Mus musculus GN=Fer1l5 PE=1 SV=1 - [FR1L5_MOUSE]	1.37	1	3	3	4	1.028	1.168	2.249	2.165	2.56	1.37	3	4	2038	235.7	7.83
Q5SW46	Protein Lpo OS=Mus musculus GN=Lpo PE=2 SV=1 - [Q5SW46_MOUSE]	1.69	1	1	1	1	0.619	0.297	2.322	2.170	2.12	1.69	1	1	710	80.1	7.11
P06683	Complement component C9 OS=Mus musculus GN=C9 PE=1 SV=2 - [CO9_MOUSE]	3.65	2	2	2	6	3.333	1.979	1.646	2.176	16.20	3.65	3	6	548	62.0	5.78
P61208	ADP-ribosylation factor-like protein 4C OS=Mus musculus GN=Arl4c PE=2 SV=1 - [ARL4C_MOUSE]	16.15	1	2	3	6	5.586	1.466	1.034	2.188	16.87	16.15	4	6	192	21.5	9.09
O08599-2	Isoform 2 of Syntaxin-binding protein 1 OS=Mus musculus GN=Stxbp1 - [STXB1_MOUSE]	75.62	1	2	50	837	0.828	0.294	1.290	2.190	2451.29	75.62	91	837	603	68.7	6.77
Q3UVH3	Protein Gm10729 OS=Mus musculus GN=Gm10729 PE=2 SV=1 - [Q3UVH3_MOUSE]	13.11	1	1	1	1	4.703	2.519	3.974	2.190	2.97	13.11	1	1	122	13.3	8.10
F6T0J8	Cytoplasmic polyadenylation element-binding protein 3 (Fragment) OS=Mus musculus GN=Cpeb3 PE=2 SV=1 - [F6T0J8_MOUSE]	50.12	1	1	13	49	1.044	0.593	1.487	2.197	117.97	50.12	22	49	415	46.9	6.93
P27005	Protein S100-A8 OS=Mus musculus GN=S100a8 PE=1 SV=3 - [S10A8_MOUSE]	46.07	1	2	2	4	3.694	2.089	1.683	2.200	17.58	46.07	3	4	89	10.3	5.68
F8VQG7	Protein N4bp2 OS=Mus musculus GN=N4bp2 PE=4 SV=1 - [F8VQG7_MOUSE]	5.07	2	5	6	20	2.022	2.582	1.800	2.212	27.67	5.07	7	20	1678	184.6	5.62
P70336	Rho-associated protein kinase 2 OS=Mus musculus GN=Rock2 PE=1 SV=1 - [ROCK2_MOUSE]	50.36	3	1	62	305	0.696	3.379	1.697	2.214	838.78	50.36	107	305	1388	160.5	5.99
Q9ET01	Glycogen phosphorylase, liver form OS=Mus musculus GN=Pygl PE=1 SV=4 - [PYGL_MOUSE]	39.88	2	22	32	114	0.820	2.019	1.448	2.220	299.24	39.88	47	114	850	97.4	7.09
Q8JZN1	Macrophage galactose N-acetyl-galactosamine specific lectin 2 OS=Mus musculus GN=Mgl2 PE=2 SV=1 - [Q8JZN1_MOUSE]	16.27	7	2	2	2	1.457	1.307	1.072	2.230	5.32	16.27	2	2	332	38.0	5.66
L7N451	Interferon-induced very large GTPase 1 OS=Mus musculus GN=Gvin1 PE=4 SV=1 - [L7N451_MOUSE]	2.43	3	4	4	8	2.830	1.465	1.787	2.264	16.34	2.43	5	8	2427	280.6	6.57
Q9Z0M1	Enamel matrix serine proteinase 1 (Precursor) OS=Mus musculus GN=Klk4 PE=2 SV=1 - [Q9Z0M1_MOUSE]	9.41	1	1	1	10	1.537	1.217	1.858	2.283	0.00	9.41	1	10	255	27.5	6.16

P35487	Pyruvate dehydrogenase E1 component subunit alpha, testis-specific form, mitochondrial OS=Mus musculus GN=Pdha2 PE=2 SV=1 - [ODPAT_MOUSE]	8.95	1	2	4	26	1.342	1.408	1.924	2.288	65.82	8.95	7	26	391	43.4	8.50
B1AW21	Myotubularin OS=Mus musculus GN=Mtm1 PE=4 SV=1 - [B1AW21_MOUSE]	12.59	3	6	6	10	1.162	0.837	1.107	2.294	24.63	12.59	9	10	572	65.9	7.77
P70121	Zinc fingers and homeoboxes protein 1 OS=Mus musculus GN=Zhx1 PE=1 SV=2 - [ZHX1_MOUSE]	5.04	2	3	4	7	2.505	2.488	2.296	2.294	12.09	5.04	6	7	873	97.5	5.99
P70689	Gap junction beta-6 protein OS=Mus musculus GN=Gjb6 PE=1 SV=1 - [CXB6_MOUSE]	10.73	2	2	2	3	0.894	1.007	1.661	2.312	8.91	10.73	3	3	261	30.3	8.38
Q6TL19	Guanylate cyclase 2G OS=Mus musculus GN=Gucy2g PE=1 SV=1 - [GUC2G_MOUSE]	2.64	1	2	3	5	3.657	1.524	2.036	2.322	12.58	2.64	3	5	1100	122.5	6.39
P61953	Guanine nucleotide-binding protein G(I)/G(S)/G(O) subunit gamma-11 OS=Mus musculus GN=Gng11 PE=1 SV=1	23.29	2	1	2	4	2.963	0.616	2.320	2.342	7.21	23.29	3	4	73	8.5	5.54
P10637	Microtubule-associated protein tau OS=Mus musculus GN=Mapt PE=1 SV=3 - [TAU_MOUSE]	51.71	5	7	40	555	2.345	0.499	3.041	2.344	1524.33	51.71	72	555	733	76.2	6.79
Q64332	Synapsin-2 OS=Mus musculus GN=Syn2 PE=1 SV=2 - [SYN2_MOUSE]	58.02	1	2	26	740	0.612	4.448	3.077	2.348	2248.94	58.02	48	740	586	63.3	8.43
Q62095	ATP-dependent RNA helicase DDX3Y OS=Mus musculus GN=Ddx3y PE=1 SV=2 - [DDX3Y_MOUSE]	44.68	1	3	30	153	1.648	1.639	1.938	2.366	445.78	44.68	50	153	658	73.4	7.53
Q6GTM0	Ifit2 protein OS=Mus musculus GN=Ifit2 PE=2 SV=1 - [Q6GTM0_MOUSE]	4.26	2	1	2	2	1.166	1.943	1.175	2.378	5.25	4.26	2	2	470	54.7	7.43
E0CZG7	Glycerate kinase (Fragment) OS=Mus musculus GN=Glyctk PE=2 SV=1 - [E0CZG7_MOUSE]	20.63	3	2	2	4	1.147	0.347	3.302	2.380	14.27	20.63	3	4	126	13.5	10.55
Q9R118	Serine protease HTRA1 OS=Mus musculus GN=Htra1 PE=1 SV=2 - [HTRA1_MOUSE]	37.50	1	12	12	34	1.465	1.180	2.400	2.386	91.09	37.50	22	34	480	51.2	7.65
Q80VQ0	Aldehyde dehydrogenase family 3 member B1 OS=Mus musculus GN=Aldh3b1 PE=2 SV=1 - [AL3B1_MOUSE]	25.21	3	7	9	40	1.111	1.011	1.807	2.400	136.92	25.21	18	40	468	52.3	7.55
E9PUK2	Uncharacterized aarF domain-containing protein kinase 5 OS=Mus musculus GN=Adck5 PE=2 SV=1 - [E9PUK2_MOUSE]	7.39	4	4	4	6	1.038	1.684	1.833	2.409	12.86	7.39	6	6	582	66.6	8.76
Q61176	Arginase-1 OS=Mus musculus GN=Arg1 PE=1 SV=1 - [ARGI1_MOUSE]	37.15	1	11	11	33	1.999	1.087	2.227	2.411	80.63	37.15	15	33	323	34.8	7.01
Q6X893	Choline transporter-like protein 1 OS=Mus musculus GN=Slc44a1 PE=1 SV=3 -	16.39	5	1	11	43	0.653	0.330	2.112	2.451	133.62	16.39	19	43	653	73.0	8.75
A2A998	Complement component C8 alpha chain OS=Mus musculus GN=C8a PE=2 SV=1 - [A2A998_MOUSE]	3.68	3	1	1	3	1.785	1.804	3.099	2.456	12.67	3.68	1	3	543	61.0	6.06
Q4U4S6	Xin actin-binding repeat-containing protein 2 OS=Mus musculus GN=Xirp2 PE=1 SV=1 - [XIRP2_MOUSE]	1.48	3	4	6	7	1.746	2.288	2.264	2.470	14.97	1.48	7	7	3784	428.0	6.18
Q99LG1	Transmembrane protein 51 OS=Mus musculus GN=Tmem51 PE=1 SV=1 - [TMM51_MOUSE]	7.23	1	1	1	2	0.673	1.221	1.831	2.481	9.17	7.23	2	2	249	27.4	6.95
Q62261-2	Isoform 2 of Spectrin beta chain, non-erythrocytic 1 OS=Mus musculus GN=Sptbn1 - [SPTB2_MOUSE]	73.26	1	1	151	1979	1.204	0.497	3.122	2.487	5527.51	73.26	276	1979	2154	251.0	5.54
P31532	Serum amyloid A-4 protein OS=Mus musculus GN=Saa4 PE=1 SV=2 - [SAA4_MOUSE]	22.31	1	2	2	4	1.857	6.658	1.238	2.489	11.18	22.31	3	4	130	15.1	9.26
P97306	SH3 and cysteine-rich domain-containing protein OS=Mus musculus GN=Stac PE=2 SV=1 - [STAC_MOUSE]	5.71	1	3	3	4	1.090	1.396	1.408	2.519	8.72	5.71	4	4	403	44.3	7.81
Q9D2P8	Myelin-associated oligodendrocyte basic protein OS=Mus musculus GN=Mobp PE=2 SV=1 - [MOBP_MOUSE]	42.94	5	8	8	62	0.593	0.401	2.181	2.538	148.51	42.94	12	62	170	19.2	11.14
Q9DBT9	Dimethylglycine dehydrogenase, mitochondrial OS=Mus musculus GN=Dmgdh PE=1 SV=1 - [M2GD_MOUSE]	3.11	1	2	2	3	1.215	0.619	2.136	2.549	3.53	3.11	3	3	869	97.2	7.88

P40237	CD82 antigen OS=Mus musculus GN=Cd82 PE=1 SV=1 - [CD82_MOUSE]	16.54	3	5	5	37	1.319	0.623	2.382	2.557	102.46	16.54	10	37	266	29.6	5.02
H3BJ97	Tubulointerstitial nephritis antigen-like OS=Mus musculus GN=Tinagl1 PE=2 SV=1 -	28.97	3	9	9	29	1.137	4.218	2.026	2.568	82.64	28.97	14	29	435	49.2	7.15
Q8K0E8	Fibrinogen beta chain OS=Mus musculus GN=Fgb PE=2 SV=1 - [FIBB_MOUSE]	51.98	2	22	23	146	1.855	1.624	1.287	2.607	474.52	51.98	43	146	481	54.7	7.08
Q8C8R3-5	Isoform 5 of Ankyrin-2 OS=Mus musculus GN=Ank2 - [ANK2_MOUSE]	53.88	3	1	167	1338	1.432	1.636	1.930	2.608	3864.34	53.88	293	1338	3844	420.2	5.12
Q9WUH7	Semaphorin-4G OS=Mus musculus GN=Sema4g PE=1 SV=1 - [SEM4G_MOUSE]	3.35	1	2	2	5	0.776	0.585	0.663	2.648	10.76	3.35	3	5	837	92.3	7.94
P13634	Carbonic anhydrase 1 OS=Mus musculus GN=Ca1 PE=2 SV=4 - [CAH1_MOUSE]	54.02	2	9	9	29	2.089	3.670	1.272	2.660	85.98	54.02	16	29	261	28.3	6.96
D3YZV8	Coiled-coil domain-containing protein 8 homolog OS=Mus musculus GN=Ccdc8 PE=3 SV=2 - [CCDC8_MOUSE]	1.17	1	1	1	4	1.755	3.801	2.546	2.689	9.23	1.17	1	4	685	73.9	9.09
Q9D8M3	Heme transporter HRG1 OS=Mus musculus GN=Slc48a1 PE=2 SV=1 - [HRG1_MOUSE]	12.33	2	2	2	2	4.082	1.476	1.735	2.708	3.95	12.33	2	2	146	16.5	9.45
Q8BIR2-2	Isoform 2 of Protein asteroid homolog 1 OS=Mus musculus GN=Aste1 -	5.24	4	2	4	7	1.050	0.169	1.843	2.714	12.91	5.24	5	7	611	69.0	7.91
Q9D8H7	Metalloendopeptidase OMA1, mitochondrial OS=Mus musculus GN=Oma1 PE=2 SV=1 - [OMA1_MOUSE]	5.18	1	3	3	3	2.021	1.781	1.599	2.731	4.11	5.18	3	3	521	58.8	9.39
Q9Z0E6	Interferon-induced guanylate-binding protein 2 OS=Mus musculus GN=Gbp2 PE=1 SV=1 - [GBP2_MOUSE]	17.15	3	9	9	16	0.943	1.862	1.306	2.732	44.11	17.15	12	16	589	66.7	5.71
B1AUC7	Rho GTPase-activating protein 36 OS=Mus musculus GN=Arhgap36 PE=2 SV=1 -	2.37	1	1	1	1	7.799	0.888	1.114	2.831	2.52	2.37	1	1	590	66.6	6.06
Q9QZ85	Interferon-inducible GTPase 1 OS=Mus musculus GN=Iigp1 PE=1 SV=2 - [IIGP1_MOUSE]	13.56	1	4	5	8	1.162	1.376	1.568	2.847	18.07	13.56	8	8	413	47.5	6.33
Q6P3Z8	Histone acetyltransferase KAT2A OS=Mus musculus GN=Kat2a PE=2 SV=1 - [Q6P3Z8_MOUSE]	7.72	2	6	6	7	1.167	1.252	1.039	2.892	12.37	7.72	7	7	829	93.3	9.07
D3YXD5	Protein Ugt2b36 (Fragment) OS=Mus musculus GN=Ugt2b36 PE=2 SV=1 -	5.05	4	1	1	1	1.825	1.472	2.079	2.923	2.20	5.05	1	1	198	22.1	7.02
Q8R1A8	Ornithine carbamoyltransferase, mitochondrial OS=Mus musculus GN=Otc PE=2 SV=1 - [Q8R1A8_MOUSE]	9.69	2	3	3	10	1.595	1.271	1.957	2.935	26.69	9.69	5	10	351	39.3	8.66
P49429	4-hydroxyphenylpyruvate dioxygenase OS=Mus musculus GN=Hpd PE=1 SV=3 - [HPPD_MOUSE]	16.03	3	6	6	8	1.369	1.090	1.842	2.946	22.98	16.03	8	8	393	45.0	7.06
Q8CB59-2	Isoform 2 of Protein FAM161B OS=Mus musculus GN=Fam161b - [F161B_MOUSE]	5.54	2	2	2	2	1.615	3.370	1.896	2.955	3.45	5.54	2	2	487	55.6	10.07
Q99K47	Fibrinogen, alpha polypeptide OS=Mus musculus GN=Fga PE=2 SV=1 - [Q99K47_MOUSE]	49.55	2	22	23	134	1.887	1.697	1.472	2.958	369.83	49.55	41	134	557	61.3	7.47
P12399	Protein CTLA-2-alpha OS=Mus musculus GN=Ctla2a PE=2 SV=2 - [CTL2A_MOUSE]	18.25	2	2	2	4	2.695	1.101	3.262	3.012	11.36	18.25	2	4	137	15.9	5.34
Q78JT3	3-hydroxyanthranilate 3,4-dioxygenase OS=Mus musculus GN=Haao PE=1 SV=1 - [3HAO_MOUSE]	23.78	1	6	6	9	1.760	1.031	2.058	3.019	24.11	23.78	9	9	286	32.8	6.54
Q6TA13	Kinesin-like protein KIF1A OS=Mus musculus GN=Kif1a PE=2 SV=1 - [Q6TA13_MOUSE]	41.17	4	1	56	229	1.046	1.546	1.488	3.031	696.81	41.17	97	229	1698	191.7	6.23
Q8VCM7	Fibrinogen gamma chain OS=Mus musculus GN=Fgg PE=2 SV=1 - [FIBG_MOUSE]	48.39	1	17	17	78	2.183	1.581	1.390	3.036	230.71	48.39	31	78	436	49.4	5.86
Q8BID8	F-box/LRR-repeat protein 14 OS=Mus musculus GN=Fbxl14 PE=2 SV=1 - [FXL14_MOUSE]	8.00	1	1	2	3	3.345	11.362	9.643	3.083	2.60	8.00	2	3	400	43.8	8.65
E9QAK1	Protein 2810007J24Rik (Fragment) OS=Mus musculus GN=2810007J24Rik PE=2 SV=1 - [E9QAK1_MOUSE]	13.76	2	2	2	4	1.390	1.192	1.927	3.095	6.42	13.76	3	4	189	22.4	8.66
Q61169-2	Isoform 2 of Transcription factor GATA-6 OS=Mus musculus GN=Gata6 - [GATA6_MOUSE]	3.16	2	1	1	1	1.107	1.543	1.196	3.150	3.09	3.16	1	1	443	44.7	8.91

Q3UUX8	Protein 4732440D04Rik (Fragment) OS=Mus musculus GN=4732440D04Rik PE=2 SV=1 - [Q3UUX8_MOUSE]	14.60	1	1	1	1	0.560	1.832	3.113	3.173	2.39	14.60	1	1	226	23.7	12.03
Q64345	Interferon-induced protein with tetratricopeptide repeats 3 OS=Mus musculus GN=Ifit3 PE=2 SV=1 - [IFIT3_MOUSE]	23.08	2	8	9	13	1.093	1.200	1.606	3.239	44.62	23.08	13	13	403	47.2	5.64
F6RLA6	Ataxin-7-like protein 1 (Fragment) OS=Mus musculus GN=Atxn7l1 PE=2 SV=1 -	14.69	4	1	1	2	13.365	4.460	4.671	3.259	5.68	14.69	1	2	143	15.0	10.87
Q6DFX2	Anthrax toxin receptor 2 OS=Mus musculus GN=Antxr2 PE=2 SV=1 - [ANTR2_MOUSE]	6.37	1	1	1	1	0.853	1.253	3.156	3.263	0.00	6.37	1	1	487	53.2	7.56
P16331	Phenylalanine-4-hydroxylase OS=Mus musculus GN=Pah PE=1 SV=4 - [PH4H_MOUSE]	4.19	2	2	2	3	0.819	1.082	2.275	3.276	10.46	4.19	3	3	453	51.9	6.30
E9PY03	Protein Tstd1 OS=Mus musculus GN=Tstd1 PE=4 SV=1 - [E9PY03_MOUSE]	35.34	1	2	2	6	5.949	1.812	1.105	3.318	22.78	35.34	3	6	133	14.3	8.48
P11714	Cytochrome P450 2D9 OS=Mus musculus GN=Cyp2d9 PE=1 SV=2 - [CP2D9_MOUSE]	15.08	1	5	5	8	1.161	1.099	2.342	3.527	13.94	15.08	8	8	504	56.9	6.21
P12246	Serum amyloid P-component OS=Mus musculus GN=Apcs PE=1 SV=2 - [SAMP_MOUSE]	13.39	1	2	2	10	1.905	1.815	1.379	3.678	26.03	13.39	3	10	224	26.2	6.35
Q9DCV7	Keratin, type II cytoskeletal 7 OS=Mus musculus GN=Krt7 PE=1 SV=1 - [K2C7_MOUSE]	8.32	3	1	4	33	1.146	2.281	2.483	3.689	65.85	8.32	7	33	457	50.7	5.87
B2RVA4	Guanine nucleotide-binding protein subunit gamma OS=Mus musculus GN=Gm5741 PE=3 SV=1 - [B2RVA4_MOUSE]	23.61	2	1	2	3	0.836	0.244	1.183	3.709	6.12	23.61	3	3	72	7.7	9.35
D3YU60	Microsomal glutathione S-transferase 1 OS=Mus musculus GN=Mgst1 PE=2 SV=1 - [D3YU60_MOUSE]	45.10	4	3	3	5	1.389	0.948	1.782	3.830	11.07	45.10	4	5	102	11.7	8.48
Q9D9R9	Protein FAM186A OS=Mus musculus GN=FAM186A PE=2 SV=2 - [F186A_MOUSE]	1.01	3	1	2	5	2.027	1.332	2.047	3.841	0.00	1.01	2	5	1790	194.4	6.34
P20917-2	Isoform S-MAG of Myelin-associated glycoprotein OS=Mus musculus GN=Mag - [MAG_MOUSE]	27.84	1	1	15	164	1.449	0.275	3.384	3.841	505.22	27.84	27	164	582	64.2	5.08
Q9D154	Leukocyte elastase inhibitor A OS=Mus musculus GN=Serpinb1a PE=1 SV=1 -	48.02	5	13	18	92	1.732	0.888	2.987	3.950	329.42	48.02	34	92	379	42.5	6.21
Q9QXJ2	Signal transducer and activator of transcription 2 OS=Mus musculus GN=Stat2 PE=1 SV=1 - [Q9QXJ2_MOUSE]	5.75	3	5	5	9	1.466	1.203	1.653	3.970	22.32	5.75	9	9	922	105.3	5.21
P35428	Transcription factor HES-1 OS=Mus musculus GN=Hes1 PE=1 SV=1 - [HES1_MOUSE]	5.67	1	1	1	1	5.264	2.008	2.355	4.065	3.29	5.67	1	1	282	29.7	9.61
Q63880-2	Isoform 2 of Carboxylesterase 3A OS=Mus musculus GN=Ces3a -	8.02	6	3	4	19	1.358	0.769	2.319	4.211	57.41	8.02	7	19	524	57.8	5.63
P16015	Carbonic anhydrase 3 OS=Mus musculus GN=Ca3 PE=1 SV=3 - [CAH3_MOUSE]	48.08	1	10	10	35	2.717	2.810	2.301	4.231	90.86	48.08	17	35	260	29.3	7.37
Q64282	Interferon-induced protein with tetratricopeptide repeats 1 OS=Mus musculus GN=Ifit1 PE=1 SV=2 - [IFIT1_MOUSE]	12.96	4	3	3	5	0.551	1.967	1.991	4.248	10.03	12.96	5	5	463	53.7	7.52
Q8BPC6	Probable isocitrate dehydrogenase [NAD] gamma 2, mitochondrial OS=Mus musculus PE=1 SV=1 - [IDHG2_MOUSE]	7.32	1	1	1	3	1.793	0.747	6.843	4.366	0.00	7.32	1	3	396	43.8	8.37
Q9D035	Sodium/potassium-transporting ATPase subunit beta-1-interacting protein 1 OS=Mus musculus GN=Nkain1 PE=1 SV=2 - [NKAI1_MOUSE]	6.28	1	1	1	2	1.975	1.002	1.826	4.524	4.12	6.28	2	2	207	23.5	5.71
Q60590	Alpha-1-acid glycoprotein 1 OS=Mus musculus GN=Orm1 PE=1 SV=1 - [A1AG1_MOUSE]	20.77	1	3	5	10	5.144	3.352	3.150	4.572	28.25	20.77	8	10	207	23.9	5.85
P23927	Alpha-crystallin B chain OS=Mus musculus GN=Cryab PE=1 SV=2 - [CRYAB_MOUSE]	57.14	1	9	9	48	2.002	0.853	4.870	4.655	137.40	57.14	16	48	175	20.1	7.33
Q91XL1	Leucine-rich HEV glycoprotein (Precursor) OS=Mus musculus GN=Lrg1 PE=2 SV=1 - [Q91XL1_MOUSE]	23.68	1	7	8	30	3.997	2.711	2.418	4.684	87.07	23.68	15	30	342	37.4	6.61
J3JS28	Protein Wnt-9a OS=Mus musculus GN=Wnt9a PE=4 SV=1 - [J3JS28_MOUSE]	2.47	2	1	1	1	1.114	1.306	1.245	4.686	0.00	2.47	1	1	365	40.4	8.65

Q8VC12	Urocanate hydratase OS=Mus musculus GN=Uroc1 PE=2 SV=2 - [HUTU_MOUSE]	2.07	2	1	1	2	13.452	1.902	3.704	4.770	5.07	2.07	2	2	676	74.5	7.53
Q64374	Regucalcin OS=Mus musculus GN=Rgn PE=1 SV=1 - [RGN_MOUSE]	17.06	1	4	4	24	2.126	1.199	2.533	4.934	75.05	17.06	8	24	299	33.4	5.34
A2AKN9	Major urinary protein 2 OS=Mus musculus GN=Mup2 PE=2 SV=1 - [A2AKN9_MOUSE]	53.89	8	2	8	26	3.596	1.994	3.437	5.426	79.82	53.89	14	26	180	20.6	5.01
E9Q1R2	Probable 4-hydroxy-2-oxoglutarate aldolase, mitochondrial OS=Mus musculus GN=Hoga1 PE=2 SV=1 - [illegible]	9.49	2	1	1	1	1.551	1.372	3.211	5.704	4.73	9.49	1	1	158	17.4	8.18
Q91X83	S-adenosylmethionine synthase isoform type-1 OS=Mus musculus GN=Mat1a PE=2 SV=1 - [METK1_MOUSE]	10.86	1	3	4	9	1.263	0.704	3.444	5.725	22.75	10.86	5	9	396	43.5	5.82
Q8C196	Carbamoyl-phosphate synthase [ammonia], mitochondrial OS=Mus musculus GN=Cps1 PE=1 SV=2 - [CPSM_MOUSE]	28.73	1	36	39	110	1.458	1.075	3.626	5.780	314.35	28.73	65	110	1500	164.5	6.92
O35490	Betaine--homocysteine S-methyltransferase 1 OS=Mus musculus GN=Bhmt PE=2 SV=1 - [BHMT1_MOUSE]	32.92	2	10	10	39	1.534	0.999	4.626	6.258	126.35	32.92	19	39	407	45.0	7.90
Q9QXD6	Fructose-1,6-bisphosphatase 1 OS=Mus musculus GN=Fbp1 PE=2 SV=3 - [F16P1_MOUSE]	14.50	4	4	4	8	3.125	1.314	4.124	6.536	33.65	14.50	6	8	338	36.9	6.57
Q9JJT0	RNA 3'-terminal phosphate cyclase-like protein OS=Mus musculus GN=Rcl1 PE=2 SV=1 - [RCL1_MOUSE]	4.02	1	1	1	1	1.081	1.219	1.496	6.561	2.85	4.02	1	1	373	40.8	9.26
Q9ESM3	Hyaluronan and proteoglycan link protein 2 OS=Mus musculus GN=Hapln2 PE=1 SV=1 - [HPLN2_MOUSE]	28.45	1	8	8	25	1.255	0.697	4.037	6.604	71.35	28.45	13	25	341	37.9	9.09
P11672	Neutrophil gelatinase-associated lipocalin OS=Mus musculus GN=Lcn2 PE=1 SV=1 - [NGAL_MOUSE]	23.00	1	4	4	7	1.364	1.389	3.962	7.083	17.80	23.00	6	7	200	22.9	8.81
Q8K4J0-3	Isoform 3 of Protein artemis OS=Mus musculus GN=Dclre1c - [DCR1C_MOUSE]	6.98	5	2	3	6	1.172	14.498	20.583	7.377	14.60	6.98	3	6	473	54.2	8.56
Q80TG1-4	Isoform 4 of KAT8 regulatory NSL complex subunit 1 OS=Mus musculus GN=Kansl1 - [KANL1_MOUSE]	5.12	5	1	2	2	1.409	2.628	8.816	7.638	6.05	5.12	2	2	430	46.2	7.58
Q91Y97	Fructose-bisphosphate aldolase B OS=Mus musculus GN=Aldob PE=1 SV=3 - [ALDOB_MOUSE]	36.54	1	12	13	87	1.841	1.077	5.378	7.660	221.31	36.54	23	87	364	39.5	8.27
B1AR34	Asialoglycoprotein receptor 1 OS=Mus musculus GN=Asgr1 PE=4 SV=1 - [B1AR34_MOUSE]	4.31	2	1	1	2	0.893	0.775	3.818	7.804	4.59	4.31	2	2	255	29.4	6.02
A2AI57	Transient receptor potential cation channel subfamily M member 7 OS=Mus musculus GN=Trpm7 PE=2 SV=1 - [illegible]	3.22	2	3	5	8	1.096	1.432	0.962	7.909	12.72	3.22	5	8	1862	212.2	7.80
B6VJS2	5-hydroxytryptamine receptor 7 OS=Mus musculus GN=Htr7 PE=2 SV=1 - [B6VJS2_MOUSE]	3.68	3	2	2	3	1.727	0.602	5.353	8.890	5.51	3.68	2	3	435	48.5	8.76
Q61646	Haptoglobin OS=Mus musculus GN=Hp PE=1 SV=1 - [HPT_MOUSE]	34.01	1	12	12	34	2.324	3.005	7.284	9.414	99.91	34.01	20	34	347	38.7	6.29
Q9QXF8	Glycine N-methyltransferase OS=Mus musculus GN=Gnmt PE=1 SV=3 - [GNMT_MOUSE]	12.63	1	2	2	14	3.932	1.115	5.973	9.609	58.40	12.63	4	14	293	32.7	7.44
G3UW77	Protein inscuteable homolog OS=Mus musculus GN=Insc PE=4 SV=1 - [G3UW77_MOUSE]	2.07	2	1	1	1	0.886	1.141	1.139	9.828	2.38	2.07	1	1	532	58.6	5.16
Q5PPR4	Zfp354a protein OS=Mus musculus GN=Zfp354a PE=2 SV=1 - [Q5PPR4_MOUSE]	1.75	3	1	1	1	1.581	2.220	2.103	10.242	1.79	1.75	1	1	571	65.4	9.38
P12710	Fatty acid-binding protein, liver OS=Mus musculus GN=Fabp1 PE=1 SV=2 - [FABPL_MOUSE]	55.91	1	6	6	26	3.311	0.384	8.583	12.127	81.76	55.91	10	26	127	14.2	8.56
Q8R366	Immunoglobulin superfamily member 8 OS=Mus musculus GN=Igsf8 PE=1 SV=2 - [IGSF8_MOUSE]	35.68	2	2	17	151	2.511	7.806	6.780	13.203	487.78	35.68	33	151	611	65.0	7.99
P05367	Serum amyloid A-2 protein OS=Mus musculus GN=Saa2 PE=1 SV=1 - [SAA2_MOUSE]	28.69	1	3	4	12	1.900	1.858	10.905	24.241	36.09	28.69	7	12	122	13.6	6.93
P05366	Serum amyloid A-1 protein OS=Mus musculus GN=Saa1 PE=2 SV=2 - [SAA1_MOUSE]	27.05	1	2	3	15	1.542	0.872	12.726	29.258	44.59	27.05	5	15	122	13.8	7.03

F6RU76	Protein 4933403O08Rik OS=Mus musculus GN=4933403O08Rik PE=4 SV=2 - [F6RU76_MOUSE]	5.68	2	1	1	1	1.717	2.046	3.050	39.352	3.10	5.68		1	1	264	29.7	6.23
