# Supplementary material for: Hypothalamus proteomics from mouse models with obesity and anorexia reveals therapeutic targets of appetite regulation
Source: Nutr Diabetes. 2016 Apr 25;6(4):e204–. doi: 10.1038/nutd.2016.10 (PMC4855256; doi:10.1038/nutd.2016.10)
Supplement: Supplementary Table 4 [file nutd201610x7.pdf]

Supplementary Table 4. Differentially expressed proteins in the HFD groups compared to controls (proteins analysed with at least two unique peptides)

Accession	Description	ΣCoverage	Σ# Proteins	Σ# Unique Pepti	Σ# Peptides	Σ# PSMs	HFD1/C1	HFD2/C1	LPS1/C2	LPS2/C2
P08032	Spectrin alpha chain, erythrocytic 1 OS=Mus musculus GN=Spta1 PE=2 SV=3 - [SPTA1_MOUSE]	43.35	3	82	84	302	1.47	0.84	-0.52	0.21
P07724	Serum albumin OS=Mus musculus GN=Alb PE=1 SV=3 - [ALBU_MOUSE]	75.49	4	49	49	2294	1.97	0.72	-1.29	-0.51
Q9Z1Q9	Valine--tRNA ligase OS=Mus musculus GN=Vars PE=2 SV=1 - [SYVC_MOUSE]	40.22	6	42	42	196	-0.69	-0.83	-0.46	-0.24
D3YW52	Alpha-2-macroglobulin OS=Mus musculus GN=Pzp PE=2 SV=1 - [D3YW52_MOUSE]	36.70	3	42	45	220	1.60	1.62	-0.32	0.69
Q91UZ1	Phospholipase C beta 4 OS=Mus musculus GN=Plcb4 PE=2 SV=1 - [Q91UZ1_MOUSE]	37.45	2	32	43	132	-0.78	-1.49	-0.75	-0.33
Q8VHX6-2	Isoform 2 of Filamin-C OS=Mus musculus GN=Flnc - [FLNC_MOUSE]	17.23	4	27	35	90	0.86	1.03	-0.32	0.13
Q6PHS9-4	Isoform 4 of Voltage-dependent calcium channel subunit alpha-2/delta-2 OS=Mus musculus GN=Cacna2d2 - [CA2D2_MOUSE]	26.42	7	26	27	134	-0.69	-1.11	-0.34	0.14
P28665	Murinoglobulin-1 OS=Mus musculus GN=Mug1 PE=1 SV=3 - [MUG1_MOUSE]	35.70	1	24	40	155	1.84	1.91	0.07	0.71
P06728	Apolipoprotein A-IV OS=Mus musculus GN=Apoa4 PE=2 SV=3 - [APOA4_MOUSE]	71.14	1	24	24	119	1.64	1.89	-0.96	-0.01
Q91X72	Hemopexin OS=Mus musculus GN=Hpx PE=1 SV=2 - [HEMO_MOUSE]	50.22	1	23	23	133	1.78	1.06	0.35	1.05
Q3UX37	Protein Plekhg1 OS=Mus musculus GN=Plekhg1 PE=2 SV=1 - [Q3UX37_MOUSE]	18.27	4	22	23	52	-0.79	-1.44	-0.68	-0.61
Q99K47	Fibrinogen, alpha polypeptide OS=Mus musculus GN=Fga PE=2 SV=1 - [Q99K47_MOUSE]	49.55	2	22	23	134	0.92	0.76	0.56	1.56
Q8K0E8	Fibrinogen beta chain OS=Mus musculus GN=Fgb PE=2 SV=1 - [FIBB_MOUSE]	51.98	2	22	23	146	0.89	0.70	0.36	1.38
P28867	Protein kinase C delta type OS=Mus musculus GN=Prkcd PE=1 SV=3 - [KPCD_MOUSE]	39.17	6	21	24	93	-0.65	-1.60	-0.73	-0.63
Q8CA95-3	Isoform 3 of cAMP and cAMP-inhibited cGMP 3',5'-cyclic phosphodiesterase 10A OS=Mus musculus GN=Pde10a - [PDE10_MOUSE]	30.53	7	21	21	81	-1.27	-1.21	1.42	0.26

Q9D8E6	60S ribosomal protein L4 OS=Mus musculus GN=Rpl4 PE=1 SV=3 - [RL4_MOUSE]	44.63	1	21	21	121	-1.03	-0.81	-0.53	-0.27
Q61282	Aggrecan core protein OS=Mus musculus GN=Acan PE=1 SV=2 - [PGCA_MOUSE]	13.56	1	21	22	73	1.89	0.67	1.12	0.72
Q8CH09	SURP and G-patch domain-containing protein 2 OS=Mus musculus GN=Sugp2 PE=2 SV=2 - [SUGP2_MOUSE]	22.21	4	20	20	87	-0.90	-0.67	-0.31	-0.23
P25444	40S ribosomal protein S2 OS=Mus musculus GN=Rps2 PE=1 SV=3 - [RS2_MOUSE]	62.46	11	20	20	91	-0.86	-0.59	-0.46	-0.33
P55096	ATP-binding cassette sub-family D member 3 OS=Mus musculus GN=Abcd3 PE=1 SV=2 - [ABCD3_MOUSE]	36.42	1	19	20	66	-1.06	-0.68	-0.54	-0.21
Q99JF8	PC4 and SFRS1-interacting protein OS=Mus musculus GN=Psip1 PE=1 SV=1 - [PSIP1_MOUSE]	39.02	4	18	20	90	-0.70	-0.71	0.01	-0.44
Q9CZW4	Long-chain-fatty-acid--CoA ligase 3 OS=Mus musculus GN=Acsl3 PE=2 SV=2 - [ACSL3_MOUSE]	35.14	3	18	20	54	-0.79	-0.58	-0.25	0.14
P21614	Vitamin D-binding protein OS=Mus musculus GN=Gc PE=1 SV=2 - [VTDB_MOUSE]	49.79	1	18	19	63	1.57	0.99	-0.48	0.09
Q9QUG9	RAS guanyl-releasing protein 2 OS=Mus musculus GN=Rasgrp2 PE=1 SV=2 - [GRP2_MOUSE]	29.61	11	17	17	61	-1.13	-0.94	0.15	-0.28
Q00623	Apolipoprotein A-I OS=Mus musculus GN=Apoa1 PE=1 SV=2 - [APOA1_MOUSE]	49.62	1	17	17	157	1.65	1.93	-0.70	0.23
P12658	Calbindin OS=Mus musculus GN=Calb1 PE=1 SV=2 - [CALB1_MOUSE]	79.69	1	17	18	347	0.90	1.06	0.40	-0.12
P20918	Plasminogen OS=Mus musculus GN=Plg PE=1 SV=3 - [PLMN_MOUSE]	26.60	2	17	18	70	1.82	0.81	-1.04	-0.21
Q8VCM7	Fibrinogen gamma chain OS=Mus musculus GN=Fgg PE=2 SV=1 - [FIBG_MOUSE]	48.39	1	17	17	78	1.13	0.66	0.47	1.60
P06880	Somatotropin OS=Mus musculus GN=Gh1 PE=2 SV=1 - [SOMA_MOUSE]	65.74	1	16	17	303	-0.93	-1.18	-1.87	-1.83
Q99KK2	N-acylneuraminate cytidylyltransferase OS=Mus musculus GN=Cmas PE=1 SV=2 - [NEUA_MOUSE]	44.91	2	16	17	72	-0.94	-0.81	-0.56	-0.19
Q9WTS6-2	Isoform 2 of Teneurin-3 OS=Mus musculus GN=Tenm3 - [TEN3_MOUSE]	10.60	5	16	21	59	-0.60	-0.61	-0.31	-0.21

O08677	Kininogen-1 OS=Mus musculus GN=Kng1 PE=1 SV=1 - [KNG1_MOUSE]	26.78	4	16	17	82	1.63	0.79	-0.46	-0.01
P01029	Complement C4-B OS=Mus musculus GN=C4b PE=1 SV=3 - [CO4B_MOUSE]	10.70	2	16	16	34	1.60	0.64	-0.51	0.08
P24529	Tyrosine 3-monooxygenase OS=Mus musculus GN=Th PE=1 SV=3 - [TY3H_MOUSE]	39.96	5	15	15	55	-1.06	-1.38	1.26	0.89
P63158	High mobility group protein B1 OS=Mus musculus GN=Hmgb1 PE=1 SV=2 - [HMGB1_MOUSE]	43.72	3	15	16	101	-1.02	-1.02	-0.02	-0.57
Q80XN0	D-beta-hydroxybutyrate dehydrogenase, mitochondrial OS=Mus musculus GN=Bdh1 PE=1 SV=2 - [BDH_MOUSE]	55.10	2	15	17	189	-0.73	-0.91	-0.65	-0.68
O70503	Estradiol 17-beta-dehydrogenase 12 OS=Mus musculus GN=Hsd17b12 PE=2 SV=1 - [DHB12_MOUSE]	44.55	2	15	15	58	-0.87	-0.73	-0.37	0.03
P19221	Prothrombin OS=Mus musculus GN=F2 PE=1 SV=1 - [THRB_MOUSE]	27.67	3	15	16	52	2.02	1.25	-1.01	-0.12
A2A5R8	Double-stranded RNA-binding protein Staufen homolog 1 OS=Mus musculus GN=Stau1 PE=2 SV=1 - [A2A5R8_MOUSE]	34.43	4	14	15	31	-0.64	-1.41	-0.17	-0.48
D3Z6C3	40S ribosomal protein S3a OS=Mus musculus GN=Rps3a2 PE=3 SV=1 - [D3Z6C3_MOUSE]	51.89	3	14	14	63	-1.01	-0.66	-0.51	-0.39
P32261	Antithrombin-III OS=Mus musculus GN=Serpinc1 PE=1 SV=1 - [ANT3_MOUSE]	35.05	1	14	14	49	1.48	1.22	-0.88	-0.03
Q9JHU2	Palmdelphin OS=Mus musculus GN=Palmd PE=1 SV=1 - [PALMD_MOUSE]	30.49	3	14	14	43	0.86	0.95	0.52	-0.13
O08842	GDNF family receptor alpha-2 OS=Mus musculus GN=Gfra2 PE=2 SV=1 - [GFRA2_MOUSE]	31.10	3	13	14	44	-0.79	-1.14	-0.67	-0.18
P62702	40S ribosomal protein S4, X isoform OS=Mus musculus GN=Rps4x PE=2 SV=2 - [RS4X_MOUSE]	55.51	2	13	15	68	-0.95	-0.87	-0.48	-0.29
P62631	Elongation factor 1-alpha 2 OS=Mus musculus GN=Eef1a2 PE=1 SV=1 - [EF1A2_MOUSE]	64.79	1	13	25	320	-0.65	-0.84	-0.11	-0.02
A3KG01	Eph receptor B2 OS=Mus musculus GN=Ephb2 PE=2 SV=2 - [A3KG01_MOUSE]	19.98	8	13	17	39	-0.87	-0.84	-0.48	-0.27

Q07235	Glia-derived nexin OS=Mus musculus GN=Serpine2 PE=2 SV=2 - [GDN_MOUSE]	37.78	1	13	14	38	-0.80	-0.78	-0.42	-0.09
O35633-2	Isoform 2 of Vesicular inhibitory amino acid transporter OS=Mus musculus GN=Slc32a1 - [VIAAT_MOUSE]	30.52	2	13	14	96	-0.80	-0.66	-0.07	0.22
Q8K4P8	E3 ubiquitin-protein ligase HECW1 OS=Mus musculus GN=Hecw1 PE=2 SV=3 - [HECW1_MOUSE]	11.91	6	13	16	37	-0.60	-0.61	0.16	-0.10
P07310	Creatine kinase M-type OS=Mus musculus GN=Ckm PE=1 SV=1 - [KCRM_MOUSE]	44.88	1	13	16	123	2.87	3.59	-0.61	-0.13
P04186	Complement factor B OS=Mus musculus GN=Cfb PE=1 SV=2 - [CFAB_MOUSE]	15.64	9	13	13	33	1.33	0.99	-0.70	0.52
P14131	40S ribosomal protein S16 OS=Mus musculus GN=Rps16 PE=2 SV=4 - [RS16_MOUSE]	67.12	1	12	12	65	-0.66	-0.94	-0.45	-0.28
A2APX7	Protein Scn1a OS=Mus musculus GN=Scn1a PE=2 SV=1 - [A2APX7_MOUSE]	12.57	13	12	24	106	-0.60	-0.88	0.23	0.38
Q64012-2	Isoform 1 of RNA-binding protein Raly OS=Mus musculus GN=Raly - [RALY_MOUSE]	45.95	5	12	12	49	-1.26	-0.86	-0.35	-0.29
F6WDS8	Regulator of G-protein-signaling 6 OS=Mus musculus GN=Rgs6 PE=4 SV=1 - [F6WDS8_MOUSE]	34.21	3	12	15	78	-1.05	-0.80	0.15	0.30
G3X9Y1	Synaptotagmin III, isoform CRA_a OS=Mus musculus GN=Syt3 PE=4 SV=1 - [G3X9Y1_MOUSE]	28.96	4	12	16	59	-0.65	-0.66	0.00	0.05
D3Z6S1	Transmembrane protein 214 OS=Mus musculus GN=Tmem214 PE=2 SV=1 - [D3Z6S1_MOUSE]	22.90	4	12	13	45	-0.81	-0.59	-0.46	-0.25
Q99L45	Eukaryotic translation initiation factor 2 subunit 2 OS=Mus musculus GN=Eif2s2 PE=1 SV=1 - [IF2B_MOUSE]	32.63	3	12	12	44	-0.68	-0.58	-0.32	-0.53
Q61646	Haptoglobin OS=Mus musculus GN=Hp PE=1 SV=1 - [HPT_MOUSE]	34.01	1	12	12	34	1.22	1.59	2.86	3.23
D3YY36	Protein 1300017J02Rik OS=Mus musculus GN=1300017J02Rik PE=2 SV=1 - [D3YY36_MOUSE]	22.67	3	12	13	40	2.19	0.86	-0.79	-0.04
P62281	40S ribosomal protein S11 OS=Mus musculus GN=Rps11 PE=2 SV=3 - [RS11_MOUSE]	49.37	1	11	11	48	-1.06	-1.21	-0.52	-0.37

Q6ZWN5	40S ribosomal protein S9 OS=Mus musculus GN=Rps9 PE=2 SV=3 - [RS9_MOUSE]	41.24	6	11	12	74	-1.34	-1.16	-0.61	-0.30
P41105	60S ribosomal protein L28 OS=Mus musculus GN=Rpl28 PE=1 SV=2 - [RL28_MOUSE]	56.93	2	11	11	103	-1.13	-1.14	-0.54	-0.23
G3XA00	Glutamate receptor, metabotropic 4 OS=Mus musculus GN=Grm4 PE=3 SV=1 - [G3XA00_MOUSE]	16.78	5	11	13	40	-0.75	-0.98	0.25	0.14
P62270	40S ribosomal protein S18 OS=Mus musculus GN=Rps18 PE=1 SV=3 - [RS18_MOUSE]	49.34	4	11	11	47	-1.16	-0.81	-0.62	-0.40
Q922Q8	Leucine-rich repeat-containing protein 59 OS=Mus musculus GN=Lrrc59 PE=2 SV=1 - [LRC59_MOUSE]	42.67	1	11	11	49	-0.71	-0.78	-0.23	-0.20
P01872	Ig mu chain C region secreted form OS=Mus musculus GN=Igh-6 PE=1 SV=2 - [IGHM_MOUSE]	32.38	2	11	12	35	1.34	1.67	0.35	0.85
Q01339	Beta-2-glycoprotein 1 OS=Mus musculus GN=Apoh PE=1 SV=1 - [APOH_MOUSE]	35.65	3	11	11	39	1.79	1.26	-0.67	-0.30
P07759	Serine protease inhibitor A3K OS=Mus musculus GN=Serpina3k PE=1 SV=2 - [SPA3K_MOUSE]	46.89	1	11	16	126	1.84	1.15	-0.30	0.81
Q5XJF6	Ribosomal protein OS=Mus musculus GN=Rpl10a PE=2 SV=1 [Q5XJF6_MOUSE]	41.94	4	10	10	58	-1.37	-1.06	-0.53	-0.38
Q8BLE7	Vesicular glutamate transporter 2 OS=Mus musculus GN=Slc17a6 PE=1 SV=1 - [VGLU2_MOUSE]	16.84	1	10	10	85	-0.71	-1.05	-0.43	0.15
Q8C4C4	Repulsive guidance molecule A OS=Mus musculus GN=Rgma PE=2 SV=1 - [Q8C4C4_MOUSE]	35.17	3	10	10	19	-0.67	-0.90	-0.76	-0.51
P62830	60S ribosomal protein L23 OS=Mus musculus GN=Rpl23 PE=1 SV=1 - [RL23_MOUSE]	71.43	2	10	10	36	-1.08	-0.79	-0.63	-0.50
I7HLV2	60S ribosomal protein L10 (Fragment) OS=Mus musculus GN=Rpl10 PE=4 SV=1 - [I7HLV2_MOUSE]	50.75	4	10	10	69	-1.16	-0.77	-0.56	-0.37
Q8K2Q7	BRO1 domain-containing protein BROX OS=Mus musculus GN=Brox PE=2 SV=1 - [BROX_MOUSE]	33.82	3	10	11	26	-0.80	-0.71	-0.47	-0.06
Q3UUQ7	GPI inositol-deacylase OS=Mus musculus GN=Pgap1 PE=1 SV=3 - [PGAP1_MOUSE]	9.22	1	10	10	29	-0.96	-0.69	-0.72	-0.23

P19253	60S ribosomal protein L13a OS=Mus musculus GN=Rpl13a PE=1 SV=4 - [RL13A_MOUSE]	37.44	3	10	10	58	-0.88	-0.62	-0.45	-0.36
Q9CXW4	60S ribosomal protein L11 OS=Mus musculus GN=Rpl11 PE=1 SV=4 - [RL11_MOUSE]	47.75	6	10	10	33	-0.68	-0.58	-0.40	-0.23
P16015	Carbonic anhydrase 3 OS=Mus musculus GN=Ca3 PE=1 SV=3 - [CAH3_MOUSE]	48.08	1	10	10	35	1.44	1.49	1.20	2.08
Q923D2	Flavin reductase (NADPH) OS=Mus musculus GN=Blvrb PE=2 SV=3 - [BLVRB_MOUSE]	77.67	3	10	10	84	1.31	0.77	-0.34	0.37
Q1RLL3	Copine-9 OS=Mus musculus GN=Cpne9 PE=2 SV=1 - [CPNE9_MOUSE]	37.43	1	9	15	100	-1.48	-2.22	-0.84	-0.22
Q8R4E6	Purine-rich element-binding protein gamma OS=Mus musculus GN=Purg PE=1 SV=1 - [PURG_MOUSE]	27.71	2	9	13	33	-1.18	-1.55	-0.77	-1.04
Q50H33	BTB/POZ domain-containing protein KCTD8 OS=Mus musculus GN=Kctd8 PE=1 SV=1 - [KCTD8_MOUSE]	23.53	2	9	11	35	-1.29	-1.32	-0.22	0.21
P27600	Guanine nucleotide-binding protein subunit alpha-12 OS=Mus musculus GN=Gna12 PE=1 SV=3 - [GNA12_MOUSE]	26.12	3	9	11	99	-0.75	-1.19	-0.36	0.17
Q04736	Tyrosine-protein kinase Yes OS=Mus musculus GN=Yes1 PE=1 SV=3 - [YES_MOUSE]	36.78	15	9	19	96	-0.60	-0.68	-0.27	0.14
Q9D1E8	1-acyl-sn-glycerol-3-phosphate acyltransferase epsilon OS=Mus musculus GN=Agpat5 PE=2 SV=2 - [PLCE_MOUSE]	23.56	2	9	9	30	-0.80	-0.62	-0.42	0.09
P62245	40S ribosomal protein S15a OS=Mus musculus GN=Rps15a PE=2 SV=2 - [RS15A_MOUSE]	66.92	4	9	9	50	-0.82	-0.59	-0.49	-0.24
P13634	Carbonic anhydrase 1 OS=Mus musculus GN=Ca1 PE=2 SV=4 - [CAH1_MOUSE]	54.02	2	9	9	29	1.06	1.88	0.35	1.41
Q91VB8	Alpha globin 1 OS=Mus musculus GN=Hba-a1 PE=2 SV=1 - [Q91VB8_MOUSE]	69.72	2	9	10	1680	1.82	1.47	-0.89	0.38
P14602	Heat shock protein beta-1 OS=Mus musculus GN=Hspb1 PE=1 SV=3 - [HSPB1_MOUSE]	61.72	4	9	9	35	1.00	1.03	0.19	0.51
P52760	Ribonuclease UK114 OS=Mus musculus GN=Hrsp12 PE=1 SV=3 - [UK114_MOUSE]	92.59	1	9	11	87	1.19	0.70	1.10	0.78

O89020	Afamin OS=Mus musculus GN=Afm PE=1 SV=2 - [AFAM_MOUSE]	16.78	3	9	9	18	1.65	0.63	-1.78	-0.45
Q9D2P8	Myelin-associated oligodendrocyte basic protein OS=Mus musculus GN=Mobp PE=2 SV=1 - [MOBP_MOUSE]	42.94	5	8	8	62	-0.75	-1.32	1.12	1.34
Q8C0M2	Abhydrolase domain-containing protein 4 OS=Mus musculus GN=Abhd4 PE=2 SV=1 - [Q8C0M2_MOUSE]	22.96	3	8	8	29	-0.64	-0.80	-0.43	-0.02
E9QAZ2	Ribosomal protein L15 OS=Mus musculus GN=Gm10020 PE=3 SV=1 - [E9QAZ2_MOUSE]	39.71	4	8	8	58	-1.19	-0.76	-0.44	-0.22
P62717	60S ribosomal protein L18a OS=Mus musculus GN=Rpl18a PE=1 SV=1 - [RL18A_MOUSE]	39.20	2	8	8	43	-1.25	-0.72	-0.63	-0.30
A2A547	60S ribosomal protein L19 OS=Mus musculus GN=Rpl19 PE=2 SV=1 - [A2A547_MOUSE]	36.60	2	8	10	82	-0.62	-0.63	-0.37	-0.15
Q9JHJ0	Tropomodulin-3 OS=Mus musculus GN=Tmod3 PE=1 SV=1 [TMOD3_MOUSE]	28.41	1	8	9	27	0.96	0.88	0.10	-0.17
G5E920	MCG114640 OS=Mus musculus GN=Gm6104 PE=4 SV=1 - [G5E920_MOUSE]	29.50	3	7	7	18	-0.81	-1.26	-0.17	-0.57
P43276	Histone H1.5 OS=Mus musculus GN=Hist1h1b PE=1 SV=2 - [H15_MOUSE]	38.57	1	7	13	68	-0.91	-0.92	-0.61	-0.82
Q9CXL6	Doublecortin, isoform CRA_a OS=Mus musculus GN=Dcx PE=2 SV=1 - [Q9CXL6_MOUSE]	30.56	3	7	11	44	-0.75	-0.88	-0.71	-1.32
Q7M759	Alpha/beta hydrolase domain-containing protein 17B OS=Mus musculus GN=Abhd17b PE=2 SV=1 - [AB17B_MOUSE]	39.58	1	7	8	19	-0.67	-0.81	0.19	0.15
O70152	Dolichol-phosphate mannosyltransferase OS=Mus musculus GN=Dpm1 PE=2 SV=1 - [DPM1_MOUSE]	25.77	3	7	7	26	-0.68	-0.80	-0.43	0.01
O55142	60S ribosomal protein L35a OS=Mus musculus GN=Rpl35a PE=2 SV=2 - [RL35A_MOUSE]	49.09	1	7	7	17	-1.18	-0.65	-0.39	-0.33
P35550	rRNA 2'-O-methyltransferase fibrillarin OS=Mus musculus GN=Fbl PE=1 SV=2 - [FBRL_MOUSE]	33.64	1	7	9	55	-0.60	-0.64	-0.36	-0.01

Q9D1R9	60S ribosomal protein L34 OS=Mus musculus GN=Rpl34 PE=3 SV=2 - [RL34_MOUSE]	38.46	2	7	7	34	-1.07	-0.61	-0.45	-0.33
Q99KI3	ER membrane protein complex subunit 3 OS=Mus musculus GN=Emc3 PE=2 SV=3 - [EMC3_MOUSE]	19.16	1	7	7	33	-0.77	-0.60	-0.50	-0.34
Q9ESB3	Histidine-rich glycoprotein OS=Mus musculus GN=Hrg PE=1 SV=2 - [HRG_MOUSE]	14.29	1	7	7	25	1.81	1.50	-0.98	-0.13
Q91XL1	Leucine-rich HEV glycoprotein (Precursor) OS=Mus musculus GN=Lrg1 PE=2 SV=1 - [Q91XL1_MOUSE]	23.68	1	7	8	30	2.00	1.44	1.27	2.23
O70250	Phosphoglycerate mutase 2 OS=Mus musculus GN=Pgam2 PE=1 SV=3 - [PGAM2_MOUSE]	50.59	1	7	12	204	1.17	1.20	-0.16	-0.32
P26262	Plasma kallikrein OS=Mus musculus GN=Klkb1 PE=1 SV=2 - [KLKB1_MOUSE]	12.07	1	7	7	18	1.14	0.88	-0.74	0.27
P29391	Ferritin light chain 1 OS=Mus musculus GN=Ftl1 PE=1 SV=2 - [FRIL1_MOUSE]	52.46	4	7	7	39	0.92	0.87	0.43	0.45
Q9CPQ0	Prolactin OS=Mus musculus GN=Prl PE=2 SV=2 - [Q9CPQ0_MOUSE]	24.00	3	6	6	34	-0.81	-1.16	-3.08	-3.27
O70174	Neuronal acetylcholine receptor subunit alpha-4 OS=Mus musculus GN=Chrna4 PE=2 SV=2 - [ACHA4_MOUSE]	11.92	2	6	6	20	-0.77	-1.09	-0.17	-0.08
P35980	60S ribosomal protein L18 OS=Mus musculus GN=Rpl18 PE=2 SV=3 - [RL18_MOUSE]	32.45	5	6	6	36	-1.11	-0.95	-0.52	-0.21
Q9CQC9	GTP-binding protein SAR1b OS=Mus musculus GN=Sar1b PE=1 SV=1 - [SAR1B_MOUSE]	55.05	1	6	9	32	-0.93	-0.74	-0.69	-0.06
Q3UVU3	Zinc transporter 10 OS=Mus musculus GN=Slc30a10 PE=1 SV=1 - [ZNT10_MOUSE]	21.06	2	6	6	19	-1.33	-0.63	-0.46	-0.20
O88602	Voltage-dependent calcium channel gamma-2 subunit OS=Mus musculus GN=Cacng2 PE=1 SV=1 - [CCG2_MOUSE]	29.72	1	6	7	51	-0.66	-0.63	0.03	0.12
Q9DCU6	39S ribosomal protein L4, mitochondrial OS=Mus musculus GN=Mrpl4 PE=2 SV=1 - [RM04_MOUSE]	22.11	1	6	6	13	-0.91	-0.60	-0.23	-0.08
Q8K4Q8	Collectin-12 OS=Mus musculus GN=Colec12 PE=1 SV=1 - [COL12_MOUSE]	8.89	1	6	7	9	1.02	1.42	-0.27	-0.17

B2RSI6	Leucine rich repeat containing 8 family, member B OS=Mus musculus GN=Lrrc8b PE=2 SV=1 - [B2RSI6_MOUSE]	8.84	2	6	6	16	2.09	0.91	0.44	-0.30
Q9WVL3-2	Isoform 2 of Solute carrier family 12 member 7 OS=Mus musculus GN=Slc12a7 - [S12A7_MOUSE]	6.74	2	6	8	17	1.17	0.88	-0.38	-0.20
O35231	Kinesin-like protein KIFC3 OS=Mus musculus GN=Kifc3 PE=1 SV=4 - [KIFC3_MOUSE]	9.34	4	6	7	10	1.09	0.68	0.26	0.86
Q9JJF0	Nucleosome assembly protein 1-like 5 OS=Mus musculus GN=Nap1l5 PE=2 SV=1 - [NP1L5_MOUSE]	21.79	1	5	5	28	-0.76	-1.77	-1.41	-1.33
Q14B80	Potassium voltage-gated channel subfamily C member 2 OS=Mus musculus GN=Kcnc2 PE=1 SV=1 - [KCNC2_MOUSE]	13.55	2	5	7	12	-0.60	-1.11	0.42	0.30
E9PWD9	DNA-directed RNA polymerase OS=Mus musculus GN=Polrmt PE=2 SV=1 - [E9PWD9_MOUSE]	5.65	4	5	5	9	-1.42	-1.04	-1.02	-0.21
Q76LL6-3	Isoform 3 of FH1/FH2 domain-containing protein 3 OS=Mus musculus GN=Fhod3 - [FHOD3_MOUSE]	4.32	4	5	5	13	-0.72	-1.00	0.00	-0.07
P60867	40S ribosomal protein S20 OS=Mus musculus GN=Rps20 PE=1 SV=1 - [RS20_MOUSE]	41.18	2	5	6	35	-0.74	-0.97	-0.45	-0.37
Q91ZD4	Vang-like protein 2 OS=Mus musculus GN=Vangl2 PE=1 SV=3 - [VANG2_MOUSE]	13.44	4	5	5	14	-0.66	-0.89	-0.84	-0.34
A2A891-2	Isoform 2 of Calmodulin-binding transcription activator 1 OS=Mus musculus GN=Camta1 - [CMTA1_MOUSE]	4.84	5	5	6	13	-0.66	-0.88	-0.25	0.08
Q99N94	39S ribosomal protein L9, mitochondrial OS=Mus musculus GN=Mrpl9 PE=2 SV=2 - [RM09_MOUSE]	21.13	2	5	5	11	-0.77	-0.81	-0.16	-0.24
P62889	60S ribosomal protein L30 OS=Mus musculus GN=Rpl30 PE=2 SV=2 - [RL30_MOUSE]	36.52	1	5	5	20	-0.64	-0.75	-0.57	-0.37
P47963	60S ribosomal protein L13 OS=Mus musculus GN=Rpl13 PE=2 SV=3 - [RL13_MOUSE]	19.43	2	5	5	25	-0.63	-0.75	-0.38	-0.12
B0R0D5	FERM domain-containing protein 5 (Fragment) OS=Mus musculus GN=Frmd5 PE=2 SV=1 - [B0R0D5_MOUSE]	15.40	10	5	5	11	-0.71	-0.74	0.21	0.53

Q3TBW2	39S ribosomal protein L10, mitochondrial OS=Mus musculus GN=Mrpl10 PE=2 SV=2 - [RM10_MOUSE]	29.01	2	5	5	13	-0.64	-0.70	-0.04	-0.16
E9Q9D6	R3H domain-containing protein 2 OS=Mus musculus GN=R3hdm2 PE=2 SV=1 - [E9Q9D6_MOUSE]	9.30	7	5	7	16	-0.62	-0.69	-0.27	-0.87
P63276	40S ribosomal protein S17 OS=Mus musculus GN=Rps17 PE=1 SV=2 - [RS17_MOUSE]	47.41	1	5	5	29	-1.49	-0.66	-0.58	-0.54
Q0PD20	Rab34 OS=Mus musculus GN=Rab34 PE=2 SV=1 - [Q0PD20_MOUSE]	22.01	5	5	6	17	-0.61	-0.63	-0.41	0.11
P01868	Ig gamma-1 chain C region secreted form OS=Mus musculus GN=Ighg1 PE=1 SV=1 - [IGHG1_MOUSE]	22.53	2	5	5	22	1.43	2.36	-0.16	0.79
P03987-2	Isoform 2 of Ig gamma-3 chain C region OS=Mus musculus - [IGHG3_MOUSE]	17.02	2	5	5	20	1.03	2.33	0.30	1.04
P01837	Ig kappa chain C region OS=Mus musculus PE=1 SV=1 - [IGKC_MOUSE]	49.06	1	5	5	25	1.34	2.30	-0.03	0.45
H7BX79	Coiled-coil domain-containing protein 162 OS=Mus musculus GN=Ccdc162 PE=2 SV=2 - [H7BX79_MOUSE]	7.76	7	5	5	9	2.99	1.65	1.06	0.11
P01867-2	Isoform 2 of Ig gamma-2B chain C region OS=Mus musculus GN=Igh-3 - [IGG2B_MOUSE]	25.97	2	5	5	9	0.87	1.61	-0.24	-0.52
E9Q1N0	Protein 4932431P20Rik OS=Mus musculus GN=4932431P20Rik PE=2 SV=1 - [E9Q1N0_MOUSE]	3.55	1	5	6	8	1.80	1.60	-0.08	-0.52
F8VQG7	Protein N4bp2 OS=Mus musculus GN=N4bp2 PE=4 SV=1 - [F8VQG7_MOUSE]	5.07	2	5	6	20	1.02	1.37	0.85	1.15
Q9CQX2	Cytochrome b5 type B OS=Mus musculus GN=Cyb5b PE=1 SV=1 - [CYB5B_MOUSE]	32.19	1	5	5	27	0.91	1.07	0.87	0.45
Q62507	Cochlin OS=Mus musculus GN=Coch PE=2 SV=2 - [COCH_MOUSE]	9.42	1	5	5	9	1.01	1.05	-0.15	0.08
G3X8P9	Aldehyde oxidase OS=Mus musculus GN=Aox1 PE=4 SV=1 - [G3X8P9_MOUSE]	3.90	2	5	6	10	1.10	0.94	0.11	0.50
D3Z4H6	Nucleolysin TIA-1 OS=Mus musculus GN=Tia1 PE=2 SV=1 - [D3Z4H6_MOUSE]	24.94	6	5	9	31	1.12	0.67	0.06	0.23

Q69ZL1	FYVE, RhoGEF and PH domain-containing protein 6 OS=Mus musculus GN=Fgd6 PE=1 SV=2 - [FGD6_MOUSE]	3.50	2	5	5	5	2.50	0.65	0.77	-0.46
P13864-2	Isoform 2 of DNA (cytosine-5)-methyltransferase 1 OS=Mus musculus GN=Dnmt1 - [DNMT1_MOUSE]	4.73	3	5	5	7	1.30	0.65	0.10	-0.18
Q00897	Alpha-1-antitrypsin 1-4 OS=Mus musculus GN=Serpina1d PE=2 SV=1 - [A1AT4_MOUSE]	49.64	1	5	18	128	1.41	0.63	0.03	-0.11
D3YTQ9	40S ribosomal protein S15 OS=Mus musculus GN=Rps15 PE=2 SV=1 - [D3YTQ9_MOUSE]	33.05	3	4	4	9	-0.62	-1.83	-0.27	-0.84
Q99NG0	Helicase ARIP4 OS=Mus musculus GN=Rad54l2 PE=1 SV=1 - [ARIP4_MOUSE]	4.09	2	4	5	11	-1.25	-1.52	-0.65	0.08
Q6XUX1-3	Isoform 3 of Dual serine/threonine and tyrosine protein kinase OS=Mus musculus GN=Dstyk - [DUSTY_MOUSE]	4.47	4	4	4	7	-1.38	-1.15	-0.25	-0.17
Q9CPW7	Zinc finger matrin-type protein 2 OS=Mus musculus GN=Zmat2 PE=2 SV=1 - [ZMAT2_MOUSE]	15.58	1	4	4	5	-0.66	-1.02	0.24	-0.53
Q9WTK3	Glycosylphosphatidylinositol anchor attachment 1 protein OS=Mus musculus GN=Gpaa1 PE=1 SV=3 - [GPAA1_MOUSE]	9.18	4	4	4	9	-1.99	-0.95	-0.35	-0.15
Q5U5I3	28S ribosomal protein S18a, mitochondrial OS=Mus musculus GN=Mrps18a PE=2 SV=1 - [Q5U5I3_MOUSE]	21.94	3	4	4	5	-1.18	-0.90	-0.37	-0.58
O09167	60S ribosomal protein L21 OS=Mus musculus GN=Rpl21 PE=2 SV=3 - [RL21_MOUSE]	45.63	3	4	9	19	-1.08	-0.85	-0.40	-0.31
Q8K4R4	Cytoplasmic phosphatidylinositol transfer protein 1 OS=Mus musculus GN=Pitpnc1 PE=1 SV=1 - [PITC1_MOUSE]	41.57	3	4	13	39	-1.08	-0.81	-0.55	-0.37
P58059	28S ribosomal protein S21, mitochondrial OS=Mus musculus GN=Mrps21 PE=2 SV=1 - [RT21_MOUSE]	48.28	3	4	4	8	-0.76	-0.77	-0.16	-0.04
Q9EP72	ER membrane protein complex subunit 7 OS=Mus musculus GN=Emc7 PE=2 SV=1 - [EMC7_MOUSE]	24.48	1	4	4	12	-0.74	-0.73	-0.31	-0.17

Q8BVA5	UPF0554 protein C2orf43 homolog OS=Mus musculus PE=2 SV=1 - [CB043_MOUSE]	14.42	5	4	5	20	-0.65	-0.72	-0.11	-0.22
D3YVE8	Solute carrier family 35 member G2 OS=Mus musculus GN=Slc35g2 PE=3 SV=1 - [S35G2_MOUSE]	9.71	1	4	4	17	-0.60	-0.71	-0.58	0.10
Q9JI44	DNA methyltransferase 1-associated protein 1 OS=Mus musculus GN=Dmap1 PE=1 SV=1 - [DMAP1_MOUSE]	11.97	2	4	4	6	-1.20	-0.59	-0.34	-0.43
P04247	Myoglobin OS=Mus musculus GN=Mb PE=1 SV=3 - [MYG_MOUSE]	36.36	1	4	4	19	2.59	3.54	-0.90	0.25
Q00898	Alpha-1-antitrypsin 1-5 OS=Mus musculus GN=Serpina1e PE=1 SV=1 - [A1AT5_MOUSE]	35.35	2	4	13	133	3.34	2.46	1.73	0.10
Q00896	Alpha-1-antitrypsin 1-3 OS=Mus musculus GN=Serpina1c PE=1 SV=2 - [A1AT3_MOUSE]	52.91	2	4	17	175	1.96	2.12	-0.50	0.94
P09803	Cadherin-1 OS=Mus musculus GN=Cdh1 PE=1 SV=1 - [CADH1_MOUSE]	9.73	1	4	4	15	2.65	1.74	-0.36	-0.62
P31725	Protein S100-A9 OS=Mus musculus GN=S100a9 PE=1 SV=3 - [S10A9_MOUSE]	38.94	1	4	4	12	1.48	1.28	0.53	0.98
Q07456	Protein AMBP OS=Mus musculus GN=Ambp PE=1 SV=2 - [AMBP_MOUSE]	14.90	1	4	4	12	1.74	1.23	-0.19	0.69
A9C437	Chloride channel protein 2 OS=Mus musculus GN=Clcn2 PE=2 SV=1 - [A9C437_MOUSE]	6.40	3	4	4	8	3.89	1.20	-0.93	-0.11
Q5S006	Leucine-rich repeat serine/threonine-protein kinase 2 OS=Mus musculus GN=Lrrk2 PE=1 SV=2 - [LRRK2_MOUSE]	3.17	1	4	6	9	0.96	1.16	-0.09	-0.62
Q059P4	Filamin A interacting protein 1 OS=Mus musculus GN=Filip1 PE=2 SV=1 - [Q059P4_MOUSE]	5.77	3	4	5	11	1.14	1.14	0.40	-0.60
E9Q784	Protein Zc3h13 OS=Mus musculus GN=Zc3h13 PE=2 SV=1 - [E9Q784_MOUSE]	2.95	1	4	6	7	1.68	1.10	-0.78	0.37
Q61635	GTP-binding protein OS=Mus musculus GN=Ifi47 PE=2 SV=1 - [Q61635_MOUSE]	14.52	6	4	7	25	1.02	1.06	0.43	1.05
Q8BH35	Complement component C8 beta chain OS=Mus musculus GN=C8b PE=1 SV=1 - [CO8B_MOUSE]	10.19	2	4	4	11	0.89	0.97	0.18	0.70

E9PZQ0	Ryanodine receptor 1 OS=Mus musculus GN=Ryr1 PE=1 SV=1 - [RYR1_MOUSE]	1.37	2	4	8	11	1.12	0.94	0.98	0.90
Q5DU14-3	Isoform 3 of Unconventional myosin-XVI OS=Mus musculus GN=Myo16 - [MYO16_MOUSE]	3.38	4	4	6	10	1.40	0.89	0.60	0.18
A6PWD2	Forkhead-associated domain-containing protein 1 OS=Mus musculus GN=Fhad1 PE=2 SV=1 - [FHAD1_MOUSE]	3.59	3	4	5	6	2.84	0.88	0.54	-0.61
P29788	Vitronectin OS=Mus musculus GN=Vtn PE=1 SV=2 - [VTNC_MOUSE]	11.09	1	4	5	13	1.22	0.86	0.52	0.91
Q8BXR5	Sodium leak channel non-selective protein OS=Mus musculus GN=Nalcn PE=1 SV=2 - [NALCN_MOUSE]	2.88	3	4	5	5	2.40	0.84	0.39	-0.01
D3YY34	Polyhomeotic-like protein 3 OS=Mus musculus GN=Phc3 PE=4 SV=1 - [D3YY34_MOUSE]	7.28	7	4	4	13	0.99	0.81	0.69	0.23
P48377	MHC class II regulatory factor RFX1 OS=Mus musculus GN=Rfx1 PE=2 SV=2 - [RFX1_MOUSE]	6.54	1	4	4	11	1.27	0.77	0.25	0.06
P14069	Protein S100-A6 OS=Mus musculus GN=S100a6 PE=1 SV=3 - [S10A6_MOUSE]	47.19	1	4	4	12	1.39	0.77	-0.95	-0.10
Q9QXC1	Fetuin-B OS=Mus musculus GN=Fetub PE=1 SV=1 - [FETUB_MOUSE]	14.18	2	4	4	14	1.92	0.75	-0.90	-0.18
P82347	Delta-sarcoglycan OS=Mus musculus GN=Sgcd PE=1 SV=1 - [SGCD_MOUSE]	13.84	2	4	4	7	2.79	0.74	0.16	0.04
P97825	Hematological and neurological expressed 1 protein OS=Mus musculus GN=Hn1 PE=1 SV=3 - [HN1_MOUSE]	41.56	1	4	4	45	0.87	0.73	0.54	0.19
P84228	Histone H3.2 OS=Mus musculus GN=Hist1h3b PE=1 SV=2 - [H32_MOUSE]	59.56	2	3	11	54	-1.09	-1.88	-1.75	-1.67
Q8CEG5	Coiled-coil domain-containing protein 28B OS=Mus musculus GN=Ccdc28b PE=2 SV=3 - [CC28B_MOUSE]	20.50	4	3	3	7	-0.91	-1.64	-0.14	-1.02
E9PWQ6	Serine/threonine-protein kinase 31 OS=Mus musculus GN=Stk31 PE=2 SV=1 - [E9PWQ6_MOUSE]	3.80	4	3	3	4	-1.14	-1.54	0.00	-0.36
Q8BHJ6	Serine incorporator 5 OS=Mus musculus GN=Serinc5 PE=2 SV=1 - [SERC5_MOUSE]	7.59	1	3	3	4	-0.63	-1.47	0.17	0.42

A2AVN2	Glycoprotein hormones alpha chain (Fragment) OS=Mus musculus GN=Cga PE=2 SV=1 - [A2AVN2_MOUSE]	12.82	2	3	3	16	-0.99	-1.31	-3.22	-3.16
P62274	40S ribosomal protein S29 OS=Mus musculus GN=Rps29 PE=2 SV=2 - [RS29_MOUSE]	25.00	2	3	3	5	-1.04	-0.98	-0.45	-0.44
Q8K2P7	Sodium-coupled neutral amino acid transporter 1 OS=Mus musculus GN=Slc38a1 PE=1 SV=1 - [S38A1_MOUSE]	5.36	2	3	3	6	-0.87	-0.96	-0.58	-0.10
Q9JKK8	Serine/threonine-protein kinase ATR OS=Mus musculus GN=Atr PE=1 SV=2 - [ATR_MOUSE]	1.21	2	3	3	3	-0.91	-0.95	-0.11	0.01
Q5U452	MCG14616 OS=Mus musculus GN=2810408M09Rik PE=2 SV=1 - [Q5U452_MOUSE]	13.52	4	3	3	5	-0.84	-0.90	-0.39	-0.13
P18872-2	Isoform Alpha-2 of Guanine nucleotide-binding protein G(o) subunit alpha OS=Mus musculus GN=Gnao1 - [GNAO_MOUSE]	64.97	5	3	23	492	-1.33	-0.87	0.10	0.26
Q9JJG9	Nitric oxide-associated protein 1 OS=Mus musculus GN=Noa1 PE=1 SV=1 - [NOA1_MOUSE]	7.07	1	3	3	6	-1.00	-0.86	-0.08	0.85
Q7TNK1	Transcription factor RFX4 OS=Mus musculus GN=Rfx4 PE=1 SV=1 - [RFX4_MOUSE]	11.16	4	3	3	4	-1.53	-0.82	0.05	-0.06
Q80SY5	Pre-mRNA-splicing factor 38B OS=Mus musculus GN=Prpf38b PE=1 SV=1 - [PR38B_MOUSE]	6.09	1	3	3	6	-0.89	-0.78	-0.20	-0.04
O35972	39S ribosomal protein L23, mitochondrial OS=Mus musculus GN=Mrpl23 PE=2 SV=1 - [RM23_MOUSE]	33.56	1	3	3	10	-0.85	-0.77	-0.34	-0.16
P58501	PAX3- and PAX7-binding protein 1 OS=Mus musculus GN=Paxbp1 PE=1 SV=3 - [PAXB1_MOUSE]	2.29	3	3	3	11	-0.84	-0.74	-0.21	-0.14
Q66JZ4	T-cell activation inhibitor, mitochondrial OS=Mus musculus GN=TCAIM PE=2 SV=1 - [TCAIM_MOUSE]	8.42	3	3	3	5	-0.66	-0.68	-0.01	0.17
Q8CFP6	DnaJ homolog subfamily C member 27 OS=Mus musculus GN=Dnajc27 PE=2 SV=1 - [DJC27_MOUSE]	11.72	1	3	3	8	-0.61	-0.67	-0.22	-0.13
Q61070	Etoposide-induced protein 2.4 OS=Mus musculus GN=Ei24 PE=1 SV=3 - [EI24_MOUSE]	6.47	1	3	3	7	-0.86	-0.67	-0.28	-0.31

Q8VDS7	Centrosomal protein CEP57L1 OS=Mus musculus GN=Cep57l1 PE=2 SV=1 - [CE57L_MOUSE]	17.50	5	3	3	6	-0.67	-0.63	0.11	-0.17
Q9D2U9	Histone H2B type 3-A OS=Mus musculus GN=Hist3h2ba PE=1 SV=3 - [H2B3A_MOUSE]	77.78	2	3	14	216	-0.65	-0.61	-0.37	-0.13
Q9ES63	Ubiquitin carboxyl-terminal hydrolase 29 OS=Mus musculus GN=Usp29 PE=2 SV=2 - [UBP29_MOUSE]	4.26	1	3	3	8	-1.54	-0.60	-0.71	0.01
Q9D023	Mitochondrial pyruvate carrier 2 OS=Mus musculus GN=Mpc2 PE=1 SV=1 - [MPC2_MOUSE]	21.26	3	3	4	25	-1.33	-0.59	-0.24	-0.04
Q9CQV5	28S ribosomal protein S24, mitochondrial OS=Mus musculus GN=Mrps24 PE=2 SV=1 - [RT24_MOUSE]	13.77	1	3	3	5	-0.66	-0.59	0.00	0.00
Q80X41-2	Isoform 2 of Serine/threonine-protein kinase VRK1 OS=Mus musculus GN=Vrk1 - [VRK1_MOUSE]	9.60	4	3	3	5	-0.69	-0.59	0.05	-0.18
Q9D1N9	39S ribosomal protein L21, mitochondrial OS=Mus musculus GN=Mrpl21 PE=2 SV=1 - [RM21_MOUSE]	18.66	2	3	3	6	-0.82	-0.58	-0.30	-0.40
P01863	Ig gamma-2A chain C region, A allele OS=Mus musculus GN=Ighg PE=1 SV=1 - [GCAA_MOUSE]	15.45	3	3	4	14	1.85	3.26	-2.50	0.10
Q0GGX2-2	Isoform 2 of Zinc finger protein 541 OS=Mus musculus GN=Znf541 - [ZN541_MOUSE]	3.69	2	3	3	6	1.88	2.17	1.21	-0.16
Q60590	Alpha-1-acid glycoprotein 1 OS=Mus musculus GN=Orm1 PE=1 SV=1 - [A1AG1_MOUSE]	20.77	1	3	5	10	2.36	1.75	1.66	2.19
Q9EQR6	Fanconi anemia group G protein homolog OS=Mus musculus GN=Fancg PE=2 SV=1 - [FANCG_MOUSE]	13.16	1	3	3	4	2.28	1.65	0.16	0.44
P07309	Transthyretin OS=Mus musculus GN=Ttr PE=1 SV=1 - [TTHY_MOUSE]	15.65	4	3	3	6	2.14	1.60	0.01	0.57
Q8BYN2	DNA segment, Chr 2, ERATO Doi 435, expressed, isoform CRA_e OS=Mus musculus GN=Tubgcp4 PE=2 SV=1 - [Q8BYN2_MOUSE]	9.16	3	3	5	14	1.10	1.51	0.08	-0.05
Q91WD8	Protein Slc24a1 OS=Mus musculus GN=Slc24a1 PE=2 SV=1 - [Q91WD8_MOUSE]	5.31	1	3	3	4	1.86	1.48	-0.44	-0.87

Q8BML1-2	Isoform 2 of Protein-methionine sulfoxide oxidase MICAL2 OS=Mus musculus GN=Mical2 - [MICA2_MOUSE]	8.17	4	3	8	14	1.73	1.47	-1.16	0.39
P04117	Fatty acid-binding protein, adipocyte OS=Mus musculus GN=Fabp4 PE=1 SV=3 - [FABP4_MOUSE]	22.73	3	3	3	11	2.95	1.44	-0.85	0.00
G3X9R0	MCG68164 OS=Mus musculus GN=Cylc1 PE=4 SV=1 - [G3X9R0_MOUSE]	5.47	1	3	3	3	1.79	1.40	0.31	-0.01
P70121	Zinc fingers and homeoboxes protein 1 OS=Mus musculus GN=Zhx1 PE=1 SV=2 - [ZHX1_MOUSE]	5.04	2	3	4	7	1.32	1.32	1.20	1.20
F7BJ98	Protein Nbeal1 (Fragment) OS=Mus musculus GN=Nbeal1 PE=2 SV=1 - [F7BJ98_MOUSE]	3.81	2	3	3	4	3.87	1.30	0.01	-0.62
P22599	Alpha-1-antitrypsin 1-2 OS=Mus musculus GN=Serpina1b PE=1 SV=2 - [A1AT2_MOUSE]	47.46	2	3	15	166	1.55	1.26	-0.70	0.10
Q64726	Zinc-alpha-2-glycoprotein OS=Mus musculus GN=Azgp1 PE=1 SV=2 - [ZA2G_MOUSE]	11.73	1	3	3	3	0.96	1.24	-0.38	0.37
Q8CII2	Cell division cycle protein 123 homolog OS=Mus musculus GN=Cdc123 PE=2 SV=2 - [CD123_MOUSE]	11.31	2	3	4	16	0.89	1.14	0.91	0.59
Q60571	Corticotropin-releasing factor-binding protein OS=Mus musculus GN=Crhbp PE=2 SV=1 - [CRHBP_MOUSE]	15.22	1	3	3	3	0.87	1.13	0.01	0.07
D3YWJ0	Protein Nuggc OS=Mus musculus GN=Nuggc PE=4 SV=1 - [D3YWJ0_MOUSE]	8.17	2	3	3	4	2.16	1.09	-0.16	0.67
A2AQ07	Tubulin beta-1 chain OS=Mus musculus GN=Tubb1 PE=1 SV=1 - [TBB1_MOUSE]	31.93	1	3	11	485	1.17	1.09	0.26	0.41
Q5DTW7-3	Isoform 3 of Uncharacterized protein KIAA1551 OS=Mus musculus GN=Kiaa1551 - [K1551_MOUSE]	2.76	5	3	3	5	2.23	1.08	0.03	0.45
E9PVX6	Protein Mki67 OS=Mus musculus GN=Mki67 PE=2 SV=1 - [E9PVX6_MOUSE]	1.07	1	3	3	3	1.67	1.03	1.94	0.59
P05367	Serum amyloid A-2 protein OS=Mus musculus GN=Saa2 PE=1 SV=1 - [SAA2_MOUSE]	28.69	1	3	4	12	0.93	0.89	3.45	4.60
P57774	Pro-neuropeptide Y OS=Mus musculus GN=Npy PE=2 SV=2 - [NPY_MOUSE]	28.87	1	3	3	23	1.15	0.86	0.39	0.02

Q9D8H7	Metalloendopeptidase OMA1, mitochondrial OS=Mus musculus GN=Oma1 PE=2 SV=1 - [OMA1_MOUSE]	5.18	1	3	3	3	1.01	0.83	0.68	1.45
Q3UU35	Ovostatin homolog OS=Mus musculus GN=Ovos PE=2 SV=2 - [OVOS_MOUSE]	4.40	1	3	3	3	1.60	0.82	-0.30	0.16
Q62361	Pro-thyrotropin-releasing hormone OS=Mus musculus GN=Trh PE=2 SV=2 - [TRH_MOUSE]	10.94	1	3	3	4	1.34	0.82	-0.72	-0.02
O88508	DNA (cytosine-5)-methyltransferase 3A OS=Mus musculus GN=Dnmt3a PE=1 SV=2 - [DNM3A_MOUSE]	5.51	6	3	3	6	1.22	0.79	0.38	-0.83
Q6NXH3	Vasculin OS=Mus musculus GN=Gpbp1 PE=1 SV=1 - [GPBP1_MOUSE]	10.15	4	3	4	11	1.20	0.77	0.44	-0.01
G3UWF6	MCG1027453 OS=Mus musculus GN=2010015L04Rik PE=4 SV=1 - [G3UWF6_MOUSE]	25.58	3	3	4	6	1.49	0.77	0.16	0.15
Q9CR27	WASH complex subunit CCDC53 OS=Mus musculus GN=Ccdc53 PE=2 SV=1 - [CCD53_MOUSE]	23.71	2	3	3	12	1.70	0.76	0.24	0.74
Q8VCE1	DnaJ homolog subfamily C member 28 OS=Mus musculus GN=Dnajc28 PE=2 SV=2 - [DJC28_MOUSE]	7.01	3	3	3	8	1.15	0.74	0.13	-0.24
Q80YC5	Coagulation factor XII OS=Mus musculus GN=F12 PE=2 SV=2 - [FA12_MOUSE]	6.87	1	3	3	8	2.21	0.74	-0.93	0.03
Q8C804	Spindle and centriole-associated protein 1 OS=Mus musculus GN=Spice1 PE=1 SV=2 - [SPICE_MOUSE]	5.35	1	3	3	4	1.39	0.70	0.40	0.09
E9Q7P2	Protein Cacna1i OS=Mus musculus GN=Cacna1i PE=2 SV=1 - [E9Q7P2_MOUSE]	3.59	3	3	5	7	2.20	0.69	0.33	-0.11
Q810D6	Glutamate-rich WD repeat-containing protein 1 OS=Mus musculus GN=Grwd1 PE=2 SV=2 - [GRWD1_MOUSE]	15.02	3	3	3	4	1.04	0.68	0.45	-0.50
Q80SZ7	Guanine nucleotide-binding protein G(I)/G(S)/G(O) subunit gamma-5 OS=Mus musculus GN=Gng5 PE=2 SV=2 - [GBG5_MOUSE]	33.82	2	3	3	12	0.99	0.68	0.54	0.55
D3Z375	Astrocytic phosphoprotein PEA-15 (Fragment) OS=Mus musculus GN=Pea15a PE=2 SV=1 - [D3Z375_MOUSE]	64.13	2	3	5	96	0.95	0.67	0.49	-0.03

Q8BSI6	R3H and coiled-coil domain-containing protein 1 OS=Mus musculus GN=R3hcc1 PE=2 SV=2 - [R3HC1_MOUSE]	8.05	2	3	3	4	1.38	0.63	-0.11	-0.45
Q8CDJ6	Protein 4932411E22Rik OS=Mus musculus GN=4932411E22Rik PE=2 SV=1 - [Q8CDJ6_MOUSE]	9.68	1	3	3	4	0.86	0.62	0.43	0.33
P08071	Lactotransferrin OS=Mus musculus GN=Ltf PE=2 SV=4 - [TRFL_MOUSE]	8.06	1	3	4	8	1.83	0.62	0.15	0.42
O88968	Transcobalamin-2 OS=Mus musculus GN=Tcn2 PE=2 SV=1 - [TCO2_MOUSE]	10.23	1	3	3	6	1.54	0.62	-0.26	-0.41
G3X9G6	Luteinizing hormone beta OS=Mus musculus GN=Lhb PE=3 SV=1 - [G3X9G6_MOUSE]	21.99	2	2	2	9	-2.30	-2.91	-4.79	-4.91
F8VQD3	Protein Vmn2r37 OS=Mus musculus GN=Vmn2r37 PE=3 SV=1 - [F8VQD3_MOUSE]	4.24	2	2	2	3	-3.39	-2.27	-2.91	-2.57
Q80VA0	N-acetylgalactosaminyltransferase 7 OS=Mus musculus GN=Galnt7 PE=2 SV=2 - [GALT7_MOUSE]	3.81	3	2	2	2	-0.61	-2.22	0.76	0.48
P68404	Protein kinase C beta type OS=Mus musculus GN=Prkcb PE=1 SV=4 - [KPCB_MOUSE]	41.73	1	2	28	140	-0.88	-1.99	-0.05	0.49
Q8CBY1-4	Isoform 4 of Protein Smaug homolog 1 OS=Mus musculus GN=Samd4a - [SMAG1_MOUSE]	11.59	4	2	2	4	-0.82	-1.80	-0.55	0.22
P13011	Acyl-CoA desaturase 2 OS=Mus musculus GN=Scd2 PE=2 SV=2 - [ACOD2_MOUSE]	3.91	3	2	2	5	-0.61	-1.32	-0.47	-0.24
D3YUR6	Transmembrane protein 169 (Fragment) OS=Mus musculus GN=Tmem169 PE=2 SV=1 - [D3YUR6_MOUSE]	21.67	2	2	2	3	-0.70	-1.31	-0.55	-1.18
Q8BG15-5	Isoform 5 of CTD small phosphatase-like protein 2 OS=Mus musculus GN=Ctdspl2 - [CTSL2_MOUSE]	5.08	6	2	2	7	-1.56	-1.29	-0.06	-0.24
Q91ZU6	Dystonin OS=Mus musculus GN=Dst PE=1 SV=1 - [DYST_MOUSE]	24.24	5	2	160	508	-1.01	-1.27	-0.23	-0.12
Q80ZI1	TMF-regulated nuclear protein 1 OS=Mus musculus GN=Trnp1 PE=1 SV=1 - [TRNP1_MOUSE]	14.80	1	2	2	3	-0.77	-1.25	0.14	-0.34
Q80TC5	Pogo transposable element with KRAB domain OS=Mus musculus GN=Pogk PE=2 SV=2 - [POGK_MOUSE]	3.13	2	2	2	2	-0.74	-1.07	-0.36	-0.30

A2A6P4	Protein Fam104a OS=Mus musculus GN=Fam104a PE=2 SV=1 - [A2A6P4_MOUSE]	15.68	1	2	2	5	-1.06	-1.06	-0.30	-0.28
Q0P5V9-3	Isoform 3 of Solute carrier family 45 member 4 OS=Mus musculus GN=Slc45a4 - [S45A4_MOUSE]	5.92	4	2	2	2	-0.96	-1.03	-0.03	-0.10
Q9CWF2	Tubulin beta-2B chain OS=Mus musculus GN=Tubb2b PE=1 SV=1 - [TBB2B_MOUSE]	83.82	2	2	33	4745	-0.72	-1.02	-1.26	-1.04
Q8BP78	Protein FRA10AC1 homolog OS=Mus musculus GN=Fra10ac1 PE=1 SV=3 - [F10C1_MOUSE]	9.21	1	2	2	3	-0.72	-1.02	-0.33	-0.54
E9QMD2	Ubiquitin-conjugating enzyme E2Q-like protein 1 OS=Mus musculus GN=Ube2ql1 PE=2 SV=1 - [E9QMD2_MOUSE]	13.16	1	2	2	7	-0.85	-1.01	-0.14	-0.69
Q9WV03	Protein FAM50A OS=Mus musculus GN=Fam50a PE=2 SV=1 - [FA50A_MOUSE]	7.37	1	2	2	6	-1.23	-0.92	-0.23	-0.43
Q9WV19	Cytochrome P450, family 2, subfamily g, polypeptide 1 OS=Mus musculus GN=Cyp2g1 PE=2 SV=1 - [Q9WV19_MOUSE]	6.48	1	2	2	2	-1.37	-0.89	-0.15	-0.17
Q8VIF5	Low affinity neurotensin receptor OS=Mus musculus GN=Ntsr2 PE=2 SV=1 - [Q8VIF5_MOUSE]	6.25	2	2	2	9	-1.05	-0.84	-0.44	0.39
Q3TXX3-2	Isoform 2 of Protrudin OS=Mus musculus GN=Zfyve27 - [ZFY27_MOUSE]	7.11	3	2	2	8	-0.64	-0.75	-0.12	-0.09
Q3TZM9-2	Isoform 2 of GDP-Man:Man(3)GlcNAc(2)-PP-Dol alpha-1,2-mannosyltransferase OS=Mus musculus GN=Alg11 - [ALG11_MOUSE]	7.56	4	2	2	8	-0.94	-0.74	-0.45	0.04
Q91WB2	Probable lipid phosphate phosphatase PPAPDC3 OS=Mus musculus GN=Ppapdc3 PE=1 SV=1 - [PPAC3_MOUSE]	6.27	1	2	2	16	-1.18	-0.74	0.36	-0.26
Q9D8B6	Protein FAM210B OS=Mus musculus GN=Fam210b PE=2 SV=3 - [F210B_MOUSE]	13.16	1	2	2	6	-1.06	-0.72	0.38	0.26
Q9EQ61	Pescadillo homolog OS=Mus musculus GN=Pes1 PE=1 SV=1 - [PESC_MOUSE]	3.60	2	2	2	3	-1.03	-0.70	-0.69	-0.14
P28740-1	Isoform 1 of Kinesin-like protein KIF2A OS=Mus musculus GN=Kif2a - [KIF2A_MOUSE]	51.82	11	2	30	176	-1.05	-0.70	0.09	-0.10

F6QP10	Sn1-specific diacylglycerol lipase beta (Fragment) OS=Mus musculus GN=Daglb PE=4 SV=2 - [F6QP10_MOUSE]	4.09	2	2	2	3	-1.85	-0.68	0.24	0.41
E9Q6K3	Protein Pibf1 OS=Mus musculus GN=Pibf1 PE=2 SV=1 - [E9Q6K3_MOUSE]	2.65	1	2	3	7	-0.65	-0.68	-0.20	-0.14
Q64522	Histone H2A type 2-B OS=Mus musculus GN=Hist2h2ab PE=1 SV=3 - [H2A2B_MOUSE]	34.62	1	2	3	92	-0.94	-0.65	-0.24	0.28
Q61037-4	Isoform C of Tuberin OS=Mus musculus GN=Tsc2 - [TSC2_MOUSE]	20.45	5	2	24	80	-1.28	-0.65	0.05	-0.06
Q9QZI8	Serine incorporator 1 OS=Mus musculus GN=Serinc1 PE=1 SV=1 - [SERC1_MOUSE]	5.74	1	2	2	2	-1.51	-0.65	-0.45	-0.87
Q8BGF8	Solute carrier family 35 member D3 OS=Mus musculus GN=Slc35d3 PE=2 SV=1 - [S35D3_MOUSE]	5.69	1	2	2	5	-0.82	-0.61	0.51	0.35
P70279	Surfeit locus protein 6 OS=Mus musculus GN=Surf6 PE=2 SV=1 - [SURF6_MOUSE]	10.14	2	2	4	4	-0.84	-0.60	0.03	-0.03
Q80TJ7	Histone lysine demethylase PHF8 OS=Mus musculus GN=Phf8 PE=1 SV=2 - [PHF8_MOUSE]	3.52	4	2	3	5	-0.76	-0.60	-0.35	-0.55
Q99N32	Beta-klotho OS=Mus musculus GN=Klb PE=1 SV=1 - [KLOTB_MOUSE]	2.01	2	2	2	2	-0.94	-0.60	0.32	0.13
Q8K0P3	TLD domain-containing protein 1 OS=Mus musculus GN=Tldc1 PE=2 SV=1 - [TLDC1_MOUSE]	7.25	1	2	2	5	-0.69	-0.60	-0.42	-0.38
Q80WW9	DDRGK domain-containing protein 1 OS=Mus musculus GN=Ddrgk1 PE=1 SV=2 - [DDRGK_MOUSE]	9.84	1	2	2	4	-0.66	-0.59	-0.43	-0.31
Q9Z1B8	PHD finger protein 1 OS=Mus musculus GN=Phf1 PE=1 SV=2 - [PHF1_MOUSE]	9.12	1	2	2	5	-0.79	-0.58	-0.28	-0.04
Q9WTL4	Insulin receptor-related protein OS=Mus musculus GN=Insrr PE=1 SV=2 - [INSRR_MOUSE]	2.54	1	2	3	4	2.33	3.59	-0.41	-0.82
Q8R366	Immunoglobulin superfamily member 8 OS=Mus musculus GN=Igsf8 PE=1 SV=2 - [IGSF8_MOUSE]	35.68	2	2	17	151	1.33	2.96	2.76	3.72
P31532	Serum amyloid A-4 protein OS=Mus musculus GN=Saa4 PE=1 SV=2 - [SAA4_MOUSE]	22.31	1	2	2	4	0.89	2.73	0.31	1.32

Q7TQE7-2	Isoform 2 of Uncharacterized protein KIAA0895 OS=Mus musculus GN=Kiaa0895 - [K0895_MOUSE]	10.55	2	2	2	2	4.21	2.35	-1.41	-2.66
P33622	Apolipoprotein C-III OS=Mus musculus GN=Apoc3 PE=2 SV=2 - [APOC3_MOUSE]	27.27	2	2	2	16	1.95	2.22	-1.05	-0.07
P57725	SAM domain-containing protein SAMSN-1 OS=Mus musculus GN=Samsn1 PE=1 SV=2 - [SAMN1_MOUSE]	8.33	2	2	2	2	1.88	2.17	0.71	0.66
P52927	High mobility group protein HMGI-C OS=Mus musculus GN=Hmga2 PE=1 SV=1 - [HMGA2_MOUSE]	32.41	2	2	2	3	1.48	2.10	2.00	0.21
Q6NXY1	WD repeat-containing protein 67 OS=Mus musculus GN=Wdr67 PE=2 SV=1 - [WDR67_MOUSE]	2.11	3	2	2	2	5.19	2.04	1.91	-0.37
G3UWX1	Replication factor C subunit 1 OS=Mus musculus GN=Rfc1 PE=2 SV=1 - [G3UWX1_MOUSE]	2.39	6	2	2	2	3.92	1.96	1.34	0.03
E9Q5X9	Protein Zfp819 OS=Mus musculus GN=Zfp819 PE=2 SV=1 - [E9Q5X9_MOUSE]	8.10	2	2	2	2	1.94	1.89	0.51	-0.30
A2A4B4	EMILIN-3 OS=Mus musculus GN=Emilin3 PE=2 SV=1 - [A2A4B4_MOUSE]	3.23	2	2	2	3	1.40	1.80	-0.98	0.09
Q80ZJ8	EF-hand calcium-binding domain-containing protein 4A OS=Mus musculus GN=Efcab4a PE=2 SV=2 - [EFC4A_MOUSE]	8.63	3	2	3	5	1.01	1.73	0.63	0.35
P56400	Platelet glycoprotein Ib beta chain OS=Mus musculus GN=Gp1bb PE=1 SV=1 - [GP1BB_MOUSE]	13.11	2	2	2	4	0.98	1.65	-0.06	0.55
P62878	E3 ubiquitin-protein ligase RBX1 OS=Mus musculus GN=Rbx1 PE=1 SV=1 - [RBX1_MOUSE]	23.15	1	2	2	8	2.57	1.56	0.57	-0.15
Q91ZD1-2	Isoform 2 of Protein odd-skipped-related 2 OS=Mus musculus GN=Osr2 - [OSR2_MOUSE]	9.42	3	2	2	2	4.74	1.56	0.29	-0.25
Q9JJ94	Sjoegren syndrome nuclear autoantigen 1 homolog OS=Mus musculus GN=Ssna1 PE=1 SV=1 - [SSNA1_MOUSE]	23.53	1	2	2	6	3.20	1.51	1.37	0.60
Q69ZB3	Testis-specific Y-encoded-like protein 5 OS=Mus musculus GN=Tspyl5 PE=2 SV=2 - [TSYL5_MOUSE]	7.88	1	2	2	3	1.67	1.50	0.53	0.25

Q8CGT2	Baculoviral IAP repeat-containing protein 1e OS=Mus musculus GN=Naip5 PE=2 SV=1 - [Q8CGT2_MOUSE]	2.21	2	2	2	2	4.19	1.49	-0.28	-1.44
Q60994	Adiponectin OS=Mus musculus GN=Adipoq PE=1 SV=2 - [ADIPO_MOUSE]	9.72	2	2	2	3	1.54	1.46	-0.89	-0.18
P58774-2	Isoform 2 of Tropomyosin beta chain OS=Mus musculus GN=Tpm2 - [TPM2_MOUSE]	48.94	2	2	16	208	0.93	1.44	0.31	0.26
A6PWB3	Protein C87977 (Fragment) OS=Mus musculus GN=C87977 PE=2 SV=1 - [A6PWB3_MOUSE]	14.81	15	2	2	3	1.20	1.43	0.53	-0.36
Q7TMA2	Zinc finger protein 503 OS=Mus musculus GN=Znf503 PE=2 SV=1 [ZN503_MOUSE]	5.67	2	2	3	5	1.99	1.38	0.21	0.50
Q9CQW3	Vitamin K-dependent protein Z OS=Mus musculus GN=Proz PE=1 SV=1 - [PROZ_MOUSE]	5.01	1	2	2	5	2.42	1.35	-0.71	0.05
Q3UVD5	Leucine-rich repeat-containing G-protein coupled receptor 6 OS=Mus musculus GN=Lgr6 PE=2 SV=1 - [LGR6_MOUSE]	3.21	1	2	2	2	4.33	1.31	1.16	-0.02
Q3UR44	Protein MRVI1 (Fragment) OS=Mus musculus GN=Mrvi1 PE=2 SV=1 - [Q3UR44_MOUSE]	5.87	4	2	3	5	1.28	1.30	0.18	0.21
Q99NF1	Beta,beta-carotene 9',10'-oxygenase OS=Mus musculus GN=Bco2 PE=1 SV=1 - [BCDO2_MOUSE]	8.27	1	2	2	2	0.96	1.29	-0.40	0.18
Q8K157	Aldose 1-epimerase OS=Mus musculus GN=Galm PE=2 SV=1 - [GALM_MOUSE]	7.31	1	2	2	8	2.06	1.27	0.32	0.24
P08905	Lysozyme C-2 OS=Mus musculus GN=Lyz2 PE=1 SV=2 - [LYZ2_MOUSE]	14.86	2	2	2	3	1.49	1.25	-0.14	0.37
Q6IQY5-2	Isoform 2 of Centrosomal protein of 70 kDa OS=Mus musculus GN=Cep70 - [CEP70_MOUSE]	3.98	2	2	2	2	0.95	1.20	-0.96	0.65
Q4VBE4-2	Isoform 2 of Pikachurin OS=Mus musculus GN=Egflam - [EGFLA_MOUSE]	2.78	2	2	2	2	1.58	1.16	-0.35	0.28
M0QWQ7	Semaphorin-3B OS=Mus musculus GN=Sema3b PE=4 SV=1 - [M0QWQ7_MOUSE]	8.05	3	2	2	4	2.31	1.14	-0.78	-0.25
Q5UAK0-5	Isoform 5 of Mesoderm induction early response protein 1 OS=Mus musculus GN=Mier1 - [MIER1_MOUSE]	10.00	5	2	3	9	2.23	1.14	0.38	0.74

Q3TG86	Cytochrome P450, family 11, subfamily b, polypeptide 1 OS=Mus musculus GN=Cyp11b1 PE=2 SV=1 - [Q3TG86_MOUSE]	9.38	1	2	2	2	3.60	1.11	0.69	0.30
E9PZ67	Calsequestrin (Fragment) OS=Mus musculus GN=Casq2 PE=2 SV=1 - [E9PZ67_MOUSE]	8.65	3	2	2	3	2.44	1.11	0.50	-0.59
E9Q0N0	Intersectin-1 OS=Mus musculus GN=Itsn1 PE=2 SV=1 - [E9Q0N0_MOUSE]	38.86	13	2	63	237	2.39	1.10	-0.01	-0.25
P09026	Homeobox protein Hox-B3 OS=Mus musculus GN=Hoxb3 PE=2 SV=4 - [HXB3_MOUSE]	4.16	2	2	2	2	2.40	1.10	0.73	0.44
R4GML6	Centriolin (Fragment) OS=Mus musculus GN=Cep110 PE=4 SV=1 - [R4GML6_MOUSE]	1.47	15	2	5	6	1.21	1.07	0.19	-0.35
E9QNK8	Kv channel-interacting protein 2 OS=Mus musculus GN=Kcnip2 PE=2 SV=1 - [E9QNK8_MOUSE]	27.78	11	2	5	13	0.93	1.07	0.62	-0.32
P27005	Protein S100-A8 OS=Mus musculus GN=S100a8 PE=1 SV=3 - [S10A8_MOUSE]	46.07	1	2	2	4	1.89	1.06	0.75	1.14
Q6A044-2	Isoform 2 of Protein FAM189A1 OS=Mus musculus GN=Fam189a1 - [F1891_MOUSE]	20.86	2	2	2	3	1.19	1.03	1.57	0.83
Q4LDF6	Protein Cfhr2 (Precursor) OS=Mus musculus GN=Cfhr2 PE=2 SV=1 - [Q4LDF6_MOUSE]	19.28	1	2	5	18	1.40	1.01	-0.48	-0.14
A2AKN9	Major urinary protein 2 OS=Mus musculus GN=Mup2 PE=2 SV=1 - [A2AKN9_MOUSE]	53.89	8	2	8	26	1.85	1.00	1.78	2.44
P06683	Complement component C9 OS=Mus musculus GN=C9 PE=1 SV=2 - [CO9_MOUSE]	3.65	2	2	2	6	1.74	0.98	0.72	1.12
P49182	Heparin cofactor 2 OS=Mus musculus GN=Serpind1 PE=1 SV=1 - [HEP2_MOUSE]	4.18	1	2	2	5	1.15	0.95	-0.91	-0.43
E5FYH1	Testis- and ovary-specific PAZ domain-containing protein 1 OS=Mus musculus GN=Topaz1 PE=2 SV=1 - [TOPZ1_MOUSE]	1.57	1	2	2	2	2.37	0.95	0.97	0.42
Q0VGM9-4	Isoform 4 of Regulator of telomere elongation helicase 1 OS=Mus musculus GN=Rtel1 - [RTEL1_MOUSE]	2.13	5	2	2	2	1.31	0.94	0.61	0.28
P35276	Ras-related protein Rab-3D OS=Mus musculus GN=Rab3d PE=1 SV=1 - [RAB3D_MOUSE]	47.03	4	2	10	125	1.45	0.93	0.11	-0.28

F7AGB5	Methyltransferase-like protein 17, mitochondrial (Fragment) OS=Mus musculus GN=Mettl17 PE=2 SV=1 - [F7AGB5_MOUSE]	6.07	4	2	2	2	2.99	0.92	-0.45	0.01
Q63870	Collagen alpha-1(VII) chain OS=Mus musculus GN=Col7a1 PE=2 SV=3 - [CO7A1_MOUSE]	0.78	1	2	2	3	2.51	0.92	0.54	0.19
E9PV60	Protein Wdfy4 OS=Mus musculus GN=Wdfy4 PE=2 SV=1 - [E9PV60_MOUSE]	1.36	2	2	4	4	0.92	0.90	0.71	-0.40
Q00519	Xanthine dehydrogenase/oxidase OS=Mus musculus GN=Xdh PE=1 SV=5 - [XDH_MOUSE]	2.70	2	2	4	10	1.01	0.90	0.65	0.61
Q8BVL9	Janus kinase and microtubule-interacting protein 1 OS=Mus musculus GN=Jakmip1 PE=1 SV=2 - [JKIP1_MOUSE]	34.82	5	2	21	77	1.18	0.90	1.02	0.52
P15508	Spectrin beta chain, erythrocytic OS=Mus musculus GN=Sptb PE=1 SV=4 - [SPTB1_MOUSE]	55.69	1	2	103	407	1.04	0.89	0.09	0.29
P12246	Serum amyloid P-component OS=Mus musculus GN=Apcs PE=1 SV=2 - [SAMP_MOUSE]	13.39	1	2	2	10	0.93	0.86	0.46	1.88
E9PY03	Protein Tstd1 OS=Mus musculus GN=Tstd1 PE=4 SV=1 - [E9PY03_MOUSE]	35.34	1	2	2	6	2.57	0.86	0.14	1.73
O88327	Alpha-catulin OS=Mus musculus GN=Ctnnal1 PE=2 SV=1 - [CTNL1_MOUSE]	2.46	1	2	2	2	1.30	0.86	0.17	0.03
Q9CPW9-2	Isoform 2 of Methionine aminopeptidase 1D, mitochondrial OS=Mus musculus GN=Metap1d - [MAP12_MOUSE]	10.14	2	2	2	3	0.90	0.85	-0.85	0.25
P58660	Caspase recruitment domain-containing protein 10 OS=Mus musculus GN=Card10 PE=2 SV=1 - [CAR10_MOUSE]	4.31	2	2	4	6	4.75	0.82	0.99	0.91
Q8BLI4	Dermatan-sulfate epimerase OS=Mus musculus GN=Dse PE=2 SV=1 - [DSE_MOUSE]	1.46	1	2	2	2	1.84	0.80	-0.19	0.15
Q9QX66-3	Isoform 3 of Zinc finger protein neuro-d4 OS=Mus musculus GN=Dpf1 - [DPF1_MOUSE]	8.73	6	2	2	2	1.51	0.77	-0.88	0.12
Q29SA9	Peripheral clock protein 1 OS=Mus musculus GN=Nckap5 PE=2 SV=1 - [Q29SA9_MOUSE]	1.74	6	2	2	2	0.87	0.77	0.19	0.27

D6RH86	Ribosomal protein S6 kinase beta-2 OS=Mus musculus GN=Rps6kb2 PE=2 SV=1 - [D6RH86_MOUSE]	14.74	5	2	3	6	1.35	0.77	0.42	-0.17
Q8K1S6-2	Isoform 2 of Protein spire homolog 2 OS=Mus musculus GN=Spire2 - [SPIR2_MOUSE]	4.69	3	2	3	9	1.67	0.75	0.66	0.33
E9PX95	Protein Abca17 OS=Mus musculus GN=Abca17 PE=3 SV=1 - [E9PX95_MOUSE]	2.31	1	2	3	4	1.14	0.74	0.23	-0.08
Q9WTK5	Nuclear factor NF-kappa-B p100 subunit OS=Mus musculus GN=Nfkb2 PE=1 SV=1 - [NFKB2_MOUSE]	3.56	1	2	2	4	1.91	0.74	0.67	0.14
J3QNA8	Protein Rnpepl1 (Fragment) OS=Mus musculus GN=Rnpepl1 PE=4 SV=1 - [J3QNA8_MOUSE]	10.53	3	2	2	3	4.16	0.74	0.08	-0.46
Q4KMS1	Tripartite motif-containing 44 OS=Mus musculus GN=Trim44 PE=2 SV=1 - [Q4KMS1_MOUSE]	12.75	3	2	2	4	0.99	0.73	0.71	0.48
Q9QZ82	Cholesterol side-chain cleavage enzyme, mitochondrial OS=Mus musculus GN=Cyp11a1 PE=2 SV=1 - [CP11A_MOUSE]	3.99	1	2	2	2	1.64	0.73	0.08	-0.26
Q60750	Ephrin type-A receptor 1 OS=Mus musculus GN=Epha1 PE=1 SV=2 - [EPHA1_MOUSE]	1.74	1	2	2	3	1.85	0.72	0.45	-0.39
P39447	Tight junction protein ZO-1 OS=Mus musculus GN=Tjp1 PE=1 SV=2 - [ZO1_MOUSE]	31.75	1	2	43	186	1.38	0.71	0.20	-0.04
Q61133	Glutathione S-transferase theta-2 OS=Mus musculus GN=Gstt2 PE=2 SV=4 - [GSTT2_MOUSE]	13.11	1	2	3	10	1.10	0.71	0.02	0.41
B7ZC21	cAMP-dependent protein kinase inhibitor gamma (Fragment) OS=Mus musculus GN=Pkig PE=2 SV=1 - [B7ZC21_MOUSE]	30.36	3	2	2	6	0.96	0.71	1.01	0.06
E9Q0F0	Protein Krt78 OS=Mus musculus GN=Krt78 PE=2 SV=1 - [E9Q0F0_MOUSE]	3.37	2	2	4	19	1.49	0.70	0.58	-0.14
Q9DBX1	Regulator of cell cycle RGCC OS=Mus musculus GN=Rgcc PE=1 SV=1 - [RGCC_MOUSE]	21.90	1	2	2	10	1.33	0.69	0.45	-0.42
Q8K1N4	Spermatogenesis-associated serine-rich protein 2 OS=Mus musculus GN=Spats2 PE=1 SV=1 - [SPAS2_MOUSE]	4.04	2	2	2	2	1.48	0.66	0.14	0.49

D3YUR7	Lipoxygenase homology domain-containing protein 1 (Fragment) OS=Mus musculus GN=Loxhd1 PE=2 SV=1 - [D3YUR7_MOUSE]	1.76	4	2	2	2	1.91	0.66	0.30	0.12
E9Q605	Inhibitor of nuclear factor kappa-B kinase subunit alpha OS=Mus musculus GN=Chuk PE=2 SV=1 - [E9Q605_MOUSE]	3.06	4	2	2	2	1.18	0.65	0.08	0.04
Q8CFB8	NAD+ ADP-ribosyltransferase 3 PARP-3 OS=Mus musculus GN=Parp3 PE=2 SV=1 - [Q8CFB8_MOUSE]	4.17	4	2	2	5	1.19	0.63	-0.17	-0.35
Q9D842-2	Isoform 2 of Aprataxin and PNK-like factor OS=Mus musculus GN=Aplf - [APLF_MOUSE]	7.42	3	2	2	4	4.02	0.63	0.21	-0.14
Q6NS45-3	Isoform 3 of Coiled-coil domain-containing protein 66 OS=Mus musculus GN=Ccdc66 - [CCD66_MOUSE]	2.11	3	2	2	2	0.87	0.62	-0.11	0.19
