# Supplementary material for: Hypothalamus proteomics from mouse models with obesity and anorexia reveals therapeutic targets of appetite regulation
Source: Nutr Diabetes. 2016 Apr 25;6(4):e204–. doi: 10.1038/nutd.2016.10 (PMC4855256; doi:10.1038/nutd.2016.10)
Supplement: Supplementary Table 5 [file nutd201610x8.pdf]

Supplementary Table 5. Differentially expressed proteins in the LPS groups compared to controls (proteins analysed with at least two unique peptides)

Accession	Description	ΣCoverage	Σ# Proteins	Σ# Unique Peptides	Σ# Peptides	Σ# PSMs	HFD1/C1	HFD2/C1	LPS1/C2	LPS2/C2
Q99PL5	Ribosome-binding protein 1 OS=Mus musculus GN=Rrbp1 PE=2 SV=2 - [RRBP1_MOUSE]	54.39	12	57	69	298	0.35	-1.06	-0.58	-0.90
P07724	Serum albumin OS=Mus musculus GN=Alb PE=1 SV=3 - [ALBU_MOUSE]	75.49	4	49	49	2294	1.97	0.72	-1.29	-0.51
Q80YX1	Tenascin OS=Mus musculus GN=Tnc PE=1 SV=1 - [TENA_MOUSE]	26.73	6	45	45	127	-0.37	-0.31	-0.51	-0.99
Q9EQF6	Dihydropyrimidinase-related protein 5 OS=Mus musculus GN=Dpysl5 PE=1 SV=1 - [DPYL5_MOUSE]	71.10	2	36	36	334	-0.55	-1.20	-0.97	-0.68
Q8C196	Carbamoyl-phosphate synthase [ammonia], mitochondrial OS=Mus musculus GN=Cps1 PE=1 SV=2 - [CPSM_MOUSE]	28.73	1	36	39	110	0.54	0.10	1.86	2.53
Q5SYD0	Unconventional myosin-Id OS=Mus musculus GN=Myo1d PE=1 SV=1 - [MYO1D_MOUSE]	41.35	2	36	39	117	-0.16	-1.24	0.61	0.89
Q9ESZ8-5	Isoform 5 of General transcription factor II-I OS=Mus musculus GN=Gtf2i - [GTF2I_MOUSE]	41.96	13	31	32	75	-0.43	-0.11	-0.55	-0.48
P46660	Alpha-internexin OS=Mus musculus GN=Ina PE=1 SV=2 - [AINX_MOUSE]	67.06	1	31	35	1067	0.75	-1.41	0.84	0.64
Q61941	NAD(P) transhydrogenase, mitochondrial OS=Mus musculus GN=Nnt PE=1 SV=2 - [NNTM_MOUSE]	25.41	4	28	28	107	-0.75	0.12	-1.61	-1.83
Q8BMK4	Cytoskeleton-associated protein 4 OS=Mus musculus GN=Ckap4 PE=2 SV=2 - [CKAP4_MOUSE]	55.30	6	28	29	164	0.07	-0.10	-0.57	-0.75
E9PWE8	Dihydropyrimidinase-related protein 3 OS=Mus musculus GN=Dpysl3 PE=2 SV=1 - [E9PWE8_MOUSE]	61.49	5	27	33	852	0.06	-0.82	-0.89	-0.81
P13020-2	Isoform 2 of Gelsolin OS=Mus musculus GN=Gsn - [GELS_MOUSE]	49.25	3	27	28	146	0.78	-1.10	-0.54	-0.46
P21619	Lamin-B2 OS=Mus musculus GN=Lmnb2 PE=1 SV=2 - [LMNB2_MOUSE]	55.20	2	26	31	185	0.38	0.37	0.57	0.47
Q61102	ATP-binding cassette sub-family B member 7, mitochondrial OS=Mus musculus GN=Abcb7 PE=1 SV=3 - [ABCB7_MOUSE]	37.63	1	23	24	75	-0.30	0.27	0.54	0.66
Q9JIA1	Leucine-rich glioma-inactivated protein 1 OS=Mus musculus GN=Lgi1 PE=1 SV=1 - [LGI1_MOUSE]	40.04	2	23	23	136	-0.70	0.43	0.51	0.46
Q3UX37	Protein Plekhg1 OS=Mus musculus GN=Plekhg1 PE=2 SV=1 - [Q3UX37_MOUSE]	18.27	4	22	23	52	-0.79	-1.44	-0.68	-0.61

Q99K47	Fibrinogen, alpha polypeptide OS=Mus musculus GN=Fga PE=2 SV=1 - [Q99K47_MOUSE]	49.55	2	22	23	134	0.92	0.76	0.56	1.56
Q9ET01	Glycogen phosphorylase, liver form OS=Mus musculus GN=Pygl PE=1 SV=4 - [PYGL_MOUSE]	39.88	2	22	32	114	-0.29	1.01	0.53	1.15
Q80U19	Disheveled-associated activator of morphogenesis 2 OS=Mus musculus GN=Daam2 PE=2 SV=4 - [DAAM2_MOUSE]	24.48	4	22	26	69	-0.01	-0.44	0.91	0.85
Q6XE40	MAGUK p55 subfamily member 3 OS=Mus musculus GN=Mpp3 PE=2 SV=1 - [Q6XE40_MOUSE]	46.15	3	22	23	84	-0.33	0.51	0.78	0.52
E9PV14	Band 4.1-like protein 1 (Fragment) OS=Mus musculus GN=Epb4.1l1 PE=2 SV=1 - [E9PV14_MOUSE]	52.07	1	22	44	254	1.18	-0.80	0.63	0.45
P28867	Protein kinase C delta type OS=Mus musculus GN=Prkcd PE=1 SV=3 - [KPCD_MOUSE]	39.17	6	21	24	93	-0.65	-1.60	-0.73	-0.63
Q62351	Transferrin receptor protein 1 OS=Mus musculus GN=Tfrc PE=1 SV=1 - [TFR1_MOUSE]	27.52	2	21	21	55	-0.22	-0.71	-0.60	-0.49
Q61282	Aggrecan core protein OS=Mus musculus GN=Acan PE=1 SV=2 - [PGCA_MOUSE]	13.56	1	21	22	73	1.89	0.67	1.12	0.72
P16014	Secretogranin-1 OS=Mus musculus GN=Chgb PE=1 SV=2 - [SCG1_MOUSE]	31.31	1	20	20	99	0.19	-0.31	-0.95	-1.49
Q9D0K2	Succinyl-CoA:3-ketoacid coenzyme A transferase 1, mitochondrial OS=Mus musculus GN=Oxct1 PE=1 SV=1 - [SCOT1_MOUSE]	54.23	2	20	20	259	-0.32	-0.57	-0.58	-0.71
P02469	Laminin subunit beta-1 OS=Mus musculus GN=Lamb1 PE=1 SV=3 - [LAMB1_MOUSE]	13.10	3	19	21	46	0.13	-0.72	-1.05	-0.98
Q8R151	NFX1-type zinc finger-containing protein 1 OS=Mus musculus GN=Znfx1 PE=2 SV=3 - [ZNFX1_MOUSE]	11.05	4	19	21	40	-0.02	0.40	0.56	0.57
Q8BNW9	Kelch repeat and BTB domain-containing protein 11 OS=Mus musculus GN=Kbtbd11 PE=1 SV=3 - [KBTBB_MOUSE]	45.66	1	19	19	99	-0.41	0.65	0.55	0.54
Q08642	Protein-arginine deiminase type-2 OS=Mus musculus GN=Padi2 PE=1 SV=2 - [PADI2_MOUSE]	31.20	1	18	18	64	0.04	-1.17	0.76	1.04
Q63912	Oligodendrocyte-myelin glycoprotein OS=Mus musculus GN=Omg PE=1 SV=1 - [OMGP_MOUSE]	32.73	2	17	17	225	0.39	0.41	0.60	0.46
P06880	Somatotropin OS=Mus musculus GN=Gh1 PE=2 SV=1 - [SOMA_MOUSE]	65.74	1	16	17	303	-0.93	-1.18	-1.87	-1.83
Q8K298	Actin-binding protein anillin OS=Mus musculus GN=Anln PE=1 SV=2 - [ANLN_MOUSE]	14.72	1	16	16	52	0.77	-1.40	0.86	0.48

Q64676	2-hydroxyacylsphingosine 1-beta-galactosyltransferase OS=Mus musculus GN=Ugt8 PE=2 SV=2 - [CGT_MOUSE]	24.77	1	15	15	37	-0.18	-2.93	-1.78	-1.39
P19324	Serpin H1 OS=Mus musculus GN=Serpinh1 PE=1 SV=3 - [SERPH_MOUSE]	37.65	1	15	15	45	-0.54	-0.34	-1.47	-1.09
D3Z6B9	Mitochondrial 10-formyltetrahydrofolate dehydrogenase OS=Mus musculus GN=Aldh1l2 PE=2 SV=1 - [D3Z6B9_MOUSE]	32.35	2	15	23	73	-0.18	-1.20	-1.39	-0.95
Q80XN0	D-beta-hydroxybutyrate dehydrogenase, mitochondrial OS=Mus musculus GN=Bdh1 PE=1 SV=2 - [BDH_MOUSE]	55.10	2	15	17	189	-0.73	-0.91	-0.65	-0.68
P09242	Alkaline phosphatase, tissue-nonspecific isozyme OS=Mus musculus GN=Alpl PE=1 SV=2 - [PPBT_MOUSE]	34.92	4	15	15	43	-0.10	0.09	-1.00	-0.53
O88492	Perilipin-4 OS=Mus musculus GN=Plin4 PE=1 SV=2 - [PLIN4_MOUSE]	32.15	2	15	15	36	0.14	0.61	1.22	0.94
P24529	Tyrosine 3-monooxygenase OS=Mus musculus GN=Th PE=1 SV=3 - [TY3H_MOUSE]	39.96	5	15	15	55	-1.06	-1.38	1.26	0.89
P34914	Bifunctional epoxide hydrolase 2 OS=Mus musculus GN=Ephx2 PE=1 SV=2 - [HYES_MOUSE]	38.27	2	15	15	47	-0.04	0.79	0.77	0.65
Q07076	Annexin A7 OS=Mus musculus GN=Anxa7 PE=2 SV=2 - [ANXA7_MOUSE]	32.83	1	15	15	104	-0.21	1.31	0.56	0.63
Q8JZK9	Hydroxymethylglutaryl-CoA synthase, cytoplasmic OS=Mus musculus GN=Hmgcs1 PE=1 SV=1 - [HMCS1_MOUSE]	28.46	1	14	15	60	-0.08	-1.41	-0.95	-0.72
P36552	Coproporphyrinogen-III oxidase, mitochondrial OS=Mus musculus GN=Cpox PE=1 SV=2 - [HEM6_MOUSE]	39.73	1	14	14	66	1.16	-0.68	-0.80	-0.59
P51655	Glypican-4 OS=Mus musculus GN=Gpc4 PE=2 SV=2 - [GPC4_MOUSE]	34.65	1	14	16	43	-0.81	1.50	-0.50	-0.59
Q8CCK0	Core histone macro-H2A.2 OS=Mus musculus GN=H2afy2 PE=1 SV=3 - [H2AW_MOUSE]	48.92	1	14	16	92	-0.89	-0.45	-0.67	-0.47
P00920	Carbonic anhydrase 2 OS=Mus musculus GN=Ca2 PE=1 SV=4 - [CAH2_MOUSE]	65.38	1	14	14	421	0.36	0.30	0.63	1.05
Q3V0K9	Plastin-1 OS=Mus musculus GN=Pls1 PE=2 SV=1 - [PLSI_MOUSE]	34.13	2	14	17	53	0.47	-0.88	0.63	0.77
P54830	Tyrosine-protein phosphatase non-receptor type 5 OS=Mus musculus GN=Ptpn5 PE=2 SV=2 - [PTN5_MOUSE]	32.90	4	14	14	47	-0.34	0.32	0.93	0.47
P01193	Pro-opiomelanocortin OS=Mus musculus GN=Pomc PE=2 SV=1 - [COLI_MOUSE]	53.62	1	13	13	150	-0.51	-0.86	-3.43	-3.22

A2AQ53	Fibrillin-1 OS=Mus musculus GN=Fbn1 PE=4 SV=1 - [A2AQ53_MOUSE]	5.01	2	13	16	27	-0.32	1.96	-0.95	-0.59
P63325	40S ribosomal protein S10 OS=Mus musculus GN=Rps10 PE=1 SV=1 - [RS10_MOUSE]	60.00	3	13	13	78	-0.20	-0.50	-0.53	-0.47
Q9D154	Leukocyte elastase inhibitor A OS=Mus musculus GN=Serpinb1a PE=1 SV=1 - [ILEUA_MOUSE]	48.02	5	13	18	92	0.79	-0.17	1.58	1.98
Q99K67	Alpha-aminoadipic semialdehyde synthase, mitochondrial OS=Mus musculus GN=Aass PE=2 SV=1 - [AASS_MOUSE]	17.93	2	13	13	24	0.01	0.85	0.86	1.08
Q9QUP5	Hyaluronan and proteoglycan link protein 1 OS=Mus musculus GN=Hapln1 PE=2 SV=1 - [HPLN1_MOUSE]	47.47	1	13	14	82	0.63	0.90	1.19	0.50
P47867-2	Isoform 2 of Secretogranin-3 OS=Mus musculus GN=Scg3 - [SCG3_MOUSE]	35.62	2	12	12	57	0.77	-0.27	-0.51	-0.68
Q61646	Haptoglobin OS=Mus musculus GN=Hp PE=1 SV=1 - [HPT_MOUSE]	34.01	1	12	12	34	1.22	1.59	2.86	3.23
Q91Y97	Fructose-bisphosphate aldolase B OS=Mus musculus GN=Aldob PE=1 SV=3 - [ALDOB_MOUSE]	36.54	1	12	13	87	0.88	0.11	2.43	2.94
Q9R118	Serine protease HTRA1 OS=Mus musculus GN=Htra1 PE=1 SV=2 - [HTRA1_MOUSE]	37.50	1	12	12	34	0.55	0.24	1.26	1.25
P60202	Myelin proteolipid protein OS=Mus musculus GN=Plp1 PE=1 SV=2 - [MYPR_MOUSE]	28.16	2	12	12	709	-0.01	-2.00	0.71	0.98
Q3UHB8	Coiled-coil domain-containing protein 177 OS=Mus musculus GN=Ccdc177 PE=1 SV=1 - [CC177_MOUSE]	23.09	1	12	12	27	-0.07	0.41	0.96	0.70
P68368	Tubulin alpha-4A chain OS=Mus musculus GN=Tuba4a PE=1 SV=1 - [TBA4A_MOUSE]	68.75	1	12	31	3452	0.00	0.69	0.51	0.55
A6H630	UPF0364 protein C6orf211 homolog OS=Mus musculus PE=2 SV=1 - [CF211_MOUSE]	29.38	5	12	12	30	-0.18	0.42	0.66	0.51
Q7TQA1-5	Isoform 5 of Immunoglobulin superfamily member 1 OS=Mus musculus GN=Igsf1 - [IGSF1_MOUSE]	12.52	4	11	11	50	-0.20	-1.00	-1.19	-0.45
Q61176	Arginase-1 OS=Mus musculus GN=Arg1 PE=1 SV=1 - [ARGI1_MOUSE]	37.15	1	11	11	33	1.00	0.12	1.16	1.27
Q9DB73	NADH-cytochrome b5 reductase 1 OS=Mus musculus GN=Cyb5r1 PE=2 SV=1 - [NB5R1_MOUSE]	34.43	5	11	11	37	-0.27	0.96	0.71	1.08
Q61885	Myelin-oligodendrocyte glycoprotein OS=Mus musculus GN=Mog PE=1 SV=1 - [MOG_MOUSE]	39.43	3	11	12	176	0.36	-1.84	0.89	1.03

Q8BVI4	Dihydropteridine reductase OS=Mus musculus GN=Qdpr PE=1 SV=2 - [DHPR_MOUSE]	61.83	4	11	11	106	-0.14	-0.40	0.52	1.00
Q99L04	Dehydrogenase/reductase SDR family member 1 OS=Mus musculus GN=Dhrs1 PE=2 SV=1 - [DHRS1_MOUSE]	41.21	1	11	11	48	-0.28	0.13	0.65	0.73
P29699	Alpha-2-HS-glycoprotein OS=Mus musculus GN=Ahsg PE=1 SV=1 - [FETUA_MOUSE]	44.06	1	10	10	63	1.41	0.48	-1.40	-0.83
F2Z469	Matrilin-4 OS=Mus musculus GN=Matn4 PE=2 SV=1 - [F2Z469_MOUSE]	20.11	5	10	10	22	-1.24	-0.02	-1.08	-0.65
P50429	Arylsulfatase B OS=Mus musculus GN=Arsb PE=2 SV=3 - [ARSB_MOUSE]	18.91	3	10	10	55	0.30	-0.05	-0.48	-0.65
P23953	Carboxylesterase 1C OS=Mus musculus GN=Ces1c PE=1 SV=4 - [EST1C_MOUSE]	40.79	2	10	16	65	1.52	0.23	-1.55	-0.58
P13595	Neural cell adhesion molecule 1 OS=Mus musculus GN=Ncam1 PE=1 SV=3 - [NCAM1_MOUSE]	42.42	4	10	42	842	-0.39	-0.91	-0.57	-0.54
Q6PB93	Polypeptide N-acetylgalactosaminyltransferase 2 OS=Mus musculus GN=Galnt2 PE=2 SV=1 - [GALT2_MOUSE]	23.51	2	10	10	24	-0.39	-0.33	-0.71	-0.51
Q8C4C4	Repulsive guidance molecule A OS=Mus musculus GN=Rgma PE=2 SV=1 - [Q8C4C4_MOUSE]	35.17	3	10	10	19	-0.67	-0.90	-0.76	-0.51
P62830	60S ribosomal protein L23 OS=Mus musculus GN=Rpl23 PE=1 SV=1 - [RL23_MOUSE]	71.43	2	10	10	36	-1.08	-0.79	-0.63	-0.50
P46978	Dolichyl-diphosphooligosaccharide--protein glycosyltransferase subunit STT3A OS=Mus musculus GN=Stt3a PE=1 SV=1 - [STT3A_MOUSE]	14.04	2	10	12	32	-0.55	-0.23	-0.64	-0.46
O35490	Betaine--homocysteine S-methyltransferase 1 OS=Mus musculus GN=Bhmt PE=2 SV=1 - [BHMT1_MOUSE]	32.92	2	10	10	39	0.62	0.00	2.21	2.65
P16015	Carbonic anhydrase 3 OS=Mus musculus GN=Ca3 PE=1 SV=3 - [CAH3_MOUSE]	48.08	1	10	10	35	1.44	1.49	1.20	2.08
P84096	Rho-related GTP-binding protein RhoG OS=Mus musculus GN=Rhog PE=2 SV=1 - [RHOG_MOUSE]	54.45	7	10	11	92	-0.10	-1.44	0.54	0.81
Q8K3A0	Iron-sulfur cluster co-chaperone protein HscB, mitochondrial OS=Mus musculus GN=Hscb PE=2 SV=2 - [HSC20_MOUSE]	45.30	3	10	10	34	0.80	0.50	1.00	0.57
P05622	Platelet-derived growth factor receptor beta OS=Mus musculus GN=Pdgfrb PE=1 SV=1 - [PGFRB_MOUSE]	9.93	9	9	10	28	1.06	-0.12	-1.66	-1.67
P97792	Coxsackievirus and adenovirus receptor homolog OS=Mus musculus GN=Cxadr PE=1 SV=1 - [CXAR_MOUSE]	26.03	3	9	9	30	-0.70	-0.54	-1.31	-1.66

Q8R4E6	Purine-rich element-binding protein gamma OS=Mus musculus GN=Purg PE=1 SV=1 - [PURG_MOUSE]	27.71	2	9	13	33	-1.18	-1.55	-0.77	-1.04
B1AU75	Nuclear autoantigenic sperm protein OS=Mus musculus GN=Nasp PE=4 SV=1 - [B1AU75_MOUSE]	16.56	4	9	10	21	0.96	-0.50	-0.50	-0.98
Q9QYS9-4	Isoform 4 of Protein quaking OS=Mus musculus GN=Qki - [QKI_MOUSE]	32.92	8	9	10	52	0.38	-1.25	-0.65	-0.73
P62071	Ras-related protein R-Ras2 OS=Mus musculus GN=Rras2 PE=1 SV=1 - [RRAS2_MOUSE]	60.78	1	9	12	74	-0.16	-1.18	-0.58	-0.60
D3Z598	Latent-transforming growth factor beta-binding protein 4 OS=Mus musculus GN=Ltbp4 PE=2 SV=1 - [D3Z598_MOUSE]	7.45	7	9	9	19	-0.30	0.40	-0.50	-0.59
P62301	40S ribosomal protein S13 OS=Mus musculus GN=Rps13 PE=1 SV=2 - [RS13_MOUSE]	47.68	1	9	9	43	-0.92	-0.51	-0.47	-0.50
Q8CIM8	Integrator complex subunit 4 OS=Mus musculus GN=Ints4 PE=2 SV=1 - [INT4_MOUSE]	14.52	3	9	9	21	-0.08	0.24	-0.60	-0.49
Q91XD7	Cysteine-rich with EGF-like domain protein 1 OS=Mus musculus GN=Creld1 PE=2 SV=1 - [CREL1_MOUSE]	20.95	1	9	9	30	-0.41	-0.36	-0.54	-0.48
O89020	Afamin OS=Mus musculus GN=Afm PE=1 SV=2 - [AFAM_MOUSE]	16.78	3	9	9	18	1.65	0.63	-1.78	-0.45
Q8BH24	Transmembrane 9 superfamily member 4 OS=Mus musculus GN=Tm9sf4 PE=2 SV=1 - [TM9S4_MOUSE]	15.86	1	9	9	27	-0.52	-0.49	-0.74	-0.45
P23927	Alpha-crystallin B chain OS=Mus musculus GN=Cryab PE=1 SV=2 - [CRYAB_MOUSE]	57.14	1	9	9	48	1.00	-0.23	2.28	2.22
H3BJ97	Tubulointerstitial nephritis antigen-like OS=Mus musculus GN=Tinagl1 PE=2 SV=1 - [H3BJ97_MOUSE]	28.97	3	9	9	29	0.18	2.08	1.02	1.36
Q8QZR5	Alanine aminotransferase 1 OS=Mus musculus GN=Gpt PE=2 SV=3 - [ALAT1_MOUSE]	24.19	1	9	10	23	1.27	0.18	0.68	0.91
P52760	Ribonuclease UK114 OS=Mus musculus GN=Hrsp12 PE=1 SV=3 - [UK114_MOUSE]	92.59	1	9	11	87	1.19	0.70	1.10	0.78
Q9D6F9	Tubulin beta-4A chain OS=Mus musculus GN=Tubb4a PE=1 SV=3 - [TBB4A_MOUSE]	84.01	1	9	31	3928	-0.06	0.21	0.59	0.59
A2AFS3	UPF0577 protein KIAA1324 OS=Mus musculus GN=Kiaa1324 PE=2 SV=1 - [K1324_MOUSE]	9.91	3	8	8	19	-0.12	-0.10	-0.94	-0.90
E9Q7T7	Protein Chadl OS=Mus musculus GN=Chadl PE=2 SV=1 - [E9Q7T7_MOUSE]	10.43	3	8	8	14	-0.47	-0.79	-0.86	-0.80

Q8BHC4	Dephospho-CoA kinase domain-containing protein OS=Mus musculus GN=Dcakd PE=2 SV=1 - [DCAKD_MOUSE]	42.86	2	8	8	31	-0.39	-0.55	-0.84	-0.63
Q8HW98	IgLON family member 5 OS=Mus musculus GN=Iglon5 PE=2 SV=2 - [IGLO5_MOUSE]	29.46	1	8	9	49	0.53	-1.41	-0.88	-0.56
Q8BGJ5	MCG13402, isoform CRA_a OS=Mus musculus GN=Ptbp1 PE=2 SV=1 - [Q8BGJ5_MOUSE]	27.22	9	8	10	43	-0.06	-0.14	-0.89	-0.48
P08030	Adenine phosphoribosyltransferase OS=Mus musculus GN=Aprt PE=2 SV=2 - [APT_MOUSE]	43.89	1	8	8	21	0.47	0.52	-0.83	-0.46
P35951	Low-density lipoprotein receptor OS=Mus musculus GN=Ldlr PE=1 SV=2 - [LDLR_MOUSE]	11.25	1	8	8	19	0.34	-0.90	-0.64	-0.45
Q9ESM3	Hyaluronan and proteoglycan link protein 2 OS=Mus musculus GN=Hapln2 PE=1 SV=1 - [HPLN2_MOUSE]	28.45	1	8	8	25	0.33	-0.52	2.01	2.72
Q64345	Interferon-induced protein with tetratricopeptide repeats 3 OS=Mus musculus GN=Ifit3 PE=2 SV=1 - [IFIT3_MOUSE]	23.08	2	8	9	13	0.13	0.26	0.68	1.70
Q9D2P8	Myelin-associated oligodendrocyte basic protein OS=Mus musculus GN=Mobp PE=2 SV=1 - [MOBP_MOUSE]	42.94	5	8	8	62	-0.75	-1.32	1.12	1.34
Q922B1	O-acetyl-ADP-ribose deacetylase MACROD1 OS=Mus musculus GN=Macrod1 PE=2 SV=2 - [MACD1_MOUSE]	26.63	1	8	8	36	0.21	0.46	0.58	0.96
Q8BQV2	Choline acetyltransferase OS=Mus musculus GN=Chat PE=2 SV=1 - [Q8BQV2_MOUSE]	18.45	2	8	9	16	-0.01	0.11	1.06	0.51
P35455	Vasopressin-neurophysin 2-copeptin OS=Mus musculus GN=Avp PE=2 SV=1 - [NEU2_MOUSE]	72.02	1	7	9	109	0.28	0.26	-3.64	-3.09
Q9CXL6	Doublecortin, isoform CRA_a OS=Mus musculus GN=Dcx PE=2 SV=1 - [Q9CXL6_MOUSE]	30.56	3	7	11	44	-0.75	-0.88	-0.71	-1.32
Q61166	Microtubule-associated protein RP/EB family member 1 OS=Mus musculus GN=Mapre1 PE=1 SV=3 - [MARE1_MOUSE]	42.54	1	7	11	88	-0.10	-0.56	-0.71	-1.01
P70677	Caspase-3 OS=Mus musculus GN=Casp3 PE=1 SV=1 - [CASP3_MOUSE]	25.63	1	7	7	18	0.54	-0.17	-0.81	-0.94
Q8K4L2	Archvillin OS=Mus musculus GN=Svil PE=2 SV=1 - [Q8K4L2_MOUSE]	4.58	5	7	8	10	0.10	-0.22	-1.02	-0.84
P43276	Histone H1.5 OS=Mus musculus GN=Hist1h1b PE=1 SV=2 - [H15_MOUSE]	38.57	1	7	13	68	-0.91	-0.92	-0.61	-0.82

Q9DBY8	Nuclear valosin-containing protein-like OS=Mus musculus GN=Nvl PE=1 SV=1 - [NVL_MOUSE]	10.53	1	7	7	12	-0.02	-0.39	-0.52	-0.61
Q5I012-4	Isoform 4 of Putative sodium-coupled neutral amino acid transporter 10 OS=Mus musculus GN=Slc38a10 - [S38AA_MOUSE]	8.14	5	7	8	10	0.43	0.03	-0.65	-0.48
Q91XL1	Leucine-rich HEV glycoprotein (Precursor) OS=Mus musculus GN=Lrg1 PE=2 SV=1 - [Q91XL1_MOUSE]	23.68	1	7	8	30	2.00	1.44	1.27	2.23
Q80VQ0	Aldehyde dehydrogenase family 3 member B1 OS=Mus musculus GN=Aldh3b1 PE=2 SV=1 - [AL3B1_MOUSE]	25.21	3	7	9	40	0.15	0.02	0.85	1.26
P10637	Microtubule-associated protein tau OS=Mus musculus GN=Mapt PE=1 SV=3 - [TAU_MOUSE]	51.71	5	7	40	555	1.23	-1.00	1.60	1.23
Q9CQR4	Acyl-coenzyme A thioesterase 13 OS=Mus musculus GN=Acot13 PE=1 SV=1 - [ACO13_MOUSE]	53.57	1	7	7	30	-0.76	0.27	0.55	0.68
A2AR81	T-box brain protein 1 OS=Mus musculus GN=Tbr1 PE=2 SV=1 - [A2AR81_MOUSE]	24.40	2	7	7	9	0.02	1.12	1.18	0.53
Q9CPQ0	Prolactin OS=Mus musculus GN=Prl PE=2 SV=2 - [Q9CPQ0_MOUSE]	24.00	3	6	6	34	-0.81	-1.16	-3.08	-3.27
P51880	Fatty acid-binding protein, brain OS=Mus musculus GN=Fabp7 PE=1 SV=2 - [FABP7_MOUSE]	69.70	2	6	7	237	0.86	-1.30	-1.91	-1.77
Q6PGH2	Hematological and neurological expressed 1-like protein OS=Mus musculus GN=Hn1l PE=2 SV=1 - [HN1L_MOUSE]	41.05	2	6	6	30	1.69	-0.94	-0.71	-0.66
Q3TVI8	Pre-B-cell leukemia transcription factor-interacting protein 1 OS=Mus musculus GN=Pbxip1 PE=1 SV=2 - [PBIP1_MOUSE]	11.69	2	6	6	11	0.03	0.08	-0.52	-0.63
Q6P549	Phosphatidylinositol 3,4,5-trisphosphate 5-phosphatase 2 OS=Mus musculus GN=Inppl1 PE=1 SV=1 - [SHIP2_MOUSE]	8.19	1	6	7	20	-0.03	-0.62	-0.67	-0.59
Q9JIS8	Solute carrier family 12 member 4 OS=Mus musculus GN=Slc12a4 PE=1 SV=2 - [S12A4_MOUSE]	13.09	3	6	12	65	-0.39	-0.84	-0.75	-0.54
Q99KF1	Transmembrane emp24 domain-containing protein 9 OS=Mus musculus GN=Tmed9 PE=2 SV=2 - [TMED9_MOUSE]	27.66	1	6	7	41	-0.62	-0.18	-0.59	-0.46
P12710	Fatty acid-binding protein, liver OS=Mus musculus GN=Fabp1 PE=1 SV=2 - [FABPL_MOUSE]	55.91	1	6	6	26	1.73	-1.38	3.10	3.60

Q78JT3	3-hydroxyanthranilate 3,4-dioxygenase OS=Mus musculus GN=Haao PE=1 SV=1 - [3HAO_MOUSE]	23.78	1	6	6	9	0.82	0.04	1.04	1.59
P49429	4-hydroxyphenylpyruvate dioxygenase OS=Mus musculus GN=Hpd PE=1 SV=3 - [HPPD_MOUSE]	16.03	3	6	6	8	0.45	0.12	0.88	1.56
Q8CIM3	D-2-hydroxyglutarate dehydrogenase, mitochondrial OS=Mus musculus GN=D2hgdh PE=2 SV=3 - [D2HDH_MOUSE]	17.94	2	6	6	18	-0.11	1.54	0.77	1.03
Q9WVT6	Carbonic anhydrase 14 OS=Mus musculus GN=Ca14 PE=1 SV=1 - [CAH14_MOUSE]	20.18	1	6	6	37	0.53	-1.16	0.80	0.95
P51910	Apolipoprotein D OS=Mus musculus GN=Apod PE=2 SV=1 - [APOD_MOUSE]	34.92	1	6	6	34	0.86	-0.68	0.56	0.89
G3UZ26	Serine hydroxymethyltransferase (Fragment) OS=Mus musculus GN=Shmt1 PE=3 SV=1 - [G3UZ26_MOUSE]	15.49	4	6	7	12	0.32	-0.01	0.53	0.78
Q61818-2	Isoform 2 of Retinoic acid-induced protein 1 OS=Mus musculus GN=Rai1 - [RAI1_MOUSE]	5.43	2	6	6	12	-0.27	-0.15	0.59	0.74
Q8VED9	Galectin-related protein OS=Mus musculus GN=Lgalsl PE=1 SV=1 - [LEGL_MOUSE]	62.79	1	6	9	75	-0.27	0.76	0.72	0.61
Q8K0L9-2	Isoform 2 of Zinc finger and BTB domain-containing protein 20 OS=Mus musculus GN=Zbtb20 - [ZBT20_MOUSE]	15.72	3	6	7	12	-0.42	-0.21	0.79	0.56
A2A5V4	SH3 domain-binding protein 1 OS=Mus musculus GN=Sh3bp1 PE=2 SV=1 - [A2A5V4_MOUSE]	12.21	4	6	6	20	-0.09	0.44	0.53	0.54
Q9JJZ2	Tubulin alpha-8 chain OS=Mus musculus GN=Tuba8 PE=1 SV=1 - [TBA8_MOUSE]	50.33	1	6	22	2388	0.20	0.63	0.92	0.49
D3Z3A9	Reticulocalbin-3 (Fragment) OS=Mus musculus GN=Rcn3 PE=2 SV=1 - [D3Z3A9_MOUSE]	22.26	4	5	5	9	0.25	0.74	-2.20	-1.93
P26339	Chromogranin-A OS=Mus musculus GN=Chga PE=1 SV=1 - [CMGA_MOUSE]	13.61	1	5	5	28	0.61	-0.67	-1.10	-1.40
P30681	High mobility group protein B2 OS=Mus musculus GN=Hmgb2 PE=1 SV=3 - [HMGB2_MOUSE]	24.76	1	5	6	16	-0.47	-1.04	-1.00	-1.36
Q9JJF0	Nucleosome assembly protein 1-like 5 OS=Mus musculus GN=Nap1l5 PE=2 SV=1 - [NP1L5_MOUSE]	21.79	1	5	5	28	-0.76	-1.77	-1.41	-1.33
P97363	Serine palmitoyltransferase 2 OS=Mus musculus GN=Sptlc2 PE=2 SV=2 - [SPTC2_MOUSE]	11.07	1	5	5	7	-0.53	-1.04	-1.09	-1.04
E0CX20	Protein BUD31 homolog OS=Mus musculus GN=Bud31 PE=4 SV=1 - [E0CX20_MOUSE]	32.64	2	5	5	9	-0.02	-0.85	-0.73	-0.73

O89103	Complement component C1q receptor OS=Mus musculus GN=Cd93 PE=1 SV=1 - [C1QR1_MOUSE]	10.25	1	5	5	12	-0.13	-0.75	-0.61	-0.70
Q8CHT3	Integrator complex subunit 5 OS=Mus musculus GN=Ints5 PE=2 SV=1 - [INT5_MOUSE]	8.74	1	5	5	9	0.57	-0.02	-0.73	-0.65
J3KMU3	Uncharacterized protein OS=Mus musculus GN=Gm2058 PE=3 SV=1 - [J3KMU3_MOUSE]	22.95	4	5	5	13	0.05	-0.03	-0.47	-0.57
A2A4H9	Peptidyl-prolyl cis-trans isomerase FKBP10 OS=Mus musculus GN=Fkbp10 PE=2 SV=1 - [A2A4H9_MOUSE]	13.86	2	5	5	12	0.43	0.46	-0.71	-0.55
P63276	40S ribosomal protein S17 OS=Mus musculus GN=Rps17 PE=1 SV=2 - [RS17_MOUSE]	47.41	1	5	5	29	-1.49	-0.66	-0.58	-0.54
Q99KK1	Receptor expression-enhancing protein 3 OS=Mus musculus GN=Reep3 PE=1 SV=1 - [REEP3_MOUSE]	19.29	2	5	5	12	0.25	-0.96	-0.70	-0.53
O88736	3-keto-steroid reductase OS=Mus musculus GN=Hsd17b7 PE=2 SV=1 - [DHB7_MOUSE]	20.06	2	5	5	16	-0.55	-1.52	-0.61	-0.51
Q8BUV8	Protein GPR107 OS=Mus musculus GN=Gpr107 PE=2 SV=2 - [GP107_MOUSE]	10.89	1	5	6	10	-0.31	-0.30	-0.56	-0.51
D3YWT0	Signal peptidase complex catalytic subunit SEC11A OS=Mus musculus GN=Sec11a PE=2 SV=1 - [D3YWT0_MOUSE]	25.29	4	5	5	21	-0.83	0.06	-0.78	-0.51
Q9ER65	Calsyntenin-2 OS=Mus musculus GN=Clstn2 PE=1 SV=2 - [CSTN2_MOUSE]	7.87	2	5	5	14	0.20	-0.20	-0.55	-0.50
Q8R3Q0	Store-operated calcium entry-associated regulatory factor OS=Mus musculus GN=Tmem66 PE=2 SV=2 - [SARAF_MOUSE]	17.37	1	5	5	9	-0.61	-0.17	-0.74	-0.49
Q07813	Apoptosis regulator BAX OS=Mus musculus GN=Bax PE=1 SV=1 - [BAX_MOUSE]	33.33	1	5	5	21	-0.19	0.02	-0.47	-0.45
Q9QXJ2	Signal transducer and activator of transcription 2 OS=Mus musculus GN=Stat2 PE=1 SV=1 - [Q9QXJ2_MOUSE]	5.75	3	5	5	9	0.55	0.27	0.73	1.99
P11714	Cytochrome P450 2D9 OS=Mus musculus GN=Cyp2d9 PE=1 SV=2 - [CP2D9_MOUSE]	15.08	1	5	5	8	0.22	0.14	1.23	1.82
P40237	CD82 antigen OS=Mus musculus GN=Cd82 PE=1 SV=1 - [CD82_MOUSE]	16.54	3	5	5	37	0.40	-0.68	1.25	1.35
F8VQG7	Protein N4bp2 OS=Mus musculus GN=N4bp2 PE=4 SV=1 - [F8VQG7_MOUSE]	5.07	2	5	6	20	1.02	1.37	0.85	1.15

Q60771	Claudin-11 OS=Mus musculus GN=Cldn11 PE=1 SV=1 - [CLD11_MOUSE]	15.94	1	5	5	245	-0.55	-1.55	0.76	1.03
O35215	D-dopachrome decarboxylase OS=Mus musculus GN=Ddt PE=1 SV=3 - [DOPD_MOUSE]	55.93	2	5	5	13	0.73	0.12	0.68	0.97
Q810U3	Neurofascin OS=Mus musculus GN=Nfasc PE=1 SV=1 - [NFASC_MOUSE]	41.69	1	5	47	346	-0.21	0.83	0.90	0.82
J3QNR5	Uncharacterized protein OS=Mus musculus GN=Tubb4b-ps1 PE=3 SV=1 - [J3QNR5_MOUSE]	70.72	2	5	28	3377	-0.12	0.50	0.65	0.80
O70209	PDZ and LIM domain protein 3 OS=Mus musculus GN=Pdlim3 PE=1 SV=1 - [PDLI3_MOUSE]	28.48	1	5	5	15	0.06	1.93	0.55	0.63
Q61016	Guanine nucleotide-binding protein G(I)/G(S)/G(O) subunit gamma-7 OS=Mus musculus GN=Gng7 PE=2 SV=2 - [GBG7_MOUSE]	67.65	3	5	6	29	-0.09	0.99	1.74	0.63
Q9JMA2	Queuine tRNA-ribosyltransferase OS=Mus musculus GN=Qtrt1 PE=1 SV=2 - [TGT_MOUSE]	19.35	1	5	6	18	0.29	0.51	0.78	0.63
Q8CJF9	Protein argonaute-3 OS=Mus musculus GN=Ago3 PE=2 SV=2 - [AGO3_MOUSE]	20.23	1	5	15	39	1.37	-0.97	0.51	0.51
Q02105	Complement C1q subcomponent subunit C OS=Mus musculus GN=C1qc PE=2 SV=2 - [C1QC_MOUSE]	25.20	1	5	5	52	0.50	0.88	0.58	0.46
Q9CQX2	Cytochrome b5 type B OS=Mus musculus GN=Cyb5b PE=1 SV=1 - [CYB5B_MOUSE]	32.19	1	5	5	27	0.91	1.07	0.87	0.45
Q9D902	General transcription factor IIE subunit 2 OS=Mus musculus GN=Gtf2e2 PE=2 SV=2 - [T2EB_MOUSE]	15.41	1	4	5	8	-0.52	-2.75	-5.79	-6.26
Q8BVR6	RING finger and SPRY domain-containing protein 1 OS=Mus musculus GN=Rspry1 PE=2 SV=1 - [RSPRY_MOUSE]	8.16	3	4	4	4	0.70	-0.56	-1.43	-2.25
Q80YN3	Breast carcinoma-amplified sequence 1 homolog OS=Mus musculus GN=Bcas1 PE=1 SV=3 - [BCAS1_MOUSE]	45.02	3	4	26	241	0.12	-3.65	-0.71	-1.68
Q19LI2	Alpha-1B-glycoprotein OS=Mus musculus GN=A1bg PE=1 SV=1 - [A1BG_MOUSE]	8.79	1	4	4	5	-0.20	-1.58	-1.74	-1.58
Q80VW5-2	Isoform 2 of Whirlin OS=Mus musculus GN=Dfnb31 - [WHRN_MOUSE]	5.38	8	4	4	5	1.60	-0.56	-1.15	-1.14
Q3TTY5	Keratin, type II cytoskeletal 2 epidermal OS=Mus musculus GN=Krt2 PE=1 SV=1 - [K22E_MOUSE]	10.47	1	4	6	26	2.37	-0.32	-0.57	-1.09
P43275	Histone H1.1 OS=Mus musculus GN=Hist1h1a PE=1 SV=2 - [H11_MOUSE]	28.64	1	4	7	36	-0.71	-0.36	-0.80	-1.07

Q8BPB5	EGF-containing fibulin-like extracellular matrix protein 1 OS=Mus musculus GN=Efemp1 PE=2 SV=1 - [FBLN3_MOUSE]	12.98	1	4	4	16	-0.22	0.90	-1.21	-1.00
Q6A078	Centrosomal protein of 290 kDa OS=Mus musculus GN=Cep290 PE=1 SV=2 - [CE290_MOUSE]	2.79	3	4	8	18	-0.28	-0.52	-1.09	-0.85
Q3UK98	Chromobox protein homolog 6 OS=Mus musculus GN=Cbx6 PE=2 SV=1 - [Q3UK98_MOUSE]	10.10	4	4	4	4	-0.43	-0.47	-0.80	-0.85
P50543	Protein S100-A11 OS=Mus musculus GN=S100a11 PE=2 SV=1 - [S10AB_MOUSE]	43.88	1	4	4	19	0.43	0.99	-1.15	-0.83
Q8VE98	CD276 antigen OS=Mus musculus GN=Cd276 PE=1 SV=1 - [CD276_MOUSE]	17.72	1	4	4	6	-0.56	0.24	-0.86	-0.80
Q61555	Fibrillin-2 OS=Mus musculus GN=Fbn2 PE=1 SV=2 - [FBN2_MOUSE]	1.79	1	4	6	9	-0.17	1.40	-0.66	-0.76
Q6PGA0-2	Isoform 2 of REST corepressor 3 OS=Mus musculus GN=Rcor3 - [RCOR3_MOUSE]	11.06	5	4	4	8	0.00	-0.35	-0.62	-0.74
Q07646-2	Isoform 2 of Mesoderm-specific transcript protein OS=Mus musculus GN=Mest - [MEST_MOUSE]	22.39	5	4	4	10	-0.57	-0.54	-1.25	-0.71
P62960	Nuclease-sensitive element-binding protein 1 OS=Mus musculus GN=Ybx1 PE=1 SV=3 - [YBOX1_MOUSE]	31.68	4	4	7	14	-0.07	-1.54	-0.66	-0.70
Q8VCH6	Delta(24)-sterol reductase OS=Mus musculus GN=Dhcr24 PE=2 SV=1 - [DHC24_MOUSE]	6.20	1	4	4	9	-0.26	-1.18	-0.79	-0.68
P26645	Myristoylated alanine-rich C-kinase substrate OS=Mus musculus GN=Marcks PE=1 SV=2 - [MARCS_MOUSE]	23.95	1	4	4	55	-0.39	-1.32	-0.52	-0.57
Q9ERR7	15 kDa selenoprotein OS=Mus musculus GN=Sep15 PE=1 SV=3 - [SEP15_MOUSE]	30.86	2	4	4	18	0.24	0.06	-0.52	-0.57
Q60764	Probable E3 ubiquitin-protein ligase makorin-3 OS=Mus musculus GN=Mkrn3 PE=2 SV=2 - [MKRN3_MOUSE]	10.29	1	4	5	8	0.10	-0.68	-0.67	-0.55
P23475	X-ray repair cross-complementing protein 6 OS=Mus musculus GN=Xrcc6 PE=1 SV=5 - [XRCC6_MOUSE]	11.51	3	4	8	13	-0.33	-0.45	-0.66	-0.48
Q99LJ6	Glutathione peroxidase 7 OS=Mus musculus GN=Gpx7 PE=2 SV=1 - [GPX7_MOUSE]	22.58	1	4	5	15	-0.38	-0.35	-0.90	-0.48
Q8R4G6	Alpha-1,6-mannosylglycoprotein 6-beta-N-acetylglucosaminyltransferase A OS=Mus musculus GN=Mgat5 PE=2 SV=1 - [MGT5A_MOUSE]	10.00	1	4	5	14	-0.43	-0.62	-0.60	-0.48
O09106	Histone deacetylase 1 OS=Mus musculus GN=Hdac1 PE=1 SV=1 - [HDAC1_MOUSE]	25.10	2	4	10	33	-0.84	-0.21	-0.90	-0.48

P60764	Ras-related C3 botulinum toxin substrate 3 OS=Mus musculus GN=Rac3 PE=1 SV=1 - [RAC3_MOUSE]	42.71	8	4	10	79	-0.72	-0.56	-0.97	-0.48
Q9CY50	Translocon-associated protein subunit alpha OS=Mus musculus GN=Ssr1 PE=1 SV=1 - [SSRA_MOUSE]	23.43	1	4	4	31	-0.75	-0.34	-0.63	-0.47
Q62086	Serum paraoxonase/arylesterase 2 OS=Mus musculus GN=Pon2 PE=1 SV=2 - [PON2_MOUSE]	17.23	1	4	4	14	-0.51	-0.69	-0.72	-0.45
P11672	Neutrophil gelatinase-associated lipocalin OS=Mus musculus GN=Lcn2 PE=1 SV=1 - [NGAL_MOUSE]	23.00	1	4	4	7	0.45	0.47	1.99	2.82
Q9QXD6	Fructose-1,6-bisphosphatase 1 OS=Mus musculus GN=Fbp1 PE=2 SV=3 - [F16P1_MOUSE]	14.50	4	4	4	8	1.64	0.39	2.04	2.71
Q64374	Regucalcin OS=Mus musculus GN=Rgn PE=1 SV=1 - [RGN_MOUSE]	17.06	1	4	4	24	1.09	0.26	1.34	2.30
Q9QZ85	Interferon-inducible GTPase 1 OS=Mus musculus GN=Iigp1 PE=1 SV=2 - [IIGP1_MOUSE]	13.56	1	4	5	8	0.22	0.46	0.65	1.51
Q4U4S6	Xin actin-binding repeat-containing protein 2 OS=Mus musculus GN=Xirp2 PE=1 SV=1 - [XIRP2_MOUSE]	1.48	3	4	6	7	0.80	1.19	1.18	1.30
E9PUK2	Uncharacterized aarF domain-containing protein kinase 5 OS=Mus musculus GN=Adck5 PE=2 SV=1 - [E9PUK2_MOUSE]	7.39	4	4	4	6	0.05	0.75	0.87	1.27
L7N451	Interferon-induced very large GTPase 1 OS=Mus musculus GN=Gvin1 PE=4 SV=1 - [L7N451_MOUSE]	2.43	3	4	4	8	1.50	0.55	0.84	1.18
Q91WH7	ATPase, H+/K+ exchanging, gastric, alpha polypeptide OS=Mus musculus GN=Atp4a PE=2 SV=1 - [Q91WH7_MOUSE]	11.41	3	4	12	339	0.90	0.50	1.29	1.10
P31725	Protein S100-A9 OS=Mus musculus GN=S100a9 PE=1 SV=3 - [S10A9_MOUSE]	38.94	1	4	4	12	1.48	1.28	0.53	0.98
F6TYB7	Myelin basic protein (Fragment) OS=Mus musculus GN=Mbp PE=4 SV=1 - [F6TYB7_MOUSE]	84.82	9	4	25	2287	0.68	-2.80	1.27	0.97
Q9QY42	Probable G-protein coupled receptor 37 OS=Mus musculus GN=Gpr37 PE=2 SV=1 - [GPR37_MOUSE]	13.00	3	4	5	13	0.52	0.16	0.87	0.92
P29788	Vitronectin OS=Mus musculus GN=Vtn PE=1 SV=2 - [VTNC_MOUSE]	11.09	1	4	5	13	1.22	0.86	0.52	0.91
E9PZQ0	Ryanodine receptor 1 OS=Mus musculus GN=Ryr1 PE=1 SV=1 - [RYR1_MOUSE]	1.37	2	4	8	11	1.12	0.94	0.98	0.90
P97760	DNA-directed RNA polymerase II subunit RPB3 OS=Mus musculus GN=Polr2c PE=2 SV=1 - [RPB3_MOUSE]	17.09	2	4	4	9	0.39	-0.58	0.82	0.84

P13597-2	Isoform 2 of Intercellular adhesion molecule 1 OS=Mus musculus GN=Icam1 - [ICAM1_MOUSE]	7.53	2	4	4	8	0.61	1.24	0.76	0.79
Q3U213-2	Isoform 2 of Protein SERAC1 OS=Mus musculus GN=Serac1 - [SRAC1_MOUSE]	6.57	3	4	4	5	-0.19	-0.39	0.56	0.66
Q9JJN2	Zinc finger homeobox protein 4 OS=Mus musculus GN=Zfhx4 PE=1 SV=1 - [ZFHX4_MOUSE]	2.08	4	4	5	6	0.51	0.49	0.64	0.59
Q9JMF3	Guanine nucleotide-binding protein G(I)/G(S)/G(O) subunit gamma-13 OS=Mus musculus GN=Gng13 PE=3 SV=1 - [GBG13_MOUSE]	46.27	1	4	4	23	-0.30	1.40	1.08	0.58
Q64446	Copper-transporting ATPase 2 OS=Mus musculus GN=Atp7b PE=1 SV=2 - [ATP7B_MOUSE]	3.76	2	4	4	4	0.35	0.34	0.62	0.55
A2BDQ4	Eph receptor A7, isoform CRA_b OS=Mus musculus GN=Epha7 PE=4 SV=1 - [A2BDQ4_MOUSE]	11.77	10	4	9	20	0.91	0.36	0.76	0.52
Q8BG16	Sodium-dependent neutral amino acid transporter B(0)AT2 OS=Mus musculus GN=Slc6a15 PE=1 SV=1 - [S6A15_MOUSE]	7.82	1	4	4	4	0.78	0.47	0.86	0.49
A2RTF1	Cation channel sperm-associated protein subunit beta OS=Mus musculus GN=Catsperb PE=1 SV=1 - [CTSRB_MOUSE]	3.97	1	3	3	6	-0.16	-1.31	-3.06	-3.46
A2AVN2	Glycoprotein hormones alpha chain (Fragment) OS=Mus musculus GN=Cga PE=2 SV=1 - [A2AVN2_MOUSE]	12.82	2	3	3	16	-0.99	-1.31	-3.22	-3.16
O88904-2	Isoform 2 of Homeodomain-interacting protein kinase 1 OS=Mus musculus GN=Hipk1 - [HIPK1_MOUSE]	4.55	4	3	3	3	0.83	0.66	-3.89	-3.09
Q14BE7	Family with sequence similarity 47, member A OS=Mus musculus GN=Fam47c PE=2 SV=1 - [Q14BE7_MOUSE]	6.98	1	3	3	3	2.72	-0.01	-2.22	-2.43
Q8BP71-5	Isoform 5 of RNA binding protein fox-1 homolog 2 OS=Mus musculus GN=Rbfox2 - [RFOX2_MOUSE]	25.06	9	3	9	25	2.38	0.35	-1.64	-1.99
P84228	Histone H3.2 OS=Mus musculus GN=Hist1h3b PE=1 SV=2 - [H32_MOUSE]	59.56	2	3	11	54	-1.09	-1.88	-1.75	-1.67
E9PYP8	Beta-crystallin B1 (Fragment) OS=Mus musculus GN=Crybb1 PE=2 SV=1 - [E9PYP8_MOUSE]	16.95	2	3	3	9	-0.22	-0.80	-0.79	-1.27
P20917	Myelin-associated glycoprotein OS=Mus musculus GN=Mag PE=1 SV=2 - [MAG_MOUSE]	31.31	1	3	17	196	0.67	-2.16	-0.63	-0.90
Q8CG48	Structural maintenance of chromosomes protein 2 OS=Mus musculus GN=Smc2 PE=1 SV=2 - [SMC2_MOUSE]	2.69	1	3	3	9	0.12	-0.37	-0.67	-0.78

E9Q4X2	Protein Uggt2 OS=Mus musculus GN=Uggt2 PE=2 SV=1 - [E9Q4X2_MOUSE]	3.72	3	3	8	9	-0.18	-0.25	-0.87	-0.75
Q9CUU3	Synaptonemal complex protein 2 OS=Mus musculus GN=Sycp2 PE=1 SV=2 - [SYCP2_MOUSE]	2.93	1	3	3	6	1.80	0.58	-1.52	-0.73
Q8R5A3	Amyloid beta A4 precursor protein-binding family B member 1-interacting protein OS=Mus musculus GN=Apbb1ip PE=1 SV=2 - [AB1IP_MOUSE]	7.01	1	3	4	9	0.38	0.01	-0.79	-0.72
Q3U0J8	TBC1 domain family member 2B OS=Mus musculus GN=Tbc1d2b PE=1 SV=2 - [TBD2B_MOUSE]	4.15	2	3	4	5	-0.12	0.16	-0.66	-0.67
P16254	Signal recognition particle 14 kDa protein OS=Mus musculus GN=Srp14 PE=1 SV=1 - [SRP14_MOUSE]	27.27	2	3	3	7	0.52	-0.86	-0.55	-0.62
Q8CAY6	Acetyl-CoA acetyltransferase, cytosolic OS=Mus musculus GN=Acat2 PE=1 SV=2 - [THIC_MOUSE]	58.69	2	3	17	204	0.70	-0.34	-0.64	-0.59
Q921I1	Serotransferrin OS=Mus musculus GN=Tf PE=1 SV=1 - [TRFE_MOUSE]	57.96	6	3	44	479	1.80	0.28	-0.92	-0.55
Q9CQY5-2	Isoform 2 of Magnesium transporter protein 1 OS=Mus musculus GN=Magt1 - [MAGT1_MOUSE]	11.76	6	3	4	7	0.06	-0.25	-0.63	-0.52
A2A9I0	Golgi SNAP receptor complex member 2 OS=Mus musculus GN=Gosr2 PE=4 SV=1 - [A2A9I0_MOUSE]	14.55	2	3	3	8	-0.49	0.16	-0.63	-0.51
Q9CR89-2	Isoform 2 of Endoplasmic reticulum-Golgi intermediate compartment protein 2 OS=Mus musculus GN=Ergic2 - [ERGI2_MOUSE]	15.56	2	3	3	4	-1.38	-0.29	-0.46	-0.48
Q3URU2	Paternally-expressed gene 3 protein OS=Mus musculus GN=Peg3 PE=1 SV=1 - [PEG3_MOUSE]	8.40	1	3	10	18	0.55	-0.30	-0.61	-0.47
Q61183-4	Isoform 4 of Poly(A) polymerase alpha OS=Mus musculus GN=Papola - [PAPOA_MOUSE]	7.46	13	3	4	5	-0.55	-0.51	-0.57	-0.47
Q9D8T4	Golgi apparatus membrane protein TVP23 homolog B OS=Mus musculus GN=Tvp23b PE=1 SV=1 - [TV23B_MOUSE]	11.71	2	3	3	4	-0.91	-0.42	-0.49	-0.46
Q9D3P8	Plasminogen receptor (KT) OS=Mus musculus GN=Plgrkt PE=1 SV=1 - [PLRKT_MOUSE]	19.05	4	3	3	13	-1.09	-0.17	-0.57	-0.46
E9Q4M8	Delta(14)-sterol reductase OS=Mus musculus GN=Tm7sf2 PE=2 SV=1 - [E9Q4M8_MOUSE]	8.77	7	3	3	4	-0.01	-0.82	-0.51	-0.46
A6H6E2	Multimerin-2 OS=Mus musculus GN=Mmrn2 PE=2 SV=1 - [MMRN2_MOUSE]	4.24	1	3	4	11	0.34	0.21	-0.68	-0.45

P05367	Serum amyloid A-2 protein OS=Mus musculus GN=Saa2 PE=1 SV=1 - [SAA2_MOUSE]	28.69	1	3	4	12	0.93	0.89	3.45	4.60
Q91X83	S-adenosylmethionine synthase isoform type-1 OS=Mus musculus GN=Mat1a PE=2 SV=1 - [METK1_MOUSE]	10.86	1	3	4	9	0.34	-0.51	1.78	2.52
Q60590	Alpha-1-acid glycoprotein 1 OS=Mus musculus GN=Orm1 PE=1 SV=1 - [A1AG1_MOUSE]	20.77	1	3	5	10	2.36	1.75	1.66	2.19
Q64282	Interferon-induced protein with tetratricopeptide repeats 1 OS=Mus musculus GN=Ifit1 PE=1 SV=2 - [IFIT1_MOUSE]	12.96	4	3	3	5	-0.86	0.98	0.99	2.09
Q63880-2	Isoform 2 of Carboxylesterase 3A OS=Mus musculus GN=Ces3a - [EST3A_MOUSE]	8.02	6	3	4	19	0.44	-0.38	1.21	2.07
D3YU60	Microsomal glutathione S-transferase 1 OS=Mus musculus GN=Mgst1 PE=2 SV=1 - [D3YU60_MOUSE]	45.10	4	3	3	5	0.47	-0.08	0.83	1.94
Q8R1A8	Ornithine carbamoyltransferase, mitochondrial OS=Mus musculus GN=Otc PE=2 SV=1 - [Q8R1A8_MOUSE]	9.69	2	3	3	10	0.67	0.35	0.97	1.55
Q9D8H7	Metalloendopeptidase OMA1, mitochondrial OS=Mus musculus GN=Oma1 PE=2 SV=1 - [OMA1_MOUSE]	5.18	1	3	3	3	1.01	0.83	0.68	1.45
Q62095	ATP-dependent RNA helicase DDX3Y OS=Mus musculus GN=Ddx3y PE=1 SV=2 - [DDX3Y_MOUSE]	44.68	1	3	30	153	0.72	0.71	0.95	1.24
P70121	Zinc fingers and homeoboxes protein 1 OS=Mus musculus GN=Zhx1 PE=1 SV=2 - [ZHX1_MOUSE]	5.04	2	3	4	7	1.32	1.32	1.20	1.20
P0DM40	Fer-1-like protein 5 OS=Mus musculus GN=Fer1l5 PE=1 SV=1 - [FR1L5_MOUSE]	1.37	1	3	3	4	0.04	0.22	1.17	1.11
Q8CFB4	Guanylate-binding protein 5 OS=Mus musculus GN=Gbp5 PE=1 SV=2 - [GBP5_MOUSE]	6.27	1	3	3	4	0.83	0.51	0.80	1.11
Q91X56	Sphingosine 1-phosphate receptor 5 OS=Mus musculus GN=S1pr5 PE=2 SV=1 - [S1PR5_MOUSE]	13.00	1	3	3	6	0.52	-0.24	1.16	1.05
Q6PGB8	Probable global transcription activator SNF2L1 OS=Mus musculus GN=Smarca1 PE=1 SV=1 - [SMCA1_MOUSE]	8.03	4	3	10	18	-0.30	-0.28	1.74	0.93
Q9WVF8	Tumor suppressor candidate 2 OS=Mus musculus GN=Tusc2 PE=1 SV=3 - [TUSC2_MOUSE]	41.82	1	3	3	8	0.28	-0.27	0.95	0.86
O88700	Bloom syndrome protein homolog OS=Mus musculus GN=Blm PE=1 SV=1 - [BLM_MOUSE]	3.25	2	3	4	4	0.56	0.29	0.51	0.84

F7D143	Uncharacterized protein KIAA0232 (Fragment) OS=Mus musculus GN=D5Ertd579e PE=2 SV=1 - [F7D143_MOUSE]	4.00	3	3	3	3	0.24	0.42	1.05	0.79
Q8VD26-2	Isoform 2 of Transmembrane protein 143 OS=Mus musculus GN=Tmem143 - [TM143_MOUSE]	8.61	4	3	3	11	-0.93	0.24	0.58	0.73
B1AS67	Pleckstrin homology domain-containing family G member 5 OS=Mus musculus GN=Plekhg5 PE=2 SV=1 - [B1AS67_MOUSE]	4.62	5	3	4	9	-0.08	0.09	0.78	0.66
Q8BJ56-3	Isoform 3 of Patatin-like phospholipase domain-containing protein 2 OS=Mus musculus GN=Pnpla2 - [PLPL2_MOUSE]	10.00	5	3	3	6	1.07	-0.21	0.58	0.61
E9PVX6	Protein Mki67 OS=Mus musculus GN=Mki67 PE=2 SV=1 - [E9PVX6_MOUSE]	1.07	1	3	3	3	1.67	1.03	1.94	0.59
Q8CII2	Cell division cycle protein 123 homolog OS=Mus musculus GN=Cdc123 PE=2 SV=2 - [CD123_MOUSE]	11.31	2	3	4	16	0.89	1.14	0.91	0.59
E9QJV4	Pre-mRNA-processing factor 39 OS=Mus musculus GN=Prpf39 PE=2 SV=1 - [E9QJV4_MOUSE]	5.56	3	3	3	6	1.22	0.40	0.52	0.58
Q80SZ7	Guanine nucleotide-binding protein G(I)/G(S)/G(O) subunit gamma-5 OS=Mus musculus GN=Gng5 PE=2 SV=2 - [GBG5_MOUSE]	33.82	2	3	3	12	0.99	0.68	0.54	0.55
Q78TU8	Family with sequence similarity 107, member A OS=Mus musculus GN=Fam107a PE=2 SV=1 - [Q78TU8_MOUSE]	24.31	4	3	4	14	-0.15	-0.34	1.18	0.54
Q3UHN9	Bifunctional heparan sulfate N-deacetylase/N-sulfotransferase 1 OS=Mus musculus GN=Ndst1 PE=1 SV=2 - [NDST1_MOUSE]	5.56	2	3	4	6	0.07	-0.52	0.90	0.50
P52963	Band 4.1-like protein 4A OS=Mus musculus GN=Epb41l4a PE=2 SV=2 - [E41LA_MOUSE]	5.83	1	3	3	4	0.38	-0.59	0.53	0.49
B1AWT2	Ras-related GTP binding D, isoform CRA_a OS=Mus musculus GN=Rragd PE=4 SV=1 - [B1AWT2_MOUSE]	13.53	4	3	5	14	-0.54	1.16	0.95	0.47
A2ALI5	Adherens junction-associated protein 1 OS=Mus musculus GN=Ajap1 PE=2 SV=1 - [AJAP1_MOUSE]	11.65	1	3	3	6	0.69	0.61	0.83	0.47
B1AVY7	Kinesin-like protein KIF16B OS=Mus musculus GN=Kif16b PE=1 SV=1 - [KI16B_MOUSE]	4.34	1	3	6	13	0.80	0.00	0.52	0.46
G3X9G6	Luteinizing hormone beta OS=Mus musculus GN=Lhb PE=3 SV=1 - [G3X9G6_MOUSE]	21.99	2	2	2	9	-2.30	-2.91	-4.79	-4.91

K4DI78	SH3 domain-binding glutamic acid-rich protein OS=Mus musculus GN=Sh3bgr PE=4 SV=1 - [K4DI78_MOUSE]	19.23	5	2	2	3	1.52	-2.00	-4.20	-4.39
Q810Y9	PRAMEl6 OS=Mus musculus GN=Pramel6 PE=2 SV=1 - [Q810Y9_MOUSE]	5.98	1	2	2	2	1.66	-1.51	-3.55	-4.01
E9Q2X6	Structural maintenance of chromosomes protein OS=Mus musculus GN=Smc4 PE=2 SV=1 - [E9Q2X6_MOUSE]	2.46	2	2	4	5	4.77	-0.13	-4.20	-3.92
Q7TQE7-2	Isoform 2 of Uncharacterized protein KIAA0895 OS=Mus musculus GN=Kiaa0895 - [K0895_MOUSE]	10.55	2	2	2	2	4.21	2.35	-1.41	-2.66
F8VQD3	Protein Vmn2r37 OS=Mus musculus GN=Vmn2r37 PE=3 SV=1 - [F8VQD3_MOUSE]	4.24	2	2	2	3	-3.39	-2.27	-2.91	-2.57
Q9DBZ1-2	Isoform 2 of Inhibitor of nuclear factor kappa-B kinase-interacting protein OS=Mus musculus GN=Ikbip - [IKIP_MOUSE]	5.51	1	2	2	5	-0.25	-1.03	-2.60	-2.46
P28667	MARCKS-related protein OS=Mus musculus GN=Marcksl1 PE=1 SV=2 - [MRP_MOUSE]	6.50	1	2	2	8	-0.58	-2.54	-2.07	-2.45
P35454	Oxytocin-neurophysin 1 OS=Mus musculus GN=Oxt PE=2 SV=1 - [NEU1_MOUSE]	50.40	1	2	4	57	0.12	0.60	-2.64	-2.42
P09813	Apolipoprotein A-II OS=Mus musculus GN=Apoa2 PE=1 SV=2 - [APOA2_MOUSE]	18.63	1	2	2	10	1.79	-1.15	-1.66	-1.78
Q8BGD6	Putative sodium-coupled neutral amino acid transporter 9 OS=Mus musculus GN=Slc38a9 PE=1 SV=1 - [S38A9_MOUSE]	4.46	1	2	2	5	2.31	-0.30	-1.33	-1.75
Q80VP5	Probable peptide chain release factor C12orf65 homolog, mitochondrial OS=Mus musculus PE=1 SV=1 - [CL065_MOUSE]	13.59	3	2	2	2	2.16	-1.24	-1.49	-1.69
G3UZJ2	Microtubule-associated protein (Fragment) OS=Mus musculus GN=Map2 PE=2 SV=1 - [G3UZJ2_MOUSE]	71.78	1	2	22	183	-0.01	-0.88	-1.27	-1.46
Q9Z0F1-2	Isoform Nesp55-2 of Neuroendocrine secretory protein 55 OS=Mus musculus GN=Gnas - [GNAS3_MOUSE]	7.91	2	2	2	7	0.23	-1.17	-1.73	-1.43
Q9ER41	Torsin-1B OS=Mus musculus GN=Tor1b PE=2 SV=2 - [TOR1B_MOUSE]	10.12	4	2	2	3	1.04	-1.11	-0.74	-1.42
Q8VI24-2	Isoform 2 of DNA-binding protein SATB2 OS=Mus musculus GN=Satb2 - [SATB2_MOUSE]	2.97	3	2	2	2	0.09	0.92	-1.32	-1.34
Q6NS65	Uracil nucleotide/cysteinyl leukotriene receptor OS=Mus musculus GN=Gpr17 PE=2 SV=1 - [GPR17_MOUSE]	5.31	1	2	2	5	-0.20	-0.75	-1.06	-1.32

Q4QRL3	Coiled-coil domain-containing protein 88B OS=Mus musculus GN=Ccdc88b PE=1 SV=2 - [CC88B_MOUSE]	3.04	2	2	3	3	0.41	-1.80	-1.84	-1.28
Q9D9X8-2	Isoform 2 of Sperm acrosome membrane-associated protein 3 OS=Mus musculus GN=Spaca3 - [SACA3_MOUSE]	32.52	2	2	2	4	0.29	-1.18	-1.07	-1.22
D3YUR6	Transmembrane protein 169 (Fragment) OS=Mus musculus GN=Tmem169 PE=2 SV=1 - [D3YUR6_MOUSE]	21.67	2	2	2	3	-0.70	-1.31	-0.55	-1.18
Q9QWK5	Baculoviral IAP repeat-containing protein 1a OS=Mus musculus GN=Naip1 PE=2 SV=3 - [BIR1A_MOUSE]	1.85	3	2	2	4	1.01	0.47	-0.94	-1.12
Q8K094	Poliovirus receptor OS=Mus musculus GN=Pvr PE=2 SV=1 - [Q8K094_MOUSE]	9.80	1	2	2	4	0.61	-0.24	-0.87	-1.12
P97378	Interleukin-12 receptor subunit beta-2 OS=Mus musculus GN=Il12rb2 PE=1 SV=1 - [I12R2_MOUSE]	3.20	2	2	2	3	1.58	0.14	-1.40	-1.09
Q6S5C2	N-acetylglucosamine-1-phosphotransferase subunit gamma OS=Mus musculus GN=Gnptg PE=2 SV=1 - [GNPTG_MOUSE]	10.42	2	2	2	4	0.60	-0.11	-1.31	-1.08
Q9CWF2	Tubulin beta-2B chain OS=Mus musculus GN=Tubb2b PE=1 SV=1 - [TBB2B_MOUSE]	83.82	2	2	33	4745	-0.72	-1.02	-1.26	-1.04
Q3V188	Poly(U)-specific endoribonuclease OS=Mus musculus GN=Endou PE=2 SV=1 - [ENDOU_MOUSE]	5.83	2	2	2	6	-0.53	-0.98	-0.47	-1.04
Q8R0K2	E3 ubiquitin-protein ligase TRIM31 OS=Mus musculus GN=Trim31 PE=1 SV=1 - [TRI31_MOUSE]	7.10	1	2	3	4	0.69	-0.36	-1.13	-1.03
O88566	Axin-2 OS=Mus musculus GN=Axin2 PE=1 SV=2 - [AXIN2_MOUSE]	2.14	2	2	2	2	-0.22	-0.58	-0.68	-0.93
Q5MPP0-2	Isoform 2 of Fatty acid 2-hydroxylase OS=Mus musculus GN=Fa2h - [FA2H_MOUSE]	8.63	2	2	2	6	1.08	-1.72	-1.05	-0.88
Q6NXL1	Protein Sec24d OS=Mus musculus GN=Sec24d PE=2 SV=1 - [Q6NXL1_MOUSE]	3.00	1	2	3	5	-0.65	0.17	-1.00	-0.84
G5E8Q8	MCG115189 OS=Mus musculus GN=Gpr116 PE=4 SV=1 - [G5E8Q8_MOUSE]	3.41	1	2	2	4	-0.05	0.10	-0.62	-0.82
P97864	Caspase-7 OS=Mus musculus GN=Casp7 PE=1 SV=2 - [CASP7_MOUSE]	7.92	1	2	2	3	0.52	-0.87	-0.61	-0.79
P70213	Friend virus susceptibility protein 1 OS=Mus musculus GN=Fv1 PE=2 SV=1 - [FV1_MOUSE]	7.41	1	2	2	3	-0.58	-0.35	-1.62	-0.78
Q9EQ08	Heparan N-sulfatase OS=Mus musculus GN=Sgsh PE=2 SV=1 - [Q9EQ08_MOUSE]	6.57	1	2	2	3	2.45	-1.06	-0.76	-0.73

D3Z4D1	Protein JTB OS=Mus musculus GN=Jtb PE=2 SV=1 - [D3Z4D1_MOUSE]	21.05	2	2	2	4	0.19	-0.40	-1.64	-0.71
Q9JHP7-3	Isoform 3 of KDEL motif-containing protein 1 OS=Mus musculus GN=Kdelc1 - [KDEL1_MOUSE]	9.93	4	2	2	3	1.06	-0.29	-1.21	-0.66
Q9D9Q0	Leucine-rich repeat-containing protein 69 OS=Mus musculus GN=Lrrc69 PE=2 SV=1 - [LRC69_MOUSE]	6.05	1	2	2	4	1.05	0.18	-0.53	-0.66
Q9QY40	Plexin-B3 OS=Mus musculus GN=Plxnb3 PE=1 SV=2 - [PLXB3_MOUSE]	2.73	1	2	6	23	0.01	-1.55	-0.68	-0.62
Q9DCD6	Gamma-aminobutyric acid receptor-associated protein OS=Mus musculus GN=Gabarap PE=1 SV=2 - [GBRAP_MOUSE]	39.32	2	2	6	14	0.42	-0.71	-0.56	-0.61
Q8R4P9-2	Isoform 2 of Multidrug resistance-associated protein 7 OS=Mus musculus GN=Abcc10 - [MRP7_MOUSE]	1.99	4	2	2	3	0.14	-0.40	-0.56	-0.60
Q64519	Syndecan-3 OS=Mus musculus GN=Sdc3 PE=2 SV=2 - [SDC3_MOUSE]	7.92	1	2	2	12	-1.26	-0.12	-0.67	-0.59
P41317	Mannose-binding protein C OS=Mus musculus GN=Mbl2 PE=2 SV=2 - [MBL2_MOUSE]	12.30	1	2	2	4	1.18	0.39	-1.37	-0.58
Q9D173	Mitochondrial import receptor subunit TOM7 homolog OS=Mus musculus GN=Tomm7 PE=3 SV=1 - [TOM7_MOUSE]	41.82	1	2	2	2	0.72	-0.38	-0.56	-0.53
Q9CZP0	Ufm1-specific protease 1 OS=Mus musculus GN=Ufsp1 PE=1 SV=1 - [UFSP1_MOUSE]	17.51	1	2	2	3	0.37	-0.20	-0.48	-0.50
E9Q8R5	TraB domain-containing protein OS=Mus musculus GN=Trabd PE=2 SV=1 - [E9Q8R5_MOUSE]	8.13	3	2	2	5	-0.30	-0.26	-0.60	-0.49
Q8BZX4-2	Isoform 2 of Splicing regulatory glutamine/lysine-rich protein 1 OS=Mus musculus GN=Srek1 - [SREK1_MOUSE]	4.43	2	2	2	8	-1.75	-0.36	-0.58	-0.48
Q923Q2	StAR-related lipid transfer protein 13 OS=Mus musculus GN=Stard13 PE=1 SV=5 - [STA13_MOUSE]	2.34	2	2	2	3	1.15	-0.27	-0.54	-0.48
Q62010	Oviduct-specific glycoprotein OS=Mus musculus GN=Ovgp1 PE=2 SV=1 - [OVGP1_MOUSE]	1.94	1	2	2	2	0.26	-1.03	-0.73	-0.48
Q3UMG5-2	Isoform 2 of Leucine-rich repeat and calponin homology domain-containing protein 2 OS=Mus musculus GN=Lrch2 - [LRCH2_MOUSE]	6.92	6	2	2	3	-0.23	0.52	-0.95	-0.48
Q8C407	Protein YIPF4 OS=Mus musculus GN=Yipf4 PE=2 SV=1 - [YIPF4_MOUSE]	8.13	2	2	2	6	-0.47	-0.23	-0.62	-0.47
O08738	Caspase-6 OS=Mus musculus GN=Casp6 PE=2 SV=1 - [CASP6_MOUSE]	7.25	1	2	2	3	0.55	0.65	-0.55	-0.45

P05366	Serum amyloid A-1 protein OS=Mus musculus GN=Saa1 PE=2 SV=2 - [SAA1_MOUSE]	27.05	1	2	3	15	0.62	-0.20	3.67	4.87
Q8R366	Immunoglobulin superfamily member 8 OS=Mus musculus GN=Igsf8 PE=1 SV=2 - [IGSF8_MOUSE]	35.68	2	2	17	151	1.33	2.96	2.76	3.72
Q9QXF8	Glycine N-methyltransferase OS=Mus musculus GN=Gnmt PE=1 SV=3 - [GNMT_MOUSE]	12.63	1	2	2	14	1.98	0.16	2.58	3.26
B6VJS2	5-hydroxytryptamine receptor 7 OS=Mus musculus GN=Htr7 PE=2 SV=1 - [B6VJS2_MOUSE]	3.68	3	2	2	3	0.79	-0.73	2.42	3.15
Q8K4J0-3	Isoform 3 of Protein artemis OS=Mus musculus GN=Dclre1c - [DCR1C_MOUSE]	6.98	5	2	3	6	0.23	3.86	4.36	2.88
A2AKN9	Major urinary protein 2 OS=Mus musculus GN=Mup2 PE=2 SV=1 - [A2AKN9_MOUSE]	53.89	8	2	8	26	1.85	1.00	1.78	2.44
P16331	Phenylalanine-4-hydroxylase OS=Mus musculus GN=Pah PE=1 SV=4 - [PH4H_MOUSE]	4.19	2	2	2	3	-0.29	0.11	1.19	1.71
E9QAK1	Protein 2810007J24Rik (Fragment) OS=Mus musculus GN=2810007J24Rik PE=2 SV=1 - [E9QAK1_MOUSE]	13.76	2	2	2	4	0.48	0.25	0.95	1.63
P12399	Protein CTLA-2-alpha OS=Mus musculus GN=Ctla2a PE=2 SV=2 - [CTL2A_MOUSE]	18.25	2	2	2	4	1.43	0.14	1.71	1.59
Q8CB59-2	Isoform 2 of Protein FAM161B OS=Mus musculus GN=Fam161b - [F161B_MOUSE]	5.54	2	2	2	2	0.69	1.75	0.92	1.56
Q8BIR2-2	Isoform 2 of Protein asteroid homolog 1 OS=Mus musculus GN=Aste1 - [ASTE1_MOUSE]	5.24	4	2	4	7	0.07	-2.57	0.88	1.44
Q9D8M3	Heme transporter HRG1 OS=Mus musculus GN=Slc48a1 PE=2 SV=1 - [HRG1_MOUSE]	12.33	2	2	2	2	2.03	0.56	0.79	1.44
Q9DBT9	Dimethylglycine dehydrogenase, mitochondrial OS=Mus musculus GN=Dmgdh PE=1 SV=1 - [M2GD_MOUSE]	3.11	1	2	2	3	0.28	-0.69	1.09	1.35
E0CZG7	Glycerate kinase (Fragment) OS=Mus musculus GN=Glyctk PE=2 SV=1 - [E0CZG7_MOUSE]	20.63	3	2	2	4	0.20	-1.53	1.72	1.25
Q64332	Synapsin-2 OS=Mus musculus GN=Syn2 PE=1 SV=2 - [SYN2_MOUSE]	58.02	1	2	26	740	-0.71	2.15	1.62	1.23
Q6TL19	Guanylate cyclase 2G OS=Mus musculus GN=Gucy2g PE=1 SV=1 - [GUC2G_MOUSE]	2.64	1	2	3	5	1.87	0.61	1.03	1.22
P70689	Gap junction beta-6 protein OS=Mus musculus GN=Gjb6 PE=1 SV=1 - [CXB6_MOUSE]	10.73	2	2	2	3	-0.16	0.01	0.73	1.21

P35487	Pyruvate dehydrogenase E1 component subunit alpha, testis-specific form, mitochondrial OS=Mus musculus GN=Pdha2 PE=2 SV=1 - [ODPAT_MOUSE]	8.95	1	2	4	26	0.42	0.49	0.94	1.19
P27005	Protein S100-A8 OS=Mus musculus GN=S100a8 PE=1 SV=3 - [S10A8_MOUSE]	46.07	1	2	2	4	1.89	1.06	0.75	1.14
P06683	Complement component C9 OS=Mus musculus GN=C9 PE=1 SV=2 - [CO9_MOUSE]	3.65	2	2	2	6	1.74	0.98	0.72	1.12
Q8C838	Tumor suppressor candidate 5 homolog OS=Mus musculus GN=Tusc5 PE=2 SV=1 - [TUSC5_MOUSE]	17.34	1	2	2	3	0.34	-0.34	0.96	0.97
P43136	Nuclear receptor subfamily 2 group F member 6 OS=Mus musculus GN=Nr2f6 PE=1 SV=2 - [NR2F6_MOUSE]	11.03	3	2	2	6	-0.51	-0.93	1.22	0.94
F6ZAW1	Protein Cald1 (Fragment) OS=Mus musculus GN=Cald1 PE=4 SV=1 - [F6ZAW1_MOUSE]	35.07	2	2	9	36	-1.13	1.45	0.85	0.93
P58660	Caspase recruitment domain-containing protein 10 OS=Mus musculus GN=Card10 PE=2 SV=1 - [CAR10_MOUSE]	4.31	2	2	4	6	4.75	0.82	0.99	0.91
Q6A044-2	Isoform 2 of Protein FAM189A1 OS=Mus musculus GN=Fam189a1 - [F1891_MOUSE]	20.86	2	2	2	3	1.19	1.03	1.57	0.83
D3Z2N8	Coiled-coil domain-containing protein 67 (Fragment) OS=Mus musculus GN=Ccdc67 PE=2 SV=1 - [D3Z2N8_MOUSE]	6.05	3	2	4	11	0.83	0.09	0.59	0.80
Q921C1	Gap junction gamma-3 protein OS=Mus musculus GN=Gjc3 PE=2 SV=2 - [CXG3_MOUSE]	8.92	1	2	2	4	-0.29	-1.78	0.56	0.79
Q3UZP4	Small VCP/p97-interacting protein OS=Mus musculus GN=Svip PE=2 SV=1 - [SVIP_MOUSE]	32.47	1	2	2	27	0.67	-0.07	0.79	0.78
Q60773	Cyclin-dependent kinase 4 inhibitor D OS=Mus musculus GN=Cdkn2d PE=1 SV=2 - [CDN2D_MOUSE]	18.67	1	2	2	3	1.06	0.12	1.12	0.78
Q8BLR5	PH and SEC7 domain-containing protein 4 OS=Mus musculus GN=Psd4 PE=2 SV=1 - [PSD4_MOUSE]	4.88	1	2	3	17	-0.49	2.79	0.69	0.72
P03911	NADH-ubiquinone oxidoreductase chain 4 OS=Mus musculus GN=Mtnd4 PE=1 SV=1 - [NU4M_MOUSE]	4.79	1	2	2	13	-0.22	-0.05	0.58	0.71
G3X919	T-box transcription factor TBX18 OS=Mus musculus GN=Tbx18 PE=4 SV=1 - [G3X919_MOUSE]	5.22	2	2	2	2	0.57	1.04	0.69	0.69
B2RPV6	Multimerin-1 OS=Mus musculus GN=Mmrn1 PE=2 SV=2 - [MMRN1_MOUSE]	4.63	3	2	4	17	-0.38	-0.17	0.66	0.69

P57725	SAM domain-containing protein SAMSN-1 OS=Mus musculus GN=Samsn1 PE=1 SV=2 - [SAMN1_MOUSE]	8.33	2	2	2	2	1.88	2.17	0.71	0.66
Q6X7S9	EP300-interacting inhibitor of differentiation 2 OS=Mus musculus GN=Eid2 PE=1 SV=1 - [EID2_MOUSE]	9.75	1	2	2	4	0.81	-0.64	1.21	0.66
Q9CZT5	Vasorin OS=Mus musculus GN=Vasn PE=2 SV=2 - [VASN_MOUSE]	6.09	1	2	2	3	0.59	-0.41	0.68	0.65
Q99PW8	Kinesin-like protein KIF17 OS=Mus musculus GN=Kif17 PE=1 SV=1 - [KIF17_MOUSE]	4.43	4	2	3	4	0.61	-0.11	0.81	0.64
Q8CBX9	Basic immunoglobulin-like variable motif-containing protein OS=Mus musculus GN=Bivm PE=2 SV=1 - [BIVM_MOUSE]	4.98	1	2	2	2	0.40	1.01	0.54	0.62
Q00519	Xanthine dehydrogenase/oxidase OS=Mus musculus GN=Xdh PE=1 SV=5 - [XDH_MOUSE]	2.70	2	2	4	10	1.01	0.90	0.65	0.61
Q9JJ94	Sjoegren syndrome nuclear autoantigen 1 homolog OS=Mus musculus GN=Ssna1 PE=1 SV=1 - [SSNA1_MOUSE]	23.53	1	2	2	6	3.20	1.51	1.37	0.60
Q8R1M0	UPF0361 protein C3orf37 homolog OS=Mus musculus PE=2 SV=1 - [CC037_MOUSE]	6.52	1	2	2	5	2.27	0.16	0.64	0.59
J3KMT3	Protein Gm6882 OS=Mus musculus GN=Gm6882 PE=4 SV=1 - [J3KMT3_MOUSE]	10.86	1	2	2	3	-0.24	0.84	0.87	0.59
P57016	Ladinin-1 OS=Mus musculus GN=Lad1 PE=1 SV=1 - [LAD1_MOUSE]	5.68	1	2	2	3	-0.52	-0.11	0.67	0.58
Q8BHW4	Protein 4933430I17Rik OS=Mus musculus GN=4933430I17Rik PE=2 SV=1 - [Q8BHW4_MOUSE]	5.25	1	2	2	2	0.38	0.26	0.99	0.58
Q8R100	Protein FAM26E OS=Mus musculus GN=Fam26e PE=2 SV=1 - [FA26E_MOUSE]	10.68	1	2	2	3	-0.17	1.08	0.61	0.58
Q8BH02	Torsin-4A OS=Mus musculus GN=Tor4a PE=2 SV=1 - [TOR4A_MOUSE]	9.62	1	2	2	2	0.58	-2.05	0.70	0.57
P98086	Complement C1q subcomponent subunit A OS=Mus musculus GN=C1qa PE=1 SV=2 - [C1QA_MOUSE]	10.20	1	2	2	13	-0.07	0.83	0.71	0.57
A6PWR8	Ubiquitin carboxyl-terminal hydrolase 43 OS=Mus musculus GN=Usp43 PE=2 SV=1 - [A6PWR8_MOUSE]	2.31	3	2	3	3	0.85	1.30	0.70	0.56
P61226	Ras-related protein Rap-2b OS=Mus musculus GN=Rap2b PE=1 SV=1 - [RAP2B_MOUSE]	50.82	1	2	7	51	-0.57	0.81	0.62	0.56
F6VUT6	Protein Gm8251 OS=Mus musculus GN=Gm8251 PE=4 SV=1 - [F6VUT6_MOUSE]	2.16	1	2	3	5	0.68	0.35	0.52	0.56

Q8VEH6	COBW domain-containing protein 1 OS=Mus musculus GN=Cbwd1 PE=2 SV=1 - [CBWD1_MOUSE]	7.38	2	2	2	6	0.73	0.54	0.64	0.55
E9Q800	Mitochondrial inner membrane protein OS=Mus musculus GN=Immt PE=2 SV=1 - [E9Q800_MOUSE]	68.63	2	2	51	290	0.42	0.67	0.90	0.53
Q8BVL9	Janus kinase and microtubule-interacting protein 1 OS=Mus musculus GN=Jakmip1 PE=1 SV=2 - [JKIP1_MOUSE]	34.82	5	2	21	77	1.18	0.90	1.02	0.52
Q52KR3	Protein prune homolog 2 OS=Mus musculus GN=Prune2 PE=2 SV=2 - [PRUN2_MOUSE]	6.65	4	2	14	47	0.38	0.72	0.53	0.50
E9Q166	Protein Atad2b OS=Mus musculus GN=Atad2b PE=2 SV=1 - [E9Q166_MOUSE]	2.40	1	2	3	3	-0.15	-0.09	0.52	0.50
Q61496	Probable ATP-dependent RNA helicase DDX4 OS=Mus musculus GN=Ddx4 PE=1 SV=2 - [DDX4_MOUSE]	7.98	2	2	5	20	0.80	1.09	0.51	0.48
Q4KMS1	Tripartite motif-containing 44 OS=Mus musculus GN=Trim44 PE=2 SV=1 - [Q4KMS1_MOUSE]	12.75	3	2	2	4	0.99	0.73	0.71	0.48
Q80VA0	N-acetylgalactosaminyltransferase 7 OS=Mus musculus GN=Galnt7 PE=2 SV=2 - [GALT7_MOUSE]	3.81	3	2	2	2	-0.61	-2.22	0.76	0.48
