# Supplementary material for: Hypothalamus proteomics from mouse models with obesity and anorexia reveals therapeutic targets of appetite regulation
Source: Nutr Diabetes. 2016 Apr 25;6(4):e204–. doi: 10.1038/nutd.2016.10 (PMC4855256; doi:10.1038/nutd.2016.10)
Supplement: Supplementary Table 6 [file nutd201610x9.pdf]

**Supplementary Table 6.** Common differentially expressed proteins in the HFD and LPS groups compared to controls.

| Accession | Description | ΣCoverage | Σ# Proteins | Σ# Unique Peptides | Σ# Peptides | Σ# PSMs | HFD1/C1 | HFD2/C1 | LPS1/C2 | LPS2/C2 |
|---|---|---|---|---|---|---|---|---|---|---|
| Q9JJ94 | Sjoegren syndrome nuclear autoantigen 1 homolog OS=Mus musculus GN=Ssna1 PE=1 SV=1 - [SSNA1_MOUSE] | 23.53 | 1 | 2 | 2 | 6 | 3.20 | 1.51 | 1.37 | 0.60 |
| Q9D8H7 | Metalloendopeptidase OMA1, mitochondrial OS=Mus musculus GN=Oma1 PE=2 SV=1 - [OMA1_MOUSE] | 5.18 | 1 | 3 | 3 | 3 | 1.01 | 0.83 | 0.68 | 1.45 |
| Q9D2P8 | Myelin-associated oligodendrocyte basic protein OS=Mus musculus GN=Mobp PE=2 SV=1 - [MOBP_MOUSE] | 42.94 | 5 | 8 | 8 | 62 | -0.75 | -1.32 | 1.12 | 1.34 |
| Q9CXL6 | Doublecortin, isoform CRA_a OS=Mus musculus GN=Dcx PE=2 SV=1 - [Q9CXL6_MOUSE] | 30.56 | 3 | 7 | 11 | 44 | -0.75 | -0.88 | -0.71 | -1.32 |
| Q9CWF2 | Tubulin beta-2B chain OS=Mus musculus GN=Tubb2b PE=1 SV=1 - [TBB2B_MOUSE] | 83.82 | 2 | 2 | 33 | 4745 | -0.72 | -1.02 | -1.26 | -1.04 |
| Q9CQX2 | Cytochrome b5 type B OS=Mus musculus GN=Cyb5b PE=1 SV=1 - [CYB5B_MOUSE] | 32.19 | 1 | 5 | 5 | 27 | 0.91 | 1.07 | 0.87 | 0.45 |
| Q9CPQ0 | Prolactin OS=Mus musculus GN=Prl PE=2 SV=2 - [Q9CPQ0_MOUSE] | 24.00 | 3 | 6 | 6 | 34 | -0.81 | -1.16 | -3.08 | -3.27 |
| Q99K47 | Fibrinogen, alpha polypeptide OS=Mus musculus GN=Fga PE=2 SV=1 - [Q99K47_MOUSE] | 49.55 | 2 | 22 | 23 | 134 | 0.92 | 0.76 | 0.56 | 1.56 |
| Q91XL1 | Leucine-rich HEV glycoprotein (Precursor) OS=Mus musculus GN=Lrg1 PE=2 SV=1 - [Q91XL1_MOUSE] | 23.68 | 1 | 7 | 8 | 30 | 2.00 | 1.44 | 1.27 | 2.23 |
| Q8R4E6 | Purine-rich element-binding protein gamma OS=Mus musculus GN=Purg PE=1 SV=1 - [PURG_MOUSE] | 27.71 | 2 | 9 | 13 | 33 | -1.18 | -1.55 | -0.77 | -1.04 |
| Q8R366 | Immunoglobulin superfamily member 8 OS=Mus musculus GN=Igsf8 PE=1 SV=2 - [IGSF8_MOUSE] | 35.68 | 2 | 2 | 17 | 151 | 1.33 | 2.96 | 2.76 | 3.72 |
| Q8CII2 | Cell division cycle protein 123 homolog OS=Mus musculus GN=Cdc123 PE=2 SV=2 - [CD123_MOUSE] | 11.31 | 2 | 3 | 4 | 16 | 0.89 | 1.14 | 0.91 | 0.59 |

| | | | | | | | | | |
|---|---|---|---|---|---|---|---|---|---|
| Q8C4C4 | Repulsive guidance molecule A OS=Mus musculus GN=Rgma PE=2 SV=1 - [Q8C4C4_MOUSE] | 35.17 | 3 | 10 | 10 | 19 | -0.67 | -0.90 | -0.76 | -0.51 |
| Q8BVL9 | Janus kinase and microtubule-interacting protein 1 OS=Mus musculus GN=Jakmip1 PE=1 SV=2 - [JKIP1_MOUSE] | 34.82 | 5 | 2 | 21 | 77 | 1.18 | 0.90 | 1.02 | 0.52 |
| Q80XN0 | D-beta-hydroxybutyrate dehydrogenase, mitochondrial OS=Mus musculus GN=Bdh1 PE=1 SV=2 - [BDH_MOUSE] | 55.10 | 2 | 15 | 17 | 189 | -0.73 | -0.91 | -0.65 | -0.68 |
| Q80VA0 | N-acetylgalactosaminyltransferase 7 OS=Mus musculus GN=Galnt7 PE=2 SV=2 - [GALT7_MOUSE] | 3.81 | 3 | 2 | 2 | 2 | -0.61 | -2.22 | 0.76 | 0.48 |
| Q80SZ7 | Guanine nucleotide-binding protein G(I)/G(S)/G(O) subunit gamma-5 OS=Mus musculus GN=Gng5 PE=2 SV=2 - [GBG5_MOUSE] | 33.82 | 2 | 3 | 3 | 12 | 0.99 | 0.68 | 0.54 | 0.55 |
| Q7TQE7-2 | Isoform 2 of Uncharacterized protein KIAA0895 OS=Mus musculus GN=Kiaa0895 - [K0895_MOUSE] | 10.55 | 2 | 2 | 2 | 2 | 4.21 | 2.35 | -1.41 | -2.66 |
| Q6A044-2 | Isoform 2 of Protein FAM189A1 OS=Mus musculus GN=Fam189a1 - [F1891_MOUSE] | 20.86 | 2 | 2 | 2 | 3 | 1.19 | 1.03 | 1.57 | 0.83 |
| Q61646 | Haptoglobin OS=Mus musculus GN=Hp PE=1 SV=1 - [HPT_MOUSE] | 34.01 | 1 | 12 | 12 | 34 | 1.22 | 1.59 | 2.86 | 3.23 |
| Q61282 | Aggrecan core protein OS=Mus musculus GN=Acan PE=1 SV=2 - [PGCA_MOUSE] | 13.56 | 1 | 21 | 22 | 73 | 1.89 | 0.67 | 1.12 | 0.72 |
| Q60590 | Alpha-1-acid glycoprotein 1 OS=Mus musculus GN=Orm1 PE=1 SV=1 - [A1AG1_MOUSE] | 20.77 | 1 | 3 | 5 | 10 | 2.36 | 1.75 | 1.66 | 2.19 |
| Q4KMS1 | Tripartite motif-containing 44 OS=Mus musculus GN=Trim44 PE=2 SV=1 - [Q4KMS1_MOUSE] | 12.75 | 3 | 2 | 2 | 4 | 0.99 | 0.73 | 0.71 | 0.48 |
| Q3UX37 | Protein Plekhg1 OS=Mus musculus GN=Plekhg1 PE=2 SV=1 - [Q3UX37_MOUSE] | 18.27 | 4 | 22 | 23 | 52 | -0.79 | -1.44 | -0.68 | -0.61 |

| | | | | | | | | | | |
|---|---|---|---|---|---|---|---|---|---|---|
| Q00519 | Xanthine dehydrogenase/oxidase OS=Mus musculus GN=Xdh PE=1 SV=5 - [XDH_MOUSE] | 2.70 | 2 | 2 | 4 | 10 | 1.01 | 0.90 | 0.65 | 0.61 |
| P84228 | Histone H3.2 OS=Mus musculus GN=Hist1h3b PE=1 SV=2 - [H32_MOUSE] | 59.56 | 2 | 3 | 11 | 54 | -1.09 | -1.88 | -1.75 | -1.67 |
| P70121 | Zinc fingers and homeoboxes protein 1 OS=Mus musculus GN=Zhx1 PE=1 SV=2 - [ZHX1_MOUSE] | 5.04 | 2 | 3 | 4 | 7 | 1.32 | 1.32 | 1.20 | 1.20 |
| P63276 | 40S ribosomal protein S17 OS=Mus musculus GN=Rps17 PE=1 SV=2 - [RS17_MOUSE] | 47.41 | 1 | 5 | 5 | 29 | -1.49 | -0.66 | -0.58 | -0.54 |
| P62830 | 60S ribosomal protein L23 OS=Mus musculus GN=Rpl23 PE=1 SV=1 - [RL23_MOUSE] | 71.43 | 2 | 10 | 10 | 36 | -1.08 | -0.79 | -0.63 | -0.50 |
| P58660 | Caspase recruitment domain-containing protein 10 OS=Mus musculus GN=Card10 PE=2 SV=1 - [CAR10_MOUSE] | 4.31 | 2 | 2 | 4 | 6 | 4.75 | 0.82 | 0.99 | 0.91 |
| P57725 | SAM domain-containing protein SAMSN-1 OS=Mus musculus GN=Samsn1 PE=1 SV=2 - [SAMN1_MOUSE] | 8.33 | 2 | 2 | 2 | 2 | 1.88 | 2.17 | 0.71 | 0.66 |
| P52760 | Ribonuclease UK114 OS=Mus musculus GN=Hrsp12 PE=1 SV=3 - [UK114_MOUSE] | 92.59 | 1 | 9 | 11 | 87 | 1.19 | 0.70 | 1.10 | 0.78 |
| P43276 | Histone H1.5 OS=Mus musculus GN=Hist1h1b PE=1 SV=2 - [H15_MOUSE] | 38.57 | 1 | 7 | 13 | 68 | -0.91 | -0.92 | -0.61 | -0.82 |
| P31725 | Protein S100-A9 OS=Mus musculus GN=S100a9 PE=1 SV=3 - [S10A9_MOUSE] | 38.94 | 1 | 4 | 4 | 12 | 1.48 | 1.28 | 0.53 | 0.98 |
| P29788 | Vitronectin OS=Mus musculus GN=Vtn PE=1 SV=2 - [VTNC_MOUSE] | 11.09 | 1 | 4 | 5 | 13 | 1.22 | 0.86 | 0.52 | 0.91 |
| P28867 | Protein kinase C delta type OS=Mus musculus GN=Prkcd PE=1 SV=3 - [KPCD_MOUSE] | 39.17 | 6 | 21 | 24 | 93 | -0.65 | -1.60 | -0.73 | -0.63 |
| P27005 | Protein S100-A8 OS=Mus musculus GN=S100a8 PE=1 SV=3 - [S10A8_MOUSE] | 46.07 | 1 | 2 | 2 | 4 | 1.89 | 1.06 | 0.75 | 1.14 |
| P24529 | Tyrosine 3-monooxygenase OS=Mus musculus GN=Th PE=1 SV=3 - [TY3H_MOUSE] | 39.96 | 5 | 15 | 15 | 55 | -1.06 | -1.38 | 1.26 | 0.89 |

| | | | | | | | | | | |
|---|---|---|---|---|---|---|---|---|---|---|
| P16015 | Carbonic anhydrase 3 OS=Mus musculus GN=Ca3 PE=1 SV=3 - [CAH3_MOUSE] | 48.08 | 1 | 10 | 10 | 35 | 1.44 | 1.49 | 1.20 | 2.08 |
| P07724 | Serum albumin OS=Mus musculus GN=Alb PE=1 SV=3 - [ALBU_MOUSE] | 75.49 | 4 | 49 | 49 | 2294 | 1.97 | 0.72 | -1.29 | -0.51 |
| P06880 | Somatotropin OS=Mus musculus GN=Gh1 PE=2 SV=1 - [SOMA_MOUSE] | 65.74 | 1 | 16 | 17 | 303 | -0.93 | -1.18 | -1.87 | -1.83 |
| P06683 | Complement component C9 OS=Mus musculus GN=C9 PE=1 SV=2 - [CO9_MOUSE] | 3.65 | 2 | 2 | 2 | 6 | 1.74 | 0.98 | 0.72 | 1.12 |
| P05367 | Serum amyloid A-2 protein OS=Mus musculus GN=Saa2 PE=1 SV=1 - [SAA2_MOUSE] | 28.69 | 1 | 3 | 4 | 12 | 0.93 | 0.89 | 3.45 | 4.60 |
| O89020 | Afamin OS=Mus musculus GN=Afm PE=1 SV=2 - [AFAM_MOUSE] | 16.78 | 3 | 9 | 9 | 18 | 1.65 | 0.63 | -1.78 | -0.45 |
