# Supplementary material for: Hypothalamus proteomics from mouse models with obesity and anorexia reveals therapeutic targets of appetite regulation
Source: Nutr Diabetes. 2016 Apr 25;6(4):e204–. doi: 10.1038/nutd.2016.10 (PMC4855256; doi:10.1038/nutd.2016.10)
Supplement: Supplementary Table 3 [file nutd201610x6.pdf]

Supplementary Table 3. Total Peptides

Sequence	PSM Ambiguity	# Proteins	# Protein Groups	Protein Group Accessions	Modifications	Activation Type	ΔScore	ΔCn	Rank	Search Engine Rank	QuanResultID	Quan Info	Quan Usage	HFD1/C1	HFD2/C1	LPS1/C2	LPS2/C2	q-Value	PEP	XCorr	# Missed Cleavages	Isolation Interference [%]	Ion Inject Time [ms]	Charge	m/z [Da]	MH+ [Da]	ΔM [ppm]
rEQGSSGLGSGSSGGGGSSSGLGSGYIGR	Unambiguous	2	1	Q3UHJ0-2	N-Term(iTRAQ8plex)	CID	1.0000	0.0000	1	1	3468847	Unique	Used	0.894	1.031	1.122	1.049	0	0	11.75	1	0	162	3	954.79706	2862.37662	-4.00
tQSSASLAASYAAQQHPQAAASYR	Unambiguous	3	1	Q8C2Q3	N-Term(iTRAQ8plex)	CID	1.0000	0.0000	1	1	3656987	Unique	Used	1.279	**0.632**	1.250	1.022	0	0	10.36	0	0	145	3	923.79462	2769.36930	-5.97
sLAGSSGPGASSGPGGDHSELIVR	Unambiguous	5	1	P57776-3	N-Term(iTRAQ8plex)	HCD	1.0000	0.0000	1	1	3207381	Unique	Used	**1.957**	0.874	0.973	0.775	0	0	10.23	0	16	136	3	833.76154	2499.27005	-1.66
lLEATSGSGGAAGTAGGAHDLSSTAR	Unambiguous	2	1	E9PX29	N-Term(iTRAQ8plex)	CID	1.0000	0.0000	1	1	3317995	Unique	Used	0.703	0.931	1.105	1.209	0	0	10.16	0	11	67	3	873.77673	2619.31565	-4.60
aQAALDSVSGLEqGHTQYLASSEDASALVAALTR	Unambiguous	1	1	Q9JKY5	N-Term(iTRAQ8plex); Q13(Deamidated)	CID	0.0380	0.0000	1	1	3623155	Unique	Used	0.667	0.892	0.786	0.861	0	0	9.99	0	0	21	3	1245.97412	3735.90781	2.62
hQGGWTDGGSGSGGGYQDGAYR	Unambiguous	1	1	Q3TJZ6	N-Term(iTRAQ8plex)	CID	1.0000	0.0000	1	1	2773464	Unique	Used	**1.609**	**0.478**	0.678	**0.642**	0	0	9.91	0	4	152	3	825.36981	2474.09488	-3.01
sLAGSSGPGASSGPGGDHSELIVR	Unambiguous	5	1	P57776-3	N-Term(iTRAQ8plex)	HCD	1.0000	0.0000	1	1	3184795	Unique	Used	**2.070**	0.893	1.009	0.959	0	0	9.87	0	0	69	3	833.76123	2499.26914	-2.02
iSAEDLIDLcELTVTGHFk	Unambiguous	1	1	E9Q1W7	N-Term(iTRAQ8plex); C10(Methylthio); K19(iTRAQ8plex)	CID	1.0000	0.0000	1	1	3561439	Unique	Used	**0.319**	0.724	0.853	1.017	0	0	9.77	0	14	60	3	920.15381	2758.44687	-5.35
fEAPEALFqPHLINVEGVGVAELLFNTIQAADIDTR	Unambiguous	1	1	P61161	N-Term(iTRAQ8plex); Q9(Deamidated)	CID	1.0000	0.0000	1	1	3727641	Unique	Used		**2.163**	1.375	**1.518**	0	0	9.73	0	22	134	3	1415.07776	4243.21872	-1.01
qHGEQLGGGGSGGGGYNTSk	Unambiguous	7	1	Q80U78-2	N-Term(iTRAQ8plex); K20(iTRAQ8plex)	CID	1.0000	0.0000	1	1	2841177	Unique	Used	0.772	0.925	1.421	1.008	0	0	9.71	0	0	200	3	819.41321	2456.22507	-5.06
vADVSISEVTTLLGnVHSPVVEGYVGENISGEVk	Unambiguous	3	1	P20357	N-Term(iTRAQ8plex); N15(Deamidated); K34(iTRAQ8plex)	CID	1.0000	0.0000	1	1	3418951	Unique	Used	1.344	**1.786**	**1.848**	1.285	0	0	9.68	0	0	19	3	1369.73828	4107.20029	1.07
gYGHGQGSYSSYSNSYnSPGGGGGSDYSYDSk	Unambiguous	2	1	Q9Z1X4	N-Term(iTRAQ8plex); N17(Deamidated); K32(iTRAQ8plex)	CID	0.0176	0.0000	1	1	2844997	Unique	Used	1.251	**0.466**	1.415	0.677	0	0	9.67	0	0	200	3	1284.56738	3851.68760	2.23

rEQGSSGLGSGSSGGGGSSSGLGSGYIGR	Unambiguous	2	1	Q3UHJ0-2	N-Term(iTRAQ8plex)	CID	1.0000	0.0000	1	1	3468992	Unique	Used	0.790	**1.691**	**1.597**	**1.964**	0	0	9.65	1	2	45	3	954.79810	2862.37973	-2.91
sNHEEEVADLLAQIQASHITVER	Unambiguous	2	1	P08553	N-Term(iTRAQ8plex)	CID	1.0000	0.0000	1	1	3260315	Unique	Used	**4.846**	**0.583**	1.152	0.810	0	0	9.62	0	3	12	3	965.16724	2893.48716	-2.99
qVLGQMVIDEELLGDGHSYSPR	Unambiguous	1	1	P99026	N-Term(iTRAQ8plex)	CID	1.0000	0.0000	1	1	3238371	Unique	Used	**1.717**	0.778	**0.660**	0.711	0	0	9.60	0	1	1	3	916.47003	2747.39554	-0.78
rPAASSAAAASAAASSPAGGGGEAQELLEHcGVcR	Unambiguous	2	1	Q62318	N-Term(iTRAQ8plex); C31(Methylthio); C34(Methylthio)	CID	1.0000	0.0000	1	1	3684294	Unique	Used	0.850	**1.635**			0	0	9.51	0	0	70	3	1202.88904	3606.65256	-8.29
gTVAAAASGAAGGGGGGAGAGAPGGGR	Unambiguous	2	1	Q7TPD0	N-Term(iTRAQ8plex)	CID	1.0000	0.0000	1	1	3611681	Unique	Used	**0.622**	0.757	0.734	0.791	0	0	9.40	0	3	72	2	1122.56775	2244.12822	-4.54
dMAIATGGAVFGEEGLNLnLEDVQAHDLGk	Unambiguous	1	1	P63038	N-Term(iTRAQ8plex); N19(Deamidated); K30(iTRAQ8plex)	CID	0.0340	0.0000	1	1	3264063	Unique	Used	**1.544**	0.686	0.821	0.695	0	0	9.40	0	0	200	3	1231.97266	3693.90342	3.86
tNHSGFLFGFGDGGGGPTQTMLDR	Unambiguous	4	1	F8WIE1	N-Term(iTRAQ8plex)	CID	1.0000	0.0000	1	1	3643363	Unique	Used	0.939	1.030	1.109	**1.566**	0	0	9.38	0	15	36	3	925.11115	2773.31888	-4.25
gNFGGSFAGSFGGAGGHAPGVAR	Unambiguous	4	1	Q9D0E1-2	N-Term(iTRAQ8plex)	CID	1.0000	0.0000	1	1	3911445	Unique	Used	1.086	0.859	0.893	1.094	0	0	9.38	0	9	91	3	780.38647	2339.14487	-5.78
inGIFEQLGAYIQESASFLEDTHR	Unambiguous	4	1	Q80U63	N-Term(iTRAQ8plex); N2(Deamidated)	CID	1.0000	0.0000	1	1	3602782	Unique	Used	**0.250**	**1.527**	1.061	1.238	0	0	9.38	0	0	38	3	1015.17780	3043.51883	-4.17
aEVEEFSFITQLSGLTDHLPVAMR	Unambiguous	1	1	Q9JJY3	N-Term(iTRAQ8plex)	CID	1.0000	0.0000	1	1	3686939	Unique	Used	**0.569**	0.772	1.418	1.364	0	0	9.33	0	3	15	3	998.85004	2994.53556	-6.47
dMAIATGGAVFGEEGLNLNLEDVQAHDLGk	Unambiguous	1	1	P63038	N-Term(iTRAQ8plex); K30(iTRAQ8plex)	CID	1.0000	0.0000	1	1	3264241	Unique	Used	1.209	0.704	1.241	1.216	0	0	9.30	0	7	200	3	1231.63379	3692.88681	-4.97
rEQGSSGLGSGSSGGGGSSSGLGSGYIGR	Unambiguous	2	1	Q3UHJ0-2	N-Term(iTRAQ8plex)	CID	1.0000	0.0000	1	1	3612059	Unique	Used	**0.511**	**1.596**	1.176	1.419	0	0	9.29	1	0	176	3	954.79547	2862.37186	-5.66
hQGGWTDGGSGSGGGYQDGAYR	Unambiguous	1	1	Q3TJZ6	N-Term(iTRAQ8plex)	CID	1.0000	0.0000	1	1	2789314	Unique	Used	1.428	0.673	0.899	0.731	0	0	9.28	0	0	200	3	825.37152	2474.10001	-0.94
iSFEEFcAVVGGLDIHk	Unambiguous	2	1	Q63810-2	N-Term(iTRAQ8plex); C7(Methylthio); K17(iTRAQ8plex)	CID	1.0000	0.0000	1	1	3689794	Unique	Used	0.951	**1.592**	**2.431**	0.882	0	0	9.27	0	0	200	3	840.11041	2518.31668	-5.08

vADALANAAGHLDDLPGALSALSDLHAHk	Unambiguous	1	1	Q91VB8	N-Term(iTRAQ8plex); K29(iTRAQ8plex)	CID	1.0000	0.0000	1	1	3665859	Unique	Used	3.925	3.748	0.567	1.505	0	0	9.23	0	14	13	3	1157.95593	3471.85324	-7.87
tTTVVNVEGDALGAGILNHLNQk	Unambiguous	2	1	O35874	N-Term(iTRAQ8plex); K23(iTRAQ8plex)	CID	1.0000	0.0000	1	1	3539108	Unique	Used	0.545	0.876	0.831	1.106	0	0	9.20	0	20	34	3	991.55524	2972.65116	-3.85
iSFEEFcAVVGGLDIHk	Unambiguous	2	1	Q63810-2	N-Term(iTRAQ8plex); C7(Methylthio); K17(iTRAQ8plex)	CID	1.0000	0.0000	1	1	3687297	Unique	Used	0.695	1.476	2.055	0.788	0	0	9.20	0	14	9	3	840.10999	2518.31540	-5.59
nMGGPYGGGNYGPGGSGGSGGYGGR	Unambiguous	3	1	O88569	N-Term(iTRAQ8plex)	CID	1.0000	0.0000	1	1	3316656	Unique	Used	2.219	0.800	1.098	0.870	0	0	9.19	0	0	9	2	1247.55457	2494.10185	-3.60
eFSVQSLTSVVnEEGFYETQSHGk	Unambiguous	3	1	Q6ZPF3-3	N-Term(iTRAQ8plex); N12(Deamidated); K24(iTRAQ8plex)	CID	1.0000	0.0000	1	1	3215696	Unique	Used	0.739	1.937	2.653	0.682	0	0	9.19	0	0	60	3	1104.56055	3311.66709	4.30
aTLQEVVGLHSDMAVEAGAPYAER	Unambiguous	1	1	G3UYZ1	N-Term(iTRAQ8plex)	CID	1.0000	0.0000	1	1	3281190	Unique	Used	0.658	0.117	1.048	0.093	0	0	9.16	0	0	12	3	940.14606	2818.42362	-3.97
sLAGSSGPGASSGPGGDHSELIVR	Unambiguous	5	1	P57776-3	N-Term(iTRAQ8plex)	HCD	1.0000	0.0000	1	1	3316909	Unique	Used	1.152	0.839	0.847	0.785	0	0	9.15	0	10	98	3	833.75916	2499.26291	-4.51
yAEDIFGELFNEAHSFSFR	Unambiguous	1	1	Q8R5H6	N-Term(iTRAQ8plex)	CID	1.0000	0.0000	1	1	3541553	Unique	Used	0.901	2.308	1.439	1.092	0	0	9.13	0	2	7	3	861.75079	2583.23783	-3.09
sNHEEEVADLLAQIQASHITVER	Unambiguous	2	1	P08553	N-Term(iTRAQ8plex)	CID	1.0000	0.0000	1	1	3282315	Unique	Used	1.765	0.984	1.314	1.016	0	0	9.13	0	14	15	3	965.16760	2893.48825	-2.61
iSFEEFcAVVGGLDIHk	Unambiguous	2	1	Q63810-2	N-Term(iTRAQ8plex); C7(Methylthio); K17(iTRAQ8plex)	CID	1.0000	0.0000	1	1	3689443	Unique	Used	0.811	1.674	1.974	0.737	0	0	9.12	0	3	200	3	840.11005	2518.31559	-5.52
hQGGWTDGGSGSGGGYQDGAYR	Unambiguous	1	1	Q3TJZ6	N-Term(iTRAQ8plex)	CID	1.0000	0.0000	1	1	2750875	Unique	Used	1.602	0.684	0.722	0.646	0	0	9.11	0	0	200	3	825.37012	2474.09580	-2.64
aAATLAAVSGTPGLGAqPAELGTHk	Unambiguous	1	1	E9Q1P8	N-Term(iTRAQ8plex); Q17(Deamidated); K25(iTRAQ8plex)	CID	1.0000	0.0000	1	1	3640406	Unique	Used	1.105	0.970	1.019	1.054	0	0	9.10	0	0	66	3	966.87659	2898.61521	0.21
dFTSEQIDEILQNHTEIVFAR	Unambiguous	2	1	Q6PIC6	N-Term(iTRAQ8plex)	CID	1.0000	0.0000	1	1	3355697	Unique	Used	0.680	0.510	1.184	1.680	0	0	9.10	0	4	109	3	937.14618	2809.42398	-2.52

qGSATSAASATTASSAAEGTLqR	Unambiguous	1	1	Q8R4V2	N-Term(iTRAQ8plex); Q22(Deamidated)	CID	1.0000	0.0000	1	1	3184223	Unique	Used	1.271	0.139	0.933	1.225	0	0	9.09	0	0	56	2	1215.11230	2429.21733	4.74
nMGGPYGGGNYGPGGSGGSGGYGGR	Unambiguous	3	1	O88569	N-Term(iTRAQ8plex)	CID	1.0000	0.0000	1	1	3316498	Unique	Used	1.906	0.838	1.197	0.755	0	0	9.09	0	0	118	2	1247.55542	2494.10356	-2.91
sMLQATAEAnNLAAVATAk	Unambiguous	1	1	Q8BH66	N-Term(iTRAQ8plex); N10(Deamidated); K19(iTRAQ8plex)	CID	0.0022	0.0000	1	1	3578778	Unique	Used	0.699	1.482	1.375	1.200	0	0	9.07	0	0	88	2	1242.68506	2484.36284	1.60
dFTSEQIDEILQNHTEIVFAR	Unambiguous	2	1	Q6PIC6	N-Term(iTRAQ8plex)	CID	1.0000	0.0000	1	1	3355907	Unique	Used	0.683	0.724	1.132	1.487	0	0	9.06	0	1	129	3	937.14563	2809.42234	-3.11
vADVSISEVTTLLGNVHSPVVEGYVGEnISGEVk	Unambiguous	3	1	P20357	N-Term(iTRAQ8plex); N28(Deamidated); K34(iTRAQ8plex)	CID	0.0298	0.0000	1	1	3421072	Unique	Used		1.460	1.679	1.113	0	0	9.06	0	0	200	3	1369.73975	4107.20469	2.14
dFTSEQIDEILQNHTEIVFAR	Unambiguous	2	1	Q6PIC6	N-Term(iTRAQ8plex)	CID	1.0000	0.0000	1	1	3356657	Unique	Used	0.652	0.458	1.164	1.257	0	0	9.05	0	3	200	3	937.14636	2809.42453	-2.33
sLAGSSGPGASSGPGGDHSELIVR	Unambiguous	5	1	P57776-3	N-Term(iTRAQ8plex)	CID	1.0000	0.0000	1	1	3317048	Unique	Used	1.036	1.020	0.837	0.873	0	0	9.05	0	11	27	3	833.76007	2499.26566	-3.41
dFTSEQIDEILQNHTEIVFAR	Unambiguous	2	1	Q6PIC6	N-Term(iTRAQ8plex)	CID	1.0000	0.0000	1	1	3357108	Unique	Used	0.705	0.670	1.242	1.555	0	0	9.03	0	4	200	3	937.14520	2809.42105	-3.57
qVLGQmVIDEELLGDGHSYSPR	Unambiguous	1	1	P99026	N-Term(iTRAQ8plex); M6(Oxidation)	CID	1.0000	0.0000	1	1	3236421	Unique	Used	2.056	0.924	0.793	1.037	0	0	9.03	0	1	8	3	921.80072	2763.38761	-1.80
vEYPVMYSTDPEnGHMFNcIQR	Unambiguous	1	1	Q9CPU4	N-Term(iTRAQ8plex); N13(Deamidated); C19(Methylthio)	CID	1.0000	0.0000	1	1	3283079	Unique	Used	0.848	0.891	0.920	0.680	0	0	9.02	0	0	6	3	994.44391	2981.31717	-2.44
iSFEEFcAVVGGLDIHk	Unambiguous	2	1	Q63810-2	N-Term(iTRAQ8plex); C7(Methylthio); K17(iTRAQ8plex)	CID	1.0000	0.0000	1	1	3687986	Unique	Used	0.735	1.493	2.309	0.899	0	0	9.00	0	2	56	3	840.11005	2518.31559	-5.52
gAYGGGYGGYDDYnGYNDGYGFGSDR	Unambiguous	2	1	O35737	N-Term(iTRAQ8plex); N14(Deamidated)	CID	1.0000	0.0000	1	1	2636804	Unique	Used		1.815			0	0	9.00	0	2	106	3	1008.07959	3022.22422	-3.30

hLQAAFEDGFDNcDAGWLSDR	Unambiguous	1	1	P55066	N-Term(iTRAQ8plex); C13(Methylthio)	CID	1.0000	0.0000	1	1	3304880	Unique	Used	2.422	2.830	0.575		0	0	8.98	0	6	8	3	906.40479	2717.19980	-1.06
IDAWDEAQVEFMASHGNEAAR	Unambiguous	1	1	E9PY16	N-Term(iTRAQ8plex)	CID	1.0000	0.0000	1	1	2959014	Unique	Used	0.847	0.549	0.545	0.583	0	0	8.97	0	10	31	3	884.42041	2651.24668	0.15
tQQAAQANITLQEQIEAIHk	Unambiguous	1	1	Q8K4Z5	N-Term(iTRAQ8plex); K20(iTRAQ8plex)	CID	1.0000	0.0000	1	1	3300202	Unique	Used	1.528	1.157	0.828	0.743	0	0	8.97	0	0	101	3	948.52997	2843.57535	-2.90
sLGSLSQGSANATVLDVAQTGGHk	Unambiguous	2	1	Q8CHG7	N-Term(iTRAQ8plex); K24(iTRAQ8plex)	CID	1.0000	0.0000	1	1	3514642	Unique	Used		1.161	1.656	1.360	0	0	8.95	0	0	124	3	969.52985	2906.57499	-1.47
dYSLQIQnVDVTDDGPYTcSVQTQHTPR	Unambiguous	4	1	A0A4W9	N-Term(iTRAQ8plex); N8(Deamidated); C19(Methylthio)	CID	0.0089	0.0000	1	1	3235688	Unique	Used	1.201	2.041	1.060	1.030	0	0	8.94	0	10	77	3	1177.88831	3531.65036	5.47
dMAIATGGAVFGEEGLnLNLEDVQAHDLGk	Unambiguous	1	1	P63038	N-Term(iTRAQ8plex); N17(Deamidated); K30(iTRAQ8plex)	CID	1.0000	0.0000	1	1	3285350	Unique	Used	0.438	1.237	0.623	1.377	0	0	8.94	0	0	200	3	1231.97498	3693.91037	5.74
iSFEEFcAVVGGLDIHk	Unambiguous	2	1	Q63810-2	N-Term(iTRAQ8plex); C7(Methylthio); K17(iTRAQ8plex)	CID	1.0000	0.0000	1	1	3688867	Unique	Used	0.644	1.534	2.202	0.816	0	0	8.93	0	1	140	3	840.10962	2518.31430	-6.03
gNDISSGTVLSDYVGSGPPSGTGLHR	Unambiguous	1	1	P70296	N-Term(iTRAQ8plex)	CID	1.0000	0.0000	1	1	3474419	Unique	Used	0.619	0.755	1.090	1.290	0	0	8.92	0	19	32	3	945.47705	2834.41660	-2.01
tFPTVNPTTGEVIGHVAEGDR	Unambiguous	2	1	Q9CZS1	N-Term(iTRAQ8plex)	CID	1.0000	0.0000	1	1	3212014	Unique	Used	2.264	1.247	1.057	1.053	0	0	8.92	0	13	86	3	834.43488	2501.29007	-1.51
nMGGPYGGGNYGPGGSGGSGGYGGR	Unambiguous	3	1	O88569	N-Term(iTRAQ8plex)	CID	1.0000	0.0000	1	1	3316738	Unique	Used	1.695	0.731	1.275	0.861	0	0	8.92	0	0	172	2	1247.55554	2494.10381	-2.81
dFTSEQIDEILQNHTEIVFAR	Unambiguous	2	1	Q6PIC6	N-Term(iTRAQ8plex)	CID	1.0000	0.0000	1	1	3356882	Unique	Used	0.959	0.793	1.228	1.257	0	0	8.91	0	2	200	3	937.14587	2809.42307	-2.85
gnDISSGTVLSDYVGSGPPSGTGLHR	Unambiguous	1	1	P70296	N-Term(iTRAQ8plex); N2(Deamidated)	CID	1.0000	0.0000	1	1	3345738	Unique	Used	1.935	1.270	0.841	1.108	0	0	8.90	0	13	6	3	945.80414	2835.39786	-2.98

nMGGPYGGGnYGPGGSGGSGGYGGR	Unambiguous	3	1	O88569	N-Term(iTRAQ8plex); N10(Deamidated)	CID	1.0000	0.0000	1	1	3296204	Unique	Used	1.824	0.924	0.843	0.575	0	0	8.89	0	18	200	2	1248.05701	2495.10674	4.77
aPPAGGSGSSAAAAAAAGGTEVRPR	Unambiguous	2	1	P84309	N-Term(iTRAQ8plex)	HCD	1.0000	0.0000	1	1	3468567	Unique	Used	0.672	0.748	2.768	1.347	0	0	8.87	0	0	200	3	814.42871	2441.27158	-3.44
iSFEEFcAVVGGLDIHk	Unambiguous	2	1	Q63810-2	N-Term(iTRAQ8plex); C7(Methylthio); K17(iTRAQ8plex)	CID	1.0000	0.0000	1	1	3690500	Unique	Used	0.710	1.582	1.960	0.928	0	0	8.86	0	0	200	3	840.10974	2518.31467	-5.88
dFTSEQIDEILQNHTEIVFAR	Unambiguous	2	1	Q6PIC6	N-Term(iTRAQ8plex)	CID	1.0000	0.0000	1	1	3356027	Unique	Used	0.627	0.649	1.262	1.470	0	0	8.86	0	5	192	3	937.14557	2809.42215	-3.17
gNDISSGTVLSDYVGSGPPSGTGLHR	Unambiguous	1	1	P70296	N-Term(iTRAQ8plex)	CID	1.0000	0.0000	1	1	3515202	Unique	Used	0.971	1.711	1.238	0.867	0	0	8.84	0	0	14	3	945.47626	2834.41422	-2.85
dFTSEQIDEILQNHTEIVFAR	Unambiguous	2	1	Q6PIC6	N-Term(iTRAQ8plex)	CID	1.0000	0.0000	1	1	3355367	Unique	Used	0.589	0.595	0.986	1.282	0	0	8.84	0	2	82	3	937.14575	2809.42270	-2.98
hQGGWTDGGSGSGGGYQDGAYR	Unambiguous	1	1	Q3TJZ6	N-Term(iTRAQ8plex)	CID	1.0000	0.0000	1	1	2789427	Unique	Used	1.447	0.373	0.806	0.562	0	0	8.83	0	0	85	3	825.37189	2474.10111	-0.50
mADPQSIQESQNLSMFLANHNR	Unambiguous	1	1	Q5SQX6	N-Term(iTRAQ8plex)	CID	1.0000	0.0000	1	1	3619941	Unique	Used	1.488		0.340	0.561	0	0	8.83	0	17	23	3	945.79401	2835.36747	-5.14
lNFLSPELPAVAEFSTNETmGHSADR	Unambiguous	1	1	Q99JY0	N-Term(iTRAQ8plex); M20(Oxidation)	CID	1.0000	0.0000	1	1	3418611	Unique	Used			0.612	1.207	0	0	8.82	0	7	6	3	1051.85120	3153.53904	-2.38
vSQAAAELLAFcETHAk	Unambiguous	1	1	P63078	N-Term(iTRAQ8plex); C12(Methylthio); K17(iTRAQ8plex)	CID	1.0000	0.0000	1	1	3745850	Unique	Used	0.197	0.180	0.652	1.916	0	0	8.82	0	0	50	3	815.09839	2443.28061	-5.24
gLYSALAGFcDIGESVEETVHR	Unambiguous	1	1	Q8JZU0	N-Term(iTRAQ8plex); C10(Methylthio)	CID	1.0000	0.0000	1	1	3480170	Unique	Used	3.487	1.796	0.931	0.712	0	0	8.82	0	20	64	3	901.77026	2703.29624	-3.64
gnFGGSFAGSFGGAGGHAPGVAR	Unambiguous	4	1	Q9D0E1-2	N-Term(iTRAQ8plex); N2(Deamidated)	CID	1.0000	0.0000	1	1	3892253	Unique	Used	0.751	0.793	0.951	0.845	0	0	8.82	0	0	200	2	1170.57471	2340.14214	-0.11
yVELFLNSTAGASGGAYEHR	Unambiguous	2	1	O35737	N-Term(iTRAQ8plex)	CID	1.0000	0.0000	1	1	3685943	Unique	Used	2.431	0.955	0.657	0.722	0	0	8.81	0	4	9	3	816.07709	2446.21671	-5.63
nLHQSGFSLSGSQADDHIAR	Unambiguous	3	1	O35098	N-Term(iTRAQ8plex)	CID	1.0000	0.0000	1	1	3315922	Unique	Used	1.359	0.603	1.016	0.767	0	0	8.81	0	0	55	3	815.41028	2444.21628	-2.36

vEYPVMYSTDPEnGHMFNcIQR	Unambiguous	1	1	Q9CPU4	N-Term(iTRAQ8plex); N13(Deamidated); C19(Methylthio)	CID	1.0000	0.0000	1	1	3303954	Unique	Used	0.872	0.894	1.300	1.100	0	0	8.80	0	5	27	3	994.44543	2981.32175	-0.90
gNDISSGTVLSDYVGSGPPSGTGLHR	Unambiguous	1	1	P70296	N-Term(iTRAQ8plex)	CID	1.0000	0.0000	1	1	3495054	Unique	Used	1.566	2.466	1.188	0.828	0	0	8.79	0	2	13	3	945.47656	2834.41513	-2.53
sGQQAcEGVGSFLLYSVHEGIR	Unambiguous	1	1	Q91ZX7	N-Term(iTRAQ8plex); C6(Methylthio)	CID	1.0000	0.0000	1	1	3785071	Unique	Used	1.462	1.006	0.853	0.779	0	0	8.79	0	15	19	3	896.44171	2687.31058	-4.39
gnDISSGTVLSDYVGSGPPSGTGLHR	Unambiguous	1	1	P70296	N-Term(iTRAQ8plex); N2(Deamidated)	CID	1.0000	0.0000	1	1	3390730	Unique	Used	0.846	1.107	1.138	0.999	0	0	8.79	0	19	31	3	945.80609	2835.40372	-0.92
aAGGAGAQVGGSISSGSSASSVTVTR	Unambiguous	1	1	P48678	N-Term(iTRAQ8plex)	CID	1.0000	0.0000	1	1	3677507	Unique	Used	1.001	1.610	0.650	0.669	0	0	8.78	0	0	200	2	1263.64709	2526.28691	-7.64
gNDISSGTVLSDYVGSGPPSGTGLHR	Unambiguous	1	1	P70296	N-Term(iTRAQ8plex)	CID	1.0000	0.0000	1	1	3495343	Unique	Used	1.242	1.202	1.036	1.129	0	0	8.78	0	0	87	3	945.47430	2834.40836	-4.92
vADVSISEVTTLLGnVHSPVVEGYVGENISGEVk	Unambiguous	3	1	P20357	N-Term(iTRAQ8plex); N15(Deamidated); K34(iTRAQ8plex)	CID	1.0000	0.0000	1	1	3420848	Unique	Used		2.347	1.279	0.749	0	0	8.78	0	0	200	3	1369.73779	4107.19883	0.72
aAGGAGAQVGGSISSGSSASSVTVTR	Unambiguous	1	1	P48678	N-Term(iTRAQ8plex)	CID	1.0000	0.0000	1	1	3656706	Unique	Used	1.792	1.968	0.702	0.804	0	0	8.77	0	0	78	2	1263.64917	2526.29106	-6.00
IQEANAQLAEQTcPHDLEMTPVQR	Unambiguous	1	1	Q80Y17	N-Term(iTRAQ8plex); C13(Methylthio)	CID	1.0000	0.0000	1	1	3214182	Unique	Used	0.798	0.274	0.717	0.766	0	0	8.77	0	0	96	3	1024.83130	3072.47934	-2.01
eQGSSGLGSGSSGGGGSSSGLGSGYIGR	Unambiguous	2	1	Q3UHJ0-2	N-Term(iTRAQ8plex)	CID	1.0000	0.0000	1	1	3185357	Unique	Used	0.602	1.387	1.343	1.078	0	0	8.76	0	0	95	2	1353.64453	2706.28179	-1.90
dFTSEQIDEILQNHTEIVFAR	Unambiguous	2	1	Q6PIC6	N-Term(iTRAQ8plex)	CID	1.0000	0.0000	1	1	3375467	Unique	Used	1.158	0.805	0.745	0.807	0	0	8.75	0	29	200	3	937.14502	2809.42051	-3.76
dYSLQIQNVDVTDDGPYTcSVQTQHTPR	Unambiguous	4	1	A0A4W9	N-Term(iTRAQ8plex); C19(Methylthio)	CID	1.0000	0.0000	1	1	3235889	Unique	Used	1.883	1.462	0.880	0.902	0	0	8.74	0	0	54	3	1177.55151	3530.63999	-1.99
vVHVLEGESGSVVVQTAPGQVVSHR	Unambiguous	1	1	Q80WM4	N-Term(iTRAQ8plex)	HCD	1.0000	0.0000	1	1	3494704	Unique	Used	1.310	0.791	1.145	1.581	0	0	8.74	0	19	150	3	958.85791	2874.55918	-5.16

dMAIATGGAVFGEEGLnLNLEDVQAHDLGk	Unambiguous	1	1	P63038	N-Term(iTRAQ8plex); N17(Deamidated); K30(iTRAQ8plex)	CID	0.0332	0.0000	1	1	3285940	Unique	Used		0.732	0.627	1.238	0	0	8.74	0	0	200	3	1231.97510	3693.91074	5.84
lWVDTcSEIFGGLDIcAVEALHGk	Unambiguous	3	1	O88935	N-Term(iTRAQ8plex); C6(Methylthio); C16(Methylthio); K24(iTRAQ8plex)	CID	1.0000	0.0000	1	1	3727767	Unique	Used	0.496	1.364	1.812	1.717	0	0	8.73	0	5	98	3	1092.87646	3276.61484	-7.27
qVLGQMVIDEELLGDGHSYSPR	Unambiguous	1	1	P99026	N-Term(iTRAQ8plex)	CID	1.0000	0.0000	1	1	3216269	Unique	Used	1.519	0.909	1.083	1.298	0	0	8.73	0	0	200	3	916.47058	2747.39719	-0.18
vAcSHIPAGSTASGTSAGAGAGPDDATR	Unambiguous	2	1	Q6PAN7	N-Term(iTRAQ8plex); C3(Methylthio)	CID	1.0000	0.0000	1	1	3317042	Unique	Used	1.045	1.071	0.874	1.183	0	0	8.73	0	0	74	3	945.78064	2835.32737	-1.08
iSFEEFcAVVGGLDIHk	Unambiguous	2	1	Q63810-2	N-Term(iTRAQ8plex); C7(Methylthio); K17(iTRAQ8plex)	CID	1.0000	0.0000	1	1	3688525	Unique	Used	0.594	1.487	2.324	0.958	0	0	8.72	0	6	58	3	840.10950	2518.31394	-6.17
aVEYYFASDASAVIEHTNR	Unambiguous	2	1	P47856-2	N-Term(iTRAQ8plex)	CID	1.0000	0.0000	1	1	3282585	Unique	Used	1.197	1.064	0.712	1.006	0	0	8.71	0	0	39	3	816.40771	2447.20859	-2.42
tTTNVLGDSLGAGIVEHLSR	Unambiguous	1	1	P56564	N-Term(iTRAQ8plex)	CID	1.0000	0.0000	1	1	3704720	Unique	Used	0.900	1.898	1.187	1.150	0	0	8.71	0	8	16	3	782.09229	2344.26230	-6.47
dMAIATGGAVFGEEGLNLNLEDVqAHDLGk	Unambiguous	1	1	P63038	N-Term(iTRAQ8plex); Q24(Deamidated); K30(iTRAQ8plex)	CID	0.0034	0.0000	1	1	3265050	Unique	Used	1.210	1.345	0.877	0.857	0	0	8.71	0	0	200	3	1231.97180	3693.90085	3.16
dFTSEQIDEILQnHTEIVFAR	Unambiguous	2	1	Q6PIC6	N-Term(iTRAQ8plex); N13(Deamidated)	CID	0.0264	0.0000	1	1	3132926	Unique	Used	0.386	0.660	1.554	1.716	0	0	8.70	0	0	2	3	937.47601	2810.41349	-0.57
dSLDDLFPNEDEQSPAPSPGGGDVAAQHGGYEIPAR	Unambiguous	3	1	D3YXZ3	N-Term(iTRAQ8plex)	CID	1.0000	0.0000	1	1	2762730	Unique	Used	4.061	1.314		0.899	0	0	8.70	0	18	200	3	1338.29626	4012.87424	0.06
cQNEQLQTAVTQQASQIQQHk	Unambiguous	3	1	Q9Z1Z0	N-Term(iTRAQ8plex); C1(Methylthio); K21(iTRAQ8plex)	CID	1.0000	0.0000	1	1	3514340	Unique	Used	0.660	0.831	1.267	0.871	0	0	8.70	0	0	30	3	1022.52344	3065.55576	-5.20

sNHEEEVADLLAQIQASHITVER	Unambiguous	2	1	P08553	N-Term(iTRAQ8plex)	CID	1.0000	0.0000	1	1	3282481	Unique	Used	5.059	0.166	1.396	1.050	0	0	8.70	0	2	7	3	965.16876	2893.49173	-1.41
iSFEEFcAVVGGLDIHk	Unambiguous	2	1	Q63810-2	N-Term(iTRAQ8plex); C7(Methylthio); K17(iTRAQ8plex)	CID	1.0000	0.0000	1	1	3688970	Unique	Used	0.556	1.353	2.343	0.848	0	0	8.69	0	0	176	3	840.11047	2518.31687	-5.01
gESPVASPFQNYSSIHSQSR	Unambiguous	2	1	P53995	N-Term(iTRAQ8plex)	CID	1.0000	0.0000	1	1	3471278	Unique	Used	1.341	0.991	1.192	1.086	0	0	8.69	0	0	126	3	828.07861	2482.22129	-2.08
iSFEEFcAVVGGLDIHk	Unambiguous	2	1	Q63810-2	N-Term(iTRAQ8plex); C7(Methylthio); K17(iTRAQ8plex)	CID	1.0000	0.0000	1	1	3689918	Unique	Used	0.617	1.278	2.152	0.705	0	0	8.69	0	1	200	3	840.11102	2518.31852	-4.35
yFHVVIAGPQDSPFEGGTFk	Unambiguous	1	1	P61089	N-Term(iTRAQ8plex); K20(iTRAQ8plex)	CID	1.0000	0.0000	1	1	3538897	Unique	Used	4.343	0.967	0.738	1.019	0	0	8.68	0	4	11	3	935.49719	2804.47702	-3.50
vDEFVTGNLSFDQINQAFDLMHSGDSIR	Unambiguous	1	1	P28474	N-Term(iTRAQ8plex)	CID	1.0000	0.0000	1	1	3541331	Unique	Used	1.421	0.789	0.669	1.408	0	0	8.67	0	28	12	3	1153.89465	3459.66941	-2.88
hQGGWTDGGSGSGGGYQDGAYR	Unambiguous	1	1	Q3TJZ6	N-Term(iTRAQ8plex)	CID	1.0000	0.0000	1	1	2789554	Unique	Used	1.600	0.852	1.015	0.784	0	0	8.67	0	23	200	3	825.37280	2474.10385	0.61
aGLQSLETSGHLEDDFDMFALTR	Unambiguous	3	1	O88746	N-Term(iTRAQ8plex)	CID	1.0000	0.0000	1	1	3262236	Unique	Used	2.282	0.966	2.061	1.667	0	0	8.65	0	0	7	3	953.13617	2857.39396	-1.44
hQGGWTDGGSGSGGGYQDGAYR	Unambiguous	1	1	Q3TJZ6	N-Term(iTRAQ8plex)	CID	1.0000	0.0000	1	1	2773363	Unique	Used	1.967	1.010	2.123	1.261	0	0	8.65	0	0	200	3	825.36945	2474.09378	-3.46
gVGSSMAQLLTcAAQGNEHYTGVAAR	Unambiguous	2	1	E9PUM4	N-Term(iTRAQ8plex); C12(Methylthio)	CID	1.0000	0.0000	1	1	3878917	Unique	Used	0.730	1.021	1.141	1.095	0	0	8.65	0	29	60	3	981.47406	2942.40763	-5.07
nMGGPYGGGnYGPGGSGGSGGYGGR	Unambiguous	3	1	O88569	N-Term(iTRAQ8plex); N10(Deamidated)	CID	1.0000	0.0000	1	1	3029121	Unique	Used	1.798	1.042	1.163	1.003	0	0	8.63	0	16	200	2	1248.04773	2495.08818	-2.67
tVYFAEEVQcEGnSFHk	Unambiguous	1	1	P97315	N-Term(iTRAQ8plex); C10(Methylthio); N13(Deamidated); K17(iTRAQ8plex)	CID	1.0000	0.0000	1	1	2938661	Unique	Used	2.177	0.715	1.793	0.969	0	0	8.62	0	2	24	3	881.75623	2643.25412	-5.24
yAEDIFGELFNEAHSFSFR	Unambiguous	1	1	Q8R5H6	N-Term(iTRAQ8plex)	CID	1.0000	0.0000	1	1	3561157	Unique	Used	0.626	2.635	1.471	0.970	0	0	8.62	0	0	29	3	861.74884	2583.23197	-5.36

nMDPLnDNVATLLHQSSDR	Unambiguous	3	1	Q5SV64	N-Term(iTRAQ8plex); N6(Deamidated)	CID	0.0360	0.0000	1	1	3109256	Unique	Used	1.323	1.282	1.518	0.977	0	0	8.61	0	27	144	3	815.73816	2445.19992	0.69
dMHGDSEYNIMFGPDIcGPGTk	Unambiguous	1	1	P14211	N-Term(iTRAQ8plex); C17(Methylthio); K22(iTRAQ8plex)	CID	1.0000	0.0000	1	1	3151626	Unique	Used	1.317	0.859	0.476	0.558	0	0	8.61	0	0	8	3	1013.46869	3038.39151	-2.05
wQHDLFDSGFGGGAGVETGGk	Unambiguous	1	1	O08583	N-Term(iTRAQ8plex); K21(iTRAQ8plex)	CID	1.0000	0.0000	1	1	3257915	Unique	Used	1.093	0.683	1.169	0.828	0	0	8.61	0	0	54	3	910.79352	2730.36600	-2.67
sLGSLSQGSANATVLDVAQTGGHk	Unambiguous	2	1	Q8CHG7	N-Term(iTRAQ8plex); K24(iTRAQ8plex)	CID	1.0000	0.0000	1	1	3514770	Unique	Used	0.534	0.809	1.175	1.288	0	0	8.61	0	7	71	3	969.52826	2906.57022	-3.11
dFTSEQIDEILQNHTEIVFAR	Unambiguous	2	1	Q6PIC6	N-Term(iTRAQ8plex)	CID	1.0000	0.0000	1	1	3355142	Unique	Used	0.547	0.505	0.962	1.209	0	0	8.61	0	1	55	3	937.14563	2809.42234	-3.11
iSFEEFcAVVGGLDIHk	Unambiguous	2	1	Q63810-2	N-Term(iTRAQ8plex); C7(Methylthio); K17(iTRAQ8plex)	CID	1.0000	0.0000	1	1	3708060	Unique	Used	0.905	1.964	1.733	0.740	0	0	8.59	0	14	200	3	840.11011	2518.31577	-5.45
iSFEEFcAVVGGLDIHk	Unambiguous	2	1	Q63810-2	N-Term(iTRAQ8plex); C7(Methylthio); K17(iTRAQ8plex)	CID	1.0000	0.0000	1	1	3689563	Unique	Used	0.800	1.695	2.540	0.812	0	0	8.59	0	2	200	3	840.11005	2518.31559	-5.52
tLALLAFDSPEESPFGDLLHMMQR	Unambiguous	1	1	Q9D7M1	N-Term(iTRAQ8plex)	CID	1.0000	0.0000	1	1	3766550	Unique	Used	2.135	1.320	0.552	0.728	0	0	8.59	0	11	57	3	1008.17816	3022.51993	-4.02
tSsFAEPGGGGGGGGGGPGGSASGPGGTGGGk	Unambiguous	2	1	D3Z7E5	N-Term(iTRAQ8plex); S3(Phospho); K32(iTRAQ8plex)	CID	0.0128	0.0000	1	1	2632443	Unique	Used		2.166	1.103	0.505	0	0	8.58	0	0	103	3	1041.14856	3121.43113	-3.92
iGGELEEQAVMLDDFSHELESTQSR	Unambiguous	2	1	Q9JKK1	N-Term(iTRAQ8plex)	CID	1.0000	0.0000	1	1	2835125	Unique	Used	0.848	1.125	1.044	1.064	0	0	8.58	0	8	200	3	1042.17029	3124.49631	-2.70
iSFEEFcAVVGGLDIHk	Unambiguous	2	1	Q63810-2	N-Term(iTRAQ8plex); C7(Methylthio); K17(iTRAQ8plex)	CID	1.0000	0.0000	1	1	3689665	Unique	Used	0.761	1.405	2.309	0.909	0	0	8.58	0	0	200	3	840.11053	2518.31705	-4.94

hLVDQMPDSSDNPAGLAEQcPGMR	Unambiguous	1	1	P46414	N-Term(iTRAQ8plex); C20(Methylthio)	CID	1.0000	0.0000	1	1	3235132	Unique	Used	1.572	1.015	1.293	1.185	0	0	8.57	0	16	25	3	973.44250	2918.31296	-2.68
iSFEEFcAVVGGLDIHk	Unambiguous	2	1	Q63810-2	N-Term(iTRAQ8plex); C7(Methylthio); K17(iTRAQ8plex)	CID	1.0000	0.0000	1	1	3689233	Unique	Used	0.779	1.629	2.068	0.765	0	0	8.57	0	2	200	3	840.10876	2518.31174	-7.05
qAAAAATQTIAASQNAAISNk	Unambiguous	2	1	E9PUM4	N-Term(iTRAQ8plex); K21(iTRAQ8plex)	CID	1.0000	0.0000	1	1	3680456	Unique	Used		0.801	0.817	0.945	0	0	8.57	0	0	108	2	1305.21729	2609.42729	-7.46
dVLTSTYGATAETLSTSTTTHVTk	Unambiguous	7	1	A2AUK8	N-Term(iTRAQ8plex); K24(iTRAQ8plex)	CID	1.0000	0.0000	1	1	3413208	Unique	Used	1.026	1.249	1.398	1.432	0	0	8.57	0	0	26	3	1031.87781	3093.61887	-7.22
dMAIATGGAVFGEEGLNLNLEDVqAHDLGk	Unambiguous	1	1	P63038	N-Term(iTRAQ8plex); Q24(Deamidated); K30(iTRAQ8plex)	CID	0.0117	0.0000	1	1	3285487	Unique	Used	1.646	0.342	1.111	1.454	0	0	8.56	0	0	200	3	1231.97229	3693.90232	3.56
tTTNVLGDSLGAGIVEHLSR	Unambiguous	1	1	P56564	N-Term(iTRAQ8plex)	CID	1.0000	0.0000	1	1	3704585	Unique	Used	0.763	1.989	0.808	0.975	0	0	8.55	0	1	2	3	782.09186	2344.26102	-7.01
vADVSISEVTTLLGnVHSPVVEGYVGENISGEVk	Unambiguous	3	1	P20357	N-Term(iTRAQ8plex); N15(Deamidated); K34(iTRAQ8plex)	CID	0.0386	0.0000	1	1	3396883	Unique	Used	1.636	1.539	1.355	0.745	0	0	8.54	0	0	200	3	1369.73694	4107.19626	0.09
iSFEEFcAVVGGLDIHk	Unambiguous	2	1	Q63810-2	N-Term(iTRAQ8plex); C7(Methylthio); K17(iTRAQ8plex)	CID	1.0000	0.0000	1	1	3687837	Unique	Used	0.698	1.616	1.896	0.726	0	0	8.54	0	1	38	3	840.10999	2518.31540	-5.59
lQEANAQLAEQTcPHDLEmTPVQR	Unambiguous	1	1	Q80Y17	N-Term(iTRAQ8plex); C13(Methylthio); M19(Oxidation)	CID	1.0000	0.0000	1	1	3212794	Unique	Used	0.961	0.273	0.718	0.825	0	0	8.53	0	30	85	3	1030.16626	3088.48423	1.22
sLIDmYSEVLDVLSDYDASYNTQDHLPR	Unambiguous	5	1	P58281-2	N-Term(iTRAQ8plex); M5(Oxidation)	CID	1.0000	0.0000	1	1	3396352	Unique	Used	1.122	1.069	2.568	1.633	0	0	8.52	0	0	34	3	1193.90356	3579.69614	-3.97
tQSSASLAASYAAQQHPQAAASYR	Unambiguous	3	1	Q8C2Q3	N-Term(iTRAQ8plex)	CID	1.0000	0.0000	1	1	3677853	Unique	Used	1.380	1.063	0.905	1.127	0	0	8.52	0	24	200	3	923.79431	2769.36838	-6.30

aAASVVSGPPVASDcASPGDSAR	Unambiguous	3	1	Q6P9K8	N-Term(iTRAQ8plex); C15(Methylthio)	CID	1.0000	0.0000	1	1	3299219	Unique	Used	1.660	0.442	1.469	1.015	0	0	8.52	0	0	72	2	1211.58313	2422.15898	-2.28
dFTSEQIDEILQNHTEIVFAR	Unambiguous	2	1	Q6PIC6	N-Term(iTRAQ8plex)	CID	1.0000	0.0000	1	1	3375827	Unique	Used	0.539	0.539	1.423	1.847	0	0	8.51	0	11	200	3	937.14484	2809.41996	-3.96
sALFAQInQGESITHALk	Unambiguous	1	1	P40124	N-Term(iTRAQ8plex); N8(Deamidated); K18(iTRAQ8plex)	CID	0.0482	0.0000	1	1	3391978	Unique	Used	0.955	1.231	1.227	0.887	0	0	8.50	0	2	14	3	846.47363	2537.40635	-4.76
dLVVLLFETALLSSGFSLEDPQTHSNR	Unambiguous	1	1	P11499	N-Term(iTRAQ8plex)	HCD	1.0000	0.0000	1	1	3825382	Unique	Used	1.036	1.026	0.716	0.659	0	0	8.50	0	28	200	3	1098.24573	3292.72263	-4.30
vADVSISEVTTLLGnVHSPVVEGYVGENISGEVk	Unambiguous	3	1	P20357	N-Term(iTRAQ8plex); N15(Deamidated); K34(iTRAQ8plex)	CID	0.0306	0.0000	1	1	3421314	Unique	Used			1.534		0	0	8.50	0	14	200	3	1369.73853	4107.20102	1.25
qDHPSSMGVYGQESGGFSGPGENR	Unambiguous	4	1	Q5SUT0	N-Term(iTRAQ8plex)	CID	1.0000	0.0000	1	1	2826260	Unique	Used	1.415	1.439	1.681	1.230	0	0	8.50	0	0	106	3	928.75513	2784.25083	-2.80
nVIIWGNHSSTQYPDVNHAk	Unambiguous	1	1	P14152	N-Term(iTRAQ8plex); K20(iTRAQ8plex)	CID	1.0000	0.0000	1	1	3431352	Unique	Used				1.050	0	0	8.49	0	26	185	3	963.50800	2888.50943	-5.87
dMAIATGGAVFGEEGLnLNLEDVQAHDLGk	Unambiguous	1	1	P63038	N-Term(iTRAQ8plex); N17(Deamidated); K30(iTRAQ8plex)	CID	0.0365	0.0000	1	1	3132849	Unique	Used	1.958	0.961	0.657	0.740	0	0	8.49	0	0	61	3	1231.97034	3693.89646	1.97
gNDISSGTVLSDYVGSGPPSGTGLHR	Unambiguous	1	1	P70296	N-Term(iTRAQ8plex)	CID	1.0000	0.0000	1	1	3576042	Unique	Used	2.010	1.640	2.022	1.711	0	0	8.49	0	0	48	3	945.47333	2834.40543	-5.95
vADVSISEVTTLLGnVHSPVVEGYVGENISGEVk	Unambiguous	3	1	P20357	N-Term(iTRAQ8plex); N15(Deamidated); K34(iTRAQ8plex)	CID	0.0071	0.0000	1	1	3440063	Unique	Used	1.502	1.677	1.748	0.858	0	0	8.48	0	0	25	3	1369.73633	4107.19443	-0.35
dVLTSTYGATAETLSTSTTTHVTk	Unambiguous	7	1	A2AUK8	N-Term(iTRAQ8plex); K24(iTRAQ8plex)	CID	1.0000	0.0000	1	1	3413038	Unique	Used	1.313	1.573	2.076	0.949	0	0	8.48	0	4	4	3	1031.88062	3093.62729	-4.50
gNDISSGTVLSDYVGSGPPSGTGLHR	Unambiguous	1	1	P70296	N-Term(iTRAQ8plex)	CID	1.0000	0.0000	1	1	3454625	Unique	Used	1.908	1.272	1.395	1.293	0	0	8.48	0	0	167	3	945.47595	2834.41330	-3.17
ILEVSDDPQVLAVAAHDVGEYVR	Unambiguous	1	1	Q8BVE3	N-Term(iTRAQ8plex)	CID	1.0000	0.0000	1	1	3240507	Unique	Used		1.167			0	0	8.47	0	15	200	3	933.83301	2799.48447	0.48

fDLGQDVIDFTGHSLALYR	Unambiguous	2	1	Q61598	N-Term(iTRAQ8plex)	CID	1.0000	0.0000	1	1	3686708	Unique	Used	0.957	0.846	0.882	1.302	0	0	8.47	0	28	23	3	824.42816	2471.26993	-7.02
fEAPEALFQPHLINVEGVGVAELLFNTIQAADIDTR	Unambiguous	1	1	P61161	N-Term(iTRAQ8plex)	CID	1.0000	0.0000	1	1	3766822	Unique	Used		1.004	0.987	1.127	0	0	8.46	0	0	71	3	1414.75073	4242.23764	-0.32
mADPQSIQESQNLSMFLANHNk	Unambiguous	4	1	Q7TMB8	N-Term(iTRAQ8plex); K22(iTRAQ8plex)	CID	1.0000	0.0000	1	1	3436450	Unique	Used	3.506	1.694	1.277	1.056	0	0	8.46	0	0	134	3	1037.86157	3111.57016	-3.56
hISQISVADDDDESLLGHLMIVGk	Unambiguous	1	1	P70349	N-Term(iTRAQ8plex); K24(iTRAQ8plex)	CID	1.0000	0.0000	1	1	3282560	Unique	Used	1.226	1.064	1.381	1.117	0	0	8.46	0	0	117	3	1067.57068	3200.69748	-3.36
nMGGPYGGGNYGPGGSGGSGGYGGR	Unambiguous	3	1	O88569	N-Term(iTRAQ8plex); N1(Deamidated)	CID	1.0000	0.0000	1	1	2971218	Unique	Used	2.532	1.118	2.121	1.239	0	0	8.46	0	0	200	2	1248.05054	2495.09380	-0.42
dFTSEQIDEILQNHTEIVFAR	Unambiguous	2	1	Q6PIC6	N-Term(iTRAQ8plex)	CID	1.0000	0.0000	1	1	3375740	Unique	Used		0.614	0.846	1.171	0	0	8.46	0	18	200	3	937.14594	2809.42325	-2.78
gNDISSGTVLSDYVGSGPPSGTGLHR	Unambiguous	1	1	P70296	N-Term(iTRAQ8plex)	CID	1.0000	0.0000	1	1	3389590	Unique	Used	3.455	1.895	1.008	0.850	0	0	8.46	0	8	26	3	945.47504	2834.41056	-4.14
aLLLVGDVAQAAEVAIEHR	Unambiguous	3	1	G3X8X7	N-Term(iTRAQ8plex)	CID	1.0000	0.0000	1	1	3520609	Unique	Used	1.775	1.036	1.037	1.261	0	0	8.45	0	8	49	3	760.43713	2279.29685	-2.51
dVATVAFcDAQSTQEIHEk	Unambiguous	2	1	O88712-2	N-Term(iTRAQ8plex); C8(Methylthio); K19(iTRAQ8plex)	CID	1.0000	0.0000	1	1	2937310	Unique	Used	0.568	1.330	1.311	1.279	0	0	8.45	0	2	19	3	916.12488	2746.36008	-1.32
nLHQSGFSLSGSQADDHIAR	Unambiguous	3	1	O35098	N-Term(iTRAQ8plex)	CID	1.0000	0.0000	1	1	3384469	Unique	Used	1.067	1.018	0.831	0.834	0	0	8.45	0	0	113	3	815.40710	2444.20676	-6.26
dFTSEQIDEILQNHTEIVFAR	Unambiguous	2	1	Q6PIC6	N-Term(iTRAQ8plex)	CID	1.0000	0.0000	1	1	3354782	Unique	Used	0.625	0.605	0.956	1.266	0	0	8.44	0	5	57	3	937.14484	2809.41996	-3.96
dMHGDSEYNImFGPDIcGPGTk	Unambiguous	1	1	P14211	N-Term(iTRAQ8plex); M11(Oxidation); C17(Methylthio); K22(iTRAQ8plex)	CID	1.0000	0.0000	1	1	3150366	Unique	Used	1.655	0.847	0.930	0.715	0	0	8.44	0	0	13	3	1018.80066	3054.38742	-1.71
nMGGPYGGGNYGPGGSGGSGGYGGR	Unambiguous	3	1	O88569	N-Term(iTRAQ8plex)	CID	1.0000	0.0000	1	1	3316810	Unique	Used	1.967	1.067	1.186	0.718	0	0	8.44	0	0	200	2	1247.55505	2494.10283	-3.20

iSFEEFcAVVGGLDIHk	Unambiguous	2	1	Q63810-2	N-Term(iTRAQ8plex); C7(Methylthio); K17(iTRAQ8plex)	CID	1.0000	0.0000	1	1	3690162	Unique	Used	0.880	1.519	1.867	0.941	0	0	8.44	0	0	200	3	840.11035	2518.31650	-5.15
qVLGQMVIDEELLGDGHSYSPR	Unambiguous	1	1	P99026	N-Term(iTRAQ8plex)	CID	1.0000	0.0000	1	1	3238225	Unique	Used	1.191	1.064	0.557	0.678	0	0	8.43	0	17	75	3	916.47021	2747.39609	-0.58
rNDYQLIcIQDGYLSLLTETGEVR	Unambiguous	1	1	Q8BGY2	N-Term(iTRAQ8plex); C8(Methylthio)	CID	1.0000	0.0000	1	1	3602001	Unique	Used	1.060	1.229	1.116	1.193	0	0	8.43	1	20	14	3	1050.52991	3149.57517	-5.14
dYSLQIqNVDVTDDGPYTcSVQTQHTPR	Unambiguous	4	1	A0A4W9	N-Term(iTRAQ8plex); Q7(Deamidated); C19(Methylthio)	CID	0.0059	0.0000	1	1	3213706	Unique	Used	5.129	1.523	1.136	0.584	0	0	8.42	0	0	187	3	1177.88708	3531.64670	4.44
iSFEEFcAVVGGLDIHk	Unambiguous	2	1	Q63810-2	N-Term(iTRAQ8plex); C7(Methylthio); K17(iTRAQ8plex)	CID	1.0000	0.0000	1	1	3688225	Unique	Used	0.542	1.384	1.969	0.802	0	0	8.42	0	1	53	3	840.10962	2518.31430	-6.03
tETGQELEcLQDHLDDLAVEcR	Unambiguous	3	1	Q61543	N-Term(iTRAQ8plex); C9(Methylthio); C21(Methylthio)	CID	1.0000	0.0000	1	1	2922311	Unique	Used	1.066	0.632	0.738	0.879	0	0	8.42	0	29	200	3	971.77222	2913.30210	-0.69
dILGVcSDPLVLAQQLTHIELER	Unambiguous	1	1	E9Q1N6	N-Term(iTRAQ8plex); C6(Methylthio)	CID	1.0000	0.0000	1	1	3746196	Unique	Used	1.209	9.003	2.379	1.643	0	0	8.41	0	4	56	3	971.52008	2912.54569	-2.44
eIELLcQEHGQENDDLVQR	Unambiguous	4	1	E9Q6X0	N-Term(iTRAQ8plex); C6(Methylthio)	CID	1.0000	0.0000	1	1	2654743	Unique	Used	1.243	1.175	0.891	1.132	0	0	8.41	0	2	33	3	873.42078	2618.24778	-0.58
fTAESYTVLGDTLIDGGEHYWEVR	Unambiguous	2	1	Q7TPM6	N-Term(iTRAQ8plex)	CID	1.0000	0.0000	1	1	3284272	Unique	Used	0.847	0.682	0.839	0.863	0	0	8.41	0	10	32	3	1021.50775	3062.50870	1.22
eQASHLGGAVFSGAGnIAAATGLVk	Unambiguous	1	1	Q91ZZ3	N-Term(iTRAQ8plex); N16(Deamidated); K25(iTRAQ8plex)	CID	1.0000	0.0000	1	1	3578741	Unique	Used	1.737	1.233	1.228	0.921	0	0	8.41	0	28	89	3	979.20459	2935.59922	-3.62
vADVSISEVTTLLGNVHSPVVEGYVGENISGEVk	Unambiguous	3	1	P20357	N-Term(iTRAQ8plex); K34(iTRAQ8plex)	CID	1.0000	0.0000	1	1	3418971	Unique	Used	1.001	1.878	2.038	1.070	0	0	8.40	0	1	3	3	1369.40430	4106.19834	-3.29

gNVVFGEPITASLGIDGTHYWSk	Unambiguous	5	1	E9PWE8	N-Term(iTRAQ8plex); K23(iTRAQ8plex)	CID	1.0000	0.0000	1	1	3622616	Unique	Used	0.625	0.298	0.389	0.403	0	0	8.40	0	8	10	3	1019.54309	3056.61472	-5.07
tVYFAEEVQcEGNSFHk	Unambiguous	1	1	P97315	N-Term(iTRAQ8plex); C10(Methylthio); K17(iTRAQ8plex)	CID	1.0000	0.0000	1	1	3258210	Unique	Used	1.139	0.797	1.757	1.172	0	0	8.40	0	11	29	3	881.43011	2642.27579	-3.09
gPAVGIDLGTTYScVGVFQHGk	Unambiguous	4	1	P63017	N-Term(iTRAQ8plex); C14(Methylthio); K22(iTRAQ8plex)	CID	1.0000	0.0000	1	1	3805355	Unique	Used	1.032	1.160	0.994	1.026	0	0	8.40	0	7	37	3	954.16742	2860.48771	-2.43
eVLGSSLPNNWQDISLDDGHMk	Unambiguous	1	1	Q8VDT9	N-Term(iTRAQ8plex); K22(iTRAQ8plex)	CID	1.0000	0.0000	1	1	3281812	Unique	Used	1.499	0.962	1.230	1.033	0	0	8.40	0	14	196	3	1021.86053	3063.56705	0.13
aGLQSLETSGHLEDDFDMFALTR	Unambiguous	3	1	O88746	N-Term(iTRAQ8plex)	CID	1.0000	0.0000	1	1	3284132	Unique	Used	1.852	0.977	1.256	0.868	0	0	8.39	0	3	11	3	953.13690	2857.39615	-0.68
iSFEEFcAVVGGLDIHk	Unambiguous	2	1	Q63810-2	N-Term(iTRAQ8plex); C7(Methylthio); K17(iTRAQ8plex)	CID	1.0000	0.0000	1	1	3689131	Unique	Used	0.722	1.496	2.019	0.807	0	0	8.39	0	0	200	3	840.10962	2518.31430	-6.03
tASNIIDVSAADSQGMEQHEYMDR	Unambiguous	1	1	Q9CQ22	N-Term(iTRAQ8plex)	CID	1.0000	0.0000	1	1	2811586	Unique	Used	0.745	0.991	0.795	0.868	0	0	8.38	0	14	64	3	991.45935	2972.36350	-1.13
dVLTSTYGATAETLSTSTTTHVTk	Unambiguous	7	1	A2AUK8	N-Term(iTRAQ8plex); K24(iTRAQ8plex)	CID	1.0000	0.0000	1	1	3495562	Unique	Used	0.897	1.726	1.385	0.845	0	0	8.38	0	8	21	3	1031.88123	3093.62912	-3.91
iSFEEFcAVVGGLDIHk	Unambiguous	2	1	Q63810-2	N-Term(iTRAQ8plex); C7(Methylthio); K17(iTRAQ8plex)	CID	1.0000	0.0000	1	1	3687492	Unique	Used	0.667	1.593	2.119	0.748	0	0	8.38	0	1	15	3	840.10980	2518.31485	-5.81
dFTSEQIDEILQNHTEIVFAR	Unambiguous	2	1	Q6PIC6	N-Term(iTRAQ8plex)	CID	1.0000	0.0000	1	1	3357332	Unique	Used	0.508	1.024	1.264	1.521	0	0	8.38	0	2	200	3	937.14606	2809.42362	-2.65
fQSAAIGALQEASEAYLVGLFEDTNLcAIHAk	Unambiguous	3	1	P84244	N-Term(iTRAQ8plex); C27(Methylthio); K32(iTRAQ8plex)	CID	1.0000	0.0000	1	1	3766875	Unique	Used		1.408	0.706	0.956	0	0	8.37	0	0	35	3	1345.69580	4035.07285	-2.20

nMGGPYGGGnYGPGGSGGSGGYGGR	Unambiguous	3	1	O88569	N-Term(iTRAQ8plex); N10(Deamidated)	CID	1.0000	0.0000	1	1	3008302	Unique	Used	1.710	0.557	1.684	1.168	0	0	8.37	0	0	200	2	1248.05017	2495.09307	-0.71
iSFEEFcAVVGGLDIHk	Unambiguous	2	1	Q63810-2	N-Term(iTRAQ8plex); C7(Methylthio); K17(iTRAQ8plex)	CID	1.0000	0.0000	1	1	3707499	Unique	Used	0.736	1.294	1.983	0.881	0	0	8.37	0	0	200	3	840.10999	2518.31540	-5.59
aLLLVGDVAQAAEVAIEHR	Unambiguous	3	1	G3X8X7	N-Term(iTRAQ8plex)	CID	1.0000	0.0000	1	1	3541735	Unique	Used	1.027	0.972	1.032	1.085	0	0	8.37	0	8	9	3	760.43658	2279.29520	-3.23
eIELLcQEHGQENDDLVQR	Unambiguous	4	1	E9Q6X0	N-Term(iTRAQ8plex); C6(Methylthio)	CID	1.0000	0.0000	1	1	2654638	Unique	Used	1.234	0.799	0.979	0.982	0	0	8.36	0	2	147	3	873.42029	2618.24631	-1.13
dFTSEQIDEILQNHTEIVFAR	Unambiguous	2	1	Q6PIC6	N-Term(iTRAQ8plex)	CID	1.0000	0.0000	1	1	3354017	Unique	Used	0.546	0.536	0.761	0.982	0	0	8.35	0	4	22	3	937.14520	2809.42105	-3.57
gESPVASPFQNYSSIHSQSR	Unambiguous	2	1	P53995	N-Term(iTRAQ8plex)	CID	1.0000	0.0000	1	1	3451852	Unique	Used	1.005	0.972	1.086	1.055	0	0	8.35	0	0	125	3	828.07574	2482.21268	-5.55
vADVSISEVTTLLGnVHSPVVEGYVGENISGEVk	Unambiguous	3	1	P20357	N-Term(iTRAQ8plex); N15(Deamidated); K34(iTRAQ8plex)	CID	0.0395	0.0000	1	1	3419380	Unique	Used	2.126	0.980	1.688	1.196	0	0	8.35	0	0	64	3	1369.74036	4107.20652	2.59
gIEFPMADLDALSPIHTPQR	Unambiguous	6	1	Q5SRX1-2	N-Term(iTRAQ8plex)	CID	1.0000	0.0000	1	1	3540879	Unique	Used	1.804	1.437	1.192	2.355	0	0	8.35	0	10	4	3	838.10645	2512.30478	-4.96
sVcTLQITDTTGSHQFPAMQR	Unambiguous	2	1	Q91Z61	N-Term(iTRAQ8plex); C3(Methylthio)	CID	1.0000	0.0000	1	1	3839610	Unique	Used	0.704	0.500	1.038	0.968	0	0	8.34	0	16	67	3	891.10205	2671.29160	-1.07
dFTSEQIDEILQNHTEIVFAR	Unambiguous	2	1	Q6PIC6	N-Term(iTRAQ8plex)	CID	1.0000	0.0000	1	1	3354137	Unique	Used	0.576	0.617	1.120	1.181	0	0	8.34	0	2	25	3	937.14520	2809.42105	-3.57
iSFEEFcAVVGGLDIHk	Unambiguous	2	1	Q63810-2	N-Term(iTRAQ8plex); C7(Methylthio); K17(iTRAQ8plex)	CID	1.0000	0.0000	1	1	3708279	Unique	Used	0.828	1.462	1.615	0.780	0	0	8.34	0	7	200	3	840.11005	2518.31559	-5.52
fLSQPFQVAEVFTGHMGk	Unambiguous	1	1	P56480	N-Term(iTRAQ8plex); K18(iTRAQ8plex)	CID	1.0000	0.0000	1	1	3862196	Unique	Used	1.285	1.057	1.242	1.308	0	0	8.34	0	9	200	3	877.80621	2631.40409	-6.58
dVQFAVQQVLQEEHFDAR	Unambiguous	2	1	Q8R146-2	N-Term(iTRAQ8plex)	CID	1.0000	0.0000	1	1	3216460	Unique	Used	1.067	1.404	0.774	1.083	0	0	8.34	0	7	37	3	821.75604	2463.25357	-1.42
gNDISSGTVLSDYVGSGPPSGTGLHR	Unambiguous	1	1	P70296	N-Term(iTRAQ8plex)	CID	1.0000	0.0000	1	1	3474553	Unique	Used	0.660	1.017	1.543	1.162	0	0	8.33	0	0	7	3	945.47668	2834.41550	-2.40

dMHGDSEYNImFGPDIcGPGTk	Unambiguous	1	1	P14211	N-Term(iTRAQ8plex); M11(Oxidation); C17(Methylthio); K22(iTRAQ8plex)	CID	1.0000	0.0000	1	1	3170433	Unique	Used	1.141	1.103	0.928	0.785	0	0	8.33	0	0	67	3	1018.80292	3054.39420	0.50
tMSIVSYNHLGNNDGQNLSAPLQFR	Unambiguous	1	1	Q9JHU9	N-Term(iTRAQ8plex)	CID	1.0000	0.0000	1	1	3840125	Unique	Used	1.279	2.094	0.860	0.741	0	0	8.33	0	4	33	3	1027.51880	3080.54184	-3.49
iSFEEFcAVVGGLDIHk	Unambiguous	2	1	Q63810-2	N-Term(iTRAQ8plex); C7(Methylthio); K17(iTRAQ8plex)	CID	1.0000	0.0000	1	1	3690030	Unique	Used	0.648	1.337	2.378	0.875	0	0	8.33	0	2	200	3	840.10968	2518.31449	-5.95
sLIDMYSEVLDVLSDYDASYNTQDHLPR	Unambiguous	5	1	P58281-2	N-Term(iTRAQ8plex)	CID	1.0000	0.0000	1	1	3374475	Unique	Used	0.927	0.898	1.040	0.843	0	0	8.33	0	0	45	3	1188.57153	3563.70005	-4.32
hTALAANTQSqQSIHTTTLAR	Unambiguous	2	1	Q920I9	N-Term(iTRAQ8plex); Q11(Deamidated)	CID	0.0012	0.0000	1	1	3832619	Unique	Used		0.714	1.381	1.027	0	0	8.33	0	29	200	3	852.45758	2555.35819	4.00
qFGFIVLTTSAGIMDHEEAR	Unambiguous	1	1	P62245	N-Term(iTRAQ8plex)	CID	1.0000	0.0000	1	1	3560601	Unique	Used		0.506		0.911	0	0	8.33	0	19	16	3	842.76782	2526.28891	-3.00
tLALLAFDSPEESPFGDLLHMMQR	Unambiguous	1	1	Q9D7M1	N-Term(iTRAQ8plex)	CID	1.0000	0.0000	1	1	3746127	Unique	Used	1.446	0.897	1.149	1.145	0	0	8.33	0	18	136	3	1008.17896	3022.52231	-3.24
aAASVVSGPPVASDcASPGDSAR	Unambiguous	3	1	Q6P9K8	N-Term(iTRAQ8plex); C15(Methylthio)	CID	1.0000	0.0000	1	1	3299382	Unique	Used	1.680	0.463	1.767	1.273	0	0	8.33	0	0	13	2	1211.58289	2422.15849	-2.48
hELLLGAAGAGPGAGPQQATPGALLQAGPPR	Unambiguous	1	1	Q6PE01	N-Term(iTRAQ8plex)	CID	1.0000	0.0000	1	1	3876383	Unique	Used	0.961	1.412	0.773	0.434	0	0	8.32	0	18	200	3	1059.91711	3177.73679	-2.14
aESSSSISSSASLSAANTPTVGVSR	Unambiguous	2	1	Q3UQN2	N-Term(iTRAQ8plex)	CID	1.0000	0.0000	1	1	3409941	Unique	Used	0.975	1.667	1.523	0.918	0	0	8.31	0	0	82	2	1329.17468	2657.34209	-4.19
vIHLSNLPHSGYSDSAVLk	Unambiguous	1	1	Q8K310	N-Term(iTRAQ8plex); K19(iTRAQ8plex)	CID	1.0000	0.0000	1	1	3659248	Unique	Used	0.417	0.663	1.002	0.918	0	0	8.31	0	13	69	3	882.49445	2645.46878	-6.94
dFTSEQIDEILQNHTEIVFAR	Unambiguous	2	1	Q6PIC6	N-Term(iTRAQ8plex)	CID	1.0000	0.0000	1	1	3352579	Unique	Used			1.094	1.879	0	0	8.31	0	0	9	3	937.14514	2809.42087	-3.63
gNDISSGTVLSDYVGSGPPSGTGLHR	Unambiguous	1	1	P70296	N-Term(iTRAQ8plex)	CID	1.0000	0.0000	1	1	3474230	Unique	Used	2.144	0.961	1.172	0.974	0	0	8.31	0	0	100	3	945.47247	2834.40287	-6.86

iSFEEFcAVVGGLDIHk	Unambiguous	2	1	Q63810-2	N-Term(iTRAQ8plex); C7(Methylthio); K17(iTRAQ8plex)	CID	1.0000	0.0000	1	1	3688341	Unique	Used	0.837	1.517	2.307	0.910	0	0	8.31	0	0	63	3	840.11029	2518.31632	-5.23
gNDISSGTVLSDYVGSGPPSGTGLHR	Unambiguous	1	1	P70296	N-Term(iTRAQ8plex)	CID	1.0000	0.0000	1	1	3390489	Unique	Used	1.173	1.174	1.056	1.387	0	0	8.31	0	6	27	3	945.47583	2834.41294	-3.30
vNFHFILFNNVDGHLYELDGR	Unambiguous	1	1	Q9R0P9	N-Term(iTRAQ8plex)	CID	1.0000	0.0000	1	1	3706148	Unique	Used	4.319	1.852	1.080	0.810	0	0	8.31	0	8	9	3	941.81519	2823.43100	-7.46
nMGGPYGGGnYGPGGSGGSGGYGGR	Unambiguous	3	1	O88569	N-Term(iTRAQ8plex); N10(Deamidated)	CID	1.0000	0.0000	1	1	3028607	Unique	Used	1.493	0.933	1.471	1.068	0	0	8.30	0	0	113	2	1248.04956	2495.09184	-1.20
tQSSASLAASYAAqQHPQAAASYR	Unambiguous	3	1	Q8C2Q3	N-Term(iTRAQ8plex); Q14(Deamidated)	CID	0.0012	0.0000	1	1	3718116	Unique	Used	0.440	0.269	1.293	1.069	0	0	8.30	0	0	200	3	924.12921	2770.37308	1.17
aEVEEFSFITQLSGLTDHLPVAmR	Unambiguous	1	1	Q9JJY3	N-Term(iTRAQ8plex); M23(Oxidation)	CID	1.0000	0.0000	1	1	3686802	Unique	Used	0.198	1.011	1.765	1.113	0	0	8.30	0	2	5	3	1004.18250	3010.53293	-5.62
aALPSHVVTMLDNFPTNLHPMSQLSAAITALnSESNFAR	Unambiguous	1	1	Q9CZU6	N-Term(iTRAQ8plex); N32(Deamidated)	CID	0.0181	0.0000	1	1	3936080	Unique	Used				1.024	0	0	8.30	0	22	31	3	1491.09631	4471.27439	1.13
gLLEPLGLAcTFIDDISAYHk	Unambiguous	1	1	Q08642	N-Term(iTRAQ8plex); C10(Methylthio); K21(iTRAQ8plex)	CID	1.0000	0.0000	1	1	3786039	Unique	Used	0.709	0.347	1.874	2.142	0	0	8.29	0	0	200	3	977.52032	2930.54642	-5.20
nMGGPYGGGNYGPGGSGGSGGYGGR	Unambiguous	3	1	O88569	N-Term(iTRAQ8plex)	CID	1.0000	0.0000	1	1	3338948	Unique	Used	1.589	0.551	1.155	0.786	0	0	8.29	0	0	36	2	1247.55554	2494.10381	-2.81
tQSSASLAASYAAQQHPQAAASYR	Unambiguous	3	1	Q8C2Q3	N-Term(iTRAQ8plex)	CID	1.0000	0.0000	1	1	3635149	Unique	Used	1.844	0.582	0.874	0.929	0	0	8.29	0	27	9	3	923.79504	2769.37058	-5.50
eQGSSGLGSGSSGGGGSSSGLGSGYIGR	Unambiguous	2	1	Q3UHJ0-2	N-Term(iTRAQ8plex)	CID	1.0000	0.0000	1	1	3185394	Unique	Used	0.349	0.922	1.382	1.065	0	0	8.28	0	0	32	2	1353.64197	2706.27666	-3.80
iSFEEFcAVVGGLDIHk	Unambiguous	2	1	Q63810-2	N-Term(iTRAQ8plex); C7(Methylthio); K17(iTRAQ8plex)	CID	1.0000	0.0000	1	1	3688679	Unique	Used	0.796	1.547	2.045	0.805	0	0	8.28	0	0	145	3	840.10980	2518.31485	-5.81

aQLGIADEnGWQEIHQAcQR	Unambiguous	3	1	D3YZU1	N-Term(iTRAQ8plex); N9(Deamidated); C18(Methylthio)	CID	1.0000	0.0000	1	1	3235951	Unique	Used	1.165	1.544	1.240	1.036	0	0	8.27	0	23	119	3	873.41626	2618.23423	-1.97
hLVDQmPDSSDNPAGLAEQcPGMR	Unambiguous	1	1	P46414	N-Term(iTRAQ8plex); M6(Oxidation); C20(Methylthio)	CID	1.0000	0.0000	1	1	3233584	Unique	Used	1.300	1.598	1.347	1.280	0	0	8.27	0	12	91	3	978.77515	2934.31089	-1.64
gPAVGIDLGTTYScVGVFQHGk	Unambiguous	4	1	P63017	N-Term(iTRAQ8plex); C14(Methylthio); K22(iTRAQ8plex)	CID	1.0000	0.0000	1	1	3808000	Unique	Used	1.160	0.784	1.438	1.077	0	0	8.27	0	17	200	3	954.16650	2860.48496	-3.39
vFVLDEADVMIATQGHQDQSIR	Unambiguous	3	1	Q61655	N-Term(iTRAQ8plex)	CID	1.0000	0.0000	1	1	3262166	Unique	Used	0.805	0.971	0.751	0.808	0	0	8.27	0	0	16	3	926.14703	2776.42655	0.83
iIEVVDAIMTTAQSHQR	Unambiguous	3	1	Q8C605	N-Term(iTRAQ8plex)	CID	1.0000	0.0000	1	1	3899031	Unique	Used	1.572	1.073	0.948	1.139	0	0	8.27	0	0	22	3	739.40137	2216.18955	-5.22
fTDTDLLDFGQcVDQNHQR	Unambiguous	1	1	Q9DCJ9	N-Term(iTRAQ8plex); C12(Methylthio)	CID	1.0000	0.0000	1	1	3304918	Unique	Used	1.401	0.674	1.199	1.125	0	0	8.26	0	9	41	3	868.07294	2602.20426	2.85
sQLQDLVEFPYVnLHNEVVGIIESR	Unambiguous	1	1	Q9Z0W3	N-Term(iTRAQ8plex); N13(Deamidated)	CID	1.0000	0.0000	1	1	3645324	Unique	Used		0.930		0.741	0	0	8.26	0	0	29	3	1068.56873	3203.69162	0.79
iTSLAQLnAANHDAAIFPGGFGAAk	Unambiguous	1	1	Q9D172	N-Term(iTRAQ8plex); N8(Deamidated); K25(iTRAQ8plex)	CID	0.0024	0.0000	1	1	3622677	Unique	Used	1.542	1.167	1.619	1.170	0	0	8.26	0	5	6	3	1022.22211	3064.65177	-5.20
nLHQSGFSLSGAQIDDNIPR	Unambiguous	1	1	O08553	N-Term(iTRAQ8plex)	CID	1.0000	0.0000	1	1	3617078	Unique	Used	2.515	0.978	1.624	2.103	0	0	8.26	0	4	7	3	825.09180	2473.26084	-5.23
kAAAELLQSQGSQAGGSQTLk	Unambiguous	3	1	Q9R1T4-2	N-Term(iTRAQ8plex); K1(iTRAQ8plex); K21(iTRAQ8plex)	CID	1.0000	0.0000	1	1	3471707	Unique	Used	1.083	1.016	1.299	0.957	0	0	8.26	1	0	174	3	995.90387	2985.69706	-4.22
iSFEEFcAVVGGLDIHk	Unambiguous	2	1	Q63810-2	N-Term(iTRAQ8plex); C7(Methylthio); K17(iTRAQ8plex)	CID	1.0000	0.0000	1	1	3690385	Unique	Used	0.449	1.877	1.941	0.647	0	0	8.26	0	3	200	3	840.11047	2518.31687	-5.01

lSDQcTGLqGFLVFHSFGGGTGSGFTSLLMER	Unambiguous	1	1	P68368	N-Term(iTRAQ8plex); C5(Methylthio); Q9(Deamidated)	CID	1.0000	0.0000	1	1	3917969	Unique	Used	0.996	1.549	1.599	1.569	0	0	8.26	0	16	59	3	1234.26514	3700.78086	1.51
vVDNPIYLSDMGAALTGAESHELQDVLEETnILk	Unambiguous	1	1	Q8CGK3	N-Term(iTRAQ8plex); N31(Deamidated); K34(iTRAQ8plex)	CID	1.0000	0.0000	1	1	3305783	Unique	Used	0.635	0.964	0.931	1.789	0	0	8.26	0	23	147	3	1432.07874	4294.22165	-1.06
aLILDLYEQIVTGVEYIHSk	Unambiguous	1	1	Q03963	N-Term(iTRAQ8plex); K20(iTRAQ8plex)	CID	1.0000	0.0000	1	1	3785964	Unique	Used	0.501	0.949	0.793	1.063	0	0	8.26	0	17	171	3	971.55469	2912.64951	-3.39
vTAGDSEVEAAAqAAGIHDAILSFPEGYETQVGER	Unambiguous	1	1	Q9DC29	N-Term(iTRAQ8plex); Q13(Deamidated)	CID	1.0000	0.0000	1	1	3132786	Unique	Used	0.702	0.720	0.797	1.017	0	0	8.25	0	18	69	3	1298.63855	3893.90110	0.69
sLSSSMqSLSSGSSPGSLTSSR	Unambiguous	1	1	Q5SXA9	N-Term(iTRAQ8plex); Q7(Deamidated)	CID	1.0000	0.0000	1	1	3893526	Unique	Used	0.500	0.995	1.155	1.011	0	0	8.25	0	0	200	2	1218.09912	2435.19097	1.47
dFTSEQIDEILQNHTEIVFAR	Unambiguous	2	1	Q6PIC6	N-Term(iTRAQ8plex)	CID	1.0000	0.0000	1	1	3375347	Unique	Used	0.610	0.897	0.921	1.140	0	0	8.25	0	0	200	3	937.14551	2809.42197	-3.24
dVQFAVQQVLQEEHFDAR	Unambiguous	2	1	Q8R146-2	N-Term(iTRAQ8plex)	CID	1.0000	0.0000	1	1	3238566	Unique	Used	1.608	1.094	0.874	1.607	0	0	8.24	0	1	13	3	821.75635	2463.25449	-1.04
nLHQSGFSLSGAQIDDNIPR	Unambiguous	1	1	O08553	N-Term(iTRAQ8plex)	CID	1.0000	0.0000	1	1	3534944	Unique	Used	0.955	1.322	1.078	1.162	0	0	8.24	0	0	1	3	825.09283	2473.26395	-3.97
aTLQEVVGLHSDMAVEAGAPYAER	Unambiguous	1	1	G3UYZ1	N-Term(iTRAQ8plex)	CID	1.0000	0.0000	1	1	3302505	Unique	Used	0.677	0.244	1.224	0.584	0	0	8.24	0	16	71	3	940.14783	2818.42893	-2.08
gTAEGAVDAMLVHGQDAITVSnGGR	Unambiguous	2	1	Q8C7X2-2	N-Term(iTRAQ8plex); N22(Deamidated)	CID	1.0000	0.0000	1	1	3109294	Unique	Used	0.989	0.826	0.699	0.803	0	0	8.24	0	17	200	3	911.12787	2731.36905	2.44
qDLLTAAPASEIAEGLNLHTIR	Unambiguous	1	1	Q9WUL7	N-Term(iTRAQ8plex)	CID	1.0000	0.0000	1	1	3560346	Unique	Used	2.677	1.313	0.729	0.992	0	0	8.23	0	5	5	3	879.81836	2637.44052	-4.13
eVNEnFAIDLIAQQPVNEVEHR	Unambiguous	2	1	P52503	N-Term(iTRAQ8plex); N5(Deamidated)	CID	1.0000	0.0000	1	1	2814917	Unique	Used	2.614	1.114	1.934	1.181	0	0	8.23	0	4	200	3	957.15875	2869.46170	-0.61
lALSQNQQSSGAAGPTGk	Unambiguous	3	1	Q5SUF2	N-Term(iTRAQ8plex); K18(iTRAQ8plex)	CID	1.0000	0.0000	1	1	3407233	Unique	Used	0.327	0.760	0.793	0.716	0	0	8.23	0	0	101	2	1162.14392	2323.28057	-0.90

eLAcDDPEAEQVALLAVDYLnNHLLQGFk	Unambiguous	1	1	P29699	N-Term(iTRAQ8plex); C4(Methylthio); N21(Deamidated); K29(iTRAQ8plex)	CID	0.0255	0.0000	1	1	3500892	Unique	Used	2.479	1.738	0.274	0.510	0	0	8.23	0	28	79	3	1295.32788	3883.96909	-0.43
eTAAAHQASSSPPIDAATAEPYGFR	Unambiguous	1	1	P14873	N-Term(iTRAQ8plex)	CID	1.0000	0.0000	1	1	3166155	Unique	Used	1.638	0.677	1.533	0.978	0	0	8.22	0	2	8	3	950.46912	2849.39280	-2.82
gNFGGSFAGSFGGAGGHAPGVAR	Unambiguous	4	1	Q9D0E1-2	N-Term(iTRAQ8plex)	CID	1.0000	0.0000	1	1	3892328	Unique	Used	0.927	0.715	0.937	0.852	0	0	8.22	0	2	18	3	780.38770	2339.14853	-4.21
yFHVVIAGPQDSPFEGGTFk	Unambiguous	1	1	P61089	N-Term(iTRAQ8plex); K20(iTRAQ8plex)	CID	1.0000	0.0000	1	1	3517963	Unique	Used	1.492	0.979	0.913	0.778	0	0	8.22	0	8	18	3	935.49780	2804.47885	-2.85
hLNYVEFTQFLQELQLEHAR	Unambiguous	1	1	Q8BH59	N-Term(iTRAQ8plex)	CID	1.0000	0.0000	1	1	3726475	Unique	Used	3.749	1.937	1.512	2.788	0	0	8.22	0	24	4	3	940.49225	2819.46219	-5.70
nSEDGLNMFDGTDScYFHSGPR	Unambiguous	3	1	Q9D6Y9	N-Term(iTRAQ8plex); C15(Methylthio)	CID	1.0000	0.0000	1	1	3054267	Unique	Used	1.321	0.706	0.960	1.345	0	0	8.21	0	26	147	3	933.72992	2799.17521	0.03
sLEAIELGLGEAQVILALSSHLGAVESEk	Unambiguous	4	1	D3YXZ3	N-Term(iTRAQ8plex); K29(iTRAQ8plex)	CID	1.0000	0.0000	1	1	3520834	Unique	Used	0.921	0.938	0.857	0.844	0	0	8.21	0	5	74	3	1191.33289	3571.98410	-5.69
kYVATLGVEVHPLVFHTNR	Unambiguous	1	1	P62827	N-Term(iTRAQ8plex); K1(iTRAQ8plex)	HCD	1.0000	0.0000	1	1	3874475	Unique	Used	0.466	1.031	0.998	1.119	0	0	8.21	1	0	192	3	930.20416	2788.59793	-3.71
tFEHVTSEIGAEEAEEVGVEHLLR	Unambiguous	1	1	P26516	N-Term(iTRAQ8plex)	CID	1.0000	0.0000	1	1	2852011	Unique	Used	1.491	1.478	0.691	1.523	0	0	8.20	0	19	200	3	995.84137	2985.50956	-0.42
cSSPVDMDHSNAEGGQSPGPEk	Unambiguous	2	1	G3X9K4	N-Term(iTRAQ8plex); C1(Methylthio); K22(iTRAQ8plex)	CID	1.0000	0.0000	1	1	2698140	Unique	Used	1.264	1.137	2.175	1.827	0	0	8.20	0	0	200	3	961.77502	2883.31052	-2.19
nMGGPYGGGNYGPGGSGGSGGyGGR	Unambiguous	3	1	O88569	N-Term(iTRAQ8plex); Y22(iTRAQ8plex)	CID	1.0000	0.0000	1	1	3428892	Unique	Used	1.268	1.244	0.867	0.608	0	0	8.20	0	0	200	3	933.43982	2798.30490	-4.03
rQPMPSPSEGSLSSGGMDQGSDAPAR	Unambiguous	7	1	E9PVY0	N-Term(iTRAQ8plex)	CID	1.0000	0.0000	1	1	3007350	Unique	Used	1.550	0.747	1.218	0.643	0	0	8.20	1	10	90	3	969.45715	2906.35691	-3.66

sHSNQLVTDcISAmNPDTVLR	Unambiguous	2	1	Q9Z0S1	N-Term(iTRAQ8plex); C10(Methylthio); M14(Oxidation)	CID	1.0000	0.0000	1	1	3782098	Unique	Used	0.734	1.027	1.768	2.422	0	0	8.20	0	0	3	3	889.76385	2667.27701	-2.73
gnDISSGTVLSDYVGSGPPSGTGLHR	Unambiguous	1	1	P70296	N-Term(iTRAQ8plex); N2(Deamidated)	CID	1.0000	0.0000	1	1	3211616	Unique	Used	1.320	1.067	1.059	1.103	0	0	8.20	0	0	200	3	945.80554	2835.40207	-1.50
nAQAIEDMVGYAQETQHEk	Unambiguous	1	1	Q3TXS7	N-Term(iTRAQ8plex); K19(iTRAQ8plex)	CID	1.0000	0.0000	1	1	2918842	Unique	Used	0.673	0.675	0.693	0.720	0	0	8.19	0	0	115	3	924.12927	2770.37326	-7.01
aAASGPASASAPAAEILLTPAR	Unambiguous	1	1	Q91VK4	N-Term(iTRAQ8plex)	CID	1.0000	0.0000	1	1	3641424	Unique	Used	0.360	1.047	0.999	1.152	0	0	8.19	0	0	10	2	1149.13562	2297.26396	-5.57
aAFGLSEAGFNTAcLTk	Unambiguous	1	1	Q8K2B3	N-Term(iTRAQ8plex); C14(Methylthio); K17(iTRAQ8plex)	CID	1.0000	0.0000	1	1	3726360	Unique	Used	1.423	0.759	0.947	1.063	0	0	8.19	0	0	16	2	1178.10889	2355.21050	-8.18
sLEMLELGLSEAQVMMALSnHLNAVESEk	Unambiguous	5	1	Q5UE59	N-Term(iTRAQ8plex); N20(Deamidated); K29(iTRAQ8plex)	CID	0.0269	0.0000	1	1	3500865	Unique	Used	1.044	0.787	0.902	1.041	0	0	8.19	0	0	79	3	1261.65796	3782.95932	3.10
vTYMVHDFEEGGGVAMGMYnQDk	Unambiguous	1	1	O88844	N-Term(iTRAQ8plex); N20(Deamidated); K23(iTRAQ8plex)	CID	0.0110	0.0000	1	1	2960204	Unique	Used	1.068	0.790	0.558	0.662	0	0	8.18	0	0	200	3	1063.16858	3187.49118	-2.63
qQADFVDLSFPTISSTGPnGAIIHYAPVPETNR	Unambiguous	1	1	Q6P1B1	N-Term(iTRAQ8plex); N19(Deamidated)	CID	1.0000	0.0000	1	1	3439683	Unique	Used	3.058	2.234	1.035	1.191	0	0	8.18	0	21	10	3	1283.31653	3847.93503	-2.48
gnDISSGTVLSDYVGSGPPSGTGLHR	Unambiguous	1	1	P70296	N-Term(iTRAQ8plex); N2(Deamidated)	CID	1.0000	0.0000	1	1	3390476	Unique	Used	1.678	1.426	1.340	1.490	0	0	8.18	0	13	27	3	945.80658	2835.40519	-0.40
lNDHFPLVVWQTGSGTQTNMnVNEVISNR	Unambiguous	2	1	P97807-2	N-Term(iTRAQ8plex); N21(Deamidated)	CID	0.0134	0.0000	1	1	3824125	Unique	Used	4.569	1.459	1.264	1.031	0	0	8.18	0	18	66	3	1192.60168	3575.79050	1.39

aADFQLHTHVNDGTEFGGSIYQk	Unambiguous	3	1	J3QPE8	N-Term(iTRAQ8plex); K23(iTRAQ8plex)	CID	1.0000	0.0000	1	1	3277709	Unique	Used	0.583	0.826	1.219	1.360	0	0	8.18	0	14	56	3	1048.53711	3143.59677	-1.25
aIQINPAFADAHSNLASIHk	Unambiguous	2	1	Q8CGY8	N-Term(iTRAQ8plex); K20(iTRAQ8plex)	CID	1.0000	0.0000	1	1	3660018	Unique	Used	0.499	1.526	1.312	0.973	0	0	8.17	0	0	53	3	909.50781	2726.50888	-4.03
iSFEEFcAVVGGLDIHk	Unambiguous	2	1	Q63810-2	N-Term(iTRAQ8plex); C7(Methylthio); K17(iTRAQ8plex)	CID	1.0000	0.0000	1	1	3707616	Unique	Used	0.811	1.292	2.120	0.833	0	0	8.17	0	4	200	3	840.10992	2518.31522	-5.66
eMQEVETNLQEVVFDYLHATAYQNTALGR	Unambiguous	1	1	Q9CXT8	N-Term(iTRAQ8plex)	CID	1.0000	0.0000	1	1	3419456	Unique	Used	1.175	1.264	0.452	0.819	0	0	8.17	0	25	29	3	1225.27234	3673.80246	-2.35
gNVVFGEPITASLGIDGTHYWSk	Unambiguous	5	1	E9PWE8	N-Term(iTRAQ8plex); K23(iTRAQ8plex)	CID	1.0000	0.0000	1	1	3644510	Unique	Used	0.841	0.329	0.308	0.370	0	0	8.17	0	3	4	3	1019.54388	3056.61710	-4.29
qISQMADDTVAELDQHLAAk	Unambiguous	1	1	Q9D6S7	N-Term(iTRAQ8plex); K20(iTRAQ8plex)	CID	1.0000	0.0000	1	1	3238762	Unique	Used	1.020	0.373	1.390	0.926	0	0	8.17	0	0	66	3	931.49396	2792.46732	-1.30
gNDISSGTVLSDYVGSGPPSGTGLHR	Unambiguous	1	1	P70296	N-Term(iTRAQ8plex)	CID	1.0000	0.0000	1	1	3556190	Unique	Used	1.640	0.927	0.744	0.553	0	0	8.17	0	23	138	3	945.47375	2834.40671	-5.50
rHHQEEVGELLGQIqGcGAAQAQAQAEAR	Unambiguous	1	1	P19246	N-Term(iTRAQ8plex); Q15(Deamidated); C17(Methylthio)	CID	0.0098	0.0000	1	1	3454581	Unique	Used	2.761	0.959	0.798	1.287	0	0	8.17	1	0	200	3	1146.23291	3436.68418	2.58
qHGEQLGGGGSGGGGYNTSk	Unambiguous	7	1	Q80U78-2	N-Term(iTRAQ8plex); K20(iTRAQ8plex)	CID	1.0000	0.0000	1	1	2841061	Unique	Used	0.892	1.084	1.115	0.892	0	0	8.16	0	29	200	3	819.41595	2456.23331	-1.70
dFTSEQIDEILQNHTEIVFAR	Unambiguous	2	1	Q6PIC6	N-Term(iTRAQ8plex)	CID	1.0000	0.0000	1	1	3354902	Unique	Used	0.574	0.684	0.968	1.314	0	0	8.16	0	1	51	3	937.14520	2809.42105	-3.57
sMLQATAEANNLAAVATAk	Unambiguous	1	1	Q8BH66	N-Term(iTRAQ8plex); K19(iTRAQ8plex)	CID	1.0000	0.0000	1	1	3578826	Unique	Used	0.600	1.003	1.009	0.962	0	0	8.16	0	0	24	2	1242.18494	2483.36260	-4.94
nYLPAInGIVFLVDcADHSR	Unambiguous	2	1	Q99JZ4	N-Term(iTRAQ8plex); N7(Deamidated); C15(Methylthio)	CID	1.0000	0.0000	1	1	3623447	Unique	Used	1.900	2.172	1.185	2.056	0	0	8.16	0	7	25	3	856.76544	2568.28177	-2.93

qETLAQQQLqLEQIQQLQQQLHQQLEEQk	Unambiguous	2	1	Q9QYX7	N-Term(iTRAQ8plex); Q10(Deamidated); K29(iTRAQ8plex)	CID	0.0061	0.0000	1	1	3458775	Unique	Used	0.583	0.339	1.683	0.938	0	0	8.16	0	29	200	3	1389.42114	4166.24887	4.47
fDLGQDVIDFTGHSLALYR	Unambiguous	2	1	Q61598	N-Term(iTRAQ8plex)	CID	1.0000	0.0000	1	1	3666100	Unique	Used	0.656	0.594	0.842	1.016	0	0	8.15	0	9	7	3	824.42999	2471.27542	-4.80
vEQIAAIAQELNELDYHDAVNVNDR	Unambiguous	1	1	Q9JI91	N-Term(iTRAQ8plex)	CID	1.0000	0.0000	1	1	3284527	Unique	Used	1.818	3.011	2.045	0.726	0	0	8.15	0	10	73	3	1048.53638	3143.59458	1.08
lDAWDEAQVEFMASHGNEAAR	Unambiguous	1	1	E9PY16	N-Term(iTRAQ8plex)	CID	1.0000	0.0000	1	1	2940144	Unique	Used	0.565	0.794	0.704	0.851	0	0	8.14	0	4	15	3	884.41937	2651.24356	-1.02
gNFGGSFAGSFGGAGGHAPGVAR	Unambiguous	4	1	Q9D0E1-2	N-Term(iTRAQ8plex)	CID	1.0000	0.0000	1	1	3911363	Unique	Used	0.642	0.781	0.931	0.908	0	0	8.14	0	0	200	2	1170.07556	2339.14385	-6.21
tIGSTYMAATGLSAVPSQEHAQEPER	Unambiguous	2	1	Q80TL1	N-Term(iTRAQ8plex)	CID	1.0000	0.0000	1	1	3278358	Unique	Used	0.806	0.790	1.121	1.050	0	0	8.14	0	0	136	3	1012.50800	3035.50943	1.57
dFTSEQIDEILQNHTEIVFAR	Unambiguous	2	1	Q6PIC6	N-Term(iTRAQ8plex)	CID	1.0000	0.0000	1	1	3354302	Unique	Used	0.667	0.599	1.214	1.500	0	0	8.14	0	5	39	3	937.14508	2809.42069	-3.70
dVQFAVQQVLQEEHFDAR	Unambiguous	2	1	Q8R146-2	N-Term(iTRAQ8plex)	CID	1.0000	0.0000	1	1	3238416	Unique	Used	1.353	0.909	0.989	1.107	0	0	8.14	0	3	23	3	821.75635	2463.25449	-1.04
iSFEEFcAVVGGLDIHk	Unambiguous	2	1	Q63810-2	N-Term(iTRAQ8plex); C7(Methylthio); K17(iTRAQ8plex)	CID	1.0000	0.0000	1	1	3690273	Unique	Used	0.816	2.022	2.336	1.034	0	0	8.14	0	3	200	3	840.11041	2518.31668	-5.08
iSFEEFcAVVGGLDIHk	Unambiguous	2	1	Q63810-2	N-Term(iTRAQ8plex); C7(Methylthio); K17(iTRAQ8plex)	CID	1.0000	0.0000	1	1	3708596	Unique	Used	0.532	1.306	1.659	0.815	0	0	8.13	0	4	200	3	840.11053	2518.31705	-4.94
lEGYWGEAQSLcQAVDEHLR	Unambiguous	2	1	Q6R891	N-Term(iTRAQ8plex); C12(Methylthio)	CID	1.0000	0.0000	1	1	3541554	Unique	Used	1.781	0.648	0.836	0.781	0	0	8.13	0	10	11	3	885.42401	2654.25748	-2.66
dFTSEQIDEILQNHTEIVFAR	Unambiguous	2	1	Q6PIC6	N-Term(iTRAQ8plex)	CID	1.0000	0.0000	1	1	3354542	Unique	Used	0.586	0.634	1.145	1.378	0	0	8.13	0	3	65	3	937.14557	2809.42215	-3.17
sNMHFTSSSTGGLSSSqSSYSPSSR	Unambiguous	2	1	Q80UY2	N-Term(iTRAQ8plex); Q17(Deamidated)	CID	1.0000	0.0000	1	1	3755853	Unique	Used	1.264	1.364	1.576	0.757	0	0	8.13	0	0	200	3	957.77460	2871.30924	3.04

gSDHTDVcGNVVGSSGGSSSSGGSDk	Unambiguous	2	1	A2AHG0	N-Term(iTRAQ8plex); C8(Methylthio); K26(iTRAQ8plex)	CID	1.0000	0.0000	1	1	2896612	Unique	Used	3.454	1.285	2.266	0.867	0	0	8.12	0	0	200	3	998.79193	2994.36124	-0.45
eVAQQAVDADVHAVGVSTLAAGHk	Unambiguous	1	1	P16332	N-Term(iTRAQ8plex); K24(iTRAQ8plex)	CID	1.0000	0.0000	1	1	3211153	Unique	Used	1.099	1.530	1.177	1.119	0	0	8.12	0	0	186	3	994.54236	2981.61252	-4.71
sALFAQINQGESITHALk	Unambiguous	1	1	P40124	N-Term(iTRAQ8plex); K18(iTRAQ8plex)	CID	1.0000	0.0000	1	1	3704145	Unique	Used	0.602	0.974	1.214	1.109	0	0	8.11	0	0	64	3	846.14526	2536.42124	-5.19
dFTSEQIDEILQNHTEIVFAR	Unambiguous	2	1	Q6PIC6	N-Term(iTRAQ8plex)	CID	1.0000	0.0000	1	1	3375933	Unique	Used	0.749	0.970	0.770	0.965	0	0	8.11	0	26	200	3	937.14539	2809.42160	-3.37
mVVNEGADGGQSVYHIHLHVLGGR	Unambiguous	1	1	P70349	N-Term(iTRAQ8plex); M1(Oxidation)	CID	1.0000	0.0000	1	1	3679142	Unique	Used	2.102	0.989	1.612	1.483	0	0	8.11	0	0	78	3	955.82214	2865.45188	-7.45
aVEPLWVEGAGHNDIELYSQYLER	Unambiguous	2	1	Q99JW1-2	N-Term(iTRAQ8plex)	CID	1.0000	0.0000	1	1	3216971	Unique	Used	0.769	0.631	1.221	0.965	0	0	8.11	0	24	29	3	1031.52515	3092.56089	-0.73
aAAASAAEAGIATPGTEGER	Unambiguous	4	1	O35226-2	N-Term(iTRAQ8plex)	CID	1.0000	0.0000	1	1	2846532	Unique	Used	1.835	0.836	1.305	1.046	0	0	8.11	0	0	106	2	1053.03772	2105.06816	-4.54
tQSSASLAASYAAQqHPQAAASYR	Unambiguous	3	1	Q8C2Q3	N-Term(iTRAQ8plex); Q15(Deamidated)	CID	0.0185	0.0000	1	1	3756843	Unique	Used	1.134	0.772	1.016	0.776	0	0	8.10	0	0	170	3	924.13141	2770.37967	3.55
dMAIATGGAVFGEEGLNLNLEDVQAHDLGk	Unambiguous	1	1	P63038	N-Term(iTRAQ8plex); K30(iTRAQ8plex)	CID	1.0000	0.0000	1	1	3265230	Unique	Used		1.060	0.980	1.468	0	0	8.10	0	0	200	3	1231.63940	3692.90366	-0.40
rGPNYTSGYGTNSELSNPSETESER	Unambiguous	6	1	Q61584	N-Term(iTRAQ8plex)	CID	1.0000	0.0000	1	1	2631547	Unique	Used	1.166		0.822	1.035	0	0	8.10	1	0	40	3	1012.80383	3036.39695	-3.80
dFTSEQIDEILQNHTEIVFAR	Unambiguous	2	1	Q6PIC6	N-Term(iTRAQ8plex)	CID	1.0000	0.0000	1	1	3356762	Unique	Used	0.804	0.840	1.333	1.602	0	0	8.10	0	0	200	3	937.14557	2809.42215	-3.17
sLNPALDGLTcGLTSHDk	Unambiguous	1	1	Q8CGF6	N-Term(iTRAQ8plex); C11(Methylthio); K18(iTRAQ8plex)	CID	1.0000	0.0000	1	1	3436580	Unique	Used	0.527	0.831	0.957	1.049	0	0	8.10	0	14	39	3	832.77020	2496.29605	-3.47
tVQTAAANAASTAATSAAQNAFk	Unambiguous	2	1	Q8K021	N-Term(iTRAQ8plex); K23(iTRAQ8plex)	CID	1.0000	0.0000	1	1	3762266	Unique	Used	0.421	0.995	1.125	1.177	0	0	8.09	0	0	13	2	1387.74231	2774.47734	-4.34
eAMEAQIQNFGQTPSQLLIEPHPPR	Unambiguous	4	1	Q9EPN1-4	N-Term(iTRAQ8plex)	CID	1.0000	0.0000	1	1	3457567	Unique	Used	0.906	1.243	0.947	0.880	0	0	8.09	0	0	195	3	1045.87354	3135.60605	-4.44

iSFEEFcAVVGGLDIHk	Unambiguous	2	1	Q63810-2	N-Term(iTRAQ8plex); C7(Methylthio); K17(iTRAQ8plex)	CID	1.0000	0.0000	1	1	3709147	Unique	Used	0.861	1.221	2.057	1.189	0	0	8.09	0	0	200	3	840.11053	2518.31705	-4.94
qALNEHFQSILQTLEEQVSGER	Unambiguous	1	1	Q03157	N-Term(iTRAQ8plex)	CID	1.0000	0.0000	1	1	3645147	Unique	Used	1.131				0	0	8.09	0	24	17	3	954.15631	2860.45438	-6.97
sNVASTAAcSSASSLGLGLAYR	Unambiguous	1	1	P46660	N-Term(iTRAQ8plex); C9(Methylthio)	CID	1.0000	0.0000	1	1	3934586	Unique	Used	1.373				0	0	8.09	0	0	200	2	1218.60437	2436.20146	-6.18
ecDEELQDTMLLLSQMGPDAHMR	Unambiguous	3	1	Q9EQZ6-3	N-Term(iTRAQ8plex); C2(Methylthio)	CID	1.0000	0.0000	1	1	3217633	Unique	Used	0.996	1.315	0.852	0.879	0	0	8.08	0	0	83	3	1004.78754	3012.34806	-2.24
fNQSQLEAGENEqITIADDHYcSSGQDQGSQVPR	Unambiguous	1	1	Q80XQ2	N-Term(iTRAQ8plex); Q13(Deamidated); C22(Methylthio)	CID	0.0074	0.0000	1	1	2831602	Unique	Used			2.263	1.885	0	0	8.08	0	0	200	3	1368.28528	4102.84128	2.81
aHDGGIYAISWSPDSTHLLSASGDk	Unambiguous	1	1	O88342	N-Term(iTRAQ8plex); K25(iTRAQ8plex)	CID	1.0000	0.0000	1	1	3345721	Unique	Used	1.496	1.646	1.654	2.263	0	0	8.08	0	18	40	3	1065.21497	3193.63034	-2.24
sVFIAQNVASLQELGGSEk	Unambiguous	4	1	A2AN08-3	N-Term(iTRAQ8plex); K19(iTRAQ8plex)	CID	1.0000	0.0000	1	1	3478841	Unique	Used	0.358	1.024	0.658	0.857	0	0	8.08	0	0	200	2	1293.22058	2585.43389	-2.20
vQNATLAVAnITNADSATR	Unambiguous	1	1	P54116	N-Term(iTRAQ8plex); N10(Deamidated)	CID	0.0037	0.0000	1	1	3702885	Unique	Used	0.925	1.677	0.579	1.326	0	0	8.08	0	26	200	2	1118.09741	2235.18755	-0.34
dYNFVnGScMLYEANHVR	Unambiguous	1	1	Q9DBB8	N-Term(iTRAQ8plex); N6(Deamidated); C9(Methylthio)	CID	1.0000	0.0000	1	1	3281750	Unique	Used	1.015	1.117	1.304	1.447	0	0	8.08	0	0	87	3	828.37384	2483.10697	-1.06
tGEMSGPVFTDSGIHIILR	Unambiguous	1	1	Q9QUR7	N-Term(iTRAQ8plex)	CID	1.0000	0.0000	1	1	3642807	Unique	Used	2.921	0.926	1.459	0.840	0	0	8.08	0	2	5	3	778.74829	2334.23032	-5.43
sLEMLELGLSEAQVMMALSnHLNAVESEk	Unambiguous	5	1	Q5UE59	N-Term(iTRAQ8plex); N20(Deamidated); K29(iTRAQ8plex)	CID	0.0087	0.0000	1	1	3480310	Unique	Used	0.814	0.950	0.877	0.938	0	0	8.08	0	13	58	3	1261.65417	3782.94797	0.10

vADVSISEVTTLLGNVHSPVVEGYVGEnISGEVk	Unambiguous	3	1	P20357	N-Term(iTRAQ8plex); N28(Deamidated); K34(iTRAQ8plex)	CID	0.0434	0.0000	1	1	3420361	Unique	Used	1.172	1.687	1.979	1.376	0	0	8.07	0	11	200	3	1369.73792	4107.19919	0.81
sNMHFTSSSTGGLSSSQSSYSPSSR	Unambiguous	2	1	Q80UY2	N-Term(iTRAQ8plex)	CID	1.0000	0.0000	1	1	3755863	Unique	Used	1.362	1.192	1.388	0.781	0	0	8.07	0	0	188	3	957.44293	2870.31424	-0.78
nVAELTVmDVYDIASLVGHEFER	Unambiguous	1	1	Q9JJC6	N-Term(iTRAQ8plex); M8(Oxidation)	CID	1.0000	0.0000	1	1	3419650	Unique	Used	1.757	2.918	1.230	1.025	0	0	8.07	0	10	94	3	976.49396	2927.46732	-3.08
gNDISSGTVLSDYVGSGPPSGTGLHR	Unambiguous	1	1	P70296	N-Term(iTRAQ8plex)	CID	1.0000	0.0000	1	1	3495327	Unique	Used	1.257	1.316	1.260	1.230	0	0	8.07	0	0	69	3	945.47614	2834.41385	-2.98
sGILEQTGATTGVLQSDTMDHYR	Unambiguous	2	1	Q9JI19	N-Term(iTRAQ8plex)	CID	1.0000	0.0000	1	1	3300642	Unique	Used	1.109	1.052	1.099	0.962	0	0	8.07	0	21	26	3	928.79523	2784.37113	-2.34
aVLASMDnENMHTPDIGGQGTTSQAIQDIIR	Unambiguous	1	1	P70404	N-Term(iTRAQ8plex); N8(Deamidated)	CID	1.0000	0.0000	1	1	3304623	Unique	Used	1.217	1.550	1.622	1.454	0	0	8.06	0	14	192	3	1196.92224	3588.75217	-1.54
aVVFETGEAGAASGAGSLPGEVR	Unambiguous	3	1	Q9QYR6	N-Term(iTRAQ8plex)	CID	1.0000	0.0000	1	1	3108259	Unique	Used	2.081	0.704	2.160	0.971	0	0	8.06	0	0	40	2	1218.63635	2436.26543	-0.78
iSFEEFcAVVGGLDIHk	Unambiguous	2	1	Q63810-2	N-Term(iTRAQ8plex); C7(Methylthio); K17(iTRAQ8plex)	CID	1.0000	0.0000	1	1	3707220	Unique	Used	0.723	1.386	1.711	0.765	0	0	8.06	0	10	115	3	840.10986	2518.31504	-5.74
hLNLAENSSYMHVYR	Unambiguous	1	1	Q61644	N-Term(iTRAQ8plex)	CID	1.0000	0.0000	1	1	3552741	Unique	Used	1.385	1.324	1.587	1.123	0	0	8.05	0	9	141	3	713.35956	2138.06412	-5.31
sNHEEEVADLLAQIQASHITVER	Unambiguous	2	1	P08553	N-Term(iTRAQ8plex)	CID	1.0000	0.0000	1	1	3303549	Unique	Used	2.485	0.609	1.262	1.117	0	0	8.05	0	0	28	3	965.16809	2893.48972	-2.10
gNDISSGTVLSDYVGSGPPSGTGLHR	Unambiguous	1	1	P70296	N-Term(iTRAQ8plex)	CID	1.0000	0.0000	1	1	3681636	Unique	Used	1.098	0.784	1.329	1.169	0	0	8.04	0	14	108	3	945.47192	2834.40122	-7.44
gNDISSGTVLSDYVGSGPPSGTGLHR	Unambiguous	1	1	P70296	N-Term(iTRAQ8plex)	CID	1.0000	0.0000	1	1	3390143	Unique	Used	2.854	1.417	1.837	1.252	0	0	8.04	0	4	3	3	945.47601	2834.41349	-3.11
vEYPVMYSTDPEnGHMFNcIQR	Unambiguous	1	1	Q9CPU4	N-Term(iTRAQ8plex); N13(Deamidated); C19(Methylthio)	CID	1.0000	0.0000	1	1	3283245	Unique	Used	0.819	1.044	1.025	0.953	0	0	8.04	0	16	57	3	994.44501	2981.32047	-1.33
tVYFAEEVQcEGNSFHk	Unambiguous	1	1	P97315	N-Term(iTRAQ8plex); C10(Methylthio); K17(iTRAQ8plex)	CID	1.0000	0.0000	1	1	3129018	Unique	Used	2.056	0.934	1.740	0.975	0	0	8.04	0	0	31	3	881.43170	2642.28055	-1.29

tGQTDPFLGMMmGLGGGMDFDSk	Unambiguous	3	1	Q8VDQ8-2	N-Term(iTRAQ8plex); M12(Oxidation); K23(iTRAQ8plex)	CID	1.0000	0.0000	1	1	3459375	Unique	Used	2.362	1.459	1.166	1.279	0	0	8.04	0	19	64	3	1006.48132	3017.42942	-1.46
iNSVSSGLAEVDLETFLQAR	Unambiguous	1	1	Q8R4N0	N-Term(iTRAQ8plex)	CID	1.0000	0.0000	1	1	3623411	Unique	Used	0.996	1.036	1.087	1.388	0	0	8.03	0	11	13	2	1227.15540	2453.30351	-6.31
vTAGDSEVEAAAQAAGIHDAILSFPEGYETQVGER	Unambiguous	1	1	Q9DC29	N-Term(iTRAQ8plex)	CID	1.0000	0.0000	1	1	3111858	Unique	Used		1.192	1.038	1.568	0	0	8.03	0	20	176	3	1298.30957	3892.91416	-0.07
aFVEVNEEGTEAAAATAGMMTVR	Unambiguous	2	1	F8WIV2	N-Term(iTRAQ8plex)	CID	1.0000	0.0000	1	1	3132576	Unique	Used	0.697	1.498	1.051	1.320	0	0	8.03	0	19	200	2	1330.15515	2659.30303	0.75
sLEAIELGLGEAQVILALSSHLGAVESEk	Unambiguous	4	1	D3YXZ3	N-Term(iTRAQ8plex); K29(iTRAQ8plex)	CID	1.0000	0.0000	1	1	3541980	Unique	Used				0.961	0	0	8.03	0	14	97	3	1191.33728	3571.99729	-2.00
gnDISSGTVLSDYVGSGPPSGTGLHR	Unambiguous	1	1	P70296	N-Term(iTRAQ8plex); N2(Deamidated)	CID	1.0000	0.0000	1	1	3233830	Unique	Used	2.911	1.057	1.212	1.111	0	0	8.03	0	0	92	3	945.80597	2835.40335	-1.04
gSSGPAQINQVVSHPSQPLTITAHDDR	Unambiguous	2	1	P58404-2	N-Term(iTRAQ8plex)	CID	1.0000	0.0000	1	1	3386765	Unique	Used	1.097	1.940	1.218	0.694	0	0	8.02	0	0	79	3	1039.53503	3116.59055	-3.91
aATFGLILDDVSLTHLTFGk	Unambiguous	2	1	P67778	N-Term(iTRAQ8plex); K20(iTRAQ8plex)	CID	1.0000	0.0000	1	1	3766134	Unique	Used	1.190	1.217	1.218	1.371	0	0	8.02	0	24	38	3	909.85297	2727.54435	-3.61
nMGGPYGGGnYGPGGSGGSGGYGGR	Unambiguous	3	1	O88569	N-Term(iTRAQ8plex); N10(Deamidated)	CID	1.0000	0.0000	1	1	3028871	Unique	Used	1.343	0.405	1.164	0.863	0	0	8.02	0	0	200	2	1248.04517	2495.08306	-4.72
yVVVDSGGGTVDLTVHQIR	Unambiguous	1	1	Q8K0U4	N-Term(iTRAQ8plex)	CID	1.0000	0.0000	1	1	3390100	Unique	Used	1.359	1.307	1.210	1.585	0	0	8.02	0	0	27	3	773.75647	2319.25486	-2.68
aVcGFHLGYLDGEVELVNSVVTR	Unambiguous	1	1	Q62465	N-Term(iTRAQ8plex); C3(Methylthio)	CID	1.0000	0.0000	1	1	3726868	Unique	Used	0.519	0.850	0.805	1.362	0	0	8.02	0	22	14	3	943.14648	2827.42490	-6.23
gnFGGSFAGSFGGAGGHAPGVAR	Unambiguous	4	1	Q9D0E1-2	N-Term(iTRAQ8plex); N2(Deamidated)	CID	1.0000	0.0000	1	1	3911277	Unique	Used	0.430	0.591	0.828	0.931	0	0	8.01	0	0	200	2	1170.57666	2340.14604	1.56
dMAIATGGAVFGEEGLNLNLEDVQAHDLGk	Unambiguous	1	1	P63038	N-Term(iTRAQ8plex); K30(iTRAQ8plex)	CID	1.0000	0.0000	1	1	3264795	Unique	Used	1.108	0.521	0.737	0.727	0	0	8.01	0	0	200	3	1231.63940	3692.90366	-0.40
eSTFLDEGPNEQEITPLQHTPR	Unambiguous	3	1	Q9QYR6	N-Term(iTRAQ8plex)	CID	1.0000	0.0000	1	1	2794900	Unique	Used	2.300	0.668	1.946	0.994	0	0	8.01	0	0	20	3	948.14276	2842.41373	-0.85

iSFEEFcAVVGGLDIHk	Unambiguous	2	1	Q63810-2	N-Term(iTRAQ8plex); C7(Methylthio); K17(iTRAQ8plex)	CID	1.0000	0.0000	1	1	3707834	Unique	Used	0.642	1.233	2.808	1.124	0	0	8.01	0	6	200	3	840.11005	2518.31559	-5.52
aTSDFAFITSSSLVATSGHSNDNR	Unambiguous	4	1	Q8BPN8-2	N-Term(iTRAQ8plex)	CID	1.0000	0.0000	1	1	3598908	Unique	Used	0.609	1.068	1.181	0.902	0	0	8.01	0	4	12	3	930.45508	2789.35068	-4.93
sGAASTGGGVYLHSQSQPSSQYR	Unambiguous	4	1	Q8BIE6	N-Term(iTRAQ8plex)	CID	1.0000	0.0000	1	1	3382984	Unique	Used	1.150	0.582	0.943	0.727	0	0	8.01	0	0	200	3	877.09949	2629.28391	-2.64
nMGGPYGGGNYGPGGSGGSGGYGGR	Unambiguous	3	1	O88569	N-Term(iTRAQ8plex); N1(Deamidated)	CID	0.0025	0.0000	1	1	3296456	Unique	Used	1.689	0.681	1.317	0.847	0	0	8.01	0	0	200	2	1248.05359	2495.09990	2.03
aAAASAAEAGIATPGTEGER	Unambiguous	4	1	O35226-2	N-Term(iTRAQ8plex)	CID	1.0000	0.0000	1	1	2827976	Unique	Used	2.157	1.113	1.447	0.766	0	0	8.00	0	0	22	2	1053.03992	2105.07256	-2.45
lLEVSDDPQVLAVAAHDVGEYVR	Unambiguous	1	1	Q8BVE3	N-Term(iTRAQ8plex)	CID	1.0000	0.0000	1	1	3240250	Unique	Used	0.861	1.521	1.100	1.348	0	0	8.00	0	12	200	3	933.83221	2799.48209	-0.37
gnDISSGTVLSDYVGSGPPSGTGLHR	Unambiguous	1	1	P70296	N-Term(iTRAQ8plex); N2(Deamidated)	CID	1.0000	0.0000	1	1	3321974	Unique	Used	1.766	1.262	0.995	0.865	0	0	8.00	0	0	56	3	945.80499	2835.40042	-2.08
sQSSNYGASSSSTmIGFHVADSLR	Unambiguous	6	1	Q8BRR9	N-Term(iTRAQ8plex); M14(Oxidation)	CID	1.0000	0.0000	1	1	3720218	Unique	Used	0.610	2.293	1.426	1.211	0	0	8.00	0	0	164	3	937.10925	2809.31321	-8.29
mkDVPAFLQQSQSSGPGQAAVWHR	Unambiguous	4	1	Q9WVJ2	N-Term(iTRAQ8plex); K2(iTRAQ8plex)	CID	1.0000	0.0000	1	1	3856526	Unique	Used	0.671	1.335	0.846	0.985	0	0	8.00	1	0	200	3	1078.56946	3233.69382	-4.98
lFDEVQEVIYcPAAVHNDLEk	Unambiguous	2	1	Q3TMH2	N-Term(iTRAQ8plex); C11(Methylthio); K21(iTRAQ8plex)	CID	1.0000	0.0000	1	1	3239477	Unique	Used	1.298	0.776	1.117	1.177	0	0	8.00	0	24	36	3	1029.52722	3086.56711	-3.78
dFTSEQIDEILQNHTEIVFAR	Unambiguous	2	1	Q6PIC6	N-Term(iTRAQ8plex)	CID	1.0000	0.0000	1	1	3353357	Unique	Used	1.244	0.756	1.221	1.322	0	0	7.99	0	0	13	3	937.14685	2809.42600	-1.81
mTTVLSESSGPSNASHSSSR	Unambiguous	1	1	Q80X80	N-Term(iTRAQ8plex); M1(Oxidation)	CID	1.0000	0.0000	1	1	3447552	Unique	Used	0.972	1.829	3.760	2.731	0	0	7.99	0	11	200	3	781.37543	2342.11173	-3.37
lELQGIQGAVDHAAAFGR	Unambiguous	5	1	Q9CPV4	N-Term(iTRAQ8plex)	CID	1.0000	0.0000	1	1	3642469	Unique	Used	1.070	0.925	1.132	1.169	0	0	7.99	0	5	1	3	719.72467	2157.15946	-5.76
gPLLVQDVVFTDEmAHFDR	Unambiguous	2	1	P24270	N-Term(iTRAQ8plex); M14(Oxidation)	CID	1.0000	0.0000	1	1	3110712	Unique	Used	2.719	1.557	1.029	1.356	0	0	7.99	0	16	200	3	837.09320	2509.26505	-1.96

dASPGGPGGPGFASTDISMYTLSR	Unambiguous	2	1	F7CZ64	N-Term(iTRAQ8plex)	CID	1.0000	0.0000	1	1	3415931	Unique	Used	0.596	2.578	2.587	1.338	0	0	7.99	0	0	40	2	1323.14233	2645.27739	-1.74
dSDMASHIQQPGGHGnPGPAPSPSPGPGPGPGASER	Unambiguous	4	1	P63115	N-Term(iTRAQ8plex); N16(Deamidated)	CID	1.0000	0.0000	1	1	3006801	Unique	Used	2.306		1.623	0.422	0	0	7.99	0	19	135	3	1235.91675	3705.73569	2.75
sGAASTGGGVYLHSQSQPSSQYR	Unambiguous	4	1	Q8BIE6	N-Term(iTRAQ8plex)	CID	1.0000	0.0000	1	1	3362022	Unique	Used	0.925	0.311	0.732	0.562	0	0	7.99	0	0	158	3	877.09723	2629.27713	-5.22
hNHDLSSYQDTIQQLEnELR	Unambiguous	2	1	P08553	N-Term(iTRAQ8plex); N17(Deamidated)	CID	0.0013	0.0000	1	1	3214778	Unique	Used	2.514	0.727	1.239	1.013	0	0	7.98	0	21	107	3	915.78937	2745.35355	5.59
gNDISSGTVLSDYVGSGPPSGTGLHR	Unambiguous	1	1	P70296	N-Term(iTRAQ8plex)	CID	1.0000	0.0000	1	1	3515334	Unique	Used	1.366	1.465	0.993	0.854	0	0	7.98	0	7	83	3	945.47552	2834.41202	-3.63
aALANEDcPHIDQALTPDEGLPFTR	Unambiguous	1	1	E9PVV3	N-Term(iTRAQ8plex); C8(Methylthio)	CID	1.0000	0.0000	1	1	3216309	Unique	Used	0.939	0.249	1.022	1.334	0	0	7.98	0	6	25	3	1015.49652	3044.47501	-0.33
dMAIATGGAVFGEEGLNLNLEDVQAHDLGk	Unambiguous	1	1	P63038	N-Term(iTRAQ8plex); K30(iTRAQ8plex)	CID	1.0000	0.0000	1	1	3263145	Unique	Used	1.744	1.081	0.962	0.917	0	0	7.98	0	18	65	3	1231.64270	3692.91355	2.27
sMLQATAEANNLAAVATAk	Unambiguous	1	1	Q8BH66	N-Term(iTRAQ8plex); K19(iTRAQ8plex)	CID	1.0000	0.0000	1	1	3578903	Unique	Used	1.087	0.976	0.976	1.037	0	0	7.98	0	0	52	2	1242.18298	2483.35869	-6.51
aAALGGPEDESGAAEAHFLPR	Unambiguous	3	1	Q6PDM1-4	N-Term(iTRAQ8plex)	CID	1.0000	0.0000	1	1	2900081	Unique	Used	1.469	0.822	1.215	0.883	0	0	7.98	0	30	200	3	790.73492	2370.19022	-3.81
vEQAEFSDLNLVAHAnGGFSVDMEVLR	Unambiguous	3	1	O88935	N-Term(iTRAQ8plex); N16(Deamidated)	CID	1.0000	0.0000	1	1	3284298	Unique	Used	1.029	1.486	1.623	1.830	0	0	7.97	0	5	16	3	1084.87390	3252.60715	-2.41
yGAGGGGSGPAAGVVVGAAGGR	Unambiguous	2	1	P97445	N-Term(iTRAQ8plex)	CID	1.0000	0.0000	1	1	3635619	Unique	Used	0.575	0.761	1.157	0.962	0	0	7.97	0	0	103	2	1025.03809	2049.06890	-4.44
vADVSISEVTTLLGNVHSPVVEGYVGENISGEVk	Unambiguous	3	1	P20357	N-Term(iTRAQ8plex); K34(iTRAQ8plex)	CID	1.0000	0.0000	1	1	3420100	Unique	Used	1.150	1.353	1.990	1.242	0	0	7.97	0	0	200	3	1369.40479	4106.19980	-2.94
qVLDLEDLVFTQGSHFMANk	Unambiguous	1	1	Q6P4T2	N-Term(iTRAQ8plex); K20(iTRAQ8plex)	CID	1.0000	0.0000	1	1	3500196	Unique	Used	0.360	0.375	0.641	0.645	0	0	7.97	0	26	19	3	967.51520	2900.53104	-4.37
iLQVAQELSGEDMHQFHR	Unambiguous	1	1	Q9QZE7	N-Term(iTRAQ8plex)	CID	1.0000	0.0000	1	1	3438871	Unique	Used	1.403	1.235	0.828	0.876	0	0	7.97	0	15	142	3	814.75317	2442.24497	-2.14

qGASGGSGGAGPAGIAGAAAGAGGR	Unambiguous	1	1	Q80ZI1	N-Term(iTRAQ8plex); Q1(Deamidated)	CID	1.0000	0.0000	1	1	3551340	Unique	Used	0.588	0.422	1.100	0.790	0	0	7.97	0	0	200	2	1094.56348	2188.11968	8.56
iSFEEFcAVVGGLDIHk	Unambiguous	2	1	Q63810-2	N-Term(iTRAQ8plex); C7(Methylthio); K17(iTRAQ8plex)	CID	1.0000	0.0000	1	1	3709367	Unique	Used	0.606	0.956	2.666	0.930	0	0	7.97	0	19	200	3	840.11005	2518.31559	-5.52
sVcTLQITDTTGSHQFPAMQR	Unambiguous	2	1	Q91Z61	N-Term(iTRAQ8plex); C3(Methylthio)	CID	1.0000	0.0000	1	1	3839738	Unique	Used	0.896	0.775	0.989	1.009	0	0	7.97	0	15	84	3	891.09869	2671.28153	-4.84
lDINLLDNVVNcLYHGEGAQqR	Unambiguous	2	1	Q6P5F9	N-Term(iTRAQ8plex); C12(Methylthio); Q21(Deamidated)	CID	0.0075	0.0000	1	1	3806437	Unique	Used	0.435	0.778	0.737	0.909	0	0	7.96	0	0	27	3	945.80835	2835.41050	1.17
sGTAHEYSScPDDAIFQSLAR	Unambiguous	1	1	Q00493	N-Term(iTRAQ8plex); C10(Methylthio)	CID	1.0000	0.0000	1	1	3280665	Unique	Used	0.840	0.759	0.629	0.843	0	0	7.96	0	0	13	3	869.06842	2605.19071	-2.22
mSSVFEDQNAATHLIR	Unambiguous	4	1	A8Y5Q0	N-Term(iTRAQ8plex)	CID	1.0000	0.0000	1	1	3413023	Unique	Used	0.922	0.651	0.920	0.875	0	0	7.96	0	6	19	3	708.36395	2123.07730	-3.98
gFcHLcDGQEAccVGLEAGlnPTDHLITAYR	Unambiguous	1	1	P35486	N-Term(iTRAQ8plex); C3(Methylthio); C6(Methylthio); C12(Methylthio); C13(Methylthio); N21(Deamidated)	CID	1.0000	0.0000	1	1	3745719	Unique	Used	1.086	0.939	1.058	1.008	0	0	7.96	0	21	40	3	1265.87891	3795.62217	0.49
aAASGPASASAPAAEILLTPAR	Unambiguous	1	1	Q91VK4	N-Term(iTRAQ8plex)	CID	1.0000	0.0000	1	1	3641270	Unique	Used	0.437	0.997	1.020	1.144	0	0	7.96	0	0	63	2	1149.13586	2297.26445	-5.36
dFTSEQIDEILQNHTEIVFAR	Unambiguous	2	1	Q6PIC6	N-Term(iTRAQ8plex)	CID	1.0000	0.0000	1	1	3356237	Unique	Used	0.592	0.828	1.124	1.159	0	0	7.96	0	3	200	3	937.14587	2809.42307	-2.85
lVQDYGLESEVAQHLAk	Unambiguous	2	1	Q64521	N-Term(iTRAQ8plex); K17(iTRAQ8plex)	CID	1.0000	0.0000	1	1	3259043	Unique	Used	0.818	1.517	1.414	1.368	0	0	7.95	0	0	102	3	836.80023	2508.38614	-2.28

gPAVGIDLGTTYScVGVFQHGk	Unambiguous	4	1	P63017	N-Term(iTRAQ8plex); C14(Methylthio); K22(iTRAQ8plex)	CID	1.0000	0.0000	1	1	3879024	Unique	Used	1.073	0.927	0.914	1.067	0	0	7.95	0	16	14	3	954.16638	2860.48459	-3.52
tLLEGSGLESIINIIHSSLAEPR	Unambiguous	1	1	P19096	N-Term(iTRAQ8plex)	CID	1.0000	0.0000	1	1	3880117	Unique	Used	0.923	0.511	0.762	1.000	0	0	7.95	0	5	7	3	918.51392	2753.52719	-2.89
dMAIATGGAVFGEEGLNLNLEDVQAHDLGk	Unambiguous	1	1	P63038	N-Term(iTRAQ8plex); K30(iTRAQ8plex)	CID	1.0000	0.0000	1	1	3263780	Unique	Used	0.741	1.040	0.969	0.893	0	0	7.95	0	5	200	3	1231.63513	3692.89084	-3.87
nLHQSGFSLSGAQIDDNIPR	Unambiguous	1	1	O08553	N-Term(iTRAQ8plex)	CID	1.0000	0.0000	1	1	3514441	Unique	Used	0.914	0.826	0.842	0.804	0	0	7.95	0	16	11	3	825.09259	2473.26322	-4.27
lGALSGAGALGLASYGAHGAqFPDAYGk	Unambiguous	1	1	Q5F285	N-Term(iTRAQ8plex); Q21(Deamidated); K28(iTRAQ8plex)	CID	1.0000	0.0000	1	1	3784685	Unique	Used	0.571	1.330	1.262	1.411	0	0	7.95	0	24	149	3	1077.24426	3229.71823	2.46
nLHQSGFSLSGAqIDDNIPR	Unambiguous	1	1	O08553	N-Term(iTRAQ8plex); Q13(Deamidated)	CID	1.0000	0.0000	1	1	3534855	Unique	Used	1.121	0.953	1.282	1.186	0	0	7.95	0	0	173	2	1237.63513	2474.26299	2.10
gNDISSGTVLSDYVGSGPPSGTGLHR	Unambiguous	1	1	P70296	N-Term(iTRAQ8plex)	CID	1.0000	0.0000	1	1	3639623	Unique	Used	1.063	1.240	1.089	1.022	0	0	7.94	0	8	48	3	945.47461	2834.40927	-4.60
vSDAISTqYPVVDHEFDAVVVGAGGAGLR	Unambiguous	1	1	Q8K2B3	N-Term(iTRAQ8plex); Q8(Deamidated)	CID	1.0000	0.0000	1	1	3354400	Unique	Used	1.483	0.942	1.125	1.149	0	0	7.94	0	0	200	3	1078.89417	3234.66794	2.90
iSFEEFcAVVGGLDIHk	Unambiguous	2	1	Q63810-2	N-Term(iTRAQ8plex); C7(Methylthio); K17(iTRAQ8plex)	CID	1.0000	0.0000	1	1	3687657	Unique	Used	0.598	1.535	2.223	0.862	0	0	7.94	0	0	21	3	840.10992	2518.31522	-5.66
icDPGLTSFEPEALGNLVEGMDFHR	Unambiguous	9	1	Q5SVJ0	N-Term(iTRAQ8plex); C2(Methylthio)	CID	1.0000	0.0000	1	1	3500539	Unique	Used	0.287	0.853	1.415	1.253	0	0	7.94	0	7	11	3	1033.15857	3097.46115	-4.02
eEGNPVASMGHSEAETPVDAYSNGqAALMGER	Unambiguous	1	1	Q9JKZ2	N-Term(iTRAQ8plex); Q25(Deamidated)	CID	0.0139	0.0000	1	1	2636953	Unique	Used		0.918	0.964	1.370	0	0	7.94	0	20	200	3	1203.88220	3609.63205	-1.55

tFHEASEDcISQGGTLGTPQSELEnEALFEYAR	Unambiguous	2	1	P43025	N-Term(iTRAQ8plex); C9(Methylthio); N25(Deamidated)	CID	1.0000	0.0000	1	1	3036952	Unique	Used	2.198	0.996	0.994	0.534	0	0	7.94	0	0	200	3	1327.61365	3980.82639	3.89
dFTSEQIDEILQNHTEIVFAR	Unambiguous	2	1	Q6PIC6	N-Term(iTRAQ8plex)	CID	1.0000	0.0000	1	1	3353177	Unique	Used	0.583	0.417	0.971	1.230	0	0	7.94	0	0	7	3	937.14612	2809.42380	-2.59
eSASSSEVTSAVYNAVSEGTR	Unambiguous	1	1	Q8C145	N-Term(iTRAQ8plex)	CID	1.0000	0.0000	1	1	3150289	Unique	Used	1.263	1.605	1.910	1.448	0	0	7.94	0	0	200	2	1218.09338	2435.17949	-1.85
gGLHTVAVMnVGAPAAGMNAAVR	Unambiguous	1	1	P47857	N-Term(iTRAQ8plex); N10(Deamidated)	CID	0.0038	0.0000	1	1	3931039	Unique	Used	2.028	0.935	1.015	1.013	0	0	7.94	0	0	200	2	1235.15979	2469.31230	4.62
qTTAAAAATFSEQVGGGSGGAGR	Unambiguous	3	1	O88935	N-Term(iTRAQ8plex)	CID	1.0000	0.0000	1	1	3472503	Unique	Used	0.462	1.324	1.432	1.370	0	0	7.94	0	0	29	2	1178.58911	2356.17095	-3.66
lQEANAQLAEqTcPHDLEMTPVQR	Unambiguous	1	1	Q80Y17	N-Term(iTRAQ8plex); Q11(Deamidated); C13(Methylthio)	CID	0.0353	0.0000	1	1	3214010	Unique	Used	0.971	0.449	0.670	0.785	0	0	7.94	0	30	143	3	1025.16882	3073.49192	7.28
gVGAAATAVTQALNELLQHVk	Unambiguous	1	1	P26039	N-Term(iTRAQ8plex); K21(iTRAQ8plex)	HCD	1.0000	0.0000	1	1	3918073	Unique	Used	0.567	1.968	0.776	0.960	0	0	7.94	0	13	39	3	900.52484	2699.55997	-2.42
gNDISSGTVLSDYVGSGPPSGTGLHR	Unambiguous	1	1	P70296	N-Term(iTRAQ8plex)	CID	1.0000	0.0000	1	1	3368174	Unique	Used	1.665	0.735	1.544	1.437	0	0	7.94	0	0	14	3	945.47418	2834.40799	-5.05
vLRPPGGGSNFSLGFDEPAEQPVR	Unambiguous	1	1	P97825	N-Term(iTRAQ8plex)	CID	1.0000	0.0000	1	1	3577462	Unique	Used	2.478	1.833	1.879	1.168	0	0	7.94	0	0	3	3	944.16064	2830.46738	-4.13
qTTAAAAATFSEQVGGGSGGAGR	Unambiguous	3	1	O88935	N-Term(iTRAQ8plex)	CID	1.0000	0.0000	1	1	3492956	Unique	Used	0.727	1.711	1.950	1.791	0	0	7.93	0	0	200	2	1178.58850	2356.16973	-4.17
lSASEALGSAALPSHSSAISQHSk	Unambiguous	1	1	Q9CQX8	N-Term(iTRAQ8plex); K24(iTRAQ8plex)	CID	1.0000	0.0000	1	1	3532724	Unique	Used	1.943	0.579	1.820	1.000	0	0	7.93	0	0	99	3	982.19977	2944.58475	-3.44
dASPGGPGGPGFASTDISMYTLSR	Unambiguous	2	1	F7CZ64	N-Term(iTRAQ8plex)	CID	1.0000	0.0000	1	1	3415775	Unique	Used	0.506	2.471	2.273	1.274	0	0	7.93	0	0	55	2	1323.13953	2645.27178	-3.86
qLSLISGcGNLAEAVEGAVHIqEcVPENLELk	Unambiguous	1	1	Q99KP3	N-Term(iTRAQ8plex); C8(Methylthio); Q22(Deamidated); C24(Methylthio); K32(iTRAQ8plex)	CID	0.0113	0.0000	1	1	3561341	Unique	Used	0.658	1.443	0.564	0.514	0	0	7.93	0	0	143	3	1355.69495	4065.07029	1.88

nLLEVSEVEQELAcQnDHSSALQNVk	Unambiguous	1	1	P97390	N-Term(iTRAQ8plex); C14(Methylthio); N16(Deamidated); K26(iTRAQ8plex)	CID	0.0013	0.0000	1	1	3110934	Unique	Used	0.808	0.705	0.897	1.022	0	0	7.93	0	12	200	3	1184.93555	3552.79209	4.21
gNDISSGTVLSDYVGSGPPSGTGLHR	Unambiguous	1	1	P70296	N-Term(iTRAQ8plex)	CID	1.0000	0.0000	1	1	3535656	Unique	Used	2.004	1.544	1.163	0.919	0	0	7.93	0	0	62	3	945.47516	2834.41092	-4.01
tTTNVLGDSLGAGIVEHLSR	Unambiguous	1	1	P56564	N-Term(iTRAQ8plex)	CID	1.0000	0.0000	1	1	3663927	Unique	Used	0.817	2.397	1.185	1.292	0	0	7.93	0	17	10	3	782.09308	2344.26468	-5.45
tQTAVASEEGPATLPEAEALLAQHAALR	Unambiguous	1	1	Q68FG2	N-Term(iTRAQ8plex)	CID	1.0000	0.0000	1	1	3303818	Unique	Used	0.784	1.579	3.779	1.464	0	0	7.92	0	0	8	3	1050.56067	3149.66745	-2.25
vNFHFILFNNVDGHLYELDGR	Unambiguous	1	1	Q9R0P9	N-Term(iTRAQ8plex)	CID	1.0000	0.0000	1	1	3686218	Unique	Used	1.777		1.568	0.857	0	0	7.92	0	5	7	3	941.81445	2823.42881	-8.24
mTTVLSESSGPSNASHSSSR	Unambiguous	1	1	Q80X80	N-Term(iTRAQ8plex)	CID	1.0000	0.0000	1	1	3338023	Unique	Used	0.919	1.075	1.426	1.151	0	0	7.92	0	9	19	3	776.04315	2326.11490	-4.22
vADVSISEVTTLLGnVHSPVVEGYVGENISGEVk	Unambiguous	3	1	P20357	N-Term(iTRAQ8plex); N15(Deamidated); K34(iTRAQ8plex)	CID	1.0000	0.0000	1	1	3420478	Unique	Used	1.584	1.715	1.519	0.740	0	0	7.92	0	0	200	3	1369.73633	4107.19443	-0.35
dGSGFLINLIDSPGHVDFSSEVTAALR	Unambiguous	1	1	P58252	N-Term(iTRAQ8plex)	CID	1.0000	0.0000	1	1	3602130	Unique	Used	0.892	0.659	1.219	0.823	0	0	7.92	0	0	31	3	1041.20447	3121.59885	-3.85
nLHQSGFSLSGAQIDDNIPR	Unambiguous	1	1	O08553	N-Term(iTRAQ8plex)	CID	1.0000	0.0000	1	1	3411616	Unique	Used	1.823	0.840	1.518	1.395	0	0	7.92	0	0	18	2	1237.13501	2473.26274	-4.46
qTIGNScGTIGLIHAVANnQDk	Unambiguous	1	1	Q9R0P9	N-Term(iTRAQ8plex); C7(Methylthio); N19(Deamidated); K22(iTRAQ8plex)	CID	0.0101	0.0000	1	1	3391518	Unique	Used	1.418	1.234	1.055	1.052	0	0	7.91	0	0	29	3	970.50427	2909.49826	-3.01
qTTAAAAATFSEqVGGGSGGAGR	Unambiguous	3	1	O88935	N-Term(iTRAQ8plex); Q13(Deamidated)	CID	1.0000	0.0000	1	1	3494656	Unique	Used	0.788	1.706	1.333	1.494	0	0	7.91	0	0	200	2	1179.08960	2357.17192	3.54
dYIQESENIASLHNQITAcDAVLER	Unambiguous	1	1	Q8C754	N-Term(iTRAQ8plex); C19(Methylthio)	CID	1.0000	0.0000	1	1	3326611	Unique	Used	0.749	1.342	0.864	0.841	0	0	7.91	0	13	12	3	1061.51697	3182.53635	-1.16
gTAEGAVDAMLVHGQDAITVSnGGR	Unambiguous	2	1	Q8C7X2-2	N-Term(iTRAQ8plex); N22(Deamidated)	CID	1.0000	0.0000	1	1	3129778	Unique	Used	1.160	0.981	0.734	1.048	0	0	7.91	0	0	50	3	911.12537	2731.36155	-0.30

gGVSAVAG GVTAVGSA VVNk	Unambiguous	1	1	Q9DAM7	N-Term(iTRAQ8plex); K20(iTRAQ8plex)	HCD	1.0000	0.0000	1	1	3762056	Unique	Used	1.304	0.889	1.366	0.827	0	0	7.91	0	0	116	2	1154.66870	2308.33013	-6.25
mGPQGSAP APSSAPLN HGEPSSFR	Unambiguous	1	1	Q7TSG2	N-Term(iTRAQ8plex)	CID	1.0000	0.0000	1	1	3364165	Unique	Used	2.872	0.895	0.986	0.882	0	0	7.91	0	0	200	3	895.10687	2683.30606	-5.23
vADVSISEV TTLLGNVH SPVVEGYV GEnISGEVk	Unambiguous	3	1	P20357	N-Term(iTRAQ8plex); N28(Deamidated); K34(iTRAQ8plex)	CID	1.0000	0.0000	1	1	3421190	Unique	Used			1.298	0.764	0	0	7.90	0	0	200	3	1369.73975	4107.20469	2.14
gGVDDEMT LSAYITMAL LESSLPATH PVVSk	Unambiguous	1	1	P28665	N-Term(iTRAQ8plex); K31(iTRAQ8plex)	CID	1.0000	0.0000	1	1	3581686	Unique	Used	4.982	8.882	0.753	1.635	0	0	7.90	0	28	113	3	1281.00940	3841.01364	-2.30
gAIQLGIGY TVGNLSSk PER	Unambiguous	4	1	F8WHW6	N-Term(iTRAQ8plex); K17(iTRAQ8plex)	CID	1.0000	0.0000	1	1	3821331	Unique	Used	0.478	1.628	1.939	1.427	0	0	7.90	0	4	66	3	890.17645	2668.51480	-3.56
qTTAAAAAT FSEQVGGG SGGAGR	Unambiguous	3	1	O88935	N-Term(iTRAQ8plex)	CID	1.0000	0.0000	1	1	3493631	Unique	Used	0.561	1.714	2.041	2.347	0	0	7.90	0	0	83	2	1178.58459	2356.16191	-7.49
dAAEAEAW MGEQELH MMGQEk	Unambiguous	1	1	Q68FG2	N-Term(iTRAQ8plex); K21(iTRAQ8plex)	CID	1.0000	0.0000	1	1	2763909	Unique	Used	0.791	3.170	4.082	1.378	0	0	7.90	0	8	200	3	1000.47766	2999.41843	0.86
vNQELAGY TGGDVSFIk	Unambiguous	1	1	Q8BGH2	N-Term(iTRAQ8plex); K17(iTRAQ8plex)	CID	1.0000	0.0000	1	1	3302365	Unique	Used	0.428	1.152	1.041	1.479	0	0	7.90	0	0	119	2	1203.65442	2406.30156	-4.57
vADVSISEV TTLLGNVH SPVVEGYV GEnISGEVk	Unambiguous	3	1	P20357	N-Term(iTRAQ8plex); N28(Deamidated); K34(iTRAQ8plex)	CID	1.0000	0.0000	1	1	3419731	Unique	Used	2.739	3.223	1.135	0.565	0	0	7.90	0	0	159	3	1369.73804	4107.19956	0.90
sSLSASLEP DSLGLGSA NSSQDSLH k	Unambiguous	2	1	B8QI34	N-Term(iTRAQ8plex); K26(iTRAQ8plex)	CID	1.0000	0.0000	1	1	3212120	Unique	Used	0.767	0.790	1.399	1.142	0	0	7.90	0	0	120	3	1065.88818	3195.65000	-2.82
eQVTNVGG AVVTGVTAV AQk	Unambiguous	2	1	O55042	N-Term(iTRAQ8plex); K20(iTRAQ8plex)	CID	1.0000	0.0000	1	1	3642246	Unique	Used	0.539	1.243	1.864	0.777	0	0	7.89	0	0	102	2	1268.72363	2536.43999	-6.14
qDHPSSMG VYGQESG GFSGPGEn R	Unambiguous	4	1	Q5SUT0	N-Term(iTRAQ8plex); N23(Deamidated)	CID	1.0000	0.0000	1	1	2649527	Unique	Used	3.203	0.843	1.228	0.877	0	0	7.89	0	0	122	3	929.08337	2785.23557	-2.53

aQELGLLQEYLLALTTEDHLLR	Unambiguous	4	1	E9Q5W5	N-Term(iTRAQ8plex)	CID	1.0000	0.0000	1	1	3666592	Unique	Used	0.514	0.831	0.628	1.181	0	0	7.89	0	0	18	3	948.52429	2843.55832	-8.36
sLAGSSGPGASSGPGGDHSELIVR	Unambiguous	5	1	P57776-3	N-Term(iTRAQ8plex)	CID	1.0000	0.0000	1	1	3339242	Unique	Used	1.191	0.970	0.887	0.861	0	0	7.88	0	8	26	3	833.75696	2499.25632	-7.15
lSASEALGSAALPSHSSAlSqHSk	Unambiguous	1	1	Q9CQX8	N-Term(iTRAQ8plex); Q21(Deamidated); K24(iTRAQ8plex)	CID	1.0000	0.0000	1	1	3532807	Unique	Used	1.834	0.540	1.723	0.879	0	0	7.88	0	0	144	3	982.53400	2945.58744	2.90
sIATLMTDEVFHDVAYk	Unambiguous	3	1	Q5DTL9	N-Term(iTRAQ8plex); K17(iTRAQ8plex)	CID	1.0000	0.0000	1	1	3305045	Unique	Used	0.154	1.022	1.097	1.034	0	0	7.88	0	2	8	3	850.12219	2548.35202	-2.26
sEIGGELAASSGVSTPGSVGHDIITEqPR	Unambiguous	3	1	P42859	N-Term(iTRAQ8plex); Q27(Deamidated)	CID	1.0000	0.0000	1	1	3257151	Unique	Used	0.921	1.192	1.435	1.189	0	0	7.88	0	0	91	3	1052.87708	3156.61667	6.44
gGLSTFFEAQDALSAIHQk	Unambiguous	1	1	Q9D4H1	N-Term(iTRAQ8plex); K19(iTRAQ8plex)	CID	1.0000	0.0000	1	1	3579982	Unique	Used	0.406	1.322	1.192	1.191	0	0	7.88	0	24	38	3	876.81061	2628.41727	-2.65
eQASHLGGAVFSGAGnIAAATGLVk	Unambiguous	1	1	Q91ZZ3	N-Term(iTRAQ8plex); N16(Deamidated); K25(iTRAQ8plex)	CID	1.0000	0.0000	1	1	3643218	Unique	Used	1.659	1.804	2.341	2.251	0	0	7.88	0	24	67	3	979.20398	2935.59739	-4.25
gPAVGIDLGTTYScVGVFQHGk	Unambiguous	4	1	P63017	N-Term(iTRAQ8plex); C14(Methylthio); K22(iTRAQ8plex)	CID	1.0000	0.0000	1	1	3808463	Unique	Used	1.671	0.951	1.079	1.457	0	0	7.87	0	30	200	3	954.16779	2860.48880	-2.04
aAASVVSGPPVASDcASPGDSAR	Unambiguous	3	1	Q6P9K8	N-Term(iTRAQ8plex); C15(Methylthio)	CID	1.0000	0.0000	1	1	3277964	Unique	Used			1.401	1.073	0	0	7.87	0	0	94	2	1211.58154	2422.15581	-3.59
eLNIDVADVESLLVQcILDNTIHGR	Unambiguous	3	1	P61202	N-Term(iTRAQ8plex); C16(Methylthio)	CID	1.0000	0.0000	1	1	3667054	Unique	Used	2.370	1.454	0.618	0.815	0	0	7.87	0	19	200	3	1043.87378	3129.60678	-5.08
fDTLPQGQAADGFFLHVGR	Unambiguous	3	1	A2AHC3	N-Term(iTRAQ8plex)	CID	1.0000	0.0000	1	1	3664825	Unique	Used	1.202	0.722	1.094	1.076	0	0	7.87	0	7	20	3	794.07867	2380.22147	-5.77
sDIAAAQQSGMSLSNSk	Unambiguous	1	1	B1AQZ0	N-Term(iTRAQ8plex); K17(iTRAQ8plex)	CID	1.0000	0.0000	1	1	3252721	Unique	Used	0.396	1.523	1.685	1.449	0	0	7.87	0	0	82	2	1152.11047	2303.21367	0.64

nLHQSGFSLSGAQIDDNIPR	Unambiguous	1	1	O08553	N-Term(iTRAQ8plex)	CID	1.0000	0.0000	1	1	3514575	Unique	Used	1.543	1.111	1.520	1.795	0	0	7.87	0	0	1	3	825.09277	2473.26377	-4.04
dFTSEQIDEILQNHTEIVFAR	Unambiguous	2	1	Q6PIC6	N-Term(iTRAQ8plex)	CID	1.0000	0.0000	1	1	3352968	Unique	Used		1.380	1.143	1.003	0	0	7.87	0	7	9	3	937.14587	2809.42307	-2.85
iSFEEFcAVVGGLDIHk	Unambiguous	2	1	Q63810-2	N-Term(iTRAQ8plex); C7(Methylthio); K17(iTRAQ8plex)	CID	1.0000	0.0000	1	1	3690607	Unique	Used	0.371	1.512	2.318	0.639	0	0	7.87	0	0	200	3	840.11188	2518.32108	-3.34
aAELIANSLATAGDGLIELR	Unambiguous	1	1	P67778	N-Term(iTRAQ8plex)	CID	1.0000	0.0000	1	1	3666520	Unique	Used	1.067	1.129	1.035	1.353	0	0	7.87	0	10	26	2	1151.64294	2302.27861	-5.85
mVAAATNNLcEAANAAVQGHASQEk	Unambiguous	1	1	P26039	N-Term(iTRAQ8plex); M1(Oxidation); C10(Methylthio); K25(iTRAQ8plex)	CID	1.0000	0.0000	1	1	3743312	Unique	Used	0.991	1.103	0.963	0.892	0	0	7.86	0	0	115	3	1057.18811	3169.54978	-4.79
qDHPSSmGVYGQESGGFSGPGENR	Unambiguous	4	1	Q5SUT0	N-Term(iTRAQ8plex); M7(Oxidation)	CID	1.0000	0.0000	1	1	2824750	Unique	Used	1.262	1.099	1.770	0.909	0	0	7.86	0	0	40	3	934.08807	2800.24967	-1.38
sNVYQPTEMAVVLnGGTIPTAPPSHTGR	Unambiguous	1	1	Q923Z0	N-Term(iTRAQ8plex); N14(Deamidated)	CID	1.0000	0.0000	1	1	3475808	Unique	Used	1.303	1.282	1.064	0.912	0	0	7.86	0	9	79	3	1067.21118	3199.61899	-5.32
hELLLGAAGAGPGAGPQqATPGALLQAGPPR	Unambiguous	1	1	Q6PE01	N-Term(iTRAQ8plex); Q18(Deamidated)	CID	0.0229	0.0000	1	1	3876552	Unique	Used	1.671	0.996	1.222	0.637	0	0	7.86	0	19	200	3	1060.24866	3178.73142	1.20
gLQLTPGIGGMQQHFFDDEDR	Unambiguous	2	1	Q7M739	N-Term(iTRAQ8plex)	CID	1.0000	0.0000	1	1	3324111	Unique	Used	2.076	0.969	1.274	1.274	0	0	7.86	0	18	80	3	889.10248	2665.29288	-2.03
lEGYWGEAQSLcQAVDEHLR	Unambiguous	2	1	Q6R891	N-Term(iTRAQ8plex); C12(Methylthio)	CID	1.0000	0.0000	1	1	3561153	Unique	Used	1.310	1.451	1.356	0.846	0	0	7.86	0	21	13	3	885.42035	2654.24649	-6.80
cPLEEGQAGAAEATAAPSVEDR	Unambiguous	6	1	Q6IR34-3	N-Term(iTRAQ8plex); C1(Methylthio)	CID	1.0000	0.0000	1	1	2636090	Unique	Used	0.610	0.819	1.315	0.874	0	0	7.86	0	0	200	2	1261.59253	2522.17778	-1.10
eHGYLGNLSAVASTEGTIEETLnEASR	Unambiguous	2	1	Q99P72	N-Term(iTRAQ8plex); N23(Deamidated)	CID	1.0000	0.0000	1	1	2922098	Unique	Used			0.548	1.361	0	0	7.86	0	6	175	3	1051.85474	3153.54966	0.20

cVHLAAMDAnGYSDPFVk	Unambiguous	1	1	P47708	N-Term(iTRAQ8plex); C1(Methylthio); N10(Deamidated); K18(iTRAQ8plex)	CID	1.0000	0.0000	1	1	3302627	Unique	Used	1.545	0.927	1.208	1.007	0	0	7.86	0	21	93	3	865.09137	2593.25956	-4.40
smLQATAEANNLAAVATAk	Unambiguous	1	1	Q8BH66	N-Term(iTRAQ8plex); M2(Oxidation); K19(iTRAQ8plex)	CID	1.0000	0.0000	1	1	3556439	Unique	Used	0.462	1.053	1.208	1.053	0	0	7.85	0	0	200	2	1250.18250	2499.35771	-4.83
nLATAYDNFVELVANLk	Unambiguous	2	1	Q9WU78	N-Term(iTRAQ8plex); K17(iTRAQ8plex)	CID	1.0000	0.0000	1	1	3785897	Unique	Used	0.748	1.369	0.983	1.229	0	0	7.85	0	19	86	2	1252.19702	2503.38677	-5.97
dMAIATGGAVFGEEGLNLNLEDVQAHDLGk	Unambiguous	1	1	P63038	N-Term(iTRAQ8plex); K30(iTRAQ8plex)	CID	1.0000	0.0000	1	1	3263675	Unique	Used	1.131	0.871	0.857	0.868	0	0	7.85	0	8	200	3	1231.63892	3692.90219	-0.80
gTGGVDTAAVGAVFDISNADR	Unambiguous	1	1	P07310	N-Term(iTRAQ8plex)	CID	1.0000	0.0000	1	1	3303758	Unique	Used	31.352	46.259	0.274	0.847	0	0	7.85	0	2	22	2	1149.08472	2297.16216	-2.37
sGSSSSSSGNPASQFYPQSR	Unambiguous	1	1	Q61771	N-Term(iTRAQ8plex)	CID	1.0000	0.0000	1	1	3490728	Unique	Used	0.980	0.670	1.240	0.646	0	0	7.85	0	0	48	2	1161.54492	2322.08257	-3.21
aDDITSLVADTTLLVHFFGk	Unambiguous	2	1	Q9CTY5	N-Term(iTRAQ8plex); K20(iTRAQ8plex)	CID	1.0000	0.0000	1	1	3707232	Unique	Used	0.641	1.010	1.009	0.905	0	0	7.85	0	8	105	3	924.51343	2771.52573	-6.60
dFTSEQIDEILQNHTEIVFAR	Unambiguous	2	1	Q6PIC6	N-Term(iTRAQ8plex)	CID	1.0000	0.0000	1	1	3373443	Unique	Used	1.008	0.599	1.041	1.336	0	0	7.85	0	16	10	3	937.14478	2809.41977	-4.02
rnDFQLIGIQDGYLSLLQDSGEVR	Unambiguous	2	1	P63242	N-Term(iTRAQ8plex); N2(Deamidated)	CID	1.0000	0.0000	1	1	3352384	Unique	Used			0.352	0.892	0	0	7.84	1	4	10	3	1014.52899	3041.57242	-4.01
dMAIATGGAVFGEEGLNLNLEDVQAHDLGk	Unambiguous	1	1	P63038	N-Term(iTRAQ8plex); K30(iTRAQ8plex)	CID	1.0000	0.0000	1	1	3264645	Unique	Used	1.804	1.099	1.228	1.180	0	0	7.84	0	0	200	3	1231.63477	3692.88974	-4.17
mTEEEVEMLVAGHEDSnGcINYEELVR	Unambiguous	1	1	Q60605-2	N-Term(iTRAQ8plex); N17(Deamidated); C19(Methylthio)	CID	0.0179	0.0000	1	1	2766291	Unique	Used	1.722	1.570	1.468	0.456	0	0	7.83	0	10	200	3	1149.84766	3447.52842	-2.41

fLSQPFQVAEVFTGHMGk	Unambiguous	1	1	P56480	N-Term(iTRAQ8plex); K18(iTRAQ8plex)	CID	1.0000	0.0000	1	1	3863201	Unique	Used	1.205	0.870	1.110	1.337	0	0	7.83	0	20	200	3	877.80817	2631.40995	-4.36
iSFEEFcAVVGGLDIHk	Unambiguous	2	1	Q63810-2	N-Term(iTRAQ8plex); C7(Methylthio); K17(iTRAQ8plex)	CID	1.0000	0.0000	1	1	3690807	Unique	Used		1.511	1.332	1.067	0	0	7.83	0	5	200	3	840.11053	2518.31705	-4.94
qVHDEEVAELLATLQASSqAAAEVDVAVAkPDLTSALR	Unambiguous	1	1	P46660	N-Term(iTRAQ8plex); Q19(Deamidated); K30(iTRAQ8plex)	CID	0.0460	0.0000	1	1	3479866	Unique	Used	1.928				0	0	7.83	0	0	14	3	1519.14807	4555.42966	-1.24
gPAVGIDLGTTYScVGVFQHGk	Unambiguous	4	1	P63017	N-Term(iTRAQ8plex); C14(Methylthio); K22(iTRAQ8plex)	CID	1.0000	0.0000	1	1	3824057	Unique	Used	1.199	1.046	1.272	1.030	0	0	7.83	0	24	29	3	954.16510	2860.48075	-4.86
iSFEEFcAVVGGLDIHk	Unambiguous	2	1	Q63810-2	N-Term(iTRAQ8plex); C7(Methylthio); K17(iTRAQ8plex)	CID	1.0000	0.0000	1	1	3691022	Unique	Used	0.943	1.217	1.737	0.895	0	0	7.83	0	7	200	3	840.10931	2518.31339	-6.39
vADVSISEVTTLLGNVHSPVVEGYVGENISGEVk	Unambiguous	3	1	P20357	N-Term(iTRAQ8plex); K34(iTRAQ8plex)	CID	1.0000	0.0000	1	1	3420654	Unique	Used	2.869	2.074	1.605	0.771	0	0	7.83	0	0	200	3	1369.40552	4106.20200	-2.40
dIGAIAQVHAENGDIIAEEQQR	Unambiguous	1	1	O08553	N-Term(iTRAQ8plex)	CID	1.0000	0.0000	1	1	2811406	Unique	Used	1.821	1.121	1.845	1.570	0	0	7.82	0	13	14	3	894.46375	2681.37668	-1.13
eQSVSWVSLEEAEPIPDIHWGIR	Unambiguous	2	1	Q8R146-2	N-Term(iTRAQ8plex)	CID	1.0000	0.0000	1	1	3284135	Unique	Used	1.576	0.903	1.729	1.301	0	0	7.82	0	7	19	3	994.51447	2981.52884	-0.77
dLVVLLFETALLSSGFSLEDPQTHSNR	Unambiguous	1	1	P11499	N-Term(iTRAQ8plex)	HCD	1.0000	0.0000	1	1	3861530	Unique	Used	0.857	0.953	0.870	1.047	0	0	7.82	0	18	31	3	1098.24585	3292.72300	-4.19
vEYPVMYSTDPEnGHMFNcIQR	Unambiguous	1	1	Q9CPU4	N-Term(iTRAQ8plex); N13(Deamidated); C19(Methylthio)	CID	1.0000	0.0000	1	1	3303819	Unique	Used	1.070	0.896	1.235	1.148	0	0	7.82	0	0	57	3	994.44531	2981.32138	-1.02
gGNVGInSFGFGGSNVHVILQPNTR	Unambiguous	1	1	P19096	N-Term(iTRAQ8plex); N7(Deamidated)	CID	1.0000	0.0000	1	1	3877148	Unique	Used	0.830	0.668	1.010	1.205	0	0	7.82	0	0	116	3	949.49438	2846.46860	-5.83

nLHQSGFSLSGAQIDDNIPR	Unambiguous	1	1	O08553	N-Term(iTRAQ8plex)	CID	1.0000	0.0000	1	1	3721054	Unique	Used	1.128	1.192	1.056	1.319	0	0	7.82	0	0	110	3	825.09064	2473.25736	-6.64
hcAYEHDIQALSTQVQQVQDDGQR	Unambiguous	1	1	Q68FG2	N-Term(iTRAQ8plex); C2(Methylthio)	CID	1.0000	0.0000	1	1	3189795	Unique	Used		1.713	1.240	0.971	0	0	7.82	0	0	43	3	1040.48755	3119.44809	-3.08
gFLWDEGFHQLVVQR	Unambiguous	1	1	Q80UM7	N-Term(iTRAQ8plex)	CID	1.0000	0.0000	1	1	3725487	Unique	Used	0.700	0.864	0.632	0.854	0	0	7.82	0	13	65	3	712.37915	2135.12290	-5.21
gGGSAAAAAAAAASGGAGSDNSVEHSDYR	Unambiguous	4	1	D9J2V6	N-Term(iTRAQ8plex)	CID	1.0000	0.0000	1	1	2863031	Unique	Used	4.915	2.682	1.622	1.424	0	0	7.82	0	12	200	3	947.10651	2839.30497	-3.38
iSGETIFVTAPHEATAGIIGVNR	Unambiguous	2	1	Q68FD5	N-Term(iTRAQ8plex)	CID	1.0000	0.0000	1	1	3704690	Unique	Used	0.574	1.031	1.419	1.366	0	0	7.82	0	2	6	3	886.48499	2657.44040	-6.06
lLEVSDDPQVLAVAAHDVGEYVR	Unambiguous	1	1	Q8BVE3	N-Term(iTRAQ8plex)	CID	1.0000	0.0000	1	1	3239301	Unique	Used		1.457	0.609	0.814	0	0	7.81	0	6	3	3	933.83221	2799.48209	-0.37
gGPPPPPPPPHSSGPPPPPAR	Unambiguous	1	1	Q91YD9	N-Term(iTRAQ8plex)	CID	1.0000	0.0000	1	1	3427650	Unique	Used	2.069	0.858	1.480	0.793	0	0	7.81	0	0	54	3	765.41278	2294.22379	-4.81
sLSSSMQSLSSGSSPGSLTSSR	Unambiguous	1	1	Q5SXA9	N-Term(iTRAQ8plex)	CID	1.0000	0.0000	1	1	3893390	Unique	Used	0.510	0.798	1.231	0.923	0	0	7.81	0	0	200	2	1217.60144	2434.19560	-3.19
sNVASTAAcSSASSLGLGLAYR	Unambiguous	1	1	P46660	N-Term(iTRAQ8plex); C9(Methylthio)	CID	1.0000	0.0000	1	1	3934938	Unique	Used	1.330	0.132	1.569	1.283	0	0	7.81	0	0	19	2	1218.60669	2436.20610	-4.27
sNVASTAAcSSASSLGLGLAYR	Unambiguous	1	1	P46660	N-Term(iTRAQ8plex); C9(Methylthio)	CID	1.0000	0.0000	1	1	3934703	Unique	Used	1.142	0.301	2.819	2.400	0	0	7.81	0	0	200	2	1218.60889	2436.21050	-2.47
lSGLTSTLPDTVLSHLFSqEELVNNTELVQSYR	Unambiguous	4	1	Q8BTG7-3	N-Term(iTRAQ8plex); Q19(Deamidated)	CID	0.0448	0.0000	1	1	3766412	Unique	Used	1.745	0.840	0.800	0.938	0	0	7.81	0	14	35	3	1332.69824	3996.08017	1.14
qFGFIVLTTSAGImDHEEAR	Unambiguous	1	1	P62245	N-Term(iTRAQ8plex); M14(Oxidation)	CID	1.0000	0.0000	1	1	3559437	Unique	Used	0.994	0.660	0.606	0.880	0	0	7.81	0	16	110	3	848.09766	2542.27842	-5.11
lYHDPNVnGGTELEPVEGNPYR	Unambiguous	2	1	Q8BH74	N-Term(iTRAQ8plex); N8(Deamidated)	CID	1.0000	0.0000	1	1	2652349	Unique	Used	1.414	1.111		0.827	0	0	7.81	0	10	200	3	925.78943	2775.35373	0.33
dMAIATGGAVFGEEGLNLNLEDVQAHDLGk	Unambiguous	1	1	P63038	N-Term(iTRAQ8plex); K30(iTRAQ8plex)	CID	1.0000	0.0000	1	1	3262859	Unique	Used	1.206	0.929	1.205	1.001	0	0	7.80	0	0	54	3	1231.64014	3692.90586	0.19
gDSALFAVNGFNILVDGGSDR	Unambiguous	3	1	Q9QYR6	N-Term(iTRAQ8plex)	CID	1.0000	0.0000	1	1	3479804	Unique	Used	0.608	1.013	1.006	0.845	0	0	7.80	0	7	7	2	1214.62109	2428.23491	-2.55

gAALDVHESEPFSFSQGPLk	Unambiguous	3	1	O88712-2	N-Term(iTRAQ8plex); K20(iTRAQ8plex)	CID	1.0000	0.0000	1	1	3234509	Unique	Used			1.306	1.034	0	0	7.80	0	13	108	3	908.81940	2724.44364	-0.64
aAGGAGAQVGGSISSGSSASSVTVTR	Unambiguous	1	1	P48678	N-Term(iTRAQ8plex)	CID	1.0000	0.0000	1	1	3634823	Unique	Used	1.135	1.823	0.772	0.778	0	0	7.80	0	0	107	2	1263.64966	2526.29204	-5.61
nLHQSGFSLSGAQIDDNIPR	Unambiguous	1	1	O08553	N-Term(iTRAQ8plex)	CID	1.0000	0.0000	1	1	3595986	Unique	Used	1.870	1.545	1.330	1.295	0	0	7.79	0	17	42	3	825.09253	2473.26303	-4.34
nLHQSGFSLSGAqIDDNIPR	Unambiguous	1	1	O08553	N-Term(iTRAQ8plex); Q13(Deamidated)	CID	0.0193	0.0000	1	1	3596033	Unique	Used	3.610	1.082	1.113	1.507	0	0	7.79	0	0	158	2	1237.63354	2474.25981	0.82
vQNATLAVANITnADSATR	Unambiguous	1	1	P54116	N-Term(iTRAQ8plex); N13(Deamidated)	CID	0.0090	0.0000	1	1	3682680	Unique	Used	1.626	1.858	0.681	1.334	0	0	7.79	0	0	200	2	1118.10095	2235.19463	2.83
gTGGVDTAATGSVFDISNLDR	Unambiguous	1	1	P30275	N-Term(iTRAQ8plex)	CID	1.0000	0.0000	1	1	3260152	Unique	Used	0.859	1.362	0.896	1.250	0	0	7.79	0	0	32	2	1179.10120	2357.19512	2.71
gGGGSGGYYGQGGMSGGGWR	Unambiguous	2	1	D3Z3N4	N-Term(iTRAQ8plex)	CID	1.0000	0.0000	1	1	3385519	Unique	Used	1.593	0.991	0.789	0.826	0	0	7.79	0	0	146	2	1054.97546	2108.94365	-3.51
iNALTAASEAAcLIVSVDETIkNPR	Unambiguous	1	1	P80313	N-Term(iTRAQ8plex); C12(Methylthio); K22(iTRAQ8plex)	HCD	1.0000	0.0000	1	1	3785848	Unique	Used	0.564	0.537	0.828	1.059	0	0	7.79	1	20	142	3	1085.25854	3253.76108	-4.14
lEPSDLSEDSEYDSIWTAHSYR	Unambiguous	6	1	Q9ES28-3	N-Term(iTRAQ8plex)	CID	1.0000	0.0000	1	1	2814015	Unique	Used	0.798	0.825	1.016	0.993	0	0	7.79	0	3	43	3	968.78815	2904.34989	0.73
vADALAnAAGHLDDLPGALSALSDLHAHk	Unambiguous	1	1	Q91VB8	N-Term(iTRAQ8plex); N7(Deamidated); K29(iTRAQ8plex)	CID	1.0000	0.0000	1	1	3706458	Unique	Used	2.829	4.173	0.563	1.266	0	0	7.79	0	0	54	3	1158.28955	3472.85410	-3.02
nLHQSGFSLSGAQIDDNIPR	Unambiguous	1	1	O08553	N-Term(iTRAQ8plex)	CID	1.0000	0.0000	1	1	3660207	Unique	Used	1.754	1.176	1.178	1.815	0	0	7.78	0	4	21	3	825.09064	2473.25736	-6.64
eGITAIGGTSSISSEGTQHSYSEEEk	Unambiguous	2	1	Q3V0K9	N-Term(iTRAQ8plex); K26(iTRAQ8plex)	CID	1.0000	0.0000	1	1	2667231	Unique	Used	1.518	0.306	1.976	3.549	0	0	7.78	0	10	200	3	1098.20984	3292.61496	-3.89
iSFEEFcAVVGGLDIHk	Unambiguous	2	1	Q63810-2	N-Term(iTRAQ8plex); C7(Methylthio); K17(iTRAQ8plex)	CID	1.0000	0.0000	1	1	3689338	Unique	Used	0.538	1.276	2.187	0.846	0	0	7.78	0	1	172	3	840.11053	2518.31705	-4.94

eQASHLGGAVFSGAGnIAAATGLVk	Unambiguous	1	1	Q91ZZ3	N-Term(iTRAQ8plex); N16(Deamidated); K25(iTRAQ8plex)	CID	1.0000	0.0000	1	1	3558570	Unique	Used	1.376	1.284	1.382	1.008	0	0	7.78	0	18	159	3	979.20490	2935.60013	-3.31
sNVASTAAcSSASSLGLGLAYR	Unambiguous	1	1	P46660	N-Term(iTRAQ8plex); C9(Methylthio)	CID	1.0000	0.0000	1	1	3935169	Unique	Used	1.036	0.244	1.379	1.155	0	0	7.78	0	23	200	2	1218.60547	2436.20366	-5.28
sGTAHEYSScPDDAIFQSLAR	Unambiguous	1	1	Q00493	N-Term(iTRAQ8plex); C10(Methylthio)	CID	1.0000	0.0000	1	1	3280830	Unique	Used	1.163	0.948	0.856	1.021	0	0	7.78	0	6	11	3	869.06915	2605.19291	-1.38
iSFEEFcAVVGGLDIHk	Unambiguous	2	1	Q63810-2	N-Term(iTRAQ8plex); C7(Methylthio); K17(iTRAQ8plex)	CID	1.0000	0.0000	1	1	3708704	Unique	Used	0.790	1.963	1.893	0.766	0	0	7.77	0	7	200	3	840.11194	2518.32126	-3.26
sVcTLQITDTTGSHQFPAMQR	Unambiguous	2	1	Q91Z61	N-Term(iTRAQ8plex); C3(Methylthio)	CID	1.0000	0.0000	1	1	3857475	Unique	Used	0.797	0.839	1.037	0.996	0	0	7.77	0	28	141	3	891.09991	2671.28519	-3.47
dFTSEQIDEILQNHTEIVFAR	Unambiguous	2	1	Q6PIC6	N-Term(iTRAQ8plex)	CID	1.0000	0.0000	1	1	3355022	Unique	Used	0.548	0.450	1.085	1.418	0	0	7.77	0	4	52	3	937.14545	2809.42179	-3.30
qHGEQLGGGGSGGGGYnTSk	Unambiguous	7	1	Q80U78-2	N-Term(iTRAQ8plex); N17(Deamidated); K20(iTRAQ8plex)	CID	1.0000	0.0000	1	1	2646029	Unique	Used	1.158	0.792	0.557	0.306	0	0	7.77	0	27	200	3	819.74420	2457.21805	-1.41
eSGcAAAGETEAAAATAFR	Unambiguous	1	1	Q8BR63	N-Term(iTRAQ8plex); C4(Methylthio)	CID	1.0000	0.0000	1	1	2937926	Unique	Used	1.343	0.100	1.247	1.461	0	0	7.77	0	0	12	2	1067.49438	2133.98149	-1.52
dFTSEQIDEILQNHTEIVFAR	Unambiguous	2	1	Q6PIC6	N-Term(iTRAQ8plex)	CID	1.0000	0.0000	1	1	3353747	Unique	Used	0.462	0.662	0.890	1.380	0	0	7.77	0	0	11	3	937.14502	2809.42051	-3.76
nMGGPYGGGNYGPGGSGGSGGYGGR	Unambiguous	3	1	O88569	N-Term(iTRAQ8plex); N1(Deamidated)	CID	1.0000	0.0000	1	1	2989604	Unique	Used	1.392	0.646	1.421	0.723	0	0	7.76	0	0	200	2	1248.04761	2495.08794	-2.77
qcGQmPATATTAAAQPPTAHGPER	Unambiguous	1	1	Q9JLV1	N-Term(iTRAQ8plex); C2(Methylthio); M5(Oxidation)	CID	1.0000	0.0000	1	1	3428460	Unique	Used	1.613	0.496	0.970	1.135	0	0	7.76	0	0	200	3	920.10132	2758.28940	-4.33
qTTAAAAATFSEQVGGGSGGAGR	Unambiguous	3	1	O88935	N-Term(iTRAQ8plex)	CID	1.0000	0.0000	1	1	3513304	Unique	Used	0.564	1.398	1.996	1.746	0	0	7.76	0	0	66	2	1178.58887	2356.17046	-3.86

qAQMFQNFGGAGGGSSGTGSSSSGR	Unambiguous	4	1	E9QKY7	N-Term(iTRAQ8plex)	CID	1.0000	0.0000	1	1	3836455	Unique	Used	1.899	0.779	1.461	0.611	0	0	7.76	0	0	200	2	1312.59998	2624.19267	-5.15
sPQEEVLQPLINLYGLLHGLqAVVVQQER	Unambiguous	8	1	H3BJU7	N-Term(iTRAQ8plex); Q21(Deamidated)	CID	1.0000	0.0000	1	1	3918455	Unique	Used	0.991	1.637	1.112	0.544	0	0	7.76	0	16	76	3	1192.66479	3575.97983	1.63
vFVLDEADVMIATQGHQDQSIR	Unambiguous	3	1	Q61655	N-Term(iTRAQ8plex)	CID	1.0000	0.0000	1	1	3238974	Unique	Used	0.594	1.157	1.049	1.039	0	0	7.76	0	10	37	3	926.14417	2776.41794	-2.27
sNVASTAAcSSASSLGLGLAYR	Unambiguous	1	1	P46660	N-Term(iTRAQ8plex); C9(Methylthio)	CID	1.0000	0.0000	1	1	3935061	Unique	Used	1.153	0.253	1.022	1.091	0	0	7.76	0	16	200	2	1218.60693	2436.20659	-4.07
aVFYAAEIccGLEDLHR	Unambiguous	3	1	O70293-2	N-Term(iTRAQ8plex); C9(Methylthio); C10(Methylthio)	CID	1.0000	0.0000	1	1	3707161	Unique	Used	1.737	1.154	0.762	1.007	0	0	7.75	0	8	135	3	769.35895	2306.06229	-5.25
sALFAQInQGESITHALk	Unambiguous	1	1	P40124	N-Term(iTRAQ8plex); N8(Deamidated); K18(iTRAQ8plex)	CID	1.0000	0.0000	1	1	3415842	Unique	Used	1.056	1.575	1.504	1.094	0	0	7.75	0	0	52	3	846.47400	2537.40744	-4.32
gNDISSGTVLSDYVGSGPPSGTGLHR	Unambiguous	1	1	P70296	N-Term(iTRAQ8plex)	CID	1.0000	0.0000	1	1	3660898	Unique	Used	2.630	1.377	1.048	1.152	0	0	7.75	0	0	153	3	945.47345	2834.40580	-5.82
iSFEEFcAVVGGLDIHk	Unambiguous	2	1	Q63810-2	N-Term(iTRAQ8plex); C7(Methylthio); K17(iTRAQ8plex)	CID	1.0000	0.0000	1	1	3687102	Unique	Used	0.583	1.295	2.194	0.998	0	0	7.75	0	1	4	3	840.10925	2518.31321	-6.46
tVYFAEEVQcEGNSFHk	Unambiguous	1	1	P97315	N-Term(iTRAQ8plex); C10(Methylthio); K17(iTRAQ8plex)	CID	1.0000	0.0000	1	1	3090894	Unique	Used	1.642	0.801	1.701	1.200	0	0	7.75	0	10	162	3	881.43140	2642.27964	-1.64
icDPGLTSFEPEALGnLVEGMDFHR	Unambiguous	9	1	Q5SVJ0	N-Term(iTRAQ8plex); C2(Methylthio); N16(Deamidated)	CID	1.0000	0.0000	1	1	3481504	Unique	Used	0.546	0.530	0.755	0.961	0	0	7.75	0	26	200	3	1033.49463	3098.46933	3.78
iSFEEFcAVVGGLDIHk	Unambiguous	2	1	Q63810-2	N-Term(iTRAQ8plex); C7(Methylthio); K17(iTRAQ8plex)	CID	1.0000	0.0000	1	1	3708808	Unique	Used	1.392	1.257	1.467	0.631	0	0	7.74	0	21	200	3	840.11127	2518.31925	-4.06

yVVVDSGGGTVDLTVHQIR	Unambiguous	1	1	Q8K0U4	N-Term(iTRAQ8plex)	CID	1.0000	0.0000	1	1	3390070	Unique	Used	1.326	1.042	1.024	1.330	0	0	7.74	0	17	20	3	773.75629	2319.25431	-2.92
vGELTQEANALAAGHPAQAPAINTR	Unambiguous	1	1	Q68FG2	N-Term(iTRAQ8plex)	HCD	1.0000	0.0000	1	1	3476498	Unique	Used	0.602	2.240	2.402	1.036	0	0	7.74	0	9	11	3	935.50153	2804.49002	-2.05
sAHGPLGDSAAAGPGGDTEYPMGk	Unambiguous	2	1	Q69Z98	N-Term(iTRAQ8plex); K24(iTRAQ8plex)	CID	1.0000	0.0000	1	1	2989676	Unique	Used	1.049	1.000	0.908	0.904	0	0	7.74	0	0	200	3	951.14001	2851.40549	-3.05
dYSGLHDVAAVGDGLPEGATAnGMk	Unambiguous	3	1	Q3UHH2	N-Term(iTRAQ8plex); N22(Deamidated); K25(iTRAQ8plex)	CID	1.0000	0.0000	1	1	2918062	Unique	Used		0.409	0.883	2.364	0	0	7.74	0	5	68	3	1018.84491	3054.52018	-3.20
rFQSVESGANNVVFIR	Unambiguous	1	1	Q99J36	N-Term(iTRAQ8plex)	HCD	1.0000	0.0000	1	1	3873842	Unique	Used	1.401	1.111	1.151	1.019	0	0	7.74	1	13	65	3	709.72290	2127.15415	-3.36
sELEAQTGLQILQTGVGQR	Unambiguous	2	1	Q60673	N-Term(iTRAQ8plex)	CID	1.0000	0.0000	1	1	3477740	Unique	Used	0.598	0.947	0.848	1.621	0	0	7.74	0	16	86	2	1166.63965	2332.27202	-2.33
vPRPEDTLTYADLDMVHLSR	Unambiguous	7	1	Q6P6I8	N-Term(iTRAQ8plex)	CID	1.0000	0.0000	1	1	3370352	Unique	Used	1.055	1.761	1.545	1.283	0	0	7.74	0	0	152	3	878.12421	2632.35807	-4.81
sLAGSSGPGASSGPGGDHSELIVR	Unambiguous	5	1	P57776-3	N-Term(iTRAQ8plex)	HCD	1.0000	0.0000	1	1	3207507	Unique	Used	1.924	0.841	1.013	1.027	0	0	7.74	0	0	29	3	833.76147	2499.26987	-1.73
aPPAGGSGSSAAAAAAAGGTEVRPR	Unambiguous	2	1	P84309	N-Term(iTRAQ8plex)	HCD	1.0000	0.0000	1	1	3468683	Unique	Used	0.743	0.733	2.747	1.331	0	0	7.73	0	5	67	3	814.42822	2441.27011	-4.04
sSGNSANDLANVAVIDGNR	Unambiguous	1	1	Q7TT37	N-Term(iTRAQ8plex)	CID	1.0000	0.0000	1	1	3125576	Unique	Used	0.759	0.991	0.892	1.240	0	0	7.73	0	0	185	2	1089.55408	2178.10088	-2.04
sIYVGnVDYGATAEELEAHFHGcGSVNR	Unambiguous	8	1	D3Z5F7	N-Term(iTRAQ8plex); N6(Deamidated); C23(Methylthio)	CID	1.0000	0.0000	1	1	3394225	Unique	Used	1.089	0.817	1.171	0.882	0	0	7.73	0	0	200	3	1116.18677	3346.54575	1.38
tLQLVVLDADTINHPAQLSk	Unambiguous	4	1	Q8CC27-4	N-Term(iTRAQ8plex); K20(iTRAQ8plex)	CID	1.0000	0.0000	1	1	3498415	Unique	Used	0.932	1.123	1.176	1.423	0	0	7.73	0	12	80	3	928.87238	2784.60257	-1.96
fDPYTLAIDVLDSPHTIR	Unambiguous	2	1	P24529	N-Term(iTRAQ8plex)	CID	1.0000	0.0000	1	1	3541348	Unique	Used		0.819	1.937	1.652	0	0	7.73	0	16	27	3	793.09113	2377.25882	-4.94
gLTIVGSVLEGTFLDNHPQAQR	Unambiguous	2	1	Q91V14	N-Term(iTRAQ8plex)	CID	1.0000	0.0000	1	1	3844484	Unique	Used	0.355	0.583	1.216	0.561	0	0	7.73	0	15	200	3	886.14764	2656.42838	-2.91
qTTAAAAATFSEQVGGGSGGAGR	Unambiguous	3	1	O88935	N-Term(iTRAQ8plex); Q1(Deamidated)	CID	1.0000	0.0000	1	1	3298735	Unique	Used	3.981	3.581	3.514	1.888	0	0	7.73	0	0	152	2	1179.08447	2357.16167	-0.81

gTVVYGEPITASLGTDGSHYWSk	Unambiguous	1	1	O08553	N-Term(iTRAQ8plex); K23(iTRAQ8plex)	CID	1.0000	0.0000	1	1	3347269	Unique	Used	2.297	1.772	0.945	0.939	0	0	7.72	0	13	13	3	1011.86163	3033.57035	-2.47
aAFGLSEAGFnTAcLTk	Unambiguous	1	1	Q8K2B3	N-Term(iTRAQ8plex); N11(Deamidated); C14(Methylthio); K17(iTRAQ8plex)	CID	1.0000	0.0000	1	1	3726468	Unique	Used	1.141	0.744	1.155	1.077	0	0	7.72	0	0	54	2	1178.61353	2356.21977	2.54
aAQGEAEcPGGEAEQGGAAAIQAHYk	Unambiguous	1	1	Q04888	N-Term(iTRAQ8plex); C8(Methylthio); K26(iTRAQ8plex)	CID	1.0000	0.0000	1	1	2809450	Unique	Used	1.126	0.302	0.454	0.269	0	0	7.72	0	0	150	3	1056.84351	3168.51596	-4.42
hQGGWTDGGSGSGGGYQDGAYR	Unambiguous	1	1	Q3TJZ6	N-Term(iTRAQ8plex)	CID	1.0000	0.0000	1	1	2789674	Unique	Used	1.738	1.040	1.003	0.793	0	0	7.72	0	9	200	3	825.37323	2474.10514	1.13
aAAASAAEAGIATPGTEGER	Unambiguous	4	1	O35226-2	N-Term(iTRAQ8plex)	CID	1.0000	0.0000	1	1	2827832	Unique	Used	1.726	1.055	1.021	0.914	0	0	7.72	0	0	71	2	1053.03955	2105.07182	-2.80
sNVASTAAcSSASSLGLGLAYR	Unambiguous	1	1	P46660	N-Term(iTRAQ8plex); C9(Methylthio)	CID	1.0000	0.0000	1	1	3915851	Unique	Used	1.821	0.504	2.465	2.155	0	0	7.72	0	0	126	2	1218.60327	2436.19927	-7.08
sMLQATAEANNLAAAASAk	Unambiguous	3	1	Q91YH5	N-Term(iTRAQ8plex); K19(iTRAQ8plex)	CID	1.0000	0.0000	1	1	3496957	Unique	Used	2.242	1.318	1.877	1.600	0	0	7.72	0	22	179	2	1221.16260	2441.31792	-4.09
fNSVVTNPQGHYNPSTGk	Unambiguous	1	1	Q02105	N-Term(iTRAQ8plex); K18(iTRAQ8plex)	CID	1.0000	0.0000	1	1	3552684	Unique	Used	1.367	1.853	1.429	1.643	0	0	7.72	0	0	200	3	852.45209	2555.34171	-1.79
dFTSEQIDEILQNHTEIVFAR	Unambiguous	2	1	Q6PIC6	N-Term(iTRAQ8plex)	CID	1.0000	0.0000	1	1	3356447	Unique	Used	0.539	0.512	1.159	0.942	0	0	7.72	0	0	200	3	937.14624	2809.42417	-2.46
sAHGPLGDSAAAGPGGDTEYPmGk	Unambiguous	2	1	Q69Z98	N-Term(iTRAQ8plex); M22(Oxidation); K24(iTRAQ8plex)	CID	1.0000	0.0000	1	1	2862476	Unique	Used	1.750	1.087	0.998	0.963	0	0	7.72	0	0	200	3	956.47253	2867.40305	-2.11
aVVFETGEAGAASGAGSLPGEVR	Unambiguous	3	1	Q9QYR6	N-Term(iTRAQ8plex)	CID	1.0000	0.0000	1	1	3129407	Unique	Used	2.530	1.444	1.789	1.863	0	0	7.72	0	0	175	2	1218.63525	2436.26323	-1.68
gNDISSGTVLSDYVGSGPPSGTGLHR	Unambiguous	1	1	P70296	N-Term(iTRAQ8plex)	CID	1.0000	0.0000	1	1	3701899	Unique	Used	0.988	1.030	0.998	1.002	0	0	7.71	0	27	200	3	945.47125	2834.39920	-8.15

aGLEQGSDAGYLAESQk	Unambiguous	1	1	Q8BMS1	N-Term(iTRAQ8plex); K17(iTRAQ8plex)	CID	1.0000	0.0000	1	1	2715201	Unique	Used	0.581	0.707	1.178	1.441	0	0	7.71	0	0	200	2	1166.61182	2332.21636	-3.33
eIGWADVGGWTGQGGSILGTk	Unambiguous	2	1	Q8C605	N-Term(iTRAQ8plex); K21(iTRAQ8plex)	CID	1.0000	0.0000	1	1	3540779	Unique	Used	1.553	1.263	0.935	1.093	0	0	7.71	0	19	13	2	1349.21899	2697.43071	-5.57
vQScPDPAVYGVVQSGDHVGR	Unambiguous	1	1	Q6ZPJ3	N-Term(iTRAQ8plex); C4(Methylthio)	CID	1.0000	0.0000	1	1	3474587	Unique	Used	1.313	1.356	1.040	1.191	0	0	7.71	0	10	81	3	840.74475	2520.21970	-3.20
nLHQSGFSLSGAQIDDNIPR	Unambiguous	1	1	O08553	N-Term(iTRAQ8plex); N1(Deamidated)	CID	0.0351	0.0000	1	1	3278903	Unique	Used	0.978	1.889	2.543	1.970	0	0	7.70	0	21	2	3	825.42249	2474.25290	-1.97
nLHQSGFSLSGAQIDDNIPR	Unambiguous	1	1	O08553	N-Term(iTRAQ8plex)	CID	1.0000	0.0000	1	1	3411773	Unique	Used	1.526	0.295	1.108	1.143	0	0	7.70	0	0	0	3	825.09320	2473.26505	-3.53
aDDITSLVADTTLLVHFFGk	Unambiguous	2	1	Q9CTY5	N-Term(iTRAQ8plex); K20(iTRAQ8plex)	CID	1.0000	0.0000	1	1	3687298	Unique	Used	0.354	0.981	1.030	1.031	0	0	7.70	0	0	14	3	924.51465	2771.52939	-5.28
gNDISSGTVLSDYVGSGPPSGTGLHR	Unambiguous	1	1	P70296	N-Term(iTRAQ8plex)	CID	1.0000	0.0000	1	1	3454799	Unique	Used	1.581	0.956	1.052	0.900	0	0	7.70	0	16	200	3	945.47583	2834.41294	-3.30
tLTQEEVEmLYEEALYTVLHR	Unambiguous	2	1	E9Q350	N-Term(iTRAQ8plex); M9(Oxidation)	CID	1.0000	0.0000	1	1	3374147	Unique	Used		0.672	0.330	1.497	0	0	7.70	0	4	23	3	963.15662	2887.45530	-5.14
sIVnNGHSFNVEFDDSQDNAVLk	Unambiguous	1	1	P00920	N-Term(iTRAQ8plex); N4(Deamidated); K23(iTRAQ8plex)	CID	0.0130	0.0000	1	1	2938288	Unique	Used	2.245	1.548	1.390	1.813	0	0	7.70	0	0	81	3	1053.53149	3158.57993	-1.65
dFTSEQIDEILQNHTEIVFAR	Unambiguous	2	1	Q6PIC6	N-Term(iTRAQ8plex)	CID	1.0000	0.0000	1	1	3355487	Unique	Used	0.679	0.801	1.101	1.295	0	0	7.70	0	2	80	3	937.14655	2809.42508	-2.13
eAmEAQIQNFGQTPSQLLIEPHPPR	Unambiguous	4	1	Q9EPN1-4	N-Term(iTRAQ8plex); M3(Oxidation)	CID	1.0000	0.0000	1	1	3455799	Unique	Used	1.211	1.078	0.758	0.859	0	0	7.70	0	10	147	3	1051.20764	3151.60837	-2.07
aAAASAAEAGIATPGTEGER	Unambiguous	4	1	O35226-2	N-Term(iTRAQ8plex)	CID	1.0000	0.0000	1	1	2846384	Unique	Used	0.998	0.795	1.099	0.846	0	0	7.70	0	16	200	2	1053.03748	2105.06767	-4.77
iGLSVSEVISGYEIAck	Unambiguous	3	1	P42932	N-Term(iTRAQ8plex); C16(Methylthio); K17(iTRAQ8plex)	CID	1.0000	0.0000	1	1	3623503	Unique	Used	0.341	0.854	0.592	0.931	0	0	7.70	0	16	9	2	1211.65588	2422.30449	-5.67

fLSQPFQVAEVFTGHMGk	Unambiguous	1	1	P56480	N-Term(iTRAQ8plex); K18(iTRAQ8plex)	CID	1.0000	0.0000	1	1	3862990	Unique	Used	1.138	0.773	1.020	1.325	0	0	7.70	0	0	200	3	877.80908	2631.41269	-3.31
lNSDPQFVLAQNVGTTHDLLDIcLR	Unambiguous	3	1	Q8R016	N-Term(iTRAQ8plex); C23(Methylthio)	CID	1.0000	0.0000	1	1	3785493	Unique	Used	1.000	1.087	0.984	1.134	0	0	7.70	0	24	32	3	1044.87317	3132.60495	-2.39
nINADEAAAMGAVYQAAALSk	Unambiguous	1	1	Q9JKR6	N-Term(iTRAQ8plex); K21(iTRAQ8plex)	CID	1.0000	0.0000	1	1	3351965	Unique	Used	0.563	0.939	0.643	0.843	0	0	7.70	0	23	11	2	1344.21240	2687.41753	-4.03
rITTIQGPEPGALQEYEQVLVSTR	Unambiguous	13	1	Q02357-5	N-Term(iTRAQ8plex)	CID	1.0000	0.0000	1	1	3539685	Unique	Used	1.592	1.184	1.412	2.728	0	0	7.69	1	20	63	3	997.20966	2989.61441	-3.91
eQVTnVGGAVVTGVTAVAQk	Unambiguous	2	1	O55042	N-Term(iTRAQ8plex); N5(Deamidated); K20(iTRAQ8plex)	CID	0.0221	0.0000	1	1	3620019	Unique	Used	0.662	1.574	1.672	0.704	0	0	7.69	0	29	200	2	1269.22827	2537.44927	3.82
tIGGGDDSFNTFFSETGAGk	Unambiguous	4	1	P68373	N-Term(iTRAQ8plex); K20(iTRAQ8plex)	CID	1.0000	0.0000	1	1	3152866	Unique	Used	0.508	1.590	1.156	1.517	0	0	7.69	0	0	200	2	1308.65479	2616.30229	-0.60
dLVILLYETALLSSGFSLEDPQTHANR	Unambiguous	1	1	P07901	N-Term(iTRAQ8plex)	HCD	1.0000	0.0000	1	1	3786119	Unique	Used	0.934	1.153	0.876	1.101	0	0	7.69	0	2	200	3	1102.92114	3306.74887	-1.07
eLAcDDPEAEQVALLAVDYLNNHLLQGFk	Unambiguous	1	1	P29699	N-Term(iTRAQ8plex); C4(Methylthio); K29(iTRAQ8plex)	CID	1.0000	0.0000	1	1	3480350	Unique	Used	2.187	1.663	0.308	0.568	0	0	7.69	0	0	47	3	1294.99854	3882.98105	-1.47
gIEFPmADLDALSPIHTPQR	Unambiguous	6	1	Q5SRX1-2	N-Term(iTRAQ8plex); M6(Oxidation)	CID	1.0000	0.0000	1	1	3539635	Unique	Used	6.745	2.092	1.394	1.248	0	0	7.69	0	5	7	3	843.43756	2528.29813	-5.55
qQQQQQQQQQEYHELFR	Unambiguous	4	1	E9PU87	N-Term(iTRAQ8plex)	CID	1.0000	0.0000	1	1	2952242	Unique	Used	0.988	0.341	1.318	1.062	0	0	7.69	0	6	22	3	860.09436	2578.26853	-0.59
aVVFETGEAGAASGAGSLPGEVR	Unambiguous	3	1	Q9QYR6	N-Term(iTRAQ8plex)	CID	1.0000	0.0000	1	1	3149526	Unique	Used	2.735	1.087	2.322	1.041	0	0	7.68	0	0	106	2	1218.63477	2436.26225	-2.08
tFLLDGDEVIITGHcQGDGYR	Unambiguous	1	1	P35505	N-Term(iTRAQ8plex); C15(Methylthio)	CID	1.0000	0.0000	1	1	3260944	Unique	Used	3.905	2.128	0.477	1.403	0	0	7.68	0	0	95	3	887.09882	2659.28189	0.77
iNVPVPQFGGGDPADIIHDFQR	Unambiguous	1	1	Q91VK4	N-Term(iTRAQ8plex)	CID	1.0000	0.0000	1	1	3580862	Unique	Used	1.387	1.019	0.986	0.703	0	0	7.68	0	5	2	3	899.47058	2696.39719	-4.71

qLLHNFPPDQLTSSGAPFWSGPk	Unambiguous	1	1	Q02053	N-Term(iTRAQ8plex); K23(iTRAQ8plex)	CID	1.0000	0.0000	1	1	3841411	Unique	Used	0.907	1.129	0.965	1.218	0	0	7.68	0	4	99	3	1044.89355	3132.66611	-2.12
lSLcGEESFGTGSDHIR	Unambiguous	2	1	Q9D0F9	N-Term(iTRAQ8plex); C4(Methylthio)	CID	1.0000	0.0000	1	1	3322886	Unique	Used	0.640	0.590	1.052	1.416	0	0	7.68	0	2	3	3	720.00958	2158.01419	-3.20
iGGILANELSVDEAALHAAVIAINEAIDR	Unambiguous	1	1	Q9JKF1	N-Term(iTRAQ8plex)	CID	1.0000	0.0000	1	1	3646209	Unique	Used	0.941	1.721	0.860	1.153	0	0	7.68	0	20	200	3	1088.26355	3262.77610	-5.79
vNQELAGYTGGDVSFIk	Unambiguous	1	1	Q8BGH2	N-Term(iTRAQ8plex); K17(iTRAQ8plex)	CID	1.0000	0.0000	1	1	3302503	Unique	Used	0.291	1.159	1.029	1.293	0	0	7.68	0	0	29	2	1203.65845	2406.30962	-1.22
dFTSEQIDEILQNHTEIVFAR	Unambiguous	2	1	Q6PIC6	N-Term(iTRAQ8plex)	CID	1.0000	0.0000	1	1	3356342	Unique	Used	0.600	0.528	0.967	1.285	0	0	7.68	0	4	200	3	937.14642	2809.42472	-2.26
eLAcDDPEAEQVALLAVDYLNNHLLQGFk	Unambiguous	1	1	P29699	N-Term(iTRAQ8plex); C4(Methylthio); K29(iTRAQ8plex)	CID	1.0000	0.0000	1	1	3440610	Unique	Used	2.406	1.383		0.355	0	0	7.68	0	14	24	3	1294.99316	3882.96494	-5.62
dFTSEQIDEILQNHTEIVFAR	Unambiguous	2	1	Q6PIC6	N-Term(iTRAQ8plex)	CID	1.0000	0.0000	1	1	3356132	Unique	Used	0.771	0.786	1.049	1.241	0	0	7.67	0	0	200	3	937.14575	2809.42270	-2.98
aGNSLAASTAEETAGSAQSR	Unambiguous	1	1	Q61292	N-Term(iTRAQ8plex)	CID	1.0000	0.0000	1	1	2844612	Unique	Used	1.201	1.799	1.294	1.449	0	0	7.67	0	0	85	2	1092.04211	2183.07695	-3.34
tNHSGFLFGFGDGGGGPTQTMLDR	Unambiguous	4	1	F8WIE1	N-Term(iTRAQ8plex)	CID	1.0000	0.0000	1	1	3643534	Unique	Used	0.793	0.935	0.848	1.109	0	0	7.67	0	0	89	3	925.11176	2773.32071	-3.59
sGSSSSSSGNPASQFYPQSR	Unambiguous	1	1	Q61771	N-Term(iTRAQ8plex)	CID	1.0000	0.0000	1	1	3510926	Unique	Used	0.729	0.467	1.544	0.907	0	0	7.67	0	0	200	2	1161.54517	2322.08306	-3.00
gPAVGIDLGTTYScVGVFQHGk	Unambiguous	4	1	P63017	N-Term(iTRAQ8plex); C14(Methylthio); K22(iTRAQ8plex)	CID	1.0000	0.0000	1	1	3807420	Unique	Used	1.238	0.778	1.089	0.914	0	0	7.67	0	24	200	3	954.16675	2860.48569	-3.13
dFTSEQIDEILQNHTEIVFAR	Unambiguous	2	1	Q6PIC6	N-Term(iTRAQ8plex)	CID	1.0000	0.0000	1	1	3356987	Unique	Used	0.681	0.777	1.294	1.200	0	0	7.67	0	2	200	3	937.14618	2809.42398	-2.52
dVALSSGSAcTSASLEPSYVLR	Unambiguous	3	1	Q8C6I5	N-Term(iTRAQ8plex); C10(Methylthio)	CID	1.0000	0.0000	1	1	3518534	Unique	Used	0.946	1.021	1.199	1.150	0	0	7.67	0	0	88	2	1282.13135	2563.25542	-5.15
vDSILLTHIGDDNLPGINSMLQR	Unambiguous	1	1	P14873	N-Term(iTRAQ8plex)	CID	1.0000	0.0000	1	1	3686487	Unique	Used	0.927	0.692	0.668	1.032	0	0	7.67	0	5	3	3	942.50378	2825.49680	-5.87
eASSPSSANPASETLQSPSSR	Unambiguous	4	1	Q9R0N8-2	N-Term(iTRAQ8plex)	CID	1.0000	0.0000	1	1	2844119	Unique	Used	1.066	0.346	1.504	1.507	0	0	7.67	0	0	200	2	1197.58374	2394.16020	-3.55

qTTAAAATFSEQVGGGSGGAGR	Unambiguous	3	1	O88935	N-Term(iTRAQ8plex)	CID	1.0000	0.0000	1	1	3554329	Unique	Used	0.536	1.363	1.707	1.480	0	0	7.67	0	0	200	2	1178.58752	2356.16777	-5.00
qDHPSSmGVYGQESGGFSGPGENR	Unambiguous	4	1	Q5SUT0	N-Term(iTRAQ8plex); M7(Oxidation)	CID	1.0000	0.0000	1	1	2824642	Unique	Used	1.745	1.619	1.342	0.847	0	0	7.67	0	21	200	3	934.09064	2800.25736	1.37
tVFEALQAPAcHENLVk	Unambiguous	1	1	P17427	N-Term(iTRAQ8plex); C11(Methylthio); K17(iTRAQ8plex)	CID	1.0000	0.0000	1	1	3497544	Unique	Used	0.516	1.265	0.998	1.020	0	0	7.67	0	18	35	3	842.11926	2524.34323	-3.18
qTTAAAATFSEQVGGGSGGAGR	Unambiguous	3	1	O88935	N-Term(iTRAQ8plex)	CID	1.0000	0.0000	1	1	3472200	Unique	Used	0.563	1.396	1.674	1.447	0	0	7.67	0	0	122	2	1178.58862	2356.16997	-4.07
aFVEVNEEGTEAAAATAGMMTVR	Unambiguous	2	1	F8WIV2	N-Term(iTRAQ8plex)	CID	1.0000	0.0000	1	1	3132564	Unique	Used	0.678	1.266	0.797	1.358	0	0	7.67	0	0	200	2	1330.15552	2659.30376	1.03
dYIQESENIASLHNQITAcDAVLER	Unambiguous	1	1	Q8C754	N-Term(iTRAQ8plex); C19(Methylthio)	CID	1.0000	0.0000	1	1	3326601	Unique	Used	1.391	1.073	1.520	1.341	0	0	7.67	0	10	31	3	1061.51526	3182.53122	-2.77
dFTSEQIDEILQnHTEIVFAR	Unambiguous	2	1	Q6PIC6	N-Term(iTRAQ8plex); N13(Deamidated)	CID	0.0130	0.0000	1	1	3153381	Unique	Used	0.543	0.741	1.377	1.686	0	0	7.67	0	0	7	3	937.47577	2810.41275	-0.83
tARPFGIQSPGTSQIEQSPMMGR	Unambiguous	4	1	Q8CC35	N-Term(iTRAQ8plex)	CID	1.0000	0.0000	1	1	3893152	Unique	Used	0.903	2.961	10.017	1.485	0	0	7.66	0	10	108	3	927.47076	2780.39774	-5.36
aVVTHSSGNHGQALTYAAk	Unambiguous	4	1	Q9QZX7-2	N-Term(iTRAQ8plex); K19(iTRAQ8plex)	CID	1.0000	0.0000	1	1	3467530	Unique	Used	0.145	2.289	2.595	1.172	0	0	7.66	0	0	118	3	840.79565	2520.37241	-2.19
dVQFAVQQVLQEEHFDAR	Unambiguous	2	1	Q8R146-2	N-Term(iTRAQ8plex)	CID	1.0000	0.0000	1	1	3216329	Unique	Used	1.735	1.197	1.723	1.072	0	0	7.66	0	11	115	3	821.75690	2463.25614	-0.37
hGSGSGGPDLVQYQPQHGPGLSAPqGLAPLR	Unambiguous	2	1	O88737	N-Term(iTRAQ8plex); Q25(Deamidated)	CID	1.0000	0.0000	1	1	3700807	Unique	Used	0.765	0.960	2.076	0.870	0	0	7.66	0	0	163	3	1128.58337	3383.73557	-1.29
eFTTAAASPGGqSVVLGSYDR	Unambiguous	1	1	Q6VH22	N-Term(iTRAQ8plex); Q12(Deamidated)	CID	1.0000	0.0000	1	1	3300330	Unique	Used	0.826	1.007	0.764	1.007	0	0	7.66	0	0	200	2	1209.61536	2418.22344	5.93
vPRPEDTLTYADLDMVHLSR	Unambiguous	7	1	Q6P6I8	N-Term(iTRAQ8plex)	CID	1.0000	0.0000	1	1	3392231	Unique	Used	0.676	2.194	2.122	1.428	0	0	7.66	0	3	22	3	878.12494	2632.36026	-3.97
aVVFETGEAGAASGAGSLPGEVR	Unambiguous	3	1	Q9QYR6	N-Term(iTRAQ8plex)	CID	1.0000	0.0000	1	1	3129094	Unique	Used	2.357	1.294	1.756	1.015	0	0	7.66	0	0	37	2	1218.63599	2436.26470	-1.08
qDHPSSmGVYGQESGGFSGPGENR	Unambiguous	4	1	Q5SUT0	N-Term(iTRAQ8plex); M7(Oxidation)	CID	1.0000	0.0000	1	1	2968686	Unique	Used	1.965	1.502	1.153	0.749	0	0	7.66	0	0	200	3	934.08575	2800.24271	-3.86

lLEVSDDPQVLAVAAHDVGEYVR	Unambiguous	1	1	Q8BVE3	N-Term(iTRAQ8plex)	CID	1.0000	0.0000	1	1	3240625	Unique	Used	1.343	1.020	1.514	1.478	0	0	7.65	0	11	200	3	933.83337	2799.48557	0.87
rAPPGPLPPAASPGPPAASPPAAPSsPR	Unambiguous	1	1	O88703	N-Term(iTRAQ8plex); S26(Phospho)	CID	0.0209	0.0000	1	1	3409393	Unique	Used	1.752	0.946	1.118	1.319	0	0	7.65	1	0	62	3	984.84766	2952.52842	-3.92
kTGQAAGFSYTDANk	Unambiguous	2	1	G3UWG1	N-Term(iTRAQ8plex); K1(iTRAQ8plex); K15(iTRAQ8plex)	HCD	1.0000	0.0000	1	1	3227001	Unique	Used	1.461	0.691	1.504	0.976	0	0	7.65	1	14	109	3	824.46014	2471.36588	0.05
qGSATSAASATTASSAAEGTLQR	Unambiguous	1	1	Q8R4V2	N-Term(iTRAQ8plex)	CID	1.0000	0.0000	1	1	3163437	Unique	Used	1.357	0.143	0.913	1.014	0	0	7.65	0	0	63	2	1214.61133	2428.21538	-2.64
gSDHTDVcGNVVGSSGGSSSSGGSDk	Unambiguous	2	1	A2AHG0	N-Term(iTRAQ8plex); C8(Methylthio); K26(iTRAQ8plex)	CID	1.0000	0.0000	1	1	2896588	Unique	Used	0.896	0.670	2.612	1.058	0	0	7.65	0	0	200	3	998.78809	2994.34970	-4.31
gNDISSGTVLSDYVGSGPPSGTGLHR	Unambiguous	1	1	P70296	N-Term(iTRAQ8plex)	CID	1.0000	0.0000	1	1	3454485	Unique	Used	1.458	0.909	1.075	1.306	0	0	7.65	0	0	200	3	945.47589	2834.41312	-3.24
qTTAAAAATFSEqVGGGSGGAGR	Unambiguous	3	1	O88935	N-Term(iTRAQ8plex); Q13(Deamidated)	CID	1.0000	0.0000	1	1	3494160	Unique	Used	0.597	1.608	1.707	1.869	0	0	7.64	0	0	104	2	1179.09009	2357.17290	3.95
nLSPAPASSSQGPPPQGPVSPGPAk	Unambiguous	3	1	Q7TQ95	N-Term(iTRAQ8plex); K25(iTRAQ8plex)	CID	1.0000	0.0000	1	1	3408178	Unique	Used	1.035	1.453	1.124	1.450	0	0	7.64	0	0	80	3	978.53082	2933.57792	-5.55
dLQSSNQLSNHISSLFR	Unambiguous	4	1	Q00612	N-Term(iTRAQ8plex)	CID	1.0000	0.0000	1	1	3875918	Unique	Used	0.998	1.011	0.969	1.096	0	0	7.64	0	22	200	3	750.72699	2250.16642	-5.17
eAVcIVLSDDTcSDEk	Unambiguous	1	1	Q01853	N-Term(iTRAQ8plex); C4(Methylthio); C12(Methylthio); K16(iTRAQ8plex)	CID	1.0000	0.0000	1	1	2815982	Unique	Used	0.324	0.556	0.869	0.850	0	0	7.64	0	4	105	2	1214.06970	2427.13213	-2.11
sNVASTAAcSSASSLGLGLAYR	Unambiguous	1	1	P46660	N-Term(iTRAQ8plex); C9(Methylthio)	CID	1.0000	0.0000	1	1	3934815	Unique	Used	1.306	0.096	1.957	1.200	0	0	7.64	0	2	14	2	1218.60535	2436.20342	-5.38
eFESVLVDAFSHVAR	Unambiguous	3	1	Q9CSU0-2	N-Term(iTRAQ8plex)	CID	1.0000	0.0000	1	1	3520063	Unique	Used	1.180	1.014	0.663	1.007	0	0	7.64	0	25	42	3	670.68793	2010.04923	-5.28

tYFPHFDVSHGSAQVk	Unambiguous	2	1	Q91VB8	N-Term(iTRAQ8plex); K16(iTRAQ8plex)	CID	1.0000	0.0000	1	1	3513052	Unique	Used	3.708	2.836	0.499	1.374	0	0	7.64	0	3	50	3	810.09589	2428.27311	-5.71
cPLEEGQAGAAEATAAPSVEDR	Unambiguous	6	1	Q6IR34-3	N-Term(iTRAQ8plex); C1(Methylthio)	CID	1.0000	0.0000	1	1	2636205	Unique	Used	0.817	0.802	1.295	0.881	0	0	7.64	0	0	99	2	1261.59082	2522.17436	-2.46
eSLFHSEHGALAQVGSPGAGR	Unambiguous	1	1	Q5HZI2	N-Term(iTRAQ8plex)	CID	1.0000	0.0000	1	1	3386164	Unique	Used	0.925	1.299	1.453	1.190	0	0	7.64	0	0	57	3	804.41455	2411.22910	-3.28
sGLGLGAASASTSGSGPADSVMR	Unambiguous	3	1	Q7TSJ2	N-Term(iTRAQ8plex)	CID	1.0000	0.0000	1	1	3513825	Unique	Used	1.056	0.691	1.652	1.532	0	0	7.63	0	0	151	2	1170.58594	2340.16460	-5.21
fTHGFQNIVDAYGIGTYR	Unambiguous	4	1	Q9Z1G4-3	N-Term(iTRAQ8plex)	CID	1.0000	0.0000	1	1	3878195	Unique	Used	0.430	1.154	1.164	1.292	0	0	7.63	0	0	0	3	788.40515	2363.20090	-3.27
qNHVVDVSVAVSTPAGLITPIVFNAHIk	Unambiguous	1	1	Q8BMF4	N-Term(iTRAQ8plex); K28(iTRAQ8plex)	HCD	1.0000	0.0000	1	1	3879129	Unique	Used	0.955	0.952	1.118	1.049	0	0	7.63	0	30	30	3	1179.00879	3535.01181	-3.94
gNDISSGTVLSDYVGSGPPSGTGLHR	Unambiguous	1	1	P70296	N-Term(iTRAQ8plex)	CID	1.0000	0.0000	1	1	3454763	Unique	Used	1.583	1.052	1.156	0.962	0	0	7.62	0	0	101	3	945.47412	2834.40781	-5.11
lVGSQEELASWGHEYVR	Unambiguous	2	1	Q8VDM4	N-Term(iTRAQ8plex)	CID	1.0000	0.0000	1	1	3280410	Unique	Used	0.876	1.012	1.147	0.919	0	0	7.62	0	2	14	3	755.39093	2264.15824	-1.38
qHGEQLGGGGSGGGGYNTSk	Unambiguous	7	1	Q80U78-2	N-Term(iTRAQ8plex); K20(iTRAQ8plex)	CID	1.0000	0.0000	1	1	2858924	Unique	Used	0.896	0.735	0.742	0.658	0	0	7.62	0	0	200	3	819.41541	2456.23166	-2.37
dMAIATGGAVFGEEGLNLNLEDVQAHDLGk	Unambiguous	1	1	P63038	N-Term(iTRAQ8plex); K30(iTRAQ8plex)	CID	1.0000	0.0000	1	1	3264915	Unique	Used	1.047	0.944	1.013	0.761	0	0	7.62	0	0	200	3	1231.63940	3692.90366	-0.40
kAEGATLSNATGAVESTSQAGDR	Unambiguous	1	1	Q61644	N-Term(iTRAQ8plex); K1(iTRAQ8plex)	CID	1.0000	0.0000	1	1	2952237	Unique	Used	8.066	0.836	1.246	0.837	0	0	7.62	1	13	200	3	943.83002	2829.47550	-1.57
hQPADLLEFALQHFTR	Unambiguous	1	1	P31324	N-Term(iTRAQ8plex)	CID	1.0000	0.0000	1	1	3879332	Unique	Used	0.524	1.378	0.993	0.813	0	0	7.62	0	0	22	3	743.06555	2227.18210	-4.71
sSGSPYGGGYGSGGGSGGYGSR	Unambiguous	7	1	J3QNY1	N-Term(iTRAQ8plex)	CID	1.0000	0.0000	1	1	2968350	Unique	Used	2.014	0.465	1.163	1.033	0	0	7.62	0	5	27	3	739.00262	2214.99332	-0.94
tIHQEDELIEIQSDTGTWYQR	Unambiguous	1	1	Q9JIS5	N-Term(iTRAQ8plex)	CID	1.0000	0.0000	1	1	2832040	Unique	Used			0.905	0.820	0	0	7.62	0	3	15	3	956.14374	2866.41666	0.18
aVGSTLQDFFNGFDALLEHIR	Unambiguous	1	1	F8VQK3	N-Term(iTRAQ8plex)	CID	1.0000	0.0000	1	1	3880478	Unique	Used	0.761	0.794	1.264	1.179	0	0	7.61	0	16	38	3	885.46423	2654.37815	-3.75
tTTNVLGDSLGAGIVEHLSR	Unambiguous	1	1	P56564	N-Term(iTRAQ8plex)	CID	1.0000	0.0000	1	1	3684480	Unique	Used	1.446	1.829	0.775	0.974	0	0	7.61	0	26	130	3	782.09259	2344.26322	-6.08
qGASPNVQDASGTSPVHDAAR	Unambiguous	1	1	Q60773	N-Term(iTRAQ8plex)	CID	1.0000	0.0000	1	1	2877903	Unique	Used	2.089	1.086	2.173	1.712	0	0	7.61	0	0	200	3	790.39380	2369.16684	-3.35

dFTSEQIDEILQNHTEIVFAR	Unambiguous	2	1	Q6PIC6	N-Term(iTRAQ8plex)	CID	1.0000	0.0000	1	1	3355592	Unique	Used	0.574	0.659	1.036	1.084	0	0	7.61	0	4	94	3	937.14587	2809.42307	-2.85
tQQAAQANITLQEQIEAIHk	Unambiguous	1	1	Q8K4Z5	N-Term(iTRAQ8plex); K20(iTRAQ8plex)	CID	1.0000	0.0000	1	1	3321361	Unique	Used	1.334	0.889	0.876	0.867	0	0	7.61	0	0	18	3	948.52930	2843.57334	-3.61
sGSSSSSSGNPASQFYPQSR	Unambiguous	1	1	Q61771	N-Term(iTRAQ8plex)	CID	1.0000	0.0000	1	1	3511043	Unique	Used	0.724	0.410	1.653	0.837	0	0	7.61	0	0	112	2	1161.54358	2322.07988	-4.37
qAYTANNSSQVQAAMk	Unambiguous	1	1	Q9CZW5	N-Term(iTRAQ8plex); K16(iTRAQ8plex)	CID	1.0000	0.0000	1	1	3227948	Unique	Used	0.341	1.079	0.977	0.696	0	0	7.60	0	0	200	2	1160.60864	2320.21001	-3.27
kDLYANNVLSGGTTMYPGIADR	Unambiguous	3	1	P62737	N-Term(iTRAQ8plex); K1(iTRAQ8plex)	CID	1.0000	0.0000	1	1	3557201	Unique	Used	0.281	5.764	0.868	1.939	0	0	7.60	1	0	36	3	988.85791	2964.55918	-3.99
aQQVFVDEGHTVQFVcR	Unambiguous	2	1	Q9D1T0	N-Term(iTRAQ8plex); C16(Methylthio)	CID	1.0000	0.0000	1	1	3476693	Unique	Used	1.275	1.664	0.842	0.605	0	0	7.60	0	17	27	3	771.71722	2313.13712	-2.21
dFTSEQIDEILQNHTEIVFAR	Unambiguous	2	1	Q6PIC6	N-Term(iTRAQ8plex)	CID	1.0000	0.0000	1	1	3357227	Unique	Used	0.599	0.625	0.964	1.730	0	0	7.60	0	3	200	3	937.14648	2809.42490	-2.20
yLQEVINVLETDGHFR	Unambiguous	3	1	Q02819	N-Term(iTRAQ8plex)	CID	1.0000	0.0000	1	1	3561072	Unique	Used	3.029	0.673	0.751	0.706	0	0	7.60	0	0	48	3	746.39551	2237.17197	-6.64
lNFLSPELPAVAEFSTNETMGHSADR	Unambiguous	1	1	Q99JY0	N-Term(iTRAQ8plex)	CID	1.0000	0.0000	1	1	3395825	Unique	Used			1.890	0.734	0	0	7.60	0	0	19	3	1046.51929	3137.54331	-2.65
aSPAGTAGGPVAGVATAGTGPVAAR	Unambiguous	1	1	Q8K0H5	N-Term(iTRAQ8plex)	CID	1.0000	0.0000	1	1	3718860	Unique	Used	1.645	0.821	1.313	0.903	0	0	7.60	0	0	119	2	1184.63672	2368.26616	-9.53
vEYPVMYSTDPENGHMFNcIQR	Unambiguous	1	1	Q9CPU4	N-Term(iTRAQ8plex); C19(Methylthio)	CID	1.0000	0.0000	1	1	3393532	Unique	Used	1.172	0.583	1.103	1.024	0	0	7.59	0	4	31	3	994.11505	2980.33060	-3.30
lcnGIQDcMDGSDEGAHcR	Unambiguous	3	1	Q91ZX7	N-Term(iTRAQ8plex); C2(Methylthio); N3(Deamidated); C8(Methylthio); C18(Methylthio)	CID	1.0000	0.0000	1	1	2886928	Unique	Used	1.241	0.828	0.645	0.723	0	0	7.59	0	12	200	3	822.97778	2466.91880	-2.57
tIGGGDDSFNTFFSETGAGk	Unambiguous	4	1	P68373	N-Term(iTRAQ8plex); K20(iTRAQ8plex)	CID	1.0000	0.0000	1	1	3152454	Unique	Used	0.494	1.413	1.291	1.322	0	0	7.59	0	0	200	2	1308.65454	2616.30181	-0.79

sVFIAQNVASLQELGGSEk	Unambiguous	4	1	A2AN08-3	N-Term(iTRAQ8plex); K19(iTRAQ8plex)	CID	1.0000	0.0000	1	1	3478783	Unique	Used	0.688	1.303	0.796	0.804	0	0	7.59	0	0	200	2	1293.21887	2585.43047	-3.52
fLSQPFQVAEVFTGHMGk	Unambiguous	1	1	P56480	N-Term(iTRAQ8plex); K18(iTRAQ8plex)	CID	1.0000	0.0000	1	1	3861923	Unique	Used	1.145	0.821	1.159	1.290	0	0	7.59	0	0	200	3	877.80634	2631.40445	-6.44
dVATVAFcDAQSTQEIHEk	Unambiguous	2	1	O88712-2	N-Term(iTRAQ8plex); C8(Methylthio); K19(iTRAQ8plex)	CID	1.0000	0.0000	1	1	2937490	Unique	Used	0.964	1.320	1.081	1.073	0	0	7.59	0	7	18	3	916.12396	2746.35733	-2.32
cTHWAEGGQGALALAQAVQR	Unambiguous	1	1	Q922D8	N-Term(iTRAQ8plex); C1(Methylthio)	CID	1.0000	0.0000	1	1	3857773	Unique	Used	0.885	1.373	1.335	1.047	0	0	7.59	0	12	26	3	806.40649	2417.20493	-2.95
gTGGVDTAATGSVFDISNLDR	Unambiguous	1	1	P30275	N-Term(iTRAQ8plex)	CID	1.0000	0.0000	1	1	3264441	Unique	Used	0.957	2.102	0.832	0.936	0	0	7.59	0	0	200	2	1179.09949	2357.19170	1.26
fTLScTETLMSELGNIAk	Unambiguous	2	1	Q9R111	N-Term(iTRAQ8plex); C5(Methylthio); K18(iTRAQ8plex)	CID	1.0000	0.0000	1	1	3766567	Unique	Used	0.760	3.727	1.142	1.058	0	0	7.59	0	0	82	2	1306.68030	2612.35332	-2.35
sLNPALDGLTcGLTSHDk	Unambiguous	1	1	Q8CGF6	N-Term(iTRAQ8plex); C11(Methylthio); K18(iTRAQ8plex)	CID	1.0000	0.0000	1	1	3436725	Unique	Used	0.512	0.793	0.916	0.960	0	0	7.59	0	3	18	3	832.76923	2496.29312	-4.64
iNPVcPADLVIDHSIQVDFNR	Unambiguous	1	1	P28271	N-Term(iTRAQ8plex); C5(Methylthio)	CID	1.0000	0.0000	1	1	3601748	Unique	Used	1.874	0.440	0.416	0.891	0	0	7.58	0	3	9	3	905.79694	2715.37625	-5.09
aIQImYQNLQQDGLENVHLEQVR	Unambiguous	2	1	Q9WVJ3-2	N-Term(iTRAQ8plex); M5(Oxidation)	CID	1.0000	0.0000	1	1	3558256	Unique	Used		1.562	1.201	1.254	0	0	7.58	0	0	195	3	1020.52911	3059.57279	-5.19
tIGGGDDSFNTFFSETGAGk	Unambiguous	4	1	P68373	N-Term(iTRAQ8plex); K20(iTRAQ8plex)	CID	1.0000	0.0000	1	1	3130464	Unique	Used	0.713	1.584	1.306	1.596	0	0	7.58	0	0	64	2	1308.65186	2616.29643	-2.84
nSLISYLEQIHR	Unambiguous	1	1	Q00493	N-Term(iTRAQ8plex)	CID	1.0000	0.0000	1	1	3840767	Unique	Used	0.540	0.973	0.759	0.827	0	0	7.58	0	4	10	3	592.99994	1776.98526	-3.23
eQVTTLEDTLEEEYVLHHF	Unambiguous	1	1	Q6ZWR6-5	N-Term(iTRAQ8plex)	CID	1.0000	0.0000	1	1	2639896	Unique	Used	0.723	0.982	2.704	1.749	0	0	7.58	0	0	135	3	879.43896	2636.30234	-0.39
gNDISSGTVLSDYVGSGPPSGTGLHR	Unambiguous	1	1	P70296	N-Term(iTRAQ8plex)	CID	1.0000	0.0000	1	1	3701932	Unique	Used	1.826	1.397	1.204	1.102	0	0	7.58	0	0	200	3	945.47058	2834.39719	-8.86

aVTLHDQGTTQWADLSSQFYLR	Unambiguous	1	1	Q02053	N-Term(iTRAQ8plex)	CID	1.0000	0.0000	1	1	3705350	Unique	Used	0.630	1.278	1.058	1.038	0	0	7.58	0	6	14	3	947.81549	2841.43192	-5.44
sNVASTAAcSSASSLGLGLAYR	Unambiguous	1	1	P46660	N-Term(iTRAQ8plex); C9(Methylthio)	CID	1.0000	0.0000	1	1	3934322	Unique	Used	1.937	0.834	2.595	1.757	0	0	7.58	0	0	200	2	1218.60754	2436.20781	-3.57
dMAIATGGAVFGEEGLNLNLEDVQAHDLGk	Unambiguous	1	1	P63038	N-Term(iTRAQ8plex); K30(iTRAQ8plex)	CID	1.0000	0.0000	1	1	3265694	Unique	Used			0.913		0	0	7.58	0	0	200	3	1231.64038	3692.90659	0.39
aVVFETGEAGAASGAGSLPGEVR	Unambiguous	3	1	Q9QYR6	N-Term(iTRAQ8plex)	CID	1.0000	0.0000	1	1	3128924	Unique	Used	2.245	1.165	1.618	0.691	0	0	7.57	0	0	14	2	1218.63489	2436.26250	-1.98
nLHQSGFSLSGAQIDDNIPR	Unambiguous	1	1	O08553	N-Term(iTRAQ8plex)	CID	1.0000	0.0000	1	1	3494623	Unique	Used	1.491	1.051	0.977	1.158	0	0	7.57	0	3	50	3	825.09363	2473.26633	-3.01
sGLGLGAASASTSGSGPADSVMR	Unambiguous	3	1	Q7TSJ2	N-Term(iTRAQ8plex)	CID	1.0000	0.0000	1	1	3534399	Unique	Used	1.243	1.146	2.037	1.543	0	0	7.57	0	0	200	2	1170.59033	2340.17339	-1.45
lSVSSNDTQESGNSSGPSPGAk	Unambiguous	2	1	G3X9K3	N-Term(iTRAQ8plex); K22(iTRAQ8plex)	CID	1.0000	0.0000	1	1	2825389	Unique	Used	4.595	1.122	0.845	0.720	0	0	7.57	0	0	200	3	905.46124	2714.36917	0.07
dMAIATGGAVFGEEGLNLNLEDVQAHDLGk	Unambiguous	1	1	P63038	N-Term(iTRAQ8plex); K30(iTRAQ8plex)	CID	1.0000	0.0000	1	1	3262499	Unique	Used	1.128	0.812	0.834	0.850	0	0	7.57	0	7	21	3	1231.64050	3692.90696	0.49
dVTFDPELVDPVVAAGALATSSGIVFFSnPAHPEFR	Unambiguous	1	1	O35657	N-Term(iTRAQ8plex); N29(Deamidated)	CID	1.0000	0.0000	1	1	3687242	Unique	Used	1.667	0.885			0	0	7.56	0	17	57	3	1360.02734	4078.06748	-1.91
hQPADLLEFALQHFTR	Unambiguous	1	1	P31324	N-Term(iTRAQ8plex)	CID	1.0000	0.0000	1	1	3898308	Unique	Used	0.378	0.853	0.974	1.050	0	0	7.56	0	11	11	3	743.06549	2227.18192	-4.79
iVFDSIDNLEAAPHDIGHVk	Unambiguous	3	1	Q9QZ08	N-Term(iTRAQ8plex); K20(iTRAQ8plex)	CID	1.0000	0.0000	1	1	3214729	Unique	Used	0.944	2.306	1.118	1.065	0	0	7.56	0	25	85	3	933.51215	2798.52188	-2.82
kGDSSSGGGGGSSGGGGSSNVGGGSSGGSGScSSSSR	Unambiguous	3	1	Q64012-2	N-Term(iTRAQ8plex); K1(iTRAQ8plex); C32(Methylthio)	CID	1.0000	0.0000	1	1	2965674	Unique	Used			0.742	0.711	0	0	7.56	1	0	200	3	1202.86719	3606.58701	0.88
dSGQWHYFDDNSVSPVNENQIESk	Unambiguous	1	1	Q99K46	N-Term(iTRAQ8plex); K24(iTRAQ8plex)	CID	1.0000	0.0000	1	1	2811854	Unique	Used	1.218	1.016	1.414	1.118	0	0	7.56	0	0	200	3	1135.21118	3403.61899	-2.87
aAQQAASSSGQGQQAQTPTGk	Unambiguous	5	1	E9Q4G7	N-Term(iTRAQ8plex); K21(iTRAQ8plex)	CID	1.0000	0.0000	1	1	3118505	Unique	Used	0.272	0.511	0.664	0.747	0	0	7.56	0	0	113	2	1305.69507	2610.38286	5.22

gNAGQTNYGFANSTMER	Unambiguous	1	1	P19096	N-Term(iTRAQ8plex)	CID	1.0000	0.0000	1	1	3143747	Unique	Used	0.520	0.575	0.829	0.951	0	0	7.56	0	4	25	2	1061.49780	2121.98833	-2.00
aSGSAGAAASPSAAAAGER	Unambiguous	2	1	P83093	N-Term(iTRAQ8plex)	CID	1.0000	0.0000	1	1	2841495	Unique	Used	0.737	1.055	1.467	1.181	0	0	7.56	0	11	200	2	932.47467	1863.94206	-2.27
fLSQPFQVAEVFTGHMGk	Unambiguous	1	1	P56480	N-Term(iTRAQ8plex); K18(iTRAQ8plex)	CID	1.0000	0.0000	1	1	3862544	Unique	Used	1.123	0.825	1.049	1.413	0	0	7.56	0	13	200	3	877.80878	2631.41178	-3.66
aSPGEQGTGGGSQGGSGGTPSGTAGSSR	Unambiguous	1	1	Q8VDU5	N-Term(iTRAQ8plex)	CID	1.0000	0.0000	1	1	2724894	Unique	Used	3.502	2.530	1.535	1.191	0	0	7.56	0	0	200	2	1327.11951	2653.23174	-1.32
aAGQGPLAQLmDPGPAFSGSPATSHTQR	Unambiguous	4	1	Q3V1Z0	N-Term(iTRAQ8plex); M11(Oxidation)	CID	1.0000	0.0000	1	1	3819895	Unique	Used	0.779	0.564	0.921	0.743	0	0	7.56	0	0	200	3	1024.17969	3070.52451	-2.41
lLEVSDDPQVLAVAAHDVGEYVR	Unambiguous	1	1	Q8BVE3	N-Term(iTRAQ8plex)	CID	1.0000	0.0000	1	1	3240429	Unique	Used	0.528	1.183	1.157	1.390	0	0	7.56	0	9	200	3	933.83270	2799.48355	0.15
fQMPDQGMTSADDFFQGTk	Unambiguous	1	1	O08553	N-Term(iTRAQ8plex); K19(iTRAQ8plex)	CID	1.0000	0.0000	1	1	3307083	Unique	Used	1.482	1.646	1.137	1.349	0	0	7.56	0	5	200	3	920.44525	2759.32120	-1.96
nLSPAPASSSQGPPPQGPVSPGPAk	Unambiguous	3	1	Q7TQ95	N-Term(iTRAQ8plex); K25(iTRAQ8plex)	CID	1.0000	0.0000	1	1	3385794	Unique	Used		1.429	1.703	0.908	0	0	7.56	0	9	22	3	978.53204	2933.58158	-4.30
kISSVQSIVPALEIANAHR	Unambiguous	1	1	P63038	N-Term(iTRAQ8plex); K1(iTRAQ8plex)	HCD	1.0000	0.0000	1	1	3875908	Unique	Used	0.997	0.933	0.993	0.971	0	0	7.56	1	10	50	3	881.18903	2641.55252	-3.21
kTGSPGSPSAGGVQSTAk	Unambiguous	1	1	Q6PDL0	N-Term(iTRAQ8plex); K1(iTRAQ8plex); K18(iTRAQ8plex)	CID	1.0000	0.0000	1	1	3246643	Unique	Used	0.967	0.729	1.216	0.918	0	0	7.56	1	0	116	3	843.81525	2529.43119	-3.48
iSFEEFcAVVGGLDIHk	Unambiguous	2	1	Q63810-2	N-Term(iTRAQ8plex); C7(Methylthio); K17(iTRAQ8plex)	CID	1.0000	0.0000	1	1	3708489	Unique	Used	0.828	1.418	1.996	0.869	0	0	7.56	0	20	200	3	840.10822	2518.31009	-7.70
tVAGIIVEPIQSEGGDNHASDDFFR	Unambiguous	2	1	P61922	N-Term(iTRAQ8plex)	CID	1.0000	0.0000	1	1	3193853	Unique	Used	1.815	0.929	0.868	1.178	0	0	7.55	0	4	23	3	993.49554	2978.47208	-2.61
vSELEAELAEQQHLGR	Unambiguous	1	1	Q8VD75	N-Term(iTRAQ8plex)	CID	1.0000	0.0000	1	1	2850954	Unique	Used	1.138	1.154	1.287	0.817	0	0	7.55	0	1	80	3	705.04254	2113.11307	-2.88

lAGTLGSEQDQNFLnHSGISLER	Unambiguous	3	1	Q80U40-2	N-Term(iTRAQ8plex); N15(Deamidated)	CID	1.0000	0.0000	1	1	3281786	Unique	Used	1.509	1.641	1.581	0.957	0	0	7.55	0	12	146	3	931.14215	2791.41190	-1.64
qTTAAAAATFSEQVGGGSGGAGR	Unambiguous	3	1	O88935	N-Term(iTRAQ8plex)	CID	1.0000	0.0000	1	1	3493479	Unique	Used	0.838	1.507	1.610	1.639	0	0	7.55	0	0	69	2	1178.58752	2356.16777	-5.00
qSSYGQQSYNNQGQQNTESSGGQGGR	Unambiguous	1	1	Q8BQ46	N-Term(iTRAQ8plex)	CID	1.0000	0.0000	1	1	2628253	Unique	Used			0.948	1.035	0	0	7.55	0	2	200	3	1017.79260	3051.36325	-1.90
aGEGGGVIQGSASEATLVALLAAR	Unambiguous	2	1	O88533	N-Term(iTRAQ8plex)	CID	1.0000	0.0000	1	1	3645036	Unique	Used	1.009	0.327	1.183	1.789	0	0	7.55	0	0	12	2	1251.68848	2502.36968	-5.34
rDYSLQIQnVDVTDDGPYTcSVQTQHTPR	Unambiguous	4	1	A0A4W9	N-Term(iTRAQ8plex); N9(Deamidated); C20(Methylthio)	CID	0.0013	0.0000	1	1	3300343	Unique	Used	1.661	1.090	1.757	1.654	0	0	7.55	1	0	200	3	1229.91736	3687.73752	1.45
gTNTGVWVGVSGSEASEALSR	Unambiguous	1	1	P19096	N-Term(iTRAQ8plex)	CID	1.0000	0.0000	1	1	3321868	Unique	Used	0.605	0.521	0.809	0.931	0	0	7.55	0	0	19	2	1184.60217	2368.19707	-3.22
tYFPHFDVSHGSAQVk	Unambiguous	2	1	Q91VB8	N-Term(iTRAQ8plex); K16(iTRAQ8plex)	CID	1.0000	0.0000	1	1	3615196	Unique	Used	2.173	2.330	0.682	1.302	0	0	7.55	0	16	65	3	810.09741	2428.27768	-3.83
iGEHTPSALAImENANVLAR	Unambiguous	3	1	A6ZI44	N-Term(iTRAQ8plex); M12(Oxidation)	CID	1.0000	0.0000	1	1	3701769	Unique	Used	1.445	1.165	1.214	1.299	0	0	7.55	0	1	5	3	809.76624	2427.28415	-5.23
aRPELTSSEGGLPLLqTQGLQYTGPSPGLAVAPR	Unambiguous	3	1	Q5SW75	N-Term(iTRAQ8plex); Q16(Deamidated)	CID	1.0000	0.0000	1	1	3859801	Unique	Used	1.278	2.196	1.347	1.069	0	0	7.54	0	0	62	3	1256.34912	3767.03281	1.21
tYFPHFDVSHGSAQVk	Unambiguous	2	1	Q91VB8	N-Term(iTRAQ8plex); K16(iTRAQ8plex)	CID	1.0000	0.0000	1	1	3452235	Unique	Used	3.161	3.230	0.382	0.826	0	0	7.54	0	17	104	3	810.09949	2428.28391	-1.27
nEAIQAAHDSVAQEGQcR	Unambiguous	1	1	Q9R0P9	N-Term(iTRAQ8plex); C17(Methylthio)	CID	1.0000	0.0000	1	1	3207985	Unique	Used	1.291	0.888	1.262	1.403	0	0	7.54	0	0	1	3	759.69208	2277.06168	-1.65
sEEITTGSAWFSFLESHnk	Unambiguous	2	1	D3Z375	N-Term(iTRAQ8plex); N18(Deamidated); K19(iTRAQ8plex)	CID	1.0000	0.0000	1	1	3216731	Unique	Used	2.246	1.800	1.282	0.855	0	0	7.54	0	6	176	3	927.14166	2779.41043	2.47
vADALANAAGHLDDLPGALSALSDLHAHk	Unambiguous	1	1	Q91VB8	N-Term(iTRAQ8plex); K29(iTRAQ8plex)	CID	1.0000	0.0000	1	1	3706464	Unique	Used	2.510	3.062	0.407	1.123	0	0	7.54	0	0	17	3	1157.95569	3471.85251	-8.08

vADVSISEVTTLLGnVHSPVVEGYVGENISGEVk	Unambiguous	3	1	P20357	N-Term(iTRAQ8plex); N15(Deamidated); K34(iTRAQ8plex)	CID	1.0000	0.0000	1	1	3420728	Unique	Used	1.641	1.361	1.487	1.456	0	0	7.54	0	0	200	3	1369.73682	4107.19590	0.00
lDNASAFQGAVISPHYDSLLVk	Unambiguous	3	1	G5E8R3	N-Term(iTRAQ8plex); K22(iTRAQ8plex)	CID	1.0000	0.0000	1	1	3600279	Unique	Used	0.464	0.632	0.892	1.143	0	0	7.54	0	0	111	3	985.20795	2953.60929	-5.12
lSPLEAcAHSFFDELR	Unambiguous	2	1	D3Z7E5	N-Term(iTRAQ8plex); C7(Methylthio)	CID	1.0000	0.0000	1	1	3686943	Unique	Used	0.599	0.722	1.184	1.084	0	0	7.54	0	0	35	3	729.02454	2185.05906	-6.11
iVLDSDAAEYGGHQR	Unambiguous	2	1	Q9D6Y9	N-Term(iTRAQ8plex)	CID	1.0000	0.0000	1	1	2808370	Unique	Used	1.367	1.196	0.952	1.153	0	0	7.54	0	6	48	3	645.66687	1934.98606	-0.69
nLHQSGFSLSGAQIDDNIPR	Unambiguous	1	1	O08553	N-Term(iTRAQ8plex)	CID	1.0000	0.0000	1	1	3494488	Unique	Used	0.941	1.024	1.172	1.295	0	0	7.53	0	0	46	2	1237.13464	2473.26201	-4.76
tQAIVcQQLDLTHLk	Unambiguous	1	1	P61222	N-Term(iTRAQ8plex); C6(Methylthio); K15(iTRAQ8plex)	CID	1.0000	0.0000	1	1	3704705	Unique	Used	0.419	0.625	0.813	0.918	0	0	7.53	0	3	15	3	789.10638	2365.30460	-6.18
iAEGFGDAALIMVDNAk	Unambiguous	3	1	O70378	N-Term(iTRAQ8plex); K17(iTRAQ8plex)	CID	1.0000	0.0000	1	1	3284330	Unique	Used	0.845	0.646	0.770	0.976	0	0	7.53	0	0	21	2	1172.14050	2343.27373	-4.35
iGTIYEGASNIQLNTIAk	Unambiguous	1	1	Q9DBL1	N-Term(iTRAQ8plex); K18(iTRAQ8plex)	CID	1.0000	0.0000	1	1	3664512	Unique	Used	0.580	1.176	0.800	1.213	0	0	7.53	0	0	91	2	1257.71313	2514.41899	-7.88
lYPSESYLQnYEVAYPDcSPVHLISEASLVDLNTR	Unambiguous	4	1	Q922Q1	N-Term(iTRAQ8plex); N10(Deamidated); C18(Methylthio)	CID	0.0080	0.0000	1	1	3602220	Unique	Used			1.448	1.719	0	0	7.53	0	24	54	3	1446.36963	4337.09433	0.16
hcDSINSDFGSESGGGGDSGPGPSAVPGPR	Unambiguous	1	1	Q8K1R7	N-Term(iTRAQ8plex); C2(Methylthio)	CID	1.0000	0.0000	1	1	2934516	Unique	Used	1.038	1.326	1.177	1.209	0	0	7.53	0	23	177	3	1050.79663	3150.37534	-1.34
aGLGHLSPEGPLSLSEGPSSVGPEGGPGGVGAGGGSSTFPR	Unambiguous	2	1	B1AS06	N-Term(iTRAQ8plex)	CID	1.0000	0.0000	1	1	3436833	Unique	Used			4.535	2.036	0	0	7.53	0	0	51	3	1326.33167	3976.98044	-3.52
qHGEQLGGGGSGGGYNTSk	Unambiguous	7	1	Q80U78-2	N-Term(iTRAQ8plex); K20(iTRAQ8plex)	CID	1.0000	0.0000	1	1	2859036	Unique	Used	0.910	0.698	0.763	0.464	0	0	7.53	0	0	200	3	819.41547	2456.23185	-2.30

fDLGQDVIDFTGHALALYR	Unambiguous	1	1	P50396	N-Term(iTRAQ8plex)	CID	1.0000	0.0000	1	1	3766324	Unique	Used	1.021	1.205	0.895	1.140	0	0	7.53	0	7	23	3	819.10004	2455.28556	-2.78
qVVSASYNSPIGLYSTSnIQDALHGQLR	Unambiguous	1	1	O70209	N-Term(iTRAQ8plex); N18(Deamidated)	CID	0.0305	0.0000	1	1	3859664	Unique	Used	0.815	2.372	1.075	1.178	0	0	7.53	0	23	117	3	1108.58118	3323.72898	3.48
iSFEEFcAVVGGLDIHk	Unambiguous	2	1	Q63810-2	N-Term(iTRAQ8plex); C7(Methylthio); K17(iTRAQ8plex)	CID	1.0000	0.0000	1	1	3708924	Unique	Used	1.106	1.969	2.235	0.479	0	0	7.53	0	0	200	3	840.11139	2518.31961	-3.92
lAASGEGGLQELSGHFENQk	Unambiguous	3	1	Q9QXS6	N-Term(iTRAQ8plex); K20(iTRAQ8plex)	CID	1.0000	0.0000	1	1	3168697	Unique	Used	0.828	0.981	1.727	0.764	0	0	7.53	0	0	45	2	1340.70825	2680.40923	-2.21
sNcNVAVInVGAPAAGMNAAVR	Unambiguous	3	1	Q8C605	N-Term(iTRAQ8plex); C3(Methylthio); N9(Deamidated)	CID	0.0186	0.0000	1	1	3915103	Unique	Used	1.505	0.852	1.282	1.147	0	0	7.53	0	0	200	2	1225.61963	2450.23198	2.57
dGHLLFGQVPLVEIDGMMLTQTR	Unambiguous	1	1	P24472	N-Term(iTRAQ8plex)	CID	1.0000	0.0000	1	1	3706606	Unique	Used			0.701	1.173	0	0	7.52	0	23	25	3	958.83746	2874.49784	-6.33
sELEAQTGLQILQTGVGQR	Unambiguous	2	1	Q60673	N-Term(iTRAQ8plex)	CID	1.0000	0.0000	1	1	3498443	Unique	Used	1.125	0.874	0.866	1.707	0	0	7.52	0	4	18	2	1166.63745	2332.26763	-4.22
fGAVFLWDTGSSVGEITGHNk	Unambiguous	1	1	O88342	N-Term(iTRAQ8plex); K21(iTRAQ8plex)	CID	1.0000	0.0000	1	1	3644720	Unique	Used	1.038	0.833	0.989	1.468	0	0	7.52	0	9	7	3	944.16528	2830.48130	-6.07
sSELRPGEFVVAIGSPFSLqNTVTTGIVSTTQR	Unambiguous	1	1	Q9R118	N-Term(iTRAQ8plex); Q20(Deamidated)	CID	0.0279	0.0000	1	1	3917376	Unique	Used	0.964	0.822	1.646	1.533	0	0	7.52	0	0	33	3	1262.00684	3784.00595	-0.32
vADVSISEVTTLLGnVHSPVVEGYVGENISGEVk	Unambiguous	3	1	P20357	N-Term(iTRAQ8plex); N15(Deamidated); K34(iTRAQ8plex)	CID	0.0146	0.0000	1	1	3419564	Unique	Used	1.465	0.768	1.067	0.622	0	0	7.52	0	0	77	3	1369.73730	4107.19736	0.36
lVAEDIPLLFSLLSDVFPGVQYHR	Unambiguous	1	1	Q9JHU4	N-Term(iTRAQ8plex)	CID	1.0000	0.0000	1	1	3880779	Unique	Used	0.830	0.849	1.148	1.007	0	0	7.52	0	27	107	3	1011.56067	3032.66745	-2.93
eHINLGcDVDFDIAGPSIR	Unambiguous	3	1	Q60932-2	N-Term(iTRAQ8plex); C7(Methylthio)	CID	1.0000	0.0000	1	1	3375148	Unique	Used	0.924	1.008	1.314	1.430	0	0	7.51	0	7	200	3	807.72980	2421.17484	-3.99

eQASHLGGAVFSGAGnIAATGLVk	Unambiguous	1	1	Q91ZZ3	N-Term(iTRAQ8plex); N16(Deamidated); K25(iTRAQ8plex)	CID	1.0000	0.0000	1	1	3558721	Unique	Used	1.208	1.158	1.639	1.158	0	0	7.51	0	5	25	3	979.20471	2935.59958	-3.50
mLSLAEQQLVDcAQAFNNHGck	Unambiguous	1	1	P49935	N-Term(iTRAQ8plex); M1(Oxidation); C12(Methylthio); C21(Methylthio); K22(iTRAQ8plex)	CID	1.0000	0.0000	1	1	3824621	Unique	Used	1.549	1.114	0.833	0.821	0	0	7.51	0	23	52	3	1046.16516	3136.48093	-5.04
qQVADLTAQLHELNR	Unambiguous	3	1	Q8BPM0	N-Term(iTRAQ8plex)	CID	1.0000	0.0000	1	1	3535830	Unique	Used	0.660	0.781	1.119	1.099	0	0	7.51	0	13	11	3	680.70642	2040.10471	-4.55
kHGcAFLVDEVQTGGGcTGk	Unambiguous	1	1	P61922	N-Term(iTRAQ8plex); K1(iTRAQ8plex); C4(Methylthio); C17(Methylthio); K20(iTRAQ8plex)	CID	1.0000	0.0000	1	1	3455012	Unique	Used	1.110	0.534	0.838	1.429	0	0	7.51	1	0	200	3	1004.51196	3011.52134	-4.17
lLGYGPGDQAGEAQHGFLLR	Unambiguous	1	1	Q3U5Q7	N-Term(iTRAQ8plex)	CID	1.0000	0.0000	1	1	3577837	Unique	Used	0.870	0.821	1.213	1.874	0	0	7.51	0	19	33	3	801.75824	2403.26017	-5.06
gGGGPGGGAPGGPGLGLGSLGEER	Unambiguous	1	1	Q69ZK9	N-Term(iTRAQ8plex)	CID	1.0000	0.0000	1	1	3232711	Unique	Used	0.527	1.144	1.119	1.181	0	0	7.51	0	0	62	2	1134.08643	2267.16557	-1.20
gFQQILAGEYDHLPEqAFYMVGPIEEAVAk	Unambiguous	1	1	P56480	N-Term(iTRAQ8plex); Q16(Deamidated); K30(iTRAQ8plex)	CID	1.0000	0.0000	1	1	3398146	Unique	Used		0.586	0.692	1.043	0	0	7.51	0	10	200	3	1320.68591	3960.04319	2.05
aMLGVQEEDGSWSAVTLSNDHNAQNER	Unambiguous	4	1	A8Y5Q0	N-Term(iTRAQ8plex)	CID	1.0000	0.0000	1	1	2937445	Unique	Used	0.819	0.704	1.063	1.210	0	0	7.50	0	0	71	3	1088.18054	3262.52707	-2.05
lLEVSDDPQVLAVAAHDVGEYVR	Unambiguous	1	1	Q8BVE3	N-Term(iTRAQ8plex)	CID	1.0000	0.0000	1	1	3242111	Unique	Used	1.691		0.860	1.534	0	0	7.50	0	0	200	3	933.83466	2799.48941	2.25
nQDEcVIALHDcnGDVNR	Unambiguous	5	1	Q80X50	N-Term(iTRAQ8plex); C5(Methylthio); C12(Methylthio); N13(Deamidated)	CID	1.0000	0.0000	1	1	2739160	Unique	Used	2.050	0.908	0.819	0.734	0	0	7.50	0	13	200	3	804.68274	2412.03366	-0.74

sQTEEDcTEELFDFLHAR	Unambiguous	1	1	P99028	N-Term(iTRAQ8plex); C7(Methylthio)	CID	1.0000	0.0000	1	1	2854316	Unique	Used			1.079	1.023	0	0	7.50	0	5	200	3	840.71405	2520.12760	-1.95
lGQHVVGMAPLSVGSLDDEPGGETR	Unambiguous	1	1	Q9CZR8	N-Term(iTRAQ8plex)	CID	1.0000	0.0000	1	1	3149826	Unique	Used	1.346	0.829	0.837	0.821	0	0	7.50	0	2	36	3	1019.18036	3055.52652	-1.44
lGTEEFPLIDQTFYPNHk	Unambiguous	3	1	O88935	N-Term(iTRAQ8plex); K18(iTRAQ8plex)	CID	1.0000	0.0000	1	1	3328150	Unique	Used	0.582	2.100	1.309	0.928	0	0	7.50	0	16	200	3	919.82544	2757.46177	-3.29
sENGLEFTSSGSANTETTk	Unambiguous	3	1	Q60932-2	N-Term(iTRAQ8plex); K19(iTRAQ8plex)	CID	1.0000	0.0000	1	1	2666422	Unique	Used	0.460	0.764	0.860	0.839	0	0	7.50	0	0	200	2	1284.64832	2568.28935	0.30
iSFEEFcAVVGGLDIHk	Unambiguous	2	1	Q63810-2	N-Term(iTRAQ8plex); C7(Methylthio); K17(iTRAQ8plex)	CID	1.0000	0.0000	1	1	3709259	Unique	Used	0.643	1.437	2.019	1.019	0	0	7.50	0	0	200	3	840.11176	2518.32071	-3.48
nMGGPYGGGNYGPGGSGGSGGYGGR	Unambiguous	3	1	O88569	N-Term(iTRAQ8plex); N1(Deamidated)	CID	1.0000	0.0000	1	1	2971229	Unique	Used	1.656		1.729	1.257	0	0	7.50	0	0	200	2	1248.04712	2495.08696	-3.16
fDTLPQGQAADGFFLHVGR	Unambiguous	3	1	A2AHC3	N-Term(iTRAQ8plex)	CID	1.0000	0.0000	1	1	3664983	Unique	Used	0.779	0.835	0.951	1.002	0	0	7.49	0	26	103	3	794.07825	2380.22019	-6.31
eVLGHSIAQLNAEnDHPFYYk	Unambiguous	4	1	O08677	N-Term(iTRAQ8plex); N14(Deamidated); K21(iTRAQ8plex)	CID	1.0000	0.0000	1	1	3234461	Unique	Used	3.545	2.106	0.726	0.916	0	0	7.49	0	10	51	3	1018.86432	3054.57840	0.08
iTGEAFVQFASQELAEk	Unambiguous	5	1	Q9Z2X1-2	N-Term(iTRAQ8plex); K17(iTRAQ8plex)	CID	1.0000	0.0000	1	1	3305256	Unique	Used	1.314	0.822	0.732	0.753	0	0	7.49	0	0	17	2	1238.67773	2476.34819	-2.52
qLNTIQNAEAVHFFcNYEESR	Unambiguous	4	1	P61922	N-Term(iTRAQ8plex); C15(Methylthio)	CID	1.0000	0.0000	1	1	3458318	Unique	Used	1.903	0.558	0.937	1.626	0	0	7.49	0	0	12	3	955.11621	2863.33408	-3.66
lLEVSDDPQVLAVAAHDVGEYVR	Unambiguous	1	1	Q8BVE3	N-Term(iTRAQ8plex)	CID	1.0000	0.0000	1	1	3262440	Unique	Used	0.813	1.797	0.954	1.069	0	0	7.49	0	9	9	3	933.83136	2799.47953	-1.28
tSPcQSPTVPEYSAPSLPIRPSR	Unambiguous	4	1	Q8CHC4	N-Term(iTRAQ8plex); C4(Methylthio)	HCD	1.0000	0.0000	1	1	3875591	Unique	Used	1.014	0.768	1.128	0.832	0	0	7.49	0	30	160	3	940.80902	2820.41251	-7.15
qTTAAAAATFSEQVGGGSGGAGR	Unambiguous	3	1	O88935	N-Term(iTRAQ8plex)	CID	1.0000	0.0000	1	1	3494107	Unique	Used		0.989	1.463	1.833	0	0	7.49	0	0	127	2	1178.58740	2356.16753	-5.11

aHAGQVTcVAASPHk	Unambiguous	2	1	Q99J09	N-Term(iTRAQ8plex); C8(Methylthio); K15(iTRAQ8plex)	CID	1.0000	0.0000	1	1	3488464	Unique	Used	1.002	0.884	0.840	0.807	0	0	7.49	0	0	200	3	711.04919	2131.13303	-1.45
sQTEEDcTEELFDFLHAR	Unambiguous	1	1	P99028	N-Term(iTRAQ8plex); C7(Methylthio)	CID	1.0000	0.0000	1	1	2853363	Unique	Used	3.514	1.566	1.659	1.179	0	0	7.49	0	0	200	3	840.71277	2520.12375	-3.48
gPAVGIDLGTTYScVGVFQHGk	Unambiguous	4	1	P63017	N-Term(iTRAQ8plex); C14(Methylthio); K22(iTRAQ8plex)	CID	1.0000	0.0000	1	1	3784711	Unique	Used	0.929	1.010	0.962	0.839	0	0	7.49	0	7	20	3	954.16595	2860.48331	-3.96
aTSSFSQSVSSLFGEDNVR	Unambiguous	1	1	Q8BXV2	N-Term(iTRAQ8plex)	CID	1.0000	0.0000	1	1	3559993	Unique	Used	0.287	0.608	0.604	0.816	0	0	7.49	0	5	21	2	1161.57385	2322.14043	-4.80
aSPGEQGTGGGSQGGSGGTPSGTAGSSR	Unambiguous	1	1	Q8VDU5	N-Term(iTRAQ8plex)	CID	1.0000	0.0000	1	1	2709304	Unique	Used	1.990	0.727	1.582	0.984	0	0	7.48	0	5	200	2	1327.11841	2653.22954	-2.15
gTAEGAVDAMLVHGQDAITVSnGGR	Unambiguous	2	1	Q8C7X2-2	N-Term(iTRAQ8plex); N22(Deamidated)	CID	1.0000	0.0000	1	1	3129916	Unique	Used	1.010	0.869	0.828	1.002	0	0	7.48	0	14	42	3	911.12469	2731.35953	-1.04
qGSTAVHPQEATQSSWQEEVTQGPHSFQR	Unambiguous	13	1	Q02357-5	N-Term(iTRAQ8plex)	CID	1.0000	0.0000	1	1	3086435	Unique	Used	2.465	0.652	0.999	1.145	0	0	7.48	0	8	200	3	1181.23596	3541.69333	-1.85
sSSADFGTFSTSQSHQTASTVSk	Unambiguous	4	1	Q8K2K6-3	N-Term(iTRAQ8plex); K23(iTRAQ8plex)	CID	1.0000	0.0000	1	1	3295698	Unique	Used	2.154	0.720	1.790	0.855	0	0	7.48	0	0	56	3	986.15771	2956.45859	-5.37
yTPSGQSGAAASESLFISNHAy	Unambiguous	2	1	A6ZI44	N-Term(iTRAQ8plex); Y22(iTRAQ8plex)	CID	1.0000	0.0000	1	1	3433633	Unique	Used	0.609	1.563	0.841	1.061	0	0	7.48	0	0	12	3	956.14990	2866.43515	-4.06
aQFETLQQLVQHYSER	Unambiguous	1	1	P39688	N-Term(iTRAQ8plex)	CID	1.0000	0.0000	1	1	3726024	Unique	Used	0.739	0.716	0.682	0.751	0	0	7.48	0	9	22	3	761.06293	2281.17423	-5.98
gGPGSAVSPYPSFnVSSDVAALHk	Unambiguous	1	1	P10107	N-Term(iTRAQ8plex); N14(Deamidated); K24(iTRAQ8plex)	CID	1.0000	0.0000	1	1	3496280	Unique	Used	0.888	2.994	0.736	1.025	0	0	7.47	0	17	112	3	985.19183	2953.56095	3.15
dMAIATGGAVFGEEGLNLNLEDVQAHDLGk	Unambiguous	1	1	P63038	N-Term(iTRAQ8plex); K30(iTRAQ8plex)	CID	1.0000	0.0000	1	1	3239026	Unique	Used	1.682	1.161	1.052	1.267	0	0	7.47	0	11	35	3	1231.64148	3692.90989	1.28

gLTIVGSVLEGTFLDNHPQAQR	Unambiguous	2	1	Q91V14	N-Term(iTRAQ8plex)	CID	1.0000	0.0000	1	1	3844159	Unique	Used	0.943	0.778	1.568	1.507	0	0	7.47	0	8	200	3	886.14703	2656.42655	-3.60
cEAFGWHTIIVDGHSVEELck	Unambiguous	1	1	P40142	N-Term(iTRAQ8plex); C1(Methylthio); C20(Methylthio); K21(iTRAQ8plex)	CID	1.0000	0.0000	1	1	3439565	Unique	Used	2.003	0.600	1.214	1.221	0	0	7.47	0	30	32	3	1025.16492	3073.48020	-2.05
lQQELDDLTVDLDHQR	Unambiguous	3	1	Q5SV64	N-Term(iTRAQ8plex)	CID	1.0000	0.0000	1	1	2834780	Unique	Used	1.804	1.300	1.684	0.877	0	0	7.47	0	12	200	3	748.05695	2242.15628	-2.44
vTTLEEQLAGAHQQVSALQQGAGIR	Unambiguous	1	1	B8QI36	N-Term(iTRAQ8plex)	HCD	1.0000	0.0000	1	1	3804943	Unique	Used	1.249	0.845	1.082	0.885	0	0	7.47	0	0	39	3	970.52618	2909.56400	-3.69
fQSAAIGALQEASEAYLVGLFEDTNLcAIHAk	Unambiguous	3	1	P84244	N-Term(iTRAQ8plex); C27(Methylthio); K32(iTRAQ8plex)	CID	1.0000	0.0000	1	1	3746360	Unique	Used	0.271	1.140	0.985	1.108	0	0	7.47	0	0	200	3	1345.69812	4035.07981	-0.47
vINLSQYGPAcFGQEHAR	Unambiguous	4	1	Q8BPN8-2	N-Term(iTRAQ8plex); C11(Methylthio)	CID	1.0000	0.0000	1	1	3858322	Unique	Used	0.165	0.781	1.035	0.904	0	0	7.47	0	2	5	3	780.71960	2340.14426	-3.78
gFGDGYnGYGGGPGGGNFGGSPGYGGGR	Unambiguous	2	1	O88569	N-Term(iTRAQ8plex); N7(Deamidated)	CID	1.0000	0.0000	1	1	3106488	Unique	Used	1.752	1.512	1.068	1.011	0	0	7.47	0	0	55	3	934.07770	2800.21854	-3.75
sNcNVAVINVGAPAAGMNAAVR	Unambiguous	3	1	Q8C605	N-Term(iTRAQ8plex); C3(Methylthio)	CID	1.0000	0.0000	1	1	3933712	Unique	Used	1.767	0.866	1.079	0.947	0	0	7.47	0	0	200	2	1225.11853	2449.22978	-4.85
dLPQPSLSPASLHSQGFER	Unambiguous	2	1	B9EJ86	N-Term(iTRAQ8plex)	CID	1.0000	0.0000	1	1	3411481	Unique	Used	1.514	1.149	1.404	1.043	0	0	7.47	0	18	46	3	790.74597	2370.22336	-5.16
aVGLISHVLEQNQEGDAAYR	Unambiguous	3	1	Q9JLZ3	N-Term(iTRAQ8plex)	CID	1.0000	0.0000	1	1	3371868	Unique	Used	1.176	1.082	1.246	1.655	0	0	7.46	0	0	1	3	825.43286	2474.28403	-4.10
qAYTANNSSQVQAAMk	Unambiguous	1	1	Q9CZW5	N-Term(iTRAQ8plex); K16(iTRAQ8plex)	CID	1.0000	0.0000	1	1	3250470	Unique	Used	0.380	1.137	0.823	0.985	0	0	7.46	0	0	25	2	1160.60938	2320.21147	-2.64
tLLGSSDNVATVSNIEk	Unambiguous	1	1	Q5XG69	N-Term(iTRAQ8plex); K17(iTRAQ8plex)	CID	1.0000	0.0000	1	1	3211703	Unique	Used		0.543	1.089	0.982	0	0	7.46	0	14	129	2	1178.65845	2356.30962	-3.57

hGcAFLVDEVQTGGGcTGk	Unambiguous	1	1	P61922	N-Term(iTRAQ8plex); C3(Methylthio); C16(Methylthio); K19(iTRAQ8plex)	CID	1.0000	0.0000	1	1	3392365	Unique	Used	1.522	0.595	0.571	1.514	0	0	7.46	0	1	7	3	860.41211	2579.22177	-4.57
qLGSVSTGSTLQqSLELTR	Unambiguous	1	1	Q921G8	N-Term(iTRAQ8plex); Q13(Deamidated)	CID	0.0013	0.0000	1	1	3683142	Unique	Used	0.431	1.064	1.006	1.198	0	0	7.46	0	0	148	2	1155.62903	2310.25078	2.30
aSPAGTAGGPVAGVATAGTGPVAAR	Unambiguous	1	1	Q8K0H5	N-Term(iTRAQ8plex)	CID	1.0000	0.0000	1	1	3698804	Unique	Used	2.192	1.111	1.212	0.792	0	0	7.46	0	0	81	2	1184.64111	2368.27495	-5.82
nLHQSGFSLSGAQIDDNIPR	Unambiguous	1	1	O08553	N-Term(iTRAQ8plex)	CID	1.0000	0.0000	1	1	3660127	Unique	Used	1.937	0.989	1.149	1.260	0	0	7.46	0	0	175	2	1237.13391	2473.26055	-5.35
gAQIASTGQLYAAWnGGLDR	Unambiguous	1	1	Q61361	N-Term(iTRAQ8plex); N15(Deamidated)	CID	1.0000	0.0000	1	1	3393150	Unique	Used	1.791	1.065	1.035	1.187	0	0	7.46	0	0	96	2	1177.60046	2354.19365	-4.52
lENTEDIEEVEQHIQTIR	Unambiguous	3	1	Q8K0B2-3	N-Term(iTRAQ8plex)	CID	1.0000	0.0000	1	1	2671858	Unique	Used	1.079	0.673	1.039	0.981	0	0	7.46	0	16	200	3	834.09961	2500.28427	0.38
nVIIWGNHSSTQYPDVNHAk	Unambiguous	1	1	P14152	N-Term(iTRAQ8plex); K20(iTRAQ8plex)	CID	1.0000	0.0000	1	1	3431487	Unique	Used	0.547	0.918	1.141	0.920	0	0	7.46	0	10	76	3	963.50970	2888.51456	-4.10
eQASHLGGAVFSGAGnIAAATGLVk	Unambiguous	1	1	Q91ZZ3	N-Term(iTRAQ8plex); N16(Deamidated); K25(iTRAQ8plex)	CID	1.0000	0.0000	1	1	3558551	Unique	Used	0.961	1.027	1.188	0.972	0	0	7.46	0	24	200	3	979.20667	2935.60544	-1.50
vNQELAGYTGGDVSFIk	Unambiguous	1	1	Q8BGH2	N-Term(iTRAQ8plex); K17(iTRAQ8plex)	CID	1.0000	0.0000	1	1	3302571	Unique	Used	0.550	1.138	1.098	1.380	0	0	7.46	0	0	125	2	1203.65723	2406.30718	-2.23
dFTSEQIDEILQNHTEIVFAR	Unambiguous	2	1	Q6PIC6	N-Term(iTRAQ8plex)	CID	1.0000	0.0000	1	1	3357347	Unique	Used	0.449	0.583	0.801	1.241	0	0	7.45	0	10	200	3	937.14728	2809.42728	-1.35
gLGPLAEAAAAGDGAAAAGAAR	Unambiguous	1	1	O55106	N-Term(iTRAQ8plex)	CID	1.0000	0.0000	1	1	3322573	Unique	Used	1.340	1.556	1.171	0.850	0	0	7.45	0	26	84	2	1057.06506	2113.12285	-3.60
aYAPGGPAYQPVVEAFGTDILHk	Unambiguous	1	1	Q9DBL7	N-Term(iTRAQ8plex); K23(iTRAQ8plex)	CID	1.0000	0.0000	1	1	3478949	Unique	Used	0.639	0.643	0.778	0.956	0	0	7.45	0	6	26	3	1003.87750	3009.61795	-3.83
aPPAGGSGSSAAAAAAGGTEVRPR	Unambiguous	2	1	P84309	N-Term(iTRAQ8plex)	HCD	1.0000	0.0000	1	1	3428770	Unique	Used	0.436	0.943	2.662	1.228	0	0	7.45	0	9	54	3	814.42810	2441.26975	-4.19

IYSSMSDTNLAEAGLNYHAQR	Unambiguous	2	1	O88737	N-Term(iTRAQ8plex)	CID	1.0000	0.0000	1	1	3390685	Unique	Used	0.861	0.753	1.422	1.066	0	0	7.45	0	0	86	3	882.43268	2645.28348	-3.66
aVQFGTGELcDAISAVEEk	Unambiguous	3	1	Q6ZPU9	N-Term(iTRAQ8plex); C10(Methylthio); K19(iTRAQ8plex)	CID	1.0000	0.0000	1	1	3239565	Unique	Used	0.944	1.078	0.919	1.052	0	0	7.45	0	13	36	2	1311.17200	2621.33672	-1.70
aTGHSGGGcISQGQSYDTDk	Unambiguous	1	1	Q8K274	N-Term(iTRAQ8plex); C9(Methylthio); K20(iTRAQ8plex)	CID	1.0000	0.0000	1	1	2843510	Unique	Used	0.646	0.738	1.173	1.068	0	0	7.45	0	4	84	3	875.07941	2623.22367	-3.83
aQQVAEQQSQQEQGGTPPAPHSPFR	Unambiguous	1	1	Q80YF9	N-Term(iTRAQ8plex)	CID	1.0000	0.0000	1	1	3122053	Unique	Used	0.709	1.563	1.036	0.349	0	0	7.45	0	0	72	3	1003.17010	3007.49576	1.10
lDAWDEAQVEFMASHGNEAAR	Unambiguous	1	1	E9PY16	N-Term(iTRAQ8plex)	CID	1.0000	0.0000	1	1	2940296	Unique	Used	0.454	0.745	0.817	0.960	0	0	7.45	0	6	34	3	884.41901	2651.24247	-1.44
sSGSPYGGGYGSGGGSGGYGSR	Unambiguous	7	1	J3QNY1	N-Term(iTRAQ8plex)	CID	1.0000	0.0000	1	1	2950494	Unique	Used	0.646		1.074	1.019	0	0	7.45	0	2	17	3	739.00317	2214.99497	-0.20
gTGGVDTAAVGGVFDVSNADR	Unambiguous	1	1	Q04447	N-Term(iTRAQ8plex)	CID	1.0000	0.0000	1	1	3128264	Unique	Used				1.234	0	0	7.45	0	0	1	3	757.04968	2269.13449	-0.80
qTTAAAAATFSEQVGGGSGGAGR	Unambiguous	3	1	O88935	N-Term(iTRAQ8plex)	CID	1.0000	0.0000	1	1	3533447	Unique	Used	0.618	1.882	1.641	1.889	0	0	7.44	0	0	131	2	1178.58801	2356.16875	-4.59
aVEAEEELTVAYGYDHSPPGk	Unambiguous	1	1	Q8VHL1	N-Term(iTRAQ8plex); K21(iTRAQ8plex)	CID	1.0000	0.0000	1	1	2686714	Unique	Used	1.847		1.501	0.799	0	0	7.44	0	10	200	3	957.49426	2870.46823	0.47
gGPPPPPPPHSSGPPPPPAR	Unambiguous	1	1	Q91YD9	N-Term(iTRAQ8plex)	CID	1.0000	0.0000	1	1	3653920	Unique	Used	1.642	0.739	1.614	1.513	0	0	7.44	0	19	200	3	765.41449	2294.22892	-2.58
qGSSSVSGSPAPSTSGTSTPR	Unambiguous	2	1	P97445	N-Term(iTRAQ8plex)	CID	1.0000	0.0000	1	1	3224376	Unique	Used	0.504	0.867	1.796	1.654	0	0	7.44	0	0	200	2	1120.05835	2239.10942	-0.45
vDSANALAnGLIAGGHAAR	Unambiguous	1	1	Q68FG2	N-Term(iTRAQ8plex); N9(Deamidated)	CID	1.0000	0.0000	1	1	3454889	Unique	Used	0.994	2.046	1.944	1.229	0	0	7.44	0	2	26	3	695.04279	2083.11380	-2.90
gTVVYGEPITASLGTDGSHYWSk	Unambiguous	1	1	O08553	N-Term(iTRAQ8plex); K23(iTRAQ8plex)	CID	1.0000	0.0000	1	1	3369717	Unique	Used	1.464	1.367	1.321	1.389	0	0	7.44	0	11	58	3	1011.85736	3033.55753	-6.69
aFVDFFSQcLHEEYk	Unambiguous	2	1	O70503	N-Term(iTRAQ8plex); C9(Methylthio); K15(iTRAQ8plex)	CID	1.0000	0.0000	1	1	3541300	Unique	Used	0.338	0.560	0.609	0.837	0	0	7.44	0	0	15	3	839.74927	2517.23325	-2.80

gPNIVLSEELAGHQTPITDIATER	Unambiguous	1	1	Q9R0D8	N-Term(iTRAQ8plex)	CID	1.0000	0.0000	1	1	3236385	Unique	Used	2.457	1.021	1.228	1.266	0	0	7.44	0	0	149	3	955.84534	2865.52146	-1.60
yTPSGQSGAAASESLFISNHAY	Unambiguous	2	1	A6ZI44	N-Term(iTRAQ8plex)	CID	1.0000	0.0000	1	1	3301163	Unique	Used	1.428	1.569	1.205	1.312	0	0	7.44	0	23	171	2	1281.62402	2562.24077	-0.26
dVLQAAAAQHQDQNQEANGEVR	Unambiguous	3	1	E9QP99	N-Term(iTRAQ8plex)	CID	1.0000	0.0000	1	1	2713424	Unique	Used	1.987	1.141	1.214	0.734	0	0	7.43	0	20	200	3	899.44623	2696.32413	-1.83
gTSFEAAATSGGSASSEk	Unambiguous	1	1	Q99LC5	N-Term(iTRAQ8plex); K18(iTRAQ8plex)	CID	1.0000	0.0000	1	1	2807097	Unique	Used	1.254	1.183	1.069	1.507	0	0	7.43	0	0	32	2	1127.07141	2253.13555	-4.44
iSFEEFcAVVGGLDIHk	Unambiguous	2	1	Q63810-2	N-Term(iTRAQ8plex); C7(Methylthio); K17(iTRAQ8plex)	CID	1.0000	0.0000	1	1	3690703	Unique	Used	0.494	1.174	2.044	0.693	0	0	7.43	0	6	200	3	840.11090	2518.31815	-4.50
dAAEAEAWMGEQELHMMGQEk	Unambiguous	1	1	Q68FG2	N-Term(iTRAQ8plex); K21(iTRAQ8plex)	CID	1.0000	0.0000	1	1	2740823	Unique	Used		2.078	3.614	1.188	0	0	7.43	0	0	200	3	1000.47546	2999.41184	-1.34
yTPSGQSGAAASESLFISNHAY	Unambiguous	2	1	A6ZI44	N-Term(iTRAQ8plex)	CID	1.0000	0.0000	1	1	3235208	Unique	Used	1.181	0.949	1.175	1.375	0	0	7.43	0	0	40	2	1281.62195	2562.23662	-1.88
tVTSFYNQSAIDVAAEkPSVR	Unambiguous	2	1	O55028	N-Term(iTRAQ8plex); K17(iTRAQ8plex)	CID	1.0000	0.0000	1	1	3454850	Unique	Used	1.113	1.705	0.494	0.666	0	0	7.43	0	0	179	3	964.52478	2891.55979	-4.35
sALFAQINQGESITHALk	Unambiguous	1	1	P40124	N-Term(iTRAQ8plex); K18(iTRAQ8plex)	CID	1.0000	0.0000	1	1	3704007	Unique	Used	0.824	1.076	1.258	0.988	0	0	7.43	0	26	17	3	846.14526	2536.42124	-5.19
eVAETSMHNLLETDIEQVSQLSALVDAQLDYHR	Unambiguous	1	1	Q62419	N-Term(iTRAQ8plex)	CID	1.0000	0.0000	1	1	3352874	Unique	Used	1.700	1.040	1.605	0.787	0	0	7.43	0	0	34	3	1353.67944	4059.02378	-1.13
gALGcPQEAATATAPGSGTAVTLR	Unambiguous	1	1	Q3UVU3	N-Term(iTRAQ8plex); C5(Methylthio)	CID	1.0000	0.0000	1	1	3640078	Unique	Used	0.312	0.644	0.539	0.802	0	0	7.42	0	0	127	2	1275.64746	2550.28765	-3.22
fDVNTSAVQVLIEHIGNLDR	Unambiguous	2	1	Q68FD5	N-Term(iTRAQ8plex)	CID	1.0000	0.0000	1	1	3766156	Unique	Used	0.598	1.334	1.202	1.387	0	0	7.42	0	0	11	3	848.79321	2544.36509	-2.89
gPAVGIDLGTTYScVGVFQHGk	Unambiguous	4	1	P63017	N-Term(iTRAQ8plex); C14(Methylthio); K22(iTRAQ8plex)	CID	1.0000	0.0000	1	1	3860043	Unique	Used	0.706	0.644	1.110	1.033	0	0	7.42	0	17	12	3	954.16583	2860.48294	-4.09

dEVQEVVYFPAVDHDAESk	Unambiguous	2	1	Q9CZC8	N-Term(iTRAQ8plex); K19(iTRAQ8plex)	CID	1.0000	0.0000	1	1	2737438	Unique	Used	1.697	1.100	1.228	1.032	0	0	7.42	0	0	200	3	929.13995	2785.40531	-3.17
rLSLESEGANEGATAAPELSALEEAFR	Unambiguous	1	1	Q7TQD2	N-Term(iTRAQ8plex)	CID	1.0000	0.0000	1	1	3152812	Unique	Used	2.920	1.348	1.342	1.086	0	0	7.42	1	4	123	3	1041.53357	3122.58615	-1.51
hAFSPVASVESASGETLHSPk	Unambiguous	1	1	P29699	N-Term(iTRAQ8plex); K21(iTRAQ8plex)	CID	1.0000	0.0000	1	1	3253008	Unique	Used	4.196	1.906	0.520	0.595	0	0	7.42	0	0	58	3	916.15497	2746.45035	-4.27
dFTSEQIDEILQNHTEIVFAR	Unambiguous	2	1	Q6PIC6	N-Term(iTRAQ8plex)	CID	1.0000	0.0000	1	1	3357672	Unique	Used			1.381	0.972	0	0	7.42	0	5	200	3	937.14612	2809.42380	-2.59
aLLTGNFESAVDLcLHDNR	Unambiguous	2	1	Q3UPL0	N-Term(iTRAQ8plex); C14(Methylthio)	CID	1.0000	0.0000	1	1	3602036	Unique	Used	0.921	1.053	1.106	1.283	0	0	7.42	0	6	34	3	813.40436	2438.19852	-5.14
fLSQPFQVAEVFTGHMGk	Unambiguous	1	1	P56480	N-Term(iTRAQ8plex); K18(iTRAQ8plex)	CID	1.0000	0.0000	1	1	3863885	Unique	Used	1.035	0.902	0.978	1.269	0	0	7.42	0	4	200	3	877.80878	2631.41178	-3.66
gTLGGLFSQILQGEDIVR	Unambiguous	1	1	O35841	N-Term(iTRAQ8plex)	CID	1.0000	0.0000	1	1	3806361	Unique	Used	0.543	1.060	0.714	0.820	0	0	7.42	0	11	32	2	1104.11755	2207.22783	-2.71
aAGGAGAQVGGSISSGSSASSVTVTR	Unambiguous	1	1	P48678	N-Term(iTRAQ8plex)	CID	1.0000	0.0000	1	1	3737416	Unique	Used	1.818	1.552	0.769	0.891	0	0	7.42	0	0	200	2	1263.65100	2526.29473	-4.55
qYPANqGQDVEYLVTGTHPYPAGPGVALTADTk	Unambiguous	2	1	Q922V4	N-Term(iTRAQ8plex); Q6(Deamidated); K33(iTRAQ8plex)	CID	0.0040	0.0000	1	1	3258992	Unique	Used	1.827	1.174	2.225	2.003	0	0	7.42	0	0	46	3	1356.70093	4068.08823	1.74
nLHQSGFSLSGAQIDDNIPR	Unambiguous	1	1	O08553	N-Term(iTRAQ8plex)	CID	1.0000	0.0000	1	1	3555516	Unique	Used	2.214	1.144	0.744	0.837	0	0	7.42	0	23	43	3	825.09210	2473.26175	-4.86
eQSGEYEcSASNDVAAPVVR	Unambiguous	5	1	D3Z396	N-Term(iTRAQ8plex); C8(Methylthio)	CID	1.0000	0.0000	1	1	2668855	Unique	Used	0.593	0.837	0.895	0.689	0	0	7.42	0	0	121	2	1231.06555	2461.12383	-1.60
eQVTNVGGAVVTGVTAVAQk	Unambiguous	2	1	O55042	N-Term(iTRAQ8plex); K20(iTRAQ8plex)	CID	1.0000	0.0000	1	1	3642336	Unique	Used	0.932	1.591	1.624	0.786	0	0	7.42	0	0	147	2	1268.71924	2536.43120	-9.60
iSFEEFcAVVGGLDIHk	Unambiguous	2	1	Q63810-2	N-Term(iTRAQ8plex); C7(Methylthio); K17(iTRAQ8plex)	CID	1.0000	0.0000	1	1	3709473	Unique	Used	0.780	0.782	2.269	0.513	0	0	7.42	0	9	200	3	840.10980	2518.31485	-5.81

qDHPSSmGVYGQESGGFSGPGENR	Unambiguous	4	1	Q5SUT0	N-Term(iTRAQ8plex); M7(Oxidation)	CID	1.0000	0.0000	1	1	2987137	Unique	Used	5.079	2.452	1.416	0.616	0	0	7.42	0	0	200	3	934.08911	2800.25278	-0.27
tGIGSGLSLSGIVHPELSR	Unambiguous	2	1	P50544	N-Term(iTRAQ8plex)	HCD	1.0000	0.0000	1	1	3822042	Unique	Used	1.403	0.836	0.814	1.155	0	0	7.42	0	5	32	3	728.74475	2184.21970	-4.28
sLQLVVLDADTINHPAQLAk	Unambiguous	2	1	D3Z3Z3	N-Term(iTRAQ8plex); K20(iTRAQ8plex)	CID	1.0000	0.0000	1	1	3518189	Unique	Used	0.669	1.192	1.190	0.887	0	0	7.41	0	25	141	3	918.86554	2754.58207	-5.59
gVVESVVTIEDDFITVVqTTTDEGESGSHSVR	Unambiguous	1	1	P20357	N-Term(iTRAQ8plex); Q18(Deamidated)	CID	1.0000	0.0000	1	1	2942600	Unique	Used			1.143	0.490	0	0	7.41	0	18	200	3	1233.28650	3697.84494	5.80
gGAFEGTLqGPFGHGYGEGAGEGIDDAEWVVAR	Unambiguous	1	1	Q9QXY6	N-Term(iTRAQ8plex); Q9(Deamidated)	CID	1.0000	0.0000	1	1	3074849	Unique	Used		4.358	1.722	1.685	0	0	7.41	0	28	200	3	1204.58008	3611.72568	7.60
dAQVGWIQIVEDLHER	Unambiguous	2	1	E9QKE4	N-Term(iTRAQ8plex)	CID	1.0000	0.0000	1	1	3111490	Unique	Used	1.212	1.219	1.349	1.070	0	0	7.41	0	6	200	3	738.06042	2212.16672	0.12
vLTLEGDLVLGGLFPVHQk	Unambiguous	1	1	Q14BI2	N-Term(iTRAQ8plex); K19(iTRAQ8plex)	CID	1.0000	0.0000	1	1	3726930	Unique	Used		1.082	0.597	0.562	0	0	7.41	0	21	19	3	881.85748	2643.55790	-4.38
tVHDMDQFGQQQYDIYER	Unambiguous	6	1	Q68FH0	N-Term(iTRAQ8plex)	CID	1.0000	0.0000	1	1	2883209	Unique	Used	1.272	1.325	1.122	1.143	0	0	7.41	0	14	100	3	859.73645	2577.19480	-1.32
sNHEEEVADLLAQIqASHITVER	Unambiguous	2	1	P08553	N-Term(iTRAQ8plex); Q15(Deamidated)	CID	1.0000	0.0000	1	1	3303599	Unique	Used	2.160	0.787	1.101	1.372	0	0	7.41	0	29	96	3	965.50348	2894.49588	5.55
sQTEEDcTEELFDFLHAR	Unambiguous	1	1	P99028	N-Term(iTRAQ8plex); C7(Methylthio)	CID	1.0000	0.0000	1	1	2835208	Unique	Used	2.322	1.343	1.759	1.468	0	0	7.41	0	24	160	3	840.71490	2520.13016	-0.93
vSALEEELAAANQEIVALR	Unambiguous	2	1	B8QI34	N-Term(iTRAQ8plex)	CID	1.0000	0.0000	1	1	3262912	Unique	Used	1.141	1.883	1.755	1.039	0	0	7.41	0	14	19	2	1165.64539	2330.28349	-1.50
sSMSVTSLEDELQASIQR	Unambiguous	1	1	Q99JR1	N-Term(iTRAQ8plex)	CID	1.0000	0.0000	1	1	3239093	Unique	Used	0.662	1.250	0.940	1.345	0	0	7.41	0	7	12	2	1143.08203	2285.15679	-1.28
vGTGEPccDWVGDEGAGHFVk	Unambiguous	1	1	Q9DCD0	N-Term(iTRAQ8plex); C7(Methylthio); C8(Methylthio); K21(iTRAQ8plex)	CID	1.0000	0.0000	1	1	3130996	Unique	Used	0.316	0.871	1.236	1.404	0	0	7.41	0	14	13	3	955.10828	2863.31028	-1.05
lATLETEAAQHQAVIDGLTR	Unambiguous	1	1	Q3TDD9	N-Term(iTRAQ8plex)	CID	1.0000	0.0000	1	1	3393751	Unique	Used	1.005	0.803	1.263	1.090	0	0	7.41	0	1	6	3	814.44525	2441.32120	-3.70

tLTQEEVEmLYEEALYTVLHR	Unambiguous	2	1	E9Q350	N-Term(iTRAQ8plex); M9(Oxidation)	CID	1.0000	0.0000	1	1	3396435	Unique	Used	0.977	1.248	1.022	1.643	0	0	7.40	0	26	11	3	963.15576	2887.45273	-6.03
mTEEEVEMLVAGHEDSnGcINYEAFVR	Unambiguous	1	1	Q60605	N-Term(iTRAQ8plex); N17(Deamidated); C19(Methylthio)	CID	0.0068	0.0000	1	1	2942150	Unique	Used	2.194	1.095	1.012	0.926	0	0	7.40	0	25	89	3	1141.84497	3423.52036	1.39
iSFEEFcAVVGGLDIHk	Unambiguous	2	1	Q63810-2	N-Term(iTRAQ8plex); C7(Methylthio); K17(iTRAQ8plex)	CID	1.0000	0.0000	1	1	3709563	Unique	Used	0.498	1.605	2.160	1.009	0	0	7.40	0	12	200	3	840.10931	2518.31339	-6.39
dFTSEQIDEILQNHTEIVFAR	Unambiguous	2	1	Q6PIC6	N-Term(iTRAQ8plex)	CID	1.0000	0.0000	1	1	3352368	Unique	Used	1.169	0.798	0.818	1.443	0	0	7.40	0	2	7	3	937.14520	2809.42105	-3.57
aISEAGAPPGSAGPLLLQPQQHR	Unambiguous	1	1	Q99MX7	N-Term(iTRAQ8plex)	CID	1.0000	0.0000	1	1	3680704	Unique	Used	0.885	1.750	1.313	0.879	0	0	7.40	0	0	76	3	867.14301	2599.41446	-4.39
mVISNPAATHQDIDFLIEEIER	Unambiguous	1	1	P48320	N-Term(iTRAQ8plex); M1(Oxidation)	CID	1.0000	0.0000	1	1	3132935	Unique	Used		0.817	1.106	1.537	0	0	7.40	0	0	32	3	954.49359	2861.46622	0.16
fGAILAQGILDAGGHNVTISLQSR	Unambiguous	1	1	Q3TXS7	N-Term(iTRAQ8plex)	CID	1.0000	0.0000	1	1	3935906	Unique	Used	1.373	0.967	2.094	2.907	0	0	7.40	0	12	48	3	914.84283	2742.51395	-2.39
aTVQGAQHVFQHVVPqEGkPVTNQk	Unambiguous	3	1	Q8R016	N-Term(iTRAQ8plex); Q16(Deamidated); K19(iTRAQ8plex); K25(iTRAQ8plex)	CID	0.0392	0.0000	1	1	3635694	Unique	Used	0.324	1.748	0.754	0.876	0	0	7.40	0	0	167	3	1214.34863	3641.03135	-0.38
gLDTVVDLLADVVLHPR	Unambiguous	2	1	Q9DC61	N-Term(iTRAQ8plex)	CID	1.0000	0.0000	1	1	3766743	Unique	Used	0.721	1.135	0.797	0.837	0	0	7.40	0	0	62	3	712.74713	2136.22684	-2.92
hHAAYVNNLnATEEk	Unambiguous	1	1	P09671	N-Term(iTRAQ8plex); N10(Deamidated); K15(iTRAQ8plex)	CID	1.0000	0.0000	1	1	2695302	Unique	Used	1.480	0.786	0.964	1.155	0	0	7.40	0	3	140	3	774.07410	2320.20774	-2.79
qNTGSATQGPAYHGVNR	Unambiguous	4	1	E9QAQ7	N-Term(iTRAQ8plex)	CID	1.0000	0.0000	1	1	3380290	Unique	Used	0.982	0.731	0.894	0.875	0	0	7.40	0	0	200	3	688.01422	2062.02811	-4.21
gNAGQTNYGFANSTMER	Unambiguous	1	1	P19096	N-Term(iTRAQ8plex)	CID	1.0000	0.0000	1	1	3122468	Unique	Used	0.582	0.441	0.644	0.891	0	0	7.39	0	0	13	2	1061.49744	2121.98760	-2.34

dmHGDSEYNIMFGPDIcGPGTk	Unambiguous	1	1	P14211	N-Term(iTRAQ8plex); M2(Oxidation); C17(Methylthio); K22(iTRAQ8plex)	CID	1.0000	0.0000	1	1	3171317	Unique	Used	1.524	0.908	1.201	0.623	0	0	7.39	0	6	31	3	1018.80133	3054.38944	-1.05
tQPTDEEMLFIYSHFk	Unambiguous	4	1	P31786	N-Term(iTRAQ8plex); K16(iTRAQ8plex)	CID	1.0000	0.0000	1	1	3287043	Unique	Used	1.514	0.783	0.978	0.909	0	0	7.39	0	0	200	3	865.45123	2594.33915	-1.15
vSHVSTGGGASLELLEGk	Unambiguous	2	1	P09411	N-Term(iTRAQ8plex); K18(iTRAQ8plex)	CID	1.0000	0.0000	1	1	3277417	Unique	Used	1.461	0.836	2.021	2.600	0	0	7.39	0	0	119	2	1175.16260	2349.31792	-2.36
hLVDQMPDSSDNPAGLAEQcPGmR	Unambiguous	1	1	P46414	N-Term(iTRAQ8plex); C20(Methylthio); M23(Oxidation)	CID	1.0000	0.0000	1	1	3211631	Unique	Used	1.670	1.404	1.267	1.189	0	0	7.39	0	15	87	3	978.77649	2934.31491	-0.27
gALGcPQEAATATAPGSGTAVTLR	Unambiguous	1	1	Q3UVU3	N-Term(iTRAQ8plex); C5(Methylthio)	CID	1.0000	0.0000	1	1	3618289	Unique	Used	0.245	0.605	0.544	0.871	0	0	7.39	0	0	86	2	1275.64380	2550.28032	-6.09
iGIIDDDIFEEDEHFFVR	Unambiguous	1	1	Q8K596	N-Term(iTRAQ8plex)	CID	1.0000	0.0000	1	1	2834980	Unique	Used	0.265	3.042	3.977	2.759	0	0	7.39	0	11	44	3	838.42047	2513.24686	-1.35
icDPGLTSFEPEALGNLVEGmDFHR	Unambiguous	9	1	Q5SVJ0	N-Term(iTRAQ8plex); C2(Methylthio); M21(Oxidation)	CID	1.0000	0.0000	1	1	3500284	Unique	Used	1.597	1.042	1.243	0.711	0	0	7.39	0	22	9	3	1038.48950	3113.45395	-4.67
sENGLEFTSSGSANTETTk	Unambiguous	3	1	Q60932-2	N-Term(iTRAQ8plex); K19(iTRAQ8plex)	CID	1.0000	0.0000	1	1	2682925	Unique	Used			4.019	3.326	0	0	7.39	0	0	200	2	1284.64941	2568.29155	1.16
lDAWDEAQVEFmASHGNEAAR	Unambiguous	1	1	E9PY16	N-Term(iTRAQ8plex); M12(Oxidation)	CID	1.0000	0.0000	1	1	2957726	Unique	Used	0.794	0.645	0.929	0.865	0	0	7.38	0	12	143	3	889.75079	2667.23783	-1.26
gGGGGGGGPGGFQPAPR	Unambiguous	1	1	O35295	N-Term(iTRAQ8plex)	CID	1.0000	0.0000	1	1	3382154	Unique	Used	1.153	0.674	0.683	0.466	0	0	7.38	0	3	3	2	872.44080	1743.87432	-4.94
sAAQAAAQTnSNAAGk	Unambiguous	4	1	Q9CY58	N-Term(iTRAQ8plex); N10(Deamidated); K16(iTRAQ8plex)	CID	0.0407	0.0000	1	1	2842286	Unique	Used	0.935	0.749	2.504	0.853	0	0	7.38	0	0	200	2	1035.55347	2070.09966	-1.91

gPAVGIDLGTTYScVGVFQHGk	Unambiguous	4	1	P63017	N-Term(iTRAQ8plex); C14(Methylthio); K22(iTRAQ8plex)	CID	1.0000	0.0000	1	1	3808215	Unique	Used	1.282	1.087	0.990	0.919	0	0	7.38	0	14	200	3	954.16504	2860.48056	-4.93
nLHQSGFSLSGAQIDDNIPR	Unambiguous	1	1	O08553	N-Term(iTRAQ8plex)	CID	1.0000	0.0000	1	1	3616928	Unique	Used	0.949	1.048	1.239	1.357	0	0	7.38	0	21	22	3	825.09082	2473.25791	-6.41
sSIDSQASLSGPLAGSAAGSGAR	Unambiguous	1	1	E9Q6B2	N-Term(iTRAQ8plex)	CID	1.0000	0.0000	1	1	3409990	Unique	Used	1.397	0.812	1.387	0.738	0	0	7.38	0	0	102	2	1176.10266	2351.19805	-5.31
dIAAGDFIEHAEFSGNLYGTSk	Unambiguous	3	1	Q564G0	N-Term(iTRAQ8plex); K22(iTRAQ8plex)	CID	1.0000	0.0000	1	1	3130459	Unique	Used				1.748	0	0	7.38	0	0	11	3	984.16992	2950.49521	-3.10
tTAASGDLAQAPGAFk	Unambiguous	2	1	P20357	N-Term(iTRAQ8plex); K16(iTRAQ8plex)	CID	1.0000	0.0000	1	1	3253687	Unique	Used	0.773	1.626	2.313	1.714	0	0	7.38	0	4	8	2	1057.58740	2114.16753	-1.29
rEEVTEPGGAGEAALWk	Unambiguous	1	1	Q80VL1	N-Term(iTRAQ8plex); K17(iTRAQ8plex)	CID	1.0000	0.0000	1	1	2809293	Unique	Used	1.007	1.109	0.893	0.867	0	0	7.37	1	3	31	3	803.43750	2408.29795	-2.13
aANAAENDFSVSQAEVSSR	Unambiguous	1	1	Q6P542	N-Term(iTRAQ8plex)	CID	1.0000	0.0000	1	1	2757262	Unique	Used	0.675	0.696	0.904	0.769	0	0	7.37	0	0	200	2	1129.05054	2257.09380	-2.70
aTLQEVVGLHSDMAVEAGAPYAER	Unambiguous	1	1	G3UYZ1	N-Term(iTRAQ8plex)	CID	1.0000	0.0000	1	1	3281359	Unique	Used	0.833	0.378	0.832	0.551	0	0	7.37	0	0	60	3	940.14734	2818.42746	-2.60
vYVGNASVAQDITQLQk	Unambiguous	3	1	Q9D7X3	N-Term(iTRAQ8plex); K17(iTRAQ8plex)	CID	1.0000	0.0000	1	1	3414727	Unique	Used	0.830	0.640	1.146	0.808	0	0	7.37	0	0	20	2	1221.68872	2442.37016	-4.56
rPEGQSAISYDYSEEELMASIER	Unambiguous	1	1	Q8R4T1	N-Term(iTRAQ8plex)	CID	1.0000	0.0000	1	1	2832545	Unique	Used	1.895	0.907	1.402	1.185	0	0	7.37	0	10	200	3	988.81445	2964.42881	3.00
dMAIATGGAVFGEEGLNLNLEDVQAHDLGk	Unambiguous	1	1	P63038	N-Term(iTRAQ8plex); K30(iTRAQ8plex)	CID	1.0000	0.0000	1	1	3265021	Unique	Used	1.042	1.330	1.627	1.517	0	0	7.37	0	0	200	3	1231.63867	3692.90146	-1.00
gHIQLSYTDGDDcGSDk	Unambiguous	1	1	Q07113	N-Term(iTRAQ8plex); C13(Methylthio); K17(iTRAQ8plex)	CID	1.0000	0.0000	1	1	2667061	Unique	Used	1.323	0.924	1.429	1.064	0	0	7.37	0	10	200	3	822.38892	2465.15219	-0.47
eSTFLDEGPNEQEITPLQHTPR	Unambiguous	3	1	Q9QYR6	N-Term(iTRAQ8plex)	CID	1.0000	0.0000	1	1	2795126	Unique	Used	2.495	1.019	1.358	0.722	0	0	7.37	0	9	164	3	948.14282	2842.41391	-0.79

gTQLYEANQLLQFENNAEDLkR	Unambiguous	1	1	P08032	N-Term(iTRAQ8plex); K21(iTRAQ8plex)	CID	1.0000	0.0000	1	1	3259996	Unique	Used	2.622	1.668	0.564	1.035	0	0	7.37	1	0	114	3	1068.23450	3202.68894	-2.00
sQAAAScPEPLSAAEAPAER	Unambiguous	1	1	B9EJI9	N-Term(iTRAQ8plex); C7(Methylthio)	CID	1.0000	0.0000	1	1	2935936	Unique	Used			1.055	1.265	0	0	7.37	0	0	200	2	1153.55444	2306.10161	-1.87
tVLGIGPGPVELIDEVTGHLk	Unambiguous	1	1	Q8R2Y8	N-Term(iTRAQ8plex); K21(iTRAQ8plex)	CID	1.0000	0.0000	1	1	3560933	Unique	Used	0.852	0.872	0.958	1.150	0	0	7.37	0	0	35	3	918.20343	2752.59574	-4.08
vGDSPcIGAGGYADNNLGAVSTTGHGESILk	Unambiguous	1	1	Q8C0M9	N-Term(iTRAQ8plex); C6(Methylthio); K31(iTRAQ8plex)	CID	1.0000	0.0000	1	1	3301706	Unique	Used	2.072	0.810	0.654	0.915	0	0	7.37	0	0	94	3	1205.59949	3614.78391	-5.56
qVLGQmVIDEELLGDGHSYSPR	Unambiguous	1	1	P99026	N-Term(iTRAQ8plex); M6(Oxidation)	CID	1.0000	0.0000	1	1	3236273	Unique	Used	1.784	1.092	0.895	1.011	0	0	7.37	0	10	49	3	921.79858	2763.38120	-4.12
lATMYSSGSPEGGSDSSESR	Unambiguous	2	1	F8WIE5	N-Term(iTRAQ8plex)	CID	1.0000	0.0000	1	1	2756379	Unique	Used	0.967	0.893	0.730	1.219	0	0	7.36	0	0	200	2	1155.02625	2309.04521	-2.31
gLTIVGSVLEGTFLDNHPQAQR	Unambiguous	2	1	Q91V14	N-Term(iTRAQ8plex)	CID	1.0000	0.0000	1	1	3844052	Unique	Used	0.673	1.220	0.891	0.901	0	0	7.36	0	21	200	3	886.14618	2656.42398	-4.56
lAQQHAAAAAAAAAATNQQGSAk	Unambiguous	1	1	Q8BHJ5	N-Term(iTRAQ8plex); K23(iTRAQ8plex)	CID	1.0000	0.0000	1	1	3717644	Unique	Used	1.933	1.382	0.977	0.929	0	0	7.36	0	0	200	3	910.49506	2729.47062	-7.24
dLGLPTEAYISVEEVHDDGTPTSk	Unambiguous	1	1	P26516	N-Term(iTRAQ8plex); K24(iTRAQ8plex)	CID	1.0000	0.0000	1	1	2832645	Unique	Used		1.006	0.790	1.028	0	0	7.36	0	17	200	3	1061.21472	3181.62961	-2.06
sSGSPYGGGYGSGGGSGGYGSR	Unambiguous	7	1	J3QNY1	N-Term(iTRAQ8plex)	CID	1.0000	0.0000	1	1	2968244	Unique	Used	1.370	0.799	1.932	0.849	0	0	7.36	0	0	200	2	1107.99854	2214.98979	-2.53
fLSQPFQVAEVFTGHMGk	Unambiguous	1	1	P56480	N-Term(iTRAQ8plex); K18(iTRAQ8plex)	CID	1.0000	0.0000	1	1	3863777	Unique	Used	1.302	0.898	1.096	1.298	0	0	7.36	0	24	200	3	877.80939	2631.41361	-2.97
gLTIVGSVLEGTFLDNHPQAQR	Unambiguous	2	1	Q91V14	N-Term(iTRAQ8plex)	CID	1.0000	0.0000	1	1	3843707	Unique	Used	0.221	0.959	0.980	0.611	0	0	7.36	0	22	200	3	886.14734	2656.42746	-3.25
gTVVYGEPITASLGTDGSHYWSk	Unambiguous	1	1	O08553	N-Term(iTRAQ8plex); K23(iTRAQ8plex)	CID	1.0000	0.0000	1	1	3414188	Unique	Used	0.962	1.555	0.981	1.157	0	0	7.36	0	0	31	3	1011.86023	3033.56614	-3.86

wGEAGAEYVVESTGVFTTMEk	Unambiguous	3	1	P16858	N-Term(iTRAQ8plex); K21(iTRAQ8plex)	CID	1.0000	0.0000	1	1	3241739	Unique	Used		1.668			0	0	7.36	0	29	200	3	967.16284	2899.47397	3.28
gnDISSGTVLSDYVGSGPPSGTGLHR	Unambiguous	1	1	P70296	N-Term(iTRAQ8plex); N2(Deamidated)	CID	1.0000	0.0000	1	1	3211650	Unique	Used	1.070	1.048	0.911	1.188	0	0	7.36	0	0	118	3	945.80475	2835.39969	-2.34
aVVFETGEAGAASGAGSLPGEVR	Unambiguous	3	1	Q9QYR6	N-Term(iTRAQ8plex)	CID	1.0000	0.0000	1	1	3129242	Unique	Used	2.054	0.899	1.306	0.902	0	0	7.36	0	0	72	2	1218.63586	2436.26445	-1.18
qTTAAAAATFSEQVGGGSGGAGR	Unambiguous	3	1	O88935	N-Term(iTRAQ8plex)	CID	1.0000	0.0000	1	1	3594700	Unique	Used	0.459	1.792	1.400	1.546	0	0	7.36	0	0	130	2	1178.58765	2356.16802	-4.90
sGLGLGAASASTSGSGPADSVMR	Unambiguous	3	1	Q7TSJ2	N-Term(iTRAQ8plex)	CID	1.0000	0.0000	1	1	3493949	Unique	Used			1.876	1.407	0	0	7.35	0	0	72	2	1170.58691	2340.16655	-4.38
gAVVGIDLGTTNScVAVmEGk	Unambiguous	1	1	P38647	N-Term(iTRAQ8plex); C14(Methylthio); M18(Oxidation); K21(iTRAQ8plex)	CID	1.0000	0.0000	1	1	3518216	Unique	Used	1.102	0.973	0.837	0.703	0	0	7.35	0	5	26	3	897.80035	2691.38651	-4.15
sQTEEDcTEELFDFLHAR	Unambiguous	1	1	P99028	N-Term(iTRAQ8plex); C7(Methylthio)	CID	1.0000	0.0000	1	1	2853803	Unique	Used	2.171	1.019	2.265	1.247	0	0	7.35	0	3	200	3	840.71552	2520.13199	-0.21
ekPELQFPFLqDEDTVATLHEck	Unambiguous	2	1	P16330	N-Term(iTRAQ8plex); K2(iTRAQ8plex); Q11(Deamidated); C22(Methylthio); K23(iTRAQ8plex)	CID	0.0299	0.0000	1	1	3215526	Unique	Used	1.153	0.477	2.033	2.422	0	0	7.35	0	13	200	3	1226.30920	3676.91306	3.38
rVVLGDSVDGGGADQLGGVAEVR	Unambiguous	1	1	P49070	N-Term(iTRAQ8plex)	CID	1.0000	0.0000	1	1	3147371	Unique	Used	1.368	1.315	0.870	1.031	0	0	7.35	1	6	56	3	844.12128	2530.34928	-1.39
tYFPHFDVSHGSAQVk	Unambiguous	2	1	Q91VB8	N-Term(iTRAQ8plex); K16(iTRAQ8plex)	CID	1.0000	0.0000	1	1	3594296	Unique	Used	1.945	1.919	0.706	1.178	0	0	7.35	0	0	76	3	810.09479	2428.26981	-7.07
qQSAPVSTLYPSTSNLLTNHQHEGR	Unambiguous	2	1	Q3UGN9	N-Term(iTRAQ8plex)	CID	1.0000	0.0000	1	1	3636521	Unique	Used	2.166	0.892	1.266	0.944	0	0	7.35	0	0	36	3	1023.85419	3069.54801	-5.73
sYHEAYEPDYGGGYSPSYDR	Unambiguous	1	1	Q9Z0U1	N-Term(iTRAQ8plex)	CID	1.0000	0.0000	1	1	2632845	Unique	Used	1.809	0.663	1.077	0.917	0	0	7.34	0	5	177	3	873.05170	2617.14054	-0.59

aATGEEVSAEDLGGADLHcR	Unambiguous	1	1	Q3ULD5	N-Term(iTRAQ8plex); C19(Methylthio)	CID	1.0000	0.0000	1	1	2635304	Unique	Used	1.688	1.209	1.293	1.037	0	0	7.34	0	29	82	3	784.36639	2351.08463	-2.71
yTPSGQSGAAASESLFISNHAy	Unambiguous	2	1	A6ZI44	N-Term(iTRAQ8plex); Y22(iTRAQ8plex)	CID	1.0000	0.0000	1	1	3514315	Unique	Used	3.007	2.448	1.969	2.867	0	0	7.34	0	0	84	3	956.14996	2866.43534	-4.00
aAASVVSGPPVASDcASPGDSAR	Unambiguous	3	1	Q6P9K8	N-Term(iTRAQ8plex); C15(Methylthio)	CID	1.0000	0.0000	1	1	3278108	Unique	Used	1.348	0.597	1.353	0.928	0	0	7.34	0	8	17	2	1211.58301	2422.15874	-2.38
sAAQAAAQTNSNAAGk	Unambiguous	4	1	Q9CY58	N-Term(iTRAQ8plex); K16(iTRAQ8plex)	CID	1.0000	0.0000	1	1	3118423	Unique	Used	0.592	0.229	1.050	0.560	0	0	7.34	0	7	86	2	1035.06104	2069.11479	-2.32
aSLDSAGASGNSPVLLPTPAVGGPR	Unambiguous	3	1	Q8C6B2-2	N-Term(iTRAQ8plex)	CID	1.0000	0.0000	1	1	3683373	Unique	Used	0.978	0.371	0.785	0.851	0	0	7.34	0	0	151	2	1298.19910	2595.39092	-5.23
iSFEEFcAVVGGLDIHk	Unambiguous	2	1	Q63810-2	N-Term(iTRAQ8plex); C7(Methylthio); K17(iTRAQ8plex)	CID	1.0000	0.0000	1	1	3709038	Unique	Used	0.567	1.116	1.669	0.686	0	0	7.34	0	0	200	3	840.10962	2518.31430	-6.03
aSDLAGSVDTGSAGSVk	Unambiguous	3	1	Q6P9K8	N-Term(iTRAQ8plex); K17(iTRAQ8plex)	CID	1.0000	0.0000	1	1	2971498	Unique	Used	0.722	0.315	1.399	0.886	0	0	7.34	0	0	200	2	1065.57483	2130.14238	-3.53
aIQINPAFADAHSNLASIHk	Unambiguous	2	1	Q8CGY8	N-Term(iTRAQ8plex); K20(iTRAQ8plex)	CID	1.0000	0.0000	1	1	3660005	Unique	Used	0.568	1.320	1.301	1.014	0	0	7.34	0	0	126	3	909.50568	2726.50248	-6.38
vQNATLAVANITNADSATR	Unambiguous	1	1	P54116	N-Term(iTRAQ8plex)	CID	1.0000	0.0000	1	1	3702903	Unique	Used	1.162	2.016	0.667	1.540	0	0	7.34	0	0	200	2	1117.59583	2234.18437	-8.91
gGPPPPPPPPHSSGPPPPPAR	Unambiguous	1	1	Q91YD9	N-Term(iTRAQ8plex)	CID	1.0000	0.0000	1	1	3631873	Unique	Used	1.156	1.689	1.521	1.072	0	0	7.33	0	0	137	3	765.41309	2294.22470	-4.41
sQTEEDcTEELFDFLHAR	Unambiguous	1	1	P99028	N-Term(iTRAQ8plex); C7(Methylthio)	CID	1.0000	0.0000	1	1	2853699	Unique	Used	2.948	1.920			0	0	7.33	0	23	200	3	840.71497	2520.13034	-0.86
sGSSSSSSGNPASQFYPQSR	Unambiguous	1	1	Q61771	N-Term(iTRAQ8plex)	CID	1.0000	0.0000	1	1	3490606	Unique	Used	0.480	0.389	1.267	0.778	0	0	7.33	0	0	200	2	1161.54370	2322.08013	-4.26
qGSATSAASATTASSAAEGTLQR	Unambiguous	1	1	Q8R4V2	N-Term(iTRAQ8plex)	CID	1.0000	0.0000	1	1	3184192	Unique	Used	1.316	0.104	0.927	1.313	0	0	7.33	0	0	98	2	1214.61389	2428.22051	-0.53
nMGGPYGGGNYGPGGSGGSGGYGGR	Unambiguous	3	1	O88569	N-Term(iTRAQ8plex); N1(Deamidated)	CID	1.0000	0.0000	1	1	2989864	Unique	Used	1.436	0.919	1.181	0.610	0	0	7.33	0	0	83	2	1248.04749	2495.08769	-2.86

sGLGLGAASASTSGSGPADSVMR	Unambiguous	3	1	Q7TSJ2	N-Term(iTRAQ8plex)	CID	1.0000	0.0000	1	1	3514104	Unique	Used	1.153	0.767	1.912	1.639	0	0	7.33	0	0	55	2	1170.58655	2340.16582	-4.69
dAAEAEAWMGEQELHMMGQEk	Unambiguous	1	1	Q68FG2	N-Term(iTRAQ8plex); K21(iTRAQ8plex)	CID	1.0000	0.0000	1	1	2764048	Unique	Used	0.320	2.174	3.299	1.440	0	0	7.33	0	11	200	3	1000.47571	2999.41257	-1.10
aDDPNSIFLIDHAWTcR	Unambiguous	1	1	Q3UDE2	N-Term(iTRAQ8plex); C16(Methylthio)	CID	1.0000	0.0000	1	1	3372895	Unique	Used	1.288	1.158	1.031	1.118	0	0	7.33	0	18	93	3	775.37207	2324.10166	-3.84
qYPANQGqDVEYLVTGTHPYPAGPGVALTADTk	Unambiguous	2	1	Q922V4	N-Term(iTRAQ8plex); Q8(Deamidated); K33(iTRAQ8plex)	CID	0.0041	0.0000	1	1	3281233	Unique	Used		1.417			0	0	7.32	0	0	171	3	1356.70068	4068.08750	1.56
sTLSATSAFAIATAGANEGPSNk	Unambiguous	1	1	P27671	N-Term(iTRAQ8plex); K23(iTRAQ8plex)	CID	1.0000	0.0000	1	1	3557880	Unique	Used	0.795	0.809	0.966	1.115	0	0	7.32	0	5	39	3	925.49298	2774.46439	-4.96
iSFEEFcAVVGGLDIHk	Unambiguous	2	1	Q63810-2	N-Term(iTRAQ8plex); C7(Methylthio); K17(iTRAQ8plex)	CID	1.0000	0.0000	1	1	3707722	Unique	Used	0.784	1.312	2.219	0.863	0	0	7.32	0	14	200	3	840.10992	2518.31522	-5.66
tANSSPVHFAEGQSGLPAFSPGR	Unambiguous	5	1	A2AIR9	N-Term(iTRAQ8plex)	CID	1.0000	0.0000	1	1	3681141	Unique	Used	1.359	1.201	1.275	1.223	0	0	7.32	0	8	68	3	873.44366	2618.31644	-3.86
sSMSVTSLEDELQASIQR	Unambiguous	1	1	Q99JR1	N-Term(iTRAQ8plex)	CID	1.0000	0.0000	1	1	3217051	Unique	Used	0.718	1.107	0.933	1.197	0	0	7.32	0	10	65	2	1143.08398	2285.16069	0.43
eAFQNAYLELGGLGER	Unambiguous	2	1	Q6PIC6	N-Term(iTRAQ8plex)	CID	1.0000	0.0000	1	1	3355699	Unique	Used	0.540	0.629	0.899	1.242	0	0	7.32	0	15	200	2	1036.03760	2071.06792	-4.01
gTGGVDTAAVGGVFDVSNADR	Unambiguous	1	1	Q04447	N-Term(iTRAQ8plex)	CID	1.0000	0.0000	1	1	3135364	Unique	Used	1.276	1.066	1.112	1.458	0	0	7.32	0	0	200	2	1135.07104	2269.13481	-0.66
qDHPSSMGVYGQESGGFSGPGENR	Unambiguous	4	1	Q5SUT0	N-Term(iTRAQ8plex)	CID	1.0000	0.0000	1	1	2844829	Unique	Used	1.654	1.858	1.016	0.726	0	0	7.32	0	0	200	3	928.75372	2784.24662	-4.31
sEIGGELAASSGVSTPGSVGHDIITEQPR	Unambiguous	3	1	P42859	N-Term(iTRAQ8plex)	CID	1.0000	0.0000	1	1	3257163	Unique	Used			1.110	0.873	0	0	7.32	0	10	56	3	1052.54016	3155.60593	-2.03
kAAAGELQEDSGLHVLAR	Unambiguous	1	1	Q8C845	N-Term(iTRAQ8plex); K1(iTRAQ8plex)	HCD	1.0000	0.0000	1	1	3186461	Unique	Used	1.069	1.180	1.328	0.882	0	0	7.32	1	20	29	3	825.13629	2473.39432	-1.64
yTPSGQSGAAASESLFISNHAY	Unambiguous	2	1	A6ZI44	N-Term(iTRAQ8plex)	CID	1.0000	0.0000	1	1	3235057	Unique	Used	1.114	0.656	1.542	1.081	0	0	7.31	0	0	26	2	1281.62256	2562.23784	-1.41

sGLGLGAASASTSGSGPADSVmR	Unambiguous	3	1	Q7TSJ2	N-Term(iTRAQ8plex); M22(Oxidation)	CID	1.0000	0.0000	1	1	3513961	Unique	Used	0.821	0.545	1.462	1.467	0	0	7.31	0	0	200	2	1178.58240	2356.15752	-6.02
lLEVSDDPQVLAVAAHDVGEYVR	Unambiguous	1	1	Q8BVE3	N-Term(iTRAQ8plex)	CID	1.0000	0.0000	1	1	3240414	Unique	Used	0.916	0.820	1.156	1.087	0	0	7.31	0	9	200	3	933.83655	2799.49509	4.28
vVIEAcDELGIVLAHTDLR	Unambiguous	1	1	Q9CWJ9	N-Term(iTRAQ8plex); C6(Methylthio)	CID	1.0000	0.0000	1	1	3542106	Unique	Used	1.545	1.208	1.124	1.173	0	0	7.31	0	13	192	3	806.09918	2416.28299	-2.17
gHAPSTAAPAPSPAPMSSSVQSDEER	Unambiguous	1	1	Q8C996	N-Term(iTRAQ8plex)	CID	1.0000	0.0000	1	1	2824743	Unique	Used	1.372	0.680	1.305	0.852	0	0	7.31	0	0	200	3	956.79456	2868.36911	-1.59
vGELTQEANALAAGHPAQAPAINTR	Unambiguous	1	1	Q68FG2	N-Term(iTRAQ8plex)	HCD	1.0000	0.0000	1	1	3456535	Unique	Used	1.029	2.186	1.930	1.045	0	0	7.31	0	12	108	3	935.50055	2804.48709	-3.09
eAGSmAGVLIAGPPGQGQAk	Unambiguous	1	1	P70206	N-Term(iTRAQ8plex); M5(Oxidation); K20(iTRAQ8plex)	CID	1.0000	0.0000	1	1	3409302	Unique	Used			0.646	0.899	0	0	7.31	0	0	130	2	1232.17163	2463.33599	-5.15
dFSGAGGIGVFSESSSVASk	Unambiguous	1	1	B1AWN6	N-Term(iTRAQ8plex); K20(iTRAQ8plex)	CID	1.0000	0.0000	1	1	3301470	Unique	Used	1.175	3.048	3.027	1.885	0	0	7.31	0	0	200	2	1249.15186	2497.29643	-2.68
nGPLEVAGAAISAGHGLPAk	Unambiguous	2	1	Q9QZQ8-2	N-Term(iTRAQ8plex); K20(iTRAQ8plex)	CID	1.0000	0.0000	1	1	3640131	Unique	Used	0.416	0.686	0.770	1.128	0	0	7.31	0	0	31	3	813.46619	2438.38401	-5.60
lSIEDFTAYGGVFGNk	Unambiguous	5	1	Q3TKX1	N-Term(iTRAQ8plex); K16(iTRAQ8plex)	CID	1.0000	0.0000	1	1	3560666	Unique	Used	0.818	1.153	0.698	0.772	0	0	7.31	0	12	29	2	1163.63000	2326.25273	-0.53
eAEAAIYHLQLFEELR	Unambiguous	1	1	P52480	N-Term(iTRAQ8plex)	CID	1.0000	0.0000	1	1	3326415	Unique	Used	1.290	0.949	0.970	1.256	0	0	7.31	0	5	23	3	746.06622	2236.18412	-3.34
nLHVVFTmNPSSEGLk	Unambiguous	1	1	Q9JHU4	N-Term(iTRAQ8plex); M8(Oxidation); K16(iTRAQ8plex)	CID	1.0000	0.0000	1	1	3637383	Unique	Used	0.660	0.943	1.119	1.117	0	0	7.31	0	12	13	3	799.76880	2397.29184	-5.78
lSVSSNDTQESGNSSGPSPGAk	Unambiguous	2	1	G3X9K3	N-Term(iTRAQ8plex); K22(iTRAQ8plex)	CID	1.0000	0.0000	1	1	2825407	Unique	Used	2.829	0.672	1.275	0.530	0	0	7.30	0	7	200	3	905.46021	2714.36606	-1.07
eNPNDLGGWAAHGDR	Unambiguous	2	1	Q9ES97-2	N-Term(iTRAQ8plex)	CID	1.0000	0.0000	1	1	2753282	Unique	Used	1.811	1.261	1.625	1.054	0	0	7.30	0	4	13	3	638.31030	1912.91635	-2.12
aGPGDTPAGAAAPEEPHGLSPLLPAR	Unambiguous	5	1	A2AHB7	N-Term(iTRAQ8plex)	HCD	1.0000	0.0000	1	1	3167701	Unique	Used	1.139	0.856	1.198	1.137	0	0	7.30	0	11	78	3	918.48761	2753.44828	-1.54

dFTSEQIDEILQNHTEIVFAR	Unambiguous	2	1	Q6PIC6	N-Term(iTRAQ8plex)	CID	1.0000	0.0000	1	1	3375361	Unique	Used	0.442	1.108	1.096	1.192	0	0	7.30	0	10	200	3	937.14496	2809.42032	-3.83
yATDTFAGLcHQLTNALVER	Unambiguous	2	1	O88543	N-Term(iTRAQ8plex); C10(Methylthio)	CID	1.0000	0.0000	1	1	3898848	Unique	Used	0.443	0.930	0.702	0.830	0	0	7.30	0	18	12	3	858.42816	2573.26993	-3.70
qTTAAAAATFSEQVGGGSGGAGR	Unambiguous	3	1	O88935	N-Term(iTRAQ8plex)	CID	1.0000	0.0000	1	1	3554315	Unique	Used	1.214	2.016	1.546	1.449	0	0	7.30	0	0	200	2	1178.58728	2356.16728	-5.21
tQPTDEEMLFIYSHFk	Unambiguous	4	1	P31786	N-Term(iTRAQ8plex); K16(iTRAQ8plex)	CID	1.0000	0.0000	1	1	3260735	Unique	Used	2.413	0.867	0.550	0.523	0	0	7.30	0	0	12	3	865.45166	2594.34043	-0.66
wQHDLFDSGFGGGAGVETGGk	Unambiguous	1	1	O08583	N-Term(iTRAQ8plex); K21(iTRAQ8plex)	CID	1.0000	0.0000	1	1	3257802	Unique	Used	1.035	0.976	0.871	0.810	0	0	7.30	0	16	15	3	910.79449	2730.36893	-1.60
hHAAYVnNLNATEEk	Unambiguous	1	1	P09671	N-Term(iTRAQ8plex); N7(Deamidated); K15(iTRAQ8plex)	CID	0.0288	0.0000	1	1	2928649	Unique	Used	1.579	0.829	1.060	1.201	0	0	7.30	0	11	200	3	774.07355	2320.20609	-3.50
lEPSDLSEDSEYDSIWTAHSYR	Unambiguous	6	1	Q9ES28-3	N-Term(iTRAQ8plex)	CID	1.0000	0.0000	1	1	2832655	Unique	Used	0.762	0.531	1.206	0.597	0	0	7.29	0	6	87	3	968.78754	2904.34806	0.10
nLSPAPASSSqGPPPQGPVSPGPAk	Unambiguous	3	1	Q7TQ95	N-Term(iTRAQ8plex); Q11(Deamidated); K25(iTRAQ8plex)	CID	0.0165	0.0000	1	1	3408134	Unique	Used	0.956	0.998	1.706	1.648	0	0	7.29	0	0	118	3	978.86322	2934.57511	-1.06
aVVFETGEAGAASGAGSLPGEVR	Unambiguous	3	1	Q9QYR6	N-Term(iTRAQ8plex)	CID	1.0000	0.0000	1	1	3108099	Unique	Used	2.556	0.967	1.578	0.888	0	0	7.29	0	0	123	2	1218.63464	2436.26201	-2.18
lAAEDVTFIGPDTHAIQAMGDk	Unambiguous	2	1	Q91ZA3	N-Term(iTRAQ8plex); K22(iTRAQ8plex)	CID	1.0000	0.0000	1	1	3192923	Unique	Used	0.768	1.267	1.221	1.230	0	0	7.29	0	4	9	3	970.18109	2908.52872	-1.67
eLAcDDPEAEQVALLAVDYLNNHLLQGFk	Unambiguous	1	1	P29699	N-Term(iTRAQ8plex); C4(Methylthio); K29(iTRAQ8plex)	CID	1.0000	0.0000	1	1	3480359	Unique	Used	3.486	1.378	0.221	0.361	0	0	7.29	0	7	15	3	1294.99658	3882.97519	-2.97
gIEFPMADLDALsPIHTPQR	Unambiguous	6	1	Q5SRX1-2	N-Term(iTRAQ8plex); S13(Phospho)	CID	1.0000	0.0000	1	1	3173615	Unique	Used	1.221	0.628	0.566	0.750	0	0	7.29	0	25	23	3	864.76465	2592.27939	-1.61
lTLYDIAHTPGVAADLSHIETR	Unambiguous	1	1	P08249	N-Term(iTRAQ8plex)	CID	1.0000	0.0000	1	1	3460703	Unique	Used	2.924	0.704	0.508	0.749	0	0	7.29	0	19	200	3	899.81677	2697.43576	-5.80

gLVHAEQGQGSGSPTGSSTR	Unambiguous	2	1	Q3URS9-2	N-Term(iTRAQ8plex)	CID	1.0000	0.0000	1	1	3269825	Unique	Used	0.987	0.973	1.089	0.924	0	0	7.29	0	0	200	3	739.70911	2217.11277	-1.55
vGELTQEAnALAAGHPAQAPAINTR	Unambiguous	1	1	Q68FG2	N-Term(iTRAQ8plex); N9(Deamidated)	HCD	1.0000	0.0000	1	1	3147592	Unique	Used	0.712	2.909	2.397	1.471	0	0	7.29	0	18	16	3	935.83014	2805.47586	-1.39
eVLGHSIAQLNAENDHPFYYk	Unambiguous	4	1	O08677	N-Term(iTRAQ8plex); K21(iTRAQ8plex)	CID	1.0000	0.0000	1	1	3345204	Unique	Used	2.885	1.569	0.505	2.440	0	0	7.29	0	12	21	3	1018.53229	3053.58231	-3.87
dILQDVLDADLSNEAFPFSTHQLVR	Unambiguous	1	1	Q99LB7	N-Term(iTRAQ8plex)	CID	1.0000	0.0000	1	1	3419145	Unique	Used				1.114	0	0	7.29	0	0	27	3	1049.87524	3147.61118	-4.87
aSGSAGAAASPSAAAAGER	Unambiguous	2	1	P83093	N-Term(iTRAQ8plex)	CID	1.0000	0.0000	1	1	2841387	Unique	Used	0.611	1.545	1.699	1.191	0	0	7.29	0	0	200	2	932.47601	1863.94475	-0.83
tTTNVLGDSLGAGIVEHLSR	Unambiguous	1	1	P56564	N-Term(iTRAQ8plex)	CID	1.0000	0.0000	1	1	3704620	Unique	Used	0.470	1.458	0.848	1.224	0	0	7.29	0	15	200	2	1172.63599	2344.26470	-5.45
gAALDVHESEPFSFSQGPLk	Unambiguous	3	1	O88712-2	N-Term(iTRAQ8plex); K20(iTRAQ8plex)	CID	1.0000	0.0000	1	1	3234683	Unique	Used	0.803	1.477	1.427	1.226	0	0	7.29	0	3	13	3	908.81866	2724.44144	-1.45
hLNLAENSSYmHVYR	Unambiguous	1	1	Q61644	N-Term(iTRAQ8plex); M11(Oxidation)	CID	1.0000	0.0000	1	1	3570705	Unique	Used	1.196	1.151	1.650	1.237	0	0	7.28	0	7	98	3	718.69135	2154.05948	-5.07
lQVLELDHVSEITQEVAAEVcR	Unambiguous	1	1	Q6NS60	N-Term(iTRAQ8plex); C21(Methylthio)	CID	1.0000	0.0000	1	1	3440445	Unique	Used	0.276	1.198	0.928	0.660	0	0	7.28	0	5	29	3	944.48767	2831.44846	-3.57
nLHQSGFSLSGAQIDDNIPR	Unambiguous	1	1	O08553	N-Term(iTRAQ8plex)	CID	1.0000	0.0000	1	1	3680849	Unique	Used	1.144	1.082	0.875	1.011	0	0	7.28	0	7	50	3	825.09015	2473.25589	-7.23
icDPGLTSFEPEALGNLVEGmDFHR	Unambiguous	9	1	Q5SVJ0	N-Term(iTRAQ8plex); C2(Methylthio); M21(Oxidation)	CID	1.0000	0.0000	1	1	3479727	Unique	Used	0.772	1.019	1.031	1.034	0	0	7.28	0	9	3	3	1038.49255	3113.46311	-1.73
kAAAGELQEDSGLHVLAR	Unambiguous	1	1	Q8C845	N-Term(iTRAQ8plex); K1(iTRAQ8plex)	HCD	1.0000	0.0000	1	1	3186429	Unique	Used	1.304	1.363	1.099	0.933	0	0	7.28	1	0	93	3	825.13770	2473.39853	0.07
tGVGYPQLSAVmEcADAAHGLk	Unambiguous	1	1	Q99L27	N-Term(iTRAQ8plex); M12(Oxidation); C14(Methylthio); K22(iTRAQ8plex)	CID	1.0000	0.0000	1	1	3579588	Unique	Used	1.544	0.664	0.714	0.962	0	0	7.28	0	13	82	3	963.48431	2888.43839	-6.29

aATTHLLQVLEDLEQAHEEFQk	Unambiguous	1	1	Q9Z268	N-Term(iTRAQ8plex); K22(iTRAQ8plex)	HCD	1.0000	0.0000	1	1	3326613	Unique	Used	0.318	2.822	1.459	1.101	0	0	7.28	0	6	16	3	1053.56702	3158.68650	-2.46
eLDPTnMTYITNQAAVHFEk	Unambiguous	1	1	Q60864	N-Term(iTRAQ8plex); N6(Deamidated); K20(iTRAQ8plex)	CID	1.0000	0.0000	1	1	3260495	Unique	Used	1.743	0.777	1.464	0.984	0	0	7.28	0	0	10	3	977.83649	2931.49491	-2.38
lDNNLDSVSLLIDHVcSR	Unambiguous	2	1	E0CYH4	N-Term(iTRAQ8plex); C16(Methylthio)	CID	1.0000	0.0000	1	1	3727062	Unique	Used	3.656	1.107	1.109	0.809	0	0	7.28	0	24	18	3	788.39972	2363.18460	-6.57
gQHAAEIqPLAQSHATk	Unambiguous	1	1	P04247	N-Term(iTRAQ8plex); Q8(Deamidated); K17(iTRAQ8plex)	CID	0.0110	0.0000	1	1	3181047	Unique	Used	4.276	6.455	0.953	1.215	0	0	7.28	0	0	200	3	799.44812	2396.32981	6.48
nQGGYGGSSSSSSyGSGR	Unambiguous	3	1	Q5EBP8	N-Term(iTRAQ8plex); Y14(iTRAQ8plex)	HCD	1.0000	0.0000	1	1	3116923	Unique	Used	1.235	0.639	0.749	0.646	0	0	7.28	0	7	200	3	768.37329	2303.10532	-2.41
fLSQPFQVAEVFTGHMGk	Unambiguous	1	1	P56480	N-Term(iTRAQ8plex); K18(iTRAQ8plex)	CID	1.0000	0.0000	1	1	3863544	Unique	Used	1.251	0.764	0.768	1.023	0	0	7.28	0	18	200	3	877.80878	2631.41178	-3.66
tVFEALQAPAcHENLVk	Unambiguous	1	1	P17427	N-Term(iTRAQ8plex); C11(Methylthio); K17(iTRAQ8plex)	CID	1.0000	0.0000	1	1	3517578	Unique	Used	0.214	1.275	1.060	1.071	0	0	7.28	0	6	2	3	842.11798	2524.33939	-4.71
dLVVLLFETALLSSGFSLEDPQTHSNR	Unambiguous	1	1	P11499	N-Term(iTRAQ8plex)	CID	1.0000	0.0000	1	1	3843356	Unique	Used	1.300	1.176	0.499	2.201	0	0	7.28	0	0	200	3	1098.25366	3292.74643	2.93
dAQVGWIQIVEDLHER	Unambiguous	2	1	E9QKE4	N-Term(iTRAQ8plex)	CID	1.0000	0.0000	1	1	3132506	Unique	Used	0.564	1.157	1.038	1.200	0	0	7.28	0	0	17	3	738.05957	2212.16416	-1.04
vNIHGGAVSLGHPIGMSGAR	Unambiguous	1	1	Q8QZT1	N-Term(iTRAQ8plex)	CID	1.0000	0.0000	1	1	3909863	Unique	Used	1.047	0.811	0.782	0.812	0	0	7.28	0	0	200	3	745.40442	2234.19870	-6.42
iGGELEEQAVmLDDFSHELESTQSR	Unambiguous	2	1	Q9JKK1	N-Term(iTRAQ8plex); M11(Oxidation)	CID	1.0000	0.0000	1	1	2816086	Unique	Used	1.194	1.065	0.978	1.280	0	0	7.28	0	2	40	3	1047.50488	3140.50010	0.14
aTSSFSQSVSSLFGEDNVR	Unambiguous	1	1	Q8BXV2	N-Term(iTRAQ8plex)	CID	1.0000	0.0000	1	1	3559871	Unique	Used	0.418	0.885	0.571	0.844	0	0	7.27	0	0	5	2	1161.57410	2322.14092	-4.59

sGILEQTGATTGVLQSDTmDHYR	Unambiguous	2	1	Q9JI19	N-Term(iTRAQ8plex); M19(Oxidation)	CID	1.0000	0.0000	1	1	3299295	Unique	Used	1.021	1.464	1.279	2.034	0	0	7.27	0	0	76	3	934.12567	2800.36246	-3.61
gTGGVDTAATGSVFDISNLDR	Unambiguous	1	1	P30275	N-Term(iTRAQ8plex)	CID	1.0000	0.0000	1	1	3260319	Unique	Used	0.973	1.097	1.174	2.217	0	0	7.27	0	0	63	2	1179.09534	2357.18340	-2.26
aLIGMTAGATGAFVGTPAEVALIR	Unambiguous	2	1	Q9CR62	N-Term(iTRAQ8plex)	CID	1.0000	0.0000	1	1	3917565	Unique	Used		1.151	1.426	1.474	0	0	7.27	0	7	17	2	1296.22498	2591.44267	-4.13
sMDSYLNQSYGMDNHSGGGGGSR	Unambiguous	1	1	O88291	N-Term(iTRAQ8plex)	CID	1.0000	0.0000	1	1	3186159	Unique	Used	1.629	1.004	1.446	1.145	0	0	7.27	0	0	84	3	894.39050	2681.15696	-1.98
gLTIVGSVLEGTFLDNHPQAQR	Unambiguous	2	1	Q91V14	N-Term(iTRAQ8plex)	CID	1.0000	0.0000	1	1	3844923	Unique	Used		0.560	1.017	1.395	0	0	7.27	0	26	200	3	886.14740	2656.42765	-3.18
lQQQAALSPTTAPAVSSVSk	Unambiguous	1	1	Q8VHR5	N-Term(iTRAQ8plex); K20(iTRAQ8plex)	CID	1.0000	0.0000	1	1	3595486	Unique	Used	0.695	0.685	1.183	0.661	0	0	7.27	0	0	136	2	1296.73889	2592.47051	-4.34
qGSSSVSGSPAPSTSGTSTPR	Unambiguous	2	1	P97445	N-Term(iTRAQ8plex)	CID	1.0000	0.0000	1	1	3224407	Unique	Used	0.788	0.730	2.188	1.934	0	0	7.27	0	0	69	2	1120.05920	2239.11113	0.31
aAEDSNLISGFGVAEGSEk	Unambiguous	2	1	Q3UHJ0-2	N-Term(iTRAQ8plex); K19(iTRAQ8plex)	CID	1.0000	0.0000	1	1	2813097	Unique	Used	1.777	1.684	1.907	0.859	0	0	7.27	0	15	200	2	1245.14771	2489.28813	-3.98
aAEAGVTEEQYGYLGTSAk	Unambiguous	1	1	P14873	N-Term(iTRAQ8plex); K19(iTRAQ8plex)	CID	1.0000	0.0000	1	1	2847865	Unique	Used	1.324	0.824	1.265	0.853	0	0	7.27	0	0	70	2	1277.16394	2553.32060	-3.41
sNVYQPTEMAVVLnGGTIPTAPPSHTGR	Unambiguous	1	1	Q923Z0	N-Term(iTRAQ8plex); N14(Deamidated)	CID	1.0000	0.0000	1	1	3497383	Unique	Used	1.351	1.170	1.113	1.174	0	0	7.27	0	0	79	3	1067.21118	3199.61899	-5.32
vADVSISEVTTLLGnVHSPVVEGYVGENISGEVk	Unambiguous	3	1	P20357	N-Term(iTRAQ8plex); N15(Deamidated); K34(iTRAQ8plex)	CID	1.0000	0.0000	1	1	3419876	Unique	Used	2.105	2.300	1.228	0.643	0	0	7.27	0	0	110	3	1369.73730	4107.19736	0.36
yEVLTAEQILQHMVEclR	Unambiguous	2	1	Q9Z1K5	N-Term(iTRAQ8plex); C16(Methylthio)	CID	1.0000	0.0000	1	1	3806605	Unique	Used	0.949	0.892	0.729	0.750	0	0	7.27	0	0	61	3	842.43030	2525.27634	-4.16
gNAGQTNYGFANSTMER	Unambiguous	1	1	P19096	N-Term(iTRAQ8plex)	CID	1.0000	0.0000	1	1	3143623	Unique	Used	0.680	0.526	0.793	1.171	0	0	7.26	0	0	147	2	1061.49792	2121.98857	-1.88
hQGGWTDGGSGSGGGYqDGAYR	Unambiguous	1	1	Q3TJZ6	N-Term(iTRAQ8plex); Q17(Deamidated)	CID	1.0000	0.0000	1	1	2789602	Unique	Used	1.193	0.419	0.892	0.813	0	0	7.26	0	0	200	3	825.70422	2475.09812	4.75

iLEAHQNVAQmSLIEAk	Unambiguous	1	1	Q8CIB5	N-Term(iTRAQ8plex); M11(Oxidation); K17(iTRAQ8plex)	CID	1.0000	0.0000	1	1	3453746	Unique	Used	0.770	1.009	1.006	1.086	0	0	7.26	0	0	145	3	840.46991	2519.39518	-6.37
vADVSISEVTTLLGNVHSPVVEGYVGENISGEVk	Unambiguous	3	1	P20357	N-Term(iTRAQ8plex); K34(iTRAQ8plex)	CID	1.0000	0.0000	1	1	3420348	Unique	Used	1.572	1.433	1.543	0.717	0	0	7.26	0	10	200	3	1369.40063	4106.18735	-5.97
hNHDLSSYQDTIQQLENELR	Unambiguous	2	1	P08553	N-Term(iTRAQ8plex)	CID	1.0000	0.0000	1	1	3236947	Unique	Used	2.330	0.506	1.059	0.840	0	0	7.26	0	0	22	3	915.45380	2744.34684	-2.68
sNVASTAAcSSASSLGLGLAYR	Unambiguous	1	1	P46660	N-Term(iTRAQ8plex); C9(Methylthio)	CID	1.0000	0.0000	1	1	3915729	Unique	Used	1.885	0.358	2.236	2.116	0	0	7.26	0	0	155	2	1218.60742	2436.20757	-3.67
tGPAGGFQAAPHTAAPYSQAAPAR	Unambiguous	1	1	P47708	N-Term(iTRAQ8plex)	CID	1.0000	0.0000	1	1	3656175	Unique	Used	1.441	0.514	1.243	0.927	0	0	7.26	0	0	48	3	867.10907	2599.31266	-7.43
sSGSPYGGGYGSGGGSGGYGSR	Unambiguous	7	1	J3QNY1	N-Term(iTRAQ8plex)	CID	1.0000	0.0000	1	1	3382905	Unique	Used	1.331	0.982	1.194	0.680	0	0	7.26	0	0	200	2	1108.00220	2214.99712	0.77
rNQDEAPQSGMnGLPk	Unambiguous	1	1	Q99M08	N-Term(iTRAQ8plex); N12(Deamidated); K16(iTRAQ8plex)	CID	1.0000	0.0000	1	1	2773847	Unique	Used	0.841	0.686	0.778	0.727	0	0	7.26	1	10	200	3	784.41571	2351.23258	3.89
qTTAAAAATFSEQVGGGSGGAGR	Unambiguous	3	1	O88935	N-Term(iTRAQ8plex)	CID	1.0000	0.0000	1	1	3494418	Unique	Used	0.833	1.773	1.839	1.496	0	0	7.26	0	0	179	2	1178.58972	2356.17217	-3.14
vATSAASGGHGSPLR	Unambiguous	4	1	A2AG50	N-Term(iTRAQ8plex)	CID	1.0000	0.0000	1	1	3631168	Unique	Used	1.209	0.494	1.646	1.116	0	0	7.26	0	13	25	3	557.97083	1671.89792	-6.05
dMAIATGGAVFGEEGLnLNLEDVQAHDLGk	Unambiguous	1	1	P63038	N-Term(iTRAQ8plex); N17(Deamidated); K30(iTRAQ8plex)	CID	0.0372	0.0000	1	1	3285714	Unique	Used	1.219	1.560	0.768	1.003	0	0	7.26	0	0	200	3	1231.97314	3693.90488	4.25
aGQAQADLETQYSALQQR	Unambiguous	2	1	E9QAH1	N-Term(iTRAQ8plex)	CID	1.0000	0.0000	1	1	3189229	Unique	Used	2.106	1.036	0.797	0.600	0	0	7.26	0	0	88	2	1141.58875	2282.17021	1.02
dIPMPLVSSGVEHGNLR	Unambiguous	2	1	Q9CZX0	N-Term(iTRAQ8plex)	CID	1.0000	0.0000	1	1	3412413	Unique	Used	0.960	0.789	0.945	1.202	0	0	7.26	0	0	47	3	709.04749	2125.12790	-4.66
aEAGAAATTASEAGAEAGAR	Unambiguous	1	1	E9PWM3	N-Term(iTRAQ8plex)	CID	1.0000	0.0000	1	1	2632108	Unique	Used	1.454	0.925	0.992	0.730	0	0	7.26	0	0	200	2	1019.00909	2037.01091	-2.07
sASSSAAGSPAGLTSLQQQk	Unambiguous	3	1	Q8CHS8	N-Term(iTRAQ8plex); K20(iTRAQ8plex)	CID	1.0000	0.0000	1	1	3408241	Unique	Used	0.574	1.099	1.119	1.157	0	0	7.26	0	0	129	2	1242.67395	2484.34062	-4.36

dHLFEESTFAPYIDELSDEQHR	Unambiguous	1	1	Q8K0T0	N-Term(iTRAQ8plex)	CID	1.0000	0.0000	1	1	2815305	Unique	Used	3.756	4.653	2.163	1.623	0	0	7.26	0	6	145	3	994.80719	2982.40702	0.35
aTSSSSSSSGVGMSVGQGR	Unambiguous	2	1	P21619	N-Term(iTRAQ8plex)	CID	1.0000	0.0000	1	1	3404788	Unique	Used	0.935	1.083	1.770	1.541	0	0	7.26	0	3	21	2	1016.99268	2032.97807	-4.47
aTSSSSSSSGVGMSVGQGR	Unambiguous	2	1	P21619	N-Term(iTRAQ8plex)	CID	1.0000	0.0000	1	1	3404623	Unique	Used	0.802	0.847	1.429	1.455	0	0	7.25	0	0	185	2	1016.99249	2032.97771	-4.65
lLEVSDDPQVLAVAAHDVGEYVR	Unambiguous	1	1	Q8BVE3	N-Term(iTRAQ8plex)	CID	1.0000	0.0000	1	1	3241578	Unique	Used	1.483	1.540	1.696	1.451	0	0	7.25	0	0	200	3	933.82922	2799.47312	-3.57
yTPSGQSGAAASESLFISNHAY	Unambiguous	2	1	A6ZI44	N-Term(iTRAQ8plex)	CID	1.0000	0.0000	1	1	3235690	Unique	Used	1.216	1.217	1.285	1.388	0	0	7.25	0	11	83	2	1281.62305	2562.23882	-1.02
dYNFVnGScMLYEANHVR	Unambiguous	1	1	Q9DBB8	N-Term(iTRAQ8plex); N6(Deamidated); C9(Methylthio)	CID	1.0000	0.0000	1	1	3281766	Unique	Used	1.260	1.141	1.119	1.408	0	0	7.25	0	21	112	3	828.37256	2483.10312	-2.60
wITDNQDSADVDPQEHSFTR	Unambiguous	1	1	E9PZP8	N-Term(iTRAQ8plex)	CID	1.0000	0.0000	1	1	2716185	Unique	Used	0.964	0.723	1.244	0.826	0	0	7.25	0	7	200	3	889.08606	2665.24363	0.14
gVVVFGEPITASLGTDGSHYWSk	Unambiguous	3	1	O35098	N-Term(iTRAQ8plex); K23(iTRAQ8plex)	CID	1.0000	0.0000	1	1	3580191	Unique	Used	0.868	0.611	0.699	0.643	0	0	7.25	0	7	48	3	1005.86853	3015.59104	-4.19
iSFEEFcAVVGGLDIHk	Unambiguous	2	1	Q63810-2	N-Term(iTRAQ8plex); C7(Methylthio); K17(iTRAQ8plex)	CID	1.0000	0.0000	1	1	3707951	Unique	Used	0.691	1.348	2.209	1.046	0	0	7.25	0	0	200	3	840.10974	2518.31467	-5.88
rAPPGPLPPAASPGPPAAsPPAAPSSPR	Unambiguous	1	1	O88703	N-Term(iTRAQ8plex); S19(Phospho)	CID	1.0000	0.0000	1	1	3387129	Unique	Used	0.999	0.745	1.062	1.028	0	0	7.25	1	0	58	3	984.84808	2952.52970	-3.48
dGDVTITnDGATILSMMDVDHQIAk	Unambiguous	2	1	P80316	N-Term(iTRAQ8plex); N8(Deamidated); K25(iTRAQ8plex)	CID	1.0000	0.0000	1	1	3111799	Unique	Used	0.927	1.370	1.104	1.015	0	0	7.25	0	0	200	3	1090.55957	3269.66416	4.61
dNLTLWTSDMQGDGEEQNk	Unambiguous	1	1	P62259	N-Term(iTRAQ8plex); K19(iTRAQ8plex)	CID	1.0000	0.0000	1	1	2670585	Unique	Used	3.738				0	0	7.25	0	28	200	3	930.45428	2789.34830	-0.93

tVYFAEEVqcEGNSFHk	Unambiguous	1	1	P97315	N-Term(iTRAQ8plex); Q9(Deamidated); C10(Methylthio); K17(iTRAQ8plex)	CID	1.0000	0.0000	1	1	3258201	Unique	Used	1.253	0.938	1.471	1.076	0	0	7.25	0	0	64	3	881.76520	2643.28104	4.94
gGPPPPPPPPHSSGPPPPPAR	Unambiguous	1	1	Q91YD9	N-Term(iTRAQ8plex)	CID	1.0000	0.0000	1	1	3448449	Unique	Used	1.306	0.655	1.432	1.164	0	0	7.24	0	0	200	3	765.41339	2294.22562	-4.01
fLSQPFQVAEVFTGHMGk	Unambiguous	1	1	P56480	N-Term(iTRAQ8plex); K18(iTRAQ8plex)	CID	1.0000	0.0000	1	1	3863998	Unique	Used	1.166	0.717	1.038	1.193	0	0	7.24	0	4	200	3	877.81104	2631.41855	-1.09
qVHDEEVAELLATLQASSQAAAEVDVAVAkPDLTSALR	Unambiguous	1	1	P46660	N-Term(iTRAQ8plex); K30(iTRAQ8plex)	CID	1.0000	0.0000	1	1	3479880	Unique	Used	1.680		2.533	1.803	0	0	7.24	0	8	13	3	1518.81897	4554.44236	-1.96
tIGGGDDSFNTFFSETGAGk	Unambiguous	4	1	P68373	N-Term(iTRAQ8plex); K20(iTRAQ8plex)	CID	1.0000	0.0000	1	1	3172148	Unique	Used		1.234	1.318	1.484	0	0	7.24	0	0	200	2	1308.65344	2616.29961	-1.63
kGSGMAEQDGGLIGAEEk	Unambiguous	1	1	Q06335	N-Term(iTRAQ8plex); K1(iTRAQ8plex); K18(iTRAQ8plex)	CID	1.0000	0.0000	1	1	2666092	Unique	Used	0.875	0.803	1.084	0.741	0	0	7.24	1	0	200	3	897.15436	2689.44852	-4.06
gNDISSGTVLSDYVGSGPPSGTGLHR	Unambiguous	1	1	P70296	N-Term(iTRAQ8plex)	CID	1.0000	0.0000	1	1	3389755	Unique	Used	1.640	1.163	1.705	1.175	0	0	7.24	0	0	31	3	945.47412	2834.40781	-5.11
gAASFGTSTAMAALELTNR	Unambiguous	2	1	Q80XK6	N-Term(iTRAQ8plex)	CID	1.0000	0.0000	1	1	3805331	Unique	Used	0.685	0.880	0.992	1.224	0	0	7.24	0	0	110	2	1087.06165	2173.11601	-3.01
yTPSGQSGAAASESLFISNHAY	Unambiguous	2	1	A6ZI44	N-Term(iTRAQ8plex)	CID	1.0000	0.0000	1	1	3257801	Unique	Used	1.064	0.809	1.346	1.215	0	0	7.24	0	0	11	2	1281.62134	2562.23540	-2.36
sEMEMAHLYSLcDAAHAQTEVAk	Unambiguous	1	1	Q9CZW5	N-Term(iTRAQ8plex); C12(Methylthio); K23(iTRAQ8plex)	CID	1.0000	0.0000	1	1	3348191	Unique	Used	0.638	1.128	1.109	1.100	0	0	7.24	0	17	13	3	1063.84460	3189.51926	-3.31
nQDEcVIALHDcnGDVNR	Unambiguous	5	1	Q80X50	N-Term(iTRAQ8plex); C5(Methylthio); C12(Methylthio); N13(Deamidated)	CID	1.0000	0.0000	1	1	2702556	Unique	Used	1.817	1.048	0.923	1.269	0	0	7.24	0	4	200	3	804.68152	2412.03000	-2.26

sAAQAAAQTNSnAAGk	Unambiguous	4	1	Q9CY58	N-Term(iTRAQ8plex); N12(Deamidated); K16(iTRAQ8plex)	CID	0.0124	0.0000	1	1	2859985	Unique	Used	0.644	0.312	1.148	0.540	0	0	7.24	0	0	107	2	1035.55078	2070.09429	-4.51
dMHGDSEYNImFGPDIcGPGTk	Unambiguous	1	1	P14211	N-Term(iTRAQ8plex); M11(Oxidation); C17(Methylthio); K22(iTRAQ8plex)	CID	1.0000	0.0000	1	1	3170581	Unique	Used	1.464	0.943	0.721	0.602	0	0	7.24	0	0	27	3	1018.80011	3054.38578	-2.25
qLNTIQNAEAVHFFcNYEESR	Unambiguous	4	1	P61922	N-Term(iTRAQ8plex); C15(Methylthio)	CID	1.0000	0.0000	1	1	3438860	Unique	Used	1.628	0.506	0.656	1.158	0	0	7.24	0	1	7	3	955.11603	2863.33353	-3.85
aGYSQGATQYTQAQQAR	Unambiguous	2	1	O88532	N-Term(iTRAQ8plex)	CID	1.0000	0.0000	1	1	3204937	Unique	Used	0.959	0.626	1.070	0.967	0	0	7.24	0	0	12	2	1067.03259	2133.05791	-2.23
sELEAQTGLQILQTGVGQR	Unambiguous	2	1	Q60673	N-Term(iTRAQ8plex)	CID	1.0000	0.0000	1	1	3477880	Unique	Used	0.785	0.864	0.801	1.562	0	0	7.24	0	9	43	2	1166.63806	2332.26885	-3.69
mkDVPAFLQQSqSSGPGQAAVWHR	Unambiguous	4	1	Q9WVJ2	N-Term(iTRAQ8plex); K2(iTRAQ8plex); Q12(Deamidated)	CID	0.0055	0.0000	1	1	3856513	Unique	Used	0.907	1.393	0.751	0.910	0	0	7.24	1	0	200	3	1078.90674	3234.70566	3.63
sLHEMSPSDSSPSPQDATSPQLATTR	Unambiguous	4	1	G3UWG2	N-Term(iTRAQ8plex)	CID	1.0000	0.0000	1	1	3029065	Unique	Used	0.933	1.983	0.969	0.818	0	0	7.23	0	0	200	3	1011.15753	3031.45804	-0.02
tALAEAELEYNPEHVSR	Unambiguous	3	1	Q8BIJ6	N-Term(iTRAQ8plex)	CID	1.0000	0.0000	1	1	2811526	Unique	Used	1.186	1.062	0.859	0.901	0	0	7.23	0	2	42	3	745.05096	2233.13834	-0.87
iSIEMnGTLEDQLSHLk	Unambiguous	1	1	P57780	N-Term(iTRAQ8plex); N6(Deamidated); K17(iTRAQ8plex)	CID	1.0000	0.0000	1	1	3238267	Unique	Used	0.911	2.814	1.380	0.776	0	0	7.23	0	9	40	3	846.46252	2537.37302	-0.46
iVLAAAGGVcHNELLELAk	Unambiguous	1	1	Q9CXT8	N-Term(iTRAQ8plex); C10(Methylthio); K19(iTRAQ8plex)	CID	1.0000	0.0000	1	1	3726777	Unique	Used	0.738	0.781	0.577	0.839	0	0	7.23	0	13	9	3	859.15265	2575.44339	-4.93
iLDIGLAYVNHLVER	Unambiguous	1	1	Q5KU39	N-Term(iTRAQ8plex)	CID	1.0000	0.0000	1	1	3879959	Unique	Used	0.443	0.712	0.606	1.074	0	0	7.23	0	0	47	3	677.06165	2029.17038	-2.18

eQSGEYEcSASNDVAAPVVR	Unambiguous	5	1	D3Z396	N-Term(iTRAQ8plex); C8(Methylthio)	CID	1.0000	0.0000	1	1	2684702	Unique	Used	0.561	1.017	1.340	1.315	0	0	7.23	0	3	200	2	1231.06677	2461.12627	-0.61
tGEMSGPVFTDSGIHIILR	Unambiguous	1	1	Q9QUR7	N-Term(iTRAQ8plex)	CID	1.0000	0.0000	1	1	3704766	Unique	Used	1.265	1.032	0.999	1.007	0	0	7.23	0	26	85	3	778.74701	2334.22647	-7.08
yHTSQSGDEmTSLSEYVSR	Unambiguous	2	1	P11499	N-Term(iTRAQ8plex); M10(Oxidation)	CID	1.0000	0.0000	1	1	3122586	Unique	Used	1.263	1.016	0.972	0.996	0	0	7.23	0	8	39	3	833.05261	2497.14328	-0.88
yTPSGQSGAAASESLFISNHAY	Unambiguous	2	1	A6ZI44	N-Term(iTRAQ8plex)	CID	1.0000	0.0000	1	1	3257457	Unique	Used	1.424	0.937	0.917	0.848	0	0	7.23	0	0	45	2	1281.62146	2562.23564	-2.26
fTLNPNTTGVQNPHIER	Unambiguous	2	1	P47809	N-Term(iTRAQ8plex)	CID	1.0000	0.0000	1	1	3594101	Unique	Used	1.450	1.098	0.965	0.873	0	0	7.23	0	25	15	3	748.06378	2242.17679	-5.10
nLHQSGFSLSGAQIDDNIPR	Unambiguous	1	1	O08553	N-Term(iTRAQ8plex)	CID	1.0000	0.0000	1	1	3514247	Unique	Used	1.080	1.221	1.070	1.112	0	0	7.23	0	16	116	3	825.09351	2473.26596	-3.16
fQPGETLTEILETPATNEQEAEHQR	Unambiguous	1	1	Q9JKF1	N-Term(iTRAQ8plex)	CID	1.0000	0.0000	1	1	2834154	Unique	Used	1.165	1.276	0.915	1.309	0	0	7.23	0	3	176	3	1058.19592	3172.57322	0.98
qTTAAAAATFSEQVGGGSGGAGR	Unambiguous	3	1	O88935	N-Term(iTRAQ8plex)	CID	1.0000	0.0000	1	1	3493768	Unique	Used	0.763	1.582	1.920	1.450	0	0	7.23	0	0	101	2	1178.58789	2356.16850	-4.69
iDHFPEDADYEQDTAEYLLR	Unambiguous	1	1	Q9JJV5	N-Term(iTRAQ8plex)	CID	1.0000	0.0000	1	1	2638680	Unique	Used		3.808	3.315	1.144	0	0	7.22	0	0	200	3	915.43878	2744.30179	0.88
kGTVVYGEPITASLGTDGSHYWSk	Unambiguous	1	1	O08553	N-Term(iTRAQ8plex); K1(iTRAQ8plex); K24(iTRAQ8plex)	CID	1.0000	0.0000	1	1	3412247	Unique	Used	1.527	1.160	1.695	2.704	0	0	7.22	1	0	63	3	1155.95862	3465.86130	-4.86
qLLHNFPPDQLTSSGAPFWSGPk	Unambiguous	1	1	Q02053	N-Term(iTRAQ8plex); K23(iTRAQ8plex)	CID	1.0000	0.0000	1	1	3841084	Unique	Used	1.059	1.364	0.820	0.872	0	0	7.22	0	6	119	3	1044.89319	3132.66501	-2.47
eHINLGcDVDFDIAGPSIR	Unambiguous	3	1	Q60932-2	N-Term(iTRAQ8plex); C7(Methylthio)	CID	1.0000	0.0000	1	1	3354737	Unique	Used	1.286	1.445	1.334	1.183	0	0	7.22	0	6	135	3	807.73016	2421.17594	-3.54
aASVAEncINYQQGTPHk	Unambiguous	1	1	P70202	N-Term(iTRAQ8plex); N7(Deamidated); C8(Methylthio); K18(iTRAQ8plex)	CID	1.0000	0.0000	1	1	3125597	Unique	Used	1.163	1.020	1.024	1.087	0	0	7.22	0	0	44	3	862.76392	2586.27719	-4.99
sENGLEFTSSGSANTETTk	Unambiguous	3	1	Q60932-2	N-Term(iTRAQ8plex); K19(iTRAQ8plex)	CID	1.0000	0.0000	1	1	2666549	Unique	Used	0.574	0.981	1.196	1.044	0	0	7.22	0	0	200	2	1284.64136	2568.27544	-5.12

sQTEEDcTEELFDFLHAR	Unambiguous	1	1	P99028	N-Term(iTRAQ8plex); C7(Methylthio)	CID	1.0000	0.0000	1	1	2871418	Unique	Used	2.645	0.959	1.621	0.853	0	0	7.22	0	7	51	3	840.71362	2520.12632	-2.46
vADVSISEVTTLLGNVHSPVVEGYVGENISGEVk	Unambiguous	3	1	P20357	N-Term(iTRAQ8plex); K34(iTRAQ8plex)	CID	1.0000	0.0000	1	1	3420594	Unique	Used	0.914	1.297	1.666	1.364	0	0	7.22	0	0	200	3	1369.40381	4106.19687	-3.65
dFTSEQIDEILQnHTEIVFAR	Unambiguous	2	1	Q6PIC6	N-Term(iTRAQ8plex); N13(Deamidated)	HCD	0.0402	0.0000	1	1	3111955	Unique	Used	0.837	1.077	1.192	1.418	0	0	7.22	0	24	65	3	937.47681	2810.41587	0.28
fTDTDLLDFGQcVDQNHQR	Unambiguous	1	1	Q9DCJ9	N-Term(iTRAQ8plex); C12(Methylthio)	CID	1.0000	0.0000	1	1	3326402	Unique	Used	1.787	1.060	0.684	0.843	0	0	7.22	0	10	11	3	868.06799	2602.18943	-2.85
dAQVGWIQIVEDLHER	Unambiguous	2	1	E9QKE4	N-Term(iTRAQ8plex)	CID	1.0000	0.0000	1	1	3132377	Unique	Used	0.914	1.007	1.256	1.128	0	0	7.22	0	10	173	3	738.05957	2212.16416	-1.04
qTTAAAAATFSEQVGGGSGGAGR	Unambiguous	3	1	O88935	N-Term(iTRAQ8plex)	CID	1.0000	0.0000	1	1	3493332	Unique	Used	0.535	1.429	2.064	1.705	0	0	7.22	0	8	40	2	1178.58789	2356.16850	-4.69
gnDISSGTVLSDYVGSGPPSGTGLHR	Unambiguous	1	1	P70296	N-Term(iTRAQ8plex); N2(Deamidated)	CID	1.0000	0.0000	1	1	3596840	Unique	Used	1.917	2.312	0.943	1.364	0	0	7.22	0	0	72	3	945.80737	2835.40757	0.44
dHFMDEFFEQVEEIR	Unambiguous	1	1	P61264	N-Term(iTRAQ8plex)	CID	1.0000	0.0000	1	1	2834981	Unique	Used	1.002	0.897	1.136	1.189	0	0	7.22	0	22	74	3	759.02533	2275.06144	-1.29
aVVFETGEAGAASGAGSLPGEVR	Unambiguous	3	1	Q9QYR6	N-Term(iTRAQ8plex)	CID	1.0000	0.0000	1	1	3108090	Unique	Used	2.519	0.963	1.657	0.923	0	0	7.22	0	0	200	2	1218.63489	2436.26250	-1.98
nLHQSGFSLSGAQIDDNIPR	Unambiguous	1	1	O08553	N-Term(iTRAQ8plex)	CID	1.0000	0.0000	1	1	3412110	Unique	Used	1.092	0.912	1.149	2.938	0	0	7.22	0	20	19	3	825.09296	2473.26432	-3.82
dFTSEQIDEILQNHTEIVFAR	Unambiguous	2	1	Q6PIC6	N-Term(iTRAQ8plex)	CID	1.0000	0.0000	1	1	3356552	Unique	Used	0.475	0.542	1.161	1.592	0	0	7.22	0	3	200	3	937.14648	2809.42490	-2.20
hSAEVAGYQDSIGQLESDLR	Unambiguous	1	1	P46660	N-Term(iTRAQ8plex)	CID	1.0000	0.0000	1	1	2901493	Unique	Used	2.793				0	0	7.21	0	0	0	3	827.08069	2479.22751	-3.71
aFVEVNEEGTEAAAATAGMMTVR	Unambiguous	2	1	F8WIV2	N-Term(iTRAQ8plex)	CID	1.0000	0.0000	1	1	3152972	Unique	Used	0.601	2.226	1.215	1.366	0	0	7.21	0	23	200	2	1330.15222	2659.29717	-1.45
vIHLSNLPHSGYSDSAVLk	Unambiguous	1	1	Q8K310	N-Term(iTRAQ8plex); K19(iTRAQ8plex)	CID	1.0000	0.0000	1	1	3659202	Unique	Used	0.443	0.733	0.922	1.041	0	0	7.21	0	0	44	3	882.49603	2645.47354	-5.14
aISEAGAPPGSAGPLLLQPQQHR	Unambiguous	1	1	Q99MX7	N-Term(iTRAQ8plex)	CID	1.0000	0.0000	1	1	3659887	Unique	Used	1.670	1.757	1.403	0.908	0	0	7.21	0	0	40	3	867.14215	2599.41190	-5.38
tNIVTASVDAINFHDk	Unambiguous	5	1	Q91V12-2	N-Term(iTRAQ8plex); K16(iTRAQ8plex)	CID	1.0000	0.0000	1	1	3346734	Unique	Used	0.783	0.678	0.791	0.922	0	0	7.21	0	0	32	2	1177.14355	2353.27983	-7.39

sLAESGLNWFSESEEk	Unambiguous	4	1	Q8CH77	N-Term(iTRAQ8plex); K16(iTRAQ8plex)	CID	1.0000	0.0000	1	1	2996469	Unique	Used	0.993	0.297	0.943	0.526	0	0	7.21	0	12	200	2	1211.12451	2421.24175	0.95
qHLNNMEAQLASQSTQR	Unambiguous	2	1	Q7M739	N-Term(iTRAQ8plex)	CID	1.0000	0.0000	1	1	3384972	Unique	Used	1.120	0.696	1.063	1.160	0	0	7.21	0	2	17	3	754.04779	2260.12882	-5.23
tIGGGDDSFNTFFSETGAGk	Unambiguous	4	1	P68373	N-Term(iTRAQ8plex); K20(iTRAQ8plex)	CID	1.0000	0.0000	1	1	3130302	Unique	Used	0.464	1.775	1.114	1.541	0	0	7.21	0	0	14	2	1308.65479	2616.30229	-0.60
kDVLPGAEGLTAVGGQAGNLAR	Unambiguous	2	1	A2AJ26	N-Term(iTRAQ8plex); K1(iTRAQ8plex)	CID	1.0000	0.0000	1	1	3556248	Unique	Used	0.484	0.516	1.000	1.226	0	0	7.21	1	0	21	3	901.51355	2702.52610	-5.53
rGAYGGGYGGYDDYGGYNDGYGFGSDR	Unambiguous	1	1	P70333	N-Term(iTRAQ8plex)	CID	1.0000	0.0000	1	1	2883007	Unique	Used	3.612	3.244	1.009	0.603	0	0	7.21	1	0	200	3	1040.77979	3120.32480	-1.61
aVQYSGAAVTSGGAAVPSGGAATPR	Unambiguous	1	1	Q6A058	N-Term(iTRAQ8plex)	CID	1.0000	0.0000	1	1	3657864	Unique	Used	1.813	0.665	0.976	1.382	0	0	7.20	0	0	71	2	1254.15515	2507.30303	-5.04
gITNLcVIGGDGSLTGADTFR	Unambiguous	1	1	P47857	N-Term(iTRAQ8plex); C6(Methylthio)	CID	1.0000	0.0000	1	1	3726746	Unique	Used	1.740	0.814	0.903	1.102	0	0	7.20	0	3	6	2	1209.10022	2417.19316	-7.26
iTQEVQGAHDGGVFALcALR	Unambiguous	5	1	E9QK48	N-Term(iTRAQ8plex); C17(Methylthio)	CID	1.0000	0.0000	1	1	3765601	Unique	Used	0.791	0.734	0.532	0.981	0	0	7.20	0	7	11	3	812.41760	2435.23825	-3.91
rHHQEEVGELLGqIQGcGAAQAQAQAEAR	Unambiguous	1	1	P19246	N-Term(iTRAQ8plex); Q13(Deamidated); C17(Methylthio)	CID	0.0431	0.0000	1	1	3434384	Unique	Used	1.716	0.711	1.038	1.051	0	0	7.20	1	0	113	3	1146.23071	3436.67759	0.67
tQPTDEEMLFIYSHFk	Unambiguous	4	1	P31786	N-Term(iTRAQ8plex); K16(iTRAQ8plex)	CID	1.0000	0.0000	1	1	3286939	Unique	Used	1.922	0.473	1.145	0.873	0	0	7.20	0	0	200	3	865.45087	2594.33805	-1.57
qEFLEGGVGHVEMLFR	Unambiguous	2	1	Q9CR39	N-Term(iTRAQ8plex)	CID	1.0000	0.0000	1	1	3560386	Unique	Used	1.625	0.848	1.052	0.993	0	0	7.20	0	25	85	3	718.04059	2152.10721	-4.24
iNMAHLcIAGSHAVnGVAR	Unambiguous	2	1	Q9WUB3	N-Term(iTRAQ8plex); C7(Methylthio); N15(Deamidated)	CID	1.0000	0.0000	1	1	3894165	Unique	Used	0.602	0.695	0.750	1.074	0	0	7.20	0	10	80	3	762.38812	2285.14981	-5.30
vQNATLAVANITNADSATR	Unambiguous	1	1	P54116	N-Term(iTRAQ8plex)	CID	1.0000	0.0000	1	1	3682704	Unique	Used	2.209	1.560	0.713	1.311	0	0	7.20	0	21	135	2	1117.59814	2234.18901	-6.84

ekQPDNFFAGGEDcVVMVAHESGR	Unambiguous	1	1	P55066	N-Term(iTRAQ8plex); K2(iTRAQ8plex); C14(Methylthio)	CID	1.0000	0.0000	1	1	3130746	Unique	Used	0.994	0.911	1.124	0.567	0	0	7.20	1	0	64	3	1092.86108	3276.56870	-0.32
kTESIDVMDAVGSNIVVSTR	Unambiguous	1	1	Q91VD9	N-Term(iTRAQ8plex); K1(iTRAQ8plex)	CID	1.0000	0.0000	1	1	3370181	Unique	Used	0.696	1.267	1.182	0.899	0	0	7.20	1	18	23	3	910.49823	2729.48014	-5.97
aTLQEVVGLHSDMAVEAGAPYAER	Unambiguous	1	1	G3UYZ1	N-Term(iTRAQ8plex)	CID	1.0000	0.0000	1	1	3302661	Unique	Used	0.694	0.393	1.066	0.657	0	0	7.20	0	0	158	3	940.14740	2818.42765	-2.54
qVDTEEAGMVTAATASNVk	Unambiguous	3	1	Q99JF8	N-Term(iTRAQ8plex); K19(iTRAQ8plex)	CID	1.0000	0.0000	1	1	2916844	Unique	Used	0.286	0.557	0.939	0.778	0	0	7.19	0	0	200	2	1265.66528	2530.32329	-1.85
lGALSGAGALGLASYGAHGAQFPDAYGk	Unambiguous	1	1	Q5F285	N-Term(iTRAQ8plex); K28(iTRAQ8plex)	HCD	1.0000	0.0000	1	1	3784696	Unique	Used	0.355	1.276	1.242	1.602	0	0	7.19	0	9	57	3	1076.90796	3228.70932	-5.25
sAATSGAGSTTSGVVSGSLGSR	Unambiguous	5	1	Q7TMY8-3	N-Term(iTRAQ8plex)	CID	1.0000	0.0000	1	1	3634354	Unique	Used	0.432	0.972	0.991	1.131	0	0	7.19	0	0	132	2	1101.06348	2201.11968	-5.23
dIGAIAQVHAEnGDIIAEEQQR	Unambiguous	1	1	O08553	N-Term(iTRAQ8plex); N12(Deamidated)	CID	0.0431	0.0000	1	1	2812096	Unique	Used	1.804	1.198	1.108	1.151	0	0	7.19	0	4	50	3	894.79193	2682.36124	-0.93
lMGSAGNATISTVSSTQR	Unambiguous	2	1	Q01815-2	N-Term(iTRAQ8plex)	CID	1.0000	0.0000	1	1	3873776	Unique	Used	0.373	1.408	1.186	0.922	0	0	7.19	0	8	164	2	1043.04614	2085.08501	-2.98
qkEVnENFAIDLIAQQPVNEVEHR	Unambiguous	2	1	P52503	N-Term(iTRAQ8plex); K2(iTRAQ8plex); N5(Deamidated)	CID	0.0292	0.0000	1	1	3129023	Unique	Used	2.838	1.197	1.451	1.154	0	0	7.19	1	0	59	3	1143.95203	3429.84153	5.59
iGIIDDDIFEEDEHFFVR	Unambiguous	1	1	Q8K596	N-Term(iTRAQ8plex)	CID	1.0000	0.0000	1	1	2816177	Unique	Used	0.799	3.219	2.687	1.764	0	0	7.19	0	4	118	3	838.42059	2513.24723	-1.21
vTVVcEPEDYAGVAAEMHGSDSk	Unambiguous	1	1	Q9CWJ9	N-Term(iTRAQ8plex); C5(Methylthio); K23(iTRAQ8plex)	CID	1.0000	0.0000	1	1	2939761	Unique	Used			0.855	1.220	0	0	7.19	0	9	137	3	1016.82166	3048.45041	-2.28
dIGAIAQVHAEnGDIIAEEQQR	Unambiguous	1	1	O08553	N-Term(iTRAQ8plex); N12(Deamidated)	CID	1.0000	0.0000	1	1	2636191	Unique	Used	1.893	1.557	1.336	1.679	0	0	7.19	0	13	142	3	894.79071	2682.35758	-2.30

IWGTSDVVQEIQEMk	Unambiguous	1	1	P32037	N-Term(iTRAQ8plex); K15(iTRAQ8plex)	CID	1.0000	0.0000	1	1	3239217	Unique	Used	0.351	1.107	1.459	1.283	0	0	7.19	0	10	26	2	1186.14197	2371.27666	-0.91
wFVVLEETPWDATSHIDNMENER	Unambiguous	1	1	Q91YM2	N-Term(iTRAQ8plex)	CID	1.0000	0.0000	1	1	3196303	Unique	Used	1.130	0.885	0.667	0.840	0	0	7.19	0	22	32	3	1041.49988	3122.48508	0.60
eQANAVSEAVVSSVNTVANk	Unambiguous	1	1	Q9Z0F7	N-Term(iTRAQ8plex); K20(iTRAQ8plex)	CID	1.0000	0.0000	1	1	3437244	Unique	Used	3.559	0.451	0.865	1.547	0	0	7.19	0	0	60	2	1313.21167	2625.41606	-5.48
mTTVLSESSGPSNASHSSSR	Unambiguous	1	1	Q80X80	N-Term(iTRAQ8plex); M1(Oxidation)	CID	1.0000	0.0000	1	1	3334267	Unique	Used	0.997	1.347	1.190	0.974	0	0	7.19	0	0	187	3	781.37634	2342.11448	-2.20
aEGATLSNATGAVESTSQAGDR	Unambiguous	1	1	Q61644	N-Term(iTRAQ8plex)	CID	1.0000	0.0000	1	1	2828142	Unique	Used	2.182	1.107	1.880	1.001	0	0	7.19	0	0	71	2	1199.08826	2397.16924	-4.33
mYGADLAPVDFLHASEDAR	Unambiguous	1	1	Q9D154	N-Term(iTRAQ8plex)	CID	1.0000	0.0000	1	1	3110618	Unique	Used	1.732	0.577	4.253	4.782	0	0	7.19	0	4	50	3	794.72729	2382.16733	-1.22
sQSVDITAPGFNPLGGAGk	Unambiguous	2	1	Q4KMM3	N-Term(iTRAQ8plex); K19(iTRAQ8plex)	CID	1.0000	0.0000	1	1	3516586	Unique	Used	0.985	1.156	2.397	1.781	0	0	7.19	0	11	42	2	1212.66589	2424.32451	-4.07
gGGGPGGGAPGGPGLGLGSLGEER	Unambiguous	1	1	Q69ZK9	N-Term(iTRAQ8plex)	CID	1.0000	0.0000	1	1	3232856	Unique	Used	0.491	1.005	0.960	1.157	0	0	7.19	0	5	25	2	1134.08740	2267.16753	-0.34
sSMVQVANITQAGLTHGINLAVAk	Unambiguous	3	1	Q8CDA1	N-Term(iTRAQ8plex); K24(iTRAQ8plex)	CID	1.0000	0.0000	1	1	3897758	Unique	Used	0.591	0.468	1.047	1.091	0	0	7.19	0	18	95	3	1011.24347	3031.71585	-0.82
nYLPAInGIVFLVDcADHER	Unambiguous	1	1	Q9CQC9	N-Term(iTRAQ8plex); N7(Deamidated); C15(Methylthio)	CID	1.0000	0.0000	1	1	3396260	Unique	Used			0.612	0.920	0	0	7.19	0	0	16	3	870.76855	2610.29111	-3.35
tTSSAIYMNLASHIQPGTVNR	Unambiguous	2	1	Q8VHR5	N-Term(iTRAQ8plex)	CID	1.0000	0.0000	1	1	3932795	Unique	Used	0.867	0.714	1.119	1.090	0	0	7.18	0	12	119	3	855.78229	2565.33231	-2.89
vGDSPcIGAGGYADNNLGAVSTTGHGESILk	Unambiguous	1	1	Q8C0M9	N-Term(iTRAQ8plex); C6(Methylthio); K31(iTRAQ8plex)	CID	1.0000	0.0000	1	1	3322942	Unique	Used	2.271	0.615	1.382	1.309	0	0	7.18	0	24	100	3	1205.60120	3614.78904	-4.15
eQAADTEAAQEVTQLLVER	Unambiguous	1	1	O54774	N-Term(iTRAQ8plex)	CID	1.0000	0.0000	1	1	2766088	Unique	Used	0.488	1.078	1.389	1.375	0	0	7.18	0	12	200	2	1203.12610	2405.24492	-0.54
vTYMVHDFEEGGGVAMGMYNQDk	Unambiguous	1	1	O88844	N-Term(iTRAQ8plex); K23(iTRAQ8plex)	CID	1.0000	0.0000	1	1	3132116	Unique	Used	0.426	0.577	0.523	0.474	0	0	7.18	0	0	11	3	1062.84143	3186.50974	-1.82

fLSqPFQVAEVFTGHMGk	Unambiguous	1	1	P56480	N-Term(iTRAQ8plex); Q4(Deamidated); K18(iTRAQ8plex)	CID	0.0237	0.0000	1	1	3881004	Unique	Used	0.843	0.956	1.038	1.176	0	0	7.18	0	0	200	3	878.14038	2632.40659	0.44
sAAQAAAQTNSNAAGk	Unambiguous	4	1	Q9CY58	N-Term(iTRAQ8plex); K16(iTRAQ8plex)	CID	1.0000	0.0000	1	1	3139928	Unique	Used	0.668	0.439	1.044	0.635	0	0	7.18	0	0	158	2	1035.06226	2069.11724	-1.14
sPSSTLSGPDLAGHSSSATSTPEELR	Unambiguous	1	1	E9PV26	N-Term(iTRAQ8plex)	CID	1.0000	0.0000	1	1	3009262	Unique	Used	2.582	1.881	2.928	1.240	0	0	7.18	0	0	153	3	959.14368	2875.41648	-2.05
sIDDLEDQLYHQLEQnR	Unambiguous	3	1	E9Q452	N-Term(iTRAQ8plex); N16(Deamidated)	CID	0.0056	0.0000	1	1	2765967	Unique	Used	2.109	1.401	1.785	0.878	0	0	7.18	0	1	116	3	807.73248	2421.18290	-0.29
mTDSFTEQADQVTADVGk	Unambiguous	3	1	Q8BVI4	N-Term(iTRAQ8plex); K18(iTRAQ8plex)	CID	1.0000	0.0000	1	1	2831913	Unique	Used	0.804	0.806	1.329	2.010	0	0	7.18	0	0	200	2	1276.14197	2551.27666	-1.58
iIEVVDAIMTTAQSHQR	Unambiguous	3	1	Q8C605	N-Term(iTRAQ8plex)	CID	1.0000	0.0000	1	1	3918103	Unique	Used	2.166	1.275	1.063	0.913	0	0	7.18	0	18	61	3	739.40106	2216.18863	-5.64
lENTEDIEEVEQHIQTIR	Unambiguous	3	1	Q8K0B2-3	N-Term(iTRAQ8plex)	CID	1.0000	0.0000	1	1	2687714	Unique	Used				2.239	0	0	7.18	0	4	200	3	834.09930	2500.28336	0.01
gNDISSGTVLSDYVGSGPPSGTGLHR	Unambiguous	1	1	P70296	N-Term(iTRAQ8plex)	CID	1.0000	0.0000	1	1	3454456	Unique	Used	1.409	1.180	1.050	1.004	0	0	7.18	0	0	200	3	945.47742	2834.41770	-1.62
tQPTDEEMLFIYSHFk	Unambiguous	4	1	P31786	N-Term(iTRAQ8plex); K16(iTRAQ8plex)	CID	1.0000	0.0000	1	1	3285937	Unique	Used	1.674	0.631	0.872	0.764	0	0	7.18	0	4	200	3	865.45056	2594.33713	-1.93
dVLTSTYGATAETLSTSTTTHVTk	Unambiguous	7	1	A2AUK8	N-Term(iTRAQ8plex); K24(iTRAQ8plex)	CID	1.0000	0.0000	1	1	3474967	Unique	Used	1.078	1.500	1.432	0.906	0	0	7.18	0	15	82	3	1031.88330	3093.63535	-1.89
yTPSGQSGAAASESLFISNHAy	Unambiguous	2	1	A6ZI44	N-Term(iTRAQ8plex); Y22(iTRAQ8plex)	CID	1.0000	0.0000	1	1	3367471	Unique	Used	0.886	1.244	1.038	1.258	0	0	7.18	0	0	65	3	956.14819	2866.43003	-5.85
eHAPSIIFmDEIDSIGSSR	Unambiguous	2	1	P62196	N-Term(iTRAQ8plex); M9(Oxidation)	CID	1.0000	0.0000	1	1	3170566	Unique	Used	1.108	1.167	1.276	1.276	0	0	7.18	0	2	34	3	808.73755	2424.19809	-1.58
tQAQHSSPGEmASSPQGLDNPALLR	Unambiguous	1	1	A2ADY9	N-Term(iTRAQ8plex); M11(Oxidation)	CID	1.0000	0.0000	1	1	3296293	Unique	Used	0.868	1.185	1.483	1.574	0	0	7.18	0	0	186	3	971.48444	2912.43875	-3.00

sIcTLQITDTTGSHQFPAMQR	Unambiguous	1	1	Q5PR73	N-Term(iTRAQ8plex); C3(Methylthio)	CID	1.0000	0.0000	1	1	3858142	Unique	Used	1.049	1.005	1.095	0.952	0	0	7.18	0	30	200	3	895.77252	2685.30301	-2.64
tLLAAGTTDSEGVATLPLSYR	Unambiguous	1	1	A2A699	N-Term(iTRAQ8plex)	CID	1.0000	0.0000	1	1	3539758	Unique	Used	0.988	1.442	1.002	0.889	0	0	7.18	0	0	92	2	1220.66016	2440.31304	-4.39
vTFLcGDAGPLAVGAVIYHk	Unambiguous	2	1	F6RJV6	N-Term(iTRAQ8plex); C5(Methylthio); K20(iTRAQ8plex)	CID	1.0000	0.0000	1	1	3879640	Unique	Used		1.392	0.976	0.789	0	0	7.17	0	18	20	3	895.82489	2685.46012	-4.33
sSGTGASVGPPQPSDQDTLVQR	Unambiguous	1	1	Q8CI51	N-Term(iTRAQ8plex)	CID	1.0000	0.0000	1	1	2952873	Unique	Used	1.684	1.360	0.951	0.722	0	0	7.17	0	0	78	2	1244.63086	2488.25444	-1.51
gLTIVGSVLEGTFLDnHPQAQR	Unambiguous	2	1	Q91V14	N-Term(iTRAQ8plex); N16(Deamidated)	CID	1.0000	0.0000	1	1	3843992	Unique	Used	1.030	1.437	1.018	0.637	0	0	7.17	0	21	200	3	886.48175	2657.43070	3.98
eHINLGcDVDFDIAGPSIR	Unambiguous	3	1	Q60932-2	N-Term(iTRAQ8plex); C7(Methylthio)	CID	1.0000	0.0000	1	1	3355113	Unique	Used	1.378	1.220	0.982	1.087	0	0	7.17	0	2	200	3	807.73071	2421.17759	-2.86
sQTEEDcTEELFDFLHAR	Unambiguous	1	1	P99028	N-Term(iTRAQ8plex); C7(Methylthio)	CID	1.0000	0.0000	1	1	2853473	Unique	Used	2.151	1.180	1.673	1.300	0	0	7.17	0	0	16	3	840.71509	2520.13071	-0.71
gVVGPGPAAIAAPGGGGAGPPAVGGGGGR	Unambiguous	1	1	Q3B7Z2	N-Term(iTRAQ8plex)	CID	1.0000	0.0000	1	1	3819295	Unique	Used	1.013	1.050	1.236	1.067	0	0	7.17	0	0	200	2	1271.69043	2542.37358	-2.26
dAAVSPDLGAGGDAPAPAPAPAHTR	Unambiguous	1	1	Q9Z2A7	N-Term(iTRAQ8plex)	CID	1.0000	0.0000	1	1	2896800	Unique	Used	0.961	0.803	0.829	0.908	0	0	7.17	0	2	136	3	862.77722	2586.31711	-1.70
sGYQQAASEHGLVVIAPDTSPR	Unambiguous	8	1	Q9R0P3	N-Term(iTRAQ8plex)	HCD	1.0000	0.0000	1	1	3276432	Unique	Used	0.928	0.841	0.808	1.149	0	0	7.17	0	11	94	3	863.11633	2587.33445	-2.86
aGESVLVHGASGGVGLATcQIAR	Unambiguous	2	1	P47199	N-Term(iTRAQ8plex); C19(Methylthio)	CID	1.0000	0.0000	1	1	3839376	Unique	Used	0.815	1.028	0.954	1.217	0	0	7.17	0	0	197	3	835.10376	2503.29673	-3.85
eTVAYLQAHALTTEGIFR	Unambiguous	2	1	A2AH25	N-Term(iTRAQ8plex)	CID	1.0000	0.0000	1	1	3725046	Unique	Used	0.272	1.898	0.814	1.110	0	0	7.17	0	1	4	3	775.41803	2324.23954	-6.76
aSPAGTAGGPVAGVATAGTGPVAAR	Unambiguous	1	1	Q8K0H5	N-Term(iTRAQ8plex)	CID	1.0000	0.0000	1	1	3698964	Unique	Used	1.958	1.360	1.218	1.166	0	0	7.17	0	0	200	2	1184.64075	2368.27422	-6.13
sDEIQNFIDTLNHTLk	Unambiguous	6	1	J3QM95	N-Term(iTRAQ8plex); K16(iTRAQ8plex)	CID	1.0000	0.0000	1	1	3439893	Unique	Used	0.784	0.955	0.847	0.794	0	0	7.17	0	3	13	3	832.78711	2496.34677	-3.48
lLENSSFEAINSQLTVETGDAHIIGR	Unambiguous	3	1	E0CYK4	N-Term(iTRAQ8plex)	CID	1.0000	0.0000	1	1	3395599	Unique	Used	2.357	1.189	1.010	0.837	0	0	7.17	0	19	37	3	1040.21362	3118.62632	-1.92

qAQMFQNFGGAGGGSSGTGSSSSGR	Unambiguous	4	1	E9QKY7	N-Term(iTRAQ8plex)	CID	1.0000	0.0000	1	1	3836308	Unique	Used	0.909	0.778	1.493	1.055	0	0	7.17	0	0	200	2	1312.60291	2624.19853	-2.92
qADVPAAVTDAAATTPAAEDAATk	Unambiguous	1	1	P06837	N-Term(iTRAQ8plex); K24(iTRAQ8plex)	CID	1.0000	0.0000	1	1	2830069	Unique	Used	0.928	0.533	0.835	0.736	0	0	7.17	0	0	200	2	1432.75562	2864.50395	-2.06
tQPTDEEMLFIYSHFk	Unambiguous	4	1	P31786	N-Term(iTRAQ8plex); K16(iTRAQ8plex)	CID	1.0000	0.0000	1	1	3285494	Unique	Used	2.293	0.683	0.780	0.806	0	0	7.16	0	21	200	3	865.45081	2594.33786	-1.64
lELQGIQGAVDHAAAFGR	Unambiguous	5	1	Q9CPV4	N-Term(iTRAQ8plex)	CID	1.0000	0.0000	1	1	3663728	Unique	Used	1.269	1.134	1.076	1.199	0	0	7.16	0	21	74	3	719.72510	2157.16074	-5.16
hLNLAEnSSYMHVYR	Unambiguous	1	1	Q61644	N-Term(iTRAQ8plex); N7(Deamidated)	CID	1.0000	0.0000	1	1	3297232	Unique	Used	1.840	1.576	1.390	1.276	0	0	7.16	0	17	39	3	713.68951	2139.05399	-2.58
eGTGSTATSSGSAGGAVGk	Unambiguous	1	1	Q91WK2	N-Term(iTRAQ8plex); K19(iTRAQ8plex)	CID	1.0000	0.0000	1	1	2822690	Unique	Used	0.631	0.816	0.728	0.707	0	0	7.16	0	0	87	2	1095.57141	2190.13555	-4.72
nASAGLNQVNNQGLTPLHLAck	Unambiguous	2	1	P97819-2	N-Term(iTRAQ8plex); C21(Methylthio); K22(iTRAQ8plex)	CID	1.0000	0.0000	1	1	3839188	Unique	Used			0.963	1.085	0	0	7.16	0	12	200	3	973.18805	2917.54959	-3.47
vGGASLGAPAAFGqSPFSQPPAAPHQNTFPPR	Unambiguous	4	1	A2AJ72	N-Term(iTRAQ8plex); Q14(Deamidated)	CID	0.0014	0.0000	1	1	3933785	Unique	Used	1.948	1.558	0.664	0.779	0	0	7.16	0	0	200	3	1155.59473	3464.76963	1.21
rAPPGPLPPAASPGPPAAsPPAAPSSPR	Unambiguous	1	1	O88703	N-Term(iTRAQ8plex); S19(Phospho)	CID	1.0000	0.0000	1	1	3365997	Unique	Used	1.359	2.136	1.040	1.521	0	0	7.16	1	16	200	3	984.84851	2952.53098	-3.05
vVHVLEGESGSVVVQTAPGQVVSHR	Unambiguous	1	1	Q80WM4	N-Term(iTRAQ8plex)	CID	1.0000	0.0000	1	1	3494805	Unique	Used	1.691	0.974	1.011	1.540	0	0	7.16	0	0	118	3	958.85986	2874.56504	-3.12
gLETIASDVVSLASk	Unambiguous	1	1	Q8BMF4	N-Term(iTRAQ8plex); K15(iTRAQ8plex)	CID	1.0000	0.0000	1	1	3479788	Unique	Used	0.587	0.905	1.231	1.283	0	0	7.16	0	13	12	2	1049.61108	2098.21489	-3.20
qLLHNFPPDQLTSSGAPFWSGPk	Unambiguous	1	1	Q02053	N-Term(iTRAQ8plex); K23(iTRAQ8plex)	CID	1.0000	0.0000	1	1	3859249	Unique	Used	1.337	1.382	1.134	1.094	0	0	7.16	0	11	121	3	1044.89209	3132.66172	-3.53
qDHPSSMGVYGQESGGFSGPGENR	Unambiguous	4	1	Q5SUT0	N-Term(iTRAQ8plex)	CID	1.0000	0.0000	1	1	2988754	Unique	Used	2.749	1.309	1.279	1.247	0	0	7.16	0	0	32	3	928.75616	2784.25394	-1.68

tETVcTFQDGALVQHQQWDGk	Unambiguous	1	1	Q05816	N-Term(iTRAQ8plex); C5(Methylthio); K21(iTRAQ8plex)	CID	1.0000	0.0000	1	1	3257730	Unique	Used	2.085	0.665	0.729	0.553	0	0	7.16	0	16	43	3	1015.83875	3045.50168	-0.08
lVQDYGLESEVAQHLAk	Unambiguous	2	1	Q64521	N-Term(iTRAQ8plex); K17(iTRAQ8plex)	HCD	1.0000	0.0000	1	1	3236050	Unique	Used	0.874	1.643	1.223	1.278	0	0	7.16	0	11	52	3	836.80023	2508.38614	-2.28
eLEGVILELQEQLTGLIPGDHAPLAQGSk	Unambiguous	3	1	O08576-2	N-Term(iTRAQ8plex); K29(iTRAQ8plex)	CID	1.0000	0.0000	1	1	3581769	Unique	Used		1.136	0.845	0.900	0	0	7.16	0	11	74	3	1222.01318	3664.02500	-4.61
sQEVDVAYIcTESSSHEDYIR	Unambiguous	4	1	Q9CY64	N-Term(iTRAQ8plex); C10(Methylthio)	CID	1.0000	0.0000	1	1	2902591	Unique	Used	1.451	1.235	0.736	1.003	0	0	7.15	0	27	179	3	927.75824	2781.26017	-1.72
tVEGAGNIAAATGFVk	Unambiguous	2	1	O55042	N-Term(iTRAQ8plex); K16(iTRAQ8plex)	CID	1.0000	0.0000	1	1	3434996	Unique	Used	0.584	1.534	2.174	0.841	0	0	7.15	0	6	35	2	1057.60217	2114.19707	-4.53
mASISDQDMDAYLVEQSR	Unambiguous	2	1	P70208	N-Term(iTRAQ8plex)	CID	1.0000	0.0000	1	1	2939903	Unique	Used	0.667	1.154	0.666	0.776	0	0	7.15	0	0	175	2	1182.05798	2363.10869	-3.15
hVPVVYVDYPGPASLTQIYGTFNR	Unambiguous	1	1	Q9JHU4	N-Term(iTRAQ8plex)	CID	1.0000	0.0000	1	1	3879157	Unique	Used	1.062	1.883	1.216	1.019	0	0	7.15	0	10	9	3	999.86047	2997.56687	-3.60
rFPLEDIPADETSAAQWLHk	Unambiguous	1	1	Q9D517	N-Term(iTRAQ8plex); K20(iTRAQ8plex)	CID	1.0000	0.0000	1	1	3347914	Unique	Used	0.539	1.215	1.322	1.710	0	0	7.15	1	15	13	3	978.19281	2932.56388	-4.75
qAAASATQTIAAAQHAASAPk	Unambiguous	1	1	P26039	N-Term(iTRAQ8plex); K21(iTRAQ8plex)	CID	1.0000	0.0000	1	1	3699513	Unique	Used	0.556	1.226	0.864	1.039	0	0	7.15	0	0	136	3	858.47369	2573.40653	-7.43
gAQIASTGQLYAAWnGGLDR	Unambiguous	1	1	Q61361	N-Term(iTRAQ8plex); N15(Deamidated)	CID	1.0000	0.0000	1	1	3370899	Unique	Used	1.770	1.067	1.102	1.090	0	0	7.15	0	4	32	2	1177.60071	2354.19414	-4.31
vSSDQLSSENLTSAVGQk	Unambiguous	1	1	Q8CC88	N-Term(iTRAQ8plex); K18(iTRAQ8plex)	CID	1.0000	0.0000	1	1	3188241	Unique	Used	0.537	0.678	0.878	1.185	0	0	7.15	0	0	34	2	1229.66284	2458.31841	-2.51
lDNNLDSVSLLIDHVcSR	Unambiguous	2	1	E0CYH4	N-Term(iTRAQ8plex); C16(Methylthio)	CID	1.0000	0.0000	1	1	3745828	Unique	Used	2.465	0.923	0.897	1.250	0	0	7.15	0	29	93	3	788.40082	2363.18790	-5.18

iSFEEFcAVVGGLDIHk	Unambiguous	2	1	Q63810-2	N-Term(iTRAQ8plex); C7(Methylthio); K17(iTRAQ8plex)	CID	1.0000	0.0000	1	1	3709070	Unique	Used	0.579	1.663	2.484	0.973	0	0	7.15	0	13	200	3	840.10889	2518.31211	-6.90
aTGHSGGGcISQGQSYDTDk	Unambiguous	1	1	Q8K274	N-Term(iTRAQ8plex); C9(Methylthio); K20(iTRAQ8plex)	CID	1.0000	0.0000	1	1	3005899	Unique	Used	0.611	0.777	0.721	0.848	0	0	7.15	0	0	200	3	875.08154	2623.23008	-1.39
nLHQSGFSLSGAQIDDNIPR	Unambiguous	1	1	O08553	N-Term(iTRAQ8plex)	CID	1.0000	0.0000	1	1	3388550	Unique	Used	2.312	1.289	1.104	1.063	0	0	7.15	0	10	40	3	825.09222	2473.26212	-4.71
gTGGVDTAATGSVFDISNLDR	Unambiguous	1	1	P30275	N-Term(iTRAQ8plex)	CID	1.0000	0.0000	1	1	3264862	Unique	Used		0.947	0.926	1.340	0	0	7.15	0	16	200	2	1179.09827	2357.18926	0.23
lVPFVVQLSGHLAQETER	Unambiguous	1	1	P06728	N-Term(iTRAQ8plex)	CID	1.0000	0.0000	1	1	3879650	Unique	Used	2.917	3.237	0.482	1.138	0	0	7.15	0	29	31	3	776.43634	2327.29447	-3.47
tQPTDEEMLFIYSHFk	Unambiguous	4	1	P31786	N-Term(iTRAQ8plex); K16(iTRAQ8plex)	CID	1.0000	0.0000	1	1	3286626	Unique	Used	1.649	0.612	0.950	0.772	0	0	7.14	0	2	200	3	865.45135	2594.33951	-1.01
gIEFPmADLDALSPIHTPQR	Unambiguous	6	1	Q5SRX1-2	N-Term(iTRAQ8plex); M6(Oxidation)	CID	1.0000	0.0000	1	1	3539479	Unique	Used	1.651	1.183	1.297	1.158	0	0	7.14	0	10	55	3	843.43982	2528.30490	-2.87
gGEATWSGSEFEISFPDSPGAQAQADHLPR	Unambiguous	1	1	Q91X51	N-Term(iTRAQ8plex)	CID	1.0000	0.0000	1	1	3151298	Unique	Used	3.122	1.096	1.519	1.206	0	0	7.14	0	0	75	3	1150.21570	3448.63254	-0.67
iVAPELYIAVGISGAIQHLAGMk	Unambiguous	1	1	Q99LC5	N-Term(iTRAQ8plex); K23(iTRAQ8plex)	CID	1.0000	0.0000	1	1	3936555	Unique	Used	1.257	1.317	0.973	1.085	0	0	7.14	0	7	47	3	987.24200	2959.71146	-5.04
vTYMVHDFEEGGGVAMGMYNQDk	Unambiguous	1	1	O88844	N-Term(iTRAQ8plex); K23(iTRAQ8plex)	CID	1.0000	0.0000	1	1	3132252	Unique	Used	0.675	0.633	0.818	0.952	0	0	7.14	0	0	36	3	1062.84204	3186.51157	-1.25
dEVQEVVYFPAVDHDAESk	Unambiguous	2	1	Q9CZC8	N-Term(iTRAQ8plex); K19(iTRAQ8plex)	CID	1.0000	0.0000	1	1	2636363	Unique	Used	2.216	0.931	1.539	1.181	0	0	7.14	0	17	200	3	929.14056	2785.40714	-2.51
lQEANAQLAEQTcPHDLEMTPVQR	Unambiguous	1	1	Q80Y17	N-Term(iTRAQ8plex); C13(Methylthio)	CID	1.0000	0.0000	1	1	3192404	Unique	Used	0.993	0.392	0.718	0.741	0	0	7.14	0	11	93	3	1024.83044	3072.47678	-2.85
nVQAEEMVEFSSGLk	Unambiguous	2	1	Q03265	N-Term(iTRAQ8plex); K15(iTRAQ8plex)	CID	1.0000	0.0000	1	1	2975333	Unique	Used	0.846	1.498	2.110	2.175	0	0	7.14	0	0	116	2	1138.60278	2276.19829	-3.09

kLASQGDSIGSQLGPIHPPPR	Unambiguous	1	1	Q3U0V1	N-Term(iTRAQ8plex); K1(iTRAQ8plex)	CID	1.0000	0.0000	1	1	3678551	Unique	Used	0.848	0.963	1.524	0.978	0	0	7.13	1	0	200	3	921.85553	2763.55204	-7.46
gITNLcVIGGDGSLTGADTFR	Unambiguous	1	1	P47857	N-Term(iTRAQ8plex); C6(Methylthio)	CID	1.0000	0.0000	1	1	3728260	Unique	Used	1.826	0.906	1.077	1.297	0	0	7.13	0	12	200	2	1209.10095	2417.19463	-6.66
kTGSPGSPSAGGVQSTAk	Unambiguous	1	1	Q6PDL0	N-Term(iTRAQ8plex); K1(iTRAQ8plex); K18(iTRAQ8plex)	CID	1.0000	0.0000	1	1	3270144	Unique	Used	0.786	0.682	1.203	0.982	0	0	7.13	1	0	200	3	843.81506	2529.43064	-3.70
vTITGSPAATQAAQYLISQR	Unambiguous	2	1	D3YVV7	N-Term(iTRAQ8plex)	CID	1.0000	0.0000	1	1	3934954	Unique	Used	1.564	0.908	1.094	1.024	0	0	7.13	0	0	200	2	1190.65662	2380.30596	-3.31
vGMDSTTVNYFALFEVInHSFVR	Unambiguous	3	1	Q3UHD6-2	N-Term(iTRAQ8plex); N18(Deamidated)	CID	1.0000	0.0000	1	1	3766481	Unique	Used		1.608			0	0	7.13	0	0	60	3	984.49951	2951.48398	-2.57
lGcEVLGVSVDSQFTHLAWINTPR	Unambiguous	2	1	Q61171	N-Term(iTRAQ8plex); C3(Methylthio)	CID	1.0000	0.0000	1	1	3879696	Unique	Used	1.570	1.175	1.329	1.303	0	0	7.13	0	28	14	3	998.17743	2992.51773	-5.01
aAMEALQSQALHATSQQPLR	Unambiguous	5	1	B1AQZ0	N-Term(iTRAQ8plex)	HCD	1.0000	0.0000	1	1	3856492	Unique	Used	2.004	1.737	1.035	1.233	0	0	7.13	0	23	104	3	819.10339	2455.29563	-2.99
lVDWIILQcAEDIEHPPPGR	Unambiguous	1	1	Q9R1Q8	N-Term(iTRAQ8plex); C9(Methylthio)	CID	1.0000	0.0000	1	1	3561559	Unique	Used	1.202	0.615	1.356	1.433	0	0	7.13	0	8	174	3	884.45544	2651.35178	-4.16
gQIQEAIALINSLHPELLDTNR	Unambiguous	1	1	Q9D7M1	N-Term(iTRAQ8plex)	CID	1.0000	0.0000	1	1	3806197	Unique	Used	0.798	1.719	0.864	0.525	0	0	7.13	0	0	33	3	917.17419	2749.50803	-2.56
qGTGSLSESSIPSISDTSTPR	Unambiguous	2	1	Q61290	N-Term(iTRAQ8plex)	CID	1.0000	0.0000	1	1	3210098	Unique	Used	0.598	1.775	3.124	1.567	0	0	7.13	0	0	73	2	1206.11475	2411.22222	0.76
vADVSISEVTTLLGNVHSPVVEGYVGEnISGEVk	Unambiguous	3	1	P20357	N-Term(iTRAQ8plex); N28(Deamidated); K34(iTRAQ8plex)	CID	0.0393	0.0000	1	1	3421672	Unique	Used			1.150	0.666	0	0	7.13	0	0	200	3	1369.73901	4107.20249	1.61
mAQALALQAGSPEDGAPPR	Unambiguous	3	1	B8QI35	N-Term(iTRAQ8plex)	CID	1.0000	0.0000	1	1	3345224	Unique	Used	1.144	1.080	1.582	1.268	0	0	7.13	0	0	53	2	1092.56982	2184.13237	-2.83
nMGGPYGGGnYGPGGSGGSGGYGGR	Unambiguous	3	1	O88569	N-Term(iTRAQ8plex); N10(Deamidated)	CID	1.0000	0.0000	1	1	3009004	Unique	Used	1.861	1.225	1.891	1.679	0	0	7.13	0	0	200	2	1248.04810	2495.08891	-2.37
gIEDEQDLVTLEVTGVVFHYR	Unambiguous	1	1	P55066	N-Term(iTRAQ8plex)	CID	1.0000	0.0000	1	1	3153501	Unique	Used	1.922	2.300	1.986	1.138	0	0	7.13	0	2	14	3	908.47754	2723.41806	-0.51

dQQNLPYGVTPASPSGHSQGR	Unambiguous	14	1	E9QMP6	N-Term(iTRAQ8plex)	CID	1.0000	0.0000	1	1	3469051	Unique	Used	1.149	1.399	0.851	0.977	0	0	7.12	0	29	200	3	834.08453	2500.23905	-3.69
sAAQAAAQTnSNAAGk	Unambiguous	4	1	Q9CY58	N-Term(iTRAQ8plex); N10(Deamidated); K16(iTRAQ8plex)	CID	1.0000	0.0000	1	1	2823392	Unique	Used	0.837	0.553	1.031	0.694	0	0	7.12	0	0	200	2	1035.54980	2070.09233	-5.45
yTPSGQSGAAASESLFISNHAY	Unambiguous	2	1	A6ZI44	N-Term(iTRAQ8plex)	CID	1.0000	0.0000	1	1	3258517	Unique	Used	0.998	0.933	1.207	1.098	0	0	7.12	0	0	99	2	1281.62244	2562.23760	-1.50
kTGQAAGFSYTDANk	Unambiguous	2	1	G3UWG1	N-Term(iTRAQ8plex); K1(iTRAQ8plex); K15(iTRAQ8plex)	HCD	1.0000	0.0000	1	1	3183573	Unique	Used	1.212	0.650	1.368	0.954	0	0	7.12	1	0	105	3	824.45886	2471.36203	-1.50
sLLAAESAASLQGAELER	Unambiguous	1	1	Q3UHK1	N-Term(iTRAQ8plex)	CID	1.0000	0.0000	1	1	3170870	Unique	Used	0.925	1.027	0.967	1.221	0	0	7.12	0	11	174	2	1060.57776	2120.14824	-0.90
dMAIATGGAVFGEEGLNLNLEDVQAHDLGk	Unambiguous	1	1	P63038	N-Term(iTRAQ8plex); K30(iTRAQ8plex)	CID	1.0000	0.0000	1	1	3264023	Unique	Used		0.702	1.126	0.720	0	0	7.12	0	0	200	3	1231.63879	3692.90183	-0.90
fAGAHLGPEGQNLVQEEVVAR	Unambiguous	2	1	Q91XI1	N-Term(iTRAQ8plex)	CID	1.0000	0.0000	1	1	3189774	Unique	Used	0.861	0.954	0.891	1.142	0	0	7.12	0	11	20	3	842.45032	2525.33640	-2.01
fVSSPEEVMDVIDEGk	Unambiguous	1	1	P28738	N-Term(iTRAQ8plex); K16(iTRAQ8plex)	CID	1.0000	0.0000	1	1	2816047	Unique	Used	0.545	1.060	1.052	1.029	0	0	7.12	0	3	72	2	1195.12122	2389.23516	-2.77
gITNLcVIGGDGSLTGADTFR	Unambiguous	1	1	P47857	N-Term(iTRAQ8plex); C6(Methylthio)	CID	1.0000	0.0000	1	1	3729034	Unique	Used	1.932	0.825	1.120	0.857	0	0	7.12	0	0	200	2	1209.10034	2417.19341	-7.16
mTTVLSESSGPSNASHSSSR	Unambiguous	1	1	Q80X80	N-Term(iTRAQ8plex)	CID	1.0000	0.0000	1	1	3510619	Unique	Used	4.706	1.371	0.988	0.691	0	0	7.12	0	0	200	3	776.04401	2326.11747	-3.11
sAAQAAAQTnSNAAGk	Unambiguous	4	1	Q9CY58	N-Term(iTRAQ8plex); N10(Deamidated); K16(iTRAQ8plex)	CID	0.0449	0.0000	1	1	2841783	Unique	Used	0.521	0.381	0.820	0.528	0	0	7.12	0	0	200	2	1035.55225	2070.09722	-3.09
aTHHPAPGPGLLGPPSAIPGLqNQQLLGER	Unambiguous	2	1	D3Z3Z3	N-Term(iTRAQ8plex); Q22(Deamidated)	CID	0.0183	0.0000	1	1	3856952	Unique	Used	0.815	2.001	1.354	1.267	0	0	7.12	0	0	165	3	1110.27734	3328.81748	3.18
hLQLEQGVENYEESIAQLSR	Unambiguous	3	1	E9PX29	N-Term(iTRAQ8plex)	CID	1.0000	0.0000	1	1	3193103	Unique	Used	1.159	1.236	0.977	0.909	0	0	7.12	0	16	16	3	883.12427	2647.35825	-1.78

iYDAQNELSAATHLTSk	Unambiguous	1	1	Q8K3H0	N-Term(iTRAQ8plex); K17(iTRAQ8plex)	CID	1.0000	0.0000	1	1	3301532	Unique	Used	1.020	1.259	1.166	1.196	0	0	7.12	0	19	43	3	824.11572	2470.33261	-2.91
lEQQVHSASSGPnSGSAINMSGVDSGEGTR	Unambiguous	1	1	Q9QYE6	N-Term(iTRAQ8plex); N13(Deamidated)	CID	0.0295	0.0000	1	1	3068563	Unique	Used	0.854	0.318	0.779	0.654	0	0	7.12	0	0	200	3	1088.85400	3264.54746	4.08
yTPSGQSGAAASESLFISNHAY	Unambiguous	2	1	A6ZI44	N-Term(iTRAQ8plex)	CID	1.0000	0.0000	1	1	3346433	Unique	Used	0.626	1.006	1.105	1.063	0	0	7.12	0	0	14	2	1281.62073	2562.23418	-2.83
gITNLcVIGGDGSLTGADTFR	Unambiguous	1	1	P47857	N-Term(iTRAQ8plex); C6(Methylthio)	CID	1.0000	0.0000	1	1	3728572	Unique	Used	1.708	0.882	0.992	1.293	0	0	7.12	0	0	200	2	1209.10022	2417.19316	-7.26
nAIHTYTQAGSHMAAk	Unambiguous	2	1	P24270	N-Term(iTRAQ8plex); K16(iTRAQ8plex)	CID	1.0000	0.0000	1	1	3488884	Unique	Used	1.405	0.644	0.657	1.036	0	0	7.11	0	0	200	3	770.41254	2309.22306	-2.18
sNVYQPTEMAVVLnGGTIPTAPPSHTGR	Unambiguous	1	1	Q923Z0	N-Term(iTRAQ8plex); N14(Deamidated)	CID	1.0000	0.0000	1	1	3476236	Unique	Used	1.038	0.857	1.061	1.199	0	0	7.11	0	10	53	3	1067.21558	3199.63218	-1.20
wDETPASQMGGSTPVLTPGk	Unambiguous	2	1	G5E866	N-Term(iTRAQ8plex); K20(iTRAQ8plex)	CID	1.0000	0.0000	1	1	3301070	Unique	Used	0.795	0.764	1.208	1.033	0	0	7.11	0	0	92	2	1334.19775	2667.38823	-1.00
lSLcGEESFGTGSDHIR	Unambiguous	2	1	Q9D0F9	N-Term(iTRAQ8plex); C4(Methylthio)	CID	1.0000	0.0000	1	1	3322739	Unique	Used	1.447	1.078	1.138	1.118	0	0	7.11	0	27	101	3	720.01056	2158.01712	-1.84
gAGQTVTIIAQYQPEDYAR	Unambiguous	9	1	Q91XM9-7	N-Term(iTRAQ8plex)	CID	1.0000	0.0000	1	1	3301351	Unique	Used	0.700	2.728	1.896	0.942	0	0	7.11	0	0	16	2	1193.11841	2385.22954	-2.39
sSGSPYGGGYGSGGGSGGYGSR	Unambiguous	7	1	J3QNY1	N-Term(iTRAQ8plex)	CID	1.0000	0.0000	1	1	2950619	Unique	Used	2.407	1.015	1.140	0.781	0	0	7.11	0	2	86	3	739.00281	2214.99387	-0.69
dDTTPAAAPAPQGLEHGk	Unambiguous	1	1	P56213	N-Term(iTRAQ8plex); K18(iTRAQ8plex)	CID	1.0000	0.0000	1	1	2681173	Unique	Used	1.506	1.252	1.330	1.217	0	0	7.11	0	24	200	3	795.42645	2384.26480	-0.79
lTLYDIAHTPGVAADLSHIETR	Unambiguous	1	1	P08249	N-Term(iTRAQ8plex)	CID	1.0000	0.0000	1	1	3458125	Unique	Used	5.172	1.183	0.896	1.074	0	0	7.11	0	7	193	3	899.81628	2697.43430	-6.34
dHIIEVVGSSMPLIGDHQDEDk	Unambiguous	3	1	O88935	N-Term(iTRAQ8plex); K22(iTRAQ8plex)	CID	1.0000	0.0000	1	1	2976788	Unique	Used	0.675	1.625	1.511	1.288	0	0	7.11	0	20	131	3	1014.85614	3042.55387	-4.09
gITNLcVIGGDGSLTGADTFR	Unambiguous	1	1	P47857	N-Term(iTRAQ8plex); C6(Methylthio)	CID	1.0000	0.0000	1	1	3728918	Unique	Used	2.864	0.846	1.005	1.391	0	0	7.11	0	0	200	2	1209.10059	2417.19390	-6.96

dAGVSPEQISYVNAHATSTPLGDAAENR	Unambiguous	1	1	Q9D404	N-Term(iTRAQ8plex)	CID	1.0000	0.0000	1	1	3031686	Unique	Used	1.534	1.159	1.200	1.418	0	0	7.11	0	0	98	3	1058.85791	3174.55918	-0.45
aMASINERPIIFALSNPTAQAEcTAEDAYTLTEGR	Unambiguous	1	1	Q99KE1	N-Term(iTRAQ8plex); C23(Methylthio)	CID	1.0000	0.0000	1	1	3500174	Unique	Used	1.685	0.863	0.686	0.791	0	0	7.11	0	14	31	3	1369.00146	4104.98984	-2.08
icTTLIGLEEHLNALDR	Unambiguous	1	1	Q8VC30	N-Term(iTRAQ8plex); C2(Methylthio)	CID	1.0000	0.0000	1	1	3706986	Unique	Used	0.638	0.673	0.712	1.045	0	0	7.11	0	0	20	3	754.39832	2261.18039	-5.84
qTGHTLLEDYQIVERPQR	Unambiguous	2	1	Q8R1X6	N-Term(iTRAQ8plex)	HCD	1.0000	0.0000	1	1	3297639	Unique	Used	1.835	0.849	1.124	1.114	0	0	7.11	0	4	68	3	829.77838	2487.32059	-2.09
sSGnSSSSLGEMVSGTFR	Unambiguous	4	1	Q8R0S4	N-Term(iTRAQ8plex); N4(Deamidated)	CID	1.0000	0.0000	1	1	3639512	Unique	Used	0.558	0.674	1.438	0.956	0	0	7.11	0	0	91	2	1048.00244	2094.99761	2.88
dHLFEESTFAPYIDELSDEQHR	Unambiguous	1	1	Q8K0T0	N-Term(iTRAQ8plex)	CID	1.0000	0.0000	1	1	2797975	Unique	Used		1.572	1.148		0	0	7.11	0	0	200	3	994.80579	2982.40281	-1.06
aAMEALQSQALHATSQQPLR	Unambiguous	5	1	B1AQZ0	N-Term(iTRAQ8plex)	HCD	1.0000	0.0000	1	1	3820626	Unique	Used	1.716	1.321	1.719	1.400	0	0	7.11	0	13	91	3	819.10284	2455.29398	-3.66
sALLDGDTQNYDLDHGFSR	Unambiguous	1	1	Q8C726	N-Term(iTRAQ8plex)	CID	1.0000	0.0000	1	1	2993364	Unique	Used	1.933	1.312	1.235	1.189	0	0	7.11	0	30	142	3	810.05762	2428.15830	-4.12
kVEEEVINDQALQALSDSLGTR	Unambiguous	6	1	P51125-3	N-Term(iTRAQ8plex); K1(iTRAQ8plex)	CID	1.0000	0.0000	1	1	3194963	Unique	Used	2.387	0.910	1.126	0.849	0	0	7.11	1	3	32	3	1008.55298	3023.64438	-0.87
tTSPATDAGHVQETSEPSLQALESR	Unambiguous	1	1	Q8R010	N-Term(iTRAQ8plex)	CID	1.0000	0.0000	1	1	2809688	Unique	Used	1.199	1.318	1.353	1.330	0	0	7.11	0	0	200	3	972.81970	2916.44455	-1.49
cQNEQLQTAVTQQASqIQQHk	Unambiguous	3	1	Q9Z1Z0	N-Term(iTRAQ8plex); C1(Methylthio); Q16(Deamidated); K21(iTRAQ8plex)	CID	0.0028	0.0000	1	1	3514369	Unique	Used	0.599	0.566	0.931	0.924	0	0	7.11	0	8	94	3	1022.85870	3066.56156	1.90
sSGNSANDLANVAVIDGNR	Unambiguous	1	1	Q7TT37	N-Term(iTRAQ8plex)	CID	1.0000	0.0000	1	1	3105391	Unique	Used	0.547	1.070	0.744	1.062	0	0	7.10	0	14	73	2	1089.55493	2178.10259	-1.25
aLDDGVGNQLGALVHQR	Unambiguous	2	1	A2AJ72	N-Term(iTRAQ8plex)	CID	1.0000	0.0000	1	1	3434730	Unique	Used	1.401	0.969	0.823	0.903	0	0	7.10	0	0	104	3	689.71185	2067.12101	-1.89
dLGAQGAVAEAELAEAQR	Unambiguous	1	1	Q61001	N-Term(iTRAQ8plex)	CID	1.0000	0.0000	1	1	2636641	Unique	Used	1.282	3.230	1.215	1.479	0	0	7.10	0	0	200	2	1052.05212	2103.09697	-0.70
smLQATAEANNLAAVATAk	Unambiguous	1	1	Q8BH66	N-Term(iTRAQ8plex); M2(Oxidation); K19(iTRAQ8plex)	CID	1.0000	0.0000	1	1	3576518	Unique	Used	0.302	1.364	1.071	1.129	0	0	7.10	0	0	167	2	1250.18213	2499.35698	-5.12

aANAAENDFSVSQAEVSSR	Unambiguous	1	1	Q6P542	N-Term(iTRAQ8plex)	CID	1.0000	0.0000	1	1	2734418	Unique	Used	0.486	0.548	0.752	0.789	0	0	7.10	0	0	200	2	1129.05176	2257.09624	-1.62
eQTVQYILTmVDDMLQENHQR	Unambiguous	1	1	Q8BVE3	N-Term(iTRAQ8plex); M10(Oxidation)	CID	1.0000	0.0000	1	1	3707048	Unique	Used	1.074	1.377	1.047	1.312	0	0	7.10	0	3	22	3	971.13977	2911.40476	-6.34
gVVGPGPAAIAAPGGGGAGPPAVGGGGGR	Unambiguous	1	1	Q3B7Z2	N-Term(iTRAQ8plex)	CID	1.0000	0.0000	1	1	3819522	Unique	Used	1.148	0.943	1.052	1.093	0	0	7.10	0	5	167	2	1271.68933	2542.37139	-3.13
iITLEEGDLILTGTPk	Unambiguous	1	1	Q8R0F8	N-Term(iTRAQ8plex); K16(iTRAQ8plex)	CID	1.0000	0.0000	1	1	3305191	Unique	Used	1.792	1.553	1.439	1.208	0	0	7.10	0	15	27	2	1161.18958	2321.37187	-3.01
lGQHVVGMAPLSVGSLDDEPGGETETR	Unambiguous	1	1	Q9CZR8	N-Term(iTRAQ8plex)	CID	1.0000	0.0000	1	1	3129118	Unique	Used	2.310		2.116	3.133	0	0	7.10	0	3	9	3	1019.18066	3055.52744	-1.14
gDPEcHLFYNEQQETGGAHAQSVQ	Unambiguous	2	1	D3YU40	N-Term(iTRAQ8plex); C5(Methylthio)	CID	1.0000	0.0000	1	1	2716309	Unique	Used	6.482	0.564	1.079	1.078	0	0	7.10	0	3	200	3	999.11237	2995.32254	-0.92
sNISSPVTPTAASSSSTTPTR	Unambiguous	6	1	A2AU91	N-Term(iTRAQ8plex)	CID	1.0000	0.0000	1	1	3612523	Unique	Used	0.551	0.429	0.633	0.847	0	0	7.10	0	0	200	2	1177.10815	2353.20903	-2.49
qLIIVHGPPEASQDLAEccR	Unambiguous	1	1	O35218	N-Term(iTRAQ8plex); C18(Methylthio); C19(Methylthio)	CID	1.0000	0.0000	1	1	3478507	Unique	Used	1.082	1.154	0.988	1.259	0	0	7.10	0	29	121	3	859.08344	2575.23575	-3.34
lDQEDALLGSYPVDDGcR	Unambiguous	1	1	Q9D1E6	N-Term(iTRAQ8plex); C17(Methylthio)	CID	1.0000	0.0000	1	1	2741707	Unique	Used	1.747	0.635	0.959	1.418	0	0	7.10	0	0	55	2	1158.54358	2316.07988	0.37
nLHQSGFSLSGAQIDDNIPR	Unambiguous	1	1	O08553	N-Term(iTRAQ8plex)	CID	1.0000	0.0000	1	1	3680995	Unique	Used	1.086	0.959	1.070	1.153	0	0	7.10	0	0	41	3	825.09058	2473.25718	-6.71
qASGGPVDIGPEYQQDLDR	Unambiguous	2	1	P97450	N-Term(iTRAQ8plex)	CID	1.0000	0.0000	1	1	2635631	Unique	Used	1.665	0.594	0.852	0.993	0	0	7.10	0	0	200	2	1175.08252	2349.15776	-2.01
qDHPSSMGVYGQESGGFSGPGENR	Unambiguous	4	1	Q5SUT0	N-Term(iTRAQ8plex)	CID	1.0000	0.0000	1	1	2988866	Unique	Used	1.921	0.838	1.333	0.821	0	0	7.10	0	15	200	3	928.75348	2784.24588	-4.57
gLTIVGSVLEGTFLDNHPQAQR	Unambiguous	2	1	Q91V14	N-Term(iTRAQ8plex)	CID	1.0000	0.0000	1	1	3844697	Unique	Used		0.794	1.464	0.811	0	0	7.09	0	23	200	3	886.14746	2656.42783	-3.11
vAAINVAHLEEADEGPEPAR	Unambiguous	1	1	Q8VBY2	N-Term(iTRAQ8plex)	CID	1.0000	0.0000	1	1	2635755	Unique	Used	1.440	1.027	1.570	1.511	0	0	7.09	0	5	52	3	798.08380	2392.23685	-1.78
nLHQSGFSLSGAQIDDNIPR	Unambiguous	1	1	O08553	N-Term(iTRAQ8plex)	CID	1.0000	0.0000	1	1	3596129	Unique	Used	1.041	0.847	1.678	1.986	0	0	7.09	0	22	7	3	825.09174	2473.26065	-5.30
eTSLHSPSNTSASHSQGGGPPTSGm	Unambiguous	2	1	O09117-2	N-Term(iTRAQ8plex); M25(Oxidation)	CID	1.0000	0.0000	1	1	3098905	Unique	Used	0.886	0.767	1.814	1.076	0	0	7.09	0	0	200	3	911.08826	2731.25022	-1.08

fTASAGIQVVGDDLTVTNPk	Unambiguous	2	1	P17182	N-Term(iTRAQ8plex); K20(iTRAQ8plex)	CID	1.0000	0.0000	1	1	3348515	Unique	Used	0.904	1.778	1.133	1.443	0	0	7.09	0	0	25	2	1321.23242	2641.45757	-3.11
vTLENFYSNLIAQHEER	Unambiguous	1	1	Q91VJ4	N-Term(iTRAQ8plex)	CID	1.0000	0.0000	1	1	3458087	Unique	Used	1.296	1.016	0.742	0.956	0	0	7.09	0	30	200	3	789.74274	2367.21366	-4.65
tTAASGDLAQAPGAFk	Unambiguous	2	1	P20357	N-Term(iTRAQ8plex); K16(iTRAQ8plex)	CID	1.0000	0.0000	1	1	3253453	Unique	Used		1.916	2.067	1.265	0	0	7.09	0	11	37	2	1057.58679	2114.16631	-1.87
gGGAAGGASGVSGAGAAGGGR	Unambiguous	2	1	Q6PAR0-2	N-Term(iTRAQ8plex)	CID	1.0000	0.0000	1	1	3202336	Unique	Used	0.514	0.727	0.666	0.759	0	0	7.09	0	0	200	2	903.46002	1805.91277	-1.62
cTHWAEGGQGALALAQAVQR	Unambiguous	1	1	Q922D8	N-Term(iTRAQ8plex); C1(Methylthio)	CID	1.0000	0.0000	1	1	3857724	Unique	Used	0.558	0.454	0.773	1.222	0	0	7.09	0	0	200	2	1209.10437	2417.20146	-4.38
lTLYDIAHTPGVAADLSHIETR	Unambiguous	1	1	P08249	N-Term(iTRAQ8plex)	CID	1.0000	0.0000	1	1	3477966	Unique	Used	2.367	0.912	0.985	1.111	0	0	7.09	0	0	2	3	899.81927	2697.44327	-3.01
sVVQTTPVAGSqSLSSGTVAk	Unambiguous	3	1	Q8BTI8	N-Term(iTRAQ8plex); Q12(Deamidated); K21(iTRAQ8plex)	CID	1.0000	0.0000	1	1	3512759	Unique	Used	0.641	0.834	0.928	0.757	0	0	7.08	0	25	200	2	1307.23682	2613.46636	4.12
lVQFQAPHLEEPHLGLESGVGGGVVDLnAFDSTLPk	Unambiguous	1	1	Q3TC72	N-Term(iTRAQ8plex); N28(Deamidated); K36(iTRAQ8plex)	CID	1.0000	0.0000	1	1	3479361	Unique	Used	1.720	1.815	0.988	1.204	0	0	7.08	0	15	23	3	1460.78564	4380.34238	1.97
tEPGVPVSLVNPQPSSGHFSTk	Unambiguous	1	1	Q8BMJ2	N-Term(iTRAQ8plex); K22(iTRAQ8plex)	CID	1.0000	0.0000	1	1	3433083	Unique	Used	2.292	1.880	0.715	1.130	0	0	7.08	0	0	41	3	958.52020	2873.54605	-5.48
qTTAAAAATFSEQVGGGSGGAGR	Unambiguous	3	1	O88935	N-Term(iTRAQ8plex)	CID	1.0000	0.0000	1	1	3502277	Unique	Used	0.595	1.066	1.931	1.279	0	0	7.08	0	0	200	2	1178.59656	2356.18584	2.66
gFLSDTPVGVAHFILER	Unambiguous	5	1	E9QAD8	N-Term(iTRAQ8plex)	CID	1.0000	0.0000	1	1	3765725	Unique	Used	0.471	1.891	2.014	1.348	0	0	7.08	0	8	17	3	721.39911	2162.18277	-3.90
tQPTDEEMLFIYSHFk	Unambiguous	4	1	P31786	N-Term(iTRAQ8plex); K16(iTRAQ8plex)	CID	1.0000	0.0000	1	1	3287416	Unique	Used	1.965	0.569	0.951	0.987	0	0	7.08	0	0	200	3	865.45050	2594.33695	-2.00
gGFGGAGMGAYVPSGFVHPQSR	Unambiguous	2	1	Q811P8	N-Term(iTRAQ8plex)	CID	1.0000	0.0000	1	1	3913654	Unique	Used	1.258	1.319	1.311	0.927	0	0	7.08	0	14	153	3	814.07361	2440.20627	-2.94
mVISNPAATHQDIDFLIEEIER	Unambiguous	1	1	P48320	N-Term(iTRAQ8plex)	CID	1.0000	0.0000	1	1	3173761	Unique	Used	0.281	0.901	1.185	1.535	0	0	7.08	0	1	5	3	949.15997	2845.46537	-1.93

nFSEEETLQQLNQLNScHQEAVLk	Unambiguous	1	1	Q9CZG3	N-Term(iTRAQ8plex); C17(Methylthio); K24(iTRAQ8plex)	CID	1.0000	0.0000	1	1	3324432	Unique	Used	1.486	1.070	1.058	0.866	0	0	7.08	0	0	102	3	1152.91321	3456.72507	-2.83
iEGSGDQVDTLELSGGAk	Unambiguous	3	1	Q8BZ98-2	N-Term(iTRAQ8plex); K18(iTRAQ8plex)	CID	1.0000	0.0000	1	1	2736291	Unique	Used	0.373	0.590	1.066	1.159	0	0	7.08	0	25	200	2	1192.63611	2384.26494	-4.88
hGcAFLVDEVQTGGGcTGk	Unambiguous	1	1	P61922	N-Term(iTRAQ8plex); C3(Methylthio); C16(Methylthio); K19(iTRAQ8plex)	CID	1.0000	0.0000	1	1	3370423	Unique	Used	1.619	0.907	0.878	1.141	0	0	7.08	0	16	43	3	860.41327	2579.22525	-3.22
sEEITTGSAWFSFLESHnk	Unambiguous	2	1	D3Z375	N-Term(iTRAQ8plex); N18(Deamidated); K19(iTRAQ8plex)	CID	1.0000	0.0000	1	1	3238853	Unique	Used	1.900	1.936	1.650	0.915	0	0	7.08	0	0	21	3	927.13751	2779.39798	-2.01
iGEHTPSALAImENANVLAR	Unambiguous	3	1	A6ZI44	N-Term(iTRAQ8plex); M12(Oxidation)	CID	1.0000	0.0000	1	1	3708702	Unique	Used	0.587	0.975	0.875	0.989	0	0	7.08	0	5	200	3	809.76727	2427.28727	-3.94
dLVILLYETALLSSGFSLEDPQTHANR	Unambiguous	1	1	P07901	N-Term(iTRAQ8plex)	CID	1.0000	0.0000	1	1	3786585	Unique	Used	1.559	0.758	0.653	1.083	0	0	7.08	0	15	200	3	1102.91736	3306.73752	-4.51
lGQmLSIQDDAFINPHLAk	Unambiguous	2	1	Q60936	N-Term(iTRAQ8plex); M4(Oxidation); K19(iTRAQ8plex)	CID	1.0000	0.0000	1	1	3578197	Unique	Used	0.803	1.556	1.089	1.442	0	0	7.07	0	0	109	3	912.50031	2735.48636	-5.40
aATSAPAASAAAASAAVAAEYk	Unambiguous	1	1	F8VQK3	N-Term(iTRAQ8plex); K22(iTRAQ8plex)	CID	1.0000	0.0000	1	1	3345737	Unique	Used	1.241	0.775	1.322	1.293	0	0	7.07	0	0	57	2	1265.18811	2529.36894	-3.17
nLHQSGFSLSGAQIDDNIPR	Unambiguous	1	1	O08553	N-Term(iTRAQ8plex)	CID	1.0000	0.0000	1	1	3494350	Unique	Used	0.866	0.944	1.008	1.217	0	0	7.07	0	6	16	3	825.09381	2473.26688	-2.79

ekPELqFPFLQDEDTVATLHEck	Unambiguous	2	1	P16330	N-Term(iTRAQ8plex); K2(iTRAQ8plex); Q6(Deamidated); C22(Methylthio); K23(iTRAQ8plex)	CID	0.0311	0.0000	1	1	3237599	Unique	Used	1.975	0.185	1.201	1.574	0	0	7.07	0	10	113	3	1226.30957	3676.91416	3.67
eSTFLDEGPNEQEITPLQHTPR	Unambiguous	3	1	Q9QYR6	N-Term(iTRAQ8plex)	CID	1.0000	0.0000	1	1	2812125	Unique	Used	2.904	1.214	1.559	1.015	0	0	7.07	0	3	62	3	948.14246	2842.41281	-1.17
tVYGGGcSEMLMAHAVTQLANR	Unambiguous	1	1	P80314	N-Term(iTRAQ8plex); C7(Methylthio)	CID	1.0000	0.0000	1	1	3897924	Unique	Used	0.861	0.805	0.372	1.001	0	0	7.07	0	24	27	3	887.09241	2659.26267	-5.28
sSGNSANDLANVAVIDGNR	Unambiguous	1	1	Q7TT37	N-Term(iTRAQ8plex)	CID	1.0000	0.0000	1	1	3105527	Unique	Used	0.607	1.078	0.825	1.079	0	0	7.07	0	9	128	2	1089.55469	2178.10210	-1.48
sQQQLHSPALSPAHSPAk	Unambiguous	2	1	O88737	N-Term(iTRAQ8plex); K18(iTRAQ8plex)	HCD	1.0000	0.0000	1	1	3382177	Unique	Used	0.978	1.024	2.591	1.009	0	0	7.07	0	0	200	3	831.46100	2492.36844	-5.85
hISqISVADDDDESLLGHLMIVGk	Unambiguous	1	1	P70349	N-Term(iTRAQ8plex); Q4(Deamidated); K24(iTRAQ8plex)	CID	1.0000	0.0000	1	1	3282548	Unique	Used	1.141	0.874	1.331	1.108	0	0	7.07	0	19	133	3	1067.90747	3201.70786	4.87
hNHDLSSYQDTIQQLENELR	Unambiguous	2	1	P08553	N-Term(iTRAQ8plex)	CID	1.0000	0.0000	1	1	3215137	Unique	Used	2.023	0.668	1.158	1.428	0	0	7.07	0	19	169	3	915.45551	2744.35196	-0.81
gITNLcVIGGDGSLTGADTFR	Unambiguous	1	1	P47857	N-Term(iTRAQ8plex); C6(Methylthio)	CID	1.0000	0.0000	1	1	3728156	Unique	Used	1.983	0.814	1.004	1.153	0	0	7.07	0	0	200	2	1209.10132	2417.19536	-6.36
dSDWPFcSDDDWNHk	Unambiguous	2	1	Q99K47	N-Term(iTRAQ8plex); C7(Methylthio); K15(iTRAQ8plex)	CID	1.0000	0.0000	1	1	2814272	Unique	Used	1.323	1.561	1.037	3.036	0	0	7.07	0	3	200	3	841.03790	2521.09916	-0.70
IAELEEFInGPNNAHIQQVGDR	Unambiguous	2	1	Q68FD5	N-Term(iTRAQ8plex); N9(Deamidated)	CID	1.0000	0.0000	1	1	3037633	Unique	Used	0.227	1.364	1.100	1.193	0	0	7.07	0	0	1	3	923.80811	2769.40976	-0.45
sQTEEDcTEELFDFLHAR	Unambiguous	1	1	P99028	N-Term(iTRAQ8plex); C7(Methylthio)	CID	1.0000	0.0000	1	1	2854631	Unique	Used	1.062	0.859	1.176	0.999	0	0	7.07	0	3	200	3	840.71326	2520.12522	-2.89
tDAVEALTALNHYQIR	Unambiguous	1	1	Q921F4	N-Term(iTRAQ8plex)	CID	1.0000	0.0000	1	1	3620930	Unique	Used	1.027	1.131	0.659	1.097	0	0	7.07	0	12	12	3	707.04968	2119.13449	-4.94

gPQAAQEQDFYTVELER	Unambiguous	4	1	Q6RHR9-3	N-Term(iTRAQ8plex)	CID	1.0000	0.0000	1	1	2717371	Unique	Used	2.254	0.986	0.883	1.062	0	0	7.07	0	0	40	2	1143.07007	2285.13286	-1.02
tGSPGGPGVGGSPGGGAAGASPSLPPSAk	Unambiguous	1	1	Q8R1Q8	N-Term(iTRAQ8plex); K29(iTRAQ8plex)	CID	1.0000	0.0000	1	1	3408592	Unique	Used	4.466	2.275	2.469	2.026	0	0	7.07	0	0	87	3	980.85205	2940.54160	-7.50
nLHQSGFSLSGAQIDDNIPR	Unambiguous	1	1	O08553	N-Term(iTRAQ8plex)	CID	1.0000	0.0000	1	1	3411928	Unique	Used	1.537	0.808	1.439	1.232	0	0	7.07	0	0	108	2	1237.13684	2473.26641	-2.98
nLHQSGFSLSGTQVDEGVR	Unambiguous	3	1	E9PWE8	N-Term(iTRAQ8plex)	CID	1.0000	0.0000	1	1	3365512	Unique	Used	1.041	0.773	0.991	0.835	0	0	7.07	0	10	32	3	779.06580	2335.18283	-4.97
mGPQGSAPAPSSAPLNHGEPSSFR	Unambiguous	1	1	Q7TSG2	N-Term(iTRAQ8plex)	CID	1.0000	0.0000	1	1	3364177	Unique	Used	4.888	2.347	1.407	0.914	0	0	7.07	0	0	200	3	895.10754	2683.30808	-4.48
dIGAIAQVHAEnGDIIAEEQQR	Unambiguous	1	1	O08553	N-Term(iTRAQ8plex); N12(Deamidated)	CID	1.0000	0.0000	1	1	2830871	Unique	Used	1.757	1.265	1.151	1.124	0	0	7.06	0	5	69	3	894.79132	2682.35941	-1.61
nLHQSGFSLSGSQADDHIAR	Unambiguous	3	1	O35098	N-Term(iTRAQ8plex)	CID	1.0000	0.0000	1	1	3363461	Unique	Used	1.058	0.657	1.073	1.079	0	0	7.06	0	0	160	3	815.40826	2444.21024	-4.84
gGHMDDGGYSmNFTMSSSR	Unambiguous	3	1	Q9WV02	N-Term(iTRAQ8plex); M11(Oxidation)	CID	1.0000	0.0000	1	1	3206726	Unique	Used	1.330	0.883	1.350	1.093	0	0	7.06	0	0	140	3	786.33575	2356.99271	1.19
fLSQPFqVAEVFTGHMGk	Unambiguous	1	1	P56480	N-Term(iTRAQ8plex); Q7(Deamidated); K18(iTRAQ8plex)	CID	1.0000	0.0000	1	1	3862708	Unique	Used	1.289	0.953	1.136	1.524	0	0	7.06	0	24	200	3	878.14215	2632.41190	2.46
iSIEMnGTLEDQLSHLk	Unambiguous	1	1	P57780	N-Term(iTRAQ8plex); N6(Deamidated); K17(iTRAQ8plex)	CID	1.0000	0.0000	1	1	3283470	Unique	Used	1.018	2.056	1.295	0.785	0	0	7.06	0	7	55	3	846.45984	2537.36496	-3.63
gTGGVDTAAVGGVFDVSNADR	Unambiguous	1	1	Q04447	N-Term(iTRAQ8plex)	HCD	1.0000	0.0000	1	1	3130830	Unique	Used	1.255	0.978	1.155	1.181	0	0	7.06	0	0	200	2	1135.07068	2269.13408	-0.98
sTmDNPTTTQYANLMHNFILk	Unambiguous	2	1	P62627	N-Term(iTRAQ8plex); M3(Oxidation); K21(iTRAQ8plex)	CID	1.0000	0.0000	1	1	3725166	Unique	Used	1.456	0.667	1.169	0.808	0	0	7.06	0	5	4	3	1022.18774	3064.54868	-6.72

kQQEGATVFSnVAGDSLNTENEPEETTk	Unambiguous	1	1	Q3UVU3	N-Term(iTRAQ8plex); K1(iTRAQ8plex); N11(Deamidated); K28(iTRAQ8plex)	CID	0.0028	0.0000	1	1	2758392	Unique	Used		0.860	0.771	0.699	0	0	7.06	1	0	200	3	1313.00867	3937.01145	0.92
gTGGVDTAAVGAVFDISNADR	Unambiguous	1	1	P07310	N-Term(iTRAQ8plex)	CID	1.0000	0.0000	1	1	3303624	Unique	Used	17.711	30.892	0.162	0.672	0	0	7.06	0	0	36	2	1149.08411	2297.16094	-2.90
tQPTDEEMLFIYSHFk	Unambiguous	4	1	P31786	N-Term(iTRAQ8plex); K16(iTRAQ8plex)	CID	1.0000	0.0000	1	1	3286731	Unique	Used	1.761	0.653	1.009	0.909	0	0	7.06	0	3	200	3	865.45087	2594.33805	-1.57
lSSQEIQQFAALLHEYR	Unambiguous	3	1	Q8K2Y9	N-Term(iTRAQ8plex)	CID	1.0000	0.0000	1	1	3765829	Unique	Used	2.392	1.593	2.528	2.059	0	0	7.06	0	0	34	3	779.75140	2337.23966	-4.64
aFTGFIVEADTPGIHIGk	Unambiguous	1	1	P45952	N-Term(iTRAQ8plex); K18(iTRAQ8plex)	CID	1.0000	0.0000	1	1	3518561	Unique	Used	0.717	1.033	0.692	0.844	0	0	7.06	0	3	32	3	827.79987	2481.38504	-4.51
vEGFNYAGMGNSTNk	Unambiguous	3	1	E9QNN1	N-Term(iTRAQ8plex); K15(iTRAQ8plex)	CID	1.0000	0.0000	1	1	3298470	Unique	Used	0.832	0.857	0.940	0.860	0	0	7.05	0	0	75	2	1099.05896	2197.11064	-2.81
tIGGGDDSFNTFFSETGAGk	Unambiguous	4	1	P68373	N-Term(iTRAQ8plex); K20(iTRAQ8plex)	CID	1.0000	0.0000	1	1	3152064	Unique	Used	0.313	1.119	1.312	1.414	0	0	7.05	0	0	95	2	1308.65442	2616.30156	-0.88
sQHVQVEEGSEPDAFWEALGGk	Unambiguous	2	1	P13020-2	N-Term(iTRAQ8plex); K22(iTRAQ8plex)	CID	1.0000	0.0000	1	1	2902127	Unique	Used	2.763	0.567	1.119	1.033	0	0	7.05	0	0	139	3	1003.50732	3008.50742	-4.54
eHGYLGNLSAVASTEGTIEETLNEASR	Unambiguous	2	1	Q99P72	N-Term(iTRAQ8plex)	CID	1.0000	0.0000	1	1	3172435	Unique	Used	1.627	0.938	1.265	0.895	0	0	7.05	0	0	200	3	1051.52087	3152.54807	-5.37
qDHPSSMGVYGQESGGFSGPGENR	Unambiguous	4	1	Q5SUT0	N-Term(iTRAQ8plex)	CID	1.0000	0.0000	1	1	3066793	Unique	Used	1.572	0.936	0.574	0.601	0	0	7.05	0	0	200	3	928.75641	2784.25467	-1.41
lAELEEFINGPnNAHIQQVGDR	Unambiguous	2	1	Q68FD5	N-Term(iTRAQ8plex); N12(Deamidated)	CID	0.0142	0.0000	1	1	3133151	Unique	Used	0.367	1.229	1.217	1.153	0	0	7.05	0	0	8	3	923.80756	2769.40812	-1.05
tALHVAAAEGHVEVVk	Unambiguous	3	1	D3Z7P3	N-Term(iTRAQ8plex); K16(iTRAQ8plex)	HCD	1.0000	0.0000	1	1	3144029	Unique	Used	0.877	1.220	1.200	1.106	0	0	7.05	0	18	22	3	747.10400	2239.29746	-2.00
aTSSSSSSGVGMSVGQGR	Unambiguous	2	1	P21619	N-Term(iTRAQ8plex)	CID	1.0000	0.0000	1	1	3610685	Unique	Used	0.636	1.430	1.222	1.297	0	0	7.05	0	0	200	2	1016.99347	2032.97966	-3.69

aDLQNHLDTAQHALQDk	Unambiguous	1	1	Q8BL66	N-Term(iTRAQ8plex); K17(iTRAQ8plex)	HCD	1.0000	0.0000	1	1	3165342	Unique	Used	1.223	0.910	1.169	0.847	0	0	7.05	0	13	78	3	842.78534	2526.34146	-4.22
lSFGDTLQVQNVHGAFNALGGADR	Unambiguous	1	1	Q9DC16	N-Term(iTRAQ8plex)	CID	1.0000	0.0000	1	1	3804584	Unique	Used	0.289	0.928	0.761	0.948	0	0	7.05	0	0	9	3	931.14899	2791.43241	-3.79
aDDITSLVADTTLLVHFFGk	Unambiguous	2	1	Q9CTY5	N-Term(iTRAQ8plex); K20(iTRAQ8plex)	CID	1.0000	0.0000	1	1	3687283	Unique	Used	0.434	1.125	1.053	1.017	0	0	7.05	0	20	66	3	924.51410	2771.52774	-5.88
dFTSEQIDEILQnHTEIVFAR	Unambiguous	2	1	Q6PIC6	N-Term(iTRAQ8plex); N13(Deamidated)	CID	0.0099	0.0000	1	1	3376135	Unique	Used	0.856		0.737	0.948	0	0	7.05	0	0	200	3	937.48090	2810.42813	4.64
aGLQEGDQVLAVNDVDFQDIEHSk	Unambiguous	1	1	Q9CZG9	N-Term(iTRAQ8plex); K24(iTRAQ8plex)	CID	1.0000	0.0000	1	1	2813944	Unique	Used	1.333	1.360	0.830	0.756	0	0	7.05	0	23	148	3	1079.22559	3235.66220	-2.17
gHAPSTAAPAPSPAPmSSSVQSDEER	Unambiguous	1	1	Q8C996	N-Term(iTRAQ8plex); M16(Oxidation)	CID	1.0000	0.0000	1	1	2842206	Unique	Used	1.647	1.258	0.959	0.887	0	0	7.05	0	0	138	3	962.12292	2884.35422	-4.98
eHHLSEVQNmASEEk	Unambiguous	1	1	Q91VA7	N-Term(iTRAQ8plex); M10(Oxidation); K15(iTRAQ8plex)	CID	1.0000	0.0000	1	1	2646207	Unique	Used	0.600	0.921	1.138	1.148	0	0	7.05	0	0	200	3	798.07001	2392.19547	-2.88
vADALANAAGHLDDLPGALSALSDLHAHk	Unambiguous	1	1	Q91VB8	N-Term(iTRAQ8plex); K29(iTRAQ8plex)	HCD	1.0000	0.0000	1	1	3745491	Unique	Used	4.625	2.819	0.540	2.108	0	0	7.05	0	5	19	3	1157.95947	3471.86386	-4.81
dFSGAGGIGVFSESSSVASk	Unambiguous	1	1	B1AWN6	N-Term(iTRAQ8plex); K20(iTRAQ8plex)	CID	1.0000	0.0000	1	1	3301455	Unique	Used	0.791	2.004	3.536	2.232	0	0	7.05	0	13	200	2	1249.15222	2497.29717	-2.39
lAALNPESNTSGLDIFAk	Unambiguous	1	1	Q9Z1Q5	N-Term(iTRAQ8plex); K18(iTRAQ8plex)	CID	1.0000	0.0000	1	1	3519862	Unique	Used	0.593	1.760	0.727	0.705	0	0	7.05	0	0	23	2	1235.18921	2469.37114	-3.98
vGELTQEANALAAGHPAQAPAINTR	Unambiguous	1	1	Q68FG2	N-Term(iTRAQ8plex)	CID	1.0000	0.0000	1	1	3476649	Unique	Used	0.488	2.731	2.840	1.796	0	0	7.05	0	0	3	3	935.50116	2804.48893	-2.44
ySEEILGPLDHDDQcGk	Unambiguous	1	1	Q80UG2	N-Term(iTRAQ8plex); C15(Methylthio); K17(iTRAQ8plex)	CID	1.0000	0.0000	1	1	2738347	Unique	Used	0.217	0.934	0.805	0.932	0	0	7.04	0	3	145	3	858.41986	2573.24503	-0.86

nATNVEQAFMTMAAEIk	Unambiguous	1	1	Q9D1G1	N-Term(iTRAQ8plex); K17(iTRAQ8plex)	CID	1.0000	0.0000	1	1	3396109	Unique	Used	0.405	1.064	1.168	1.414	0	0	7.04	0	11	15	2	1239.14746	2477.28765	-4.55
gADQADVADVLGTALEELNR	Unambiguous	2	1	Q6IQX7	N-Term(iTRAQ8plex)	CID	1.0000	0.0000	1	1	3133546	Unique	Used	0.712	0.708	0.761	1.029	0	0	7.04	0	7	138	2	1181.11096	2361.21465	-2.28
hGIPDETcnNYQAk	Unambiguous	1	1	Q9WUU7	N-Term(iTRAQ8plex); C8(Methylthio); N9(Deamidated); K14(iTRAQ8plex)	CID	0.0355	0.0000	1	1	2713512	Unique	Used	2.520	1.726	0.986	0.958	0	0	7.04	0	0	200	3	749.02905	2245.07260	-4.98
vAHSFNcTPIEGMLSHQLk	Unambiguous	2	1	P50580	N-Term(iTRAQ8plex); C7(Methylthio); K19(iTRAQ8plex)	CID	1.0000	0.0000	1	1	3803351	Unique	Used	0.529	0.555	0.605	0.698	0	0	7.04	0	15	34	3	922.81366	2766.42643	-3.11
sQPTAMNSIMETGNTk	Unambiguous	3	1	Q922V4	N-Term(iTRAQ8plex); K16(iTRAQ8plex)	CID	1.0000	0.0000	1	1	3230413	Unique	Used	0.859	0.669	0.749	0.658	0	0	7.04	0	0	122	2	1159.59619	2318.18511	-3.88
dHQPcIIFMDEIDAIGGR	Unambiguous	1	1	P62334	N-Term(iTRAQ8plex); C5(Methylthio)	CID	1.0000	0.0000	1	1	3326911	Unique	Used	0.792	0.599	0.987	0.794	0	0	7.04	0	8	13	3	794.04883	2380.13193	-3.48
dSIPVAELSASGPFESHDLLR	Unambiguous	1	1	Q9CQT5	N-Term(iTRAQ8plex)	CID	1.0000	0.0000	1	1	3282170	Unique	Used	2.183	1.381	0.969	0.965	0	0	7.04	0	13	69	3	848.77917	2544.32297	-0.73
eAAIqGSLASLEAEQASIR	Unambiguous	1	1	Q8VDC1	N-Term(iTRAQ8plex); Q5(Deamidated)	CID	0.0099	0.0000	1	1	3216153	Unique	Used	1.854	0.968	1.039	0.719	0	0	7.04	0	25	200	2	1125.10925	2249.21123	8.22
aAEDSNLISGFGVAEGSEk	Unambiguous	2	1	Q3UHJ0-2	N-Term(iTRAQ8plex); K19(iTRAQ8plex)	CID	1.0000	0.0000	1	1	2812920	Unique	Used	1.568	1.422	2.065	1.041	0	0	7.03	0	2	63	2	1245.15271	2489.29814	0.04
aTSDFAFITSSSLVATSGHSNDNR	Unambiguous	4	1	Q8BPN8-2	N-Term(iTRAQ8plex)	CID	1.0000	0.0000	1	1	3663064	Unique	Used	0.770	1.615	1.165	1.109	0	0	7.03	0	24	68	3	930.45239	2789.34262	-7.82
eHANIDAQSGSQAPNPSTTISPGk	Unambiguous	3	1	Q8VDQ8-2	N-Term(iTRAQ8plex); K24(iTRAQ8plex)	CID	1.0000	0.0000	1	1	3007154	Unique	Used	0.759		0.947	1.572	0	0	7.03	0	1	10	3	1005.85510	3015.55075	-2.81
lDINLLDNVVNcLYHGEGAQQR	Unambiguous	2	1	Q6P5F9	N-Term(iTRAQ8plex); C12(Methylthio)	CID	1.0000	0.0000	1	1	3785719	Unique	Used	0.605	0.821	0.628	0.970	0	0	7.03	0	10	37	3	945.47534	2834.41147	-4.12

gVIALcIEDGSIHR	Unambiguous	1	1	Q8K2B3	N-Term(iTRAQ8plex); C6(Methylthio)	CID	1.0000	0.0000	1	1	3621473	Unique	Used	1.187	0.829	1.422	0.890	0	0	7.03	0	0	55	3	611.65765	1832.95841	-4.40
gFDQTINLILDESHER	Unambiguous	1	1	Q6ZWM4	N-Term(iTRAQ8plex)	CID	1.0000	0.0000	1	1	3132326	Unique	Used	2.275	0.903	0.894	0.619	0	0	7.03	0	16	4	3	731.04736	2191.12754	-1.00
dGTVTAGnASGVSDGAGAVIIASEDAVk	Unambiguous	1	1	Q8BWT1	N-Term(iTRAQ8plex); N8(Deamidated); K28(iTRAQ8plex)	CID	1.0000	0.0000	1	1	2815125	Unique	Used		2.629			0	0	7.03	0	2	61	3	1047.88269	3141.63352	-1.19
eGSSLQNQALQTLQER	Unambiguous	1	1	Q9QYE6	N-Term(iTRAQ8plex)	CID	1.0000	0.0000	1	1	3255701	Unique	Used	1.554		0.932	0.835	0	0	7.03	0	0	137	2	1053.55505	2106.10283	-3.08
qDHPSSMGVYGQESGGFSGPGENR	Unambiguous	4	1	Q5SUT0	N-Term(iTRAQ8plex)	CID	1.0000	0.0000	1	1	2970370	Unique	Used	0.920	1.158	1.643	1.165	0	0	7.03	0	0	191	3	928.75598	2784.25339	-1.87
lVHEALSEELAGPVHALAIQAk	Unambiguous	1	1	Q9D8S9	N-Term(iTRAQ8plex); K22(iTRAQ8plex)	CID	1.0000	0.0000	1	1	3457488	Unique	Used	1.498	0.843	1.204	0.829	0	0	7.03	0	19	44	3	968.89368	2904.66648	-3.55
vEIAGPGFINVHLR	Unambiguous	1	1	Q9D0I9	N-Term(iTRAQ8plex)	CID	1.0000	0.0000	1	1	3802304	Unique	Used	1.108	2.085	1.052	1.062	0	0	7.03	0	5	45	3	609.35583	1826.05295	-3.35
dLSEGHSAQANPPSGPAGAGnALHck	Unambiguous	3	1	B2RQX9	N-Term(iTRAQ8plex); N21(Deamidated); C25(Methylthio); K26(iTRAQ8plex)	CID	0.0114	0.0000	1	1	3163749	Unique	Used	2.965				0	0	7.03	0	0	200	3	1047.85034	3141.53647	1.97
sENGLEFTSSGSANTETTk	Unambiguous	3	1	Q60932-2	N-Term(iTRAQ8plex); K19(iTRAQ8plex)	CID	1.0000	0.0000	1	1	2682911	Unique	Used		0.986	1.054	1.295	0	0	7.03	0	0	200	2	1284.64441	2568.28154	-2.74
aFYSLGNYLVASEIAk	Unambiguous	1	1	Q3U5Q7	N-Term(iTRAQ8plex); K16(iTRAQ8plex)	CID	1.0000	0.0000	1	1	3706523	Unique	Used	0.416	1.148	1.118	1.700	0	0	7.02	0	0	19	2	1177.65601	2354.30474	-7.18
sSSEAGDGSGVGAVGGIPSDSR	Unambiguous	3	1	P55144-3	N-Term(iTRAQ8plex)	CID	1.0000	0.0000	1	1	2649346	Unique	Used	1.240	0.729	1.623	0.789	0	0	7.02	0	0	200	2	1127.04675	2253.08623	-1.56
lYSSmSDTNLAEAGLNYHAQR	Unambiguous	2	1	O88737	N-Term(iTRAQ8plex); M5(Oxidation)	CID	1.0000	0.0000	1	1	3410988	Unique	Used	1.264	1.526	1.703	1.101	0	0	7.02	0	18	48	3	887.76422	2661.27811	-3.75
dLQSSNQLSNHISSLFR	Unambiguous	4	1	Q00612	N-Term(iTRAQ8plex)	CID	1.0000	0.0000	1	1	3876037	Unique	Used	0.570	0.643	1.089	1.244	0	0	7.02	0	0	10	3	750.72797	2250.16935	-3.87
iDSAATSTYEVGNPPDYR	Unambiguous	1	1	Q9D358	N-Term(iTRAQ8plex)	CID	1.0000	0.0000	1	1	2899405	Unique	Used	2.006	1.024	0.945	1.080	0	0	7.02	0	0	200	2	1130.55042	2260.09355	-4.42

rPITGGSGGGEGAGLGGGk	Unambiguous	1	1	Q9R059	N-Term(iTRAQ8plex); K19(iTRAQ8plex)	HCD	1.0000	0.0000	1	1	3271120	Unique	Used	1.258	2.045	0.894	0.798	0	0	7.02	0	0	92	3	731.74438	2193.21860	-0.50
kTGQAAGFSYTDANk	Unambiguous	2	1	G3UWG1	N-Term(iTRAQ8plex); K1(iTRAQ8plex); K15(iTRAQ8plex)	HCD	1.0000	0.0000	1	1	3205744	Unique	Used	1.132	0.621	1.527	0.949	0	0	7.02	1	0	77	3	824.45966	2471.36441	-0.54
sQTEEDcTEELFDFLHAR	Unambiguous	1	1	P99028	N-Term(iTRAQ8plex); C7(Methylthio)	CID	1.0000	0.0000	1	1	2854211	Unique	Used	3.050	1.112	1.155	0.908	0	0	7.02	0	19	200	3	840.71417	2520.12796	-1.80
vVDnPIYLSDMGAALTGAESHELQDVLEETNILk	Unambiguous	1	1	Q8CGK3	N-Term(iTRAQ8plex); N4(Deamidated); K34(iTRAQ8plex)	CID	0.0085	0.0000	1	1	3284814	Unique	Used	0.482	0.775	0.523	0.835	0	0	7.02	0	11	92	3	1432.08276	4294.23374	1.75
hcAYEHDIQALSTQVQQVqDDGQR	Unambiguous	1	1	Q68FG2	N-Term(iTRAQ8plex); C2(Methylthio); Q19(Deamidated)	CID	0.0328	0.0000	1	1	3211941	Unique	Used	1.854	1.244	1.739	1.537	0	0	7.02	0	0	177	3	1040.82397	3120.45737	5.02
aVGLISHVLEQNQEGDAAYR	Unambiguous	3	1	Q9JLZ3	N-Term(iTRAQ8plex)	CID	1.0000	0.0000	1	1	3371736	Unique	Used	1.448	1.174	1.334	1.588	0	0	7.02	0	14	28	3	825.43347	2474.28586	-3.36
sDIAAAQQSGMSLSNSk	Unambiguous	1	1	B1AQZ0	N-Term(iTRAQ8plex); K17(iTRAQ8plex)	CID	1.0000	0.0000	1	1	3252736	Unique	Used	1.711	1.235	0.903	1.004	0	0	7.02	0	8	46	3	768.40851	2303.21097	-0.53
eVnENFAIDLIAQQPVNEVEHR	Unambiguous	2	1	P52503	N-Term(iTRAQ8plex); N3(Deamidated)	CID	1.0000	0.0000	1	1	2870716	Unique	Used	2.029	1.767	1.458	1.169	0	0	7.02	0	23	200	3	957.15656	2869.45511	-2.90
iGEHTPSALAImENANVLAR	Unambiguous	3	1	A6ZI44	N-Term(iTRAQ8plex); M12(Oxidation)	CID	1.0000	0.0000	1	1	3701634	Unique	Used	0.988	1.625	0.858	1.075	0	0	7.02	0	0	0	3	809.76599	2427.28342	-5.53
vDDkVNFHFILFnNVDGHLYELDGR	Unambiguous	1	1	Q9R0P9	N-Term(iTRAQ8plex); K4(iTRAQ8plex); N13(Deamidated)	CID	0.0114	0.0000	1	1	3417259	Unique	Used	1.555	0.971	1.340	1.164	0	0	7.02	1	0	126	3	1195.95862	3585.86130	0.72
sWGSGGGQEPGGSQAAGGGQPR	Unambiguous	5	1	Q8CFT0	N-Term(iTRAQ8plex)	CID	1.0000	0.0000	1	1	3005231	Unique	Used	0.641	0.361	1.303	1.273	0	0	7.02	0	0	132	2	1145.04626	2289.08525	-2.55

tQPTDEEMLFIYSHFk	Unambiguous	4	1	P31786	N-Term(iTRAQ8plex); K16(iTRAQ8plex)	CID	1.0000	0.0000	1	1	3287148	Unique	Used	2.056	0.749	0.894	0.811	0	0	7.02	0	0	200	3	865.45074	2594.33768	-1.72
ekPELQFPFLqDEDTVATLHEck	Unambiguous	2	1	P16330	N-Term(iTRAQ8plex); K2(iTRAQ8plex); Q11(Deamidated); C22(Methylthio); K23(iTRAQ8plex)	CID	1.0000	0.0000	1	1	3194391	Unique	Used	1.875	0.512	1.072	1.054	0	0	7.01	0	0	200	3	1226.30750	3676.90793	1.98
iLFIFIDSDHTDNQR	Unambiguous	1	1	P09103	N-Term(iTRAQ8plex)	CID	1.0000	0.0000	1	1	3541194	Unique	Used	1.850	0.833	1.024	0.809	0	0	7.01	0	8	14	3	713.37561	2138.11228	-2.87
kGDSSSGGGGGSSGGGGSSnVGGGSSGGSGScSSSSR	Unambiguous	3	1	Q64012-2	K1(iTRAQ8plex); N-Term(iTRAQ8plex); N20(Deamidated); C32(Methylthio)	CID	1.0000	0.0000	1	1	2984069	Unique	Used		0.510	1.512	1.691	0	0	7.01	1	0	200	3	1203.19678	3607.57578	2.20
gPPDSDFDGGVYHGR	Unambiguous	2	1	F6SPK0	N-Term(iTRAQ8plex)	CID	1.0000	0.0000	1	1	2631620	Unique	Used	1.127	0.652	0.610	1.003	0	0	7.01	0	17	28	3	627.29926	1879.88321	-2.38
gESPELDLSYSHSDLGk	Unambiguous	1	1	P20917	N-Term(iTRAQ8plex); K17(iTRAQ8plex)	CID	1.0000	0.0000	1	1	2828774	Unique	Used	1.719	0.144	0.483	0.424	0	0	7.01	0	0	176	2	1221.62976	2442.25224	-3.54
tTLYDIAHTPGVAADLSHIETR	Unambiguous	1	1	P08249	N-Term(iTRAQ8plex)	CID	1.0000	0.0000	1	1	3457828	Unique	Used	2.529	1.062	0.920	1.009	0	0	7.01	0	7	22	3	899.81854	2697.44107	-3.83
qTTAAAAATFSEQVGGGSGGAGR	Unambiguous	3	1	O88935	N-Term(iTRAQ8plex)	CID	1.0000	0.0000	1	1	3502651	Unique	Used		2.524	1.493	1.805	0	0	7.01	0	16	200	2	1178.59131	2356.17534	-1.79
lTEMETMQSQLMAEk	Unambiguous	1	1	Q8VDD5	N-Term(iTRAQ8plex); K15(iTRAQ8plex)	CID	1.0000	0.0000	1	1	3172593	Unique	Used	0.911	0.835	0.908	0.991	0	0	7.01	0	0	117	2	1189.61218	2378.21709	-2.33
eYGFSQEEILQLHSDR	Unambiguous	1	1	O09174	N-Term(iTRAQ8plex)	CID	1.0000	0.0000	1	1	2956332	Unique	Used	2.025	0.691	1.279	1.947	0	0	7.01	0	14	115	3	752.37848	2255.12088	-1.66
yIPVQQGPVGVNVTYGGDHIPk	Unambiguous	3	1	B7FAU9	N-Term(iTRAQ8plex); K22(iTRAQ8plex)	CID	1.0000	0.0000	1	1	3474756	Unique	Used	0.391	2.292	0.794	0.908	0	0	7.00	0	21	18	3	982.87793	2946.61924	-3.59
lSSGEHIAAFcLTEPASGSDAASIQTR	Unambiguous	1	1	Q8JZN5	N-Term(iTRAQ8plex); C11(Methylthio)	CID	1.0000	0.0000	1	1	3457906	Unique	Used	2.220	1.521	1.602	1.121	0	0	7.00	0	3	74	3	1023.83276	3069.48374	-2.82

dSDWPFcSDDDWNHk	Unambiguous	2	1	Q99K47	N-Term(iTRAQ8plex); C7(Methylthio); K15(iTRAQ8plex)	CID	1.0000	0.0000	1	1	2814153	Unique	Used	1.207	1.766	1.271	3.305	0	0	7.00	0	4	76	3	841.03455	2521.08908	-4.70
tIGSTYMAATGLSAVPSQEHAQEPER	Unambiguous	2	1	Q80TL1	N-Term(iTRAQ8plex)	CID	1.0000	0.0000	1	1	3278329	Unique	Used	1.243	0.917	1.599	1.211	0	0	7.00	0	9	98	3	1012.50415	3035.49790	-2.23
lQELTGSEGQVFmENVTQLLQSSHk	Unambiguous	2	1	Q8WTY4	N-Term(iTRAQ8plex); M13(Oxidation); K25(iTRAQ8plex)	CID	1.0000	0.0000	1	1	3686483	Unique	Used	1.738	0.488	1.027	0.697	0	0	7.00	0	14	15	3	1143.26233	3427.77243	-7.70
eGITVYSTQFGGYAk	Unambiguous	1	1	Q9R257	N-Term(iTRAQ8plex); K15(iTRAQ8plex)	CID	1.0000	0.0000	1	1	3344879	Unique	Used	1.248	2.832	1.411	0.659	0	0	7.00	0	11	17	2	1115.09912	2229.19097	-4.58
tYFPHFDVSHGSAQVk	Unambiguous	2	1	Q91VB8	N-Term(iTRAQ8plex); K16(iTRAQ8plex)	CID	1.0000	0.0000	1	1	3473330	Unique	Used	2.960	2.241	0.646	1.345	0	0	7.00	0	0	90	3	810.09674	2428.27567	-4.66
aFYSLGNYLVASEIAk	Unambiguous	1	1	Q3U5Q7	N-Term(iTRAQ8plex); K16(iTRAQ8plex)	CID	1.0000	0.0000	1	1	3706514	Unique	Used	0.494	1.173	0.967	1.592	0	0	7.00	0	0	48	2	1177.65625	2354.30522	-6.97
sAATSGAGSTTSGVVSGSLGSR	Unambiguous	5	1	Q7TMY8-3	N-Term(iTRAQ8plex)	CID	1.0000	0.0000	1	1	3634209	Unique	Used	0.402	0.970	1.059	1.274	0	0	7.00	0	0	80	2	1101.06360	2201.11992	-5.12
lGADVTAVEFLGHVGGIGIDmEISk	Unambiguous	1	1	O08749	N-Term(iTRAQ8plex); M21(Oxidation); K25(iTRAQ8plex)	CID	1.0000	0.0000	1	1	3479535	Unique	Used	2.251	1.888	1.784	0.981	0	0	7.00	0	20	18	3	1051.57532	3152.71140	-0.28
gGLSTFFEAQDALSAIHQk	Unambiguous	1	1	Q9D4H1	N-Term(iTRAQ8plex); K19(iTRAQ8plex)	CID	1.0000	0.0000	1	1	3579804	Unique	Used	0.391	1.237	0.923	1.005	0	0	7.00	0	0	45	3	876.80756	2628.40812	-6.14
aEGATLSNATGAVESTSQAGDR	Unambiguous	1	1	Q61644	N-Term(iTRAQ8plex)	CID	1.0000	0.0000	1	1	2846527	Unique	Used	2.089	1.315	1.898	1.131	0	0	7.00	0	0	200	2	1199.09045	2397.17363	-2.49
gTGGVDTAAVGGVFDVSNADR	Unambiguous	1	1	Q04447	N-Term(iTRAQ8plex)	CID	1.0000	0.0000	1	1	3134945	Unique	Used	1.324	1.144	1.210	1.297	0	0	7.00	0	0	200	2	1135.06860	2269.12993	-2.81
qTTAAAAATFSEQVGGGSGGAGR	Unambiguous	3	1	O88935	N-Term(iTRAQ8plex)	CID	1.0000	0.0000	1	1	3502558	Unique	Used	1.106	1.720	1.267	1.634	0	0	7.00	0	0	200	2	1178.59070	2356.17412	-2.31
vSHVSTGGGASLELLEGk	Unambiguous	2	1	P09411	N-Term(iTRAQ8plex); K18(iTRAQ8plex)	CID	1.0000	0.0000	1	1	3254749	Unique	Used	0.881	0.851	1.187	1.168	0	0	7.00	0	0	57	2	1175.16211	2349.31694	-2.77

gTVVYGEPITASLGTDGSHYWSk	Unambiguous	1	1	O08553	N-Term(iTRAQ8plex); K23(iTRAQ8plex)	CID	1.0000	0.0000	1	1	3369971	Unique	Used	1.311	1.465	1.219	1.248	0	0	6.99	0	11	24	3	1011.85846	3033.56083	-5.61
eTAAAHQASSSPPIDAATAEPYGFR	Unambiguous	1	1	P14873	N-Term(iTRAQ8plex)	CID	1.0000	0.0000	1	1	3010000	Unique	Used	2.871	0.692	1.011	0.773	0	0	6.99	0	20	55	3	950.46985	2849.39499	-2.05
eHINLGcDVDFDIAGPSIR	Unambiguous	3	1	Q60932-2	N-Term(iTRAQ8plex); C7(Methylthio)	CID	1.0000	0.0000	1	1	3357577	Unique	Used	1.000	0.979	1.431	0.914	0	0	6.99	0	2	200	3	807.73035	2421.17649	-3.31
iIGLDQVAGMSETALPGAFk	Unambiguous	1	1	Q8VDD5	N-Term(iTRAQ8plex); K20(iTRAQ8plex)	CID	1.0000	0.0000	1	1	3645023	Unique	Used	0.508	1.013	0.828	1.004	0	0	6.99	0	0	44	2	1313.73413	2626.46099	-4.78
lVAPLLATVTILDDDHAGIFSFQDR	Unambiguous	1	1	Q8K596	N-Term(iTRAQ8plex)	CID	1.0000	0.0000	1	1	3727531	Unique	Used	0.467	1.931	1.920	1.322	0	0	6.99	0	22	117	3	1011.21252	3031.62302	-5.83
sITIANQTNcPLYVTk	Unambiguous	1	1	O08553	N-Term(iTRAQ8plex); C10(Methylthio); K16(iTRAQ8plex)	CID	1.0000	0.0000	1	1	3803570	Unique	Used	1.157	1.149	1.088	1.566	0	0	6.99	0	2	9	2	1210.65552	2420.30376	-4.15
gAIQLGIGYTVGNLSSkPER	Unambiguous	4	1	F8WHW6	N-Term(iTRAQ8plex); K17(iTRAQ8plex)	CID	1.0000	0.0000	1	1	3821211	Unique	Used	0.352	1.519	1.529	1.136	0	0	6.99	0	4	53	3	890.17609	2668.51371	-3.97
qTTAAAAATFSEQVGGGSGGAGR	Unambiguous	3	1	O88935	N-Term(iTRAQ8plex)	CID	1.0000	0.0000	1	1	3493966	Unique	Used	0.342	1.260	1.797	1.640	0	0	6.99	0	0	200	2	1178.58899	2356.17070	-3.76
sNcNVAVINVGAPAAGMNAAVR	Unambiguous	3	1	Q8C605	N-Term(iTRAQ8plex); C3(Methylthio)	CID	1.0000	0.0000	1	1	3933825	Unique	Used	1.767	0.969	1.017	0.919	0	0	6.99	0	0	18	2	1225.11890	2449.23052	-4.55
nVGSTALSPYPTAISGLQSQR	Unambiguous	4	1	Q8R0S4	N-Term(iTRAQ8plex)	CID	1.0000	0.0000	1	1	3875660	Unique	Used	0.802	0.984	1.792	1.155	0	0	6.99	0	0	200	2	1226.15662	2451.30596	-3.51
vYFQSPPGAAGEGPGGADDDGPVR	Unambiguous	1	1	Q62084	N-Term(iTRAQ8plex)	CID	1.0000	0.0000	1	1	2758915	Unique	Used	1.501		0.881		0	0	6.99	0	0	200	2	1310.62769	2620.24809	-3.86
sIDDLEDQLYHQLEQnR	Unambiguous	3	1	E9Q452	N-Term(iTRAQ8plex); N16(Deamidated)	CID	0.0372	0.0000	1	1	2766072	Unique	Used	2.568	1.487	1.784	1.109	0	0	6.99	0	7	200	3	807.73163	2421.18033	-1.35
gVMGGQSAGPQHTEAETIQk	Unambiguous	3	1	Q9Z1Z0	N-Term(iTRAQ8plex); K20(iTRAQ8plex)	CID	1.0000	0.0000	1	1	2912562	Unique	Used	0.751	0.861	0.835	0.801	0	0	6.99	0	0	200	3	878.79352	2634.36600	-4.04
vLAGDVETHAEMVHGAFQAQR	Unambiguous	2	1	Q9CYT6	N-Term(iTRAQ8plex)	CID	1.0000	0.0000	1	1	3388888	Unique	Used	0.754	1.526	1.421	0.990	0	0	6.98	0	15	43	3	857.43793	2570.29923	-3.72

sNcNVAVINVGAPAAGMNAAVR	Unambiguous	3	1	Q8C605	N-Term(iTRAQ8plex); C3(Methylthio)	CID	1.0000	0.0000	1	1	3915112	Unique	Used	1.902	1.097	1.373	1.376	0	0	6.98	0	0	98	2	1225.11914	2449.23100	-4.35
qSESSFITGDINSSASLNR	Unambiguous	22	1	Q80TS3-5	N-Term(iTRAQ8plex)	HCD	1.0000	0.0000	1	1	3211123	Unique	Used	0.563	1.665	1.478	1.328	0	0	6.98	0	0	194	2	1159.08179	2317.15630	-0.47
tETVcTFQDGALVQHQQWDGk	Unambiguous	1	1	Q05816	N-Term(iTRAQ8plex); C5(Methylthio); K21(iTRAQ8plex)	CID	1.0000	0.0000	1	1	3280142	Unique	Used	1.762	0.399	0.790	0.634	0	0	6.98	0	2	11	3	1015.83636	3045.49454	-2.42
fTASAGIqVVGDDLTVTNPk	Unambiguous	2	1	P17182	N-Term(iTRAQ8plex); Q8(Deamidated); K20(iTRAQ8plex)	CID	1.0000	0.0000	1	1	3349813	Unique	Used	0.752	1.433	1.113	1.222	0	0	6.98	0	13	34	2	1321.73340	2642.45952	3.68
sGLGLGAASASTSGSGPADSVMR	Unambiguous	3	1	Q7TSJ2	N-Term(iTRAQ8plex)	CID	1.0000	0.0000	1	1	3534250	Unique	Used	1.311	0.675	1.344	1.289	0	0	6.98	0	0	111	2	1170.58569	2340.16411	-5.42
iLTEAEIDAHLVALAERD	Unambiguous	2	1	Q9QUM9	N-Term(iTRAQ8plex)	HCD	1.0000	0.0000	1	1	3111939	Unique	Used	2.523	1.107	0.751	1.029	0	0	6.98	1	0	27	3	761.75403	2283.24753	-1.02
sQTEEDcTEELFDFLHAR	Unambiguous	1	1	P99028	N-Term(iTRAQ8plex); C7(Methylthio)	CID	1.0000	0.0000	1	1	2853594	Unique	Used	1.480	0.956	1.486	1.213	0	0	6.98	0	28	200	3	840.71417	2520.12796	-1.80
nVIIWGNHSSTQYPDVnHAk	Unambiguous	1	1	P14152	N-Term(iTRAQ8plex); N17(Deamidated); K20(iTRAQ8plex)	CID	0.0229	0.0000	1	1	3553396	Unique	Used	0.520	1.306	1.036	1.147	0	0	6.98	0	0	200	3	963.84521	2889.52109	3.70
sTMDNPTTTQYANLMHNFILk	Unambiguous	2	1	P62627	N-Term(iTRAQ8plex); K21(iTRAQ8plex)	CID	1.0000	0.0000	1	1	3726073	Unique	Used	1.640	1.014	1.177	2.216	0	0	6.98	0	18	123	3	1016.85760	3048.55826	-5.28
aAELIAnSLATAGDGLIELR	Unambiguous	1	1	P67778	N-Term(iTRAQ8plex); N7(Deamidated)	CID	1.0000	0.0000	1	1	3667487	Unique	Used	0.731	0.607	1.352	1.588	0	0	6.98	0	21	200	2	1152.14124	2303.27519	-0.39
tVAGIIVEPIQSEGGDnHASDDFFR	Unambiguous	2	1	P61922	N-Term(iTRAQ8plex); N17(Deamidated)	CID	1.0000	0.0000	1	1	2921197	Unique	Used	3.873	0.783	0.777	1.042	0	0	6.98	0	0	200	3	993.82520	2979.46103	-0.95
hGEASSANSAGGPTSDAGAAVAPNPGPR	Unambiguous	1	1	Q80W04	N-Term(iTRAQ8plex)	CID	1.0000	0.0000	1	1	2842278	Unique	Used	2.136	1.510	1.428	0.861	0	0	6.98	0	0	200	3	936.45642	2807.35471	-2.29
aEGATLSNATGAVESTSQAGDR	Unambiguous	1	1	Q61644	N-Term(iTRAQ8plex)	CID	1.0000	0.0000	1	1	2846664	Unique	Used	1.919	1.170	2.236	1.377	0	0	6.98	0	4	30	2	1199.08813	2397.16899	-4.43

sDALETLGFLNHYQMk	Unambiguous	3	1	G5E924	N-Term(iTRAQ8plex); K16(iTRAQ8plex)	CID	1.0000	0.0000	1	1	3518426	Unique	Used	0.734	0.820	0.942	0.726	0	0	6.98	0	16	29	3	825.77386	2475.30704	-3.72
mVQASSQSLLPPAQDRPR	Unambiguous	3	1	Q8BTI8	N-Term(iTRAQ8plex)	HCD	1.0000	0.0000	1	1	3891706	Unique	Used	1.157	1.072	1.497	1.194	0	0	6.98	0	11	113	3	762.41083	2285.21793	-6.96
eHGYLGNLSAVASTEGTIEETLNEASR	Unambiguous	2	1	Q99P72	N-Term(iTRAQ8plex)	CID	1.0000	0.0000	1	1	3152261	Unique	Used	1.463	0.867	1.536	1.009	0	0	6.97	0	20	200	3	1051.52576	3152.56272	-0.73
gPNIVLSEELAGHQTPITDIATER	Unambiguous	1	1	Q9R0D8	N-Term(iTRAQ8plex)	CID	1.0000	0.0000	1	1	3214344	Unique	Used	2.719	1.006	1.303	1.255	0	0	6.97	0	0	115	3	955.84546	2865.52182	-1.48
vIHGNPSGVDNAVSTWGGALR	Unambiguous	3	1	Q9R008	N-Term(iTRAQ8plex)	CID	1.0000	0.0000	1	1	3760655	Unique	Used	0.725	0.556	0.598	0.867	0	0	6.97	0	6	14	3	804.42731	2411.26737	-2.50
gAHGIIVVYDVTDQESYANVk	Unambiguous	1	1	Q9D1G1	N-Term(iTRAQ8plex); K21(iTRAQ8plex)	CID	1.0000	0.0000	1	1	3148983	Unique	Used	1.081	0.871	1.200	1.076	0	0	6.97	0	0	30	3	962.85144	2886.53977	-2.09
aTLDcLnGDYNVEEGHGk	Unambiguous	1	1	P97490	N-Term(iTRAQ8plex); C5(Methylthio); N7(Deamidated); K18(iTRAQ8plex)	CID	1.0000	0.0000	1	1	2760060	Unique	Used	0.409	1.107	1.148	1.283	0	0	6.97	0	24	171	3	864.08234	2590.23246	-1.92
gPTGPPSTLPLGTQTSSGSSTLSk	Unambiguous	7	1	H3BJY2	N-Term(iTRAQ8plex); K24(iTRAQ8plex)	CID	1.0000	0.0000	1	1	3595514	Unique	Used	0.976	1.295	0.993	1.337	0	0	6.97	0	0	60	3	956.18738	2866.54758	-4.98
eDDGVPVIcQVEHPAVTGNLQTQR	Unambiguous	9	1	Q8R5M8-4	N-Term(iTRAQ8plex); C9(Methylthio)	CID	1.0000	0.0000	1	1	3191603	Unique	Used	0.763	1.561	1.357	1.335	0	0	6.97	0	0	11	3	985.82379	2955.45682	-1.31
qVHDEEVAELLATLqASSQAAAEVDVAVAkPDLTSALR	Unambiguous	1	1	P46660	N-Term(iTRAQ8plex); Q15(Deamidated); K30(iTRAQ8plex)	CID	0.0230	0.0000	1	1	3459479	Unique	Used	1.843				0	0	6.97	0	0	50	3	1519.14697	4555.42636	-1.97
aITVASQTNcPLYVTk	Unambiguous	5	1	E9PWE8	N-Term(iTRAQ8plex); C10(Methylthio); K16(iTRAQ8plex)	CID	1.0000	0.0000	1	1	3840585	Unique	Used	0.803	0.412	0.449	0.350	0	0	6.97	0	0	200	2	1182.14905	2363.29082	-0.64
sVQLQPGSYVAAGVEPGESAAk	Unambiguous	3	1	Q3U7U3	N-Term(iTRAQ8plex); K22(iTRAQ8plex)	CID	1.0000	0.0000	1	1	3126594	Unique	Used	0.491	1.150	1.018	1.376	0	0	6.97	0	0	111	2	1377.24658	2753.48589	-2.61

aVVFETGEAGAASGAGSLPGEVR	Unambiguous	3	1	Q9QYR6	N-Term(iTRAQ8plex)	CID	1.0000	0.0000	1	1	3129393	Unique	Used	2.179	0.856	1.318	1.996	0	0	6.97	0	0	101	2	1218.63611	2436.26494	-0.98
aTSSSSSSGVGMSVGQGR	Unambiguous	2	1	P21619	N-Term(iTRAQ8plex)	CID	1.0000	0.0000	1	1	3427583	Unique	Used	0.832	0.896	1.490	1.566	0	0	6.97	0	0	200	2	1016.99261	2032.97795	-4.53
vLDGLTAGSSSASQQQQQQHPPGNR	Unambiguous	3	1	D3Z2Q2	N-Term(iTRAQ8plex)	HCD	1.0000	0.0000	1	1	3552749	Unique	Used	0.480	1.099	1.338	1.294	0	0	6.97	0	0	33	3	1008.50690	3023.50614	-4.49
aSSGGGGGGLMEEMNk	Unambiguous	4	1	P70429-2	N-Term(iTRAQ8plex); K16(iTRAQ8plex)	CID	1.0000	0.0000	1	1	2846075	Unique	Used	0.927	0.939	0.863	0.872	0	0	6.97	0	0	200	2	1045.52698	2090.04668	-0.03
eVSSGSEETFSYSDFLR	Unambiguous	1	1	O70200	N-Term(iTRAQ8plex)	CID	1.0000	0.0000	1	1	2939717	Unique	Used	1.731	1.353	1.178	0.809	0	0	6.97	0	12	67	2	1122.53308	2244.05889	-0.95
tPSAAYLWVGAGASEAEk	Unambiguous	2	1	P13020-2	N-Term(iTRAQ8plex); K18(iTRAQ8plex)	CID	1.0000	0.0000	1	1	3258155	Unique	Used	1.907	0.672	0.415	0.615	0	0	6.97	0	19	99	2	1208.65137	2416.29546	-0.60
dFLSNETSLGAIGSSLPSR	Unambiguous	1	1	Q6ZPS6	N-Term(iTRAQ8plex)	CID	1.0000	0.0000	1	1	3579627	Unique	Used			0.987	1.056	0	0	6.97	0	0	101	2	1128.08728	2255.16728	-6.60
sQTEEDcTEELFDFLHAR	Unambiguous	1	1	P99028	N-Term(iTRAQ8plex); C7(Methylthio)	CID	1.0000	0.0000	1	1	2835068	Unique	Used	2.712	1.395	1.691	1.019	0	0	6.97	0	1	10	3	840.71490	2520.13016	-0.93
tANcTLAnTNYVNITk	Unambiguous	1	1	P63141	N-Term(iTRAQ8plex); C4(Methylthio); N8(Deamidated); K16(iTRAQ8plex)	CID	0.0115	0.0000	1	1	3702625	Unique	Used	0.471	0.338	1.150	1.126	0	0	6.97	0	0	200	2	1198.62354	2396.23979	-0.51
dMAIATGGAVFGEEGLNLNLEDVQAHDLGk	Unambiguous	1	1	P63038	N-Term(iTRAQ8plex); K30(iTRAQ8plex)	CID	1.0000	0.0000	1	1	3285827	Unique	Used	0.660	0.814	0.823	1.223	0	0	6.97	0	17	200	3	1231.63647	3692.89487	-2.78
yEGSGDGGAAAQSLYIANHAY	Unambiguous	1	1	P05063	N-Term(iTRAQ8plex)	CID	1.0000	0.0000	1	1	2957309	Unique	Used	1.267	0.557	1.167	1.537	0	0	6.96	0	0	139	2	1210.07361	2419.13994	-2.84
kTGSPGGPGVGGSPGGGAAGASPSLPPSAk	Unambiguous	1	1	Q8R1Q8	N-Term(iTRAQ8plex); K1(iTRAQ8plex); K30(iTRAQ8plex)	CID	1.0000	0.0000	1	1	3450415	Unique	Used	0.794	0.989	1.133	0.899	0	0	6.96	1	0	200	3	1124.95349	3372.84592	-5.35
yDGQVAVFGSDFQEk	Unambiguous	1	1	Q02053	N-Term(iTRAQ8plex); K15(iTRAQ8plex)	CID	1.0000	0.0000	1	1	2938059	Unique	Used	1.032	1.409	1.002	1.475	0	0	6.96	0	1	6	3	766.73224	2298.18216	-1.79

tIGGGDDSFNTFFSETGAGk	Unambiguous	4	1	P68373	N-Term(iTRAQ8plex); K20(iTRAQ8plex)	CID	1.0000	0.0000	1	1	3150817	Unique	Used	0.606	1.607	0.952	1.233	0	0	6.96	0	0	49	2	1308.65320	2616.29912	-1.81
dIkPQNLLVDPDTAVLk	Unambiguous	2	1	D3Z7E5	N-Term(iTRAQ8plex); K3(iTRAQ8plex); K17(iTRAQ8plex)	HCD	1.0000	0.0000	1	1	3280334	Unique	Used	0.557	1.090	1.071	1.052	0	0	6.96	0	19	118	3	931.22644	2791.66477	-1.76
dLVILLYETALLSSGFSLEDPQTHANR	Unambiguous	1	1	P07901	N-Term(iTRAQ8plex)	CID	1.0000	0.0000	1	1	3786482	Unique	Used	1.290	2.025	0.746	0.741	0	0	6.96	0	0	200	3	1102.91919	3306.74302	-2.84
sSGSPYGGGYGSGGGSGGYGSR	Unambiguous	7	1	J3QNY1	N-Term(iTRAQ8plex)	CID	1.0000	0.0000	1	1	3226210	Unique	Used	0.718	0.858	1.221	0.830	0	0	6.96	0	0	200	2	1108.00073	2214.99419	-0.55
kGAAALGIGTDSVILk	Unambiguous	1	1	P48320	N-Term(iTRAQ8plex); K1(iTRAQ8plex); K17(iTRAQ8plex)	CID	1.0000	0.0000	1	1	3662138	Unique	Used	0.759	0.738	1.089	1.148	0	0	6.96	1	14	176	3	847.19604	2539.57358	-8.46
sASAFTYSSSSISAk	Unambiguous	1	1	E9Q6Y8	N-Term(iTRAQ8plex); K15(iTRAQ8plex)	CID	1.0000	0.0000	1	1	3431763	Unique	Used	0.677	0.520	1.604	0.986	0	0	6.96	0	0	200	2	1051.55713	2102.10698	-7.43
fLSQPFQVAEVFTGHMGk	Unambiguous	1	1	P56480	N-Term(iTRAQ8plex); K18(iTRAQ8plex)	CID	1.0000	0.0000	1	1	3862874	Unique	Used	1.649	1.178	1.098	1.312	0	0	6.96	0	17	200	3	877.80695	2631.40628	-5.75
mAAAGGTVSGPSSAckPGR	Unambiguous	2	1	F8WIE5	N-Term(iTRAQ8plex); C15(Methylthio); K16(iTRAQ8plex)	CID	1.0000	0.0000	1	1	3816703	Unique	Used	0.462	0.703	0.804	0.917	0	0	6.96	0	0	200	3	787.07324	2359.20517	-3.81
lQEANAQLAEQTcPHDLEMTPVQR	Unambiguous	1	1	Q80Y17	N-Term(iTRAQ8plex); C13(Methylthio)	CID	1.0000	0.0000	1	1	3192389	Unique	Used	0.840	0.317	0.631	0.749	0	0	6.96	0	11	56	3	1024.83118	3072.47898	-2.13
lNITPNSGATGnNAGPk	Unambiguous	1	1	Q62433	N-Term(iTRAQ8plex); N12(Deamidated); K17(iTRAQ8plex)	CID	0.0244	0.0000	1	1	3185341	Unique	Used	0.948	0.605	1.375	1.664	0	0	6.96	0	0	71	2	1118.11121	2235.21514	-1.72
tYFPHFDVSHGSAQVk	Unambiguous	2	1	Q91VB8	N-Term(iTRAQ8plex); K16(iTRAQ8plex)	CID	1.0000	0.0000	1	1	3738928	Unique	Used	1.584	1.902	0.731	1.868	0	0	6.96	0	20	139	3	810.09723	2428.27713	-4.06

gLTIVGSVLEGTFLDnHPQAQR	Unambiguous	2	1	Q91V14	N-Term(iTRAQ8plex); N16(Deamidated)	CID	1.0000	0.0000	1	1	3843121	Unique	Used	0.663	1.175	1.057	0.658	0	0	6.96	0	11	200	3	886.48169	2657.43052	3.91
gSVAAGLASAGGGGSGAGVGAYGGAAGAAGGGGAGSER	Unambiguous	2	1	F7CZ64	N-Term(iTRAQ8plex)	CID	1.0000	0.0000	1	1	3493408	Unique	Used			3.002		0	0	6.95	0	0	41	3	1061.18628	3181.54428	-2.61
gTVVYGEPITASLGTDGSHYWSk	Unambiguous	1	1	O08553	N-Term(iTRAQ8plex); K23(iTRAQ8plex)	CID	1.0000	0.0000	1	1	3436090	Unique	Used	0.875	1.528	1.250	1.508	0	0	6.95	0	0	24	3	1011.86115	3033.56888	-2.95
kGTVLcGPPPAVENASLVGVR	Unambiguous	1	1	P55066	N-Term(iTRAQ8plex); K1(iTRAQ8plex); C6(Methylthio)	HCD	1.0000	0.0000	1	1	3801902	Unique	Used	1.046	1.014	1.131	0.536	0	0	6.95	1	0	38	3	906.84552	2718.52201	-1.32
sQSVDITAPGFNPLGGAGk	Unambiguous	2	1	Q4KMM3	N-Term(iTRAQ8plex); K19(iTRAQ8plex)	CID	1.0000	0.0000	1	1	3516657	Unique	Used	0.925	0.978	1.817	1.568	0	0	6.95	0	18	200	2	1212.66565	2424.32402	-4.27
eSNPALGIDcLHk	Unambiguous	1	1	P80316	N-Term(iTRAQ8plex); C10(Methylthio); K13(iTRAQ8plex)	CID	1.0000	0.0000	1	1	3255665	Unique	Used	0.622	1.062	0.805	0.863	0	0	6.95	0	7	11	3	684.36615	2051.08390	-1.72
iGEHTPSALAImENANVLAR	Unambiguous	3	1	A6ZI44	N-Term(iTRAQ8plex); M12(Oxidation)	CID	1.0000	0.0000	1	1	3660361	Unique	Used	1.345	0.928	1.012	0.959	0	0	6.95	0	15	37	3	809.76599	2427.28342	-5.53
lLEVSDDPQVLAVAAHDVGEYVR	Unambiguous	1	1	Q8BVE3	N-Term(iTRAQ8plex)	CID	1.0000	0.0000	1	1	3241481	Unique	Used	0.846		1.504	1.118	0	0	6.95	0	0	200	3	933.83429	2799.48832	1.86
qTTAAAAATFSEQVGGGSGGAGR	Unambiguous	3	1	O88935	N-Term(iTRAQ8plex)	CID	1.0000	0.0000	1	1	3495261	Unique	Used	1.046	1.408	1.757	1.699	0	0	6.95	0	0	200	2	1178.59204	2356.17681	-1.17
tGDFQLHTNVnNGTEFGGSIYQk	Unambiguous	2	1	G3UX26	N-Term(iTRAQ8plex); N11(Deamidated); K23(iTRAQ8plex)	CID	0.0101	0.0000	1	1	3233634	Unique	Used	0.663	1.173	1.174	1.328	0	0	6.95	0	0	23	3	1046.19812	3136.57981	0.06
lcDFGISGQLVDSIAk	Unambiguous	2	1	P47809	N-Term(iTRAQ8plex); C2(Methylthio); K16(iTRAQ8plex)	CID	1.0000	0.0000	1	1	3645206	Unique	Used		0.955	0.919	1.118	0	0	6.95	0	18	20	2	1160.62268	2320.23808	-5.21

qFMDIFSLPEMTLLScVNEHFLk	Unambiguous	1	1	Q3UHB1	N-Term(iTRAQ8plex); C16(Methylthio); K23(iTRAQ8plex)	CID	1.0000	0.0000	1	1	3861441	Unique	Used	0.369	0.731	0.947	0.969	0	0	6.95	0	0	61	3	1132.91284	3396.72397	-2.53
yTPSGQSGAAASESLFISNHAy	Unambiguous	2	1	A6ZI44	N-Term(iTRAQ8plex); Y22(iTRAQ8plex)	CID	1.0000	0.0000	1	1	3514248	Unique	Used	2.471	1.787	1.606	2.038	0	0	6.95	0	10	189	3	956.14972	2866.43460	-4.26
iGEHTPSALAImENANVLAR	Unambiguous	3	1	A6ZI44	N-Term(iTRAQ8plex); M12(Oxidation)	CID	1.0000	0.0000	1	1	3709145	Unique	Used	1.676	0.855	1.347	1.680	0	0	6.95	0	12	200	3	809.76685	2427.28598	-4.47
aVAQGNLSSADVQAAk	Unambiguous	1	1	Q9DB77	N-Term(iTRAQ8plex); K16(iTRAQ8plex)	CID	1.0000	0.0000	1	1	3122340	Unique	Used	1.079	1.869	1.280	1.129	0	0	6.95	0	0	157	2	1069.60522	2138.20317	0.26
ySSQFGGGSqYAYFHEEDETSFQLVDTAR	Unambiguous	1	1	O70194	N-Term(iTRAQ8plex); Q10(Deamidated)	CID	1.0000	0.0000	1	1	3015218	Unique	Used	0.770	0.438	0.825	1.281	0	0	6.95	0	0	143	3	1208.88782	3624.64890	3.96
eScTQEAAALQQQYEEILHk	Unambiguous	2	1	Q6ZWR6-4	N-Term(iTRAQ8plex); C3(Methylthio); K20(iTRAQ8plex)	CID	1.0000	0.0000	1	1	3195583	Unique	Used	0.623	0.809	1.106	1.137	0	0	6.94	0	14	95	3	991.83173	2973.48062	-3.38
kLASQGDSIGSqLGPIHPPPR	Unambiguous	1	1	Q3U0V1	N-Term(iTRAQ8plex); K1(iTRAQ8plex); Q12(Deamidated)	CID	1.0000	0.0000	1	1	3678584	Unique	Used	0.954	0.814	1.159	0.812	0	0	6.94	1	0	200	3	922.18896	2764.55234	-1.56
eHAPSIIFMDEIDSIGSSR	Unambiguous	2	1	P62196	N-Term(iTRAQ8plex)	CID	1.0000	0.0000	1	1	3152182	Unique	Used	0.879	1.229	0.831	0.873	0	0	6.94	0	2	54	3	803.40613	2408.20383	-1.32
vQHDLSSGVFnNQENAIIQEIVk	Unambiguous	1	1	O88703	N-Term(iTRAQ8plex); N11(Deamidated); K23(iTRAQ8plex)	CID	1.0000	0.0000	1	1	3172672	Unique	Used	2.631	1.006	1.190	0.959	0	0	6.94	0	0	200	3	1064.57471	3191.70957	-1.94
gLVHAEQGQGSGSPTGSSTR	Unambiguous	2	1	Q3URS9-2	N-Term(iTRAQ8plex)	CID	1.0000	0.0000	1	1	3098656	Unique	Used	1.002	1.069	1.902	1.601	0	0	6.94	0	8	200	3	739.70917	2217.11295	-1.47

lGLALNYSVFYYEIQNAPEQAcHLAk	Unambiguous	1	1	P61982	N-Term(iTRAQ8plex); C22(Methylthio); K26(iTRAQ8plex)	CID	1.0000	0.0000	1	1	3806238	Unique	Used	2.726	1.115	0.784	1.170	0	0	6.94	0	11	19	3	1203.95605	3609.85361	-4.39
yMSVNQAAQQLLEIVQNHR	Unambiguous	1	1	Q9CWQ0	N-Term(iTRAQ8plex)	CID	1.0000	0.0000	1	1	3935978	Unique	Used	1.361	0.911	0.923	1.214	0	0	6.94	0	0	36	3	849.45135	2546.33951	-2.21
tHnSALEYNIFEGMEcR	Unambiguous	1	1	O08553	N-Term(iTRAQ8plex); N3(Deamidated); C16(Methylthio)	CID	1.0000	0.0000	1	1	3152121	Unique	Used	1.488	0.999	1.409	1.382	0	0	6.94	0	9	13	3	789.02277	2365.05375	-1.16
tQPTDEEMLFIYSHFk	Unambiguous	4	1	P31786	N-Term(iTRAQ8plex); K16(iTRAQ8plex)	CID	1.0000	0.0000	1	1	3286156	Unique	Used	2.074	0.719	1.184	0.842	0	0	6.94	0	6	200	3	865.45081	2594.33786	-1.64
aMVALIDVFHQYSGR	Unambiguous	1	1	P50114	N-Term(iTRAQ8plex)	CID	1.0000	0.0000	1	1	3936149	Unique	Used	2.507	0.633	0.947	1.083	0	0	6.94	0	26	68	3	671.02637	2011.06455	-4.56
qTTAAAAATFSEQVGGGSGGAGR	Unambiguous	3	1	O88935	N-Term(iTRAQ8plex)	CID	1.0000	0.0000	1	1	3495002	Unique	Used	0.693	1.300	1.651	1.539	0	0	6.94	0	0	200	2	1178.59082	2356.17436	-2.21
tQPTDEEMLFIYSHFk	Unambiguous	4	1	P31786	N-Term(iTRAQ8plex); K16(iTRAQ8plex)	CID	1.0000	0.0000	1	1	3287529	Unique	Used	1.722	0.790	0.926	0.719	0	0	6.94	0	0	200	3	865.45209	2594.34171	-0.16
lDQEDALLGSYPVDDGcR	Unambiguous	1	1	Q9D1E6	N-Term(iTRAQ8plex); C17(Methylthio)	CID	1.0000	0.0000	1	1	2741497	Unique	Used	1.425	0.581	0.758	1.002	0	0	6.94	0	0	88	2	1158.54285	2316.07842	-0.26
hHAAYVnNLNATEEk	Unambiguous	1	1	P09671	N-Term(iTRAQ8plex); N7(Deamidated); K15(iTRAQ8plex)	CID	1.0000	0.0000	1	1	2663195	Unique	Used	1.283	1.141	1.187	0.935	0	0	6.94	0	19	158	3	774.07355	2320.20609	-3.50
vSETLQEVYSSDWDGHEDLk	Unambiguous	1	1	D3Z6Q9	N-Term(iTRAQ8plex); K20(iTRAQ8plex)	CID	1.0000	0.0000	1	1	2814503	Unique	Used	0.877	1.258	0.626	0.826	0	0	6.94	0	7	200	3	982.49359	2945.46622	1.25
kAAMEALQSQALHATSQQPLR	Unambiguous	5	1	B1AQZ0	N-Term(iTRAQ8plex); K1(iTRAQ8plex)	HCD	1.0000	0.0000	1	1	3856088	Unique	Used	1.199	0.755	1.646	1.457	0	0	6.94	1	0	200	3	963.20221	2887.59208	-3.89
nLHQSGFSLSGAQIDDNIPR	Unambiguous	1	1	O08553	N-Term(iTRAQ8plex)	CID	1.0000	0.0000	1	1	3473867	Unique	Used	1.915	1.847	0.805	1.140	0	0	6.94	0	26	31	3	825.09406	2473.26761	-2.49

fYPGQAPSLAENFAEHVLk	Unambiguous	1	1	Q9CZP5	N-Term(iTRAQ8plex); K19(iTRAQ8plex)	CID	1.0000	0.0000	1	1	3479292	Unique	Used	0.497	1.041	1.170	1.180	0	0	6.94	0	0	12	3	909.49347	2726.46585	-3.82
aFTDEETEAQAGQASWPR	Unambiguous	1	1	Q08274	N-Term(iTRAQ8plex)	CID	1.0000	0.0000	1	1	2668989	Unique	Used	0.946	2.471	2.107	2.067	0	0	6.94	0	0	200	2	1149.54797	2298.08867	-2.35
qASGGPVDIGPEYQQDLDR	Unambiguous	2	1	P97450	N-Term(iTRAQ8plex)	CID	1.0000	0.0000	1	1	2668608	Unique	Used	2.747	0.928	2.189	1.312	0	0	6.93	0	0	200	2	1175.08435	2349.16142	-0.45
yGAATANYmEVVSLLk	Unambiguous	2	1	Q64521	N-Term(iTRAQ8plex); M9(Oxidation); K16(iTRAQ8plex)	CID	1.0000	0.0000	1	1	3665603	Unique	Used	0.606	1.640	1.328	1.443	0	0	6.93	0	20	12	2	1177.64246	2354.27764	-4.67
mTTVLSESSGPSNASHSSSR	Unambiguous	1	1	Q80X80	N-Term(iTRAQ8plex); M1(Oxidation)	CID	1.0000	0.0000	1	1	3588926	Unique	Used	0.881	1.277	0.874	0.839	0	0	6.93	0	0	200	3	781.37543	2342.11173	-3.37
yTPSGQSGAAASESLFISNHAY	Unambiguous	2	1	A6ZI44	N-Term(iTRAQ8plex)	CID	1.0000	0.0000	1	1	3301230	Unique	Used	1.604	1.127	1.137	1.348	0	0	6.93	0	14	94	2	1281.62317	2562.23906	-0.93
hGGSPTSLGTWGSWAGPDHDk	Unambiguous	2	1	O08795-2	N-Term(iTRAQ8plex); K21(iTRAQ8plex)	CID	1.0000	0.0000	1	1	3297195	Unique	Used	1.561	0.765	1.215	0.933	0	0	6.93	0	0	200	3	920.12915	2758.37290	-2.36
wNPGASPADTGDVNVHSGIIAAR	Unambiguous	3	1	F8VPN4	N-Term(iTRAQ8plex)	CID	1.0000	0.0000	1	1	3388652	Unique	Used	0.801	1.100	1.047	1.307	0	0	6.93	0	0	13	3	870.44769	2609.32853	-3.42
iSFEEFcAVVGGLDIHk	Unambiguous	2	1	Q63810-2	N-Term(iTRAQ8plex); C7(Methylthio); K17(iTRAQ8plex)	CID	1.0000	0.0000	1	1	3709925	Unique	Used		1.056	2.560	0.917	0	0	6.93	0	0	200	3	840.10986	2518.31504	-5.74
yTPSGQSGAAASESLFISNHAY	Unambiguous	2	1	A6ZI44	N-Term(iTRAQ8plex)	CID	1.0000	0.0000	1	1	3258352	Unique	Used	1.014	1.127	0.987	0.974	0	0	6.93	0	0	85	2	1281.62354	2562.23979	-0.64
sAHGPLGDSAAAGPGGDTEYPMGk	Unambiguous	2	1	Q69Z98	N-Term(iTRAQ8plex); K24(iTRAQ8plex)	CID	1.0000	0.0000	1	1	2989738	Unique	Used	1.168	0.971	0.859	1.040	0	0	6.93	0	13	200	3	951.14075	2851.40769	-2.28
gTGGVDTAAVGGVFDVSNADR	Unambiguous	1	1	Q04447	N-Term(iTRAQ8plex)	CID	1.0000	0.0000	1	1	3128102	Unique	Used	1.486	0.969	1.368	1.393	0	0	6.93	0	0	36	2	1135.07092	2269.13457	-0.76
sIATLMTDEVFHDVAYk	Unambiguous	3	1	Q5DTL9	N-Term(iTRAQ8plex); K17(iTRAQ8plex)	CID	1.0000	0.0000	1	1	3326521	Unique	Used	0.196	0.889	1.216	0.865	0	0	6.93	0	0	6	3	850.12079	2548.34781	-3.92
tVIQAEIDAAAELIDFFR	Unambiguous	1	1	Q8CHT0	N-Term(iTRAQ8plex)	CID	1.0000	0.0000	1	1	3501272	Unique	Used	0.592	1.883	0.865	1.529	0	0	6.93	0	8	87	2	1163.62964	2326.25200	-3.31

qAEGVLSSQEYVLSHTEMPGTLQAADAAIk	Unambiguous	1	1	Q68FG2	N-Term(iTRAQ8plex); K30(iTRAQ8plex)	CID	1.0000	0.0000	1	1	3304168	Unique	Used	1.473	1.805	1.845	1.269	0	0	6.93	0	0	115	3	1251.65161	3752.94028	-5.96
eGYVLQTEVATSPSGAWQR	Unambiguous	1	1	Q5SWZ5	N-Term(iTRAQ8plex)	CID	1.0000	0.0000	1	1	3279714	Unique	Used			1.558	0.878	0	0	6.93	0	0	110	2	1192.11084	2383.21440	-2.18
aGQVFLEELGNHk	Unambiguous	2	1	P16330	N-Term(iTRAQ8plex); K13(iTRAQ8plex)	CID	1.0000	0.0000	1	1	3257293	Unique	Used	0.726	0.169	1.190	1.411	0	0	6.93	0	18	26	3	684.05682	2050.15592	0.84
eAFQNAYLELGGLGER	Unambiguous	2	1	Q6PIC6	N-Term(iTRAQ8plex)	CID	1.0000	0.0000	1	1	3354905	Unique	Used	0.611	0.591	1.096	1.483	0	0	6.93	0	15	200	2	1036.03760	2071.06792	-4.01
lILPYVELDLHSYDLGIENR	Unambiguous	2	1	O88844	N-Term(iTRAQ8plex)	CID	1.0000	0.0000	1	1	3541240	Unique	Used	0.830	0.841	0.576	0.993	0	0	6.92	0	24	8	3	892.82001	2676.44547	-3.59
nLHQSGFSLSGAQIDDNIPR	Unambiguous	1	1	O08553	N-Term(iTRAQ8plex)	CID	1.0000	0.0000	1	1	3514630	Unique	Used	1.445	1.149	0.960	1.383	0	0	6.92	0	0	200	2	1237.13501	2473.26274	-4.46
qLNTITLPDFSDPETGSVHATSVAASR	Unambiguous	1	1	E9Q6P5	N-Term(iTRAQ8plex)	CID	1.0000	0.0000	1	1	3415036	Unique	Used	1.599	1.100	1.398	1.752	0	0	6.92	0	0	60	3	1040.19788	3118.57907	-5.40
yTPSGQSGAAASESLFISNHAY	Unambiguous	2	1	A6ZI44	N-Term(iTRAQ8plex)	CID	1.0000	0.0000	1	1	3279967	Unique	Used	0.949	1.116	1.362	1.096	0	0	6.92	0	0	93	2	1281.62329	2562.23931	-0.83
qHLNNmEAQLASQSTQR	Unambiguous	2	1	Q7M739	N-Term(iTRAQ8plex); M6(Oxidation)	CID	1.0000	0.0000	1	1	3382060	Unique	Used	1.761	0.837	1.261	0.988	0	0	6.92	0	0	200	3	759.38245	2276.13279	-1.21
gLTIVGSVLEGTFLDNHPQAQR	Unambiguous	2	1	Q91V14	N-Term(iTRAQ8plex)	CID	1.0000	0.0000	1	1	3845026	Unique	Used	0.527	0.678	2.587	2.506	0	0	6.92	0	0	200	3	886.14948	2656.43387	-0.84
gGGTSSGGSSSTNSLTGSRPPk	Unambiguous	1	1	Q78T81	N-Term(iTRAQ8plex); K22(iTRAQ8plex)	CID	1.0000	0.0000	1	1	3447455	Unique	Used	0.493	0.721	1.075	0.921	0	0	6.92	0	0	190	3	863.12067	2587.34745	-2.25
sMQSVAASPEQEAPDSQDcTHk	Unambiguous	1	1	Q91VW5	N-Term(iTRAQ8plex); C19(Methylthio); K22(iTRAQ8plex)	CID	1.0000	0.0000	1	1	2666454	Unique	Used	0.989	0.688	0.905	0.683	0	0	6.92	0	14	200	3	1000.79858	3000.38120	-4.87
eSGcAAAGETEAAAATAFR	Unambiguous	1	1	Q8BR63	N-Term(iTRAQ8plex); C4(Methylthio)	CID	1.0000	0.0000	1	1	2937748	Unique	Used	1.274	0.289	1.078	1.260	0	0	6.92	0	0	53	2	1067.49207	2133.97685	-3.70
gYTVNVFDIHQGFDNPR	Unambiguous	1	1	Q9R1J0	N-Term(iTRAQ8plex)	CID	1.0000	0.0000	1	1	3496039	Unique	Used	1.276	0.824	0.760	1.002	0	0	6.92	0	22	25	3	761.71796	2283.13932	-2.94
gLGPSLTEDqPGPYLAPGLLGSNIHQQGR	Unambiguous	4	1	D3YZR8	N-Term(iTRAQ8plex); Q10(Deamidated)	CID	1.0000	0.0000	1	1	3704645	Unique	Used	0.588	0.690	0.923	0.743	0	0	6.92	0	13	143	3	1093.24146	3277.70981	-0.66

vSLDGLQEPTHSVLQALNDLQESLTGSR	Unambiguous	3	1	Q8BNU0	N-Term(iTRAQ8plex)	HCD	1.0000	0.0000	1	1	3824983	Unique	Used	1.105	0.988	1.005	1.052	0	0	6.92	0	11	25	3	1104.58142	3311.72971	-2.68
aGLEQGSDAGYLAESQk	Unambiguous	1	1	Q8BMS1	N-Term(iTRAQ8plex); K17(iTRAQ8plex)	CID	1.0000	0.0000	1	1	2699496	Unique	Used	0.481	0.604	0.651	0.984	0	0	6.92	0	5	183	2	1166.61279	2332.21831	-2.50
tATYNGPPASPSLSHEATPLSQTR	Unambiguous	3	1	Q03141-3	N-Term(iTRAQ8plex)	CID	1.0000	0.0000	1	1	3408596	Unique	Used	0.821	0.574	1.221	0.904	0	0	6.92	0	0	134	3	929.80792	2787.40921	-4.43
qAEGVLSSqEYVLSHTEMPGTLQAADAAIk	Unambiguous	1	1	Q68FG2	N-Term(iTRAQ8plex); Q9(Deamidated); K30(iTRAQ8plex)	CID	1.0000	0.0000	1	1	3304197	Unique	Used	0.381	1.786	2.478	1.505	0	0	6.91	0	0	116	3	1251.99158	3753.96018	3.60
vNIHGGAVSLGHPIGMSGAR	Unambiguous	1	1	Q8QZT1	N-Term(iTRAQ8plex)	CID	1.0000	0.0000	1	1	3909732	Unique	Used	0.774	0.602	0.941	0.903	0	0	6.91	0	0	83	3	745.40521	2234.20108	-5.35
dFGNIPGIDLAFIEnGYIYHTk	Unambiguous	2	1	Q3UVK0	N-Term(iTRAQ8plex); N15(Deamidated); K22(iTRAQ8plex)	CID	1.0000	0.0000	1	1	3373653	Unique	Used	1.387	1.013			0	0	6.91	0	26	14	3	1036.21228	3106.62229	-3.97
gDTSGETLHSLIQQGQQIASk	Unambiguous	1	1	Q91YM2	N-Term(iTRAQ8plex); K21(iTRAQ8plex)	CID	1.0000	0.0000	1	1	3475993	Unique	Used	1.607	1.505	0.970	0.880	0	0	6.91	0	0	83	3	936.17413	2806.50785	-2.75
sAQSQAGLETGPESALcGDR	Unambiguous	2	1	Q8CBC4	N-Term(iTRAQ8plex); C17(Methylthio)	CID	1.0000	0.0000	1	1	2917638	Unique	Used	1.411	0.740	2.186	1.218	0	0	6.91	0	0	200	2	1164.04541	2327.08354	-3.20
dVLQAETSQQLccQk	Unambiguous	1	1	Q6P8X1	N-Term(iTRAQ8plex); C12(Methylthio); C13(Methylthio); K15(iTRAQ8plex)	CID	1.0000	0.0000	1	1	3301721	Unique	Used	0.436	0.639	0.807	1.019	0	0	6.91	0	0	150	2	1197.58826	2394.16924	-2.25
gAAGSGPVVLPAGMINPSVPIR	Unambiguous	3	1	Q9EPN1-4	N-Term(iTRAQ8plex)	CID	1.0000	0.0000	1	1	3934957	Unique	Used	0.927	2.073	1.196	1.178	0	0	6.91	0	23	200	2	1182.66895	2364.33061	-2.96
gFQQILAGEYDHLPEQAFYMVGPIEEAVAk	Unambiguous	1	1	P56480	N-Term(iTRAQ8plex); K30(iTRAQ8plex)	CID	1.0000	0.0000	1	1	3396603	Unique	Used	1.275	1.164	1.464	2.330	0	0	6.91	0	9	51	3	1320.35132	3959.03940	-2.95
rVELLQDELALSEPR	Unambiguous	2	1	Q3TDD9	N-Term(iTRAQ8plex)	HCD	1.0000	0.0000	1	1	3108046	Unique	Used	3.188	1.245	0.851	0.936	0	0	6.91	1	17	78	3	691.39081	2072.15787	-3.63

dSEGLGISIIGMGAGADMGLEk	Unambiguous	2	1	Q6R891	N-Term(iTRAQ8plex); K22(iTRAQ8plex)	CID	1.0000	0.0000	1	1	3217114	Unique	Used	1.366	0.904	0.749		0	0	6.91	0	12	55	3	910.47778	2729.41880	-4.51
sGMDAALDDLIDTLGGHEDTNR	Unambiguous	6	1	P51125-3	N-Term(iTRAQ8plex)	CID	1.0000	0.0000	1	1	2942364	Unique	Used	2.658	0.570	1.511	0.952	0	0	6.91	0	26	21	3	874.08575	2620.24271	-1.38
gTVVYGEPITASLGTDGSHYWSk	Unambiguous	1	1	O08553	N-Term(iTRAQ8plex); K23(iTRAQ8plex)	CID	1.0000	0.0000	1	1	3391606	Unique	Used	1.503	1.663	1.352	1.344	0	0	6.91	0	8	32	3	1011.86017	3033.56595	-3.92
iGEHTPSALAImENANVLAR	Unambiguous	3	1	A6ZI44	N-Term(iTRAQ8plex); M12(Oxidation)	CID	1.0000	0.0000	1	1	3709986	Unique	Used	1.163				0	0	6.91	0	0	200	3	809.76514	2427.28086	-6.58
qQLSDSVSDTVALSAAVPR	Unambiguous	2	1	Q3V3Q7	N-Term(iTRAQ8plex)	CID	1.0000	0.0000	1	1	3476757	Unique	Used	1.346	0.317	0.859	0.905	0	0	6.91	0	0	90	2	1124.60400	2248.20073	-3.55
aGNSLAASTAEETAGSAQSR	Unambiguous	1	1	Q61292	N-Term(iTRAQ8plex)	CID	1.0000	0.0000	1	1	2844493	Unique	Used	1.382	1.912	1.278	1.074	0	0	6.91	0	0	178	2	1092.04553	2183.08379	-0.21
tVEGAGNIAAATGFVk	Unambiguous	2	1	O55042	N-Term(iTRAQ8plex); K16(iTRAQ8plex)	CID	1.0000	0.0000	1	1	3413054	Unique	Used	0.670	1.435	1.807	0.616	0	0	6.91	0	0	16	2	1057.60352	2114.19975	-3.26
eNTLNQLVGAAFGAAGQR	Unambiguous	1	1	Q9EQ20	N-Term(iTRAQ8plex)	CID	1.0000	0.0000	1	1	3898437	Unique	Used	0.779	1.561	0.976	1.554	0	0	6.91	0	12	30	2	1061.06616	2121.12505	-4.92
vGDSMSMGNDFDVFAHIGNDTSETR	Unambiguous	1	1	P21981	N-Term(iTRAQ8plex)	CID	1.0000	0.0000	1	1	3152157	Unique	Used	1.242	2.326	1.320	1.775	0	0	6.90	0	0	200	3	1002.78876	3006.35172	0.17
vGFYVnTFQSIAGLEENFHk	Unambiguous	3	1	O08539	N-Term(iTRAQ8plex); N6(Deamidated); K20(iTRAQ8plex)	CID	1.0000	0.0000	1	1	3373440	Unique	Used	1.538	1.155	1.693	0.762	0	0	6.90	0	10	5	3	970.51135	2909.51950	-3.41
gNDISSGTVLSDYVGSGPPSGTGLHR	Unambiguous	1	1	P70296	N-Term(iTRAQ8plex)	CID	1.0000	0.0000	1	1	3434439	Unique	Used	1.241	1.234	1.223	1.225	0	0	6.90	0	0	143	3	945.47656	2834.41513	-2.53
eTAGVLSNDTSDSGVELQGGSR	Unambiguous	1	1	F8VQ95	N-Term(iTRAQ8plex)	CID	1.0000	0.0000	1	1	2634133	Unique	Used	1.381	0.379	1.068	0.709	0	0	6.90	0	0	200	2	1242.10962	2483.21196	-1.78
vAAYFGLDHNVDQTGk	Unambiguous	13	1	E9Q4A0	N-Term(iTRAQ8plex); K16(iTRAQ8plex)	CID	1.0000	0.0000	1	1	3321634	Unique	Used	0.818	0.953	1.088	0.745	0	0	6.90	0	27	54	3	781.75659	2343.25522	-0.06
gLTIVGSVLEGTFLDNHPQAQR	Unambiguous	2	1	Q91V14	N-Term(iTRAQ8plex)	CID	1.0000	0.0000	1	1	3842612	Unique	Used	0.926	0.737	1.428	1.260	0	0	6.90	0	19	92	3	886.14648	2656.42490	-4.22
lQGTHYSVQSDIWSMGLSLVEMAVGR	Unambiguous	1	1	P31938	N-Term(iTRAQ8plex)	CID	1.0000	0.0000	1	1	3918309	Unique	Used		1.321	0.878	1.145	0	0	6.90	0	9	13	3	1056.87207	3168.60166	-3.41
sMLQATAEANNLAAVAGAR	Unambiguous	3	1	Q6PA06	N-Term(iTRAQ8plex)	CID	1.0000	0.0000	1	1	3724280	Unique	Used	0.610	0.776	1.103	1.147	0	0	6.90	0	3	22	2	1082.07153	2163.13579	-6.31

sAAQAAAQTnSNAAGk	Unambiguous	4	1	Q9CY58	N-Term(iTRAQ8plex); N10(Deamidated); K16(iTRAQ8plex)	CID	0.0420	0.0000	1	1	2841772	Unique	Used	0.576	0.251	1.152	0.618	0	0	6.90	0	0	200	2	1035.55054	2070.09380	-4.74
qLLHNFPPDQLTSSGAPFWSGPk	Unambiguous	1	1	Q02053	N-Term(iTRAQ8plex); K23(iTRAQ8plex)	CID	1.0000	0.0000	1	1	3841572	Unique	Used	0.742	1.294	0.888	0.928	0	0	6.90	0	12	200	3	1044.89417	3132.66794	-1.54
sGTGQQLGQAAEESNccAR	Unambiguous	2	1	Q5F284	N-Term(iTRAQ8plex); C16(Methylthio); C17(Methylthio)	CID	1.0000	0.0000	1	1	3069394	Unique	Used	0.427	1.174	0.816	1.501	0	0	6.90	0	0	200	2	1153.49939	2305.99150	-0.91
eHINLGcDVDFDIAGPSIR	Unambiguous	3	1	Q60932-2	N-Term(iTRAQ8plex); C7(Methylthio)	CID	1.0000	0.0000	1	1	3351872	Unique	Used	0.851	0.597	2.096	1.409	0	0	6.90	0	1	2	3	807.73029	2421.17630	-3.39
sAAGSSSGFHSWAR	Unambiguous	1	1	P19246	N-Term(iTRAQ8plex)	CID	1.0000	0.0000	1	1	3869220	Unique	Used	3.860	0.489	1.483	1.492	0	0	6.90	0	12	119	3	571.28381	1711.83689	-4.99
sLAGSSGPGASSGPGGDHSELIVR	Unambiguous	5	1	P57776-3	N-Term(iTRAQ8plex)	CID	1.0000	0.0000	1	1	3184942	Unique	Used	2.304	0.586	0.704	0.486	0	0	6.90	0	0	39	2	1250.13867	2499.27007	-1.65
yEGSGDGGAAAqSLYIANHAY	Unambiguous	1	1	P05063	N-Term(iTRAQ8plex); Q12(Deamidated)	CID	0.0217	0.0000	1	1	2779887	Unique	Used	0.845	1.106	2.490	1.072	0	0	6.90	0	0	200	2	1210.56848	2420.12969	-0.47
sHPPANFFGSGPILQNSPPLSSk	Unambiguous	2	1	F8VQ95	N-Term(iTRAQ8plex); K23(iTRAQ8plex)	CID	1.0000	0.0000	1	1	3856892	Unique	Used	1.000	1.105	0.958	0.795	0	0	6.90	0	0	104	3	996.54352	2987.61600	-1.34
nVIIWGNHSSTQYPDVNHAk	Unambiguous	1	1	P14152	N-Term(iTRAQ8plex); K20(iTRAQ8plex)	CID	1.0000	0.0000	1	1	3452051	Unique	Used	0.552	0.936	0.986	1.063	0	0	6.90	0	0	200	3	963.51056	2888.51712	-3.21
qADVPAAVTDAAATTPAAEDAATk	Unambiguous	1	1	P06837	N-Term(iTRAQ8plex); K24(iTRAQ8plex)	CID	1.0000	0.0000	1	1	2667783	Unique	Used	2.970	2.072	2.151	2.692	0	0	6.89	0	16	200	3	955.50787	2864.50907	-0.28
gGPPPPPPPPHSSGPPPPPAR	Unambiguous	1	1	Q91YD9	N-Term(iTRAQ8plex)	CID	1.0000	0.0000	1	1	3589743	Unique	Used	2.903	2.042	1.593	1.152	0	0	6.89	0	0	200	3	765.41302	2294.22452	-4.49
dLGLPTEAYISVEEVHDDGTPTSk	Unambiguous	1	1	P26516	N-Term(iTRAQ8plex); K24(iTRAQ8plex)	CID	1.0000	0.0000	1	1	2814001	Unique	Used	0.668	0.930	0.986	1.183	0	0	6.89	0	12	93	3	1061.21667	3181.63547	-0.22
tQTAVASEEGPATLPEAEALLAQHAALR	Unambiguous	1	1	Q68FG2	N-Term(iTRAQ8plex)	CID	1.0000	0.0000	1	1	3303625	Unique	Used	1.267	1.332	1.343	1.097	0	0	6.89	0	6	65	3	1050.55566	3149.65244	-7.01

eHINLGcDVDFDIAGPSIR	Unambiguous	3	1	Q60932-2	N-Term(iTRAQ8plex); C7(Methylthio)	CID	1.0000	0.0000	1	1	3357473	Unique	Used		1.400	1.504	1.350	0	0	6.89	0	0	200	3	807.73126	2421.17923	-2.18
lARPDDLETcASPPTLAGR	Unambiguous	1	1	Q80XU8	N-Term(iTRAQ8plex); C10(Methylthio)	HCD	1.0000	0.0000	1	1	3413298	Unique	Used	1.005	0.914	0.887	0.874	0	0	6.89	0	21	62	3	778.39728	2333.17728	-5.28
sGAAQGLAEVmAGLGVEk	Unambiguous	2	1	E9PVA8	N-Term(iTRAQ8plex); M11(Oxidation); K18(iTRAQ8plex)	CID	1.0000	0.0000	1	1	3516207	Unique	Used	0.497	0.640	0.689	0.872	0	0	6.89	0	21	200	2	1156.63525	2312.26323	-4.70
eQANAVSEAVVSSVNTVANk	Unambiguous	1	1	Q9Z0F7	N-Term(iTRAQ8plex); K20(iTRAQ8plex)	CID	1.0000	0.0000	1	1	3415688	Unique	Used	3.254	0.334	0.966	1.591	0	0	6.89	0	0	20	2	1313.21216	2625.41704	-5.11
vADNLAIQLTTVTEDkYEILqSVDDAAIVIk	Unambiguous	4	1	Q9Z1X4	N-Term(iTRAQ8plex); K16(iTRAQ8plex); Q21(Deamidated); K31(iTRAQ8plex)	CID	1.0000	0.0000	1	1	3374258	Unique	Used	0.651	0.869	0.753	0.789	0	0	6.89	1	13	72	3	1434.80933	4302.41343	0.79
eQSGEYEcSASNDVAAPVVR	Unambiguous	5	1	D3Z396	N-Term(iTRAQ8plex); C8(Methylthio)	CID	1.0000	0.0000	1	1	2668615	Unique	Used	0.628	1.216	0.740	0.928	0	0	6.89	0	2	2	2	1231.06628	2461.12529	-1.00
qASAAENHIHELEEALAGER	Unambiguous	2	1	P21619	N-Term(iTRAQ8plex)	CID	1.0000	0.0000	1	1	2831753	Unique	Used	1.445	1.249	1.749	1.807	0	0	6.89	0	2	39	3	827.08759	2479.24820	0.10
gnAGQTNYGFANSTMER	Unambiguous	1	1	P19096	N-Term(iTRAQ8plex); N2(Deamidated)	CID	0.0276	0.0000	1	1	2897883	Unique	Used	0.574	0.697	0.702	1.027	0	0	6.89	0	8	112	2	1061.98706	2122.96684	-4.59
rEHANIDAQSGSQAPNPSTTISPGk	Unambiguous	3	1	Q8VDQ8-2	N-Term(iTRAQ8plex); K25(iTRAQ8plex)	CID	1.0000	0.0000	1	1	3182032	Unique	Used	1.217	0.160	1.074	1.080	0	0	6.89	1	0	25	3	1057.88892	3171.65219	-2.57
aVGTPGANAGGAGPGISAMSR	Unambiguous	2	1	Q7TSC1	N-Term(iTRAQ8plex)	CID	1.0000	0.0000	1	1	3698358	Unique	Used	0.600	0.461	1.274	0.773	0	0	6.89	0	0	118	2	1052.04187	2103.07646	-7.36
dHFmDEFFEQVEEIR	Unambiguous	1	1	P61264	N-Term(iTRAQ8plex); M4(Oxidation)	CID	1.0000	0.0000	1	1	2833408	Unique	Used	1.351	0.870	1.418	1.370	0	0	6.89	0	23	150	3	764.35699	2291.05643	-1.24
lEVSSLAQTSSAVASSTDGSIHTESVDGIPDPqR	Unambiguous	3	1	Q69ZZ6	N-Term(iTRAQ8plex); Q33(Deamidated)	CID	0.0174	0.0000	1	1	3192845	Unique	Used	1.968	0.846	1.525	1.184	0	0	6.89	0	0	128	3	1249.62524	3746.86118	2.69

nIANPTATLLAScMMLDHLk	Unambiguous	1	1	P70404	N-Term(iTRAQ8plex); C13(Methylthio); K20(iTRAQ8plex)	CID	1.0000	0.0000	1	1	3898803	Unique	Used	0.328	0.611	0.914	1.023	0	0	6.89	0	16	9	3	937.82904	2811.47257	-4.43
qQLSDSVSDTVALSAAVPR	Unambiguous	2	1	Q3V3Q7	N-Term(iTRAQ8plex)	CID	1.0000	0.0000	1	1	3476903	Unique	Used	1.154	0.241	0.766	1.014	0	0	6.89	0	0	18	2	1124.60376	2248.20024	-3.77
nQEQLAAELAEFTAk	Unambiguous	1	1	P26043	N-Term(iTRAQ8plex); K15(iTRAQ8plex)	CID	1.0000	0.0000	1	1	3056018	Unique	Used	1.324	0.304	0.737	0.720	0	0	6.89	0	14	122	2	1136.12305	2271.23882	-2.34
eHAnIDAQSGSQAPNPSTTISPGk	Unambiguous	3	1	Q8VDQ8-2	N-Term(iTRAQ8plex); N4(Deamidated); K24(iTRAQ8plex)	CID	1.0000	0.0000	1	1	2844533	Unique	Used	1.442	1.082	1.476	1.123	0	0	6.89	0	0	200	3	1006.18292	3016.53421	-3.00
aGAQGAAAITTSGSTkPPGAR	Unambiguous	2	1	Q5SWP3	N-Term(iTRAQ8plex); K16(iTRAQ8plex)	CID	1.0000	0.0000	1	1	3654478	Unique	Used	1.569	0.245	0.809	0.507	0	0	6.89	0	0	17	3	826.79675	2478.37571	-5.18
yTPSGQSGAAASESLFISnHAY	Unambiguous	2	1	A6ZI44	N-Term(iTRAQ8plex); N19(Deamidated)	CID	1.0000	0.0000	1	1	2994946	Unique	Used	0.974	1.148	1.713	1.837	0	0	6.89	0	2	33	2	1282.11401	2563.22075	-1.84
gQLLPPPPVLPAAAVETPDHAELR	Unambiguous	4	1	Q0PMG2	N-Term(iTRAQ8plex)	CID	1.0000	0.0000	1	1	3393293	Unique	Used	1.303	1.004	0.903	0.842	0	0	6.88	0	25	63	3	931.52155	2792.55008	-4.03
nLHQSGFSLSGAQIDDNIPR	Unambiguous	1	1	O08553	N-Term(iTRAQ8plex)	CID	1.0000	0.0000	1	1	3433764	Unique	Used	1.689	1.334	0.921	0.902	0	0	6.88	0	10	26	3	825.09344	2473.26578	-3.23
wGEAGAEYVVESTGVFTTmEk	Unambiguous	3	1	P16858	N-Term(iTRAQ8plex); M19(Oxidation); K21(iTRAQ8plex)	CID	1.0000	0.0000	1	1	3240713	Unique	Used	1.151	1.402	0.909	1.171	0	0	6.88	0	0	200	3	972.49023	2915.45615	-1.11
gLGPLAEAAAAGDGAAAAGAAR	Unambiguous	1	1	O55106	N-Term(iTRAQ8plex)	CID	1.0000	0.0000	1	1	3322285	Unique	Used	0.856	1.009	1.105	0.839	0	0	6.88	0	0	132	2	1057.06616	2113.12505	-2.56
lSSTETSQSQSSHEEFR	Unambiguous	3	1	Q80YA9	N-Term(iTRAQ8plex)	CID	1.0000	0.0000	1	1	2823889	Unique	Used	1.628	1.155	1.569	1.093	0	0	6.88	0	20	144	3	748.69312	2244.06479	-1.53
gATVVELFPYAVNPDHYTPYk	Unambiguous	2	1	Q8BW41-2	N-Term(iTRAQ8plex); K21(iTRAQ8plex)	CID	1.0000	0.0000	1	1	3539966	Unique	Used	0.858	0.779	0.650	1.143	0	0	6.88	0	6	44	3	997.19873	2989.58164	-3.47
gALETESSSSSAqVSTVGQASR	Unambiguous	2	1	Q8BKI2	N-Term(iTRAQ8plex); Q13(Deamidated)	CID	1.0000	0.0000	1	1	2931740	Unique	Used	1.932	0.727	0.857	0.621	0	0	6.88	0	0	200	2	1222.61157	2444.21587	4.25

aTSSSSSSSGVGMSVGQGR	Unambiguous	2	1	P21619	N-Term(iTRAQ8plex)	CID	1.0000	0.0000	1	1	3610661	Unique	Used	0.834	1.527	0.940	1.232	0	0	6.88	0	0	200	2	1016.99158	2032.97588	-5.55
eHANIDAQSGSQAPNPSTTISPGk	Unambiguous	3	1	Q8VDQ8-2	N-Term(iTRAQ8plex); K24(iTRAQ8plex)	CID	1.0000	0.0000	1	1	3121289	Unique	Used	0.522	0.154	0.639	0.866	0	0	6.88	0	0	32	3	1005.85480	3015.54984	-3.12
gLVHAEQGQGSGSPTGSSTR	Unambiguous	2	1	Q3URS9-2	N-Term(iTRAQ8plex)	CID	1.0000	0.0000	1	1	3269848	Unique	Used	0.611	0.968	1.145	1.127	0	0	6.88	0	0	146	3	739.70929	2217.11332	-1.30
tIGGGDDSFNTFFSETGAGk	Unambiguous	4	1	P68373	N-Term(iTRAQ8plex); K20(iTRAQ8plex)	CID	1.0000	0.0000	1	1	3155223	Unique	Used	0.357	1.492	1.113	1.501	0	0	6.88	0	0	200	2	1308.65283	2616.29839	-2.09
qSQANAPVYQQNVLHTGk	Unambiguous	14	1	E9Q6Q2	N-Term(iTRAQ8plex); K18(iTRAQ8plex)	CID	1.0000	0.0000	1	1	3363261	Unique	Used	0.645	0.718	1.522	0.907	0	0	6.88	0	0	110	3	864.47473	2591.40964	-2.08
wRPSIGVQVETISDSDTENR	Unambiguous	2	1	B1AS06	N-Term(iTRAQ8plex)	HCD	1.0000	0.0000	1	1	3211376	Unique	Used	1.291	1.590	2.122	1.099	0	0	6.88	0	9	117	3	865.10895	2593.31229	-1.45
iSFEEFcAVVGGLDIHk	Unambiguous	2	1	Q63810-2	N-Term(iTRAQ8plex); C7(Methylthio); K17(iTRAQ8plex)	CID	1.0000	0.0000	1	1	3745853	Unique	Used	0.732	1.431	2.006	0.871	0	0	6.88	0	10	65	3	840.11157	2518.32016	-3.70
lELQGIQGAVDHAAAFGR	Unambiguous	5	1	Q9CPV4	N-Term(iTRAQ8plex)	CID	1.0000	0.0000	1	1	3642311	Unique	Used	0.904	1.219	1.053	1.204	0	0	6.88	0	8	22	3	719.72437	2157.15854	-6.18
dAYSGGAVNLYHVR	Unambiguous	1	1	O55234	N-Term(iTRAQ8plex)	CID	1.0000	0.0000	1	1	3317139	Unique	Used	1.513	0.932	1.460	1.343	0	0	6.88	0	21	21	3	609.31946	1825.94382	-3.31
dVQFAVQQVLQEEHFDAR	Unambiguous	2	1	Q8R146-2	N-Term(iTRAQ8plex)	CID	1.0000	0.0000	1	1	3238393	Unique	Used	1.400	1.110	1.211	0.786	0	0	6.88	0	15	96	3	821.75397	2463.24735	-3.94
vSALTQMESAcVSIHEALk	Unambiguous	2	1	E9Q7G1	N-Term(iTRAQ8plex); C11(Methylthio); K19(iTRAQ8plex)	CID	1.0000	0.0000	1	1	3706581	Unique	Used	0.676	0.641	0.478	0.721	0	0	6.88	0	5	14	3	891.13544	2671.39176	-6.01
iVITGDAALDHDQALAQAIR	Unambiguous	1	1	O70318	N-Term(iTRAQ8plex)	CID	1.0000	0.0000	1	1	3347427	Unique	Used	1.154	1.447	1.090	1.055	0	0	6.88	0	26	38	2	1198.16211	2395.31694	-3.26
lPDFQDSIFEYFNTAPLAHDLTFR	Unambiguous	1	1	Q7TT50	N-Term(iTRAQ8plex)	CID	1.0000	0.0000	1	1	3746044	Unique	Used	0.666	1.145	1.363	1.084	0	0	6.88	0	12	50	3	1054.53320	3161.58506	-1.13
lASQGDSIGSQLGPIHPPPR	Unambiguous	1	1	Q3U0V1	N-Term(iTRAQ8plex)	CID	1.0000	0.0000	1	1	3637974	Unique	Used	1.634	0.762	1.236	0.793	0	0	6.88	0	0	104	2	1166.12915	2331.25102	-9.14
vSELEAELAEQQHLGR	Unambiguous	1	1	Q8VD75	N-Term(iTRAQ8plex)	CID	1.0000	0.0000	1	1	2832424	Unique	Used	1.709	1.038	1.025	1.282	0	0	6.88	0	15	200	3	705.04315	2113.11490	-2.02

vTAQGPGLEPSGNIANk	Unambiguous	4	1	B7FAU9	N-Term(iTRAQ8plex); K17(iTRAQ8plex)	CID	1.0000	0.0000	1	1	3230090	Unique	Used	0.657	4.276	0.809	1.217	0	0	6.88	0	0	104	2	1131.13708	2261.26689	-1.83
gNLAYATSTGGIVNk	Unambiguous	1	1	Q8C0M9	N-Term(iTRAQ8plex); K15(iTRAQ8plex)	CID	1.0000	0.0000	1	1	3513176	Unique	Used	1.463	0.718	0.701	0.809	0	0	6.87	0	8	48	2	1037.58618	2074.16509	-4.93
fSVcVLGDQQHcDEAk	Unambiguous	1	1	Q5XJF6	N-Term(iTRAQ8plex); C4(Methylthio); C12(Methylthio); K16(iTRAQ8plex)	CID	1.0000	0.0000	1	1	3213423	Unique	Used	0.235	0.332	0.694	0.807	0	0	6.87	0	4	6	3	827.06061	2479.16727	-1.05
yIQDIYScGETVEHLEESTAFR	Unambiguous	3	1	G3X8W7	N-Term(iTRAQ8plex); C8(Methylthio)	CID	1.0000	0.0000	1	1	3196413	Unique	Used	0.676	1.068	0.748	0.908	0	0	6.87	0	8	64	3	980.79413	2940.36783	-0.66
gDTSGETLHSLIQQGQQIASk	Unambiguous	1	1	Q91YM2	N-Term(iTRAQ8plex); K21(iTRAQ8plex)	CID	1.0000	0.0000	1	1	3455984	Unique	Used	3.745	1.035	1.086	0.980	0	0	6.87	0	23	147	3	936.17365	2806.50638	-3.27
kVSQPIEGHAASFAQFk	Unambiguous	2	1	Q68FD5	N-Term(iTRAQ8plex); K1(iTRAQ8plex); K17(iTRAQ8plex)	HCD	1.0000	0.0000	1	1	3532034	Unique	Used	0.425	1.052	1.440	1.185	0	0	6.87	1	0	55	3	919.86322	2757.57511	-2.33
lQQELDDLTVDLDHQR	Unambiguous	3	1	Q5SV64	N-Term(iTRAQ8plex)	CID	1.0000	0.0000	1	1	2815964	Unique	Used	1.670	1.174	1.690	0.858	0	0	6.87	0	0	200	3	748.05963	2242.16434	1.15
iQNTGDYYDLYGGEk	Unambiguous	2	1	P35235	N-Term(iTRAQ8plex); K15(iTRAQ8plex)	CID	1.0000	0.0000	1	1	2883843	Unique	Used	1.419	1.122	1.116	0.768	0	0	6.87	0	24	200	2	1172.59424	2344.18120	-4.49
fLSQPFQVAEVFTGHMGk	Unambiguous	1	1	P56480	N-Term(iTRAQ8plex); K18(iTRAQ8plex)	CID	1.0000	0.0000	1	1	3881567	Unique	Used	1.341	0.984	0.895	1.142	0	0	6.87	0	12	200	3	877.80927	2631.41324	-3.10
eSASSSEVTSAVYNAVSEGTR	Unambiguous	1	1	Q8C145	N-Term(iTRAQ8plex)	CID	1.0000	0.0000	1	1	3170531	Unique	Used	0.970	1.391	1.211	1.048	0	0	6.87	0	0	200	2	1218.09387	2435.18047	-1.45
fLSQPFQVAEVFTGHMGk	Unambiguous	1	1	P56480	N-Term(iTRAQ8plex); K18(iTRAQ8plex)	CID	1.0000	0.0000	1	1	3865237	Unique	Used		1.118			0	0	6.87	0	7	200	3	877.80853	2631.41104	-3.94
lHDINAQmVEDQGFLDSLR	Unambiguous	5	1	Q9DBG3	N-Term(iTRAQ8plex); M8(Oxidation)	CID	1.0000	0.0000	1	1	3214286	Unique	Used	1.209	1.732	1.419	1.298	0	0	6.87	0	16	32	3	841.09369	2521.26651	0.23

gTGGVDTAATGSVFDISNLDR	Unambiguous	1	1	P30275	N-Term(iTRAQ8plex)	CID	1.0000	0.0000	1	1	3236586	Unique	Used	1.489	1.262	1.024	1.282	0	0	6.87	0	0	24	2	1179.09534	2357.18340	-2.26
aSGAVGLSYGAHSNLcVNQIVR	Unambiguous	1	1	Q9JHI5	N-Term(iTRAQ8plex); C16(Methylthio)	CID	1.0000	0.0000	1	1	3932522	Unique	Used	1.804	1.172	0.883	0.981	0	0	6.87	0	21	70	3	856.10754	2566.30808	-3.57
gTGLTAVAVQEDNSDVIEVR	Unambiguous	3	1	Q9DBX3	N-Term(iTRAQ8plex)	CID	1.0000	0.0000	1	1	2812580	Unique	Used	0.712	1.594	0.897	0.970	0	0	6.86	0	7	200	2	1189.12878	2377.25029	-0.43
gTNTGVWVGVSGSEASEALSR	Unambiguous	1	1	P19096	N-Term(iTRAQ8plex)	CID	1.0000	0.0000	1	1	3322023	Unique	Used	0.764	0.575	0.890	1.052	0	0	6.86	0	15	163	2	1184.60156	2368.19585	-3.74
gGHMDDGGYSMNFTmSSSR	Unambiguous	3	1	Q9WV02	N-Term(iTRAQ8plex); M15(Oxidation)	CID	1.0000	0.0000	1	1	3207296	Unique	Used	1.542	0.813	1.457	1.170	0	0	6.86	0	12	76	3	786.33276	2356.98374	-2.61
dLVILLYETALLSSGFSLEDPQTHANR	Unambiguous	1	1	P07901	N-Term(iTRAQ8plex)	HCD	1.0000	0.0000	1	1	3746159	Unique	Used	1.158	1.084	1.313	1.882	0	0	6.86	0	17	153	3	1102.91699	3306.73642	-4.84
tVEGAGNIAAATGFVk	Unambiguous	2	1	O55042	N-Term(iTRAQ8plex); K16(iTRAQ8plex)	CID	1.0000	0.0000	1	1	3455015	Unique	Used	1.049	1.699	2.001	0.816	0	0	6.86	0	0	200	2	1057.60413	2114.20098	-2.68
eAAEAIEAEGATAPLTELLHSR	Unambiguous	2	1	Q02248	N-Term(iTRAQ8plex)	CID	1.0000	0.0000	1	1	3036945	Unique	Used	0.580	1.008	0.950	0.816	0	0	6.86	0	22	29	3	861.78967	2583.35447	-0.93
lLEVSDDPQVLAVAAHDVGEYVR	Unambiguous	1	1	Q8BVE3	N-Term(iTRAQ8plex)	CID	1.0000	0.0000	1	1	3241160	Unique	Used	0.905	1.258	1.022	1.065	0	0	6.86	0	12	200	3	933.83588	2799.49308	3.56
rHHQEEVGELLGQIQGcGAAQAQAQAEAR	Unambiguous	1	1	P19246	N-Term(iTRAQ8plex); C17(Methylthio)	CID	1.0000	0.0000	1	1	3434326	Unique	Used	1.757	1.082	0.747	0.619	0	0	6.86	1	12	101	3	1145.89893	3435.68222	-2.64
dLIDQSQSSGSGSGLPLLVQR	Unambiguous	2	1	Q60607	N-Term(iTRAQ8plex)	CID	1.0000	0.0000	1	1	3477249	Unique	Used	0.665	0.805	0.875	0.983	0	0	6.86	0	0	148	2	1231.16174	2461.31621	-1.56
vNIHGGAVSLGHPIGMSGAR	Unambiguous	1	1	Q8QZT1	N-Term(iTRAQ8plex)	CID	1.0000	0.0000	1	1	3909722	Unique	Used	1.130	0.854	0.935	0.842	0	0	6.86	0	0	200	3	745.40515	2234.20090	-5.43
aEGATLSNATGAVESTSQAGDR	Unambiguous	1	1	Q61644	N-Term(iTRAQ8plex)	CID	1.0000	0.0000	1	1	2846538	Unique	Used	2.223	1.375	2.190	1.132	0	0	6.86	0	0	186	2	1199.08643	2397.16557	-5.86
eHINLGcDVDFDIAGPSIR	Unambiguous	3	1	Q60932-2	N-Term(iTRAQ8plex); C7(Methylthio)	CID	1.0000	0.0000	1	1	3353717	Unique	Used	0.837	0.869	1.003	1.505	0	0	6.86	0	12	28	3	807.73108	2421.17868	-2.40
vLDGLTAGSSSASQQQQQQQHPPGNR	Unambiguous	3	1	D3Z2Q2	N-Term(iTRAQ8plex)	CID	1.0000	0.0000	1	1	3552605	Unique	Used	0.517	1.270	1.215	1.018	0	0	6.86	0	0	200	3	1008.50531	3023.50138	-6.07
dGPGPPGVTEnVGQSLWEISESHFk	Unambiguous	1	1	Q9JIP4	N-Term(iTRAQ8plex); N11(Deamidated); K25(iTRAQ8plex)	CID	0.0219	0.0000	1	1	3261378	Unique	Used	1.054	0.820	0.464	0.500	0	0	6.86	0	0	181	3	1092.89844	3276.68076	5.30

gGFGGAGMGAYVPSGFVHPQSR	Unambiguous	2	1	Q811P8	N-Term(iTRAQ8plex)	CID	1.0000	0.0000	1	1	3913775	Unique	Used	1.577	1.290	1.528	1.111	0	0	6.86	0	25	111	3	814.07208	2440.20169	-4.81
gPAVGIDLGTTYScVGVFQHGk	Unambiguous	4	1	P63017	N-Term(iTRAQ8plex); C14(Methylthio); K22(iTRAQ8plex)	CID	1.0000	0.0000	1	1	3860018	Unique	Used	0.690	1.190	0.677	0.607	0	0	6.86	0	22	53	3	954.16736	2860.48752	-2.49
iEGSGDQVDTLELSGGAk	Unambiguous	3	1	Q8BZ98-2	N-Term(iTRAQ8plex); K18(iTRAQ8plex)	CID	1.0000	0.0000	1	1	2716768	Unique	Used	0.432	0.826	1.076	1.607	0	0	6.86	0	12	200	2	1192.63953	2384.27178	-2.02
dcFIVYQGHHGDVGAPMADVILPGAAYTEk	Unambiguous	1	1	Q91VD9	N-Term(iTRAQ8plex); C2(Methylthio); K30(iTRAQ8plex)	CID	1.0000	0.0000	1	1	3372376	Unique	Used		0.586			0	0	6.86	0	13	140	3	1276.96509	3828.88071	-5.27
hSPLDVPVAGDGTEDPSLTALR	Unambiguous	1	1	Q9JHU2	N-Term(iTRAQ8plex)	CID	1.0000	0.0000	1	1	2956899	Unique	Used	2.863	2.289	1.778	0.776	0	0	6.85	0	3	26	3	851.11456	2551.32914	-0.59
dAYGTSSLSSSSNSGSck	Unambiguous	2	1	Q80U23	N-Term(iTRAQ8plex); C17(Methylthio); K18(iTRAQ8plex)	CID	1.0000	0.0000	1	1	3028294	Unique	Used	0.583	0.313	1.297	1.272	0	0	6.85	0	0	110	2	1196.56226	2392.11724	-1.86
yEGSGDGGAAAQSLYIAnHAY	Unambiguous	1	1	P05063	N-Term(iTRAQ8plex); N18(Deamidated)	CID	1.0000	0.0000	1	1	2779780	Unique	Used	1.331	0.659	1.755	2.095	0	0	6.85	0	0	200	2	1210.56604	2420.12480	-2.49
icDPGLTSFEPEALGNLVEGMDFHR	Unambiguous	9	1	Q5SVJ0	N-Term(iTRAQ8plex); C2(Methylthio)	CID	1.0000	0.0000	1	1	3481200	Unique	Used		0.703	2.325	1.633	0	0	6.85	0	27	200	3	1033.16077	3097.46775	-1.89
gITNLcVIGGDGSLTGADTFR	Unambiguous	1	1	P47857	N-Term(iTRAQ8plex); C6(Methylthio)	CID	1.0000	0.0000	1	1	3727934	Unique	Used	1.989	0.676	1.015	1.275	0	0	6.85	0	0	200	2	1209.10083	2417.19438	-6.76
yTPSGQSGAAASESLFISNHAY	Unambiguous	2	1	A6ZI44	N-Term(iTRAQ8plex)	CID	1.0000	0.0000	1	1	3322403	Unique	Used	4.131	1.135	0.713	1.609	0	0	6.85	0	0	145	2	1281.62012	2562.23296	-3.31
hTGMASIDSSAPETTSDSSPTLSR	Unambiguous	4	1	Q3UHH1-2	N-Term(iTRAQ8plex)	CID	1.0000	0.0000	1	1	2913603	Unique	Used	1.776	1.195	1.491	1.722	0	0	6.85	0	0	200	3	913.77063	2739.29734	-2.63
aPAFLSAEEVQDHLR	Unambiguous	1	1	O54983	N-Term(iTRAQ8plex)	CID	1.0000	0.0000	1	1	3234202	Unique	Used	1.048	3.254	0.923	0.943	0	0	6.85	0	2	1	3	663.02271	1987.05356	-0.77
gVIALcIEDGSIHR	Unambiguous	1	1	Q8K2B3	N-Term(iTRAQ8plex); C6(Methylthio)	CID	1.0000	0.0000	1	1	3518340	Unique	Used	1.585	0.662	1.153	1.138	0	0	6.85	0	14	59	3	611.65778	1832.95877	-4.21

vIVVGNPAnTNcLTASk	Unambiguous	2	1	P14152	N-Term(iTRAQ8plex); N9(Deamidated); C12(Methylthio); K17(iTRAQ8plex)	CID	1.0000	0.0000	1	1	3537685	Unique	Used	0.400	0.870	1.111	1.409	0	0	6.85	0	1	3	3	786.09583	2356.27292	-4.07
yVIHTVGPIAVGQPTASQAAELR	Unambiguous	1	1	Q922B1	N-Term(iTRAQ8plex)	CID	1.0000	0.0000	1	1	3781041	Unique	Used	0.675	1.530	1.080	1.044	0	0	6.85	0	0	75	3	894.83105	2682.47861	-3.55
sIQDLTVTGTEPGQVSSR	Unambiguous	4	1	Q62073	N-Term(iTRAQ8plex)	CID	1.0000	0.0000	1	1	3232213	Unique	Used	1.634		1.758	1.236	0	0	6.85	0	0	81	2	1090.07715	2179.14702	-1.76
gFDQTINLILDESHER	Unambiguous	1	1	Q6ZWM4	N-Term(iTRAQ8plex)	CID	1.0000	0.0000	1	1	3111503	Unique	Used	2.299	1.158	0.983	0.748	0	0	6.85	0	2	60	3	731.04749	2191.12790	-0.83
eHINLGcDVDFDIAGPSIR	Unambiguous	3	1	Q60932-2	N-Term(iTRAQ8plex); C7(Methylthio)	CID	1.0000	0.0000	1	1	3375767	Unique	Used	1.360	1.853	1.737	1.564	0	0	6.85	0	4	200	3	807.73022	2421.17612	-3.46
dFLGQAFLALGEVIGGQGSR	Unambiguous	1	1	Q1RLL3	N-Term(iTRAQ8plex)	CID	1.0000	0.0000	1	1	3918386	Unique	Used	0.270	0.135	0.524	0.879	0	0	6.85	0	29	41	2	1170.13318	2339.25908	-3.04
fYLLVVVGETVTEEHLR	Unambiguous	1	1	P14873	N-Term(iTRAQ8plex)	CID	1.0000	0.0000	1	1	3666536	Unique	Used	1.317	0.801	1.163	0.998	0	0	6.85	0	18	21	3	770.09583	2308.27292	-5.45
gEETAMWISGPGPAPSk	Unambiguous	6	1	Q8CA95-3	N-Term(iTRAQ8plex); K17(iTRAQ8plex)	CID	1.0000	0.0000	1	1	3127067	Unique	Used	0.214	0.224	4.458	1.493	0	0	6.85	0	0	29	2	1162.11145	2323.21562	-2.45
qIDHFPEDADYEADTAEYFLR	Unambiguous	1	1	O88602	N-Term(iTRAQ8plex)	CID	1.0000	0.0000	1	1	2672801	Unique	Used		1.043	3.018	3.043	0	0	6.84	0	2	200	3	950.44513	2849.32083	0.00
aGYSQGATQYTQAQQAR	Unambiguous	2	1	O88532	N-Term(iTRAQ8plex)	CID	1.0000	0.0000	1	1	3182054	Unique	Used	1.042	0.541	0.974	0.707	0	0	6.84	0	0	170	2	1067.03210	2133.05693	-2.69
iSFEEFcAVVGGLDIHk	Unambiguous	2	1	Q63810-2	N-Term(iTRAQ8plex); C7(Methylthio); K17(iTRAQ8plex)	CID	1.0000	0.0000	1	1	3709581	Unique	Used	1.466	2.450	1.492	0.947	0	0	6.84	0	12	200	3	840.11182	2518.32090	-3.41
iGEHTPSALAImENANVLAR	Unambiguous	3	1	A6ZI44	N-Term(iTRAQ8plex); M12(Oxidation)	CID	1.0000	0.0000	1	1	3707877	Unique	Used	0.878	1.070	0.760	1.107	0	0	6.84	0	15	200	3	809.76709	2427.28672	-4.17
lATMYSSGSPEGGSDSSESR	Unambiguous	2	1	F8WIE5	N-Term(iTRAQ8plex)	CID	1.0000	0.0000	1	1	2756264	Unique	Used	1.971	0.922	0.685	1.013	0	0	6.84	0	10	200	2	1155.02637	2309.04546	-2.21
yHDLEcQLIQEFTSAQR	Unambiguous	3	1	Q3TPX4	N-Term(iTRAQ8plex); C6(Methylthio)	CID	1.0000	0.0000	1	1	3459237	Unique	Used	0.785	0.855	1.006	1.081	0	0	6.84	0	6	19	3	811.05963	2431.16434	-1.84

nmGGPYGGGNYGPGGSGGSGGYGGR	Unambiguous	3	1	O88569	N-Term(iTRAQ8plex); M2(Oxidation)	CID	1.0000	0.0000	1	1	3428628	Unique	Used	1.035	0.950	1.101	0.853	0	0	6.84	0	0	200	3	837.36932	2510.09342	-4.91
iIAEEAIAAGAPVEALk	Unambiguous	1	1	Q9ERL9	N-Term(iTRAQ8plex); K17(iTRAQ8plex)	CID	1.0000	0.0000	1	1	3195579	Unique	Used	0.420	1.708	1.484	1.334	0	0	6.84	0	3	40	2	1137.67712	2274.34697	-2.65
nVLGHMQQGGSPTPFDR	Unambiguous	1	1	P47857	N-Term(iTRAQ8plex)	CID	1.0000	0.0000	1	1	3658001	Unique	Used	1.814	0.770	0.954	1.148	0	0	6.84	0	1	4	3	715.69379	2145.06681	-6.78
eHINLGcDVDFDIAGPSIR	Unambiguous	3	1	Q60932-2	N-Term(iTRAQ8plex); C7(Methylthio)	CID	1.0000	0.0000	1	1	3374925	Unique	Used	1.159	1.157	1.129	1.036	0	0	6.84	0	0	200	3	807.73065	2421.17740	-2.93
sGASTATAVTDVPSGnLAGAGEAGk	Unambiguous	1	1	Q9WUM3	N-Term(iTRAQ8plex); N16(Deamidated); K25(iTRAQ8plex)	CID	1.0000	0.0000	1	1	3009889	Unique	Used	0.600	0.917	1.416	1.272	0	0	6.84	0	0	200	2	1399.74097	2798.47466	4.20
qTSAYNISNSSTFTk	Unambiguous	1	1	P70206	N-Term(iTRAQ8plex); K15(iTRAQ8plex)	CID	1.0000	0.0000	1	1	3365183	Unique	Used	0.359	0.751	0.480	0.853	0	0	6.84	0	0	200	2	1129.09192	2257.17656	-6.87
sQTEEDcTEELFDFLHAR	Unambiguous	1	1	P99028	N-Term(iTRAQ8plex); C7(Methylthio)	CID	1.0000	0.0000	1	1	2854028	Unique	Used	2.347	1.390	3.322	1.257	0	0	6.84	0	14	200	3	840.71362	2520.12632	-2.46
gFGFVTFENEDVVEk	Unambiguous	3	1	Q920Q6-3	N-Term(iTRAQ8plex); K15(iTRAQ8plex)	CID	1.0000	0.0000	1	1	2940500	Unique	Used	0.924	0.703	0.764	0.849	0	0	6.84	0	12	200	2	1163.10803	2325.20879	-5.83
nLHqSGFSLSGAQIDDNIPR	Unambiguous	1	1	O08553	N-Term(iTRAQ8plex); Q4(Deamidated)	CID	0.0190	0.0000	1	1	3299858	Unique	Used	3.113	1.341	1.347	1.146	0	0	6.84	0	12	67	3	825.42114	2474.24887	-3.60
nSVQTSVEDSSSNSQHQAk	Unambiguous	1	1	Q9JJY4	N-Term(iTRAQ8plex); K19(iTRAQ8plex)	CID	1.0000	0.0000	1	1	2662471	Unique	Used	0.570	0.749	1.397	0.987	0	0	6.84	0	0	200	3	881.11127	2641.31925	-3.09
nTSSEPPEYQLMTGEQIHQER	Unambiguous	1	1	Q9CZD5	N-Term(iTRAQ8plex)	CID	1.0000	0.0000	1	1	2756647	Unique	Used	1.525			0.900	0	0	6.84	0	0	200	3	926.77893	2778.32224	-3.04
aVLDVAETGTEAAAATGVIGGIR	Unambiguous	1	1	P07759	N-Term(iTRAQ8plex)	CID	1.0000	0.0000	1	1	3420595	Unique	Used	4.031	2.024	1.052	1.385	0	0	6.84	0	0	200	2	1223.67114	2446.33501	-4.32
rVDHTTVTGNMQTSQk	Unambiguous	1	1	Q3UNH4	N-Term(iTRAQ8plex); K16(iTRAQ8plex)	CID	1.0000	0.0000	1	1	3381075	Unique	Used	1.209	1.945	1.459	0.491	0	0	6.84	1	0	200	3	804.43195	2411.28128	-4.51

aPAFLSAEEVQDHLR	Unambiguous	1	1	O54983	N-Term(iTRAQ8plex)	CID	1.0000	0.0000	1	1	3234052	Unique	Used	1.032	2.528	1.046	0.827	0	0	6.83	0	2	10	3	663.02240	1987.05265	-1.24
sWIEEQEMGSFLSVAk	Unambiguous	2	1	Q9CPY7	N-Term(iTRAQ8plex); K16(iTRAQ8plex)	CID	1.0000	0.0000	1	1	3351844	Unique	Used	1.635	0.650	0.924	1.223	0	0	6.83	0	5	8	2	1225.14404	2449.28081	-3.50
aLQDILDFTGPPAFPk	Unambiguous	2	1	A2AG50	N-Term(iTRAQ8plex); K16(iTRAQ8plex)	CID	1.0000	0.0000	1	1	3666177	Unique	Used	1.749	0.418	1.059	0.787	0	0	6.83	0	0	18	2	1169.65820	2338.30913	-7.54
hQSGGnFFEPTLLSNVTR	Unambiguous	1	1	Q8BWF0	N-Term(iTRAQ8plex); N6(Deamidated)	CID	1.0000	0.0000	1	1	3456335	Unique	Used	1.441	0.913	0.945	1.126	0	0	6.83	0	2	41	3	770.39691	2309.17618	-2.87
tTQAPLHSVVQqLHGk	Unambiguous	6	1	E9PWV3	N-Term(iTRAQ8plex); Q12(Deamidated); K16(iTRAQ8plex)	CID	0.0278	0.0000	1	1	3776918	Unique	Used	0.643	0.733	0.873	1.050	0	0	6.83	0	0	200	3	785.12384	2353.35697	5.15
dFTSEQIDEILQNHTEIVFAR	Unambiguous	2	1	Q6PIC6	N-Term(iTRAQ8plex)	HCD	1.0000	0.0000	1	1	3355802	Unique	Used	0.650	0.623	0.965	1.130	0	0	6.83	0	3	116	3	937.14551	2809.42197	-3.24
hLNLAENSSYmHVYR	Unambiguous	1	1	Q61644	N-Term(iTRAQ8plex); M11(Oxidation)	CID	1.0000	0.0000	1	1	3551246	Unique	Used	1.318	0.878	1.791	1.218	0	0	6.83	0	0	35	3	718.69147	2154.05985	-4.90
gTGGVDTAAVGGVFDVSNADR	Unambiguous	1	1	Q04447	N-Term(iTRAQ8plex)	CID	1.0000	0.0000	1	1	3129088	Unique	Used	1.300	1.230	2.157	1.741	0	0	6.83	0	0	18	2	1135.06970	2269.13213	-1.84
mYSPSPSDGPASQQPLPNHPR	Unambiguous	2	1	E9QAT4	N-Term(iTRAQ8plex)	CID	1.0000	0.0000	1	1	3338162	Unique	Used	1.601	1.005	1.308	1.057	0	0	6.83	0	0	48	3	856.42303	2567.25455	-2.70
qQLSAEELDAQLDAYnAR	Unambiguous	3	1	O08583	N-Term(iTRAQ8plex); N16(Deamidated)	CID	1.0000	0.0000	1	1	2654372	Unique	Used	1.557	0.438	1.251	0.445	0	0	6.83	0	0	200	2	1170.58276	2340.15825	-1.67
eLAcDDPEAEQVALLAVDYLNNHLLQGFk	Unambiguous	1	1	P29699	N-Term(iTRAQ8plex); C4(Methylthio); K29(iTRAQ8plex)	CID	1.0000	0.0000	1	1	3459854	Unique	Used	3.281	0.794	0.365	2.945	0	0	6.83	0	16	200	3	1294.99646	3882.97483	-3.07
vFYEEELDVPLVLFNEVLDHVLR	Unambiguous	1	1	Q9JHU4	N-Term(iTRAQ8plex)	CID	1.0000	0.0000	1	1	3542324	Unique	Used	0.925	1.143	1.454	1.217	0	0	6.83	0	29	200	3	1031.22339	3091.65561	-3.31
gTGGVDTAATGSVFDISNLDR	Unambiguous	1	1	P30275	N-Term(iTRAQ8plex)	CID	1.0000	0.0000	1	1	3264963	Unique	Used	0.678	1.178	0.868	1.157	0	0	6.83	0	0	200	2	1179.09766	2357.18804	-0.29

iSIEMnGTLEDQLSHLk	Unambiguous	1	1	P57780	N-Term(iTRAQ8plex); N6(Deamidated); K17(iTRAQ8plex)	HCD	1.0000	0.0000	1	1	3283622	Unique	Used	0.948	2.065	1.085	0.713	0	0	6.83	0	16	67	3	846.46088	2537.36808	-2.40
gHQAALGLMEDEQEAQQLR	Unambiguous	1	1	Q8VCA8	N-Term(iTRAQ8plex)	CID	1.0000	0.0000	1	1	2810640	Unique	Used	1.918	1.349	1.665	1.179	0	0	6.83	0	12	55	3	852.76398	2556.27738	-0.20
aSLLQNESTNEQLQIHYk	Unambiguous	4	1	O88544	N-Term(iTRAQ8plex); K18(iTRAQ8plex)	CID	1.0000	0.0000	1	1	3299517	Unique	Used	1.782	1.459	0.691	0.818	0	0	6.83	0	24	56	3	908.82898	2724.47239	-1.95
tYPGVmHSScPQEMAAVk	Unambiguous	1	1	Q9WTL7	N-Term(iTRAQ8plex); M6(Oxidation); C10(Methylthio); K18(iTRAQ8plex)	CID	1.0000	0.0000	1	1	3365562	Unique	Used	0.528	0.783	1.128	1.286	0	0	6.83	0	13	91	3	869.42371	2606.25656	-5.00
sSQEAPSAEAGFATADLSGR	Unambiguous	1	1	Q9CQ13	N-Term(iTRAQ8plex)	CID	1.0000	0.0000	1	1	2828841	Unique	Used	1.723	0.787	0.784	0.878	0	0	6.82	0	17	200	2	1128.55591	2256.10454	-0.05
aAADLMAYcEAHAk	Unambiguous	1	1	P63213	N-Term(iTRAQ8plex); C9(Methylthio); K14(iTRAQ8plex)	CID	1.0000	0.0000	1	1	3416999	Unique	Used	0.286	0.867	1.016	0.955	0	0	6.82	0	5	2	3	707.02185	2119.05100	-4.02
iGEHTPSALAIMENANVLAR	Unambiguous	3	1	A6ZI44	N-Term(iTRAQ8plex)	CID	1.0000	0.0000	1	1	3705215	Unique	Used	1.621	1.596	1.077	2.098	0	0	6.82	0	0	1	3	804.43457	2411.28916	-5.29
aIEPPPLDAVIEAEHTLR	Unambiguous	1	1	E9QNN1	N-Term(iTRAQ8plex)	CID	1.0000	0.0000	1	1	3111008	Unique	Used	0.582	0.650	0.716	1.097	0	0	6.82	0	7	37	3	759.09131	2275.25937	-0.29
nQGGYGGSSSSSSYGSGR	Unambiguous	3	1	Q5EBP8	N-Term(iTRAQ8plex)	CID	1.0000	0.0000	1	1	2787323	Unique	Used	2.168	0.591	1.084	0.691	0	0	6.82	0	3	200	2	999.95392	1998.90056	-2.47
tYFPHFDVSHGSAQVk	Unambiguous	2	1	Q91VB8	N-Term(iTRAQ8plex); K16(iTRAQ8plex)	CID	1.0000	0.0000	1	1	3533153	Unique	Used	2.947	1.965	0.555	1.196	0	0	6.82	0	15	32	3	810.09705	2428.27658	-4.28
hHAAYVnNLNATEEk	Unambiguous	1	1	P09671	N-Term(iTRAQ8plex); N7(Deamidated); K15(iTRAQ8plex)	CID	0.0367	0.0000	1	1	2728315	Unique	Used	1.291	0.793	1.211	1.007	0	0	6.82	0	14	200	3	774.07611	2320.21378	-0.18
gHAPSTAAPAPSPAPMSSSVQSDEER	Unambiguous	1	1	Q8C996	N-Term(iTRAQ8plex)	CID	1.0000	0.0000	1	1	2968885	Unique	Used		1.147	0.939	0.968	0	0	6.82	0	0	104	3	956.79517	2868.37094	-0.95

tGISEGHTVYIVHDGFEGLAk	Unambiguous	1	1	P12382	N-Term(iTRAQ8plex); K21(iTRAQ8plex)	CID	1.0000	0.0000	1	1	3255150	Unique	Used	1.108	1.422	1.437	1.686	0	0	6.82	0	0	27	3	946.84576	2838.52274	-0.68
gLGPLAEAAAAGDGAAAAGAAR	Unambiguous	1	1	O55106	N-Term(iTRAQ8plex)	CID	1.0000	0.0000	1	1	3322422	Unique	Used	1.121	0.868	1.142	0.625	0	0	6.82	0	13	8	2	1057.06519	2113.12309	-3.48
iHYVGQNEPELLVAHAYTR	Unambiguous	2	1	O70252	N-Term(iTRAQ8plex)	CID	1.0000	0.0000	1	1	3411374	Unique	Used	0.563	1.048	1.683	1.291	0	0	6.82	0	15	13	3	838.78143	2514.32975	-4.36
iLAVNSVGLEDVMHEDAVAALk	Unambiguous	4	1	Q62108-3	N-Term(iTRAQ8plex); K22(iTRAQ8plex)	CID	1.0000	0.0000	1	1	3352187	Unique	Used	0.482	1.940	1.864	1.080	0	0	6.82	0	25	6	3	968.20721	2902.60709	-3.38
vEQAEFSDLNLVAHAnGGFSVDMEVLR	Unambiguous	3	1	O88935	N-Term(iTRAQ8plex); N16(Deamidated)	CID	1.0000	0.0000	1	1	3262366	Unique	Used	1.206	1.431	1.779	1.444	0	0	6.82	0	30	19	3	1084.87512	3252.61081	-1.28
lTLEQGQQLVAEGHPGANQASTR	Unambiguous	1	1	Q68FG2	N-Term(iTRAQ8plex)	CID	1.0000	0.0000	1	1	3410191	Unique	Used	1.307	2.202	1.377	0.813	0	0	6.81	0	0	49	3	903.80914	2709.41288	-3.46
iALMGHSFGGATVLQALSEDQR	Unambiguous	2	1	Q60963	N-Term(iTRAQ8plex)	CID	1.0000	0.0000	1	1	3842038	Unique	Used			0.584		0	0	6.81	0	6	23	3	869.12659	2605.36521	-2.25
hTQEScETWGVnGETGTLVDMk	Unambiguous	3	1	P80318	N-Term(iTRAQ8plex); C6(Methylthio); N12(Deamidated); K22(iTRAQ8plex)	CID	1.0000	0.0000	1	1	2850314	Unique	Used	1.740	1.315	1.368	1.931	0	0	6.81	0	16	200	3	1026.48657	3077.44516	-0.77
aIQTLGYFPVGDGVFPHQk	Unambiguous	4	1	Q6PDI5	N-Term(iTRAQ8plex); K19(iTRAQ8plex)	CID	1.0000	0.0000	1	1	3725186	Unique	Used	0.896	0.826	0.805	0.815	0	0	6.81	0	10	94	3	894.82562	2682.46231	-8.99
aDALQAGASqFESSAAk	Unambiguous	3	1	Q62442-2	N-Term(iTRAQ8plex); Q10(Deamidated); K17(iTRAQ8plex)	CID	0.0176	0.0000	1	1	3127284	Unique	Used	0.724	0.466	0.868	1.186	0	0	6.81	0	0	137	2	1131.10437	2261.20146	6.38
gNAGQTNYGFANSTMER	Unambiguous	1	1	P19096	N-Term(iTRAQ8plex)	CID	1.0000	0.0000	1	1	3122313	Unique	Used	0.626	0.685	0.896	0.959	0	0	6.81	0	0	35	2	1061.49927	2121.99126	-0.62
ySSIDTLAEDLTHLLIR	Unambiguous	2	1	Q4QQM5	N-Term(iTRAQ8plex)	CID	1.0000	0.0000	1	1	3666578	Unique	Used	0.589	1.534	0.801	0.899	0	0	6.81	0	6	25	3	755.41528	2264.23130	-5.62
sFNLTAGSLESTEYTYVHk	Unambiguous	4	1	Q99K01	N-Term(iTRAQ8plex); K19(iTRAQ8plex)	CID	1.0000	0.0000	1	1	3346295	Unique	Used	0.756	0.710	1.098	1.819	0	0	6.81	0	8	25	3	919.14996	2755.43534	-1.66

iGEHTPSALAImENANVLAR	Unambiguous	3	1	A6ZI44	N-Term(iTRAQ8plex); M12(Oxidation)	CID	1.0000	0.0000	1	1	3707498	Unique	Used	1.277	0.939	1.150	1.392	0	0	6.81	0	0	200	3	809.76550	2427.28196	-6.13
gAEAANVTGPGGVPVQGSk	Unambiguous	2	1	P62960	N-Term(iTRAQ8plex); K19(iTRAQ8plex)	CID	1.0000	0.0000	1	1	3122262	Unique	Used	0.955	0.186	0.543	0.308	0	0	6.81	0	0	132	2	1152.64514	2304.28301	2.67
kVTITGTPAATQAAQYLITQR	Unambiguous	1	1	Q9JKN6	N-Term(iTRAQ8plex); K1(iTRAQ8plex)	CID	1.0000	0.0000	1	1	3914202	Unique	Used	1.326	0.898	0.901	1.048	0	0	6.81	1	23	180	3	947.54791	2840.62918	-5.73
lAVcGNPATSQPVTSSNHSSR	Unambiguous	1	1	P46935	N-Term(iTRAQ8plex); C4(Methylthio)	CID	1.0000	0.0000	1	1	3929449	Unique	Used	1.131	0.823	1.153	0.916	0	0	6.81	0	0	91	3	821.73602	2463.19352	-3.55
eYNLNFSGSSTVqEVk	Unambiguous	1	1	P54731	N-Term(iTRAQ8plex); Q13(Deamidated); K16(iTRAQ8plex)	CID	0.0382	0.0000	1	1	3106478	Unique	Used	0.930	0.811	0.976	1.288	0	0	6.81	0	25	81	2	1206.13818	2411.26909	5.81
dIGAIAQVHAENGDIIAEEQQR	Unambiguous	1	1	O08553	N-Term(iTRAQ8plex)	CID	1.0000	0.0000	1	1	2830238	Unique	Used	2.245	1.812	1.729	1.677	0	0	6.81	0	8	2	3	894.46375	2681.37668	-1.13
rAPPGPLPPAASPGPPAAsPPAAPSSPR	Unambiguous	1	1	O88703	N-Term(iTRAQ8plex); S19(Phospho)	CID	1.0000	0.0000	1	1	3387101	Unique	Used	1.364	0.866	1.073	1.158	0	0	6.80	1	0	69	3	984.84827	2952.53025	-3.30
tALAEAELEYNPEHVSR	Unambiguous	3	1	Q8BIJ6	N-Term(iTRAQ8plex)	CID	1.0000	0.0000	1	1	2811380	Unique	Used	1.752	1.084	1.176	1.043	0	0	6.80	0	11	124	3	745.04883	2233.13193	-3.74
aSAGPQVQPSTSASSANLDAAR	Unambiguous	1	1	Q8R1N0	N-Term(iTRAQ8plex)	CID	1.0000	0.0000	1	1	3183858	Unique	Used	1.414	0.512	0.974	0.649	0	0	6.80	0	0	57	2	1195.61316	2390.21904	-0.99
gEVGSGAGPGTQAGPSAk	Unambiguous	4	1	E9Q9M5	N-Term(iTRAQ8plex); K18(iTRAQ8plex)	CID	1.0000	0.0000	1	1	2860242	Unique	Used			0.840	0.721	0	0	6.80	0	0	200	2	1068.57397	2136.14067	-4.64
aVTLHDQGTTQWADLSSQFYLR	Unambiguous	1	1	Q02053	N-Term(iTRAQ8plex)	CID	1.0000	0.0000	1	1	3705216	Unique	Used	0.510	1.222	0.799	0.950	0	0	6.80	0	11	39	3	947.81445	2841.42881	-6.53
tQPTDEEMLFIYSHFk	Unambiguous	4	1	P31786	N-Term(iTRAQ8plex); K16(iTRAQ8plex)	CID	1.0000	0.0000	1	1	3285819	Unique	Used	1.908	0.732	0.835	0.776	0	0	6.80	0	7	200	3	865.45032	2594.33640	-2.21
iHLYIQQDDQFQk	Unambiguous	1	1	Q91WG4	N-Term(iTRAQ8plex); K13(iTRAQ8plex)	CID	1.0000	0.0000	1	1	3254992	Unique	Used	1.729	0.834	0.803	1.086	0	0	6.80	0	12	50	3	762.08905	2284.25260	-0.88

sSSADFGTFSTSQSHQTASTVSk	Unambiguous	4	1	Q8K2K6-3	N-Term(iTRAQ8plex); K23(iTRAQ8plex)	CID	1.0000	0.0000	1	1	3274195	Unique	Used	2.333	0.757	1.686	0.829	0	0	6.80	0	0	66	3	986.16315	2956.47489	0.14
lAVcGNPATSQPVTSSNHSSR	Unambiguous	1	1	P46935	N-Term(iTRAQ8plex); C4(Methylthio)	CID	1.0000	0.0000	1	1	3929329	Unique	Used	0.619	0.643	0.789	0.714	0	0	6.80	0	0	116	3	821.73657	2463.19516	-2.88
vTITGSPAATQAAQYLISQR	Unambiguous	2	1	D3YVV7	N-Term(iTRAQ8plex)	CID	1.0000	0.0000	1	1	3916418	Unique	Used	1.924	1.274	0.927	0.640	0	0	6.80	0	13	124	2	1190.65515	2380.30303	-4.54
sTVDGLQVTqLNMDHTTENEDELFR	Unambiguous	1	1	Q8CF89	N-Term(iTRAQ8plex); Q10(Deamidated)	CID	0.0176	0.0000	1	1	2902548	Unique	Used	1.070	1.125	0.861	1.236	0	0	6.80	0	0	200	3	1066.51794	3197.53928	5.68
nLHQSGFSLSGAQIDDNIPR	Unambiguous	1	1	O08553	N-Term(iTRAQ8plex)	CID	1.0000	0.0000	1	1	3534800	Unique	Used	0.827	0.848	1.017	0.909	0	0	6.80	0	28	46	3	825.09216	2473.26194	-4.79
eAFQNAYLELGGLGER	Unambiguous	2	1	Q6PIC6	N-Term(iTRAQ8plex)	CID	1.0000	0.0000	1	1	3355341	Unique	Used	0.761	0.875	0.997	1.071	0	0	6.80	0	14	200	2	1036.03821	2071.06914	-3.42
sLPDPNLITFLFLLEHLk	Unambiguous	5	1	Q5SSL4	N-Term(iTRAQ8plex); K18(iTRAQ8plex)	CID	1.0000	0.0000	1	1	3880263	Unique	Used		0.925	0.676	0.929	0	0	6.80	0	20	45	3	906.86902	2718.59250	-4.78
qGTGSLSESSIPSISDTSTPR	Unambiguous	2	1	Q61290	N-Term(iTRAQ8plex)	CID	1.0000	0.0000	1	1	3210232	Unique	Used	0.662	1.314	2.918	1.533	0	0	6.80	0	0	90	2	1206.11328	2411.21929	-0.46
vVELLADIVQNSSLEDSQIEk	Unambiguous	1	1	Q9CZ13	N-Term(iTRAQ8plex); K21(iTRAQ8plex)	CID	1.0000	0.0000	1	1	3374577	Unique	Used	1.051	0.799	1.004	0.658	0	0	6.80	0	23	200	3	979.87714	2937.61686	-2.48
eLGGESNFGDALLDAGESMk	Unambiguous	1	1	Q62419	N-Term(iTRAQ8plex); K20(iTRAQ8plex)	CID	1.0000	0.0000	1	1	2852982	Unique	Used	0.587	1.003	1.150	0.651	0	0	6.80	0	0	200	2	1324.66663	2648.32598	-2.83
sENGLEFTSSGSANTETTk	Unambiguous	3	1	Q60932-2	N-Term(iTRAQ8plex); K19(iTRAQ8plex)	CID	1.0000	0.0000	1	1	2698378	Unique	Used	0.723	1.080	1.554	1.551	0	0	6.80	0	0	200	2	1284.64355	2568.27983	-3.41
hVSIAASEVEQNPASFLk	Unambiguous	1	1	Q8C419	N-Term(iTRAQ8plex); K18(iTRAQ8plex)	CID	1.0000	0.0000	1	1	3344687	Unique	Used	0.657	0.864	1.871	0.726	0	0	6.80	0	16	21	2	1268.20227	2535.39726	-2.17
sVGVSAPAGGQEAEGSAPTR	Unambiguous	4	1	P41242-3	N-Term(iTRAQ8plex)	CID	1.0000	0.0000	1	1	2824740	Unique	Used		0.952	1.037	1.031	0	0	6.80	0	21	200	2	1066.54785	2132.08843	-0.09
iGEHTPSALAlmENANVLAR	Unambiguous	3	1	A6ZI44	N-Term(iTRAQ8plex); M12(Oxidation)	CID	1.0000	0.0000	1	1	3707736	Unique	Used	1.463	0.880	0.811	1.192	0	0	6.80	0	9	200	3	809.76532	2427.28141	-6.36

gSEHVAYGAITLELASHPVR	Unambiguous	1	1	Q9QYS2	N-Term(iTRAQ8plex)	CID	1.0000	0.0000	1	1	3473679	Unique	Used		1.071	1.319	1.216	0	0	6.80	0	17	144	3	804.43695	2411.29630	-0.92
tGEMSGPVFTDSGIHIILR	Unambiguous	1	1	Q9QUR7	N-Term(iTRAQ8plex)	CID	1.0000	0.0000	1	1	3684687	Unique	Used	1.782	1.185	1.152	1.052	0	0	6.80	0	22	120	3	778.74768	2334.22849	-6.22
gITNLcVIGGDGSLTGADTFR	Unambiguous	1	1	P47857	N-Term(iTRAQ8plex); C6(Methylthio)	CID	1.0000	0.0000	1	1	3728050	Unique	Used	1.922	0.779	0.693	0.813	0	0	6.80	0	16	200	2	1209.10168	2417.19609	-6.05
gSQSVEGAGVmAGEPPSHSSGGR	Unambiguous	2	1	Q6PGL7	N-Term(iTRAQ8plex); M11(Oxidation)	CID	1.0000	0.0000	1	1	2910260	Unique	Used	1.317	1.086	1.216	0.963	0	0	6.80	0	7	200	3	821.05743	2461.15775	-4.17
iIEVVDAIMTTAQSHqR	Unambiguous	3	1	Q8C605	N-Term(iTRAQ8plex); Q16(Deamidated)	CID	0.0250	0.0000	1	1	3918086	Unique	Used	1.453	0.969	1.309	1.166	0	0	6.80	0	0	39	3	739.73688	2217.19608	4.93
yEGSGDGGAAAQSLYIAnHAY	Unambiguous	1	1	P05063	N-Term(iTRAQ8plex); N18(Deamidated)	CID	1.0000	0.0000	1	1	2761186	Unique	Used	1.560	0.839	0.923	1.806	0	0	6.79	0	9	200	2	1210.56763	2420.12798	-1.18
gTVVYGEPITASLGTDGSHYWSk	Unambiguous	1	1	O08553	N-Term(iTRAQ8plex); K23(iTRAQ8plex)	CID	1.0000	0.0000	1	1	3436189	Unique	Used	1.180	1.365	1.055	1.129	0	0	6.79	0	21	98	3	1011.85919	3033.56302	-4.88
nGVQAMVEFDSVQSAQR	Unambiguous	5	1	G5E924	N-Term(iTRAQ8plex)	CID	1.0000	0.0000	1	1	3256529	Unique	Used	0.855	0.687	0.913	0.902	0	0	6.79	0	0	129	2	1085.54346	2170.07964	-3.16
dMSEFENEGFFALHQR	Unambiguous	3	1	Q8CAV6	N-Term(iTRAQ8plex)	CID	1.0000	0.0000	1	1	3054302	Unique	Used	0.936	0.830	0.934	1.381	0	0	6.79	0	14	200	3	754.35907	2261.06266	1.20
gTGGVDTAAVGGVFDVSNADR	Unambiguous	1	1	Q04447	N-Term(iTRAQ8plex)	CID	1.0000	0.0000	1	1	3149048	Unique	Used	1.280	1.128	1.424	1.571	0	0	6.79	0	0	78	2	1135.06812	2269.12895	-3.24
aVqYSGAAVTSGGAAVPSGGAATPR	Unambiguous	1	1	Q6A058	N-Term(iTRAQ8plex); Q3(Deamidated)	CID	1.0000	0.0000	1	1	3657835	Unique	Used	1.285	0.594	1.259	1.316	0	0	6.79	0	0	186	2	1254.65405	2508.30083	0.46
iGEHTPSALAImENANVLAR	Unambiguous	3	1	A6ZI44	N-Term(iTRAQ8plex); M12(Oxidation)	CID	1.0000	0.0000	1	1	3707634	Unique	Used	1.294	1.285	0.797	0.983	0	0	6.79	0	0	200	3	809.76630	2427.28434	-5.15
lIEESAIDEVVVTNTIPHEIQk	Unambiguous	1	1	Q8R574	N-Term(iTRAQ8plex); K22(iTRAQ8plex)	CID	1.0000	0.0000	1	1	3111500	Unique	Used	0.933	1.794	1.813	1.887	0	0	6.79	0	0	200	3	1029.24536	3085.72153	-0.86
sTAGSPPAAAGSGTGPAGScAPAAGAGR	Unambiguous	2	1	Q8K3I9	N-Term(iTRAQ8plex); C20(Methylthio)	CID	1.0000	0.0000	1	1	3592169	Unique	Used	1.137	0.823	2.730	1.660	0	0	6.79	0	0	187	2	1303.11865	2605.23003	-3.89

sSGAASSAPGGGDGVEYk	Unambiguous	1	1	Q0VBL3	N-Term(iTRAQ8plex); K18(iTRAQ8plex)	CID	1.0000	0.0000	1	1	2630528	Unique	Used	0.341	0.641	0.827	0.835	0	0	6.79	0	0	200	2	1103.06189	2205.11650	-3.59
eQASHLGGAVFSGAGNIAAATGLVk	Unambiguous	1	1	Q91ZZ3	N-Term(iTRAQ8plex); K25(iTRAQ8plex)	CID	1.0000	0.0000	1	1	3803735	Unique	Used	0.255	1.101	2.098	1.102	0	0	6.79	0	2	6	2	1467.81165	2934.61601	-3.35
aFSSSSDLTGSSSSAQLnGR	Unambiguous	2	1	Q0V8T8-2	N-Term(iTRAQ8plex); N18(Deamidated)	CID	0.0118	0.0000	1	1	3145742	Unique	Used	0.398	0.875	0.882	0.818	0	0	6.79	0	0	180	2	1132.54822	2264.08916	-2.34
mAASLNAGETTYNLEHANDLR	Unambiguous	1	1	Q80Y56	N-Term(iTRAQ8plex)	HCD	1.0000	0.0000	1	1	3168465	Unique	Used	1.241	0.850	1.167	1.125	0	0	6.79	0	26	70	3	865.76111	2595.26877	-3.38
iVEIVDAITTTAQSHQR	Unambiguous	1	1	P47857	N-Term(iTRAQ8plex)	CID	1.0000	0.0000	1	1	3824698	Unique	Used	2.219	1.055	0.913	2.385	0	0	6.79	0	13	27	3	729.40430	2186.19834	-4.56
aYALLQEQTGGIIDAER	Unambiguous	1	1	D3YXK0	N-Term(iTRAQ8plex)	CID	1.0000	0.0000	1	1	3304375	Unique	Used	0.608	0.801	1.238	0.886	0	0	6.79	0	9	142	2	1076.57800	2152.14873	-3.01
aGGGGGGGGGSSGDYGLVTAGcGFGk	Unambiguous	1	1	Q6NVE9	N-Term(iTRAQ8plex); C22(Methylthio); K26(iTRAQ8plex)	CID	1.0000	0.0000	1	1	3414643	Unique	Used	0.876	0.430	1.249	1.173	0	0	6.79	0	0	82	2	1379.15564	2757.30400	-5.14
aMGIETSDIADVPSDTSk	Unambiguous	2	1	Q91UZ1	N-Term(iTRAQ8plex); K18(iTRAQ8plex)	CID	1.0000	0.0000	1	1	2811859	Unique	Used	0.317	0.059	0.524	0.788	0	0	6.79	0	0	200	2	1223.13232	2445.25737	-2.70
nmGGPYGGGNYGPGGSGGSGGYGGR	Unambiguous	3	1	O88569	N-Term(iTRAQ8plex); M2(Oxidation)	CID	1.0000	0.0000	1	1	3046317	Unique	Used	1.978	0.943	1.439	0.686	0	0	6.79	0	10	133	3	837.37170	2510.10056	-2.06
eDGSIIIVPATQTAIVQLLQGEHMLR	Unambiguous	2	1	Q920I9	N-Term(iTRAQ8plex)	CID	1.0000	0.0000	1	1	3806208	Unique	Used	1.038	1.356	1.011	1.391	0	0	6.79	0	30	11	3	1046.24719	3136.72702	-2.32
nQVAMNPTNTVFDAkR	Unambiguous	4	1	P63017	N-Term(iTRAQ8plex); K15(iTRAQ8plex)	CID	1.0000	0.0000	1	1	3593666	Unique	Used	0.887	0.723	1.023	0.937	0	0	6.79	1	0	182	3	805.43933	2414.30344	-1.52
gPNIVLSEELAGHQTPITDIATER	Unambiguous	1	1	Q9R0D8	N-Term(iTRAQ8plex)	CID	1.0000	0.0000	1	1	3214358	Unique	Used	2.894	1.058	1.275	1.026	0	0	6.79	0	7	42	3	955.84430	2865.51834	-2.69
vELSDVQNPAISITDNVLHFk	Unambiguous	1	1	Q8K2C9	N-Term(iTRAQ8plex); K21(iTRAQ8plex)	CID	1.0000	0.0000	1	1	3418536	Unique	Used	0.799	0.783	0.590	0.776	0	0	6.79	0	5	4	3	983.21271	2947.62357	-3.87
aGYSQGATQYTQAQQAR	Unambiguous	2	1	O88532	N-Term(iTRAQ8plex)	CID	1.0000	0.0000	1	1	3226297	Unique	Used	1.129	0.682	1.189	0.838	0	0	6.79	0	0	200	2	1067.03333	2133.05937	-1.55

lQATLDMQSGIIHAAEEk	Unambiguous	2	1	Q8BG32	N-Term(iTRAQ8plex); K18(iTRAQ8plex)	CID	1.0000	0.0000	1	1	3236889	Unique	Used	0.613	0.962	0.774	0.835	0	0	6.79	0	14	47	3	855.13885	2563.40201	0.37
lEFENLVEETSHFVR	Unambiguous	2	1	Q9R087	N-Term(iTRAQ8plex)	CID	1.0000	0.0000	1	1	3305070	Unique	Used	0.405	0.649	0.521	0.575	0	0	6.79	0	29	30	3	718.37683	2153.11594	-1.00
qADVPAAVTDAAATTPAAEDAATk	Unambiguous	1	1	P06837	N-Term(iTRAQ8plex); K24(iTRAQ8plex)	CID	1.0000	0.0000	1	1	2811169	Unique	Used		0.904	1.075	1.257	0	0	6.78	0	0	44	2	1432.75354	2864.49980	-3.51
dHIIEVVGSSMPLIGDHQDEDk	Unambiguous	3	1	O88935	N-Term(iTRAQ8plex); K22(iTRAQ8plex)	CID	1.0000	0.0000	1	1	2995839	Unique	Used	0.958	1.588	1.644	1.344	0	0	6.78	0	14	188	3	1014.85760	3042.55826	-2.65
hLNLAENSSYmHVYR	Unambiguous	1	1	Q61644	N-Term(iTRAQ8plex); M11(Oxidation)	CID	1.0000	0.0000	1	1	3655371	Unique	Used	1.126	1.410	1.469	1.338	0	0	6.78	0	5	62	3	718.69159	2154.06021	-4.73
qISQMADDTVAELDQHLAAk	Unambiguous	1	1	Q9D6S7	N-Term(iTRAQ8plex); K20(iTRAQ8plex)	CID	1.0000	0.0000	1	1	3216700	Unique	Used	0.942	0.354	1.271	1.061	0	0	6.78	0	8	45	3	931.49445	2792.46878	-0.78
eGSSGFGGPVVSSLYHEETIR	Unambiguous	1	1	Q9D061	N-Term(iTRAQ8plex)	CID	1.0000	0.0000	1	1	3107320	Unique	Used	1.378	1.227	1.013	0.883	0	0	6.78	0	11	121	3	838.09015	2512.25589	-2.51
dLVVLLFETALLSSGFSLEDPQTHSNR	Unambiguous	1	1	P11499	N-Term(iTRAQ8plex)	CID	1.0000	0.0000	1	1	3843717	Unique	Used	1.524	1.273	1.144	1.544	0	0	6.78	0	25	200	3	1098.25037	3292.73655	-0.07
sGYQQAASEHGLVVIAPDTSPR	Unambiguous	8	1	Q9R0P3	N-Term(iTRAQ8plex)	HCD	1.0000	0.0000	1	1	3276595	Unique	Used	0.679	0.537	0.903	0.854	0	0	6.78	0	0	8	3	863.11755	2587.33811	-1.45
gPAVGIDLGTTYScVGVFQHGk	Unambiguous	4	1	P63017	N-Term(iTRAQ8plex); C14(Methylthio); K22(iTRAQ8plex)	CID	1.0000	0.0000	1	1	3807284	Unique	Used	1.191	1.180	1.079	1.024	0	0	6.78	0	29	200	3	954.16748	2860.48789	-2.36
eVNENFAIDLIAQQPVNEVEHR	Unambiguous	2	1	P52503	N-Term(iTRAQ8plex)	CID	1.0000	0.0000	1	1	3016873	Unique	Used	2.265	1.346	1.230	0.904	0	0	6.78	0	0	200	3	956.82990	2868.47513	-1.50
sSEYHLEDNAAYYLNDLER	Unambiguous	1	1	O70443	N-Term(iTRAQ8plex)	CID	1.0000	0.0000	1	1	2938992	Unique	Used	1.413	1.579	1.080	1.052	0	0	6.78	0	30	83	3	869.41412	2606.22782	-1.32
lLGGQLGLEDFIFAHVk	Unambiguous	2	1	Q9Z0G0	N-Term(iTRAQ8plex); K17(iTRAQ8plex)	CID	1.0000	0.0000	1	1	3766028	Unique	Used	0.976	1.819	0.771	0.839	0	0	6.78	0	12	32	3	822.48108	2465.42868	-3.66
nmGGPYGGGNYGPGGSGGSGGYGGR	Unambiguous	3	1	O88569	N-Term(iTRAQ8plex); M2(Oxidation)	CID	1.0000	0.0000	1	1	3295674	Unique	Used	1.868	0.900	1.228	1.019	0	0	6.78	0	0	163	2	1255.54907	2510.09087	-5.92

qcGQMPATATTAAAQPPTAHGPER	Unambiguous	1	1	Q9JLV1	N-Term(iTRAQ8plex); C2(Methylthio)	CID	1.0000	0.0000	1	1	3430278	Unique	Used	1.833	0.609	1.250	1.123	0	0	6.78	0	0	75	3	914.76923	2742.29312	-4.86
aTDVMAHVAGFTVAHDVSAR	Unambiguous	1	1	Q3TC72	N-Term(iTRAQ8plex)	CID	1.0000	0.0000	1	1	3682093	Unique	Used	1.451	1.116	0.956	1.269	0	0	6.78	0	12	24	3	787.07068	2359.19748	-6.62
sGPLPSSSGSSSSSSQLSVATLGR	Unambiguous	11	1	Q6PDH0	N-Term(iTRAQ8plex)	CID	1.0000	0.0000	1	1	3799589	Unique	Used	0.808	0.300	0.946	0.901	0	0	6.78	0	0	178	2	1270.65674	2540.30620	-1.74
kHGcAFLVDEVQTGGGcTGk	Unambiguous	1	1	P61922	N-Term(iTRAQ8plex); K1(iTRAQ8plex); C4(Methylthio); C17(Methylthio); K20(iTRAQ8plex)	CID	1.0000	0.0000	1	1	3455145	Unique	Used	1.075	0.471	0.807	1.229	0	0	6.77	1	0	96	3	1004.51123	3011.51914	-4.90
eAFEEMEGTSPSSPPHSVAR	Unambiguous	1	1	E9Q0S6	N-Term(iTRAQ8plex)	CID	1.0000	0.0000	1	1	2683099	Unique	Used	3.627	3.049	1.211	1.141	0	0	6.77	0	10	200	3	817.05713	2449.15683	-1.62
iDSNLDELSVGLGHLk	Unambiguous	1	1	Q9ERB0	N-Term(iTRAQ8plex); K16(iTRAQ8plex)	CID	1.0000	0.0000	1	1	3324484	Unique	Used	1.790	0.719	0.793	0.547	0	0	6.77	0	10	28	3	773.44043	2318.30674	-4.70
eHINLGcDVDFDIAGPSIR	Unambiguous	3	1	Q60932-2	N-Term(iTRAQ8plex); C7(Methylthio)	CID	1.0000	0.0000	1	1	3354498	Unique	Used	0.820	1.248	1.019	0.823	0	0	6.77	0	6	139	3	807.73065	2421.17740	-2.93
aNcIDSTASAEAVFASEVR	Unambiguous	1	1	Q80WS3	N-Term(iTRAQ8plex); C3(Methylthio)	CID	1.0000	0.0000	1	1	3238616	Unique	Used	0.324	0.480	0.670	0.989	0	0	6.77	0	10	119	2	1146.04956	2291.09184	-1.38
mAAAGGTVSGPSSAckPGR	Unambiguous	2	1	F8WIE5	N-Term(iTRAQ8plex); C15(Methylthio); K16(iTRAQ8plex)	CID	1.0000	0.0000	1	1	3834766	Unique	Used	0.398	0.673	0.833	1.057	0	0	6.77	0	25	200	3	787.07275	2359.20371	-4.43
hGEASSAnSAGGPTSDAGAAVAPNPGPR	Unambiguous	1	1	Q80W04	N-Term(iTRAQ8plex); N8(Deamidated)	CID	1.0000	0.0000	1	1	2647154	Unique	Used	0.769	0.555	0.862	0.720	0	0	6.77	0	26	200	3	936.78479	2808.33982	-1.90
lTLYDIAHTPGVAADLSHIETR	Unambiguous	1	1	P08249	N-Term(iTRAQ8plex)	CID	1.0000	0.0000	1	1	3460472	Unique	Used	1.850	0.860	0.705	0.850	0	0	6.77	0	7	200	3	899.82166	2697.45041	-0.37
gLESVAAGGSQSGGPVEASGPAEAGScDPDHSAEATVk	Unambiguous	4	1	H3BKJ6	N-Term(iTRAQ8plex); C27(Methylthio); K38(iTRAQ8plex)	CID	1.0000	0.0000	1	1	2811159	Unique	Used			1.441	0.574	0	0	6.77	0	0	200	3	1379.65491	4136.95017	-2.34

sIDDLEDQLYHQLEqNR	Unambiguous	3	1	E9Q452	N-Term(iTRAQ8plex); Q15(Deamidated)	CID	0.0103	0.0000	1	1	2766182	Unique	Used	2.325	1.046	1.854	0.779	0	0	6.77	0	12	200	3	807.73212	2421.18180	-0.74
dDAVPNLIQLITNSVEmHAYTVQR	Unambiguous	2	1	P22892	N-Term(iTRAQ8plex); M17(Oxidation)	CID	1.0000	0.0000	1	1	3824970	Unique	Used	0.729	0.738	0.849	0.962	0	0	6.77	0	12	68	3	1016.52765	3047.56839	-2.96
qTTAAAAATFSEQVGGGSGGAGR	Unambiguous	3	1	O88935	N-Term(iTRAQ8plex)	CID	1.0000	0.0000	1	1	3502412	Unique	Used	2.007	2.757	1.731	1.317	0	0	6.77	0	0	200	2	1178.59216	2356.17705	-1.07
iGVGAMTWSPLAcGIVSGk	Unambiguous	3	1	P62482	N-Term(iTRAQ8plex); C13(Methylthio); K19(iTRAQ8plex)	CID	1.0000	0.0000	1	1	3917668	Unique	Used	0.533	0.507	1.374	1.034	0	0	6.77	0	19	21	2	1251.17334	2501.33940	-5.81
eRPGEQSEVAQLIQQTLEQER	Unambiguous	2	1	Q6R891	N-Term(iTRAQ8plex)	CID	1.0000	0.0000	1	1	3110888	Unique	Used	1.283	1.217	1.562	1.099	0	0	6.77	0	22	24	3	924.81873	2772.44162	-0.51
sFADEnNVFHDPVDGWQNETPSGSLESPAQAR	Unambiguous	3	1	G5E8K5-2	N-Term(iTRAQ8plex); N6(Deamidated)	CID	0.0148	0.0000	1	1	2832888	Unique	Used	2.283	1.061	2.008	0.778	0	0	6.77	0	12	200	3	1269.58630	3806.74436	-0.78
vTTLEEQLAGAHQQVSALQQGAGIR	Unambiguous	1	1	B8QI36	N-Term(iTRAQ8plex)	HCD	1.0000	0.0000	1	1	3804793	Unique	Used	1.339	1.073	1.061	0.847	0	0	6.77	0	5	26	3	970.52814	2909.56986	-1.67
hNHDLSSYQDTIQQLENELR	Unambiguous	2	1	P08553	N-Term(iTRAQ8plex)	CID	1.0000	0.0000	1	1	3259854	Unique	Used	0.456	0.662	1.045	1.427	0	0	6.77	0	15	29	3	915.45532	2744.35141	-1.01
aGQVFLEELGNHk	Unambiguous	2	1	P16330	N-Term(iTRAQ8plex); K13(iTRAQ8plex)	CID	1.0000	0.0000	1	1	3300673	Unique	Used	0.900	0.166	1.106	1.305	0	0	6.77	0	29	80	3	684.05505	2050.15061	-1.75
gGLHTVAVmNVGAPAAGMNAAVR	Unambiguous	1	1	P47857	N-Term(iTRAQ8plex); M9(Oxidation)	CID	1.0000	0.0000	1	1	3929331	Unique	Used	2.098	1.119	1.066	0.954	0	0	6.76	0	19	200	3	828.77167	2484.30045	-4.57
tTSPATDAGHVQETSEPSLQALESR	Unambiguous	1	1	Q8R010	N-Term(iTRAQ8plex)	CID	1.0000	0.0000	1	1	2809815	Unique	Used	0.826	0.583	1.594	1.597	0	0	6.76	0	0	12	3	972.81866	2916.44144	-2.56
mVISNPAATHQDIDFLIEEIER	Unambiguous	1	1	P48320	N-Term(iTRAQ8plex)	CID	1.0000	0.0000	1	1	3133106	Unique	Used	0.658	0.836	1.255	1.713	0	0	6.76	0	1	8	3	949.16052	2845.46701	-1.35
eGSSLQNQALQTLQER	Unambiguous	1	1	Q9QYE6	N-Term(iTRAQ8plex)	CID	1.0000	0.0000	1	1	3255877	Unique	Used	1.898	0.795	0.920	0.766	0	0	6.76	0	0	151	2	1053.55627	2106.10527	-1.92
gTnTGVWVGVSGSEASEALSR	Unambiguous	1	1	P19096	N-Term(iTRAQ8plex); N3(Deamidated)	CID	1.0000	0.0000	1	1	3345475	Unique	Used	1.015		0.828	0.915	0	0	6.76	0	0	34	2	1185.10437	2369.20146	5.38

gAVPYVQAFDSLLANPVAEYLk	Unambiguous	2	1	P40124	N-Term(iTRAQ8plex); K22(iTRAQ8plex)	CID	1.0000	0.0000	1	1	3727285	Unique	Used			2.749		0	0	6.76	0	23	9	3	991.88287	2973.63407	-6.92
vLDGLTAGSSSASqQQQQQQHPPGNR	Unambiguous	3	1	D3Z2Q2	N-Term(iTRAQ8plex); Q14(Deamidated)	HCD	1.0000	0.0000	1	1	3531550	Unique	Used	0.496	1.144	1.120	1.299	0	0	6.76	0	0	200	3	1008.84479	3024.51981	5.31
eHINLGcDVDFDIAGPSIR	Unambiguous	3	1	Q60932-2	N-Term(iTRAQ8plex); C7(Methylthio)	CID	1.0000	0.0000	1	1	3355338	Unique	Used	1.125	1.195	1.298	1.180	0	0	6.76	0	2	200	3	807.73041	2421.17667	-3.23
tVGLGDAISAEGLFYSEARPD	Unambiguous	2	1	Q8VDL4-3	N-Term(iTRAQ8plex)	CID	1.0000	0.0000	1	1	3217143	Unique	Used	1.058	0.777	0.774	1.168	0	0	6.76	0	10	61	2	1236.63025	2472.25322	-1.15
gGAFEGTLqGPFGHGYGEGAGEGIDDAEWVVAR	Unambiguous	1	1	Q9QXY6	N-Term(iTRAQ8plex); Q9(Deamidated)	CID	1.0000	0.0000	1	1	3036862	Unique	Used	1.091	1.282	1.740	1.477	0	0	6.76	0	0	200	3	1204.57410	3611.70774	2.64
tSLPqHVMEPPVGAAAAGTSGSSLDSFSk	Unambiguous	1	1	Q6PE13	N-Term(iTRAQ8plex); Q5(Deamidated); K29(iTRAQ8plex)	CID	1.0000	0.0000	1	1	3413133	Unique	Used	2.839	1.152	1.407	0.991	0	0	6.76	0	0	44	3	1146.92932	3438.77341	1.80
aPPAGGSGSSAAAAAAAGGTEVRPR	Unambiguous	2	1	P84309	N-Term(iTRAQ8plex)	HCD	1.0000	0.0000	1	1	3449451	Unique	Used	0.350	0.748	2.976	1.187	0	0	6.76	0	0	9	3	814.42889	2441.27213	-3.21
eAFQNAYLELGGLGER	Unambiguous	2	1	Q6PIC6	N-Term(iTRAQ8plex)	CID	1.0000	0.0000	1	1	3350327	Unique	Used	0.678	0.651	0.994	1.398	0	0	6.76	0	11	0	2	1036.03870	2071.07012	-2.95
aGAVTSSASLSSPALAk	Unambiguous	4	1	O08784	N-Term(iTRAQ8plex); K17(iTRAQ8plex)	CID	1.0000	0.0000	1	1	3573951	Unique	Used	0.672	0.540	1.047	0.817	0	0	6.76	0	0	198	2	1063.61145	2126.21562	-5.71
tVEGAGNIAAATGFVk	Unambiguous	2	1	O55042	N-Term(iTRAQ8plex); K16(iTRAQ8plex)	CID	1.0000	0.0000	1	1	3413148	Unique	Used	0.964	1.461	2.032	0.804	0	0	6.76	0	12	76	2	1057.60059	2114.19390	-6.03
aGLAEAPLVTGQPGPGHGk	Unambiguous	5	1	F8WHW6	N-Term(iTRAQ8plex); K19(iTRAQ8plex)	CID	1.0000	0.0000	1	1	3340079	Unique	Used	0.342	1.473	1.771	1.309	0	0	6.76	0	0	30	2	1183.17273	2365.33818	-2.83
hHAAYVNNLnATEEk	Unambiguous	1	1	P09671	N-Term(iTRAQ8plex); N10(Deamidated); K15(iTRAQ8plex)	CID	1.0000	0.0000	1	1	2727791	Unique	Used	1.752	1.127	1.156	1.224	0	0	6.75	0	0	179	3	774.07416	2320.20792	-2.71

dMAIATGGAVFGEEGLNLNLEDVQAHDLGk	Unambiguous	1	1	P63038	N-Term(iTRAQ8plex); K30(iTRAQ8plex)	CID	1.0000	0.0000	1	1	3264136	Unique	Used	1.672	1.367	1.170	1.501	0	0	6.75	0	9	200	3	1231.63611	3692.89377	-3.08
vLGILAMIDEGETDWk	Unambiguous	1	1	Q9D819	N-Term(iTRAQ8plex); K16(iTRAQ8plex)	CID	1.0000	0.0000	1	1	3353163	Unique	Used	1.406	0.724	0.752	0.941	0	0	6.75	0	4	11	2	1199.65759	2398.30791	-2.91
aVGLISHVLEQNQEGDAAYR	Unambiguous	3	1	Q9JLZ3	N-Term(iTRAQ8plex)	CID	1.0000	0.0000	1	1	3372004	Unique	Used	1.155	0.883	1.020	1.302	0	0	6.75	0	9	23	3	825.43359	2474.28623	-3.21
eVLGSSLPNNWQDISLDDGHMk	Unambiguous	1	1	Q8VDT9	N-Term(iTRAQ8plex); K22(iTRAQ8plex)	CID	1.0000	0.0000	1	1	3259645	Unique	Used	0.930	1.154	1.062	0.808	0	0	6.75	0	6	23	3	1021.85797	3063.55936	-2.38
sSGSPYGGGYGSGGGSGGYGSR	Unambiguous	7	1	J3QNY1	N-Term(iTRAQ8plex)	CID	1.0000	0.0000	1	1	3026125	Unique	Used	1.237	0.790	1.635	1.029	0	0	6.75	0	0	200	2	1107.99854	2214.98979	-2.53
gTGGVDTAAVGGVFDVSNADR	Unambiguous	1	1	Q04447	N-Term(iTRAQ8plex)	CID	1.0000	0.0000	1	1	3169256	Unique	Used	1.425	0.943	1.257	1.576	0	0	6.75	0	0	83	2	1135.06958	2269.13188	-1.95
gTGGVDTAAVGGVFDVSNADR	Unambiguous	1	1	Q04447	N-Term(iTRAQ8plex)	HCD	1.0000	0.0000	1	1	3130133	Unique	Used	2.551	1.276	1.299	1.251	0	0	6.75	0	0	152	2	1135.07312	2269.13896	1.17
hHQEEVGELLGqIQGcGAAQAQAQAEAR	Unambiguous	1	1	P19246	N-Term(iTRAQ8plex); Q12(Deamidated); C16(Methylthio)	CID	0.0326	0.0000	1	1	3345936	Unique	Used	2.140	0.435	1.139	1.040	0	0	6.75	0	0	10	3	1094.19812	3280.57981	1.72
tVTSFYNQSAIDVAAEkPSVR	Unambiguous	2	1	O55028	N-Term(iTRAQ8plex); K17(iTRAQ8plex)	CID	1.0000	0.0000	1	1	3454966	Unique	Used	0.710	0.778	0.704	0.891	0	0	6.75	0	0	143	3	964.52496	2891.56034	-4.16
gFQQILAGEYDHLPEqAFYMVGPIEEAVAk	Unambiguous	1	1	P56480	N-Term(iTRAQ8plex); Q16(Deamidated); K30(iTRAQ8plex)	CID	1.0000	0.0000	1	1	3420139	Unique	Used	0.775	1.568	1.345	1.198	0	0	6.75	0	19	200	3	1320.68347	3960.03586	0.20
aAVQELSGSILTSEDPEER	Unambiguous	1	1	P49769	N-Term(iTRAQ8plex)	CID	1.0000	0.0000	1	1	2813798	Unique	Used	0.908	0.772	0.650	0.749	0	0	6.75	0	0	200	2	1168.09912	2335.19097	-0.93
vAHSFNcTPIEGMLSHQLk	Unambiguous	2	1	P50580	N-Term(iTRAQ8plex); C7(Methylthio); K19(iTRAQ8plex)	CID	1.0000	0.0000	1	1	3803379	Unique	Used	0.485	0.563	0.697	0.749	0	0	6.75	0	0	52	3	922.81366	2766.42643	-3.11
eIPIEGLEFMGHGk	Unambiguous	1	1	Q9D8Z2	N-Term(iTRAQ8plex); K14(iTRAQ8plex)	CID	1.0000	0.0000	1	1	3213775	Unique	Used	1.602	1.222	1.353	0.936	0	0	6.74	0	11	16	3	722.40076	2165.18772	-0.39

tQTAVASEEGPATLPEAEALLAQHAALR	Unambiguous	1	1	Q68FG2	N-Term(iTRAQ8plex)	HCD	1.0000	0.0000	1	1	3325202	Unique	Used	1.530	2.643	1.841	1.108	0	0	6.74	0	9	7	3	1050.56067	3149.66745	-2.25
yTPSGQSGAAASESLFISNHAY	Unambiguous	2	1	A6ZI44	N-Term(iTRAQ8plex)	CID	1.0000	0.0000	1	1	3346233	Unique	Used	0.678	1.282	0.855	1.034	0	0	6.74	0	0	2	2	1281.61792	2562.22856	-5.03
iPNIYAIGDVVAGPmLAHk	Unambiguous	1	1	O08749	N-Term(iTRAQ8plex); M15(Oxidation); K19(iTRAQ8plex)	CID	1.0000	0.0000	1	1	3765084	Unique	Used	2.438	2.328	1.589	1.057	0	0	6.74	0	4	20	3	868.49493	2603.47025	-5.29
vVIEAcDELGIVLAHTDLR	Unambiguous	1	1	Q9CWJ9	N-Term(iTRAQ8plex); C6(Methylthio)	CID	1.0000	0.0000	1	1	3561529	Unique	Used	1.047	1.556	0.815	1.274	0	0	6.74	0	12	64	3	806.09760	2416.27823	-4.14
yHTSQSGDEMTSLSEYVSR	Unambiguous	2	1	P11499	N-Term(iTRAQ8plex)	CID	1.0000	0.0000	1	1	3146616	Unique	Used	1.236	1.494	1.451	0.940	0	0	6.74	0	15	41	3	827.71918	2481.14298	-3.06
cFSIDnPGYEPEVVAVHPGGDTVAVGGTDGNVR	Unambiguous	1	1	O88342	N-Term(iTRAQ8plex); C1(Methylthio); N6(Deamidated)	CID	1.0000	0.0000	1	1	3193910	Unique	Used	4.050	1.067	1.004	1.057	0	0	6.74	0	13	173	3	1227.24670	3679.72556	-1.52
dFTSEQIDEILQNHTEIVFAR	Unambiguous	2	1	Q6PIC6	N-Term(iTRAQ8plex)	CID	1.0000	0.0000	1	1	3376712	Unique	Used		0.667	2.273	2.655	0	0	6.74	0	7	200	3	937.14563	2809.42234	-3.11
yEGSGDGGAAAQSLYIAnHAY	Unambiguous	1	1	P05063	N-Term(iTRAQ8plex); N18(Deamidated)	CID	1.0000	0.0000	1	1	2761547	Unique	Used	1.396	0.683	1.519	1.284	0	0	6.74	0	0	200	2	1210.56592	2420.12456	-2.59
iSVAGVTSGNVGYLAHAIHQVTk	Unambiguous	1	1	P05202	N-Term(iTRAQ8plex); K23(iTRAQ8plex)	CID	1.0000	0.0000	1	1	3896002	Unique	Used	1.690	1.324	0.880	1.116	0	0	6.74	0	16	17	3	977.55481	2930.64988	-5.93
qTVNRGPGLGSTQGQTIALPAQGLIEFR	Unambiguous	1	1	P42669	N-Term(iTRAQ8plex)	HCD	1.0000	0.0000	1	1	3934240	Unique	Used	1.808	0.795	0.922	0.997	0	0	6.74	1	23	199	3	1071.92102	3213.74851	-5.03
fWNAAFFDAVHcER	Unambiguous	2	1	Q3TA40	N-Term(iTRAQ8plex); C12(Methylthio)	CID	1.0000	0.0000	1	1	3765703	Unique	Used	0.731	1.529	0.868	1.091	0	0	6.74	0	0	33	3	688.32086	2062.94803	-4.34
lAQGPELAEDDADLLHk	Unambiguous	1	1	Q8R5H6	N-Term(iTRAQ8plex); K17(iTRAQ8plex)	CID	1.0000	0.0000	1	1	2653889	Unique	Used	0.678	1.193	1.477	0.703	0	0	6.74	0	16	200	3	815.11420	2443.32804	-0.37
sFGSAQEFAWAHDSSEYAIR	Unambiguous	1	1	O55029	N-Term(iTRAQ8plex)	CID	1.0000	0.0000	1	1	3301139	Unique	Used	1.354	0.972	0.839	0.702	0	0	6.74	0	15	29	3	855.07300	2563.20444	-4.35
lAAAAPVAPTAIVTAHADAIPTVEATAAR	Unambiguous	2	1	Q9QYX7	N-Term(iTRAQ8plex)	CID	1.0000	0.0000	1	1	3705185	Unique	Used	0.134	0.723	1.691	0.848	0	0	6.74	0	6	19	3	1015.56665	3044.68540	-6.35

aAEAGVTEEQYGYLGTSAk	Unambiguous	1	1	P14873	N-Term(iTRAQ8plex); K19(iTRAQ8plex)	CID	1.0000	0.0000	1	1	2847747	Unique	Used	1.171	0.747	1.487	0.997	0	0	6.74	0	0	191	2	1277.16467	2553.32207	-2.84
eAIVNScVFVHQTLHQANAR	Unambiguous	1	1	Q9JHU4	N-Term(iTRAQ8plex); C7(Methylthio)	CID	1.0000	0.0000	1	1	3933315	Unique	Used	1.065	0.930	1.064	1.066	0	0	6.74	0	21	154	3	863.10791	2587.30918	-3.24
mTVAHMWFDNQIHEADTTEDqSGTSFDk	Unambiguous	2	1	Q6PIC6	N-Term(iTRAQ8plex); Q21(Deamidated); K28(iTRAQ8plex)	CID	1.0000	0.0000	1	1	3014741	Unique	Used	0.709	0.775	1.352	1.287	0	0	6.74	0	0	142	3	1284.26917	3850.79294	3.73
qNTGSATQGPAYHGVNR	Unambiguous	4	1	E9QAQ7	N-Term(iTRAQ8plex)	HCD	1.0000	0.0000	1	1	3202211	Unique	Used	0.964	0.881	1.099	1.098	0	0	6.74	0	0	88	3	688.01556	2062.03214	-2.26
vSQAASDLLAYcEAHVR	Unambiguous	1	1	P50153	N-Term(iTRAQ8plex); C12(Methylthio)	CID	1.0000	0.0000	1	1	3862077	Unique	Used	0.758	0.949	0.672	1.338	0	0	6.73	0	18	200	3	728.36359	2183.07621	-5.91
yIEESIGESANLLGAMEk	Unambiguous	1	1	Q8BLE7	N-Term(iTRAQ8plex); K18(iTRAQ8plex)	CID	1.0000	0.0000	1	1	3217316	Unique	Used	0.402	0.432	0.742	1.267	0	0	6.73	0	6	20	2	1281.68091	2562.35454	-1.43
lVQDYGLESEVAQHLAk	Unambiguous	2	1	Q64521	N-Term(iTRAQ8plex); K17(iTRAQ8plex)	CID	1.0000	0.0000	1	1	3236206	Unique	Used	0.831	1.427	1.065	1.255	0	0	6.73	0	0	136	2	1254.69775	2508.38823	-1.45
sWGSGGGQEPGGSQAAGGGQPR	Unambiguous	5	1	Q8CFT0	N-Term(iTRAQ8plex)	CID	1.0000	0.0000	1	1	3005361	Unique	Used	0.603	0.299	1.234	1.121	0	0	6.73	0	0	43	2	1145.04382	2289.08037	-4.68
eVYMGNVIQGGEGQAPTR	Unambiguous	1	1	Q8QZT1	N-Term(iTRAQ8plex)	CID	1.0000	0.0000	1	1	3125985	Unique	Used	1.007	0.989	0.847	1.040	0	0	6.73	0	0	0	2	1105.56140	2210.11553	-1.02
hNIQFSSFDIFSDEEVR	Unambiguous	1	1	Q9CQM9	N-Term(iTRAQ8plex)	CID	1.0000	0.0000	1	1	3236751	Unique	Used	2.120	0.850	0.862	0.881	0	0	6.73	0	0	2	3	792.05719	2374.15702	-1.99
sINTDNcVDIYSHAk	Unambiguous	4	1	Q6GQW0	N-Term(iTRAQ8plex); C7(Methylthio); K15(iTRAQ8plex)	CID	1.0000	0.0000	1	1	3255378	Unique	Used	0.624	0.866	0.695	0.967	0	0	6.73	0	30	42	3	778.72369	2334.15653	-4.85
qIQQLQGELQHLR	Unambiguous	6	1	Q3TVA9	N-Term(iTRAQ8plex)	CID	1.0000	0.0000	1	1	3640143	Unique	Used	1.106	0.588	0.820	0.947	0	0	6.73	0	10	8	3	632.36023	1895.06614	-5.46
rLQLQLEEAAAQNQR	Unambiguous	4	1	O08576-2	N-Term(iTRAQ8plex)	HCD	1.0000	0.0000	1	1	3532693	Unique	Used	0.920	0.899	0.898	0.943	0	0	6.73	1	27	24	3	691.38550	2072.14194	-4.59
rGDQVALANWQSHPSLR	Unambiguous	3	1	Q61749	N-Term(iTRAQ8plex)	CID	1.0000	0.0000	1	1	3852583	Unique	Used	1.995	1.394	1.629	1.055	0	0	6.73	1	0	200	3	747.06940	2239.19364	-2.76

nISLSSEEEAEGLAGHPR	Unambiguous	1	1	Q5U3K5	N-Term(iTRAQ8plex)	CID	1.0000	0.0000	1	1	2667469	Unique	Used	1.333	0.891	1.144	1.032	0	0	6.73	0	17	200	3	734.04187	2200.11106	-1.71
gSDHTDVcGNVVGSSGGSSSSGGSDk	Unambiguous	2	1	A2AHG0	N-Term(iTRAQ8plex); C8(Methylthio); K26(iTRAQ8plex)	CID	1.0000	0.0000	1	1	2880237	Unique	Used		0.820	2.825	1.790	0	0	6.73	0	0	200	3	998.79047	2994.35685	-1.92
eSGcAAAGETEAAAATAFR	Unambiguous	1	1	Q8BR63	N-Term(iTRAQ8plex); C4(Methylthio)	CID	1.0000	0.0000	1	1	2957237	Unique	Used	1.806	0.485	1.620	1.243	0	0	6.73	0	0	200	2	1067.49451	2133.98174	-1.41
gLcSGPGAGEESPAATLPR	Unambiguous	1	1	Q80UU9	N-Term(iTRAQ8plex); C3(Methylthio)	CID	1.0000	0.0000	1	1	3321141	Unique	Used	0.608	0.990	0.991	1.130	0	0	6.73	0	0	138	2	1060.52148	2120.03569	-2.91
aANAAENDFSVSQAEVSSR	Unambiguous	1	1	Q6P542	N-Term(iTRAQ8plex)	CID	1.0000	0.0000	1	1	2734302	Unique	Used	0.808	0.452	0.871	0.684	0	0	6.73	0	13	200	2	1129.04956	2257.09184	-3.57
sNISSPVTPTAASSSTTPTR	Unambiguous	6	1	A2AU91	N-Term(iTRAQ8plex)	CID	1.0000	0.0000	1	1	3612648	Unique	Used	0.407	0.399	0.571	0.923	0	0	6.73	0	0	106	2	1177.10522	2353.20317	-4.98
iQEGVFDINNEAnGlk	Unambiguous	1	1	P53994	N-Term(iTRAQ8plex); N13(Deamidated); K16(iTRAQ8plex)	CID	0.0297	0.0000	1	1	2850591	Unique	Used	0.764	1.062	0.917	1.135	0	0	6.73	0	0	200	2	1185.63855	2370.26982	-2.68
tLTGTAALTVQSQEENLR	Unambiguous	1	1	P24527	N-Term(iTRAQ8plex)	CID	1.0000	0.0000	1	1	3278907	Unique	Used	1.038	1.714	0.946	1.164	0	0	6.73	0	0	49	2	1118.60522	2236.20317	-2.47
vLVETEGPAGVAVMk	Unambiguous	1	1	P42125	N-Term(iTRAQ8plex); K15(iTRAQ8plex)	CID	1.0000	0.0000	1	1	3257816	Unique	Used	2.003	1.300	0.811	1.241	0	0	6.72	0	4	19	2	1054.61340	2108.21953	-2.41
kMEDGVGcLETAEEAk	Unambiguous	1	1	Q8VDD5	N-Term(iTRAQ8plex); K1(iTRAQ8plex); C8(Methylthio); K16(iTRAQ8plex)	CID	1.0000	0.0000	1	1	2685767	Unique	Used	0.865	0.839	1.301	0.767	0	0	6.72	1	14	200	3	890.12799	2668.36942	-2.46
lGQHVVGMAPLSVGSLDDEPGGETETR	Unambiguous	1	1	Q9CZR8	N-Term(iTRAQ8plex)	CID	1.0000	0.0000	1	1	3128968	Unique	Used	0.692	1.342	1.163	1.123	0	0	6.72	0	0	10	3	1019.18036	3055.52652	-1.44
nLHQSGFSLSGAQIDDNIPR	Unambiguous	1	1	O08553	N-Term(iTRAQ8plex)	CID	1.0000	0.0000	1	1	3366961	Unique	Used	1.670	1.139	1.035	1.073	0	0	6.72	0	13	56	3	825.09290	2473.26413	-3.90
eHINLGcDVDFDIAGPSIR	Unambiguous	3	1	Q60932-2	N-Term(iTRAQ8plex); C7(Methylthio)	CID	1.0000	0.0000	1	1	3355757	Unique	Used	0.837	1.202	0.704	1.228	0	0	6.72	0	11	200	3	807.73108	2421.17868	-2.40

nSLcPSGGNILTPLLQQDcHQk	Unambiguous	1	1	P35762	N-Term(iTRAQ8plex); N1(Deamidated); C4(Methylthio); C19(Methylthio); K22(iTRAQ8plex)	CID	1.0000	0.0000	1	1	3644706	Unique	Used		1.603	1.387	2.211	0	0	6.72	0	18	11	3	1023.17657	3067.51517	-4.64
hHAAYVnNLNATEEk	Unambiguous	1	1	P09671	N-Term(iTRAQ8plex); N7(Deamidated); K15(iTRAQ8plex)	CID	1.0000	0.0000	1	1	2750434	Unique	Used	1.379	0.863	1.145	0.911	0	0	6.72	0	0	59	3	774.07269	2320.20353	-4.60
gEGGGATTSAQVMVIk	Unambiguous	2	1	Q9Z2D6	N-Term(iTRAQ8plex); K16(iTRAQ8plex)	CID	1.0000	0.0000	1	1	3186059	Unique	Used	0.780	1.492	1.366	1.432	0	0	6.72	0	0	62	2	1057.58875	2114.17021	-1.62
yEGSGDGGAAAQSLYIANHAY	Unambiguous	1	1	P05063	N-Term(iTRAQ8plex)	CID	1.0000	0.0000	1	1	3107304	Unique	Used	0.697	0.765	0.854	1.329	0	0	6.72	0	0	85	2	1210.07629	2419.14531	-0.62
tGSPGGPGVGGSPGGGAAGASPSLPPSAk	Unambiguous	1	1	Q8R1Q8	N-Term(iTRAQ8plex); K29(iTRAQ8plex)	CID	1.0000	0.0000	1	1	3385856	Unique	Used	2.960	1.158	1.506	1.418	0	0	6.72	0	0	55	3	980.85626	2940.55423	-3.20
sQSSNYGASSSSTmIGFHVADSLR	Unambiguous	6	1	Q8BRR9	N-Term(iTRAQ8plex); M14(Oxidation)	CID	1.0000	0.0000	1	1	3720235	Unique	Used	0.558	2.087	1.144	1.213	0	0	6.72	0	13	116	3	937.10986	2809.31504	-7.64
sIDDLEDQLYHQLEQnR	Unambiguous	3	1	E9Q452	N-Term(iTRAQ8plex); N16(Deamidated)	CID	0.0164	0.0000	1	1	2782547	Unique	Used	2.831	2.277	2.190	0.917	0	0	6.72	0	17	200	3	807.73254	2421.18308	-0.21
gFQQILAGEYDHLPEqAFYMVGPIEEAVAk	Unambiguous	1	1	P56480	N-Term(iTRAQ8plex); Q16(Deamidated); K30(iTRAQ8plex)	CID	1.0000	0.0000	1	1	3398720	Unique	Used	1.381	1.205	1.201	1.011	0	0	6.72	0	0	200	3	1320.68408	3960.03769	0.66
gNAGQTNYGFAnSTMER	Unambiguous	1	1	P19096	N-Term(iTRAQ8plex); N12(Deamidated)	CID	1.0000	0.0000	1	1	2826800	Unique	Used	0.721	0.812	0.736	0.824	0	0	6.72	0	0	200	2	1061.98901	2122.97075	-2.75
lAQQHAAAAAAAAAATnQQGSAk	Unambiguous	1	1	Q8BHJ5	N-Term(iTRAQ8plex); N17(Deamidated); K23(iTRAQ8plex)	CID	0.0253	0.0000	1	1	3717620	Unique	Used	2.073	1.061	0.933	0.887	0	0	6.72	0	30	200	3	910.83246	2730.48282	3.09

eDDGVPVIcQVEHPAVTGNLQTQR	Unambiguous	9	1	Q8R5M8-4	N-Term(iTRAQ8plex); C9(Methylthio)	CID	1.0000	0.0000	1	1	3213439	Unique	Used	0.729	1.439	1.070	1.216	0	0	6.72	0	14	38	3	985.82233	2955.45243	-2.80
eSGcAAAGETEAAAATAFR	Unambiguous	1	1	Q8BR63	N-Term(iTRAQ8plex); C4(Methylthio)	CID	1.0000	0.0000	1	1	2938077	Unique	Used	2.160	0.463	1.051	1.177	0	0	6.72	0	15	82	2	1067.49646	2133.98564	0.42
qNAVLQAAQDDLGHLR	Unambiguous	7	1	O35551	N-Term(iTRAQ8plex)	CID	1.0000	0.0000	1	1	3280246	Unique	Used	1.193	0.575	1.019	1.033	0	0	6.71	0	13	25	3	685.04053	2053.10703	-1.09
dMAIATGGAVFGEEGLNLNLEDVQAHDLGk	Unambiguous	1	1	P63038	N-Term(iTRAQ8plex); K30(iTRAQ8plex)	CID	1.0000	0.0000	1	1	3304991	Unique	Used	1.040	0.544	0.994	1.110	0	0	6.71	0	23	19	3	1231.63586	3692.89304	-3.28
aDIGIAMGISGSDVSk	Unambiguous	2	1	Q6PIE5	N-Term(iTRAQ8plex); K16(iTRAQ8plex)	CID	1.0000	0.0000	1	1	3257756	Unique	Used	0.488	0.601	1.043	1.511	0	0	6.71	0	0	9	2	1065.08752	2129.16777	-2.60
aTSSSSSSSGVGmSVGQGR	Unambiguous	2	1	P21619	N-Term(iTRAQ8plex); M13(Oxidation)	CID	1.0000	0.0000	1	1	3402859	Unique	Used	0.933	1.319	1.265	1.477	0	0	6.71	0	0	171	3	683.66223	2048.97214	-4.85
aLEAAHGPGQANqEIAAMIDTEFAk	Unambiguous	1	1	Q8CF89	N-Term(iTRAQ8plex); Q13(Deamidated); K25(iTRAQ8plex)	CID	0.0387	0.0000	1	1	3172312	Unique	Used	1.641	1.023	0.978	0.975	0	0	6.71	0	0	200	3	1064.89014	3192.65586	3.19
gAQIASTGQLYAAWnGGLDR	Unambiguous	1	1	Q61361	N-Term(iTRAQ8plex); N15(Deamidated)	CID	1.0000	0.0000	1	1	3393456	Unique	Used	2.400	1.402	1.332	1.558	0	0	6.71	0	0	39	2	1177.60168	2354.19609	-3.49
dEVQEVVYFPAVDHDAESk	Unambiguous	2	1	Q9CZC8	N-Term(iTRAQ8plex); K19(iTRAQ8plex)	CID	1.0000	0.0000	1	1	2737546	Unique	Used	0.536	1.180	0.891	1.192	0	0	6.71	0	13	200	3	929.14014	2785.40586	-2.97
gGGGPGGGGPGGGGASGGPSQPPGGGGPGIR	Unambiguous	1	1	Q3U0V1	N-Term(iTRAQ8plex)	CID	1.0000	0.0000	1	1	3361860	Unique	Used	1.755		2.158	1.035	0	0	6.71	0	0	200	3	877.76587	2631.28305	-3.67
yGAATANYMEVVSLLk	Unambiguous	2	1	Q64521	N-Term(iTRAQ8plex); K16(iTRAQ8plex)	CID	1.0000	0.0000	1	1	3665952	Unique	Used	0.700	1.822	1.094	1.219	0	0	6.71	0	0	25	2	1169.64539	2338.28349	-4.37
lAASGEGGLQELSGHFENQk	Unambiguous	3	1	Q9QXS6	N-Term(iTRAQ8plex); K20(iTRAQ8plex)	CID	1.0000	0.0000	1	1	3148552	Unique	Used	1.400	1.512	1.090	0.649	0	0	6.71	0	14	72	3	894.14172	2680.41062	-1.69
aDAVQDSEMVELVELEIR	Unambiguous	3	1	Q8BFR5	N-Term(iTRAQ8plex)	CID	1.0000	0.0000	1	1	2689619	Unique	Used	0.876	0.855	0.889	1.549	0	0	6.71	0	8	200	2	1175.60876	2350.21025	-0.51

gITNLcVIGGDGSLTGADTFR	Unambiguous	1	1	P47857	N-Term(iTRAQ8plex); C6(Methylthio)	CID	1.0000	0.0000	1	1	3729147	Unique	Used	1.917	0.766	0.804	1.111	0	0	6.71	0	0	200	2	1209.10156	2417.19585	-6.15
akAGAGSATLSMAYAGAR	Unambiguous	1	1	P08249	N-Term(iTRAQ8plex); K2(iTRAQ8plex)	HCD	1.0000	0.0000	1	1	3909533	Unique	Used	2.428	1.765	0.616	0.628	0	0	6.71	1	0	200	3	754.75140	2262.23966	-3.92
sTGGPPGPGPLPGPQGLSSSSGSR	Unambiguous	2	1	Q3UHD9	N-Term(iTRAQ8plex)	CID	1.0000	0.0000	1	1	3757980	Unique	Used	0.588	1.323	1.211	0.970	0	0	6.71	0	0	182	2	1219.62903	2438.25078	-2.88
dHQPcIIFmDEIDAIGGR	Unambiguous	1	1	P62334	N-Term(iTRAQ8plex); C5(Methylthio); M9(Oxidation)	CID	1.0000	0.0000	1	1	3326538	Unique	Used	1.801	0.967	0.721	0.766	0	0	6.71	0	0	18	3	799.38092	2396.12821	-2.88
dEVQEVVYFPAVDHDAESk	Unambiguous	2	1	Q9CZC8	N-Term(iTRAQ8plex); K19(iTRAQ8plex)	CID	1.0000	0.0000	1	1	2670051	Unique	Used	1.922	1.098	1.709	0.700	0	0	6.71	0	7	200	3	929.14142	2785.40970	-1.59
eAAGTTAAAGTGGTTEQPPR	Unambiguous	1	1	Q9Z315	N-Term(iTRAQ8plex)	CID	1.0000	0.0000	1	1	2646722	Unique	Used		1.097	0.958	0.959	0	0	6.71	0	0	200	2	1074.54333	2148.07939	-1.92
yEGSGDGGAAAQSLYIANHAY	Unambiguous	1	1	P05063	N-Term(iTRAQ8plex)	CID	1.0000	0.0000	1	1	2937129	Unique	Used	1.133	0.519	0.987	1.373	0	0	6.71	0	11	1	2	1210.07458	2419.14189	-2.03
fQMPDQGMTSADDFFQGTk	Unambiguous	1	1	O08553	N-Term(iTRAQ8plex); K19(iTRAQ8plex)	CID	1.0000	0.0000	1	1	3306772	Unique	Used	0.629	1.176	1.748	1.255	0	0	6.71	0	7	200	3	920.44519	2759.32102	-2.02
yQELAVALDSSYVTNk	Unambiguous	4	1	E9PUQ5	N-Term(iTRAQ8plex); K16(iTRAQ8plex)	CID	1.0000	0.0000	1	1	3325962	Unique	Used	1.564	0.577	0.923	0.715	0	0	6.70	0	0	195	2	1205.15320	2409.29912	-5.43
tDSYSAGQSVEILDGVELGEPAHk	Unambiguous	1	1	Q9JLN9	N-Term(iTRAQ8plex); K24(iTRAQ8plex)	CID	1.0000	0.0000	1	1	2868335	Unique	Used	0.994	1.108	1.356	1.642	0	0	6.70	0	24	200	3	1037.53430	3110.58835	-7.05
qTIGNScGTIGLIHAVANNQDk	Unambiguous	1	1	Q9R0P9	N-Term(iTRAQ8plex); C7(Methylthio); K22(iTRAQ8plex)	CID	1.0000	0.0000	1	1	3702938	Unique	Used	1.426	0.916	1.551	1.228	0	0	6.70	0	0	38	3	970.17249	2908.50290	-6.91
vPRPEDTLTYADLDMVHLSR	Unambiguous	7	1	Q6P6I8	N-Term(iTRAQ8plex)	HCD	1.0000	0.0000	1	1	3370365	Unique	Used	1.166	2.043	1.836	1.243	0	0	6.70	0	17	157	3	878.12457	2632.35917	-4.39

iPNIYAIGDVVAGPmLAHk	Unambiguous	1	1	O08749	N-Term(iTRAQ8plex); M15(Oxidation); K19(iTRAQ8plex)	CID	1.0000	0.0000	1	1	3825485	Unique	Used	2.060	1.165	1.411	1.179	0	0	6.70	0	27	200	3	868.49799	2603.47940	-1.77
hAVVNLINYQDDAELATR	Unambiguous	3	1	Q02248	N-Term(iTRAQ8plex)	CID	1.0000	0.0000	1	1	3256533	Unique	Used	0.733	1.337	1.237	1.263	0	0	6.70	0	10	1	3	782.74927	2346.23325	-0.99
fGNVDADSPVEETIQDHSALk	Unambiguous	5	1	Q8BPN8-2	N-Term(iTRAQ8plex); K21(iTRAQ8plex)	CID	1.0000	0.0000	1	1	2812595	Unique	Used	0.698	0.989	1.070	0.960	0	0	6.70	0	7	200	3	960.83447	2880.48886	1.82
gTGGVDTAAVGGVFDVSNADR	Unambiguous	1	1	Q04447	N-Term(iTRAQ8plex)	CID	1.0000	0.0000	1	1	3135673	Unique	Used	1.186	1.003	1.322	1.875	0	0	6.70	0	0	200	2	1135.08008	2269.15288	7.31
qFYDQALQQAVMDDDANnAk	Unambiguous	1	1	P35762	N-Term(iTRAQ8plex); N18(Deamidated); K20(iTRAQ8plex)	CID	0.0209	0.0000	1	1	2719128	Unique	Used	1.083	0.827	0.699		0	0	6.70	0	16	200	3	965.47369	2894.40653	-0.76
lLYDLADQLHAAVGASR	Unambiguous	1	1	Q99LC5	N-Term(iTRAQ8plex)	CID	1.0000	0.0000	1	1	3879667	Unique	Used	1.105	0.721	1.177	1.492	0	0	6.70	0	2	10	3	706.39050	2117.15696	-4.14
yLQEVINVLETDGHFR	Unambiguous	3	1	Q02819	N-Term(iTRAQ8plex)	CID	1.0000	0.0000	1	1	3581372	Unique	Used	2.705	0.526	0.923	0.696	0	0	6.70	0	0	7	3	746.39679	2237.17582	-4.92
iYELAAGGTAVGTGLnTR	Unambiguous	2	1	P97807-2	N-Term(iTRAQ8plex); N16(Deamidated)	CID	1.0000	0.0000	1	1	3416693	Unique	Used	2.303	1.924	0.791	1.219	0	0	6.69	0	0	14	2	1035.05701	2069.10674	-5.49
fVTDQSAcAGSPSLVAAAR	Unambiguous	2	1	Q8VCY8-2	N-Term(iTRAQ8plex); C8(Methylthio)	CID	1.0000	0.0000	1	1	3723696	Unique	Used	0.389	1.639	1.131	0.784	0	0	6.69	0	0	172	2	1101.05017	2201.09307	-3.02
eHANIDAQSGSQAPNPSTTISPGk	Unambiguous	3	1	Q8VDQ8-2	N-Term(iTRAQ8plex); K24(iTRAQ8plex)	CID	1.0000	0.0000	1	1	3227561	Unique	Used	1.055	1.040	0.458	0.618	0	0	6.69	0	0	200	3	1005.85638	3015.55460	-1.54
fTLNPnTTGVQNPHIER	Unambiguous	2	1	P47809	N-Term(iTRAQ8plex); N6(Deamidated)	CID	0.0493	0.0000	1	1	3341894	Unique	Used	1.290	1.230	1.231	1.073	0	0	6.69	0	16	17	3	748.39203	2243.16153	-4.78
sIEALLEAGQAQDTQASHAEAnQQQTR	Unambiguous	2	1	Q99PL5	N-Term(iTRAQ8plex); N22(Deamidated)	CID	0.0389	0.0000	1	1	2850161	Unique	Used	1.857	0.937	1.197	0.935	0	0	6.69	0	0	200	3	1067.52405	3200.55759	-4.57

rEHANIDAQSGSQAPnPSTTISPGk	Unambiguous	3	1	Q8VDQ8-2	N-Term(iTRAQ8plex); N16(Deamidated); K25(iTRAQ8plex)	CID	0.0045	0.0000	1	1	3271971	Unique	Used	0.429	0.300	0.755	1.002	0	0	6.69	1	0	141	3	1058.22070	3172.64756	1.00
sSEYHLEDNAAYYLNDLER	Unambiguous	1	1	O70443	N-Term(iTRAQ8plex)	CID	1.0000	0.0000	1	1	2919606	Unique	Used	0.765	1.407	1.315	1.261	0	0	6.69	0	9	121	3	869.41498	2606.23038	-0.34
sAGFHPSGSVLAVGTVTGR	Unambiguous	4	1	E9QK48	N-Term(iTRAQ8plex)	CID	1.0000	0.0000	1	1	3911155	Unique	Used	1.007	0.630	0.961	0.986	0	0	6.69	0	0	29	3	702.05005	2104.13559	-4.62
gTSFEAAATSGGSASSEk	Unambiguous	1	1	Q99LC5	N-Term(iTRAQ8plex); K18(iTRAQ8plex)	CID	1.0000	0.0000	1	1	2806982	Unique	Used	1.311	1.360	1.267	1.211	0	0	6.69	0	0	200	2	1127.07239	2253.13750	-3.57
aVDEAVILLQEIGVLDQR	Unambiguous	4	1	D3Z2J3	N-Term(iTRAQ8plex)	CID	1.0000	0.0000	1	1	3440793	Unique	Used	0.553	0.626	0.916	0.986	0	0	6.69	0	0	160	2	1143.15100	2285.29473	-3.14
sQTEEDcTEELFDFLHAR	Unambiguous	1	1	P99028	N-Term(iTRAQ8plex); C7(Methylthio)	CID	1.0000	0.0000	1	1	2835908	Unique	Used			2.708		0	0	6.69	0	5	200	3	840.71704	2520.13657	1.61
fLSQPFQVAEVFTGHMGk	Unambiguous	1	1	P56480	N-Term(iTRAQ8plex); K18(iTRAQ8plex)	CID	1.0000	0.0000	1	1	3882794	Unique	Used	0.956	0.878	1.221	1.670	0	0	6.69	0	21	200	3	877.80896	2631.41233	-3.45
lAELEEFINGPnNAHIQQVGDR	Unambiguous	2	1	Q68FD5	N-Term(iTRAQ8plex); N12(Deamidated)	CID	0.0179	0.0000	1	1	3173645	Unique	Used	1.103	2.333	1.101	1.660	0	0	6.69	0	23	26	3	923.80725	2769.40720	-1.38
aFTGFIVEADTPGIHIGk	Unambiguous	1	1	P45952	N-Term(iTRAQ8plex); K18(iTRAQ8plex)	CID	1.0000	0.0000	1	1	3518714	Unique	Used	0.787	1.212	0.685	1.177	0	0	6.69	0	13	49	3	827.79999	2481.38541	-4.36
mVLAAAGGVEHQQLLDLAQk	Unambiguous	1	1	Q9CZ13	N-Term(iTRAQ8plex); M1(Oxidation); K20(iTRAQ8plex)	HCD	1.0000	0.0000	1	1	3475901	Unique	Used	1.131	1.614	1.189	1.359	0	0	6.69	0	18	191	3	906.17682	2716.51590	-4.34
vEGYSGTAGDSMNYHnGR	Unambiguous	5	1	Q80YX1	N-Term(iTRAQ8plex); N16(Deamidated)	CID	1.0000	0.0000	1	1	2681616	Unique	Used	1.623	1.261	0.914	0.772	0	0	6.69	0	28	200	3	740.66876	2219.99173	-0.55
tIGGGDDSFNTFFSETGAGk	Unambiguous	4	1	P68373	N-Term(iTRAQ8plex); K20(iTRAQ8plex)	CID	1.0000	0.0000	1	1	3131060	Unique	Used	0.616	2.528	2.035	2.486	0	0	6.69	0	11	41	2	1308.65417	2616.30107	-1.07
gITNLcVIGGDGSLTGADTFR	Unambiguous	1	1	P47857	N-Term(iTRAQ8plex); C6(Methylthio)	CID	1.0000	0.0000	1	1	3729679	Unique	Used	1.247	0.718	1.200	0.987	0	0	6.69	0	0	200	2	1209.10107	2417.19487	-6.56

dGDVTITNDGATILSMMDVDHQIAk	Unambiguous	2	1	P80316	N-Term(iTRAQ8plex); K25(iTRAQ8plex)	HCD	1.0000	0.0000	1	1	3132717	Unique	Used	0.731	1.081	0.917	1.082	0	0	6.69	0	2	16	3	1090.22534	3268.66147	-1.10
gQVQEVGWHDVAGWLGR	Unambiguous	1	1	P12382	N-Term(iTRAQ8plex)	CID	1.0000	0.0000	1	1	3663532	Unique	Used	1.319	1.392	1.388	2.021	0	0	6.69	0	17	35	3	733.38208	2198.13169	-4.20
mLTGPVYSQSTALTHk	Unambiguous	1	1	Q6ZQ38	N-Term(iTRAQ8plex); M1(Oxidation); K16(iTRAQ8plex)	CID	1.0000	0.0000	1	1	3698701	Unique	Used	0.445	1.153	0.914	1.140	0	0	6.69	0	1	13	3	786.76337	2358.27555	-8.15
nLHQSGFSLSGAQIDDNIPR	Unambiguous	1	1	O08553	N-Term(iTRAQ8plex)	CID	1.0000	0.0000	1	1	3433899	Unique	Used	1.295	1.409	1.042	1.153	0	0	6.69	0	14	38	3	825.09265	2473.26340	-4.19
eSGVINGLFSDDMLFLR	Unambiguous	2	1	Q8CGF6	N-Term(iTRAQ8plex)	CID	1.0000	0.0000	1	1	3541529	Unique	Used	0.647	1.119	0.648	0.658	0	0	6.69	0	20	25	2	1109.07642	2217.14556	-3.26
lAALNPESNTSGLDIFAk	Unambiguous	1	1	Q9Z1Q5	N-Term(iTRAQ8plex); K18(iTRAQ8plex)	CID	1.0000	0.0000	1	1	3499988	Unique	Used	0.549	1.858	0.546	0.707	0	0	6.69	0	0	17	2	1235.19019	2469.37309	-3.19
skEPSSPGTDDVFTPGSSDSPSSQR	Unambiguous	1	1	Q61165	N-Term(iTRAQ8plex); K2(iTRAQ8plex)	CID	1.0000	0.0000	1	1	2774902	Unique	Used	1.335	0.892	2.676	2.153	0	0	6.68	1	0	200	3	1054.18298	3160.53440	-4.67
nVIIWGNHSSTQYPDVNHAk	Unambiguous	1	1	P14152	N-Term(iTRAQ8plex); K20(iTRAQ8plex)	CID	1.0000	0.0000	1	1	3451880	Unique	Used	0.643	0.886	1.172	1.104	0	0	6.68	0	12	198	3	963.51007	2888.51566	-3.72
rLSQIGVENTEENRR	Unambiguous	1	1	P05063	N-Term(iTRAQ8plex)	HCD	1.0000	0.0000	1	1	3270964	Unique	Used	1.198	0.438	1.105	1.131	0	0	6.68	2	20	74	3	702.38171	2105.13059	-2.83
fPQASASDVVVVHGR	Unambiguous	5	1	Q921H8	N-Term(iTRAQ8plex)	CID	1.0000	0.0000	1	1	3512482	Unique	Used	0.931	1.050	0.802	1.039	0	0	6.68	0	8	12	3	625.01062	1873.01731	-3.25
eQASHLGGAVFSGAGNIAAATGLVk	Unambiguous	1	1	Q91ZZ3	N-Term(iTRAQ8plex); K25(iTRAQ8plex)	CID	1.0000	0.0000	1	1	3822878	Unique	Used	0.475	0.690	1.722	1.147	0	0	6.68	0	6	33	3	978.87714	2934.61686	-3.06
rPQPEEGATYEGIQk	Unambiguous	1	1	Q8R0Y6	N-Term(iTRAQ8plex); K15(iTRAQ8plex)	CID	1.0000	0.0000	1	1	2806333	Unique	Used	0.835	0.858	0.838	1.064	0	0	6.68	0	15	140	3	771.08801	2311.24948	-0.34
aQEAEALDVSFGHSSSAR	Unambiguous	2	1	Q9QYX7	N-Term(iTRAQ8plex)	CID	1.0000	0.0000	1	1	2865075	Unique	Used	1.049	1.023	1.706	1.075	0	0	6.68	0	14	33	3	751.70496	2253.10031	-2.07
mATEVAADALGEEWk	Unambiguous	1	1	P62754	N-Term(iTRAQ8plex); K15(iTRAQ8plex)	CID	1.0000	0.0000	1	1	2903670	Unique	Used	0.372	0.660	0.765	0.860	0	0	6.68	0	7	200	2	1115.08521	2229.16313	-2.28

mVISNPAATQSDIDFLIEEIER	Unambiguous	1	1	P48318	N-Term(iTRAQ8plex); M1(Oxidation)	CID	1.0000	0.0000	1	1	3133452	Unique	Used	1.806	1.201	0.477	1.007	0	0	6.68	0	13	23	3	937.81805	2811.43961	0.26
kTGQAAGFSYTDANk	Unambiguous	2	1	G3UWG1	N-Term(iTRAQ8plex); K1(iTRAQ8plex); K15(iTRAQ8plex)	HCD	1.0000	0.0000	1	1	3161875	Unique	Used	1.606	1.092	1.065	0.740	0	0	6.68	1	0	28	3	824.45831	2471.36039	-2.17
nTSSQTVGVATVGTQTIR	Unambiguous	2	1	E1U8D0	N-Term(iTRAQ8plex)	CID	1.0000	0.0000	1	1	3657101	Unique	Used	0.943	0.349	0.523	0.558	0	0	6.68	0	13	200	2	1062.57532	2124.14336	-6.07
hLNEIDLFHcIDPNDSk	Unambiguous	2	1	Q9R0Q7	N-Term(iTRAQ8plex); C10(Methylthio); K17(iTRAQ8plex)	CID	1.0000	0.0000	1	1	3236737	Unique	Used	1.112	0.863	1.076	0.809	0	0	6.68	0	0	6	3	888.78174	2664.33066	-2.41
aDIGVAMGIAGSDVSk	Unambiguous	2	1	Q6PIC6	N-Term(iTRAQ8plex); K16(iTRAQ8plex)	CID	1.0000	0.0000	1	1	3280040	Unique	Used	0.459	0.994	1.292	1.270	0	0	6.68	0	23	77	2	1050.08557	2099.16387	0.54
eQASHLGGAVFSGAGNIAAATGLVk	Unambiguous	1	1	Q91ZZ3	N-Term(iTRAQ8plex); K25(iTRAQ8plex)	CID	1.0000	0.0000	1	1	3803915	Unique	Used	1.148	0.814	1.538	0.911	0	0	6.68	0	0	24	3	978.87714	2934.61686	-3.06
nAScTLSSAVHSQGVTR	Unambiguous	2	1	Q99J09	N-Term(iTRAQ8plex); C4(Methylthio)	CID	1.0000	0.0000	1	1	3871202	Unique	Used	1.294	1.002	0.781	0.716	0	0	6.68	0	0	200	3	690.01038	2068.01657	-2.50
dLIDQSQSSGSGSGLPLLVQR	Unambiguous	2	1	Q60607	N-Term(iTRAQ8plex)	CID	1.0000	0.0000	1	1	3477290	Unique	Used	0.470	0.766	1.037	1.069	0	0	6.68	0	29	101	2	1231.15906	2461.31084	-3.75
vADALANAAGHLDDLPGALSALSDLHAHk	Unambiguous	1	1	Q91VB8	N-Term(iTRAQ8plex); K29(iTRAQ8plex)	CID	1.0000	0.0000	1	1	3686502	Unique	Used	2.769	3.509	0.466	1.334	0	0	6.68	0	10	7	3	1157.95801	3471.85947	-6.07
gETGPAGPAGPIGPAGAR	Unambiguous	2	1	P11087-2	N-Term(iTRAQ8plex)	CID	1.0000	0.0000	1	1	3162756	Unique	Used	0.718	7.304	1.418	1.235	0	0	6.68	0	0	68	2	918.99469	1836.98210	-2.69
qAHDAVNTEEcSLAQQNk	Unambiguous	2	1	Q6PNC0	N-Term(iTRAQ8plex); C11(Methylthio); K18(iTRAQ8plex)	CID	1.0000	0.0000	1	1	2845199	Unique	Used	1.267	1.298	1.110	1.204	0	0	6.68	0	0	155	3	880.76923	2640.29312	-1.34
gVSSQETAGIGASAHLVNFk	Unambiguous	1	1	Q99KQ4	N-Term(iTRAQ8plex); K20(iTRAQ8plex)	HCD	1.0000	0.0000	1	1	3433264	Unique	Used	1.008	1.144	0.961	0.950	0	0	6.68	0	0	138	3	861.14252	2581.41300	-2.52

sNLNNIDFQMAAEEEk	Unambiguous	4	1	E9Q242	N-Term(iTRAQ8plex); K16(iTRAQ8plex)	CID	1.0000	0.0000	1	1	2795107	Unique	Used			0.720	0.995	0	0	6.68	0	13	200	2	1231.12744	2461.24761	-0.56
eSGcAAAGETEAAAATAFR	Unambiguous	1	1	Q8BR63	N-Term(iTRAQ8plex); C4(Methylthio)	CID	1.0000	0.0000	1	1	2957118	Unique	Used	1.671	0.657	1.305	1.499	0	0	6.67	0	0	200	2	1067.49585	2133.98442	-0.15
mALVSGISLDPEAAIGVTk	Unambiguous	6	1	Q8BVI5	N-Term(iTRAQ8plex); K19(iTRAQ8plex)	CID	1.0000	0.0000	1	1	3623153	Unique	Used	0.539	0.805	1.034	1.134	0	0	6.67	0	30	16	2	1240.70764	2480.40801	-7.06
nQDEcVIALHDcnGDVNR	Unambiguous	5	1	Q80X50	N-Term(iTRAQ8plex); C5(Methylthio); C12(Methylthio); N13(Deamidated)	CID	1.0000	0.0000	1	1	2702449	Unique	Used	2.055	0.909	1.222	1.058	0	0	6.67	0	6	200	3	804.68311	2412.03476	-0.29
lATPTYGDLNHLVSATMSGVTTSLR	Unambiguous	1	1	Q9ERD7	N-Term(iTRAQ8plex)	CID	1.0000	0.0000	1	1	3938160	Unique	Used	0.673	0.366	0.831	1.107	0	0	6.67	0	11	200	3	970.51373	2909.52665	-2.70
mAESTGPEPTQLHDSASFAQVSR	Unambiguous	2	1	F6ZGR6	N-Term(iTRAQ8plex)	CID	1.0000	0.0000	1	1	3087167	Unique	Used	2.275	1.123	1.810	1.138	0	0	6.67	0	18	200	3	917.45203	2750.34153	2.08
sISQTVTGRPLqAPPTSAAQAPAQGLSk	Unambiguous	2	1	Q9QYX7	N-Term(iTRAQ8plex); Q12(Deamidated); K28(iTRAQ8plex)	CID	1.0000	0.0000	1	1	3873222	Unique	Used	0.869	0.791	2.254	0.915	0	0	6.67	0	15	200	3	1124.63147	3371.87986	1.62
eHAPSIIFMDEIDSIGSSR	Unambiguous	2	1	P62196	N-Term(iTRAQ8plex)	CID	1.0000	0.0000	1	1	3172428	Unique	Used	1.092	1.714	0.703	0.791	0	0	6.67	0	2	18	3	803.40594	2408.20328	-1.55
aYHLQEEFGSTFPSDSMLLDLER	Unambiguous	2	1	Q8BK03	N-Term(iTRAQ8plex)	CID	1.0000	0.0000	1	1	3262237	Unique	Used	1.139	0.964		1.770	0	0	6.67	0	3	10	3	997.15472	2989.44962	-1.99
gVGAAATAVTQALNELLQHVk	Unambiguous	1	1	P26039	N-Term(iTRAQ8plex); K21(iTRAQ8plex)	HCD	1.0000	0.0000	1	1	3936429	Unique	Used	0.367	1.395	0.832	1.037	0	0	6.67	0	20	74	3	900.52368	2699.55649	-3.71
fLSQPFQVAEVFTGHMGk	Unambiguous	1	1	P56480	N-Term(iTRAQ8plex); K18(iTRAQ8plex)	CID	1.0000	0.0000	1	1	3863094	Unique	Used	1.283	0.848	1.233	1.518	0	0	6.67	0	6	200	3	877.80933	2631.41343	-3.03
eQASHLGGAVFSGAGNIAAATGLVk	Unambiguous	1	1	Q91ZZ3	N-Term(iTRAQ8plex); K25(iTRAQ8plex)	CID	1.0000	0.0000	1	1	3804260	Unique	Used	1.265	0.585	1.768	1.035	0	0	6.67	0	26	48	3	978.87579	2934.61283	-4.43

tGEmSGPVFTDSGIHIILR	Unambiguous	1	1	Q9QUR7	N-Term(iTRAQ8plex); M4(Oxidation)	CID	1.0000	0.0000	1	1	3641169	Unique	Used	3.362	1.216	1.328	0.794	0	0	6.67	0	6	6	3	784.07959	2350.22422	-5.83
gFGDGYnGYGGGPGGGNFGGSPGYGGGR	Unambiguous	2	1	O88569	N-Term(iTRAQ8plex); N7(Deamidated)	CID	1.0000	0.0000	1	1	3088589	Unique	Used	2.856	2.085	1.074	0.889	0	0	6.67	0	0	141	3	934.08063	2800.22733	-0.61
gGGGGGGGPGGFQPAPR	Unambiguous	1	1	O35295	N-Term(iTRAQ8plex)	CID	1.0000	0.0000	1	1	3404327	Unique	Used	1.120	0.803	0.797	0.665	0	0	6.66	0	0	189	2	872.44299	1743.87871	-2.42
lHDINAQLVEDQGFLDTLk	Unambiguous	3	1	Q5SVG5	N-Term(iTRAQ8plex); K19(iTRAQ8plex)	CID	1.0000	0.0000	1	1	3283966	Unique	Used	1.808	1.306	0.534	0.809	0	0	6.66	0	11	25	3	926.51501	2777.53049	0.38
aSAGPVPGAVVIAEGLHPSLPSPTGNSTPLGTSk	Unambiguous	1	1	Q8BHL3	N-Term(iTRAQ8plex); K34(iTRAQ8plex)	CID	1.0000	0.0000	1	1	3725860	Unique	Used	0.582	0.875	1.154	1.006	0	0	6.66	0	0	95	3	1259.02307	3775.05466	-8.08
lAPITSDPTEAAAVGAVEASFk	Unambiguous	1	1	P52480	N-Term(iTRAQ8plex); K22(iTRAQ8plex)	CID	1.0000	0.0000	1	1	3284155	Unique	Used	1.335	0.972	0.962	1.068	0	0	6.66	0	0	39	2	1377.26160	2753.51592	-0.84
qLADHLGFEFFEASAk	Unambiguous	1	1	P63011	N-Term(iTRAQ8plex); K16(iTRAQ8plex)	CID	1.0000	0.0000	1	1	3349203	Unique	Used	0.329	1.542	1.403	1.197	0	0	6.66	0	0	4	2	1209.64490	2418.28252	-3.69
yTPSGQSGAAASESLFISnHAY	Unambiguous	2	1	A6ZI44	N-Term(iTRAQ8plex); N19(Deamidated)	CID	1.0000	0.0000	1	1	3129104	Unique	Used	1.280	0.716	0.794	0.872	0	0	6.66	0	0	40	2	1282.11462	2563.22197	-1.36
sSGAGSTSDAYTVIQVGR	Unambiguous	1	1	Q8R361	N-Term(iTRAQ8plex)	CID	1.0000	0.0000	1	1	3318680	Unique	Used	0.786	0.607	1.018	1.177	0	0	6.66	0	0	143	2	1030.52820	2060.04912	-3.44
iGEHTPSALAImENANVLAR	Unambiguous	3	1	A6ZI44	N-Term(iTRAQ8plex); M12(Oxidation)	CID	1.0000	0.0000	1	1	3707980	Unique	Used	1.722	1.074	1.208	1.285	0	0	6.66	0	3	200	3	809.76514	2427.28086	-6.58
dAGYGGLSLSIEGPSk	Unambiguous	4	1	B7FAU9	N-Term(iTRAQ8plex); K16(iTRAQ8plex)	CID	1.0000	0.0000	1	1	3212534	Unique	Used	0.986	3.867	0.871	1.247	0	0	6.66	0	13	200	2	1080.09326	2159.17925	-0.57
qFGFIVLTTSAGImDHEEAR	Unambiguous	1	1	P62245	N-Term(iTRAQ8plex); M14(Oxidation)	CID	1.0000	0.0000	1	1	3539465	Unique	Used	1.278	1.306	0.669	0.838	0	0	6.66	0	0	100	3	848.10016	2542.28592	-2.16
sQTEEDcTEELFDFLHAR	Unambiguous	1	1	P99028	N-Term(iTRAQ8plex); C7(Methylthio)	CID	1.0000	0.0000	1	1	2853908	Unique	Used	1.703	1.077			0	0	6.66	0	0	200	3	840.71484	2520.12998	-1.01

tDQVIQSLIALVNDPQPEHPLR	Unambiguous	1	1	P68037	N-Term(iTRAQ8plex)	CID	1.0000	0.0000	1	1	3785557	Unique	Used	1.637	0.939	0.897	1.260	0	0	6.66	0	13	40	3	929.84552	2787.52201	-3.13
eHAPSIIFMDEIDSIGSSR	Unambiguous	2	1	P62196	N-Term(iTRAQ8plex)	CID	1.0000	0.0000	1	1	3152301	Unique	Used	1.445	0.856	0.594	0.835	0	0	6.66	0	13	88	3	803.40570	2408.20255	-1.85
iSnGGLEEGQPVDLVLScVDNFEAR	Unambiguous	2	1	Q8VE47	N-Term(iTRAQ8plex); N3(Deamidated); C18(Methylthio)	CID	1.0000	0.0000	1	1	3285135	Unique	Used		1.265	1.004	1.114	0	0	6.66	0	1	65	3	1004.82355	3012.45609	-1.20
tGIGSGLSLSGIVHPELSR	Unambiguous	2	1	P50544	N-Term(iTRAQ8plex)	HCD	1.0000	0.0000	1	1	3802747	Unique	Used	1.374	1.203	0.959	1.017	0	0	6.66	0	18	169	3	728.74561	2184.22226	-3.11
aPPAGGSGSSAAAAAAAGGTEVRPR	Unambiguous	2	1	P84309	N-Term(iTRAQ8plex)	HCD	1.0000	0.0000	1	1	3489503	Unique	Used	0.652	1.060	2.449	1.100	0	0	6.66	0	19	154	3	814.42957	2441.27414	-2.39
nIINSSEGSSSGSGHk	Unambiguous	1	1	Q8C739	N-Term(iTRAQ8plex); K16(iTRAQ8plex)	CID	1.0000	0.0000	1	1	2966874	Unique	Used	1.708	0.936	2.144	1.189	0	0	6.66	0	13	200	3	723.71503	2169.13053	-2.35
vHAVIGVLMDDTDPLVTVmk	Unambiguous	1	1	P62192	N-Term(iTRAQ8plex); M19(Oxidation); K20(iTRAQ8plex)	CID	1.0000	0.0000	1	1	3560734	Unique	Used	2.032	1.904	0.383	1.681	0	0	6.66	0	19	17	3	926.51495	2777.53031	-3.57
hELLLGAAGAGPGAGPQQATPGALLQAGPPR	Unambiguous	1	1	Q6PE01	N-Term(iTRAQ8plex)	CID	1.0000	0.0000	1	1	3876509	Unique	Used	0.790	0.511	0.943	0.838	0	0	6.66	0	0	48	3	1059.91602	3177.73349	-3.17
gNAGQTNYGFAnSTMER	Unambiguous	1	1	P19096	N-Term(iTRAQ8plex); N12(Deamidated)	CID	1.0000	0.0000	1	1	2827023	Unique	Used	0.837	0.704	0.901	1.080	0	0	6.66	0	0	200	2	1061.98914	2122.97099	-2.63
nQGGYGGSSSSSSYGSGR	Unambiguous	3	1	Q5EBP8	N-Term(iTRAQ8plex)	CID	1.0000	0.0000	1	1	2787215	Unique	Used	1.842	0.414	0.977	0.794	0	0	6.66	0	0	200	2	999.95422	1998.90117	-2.17
hSAEVAGYQDSIGQLESDLR	Unambiguous	1	1	P46660	N-Term(iTRAQ8plex)	CID	1.0000	0.0000	1	1	2919127	Unique	Used	1.962	0.647	1.245	1.149	0	0	6.66	0	17	34	3	827.08154	2479.23008	-2.68
aNTGSAMLEQVAMTER	Unambiguous	1	1	Q8JZP2	N-Term(iTRAQ8plex)	CID	1.0000	0.0000	1	1	3279681	Unique	Used	0.656	1.024	1.217	1.115	0	0	6.66	0	25	47	2	1007.00488	2013.00249	-1.12
lAQGPELAEDDADLLHk	Unambiguous	1	1	Q8R5H6	N-Term(iTRAQ8plex); K17(iTRAQ8plex)	CID	1.0000	0.0000	1	1	2636835	Unique	Used	0.458	1.298	1.931	0.773	0	0	6.66	0	5	127	3	815.11377	2443.32676	-0.90
nmGGPYGGGNYGPGGSGGSGGYGGR	Unambiguous	3	1	O88569	N-Term(iTRAQ8plex); M2(Oxidation)	CID	1.0000	0.0000	1	1	3428586	Unique	Used	1.515	1.156	0.953	1.313	0	0	6.66	0	0	200	3	837.36975	2510.09470	-4.40
dLVILLYETALLSSGFSLEDPQTHANR	Unambiguous	1	1	P07901	N-Term(iTRAQ8plex)	HCD	1.0000	0.0000	1	1	3786242	Unique	Used	0.832	1.081	1.259	1.356	0	0	6.66	0	6	200	3	1102.91858	3306.74118	-3.40

ySVEVAASTGAGPGVk	Unambiguous	2	1	G5E843	N-Term(iTRAQ8plex); K16(iTRAQ8plex)	CID	1.0000	0.0000	1	1	3186755	Unique	Used	3.669	0.537	0.768	0.727	0	0	6.66	0	0	100	2	1051.09290	2101.17851	1.68
gFLGVMPAYSAAEDALTTk	Unambiguous	1	1	O54983	N-Term(iTRAQ8plex); K19(iTRAQ8plex)	CID	1.0000	0.0000	1	1	3418570	Unique	Used	0.772	2.912	1.283	0.917	0	0	6.66	0	23	14	2	1275.68372	2550.36016	-5.22
gITNLcVIGGDGSLTGADTFR	Unambiguous	1	1	P47857	N-Term(iTRAQ8plex); C6(Methylthio)	CID	1.0000	0.0000	1	1	3728677	Unique	Used	1.904	1.142	0.664	1.073	0	0	6.65	0	7	200	2	1209.10352	2417.19975	-4.54
dGNLANNPYSGDVTk	Unambiguous	2	1	A2AMY5	N-Term(iTRAQ8plex); K15(iTRAQ8plex)	CID	1.0000	0.0000	1	1	2827024	Unique	Used	1.292	0.618	0.901	0.579	0	0	6.65	0	0	200	2	1087.06909	2173.13091	-1.69
sIEDQLQPNSASGSGALEHDPSSIYLR	Unambiguous	3	1	Q8C547	N-Term(iTRAQ8plex)	CID	1.0000	0.0000	1	1	3127820	Unique	Used	0.873	1.242	1.162	1.146	0	0	6.65	0	8	32	3	1059.19409	3175.56772	-4.18
aSAELALGENNEVLkSGR	Unambiguous	1	1	P05202	N-Term(iTRAQ8plex); K15(iTRAQ8plex)	CID	1.0000	0.0000	1	1	3911095	Unique	Used	2.341	0.483	1.117	1.302	0	0	6.65	1	0	200	3	822.79364	2466.36637	-4.43
lLEVSDDPQVLAVAAHDVGEYVR	Unambiguous	1	1	Q8BVE3	N-Term(iTRAQ8plex)	CID	1.0000	0.0000	1	1	3240533	Unique	Used	0.630	1.149	1.898	2.852	0	0	6.65	0	23	200	3	933.83362	2799.48630	1.14
nQQITHAnNTVSSFk	Unambiguous	4	1	Q61699-2	N-Term(iTRAQ8plex); N8(Deamidated); K15(iTRAQ8plex)	CID	0.0105	0.0000	1	1	3405970	Unique	Used	0.535	0.838	1.107	1.130	0	0	6.65	0	27	200	2	1149.61890	2298.23052	0.29
dELHIVEAEAmNYEGSPIk	Unambiguous	5	1	Q61937	N-Term(iTRAQ8plex); M11(Oxidation); K19(iTRAQ8plex)	CID	1.0000	0.0000	1	1	2813285	Unique	Used	2.010	2.244	1.901	0.760	0	0	6.65	0	14	114	3	923.81128	2769.41928	-1.20
sSGSPyGGGYGSGGGSGGYGSR	Unambiguous	7	1	J3QNY1	N-Term(iTRAQ8plex); Y6(iTRAQ8plex)	CID	1.0000	0.0000	1	1	3247411	Unique	Used		1.018	1.424		0	0	6.65	0	0	86	3	840.40149	2519.18991	-4.31
sTGEALVQGLMGAAVTLk	Unambiguous	1	1	Q8K1Z0	N-Term(iTRAQ8plex); K18(iTRAQ8plex)	CID	1.0000	0.0000	1	1	3806046	Unique	Used	0.945	1.649	1.047	1.174	0	0	6.65	0	0	17	2	1177.67737	2354.34746	-4.23
iGEHTPSALAImENANVLAR	Unambiguous	3	1	A6ZI44	N-Term(iTRAQ8plex); M12(Oxidation)	CID	1.0000	0.0000	1	1	3721214	Unique	Used	1.068	1.232	1.157	1.217	0	0	6.65	0	8	57	3	809.76294	2427.27427	-9.30

gQETSTNPIASIFAWSR	Unambiguous	1	1	O88844	N-Term(iTRAQ8plex)	CID	1.0000	0.0000	1	1	3898533	Unique	Used	0.774	1.279	0.554	0.835	0	0	6.65	0	0	9	2	1085.06055	2169.11382	-4.81
gAAGGALPTSPSPALGAk	Unambiguous	1	1	Q8K394	N-Term(iTRAQ8plex); K18(iTRAQ8plex)	CID	1.0000	0.0000	1	1	3492433	Unique	Used	0.455	0.643	1.338	1.039	0	0	6.65	0	0	172	2	1066.11731	2131.22734	-2.75
qTTAAAAATFSEQVGGGSGGAGR	Unambiguous	3	1	O88935	N-Term(iTRAQ8plex)	CID	1.0000	0.0000	1	1	3472784	Unique	Used	0.758	2.150	1.495	4.517	0	0	6.65	0	0	200	2	1178.58887	2356.17046	-3.86
nFEVVAGTGDqcLPFDQSHcGDGGk	Unambiguous	2	1	Q9WTS4	N-Term(iTRAQ8plex); Q11(Deamidated); C12(Methylthio); C20(Methylthio); K25(iTRAQ8plex)	CID	1.0000	0.0000	1	1	3236856	Unique	Used	0.620	0.944	0.880	1.000	0	0	6.65	0	10	89	3	1094.83545	3282.49179	4.04
lLAALMDDDVGSGEDLLR	Unambiguous	2	1	E9PUM4	N-Term(iTRAQ8plex)	CID	1.0000	0.0000	1	1	3196586	Unique	Used	0.624	0.901	0.810	0.959	0	0	6.65	0	0	42	2	1104.07898	2207.15068	-1.14
tAAVLLQNDPNPDVLSk	Unambiguous	15	1	Q02357-5	N-Term(iTRAQ8plex); K17(iTRAQ8plex)	CID	1.0000	0.0000	1	1	3301718	Unique	Used				1.350	0	0	6.65	0	0	118	2	1202.18237	2403.35747	-5.38
nQGGYGGSSSSSSYGSGR	Unambiguous	3	1	Q5EBP8	N-Term(iTRAQ8plex)	CID	1.0000	0.0000	1	1	2771476	Unique	Used	2.296	0.477	0.854	0.701	0	0	6.65	0	3	200	2	999.95343	1998.89958	-2.96
kDSAIQQQVANLQmk	Unambiguous	1	1	Q9JHU4	N-Term(iTRAQ8plex); K1(iTRAQ8plex); M14(Oxidation); K15(iTRAQ8plex)	CID	1.0000	0.0000	1	1	3471608	Unique	Used	0.312	0.640	0.764	0.849	0	0	6.65	1	0	34	3	877.50439	2630.49863	-2.92
nmGGPYGGGNYGPGGSGGSGGYGGR	Unambiguous	3	1	O88569	N-Term(iTRAQ8plex); M2(Oxidation)	CID	1.0000	0.0000	1	1	3294270	Unique	Used	2.231	1.457	1.232	1.405	0	0	6.65	0	0	71	3	837.37207	2510.10166	-1.62
gITNLcVIGGDGSLTGADTFR	Unambiguous	1	1	P47857	N-Term(iTRAQ8plex); C6(Methylthio)	CID	1.0000	0.0000	1	1	3728801	Unique	Used	1.248	0.817	1.162	1.115	0	0	6.64	0	10	200	2	1209.10120	2417.19512	-6.46
aGQTVTVWAAGAGATHSPPSTLVWk	Unambiguous	2	1	P21619	N-Term(iTRAQ8plex); K25(iTRAQ8plex)	HCD	1.0000	0.0000	1	1	3876554	Unique	Used	0.791	0.843	1.276	1.453	0	0	6.64	0	0	143	3	1034.56543	3101.68174	-5.67
ySDPGPVYDcLVWHDGETWR	Unambiguous	2	1	Q64514-2	N-Term(iTRAQ8plex); C10(Methylthio)	CID	1.0000	0.0000	1	1	3305084	Unique	Used	0.351	0.441	1.032	1.011	0	0	6.64	0	0	56	3	915.74963	2745.23435	-1.32

lELQGIQGAVDHAAAFGR	Unambiguous	5	1	Q9CPV4	N-Term(iTRAQ8plex)	CID	1.0000	0.0000	1	1	3663583	Unique	Used	1.387	1.136	0.867	1.195	0	0	6.64	0	0	73	3	719.72437	2157.15854	-6.18
iGEHTPSALAImENANVLAR	Unambiguous	3	1	A6ZI44	N-Term(iTRAQ8plex); M12(Oxidation)	CID	1.0000	0.0000	1	1	3708428	Unique	Used	1.245	1.165	1.492	1.212	0	0	6.64	0	0	200	3	809.76599	2427.28342	-5.53
yHVPVVVVPEGSTSDTQEQAILR	Unambiguous	2	1	Q9DBG6	N-Term(iTRAQ8plex)	CID	1.0000	0.0000	1	1	3234578	Unique	Used	1.428	1.162	1.067	1.206	0	0	6.64	0	3	8	3	943.50702	2828.50650	-1.11
aLMLQGVDLLADAVAVTmGPk	Unambiguous	3	1	P63038	N-Term(iTRAQ8plex); M18(Oxidation); K21(iTRAQ8plex)	CID	1.0000	0.0000	1	1	3766564	Unique	Used	2.342	0.973		1.730	0	0	6.64	0	11	32	3	913.18530	2737.54135	-1.45
lVSPGSANETSSILVESVTR	Unambiguous	1	1	Q80X90	N-Term(iTRAQ8plex)	CID	1.0000	0.0000	1	1	3477616	Unique	Used	1.316	1.751	0.864	0.983	0	0	6.64	0	26	101	2	1175.63940	2350.27153	-2.24
lEDkVSGELFAQAPVEQYPGIAVETVTDSSR	Unambiguous	1	1	Q9CR95	N-Term(iTRAQ8plex); K4(iTRAQ8plex)	CID	1.0000	0.0000	1	1	3284037	Unique	Used	2.337	1.764	0.649	1.071	0	0	6.64	1	0	71	3	1315.35986	3944.06504	-2.53
tYFPHFDVSHGSAQVk	Unambiguous	2	1	Q91VB8	N-Term(iTRAQ8plex); K16(iTRAQ8plex)	CID	1.0000	0.0000	1	1	3473362	Unique	Used	2.775	2.134	0.549	1.212	0	0	6.64	0	17	117	3	810.09814	2428.27988	-2.92
sSTSSSVGTEVTETPAHSVPHTR	Unambiguous	2	1	Q8CH77	N-Term(iTRAQ8plex)	CID	1.0000	0.0000	1	1	2968068	Unique	Used	1.229	0.545	1.592	0.795	0	0	6.64	0	18	200	3	886.77948	2658.32389	-1.29
eVAnAVVRPLGQALGTSSVAGSESLLk	Unambiguous	5	1	A2ACC6	N-Term(iTRAQ8plex); N4(Deamidated); K27(iTRAQ8plex)	CID	1.0000	0.0000	1	1	3804362	Unique	Used	0.468	0.906	0.903	1.090	0	0	6.64	0	0	118	3	1088.29199	3262.86142	4.48
vNInGGAIALGHPLGASGcR	Selected	1	1	Q80X81	N-Term(iTRAQ8plex); N4(Deamidated); C19(Methylthio)	HCD	0.0000	0.0000	1	1	3801628	Unique	Used	2.692	0.752	0.633	0.746	0	0	6.64	0	20	9	3	743.38763	2228.14835	-4.43
lGDVYVnDAFGTAHR	Unambiguous	2	1	P09411	N-Term(iTRAQ8plex); N7(Deamidated)	CID	1.0000	0.0000	1	1	3051743	Unique	Used	0.888	0.858	1.154	1.067	0	0	6.64	0	14	35	3	647.33148	1939.97989	-0.87
tALHVAAAEGHVEVVk	Unambiguous	3	1	D3Z7P3	N-Term(iTRAQ8plex); K16(iTRAQ8plex)	CID	1.0000	0.0000	1	1	3144017	Unique	Used	1.224	1.387	1.043	1.073	0	0	6.64	0	19	14	3	747.10480	2239.29984	-0.93
dLVVLLFETALLSSGFSLEDPQTHSNR	Unambiguous	1	1	P11499	N-Term(iTRAQ8plex)	CID	1.0000	0.0000	1	1	3843476	Unique	Used		0.960	0.750	0.659	0	0	6.64	0	12	200	3	1098.24719	3292.72702	-2.96

eHINLGcDVDFDIAGPSIR	Unambiguous	3	1	Q60932-2	N-Term(iTRAQ8plex); C7(Methylthio)	CID	1.0000	0.0000	1	1	3375464	Unique	Used	0.961	1.313	1.281	1.021	0	0	6.64	0	5	200	3	807.73041	2421.17667	-3.23
qVDDLEVWSHTNDTIGSVR	Unambiguous	2	1	Q4FE56	N-Term(iTRAQ8plex)	CID	1.0000	0.0000	1	1	3090005	Unique	Used	0.778	0.857	0.829	1.044	0	0	6.64	0	9	27	3	825.75208	2475.24167	-0.05
gGPPPPPPPPHSSGPPPPPAR	Unambiguous	1	1	Q91YD9	N-Term(iTRAQ8plex)	CID	1.0000	0.0000	1	1	3528526	Unique	Used	1.008	1.093	0.957	0.946	0	0	6.63	0	0	200	3	765.41193	2294.22123	-5.93
iLTEAEIDAHLVALAER	Unambiguous	2	1	Q9QUM9	N-Term(iTRAQ8plex)	CID	1.0000	0.0000	1	1	3326621	Unique	Used	2.093	0.573	1.000	1.188	0	0	6.63	0	23	34	3	723.40979	2168.21482	-3.73
ySTIGLAQQWDQLHQLGMR	Unambiguous	1	1	P08032	N-Term(iTRAQ8plex)	CID	1.0000	0.0000	1	1	3878345	Unique	Used	1.585	1.632	0.766	1.206	0	0	6.63	0	13	107	3	850.44312	2549.31479	-3.49
kLPEPGAVIGALILEFADR	Unambiguous	1	1	Q9CPS8	N-Term(iTRAQ8plex); K1(iTRAQ8plex)	HCD	1.0000	0.0000	1	1	3806548	Unique	Used	0.444	0.670	1.379	1.268	0	0	6.63	1	16	19	3	873.18719	2617.54703	-2.60
sQPTAMNSIMETGNTk	Unambiguous	3	1	Q922V4	N-Term(iTRAQ8plex); K16(iTRAQ8plex)	CID	1.0000	0.0000	1	1	3230430	Unique	Used	0.891	0.825	0.696	0.780	0	0	6.63	0	0	119	2	1159.59778	2318.18828	-2.51
fTASAGIQVVGDDLTVTNPk	Unambiguous	2	1	P17182	N-Term(iTRAQ8plex); K20(iTRAQ8plex)	CID	1.0000	0.0000	1	1	3348532	Unique	Used	0.881	1.482	1.172	1.712	0	0	6.63	0	0	17	2	1321.23071	2641.45415	-4.40
lNATNIELATVQPGQNFHmFTk	Unambiguous	1	1	Q9Z2U1	N-Term(iTRAQ8plex); M19(Oxidation); K22(iTRAQ8plex)	CID	1.0000	0.0000	1	1	3762357	Unique	Used	1.406	0.983	0.543	0.722	0	0	6.63	0	0	34	3	1033.55298	3098.64438	-3.55
gTVVYGEPITASLGTDGSHYWSk	Unambiguous	1	1	O08553	N-Term(iTRAQ8plex); K23(iTRAQ8plex)	CID	1.0000	0.0000	1	1	3414349	Unique	Used	1.149	1.591	1.132	1.113	0	0	6.63	0	0	18	3	1011.86017	3033.56595	-3.92
gTGGVDTAAVGGVFDVSNADR	Unambiguous	1	1	Q04447	N-Term(iTRAQ8plex)	CID	1.0000	0.0000	1	1	3128939	Unique	Used	1.401	0.909	1.228	1.432	0	0	6.63	0	0	10	2	1135.06970	2269.13213	-1.84
tALPTSGSSTGELELLAGEVPAR	Unambiguous	2	1	Q80VP1	N-Term(iTRAQ8plex)	CID	1.0000	0.0000	1	1	3283066	Unique	Used	1.480	1.993	2.603	1.384	0	0	6.63	0	0	8	2	1280.69031	2560.37334	-1.53
iQAAASPPANATAASDTNAGDR	Unambiguous	2	1	E9QAQ5	N-Term(iTRAQ8plex)	CID	1.0000	0.0000	1	1	2952071	Unique	Used	0.464	0.937	0.927	0.952	0	0	6.63	0	0	200	2	1187.59595	2374.18462	-2.32
eQELEVAAInPNHPLAQMPLPPSMk	Unambiguous	5	1	A2ACM0	N-Term(iTRAQ8plex); N10(Deamidated); K25(iTRAQ8plex)	CID	0.0090	0.0000	1	1	3393822	Unique	Used	0.812	0.799	0.860	0.839	0	0	6.63	0	26	126	3	1121.93579	3363.79282	0.77

sATLSSTESTASGMQDEVR	Unambiguous	2	1	Q9WTQ5	N-Term(iTRAQ8plex)	CID	1.0000	0.0000	1	1	2733562	Unique	Used	2.300	0.432	1.115	1.014	0	0	6.63	0	3	37	2	1131.04504	2261.08281	-1.83
qSESSFITGDINSSASLNR	Unambiguous	22	1	Q80TS3-5	N-Term(iTRAQ8plex)	CID	1.0000	0.0000	1	1	3211111	Unique	Used	0.506	1.520	1.255	1.310	0	0	6.63	0	13	67	2	1159.08240	2317.15752	0.05
yGAATANYMEVVSLLk	Unambiguous	2	1	Q64521	N-Term(iTRAQ8plex); K16(iTRAQ8plex)	CID	1.0000	0.0000	1	1	3686552	Unique	Used	0.528	1.372	1.187	1.447	0	0	6.63	0	0	30	2	1169.64197	2338.27666	-7.30
dSLGDFIEHYAQLGPSSPEQLAAGAEEGGGPR	Unambiguous	1	1	O35295	N-Term(iTRAQ8plex)	HCD	1.0000	0.0000	1	1	3195866	Unique	Used	1.358	0.961	1.175	0.624	0	0	6.62	0	7	15	3	1187.24646	3559.72483	0.12
sSEYHLEDNAAYYLNDLER	Unambiguous	1	1	O70443	N-Term(iTRAQ8plex)	CID	1.0000	0.0000	1	1	2762682	Unique	Used	0.836	1.338	1.248	1.118	0	0	6.62	0	4	100	3	869.41412	2606.22782	-1.32
eEEVTGPVIAPLFPQk	Unambiguous	1	1	Q6P4T2	N-Term(iTRAQ8plex); K16(iTRAQ8plex)	CID	1.0000	0.0000	1	1	3130998	Unique	Used	0.303	0.814	0.826	0.901	0	0	6.62	0	8	92	2	1181.67529	2362.34331	-1.94
gVLTGPEYEALDALPDAER	Unambiguous	1	1	Q9D1X0	N-Term(iTRAQ8plex)	CID	1.0000	0.0000	1	1	2797960	Unique	Used	2.057	1.465	1.393	1.167	0	0	6.62	0	0	67	2	1160.60144	2320.19560	-0.81
mVISNPAATHQDIDFLIEEIER	Unambiguous	1	1	P48320	N-Term(iTRAQ8plex); M1(Oxidation)	HCD	1.0000	0.0000	1	1	3153351	Unique	Used	0.652	0.960	1.127	1.867	0	0	6.62	0	0	6	3	954.49176	2861.46073	-1.76
yTPSGQSGAAASESLFISNHAY	Unambiguous	2	1	A6ZI44	N-Term(iTRAQ8plex)	CID	1.0000	0.0000	1	1	3346416	Unique	Used	0.562	1.170	1.061	0.888	0	0	6.62	0	22	22	2	1281.61877	2562.23027	-4.36
dNLTLWTSDMQGDGEEQNk	Unambiguous	1	1	P62259	N-Term(iTRAQ8plex); K19(iTRAQ8plex)	CID	1.0000	0.0000	1	1	2738351	Unique	Used	1.924				0	0	6.62	0	0	200	3	930.45331	2789.34537	-1.98
eHINLGcDVDFDIAGPSIR	Unambiguous	3	1	Q60932-2	N-Term(iTRAQ8plex); C7(Methylthio)	CID	1.0000	0.0000	1	1	3375358	Unique	Used	0.930	1.029	1.449	1.246	0	0	6.62	0	14	200	3	807.73096	2421.17832	-2.55
eTAAVIFLHGLGDTGHSWADALSTIR	Unambiguous	1	1	Q9WTL7	N-Term(iTRAQ8plex)	CID	1.0000	0.0000	1	1	3805982	Unique	Used			1.617		0	0	6.62	0	0	16	3	1014.86548	3042.58188	-4.36
tVQSPGAAVHPVAAQSTGVVVPPR	Unambiguous	1	1	Q6A058	N-Term(iTRAQ8plex)	CID	1.0000	0.0000	1	1	3817644	Unique	Used	1.415	1.145	1.332	1.761	0	0	6.62	0	0	200	3	877.16010	2629.46573	-2.70
iVFDSIDNLEAAPHDIGHVk	Unambiguous	3	1	Q9QZ08	N-Term(iTRAQ8plex); K20(iTRAQ8plex)	CID	1.0000	0.0000	1	1	3214699	Unique	Used	1.181	1.991	0.967	1.152	0	0	6.62	0	19	170	3	933.51404	2798.52756	-0.79
hHAAYVNNLnATEEk	Unambiguous	1	1	P09671	N-Term(iTRAQ8plex); N10(Deamidated); K15(iTRAQ8plex)	CID	1.0000	0.0000	1	1	2711308	Unique	Used	1.872	0.890	1.205	1.396	0	0	6.62	0	8	200	3	774.07471	2320.20957	-2.00

sHTVAAQGGINAALGNMEEDNWR	Unambiguous	1	1	Q8K2B3	N-Term(iTRAQ8plex)	CID	1.0000	0.0000	1	1	3233527	Unique	Used	1.321	1.206	0.976	1.003	0	0	6.62	0	12	129	3	915.78125	2745.32920	-0.92
rVDHTTVTGNMQTSQk	Unambiguous	1	1	Q3UNH4	N-Term(iTRAQ8plex); K16(iTRAQ8plex)	CID	1.0000	0.0000	1	1	3381061	Unique	Used	0.935	1.428	1.917	0.769	0	0	6.62	1	16	200	3	804.42957	2411.27414	-7.47
ySEEILGPLDHDDQcGk	Unambiguous	1	1	Q80UG2	N-Term(iTRAQ8plex); C15(Methylthio); K17(iTRAQ8plex)	CID	1.0000	0.0000	1	1	2738455	Unique	Used	0.310	0.884	0.663	0.816	0	0	6.62	0	0	200	3	858.42041	2573.24668	-0.22
vTITGSPAATQAAQYLISQR	Unambiguous	2	1	D3YVV7	N-Term(iTRAQ8plex)	CID	1.0000	0.0000	1	1	3916304	Unique	Used	2.144	1.625	0.873	0.779	0	0	6.62	0	0	200	2	1190.65576	2380.30425	-4.03
eHINLGcDVDFDIAGPSIR	Unambiguous	3	1	Q60932-2	N-Term(iTRAQ8plex); C7(Methylthio)	CID	1.0000	0.0000	1	1	3353852	Unique	Used	0.927	1.057	1.736	1.582	0	0	6.62	0	5	33	3	807.73041	2421.17667	-3.23
mEAGSGPPGGPGSESPNR	Unambiguous	4	1	E9QAD8	N-Term(iTRAQ8plex)	CID	1.0000	0.0000	1	1	2711706	Unique	Used	1.105	2.285	3.634	1.164	0	0	6.62	0	0	200	2	994.47333	1987.93938	-2.62
eHINLGcDVDFDIAGPSIR	Unambiguous	3	1	Q60932-2	N-Term(iTRAQ8plex); C7(Methylthio)	CID	1.0000	0.0000	1	1	3375253	Unique	Used	0.954	0.818	1.559	1.521	0	0	6.62	0	4	200	3	807.72937	2421.17356	-4.52
sALQSINEWASQTTDGk	Unambiguous	1	1	P19324	N-Term(iTRAQ8plex); K17(iTRAQ8plex)	CID	1.0000	0.0000	1	1	3347461	Unique	Used	0.535	0.713	0.360	0.393	0	0	6.62	0	0	39	2	1222.64209	2444.27690	-4.45
yLLETSGNLDGLEYk	Unambiguous	1	1	Q99KQ4	N-Term(iTRAQ8plex); K15(iTRAQ8plex)	CID	1.0000	0.0000	1	1	3130684	Unique	Used	0.457	1.072	0.879	0.989	0	0	6.62	0	0	28	2	1162.13257	2323.25786	-2.72
dAAVSPDLGAGGDAPAPAPAPAHTR	Unambiguous	1	1	Q9Z2A7	N-Term(iTRAQ8plex)	CID	1.0000	0.0000	1	1	2880440	Unique	Used	1.251	1.202	1.088	1.441	0	0	6.62	0	0	200	3	862.77545	2586.31180	-3.75
vTTADELLGGLHEQLTQR	Unambiguous	4	1	E9QQ10	N-Term(iTRAQ8plex)	CID	1.0000	0.0000	1	1	3518564	Unique	Used	2.176	1.219	1.176	0.809	0	0	6.62	0	12	72	3	762.41510	2285.23075	-4.20
smDSYLNQSYGMDNHSGGGGGSR	Unambiguous	1	1	O88291	N-Term(iTRAQ8plex); M2(Oxidation)	CID	1.0000	0.0000	1	1	3183282	Unique	Used	1.612	0.880	1.563	1.022	0	0	6.61	0	0	198	3	899.72369	2697.15653	-0.24
vEGAFPVTMLPGDGVGPELMHAVk	Unambiguous	1	1	Q91VA7	N-Term(iTRAQ8plex); K24(iTRAQ8plex)	CID	1.0000	0.0000	1	1	3441186	Unique	Used	0.645	0.837	0.947	1.194	0	0	6.61	0	29	200	3	1020.54871	3059.63156	-6.66

rEHANIDAQSGSqAPNPSTTISPGk	Unambiguous	3	1	Q8VDQ8-2	N-Term(iTRAQ8plex); Q13(Deamidated); K25(iTRAQ8plex)	CID	0.0106	0.0000	1	1	3161698	Unique	Used	0.892	0.173	1.666	1.617	0	0	6.61	1	0	118	3	1058.22229	3172.65232	2.50
lAASGEGGLQELSGHFENQk	Unambiguous	3	1	Q9QXS6	N-Term(iTRAQ8plex); K20(iTRAQ8plex)	CID	1.0000	0.0000	1	1	3189863	Unique	Used	1.127	1.439	1.510	0.697	0	0	6.61	0	0	15	3	894.14215	2680.41190	-1.21
gTVGEPTYDAEFQHFLR	Unambiguous	1	1	Q9JHU4	N-Term(iTRAQ8plex)	CID	1.0000	0.0000	1	1	3190973	Unique	Used	0.953	0.600	0.589	0.755	0	0	6.61	0	19	25	3	757.71503	2271.13053	-1.88
kQQEEAQAAAAAASAESR	Unambiguous	1	1	B1AWN6	N-Term(iTRAQ8plex); K1(iTRAQ8plex)	CID	1.0000	0.0000	1	1	2791396	Unique	Used	0.490	1.331	1.434	1.266	0	0	6.61	1	3	42	3	809.09821	2425.28006	-3.77
gPAVGIDLGTTYScVGVFQHGk	Unambiguous	4	1	P63017	N-Term(iTRAQ8plex); C14(Methylthio); K22(iTRAQ8plex)	CID	1.0000	0.0000	1	1	3807667	Unique	Used	1.438	1.152	1.065	0.731	0	0	6.61	0	30	200	3	954.16608	2860.48368	-3.84
eLGLSSVPASGGLVGVYnGk	Unambiguous	1	1	Q9CQF9	N-Term(iTRAQ8plex); N18(Deamidated); K20(iTRAQ8plex)	CID	1.0000	0.0000	1	1	3558342	Unique	Used	0.824	1.177	0.832	1.129	0	0	6.61	0	0	173	2	1257.20850	2513.40972	1.00
eAVPLEDLVLPEPVGFLLVLLGPEAPHVDYTqLGR	Unambiguous	2	1	P04919-2	N-Term(iTRAQ8plex); Q32(Deamidated)	CID	1.0000	0.0000	1	1	3746526	Unique	Used			0.863		0	0	6.61	0	11	200	3	1367.75403	4101.24753	0.20
tGQTDPFLGMmMGLGGGMDFDSk	Unambiguous	3	1	Q8VDQ8-2	N-Term(iTRAQ8plex); M11(Oxidation); K23(iTRAQ8plex)	CID	1.0000	0.0000	1	1	3459177	Unique	Used	3.174	1.907	0.833	4.726	0	0	6.61	0	14	48	3	1006.47797	3017.41935	-4.80
sDPESQPLLGGGAGGPSAGAPTAPPPR	Unambiguous	1	1	Q03391	N-Term(iTRAQ8plex)	CID	1.0000	0.0000	1	1	3104924	Unique	Used	0.494	0.571	0.720	1.335	0	0	6.61	0	12	200	2	1373.20740	2745.40752	-1.29
lGTEEFPLIDQTFYPNHk	Unambiguous	3	1	O88935	N-Term(iTRAQ8plex); K18(iTRAQ8plex)	CID	1.0000	0.0000	1	1	3327776	Unique	Used	0.585	1.210	2.165	1.146	0	0	6.61	0	29	200	3	919.82794	2757.46927	-0.57
lGNDFMGITLASSQAVSNAR	Unambiguous	6	1	Q9CPV4	N-Term(iTRAQ8plex)	CID	1.0000	0.0000	1	1	3860233	Unique	Used	1.551	1.324	1.038	1.081	0	0	6.61	0	30	17	2	1178.60986	2356.21245	-4.62
nMGGPYGGGNYGPGGSGGSGGYGGR	Unambiguous	3	1	O88569	N-Term(iTRAQ8plex)	CID	1.0000	0.0000	1	1	3295661	Unique	Used			1.896	1.706	0	0	6.61	0	0	1	3	832.03979	2494.10483	-2.40

gFGDGYnGYGGGPGGGNFGGSPGYGGGR	Unambiguous	2	1	O88569	N-Term(iTRAQ8plex); N7(Deamidated)	CID	1.0000	0.0000	1	1	3088988	Unique	Used	2.081	1.226	0.994	1.054	0	0	6.61	0	9	125	3	934.08118	2800.22898	-0.02
iGEHTPSALAImENANVLAR	Unambiguous	3	1	A6ZI44	N-Term(iTRAQ8plex); M12(Oxidation)	CID	1.0000	0.0000	1	1	3702055	Unique	Used	1.844	0.627	0.821	0.831	0	0	6.61	0	3	18	3	809.76514	2427.28086	-6.58
wGEAGAEYVVESTGVFTTMEk	Unambiguous	3	1	P16858	N-Term(iTRAQ8plex); K21(iTRAQ8plex)	CID	1.0000	0.0000	1	1	3218659	Unique	Used	0.952	2.336	1.736	1.405	0	0	6.61	0	0	200	3	967.15802	2899.45951	-1.71
yTPSGQSGAAASESLFISNHAY	Unambiguous	2	1	A6ZI44	N-Term(iTRAQ8plex)	CID	1.0000	0.0000	1	1	3257970	Unique	Used	1.142	0.613	1.283	0.948	0	0	6.61	0	0	25	2	1281.62573	2562.24419	1.07
sPSGGAAGPLLTPSQSLDGSR	Unambiguous	1	1	Q6WVG3	N-Term(iTRAQ8plex)	CID	1.0000	0.0000	1	1	3432965	Unique	Used	1.183	1.319	0.984	0.808	0	0	6.61	0	0	68	2	1130.09375	2259.18022	-3.58
lQNQLQGAIVIERPNVk	Unambiguous	1	1	P46467	N-Term(iTRAQ8plex); K17(iTRAQ8plex)	HCD	1.0000	0.0000	1	1	3760586	Unique	Used	0.896	0.948	0.823	1.068	0	0	6.61	0	11	100	3	843.50592	2528.50321	-4.00
lEDTLWAGLTDQHVk	Unambiguous	1	1	Q8BWT1	N-Term(iTRAQ8plex); K15(iTRAQ8plex)	CID	1.0000	0.0000	1	1	3260572	Unique	Used	1.353	1.326	0.966	1.307	0	0	6.61	0	25	72	3	778.76648	2334.28489	-2.80
iGEHTPSALAImENANVLAR	Unambiguous	3	1	A6ZI44	N-Term(iTRAQ8plex); M12(Oxidation)	CID	1.0000	0.0000	1	1	3709379	Unique	Used	1.905	1.236	1.557	0.922	0	0	6.60	0	0	200	3	809.76605	2427.28360	-5.45
dLESDIIGDTSGHFQk	Unambiguous	2	1	P14824	N-Term(iTRAQ8plex); K16(iTRAQ8plex)	CID	1.0000	0.0000	1	1	2918240	Unique	Used	1.020	1.140	0.961	0.858	0	0	6.60	0	19	69	3	790.74908	2370.23270	-2.99
qLQNISQAAASGGAk	Unambiguous	1	1	Q9JHU4	N-Term(iTRAQ8plex); K15(iTRAQ8plex)	CID	1.0000	0.0000	1	1	3511894	Unique	Used	0.399	0.679	0.718	1.188	0	0	6.60	0	0	74	2	1026.58093	2052.15459	-5.47
qPGTSLVDADTFHHQVR	Unambiguous	3	1	O08579	N-Term(iTRAQ8plex)	HCD	1.0000	0.0000	1	1	3250617	Unique	Used	1.098	1.119	0.969	0.999	0	0	6.60	0	0	58	3	738.04889	2212.13211	-4.14
dQTNDQVTIDSALATQk	Unambiguous	2	1	P54071	N-Term(iTRAQ8plex); K17(iTRAQ8plex)	CID	1.0000	0.0000	1	1	2829080	Unique	Used	0.860	1.862	0.874	1.111	0	0	6.60	0	13	200	2	1228.65515	2456.30303	-2.40
eHINLGcDVDFDIAGPSIR	Unambiguous	3	1	Q60932-2	N-Term(iTRAQ8plex); C7(Methylthio)	CID	1.0000	0.0000	1	1	3353988	Unique	Used	1.192	1.212	0.997	0.862	0	0	6.60	0	10	48	3	807.73022	2421.17612	-3.46

sSDSWDVWGSGSASnNk	Unambiguous	3	1	Q9EPJ9	N-Term(iTRAQ8plex); N15(Deamidated); K17(iTRAQ8plex)	CID	0.0455	0.0000	1	1	2848329	Unique	Used	1.318	1.432	2.424	1.140	0	0	6.60	0	0	179	2	1197.07239	2393.13750	-3.81
sENGLEFTSSGSANTETTk	Unambiguous	3	1	Q60932-2	N-Term(iTRAQ8plex); K19(iTRAQ8plex)	CID	1.0000	0.0000	1	1	2682796	Unique	Used	0.513	0.923	1.053	0.889	0	0	6.60	0	0	200	2	1284.64282	2568.27837	-3.98
kDVLPGAEGLTAVGGQAGNLAR	Unambiguous	2	1	A2AJ26	N-Term(iTRAQ8plex); K1(iTRAQ8plex)	CID	1.0000	0.0000	1	1	3535643	Unique	Used	1.048	0.682	0.794	0.924	0	0	6.60	1	0	65	3	901.51233	2702.52243	-6.89
qPSQPTTSQSLGQLQAHAASAPGSNPR	Unambiguous	1	1	E9PZA8	N-Term(iTRAQ8plex)	CID	1.0000	0.0000	1	1	3737152	Unique	Used	0.386	1.528	2.054	1.424	0	0	6.60	0	0	87	3	1007.51752	3020.53800	-2.39
qLASEDISHITPTQGFNIk	Unambiguous	1	1	Q9WUL7	N-Term(iTRAQ8plex); K19(iTRAQ8plex)	CID	1.0000	0.0000	1	1	3368974	Unique	Used	1.934	1.082	0.973	0.937	0	0	6.60	0	21	83	3	903.16510	2707.48075	-2.52
dFPLNDLLSATELDk	Unambiguous	1	1	Q9JHU4	N-Term(iTRAQ8plex); K15(iTRAQ8plex)	CID	1.0000	0.0000	1	1	3196148	Unique	Used	0.351	0.595	0.746	0.953	0	0	6.60	0	9	11	2	1150.13428	2299.26128	-1.28
tASVSGASAEASGEQVR	Unambiguous	1	1	Q80U49	N-Term(iTRAQ8plex)	CID	1.0000	0.0000	1	1	2680618	Unique	Used	0.811	0.588	1.340	1.192	0	0	6.60	0	0	200	2	955.98969	1910.97209	-0.04
qLNTIQNAEAVHFFcNYEESR	Unambiguous	4	1	P61922	N-Term(iTRAQ8plex); C15(Methylthio)	CID	1.0000	0.0000	1	1	3458204	Unique	Used	1.658	0.741	0.843	1.162	0	0	6.60	0	22	200	3	955.11725	2863.33719	-2.57
sSGSPYGGGYGSGGGSGGYGSR	Unambiguous	7	1	J3QNY1	N-Term(iTRAQ8plex)	CID	1.0000	0.0000	1	1	2950759	Unique	Used	1.106	1.097	1.535	0.801	0	0	6.59	0	0	200	2	1107.99829	2214.98931	-2.75
gGGTSSGGSSSTnSLTGSRPPk	Unambiguous	1	1	Q78T81	N-Term(iTRAQ8plex); N13(Deamidated); K22(iTRAQ8plex)	CID	1.0000	0.0000	1	1	3099103	Unique	Used	0.667	0.807	1.086	0.912	0	0	6.59	0	0	200	3	863.44971	2588.33457	-1.05
gTQGATAGASSELDASk	Unambiguous	2	1	F8VQC1	N-Term(iTRAQ8plex); K17(iTRAQ8plex)	CID	1.0000	0.0000	1	1	2664695	Unique	Used	0.528	0.840	0.722	0.858	0	0	6.59	0	0	128	2	1080.07214	2159.13701	-1.42
nYGSYSTQASAAAATAELLk	Unambiguous	3	1	E9Q855	N-Term(iTRAQ8plex); K20(iTRAQ8plex)	CID	1.0000	0.0000	1	1	3347430	Unique	Used	0.626	0.862	1.105	1.045	0	0	6.59	0	0	46	2	1313.19995	2625.39263	-2.07

sHLGQNSSSLnGGDVINLRPNk	Unambiguous	1	1	Q9D4I9	N-Term(iTRAQ8plex); N11(Deamidated); K22(iTRAQ8plex)	CID	1.0000	0.0000	1	1	3386173	Unique	Used	1.051	1.313	1.174	0.702	0	0	6.59	0	0	200	3	972.85431	2916.54837	-9.07
lVQDYGLESEVAQHLAk	Unambiguous	2	1	Q64521	N-Term(iTRAQ8plex); K17(iTRAQ8plex)	CID	1.0000	0.0000	1	1	3236038	Unique	Used		1.570	1.363	1.400	0	0	6.59	0	20	116	3	836.80072	2508.38761	-1.70
hTQEScETWGVnGETGTLVDMk	Unambiguous	3	1	P80318	N-Term(iTRAQ8plex); C6(Methylthio); N12(Deamidated); K22(iTRAQ8plex)	CID	1.0000	0.0000	1	1	2850236	Unique	Used	0.926	0.946	0.948	0.804	0	0	6.59	0	1	71	3	1026.48523	3077.44114	-2.07
aPSMVAAAVTYSkPR	Unambiguous	1	1	Q9CPQ8	N-Term(iTRAQ8plex); K13(iTRAQ8plex)	HCD	1.0000	0.0000	1	1	3930572	Unique	Used	0.580	1.142	1.184	1.247	0	0	6.59	0	2	42	3	719.74530	2157.22135	-4.51
qAEIENPLEDPVTGDYVHk	Unambiguous	4	1	Q9Z1G4-3	N-Term(iTRAQ8plex); K19(iTRAQ8plex)	CID	1.0000	0.0000	1	1	2760748	Unique	Used	0.524	1.272	1.041	1.119	0	0	6.59	0	0	200	3	921.48590	2762.44315	-0.95
tGIGSGLSLSGIVHPELSR	Unambiguous	2	1	P50544	N-Term(iTRAQ8plex)	HCD	1.0000	0.0000	1	1	3839821	Unique	Used	0.944	1.100	0.811	0.918	0	0	6.59	0	0	200	3	728.74683	2184.22593	-1.43
tDAVEALTALNHYQIR	Unambiguous	1	1	Q921F4	N-Term(iTRAQ8plex)	CID	1.0000	0.0000	1	1	3620766	Unique	Used	0.865	1.135	0.866	0.976	0	0	6.59	0	6	20	3	707.04791	2119.12918	-7.44
lLEVSDDPQVLAVAAHDVGEYVR	Unambiguous	1	1	Q8BVE3	N-Term(iTRAQ8plex)	CID	1.0000	0.0000	1	1	3242006	Unique	Used			1.036	1.034	0	0	6.59	0	0	200	3	933.83551	2799.49198	3.16
yHTSQSGDEMTSLSEYVSR	Unambiguous	2	1	P11499	N-Term(iTRAQ8plex)	CID	1.0000	0.0000	1	1	2991555	Unique	Used	1.189	1.507	0.801	1.193	0	0	6.59	0	7	10	3	827.71979	2481.14481	-2.32
aAQIGAHTLSGAcLDPAAFk	Unambiguous	2	1	Q921G7	N-Term(iTRAQ8plex); C13(Methylthio); K20(iTRAQ8plex)	CID	1.0000	0.0000	1	1	3724473	Unique	Used	1.029	1.056	1.037	1.108	0	0	6.59	0	13	31	3	866.12640	2596.36466	-7.32
nSEVVAGTGDQcLPFDDTR	Unambiguous	4	1	Q3UHK6	N-Term(iTRAQ8plex); C12(Methylthio)	CID	1.0000	0.0000	1	1	2832108	Unique	Used	0.793	0.920	0.954	0.944	0	0	6.59	0	30	200	2	1187.54395	2374.08061	-6.37
sAPAQGGSAPAPPPPPTHTVQHEGFLLR	Unambiguous	3	1	E9PX29	N-Term(iTRAQ8plex)	CID	1.0000	0.0000	1	1	3614064	Unique	Used	0.678	0.688	1.404	1.135	0	0	6.59	0	0	200	3	1041.21277	3121.62375	-7.95

dHFmDEFFEQVEEIR	Unambiguous	1	1	P61264	N-Term(iTRAQ8plex); M4(Oxidation)	CID	1.0000	0.0000	1	1	2833645	Unique	Used	0.723	0.831	1.516	1.302	0	0	6.58	0	1	97	3	764.35645	2291.05478	-1.96
tVYFAEEVQcEGNSFHk	Unambiguous	1	1	P97315	N-Term(iTRAQ8plex); C10(Methylthio); K17(iTRAQ8plex)	CID	1.0000	0.0000	1	1	3149910	Unique	Used	1.834	1.118	1.446	1.192	0	0	6.58	0	11	184	3	881.43140	2642.27964	-1.64
tPSAAYLWVGAGASEAEk	Unambiguous	2	1	P13020-2	N-Term(iTRAQ8plex); K18(iTRAQ8plex)	CID	1.0000	0.0000	1	1	3235386	Unique	Used	1.066	0.809	0.485	0.452	0	0	6.58	0	10	6	3	806.10217	2416.29197	-2.05
gTGGVDTAAVGGVFDVSNADR	Unambiguous	1	1	Q04447	N-Term(iTRAQ8plex)	CID	1.0000	0.0000	1	1	3149521	Unique	Used	1.204	0.952	1.189	1.140	0	0	6.58	0	0	43	2	1135.06934	2269.13140	-2.16
iPNIYAIGDVVAGPmLAHk	Unambiguous	1	1	O08749	N-Term(iTRAQ8plex); M15(Oxidation); K19(iTRAQ8plex)	CID	1.0000	0.0000	1	1	3823983	Unique	Used	1.330	1.074	1.238	1.056	0	0	6.58	0	0	1	3	868.49634	2603.47446	-3.67
aVVTHSSGNHGQALTYAAk	Unambiguous	4	1	Q9QZX7-2	N-Term(iTRAQ8plex); K19(iTRAQ8plex)	CID	1.0000	0.0000	1	1	3569408	Unique	Used	0.387	2.958	1.650	1.017	0	0	6.58	0	0	200	3	840.79352	2520.36600	-4.73
rGGGGSGGGGTQGAEEEPPPPLQAVLVADSFNR	Unambiguous	1	1	Q8CHW4	N-Term(iTRAQ8plex)	CID	1.0000	0.0000	1	1	3259407	Unique	Used	1.363	1.082	0.961	0.747	0	0	6.58	1	0	45	3	1156.91821	3468.74009	-0.29
lGQHVVGmAPLSVGSLDDEPGGETETR	Unambiguous	1	1	Q9CZR8	N-Term(iTRAQ8plex); M8(Oxidation)	CID	1.0000	0.0000	1	1	3148149	Unique	Used	0.583	0.612	0.971	0.891	0	0	6.58	0	14	23	3	1024.51233	3071.52243	-1.10
fYYSSGSSSPTHAk	Unambiguous	1	1	Q60771	N-Term(iTRAQ8plex); K14(iTRAQ8plex)	CID	1.0000	0.0000	1	1	3675850	Unique	Used	0.961	0.274	1.900	2.687	0	0	6.58	0	7	71	3	709.69965	2127.08438	-5.79
gETDEEYLWcIEQTLHFk	Unambiguous	1	1	P50247	N-Term(iTRAQ8plex); C10(Methylthio); K18(iTRAQ8plex)	CID	1.0000	0.0000	1	1	3133129	Unique	Used	0.733	0.986	0.821	1.180	0	0	6.58	0	14	35	3	965.80914	2895.41288	-0.87
vPFSPGPAPPPHMGELDQER	Unambiguous	6	1	G3UYG6	N-Term(iTRAQ8plex)	HCD	1.0000	0.0000	1	1	3211054	Unique	Used	1.499	0.937	1.252	0.995	0	0	6.58	0	13	107	3	821.41888	2462.24210	-0.80
sIVTDLVSQMDPHGR	Unambiguous	4	1	P58281-2	N-Term(iTRAQ8plex)	CID	1.0000	0.0000	1	1	3643167	Unique	Used	0.315	1.108	1.278	1.396	0	0	6.58	0	3	4	3	653.67712	1959.01682	-5.28

fLSQPFQVAEVFTGHMGk	Unambiguous	1	1	P56480	N-Term(iTRAQ8plex); K18(iTRAQ8plex)	CID	1.0000	0.0000	1	1	3882691	Unique	Used	0.973	0.811	1.339	1.366	0	0	6.58	0	8	200	3	877.80939	2631.41361	-2.97
vADYcENNYIQATDk	Unambiguous	11	1	Q8CBW3-2	N-Term(iTRAQ8plex); C5(Methylthio); K15(iTRAQ8plex)	CID	1.0000	0.0000	1	1	2976235	Unique	Used	0.228	0.805	1.032	0.768	0	0	6.58	0	7	180	2	1201.08484	2401.16240	-0.01
gPAVGIDLGTTYScVGVFQHGk	Unambiguous	4	1	P63017	N-Term(iTRAQ8plex); C14(Methylthio); K22(iTRAQ8plex)	CID	1.0000	0.0000	1	1	3808916	Unique	Used	1.510	1.155	1.214	0.935	0	0	6.58	0	0	200	3	954.16510	2860.48075	-4.86
gHAPSTAAPAPSPAPMSSSVqSDEER	Unambiguous	1	1	Q8C996	N-Term(iTRAQ8plex); Q21(Deamidated)	CID	1.0000	0.0000	1	1	2843558	Unique	Used	1.607	0.641	0.986	0.661	0	0	6.58	0	0	200	3	957.12769	2869.36850	3.77
gmFLISSGPPEMYEVHAASR	Unambiguous	10	1	H3BJU7	N-Term(iTRAQ8plex); M2(Oxidation)	CID	1.0000	0.0000	1	1	3597428	Unique	Used	0.626	1.154	1.345	0.911	0	0	6.58	0	6	26	3	833.74371	2499.21659	-5.95
nIDNPAIADIYTEHAHqVVVAk	Unambiguous	1	1	O88342	N-Term(iTRAQ8plex); Q17(Deamidated); K22(iTRAQ8plex)	CID	1.0000	0.0000	1	1	3299398	Unique	Used	1.474	1.234	1.033	1.288	0	0	6.58	0	13	97	3	1009.88605	3027.64359	2.50
dILcGAADEVLAVLk	Unambiguous	1	1	Q6P4T2	N-Term(iTRAQ8plex); C4(Methylthio); K15(iTRAQ8plex)	CID	1.0000	0.0000	1	1	3500929	Unique	Used	0.468	0.456	0.912	0.850	0	0	6.58	0	3	29	2	1092.61035	2184.21343	-4.33
eALELTDTGLLSGSEER	Unambiguous	1	1	Q9JHU4	N-Term(iTRAQ8plex)	CID	1.0000	0.0000	1	1	2703605	Unique	Used	0.345	0.644	1.134	0.935	0	0	6.58	0	2	200	2	1062.55090	2124.09453	-1.37
lATMYSSGSPEGGSDSSESR	Unambiguous	2	1	F8WIE5	N-Term(iTRAQ8plex)	CID	1.0000	0.0000	1	1	2756393	Unique	Used	1.390	1.201	0.913	0.981	0	0	6.57	0	0	200	2	1155.02673	2309.04619	-1.89
sLASVPQQnGFSGVSETAGAQR	Unambiguous	4	1	D3YVV9	N-Term(iTRAQ8plex); N9(Deamidated)	CID	0.0335	0.0000	1	1	3254815	Unique	Used	0.357	0.477	0.775	0.533	0	0	6.57	0	0	90	2	1248.63525	2496.26323	-0.02
vNNININQGSITFAGGPGR	Unambiguous	3	1	Q9WTI7-3	N-Term(iTRAQ8plex)	CID	1.0000	0.0000	1	1	3913872	Unique	Used	0.578	1.220	0.697	0.826	0	0	6.57	0	12	200	2	1117.09839	2233.18950	-4.32
sSGSPYGGGYGSGGGSGGYGSR	Unambiguous	7	1	J3QNY1	N-Term(iTRAQ8plex)	CID	1.0000	0.0000	1	1	2968490	Unique	Used	1.368	0.861	1.709	0.778	0	0	6.57	0	0	200	2	1107.99976	2214.99224	-1.43

sAELGcTVDEVESLIk	Unambiguous	1	1	Q68FG2	N-Term(iTRAQ8plex); C6(Methylthio); K16(iTRAQ8plex)	CID	1.0000	0.0000	1	1	3037832	Unique	Used	0.457	2.021	2.224	1.009	0	0	6.57	0	6	89	2	1174.12048	2347.23369	-0.38
mMVDkDGDVTITNDGATILSMMDVDHqIAk	Unambiguous	2	1	P80316	N-Term(iTRAQ8plex); K5(iTRAQ8plex); Q27(Deamidated); K30(iTRAQ8plex)	CID	1.0000	0.0000	1	1	3283937	Unique	Used	0.773	0.892	0.748	0.730	0	0	6.57	1	0	49	3	1393.38184	4178.13095	1.24
aIGFAAADSFDHk	Unambiguous	1	1	P32848	N-Term(iTRAQ8plex); K14(iTRAQ8plex)	CID	1.0000	0.0000	1	1	3189487	Unique	Used	2.890	0.591	1.568	0.772	0	0	6.57	0	2	2	3	677.03448	2029.08890	-3.67
lTHFPTVSGSPASLADSMQQk	Unambiguous	2	1	A3KGR9	N-Term(iTRAQ8plex); K21(iTRAQ8plex)	CID	1.0000	0.0000	1	1	3661563	Unique	Used	0.792	1.129	0.822	0.995	0	0	6.57	0	8	56	3	937.49829	2810.48032	-5.85
nLHQSGFSLSGAQIDDNIPR	Unambiguous	1	1	O08553	N-Term(iTRAQ8plex)	CID	1.0000	0.0000	1	1	3660062	Unique	Used	1.391	1.023	1.053	1.264	0	0	6.57	0	19	47	3	825.09247	2473.26285	-4.42
kVADALANAAGHLDDLPGALSALSDLHAHk	Unambiguous	1	1	Q91VB8	N-Term(iTRAQ8plex); K1(iTRAQ8plex); K30(iTRAQ8plex)	HCD	1.0000	0.0000	1	1	3765542	Unique	Used	1.723	3.340	0.407	1.225	0	0	6.57	1	13	25	3	1302.06091	3904.16819	-3.25
vADVSISEVTTLLGnVHSPVVEGYVGENISGEVk	Unambiguous	3	1	P20357	N-Term(iTRAQ8plex); N15(Deamidated); K34(iTRAQ8plex)	CID	0.0426	0.0000	1	1	3397756	Unique	Used	2.844	2.326	1.638	1.001	0	0	6.57	0	0	200	3	1369.73755	4107.19809	0.54
tNVSGGAIALGHPLGGSGSR	Unambiguous	1	1	Q8BWT1	N-Term(iTRAQ8plex)	CID	1.0000	0.0000	1	1	3871752	Unique	Used	1.203	1.313	0.989	1.268	0	0	6.57	0	0	52	3	704.71899	2112.14243	-1.88
eAGPRPPGSGPGPGPTPGAk	Unambiguous	2	1	O88737	N-Term(iTRAQ8plex); K20(iTRAQ8plex)	CID	1.0000	0.0000	1	1	3225622	Unique	Used		1.336	1.846	0.757	0	0	6.57	0	0	200	2	1196.66077	2392.31426	-2.15
aLIQYFSPYVSADmHk	Unambiguous	5	1	G3UXW9	N-Term(iTRAQ8plex); M14(Oxidation); K16(iTRAQ8plex)	CID	1.0000	0.0000	1	1	3763901	Unique	Used	0.717	1.007	1.070	1.004	0	0	6.57	0	0	53	3	832.11194	2494.32126	-1.93

aAMEALQSQALHATSQQPLR	Unambiguous	5	1	B1AQZ0	N-Term(iTRAQ8plex)	CID	1.0000	0.0000	1	1	3856367	Unique	Used	1.137	1.301	1.098	1.069	0	0	6.57	0	22	111	3	819.10199	2455.29142	-4.71
dVLGTGAFSEVILAEDkR	Unambiguous	1	1	Q91YS8	N-Term(iTRAQ8plex); K17(iTRAQ8plex)	HCD	1.0000	0.0000	1	1	3236901	Unique	Used	0.854	1.671	0.847	1.007	0	0	6.57	1	12	17	3	843.47662	2528.41532	-1.11
qTTAAAAATFSEQVGGGSGGAGR	Unambiguous	3	1	O88935	N-Term(iTRAQ8plex)	CID	1.0000	0.0000	1	1	3501752	Unique	Used	1.452	2.086	2.182	1.280	0	0	6.57	0	22	200	2	1178.58533	2356.16338	-6.87
eVnENFAIDLIAQQPVNEVEHR	Unambiguous	2	1	P52503	N-Term(iTRAQ8plex); N3(Deamidated)	CID	0.0046	0.0000	1	1	2815050	Unique	Used	3.187	1.128	1.579	0.945	0	0	6.57	0	2	51	3	957.15820	2869.46006	-1.18
mQDTSASFGYQLDLPk	Unambiguous	2	1	Q9QYA2	N-Term(iTRAQ8plex); K16(iTRAQ8plex)	CID	1.0000	0.0000	1	1	3323895	Unique	Used	0.340	0.610	0.688	0.877	0	0	6.57	0	0	86	2	1205.12891	2409.25054	-3.13
eFPDVLEcTMSHAVEk	Unambiguous	3	1	O09172	N-Term(iTRAQ8plex); C8(Methylthio); K16(iTRAQ8plex)	CID	1.0000	0.0000	1	1	3172127	Unique	Used	0.830	0.711	0.930	1.208	0	0	6.57	0	16	42	3	830.41364	2489.22635	-2.88
qTDVLQQLSIQmANAk	Unambiguous	1	1	Q9JHU4	N-Term(iTRAQ8plex); M12(Oxidation); K16(iTRAQ8plex)	CID	1.0000	0.0000	1	1	3661274	Unique	Used	0.299	0.591	0.899	1.020	0	0	6.57	0	0	78	2	1206.66479	2412.32231	-6.39
yHVPVVVVPEGSTSDTQEQAILR	Unambiguous	2	1	Q9DBG6	N-Term(iTRAQ8plex)	CID	1.0000	0.0000	1	1	3257306	Unique	Used	0.686		0.497	0.615	0	0	6.56	0	0	5	3	943.50806	2828.50962	-0.01
tGAMLGQSEELQPLSQR	Unambiguous	4	1	Q5NCM5	N-Term(iTRAQ8plex)	CID	1.0000	0.0000	1	1	3278684	Unique	Used	1.213	1.540	1.817	1.077	0	0	6.56	0	0	46	2	1075.06128	2149.11528	-3.38
iGEHTPSALAImENANVLAR	Unambiguous	3	1	A6ZI44	N-Term(iTRAQ8plex); M12(Oxidation)	CID	1.0000	0.0000	1	1	3740804	Unique	Used	0.304	1.667	0.956	0.990	0	0	6.56	0	0	3	3	809.76740	2427.28763	-3.79
vYSPSVLFLHPDMGIGGAER	Unambiguous	1	1	Q9DBE8	N-Term(iTRAQ8plex)	CID	1.0000	0.0000	1	1	3705869	Unique	Used	0.863	0.824	0.864	1.120	0	0	6.56	0	17	20	3	817.09515	2449.27091	-5.84
eHINLGcDVDFDIAGPSIR	Unambiguous	3	1	Q60932-2	N-Term(iTRAQ8plex); C7(Methylthio)	CID	1.0000	0.0000	1	1	3357684	Unique	Used	0.569	0.558	1.460	1.424	0	0	6.56	0	3	200	3	807.73108	2421.17868	-2.40
eDPTFQALEDFGTELIDSGHR	Unambiguous	1	1	P08032	N-Term(iTRAQ8plex)	CID	1.0000	0.0000	1	1	2922440	Unique	Used	3.813	1.053	0.469	1.463	0	0	6.56	0	5	77	3	894.43713	2681.29685	-1.08

kFVEVMTEYnATQSk	Unambiguous	1	1	P61264	N-Term(iTRAQ8plex); K1(iTRAQ8plex); N10(Deamidated); K15(iTRAQ8plex)	CID	1.0000	0.0000	1	1	3148005	Unique	Used	0.749	1.295	1.023	1.280	0	0	6.56	1	0	51	3	896.82660	2688.46524	-1.09
eTAAAHQASSsPPIDAATAEPYGFR	Unambiguous	1	1	P14873	N-Term(iTRAQ8plex); S11(Phospho)	CID	0.0442	0.0000	1	1	2716967	Unique	Used			1.068	1.974	0	0	6.56	0	0	200	3	977.12488	2929.36008	-2.42
nLHQSGFSLSGAQIDDNIPR	Unambiguous	1	1	O08553	N-Term(iTRAQ8plex)	CID	1.0000	0.0000	1	1	3411934	Unique	Used	1.664	0.861	1.120	1.428	0	0	6.56	0	2	8	3	825.09271	2473.26358	-4.12
eGSPALGLLGGSqAAQPGAR	Unambiguous	1	1	B2RXC2	N-Term(iTRAQ8plex); Q13(Deamidated)	CID	0.0229	0.0000	1	1	3596216	Unique	Used	1.702	0.510	0.677	0.888	0	0	6.56	0	0	188	2	1071.57715	2142.14702	0.60
eQAADTEAAQEVTQLLVER	Unambiguous	1	1	O54774	N-Term(iTRAQ8plex)	CID	1.0000	0.0000	1	1	2766192	Unique	Used	0.384	0.994	0.946	1.107	0	0	6.56	0	0	29	2	1203.12476	2405.24224	-1.66
aTGHSGGGcISQGQSYDTDk	Unambiguous	1	1	Q8K274	N-Term(iTRAQ8plex); C9(Methylthio); K20(iTRAQ8plex)	CID	1.0000	0.0000	1	1	2987015	Unique	Used	0.528	0.934	0.878	1.092	0	0	6.56	0	16	200	3	875.08173	2623.23062	-1.18
gINSNNLESQLQATQAAR	Unambiguous	4	1	P52293	N-Term(iTRAQ8plex)	CID	1.0000	0.0000	1	1	3410816	Unique	Used	1.006	0.583	0.932	1.250	0	0	6.55	0	0	92	2	1110.08264	2219.15801	-4.61
qAGIEPGPDTYLALLnAHAER	Unambiguous	1	1	Q6PB66	N-Term(iTRAQ8plex); N16(Deamidated)	CID	1.0000	0.0000	1	1	3130580	Unique	Used	1.440	1.165	1.074	1.273	0	0	6.55	0	11	83	3	847.77905	2541.32260	-1.00
qTTAAAATFSEQVGGGSGGAGR	Unambiguous	3	1	O88935	N-Term(iTRAQ8plex); Q1(Deamidated)	CID	1.0000	0.0000	1	1	3298796	Unique	Used	4.372	2.744	2.058	1.346	0	0	6.55	0	0	200	2	1179.08313	2357.15898	-1.95
vLVETEGPAGVAVMk	Unambiguous	1	1	P42125	N-Term(iTRAQ8plex); K15(iTRAQ8plex)	CID	1.0000	0.0000	1	1	3234999	Unique	Used	1.609	0.879	0.864	1.479	0	0	6.55	0	10	20	2	1054.61328	2108.21929	-2.53
vVYTGIYDTEGVAPTk	Unambiguous	2	1	Q5M8N0	N-Term(iTRAQ8plex); K16(iTRAQ8plex)	CID	1.0000	0.0000	1	1	3148914	Unique	Used	1.183	1.823	0.907	0.732	0	0	6.55	0	0	72	2	1161.14148	2321.27568	-3.98
gQIQEAIALINSLHPELLDTNR	Unambiguous	1	1	Q9D7M1	N-Term(iTRAQ8plex)	CID	1.0000	0.0000	1	1	3785491	Unique	Used	1.861	1.149	0.886	0.802	0	0	6.55	0	17	24	3	917.17413	2749.50785	-2.63

gQGAGSVSQAnGVAIDPTMk	Unambiguous	4	1	Q9Z0J4	N-Term(iTRAQ8plex); N11(Deamidated); K20(iTRAQ8plex)	CID	0.0412	0.0000	1	1	2954395	Unique	Used	0.465	1.804	0.858	1.015	0	0	6.55	0	0	200	2	1249.16089	2497.31450	-1.30
eESGAVAAAASVPAQSTAR	Unambiguous	1	1	Q9EPB4	N-Term(iTRAQ8plex)	CID	1.0000	0.0000	1	1	2970809	Unique	Used	2.531	1.325	0.737	0.884	0	0	6.55	0	11	200	2	1039.04089	2077.07451	-3.99
iLLLDEATSALDAENEHLVQEALDR	Unambiguous	1	1	Q9JI39	N-Term(iTRAQ8plex)	CID	1.0000	0.0000	1	1	3018240	Unique	Used	0.585	0.870	0.912	1.462	0	0	6.55	0	5	194	3	1028.21289	3082.62412	0.98
sIDDLEDQLYHQLEQNR	Unambiguous	3	1	E9Q452	N-Term(iTRAQ8plex)	CID	1.0000	0.0000	1	1	2979518	Unique	Used	1.836	1.455	0.764	0.841	0	0	6.55	0	0	200	3	807.40240	2420.19266	-2.86
lQQELDDLTVDLDHQR	Unambiguous	3	1	Q5SV64	N-Term(iTRAQ8plex)	CID	1.0000	0.0000	1	1	2816089	Unique	Used	2.719	1.013	1.417	0.741	0	0	6.55	0	4	115	3	748.05774	2242.15866	-1.38
tLTLTGVSSLEDVk	Unambiguous	1	1	Q8CHP8	N-Term(iTRAQ8plex); K15(iTRAQ8plex)	CID	1.0000	0.0000	1	1	3417897	Unique	Used	1.379	0.965	0.993	1.261	0	0	6.55	0	8	64	2	1092.64746	2184.28765	-3.26
iHVSDQELQSANASVDDSR	Unambiguous	1	1	Q02053	N-Term(iTRAQ8plex)	CID	1.0000	0.0000	1	1	2650363	Unique	Used	0.470	0.510	0.802	0.896	0	0	6.55	0	2	16	3	792.39526	2375.17124	-1.21
hVSIQEAESYAESVGAk	Unambiguous	1	1	P35282	N-Term(iTRAQ8plex); K17(iTRAQ8plex)	CID	1.0000	0.0000	1	1	3012529	Unique	Used	1.044	0.786	0.986	1.269	0	0	6.55	0	23	200	2	1207.14001	2413.27275	-3.82
nMGGPYGGGNYGPGGSGGSGGYGGR	Unambiguous	3	1	O88569	N-Term(iTRAQ8plex)	CID	1.0000	0.0000	1	1	3295807	Unique	Used	2.211	0.896	1.166	1.023	0	0	6.55	0	0	41	3	832.03967	2494.10447	-2.55
dIGAIAQVHAEnGDIIAEEQQR	Unambiguous	1	1	O08553	N-Term(iTRAQ8plex); N12(Deamidated)	CID	1.0000	0.0000	1	1	2760258	Unique	Used	2.672	1.254	1.109	0.786	0	0	6.55	0	28	200	3	894.78961	2682.35428	-3.52
hQSGGnFFEPTLLSNVTR	Unambiguous	1	1	Q8BWF0	N-Term(iTRAQ8plex); N6(Deamidated)	CID	1.0000	0.0000	1	1	3456207	Unique	Used	1.890	1.269	1.081	1.076	0	0	6.55	0	17	78	3	770.39484	2309.16996	-5.57
sSGAGSTSDAYTVIQVGR	Unambiguous	1	1	Q8R361	N-Term(iTRAQ8plex)	CID	1.0000	0.0000	1	1	3318812	Unique	Used	0.760	0.573	0.809	1.206	0	0	6.55	0	0	76	2	1030.52844	2060.04961	-3.21
lELNENPDTLHQDIQEVR	Unambiguous	2	1	Q8BG92	N-Term(iTRAQ8plex)	CID	1.0000	0.0000	1	1	2685336	Unique	Used	1.460	1.220	1.094	0.844	0	0	6.55	0	14	200	3	823.09564	2467.27237	-0.30
aLLADAQIMLDHLk	Unambiguous	12	1	B2RRE2	N-Term(iTRAQ8plex); K14(iTRAQ8plex)	CID	1.0000	0.0000	1	1	3601970	Unique	Used	0.889	0.541	1.183	0.588	0	0	6.55	0	3	5	3	720.75665	2160.25541	-5.43
sAPDFTATAVVDGAFk	Unambiguous	2	1	Q61171	N-Term(iTRAQ8plex); K16(iTRAQ8plex)	CID	1.0000	0.0000	1	1	3302607	Unique	Used	1.545	1.269	0.877	0.907	0	0	6.55	0	0	5	2	1103.10144	2205.19560	-2.55

mLDLYSQISSVPIGYNHPALAk	Unambiguous	3	1	P61922	N-Term(iTRAQ8plex); M1(Oxidation); K22(iTRAQ8plex)	CID	1.0000	0.0000	1	1	3783468	Unique	Used	1.201	0.553	0.897	1.327	0	0	6.55	0	22	172	3	1014.55316	3041.64493	-4.65
sHTVAAQGGINAALGNMEEDNWR	Unambiguous	1	1	Q8K2B3	N-Term(iTRAQ8plex)	CID	1.0000	0.0000	1	1	3233545	Unique	Used	1.651	1.424	1.093	1.214	0	0	6.55	0	0	49	3	915.77917	2745.32297	-3.19
gNAGQTNYGFAnSTMER	Unambiguous	1	1	P19096	N-Term(iTRAQ8plex); N12(Deamidated)	CID	1.0000	0.0000	1	1	2826953	Unique	Used	0.514	0.442	0.772	0.891	0	0	6.55	0	0	122	2	1061.98962	2122.97197	-2.17
eAAWAITNATSGGSAEQIk	Unambiguous	1	1	Q60960	N-Term(iTRAQ8plex); K19(iTRAQ8plex)	CID	1.0000	0.0000	1	1	3256708	Unique	Used	0.580	1.083	0.904	0.984	0	0	6.55	0	0	105	2	1257.17346	2513.33965	-2.39
aGSHIQTLMGSQSLQHR	Unambiguous	2	1	Q80TE7	N-Term(iTRAQ8plex)	CID	1.0000	0.0000	1	1	3908446	Unique	Used	0.557	1.361	2.627	0.794	0	0	6.55	0	0	200	3	719.04688	2155.12607	-3.87
aIYPFDGHNEGTLAMk	Unambiguous	4	1	E9PUI5	N-Term(iTRAQ8plex); K16(iTRAQ8plex)	CID	1.0000	0.0000	1	1	3278501	Unique	Used	1.026	1.022	1.129	0.821	0	0	6.55	0	5	24	3	791.42120	2372.24906	-1.63
aFMEEFGAEPELAVSAPGR	Unambiguous	1	1	Q9JIA6	N-Term(iTRAQ8plex)	CID	1.0000	0.0000	1	1	3111189	Unique	Used	0.306	0.937	0.745	1.042	0	0	6.55	0	0	66	2	1156.57959	2312.15190	-0.71
tALPTSGSSTGELELLAGEVPAR	Unambiguous	2	1	Q80VP1	N-Term(iTRAQ8plex)	CID	1.0000	0.0000	1	1	3260845	Unique	Used	1.641	1.639	2.788	1.185	0	0	6.55	0	0	37	2	1280.68945	2560.37163	-2.20
eHINLGcDVDFDIAGPSIR	Unambiguous	3	1	Q60932-2	N-Term(iTRAQ8plex); C7(Methylthio)	CID	1.0000	0.0000	1	1	3355547	Unique	Used	1.202	1.231	0.685	1.128	0	0	6.54	0	13	200	3	807.73022	2421.17612	-3.46
lAPITSDPTEAAAVGAVEASFk	Unambiguous	1	1	P52480	N-Term(iTRAQ8plex); K22(iTRAQ8plex)	CID	1.0000	0.0000	1	1	3262270	Unique	Used	1.300	0.623	0.689	1.153	0	0	6.54	0	0	11	2	1377.26025	2753.51323	-1.82
vnEVDVSEVVHSR	Unambiguous	3	1	Q52KF7	N-Term(iTRAQ8plex); N2(Deamidated)	CID	1.0000	0.0000	1	1	2667876	Unique	Used	1.076	1.666	1.328	0.943	0	0	6.54	0	29	200	3	591.98004	1773.92557	-1.64
tVQSPGAAVHPVAAQSTGVVVPPR	Unambiguous	1	1	Q6A058	N-Term(iTRAQ8plex)	CID	1.0000	0.0000	1	1	3817550	Unique	Used	1.473	0.733	1.176	1.221	0	0	6.54	0	23	200	3	877.15985	2629.46500	-2.98
gYGYGQGAGTLSTDk	Unambiguous	1	1	P97315	N-Term(iTRAQ8plex); K15(iTRAQ8plex)	CID	1.0000	0.0000	1	1	2970474	Unique	Used	2.000	0.862	1.823	1.241	0	0	6.54	0	0	200	2	1042.05176	2083.09624	2.21

sDIAAAQQSGMSLSnSk	Unambiguous	1	1	B1AQZ0	N-Term(iTRAQ8plex); N15(Deamidated); K17(iTRAQ8plex)	CID	1.0000	0.0000	1	1	2990659	Unique	Used	0.467	3.001	2.496	2.459	0	0	6.54	0	0	200	2	1152.59851	2304.18974	-2.80
gITNLcVIGGDGSLTGADTFR	Unambiguous	1	1	P47857	N-Term(iTRAQ8plex); C6(Methylthio)	CID	1.0000	0.0000	1	1	3728364	Unique	Used	1.774	0.795	0.755	1.181	0	0	6.54	0	14	200	2	1209.10144	2417.19560	-6.25
mADPQSIQESQNLSMFLANHNk	Unambiguous	4	1	Q7TMB8	N-Term(iTRAQ8plex); K22(iTRAQ8plex)	CID	1.0000	0.0000	1	1	3436582	Unique	Used	0.584	1.334	0.977	1.082	0	0	6.54	0	16	52	3	1037.86316	3111.57492	-2.03
tVTYEDPQAVGGLASALDnR	Unambiguous	8	1	Q5SRX1-2	N-Term(iTRAQ8plex); N19(Deamidated)	CID	1.0000	0.0000	1	1	2939603	Unique	Used	1.675	1.221	1.499	1.126	0	0	6.54	0	30	118	2	1191.60559	2382.20391	-2.18
aVQSIGNLGQQLGQGk	Unambiguous	1	1	Q9Z268	N-Term(iTRAQ8plex); K16(iTRAQ8plex)	CID	1.0000	0.0000	1	1	3761077	Unique	Used	0.292	2.438	1.066	0.976	0	0	6.54	0	0	120	2	1103.63684	2206.26641	-4.55
iGGVQQDTILAEGLHFR	Unambiguous	1	1	O35129	N-Term(iTRAQ8plex)	CID	1.0000	0.0000	1	1	3538360	Unique	Used	1.154	1.204	1.206	1.410	0	0	6.54	0	18	30	3	720.06415	2158.17789	-6.66
vEQIAAIAQELNELDYHDAVNVNDR	Unambiguous	1	1	Q9JI91	N-Term(iTRAQ8plex)	CID	1.0000	0.0000	1	1	3262670	Unique	Used		2.047			0	0	6.54	0	0	16	3	1048.53650	3143.59494	1.20
tIGGGDDSFnTFFSETGAGk	Unambiguous	4	1	P68373	N-Term(iTRAQ8plex); N10(Deamidated); K20(iTRAQ8plex)	CID	1.0000	0.0000	1	1	2902296	Unique	Used	0.953	1.526	1.289	1.453	0	0	6.54	0	0	200	2	1309.14600	2617.28472	-1.21
nQEALPVLQInPVIHLQPTAGLEEk	Unambiguous	1	1	Q8BZB3	N-Term(iTRAQ8plex); N11(Deamidated); K25(iTRAQ8plex)	CID	0.0076	0.0000	1	1	3439196	Unique	Used			0.979	0.871	0	0	6.54	0	0	200	3	1120.97083	3360.89792	-0.27
aSANLGQGSGSQLVPTVcnGR	Unambiguous	1	1	Q14BI2	N-Term(iTRAQ8plex); C18(Methylthio); N19(Deamidated)	CID	1.0000	0.0000	1	1	3662078	Unique	Used	0.400	0.792	0.923	0.894	0	0	6.54	0	0	122	2	1184.07837	2367.14946	-8.64

tTSQSPDAQAScGHPqTDSVAGPAMATTk	Unambiguous	1	1	D3YXJ0	N-Term(iTRAQ8plex); C12(Methylthio); Q16(Deamidated); K29(iTRAQ8plex)	CID	1.0000	0.0000	1	1	3085888	Unique	Used	0.768		2.753	1.284	0	0	6.54	0	0	200	3	1167.56152	3500.67002	4.20
kGVAGQQQASVMASk	Unambiguous	6	1	Q9WUU8-2	N-Term(iTRAQ8plex); K1(iTRAQ8plex); K15(iTRAQ8plex)	HCD	1.0000	0.0000	1	1	3405367	Unique	Used	0.919	0.818	1.125	0.861	0	0	6.54	1	0	200	3	801.46637	2402.38456	-4.47
lYSSMSDTNLAEAGLNYHAQR	Unambiguous	2	1	O88737	N-Term(iTRAQ8plex)	CID	1.0000	0.0000	1	1	3413266	Unique	Used	1.141	1.495	1.529	1.021	0	0	6.54	0	18	14	3	882.43335	2645.28550	-2.90
eQVDAALGAEEMVEMLTDR	Unambiguous	5	1	E9Q3M3	N-Term(iTRAQ8plex)	CID	1.0000	0.0000	1	1	2816421	Unique	Used	1.044	0.582	1.059	0.972	0	0	6.54	0	15	200	2	1206.08643	2411.16557	-3.37
qNTGSATQGPAYHGVNR	Unambiguous	4	1	E9QAQ7	N-Term(iTRAQ8plex)	CID	1.0000	0.0000	1	1	3223461	Unique	Used	0.801	0.980	1.191	0.966	0	0	6.54	0	14	200	3	688.01593	2062.03324	-1.73
lSQSVHHGASSEGSLSPDTR	Unambiguous	1	1	Q8VHI6	N-Term(iTRAQ8plex)	HCD	1.0000	0.0000	1	1	3203873	Unique	Used	0.946	0.777	1.373	1.104	0	0	6.54	0	0	190	3	786.06464	2356.17936	-0.07
vSELEAELAEQQHLGR	Unambiguous	1	1	Q8VD75	N-Term(iTRAQ8plex)	CID	1.0000	0.0000	1	1	2851129	Unique	Used	1.797	0.989	1.015	1.066	0	0	6.54	0	11	200	3	705.04382	2113.11692	-1.06
sSGSPYGGGYGSGGGSGGYGSR	Unambiguous	7	1	J3QNY1	N-Term(iTRAQ8plex)	CID	1.0000	0.0000	1	1	2986772	Unique	Used	1.082	0.573	1.462	0.725	0	0	6.54	0	0	200	2	1107.99744	2214.98760	-3.52
fLHDPSATQGFVGcALSSNIQR	Unambiguous	3	1	P17563	N-Term(iTRAQ8plex); C14(Methylthio)	CID	1.0000	0.0000	1	1	3897323	Unique	Used	1.517	0.773	1.120	1.536	0	0	6.54	0	13	37	3	900.11346	2698.32584	-4.64
sGNFGGSRnMGGPYGGGNYGPGGSGGSGGYGGR	Unambiguous	3	1	O88569	N-Term(iTRAQ8plex); N9(Deamidated)	CID	1.0000	0.0000	1	1	3385508	Unique	Used	1.458	0.832	1.290	1.186	0	0	6.53	1	0	158	3	1086.47998	3257.42539	-3.19
aFMEEFGAEPELAVSAPGR	Unambiguous	1	1	Q9JIA6	N-Term(iTRAQ8plex)	CID	1.0000	0.0000	1	1	3092938	Unique	Used	0.721	1.173	1.191	1.385	0	0	6.53	0	11	200	2	1156.57776	2312.14824	-2.29
sVFDIPIFTEEFLNHSk	Unambiguous	2	1	Q9DC33	N-Term(iTRAQ8plex); K17(iTRAQ8plex)	CID	1.0000	0.0000	1	1	3479763	Unique	Used		0.434	1.031	0.522	0	0	6.53	0	15	18	3	877.81146	2631.41983	-3.07
gTGGVDTAATGSVFDISNLDR	Unambiguous	1	1	P30275	N-Term(iTRAQ8plex)	CID	1.0000	0.0000	1	1	3260333	Unique	Used	1.389	1.539	1.140	1.577	0	0	6.53	0	0	69	2	1179.09570	2357.18413	-1.95
dLGAQGAVAEAELAEAQR	Unambiguous	1	1	Q61001	N-Term(iTRAQ8plex)	CID	1.0000	0.0000	1	1	2636760	Unique	Used	1.819	2.630	1.208	1.329	0	0	6.53	0	9	200	2	1052.05042	2103.09355	-2.33

hHQEEVGELLGQIQGcGAAQAQAQAEAR	Unambiguous	1	1	P19246	N-Term(iTRAQ8plex); C16(Methylthio)	CID	1.0000	0.0000	1	1	3346133	Unique	Used	2.215	1.020	1.000	0.990	0	0	6.53	0	0	39	3	1093.86328	3279.57529	-4.53
sLWDSASGGGVVAIDNk	Unambiguous	1	1	E9Q7M2	N-Term(iTRAQ8plex); K17(iTRAQ8plex)	CID	1.0000	0.0000	1	1	3322405	Unique	Used	1.451	0.582	1.304	0.919	0	0	6.53	0	13	138	2	1142.61829	2284.22930	-4.42
iGNSTNPGPALATNPQNAASR	Unambiguous	2	1	P97445	N-Term(iTRAQ8plex)	CID	1.0000	0.0000	1	1	3698086	Unique	Used	0.593	0.555	1.057	0.508	0	0	6.53	0	0	200	2	1178.11133	2355.21538	-7.02
eTQDLIEQGALLQAHR	Unambiguous	1	1	Q6KAR6	N-Term(iTRAQ8plex)	CID	1.0000	0.0000	1	1	3126930	Unique	Used	0.693	1.646	0.996	1.239	0	0	6.53	0	6	5	3	709.38733	2126.14743	-1.58
tFVSITPAEVGVLVGkDR	Unambiguous	2	1	P62962	N-Term(iTRAQ8plex); K16(iTRAQ8plex)	CID	1.0000	0.0000	1	1	3725744	Unique	Used	0.485	1.276	0.881	0.842	0	0	6.53	1	24	50	3	832.82172	2496.45060	-5.63
dLVILLYETALLSSGFSLEDPQTHANR	Unambiguous	1	1	P07901	N-Term(iTRAQ8plex)	HCD	1.0000	0.0000	1	1	3786360	Unique	Used	1.248	1.048	1.275	1.488	0	0	6.53	0	17	200	3	1102.91980	3306.74485	-2.29
aLMSSSQATASSSNk	Unambiguous	4	1	Q6PDI5	N-Term(iTRAQ8plex); K15(iTRAQ8plex)	CID	1.0000	0.0000	1	1	3362520	Unique	Used	0.360	0.679	0.528	0.803	0	0	6.53	0	0	134	2	1039.55103	2078.09477	-2.91
vAEkLDEIYVAGLVAHSDLDER	Unambiguous	7	1	G3V018	N-Term(iTRAQ8plex); K4(iTRAQ8plex)	CID	1.0000	0.0000	1	1	3153128	Unique	Used	1.315	0.862	1.156	0.787	0	0	6.53	1	18	200	3	1017.56018	3050.66599	1.33
kAEGATLSnATGAVESTSQAGDR	Unambiguous	1	1	Q61644	N-Term(iTRAQ8plex); K1(iTRAQ8plex); N9(Deamidated)	CID	1.0000	0.0000	1	1	2845902	Unique	Used	1.473	1.662	2.786	1.478	0	0	6.53	1	0	76	3	944.15851	2830.46097	-1.05
scQTAGIATAESSAQAR	Unambiguous	2	1	Q5U4F6	N-Term(iTRAQ8plex); C2(Methylthio)	CID	1.0000	0.0000	1	1	3274892	Unique	Used	0.491	1.045	0.943	1.147	0	0	6.53	0	0	200	2	1001.48419	2001.96111	-1.24
eHINLGcDVDFDIAGPSIR	Unambiguous	3	1	Q60932-2	N-Term(iTRAQ8plex); C7(Methylthio)	CID	1.0000	0.0000	1	1	3375871	Unique	Used	0.910	1.030	1.227	1.367	0	0	6.53	0	15	200	3	807.72974	2421.17466	-4.07
aQNVGDSEVAAIGQLTFLR	Unambiguous	2	1	F8VQK6	N-Term(iTRAQ8plex)	CID	1.0000	0.0000	1	1	3580913	Unique	Used	0.632	0.961	0.867	0.715	0	0	6.53	0	24	25	2	1147.11841	2293.22954	-6.93
rPASVSSAAAEHEAR	Unambiguous	1	1	E9Q1P8	N-Term(iTRAQ8plex)	CID	1.0000	0.0000	1	1	2892102	Unique	Used	2.091	0.949	0.926	0.838	0	0	6.52	0	11	200	3	614.99432	1842.96842	-2.18
tALPTSGSSTGELELLAGEVPAR	Unambiguous	2	1	Q80VP1	N-Term(iTRAQ8plex)	CID	1.0000	0.0000	1	1	3282924	Unique	Used	1.677	1.535	1.765	1.156	0	0	6.52	0	0	135	2	1280.69006	2560.37285	-1.72

gPAVGIDLGTTYScVGVFQHGk	Unambiguous	4	1	P63017	N-Term(iTRAQ8plex); C14(Methylthio); K22(iTRAQ8plex)	CID	1.0000	0.0000	1	1	3808245	Unique	Used	1.106	0.910	0.918	0.819	0	0	6.52	0	23	200	3	954.16852	2860.49100	-1.28
nITLSLVANPSHLEAVDPVVQGk	Unambiguous	1	1	E9Q7L0	N-Term(iTRAQ8plex); K23(iTRAQ8plex)	CID	1.0000	0.0000	1	1	3498238	Unique	Used	0.780	0.620	1.300	1.342	0	0	6.52	0	13	84	3	1003.90582	3009.70292	-5.47
fTLGSVAGAVGATAVYPIDLVk	Unambiguous	1	1	Q8BH59	N-Term(iTRAQ8plex); K22(iTRAQ8plex)	CID	1.0000	0.0000	1	1	3842456	Unique	Used	0.332	1.046	1.197	1.197	0	0	6.52	0	13	33	2	1379.29846	2757.58965	-4.18
gPLLVQDVVFTDEMAHFDR	Unambiguous	2	1	P24270	N-Term(iTRAQ8plex)	CID	1.0000	0.0000	1	1	3262295	Unique	Used		1.009			0	0	6.52	0	3	6	3	831.76245	2493.27280	-0.90
nQGGYGGSSSSSSYGSGR	Unambiguous	3	1	Q5EBP8	N-Term(iTRAQ8plex)	CID	1.0000	0.0000	1	1	2771584	Unique	Used	2.379	0.588	0.715	0.578	0	0	6.52	0	0	200	2	999.95416	1998.90105	-2.23
tLTGTAALTVQSQEENLR	Unambiguous	1	1	P24527	N-Term(iTRAQ8plex)	CID	1.0000	0.0000	1	1	3278757	Unique	Used	0.873	1.381	1.099	1.388	0	0	6.52	0	0	50	2	1118.60474	2236.20220	-2.91
lATPTYGDLNHLVSATMSGVTTSLR	Unambiguous	1	1	Q9ERD7	N-Term(iTRAQ8plex)	CID	1.0000	0.0000	1	1	3937614	Unique	Used	0.429	0.765	0.816	0.951	0	0	6.52	0	15	200	3	970.51434	2909.52848	-2.07
sAAQAAAQTNSNAAGk	Unambiguous	4	1	Q9CY58	N-Term(iTRAQ8plex); K16(iTRAQ8plex)	CID	1.0000	0.0000	1	1	3140071	Unique	Used	0.665	0.233	1.153	0.623	0	0	6.52	0	3	52	2	1035.06152	2069.11577	-1.85
aAAQLLQSQAQQSGAQQTk	Unambiguous	3	1	Q8C1B7-3	N-Term(iTRAQ8plex); K19(iTRAQ8plex)	CID	1.0000	0.0000	1	1	3657833	Unique	Used	1.353	1.368	1.510	1.006	0	0	6.52	0	15	187	3	855.80695	2565.40628	-5.56
aDIGVAMGIAGSDVSk	Unambiguous	2	1	Q6PIC6	N-Term(iTRAQ8plex); K16(iTRAQ8plex)	CID	1.0000	0.0000	1	1	3257534	Unique	Used	0.561	0.722	1.186	1.316	0	0	6.52	0	0	26	2	1050.08252	2099.15776	-2.37
aHLQGPTGTSVAGPQTSEAFVITVR	Unambiguous	2	1	Q8VHY0	N-Term(iTRAQ8plex)	CID	1.0000	0.0000	1	1	3721782	Unique	Used	1.020	1.925	1.364	1.277	0	0	6.52	0	14	121	3	943.50629	2828.50431	-5.87
nLHQSGFSLSGSQADDHIAR	Unambiguous	3	1	O35098	N-Term(iTRAQ8plex)	CID	1.0000	0.0000	1	1	3384507	Unique	Used	0.712	0.890	0.800	0.896	0	0	6.52	0	12	93	3	815.40875	2444.21170	-4.24
sGFEPPGDFPFEDYSQHIYR	Unambiguous	4	1	E9PUI5	N-Term(iTRAQ8plex)	CID	1.0000	0.0000	1	1	3303057	Unique	Used	0.695	0.863	0.878	0.818	0	0	6.52	0	17	92	3	898.09100	2692.25846	-1.39
eHINLGcDVDFDIAGPSIR	Unambiguous	3	1	Q60932-2	N-Term(iTRAQ8plex); C7(Methylthio)	CID	1.0000	0.0000	1	1	3372678	Unique	Used		1.761	0.815	0.687	0	0	6.52	0	5	44	3	807.72937	2421.17356	-4.52
lQELGATLEHR	Unambiguous	1	1	Q8JZL3	N-Term(iTRAQ8plex)	CID	1.0000	0.0000	1	1	3321241	Unique	Used	1.738	0.856	0.930	0.971	0	0	6.51	0	3	23	3	524.29865	1570.88138	-2.62

eYQLNDSAAYYLnDLER	Unambiguous	1	1	P08752	N-Term(iTRAQ8plex); N13(Deamidated)	CID	1.0000	0.0000	1	1	2741755	Unique	Used			1.779	2.862	0	0	6.51	0	0	200	2	1191.57410	2382.14092	0.25
vSDAISTqYPVVDHEFDAVVVGAGGAGLR	Unambiguous	1	1	Q8K2B3	N-Term(iTRAQ8plex); Q8(Deamidated)	CID	1.0000	0.0000	1	1	3355054	Unique	Used	1.350	0.984	1.249	1.086	0	0	6.51	0	0	200	3	1078.89294	3234.66428	1.77
aPPQTQLPSGASSGTGSASATHGGAPLPGTR	Unambiguous	8	1	Q3UF95	N-Term(iTRAQ8plex)	CID	1.0000	0.0000	1	1	3697607	Unique	Used	0.595	0.631	1.253	1.150	0	0	6.51	0	0	17	3	1040.86279	3120.57383	-7.64
aASSGGPPLTTAAPQR	Unambiguous	2	1	B2RY58	N-Term(iTRAQ8plex)	CID	1.0000	0.0000	1	1	3529325	Unique	Used	0.914	0.436	0.900	0.756	0	0	6.51	0	4	39	2	893.48700	1785.96672	-5.26
hSFMSTEPLSAEASLSSDSQR	Unambiguous	4	1	O08576-2	N-Term(iTRAQ8plex)	CID	1.0000	0.0000	1	1	3087984	Unique	Used	1.426	1.609	0.899	0.874	0	0	6.51	0	4	33	3	857.74927	2571.23325	1.29
vFEVSLADLQNDEVAFR	Unambiguous	3	1	D3Z6C3	N-Term(iTRAQ8plex)	CID	1.0000	0.0000	1	1	3326960	Unique	Used	0.440	0.593	0.657	0.830	0	0	6.51	0	0	25	2	1128.59082	2256.17436	-3.14
sGGPHGAGVLGVHLEGPFISR	Unambiguous	2	1	Q8JZV7	N-Term(iTRAQ8plex)	CID	1.0000	0.0000	1	1	3820003	Unique	Used	1.957	1.068	1.259	1.262	0	0	6.51	0	17	200	3	783.43005	2348.27561	-0.91
vGSHITGGDIYGIVnENSLIk	Unambiguous	4	1	P50516	N-Term(iTRAQ8plex); N15(Deamidated); K21(iTRAQ8plex)	CID	0.0415	0.0000	1	1	3302099	Unique	Used	0.723	1.031	1.390	1.095	0	0	6.51	0	3	11	3	932.51526	2795.53122	-3.14
lHQLSGSDQLEATAHSR	Unambiguous	4	1	Q58A65-2	N-Term(iTRAQ8plex)	CID	1.0000	0.0000	1	1	3227172	Unique	Used	1.428	1.129	1.168	1.001	0	0	6.51	0	0	81	3	718.70953	2154.11405	-3.01
eAPVPVVVSSADGDTVRPLR	Unambiguous	2	1	Q5XJV6	N-Term(iTRAQ8plex)	HCD	1.0000	0.0000	1	1	3298832	Unique	Used	1.780	1.359	1.577	1.060	0	0	6.51	0	17	66	3	790.10706	2368.30661	-3.06
lFQEGSTADALGTGADGQMER	Unambiguous	1	1	Q00560	N-Term(iTRAQ8plex)	CID	1.0000	0.0000	1	1	2867952	Unique	Used	1.370	0.946	1.068	0.983	0	0	6.51	0	14	200	2	1229.59143	2458.17558	-2.72
qGTGSLSESSIPSISDTSTPR	Unambiguous	2	1	Q61290	N-Term(iTRAQ8plex); Q1(Deamidated)	CID	1.0000	0.0000	1	1	3210092	Unique	Used	0.675	1.904	3.485	1.875	0	0	6.51	0	30	200	2	1206.61340	2412.21953	6.27
tGPAGGFQAAPHTAAPYSQAAPAR	Unambiguous	1	1	P47708	N-Term(iTRAQ8plex)	HCD	1.0000	0.0000	1	1	3736845	Unique	Used	0.968	0.629	1.165	0.849	0	0	6.51	0	0	200	3	867.11249	2599.32291	-3.49
eLLLPNWQGSGSHGLTIAQR	Unambiguous	2	1	E9Q616	N-Term(iTRAQ8plex)	CID	1.0000	0.0000	1	1	3876756	Unique	Used	1.608	1.946	1.009	1.006	0	0	6.51	0	0	200	3	827.78339	2481.33561	-6.46
qPYAVSELAGHQTSAESWGTGR	Unambiguous	1	1	Q9D8E6	N-Term(iTRAQ8plex)	CID	1.0000	0.0000	1	1	3298338	Unique	Used	10.301	0.470	0.565	0.517	0	0	6.51	0	25	10	3	879.43628	2636.29428	-2.44

iSIEMnGTLEDQLSHLk	Unambiguous	1	1	P57780	N-Term(iTRAQ8plex); N6(Deamidated); K17(iTRAQ8plex)	HCD	1.0000	0.0000	1	1	3261605	Unique	Used	1.097	2.295	1.367	0.780	0	0	6.51	0	2	5	3	846.46222	2537.37210	-0.82
dLVILLYETALLSSGFSLEDPQTHANR	Unambiguous	1	1	P07901	N-Term(iTRAQ8plex)	CID	1.0000	0.0000	1	1	3786820	Unique	Used		0.558	1.141	1.292	0	0	6.51	0	16	200	3	1102.91931	3306.74338	-2.73
gGADAPGPAGAFPASAASSPR	Unambiguous	1	1	Q01721	N-Term(iTRAQ8plex)	CID	1.0000	0.0000	1	1	3296349	Unique	Used	1.008	1.306	0.834	0.654	0	0	6.51	0	0	200	2	1058.54004	2116.07280	0.10
tLETQSALGPALQAAFk	Unambiguous	2	1	A2AA71	N-Term(iTRAQ8plex); K17(iTRAQ8plex)	CID	1.0000	0.0000	1	1	3643515	Unique	Used	0.724	0.610	0.922	0.996	0	0	6.51	0	0	83	2	1177.67358	2354.33989	-6.01
qSIAIDDcTFHQcVR	Unambiguous	2	1	Q3TWV4	N-Term(iTRAQ8plex); C8(Methylthio); C13(Methylthio)	CID	1.0000	0.0000	1	1	3557541	Unique	Used	0.668	1.149	1.252	1.411	0	0	6.51	0	7	26	3	711.32538	2131.96158	-3.91
qAQLQDAGIYEcESk	Unambiguous	2	1	P29533	N-Term(iTRAQ8plex); C12(Methylthio); K15(iTRAQ8plex)	CID	1.0000	0.0000	1	1	2848938	Unique	Used	0.629	1.210	1.034	1.184	0	0	6.51	0	24	200	2	1169.08813	2337.16899	0.62
qSTSEPASAPFVPFSGGGQR	Unambiguous	4	1	Q8VBT9	N-Term(iTRAQ8plex)	CID	1.0000	0.0000	1	1	3494448	Unique	Used	1.155	0.445	0.813	0.818	0	0	6.51	0	0	29	2	1156.07959	2311.15190	-4.41
aVVTHSSGNHGQALTYAAk	Unambiguous	4	1	Q9QZX7-2	N-Term(iTRAQ8plex); K19(iTRAQ8plex)	HCD	1.0000	0.0000	1	1	3467522	Unique	Used	0.301	2.183	2.501	1.188	0	0	6.51	0	0	200	3	840.79590	2520.37314	-1.90
cGQSAAVASPGGSIDSR	Unambiguous	3	1	B7FAU9	N-Term(iTRAQ8plex); C1(Methylthio)	CID	1.0000	0.0000	1	1	3316957	Unique	Used	0.314	3.448	0.877	1.979	0	0	6.51	0	0	143	2	956.96240	1912.91753	0.84
qGTGSLSESSIPSISDTSTPR	Unambiguous	2	1	Q61290	N-Term(iTRAQ8plex)	CID	1.0000	0.0000	1	1	3210273	Unique	Used	1.004	1.485	2.449	1.265	0	0	6.50	0	0	200	2	1206.11328	2411.21929	-0.46
lLEVSDDPQVLAVAAHDVGEYVR	Unambiguous	1	1	Q8BVE3	N-Term(iTRAQ8plex)	CID	1.0000	0.0000	1	1	3241203	Unique	Used	0.419	1.146	1.214	1.092	0	0	6.50	0	14	200	3	933.83173	2799.48062	-0.89
eAEAQAAMEANSEGSLTRPk	Unambiguous	1	1	P63040	N-Term(iTRAQ8plex); K20(iTRAQ8plex)	CID	1.0000	0.0000	1	1	2809138	Unique	Used	1.888	0.476	1.856	1.034	0	0	6.50	0	0	132	2	1349.69568	2698.38408	-3.20
sIPISPSASQIQIQHR	Unambiguous	4	1	E9PU87	N-Term(iTRAQ8plex)	CID	1.0000	0.0000	1	1	3853987	Unique	Used	0.675	0.796	1.035	1.059	0	0	6.50	0	0	27	3	689.39038	2066.15659	-4.57

eNLETSPFITPGkPWEEAR	Unambiguous	1	1	Q91YM2	N-Term(iTRAQ8plex); K13(iTRAQ8plex)	HCD	1.0000	0.0000	1	1	3212531	Unique	Used	0.705	0.997	0.900	1.000	0	0	6.50	0	15	173	3	937.16925	2809.49320	-1.76
aGnSLAASTAEETAGSAQSR	Unambiguous	1	1	Q61292	N-Term(iTRAQ8plex); N3(Deamidated)	CID	1.0000	0.0000	1	1	2632451	Unique	Used	1.471	1.938	1.758	1.796	0	0	6.50	0	22	200	2	1092.53296	2184.05864	-4.40
gPVTDVAYSHDGAFLAVcDASk	Unambiguous	1	1	O88342	N-Term(iTRAQ8plex); C18(Methylthio); K22(iTRAQ8plex)	CID	1.0000	0.0000	1	1	3324767	Unique	Used	0.723	1.152	0.895	0.896	0	0	6.50	0	8	14	3	959.81372	2877.42661	-3.68
iGEHTPSALAImENANVLAR	Unambiguous	3	1	A6ZI44	N-Term(iTRAQ8plex); M12(Oxidation)	CID	1.0000	0.0000	1	1	3721360	Unique	Used	1.219	1.229	0.952	1.038	0	0	6.50	0	0	27	3	809.76385	2427.27701	-8.17
iQAAASPPAnATAASDTNAGDR	Unambiguous	2	1	E9QAQ5	N-Term(iTRAQ8plex); N10(Deamidated)	CID	1.0000	0.0000	1	1	2754540	Unique	Used	0.862	0.945	0.966	0.684	0	0	6.50	0	0	200	2	1188.08997	2375.17266	-0.62
kAAAQLLQSQAQQSGAQQTk	Unambiguous	3	1	Q8C1B7-3	N-Term(iTRAQ8plex); K1(iTRAQ8plex); K20(iTRAQ8plex)	CID	1.0000	0.0000	1	1	3757170	Unique	Used	0.926	1.972	1.563	1.071	0	0	6.50	1	0	77	3	999.90887	2997.71207	-2.94
qTTAAAAATFSEQVGGGSGGAGR	Unambiguous	3	1	O88935	N-Term(iTRAQ8plex)	CID	1.0000	0.0000	1	1	3365554	Unique	Used	1.108	2.607	2.524	2.107	0	0	6.50	0	0	196	2	1178.59045	2356.17363	-2.52
nLHQSGFSLSGAQIDDNIPR	Unambiguous	1	1	O08553	N-Term(iTRAQ8plex)	CID	1.0000	0.0000	1	1	3389050	Unique	Used	2.250	1.254	1.043	1.299	0	0	6.50	0	26	52	3	825.09326	2473.26523	-3.45
aLETMAEqTTDVVHSPSTDTTPGPDTEAALAk	Unambiguous	4	1	P27546-2	N-Term(iTRAQ8plex); Q8(Deamidated); K32(iTRAQ8plex)	CID	1.0000	0.0000	1	1	2995614	Unique	Used	0.931	0.711	0.962	0.627	0	0	6.50	0	0	200	3	1298.65820	3893.96006	4.54
wGEAGAEYVVESTGVFTTmEk	Unambiguous	3	1	P16858	N-Term(iTRAQ8plex); M19(Oxidation); K21(iTRAQ8plex)	CID	1.0000	0.0000	1	1	3240646	Unique	Used			0.537	0.323	0	0	6.50	0	0	200	3	972.49213	2915.46183	0.84
aPSMVAAAVTYSkPR	Unambiguous	1	1	Q9CPQ8	N-Term(iTRAQ8plex); K13(iTRAQ8plex)	HCD	1.0000	0.0000	1	1	3911939	Unique	Used	0.673	1.216	1.069	1.144	0	0	6.50	0	0	84	3	719.74451	2157.21897	-5.61

aHLQGPTGTSVAGPqTSEAFVITVR	Unambiguous	2	1	Q8VHY0	N-Term(iTRAQ8plex); Q15(Deamidated)	CID	1.0000	0.0000	1	1	3721900	Unique	Used	1.460	1.766	1.103	0.912	0	0	6.50	0	0	116	3	943.84003	2829.50553	0.22
gLcSGPGAGEESPAATLPR	Unambiguous	1	1	Q80UU9	N-Term(iTRAQ8plex); C3(Methylthio)	CID	1.0000	0.0000	1	1	3321270	Unique	Used	0.676	1.134	0.856	1.092	0	0	6.50	0	0	21	2	1060.52124	2120.03520	-3.14
aTLQEVVGLHSDMAVEAGAPYAER	Unambiguous	1	1	G3UYZ1	N-Term(iTRAQ8plex)	HCD	1.0000	0.0000	1	1	3302412	Unique	Used	0.637	0.113	0.890	0.113	0	0	6.50	0	0	14	3	940.14850	2818.43094	-1.37
lSLPPHEGPGGNLVAGALYR	Unambiguous	5	1	E9Q3M3	N-Term(iTRAQ8plex)	CID	1.0000	0.0000	1	1	3839141	Unique	Used	0.966	1.164	1.052	1.194	0	0	6.49	0	26	200	3	774.76483	2322.27994	-3.14
lSFGDTLQVQNVHGAFNALGGADR	Unambiguous	1	1	Q9DC16	N-Term(iTRAQ8plex)	HCD	1.0000	0.0000	1	1	3804412	Unique	Used	0.704	1.164	0.746	0.973	0	0	6.49	0	0	30	3	931.15082	2791.43790	-1.82
tGTkPSAEPQSEAVTNVESQNTNR	Unambiguous	2	1	Q8BXA0	N-Term(iTRAQ8plex); K4(iTRAQ8plex)	CID	1.0000	0.0000	1	1	2824813	Unique	Used	0.621	0.613	0.817	0.758	0	0	6.49	0	0	200	3	1051.87891	3153.62217	-0.36
hPLEPDSSAScFQQLR	Unambiguous	3	1	D3YXA8	N-Term(iTRAQ8plex); C11(Methylthio)	CID	1.0000	0.0000	1	1	3367469	Unique	Used	2.413	0.976	0.865	1.054	0	0	6.49	0	15	38	3	722.34857	2165.03116	-5.08
iHVSDQELQSANASVDDSR	Unambiguous	1	1	Q02053	N-Term(iTRAQ8plex)	CID	1.0000	0.0000	1	1	2649831	Unique	Used	2.291	1.110	1.221	0.815	0	0	6.49	0	16	200	3	792.39642	2375.17472	0.25
gAALDVHESEPFSFSQGPLk	Unambiguous	3	1	O88712-2	N-Term(iTRAQ8plex); K20(iTRAQ8plex)	CID	1.0000	0.0000	1	1	3234517	Unique	Used	1.179	1.746	1.390	1.153	0	0	6.49	0	0	32	3	908.81793	2724.43924	-2.25
cQNEQLQTAVTQQASQIQQHk	Unambiguous	3	1	Q9Z1Z0	N-Term(iTRAQ8plex); C1(Methylthio); K21(iTRAQ8plex)	CID	1.0000	0.0000	1	1	3514210	Unique	Used	1.242	1.102	0.929	0.955	0	0	6.49	0	12	124	3	1022.52399	3065.55741	-4.67
vSTHSQEMDSGAEYGIGSSTk	Unambiguous	8	1	B9EKN8	N-Term(iTRAQ8plex); K21(iTRAQ8plex)	CID	1.0000	0.0000	1	1	2807405	Unique	Used	3.153	2.186	0.972	0.971	0	0	6.49	0	12	157	3	927.12463	2779.35935	-2.58
vETIGDAYcVASGLHR	Unambiguous	1	1	F8VQK3	N-Term(iTRAQ8plex); C9(Methylthio)	CID	1.0000	0.0000	1	1	3537955	Unique	Used		0.839	0.943	0.660	0	0	6.49	0	7	63	3	681.00732	2041.00742	-3.66
kQPPVSPGTALVGSQk	Unambiguous	1	1	P17095	N-Term(iTRAQ8plex); K1(iTRAQ8plex); K16(iTRAQ8plex)	CID	1.0000	0.0000	1	1	3571429	Unique	Used	0.394	0.819	0.836	0.769	0	0	6.49	1	11	164	3	836.17267	2506.50345	-3.43

gIEEEEILPGFILcDLNNLcHSGR	Unambiguous	2	1	Q8R050-2	N-Term(iTRAQ8plex); C14(Methylthio); C20(Methylthio)	CID	1.0000	0.0000	1	1	3480392	Unique	Used	1.487	1.277	0.961	1.117	0	0	6.49	0	25	73	3	1023.16046	3067.46683	0.15
sQVFSTAADGQTQVEIk	Unambiguous	1	1	P38647	N-Term(iTRAQ8plex); K17(iTRAQ8plex)	CID	1.0000	0.0000	1	1	3147373	Unique	Used	0.942	0.871	1.322	1.146	0	0	6.49	0	0	91	2	1209.15833	2417.30937	-1.62
sNISSPVTPTAASSSSTTPTR	Unambiguous	6	1	A2AU91	N-Term(iTRAQ8plex)	CID	1.0000	0.0000	1	1	3591606	Unique	Used	0.532	0.392	0.528	0.745	0	0	6.49	0	0	140	2	1177.10754	2353.20781	-3.01
rTNPFGESGGSTk	Unambiguous	1	1	Q8K3H0	N-Term(iTRAQ8plex); K13(iTRAQ8plex)	HCD	1.0000	0.0000	1	1	3292134	Unique	Used	0.573	1.064	1.101	1.162	0	0	6.49	1	0	95	3	649.35541	1946.05167	-1.81
sYSPELIVHPVLDSSnAVEEVEk	Unambiguous	6	1	J3QMM7	N-Term(iTRAQ8plex); N16(Deamidated); K23(iTRAQ8plex)	CID	1.0000	0.0000	1	1	2813928	Unique	Used	1.511	1.175	0.587	0.798	0	0	6.49	0	18	200	3	1050.89331	3150.66538	-0.43
yEGSGDGGAAAQSLYIAnHAY	Unambiguous	1	1	P05063	N-Term(iTRAQ8plex); N18(Deamidated)	CID	1.0000	0.0000	1	1	2760674	Unique	Used	1.139	0.591	1.102	1.126	0	0	6.49	0	0	200	2	1210.56934	2420.13140	0.23
aAmEALQSQALHATSQQPLR	Unambiguous	5	1	B1AQZ0	N-Term(iTRAQ8plex); M3(Oxidation)	HCD	1.0000	0.0000	1	1	3818195	Unique	Used	2.145	0.964	1.664	1.355	0	0	6.49	0	1	24	3	824.43524	2471.29117	-2.72
aQPFVAAANIDDkR	Unambiguous	1	1	O70209	N-Term(iTRAQ8plex); K13(iTRAQ8plex)	HCD	1.0000	0.0000	1	1	3252569	Unique	Used	1.240	2.879	1.384	1.355	0	0	6.49	1	17	52	3	708.73767	2124.19846	-1.77
qcnNFLLHNLILDTGITQQLVER	Unambiguous	7	1	Q6PHS9-4	N-Term(iTRAQ8plex); C2(Methylthio); N3(Deamidated)	CID	0.0216	0.0000	1	1	3766274	Unique	Used	1.637	1.456	0.301	0.632	0	0	6.49	0	12	17	3	1011.86334	3033.57547	-1.62
gETGPAGPAGPIGPAGAR	Unambiguous	2	1	P11087-2	N-Term(iTRAQ8plex)	CID	1.0000	0.0000	1	1	3142772	Unique	Used	0.598	7.612	1.020	0.968	0	0	6.49	0	3	1	2	918.99597	1836.98467	-1.30
aAEFLAAcAGGDLDEAR	Unambiguous	1	1	Q3UMT1	N-Term(iTRAQ8plex); C8(Methylthio)	CID	1.0000	0.0000	1	1	3239554	Unique	Used	1.106	0.711	1.282	0.927	0	0	6.49	0	0	39	2	1015.48132	2029.95537	-3.54
lAAEDVTFIGPDTHAIQAMGDk	Unambiguous	2	1	Q91ZA3	N-Term(iTRAQ8plex); K22(iTRAQ8plex)	CID	1.0000	0.0000	1	1	3171182	Unique	Used	0.770	1.372	1.346	1.368	0	0	6.48	0	5	74	3	970.17926	2908.52323	-3.56

aVAQGNLSSADVQAAk	Unambiguous	1	1	Q9DB77	N-Term(iTRAQ8plex); K16(iTRAQ8plex)	CID	1.0000	0.0000	1	1	3184808	Unique	Used	0.983	1.335	1.248	1.748	0	0	6.48	0	0	133	2	1069.60315	2138.19902	-1.68
hNHDLSSYQDTIQQLENELR	Unambiguous	2	1	P08553	N-Term(iTRAQ8plex)	CID	1.0000	0.0000	1	1	3215072	Unique	Used	1.686	0.889	0.893	1.020	0	0	6.48	0	27	100	3	915.45471	2744.34958	-1.68
vSHVSTGGGASLELLEGk	Unambiguous	2	1	P09411	N-Term(iTRAQ8plex); K18(iTRAQ8plex)	CID	1.0000	0.0000	1	1	3254931	Unique	Used	0.950	0.958	1.418	1.158	0	0	6.48	0	0	164	2	1175.16260	2349.31792	-2.36
qkPAPLPPASNAPQPGSSQQIQQR	Unambiguous	1	1	Q62415	N-Term(iTRAQ8plex); K2(iTRAQ8plex)	CID	1.0000	0.0000	1	1	3655374	Unique	Used	0.930	0.835	1.561	0.651	0	0	6.48	0	0	106	3	1045.24329	3133.71531	-5.56
aGQVFLEELGNHk	Unambiguous	2	1	P16330	N-Term(iTRAQ8plex); K13(iTRAQ8plex)	CID	1.0000	0.0000	1	1	3256460	Unique	Used	0.794	0.309	0.769	0.996	0	0	6.48	0	0	23	3	684.05591	2050.15317	-0.50
lVAGEMGQNEPDQGGQR	Unambiguous	3	1	Q99N15	N-Term(iTRAQ8plex)	CID	1.0000	0.0000	1	1	2632711	Unique	Used	0.882	1.178	1.275	1.187	0	0	6.48	0	7	165	2	1045.51270	2090.01811	-2.77
tGEGGLLQVTLEGINLk	Unambiguous	15	1	A2AGR0	N-Term(iTRAQ8plex); K17(iTRAQ8plex)	CID	1.0000	0.0000	1	1	3519952	Unique	Used	0.501	1.024	1.009	1.125	0	0	6.48	0	0	28	2	1175.68591	2350.36455	-6.68
ePcGGTGSTGAGGGTnGGVR	Unambiguous	1	1	Q80WM4	N-Term(iTRAQ8plex); C3(Methylthio); N16(Deamidated)	CID	1.0000	0.0000	1	1	2752205	Unique	Used	0.795	0.188	1.012	1.599	0	0	6.48	0	5	148	2	1021.95966	2042.91203	-2.62
lALTEWLQEFGVPHQYSSR	Unambiguous	2	1	Q9EQQ9-3	N-Term(iTRAQ8plex)	CID	1.0000	0.0000	1	1	3860586	Unique	Used	1.327	1.435	1.588	0.975	0	0	6.48	0	22	68	3	855.78284	2565.33396	-2.50
gGGAAGLTSGGGGQPQWQk	Unambiguous	6	1	Q91ZM2	N-Term(iTRAQ8plex); K19(iTRAQ8plex)	CID	1.0000	0.0000	1	1	3408105	Unique	Used	0.719	1.175	1.151	0.870	0	0	6.48	0	0	200	2	1161.62402	2322.24077	-0.16
vQDDEVGDGTTSVTVLAAELLR	Unambiguous	1	1	P80314	N-Term(iTRAQ8plex)	CID	1.0000	0.0000	1	1	3133376	Unique	Used	0.693	1.138	1.072	1.224	0	0	6.48	0	0	10	2	1296.68555	2592.36382	-1.26
yPSLSLHGIEGAFSGSGAk	Unambiguous	1	1	Q9D1A2	N-Term(iTRAQ8plex); K19(iTRAQ8plex)	CID	1.0000	0.0000	1	1	3641499	Unique	Used	0.829	1.103	1.133	0.976	0	0	6.48	0	11	20	3	829.45203	2486.34153	-3.41
sATLSSTESTASGMQDEVR	Unambiguous	2	1	Q9WTQ5	N-Term(iTRAQ8plex)	CID	1.0000	0.0000	1	1	2733683	Unique	Used	1.917	0.216	1.356	1.431	0	0	6.48	0	0	200	2	1131.04602	2261.08476	-0.96
eTAGVLSNDTSDSGVELQGGSR	Unambiguous	1	1	F8VQ95	N-Term(iTRAQ8plex)	CID	1.0000	0.0000	1	1	2634251	Unique	Used	1.803		1.048	0.335	0	0	6.48	0	0	200	2	1242.10852	2483.20976	-2.67

tGIGSGLSLSGIVHPELSR	Unambiguous	2	1	P50544	N-Term(iTRAQ8plex)	CID	1.0000	0.0000	1	1	3821913	Unique	Used	1.106	0.938	0.800	1.227	0	0	6.48	0	14	200	3	728.74481	2184.21988	-4.20
aGQAQADLETQYSALQQR	Unambiguous	2	1	E9QAH1	N-Term(iTRAQ8plex)	CID	1.0000	0.0000	1	1	3189213	Unique	Used	2.149	1.032	1.006	0.872	0	0	6.48	0	0	74	2	1141.58496	2282.16265	-2.30
dSLGDFIEHYAQLGPSSPEQLAAGAEEGGGPR	Unambiguous	1	1	O35295	N-Term(iTRAQ8plex)	HCD	1.0000	0.0000	1	1	3173553	Unique	Used	1.551	0.910	1.088	1.109	0	0	6.48	0	16	69	3	1187.24634	3559.72446	0.01
tALHVAAAEGHVEVVk	Unambiguous	3	1	D3Z7P3	N-Term(iTRAQ8plex); K16(iTRAQ8plex)	HCD	1.0000	0.0000	1	1	3143852	Unique	Used	6.358	1.102	1.204	0.948	0	0	6.47	0	29	24	3	747.10498	2239.30039	-0.69
dQQNLPYGVTPASPSGHSQGR	Unambiguous	14	1	E9QMP6	N-Term(iTRAQ8plex)	CID	1.0000	0.0000	1	1	3227464	Unique	Used	2.652	1.227	1.557	1.372	0	0	6.47	0	0	153	3	834.08533	2500.24143	-2.74
hNNcMASHLTPAVYAR	Unambiguous	5	1	P30275	N-Term(iTRAQ8plex); C4(Methylthio)	CID	1.0000	0.0000	1	1	3870888	Unique	Used	1.299	0.769	1.383	1.449	0	0	6.47	0	0	200	3	712.34406	2135.01761	-3.49
qISQMADDTVAELDQHLAAk	Unambiguous	1	1	Q9D6S7	N-Term(iTRAQ8plex); K20(iTRAQ8plex)	CID	1.0000	0.0000	1	1	3238627	Unique	Used	1.024	0.507	1.390	0.999	0	0	6.47	0	15	108	3	931.49384	2792.46695	-1.43
iGEHTPSALAImENANVLAR	Unambiguous	3	1	A6ZI44	N-Term(iTRAQ8plex); M12(Oxidation)	CID	1.0000	0.0000	1	1	3708147	Unique	Used	1.402	1.261	1.021	1.347	0	0	6.47	0	6	200	3	809.76630	2427.28434	-5.15
eSASSSEVTSAVYNAVSEGTR	Unambiguous	1	1	Q8C145	N-Term(iTRAQ8plex)	CID	1.0000	0.0000	1	1	3170400	Unique	Used	0.890	1.283	1.495	1.307	0	0	6.47	0	0	200	2	1218.09460	2435.18193	-0.85
aAFTVTTPQQQPASQEYGAHLEEER	Unambiguous	5	1	Q5NBX1-2	N-Term(iTRAQ8plex)	CID	1.0000	0.0000	1	1	2916015	Unique	Used	5.554	4.911	3.069	1.429	0	0	6.47	0	12	200	3	1031.50928	3092.51328	-3.06
tVYFAEEVQcEGNSFHk	Unambiguous	1	1	P97315	N-Term(iTRAQ8plex); C10(Methylthio); K17(iTRAQ8plex)	CID	1.0000	0.0000	1	1	3108800	Unique	Used	1.543	0.825	1.425	1.153	0	0	6.47	0	22	200	3	881.43134	2642.27945	-1.71
hEQNIDcGGGYVk	Unambiguous	1	1	P14211	N-Term(iTRAQ8plex); C7(Methylthio); K13(iTRAQ8plex)	CID	1.0000	0.0000	1	1	2931421	Unique	Used	1.111	0.959	0.764	0.662	0	0	6.47	0	0	38	3	692.01343	2074.02573	-2.36
gTGGVDTAAVGGVFDVSNADR	Unambiguous	1	1	Q04447	N-Term(iTRAQ8plex)	CID	1.0000	0.0000	1	1	3149191	Unique	Used		1.428			0	0	6.47	0	0	2	3	757.04950	2269.13395	-1.04

tHnSALEYNIFEGMEcR	Unambiguous	1	1	O08553	N-Term(iTRAQ8plex); N3(Deamidated); C16(Methylthio)	CID	1.0000	0.0000	1	1	3151350	Unique	Used	2.550	1.331	1.171	1.121	0	0	6.47	0	16	157	3	789.02374	2365.05667	0.07
aVAQGNLSSADVQAAk	Unambiguous	1	1	Q9DB77	N-Term(iTRAQ8plex); K16(iTRAQ8plex)	CID	1.0000	0.0000	1	1	3122929	Unique	Used	1.185	1.400	1.500	1.212	0	0	6.47	0	0	65	2	1069.60278	2138.19829	-2.02
sSEYHLEDNAAYYLNDLER	Unambiguous	1	1	O70443	N-Term(iTRAQ8plex)	CID	1.0000	0.0000	1	1	2762562	Unique	Used	0.390	1.424	1.174	1.075	0	0	6.47	0	0	28	3	869.41510	2606.23075	-0.20
dAYGTSSLSSSSnSGSck	Unambiguous	2	1	Q80U23	N-Term(iTRAQ8plex); N13(Deamidated); C17(Methylthio); K18(iTRAQ8plex)	CID	1.0000	0.0000	1	1	2845617	Unique	Used	0.391	0.263	1.424	1.382	0	0	6.47	0	0	200	2	1197.05225	2393.09722	-3.55
mTnGAMNVEIGNPTYk	Unambiguous	1	1	Q91ZX7	N-Term(iTRAQ8plex); N3(Deamidated); K16(iTRAQ8plex)	CID	0.0372	0.0000	1	1	3168231	Unique	Used	0.478	0.939	0.762	0.922	0	0	6.46	0	0	112	2	1175.10242	2349.19756	-2.72
sEGVQDAEPQSLVGSPSTR	Unambiguous	1	1	Q8R4H2	N-Term(iTRAQ8plex)	CID	1.0000	0.0000	1	1	2776331	Unique	Used	0.683	0.704	1.169	0.998	0	0	6.46	0	0	200	2	1124.57141	2248.13555	-0.18
gTGGVDTAAVGGVFDVSNADR	Unambiguous	1	1	Q04447	N-Term(iTRAQ8plex)	CID	1.0000	0.0000	1	1	3130498	Unique	Used	1.728	1.080	1.059	1.352	0	0	6.46	0	0	151	2	1135.07104	2269.13481	-0.66
sIHNLGTILTTnAVASETVATGEGLR	Unambiguous	3	1	Q9ESN6	N-Term(iTRAQ8plex); N12(Deamidated)	CID	1.0000	0.0000	1	1	3392840	Unique	Used	2.192	1.079	1.050	0.794	0	0	6.46	0	0	27	3	977.52606	2930.56363	-3.44
qASHAQLGDAYDQEIR	Unambiguous	2	1	P08553	N-Term(iTRAQ8plex)	CID	1.0000	0.0000	1	1	2681478	Unique	Used	2.255	0.386	1.250	0.834	0	0	6.46	0	5	51	3	702.68616	2106.04392	-3.74
lLEVSDDPQVLVAVAAHDVGEYVR	Unambiguous	1	1	Q8BVE3	N-Term(iTRAQ8plex)	CID	1.0000	0.0000	1	1	3240326	Unique	Used	0.929	1.356	1.782	1.118	0	0	6.46	0	24	200	3	933.83289	2799.48410	0.35
aNNLHSGDNFQLNDSEIER	Unambiguous	2	1	B9EJ86	N-Term(iTRAQ8plex)	CID	1.0000	0.0000	1	1	2698961	Unique	Used	1.404	1.012	1.033	0.691	0	0	6.46	0	25	200	3	826.40167	2477.19046	-2.20
lADELHMPSLPEMMFGDNVLR	Unambiguous	1	1	Q8BH58	N-Term(iTRAQ8plex)	CID	1.0000	0.0000	1	1	3520225	Unique	Used		1.322	1.870	1.629	0	0	6.46	0	20	36	3	907.12012	2719.34580	-3.77
lTLYDIAHTPGVAADLSHIETR	Unambiguous	1	1	P08249	N-Term(iTRAQ8plex)	CID	1.0000	0.0000	1	1	3458139	Unique	Used	4.824	1.236	1.088	1.146	0	0	6.46	0	10	179	3	899.81873	2697.44162	-3.62

dYFSTFGEVLMVQVk	Unambiguous	5	1	Q921F2	N-Term(iTRAQ8plex); K15(iTRAQ8plex)	CID	1.0000	0.0000	1	1	3623300	Unique	Used		1.266	0.859	1.099	0	0	6.46	0	23	23	2	1186.14307	2371.27886	-1.68
yLGTQPEPDIVGLDSGHIR	Unambiguous	1	1	P52196	N-Term(iTRAQ8plex)	CID	1.0000	0.0000	1	1	3169248	Unique	Used	0.808	0.481	0.853	1.392	0	0	6.46	0	1	2	3	791.08905	2371.25260	-1.43
nWRDPDQTDGPGLGYLSSHIAnVER	Unambiguous	2	1	P13020-2	N-Term(iTRAQ8plex); N22(Deamidated)	CID	1.0000	0.0000	1	1	3345277	Unique	Used	2.365	0.763	0.578	0.770	0	0	6.46	1	21	15	3	1034.84460	3102.51926	0.30
rnGSPTPAGALAGGAVGPPGGPGSR	Unambiguous	1	1	Q3U3E2	N-Term(iTRAQ8plex); N2(Deamidated)	CID	1.0000	0.0000	1	1	3717946	Unique	Used	1.443	0.978	1.096	1.138	0	0	6.46	1	0	82	3	821.76483	2463.27994	-8.43
rPAASSAAAASAAASSPAGGGGEAqELLEHcGVcR	Unambiguous	2	1	Q62318	N-Term(iTRAQ8plex); Q25(Deamidated); C31(Methylthio); C34(Methylthio)	CID	1.0000	0.0000	1	1	3684449	Unique	Used	1.615	1.029	0.880	0.963	0	0	6.46	0	29	178	3	1203.22534	3607.66147	-1.39
eRPGEQSEVAQLIQQTLEQER	Unambiguous	2	1	Q6R891	N-Term(iTRAQ8plex)	HCD	1.0000	0.0000	1	1	3110740	Unique	Used	0.968	1.321	1.735	1.281	0	0	6.46	0	0	110	3	924.81793	2772.43924	-1.37
vAVLISGTGSNLQALIDSTR	Unambiguous	1	1	Q64737	N-Term(iTRAQ8plex)	CID	1.0000	0.0000	1	1	3936257	Unique	Used	0.721	0.863	0.904	1.356	0	0	6.46	0	0	49	2	1160.15771	2319.30815	-4.50
sLSTSSSPSSGTPGPSGLAR	Unambiguous	4	1	D3YUI2	N-Term(iTRAQ8plex)	CID	1.0000	0.0000	1	1	3634227	Unique	Used	1.012	0.957	1.763	0.909	0	0	6.46	0	0	125	2	1069.05054	2137.09380	-4.73
aAGALGSASSGSMPNLAAR	Unambiguous	4	1	Q8BIE6	N-Term(iTRAQ8plex)	CID	1.0000	0.0000	1	1	3855095	Unique	Used	0.741	0.421	1.028	0.801	0	0	6.45	0	0	115	2	997.02118	1993.03508	-4.43
gTGGVDTAAVGGVFDVSNADR	Unambiguous	1	1	Q04447	N-Term(iTRAQ8plex)	CID	1.0000	0.0000	1	1	3134738	Unique	Used	0.925	0.966	1.250	1.224	0	0	6.45	0	0	200	2	1135.07043	2269.13359	-1.19
aAMEALQSQALHATSQQPLR	Unambiguous	5	1	B1AQZ0	N-Term(iTRAQ8plex)	CID	1.0000	0.0000	1	1	3838567	Unique	Used	1.321	1.282	1.968	1.307	0	0	6.45	0	7	25	3	819.10248	2455.29288	-4.11
gNVSTISGLSSQTAGAk	Unambiguous	3	1	Q9EPN1-4	N-Term(iTRAQ8plex); K17(iTRAQ8plex)	CID	1.0000	0.0000	1	1	3470888	Unique	Used	0.323	1.391	1.200	0.996	0	0	6.45	0	0	82	2	1093.61365	2186.22002	-1.70
rGQEQLQSAWTGLR	Unambiguous	3	1	E9PX29	N-Term(iTRAQ8plex)	HCD	1.0000	0.0000	1	1	3835660	Unique	Used	1.195	0.869	1.987	1.364	0	0	6.45	1	4	39	3	645.35352	1934.04599	-2.59
yTPSGQSGAAASESLFISNHAY	Unambiguous	2	1	A6ZI44	N-Term(iTRAQ8plex)	CID	1.0000	0.0000	1	1	3258298	Unique	Used	1.562	0.840	1.003	0.982	0	0	6.45	0	0	57	2	1281.62097	2562.23467	-2.64
gDNFETVTWLSDSEVVR	Unambiguous	2	1	G3X997	N-Term(iTRAQ8plex)	CID	1.0000	0.0000	1	1	2977986	Unique	Used	0.501	1.329	0.656	0.906	0	0	6.45	0	0	55	2	1129.56470	2258.12212	-0.97
aGLNEMVEYITHSR	Unambiguous	1	1	Q91V89	N-Term(iTRAQ8plex)	CID	1.0000	0.0000	1	1	3600305	Unique	Used	0.583	0.906	0.871	0.841	0	0	6.45	0	0	43	3	641.99811	1923.97977	-5.34

sSASVTGEPPLYPTGSDSSR	Unambiguous	1	1	Q80U93	N-Term(iTRAQ8plex)	CID	1.0000	0.0000	1	1	2953621	Unique	Used	1.121	0.925	1.200	1.165	0	0	6.45	0	0	200	2	1150.06860	2299.12993	-2.46
aEHIcTYSLSPSELPVASVEk	Unambiguous	2	1	Q9ES97-2	N-Term(iTRAQ8plex); C5(Methylthio); K21(iTRAQ8plex)	CID	1.0000	0.0000	1	1	3348051	Unique	Used	1.189	0.961	1.006	0.789	0	0	6.45	0	14	16	3	972.17206	2914.50162	-4.63
aMVALIDVFHQySGR	Unambiguous	1	1	P50114	N-Term(iTRAQ8plex); Y12(iTRAQ8plex)	HCD	1.0000	0.0000	1	1	3935445	Unique	Used	1.793	0.841	1.564	1.123	0	0	6.45	0	22	37	3	772.42841	2315.27066	-3.64
eVINYEDIEALIGPPPHGPk	Unambiguous	4	1	D3YZN4	N-Term(iTRAQ8plex); K20(iTRAQ8plex)	CID	1.0000	0.0000	1	1	3283845	Unique	Used	0.594	1.162	1.069	1.180	0	0	6.45	0	30	28	3	932.84790	2796.52915	-3.62
kAVLGPLVGAVDQGTSSTR	Unambiguous	4	1	Q64516	N-Term(iTRAQ8plex); K1(iTRAQ8plex)	HCD	1.0000	0.0000	1	1	3857078	Unique	Used	0.728	1.320	1.339	1.313	0	0	6.45	1	27	156	3	822.14771	2464.42856	-2.38
eQASHLGGAVFSGAGNIAAATGLVk	Unambiguous	1	1	Q91ZZ3	N-Term(iTRAQ8plex); K25(iTRAQ8plex)	CID	1.0000	0.0000	1	1	3762774	Unique	Used	1.275	1.063	1.978	1.684	0	0	6.45	0	0	26	3	978.87616	2934.61393	-4.06
vAEFTTNLMEEEEk	Unambiguous	1	1	Q8VDD5	N-Term(iTRAQ8plex); K14(iTRAQ8plex)	CID	1.0000	0.0000	1	1	2639436	Unique	Used	1.697	1.560	1.239	0.664	0	0	6.45	0	14	200	2	1139.58984	2278.17241	-0.41
gTGGVDTAAVGGVFDVSnADR	Unambiguous	1	1	Q04447	N-Term(iTRAQ8plex); N18(Deamidated)	CID	1.0000	0.0000	1	1	2902045	Unique	Used	1.162	0.380	0.822	0.760	0	0	6.45	0	0	200	2	1135.56067	2270.11406	-2.76
aITVASQTNcPLYVTk	Unambiguous	5	1	E9PWE8	N-Term(iTRAQ8plex); C10(Methylthio); K16(iTRAQ8plex)	CID	1.0000	0.0000	1	1	3858350	Unique	Used	0.725	0.342	0.396	0.388	0	0	6.45	0	0	142	2	1182.14868	2363.29009	-0.95
qTTAAAAATFSEQVGGGSGGAGR	Unambiguous	3	1	O88935	N-Term(iTRAQ8plex)	CID	1.0000	0.0000	1	1	3501389	Unique	Used	0.658	1.586	1.435	1.865	0	0	6.45	0	0	200	2	1178.58984	2356.17241	-3.03
qSEDLGSQFTEIFIk	Unambiguous	3	1	Q9Z1Z0	N-Term(iTRAQ8plex); K15(iTRAQ8plex)	CID	1.0000	0.0000	1	1	3216467	Unique	Used	0.468	1.219	0.770	0.999	0	0	6.45	0	11	200	2	1175.63855	2350.26982	-2.25
gDGAALQEMTSQINQk	Unambiguous	2	1	Q8C8T8	N-Term(iTRAQ8plex); K16(iTRAQ8plex)	CID	1.0000	0.0000	1	1	3126745	Unique	Used	2.180	1.643	1.787	1.312	0	0	6.45	0	0	101	2	1150.10852	2299.20976	-3.27

eTGANLAIcQWGFDDEANHLLLQnGLPAVR	Unambiguous	1	1	P80316	N-Term(iTRAQ8plex); C9(Methylthio); N24(Deamidated)	HCD	0.0264	0.0000	1	1	3440100	Unique	Used	0.817	0.803	0.829	0.874	0	0	6.44	0	4	9	3	1206.26025	3616.76621	-4.68
sDGGYTYDTSDLAAIk	Unambiguous	1	1	Q9D0I9	N-Term(iTRAQ8plex); K16(iTRAQ8plex)	CID	1.0000	0.0000	1	1	2884067	Unique	Used	0.247	0.805	0.812	0.918	0	0	6.44	0	0	200	2	1143.08704	2285.16680	-3.92
fGIVTSSAGTGTTEDTEAk	Unambiguous	1	1	Q9D1J3	N-Term(iTRAQ8plex); K19(iTRAQ8plex)	CID	1.0000	0.0000	1	1	2917035	Unique	Used	0.504	0.264	1.107	0.681	0	0	6.44	0	0	200	2	1240.65027	2480.29326	-1.79
aPPQTQLPSGASSGTGSASATHGGAPLPGTR	Unambiguous	8	1	Q3UF95	N-Term(iTRAQ8plex)	CID	1.0000	0.0000	1	1	3697480	Unique	Used	0.787	0.554	1.345	1.133	0	0	6.44	0	5	74	3	1040.86182	3120.57090	-8.58
vAAGHELQPLAIVDQRPSSR	Unambiguous	1	1	P23242	N-Term(iTRAQ8plex)	HCD	1.0000	0.0000	1	1	3699636	Unique	Used	1.073	1.084	1.391	1.742	0	0	6.44	0	0	200	3	816.78723	2448.34714	-6.29
gTGGVDTAATGSVFDISnLDR	Unambiguous	1	1	P30275	N-Term(iTRAQ8plex); N18(Deamidated)	CID	1.0000	0.0000	1	1	2958991	Unique	Used	1.086	1.115	1.575	1.346	0	0	6.44	0	28	200	2	1179.58618	2358.16509	-3.24
fLSQPFQVAEVFTGHMGk	Unambiguous	1	1	P56480	N-Term(iTRAQ8plex); K18(iTRAQ8plex)	CID	1.0000	0.0000	1	1	3881249	Unique	Used	1.183	1.101	0.923	1.058	0	0	6.44	0	0	200	3	877.80933	2631.41343	-3.03
sAVGFNEMEAPTTAYk	Unambiguous	1	1	P49710	N-Term(iTRAQ8plex); K16(iTRAQ8plex)	CID	1.0000	0.0000	1	1	3106317	Unique	Used	1.446	0.784	1.082	0.755	0	0	6.44	0	0	200	2	1162.60425	2324.20122	-1.76
gGPPPPPPPPHSSGPPPPAR	Unambiguous	1	1	Q91YD9	N-Term(iTRAQ8plex)	CID	1.0000	0.0000	1	1	3404677	Unique	Used	1.320	1.297	1.137	0.760	0	0	6.44	0	5	31	3	765.41254	2294.22306	-5.13
aVVTHSSGNHGQALTYAAk	Unambiguous	4	1	Q9QZX7-2	N-Term(iTRAQ8plex); K19(iTRAQ8plex)	CID	1.0000	0.0000	1	1	3569429	Unique	Used	0.274	3.220	1.441	0.859	0	0	6.44	0	27	200	3	840.79364	2520.36637	-4.59
tVYFAEEVQcEGNSFHk	Unambiguous	1	1	P97315	N-Term(iTRAQ8plex); C10(Methylthio); K17(iTRAQ8plex)	CID	1.0000	0.0000	1	1	3129437	Unique	Used	1.806	0.815	1.487	0.996	0	0	6.44	0	16	123	3	881.43323	2642.28513	0.44
tLNAHEHFVTSLDFHk	Unambiguous	1	1	P63005	N-Term(iTRAQ8plex); K16(iTRAQ8plex)	CID	1.0000	0.0000	1	1	3386675	Unique	Used	1.451	0.859	0.791	0.810	0	0	6.44	0	0	75	3	835.45026	2504.33622	-5.76

dIGAIAQVHAEnGDIIAEEQQR	Unambiguous	1	1	O08553	N-Term(iTRAQ8plex); N12(Deamidated)	CID	1.0000	0.0000	1	1	2760403	Unique	Used	1.885	1.008	1.118	1.472	0	0	6.44	0	3	86	3	894.79199	2682.36142	-0.86
ILEVSDDPQVLAVAAHDVGEYVR	Unambiguous	1	1	Q8BVE3	N-Term(iTRAQ8plex)	CID	1.0000	0.0000	1	1	3240748	Unique	Used	1.008	0.990	1.430	1.179	0	0	6.44	0	0	200	3	933.83386	2799.48703	1.40
dGPMcMHDNQGGAPNYYPnSFSAPEQQR	Unambiguous	1	1	P24270	N-Term(iTRAQ8plex); C5(Methylthio); N19(Deamidated)	CID	1.0000	0.0000	1	1	2974156	Unique	Used	3.634	1.215	1.121	1.136	0	0	6.44	0	0	200	3	1154.82068	3462.44748	-2.18
tGPAGGFQAAPHTAAPYSQAAPAR	Unambiguous	1	1	P47708	N-Term(iTRAQ8plex)	CID	1.0000	0.0000	1	1	3634110	Unique	Used	1.650	0.611	1.466	0.833	0	0	6.43	0	0	78	3	867.11163	2599.32035	-4.47
sEDTTGPITGLALTSVNk	Unambiguous	2	1	Q6DFZ1	N-Term(iTRAQ8plex); K18(iTRAQ8plex)	CID	1.0000	0.0000	1	1	3301363	Unique	Used	0.408	0.456	0.649	0.733	0	0	6.43	0	20	200	2	1206.67456	2412.34184	-1.00
gTGGVDTAAVGGVFDVSNADR	Unambiguous	1	1	Q04447	N-Term(iTRAQ8plex)	CID	1.0000	0.0000	1	1	3149664	Unique	Used	1.153	1.388	1.216	1.231	0	0	6.43	0	0	143	2	1135.06995	2269.13262	-1.62
aAAQLLQSQAQQSGAQQTk	Unambiguous	3	1	Q8C1B7-3	N-Term(iTRAQ8plex); K19(iTRAQ8plex)	CID	1.0000	0.0000	1	1	3658488	Unique	Used	1.019	1.057	1.118	1.136	0	0	6.43	0	27	150	3	855.80811	2565.40976	-4.20
dFTSEQIDEILQNHTEIVFAR	Unambiguous	2	1	Q6PIC6	N-Term(iTRAQ8plex)	CID	1.0000	0.0000	1	1	3375257	Unique	Used	0.624	0.976	1.167	1.311	0	0	6.43	0	0	200	3	937.14587	2809.42307	-2.85
eQASHLGGAVFSGAGNIAAATGLVk	Unambiguous	1	1	Q91ZZ3	N-Term(iTRAQ8plex); K25(iTRAQ8plex)	CID	1.0000	0.0000	1	1	3840697	Unique	Used	0.544	0.456	0.855	0.872	0	0	6.43	0	11	193	3	978.87769	2934.61850	-2.50
gDAVGFQEASLAQYSATTAR	Unambiguous	2	1	O88737	N-Term(iTRAQ8plex)	CID	1.0000	0.0000	1	1	3256855	Unique	Used	0.704	1.257	1.608	1.096	0	0	6.43	0	0	98	2	1174.09546	2347.18364	0.18
vVDPLAFADVFVALGPQHR	Unambiguous	1	1	Q80WM4	N-Term(iTRAQ8plex)	CID	1.0000	0.0000	1	1	3879900	Unique	Used	0.968	0.350	0.746	0.926	0	0	6.43	0	11	32	3	785.77405	2355.30759	-2.19
sIEDQLQPNSASGSGALEHDPSSIYLR	Unambiguous	3	1	Q8C547	N-Term(iTRAQ8plex)	CID	1.0000	0.0000	1	1	3089609	Unique	Used	0.908	0.763	1.118	0.875	0	0	6.43	0	10	200	3	1059.19885	3175.58200	0.32
gGPPPPPPPHSSGPPPPAR	Unambiguous	1	1	Q91YD9	N-Term(iTRAQ8plex)	CID	1.0000	0.0000	1	1	3610691	Unique	Used	1.044	1.232	1.211	0.977	0	0	6.43	0	3	52	3	765.41333	2294.22544	-4.09
sAQGTGFELGQLQSIR	Unambiguous	2	1	Q8VDZ4	N-Term(iTRAQ8plex)	CID	1.0000	0.0000	1	1	3640444	Unique	Used	1.087	1.036	1.393	1.096	0	0	6.43	0	0	19	2	998.53693	1996.06658	-5.00
eFEPPSAGAAVTAkPGQEIR	Unambiguous	4	1	Q7TQ95	N-Term(iTRAQ8plex); K14(iTRAQ8plex)	HCD	1.0000	0.0000	1	1	3104559	Unique	Used	1.167	0.831	0.898	0.963	0	0	6.43	0	0	177	3	888.48663	2663.44535	-6.02

sSGPYGGGGQYFAkPR	Unambiguous	3	1	Q5EBP8	N-Term(iTRAQ8plex); K14(iTRAQ8plex)	HCD	1.0000	0.0000	1	1	3633921	Unique	Used	1.635	0.682	0.942	0.739	0	0	6.43	0	24	69	3	746.39722	2237.17710	-6.82
gTGGVDTAATGSVFDISNLDR	Unambiguous	1	1	P30275	N-Term(iTRAQ8plex)	CID	1.0000	0.0000	1	1	3214232	Unique	Used	3.743	1.546	1.061	1.169	0	0	6.43	0	0	100	2	1179.09558	2357.18389	-2.05
gTGGVDTAATGSVFDISNLDR	Unambiguous	1	1	P30275	N-Term(iTRAQ8plex)	CID	1.0000	0.0000	1	1	3214891	Unique	Used	2.218	3.347	3.592	2.747	0	0	6.43	0	0	88	2	1179.09595	2357.18462	-1.74
dLGLPTEAYISVEEVHDDGTPTSk	Unambiguous	1	1	P26516	N-Term(iTRAQ8plex); K24(iTRAQ8plex)	CID	1.0000	0.0000	1	1	2832664	Unique	Used	0.710	0.703	0.714	0.719	0	0	6.43	0	0	200	3	1061.21436	3181.62851	-2.40
sImAAAGVPVVEGYHGk	Unambiguous	2	1	Q99MR8	N-Term(iTRAQ8plex); M3(Oxidation); K17(iTRAQ8plex)	HCD	1.0000	0.0000	1	1	3512786	Unique	Used	0.721	1.101	0.992	1.511	0	0	6.43	0	0	68	3	770.75787	2310.25907	-6.32
gTVVYGEPITASLGTDGSHYWSk	Unambiguous	1	1	O08553	N-Term(iTRAQ8plex); K23(iTRAQ8plex)	CID	1.0000	0.0000	1	1	3370243	Unique	Used	1.387	1.368	1.200	1.178	0	0	6.43	0	11	82	3	1011.85773	3033.55863	-6.33
nLHQSGFSLSGTQVDEGVR	Unambiguous	3	1	E9PWE8	N-Term(iTRAQ8plex)	CID	1.0000	0.0000	1	1	3365381	Unique	Used	2.464	0.787	1.118	0.856	0	0	6.43	0	15	112	3	779.06580	2335.18283	-4.97
vADVSISEVTTLLGnVHSPVVEGYVGENISGEVk	Unambiguous	3	1	P20357	N-Term(iTRAQ8plex); N15(Deamidated); K34(iTRAQ8plex)	CID	0.0124	0.0000	1	1	3420251	Unique	Used	2.627	2.132	2.248	1.308	0	0	6.43	0	0	200	3	1369.73877	4107.20176	1.43
iVLAAAGGVcHNELLELAk	Unambiguous	1	1	Q9CXT8	N-Term(iTRAQ8plex); C10(Methylthio); K19(iTRAQ8plex)	CID	1.0000	0.0000	1	1	3726770	Unique	Used	0.602	0.894	0.675	0.897	0	0	6.43	0	15	38	3	859.15228	2575.44229	-5.36
kTTAASGDLAQAPGAFk	Unambiguous	2	1	P20357	N-Term(iTRAQ8plex); K1(iTRAQ8plex); K17(iTRAQ8plex)	CID	1.0000	0.0000	1	1	3339298	Unique	Used	0.300	0.180	0.190	0.222	0	0	6.43	1	8	8	3	849.49377	2546.46677	-1.50
fQNPQAEGVNAVEDEIk	Unambiguous	2	1	Q3UHJ0-2	N-Term(iTRAQ8plex); K17(iTRAQ8plex)	CID	1.0000	0.0000	1	1	2636864	Unique	Used	0.480	1.048	0.872	1.032	0	0	6.43	0	10	186	3	832.77679	2496.31583	-1.31

gSSGPPVYVDLAYIPNHcSGk	Unambiguous	3	1	Q9QYR6	N-Term(iTRAQ8plex); C18(Methylthio); K21(iTRAQ8plex)	CID	1.0000	0.0000	1	1	3642014	Unique	Used	0.579	0.352	1.853	0.930	0	0	6.43	0	0	12	3	939.14508	2815.42069	-5.72
gTGGVDTAAVGGVFDVSNADR	Unambiguous	1	1	Q04447	N-Term(iTRAQ8plex)	HCD	1.0000	0.0000	1	1	3129388	Unique	Used	1.233	1.118	1.403	1.403	0	0	6.42	0	8	31	2	1135.07043	2269.13359	-1.19
eVLGSSLPNNWQDISLDDGHMk	Unambiguous	1	1	Q8VDT9	N-Term(iTRAQ8plex); K22(iTRAQ8plex)	CID	1.0000	0.0000	1	1	3259608	Unique	Used	1.196	1.249	1.007	0.848	0	0	6.42	0	0	101	3	1021.85541	3063.55167	-4.89
gPPLSEGEELVmDEEAYVLYHR	Unambiguous	3	1	Q810D6	N-Term(iTRAQ8plex); M12(Oxidation)	CID	1.0000	0.0000	1	1	2766231	Unique	Used	2.055	1.605	1.366	0.705	0	0	6.42	0	6	200	3	951.80054	2853.38706	-1.70
aAADLMAYcEAHAk	Unambiguous	1	1	P63213	N-Term(iTRAQ8plex); C9(Methylthio); K14(iTRAQ8plex)	CID	1.0000	0.0000	1	1	3420059	Unique	Used	0.739	1.076	0.966	1.057	0	0	6.42	0	17	200	3	707.02240	2119.05265	-3.24
hIVLSGGSTMYPGLPSR	Unambiguous	1	1	P61161	N-Term(iTRAQ8plex)	CID	1.0000	0.0000	1	1	3912112	Unique	Used	1.909	1.805	1.278	0.827	0	0	6.42	0	10	3	3	692.70911	2076.11277	-4.16
fSHLAADTIVnGAATSHLAPTDPADR	Unambiguous	1	1	Q9JKY5	N-Term(iTRAQ8plex); N11(Deamidated)	CID	1.0000	0.0000	1	1	3257444	Unique	Used	2.751	1.604	1.035	0.783	0	0	6.42	0	0	72	3	985.16931	2953.49338	-0.82
yLVQEMMYEAELEHLQQQR	Unambiguous	1	1	A2AHL1	N-Term(iTRAQ8plex)	CID	1.0000	0.0000	1	1	3216647	Unique	Used	1.389	1.303	1.855	1.187	0	0	6.42	0	26	200	3	914.78851	2742.35099	-0.86
tVHDMDQFGQQQYDIYER	Unambiguous	6	1	Q68FH0	N-Term(iTRAQ8plex)	CID	1.0000	0.0000	1	1	2866183	Unique	Used	1.233	1.244	1.474	1.039	0	0	6.42	0	1	29	3	859.73505	2577.19059	-2.95
dLQQLDDTTqLPLLSHFAETVEGLTTIR	Unambiguous	1	1	B2RUS7	N-Term(iTRAQ8plex); Q10(Deamidated)	CID	0.0249	0.0000	1	1	3623752	Unique	Used	1.040	1.085	0.935	1.304	0	0	6.42	0	0	75	3	1153.94275	3459.81369	-0.71
aASTLGSPLEAQVAPGFAR	Unambiguous	2	1	Q68EF6	N-Term(iTRAQ8plex)	CID	1.0000	0.0000	1	1	3683415	Unique	Used	0.838	1.306	2.117	1.137	0	0	6.42	0	0	15	2	1074.08618	2147.16509	-5.22
eNVNATENcISAVGk	Unambiguous	2	1	Q8BKC5	N-Term(iTRAQ8plex); C9(Methylthio); K15(iTRAQ8plex)	CID	1.0000	0.0000	1	1	3088743	Unique	Used	0.361	0.970	1.076	1.022	0	0	6.42	0	0	200	2	1102.06470	2203.12212	-3.92
gQAGPPESLGVPTAQETSVLR	Unambiguous	4	1	F8VQL9	N-Term(iTRAQ8plex)	HCD	1.0000	0.0000	1	1	3256426	Unique	Used	1.275	0.835	0.864	0.719	0	0	6.42	0	19	123	2	1199.64746	2398.28765	-0.17

sYVVAGSTGSPSVSYYk	Unambiguous	1	1	Q8VD65	N-Term(iTRAQ8plex); K17(iTRAQ8plex)	CID	1.0000	0.0000	1	1	3432782	Unique	Used	0.377	1.198	1.079	1.464	0	0	6.42	0	0	200	2	1180.62915	2360.25102	-3.55
aAADLMAYcEAHAk	Unambiguous	1	1	P63213	N-Term(iTRAQ8plex); C9(Methylthio); K14(iTRAQ8plex)	CID	1.0000	0.0000	1	1	3421070	Unique	Used	1.531	1.771	1.041	1.228	0	0	6.42	0	30	200	3	707.02277	2119.05375	-2.72
hDYVEDSSEGGLSPLPPQPPAR	Unambiguous	2	1	O88737	N-Term(iTRAQ8plex)	CID	1.0000	0.0000	1	1	2809578	Unique	Used	6.219	2.902	1.204	0.983	0	0	6.42	0	1	34	3	884.77814	2652.31986	-0.35
qGSTAVHPQEATQSSWQEEVTQGPHSFQR	Unambiguous	13	1	Q02357-5	N-Term(iTRAQ8plex)	CID	1.0000	0.0000	1	1	3103885	Unique	Used	2.097	0.533	1.279	1.726	0	0	6.42	0	0	140	3	1181.23389	3541.68711	-3.61
qIDHFPEDADYEADTAEYFLR	Unambiguous	1	1	O88602	N-Term(iTRAQ8plex)	CID	1.0000	0.0000	1	1	2672695	Unique	Used	0.323	0.817	0.945	1.075	0	0	6.42	0	0	140	3	950.44427	2849.31827	-0.90
tLVTQNSGVEALIHAILR	Unambiguous	1	1	Q02257	N-Term(iTRAQ8plex)	CID	1.0000	0.0000	1	1	3936074	Unique	Used	0.790	0.762	0.740	0.763	0	0	6.42	0	9	42	3	747.10492	2239.30021	-3.32
sGLGLGAASASTSGSGPADSVmR	Unambiguous	3	1	Q7TSJ2	N-Term(iTRAQ8plex); M22(Oxidation)	CID	1.0000	0.0000	1	1	3614443	Unique	Used	0.710	0.928	0.919	1.031	0	0	6.42	0	0	200	2	1178.58154	2356.15581	-6.75
aFIANFQDNLHSVNR	Unambiguous	2	1	Q9DB40-2	N-Term(iTRAQ8plex)	CID	1.0000	0.0000	1	1	3820499	Unique	Used	0.801	1.072	0.800	0.915	0	0	6.41	0	22	200	3	684.02850	2050.07096	-3.06
vGFGNLEDEFWLGLDNIHR	Unambiguous	2	1	Q8BYI9	N-Term(iTRAQ8plex)	CID	1.0000	0.0000	1	1	3373830	Unique	Used	1.204	1.176	1.356	1.134	0	0	6.41	0	2	2	3	845.76599	2535.28342	-3.96
scSGVEFSTSGHAYTDTGk	Unambiguous	2	1	J3QPE8	N-Term(iTRAQ8plex); C2(Methylthio); K19(iTRAQ8plex)	CID	1.0000	0.0000	1	1	3145230	Unique	Used	1.154	1.001	0.667	0.958	0	0	6.41	0	0	72	3	863.41199	2588.22141	-0.13
lNITPNSGATGnNAGPk	Unambiguous	1	1	Q62433	N-Term(iTRAQ8plex); N12(Deamidated); K17(iTRAQ8plex)	CID	0.0125	0.0000	1	1	3185495	Unique	Used	1.121	0.478	1.439	1.687	0	0	6.41	0	0	18	2	1118.11279	2235.21831	-0.30
lVVTPASSGGDAAGVSFQk	Unambiguous	1	1	Q9DB72	N-Term(iTRAQ8plex); K19(iTRAQ8plex)	CID	1.0000	0.0000	1	1	3412612	Unique	Used	0.542	0.556	1.317	1.488	0	0	6.41	0	28	53	2	1200.17065	2399.33403	-2.12
vTLENFYSnLIAQHEER	Unambiguous	1	1	Q91VJ4	N-Term(iTRAQ8plex); N9(Deamidated)	CID	1.0000	0.0000	1	1	3458097	Unique	Used	1.455	1.184	0.727	0.762	0	0	6.41	0	19	200	3	790.07849	2368.22092	5.17

vNNININQGSITFAGGPGR	Unambiguous	3	1	Q9WTI7-3	N-Term(iTRAQ8plex)	CID	1.0000	0.0000	1	1	3913860	Unique	Used	0.424	1.215	0.667	0.953	0	0	6.41	0	0	200	2	1117.09790	2233.18852	-4.76
gGQTGLQGPGLSVSGPQGHLESESGk	Unambiguous	1	1	E9Q616	N-Term(iTRAQ8plex); K26(iTRAQ8plex)	CID	1.0000	0.0000	1	1	3145063	Unique	Used	1.836	1.099	1.042	0.876	0	0	6.40	0	0	76	3	1024.87756	3072.61814	0.33
aHDLELPALDPDLENDDFFVR	Unambiguous	5	1	E9PYI7	N-Term(iTRAQ8plex)	CID	1.0000	0.0000	1	1	2942054	Unique	Used		3.402	3.004	1.237	0	0	6.40	0	5	48	3	915.79437	2745.36856	0.41
aVAQGNLSSADVQAAk	Unambiguous	1	1	Q9DB77	N-Term(iTRAQ8plex); K16(iTRAQ8plex)	CID	1.0000	0.0000	1	1	3122489	Unique	Used	1.017	1.726	1.065	0.999	0	0	6.40	0	0	59	2	1069.60327	2138.19927	-1.56
ISSGEHIAAFcLTEPASGSDAASIQTR	Unambiguous	1	1	Q8JZN5	N-Term(iTRAQ8plex); C11(Methylthio)	CID	1.0000	0.0000	1	1	3478055	Unique	Used	1.386	1.317	2.774	2.921	0	0	6.40	0	7	62	3	1023.83301	3069.48447	-2.58
nLHQSGFSLSGTQVDEGVR	Unambiguous	3	1	E9PWE8	N-Term(iTRAQ8plex)	CID	1.0000	0.0000	1	1	3318072	Unique	Used	1.592	0.997	0.860	0.903	0	0	6.40	0	30	107	3	779.06659	2335.18521	-3.96
aMVALIDVFHQYSGR	Unambiguous	1	1	P50114	N-Term(iTRAQ8plex)	CID	1.0000	0.0000	1	1	3936936	Unique	Used	3.194	0.847	1.411	1.429	0	0	6.40	0	19	200	3	671.02753	2011.06803	-2.83
kVEEEVInDQALQALSDSLGTR	Unambiguous	6	1	P51125-3	N-Term(iTRAQ8plex); K1(iTRAQ8plex); N8(Deamidated)	CID	1.0000	0.0000	1	1	2941067	Unique	Used	1.530	0.966	1.410	1.183	0	0	6.40	1	9	200	3	1008.87958	3024.62418	-2.26
eAAIQGSLASLEAEQASIR	Unambiguous	1	1	Q8VDC1	N-Term(iTRAQ8plex)	CID	1.0000	0.0000	1	1	3216206	Unique	Used	1.307	0.722	1.316	0.752	0	0	6.40	0	25	104	2	1124.60730	2248.20732	-0.62
aGQAQADLETQYSALQQR	Unambiguous	2	1	E9QAH1	N-Term(iTRAQ8plex)	CID	1.0000	0.0000	1	1	3211364	Unique	Used	2.198	0.898	1.099	0.759	0	0	6.40	0	0	200	2	1141.58752	2282.16777	-0.05
aDIGVAmGIAGSDVSk	Unambiguous	2	1	Q6PIC6	N-Term(iTRAQ8plex); M7(Oxidation); K16(iTRAQ8plex)	CID	1.0000	0.0000	1	1	3277018	Unique	Used	0.645	0.938	1.204	1.170	0	0	6.40	0	0	89	2	1058.07751	2115.14775	-4.68
yTPSGQSGAAASESLFISNHAY	Unambiguous	2	1	A6ZI44	N-Term(iTRAQ8plex)	CID	1.0000	0.0000	1	1	3257305	Unique	Used	1.126	1.121	1.220	1.226	0	0	6.40	0	0	145	2	1281.62317	2562.23906	-0.93
tAAANAAAGAAENAFR	Unambiguous	3	1	E9Q855	N-Term(iTRAQ8plex)	CID	1.0000	0.0000	1	1	3514942	Unique	Used	0.677	0.755	0.884	0.975	0	0	6.40	0	4	11	2	890.96118	1780.91509	-5.25
vSDFGQMASGMnVDAGk	Unambiguous	1	1	P20357	N-Term(iTRAQ8plex); N12(Deamidated); K17(iTRAQ8plex)	CID	1.0000	0.0000	1	1	3034194	Unique	Used	1.590	1.965	2.185	1.149	0	0	6.40	0	0	200	2	1162.07800	2323.14873	-1.38

fGGNPGGFGNQGGFGNSR	Unambiguous	1	1	Q921F2	N-Term(iTRAQ8plex)	CID	1.0000	0.0000	1	1	3873447	Unique	Used			0.706	0.854	0	0	6.40	0	0	53	2	1015.98822	2030.96916	-2.14
gHAPSTAAPAPSPAPMSSSVQSDEER	Unambiguous	1	1	Q8C996	N-Term(iTRAQ8plex)	CID	1.0000	0.0000	1	1	3006170	Unique	Used	1.080	1.162	1.363	1.233	0	0	6.40	0	0	200	3	956.79303	2868.36454	-3.18
wIQDEIEFGYIEAPHk	Unambiguous	1	1	Q08642	N-Term(iTRAQ8plex); K16(iTRAQ8plex)	CID	1.0000	0.0000	1	1	3152198	Unique	Used	0.978	0.409	1.929	2.058	0	0	6.40	0	2	51	3	861.79150	2583.35996	-4.04
gITNLcVIGGDGSLTGADTFR	Unambiguous	1	1	P47857	N-Term(iTRAQ8plex); C6(Methylthio)	CID	1.0000	0.0000	1	1	3727692	Unique	Used	2.245	1.056	0.751	0.970	0	0	6.40	0	12	162	2	1209.10107	2417.19487	-6.56
gTGGVDTAAVGGVFDVSNADR	Unambiguous	1	1	Q04447	N-Term(iTRAQ8plex)	CID	1.0000	0.0000	1	1	3132007	Unique	Used	1.474	1.114	1.033	1.226	0	0	6.40	0	13	200	2	1135.06946	2269.13164	-2.05
dTnGENIAESLVAEGLATR	Unambiguous	2	1	Q78PY7	N-Term(iTRAQ8plex); N3(Deamidated)	CID	1.0000	0.0000	1	1	2782555	Unique	Used	0.562	0.884	0.609	0.765	0	0	6.39	0	0	121	2	1133.07800	2265.14873	-1.12
lLEVSDDPQVLAVAAHDVGEYVR	Unambiguous	1	1	Q8BVE3	N-Term(iTRAQ8plex)	CID	1.0000	0.0000	1	1	3241681	Unique	Used	2.010	1.926	4.384	2.691	0	0	6.39	0	8	200	3	933.83533	2799.49143	2.97
aLLnNSHYYHMAHGk	Unambiguous	1	1	O08749	N-Term(iTRAQ8plex); N4(Deamidated); K15(iTRAQ8plex)	CID	0.0141	0.0000	1	1	3363552	Unique	Used	1.608	1.126	1.439	1.020	0	0	6.39	0	30	200	3	789.07831	2365.22037	-5.41
tNVSGGAIALGHPLGGSGSR	Unambiguous	1	1	Q8BWT1	N-Term(iTRAQ8plex)	CID	1.0000	0.0000	1	1	3909747	Unique	Used	0.910	1.569	0.987	1.333	0	0	6.39	0	16	69	3	704.71741	2112.13767	-4.13
vTVVcEPEDYAGVAAEMHGSDSk	Unambiguous	1	1	Q9CWJ9	N-Term(iTRAQ8plex); C5(Methylthio); K23(iTRAQ8plex)	CID	1.0000	0.0000	1	1	2939720	Unique	Used	0.735	1.095	0.692	1.247	0	0	6.39	0	0	146	3	1016.82202	3048.45151	-1.92
iNAMAAMQNLYETEDEETDkR	Unambiguous	2	1	Q80U19	N-Term(iTRAQ8plex); K20(iTRAQ8plex)	CID	1.0000	0.0000	1	1	2869391	Unique	Used	0.650	0.613	2.679	1.740	0	0	6.39	1	9	200	3	1027.50745	3080.50779	-1.55
tGSPGGPGVGGSPGGGAAGASPSLPPSAk	Unambiguous	1	1	Q8R1Q8	N-Term(iTRAQ8plex); K29(iTRAQ8plex)	CID	1.0000	0.0000	1	1	3364770	Unique	Used				0.523	0	0	6.39	0	0	28	3	980.85553	2940.55204	-3.95
qETSVQGGGALAMNDR	Unambiguous	4	1	E9QP59	N-Term(iTRAQ8plex)	CID	1.0000	0.0000	1	1	2844390	Unique	Used	0.921	0.627	0.917	0.720	0	0	6.39	0	30	200	2	969.48608	1937.96489	-0.22
nVGSTALSPYPTAISGLQSQR	Unambiguous	4	1	Q8R0S4	N-Term(iTRAQ8plex)	CID	1.0000	0.0000	1	1	3875776	Unique	Used	0.703	0.583	1.478	1.469	0	0	6.39	0	0	19	2	1226.15576	2451.30425	-4.21

nLHQSGFSLSGTQVDEGVR	Unambiguous	3	1	E9PWE8	N-Term(iTRAQ8plex)	CID	1.0000	0.0000	1	1	3451985	Unique	Used	1.341	0.986	0.793	0.864	0	0	6.39	0	0	200	3	779.06732	2335.18741	-3.01
vGELTQEAnALAAGHPAQAPAINTR	Unambiguous	1	1	Q68FG2	N-Term(iTRAQ8plex); N9(Deamidated)	HCD	1.0000	0.0000	1	1	3167896	Unique	Used	1.340	1.991	1.977	1.094	0	0	6.39	0	8	92	3	935.83038	2805.47660	-1.13
sIVTDLVSQMDPHGR	Unambiguous	4	1	P58281-2	N-Term(iTRAQ8plex)	CID	1.0000	0.0000	1	1	3643005	Unique	Used	1.001	1.306	0.770	1.093	0	0	6.39	0	7	68	3	653.67682	1959.01590	-5.75
sAVFQQPVIFLGADVTHPPAGDGk	Unambiguous	2	1	Q8CJG1	N-Term(iTRAQ8plex); K24(iTRAQ8plex)	CID	1.0000	0.0000	1	1	3539511	Unique	Used	1.528	1.290	0.783	0.799	0	0	6.39	0	21	78	3	1020.56158	3059.67020	-2.40
aGQQGLSVAFDLATHR	Unambiguous	1	1	P16332	N-Term(iTRAQ8plex)	CID	1.0000	0.0000	1	1	3722805	Unique	Used	0.861	1.363	0.943	1.250	0	0	6.39	0	0	12	3	659.02203	1975.05155	-7.49
tESAYDWTSLSSSSIk	Unambiguous	4	1	F2Z4B7	N-Term(iTRAQ8plex); K16(iTRAQ8plex)	CID	1.0000	0.0000	1	1	3280162	Unique	Used		0.844	1.092	1.018	0	0	6.39	0	0	109	2	1185.61536	2370.22344	-2.15
aESPTPGLTQGTEPGAGQEGAMFVHTR	Unambiguous	2	1	Q6PDG8-2	N-Term(iTRAQ8plex)	CID	1.0000	0.0000	1	1	3030772	Unique	Used	1.213	1.126	0.929	1.045	0	0	6.39	0	0	159	3	1010.83282	3030.48392	-1.80
aYALLQEQTGGIIDAER	Unambiguous	1	1	D3YXK0	N-Term(iTRAQ8plex)	CID	1.0000	0.0000	1	1	3304243	Unique	Used	0.518	0.784	1.204	0.826	0	0	6.39	0	0	155	2	1076.57922	2152.15117	-1.88
fTAGDFSTTVIQNVNk	Unambiguous	1	1	P57746	N-Term(iTRAQ8plex); K16(iTRAQ8plex)	CID	1.0000	0.0000	1	1	3599113	Unique	Used	0.542	2.124	1.144	1.015	0	0	6.39	0	10	34	2	1175.64185	2350.27641	-4.22
iHVIEQTEFIDDQTFLPHR	Unambiguous	2	1	P97393	N-Term(iTRAQ8plex)	HCD	1.0000	0.0000	1	1	3302744	Unique	Used	1.150	0.909	0.900	0.606	0	0	6.39	0	25	106	3	881.46576	2642.38272	-2.02
sVQTNSVQNQGSSSQNTTTPTTk	Unambiguous	3	1	Q0GNC1-3	N-Term(iTRAQ8plex); K23(iTRAQ8plex)	CID	1.0000	0.0000	1	1	3140731	Unique	Used	1.854		0.964	0.764	0	0	6.38	0	0	192	3	1001.84979	3003.53482	-3.04
dLESDIIGDTSGHFQk	Unambiguous	2	1	P14824	N-Term(iTRAQ8plex); K16(iTRAQ8plex)	CID	1.0000	0.0000	1	1	2918126	Unique	Used	1.236	1.202	0.852	0.904	0	0	6.38	0	0	200	2	1185.62402	2370.24077	0.42
sTSIIIMLTDGDANTGESRPEk	Unambiguous	2	1	Q61704	N-Term(iTRAQ8plex); K22(iTRAQ8plex)	CID	1.0000	0.0000	1	1	3150563	Unique	Used	1.058	0.890	0.968	2.030	0	0	6.38	0	10	186	3	981.85474	2943.54966	-1.96
gIQQATAGVSQDTSENANk	Unambiguous	6	1	P06537-3	N-Term(iTRAQ8plex); K19(iTRAQ8plex)	CID	1.0000	0.0000	1	1	2844728	Unique	Used	0.507	0.459	0.439	0.668	0	0	6.38	0	0	200	2	1264.16089	2527.31450	-2.53

akAGAGSATLSMAYAGAR	Unambiguous	1	1	P08249	N-Term(iTRAQ8plex); K2(iTRAQ8plex)	HCD	1.0000	0.0000	1	1	3890487	Unique	Used	1.871	1.301	0.437	0.432	0	0	6.38	1	10	128	3	754.75244	2262.24277	-2.55
gDSALFAVnGFNILVDGGSDR	Unambiguous	3	1	Q9QYR6	N-Term(iTRAQ8plex); N9(Deamidated)	CID	0.0439	0.0000	1	1	3284475	Unique	Used	0.687	1.318	1.384	1.204	0	0	6.38	0	5	18	2	1215.11389	2429.22051	-1.90
gTGGVDTAAVGGVFDVSnADR	Unambiguous	1	1	Q04447	N-Term(iTRAQ8plex); N18(Deamidated)	CID	1.0000	0.0000	1	1	2901945	Unique	Used	1.510	1.011	1.311	1.552	0	0	6.38	0	7	200	2	1135.56018	2270.11308	-3.19
lYGVQFHPEVGLTEnGk	Unambiguous	1	1	Q3THK7	N-Term(iTRAQ8plex); N15(Deamidated); K17(iTRAQ8plex)	CID	1.0000	0.0000	1	1	3169509	Unique	Used	0.958	1.321	1.046	0.878	0	0	6.38	0	26	128	3	833.12213	2497.35184	-1.16
eAFQNAYLELGGLGER	Unambiguous	2	1	Q6PIC6	N-Term(iTRAQ8plex)	CID	1.0000	0.0000	1	1	3354743	Unique	Used	0.719	0.825	1.265	1.340	0	0	6.38	0	22	200	2	1036.03992	2071.07256	-1.77
fLSQPFQVAEVFTGHmGk	Unambiguous	1	1	P56480	N-Term(iTRAQ8plex); M16(Oxidation); K18(iTRAQ8plex)	CID	1.0000	0.0000	1	1	3879247	Unique	Used	0.900	0.842	0.903	1.320	0	0	6.38	0	25	15	3	883.13879	2647.40183	-5.48
eQGYDVIAYLANIGQk	Unambiguous	2	1	P16460	N-Term(iTRAQ8plex); K16(iTRAQ8plex)	CID	1.0000	0.0000	1	1	3560779	Unique	Used	1.055	1.183	1.143	1.510	0	0	6.38	0	4	17	2	1195.65588	2390.30449	-5.49
iEPDSESQEEIIHNIAR	Unambiguous	1	1	P70444	N-Term(iTRAQ8plex)	CID	1.0000	0.0000	1	1	2635832	Unique	Used	1.721	1.189	1.149	1.207	0	0	6.38	0	7	200	3	762.06122	2284.16910	-1.41
gSAGSAAAGPVEAAIR	Unambiguous	1	1	Q9D8S9	N-Term(iTRAQ8plex)	CID	1.0000	0.0000	1	1	3207641	Unique	Used	2.455	0.945	1.544	1.039	0	0	6.38	0	3	4	2	844.96338	1688.91948	-2.30
eALELTDTGLLSGSEER	Unambiguous	1	1	Q9JHU4	N-Term(iTRAQ8plex)	CID	1.0000	0.0000	1	1	2719066	Unique	Used	0.495	0.623	1.022	0.927	0	0	6.38	0	6	200	2	1062.54968	2124.09209	-2.52
nSPSNLSSSSETGSGGGTYR	Unambiguous	2	1	Q8C0T5	N-Term(iTRAQ8plex)	CID	1.0000	0.0000	1	1	2823454	Unique	Used	1.239	2.060	3.090	0.839	0	0	6.38	0	0	179	2	1125.02710	2249.04692	-5.10
qATLGAGLPISTPcTTVNk	Unambiguous	1	1	Q8QZT1	N-Term(iTRAQ8plex); C14(Methylthio); K19(iTRAQ8plex)	CID	1.0000	0.0000	1	1	3841041	Unique	Used	0.572	0.636	0.957	0.885	0	0	6.38	0	0	166	2	1263.69360	2526.37993	-3.21
tTTNVLGDSLGAGIVEHLSR	Unambiguous	1	1	P56564	N-Term(iTRAQ8plex)	CID	1.0000	0.0000	1	1	3663765	Unique	Used	0.852	1.350	1.085	1.396	0	0	6.37	0	0	12	2	1172.63391	2344.26055	-7.22

ecPSDEcGAGVFMGSHFDR	Unambiguous	1	1	P62983	N-Term(iTRAQ8plex); C2(Methylthio); C7(Methylthio)	CID	1.0000	0.0000	1	1	3150577	Unique	Used	1.844	1.125	0.659	0.677	0	0	6.37	0	21	22	3	813.99646	2439.97483	-2.23
gFGDGYnGYGGGPGGGNFGGSPGYGGGR	Unambiguous	2	1	O88569	N-Term(iTRAQ8plex); N7(Deamidated)	CID	1.0000	0.0000	1	1	3070364	Unique	Used	1.116	1.050	1.723	1.704	0	0	6.37	0	28	132	3	934.08026	2800.22623	-1.00
gQPASLLAYLEQAGASPHR	Unambiguous	1	1	Q8R307	N-Term(iTRAQ8plex)	HCD	1.0000	0.0000	1	1	3841640	Unique	Used	0.946	1.012	0.964	1.282	0	0	6.37	0	10	65	3	757.41125	2270.21921	-0.15
tGIGSGLSLSGIVHPELSR	Unambiguous	2	1	P50544	N-Term(iTRAQ8plex)	HCD	1.0000	0.0000	1	1	3802606	Unique	Used	1.125	0.795	0.691	0.899	0	0	6.37	0	0	65	3	728.74475	2184.21970	-4.28
sIVTDLVSQmDPHGR	Unambiguous	4	1	P58281-2	N-Term(iTRAQ8plex); M10(Oxidation)	CID	1.0000	0.0000	1	1	3638158	Unique	Used	0.559	1.085	1.563	1.637	0	0	6.37	0	3	3	3	659.00842	1975.01072	-5.76
sPSGGAAGPLLTPSQSLDGSR	Unambiguous	1	1	Q6WVG3	N-Term(iTRAQ8plex)	CID	1.0000	0.0000	1	1	3433125	Unique	Used	1.275	1.360	0.882	0.681	0	0	6.37	0	0	15	2	1130.09326	2259.17925	-4.02
nASNPLAHYDDTAEEILQQcDGk	Unambiguous	2	1	Q91WT9-2	N-Term(iTRAQ8plex); C20(Methylthio); K23(iTRAQ8plex)	CID	1.0000	0.0000	1	1	2978814	Unique	Used	0.963	0.787	1.087	0.864	0	0	6.37	0	0	89	3	1062.84631	3186.52439	-1.53
dVQTPFLVELEVLDGHEPDGGQR	Unambiguous	1	1	Q9QYR9	N-Term(iTRAQ8plex)	CID	1.0000	0.0000	1	1	2904841	Unique	Used	0.809	1.437	1.215	1.548	0	0	6.37	0	3	157	3	952.15326	2854.44522	-2.57
tVTYEDPQAVGGLASALDNR	Unambiguous	8	1	Q5SRX1-2	N-Term(iTRAQ8plex)	CID	1.0000	0.0000	1	1	3192362	Unique	Used	2.681	1.236	1.620	1.566	0	0	6.37	0	0	69	2	1191.11401	2381.22075	-1.82
tLVPLVEQLFSHLLk	Unambiguous	3	1	P42859	N-Term(iTRAQ8plex); K15(iTRAQ8plex)	HCD	1.0000	0.0000	1	1	3936514	Unique	Used	0.210	0.584	0.959	1.066	0	0	6.37	0	20	124	3	782.48169	2345.43052	-4.77
lHDVNNDGFLDEQELEALFTR	Unambiguous	1	1	P81117	N-Term(iTRAQ8plex)	CID	1.0000	0.0000	1	1	3037650	Unique	Used		2.300	0.890	1.165	0	0	6.37	0	17	24	3	927.13092	2779.37821	-2.13
gLLEPLGLAcTFIDDISAYHk	Unambiguous	1	1	Q08642	N-Term(iTRAQ8plex); C10(Methylthio); K21(iTRAQ8plex)	CID	1.0000	0.0000	1	1	3766848	Unique	Used	0.794	0.337	1.939	1.916	0	0	6.37	0	11	164	3	977.51813	2930.53983	-7.45
aAGALGSASSGSMPNLAAR	Unambiguous	4	1	Q8BIE6	N-Term(iTRAQ8plex)	CID	1.0000	0.0000	1	1	3854973	Unique	Used	0.656	0.270	0.978	0.778	0	0	6.37	0	0	200	2	997.02380	1993.04033	-1.80
eLLALEVFQVSHPR	Unambiguous	1	1	Q9WVL0	N-Term(iTRAQ8plex)	CID	1.0000	0.0000	1	1	3706088	Unique	Used	1.303	1.471	1.460	2.108	0	0	6.37	0	3	4	3	648.03601	1942.09348	-6.65

eAQVSAQHLEVHLk	Unambiguous	8	1	P49025-3	N-Term(iTRAQ8plex); K14(iTRAQ8plex)	HCD	1.0000	0.0000	1	1	3143572	Unique	Used	1.159	0.904	1.295	1.005	0	0	6.37	0	20	149	3	733.08820	2197.25003	-2.24
hQADIASYQDAIQQLDSELR	Unambiguous	1	1	P19246	N-Term(iTRAQ8plex)	CID	1.0000	0.0000	1	1	3131315	Unique	Used	2.466	1.303	1.109	1.084	0	0	6.37	0	21	103	3	869.10919	2605.31302	-1.15
qTTAAAAATFSEQVGGGSGGAGR	Unambiguous	3	1	O88935	N-Term(iTRAQ8plex)	CID	1.0000	0.0000	1	1	3501266	Unique	Used	0.886	1.473	1.536	1.567	0	0	6.37	0	19	200	2	1178.59363	2356.17998	0.18
aIEPPPLDAVIEAEHTLR	Unambiguous	1	1	E9QNN1	N-Term(iTRAQ8plex)	HCD	1.0000	0.0000	1	1	3172405	Unique	Used	0.958	0.971	0.730	0.915	0	0	6.37	0	20	200	3	759.08942	2275.25370	-2.78
aAVQELSGSILTSEDPEER	Unambiguous	1	1	P49769	N-Term(iTRAQ8plex)	CID	1.0000	0.0000	1	1	2813911	Unique	Used	0.579	0.936	0.885	0.908	0	0	6.37	0	7	142	2	1168.09888	2335.19048	-1.14
iYGAPGVEFTGLHR	Unambiguous	1	1	Q80Y17	N-Term(iTRAQ8plex)	CID	1.0000	0.0000	1	1	3681259	Unique	Used	1.001	0.363	0.766	1.061	0	0	6.37	0	10	32	3	607.66644	1820.98478	-6.23
tVTYEDPQAVGGLASALDNR	Unambiguous	8	1	Q5SRX1-2	N-Term(iTRAQ8plex)	CID	1.0000	0.0000	1	1	3150371	Unique	Used	2.869	1.431	1.510	1.126	0	0	6.37	0	0	122	2	1191.11414	2381.22099	-1.72
sGYQQAASEHGLVVIAPDTSPR	Unambiguous	8	1	Q9R0P3	N-Term(iTRAQ8plex)	HCD	1.0000	0.0000	1	1	3253867	Unique	Used	0.818	0.715	1.022	0.905	0	0	6.37	0	4	9	3	863.11804	2587.33957	-0.88
eHINLGcDVDFDIAGPSIR	Unambiguous	3	1	Q60932-2	N-Term(iTRAQ8plex); C7(Methylthio)	CID	1.0000	0.0000	1	1	3353195	Unique	Used	1.515	1.003	1.269	1.105	0	0	6.37	0	4	20	3	807.73047	2421.17685	-3.16
eASSPSSANPASETLQSPSSR	Unambiguous	4	1	Q9R0N8-2	N-Term(iTRAQ8plex)	CID	1.0000	0.0000	1	1	2844112	Unique	Used	0.862		2.077	1.825	0	0	6.37	0	0	200	2	1197.58545	2394.16362	-2.12
nQGGYGGSSSSSSYGSGR	Unambiguous	3	1	Q5EBP8	N-Term(iTRAQ8plex); N1(Deamidated)	CID	0.0157	0.0000	1	1	2840810	Unique	Used	1.186		0.671	0.760	0	0	6.37	0	0	200	2	1000.44556	1999.88384	-2.84
dAAVSPDLGAGGDAPAPAPAPAHTR	Unambiguous	1	1	Q9Z2A7	N-Term(iTRAQ8plex)	CID	1.0000	0.0000	1	1	2880580	Unique	Used	0.720	0.658	0.782	0.836	0	0	6.37	0	0	180	3	862.77704	2586.31656	-1.91
kLDLFANVVHVk	Unambiguous	1	1	Q91VA7	N-Term(iTRAQ8plex); K1(iTRAQ8plex); K12(iTRAQ8plex)	HCD	1.0000	0.0000	1	1	3741659	Unique	Used	0.393	0.787	1.143	1.190	0	0	6.36	1	20	18	3	765.81323	2295.42514	-2.81
eALLSSAVDHGSDEAR	Unambiguous	1	1	P80314	N-Term(iTRAQ8plex)	CID	1.0000	0.0000	1	1	2700351	Unique	Used	0.978	1.067	0.791	1.205	0	0	6.36	0	10	200	3	654.33350	1960.98594	-0.96
yEGSGDGGAAAQSLYIANHAY	Unambiguous	1	1	P05063	N-Term(iTRAQ8plex)	CID	1.0000	0.0000	1	1	2957065	Unique	Used	1.083	0.936	1.236	1.732	0	0	6.36	0	8	200	2	1210.07520	2419.14311	-1.53
eHINLGcDVDFDIAGPSIR	Unambiguous	3	1	Q60932-2	N-Term(iTRAQ8plex); C7(Methylthio)	CID	1.0000	0.0000	1	1	3397174	Unique	Used	3.729	2.216	1.211	1.258	0	0	6.36	0	14	200	3	807.72949	2421.17392	-4.37

eAVcIVLSDDTcSDEk	Unambiguous	1	1	Q01853	N-Term(iTRAQ8plex); C4(Methylthio); C12(Methylthio); K16(iTRAQ8plex)	CID	1.0000	0.0000	1	1	2798575	Unique	Used	0.453	0.434	0.721	1.134	0	0	6.36	0	6	33	3	809.71613	2427.13382	-1.41
gTGGVDTAATGSVFDISNLDR	Unambiguous	1	1	P30275	N-Term(iTRAQ8plex)	CID	1.0000	0.0000	1	1	3263785	Unique	Used	1.689	0.929	1.538	1.462	0	0	6.36	0	27	200	2	1179.09729	2357.18730	-0.60
vETLHDFEAAnSDELNLQR	Unambiguous	1	1	Q7TQF7	N-Term(iTRAQ8plex); N11(Deamidated)	CID	1.0000	0.0000	1	1	2654370	Unique	Used	2.158	1.729	1.973	1.726	0	0	6.36	0	2	200	3	836.08398	2506.23740	0.40
sSGSPYGGGYGSGGGSGGYGSR	Unambiguous	7	1	J3QNY1	N-Term(iTRAQ8plex)	CID	1.0000	0.0000	1	1	2929651	Unique	Used	1.154	0.680	1.469	0.731	0	0	6.36	0	0	166	2	1107.99841	2214.98955	-2.64
mLTGPVYSQSTALTHk	Unambiguous	1	1	Q6ZQ38	N-Term(iTRAQ8plex); K16(iTRAQ8plex)	CID	1.0000	0.0000	1	1	3759117	Unique	Used	0.811	1.463	0.645	0.826	0	0	6.36	0	14	42	3	781.43500	2342.29044	-4.02
yHTSQSGDEMTSLSEYVSR	Unambiguous	2	1	P11499	N-Term(iTRAQ8plex)	CID	1.0000	0.0000	1	1	2991841	Unique	Used	1.379	1.574	1.478	0.983	0	0	6.36	0	15	16	3	827.71960	2481.14426	-2.54
sGLGLGAASASTSGSGPADSVmR	Unambiguous	3	1	Q7TSJ2	N-Term(iTRAQ8plex); M22(Oxidation)	CID	1.0000	0.0000	1	1	3491850	Unique	Used	1.230	1.245	1.834	1.516	0	0	6.36	0	0	151	2	1178.58667	2356.16606	-2.40
vRPcVVYGGADTVQQIR	Unambiguous	1	1	Q62095	N-Term(iTRAQ8plex); C4(Methylthio)	HCD	1.0000	0.0000	1	1	3873962	Unique	Used	1.648	1.639	1.938	2.366	0	0	6.36	0	11	200	3	737.72461	2211.15927	-3.97
mSSTFIGnSTAIQELFk	Unambiguous	1	1	Q9ERD7	N-Term(iTRAQ8plex); N8(Deamidated); K17(iTRAQ8plex)	CID	1.0000	0.0000	1	1	3583712	Unique	Used		4.463	0.972	1.272	0	0	6.36	0	6	200	3	828.44611	2483.32376	-3.01
lENTEDIEEVEQHIQTIR	Unambiguous	3	1	Q8K0B2-3	N-Term(iTRAQ8plex)	CID	1.0000	0.0000	1	1	2671750	Unique	Used	0.737	0.462	1.143	1.105	0	0	6.36	0	1	82	3	834.09937	2500.28354	0.08
eAGFPPGVVnIVPGFGPTAGAAIASHEGVDk	Unambiguous	1	1	P47738	N-Term(iTRAQ8plex); N10(Deamidated); K31(iTRAQ8plex)	CID	1.0000	0.0000	1	1	3458848	Unique	Used	1.287	2.564	0.651	1.035	0	0	6.36	0	0	63	3	1190.97485	3570.91001	1.29
vSTHSQEMDSGAEYGIGSSTk	Unambiguous	8	1	B9EKN8	N-Term(iTRAQ8plex); K21(iTRAQ8plex)	CID	1.0000	0.0000	1	1	2931840	Unique	Used	1.764	1.013	1.108	0.901	0	0	6.36	0	0	163	3	927.12457	2779.35917	-2.65

gGPPPPPPPPHSSGPPPPPAR	Unambiguous	1	1	Q91YD9	N-Term(iTRAQ8plex)	CID	1.0000	0.0000	1	1	3631952	Unique	Used	1.390	1.343	1.328	1.100	0	0	6.36	0	0	200	3	765.41284	2294.22397	-4.73
rGTGGVDTAAVGGVFDVSNADR	Unambiguous	1	1	Q04447	N-Term(iTRAQ8plex)	CID	1.0000	0.0000	1	1	3319574	Unique	Used	1.266		1.492	1.465	0	0	6.36	1	0	0	3	809.08252	2425.23301	-1.82
lAELEEFINGPNnAHIQQVGDR	Unambiguous	2	1	Q68FD5	N-Term(iTRAQ8plex); N13(Deamidated)	CID	0.0283	0.0000	1	1	3153563	Unique	Used	1.265	1.224	1.531	1.605	0	0	6.36	0	3	48	3	923.80713	2769.40683	-1.51
iSGWTQALPDMVVSHLFGk	Unambiguous	1	1	Q62433	N-Term(iTRAQ8plex); K19(iTRAQ8plex)	CID	1.0000	0.0000	1	1	3898746	Unique	Used	0.824	0.583	1.296	1.745	0	0	6.36	0	17	10	3	898.83081	2694.47788	-4.44
sLLAAESAASLQGAELER	Unambiguous	1	1	Q3UHK1	N-Term(iTRAQ8plex)	CID	1.0000	0.0000	1	1	3150792	Unique	Used	0.752	1.094	1.050	1.656	0	0	6.36	0	21	163	2	1060.57739	2120.14751	-1.24
lATPTYGDLNHLVSATMSGVTTSLR	Unambiguous	1	1	Q9ERD7	N-Term(iTRAQ8plex)	CID	1.0000	0.0000	1	1	3917516	Unique	Used	1.359	0.535	0.915	0.708	0	0	6.36	0	13	15	3	970.51276	2909.52372	-3.70
aQGATGEVYcPSGEk	Unambiguous	1	1	Q8K3X4	N-Term(iTRAQ8plex); C10(Methylthio); K15(iTRAQ8plex)	CID	1.0000	0.0000	1	1	2897732	Unique	Used	0.955	1.194	0.863	0.611	0	0	6.36	0	0	200	2	1076.03442	2151.06157	-2.57
aIGFQSQLSEFQVSLHDLEk	Unambiguous	2	1	Q8C163	N-Term(iTRAQ8plex); K20(iTRAQ8plex)	CID	1.0000	0.0000	1	1	3418537	Unique	Used	0.702	1.293	1.485	1.178	0	0	6.36	0	4	6	3	962.19031	2884.55637	-3.53
dNFQDTLQVVTAHYR	Unambiguous	4	1	Q4PZA2-3	N-Term(iTRAQ8plex)	CID	1.0000	0.0000	1	1	3495999	Unique	Used	1.058	1.442	0.898	1.306	0	0	6.36	0	15	61	3	704.36401	2111.07749	-2.30
vSQAASDLLAYcEAHVR	Unambiguous	1	1	P50153	N-Term(iTRAQ8plex); C12(Methylthio)	CID	1.0000	0.0000	1	1	3863324	Unique	Used		1.222	1.364	0.451	0	0	6.36	0	14	200	3	728.36530	2183.08133	-3.56
fLSQPFQVAEVFTGHMGk	Unambiguous	1	1	P56480	N-Term(iTRAQ8plex); K18(iTRAQ8plex)	CID	1.0000	0.0000	1	1	3883223	Unique	Used	1.538	2.416	2.016	3.249	0	0	6.36	0	0	200	3	877.80780	2631.40885	-4.77
sATLSSTESTASGMQDEVR	Unambiguous	2	1	Q9WTQ5	N-Term(iTRAQ8plex)	CID	1.0000	0.0000	1	1	2733842	Unique	Used	2.452	0.300	0.587	0.663	0	0	6.36	0	0	200	2	1131.04297	2261.07866	-3.66
vFEANEEALGVVLHk	Unambiguous	1	1	O35857	N-Term(iTRAQ8plex); K15(iTRAQ8plex)	HCD	1.0000	0.0000	1	1	3260317	Unique	Used	0.528	0.746	0.893	0.972	0	0	6.35	0	7	34	3	755.10144	2263.28977	-0.41
vTGEADVEFATHEDAVAAMAk	Unambiguous	1	1	P70333	N-Term(iTRAQ8plex); K21(iTRAQ8plex)	CID	1.0000	0.0000	1	1	2831961	Unique	Used	1.123	1.096	1.237	0.944	0	0	6.35	0	25	200	3	924.14130	2770.40934	-3.08

rVVDDWANDGWGLk	Unambiguous	1	1	P35564	N-Term(iTRAQ8plex); K14(iTRAQ8plex)	CID	1.0000	0.0000	1	1	3321786	Unique	Used				0.985	0	0	6.35	1	0	92	3	747.07190	2239.20114	-3.08
kGDSSSGGGGGSSGGGGSSnVGGGSSGGSGScSSSSR	Unambiguous	3	1	Q64012-2	K1(iTRAQ8plex); N-Term(iTRAQ8plex); N20(Deamidated); C32(Methylthio)	CID	1.0000	0.0000	1	1	3179198	Unique	Used	0.273	0.355	0.862	1.033	0	0	6.35	1	0	200	3	1203.20178	3607.59079	6.36
sSGNSSSSLGEMVSGTFR	Unambiguous	4	1	Q8R0S4	N-Term(iTRAQ8plex)	CID	1.0000	0.0000	1	1	3639474	Unique	Used	0.518	0.696	1.520	1.034	0	0	6.35	0	0	140	2	1047.50220	2093.99712	-4.99
vSDAISTQYPVVDHEFDAVVVGAGGAGLR	Unambiguous	1	1	Q8K2B3	N-Term(iTRAQ8plex)	CID	1.0000	0.0000	1	1	3355432	Unique	Used	1.346	1.165	1.284	1.293	0	0	6.35	0	15	200	3	1078.55969	3233.66452	-3.09
sQSNESAGSQVGTEFk	Unambiguous	1	1	Q9ERU9	N-Term(iTRAQ8plex); K16(iTRAQ8plex)	CID	1.0000	0.0000	1	1	2730172	Unique	Used	0.774	0.936	1.067	0.756	0	0	6.35	0	0	200	2	1132.58191	2264.15654	-2.20
lMNESLMLVTALNPHIGYDk	Unambiguous	3	1	P97807-2	N-Term(iTRAQ8plex); K20(iTRAQ8plex)	CID	1.0000	0.0000	1	1	3745635	Unique	Used	1.424	0.911	0.817	1.190	0	0	6.35	0	8	53	3	956.51886	2867.54203	-6.97
aVVFETGEAGAASGAGSLPGEVR	Unambiguous	3	1	Q9QYR6	N-Term(iTRAQ8plex)	CID	1.0000	0.0000	1	1	3169783	Unique	Used	2.483	0.952	1.527	1.345	0	0	6.35	0	0	154	2	1218.63550	2436.26372	-1.48
sLAESIDEALncHPSGPMSEEPGSAQLEk	Unambiguous	5	1	E9QAD8	N-Term(iTRAQ8plex); N11(Deamidated); C12(Methylthio); K29(iTRAQ8plex)	CID	1.0000	0.0000	1	1	3037148	Unique	Used		1.568	3.718	2.011	0	0	6.35	0	0	200	3	1227.93079	3681.77781	6.37
vTAGDSEVEAAAqAAGIHDAILSFPEGYETQVGER	Unambiguous	1	1	Q9DC29	N-Term(iTRAQ8plex); Q13(Deamidated)	CID	1.0000	0.0000	1	1	3093574	Unique	Used		1.287		0.865	0	0	6.35	0	0	200	3	1298.64136	3893.90952	2.85
tALHVAAAEGHVEVVk	Unambiguous	3	1	D3Z7P3	N-Term(iTRAQ8plex); K16(iTRAQ8plex)	HCD	1.0000	0.0000	1	1	3164059	Unique	Used	1.110	1.483	1.177	1.041	0	0	6.35	0	0	26	3	747.10321	2239.29508	-3.06
aIEPPPLDAVIEAEHTLR	Unambiguous	1	1	E9QNN1	N-Term(iTRAQ8plex)	CID	1.0000	0.0000	1	1	3152376	Unique	Used	0.226	0.712	0.876	1.257	0	0	6.35	0	0	3	3	759.09070	2275.25754	-1.09
dLESDIIGDTSGHFQk	Unambiguous	2	1	P14824	N-Term(iTRAQ8plex); K16(iTRAQ8plex)	CID	1.0000	0.0000	1	1	2918113	Unique	Used	0.751	0.831	0.915	1.110	0	0	6.35	0	0	200	2	1185.62256	2370.23784	-0.82

kGNVSTISGLSSqTAGAk	Unambiguous	3	1	Q9EPN1-4	N-Term(iTRAQ8plex); K1(iTRAQ8plex); Q13(Deamidated); K18(iTRAQ8plex)	CID	1.0000	0.0000	1	1	3571366	Unique	Used	0.513	1.103	0.919	0.849	0	0	6.35	1	21	200	3	873.84302	2619.51450	2.45
vTQATSNASNDMLPVITGNR	Unambiguous	7	1	F6R4N2	N-Term(iTRAQ8plex)	CID	1.0000	0.0000	1	1	3661971	Unique	Used	0.785	0.808	0.914	0.953	0	0	6.35	0	0	182	2	1197.11584	2393.22441	-6.39
aDIGVAmGIAGSDVSk	Unambiguous	2	1	Q6PIC6	N-Term(iTRAQ8plex); M7(Oxidation); K16(iTRAQ8plex)	CID	1.0000	0.0000	1	1	3254267	Unique	Used	0.308	0.607	1.188	1.429	0	0	6.35	0	0	12	2	1058.08008	2115.15288	-2.26
qFASQANMVGPWIQTk	Unambiguous	1	1	P57780	N-Term(iTRAQ8plex); K16(iTRAQ8plex)	CID	1.0000	0.0000	1	1	3802730	Unique	Used	1.195	1.881	1.682	0.945	0	0	6.35	0	0	160	2	1207.65259	2414.29790	-5.48
tVLSANADHMAQIEGLmDDVDFk	Unambiguous	1	1	Q9JKR6	N-Term(iTRAQ8plex); M17(Oxidation); K23(iTRAQ8plex)	CID	1.0000	0.0000	1	1	3214707	Unique	Used	1.212	1.077	1.087	1.104	0	0	6.35	0	10	141	3	1048.86218	3144.57199	-2.63
lGEHNInVLEGNEQFIDAAk	Unambiguous	1	1	Q9Z1R9	N-Term(iTRAQ8plex); N7(Deamidated); K20(iTRAQ8plex)	CID	1.0000	0.0000	1	1	3107912	Unique	Used	2.317	1.196	0.877	0.723	0	0	6.34	0	0	200	3	940.84283	2820.51395	5.34
yEFTDALLcHDDELEGR	Unambiguous	4	1	D3YTU2	N-Term(iTRAQ8plex); C9(Methylthio)	CID	1.0000	0.0000	1	1	2656276	Unique	Used	1.113	1.076	0.512	1.190	0	0	6.34	0	29	200	3	792.69714	2376.07688	-0.90
sGGPHGAGVLGVHLEGPFISR	Unambiguous	2	1	Q8JZV7	N-Term(iTRAQ8plex)	HCD	1.0000	0.0000	1	1	3819955	Unique	Used	2.163	1.190	1.285	1.425	0	0	6.34	0	25	200	3	783.42828	2348.27030	-3.17
nVVEELLSGNPHIEk	Unambiguous	1	1	Q9JKL5	N-Term(iTRAQ8plex); K15(iTRAQ8plex)	CID	1.0000	0.0000	1	1	3302984	Unique	Used	1.124	1.801	2.069	1.178	0	0	6.34	0	10	128	3	762.76538	2286.28159	-4.30
hVVLPVDDDSDLNVVASFDR	Unambiguous	2	1	Q8BX09-2	N-Term(iTRAQ8plex)	CID	1.0000	0.0000	1	1	3015830	Unique	Used	2.104	0.778	0.865	1.158	0	0	6.34	0	16	61	3	839.43506	2516.29062	-1.15
aVVnGYSASDTVGAGFAQAk	Unambiguous	1	1	Q8K0U4	N-Term(iTRAQ8plex); N4(Deamidated); K20(iTRAQ8plex)	CID	1.0000	0.0000	1	1	3168085	Unique	Used	0.365	0.665	0.651	1.033	0	0	6.34	0	0	18	2	1261.66760	2522.32793	-2.71

fGGNPGGFGNQGGFGNSR	Unambiguous	1	1	Q921F2	N-Term(iTRAQ8plex)	CID	1.0000	0.0000	1	1	3873305	Unique	Used	0.683	0.891	0.692	0.841	0	0	6.34	0	6	31	2	1015.98560	2030.96391	-4.73
hSAEVAGYQDSIGQLESDLR	Unambiguous	1	1	P46660	N-Term(iTRAQ8plex)	CID	1.0000	0.0000	1	1	2901373	Unique	Used	1.114	0.974	1.414	1.268	0	0	6.34	0	2	53	3	827.08209	2479.23172	-2.01
sAYSSYSAPVSSSLSVR	Unambiguous	1	1	P08551	N-Term(iTRAQ8plex)	CID	1.0000	0.0000	1	1	3637114	Unique	Used	3.293	0.243	2.691	2.426	0	0	6.34	0	0	75	2	1026.53162	2052.05596	0.41
dAQVGWIQIVEDLHER	Unambiguous	2	1	E9QKE4	N-Term(iTRAQ8plex)	CID	1.0000	0.0000	1	1	3111616	Unique	Used	1.277	1.009	1.252	1.026	0	0	6.34	0	7	200	3	738.06024	2212.16617	-0.13
rEHANIDAQSGSqAPNPSTTISPGk	Unambiguous	3	1	Q8VDQ8-2	N-Term(iTRAQ8plex); Q13(Deamidated); K25(iTRAQ8plex)	CID	0.0457	0.0000	1	1	3141447	Unique	Used	0.861	0.213	0.983	1.238	0	0	6.34	1	0	200	3	1058.22546	3172.66184	5.50
iQAAASPPANATAASDTNAGDR	Unambiguous	2	1	E9QAQ5	N-Term(iTRAQ8plex)	CID	1.0000	0.0000	1	1	2952046	Unique	Used	0.957	0.782	0.971	0.573	0	0	6.34	0	0	200	2	1187.59937	2374.19145	0.56
dIGAIAQVHAEnGDIIAEEQQR	Unambiguous	1	1	O08553	N-Term(iTRAQ8plex); N12(Deamidated)	CID	0.0473	0.0000	1	1	2760538	Unique	Used	1.813	0.929	1.119	1.310	0	0	6.34	0	10	166	3	894.79156	2682.36014	-1.34
vNIHGGAVSLGHPIGMSGAR	Unambiguous	1	1	Q8QZT1	N-Term(iTRAQ8plex)	HCD	1.0000	0.0000	1	1	3928568	Unique	Used	0.729	0.659	0.769	0.878	0	0	6.34	0	0	196	3	745.40552	2234.20200	-4.94
sGAQATWTEVSWPHEk	Unambiguous	1	1	Q91X72	N-Term(iTRAQ8plex); K16(iTRAQ8plex)	CID	1.0000	0.0000	1	1	3231850	Unique	Used	5.027	2.692	1.368	2.379	0	0	6.34	0	3	12	3	808.08923	2422.25315	-3.32
eQVTNVGGAVVTGVTAVAQk	Unambiguous	2	1	O55042	N-Term(iTRAQ8plex); K20(iTRAQ8plex)	CID	1.0000	0.0000	1	1	3642628	Unique	Used	0.961	2.272	2.513	1.148	0	0	6.34	0	0	103	2	1268.72351	2536.43974	-6.23
fLSQPFQVAEVFTGHMGk	Unambiguous	1	1	P56480	N-Term(iTRAQ8plex); K18(iTRAQ8plex)	CID	1.0000	0.0000	1	1	3882706	Unique	Used	1.113	0.987	1.072	1.264	0	0	6.34	0	22	200	3	877.80865	2631.41141	-3.80
sIGSASPGPGQPPLSSPTR	Unambiguous	1	1	Q8VDZ4	N-Term(iTRAQ8plex)	CID	1.0000	0.0000	1	1	3677746	Unique	Used	1.692	1.071	1.886	0.973	0	0	6.34	0	0	102	2	1049.05872	2097.11016	-6.72
gWDQGLLGMcEGEk	Unambiguous	1	1	P45878	N-Term(iTRAQ8plex); C10(Methylthio); K14(iTRAQ8plex)	CID	1.0000	0.0000	1	1	3262254	Unique	Used		1.433	1.040	0.856	0	0	6.34	0	3	13	3	726.35962	2177.06430	-0.33

vGDSPcIGAGGYADnNLGAVSTTGHGESILk	Unambiguous	1	1	Q8C0M9	N-Term(iTRAQ8plex); C6(Methylthio); N15(Deamidated); K31(iTRAQ8plex)	CID	0.0457	0.0000	1	1	3323166	Unique	Used	2.543	0.723	1.218	1.424	0	0	6.34	0	0	150	3	1205.93311	3615.78476	-0.91
gAIQLGIGYTVGNLSSkPER	Unambiguous	4	1	F8WHW6	N-Term(iTRAQ8plex); K17(iTRAQ8plex)	CID	1.0000	0.0000	1	1	3857027	Unique	Used	0.604	1.258	1.428	1.148	0	0	6.34	0	24	200	3	890.17590	2668.51316	-4.17
hHAAYVNNLNATEEk	Unambiguous	1	1	P09671	N-Term(iTRAQ8plex); K15(iTRAQ8plex)	CID	1.0000	0.0000	1	1	2928243	Unique	Used	0.992	0.660	0.823	0.722	0	0	6.34	0	2	42	3	773.74561	2319.22226	-3.42
dGETIHQQLAQMGDLEAIDVAR	Unambiguous	1	1	P54923	N-Term(iTRAQ8plex)	CID	1.0000	0.0000	1	1	3073395	Unique	Used	0.762	0.845	0.782	0.778	0	0	6.34	0	0	12	3	905.46234	2714.37247	0.10
hQADIASYQDAIQQLDSELR	Unambiguous	1	1	P19246	N-Term(iTRAQ8plex)	CID	1.0000	0.0000	1	1	3073717	Unique	Used	2.591		1.268	0.968	0	0	6.34	0	0	200	3	869.11011	2605.31577	-0.10
tQPTDEEMLFIYSHFk	Unambiguous	4	1	P31786	N-Term(iTRAQ8plex); K16(iTRAQ8plex)	CID	1.0000	0.0000	1	1	3288009	Unique	Used			1.413	1.220	0	0	6.33	0	0	200	3	865.45178	2594.34079	-0.52
sFEAPATINSASLHPEk	Unambiguous	1	1	Q9Z1Z2	N-Term(iTRAQ8plex); K17(iTRAQ8plex)	HCD	1.0000	0.0000	1	1	3230780	Unique	Used	3.273	1.036	0.707	0.628	0	0	6.33	0	11	37	3	803.10461	2407.29929	-3.54
ITLEQGQQLVAEGHPGAnQASTR	Unambiguous	1	1	Q68FG2	N-Term(iTRAQ8plex); N18(Deamidated)	HCD	1.0000	0.0000	1	1	3166959	Unique	Used	1.754	2.190	3.010	1.479	0	0	6.33	0	0	52	3	904.13812	2710.39981	-2.38
gLTIVGSVLEGTFLDNHPQAQR	Unambiguous	2	1	Q91V14	N-Term(iTRAQ8plex)	CID	1.0000	0.0000	1	1	3845257	Unique	Used	1.177	0.772	1.265	1.212	0	0	6.33	0	18	200	3	886.14740	2656.42765	-3.18
gAAALGIGTDSVILIk	Unambiguous	1	1	P48320	N-Term(iTRAQ8plex); K16(iTRAQ8plex)	CID	1.0000	0.0000	1	1	3621985	Unique	Used	0.780	1.101	1.231	1.616	0	0	6.33	0	0	95	2	1054.14258	2107.27788	-8.00
lAQGFGVDHGQVAEQSAGVLQLR	Unambiguous	2	1	Q99J39-2	N-Term(iTRAQ8plex)	CID	1.0000	0.0000	1	1	3662379	Unique	Used	0.918	1.593	1.105	1.112	0	0	6.33	0	11	23	3	895.47919	2684.42301	-7.17
sAHGYIAAHQSNTk	Unambiguous	4	1	Q61036-2	N-Term(iTRAQ8plex); K14(iTRAQ8plex)	HCD	1.0000	0.0000	1	1	3380001	Unique	Used	0.753	0.848	1.050	1.015	0	0	6.33	0	0	200	3	698.37933	2093.12345	-5.43
nmGGPYGGGNYGPGGSGGSGGYGGR	Unambiguous	3	1	O88569	N-Term(iTRAQ8plex); M2(Oxidation)	CID	1.0000	0.0000	1	1	3315010	Unique	Used	0.920	0.773	1.085	1.106	0	0	6.33	0	2	24	3	837.37073	2510.09763	-3.23

qAEIENPLEDPVTGDYVHk	Unambiguous	4	1	Q9Z1G4-3	N-Term(iTRAQ8plex); K19(iTRAQ8plex)	CID	1.0000	0.0000	1	1	2737785	Unique	Used	1.209	1.591	1.022	0.751	0	0	6.33	0	15	200	3	921.48486	2762.44004	-2.07
iQAAASPPAnATAASDTNAGDR	Unambiguous	2	1	E9QAQ5	N-Term(iTRAQ8plex); N10(Deamidated)	CID	1.0000	0.0000	1	1	2754629	Unique	Used	0.849	1.175	0.899	0.767	0	0	6.33	0	16	200	2	1188.08679	2375.16631	-3.30
tESIDVMDAVGSNIVVSTR	Unambiguous	1	1	Q91VD9	N-Term(iTRAQ8plex)	CID	1.0000	0.0000	1	1	3350761	Unique	Used	1.204	0.842	1.016	1.191	0	0	6.33	0	0	49	2	1149.09680	2297.18633	-4.26
dAYSGGAVNLYHVR	Unambiguous	1	1	O55234	N-Term(iTRAQ8plex)	CID	1.0000	0.0000	1	1	3317289	Unique	Used	1.179	0.638	0.800	0.670	0	0	6.33	0	12	4	3	609.31940	1825.94364	-3.41
aYGNLGNTHYLLGnFTEATTFHk	Unambiguous	6	1	Q6IR34-3	N-Term(iTRAQ8plex); N14(Deamidated); K23(iTRAQ8plex)	CID	1.0000	0.0000	1	1	3742677	Unique	Used	1.086	1.062	1.237	1.609	0	0	6.33	0	0	200	3	1060.22168	3178.65049	2.73
rEPVGnDVATILSR	Unambiguous	1	1	Q8VHI6	N-Term(iTRAQ8plex); N6(Deamidated)	HCD	1.0000	0.0000	1	1	3146056	Unique	Used	1.270	1.014	1.510	1.170	0	0	6.33	1	27	37	3	611.34247	1832.01285	-2.80
sYVVAGSTGSPSVSYYk	Unambiguous	1	1	Q8VD65	N-Term(iTRAQ8plex); K17(iTRAQ8plex)	CID	1.0000	0.0000	1	1	3432872	Unique	Used	0.603	1.070	0.983	1.202	0	0	6.33	0	0	200	2	1180.62988	2360.25249	-2.93
yEGSGDGGAAAQSLYIANHAY	Unambiguous	1	1	P05063	N-Term(iTRAQ8plex)	CID	1.0000	0.0000	1	1	3071276	Unique	Used	0.677	0.708	0.700	0.895	0	0	6.33	0	0	200	2	1210.07581	2419.14433	-1.02
qADLYISEGLHPR	Unambiguous	4	1	P80317	N-Term(iTRAQ8plex)	CID	1.0000	0.0000	1	1	3165963	Unique	Used	0.998	1.165	0.914	1.199	0	0	6.33	0	3	2	3	601.66058	1802.96720	-1.71
lMGSAGNATISTVSSTQR	Unambiguous	2	1	Q01815-2	N-Term(iTRAQ8plex)	CID	1.0000	0.0000	1	1	3892685	Unique	Used	0.580	1.738	1.272	0.896	0	0	6.32	0	0	200	2	1043.04419	2085.08110	-4.86
sImAAAGVPVVEGYHGk	Unambiguous	2	1	Q99MR8	N-Term(iTRAQ8plex); M3(Oxidation); K17(iTRAQ8plex)	CID	1.0000	0.0000	1	1	3532866	Unique	Used	0.775	1.800	1.722	1.268	0	0	6.32	0	17	27	3	770.76038	2310.26657	-3.07
nLSNDDSVTDSQMLTAISR	Unambiguous	2	1	M0QWN7	N-Term(iTRAQ8plex)	CID	1.0000	0.0000	1	1	3053341	Unique	Used	0.801	0.618	0.825	1.044	0	0	6.32	0	0	200	2	1186.08594	2371.16460	-2.84
gQGTASPGSVSDLAQTVk	Unambiguous	2	1	P51830	N-Term(iTRAQ8plex); K18(iTRAQ8plex)	CID	1.0000	0.0000	1	1	3253722	Unique	Used	0.716	1.185	2.391	1.684	0	0	6.32	0	0	82	2	1156.13757	2311.26787	-1.54
nEEDATELVGLAQAVnAR	Unambiguous	1	1	Q02053	N-Term(iTRAQ8plex); N16(Deamidated)	CID	0.0396	0.0000	1	1	2741332	Unique	Used	0.755	1.307	0.941	1.003	0	0	6.32	0	3	132	2	1103.06799	2205.12871	-0.65

aTASSSAQEMEQQLAER	Unambiguous	1	1	Q8BV13	N-Term(iTRAQ8plex)	CID	1.0000	0.0000	1	1	2700526	Unique	Used	1.011	0.556	0.592	0.799	0	0	6.32	0	0	200	2	1071.02051	2141.03374	-5.11
vSYFAVFDGHGGIR	Unambiguous	2	1	Q8R0F6	N-Term(iTRAQ8plex)	CID	1.0000	0.0000	1	1	3802622	Unique	Used	0.781	0.946	0.878	0.904	0	0	6.32	0	13	54	3	610.32440	1828.95865	-3.37
aLIFVSHGAGEHcGR	Unambiguous	5	1	O35678	N-Term(iTRAQ8plex); C13(Methylthio)	CID	1.0000	0.0000	1	1	3818391	Unique	Used	0.499	0.775	1.059	0.786	0	0	6.32	0	9	36	3	635.32196	1903.95133	-3.14
sLQDIIAILGMDELSEEDkLTVSR	Unambiguous	1	1	P56480	N-Term(iTRAQ8plex); K19(iTRAQ8plex)	CID	1.0000	0.0000	1	1	3374085	Unique	Used	0.821	1.228	1.201	1.194	0	0	6.32	1	12	17	3	1095.26392	3283.77719	-4.40
nEAIQAAHDSVAQEGGQcR	Unambiguous	1	1	Q9R0P9	N-Term(iTRAQ8plex); C17(Methylthio)	CID	1.0000	0.0000	1	1	3029627	Unique	Used	0.977	0.882	1.159	1.080	0	0	6.32	0	18	68	3	759.69244	2277.06278	-1.17
nSTGSQDGPVSNPSSSNSSQDSLHk	Unambiguous	1	1	B2RXW8	N-Term(iTRAQ8plex); K25(iTRAQ8plex)	CID	1.0000	0.0000	1	1	2773080	Unique	Used			1.132	0.931	0	0	6.32	0	0	200	3	1042.51147	3125.51987	0.21
iASTGGAALIADYGHDGTk	Unambiguous	2	1	Q9CWG8	N-Term(iTRAQ8plex); K19(iTRAQ8plex)	CID	1.0000	0.0000	1	1	3188688	Unique	Used	0.865	0.978	1.174	1.400	0	0	6.32	0	21	33	3	809.44055	2426.30710	-2.69
aVVFETGEAGAASGAGSLPGEVR	Unambiguous	3	1	Q9QYR6	N-Term(iTRAQ8plex)	CID	1.0000	0.0000	1	1	3129865	Unique	Used	13.754	1.169	1.400	1.345	0	0	6.32	0	21	200	2	1218.63281	2436.25835	-3.69
eHGYLGNLSAVASTEGTIEETLnEASR	Unambiguous	2	1	Q99P72	N-Term(iTRAQ8plex); N23(Deamidated)	CID	1.0000	0.0000	1	1	2833795	Unique	Used	3.455		3.120	2.794	0	0	6.32	0	0	94	3	1051.85437	3153.54856	-0.15
ikTDGAAPNVAPSDVGGGGGTNR	Unambiguous	1	1	P12960	N-Term(iTRAQ8plex); K2(iTRAQ8plex)	HCD	1.0000	0.0000	1	1	3206321	Unique	Used	1.003	1.136	1.080	1.016	0	0	6.32	1	13	190	3	907.15454	2719.44907	-3.44
kEELNAISGPNEFAEFYNR	Unambiguous	1	1	Q9D554	N-Term(iTRAQ8plex); K1(iTRAQ8plex)	HCD	1.0000	0.0000	1	1	3213612	Unique	Used	1.282	0.890	0.945	0.681	0	0	6.32	1	4	15	3	946.16083	2836.46793	-1.66
aSNMGMGTDSGTQETk	Unambiguous	1	1	Q8R4U7	N-Term(iTRAQ8plex); K16(iTRAQ8plex)	CID	1.0000	0.0000	1	1	2696374	Unique	Used	0.776		1.511	0.962	0	0	6.32	0	0	200	2	1112.04333	2223.07939	-2.17
qAEGVLSSQEYVLSHTEMPGTLQAADAAlk	Unambiguous	1	1	Q68FG2	N-Term(iTRAQ8plex); K30(iTRAQ8plex)	CID	1.0000	0.0000	1	1	3283067	Unique	Used	0.555	2.312	3.362	1.566	0	0	6.32	0	0	19	3	1251.65942	3752.96372	0.29

gYGYGQGAGTLSTDk	Unambiguous	1	1	P97315	N-Term(iTRAQ8plex); K15(iTRAQ8plex)	CID	1.0000	0.0000	1	1	2970767	Unique	Used	1.992	0.945	1.571	0.837	0	0	6.32	0	0	200	2	1042.04553	2083.08379	-3.77
aQMEQLnGVLLHLESELAQTR	Unambiguous	1	1	P05784	N-Term(iTRAQ8plex); N7(Deamidated)	CID	1.0000	0.0000	1	1	3305260	Unique	Used	0.681	1.477	0.573	0.960	0	0	6.32	0	0	59	3	895.80890	2685.41214	-2.33
dIGAIAQVHAEnGDIIAEEQQR	Unambiguous	1	1	O08553	N-Term(iTRAQ8plex); N12(Deamidated)	CID	1.0000	0.0000	1	1	2669864	Unique	Used	1.188	1.456	0.860	1.327	0	0	6.32	0	23	200	3	894.78961	2682.35428	-3.52
aSAELALGEnNEVLk	Unambiguous	1	1	P05202	N-Term(iTRAQ8plex); N10(Deamidated); K15(iTRAQ8plex)	CID	0.0443	0.0000	1	1	2669936	Unique	Used	1.411	1.550	0.799	1.056	0	0	6.32	0	0	138	2	1084.10486	2167.20244	-1.96
iGEHTPSALAImENANVLAR	Unambiguous	3	1	A6ZI44	N-Term(iTRAQ8plex); M12(Oxidation)	CID	1.0000	0.0000	1	1	3638964	Unique	Used	1.459	1.287	0.851	1.140	0	0	6.31	0	19	53	3	809.76605	2427.28360	-5.45
sPSGGAAGPLLTPSQSLDGSR	Unambiguous	1	1	Q6WVG3	N-Term(iTRAQ8plex)	CID	1.0000	0.0000	1	1	3453390	Unique	Used	0.780	1.475	0.853	0.694	0	0	6.31	0	0	200	2	1130.09717	2259.18706	-0.56
ekQPDNFFAGGEDcVVMVAHESGR	Unambiguous	1	1	P55066	N-Term(iTRAQ8plex); K2(iTRAQ8plex); C14(Methylthio)	HCD	1.0000	0.0000	1	1	3130729	Unique	Used	0.876	0.919	1.351	0.503	0	0	6.31	1	0	78	3	1092.86194	3276.57126	0.47
dFTSEQIDEILQNHTEIVFAR	Unambiguous	2	1	Q6PIC6	N-Term(iTRAQ8plex)	CID	1.0000	0.0000	1	1	3376034	Unique	Used	1.353	0.584	1.072	1.113	0	0	6.31	0	21	200	3	937.14398	2809.41739	-4.87
eHINLGcDVDFDIAGPSIR	Unambiguous	3	1	Q60932-2	N-Term(iTRAQ8plex); C7(Methylthio)	CID	1.0000	0.0000	1	1	3351513	Unique	Used	1.850		0.791	1.374	0	0	6.31	0	7	6	3	807.72998	2421.17539	-3.76
aLVGGAVGGLAGAASk	Unambiguous	2	1	Q5H8C4-2	N-Term(iTRAQ8plex); K16(iTRAQ8plex)	HCD	1.0000	0.0000	1	1	3760437	Unique	Used	0.386	1.075	1.134	1.091	0	0	6.31	0	0	96	2	954.07788	1907.14849	-2.62
nETLGGTcLNVGcIPSk	Unambiguous	1	1	O08749	N-Term(iTRAQ8plex); C8(Methylthio); C13(Methylthio); K17(iTRAQ8plex)	CID	1.0000	0.0000	1	1	3664909	Unique	Used	1.605	1.077	1.234	1.276	0	0	6.31	0	13	91	2	1203.60364	2406.20000	-4.58

iYDAVSGIDTQIIYHk	Unambiguous	1	1	P97927	N-Term(iTRAQ8plex); K16(iTRAQ8plex)	CID	1.0000	0.0000	1	1	3416655	Unique	Used	1.037	0.999	0.835	0.910	0	0	6.31	0	28	72	3	815.45715	2444.35691	-3.13
cEEHDVSLPLSEHNEPWFMR	Unambiguous	2	1	O88741	N-Term(iTRAQ8plex); C1(Methylthio)	CID	1.0000	0.0000	1	1	3192313	Unique	Used	1.140	1.201	1.095	1.405	0	0	6.31	0	0	96	3	935.76251	2805.27298	-0.27
eVYMGNVIQGGEGQAPTR	Unambiguous	1	1	Q8QZT1	N-Term(iTRAQ8plex)	CID	1.0000	0.0000	1	1	3126151	Unique	Used	0.965	0.775	0.868	1.003	0	0	6.31	0	0	7	2	1105.56055	2210.11382	-1.80
gTGGVDTAAVGGVFDVSNADR	Unambiguous	1	1	Q04447	N-Term(iTRAQ8plex)	HCD	1.0000	0.0000	1	1	3149386	Unique	Used	1.246	1.171	1.336	1.399	0	0	6.31	0	0	16	2	1135.06946	2269.13164	-2.05
nEEDATELVGLAQAVNAR	Unambiguous	1	1	Q02053	N-Term(iTRAQ8plex)	CID	1.0000	0.0000	1	1	2959648	Unique	Used	0.971	1.333	1.216	1.156	0	0	6.31	0	0	200	2	1102.57581	2204.14433	-0.81
hEAPMQmASAQDAR	Unambiguous	1	1	P62823	N-Term(iTRAQ8plex); M7(Oxidation)	CID	1.0000	0.0000	1	1	2892157	Unique	Used	0.636	1.003	1.267	1.184	0	0	6.31	0	6	71	3	621.62903	1862.87253	-3.55
nITLSLVANPSHLEAVDPVVQGk	Unambiguous	1	1	E9Q7L0	N-Term(iTRAQ8plex); K23(iTRAQ8plex)	CID	1.0000	0.0000	1	1	3498118	Unique	Used	0.564	0.615	1.120	1.380	0	0	6.31	0	22	15	3	1003.90735	3009.70749	-3.95
aqDIEAGDGTTSVVIIAGSLLDScTkLLQk	Unambiguous	2	1	P80315	N-Term(iTRAQ8plex); Q2(Deamidated); C24(Methylthio); K26(iTRAQ8plex); K30(iTRAQ8plex)	CID	0.0333	0.0000	1	1	3786128	Unique	Used	0.659	1.003	0.924	1.227	0	0	6.30	1	12	200	3	1336.07007	4006.19565	2.50
tGSPGGPGVGGSPGGGAAGASPSLPPSAk	Unambiguous	1	1	Q8R1Q8	N-Term(iTRAQ8plex); K29(iTRAQ8plex)	CID	1.0000	0.0000	1	1	3364637	Unique	Used	1.353	1.181	0.974	0.880	0	0	6.30	0	0	200	3	980.85394	2940.54728	-5.57
aVVTHSSGNHGQALTYAAk	Unambiguous	4	1	Q9QZX7-2	N-Term(iTRAQ8plex); K19(iTRAQ8plex)	CID	1.0000	0.0000	1	1	3654045	Unique	Used	0.612	2.783	2.017	1.658	0	0	6.30	0	29	200	3	840.79187	2520.36106	-6.70
gDVGMAGVAIDTVEDTk	Unambiguous	1	1	P16332	N-Term(iTRAQ8plex); K17(iTRAQ8plex)	CID	1.0000	0.0000	1	1	2762654	Unique	Used	0.506	1.913	1.474	2.094	0	0	6.30	0	9	200	2	1143.60498	2286.20268	-3.56
nLHQSGFSLSGAQIDDNIPR	Unambiguous	1	1	O08553	N-Term(iTRAQ8plex)	CID	1.0000	0.0000	1	1	3453999	Unique	Used	0.847	0.500	0.826	1.104	0	0	6.30	0	15	115	3	825.09290	2473.26413	-3.90

vDISAPDVDVQGPDWHLk	Unambiguous	1	1	E9Q616	N-Term(iTRAQ8plex); K18(iTRAQ8plex)	CID	1.0000	0.0000	1	1	3170286	Unique	Used	1.428	2.273	0.884	0.887	0	0	6.30	0	0	125	3	867.13580	2599.39286	-1.86
nSPPPSSYPSVEnTGGAVAGPHPGGSLLPTDEGLPk	Unambiguous	1	1	Q3U422	N-Term(iTRAQ8plex); N13(Deamidated); K36(iTRAQ8plex)	CID	1.0000	0.0000	1	1	3212487	Unique	Used	3.291	0.817	1.193	0.930	0	0	6.30	0	0	200	3	1365.04333	4093.11545	4.37
tQTAVASEEGPATLPEAEALLAQHAALR	Unambiguous	1	1	Q68FG2	N-Term(iTRAQ8plex)	CID	1.0000	0.0000	1	1	3325068	Unique	Used	1.482	1.211	1.105	1.183	0	0	6.30	0	20	31	3	1050.55884	3149.66196	-3.99
sDIAAAQQSGMSLSnSk	Unambiguous	1	1	B1AQZ0	N-Term(iTRAQ8plex); N15(Deamidated); K17(iTRAQ8plex)	CID	1.0000	0.0000	1	1	2990394	Unique	Used	0.339	1.477	1.729	1.295	0	0	6.30	0	0	193	2	1152.59644	2304.18559	-4.61
hNNcMASHLTPAVYAR	Unambiguous	5	1	P30275	N-Term(iTRAQ8plex); C4(Methylthio)	CID	1.0000	0.0000	1	1	3870905	Unique	Used	1.336	0.734	1.385	1.217	0	0	6.30	0	0	200	3	712.34363	2135.01633	-4.09
nFVAPHLAQPVGNQPLPPGAk	Unambiguous	2	1	Q91YM4-2	N-Term(iTRAQ8plex); K21(iTRAQ8plex)	CID	1.0000	0.0000	1	1	3739721	Unique	Used	0.688	0.865	0.955	0.899	0	0	6.30	0	0	200	3	920.86115	2760.56888	-2.94
vETLHDFEAANSDELNLQR	Unambiguous	1	1	Q7TQF7	N-Term(iTRAQ8plex)	CID	1.0000	0.0000	1	1	2851122	Unique	Used	2.063	1.007	1.306	1.194	0	0	6.30	0	4	127	3	835.75415	2505.24790	-1.79
gVIALcIEDGSIHR	Unambiguous	1	1	Q8K2B3	N-Term(iTRAQ8plex); C6(Methylthio)	CID	1.0000	0.0000	1	1	3518471	Unique	Used	2.087	0.615	0.916	1.243	0	0	6.30	0	5	13	3	611.65741	1832.95768	-4.80
mDTSGTPQTTADSATSPk	Unambiguous	2	1	Q3UX37	N-Term(iTRAQ8plex); K18(iTRAQ8plex)	CID	1.0000	0.0000	1	1	2825100	Unique	Used	0.231		0.497	0.638	0	0	6.30	0	0	200	2	1202.60754	2404.20781	-1.85
nSGVLFENQLLQIGVk	Unambiguous	3	1	P17426-2	N-Term(iTRAQ8plex); K16(iTRAQ8plex)	CID	1.0000	0.0000	1	1	3686268	Unique	Used	0.492	1.165	0.851	0.734	0	0	6.30	0	0	32	2	1184.18945	2367.37163	-5.93
kPIDYTVLDDVGHGVk	Unambiguous	11	1	Q8CBW3-2	N-Term(iTRAQ8plex); K1(iTRAQ8plex); K16(iTRAQ8plex)	CID	1.0000	0.0000	1	1	3188317	Unique	Used	0.518	0.971	1.234	0.806	0	0	6.30	0	9	34	3	890.18488	2668.54007	-1.37
gHAPSTAAPAPSPAPMSSSVQSDEER	Unambiguous	1	1	Q8C996	N-Term(iTRAQ8plex)	CID	1.0000	0.0000	1	1	2843584	Unique	Used	1.178	1.465	0.667	0.972	0	0	6.30	0	0	200	3	956.79242	2868.36271	-3.82

vLHEAEGHIVTcETNTGEVYR	Unambiguous	1	1	P62320	N-Term(iTRAQ8plex); C12(Methylthio)	CID	1.0000	0.0000	1	1	3070495	Unique	Used	1.266	0.663	1.229	1.060	0	0	6.30	0	29	29	3	903.10840	2707.31064	-0.58
sGASTATAVTDVPSGNLAGAGEAGk	Unambiguous	1	1	Q9WUM3	N-Term(iTRAQ8plex); K25(iTRAQ8plex)	CID	1.0000	0.0000	1	1	3010014	Unique	Used	0.915	1.005	1.334	0.930	0	0	6.30	0	3	64	2	1399.23755	2797.46782	-3.96
iLYEENDESEVEIIHVTSPMLETR	Unambiguous	1	1	Q80YA7	N-Term(iTRAQ8plex)	CID	1.0000	0.0000	1	1	2853054	Unique	Used	2.328	1.208	1.467	1.716	0	0	6.30	0	24	200	3	1050.86292	3150.57419	-2.45
sIATLMTDEVFHDVAYk	Unambiguous	3	1	Q5DTL9	N-Term(iTRAQ8plex); K17(iTRAQ8plex)	CID	1.0000	0.0000	1	1	3326516	Unique	Used	0.119	0.946	1.241	0.964	0	0	6.30	0	23	54	3	850.12213	2548.35184	-2.34
gTGGVDTAAVGGVFDVSNADR	Unambiguous	1	1	Q04447	N-Term(iTRAQ8plex)	CID	1.0000	0.0000	1	1	3107749	Unique	Used	2.168	1.159	4.184	7.173	0	0	6.30	0	0	106	2	1135.07056	2269.13384	-1.09
iASLPQEVQDVSLLEk	Unambiguous	5	1	Q9CSU0-2	N-Term(iTRAQ8plex); K16(iTRAQ8plex)	CID	1.0000	0.0000	1	1	3284074	Unique	Used	1.002	0.674	0.903	0.899	0	0	6.30	0	15	25	2	1189.18933	2377.37139	-3.59
cMLDAALATLNAHGk	Unambiguous	1	1	E9PVA8	N-Term(iTRAQ8plex); C1(Methylthio); K15(iTRAQ8plex)	CID	1.0000	0.0000	1	1	3841834	Unique	Used	0.423	0.606	0.604	0.903	0	0	6.29	0	0	77	3	728.38861	2183.15128	-3.80
aLIGMTAGATGAFVGTPAEVALIR	Unambiguous	2	1	Q9CR62	N-Term(iTRAQ8plex)	CID	1.0000	0.0000	1	1	3898583	Unique	Used	0.711	1.421	1.600	1.929	0	0	6.29	0	3	10	2	1296.22473	2591.44219	-4.32
nISVNVLDcDTIGQAk	Unambiguous	1	1	Q9QZC2	N-Term(iTRAQ8plex); C9(Methylthio); K16(iTRAQ8plex)	CID	1.0000	0.0000	1	1	3371761	Unique	Used	0.211	1.569	0.766	0.802	0	0	6.29	0	0	200	2	1172.62231	2344.23735	-3.75
qPIGQPEGPSSEGPGAkPFR	Unambiguous	4	1	A2AFI8	N-Term(iTRAQ8plex); K17(iTRAQ8plex)	HCD	1.0000	0.0000	1	1	3206766	Unique	Used	0.918	1.346	1.528	1.162	0	0	6.29	0	0	142	3	882.14655	2644.42508	-2.01
hIVLSGGSTMYPGLPSR	Unambiguous	1	1	P61161	N-Term(iTRAQ8plex)	CID	1.0000	0.0000	1	1	3930691	Unique	Used	1.831	0.743	0.706	0.764	0	0	6.29	0	3	16	3	692.71014	2076.11588	-2.66
sGTGIAAMSVMRPELIMk	Unambiguous	3	1	D3Z3B2	N-Term(iTRAQ8plex); K18(iTRAQ8plex)	HCD	1.0000	0.0000	1	1	3857896	Unique	Used	0.329	2.978	1.031	0.927	0	0	6.29	0	9	62	3	834.13080	2500.37784	-5.29
qLADHLGFEFFEASAk	Unambiguous	1	1	P63011	N-Term(iTRAQ8plex); K16(iTRAQ8plex)	CID	1.0000	0.0000	1	1	3348989	Unique	Used	0.403	1.346	1.320	1.160	0	0	6.29	0	0	32	2	1209.64087	2418.27446	-7.02

iGEHTPSALAImENANVLAR	Unambiguous	3	1	A6ZI44	N-Term(iTRAQ8plex); M12(Oxidation)	CID	1.0000	0.0000	1	1	3707147	Unique	Used	1.240	1.055	0.973	0.890	0	0	6.29	0	26	138	3	809.76569	2427.28250	-5.90
sIDDLEDQLYHQLEQNR	Unambiguous	3	1	E9Q452	N-Term(iTRAQ8plex)	CID	1.0000	0.0000	1	1	2979258	Unique	Used	3.227	1.782	2.263	0.945	0	0	6.29	0	7	200	3	807.40564	2420.20237	1.15
lLqTAATAAQQGGQANHPTAAVVTEk	Unambiguous	3	1	P42227-2	N-Term(iTRAQ8plex); Q3(Deamidated); K26(iTRAQ8plex)	CID	1.0000	0.0000	1	1	3617519	Unique	Used	0.753	1.169	0.695	1.262	0	0	6.29	0	0	120	3	1062.58447	3185.73886	0.41
tQRPAMVQTEDQYQFcYR	Unambiguous	20	1	Q64487-7	N-Term(iTRAQ8plex); C16(Methylthio)	HCD	1.0000	0.0000	1	1	3473150	Unique	Used	1.165	0.999	1.160	1.075	0	0	6.29	0	0	82	3	872.07428	2614.20829	-2.74
aALSSSELLELGDSLMR	Unambiguous	1	1	Q6ZPS6	N-Term(iTRAQ8plex)	CID	1.0000	0.0000	1	1	3440095	Unique	Used	1.310	1.146	1.185	0.896	0	0	6.29	0	9	28	2	1048.56079	2096.11431	-3.27
sQAAAScPEPLSAAEAPAER	Unambiguous	1	1	B9EJI9	N-Term(iTRAQ8plex); C7(Methylthio)	CID	1.0000	0.0000	1	1	2935968	Unique	Used	1.053	0.668	1.078	1.211	0	0	6.29	0	0	126	2	1153.55273	2306.09819	-3.35
sQLQQAAALNAQHk	Unambiguous	2	1	O70279	N-Term(iTRAQ8plex); K14(iTRAQ8plex)	CID	1.0000	0.0000	1	1	3449567	Unique	Used	1.124	0.879	1.048	0.844	0	0	6.29	0	9	200	3	706.07043	2116.19675	-5.48
gSSGPPVYVDLAYIPNHcSGk	Unambiguous	3	1	Q9QYR6	N-Term(iTRAQ8plex); C18(Methylthio); K21(iTRAQ8plex)	CID	1.0000	0.0000	1	1	3642168	Unique	Used	0.677	0.442	1.418	0.853	0	0	6.29	0	13	21	3	939.14264	2815.41336	-8.32
vSDFGQmASGMNVDAGk	Unambiguous	1	1	P20357	N-Term(iTRAQ8plex); M7(Oxidation); K17(iTRAQ8plex)	CID	1.0000	0.0000	1	1	3105426	Unique	Used	1.897	2.808	1.738	0.886	0	0	6.29	0	0	141	2	1169.58472	2338.16216	-0.29
aLIQYFSPYVSADMHk	Unambiguous	5	1	G3UXW9	N-Term(iTRAQ8plex); K16(iTRAQ8plex)	CID	1.0000	0.0000	1	1	3765010	Unique	Used	1.227	1.350	0.805	1.166	0	0	6.29	0	23	56	3	826.77979	2478.32480	-2.57
aVLDVAETGTEAAAATGVIGGIR	Unambiguous	1	1	P07759	N-Term(iTRAQ8plex)	CID	1.0000	0.0000	1	1	3420540	Unique	Used	2.362	1.903	0.755	1.917	0	0	6.28	0	30	200	2	1223.67505	2446.34282	-1.13
eVGDVIALSDISPSGAADHSQEPSPVGSR	Unambiguous	4	1	F8WJ74	N-Term(iTRAQ8plex)	CID	1.0000	0.0000	1	1	2976073	Unique	Used	1.483	1.006	0.938	0.833	0	0	6.28	0	19	200	3	1061.20056	3181.58713	-1.40
mVISNPAATHQDIDFLIEEIER	Unambiguous	1	1	P48320	N-Term(iTRAQ8plex)	CID	1.0000	0.0000	1	1	3262490	Unique	Used			1.266	1.692	0	0	6.28	0	13	5	3	949.16016	2845.46592	-1.73

hHAAYVNnLNATEEk	Unambiguous	1	1	P09671	N-Term(iTRAQ8plex); N8(Deamidated); K15(iTRAQ8plex)	CID	1.0000	0.0000	1	1	2751061	Unique	Used	1.814	0.931	1.005	0.827	0	0	6.28	0	15	200	3	774.07495	2320.21030	-1.68
sEALSSVLDQEEAMDTk	Unambiguous	5	1	A2AU91	N-Term(iTRAQ8plex); K17(iTRAQ8plex)	CID	1.0000	0.0000	1	1	2638186	Unique	Used	1.219	0.709	1.030	0.724	0	0	6.28	0	15	200	2	1231.13098	2461.25469	-1.70
dLPITEAVFSALVTGHAR	Unambiguous	1	1	Q6PB66	N-Term(iTRAQ8plex)	CID	1.0000	0.0000	1	1	3824592	Unique	Used	0.898	0.695	0.822	1.462	0	0	6.28	0	8	11	3	734.41034	2201.21646	-3.08
gFAFVYFENVDDAk	Unambiguous	2	1	F8WJG3	N-Term(iTRAQ8plex); K14(iTRAQ8plex)	CID	1.0000	0.0000	1	1	3173073	Unique	Used	0.883	1.532	0.925	1.050	0	0	6.28	0	3	94	3	744.05872	2230.16159	-1.12
nIDnPAIADIYTEHAHQVVVAk	Unambiguous	1	1	O88342	N-Term(iTRAQ8plex); N4(Deamidated); K22(iTRAQ8plex)	CID	1.0000	0.0000	1	1	3299532	Unique	Used	1.319	1.114	0.897	0.869	0	0	6.28	0	0	101	3	1009.88531	3027.64139	1.77
tFHETLNccGSnALTTLTTTILR	Unambiguous	1	1	P35762	N-Term(iTRAQ8plex); C8(Methylthio); C9(Methylthio); N12(Deamidated)	CID	0.0414	0.0000	1	1	3898807	Unique	Used			0.669		0	0	6.28	0	8	13	3	969.80096	2907.38834	-4.93
ePcGGTGSTGAGGGTnGGVR	Unambiguous	1	1	Q80WM4	N-Term(iTRAQ8plex); C3(Methylthio); N16(Deamidated)	CID	1.0000	0.0000	1	1	2752333	Unique	Used	0.586	0.269	0.755	1.631	0	0	6.28	0	0	200	2	1021.96088	2042.91448	-1.43
iEYGAVEDGATMTFFk	Unambiguous	3	1	P39087	N-Term(iTRAQ8plex); K16(iTRAQ8plex)	CID	1.0000	0.0000	1	1	3194615	Unique	Used	0.726	1.392	1.089	0.887	0	0	6.28	0	0	185	2	1194.12341	2387.23955	-0.76
lLEVSDDPQVLAVAAHDVGEYVR	Unambiguous	1	1	Q8BVE3	N-Term(iTRAQ8plex)	CID	1.0000	0.0000	1	1	3240016	Unique	Used	1.004	1.084	1.164	1.471	0	0	6.28	0	18	158	3	933.83282	2799.48392	0.29
aDAVQDSEMVELVELEIR	Unambiguous	3	1	Q8BFR5	N-Term(iTRAQ8plex)	CID	1.0000	0.0000	1	1	2673708	Unique	Used	1.632	1.075	1.214	1.343	0	0	6.28	0	0	200	2	1175.60706	2350.20683	-1.96
qAGEVTFADAHRPk	Unambiguous	2	1	O35326	N-Term(iTRAQ8plex); K14(iTRAQ8plex)	HCD	1.0000	0.0000	1	1	3100176	Unique	Used	0.772	1.044	1.067	1.153	0	0	6.28	0	0	161	3	712.39850	2135.18094	-0.41

sGMAQQIAYEMHLEQQSEGELQk	Unambiguous	5	1	A2ACC6	N-Term(iTRAQ8plex); K23(iTRAQ8plex)	CID	1.0000	0.0000	1	1	2995431	Unique	Used	1.093	1.109	1.416	0.647	0	0	6.28	0	0	119	3	1081.87634	3243.61448	-2.79
iMVVGSTINDVLAVNTPk	Unambiguous	1	1	Q78JW9	N-Term(iTRAQ8plex); K18(iTRAQ8plex)	CID	1.0000	0.0000	1	1	3860664	Unique	Used	1.476	0.625	0.727	0.628	0	0	6.28	0	23	11	2	1240.22119	2479.43511	-2.57
gTGGVDTAAVGGVFDVSNADR	Unambiguous	1	1	Q04447	N-Term(iTRAQ8plex)	CID	1.0000	0.0000	1	1	3130805	Unique	Used	1.538	1.018	1.520	1.410	0	0	6.28	0	0	122	2	1135.06885	2269.13042	-2.59
lLSDSLPPSTGTFQEAQSR	Unambiguous	1	1	P26039	N-Term(iTRAQ8plex)	CID	1.0000	0.0000	1	1	3391900	Unique	Used	0.943	1.622	0.671	0.885	0	0	6.28	0	0	96	2	1169.61145	2338.21562	-1.56
vSHALAEGLGVIAcIGEk	Unambiguous	2	1	P17751	N-Term(iTRAQ8plex); C14(Methylthio); K18(iTRAQ8plex)	CID	1.0000	0.0000	1	1	3707921	Unique	Used	1.053	1.500	0.794	1.183	0	0	6.28	0	10	200	3	807.78217	2421.33194	-5.59
yFATVSGSASEANVEEk	Unambiguous	3	1	D3YZ62	N-Term(iTRAQ8plex); K17(iTRAQ8plex)	CID	1.0000	0.0000	1	1	2848270	Unique	Used	0.333	0.565	0.857	0.872	0	0	6.28	0	0	98	2	1199.11902	2397.23076	-3.62
lATPTYGDLNHLVSATMSGVTTSLR	Unambiguous	1	1	Q9ERD7	N-Term(iTRAQ8plex)	CID	1.0000	0.0000	1	1	3935970	Unique	Used	2.027	0.701	0.555	0.614	0	0	6.28	0	10	7	3	970.51208	2909.52170	-4.40
gLTIVGSVLEGTFLDNHPQAQR	Unambiguous	2	1	Q91V14	N-Term(iTRAQ8plex)	CID	1.0000	0.0000	1	1	3845151	Unique	Used		0.544	1.879	1.070	0	0	6.28	0	5	200	3	886.14795	2656.42929	-2.56
cFSIDNPGYEPEVVAVHPGGDTVAVGGTDGNVR	Unambiguous	1	1	O88342	N-Term(iTRAQ8plex); C1(Methylthio)	CID	1.0000	0.0000	1	1	3172054	Unique	Used	1.122	0.528	1.034	1.321	0	0	6.28	0	22	200	3	1226.91895	3678.74228	-1.32
gHAPSTAAPAPSPAPmSSSVQSDEER	Unambiguous	1	1	Q8C996	N-Term(iTRAQ8plex); M16(Oxidation)	CID	1.0000	0.0000	1	1	2842336	Unique	Used	1.496	0.806	0.877	0.329	0	0	6.28	0	0	75	3	962.12421	2884.35807	-3.64
gVQGIIVVNTEGIPlk	Unambiguous	2	1	P62627	N-Term(iTRAQ8plex); K16(iTRAQ8plex)	CID	1.0000	0.0000	1	1	3599910	Unique	Used	1.776	1.155	1.220	1.955	0	0	6.28	0	0	161	2	1123.18127	2245.35527	-8.36
aTGGPYGSLGVDYEFEk	Unambiguous	1	1	Q8K0U4	N-Term(iTRAQ8plex); K17(iTRAQ8plex)	CID	1.0000	0.0000	1	1	3013559	Unique	Used	0.636	1.065	1.168	1.162	0	0	6.28	0	0	139	2	1199.62085	2398.23442	-1.79
iPNIYAIGDVVAGPMLAHk	Unambiguous	1	1	O08749	N-Term(iTRAQ8plex); K19(iTRAQ8plex)	CID	1.0000	0.0000	1	1	3842140	Unique	Used	1.623	1.523	1.258	0.953	0	0	6.28	0	7	12	3	863.16492	2587.48020	-3.44
gSQSVEGAGVMAGEPPSHSSGGR	Unambiguous	2	1	Q6PGL7	N-Term(iTRAQ8plex)	CID	1.0000	0.0000	1	1	2911638	Unique	Used	1.365	0.762	1.278	0.954	0	0	6.28	0	7	74	3	815.72711	2445.16678	-2.58

aLLNNSHYYHMAHGk	Unambiguous	1	1	O08749	N-Term(iTRAQ8plex); K15(iTRAQ8plex)	CID	1.0000	0.0000	1	1	3655037	Unique	Used	1.231	0.891	1.387	1.167	0	0	6.28	0	0	101	3	788.75098	2364.23838	-4.55
hDIAFVEFENDGQAGAAR	Unambiguous	1	1	Q9CQI7	N-Term(iTRAQ8plex)	CID	1.0000	0.0000	1	1	2777669	Unique	Used	3.106	1.291	1.127	0.888	0	0	6.28	0	22	200	3	751.03961	2251.10428	-0.14
eYLQTHWEEEEcQQDVSLLR	Unambiguous	1	1	Q8BGB7	N-Term(iTRAQ8plex); C12(Methylthio)	CID	1.0000	0.0000	1	1	2852138	Unique	Used	0.960	1.504	1.628	1.195	0	0	6.28	0	16	91	3	962.45056	2885.33713	-0.58
lHDVNNDGFLDEQELEALFTR	Unambiguous	1	1	P81117	N-Term(iTRAQ8plex)	CID	1.0000	0.0000	1	1	3017769	Unique	Used			0.487	0.988	0	0	6.27	0	13	38	3	927.13184	2779.38095	-1.14
lVAEDIPLLFSLLSDVFPGVQYHR	Unambiguous	1	1	Q9JHU4	N-Term(iTRAQ8plex)	HCD	1.0000	0.0000	1	1	3899703	Unique	Used	0.681	0.870	0.932	0.951	0	0	6.27	0	19	96	3	1011.56049	3032.66690	-3.11
lSAQAALSHEYFSDLPPR	Unambiguous	3	1	O35495	N-Term(iTRAQ8plex)	HCD	1.0000	0.0000	1	1	3496406	Unique	Used	1.260	1.232	1.105	1.103	0	0	6.27	0	0	200	3	769.40497	2306.20035	-3.45
tNIVTASVDAInFHDk	Unambiguous	5	1	Q91V12-2	N-Term(iTRAQ8plex); N12(Deamidated); K16(iTRAQ8plex)	CID	1.0000	0.0000	1	1	3129571	Unique	Used	0.819	0.480	1.117	1.099	0	0	6.27	0	0	50	3	785.42975	2354.27469	-2.78
lVTLPEAIHFLTEIQVLDVR	Unambiguous	1	1	Q9JJ28	N-Term(iTRAQ8plex)	CID	1.0000	0.0000	1	1	3861232	Unique	Used	0.452	0.853	1.417	1.364	0	0	6.27	0	0	20	3	870.84100	2610.50846	-3.39
mDHFEGPPPNYVNTYPTDLSVGAGPAILR	Unambiguous	4	1	Q8BPN8-2	N-Term(iTRAQ8plex)	CID	1.0000	0.0000	1	1	3478524	Unique	Used	0.978	1.101	0.849	0.903	0	0	6.27	0	0	186	3	1144.90991	3432.71518	-1.43
qPDNFFAGGEDcVVMVAHESGR	Unambiguous	1	1	P55066	N-Term(iTRAQ8plex); C12(Methylthio)	CID	1.0000	0.0000	1	1	3326500	Unique	Used	1.376	1.241	1.279	1.184	0	0	6.27	0	14	46	3	905.74591	2715.22318	-1.33
tIGGGDDSFNTFFSETGAGk	Unambiguous	4	1	P68373	N-Term(iTRAQ8plex); K20(iTRAQ8plex)	CID	1.0000	0.0000	1	1	3072790	Unique	Used	0.743	2.201	1.004	1.448	0	0	6.27	0	14	200	2	1308.65698	2616.30669	1.08
aISFVGSNQAGEYIFER	Unambiguous	1	1	Q9EQ20	N-Term(iTRAQ8plex)	CID	1.0000	0.0000	1	1	3438319	Unique	Used	0.491	1.518	1.127	1.452	0	0	6.27	0	0	3	2	1096.56396	2192.12065	-3.80
lLIQVGHEPMPPTLGTNVLGR	Unambiguous	2	1	Q791T5-2	N-Term(iTRAQ8plex)	HCD	1.0000	0.0000	1	1	3897505	Unique	Used	0.920	1.123	0.901	0.968	0	0	6.27	0	0	86	3	849.48175	2546.43070	-4.87
dGNVLLHEMQIQHPTASLIAk	Unambiguous	4	1	P80317	N-Term(iTRAQ8plex); K21(iTRAQ8plex)	CID	1.0000	0.0000	1	1	3495862	Unique	Used	0.808	0.936	0.897	0.989	0	0	6.27	0	14	78	3	975.20966	2923.61441	-4.80
iDQYQGADAVGLEEk	Unambiguous	1	1	Q8CDN6	N-Term(iTRAQ8plex); K15(iTRAQ8plex)	CID	1.0000	0.0000	1	1	2635164	Unique	Used	1.363	1.140	0.963	0.789	0	0	6.27	0	22	200	2	1122.60291	2244.19853	0.75

sQPPAAHAGQVYGEQPPk	Unambiguous	3	1	Q6P9K8	N-Term(iTRAQ8plex); K18(iTRAQ8plex)	CID	1.0000	0.0000	1	1	2950242	Unique	Used	0.665	0.589	1.013	0.964	0	0	6.27	0	0	84	3	824.11395	2470.32730	-1.06
aVGQPAAAAGHFQR	Unambiguous	2	1	Q00558	N-Term(iTRAQ8plex)	CID	1.0000	0.0000	1	1	3653953	Unique	Used	0.663	1.161	1.428	1.103	0	0	6.27	0	15	39	3	562.30774	1684.90866	-5.86
vSSDQLSSENLTSAVGQk	Unambiguous	1	1	Q8CC88	N-Term(iTRAQ8plex); K18(iTRAQ8plex)	CID	1.0000	0.0000	1	1	3188212	Unique	Used			0.770	1.085	0	0	6.27	0	0	77	2	1229.66687	2458.32646	0.77
yFDSFGDLSSASAIMGNPk	Unambiguous	1	1	P02089	N-Term(iTRAQ8plex); K19(iTRAQ8plex)	CID	1.0000	0.0000	1	1	3540871	Unique	Used	2.715	21.141	0.568	0.676	0	0	6.26	0	10	25	2	1308.16333	2615.31938	-3.00
mLTGPVYSQSTALTHk	Unambiguous	1	1	Q6ZQ38	N-Term(iTRAQ8plex); M1(Oxidation); K16(iTRAQ8plex)	CID	1.0000	0.0000	1	1	3698570	Unique	Used	1.191	1.373	0.957	1.267	0	0	6.26	0	23	140	3	786.76337	2358.27555	-8.15
yEGSGDGGAAAQSLYIANHAY	Unambiguous	1	1	P05063	N-Term(iTRAQ8plex)	CID	1.0000	0.0000	1	1	2975454	Unique	Used	1.221	0.668	0.859	1.493	0	0	6.26	0	4	97	2	1210.07593	2419.14458	-0.92
nYLPAINGIVFLVDcADHSR	Unambiguous	2	1	Q99JZ4	N-Term(iTRAQ8plex); C15(Methylthio)	CID	1.0000	0.0000	1	1	3861060	Unique	Used			1.071	1.223	0	0	6.26	0	0	64	3	856.43451	2567.28897	-6.35
eITENLmATGDLDQDGk	Unambiguous	5	1	Q61233	N-Term(iTRAQ8plex); M7(Oxidation); K17(iTRAQ8plex)	CID	1.0000	0.0000	1	1	2635353	Unique	Used	2.234	1.360	1.706	1.478	0	0	6.26	0	0	200	3	825.42053	2474.24704	-2.87
eTAAAHQASSSPPIDAATAEPYGFR	Unambiguous	1	1	P14873	N-Term(iTRAQ8plex)	CID	1.0000	0.0000	1	1	3187081	Unique	Used	6.675	1.125	1.118	1.035	0	0	6.26	0	25	115	3	950.47144	2849.39975	-0.38
vTAQGPGLEPSGNIANk	Unambiguous	4	1	B7FAU9	N-Term(iTRAQ8plex); K17(iTRAQ8plex)	CID	1.0000	0.0000	1	1	3230242	Unique	Used	0.537	3.826	0.653	1.068	0	0	6.26	0	0	37	2	1131.13623	2261.26518	-2.59
vFEANEEALGVVLHk	Unambiguous	1	1	O35857	N-Term(iTRAQ8plex); K15(iTRAQ8plex)	CID	1.0000	0.0000	1	1	3260497	Unique	Used	0.444	0.720	0.744	0.900	0	0	6.26	0	23	22	3	755.10162	2263.29032	-0.17
sVVEFLQGYIGIPHGGFPEPFR	Unambiguous	3	1	G5E8R3	N-Term(iTRAQ8plex)	CID	1.0000	0.0000	1	1	3898713	Unique	Used	0.648	1.490	0.749	1.379	0	0	6.26	0	16	2	3	917.48828	2750.45029	-3.84

sQPNHTPSGPPGPSSNPVSnGk	Unambiguous	2	1	Q8R2Y9	N-Term(iTRAQ8plex); N20(Deamidated); K22(iTRAQ8plex)	CID	0.0415	0.0000	1	1	2967756	Unique	Used	0.940	0.705	0.949	0.644	0	0	6.26	0	0	200	3	918.14240	2752.41263	0.55
gTGGVDTAAVGGVFDVSNADR	Unambiguous	1	1	Q04447	N-Term(iTRAQ8plex)	CID	1.0000	0.0000	1	1	3134530	Unique	Used	1.088	1.185	1.507	1.685	0	0	6.26	0	8	200	2	1135.07043	2269.13359	-1.19
yEGSGDGGAAAQSLYIANHAY	Unambiguous	1	1	P05063	N-Term(iTRAQ8plex)	CID	1.0000	0.0000	1	1	2937777	Unique	Used	1.793	0.393	1.124	2.044	0	0	6.26	0	5	29	2	1210.07202	2419.13677	-4.15
lEVGIQAMEHLIHVLQTDR	Unambiguous	3	1	Q9Z1Z0	N-Term(iTRAQ8plex)	CID	1.0000	0.0000	1	1	3861042	Unique	Used	0.590	1.358	0.772	0.881	0	0	6.26	0	3	12	3	836.12646	2506.36484	-4.22
eLQLESLLGAFESLGk	Unambiguous	2	1	Q6PDI5	N-Term(iTRAQ8plex); K16(iTRAQ8plex)	CID	1.0000	0.0000	1	1	3561326	Unique	Used	1.482	1.385	0.610	0.857	0	0	6.26	0	24	139	2	1171.66748	2342.32768	-6.45
gTGGVDTAATGSVFDISNLDR	Unambiguous	1	1	P30275	N-Term(iTRAQ8plex)	CID	1.0000	0.0000	1	1	3263896	Unique	Used	1.013	0.901	1.141	1.210	0	0	6.26	0	0	200	2	1179.09863	2357.18999	0.54
eHINLGcDVDFDIAGPSIR	Unambiguous	3	1	Q60932-2	N-Term(iTRAQ8plex); C7(Methylthio)	CID	1.0000	0.0000	1	1	3374560	Unique	Used	1.441	1.164	1.050	1.041	0	0	6.26	0	11	200	3	807.73181	2421.18088	-1.50
gHIQLSYTDGDDcGSDk	Unambiguous	1	1	Q07113	N-Term(iTRAQ8plex); C13(Methylthio); K17(iTRAQ8plex)	CID	1.0000	0.0000	1	1	2667178	Unique	Used	1.822	1.185	1.129	0.884	0	0	6.26	0	0	200	3	822.38818	2465.15000	-1.36
fLSQPFQVAEVFTGHmGk	Unambiguous	1	1	P56480	N-Term(iTRAQ8plex); M16(Oxidation); K18(iTRAQ8plex)	CID	1.0000	0.0000	1	1	3805680	Unique	Used	1.773	1.468	0.934	1.112	0	0	6.26	0	8	12	3	883.14093	2647.40824	-3.06
tIHQEDELIEIQSDTGTWYQR	Unambiguous	1	1	Q9JIS5	N-Term(iTRAQ8plex)	CID	1.0000	0.0000	1	1	2832158	Unique	Used	0.924	2.485	1.116	1.058	0	0	6.26	0	2	59	3	956.14233	2866.41245	-1.28
eIPIEGLEFMGHGk	Unambiguous	1	1	Q9D8Z2	N-Term(iTRAQ8plex); K14(iTRAQ8plex)	HCD	1.0000	0.0000	1	1	3213615	Unique	Used	1.797	1.026	1.378	0.858	0	0	6.26	0	22	54	3	722.39990	2165.18515	-1.57
dLQSSNQLSNHISSLFR	Unambiguous	4	1	Q00612	N-Term(iTRAQ8plex)	HCD	1.0000	0.0000	1	1	3894968	Unique	Used	0.869	0.873	0.998	1.337	0	0	6.26	0	13	75	3	750.72662	2250.16532	-5.66
vRPcVVYGGAEIGQQIR	Unambiguous	1	1	Q62167	N-Term(iTRAQ8plex); C4(Methylthio)	HCD	1.0000	0.0000	1	1	3893832	Unique	Used	0.843	1.031	0.845	0.915	0	0	6.25	0	14	196	3	732.39362	2195.16629	-3.12

cELLYEGPPDDEAAmGIk	Unambiguous	1	1	P58252	N-Term(iTRAQ8plex); C1(Methylthio); M15(Oxidation); K18(iTRAQ8plex)	CID	1.0000	0.0000	1	1	2903983	Unique	Used	0.918	0.666	0.797	0.880	0	0	6.25	0	2	101	3	874.42914	2621.27286	-1.12
tVTYEDPQAVGGLASALDNR	Unambiguous	8	1	Q5SRX1-2	N-Term(iTRAQ8plex)	CID	1.0000	0.0000	1	1	3192351	Unique	Used	2.311	1.302	1.803	1.553	0	0	6.25	0	0	92	2	1191.11157	2381.21587	-3.87
cLLDDDLVMGVYIHR	Unambiguous	3	1	E9QP54	N-Term(iTRAQ8plex); C1(Methylthio)	CID	1.0000	0.0000	1	1	3439741	Unique	Used			0.974	1.658	0	0	6.25	0	13	14	3	704.68896	2112.05234	-3.34
cVFHYVEnGDSAYWEk	Unambiguous	4	1	P06909	N-Term(iTRAQ8plex); C1(Methylthio); N8(Deamidated); K16(iTRAQ8plex)	CID	1.0000	0.0000	1	1	3034581	Unique	Used	3.001	1.926	0.899	0.932	0	0	6.25	0	19	200	3	868.07220	2602.20206	-7.00
dLGLPTEAYISVEEVHDDGTPTSk	Unambiguous	1	1	P26516	N-Term(iTRAQ8plex); K24(iTRAQ8plex)	CID	1.0000	0.0000	1	1	2813886	Unique	Used	0.552	0.901	0.781	0.903	0	0	6.25	0	10	200	3	1061.21875	3181.64170	1.74
lADDVDLEQVANETHGHVGADLAALcSEAALQAIR	Unambiguous	1	1	Q01853	N-Term(iTRAQ8plex); C26(Methylthio)	CID	1.0000	0.0000	1	1	3440193	Unique	Used	0.972	0.634	0.653	0.783	0	0	6.25	0	0	33	3	1322.65649	3965.95493	-2.30
eSSPLYSPGFSDSTSAAk	Unambiguous	1	1	P14873	N-Term(iTRAQ8plex); K18(iTRAQ8plex)	CID	1.0000	0.0000	1	1	2973038	Unique	Used	1.646	1.261	1.968	1.061	0	0	6.25	0	0	52	2	1220.12378	2439.24028	-3.98
gNDISSGTVLSDYVGSGPPSGTGLHR	Unambiguous	1	1	P70296	N-Term(iTRAQ8plex)	CID	1.0000	0.0000	1	1	3390308	Unique	Used	0.687	0.725	0.982	0.810	0	0	6.25	0	24	17	3	945.47546	2834.41184	-3.69
nETELSLVLSHcTGATDGAIADk	Unambiguous	3	1	G3X8X7	N-Term(iTRAQ8plex); C12(Methylthio); K23(iTRAQ8plex)	CID	1.0000	0.0000	1	1	3283834	Unique	Used	0.613	0.941	1.049	1.154	0	0	6.25	0	9	61	3	1000.51093	2999.51822	-3.09
gTNTGVWVGVSGSEASEALSR	Unambiguous	1	1	P19096	N-Term(iTRAQ8plex)	CID	1.0000	0.0000	1	1	3345493	Unique	Used	0.767	0.727	0.870	0.988	0	0	6.25	0	0	22	2	1184.60034	2368.19341	-4.77
kTMQGEGPQLLLSEAVSR	Unambiguous	2	1	Q3UF95	N-Term(iTRAQ8plex); K1(iTRAQ8plex)	HCD	1.0000	0.0000	1	1	3412951	Unique	Used	0.941	0.984	1.129	1.041	0	0	6.25	1	0	58	3	851.47809	2552.41971	-5.09

dIFGLLQAYADGVDLTEk	Unambiguous	4	1	E9PX52	N-Term(iTRAQ8plex); K18(iTRAQ8plex)	CID	1.0000	0.0000	1	1	3419445	Unique	Used	1.844	1.371	0.547	1.325	0	0	6.25	0	26	84	3	859.46991	2576.39518	-4.54
hLVDQmPDSSDNPAGLAEQcPGMR	Unambiguous	1	1	P46414	N-Term(iTRAQ8plex); M6(Oxidation); C20(Methylthio)	CID	1.0000	0.0000	1	1	3211793	Unique	Used		2.114	1.896	1.735	0	0	6.25	0	14	69	3	978.77557	2934.31217	-1.21
gEILIGGQNVTMGYYk	Unambiguous	2	1	Q9CZW4	N-Term(iTRAQ8plex); K16(iTRAQ8plex)	CID	1.0000	0.0000	1	1	3476429	Unique	Used	0.433	0.700	0.950	1.119	0	0	6.25	0	0	116	2	1176.14624	2351.28520	-1.60
gGNVGInSFGFGGSNVHVILQPNTR	Unambiguous	1	1	P19096	N-Term(iTRAQ8plex); N7(Deamidated)	HCD	1.0000	0.0000	1	1	3877017	Unique	Used	0.962	0.884	0.732	1.021	0	0	6.25	0	8	101	3	949.49622	2846.47409	-3.90
yEVALAGPALELHNcmAk	Unambiguous	1	1	Q3UI43	N-Term(iTRAQ8plex); C15(Methylthio); M16(Oxidation); K18(iTRAQ8plex)	CID	1.0000	0.0000	1	1	3578587	Unique	Used	0.641	0.662	0.976	0.993	0	0	6.25	0	30	40	3	867.44855	2600.33109	-7.11
tSDIFGSPVTATAPLAHPNkPk	Unambiguous	1	1	Q6PGH2	N-Term(iTRAQ8plex); K20(iTRAQ8plex); K22(iTRAQ8plex)	CID	1.0000	0.0000	1	1	3594981	Unique	Used	2.114	0.343	0.617	0.632	0	0	6.25	0	0	111	3	1054.60120	3161.78904	-6.20
vEAVNMAEGIIHDTETk	Unambiguous	1	1	P38647	N-Term(iTRAQ8plex); K17(iTRAQ8plex)	CID	1.0000	0.0000	1	1	2959617	Unique	Used	1.606	0.764	1.612	1.157	0	0	6.25	0	18	200	2	1233.16113	2465.31499	-0.68
kLISPASAPGSMGGPVPGNnSSSSFSAAIPTR	Unambiguous	1	1	Q80XQ2	N-Term(iTRAQ8plex); K1(iTRAQ8plex); N20(Deamidated)	CID	0.0096	0.0000	1	1	3913614	Unique	Used		1.253	1.270	1.242	0	0	6.24	1	0	200	3	1217.98193	3651.93125	1.38
tNVLGHLQQGGAPTPFDR	Unambiguous	1	1	P12382	N-Term(iTRAQ8plex)	HCD	1.0000	0.0000	1	1	3779929	Unique	Used	1.055	1.337	0.921	1.223	0	0	6.24	0	13	147	3	738.06268	2212.17350	-1.89
nLHQSGFSLSGAQIDDNIPR	Unambiguous	1	1	O08553	N-Term(iTRAQ8plex)	CID	1.0000	0.0000	1	1	3367099	Unique	Used	2.019	1.314	1.016	0.865	0	0	6.24	0	0	23	3	825.09241	2473.26267	-4.49
yVAVMPPHIGDQPLTGAYTVTLDGR	Unambiguous	3	1	Q923D2	N-Term(iTRAQ8plex)	CID	1.0000	0.0000	1	1	3685824	Unique	Used	2.125	2.265	1.084	1.717	0	0	6.24	0	4	48	3	992.52002	2975.54551	-4.98

aSLSTDIcSGLASDGGGAR	Unambiguous	13	1	E9Q6Q2	N-Term(iTRAQ8plex); C8(Methylthio)	CID	1.0000	0.0000	1	1	3477745	Unique	Used	0.480	0.645	1.192	0.952	0	0	6.24	0	8	61	2	1044.50037	2087.99346	-3.32
mQDTSASFGYQLDLPk	Unambiguous	2	1	Q9QYA2	N-Term(iTRAQ8plex); K16(iTRAQ8plex)	CID	1.0000	0.0000	1	1	3348127	Unique	Used	0.592	0.925	0.845	1.006	0	0	6.24	0	0	24	2	1205.12646	2409.24565	-5.15
qSESSFITGDINSSASLNR	Unambiguous	22	1	Q80TS3-5	N-Term(iTRAQ8plex)	CID	1.0000	0.0000	1	1	3211254	Unique	Used	0.930	1.385	1.137	0.839	0	0	6.24	0	0	131	2	1159.07983	2317.15239	-2.16
ITAVSVGVQGSGWGWLGFNk	Unambiguous	1	1	P09671	N-Term(iTRAQ8plex); K20(iTRAQ8plex)	CID	1.0000	0.0000	1	1	3917564	Unique	Used	1.781	1.368	1.195	1.011	0	0	6.24	0	12	25	2	1336.23572	2671.46416	-6.57
qSSGPGASSGPGGDHSELIVR	Unambiguous	3	1	F6ZFU0	N-Term(iTRAQ8plex)	HCD	1.0000	0.0000	1	1	3100875	Unique	Used	1.042	1.080	0.732	0.896	0	0	6.24	0	0	144	3	767.05499	2299.15042	-3.33
hSMNPFcEIAVEEAVR	Unambiguous	1	1	Q9DCW4	N-Term(iTRAQ8plex); C7(Methylthio)	CID	1.0000	0.0000	1	1	3325187	Unique	Used	1.901	1.320	1.220	1.168	0	0	6.24	0	23	27	3	728.01563	2182.03232	-3.40
eALLGVQEDVDEYVk	Unambiguous	1	1	Q8BP92	N-Term(iTRAQ8plex); K15(iTRAQ8plex)	CID	1.0000	0.0000	1	1	2639099	Unique	Used	1.471	0.726	1.004	0.837	0	0	6.24	0	15	200	2	1158.13086	2315.25444	-2.02
gDAFDFNVALQDHFk	Unambiguous	2	1	Q9D1J1	N-Term(iTRAQ8plex); K15(iTRAQ8plex)	CID	1.0000	0.0000	1	1	3302387	Unique	Used	1.189	0.729	0.772	0.845	0	0	6.24	0	18	99	3	778.07623	2332.21415	-1.76
tDSNDLGPLPPGWEER	Unambiguous	1	1	P46935	N-Term(iTRAQ8plex)	CID	1.0000	0.0000	1	1	2760374	Unique	Used	1.040	0.978	0.767	0.970	0	0	6.24	0	0	200	2	1044.02002	2087.03276	-0.96
gLVHAEQGQGSGSPTGSSTR	Unambiguous	2	1	Q3URS9-2	N-Term(iTRAQ8plex)	CID	1.0000	0.0000	1	1	3081757	Unique	Used	1.072	0.995	1.366	1.111	0	0	6.24	0	11	200	3	739.71021	2217.11606	-0.06
aVVHAVDGIAEnGIQPLSSSEVDELIHk	Unambiguous	1	1	Q9Z0P4-2	N-Term(iTRAQ8plex); N12(Deamidated); K28(iTRAQ8plex)	HCD	1.0000	0.0000	1	1	3111059	Unique	Used	0.655	1.096	0.713	0.546	0	0	6.24	0	0	200	3	1179.64038	3536.90659	0.23
dITAEATPALAQTLPGGk	Unambiguous	2	1	Q9WTQ5	N-Term(iTRAQ8plex); K18(iTRAQ8plex)	CID	1.0000	0.0000	1	1	3279768	Unique	Used	1.639	0.274	0.877	0.961	0	0	6.24	0	3	19	2	1181.67334	2362.33940	-1.89
eHINLGcDVDFDIAGPSIR	Unambiguous	3	1	Q60932-2	N-Term(iTRAQ8plex); C7(Methylthio)	CID	1.0000	0.0000	1	1	3375043	Unique	Used	1.281	0.985	1.239	1.479	0	0	6.24	0	17	175	3	807.73096	2421.17832	-2.55
IGGDLLQALNEITESGR	Unambiguous	4	1	Q542I8	N-Term(iTRAQ8plex)	CID	1.0000	0.0000	1	1	3602531	Unique	Used	0.724	1.255	1.155	1.222	0	0	6.24	0	0	45	2	1045.56860	2090.12993	-4.61

gNAGQTNYGFAnSTMER	Unambiguous	1	1	P19096	N-Term(iTRAQ8plex); N12(Deamidated)	CID	1.0000	0.0000	1	1	2845685	Unique	Used	0.772	0.497	0.820	1.008	0	0	6.24	0	0	200	2	1061.98938	2122.97148	-2.40
sLSDEEPFLILADQHEIR	Unambiguous	3	1	Q9JI18	N-Term(iTRAQ8plex)	CID	1.0000	0.0000	1	1	2903653	Unique	Used	1.630	1.273	1.471	1.122	0	0	6.24	0	3	200	3	806.09229	2416.26230	-1.63
sQSLTNSLSTSDTSHR	Unambiguous	1	1	Q8JZP2	N-Term(iTRAQ8plex)	CID	1.0000	0.0000	1	1	3405302	Unique	Used	1.168	0.976	1.159	0.901	0	0	6.24	0	5	45	3	675.67444	2025.00876	-3.11
sSPPPLSGASEVDAGELGSER	Unambiguous	1	1	A6X919	N-Term(iTRAQ8plex)	CID	1.0000	0.0000	1	1	2668438	Unique	Used	0.635	0.234	1.022	1.319	0	0	6.24	0	0	200	2	1173.58679	2346.16631	-2.74
ySEVFEAINITNNEk	Unambiguous	3	1	Q60737	N-Term(iTRAQ8plex); K15(iTRAQ8plex)	CID	1.0000	0.0000	1	1	3215938	Unique	Used	0.895	1.054	1.545	1.393	0	0	6.24	0	0	117	2	1190.13635	2379.26543	0.07
iMETSPTLLQISPGPPk	Unambiguous	1	1	Q8R361	N-Term(iTRAQ8plex); K17(iTRAQ8plex)	CID	1.0000	0.0000	1	1	3643640	Unique	Used	1.575	1.158	1.224	1.070	0	0	6.24	0	0	80	2	1209.19556	2417.38384	-3.98
cHAPLAQAQALVTSELER	Unambiguous	1	1	Q9CR98	N-Term(iTRAQ8plex); C1(Methylthio)	CID	1.0000	0.0000	1	1	3685883	Unique	Used	1.574	1.042	1.934	1.200	0	0	6.23	0	11	22	3	763.06232	2287.17240	-5.11
rPAEPPLPSPAVPGGGSGSNnGNk	Unambiguous	1	1	Q9CW46	N-Term(iTRAQ8plex); N21(Deamidated); K24(iTRAQ8plex)	CID	0.0144	0.0000	1	1	3228797	Unique	Used	1.539	1.412	0.853	0.748	0	0	6.23	0	0	188	3	956.17932	2866.52341	-1.20
ePPLSLTIHLTSPVVR	Unambiguous	4	1	Q9Z1X4	N-Term(iTRAQ8plex)	HCD	1.0000	0.0000	1	1	3857864	Unique	Used	1.026	0.946	0.868	1.018	0	0	6.23	0	23	49	3	688.40851	2063.21097	-2.77
sMLQATAEAnNLAAVAGAR	Unambiguous	3	1	Q6PA06	N-Term(iTRAQ8plex); N10(Deamidated)	CID	0.0321	0.0000	1	1	3393251	Unique	Used	0.793	0.881	0.960	1.063	0	0	6.23	0	0	145	2	1082.56433	2164.12139	-5.58
aAVEEGIVLGGGcALLR	Unambiguous	2	1	P63038	N-Term(iTRAQ8plex); C13(Methylthio)	CID	1.0000	0.0000	1	1	3647974	Unique	Used	1.276	0.589	1.116	0.934	0	0	6.23	0	10	200	2	989.53717	1978.06706	-4.92
kGNVSTISGLSSQTAGAk	Unambiguous	3	1	Q9EPN1-4	N-Term(iTRAQ8plex); K1(iTRAQ8plex); K18(iTRAQ8plex)	CID	1.0000	0.0000	1	1	3591912	Unique	Used	0.439	1.251	1.085	0.998	0	0	6.23	1	0	158	3	873.50787	2618.50907	-5.73
sTAIIFDDDEGDLFk	Unambiguous	2	1	Q6PGL7	N-Term(iTRAQ8plex); K15(iTRAQ8plex)	CID	1.0000	0.0000	1	1	2741724	Unique	Used	1.543	1.044	0.895	0.972	0	0	6.23	0	3	200	2	1147.60486	2294.20244	0.51

yVAVMPPHIGDQPLTGAYTVTLDGR	Unambiguous	3	1	Q923D2	N-Term(iTRAQ8plex)	CID	1.0000	0.0000	1	1	3685691	Unique	Used	3.028	2.436	0.775	1.436	0	0	6.23	0	4	13	3	992.51776	2975.53873	-7.26
ePcGGTGSTGAGGGTnGGVR	Unambiguous	1	1	Q80WM4	N-Term(iTRAQ8plex); C3(Methylthio); N16(Deamidated)	CID	1.0000	0.0000	1	1	2774345	Unique	Used	0.431		1.360	2.032	0	0	6.23	0	0	200	2	1021.96051	2042.91374	-1.78
fTASAGIQVVGDDLTVTNPk	Unambiguous	2	1	P17182	N-Term(iTRAQ8plex); K20(iTRAQ8plex)	CID	1.0000	0.0000	1	1	3349932	Unique	Used	1.529	1.193	1.096	1.174	0	0	6.23	0	0	39	2	1321.22961	2641.45195	-5.24
hVGMAVAGLLADAR	Unambiguous	1	1	O70435	N-Term(iTRAQ8plex)	CID	1.0000	0.0000	1	1	3875773	Unique	Used	2.737	0.706	0.695	0.443	0	0	6.23	0	3	3	3	562.31781	1684.93888	-4.87
sLDGLSGAcGGGGSSSGETGAGGGR	Unambiguous	1	1	Q9D6W8	N-Term(iTRAQ8plex); C9(Methylthio)	CID	1.0000	0.0000	1	1	3319748	Unique	Used	2.124	1.271	1.483	1.540	0	0	6.23	0	0	143	2	1246.05469	2491.10210	-2.85
sLSDEEPFLILADQHEIR	Unambiguous	3	1	Q9JI18	N-Term(iTRAQ8plex)	CID	1.0000	0.0000	1	1	2920926	Unique	Used	1.536	1.577	1.439	1.007	0	0	6.23	0	17	145	3	806.09259	2416.26322	-1.25
iQEGVFDInNEANGIk	Unambiguous	1	1	P53994	N-Term(iTRAQ8plex); N9(Deamidated); K16(iTRAQ8plex)	CID	0.0305	0.0000	1	1	2868747	Unique	Used	1.276	0.893	0.928	0.885	0	0	6.23	0	12	200	2	1185.63757	2370.26787	-3.50
gVEITGFPEAQALGLQVFHAGTALk	Unambiguous	2	1	Q64737	N-Term(iTRAQ8plex); K25(iTRAQ8plex)	CID	1.0000	0.0000	1	1	3765749	Unique	Used	0.945	0.967	1.016	1.245	0	0	6.22	0	6	12	3	1054.92578	3162.76279	-4.57
tAVTAFSSTEPGTSEGFk	Unambiguous	4	1	F8WHT3	N-Term(iTRAQ8plex); K18(iTRAQ8plex)	CID	1.0000	0.0000	1	1	3126464	Unique	Used	1.098	0.628	1.171	0.713	0	0	6.22	0	0	109	2	1213.13599	2425.26470	-2.50
yNIIPVLSDILQESVk	Unambiguous	1	1	Q8BVE3	N-Term(iTRAQ8plex); K16(iTRAQ8plex)	CID	1.0000	0.0000	1	1	3707132	Unique	Used		1.040	1.014	0.781	0	0	6.22	0	22	117	2	1220.21228	2439.41728	-6.00
vAAGHELQPLAIVDQRPSSR	Unambiguous	1	1	P23242	N-Term(iTRAQ8plex)	HCD	1.0000	0.0000	1	1	3679361	Unique	Used	1.011	1.094	1.278	1.414	0	0	6.22	0	0	200	3	816.78735	2448.34751	-6.14
fVDDLFETLFSTVHR	Unambiguous	1	1	P70207	N-Term(iTRAQ8plex)	CID	1.0000	0.0000	1	1	3825194	Unique	Used	0.472	2.122	1.744	0.985	0	0	6.22	0	0	90	3	710.70862	2130.11130	-2.84
qVLDLEDLVFTQGSHFmANk	Unambiguous	1	1	Q6P4T2	N-Term(iTRAQ8plex); M17(Oxidation); K20(iTRAQ8plex)	CID	1.0000	0.0000	1	1	3499908	Unique	Used	0.695		1.032	0.656	0	0	6.22	0	22	14	3	972.85175	2916.54068	0.70

iGEHTPSALAImENANVLAR	Unambiguous	3	1	A6ZI44	N-Term(iTRAQ8plex); M12(Oxidation)	CID	1.0000	0.0000	1	1	3708536	Unique	Used	0.816	0.798	0.712	1.011	0	0	6.22	0	12	200	3	809.76581	2427.28287	-5.75
mFDYTDDPEGPVMPGSHSVER	Unambiguous	1	1	Q3UYV9	N-Term(iTRAQ8plex)	CID	1.0000	0.0000	1	1	2812250	Unique	Used	1.398	1.215	0.870	0.903	0	0	6.22	0	10	125	3	890.74152	2670.21000	-0.70
eAAASGLPLMVIIHk	Unambiguous	1	1	Q9CQU0	N-Term(iTRAQ8plex); K15(iTRAQ8plex)	CID	1.0000	0.0000	1	1	3684738	Unique	Used	1.593	0.613	0.803	0.643	0	0	6.22	0	12	13	3	720.09583	2158.27292	-6.93
aVVVNAAQLASYSQSk	Unambiguous	2	1	Q9CR62	N-Term(iTRAQ8plex); K16(iTRAQ8plex)	CID	1.0000	0.0000	1	1	3761366	Unique	Used	0.590	1.301	1.237	1.371	0	0	6.22	0	0	162	2	1122.64038	2244.27349	-3.28
kQQEGATVFSNVAGDSLNTENEPEETTk	Unambiguous	1	1	Q3UVU3	N-Term(iTRAQ8plex); K1(iTRAQ8plex); K28(iTRAQ8plex)	CID	1.0000	0.0000	1	1	2758364	Unique	Used		0.617	0.643	0.896	0	0	6.22	1	0	200	3	1312.67810	3936.01975	-1.03
aSGSAGAAASPSAAAAGER	Unambiguous	2	1	P83093	N-Term(iTRAQ8plex)	CID	1.0000	0.0000	1	1	2859310	Unique	Used	0.350	1.464	2.867	1.372	0	0	6.22	0	0	200	2	932.47369	1863.94011	-3.32
iMDPNIVGnEHYDVAR	Unambiguous	1	1	P56480	N-Term(iTRAQ8plex); N9(Deamidated)	CID	1.0000	0.0000	1	1	2849318	Unique	Used	1.326	1.113	0.743	0.947	0	0	6.22	0	1	5	3	716.69287	2148.06406	-2.66
dVLQAETSQQLccQk	Unambiguous	1	1	Q6P8X1	N-Term(iTRAQ8plex); C12(Methylthio); C13(Methylthio); K15(iTRAQ8plex)	CID	1.0000	0.0000	1	1	3301686	Unique	Used	0.509	0.790	0.853	1.078	0	0	6.22	0	0	200	2	1197.58923	2394.17119	-1.43
kMEDGVGcLETAEEAk	Unambiguous	1	1	Q8VDD5	N-Term(iTRAQ8plex); K1(iTRAQ8plex); C8(Methylthio); K16(iTRAQ8plex)	CID	1.0000	0.0000	1	1	2701604	Unique	Used	1.036	0.738	1.191	0.688	0	0	6.22	1	12	200	3	890.13000	2668.37546	-0.19
gAPSPGVLGPHASEPQLAPPAR	Unambiguous	1	1	Q8BTW9	N-Term(iTRAQ8plex)	HCD	1.0000	0.0000	1	1	3471356	Unique	Used	1.754	1.178	1.188	1.264	0	0	6.22	0	0	134	3	804.10742	2410.30771	-2.83
vETLHDFEAANSDELNLQR	Unambiguous	1	1	Q7TQF7	N-Term(iTRAQ8plex)	CID	1.0000	0.0000	1	1	2850999	Unique	Used	2.664	1.206	1.600	0.915	0	0	6.22	0	6	43	3	835.75262	2505.24332	-3.61

lVDWIILQcAEDIEHPPPGR	Unambiguous	1	1	Q9R1Q8	N-Term(iTRAQ8plex); C9(Methylthio)	CID	1.0000	0.0000	1	1	3542034	Unique	Used	1.371	0.693	1.145	1.252	0	0	6.22	0	4	25	3	884.45648	2651.35489	-2.99
ILEVSDDPQVLAVAAHDVGEYVR	Unambiguous	1	1	Q8BVE3	N-Term(iTRAQ8plex)	CID	1.0000	0.0000	1	1	3241500	Unique	Used	1.087	0.917	1.479	1.217	0	0	6.21	0	17	200	3	933.83466	2799.48941	2.25
qEAIDVLLLGNTLNSDLADk	Unambiguous	5	1	Q8CHC4	N-Term(iTRAQ8plex); K20(iTRAQ8plex)	CID	1.0000	0.0000	1	1	3305438	Unique	Used	0.712	1.562	1.168	1.550	0	0	6.21	0	0	19	3	917.51569	2750.53250	-2.61
eFEPPSAGAAVTAkPGQEIR	Unambiguous	4	1	Q7TQ95	N-Term(iTRAQ8plex); K14(iTRAQ8plex)	HCD	1.0000	0.0000	1	1	3124416	Unique	Used	0.584	0.858	0.863	0.875	0	0	6.21	0	0	79	3	888.48883	2663.45194	-3.55
eSAPFAVIGSNTVVEAk	Unambiguous	1	1	Q9Z2Q6	N-Term(iTRAQ8plex); K17(iTRAQ8plex)	CID	1.0000	0.0000	1	1	3258026	Unique	Used	0.603	2.094	1.607	1.105	0	0	6.21	0	3	7	2	1164.15503	2327.30278	-1.71
iVLDSDAAEYGGHQR	Unambiguous	2	1	Q9D6Y9	N-Term(iTRAQ8plex)	CID	1.0000	0.0000	1	1	2808533	Unique	Used	1.289	0.721	0.875	1.262	0	0	6.21	0	22	69	3	645.66595	1934.98331	-2.11
aTDVMAHVAGFTVAHDVSAR	Unambiguous	1	1	Q3TC72	N-Term(iTRAQ8plex)	CID	1.0000	0.0000	1	1	3661323	Unique	Used	1.235	1.042	0.986	0.954	0	0	6.21	0	0	101	3	787.06995	2359.19529	-7.55
fGIPSSSSGLSQTLTSTGNFk	Unambiguous	1	1	E9Q3G8	N-Term(iTRAQ8plex); K21(iTRAQ8plex)	CID	1.0000	0.0000	1	1	3840818	Unique	Used	0.708	0.806	1.322	0.796	0	0	6.21	0	17	200	2	1362.73486	2724.46245	-1.48
lAAALTAEPEPERPLR	Unambiguous	1	1	A2BDX3	N-Term(iTRAQ8plex)	HCD	1.0000	0.0000	1	1	3299840	Unique	Used	0.946	0.948	0.968	1.270	0	0	6.21	0	10	34	3	680.05652	2038.15500	-2.41
gEENTDYVNASFIDGYR	Unambiguous	3	1	Q91V35	N-Term(iTRAQ8plex)	CID	1.0000	0.0000	1	1	2795531	Unique	Used	0.500	1.056	1.088	1.131	0	0	6.21	0	0	145	2	1127.52942	2254.05156	-2.24
nMDPLnDNVATLLHQSSDR	Unambiguous	3	1	Q5SV64	N-Term(iTRAQ8plex); N6(Deamidated)	CID	1.0000	0.0000	1	1	3091246	Unique	Used	1.396	1.072	0.972	1.086	0	0	6.21	0	25	200	3	815.73761	2445.19828	0.02
lkPEQLGDISVGNVLEPGAGAVMAR	Unambiguous	4	1	Q921H8	N-Term(iTRAQ8plex); K2(iTRAQ8plex)	CID	1.0000	0.0000	1	1	3539463	Unique	Used	0.994	0.962	0.729	1.034	0	0	6.21	0	0	30	3	1043.91833	3129.74045	-4.70
hTGPGILSMANAGPNTnGSQFFIcTAk	Unambiguous	1	1	P17742	N-Term(iTRAQ8plex); N17(Deamidated); C24(Methylthio); K27(iTRAQ8plex)	CID	0.0209	0.0000	1	1	3805574	Unique	Used	1.178	0.820	1.516	1.041	0	0	6.21	0	11	8	3	1130.56482	3389.67990	-3.03

vNNSSLIGVGYTQTLRPGVk	Unambiguous	2	1	G3UX26	N-Term(iTRAQ8plex); K20(iTRAQ8plex)	HCD	1.0000	0.0000	1	1	3931169	Unique	Used	0.558	1.031	1.032	1.301	0	0	6.21	0	0	200	3	904.52203	2711.55155	-5.51
dMHGDSEYNIMFGPDIcGPGTk	Unambiguous	1	1	P14211	N-Term(iTRAQ8plex); C17(Methylthio); K22(iTRAQ8plex)	CID	1.0000	0.0000	1	1	3171622	Unique	Used	1.492	1.223	1.050	0.810	0	0	6.21	0	9	146	3	1013.46863	3038.39133	-2.11
qIPGGGSGGGGSGSGGGGGGSGGGR	Unambiguous	2	1	Q8BMA6	N-Term(iTRAQ8plex)	CID	1.0000	0.0000	1	1	3290783	Unique	Used	0.675	0.468	0.942	0.996	0	0	6.21	0	0	200	2	1068.00635	2135.00542	-3.48
aLQDLVDLLDTGQEk	Unambiguous	2	1	Q6ZWR6-4	N-Term(iTRAQ8plex); K15(iTRAQ8plex)	CID	1.0000	0.0000	1	1	3284732	Unique	Used	0.480	1.467	1.272	1.184	0	0	6.21	0	17	68	2	1133.63928	2266.27129	-1.69
sAAAADGGDQPLQPPGAAGR	Unambiguous	2	1	E9Q6C8	N-Term(iTRAQ8plex)	CID	1.0000	0.0000	1	1	2844402	Unique	Used	0.618	0.347	0.475	1.078	0	0	6.21	0	0	138	2	1056.04016	2111.07305	-2.53
sSQEAPSAEAGFATADLSGR	Unambiguous	1	1	Q9CQ13	N-Term(iTRAQ8plex)	CID	1.0000	0.0000	1	1	2828967	Unique	Used	2.413	1.064	0.757	0.858	0	0	6.20	0	0	95	2	1128.55273	2256.09819	-2.86
qEIIEQLLSNIFHk	Unambiguous	5	1	Q922D4-2	N-Term(iTRAQ8plex); K14(iTRAQ8plex)	CID	1.0000	0.0000	1	1	3861115	Unique	Used	0.393	1.565	0.731	0.886	0	0	6.20	0	5	22	3	774.11792	2320.33921	-4.02
gGGTSSGGSSSTnSLTGSRPPk	Unambiguous	1	1	Q78T81	N-Term(iTRAQ8plex); N13(Deamidated); K22(iTRAQ8plex)	CID	1.0000	0.0000	1	1	3099089	Unique	Used		0.775	1.054	0.965	0	0	6.20	0	0	200	3	863.44983	2588.33493	-0.91
lQPHEFQGGTFTISNLGMFGIk	Unambiguous	1	1	Q8BMF4	N-Term(iTRAQ8plex); K22(iTRAQ8plex)	CID	1.0000	0.0000	1	1	3860188	Unique	Used		0.947			0	0	6.20	0	9	9	3	1010.87860	3030.62125	-3.95
vAASYGNAVcIFEPLGVNSHk	Unambiguous	4	1	Q8BPN8-2	N-Term(iTRAQ8plex); C10(Methylthio); K21(iTRAQ8plex)	CID	1.0000	0.0000	1	1	3744797	Unique	Used	0.531	1.021	1.227	1.216	0	0	6.20	0	8	72	3	944.16437	2830.47855	-1.95
nEEDATELVGLAQAVNAR	Unambiguous	1	1	Q02053	N-Term(iTRAQ8plex); N1(Deamidated)	CID	1.0000	0.0000	1	1	2764785	Unique	Used	0.764	1.049	1.166	1.163	0	0	6.20	0	0	200	2	1103.06555	2205.12383	-2.86
lLEGVGLENLDSPAATk	Unambiguous	1	1	G3X922	N-Term(iTRAQ8plex); K17(iTRAQ8plex)	CID	1.0000	0.0000	1	1	3261070	Unique	Used	0.373	1.076	0.938	1.040	0	0	6.20	0	14	50	2	1168.16650	2335.32573	-3.10

sVVQTTPVAGSQSLSSGTVAk	Unambiguous	3	1	Q8BTI8	N-Term(iTRAQ8plex); K21(iTRAQ8plex)	CID	1.0000	0.0000	1	1	3532980	Unique	Used	0.837	1.073	1.217	1.162	0	0	6.20	0	0	179	2	1306.73669	2612.46611	-2.09
tkSENGLEFTSSGSANTETTk	Unambiguous	3	1	Q60932-2	N-Term(iTRAQ8plex); K2(iTRAQ8plex); K21(iTRAQ8plex)	CID	1.0000	0.0000	1	1	2862300	Unique	Used	0.494	0.696	0.997	1.240	0	0	6.20	1	0	200	3	1034.54602	3101.62351	-4.22
eAQVSAQHLEVHLk	Unambiguous	8	1	P49025-3	N-Term(iTRAQ8plex); K14(iTRAQ8plex)	HCD	1.0000	0.0000	1	1	3143586	Unique	Used	1.057	0.647	1.330	0.895	0	0	6.20	0	7	59	3	733.08856	2197.25113	-1.74
gLGPSLTEDQPGPYLAPGLLGSNIHQQGR	Unambiguous	4	1	D3YZR8	N-Term(iTRAQ8plex)	HCD	1.0000	0.0000	1	1	3642634	Unique	Used	1.763	0.940	1.408	1.377	0	0	6.20	0	8	51	3	1092.90967	3276.71445	-4.12
vADVSISEVTTLLGNVHSPVVEGYVGENISGEVk	Unambiguous	3	1	P20357	N-Term(iTRAQ8plex); K34(iTRAQ8plex)	CID	1.0000	0.0000	1	1	3421994	Unique	Used			1.137		0	0	6.20	0	11	200	3	1369.40259	4106.19321	-4.54
dFTSEQIDEILQNHTEIVFAR	Unambiguous	2	1	Q6PIC6	N-Term(iTRAQ8plex)	HCD	1.0000	0.0000	1	1	3354422	Unique	Used	0.686	0.583	0.968	1.287	0	0	6.19	0	0	61	3	937.14575	2809.42270	-2.98
hYPAPEGPAPAPPGPLPPAPnSGTGPSGVAGGR	Unambiguous	5	1	E9QAD8	N-Term(iTRAQ8plex); N21(Deamidated)	CID	1.0000	0.0000	1	1	3472229	Unique	Used	0.759	1.045	1.871	1.055	0	0	6.19	0	0	200	3	1111.57129	3332.69931	0.79
dSDTPLSLMSVNHEELER	Unambiguous	1	1	Q8K3F6	N-Term(iTRAQ8plex)	CID	1.0000	0.0000	1	1	2795130	Unique	Used	1.258	1.103	2.417	1.205	0	0	6.19	0	29	200	3	792.72607	2376.16367	-0.78
iMDQVQQASSTSSGANk	Unambiguous	6	1	B2RUG9	N-Term(iTRAQ8plex); K17(iTRAQ8plex)	CID	1.0000	0.0000	1	1	3341318	Unique	Used	0.642	0.483	1.209	0.699	0	0	6.19	0	0	67	2	1180.61731	2360.22734	-2.67
aVAIETQPAQGESDAVAR	Unambiguous	3	1	Q7TSJ2	N-Term(iTRAQ8plex)	CID	1.0000	0.0000	1	1	2666860	Unique	Used	1.266	0.724	1.429	1.222	0	0	6.19	0	0	53	2	1059.05811	2117.10893	-2.44
eHSIAQLIYLQNADGSWDMNEDLAk	Unambiguous	1	1	Q8BGF0	N-Term(iTRAQ8plex); K25(iTRAQ8plex)	CID	1.0000	0.0000	1	1	3216524	Unique	Used	1.389	0.907	0.866	0.891	0	0	6.19	0	0	113	3	1157.25391	3469.74717	-1.36
nASAGLNQVNNQGLTPLHLAck	Unambiguous	2	1	P97819-2	N-Term(iTRAQ8plex); C21(Methylthio); K22(iTRAQ8plex)	CID	1.0000	0.0000	1	1	3839147	Unique	Used	0.620	0.858	0.876	1.063	0	0	6.19	0	16	200	3	973.18896	2917.55234	-2.53
gTGGVDTAAVGGVFDVSNADR	Unambiguous	1	1	Q04447	N-Term(iTRAQ8plex)	CID	1.0000	0.0000	1	1	3133716	Unique	Used	1.260	1.005	1.417	1.433	0	0	6.19	0	12	200	2	1135.07153	2269.13579	-0.23

lFSQQGYFLQMHPDGTIDGTk	Unambiguous	2	1	P61329-2	N-Term(iTRAQ8plex); K21(iTRAQ8plex)	CID	1.0000	0.0000	1	1	3518252	Unique	Used	1.175	0.703	0.828	1.038	0	0	6.19	0	11	66	3	997.85211	2991.54178	-2.59
vTITGSPAATQAAQYLISQR	Unambiguous	2	1	D3YVV7	N-Term(iTRAQ8plex)	CID	1.0000	0.0000	1	1	3934892	Unique	Used	1.063	0.791	1.202	1.191	0	0	6.19	0	0	200	2	1190.65381	2380.30034	-5.67
gTGGVDTAAVGGVFDVSNADR	Unambiguous	1	1	Q04447	N-Term(iTRAQ8plex)	CID	1.0000	0.0000	1	1	3136184	Unique	Used	0.934	0.575		0.496	0	0	6.19	0	0	200	2	1135.06995	2269.13262	-1.62
nLTEDNSQNQDVIAk	Unambiguous	1	1	P28658	N-Term(iTRAQ8plex); K15(iTRAQ8plex)	CID	1.0000	0.0000	1	1	2632474	Unique	Used	0.587	0.945	0.714	1.566	0	0	6.19	0	0	200	2	1149.11304	2297.21880	-0.25
iVQVTAADAFLDLIR	Unambiguous	1	1	P56564	N-Term(iTRAQ8plex)	CID	1.0000	0.0000	1	1	3825012	Unique	Used		1.495			0	0	6.19	0	17	32	3	650.38190	1949.13114	-3.20
sAYSSYSAPVSSSLSVR	Unambiguous	1	1	P08551	N-Term(iTRAQ8plex)	CID	1.0000	0.0000	1	1	3637385	Unique	Used	3.601	0.187	2.293	1.747	0	0	6.19	0	0	38	2	1026.52295	2052.03862	-8.04
cSSPVDmDHSNAEGGQSPGPEk	Unambiguous	2	1	G3X9K4	N-Term(iTRAQ8plex); C1(Methylthio); M7(Oxidation); K22(iTRAQ8plex)	CID	1.0000	0.0000	1	1	2696837	Unique	Used	1.375	1.272	1.471	1.116	0	0	6.19	0	0	200	3	967.10541	2899.30167	-3.48
nLHQSGFSLSGAQIDDNIPR	Unambiguous	1	1	O08553	N-Term(iTRAQ8plex); N1(Deamidated)	CID	0.0323	0.0000	1	1	3300957	Unique	Used	0.719	1.209	0.854	1.276	0	0	6.19	0	30	44	3	825.42242	2474.25272	-2.05
lLETLGDVEIAELHR	Unambiguous	3	1	P05622	N-Term(iTRAQ8plex)	CID	1.0000	0.0000	1	1	3305244	Unique	Used	0.838	1.705	0.815	1.471	0	0	6.19	0	18	31	3	671.38055	2012.12711	-2.94
tNVLYELAQYASEPSEQEHLHk	Unambiguous	2	1	P37040	N-Term(iTRAQ8plex); K22(iTRAQ8plex)	CID	1.0000	0.0000	1	1	3237506	Unique	Used	1.207	0.924	1.111	1.240	0	0	6.19	0	0	145	3	1065.55371	3194.64658	-3.53
aPPAGGSGSSAAAAAAAGGTEVRPR	Unambiguous	2	1	P84309	N-Term(iTRAQ8plex)	HCD	1.0000	0.0000	1	1	3449343	Unique	Used	0.641	1.036	2.643	1.148	0	0	6.19	0	6	200	3	814.42847	2441.27085	-3.74
nMGGPYGGGnYGPGGSGGSGGYGGR	Unambiguous	3	1	O88569	N-Term(iTRAQ8plex); N10(Deamidated)	CID	1.0000	0.0000	1	1	3008425	Unique	Used	1.590	0.999	1.432	0.957	0	0	6.19	0	0	28	3	832.36786	2495.08902	-2.33
lTIVSTVGFGDQINk	Unambiguous	4	1	Q9R1T4-2	N-Term(iTRAQ8plex); K15(iTRAQ8plex)	CID	1.0000	0.0000	1	1	3664735	Unique	Used	0.490	0.968	1.746	1.449	0	0	6.19	0	3	24	2	1100.63733	2200.26738	-5.64
lAEENPDLQEAYIAk	Unambiguous	1	1	O88741	N-Term(iTRAQ8plex); K15(iTRAQ8plex)	CID	1.0000	0.0000	1	1	2737432	Unique	Used	0.379	0.693	0.815	0.983	0	0	6.19	0	0	156	2	1156.63184	2312.25640	-1.32

gETGPAGPAGPIGPAGAR	Unambiguous	2	1	P11087-2	N-Term(iTRAQ8plex)	HCD	1.0000	0.0000	1	1	3142630	Unique	Used	0.570	5.116	1.138	1.293	0	0	6.18	0	18	146	2	918.99554	1836.98381	-1.76
sVSGASTGLSSSPLSSPR	Unambiguous	2	1	Q5RJI5	N-Term(iTRAQ8plex)	CID	1.0000	0.0000	1	1	3757308	Unique	Used	0.623	0.746	1.336	1.184	0	0	6.18	0	0	22	2	991.02448	1981.04167	-4.41
mVLAAAGGVEHQQLLDLAQk	Unambiguous	1	1	Q9CZ13	N-Term(iTRAQ8plex); K20(iTRAQ8plex)	CID	1.0000	0.0000	1	1	3621247	Unique	Used	0.947	1.168	0.909	1.040	0	0	6.18	0	22	75	3	900.84406	2700.51761	-5.61
sVVEFLQGYIGIPHGGFPEPFR	Unambiguous	3	1	G5E8R3	N-Term(iTRAQ8plex)	CID	1.0000	0.0000	1	1	3917721	Unique	Used	0.797	1.106	2.201	1.697	0	0	6.18	0	0	25	3	917.48535	2750.44150	-7.04
nLHQSGFSLSGAQIDDNIPR	Unambiguous	1	1	O08553	N-Term(iTRAQ8plex); N1(Deamidated)	CID	0.0210	0.0000	1	1	3278755	Unique	Used	0.961	0.996	0.999	0.932	0	0	6.18	0	15	49	3	825.42218	2474.25199	-2.34
gTGGVDTAAVGGVFDVSNADR	Unambiguous	1	1	Q04447	N-Term(iTRAQ8plex)	HCD	1.0000	0.0000	1	1	3128774	Unique	Used	1.062	0.924	1.318	1.384	0	0	6.18	0	8	9	2	1135.06934	2269.13140	-2.16
eTAAAHQASSSPPIDAATAEPYGFR	Unambiguous	1	1	P14873	N-Term(iTRAQ8plex)	CID	1.0000	0.0000	1	1	3010532	Unique	Used	1.435	1.042	1.360	1.169	0	0	6.18	0	0	200	3	950.46783	2849.38895	-4.17
eYAESIGAIVVETSAk	Unambiguous	2	1	Q921E2	N-Term(iTRAQ8plex); K16(iTRAQ8plex)	CID	1.0000	0.0000	1	1	3171737	Unique	Used	0.572	0.646	0.914	0.957	0	0	6.18	0	11	96	2	1138.13025	2275.25322	-4.83
fSPDGELYASGSEDGTLR	Unambiguous	1	1	Q9Z1Z2	N-Term(iTRAQ8plex)	CID	1.0000	0.0000	1	1	2685794	Unique	Used	1.946	1.120	0.785	0.756	0	0	6.18	0	0	29	2	1103.03369	2205.06011	-0.57
dVLEPTQALAAGAVPILAk	Unambiguous	2	1	Q9WTQ5	N-Term(iTRAQ8plex); K19(iTRAQ8plex)	CID	1.0000	0.0000	1	1	3540864	Unique	Used	1.711	0.383	0.986	0.867	0	0	6.18	0	0	11	2	1243.24231	2485.47734	-3.11
lATPTYGDLNHLVSATMSGVTTSLR	Unambiguous	1	1	Q9ERD7	N-Term(iTRAQ8plex)	CID	1.0000	0.0000	1	1	3938375	Unique	Used	0.425	0.725	1.339	1.679	0	0	6.18	0	7	200	3	970.51306	2909.52463	-3.39
fQLQATTQQGPGEAIVR	Unambiguous	3	1	A2AFG8	N-Term(iTRAQ8plex)	CID	1.0000	0.0000	1	1	3535354	Unique	Used	0.679	1.045	0.651	0.803	0	0	6.18	0	0	38	2	1074.58533	2148.16338	-3.80
sGLGLGAASASTSGSGPADSVMR	Unambiguous	3	1	Q7TSJ2	N-Term(iTRAQ8plex)	CID	1.0000	0.0000	1	1	3514361	Unique	Used	1.735	0.931	1.545	1.569	0	0	6.18	0	0	200	2	1170.58789	2340.16850	-3.54
eHINLGcDVDFDIAGPSIR	Unambiguous	3	1	Q60932-2	N-Term(iTRAQ8plex); C7(Methylthio)	CID	1.0000	0.0000	1	1	3354227	Unique	Used	0.929	1.415	1.224	1.301	0	0	6.18	0	5	71	3	807.73071	2421.17759	-2.86
qkPDGVFQEDGPVIHQEMIGGFR	Unambiguous	1	1	P01027	N-Term(iTRAQ8plex); K2(iTRAQ8plex)	CID	1.0000	0.0000	1	1	3278752	Unique	Used	2.636	1.796	0.609	0.831	0	0	6.18	0	21	170	3	1064.89270	3192.66355	-2.69
gSGSQSSVPSVDQFTGVGIR	Unambiguous	1	1	Q9CW03	N-Term(iTRAQ8plex)	CID	1.0000	0.0000	1	1	3618239	Unique	Used	0.554	0.727	0.776	0.855	0	0	6.18	0	0	74	2	1135.08325	2269.15923	-5.92

nMGGPYGGGNYGPGGSGGSGGYGGR	Unambiguous	3	1	O88569	N-Term(iTRAQ8plex); N1(Deamidated)	CID	1.0000	0.0000	1	1	2990007	Unique	Used	1.633	0.857	1.509	0.476	0	0	6.18	0	0	200	2	1248.04822	2495.08916	-2.28
lVANHVASDISLTGGSVVQR	Unambiguous	3	1	Q9Z2G6	N-Term(iTRAQ8plex)	CID	1.0000	0.0000	1	1	3740668	Unique	Used	1.192	1.306	0.766	0.872	0	0	6.18	0	7	24	3	776.43585	2327.29300	-2.38
sEIGIAMGSGTAVAk	Unambiguous	3	1	O55143	N-Term(iTRAQ8plex); K15(iTRAQ8plex)	HCD	1.0000	0.0000	1	1	3319614	Unique	Used	0.522	0.980	1.198	0.974	0	0	6.18	0	0	99	2	1000.56720	2000.12712	-1.79
nLEVSScVSSEGPSEAR	Unambiguous	1	1	P58871	N-Term(iTRAQ8plex); C7(Methylthio)	CID	1.0000	0.0000	1	1	2935304	Unique	Used	1.337	1.153	1.750	0.638	0	0	6.18	0	0	108	2	1050.99377	2100.98027	-1.96
aFEDWLnDDLGSYQGAQENR	Unambiguous	3	1	Q9EPJ9	N-Term(iTRAQ8plex); N7(Deamidated)	CID	1.0000	0.0000	1	1	2673400	Unique	Used		0.599			0	0	6.18	0	12	200	3	878.40723	2633.20713	0.50
tLDGGLNVIQLETAVGAAlk	Unambiguous	2	1	Q91ZJ5	N-Term(iTRAQ8plex); K20(iTRAQ8plex)	CID	1.0000	0.0000	1	1	3541298	Unique	Used	0.745	1.480	0.921	1.170	0	0	6.18	0	11	4	2	1296.26001	2591.51274	-3.92
dLVLGPSGVLQGIRPGk	Unambiguous	2	1	Q922P9	N-Term(iTRAQ8plex); K17(iTRAQ8plex)	HCD	1.0000	0.0000	1	1	3876303	Unique	Used	0.406	0.826	0.795	0.920	0	0	6.18	0	20	92	3	772.13770	2314.39853	-3.55
kEEAPAAGGQSAVQETFSR	Unambiguous	1	1	A2A7B5	N-Term(iTRAQ8plex); K1(iTRAQ8plex)	CID	1.0000	0.0000	1	1	2913318	Unique	Used	1.523	0.973	1.079	0.778	0	0	6.18	1	0	200	3	857.78937	2571.35355	-3.43
cHAPLAQAQALVTSELER	Unambiguous	1	1	Q9CR98	N-Term(iTRAQ8plex); C1(Methylthio)	CID	1.0000	0.0000	1	1	3686031	Unique	Used	1.396	1.094	0.480	0.776	0	0	6.18	0	15	7	3	763.05981	2287.16489	-8.40
iQEGVFDINNEAnGlk	Unambiguous	1	1	P53994	N-Term(iTRAQ8plex); N13(Deamidated); K16(iTRAQ8plex)	CID	1.0000	0.0000	1	1	2832183	Unique	Used	0.985	0.996	1.106	1.084	0	0	6.18	0	0	200	2	1185.64014	2370.27300	-1.34
nSYVAGQYDDAASYk	Unambiguous	2	1	Q00612	N-Term(iTRAQ8plex); K15(iTRAQ8plex)	CID	1.0000	0.0000	1	1	2827838	Unique	Used	0.485	0.605	1.026	1.569	0	0	6.17	0	0	25	2	1130.56787	2260.12847	-2.54
fGIVTSSAGTGTTEDTEAk	Unambiguous	1	1	Q9D1J3	N-Term(iTRAQ8plex); K19(iTRAQ8plex)	CID	1.0000	0.0000	1	1	2916809	Unique	Used	0.476	0.156	1.173	1.114	0	0	6.17	0	17	200	2	1240.65027	2480.29326	-1.79

gkGTVAAAASGAAGGGGGAGAGAPGGGR	Unambiguous	2	1	Q7TPD0	N-Term(iTRAQ8plex); K2(iTRAQ8plex)	CID	1.0000	0.0000	1	1	3695764	Unique	Used			2.436		0	0	6.17	1	0	200	3	911.82147	2733.44986	-3.79
fNALFAQGNYSEAAk	Unambiguous	2	1	Q68FD5	N-Term(iTRAQ8plex); K15(iTRAQ8plex)	CID	1.0000	0.0000	1	1	3517307	Unique	Used	0.585	1.118	1.347	1.244	0	0	6.17	0	0	62	2	1120.09668	2239.18608	-4.78
eSTLEAIGYIcQDIDPEQLQDk	Unambiguous	1	1	P70168	N-Term(iTRAQ8plex); C11(Methylthio); K22(iTRAQ8plex)	CID	1.0000	0.0000	1	1	3112434	Unique	Used			1.390		0	0	6.17	0	3	97	3	1054.86499	3162.58042	0.27
mPFPVNHGASSEDSLLQDAAk	Unambiguous	1	1	Q9R0P9	N-Term(iTRAQ8plex); K21(iTRAQ8plex)	CID	1.0000	0.0000	1	1	3212329	Unique	Used	3.316	1.458	1.385	1.324	0	0	6.17	0	0	58	3	941.49097	2822.45835	-0.72
rSPASPSQAPYAQR	Unambiguous	7	1	D3Z5R9	N-Term(iTRAQ8plex)	HCD	1.0000	0.0000	1	1	3791794	Unique	Used	1.191	0.992	1.296	1.208	0	0	6.17	1	0	200	3	607.32727	1819.96726	-2.43
eSQASYSLELVAQDGGRPPR	Unambiguous	3	1	Q7TSK3-3	N-Term(iTRAQ8plex)	HCD	1.0000	0.0000	1	1	3105681	Unique	Used	1.677	1.811	0.942	0.824	0	0	6.17	0	0	129	3	822.09497	2464.27036	-1.25
kEDGSEVGVGGAQVTGSNTR	Unambiguous	3	1	Q9JLV5	N-Term(iTRAQ8plex); K1(iTRAQ8plex)	CID	1.0000	0.0000	1	1	2773506	Unique	Used	0.316	0.582	0.998	1.183	0	0	6.17	1	0	200	3	852.78607	2556.34366	-1.49
mYGADLAPVDFLHASEDAR	Unambiguous	1	1	Q9D154	N-Term(iTRAQ8plex)	CID	1.0000	0.0000	1	1	3131431	Unique	Used	2.090	0.713	5.583	6.707	0	0	6.17	0	2	24	3	794.72705	2382.16660	-1.52
qTVLTAAGSIGQASGDLLR	Unambiguous	2	1	E9PUM4	N-Term(iTRAQ8plex)	CID	1.0000	0.0000	1	1	3841711	Unique	Used	0.734	1.058	1.063	1.049	0	0	6.17	0	5	52	2	1081.60498	2162.20268	-2.61
gTGGVDTAAVGGVFDVSNADR	Unambiguous	1	1	Q04447	N-Term(iTRAQ8plex)	CID	1.0000	0.0000	1	1	3132594	Unique	Used	1.535	1.259	1.384	1.340	0	0	6.17	0	0	200	2	1135.06909	2269.13091	-2.38
tGEmSGPVFTDSGIHIILR	Unambiguous	1	1	Q9QUR7	N-Term(iTRAQ8plex); M4(Oxidation)	CID	1.0000	0.0000	1	1	3641334	Unique	Used	1.943	1.127	0.982	0.945	0	0	6.17	0	19	11	3	784.07971	2350.22458	-5.67
lLEIPAVSHVcADLLER	Unambiguous	4	1	O88939-2	N-Term(iTRAQ8plex); C11(Methylthio)	CID	1.0000	0.0000	1	1	3766414	Unique	Used	0.399	0.566	0.808	0.850	0	0	6.17	0	10	24	3	743.40546	2228.20182	-3.01
dMAIATGGAVFGEEGLNLNLEDVQAHDLGk	Unambiguous	1	1	P63038	N-Term(iTRAQ8plex); K30(iTRAQ8plex)	CID	1.0000	0.0000	1	1	3285585	Unique	Used	1.494	0.856	2.899	3.330	0	0	6.17	0	0	200	3	1231.63989	3692.90512	-0.01
gTGGVDTAAVGGVFDVSNADR	Unambiguous	1	1	Q04447	N-Term(iTRAQ8plex)	HCD	1.0000	0.0000	1	1	3130187	Unique	Used	2.176	1.156	1.225	1.233	0	0	6.17	0	10	76	2	1135.07104	2269.13481	-0.66

eDDGVPVIcQVEHPAVTGNLQTQR	Unambiguous	9	1	Q8R5M8-4	N-Term(iTRAQ8plex); C9(Methylthio)	CID	1.0000	0.0000	1	1	3170147	Unique	Used	1.174	1.762	1.247	0.897	0	0	6.17	0	5	39	3	985.82288	2955.45407	-2.24
yEGSGDGGAAAQSLYIANHAY	Unambiguous	1	1	P05063	N-Term(iTRAQ8plex)	CID	1.0000	0.0000	1	1	2937459	Unique	Used	1.247	0.440	0.800	1.171	0	0	6.17	0	3	19	2	1210.07544	2419.14360	-1.33
eVSELSETFISGSEk	Unambiguous	4	1	E9PWI6	N-Term(iTRAQ8plex); K15(iTRAQ8plex)	CID	1.0000	0.0000	1	1	2832525	Unique	Used	1.500	0.567	0.947	0.609	0	0	6.17	0	14	200	2	1125.60364	2250.20000	1.70
nSYVAGQYDDAASYk	Unambiguous	2	1	Q00612	N-Term(iTRAQ8plex); K15(iTRAQ8plex)	CID	1.0000	0.0000	1	1	2827702	Unique	Used	0.731	0.768	0.798	1.044	0	0	6.17	0	0	200	2	1130.56543	2260.12358	-4.70
sLEQSEAAVAQVLEER	Unambiguous	3	1	A2AHG0	N-Term(iTRAQ8plex)	CID	1.0000	0.0000	1	1	2871118	Unique	Used	1.002	1.949	2.227	0.847	0	0	6.16	0	9	56	2	1032.04810	2063.08891	-1.63
kEPVANQDPVSPSLVQGR	Unambiguous	1	1	Q9ERZ4	N-Term(iTRAQ8plex); K1(iTRAQ8plex)	HCD	1.0000	0.0000	1	1	3184684	Unique	Used	0.645	0.456	1.076	1.353	0	0	6.16	1	0	37	3	843.80939	2529.41361	-4.34
gGPPPPPPPPHSSGPPPPAR	Unambiguous	1	1	Q91YD9	N-Term(iTRAQ8plex)	HCD	1.0000	0.0000	1	1	3404826	Unique	Used	0.884	1.138	1.203	0.818	0	0	6.16	0	9	187	3	765.41406	2294.22763	-3.14
cGQSAAVASPGGSIDSR	Unambiguous	3	1	B7FAU9	N-Term(iTRAQ8plex); C1(Methylthio)	CID	1.0000	0.0000	1	1	3295987	Unique	Used	0.326	3.025	0.886	1.853	0	0	6.16	0	0	130	2	956.95850	1912.90972	-3.24
kLPSVSQGANDVALAk	Unambiguous	1	1	Q2KN98	N-Term(iTRAQ8plex); K1(iTRAQ8plex); K16(iTRAQ8plex)	HCD	1.0000	0.0000	1	1	3365787	Unique	Used	0.706	0.660	0.909	0.699	0	0	6.16	1	11	168	3	837.50031	2510.48636	-8.21
rPEGQSAISYDYSEEELMASIER	Unambiguous	1	1	Q8R4T1	N-Term(iTRAQ8plex)	CID	1.0000	0.0000	1	1	2850913	Unique	Used	2.283	2.523	0.941	0.944	0	0	6.16	0	0	200	3	988.80707	2964.40665	-4.48
iQEGVFDINNEAnGIk	Unambiguous	1	1	P53994	N-Term(iTRAQ8plex); N13(Deamidated); K16(iTRAQ8plex)	CID	0.0357	0.0000	1	1	2832506	Unique	Used	1.010	1.091	0.689	1.061	0	0	6.16	0	5	174	2	1185.63721	2370.26714	-3.81
sSGSPYGGGYGSGGGSGGYGSR	Unambiguous	7	1	J3QNY1	N-Term(iTRAQ8plex)	CID	1.0000	0.0000	1	1	3005561	Unique	Used	1.644	0.722	1.909	0.995	0	0	6.16	0	0	200	2	1107.99890	2214.99053	-2.20
vFSSSQGVEQVVLGLYIVR	Unambiguous	1	1	Q6ZWM4	N-Term(iTRAQ8plex)	CID	1.0000	0.0000	1	1	3917889	Unique	Used	2.534	0.552	0.967	0.669	0	0	6.16	0	8	23	2	1192.67236	2384.33745	-4.91
gLLGALTSTPYSPTQHLER	Unambiguous	1	1	Q921M7	N-Term(iTRAQ8plex)	CID	1.0000	0.0000	1	1	3876793	Unique	Used	1.002	1.248	1.107	1.119	0	0	6.16	0	26	95	3	782.42786	2345.26902	-3.28

tHnSALEYNIFEGMEcR	Unambiguous	1	1	O08553	N-Term(iTRAQ8plex); N3(Deamidated); C16(Methylthio)	CID	1.0000	0.0000	1	1	3130788	Unique	Used	2.596	1.448	1.215	1.435	0	0	6.16	0	12	74	3	789.02362	2365.05631	-0.08
eDGLDGETIFMYMFLAGLGLLVVVGLHQLLESR	Unambiguous	1	1	Q9CY50	N-Term(iTRAQ8plex)	CID	1.0000	0.0000	1	1	3919632	Unique	Used		1.962	0.325	0.442	0	0	6.16	0	11	200	3	1314.02844	3940.07077	-5.07
sGEEDFESLASQFSDcSSAk	Unambiguous	1	1	Q9QUR7	N-Term(iTRAQ8plex); C16(Methylthio); K20(iTRAQ8plex)	CID	1.0000	0.0000	1	1	2960531	Unique	Used		1.361	0.917	0.538	0	0	6.16	0	28	48	3	926.76196	2778.27134	0.65
hGEASSANSAGGPTSDAGAAVAPnPGPR	Unambiguous	1	1	Q80W04	N-Term(iTRAQ8plex); N24(Deamidated)	CID	1.0000	0.0000	1	1	2985928	Unique	Used	1.575	0.976	1.379	1.331	0	0	6.16	0	0	200	3	936.78790	2808.34916	1.43
mYGADLAPVDFLHASEDAR	Unambiguous	1	1	Q9D154	N-Term(iTRAQ8plex); M1(Oxidation)	CID	1.0000	0.0000	1	1	3109362	Unique	Used	1.833	0.814	3.992	6.636	0	0	6.16	0	4	85	3	800.05774	2398.15866	-2.70
nLHQSGFSLSGAQIDDNIPR	Unambiguous	1	1	O08553	N-Term(iTRAQ8plex)	CID	1.0000	0.0000	1	1	3493905	Unique	Used	1.643	1.348	0.910	1.185	0	0	6.16	0	0	77	3	825.09302	2473.26450	-3.75
eLGDDcNFGPALGEVGEAMR	Unambiguous	5	1	Q62420	N-Term(iTRAQ8plex); C6(Methylthio)	CID	1.0000	0.0000	1	1	3037603	Unique	Used	0.794	0.915	1.205	0.659	0	0	6.16	0	6	17	2	1215.55396	2430.10063	-1.48
nQNPDLTGLcEPTPQDHIk	Unambiguous	1	1	P22682	N-Term(iTRAQ8plex); C10(Methylthio); K19(iTRAQ8plex)	CID	1.0000	0.0000	1	1	3051863	Unique	Used	0.747	1.160	1.038	1.292	0	0	6.16	0	10	34	3	925.47412	2774.40781	0.57
eHINLGcDVDFDIAGPSIR	Unambiguous	3	1	Q60932-2	N-Term(iTRAQ8plex); C7(Methylthio)	CID	1.0000	0.0000	1	1	3398410	Unique	Used	1.938		1.121	0.712	0	0	6.16	0	24	200	3	807.73059	2421.17722	-3.01
gPAVGIDLGTTYScVGVFQHGk	Unambiguous	4	1	P63017	N-Term(iTRAQ8plex); C14(Methylthio); K22(iTRAQ8plex)	CID	1.0000	0.0000	1	1	3917004	Unique	Used	0.743	0.873	0.875	0.737	0	0	6.16	0	25	12	3	954.16522	2860.48111	-4.73
tFTTQETITnAETAk	Unambiguous	1	1	P06745	N-Term(iTRAQ8plex); N10(Deamidated); K15(iTRAQ8plex)	CID	1.0000	0.0000	1	1	2715004	Unique	Used	1.679	1.569	1.362	1.170	0	0	6.16	0	0	200	2	1133.10535	2265.20342	-1.61

aLEVFPEFAAAHSNLASVLQQQGk	Unambiguous	2	1	Q8CGY8	N-Term(iTRAQ8plex); K24(iTRAQ8plex)	CID	1.0000	0.0000	1	1	3785068	Unique	Used	1.359	1.382	0.713	0.576	0	0	6.16	0	24	54	3	1055.25122	3163.73911	0.95
gDGAALQEMTSQINQk	Unambiguous	2	1	Q8C8T8	N-Term(iTRAQ8plex); K16(iTRAQ8plex)	CID	1.0000	0.0000	1	1	3147833	Unique	Used	1.438	1.714	1.526	1.166	0	0	6.15	0	0	12	2	1150.10889	2299.21050	-2.95
rEHANIDAQSGSQAPNPSTTISPGk	Unambiguous	3	1	Q8VDQ8-2	N-Term(iTRAQ8plex); K25(iTRAQ8plex)	CID	1.0000	0.0000	1	1	3141473	Unique	Used	1.106	0.281	1.187	1.094	0	0	6.15	1	0	90	3	1057.88879	3171.65183	-2.69
yFATVSGSASEANVEEk	Unambiguous	3	1	D3YZ62	N-Term(iTRAQ8plex); K17(iTRAQ8plex)	CID	1.0000	0.0000	1	1	2848158	Unique	Used	0.436	0.809	1.018	1.261	0	0	6.15	0	15	200	2	1199.11621	2397.22515	-5.96
vNAGDQPGADLGPLITPQAk	Unambiguous	1	1	Q9EQ20	N-Term(iTRAQ8plex); K20(iTRAQ8plex)	CID	1.0000	0.0000	1	1	3279947	Unique	Used	0.490	1.363	0.830	1.195	0	0	6.15	0	7	24	2	1285.72119	2570.43511	-1.87
kGALETESSSSSAQVSTVGQASR	Unambiguous	2	1	Q8BKI2	N-Term(iTRAQ8plex); K1(iTRAQ8plex)	CID	1.0000	0.0000	1	1	3142914	Unique	Used			1.250		0	0	6.15	1	0	200	3	959.17639	2875.51462	-2.49
tHnSALEYNIFEGMEcR	Unambiguous	1	1	O08553	N-Term(iTRAQ8plex); N3(Deamidated); C16(Methylthio)	CID	1.0000	0.0000	1	1	3172171	Unique	Used	1.299	1.167	1.025	0.953	0	0	6.15	0	26	9	3	789.02228	2365.05228	-1.78
fSGWYDADLSPAGHEEAk	Unambiguous	1	1	Q9DBJ1	N-Term(iTRAQ8plex); K18(iTRAQ8plex)	CID	1.0000	0.0000	1	1	2918000	Unique	Used	2.869	1.223	0.748	1.131	0	0	6.15	0	21	71	3	863.42932	2588.27341	-5.56
aLIFVSHGAGEHcGR	Unambiguous	5	1	O35678	N-Term(iTRAQ8plex); C13(Methylthio)	CID	1.0000	0.0000	1	1	3818273	Unique	Used	0.600	0.769	0.988	0.779	0	0	6.15	0	20	97	3	635.32153	1903.95005	-3.82
gTGGVDTAAVGGVFDVSNADR	Unambiguous	1	1	Q04447	N-Term(iTRAQ8plex)	CID	1.0000	0.0000	1	1	3129887	Unique	Used	1.553	1.390	2.001	1.412	0	0	6.15	0	0	58	2	1135.06836	2269.12944	-3.02
gTGGVDTAAVGGVFDVSNADR	Unambiguous	1	1	Q04447	N-Term(iTRAQ8plex)	CID	1.0000	0.0000	1	1	3134203	Unique	Used	1.407	1.299	1.236	1.220	0	0	6.15	0	18	200	2	1135.06970	2269.13213	-1.84
sFNLTAGSLESTEYTYVHk	Unambiguous	4	1	Q99K01	N-Term(iTRAQ8plex); K19(iTRAQ8plex)	CID	1.0000	0.0000	1	1	3346310	Unique	Used	0.620	0.955	0.664	1.715	0	0	6.15	0	26	17	3	919.14789	2755.42911	-3.92

qFLEcAQNQSDVk	Unambiguous	3	1	B2RPU8	N-Term(iTRAQ8plex); C5(Methylthio); K13(iTRAQ8plex)	CID	1.0000	0.0000	1	1	3167923	Unique	Used	1.922	0.870	1.157	0.962	0	0	6.15	0	12	13	3	722.03607	2164.09366	-2.34
sLEDALSSDTSGHFR	Unambiguous	2	1	P14824	N-Term(iTRAQ8plex)	CID	1.0000	0.0000	1	1	3231895	Unique	Used	1.212	1.395	0.892	1.090	0	0	6.14	0	12	18	3	642.65454	1925.94907	-0.84
aTAAFILANEHNVALFk	Unambiguous	2	1	Q8BKC5	N-Term(iTRAQ8plex); K17(iTRAQ8plex)	HCD	1.0000	0.0000	1	1	3804704	Unique	Used	0.367	1.337	0.889	1.104	0	0	6.14	0	4	19	3	813.47052	2438.39701	-1.90
gGLHTVAVmNVGAPAAGMNAAVR	Unambiguous	1	1	P47857	N-Term(iTRAQ8plex); M9(Oxidation)	CID	1.0000	0.0000	1	1	3891707	Unique	Used	1.681	1.329	1.082	1.072	0	0	6.14	0	0	141	3	828.77142	2484.29972	-4.87
tNVSGGAIALGHPLGGSGSR	Unambiguous	1	1	Q8BWT1	N-Term(iTRAQ8plex)	HCD	1.0000	0.0000	1	1	3890800	Unique	Used	0.965	1.083	1.264	1.225	0	0	6.14	0	14	200	3	704.71747	2112.13785	-4.04
gcITIIGGGDTATccAk	Unambiguous	1	1	P09411	N-Term(iTRAQ8plex); C2(Methylthio); C14(Methylthio); C15(Methylthio); K17(iTRAQ8plex)	CID	1.0000	0.0000	1	1	3726379	Unique	Used	0.265	0.803	1.049	1.239	0	0	6.14	0	0	48	2	1165.54419	2330.08110	-6.64
nLHQSGFSLSGTQVDEGVR	Unambiguous	3	1	E9PWE8	N-Term(iTRAQ8plex)	CID	1.0000	0.0000	1	1	3492253	Unique	Used	2.414	0.976	0.930	0.942	0	0	6.14	0	12	42	3	779.06506	2335.18064	-5.92
lGPSDYFGEIALLLNRPR	Unambiguous	2	1	P12849	N-Term(iTRAQ8plex)	HCD	1.0000	0.0000	1	1	3917653	Unique	Used	0.531	0.979	1.006	1.070	0	0	6.14	0	0	16	3	779.10339	2335.29563	-5.14
gTGGVDTAAVGGVFDVSNADR	Unambiguous	1	1	Q04447	N-Term(iTRAQ8plex)	CID	1.0000	0.0000	1	1	3155148	Unique	Used	0.551	1.100	1.595	2.132	0	0	6.14	0	0	200	2	1135.07043	2269.13359	-1.19
smSQIYcIENAHGQLVR	Unambiguous	1	1	Q8K0G5	N-Term(iTRAQ8plex); M2(Oxidation); C7(Methylthio)	CID	1.0000	0.0000	1	1	3739491	Unique	Used	1.129	1.022	0.808	0.954	0	0	6.14	0	22	88	3	772.37665	2315.11539	-4.08
eGYVLTAVEGTVGDFk	Unambiguous	1	1	Q6GQT9	N-Term(iTRAQ8plex); K16(iTRAQ8plex)	CID	1.0000	0.0000	1	1	3173644	Unique	Used	0.890	0.814	0.691	0.712	0	0	6.14	0	11	21	2	1147.12744	2293.24761	-2.63
yLLSQSSPAPLTAAEEELR	Unambiguous	1	1	Q91YR1	N-Term(iTRAQ8plex)	HCD	1.0000	0.0000	1	1	3172362	Unique	Used	0.945	0.864	0.937	1.041	0	0	6.14	0	0	200	2	1190.13831	2379.26933	-0.69
gLGQMTTNADTDGDSYk	Unambiguous	2	1	Q9QYB8	N-Term(iTRAQ8plex); K17(iTRAQ8plex)	CID	1.0000	0.0000	1	1	2733374	Unique	Used			1.784	1.049	0	0	6.14	0	11	200	2	1191.58521	2382.16313	-3.04

sQSLTnSLSTSDTSHR	Unambiguous	1	1	Q8JZP2	N-Term(iTRAQ8plex); N6(Deamidated)	CID	1.0000	0.0000	1	1	2969146	Unique	Used	1.253	0.665	1.147	1.112	0	0	6.14	0	18	44	3	676.00323	2025.99515	-1.93
lALSMLGQSDAYGAADAR	Unambiguous	3	1	O09012	N-Term(iTRAQ8plex)	CID	1.0000	0.0000	1	1	3518238	Unique	Used			0.746	0.803	0	0	6.14	0	0	117	2	1057.54114	2114.07500	-4.93
qHLNNMEAQLASQSTQR	Unambiguous	2	1	Q7M739	N-Term(iTRAQ8plex)	CID	1.0000	0.0000	1	1	3384912	Unique	Used	1.113	0.944	1.018	1.336	0	0	6.14	0	11	112	3	754.04761	2260.12827	-5.47
iFHAPNSSAPSSSLSTFk	Unambiguous	1	1	A2BDX0	N-Term(iTRAQ8plex); K18(iTRAQ8plex)	CID	1.0000	0.0000	1	1	3779766	Unique	Used	0.883	1.175	0.902	0.823	0	0	6.14	0	26	200	3	829.45264	2486.34336	-2.67
hLNEIDLFHcIDPNDSk	Unambiguous	2	1	Q9R0Q7	N-Term(iTRAQ8plex); C10(Methylthio); K17(iTRAQ8plex)	CID	1.0000	0.0000	1	1	3214770	Unique	Used	1.321	0.822	0.898	0.778	0	0	6.13	0	2	30	3	888.78223	2664.33213	-1.86
lATPTYGDLNHLVSATMSGVTTSLR	Unambiguous	1	1	Q9ERD7	N-Term(iTRAQ8plex)	CID	1.0000	0.0000	1	1	3939044	Unique	Used		0.171	1.168	1.317	0	0	6.13	0	6	200	3	970.51312	2909.52481	-3.33
aASGGSSGENVGPPAAVPGPLSR	Unambiguous	1	1	Q6ZQ29-2	N-Term(iTRAQ8plex)	CID	1.0000	0.0000	1	1	3341414	Unique	Used	0.940	0.613	0.988	0.930	0	0	6.13	0	0	28	2	1170.10962	2339.21196	-5.91
yEGSGDGGAAAQSLYIANHAY	Unambiguous	1	1	P05063	N-Term(iTRAQ8plex)	CID	1.0000	0.0000	1	1	2937616	Unique	Used	1.335	0.632	0.903	1.147	0	0	6.13	0	0	60	2	1210.07458	2419.14189	-2.03
aVGQPAAAAGHFQR	Unambiguous	2	1	Q00558	N-Term(iTRAQ8plex)	CID	1.0000	0.0000	1	1	3653831	Unique	Used	0.711	1.188	1.566	1.054	0	0	6.13	0	18	54	3	562.30817	1684.90995	-5.10
aAEFLAAcAGGDLDEAR	Unambiguous	1	1	Q3UMT1	N-Term(iTRAQ8plex); C8(Methylthio)	CID	1.0000	0.0000	1	1	3196302	Unique	Used	0.872	0.636	1.143	1.089	0	0	6.13	0	0	28	2	1015.48590	2029.96453	0.97
vLLVIDEPHTDWAk	Unambiguous	3	1	O88935	N-Term(iTRAQ8plex); K14(iTRAQ8plex)	CID	1.0000	0.0000	1	1	3263651	Unique	Used	1.045	1.939	1.355	1.131	0	0	6.13	0	30	200	3	748.76532	2244.28141	-1.55
lIEEcVSQIVLHk	Unambiguous	5	1	D3Z074	N-Term(iTRAQ8plex); C5(Methylthio); K13(iTRAQ8plex)	CID	1.0000	0.0000	1	1	3727095	Unique	Used	0.425	1.039	1.037	1.026	0	0	6.13	0	0	21	3	722.41010	2165.21573	-5.80
qLLHNFPPDQLTSSGAPFWSGPk	Unambiguous	1	1	Q02053	N-Term(iTRAQ8plex); K23(iTRAQ8plex)	CID	1.0000	0.0000	1	1	3841043	Unique	Used			0.903	1.054	0	0	6.13	0	8	200	3	1044.89050	3132.65696	-5.05
nMQLSNTPDSGISLLHk	Unambiguous	3	1	E9QNG1	N-Term(iTRAQ8plex); K17(iTRAQ8plex)	CID	1.0000	0.0000	1	1	3494508	Unique	Used	1.460	0.872	1.224	1.169	0	0	6.13	0	23	121	3	821.78510	2463.34073	-3.20

sLQDIIAILGMDELSEEDkLTVSR	Unambiguous	1	1	P56480	N-Term(iTRAQ8plex); K19(iTRAQ8plex)	HCD	1.0000	0.0000	1	1	3353809	Unique	Used	1.040	1.131	1.197	1.153	0	0	6.13	1	0	43	3	1095.26489	3283.78012	-3.51
aILITGcDSGFGFSLAk	Unambiguous	2	1	Q80XN0	N-Term(iTRAQ8plex); C7(Methylthio); K17(iTRAQ8plex)	CID	1.0000	0.0000	1	1	3860665	Unique	Used	0.663	0.264	0.533	0.513	0	0	6.13	0	27	16	2	1177.63379	2354.26030	-4.50
tLPTLSEDPSGAGITGYk	Unambiguous	1	1	Q9WTR1	N-Term(iTRAQ8plex); K18(iTRAQ8plex)	CID	1.0000	0.0000	1	1	3257347	Unique	Used	0.408	1.243	0.991	1.087	0	0	6.13	0	0	91	2	1208.16333	2415.31938	-1.41
fLSQPFQVAEVFTGHMGk	Unambiguous	1	1	P56480	N-Term(iTRAQ8plex); K18(iTRAQ8plex)	CID	1.0000	0.0000	1	1	3864343	Unique	Used	1.028	0.932	1.255	1.801	0	0	6.13	0	7	200	3	877.80841	2631.41068	-4.08
kDDEENYLDLFSHk	Unambiguous	1	1	Q60749	N-Term(iTRAQ8plex); K1(iTRAQ8plex); K14(iTRAQ8plex)	CID	1.0000	0.0000	1	1	2830039	Unique	Used	0.995	0.734	0.773	0.756	0	0	6.13	1	29	200	3	889.14569	2665.42252	-0.43
rSPATNPVSSSGGGMSDDDk	Unambiguous	1	1	Q921L5	N-Term(iTRAQ8plex); K20(iTRAQ8plex)	CID	1.0000	0.0000	1	1	2750296	Unique	Used	0.904	0.993	0.874	1.208	0	0	6.13	1	15	200	3	858.42615	2573.26389	-3.25
vQPGENSVLQLPDAPPAAPAPPPkPGR	Unambiguous	1	1	A2ACD2	N-Term(iTRAQ8plex); K24(iTRAQ8plex)	HCD	1.0000	0.0000	1	1	3515375	Unique	Used	1.766	1.284	0.861	0.869	0	0	6.13	0	0	200	3	1103.62036	3308.84653	-3.36
fLSQPFQVAEVFTGHmGk	Unambiguous	1	1	P56480	N-Term(iTRAQ8plex); M16(Oxidation); K18(iTRAQ8plex)	CID	1.0000	0.0000	1	1	3824277	Unique	Used	1.479	1.091	1.190	1.317	0	0	6.13	0	5	2	3	883.13983	2647.40494	-4.30
dLVILLYETALLSSGFSLEDPQTHANR	Unambiguous	1	1	P07901	N-Term(iTRAQ8plex)	CID	1.0000	0.0000	1	1	3786689	Unique	Used	1.450	0.755	0.763	0.811	0	0	6.13	0	10	200	3	1102.91943	3306.74375	-2.62
lYHDPNVnGGTELEPVEGNPYR	Unambiguous	2	1	Q8BH74	N-Term(iTRAQ8plex); N8(Deamidated)	CID	1.0000	0.0000	1	1	2635291	Unique	Used	1.002	1.078	1.247	0.984	0	0	6.13	0	7	200	3	925.78882	2775.35190	-0.33
lTLYDIAHTPGVAADLSHIETR	Unambiguous	1	1	P08249	N-Term(iTRAQ8plex)	CID	1.0000	0.0000	1	1	3460243	Unique	Used	3.248	1.195	0.802	1.103	0	0	6.13	0	20	200	3	899.81958	2697.44419	-2.67
rLQQTQAQVDEVVDIMR	Unambiguous	2	1	P63044	N-Term(iTRAQ8plex)	HCD	1.0000	0.0000	1	1	3370300	Unique	Used	1.253	2.935	1.520	1.053	0	0	6.13	1	8	15	3	778.41943	2333.24375	-4.81

lAMQEFMILPVGAESFR	Unambiguous	1	1	P17183	N-Term(iTRAQ8plex)	CID	1.0000	0.0000	1	1	3809155	Unique	Used	1.481	1.179	1.435	1.640	0	0	6.13	0	21	200	2	1122.09485	2243.18242	-2.08
gETGPAGPAGPIGPAGAR	Unambiguous	2	1	P11087-2	N-Term(iTRAQ8plex)	CID	1.0000	0.0000	1	1	3142910	Unique	Used	0.554	6.948	1.050	1.065	0	0	6.13	0	0	74	2	918.99506	1836.98284	-2.29
lTmQVTTLNENLGTVk	Unambiguous	1	1	Q8BL66	N-Term(iTRAQ8plex); M3(Oxidation); K16(iTRAQ8plex)	CID	1.0000	0.0000	1	1	3576841	Unique	Used			1.151		0	0	6.13	0	0	200	2	1193.67126	2386.33525	-5.02
iPNIYAIGDVVAGPMLAHk	Unambiguous	1	1	O08749	N-Term(iTRAQ8plex); K19(iTRAQ8plex)	CID	1.0000	0.0000	1	1	3824353	Unique	Used	1.639	1.247	1.445	1.178	0	0	6.13	0	0	8	3	863.16339	2587.47562	-5.21
gTGGVDTAAVGGVFDVSNADR	Unambiguous	1	1	Q04447	N-Term(iTRAQ8plex)	CID	1.0000	0.0000	1	1	3134311	Unique	Used	1.091	1.056	1.487	1.525	0	0	6.13	0	0	200	2	1135.07080	2269.13432	-0.87
aTDLLLDDSLVSLFGNR	Unambiguous	4	1	P99029-2	N-Term(iTRAQ8plex)	CID	1.0000	0.0000	1	1	3561232	Unique	Used	2.073	2.313	2.095	2.527	0	0	6.12	0	0	58	2	1077.08447	2153.16167	-6.48
aTLSDSEIETnSATSAIFGk	Unambiguous	24	1	A2AGR0	N-Term(iTRAQ8plex); N11(Deamidated); K20(iTRAQ8plex)	CID	1.0000	0.0000	1	1	3170623	Unique	Used	0.681	1.329	1.429	1.451	0	0	6.12	0	0	200	2	1326.19922	2651.39116	1.48
gDAFDFNVALQDHFk	Unambiguous	2	1	Q9D1J1	N-Term(iTRAQ8plex); K15(iTRAQ8plex)	CID	1.0000	0.0000	1	1	3302238	Unique	Used	1.155	0.670	0.660	0.805	0	0	6.12	0	13	77	3	778.07605	2332.21360	-2.00
fLSqPFQVAEVFTGHMGk	Unambiguous	1	1	P56480	N-Term(iTRAQ8plex); Q4(Deamidated); K18(iTRAQ8plex)	CID	0.0147	0.0000	1	1	3881929	Unique	Used	1.260	1.053	1.103	1.718	0	0	6.12	0	16	200	3	878.14349	2632.41593	3.99
aASGGSSGEnVGPPAAVPGPLSR	Unambiguous	1	1	Q6ZQ29-2	N-Term(iTRAQ8plex); N10(Deamidated)	CID	1.0000	0.0000	1	1	3366039	Unique	Used	0.847	1.133	1.179	0.681	0	0	6.12	0	0	200	2	1170.61255	2340.21782	3.42
qTTAAAAATFSEQVGGGSGGAGRGGAAAR	Unambiguous	3	1	O88935	N-Term(iTRAQ8plex)	HCD	1.0000	0.0000	1	1	3797018	Unique	Used	0.522	0.545	1.826	2.162	0	0	6.12	1	20	84	3	947.14642	2839.42472	-3.62
iNVYYNEATGnkYVPR	Unambiguous	1	1	Q9CWF2	N-Term(iTRAQ8plex); N11(Deamidated); K12(iTRAQ8plex)	HCD	1.0000	0.0000	1	1	3320257	Unique	Used	0.509	0.493	0.485	0.616	0	0	6.12	1	0	39	3	837.45392	2510.34720	-1.10

sTFQAHVAPVVcPEQVk	Unambiguous	1	1	O55091	N-Term(iTRAQ8plex); C12(Methylthio); K17(iTRAQ8plex)	CID	1.0000	0.0000	1	1	3535002	Unique	Used	0.511	0.476	0.701	1.155	0	0	6.12	0	3	7	3	832.11505	2494.33060	-4.05
qQLSAEELDAQLDAYNAR	Unambiguous	3	1	O08583	N-Term(iTRAQ8plex)	CID	1.0000	0.0000	1	1	2885331	Unique	Used	1.343	0.590	1.137	0.956	0	0	6.12	0	0	200	2	1170.08936	2339.17143	-2.86
vADVSISEVTTLLGnVHSPVVEGYVGENISGEVk	Unambiguous	3	1	P20357	N-Term(iTRAQ8plex); N15(Deamidated); K34(iTRAQ8plex)	CID	1.0000	0.0000	1	1	3397638	Unique	Used	1.538	2.457	1.596	1.104	0	0	6.12	0	30	200	3	1369.74011	4107.20578	2.41
tVQDALSSVQESDIAVVAR	Unambiguous	2	1	P33622	N-Term(iTRAQ8plex)	CID	1.0000	0.0000	1	1	3349716	Unique	Used	3.635	4.269	0.386	0.878	0	0	6.12	0	0	13	2	1146.61597	2292.22466	-4.48
aAVEEGIVLGGGcALLR	Unambiguous	2	1	P63038	N-Term(iTRAQ8plex); C13(Methylthio)	CID	1.0000	0.0000	1	1	3645883	Unique	Used	0.922	0.865	0.996	0.964	0	0	6.12	0	11	47	2	989.53711	1978.06694	-4.98
gFDPLLVEEALEMHQcSEEk	Unambiguous	3	1	Q8BH48-2	N-Term(iTRAQ8plex); C16(Methylthio); K20(iTRAQ8plex)	CID	1.0000	0.0000	1	1	2922118	Unique	Used	2.219	0.622	1.398	0.770	0	0	6.12	0	14	116	3	986.82086	2958.44803	-0.94
vSHVSTGGGASLELLEGk	Unambiguous	2	1	P09411	N-Term(iTRAQ8plex); K18(iTRAQ8plex)	CID	1.0000	0.0000	1	1	3277563	Unique	Used	0.722	0.955	1.493	1.104	0	0	6.12	0	0	26	2	1175.16296	2349.31865	-2.05
aFTGFIVEADTPGIHIGk	Unambiguous	1	1	P45952	N-Term(iTRAQ8plex); K18(iTRAQ8plex)	CID	1.0000	0.0000	1	1	3518740	Unique	Used	0.625	1.027	0.533	1.382	0	0	6.12	0	27	87	3	827.79999	2481.38541	-4.36
tSLPQHVmEPPVGAAAAGTSGSSLDSFSk	Unambiguous	1	1	Q6PE13	N-Term(iTRAQ8plex); M8(Oxidation); K29(iTRAQ8plex)	CID	1.0000	0.0000	1	1	3388995	Unique	Used	1.571	1.215	2.022	1.315	0	0	6.12	0	0	94	3	1151.92688	3453.76609	-3.49
lSLPPHEGPGGNLVAGALYR	Unambiguous	5	1	E9Q3M3	N-Term(iTRAQ8plex)	CID	1.0000	0.0000	1	1	3821409	Unique	Used	0.639	1.153	0.854	1.007	0	0	6.12	0	0	108	3	774.76495	2322.28031	-2.99
qLLHNFPPDQLTSSGAPFWSGPk	Unambiguous	1	1	Q02053	N-Term(iTRAQ8plex); K23(iTRAQ8plex)	CID	1.0000	0.0000	1	1	3841528	Unique	Used	0.968	1.541	0.773	0.936	0	0	6.11	0	12	200	3	1044.89355	3132.66611	-2.12

gTGGVDTAAVGGVFDVSnADR	Unambiguous	1	1	Q04447	N-Term(iTRAQ8plex); N18(Deamidated)	CID	1.0000	0.0000	1	1	2919449	Unique	Used	1.748	1.460	1.134	1.341	0	0	6.11	0	0	200	2	1135.55981	2270.11235	-3.51
mVQASSQSLLPPAQDRPR	Unambiguous	3	1	Q8BTI8	N-Term(iTRAQ8plex)	HCD	1.0000	0.0000	1	1	3872615	Unique	Used	0.972	1.264	1.556	1.116	0	0	6.11	0	6	174	3	762.41327	2285.22525	-3.75
qEEcSPQDNALGLMHLR	Unambiguous	3	1	Q6VNB8	N-Term(iTRAQ8plex); C4(Methylthio)	CID	1.0000	0.0000	1	1	3212876	Unique	Used	0.956	0.913	1.095	1.116	0	0	6.11	0	3	18	3	764.36725	2291.08719	-0.56
sMATIMTDEIFHDVAYk	Unambiguous	2	1	Q8JZR6	N-Term(iTRAQ8plex); K17(iTRAQ8plex)	CID	1.0000	0.0000	1	1	3284080	Unique	Used	0.475	0.314	1.083	1.385	0	0	6.11	0	0	24	3	860.78076	2580.32773	-0.83
vnNASLIGLGYTQTLRPGVk	Unambiguous	3	1	J3QPE8	N-Term(iTRAQ8plex); N2(Deamidated); K20(iTRAQ8plex)	HCD	1.0000	0.0000	1	1	3781985	Unique	Used	0.536	1.298	1.245	1.422	0	0	6.11	0	0	117	3	904.19092	2710.55820	-4.81
qmAEIAVNAVLTVADMER	Unambiguous	2	1	P80316	N-Term(iTRAQ8plex); M2(Oxidation)	CID	1.0000	0.0000	1	1	3666077	Unique	Used	1.686	0.990	0.874	1.093	0	0	6.11	0	0	20	2	1141.09009	2281.17290	-4.63
yEGSGDGGAAAQSLYIAnHAY	Unambiguous	1	1	P05063	N-Term(iTRAQ8plex); N18(Deamidated)	CID	1.0000	0.0000	1	1	2760567	Unique	Used	1.544	0.619	1.088	1.050	0	0	6.11	0	0	163	2	1210.56799	2420.12871	-0.87
gGGGGGGGPGGFQPAPR	Unambiguous	1	1	O35295	N-Term(iTRAQ8plex)	CID	1.0000	0.0000	1	1	3382293	Unique	Used	1.082	0.638	0.726	0.526	0	0	6.11	0	4	14	2	872.44092	1743.87456	-4.80
acDSSSSSSLGSGEAAGLGR	Unambiguous	1	1	Q80TG9	N-Term(iTRAQ8plex); C2(Methylthio)	CID	1.0000	0.0000	1	1	3068525	Unique	Used	0.737	0.805	1.394	1.523	0	0	6.11	0	0	200	2	1074.99243	2148.97759	-1.30
qAEIENPLEDPVTGDYVHk	Unambiguous	4	1	Q9Z1G4-3	N-Term(iTRAQ8plex); K19(iTRAQ8plex)	CID	1.0000	0.0000	1	1	2737815	Unique	Used		2.219	1.043	1.248	0	0	6.11	0	0	200	3	921.48596	2762.44333	-0.88
aGQTVTVWAAGAGATHSPPSTLVWk	Unambiguous	2	1	P21619	N-Term(iTRAQ8plex); K25(iTRAQ8plex)	CID	1.0000	0.0000	1	1	3857561	Unique	Used	0.690	1.113	1.323	1.213	0	0	6.11	0	0	77	3	1034.56677	3101.68576	-4.37
hEGLDVIENAVDNLDSR	Unambiguous	3	1	E9QN14	N-Term(iTRAQ8plex)	CID	1.0000	0.0000	1	1	2814841	Unique	Used	0.356	1.026	0.922	1.258	0	0	6.11	0	1	27	3	734.04266	2200.11344	-0.62
eVEHVLDADPELcAPFAESk	Unambiguous	2	1	F6ZGR6	N-Term(iTRAQ8plex); C13(Methylthio); K20(iTRAQ8plex)	CID	1.0000	0.0000	1	1	2871109	Unique	Used		0.604	2.305	1.067	0	0	6.10	0	15	200	3	951.81055	2853.41709	-3.12

eQGVTFPAIGSQAAEQAk	Unambiguous	3	1	Q3UGN9	N-Term(iTRAQ8plex); K18(iTRAQ8plex)	CID	1.0000	0.0000	1	1	3126366	Unique	Used	1.742	0.721	1.154	1.073	0	0	6.10	0	16	74	2	1220.67029	2440.33330	1.65
dINQEVYNFLATAGAk	Unambiguous	1	1	Q99KI0	N-Term(iTRAQ8plex); K16(iTRAQ8plex)	CID	1.0000	0.0000	1	1	3543546	Unique	Used	0.802	1.086	1.296	1.344	0	0	6.10	0	0	200	2	1181.63916	2362.27104	-6.47
eLDPTnMTYITNQAAVHFEk	Unambiguous	1	1	Q60864	N-Term(iTRAQ8plex); N6(Deamidated); K20(iTRAQ8plex)	CID	1.0000	0.0000	1	1	3348317	Unique	Used	1.471	0.765	1.334	1.128	0	0	6.10	0	17	46	3	977.84302	2931.51450	4.30
gSQPAGELLAAFELIQR	Unambiguous	6	1	Q9ESD7-3	N-Term(iTRAQ8plex)	CID	1.0000	0.0000	1	1	3727130	Unique	Used	1.524	0.873	0.701	0.766	0	0	6.10	0	0	44	2	1052.58142	2104.15557	-7.09
fVTDQSAcAGSPSLVAAAR	Unambiguous	2	1	Q8VCY8-2	N-Term(iTRAQ8plex); C8(Methylthio)	CID	1.0000	0.0000	1	1	3723852	Unique	Used	0.209	1.650	1.391	0.958	0	0	6.10	0	6	24	2	1101.04504	2201.08281	-7.67
aATAGSQAGPGGLVAGk	Unambiguous	2	1	Q8C4B4	N-Term(iTRAQ8plex); K17(iTRAQ8plex)	CID	1.0000	0.0000	1	1	3363964	Unique	Used	0.928	0.900	0.858	0.776	0	0	6.10	0	0	200	2	1011.07990	2021.15251	-3.72
aSTVAYLTSQLHAAk	Unambiguous	1	1	Q8VDN4	N-Term(iTRAQ8plex); K15(iTRAQ8plex)	HCD	1.0000	0.0000	1	1	3876159	Unique	Used	1.589	0.482	1.047	0.926	0	0	6.10	0	7	32	3	723.75104	2169.23856	-4.72
kVFVGGLSPDTSEEQIk	Unambiguous	3	1	D3YTQ3	N-Term(iTRAQ8plex); K1(iTRAQ8plex); K17(iTRAQ8plex)	CID	1.0000	0.0000	1	1	3105796	Unique	Used	1.027	0.740	1.248	0.760	0	0	6.10	1	8	44	3	916.19531	2746.57138	-1.48
sSEYHLEDNAAYYLNDLER	Unambiguous	1	1	O70443	N-Term(iTRAQ8plex)	CID	1.0000	0.0000	1	1	2780657	Unique	Used	0.958	1.633	1.496	1.371	0	0	6.10	0	26	200	3	869.41541	2606.23166	0.15
yEGSGDGGAAAQSLYIANHAY	Unambiguous	1	1	P05063	N-Term(iTRAQ8plex)	CID	1.0000	0.0000	1	1	2937294	Unique	Used	1.163	0.389	0.807	1.298	0	0	6.10	0	4	1	2	1210.07446	2419.14165	-2.13
mVISNPAATHQDIDFLIEEIER	Unambiguous	1	1	P48320	N-Term(iTRAQ8plex); M1(Oxidation)	CID	1.0000	0.0000	1	1	3195962	Unique	Used			1.617	1.100	0	0	6.10	0	6	27	3	954.49371	2861.46659	0.29
dLVILLYETALLSSGFSLEDPQTHANR	Unambiguous	1	1	P07901	N-Term(iTRAQ8plex)	CID	1.0000	0.0000	1	1	3787029	Unique	Used	0.916	0.759	0.536	0.609	0	0	6.10	0	9	200	3	1102.91907	3306.74265	-2.96
iEVGLVVGNSQVAFEk	Unambiguous	2	1	P46460	N-Term(iTRAQ8plex); K16(iTRAQ8plex)	CID	1.0000	0.0000	1	1	3393978	Unique	Used	0.448	1.415	1.457	1.306	0	0	6.10	0	0	18	2	1149.16504	2297.32280	-4.25

qVLYQQSSGHNNR	Unambiguous	2	1	Q99M71	N-Term(iTRAQ8plex)	CID	1.0000	0.0000	1	1	3792101	Unique	Used	3.929	0.776	1.161	1.478	0	0	6.10	0	7	200	3	612.31732	1834.93741	-4.77
sGYQQAASEHGLVVIAPDTSPR	Unambiguous	8	1	Q9R0P3	N-Term(iTRAQ8plex)	HCD	1.0000	0.0000	1	1	3253690	Unique	Used	1.032	0.751	0.821	0.784	0	0	6.10	0	7	17	3	863.11792	2587.33921	-1.02
eHHLSEVQNMASEEk	Unambiguous	1	1	Q91VA7	N-Term(iTRAQ8plex); K15(iTRAQ8plex)	CID	1.0000	0.0000	1	1	2648011	Unique	Used	0.472	0.621	1.484	1.398	0	0	6.10	0	0	97	2	1188.60168	2376.19609	-4.77
tVYFAEEVQcEGNSFHk	Unambiguous	1	1	P97315	N-Term(iTRAQ8plex); C10(Methylthio); K17(iTRAQ8plex)	CID	1.0000	0.0000	1	1	3322969	Unique	Used	3.595	1.036	1.784	1.775	0	0	6.10	0	18	179	3	881.42963	2642.27433	-3.65
qVLYQQSSGHNNR	Unambiguous	2	1	Q99M71	N-Term(iTRAQ8plex)	CID	1.0000	0.0000	1	1	3446668	Unique	Used	1.267	1.115	1.291	1.201	0	0	6.10	0	0	200	3	612.31866	1834.94144	-2.58
tSVAQTHLEDAGAAIAAAEAAVQqLR	Unambiguous	2	1	Q9QYX7	N-Term(iTRAQ8plex); Q24(Deamidated)	CID	0.0279	0.0000	1	1	3645021	Unique	Used	4.902	3.588	0.486	1.147	0	0	6.10	0	8	21	3	966.51544	2897.53177	1.60
gHAPSTAAPAPSPAPMSSSVQSDEER	Unambiguous	1	1	Q8C996	N-Term(iTRAQ8plex)	CID	1.0000	0.0000	1	1	2987341	Unique	Used	1.336	0.735	0.656	1.538	0	0	6.10	0	0	96	3	956.79120	2868.35904	-5.10
fYTAEIVSALEYLHGk	Unambiguous	4	1	F2Z3X6	N-Term(iTRAQ8plex); K16(iTRAQ8plex)	CID	1.0000	0.0000	1	1	3686642	Unique	Used	0.755	0.898	0.885	1.395	0	0	6.10	0	14	18	3	817.11865	2449.34140	-7.08
vDVYDEGSYTcSVQTQHEPk	Unambiguous	2	1	Q3TYE5	N-Term(iTRAQ8plex); C11(Methylthio); K20(iTRAQ8plex)	CID	1.0000	0.0000	1	1	2848358	Unique	Used	0.694	0.764	0.859	0.857	0	0	6.10	0	3	52	3	980.46936	2939.39353	-2.60
vGSDASSQQEFTScLk	Unambiguous	1	1	Q8VCI5	N-Term(iTRAQ8plex); C14(Methylthio); K16(iTRAQ8plex)	CID	1.0000	0.0000	1	1	3190260	Unique	Used	1.174	0.512	0.909	0.436	0	0	6.10	0	0	92	2	1171.08179	2341.15630	-2.63
wGEAGAEYVVESTGVFTTmEk	Unambiguous	3	1	P16858	N-Term(iTRAQ8plex); M19(Oxidation); K21(iTRAQ8plex)	CID	1.0000	0.0000	1	1	3219095	Unique	Used			1.477	1.052	0	0	6.10	0	20	200	3	972.48865	2915.45139	-2.74
lHQDPTQEcQPPAAGDR	Unambiguous	2	1	Q5SVR0-2	N-Term(iTRAQ8plex); C9(Methylthio)	CID	1.0000	0.0000	1	1	2880444	Unique	Used	0.927	0.872	0.856	1.193	0	0	6.10	0	4	79	3	738.34998	2213.03537	-1.26

tASRPEDTPDSPSGPSSPk	Unambiguous	4	1	Q6EDY6	N-Term(iTRAQ8plex); K19(iTRAQ8plex)	CID	1.0000	0.0000	1	1	2727428	Unique	Used	0.829	0.260	1.013	0.925	0	0	6.09	0	0	200	3	841.10388	2521.29709	-0.80
vVAGVAAALAHkYH	Unambiguous	1	1	A8DUK4	N-Term(iTRAQ8plex); K12(iTRAQ8plex)	HCD	1.0000	0.0000	1	1	3914625	Unique	Used	5.009	0.344	0.394	1.508	0	0	6.09	1	23	97	3	672.39917	2015.18296	-8.99
eSVNAAFEMTLTEGNk	Unambiguous	2	1	Q8BH95	N-Term(iTRAQ8plex); K16(iTRAQ8plex)	CID	1.0000	0.0000	1	1	3014088	Unique	Used	1.914	1.894	1.478	1.732	0	0	6.09	0	0	55	2	1175.11108	2349.21489	-2.90
qQIDFLDVYHGLR	Unambiguous	3	1	Q924L1	N-Term(iTRAQ8plex)	CID	1.0000	0.0000	1	1	3496998	Unique	Used	1.192	1.527	0.964	0.976	0	0	6.09	0	14	100	3	636.68036	1908.02652	-0.84
lGTEEFPLIDQTFYPNHk	Unambiguous	3	1	O88935	N-Term(iTRAQ8plex); K18(iTRAQ8plex)	CID	1.0000	0.0000	1	1	3303326	Unique	Used	1.247	1.464	1.075	1.056	0	0	6.09	0	0	129	3	919.82794	2757.46927	-0.57
tVAGIIVEPIQSEGGDNHASDDFFR	Unambiguous	2	1	P61922	N-Term(iTRAQ8plex)	CID	1.0000	0.0000	1	1	3035805	Unique	Used	2.238	0.943	0.926	1.147	0	0	6.09	0	13	106	3	993.49878	2978.48178	0.65
rLSLESEGANEGATAAPELSALEEAFR	Unambiguous	1	1	Q7TQD2	N-Term(iTRAQ8plex)	HCD	1.0000	0.0000	1	1	3195413	Unique	Used	2.137	0.837	1.661	1.377	0	0	6.09	1	0	22	3	1041.53479	3122.58982	-0.34
eITELATcAAVSEADIGR	Unambiguous	7	1	Q9QX47-3	N-Term(iTRAQ8plex); C8(Methylthio)	CID	1.0000	0.0000	1	1	3153527	Unique	Used	0.576	0.638	1.023	0.869	0	0	6.09	0	18	87	2	1100.04639	2199.08550	-3.84
mHIVEQTEFIDDQTFQPHR	Unambiguous	1	1	Q91YM2	N-Term(iTRAQ8plex)	CID	1.0000	0.0000	1	1	3148587	Unique	Used	1.670	1.315	1.181	1.001	0	0	6.09	0	28	77	3	892.44263	2675.31333	-2.12
vQNHLPASGPPQPPAASPTR	Unambiguous	10	1	Q62219-2	N-Term(iTRAQ8plex)	CID	1.0000	0.0000	1	1	3716580	Unique	Used	1.494	2.386	0.886	1.076	0	0	6.09	0	0	29	3	776.08527	2326.24125	-6.77
gTGGVDTAAVGGVFDVSNADR	Unambiguous	1	1	Q04447	N-Term(iTRAQ8plex)	CID	1.0000	0.0000	1	1	3130661	Unique	Used	1.488	1.124	1.270	1.357	0	0	6.09	0	0	121	2	1135.06860	2269.12993	-2.81
eLSSAVSPPNLHSDTPTFSYASLAGPTIPPR	Unambiguous	3	1	Q9QYR6	N-Term(iTRAQ8plex)	CID	1.0000	0.0000	1	1	3764235	Unique	Used	1.927	1.084	1.380	1.164	0	0	6.09	0	0	95	3	1171.93848	3513.80088	-4.54
sEAAAAAAGAASDPAAAR	Unambiguous	2	1	Q8CES0	N-Term(iTRAQ8plex)	CID	1.0000	0.0000	1	1	2952456	Unique	Used	1.635	1.035	1.001	0.841	0	0	6.09	0	10	200	2	916.97198	1832.93669	-2.07
tMIQQGGSIVNVGSIIGLk	Unambiguous	1	1	Q91VT4	N-Term(iTRAQ8plex); K19(iTRAQ8plex)	CID	1.0000	0.0000	1	1	3898282	Unique	Used	0.491	0.988	1.120	1.203	0	0	6.09	0	0	19	2	1262.23511	2523.46294	-6.34
vLAQNSGFDLQETLVk	Unambiguous	1	1	P80317	N-Term(iTRAQ8plex); K16(iTRAQ8plex)	CID	1.0000	0.0000	1	1	3349012	Unique	Used	0.629	1.145	0.916	1.189	0	0	6.09	0	0	22	2	1185.67480	2370.34233	-2.79

iSQAEEDDQQLLGHLLLVAk	Unambiguous	1	1	Q9D0S9	N-Term(iTRAQ8plex); K20(iTRAQ8plex)	CID	1.0000	0.0000	1	1	3262359	Unique	Used	0.983	1.515	1.134	1.219	0	0	6.09	0	17	8	3	943.53687	2828.59604	-0.64
iVHELNTTVPTASFAGk	Unambiguous	2	1	F8WJI3	N-Term(iTRAQ8plex); K17(iTRAQ8plex)	CID	1.0000	0.0000	1	1	3617836	Unique	Used	1.392	1.162	1.158	0.767	0	0	6.09	0	0	185	2	1197.18262	2393.35796	-2.91
aYSPLGGGAGSYcASSNADSR	Unambiguous	1	1	Q8R3Q0	N-Term(iTRAQ8plex); C13(Methylthio)	CID	1.0000	0.0000	1	1	3411533	Unique	Used	0.603	0.933	0.630	0.716	0	0	6.09	0	0	92	2	1171.02258	2341.03789	-4.79
hHAAYVNnLNATEEk	Unambiguous	1	1	P09671	N-Term(iTRAQ8plex); N8(Deamidated); K15(iTRAQ8plex)	CID	0.0016	0.0000	1	1	2728037	Unique	Used	2.395	0.891	1.136	0.822	0	0	6.09	0	24	200	3	774.07330	2320.20536	-3.81
qANVSEVMGPVEAAPEYR	Unambiguous	5	1	Q8CCF0-3	N-Term(iTRAQ8plex)	CID	1.0000	0.0000	1	1	2936687	Unique	Used	0.884	1.058	0.877	0.873	0	0	6.08	0	9	80	2	1126.06970	2251.13213	-0.44
yLEVqYkPQVHIQMTYPLQGLTR	Unambiguous	9	1	Q8R5M8-4	N-Term(iTRAQ8plex); Q5(Deamidated); K7(iTRAQ8plex)	HCD	0.0066	0.0000	1	1	3877615	Unique	Used	0.548	2.409	1.546	1.239	0	0	6.08	0	0	132	3	1138.96338	3414.87558	1.50
gFGTDEQAIVDVVSNR	Unambiguous	1	1	Q07076	N-Term(iTRAQ8plex)	CID	1.0000	0.0000	1	1	3110153	Unique	Used	0.607	2.584	1.620	1.682	0	0	6.08	0	0	5	2	1006.02313	2011.03899	-0.43
eHINLGcDVDFDIAGPSIR	Unambiguous	3	1	Q60932-2	N-Term(iTRAQ8plex); C7(Methylthio)	CID	1.0000	0.0000	1	1	3354617	Unique	Used	0.762	1.004	0.940	1.082	0	0	6.08	0	9	107	3	807.72998	2421.17539	-3.76
lDHALSSPSSPcEElk	Unambiguous	2	1	Q8BGT8	N-Term(iTRAQ8plex); C12(Methylthio); K16(iTRAQ8plex)	CID	1.0000	0.0000	1	1	3106822	Unique	Used	1.322	0.886	1.363	1.177	0	0	6.08	0	6	16	3	789.74182	2367.21091	-1.51
mATEVAADALGEEWk	Unambiguous	1	1	P62754	N-Term(iTRAQ8plex); K15(iTRAQ8plex)	CID	1.0000	0.0000	1	1	2886987	Unique	Used	0.459	0.612	0.588	1.006	0	0	6.08	0	7	80	3	743.72626	2229.16422	-1.80
dHIIEVVGSSMPLIGDHQDEDk	Unambiguous	3	1	O88935	N-Term(iTRAQ8plex); K22(iTRAQ8plex)	CID	1.0000	0.0000	1	1	2995683	Unique	Used	0.552	1.654	1.745	1.419	0	0	6.08	0	8	103	3	1014.85822	3042.56009	-2.05
mVTGDTLPSSGEAVLAGHNVAQER	Unambiguous	2	1	Q91V24	N-Term(iTRAQ8plex)	HCD	1.0000	0.0000	1	1	3255634	Unique	Used	1.365	0.881	1.136	1.256	0	0	6.08	0	20	91	3	915.13580	2743.39286	-2.15

vSPANAAQISSQATANR	Unambiguous	3	1	E9Q945	N-Term(iTRAQ8plex)	CID	1.0000	0.0000	1	1	3677474	Unique	Used	0.917	0.479	0.874	0.786	0	0	6.08	0	0	200	2	995.52759	1990.04790	-7.07
sIEDQLQPNSASGSGALEHDPSSIYLR	Unambiguous	3	1	Q8C547	N-Term(iTRAQ8plex)	CID	1.0000	0.0000	1	1	3107443	Unique	Used	0.835	0.946	1.041	1.014	0	0	6.08	0	0	200	3	1059.19751	3175.57798	-0.95
hTVTMIPGDGIGPELMLHVk	Unambiguous	1	1	P70404	N-Term(iTRAQ8plex); K20(iTRAQ8plex)	CID	1.0000	0.0000	1	1	3516807	Unique	Used	0.758	0.946	0.805	0.892	0	0	6.08	0	10	109	3	918.51044	2753.51676	-4.93
sNcNVAVINVGAPAAGMNAAVR	Unambiguous	3	1	Q8C605	N-Term(iTRAQ8plex); C3(Methylthio)	CID	1.0000	0.0000	1	1	3915235	Unique	Used	1.550	0.893	0.927	0.800	0	0	6.08	0	2	15	2	1225.11829	2449.22930	-5.05
qAEMEGAVQSIQGELSk	Unambiguous	1	1	O54724	N-Term(iTRAQ8plex); K17(iTRAQ8plex)	CID	1.0000	0.0000	1	1	3109877	Unique	Used	1.282	1.651	1.226	1.253	0	0	6.08	0	0	200	2	1207.14429	2413.28130	-1.69
vIVVGNPANTncLTASk	Unambiguous	2	1	P14152	N-Term(iTRAQ8plex); N11(Deamidated); C12(Methylthio); K17(iTRAQ8plex)	CID	0.0362	0.0000	1	1	3516916	Unique	Used	0.669	1.112	1.093	1.388	0	0	6.08	0	0	44	2	1178.63916	2356.27104	-4.87
yVIHTVGPIAVGQPTASQAAELR	Unambiguous	1	1	Q922B1	N-Term(iTRAQ8plex)	CID	1.0000	0.0000	1	1	3781064	Unique	Used	0.764	1.363	1.096	1.293	0	0	6.07	0	10	129	3	894.82947	2682.47385	-5.32
yPNAELAWcQEEHk	Unambiguous	4	1	Q60597	N-Term(iTRAQ8plex); C9(Methylthio); K14(iTRAQ8plex)	CID	1.0000	0.0000	1	1	2974641	Unique	Used	0.714	0.653	0.852	1.016	0	0	6.07	0	10	66	3	791.39008	2372.15567	-2.83
sVTNEDVTQEQLGGAk	Unambiguous	3	1	Q99MN9	N-Term(iTRAQ8plex); K16(iTRAQ8plex)	CID	1.0000	0.0000	1	1	2649735	Unique	Used	0.483	0.926	1.148	1.279	0	0	6.07	0	5	157	2	1142.61218	2284.21709	-3.08
lSPGTPALYSEAAAHLk	Unambiguous	1	1	A2ALK6	N-Term(iTRAQ8plex); K17(iTRAQ8plex)	CID	1.0000	0.0000	1	1	3474802	Unique	Used	0.778	1.140	1.089	1.148	0	0	6.07	0	27	52	3	778.77899	2334.32242	-2.30
iEGSITGPSVEIGTPDVDVHGLGGk	Unambiguous	1	1	E9Q616	N-Term(iTRAQ8plex); K25(iTRAQ8plex)	CID	1.0000	0.0000	1	1	3258976	Unique	Used	1.704	2.413	0.685	0.700	0	0	6.07	0	0	63	3	1014.88824	3042.65018	-2.20
iHVSDQELQSAnASVDDSR	Unambiguous	1	1	Q02053	N-Term(iTRAQ8plex); N12(Deamidated)	CID	1.0000	0.0000	1	1	2632862	Unique	Used	1.115	1.379	0.946	1.085	0	0	6.07	0	24	66	3	792.72467	2376.15946	0.56

sLQDIIAILGMDELSEEDkLTVSR	Unambiguous	1	1	P56480	N-Term(iTRAQ8plex); K19(iTRAQ8plex)	HCD	1.0000	0.0000	1	1	3374306	Unique	Used	1.054	1.123	1.006	1.137	0	0	6.07	1	0	96	3	1095.26282	3283.77390	-5.41
nSAGGTSVGWDSPPASPLQR	Unambiguous	2	1	A2AKD7	N-Term(iTRAQ8plex)	CID	1.0000	0.0000	1	1	3433133	Unique	Used	1.193	0.794	0.876	1.049	0	0	6.07	0	14	137	2	1144.57764	2288.14800	-4.09
lFQEGSTADALGTGADGQMER	Unambiguous	1	1	Q00560	N-Term(iTRAQ8plex)	CID	1.0000	0.0000	1	1	2867937	Unique	Used	1.253	0.773	0.853	0.994	0	0	6.07	0	0	200	2	1229.58862	2458.16997	-5.00
iPNIYAIGDVVAGPmLAHk	Unambiguous	1	1	O08749	N-Term(iTRAQ8plex); M15(Oxidation); K19(iTRAQ8plex)	CID	1.0000	0.0000	1	1	3744730	Unique	Used	1.798	1.690	1.102	0.929	0	0	6.07	0	22	200	3	868.49646	2603.47483	-3.53
fNFQQTNEDELSFSk	Unambiguous	9	1	Q9ES28-3	N-Term(iTRAQ8plex); K15(iTRAQ8plex)	CID	1.0000	0.0000	1	1	3053763	Unique	Used	1.704	1.075	0.962	0.831	0	0	6.07	0	0	200	2	1221.61902	2442.23076	-3.69
fQFVGIENIHVMR	Unambiguous	1	1	Q8VE11	N-Term(iTRAQ8plex)	CID	1.0000	0.0000	1	1	3877387	Unique	Used	1.059	1.176	0.637	0.791	0	0	6.07	0	9	107	3	632.01251	1894.02298	-4.30
kLGLVFDDVVGIVEIINSR	Unambiguous	1	1	P40124	N-Term(iTRAQ8plex); K1(iTRAQ8plex)	HCD	1.0000	0.0000	1	1	3825223	Unique	Used	0.775	1.043	1.409	0.872	0	0	6.07	1	16	137	3	898.86847	2694.59085	-3.95
ySEATEHPGAPPqPPAPLQPALAPPSR	Unambiguous	3	1	Q7TSJ2	N-Term(iTRAQ8plex); Q13(Deamidated)	HCD	1.0000	0.0000	1	1	3319973	Unique	Used	5.984	1.020	1.003	0.865	0	0	6.07	0	0	118	3	1027.87439	3081.60862	4.48
gLTIVGSVLEGTFLDNHPQAQR	Unambiguous	2	1	Q91V14	N-Term(iTRAQ8plex)	CID	1.0000	0.0000	1	1	3844813	Unique	Used		0.987			0	0	6.07	0	5	200	3	886.14923	2656.43314	-1.11
gLTIVGSVLEGTFLDNHPQAQR	Unambiguous	2	1	Q91V14	N-Term(iTRAQ8plex)	CID	1.0000	0.0000	1	1	3843951	Unique	Used	0.601	0.975	1.108	0.999	0	0	6.07	0	18	200	3	886.14685	2656.42600	-3.80
gTGGVDTAATGSVFDISNLDR	Unambiguous	1	1	P30275	N-Term(iTRAQ8plex)	CID	1.0000	0.0000	1	1	3237436	Unique	Used	1.195	0.993	1.258	1.319	0	0	6.07	0	14	172	2	1179.09436	2357.18144	-3.09
hALIIYDDLSkQAVAYR	Unambiguous	2	1	Q03265	N-Term(iTRAQ8plex); K11(iTRAQ8plex)	HCD	1.0000	0.0000	1	1	3576261	Unique	Used	0.838	2.801	0.682	0.628	0	0	6.06	1	0	117	3	862.15778	2584.45877	-4.64
iYELAAGGTAVGTGLNTR	Unambiguous	2	1	P97807-2	N-Term(iTRAQ8plex)	CID	1.0000	0.0000	1	1	3744017	Unique	Used	1.959	1.202	0.941	1.109	0	0	6.06	0	3	14	2	1034.56677	2068.12627	-3.78
tASVSGASAEASGEQVR	Unambiguous	1	1	Q80U49	N-Term(iTRAQ8plex)	CID	1.0000	0.0000	1	1	2696366	Unique	Used	1.314	0.557	1.352	0.960	0	0	6.06	0	0	200	2	955.98767	1910.96807	-2.14
aYSGSQIDILIDQGk	Unambiguous	6	1	Q9R0P3	N-Term(iTRAQ8plex); K15(iTRAQ8plex)	CID	1.0000	0.0000	1	1	3149994	Unique	Used	0.429	0.507	0.822	0.778	0	0	6.06	0	0	75	2	1108.62061	2216.23393	-1.97

sSSADFGTFSTSQSHQTASTVSk	Unambiguous	4	1	Q8K2K6-3	N-Term(iTRAQ8plex); K23(iTRAQ8plex)	CID	1.0000	0.0000	1	1	3338929	Unique	Used			1.112	0.609	0	0	6.06	0	0	49	3	986.15991	2956.46518	-3.15
iGEHTPSALAIMENANVLAR	Unambiguous	3	1	A6ZI44	N-Term(iTRAQ8plex)	CID	1.0000	0.0000	1	1	3744181	Unique	Used	0.780	0.709	0.334	1.079	0	0	6.06	0	2	6	3	804.43695	2411.29630	-2.33
dLESDIIGDTSGHFQk	Unambiguous	2	1	P14824	N-Term(iTRAQ8plex); K16(iTRAQ8plex)	CID	1.0000	0.0000	1	1	2901089	Unique	Used	1.427	1.292	0.956	0.859	0	0	6.06	0	6	200	3	790.74976	2370.23471	-2.14
sVSSNVASVSPIPAGSk	Unambiguous	1	1	Q8R1Q8	N-Term(iTRAQ8plex); K17(iTRAQ8plex)	CID	1.0000	0.0000	1	1	3533025	Unique	Used	0.535	0.981	1.032	0.934	0	0	6.06	0	6	33	2	1098.12329	2195.23931	-4.52
gTSFEAAATSGGSASSEk	Unambiguous	1	1	Q99LC5	N-Term(iTRAQ8plex); K18(iTRAQ8plex)	CID	1.0000	0.0000	1	1	2825695	Unique	Used	1.530	0.912	1.189	1.096	0	0	6.06	0	0	200	2	1127.07678	2253.14629	0.33
eALLSSAVDHGSDEAR	Unambiguous	1	1	P80314	N-Term(iTRAQ8plex)	CID	1.0000	0.0000	1	1	2699991	Unique	Used	1.684	1.209	1.224	1.338	0	0	6.06	0	28	197	3	654.33258	1960.98319	-2.36
aTDLLLDDSLVSLFGnR	Unambiguous	4	1	P99029-2	N-Term(iTRAQ8plex); N16(Deamidated)	CID	1.0000	0.0000	1	1	3352969	Unique	Used	2.195	1.013	1.313	1.177	0	0	6.06	0	16	12	2	1077.57935	2154.15141	-3.82
aLAGLQSSSATPSPVPSPAR	Unambiguous	2	1	Q6P9K8	N-Term(iTRAQ8plex)	CID	1.0000	0.0000	1	1	3912459	Unique	Used	1.236	0.561	1.191	1.213	0	0	6.06	0	0	141	2	1099.60400	2198.20073	-3.45
aMVALIDVFHQYSGR	Unambiguous	1	1	P50114	N-Term(iTRAQ8plex)	CID	1.0000	0.0000	1	1	3938099	Unique	Used	3.192	0.936	1.067	1.082	0	0	6.06	0	24	200	3	671.02722	2011.06711	-3.29
tASNIIDVSAADSQGmEQHEYMDR	Unambiguous	1	1	Q9CQ22	N-Term(iTRAQ8plex); M16(Oxidation)	CID	1.0000	0.0000	1	1	2810168	Unique	Used	1.266	0.999	0.796	0.979	0	0	6.06	0	0	194	3	996.79156	2988.36014	-0.54
tVAGIIVEPIQSEGGDnHASDDFFR	Unambiguous	2	1	P61922	N-Term(iTRAQ8plex); N17(Deamidated)	CID	1.0000	0.0000	1	1	2887113	Unique	Used				1.651	0	0	6.06	0	4	200	3	993.82404	2979.45755	-2.12
aASVAEncINYQQGTPHk	Unambiguous	1	1	P70202	N-Term(iTRAQ8plex); N7(Deamidated); C8(Methylthio); K18(iTRAQ8plex)	CID	1.0000	0.0000	1	1	3068828	Unique	Used	1.164	0.971	0.733	0.884	0	0	6.06	0	9	23	3	862.76776	2586.28873	-0.53
iDQYQGADAVGLEEk	Unambiguous	1	1	Q8CDN6	N-Term(iTRAQ8plex); K15(iTRAQ8plex)	CID	1.0000	0.0000	1	1	2635319	Unique	Used	0.886	1.078	0.850	0.928	0	0	6.06	0	4	170	2	1122.59937	2244.19145	-2.41

nMVLQEDAILHSEDSLR	Unambiguous	1	1	Q9CRC6	N-Term(iTRAQ8plex)	CID	1.0000	0.0000	1	1	2849829	Unique	Used	2.374	1.123	1.323	0.890	0	0	6.06	0	18	111	3	758.72632	2274.16440	-2.56
sLQSLEASLHAMESTR	Unambiguous	1	1	Q9CW03	N-Term(iTRAQ8plex)	HCD	1.0000	0.0000	1	1	3416064	Unique	Used	0.936	1.081	0.946	1.117	0	0	6.06	0	0	53	3	688.69165	2064.06040	-4.54
eDkDGSLEVTGQLGDVmk	Unambiguous	1	1	Q8CGK3	N-Term(iTRAQ8plex); K3(iTRAQ8plex); M17(Oxidation); K18(iTRAQ8plex)	CID	1.0000	0.0000	1	1	2828343	Unique	Used	1.353	0.757	0.691	0.786	0	0	6.06	1	0	200	3	950.51666	2849.53543	0.85
tGqTDPFLGMMMGLGGGMDFDSk	Unambiguous	3	1	Q8VDQ8-2	N-Term(iTRAQ8plex); Q3(Deamidated); K23(iTRAQ8plex)	CID	1.0000	0.0000	1	1	3459584	Unique	Used	1.633	0.534	0.886	0.583	0	0	6.06	0	11	91	3	1001.48029	3002.42630	1.12
gAPVSVYQIVVEEERPR	Unambiguous	1	1	P28828	N-Term(iTRAQ8plex)	HCD	1.0000	0.0000	1	1	3258103	Unique	Used	1.452	1.015	0.777	0.823	0	0	6.06	0	0	21	3	744.74744	2232.22776	-0.58
eTTGAQVQVAGDLLPNSTER	Unambiguous	1	1	P57724	N-Term(iTRAQ8plex)	CID	1.0000	0.0000	1	1	3032257	Unique	Used	0.769	0.324	0.732	0.766	0	0	6.06	0	12	200	2	1195.62292	2390.23857	-3.34
gTQGATAGASSELDASk	Unambiguous	2	1	F8VQC1	N-Term(iTRAQ8plex); K17(iTRAQ8plex)	CID	1.0000	0.0000	1	1	2648209	Unique	Used	0.713	0.473	0.911	0.750	0	0	6.06	0	0	200	2	1080.07031	2159.13335	-3.11
aLQLGTLFSPAEALk	Unambiguous	1	1	P42125	N-Term(iTRAQ8plex); K15(iTRAQ8plex)	CID	1.0000	0.0000	1	1	3745488	Unique	Used	1.170	0.877	0.639	1.100	0	0	6.06	0	16	9	3	723.09985	2167.28501	-4.48
eDDGVPVIcQVEHPAVTGNLQTQR	Unambiguous	9	1	Q8R5M8-4	N-Term(iTRAQ8plex); C9(Methylthio)	CID	1.0000	0.0000	1	1	3191788	Unique	Used	0.724	1.643	0.915	1.020	0	0	6.06	0	5	12	3	985.82404	2955.45755	-1.06
sSGNSANDLANVAVIDGNR	Unambiguous	1	1	Q7TT37	N-Term(iTRAQ8plex)	CID	1.0000	0.0000	1	1	3087806	Unique	Used	0.436	1.117	0.864	1.213	0	0	6.05	0	28	185	2	1089.55640	2178.10552	0.09
gTGGVDTAAVGGVFDVSNADR	Unambiguous	1	1	Q04447	N-Term(iTRAQ8plex)	CID	1.0000	0.0000	1	1	3130356	Unique	Used	2.601	2.213	2.119	1.201	0	0	6.05	0	24	124	2	1135.06909	2269.13091	-2.38
fQEYHIQQNEALAAk	Unambiguous	1	1	Q9WV98	N-Term(iTRAQ8plex); K15(iTRAQ8plex)	CID	1.0000	0.0000	1	1	3104721	Unique	Used	1.532	0.856	1.025	0.828	0	0	6.05	0	0	112	2	1199.65088	2398.29448	-1.28
tLHWDTDPSVLQLHSDSDLGR	Unambiguous	3	1	Q3TPJ8	N-Term(iTRAQ8plex)	CID	1.0000	0.0000	1	1	3257015	Unique	Used	1.333	1.251	1.028	1.030	0	0	6.05	0	16	125	3	899.45587	2696.35306	-1.92
vLDGLTAGSSSASQQQQQQHPPGNR	Unambiguous	3	1	D3Z2Q2	N-Term(iTRAQ8plex)	CID	1.0000	0.0000	1	1	3531572	Unique	Used	0.706	0.688	1.584	1.665	0	0	6.05	0	0	116	3	1008.51178	3023.52079	0.35

kAEGATLSnATGAVESTSQAGDR	Unambiguous	1	1	Q61644	N-Term(iTRAQ8plex); K1(iTRAQ8plex); N9(Deamidated)	CID	1.0000	0.0000	1	1	2807343	Unique	Used	5.485	2.010	2.182	1.168	0	0	6.05	1	0	200	3	944.15698	2830.45639	-2.67
qQGASGEVYcPSGEk	Unambiguous	1	1	E9Q1P8	N-Term(iTRAQ8plex); C10(Methylthio); K15(iTRAQ8plex)	CID	1.0000	0.0000	1	1	2826607	Unique	Used	1.526	0.933	1.186	0.879	0	0	6.05	0	0	200	2	1097.53857	2194.06987	-1.38
eAGYSAAVGVGQRPPR	Unambiguous	4	1	E9QAD8	N-Term(iTRAQ8plex)	HCD	1.0000	0.0000	1	1	3468256	Unique	Used	1.595	1.323	1.511	1.293	0	0	6.05	0	17	200	3	640.35010	1919.03574	-2.28
aAVEEGIVLGGGcALLR	Unambiguous	2	1	P63038	N-Term(iTRAQ8plex); C13(Methylthio)	CID	1.0000	0.0000	1	1	3646887	Unique	Used	0.793	0.671	0.980	1.059	0	0	6.05	0	24	200	2	989.53870	1978.07012	-3.38
vEAVNMAEGIIHDTETk	Unambiguous	1	1	P38647	N-Term(iTRAQ8plex); K17(iTRAQ8plex)	CID	1.0000	0.0000	1	1	2959930	Unique	Used	1.825	0.984	1.356	1.156	0	0	6.05	0	18	200	3	822.44238	2465.31260	-1.65
aGLEQGSDAGYLAESQk	Unambiguous	1	1	Q8BMS1	N-Term(iTRAQ8plex); K17(iTRAQ8plex)	CID	1.0000	0.0000	1	1	2699622	Unique	Used		0.770	0.741	0.803	0	0	6.05	0	0	200	2	1166.61169	2332.21611	-3.44
dAYGTSSLSSSSnSGSck	Unambiguous	2	1	Q80U23	N-Term(iTRAQ8plex); N13(Deamidated); C17(Methylthio); K18(iTRAQ8plex)	CID	1.0000	0.0000	1	1	2845459	Unique	Used	0.297	0.457	1.714	1.660	0	0	6.05	0	0	200	2	1197.05457	2393.10185	-1.61
eVFTEQADLSGITETk	Unambiguous	1	1	P07759	N-Term(iTRAQ8plex); K16(iTRAQ8plex)	CID	1.0000	0.0000	1	1	2850043	Unique	Used	4.721	3.498	0.126	2.772	0	0	6.05	0	12	93	2	1188.63696	2376.26665	-3.73
gLFSDEEDSEDLFSSQSSSkPk	Unambiguous	2	1	Q6PGL7	N-Term(iTRAQ8plex); K20(iTRAQ8plex); K22(iTRAQ8plex)	CID	1.0000	0.0000	1	1	2812030	Unique	Used	1.813	1.332	1.600	0.935	0	0	6.05	0	0	200	3	1111.23193	3331.68125	-3.98
vSDFGQMASGmNVDAGk	Unambiguous	1	1	P20357	N-Term(iTRAQ8plex); M11(Oxidation); K17(iTRAQ8plex)	CID	1.0000	0.0000	1	1	3298859	Unique	Used	1.105	1.297	1.684	0.814	0	0	6.05	0	2	18	2	1169.58167	2338.15605	-2.90

iGGVQQDTILAEGLHFR	Unambiguous	1	1	O35129	N-Term(iTRAQ8plex)	CID	1.0000	0.0000	1	1	3517577	Unique	Used	0.417	0.817	0.965	1.256	0	0	6.05	0	0	1	3	720.06598	2158.18338	-4.12
kAEGATLSNATGAVESTSQAGDR	Unambiguous	1	1	Q61644	N-Term(iTRAQ8plex); K1(iTRAQ8plex)	CID	1.0000	0.0000	1	1	2970279	Unique	Used			1.574	0.975	0	0	6.05	1	0	170	3	943.82806	2829.46964	-3.64
gTGGVDTAAVGGVFDVSNADR	Unambiguous	1	1	Q04447	N-Term(iTRAQ8plex)	CID	1.0000	0.0000	1	1	3135873	Unique	Used	1.602	1.007	1.273	1.986	0	0	6.05	0	0	200	2	1135.07043	2269.13359	-1.19
dGRDHIIEVVGSSMPLIGDHQDEDk	Unambiguous	3	1	O88935	N-Term(iTRAQ8plex); K25(iTRAQ8plex)	CID	1.0000	0.0000	1	1	2937522	Unique	Used	1.364	1.103	1.675	1.141	0	0	6.05	1	0	60	3	1124.24060	3370.70725	-2.56
amVALIDVFHQYSGR	Unambiguous	1	1	P50114	N-Term(iTRAQ8plex); M2(Oxidation)	CID	1.0000	0.0000	1	1	3935022	Unique	Used	4.390	0.629	1.536	1.415	0	0	6.05	0	2	10	3	676.35852	2027.06101	-3.76
qYPANQGQDVEYLVTGTHPYPAGPGVALTADTk	Unambiguous	2	1	Q922V4	N-Term(iTRAQ8plex); K33(iTRAQ8plex)	CID	1.0000	0.0000	1	1	3259007	Unique	Used	1.623	1.581	1.851	1.549	0	0	6.05	0	0	56	3	1356.36731	4067.08738	-2.40
fGGNPGGFGNQGGFGnSR	Unambiguous	1	1	Q921F2	N-Term(iTRAQ8plex); N16(Deamidated)	CID	0.0116	0.0000	1	1	3493934	Unique	Used	0.716	0.732	0.912	1.025	0	0	6.05	0	0	71	2	1016.47717	2031.94707	-5.15
gSVHDFPEFDAnQDAEALYTAMk	Unambiguous	2	1	P14824	N-Term(iTRAQ8plex); N12(Deamidated); K23(iTRAQ8plex)	CID	0.0132	0.0000	1	1	2941414	Unique	Used	1.360	0.682	1.068	0.975	0	0	6.05	0	0	200	3	1055.84583	3165.52292	-2.11
aLPSSEDLASLAPDLDk	Unambiguous	5	1	P58281-2	N-Term(iTRAQ8plex); K17(iTRAQ8plex)	CID	1.0000	0.0000	1	1	3131506	Unique	Used	0.706	0.813	1.194	1.143	0	0	6.05	0	0	75	2	1175.64746	2350.28765	-3.66
aVVFETGEAGAASGAGSLPGEVR	Unambiguous	3	1	Q9QYR6	N-Term(iTRAQ8plex)	CID	1.0000	0.0000	1	1	3149661	Unique	Used	1.875	1.217	2.055	0.555	0	0	6.05	0	4	22	2	1218.63660	2436.26592	-0.58
eAGPRPPGSGPGPGPTPGAk	Unambiguous	2	1	O88737	N-Term(iTRAQ8plex); K20(iTRAQ8plex)	HCD	1.0000	0.0000	1	1	3225484	Unique	Used	0.985	1.520	2.182	0.929	0	0	6.05	0	0	133	3	798.11066	2392.31742	-0.83
nIANPTATLLAScMMLDHLk	Unambiguous	1	1	P70404	N-Term(iTRAQ8plex); C13(Methylthio); K20(iTRAQ8plex)	CID	1.0000	0.0000	1	1	3879885	Unique	Used	0.456	0.973	0.997	1.243	0	0	6.05	0	17	17	3	937.82880	2811.47184	-4.69

sQIFSTASDnQPTVTIk	Unambiguous	1	1	P20029	N-Term(iTRAQ8plex); N10(Deamidated); K17(iTRAQ8plex)	CID	1.0000	0.0000	1	1	3256915	Unique	Used	1.376	0.808	0.935	0.470	0	0	6.05	0	0	155	2	1223.66333	2446.31938	-3.76
gESPELDLSYSHSDLGk	Unambiguous	1	1	P20917	N-Term(iTRAQ8plex); K17(iTRAQ8plex)	CID	1.0000	0.0000	1	1	2828760	Unique	Used	1.574		0.481	0.354	0	0	6.05	0	0	136	2	1221.62903	2442.25078	-4.14
eSAPFAVIGSNTVVEAk	Unambiguous	1	1	Q9Z2Q6	N-Term(iTRAQ8plex); K17(iTRAQ8plex)	CID	1.0000	0.0000	1	1	3235118	Unique	Used	0.408	2.098	1.576	1.420	0	0	6.04	0	2	14	2	1164.15479	2327.30229	-1.92
aHcLSEVEHDTmPADLPAIAADFVEDQEVck	Unambiguous	1	1	P07724	N-Term(iTRAQ8plex); C3(Methylthio); M12(Oxidation); C30(Methylthio); K31(iTRAQ8plex)	CID	1.0000	0.0000	1	1	3057166	Unique	Used	4.248	2.600	0.423	1.868	0	0	6.04	0	18	74	3	1367.29932	4099.88340	-5.05
kVPGVTAIELDEDTGTFR	Unambiguous	7	1	Q61584	N-Term(iTRAQ8plex); K1(iTRAQ8plex)	HCD	1.0000	0.0000	1	1	3107435	Unique	Used	0.791	0.602	0.645	0.828	0	0	6.04	1	0	16	3	852.80798	2556.40940	-1.42
aGGGAGGSGGSGPSAILR	Unambiguous	2	1	F7CZ64	N-Term(iTRAQ8plex)	CID	1.0000	0.0000	1	1	3612167	Unique	Used	0.246	2.271	1.388	1.053	0	0	6.04	0	9	13	2	866.96130	1732.91533	-5.26
mLLEYTDSSYDEk	Unambiguous	2	1	P10649	N-Term(iTRAQ8plex); K13(iTRAQ8plex)	CID	1.0000	0.0000	1	1	2779871	Unique	Used	1.200	3.060	2.139	1.533	0	0	6.04	0	5	200	3	734.70697	2202.10636	-1.50
sAAPSTLDSSSTAPAQLGk	Unambiguous	2	1	E9Q035	N-Term(iTRAQ8plex); K19(iTRAQ8plex)	CID	1.0000	0.0000	1	1	3185535	Unique	Used	0.416	0.765	0.517	0.735	0	0	6.04	0	0	41	2	1199.15942	2397.31157	1.41
wGEAGAEYVVESTGVFTTmEk	Unambiguous	3	1	P16858	N-Term(iTRAQ8plex); M19(Oxidation); K21(iTRAQ8plex)	CID	1.0000	0.0000	1	1	3241366	Unique	Used		0.942	2.941	1.687	0	0	6.04	0	0	200	3	972.49188	2915.46109	0.59
aAVEEGIVLGGGcALLR	Unambiguous	2	1	P63038	N-Term(iTRAQ8plex); C13(Methylthio)	CID	1.0000	0.0000	1	1	3647001	Unique	Used	1.193	0.976	0.944	0.895	0	0	6.04	0	24	200	2	989.53760	1978.06792	-4.49

eGPSLAELLANVASSILSHIk	Unambiguous	2	1	Q3UVL4	N-Term(iTRAQ8plex); K21(iTRAQ8plex)	HCD	1.0000	0.0000	1	1	3918602	Unique	Used	0.543	0.555	1.214	1.192	0	0	6.04	0	18	134	3	919.86493	2757.58023	-6.13
aVAIETQPAQGESDAVAR	Unambiguous	3	1	Q7TSJ2	N-Term(iTRAQ8plex)	CID	1.0000	0.0000	1	1	2666621	Unique	Used	1.340	1.018	1.410	1.266	0	0	6.04	0	0	1	2	1059.05847	2117.10967	-2.09
eQASHLGGAVFSGAGnIAAATGLVk	Unambiguous	1	1	Q91ZZ3	N-Term(iTRAQ8plex); N16(Deamidated); K25(iTRAQ8plex)	CID	1.0000	0.0000	1	1	3807911	Unique	Used	2.057	0.796	0.891	0.500	0	0	6.04	0	29	200	3	979.20703	2935.60654	-1.13
vIVVGNPANTncLTASk	Unambiguous	2	1	P14152	N-Term(iTRAQ8plex); N11(Deamidated); C12(Methylthio); K17(iTRAQ8plex)	CID	1.0000	0.0000	1	1	3538030	Unique	Used	0.570	0.888	0.988	1.065	0	0	6.04	0	0	61	2	1178.63879	2356.27031	-5.18
gTGSSSSGVLMVGPNFR	Unambiguous	1	1	Q8C4X2	N-Term(iTRAQ8plex)	CID	1.0000	0.0000	1	1	3874627	Unique	Used	0.476	0.516	0.853	0.899	0	0	6.04	0	8	168	2	979.00671	1957.00615	-2.75
gQFSTDELVAEVEkR	Unambiguous	2	1	Q68FD5	N-Term(iTRAQ8plex); K14(iTRAQ8plex)	CID	1.0000	0.0000	1	1	2937476	Unique	Used	0.457	1.176	1.704	1.578	0	0	6.04	1	14	40	3	772.75800	2316.25943	-2.67
lNMEAINYMAADGDFk	Unambiguous	1	1	P16045	N-Term(iTRAQ8plex); K16(iTRAQ8plex)	CID	1.0000	0.0000	1	1	3216174	Unique	Used	2.307	2.192	1.217	1.306	0	0	6.04	0	1	10	3	804.41010	2411.21573	-1.60
tIHQEDELIEIQSDTGTWYQR	Unambiguous	1	1	Q9JIS5	N-Term(iTRAQ8plex)	CID	1.0000	0.0000	1	1	2832279	Unique	Used	3.585	2.333	1.999	1.512	0	0	6.04	0	0	171	3	956.14148	2866.40989	-2.18
vTYMVHDFEEGGGVAMGmYNQDk	Unambiguous	1	1	O88844	N-Term(iTRAQ8plex); M18(Oxidation); K23(iTRAQ8plex)	CID	1.0000	0.0000	1	1	3130743	Unique	Used	0.652	0.913	0.786	1.161	0	0	6.04	0	0	23	3	1068.17456	3202.50913	-0.42
gYQGYPGGDYGGGPQDGGAGk	Unambiguous	3	1	E9QAQ7	N-Term(iTRAQ8plex); K21(iTRAQ8plex)	CID	1.0000	0.0000	1	1	2775463	Unique	Used	0.904	0.375	1.539	0.762	0	0	6.04	0	0	200	2	1283.62219	2566.23711	-1.87
eDQASTPGLSQASSGScFPR	Unambiguous	2	1	Q8CH09	N-Term(iTRAQ8plex); C17(Methylthio)	CID	1.0000	0.0000	1	1	3255631	Unique	Used	0.675	0.394	0.979	0.790	0	0	6.04	0	0	68	2	1188.04785	2375.08843	-1.08

iNMAHLcVIGSHAVnGVAR	Unambiguous	1	1	Q8CI94	N-Term(iTRAQ8plex); C7(Methylthio); N15(Deamidated)	CID	1.0000	0.0000	1	1	3914258	Unique	Used	0.823	1.053	0.844	1.213	0	0	6.04	0	18	172	3	771.73260	2313.18326	-4.31
dLVDFASTSQAFGEDPSSHEk	Unambiguous	4	1	Q8C5W0-4	N-Term(iTRAQ8plex); K21(iTRAQ8plex)	CID	1.0000	0.0000	1	1	2867653	Unique	Used	0.938	0.621	1.119	0.873	0	0	6.04	0	5	76	3	959.14264	2875.41336	-2.54
aAADLMAYcEAHAk	Unambiguous	1	1	P63213	N-Term(iTRAQ8plex); C9(Methylthio); K14(iTRAQ8plex)	CID	1.0000	0.0000	1	1	3420744	Unique	Used	0.496	1.101	1.041	1.010	0	0	6.03	0	0	200	3	707.02203	2119.05155	-3.76
eIVSFGSGYGGNSLLGk	Unambiguous	1	1	Q8BH04	N-Term(iTRAQ8plex); K17(iTRAQ8plex)	CID	1.0000	0.0000	1	1	3578559	Unique	Used	2.476	1.521	0.987	1.358	0	0	6.03	0	0	166	2	1147.13159	2293.25591	-3.91
hVVFTAETHNFPTGVAPFSGATTGTGGR	Unambiguous	1	1	Q5SUR0	N-Term(iTRAQ8plex)	HCD	1.0000	0.0000	1	1	3855799	Unique	Used	0.867	0.794	0.961	1.288	0	0	6.03	0	0	184	3	1040.85999	3120.56540	-4.85
dHVYLQLHHLPPEQLATR	Unambiguous	1	1	Q8K2B3	N-Term(iTRAQ8plex)	HCD	1.0000	0.0000	1	1	3451715	Unique	Used	1.966	0.844	0.982	1.013	0	0	6.03	0	20	200	3	824.44989	2471.33512	-4.45
tSFEEGTGEcEVFSk	Unambiguous	3	1	P48722	N-Term(iTRAQ8plex); C10(Methylthio); K15(iTRAQ8plex)	CID	1.0000	0.0000	1	1	2832099	Unique	Used	0.655	0.571	1.094	1.394	0	0	6.03	0	7	200	2	1152.55066	2304.09404	-1.92
iATEFNQLQFHAVQSk	Unambiguous	1	1	Q921L5	N-Term(iTRAQ8plex); K16(iTRAQ8plex)	CID	1.0000	0.0000	1	1	3618833	Unique	Used	0.743	1.139	0.649	0.803	0	0	6.03	0	25	92	3	823.78778	2469.34879	-9.02
vVYEnGVFMcAEGTGk	Unambiguous	1	1	Q9EQF6	N-Term(iTRAQ8plex); N5(Deamidated); C10(Methylthio); K16(iTRAQ8plex)	CID	1.0000	0.0000	1	1	3131179	Unique	Used	0.445	0.246	0.488	0.585	0	0	6.03	0	0	65	2	1180.08142	2359.15557	-1.58
gVMGGQSAGPQHTEAETIQk	Unambiguous	3	1	Q9Z1Z0	N-Term(iTRAQ8plex); K20(iTRAQ8plex)	CID	1.0000	0.0000	1	1	3026945	Unique	Used	0.454	1.163	0.504	0.657	0	0	6.03	0	0	200	3	878.79730	2634.37735	0.27

sLQDIIAILGMDELSEEDkLTVSR	Unambiguous	1	1	P56480	N-Term(iTRAQ8plex); K19(iTRAQ8plex)	HCD	1.0000	0.0000	1	1	3374320	Unique	Used	1.041	1.165	1.167	1.376	0	0	6.03	1	11	132	3	1095.26721	3283.78708	-1.39
lLVDVDESTLSPEEQk	Unambiguous	2	1	G5E866	N-Term(iTRAQ8plex); K16(iTRAQ8plex)	CID	1.0000	0.0000	1	1	2638113	Unique	Used	0.590	0.704	1.082	0.959	0	0	6.03	0	0	200	2	1205.66052	2410.31377	-1.49
eYQLNDSAAYYLnDLER	Unambiguous	1	1	P08752	N-Term(iTRAQ8plex); N13(Deamidated)	CID	1.0000	0.0000	1	1	2741652	Unique	Used				1.760	0	0	6.03	0	0	200	2	1191.57153	2382.13579	-1.90
qVDTEEAGMVTAATASNVk	Unambiguous	3	1	Q99JF8	N-Term(iTRAQ8plex); K19(iTRAQ8plex)	CID	1.0000	0.0000	1	1	2899687	Unique	Used	0.359	0.463	0.941	0.677	0	0	6.03	0	0	200	2	1265.66333	2530.31938	-3.39
vADVSISEVTTLLGNVHSPVVEGYVGENISGEVk	Unambiguous	3	1	P20357	N-Term(iTRAQ8plex); K34(iTRAQ8plex)	CID	1.0000	0.0000	1	1	3397652	Unique	Used	0.979	1.271	1.402	1.259	0	0	6.03	0	0	200	3	1369.40405	4106.19760	-3.47
vAPGTATAAAGPVADLFR	Unambiguous	2	1	A2AFI8	N-Term(iTRAQ8plex)	CID	1.0000	0.0000	1	1	3783890	Unique	Used	0.966	1.403	1.302	1.077	0	0	6.03	0	4	11	2	995.05402	1989.10076	-3.22
eAFEEMEGTSPSSPPHSVAR	Unambiguous	1	1	E9Q0S6	N-Term(iTRAQ8plex)	CID	1.0000	0.0000	1	1	2698910	Unique	Used	1.848	2.605	1.537	1.097	0	0	6.03	0	3	183	3	817.05646	2449.15482	-2.44
gSASGMGTPATAEPAPPSNk	Unambiguous	4	1	P00520-2	N-Term(iTRAQ8plex); K20(iTRAQ8plex)	CID	1.0000	0.0000	1	1	2969547	Unique	Used	0.809	0.633	1.185	0.925	0	0	6.03	0	0	200	2	1218.63574	2436.26421	-0.32
tQPTDEEMLFIYSHFk	Unambiguous	4	1	P31786	N-Term(iTRAQ8plex); K16(iTRAQ8plex)	CID	1.0000	0.0000	1	1	3192715	Unique	Used	1.995	0.716	1.208	0.711	0	0	6.03	0	13	63	3	865.45111	2594.33878	-1.29
tSDVETMSSQSALALDER	Unambiguous	1	1	P14873	N-Term(iTRAQ8plex)	CID	1.0000	0.0000	1	1	2759803	Unique	Used	2.360	0.653	1.858	0.904	0	0	6.03	0	0	30	2	1122.54944	2244.09160	-2.31
vTYMVHDFEEGGGVAMGMYNQDk	Unambiguous	1	1	O88844	N-Term(iTRAQ8plex); K23(iTRAQ8plex)	CID	1.0000	0.0000	1	1	3132287	Unique	Used	0.835	0.965	0.875	0.771	0	0	6.03	0	0	186	3	1062.84106	3186.50864	-2.17
vEFAALQEALAHALTEk	Unambiguous	1	1	E9Q7G0	N-Term(iTRAQ8plex); K17(iTRAQ8plex)	CID	1.0000	0.0000	1	1	3541242	Unique	Used	1.310	0.879	0.811	0.725	0	0	6.03	0	23	10	3	817.13190	2449.38114	-4.09
fIGLESVFQAVDYmYTGk	Unambiguous	1	1	Q8BGC4	N-Term(iTRAQ8plex); M14(Oxidation); K18(iTRAQ8plex)	CID	1.0000	0.0000	1	1	3785730	Unique	Used	0.657	0.894	1.009	0.712	0	0	6.03	0	5	26	3	898.14050	2692.40696	-3.10

hALIIYDDLSkQAVAYR	Unambiguous	2	1	Q03265	N-Term(iTRAQ8plex); K11(iTRAQ8plex)	HCD	1.0000	0.0000	1	1	3894156	Unique	Used	0.682	0.799	1.091	1.198	0	0	6.03	1	18	136	3	862.15955	2584.46408	-2.59
qAEIENPLEDPVTGDYVHk	Unambiguous	4	1	Q9Z1G4-3	N-Term(iTRAQ8plex); K19(iTRAQ8plex)	CID	1.0000	0.0000	1	1	2761029	Unique	Used	1.777	2.238	0.874	0.607	0	0	6.03	0	0	200	3	921.48590	2762.44315	-0.95
iMDPNIVGnEHYDVAR	Unambiguous	1	1	P56480	N-Term(iTRAQ8plex); N9(Deamidated)	CID	1.0000	0.0000	1	1	2849198	Unique	Used	1.398	1.088	1.201	1.366	0	0	6.03	0	3	4	3	716.69238	2148.06260	-3.34
nAQAIEDMVGYAQETQHEk	Unambiguous	1	1	Q3TXS7	N-Term(iTRAQ8plex); K19(iTRAQ8plex)	CID	1.0000	0.0000	1	1	2918723	Unique	Used	0.636	0.779	0.658	0.857	0	0	6.02	0	19	120	3	924.13245	2770.38279	-3.57
aAADLMAYcEAHAk	Unambiguous	1	1	P63213	N-Term(iTRAQ8plex); C9(Methylthio); K14(iTRAQ8plex)	CID	1.0000	0.0000	1	1	3416826	Unique	Used	0.308	1.129	1.062	1.059	0	0	6.02	0	5	1	3	707.02191	2119.05118	-3.93
imETSPTLLQISPGPPk	Unambiguous	1	1	Q8R361	N-Term(iTRAQ8plex); M2(Oxidation); K17(iTRAQ8plex)	CID	1.0000	0.0000	1	1	3662511	Unique	Used	1.040	0.513	0.884	0.695	0	0	6.02	0	0	129	2	1217.18945	2433.37163	-6.88
dVEPEDPMFLMDPFAIHR	Unambiguous	1	1	Q99KX1	N-Term(iTRAQ8plex)	CID	1.0000	0.0000	1	1	3037708	Unique	Used		2.044	3.089	1.541	0	0	6.02	0	0	2	3	821.73663	2463.19535	-1.53
aYAPGGPAYQPVVEAFGTDILHk	Unambiguous	1	1	Q9DBL7	N-Term(iTRAQ8plex); K23(iTRAQ8plex)	CID	1.0000	0.0000	1	1	3479098	Unique	Used	0.723	0.624	0.890	0.997	0	0	6.02	0	15	54	3	1003.87976	3009.62473	-1.58
iGEHTPSALAImENANVLAR	Unambiguous	3	1	A6ZI44	N-Term(iTRAQ8plex); M12(Oxidation)	CID	1.0000	0.0000	1	1	3708804	Unique	Used	1.197	1.472	1.584	1.242	0	0	6.02	0	6	200	3	809.76630	2427.28434	-5.15
aAFGISDSYVDGSSFDPQR	Unambiguous	3	1	Q8BTI8	N-Term(iTRAQ8plex)	CID	1.0000	0.0000	1	1	3213997	Unique	Used	0.662	1.096	1.163	1.041	0	0	6.02	0	17	200	2	1162.05786	2323.10845	-2.59
sIDDLEDQLYHQLEQNR	Unambiguous	3	1	E9Q452	N-Term(iTRAQ8plex)	CID	1.0000	0.0000	1	1	2961324	Unique	Used	3.188				0	0	6.02	0	26	200	3	807.40472	2420.19962	0.02
tVEGAGNIAAATGFVk	Unambiguous	2	1	O55042	N-Term(iTRAQ8plex); K16(iTRAQ8plex)	CID	1.0000	0.0000	1	1	3413437	Unique	Used	1.459	1.401	1.535	0.901	0	0	6.02	0	0	101	2	1057.60217	2114.19707	-4.53
sVTIQAPAGEPLLANDSAR	Unambiguous	4	1	Q3UXU7	N-Term(iTRAQ8plex)	CID	1.0000	0.0000	1	1	3300166	Unique	Used	0.677	0.542	0.616	0.790	0	0	6.02	0	0	200	2	1107.60181	2214.19634	-3.12

tVEGAGnIAAATGFVk	Unambiguous	2	1	O55042	N-Term(iTRAQ8plex); N7(Deamidated); K16(iTRAQ8plex)	CID	1.0000	0.0000	1	1	3280085	Unique	Used	1.285	2.364	2.098	0.968	0	0	6.02	0	14	80	2	1058.09814	2115.18901	-0.78
aAAGELQEDSGLHVLAR	Unambiguous	1	1	Q8C845	N-Term(iTRAQ8plex)	CID	1.0000	0.0000	1	1	2972750	Unique	Used	1.218	0.784	1.289	1.126	0	0	6.02	0	3	7	3	681.03625	2041.09421	-1.88
gLTIVGSVLEGTFLDNHPQAQR	Unambiguous	2	1	Q91V14	N-Term(iTRAQ8plex)	CID	1.0000	0.0000	1	1	3845601	Unique	Used	0.793				0	0	6.02	0	7	200	3	886.14685	2656.42600	-3.80
vTnGAFTGEISPGMIk	Unambiguous	1	1	P17751	N-Term(iTRAQ8plex); N3(Deamidated); K16(iTRAQ8plex)	CID	1.0000	0.0000	1	1	3301966	Unique	Used	1.443	1.394	1.592	1.127	0	0	6.02	0	0	10	2	1116.10876	2231.21025	-4.48
aADLVSENcETYEAHMR	Unambiguous	1	1	Q9JLI6	N-Term(iTRAQ8plex); C9(Methylthio)	CID	1.0000	0.0000	1	1	2938483	Unique	Used	1.404	0.882	1.131	0.878	0	0	6.02	0	20	109	3	763.67969	2289.02451	-0.30
lELVESLDSDEVDLk	Unambiguous	3	1	Q8CH77	N-Term(iTRAQ8plex); K15(iTRAQ8plex)	CID	1.0000	0.0000	1	1	2766381	Unique	Used	0.630	0.639	1.191	0.426	0	0	6.02	0	25	200	2	1156.63818	2312.26909	-0.12
sTTTTDMITEVGTAFSR	Unambiguous	5	1	J3QMM7	N-Term(iTRAQ8plex)	CID	1.0000	0.0000	1	1	3457952	Unique	Used	1.499	1.059	0.786	1.054	0	0	6.02	0	0	183	2	1061.53381	2122.06035	-1.73
aSVASEVSTTSSTSkPPTGR	Unambiguous	2	1	H7BX26	N-Term(iTRAQ8plex); K15(iTRAQ8plex)	HCD	1.0000	0.0000	1	1	3315208	Unique	Used	0.548	0.391	1.255	1.552	0	0	6.02	0	15	107	3	853.46350	2558.37595	-4.80
eAAASGLPLMVIIHk	Unambiguous	1	1	Q9CQU0	N-Term(iTRAQ8plex); K15(iTRAQ8plex)	CID	1.0000	0.0000	1	1	3684608	Unique	Used	1.799	1.000	0.899	0.813	0	0	6.02	0	12	100	3	720.09564	2158.27237	-7.18
yEGSGDGGAAAQSLYIANHAY	Unambiguous	1	1	P05063	N-Term(iTRAQ8plex)	CID	1.0000	0.0000	1	1	2956825	Unique	Used	1.338	0.858	1.204	1.377	0	0	6.02	0	20	66	2	1210.07507	2419.14287	-1.63
gTGGVDTAAVGGVFDVSNADR	Unambiguous	1	1	Q04447	N-Term(iTRAQ8plex)	CID	1.0000	0.0000	1	1	3132472	Unique	Used	1.141	1.122	1.545	1.435	0	0	6.02	0	0	200	2	1135.06873	2269.13017	-2.70
nWETDVVLTDTLHLk	Unambiguous	5	1	Q9Z0J4	N-Term(iTRAQ8plex); K15(iTRAQ8plex)	CID	1.0000	0.0000	1	1	3260457	Unique	Used		1.607	1.155	1.310	0	0	6.01	0	10	109	3	798.11310	2392.32474	-3.57
gLTIVGSVLEGTFLDNHPQAQR	Unambiguous	2	1	Q91V14	N-Term(iTRAQ8plex)	CID	1.0000	0.0000	1	1	3843594	Unique	Used	0.805	1.136	1.153	0.796	0	0	6.01	0	23	200	3	886.14752	2656.42801	-3.04
lATPTYGDLNHLVSATMSGVTTSLR	Unambiguous	1	1	Q9ERD7	N-Term(iTRAQ8plex)	CID	1.0000	0.0000	1	1	3939367	Unique	Used			1.221	1.249	0	0	6.01	0	0	200	3	970.51373	2909.52665	-2.70

dQQNLPYGVTPASPSGHSQGR	Unambiguous	14	1	E9QMP6	N-Term(iTRAQ8plex)	CID	1.0000	0.0000	1	1	3337557	Unique	Used	1.353	1.081	1.529	1.157	0	0	6.01	0	0	39	3	834.08582	2500.24289	-2.15
wITDNQDSADVDPQEHSFTR	Unambiguous	1	1	E9PZP8	N-Term(iTRAQ8plex)	CID	1.0000	0.0000	1	1	2716278	Unique	Used	0.537	0.611	1.151	1.067	0	0	6.01	0	0	169	3	889.08563	2665.24234	-0.34
iEGYEDQVLITEHGDLGNGk	Unambiguous	2	1	P47754	N-Term(iTRAQ8plex); K20(iTRAQ8plex)	CID	1.0000	0.0000	1	1	2812849	Unique	Used	0.670	1.394	1.215	0.793	0	0	6.01	0	19	200	3	932.49304	2795.46457	-0.95
qDVSPSPAPALAPASTSVEQLR	Unambiguous	1	1	Q8K382	N-Term(iTRAQ8plex)	CID	1.0000	0.0000	1	1	3256121	Unique	Used	0.819	1.262	1.272	0.988	0	0	6.01	0	0	50	2	1263.17969	2525.35210	0.29
iLHGGMVAQQGLLHVGDIIk	Unambiguous	2	1	Q9WV34	N-Term(iTRAQ8plex); K20(iTRAQ8plex)	HCD	1.0000	0.0000	1	1	3803090	Unique	Used	0.642	1.565	1.214	0.937	0	0	6.01	0	24	85	3	903.19952	2707.58402	-2.29
nSLHLLMETLNATTPHYVR	Unambiguous	3	1	D3YZ62	N-Term(iTRAQ8plex)	CID	1.0000	0.0000	1	1	3916310	Unique	Used	1.065	0.907	1.061	1.111	0	0	6.01	0	22	97	3	838.78247	2514.33286	-4.46
nIDNPAIADIYTEHAHQVVVAk	Unambiguous	1	1	O88342	N-Term(iTRAQ8plex); K22(iTRAQ8plex)	CID	1.0000	0.0000	1	1	3278173	Unique	Used	0.909	0.812	0.952	0.930	0	0	6.01	0	0	85	3	1009.55255	3026.64310	-2.94
vFLTTAEVISQQVSDk	Unambiguous	1	1	P06801	N-Term(iTRAQ8plex); K16(iTRAQ8plex)	CID	1.0000	0.0000	1	1	3500263	Unique	Used	0.916	0.797	0.737	0.933	0	0	6.01	0	16	43	2	1187.17200	2373.33672	-5.01
sVISGGLDALEFIGk	Unambiguous	2	1	Q8VE88-2	N-Term(iTRAQ8plex); K15(iTRAQ8plex)	CID	1.0000	0.0000	1	1	3479617	Unique	Used	0.675	1.555	1.069	0.894	0	0	6.01	0	9	24	2	1057.61438	2114.22148	-4.88
sTMDNPTTTQYANLMHNFILk	Unambiguous	2	1	P62627	N-Term(iTRAQ8plex); K21(iTRAQ8plex)	CID	1.0000	0.0000	1	1	3726025	Unique	Used	1.476	0.738	1.306	0.840	0	0	6.01	0	3	22	3	1016.85565	3048.55240	-7.20
aHLQGPTGTSVAGPqTSEAFVITVR	Unambiguous	2	1	Q8VHY0	N-Term(iTRAQ8plex); Q15(Deamidated)	CID	1.0000	0.0000	1	1	3760666	Unique	Used	1.158	1.005	1.262	1.102	0	0	6.01	0	0	119	3	943.84357	2829.51615	3.97
aFMEEFGAEPELAVSAPGR	Unambiguous	1	1	Q9JIA6	N-Term(iTRAQ8plex)	CID	1.0000	0.0000	1	1	3074955	Unique	Used	0.531	1.400	0.760	0.871	0	0	6.01	0	0	71	2	1156.58118	2312.15508	0.66
tPYLGDVAIVVHPGk	Unambiguous	1	1	Q9EQF6	N-Term(iTRAQ8plex); K15(iTRAQ8plex)	CID	1.0000	0.0000	1	1	3495534	Unique	Used	0.723	0.545	0.801	0.715	0	0	6.01	0	11	68	3	725.43024	2174.27616	-1.49
kEFGGVGAPPSPGVAR	Unambiguous	4	1	Q8CHC4	N-Term(iTRAQ8plex); K1(iTRAQ8plex)	HCD	1.0000	0.0000	1	1	3450529	Unique	Used	0.631	1.757	1.717	0.980	0	0	6.01	1	0	134	3	712.07648	2134.21488	-3.79

tNIVTASVDAInFHDk	Unambiguous	5	1	Q91V12-2	N-Term(iTRAQ8plex); N12(Deamidated); K16(iTRAQ8plex)	CID	1.0000	0.0000	1	1	3150324	Unique	Used	0.755	0.650	0.873	1.068	0	0	6.01	0	15	125	3	785.43097	2354.27835	-1.23
eHINLGcDVDFDIAGPSIR	Unambiguous	3	1	Q60932-2	N-Term(iTRAQ8plex); C7(Methylthio)	CID	1.0000	0.0000	1	1	3378558	Unique	Used	1.014	1.084	0.859	0.452	0	0	6.00	0	17	200	3	807.73047	2421.17685	-3.16
eANSIVITPGYGLcAAk	Unambiguous	4	1	Q61941	N-Term(iTRAQ8plex); C14(Methylthio); K17(iTRAQ8plex)	CID	1.0000	0.0000	1	1	3559171	Unique	Used	0.469	0.992	0.204	4.121	0	0	6.00	0	0	179	2	1181.13635	2361.26543	-4.77
gITNLcVIGGDGSLTGADTFR	Unambiguous	1	1	P47857	N-Term(iTRAQ8plex); C6(Methylthio)	CID	1.0000	0.0000	1	1	3730012	Unique	Used	1.174		1.200	0.949	0	0	6.00	0	0	200	2	1209.10107	2417.19487	-6.56
aIESGDTDLVFTVLLHLk	Unambiguous	3	1	G3X8X7	N-Term(iTRAQ8plex); K18(iTRAQ8plex)	CID	1.0000	0.0000	1	1	3561056	Unique	Used	0.314	0.753	0.832	1.071	0	0	6.00	0	7	52	3	860.49628	2579.47428	-6.30
iHVSDQELQSANASVDDSR	Unambiguous	1	1	Q02053	N-Term(iTRAQ8plex)	CID	1.0000	0.0000	1	1	2650486	Unique	Used	2.026	0.878	1.343	1.144	0	0	6.00	0	7	110	3	792.39600	2375.17344	-0.28
qVQEAIAGISSAAQATsPTDEAk	Unambiguous	5	1	Q61301-2	N-Term(iTRAQ8plex); S17(Phospho); K23(iTRAQ8plex)	CID	0.0283	0.0000	1	1	2920401	Unique	Used				0.826	0	0	6.00	0	0	146	3	987.83899	2961.50241	-0.10
sIVTDLVSQMDPHGR	Unambiguous	4	1	P58281-2	N-Term(iTRAQ8plex)	CID	1.0000	0.0000	1	1	3643320	Unique	Used	1.292	1.121	0.766	0.734	0	0	6.00	0	12	41	3	653.67651	1959.01499	-6.22
vDISAPDVDVQGPDWHLk	Unambiguous	1	1	E9Q616	N-Term(iTRAQ8plex); K18(iTRAQ8plex)	CID	1.0000	0.0000	1	1	3170436	Unique	Used	1.264	1.942	1.003	0.898	0	0	6.00	0	18	123	3	867.13452	2599.38901	-3.34
sSGNSSSSLGEMVSGTFR	Unambiguous	4	1	Q8R0S4	N-Term(iTRAQ8plex)	CID	1.0000	0.0000	1	1	3639633	Unique	Used	0.605	0.692	1.457	1.039	0	0	6.00	0	0	141	2	1047.50342	2093.99956	-3.82
eHINLGcDVDFDIAGPSIR	Unambiguous	3	1	Q60932-2	N-Term(iTRAQ8plex); C7(Methylthio)	CID	1.0000	0.0000	1	1	3357910	Unique	Used		1.284			0	0	6.00	0	5	200	3	807.73096	2421.17832	-2.55
sGLGLGAASASTSGSGPADSVmR	Unambiguous	3	1	Q7TSJ2	N-Term(iTRAQ8plex); M22(Oxidation)	CID	1.0000	0.0000	1	1	3512426	Unique	Used	0.682	0.634	1.433	1.565	0	0	6.00	0	0	148	2	1178.58337	2356.15947	-5.19

eEQQLLASTSkPVVk	Unambiguous	1	1	Q8BGV7	N-Term(iTRAQ8plex); K11(iTRAQ8plex); K15(iTRAQ8plex)	HCD	1.0000	0.0000	1	1	3165767	Unique	Used	0.559	0.964	1.185	1.019	0	0	6.00	0	0	157	3	857.17987	2569.52506	-3.02
vGVFSYGSGLAATLYSLk	Unambiguous	1	1	Q8JZK9	N-Term(iTRAQ8plex); K18(iTRAQ8plex)	CID	1.0000	0.0000	1	1	3917730	Unique	Used	0.723	0.372		0.554	0	0	6.00	0	16	15	2	1221.19397	2441.38066	-3.85
iYELAAGGTAVGTGLNTR	Unambiguous	2	1	P97807-2	N-Term(iTRAQ8plex)	CID	1.0000	0.0000	1	1	3743839	Unique	Used	2.173	1.130	0.764	1.078	0	0	6.00	0	0	2	2	1034.56726	2068.12724	-3.31
mTVVQAYVGDVHYk	Unambiguous	2	1	Q9R0K7	N-Term(iTRAQ8plex); K14(iTRAQ8plex)	CID	1.0000	0.0000	1	1	3577764	Unique	Used	0.481	0.631	1.267	0.988	0	0	6.00	0	4	59	3	740.07349	2218.20591	-4.13
aVLDVAETGTEAAAATGVIGGIR	Unambiguous	1	1	P07759	N-Term(iTRAQ8plex)	CID	1.0000	0.0000	1	1	3395789	Unique	Used			1.090	1.713	0	0	6.00	0	16	4	2	1223.67004	2446.33281	-5.22
kVQHQDALQISDVVMASLLR	Unambiguous	1	1	P70168	N-Term(iTRAQ8plex); K1(iTRAQ8plex)	HCD	1.0000	0.0000	1	1	3804992	Unique	Used	0.621	1.688	0.894	0.998	0	0	6.00	1	21	51	3	953.87994	2859.62528	-2.89
sLQDIIAILGMDELSEEDkLTVSR	Unambiguous	1	1	P56480	N-Term(iTRAQ8plex); K19(iTRAQ8plex)	HCD	1.0000	0.0000	1	1	3355068	Unique	Used	0.888	1.051	1.047	1.137	0	0	6.00	1	0	200	3	1095.26453	3283.77903	-3.84
lVSSPEVGHDEFSTQALAR	Unambiguous	1	1	Q68FG2	N-Term(iTRAQ8plex)	HCD	1.0000	0.0000	1	1	3188680	Unique	Used	0.794	2.408	2.744	1.436	0	0	6.00	0	0	6	3	783.07739	2347.21762	-0.85
yHVPVVVVPEGSTSDTQEQAILR	Unambiguous	2	1	Q9DBG6	N-Term(iTRAQ8plex)	CID	1.0000	0.0000	1	1	3234415	Unique	Used	1.252	0.818	0.816	0.771	0	0	5.99	0	3	17	3	943.50726	2828.50724	-0.85
gTGGVDTAAVGGVFDVSNADR	Unambiguous	1	1	Q04447	N-Term(iTRAQ8plex)	CID	1.0000	0.0000	1	1	3135156	Unique	Used	1.418	0.700	1.723	1.765	0	0	5.99	0	6	200	2	1135.07141	2269.13555	-0.33
dLESDIIGDTSGHFQk	Unambiguous	2	1	P14824	N-Term(iTRAQ8plex); K16(iTRAQ8plex)	CID	1.0000	0.0000	1	1	2900969	Unique	Used	0.911	1.077	0.961	0.852	0	0	5.99	0	5	94	3	790.74957	2370.23417	-2.37
aAVEEGIVLGGGcALLR	Unambiguous	2	1	P63038	N-Term(iTRAQ8plex); C13(Methylthio)	CID	1.0000	0.0000	1	1	3647217	Unique	Used	1.247	0.882	0.938	0.972	0	0	5.99	0	22	200	2	989.53754	1978.06780	-4.55
iELPQFSIVEHR	Unambiguous	2	1	P63080	N-Term(iTRAQ8plex)	CID	1.0000	0.0000	1	1	3578031	Unique	Used	0.786	1.444	1.185	0.929	0	0	5.99	0	4	19	3	591.33582	1771.99289	-4.50

dcFIVYqGHHGDVGAPMADVILPGAAYTEk	Unambiguous	1	1	Q91VD9	N-Term(iTRAQ8plex); C2(Methylthio); Q7(Deamidated); K30(iTRAQ8plex)	CID	1.0000	0.0000	1	1	3372531	Unique	Used	0.348	0.666	1.144	1.120	0	0	5.99	0	0	127	3	1277.29956	3829.88413	-0.21
gGAFEGTLqGPFGHGYGEGAGEGIDDAEWVVAR	Unambiguous	1	1	Q9QXY6	N-Term(iTRAQ8plex); Q9(Deamidated)	CID	1.0000	0.0000	1	1	3056672	Unique	Used	1.771	2.296	1.708	2.795	0	0	5.99	0	0	200	3	1204.57776	3611.71872	5.68
tTIVENVGSVEGLAYHR	Unambiguous	1	1	Q91ZX7	N-Term(iTRAQ8plex)	CID	1.0000	0.0000	1	1	3369002	Unique	Used	1.179	1.044	0.860	0.728	0	0	5.99	0	15	69	3	717.05383	2149.14695	-3.99
dHIIEVVGSSmPLIGDHQDEDk	Unambiguous	3	1	O88935	N-Term(iTRAQ8plex); M11(Oxidation); K22(iTRAQ8plex)	CID	1.0000	0.0000	1	1	2811098	Unique	Used	1.476	1.791	1.490	1.099	0	0	5.99	0	18	176	3	1020.19061	3058.55729	-1.29
yQQETNVSQLPGRPk	Unambiguous	3	1	A2A690	N-Term(iTRAQ8plex); K15(iTRAQ8plex)	HCD	1.0000	0.0000	1	1	3315713	Unique	Used	0.800	1.079	1.451	1.016	0	0	5.99	0	0	200	3	785.10571	2353.30259	-2.49
iNAMAAMQnLYETEDEETDkR	Unambiguous	2	1	Q80U19	N-Term(iTRAQ8plex); N9(Deamidated); K20(iTRAQ8plex)	CID	0.0033	0.0000	1	1	2869377	Unique	Used	1.488	0.601	3.818	2.413	0	0	5.99	1	28	200	3	1027.83936	3081.50351	2.25
iANDNSLNHEYLPILGLAEFR	Unambiguous	2	1	P05201	N-Term(iTRAQ8plex)	CID	1.0000	0.0000	1	1	3604131	Unique	Used	1.686	0.647	1.078	1.175	0	0	5.99	0	5	200	3	901.81567	2703.43247	-3.10
tWVTSSADPVSEHGGSTSAVR	Unambiguous	1	1	P58871	N-Term(iTRAQ8plex)	HCD	1.0000	0.0000	1	1	3184973	Unique	Used	1.614	1.226	1.358	0.743	0	0	5.99	0	0	38	3	812.40503	2435.20053	-4.09
qLLLTEGPHLYYVDPVNk	Unambiguous	4	1	F2Z3X6	N-Term(iTRAQ8plex); K18(iTRAQ8plex)	CID	1.0000	0.0000	1	1	3437360	Unique	Used	0.451	1.103	0.994	1.047	0	0	5.99	0	8	23	3	903.17859	2707.52121	-2.49
nQLEESEFTcAAAVk	Unambiguous	12	1	B2RRE2	N-Term(iTRAQ8plex); C10(Methylthio); K15(iTRAQ8plex)	CID	1.0000	0.0000	1	1	2975959	Unique	Used	0.881	0.661	1.416	0.939	0	0	5.99	0	25	188	2	1147.58032	2294.15337	-3.65
eDPAASLDAYAHALLAGR	Unambiguous	1	1	Q8BGD5	N-Term(iTRAQ8plex)	CID	1.0000	0.0000	1	1	3373504	Unique	Used	0.718	1.208	1.365	1.425	0	0	5.99	0	9	31	3	715.70935	2145.11350	-5.01

gTLTLQGDALSQADVNLk	Unambiguous	2	1	Q3TDK6	N-Term(iTRAQ8plex); K18(iTRAQ8plex)	CID	1.0000	0.0000	1	1	3323102	Unique	Used	0.522	1.701	1.281	1.298	0	0	5.99	0	0	120	2	1226.69312	2452.37895	-3.20
aYSGSDLPSGTGSGGGGADGAR	Unambiguous	1	1	Q71FD5	N-Term(iTRAQ8plex)	CID	1.0000	0.0000	1	1	2790185	Unique	Used	1.626	1.184	1.189	0.737	0	0	5.99	0	0	200	2	1101.01965	2201.03203	-2.39
sDIAAAQQSGMSLSnSk	Unambiguous	1	1	B1AQZ0	N-Term(iTRAQ8plex); N15(Deamidated); K17(iTRAQ8plex)	CID	1.0000	0.0000	1	1	2990525	Unique	Used	0.317	1.799	1.918	1.648	0	0	5.99	0	0	166	2	1152.59790	2304.18852	-3.33
gHAYSVTGAEEVESSGSLQk	Unambiguous	1	1	O08529	N-Term(iTRAQ8plex); K20(iTRAQ8plex)	CID	1.0000	0.0000	1	1	2808072	Unique	Used	1.608	1.211	1.250	0.968	0	0	5.98	0	12	51	3	882.12543	2644.36173	-2.18
gVLTGPEYEALDALPDAER	Unambiguous	1	1	Q9D1X0	N-Term(iTRAQ8plex)	CID	1.0000	0.0000	1	1	2798065	Unique	Used	1.735	0.889	1.156	1.477	0	0	5.98	0	0	200	2	1160.60144	2320.19560	-0.81
eNTLNQLVGAAFGAAGQR	Unambiguous	1	1	Q9EQ20	N-Term(iTRAQ8plex)	CID	1.0000	0.0000	1	1	3917461	Unique	Used	0.744	1.129	0.988	1.271	0	0	5.98	0	10	25	2	1061.06689	2121.12651	-4.23
eALLSSAVDHGSDEAR	Unambiguous	1	1	P80314	N-Term(iTRAQ8plex)	CID	1.0000	0.0000	1	1	2700229	Unique	Used	1.066	1.158	1.026	1.119	0	0	5.98	0	5	86	3	654.33344	1960.98575	-1.05
aEGATLSnATGAVESTSQAGDR	Unambiguous	1	1	Q61644	N-Term(iTRAQ8plex); N8(Deamidated)	CID	1.0000	0.0000	1	1	2668216	Unique	Used	2.104	2.371	4.161	2.331	0	0	5.98	0	8	200	2	1199.58179	2398.15630	-3.06
vIPAADLSEQISTAGTEASGTGNmk	Unambiguous	2	1	Q9WUB3	N-Term(iTRAQ8plex); M24(Oxidation); K25(iTRAQ8plex)	CID	1.0000	0.0000	1	1	3235253	Unique	Used	1.916	1.530	0.685	0.630	0	0	5.98	0	4	30	3	1024.86963	3072.59433	-1.20
lAQGPELAEDDADLLHk	Unambiguous	1	1	Q8R5H6	N-Term(iTRAQ8plex); K17(iTRAQ8plex)	CID	1.0000	0.0000	1	1	2636571	Unique	Used	0.935	1.729	1.682	0.608	0	0	5.98	0	6	200	3	815.11487	2443.33005	0.45
kGNVSTISGLSSQTAGAk	Unambiguous	3	1	Q9EPN1-4	N-Term(iTRAQ8plex); K1(iTRAQ8plex); K18(iTRAQ8plex)	HCD	1.0000	0.0000	1	1	3571372	Unique	Used	0.452	1.013	1.108	0.873	0	0	5.98	1	0	156	3	873.50964	2618.51438	-3.70
aEGATLSnATGAVESTSQAGDR	Unambiguous	1	1	Q61644	N-Term(iTRAQ8plex); N8(Deamidated)	CID	1.0000	0.0000	1	1	2634139	Unique	Used	1.009	1.871			0	0	5.98	0	0	200	2	1199.58350	2398.15972	-1.63

vLPMNTGVEAGETAck	Unambiguous	1	1	P29758	N-Term(iTRAQ8plex); C15(Methylthio); K16(iTRAQ8plex)	CID	1.0000	0.0000	1	1	3234529	Unique	Used	2.476	2.900	1.122	1.258	0	0	5.98	0	0	139	2	1137.58777	2274.16826	-3.09
sIEALLEAGQAQDTQASHAEANQQQTR	Unambiguous	2	1	Q99PL5	N-Term(iTRAQ8plex)	CID	1.0000	0.0000	1	1	3013038	Unique	Used	3.636	1.775	1.927	1.093	0	0	5.98	0	20	25	3	1067.19812	3199.57981	-2.63
iDSAATSTYEVGNPPDYR	Unambiguous	1	1	Q9D358	N-Term(iTRAQ8plex)	CID	1.0000	0.0000	1	1	2916585	Unique	Used	1.877	0.975	1.080	1.062	0	0	5.98	0	0	200	2	1130.55481	2260.10234	-0.53
qLYRPEPLPEVALPGSFVVR	Unambiguous	3	1	E9PVD3	N-Term(iTRAQ8plex)	HCD	1.0000	0.0000	1	1	3841493	Unique	Used	1.090	0.766	0.901	1.209	0	0	5.98	0	0	200	3	857.82239	2571.45261	-2.92
fGQAATMEGIGAIGGTPPAFNRPAPGAEFAPNk	Unambiguous	1	1	Q99K48	N-Term(iTRAQ8plex); K33(iTRAQ8plex)	CID	1.0000	0.0000	1	1	3744044	Unique	Used	1.053	0.833	0.823	0.659	0	0	5.98	0	0	200	3	1284.34094	3851.00827	-2.02
gVVGPGPAAIAAPGGGGAGPPAVGGGGGR	Unambiguous	1	1	Q3B7Z2	N-Term(iTRAQ8plex)	CID	1.0000	0.0000	1	1	3819399	Unique	Used		0.851	1.408	2.552	0	0	5.98	0	8	4	3	848.12891	2542.37217	-2.82
dLIALANTTGEVLLHR	Unambiguous	2	1	Q91W96	N-Term(iTRAQ8plex)	CID	1.0000	0.0000	1	1	3765802	Unique	Used			1.318	1.260	0	0	5.98	0	9	28	3	680.72845	2040.17081	-2.32
fTAGDFSTTVIQNVNk	Unambiguous	1	1	P57746	N-Term(iTRAQ8plex); K16(iTRAQ8plex)	CID	1.0000	0.0000	1	1	3578398	Unique	Used	0.408	2.002	0.939	0.874	0	0	5.98	0	0	14	2	1175.64087	2350.27446	-5.05
aAVEEGIVLGGGcALLR	Unambiguous	2	1	P63038	N-Term(iTRAQ8plex); C13(Methylthio)	CID	1.0000	0.0000	1	1	3647445	Unique	Used	1.127	0.916	1.242	1.161	0	0	5.97	0	6	200	2	989.53699	1978.06670	-5.10
hHAAYVnNLNATEEk	Unambiguous	1	1	P09671	N-Term(iTRAQ8plex); N7(Deamidated); K15(iTRAQ8plex)	CID	0.0302	0.0000	1	1	2750291	Unique	Used	1.744	0.898	0.903	0.845	0	0	5.97	0	0	200	3	774.07501	2320.21048	-1.60
vAPGPSSGcTPGQVPGSSALSSPR	Unambiguous	2	1	Q8CH77	N-Term(iTRAQ8plex); C9(Methylthio)	CID	1.0000	0.0000	1	1	3837828	Unique	Used	0.376	0.386	0.922	0.615	0	0	5.97	0	0	200	2	1273.63208	2546.25688	-3.01
scSGVEFSTSGSSNTDTGk	Unambiguous	4	1	G3UX26	N-Term(iTRAQ8plex); C2(Methylthio); K19(iTRAQ8plex)	CID	1.0000	0.0000	1	1	3009696	Unique	Used	0.566	0.777	1.160	1.198	0	0	5.97	0	0	35	2	1253.08630	2505.16533	-1.62
fIADGGPEVETIALQNNR	Unambiguous	1	1	Q8CH02	N-Term(iTRAQ8plex)	CID	1.0000	0.0000	1	1	3214568	Unique	Used	1.587	0.609	1.100	0.781	0	0	5.97	0	0	129	2	1124.59656	2248.18584	-0.78

lISENTEGSAQAPQLPR	Unambiguous	4	1	E9PX30	N-Term(iTRAQ8plex)	CID	1.0000	0.0000	1	1	3210385	Unique	Used	1.327	0.967	0.877	0.951	0	0	5.97	0	0	140	2	1058.07019	2115.13310	-0.81
gTGGVDTAATGSVFDISNLDR	Unambiguous	1	1	P30275	N-Term(iTRAQ8plex)	CID	1.0000	0.0000	1	1	3214540	Unique	Used	8.688	1.409	1.330	1.175	0	0	5.97	0	18	186	2	1179.09790	2357.18852	-0.08
aYFILDEFLLGGEVQETSk	Unambiguous	4	1	Q3TIV9	N-Term(iTRAQ8plex); K19(iTRAQ8plex)	CID	1.0000	0.0000	1	1	3305896	Unique	Used	1.654	1.023	1.111	1.225	0	0	5.97	0	10	200	3	923.17078	2767.49778	-1.34
sAAAADGGDQPLQPPGAAGR	Unambiguous	2	1	E9Q6C8	N-Term(iTRAQ8plex)	CID	1.0000	0.0000	1	1	2844293	Unique	Used	0.947	0.498	0.603	1.249	0	0	5.97	0	0	200	2	1056.04126	2111.07524	-1.49
qTVLTAAGSIGQASGDLLR	Unambiguous	2	1	E9PUM4	N-Term(iTRAQ8plex)	CID	1.0000	0.0000	1	1	3841483	Unique	Used	0.493	0.987	0.930	0.945	0	0	5.97	0	0	200	2	1081.60229	2162.19731	-5.09
iDINMSGFNETDDLk	Unambiguous	2	1	B1AX58	N-Term(iTRAQ8plex); K15(iTRAQ8plex)	CID	1.0000	0.0000	1	1	2939770	Unique	Used	0.595	0.554	0.813	1.081	0	0	5.97	0	0	47	2	1160.59766	2320.18804	-3.07
kFDVNTSAVQVLIEHIGNLDR	Unambiguous	2	1	Q68FD5	N-Term(iTRAQ8plex); K1(iTRAQ8plex)	CID	1.0000	0.0000	1	1	3785027	Unique	Used	0.350	1.254	1.039	1.058	0	0	5.97	1	15	17	3	992.89386	2976.66703	-1.93
gGPPPPPPPHSSGPPPPPAR	Unambiguous	1	1	Q91YD9	N-Term(iTRAQ8plex)	CID	1.0000	0.0000	1	1	3404552	Unique	Used	1.365	1.077	1.616	1.043	0	0	5.97	0	0	95	3	765.41357	2294.22617	-3.78
gVSSVVGPSAVGASPGDk	Unambiguous	2	1	Q8CI75	N-Term(iTRAQ8plex); K18(iTRAQ8plex)	CID	1.0000	0.0000	1	1	3252634	Unique	Used	0.572	0.565	1.206	1.148	0	0	5.97	0	10	98	2	1090.11462	2179.22197	1.85
tVmDDFAQFLDTcck	Unambiguous	1	1	P07724	N-Term(iTRAQ8plex); M3(Oxidation); C13(Methylthio); C14(Methylthio); K15(iTRAQ8plex)	CID	1.0000	0.0000	1	1	3626656	Unique	Used	2.792	1.981	0.630	0.878	0	0	5.97	0	0	200	3	818.37207	2453.10166	-5.03
yEGSGDGGAAAQSLYIAnHAY	Unambiguous	1	1	P05063	N-Term(iTRAQ8plex); N18(Deamidated)	CID	1.0000	0.0000	1	1	2760168	Unique	Used	1.604	1.147	1.398	1.432	0	0	5.97	0	16	200	2	1210.56616	2420.12505	-2.39
hVPVVYVDYPGPASLTQIYGTFNR	Unambiguous	1	1	Q9JHU4	N-Term(iTRAQ8plex)	CID	1.0000	0.0000	1	1	3898119	Unique	Used	0.600	0.721	0.895	0.933	0	0	5.97	0	27	18	3	999.86011	2997.56577	-3.96
eVcAGAFEHLPGLR	Unambiguous	1	1	Q9Z0L0	N-Term(iTRAQ8plex); C3(Methylthio)	CID	1.0000	0.0000	1	1	3704631	Unique	Used	0.598	0.761	0.693	0.937	0	0	5.97	0	12	23	3	616.98132	1848.92942	-5.87

nLPMSVTDIVkPGMR	Unambiguous	1	1	Q9D832	N-Term(iTRAQ8plex); K11(iTRAQ8plex)	HCD	1.0000	0.0000	1	1	3838936	Unique	Used	0.697	0.999	0.910	0.991	0	0	5.97	0	7	166	3	756.09937	2266.28354	-1.63
tATPQQAQEVHEk	Unambiguous	2	1	P17751	N-Term(iTRAQ8plex); K13(iTRAQ8plex)	CID	1.0000	0.0000	1	1	2628118	Unique	Used	2.239	1.111	1.631	1.323	0	0	5.97	0	3	200	3	692.38165	2075.13041	-1.81
tLASENIPSLPPGGELASk	Unambiguous	1	1	P49443	N-Term(iTRAQ8plex); K19(iTRAQ8plex)	CID	1.0000	0.0000	1	1	3322572	Unique	Used	0.789	0.863	0.964	0.902	0	0	5.97	0	3	24	2	1245.20325	2489.39922	-3.21
dINQEVYNFLATAGAk	Unambiguous	1	1	Q99KI0	N-Term(iTRAQ8plex); K16(iTRAQ8plex)	CID	1.0000	0.0000	1	1	3543172	Unique	Used	0.457	0.763	0.850	0.831	0	0	5.97	0	26	200	2	1181.64404	2362.28081	-2.34
sLSESELIGELSADFDR	Unambiguous	8	1	P51125-3	N-Term(iTRAQ8plex)	CID	1.0000	0.0000	1	1	2904973	Unique	Used	3.769	0.829	1.197	1.040	0	0	5.97	0	0	109	2	1086.55017	2172.09307	-2.01
tNVSGGAIALGHPLGGSGSR	Unambiguous	1	1	Q8BWT1	N-Term(iTRAQ8plex)	CID	1.0000	0.0000	1	1	3890696	Unique	Used	1.150	1.248	1.217	1.745	0	0	5.97	0	0	4	3	704.71686	2112.13602	-4.91
lTPIPSTSPNLQAYHQGNTQVSR	Unambiguous	1	1	Q8BIW1	N-Term(iTRAQ8plex)	CID	1.0000	0.0000	1	1	3836594	Unique	Used	0.588	0.960	1.139	1.418	0	0	5.97	0	4	91	3	938.49768	2813.47849	-2.25
gIGTDEATIIDIVTHR	Unambiguous	2	1	P14824	N-Term(iTRAQ8plex)	CID	1.0000	0.0000	1	1	3283604	Unique	Used	0.913	1.104	0.661	0.837	0	0	5.97	0	0	1	3	672.37286	2015.10404	-1.73
gPGTGGDEASAAGGSPPQPPR	Unambiguous	1	1	P81122	N-Term(iTRAQ8plex)	CID	1.0000	0.0000	1	1	2751171	Unique	Used		0.568			0	0	5.96	0	0	200	2	1084.03479	2167.06230	-2.73
lGVADGmLQTGVTVGTk	Unambiguous	2	1	Q924D0	N-Term(iTRAQ8plex); M7(Oxidation); K17(iTRAQ8plex)	CID	1.0000	0.0000	1	1	3596659	Unique	Used	0.507	1.073	0.674	0.944	0	0	5.96	0	0	135	2	1136.14099	2271.27471	-4.06
aAVEEGIVLGGGcALLR	Unambiguous	2	1	P63038	N-Term(iTRAQ8plex); C13(Methylthio)	CID	1.0000	0.0000	1	1	3647111	Unique	Used	0.976	0.726	1.126	0.995	0	0	5.96	0	6	200	2	989.53711	1978.06694	-4.98
lALSMLGqSDAYGAADAR	Unambiguous	3	1	O09012	N-Term(iTRAQ8plex); Q8(Deamidated)	CID	1.0000	0.0000	1	1	3518210	Unique	Used	1.088	1.089	0.732	0.817	0	0	5.96	0	0	200	2	1058.04407	2115.08086	5.39
gTGGVDTAAVGGVFDVSNADR	Unambiguous	1	1	Q04447	N-Term(iTRAQ8plex)	CID	1.0000	0.0000	1	1	3130036	Unique	Used	1.883	1.061	1.288	1.723	0	0	5.96	0	15	103	2	1135.07263	2269.13799	0.74
aQFPHGQETQDSTDLSEAVPHAGLqPELSAEAR	Unambiguous	3	1	Q6NZK8	N-Term(iTRAQ8plex); Q25(Deamidated)	CID	0.0151	0.0000	1	1	2936345	Unique	Used	1.950	1.224	1.905	1.899	0	0	5.96	0	25	200	3	1274.62317	3821.85495	0.74

lSmPQSAAVNTTPPHNR	Unambiguous	6	1	A2ACC6	N-Term(iTRAQ8plex); M3(Oxidation)	HCD	1.0000	0.0000	1	1	3793743	Unique	Used	1.649	1.130	0.984	1.217	0	0	5.96	0	0	106	3	714.37244	2141.10276	-2.24
iHEGcEEPATHNALAk	Unambiguous	2	1	Q68FD5	N-Term(iTRAQ8plex); C5(Methylthio); K16(iTRAQ8plex)	CID	1.0000	0.0000	1	1	2895793	Unique	Used	0.740	1.009	1.341	1.273	0	0	5.96	0	0	200	3	792.07288	2374.20407	-2.67
eQASHLGGAVFSGAGNIAAATGLVk	Unambiguous	1	1	Q91ZZ3	N-Term(iTRAQ8plex); K25(iTRAQ8plex)	CID	1.0000	0.0000	1	1	3822735	Unique	Used	0.884	1.400	1.337	0.826	0	0	5.96	0	0	200	2	1467.81152	2934.61577	-3.43
eDQDEALPSSIQELHLLVQNTR	Unambiguous	3	1	Q91WG2	N-Term(iTRAQ8plex)	HCD	1.0000	0.0000	1	1	3152332	Unique	Used	2.249	0.960	1.365	0.942	0	0	5.96	0	22	121	3	947.16205	2839.47159	-0.85
lHDVnNDGFLDEQELEALFTR	Unambiguous	1	1	P81117	N-Term(iTRAQ8plex); N5(Deamidated)	CID	0.0084	0.0000	1	1	2942270	Unique	Used	1.212	0.983	1.033	1.165	0	0	5.96	0	21	194	3	927.46130	2780.36936	0.44
fLSQPFQVAEVFTGHMGk	Unambiguous	1	1	P56480	N-Term(iTRAQ8plex); K18(iTRAQ8plex)	CID	1.0000	0.0000	1	1	3863651	Unique	Used	1.225	0.704	1.126	1.434	0	0	5.96	0	6	200	3	877.80823	2631.41013	-4.29
hHAAYVNNLnATEEk	Unambiguous	1	1	P09671	N-Term(iTRAQ8plex); N10(Deamidated); K15(iTRAQ8plex)	CID	1.0000	0.0000	1	1	2711093	Unique	Used	1.803	0.970	1.265	1.087	0	0	5.96	0	11	200	3	774.07703	2320.21653	1.00
hLLQAPLDDAQEILQAR	Unambiguous	1	1	Q9D662	N-Term(iTRAQ8plex)	CID	1.0000	0.0000	1	1	3394038	Unique	Used	1.134	0.820	0.776	1.001	0	0	5.96	0	12	14	3	745.74805	2235.22959	-4.64
tHnSALEYNIFEGMEcR	Unambiguous	1	1	O08553	N-Term(iTRAQ8plex); N3(Deamidated); C16(Methylthio)	HCD	1.0000	0.0000	1	1	3172292	Unique	Used	2.171	1.193	1.321	1.016	0	0	5.95	0	6	7	3	789.02283	2365.05393	-1.09
sPLDPQAGSSSGLVLHAAFPGHSQR	Unambiguous	6	1	Q8VBU5	N-Term(iTRAQ8plex)	HCD	1.0000	0.0000	1	1	3799646	Unique	Used	0.891	0.759	1.307	1.170	0	0	5.95	0	0	177	3	940.82672	2820.46561	-1.39
iMDPNIVGNEHYDVAR	Unambiguous	1	1	P56480	N-Term(iTRAQ8plex)	CID	1.0000	0.0000	1	1	3051203	Unique	Used	1.375	1.021	1.100	1.162	0	0	5.95	0	0	23	3	716.36578	2147.08280	-1.38
mLIDAQAEAAEQLGk	Unambiguous	2	1	E9Q912	N-Term(iTRAQ8plex); K15(iTRAQ8plex)	CID	1.0000	0.0000	1	1	3109796	Unique	Used	0.446	1.059	0.975	1.008	0	0	5.95	0	0	186	2	1098.61145	2196.21562	0.06

iGEHTPSALAImENANVLAR	Unambiguous	3	1	A6ZI44	N-Term(iTRAQ8plex); M12(Oxidation)	CID	1.0000	0.0000	1	1	3705205	Unique	Used	1.435	1.192	1.443	1.531	0	0	5.95	0	23	86	3	809.76538	2427.28159	-6.28
tHSAITLGDWSDPLDPDAEAQAVYR	Unambiguous	1	1	Q9Z268	N-Term(iTRAQ8plex)	CID	1.0000	0.0000	1	1	2996702	Unique	Used	2.605		1.506	0.830	0	0	5.95	0	8	58	3	1011.49933	3032.48343	-2.29
tSDVETMSSQSALALDER	Unambiguous	1	1	P14873	N-Term(iTRAQ8plex)	CID	1.0000	0.0000	1	1	2778824	Unique	Used	2.707	0.668	1.283	0.729	0	0	5.95	0	2	96	2	1122.54871	2244.09014	-2.96
iISGGEPQGALTSTMTHNEDLDR	Unambiguous	2	1	E9QAD4	N-Term(iTRAQ8plex)	CID	1.0000	0.0000	1	1	2992849	Unique	Used	1.281	0.863	1.107	1.193	0	0	5.95	0	24	86	3	916.12262	2746.35331	-3.17
sQGSGLSSGGAGEGQGPk	Unambiguous	1	1	Q8CGC7	N-Term(iTRAQ8plex); K18(iTRAQ8plex)	CID	1.0000	0.0000	1	1	2822762	Unique	Used	0.675	0.621	0.708	0.890	0	0	5.95	0	6	25	2	1085.06775	2169.12822	-3.43
gPAVGIDLGTTYScVGVFQHGk	Unambiguous	4	1	P63017	N-Term(iTRAQ8plex); C14(Methylthio); K22(iTRAQ8plex)	CID	1.0000	0.0000	1	1	3807525	Unique	Used	0.925	1.163	0.931	1.050	0	0	5.95	0	21	200	3	954.16736	2860.48752	-2.49
tSDVETMSSQSALALDER	Unambiguous	1	1	P14873	N-Term(iTRAQ8plex)	CID	1.0000	0.0000	1	1	2778545	Unique	Used	2.259	1.162	1.255	0.764	0	0	5.95	0	0	200	2	1122.54932	2244.09136	-2.42
fNPLPLnPQDFSGVHGINEk	Unambiguous	2	1	F7B8J4	N-Term(iTRAQ8plex); N7(Deamidated); K20(iTRAQ8plex)	CID	0.0017	0.0000	1	1	3559172	Unique	Used	0.987	1.462	0.957	1.192	0	0	5.95	0	0	200	3	944.84320	2832.51505	0.33
nMGGPYGGGnYGPGGSGGSGGYGGR	Unambiguous	3	1	O88569	N-Term(iTRAQ8plex); N10(Deamidated)	CID	0.0286	0.0000	1	1	3008559	Unique	Used	0.772	1.120	0.791	1.469	0	0	5.95	0	0	14	3	832.36749	2495.08792	-2.77
vSASNILGTGEPSGPSSR	Unambiguous	1	1	Q61330	N-Term(iTRAQ8plex)	CID	1.0000	0.0000	1	1	3472294	Unique	Used	1.218	0.628	1.839	2.296	0	0	5.94	0	0	45	2	1010.53076	2020.05425	-3.50
qALLGDSSSQSWNAGVADR	Unambiguous	2	1	Q9JKK1	N-Term(iTRAQ8plex)	CID	1.0000	0.0000	1	1	3344536	Unique	Used	0.960	1.050	0.940	1.103	0	0	5.94	0	10	28	2	1133.56750	2266.12773	-3.92
kTGSPGGPGVGGSPGGGAAGASPSLPPSAk	Unambiguous	1	1	Q8R1Q8	N-Term(iTRAQ8plex); K1(iTRAQ8plex); K30(iTRAQ8plex)	CID	1.0000	0.0000	1	1	3469419	Unique	Used	0.935	0.886	0.632	0.559	0	0	5.94	1	28	163	3	1124.95483	3372.84995	-4.16
tVDDLEIIYDELLHIk	Unambiguous	4	1	Q9EQZ6-3	N-Term(iTRAQ8plex); K16(iTRAQ8plex)	CID	1.0000	0.0000	1	1	3133454	Unique	Used	0.316	1.243	1.154	1.254	0	0	5.94	0	4	40	3	846.48193	2537.43125	-0.43

rGAGGAGTGPGGGGSGGVAk	Unambiguous	2	1	A2AQ87	N-Term(iTRAQ8plex); K20(iTRAQ8plex)	CID	1.0000	0.0000	1	1	3359183	Unique	Used	0.536	0.716	1.672	1.181	0	0	5.94	1	0	200	3	712.72681	2136.16587	-3.38
kGPGPGGPGGAGGARGGAGGGPSGD	Unambiguous	1	1	P60761	N-Term(iTRAQ8plex); K1(iTRAQ8plex)	CID	1.0000	0.0000	1	1	3098209	Unique	Used	0.668	2.106	2.776	0.664	0	0	5.94	2	3	200	3	838.43958	2513.30417	-0.99
sLAASGAQQPDSPGGPQPPGQk	Unambiguous	2	1	Q9JKS5	N-Term(iTRAQ8plex); K22(iTRAQ8plex)	CID	1.0000	0.0000	1	1	3102099	Unique	Used			2.980	1.570	0	0	5.94	0	4	35	3	895.14520	2683.42105	-1.86
aTDLLLDDSLVSLFGNRR	Unambiguous	4	1	P99029-2	N-Term(iTRAQ8plex)	HCD	1.0000	0.0000	1	1	3785354	Unique	Used	0.821	0.512	1.122	1.133	0	0	5.94	1	2	5	3	770.42816	2309.26993	-2.95
iGEHTPSALAImENANVLAR	Unambiguous	3	1	A6ZI44	N-Term(iTRAQ8plex); M12(Oxidation)	CID	1.0000	0.0000	1	1	3709498	Unique	Used	2.645	1.302	1.244	1.531	0	0	5.94	0	4	200	3	809.76654	2427.28507	-4.85
eHINLGcDVDFDIAGPSIR	Unambiguous	3	1	Q60932-2	N-Term(iTRAQ8plex); C7(Methylthio)	CID	1.0000	0.0000	1	1	3376078	Unique	Used	0.915	1.778	1.594	1.111	0	0	5.94	0	3	200	3	807.73047	2421.17685	-3.16
kETISLQMEEFnTAIYSNDDLLSSk	Unambiguous	2	1	Q99P72	N-Term(iTRAQ8plex); K1(iTRAQ8plex); N12(Deamidated); K25(iTRAQ8plex)	CID	0.0051	0.0000	1	1	3326338	Unique	Used	8.626	1.618	0.811	1.450	0	0	5.94	1	0	150	3	1264.00232	3789.99240	1.37
aGQVFLEELGNHk	Unambiguous	2	1	P16330	N-Term(iTRAQ8plex); K13(iTRAQ8plex)	CID	1.0000	0.0000	1	1	3212030	Unique	Used	0.926	0.275	1.236	1.281	0	0	5.94	0	18	93	3	684.05457	2050.14914	-2.46
gQIHLDSQQDYQLLQAQR	Unambiguous	2	1	Q64237	N-Term(iTRAQ8plex)	HCD	1.0000	0.0000	1	1	3298624	Unique	Used	2.232	1.246	1.115	1.053	0	0	5.94	0	0	122	3	815.76410	2445.27774	-0.44
fLSQPFQVAEVFTGHMGk	Unambiguous	1	1	P56480	N-Term(iTRAQ8plex); K18(iTRAQ8plex)	CID	1.0000	0.0000	1	1	3862652	Unique	Used	1.156	0.946	1.577	1.704	0	0	5.94	0	29	200	3	877.80945	2631.41379	-2.90
kPPGPPPGPPPPQVLQMYGR	Unambiguous	1	1	Q923D5	N-Term(iTRAQ8plex); K1(iTRAQ8plex)	HCD	1.0000	0.0000	1	1	3874278	Unique	Used	0.581	0.700	1.020	0.843	0	0	5.93	0	25	100	3	906.84680	2718.52585	-4.27
aTDLFDELVAHEVAR	Unambiguous	2	1	Q6ZPE2-2	N-Term(iTRAQ8plex)	CID	1.0000	0.0000	1	1	3075505	Unique	Used	0.888	1.057	1.780	1.409	0	0	5.93	0	16	200	3	664.02264	1990.05338	-0.70

nLALFEEEL DIRPk	Unambiguous	3	1	Q9JIS8	N-Term(iTRAQ8plex); K14(iTRAQ8plex)	HCD	1.0000	0.0000	1	1	3215423	Unique	Used	0.761	0.560	0.595	0.688	0	0	5.93	0	24	200	3	765.77454	2295.30906	-3.43
lLAEQLGFD FFEASAk	Unambiguous	1	1	Q9CZT8	N-Term(iTRAQ8plex); K16(iTRAQ8plex)	CID	1.0000	0.0000	1	1	3419234	Unique	Used		3.601	1.079	1.007	0	0	5.93	0	0	34	2	1197.65601	2394.30474	-4.95
aSDDLGEP DVFATAPFR	Unambiguous	1	1	Q6PIU9	N-Term(iTRAQ8plex)	CID	1.0000	0.0000	1	1	2977513	Unique	Used	2.178	1.209	0.914	0.789	0	0	5.93	0	18	200	2	1056.52686	2112.04643	-4.14
hQADIASYQ DAIQQLDS ELR	Unambiguous	1	1	P19246	N-Term(iTRAQ8plex)	CID	1.0000	0.0000	1	1	3151827	Unique	Used	2.217	0.975	1.330	1.206	0	0	5.93	0	21	158	3	869.10907	2605.31266	-1.29
aVAIETQPA QGESDAVA R	Unambiguous	3	1	Q7TSJ2	N-Term(iTRAQ8plex)	CID	1.0000	0.0000	1	1	2649896	Unique	Used	0.839	0.815	1.923	2.086	0	0	5.93	0	0	69	2	1059.05725	2117.10723	-3.24
kLAAGAES PQPASGNS PSEDDR	Unambiguous	1	1	P16054	N-Term(iTRAQ8plex); K1(iTRAQ8plex)	CID	1.0000	0.0000	1	1	2696099	Unique	Used		1.698	1.275	0.768	0	0	5.93	1	0	200	3	931.47681	2792.41587	-4.05
gIGTDEATII DIVTHR	Unambiguous	2	1	P14824	N-Term(iTRAQ8plex)	CID	1.0000	0.0000	1	1	3261620	Unique	Used	0.878	0.863	0.732	0.864	0	0	5.93	0	0	3	3	672.37354	2015.10605	-0.73
aAGTTVVE VEEIVDIGS FAPEDIHIPk	Unambiguous	2	1	Q9D0K2	N-Term(iTRAQ8plex); K27(iTRAQ8plex)	CID	1.0000	0.0000	1	1	3217450	Unique	Used	0.582	0.458	0.556	0.468	0	0	5.93	0	2	13	3	1148.96045	3444.86679	-1.61
gTVcAANDF NPDADAk	Unambiguous	2	1	P14824	N-Term(iTRAQ8plex); C4(Methylthio); K16(iTRAQ8plex)	CID	1.0000	0.0000	1	1	2829684	Unique	Used	0.609	1.051	0.861	1.021	0	0	5.93	0	0	49	2	1132.04907	2263.09087	-1.56
eAYPGDVF YLHSR	Unambiguous	2	1	Q03265	N-Term(iTRAQ8plex)	CID	1.0000	0.0000	1	1	3240504	Unique	Used	1.365	0.832	1.083	1.191	0	0	5.93	0	0	200	3	619.98523	1857.94114	-1.39
hLNEIDLFH cIDPNDSk	Unambiguous	2	1	Q9R0Q7	N-Term(iTRAQ8plex); C10(Methylthio); K17(iTRAQ8plex)	CID	1.0000	0.0000	1	1	3236886	Unique	Used	0.781	0.647	1.248	0.910	0	0	5.93	0	0	10	3	888.78094	2664.32828	-3.30
aGAAGAGG GcGGAMAE R	Unambiguous	1	1	E9QK48	N-Term(iTRAQ8plex); C10(Methylthio)	CID	1.0000	0.0000	1	1	3162542	Unique	Used	0.784	0.295	0.710	0.962	0	0	5.93	0	0	144	2	857.39172	1713.77617	-0.68
iGEHTPSAL AImENANV LAR	Unambiguous	3	1	A6ZI44	N-Term(iTRAQ8plex); M12(Oxidation)	CID	1.0000	0.0000	1	1	3474256	Unique	Used	2.294	1.592	0.795	1.273	0	0	5.92	0	13	30	3	809.76892	2427.29221	-1.91

gGGTSSGGSSSTNSLTGSRPPk	Unambiguous	1	1	Q78T81	N-Term(iTRAQ8plex); K22(iTRAQ8plex)	CID	1.0000	0.0000	1	1	3466452	Unique	Used		0.922	1.054	1.062	0	0	5.92	0	0	200	3	863.12042	2587.34671	-2.53
qASGGPVDIGPEYQQDLDR	Unambiguous	2	1	P97450	N-Term(iTRAQ8plex)	CID	1.0000	0.0000	1	1	2635259	Unique	Used	2.846	0.990	1.793	0.926	0	0	5.92	0	0	129	2	1175.08081	2349.15434	-3.47
aSDLAGSVDTGSAGSVk	Unambiguous	3	1	Q6P9K8	N-Term(iTRAQ8plex); K17(iTRAQ8plex)	CID	1.0000	0.0000	1	1	2971657	Unique	Used	0.824	0.303	1.088	0.661	0	0	5.92	0	8	54	2	1065.57581	2130.14433	-2.62
kEELNAISGPNEFAEFYNR	Unambiguous	1	1	Q9D554	N-Term(iTRAQ8plex); K1(iTRAQ8plex)	HCD	1.0000	0.0000	1	1	3213747	Unique	Used	0.945	1.115	0.804	0.811	0	0	5.92	1	4	26	3	946.16010	2836.46573	-2.44
aVQSIGNLGQQLGQGk	Unambiguous	1	1	Q9Z268	N-Term(iTRAQ8plex); K16(iTRAQ8plex)	HCD	1.0000	0.0000	1	1	3760975	Unique	Used	0.234	3.647	1.099	1.026	0	0	5.92	0	0	14	2	1103.63867	2206.27007	-2.89
aVEAEEELTVAYGYDHSPPGk	Unambiguous	1	1	Q8VHL1	N-Term(iTRAQ8plex); K21(iTRAQ8plex)	CID	1.0000	0.0000	1	1	2686834	Unique	Used	1.386	0.532	1.419	0.524	0	0	5.92	0	0	200	3	957.49408	2870.46769	0.28
eALELTDTGLLSGSEER	Unambiguous	1	1	Q9JHU4	N-Term(iTRAQ8plex)	CID	1.0000	0.0000	1	1	2740000	Unique	Used	0.573	0.888	0.967	1.026	0	0	5.92	0	9	200	2	1062.55347	2124.09966	1.05
fQEYHIQQNEALAAk	Unambiguous	1	1	Q9WV98	N-Term(iTRAQ8plex); K15(iTRAQ8plex)	CID	1.0000	0.0000	1	1	3124781	Unique	Used	1.548	1.060	0.932	0.833	0	0	5.92	0	5	4	3	800.10291	2398.29416	-1.41
mAAAGGTVSGPSSAckPGR	Unambiguous	2	1	F8WIE5	N-Term(iTRAQ8plex); M1(Oxidation); C15(Methylthio); K16(iTRAQ8plex)	HCD	1.0000	0.0000	1	1	3815093	Unique	Used	0.380	0.612	0.720	0.888	0	0	5.92	0	0	200	3	792.40405	2375.19760	-4.83
sAYSSYSAPVSSSLSVR	Unambiguous	1	1	P08551	N-Term(iTRAQ8plex)	CID	1.0000	0.0000	1	1	3739412	Unique	Used	2.070	0.104	1.141	1.330	0	0	5.92	0	17	200	2	1026.52820	2052.04912	-2.92
gPNIVLSEELAGHQTPITDIATER	Unambiguous	1	1	Q9R0D8	N-Term(iTRAQ8plex)	HCD	1.0000	0.0000	1	1	3214486	Unique	Used	2.712	1.017	1.255	1.115	0	0	5.92	0	0	58	3	955.84528	2865.52127	-1.67
nmTVEQLLTGSPTSPTVEPEkPTR	Unambiguous	1	1	Q6ZPJ3	N-Term(iTRAQ8plex); M2(Oxidation); K21(iTRAQ8plex)	HCD	1.0000	0.0000	1	1	3368598	Unique	Used	1.500	1.142	1.097	1.202	0	0	5.92	0	0	30	3	1079.57605	3236.71360	-4.87

dLLVEALAEILHQk	Unambiguous	2	1	Q3UHQ6-2	N-Term(iTRAQ8plex); K14(iTRAQ8plex)	CID	1.0000	0.0000	1	1	3541587	Unique	Used	0.341	0.918	0.591	0.725	0	0	5.92	0	0	26	3	734.10742	2200.30771	-3.85
aAQQAASSSGQGQQAQTPTGk	Unambiguous	5	1	E9Q4G7	N-Term(iTRAQ8plex); K21(iTRAQ8plex)	CID	1.0000	0.0000	1	1	3118371	Unique	Used	1.174	1.332	1.789	1.130	0	0	5.92	0	29	200	3	870.79370	2610.36655	-1.03
nSQSFFSGLFGGSSk	Unambiguous	1	1	Q9DB05	N-Term(iTRAQ8plex); K15(iTRAQ8plex)	CID	1.0000	0.0000	1	1	3878311	Unique	Used	0.559	1.200	0.939	1.037	0	0	5.92	0	3	15	2	1079.56787	2158.12847	-4.85
lLPEPPPSQAAATHSVQnGR	Unambiguous	5	1	Q8CFT0	N-Term(iTRAQ8plex); N18(Deamidated)	HCD	0.0169	0.0000	1	1	3185509	Unique	Used	1.647	0.944	0.992	0.862	0	0	5.92	0	23	13	3	792.42371	2375.25656	-2.35
iQAAASPPANATAASDTNAGDR	Unambiguous	2	1	E9QAQ5	N-Term(iTRAQ8plex)	CID	1.0000	0.0000	1	1	2931894	Unique	Used	0.730	0.873	1.168	1.091	0	0	5.91	0	0	180	2	1187.59705	2374.18682	-1.39
dINQEVYNFLATAGAk	Unambiguous	1	1	Q99KI0	N-Term(iTRAQ8plex); K16(iTRAQ8plex)	CID	1.0000	0.0000	1	1	3542785	Unique	Used	0.467	0.954	0.911	1.148	0	0	5.91	0	21	200	2	1181.64368	2362.28008	-2.65
dkLVGAIVYYDGQHNDAR	Unambiguous	2	1	Q64521	N-Term(iTRAQ8plex); K2(iTRAQ8plex)	HCD	1.0000	0.0000	1	1	3299445	Unique	Used	0.802	1.638	1.331	1.357	0	0	5.91	1	0	112	3	881.47595	2642.41330	-0.54
sLHGEFSASDSSLTDIQETR	Unambiguous	2	1	Q8C0T5	N-Term(iTRAQ8plex)	CID	1.0000	0.0000	1	1	2847375	Unique	Used	2.055	1.115	1.291	1.010	0	0	5.91	0	28	200	3	828.74054	2484.20707	-3.45
dYAVSTVPVADSLHLk	Unambiguous	2	1	Q9CWS0	N-Term(iTRAQ8plex); K16(iTRAQ8plex)	CID	1.0000	0.0000	1	1	3279188	Unique	Used	1.144	1.286	1.374	1.044	0	0	5.91	0	19	25	3	775.10822	2323.31009	-0.74
sEESLSSQASGAGLQR	Unambiguous	1	1	Q80YF9	N-Term(iTRAQ8plex)	CID	1.0000	0.0000	1	1	2862175	Unique	Used	0.414	2.680	3.607	1.757	0	0	5.91	0	0	200	2	955.98737	1910.96745	-2.46
aALAGGTTMIIDHVVPEPESSLTEAYEk	Unambiguous	3	1	E9PWE8	N-Term(iTRAQ8plex); K28(iTRAQ8plex)	CID	1.0000	0.0000	1	1	3216539	Unique	Used	0.630	0.534	0.541	0.440	0	0	5.91	0	12	198	3	1179.95581	3537.85288	-2.24
eHLLmALADLDELLVR	Unambiguous	3	1	E9PZ16	N-Term(iTRAQ8plex); M5(Oxidation)	CID	1.0000	0.0000	1	1	3520534	Unique	Used	2.912	1.889	1.066	0.762	0	0	5.91	0	11	37	3	724.40369	2171.19651	-3.83
hDSATSFVDAGNAYk	Unambiguous	2	1	P28663	N-Term(iTRAQ8plex); K15(iTRAQ8plex)	CID	1.0000	0.0000	1	1	2970569	Unique	Used	0.572	1.464	1.376	1.067	0	0	5.91	0	0	200	2	1096.05969	2191.11211	-5.43

nPTEVQmSQLVLPcHTNHR	Unambiguous	6	1	Q8VHQ9	N-Term(iTRAQ8plex); M7(Oxidation); C14(Methylthio)	CID	1.0000	0.0000	1	1	3818031	Unique	Used	1.217	0.657	0.958	1.272	0	0	5.91	0	8	200	3	857.42218	2570.25199	-2.34
gETGPAGPAGPIGPAGAR	Unambiguous	2	1	P11087-2	N-Term(iTRAQ8plex)	CID	1.0000	0.0000	1	1	3162918	Unique	Used	0.635	6.994	1.348	1.298	0	0	5.91	0	0	25	2	918.99573	1836.98418	-1.56
aWSSAFDNLIGNHISR	Unambiguous	1	1	P70423	N-Term(iTRAQ8plex)	CID	1.0000	0.0000	1	1	3897290	Unique	Used	0.792	1.025	1.216	1.220	0	0	5.91	0	17	172	3	698.03101	2092.07846	-4.46
nVGTGLVGAPAcGDVMk	Unambiguous	3	1	Q9D7P6	N-Term(iTRAQ8plex); C12(Methylthio); K17(iTRAQ8plex)	CID	1.0000	0.0000	1	1	3476246	Unique	Used	1.182	0.650	1.115	0.986	0	0	5.91	0	20	27	2	1122.08997	2243.17266	-3.60
eSTFLDEGPNEQEITPLQHTPR	Unambiguous	3	1	Q9QYR6	N-Term(iTRAQ8plex)	CID	1.0000	0.0000	1	1	2778900	Unique	Used	4.180		1.186	1.403	0	0	5.91	0	15	200	3	948.14362	2842.41629	0.05
yPVGIEVGPQPHGVLR	Unambiguous	1	1	Q8R3P0	N-Term(iTRAQ8plex)	CID	1.0000	0.0000	1	1	3616262	Unique	Used	3.277	0.769	0.933	1.259	0	0	5.91	0	18	82	3	674.71552	2022.13199	-5.86
dIGAIAQVHAENGDIIAEEQQR	Unambiguous	1	1	O08553	N-Term(iTRAQ8plex)	CID	1.0000	0.0000	1	1	2811691	Unique	Used	2.028	1.328	0.896	2.047	0	0	5.90	0	1	8	3	894.46332	2681.37540	-1.61
vSASNILGTGEPSGPSSR	Unambiguous	1	1	Q61330	N-Term(iTRAQ8plex)	CID	1.0000	0.0000	1	1	3472437	Unique	Used	0.879	0.390	1.601	2.002	0	0	5.90	0	0	10	2	1010.53040	2020.05351	-3.86
gAESITTYTFNTHk	Unambiguous	1	1	Q9CXS4	N-Term(iTRAQ8plex); K14(iTRAQ8plex)	CID	1.0000	0.0000	1	1	3340563	Unique	Used	0.904	0.934	1.073	1.178	0	0	5.90	0	23	10	3	726.72394	2178.15726	-3.61
nPPVLILDEATSALDAESEYLIQQAIHGNLQR	Unambiguous	1	1	Q9JJ59	N-Term(iTRAQ8plex)	CID	1.0000	0.0000	1	1	3459980	Unique	Used	1.230	0.478	0.895	1.563	0	0	5.90	0	24	200	3	1275.00562	3823.00229	-4.12
gPGTSFEFALAIVEALVGkDMAnQVk	Unambiguous	1	1	Q99LX0	N-Term(iTRAQ8plex); K19(iTRAQ8plex); N23(Deamidated); K26(iTRAQ8plex)	CID	0.0339	0.0000	1	1	3843087	Unique	Used	0.466	0.956	1.184	1.447	0	0	5.90	1	20	169	3	1202.67578	3606.01279	3.07
gHYTEGAELVDAVLDVVR	Unambiguous	1	1	Q9D6F9	N-Term(iTRAQ8plex)	CID	1.0000	0.0000	1	1	3355971	Unique	Used	0.981	0.997	0.821	0.946	0	0	5.90	0	11	200	3	749.73322	2247.18509	-3.22
dYAVSTVPVADSLHLk	Unambiguous	2	1	Q9CWS0	N-Term(iTRAQ8plex); K16(iTRAQ8plex)	CID	1.0000	0.0000	1	1	3300558	Unique	Used	1.192	0.905	1.421	0.935	0	0	5.90	0	12	9	3	775.10693	2323.30625	-2.39
eHINLGcDVDFDIAGPSIR	Unambiguous	3	1	Q60932-2	N-Term(iTRAQ8plex); C7(Methylthio)	CID	1.0000	0.0000	1	1	3375651	Unique	Used	1.066	1.261	1.477	0.964	0	0	5.90	0	17	200	3	807.73120	2421.17905	-2.25

iQAAASPPAnATAASDTNAGDR	Unambiguous	2	1	E9QAQ5	N-Term(iTRAQ8plex); N10(Deamidated)	CID	0.0356	0.0000	1	1	2931984	Unique	Used	0.258	0.821	0.850	0.948	0	0	5.90	0	30	200	2	1188.09619	2375.18511	4.62
sEALSSVLDQEEAMDTk	Unambiguous	5	1	A2AU91	N-Term(iTRAQ8plex); K17(iTRAQ8plex)	CID	1.0000	0.0000	1	1	2638091	Unique	Used	0.958	0.632	0.830	0.773	0	0	5.90	0	26	200	2	1231.13257	2461.25786	-0.41
gEAGTPVAAGTTAAAIPR	Unambiguous	1	1	O08919	N-Term(iTRAQ8plex)	CID	1.0000	0.0000	1	1	3275177	Unique	Used	0.624	1.424	1.492	1.066	0	0	5.90	0	3	18	2	958.02954	1915.05181	-1.73
dGFTSEGEILEISDIQR	Unambiguous	1	1	Q8HW98	N-Term(iTRAQ8plex)	CID	1.0000	0.0000	1	1	2688434	Unique	Used	0.953		0.830	1.400	0	0	5.90	0	2	200	2	1107.06396	2213.12065	-1.50
lGEHNInVLEGNEQFIDAAk	Unambiguous	1	1	Q9Z1R9	N-Term(iTRAQ8plex); N7(Deamidated); K20(iTRAQ8plex)	CID	0.0356	0.0000	1	1	2901991	Unique	Used	1.066	1.054	0.725	0.750	0	0	5.90	0	19	161	3	940.83606	2820.49363	-1.86
dHGLSDEELNAFQk	Unambiguous	1	1	Q8JZN7	N-Term(iTRAQ8plex); K14(iTRAQ8plex)	CID	1.0000	0.0000	1	1	2699767	Unique	Used	1.646	1.138	1.162	1.156	0	0	5.90	0	17	200	3	737.72046	2211.14682	-1.54
vPATDTYSISGYGAAGGk	Unambiguous	1	1	P97793	N-Term(iTRAQ8plex); K18(iTRAQ8plex)	CID	1.0000	0.0000	1	1	3254591	Unique	Used	0.569	0.808	0.858	0.898	0	0	5.90	0	0	84	2	1162.11853	2323.22978	-3.98
lASIFSGTGADPVVSAk	Unambiguous	2	1	O08842	N-Term(iTRAQ8plex); K17(iTRAQ8plex)	CID	1.0000	0.0000	1	1	3665432	Unique	Used	0.450	0.618	1.003	1.345	0	0	5.90	0	0	82	2	1114.63208	2228.25688	-8.01
vEELEENISHLSEk	Unambiguous	5	1	A2APV2-3	N-Term(iTRAQ8plex); K14(iTRAQ8plex)	CID	1.0000	0.0000	1	1	2763989	Unique	Used	1.158	0.773	1.332	1.250	0	0	5.90	0	4	200	3	755.41193	2264.22123	-0.81
aVAQGNLSSADVQAAk	Unambiguous	1	1	Q9DB77	N-Term(iTRAQ8plex); K16(iTRAQ8plex)	CID	1.0000	0.0000	1	1	3122761	Unique	Used	0.558	1.428	1.378	1.522	0	0	5.90	0	0	8	2	1069.60400	2138.20073	-0.88
fGGNPGGFGnQGGFGNSR	Unambiguous	1	1	Q921F2	N-Term(iTRAQ8plex); N10(Deamidated)	CID	0.0424	0.0000	1	1	3513897	Unique	Used	0.592	0.913	0.967	1.404	0	0	5.90	0	22	33	2	1016.47870	2031.95012	-3.65
gSVHDFPEFDANQDAEALYTAMk	Unambiguous	2	1	P14824	N-Term(iTRAQ8plex); K23(iTRAQ8plex)	CID	1.0000	0.0000	1	1	3074498	Unique	Used	1.499	1.803	0.739	1.138	0	0	5.90	0	15	200	3	1055.52502	3164.56052	4.72

tSPDAFIQLALQLAHYk	Unambiguous	1	1	P97742	N-Term(iTRAQ8plex); K17(iTRAQ8plex)	CID	1.0000	0.0000	1	1	3879843	Unique	Used	0.837	0.925	0.534	0.774	0	0	5.89	0	11	46	3	842.14703	2524.42655	-4.70
sHAEDLGNLEGVkPAVLTR	Unambiguous	2	1	P04919-2	N-Term(iTRAQ8plex); K13(iTRAQ8plex)	HCD	1.0000	0.0000	1	1	3319106	Unique	Used	1.218	1.845	0.468	1.260	0	0	5.89	0	13	53	3	872.15997	2614.46537	-4.58
aDNFEYSDPVDGSISk	Unambiguous	2	1	Q9D0F9	N-Term(iTRAQ8plex); K16(iTRAQ8plex)	CID	1.0000	0.0000	1	1	2685122	Unique	Used	0.586	0.890	0.811	1.357	0	0	5.89	0	0	200	2	1176.59204	2352.17681	-2.03
hALIIYDDLSkQAVAYR	Unambiguous	2	1	Q03265	N-Term(iTRAQ8plex); K11(iTRAQ8plex)	HCD	1.0000	0.0000	1	1	3913392	Unique	Used	0.730	0.841	0.982	1.070	0	0	5.89	1	17	198	3	862.15613	2584.45383	-6.55
sAYALGGLGSGIcPNk	Unambiguous	1	1	Q8C196	N-Term(iTRAQ8plex); C13(Methylthio); K16(iTRAQ8plex)	CID	1.0000	0.0000	1	1	3803404	Unique	Used	1.706	1.172	4.392	7.456	0	0	5.89	0	0	123	2	1081.57715	2162.14702	-4.08
gTGSSSSGVLMVGPNFR	Unambiguous	1	1	Q8C4X2	N-Term(iTRAQ8plex)	CID	1.0000	0.0000	1	1	3874506	Unique	Used	0.504	0.523	1.137	1.029	0	0	5.89	0	0	127	2	979.00562	1957.00395	-3.87
iQDALcALDDSGALEPk	Unambiguous	1	1	Q6P4T1	N-Term(iTRAQ8plex); C6(Methylthio); K17(iTRAQ8plex)	CID	1.0000	0.0000	1	1	3037730	Unique	Used		0.537	0.954	0.889	0	0	5.89	0	17	70	2	1207.13049	2413.25371	-1.11
iTnGQPQQTTGAASGGYIk	Unambiguous	3	1	O09044	N-Term(iTRAQ8plex); N3(Deamidated); K19(iTRAQ8plex)	CID	0.0390	0.0000	1	1	3103845	Unique	Used	0.976	0.959	0.782	0.934	0	0	5.89	0	0	200	2	1251.17603	2501.34477	-0.35
aLNAADPGSDLLSVQALQR	Unambiguous	1	1	P08032	N-Term(iTRAQ8plex)	CID	1.0000	0.0000	1	1	3477809	Unique	Used	4.291	2.808	0.623	1.318	0	0	5.89	0	7	50	2	1122.11389	2243.22051	-4.14
kAAMEALQSQALHATSQQPLR	Unambiguous	5	1	B1AQZ0	N-Term(iTRAQ8plex); K1(iTRAQ8plex)	CID	1.0000	0.0000	1	1	3856104	Unique	Used	1.272	0.971	1.611	1.216	0	0	5.89	1	7	150	3	963.20001	2887.58548	-6.17
vDAILcVAGGWAGGNAk	Unambiguous	4	1	Q8BVI4	N-Term(iTRAQ8plex); C6(Methylthio); K17(iTRAQ8plex)	CID	1.0000	0.0000	1	1	3842411	Unique	Used	0.779	0.516	1.538	2.400	0	0	5.89	0	25	24	2	1128.60364	2256.20000	-3.98

aSSEGGAATGAGLDSLHk	Unambiguous	2	1	Q9WV32	N-Term(iTRAQ8plex); K18(iTRAQ8plex)	CID	1.0000	0.0000	1	1	2931555	Unique	Used	1.955	0.750	1.124	0.858	0	0	5.89	0	16	108	3	746.40369	2237.19651	-0.78
eTTGAQVQVAGDLLPNSTER	Unambiguous	1	1	P57724	N-Term(iTRAQ8plex)	CID	1.0000	0.0000	1	1	3032290	Unique	Used	0.620	0.377	0.683	0.958	0	0	5.89	0	0	178	2	1195.62476	2390.24224	-1.81
eVHGISmMPGSSETLPAGFR	Unambiguous	2	1	B8QI34	N-Term(iTRAQ8plex); M7(Oxidation)	CID	1.0000	0.0000	1	1	3514117	Unique	Used	1.292	1.241	1.197	1.178	0	0	5.89	0	13	95	3	808.40326	2423.19522	-2.04
tSDVETMSSQSALALDER	Unambiguous	1	1	P14873	N-Term(iTRAQ8plex)	CID	1.0000	0.0000	1	1	2794753	Unique	Used	2.921	1.227	1.117	0.930	0	0	5.88	0	0	200	2	1122.55054	2244.09380	-1.33
lVTLSATVTELEEDLGTAR	Unambiguous	1	1	B8QI36	N-Term(iTRAQ8plex)	HCD	1.0000	0.0000	1	1	3263270	Unique	Used	1.116	0.842	0.828	0.842	0	0	5.88	0	0	29	2	1161.63818	2322.26909	-0.67
sTTEPGPPGYSVSPPVPGR	Unambiguous	1	1	Q640R3	N-Term(iTRAQ8plex)	CID	1.0000	0.0000	1	1	3297712	Unique	Used	0.687	0.442	1.289	1.349	0	0	5.88	0	0	3	2	1093.57019	2186.13310	-2.95
sTQDLSDVSTDEVGIPLR	Unambiguous	4	1	D3Z5J3	N-Term(iTRAQ8plex)	CID	1.0000	0.0000	1	1	2919129	Unique	Used	1.844	0.281	1.802	1.145	0	0	5.88	0	0	94	2	1118.58105	2236.15483	-2.80
gGLHTVAVMNVGAPAAGMNAAVR	Unambiguous	1	1	P47857	N-Term(iTRAQ8plex)	CID	1.0000	0.0000	1	1	3931125	Unique	Used	2.828				0	0	5.88	0	0	1	3	823.44116	2468.30893	-3.22
dHIIEVVGSSMPLIGDHQDEDk	Unambiguous	3	1	O88935	N-Term(iTRAQ8plex); K22(iTRAQ8plex)	CID	1.0000	0.0000	1	1	3014590	Unique	Used		1.731	1.579	1.309	0	0	5.88	0	0	200	3	1014.85724	3042.55716	-3.01
lQGAVDVDTLEPVDAASk	Unambiguous	2	1	E9Q350	N-Term(iTRAQ8plex); K18(iTRAQ8plex)	CID	1.0000	0.0000	1	1	2832150	Unique	Used			0.567	1.124	0	0	5.88	0	0	200	2	1218.67603	2436.34477	0.21
nQVLSQQLQLLEAEHNTLk	Unambiguous	2	1	E9QAH1	N-Term(iTRAQ8plex); K19(iTRAQ8plex)	CID	1.0000	0.0000	1	1	3392683	Unique	Used	2.381	1.010	1.039	0.801	0	0	5.88	0	0	86	3	938.86475	2814.57969	-4.88
qAQLQDAGIYEcESk	Unambiguous	2	1	P29533	N-Term(iTRAQ8plex); C12(Methylthio); K15(iTRAQ8plex)	CID	1.0000	0.0000	1	1	2848895	Unique	Used	0.639	0.846	0.977	0.801	0	0	5.88	0	18	200	2	1169.08472	2337.16216	-2.30
gTGGVDTAAVGGVFDVSNADR	Unambiguous	1	1	Q04447	N-Term(iTRAQ8plex)	CID	1.0000	0.0000	1	1	3134423	Unique	Used	1.674	0.871	1.409	1.146	0	0	5.88	0	0	200	2	1135.06946	2269.13164	-2.05
nEEDATELVGLAQAVnAR	Unambiguous	1	1	Q02053	N-Term(iTRAQ8plex); N16(Deamidated)	CID	1.0000	0.0000	1	1	2741558	Unique	Used	0.682	1.356	1.174	1.533	0	0	5.88	0	0	200	2	1103.06689	2205.12651	-1.64

qADVPAAVTDAAATTPAAEDAATk	Unambiguous	1	1	P06837	N-Term(iTRAQ8plex); K24(iTRAQ8plex)	CID	1.0000	0.0000	1	1	2811956	Unique	Used			0.653	0.807	0	0	5.88	0	0	200	2	1432.75659	2864.50591	-1.38
gIGTDEATIIDIVTHR	Unambiguous	2	1	P14824	N-Term(iTRAQ8plex)	CID	1.0000	0.0000	1	1	3283739	Unique	Used	0.882	1.249	0.981	1.198	0	0	5.88	0	5	34	3	672.37292	2015.10422	-1.64
cSLLEEELGATHk	Unambiguous	1	1	B2RXW8	N-Term(iTRAQ8plex); C1(Methylthio); K13(iTRAQ8plex)	HCD	1.0000	0.0000	1	1	3110201	Unique	Used	0.955	0.815	1.105	0.948	0	0	5.88	0	21	160	3	695.37085	2084.09800	0.16
tTYDSSLSSYTVPLEk	Unambiguous	5	1	Q7TQH0-3	N-Term(iTRAQ8plex); K16(iTRAQ8plex)	CID	1.0000	0.0000	1	1	3169387	Unique	Used	1.047	0.699	1.139	0.791	0	0	5.88	0	0	41	2	1200.13989	2399.27251	-3.21
aASAQAPSSSPGNIRPVk	Unambiguous	2	1	D3YWN7	N-Term(iTRAQ8plex); K18(iTRAQ8plex)	HCD	1.0000	0.0000	1	1	3632531	Unique	Used	1.135	1.113	1.717	0.786	0	0	5.88	0	0	200	3	782.77808	2346.31968	-6.54
dkPHVnVGTIGHVDHGk	Unambiguous	3	1	Q8BFR5	N-Term(iTRAQ8plex); K2(iTRAQ8plex); N6(Deamidated); K17(iTRAQ8plex)	CID	1.0000	0.0000	1	1	3225858	Unique	Used	0.859	0.973	1.238	0.934	0	0	5.88	0	0	74	3	908.52130	2723.54935	5.04
gGVSAVAGGVTAVGSAVVNk	Unambiguous	1	1	Q9DAM7	N-Term(iTRAQ8plex); K20(iTRAQ8plex)	HCD	1.0000	0.0000	1	1	3762083	Unique	Used	1.201	0.851	1.382	0.869	0	0	5.88	0	20	65	2	1154.66907	2308.33086	-5.93
dVGSSAWAAGPSVVEEk	Unambiguous	2	1	G3X9K4	N-Term(iTRAQ8plex); K17(iTRAQ8plex)	CID	1.0000	0.0000	1	1	2917406	Unique	Used	0.893	1.213	1.400	1.021	0	0	5.88	0	0	200	2	1149.11304	2297.21880	-2.01
iQEGVFDINNEAnGIk	Unambiguous	1	1	P53994	N-Term(iTRAQ8plex); N13(Deamidated); K16(iTRAQ8plex)	CID	0.0187	0.0000	1	1	2832370	Unique	Used	0.959	1.477	0.997	1.203	0	0	5.88	0	4	29	2	1185.63770	2370.26811	-3.40
sQHVQVEEGSEPDAFWEALGGk	Unambiguous	2	1	P13020-2	N-Term(iTRAQ8plex); K22(iTRAQ8plex)	CID	1.0000	0.0000	1	1	2902246	Unique	Used	1.063	0.354	1.548	1.073	0	0	5.88	0	3	36	3	1003.50812	3008.50980	-3.75
hQSVQTEDGPGGVAR	Unambiguous	1	1	Q9R1L5	N-Term(iTRAQ8plex)	CID	1.0000	0.0000	1	1	2661182	Unique	Used	2.252	0.706	1.274	0.963	0	0	5.88	0	2	64	3	614.65009	1841.93570	-2.76

hINNnLSPSLGDAQEEk	Unambiguous	2	1	Q14CH0-2	N-Term(iTRAQ8plex); N5(Deamidated); K17(iTRAQ8plex)	CID	0.0442	0.0000	1	1	2632586	Unique	Used	1.375	1.054	1.188	1.123	0	0	5.88	0	28	200	3	825.76947	2475.29386	0.10
enPGFDFSGAEISGNYTk	Unambiguous	2	1	Q9CQ48	N-Term(iTRAQ8plex); N2(Deamidated); K18(iTRAQ8plex)	CID	0.0442	0.0000	1	1	3033430	Unique	Used	0.963	0.789	0.736	0.717	0	0	5.88	0	0	200	2	1271.63647	2542.26567	3.87
yEGSGDGGAAAQSLYIANHAY	Unambiguous	1	1	P05063	N-Term(iTRAQ8plex)	CID	1.0000	0.0000	1	1	2975334	Unique	Used	1.855	0.706	1.063	1.383	0	0	5.88	0	6	123	2	1210.07544	2419.14360	-1.33
yFDSFGDLSSASAImGNPk	Unambiguous	1	1	P02089	N-Term(iTRAQ8plex); M15(Oxidation); K19(iTRAQ8plex)	CID	1.0000	0.0000	1	1	3559421	Unique	Used	1.445	8.703			0	0	5.88	0	2	17	3	877.77557	2631.31217	-3.79
qGEQGPPGGPGNIATRPk	Unambiguous	2	1	Q5FW52-2	N-Term(iTRAQ8plex); K18(iTRAQ8plex)	HCD	1.0000	0.0000	1	1	3225755	Unique	Used	0.932	1.650	3.062	0.893	0	0	5.88	0	10	136	3	790.44141	2369.30967	-2.10
nSPSNLSSSSETGSGGGTYR	Unambiguous	2	1	Q8C0T5	N-Term(iTRAQ8plex)	CID	1.0000	0.0000	1	1	2823340	Unique	Used	0.869	2.588	2.108	0.583	0	0	5.88	0	0	200	2	1125.03015	2249.05303	-2.39
sIEALLEAGQAQDTQASHAEAnQQQTR	Unambiguous	2	1	Q99PL5	N-Term(iTRAQ8plex); N22(Deamidated)	CID	0.0068	0.0000	1	1	2849867	Unique	Used	1.685	0.791	1.366	0.797	0	0	5.88	0	0	200	3	1067.52710	3200.56675	-1.71
sLSSSPQAQPPRPAELSDEEVAELFQR	Unambiguous	3	1	A2AEW8	N-Term(iTRAQ8plex)	HCD	1.0000	0.0000	1	1	3193104	Unique	Used	2.790	1.569	2.124	1.159	0	0	5.88	0	0	19	3	1091.55896	3272.66233	-2.40
aAVEEGIVLGGGcALLR	Unambiguous	2	1	P63038	N-Term(iTRAQ8plex); C13(Methylthio)	CID	1.0000	0.0000	1	1	3647559	Unique	Used	0.772	0.783	0.972	1.011	0	0	5.87	0	19	200	2	989.53754	1978.06780	-4.55
tVSGVNGPLVILDHVk	Unambiguous	1	1	P62814	N-Term(iTRAQ8plex); K16(iTRAQ8plex)	CID	1.0000	0.0000	1	1	3598539	Unique	Used	0.487	0.693	1.026	1.175	0	0	5.87	0	0	2	3	752.78491	2256.34018	-5.96
kLQNQLQTDLSVIPVINR	Unambiguous	1	1	P97449	N-Term(iTRAQ8plex); K1(iTRAQ8plex)	HCD	1.0000	0.0000	1	1	3859191	Unique	Used	0.817	2.489	0.739	0.804	0	0	5.87	1	12	59	3	896.53528	2687.59128	-4.31
lGSSGLGSASSIQAAVR	Unambiguous	4	1	Q7TMY8-3	N-Term(iTRAQ8plex)	CID	1.0000	0.0000	1	1	3855651	Unique	Used	0.456	0.768	0.829	1.070	0	0	5.87	0	0	54	2	933.02032	1865.03337	-3.26
aASTLGSPLEAQVAPGFAR	Unambiguous	2	1	Q68EF6	N-Term(iTRAQ8plex)	CID	1.0000	0.0000	1	1	3683269	Unique	Used	0.829	1.271	1.844	1.352	0	0	5.87	0	0	60	2	1074.08630	2147.16533	-5.11

sENVQDLLLLDVTPLSLGIETAGGVMTVLIkR	Unambiguous	2	1	P63017	N-Term(iTRAQ8plex); K31(iTRAQ8plex)	HCD	1.0000	0.0000	1	1	3880538	Unique	Used	0.548	0.619	1.098	0.961	0	0	5.87	1	21	32	3	1335.09814	4003.27988	-4.39
aVVnGYSASDTVGAGFAQAk	Unambiguous	1	1	Q8K0U4	N-Term(iTRAQ8plex); N4(Deamidated); K20(iTRAQ8plex)	CID	1.0000	0.0000	1	1	3189496	Unique	Used	0.527	1.350	1.668	1.411	0	0	5.87	0	0	35	2	1261.66919	2522.33110	-1.45
aLPSSEDLASLAPDLDk	Unambiguous	5	1	P58281-2	N-Term(iTRAQ8plex); K17(iTRAQ8plex)	CID	1.0000	0.0000	1	1	3131519	Unique	Used	0.681	0.738	0.929	0.859	0	0	5.87	0	23	71	2	1175.64990	2350.29253	-1.58
eSQISAEIELLPTNDk	Unambiguous	2	1	Q3TWV4	N-Term(iTRAQ8plex); K16(iTRAQ8plex)	CID	1.0000	0.0000	1	1	2832722	Unique	Used	0.514	0.934	1.177	1.246	0	0	5.87	0	0	200	2	1198.16223	2395.31719	-0.22
iSVSLQEEASGGPQTASPR	Unambiguous	4	1	Q3TRM4-2	N-Term(iTRAQ8plex)	CID	1.0000	0.0000	1	1	3125439	Unique	Used	1.085	0.497	0.605	0.895	0	0	5.87	0	0	96	2	1109.58484	2218.16240	0.29
qAEMEGAVQSIQGELSk	Unambiguous	1	1	O54724	N-Term(iTRAQ8plex); K17(iTRAQ8plex)	CID	1.0000	0.0000	1	1	3091969	Unique	Used	0.677	1.682	1.077	0.997	0	0	5.87	0	26	200	2	1207.14526	2413.28325	-0.88
lGPSDYFGEIALLMNRPR	Unambiguous	1	1	Q9DBC7	N-Term(iTRAQ8plex)	HCD	1.0000	0.0000	1	1	3898487	Unique	Used	0.715	0.821	1.006	1.204	0	0	5.87	0	0	39	3	785.09039	2353.25663	-3.16
mSESLDTADPAVTGAk	Unambiguous	1	1	P63011	N-Term(iTRAQ8plex); K16(iTRAQ8plex)	CID	1.0000	0.0000	1	1	2793288	Unique	Used	0.580	1.340	1.292	1.115	0	0	5.87	0	12	200	2	1101.08008	2201.15288	-2.34
lGEHnINVLEGNEQFIDAAk	Unambiguous	1	1	Q9Z1R9	N-Term(iTRAQ8plex); N5(Deamidated); K20(iTRAQ8plex)	CID	0.0068	0.0000	1	1	2919365	Unique	Used	1.163	1.562	0.837	0.915	0	0	5.87	0	0	102	3	940.83521	2820.49106	-2.77
eTLLnGVGYLHEGLSPMER	Unambiguous	1	1	Q6P4T2	N-Term(iTRAQ8plex); N5(Deamidated)	CID	1.0000	0.0000	1	1	3150582	Unique	Used	0.845	0.861	0.904	1.219	0	0	5.87	0	0	89	3	807.41833	2420.24045	-1.21
iGQLQGEIIPTSFYHQGR	Unambiguous	7	1	Q9R1V7	N-Term(iTRAQ8plex)	HCD	1.0000	0.0000	1	1	3803224	Unique	Used	0.840	0.485	1.439	1.456	0	0	5.87	0	0	140	2	1174.63391	2348.26055	-2.53
iDSAATSTYEVGNPPDYR	Unambiguous	1	1	Q9D358	N-Term(iTRAQ8plex)	CID	1.0000	0.0000	1	1	2916815	Unique	Used	1.091	0.873	0.748	0.715	0	0	5.87	0	16	80	2	1130.55298	2260.09868	-2.16
vAAINVAHLEEADEGPEPAR	Unambiguous	1	1	Q8VBY2	N-Term(iTRAQ8plex)	CID	1.0000	0.0000	1	1	2635638	Unique	Used	2.365	1.412	1.876	1.665	0	0	5.87	0	26	189	3	798.08234	2392.23246	-3.61

qSGTPPGMGAPGIPGASIVGGAGGR	Unambiguous	5	1	G3X9J4	N-Term(iTRAQ8plex)	CID	1.0000	0.0000	1	1	3894428	Unique	Used	1.076	0.827	1.229	1.210	0	0	5.86	0	16	200	2	1227.14355	2453.27983	-3.07
vNAGDQPGADLGPLITPQAk	Unambiguous	1	1	Q9EQ20	N-Term(iTRAQ8plex); K20(iTRAQ8plex)	CID	1.0000	0.0000	1	1	3257320	Unique	Used	0.929	1.605	1.026	1.158	0	0	5.86	0	0	127	2	1285.72217	2570.43706	-1.11
tVTDVSTIDGnAAELVSGRPLSDTTEPSSPEGk	Unambiguous	1	1	Q8BR92	N-Term(iTRAQ8plex); N11(Deamidated); K33(iTRAQ8plex)	CID	1.0000	0.0000	1	1	2994957	Unique	Used	0.983	0.860	1.034	0.886	0	0	5.86	0	0	200	3	1314.01208	3940.02170	2.15
iSGTAFSVGSGSVYAYGVmDR	Unambiguous	1	1	O55234	N-Term(iTRAQ8plex); M19(Oxidation)	CID	1.0000	0.0000	1	1	3743137	Unique	Used	2.414	0.754	0.841	0.795	0	0	5.86	0	0	200	2	1222.60205	2444.19682	-4.16
sDIAAAQQSGmSLSNSk	Unambiguous	1	1	B1AQZ0	N-Term(iTRAQ8plex); M11(Oxidation); K17(iTRAQ8plex)	CID	1.0000	0.0000	1	1	3250912	Unique	Used	0.254	1.115	1.622	1.266	0	0	5.86	0	0	94	2	1160.10254	2319.19780	-4.01
fGWDcHGLPVEYEIDk	Unambiguous	1	1	Q8BU30	N-Term(iTRAQ8plex); C5(Methylthio); K16(iTRAQ8plex)	CID	1.0000	0.0000	1	1	3283298	Unique	Used	0.493	0.601	0.686	0.807	0	0	5.86	0	0	88	3	854.75555	2562.25211	-3.78
kYNTDcVQGLTHSk	Unambiguous	2	1	Q6PIC6	N-Term(iTRAQ8plex); K1(iTRAQ8plex); C6(Methylthio); K14(iTRAQ8plex)	CID	1.0000	0.0000	1	1	3385298	Unique	Used	0.249	0.423	0.966	1.167	0	0	5.86	1	0	26	3	851.45636	2552.35453	-7.16
gTGGVDTAAVGGVFDVSNADR	Unambiguous	1	1	Q04447	N-Term(iTRAQ8plex)	CID	1.0000	0.0000	1	1	3137484	Unique	Used	1.596	0.702	0.760	0.885	0	0	5.86	0	0	200	2	1135.07117	2269.13506	-0.55
lGLVFDDVVGIVEIINSR	Unambiguous	1	1	P40124	N-Term(iTRAQ8plex)	HCD	1.0000	0.0000	1	1	3807373	Unique	Used	1.401	1.381	1.359	0.994	0	0	5.86	0	7	54	2	1131.65125	2262.29521	-2.63
qAEIENPLEDPVTGDYVHk	Unambiguous	4	1	Q9Z1G4-3	N-Term(iTRAQ8plex); K19(iTRAQ8plex)	CID	1.0000	0.0000	1	1	2760171	Unique	Used	0.586	1.415	1.547	1.142	0	0	5.86	0	9	200	3	921.48450	2762.43894	-2.47
sTnGDTFLGGEDFDQALLR	Unambiguous	1	1	P38647	N-Term(iTRAQ8plex); N3(Deamidated)	CID	1.0000	0.0000	1	1	2815832	Unique	Used	2.051	1.285	1.551	1.335	0	0	5.86	0	6	159	2	1181.07703	2361.14678	-1.90

tAAANAAAGAAENAFR	Unambiguous	3	1	E9Q855	N-Term(iTRAQ8plex)	CID	1.0000	0.0000	1	1	3514796	Unique	Used	0.714	0.881	1.027	1.316	0	0	5.86	0	14	41	2	890.96198	1780.91667	-4.36
vDFHDVQDYADNIk	Unambiguous	1	1	P35290	N-Term(iTRAQ8plex); K14(iTRAQ8plex)	CID	1.0000	0.0000	1	1	2918376	Unique	Used	0.908	1.099	0.826	0.847	0	0	5.86	0	5	81	3	763.06470	2287.17954	-0.87
tTcSSSEEDDcISLSk	Unambiguous	2	1	Q9CWE0	N-Term(iTRAQ8plex); C3(Methylthio); C11(Methylthio); K16(iTRAQ8plex)	CID	1.0000	0.0000	1	1	2760495	Unique	Used	0.848	0.715	1.277	1.046	0	0	5.86	0	8	200	2	1203.04150	2405.07573	-1.82
aQPGSPQALGGPHGPATAk	Unambiguous	3	1	Q6P9K8	N-Term(iTRAQ8plex); K19(iTRAQ8plex)	HCD	1.0000	0.0000	1	1	3404984	Unique	Used	0.522	0.716	1.098	0.886	0	0	5.86	0	0	70	3	784.10107	2350.28867	-8.57
qAQIEVVPSASALIIk	Unambiguous	2	1	P35979	N-Term(iTRAQ8plex); K16(iTRAQ8plex)	CID	1.0000	0.0000	1	1	3558857	Unique	Used	0.495	0.794	0.836	0.809	0	0	5.86	0	20	32	2	1138.18909	2275.37090	-6.02
iPNIYAIGDVVAGPmLAHk	Unambiguous	1	1	O08749	N-Term(iTRAQ8plex); M15(Oxidation); K19(iTRAQ8plex)	CID	1.0000	0.0000	1	1	3825889	Unique	Used	1.998	1.134	1.390	0.929	0	0	5.86	0	19	200	3	868.49811	2603.47977	-1.63
lVSPGSANETSSILVESVTR	Unambiguous	1	1	Q80X90	N-Term(iTRAQ8plex)	CID	1.0000	0.0000	1	1	3477759	Unique	Used	1.370	1.825	1.112	0.996	0	0	5.86	0	0	46	2	1175.63855	2350.26982	-2.96
gPELSAQEQMEGMLcR	Unambiguous	1	1	Q68FG2	N-Term(iTRAQ8plex); C15(Methylthio)	CID	1.0000	0.0000	1	1	3193965	Unique	Used	0.900	1.656	2.279	1.311	0	0	5.86	0	12	130	2	1064.99329	2128.97930	-0.42
hALQSASAGSGSFTDVR	Unambiguous	1	1	P11276	N-Term(iTRAQ8plex)	CID	1.0000	0.0000	1	1	3383888	Unique	Used	0.713	0.941	0.999	1.053	0	0	5.86	0	6	36	3	665.67395	1995.00730	-6.25
aSTDIAGQAVTISLVPVEEqTkPYR	Unambiguous	1	1	Q69Z38	N-Term(iTRAQ8plex); Q20(Deamidated); K22(iTRAQ8plex)	HCD	1.0000	0.0000	1	1	3370999	Unique	Used	0.762	1.088	1.009	0.779	0	0	5.86	0	0	130	3	1094.94141	3282.80967	1.65
lATPTYGDLNHLVSATMSGVTTSLR	Unambiguous	1	1	Q9ERD7	N-Term(iTRAQ8plex)	CID	1.0000	0.0000	1	1	3939570	Unique	Used	0.796		0.889		0	0	5.86	0	3	200	3	970.51440	2909.52866	-2.00

eQANAVSEAVVSSVnTVANk	Unambiguous	1	1	Q9Z0F7	N-Term(iTRAQ8plex); N15(Deamidated); K20(iTRAQ8plex)	CID	1.0000	0.0000	1	1	2992874	Unique	Used	2.900	0.251	0.779	1.692	0	0	5.86	0	0	200	2	1313.70178	2626.39629	-6.92
sMkEEGVTFPSAGSQTVAAAAk	Unambiguous	2	1	O88811	N-Term(iTRAQ8plex); K3(iTRAQ8plex); K22(iTRAQ8plex)	CID	1.0000	0.0000	1	1	3276371	Unique	Used	1.179	1.016	0.170	0.183	0	0	5.85	1	0	87	3	1027.23120	3079.67905	-2.31
gHAPSTAAPAPSPAPmSSSVQSDEER	Unambiguous	1	1	Q8C996	N-Term(iTRAQ8plex); M16(Oxidation)	CID	1.0000	0.0000	1	1	2967562	Unique	Used		2.067	0.878		0	0	5.85	0	0	200	3	962.12769	2884.36850	-0.03
nLSDTVVGLESFIGSER	Unambiguous	2	1	Q9ER73	N-Term(iTRAQ8plex)	CID	1.0000	0.0000	1	1	3373761	Unique	Used	0.690	0.854	1.021	1.164	0	0	5.85	0	0	26	2	1064.06189	2127.11650	-3.33
tGNELmEILDEMDQSHR	Unambiguous	4	1	Q9QXA1-2	N-Term(iTRAQ8plex); M6(Oxidation)	CID	1.0000	0.0000	1	1	2904287	Unique	Used	1.005	1.287	0.634	0.586	0	0	5.85	0	19	200	3	780.03448	2338.08890	-2.93
sVAGEIVLITGAGHGIGR	Unambiguous	2	1	Q9EQ06	N-Term(iTRAQ8plex)	CID	1.0000	0.0000	1	1	3782827	Unique	Used	1.179	1.172	1.007	1.525	0	0	5.85	0	0	138	3	671.05634	2011.15445	-2.88
vETLHDFEAANSDELNLQR	Unambiguous	1	1	Q7TQF7	N-Term(iTRAQ8plex)	CID	1.0000	0.0000	1	1	2851241	Unique	Used	2.026	1.228	0.963	1.188	0	0	5.85	0	8	138	3	835.75452	2505.24900	-1.35
lAGGQTSQPTTPLTSPQR	Unambiguous	3	1	Q9ES00	N-Term(iTRAQ8plex)	CID	1.0000	0.0000	1	1	3756990	Unique	Used	1.361	1.332	1.023	1.050	0	0	5.85	0	0	126	2	1072.57910	2144.15093	-4.86
lQGAVDVDTLEPVDAASk	Unambiguous	2	1	E9Q350	N-Term(iTRAQ8plex); K18(iTRAQ8plex)	CID	1.0000	0.0000	1	1	2850623	Unique	Used	0.240	0.570	0.369	1.064	0	0	5.85	0	2	115	2	1218.67114	2436.33501	-3.80
gTGGVDTAAVGGVFDVSNADR	Unambiguous	1	1	Q04447	N-Term(iTRAQ8plex)	CID	1.0000	0.0000	1	1	3131364	Unique	Used	1.973	1.111	1.945	1.665	0	0	5.85	0	0	194	2	1135.07068	2269.13408	-0.98
qDHPSSmGVYGQESGGFSGPGENR	Unambiguous	4	1	Q5SUT0	N-Term(iTRAQ8plex); M7(Oxidation)	CID	1.0000	0.0000	1	1	2968700	Unique	Used	1.425	1.317	0.872	0.682	0	0	5.85	0	26	200	3	934.08832	2800.25040	-1.12
yIEESIGESANLLGAmEk	Unambiguous	1	1	Q8BLE7	N-Term(iTRAQ8plex); M16(Oxidation); K18(iTRAQ8plex)	CID	1.0000	0.0000	1	1	3194877	Unique	Used	0.527	0.438	0.529	1.112	0	0	5.85	0	0	98	2	1289.67554	2578.34380	-3.61
tVQDALSSVQESDIAVVAR	Unambiguous	2	1	P33622	N-Term(iTRAQ8plex)	CID	1.0000	0.0000	1	1	3349538	Unique	Used	4.792	5.175	0.510	0.911	0	0	5.85	0	0	13	2	1146.61572	2292.22417	-4.70

vALVTFnSAAHNkPSLIR	Unambiguous	1	1	Q6ZQ38	N-Term(iTRAQ8plex); N7(Deamidated); K13(iTRAQ8plex)	HCD	1.0000	0.0000	1	1	3855959	Unique	Used	0.510	1.259	1.156	1.260	0	0	5.85	0	19	90	3	849.83057	2547.47715	-3.78
lLYDLADQLHAAVGASR	Unambiguous	1	1	Q99LC5	N-Term(iTRAQ8plex)	CID	1.0000	0.0000	1	1	3898642	Unique	Used	0.884	1.058	0.997	1.428	0	0	5.85	0	0	11	3	706.39117	2117.15897	-3.18
kATVGQAEEATIDHTEk	Unambiguous	2	1	D3YVF0	N-Term(iTRAQ8plex); K1(iTRAQ8plex); K17(iTRAQ8plex)	CID	1.0000	0.0000	1	1	2665835	Unique	Used	0.853	3.462	3.888	0.648	0	0	5.85	1	9	200	3	914.18005	2740.52561	0.42
lRPQSVqSSFPSPPSPSSDVQLTTLAHR	Unambiguous	2	1	P70295	N-Term(iTRAQ8plex); Q7(Deamidated)	CID	0.0188	0.0000	1	1	3913304	Unique	Used	1.280	1.152		2.788	0	0	5.85	0	0	159	3	1108.92139	3324.74961	0.16
aAQLcGAGmAAVVEk	Unambiguous	5	1	G3UVV4	N-Term(iTRAQ8plex); C5(Methylthio); M9(Oxidation); K15(iTRAQ8plex)	CID	1.0000	0.0000	1	1	3597265	Unique	Used	0.661	0.880	1.084	1.142	0	0	5.85	0	11	3	3	697.03656	2089.09513	-5.44
vTEGLVDVILYHQPDDk	Unambiguous	1	1	Q8VHM5	N-Term(iTRAQ8plex); K17(iTRAQ8plex)	HCD	1.0000	0.0000	1	1	3152888	Unique	Used	0.479	0.651	0.886	0.914	0	0	5.85	0	16	175	3	850.47363	2549.40635	-0.33
hHAAYVnNLNATEEk	Unambiguous	1	1	P09671	N-Term(iTRAQ8plex); N7(Deamidated); K15(iTRAQ8plex)	CID	0.0393	0.0000	1	1	2750307	Unique	Used	2.009	1.058	0.954	0.919	0	0	5.85	0	19	200	3	774.07501	2320.21048	-1.60
sLEQSEAAVAQVLEER	Unambiguous	3	1	A2AHG0	N-Term(iTRAQ8plex)	CID	1.0000	0.0000	1	1	2887833	Unique	Used			1.453	1.071	0	0	5.84	0	15	200	2	1032.04968	2063.09209	-0.09
gGLHTVAVMNVGAPAAGMNAAVR	Unambiguous	1	1	P47857	N-Term(iTRAQ8plex)	CID	1.0000	0.0000	1	1	3930994	Unique	Used	1.664	1.292	1.607	1.168	0	0	5.84	0	0	67	3	823.44141	2468.30967	-2.93
hQGGWTDGGSGSGGGYQDGAYR	Unambiguous	1	1	Q3TJZ6	N-Term(iTRAQ8plex)	CID	1.0000	0.0000	1	1	2789824	Unique	Used	2.397	1.147	1.389	1.420	0	0	5.84	0	0	200	3	825.37030	2474.09635	-2.42
nSSVGLIQLNRPk	Unambiguous	1	1	Q8BH95	N-Term(iTRAQ8plex); K13(iTRAQ8plex)	HCD	1.0000	0.0000	1	1	3909784	Unique	Used	1.337	1.155	1.057	1.429	0	0	5.84	0	0	82	3	678.74347	2034.21585	-5.98
eQAAALPPALSHTPEVQGR	Unambiguous	1	1	Q68FG2	N-Term(iTRAQ8plex)	CID	1.0000	0.0000	1	1	3253050	Unique	Used	0.987	2.053	1.994	1.974	0	0	5.84	0	8	5	3	759.41449	2276.22892	-0.53

aMGVVVATGVNTEIGk	Unambiguous	3	1	O55143	N-Term(iTRAQ8plex); K16(iTRAQ8plex)	CID	1.0000	0.0000	1	1	3475613	Unique	Used	1.443	1.199	0.999	1.002	0	0	5.84	0	0	102	3	718.74811	2154.22977	-5.36
eGSYSISVLYGEEEVPR	Unambiguous	3	1	B7FAU9	N-Term(iTRAQ8plex)	CID	1.0000	0.0000	1	1	2796342	Unique	Used	0.954	4.155	0.955	1.042	0	0	5.84	0	0	200	2	1109.56055	2218.11382	-1.95
sGLGLGAASASTSGSGPADSVmR	Unambiguous	3	1	Q7TSJ2	N-Term(iTRAQ8plex); M22(Oxidation)	CID	1.0000	0.0000	1	1	3512130	Unique	Used	0.953	0.590	1.333	1.233	0	0	5.84	0	0	130	2	1178.58154	2356.15581	-6.75
gNPSGIQPDLLISLTAPk	Unambiguous	1	1	Q8K4Z3	N-Term(iTRAQ8plex); K18(iTRAQ8plex)	CID	1.0000	0.0000	1	1	3686172	Unique	Used	0.676	1.104	0.971	1.466	0	0	5.84	0	0	6	2	1215.20654	2429.40581	-6.85
nLHQSGFSLSGTQVDEGVR	Unambiguous	3	1	E9PWE8	N-Term(iTRAQ8plex)	CID	1.0000	0.0000	1	1	3275030	Unique	Used	2.086	2.856	0.982	1.254	0	0	5.84	0	13	75	3	779.06903	2335.19254	-0.82
dSSSLSSYTSGVLEER	Unambiguous	1	1	Q9JM96	N-Term(iTRAQ8plex)	CID	1.0000	0.0000	1	1	2974446	Unique	Used	1.808	0.970	1.556	1.177	0	0	5.84	0	6	102	2	1010.99902	2020.99077	-3.42
eAGYSAAVGVGQRPPR	Unambiguous	4	1	E9QAD8	N-Term(iTRAQ8plex)	HCD	1.0000	0.0000	1	1	3449242	Unique	Used	1.493	1.494	1.846	1.197	0	0	5.84	0	0	78	3	640.34894	1919.03226	-4.10
sETSSNPSSPEIcPNk	Unambiguous	2	1	Q5SVL6	N-Term(iTRAQ8plex); C13(Methylthio); K16(iTRAQ8plex)	CID	1.0000	0.0000	1	1	2914395	Unique	Used	1.712	1.124	3.022	1.577	0	0	5.84	0	13	200	2	1166.07434	2331.14141	-0.13
vNIHGGAVSLGHPIGMSGAR	Unambiguous	1	1	Q8QZT1	N-Term(iTRAQ8plex)	CID	1.0000	0.0000	1	1	3928559	Unique	Used	0.828	0.890	0.894	1.186	0	0	5.84	0	0	200	3	745.40552	2234.20200	-4.94
iQAAASPPANATAASDTnAGDR	Unambiguous	2	1	E9QAQ5	N-Term(iTRAQ8plex); N18(Deamidated)	CID	1.0000	0.0000	1	1	2731602	Unique	Used	0.501	0.846	1.413	1.104	0	0	5.84	0	6	186	2	1188.08691	2375.16655	-3.19
lLEVSDDPQVLAVAAHDVGEYVR	Unambiguous	1	1	Q8BVE3	N-Term(iTRAQ8plex)	CID	1.0000	0.0000	1	1	3241367	Unique	Used	0.628	1.194	1.988	1.739	0	0	5.83	0	17	200	3	933.83228	2799.48227	-0.30
gTGSSSSGVLMVGPNFR	Unambiguous	1	1	Q8C4X2	N-Term(iTRAQ8plex)	CID	1.0000	0.0000	1	1	3893593	Unique	Used	0.512	0.617	1.007	1.150	0	0	5.83	0	0	33	2	979.00513	1957.00298	-4.37
lHLPTAPPPLNEGESLVSR	Unambiguous	1	1	Q8C8R3-2	N-Term(iTRAQ8plex)	HCD	1.0000	0.0000	1	1	3516975	Unique	Used	0.906	1.177	0.880	0.998	0	0	5.83	0	0	18	3	777.76758	2331.28818	-3.98
hIVLSGGSTMYPGLPSR	Unambiguous	1	1	P61161	N-Term(iTRAQ8plex)	CID	1.0000	0.0000	1	1	3911978	Unique	Used	1.702	0.865	0.994	1.003	0	0	5.83	0	21	30	3	692.70911	2076.11277	-4.16
gTGGVDTAATGSVFDISNLDR	Unambiguous	1	1	P30275	N-Term(iTRAQ8plex)	CID	1.0000	0.0000	1	1	3260524	Unique	Used	1.088	1.386	1.101	1.494	0	0	5.83	0	0	116	2	1179.09607	2357.18486	-1.64
lTLYDIAHTPGVAADLSHIETR	Unambiguous	1	1	P08249	N-Term(iTRAQ8plex)	CID	1.0000	0.0000	1	1	3457687	Unique	Used	2.091	1.121	0.686	0.996	0	0	5.83	0	0	1	3	899.81787	2697.43906	-4.58

vYVGNASVAQDITQLQk	Unambiguous	3	1	Q9D7X3	N-Term(iTRAQ8plex); K17(iTRAQ8plex)	CID	1.0000	0.0000	1	1	3414775	Unique	Used			1.090	0.919	0	0	5.83	0	0	70	2	1221.68750	2442.36772	-5.56
eALELTDTGLLSGSEER	Unambiguous	1	1	Q9JHU4	N-Term(iTRAQ8plex)	CID	1.0000	0.0000	1	1	2739772	Unique	Used	0.496	0.586	0.756	0.908	0	0	5.83	0	0	52	2	1062.55176	2124.09624	-0.56
iEPDSESQEEIIHNIAR	Unambiguous	1	1	P70444	N-Term(iTRAQ8plex)	CID	1.0000	0.0000	1	1	2635709	Unique	Used	2.249	1.097	1.589	1.028	0	0	5.83	0	0	55	3	762.06061	2284.16727	-2.21
gDAAVYcYQAEAGEWR	Unambiguous	1	1	Q8BNW9	N-Term(iTRAQ8plex); C7(Methylthio)	CID	1.0000	0.0000	1	1	2996055	Unique	Used	0.387	1.194	1.422	1.446	0	0	5.83	0	12	55	2	1069.98059	2138.95391	-1.80
nMTVEQLLTGSPTSPTVEPEkPTR	Unambiguous	1	1	Q6ZPJ3	N-Term(iTRAQ8plex); K21(iTRAQ8plex)	HCD	1.0000	0.0000	1	1	3369866	Unique	Used	0.981	1.070	1.409	1.216	0	0	5.83	0	3	21	3	1074.24500	3220.72043	-4.35
nTNAGAPPGTAYQSPLSLSR	Unambiguous	5	1	Q60597	N-Term(iTRAQ8plex)	CID	1.0000	0.0000	1	1	3739522	Unique	Used	1.011	1.129	1.223	1.578	0	0	5.83	0	0	197	2	1153.60120	2306.19512	-3.97
qVLYQQSSGHNNR	Unambiguous	2	1	Q99M71	N-Term(iTRAQ8plex)	CID	1.0000	0.0000	1	1	3507329	Unique	Used	1.884	0.748	1.284	0.997	0	0	5.83	0	4	116	3	612.31775	1834.93869	-4.08
gIVPGVSPLVLHIk	Unambiguous	1	1	Q3USB7	N-Term(iTRAQ8plex); K14(iTRAQ8plex)	HCD	1.0000	0.0000	1	1	3876896	Unique	Used	0.621	0.576	1.186	1.447	0	0	5.83	0	17	73	3	679.77136	2037.29953	-2.43
tVEGAGNIAAATGFVk	Unambiguous	2	1	O55042	N-Term(iTRAQ8plex); K16(iTRAQ8plex)	CID	1.0000	0.0000	1	1	3413350	Unique	Used	1.246	1.687	1.868	0.798	0	0	5.83	0	21	130	2	1057.60303	2114.19878	-3.72
gQVPGHASQGTQAVESScSk	Unambiguous	1	1	Q8R4U7	N-Term(iTRAQ8plex); C18(Methylthio); K20(iTRAQ8plex)	CID	1.0000	0.0000	1	1	3183178	Unique	Used	1.160	0.182	1.543	0.924	0	0	5.83	0	0	103	3	871.43805	2612.29959	-0.83
tVEGAGNIAAATGFVk	Unambiguous	2	1	O55042	N-Term(iTRAQ8plex); K16(iTRAQ8plex)	CID	1.0000	0.0000	1	1	3413221	Unique	Used	1.227	1.844	2.000	0.785	0	0	5.83	0	0	50	2	1057.60254	2114.19780	-4.18
tkEQASHLGGAVFSGAGnIAAATGLVk	Unambiguous	1	1	Q91ZZ3	N-Term(iTRAQ8plex); K2(iTRAQ8plex); N18(Deamidated); K27(iTRAQ8plex)	CID	0.0172	0.0000	1	1	3839848	Unique	Used	0.791	1.196	1.572	0.959	0	0	5.83	1	0	200	3	1156.99158	3468.96018	0.67
tGIGSGLSLSGIVHPELSR	Unambiguous	2	1	P50544	N-Term(iTRAQ8plex)	HCD	1.0000	0.0000	1	1	3839940	Unique	Used	1.103	1.169	0.768	0.986	0	0	5.83	0	0	200	3	728.74750	2184.22794	-0.51

eQASHLGGAVFSGAGNIAAATGLVk	Unambiguous	1	1	Q91ZZ3	N-Term(iTRAQ8plex); K25(iTRAQ8plex)	CID	1.0000	0.0000	1	1	3782820	Unique	Used	2.026	0.901	1.046	0.839	0	0	5.82	0	0	66	3	978.87610	2934.61374	-4.12
qSHLLLQSSLPDQQLLk	Unambiguous	2	1	Q8K2Q9-2	N-Term(iTRAQ8plex); K17(iTRAQ8plex)	CID	1.0000	0.0000	1	1	3597434	Unique	Used	1.339	1.061	1.312	1.139	0	0	5.82	0	0	49	3	852.83173	2556.48062	-6.40
lNELADQDFPLHPR	Unambiguous	3	1	Q9ESN6	N-Term(iTRAQ8plex)	CID	1.0000	0.0000	1	1	3235372	Unique	Used	1.149	0.862	1.183	1.201	0	0	5.82	0	1	2	3	657.01904	1969.04258	-0.99
gHTDSVQDISFDHSGk	Unambiguous	2	1	P63005	N-Term(iTRAQ8plex); K16(iTRAQ8plex)	CID	1.0000	0.0000	1	1	2824753	Unique	Used	2.351	0.815	0.959	0.988	0	0	5.82	0	12	200	3	780.06592	2338.18320	-2.22
gAGIGGLGITVEGPSESk	Unambiguous	1	1	Q80X90	N-Term(iTRAQ8plex); K18(iTRAQ8plex)	CID	1.0000	0.0000	1	1	3233845	Unique	Used	1.105	1.798	1.097	0.858	0	0	5.82	0	24	84	2	1119.13098	2237.25469	-2.29
eSPVSSSSGNQEPPAVk	Unambiguous	2	1	Q6PNC0	N-Term(iTRAQ8plex); K17(iTRAQ8plex)	CID	1.0000	0.0000	1	1	2806359	Unique	Used	0.275	0.593	1.100	1.375	0	0	5.82	0	0	200	2	1154.61169	2308.21611	-3.47
fYYSSGSSSPTHAk	Unambiguous	1	1	Q60771	N-Term(iTRAQ8plex); K14(iTRAQ8plex)	CID	1.0000	0.0000	1	1	3590853	Unique	Used	0.977	0.312	1.497	1.984	0	0	5.82	0	3	23	3	709.69940	2127.08365	-6.13
aAAPSAASSPAEVQSLk	Unambiguous	2	1	E9QAH1	N-Term(iTRAQ8plex); K17(iTRAQ8plex)	CID	1.0000	0.0000	1	1	3164689	Unique	Used	0.801	0.751	0.725	0.602	0	0	5.82	0	0	68	2	1097.11829	2193.22930	-1.95
tGSPGGPGVGGSPGGGAAGASPSLPPSAk	Unambiguous	1	1	Q8R1Q8	N-Term(iTRAQ8plex); K29(iTRAQ8plex)	CID	1.0000	0.0000	1	1	3385845	Unique	Used	2.567	1.439	1.269	1.325	0	0	5.82	0	0	110	3	980.85541	2940.55167	-4.07
aSNmGMGTDSGTQETk	Unambiguous	1	1	Q8R4U7	N-Term(iTRAQ8plex); M4(Oxidation); K16(iTRAQ8plex)	CID	1.0000	0.0000	1	1	2694737	Unique	Used	0.926	1.107	1.144	1.172	0	0	5.82	0	7	200	3	747.03021	2239.07608	-1.36
aGGGAGGSGGSGPSAILR	Unambiguous	2	1	F7CZ64	N-Term(iTRAQ8plex)	CID	1.0000	0.0000	1	1	3591107	Unique	Used	0.392	2.498	1.520	1.159	0	0	5.82	0	0	200	2	866.96167	1732.91606	-4.83
sSGPPPPSGSSGSEAAAGAAAPASQHPATGTGAVQTEAMk	Unambiguous	1	1	Q60865	N-Term(iTRAQ8plex); K40(iTRAQ8plex)	CID	1.0000	0.0000	1	1	3007439	Unique	Used	1.946		1.195	0.681	0	0	5.82	0	17	147	3	1395.36145	4184.06980	-1.87
hDADGQATLLNLLLR	Unambiguous	2	1	P14685	N-Term(iTRAQ8plex)	CID	1.0000	0.0000	1	1	3686262	Unique	Used			0.533	1.679	0	0	5.82	0	8	5	3	652.03436	1954.08854	-7.09

gTGSSSSGVLMVGPNFR	Unambiguous	1	1	Q8C4X2	N-Term(iTRAQ8plex)	CID	1.0000	0.0000	1	1	3893480	Unique	Used	0.978	0.542	0.951	1.088	0	0	5.82	0	0	200	2	979.00415	1957.00102	-5.37
kDLYANNVLSGGTTmYPGIADR	Unambiguous	3	1	P62737	N-Term(iTRAQ8plex); K1(iTRAQ8plex); M15(Oxidation)	CID	1.0000	0.0000	1	1	3454452	Unique	Used	1.101	4.840	1.301	1.693	0	0	5.82	1	19	200	3	994.18640	2980.54465	-7.13
dGFTSEGEILEISDIQR	Unambiguous	1	1	Q8HW98	N-Term(iTRAQ8plex)	CID	1.0000	0.0000	1	1	2672455	Unique	Used	1.254	0.174	0.365	0.434	0	0	5.82	0	0	63	2	1107.06506	2213.12285	-0.51
vQDINDNPPEFLHEIYHANVPER	Unambiguous	1	1	P55288	N-Term(iTRAQ8plex)	HCD	1.0000	0.0000	1	1	3150134	Unique	Used	1.314	1.006	1.098	0.868	0	0	5.82	0	15	173	3	1017.51154	3050.52005	-2.42
kEFGGVGAPPSPGVAR	Unambiguous	4	1	Q8CHC4	N-Term(iTRAQ8plex); K1(iTRAQ8plex)	HCD	1.0000	0.0000	1	1	3450654	Unique	Used	0.643	1.673	1.831	0.999	0	0	5.82	1	0	91	3	712.07507	2134.21067	-5.77
aGQDISADLDGPPHR	Unambiguous	2	1	Q5MPP0-2	N-Term(iTRAQ8plex)	CID	1.0000	0.0000	1	1	2665787	Unique	Used	1.650	0.235	0.425	0.538	0	0	5.81	0	14	91	3	618.31952	1852.94400	-0.84
rVTVNTAYGSnGVSVLR	Unambiguous	1	1	P97300	N-Term(iTRAQ8plex); N11(Deamidated)	HCD	1.0000	0.0000	1	1	3757596	Unique	Used	0.882	1.820	1.652	1.228	0	0	5.81	1	28	40	3	700.05408	2098.14768	-3.90
gNDISSGTVLSDYVGSGPPSGTGLHR	Unambiguous	1	1	P70296	N-Term(iTRAQ8plex)	CID	1.0000	0.0000	1	1	3412988	Unique	Used	1.769	0.989	1.298	0.930	0	0	5.81	0	11	86	3	945.47711	2834.41678	-1.95
lAAEFnGLTLTGRPR	Unambiguous	1	1	B2RW11	N-Term(iTRAQ8plex); N6(Deamidated)	HCD	1.0000	0.0000	1	1	3741986	Unique	Used	0.912	1.099	1.260	0.828	0	0	5.81	0	11	79	3	641.02960	1921.07425	-3.47
lAQAEAAGLHk	Unambiguous	3	1	Q64511	N-Term(iTRAQ8plex); K11(iTRAQ8plex)	HCD	1.0000	0.0000	1	1	3207534	Unique	Used	0.783	0.917	0.870	0.921	0	0	5.81	0	22	172	3	573.01160	1717.02024	-0.86
gSTSPDLLMHQGPPDTAEIIk	Unambiguous	3	1	Q9CQX8	N-Term(iTRAQ8plex); K21(iTRAQ8plex)	CID	1.0000	0.0000	1	1	3107046	Unique	Used	1.747	0.860	1.703	0.668	0	0	5.81	0	11	36	3	939.17401	2815.50748	-1.64
iSQAEEDDQQLLGHLLLVAk	Unambiguous	1	1	Q9D0S9	N-Term(iTRAQ8plex); K20(iTRAQ8plex)	CID	1.0000	0.0000	1	1	3284234	Unique	Used	1.288	1.319	0.954	1.108	0	0	5.81	0	21	5	3	943.53589	2828.59311	-1.68
lVGAIVYYDGQHNDAR	Unambiguous	2	1	Q64521	N-Term(iTRAQ8plex)	CID	1.0000	0.0000	1	1	3369595	Unique	Used	1.300	1.689	0.829	1.138	0	0	5.81	0	16	23	3	699.03070	2095.07755	-4.72
qQQMVAAHQYSFASDGEAR	Unambiguous	1	1	Q5SSZ5	N-Term(iTRAQ8plex)	CID	1.0000	0.0000	1	1	3066754	Unique	Used	1.735	1.595	0.625	0.689	0	0	5.81	0	19	200	3	810.05792	2428.15921	-1.05

aRPSQLPEQSSSAQQnAR	Unambiguous	1	1	P28740-2	N-Term(iTRAQ8plex); N16(Deamidated)	CID	0.0327	0.0000	1	1	2927580	Unique	Used	1.263	0.476	0.907	0.921	0	0	5.81	0	0	71	3	754.05524	2260.15116	-3.20
gGPPPPPPPPHSSGPPPPPAR	Unambiguous	1	1	Q91YD9	N-Term(iTRAQ8plex)	HCD	1.0000	0.0000	1	1	3467447	Unique	Used	1.499	1.578	1.526	1.020	0	0	5.81	0	0	200	3	765.41479	2294.22983	-2.18
lGTIGPEALLEkPGR	Unambiguous	1	1	Q9Z1R4	N-Term(iTRAQ8plex); K12(iTRAQ8plex)	HCD	1.0000	0.0000	1	1	3577435	Unique	Used	0.643	0.720	1.026	1.244	0	0	5.81	0	16	68	3	720.43341	2159.28568	-7.04
fGVPVIADGGIQNVGHIAk	Unambiguous	1	1	P24547	N-Term(iTRAQ8plex); K19(iTRAQ8plex)	CID	1.0000	0.0000	1	1	3663058	Unique	Used	1.067	1.044	0.606	0.907	0	0	5.81	0	16	43	3	834.14941	2500.43369	-6.40
fPQASASDVVVVHGR	Unambiguous	5	1	Q921H8	N-Term(iTRAQ8plex)	CID	1.0000	0.0000	1	1	3512359	Unique	Used	0.958	1.225	0.939	1.053	0	0	5.81	0	28	18	3	625.01031	1873.01639	-3.74
gSQSVEGAGVMAGEPPSHSSGGR	Unambiguous	2	1	Q6PGL7	N-Term(iTRAQ8plex)	CID	1.0000	0.0000	1	1	2911523	Unique	Used	1.267	1.504	1.138	1.160	0	0	5.81	0	29	200	3	815.72455	2445.15909	-5.73
lScnGLGPSDLLGkPLAR	Unambiguous	3	1	Q5PR72	N-Term(iTRAQ8plex); C3(Methylthio); N4(Deamidated); K14(iTRAQ8plex)	HCD	1.0000	0.0000	1	1	3744646	Unique	Used	0.109	1.733	1.945	1.225	0	0	5.81	0	3	46	3	822.79102	2466.35849	-3.42
gTGGVDTAAVGGVFDVSNADR	Unambiguous	1	1	Q04447	N-Term(iTRAQ8plex)	CID	1.0000	0.0000	1	1	3234559	Unique	Used	1.406	0.792	1.201	1.076	0	0	5.81	0	30	161	2	1135.06726	2269.12724	-3.99
aAEDSNLISGFGVAEGSEk	Unambiguous	2	1	Q3UHJ0-2	N-Term(iTRAQ8plex); K19(iTRAQ8plex)	CID	1.0000	0.0000	1	1	2813053	Unique	Used	1.034	1.114	1.905	0.890	0	0	5.81	0	0	200	2	1245.15198	2489.29668	-0.55
aAADLMAYcEAHAk	Unambiguous	1	1	P63213	N-Term(iTRAQ8plex); C9(Methylthio); K14(iTRAQ8plex)	CID	1.0000	0.0000	1	1	3420442	Unique	Used	0.812	0.967	1.423	1.308	0	0	5.81	0	17	200	3	707.02240	2119.05265	-3.24
vAVLGASGGIGQPLSLLLk	Unambiguous	1	1	P08249	N-Term(iTRAQ8plex); K19(iTRAQ8plex)	CID	1.0000	0.0000	1	1	3936196	Unique	Used	1.416	1.219	0.881	1.101	0	0	5.80	0	9	15	2	1201.24768	2401.48808	-5.10
sQPPAAHAGQVYGEQPPk	Unambiguous	3	1	Q6P9K8	N-Term(iTRAQ8plex); K18(iTRAQ8plex)	CID	1.0000	0.0000	1	1	2968134	Unique	Used	0.715	0.487	1.203	1.024	0	0	5.80	0	0	105	3	824.11365	2470.32639	-1.43

iIAcDGGGGALGHPk	Unambiguous	2	1	P52503	N-Term(iTRAQ8plex); C4(Methylthio); K15(iTRAQ8plex)	CID	1.0000	0.0000	1	1	3432609	Unique	Used	1.139	1.224	1.373	0.851	0	0	5.80	0	22	16	3	674.03326	2020.08524	-3.78
lcALDGGQEQQYHSk	Unambiguous	8	1	Q8BYR5	N-Term(iTRAQ8plex); C2(Methylthio); K15(iTRAQ8plex)	CID	1.0000	0.0000	1	1	3104914	Unique	Used	0.788	2.057	1.213	1.477	0	0	5.80	0	5	101	3	777.72632	2331.16440	-1.63
aFVDLTNQVScSGDVk	Unambiguous	2	1	Q8BWW3	N-Term(iTRAQ8plex); C11(Methylthio); K16(iTRAQ8plex)	CID	1.0000	0.0000	1	1	3371218	Unique	Used	0.641	1.268	1.345	1.450	0	0	5.80	0	28	110	2	1169.09534	2337.18340	-8.79
tVAGIIVEPIQSEGGDnHASDDFFR	Unambiguous	2	1	P61922	N-Term(iTRAQ8plex); N17(Deamidated)	CID	1.0000	0.0000	1	1	2903910	Unique	Used	1.332		0.967	1.773	0	0	5.80	0	5	200	3	993.82465	2979.45938	-1.50
hEAPmQMASAQDAR	Unambiguous	1	1	P62823	N-Term(iTRAQ8plex); M5(Oxidation)	CID	1.0000	0.0000	1	1	2858228	Unique	Used	0.409	0.968	1.728	1.156	0	0	5.80	0	6	52	3	621.62921	1862.87308	-3.25
gIHVEIPGAQAESLGPLQVAR	Unambiguous	1	1	Q9DCW4	N-Term(iTRAQ8plex)	CID	1.0000	0.0000	1	1	3619836	Unique	Used		1.724	1.029	1.666	0	0	5.80	0	0	0	3	816.12512	2446.36081	-4.59
eITENLMATGDLDQDGk	Unambiguous	5	1	Q61233	N-Term(iTRAQ8plex); K17(iTRAQ8plex)	CID	1.0000	0.0000	1	1	2653939	Unique	Used	1.965	1.279	1.307	1.396	0	0	5.80	0	17	200	3	820.08954	2458.25406	-2.10
hLEINPDHPIVETLR	Unambiguous	1	1	P11499	N-Term(iTRAQ8plex)	HCD	1.0000	0.0000	1	1	3297491	Unique	Used	1.177	0.971	1.002	1.111	0	0	5.80	0	25	151	3	696.38910	2087.15274	-1.15
lSLPPHEGPGGNLVAGALYR	Unambiguous	5	1	E9Q3M3	N-Term(iTRAQ8plex)	HCD	1.0000	0.0000	1	1	3801912	Unique	Used	0.592	0.656	1.204	1.386	0	0	5.80	0	4	9	3	774.76410	2322.27774	-4.09
lQQELDDLTVDLDHQR	Unambiguous	3	1	Q5SV64	N-Term(iTRAQ8plex)	CID	1.0000	0.0000	1	1	2816729	Unique	Used	2.223	0.866	1.610	1.062	0	0	5.80	0	17	200	3	748.05853	2242.16104	-0.32
fLSqPFQVAEVFTGHMGk	Unambiguous	1	1	P56480	N-Term(iTRAQ8plex); Q4(Deamidated); K18(iTRAQ8plex)	CID	0.0500	0.0000	1	1	3861727	Unique	Used	1.249	0.880	1.085	1.127	0	0	5.80	0	0	188	3	878.14191	2632.41117	2.18
eDSAPVTTVAAAGQVQQQQR	Unambiguous	1	1	Q8R4S0	N-Term(iTRAQ8plex)	CID	1.0000	0.0000	1	1	2989344	Unique	Used	1.478	0.424	0.823	0.533	0	0	5.80	0	15	200	2	1194.61987	2388.23247	-4.05
gTGGVDTAAVGGVFDVSNADR	Unambiguous	1	1	Q04447	N-Term(iTRAQ8plex)	CID	1.0000	0.0000	1	1	3134632	Unique	Used	0.981	1.066	1.433	1.772	0	0	5.80	0	0	200	2	1135.07043	2269.13359	-1.19

fYYSSGSSSPTHAk	Unambiguous	1	1	Q60771	N-Term(iTRAQ8plex); K14(iTRAQ8plex)	CID	1.0000	0.0000	1	1	3570328	Unique	Used	1.507	0.366	1.662	2.425	0	0	5.80	0	0	133	3	709.69977	2127.08475	-5.61
vQnHLPASGPPQPPAASPTR	Unambiguous	10	1	Q62219-2	N-Term(iTRAQ8plex); N3(Deamidated)	HCD	0.0328	0.0000	1	1	3363626	Unique	Used	1.461	1.232	1.258	1.485	0	0	5.80	0	25	160	3	776.41650	2327.23496	-2.60
tNVSGGAIALGHPLGGSGSR	Unambiguous	1	1	Q8BWT1	N-Term(iTRAQ8plex)	HCD	1.0000	0.0000	1	1	3909633	Unique	Used	1.028	1.561	0.977	1.256	0	0	5.80	0	0	200	3	704.71619	2112.13401	-5.86
kEFGGVGAPPSPGVAR	Unambiguous	4	1	Q8CHC4	N-Term(iTRAQ8plex); K1(iTRAQ8plex)	HCD	1.0000	0.0000	1	1	3429838	Unique	Used	0.890	2.038	1.828	1.036	0	0	5.80	1	6	36	3	712.07648	2134.21488	-3.79
nLHqSGFSLSGTQVDEGVR	Unambiguous	3	1	E9PWE8	N-Term(iTRAQ8plex); Q4(Deamidated)	CID	0.0034	0.0000	1	1	3069083	Unique	Used	1.596	0.717	1.103	0.833	0	0	5.80	0	15	59	3	779.39807	2336.17966	0.51
gAPSSSGGSSISGPk	Unambiguous	6	1	Q6PGN3-3	N-Term(iTRAQ8plex); K15(iTRAQ8plex)	CID	1.0000	0.0000	1	1	3140220	Unique	Used	0.452	0.659	1.142	1.039	0	0	5.80	0	0	200	2	942.51508	1884.02287	-2.89
gLGQMTTNADTDGDSYk	Unambiguous	2	1	Q9QYB8	N-Term(iTRAQ8plex); K17(iTRAQ8plex)	CID	1.0000	0.0000	1	1	2733249	Unique	Used	0.410	0.706	2.501	1.026	0	0	5.80	0	3	200	2	1191.58740	2382.16753	-1.20
iNSENAEALAQLLTLAR	Unambiguous	1	1	O70481	N-Term(iTRAQ8plex)	HCD	1.0000	0.0000	1	1	3785460	Unique	Used	0.605	0.628	0.896	1.167	0	0	5.80	0	0	22	2	1066.10217	2131.19707	-2.55
dINQEVYNFLATAGAk	Unambiguous	1	1	Q99KI0	N-Term(iTRAQ8plex); K16(iTRAQ8plex)	CID	1.0000	0.0000	1	1	3542708	Unique	Used	0.649	0.679	0.790	0.992	0	0	5.79	0	16	200	2	1181.64160	2362.27593	-4.40
gLAEAQGAVGVAPFcSR	Unambiguous	4	1	E9PUM9	N-Term(iTRAQ8plex); C15(Methylthio)	HCD	1.0000	0.0000	1	1	3783354	Unique	Used	0.463	0.906	0.758	0.977	0	0	5.79	0	14	75	2	992.00476	1983.00224	-3.62
lAEENPDLQEAYIAk	Unambiguous	1	1	O88741	N-Term(iTRAQ8plex); K15(iTRAQ8plex)	CID	1.0000	0.0000	1	1	2737540	Unique	Used	0.641	0.466	0.786	1.174	0	0	5.79	0	7	200	2	1156.63196	2312.25664	-1.22
hHAAYVNNLNATEEk	Unambiguous	1	1	P09671	N-Term(iTRAQ8plex); K15(iTRAQ8plex)	CID	1.0000	0.0000	1	1	2928384	Unique	Used	1.364	0.563	0.762	0.952	0	0	5.79	0	2	23	3	773.74615	2319.22391	-2.71
yTLSGDGAGTVFTIDETTGDIHAIR	Unambiguous	1	1	Q5RJH3	N-Term(iTRAQ8plex)	CID	1.0000	0.0000	1	1	3193154	Unique	Used	1.295	1.095	1.069	0.963	0	0	5.79	0	0	65	3	972.16364	2914.47635	0.92

cSSPVDMDHSNAEGGQSPGPEk	Unambiguous	2	1	G3X9K4	N-Term(iTRAQ8plex); C1(Methylthio); K22(iTRAQ8plex)	CID	1.0000	0.0000	1	1	2698260	Unique	Used	1.431	1.020	1.971	1.079	0	0	5.79	0	12	200	3	961.78027	2883.32627	3.27
rnGLLNEALELTGTPEEEQMGk	Unambiguous	3	1	Q9Z0V1-2	N-Term(iTRAQ8plex); N2(Deamidated); K22(iTRAQ8plex)	CID	1.0000	0.0000	1	1	2870207	Unique	Used	0.813	1.181	0.766	0.970	0	0	5.79	1	19	200	3	1013.53186	3038.58103	-3.79
fYYSSGSSSPTHAk	Unambiguous	1	1	Q60771	N-Term(iTRAQ8plex); K14(iTRAQ8plex)	HCD	1.0000	0.0000	1	1	3405771	Unique	Used	1.198	0.561	1.306	1.437	0	0	5.79	0	27	112	3	709.70068	2127.08750	-4.32
vFLMDPVIFTVAHLEAk	Unambiguous	5	1	E9Q3V9	N-Term(iTRAQ8plex); K17(iTRAQ8plex)	CID	1.0000	0.0000	1	1	3766332	Unique	Used	0.641	1.255	1.048	0.849	0	0	5.79	0	6	29	3	846.82275	2538.45371	-3.07
kEPQPSGPQPSGGAGGGGGk	Unambiguous	1	1	Q8JZQ2	N-Term(iTRAQ8plex); K1(iTRAQ8plex); K20(iTRAQ8plex)	CID	1.0000	0.0000	1	1	2909663	Unique	Used	0.401	0.890	1.157	1.051	0	0	5.79	1	0	200	3	888.15765	2662.45841	-3.47
rAPPGPLPPAASPGPPAAsPPAAPSSPR	Unambiguous	1	1	O88703	N-Term(iTRAQ8plex); S19(Phospho)	CID	1.0000	0.0000	1	1	3365981	Unique	Used	1.216	1.673	1.296	1.341	0	0	5.79	1	0	178	3	984.84662	2952.52530	-4.97
gVLTGPEYEALDALPDAER	Unambiguous	1	1	Q9D1X0	N-Term(iTRAQ8plex)	CID	1.0000	0.0000	1	1	2798172	Unique	Used			1.802	1.151	0	0	5.79	0	13	200	2	1160.60034	2320.19341	-1.76
gTGGVDTAATGSVFDISnLDR	Unambiguous	1	1	P30275	N-Term(iTRAQ8plex); N18(Deamidated)	CID	1.0000	0.0000	1	1	2996351	Unique	Used	0.876	1.193	1.333	1.281	0	0	5.79	0	9	200	2	1179.58826	2358.16924	-1.48
gTGGVDTAAVGGVFDVSNADR	Unambiguous	1	1	Q04447	N-Term(iTRAQ8plex)	CID	1.0000	0.0000	1	1	3300968	Unique	Used	1.248	1.079	0.968	1.191	0	0	5.79	0	0	200	2	1135.06982	2269.13237	-1.73
aAVEEGIVLGGGcALLR	Unambiguous	2	1	P63038	N-Term(iTRAQ8plex); C13(Methylthio)	CID	1.0000	0.0000	1	1	3646025	Unique	Used	0.974	0.831	0.987	1.085	0	0	5.79	0	26	88	2	989.53662	1978.06597	-5.47
eLHLNDNPMGDAGLk	Unambiguous	1	1	Q91VI7	N-Term(iTRAQ8plex); K15(iTRAQ8plex)	CID	1.0000	0.0000	1	1	3011021	Unique	Used	1.449	1.209	0.953	1.017	0	0	5.79	0	9	69	3	744.73492	2232.19022	-0.05

aVAQGNLSSADVQAAk	Unambiguous	1	1	Q9DB77	N-Term(iTRAQ8plex); K16(iTRAQ8plex)	CID	1.0000	0.0000	1	1	3122869	Unique	Used		1.301	1.206	1.345	0	0	5.78	0	0	63	2	1069.60327	2138.19927	-1.56
aVAQGNLSSADVQAAk	Unambiguous	1	1	Q9DB77	N-Term(iTRAQ8plex); K16(iTRAQ8plex)	CID	1.0000	0.0000	1	1	3144159	Unique	Used		1.547			0	0	5.78	0	0	66	2	1069.60425	2138.20122	-0.65
iSMADYVSGELEHVTR	Unambiguous	1	1	P12382	N-Term(iTRAQ8plex)	CID	1.0000	0.0000	1	1	3349728	Unique	Used	1.162	1.629	0.769	1.138	0	0	5.78	0	2	6	3	704.36108	2111.06870	-2.75
aQATISVLQkPR	Unambiguous	1	1	P97300	N-Term(iTRAQ8plex); K10(iTRAQ8plex)	HCD	1.0000	0.0000	1	1	3890742	Unique	Used	0.390	2.927	2.544	0.933	0	0	5.78	0	20	96	3	640.73083	1920.17795	-3.72
lSVIEVAASEMAADDQR	Unambiguous	3	1	Q5SW75	N-Term(iTRAQ8plex)	CID	1.0000	0.0000	1	1	3037875	Unique	Used	0.914	0.913	1.279	0.745	0	0	5.78	0	7	74	2	1055.04175	2109.07622	-1.80
lYSMEGVGTDAVIYLk	Unambiguous	1	1	Q922H2	N-Term(iTRAQ8plex); K16(iTRAQ8plex)	CID	1.0000	0.0000	1	1	3395737	Unique	Used	0.919	1.260	1.102	1.194	0	0	5.78	0	0	23	2	1184.15332	2367.29936	-4.09
fGGNPGGFGNQGGFGnSR	Unambiguous	1	1	Q921F2	N-Term(iTRAQ8plex); N16(Deamidated)	CID	0.0260	0.0000	1	1	3514163	Unique	Used	0.555	0.807	0.871	0.842	0	0	5.78	0	13	37	2	1016.47742	2031.94756	-4.91
aLGQAASDSSGPEDMk	Unambiguous	1	1	Q6GQT9	N-Term(iTRAQ8plex); K16(iTRAQ8plex)	CID	1.0000	0.0000	1	1	2632895	Unique	Used	0.932	0.678	0.591	0.623	0	0	5.78	0	10	200	2	1086.55225	2172.09722	-4.20
tVAVYSEQDTGQMHR	Unambiguous	3	1	G5E8R3	N-Term(iTRAQ8plex)	CID	1.0000	0.0000	1	1	2879344	Unique	Used	2.311	1.256	1.615	1.084	0	0	5.78	0	3	20	3	676.00201	2025.99149	-2.51
vQASDGGSPQLSSNALVQVR	Unambiguous	3	1	Q7TSK3-3	N-Term(iTRAQ8plex)	CID	1.0000	0.0000	1	1	3473289	Unique	Used	0.675	2.604	0.777	0.508	0	0	5.78	0	0	150	2	1159.11804	2317.22881	-5.44
iIVNSHSPASSAAISqQDFHPk	Unambiguous	2	1	B1AY13	N-Term(iTRAQ8plex); Q16(Deamidated); K22(iTRAQ8plex)	CID	0.0415	0.0000	1	1	3451832	Unique	Used	0.486	0.815	1.102	1.444	0	0	5.78	0	0	200	3	981.86365	2943.57639	-0.72
lAmLQEHVGNLGSSSDLASTVR	Unambiguous	1	1	Q8BMK4	N-Term(iTRAQ8plex); M3(Oxidation)	HCD	1.0000	0.0000	1	1	3801823	Unique	Used	1.286	0.837	0.688	0.492	0	0	5.78	0	8	12	3	869.12061	2605.34726	-3.28
lDSFSQVGPGSETVTQk	Unambiguous	3	1	E9QML5	N-Term(iTRAQ8plex); K17(iTRAQ8plex)	CID	1.0000	0.0000	1	1	3211570	Unique	Used	0.693	0.221	1.003	0.627	0	0	5.78	0	0	130	2	1194.64563	2388.28398	-1.15

lALSmLGQSDAYGAADAR	Unambiguous	3	1	O09012	N-Term(iTRAQ8plex); M5(Oxidation)	CID	1.0000	0.0000	1	1	3515643	Unique	Used	1.740	1.005	0.739	1.081	0	0	5.78	0	0	156	2	1065.53967	2130.07207	-3.89
mLLEYTDSSYDEk	Unambiguous	2	1	P10649	N-Term(iTRAQ8plex); M1(Oxidation); K13(iTRAQ8plex)	CID	1.0000	0.0000	1	1	2795172	Unique	Used	2.669	2.820	2.178	1.965	0	0	5.78	0	3	200	3	740.03888	2218.10208	-1.12
qPAPAEEnGSLPPGDAAASAPLGGR	Unambiguous	1	1	Q3UHH2	N-Term(iTRAQ8plex); N8(Deamidated)	CID	1.0000	0.0000	1	1	2651285	Unique	Used			0.824	0.825	0	0	5.78	0	0	200	2	1318.16650	2635.32573	-0.35
ySEATEHPGAPPQPPAPLQPALAPPSR	Unambiguous	3	1	Q7TSJ2	N-Term(iTRAQ8plex)	HCD	1.0000	0.0000	1	1	3343149	Unique	Used	0.922	0.967	1.702	1.601	0	0	5.77	0	3	2	3	1027.53845	3080.60080	-3.24
rLEEDTEETSGLLEQLR	Unambiguous	3	1	E9QP99	N-Term(iTRAQ8plex)	CID	1.0000	0.0000	1	1	2738737	Unique	Used	1.809	0.794	1.144	0.833	0	0	5.77	1	0	81	3	774.74036	2322.20652	-1.12
aTASSSAQEmEQQLAER	Unambiguous	1	1	Q8BV13	N-Term(iTRAQ8plex); M10(Oxidation)	CID	1.0000	0.0000	1	1	2696784	Unique	Used	1.124	0.751	1.002	0.555	0	0	5.77	0	0	200	2	1079.02026	2157.03325	-2.94
dSVSAAEVGTGQYATTk	Unambiguous	6	1	Q9WV92-2	N-Term(iTRAQ8plex); K17(iTRAQ8plex)	CID	1.0000	0.0000	1	1	2845615	Unique	Used	0.466	0.881	1.548	0.938	0	0	5.77	0	4	125	2	1147.10559	2293.20391	-4.06
vIESTQGLGNDLAGVLALQR	Unambiguous	1	1	Q68FG2	N-Term(iTRAQ8plex)	CID	1.0000	0.0000	1	1	3785294	Unique	Used	0.601	2.297	2.458	1.199	0	0	5.77	0	4	5	2	1179.66565	2358.32402	-2.33
gLGTDEDTLIEILTTR	Unambiguous	1	1	P10107	N-Term(iTRAQ8plex)	HCD	1.0000	0.0000	1	1	3153726	Unique	Used	0.620	6.231	0.542	1.228	0	0	5.77	0	12	46	2	1026.06091	2051.11455	-1.40
aEQVDGVINLGNSLIER	Unambiguous	1	1	P08032	N-Term(iTRAQ8plex)	CID	1.0000	0.0000	1	1	3373057	Unique	Used	4.020	2.042	0.693	1.162	0	0	5.77	0	14	141	2	1066.08057	2131.15386	-5.76
gFESPSDNSSAVLLQWHEk	Unambiguous	1	1	Q9CPW4	N-Term(iTRAQ8plex); K19(iTRAQ8plex)	CID	1.0000	0.0000	1	1	3212182	Unique	Used	1.176	2.318	1.053	0.719	0	0	5.77	0	20	101	3	913.80920	2739.41306	-2.49
vQHQDALQISDVVmASLLR	Unambiguous	1	1	P70168	N-Term(iTRAQ8plex); M14(Oxidation)	CID	1.0000	0.0000	1	1	3804441	Unique	Used		1.420	1.463	0.852	0	0	5.77	0	21	16	3	815.11151	2443.31998	-3.33
rGAYGGGYGGYDDYGGYnDGYGFGSDR	Unambiguous	1	1	P70333	N-Term(iTRAQ8plex); N18(Deamidated)	CID	1.0000	0.0000	1	1	2882992	Unique	Used	2.104	1.884	0.754	0.903	0	0	5.77	1	0	200	3	1041.11304	3121.32456	3.43
vSDFGQMASGMNVDAGk	Unambiguous	1	1	P20357	N-Term(iTRAQ8plex); K17(iTRAQ8plex)	CID	1.0000	0.0000	1	1	3301294	Unique	Used	0.928	1.167	1.752	1.238	0	0	5.76	0	0	4	3	774.72522	2322.16111	-2.93

sVSGASTGLSSSPLSSPR	Unambiguous	2	1	Q5RJI5	N-Term(iTRAQ8plex)	CID	1.0000	0.0000	1	1	3835625	Unique	Used	0.632	0.943	0.696	0.983	0	0	5.76	0	0	200	2	991.02551	1981.04375	-3.36
iEGDMIVcAAYAHELPk	Unambiguous	2	1	P47962	N-Term(iTRAQ8plex); C8(Methylthio); K17(iTRAQ8plex)	CID	1.0000	0.0000	1	1	3305033	Unique	Used	0.495	0.806	0.668	0.734	0	0	5.76	0	7	21	3	838.76691	2514.28617	-6.12
gNAGQTNYGFANSTmER	Unambiguous	1	1	P19096	N-Term(iTRAQ8plex); M15(Oxidation)	CID	1.0000	0.0000	1	1	3141990	Unique	Used	0.670	0.587	0.825	1.062	0	0	5.76	0	0	194	2	1069.49683	2137.98638	-0.52
sPAASAPSLPPAAAVLPSk	Unambiguous	3	1	Q99P72	N-Term(iTRAQ8plex); K19(iTRAQ8plex)	CID	1.0000	0.0000	1	1	3722686	Unique	Used	1.403	0.793	1.452	0.878	0	0	5.76	0	4	11	2	1170.68188	2340.35649	-7.81
iDINMSGFnETDDLk	Unambiguous	2	1	B1AX58	N-Term(iTRAQ8plex); N9(Deamidated); K15(iTRAQ8plex)	CID	1.0000	0.0000	1	1	2814654	Unique	Used	1.556	0.964	1.439	0.796	0	0	5.76	0	11	200	3	774.39844	2321.18076	0.69
aAELIAnSLATAGDGLIELR	Unambiguous	1	1	P67778	N-Term(iTRAQ8plex); N7(Deamidated)	CID	1.0000	0.0000	1	1	3396675	Unique	Used	2.709	2.492	1.015	0.843	0	0	5.76	0	14	60	2	1152.13745	2303.26763	-3.67
tIGTGLVTDVPAMTEEDk	Unambiguous	2	1	Q8BFR5	N-Term(iTRAQ8plex); K18(iTRAQ8plex)	CID	1.0000	0.0000	1	1	2919653	Unique	Used	0.840	0.660	1.017	1.581	0	0	5.76	0	9	200	2	1243.16907	2485.33086	-0.32
lTLYDIAHTPGVAADLSHIETR	Unambiguous	1	1	P08249	N-Term(iTRAQ8plex)	CID	1.0000	0.0000	1	1	3460341	Unique	Used	1.647	1.062	0.786	0.853	0	0	5.76	0	8	200	3	899.81964	2697.44437	-2.61
qAEIENPLEDPVTGDYVHk	Unambiguous	4	1	Q9Z1G4-3	N-Term(iTRAQ8plex); K19(iTRAQ8plex)	CID	1.0000	0.0000	1	1	2760857	Unique	Used	1.429	2.088	1.213	1.100	0	0	5.76	0	5	200	3	921.48590	2762.44315	-0.95
tSDVETMSSQSALALDER	Unambiguous	1	1	P14873	N-Term(iTRAQ8plex)	CID	1.0000	0.0000	1	1	2778942	Unique	Used	3.075	0.939	1.415	0.832	0	0	5.76	0	0	200	2	1122.55054	2244.09380	-1.33
vGIGAFPTEQDNEIGELLQTR	Unambiguous	1	1	P46664	N-Term(iTRAQ8plex)	HCD	1.0000	0.0000	1	1	3284195	Unique	Used	0.914	1.096	1.006	1.168	0	0	5.76	0	15	17	2	1296.18311	2591.35893	-1.16
iTVAALDAANLLASVPASQR	Unambiguous	2	1	Q8VHY0	N-Term(iTRAQ8plex)	CID	1.0000	0.0000	1	1	3917918	Unique	Used	0.759	0.584	0.699	0.801	0	0	5.76	0	0	9	2	1143.15601	2285.30474	-3.67
sWGSGGGQEPGGSQAAGGGQPR	Unambiguous	5	1	Q8CFT0	N-Term(iTRAQ8plex)	CID	1.0000	0.0000	1	1	3025854	Unique	Used	0.803	0.301	1.143	0.920	0	0	5.75	0	0	200	2	1145.04895	2289.09062	-0.20

lGSASSSHTSIQENHk	Unambiguous	2	1	P08414	N-Term(iTRAQ8plex); K16(iTRAQ8plex)	CID	1.0000	0.0000	1	1	3224034	Unique	Used	0.449	1.230	1.629	0.691	0	0	5.75	0	0	200	3	764.41071	2291.21756	-1.07
eLLDVGNIGHLEQR	Unambiguous	3	1	Q9Z1B3-3	N-Term(iTRAQ8plex)	CID	1.0000	0.0000	1	1	3257009	Unique	Used	0.552	1.749	1.971	1.112	0	0	5.75	0	13	6	3	633.01947	1897.04386	-0.36
qASTDAGTAGALTPQHVR	Unambiguous	5	1	P46938-2	N-Term(iTRAQ8plex)	CID	1.0000	0.0000	1	1	3204615	Unique	Used	2.122	1.810	1.309	1.114	0	0	5.75	0	16	71	3	695.70367	2085.09647	-1.26
eSEGLYGQPGDETYQDIFR	Unambiguous	2	1	Q6ZQK5	N-Term(iTRAQ8plex)	CID	1.0000	0.0000	1	1	2687594	Unique	Used	0.910		1.082		0	0	5.75	0	0	200	2	1254.59326	2508.17925	-1.60
aSESAPSGSPSATPLEFDSR	Unambiguous	3	1	Q80XI3-2	N-Term(iTRAQ8plex)	CID	1.0000	0.0000	1	1	2828686	Unique	Used	0.900	0.975	1.015	1.226	0	0	5.75	0	0	122	2	1149.06482	2297.12236	1.05
vATSAASGGHGSPLR	Unambiguous	4	1	A2AG50	N-Term(iTRAQ8plex)	CID	1.0000	0.0000	1	1	3813729	Unique	Used	1.076	0.687	1.186	1.181	0	0	5.75	0	21	200	3	557.97174	1671.90067	-4.40
yVIGVGNAFNkPQSR	Unambiguous	7	1	Q3U1U4	N-Term(iTRAQ8plex); K11(iTRAQ8plex)	HCD	1.0000	0.0000	1	1	3911422	Unique	Used	0.588	1.227	1.086	1.140	0	0	5.75	0	19	153	3	753.43311	2258.28476	-0.81
gTGGVDTAAVGGVFDVSNADR	Unambiguous	1	1	Q04447	N-Term(iTRAQ8plex)	CID	1.0000	0.0000	1	1	3136083	Unique	Used	0.807		1.401	0.813	0	0	5.75	0	0	200	2	1135.07239	2269.13750	0.53
aAEDSNLISGFGVAEGSEk	Unambiguous	2	1	Q3UHJ0-2	N-Term(iTRAQ8plex); K19(iTRAQ8plex)	CID	1.0000	0.0000	1	1	2812711	Unique	Used	1.223	1.898	1.863	0.619	0	0	5.75	0	5	160	2	1245.15088	2489.29448	-1.43
tYFPHFDVSHGSAQVk	Unambiguous	2	1	Q91VB8	N-Term(iTRAQ8plex); K16(iTRAQ8plex)	CID	1.0000	0.0000	1	1	3452578	Unique	Used	1.440	1.818	0.901	1.223	0	0	5.75	0	0	187	3	810.09515	2428.27091	-6.62
mQINAEEVVVGDLVEVk	Unambiguous	3	1	Q6PIE5	N-Term(iTRAQ8plex); M1(Oxidation); K17(iTRAQ8plex)	CID	1.0000	0.0000	1	1	2921602	Unique	Used	1.536	0.890	0.810	1.076	0	0	5.75	0	12	200	3	832.79938	2496.38358	-0.18
kLLEAHEEQNSEAYTEAVk	Unambiguous	1	1	P28663	N-Term(iTRAQ8plex); K1(iTRAQ8plex); K19(iTRAQ8plex)	CID	1.0000	0.0000	1	1	2791866	Unique	Used	0.790	2.119	1.330	1.047	0	0	5.75	1	0	200	3	1034.56799	3101.68943	0.39
kLFIGGLSFETTDDSLR	Unambiguous	6	1	J3QNY1	N-Term(iTRAQ8plex); K1(iTRAQ8plex)	HCD	1.0000	0.0000	1	1	3457358	Unique	Used	1.123	0.778	0.952	0.672	0	0	5.75	1	0	138	3	836.47028	2507.39627	-0.14
gSHSQSSSPGALTLGR	Unambiguous	3	1	O88935	N-Term(iTRAQ8plex)	HCD	1.0000	0.0000	1	1	3887653	Unique	Used	4.757	4.536	2.857	3.537	0	0	5.75	0	3	111	3	615.99286	1845.96402	-4.37

eHINLGcDVDFDIAGPSIR	Unambiguous	3	1	Q60932-2	N-Term(iTRAQ8plex); C7(Methylthio)	CID	1.0000	0.0000	1	1	3356283	Unique	Used	4.013	3.414	0.830	1.274	0	0	5.74	0	15	200	3	807.73163	2421.18033	-1.72
eVFTEQADLSGITETk	Unambiguous	1	1	P07759	N-Term(iTRAQ8plex); K16(iTRAQ8plex)	CID	1.0000	0.0000	1	1	2868292	Unique	Used	3.794	2.592	0.176	2.796	0	0	5.74	0	0	200	2	1188.63342	2376.25957	-6.70
vATSAASGGHGSPLR	Unambiguous	4	1	A2AG50	N-Term(iTRAQ8plex)	HCD	1.0000	0.0000	1	1	3753031	Unique	Used	1.109	0.541	1.153	1.128	0	0	5.74	0	18	200	3	557.97217	1671.90195	-3.64
gQVPGHASQGTQAVESScSk	Unambiguous	1	1	Q8R4U7	N-Term(iTRAQ8plex); C18(Methylthio); K20(iTRAQ8plex)	CID	1.0000	0.0000	1	1	3272993	Unique	Used	0.657	0.426	0.922	0.948	0	0	5.74	0	16	179	3	871.43750	2612.29795	-1.46
iPNIYAIGDVVAGPmLAHk	Unambiguous	1	1	O08749	N-Term(iTRAQ8plex); M15(Oxidation); K19(iTRAQ8plex)	CID	1.0000	0.0000	1	1	3805080	Unique	Used	1.869	1.274	1.397	1.098	0	0	5.74	0	27	50	3	868.49640	2603.47464	-3.60
vSAADSTQVDGGTPMVk	Unambiguous	6	1	A2AUK8	N-Term(iTRAQ8plex); K17(iTRAQ8plex)	CID	1.0000	0.0000	1	1	2915048	Unique	Used	0.600	1.161	1.456	1.050	0	0	5.74	0	0	183	2	1136.10461	2271.20195	-4.05
vSDFGQMASGmNVDAGk	Unambiguous	1	1	P20357	N-Term(iTRAQ8plex); M11(Oxidation); K17(iTRAQ8plex)	CID	1.0000	0.0000	1	1	3277578	Unique	Used	1.473	1.559	1.833	0.833	0	0	5.74	0	0	38	2	1169.58350	2338.15972	-1.33
vLDHAEENNAAMAk	Unambiguous	1	1	B1AVZ0	N-Term(iTRAQ8plex); K14(iTRAQ8plex)	CID	1.0000	0.0000	1	1	2730808	Unique	Used	1.498	1.097	0.937	0.877	0	0	5.74	0	16	200	3	707.71014	2121.11588	-2.85
qDVSPSPAPALAPASTSVEQLR	Unambiguous	1	1	Q8K382	N-Term(iTRAQ8plex)	CID	1.0000	0.0000	1	1	3256072	Unique	Used	0.922	1.252	1.435	0.606	0	0	5.74	0	0	73	2	1263.17700	2525.34673	-1.84
scSGVEFSTSGSSNTDTGk	Unambiguous	4	1	G3UX26	N-Term(iTRAQ8plex); C2(Methylthio); K19(iTRAQ8plex)	CID	1.0000	0.0000	1	1	3029921	Unique	Used	0.379	0.686	1.222	1.251	0	0	5.74	0	0	200	2	1253.08936	2505.17143	0.82
sAAAPSASSTPTAIASPk	Unambiguous	2	1	P26049	N-Term(iTRAQ8plex); K18(iTRAQ8plex)	CID	1.0000	0.0000	1	1	3338395	Unique	Used	0.414	1.889	1.503	1.194	0	0	5.74	0	0	25	2	1112.11926	2223.23125	-5.80

aAEFLAAcAGGDLDEAR	Unambiguous	1	1	Q3UMT1	N-Term(iTRAQ8plex); C8(Methylthio)	CID	1.0000	0.0000	1	1	3217483	Unique	Used	1.163	0.675	1.065	0.985	0	0	5.74	0	6	61	2	1015.48517	2029.96306	0.25
dHIIEVVGSSMPLIGDHQDEDk	Unambiguous	3	1	O88935	N-Term(iTRAQ8plex); K22(iTRAQ8plex)	CID	1.0000	0.0000	1	1	3014765	Unique	Used	1.469	1.861	1.723	1.248	0	0	5.74	0	9	97	3	1014.86090	3042.56815	0.60
vHAPSATPSTSAQPASPVAR	Unambiguous	2	1	Q9QZS8-2	N-Term(iTRAQ8plex)	CID	1.0000	0.0000	1	1	3569664	Unique	Used	1.206	1.064	1.746	1.382	0	0	5.74	0	10	200	3	746.06891	2236.19217	-2.97
yEGSGDGGAAAQSLYIANHAY	Unambiguous	1	1	P05063	N-Term(iTRAQ8plex)	CID	1.0000	0.0000	1	1	2936613	Unique	Used	1.970	0.842	0.984	1.094	0	0	5.73	0	0	166	2	1210.07300	2419.13872	-3.34
mLDLYSQISSVPIGYNHPALAk	Unambiguous	3	1	P61922	N-Term(iTRAQ8plex); M1(Oxidation); K22(iTRAQ8plex)	CID	1.0000	0.0000	1	1	3858941	Unique	Used	1.306	0.559	0.868	1.362	0	0	5.73	0	19	63	3	1014.55084	3041.63797	-6.93
aALAGGTTMIIDHVVPEPGTSLLAAFDqWR	Unambiguous	1	1	O08553	N-Term(iTRAQ8plex); Q28(Deamidated)	CID	1.0000	0.0000	1	1	3842559	Unique	Used	1.951	1.272	1.117	1.447	0	0	5.73	0	12	42	3	1148.27246	3442.80283	1.29
vLSVDTALSIQAHPNk	Unambiguous	2	1	Q924M7	N-Term(iTRAQ8plex); K16(iTRAQ8plex)	CID	1.0000	0.0000	1	1	3576101	Unique	Used	0.725	1.125	1.011	0.946	0	0	5.73	0	29	77	3	767.78357	2301.33615	-1.11
dHFmDEFFEQVEEIR	Unambiguous	1	1	P61264	N-Term(iTRAQ8plex); M4(Oxidation)	CID	1.0000	0.0000	1	1	2815373	Unique	Used	3.312	1.285	1.345	0.891	0	0	5.73	0	19	200	3	764.35773	2291.05863	-0.28
nTTPDELLSAVLTAVLQDVR	Unambiguous	5	1	Q921H8	N-Term(iTRAQ8plex)	HCD	1.0000	0.0000	1	1	3880897	Unique	Used	1.068	0.806	0.739	1.177	0	0	5.73	0	24	151	2	1230.18701	2459.36675	0.32
gSHSQSSSPGALTLGR	Unambiguous	3	1	O88935	N-Term(iTRAQ8plex)	HCD	1.0000	0.0000	1	1	3832099	Unique	Used	0.593	1.927	1.281	1.173	0	0	5.73	0	3	200	3	615.99432	1845.96842	-1.98
vEAVNMAEGIIHDTETk	Unambiguous	1	1	P38647	N-Term(iTRAQ8plex); K17(iTRAQ8plex)	CID	1.0000	0.0000	1	1	2959495	Unique	Used	1.679	0.933	1.526	1.107	0	0	5.73	0	12	200	3	822.44312	2465.31479	-0.76
mASTPASYGNTTTkPMGLLSR	Unambiguous	2	1	Q8BRF7-3	N-Term(iTRAQ8plex); K14(iTRAQ8plex)	HCD	1.0000	0.0000	1	1	3893108	Unique	Used	0.417	0.592	0.760	1.037	0	0	5.73	0	0	156	3	931.49506	2792.47062	-6.79
gASQQDLPSGLANScQQDR	Unambiguous	3	1	Q3UX37	N-Term(iTRAQ8plex); C15(Methylthio)	CID	1.0000	0.0000	1	1	3209971	Unique	Used	0.409	0.134	0.523	0.483	0	0	5.73	0	0	141	2	1163.04382	2325.08037	-2.66

amGVVVATGVNTEIGk	Unambiguous	3	1	O55143	N-Term(iTRAQ8plex); M2(Oxidation); K16(iTRAQ8plex)	CID	1.0000	0.0000	1	1	3473210	Unique	Used	0.415	0.866	1.233	0.833	0	0	5.73	0	0	58	2	1085.61658	2170.22588	-4.77
vVYTGIYDTEGVAPTk	Unambiguous	2	1	Q5M8N0	N-Term(iTRAQ8plex); K16(iTRAQ8plex)	CID	1.0000	0.0000	1	1	3128175	Unique	Used	0.874	1.744	0.652	0.554	0	0	5.73	0	5	16	2	1161.14331	2321.27934	-2.40
kEFGGVGAPPSPGVAR	Unambiguous	4	1	Q8CHC4	N-Term(iTRAQ8plex); K1(iTRAQ8plex)	HCD	1.0000	0.0000	1	1	3429990	Unique	Used	0.715	1.920	1.630	0.970	0	0	5.73	1	1	5	3	712.07581	2134.21286	-4.74
vSHQGYGSTTEFEEPR	Unambiguous	2	1	P55302	N-Term(iTRAQ8plex)	CID	1.0000	0.0000	1	1	2648200	Unique	Used	2.150	1.139	1.430	1.141	0	0	5.73	0	16	141	3	710.01208	2128.02170	-1.50
tSVAQTHLEDAGAAIAAAEAAVQQLR	Unambiguous	2	1	Q9QYX7	N-Term(iTRAQ8plex)	CID	1.0000	0.0000	1	1	3645035	Unique	Used	2.233	0.976	1.052	2.129	0	0	5.73	0	6	10	3	966.18134	2896.52945	-4.72
aIAVLTSGGDAQGMnAAVR	Unambiguous	1	1	P47857	N-Term(iTRAQ8plex); N15(Deamidated)	CID	0.0436	0.0000	1	1	3370443	Unique	Used	2.723	1.931	1.154	1.110	0	0	5.73	0	0	193	2	1054.05542	2107.10356	-4.00
aPSSSSVGISEWLDQk	Unambiguous	1	1	Q99LC5	N-Term(iTRAQ8plex); K16(iTRAQ8plex)	CID	1.0000	0.0000	1	1	3258550	Unique	Used	0.806	1.067	0.774	0.938	0	0	5.73	0	0	20	2	1150.11938	2299.23149	-3.29
ePcGGTGSTGAGGGTnGGVR	Unambiguous	1	1	Q80WM4	N-Term(iTRAQ8plex); C3(Methylthio); N16(Deamidated)	CID	1.0000	0.0000	1	1	2751946	Unique	Used			1.097	1.556	0	0	5.73	0	0	200	2	1021.95959	2042.91191	-2.68
tGAMLGQSEELQPLSQR	Unambiguous	4	1	Q5NCM5	N-Term(iTRAQ8plex)	CID	1.0000	0.0000	1	1	3278536	Unique	Used		1.237	1.937	1.064	0	0	5.73	0	0	135	2	1075.06323	2149.11919	-1.56
dQEGQDVLLFIDNIFR	Unambiguous	1	1	P56480	N-Term(iTRAQ8plex)	HCD	1.0000	0.0000	1	1	3353855	Unique	Used	1.422	1.657	1.050	1.263	0	0	5.73	0	0	87	2	1113.58423	2226.16118	-4.35
rPSAncDPYAVTEAIVR	Unambiguous	1	1	P15105	N-Term(iTRAQ8plex); N5(Deamidated); C6(Methylthio)	CID	1.0000	0.0000	1	1	3370120	Unique	Used	0.853	0.901	1.876	2.197	0	0	5.72	0	0	1	3	738.36841	2213.09067	-4.07
dTPLDAIVIHEVYEEGAAAR	Unambiguous	4	1	Q63ZW7-2	N-Term(iTRAQ8plex)	CID	1.0000	0.0000	1	1	2834470	Unique	Used	0.963	0.259	0.549	0.759	0	0	5.72	0	8	169	3	825.10046	2473.28684	-0.35
mLAPEGALNIHEk	Unambiguous	4	1	P53810	N-Term(iTRAQ8plex); K13(iTRAQ8plex)	CID	1.0000	0.0000	1	1	3231804	Unique	Used	0.861	0.986	0.969	0.934	0	0	5.72	0	21	16	3	677.72107	2031.14865	-1.53

IGYAVYETPTAHNGAk	Unambiguous	4	1	Q9QZ06	N-Term(iTRAQ8plex); K16(iTRAQ8plex)	CID	1.0000	0.0000	1	1	3229622	Unique	Used	1.096	1.062	1.029	1.125	0	0	5.72	0	7	63	3	767.41833	2300.24045	-3.95
aSSSILnEAEPTTNIQIR	Unambiguous	3	1	Q9CZ44	N-Term(iTRAQ8plex); N8(Deamidated)	HCD	1.0000	0.0000	1	1	3129424	Unique	Used	2.019	1.586	0.928	0.900	0	0	5.72	0	0	105	2	1181.63977	2362.27226	-1.91
gTGGVDTAAVGGVFDVSnADR	Unambiguous	1	1	Q04447	N-Term(iTRAQ8plex); N18(Deamidated)	CID	1.0000	0.0000	1	1	2938541	Unique	Used	1.830	1.239	1.483	1.421	0	0	5.72	0	3	30	2	1135.56177	2270.11626	-1.79
lMGPLGPPGGPAHELEVEAR	Unambiguous	3	1	E9PVD3	N-Term(iTRAQ8plex)	HCD	1.0000	0.0000	1	1	3257675	Unique	Used	1.270	1.257	0.949	1.149	0	0	5.72	0	0	66	3	777.75171	2331.24057	-1.20
aSLENQTLSTQVEELSQTLHSR	Unambiguous	4	1	E9PWI6	N-Term(iTRAQ8plex)	HCD	1.0000	0.0000	1	1	3393903	Unique	Used	2.274	1.127	1.227	1.072	0	0	5.72	0	0	73	3	925.81110	2775.41874	-8.63
tSDVETMSSQSALALDER	Unambiguous	1	1	P14873	N-Term(iTRAQ8plex)	CID	1.0000	0.0000	1	1	2759429	Unique	Used	1.922	0.424	1.861	1.058	0	0	5.72	0	0	200	2	1122.55017	2244.09307	-1.66
vTYVDFLVYDVLDQHR	Unambiguous	2	1	P15626	N-Term(iTRAQ8plex)	CID	1.0000	0.0000	1	1	3520421	Unique	Used	0.658	4.066	0.795	0.949	0	0	5.72	0	8	65	3	762.73773	2286.19864	-3.75
aIEPPPLDAVIEAEHTLR	Unambiguous	1	1	E9QNN1	N-Term(iTRAQ8plex)	CID	1.0000	0.0000	1	1	3132003	Unique	Used				0.883	0	0	5.72	0	7	161	3	759.09027	2275.25626	-1.66
tYFPHFDVSHGSAQVk	Unambiguous	2	1	Q91VB8	N-Term(iTRAQ8plex); K16(iTRAQ8plex)	CID	1.0000	0.0000	1	1	3481074	Unique	Used	2.829	2.337	0.732	1.603	0	0	5.72	0	0	200	3	810.09607	2428.27365	-5.49
lEQHSQQPQLSPATSGR	Unambiguous	5	1	Q921T2-2	N-Term(iTRAQ8plex)	CID	1.0000	0.0000	1	1	3271855	Unique	Used	1.699	0.930	0.913	0.980	0	0	5.72	0	0	15	3	723.38232	2168.13242	-1.74
lGEHnINVLEGNEQFIDAAk	Unambiguous	1	1	Q9Z1R9	N-Term(iTRAQ8plex); N5(Deamidated); K20(iTRAQ8plex)	CID	0.0105	0.0000	1	1	2939624	Unique	Used	2.447	1.529	1.767	1.223	0	0	5.72	0	13	48	3	940.83636	2820.49454	-1.54
yEGSGDGGAAAQSLYIANHAY	Unambiguous	1	1	P05063	N-Term(iTRAQ8plex)	CID	1.0000	0.0000	1	1	2993500	Unique	Used	1.523	0.441	1.198	1.514	0	0	5.72	0	25	200	2	1210.07349	2419.13970	-2.94
dDVDGAQNIGMLGILVk	Unambiguous	1	1	Q3UGR5	N-Term(iTRAQ8plex); K17(iTRAQ8plex)	CID	1.0000	0.0000	1	1	3262146	Unique	Used	2.399	0.648	0.727	0.854	0	0	5.72	0	19	12	2	1183.66089	2366.31450	-2.76
eHINLGcDVDFDIAGPSIR	Unambiguous	3	1	Q60932-2	N-Term(iTRAQ8plex); C7(Methylthio)	CID	1.0000	0.0000	1	1	3353552	Unique	Used	0.873	1.010	1.119	1.320	0	0	5.72	0	5	21	3	807.73022	2421.17612	-3.46

sATTVPSGLGAGEQTQEGLTGk	Unambiguous	2	1	O88737	N-Term(iTRAQ8plex); K22(iTRAQ8plex)	CID	1.0000	0.0000	1	1	3030513	Unique	Used	0.625	1.190	3.067	1.151	0	0	5.72	0	0	103	2	1349.22900	2697.45073	-0.32
gGGTSSGGSSSTNSLTGSRPPk	Unambiguous	1	1	Q78T81	N-Term(iTRAQ8plex); K22(iTRAQ8plex)	CID	1.0000	0.0000	1	1	3426560	Unique	Used	0.509	0.758	1.083	0.851	0	0	5.72	0	0	200	3	863.12036	2587.34653	-2.60
gSHSQSSSPGALTLGR	Unambiguous	3	1	O88935	N-Term(iTRAQ8plex)	HCD	1.0000	0.0000	1	1	3868758	Unique	Used	6.490	3.434	3.278	4.071	0	0	5.72	0	4	59	3	615.99274	1845.96366	-4.56
eVNENFAIDLIAQQPVNEVEHR	Unambiguous	2	1	P52503	N-Term(iTRAQ8plex)	CID	1.0000	0.0000	1	1	3016143	Unique	Used	1.718	1.074	1.460	0.877	0	0	5.72	0	0	3	3	956.83099	2868.47843	-0.35
nMGGPYGGGnYGPGGSGGSGGYGGR	Unambiguous	3	1	O88569	N-Term(iTRAQ8plex); N10(Deamidated)	CID	1.0000	0.0000	1	1	3008693	Unique	Used	1.846	1.229	2.195	2.758	0	0	5.72	0	2	12	3	832.36841	2495.09067	-1.67
qNTLVNNVSSPLPGEGk	Unambiguous	2	1	Q923S9	N-Term(iTRAQ8plex); K17(iTRAQ8plex)	CID	1.0000	0.0000	1	1	3298189	Unique	Used	0.576	0.525	0.775	1.271	0	0	5.71	0	0	200	2	1181.65784	2362.30840	-4.36
eDSAPVTTVAAAGQVQQQQR	Unambiguous	1	1	Q8R4S0	N-Term(iTRAQ8plex)	CID	1.0000	0.0000	1	1	3008492	Unique	Used	1.962	0.576	0.951	0.890	0	0	5.71	0	0	200	2	1194.62244	2388.23760	-1.90
sSGPYGGGGQYFAkPR	Unambiguous	3	1	Q5EBP8	N-Term(iTRAQ8plex); K14(iTRAQ8plex)	HCD	1.0000	0.0000	1	1	3634061	Unique	Used	1.275	0.734	0.988	0.809	0	0	5.71	0	24	10	3	746.39771	2237.17856	-6.17
vADVSISEVTTLLGNVHSPVVEGYVGENISGEVk	Unambiguous	3	1	P20357	N-Term(iTRAQ8plex); K34(iTRAQ8plex)	CID	1.0000	0.0000	1	1	3421845	Unique	Used			2.625		0	0	5.71	0	0	200	3	1369.40649	4106.20493	-1.69
vVGVQEDGSVVDVLESVEcR	Unambiguous	1	1	F7BAB2	N-Term(iTRAQ8plex); C19(Methylthio)	CID	1.0000	0.0000	1	1	2998312	Unique	Used			1.952	0.957	0	0	5.71	0	12	91	2	1234.61865	2468.23003	-0.61
lVTLSATVTELEEDLGTAR	Unambiguous	1	1	B8QI36	N-Term(iTRAQ8plex)	HCD	1.0000	0.0000	1	1	3240112	Unique	Used	1.298	0.957	0.922	0.874	0	0	5.71	0	14	75	2	1161.63867	2322.27007	-0.25
sENVQDLLLLDVTPLSLGIETAGGVMTVLIkR	Unambiguous	2	1	P63017	N-Term(iTRAQ8plex); K31(iTRAQ8plex)	HCD	1.0000	0.0000	1	1	3861483	Unique	Used	0.360	0.989	0.461	0.460	0	0	5.71	1	17	8	3	1335.09949	4003.28391	-3.39
iPNIYAIGDVVAGPmLAHk	Unambiguous	1	1	O08749	N-Term(iTRAQ8plex); M15(Oxidation); K19(iTRAQ8plex)	CID	1.0000	0.0000	1	1	3826246	Unique	Used	1.996	1.423	1.430	1.224	0	0	5.71	0	20	200	3	868.49786	2603.47904	-1.91

vAAGHELQPLAIVDQRPSSR	Unambiguous	1	1	P23242	N-Term(iTRAQ8plex)	HCD	1.0000	0.0000	1	1	3679489	Unique	Used	1.190	2.000	1.655	1.568	0	0	5.71	0	4	63	3	816.78760	2448.34824	-5.84
gGGLTGLLVGTLDVVLDSSAR	Unambiguous	2	1	Q5SVR0-2	N-Term(iTRAQ8plex)	CID	1.0000	0.0000	1	1	3880642	Unique	Used	0.505	0.605	0.654	0.954	0	0	5.71	0	26	72	2	1152.65295	2304.29863	-3.94
gRDPSPGLSSQEAnGQPTk	Unambiguous	4	1	Q8CFT0	N-Term(iTRAQ8plex); N14(Deamidated); K19(iTRAQ8plex)	CID	0.0175	0.0000	1	1	2646821	Unique	Used	1.069	0.351	0.901	0.771	0	0	5.71	1	0	50	3	845.77777	2535.31876	-2.85
dSFSQESSPSSPSDLAk	Unambiguous	2	1	Q9QYX7	N-Term(iTRAQ8plex); K17(iTRAQ8plex)	CID	1.0000	0.0000	1	1	2755657	Unique	Used	2.774	1.451	1.885	1.107	0	0	5.71	0	28	200	2	1189.09521	2377.18315	-6.23
kHLNYVEFTQFLQELQLEHAR	Unambiguous	1	1	Q8BH59	N-Term(iTRAQ8plex); K1(iTRAQ8plex)	HCD	1.0000	0.0000	1	1	3744380	Unique	Used	0.719	1.201	0.983	1.121	0	0	5.71	1	13	89	3	1084.59338	3251.76560	-4.00
qETSVQGGGALAMNDR	Unambiguous	4	1	E9QP59	N-Term(iTRAQ8plex)	CID	1.0000	0.0000	1	1	2825611	Unique	Used	0.728	0.571	1.092	1.095	0	0	5.71	0	0	200	2	969.48425	1937.96123	-2.11
hQADIASYQDAIQQLDSELR	Unambiguous	1	1	P19246	N-Term(iTRAQ8plex)	CID	1.0000	0.0000	1	1	3152362	Unique	Used	2.374	0.863	1.486	0.804	0	0	5.71	0	24	98	3	869.10852	2605.31101	-1.92
eGGSIPVTLTFQEATGk	Unambiguous	1	1	Q9D1A2	N-Term(iTRAQ8plex); K17(iTRAQ8plex)	CID	1.0000	0.0000	1	1	3280921	Unique	Used	0.700	1.181	1.123	1.174	0	0	5.71	0	6	17	2	1172.15173	2343.29619	-2.33
lSQVSDSVSGQTVVDPk	Unambiguous	2	1	Q91YR7	N-Term(iTRAQ8plex); K17(iTRAQ8plex)	CID	1.0000	0.0000	1	1	3167615	Unique	Used	0.572	0.826	0.868	0.830	0	0	5.71	0	0	99	2	1177.65186	2354.29643	-2.53
kDGLSYQIFPDPSDFDR	Unambiguous	1	1	Q9CX60	N-Term(iTRAQ8plex); K1(iTRAQ8plex)	HCD	1.0000	0.0000	1	1	3236211	Unique	Used	1.951	0.624	1.057	1.039	0	0	5.70	1	16	25	3	870.12000	2608.34543	-1.91
gPELSAQEQMEGMLcR	Unambiguous	1	1	Q68FG2	N-Term(iTRAQ8plex); C15(Methylthio)	CID	1.0000	0.0000	1	1	3215475	Unique	Used	0.401	1.499	2.631	1.436	0	0	5.70	0	27	69	2	1064.99304	2128.97881	-0.65
vRPLNTEGSLNLLGLETIR	Unambiguous	8	1	E9PVY0	N-Term(iTRAQ8plex)	HCD	1.0000	0.0000	1	1	3859152	Unique	Used	1.397	0.933	1.089	0.994	0	0	5.70	0	0	82	3	800.46460	2399.37925	-5.50
lNATNIELATVQPGQNFHMFTk	Unambiguous	1	1	Q9Z2U1	N-Term(iTRAQ8plex); K22(iTRAQ8plex)	CID	1.0000	0.0000	1	1	3764024	Unique	Used	1.437	1.114	0.942	0.993	0	0	5.70	0	0	78	3	1028.22205	3082.65158	-2.89

aAADLMAYcEAHAk	Unambiguous	1	1	P63213	N-Term(iTRAQ8plex); C9(Methylthio); K14(iTRAQ8plex)	CID	1.0000	0.0000	1	1	3420580	Unique	Used	0.840	1.336	1.103	1.158	0	0	5.70	0	25	200	3	707.02136	2119.04953	-4.71
eHANIDAQSGSQAPnPSTTISPGk	Unambiguous	3	1	Q8VDQ8-2	N-Term(iTRAQ8plex); N15(Deamidated); K24(iTRAQ8plex)	CID	1.0000	0.0000	1	1	2987886	Unique	Used	1.073	0.777	1.485	2.597	0	0	5.70	0	0	171	3	1006.18756	3016.54813	1.62
ePcGGTGSTGAGGGTnGGVR	Unambiguous	1	1	Q80WM4	N-Term(iTRAQ8plex); C3(Methylthio); N16(Deamidated)	CID	1.0000	0.0000	1	1	2752064	Unique	Used	0.852	0.144	1.016	1.839	0	0	5.70	0	0	200	2	1021.96021	2042.91313	-2.08
lGSSGLGSASSIQAAVR	Unambiguous	4	1	Q7TMY8-3	N-Term(iTRAQ8plex)	CID	1.0000	0.0000	1	1	3855531	Unique	Used	0.674	0.744	1.102	1.131	0	0	5.70	0	12	108	2	933.02002	1865.03276	-3.59
nEEDATELVGLAQAVNAR	Unambiguous	1	1	Q02053	N-Term(iTRAQ8plex); N1(Deamidated)	CID	1.0000	0.0000	1	1	2764685	Unique	Used	0.825	0.965	0.982	1.640	0	0	5.70	0	0	200	2	1103.06860	2205.12993	-0.09
iETMLGDVAVAVHPk	Unambiguous	2	1	Q9Z1Q9	N-Term(iTRAQ8plex); K15(iTRAQ8plex)	CID	1.0000	0.0000	1	1	3417493	Unique	Used	0.683	0.513	0.596	0.673	0	0	5.70	0	21	66	3	730.08765	2188.24839	-6.24
aVAQGnLSSADVQAAk	Unambiguous	1	1	Q9DB77	N-Term(iTRAQ8plex); N6(Deamidated); K16(iTRAQ8plex)	CID	0.0140	0.0000	1	1	2932982	Unique	Used	1.536	2.549	1.015	0.941	0	0	5.70	0	0	185	2	1070.09424	2139.18120	-2.54
eHINLGcDVDFDIAGPSIR	Unambiguous	3	1	Q60932-2	N-Term(iTRAQ8plex); C7(Methylthio)	CID	1.0000	0.0000	1	1	3375976	Unique	Used	0.937	1.250	1.387	1.119	0	0	5.70	0	3	200	3	807.73163	2421.18033	-1.72
tPGAAASGAAAASEDGScGcLPnPGTFEEcHR	Unambiguous	1	1	Q9QYA2	N-Term(iTRAQ8plex); C18(Methylthio); C20(Methylthio); N23(Deamidated); C30(Methylthio)	CID	1.0000	0.0000	1	1	3260178	Unique	Used	1.402	1.000	0.921	1.430	0	0	5.70	0	18	135	3	1159.81763	3477.43833	3.80

tASRPEDTPDSPSGPSSPk	Unambiguous	4	1	Q6EDY6	N-Term(iTRAQ8plex); K19(iTRAQ8plex)	CID	1.0000	0.0000	1	1	2727347	Unique	Used	0.786	0.320	0.669	0.605	0	0	5.70	0	0	89	3	841.10187	2521.29105	-3.19
sAEDLDLLNASFQDESk	Unambiguous	4	1	Q8CHC4	N-Term(iTRAQ8plex); K17(iTRAQ8plex)	CID	1.0000	0.0000	1	1	2740448	Unique	Used	0.829	1.092	1.153	1.012	0	0	5.70	0	0	200	3	830.76495	2490.28031	-0.70
gPPMmGAGYGHGAAR	Unambiguous	3	1	E9Q6C8	N-Term(iTRAQ8plex); M5(Oxidation)	CID	1.0000	0.0000	1	1	3588489	Unique	Used	0.990	1.005	0.954	0.974	0	0	5.70	0	22	200	3	583.95123	1749.83915	-4.33
rDDPNPVFNEAmIFSVPAIVLQDLSLR	Unambiguous	1	1	Q920N7	N-Term(iTRAQ8plex); M12(Oxidation)	HCD	1.0000	0.0000	1	1	3825093	Unique	Used	1.045	0.459	1.045	1.232	0	0	5.70	1	14	89	3	1126.26050	3376.76694	-6.18
lQQVLQmESHIQSTSDR	Unambiguous	1	1	P70168	N-Term(iTRAQ8plex); M7(Oxidation)	CID	1.0000	0.0000	1	1	3494860	Unique	Used	0.723	1.280	0.869	1.155	0	0	5.70	0	5	14	3	774.06494	2320.18027	-2.86
dVQDSVTVSNEIQTTk	Unambiguous	1	1	Q5RKN9	N-Term(iTRAQ8plex); K16(iTRAQ8plex)	CID	1.0000	0.0000	1	1	2809705	Unique	Used	1.124	1.201	1.325	1.804	0	0	5.70	0	0	200	2	1186.64026	2372.27324	-1.40
tWGDAGAAAGGGIPSk	Unambiguous	1	1	P31324	N-Term(iTRAQ8plex); K16(iTRAQ8plex)	CID	1.0000	0.0000	1	1	3297530	Unique	Used	0.425	2.151	1.832	1.500	0	0	5.69	0	20	200	2	1012.55261	2024.09795	-2.09
nAIHTYTQAGSHmAAk	Unambiguous	2	1	P24270	N-Term(iTRAQ8plex); M13(Oxidation); K16(iTRAQ8plex)	CID	1.0000	0.0000	1	1	3487151	Unique	Used	1.410	0.661	0.660	1.025	0	0	5.69	0	0	200	3	775.74304	2325.21457	-3.63
tASEPYHVDNFQDk	Unambiguous	2	1	P38585	N-Term(iTRAQ8plex); K14(iTRAQ8plex)	CID	1.0000	0.0000	1	1	2665791	Unique	Used	0.738	0.780	0.801	0.854	0	0	5.69	0	28	135	3	753.72113	2259.14884	-0.61
iYELAAGGTAVGTGLNTR	Unambiguous	2	1	P97807-2	N-Term(iTRAQ8plex)	CID	1.0000	0.0000	1	1	3743671	Unique	Used	2.185	3.919	2.141	3.117	0	0	5.69	0	0	49	2	1034.56506	2068.12285	-5.43
dGPAGGTGGSGGPGGSLGSR	Unambiguous	3	1	D3YZU1	N-Term(iTRAQ8plex)	CID	1.0000	0.0000	1	1	2928469	Unique	Used	0.175	0.917	1.121	0.772	0	0	5.69	0	0	200	2	952.96875	1904.93022	-3.28
vVAGVAAALAHk	Unambiguous	1	1	A8DUK4	N-Term(iTRAQ8plex); K12(iTRAQ8plex)	HCD	1.0000	0.0000	1	1	3875912	Unique	Used	6.774	0.838	0.294	0.916	0	0	5.69	0	9	192	3	572.36102	1715.06852	-6.02
aALAGGTTMIIDHVVPEPGTSLLAAFDQWR	Unambiguous	1	1	O08553	N-Term(iTRAQ8plex)	HCD	1.0000	0.0000	1	1	3862453	Unique	Used	1.384	1.518	1.336	1.797	0	0	5.69	0	10	111	3	1147.93970	3441.80454	-2.85

dHPLMDDSVTPIDNRPk	Unambiguous	1	1	O09126	N-Term(iTRAQ8plex); K17(iTRAQ8plex)	CID	1.0000	0.0000	1	1	2844844	Unique	Used	0.806	1.093	1.072	1.031	0	0	5.69	0	8	200	3	853.44971	2558.33457	-5.79
qGPTDEAQMAAAAALAR	Unambiguous	1	1	Q99PL6	N-Term(iTRAQ8plex)	CID	1.0000	0.0000	1	1	3211981	Unique	Used	0.634	1.075	1.735	1.447	0	0	5.69	0	2	13	2	988.51031	1976.01335	-2.03
iYELAAGGTAVGTGLNTR	Unambiguous	2	1	P97807-2	N-Term(iTRAQ8plex)	CID	1.0000	0.0000	1	1	3724795	Unique	Used	1.973	1.841	1.302	1.692	0	0	5.69	0	0	114	2	1034.56470	2068.12212	-5.78
sAAQAAAQTnSNAAGk	Unambiguous	4	1	Q9CY58	N-Term(iTRAQ8plex); N10(Deamidated); K16(iTRAQ8plex)	CID	1.0000	0.0000	1	1	2822858	Unique	Used	0.645	0.342	1.077	0.555	0	0	5.69	0	10	27	2	1035.55225	2070.09722	-3.09
lAEMPADSGYPAYLGAR	Unambiguous	2	1	P50516	N-Term(iTRAQ8plex)	CID	1.0000	0.0000	1	1	3302158	Unique	Used	0.547	1.321	0.950	1.058	0	0	5.69	0	0	0	2	1043.53113	2086.05498	-1.52
hDIAFVEFDNEVQAGAAR	Unambiguous	2	1	Q62189	N-Term(iTRAQ8plex)	CID	1.0000	0.0000	1	1	2917910	Unique	Used	1.579	0.627	1.085	1.123	0	0	5.69	0	20	137	3	765.05566	2293.15244	0.39
gAGEGYQPPNIILDIQPGGnHVIEDSHk	Unambiguous	3	1	Q8C167	N-Term(iTRAQ8plex); N20(Deamidated); K28(iTRAQ8plex)	CID	0.0316	0.0000	1	1	3148967	Unique	Used	1.031	0.815	0.765	1.297	0	0	5.69	0	0	150	3	1188.95898	3564.86240	2.25
iHVSDQELQSANASVDDSR	Unambiguous	1	1	Q02053	N-Term(iTRAQ8plex)	CID	1.0000	0.0000	1	1	2650607	Unique	Used	2.572	1.060	1.288	1.129	0	0	5.69	0	18	200	3	792.39587	2375.17307	-0.44
nAGVGSTTNELTADSTPk	Unambiguous	1	1	Q6A037	N-Term(iTRAQ8plex); K18(iTRAQ8plex)	CID	1.0000	0.0000	1	1	2826397	Unique	Used	0.447	0.818	1.225	1.107	0	0	5.69	0	0	200	2	1186.12793	2371.24858	-3.20
aTWGDGGDNSPSNVVSk	Unambiguous	3	1	O09044	N-Term(iTRAQ8plex); K17(iTRAQ8plex)	CID	1.0000	0.0000	1	1	2953387	Unique	Used	0.869	0.662	0.886	0.731	0	0	5.69	0	9	200	2	1150.08923	2299.17119	-2.75
eYSIAGDDSVTEGVTDLVR	Unambiguous	3	1	Q8VCZ6	N-Term(iTRAQ8plex)	CID	1.0000	0.0000	1	1	2833411	Unique	Used	0.619	0.755	0.753	1.297	0	0	5.69	0	16	200	2	1165.58472	2330.16216	-1.89
tDGAAPNVAPSDVGGGGTNR	Unambiguous	1	1	P12960	N-Term(iTRAQ8plex)	CID	1.0000	0.0000	1	1	2844149	Unique	Used	0.921	0.827	0.712	0.943	0	0	5.68	0	7	200	2	1087.53967	2174.07207	-0.90
nMTVEQLLTGSPTSPTVEPEkPTR	Unambiguous	1	1	Q6ZPJ3	N-Term(iTRAQ8plex); K21(iTRAQ8plex)	HCD	1.0000	0.0000	1	1	3369746	Unique	Used	1.085	1.068	0.805	0.866	0	0	5.68	0	0	56	3	1074.24500	3220.72043	-4.35
dSLGDFIEHYAQLGPSSPEQLAAGAEEGGGPR	Unambiguous	1	1	O35295	N-Term(iTRAQ8plex)	HCD	1.0000	0.0000	1	1	3173577	Unique	Used	1.318	0.942	0.893	0.642	0	0	5.68	0	12	81	3	1187.24341	3559.71567	-2.46
gTVISPAELEAPILVPHTAQR	Unambiguous	3	1	Q8C754	N-Term(iTRAQ8plex)	CID	1.0000	0.0000	1	1	3538073	Unique	Used	1.047	1.429	1.082	0.816	0	0	5.68	0	24	21	3	835.14160	2503.41025	-3.36

yTYDYDGDGQLQSVAVNDRPTWR	Unambiguous	1	1	Q9WTS5	N-Term(iTRAQ8plex)	HCD	1.0000	0.0000	1	1	3127538	Unique	Used	0.643	0.846	0.688	1.009	0	0	5.68	0	28	88	3	1008.48474	3023.43967	-1.35
eVAEYYAQSGNSAEk	Unambiguous	2	1	Q80XL6	N-Term(iTRAQ8plex); K15(iTRAQ8plex)	CID	1.0000	0.0000	1	1	2698702	Unique	Used	3.672	1.314	1.446	0.941	0	0	5.68	0	6	200	3	752.05164	2254.14035	-1.97
gFGFVTFENEDVVEk	Unambiguous	3	1	Q920Q6-3	N-Term(iTRAQ8plex); K15(iTRAQ8plex)	CID	1.0000	0.0000	1	1	2940639	Unique	Used	0.857	0.587	0.737	0.690	0	0	5.68	0	30	105	2	1163.11218	2325.21709	-2.26
sNVASTAAcSSASSLGLGLAYR	Unambiguous	1	1	P46660	N-Term(iTRAQ8plex); C9(Methylthio)	CID	1.0000	0.0000	1	1	3937692	Unique	Used	0.948	0.406			0	0	5.68	0	18	200	2	1218.60486	2436.20244	-5.78
gPMGFGFTIADSPGGGGQR	Unambiguous	4	1	Q6RHR9-3	N-Term(iTRAQ8plex)	CID	1.0000	0.0000	1	1	3743149	Unique	Used		1.342	1.227	1.053	0	0	5.68	0	0	200	2	1057.02148	2113.03569	-3.89
nLPMSVTDIVkPGMR	Unambiguous	1	1	Q9D832	N-Term(iTRAQ8plex); K11(iTRAQ8plex)	HCD	1.0000	0.0000	1	1	3839063	Unique	Used	0.366	0.955	0.812	0.918	0	0	5.68	0	3	18	3	756.09808	2266.27970	-3.32
sEDLLDVDTAAGGFQQR	Unambiguous	4	1	Q9DC63	N-Term(iTRAQ8plex)	CID	1.0000	0.0000	1	1	2850208	Unique	Used	0.698	0.832	0.724	1.053	0	0	5.68	0	12	161	2	1063.53491	2126.06255	-2.00
lAASGEGGLQELSGHFENQk	Unambiguous	3	1	Q9QXS6	N-Term(iTRAQ8plex); K20(iTRAQ8plex)	CID	1.0000	0.0000	1	1	3279068	Unique	Used	0.465	1.419	1.174	0.703	0	0	5.68	0	2	22	3	894.14148	2680.40989	-1.96
vSDFGQmASGMNVDAGk	Unambiguous	1	1	P20357	N-Term(iTRAQ8plex); M7(Oxidation); K17(iTRAQ8plex)	CID	1.0000	0.0000	1	1	3298349	Unique	Used	1.221	2.491	2.539	1.001	0	0	5.68	0	0	114	2	1169.58276	2338.15825	-1.96
sLQDIIAILGMDELSEEDkLTVSR	Unambiguous	1	1	P56480	N-Term(iTRAQ8plex); K19(iTRAQ8plex)	HCD	1.0000	0.0000	1	1	3374663	Unique	Used	1.072	1.346	1.011	1.115	0	0	5.67	1	10	200	3	1095.27014	3283.79587	1.29
iYELAAGGTAVGTGLNTR	Unambiguous	2	1	P97807-2	N-Term(iTRAQ8plex)	CID	1.0000	0.0000	1	1	3743639	Unique	Used	2.258	2.814	1.546	1.933	0	0	5.67	0	16	101	2	1034.56726	2068.12724	-3.31
gPGTGGDEASAAGGSPPQPPR	Unambiguous	1	1	P81122	N-Term(iTRAQ8plex)	CID	1.0000	0.0000	1	1	2728497	Unique	Used	1.113		0.687	0.817	0	0	5.67	0	0	200	2	1084.03564	2167.06401	-1.94
gPGGSSLLIEALSNSGPk	Unambiguous	1	1	Q8K0Z7	N-Term(iTRAQ8plex); K18(iTRAQ8plex)	CID	1.0000	0.0000	1	1	3579316	Unique	Used	0.980	0.832	0.852	0.887	0	0	5.67	0	27	172	2	1146.65149	2292.29570	-2.75

iPNIYAIGDVVAGPmLAHk	Unambiguous	1	1	O08749	N-Term(iTRAQ8plex); M15(Oxidation); K19(iTRAQ8plex)	CID	1.0000	0.0000	1	1	3825266	Unique	Used	1.401	0.992	1.291	0.856	0	0	5.67	0	15	200	3	868.49609	2603.47373	-3.95
aMVALIDVFHQYSGR	Unambiguous	1	1	P50114	N-Term(iTRAQ8plex)	CID	1.0000	0.0000	1	1	3937621	Unique	Used	2.301	0.730	1.181	0.773	0	0	5.67	0	0	200	3	671.02686	2011.06601	-3.83
gASSPDMEPSYGGGLFDMVk	Unambiguous	1	1	Q80TZ3	N-Term(iTRAQ8plex); K20(iTRAQ8plex)	CID	1.0000	0.0000	1	1	3151776	Unique	Used	2.200	1.422	1.003	0.976	0	0	5.67	0	2	41	3	885.10736	2653.30753	-0.89
iVIGYQSHADTATk	Unambiguous	1	1	P63073	N-Term(iTRAQ8plex); K14(iTRAQ8plex)	CID	1.0000	0.0000	1	1	3251103	Unique	Used	0.938	1.040	0.911	0.862	0	0	5.66	0	8	20	3	704.73425	2112.18821	-1.30
gEGGTTNPHVFPEGSEPk	Unambiguous	2	1	Q7TQI3	N-Term(iTRAQ8plex); K18(iTRAQ8plex)	CID	1.0000	0.0000	1	1	2648511	Unique	Used	0.477	1.120	0.956	0.941	0	0	5.66	0	0	200	2	1224.62891	2448.25054	-4.52
tNATSPGVnSSASPQATDk	Unambiguous	1	1	Q91Z67	N-Term(iTRAQ8plex); N9(Deamidated); K19(iTRAQ8plex)	CID	1.0000	0.0000	1	1	2728697	Unique	Used	0.775		0.994	0.470	0	0	5.66	0	0	200	2	1221.62671	2442.24614	-4.40
dSSATDGPcAEVLAQQR	Unambiguous	1	1	Q9Z268	N-Term(iTRAQ8plex); C9(Methylthio)	CID	1.0000	0.0000	1	1	2994045	Unique	Used	0.732	2.441	1.204	0.982	0	0	5.66	0	5	15	2	1049.49426	2097.98125	-1.66
qTISNAcGTIGLIHAIANNk	Unambiguous	1	1	Q9JKB1	N-Term(iTRAQ8plex); C7(Methylthio); K20(iTRAQ8plex)	CID	1.0000	0.0000	1	1	3916992	Unique	Used	1.069	0.788	0.612	0.512	0	0	5.66	0	6	26	3	898.49377	2693.46677	-0.74
lSLPPHEGPGGNLVAGALYR	Unambiguous	5	1	E9Q3M3	N-Term(iTRAQ8plex)	HCD	1.0000	0.0000	1	1	3839276	Unique	Used	0.701	0.993	0.885	1.076	0	0	5.66	0	27	200	3	774.76483	2322.27994	-3.14
gLSTTGNSSLnSTSNTk	Unambiguous	1	1	O88532	N-Term(iTRAQ8plex); N11(Deamidated); K17(iTRAQ8plex)	CID	1.0000	0.0000	1	1	2987504	Unique	Used			1.022	0.697	0	0	5.66	0	0	200	2	1139.59778	2278.18828	-4.39
gNDISSGTVLSDYVGSGPPSGTGLHR	Unambiguous	1	1	P70296	N-Term(iTRAQ8plex)	CID	1.0000	0.0000	1	1	3397443	Unique	Used	1.421	0.812	0.961	1.322	0	0	5.66	0	0	200	3	945.47284	2834.40396	-6.47
aIAVLTSGGDAQGMNAAVR	Unambiguous	1	1	P47857	N-Term(iTRAQ8plex)	CID	1.0000	0.0000	1	1	3762654	Unique	Used	2.580	0.818	1.225	1.147	0	0	5.66	0	2	10	2	1053.56372	2106.12016	-3.71

gGNVGInSFGFGGSNVHVILQPNTR	Unambiguous	1	1	P19096	N-Term(iTRAQ8plex); N7(Deamidated)	CID	0.0265	0.0000	1	1	3858012	Unique	Used	1.044	0.580	1.050	1.072	0	0	5.66	0	13	73	3	949.49506	2846.47062	-5.12
vNnASLIGLGYTQTLRPGVk	Unambiguous	3	1	J3QPE8	N-Term(iTRAQ8plex); N3(Deamidated); K20(iTRAQ8plex)	HCD	0.0106	0.0000	1	1	3802793	Unique	Used	0.877	1.188	1.093	1.315	0	0	5.66	0	14	111	3	904.18958	2710.55417	-6.30
eLTGSNLLLQSPGVLTAAR	Unambiguous	1	1	P20934	N-Term(iTRAQ8plex)	HCD	1.0000	0.0000	1	1	3841740	Unique	Used	2.127	0.817	3.898	3.146	0	0	5.66	0	30	124	2	1122.64221	2244.27715	-4.20
aAASGNENIQPPPLAYk	Unambiguous	2	1	E9QKE4	N-Term(iTRAQ8plex); K17(iTRAQ8plex)	CID	1.0000	0.0000	1	1	3254448	Unique	Used	0.660	0.821	0.765	1.044	0	0	5.65	0	0	85	2	1175.15723	2349.30718	2.07
qGSGSSQPMEVqEGYGFGSDDPYSSAEPHVSGMk	Unambiguous	2	1	Q62318	N-Term(iTRAQ8plex); Q12(Deamidated); K34(iTRAQ8plex)	CID	1.0000	0.0000	1	1	2917696	Unique	Used		0.857	0.404	0.274	0	0	5.65	0	0	200	3	1381.29980	4141.88486	-0.10
sIQGSSTSSSASSTLSHGEVk	Unambiguous	1	1	Q3UH60	N-Term(iTRAQ8plex); K21(iTRAQ8plex)	CID	1.0000	0.0000	1	1	3226683	Unique	Used	0.805	0.492	1.271	0.915	0	0	5.65	0	13	100	3	882.46765	2645.38840	1.71
vTGEADVEFATHEDAVAAMSk	Unambiguous	2	1	O35737	N-Term(iTRAQ8plex); K21(iTRAQ8plex)	CID	0.0265	0.0000	1	1	2830931	Unique	Used	0.976	0.913	0.891	0.857	0	0	5.65	0	0	89	3	929.47430	2786.40836	-1.59
aPSmVAAAVTYSkPR	Unambiguous	1	1	Q9CPQ8	N-Term(iTRAQ8plex); M4(Oxidation); K13(iTRAQ8plex)	HCD	1.0000	0.0000	1	1	3928404	Unique	Used	0.573	1.025	1.222	1.375	0	0	5.65	0	6	50	3	725.07745	2173.21781	-3.76
vLNnMEIGTSLYDEEGAk	Unambiguous	1	1	P09411	N-Term(iTRAQ8plex); N4(Deamidated); K18(iTRAQ8plex)	CID	1.0000	0.0000	1	1	2833673	Unique	Used	3.774	1.992	0.738	1.045	0	0	5.65	0	5	72	3	864.78174	2592.33066	-0.66
nLHQSGFSLSGAQIDDNIPR	Unambiguous	1	1	O08553	N-Term(iTRAQ8plex)	CID	1.0000	0.0000	1	1	3411448	Unique	Used	2.569	1.406	1.204	1.110	0	0	5.65	0	2	13	3	825.09290	2473.26413	-3.90
qHAVINYDASMDEHLVk	Unambiguous	3	1	H7BX26	N-Term(iTRAQ8plex); K17(iTRAQ8plex)	CID	1.0000	0.0000	1	1	2990120	Unique	Used	1.205	0.733	1.183	0.919	0	0	5.65	0	23	78	3	860.12219	2578.35202	-0.93

lSLPPHEGPGGNLVAGALYR	Unambiguous	5	1	E9Q3M3	N-Term(iTRAQ8plex)	HCD	1.0000	0.0000	1	1	3821287	Unique	Used	0.515	1.476	0.927	1.002	0	0	5.65	0	4	53	3	774.76434	2322.27848	-3.77
qGIGQAPVSATEDSEIR	Unambiguous	2	1	Q9D0L4	N-Term(iTRAQ8plex)	CID	1.0000	0.0000	1	1	2650154	Unique	Used	1.056	1.043	0.724	1.105	0	0	5.65	0	0	200	2	1031.53577	2062.06426	-3.70
gAAALGIGTDSVILIk	Unambiguous	1	1	P48320	N-Term(iTRAQ8plex); K16(iTRAQ8plex)	CID	1.0000	0.0000	1	1	3622138	Unique	Used	0.267	0.838	0.929	1.088	0	0	5.65	0	9	12	2	1054.14478	2107.28227	-5.91
iGEHTPSALAImENANVLAR	Unambiguous	3	1	A6ZI44	N-Term(iTRAQ8plex); M12(Oxidation)	CID	1.0000	0.0000	1	1	3740663	Unique	Used	0.927	2.310	0.647	1.068	0	0	5.65	0	0	2	3	809.76819	2427.29001	-2.81
vLNNMEIGTSLYDEEGAk	Unambiguous	1	1	P09411	N-Term(iTRAQ8plex); K18(iTRAQ8plex)	CID	1.0000	0.0000	1	1	2959196	Unique	Used	1.147	1.465	1.081	1.250	0	0	5.65	0	6	159	3	864.45428	2591.34830	-0.02
dTNGENIAESLVAEGLATR	Unambiguous	2	1	Q78PY7	N-Term(iTRAQ8plex)	CID	1.0000	0.0000	1	1	2941728	Unique	Used	0.518	0.679	0.667	0.955	0	0	5.65	0	21	200	2	1132.58252	2264.15776	-4.19
gTGGVDTAAVGGVFDVSNADR	Unambiguous	1	1	Q04447	N-Term(iTRAQ8plex)	CID	1.0000	0.0000	1	1	3133835	Unique	Used	1.101	1.052	1.062	1.269	0	0	5.65	0	0	200	2	1135.07129	2269.13530	-0.44
hYDLSYDTGDk	Unambiguous	1	1	P48320	N-Term(iTRAQ8plex); K11(iTRAQ8plex)	CID	1.0000	0.0000	1	1	2631546	Unique	Used	0.569	0.772	0.940	1.466	0	0	5.65	0	16	23	3	641.32825	1921.97019	-2.60
nYGSYSTQASAAAATAELLk	Unambiguous	3	1	E9Q855	N-Term(iTRAQ8plex); K20(iTRAQ8plex)	CID	1.0000	0.0000	1	1	3347444	Unique	Used	0.597	1.054	0.972	0.703	0	0	5.64	0	0	28	2	1313.19995	2625.39263	-2.07
gFGTDEQAIVDVVSNR	Unambiguous	1	1	Q07076	N-Term(iTRAQ8plex)	HCD	1.0000	0.0000	1	1	3110034	Unique	Used	0.981	2.341	1.572	1.455	0	0	5.64	0	6	177	2	1006.02216	2011.03703	-1.40
sScSLPPSLTTSTLASPSPPSSGHSTPR	Unambiguous	3	1	B8QI35	N-Term(iTRAQ8plex); C3(Methylthio)	CID	1.0000	0.0000	1	1	3913227	Unique	Used	1.091	0.567	1.167	0.839	0	0	5.64	0	14	200	3	1030.50659	3089.50522	-4.32
fLSQPFQVAEVFTGHMGk	Unambiguous	1	1	P56480	N-Term(iTRAQ8plex); K18(iTRAQ8plex)	CID	1.0000	0.0000	1	1	3864241	Unique	Used	1.808	0.651	1.063	1.325	0	0	5.64	0	9	200	3	877.80920	2631.41306	-3.17
imETSPTLLQISPGPPk	Unambiguous	1	1	Q8R361	N-Term(iTRAQ8plex); M2(Oxidation); K17(iTRAQ8plex)	CID	1.0000	0.0000	1	1	3641341	Unique	Used			0.911	0.924	0	0	5.64	0	0	47	2	1217.19043	2433.37358	-6.08

vLSVDTALSIqAHPNk	Unambiguous	2	1	Q924M7	N-Term(iTRAQ8plex); Q11(Deamidated); K16(iTRAQ8plex)	CID	1.0000	0.0000	1	1	3575912	Unique	Used	0.584	1.091	1.140	0.955	0	0	5.64	0	26	65	3	768.11487	2302.33005	3.19
gTGGVDTAAVGGVFDVSnADR	Unambiguous	1	1	Q04447	N-Term(iTRAQ8plex); N18(Deamidated)	CID	1.0000	0.0000	1	1	2919307	Unique	Used	1.698	1.466	1.285	1.335	0	0	5.64	0	0	74	2	1135.56006	2270.11284	-3.29
gcITIIGGGDTATccAk	Unambiguous	1	1	P09411	N-Term(iTRAQ8plex); C2(Methylthio); C14(Methylthio); C15(Methylthio); K17(iTRAQ8plex)	CID	1.0000	0.0000	1	1	3706195	Unique	Used	0.347	0.686	1.147	1.559	0	0	5.64	0	0	23	2	1165.54382	2330.08037	-6.96
sLQDIIAILGMDELSEEDkLTVSR	Unambiguous	1	1	P56480	N-Term(iTRAQ8plex); K19(iTRAQ8plex)	HCD	1.0000	0.0000	1	1	3396434	Unique	Used	0.755	0.893	1.311	1.542	0	0	5.64	1	0	7	3	1095.26416	3283.77793	-4.18
iAVSNIVTQPPQPGAIGk	Unambiguous	2	1	E9PWG2	N-Term(iTRAQ8plex); K18(iTRAQ8plex)	CID	1.0000	0.0000	1	1	3781722	Unique	Used	0.443	1.019	0.764	1.074	0	0	5.64	0	0	200	2	1199.71130	2398.41533	-5.23
sTGFSNPTSQSASQRPk	Unambiguous	2	1	Q3UFK1	N-Term(iTRAQ8plex); K17(iTRAQ8plex)	CID	1.0000	0.0000	1	1	3589353	Unique	Used	0.423	0.292	1.662	0.760	0	0	5.64	0	0	79	3	796.75812	2388.25980	-5.44
yYGGGGGGAAGGSPGDk	Unambiguous	2	1	Q68EF6	N-Term(iTRAQ8plex); K19(iTRAQ8plex)	CID	1.0000	0.0000	1	1	2773366	Unique	Used	1.362	1.827	1.621	1.044	0	0	5.64	0	0	200	2	1075.53650	2150.06572	-3.07
iTEGDAVIGVASSGLHSnGFSLVR	Unambiguous	1	1	Q64737	N-Term(iTRAQ8plex); N18(Deamidated)	CID	1.0000	0.0000	1	1	3499596	Unique	Used	1.598	1.016	1.065	1.339	0	0	5.64	0	29	52	3	897.81091	2691.41819	-2.76
gPMGFGFTIADSPGGGGqR	Unambiguous	4	1	Q6RHR9-3	N-Term(iTRAQ8plex); Q18(Deamidated)	CID	1.0000	0.0000	1	1	3743134	Unique	Used	1.528	1.303	1.399	1.180	0	0	5.64	0	27	200	2	1057.52441	2114.04155	6.44
tTQAPLHSVVQQLHGk	Unambiguous	6	1	E9PWV3	N-Term(iTRAQ8plex); K16(iTRAQ8plex)	CID	1.0000	0.0000	1	1	3776907	Unique	Used	0.622	0.769	0.843	1.016	0	0	5.64	0	11	200	3	784.78857	2352.35117	-4.10

lDIETSDVSLEGPEGk	Unambiguous	1	1	E9Q616	N-Term(iTRAQ8plex); K16(iTRAQ8plex)	CID	1.0000	0.0000	1	1	2670552	Unique	Used	1.689	3.786	0.883	0.804	0	0	5.63	0	7	200	2	1149.11682	2297.22637	-3.02
fLSQPFQVAEVFTGHMGk	Unambiguous	1	1	P56480	N-Term(iTRAQ8plex); K18(iTRAQ8plex)	CID	1.0000	0.0000	1	1	3883009	Unique	Used	0.859	0.855	1.015	1.108	0	0	5.63	0	15	200	3	877.80841	2631.41068	-4.08
aVAIETQPAQGESDAVAR	Unambiguous	3	1	Q7TSJ2	N-Term(iTRAQ8plex)	CID	1.0000	0.0000	1	1	2667104	Unique	Used	2.387	1.132	1.613	1.018	0	0	5.63	0	7	200	2	1059.05750	2117.10771	-3.01
aGDIITVLEQHPDGR	Unambiguous	4	1	Q6P9K8	N-Term(iTRAQ8plex)	CID	1.0000	0.0000	1	1	3108825	Unique	Used	0.628	0.733	0.860	1.036	0	0	5.63	0	4	57	3	642.34979	1925.03482	-2.40
nTALETLHQnSITQVSIYEVDk	Unambiguous	1	1	Q9R0Q6	N-Term(iTRAQ8plex); N10(Deamidated); K22(iTRAQ8plex)	CID	0.0142	0.0000	1	1	3149847	Unique	Used	0.943	1.613	1.167	1.053	0	0	5.63	0	0	138	3	1038.22290	3112.65415	-2.61
eAnHDGDFGITLTELR	Unambiguous	1	1	G5E829	N-Term(iTRAQ8plex); N3(Deamidated)	CID	1.0000	0.0000	1	1	2670731	Unique	Used	0.567	1.316	1.723	0.996	0	0	5.63	0	5	79	3	698.35291	2093.04416	-0.56
ySPAYAHLVAPGEEGATGAGPSk	Unambiguous	3	1	O09012	N-Term(iTRAQ8plex); K23(iTRAQ8plex)	CID	1.0000	0.0000	1	1	3068928	Unique	Used	1.874	1.148	1.034	1.007	0	0	5.63	0	0	200	3	946.83234	2838.48246	-2.06
yPNAELAWcQEEHk	Unambiguous	4	1	Q60597	N-Term(iTRAQ8plex); C9(Methylthio); K14(iTRAQ8plex)	CID	1.0000	0.0000	1	1	2993176	Unique	Used	0.527	0.573	0.964	1.073	0	0	5.63	0	5	13	3	791.39050	2372.15696	-2.29
tYFPHFDVSHGSAqVk	Unambiguous	2	1	Q91VB8	N-Term(iTRAQ8plex); Q14(Deamidated); K16(iTRAQ8plex)	CID	1.0000	0.0000	1	1	3387752	Unique	Used	2.214	2.910	0.543	0.950	0	0	5.63	0	9	46	3	810.42383	2429.25693	-5.79
nSLHLLMETLNATTPHYVR	Unambiguous	3	1	D3YZ62	N-Term(iTRAQ8plex)	HCD	1.0000	0.0000	1	1	3916445	Unique	Used	2.163	0.938	0.842	1.039	0	0	5.63	0	19	129	3	838.78265	2514.33341	-4.24
tQVEASEESALNHLQGAGGAEPR	Unambiguous	2	1	J3QP80	N-Term(iTRAQ8plex)	CID	1.0000	0.0000	1	1	2829041	Unique	Used	1.595	0.987	1.652	1.132	0	0	5.63	0	23	114	3	885.78156	2655.33011	0.92

eHSAGScTNFQck	Unambiguous	1	1	P70663	N-Term(iTRAQ8plex); C7(Methylthio); C12(Methylthio); K13(iTRAQ8plex)	CID	1.0000	0.0000	1	1	3339238	Unique	Used	1.159	0.717	0.937	0.839	0	0	5.63	0	19	13	3	704.65521	2111.95108	-3.81
mSDSMDTDPSVLGASk	Unambiguous	2	1	Q9CZT8	N-Term(iTRAQ8plex); K16(iTRAQ8plex)	CID	1.0000	0.0000	1	1	2866316	Unique	Used	0.545	2.078	1.041	1.026	0	0	5.63	0	0	200	2	1125.06067	2249.11406	-4.88
iPNIYAIGDVVAGPmLAHk	Unambiguous	1	1	O08749	N-Term(iTRAQ8plex); M15(Oxidation); K19(iTRAQ8plex)	CID	1.0000	0.0000	1	1	3826663	Unique	Used	1.135	0.952	1.275	1.171	0	0	5.63	0	17	200	3	868.49908	2603.48270	-0.51
nTADSPTGEAAAAPGPSSSAcLDAGR	Unambiguous	2	1	E9Q0V6	N-Term(iTRAQ8plex); C21(Methylthio)	CID	1.0000	0.0000	1	1	2865246	Unique	Used	0.797	1.011	2.411	0.893	0	0	5.63	0	15	200	2	1362.62476	2724.24224	-3.13
tSLSDSTTSAYPGDAGk	Unambiguous	1	1	Q80UJ7	N-Term(iTRAQ8plex); K17(iTRAQ8plex)	CID	1.0000	0.0000	1	1	2845085	Unique	Used	0.291	1.139	0.905	0.947	0	0	5.63	0	0	200	2	1133.57898	2266.15068	-6.73
vAGTQAcATETIDTNR	Unambiguous	2	1	Q8VDL4-3	N-Term(iTRAQ8plex); C7(Methylthio)	CID	1.0000	0.0000	1	1	3069189	Unique	Used	0.973	0.692	0.692	0.961	0	0	5.63	0	5	163	2	1000.98566	2000.96404	-2.15
aPPQTQLPSGASSGTGSASATHGGAPLPGTR	Unambiguous	8	1	Q3UF95	N-Term(iTRAQ8plex)	HCD	1.0000	0.0000	1	1	3756304	Unique	Used	0.587	1.023	0.912	0.936	0	0	5.63	0	0	103	3	1040.86646	3120.58481	-4.12
rAPAFLSAEEVQDHLR	Unambiguous	1	1	O54983	N-Term(iTRAQ8plex)	HCD	1.0000	0.0000	1	1	3341694	Unique	Used	1.444	2.068	1.019	0.852	0	0	5.63	1	4	3	3	715.05389	2143.14713	-4.24
nSSTTAAPLGTTLAVQAVPTAHSIVQATR	Unambiguous	1	1	Q7TT18	N-Term(iTRAQ8plex)	CID	1.0000	0.0000	1	1	3933312	Unique	Used	2.017	0.959	1.390	1.278	0	0	5.63	0	30	200	3	1056.57996	3167.72531	-2.33
kLASQGDSIGSQLGPIHPPPR	Unambiguous	1	1	Q3U0V1	N-Term(iTRAQ8plex); K1(iTRAQ8plex)	CID	1.0000	0.0000	1	1	3698820	Unique	Used	0.858	0.852	1.068	0.730	0	0	5.63	1	0	52	3	921.85468	2763.54947	-8.39
aVAIETQPAQGESDAVAR	Unambiguous	3	1	Q7TSJ2	N-Term(iTRAQ8plex)	CID	1.0000	0.0000	1	1	2666981	Unique	Used	1.947	0.769	1.581	1.388	0	0	5.63	0	0	184	2	1059.05994	2117.11260	-0.71
dENSDYTLFNLIPVGLR	Unambiguous	2	1	P61329-2	N-Term(iTRAQ8plex)	HCD	1.0000	0.0000	1	1	3561183	Unique	Used	1.305	0.789	1.118	1.157	0	0	5.62	0	19	23	2	1135.59668	2270.18608	-4.84
eHINLGcDVDFDIAGPSIR	Unambiguous	3	1	Q60932-2	N-Term(iTRAQ8plex); C7(Methylthio)	CID	1.0000	0.0000	1	1	3353007	Unique	Used		0.994	0.695		0	0	5.62	0	16	23	3	807.73047	2421.17685	-3.16

tVYFAEEVQcEGNSFHk	Unambiguous	1	1	P97315	N-Term(iTRAQ8plex); C10(Methylthio); K17(iTRAQ8plex)	CID	1.0000	0.0000	1	1	3129484	Unique	Used	1.489	0.744	1.625	1.170	0	0	5.62	0	27	87	3	881.43329	2642.28531	0.51
hHAAYVNnLNATEEk	Unambiguous	1	1	P09671	N-Term(iTRAQ8plex); N8(Deamidated); K15(iTRAQ8plex)	CID	1.0000	0.0000	1	1	2773452	Unique	Used	2.187	1.155	1.176	1.091	0	0	5.62	0	15	200	3	774.07471	2320.20957	-2.00
eQVTNVGGAVVTGVTAVAQk	Unambiguous	2	1	O55042	N-Term(iTRAQ8plex); K20(iTRAQ8plex)	CID	1.0000	0.0000	1	1	3646651	Unique	Used	1.964	1.016	0.880	0.978	0	0	5.62	0	20	200	3	846.15143	2536.43973	-6.24
kESLAESAAAYTk	Unambiguous	1	1	Q9CT10	N-Term(iTRAQ8plex); K1(iTRAQ8plex); K13(iTRAQ8plex)	HCD	1.0000	0.0000	1	1	3164962	Unique	Used	1.057	0.756	0.967	0.817	0	0	5.62	1	19	16	3	761.10938	2281.31357	-1.36
eHHLSEVQNmASEEk	Unambiguous	1	1	Q91VA7	N-Term(iTRAQ8plex); M10(Oxidation); K15(iTRAQ8plex)	CID	1.0000	0.0000	1	1	2646321	Unique	Used	0.637	0.599	1.127	1.076	0	0	5.62	0	0	200	3	798.07001	2392.19547	-2.88
aVTNmTLNFGPQHPAAHGVLR	Unambiguous	2	1	Q91WD5	N-Term(iTRAQ8plex); M5(Oxidation)	HCD	1.0000	0.0000	1	1	3909777	Unique	Used	0.824	1.542	0.914	1.131	0	0	5.62	0	0	200	3	851.11664	2551.33536	-5.97
sVEScHNVGLLAHDGQVnEDGQPDLGk	Unambiguous	1	1	Q921H9	N-Term(iTRAQ8plex); C5(Methylthio); N18(Deamidated); K27(iTRAQ8plex)	CID	0.0302	0.0000	1	1	2973270	Unique	Used	1.804	0.605	0.882	0.658	0	0	5.62	0	0	200	3	1158.57471	3473.70957	6.02
iPNIYAIGDVVAGPmLAHk	Unambiguous	1	1	O08749	N-Term(iTRAQ8plex); M15(Oxidation); K19(iTRAQ8plex)	CID	1.0000	0.0000	1	1	3826348	Unique	Used	1.543	1.212	1.567	1.038	0	0	5.62	0	5	200	3	868.49585	2603.47300	-4.23
sTGFSNPTSQSASQRPk	Unambiguous	2	1	Q3UFK1	N-Term(iTRAQ8plex); K17(iTRAQ8plex)	CID	1.0000	0.0000	1	1	3589240	Unique	Used	0.489	0.237	1.731	0.902	0	0	5.62	0	0	200	3	796.75751	2388.25797	-6.21

sLLAAESAASLQGAELER	Unambiguous	1	1	Q3UHK1	N-Term(iTRAQ8plex)	CID	1.0000	0.0000	1	1	3130063	Unique	Used	0.773	1.099	1.025	2.754	0	0	5.62	0	25	63	2	1060.57776	2120.14824	-0.90
qLGDNEAVTQEIVGcAHLENYALk	Unambiguous	2	1	Q9CR26	N-Term(iTRAQ8plex); C15(Methylthio); K24(iTRAQ8plex)	CID	1.0000	0.0000	1	1	3326494	Unique	Used	0.320	0.520	0.876	0.724	0	0	5.62	0	0	25	3	1090.55750	3269.65793	-5.39
tEGRPPNILQTTPQNTGFLR	Unambiguous	4	1	Q8BJL0-2	N-Term(iTRAQ8plex)	HCD	1.0000	0.0000	1	1	3893487	Unique	Used	0.678	1.005	0.869	1.148	0	0	5.62	0	0	87	3	848.79675	2544.37571	-3.13
gVNQATAAVVASTISGk	Unambiguous	1	1	Q8VD75	N-Term(iTRAQ8plex); K17(iTRAQ8plex)	CID	1.0000	0.0000	1	1	3761673	Unique	Used		1.023	0.859	0.833	0	0	5.62	0	0	51	2	1091.63269	2182.25810	-3.26
eHAPSIIFMDEIDSIGSSR	Unambiguous	2	1	P62196	N-Term(iTRAQ8plex)	CID	1.0000	0.0000	1	1	3172307	Unique	Used	1.619	2.611	0.695	0.656	0	0	5.62	0	0	13	3	803.40607	2408.20365	-1.40
lADAQSEAAMEEIEATVR	Unambiguous	1	1	Q8K3A0	N-Term(iTRAQ8plex)	CID	1.0000	0.0000	1	1	2742910	Unique	Used			1.246		0	0	5.61	0	24	200	2	1119.56409	2238.12090	-0.77
aGGGAGGSGGSGPSAILR	Unambiguous	2	1	F7CZ64	N-Term(iTRAQ8plex)	CID	1.0000	0.0000	1	1	3612023	Unique	Used	0.772	2.153	1.437	1.030	0	0	5.61	0	0	148	2	866.96240	1732.91753	-3.99
eTAcTTGVLQTLTLFk	Unambiguous	1	1	Q9Z2W0	N-Term(iTRAQ8plex); C4(Methylthio); K16(iTRAQ8plex)	CID	1.0000	0.0000	1	1	3842531	Unique	Used	1.184	0.839	0.603	0.607	0	0	5.61	0	24	31	2	1190.65918	2380.31108	1.44
kVPGVTAIELDEDTGTFR	Unambiguous	7	1	Q61584	N-Term(iTRAQ8plex); K1(iTRAQ8plex)	HCD	1.0000	0.0000	1	1	3127815	Unique	Used	0.567	0.583	0.610	0.734	0	0	5.61	1	1	6	3	852.80829	2556.41031	-1.06
scSSTPSSGATVDSGQR	Unambiguous	4	1	Q8CJ19	N-Term(iTRAQ8plex); C2(Methylthio)	CID	1.0000	0.0000	1	1	3025603	Unique	Used	0.508	0.568	1.053	1.109	0	0	5.61	0	21	200	2	988.95154	1976.89580	0.13
vPVASPSAHNISSSSGAPDR	Unambiguous	14	1	E9QMP6	N-Term(iTRAQ8plex)	HCD	1.0000	0.0000	1	1	3468479	Unique	Used	0.445	1.162	1.579	1.074	0	0	5.61	0	0	172	3	747.38788	2240.14908	-3.69
qAGEVTFADAHRPk	Unambiguous	2	1	O35326	N-Term(iTRAQ8plex); K14(iTRAQ8plex)	HCD	1.0000	0.0000	1	1	3100163	Unique	Used	1.042	1.201	1.041	1.129	0	0	5.61	0	0	200	3	712.39862	2135.18131	-0.24
qQLLGAEEQmQDMQDk	Unambiguous	6	1	Q3TVA9	N-Term(iTRAQ8plex); M10(Oxidation); K16(iTRAQ8plex)	CID	1.0000	0.0000	1	1	2699871	Unique	Used	1.357	0.986	1.379	1.279	0	0	5.61	0	0	200	3	839.42090	2516.24814	-3.99

aALAGGTTMIIDHVVPEPGTSLLAAFDQWR	Unambiguous	1	1	O08553	N-Term(iTRAQ8plex)	CID	1.0000	0.0000	1	1	3862178	Unique	Used	1.491	1.541	1.449	1.406	0	0	5.61	0	14	84	3	1147.93750	3441.79795	-4.77
dDFLGQVDVPLYPLPTENPR	Unambiguous	1	1	P46935	N-Term(iTRAQ8plex)	HCD	1.0000	0.0000	1	1	3305243	Unique	Used	0.695	1.107	0.956	1.113	0	0	5.61	0	0	19	2	1295.17529	2589.34331	-2.70
eHINLGcDVDFDIAGPSIR	Unambiguous	3	1	Q60932-2	N-Term(iTRAQ8plex); C7(Methylthio)	CID	1.0000	0.0000	1	1	3376411	Unique	Used	0.685	0.954	1.339	2.267	0	0	5.61	0	10	200	3	807.73102	2421.17850	-2.48
ILEVSDDPQVLAVAAHDVGEYVR	Unambiguous	1	1	Q8BVE3	N-Term(iTRAQ8plex)	CID	1.0000	0.0000	1	1	3241054	Unique	Used	0.703	1.315	2.303	1.844	0	0	5.61	0	16	200	3	933.83356	2799.48612	1.07
kEPQPSGPQPSGGAGGGGGk	Unambiguous	1	1	Q8JZQ2	N-Term(iTRAQ8plex); K1(iTRAQ8plex); K20(iTRAQ8plex)	CID	1.0000	0.0000	1	1	2909777	Unique	Used	0.416	1.017	1.153	0.929	0	0	5.61	1	0	200	3	888.15826	2662.46024	-2.78
tALQQSFPQMLHQR	Unambiguous	4	1	Q91VU6	N-Term(iTRAQ8plex)	CID	1.0000	0.0000	1	1	3930417	Unique	Used	0.922	0.849	0.937	1.093	0	0	5.60	0	0	163	3	663.68970	1989.05454	-4.86
vINEPTAAALAYGLDk	Unambiguous	1	1	P38647	N-Term(iTRAQ8plex); K16(iTRAQ8plex)	CID	1.0000	0.0000	1	1	3348831	Unique	Used	1.432	1.196	1.151	1.165	0	0	5.60	0	9	20	2	1127.64221	2254.27715	-5.86
lHLPTAPPPLNEGESLVSR	Unambiguous	1	1	Q8C8R3-2	N-Term(iTRAQ8plex)	HCD	1.0000	0.0000	1	1	3537385	Unique	Used	0.583	1.079	0.995	1.140	0	0	5.60	0	1	6	3	777.76758	2331.28818	-3.98
eHINLGcDVDFDIAGPSIR	Unambiguous	3	1	Q60932-2	N-Term(iTRAQ8plex); C7(Methylthio)	CID	1.0000	0.0000	1	1	3354347	Unique	Used	0.930	1.116	1.486	1.619	0	0	5.60	0	3	74	3	807.73022	2421.17612	-3.46
dYNASASTISPPSSmEEDk	Unambiguous	1	1	P14873	N-Term(iTRAQ8plex); M15(Oxidation); K19(iTRAQ8plex)	CID	1.0000	0.0000	1	1	2632556	Unique	Used	3.311		0.870		0	0	5.60	0	0	200	2	1327.14209	2653.27690	0.35
rPLHSAQAVDVASASSFR	Unambiguous	2	1	Q8VCT3	N-Term(iTRAQ8plex)	CID	1.0000	0.0000	1	1	3816099	Unique	Used	1.014	1.170	0.989	1.339	0	0	5.60	0	7	200	3	735.06592	2203.18320	-2.44
gGGDGNVDDTGNNPSVSLk	Unambiguous	1	1	Q61687	N-Term(iTRAQ8plex); K19(iTRAQ8plex)	CID	1.0000	0.0000	1	1	2632213	Unique	Used		0.829	1.372	0.800	0	0	5.60	0	0	200	2	1206.11633	2411.22539	-0.23
lSTLGSGGESGGDGSPGGAGATAAR	Unambiguous	1	1	Q80UU9	N-Term(iTRAQ8plex)	CID	1.0000	0.0000	1	1	3008661	Unique	Used	0.567	1.281	0.920	0.753	0	0	5.60	0	0	138	2	1197.59119	2394.17510	-2.04
iSSVQSIVPALEIANAHR	Unambiguous	1	1	P63038	N-Term(iTRAQ8plex)	CID	1.0000	0.0000	1	1	3897456	Unique	Used	1.346	1.339	1.038	1.285	0	0	5.60	0	1	8	3	737.08881	2209.25187	-3.99
aGGGAGGSGGSGPSAILR	Unambiguous	2	1	F7CZ64	N-Term(iTRAQ8plex)	CID	1.0000	0.0000	1	1	3591229	Unique	Used	0.979	1.026	1.556	1.380	0	0	5.60	0	4	26	3	578.30994	1732.91526	-5.30

fGGNPGGFGNQGGFGNSR	Unambiguous	1	1	Q921F2	N-Term(iTRAQ8plex)	CID	1.0000	0.0000	1	1	3854711	Unique	Used	0.637	0.812	0.763	0.983	0	0	5.60	0	0	95	2	1015.98718	2030.96709	-3.16
aLTVPELTQQVFDAk	Unambiguous	1	1	P99024	N-Term(iTRAQ8plex); K15(iTRAQ8plex)	CID	1.0000	0.0000	1	1	3399629	Unique	Used	2.103	1.088	1.284	1.760	0	0	5.59	0	12	200	3	756.77222	2268.30210	-1.73
aAVEEGIVLGGGcALLR	Unambiguous	2	1	P63038	N-Term(iTRAQ8plex); C13(Methylthio)	CID	1.0000	0.0000	1	1	3646782	Unique	Used	1.071	0.759	1.110	1.306	0	0	5.59	0	6	200	2	989.53687	1978.06645	-5.23
vSHALAEGLGVIAcIGEk	Unambiguous	2	1	P17751	N-Term(iTRAQ8plex); C14(Methylthio); K18(iTRAQ8plex)	CID	1.0000	0.0000	1	1	3708854	Unique	Used	1.985	1.549	1.392	1.005	0	0	5.59	0	12	200	3	807.78253	2421.33304	-5.13
iGEHTPSALAImENANVLAR	Unambiguous	3	1	A6ZI44	N-Term(iTRAQ8plex); M12(Oxidation)	CID	1.0000	0.0000	1	1	3708909	Unique	Used	1.317	1.466	0.932	0.713	0	0	5.59	0	4	200	3	809.76752	2427.28800	-3.64
iPNIYAIGDVVAGPmLAHk	Unambiguous	1	1	O08749	N-Term(iTRAQ8plex); M15(Oxidation); K19(iTRAQ8plex)	CID	1.0000	0.0000	1	1	3826334	Unique	Used	1.511	0.808	1.500	0.974	0	0	5.59	0	23	200	3	868.49640	2603.47464	-3.60
tLISGMIDEPHAIVVDPLR	Unambiguous	1	1	Q91ZX7	N-Term(iTRAQ8plex)	HCD	1.0000	0.0000	1	1	3499593	Unique	Used	0.784	1.174	0.868	0.997	0	0	5.59	0	5	14	3	794.10992	2380.31522	-2.54
gIEFPMADLDALsPIHTPQR	Unambiguous	6	1	Q5SRX1-2	N-Term(iTRAQ8plex); S13(Phospho)	CID	0.0376	0.0000	1	1	3153383	Unique	Used	0.567	0.864	0.733	0.923	0	0	5.59	0	18	18	3	864.76495	2592.28031	-1.26
iHVSDQELQSANASVDDSR	Unambiguous	1	1	Q02053	N-Term(iTRAQ8plex)	CID	1.0000	0.0000	1	1	2650243	Unique	Used	0.628	0.857	1.108	1.106	0	0	5.59	0	0	78	2	1188.08521	2375.16313	-4.62
gLTIVGSVLEGTFLDNHPQAQR	Unambiguous	2	1	Q91V14	N-Term(iTRAQ8plex)	CID	1.0000	0.0000	1	1	3844370	Unique	Used		1.440	0.541	1.182	0	0	5.59	0	30	200	3	886.14941	2656.43369	-0.91
gFGTSSSLNPAPSAFk	Unambiguous	1	1	E9Q3G8	N-Term(iTRAQ8plex); K16(iTRAQ8plex)	CID	1.0000	0.0000	1	1	3638702	Unique	Used	1.006	0.797	1.021	0.642	0	0	5.59	0	0	76	2	1088.58899	2176.17070	-6.98
gQGFVSEDEYLEISDIkR	Unambiguous	1	1	Q6DFY2	N-Term(iTRAQ8plex); K17(iTRAQ8plex)	CID	1.0000	0.0000	1	1	2918870	Unique	Used	0.839	1.203	1.065	1.018	0	0	5.59	1	12	38	3	898.47852	2693.42099	-1.23
dMATETDDSLASILTETk	Unambiguous	1	1	Q80VL1	N-Term(iTRAQ8plex); K18(iTRAQ8plex)	CID	1.0000	0.0000	1	1	2765652	Unique	Used		0.883	0.744	1.540	0	0	5.59	0	0	200	3	850.44257	2549.31314	0.72

tVYFAEEVQcEGNSFHk	Unambiguous	1	1	P97315	N-Term(iTRAQ8plex); C10(Methylthio); K17(iTRAQ8plex)	CID	1.0000	0.0000	1	1	3090773	Unique	Used	1.596	0.621	1.831	1.071	0	0	5.59	0	2	45	3	881.43268	2642.28348	-0.18
kTQPYDVYDQVEFDVPIGSR	Unambiguous	3	1	Q91WD5	N-Term(iTRAQ8plex); K1(iTRAQ8plex)	HCD	1.0000	0.0000	1	1	3170252	Unique	Used	0.628	1.095	1.259	1.012	0	0	5.59	1	0	36	3	988.85364	2964.54636	-3.38
gHTGIGELAAALNEFDNk	Unambiguous	5	1	Q61301-2	N-Term(iTRAQ8plex); K18(iTRAQ8plex)	CID	1.0000	0.0000	1	1	3260691	Unique	Used	0.822	1.144	0.919	0.933	0	0	5.58	0	5	9	3	822.44604	2465.32358	-0.39
eTLGTGAFSEVVLAEEk	Unambiguous	2	1	Q8BW96	N-Term(iTRAQ8plex); K17(iTRAQ8plex)	CID	1.0000	0.0000	1	1	2903354	Unique	Used	1.094	2.261	2.150	0.926	0	0	5.58	0	3	200	2	1194.65674	2388.30620	-2.39
eHINLGcDVDFDIAGPSIR	Unambiguous	3	1	Q60932-2	N-Term(iTRAQ8plex); C7(Methylthio)	CID	1.0000	0.0000	1	1	3351318	Unique	Used			1.648		0	0	5.58	0	3	9	3	807.73035	2421.17649	-3.31
iGGVQQDTILAEGLHFR	Unambiguous	1	1	O35129	N-Term(iTRAQ8plex)	CID	1.0000	0.0000	1	1	3517744	Unique	Used	0.722	0.755	0.857	1.132	0	0	5.58	0	8	22	3	720.06525	2158.18119	-5.14
tYFPHFDVSHGSAQVk	Unambiguous	2	1	Q91VB8	N-Term(iTRAQ8plex); K16(iTRAQ8plex)	CID	1.0000	0.0000	1	1	3481307	Unique	Used	2.117	1.924	0.631	1.366	0	0	5.58	0	17	200	3	810.09869	2428.28153	-2.25
tQAYPDQkPGTSGLR	Unambiguous	1	1	Q9D0F9	N-Term(iTRAQ8plex); K8(iTRAQ8plex)	HCD	1.0000	0.0000	1	1	3226319	Unique	Used	0.730	1.011	0.731	1.278	0	0	5.58	0	0	188	3	743.07703	2227.21653	-5.66
lVLEVAQHLGESTVR	Unambiguous	1	1	P56480	N-Term(iTRAQ8plex)	HCD	1.0000	0.0000	1	1	3746258	Unique	Used	1.332	1.063	1.110	1.266	0	0	5.58	0	8	195	3	652.37732	1955.11740	-2.75
tPVGFIGLGNMGNPMAk	Unambiguous	1	1	Q99L13	N-Term(iTRAQ8plex); K17(iTRAQ8plex)	CID	1.0000	0.0000	1	1	3878724	Unique	Used	1.068	1.660	1.060	1.229	0	0	5.58	0	28	11	3	771.42462	2312.25931	-5.31
vASVSQNAIVSAAGNIAR	Unambiguous	1	1	Q99P88	N-Term(iTRAQ8plex)	CID	1.0000	0.0000	1	1	3914966	Unique	Used	0.346	1.070	0.821	1.069	0	0	5.58	0	18	72	2	1016.57056	2032.13384	-5.65
gLIQQFTAITGASESVGk	Unambiguous	2	1	G5E8R8	N-Term(iTRAQ8plex); K18(iTRAQ8plex)	CID	1.0000	0.0000	1	1	3745222	Unique	Used	1.013	1.091	0.956	0.801	0	0	5.57	0	27	48	2	1208.18286	2415.35845	-4.95
sPAASAPSLPPAAAVLPSk	Unambiguous	3	1	Q99P72	N-Term(iTRAQ8plex); K19(iTRAQ8plex)	CID	1.0000	0.0000	1	1	3702785	Unique	Used	1.490	1.056	1.189	0.868	0	0	5.57	0	0	200	2	1170.68408	2340.36089	-5.94

cILLSNLSNTSHAPk	Unambiguous	1	1	P19096	N-Term(iTRAQ8plex); C1(Methylthio); K15(iTRAQ8plex)	HCD	1.0000	0.0000	1	1	3914686	Unique	Used	0.714	0.520	0.828	1.131	0	0	5.57	0	24	99	3	751.41376	2252.22672	-3.74
qAEMEGAVQSIQGELSk	Unambiguous	1	1	O54724	N-Term(iTRAQ8plex); K17(iTRAQ8plex)	CID	1.0000	0.0000	1	1	3091841	Unique	Used	0.560	2.150	1.184	1.091	0	0	5.57	0	21	200	2	1207.14563	2413.28398	-0.58
aGADAASGDAPPPALGGR	Unambiguous	1	1	Q3USB7	N-Term(iTRAQ8plex)	CID	1.0000	0.0000	1	1	2806750	Unique	Used	1.418	0.369	1.418	1.295	0	0	5.57	0	3	23	2	927.98175	1854.95622	-2.70
qELQVLHSNLETSAQSEAk	Unambiguous	1	1	Q8VD75	N-Term(iTRAQ8plex); K19(iTRAQ8plex)	CID	1.0000	0.0000	1	1	3010181	Unique	Used	0.853	0.872	1.123	0.977	0	0	5.57	0	26	60	3	907.49426	2720.46823	0.25
vINPDVGSGLSHLLPPAMSAR	Unambiguous	1	1	Q9JIG7	N-Term(iTRAQ8plex)	HCD	1.0000	0.0000	1	1	3897712	Unique	Used	0.704	1.333	0.929	1.009	0	0	5.57	0	23	103	3	812.44885	2435.33200	-2.59
qFYDQALQQAVMDDDANnAk	Unambiguous	1	1	P35762	N-Term(iTRAQ8plex); N18(Deamidated); K20(iTRAQ8plex)	CID	0.0018	0.0000	1	1	2739098	Unique	Used			1.167	1.902	0	0	5.57	0	3	174	3	965.47198	2894.40140	-2.53
aSESAPSGSPSATPLEFDSR	Unambiguous	3	1	Q80XI3-2	N-Term(iTRAQ8plex)	CID	1.0000	0.0000	1	1	2828548	Unique	Used	0.990	1.106	1.259	1.319	0	0	5.57	0	12	114	2	1149.06213	2297.11699	-1.29
sLSAASAPLVETSTPLR	Unambiguous	1	1	Q9QXV0	N-Term(iTRAQ8plex)	CID	1.0000	0.0000	1	1	3618063	Unique	Used	2.264	0.686	1.010	1.595	0	0	5.57	0	0	89	2	1002.56140	2004.11553	-6.19
dRYALTFAEAQEAcR	Unambiguous	1	1	P55066	N-Term(iTRAQ8plex); C14(Methylthio)	HCD	1.0000	0.0000	1	1	3322068	Unique	Used	2.219	1.518	1.129	1.116	0	0	5.57	1	25	78	3	698.67267	2094.00345	-0.76
yGISSMIQSQEkPDR	Unambiguous	2	1	Q922B2	N-Term(iTRAQ8plex); K12(iTRAQ8plex)	HCD	1.0000	0.0000	1	1	3318632	Unique	Used	0.689	0.759	0.799	0.848	0	0	5.57	0	6	51	3	783.08710	2347.24674	-2.95
hIAQLAGNSDLILPVPAFNVINGGSHAGNk	Unambiguous	2	1	P17183	N-Term(iTRAQ8plex); K30(iTRAQ8plex)	CID	1.0000	0.0000	1	1	3878431	Unique	Used	1.131	1.121	0.757	0.807	0	0	5.56	0	0	58	3	1211.67078	3632.99778	-3.98
hGAGDDYFIPSQEFLEAER	Unambiguous	1	1	P70663	N-Term(iTRAQ8plex)	CID	1.0000	0.0000	1	1	2779975	Unique	Used	1.852	0.718	0.947	0.719	0	0	5.56	0	0	21	3	829.06964	2485.19437	0.23
tVAGIIVEPIQSEGGDNHASDDFFR	Unambiguous	2	1	P61922	N-Term(iTRAQ8plex)	CID	1.0000	0.0000	1	1	3036240	Unique	Used	2.728	0.584	1.366	1.336	0	0	5.56	0	2	53	3	993.49689	2978.47611	-1.25
aALAAAGGAGGGGGGAGSR	Unambiguous	1	1	E9Q6B2	N-Term(iTRAQ8plex)	CID	1.0000	0.0000	1	1	3695686	Unique	Used	1.530	0.785	1.235	0.723	0	0	5.56	0	4	49	2	845.44458	1689.88188	-6.87

iIGATDScGDLMFLmk	Unambiguous	1	1	Q61686	N-Term(iTRAQ8plex); C8(Methylthio); M15(Oxidation); K16(iTRAQ8plex)	CID	1.0000	0.0000	1	1	3666085	Unique	Used	0.537	0.400	0.926	0.713	0	0	5.56	0	0	16	2	1193.10547	2385.20366	-4.64
tPYLGDVAIVVHPGk	Unambiguous	1	1	Q9EQF6	N-Term(iTRAQ8plex); K15(iTRAQ8plex)	CID	1.0000	0.0000	1	1	3515776	Unique	Used	0.577	0.523	0.485	0.608	0	0	5.56	0	13	41	3	725.42914	2174.27286	-3.00
vSASSPSVAAASQADSk	Unambiguous	8	1	P06537-3	N-Term(iTRAQ8plex); K17(iTRAQ8plex)	CID	1.0000	0.0000	1	1	2988066	Unique	Used	1.317	1.001	0.804	0.571	0	0	5.56	0	0	200	2	1086.08826	2171.16924	-3.32
gAISAEVYTEEDAASYVR	Unambiguous	2	1	Q9DBC7	N-Term(iTRAQ8plex)	CID	1.0000	0.0000	1	1	2779690	Unique	Used	0.675	0.555	1.042	0.837	0	0	5.56	0	0	200	2	1118.05676	2235.10625	-0.92
vVnGGVPWPSPcPSPSSRPPSR	Unambiguous	2	1	E9QM77	N-Term(iTRAQ8plex); N3(Deamidated); C12(Methylthio)	HCD	1.0000	0.0000	1	1	3933314	Unique	Used	1.085	0.532	1.187	0.862	0	0	5.56	0	8	99	3	871.10474	2611.29966	-2.57
gTGGVDTAAVGGVFDVSNADR	Unambiguous	1	1	Q04447	N-Term(iTRAQ8plex)	CID	1.0000	0.0000	1	1	3150209	Unique	Used	1.467	1.207	1.449	1.184	0	0	5.56	0	0	200	2	1135.07104	2269.13481	-0.66
aVVQVFEGTSGIDAk	Unambiguous	1	1	P62814	N-Term(iTRAQ8plex); K15(iTRAQ8plex)	CID	1.0000	0.0000	1	1	3281205	Unique	Used	0.819	0.885	1.034	0.875	0	0	5.56	0	10	19	3	710.40552	2129.20200	-2.02
gGLHTVAVMNVGAPAAGmNAAVR	Unambiguous	1	1	P47857	N-Term(iTRAQ8plex); M18(Oxidation)	CID	1.0000	0.0000	1	1	3910904	Unique	Used	1.390	1.587	1.650	0.968	0	0	5.56	0	6	95	3	828.77112	2484.29880	-5.23
lAEELPSLTHLNLSGNNLk	Unambiguous	2	1	Q9EST5-2	N-Term(iTRAQ8plex); K19(iTRAQ8plex)	CID	1.0000	0.0000	1	1	3560096	Unique	Used	1.009	1.311	0.725	0.635	0	0	5.56	0	13	55	3	891.17694	2671.51627	-2.88
gTGGVDTAAVGGVFDVSNADR	Unambiguous	1	1	Q04447	N-Term(iTRAQ8plex)	CID	1.0000	0.0000	1	1	3149804	Unique	Used	1.861	1.198	1.602	1.961	0	0	5.56	0	0	166	2	1135.07056	2269.13384	-1.09
yGGISTASVDFEQPTR	Unambiguous	3	1	Q99KG3-3	N-Term(iTRAQ8plex)	HCD	1.0000	0.0000	1	1	3189447	Unique	Used	0.998	0.419	0.924	0.728	0	0	5.56	0	8	44	2	1016.51648	2032.02568	-1.60
qMcGGSETQGPAPSQQGGR	Unambiguous	4	1	Q8C5J7	N-Term(iTRAQ8plex); C3(Methylthio)	CID	1.0000	0.0000	1	1	3227073	Unique	Used	0.547	1.113	1.806	0.962	0	0	5.56	0	0	127	2	1113.50134	2225.99541	-2.24

aTVVnGTGTPGQSPGAGR	Unambiguous	3	1	F8WHY8	N-Term(iTRAQ8plex); N5(Deamidated)	CID	1.0000	0.0000	1	1	3026423	Unique	Used	0.456	0.563	0.758	0.915	0	0	5.55	0	0	200	2	966.50684	1932.00640	-1.29
qTISNAcGTIGLIHAIANNk	Unambiguous	1	1	Q9JKB1	N-Term(iTRAQ8plex); C7(Methylthio); K20(iTRAQ8plex)	CID	1.0000	0.0000	1	1	3935416	Unique	Used	1.065	0.962	1.091	0.948	0	0	5.55	0	26	22	3	898.48981	2693.45487	-5.16
sGLGLGAASASTSGSGPADSVmR	Unambiguous	3	1	Q7TSJ2	N-Term(iTRAQ8plex); M22(Oxidation)	CID	1.0000	0.0000	1	1	3365258	Unique	Used	1.939	1.377	1.310	1.186	0	0	5.55	0	0	57	2	1178.58301	2356.15874	-5.50
vAQTDGVNVEMHLk	Unambiguous	1	1	P23242	N-Term(iTRAQ8plex); K14(iTRAQ8plex)	HCD	1.0000	0.0000	1	1	3145681	Unique	Used	0.681	0.944	1.464	1.909	0	0	5.55	0	0	92	2	1075.09717	2149.18706	-1.18
hVDLLEVAQETDGFSGSDLk	Unambiguous	1	1	Q9D5T0	N-Term(iTRAQ8plex); K20(iTRAQ8plex)	CID	1.0000	0.0000	1	1	2939712	Unique	Used	1.017	1.293	0.966	1.023	0	0	5.55	0	19	49	3	923.48883	2768.45194	-1.59
gTGGVDTAAVGGVFDVSnADR	Unambiguous	1	1	Q04447	N-Term(iTRAQ8plex); N18(Deamidated)	CID	1.0000	0.0000	1	1	2918831	Unique	Used	1.997	1.153	1.009	1.117	0	0	5.55	0	0	200	2	1135.56165	2270.11601	-1.90
fLSQPFQVAEVFTGHMGk	Unambiguous	1	1	P56480	N-Term(iTRAQ8plex); K18(iTRAQ8plex)	CID	1.0000	0.0000	1	1	3884201	Unique	Used		2.099		2.588	0	0	5.55	0	7	200	3	877.80737	2631.40757	-5.26
gFGTDEQAIVDVVSNR	Unambiguous	1	1	Q07076	N-Term(iTRAQ8plex)	CID	1.0000	0.0000	1	1	3091985	Unique	Used	0.787	2.729	1.673	1.714	0	0	5.55	0	11	126	2	1006.02332	2011.03935	-0.25
vNAGDQPGADLGPLITPQAk	Unambiguous	1	1	Q9EQ20	N-Term(iTRAQ8plex); K20(iTRAQ8plex)	CID	1.0000	0.0000	1	1	3279751	Unique	Used	0.331	1.643	1.825	1.867	0	0	5.55	0	0	181	2	1285.72180	2570.43633	-1.39
aLDAEEEAcLHScAGk	Unambiguous	3	1	Q9WV96	N-Term(iTRAQ8plex); C9(Methylthio); C13(Methylthio); K16(iTRAQ8plex)	CID	1.0000	0.0000	1	1	2851628	Unique	Used	0.902	1.103	1.119	1.174	0	0	5.55	0	15	149	3	783.03699	2347.09641	-2.01
gIGTDEATIIDIVTHR	Unambiguous	2	1	P14824	N-Term(iTRAQ8plex)	CID	1.0000	0.0000	1	1	3261463	Unique	Used	0.977	1.162	1.055	1.008	0	0	5.55	0	6	50	3	672.37183	2015.10093	-3.28
vQDDEVGDGTTSVTVLAAELLR	Unambiguous	1	1	P80314	N-Term(iTRAQ8plex)	HCD	1.0000	0.0000	1	1	3112288	Unique	Used	0.625	1.076	0.969	1.250	0	0	5.55	0	0	119	2	1296.68677	2592.36626	-0.32

nGTVMAPDLPEMLDLAGTR	Unambiguous	1	1	P20357	N-Term(iTRAQ8plex); N1(Deamidated)	HCD	1.0000	0.0000	1	1	3262520	Unique	Used	1.824	2.041	1.949	1.084	0	0	5.54	0	0	1	2	1153.58630	2306.16533	-0.93
qIQELVEAIVLPMnHk	Unambiguous	3	1	O88685	N-Term(iTRAQ8plex); N14(Deamidated); K16(iTRAQ8plex)	CID	1.0000	0.0000	1	1	3479782	Unique	Used	0.547	1.071	1.856	1.960	0	0	5.54	0	14	34	3	824.47418	2471.40799	-2.94
iDGDITLGGLFPVHGR	Unambiguous	4	1	G3XA00	N-Term(iTRAQ8plex)	CID	1.0000	0.0000	1	1	3685518	Unique	Used	0.594	0.479	1.277	1.114	0	0	5.54	0	6	35	3	657.69952	1971.08402	-6.37
hVGMAVAGLLADAR	Unambiguous	1	1	O70435	N-Term(iTRAQ8plex)	CID	1.0000	0.0000	1	1	3894731	Unique	Used	0.948	1.220	1.243	1.110	0	0	5.54	0	11	140	3	562.31757	1684.93814	-5.30
gQNLLLTNLQTIQGILER	Unambiguous	2	1	Q7M739	N-Term(iTRAQ8plex)	HCD	1.0000	0.0000	1	1	3918056	Unique	Used	1.775	1.062	1.223	0.822	0	0	5.54	0	12	20	2	1164.67676	2328.34624	-3.90
ILEVSDDPqVLAVAAHDVGEYVR	Unambiguous	1	1	Q8BVE3	N-Term(iTRAQ8plex); Q9(Deamidated)	CID	1.0000	0.0000	1	1	3241858	Unique	Used		1.677			0	0	5.54	0	0	200	3	934.16583	2800.48294	5.64
aVAENQPFLIEAMTYR	Unambiguous	2	1	Q3U3J1	N-Term(iTRAQ8plex)	HCD	1.0000	0.0000	1	1	3458735	Unique	Used	0.659	0.894	0.723	0.916	0	0	5.54	0	12	116	2	1079.06848	2157.12969	-0.91
gANIQLLDLPGIIEGAAQGR	Unambiguous	1	1	Q9QXB9	N-Term(iTRAQ8plex)	CID	1.0000	0.0000	1	1	3806251	Unique	Used		0.457	1.159	1.083	0	0	5.54	0	12	8	2	1155.65417	2310.30107	-3.17
fSGWYDADLSPAGHEEAk	Unambiguous	1	1	Q9DBJ1	N-Term(iTRAQ8plex); K18(iTRAQ8plex)	CID	1.0000	0.0000	1	1	2937297	Unique	Used	2.505	1.264	0.941	0.970	0	0	5.54	0	4	33	3	863.43188	2588.28110	-2.59
vENALNNLDDNPSPGDR	Unambiguous	3	1	A2ALU4	N-Term(iTRAQ8plex)	CID	1.0000	0.0000	1	1	2635579	Unique	Used	2.081	0.895	1.245	0.764	0	0	5.54	0	0	200	2	1072.52820	2144.04912	-1.44
sAASGIPYHSEVPVSLk	Unambiguous	1	1	Q62261	N-Term(iTRAQ8plex); K17(iTRAQ8plex)	CID	1.0000	0.0000	1	1	3387994	Unique	Used	0.753	0.529	1.694	0.944	0	0	5.54	0	0	18	2	1175.65955	2350.31182	-4.64
lGEHNInVLEGNEQFIDAAk	Unambiguous	1	1	Q9Z1R9	N-Term(iTRAQ8plex); N7(Deamidated); K20(iTRAQ8plex)	CID	0.0108	0.0000	1	1	2919233	Unique	Used	1.032	0.900	1.246	0.953	0	0	5.53	0	21	200	3	940.83643	2820.49472	-1.47
yEGSGDGGAAAQSLYIANHAY	Unambiguous	1	1	P05063	N-Term(iTRAQ8plex)	CID	1.0000	0.0000	1	1	2993515	Unique	Used	1.544	0.550	1.203	1.753	0	0	5.53	0	0	200	2	1210.07373	2419.14018	-2.74
iEEGVPQFLVLISSGk	Unambiguous	3	1	J3QQ16	N-Term(iTRAQ8plex); K16(iTRAQ8plex)	CID	1.0000	0.0000	1	1	3645369	Unique	Used	0.439	3.614	0.865	1.253	0	0	5.53	0	28	27	2	1162.68079	2324.35430	-6.16

aGADAASGDAPPPALGGR	Unambiguous	1	1	Q3USB7	N-Term(iTRAQ8plex)	CID	1.0000	0.0000	1	1	2806872	Unique	Used	1.902	0.497	1.377	1.443	0	0	5.53	0	0	180	2	927.98450	1854.96172	0.26
aALAAAGGAGGGGGGAGSR	Unambiguous	1	1	E9Q6B2	N-Term(iTRAQ8plex)	CID	1.0000	0.0000	1	1	3695575	Unique	Used	1.575	0.910	1.432	0.742	0	0	5.53	0	0	200	2	845.44342	1689.87956	-8.24
dSSTPGPGEGIPLSnGGGGSTSR	Unambiguous	5	1	Q91ZS8-5	N-Term(iTRAQ8plex); N15(Deamidated)	CID	1.0000	0.0000	1	1	2698710	Unique	Used			0.510	0.689	0	0	5.53	0	0	200	2	1196.57593	2392.14458	-3.55
tSDVETMSSQSALALDER	Unambiguous	1	1	P14873	N-Term(iTRAQ8plex)	CID	1.0000	0.0000	1	1	2759925	Unique	Used	1.913	0.741	1.345	1.067	0	0	5.53	0	0	87	2	1122.54980	2244.09233	-1.98
qIPGGGSGGGGSGSGGGGGGSGGGR	Unambiguous	2	1	Q8BMA6	N-Term(iTRAQ8plex)	CID	1.0000	0.0000	1	1	3290788	Unique	Used	0.537	0.599	0.636	0.614	0	0	5.53	0	3	108	2	1068.00720	2135.00713	-2.68
rPSAncDPYAVTEAIVR	Unambiguous	1	1	P15105	N-Term(iTRAQ8plex); N5(Deamidated); C6(Methylthio)	HCD	1.0000	0.0000	1	1	3370255	Unique	Used	0.828	0.929	1.304	1.755	0	0	5.53	0	0	7	3	738.36804	2213.08957	-4.57
kSTASPSnNVYSGGSMAR	Unambiguous	1	1	Q8VHJ5	N-Term(iTRAQ8plex); K1(iTRAQ8plex); N8(Deamidated)	HCD	1.0000	0.0000	1	1	3204644	Unique	Used	0.709	0.856	1.362	1.251	0	0	5.53	1	0	200	3	808.41821	2423.24009	-1.84
gSHSQSSSPGALTLGR	Unambiguous	3	1	O88935	N-Term(iTRAQ8plex)	HCD	1.0000	0.0000	1	1	3773845	Unique	Used	0.676	2.027	1.546	1.493	0	0	5.53	0	0	92	3	615.99341	1845.96567	-3.47
lGEHnINVLEGNEQFIDAAk	Unambiguous	1	1	Q9Z1R9	N-Term(iTRAQ8plex); N5(Deamidated); K20(iTRAQ8plex)	CID	0.0181	0.0000	1	1	2919113	Unique	Used	0.906	1.536	0.780	0.778	0	0	5.53	0	0	64	3	940.83716	2820.49692	-0.69
vADGQISTEVSEAPVASDkPk	Unambiguous	1	1	P31648	N-Term(iTRAQ8plex); K19(iTRAQ8plex); K21(iTRAQ8plex)	CID	1.0000	0.0000	1	1	2847117	Unique	Used	0.663	1.107	0.997	1.367	0	0	5.52	0	17	200	3	1014.23267	3040.68344	-3.15
sITDIINIGIGGSDLGPLMVTEALkPYSk	Unambiguous	1	1	P06745	N-Term(iTRAQ8plex); K25(iTRAQ8plex); K29(iTRAQ8plex)	CID	1.0000	0.0000	1	1	3806310	Unique	Used	1.637	1.442	1.389	1.697	0	0	5.52	0	0	7	3	1305.74585	3915.22300	-1.30
iLQVAQELSGEDmHQFHR	Unambiguous	1	1	Q9QZE7	N-Term(iTRAQ8plex); M13(Oxidation)	CID	1.0000	0.0000	1	1	3437533	Unique	Used	0.921	0.924	0.900	0.995	0	0	5.52	0	21	151	3	820.08276	2458.23374	-4.63

sVAGEIVLITGAGHGIGR	Unambiguous	2	1	Q9EQ06	N-Term(iTRAQ8plex)	CID	1.0000	0.0000	1	1	3782954	Unique	Used	0.877	1.289	1.250	1.296	0	0	5.52	0	19	59	3	671.05658	2011.15519	-2.52
dTVDSAGTSPTAVLAAGEDAGAGRPGAGTPLR	Unambiguous	2	1	Q9DBS2	N-Term(iTRAQ8plex)	HCD	1.0000	0.0000	1	1	3147997	Unique	Used	0.958	2.200	1.774	2.177	0	0	5.52	0	9	2	3	1081.55322	3242.64511	-3.24
eHQQALDILDMEEPINR	Unambiguous	1	1	Q8R349	N-Term(iTRAQ8plex)	CID	1.0000	0.0000	1	1	2779645	Unique	Used	1.568	2.098	0.640	0.761	0	0	5.52	0	2	129	3	785.73456	2355.18912	-1.09
nIINSSEGSSSGSGHk	Unambiguous	1	1	Q8C739	N-Term(iTRAQ8plex); K16(iTRAQ8plex)	CID	1.0000	0.0000	1	1	2966893	Unique	Used	1.452	0.772	1.664	1.150	0	0	5.52	0	0	200	3	723.71503	2169.13053	-2.35
gTGGVDTAAVGGVFDVSNADR	Unambiguous	1	1	Q04447	N-Term(iTRAQ8plex)	CID	1.0000	0.0000	1	1	3135050	Unique	Used	1.495	0.807	0.951	1.106	0	0	5.52	0	0	200	2	1135.07019	2269.13310	-1.41
fQLTDSQIYEVLSVIR	Unambiguous	1	1	O08553	N-Term(iTRAQ8plex)	HCD	1.0000	0.0000	1	1	3843947	Unique	Used	1.377	1.950	1.172	1.399	0	0	5.52	0	0	200	2	1108.11353	2215.21977	-3.55
sSSEAGDGSGVGAVGGIPSDSR	Unambiguous	3	1	P55144-3	N-Term(iTRAQ8plex)	CID	1.0000	0.0000	1	1	2649471	Unique	Used	1.981	1.875	1.033	0.730	0	0	5.52	0	0	200	2	1127.04626	2253.08525	-1.99
aTWGDGGDNSPSNVVSk	Unambiguous	3	1	O09044	N-Term(iTRAQ8plex); K17(iTRAQ8plex)	CID	1.0000	0.0000	1	1	2971488	Unique	Used	0.882	0.737	0.717	0.775	0	0	5.52	0	0	200	2	1150.09045	2299.17363	-1.69
nFAEkAPSMVAAAVTYSkPR	Unambiguous	1	1	Q9CPQ8	N-Term(iTRAQ8plex); K5(iTRAQ8plex); K18(iTRAQ8plex)	HCD	1.0000	0.0000	1	1	3875477	Unique	Used	0.336	0.739	0.816	0.901	0	0	5.52	1	0	200	3	1017.57452	3050.70902	-4.41
hINnNLSPSLGDAQEEk	Unambiguous	2	1	Q14CH0-2	N-Term(iTRAQ8plex); N4(Deamidated); K17(iTRAQ8plex)	CID	0.0290	0.0000	1	1	2666552	Unique	Used	1.598	1.146	1.391	1.000	0	0	5.52	0	0	200	3	825.76709	2475.28672	-2.78
fQDLGAAYEVLSDSEk	Unambiguous	1	1	Q99KV1	N-Term(iTRAQ8plex); K16(iTRAQ8plex)	CID	1.0000	0.0000	1	1	2978931	Unique	Used	0.774	0.674	0.775	0.726	0	0	5.52	0	4	11	2	1190.62659	2380.24590	-1.42
eTVTQRPGATVPTDFATFPSSAFLR	Unambiguous	5	1	E9Q3M3	N-Term(iTRAQ8plex)	HCD	1.0000	0.0000	1	1	3858985	Unique	Used	0.581	0.794	0.972	1.127	0	0	5.52	0	2	20	3	1000.85748	3000.55790	-5.15
fLSQPFqVAEVFTGHMGk	Unambiguous	1	1	P56480	N-Term(iTRAQ8plex); Q7(Deamidated); K18(iTRAQ8plex)	CID	0.0054	0.0000	1	1	3883296	Unique	Used	2.769	2.007	0.635	0.909	0	0	5.51	0	18	200	3	878.14496	2632.42032	5.66

aSSATDGQLASPGEnGEVRPGQAk	Unambiguous	1	1	P16054	N-Term(iTRAQ8plex); N15(Deamidated); K24(iTRAQ8plex)	CID	1.0000	0.0000	1	1	2696920	Unique	Used	0.462	1.784	1.687	1.043	0	0	5.51	0	0	200	3	979.51038	2936.51657	-0.17
aTSETLAADPTPAATIVHFk	Unambiguous	1	1	E9Q0S6	N-Term(iTRAQ8plex); K20(iTRAQ8plex)	CID	1.0000	0.0000	1	1	3321529	Unique	Used	0.908	1.523	1.121	1.487	0	0	5.51	0	0	58	3	883.82617	2649.46396	-2.60
yEGSGDGGAAAQSLYIAnHAY	Unambiguous	1	1	P05063	N-Term(iTRAQ8plex); N18(Deamidated)	CID	1.0000	0.0000	1	1	2760332	Unique	Used	2.571	1.105	1.037	1.508	0	0	5.51	0	14	200	2	1210.56519	2420.12309	-3.20
sIEALLEAGQAQDTQASHAEANQQQTR	Unambiguous	2	1	Q99PL5	N-Term(iTRAQ8plex)	CID	1.0000	0.0000	1	1	2993910	Unique	Used	1.724	0.850	1.192	0.564	0	0	5.51	0	2	48	3	1067.19849	3199.58091	-2.28
eALLGVQEDVDEYVk	Unambiguous	1	1	Q8BP92	N-Term(iTRAQ8plex); K15(iTRAQ8plex)	CID	1.0000	0.0000	1	1	2639220	Unique	Used	3.188	0.960	0.954	0.569	0	0	5.51	0	10	200	3	772.42383	2315.25693	-0.95
lQQELDDLTVDLDHQR	Unambiguous	3	1	Q5SV64	N-Term(iTRAQ8plex)	CID	1.0000	0.0000	1	1	2853147	Unique	Used	1.583	0.971	1.065	0.739	0	0	5.51	0	11	200	3	748.05640	2242.15464	-3.17
eGSYSISVLYGEEEVPR	Unambiguous	3	1	B7FAU9	N-Term(iTRAQ8plex)	CID	1.0000	0.0000	1	1	2796447	Unique	Used		4.701	0.468	0.765	0	0	5.51	0	0	200	2	1109.56055	2218.11382	-1.95
gDEEGIPAVVIDMSGLR	Unambiguous	1	1	Q61233	N-Term(iTRAQ8plex)	HCD	1.0000	0.0000	1	1	3057171	Unique	Used	2.821	1.073	0.776	0.963	0	0	5.51	0	24	134	2	1031.54224	2062.07720	-1.02
lTGVSISQVNHNkPLR	Unambiguous	2	1	O08917	N-Term(iTRAQ8plex); K13(iTRAQ8plex)	HCD	1.0000	0.0000	1	1	3890423	Unique	Used	0.790	1.184	0.983	0.916	0	0	5.51	0	16	200	3	791.13574	2371.39267	-4.37
vEAVNMAEGIIHDTETk	Unambiguous	1	1	P38647	N-Term(iTRAQ8plex); K17(iTRAQ8plex)	CID	1.0000	0.0000	1	1	2960054	Unique	Used	1.660	0.922	1.400	1.438	0	0	5.51	0	26	200	3	822.44031	2465.30637	-4.18
gTGGVDTAAVGGVFDVSNADR	Unambiguous	1	1	Q04447	N-Term(iTRAQ8plex)	CID	1.0000	0.0000	1	1	3135574	Unique	Used	1.468	0.921	1.194	1.308	0	0	5.51	0	0	200	2	1135.07031	2269.13335	-1.30
tSPAGGTWSSVVSGVPR	Unambiguous	5	1	E9QM77	N-Term(iTRAQ8plex)	CID	1.0000	0.0000	1	1	3874920	Unique	Used	1.476	0.286	0.911	0.641	0	0	5.51	0	2	20	2	975.01831	1949.02934	-5.19
tFTTQETITNAETAk	Unambiguous	1	1	P06745	N-Term(iTRAQ8plex); K15(iTRAQ8plex)	CID	1.0000	0.0000	1	1	2934296	Unique	Used	1.141	1.017	1.309	1.412	0	0	5.51	0	0	20	2	1132.61047	2264.21367	-4.14

lYLDELEGSGNSGASck	Unambiguous	3	1	Q8BH60	N-Term(iTRAQ8plex); C16(Methylthio); K17(iTRAQ8plex)	CID	1.0000	0.0000	1	1	2976032	Unique	Used	0.968	0.652	0.958	0.648	0	0	5.51	0	0	200	2	1199.09631	2397.18535	-1.39
sTAAmTSTVLTNTAPSk	Unambiguous	1	1	Q8VCF0	N-Term(iTRAQ8plex); M5(Oxidation); K17(iTRAQ8plex)	CID	1.0000	0.0000	1	1	3634863	Unique	Used	0.643	0.839	0.604	0.979	0	0	5.51	0	0	97	2	1153.12366	2305.24004	-5.62
sGLLSVSSGAAAHR	Unambiguous	1	1	P97927	N-Term(iTRAQ8plex)	CID	1.0000	0.0000	1	1	3851953	Unique	Used	1.120	1.013	0.920	1.029	0	0	5.51	0	22	183	3	539.63702	1616.89652	-3.52
vAAGHELQPLAIVDQRPSSR	Unambiguous	1	1	P23242	N-Term(iTRAQ8plex)	HCD	1.0000	0.0000	1	1	3699790	Unique	Used	0.975	1.158	1.523	1.837	0	0	5.51	0	4	19	3	816.78729	2448.34732	-6.22
gTGGVDTAAVGGVFDVSNADR	Unambiguous	1	1	Q04447	N-Term(iTRAQ8plex)	CID	1.0000	0.0000	1	1	3137342	Unique	Used	1.158	1.365	3.296	1.108	0	0	5.51	0	16	200	2	1135.07251	2269.13774	0.64
vDcAVLDcEDGVAENk	Unambiguous	1	1	Q8R4N0	N-Term(iTRAQ8plex); C3(Methylthio); C8(Methylthio); K16(iTRAQ8plex)	CID	1.0000	0.0000	1	1	2765295	Unique	Used		1.301	0.943	1.041	0	0	5.50	0	10	200	3	794.04077	2380.10776	-1.52
qADVPAAVTDAAATTPAAEDAATk	Unambiguous	1	1	P06837	N-Term(iTRAQ8plex); K24(iTRAQ8plex)	CID	1.0000	0.0000	1	1	2812047	Unique	Used			0.802	1.180	0	0	5.50	0	19	200	2	1432.75647	2864.50566	-1.47
iGGHGAEYGAEALER	Unambiguous	2	1	Q91VB8	N-Term(iTRAQ8plex)	CID	1.0000	0.0000	1	1	2786035	Unique	Used				0.748	0	0	5.50	0	4	200	3	611.98456	1833.93912	-0.33
dGFTSEGEILEISDIQR	Unambiguous	1	1	Q8HW98	N-Term(iTRAQ8plex)	CID	1.0000	0.0000	1	1	2672683	Unique	Used	1.993		0.896	0.719	0	0	5.50	0	0	200	2	1107.06372	2213.12016	-1.72
tVEEAENIVVTTGVVR	Unambiguous	1	1	Q9Z0F7	N-Term(iTRAQ8plex)	HCD	1.0000	0.0000	1	1	3131334	Unique	Used	4.271	0.528	1.287	1.889	0	0	5.50	0	13	149	2	1010.56317	2020.11907	-1.87
aVGTPGANAGGAGPGISAmSR	Unambiguous	2	1	Q7TSC1	N-Term(iTRAQ8plex); M19(Oxidation)	CID	1.0000	0.0000	1	1	3696664	Unique	Used	1.102	0.532	1.402	0.727	0	0	5.50	0	0	200	2	1060.03955	2119.07182	-7.09
aYSGSDLPSGTGSGGGGADGAR	Unambiguous	1	1	Q71FD5	N-Term(iTRAQ8plex)	CID	1.0000	0.0000	1	1	2774338	Unique	Used	2.075	0.669	1.307	0.816	0	0	5.50	0	8	200	2	1101.02051	2201.03374	-1.62
eVFTEQADLSGITETk	Unambiguous	1	1	P07759	N-Term(iTRAQ8plex); K16(iTRAQ8plex)	CID	1.0000	0.0000	1	1	2868164	Unique	Used	5.439	3.250	0.060	2.474	0	0	5.50	0	0	67	2	1188.63245	2376.25762	-7.53
qASHAQLGDAYDQEIR	Unambiguous	2	1	P08553	N-Term(iTRAQ8plex)	CID	1.0000	0.0000	1	1	2664918	Unique	Used	3.269	0.891	1.637	1.036	0	0	5.50	0	3	28	3	702.68549	2106.04190	-4.69

mVMIQDGPLPTGADkPLR	Unambiguous	2	1	A2AJ72	N-Term(iTRAQ8plex); M1(Oxidation); K15(iTRAQ8plex)	HCD	1.0000	0.0000	1	1	3534234	Unique	Used	1.381	0.875	0.707	0.885	0	0	5.50	0	15	65	3	855.14178	2563.41080	-3.48
kLIAEGPGETVLVAEEEAAR	Unambiguous	1	1	Q9CY73	N-Term(iTRAQ8plex); K1(iTRAQ8plex)	CID	1.0000	0.0000	1	1	2813337	Unique	Used	0.917	0.779	1.052	1.069	0	0	5.50	1	16	200	3	897.50848	2690.51090	-2.85
vIDIFDTDGnGEVDFk	Unambiguous	2	1	Q63810-2	N-Term(iTRAQ8plex); N10(Deamidated); K16(iTRAQ8plex)	CID	1.0000	0.0000	1	1	2766338	Unique	Used	0.971	1.491	2.332	0.724	0	0	5.49	0	16	200	2	1197.11719	2393.22710	-2.60
qSLSSADNLEPDVQGHQVAAR	Unambiguous	2	1	A2A5R2	N-Term(iTRAQ8plex)	CID	1.0000	0.0000	1	1	2880747	Unique	Used	1.572	1.129	1.194	1.494	0	0	5.49	0	11	200	3	842.76343	2526.27573	-3.70
nFAEkAPSMVAAAVTYSkPR	Unambiguous	1	1	Q9CPQ8	N-Term(iTRAQ8plex); K5(iTRAQ8plex); K18(iTRAQ8plex)	CID	1.0000	0.0000	1	1	3875515	Unique	Used	0.361	0.656	0.935	0.879	0	0	5.49	1	8	167	3	1017.57483	3050.70993	-4.11
dFLSNETSLGAIGSSLPSR	Unambiguous	1	1	Q6ZPS6	N-Term(iTRAQ8plex)	CID	1.0000	0.0000	1	1	3559339	Unique	Used	0.642	1.039	0.787	0.983	0	0	5.49	0	0	146	2	1128.09082	2255.17436	-3.46
gNAGQTNYGFANSTmER	Unambiguous	1	1	P19096	N-Term(iTRAQ8plex); M15(Oxidation)	CID	1.0000	0.0000	1	1	3120513	Unique	Used	0.575	0.507	0.752	0.887	0	0	5.49	0	0	180	2	1069.49683	2137.98638	-0.52
iVYGHLDDPANQEIER	Unambiguous	1	1	Q99KI0	N-Term(iTRAQ8plex)	CID	1.0000	0.0000	1	1	2757945	Unique	Used	0.552	0.650	1.205	1.400	0	0	5.49	0	0	13	3	725.04358	2173.11618	-1.37
vLHYLAMQkPADLAR	Unambiguous	1	1	Q80UJ7	N-Term(iTRAQ8plex); K9(iTRAQ8plex)	HCD	1.0000	0.0000	1	1	3894309	Unique	Used	0.550	1.111	0.779	0.842	0	0	5.49	0	28	47	3	778.78766	2334.34842	-3.96
hLEVSSASMAEDLcR	Unambiguous	3	1	A2AEW8	N-Term(iTRAQ8plex); C14(Methylthio)	CID	1.0000	0.0000	1	1	3234310	Unique	Used	2.395	1.538	1.237	1.706	0	0	5.49	0	28	38	3	666.65082	1997.93790	-0.89
iSSVQSIVPALEIAnAHR	Unambiguous	1	1	P63038	N-Term(iTRAQ8plex); N15(Deamidated)	CID	1.0000	0.0000	1	1	3685868	Unique	Used	2.370	0.951	1.124	1.207	0	0	5.49	0	0	4	3	737.41486	2210.23001	-6.64
eTQAPAYLIDLMHDk	Unambiguous	2	1	P70188-2	N-Term(iTRAQ8plex); K15(iTRAQ8plex)	CID	1.0000	0.0000	1	1	3130536	Unique	Used	0.473	1.039	0.849	0.699	0	0	5.49	0	7	30	3	785.09314	2353.26487	-1.43

vQLVGLDEESSEFIcR	Unambiguous	1	1	P61963	N-Term(iTRAQ8plex); C15(Methylthio)	CID	1.0000	0.0000	1	1	3217198	Unique	Used	1.382	1.023	0.491	0.883	0	0	5.49	0	10	25	2	1087.54114	2174.07500	-1.18
aGIEDIETLENLSLDQHSk	Unambiguous	1	1	B9EJ80	N-Term(iTRAQ8plex); K19(iTRAQ8plex)	CID	1.0000	0.0000	1	1	2886481	Unique	Used	1.974		1.066	1.224	0	0	5.49	0	15	200	3	907.48743	2720.44773	-3.16
amVALIDVFHQYSGR	Unambiguous	1	1	P50114	N-Term(iTRAQ8plex); M2(Oxidation)	HCD	1.0000	0.0000	1	1	3935133	Unique	Used	3.957	0.630	1.899	1.303	0	0	5.49	0	0	34	3	676.35803	2027.05954	-4.49
sTNVVYQAHHVSR	Unambiguous	1	1	O88428	N-Term(iTRAQ8plex)	CID	1.0000	0.0000	1	1	3526903	Unique	Used	1.309	0.997	1.110	1.059	0	0	5.49	0	0	200	3	601.32251	1801.95298	-4.50
aSVASEVSTTSSTSkPPTGR	Unambiguous	2	1	H7BX26	N-Term(iTRAQ8plex); K15(iTRAQ8plex)	HCD	1.0000	0.0000	1	1	3315331	Unique	Used	0.331	0.390	1.114	0.833	0	0	5.49	0	0	26	3	853.46503	2558.38053	-3.02
yFAGTMAEETAPAVLER	Unambiguous	1	1	Q64374	N-Term(iTRAQ8plex)	CID	1.0000	0.0000	1	1	3073501	Unique	Used	3.500	0.877	4.294	9.003	0	0	5.49	0	0	77	2	1080.54871	2160.09014	-2.22
eAPcILIYIPDGHTk	Unambiguous	1	1	Q8CGC7	N-Term(iTRAQ8plex); C4(Methylthio); K15(iTRAQ8plex)	CID	1.0000	0.0000	1	1	3393916	Unique	Used	0.374	0.679	0.855	0.810	0	0	5.49	0	4	15	3	775.42126	2324.24924	-4.76
vNLEESTGVENSPAGARPk	Unambiguous	3	1	Q8BRF7-3	N-Term(iTRAQ8plex); K19(iTRAQ8plex)	CID	1.0000	0.0000	1	1	2970334	Unique	Used	0.494	0.841	0.825	1.020	0	0	5.48	0	7	60	3	855.13361	2563.38626	-2.89
nQGGYGGSSSSSSYGSGR	Unambiguous	3	1	Q5EBP8	N-Term(iTRAQ8plex)	CID	1.0000	0.0000	1	1	2787442	Unique	Used	1.723	0.242	1.018	0.454	0	0	5.48	0	0	200	2	999.95331	1998.89934	-3.08
sAAMLGNSEDHTALSR	Unambiguous	1	1	Q9CWK8	N-Term(iTRAQ8plex)	CID	1.0000	0.0000	1	1	3046982	Unique	Used	1.158	0.880	1.439	1.122	0	0	5.48	0	25	21	3	655.33069	1963.97751	-1.75
vVnGGVPWPSPcPSPSSRPPSR	Unambiguous	2	1	E9QM77	N-Term(iTRAQ8plex); N3(Deamidated); C12(Methylthio)	HCD	1.0000	0.0000	1	1	3933194	Unique	Used	1.042	0.373	0.990	0.772	0	0	5.48	0	1	27	3	871.10358	2611.29618	-3.90
smLQATAEANNLAAAASAk	Unambiguous	3	1	Q91YH5	N-Term(iTRAQ8plex); M2(Oxidation); K19(iTRAQ8plex)	CID	1.0000	0.0000	1	1	3494352	Unique	Used	0.434	0.989	1.116	1.139	0	0	5.48	0	13	89	2	1229.16370	2457.32012	-1.10
aALAGGTTMIIDHVVPEPGTSLLAAFDQWR	Unambiguous	1	1	O08553	N-Term(iTRAQ8plex)	HCD	1.0000	0.0000	1	1	3863127	Unique	Used	1.286	1.279	1.148	1.451	0	0	5.48	0	7	170	3	1147.93921	3441.80307	-3.28

yGGEEEDQPIYLAVk	Unambiguous	1	1	Q9CQ45	N-Term(iTRAQ8plex); K15(iTRAQ8plex)	CID	1.0000	0.0000	1	1	2737762	Unique	Used	1.326	1.350	1.046	0.751	0	0	5.48	0	4	200	2	1160.11719	2319.22710	-2.50
gGGGGGGGPGGFQPAPR	Unambiguous	1	1	O35295	N-Term(iTRAQ8plex)	CID	1.0000	0.0000	1	1	3382600	Unique	Used	1.034	0.708	0.963	0.802	0	0	5.48	0	0	200	2	872.44214	1743.87700	-3.40
vDcAVLDcEDGVAENk	Unambiguous	1	1	Q8R4N0	N-Term(iTRAQ8plex); C3(Methylthio); C8(Methylthio); K16(iTRAQ8plex)	CID	1.0000	0.0000	1	1	2782360	Unique	Used	1.251	0.860	1.135	1.498	0	0	5.48	0	9	200	3	794.04089	2380.10813	-1.36
lHQYDGSIVVIQNPAR	Unambiguous	1	1	Q8R464	N-Term(iTRAQ8plex)	CID	1.0000	0.0000	1	1	3637701	Unique	Used	1.207	0.743	0.656	2.455	0	0	5.48	0	10	6	3	705.38959	2114.15421	-5.59
hATALEELSEQLEQAk	Unambiguous	3	1	Q5SV64	N-Term(iTRAQ8plex); K16(iTRAQ8plex)	CID	1.0000	0.0000	1	1	2939046	Unique	Used	1.499	0.660	2.274	1.084	0	0	5.48	0	0	200	2	1203.15515	2405.30303	-4.26
lATPTYGDLNHLVSATMSGVTTSLR	Unambiguous	1	1	Q9ERD7	N-Term(iTRAQ8plex)	CID	1.0000	0.0000	1	1	3938055	Unique	Used	0.787	0.935	0.893	1.060	0	0	5.48	0	2	200	3	970.51300	2909.52445	-3.45
rLSLESEGANEGATAAPELSALEEAFR	Unambiguous	1	1	Q7TQD2	N-Term(iTRAQ8plex)	HCD	1.0000	0.0000	1	1	3153069	Unique	Used	1.554	0.765	1.372	1.124	0	0	5.48	1	0	142	3	1041.53516	3122.59092	0.01
gTGGVDTAAVGGVFDVSNADR	Unambiguous	1	1	Q04447	N-Term(iTRAQ8plex)	CID	1.0000	0.0000	1	1	3135774	Unique	Used	1.061	0.472			0	0	5.48	0	0	200	2	1135.07227	2269.13725	0.42
gTGGVDTAAVGGVFDVSNADR	Unambiguous	1	1	Q04447	N-Term(iTRAQ8plex)	CID	1.0000	0.0000	1	1	3107888	Unique	Used	1.474	1.091	2.015	2.258	0	0	5.48	0	8	34	2	1135.07031	2269.13335	-1.30
vVYEnGVFMcAEGTGk	Unambiguous	1	1	Q9EQF6	N-Term(iTRAQ8plex); N5(Deamidated); C10(Methylthio); K16(iTRAQ8plex)	CID	1.0000	0.0000	1	1	3110425	Unique	Used	0.578	0.316	0.407	0.638	0	0	5.48	0	3	75	2	1180.08264	2359.15801	-0.55
fLSQPFQVAEVFTGHMGk	Unambiguous	1	1	P56480	N-Term(iTRAQ8plex); K18(iTRAQ8plex)	CID	1.0000	0.0000	1	1	3864547	Unique	Used	1.432	1.200	0.721	1.048	0	0	5.48	0	3	200	3	877.80658	2631.40519	-6.17
lDEIYVAGLVAHSDLDER	Unambiguous	7	1	G3V018	N-Term(iTRAQ8plex)	CID	1.0000	0.0000	1	1	2997615	Unique	Used	1.619	0.880	0.805	0.612	0	0	5.48	0	2	62	3	773.74182	2319.21091	-1.10
sIQGSSTSSSASSTLSHGEVk	Unambiguous	1	1	Q3UH60	N-Term(iTRAQ8plex); K21(iTRAQ8plex)	CID	1.0000	0.0000	1	1	3226642	Unique	Used	0.792	0.578	1.248	0.862	0	0	5.48	0	0	152	3	882.46692	2645.38620	0.88

sQPPAAHAGQVYGEQPPk	Unambiguous	3	1	Q6P9K8	N-Term(iTRAQ8plex); K18(iTRAQ8plex)	CID	1.0000	0.0000	1	1	2967993	Unique	Used	0.966	0.591	0.913	0.675	0	0	5.48	0	0	200	3	824.11316	2470.32492	-2.03
gEGVSQVGPGTPPAPESPR	Unambiguous	1	1	P58871	N-Term(iTRAQ8plex)	CID	1.0000	0.0000	1	1	2931640	Unique	Used	2.612	2.052	3.135	1.455	0	0	5.48	0	0	200	2	1062.05286	2123.09844	-2.40
gLSTTGnSSLNSTSNTk	Unambiguous	1	1	O88532	N-Term(iTRAQ8plex); N7(Deamidated); K17(iTRAQ8plex)	CID	0.0420	0.0000	1	1	2987680	Unique	Used	0.582	0.303	0.939	0.694	0	0	5.48	0	0	111	2	1139.59607	2278.18486	-5.89
aGAGSATLSMAYAGAR	Unambiguous	1	1	P08249	N-Term(iTRAQ8plex)	CID	1.0000	0.0000	1	1	3719634	Unique	Used	2.136	1.129	0.983	1.064	0	0	5.48	0	0	65	2	879.95184	1758.89641	-8.34
qQQFNVQALVAVGDHASk	Unambiguous	1	1	Q922B2	N-Term(iTRAQ8plex); K18(iTRAQ8plex)	HCD	1.0000	0.0000	1	1	3515450	Unique	Used	0.780	0.836	0.924	0.882	0	0	5.48	0	0	122	3	850.13708	2548.39670	-4.92
vETTTSLEnGGLPSSAETk	Unambiguous	3	1	Q5SW19	N-Term(iTRAQ8plex); N9(Deamidated); K19(iTRAQ8plex)	CID	1.0000	0.0000	1	1	2668251	Unique	Used	0.472	0.472	0.532	0.592	0	0	5.47	0	0	200	2	1265.67273	2530.33818	1.46
nGDRGETGPAGPAGPIGPAGAR	Unambiguous	2	1	P11087-2	N-Term(iTRAQ8plex); N1(Deamidated)	CID	1.0000	0.0000	1	1	2824868	Unique	Used	0.757	6.955	1.284	1.175	0	0	5.47	1	0	15	3	760.72491	2280.16019	-1.47
gEAIETDDTMGGIPAVNGR	Unambiguous	4	1	F8WHW6	N-Term(iTRAQ8plex)	CID	1.0000	0.0000	1	1	2736272	Unique	Used	0.493	0.696	1.702	0.992	0	0	5.47	0	0	200	2	1104.04419	2207.08110	-4.78
aDmVIEAVFEDLGVk	Unambiguous	1	1	Q8BMS1	N-Term(iTRAQ8plex); M3(Oxidation); K15(iTRAQ8plex)	CID	1.0000	0.0000	1	1	3239985	Unique	Used	0.843	0.908	0.758	1.147	0	0	5.47	0	0	95	3	754.08191	2260.23117	-1.95
gRPITMFIPSDVDNYDDIR	Unambiguous	5	1	F8WJ93	N-Term(iTRAQ8plex)	HCD	1.0000	0.0000	1	1	3302655	Unique	Used	1.301	0.738	1.086	0.824	0	0	5.47	0	21	120	3	843.42853	2528.27103	-1.87
vVYEnGVFMcAEGTGk	Unambiguous	1	1	Q9EQF6	N-Term(iTRAQ8plex); N5(Deamidated); C10(Methylthio); K16(iTRAQ8plex)	CID	1.0000	0.0000	1	1	3131050	Unique	Used	0.562	0.342	0.497	0.631	0	0	5.47	0	0	145	2	1180.08118	2359.15508	-1.79

yIEESIGESANLLGAMEk	Unambiguous	1	1	Q8BLE7	N-Term(iTRAQ8plex); K18(iTRAQ8plex)	CID	1.0000	0.0000	1	1	3196103	Unique	Used	1.341	1.192	0.524	0.787	0	0	5.47	0	3	11	3	854.79004	2562.35556	-1.03
iQAAASPPAnATAASDTNAGDR	Unambiguous	2	1	E9QAQ5	N-Term(iTRAQ8plex); N10(Deamidated)	CID	1.0000	0.0000	1	1	2754886	Unique	Used	0.934	0.779	1.037	1.226	0	0	5.47	0	0	24	2	1188.08801	2375.16875	-2.27
sAAQAAAQTnSNAAGk	Unambiguous	4	1	Q9CY58	N-Term(iTRAQ8plex); N10(Deamidated); K16(iTRAQ8plex)	CID	0.0110	0.0000	1	1	2841908	Unique	Used	0.545	0.422	0.870	0.613	0	0	5.47	0	0	200	2	1035.54626	2070.08525	-8.87
yALSGGNWDSISDAAk	Unambiguous	1	1	Q9WUT3	N-Term(iTRAQ8plex); K16(iTRAQ8plex)	CID	1.0000	0.0000	1	1	3190502	Unique	Used	1.093	1.353	1.261	2.148	0	0	5.47	0	0	158	2	1132.08984	2263.17241	-4.03
gTGGVDTAAVGGVFDVSNADR	Unambiguous	1	1	Q04447	N-Term(iTRAQ8plex)	CID	1.0000	0.0000	1	1	3130510	Unique	Used	1.435	0.918	0.957	1.314	0	0	5.47	0	14	77	2	1135.07043	2269.13359	-1.19
lQSTEFSPSGSEAGSPGLQnGEGQR	Unambiguous	2	1	Q9WV69	N-Term(iTRAQ8plex); N20(Deamidated)	CID	0.0110	0.0000	1	1	2777926	Unique	Used	0.970	1.239	1.719	2.363	0	0	5.47	0	6	200	3	942.45264	2825.34336	-2.07
lHLPTAPPPLNEGESLVSR	Unambiguous	1	1	Q8C8R3-2	N-Term(iTRAQ8plex)	HCD	1.0000	0.0000	1	1	3516840	Unique	Used		1.655	1.073	1.396	0	0	5.47	0	0	1	3	777.76776	2331.28873	-3.75
iNEAIVAVQAIIADPk	Unambiguous	2	1	Q9CQE8	N-Term(iTRAQ8plex); K16(iTRAQ8plex)	CID	1.0000	0.0000	1	1	3666403	Unique	Used	1.053	0.780	0.786	0.858	0	0	5.47	0	0	28	2	1137.18030	2273.35332	-6.88
lQSTEFSPSGSEAGSPGLQnGEGQR	Unambiguous	2	1	Q9WV69	N-Term(iTRAQ8plex); N20(Deamidated)	CID	0.0092	0.0000	1	1	2758339	Unique	Used	3.822	1.293		0.691	0	0	5.46	0	0	82	3	942.45294	2825.34427	-1.75
aGWVDNSRPLPPPPQPLGAQQR	Unambiguous	2	1	Q5XJV6	N-Term(iTRAQ8plex)	HCD	1.0000	0.0000	1	1	3873986	Unique	Used	1.322	1.413	1.704	0.916	0	0	5.46	0	5	76	3	895.81824	2685.44016	-4.70
aLIQYFSPYVSADmHk	Unambiguous	5	1	G3UXW9	N-Term(iTRAQ8plex); M14(Oxidation); K16(iTRAQ8plex)	CID	1.0000	0.0000	1	1	3763716	Unique	Used	0.292	0.401	0.765	1.071	0	0	5.46	0	27	51	3	832.11139	2494.31961	-2.59
mLINQHTLLFGGDTNPAHPk	Unambiguous	1	1	Q922H2	N-Term(iTRAQ8plex); M1(Oxidation); K20(iTRAQ8plex)	CID	1.0000	0.0000	1	1	3720083	Unique	Used	0.567	1.436	1.203	1.201	0	0	5.46	0	0	200	3	943.50946	2828.51383	-7.07

sGAAQGLAEVMAGLGVEk	Unambiguous	2	1	E9PVA8	N-Term(iTRAQ8plex); K18(iTRAQ8plex)	CID	1.0000	0.0000	1	1	3519923	Unique	Used		0.825	0.755	1.056	0	0	5.46	0	19	30	2	1148.63892	2296.27056	-3.76
yIQLPFGDEDALk	Unambiguous	2	1	O70370	N-Term(iTRAQ8plex); K13(iTRAQ8plex)	CID	1.0000	0.0000	1	1	3152244	Unique	Used	1.505	1.340	1.631	1.405	0	0	5.46	0	10	129	3	706.39569	2117.17252	-0.66
gEGGTTNPHVFPEGSEPk	Unambiguous	2	1	Q7TQI3	N-Term(iTRAQ8plex); K18(iTRAQ8plex)	CID	1.0000	0.0000	1	1	2648527	Unique	Used	0.277	0.891	0.887	0.723	0	0	5.46	0	0	200	2	1224.63196	2448.25664	-2.02
qIPGGGSGGGGSGSGGGGGGSGGGR	Unambiguous	2	1	Q8BMA6	N-Term(iTRAQ8plex)	CID	1.0000	0.0000	1	1	3290898	Unique	Used	0.616	0.236	0.429	0.476	0	0	5.46	0	0	200	2	1068.01062	2135.01396	0.52
dSVSAAEVGTGQYATTk	Unambiguous	6	1	Q9WV92-2	N-Term(iTRAQ8plex); K17(iTRAQ8plex)	CID	1.0000	0.0000	1	1	2845762	Unique	Used	0.411	0.804	1.311	0.940	0	0	5.46	0	0	200	2	1147.10803	2293.20879	-1.93
rPSAncDPYAVTEAIVR	Unambiguous	1	1	P15105	N-Term(iTRAQ8plex); N5(Deamidated); C6(Methylthio)	HCD	1.0000	0.0000	1	1	3370421	Unique	Used	1.531	1.153	1.429	1.285	0	0	5.46	0	13	21	3	738.36749	2213.08792	-5.32
hSQAVEELADQLEQTk	Unambiguous	1	1	Q8VDD5	N-Term(iTRAQ8plex); K16(iTRAQ8plex)	CID	1.0000	0.0000	1	1	2830724	Unique	Used	1.194	0.964	1.082	0.701	0	0	5.46	0	9	200	2	1217.65234	2434.29741	-2.48
eFGASEcISPQDFSk	Unambiguous	1	1	P28474	N-Term(iTRAQ8plex); C7(Methylthio); K15(iTRAQ8plex)	CID	1.0000	0.0000	1	1	3054292	Unique	Used	1.208	0.906	0.728	0.951	0	0	5.46	0	10	16	2	1150.06067	2299.11406	-2.38
rLSLESEGANEGATAAPELSALEEAFR	Unambiguous	1	1	Q7TQD2	N-Term(iTRAQ8plex)	HCD	1.0000	0.0000	1	1	3173221	Unique	Used	1.514	0.415	1.398	0.927	0	0	5.46	1	0	32	3	1041.53357	3122.58615	-1.51
fTVLTESAAkNTDAVR	Unambiguous	1	1	P08551	N-Term(iTRAQ8plex); K10(iTRAQ8plex)	HCD	1.0000	0.0000	1	1	3411495	Unique	Used	2.491	0.365	1.358	1.471	0	0	5.46	1	24	22	3	777.76959	2331.29422	-8.01
lGDGELLFEcAGHk	Unambiguous	2	1	Q3TJ22	N-Term(iTRAQ8plex); C10(Methylthio); K14(iTRAQ8plex)	CID	1.0000	0.0000	1	1	3215196	Unique	Used	1.539	1.045	0.783	0.805	0	0	5.46	0	26	156	3	715.04205	2143.11161	-0.96

nDSVVAGGGAIEMELSk	Unambiguous	1	1	P80313	N-Term(iTRAQ8plex); K17(iTRAQ8plex)	CID	1.0000	0.0000	1	1	2937722	Unique	Used	0.739	1.025	0.751	0.907	0	0	5.46	0	17	66	2	1143.11340	2285.21953	-3.18
yEGSGDGGAAAQSLYIAnHAY	Unambiguous	1	1	P05063	N-Term(iTRAQ8plex); N18(Deamidated)	CID	1.0000	0.0000	1	1	2737165	Unique	Used	2.268	0.555	0.613	1.160	0	0	5.46	0	3	200	2	1210.56628	2420.12529	-2.29
sQGSGLSSGGAGEGQGPk	Unambiguous	1	1	Q8CGC7	N-Term(iTRAQ8plex); K18(iTRAQ8plex)	CID	1.0000	0.0000	1	1	2822914	Unique	Used	0.655	0.477	0.594	0.567	0	0	5.46	0	0	200	2	1085.06726	2169.12724	-3.88
dTnGENIAESLVAEGLATR	Unambiguous	2	1	Q78PY7	N-Term(iTRAQ8plex); N3(Deamidated)	CID	0.0293	0.0000	1	1	2798576	Unique	Used	0.670	0.851	0.572	0.666	0	0	5.46	0	4	200	2	1133.07825	2265.14922	-0.90
gHAPSTAAPAPSPAPMSSSVQSDEER	Unambiguous	1	1	Q8C996	N-Term(iTRAQ8plex)	CID	1.0000	0.0000	1	1	2987192	Unique	Used	0.407	1.197	1.005	1.256	0	0	5.46	0	0	188	3	956.79486	2868.37003	-1.27
qkEVNENFAIDLIAQQPVNEVEHR	Unambiguous	2	1	P52503	N-Term(iTRAQ8plex); K2(iTRAQ8plex)	HCD	1.0000	0.0000	1	1	3129148	Unique	Used	3.022	1.197	1.423	0.999	0	0	5.45	1	0	32	3	1143.61487	3428.83005	-2.42
dSSSLSSYTSGVLEER	Unambiguous	1	1	Q9JM96	N-Term(iTRAQ8plex)	CID	1.0000	0.0000	1	1	2974688	Unique	Used	1.995	0.901	1.593	0.899	0	0	5.45	0	18	83	2	1011.00104	2020.99480	-1.43
gGAAASSVGAPSGGk	Unambiguous	1	1	O35126	N-Term(iTRAQ8plex); K15(iTRAQ8plex)	CID	1.0000	0.0000	1	1	3140238	Unique	Used	1.302	0.719	1.189	0.742	0	0	5.45	0	0	200	2	891.50256	1781.99785	0.68
rAPVIDADkPVSSQLR	Unambiguous	1	1	Q9JHU4	N-Term(iTRAQ8plex); K9(iTRAQ8plex)	HCD	1.0000	0.0000	1	1	3429175	Unique	Used	0.514	0.735	1.009	0.944	0	0	5.45	1	7	200	3	787.46393	2360.37723	-4.17
gTGGVDTAAVGGVFDVSNADR	Unambiguous	1	1	Q04447	N-Term(iTRAQ8plex)	HCD	1.0000	0.0000	1	1	3131766	Unique	Used	2.342	1.171	1.107	1.271	0	0	5.45	0	16	200	2	1135.06763	2269.12798	-3.67
rLPASDGLDLSQAAAR	Unambiguous	1	1	P46660	N-Term(iTRAQ8plex)	HCD	1.0000	0.0000	1	1	3512616	Unique	Used	1.472	0.424	1.265	1.288	0	0	5.45	1	14	11	3	649.02789	1945.06913	-4.00
iGQLQGEIIPTSFYHQGR	Unambiguous	7	1	Q9R1V7	N-Term(iTRAQ8plex)	CID	1.0000	0.0000	1	1	3803300	Unique	Used	1.353	0.710	1.503	1.530	0	0	5.45	0	3	5	3	783.42480	2348.25986	-2.82
rPVVTTHDLEAPSR	Unambiguous	1	1	Q8C052	N-Term(iTRAQ8plex)	HCD	1.0000	0.0000	1	1	3382370	Unique	Used	1.148	0.995	1.086	1.317	0	0	5.45	0	0	200	3	628.01581	1882.03287	-6.37
sQNNFVAILDLPEGEHQYk	Unambiguous	1	1	Q9R078	N-Term(iTRAQ8plex); K19(iTRAQ8plex)	CID	1.0000	0.0000	1	1	3192088	Unique	Used	0.750	1.148	1.149	1.407	0	0	5.45	0	12	38	3	937.50018	2810.48600	-2.62

yFDSFGDLSSASAImGNPk	Unambiguous	1	1	P02089	N-Term(iTRAQ8plex); M15(Oxidation); K19(iTRAQ8plex)	CID	1.0000	0.0000	1	1	3371827	Unique	Used			2.016	0.982	0	0	5.45	0	0	200	2	1316.16321	2631.31914	-1.14
vQLQMAELATSHk	Unambiguous	3	1	Q91WG2	N-Term(iTRAQ8plex); K13(iTRAQ8plex)	CID	1.0000	0.0000	1	1	3411151	Unique	Used	1.489	1.034	1.221	0.922	0	0	5.45	0	24	28	3	688.72699	2064.16642	-3.29
vVSAAVQAQHSk	Unambiguous	3	1	E9QKE4	N-Term(iTRAQ8plex); K12(iTRAQ8plex)	HCD	1.0000	0.0000	1	1	3509955	Unique	Used	0.531	1.086	0.902	1.088	0	0	5.45	0	25	157	3	611.69586	1833.07303	-3.95
lVEPQNQTLLHAQPIVSIR	Unambiguous	2	1	Q9QXJ1-2	N-Term(iTRAQ8plex)	CID	1.0000	0.0000	1	1	3801782	Unique	Used	0.914	1.203	0.812	0.862	0	0	5.44	0	9	52	3	820.80988	2460.41507	-3.65
lDQEDALLGSYPVDDGcR	Unambiguous	1	1	Q9D1E6	N-Term(iTRAQ8plex); C17(Methylthio)	CID	1.0000	0.0000	1	1	2742041	Unique	Used	5.798		1.319	0.475	0	0	5.44	0	0	200	2	1158.54089	2316.07451	-1.95
eGDTLVLTcAVTGNPRPnQIR	Unambiguous	1	1	Q8R464	N-Term(iTRAQ8plex); C9(Methylthio); N18(Deamidated)	HCD	1.0000	0.0000	1	1	3476543	Unique	Used	1.333	1.043	2.364	3.060	0	0	5.44	0	18	17	3	869.11365	2605.32639	-4.48
dGSGYIDENELDALLk	Unambiguous	1	1	Q08331	N-Term(iTRAQ8plex); K16(iTRAQ8plex)	CID	1.0000	0.0000	1	1	2765683	Unique	Used	1.651	1.051	0.879	1.127	0	0	5.44	0	4	200	2	1180.62341	2360.23955	-1.97
rPAVAPAANFPDSGR	Unambiguous	1	1	Q99MS8	N-Term(iTRAQ8plex)	CID	1.0000	0.0000	1	1	3655135	Unique	Used	0.829	1.567	1.127	1.047	0	0	5.44	0	26	32	3	610.66528	1829.98130	-6.10
eEFQQFAGLLQAGIEk	Unambiguous	1	1	P47199	N-Term(iTRAQ8plex); K16(iTRAQ8plex)	CID	1.0000	0.0000	1	1	3373667	Unique	Used	0.659	1.452	1.467	1.423	0	0	5.44	0	6	14	2	1208.66589	2416.32451	-3.64
sIDDLEDQLYHQLEQNR	Unambiguous	3	1	E9Q452	N-Term(iTRAQ8plex)	CID	1.0000	0.0000	1	1	2980309	Unique	Used	1.790	1.574	2.319	1.986	0	0	5.44	0	6	200	3	807.40454	2420.19907	-0.21
vTLAPAGGALQHSR	Unambiguous	1	1	P81122	N-Term(iTRAQ8plex)	CID	1.0000	0.0000	1	1	3816118	Unique	Used	1.137	1.151	1.037	1.189	0	0	5.44	0	5	92	3	561.32428	1681.95829	-4.08
lVANHVASDISLTGGSVVQR	Unambiguous	3	1	Q9Z2G6	N-Term(iTRAQ8plex)	CID	1.0000	0.0000	1	1	3780271	Unique	Used	1.409	1.913	0.671	1.021	0	0	5.44	0	30	200	3	776.43475	2327.28971	-3.79
lLDDNGNIAEELSILk	Unambiguous	1	1	Q9ERR7	N-Term(iTRAQ8plex); K16(iTRAQ8plex)	CID	1.0000	0.0000	1	1	3133155	Unique	Used	1.179	0.851	0.852	0.675	0	0	5.44	0	0	50	2	1183.17529	2365.34331	-0.09

aEGATLSnATGAVESTSQAGDR	Unambiguous	1	1	Q61644	N-Term(iTRAQ8plex); N8(Deamidated)	CID	1.0000	0.0000	1	1	2633772	Unique	Used	3.240	1.795	4.555	3.101	0	0	5.43	0	0	200	2	1199.58325	2398.15923	-1.83
sImAAAGVPVVEGYHGk	Unambiguous	2	1	Q99MR8	N-Term(iTRAQ8plex); M3(Oxidation); K17(iTRAQ8plex)	CID	1.0000	0.0000	1	1	3512653	Unique	Used	1.065	1.372	1.120	1.233	0	0	5.43	0	19	200	3	770.75818	2310.25998	-5.93
eVGDGTTSVVIIAAELLk	Unambiguous	2	1	P11983	N-Term(iTRAQ8plex); K18(iTRAQ8plex)	CID	1.0000	0.0000	1	1	3352878	Unique	Used	0.501	0.756	0.867	1.503	0	0	5.43	0	28	12	2	1212.21033	2423.41338	-3.47
vGSAGAAALAAGGAGGAER	Unambiguous	2	1	Q5U4F6	N-Term(iTRAQ8plex)	CID	1.0000	0.0000	1	1	3386258	Unique	Used	1.467	0.924	1.031	0.966	0	0	5.43	0	0	164	2	909.48938	1817.97148	-3.16
eHAPSIIFMDEIDSIGSSR	Unambiguous	2	1	P62196	N-Term(iTRAQ8plex)	CID	1.0000	0.0000	1	1	3194456	Unique	Used	0.948	1.524	1.065	0.841	0	0	5.43	0	14	30	3	803.40649	2408.20493	-0.86
aAELIAnSLATAGDGLIELR	Unambiguous	1	1	P67778	N-Term(iTRAQ8plex); N7(Deamidated)	CID	1.0000	0.0000	1	1	3262428	Unique	Used	1.179	1.767	0.872	0.974	0	0	5.43	0	21	28	2	1152.14075	2303.27422	-0.81
sSGSPYGGGYGSGGGSGGYGSR	Unambiguous	7	1	J3QNY1	N-Term(iTRAQ8plex)	CID	1.0000	0.0000	1	1	3100595	Unique	Used	2.302	0.435	1.097	0.717	0	0	5.43	0	0	200	2	1107.99841	2214.98955	-2.64
aTDLLLDDSLVSLFGNR	Unambiguous	4	1	P99029-2	N-Term(iTRAQ8plex)	CID	1.0000	0.0000	1	1	3605702	Unique	Used	2.302	1.783	0.874	0.816	0	0	5.43	0	0	200	2	1077.08691	2153.16655	-4.21
sQPNHTPSGPPGPSSNPVSnGk	Unambiguous	2	1	Q8R2Y9	N-Term(iTRAQ8plex); N20(Deamidated); K22(iTRAQ8plex)	CID	1.0000	0.0000	1	1	2986264	Unique	Used	0.776	0.780	1.189	1.026	0	0	5.43	0	12	200	3	918.14044	2752.40677	-1.57
gTGATADFADVAEDFENYHk	Unambiguous	1	1	Q8R4I7	N-Term(iTRAQ8plex); K20(iTRAQ8plex)	CID	1.0000	0.0000	1	1	2740054	Unique	Used		3.241	2.385	1.151	0	0	5.43	0	16	200	3	922.78802	2766.34952	0.99
vVYEnGVFMcAEGTGk	Unambiguous	1	1	Q9EQF6	N-Term(iTRAQ8plex); N5(Deamidated); C10(Methylthio); K16(iTRAQ8plex)	CID	1.0000	0.0000	1	1	3074691	Unique	Used	0.536	0.800	0.485	0.593	0	0	5.43	0	21	200	2	1180.08142	2359.15557	-1.58
aTSSSSSSSGVGmSVGQGR	Unambiguous	2	1	P21619	N-Term(iTRAQ8plex); M13(Oxidation)	CID	1.0000	0.0000	1	1	3608934	Unique	Used	0.517	1.011	1.077	1.286	0	0	5.43	0	0	200	2	1024.98926	2048.97124	-5.29

iILQGSPVVQMAEDAVDGER	Unambiguous	1	1	P01027	N-Term(iTRAQ8plex)	HCD	1.0000	0.0000	1	1	3217479	Unique	Used	3.006	1.655	0.345	0.874	0	0	5.42	0	12	17	2	1216.14160	2431.27593	-1.89
gPLLVQDVVFTDEmAHFDR	Unambiguous	2	1	P24270	N-Term(iTRAQ8plex); M14(Oxidation)	CID	1.0000	0.0000	1	1	3282992	Unique	Used	1.579		0.842	1.047	0	0	5.42	0	0	48	3	837.09387	2509.26706	-1.15
iAPALISSGISVVEQEk	Unambiguous	4	1	P17183	N-Term(iTRAQ8plex); K17(iTRAQ8plex)	CID	1.0000	0.0000	1	1	3397535	Unique	Used	1.895	1.380	1.247	1.397	0	0	5.42	0	24	200	2	1175.19250	2349.37773	-3.09
sVSPGVTQAVVEEHcASPEEk	Unambiguous	1	1	P14873	N-Term(iTRAQ8plex); C15(Methylthio); K21(iTRAQ8plex)	CID	1.0000	0.0000	1	1	2865886	Unique	Used	2.481	0.991	1.384	1.036	0	0	5.42	0	24	198	3	946.47833	2837.42044	-2.32
tNVLPTQQSDSPSHcQR	Unambiguous	2	1	Q6P6I6	N-Term(iTRAQ8plex); C15(Methylthio)	CID	1.0000	0.0000	1	1	3449589	Unique	Used	1.378	1.988	1.186	1.137	0	0	5.42	0	18	200	3	750.02802	2248.06949	-2.56
kLLEAHEEQNSEAYTEAVk	Unambiguous	1	1	P28663	N-Term(iTRAQ8plex); K1(iTRAQ8plex); K19(iTRAQ8plex)	CID	1.0000	0.0000	1	1	2808353	Unique	Used	0.420	1.488	1.094	1.022	0	0	5.42	1	0	38	3	1034.56519	3101.68100	-2.33
eHANIDAQSGSQAPNPSTTISPGk	Unambiguous	3	1	Q8VDQ8-2	N-Term(iTRAQ8plex); K24(iTRAQ8plex)	CID	1.0000	0.0000	1	1	3142660	Unique	Used	0.431	0.284	0.933	1.193	0	0	5.42	0	0	84	3	1005.85712	3015.55680	-0.81
yVIHTVGPIAVGQPTASQAAELR	Unambiguous	1	1	Q922B1	N-Term(iTRAQ8plex)	CID	1.0000	0.0000	1	1	3760992	Unique	Used	1.569	1.537	1.300	1.210	0	0	5.42	0	16	48	3	894.83130	2682.47934	-3.28
mGFPSNIVDSAAENMIk	Unambiguous	1	1	Q9Z2I9	N-Term(iTRAQ8plex); K17(iTRAQ8plex)	CID	1.0000	0.0000	1	1	3395684	Unique	Used	0.317	0.942	1.101	1.277	0	0	5.42	0	7	8	2	1216.63684	2432.26641	-4.55
aGQVFLEELGNHk	Unambiguous	2	1	P16330	N-Term(iTRAQ8plex); K13(iTRAQ8plex)	CID	1.0000	0.0000	1	1	3279617	Unique	Used	0.658	0.137	1.306	1.524	0	0	5.42	0	7	28	3	684.05334	2050.14548	-4.25
aAAGELQEDSGLHVLAR	Unambiguous	1	1	Q8C845	N-Term(iTRAQ8plex)	CID	1.0000	0.0000	1	1	2972890	Unique	Used	1.416	1.004	1.203	0.854	0	0	5.42	0	9	38	3	681.03473	2041.08963	-4.12
iTnGQPQQTTGAASGGYIk	Unambiguous	3	1	O09044	N-Term(iTRAQ8plex); N3(Deamidated); K19(iTRAQ8plex)	CID	0.0480	0.0000	1	1	3103548	Unique	Used	1.179	0.866	0.731	0.991	0	0	5.42	0	0	180	2	1251.17371	2501.34014	-2.20

fFDHSGTLVMDAYEPEISR	Unambiguous	1	1	Q9CYN2	N-Term(iTRAQ8plex)	CID	1.0000	0.0000	1	1	3215293	Unique	Used	0.674	0.725	0.622	0.992	0	0	5.42	0	0	8	3	840.07922	2518.22312	0.19
aGWVDNSRPLPPPPQPLGAQQR	Unambiguous	2	1	Q5XJV6	N-Term(iTRAQ8plex)	HCD	1.0000	0.0000	1	1	3874105	Unique	Used	1.012	1.281	1.836	0.983	0	0	5.42	0	2	22	3	895.81781	2685.43888	-5.17
tYFPHFDVSHGSAQVk	Unambiguous	2	1	Q91VB8	N-Term(iTRAQ8plex); K16(iTRAQ8plex)	CID	1.0000	0.0000	1	1	3481185	Unique	Used	2.622	1.873	0.556	1.583	0	0	5.42	0	23	200	3	810.09637	2428.27457	-5.11
aPPAGGSGSSAAAAAAAGGTEVRPR	Unambiguous	2	1	P84309	N-Term(iTRAQ8plex)	HCD	1.0000	0.0000	1	1	3633131	Unique	Used	0.820	0.998	2.017	1.145	0	0	5.42	0	0	145	3	814.42761	2441.26828	-4.79
iEGDMIVcAAYAHELPk	Unambiguous	2	1	P47962	N-Term(iTRAQ8plex); C8(Methylthio); K17(iTRAQ8plex)	CID	1.0000	0.0000	1	1	3326520	Unique	Used	0.328	0.743	0.684	0.748	0	0	5.42	0	19	6	3	838.76880	2514.29184	-3.86
gSHSQSSSPGALTLGR	Unambiguous	3	1	O88935	N-Term(iTRAQ8plex)	HCD	1.0000	0.0000	1	1	3832307	Unique	Used	0.395	1.403	1.503	1.223	0	0	5.41	0	3	28	3	615.99426	1845.96823	-2.08
iEVGLVVGnSQVAFEk	Unambiguous	2	1	P46460	N-Term(iTRAQ8plex); N9(Deamidated); K16(iTRAQ8plex)	CID	0.0407	0.0000	1	1	3260933	Unique	Used		1.487	1.164	1.332	0	0	5.41	0	8	38	3	766.77673	2298.31565	-0.41
lNEHFLNTTDFLDTlk	Unambiguous	1	1	P54071	N-Term(iTRAQ8plex); K16(iTRAQ8plex)	CID	1.0000	0.0000	1	1	3350447	Unique	Used		2.064	0.752	1.182	0	0	5.41	0	2	4	3	843.79663	2529.37534	-2.22
iMDPNIVGNEHYDVAR	Unambiguous	1	1	P56480	N-Term(iTRAQ8plex)	CID	1.0000	0.0000	1	1	3051502	Unique	Used	1.471	1.114	1.240	1.355	0	0	5.41	0	0	1	3	716.36426	2147.07822	-3.51
lGYAVYETPTAHnGAk	Unambiguous	4	1	Q9QZ06	N-Term(iTRAQ8plex); N13(Deamidated); K16(iTRAQ8plex)	CID	1.0000	0.0000	1	1	2990310	Unique	Used	1.218	1.101	1.090	1.138	0	0	5.41	0	20	50	3	767.74841	2301.23069	-1.24
hLAQIGDEMDHNIQPTLVR	Unambiguous	1	1	P70444	N-Term(iTRAQ8plex)	HCD	1.0000	0.0000	1	1	3299254	Unique	Used	1.467	1.613	1.502	1.214	0	0	5.41	0	0	107	3	831.10339	2491.29563	-2.95
vSDFGQmASGMNVDAGk	Unambiguous	1	1	P20357	N-Term(iTRAQ8plex); M7(Oxidation); K17(iTRAQ8plex)	CID	1.0000	0.0000	1	1	3277066	Unique	Used	1.402	1.479	1.808	0.821	0	0	5.41	0	0	105	2	1169.58008	2338.15288	-4.25
gTGGVDTAAVGGVFDVSNADR	Unambiguous	1	1	Q04447	N-Term(iTRAQ8plex)	CID	1.0000	0.0000	1	1	3212698	Unique	Used	1.594	1.317	1.044	1.061	0	0	5.41	0	21	125	2	1135.06885	2269.13042	-2.59

ikAEYEGDGIPTVFVSVAGR	Unambiguous	1	1	Q9DCL9	N-Term(iTRAQ8plex); K2(iTRAQ8plex)	HCD	1.0000	0.0000	1	1	3392785	Unique	Used	0.909	0.796	0.825	1.018	0	0	5.41	1	30	63	3	906.17365	2716.50638	-2.46
lEEGPPVTTVLTR	Unambiguous	1	1	P35486	N-Term(iTRAQ8plex)	CID	1.0000	0.0000	1	1	3300808	Unique	Used	2.154	1.676	0.938	1.292	0	0	5.41	0	10	23	3	572.66467	1715.97947	-2.96
tVTYEDPQAVGGLASALDNR	Unambiguous	8	1	Q5SRX1-2	N-Term(iTRAQ8plex)	CID	1.0000	0.0000	1	1	3170503	Unique	Used	2.150	2.062	2.820	2.343	0	0	5.41	0	0	200	2	1191.11536	2381.22344	-0.70
qQLLGAEEQMQDMQDk	Unambiguous	6	1	Q3TVA9	N-Term(iTRAQ8plex); K16(iTRAQ8plex)	CID	1.0000	0.0000	1	1	2701999	Unique	Used	0.536	0.181	0.618	1.021	0	0	5.41	0	0	173	3	834.09222	2500.26212	-0.46
gTGEPASAGTSTGTSTGAGSSSR	Unambiguous	1	1	Q8BUV6	N-Term(iTRAQ8plex)	CID	1.0000	0.0000	1	1	2747565	Unique	Used				1.063	0	0	5.41	0	0	200	2	1144.54407	2288.08086	-4.19
eHGYLGNLSAVASTEGTIEETLnEASR	Unambiguous	2	1	Q99P72	N-Term(iTRAQ8plex); N23(Deamidated)	CID	1.0000	0.0000	1	1	2833934	Unique	Used			1.609	0.948	0	0	5.41	0	0	200	3	1051.85535	3153.55149	0.78
kAEDLSSLnGQLQSEVTLLR	Unambiguous	3	1	P16951-2	N-Term(iTRAQ8plex); K1(iTRAQ8plex); N9(Deamidated)	HCD	1.0000	0.0000	1	1	3236334	Unique	Used	1.021	0.902	1.120	0.720	0	0	5.41	1	0	68	3	937.52905	2810.57260	0.20
aTDLLLDDSLVSLFGNR	Unambiguous	4	1	P99029-2	N-Term(iTRAQ8plex)	CID	1.0000	0.0000	1	1	3605996	Unique	Used	1.548	1.280	0.865	1.557	0	0	5.41	0	0	200	2	1077.08911	2153.17095	-2.17
aSESSSRPPPGPLPSTTTQPSQASR	Unambiguous	1	1	Q6PAJ3	N-Term(iTRAQ8plex)	HCD	1.0000	0.0000	1	1	3467940	Unique	Used	1.000	0.540	1.200	1.005	0	0	5.40	0	0	194	3	943.15125	2827.43918	-3.42
gTVcAANDFNPDADAk	Unambiguous	2	1	P14824	N-Term(iTRAQ8plex); C4(Methylthio); K16(iTRAQ8plex)	CID	1.0000	0.0000	1	1	2829542	Unique	Used	0.778	0.997	0.984	0.785	0	0	5.40	0	12	166	2	1132.04871	2263.09014	-1.88
vAYISFGPHAGk	Unambiguous	1	1	Q9CR57	N-Term(iTRAQ8plex); K12(iTRAQ8plex)	CID	1.0000	0.0000	1	1	3719854	Unique	Used	0.445	0.663	0.708	0.802	0	0	5.40	0	29	25	3	619.02417	1855.05796	-5.77
dLLDSVLPHLYnETk	Unambiguous	1	1	Q6ZQ38	N-Term(iTRAQ8plex); N12(Deamidated); K15(iTRAQ8plex)	CID	1.0000	0.0000	1	1	3216733	Unique	Used	0.713	1.522	1.175	1.396	0	0	5.40	0	20	200	3	789.43945	2366.30381	-1.09

mLLEYTDSSYDEk	Unambiguous	2	1	P10649	N-Term(iTRAQ8plex); M1(Oxidation); K13(iTRAQ8plex)	CID	1.0000	0.0000	1	1	2794647	Unique	Used	0.969	1.800	1.786	1.592	0	0	5.40	0	4	186	3	740.03827	2218.10025	-1.94
imRPDDANVAGNVHGGTILk	Unambiguous	4	1	Q91V12-2	N-Term(iTRAQ8plex); M2(Oxidation); K20(iTRAQ8plex)	CID	1.0000	0.0000	1	1	3430262	Unique	Used	0.550	0.713	0.827	0.835	0	0	5.40	0	0	200	3	901.49548	2702.47190	-5.55
gTGGVDTAAVGGVFDVSNADR	Unambiguous	1	1	Q04447	N-Term(iTRAQ8plex)	CID	1.0000	0.0000	1	1	3149941	Unique	Used	1.102	0.865	1.522	1.595	0	0	5.40	0	0	200	2	1135.07104	2269.13481	-0.66
lGEHNInVLEGNEQFIDAAk	Unambiguous	1	1	Q9Z1R9	N-Term(iTRAQ8plex); N7(Deamidated); K20(iTRAQ8plex)	CID	0.0259	0.0000	1	1	3013625	Unique	Used	1.304	1.770	0.830	0.869	0	0	5.40	0	0	27	3	940.83826	2820.50022	0.48
nmGGPYGGGNYGPGGSGGSGGYGGR	Unambiguous	3	1	O88569	N-Term(iTRAQ8plex); M2(Oxidation)	CID	1.0000	0.0000	1	1	3314874	Unique	Used	1.268	1.028	1.082	1.676	0	0	5.39	0	2	16	3	837.37073	2510.09763	-3.23
lAAEFnGLTLTGRPR	Unambiguous	1	1	B2RW11	N-Term(iTRAQ8plex); N6(Deamidated)	HCD	1.0000	0.0000	1	1	3762853	Unique	Used	1.225	1.248	1.811	1.200	0	0	5.39	0	18	99	3	641.02832	1921.07041	-5.48
gSTLSQWSLGNGIPVTSR	Unambiguous	1	1	Q3UVK0	N-Term(iTRAQ8plex)	CID	1.0000	0.0000	1	1	3933847	Unique	Used	0.774	0.696	0.795	0.955	0	0	5.39	0	0	200	2	1082.58362	2164.15996	-2.99
vADGLPLAASMQEDEQSGR	Unambiguous	2	1	O08547	N-Term(iTRAQ8plex)	CID	1.0000	0.0000	1	1	2812995	Unique	Used	0.599	1.078	0.915	0.840	0	0	5.39	0	0	45	2	1139.56628	2278.12529	-1.53
lAGGQTSQPTTPLTSPQR	Unambiguous	3	1	Q9ES00	N-Term(iTRAQ8plex)	CID	1.0000	0.0000	1	1	3756829	Unique	Used	1.320	1.181	1.079	1.075	0	0	5.39	0	0	125	2	1072.57922	2144.15117	-4.75
aIAVLTSGGDAQGMNAAVR	Unambiguous	1	1	P47857	N-Term(iTRAQ8plex)	CID	1.0000	0.0000	1	1	3762516	Unique	Used	2.300	0.449	0.914	1.059	0	0	5.39	0	29	123	2	1053.56299	2106.11870	-4.41
lTLEQGQQLVAEGHPGANQASTR	Unambiguous	1	1	Q68FG2	N-Term(iTRAQ8plex)	HCD	1.0000	0.0000	1	1	3341905	Unique	Used	0.451	2.559	2.602	1.545	0	0	5.39	0	7	5	3	903.80872	2709.41159	-3.93
iEGSGDQVDTLELSGGAk	Unambiguous	3	1	Q8BZ98-2	N-Term(iTRAQ8plex); K18(iTRAQ8plex)	CID	1.0000	0.0000	1	1	2716739	Unique	Used		1.403			0	0	5.39	0	0	200	2	1192.63916	2384.27104	-2.32
yILSGDGAGTLFIIDEk	Unambiguous	2	1	P70408	N-Term(iTRAQ8plex); K17(iTRAQ8plex)	CID	1.0000	0.0000	1	1	3326646	Unique	Used	1.034	1.774	1.504	0.820	0	0	5.39	0	0	25	2	1210.67908	2420.35088	-1.03

kTGSPGGPGVGGSPGGGAAGASPSLPPSAk	Unambiguous	1	1	Q8R1Q8	N-Term(iTRAQ8plex); K1(iTRAQ8plex); K30(iTRAQ8plex)	CID	1.0000	0.0000	1	1	3490464	Unique	Used	0.485	1.366	1.115	0.809	0	0	5.39	1	0	115	3	1124.95520	3372.85105	-3.83
gDPYPQEVGATVHk	Unambiguous	2	1	P22315	N-Term(iTRAQ8plex); K14(iTRAQ8plex)	CID	1.0000	0.0000	1	1	2825657	Unique	Used	0.918	1.147	1.113	1.072	0	0	5.39	0	6	66	3	702.71790	2106.13913	-2.32
aSDDLGEPDVFATAPFR	Unambiguous	1	1	Q6PIU9	N-Term(iTRAQ8plex)	CID	1.0000	0.0000	1	1	2959315	Unique	Used	2.009	0.915	1.212	0.852	0	0	5.38	0	10	200	2	1056.52771	2112.04814	-3.33
sGLGLGAASASTSGSGPADSVmR	Unambiguous	3	1	Q7TSJ2	N-Term(iTRAQ8plex); M22(Oxidation)	CID	1.0000	0.0000	1	1	3512297	Unique	Used	1.756	1.497	1.194	1.240	0	0	5.38	0	2	7	3	786.05975	2356.16471	-2.97
fccEPAGGLTSLTEPPk	Unambiguous	2	1	Q9D6J6	N-Term(iTRAQ8plex); C2(Methylthio); C3(Methylthio); K17(iTRAQ8plex)	CID	1.0000	0.0000	1	1	3499937	Unique	Used	1.090	0.601	1.074	0.895	0	0	5.38	0	3	6	2	1225.60193	2450.19658	-3.38
wGDEVEYMLVSFDHENR	Unambiguous	1	1	P97494	N-Term(iTRAQ8plex)	CID	1.0000	0.0000	1	1	3017900	Unique	Used	1.046	1.464	1.079	1.490	0	0	5.38	0	6	31	3	810.71539	2430.13163	-0.91
aGHLTSVHSPEEHk	Unambiguous	1	1	P55066	N-Term(iTRAQ8plex); K14(iTRAQ8plex)	CID	1.0000	0.0000	1	1	3003090	Unique	Used	0.800	0.958	1.382	0.459	0	0	5.38	0	6	104	3	713.05841	2137.16068	-0.17
gIVnGAAPELPVPTGGPMAGAR	Unambiguous	1	1	P62814	N-Term(iTRAQ8plex); N4(Deamidated)	CID	1.0000	0.0000	1	1	3415388	Unique	Used	0.795	0.887	1.017	1.043	0	0	5.38	0	0	2	2	1169.12524	2337.24321	-4.59
vTnGAFTGEISPGMIk	Unambiguous	1	1	P17751	N-Term(iTRAQ8plex); N3(Deamidated); K16(iTRAQ8plex)	CID	1.0000	0.0000	1	1	3258504	Unique	Used	1.338	1.284	1.689	1.053	0	0	5.38	0	4	13	2	1116.11279	2231.21831	-0.87
aDALQAGASQFESSAAk	Unambiguous	3	1	Q62442-2	N-Term(iTRAQ8plex); K17(iTRAQ8plex)	CID	1.0000	0.0000	1	1	3147858	Unique	Used	0.740	0.458	0.847	1.142	0	0	5.38	0	0	166	2	1130.60339	2260.19951	-1.55
qPIAVSSMPQQIGVPTSSLTQVVHSAGR	Unambiguous	5	1	P83741-2	N-Term(iTRAQ8plex)	HCD	1.0000	0.0000	1	1	3934239	Unique	Used	1.376	0.484	1.646	1.291	0	0	5.38	0	4	88	3	1060.57336	3179.70554	-2.96

sDkDLETQVIQLNEQVHSLk	Unambiguous	4	1	E9Q6X0	N-Term(iTRAQ8plex); K3(iTRAQ8plex); K20(iTRAQ8plex)	CID	1.0000	0.0000	1	1	3257978	Unique	Used	0.902	1.091	1.018	0.945	0	0	5.38	1	0	71	3	1079.60852	3236.81101	-4.45
fVDGLMIHSGDPVNYYVDTAVR	Unambiguous	1	1	P62908	N-Term(iTRAQ8plex)	CID	1.0000	0.0000	1	1	3479247	Unique	Used				1.148	0	0	5.38	0	2	5	3	924.80109	2772.38871	-2.97
yTPSGQSGAAASESLFISNHAY	Unambiguous	2	1	A6ZI44	N-Term(iTRAQ8plex)	CID	1.0000	0.0000	1	1	3240988	Unique	Used	3.361	1.568			0	0	5.38	0	0	200	2	1281.62378	2562.24028	-0.45
mILIQDGSQNTNVDkPLR	Unambiguous	1	1	Q3U0V1	N-Term(iTRAQ8plex); M1(Oxidation); K15(iTRAQ8plex)	HCD	1.0000	0.0000	1	1	3452356	Unique	Used	0.900	1.018	1.075	0.748	0	0	5.38	0	0	200	3	889.49286	2666.46402	-4.36
dDMIFEDcGNVPSEPk	Unambiguous	1	1	P14094	N-Term(iTRAQ8plex); C8(Methylthio); K16(iTRAQ8plex)	CID	1.0000	0.0000	1	1	2655929	Unique	Used		0.383	2.072	1.664	0	0	5.38	0	0	200	2	1225.57715	2450.14702	-1.16
tEGDLLLSVLLYGQPVR	Unambiguous	2	1	Q3TDT0	N-Term(iTRAQ8plex)	HCD	1.0000	0.0000	1	1	3806493	Unique	Used	0.615	0.743	0.867	0.991	0	0	5.38	0	10	30	2	1089.12427	2177.24126	-3.27
aGQQGLSVAFDLATHR	Unambiguous	1	1	P16332	N-Term(iTRAQ8plex)	CID	1.0000	0.0000	1	1	3722955	Unique	Used	1.190	1.481	1.207	1.370	0	0	5.38	0	26	53	3	659.02258	1975.05320	-6.65
qmNPYIQVTSHQNR	Unambiguous	1	1	Q02053	N-Term(iTRAQ8plex); M2(Oxidation)	CID	1.0000	0.0000	1	1	3774552	Unique	Used	0.783	1.115	1.127	0.917	0	0	5.37	0	12	200	3	679.34613	2036.02384	-2.31
sVAGEIVLITGAGHGIGR	Unambiguous	2	1	Q9EQ06	N-Term(iTRAQ8plex)	CID	1.0000	0.0000	1	1	3803246	Unique	Used	0.989	1.036	0.845	0.960	0	0	5.37	0	15	64	3	671.05713	2011.15683	-1.70
iHEGcEEPATHNALAk	Unambiguous	2	1	Q68FD5	N-Term(iTRAQ8plex); C5(Methylthio); K16(iTRAQ8plex)	CID	1.0000	0.0000	1	1	2896009	Unique	Used	0.534	0.909	1.254	1.266	0	0	5.37	0	0	115	3	792.07141	2374.19968	-4.52
lQNQLQGAIVIERPNVk	Unambiguous	1	1	P46467	N-Term(iTRAQ8plex); K17(iTRAQ8plex)	HCD	1.0000	0.0000	1	1	3760512	Unique	Used	0.944	1.069	0.974	1.190	0	0	5.37	0	11	61	3	843.50574	2528.50266	-4.22
cLLDDDLVmGVYIHR	Unambiguous	3	1	E9QP54	N-Term(iTRAQ8plex); C1(Methylthio); M9(Oxidation)	CID	1.0000	0.0000	1	1	3458193	Unique	Used	0.958	1.394	0.813	1.099	0	0	5.37	0	18	200	3	710.02106	2128.04862	-2.68

sAAAEAEAILQDAVSk	Unambiguous	1	1	Q9JKY5	N-Term(iTRAQ8plex); K16(iTRAQ8plex)	CID	1.0000	0.0000	1	1	3093641	Unique	Used			0.918		0	0	5.37	0	29	200	2	1091.61121	2182.21514	-1.13
sGGPHGAGVLGVHLEGPFISR	Unambiguous	2	1	Q8JZV7	N-Term(iTRAQ8plex)	HCD	1.0000	0.0000	1	1	3837947	Unique	Used	1.688	1.153	0.942	1.078	0	0	5.37	0	12	200	3	783.42773	2348.26865	-3.88
tGkPEAqGGPWPYPSSQTQQAVR	Unambiguous	4	1	E9QL31	N-Term(iTRAQ8plex); K3(iTRAQ8plex); Q7(Deamidated)	HCD	1.0000	0.0000	1	1	3408271	Unique	Used	1.235	1.546	0.967	0.981	0	0	5.37	0	0	141	3	1027.21118	3079.61899	4.28
eNAGGQTEIcTTYSk	Unambiguous	1	1	Q922R1	N-Term(iTRAQ8plex); C10(Methylthio); K15(iTRAQ8plex)	CID	1.0000	0.0000	1	1	2933808	Unique	Used		0.683	1.057	0.943	0	0	5.37	0	0	96	2	1128.55530	2256.10332	-2.82
yVLHmIEDGEIFASINQk	Unambiguous	1	1	O88543	N-Term(iTRAQ8plex); M5(Oxidation); K18(iTRAQ8plex)	CID	1.0000	0.0000	1	1	3439011	Unique	Used	0.834	1.040	1.085	0.831	0	0	5.37	0	6	46	3	911.15460	2731.44925	-3.41
mLQDDEDTVHLPFER	Unambiguous	2	1	Q8BTY2	N-Term(iTRAQ8plex)	CID	1.0000	0.0000	1	1	2739023	Unique	Used	0.470	0.810	1.052	0.913	0	0	5.36	0	19	195	3	717.02167	2149.05045	-1.56
iPNIYAIGDVVAGPmLAHk	Unambiguous	1	1	O08749	N-Term(iTRAQ8plex); M15(Oxidation); K19(iTRAQ8plex)	CID	1.0000	0.0000	1	1	3827341	Unique	Used	1.841	1.350	0.720	0.724	0	0	5.36	0	6	200	3	868.49768	2603.47849	-2.12
iMESTQEDALHR	Unambiguous	4	1	E9QAH1	N-Term(iTRAQ8plex)	CID	1.0000	0.0000	1	1	2682362	Unique	Used	1.973	0.993	0.945	0.675	0	0	5.36	0	11	144	3	578.63013	1733.87583	-2.08
vAVLGASGGIGQPLSLLLk	Unambiguous	1	1	P08249	N-Term(iTRAQ8plex); K19(iTRAQ8plex)	CID	1.0000	0.0000	1	1	3938449	Unique	Used	1.088	1.051	0.878	0.955	0	0	5.36	0	10	200	3	801.16925	2401.49320	-2.97
eESGAVAAAASVPAQSTAR	Unambiguous	1	1	Q9EPB4	N-Term(iTRAQ8plex)	CID	1.0000	0.0000	1	1	2952714	Unique	Used	1.613	0.852	0.560	0.881	0	0	5.36	0	0	200	2	1039.04187	2077.07646	-3.05
dITAEATPALAQTLPGGk	Unambiguous	2	1	Q9WTQ5	N-Term(iTRAQ8plex); K18(iTRAQ8plex)	CID	1.0000	0.0000	1	1	3279627	Unique	Used	2.236	0.653	1.043	0.888	0	0	5.36	0	14	198	2	1181.67480	2362.34233	-0.65
eNPGFDFSGAEISGNYTk	Unambiguous	2	1	Q9CQ48	N-Term(iTRAQ8plex); K18(iTRAQ8plex)	CID	1.0000	0.0000	1	1	3033596	Unique	Used	1.061	0.643	0.751	0.749	0	0	5.36	0	0	175	2	1271.13623	2541.26518	-2.61

aTVAAFTASEGHAHPR	Unambiguous	1	1	O88951	N-Term(iTRAQ8plex)	CID	1.0000	0.0000	1	1	3405441	Unique	Used	1.978	1.587	0.998	0.952	0	0	5.36	0	0	74	3	643.00507	1927.00064	-4.24
hYAHTDcPGHADYVk	Unambiguous	3	1	Q8BFR5	N-Term(iTRAQ8plex); C7(Methylthio); K15(iTRAQ8plex)	CID	1.0000	0.0000	1	1	2967679	Unique	Used	1.505	0.776	0.978	0.947	0	0	5.36	0	22	200	3	790.05115	2368.13889	-1.44
sAAPHPGDIGDFINEGLk	Unambiguous	2	1	P15116	N-Term(iTRAQ8plex); K18(iTRAQ8plex)	CID	1.0000	0.0000	1	1	3190687	Unique	Used	0.634	1.191	1.200	1.080	0	0	5.35	0	30	29	3	816.11096	2446.31833	-0.16
iNEAIVAVQAIIADPk	Unambiguous	2	1	Q9CQE8	N-Term(iTRAQ8plex); K16(iTRAQ8plex)	CID	1.0000	0.0000	1	1	3687047	Unique	Used	1.199	0.757	0.795	0.939	0	0	5.35	0	0	35	2	1137.18323	2273.35918	-4.30
sPEEGAETPVYLALLPPDAEGPHGqFVQDk	Unambiguous	1	1	P48758	N-Term(iTRAQ8plex); Q25(Deamidated); K30(iTRAQ8plex)	CID	1.0000	0.0000	1	1	2977697	Unique	Used	0.754	1.311	0.772	1.119	0	0	5.35	0	0	200	3	1267.65784	3800.95896	2.87
iASQVAALDLGYkPGVEAIR	Unambiguous	1	1	Q91VD9	N-Term(iTRAQ8plex); K13(iTRAQ8plex)	CID	1.0000	0.0000	1	1	3684679	Unique	Used	0.626	1.189	1.104	1.204	0	0	5.35	0	0	34	3	893.85260	2679.54325	-8.28
aVQQPDGLAVLGIFLk	Unambiguous	1	1	P00920	N-Term(iTRAQ8plex); K16(iTRAQ8plex)	CID	1.0000	0.0000	1	1	3806200	Unique	Used	1.141	1.107	1.537	1.900	0	0	5.35	0	26	35	2	1139.18872	2277.37016	-3.94
lDEDLcAAPAnGGSVSRPPVTLR	Unambiguous	1	1	P57724	N-Term(iTRAQ8plex); C6(Methylthio); N11(Deamidated)	HCD	1.0000	0.0000	1	1	3323522	Unique	Used	0.913	0.400	0.803	0.945	0	0	5.35	0	0	127	3	897.12231	2689.35239	-2.53
aLVTLGnNAAFSTNQAIIR	Unambiguous	3	1	Q9D0L7	N-Term(iTRAQ8plex); N7(Deamidated)	CID	0.0336	0.0000	1	1	3804903	Unique	Used	0.723	1.480	1.201	1.229	0	0	5.35	0	15	102	2	1140.13391	2279.26055	-2.47
lLGHWEEAAHDLALAck	Unambiguous	2	1	F8WJK8	N-Term(iTRAQ8plex); C16(Methylthio); K17(iTRAQ8plex)	CID	1.0000	0.0000	1	1	3479569	Unique	Used			1.122	0.852	0	0	5.35	0	15	13	3	844.44965	2531.33438	-0.62

aAGDTVVIENSDISPEMSEVIGk	Unambiguous	8	1	Q61595-3	N-Term(iTRAQ8plex); K23(iTRAQ8plex)	CID	1.0000	0.0000	1	1	2886688	Unique	Used		1.237			0	0	5.35	0	0	200	3	990.52332	2969.55539	-1.50
nEGITALYSGLkPTMIR	Unambiguous	1	1	Q9WVD5	N-Term(iTRAQ8plex); K12(iTRAQ8plex)	HCD	1.0000	0.0000	1	1	3857876	Unique	Used	0.881	0.967	0.998	1.388	0	0	5.35	0	29	200	3	824.80432	2472.39841	-4.89
gTGGVDTAAVGGVFDVSNADR	Unambiguous	1	1	Q04447	N-Term(iTRAQ8plex)	CID	1.0000	0.0000	1	1	3135475	Unique	Used	1.003	0.704	1.386	1.080	0	0	5.35	0	0	200	2	1135.07068	2269.13408	-0.98
iMVVGSTInDVLAVNTPk	Unambiguous	1	1	Q78JW9	N-Term(iTRAQ8plex); N9(Deamidated); K18(iTRAQ8plex)	CID	1.0000	0.0000	1	1	3417948	Unique	Used	1.835	0.594	0.790	0.844	0	0	5.34	0	0	137	2	1240.71362	2480.41997	-2.23
dNHLLGTFDLTGIPPAPR	Unambiguous	1	1	P20029	N-Term(iTRAQ8plex)	CID	1.0000	0.0000	1	1	3684708	Unique	Used	1.107	1.070	0.841	0.819	0	0	5.34	0	2	10	3	746.73975	2238.20469	-6.17
tSSFAEPGGGGGGGGGPGGSASGPGGTGGGk	Unambiguous	2	1	D3Z7E5	N-Term(iTRAQ8plex); K32(iTRAQ8plex)	CID	1.0000	0.0000	1	1	2987993	Unique	Used	5.007				0	0	5.34	0	0	97	3	1014.49457	3041.46915	-2.59
gTGGVDTAAVGGVFDVSnADR	Unambiguous	1	1	Q04447	N-Term(iTRAQ8plex); N18(Deamidated)	CID	1.0000	0.0000	1	1	2901823	Unique	Used	1.279	1.036	1.476	1.416	0	0	5.34	0	0	52	2	1135.56042	2270.11357	-2.97
aLEQHDcEIAQEIQEk	Unambiguous	4	1	F6UK66	N-Term(iTRAQ8plex); C7(Methylthio); K16(iTRAQ8plex)	CID	1.0000	0.0000	1	1	2653172	Unique	Used	1.236	0.500	1.146	1.320	0	0	5.34	0	0	200	3	846.76605	2538.28360	1.86
iDQVNQLLELDHQk	Unambiguous	3	1	P61202	N-Term(iTRAQ8plex); K14(iTRAQ8plex)	CID	1.0000	0.0000	1	1	3258789	Unique	Used	0.527	1.034	0.885	1.102	0	0	5.34	0	0	3	3	767.77148	2301.29990	-1.05
iDDLQMVLNQTEDHR	Unambiguous	4	1	Q9Z1G4-3	N-Term(iTRAQ8plex)	CID	1.0000	0.0000	1	1	3036538	Unique	Used	0.471	1.172	1.458	1.355	0	0	5.34	0	3	31	3	711.02905	2131.07260	-1.40
tHSAITLGDWSDPLDPDAEAQAVYR	Unambiguous	1	1	Q9Z268	N-Term(iTRAQ8plex)	CID	1.0000	0.0000	1	1	3016188	Unique	Used		0.923	0.949	0.694	0	0	5.34	0	4	20	3	1011.50146	3032.48984	-0.18
rEVVPPSIImSSQk	Unambiguous	1	1	Q9Z1P6	N-Term(iTRAQ8plex); M10(Oxidation); K14(iTRAQ8plex)	CID	1.0000	0.0000	1	1	3657094	Unique	Used	1.019	1.125	1.363	1.132	0	0	5.34	1	7	25	3	732.42285	2195.25400	-6.31

fLSQPFQVAEVFTGHMGk	Unambiguous	1	1	P56480	N-Term(iTRAQ8plex); K18(iTRAQ8plex)	CID	1.0000	0.0000	1	1	3864111	Unique	Used	1.296	0.704	1.142	1.130	0	0	5.34	0	28	200	3	877.80865	2631.41141	-3.80
sNIAATNSGSNSnGATR	Unambiguous	1	1	Q5GH67	N-Term(iTRAQ8plex); N13(Deamidated)	CID	0.0112	0.0000	1	1	2840537	Unique	Used			1.131		0	0	5.34	0	0	200	2	963.97156	1926.93584	-3.15
lAIQEMVSLTSPSAPASSR	Unambiguous	3	1	B8QI35	N-Term(iTRAQ8plex)	CID	1.0000	0.0000	1	1	3841937	Unique	Used	0.499	0.998	1.528	1.007	0	0	5.34	0	11	29	2	1125.10535	2249.20342	-3.53
lTEAVVTDYLNSGNANDAVSGVR	Unambiguous	4	1	G3XA17	N-Term(iTRAQ8plex)	HCD	1.0000	0.0000	1	1	3351236	Unique	Used	1.457	1.060	0.800	0.908	0	0	5.34	0	0	38	2	1335.18298	2669.35869	-3.66
lGSSGLGSASSIQAAVR	Unambiguous	4	1	Q7TMY8-3	N-Term(iTRAQ8plex)	CID	1.0000	0.0000	1	1	3874442	Unique	Used	0.782	0.980	1.127	1.225	0	0	5.34	0	0	200	2	933.02002	1865.03276	-3.59
lFQEGSTADALGTGADGQMER	Unambiguous	1	1	Q00560	N-Term(iTRAQ8plex)	CID	1.0000	0.0000	1	1	2849975	Unique	Used	1.301	1.283	1.508	1.551	0	0	5.34	0	0	194	2	1229.58960	2458.17192	-4.21
fQLTDSQIYEVLSVIR	Unambiguous	1	1	O08553	N-Term(iTRAQ8plex)	HCD	1.0000	0.0000	1	1	3843398	Unique	Used	1.570	2.172	1.222	1.417	0	0	5.34	0	12	143	2	1108.11462	2215.22197	-2.55
dSSTSPGDYVLSVSEnSR	Unambiguous	5	1	Q64010	N-Term(iTRAQ8plex); N16(Deamidated)	CID	1.0000	0.0000	1	1	2635536	Unique	Used	2.576				0	0	5.34	0	19	200	2	1103.02490	2205.04253	-1.62
iLEVnGVNVEGATHk	Unambiguous	2	1	Q3UHD6-2	N-Term(iTRAQ8plex); N5(Deamidated); K15(iTRAQ8plex)	CID	0.0432	0.0000	1	1	3011798	Unique	Used	0.834	0.952	0.884	0.880	0	0	5.33	0	21	29	3	730.41595	2189.23331	-2.44
cLAFHDISPQAPTHFLVIPk	Unambiguous	2	1	P70349	N-Term(iTRAQ8plex); C1(Methylthio); K20(iTRAQ8plex)	CID	1.0000	0.0000	1	1	3878546	Unique	Used	0.792	0.870	1.186	1.193	0	0	5.33	0	22	25	3	963.52832	2888.57041	-2.49
aLSSAPASFPPPPAAAR	Unambiguous	1	1	Q99MX7	N-Term(iTRAQ8plex)	HCD	1.0000	0.0000	1	1	3874001	Unique	Used	0.317	1.442	1.978	0.596	0	0	5.33	0	0	119	2	956.53021	1912.05315	-3.31
amVALIDVFHQYSGR	Unambiguous	1	1	P50114	N-Term(iTRAQ8plex); M2(Oxidation)	HCD	1.0000	0.0000	1	1	3859348	Unique	Used	5.332	1.226	1.615	1.448	0	0	5.33	0	9	58	3	676.35748	2027.05790	-5.30
dNHLLGTFDLTGIPPAPR	Unambiguous	1	1	P20029	N-Term(iTRAQ8plex)	CID	1.0000	0.0000	1	1	3704721	Unique	Used	1.101	1.656	1.076	1.067	0	0	5.33	0	10	23	3	746.73981	2238.20487	-6.09
vGEGFEETLGEAVISTk	Unambiguous	1	1	P46660	N-Term(iTRAQ8plex); K17(iTRAQ8plex)	CID	1.0000	0.0000	1	1	2959988	Unique	Used	1.552	0.239	2.315	1.536	0	0	5.33	0	7	156	2	1187.65039	2374.29350	-1.16

vNnSSLIGVGYTQTLRPGVk	Unambiguous	2	1	G3UX26	N-Term(iTRAQ8plex); N3(Deamidated); K20(iTRAQ8plex)	CID	0.0131	0.0000	1	1	3721678	Unique	Used	0.706	1.442	1.284	1.444	0	0	5.33	0	0	73	3	904.84808	2712.52970	-7.67
rIPQSTLSEFYPR	Unambiguous	1	1	P62814	N-Term(iTRAQ8plex)	HCD	1.0000	0.0000	1	1	3874206	Unique	Used	0.644	1.166	1.063	1.278	0	0	5.33	1	5	6	3	633.35004	1898.03556	-4.33
sSScGDAELLGqATLPVGSPSRPMSR	Unambiguous	1	1	Q80X80	N-Term(iTRAQ8plex); C4(Methylthio); Q12(Deamidated)	HCD	1.0000	0.0000	1	1	3723217	Unique	Used	0.820	1.388	1.625	1.376	0	0	5.33	0	0	162	3	985.48322	2954.43509	0.90
yVIHTVGPIAVGQPTASQAAELR	Unambiguous	1	1	Q922B1	N-Term(iTRAQ8plex)	CID	1.0000	0.0000	1	1	3760981	Unique	Used			1.395	0.999	0	0	5.33	0	0	36	3	894.83081	2682.47788	-3.82
fLSQPFQVAEVFTGHMGk	Unambiguous	1	1	P56480	N-Term(iTRAQ8plex); K18(iTRAQ8plex)	CID	1.0000	0.0000	1	1	3862767	Unique	Used	1.191	0.899	0.935	1.029	0	0	5.32	0	18	200	3	877.80811	2631.40976	-4.43
iNQDPqVMLAPLISIALkVSQLQER	Unambiguous	2	1	Q9D0F9	N-Term(iTRAQ8plex); Q6(Deamidated); K18(iTRAQ8plex)	HCD	1.0000	0.0000	1	1	3936545	Unique	Used	1.017	0.492	0.952	1.407	0	0	5.32	1	11	138	3	1138.65991	3413.96518	0.01
tSPTVATQTGASVTSTR	Unambiguous	1	1	Q3U3E2	N-Term(iTRAQ8plex)	CID	1.0000	0.0000	1	1	3449189	Unique	Used	1.050	0.599	1.610	1.034	0	0	5.32	0	0	200	2	985.02411	1969.04094	-4.80
vATVPQHATSGPGPADVSk	Unambiguous	3	1	B7FAU9	N-Term(iTRAQ8plex); K19(iTRAQ8plex)	HCD	1.0000	0.0000	1	1	3337687	Unique	Used	0.839	2.692	0.964	0.912	0	0	5.32	0	11	37	3	809.78424	2427.33817	-2.92
eNIVEAIIHSPELIR	Unambiguous	1	1	Q9EPL8	N-Term(iTRAQ8plex)	CID	1.0000	0.0000	1	1	3418691	Unique	Used	0.226	1.897	0.851	1.123	0	0	5.32	0	0	13	3	679.72467	2037.15946	-2.55
hGYIGEFEIIDDHR	Unambiguous	4	1	P62245	N-Term(iTRAQ8plex)	CID	1.0000	0.0000	1	1	2810283	Unique	Used	0.828	0.607	0.909	0.802	0	0	5.32	0	15	32	3	669.00677	2005.00577	-1.18
tDGAAPNVAPSDVGGGGTNR	Unambiguous	1	1	P12960	N-Term(iTRAQ8plex)	CID	1.0000	0.0000	1	1	2844027	Unique	Used	0.689	0.845	0.608	0.822	0	0	5.32	0	4	110	2	1087.53857	2174.06987	-1.91
yQQETNVSQLPGRPk	Unambiguous	3	1	A2A690	N-Term(iTRAQ8plex); K15(iTRAQ8plex)	HCD	1.0000	0.0000	1	1	3315543	Unique	Used	0.717	1.069	1.446	1.028	0	0	5.32	0	0	200	3	785.10590	2353.30313	-2.26
sSSSSSSGVGSPAVTPTEk	Unambiguous	3	1	E9QN14	N-Term(iTRAQ8plex); K19(iTRAQ8plex)	CID	1.0000	0.0000	1	1	2930026	Unique	Used	1.169	1.303	1.654	1.318	0	0	5.32	0	0	58	2	1180.61694	2360.22661	-5.75

dLIQSDIAALHQFYSk	Unambiguous	1	1	Q8R5A0	N-Term(iTRAQ8plex); K16(iTRAQ8plex)	CID	1.0000	0.0000	1	1	3478797	Unique	Used	0.927	0.987	0.877	1.026	0	0	5.32	0	16	200	3	819.78925	2457.35318	-2.70
eGQGFVSEDEYLEISDIkR	Unambiguous	1	1	G5E8G3	N-Term(iTRAQ8plex); K18(iTRAQ8plex)	CID	1.0000	0.0000	1	1	2795890	Unique	Used	0.501	0.691	1.067	0.904	0	0	5.31	1	10	200	3	941.49445	2822.46878	0.67
qGIGQAPVSATEDSEIR	Unambiguous	2	1	Q9D0L4	N-Term(iTRAQ8plex)	CID	1.0000	0.0000	1	1	2666667	Unique	Used	0.566	0.957	0.980	1.388	0	0	5.31	0	0	118	2	1031.53711	2062.06694	-2.40
hnHDLSSYQDTIQQLENELR	Unambiguous	2	1	P08553	N-Term(iTRAQ8plex); N2(Deamidated)	CID	1.0000	0.0000	1	1	3036047	Unique	Used	2.276	1.204	0.975	0.929	0	0	5.31	0	26	59	3	915.78333	2745.33542	-1.01
gSHSqSSSPGALTLGR	Unambiguous	3	1	O88935	N-Term(iTRAQ8plex); Q5(Deamidated)	HCD	1.0000	0.0000	1	1	3225552	Unique	Used	1.832	1.231	1.092	1.191	0	0	5.31	0	0	151	3	616.32184	1846.95096	-2.78
yMADMDELFSQVDEk	Unambiguous	1	1	Q9WUD1	N-Term(iTRAQ8plex); K15(iTRAQ8plex)	CID	1.0000	0.0000	1	1	2853324	Unique	Used	1.264	1.093	0.948	0.805	0	0	5.31	0	3	55	3	810.39703	2429.17655	-2.47
fYYSSGSSSPTHAk	Unambiguous	1	1	Q60771	N-Term(iTRAQ8plex); K14(iTRAQ8plex)	CID	1.0000	0.0000	1	1	3428754	Unique	Used	0.640	0.342	1.882	2.311	0	0	5.31	0	8	87	3	709.69946	2127.08384	-6.04
sLQDIIAILGMDELSEEDkLTVSR	Unambiguous	1	1	P56480	N-Term(iTRAQ8plex); K19(iTRAQ8plex)	HCD	1.0000	0.0000	1	1	3354077	Unique	Used	0.957	0.948	1.111	1.225	0	0	5.31	1	0	87	3	1095.26379	3283.77683	-4.51
aPPAGGSGSSAAAAAAAGGTEVRPR	Unambiguous	2	1	P84309	N-Term(iTRAQ8plex)	HCD	1.0000	0.0000	1	1	3428622	Unique	Used	0.741	0.984	2.422	1.272	0	0	5.31	0	0	158	3	814.42871	2441.27158	-3.44
kAGLAEAPLVTGQPGPGHGk	Unambiguous	5	1	F8WHW6	N-Term(iTRAQ8plex); K1(iTRAQ8plex); K20(iTRAQ8plex)	HCD	1.0000	0.0000	1	1	3384942	Unique	Used	0.446	1.269	1.675	1.415	0	0	5.31	1	0	107	3	933.21588	2797.63309	-4.33
aAASGNENIQPPPLAYk	Unambiguous	2	1	E9QKE4	N-Term(iTRAQ8plex); K17(iTRAQ8plex)	CID	1.0000	0.0000	1	1	3277049	Unique	Used	0.855	1.364	1.062	2.203	0	0	5.31	0	0	93	2	1175.15198	2349.29668	-2.40
fNPFDLISDSEAVQEETTk	Unambiguous	2	1	Q9QYX7	N-Term(iTRAQ8plex); K19(iTRAQ8plex)	CID	1.0000	0.0000	1	1	2979151	Unique	Used	2.719	2.264	2.476	3.309	0	0	5.31	0	0	200	3	926.81470	2778.42954	0.03
iDGITIVQSLAIMEYLEETRPIPR	Unambiguous	1	1	Q9WVL0	N-Term(iTRAQ8plex)	HCD	1.0000	0.0000	1	1	3806458	Unique	Used	0.426	0.914	1.194	1.518	0	0	5.31	0	8	19	3	1021.23260	3061.68326	-2.53

lVnGATQSFIIRPQk	Unambiguous	7	1	P34152-3	N-Term(iTRAQ8plex); N3(Deamidated); K15(iTRAQ8plex)	HCD	1.0000	0.0000	1	1	3780352	Unique	Used	0.566	1.193	1.232	0.987	0	0	5.31	0	29	200	3	761.11908	2281.34269	-2.71
gTGGVDTAAVGGVFDVSNADR	Unambiguous	1	1	Q04447	N-Term(iTRAQ8plex)	CID	1.0000	0.0000	1	1	3154616	Unique	Used	1.401	1.061	0.695	0.841	0	0	5.31	0	18	200	2	1135.06873	2269.13017	-2.70
gTGGVDTAAVGGVFDVSNADR	Unambiguous	1	1	Q04447	N-Term(iTRAQ8plex)	CID	1.0000	0.0000	1	1	3134097	Unique	Used	1.579	1.129	1.153	1.169	0	0	5.31	0	0	200	2	1135.07007	2269.13286	-1.52
kAEGATLSnATGAVESTSQAGDR	Unambiguous	1	1	Q61644	N-Term(iTRAQ8plex); K1(iTRAQ8plex); N9(Deamidated)	CID	1.0000	0.0000	1	1	2881187	Unique	Used	1.542	2.658	2.371	2.959	0	0	5.30	1	0	200	3	944.15814	2830.45987	-1.44
vEAVNMAEGIIHDTETk	Unambiguous	1	1	P38647	N-Term(iTRAQ8plex); K17(iTRAQ8plex)	CID	1.0000	0.0000	1	1	2959808	Unique	Used	1.975	0.724	1.539	1.335	0	0	5.30	0	14	98	3	822.44135	2465.30948	-2.92
gGSEPATSTLPAAAAATnGPR	Unambiguous	3	1	Q8BNU1	N-Term(iTRAQ8plex); N18(Deamidated)	CID	1.0000	0.0000	1	1	2932596	Unique	Used	0.615	0.805	0.765	0.572	0	0	5.30	0	0	200	2	1101.56567	2202.12407	-2.91
eLAAQGELDLHk	Unambiguous	3	1	Q8BRR9	N-Term(iTRAQ8plex); K12(iTRAQ8plex)	CID	1.0000	0.0000	1	1	2846488	Unique	Used	0.388	2.601	1.627	1.066	0	0	5.30	0	5	46	3	644.70490	1932.10013	-0.50
eFTMAEIEHFVDPTEk	Unambiguous	1	1	Q9CZD3	N-Term(iTRAQ8plex); K16(iTRAQ8plex)	CID	1.0000	0.0000	1	1	2887965	Unique	Used	0.533	0.893	0.832	0.935	0	0	5.30	0	7	200	3	844.43652	2531.29502	0.06
kLAQQYYLVYQEPIPTAQLVQR	Unambiguous	1	1	P49722	N-Term(iTRAQ8plex); K1(iTRAQ8plex)	HCD	1.0000	0.0000	1	1	3840830	Unique	Used	1.106	0.887	0.948	0.992	0	0	5.30	1	0	119	3	1086.61804	3257.83957	-3.41
eTIELVHTEPTNVAQLPSR	Unambiguous	2	1	Q9Z0P5-2	N-Term(iTRAQ8plex)	HCD	1.0000	0.0000	1	1	3188620	Unique	Used	0.653	1.130	1.072	1.184	0	0	5.30	0	0	75	2	1219.66223	2438.31719	-0.88
eYGGLDVLVNNAGIAFk	Unambiguous	1	1	P48758	N-Term(iTRAQ8plex); K17(iTRAQ8plex)	CID	1.0000	0.0000	1	1	3541043	Unique	Used			0.764	0.934	0	0	5.30	0	18	6	2	1194.66797	2388.32866	-4.07
tVQLYGPTNFAPVVTHVAR	Unambiguous	1	1	Q8JZW4	N-Term(iTRAQ8plex)	CID	1.0000	0.0000	1	1	3932940	Unique	Used	1.216	1.662	2.034	0.935	0	0	5.29	0	5	13	3	792.10803	2374.30954	-3.78
yITGDQLGALYQDFVR	Unambiguous	2	1	P17183	N-Term(iTRAQ8plex)	CID	1.0000	0.0000	1	1	3648859	Unique	Used	2.193	1.206	1.198	1.321	0	0	5.29	0	8	200	2	1082.06702	2163.12676	-5.57

sTDSSSSVSGSLQQETk	Unambiguous	1	1	Q8VDY9	N-Term(iTRAQ8plex); K17(iTRAQ8plex)	CID	1.0000	0.0000	1	1	2729628	Unique	Used			0.333	0.246	0	0	5.29	0	0	200	2	1168.60388	2336.20049	-1.41
aTSSSSSSGVGmSVGQGR	Unambiguous	2	1	P21619	N-Term(iTRAQ8plex); M13(Oxidation)	CID	1.0000	0.0000	1	1	3402744	Unique	Used	0.908	0.769	1.467	1.674	0	0	5.29	0	0	200	2	1024.98938	2048.97148	-5.17
nEEDATELVGLAQAVNAR	Unambiguous	1	1	Q02053	N-Term(iTRAQ8plex)	CID	1.0000	0.0000	1	1	2959403	Unique	Used	0.483	0.782	1.035	1.135	0	0	5.29	0	2	72	2	1102.57617	2204.14507	-0.48
kGVLETFSGTETNk	Unambiguous	1	1	Q9WTP7	N-Term(iTRAQ8plex); K1(iTRAQ8plex); K14(iTRAQ8plex)	HCD	1.0000	0.0000	1	1	3276170	Unique	Used	0.812	1.191	1.020	1.062	0	0	5.29	1	16	136	3	808.46893	2423.39225	0.55
lSTLVETTLkETEk	Unambiguous	2	1	G5E819	N-Term(iTRAQ8plex); K10(iTRAQ8plex); K14(iTRAQ8plex)	HCD	1.0000	0.0000	1	1	3237584	Unique	Used	0.511	0.958	1.014	1.155	0	0	5.29	1	21	180	3	835.50214	2504.49186	-1.27
sMPAGLTLQAVSPQSLQGR	Unambiguous	1	1	E9Q6H8	N-Term(iTRAQ8plex)	HCD	1.0000	0.0000	1	1	3933989	Unique	Used	0.437	0.721	1.162	0.875	0	0	5.29	0	0	115	2	1123.11206	2245.21684	-4.83
lQVELDSVTGLLSQSDSk	Unambiguous	1	1	Q8VDD5	N-Term(iTRAQ8plex); K18(iTRAQ8plex)	HCD	1.0000	0.0000	1	1	3352451	Unique	Used	1.144	1.051	1.296	0.738	0	0	5.29	0	17	31	2	1264.20093	2527.39458	-5.14
nQGGYGGSSSSSSYGSGR	Unambiguous	3	1	Q5EBP8	N-Term(iTRAQ8plex); N1(Deamidated)	CID	0.0151	0.0000	1	1	2841045	Unique	Used	0.912	0.924	0.747	0.714	0	0	5.29	0	0	200	2	1000.44550	1999.88371	-2.90
fGVVTEVVMIYDAEk	Unambiguous	4	1	Q9JII5-2	N-Term(iTRAQ8plex); K15(iTRAQ8plex)	CID	1.0000	0.0000	1	1	3352751	Unique	Used	2.293	2.219	0.770	0.652	0	0	5.29	0	24	27	2	1154.63757	2308.26787	-1.76
gkLPIVNEnDELVAIIAR	Unambiguous	1	1	P24547	N-Term(iTRAQ8plex); K2(iTRAQ8plex); N9(Deamidated)	HCD	0.0435	0.0000	1	1	3303981	Unique	Used	1.857	1.037	0.704	0.832	0	0	5.29	1	11	200	3	858.50659	2573.50522	-2.77
vAVLGASGGIGQPLSLLLk	Unambiguous	1	1	P08249	N-Term(iTRAQ8plex); K19(iTRAQ8plex)	CID	1.0000	0.0000	1	1	3939917	Unique	Used	2.043		0.778	1.124	0	0	5.28	0	13	200	2	1201.24841	2401.48955	-4.49
sSPPPLSGASEVDAGELGSER	Unambiguous	1	1	A6X919	N-Term(iTRAQ8plex)	CID	1.0000	0.0000	1	1	2651867	Unique	Used		0.431	0.863	0.989	0	0	5.28	0	0	200	2	1173.58923	2346.17119	-0.66

sGLGLGAASASTSGSGPADSVmR	Unambiguous	3	1	Q7TSJ2	N-Term(iTRAQ8plex); M22(Oxidation)	CID	1.0000	0.0000	1	1	3532310	Unique	Used	1.600	0.757	1.599	1.260	0	0	5.28	0	0	132	2	1178.58142	2356.15557	-6.85
sQIFSTASDNQPTVTIk	Unambiguous	1	1	P20029	N-Term(iTRAQ8plex); K17(iTRAQ8plex)	CID	1.0000	0.0000	1	1	3412354	Unique	Used	1.685	0.994	0.858	0.531	0	0	5.28	0	0	76	2	1223.16895	2445.33061	-5.71
sVIGMGTGAGAYILTR	Unambiguous	5	1	Q62433	N-Term(iTRAQ8plex)	HCD	1.0000	0.0000	1	1	3915473	Unique	Used	0.943	0.797	0.997	1.696	0	0	5.28	0	7	8	2	936.01678	1871.02629	-5.34
iGIIGGTGLDDPEILEGR	Unambiguous	1	1	Q9CQ65	N-Term(iTRAQ8plex)	CID	1.0000	0.0000	1	1	3132867	Unique	Used	3.020	0.653	0.658	0.897	0	0	5.28	0	15	18	2	1065.08960	2129.17192	-1.75
lALQQDLTSMAPGLVIQAVR	Unambiguous	1	1	Q8BFZ9	N-Term(iTRAQ8plex)	HCD	1.0000	0.0000	1	1	3898684	Unique	Used	0.820	0.982	0.721	0.961	0	0	5.28	0	12	6	2	1214.69421	2428.38115	-3.65
hVESIGDPEHISR	Unambiguous	2	1	P55302	N-Term(iTRAQ8plex)	CID	1.0000	0.0000	1	1	2728014	Unique	Used	2.540	1.194	1.603	1.114	0	0	5.28	0	21	168	3	593.98004	1779.92557	-2.01
gSLLIDSSTIDPSVSk	Unambiguous	1	1	Q99L13	N-Term(iTRAQ8plex); K16(iTRAQ8plex)	CID	1.0000	0.0000	1	1	3258043	Unique	Used	1.258	0.738	1.056	1.326	0	0	5.28	0	10	9	2	1114.13354	2227.25981	-1.97
gEAIEAILAALEVVSEPFR	Unambiguous	1	1	Q8VDM4	N-Term(iTRAQ8plex)	CID	1.0000	0.0000	1	1	3825721	Unique	Used	0.894	1.036	0.749	0.524	0	0	5.28	0	0	200	2	1159.64441	2318.28154	-4.08
lIEESPIDEVVVTNTVPHELQk	Unambiguous	2	1	Q9D0M1	N-Term(iTRAQ8plex); K22(iTRAQ8plex)	CID	1.0000	0.0000	1	1	3036257	Unique	Used	0.471	1.014	0.842	1.261	0	0	5.28	0	3	61	3	1033.24377	3097.71677	-2.40
mPLPAPALGHQPPPVPR	Unambiguous	4	1	D3YWX2	N-Term(iTRAQ8plex)	HCD	1.0000	0.0000	1	1	3930864	Unique	Used	1.147	0.741	1.578	0.917	0	0	5.28	0	20	188	3	693.73059	2079.17722	-3.25
kGDSSSGGGGGSSGGGGSSNVGGGSSGGSGScSSSSR	Unambiguous	3	1	Q64012-2	N-Term(iTRAQ8plex); K1(iTRAQ8plex); C32(Methylthio)	CID	1.0000	0.0000	1	1	2984038	Unique	Used		0.173	1.225	0.654	0	0	5.28	1	0	200	3	1202.86267	3606.57346	-2.88
sLQDIIAILGMDELSEEDkLTVSR	Unambiguous	1	1	P56480	N-Term(iTRAQ8plex); K19(iTRAQ8plex)	HCD	1.0000	0.0000	1	1	3354948	Unique	Used	0.975	0.937	1.169	0.993	0	0	5.28	1	0	194	3	1095.26477	3283.77976	-3.62
vLGEGEFGSVMEGNLk	Unambiguous	1	1	Q60805	N-Term(iTRAQ8plex); K16(iTRAQ8plex)	CID	1.0000	0.0000	1	1	3193489	Unique	Used	0.918	1.938	1.714	1.228	0	0	5.28	0	19	91	2	1137.61365	2274.22002	-2.66

dINQEVYnFLATAGAk	Unambiguous	1	1	Q99KI0	N-Term(iTRAQ8plex); N8(Deamidated); K16(iTRAQ8plex)	CID	1.0000	0.0000	1	1	3034831	Unique	Used	0.759	0.854	1.252	1.277	0	0	5.28	0	6	93	2	1182.13855	2363.26982	-0.22
hFIDVGAGVIDEDYR	Unambiguous	2	1	Q8VCG1	N-Term(iTRAQ8plex)	CID	1.0000	0.0000	1	1	2937131	Unique	Used	2.245	1.085	1.206	1.104	0	0	5.28	0	23	55	3	670.67749	2010.01792	-2.76
lGYAVYETPTAHnGAk	Unambiguous	4	1	Q9QZ06	N-Term(iTRAQ8plex); N13(Deamidated); K16(iTRAQ8plex)	CID	1.0000	0.0000	1	1	2990670	Unique	Used	1.033	0.961	1.472	1.450	0	0	5.27	0	5	26	3	767.74805	2301.22959	-1.72
gSSGGYGPGAAALQPk	Unambiguous	2	1	Q6PEV3	N-Term(iTRAQ8plex); K16(iTRAQ8plex)	CID	1.0000	0.0000	1	1	3295441	Unique	Used	1.487	0.672	1.249	0.961	0	0	5.27	0	0	200	2	1013.56165	2026.11601	-0.89
vETTTSLEnGGLPSSAETk	Unambiguous	3	1	Q5SW19	N-Term(iTRAQ8plex); N9(Deamidated); K19(iTRAQ8plex)	CID	1.0000	0.0000	1	1	2668291	Unique	Used	0.158	0.687	0.469	0.654	0	0	5.27	0	0	200	2	1265.67102	2530.33476	0.11
eALLGVQEDVDEYVk	Unambiguous	1	1	Q8BP92	N-Term(iTRAQ8plex); K15(iTRAQ8plex)	CID	1.0000	0.0000	1	1	2656199	Unique	Used	2.509	0.894	0.923	0.773	0	0	5.27	0	3	200	3	772.42389	2315.25711	-0.87
gDPYPQEVGATVHk	Unambiguous	2	1	P22315	N-Term(iTRAQ8plex); K14(iTRAQ8plex)	CID	1.0000	0.0000	1	1	2825646	Unique	Used	1.119	1.194	1.103	1.108	0	0	5.27	0	17	200	3	702.71851	2106.14096	-1.45
qSLSSADNLEPDVQGHQVAAR	Unambiguous	2	1	A2A5R2	N-Term(iTRAQ8plex)	CID	1.0000	0.0000	1	1	2880865	Unique	Used	1.168	1.093	1.452	1.379	0	0	5.27	0	0	200	3	842.76349	2526.27591	-3.63
gSETLGGGGGGAAGTR	Unambiguous	1	1	P31650	N-Term(iTRAQ8plex)	CID	1.0000	0.0000	1	1	2840947	Unique	Used	0.443	0.227	0.638	1.476	0	0	5.27	0	2	4	2	804.91290	1608.81853	-3.64
rLPASDGLDLSQAAAR	Unambiguous	1	1	P46660	N-Term(iTRAQ8plex)	HCD	1.0000	0.0000	1	1	3533040	Unique	Used	1.110	1.043	1.808	1.713	0	0	5.27	1	19	20	3	649.02716	1945.06693	-5.13
iPNIYAIGDVVAGPmLAHk	Unambiguous	1	1	O08749	N-Term(iTRAQ8plex); M15(Oxidation); K19(iTRAQ8plex)	CID	1.0000	0.0000	1	1	3827655	Unique	Used	1.613	0.810	2.076	1.075	0	0	5.27	0	6	200	3	868.49738	2603.47757	-2.47
tWGDAGAAAGGGIPSk	Unambiguous	1	1	P31324	N-Term(iTRAQ8plex); K16(iTRAQ8plex)	CID	1.0000	0.0000	1	1	3276310	Unique	Used	0.394	1.690	1.907	1.117	0	0	5.27	0	12	42	2	1012.55182	2024.09636	-2.88

iPNIYAIGDVVAGPmLAHk	Unambiguous	1	1	O08749	N-Term(iTRAQ8plex); M15(Oxidation); K19(iTRAQ8plex)	CID	1.0000	0.0000	1	1	3825987	Unique	Used	1.555	1.128	1.097	0.862	0	0	5.27	0	30	200	3	868.49622	2603.47409	-3.81
gPEPDTIIGYSGEDYPR	Unambiguous	2	1	E9PW06	N-Term(iTRAQ8plex)	CID	1.0000	0.0000	1	1	2717356	Unique	Used	1.723	0.182	1.003	1.473	0	0	5.27	0	0	112	2	1085.53235	2170.05742	-1.48
aALDGTPGMIGYGMAk	Unambiguous	3	1	Q8BVI4	N-Term(iTRAQ8plex); K16(iTRAQ8plex)	CID	1.0000	0.0000	1	1	3390643	Unique	Used	7.651	1.183	1.599	1.494	0	0	5.27	0	14	47	2	1081.07776	2161.14824	-5.73
aIAFADQLHAVDTDGVEPLESVLEDR	Unambiguous	1	1	Q8CBY0	N-Term(iTRAQ8plex)	CID	1.0000	0.0000	1	1	2979096	Unique	Used	1.970	0.583	1.338	0.722	0	0	5.27	0	7	34	3	1038.86597	3114.58335	-2.07
vVDPLAFADVFVALGPQHR	Unambiguous	1	1	Q80WM4	N-Term(iTRAQ8plex)	CID	1.0000	0.0000	1	1	3860911	Unique	Used	1.853		0.626	0.882	0	0	5.26	0	0	48	3	785.77386	2355.30704	-2.42
vDcTASGAENSGSLGk	Unambiguous	1	1	Q3UNH4	N-Term(iTRAQ8plex); C3(Methylthio); K16(iTRAQ8plex)	CID	1.0000	0.0000	1	1	2972042	Unique	Used	0.893	1.346	1.504	0.730	0	0	5.26	0	0	200	2	1075.53430	2150.06133	-3.03
dQVANSAFVERLR	Unambiguous	1	1	P07901	N-Term(iTRAQ8plex)	HCD	1.0000	0.0000	1	1	3431529	Unique	Used	0.860	0.505	1.068	1.158	0	0	5.26	1	15	111	3	603.66608	1808.98368	-4.66
iGGHGAEYGAEALER	Unambiguous	2	1	Q91VB8	N-Term(iTRAQ8plex)	CID	1.0000	0.0000	1	1	2783763	Unique	Used	4.457	1.862	0.244	1.129	0	0	5.26	0	2	200	3	611.98383	1833.93692	-1.53
gENVSTTEVEAVLSR	Unambiguous	1	1	Q60714	N-Term(iTRAQ8plex)	HCD	1.0000	0.0000	1	1	2937486	Unique	Used	0.954	1.408	1.121	1.406	0	0	5.26	0	16	134	2	948.00122	1894.99516	-3.81
sSSSAGSEVGGQSTGSNHk	Unambiguous	2	1	Q8BKI2	N-Term(iTRAQ8plex); K19(iTRAQ8plex)	CID	1.0000	0.0000	1	1	2926579	Unique	Used	1.613	0.967	1.313	0.921	0	0	5.26	0	0	200	3	791.40002	2372.18552	-1.83
gQAQSDAAGLPGAESR	Unambiguous	1	1	Q9D3L0	N-Term(iTRAQ8plex)	CID	1.0000	0.0000	1	1	2663811	Unique	Used	0.927	0.827	1.011	0.641	0	0	5.26	0	0	200	2	909.96301	1818.91875	-3.34
aSLNPNDLGPLPPGWEER	Unambiguous	5	1	E9PXB7	N-Term(iTRAQ8plex)	HCD	1.0000	0.0000	1	1	3281630	Unique	Used	0.642	1.446	1.442	1.115	0	0	5.26	0	0	49	2	1133.59021	2266.17314	-1.72
eQLDPDELETITMHk	Unambiguous	2	1	Q6P5E4	N-Term(iTRAQ8plex); K15(iTRAQ8plex)	CID	1.0000	0.0000	1	1	2653892	Unique	Used		1.054	0.687	0.581	0	0	5.26	0	4	200	3	803.09265	2407.26340	-0.07
aPkPDGPGGGPGGSHMGGNYGDDR	Unambiguous	5	1	Q8CFQ9	N-Term(iTRAQ8plex); K3(iTRAQ8plex)	CID	1.0000	0.0000	1	1	2842072	Unique	Used	1.363	0.457	1.277	0.752	0	0	5.26	0	14	153	3	954.46295	2861.37430	-3.61
gEAQAAVGSGGGVSVFR	Unambiguous	1	1	Q8BNW9	N-Term(iTRAQ8plex)	CID	1.0000	0.0000	1	1	3387018	Unique	Used	1.090	1.867	1.796	1.492	0	0	5.26	0	0	71	2	926.99103	1852.97478	-3.87

lNITPNSGATGnNAGPk	Unambiguous	1	1	Q62433	N-Term(iTRAQ8plex); N12(Deamidated); K17(iTRAQ8plex)	CID	0.0380	0.0000	1	1	3185510	Unique	Used	1.160	0.462	1.138	1.373	0	0	5.26	0	0	16	2	1118.11072	2235.21416	-2.16
lDITTLTGVPEEHIk	Unambiguous	2	1	E9QPX3	N-Term(iTRAQ8plex); K15(iTRAQ8plex)	HCD	1.0000	0.0000	1	1	3213425	Unique	Used	1.272	0.424	1.465	0.889	0	0	5.26	0	0	21	2	1137.65979	2274.31230	-1.88
aDIGVAMGIAGSDVSk	Unambiguous	2	1	Q6PIC6	N-Term(iTRAQ8plex); K16(iTRAQ8plex)	CID	1.0000	0.0000	1	1	3258227	Unique	Used	0.587	0.623	1.208	1.288	0	0	5.26	0	12	84	2	1050.08118	2099.15508	-3.65
tVSGVnGPLVILDHVk	Unambiguous	1	1	P62814	N-Term(iTRAQ8plex); N6(Deamidated); K16(iTRAQ8plex)	HCD	1.0000	0.0000	1	1	3393412	Unique	Used	0.760	1.182	1.034	0.862	0	0	5.26	0	27	26	3	753.11316	2257.32492	-5.64
eTTDTDTADQVIASFk	Unambiguous	1	1	P57780	N-Term(iTRAQ8plex); K16(iTRAQ8plex)	CID	1.0000	0.0000	1	1	2832686	Unique	Used	1.054	1.368	1.529	1.032	0	0	5.26	0	12	186	2	1175.61255	2350.21782	-2.40
yPNAELAWcQEEHk	Unambiguous	4	1	Q60597	N-Term(iTRAQ8plex); C9(Methylthio); K14(iTRAQ8plex)	CID	1.0000	0.0000	1	1	2974372	Unique	Used	0.870	0.877	1.022	1.119	0	0	5.26	0	25	139	3	791.39221	2372.16208	-0.13
dEQVVVSHDANLk	Unambiguous	1	1	Q9CRY7	N-Term(iTRAQ8plex); K13(iTRAQ8plex)	CID	1.0000	0.0000	1	1	2648674	Unique	Used	0.400	0.815	0.830	0.920	0	0	5.25	0	4	11	3	688.04926	2062.13321	-2.77
dTnGENIAESLVAEGLATR	Unambiguous	2	1	Q78PY7	N-Term(iTRAQ8plex); N3(Deamidated)	CID	1.0000	0.0000	1	1	2766181	Unique	Used	0.505	0.898	0.691	0.940	0	0	5.25	0	0	200	2	1133.07751	2265.14775	-1.55
gEAIEAILAALEVVSEPFR	Unambiguous	1	1	Q8VDM4	N-Term(iTRAQ8plex)	CID	1.0000	0.0000	1	1	3807359	Unique	Used	0.898	1.184	0.958	1.057	0	0	5.25	0	27	200	2	1159.64600	2318.28472	-2.72
eHINLGcDVDFDIAGPSIR	Unambiguous	3	1	Q60932-2	N-Term(iTRAQ8plex); C7(Methylthio)	CID	1.0000	0.0000	1	1	3397640	Unique	Used	1.200	1.179	1.186	1.248	0	0	5.25	0	29	200	3	807.72961	2421.17429	-4.22
yITGDQLGALYQDFVR	Unambiguous	2	1	P17183	N-Term(iTRAQ8plex)	CID	1.0000	0.0000	1	1	3670340	Unique	Used	2.370	1.147	2.174	2.916	0	0	5.25	0	6	200	2	1082.06653	2163.12578	-6.02

hIVLSGGSTMyPGLPSR	Unambiguous	1	1	P61161	N-Term(iTRAQ8plex); Y11(iTRAQ8plex)	HCD	1.0000	0.0000	1	1	3929328	Unique	Used	0.231	1.719	0.889	0.838	0	0	5.25	0	5	105	3	794.11139	2380.31961	-3.00
sAAPHPGDIGDFINEGLk	Unambiguous	2	1	P15116	N-Term(iTRAQ8plex); K18(iTRAQ8plex)	HCD	1.0000	0.0000	1	1	3169144	Unique	Used	1.295	1.137	1.296	1.093	0	0	5.25	0	29	43	3	816.10651	2446.30497	-5.62
vPVASPSAHNISSSSGAPDR	Unambiguous	14	1	E9QMP6	N-Term(iTRAQ8plex)	HCD	1.0000	0.0000	1	1	3468498	Unique	Used	0.623	1.562	1.394	1.007	0	0	5.25	0	0	61	3	747.38763	2240.14835	-4.01
aSSATDGQLASPGEnGEVRPGQAk	Unambiguous	1	1	P16054	N-Term(iTRAQ8plex); N15(Deamidated); K24(iTRAQ8plex)	CID	1.0000	0.0000	1	1	2665151	Unique	Used	0.395	2.250	1.906	1.759	0	0	5.25	0	0	200	3	979.50854	2936.51108	-2.04
qLVMNHMHHEDQQVR	Unambiguous	1	1	Q8BVE3	N-Term(iTRAQ8plex)	CID	1.0000	0.0000	1	1	3887662	Unique	Used		0.596	0.846	1.224	0	0	5.25	0	0	200	3	736.03326	2206.08524	-2.69
kLFIGGLSFETTDDSLR	Unambiguous	6	1	J3QNY1	N-Term(iTRAQ8plex); K1(iTRAQ8plex)	HCD	1.0000	0.0000	1	1	3498076	Unique	Used	0.981	0.676	1.058	0.877	0	0	5.25	1	15	32	3	836.46802	2507.38950	-2.84
wLAESPVGLPPEEEDk	Unambiguous	3	1	Q9QYR6	N-Term(iTRAQ8plex); K16(iTRAQ8plex)	CID	1.0000	0.0000	1	1	2797391	Unique	Used	2.014	1.174	1.881	0.966	0	0	5.25	0	10	200	2	1202.64526	2404.28325	-1.02
fLSQPFQVAEVFTGHMGk	Unambiguous	1	1	P56480	N-Term(iTRAQ8plex); K18(iTRAQ8plex)	CID	1.0000	0.0000	1	1	3881538	Unique	Used	1.655	1.155	1.239	1.207	0	0	5.25	0	0	200	3	877.80817	2631.40995	-4.36
lSPPGGSGSGVPGGGPLLPTAGR	Unambiguous	1	1	Q69ZK9	N-Term(iTRAQ8plex)	HCD	1.0000	0.0000	1	1	3894683	Unique	Used	0.498	1.534	1.049	1.251	0	0	5.25	0	0	161	2	1146.62744	2292.24761	-6.04
gTGGVDTAAVGGVFDVSNADR	Unambiguous	1	1	Q04447	N-Term(iTRAQ8plex)	CID	1.0000	0.0000	1	1	3089975	Unique	Used	1.837	1.438	1.035	1.800	0	0	5.24	0	9	75	2	1135.07141	2269.13555	-0.33
iGGHGAEYGAEALER	Unambiguous	2	1	Q91VB8	N-Term(iTRAQ8plex)	CID	1.0000	0.0000	1	1	2784486	Unique	Used	6.694	5.744	0.496	1.593	0	0	5.24	0	3	200	3	611.98401	1833.93747	-1.23
lQSVcTEGSYTAEVLGPPRPVPGR	Unambiguous	1	1	Q8C8T7	N-Term(iTRAQ8plex); C5(Methylthio)	HCD	1.0000	0.0000	1	1	3804592	Unique	Used	0.571	1.303	1.276	1.228	0	0	5.24	0	9	80	3	955.16217	2863.47196	-1.02
aGSAGGPSPGGGAPWHLR	Unambiguous	2	1	Q3UHE1-2	N-Term(iTRAQ8plex)	CID	1.0000	0.0000	1	1	3795898	Unique	Used	0.962	0.950	0.990	1.226	0	0	5.24	0	10	55	3	646.00684	1936.00595	-1.66
vGLSAVPADAcSGAQk	Unambiguous	2	1	Q99KK2	N-Term(iTRAQ8plex); C11(Methylthio); K16(iTRAQ8plex)	CID	1.0000	0.0000	1	1	3390098	Unique	Used	0.312	0.457	0.522	0.810	0	0	5.24	0	0	11	2	1064.56653	2128.12578	-4.39

sLEDALSSDTSGHFR	Unambiguous	2	1	P14824	N-Term(iTRAQ8plex)	CID	1.0000	0.0000	1	1	3050681	Unique	Used	1.506	1.365	1.152	1.187	0	0	5.24	0	19	64	3	642.65369	1925.94651	-2.17
tSRPENAIIYSNNEDFQVGQAk	Unambiguous	1	1	P40142	N-Term(iTRAQ8plex); K22(iTRAQ8plex)	CID	1.0000	0.0000	1	1	3087266	Unique	Used	2.439	0.627	0.732	0.914	0	0	5.24	0	4	143	3	1030.54004	3089.60556	-1.85
tVAGIIVEPIQSEGGDNHASDDFFR	Unambiguous	2	1	P61922	N-Term(iTRAQ8plex)	CID	1.0000	0.0000	1	1	3036376	Unique	Used	1.754	0.824	1.428	1.498	0	0	5.24	0	7	177	3	993.49744	2978.47776	-0.70
aDNFEYSDPVDGSISk	Unambiguous	2	1	Q9D0F9	N-Term(iTRAQ8plex); K16(iTRAQ8plex)	CID	1.0000	0.0000	1	1	2701149	Unique	Used	0.686	1.164	0.873	1.244	0	0	5.24	0	11	200	2	1176.59021	2352.17314	-3.59
qGFNVVVESGAGEASk	Unambiguous	4	1	Q61941	N-Term(iTRAQ8plex); K16(iTRAQ8plex)	CID	1.0000	0.0000	1	1	2991162	Unique	Used	0.361	1.230	0.069	0.063	0	0	5.24	0	0	123	2	1094.09570	2187.18413	-1.14
amGVVVATGVNTEIGk	Unambiguous	3	1	O55143	N-Term(iTRAQ8plex); M2(Oxidation); K16(iTRAQ8plex)	CID	1.0000	0.0000	1	1	3473058	Unique	Used	0.480	0.913	1.367	0.841	0	0	5.24	0	0	118	2	1085.61987	2170.23247	-1.74
kAAMEALQSQALHATSQQPLR	Unambiguous	5	1	B1AQZ0	N-Term(iTRAQ8plex); K1(iTRAQ8plex)	CID	1.0000	0.0000	1	1	3874980	Unique	Used	1.352	0.732	1.562	1.453	0	0	5.23	1	0	200	3	963.20038	2887.58658	-5.79
lHIIEVGTPPTGNQPFPk	Unambiguous	2	1	Q68FD5	N-Term(iTRAQ8plex); K18(iTRAQ8plex)	CID	1.0000	0.0000	1	1	3640417	Unique	Used	0.683	0.909	1.312	1.397	0	0	5.23	0	6	13	3	851.82129	2553.44931	-6.14
aGSHIQTLMGSQSLQHR	Unambiguous	2	1	Q80TE7	N-Term(iTRAQ8plex)	CID	1.0000	0.0000	1	1	3927305	Unique	Used	0.685	1.649	1.066	0.712	0	0	5.23	0	0	200	3	719.04614	2155.12387	-4.89
kLIGSVDDLLPSLPASSR	Unambiguous	4	1	Q9QVP9	N-Term(iTRAQ8plex); K1(iTRAQ8plex)	HCD	1.0000	0.0000	1	1	3764783	Unique	Used	0.464	2.440	1.864	1.045	0	0	5.23	1	12	19	3	826.15491	2476.45017	-3.80
vDQSILTGESVSVIk	Unambiguous	4	1	O55143	N-Term(iTRAQ8plex); K15(iTRAQ8plex)	HCD	1.0000	0.0000	1	1	3280801	Unique	Used	0.333	0.988	1.298	1.016	0	0	5.23	0	0	9	2	1092.13928	2183.27129	-1.41
sVIGMGTGAGAYILTR	Unambiguous	5	1	Q62433	N-Term(iTRAQ8plex)	HCD	1.0000	0.0000	1	1	3934106	Unique	Used	0.908	0.698	1.191	1.794	0	0	5.22	0	5	25	2	936.01776	1871.02825	-4.29
tSDVETMSSQSALALDER	Unambiguous	1	1	P14873	N-Term(iTRAQ8plex)	CID	1.0000	0.0000	1	1	2794545	Unique	Used	2.272	0.551	1.454	0.786	0	0	5.22	0	0	200	2	1122.54980	2244.09233	-1.98
aIEPPPLDAVIEAEHTLR	Unambiguous	1	1	E9QNN1	N-Term(iTRAQ8plex)	CID	1.0000	0.0000	1	1	3152685	Unique	Used	1.506	1.431	0.791	1.340	0	0	5.22	0	0	200	3	759.09161	2275.26029	0.11

eQPGSPHSVSGEILGQEkPTYR	Unambiguous	2	1	Q9QYX7	N-Term(iTRAQ8plex); K18(iTRAQ8plex)	CID	1.0000	0.0000	1	1	3085544	Unique	Used	0.889	0.808	1.763	0.758	0	0	5.22	0	0	200	3	1002.19830	3004.58036	-4.84
iQAAASPPANATAASDTNAGDR	Unambiguous	2	1	E9QAQ5	N-Term(iTRAQ8plex)	CID	1.0000	0.0000	1	1	2931620	Unique	Used	0.512	0.740	0.787	0.701	0	0	5.22	0	0	196	2	1187.59363	2374.17998	-4.27
iEVGLVVGnSQVAFEk	Unambiguous	2	1	P46460	N-Term(iTRAQ8plex); N9(Deamidated); K16(iTRAQ8plex)	CID	0.0268	0.0000	1	1	3261098	Unique	Used				0.963	0	0	5.22	0	9	25	3	766.77325	2298.30521	-4.95
ikTDGAAPNVAPSDVGGGGGTnR	Unambiguous	1	1	P12960	N-Term(iTRAQ8plex); K2(iTRAQ8plex); N22(Deamidated)	CID	1.0000	0.0000	1	1	2988494	Unique	Used	1.105	1.422	1.059	1.207	0	0	5.22	1	27	200	3	907.48297	2720.43436	-2.97
eGGSIPVTLTFQEATGk	Unambiguous	1	1	Q9D1A2	N-Term(iTRAQ8plex); K17(iTRAQ8plex)	CID	1.0000	0.0000	1	1	3301947	Unique	Used	0.645	1.188	1.163	1.025	0	0	5.21	0	0	73	2	1172.15076	2343.29424	-3.17
vAVLGASGGIGQPLSLLLk	Unambiguous	1	1	P08249	N-Term(iTRAQ8plex); K19(iTRAQ8plex)	CID	1.0000	0.0000	1	1	3937789	Unique	Used	1.538	1.208	0.878	1.067	0	0	5.21	0	6	200	3	801.16919	2401.49302	-3.04
lVHLWSSETHQPVWSR	Unambiguous	5	1	E9QK48	N-Term(iTRAQ8plex)	HCD	1.0000	0.0000	1	1	3894284	Unique	Used	1.321	0.874	1.207	1.280	0	0	5.21	0	22	143	3	756.06653	2266.18503	-8.14
dAAVSPDLGAGGDAPAPAPAPAHTR	Unambiguous	1	1	Q9Z2A7	N-Term(iTRAQ8plex)	CID	1.0000	0.0000	1	1	2896930	Unique	Used	1.849	1.154	1.110	0.902	0	0	5.21	0	0	200	3	862.77722	2586.31711	-1.70
vNEGLEEINAFLTDHHk	Unambiguous	2	1	G3X9A7	N-Term(iTRAQ8plex); K17(iTRAQ8plex)	CID	1.0000	0.0000	1	1	3172585	Unique	Used	0.852	1.509	1.171	1.312	0	0	5.21	0	16	200	3	858.79425	2574.36820	-3.53
aGQVFLEELGNHk	Unambiguous	2	1	P16330	N-Term(iTRAQ8plex); K13(iTRAQ8plex)	CID	1.0000	0.0000	1	1	3279264	Unique	Used	0.945	0.359	0.922	1.018	0	0	5.21	0	10	26	3	684.05597	2050.15335	-0.41
dIILATDLAHHLR	Unambiguous	3	1	Q5PR72	N-Term(iTRAQ8plex)	CID	1.0000	0.0000	1	1	3576519	Unique	Used	0.555	0.873	1.020	1.209	0	0	5.21	0	18	156	3	598.01599	1792.03342	-2.73
aSAELALGEnNEVLk	Unambiguous	1	1	P05202	N-Term(iTRAQ8plex); N10(Deamidated); K15(iTRAQ8plex)	CID	0.0307	0.0000	1	1	2653217	Unique	Used	2.508	1.161	0.715	0.628	0	0	5.21	0	13	200	3	723.07434	2167.20847	0.82

eGGQLMQEkPELAASVR	Unambiguous	3	1	E9PX29	N-Term(iTRAQ8plex); K9(iTRAQ8plex)	HCD	1.0000	0.0000	1	1	3125341	Unique	Used	0.647	0.677	1.613	0.994	0	0	5.21	0	9	22	3	841.46545	2522.38181	-1.57
hVISYSLSPFEQR	Unambiguous	1	1	Q9CQ69	N-Term(iTRAQ8plex)	CID	1.0000	0.0000	1	1	3740011	Unique	Used	0.296	0.553	1.366	2.353	0	0	5.21	0	2	1	3	623.00348	1866.99588	-3.05
vETPGPPAPPPDSGPPASSDR	Unambiguous	3	1	A2AQ87	N-Term(iTRAQ8plex)	CID	1.0000	0.0000	1	1	2808083	Unique	Used	0.905	1.001	1.550	0.811	0	0	5.21	0	0	200	2	1166.58911	2332.17095	-0.60
gEGGTTNPHVFPEGSEPk	Unambiguous	2	1	Q7TQI3	N-Term(iTRAQ8plex); K18(iTRAQ8plex)	CID	1.0000	0.0000	1	1	2648369	Unique	Used	1.485	1.073	1.068	1.109	0	0	5.20	0	0	200	3	816.75885	2448.26200	0.17
dSSSLSSYTSGVLEER	Unambiguous	1	1	Q9JM96	N-Term(iTRAQ8plex)	CID	1.0000	0.0000	1	1	2974565	Unique	Used	1.404	0.682	1.450	0.804	0	0	5.20	0	2	16	2	1011.00269	2020.99809	0.20
iMRPDDANVAGNVHGGTILk	Unambiguous	4	1	Q91V12-2	N-Term(iTRAQ8plex); K20(iTRAQ8plex)	HCD	1.0000	0.0000	1	1	3431695	Unique	Used	0.633	0.684	0.840	0.966	0	0	5.20	0	0	25	3	896.16425	2686.47818	-5.13
kAEGATLSNATGAVESTSQAGDR	Unambiguous	1	1	Q61644	N-Term(iTRAQ8plex); K1(iTRAQ8plex)	CID	1.0000	0.0000	1	1	2988720	Unique	Used	1.289	1.656	1.646	1.243	0	0	5.20	1	0	200	3	943.82947	2829.47385	-2.15
iDSNLDELSVGLGHLk	Unambiguous	1	1	Q9ERB0	N-Term(iTRAQ8plex); K16(iTRAQ8plex)	CID	1.0000	0.0000	1	1	3324336	Unique	Used	1.113	0.761	0.850	0.305	0	0	5.20	0	0	66	3	773.44177	2318.31076	-2.97
gTGGVDTAAVGGVFDVSNADR	Unambiguous	1	1	Q04447	N-Term(iTRAQ8plex)	CID	1.0000	0.0000	1	1	3137386	Unique	Used			1.059	0.476	0	0	5.20	0	0	200	2	1135.06921	2269.13115	-2.27
rAPVIDADkPVSSQLR	Unambiguous	1	1	Q9JHU4	N-Term(iTRAQ8plex); K9(iTRAQ8plex)	HCD	1.0000	0.0000	1	1	3449922	Unique	Used	0.437	0.802	0.894	0.974	0	0	5.20	1	0	111	3	787.46423	2360.37815	-3.78
lVAPLLATVTILDDDHAGIFSFQDR	Unambiguous	1	1	Q8K596	N-Term(iTRAQ8plex)	CID	1.0000	0.0000	1	1	3766694	Unique	Used	0.747	1.977	1.779	1.122	0	0	5.20	0	13	32	3	1011.21454	3031.62906	-3.84
gTGGVDTAAVGGVFDVSNADR	Unambiguous	1	1	Q04447	N-Term(iTRAQ8plex)	CID	1.0000	0.0000	1	1	3132226	Unique	Used	2.682	1.204	0.554	0.686	0	0	5.19	0	0	200	2	1135.07214	2269.13701	0.31
aAPEPAAGGGGAAAQAPSAR	Unambiguous	3	1	E9PUW6	N-Term(iTRAQ8plex)	CID	1.0000	0.0000	1	1	2928773	Unique	Used	0.735	0.943	0.951	1.092	0	0	5.19	0	0	200	2	991.51898	1982.03069	-2.58
hSAEVAGYQDSIGQLESDLR	Unambiguous	1	1	P46660	N-Term(iTRAQ8plex)	CID	1.0000	0.0000	1	1	2901614	Unique	Used	2.202		2.575	1.722	0	0	5.19	0	0	2	3	827.08069	2479.22751	-3.71
dALRDEYDDLSDLNAVQmESVR	Unambiguous	1	1	Q80UU9	N-Term(iTRAQ8plex); M18(Oxidation)	CID	1.0000	0.0000	1	1	2762593	Unique	Used		0.887	0.742	0.858	0	0	5.19	1	14	200	3	958.79602	2874.37351	0.18

vQGHDYEAGGSVIHPLnLHMk	Unambiguous	1	1	Q9CQF9	N-Term(iTRAQ8plex); N17(Deamidated); K21(iTRAQ8plex)	HCD	1.0000	0.0000	1	1	3340733	Unique	Used	1.124	1.210	0.872	1.086	0	0	5.19	0	0	49	3	971.18390	2911.53714	0.90
wQQEVAETVFDTINAETDRLEAR	Unambiguous	2	1	Q99MK8	N-Term(iTRAQ8plex)	HCD	1.0000	0.0000	1	1	3327272	Unique	Used	0.567	1.148	0.908	1.185	0	0	5.19	1	7	46	3	1009.17554	3025.51206	-1.62
qDLAQLmNSSGSHk	Unambiguous	2	1	O88544	N-Term(iTRAQ8plex); M7(Oxidation); K14(iTRAQ8plex)	CID	1.0000	0.0000	1	1	3362555	Unique	Used	0.954	0.973	0.991	0.965	0	0	5.19	0	15	28	3	714.04315	2140.11490	-5.99
iASQVAALDLGYkPGVEAIR	Unambiguous	1	1	Q91VD9	N-Term(iTRAQ8plex); K13(iTRAQ8plex)	CID	1.0000	0.0000	1	1	3684917	Unique	Used	0.628	1.303	1.069	1.287	0	0	5.19	0	0	27	3	893.85358	2679.54618	-7.19
nVPSAGGGIGDGGQEPTTGNWR	Unambiguous	3	1	Q8K1M6	N-Term(iTRAQ8plex)	CID	1.0000	0.0000	1	1	3068631	Unique	Used			1.454	2.073	0	0	5.19	0	0	200	2	1216.10120	2431.19512	1.91
lEGSEVQLVEYEASAAGLIR	Unambiguous	1	1	Q99KK7	N-Term(iTRAQ8plex)	HCD	1.0000	0.0000	1	1	3173896	Unique	Used	0.751	0.887	0.826	0.817	0	0	5.19	0	0	12	2	1219.65527	2438.30327	-1.98
tQQAAQANITLQEQIEAIHk	Unambiguous	1	1	Q8K4Z5	N-Term(iTRAQ8plex); K20(iTRAQ8plex)	CID	1.0000	0.0000	1	1	3300189	Unique	Used	1.402	1.022	0.988	0.869	0	0	5.19	0	0	132	3	948.52966	2843.57444	-3.22
gGLHTVAVMNVGAPAAGmNAAVR	Unambiguous	1	1	P47857	N-Term(iTRAQ8plex); M18(Oxidation)	CID	1.0000	0.0000	1	1	3929572	Unique	Used	2.050	0.695	2.167	1.871	0	0	5.18	0	0	8	3	828.77185	2484.30100	-4.35
eEFVETcPGELk	Unambiguous	1	1	P20357	N-Term(iTRAQ8plex); C7(Methylthio); K12(iTRAQ8plex)	CID	1.0000	0.0000	1	1	2780125	Unique	Used		0.370	1.060	0.605	0	0	5.18	0	8	18	3	679.01544	2035.03177	-0.93
aHLMEIQVnGGTVAEk	Unambiguous	1	1	P27659	N-Term(iTRAQ8plex); N9(Deamidated); K16(iTRAQ8plex)	CID	1.0000	0.0000	1	1	3010695	Unique	Used	0.421	0.519	0.723	1.048	0	0	5.18	0	23	200	2	1153.63269	2306.25810	-2.34
gYVVGAIHPVIQPVGQR	Unambiguous	2	1	Q8CHX7	N-Term(iTRAQ8plex)	HCD	1.0000	0.0000	1	1	3930931	Unique	Used	0.941	0.897	0.912	1.199	0	0	5.18	0	0	70	3	698.73834	2094.20047	-5.79
aASDIAMTELPPTHPIR	Unambiguous	1	1	P62259	N-Term(iTRAQ8plex)	HCD	1.0000	0.0000	1	1	3343130	Unique	Used	1.630	1.490	1.118	1.022	0	0	5.18	0	0	34	2	1062.57104	2124.13481	-3.64

ySPAYAHLVAPGEEGATGAGPSk	Unambiguous	3	1	O09012	N-Term(iTRAQ8plex); K23(iTRAQ8plex)	CID	1.0000	0.0000	1	1	3069042	Unique	Used	0.942	1.176	0.599	0.763	0	0	5.18	0	0	200	3	946.83380	2838.48685	-0.51
vVDALGnAIDGk	Unambiguous	2	1	Q03265	N-Term(iTRAQ8plex); N7(Deamidated); K12(iTRAQ8plex)	CID	1.0000	0.0000	1	1	2850431	Unique	Used	1.081	0.794	1.628	1.593	0	0	5.18	0	8	82	3	594.34503	1781.02054	-3.37
dSSTPGPGEGIPLSnGGGGSTSR	Unambiguous	5	1	Q91ZS8-5	N-Term(iTRAQ8plex); N15(Deamidated)	CID	1.0000	0.0000	1	1	2698827	Unique	Used	0.576	0.347	1.239	0.852	0	0	5.18	0	0	200	2	1196.57495	2392.14263	-4.36
sSASVTGEPPLYPTGSDSSR	Unambiguous	1	1	Q80U93	N-Term(iTRAQ8plex)	CID	1.0000	0.0000	1	1	2953739	Unique	Used	1.045	0.833	1.025	0.733	0	0	5.18	0	0	200	2	1150.06934	2299.13140	-1.83
tSEANHDSSLkPLSISYNPATAk	Unambiguous	1	1	P13634	N-Term(iTRAQ8plex); K11(iTRAQ8plex); K23(iTRAQ8plex)	HCD	1.0000	0.0000	1	1	3297094	Unique	Used	2.504	8.307	1.735	3.950	0	0	5.17	0	0	195	3	1115.27747	3343.81784	-2.48
nEAIQAAHDSVAQEGQcR	Unambiguous	1	1	Q9R0P9	N-Term(iTRAQ8plex); C17(Methylthio)	CID	1.0000	0.0000	1	1	3029490	Unique	Used	2.655	1.138	1.576	0.923	0	0	5.17	0	8	15	3	759.69165	2277.06040	-2.21
aGPPVPASHIAPTPVQPPMIR	Unambiguous	5	1	E9QAQ7	N-Term(iTRAQ8plex)	HCD	1.0000	0.0000	1	1	3893161	Unique	Used	0.702	1.062	0.873	0.827	0	0	5.17	0	25	69	3	813.12451	2437.35898	-4.20
vETLHDFEAAnSDELNLQR	Unambiguous	1	1	Q7TQF7	N-Term(iTRAQ8plex); N11(Deamidated)	CID	1.0000	0.0000	1	1	2654476	Unique	Used	1.636	1.555	1.505	0.933	0	0	5.17	0	0	195	3	836.08215	2506.23191	-1.79
tLLGSSDNVATVSNIEk	Unambiguous	1	1	Q5XG69	N-Term(iTRAQ8plex); K17(iTRAQ8plex)	CID	1.0000	0.0000	1	1	3233643	Unique	Used	0.566	0.557	0.995	0.775	0	0	5.17	0	0	56	2	1178.65967	2356.31206	-2.53
eAEAQAAMEANSEGSLTRPk	Unambiguous	1	1	P63040	N-Term(iTRAQ8plex); K20(iTRAQ8plex)	CID	1.0000	0.0000	1	1	2792262	Unique	Used	1.979	0.655	1.785	1.217	0	0	5.17	0	8	198	3	900.13367	2698.38645	-2.32
mGFPSNIVDSAAENMIk	Unambiguous	1	1	Q9Z2I9	N-Term(iTRAQ8plex); M1(Oxidation); K17(iTRAQ8plex)	CID	1.0000	0.0000	1	1	3394386	Unique	Used	0.354	0.881	1.433	1.329	0	0	5.17	0	0	111	2	1224.63403	2448.26079	-4.73

kASGTYAGPPSSALPAQR	Unambiguous	1	1	Q6PAL7	N-Term(iTRAQ8plex); K1(iTRAQ8plex)	HCD	1.0000	0.0000	1	1	3633149	Unique	Used	0.761	0.892	1.043	1.016	0	0	5.17	1	0	192	3	789.77539	2367.31162	-5.28
tNIVTASVDAINFHDk	Unambiguous	5	1	Q91V12-2	N-Term(iTRAQ8plex); K16(iTRAQ8plex)	CID	1.0000	0.0000	1	1	3346649	Unique	Used	0.661	0.561	0.853	0.918	0	0	5.17	0	0	26	2	1177.14673	2353.28618	-4.70
vGVVDEVVPEDQVHSk	Unambiguous	1	1	P42125	N-Term(iTRAQ8plex); K16(iTRAQ8plex)	CID	1.0000	0.0000	1	1	2716264	Unique	Used	2.268	1.181	0.857	1.063	0	0	5.17	0	15	200	3	782.10242	2344.29270	-1.80
eHLTVWGNANSEIVDk	Unambiguous	1	1	Q9CRY7	N-Term(iTRAQ8plex); K16(iTRAQ8plex)	CID	1.0000	0.0000	1	1	3010778	Unique	Used	1.220	0.883	0.952	0.836	0	0	5.17	0	27	33	3	807.43689	2420.29612	-2.87
ySMVGGNLVINNPDkQk	Unambiguous	1	1	P12960	N-Term(iTRAQ8plex); K15(iTRAQ8plex); K17(iTRAQ8plex)	CID	1.0000	0.0000	1	1	3453186	Unique	Used	0.547	0.933	0.959	0.958	0	0	5.16	1	0	77	3	930.52594	2789.56327	-4.11
aISESGVVINTNVGk	Unambiguous	2	1	P23953	N-Term(iTRAQ8plex); K15(iTRAQ8plex)	HCD	1.0000	0.0000	1	1	3342802	Unique	Used	3.152	1.957	0.318	0.659	0	0	5.16	0	26	30	2	1048.60706	2096.20683	-4.94
tVTNAVVTVPAYFnDSQR	Unambiguous	1	1	P63017	N-Term(iTRAQ8plex); N14(Deamidated)	HCD	1.0000	0.0000	1	1	3392675	Unique	Used	0.973	0.993	0.840	1.017	0	0	5.16	0	0	1	2	1144.09155	2287.17583	-4.98
eAIVAEIEDFHAR	Unambiguous	6	1	Q9WVS8-5	N-Term(iTRAQ8plex)	CID	1.0000	0.0000	1	1	2886437	Unique	Used				0.474	0	0	5.16	0	14	200	3	601.98853	1803.95102	-1.83
nVPSAGGGIGDGGqEPTTGNWR	Unambiguous	3	1	Q8K1M6	N-Term(iTRAQ8plex); Q14(Deamidated)	CID	0.0116	0.0000	1	1	3049248	Unique	Used	0.377	1.491	0.997	1.156	0	0	5.16	0	25	200	2	1216.59692	2432.18657	4.97
vVAGVAAALAHk	Unambiguous	1	1	A8DUK4	N-Term(iTRAQ8plex); K12(iTRAQ8plex)	HCD	1.0000	0.0000	1	1	3876046	Unique	Used	6.040	0.511	0.456	1.366	0	0	5.16	0	21	128	3	572.36322	1715.07511	-2.18
aGAQGAAAITTSGSTkPPGAR	Unambiguous	2	1	Q5SWP3	N-Term(iTRAQ8plex); K16(iTRAQ8plex)	CID	1.0000	0.0000	1	1	3675323	Unique	Used	0.908	0.199	1.002	0.943	0	0	5.16	0	22	200	3	826.79333	2478.36545	-9.32
aGTGPASPSADAMTMkPPPR	Unambiguous	1	1	Q61330	N-Term(iTRAQ8plex); K16(iTRAQ8plex)	HCD	1.0000	0.0000	1	1	3384766	Unique	Used	0.872	0.510	1.290	1.688	0	0	5.16	0	0	72	3	850.11566	2548.33243	-5.83

sSGNSANDLANVAVIDGNR	Unambiguous	1	1	Q7TT37	N-Term(iTRAQ8plex)	CID	1.0000	0.0000	1	1	3087925	Unique	Used	0.515	1.010	0.814	1.063	0	0	5.16	0	0	200	2	1089.55518	2178.10307	-1.03
aHYGGFTVQNEASk	Unambiguous	1	1	Q8K0E8	N-Term(iTRAQ8plex); K14(iTRAQ8plex)	CID	1.0000	0.0000	1	1	3084345	Unique	Used	1.306	1.453	1.257	1.861	0	0	5.16	0	8	200	3	706.37708	2117.11667	-3.27
gLTIVGSVLEGTFLDNHPQAQR	Unambiguous	2	1	Q91V14	N-Term(iTRAQ8plex)	CID	1.0000	0.0000	1	1	3844097	Unique	Used	0.451	0.920	1.276	1.110	0	0	5.16	0	15	200	3	886.14581	2656.42289	-4.97
eRPETVLIDLIQR	Unambiguous	1	1	Q5F2E8	N-Term(iTRAQ8plex)	HCD	1.0000	0.0000	1	1	3497941	Unique	Used	0.551	0.877	0.857	0.914	0	0	5.16	0	16	77	3	629.36957	1886.09415	-3.80
sHTVAAQGGINAALGNmEEDNWR	Unambiguous	1	1	Q8K2B3	N-Term(iTRAQ8plex); M17(Oxidation)	CID	1.0000	0.0000	1	1	3087033	Unique	Used	0.897	0.959	1.423	1.189	0	0	5.15	0	0	200	3	921.11292	2761.32419	-0.89
vVYEnGVFMcAEGTGk	Unambiguous	1	1	Q9EQF6	N-Term(iTRAQ8plex); N5(Deamidated); C10(Methylthio); K16(iTRAQ8plex)	CID	1.0000	0.0000	1	1	3092332	Unique	Used	0.342	0.232	0.363	0.539	0	0	5.15	0	0	152	2	1180.08276	2359.15825	-0.44
aAVEEGIVLGGGcALLR	Unambiguous	2	1	P63038	N-Term(iTRAQ8plex); C13(Methylthio)	CID	1.0000	0.0000	1	1	3645059	Unique	Used	1.062	1.021	0.965	1.146	0	0	5.15	0	25	28	2	989.53583	1978.06438	-6.28
qRPFQQPPPAAVQQAQPMSQITGLk	Unambiguous	3	1	O89084	N-Term(iTRAQ8plex); K25(iTRAQ8plex)	CID	1.0000	0.0000	1	1	3893672	Unique	Used		1.884	1.211	1.246	0	0	5.15	0	0	148	3	1118.95264	3354.84336	-3.94
kLETAVNLAWTAGNSNTR	Unambiguous	3	1	Q60932-2	N-Term(iTRAQ8plex); K1(iTRAQ8plex)	HCD	1.0000	0.0000	1	1	3914746	Unique	Used	0.599	1.151	0.953	1.142	0	0	5.15	1	15	62	3	852.14185	2554.41098	-3.46
sQDNALSASSETSSTLAGk	Unambiguous	3	1	Q8C170	N-Term(iTRAQ8plex); K19(iTRAQ8plex)	CID	1.0000	0.0000	1	1	2914470	Unique	Used	0.457	0.570	0.975	0.698	0	0	5.15	0	0	200	2	1231.63660	2462.26592	-6.98
iETmLGDVAVAVHPk	Unambiguous	2	1	Q9Z1Q9	N-Term(iTRAQ8plex); M4(Oxidation); K15(iTRAQ8plex)	CID	1.0000	0.0000	1	1	3434636	Unique	Used	0.839	0.559	0.858	1.155	0	0	5.15	0	17	136	3	735.41724	2204.23716	-8.99
vYSLGEENSTNNSTGQSR	Unambiguous	2	1	Q91ZD4	N-Term(iTRAQ8plex)	CID	1.0000	0.0000	1	1	2807719	Unique	Used	0.656	0.599	0.379	0.628	0	0	5.15	0	0	200	2	1124.04089	2247.07451	-2.05
vAAINVAHLEEADEGPEPAR	Unambiguous	1	1	Q8VBY2	N-Term(iTRAQ8plex)	CID	1.0000	0.0000	1	1	2635875	Unique	Used	1.976	1.161	1.082	1.238	0	0	5.15	0	17	200	3	798.08258	2392.23319	-3.31

gAPSSSGG SSISGPk	Unambiguous	6	1	Q6PGN3-3	N-Term(iTRAQ8plex); K15(iTRAQ8plex)	CID	1.0000	0.0000	1	1	3118681	Unique	Used	0.444	0.532	1.077	0.946	0	0	5.15	0	0	35	2	942.51636	1884.02544	-1.53
vLVTHQTG PDEDNPk	Unambiguous	1	1	Q80UG2	N-Term(iTRAQ8plex); K15(iTRAQ8plex)	CID	1.0000	0.0000	1	1	2696699	Unique	Used	0.981	0.915	1.202	1.228	0	0	5.15	0	9	200	3	753.40985	2258.21500	-3.87
lQQTQAQV DEVVDIMR	Unambiguous	2	1	P63044	N-Term(iTRAQ8plex)	CID	1.0000	0.0000	1	1	3288028	Unique	Used		1.529			0	0	5.14	0	0	200	2	1089.07910	2177.15093	-1.34
dDITASLVT TDHSEMk	Unambiguous	2	1	Q6PB44-2	N-Term(iTRAQ8plex); K16(iTRAQ8plex)	CID	1.0000	0.0000	1	1	2810208	Unique	Used	1.569	1.070	0.807	0.994	0	0	5.14	0	25	114	3	791.07874	2371.22165	-2.33
nAPAIIFIDE LDAIAPk	Unambiguous	1	1	Q01853	N-Term(iTRAQ8plex); K17(iTRAQ8plex)	CID	1.0000	0.0000	1	1	3352772	Unique	Used	0.376	0.579	0.868	0.985	0	0	5.14	0	1	4	2	1210.20129	2419.39531	-4.32
vGEGFEET LGEAVISTk	Unambiguous	1	1	P46660	N-Term(iTRAQ8plex); K17(iTRAQ8plex)	CID	1.0000	0.0000	1	1	2941797	Unique	Used	1.513	0.265	1.962	1.545	0	0	5.14	0	0	200	2	1187.65137	2374.29546	-0.33
tNkFTHGFQ NIVDAYGIG TYR	Unambiguous	4	1	Q9Z1G4-3	N-Term(iTRAQ8plex); K3(iTRAQ8plex)	HCD	1.0000	0.0000	1	1	3876811	Unique	Used	0.549	1.559	1.116	1.167	0	0	5.14	1	17	160	3	1004.20111	3010.58878	-3.58
iGGHGAEY GAEALER	Unambiguous	2	1	Q91VB8	N-Term(iTRAQ8plex)	CID	1.0000	0.0000	1	1	2762639	Unique	Used	1.593	1.654	0.665	1.336	0	0	5.14	0	8	180	3	611.98285	1833.93399	-3.13
nTVANSPQ TLLAGmNk	Unambiguous	1	1	P61222	N-Term(iTRAQ8plex); M14(Oxidation); K16(iTRAQ8plex)	CID	1.0000	0.0000	1	1	3719198	Unique	Used	0.708	1.006	0.537	0.702	0	0	5.14	0	29	200	2	1142.12378	2283.24028	-8.09
tVQEPPGS MSIAASLVS EDTk	Unambiguous	4	1	G3X9J4	N-Term(iTRAQ8plex); K21(iTRAQ8plex)	CID	1.0000	0.0000	1	1	3073280	Unique	Used	0.559	1.487	0.779	0.763	0	0	5.14	0	0	132	2	1378.23499	2755.46269	-0.64
lEGEVAQLN EAHGk	Unambiguous	3	1	F6TU77	N-Term(iTRAQ8plex); K14(iTRAQ8plex)	CID	1.0000	0.0000	1	1	2828397	Unique	Used	1.296	0.989	1.257	0.908	0	0	5.14	0	20	57	3	701.72516	2103.16092	-2.17
iAPALISSGI SVVEQEk	Unambiguous	4	1	P17183	N-Term(iTRAQ8plex); K17(iTRAQ8plex)	CID	1.0000	0.0000	1	1	3398732	Unique	Used	1.095	0.908	0.904	0.710	0	0	5.14	0	0	200	2	1175.19299	2349.37871	-2.68

rDPQELYDVYcFAR	Unambiguous	1	1	P55066	N-Term(iTRAQ8plex); C11(Methylthio)	HCD	1.0000	0.0000	1	1	3456616	Unique	Used	1.295	0.868	1.497	1.160	0	0	5.14	1	27	200	3	709.00720	2125.00705	-3.68
dYPLPDVAHVTmLSASQk	Unambiguous	1	1	P19783	N-Term(iTRAQ8plex); M12(Oxidation); K18(iTRAQ8plex)	CID	1.0000	0.0000	1	1	3412701	Unique	Used	1.152	1.139	1.270	1.248	0	0	5.14	0	13	60	3	866.13104	2596.37857	-4.46
aTDFIDQALAHk	Unambiguous	5	1	Q9D7X3	N-Term(iTRAQ8plex); K12(iTRAQ8plex)	CID	1.0000	0.0000	1	1	3323026	Unique	Used	0.906	0.818	1.210	0.818	0	0	5.14	0	20	22	3	646.70001	1938.08548	-2.60
sELSSADSTASTSQAASPR	Unambiguous	3	1	G5E832	N-Term(iTRAQ8plex)	CID	1.0000	0.0000	1	1	2751499	Unique	Used		0.434	0.635	0.749	0	0	5.13	0	0	200	2	1079.03040	2157.05351	-1.77
hPASLSSSGSSGSPSSSIk	Unambiguous	2	1	E9QAF9	N-Term(iTRAQ8plex); K19(iTRAQ8plex)	CID	1.0000	0.0000	1	1	3448106	Unique	Used	1.285	1.143	1.394	1.096	0	0	5.13	0	0	200	3	790.08704	2368.24656	-4.19
gNAGQTNYGFANSTmER	Unambiguous	1	1	P19096	N-Term(iTRAQ8plex); M15(Oxidation)	CID	1.0000	0.0000	1	1	3142122	Unique	Used	0.530	0.498	0.835	0.766	0	0	5.13	0	0	47	2	1069.49561	2137.98393	-1.66
aGPNAAGGPATQPTAGGGSGSGSASGSASSGERPk	Unambiguous	1	1	O35066	N-Term(iTRAQ8plex); K35(iTRAQ8plex)	CID	1.0000	0.0000	1	1	3044157	Unique	Used		0.492	0.618	0.718	0	0	5.13	0	0	200	3	1183.92920	3549.77304	-0.26
eRPGEQSEVAQLIQQTLEQER	Unambiguous	2	1	Q6R891	N-Term(iTRAQ8plex)	HCD	1.0000	0.0000	1	1	3131670	Unique	Used	0.757	1.287	1.369	0.791	0	0	5.13	0	0	103	2	1386.72888	2772.45049	2.69
gLSSVPEVAEVEPTTk	Unambiguous	1	1	P20357	N-Term(iTRAQ8plex); K16(iTRAQ8plex)	CID	1.0000	0.0000	1	1	2884006	Unique	Used	1.441	1.682	1.780	1.105	0	0	5.13	0	9	200	2	1126.13379	2251.26030	-1.74
aFTGFIVEADTPGIHIGk	Unambiguous	1	1	P45952	N-Term(iTRAQ8plex); K18(iTRAQ8plex)	CID	1.0000	0.0000	1	1	3498822	Unique	Used	0.889	0.899	0.744	1.055	0	0	5.13	0	18	87	3	827.80084	2481.38797	-3.33
nGLGGEDDAAGSLWNEPFR	Unambiguous	2	1	Q69ZF3	N-Term(iTRAQ8plex)	CID	1.0000	0.0000	1	1	2939811	Unique	Used	0.704	1.187	0.971	0.867	0	0	5.12	0	0	191	2	1155.05762	2309.10796	-0.92
aGLVVEDALFETLPSDVR	Unambiguous	1	1	Q8R502	N-Term(iTRAQ8plex)	HCD	1.0000	0.0000	1	1	3196379	Unique	Used	0.829	0.998	0.941	0.997	0	0	5.12	0	20	51	2	1118.10999	2235.21269	-2.15
lTGVSISQVnHNkPLR	Unambiguous	2	1	O08917	N-Term(iTRAQ8plex); N10(Deamidated); K13(iTRAQ8plex)	HCD	1.0000	0.0000	1	1	3678276	Unique	Used	0.931	2.702	2.118	2.144	0	0	5.12	0	0	63	3	791.46143	2372.36972	-7.30

aGQVFLEELGNHk	Unambiguous	2	1	P16330	N-Term(iTRAQ8plex); K13(iTRAQ8plex)	CID	1.0000	0.0000	1	1	3257085	Unique	Used	0.619	0.174	1.155	1.345	0	0	5.12	0	15	11	3	684.05530	2050.15134	-1.39
mSLQTDEDEHSIEMHLPYTAk	Unambiguous	1	1	Q91VH6	N-Term(iTRAQ8plex); K21(iTRAQ8plex)	CID	1.0000	0.0000	1	1	2956351	Unique	Used	1.388	1.280	0.719	1.318	0	0	5.12	0	30	200	3	1028.51318	3083.52500	-0.80
gPQNPSVSGAPGPTPHPVR	Unambiguous	1	1	Q91WJ0	N-Term(iTRAQ8plex)	CID	1.0000	0.0000	1	1	3488388	Unique	Used	1.131	0.742	0.776	1.018	0	0	5.12	0	0	49	3	719.38489	2156.14011	-5.27
lPSGSGPASPTTGSAVDIR	Unambiguous	1	1	E9Q616	N-Term(iTRAQ8plex)	CID	1.0000	0.0000	1	1	3387304	Unique	Used	1.535	2.861	0.869	0.745	0	0	5.12	0	0	123	2	1037.55396	2074.10063	-3.68
eQASHLGGAVFSGAGNIAAATGLVk	Unambiguous	1	1	Q91ZZ3	N-Term(iTRAQ8plex); K25(iTRAQ8plex)	CID	1.0000	0.0000	1	1	3762924	Unique	Used	1.916	1.179	1.231	1.674	0	0	5.12	0	20	96	3	978.87543	2934.61173	-4.81
gEILIGGQNVTMGYYk	Unambiguous	2	1	Q9CZW4	N-Term(iTRAQ8plex); K16(iTRAQ8plex)	CID	1.0000	0.0000	1	1	3476440	Unique	Used	0.577	0.740	0.971	1.233	0	0	5.12	0	0	88	2	1176.14368	2351.28008	-3.78
kAEGATLSnATGAVESTSQAGDR	Unambiguous	1	1	Q61644	N-Term(iTRAQ8plex); K1(iTRAQ8plex); N9(Deamidated)	CID	1.0000	0.0000	1	1	2881318	Unique	Used	1.641	1.566	3.254	2.317	0	0	5.12	1	28	200	3	944.15662	2830.45530	-3.06
qIEETLSHFR	Unambiguous	5	1	G3UVV4	N-Term(iTRAQ8plex)	CID	1.0000	0.0000	1	1	3412466	Unique	Used	0.533	1.039	1.004	1.290	0	0	5.11	0	0	163	3	521.95081	1563.83786	-3.46
fLSQPFQVAEVFTGHMGk	Unambiguous	1	1	P56480	N-Term(iTRAQ8plex); K18(iTRAQ8plex)	CID	1.0000	0.0000	1	1	3881676	Unique	Used	1.185	0.982	1.066	1.390	0	0	5.11	0	24	200	3	877.80859	2631.41123	-3.87
iVNDDQSFHADLYVEDGLIk	Unambiguous	3	1	O35098	N-Term(iTRAQ8plex); K20(iTRAQ8plex)	CID	1.0000	0.0000	1	1	2940010	Unique	Used	1.179	0.619	0.683	0.540	0	0	5.11	0	8	82	3	967.18091	2899.52817	-0.57
fQEYILELADVPSSPHGVk	Unambiguous	2	1	O35136	N-Term(iTRAQ8plex); K19(iTRAQ8plex)	CID	1.0000	0.0000	1	1	3352038	Unique	Used	0.759	0.752	1.927	1.977	0	0	5.11	0	16	6	3	913.16974	2737.49466	-2.74
lQQELDDLLVDLDHQR	Unambiguous	1	1	Q8VDD5	N-Term(iTRAQ8plex)	CID	1.0000	0.0000	1	1	2979275	Unique	Used	1.440	1.187	1.337	0.573	0	0	5.11	0	0	8	3	752.06995	2254.19529	-1.27
dASEAPAGkPALQVPSSR	Unambiguous	2	1	Q6PEV3	N-Term(iTRAQ8plex); K9(iTRAQ8plex)	HCD	1.0000	0.0000	1	1	3228307	Unique	Used	1.287	0.703	1.370	1.154	0	0	5.11	0	0	159	3	797.11127	2389.31925	-4.34

dkPHVNVGTIGHVDHGk	Unambiguous	3	1	Q8BFR5	N-Term(iTRAQ8plex); K2(iTRAQ8plex); K17(iTRAQ8plex)	CID	1.0000	0.0000	1	1	3225868	Unique	Used	0.989	0.943	1.269	0.987	0	0	5.11	0	0	62	3	908.18610	2722.54374	-2.89
tIHSYQGQVISHEk	Unambiguous	12	1	Q8R311	N-Term(iTRAQ8plex); K14(iTRAQ8plex)	CID	1.0000	0.0000	1	1	3181558	Unique	Used	1.134	0.889	1.068	0.966	0	0	5.11	0	13	170	3	745.74969	2235.23453	0.15
sDVWSLGITLYELATGR	Unambiguous	1	1	P47809	N-Term(iTRAQ8plex)	HCD	1.0000	0.0000	1	1	3899508	Unique	Used	0.636	1.138	1.060	1.102	0	0	5.11	0	14	42	2	1093.08813	2185.16899	-5.35
nNYALNTAATYAEPYRPVQYR	Unambiguous	6	1	Q68FH0	N-Term(iTRAQ8plex); N1(Deamidated)	HCD	1.0000	0.0000	1	1	3474408	Unique	Used	0.983	1.770	1.622	1.190	0	0	5.10	0	9	135	3	927.46521	2780.38108	-4.86
gSTPYSQDSAYSSSTTSTSFkPR	Unambiguous	1	1	E9PYH6	N-Term(iTRAQ8plex); K21(iTRAQ8plex)	CID	1.0000	0.0000	1	1	3316518	Unique	Used	0.724	1.133	1.057	0.923	0	0	5.10	0	0	152	3	1017.50793	3050.50925	-2.32
kLIAEGPGETVLVAEEEAAR	Unambiguous	1	1	Q9CY73	N-Term(iTRAQ8plex); K1(iTRAQ8plex)	CID	1.0000	0.0000	1	1	2832084	Unique	Used	1.170	0.892	0.863	0.850	0	0	5.10	1	12	183	3	897.51105	2690.51859	0.01
mDSAVQASLSLPATPVGk	Unambiguous	2	1	Q9ERR1-2	N-Term(iTRAQ8plex); M1(Oxidation); K18(iTRAQ8plex)	CID	1.0000	0.0000	1	1	3597230	Unique	Used	0.998	0.831	1.481	1.323	0	0	5.10	0	0	200	2	1198.66248	2396.31767	-5.81
nSLPFDAPDSSQEGQk	Unambiguous	2	1	Q8C729-2	N-Term(iTRAQ8plex); K16(iTRAQ8plex)	CID	1.0000	0.0000	1	1	2776861	Unique	Used	0.425	0.940	1.194	1.486	0	0	5.10	0	0	200	2	1164.59631	2328.18535	-3.21
tVYVLPVGDADAAVVATTSYR	Unambiguous	1	1	Q14BB9	N-Term(iTRAQ8plex)	CID	1.0000	0.0000	1	1	3439159	Unique	Used	1.815	0.824	3.310	1.423	0	0	5.10	0	8	25	2	1236.66321	2472.31914	-3.92
qRPFQQPPPAAVQqAQPMSQITGLk	Unambiguous	3	1	O89084	N-Term(iTRAQ8plex); Q14(Deamidated); K25(iTRAQ8plex)	HCD	0.0078	0.0000	1	1	3893658	Unique	Used	0.546	0.974	1.297	0.893	0	0	5.10	0	0	175	3	1119.28857	3355.85117	3.15
vSDFGQMASGMnVDAGk	Unambiguous	1	1	P20357	N-Term(iTRAQ8plex); N12(Deamidated); K17(iTRAQ8plex)	CID	1.0000	0.0000	1	1	3013665	Unique	Used	1.235	1.410	1.513	1.080	0	0	5.10	0	0	172	2	1162.07654	2323.14580	-2.64

nSSGSSESSLPNLAR	Unambiguous	2	1	Q3UHJ0-2	N-Term(iTRAQ8plex)	HCD	1.0000	0.0000	1	1	3227944	Unique	Used	1.714	1.109	1.697	0.973	0	0	5.10	0	0	145	2	905.46381	1809.92034	-2.28
sPSGGAAGPLLTPSQSLDGSR	Unambiguous	1	1	Q6WVG3	N-Term(iTRAQ8plex)	CID	1.0000	0.0000	1	1	3410984	Unique	Used	1.390	1.166	0.746	0.832	0	0	5.09	0	0	125	2	1130.09607	2259.18486	-1.53
lIASVADDEAAVPnNk	Unambiguous	2	1	P16125	N-Term(iTRAQ8plex); N14(Deamidated); K16(iTRAQ8plex)	CID	1.0000	0.0000	1	1	2670866	Unique	Used	0.827	1.057	1.482	1.270	0	0	5.09	0	4	200	2	1118.61536	2236.22344	-2.12
yITGDQLGALYQDFVR	Unambiguous	2	1	P17183	N-Term(iTRAQ8plex)	CID	1.0000	0.0000	1	1	3670240	Unique	Used	1.842	1.532	1.074	1.614	0	0	5.09	0	0	200	2	1082.06848	2163.12969	-4.22
lATPTYGDLNHLVSATMSGVTTSLR	Unambiguous	1	1	Q9ERD7	N-Term(iTRAQ8plex)	CID	1.0000	0.0000	1	1	3937730	Unique	Used	1.183	0.343	1.272	1.108	0	0	5.09	0	9	200	3	970.51349	2909.52591	-2.95
aLGQAASDSSGPEDMk	Unambiguous	1	1	Q6GQT9	N-Term(iTRAQ8plex); K16(iTRAQ8plex)	CID	1.0000	0.0000	1	1	2632743	Unique	Used	0.837	0.697	0.599	0.581	0	0	5.08	0	0	200	2	1086.55505	2172.10283	-1.62
iYGNFFPYGDASk	Unambiguous	4	1	Q91X97	N-Term(iTRAQ8plex); K13(iTRAQ8plex)	CID	1.0000	0.0000	1	1	3477188	Unique	Used	0.651	1.693	0.847	0.498	0	0	5.08	0	0	3	3	696.37134	2087.09946	-3.05
eAAASGLPLmVIIHk	Unambiguous	1	1	Q9CQU0	N-Term(iTRAQ8plex); M10(Oxidation); K15(iTRAQ8plex)	CID	1.0000	0.0000	1	1	3682623	Unique	Used	1.060	0.767	0.639	0.787	0	0	5.08	0	20	8	3	725.42700	2174.26645	-7.51
mSESLDTADPAVTGAk	Unambiguous	1	1	P63011	N-Term(iTRAQ8plex); K16(iTRAQ8plex)	CID	1.0000	0.0000	1	1	2777523	Unique	Used	0.415	1.301	1.153	1.033	0	0	5.08	0	4	200	2	1101.08032	2201.15337	-2.12
sSSSSSSGVGSPAVTPTEk	Unambiguous	3	1	E9QN14	N-Term(iTRAQ8plex); K19(iTRAQ8plex)	CID	1.0000	0.0000	1	1	2930179	Unique	Used	1.222	1.006	1.215	1.162	0	0	5.08	0	0	85	2	1180.61914	2360.23100	-3.88
eTnLESLPLVDTHSk	Unambiguous	1	1	P20152	N-Term(iTRAQ8plex); N3(Deamidated); K15(iTRAQ8plex)	CID	1.0000	0.0000	1	1	2811490	Unique	Used	1.633	2.847	1.100	1.532	0	0	5.08	0	25	200	3	764.75287	2292.24405	-4.50
tSSPAQGVGASVSALRPDLGFVR	Unambiguous	1	1	Q9DCU2	N-Term(iTRAQ8plex)	HCD	1.0000	0.0000	1	1	3933555	Unique	Used	0.898	0.460	1.205	1.716	0	0	5.08	0	2	33	3	859.47113	2576.39884	-4.29
gSETLGGGGGGAAGTR	Unambiguous	1	1	P31650	N-Term(iTRAQ8plex)	CID	1.0000	0.0000	1	1	2858905	Unique	Used	0.642	0.261	0.742	1.451	0	0	5.08	0	0	200	2	804.91321	1608.81914	-3.26

nPPGPISSEPASPLHTIVIR	Unambiguous	3	1	E9QN14	N-Term(iTRAQ8plex)	HCD	1.0000	0.0000	1	1	3759955	Unique	Used	1.512	1.477	0.921	1.054	0	0	5.08	0	28	105	3	796.11597	2386.33335	-2.65
vIDIFDTDGnGEVDFk	Unambiguous	2	1	Q63810-2	N-Term(iTRAQ8plex); N10(Deamidated); K16(iTRAQ8plex)	CID	1.0000	0.0000	1	1	2743194	Unique	Used	1.603	2.448	2.468	0.700	0	0	5.08	0	5	200	2	1197.12024	2393.23320	-0.05
vDcTASGAEnSGSLGk	Unambiguous	1	1	Q3UNH4	N-Term(iTRAQ8plex); C3(Methylthio); N10(Deamidated); K16(iTRAQ8plex)	CID	1.0000	0.0000	1	1	2828352	Unique	Used	0.650	1.614	1.431	0.715	0	0	5.07	0	0	78	2	1076.02759	2151.04790	-1.84
lDEIYVAGLVAHSDLDER	Unambiguous	7	1	G3V018	N-Term(iTRAQ8plex)	CID	1.0000	0.0000	1	1	2978765	Unique	Used	1.830	1.141	1.907	0.751	0	0	5.07	0	21	160	3	773.74048	2319.20688	-2.84
sASQDcIETTPGAQEGk	Unambiguous	2	1	P98084	N-Term(iTRAQ8plex); C6(Methylthio); K17(iTRAQ8plex)	CID	1.0000	0.0000	1	1	2632752	Unique	Used	1.881	1.399	0.938	0.732	0	0	5.07	0	0	200	2	1188.58301	2376.15874	-1.87
gSETLGGGGGAAGTR	Unambiguous	1	1	P31650	N-Term(iTRAQ8plex)	CID	1.0000	0.0000	1	1	2841485	Unique	Used	0.635	0.258	0.714	1.369	0	0	5.07	0	0	200	2	804.91302	1608.81877	-3.49
fccEPAGGLTSLTEPPk	Unambiguous	2	1	Q9D6J6	N-Term(iTRAQ8plex); C2(Methylthio); C3(Methylthio); K17(iTRAQ8plex)	CID	1.0000	0.0000	1	1	3479395	Unique	Used	1.113	0.791	1.468	1.108	0	0	5.07	0	9	24	2	1225.59851	2450.18974	-6.17
aAAGELQEDSGLHVLAR	Unambiguous	1	1	Q8C845	N-Term(iTRAQ8plex)	CID	1.0000	0.0000	1	1	2973026	Unique	Used	1.312	1.277	1.221	1.158	0	0	5.07	0	26	137	3	681.03607	2041.09366	-2.15
nLPPQEQMISALPDIk	Unambiguous	1	1	Q61074	N-Term(iTRAQ8plex); K16(iTRAQ8plex)	CID	1.0000	0.0000	1	1	3348645	Unique	Used	1.187	1.098	0.881	0.742	0	0	5.07	0	0	33	2	1201.67639	2402.34551	-4.96
lGIAVIHGEAQDAESDLVDGR	Unambiguous	1	1	Q8R574	N-Term(iTRAQ8plex)	CID	1.0000	0.0000	1	1	2796552	Unique	Used	0.526	1.393	0.811	1.317	0	0	5.07	0	11	70	3	823.76837	2469.29056	0.72
hVLSGTLGIPEHTYR	Unambiguous	1	1	P01831	N-Term(iTRAQ8plex)	HCD	1.0000	0.0000	1	1	3756881	Unique	Used	1.057	1.343	1.013	0.801	0	0	5.06	0	2	17	3	662.03271	1984.08359	-4.13
gSETLGGGGGAAGTR	Unambiguous	1	1	P31650	N-Term(iTRAQ8plex)	CID	1.0000	0.0000	1	1	2840708	Unique	Used	0.607	0.372	0.749	1.520	0	0	5.06	0	4	158	2	804.91290	1608.81853	-3.64
qPPLGGSGLLHGSR	Unambiguous	1	1	Q9D0E3	N-Term(iTRAQ8plex)	CID	1.0000	0.0000	1	1	3834284	Unique	Used	1.113	0.708	1.197	1.074	0	0	5.06	0	8	200	3	560.65295	1679.94431	-3.09

nSSVGLIQLNRPk	Unambiguous	1	1	Q8BH95	N-Term(iTRAQ8plex); K13(iTRAQ8plex)	HCD	1.0000	0.0000	1	1	3890617	Unique	Used	1.311	1.283	1.191	1.404	0	0	5.06	0	0	189	3	678.74493	2034.22025	-3.82
dETAAQTPGSGEAVLSk	Unambiguous	3	1	Q8BI84	N-Term(iTRAQ8plex); K17(iTRAQ8plex)	CID	1.0000	0.0000	1	1	2649870	Unique	Used	1.369	1.135	1.004	0.675	0	0	5.06	0	5	200	2	1135.10754	2269.20781	-2.39
sYqAEHGPDFPWSVGEDMSADGR	Unambiguous	1	1	Q9Z1Q2	N-Term(iTRAQ8plex); Q3(Deamidated)	CID	1.0000	0.0000	1	1	2850051	Unique	Used	1.678	1.095	0.895	1.063	0	0	5.06	0	0	200	3	948.42462	2843.25931	2.52
gEAGTPVAAGTTAAAIPR	Unambiguous	1	1	O08919	N-Term(iTRAQ8plex)	CID	1.0000	0.0000	1	1	3296511	Unique	Used	0.886	1.450	1.413	0.986	0	0	5.05	0	17	200	2	958.02887	1915.05046	-2.43
vGVVDEVVPEDQVHSk	Unambiguous	1	1	P42125	N-Term(iTRAQ8plex); K16(iTRAQ8plex)	CID	1.0000	0.0000	1	1	2684729	Unique	Used	1.635	0.930	0.715	1.132	0	0	5.05	0	0	170	3	782.10199	2344.29142	-2.34
tDFFIGGEEGmAEk	Unambiguous	1	1	O35685	N-Term(iTRAQ8plex); M11(Oxidation); K14(iTRAQ8plex)	CID	1.0000	0.0000	1	1	2759822	Unique	Used	1.559	1.306	0.502	0.624	0	0	5.05	0	9	200	3	719.03375	2155.08670	1.34
dAQVGWIQIVEDLHER	Unambiguous	2	1	E9QKE4	N-Term(iTRAQ8plex)	CID	1.0000	0.0000	1	1	3093443	Unique	Used	1.442	1.141	1.447	1.121	0	0	5.05	0	11	200	3	738.06104	2212.16855	0.95
vTGAVDVTLGAVQnSVDk	Unambiguous	1	1	Q9DBG5	N-Term(iTRAQ8plex); N14(Deamidated); K18(iTRAQ8plex)	CID	0.0198	0.0000	1	1	3350231	Unique	Used	1.193	0.905	0.883	0.956	0	0	5.05	0	9	28	2	1191.67676	2382.34624	5.26
aLGQAASDSSGPEDmk	Unambiguous	1	1	Q6GQT9	N-Term(iTRAQ8plex); M15(Oxidation); K16(iTRAQ8plex)	CID	1.0000	0.0000	1	1	2631244	Unique	Used	0.680	0.622	0.684	0.822	0	0	5.04	0	0	200	2	1094.55640	2188.10552	1.95
sQmSADSGVSLTSASQR	Unambiguous	23	1	A2AGR0	N-Term(iTRAQ8plex); M3(Oxidation)	CID	1.0000	0.0000	1	1	3271910	Unique	Used	0.576	0.929	1.250	1.272	0	0	5.04	0	0	104	2	1016.49774	2031.98821	-1.82
mQEVVANLQYDDGSGMk	Unambiguous	1	1	Q3U1J4	N-Term(iTRAQ8plex); K17(iTRAQ8plex)	CID	1.0000	0.0000	1	1	3073846	Unique	Used	0.935	0.804	0.647	0.869	0	0	5.04	0	4	59	3	831.75629	2493.25431	-1.26
iADYEAASAAPGIPAEQTR	Unambiguous	2	1	Q9WV96	N-Term(iTRAQ8plex)	CID	1.0000	0.0000	1	1	2936597	Unique	Used	1.495	0.980	0.953	0.985	0	0	5.04	0	9	98	2	1118.08032	2235.15337	-1.16

gLVAVVTGGASGLGLATAk	Unambiguous	2	1	Q99N15	N-Term(iTRAQ8plex); K19(iTRAQ8plex)	CID	1.0000	0.0000	1	1	3898101	Unique	Used	0.639	0.634	0.883	0.911	0	0	5.04	0	0	32	2	1125.68018	2250.35307	-4.95
lLASLFQDLQVEALHk	Unambiguous	3	1	A2AN08-3	N-Term(iTRAQ8plex); K16(iTRAQ8plex)	CID	1.0000	0.0000	1	1	3785679	Unique	Used	0.709	0.717	1.076	0.917	0	0	5.04	0	28	58	3	811.81403	2433.42752	-2.09
eLDPTNMTYITnQAAVHFEk	Unambiguous	1	1	Q60864	N-Term(iTRAQ8plex); N12(Deamidated); K20(iTRAQ8plex)	CID	0.0417	0.0000	1	1	3150219	Unique	Used	3.176	0.632	1.411	0.931	0	0	5.04	0	6	58	3	977.83630	2931.49436	-2.57
nLDYVATSIHEAVTk	Unambiguous	1	1	P05201	N-Term(iTRAQ8plex); K15(iTRAQ8plex)	CID	1.0000	0.0000	1	1	3435533	Unique	Used	0.972	1.187	0.716	1.201	0	0	5.03	0	0	63	3	757.09210	2269.26175	-1.37
aGHLTSVHSPEEHk	Unambiguous	1	1	P55066	N-Term(iTRAQ8plex); K14(iTRAQ8plex)	CID	1.0000	0.0000	1	1	2858415	Unique	Used	1.200	0.801	1.500	0.436	0	0	5.03	0	0	156	3	713.05658	2137.15519	-2.74
vEISPEQLSAASTEAER	Unambiguous	4	1	P46737	N-Term(iTRAQ8plex)	CID	1.0000	0.0000	1	1	2794802	Unique	Used		1.503	0.854	1.124	0	0	5.03	0	0	200	2	1061.05115	2121.09502	-1.29
aNTNEVLWAVVAAFTk	Unambiguous	1	1	Q9CRA5	N-Term(iTRAQ8plex); K16(iTRAQ8plex)	CID	1.0000	0.0000	1	1	3899174	Unique	Used	0.515	1.141	0.640	0.759	0	0	5.03	0	21	87	2	1171.66077	2342.31426	-7.96
yVASYLLAALGGnSSPSAk	Unambiguous	1	1	P99027	N-Term(iTRAQ8plex); N13(Deamidated); K19(iTRAQ8plex)	CID	1.0000	0.0000	1	1	3879787	Unique	Used	1.171	0.793	0.990	0.929	0	0	5.03	0	17	14	2	1239.68164	2478.35600	-5.67
aQSTDSLGTSSSLQSk	Unambiguous	6	1	O35551	N-Term(iTRAQ8plex); K16(iTRAQ8plex)	CID	1.0000	0.0000	1	1	3007138	Unique	Used	2.194	1.332	1.359	0.865	0	0	5.03	0	0	22	2	1103.09155	2205.17583	-2.76
tDFFIGGEEGMAEk	Unambiguous	1	1	O35685	N-Term(iTRAQ8plex); K14(iTRAQ8plex)	CID	1.0000	0.0000	1	1	2762025	Unique	Used	1.702	0.964	0.897	0.957	0	0	5.03	0	20	200	3	713.69916	2139.08292	-2.80
eTASGQTAPDVGQAAPVR	Unambiguous	2	1	Q3UHD3-2	N-Term(iTRAQ8plex)	CID	1.0000	0.0000	1	1	2861836	Unique	Used	1.365	1.105	1.979	0.854	0	0	5.03	0	0	200	2	1030.03857	2059.06987	-1.14
tTDDTTIDIYSAQGk	Unambiguous	2	1	A2RSY6	N-Term(iTRAQ8plex); K15(iTRAQ8plex)	CID	1.0000	0.0000	1	1	2793257	Unique	Used		1.088	0.794	0.902	0	0	5.03	0	14	200	2	1119.09070	2237.17412	-0.73

lAALSGGQGSGAAPDGPNER	Unambiguous	1	1	A2AI08	N-Term(iTRAQ8plex)	CID	1.0000	0.0000	1	1	3187122	Unique	Used	1.960		1.286	0.602	0	0	5.03	0	0	193	2	1065.04724	2129.08721	-0.82
sSGNSSSSLGEmVSGTFR	Unambiguous	4	1	Q8R0S4	N-Term(iTRAQ8plex); M12(Oxidation)	CID	1.0000	0.0000	1	1	3635498	Unique	Used	0.355	0.519	1.811	1.199	0	0	5.03	0	0	77	2	1055.49805	2109.98882	-6.47
iSELGSQLSDEAVEDGLFHEFk	Unambiguous	1	1	Q0VBL3	N-Term(iTRAQ8plex); K22(iTRAQ8plex)	CID	1.0000	0.0000	1	1	2960682	Unique	Used	0.441	0.487	0.820	0.681	0	0	5.03	0	11	86	3	1020.19843	3058.58072	-0.75
lTSTSTSDTQNINNSAQR	Unambiguous	1	1	Q8C180	N-Term(iTRAQ8plex)	CID	1.0000	0.0000	1	1	3083681	Unique	Used	1.757	0.908	0.951	0.696	0	0	5.03	0	0	200	2	1121.56311	2242.11894	-1.06
iIAEGANGPTTPEADk	Unambiguous	2	1	P26443	N-Term(iTRAQ8plex); K16(iTRAQ8plex)	CID	1.0000	0.0000	1	1	2776531	Unique	Used	0.705	0.651	0.824	1.093	0	0	5.02	0	6	200	2	1096.60303	2192.19878	-1.45
nAScTLSSAVHSQGVTR	Unambiguous	2	1	Q99J09	N-Term(iTRAQ8plex); C4(Methylthio)	CID	1.0000	0.0000	1	1	3890145	Unique	Used	1.285	0.999	0.833	0.989	0	0	5.02	0	0	200	3	690.00970	2068.01456	-3.48
vDcAVLDcEDGVAENk	Unambiguous	1	1	Q8R4N0	N-Term(iTRAQ8plex); C3(Methylthio); C8(Methylthio); K16(iTRAQ8plex)	CID	1.0000	0.0000	1	1	2765401	Unique	Used	0.417	1.126	1.596	1.255	0	0	5.02	0	22	200	2	1190.55554	2380.10381	-3.18
eVNENFAIDLIAQQPVnEVEHR	Unambiguous	2	1	P52503	N-Term(iTRAQ8plex); N17(Deamidated)	CID	0.0418	0.0000	1	1	2852048	Unique	Used	3.346	1.519	1.320	0.815	0	0	5.02	0	7	100	3	957.15808	2869.45969	-1.31
lYLDELEGSGNSGASck	Unambiguous	3	1	Q8BH60	N-Term(iTRAQ8plex); C16(Methylthio); K17(iTRAQ8plex)	CID	1.0000	0.0000	1	1	2994944	Unique	Used	1.217	0.668	0.859	0.620	0	0	5.01	0	14	200	2	1199.09595	2397.18462	-1.69
gTEDITSPHGIPLDLLDR	Unambiguous	1	1	P60122	N-Term(iTRAQ8plex)	HCD	1.0000	0.0000	1	1	3170733	Unique	Used	0.980	1.147	0.963	1.081	0	0	5.01	0	14	67	3	751.73651	2253.19498	-3.52
vTYMVHDFEEGGGVAMGMYNQDk	Unambiguous	1	1	O88844	N-Term(iTRAQ8plex); K23(iTRAQ8plex)	CID	1.0000	0.0000	1	1	3152578	Unique	Used			0.727	0.951	0	0	5.01	0	12	200	3	1062.84058	3186.50718	-2.63
gTAEGAVDAMLVHGQDAITVSnGGR	Unambiguous	2	1	Q8C7X2-2	N-Term(iTRAQ8plex); N22(Deamidated)	HCD	1.0000	0.0000	1	1	3129989	Unique	Used	1.215	0.920	0.917	1.144	0	0	5.01	0	25	155	3	911.12329	2731.35532	-2.58

sHSTEPNVSSFPNDPSPMk	Unambiguous	6	1	Q3UH68	N-Term(iTRAQ8plex); K19(iTRAQ8plex)	CID	1.0000	0.0000	1	1	2989356	Unique	Used	1.773	1.874	1.369	0.949	0	0	5.01	0	11	200	3	889.44592	2666.32322	-4.08
nGDRGETGPAGPAGPIGPAGAR	Unambiguous	2	1	P11087-2	N-Term(iTRAQ8plex); N1(Deamidated)	CID	1.0000	0.0000	1	1	2805844	Unique	Used	1.656	3.604	1.027	0.943	0	0	5.01	1	10	200	3	760.72107	2280.14865	-6.53
eAnHDGDFGITLTELR	Unambiguous	1	1	G5E829	N-Term(iTRAQ8plex); N3(Deamidated)	CID	1.0000	0.0000	1	1	2670625	Unique	Used	0.237	0.949	1.350	0.601	0	0	5.01	0	2	43	3	698.35284	2093.04398	-0.64
lAEDAPNFDGPAAEGQPGQk	Unambiguous	5	1	G3XA17	N-Term(iTRAQ8plex); K20(iTRAQ8plex)	CID	1.0000	0.0000	1	1	2700768	Unique	Used	0.836	0.541	1.028	0.681	0	0	5.01	0	7	200	3	874.11993	2620.34525	-0.44
gTGGVDTAAVGGVFDVSNADR	Unambiguous	1	1	Q04447	N-Term(iTRAQ8plex)	CID	1.0000	0.0000	1	1	3154736	Unique	Used	1.379	0.961	1.073	1.600	0	0	5.01	0	14	200	2	1135.07019	2269.13310	-1.41
rHEAAVPPLAVSSSRPEk	Unambiguous	3	1	Q8BJ05-2	N-Term(iTRAQ8plex); K18(iTRAQ8plex)	CID	1.0000	0.0000	1	1	3404526	Unique	Used	0.899	0.758	1.050	0.782	0	0	5.01	1	0	189	3	847.15302	2539.44449	-4.75
gQESAGIVTSDGSAVPk	Unambiguous	1	1	Q8CIH9	N-Term(iTRAQ8plex); K17(iTRAQ8plex)	CID	1.0000	0.0000	1	1	2826589	Unique	Used	1.265	0.924	0.909	0.715	0	0	5.00	0	0	96	2	1106.10400	2211.20073	-3.18
rHQSGGNFFEPTLLSNVTR	Unambiguous	1	1	Q8BWF0	N-Term(iTRAQ8plex)	HCD	1.0000	0.0000	1	1	3930612	Unique	Used	0.993	0.832	1.007	1.241	0	0	5.00	1	12	191	3	822.10297	2464.29435	-2.26
gVDLVLNSLAEEk	Unambiguous	1	1	P19096	N-Term(iTRAQ8plex); K13(iTRAQ8plex)	HCD	1.0000	0.0000	1	1	3152707	Unique	Used	0.427	0.547	0.711	0.936	0	0	5.00	0	4	62	2	998.08148	1995.15569	-1.30
sSGNSSSSLGEmVSGTFR	Unambiguous	4	1	Q8R0S4	N-Term(iTRAQ8plex); M12(Oxidation)	CID	1.0000	0.0000	1	1	3614137	Unique	Used	0.595	0.556	1.318	1.007	0	0	5.00	0	20	200	2	1055.49902	2109.99077	-5.55
nVGTGLVGAPAcGDVmk	Unambiguous	3	1	Q9D7P6	N-Term(iTRAQ8plex); C12(Methylthio); M16(Oxidation); K17(iTRAQ8plex)	CID	1.0000	0.0000	1	1	3474538	Unique	Used	1.607	1.086	1.041	0.913	0	0	5.00	0	0	13	3	753.72797	2259.16935	-2.78
eFADSLGVPFLETSAk	Unambiguous	1	1	Q9D1G1	N-Term(iTRAQ8plex); K16(iTRAQ8plex)	CID	1.0000	0.0000	1	1	3283992	Unique	Used	0.601	0.925	1.005	1.064	0	0	5.00	0	25	50	2	1160.13843	2319.26958	0.12

gGGGGGGGPGGFQPAPR	Unambiguous	1	1	O35295	N-Term(iTRAQ8plex)	CID	1.0000	0.0000	1	1	3404450	Unique	Used	0.879	0.908	1.395	1.071	0	0	5.00	0	8	22	3	581.96362	1743.87632	-3.79
vSDFGQmASGMNVDAGk	Unambiguous	1	1	P20357	N-Term(iTRAQ8plex); M7(Oxidation); K17(iTRAQ8plex)	CID	1.0000	0.0000	1	1	3069583	Unique	Used	1.385	2.922	1.732	0.847	0	0	5.00	0	0	200	2	1169.58398	2338.16069	-0.91
IYPSLANIQEVSANIAIk	Unambiguous	1	1	Q99KE1	N-Term(iTRAQ8plex); K18(iTRAQ8plex)	CID	1.0000	0.0000	1	1	3765784	Unique	Used	0.977	1.098	0.992	1.418	0	0	5.00	0	15	11	2	1276.74426	2552.48125	-3.76
mAPYQGPDAAPGALDYk	Unambiguous	1	1	P57780	N-Term(iTRAQ8plex); M1(Oxidation); K17(iTRAQ8plex)	CID	1.0000	0.0000	1	1	3030791	Unique	Used	1.109	1.428	1.231	0.733	0	0	5.00	0	0	200	2	1195.11938	2389.23149	-0.16
gAIQLGIGYTVGNLSSkPER	Unambiguous	4	1	F8WHW6	N-Term(iTRAQ8plex); K17(iTRAQ8plex)	CID	1.0000	0.0000	1	1	3839265	Unique	Used	0.741	1.681	1.518	1.140	0	0	5.00	0	16	200	3	890.17865	2668.52140	-1.08
kVTITGTPAATQAAQYLITQR	Unambiguous	1	1	Q9JKN6	N-Term(iTRAQ8plex); K1(iTRAQ8plex)	CID	1.0000	0.0000	1	1	3914359	Unique	Used	1.313	0.461	0.842	1.117	0	0	5.00	1	0	50	3	947.54950	2840.63395	-4.05
gHQAALGLmEDEQEQAQQLR	Unambiguous	1	1	Q8VCA8	N-Term(iTRAQ8plex); M9(Oxidation)	CID	1.0000	0.0000	1	1	2808033	Unique	Used	2.362	1.249	0.997	0.646	0	0	5.00	0	0	200	3	858.09314	2572.26487	-3.09
eGQQLMQEkPELAASVR	Unambiguous	3	1	E9PX29	N-Term(iTRAQ8plex); K9(iTRAQ8plex)	HCD	1.0000	0.0000	1	1	3105250	Unique	Used	0.888	0.815	1.516	1.148	0	0	4.99	0	22	200	3	841.46490	2522.38016	-2.22
vIDIFDTDGnGEVDFk	Unambiguous	2	1	Q63810-2	N-Term(iTRAQ8plex); N10(Deamidated); K16(iTRAQ8plex)	CID	1.0000	0.0000	1	1	2721032	Unique	Used			1.484		0	0	4.99	0	6	200	2	1197.11804	2393.22881	-1.88
sSEDLAGPLPSSVPSSTTSSkPk	Unambiguous	6	1	Q91ZM2	N-Term(iTRAQ8plex); K21(iTRAQ8plex); K23(iTRAQ8plex)	CID	1.0000	0.0000	1	1	3208169	Unique	Used	0.381	1.110	0.880	0.776	0	0	4.99	0	0	75	3	1057.92261	3171.75327	0.64
iGDVVGSSGANQQTSGk	Unambiguous	2	1	P27612	N-Term(iTRAQ8plex); K17(iTRAQ8plex)	CID	1.0000	0.0000	1	1	3185102	Unique	Used	1.001	0.838	0.714	0.687	0	0	4.99	0	0	25	2	1107.10046	2213.19365	-2.08

vLGGGGTGGGLGGPGSVPFk	Unambiguous	1	1	E9PXF0	N-Term(iTRAQ8plex); K20(iTRAQ8plex)	CID	1.0000	0.0000	1	1	3761898	Unique	Used	0.683	0.965	0.451	0.812	0	0	4.99	0	17	55	2	1140.14868	2279.29009	-2.98
kEPQPSGPQPSGGAGGGGGk	Unambiguous	1	1	Q8JZQ2	N-Term(iTRAQ8plex); K1(iTRAQ8plex); K20(iTRAQ8plex)	CID	1.0000	0.0000	1	1	2927556	Unique	Used	0.501	0.875	1.038	1.252	0	0	4.99	1	0	158	3	888.16113	2662.46885	0.45
sSEDLAGPLPSSVPSSTTSSkPk	Unambiguous	6	1	Q91ZM2	N-Term(iTRAQ8plex); K21(iTRAQ8plex); K23(iTRAQ8plex)	CID	1.0000	0.0000	1	1	3208191	Unique	Used				0.942	0	0	4.99	0	24	200	3	1057.92029	3171.74631	-1.55
dTAEGVSQELISAGLVDGR	Unambiguous	1	1	Q6P9R2	N-Term(iTRAQ8plex)	CID	1.0000	0.0000	1	1	2940612	Unique	Used	1.963	1.224	1.017	0.906	0	0	4.98	0	0	120	2	1111.08252	2221.15776	-1.66
sIDDLEDQLYHQLEQNR	Unambiguous	3	1	E9Q452	N-Term(iTRAQ8plex)	CID	1.0000	0.0000	1	1	2979622	Unique	Used	2.853	0.890	2.252	1.150	0	0	4.98	0	18	200	3	807.40387	2420.19706	-1.04
sLSESELIGELSADFDR	Unambiguous	8	1	P51125-3	N-Term(iTRAQ8plex)	CID	1.0000	0.0000	1	1	2888202	Unique	Used	3.932	0.870	1.506	0.932	0	0	4.98	0	0	200	2	1086.54785	2172.08843	-4.15
aGADAASGDAPPPALGGR	Unambiguous	1	1	Q3USB7	N-Term(iTRAQ8plex)	CID	1.0000	0.0000	1	1	2825323	Unique	Used	2.052	0.400	1.315	0.942	0	0	4.98	0	0	200	2	927.98309	1854.95891	-1.25
dHIIEVVGSSmPLIGDHQDEDk	Unambiguous	3	1	O88935	N-Term(iTRAQ8plex); M11(Oxidation); K22(iTRAQ8plex)	CID	1.0000	0.0000	1	1	2992581	Unique	Used	1.077	1.340	1.498	1.445	0	0	4.98	0	0	200	3	1020.19067	3058.55747	-1.23
iVYGHLDDPANQEIER	Unambiguous	1	1	Q99KI0	N-Term(iTRAQ8plex)	CID	1.0000	0.0000	1	1	2757705	Unique	Used	0.592	0.747	1.203	1.513	0	0	4.98	0	0	3	3	725.04346	2173.11582	-1.53
vSELEAELAEQQHLGR	Unambiguous	1	1	Q8VD75	N-Term(iTRAQ8plex)	CID	1.0000	0.0000	1	1	2850759	Unique	Used	1.645	1.346	0.922	0.938	0	0	4.98	0	11	200	3	705.04279	2113.11380	-2.54
aLLADAQImLDHLk	Unambiguous	12	1	B2RRE2	N-Term(iTRAQ8plex); M9(Oxidation); K14(iTRAQ8plex)	CID	1.0000	0.0000	1	1	3599944	Unique	Used	1.014	0.555	1.428	0.729	0	0	4.98	0	8	13	3	726.08826	2176.25022	-5.44
vLAQnSGFDLQETLVk	Unambiguous	1	1	P80317	N-Term(iTRAQ8plex); N5(Deamidated); K16(iTRAQ8plex)	CID	0.0361	0.0000	1	1	3237826	Unique	Used	0.687	1.116	0.882	0.967	0	0	4.98	0	18	73	2	1186.16504	2371.32280	-4.28

lTGVSISQVNHNkPLR	Unambiguous	2	1	O08917	N-Term(iTRAQ8plex); K13(iTRAQ8plex)	HCD	1.0000	0.0000	1	1	3928225	Unique	Used	0.434	1.393	0.887	0.948	0	0	4.98	0	11	200	3	791.13751	2371.39798	-2.13
ySMVGGNLVINNPDkQk	Unambiguous	1	1	P12960	N-Term(iTRAQ8plex); K15(iTRAQ8plex); K17(iTRAQ8plex)	CID	1.0000	0.0000	1	1	3453166	Unique	Used	0.810	1.030	0.951	0.884	0	0	4.97	1	0	128	3	930.52747	2789.56784	-2.47
lGHDESGQEGLQQELELLR	Unambiguous	2	1	E9QAH1	N-Term(iTRAQ8plex)	CID	1.0000	0.0000	1	1	2813807	Unique	Used	4.030	1.380	1.297	1.054	0	0	4.97	0	7	51	3	819.09552	2455.27201	-0.45
gLSSVPEVAEVEPTTk	Unambiguous	1	1	P20357	N-Term(iTRAQ8plex); K16(iTRAQ8plex)	CID	1.0000	0.0000	1	1	2917656	Unique	Used	1.540	1.706	1.668	0.707	0	0	4.97	0	7	104	2	1126.13184	2251.25640	-3.47
aVVFETGEAGAASGAGSLPGEVR	Unambiguous	3	1	Q9QYR6	N-Term(iTRAQ8plex)	CID	1.0000	0.0000	1	1	3129818	Unique	Used	4.035	1.033	1.395	1.035	0	0	4.97	0	0	200	2	1218.63489	2436.26250	-1.98
qTQVSVLPEGGETPLFk	Unambiguous	2	1	P13020-2	N-Term(iTRAQ8plex); K17(iTRAQ8plex)	CID	1.0000	0.0000	1	1	3258438	Unique	Used	1.490	0.471	0.688	0.626	0	0	4.97	0	24	58	2	1219.69092	2438.37456	-0.25
qASPLISPLLNDQAcPR	Unambiguous	1	1	F8VPU2	N-Term(iTRAQ8plex); C15(Methylthio)	HCD	1.0000	0.0000	1	1	3804781	Unique	Used	0.277	0.679	0.760	0.891	0	0	4.97	0	14	40	2	1087.07117	2173.13506	-2.81
fQLTDSQIYEVLSVIR	Unambiguous	1	1	O08553	N-Term(iTRAQ8plex)	HCD	1.0000	0.0000	1	1	3844050	Unique	Used	1.506	1.908	1.039	1.103	0	0	4.97	0	0	200	2	1108.11267	2215.21807	-4.32
vTLSFPSTLQTGTGTLk	Unambiguous	5	1	Q11011	N-Term(iTRAQ8plex); K17(iTRAQ8plex)	CID	1.0000	0.0000	1	1	3840843	Unique	Used	0.427	1.257	1.001	0.883	0	0	4.97	0	0	55	2	1180.18396	2359.36064	-3.68
dYSGLHDVAAVGDGLPEGATAnGMk	Unambiguous	3	1	Q3UHH2	N-Term(iTRAQ8plex); N22(Deamidated); K25(iTRAQ8plex)	CID	1.0000	0.0000	1	1	2900789	Unique	Used	0.980	0.639	0.596	1.090	0	0	4.97	0	0	134	3	1018.84418	3054.51798	-3.92
lVLPMDDSEPALNSLDDTR	Unambiguous	2	1	A1L3P4	N-Term(iTRAQ8plex)	CID	1.0000	0.0000	1	1	2870953	Unique	Used	0.510	0.826	1.498	1.306	0	0	4.97	0	8	38	2	1203.10925	2405.21123	-2.50
hLLVSNVGGDGEEIER	Unambiguous	1	1	Q99P88	N-Term(iTRAQ8plex)	CID	1.0000	0.0000	1	1	2714476	Unique	Used	5.099	1.055	1.638	1.038	0	0	4.97	0	10	117	3	676.69177	2028.06076	-2.78
aSSATDGQLASPGEnGEVRPGQAk	Unambiguous	1	1	P16054	N-Term(iTRAQ8plex); N15(Deamidated); K24(iTRAQ8plex)	CID	1.0000	0.0000	1	1	2712927	Unique	Used		2.687	1.527	1.532	0	0	4.96	0	0	200	3	979.50574	2936.50266	-4.91

gGSSAGGPAPSTmSk	Unambiguous	1	1	P47968	N-Term(iTRAQ8plex); M13(Oxidation); K15(iTRAQ8plex)	CID	1.0000	0.0000	1	1	3117134	Unique	Used	1.941	0.849	0.878	0.736	0	0	4.96	0	0	200	2	958.50085	1915.99443	-3.12
lLGPDAAINLADPDGALAk	Unambiguous	1	1	Q99KJ8	N-Term(iTRAQ8plex); K19(iTRAQ8plex)	CID	1.0000	0.0000	1	1	3326443	Unique	Used	1.377	1.326	1.047	1.798	0	0	4.96	0	0	82	2	1222.19885	2443.39043	-4.63
qDLAQLmNSSGSHk	Unambiguous	2	1	O88544	N-Term(iTRAQ8plex); M7(Oxidation); K14(iTRAQ8plex)	CID	1.0000	0.0000	1	1	3362412	Unique	Used			1.075	1.070	0	0	4.96	0	14	142	3	714.04230	2140.11234	-7.19
tESNQEVANPEHYIk	Unambiguous	1	1	P63073	N-Term(iTRAQ8plex); K15(iTRAQ8plex)	CID	1.0000	0.0000	1	1	2754382	Unique	Used	1.026	0.846	0.895	0.790	0	0	4.96	0	15	15	3	789.74878	2367.23178	-3.51
nELEcVTNISLANIIR	Unambiguous	1	1	Q8R5H6	N-Term(iTRAQ8plex); C5(Methylthio)	HCD	1.0000	0.0000	1	1	3766083	Unique	Used	0.338	1.766	1.109	0.795	0	0	4.96	0	12	15	2	1076.57043	2152.13359	-3.36
lSEISGIPLEDIEFAk	Unambiguous	2	1	Q8BY87-2	N-Term(iTRAQ8plex); K16(iTRAQ8plex)	CID	1.0000	0.0000	1	1	3262492	Unique	Used	0.578	1.085	0.804	1.074	0	0	4.96	0	6	7	2	1185.17188	2369.33647	-2.53
aAIVHNVDSDDLISMGSNDIDTLk	Unambiguous	2	1	O54965	N-Term(iTRAQ8plex); K24(iTRAQ8plex)	CID	1.0000	0.0000	1	1	3035901	Unique	Used	2.530	1.156	0.549	0.531	0	0	4.96	0	8	200	3	1051.21680	3151.63584	-1.39
aTHHPAPGPGLLGPPSAIPGLQNQQLLGER	Unambiguous	2	1	D3Z3Z3	N-Term(iTRAQ8plex)	HCD	1.0000	0.0000	1	1	3856981	Unique	Used	0.890	2.235	1.531	1.268	0	0	4.96	0	0	87	3	1109.94067	3327.80747	-4.63
gIVIGTGENSEFGEVFk	Unambiguous	7	1	H3BIX7	N-Term(iTRAQ8plex); K17(iTRAQ8plex)	CID	1.0000	0.0000	1	1	3151351	Unique	Used	0.632	1.030	0.922	0.894	0	0	4.95	0	0	200	2	1196.15137	2391.29546	-2.60
dEAEAPAAGTPQLSEVSLGR	Unambiguous	2	1	Q3UHE1-2	N-Term(iTRAQ8plex)	CID	1.0000	0.0000	1	1	2794371	Unique	Used		1.137	0.400	0.948	0	0	4.95	0	0	200	2	1151.59192	2302.17656	-2.76
lGSASSSHTSIQENHk	Unambiguous	2	1	P08414	N-Term(iTRAQ8plex); K16(iTRAQ8plex)	CID	1.0000	0.0000	1	1	3024116	Unique	Used	0.687	0.916	2.203	0.739	0	0	4.95	0	0	200	3	764.40887	2291.21207	-3.47
iVAERPGTNSTGPGPMAPPR	Unambiguous	2	1	Q62318	N-Term(iTRAQ8plex)	HCD	1.0000	0.0000	1	1	3717544	Unique	Used	0.776	1.129	0.716	0.824	0	0	4.95	0	0	31	3	770.41168	2309.22049	-5.78

vDEFPLcGHmVSDEYEQLSSEALEAAR	Unambiguous	4	1	I7HLV2	N-Term(iTRAQ8plex); C7(Methylthio); M10(Oxidation)	HCD	1.0000	0.0000	1	1	3112092	Unique	Used	0.600	0.549	0.721	0.773	0	0	4.95	0	0	89	3	1131.18347	3391.53586	-2.26
aVAQGNLSSADVQAAk	Unambiguous	1	1	Q9DB77	N-Term(iTRAQ8plex); K16(iTRAQ8plex)	CID	1.0000	0.0000	1	1	3143987	Unique	Used	0.701	1.797	1.445	1.288	0	0	4.94	0	5	11	2	1069.60327	2138.19927	-1.56
kDGQMLPGEDEPLHALVTAnTMENVk	Unambiguous	7	1	E9Q4Q2	N-Term(iTRAQ8plex); K1(iTRAQ8plex); N20(Deamidated); K26(iTRAQ8plex)	CID	1.0000	0.0000	1	1	3090014	Unique	Used	0.964	0.580	0.990	0.760	0	0	4.94	1	0	200	3	1251.00427	3750.99826	4.59
sHSEPAEFSAQLDPSFIk	Unambiguous	2	1	Q3UW66	N-Term(iTRAQ8plex); K18(iTRAQ8plex)	CID	1.0000	0.0000	1	1	3107181	Unique	Used	1.360	0.923	1.309	0.976	0	0	4.94	0	6	15	3	866.79346	2598.36582	-0.09
gVSSVVGPSAVGASPGDk	Unambiguous	2	1	Q8CI75	N-Term(iTRAQ8plex); K18(iTRAQ8plex)	CID	1.0000	0.0000	1	1	3252604	Unique	Used	0.904	0.683	1.204	1.106	0	0	4.94	0	19	97	2	1090.11047	2179.21367	-1.96
lADPTGEDQALAAAAAEGGR	Unambiguous	1	1	Q8CHE4	N-Term(iTRAQ8plex)	CID	1.0000	0.0000	1	1	2636859	Unique	Used	1.140	0.483	0.840	1.176	0	0	4.94	0	0	200	2	1094.55786	2188.10845	-2.92
eHAnIDAQSGSQAPNPSTTISPGk	Unambiguous	3	1	Q8VDQ8-2	N-Term(iTRAQ8plex); N4(Deamidated); K24(iTRAQ8plex)	CID	1.0000	0.0000	1	1	2844547	Unique	Used	1.178	0.429	0.749	1.193	0	0	4.93	0	0	200	3	1006.18317	3016.53495	-2.76
gFSLSDVPQAEISGEHLR	Unambiguous	1	1	Q9QZF2	N-Term(iTRAQ8plex)	CID	1.0000	0.0000	1	1	3300572	Unique	Used	1.177	1.038	1.299	1.277	0	0	4.93	0	23	6	3	749.39233	2246.16245	-4.22
gEAGTPVAAGTTAAAIPR	Unambiguous	1	1	O08919	N-Term(iTRAQ8plex)	CID	1.0000	0.0000	1	1	3275018	Unique	Used	0.774	1.484	1.395	1.067	0	0	4.93	0	0	101	2	958.02820	1915.04912	-3.13
sLAASGAQQPDSPGGPQPPGQk	Unambiguous	2	1	Q9JKS5	N-Term(iTRAQ8plex); K22(iTRAQ8plex)	CID	1.0000	0.0000	1	1	3066582	Unique	Used	2.518			1.013	0	0	4.93	0	0	200	3	895.14667	2683.42545	-0.22
iGRPSETGIIGIIDPEcR	Unambiguous	1	1	Q3U1J4	N-Term(iTRAQ8plex); C17(Methylthio)	HCD	1.0000	0.0000	1	1	3519205	Unique	Used	1.227	1.063	0.963	1.207	0	0	4.93	0	0	59	3	759.40320	2276.19504	-4.16

aLGSNDPMQSILSSLEQQHEEEk	Unambiguous	1	1	E9Q4K7	N-Term(iTRAQ8plex); K23(iTRAQ8plex)	CID	1.0000	0.0000	1	1	3075139	Unique	Used	0.771	0.719	0.716	0.990	0	0	4.93	0	0	200	3	1060.21216	3178.62192	2.27
tDGAAPNVAPSDVGGGGTNR	Unambiguous	1	1	P12960	N-Term(iTRAQ8plex)	CID	1.0000	0.0000	1	1	2825075	Unique	Used	1.230	1.022	0.697	0.813	0	0	4.93	0	3	86	2	1087.53821	2174.06914	-2.25
dVVGSGTDTALDVAVk	Unambiguous	3	1	B1AR76	N-Term(iTRAQ8plex); K16(iTRAQ8plex)	CID	1.0000	0.0000	1	1	2936610	Unique	Used	0.797	0.908	1.149	1.098	0	0	4.93	0	0	145	2	1078.10510	2155.20293	-1.75
aSVTSTSTSkPSSPR	Unambiguous	1	1	E9Q6Y8	N-Term(iTRAQ8plex); K10(iTRAQ8plex)	HCD	1.0000	0.0000	1	1	3487061	Unique	Used	0.619	0.720	1.708	1.063	0	0	4.93	0	0	200	3	701.06036	2101.16654	-2.10
iDSAATSTYEVGNPPDYR	Unambiguous	1	1	Q9D358	N-Term(iTRAQ8plex)	CID	1.0000	0.0000	1	1	2883071	Unique	Used	2.484	1.352	0.698	1.011	0	0	4.93	0	0	200	2	1130.55090	2260.09453	-3.99
lGEHNInVLEGNEQFIDAAk	Unambiguous	1	1	Q9Z1R9	N-Term(iTRAQ8plex); N7(Deamidated); K20(iTRAQ8plex)	CID	1.0000	0.0000	1	1	2888967	Unique	Used	0.833	1.231	0.865	0.743	0	0	4.92	0	0	200	3	940.83081	2820.47788	-7.45
vNNSSLIGVGYTQTLRPGVk	Unambiguous	2	1	G3UX26	N-Term(iTRAQ8plex); K20(iTRAQ8plex)	HCD	1.0000	0.0000	1	1	3912552	Unique	Used	0.455	0.992	1.141	1.404	0	0	4.92	0	0	171	3	904.52478	2711.55979	-2.47
gTGGVDTAAVGGVFDVSNADR	Unambiguous	1	1	Q04447	N-Term(iTRAQ8plex)	CID	1.0000	0.0000	1	1	3089582	Unique	Used	2.016	1.311	0.874	0.936	0	0	4.92	0	0	200	2	1135.07227	2269.13725	0.42
lEQmFLVALADTVATTSTELDENR	Unambiguous	4	1	Q8BPN8-2	N-Term(iTRAQ8plex); M4(Oxidation)	CID	1.0000	0.0000	1	1	3374687	Unique	Used	2.026	1.455	0.681	1.172	0	0	4.92	0	10	143	3	996.50934	2987.51346	-1.72
vFLMDPVIFTVAHLEAk	Unambiguous	5	1	E9Q3V9	N-Term(iTRAQ8plex); K17(iTRAQ8plex)	CID	1.0000	0.0000	1	1	3766277	Unique	Used	0.327	1.294	1.028	0.974	0	0	4.92	0	15	22	3	846.82202	2538.45151	-3.93
gTGGVDTAAVGGVFDVSNADR	Unambiguous	1	1	Q04447	N-Term(iTRAQ8plex)	CID	1.0000	0.0000	1	1	3135707	Unique	Used	1.460	0.727	1.197	1.474	0	0	4.92	0	0	200	2	1135.08081	2269.15434	7.95
aIAVLTSGGDAQGMNAAVR	Unambiguous	1	1	P47857	N-Term(iTRAQ8plex)	CID	1.0000	0.0000	1	1	3762792	Unique	Used	2.504	1.038	1.066	1.013	0	0	4.91	0	0	49	2	1053.56433	2106.12139	-3.13
gTGGVDTAAVGGVFDVSNADR	Unambiguous	1	1	Q04447	N-Term(iTRAQ8plex)	CID	1.0000	0.0000	1	1	3155482	Unique	Used	1.112		0.775	0.869	0	0	4.91	0	25	200	2	1135.07068	2269.13408	-0.98
lTAVELVDILDHYTk	Unambiguous	1	1	Q64676	N-Term(iTRAQ8plex); K15(iTRAQ8plex)	CID	1.0000	0.0000	1	1	3352408	Unique	Used	0.557	0.029	0.154	0.200	0	0	4.91	0	11	22	3	780.11713	2338.33683	-4.72

dSGAGFSASEPDAVHVVLDR	Unambiguous	1	1	Q8BFQ8	N-Term(iTRAQ8plex)	CID	1.0000	0.0000	1	1	2848873	Unique	Used	0.955	0.936	0.931	0.940	0	0	4.91	0	6	79	3	778.39270	2333.16355	-1.73
gSHSQSSSPGALTLGR	Unambiguous	3	1	O88935	N-Term(iTRAQ8plex)	HCD	1.0000	0.0000	1	1	3674316	Unique	Used	1.146	1.183	1.517	1.540	0	0	4.91	0	0	200	3	615.99274	1845.96366	-4.56
vTEGLVDVILYHQPDDk	Unambiguous	1	1	Q8VHM5	N-Term(iTRAQ8plex); K17(iTRAQ8plex)	CID	1.0000	0.0000	1	1	3111518	Unique	Used	0.309	0.478	0.811	0.822	0	0	4.91	0	2	21	3	850.47363	2549.40635	-0.33
lGEHNInVLEGNEQFIDAAk	Unambiguous	1	1	Q9Z1R9	N-Term(iTRAQ8plex); N7(Deamidated); K20(iTRAQ8plex)	CID	0.0183	0.0000	1	1	3149415	Unique	Used	2.618	2.792	1.384	1.309	0	0	4.91	0	0	36	3	940.83575	2820.49271	-2.19
gGSEPATSTLPAAAAATnGPR	Unambiguous	3	1	Q8BNU1	N-Term(iTRAQ8plex); N18(Deamidated)	CID	1.0000	0.0000	1	1	2932525	Unique	Used	0.828	0.783	0.785	0.605	0	0	4.91	0	0	176	2	1101.56702	2202.12676	-1.69
dLGLSESGEDVNAAILDESGkk	Unambiguous	1	1	P08003	N-Term(iTRAQ8plex); K21(iTRAQ8plex); K22(iTRAQ8plex)	CID	1.0000	0.0000	1	1	2761093	Unique	Used	1.174	0.587	0.473	0.436	0	0	4.90	1	0	113	3	1053.90686	3159.70603	-2.80
lSTLGSGGESGGDGSPGGAGATAAR	Unambiguous	1	1	Q80UU9	N-Term(iTRAQ8plex)	CID	1.0000	0.0000	1	1	3029104	Unique	Used	0.518	1.522	0.993	1.032	0	0	4.90	0	0	86	2	1197.58936	2394.17143	-3.57
vAIVEELVVGYETSLk	Unambiguous	2	1	G3X8Y3	N-Term(iTRAQ8plex); K16(iTRAQ8plex)	CID	1.0000	0.0000	1	1	3353093	Unique	Used	0.687	0.435	0.687	1.006	0	0	4.90	0	0	36	2	1179.18762	2357.36797	-4.61
sPAASAPSLPPAAAVLPSk	Unambiguous	3	1	Q99P72	N-Term(iTRAQ8plex); K19(iTRAQ8plex)	CID	1.0000	0.0000	1	1	3702457	Unique	Used	1.867	0.962	1.280	0.868	0	0	4.90	0	16	65	2	1170.68201	2340.35674	-7.71
gSETLGGGGGGAAGTR	Unambiguous	1	1	P31650	N-Term(iTRAQ8plex)	CID	1.0000	0.0000	1	1	2841730	Unique	Used				1.386	0	0	4.90	0	0	174	2	804.91107	1608.81487	-5.91
eQLLLDELVALVDkR	Unambiguous	2	1	Q69ZW3-2	N-Term(iTRAQ8plex); K14(iTRAQ8plex)	CID	1.0000	0.0000	1	1	3396415	Unique	Used	0.962	1.197	1.220	0.873	0	0	4.89	1	11	50	3	788.14172	2362.41062	-2.55
gSETLGGGGGGAAGTR	Unambiguous	1	1	P31650	N-Term(iTRAQ8plex)	CID	1.0000	0.0000	1	1	2841360	Unique	Used	0.644	0.304	0.721	1.553	0	0	4.89	0	0	66	2	804.91174	1608.81621	-5.08
gGLHTVAVmNVGAPAAGMNAAVR	Unambiguous	1	1	P47857	N-Term(iTRAQ8plex); M9(Oxidation)	CID	1.0000	0.0000	1	1	3929457	Unique	Used	0.659	0.542	2.360	2.059	0	0	4.89	0	0	18	3	828.77081	2484.29789	-5.60

sSDQAVPAIPTSNSYSk	Unambiguous	10	1	E9QMP6	N-Term(iTRAQ8plex); K17(iTRAQ8plex)	CID	1.0000	0.0000	1	1	3165265	Unique	Used	1.088	0.958	1.659	1.018	0	0	4.89	0	0	73	2	1180.62952	2360.25176	-1.55
vSLPTANPDVSSGITQIk	Unambiguous	1	1	Q8C419	N-Term(iTRAQ8plex); K18(iTRAQ8plex)	CID	1.0000	0.0000	1	1	3496218	Unique	Used	1.360	0.524	1.227	0.627	0	0	4.89	0	8	82	2	1218.19849	2435.38970	-2.84
sETSGSFEDALLAIVk	Unambiguous	3	1	P97429	N-Term(iTRAQ8plex); K16(iTRAQ8plex)	CID	1.0000	0.0000	1	1	3262554	Unique	Used		2.500		0.715	0	0	4.89	0	19	19	2	1138.13611	2275.26494	0.32
tIAEcLADELINAAk	Unambiguous	2	1	Q91V55	N-Term(iTRAQ8plex); C5(Methylthio); K15(iTRAQ8plex)	CID	1.0000	0.0000	1	1	3352788	Unique	Used	0.529	0.603	0.957	0.988	0	0	4.89	0	0	10	2	1115.10278	2229.19829	-4.34
lScnGLGPSDLLGkPLAR	Unambiguous	3	1	Q5PR72	N-Term(iTRAQ8plex); C3(Methylthio); N4(Deamidated); K14(iTRAQ8plex)	HCD	1.0000	0.0000	1	1	3765065	Unique	Used	0.411	1.782	1.625	1.310	0	0	4.88	0	11	122	3	822.79120	2466.35904	-3.20
mqINAEEVVVGDLVEVk	Unambiguous	3	1	Q6PIE5	N-Term(iTRAQ8plex); Q2(Deamidated); K17(iTRAQ8plex)	CID	0.0410	0.0000	1	1	2853149	Unique	Used	1.624	1.010	0.964	0.929	0	0	4.88	0	12	200	3	827.79468	2481.36948	-1.47
vLESELEDQLVQHR	Unambiguous	4	1	P48725-3	N-Term(iTRAQ8plex)	CID	1.0000	0.0000	1	1	2850749	Unique	Used	0.893	1.046	1.181	0.535	0	0	4.88	0	28	200	3	667.03033	1999.07645	0.11
fQVDFQLGNSLkPR	Unambiguous	2	1	Q9JL15	N-Term(iTRAQ8plex); K12(iTRAQ8plex)	HCD	1.0000	0.0000	1	1	3857414	Unique	Used	1.105	0.822	0.809	1.104	0	0	4.88	0	8	115	3	753.09869	2257.28153	-4.36
qPHILLLDEATSALDTESEk	Unambiguous	2	1	P21447	N-Term(iTRAQ8plex); K20(iTRAQ8plex)	CID	1.0000	0.0000	1	1	2959404	Unique	Used			0.960	0.998	0	0	4.88	0	7	168	3	940.17914	2818.52286	-2.36
gSETLGGGGGGAAGTR	Unambiguous	1	1	P31650	N-Term(iTRAQ8plex)	CID	1.0000	0.0000	1	1	2876633	Unique	Used	0.781	0.387	1.076	1.345	0	0	4.87	0	10	200	2	804.91290	1608.81853	-3.64
hQISMQVAQAk	Unambiguous	1	1	Q8K4X7	N-Term(iTRAQ8plex); K11(iTRAQ8plex)	CID	1.0000	0.0000	1	1	3448902	Unique	Used	0.796	1.061	1.187	1.224	0	0	4.87	0	17	158	3	617.02264	1849.05338	-2.20

lAAALTAEPEPERPLR	Unambiguous	1	1	A2BDX3	N-Term(iTRAQ8plex)	HCD	1.0000	0.0000	1	1	3299886	Unique	Used	0.889	1.038	0.877	1.052	0	0	4.87	0	22	133	3	680.05823	2038.16013	0.10
gLDHLNVTVGVDGTLYk	Unambiguous	5	1	G3UVV4	N-Term(iTRAQ8plex); K17(iTRAQ8plex)	CID	1.0000	0.0000	1	1	3345888	Unique	Used	0.795	1.852	1.100	1.021	0	0	4.87	0	5	2	3	803.78821	2409.35007	-4.05
qPSGAGLcDITEGTVVPEDR	Unambiguous	3	1	D3Z2Q2	N-Term(iTRAQ8plex); C8(Methylthio)	CID	1.0000	0.0000	1	1	2939994	Unique	Used	0.806	1.216	1.049	1.529	0	0	4.87	0	3	35	2	1197.58081	2394.15434	-1.67
aIAVLTSGGDAQGmNAAVR	Unambiguous	1	1	P47857	N-Term(iTRAQ8plex); M14(Oxidation)	CID	1.0000	0.0000	1	1	3760566	Unique	Used	2.607	0.918	1.353	1.381	0	0	4.87	0	4	2	2	1061.56006	2122.11284	-4.74
tGFDFNImVVGQSGLGk	Unambiguous	2	1	Q9Z1S5-2	N-Term(iTRAQ8plex); M8(Oxidation); K17(iTRAQ8plex)	CID	1.0000	0.0000	1	1	3744647	Unique	Used	0.431	1.427	1.204	1.226	0	0	4.87	0	14	65	2	1197.64673	2394.28618	-3.61
tDPPIIEGNMESAk	Unambiguous	1	1	P12960	N-Term(iTRAQ8plex); K14(iTRAQ8plex)	CID	1.0000	0.0000	1	1	2757315	Unique	Used	2.237	1.440	1.055	1.002	0	0	4.86	0	5	74	3	704.04810	2110.12973	-0.64
gLEELLIGGSHLk	Unambiguous	1	1	Q04857	N-Term(iTRAQ8plex); K13(iTRAQ8plex)	CID	1.0000	0.0000	1	1	3370916	Unique	Used	0.461	6.864	0.751	1.173	0	0	4.86	0	0	94	3	658.72919	1974.17301	-5.79
dVESDLASYPPPTVSSSLTSR	Unambiguous	2	1	O88737	N-Term(iTRAQ8plex)	CID	1.0000	0.0000	1	1	3090556	Unique	Used	0.726	0.676	2.391	1.092	0	0	4.86	0	0	121	2	1256.63904	2512.27080	-0.51
tDGAAPNVAPSDVGGGGTnR	Unambiguous	1	1	P12960	N-Term(iTRAQ8plex); N20(Deamidated)	CID	1.0000	0.0000	1	1	2631357	Unique	Used	1.157	1.428	1.195	1.029	0	0	4.86	0	0	200	2	1088.02966	2175.05205	-2.76
gDmSEAELLILDEFTTLAR	Unambiguous	2	1	E9Q401	N-Term(iTRAQ8plex); M3(Oxidation)	HCD	1.0000	0.0000	1	1	3153936	Unique	Used	0.312	2.125	2.384	1.580	0	0	4.86	0	0	110	2	1222.62952	2444.25176	-0.63
nVPSAGGGIGDGGQEPTTGNWR	Unambiguous	3	1	Q8K1M6	N-Term(iTRAQ8plex)	CID	1.0000	0.0000	1	1	3049254	Unique	Used	0.810	1.951	1.223	1.252	0	0	4.86	0	0	104	2	1216.09790	2431.18852	-0.80
tIGTGLVTDVPAMTEEDk	Unambiguous	2	1	Q8BFR5	N-Term(iTRAQ8plex); K18(iTRAQ8plex)	CID	1.0000	0.0000	1	1	2919532	Unique	Used	0.583	0.462	1.323	1.155	0	0	4.86	0	6	182	2	1243.16406	2485.32085	-4.35
kPLVLcGDLNVAHEEIDLR	Unambiguous	2	1	F6QA74	N-Term(iTRAQ8plex); K1(iTRAQ8plex); C6(Methylthio)	CID	1.0000	0.0000	1	1	3348953	Unique	Used	0.420	1.017	1.012	1.050	0	0	4.86	0	7	21	3	930.17877	2788.52176	-3.33

gESPELDLSYSHSDLGk	Unambiguous	1	1	P20917	N-Term(iTRAQ8plex); K17(iTRAQ8plex)	CID	1.0000	0.0000	1	1	2847215	Unique	Used	2.045	0.328	0.745	0.543	0	0	4.86	0	28	200	3	814.75403	2442.24753	-5.47
nGDRGETGPAGPAGPIGPAGAR	Unambiguous	2	1	P11087-2	N-Term(iTRAQ8plex); N1(Deamidated)	CID	1.0000	0.0000	1	1	2824636	Unique	Used	1.335	3.143	1.156	1.064	0	0	4.85	1	0	200	3	760.72498	2280.16037	-1.39
iPNIYAIGDVVAGPmLAHk	Unambiguous	1	1	O08749	N-Term(iTRAQ8plex); M15(Oxidation); K19(iTRAQ8plex)	CID	1.0000	0.0000	1	1	3826559	Unique	Used	1.491	1.318	0.862	0.766	0	0	4.85	0	0	200	3	868.49628	2603.47428	-3.74
qLEEDLYDGQVLQk	Unambiguous	1	1	Q9ES46	N-Term(iTRAQ8plex); K14(iTRAQ8plex)	CID	1.0000	0.0000	1	1	2779991	Unique	Used		0.989	0.769	1.003	0	0	4.85	0	16	200	3	762.75177	2286.24076	-1.33
lYPAAVDTIVAImAEGk	Unambiguous	2	1	Q9DB27	N-Term(iTRAQ8plex); M13(Oxidation); K17(iTRAQ8plex)	CID	1.0000	0.0000	1	1	3686528	Unique	Used	0.916	1.049	0.815	0.932	0	0	4.85	0	26	51	2	1193.67383	2386.34038	-4.56
gSVGPAPSLSELDLRPPSPINLSR	Unambiguous	1	1	Q6PE13	N-Term(iTRAQ8plex)	HCD	1.0000	0.0000	1	1	3841129	Unique	Used	0.993	2.468	2.811	2.263	0	0	4.85	0	7	200	3	921.84442	2763.51871	-4.36
aFGSGIDIkPGTPPIGGR	Unambiguous	2	1	Q3TLH4	N-Term(iTRAQ8plex); K9(iTRAQ8plex)	HCD	1.0000	0.0000	1	1	3740464	Unique	Used	1.641	1.068	1.285	0.885	0	0	4.85	0	5	52	3	783.45459	2348.34922	-2.34
gLQDHSASVDLDSSTSSTLSNTSk	Unambiguous	4	1	E9Q6L4	N-Term(iTRAQ8plex); K24(iTRAQ8plex)	CID	1.0000	0.0000	1	1	3009026	Unique	Used	1.011	1.065	1.113	0.871	0	0	4.85	0	0	170	3	1015.85266	3045.54343	0.04
aEALPAASVSQTHESk	Unambiguous	2	1	Q6PGL7	N-Term(iTRAQ8plex); K16(iTRAQ8plex)	CID	1.0000	0.0000	1	1	2930832	Unique	Used	4.500	0.837	1.592	0.922	0	0	4.85	0	9	75	3	745.40991	2234.21518	-3.83
iGGHGAEYGAEALER	Unambiguous	2	1	Q91VB8	N-Term(iTRAQ8plex)	CID	1.0000	0.0000	1	1	2755800	Unique	Used	5.506	3.671	0.383	1.644	0	0	4.85	0	3	19	3	611.98358	1833.93619	-1.93
gSETLGGGGGGAAGTR	Unambiguous	1	1	P31650	N-Term(iTRAQ8plex)	CID	1.0000	0.0000	1	1	2859019	Unique	Used	0.781	0.464	0.696	1.426	0	0	4.84	0	0	200	2	804.91211	1608.81694	-4.62
lIDRNDNAPSIGFVSVR	Unambiguous	1	1	E9PXF0	N-Term(iTRAQ8plex)	HCD	1.0000	0.0000	1	1	3722186	Unique	Used	1.245	1.237	0.878	1.045	0	0	4.84	1	26	198	3	726.40070	2177.18753	-4.85
gSAGSAAAGPVEAAIR	Unambiguous	1	1	Q9D8S9	N-Term(iTRAQ8plex)	HCD	1.0000	0.0000	1	1	3185011	Unique	Used	1.698	1.145	1.295	0.955	0	0	4.84	0	0	177	2	844.96307	1688.91887	-2.66

aSSLNENVDHSALLk	Unambiguous	1	1	Q6R891	N-Term(iTRAQ8plex); K15(iTRAQ8plex)	HCD	1.0000	0.0000	1	1	3103850	Unique	Used	0.931	0.880	1.848	0.719	0	0	4.84	0	0	106	2	1103.61499	2206.22270	-2.77
vVELLADIVQnSSLEDSQIEk	Unambiguous	1	1	Q9CZ13	N-Term(iTRAQ8plex); N11(Deamidated); K21(iTRAQ8plex)	CID	1.0000	0.0000	1	1	3038249	Unique	Used		1.614			0	0	4.84	0	7	187	3	980.20740	2938.60764	-0.18
yVIHTVGPIAVGGQPTASQAAELR	Unambiguous	1	1	Q922B1	N-Term(iTRAQ8plex)	CID	1.0000	0.0000	1	1	3780912	Unique	Used	1.314	1.213	0.964	0.965	0	0	4.84	0	26	131	3	894.83142	2682.47971	-3.14
gSETLGGGGGGAAGTR	Unambiguous	1	1	P31650	N-Term(iTRAQ8plex)	CID	1.0000	0.0000	1	1	2822151	Unique	Used	0.536	0.300	0.661	1.351	0	0	4.84	0	6	46	2	804.91327	1608.81926	-3.18
sLSSSPQAQPPRPAELSDEEVAELFQR	Unambiguous	3	1	A2AEW8	N-Term(iTRAQ8plex)	HCD	1.0000	0.0000	1	1	3214652	Unique	Used	2.427	1.701	2.143	0.948	0	0	4.84	0	0	39	3	1091.55835	3272.66050	-2.96
vTGEADVEFATHEDAVAAMSk	Unambiguous	2	1	O35737	N-Term(iTRAQ8plex); K21(iTRAQ8plex)	CID	1.0000	0.0000	1	1	2849455	Unique	Used	1.248	1.056	0.817	0.827	0	0	4.84	0	20	169	3	929.47382	2786.40689	-2.11
dHIIEVVGSSmPLIGDHQDEDk	Unambiguous	3	1	O88935	N-Term(iTRAQ8plex); M11(Oxidation); K22(iTRAQ8plex)	CID	1.0000	0.0000	1	1	2973831	Unique	Used	0.631	1.539	1.683	1.343	0	0	4.84	0	3	32	3	1020.19135	3058.55948	-0.57
vAASTGIDLLLLDDFk	Unambiguous	1	1	Q3UMR5	N-Term(iTRAQ8plex); K16(iTRAQ8plex)	CID	1.0000	0.0000	1	1	3352684	Unique	Used	0.848	1.586	1.747	1.669	0	0	4.84	0	5	10	2	1150.16699	2299.32671	-4.47
kTLDNVAIAEEEk	Unambiguous	1	1	Q6ZPJ3	N-Term(iTRAQ8plex); K1(iTRAQ8plex); K13(iTRAQ8plex)	CID	1.0000	0.0000	1	1	2714530	Unique	Used	0.789	1.210	0.864	0.924	0	0	4.84	1	30	200	3	791.46423	2372.37815	-0.79
iHEGcEEPATHNALAk	Unambiguous	2	1	Q68FD5	N-Term(iTRAQ8plex); C5(Methylthio); K16(iTRAQ8plex)	CID	1.0000	0.0000	1	1	2879539	Unique	Used	0.653	0.920	1.083	1.047	0	0	4.84	0	0	200	3	792.07379	2374.20682	-1.51
sVASGGWQEPENPHTQR	Unambiguous	2	1	Q8CBY8-2	N-Term(iTRAQ8plex)	CID	1.0000	0.0000	1	1	2929715	Unique	Used	0.822	0.756	1.153	1.025	0	0	4.84	0	14	5	3	728.69415	2184.06791	-2.61
lPSIVVEPTEGEVESGELR	Unambiguous	1	1	Q9CX60	N-Term(iTRAQ8plex)	CID	1.0000	0.0000	1	1	2781340	Unique	Used	2.324	0.294	0.771	0.843	0	0	4.83	0	1	105	2	1172.62842	2344.24956	-2.33

eHINLGcDVDFDIAGPSIR	Unambiguous	3	1	Q60932-2	N-Term(iTRAQ8plex); C7(Methylthio)	CID	1.0000	0.0000	1	1	3356496	Unique	Used		1.145	1.451	1.423	0	0	4.83	0	8	200	3	807.73169	2421.18052	-1.65
dTAEGVSQELISAGLVDGR	Unambiguous	1	1	Q6P9R2	N-Term(iTRAQ8plex)	CID	1.0000	0.0000	1	1	2959557	Unique	Used	1.505	1.155	0.860	0.817	0	0	4.83	0	15	200	2	1111.08374	2221.16020	-0.56
dHIIEVVGSSMPLIGDHQDEDk	Unambiguous	3	1	O88935	N-Term(iTRAQ8plex); K22(iTRAQ8plex)	CID	1.0000	0.0000	1	1	3014616	Unique	Used	0.951	2.213	1.716	1.055	0	0	4.83	0	0	64	3	1014.85846	3042.56083	-1.81
vIASLPDLTFMHAk	Unambiguous	2	1	Q80WT5-2	N-Term(iTRAQ8plex); K14(iTRAQ8plex)	CID	1.0000	0.0000	1	1	3805115	Unique	Used	1.253	0.948	1.078	0.892	0	0	4.83	0	9	70	3	717.75226	2151.24222	-1.60
tVAVYSEQDTGQmHR	Unambiguous	3	1	G5E8R3	N-Term(iTRAQ8plex); M13(Oxidation)	CID	1.0000	0.0000	1	1	2750276	Unique	Used	2.954	1.268	2.099	1.584	0	0	4.83	0	18	200	3	681.33667	2041.99546	1.94
aGQVFLEELGnHk	Unambiguous	2	1	P16330	N-Term(iTRAQ8plex); N11(Deamidated); K13(iTRAQ8plex)	CID	1.0000	0.0000	1	1	2957184	Unique	Used	0.665	0.193	1.238	1.370	0	0	4.83	0	28	113	3	684.38440	2051.13864	0.21
qNTVGcGAEGAEPQGVTk	Unambiguous	3	1	Q9ER35	N-Term(iTRAQ8plex); C6(Methylthio); K18(iTRAQ8plex)	CID	1.0000	0.0000	1	1	2897168	Unique	Used	0.710	0.582	0.875	0.910	0	0	4.82	0	0	200	2	1200.60486	2400.20244	-3.49
vSLVELcDGQADLASk	Unambiguous	2	1	Q8C3F2	N-Term(iTRAQ8plex); C7(Methylthio); K16(iTRAQ8plex)	CID	1.0000	0.0000	1	1	3326768	Unique	Used	0.693	0.416	0.668	0.970	0	0	4.82	0	0	38	2	1151.61011	2302.21294	-4.95
eAIESAIGGNAYQHSk	Unambiguous	1	1	P51807	N-Term(iTRAQ8plex); K16(iTRAQ8plex)	CID	1.0000	0.0000	1	1	3049004	Unique	Used	0.967	0.622	0.710	0.752	0	0	4.82	0	24	200	3	761.74225	2283.21219	-2.97
sAAGSSSGFHSWAR	Unambiguous	1	1	P19246	N-Term(iTRAQ8plex)	CID	1.0000	0.0000	1	1	3907139	Unique	Used	2.384	0.971	0.980	1.120	0	0	4.82	0	23	200	3	571.28308	1711.83469	-6.27
iPcVDAGLISPLVQLLNSk	Unambiguous	2	1	E9Q912	N-Term(iTRAQ8plex); C3(Methylthio); K19(iTRAQ8plex)	CID	1.0000	0.0000	1	1	3936602	Unique	Used	0.335	0.697	1.017	0.867	0	0	4.82	0	0	94	3	878.84143	2634.50974	-3.27

sPEDLEQISEVLIHk	Unambiguous	8	1	E9QQ02	N-Term(iTRAQ8plex); K15(iTRAQ8plex)	CID	1.0000	0.0000	1	1	3016646	Unique	Used	1.053	1.307	0.989	0.784	0	0	4.82	0	22	200	3	782.44318	2345.31498	-0.99
mASTFIGNSTAIQELFk	Unambiguous	1	1	Q922F4	N-Term(iTRAQ8plex); M1(Oxidation); K17(iTRAQ8plex)	CID	0.0290	0.0000	1	1	3788823	Unique	Used	0.437	1.907	0.537	1.091	0	0	4.82	0	11	200	3	828.11908	2482.34269	-1.83
iQAAASPPANATAASDTNAGDR	Unambiguous	2	1	E9QAQ5	N-Term(iTRAQ8plex)	CID	1.0000	0.0000	1	1	2952182	Unique	Used	0.598	0.860	1.338	1.123	0	0	4.82	0	0	84	2	1187.59631	2374.18535	-2.01
lIASVADDEAAVPnNk	Unambiguous	2	1	P16125	N-Term(iTRAQ8plex); N14(Deamidated); K16(iTRAQ8plex)	CID	0.0416	0.0000	1	1	2637376	Unique	Used	0.933	0.797	1.474	1.107	0	0	4.81	0	3	146	2	1118.61584	2236.22441	-1.68
dGFTSEGEILEISDIQR	Unambiguous	1	1	Q8HW98	N-Term(iTRAQ8plex)	CID	1.0000	0.0000	1	1	2688749	Unique	Used	1.691			0.874	0	0	4.81	0	0	200	2	1107.06372	2213.12016	-1.72
fQLTDSQIYEVLSVIR	Unambiguous	1	1	O08553	N-Term(iTRAQ8plex)	HCD	1.0000	0.0000	1	1	3844513	Unique	Used	1.746	2.144	1.149	1.406	0	0	4.81	0	0	200	2	1108.11426	2215.22124	-2.89
gALQDGPTADAnGLASVAASSAER	Unambiguous	1	1	Q8CJ19	N-Term(iTRAQ8plex); N12(Deamidated)	CID	1.0000	0.0000	1	1	2918459	Unique	Used		0.817	1.418	1.104	0	0	4.81	0	13	200	2	1267.63672	2534.26616	0.97
gSETLGGGGGGAAGTR	Unambiguous	1	1	P31650	N-Term(iTRAQ8plex)	CID	1.0000	0.0000	1	1	2822025	Unique	Used	0.605	0.248	0.846	1.550	0	0	4.81	0	14	20	2	804.91345	1608.81963	-2.95
qQQLQQQQQQGNk	Unambiguous	1	1	Q6NZL0	N-Term(iTRAQ8plex); K13(iTRAQ8plex)	CID	1.0000	0.0000	1	1	2984392	Unique	Used	1.559	0.397	1.216	0.665	0	0	4.81	0	16	200	3	731.40417	2192.19797	-0.57
iADYEAASAAPGIPAEQTR	Unambiguous	2	1	Q9WV96	N-Term(iTRAQ8plex)	CID	1.0000	0.0000	1	1	2936754	Unique	Used	1.276	0.848	0.849	0.819	0	0	4.81	0	2	18	2	1118.08032	2235.15337	-1.16
tETTGATGQASSLLSGR	Unambiguous	4	1	P39061-2	N-Term(iTRAQ8plex)	HCD	1.0000	0.0000	1	1	3365202	Unique	Used	0.884	3.394	1.116	1.659	0	0	4.80	0	0	200	2	971.00854	1941.00981	-4.79
eTSPETSLIQDEVALk	Unambiguous	1	1	P20357	N-Term(iTRAQ8plex); K16(iTRAQ8plex)	CID	1.0000	0.0000	1	1	2739159	Unique	Used	1.571	2.918	1.641	0.699	0	0	4.80	0	3	200	2	1184.65405	2368.30083	-2.52
vDGVSQDGTTTYIHNk	Unambiguous	2	1	E9Q6H7	N-Term(iTRAQ8plex); K16(iTRAQ8plex)	CID	1.0000	0.0000	1	1	2931782	Unique	Used	0.992	1.246	0.895	1.028	0	0	4.80	0	23	43	3	781.75012	2343.23581	-1.83

rPADVSPASVSGELnAGk	Unambiguous	2	1	Q80U87	N-Term(iTRAQ8plex); N15(Deamidated); K18(iTRAQ8plex)	CID	1.0000	0.0000	1	1	2952870	Unique	Used	1.636	1.071	1.101	1.140	0	0	4.80	0	22	200	3	788.77130	2364.29935	0.58
gLSSVPEVAEVEPTTk	Unambiguous	1	1	P20357	N-Term(iTRAQ8plex); K16(iTRAQ8plex)	CID	1.0000	0.0000	1	1	2900625	Unique	Used	1.149	1.674	1.770	0.958	0	0	4.80	0	0	195	2	1126.13293	2251.25859	-2.50
aLETMAEqTTDVVHSPSTDTTPGPDTEAALAk	Unambiguous	4	1	P27546-2	N-Term(iTRAQ8plex); Q8(Deamidated); K32(iTRAQ8plex)	CID	1.0000	0.0000	1	1	2939764	Unique	Used	2.451	0.922	1.109	0.739	0	0	4.79	0	19	160	3	1298.65771	3893.95859	4.17
sTGEALVQGLmGAAVTLk	Unambiguous	1	1	Q8K1Z0	N-Term(iTRAQ8plex); M11(Oxidation); K18(iTRAQ8plex)	CID	1.0000	0.0000	1	1	3805279	Unique	Used	1.109	1.789	1.132	1.213	0	0	4.79	0	19	79	2	1185.67615	2370.34502	-3.09
sQPPAAHAGQVYGEQPPk	Unambiguous	3	1	Q6P9K8	N-Term(iTRAQ8plex); K18(iTRAQ8plex)	CID	1.0000	0.0000	1	1	2950106	Unique	Used	0.745	0.593	1.050	1.067	0	0	4.79	0	0	200	3	824.11371	2470.32657	-1.36
dDGGIVIcEAQNQALPSGHSk	Unambiguous	1	1	Q8R464	N-Term(iTRAQ8plex); C8(Methylthio); K21(iTRAQ8plex)	CID	1.0000	0.0000	1	1	3031965	Unique	Used	1.672	1.552	0.775	1.223	0	0	4.79	0	30	65	3	931.80603	2793.40354	-3.05
nWASGQDLQAkLQLR	Unambiguous	1	1	Q9Z1P6	N-Term(iTRAQ8plex); K11(iTRAQ8plex)	HCD	1.0000	0.0000	1	1	3893990	Unique	Used	0.647	0.339	1.373	1.278	0	0	4.79	1	15	195	3	779.44397	2336.31736	-5.22
lASTLVHLGEYQAAVDGAR	Unambiguous	2	1	Q68FD5	N-Term(iTRAQ8plex)	CID	1.0000	0.0000	1	1	3664631	Unique	Used	1.230	1.234	1.308	1.112	0	0	4.79	0	16	43	3	759.07855	2275.22110	-6.05
yPVPFLVEMLLFHPk	Unambiguous	2	1	Q9EPR5	N-Term(iTRAQ8plex); K15(iTRAQ8plex)	CID	1.0000	0.0000	1	1	3899006	Unique	Used	0.344	1.343	0.999	1.083	0	0	4.78	0	0	41	3	813.47247	2438.40287	-4.18
aVVFETGEAGAASGAGSLPGEVR	Unambiguous	3	1	Q9QYR6	N-Term(iTRAQ8plex)	CID	1.0000	0.0000	1	1	3090168	Unique	Used	1.853	1.153	1.460	1.085	0	0	4.78	0	0	200	2	1218.63647	2436.26567	-0.68
hQSVQTEDGPGGVAR	Unambiguous	1	1	Q9R1L5	N-Term(iTRAQ8plex)	CID	1.0000	0.0000	1	1	2661081	Unique	Used	2.448	1.144	1.058	0.934	0	0	4.78	0	11	200	3	614.65057	1841.93717	-1.97

vAVLGASGGIGQPLSLLLk	Unambiguous	1	1	P08249	N-Term(iTRAQ8plex); K19(iTRAQ8plex)	CID	1.0000	0.0000	1	1	3938784	Unique	Used	1.634	1.287	1.283	1.192	0	0	4.78	0	8	200	3	801.16846	2401.49082	-3.96
gLGTDEDTLIEILTTR	Unambiguous	1	1	P10107	N-Term(iTRAQ8plex)	HCD	1.0000	0.0000	1	1	3173972	Unique	Used	0.720	6.700	0.425	1.171	0	0	4.78	0	7	43	2	1026.05969	2051.11211	-2.60
gLTPTGTLPLGVLSGGk	Unambiguous	1	1	P48455	N-Term(iTRAQ8plex); K17(iTRAQ8plex)	CID	1.0000	0.0000	1	1	3878163	Unique	Used	0.564	1.398	1.139	0.930	0	0	4.78	0	0	178	2	1088.65698	2176.30669	-4.37
IQLALDVEHLGEQIQR	Unambiguous	1	1	Q921L5	N-Term(iTRAQ8plex)	CID	1.0000	0.0000	1	1	3395763	Unique	Used		2.184	0.736	1.006	0	0	4.78	0	16	13	3	722.74188	2166.21109	-3.41
aFIITINSFGTELSk	Unambiguous	1	1	P51863	N-Term(iTRAQ8plex); K15(iTRAQ8plex)	CID	1.0000	0.0000	1	1	3805951	Unique	Used	1.266	2.439	1.107	0.994	0	0	4.78	0	23	13	2	1125.14990	2249.29253	-3.41
gSLWPQPSDSQQYFSAPAPLSPSSRPR	Unambiguous	6	1	P28661-2	N-Term(iTRAQ8plex)	HCD	1.0000	0.0000	1	1	3915057	Unique	Used	2.057	0.629	2.219	2.626	0	0	4.77	0	0	68	3	1083.21460	3247.62925	-4.50
wGQTLPTTSSTDEGNPk	Unambiguous	4	1	E9PX29	N-Term(iTRAQ8plex); K17(iTRAQ8plex)	CID	1.0000	0.0000	1	1	3009990	Unique	Used	0.341	0.710	1.511	0.916	0	0	4.77	0	0	200	2	1214.13232	2427.25737	-1.59
wAEVVLSDPEAAk	Unambiguous	1	1	P17439	N-Term(iTRAQ8plex); K13(iTRAQ8plex)	CID	1.0000	0.0000	1	1	2977214	Unique	Used	1.480	0.765	0.712	0.998	0	0	4.77	0	16	200	3	675.04871	2023.13156	-0.25
mGAPEAGMAEYLFDk	Unambiguous	1	1	P09528	N-Term(iTRAQ8plex); K15(iTRAQ8plex)	CID	1.0000	0.0000	1	1	3074865	Unique	Used	1.121	0.911	1.532	1.602	0	0	4.77	0	0	15	3	746.71747	2238.13785	-0.76
eAGPRPPGSGPGPGTPGAk	Unambiguous	2	1	O88737	N-Term(iTRAQ8plex); K20(iTRAQ8plex)	HCD	1.0000	0.0000	1	1	3225750	Unique	Used	0.855	1.269	2.372	1.083	0	0	4.77	0	5	33	3	798.11011	2392.31577	-1.52
eQASHLGGAVFSGAGNIAAATGLVk	Unambiguous	1	1	Q91ZZ3	N-Term(iTRAQ8plex); K25(iTRAQ8plex)	CID	1.0000	0.0000	1	1	3840576	Unique	Used	0.719	0.655	1.694	1.445	0	0	4.77	0	9	200	3	978.87714	2934.61686	-3.06
dDSQLNGDPSALLNPSk	Unambiguous	2	1	Q8VEK0	N-Term(iTRAQ8plex); K17(iTRAQ8plex)	CID	1.0000	0.0000	1	1	2956054	Unique	Used	0.573	1.201	1.338	1.142	0	0	4.77	0	0	200	2	1190.13501	2379.26274	0.63
gYIFLEYASPAHAVDAVk	Unambiguous	1	1	Q8JZQ9	N-Term(iTRAQ8plex); K18(iTRAQ8plex)	CID	1.0000	0.0000	1	1	3393686	Unique	Used	0.588	0.815	0.807	0.681	0	0	4.77	0	0	15	3	853.80457	2559.39914	-2.98

dLLDSVLPHLYnETk	Unambiguous	1	1	Q6ZQ38	N-Term(iTRAQ8plex); N12(Deamidated); K15(iTRAQ8plex)	CID	1.0000	0.0000	1	1	3195587	Unique	Used	0.849	1.471	1.199	1.427	0	0	4.77	0	14	169	3	789.43951	2366.30399	-1.01
dLAILQcHGELDPMVPVR	Unambiguous	1	1	Q9WTL7	N-Term(iTRAQ8plex); C7(Methylthio)	HCD	1.0000	0.0000	1	1	3499510	Unique	Used	0.949	1.307	0.883	1.171	0	0	4.76	0	8	147	3	786.07477	2356.20975	-1.37
iIAEGANGPTTPEADk	Unambiguous	2	1	P26443	N-Term(iTRAQ8plex); K16(iTRAQ8plex)	CID	1.0000	0.0000	1	1	2792696	Unique	Used	0.416	0.620	0.837	1.566	0	0	4.76	0	0	200	2	1096.60193	2192.19658	-2.45
gLHGSALIINTQEQGPDEGEEk	Unambiguous	2	1	Q9QXJ1-2	N-Term(iTRAQ8plex); K22(iTRAQ8plex)	CID	1.0000	0.0000	1	1	2650731	Unique	Used	1.031	1.265	1.019	1.217	0	0	4.76	0	0	200	3	977.51154	2930.52005	-3.95
nIDNPAIADIYTEHAHQVVVAk	Unambiguous	1	1	O88342	N-Term(iTRAQ8plex); K22(iTRAQ8plex)	CID	1.0000	0.0000	1	1	3278202	Unique	Used	0.871	0.996	0.836	0.905	0	0	4.75	0	0	89	3	1009.55481	3026.64988	-0.71
eFLVAGGEDFk	Unambiguous	1	1	Q9Z1Z2	N-Term(iTRAQ8plex); K11(iTRAQ8plex)	CID	1.0000	0.0000	1	1	2884593	Unique	Used	1.908	1.152	1.129	0.502	0	0	4.75	0	10	200	3	607.33917	1820.00296	-1.16
mAATFIGNSTAIQELFk	Unambiguous	1	1	Q9D6F9	N-Term(iTRAQ8plex); K17(iTRAQ8plex)	CID	1.0000	0.0000	1	1	3844603	Unique	Used	0.961	0.776	1.352	1.891	0	0	4.75	0	6	200	3	817.45654	2450.35508	-0.95
yIPVQQGPVGVNVTYGGDHIPk	Unambiguous	3	1	B7FAU9	N-Term(iTRAQ8plex); K22(iTRAQ8plex)	CID	1.0000	0.0000	1	1	3454993	Unique	Used	0.438	3.853	0.999	1.133	0	0	4.75	0	0	43	3	982.87756	2946.61814	-3.96
vAHSDkPGSTSAVSFR	Unambiguous	1	1	Q9WV55	N-Term(iTRAQ8plex); K6(iTRAQ8plex)	HCD	1.0000	0.0000	1	1	3382367	Unique	Used	0.437	0.983	1.024	0.892	0	0	4.75	0	0	173	3	752.08026	2254.22623	-6.13
lDNLMLELDGTENk	Unambiguous	4	1	P17183	N-Term(iTRAQ8plex); K14(iTRAQ8plex)	CID	1.0000	0.0000	1	1	2943895	Unique	Used	1.922	1.416	0.800	1.222	0	0	4.75	0	0	200	3	738.40265	2213.19339	-0.46
tSDVETmSSQSALALDER	Unambiguous	1	1	P14873	N-Term(iTRAQ8plex); M7(Oxidation)	CID	1.0000	0.0000	1	1	2776503	Unique	Used	2.226	0.766	1.290	0.798	0	0	4.75	0	0	200	2	1130.54651	2260.08574	-2.64
vSAADSTQVDGGTPMVk	Unambiguous	6	1	A2AUK8	N-Term(iTRAQ8plex); K17(iTRAQ8plex)	CID	1.0000	0.0000	1	1	2933622	Unique	Used	0.818	1.479	1.158	0.577	0	0	4.74	0	4	16	2	1136.10632	2271.20537	-2.54

vETPGPPAPPPDSGPPASSDR	Unambiguous	3	1	A2AQ87	N-Term(iTRAQ8plex)	CID	1.0000	0.0000	1	1	2791649	Unique	Used	1.054	1.260	1.499	0.874	0	0	4.74	0	0	200	2	1166.58862	2332.16997	-1.02
mATEVAADALGEEWk	Unambiguous	1	1	P62754	N-Term(iTRAQ8plex); M1(Oxidation); K15(iTRAQ8plex)	CID	1.0000	0.0000	1	1	2901820	Unique	Used	1.994	0.853	1.069	1.149	0	0	4.74	0	22	200	3	749.05737	2245.15757	-2.48
vAVLGASGGIGQPLSLLLk	Unambiguous	1	1	P08249	N-Term(iTRAQ8plex); K19(iTRAQ8plex)	CID	1.0000	0.0000	1	1	3937568	Unique	Used	1.212	1.045	0.974	1.112	0	0	4.73	0	2	164	3	801.16901	2401.49247	-3.27
lSEENIDSSAGnGVLTk	Unambiguous	5	1	G3X9J4	N-Term(iTRAQ8plex); N12(Deamidated); K17(iTRAQ8plex)	CID	1.0000	0.0000	1	1	2668998	Unique	Used	0.399	0.919	0.782	0.787	0	0	4.73	0	0	200	2	1172.12659	2343.24590	-1.76
iEGSGDQVDTLELSGGAk	Unambiguous	3	1	Q8BZ98-2	N-Term(iTRAQ8plex); K18(iTRAQ8plex)	CID	1.0000	0.0000	1	1	2716881	Unique	Used		0.870	0.720	1.295	0	0	4.73	0	0	200	2	1192.63855	2384.26982	-2.83
gTEDITSPHGIPLDLLDR	Unambiguous	1	1	P60122	N-Term(iTRAQ8plex)	HCD	1.0000	0.0000	1	1	3150654	Unique	Used	0.949	1.431	0.879	0.900	0	0	4.73	0	28	65	3	751.73932	2253.20340	0.22
vAVLGASGGIGQPLSLLLk	Unambiguous	1	1	P08249	N-Term(iTRAQ8plex); K19(iTRAQ8plex)	CID	1.0000	0.0000	1	1	3938999	Unique	Used	1.360	1.042	0.806	0.963	0	0	4.72	0	7	200	3	801.16864	2401.49137	-3.73
tLPAAPASANTAATTTAPAk	Unambiguous	3	1	Q501J7-3	N-Term(iTRAQ8plex); K20(iTRAQ8plex)	CID	1.0000	0.0000	1	1	3385772	Unique	Used	1.297	0.337	0.746	0.692	0	0	4.72	0	0	132	2	1217.68774	2434.36821	-3.29
gLGTDEDSILNLLTSR	Unambiguous	1	1	P48036	N-Term(iTRAQ8plex)	HCD	1.0000	0.0000	1	1	3396817	Unique	Used	1.711	1.442	0.733	1.089	0	0	4.72	0	9	200	2	1004.54150	2008.07573	-5.34
kNPGVGnGDDEAAELMQQVk	Unambiguous	1	1	Q61166	N-Term(iTRAQ8plex); K1(iTRAQ8plex); N7(Deamidated); K20(iTRAQ8plex)	CID	1.0000	0.0000	1	1	2794351	Unique	Used	0.691	0.596	0.560	0.486	0	0	4.72	1	9	200	3	1005.20441	3013.59867	-1.38
yALSGGNWDSISDAAk	Unambiguous	1	1	Q9WUT3	N-Term(iTRAQ8plex); K16(iTRAQ8plex)	CID	1.0000	0.0000	1	1	3190665	Unique	Used	1.161	1.253	2.978	0.859	0	0	4.72	0	0	118	2	1132.09021	2263.17314	-3.71
lTPIPSTSPNLQAYHQGNTQVSR	Unambiguous	1	1	Q8BIW1	N-Term(iTRAQ8plex)	HCD	1.0000	0.0000	1	1	3854340	Unique	Used	0.640	0.838	0.874	1.136	0	0	4.72	0	0	158	3	938.49664	2813.47538	-3.35

vGEGFEETLGEAVISTk	Unambiguous	1	1	P46660	N-Term(iTRAQ8plex); K17(iTRAQ8plex)	CID	1.0000	0.0000	1	1	2941539	Unique	Used	1.309	0.291	1.974	1.466	0	0	4.71	0	5	61	2	1187.64868	2374.29009	-2.60
tVSGVnGPLVILDHVk	Unambiguous	1	1	P62814	N-Term(iTRAQ8plex); N6(Deamidated); K16(iTRAQ8plex)	HCD	1.0000	0.0000	1	1	3347899	Unique	Used	0.860	1.274	1.084	1.030	0	0	4.71	0	0	5	2	1129.17017	2257.33306	-2.03
aLEVFPEFAAAHSNLASVLQQQGk	Unambiguous	2	1	Q8CGY8	N-Term(iTRAQ8plex); K24(iTRAQ8plex)	CID	1.0000	0.0000	1	1	3805739	Unique	Used	0.502	1.853	1.224	0.940	0	0	4.71	0	4	9	3	1055.24731	3163.72739	-2.75
eAGYSAAVGVGQRPPR	Unambiguous	4	1	E9QAD8	N-Term(iTRAQ8plex)	HCD	1.0000	0.0000	1	1	3449126	Unique	Used	1.369	1.315	1.533	1.023	0	0	4.70	0	10	62	3	640.34924	1919.03318	-3.62
fEDGDLTLYQSNAILR	Unambiguous	2	1	P19157	N-Term(iTRAQ8plex)	HCD	1.0000	0.0000	1	1	3261035	Unique	Used	0.581	1.528	1.616	2.338	0	0	4.70	0	0	0	2	1080.06689	2159.12651	-0.99
aAAGELQEDSGLHVLAR	Unambiguous	1	1	Q8C845	N-Term(iTRAQ8plex)	CID	1.0000	0.0000	1	1	2972615	Unique	Used	0.855	1.008	1.477	1.261	0	0	4.70	0	17	30	3	681.03632	2041.09439	-1.79
dLncVPEIADTLGAVAk	Unambiguous	1	1	Q8CIG8	N-Term(iTRAQ8plex); N3(Deamidated); C4(Methylthio); K17(iTRAQ8plex)	CID	1.0000	0.0000	1	1	3196128	Unique	Used	0.536	0.812	0.766	0.816	0	0	4.70	0	10	43	2	1192.63501	2384.26274	-1.46
iGGHGAEYGAEALER	Unambiguous	2	1	Q91VB8	N-Term(iTRAQ8plex)	CID	1.0000	0.0000	1	1	2757467	Unique	Used	5.325	4.528	0.432	1.416	0	0	4.69	0	7	64	3	611.98322	1833.93509	-2.53
eTAAAHQASSSPPIDAATAEPYGFR	Unambiguous	1	1	P14873	N-Term(iTRAQ8plex)	CID	1.0000	0.0000	1	1	3010296	Unique	Used	2.606	0.847	2.263	1.479	0	0	4.69	0	4	23	3	950.46857	2849.39115	-3.40
dNFHGLAIFLDTYPNDETTER	Unambiguous	1	1	Q9DBH5	N-Term(iTRAQ8plex)	CID	1.0000	0.0000	1	1	3074641	Unique	Used	1.109	1.199	1.035	1.542	0	0	4.69	0	5	93	3	924.78448	2772.33890	-1.09
eNAPAIIFIDEIDAIATk	Unambiguous	1	1	P54775	N-Term(iTRAQ8plex); K18(iTRAQ8plex)	HCD	1.0000	0.0000	1	1	3174211	Unique	Used	0.472	0.991	0.706	0.814	0	0	4.69	0	7	112	2	1276.72278	2552.43828	-1.95
qVIEVLETDPHFR	Unambiguous	1	1	P81117	N-Term(iTRAQ8plex)	CID	1.0000	0.0000	1	1	3128973	Unique	Used	2.064	1.650	1.066	0.645	0	0	4.69	0	0	57	3	629.68024	1887.02616	-0.88
nLDYVATSIHEAVTk	Unambiguous	1	1	P05201	N-Term(iTRAQ8plex); K15(iTRAQ8plex)	CID	1.0000	0.0000	1	1	3369731	Unique	Used	1.389	1.020	0.899	1.093	0	0	4.69	0	0	50	3	757.08881	2269.25187	-5.72

aDIGVAmGIAGSDVSk	Unambiguous	2	1	Q6PIC6	N-Term(iTRAQ8plex); M7(Oxidation); K16(iTRAQ8plex)	CID	1.0000	0.0000	1	1	3031010	Unique	Used		0.991	1.002	1.168	0	0	4.69	0	0	107	2	1058.07898	2115.15068	-3.30
ePQPSGPqPSGGAGGGGGk	Unambiguous	1	1	Q8JZQ2	N-Term(iTRAQ8plex); Q8(Deamidated); K19(iTRAQ8plex)	CID	0.0214	0.0000	1	1	2749057	Unique	Used	0.670	0.686	1.322	1.060	0	0	4.68	0	0	200	2	1116.08508	2231.16289	5.18
qAEMEGAVQSIQGELSk	Unambiguous	1	1	O54724	N-Term(iTRAQ8plex); K17(iTRAQ8plex)	CID	1.0000	0.0000	1	1	3091996	Unique	Used	1.441	2.684	1.152	1.087	0	0	4.68	0	22	200	2	1207.14880	2413.29033	2.06
gLVPHnGSLINVGSLLQR	Unambiguous	1	1	Q8VDP4	N-Term(iTRAQ8plex); N6(Deamidated)	HCD	1.0000	0.0000	1	1	3896846	Unique	Used	0.847	0.904	0.823	1.256	0	0	4.68	0	15	55	3	727.08673	2179.24564	-2.05
lEATLQEEAAIQQEHLEELk	Unambiguous	1	1	Q9Z2I0	N-Term(iTRAQ8plex); K20(iTRAQ8plex)	CID	1.0000	0.0000	1	1	2766075	Unique	Used	0.617	0.800	1.235	1.475	0	0	4.68	0	28	200	3	977.53302	2930.58451	-2.96
mLIDAQAEAAEQLGk	Unambiguous	2	1	E9Q912	N-Term(iTRAQ8plex); K15(iTRAQ8plex)	CID	1.0000	0.0000	1	1	3073605	Unique	Used	0.421	0.927	0.849	0.732	0	0	4.68	0	4	57	2	1098.60974	2196.21221	-1.50
lGDVYVnDAFGTAHR	Unambiguous	2	1	P09411	N-Term(iTRAQ8plex); N7(Deamidated)	CID	1.0000	0.0000	1	1	3052582	Unique	Used	0.829	1.425	1.303	1.099	0	0	4.68	0	18	32	3	647.33118	1939.97898	-1.35
gEVGSGAGPGTQAGPSAk	Unambiguous	4	1	E9Q9M5	N-Term(iTRAQ8plex); K18(iTRAQ8plex)	CID	1.0000	0.0000	1	1	2878000	Unique	Used	0.418	0.130	0.868	0.714	0	0	4.67	0	0	200	2	1068.57458	2136.14189	-4.07
lATPTYGDLNHLVSATMSGVTTSLR	Unambiguous	1	1	Q9ERD7	N-Term(iTRAQ8plex)	CID	1.0000	0.0000	1	1	3937270	Unique	Used	0.432	0.552	1.079	1.338	0	0	4.67	0	25	200	3	970.51404	2909.52756	-2.38
hWILPQDYDHAQAEAR	Unambiguous	1	1	Q05186	N-Term(iTRAQ8plex)	CID	1.0000	0.0000	1	1	3048687	Unique	Used	0.805	1.125	0.863	0.979	0	0	4.67	0	29	35	3	752.04791	2254.12918	-0.68
sLAYLSAATHGLDEEAESLk	Unambiguous	2	1	Q8CIE6	N-Term(iTRAQ8plex); K20(iTRAQ8plex)	CID	1.0000	0.0000	1	1	3016670	Unique	Used	0.520	0.118	0.314	0.071	0	0	4.67	0	25	88	3	905.15497	2713.45035	-0.06
aPAAQPPAAAAPSAVGSPAAAPR	Unambiguous	3	1	B2RPU8	N-Term(iTRAQ8plex)	CID	1.0000	0.0000	1	1	3592517	Unique	Used	1.984	0.699	1.257	0.926	0	0	4.67	0	0	200	2	1151.13135	2301.25542	-2.77

dESLAYYIENQFHEFk	Unambiguous	2	1	Q62351	N-Term(iTRAQ8plex); K16(iTRAQ8plex)	CID	1.0000	0.0000	1	1	3093606	Unique	Used	0.582	0.608	0.566	0.611	0	0	4.67	0	30	112	3	881.11780	2641.33884	-0.24
vAVLGASGGIGQPLSLLLk	Unambiguous	1	1	P08249	N-Term(iTRAQ8plex); K19(iTRAQ8plex)	CID	1.0000	0.0000	1	1	3938568	Unique	Used	1.958	0.974	0.826	0.919	0	0	4.67	0	14	200	2	1201.24646	2401.48564	-6.11
vGnGFEEGTTQGPLINEk	Unambiguous	1	1	Q8BWF0	N-Term(iTRAQ8plex); N3(Deamidated); K18(iTRAQ8plex)	CID	1.0000	0.0000	1	1	2717305	Unique	Used		2.676			0	0	4.67	0	0	200	2	1250.15869	2499.31011	-3.47
gVSAIVYMVDAADQEk	Unambiguous	2	1	F6QKK2	N-Term(iTRAQ8plex); K16(iTRAQ8plex)	CID	1.0000	0.0000	1	1	3037606	Unique	Used	0.927	0.656	0.984	0.917	0	0	4.67	0	20	35	2	1152.61951	2304.23174	-2.12
lGNTISSLFGGGTSSDAk	Unambiguous	1	1	Q9JKR6	N-Term(iTRAQ8plex); K18(iTRAQ8plex)	CID	1.0000	0.0000	1	1	3765485	Unique	Used	0.896	0.868	0.815	0.816	0	0	4.67	0	0	45	2	1160.62927	2320.25127	-3.99
lGFSEVELVQmVVDGVk	Unambiguous	1	1	Q04447	N-Term(iTRAQ8plex); M11(Oxidation); K17(iTRAQ8plex)	CID	1.0000	0.0000	1	1	3462604	Unique	Used		2.020	2.194	0.629	0	0	4.67	0	9	200	3	825.13007	2473.37564	-3.10
fEDGDLTLYQSNAILR	Unambiguous	2	1	P19157	N-Term(iTRAQ8plex)	HCD	1.0000	0.0000	1	1	3261201	Unique	Used	0.691	1.211	1.531	2.070	0	0	4.66	0	0	7	2	1080.06616	2159.12505	-1.67
iDSVSLVDYTPTDQDLLR	Unambiguous	2	1	Q8CGK7	N-Term(iTRAQ8plex)	HCD	1.0000	0.0000	1	1	3195678	Unique	Used	1.219	1.528	3.973	1.953	0	0	4.66	0	18	131	2	1177.62073	2354.23418	-2.19
tHSQGGYGSQGYk	Unambiguous	2	1	Q8C0C7	N-Term(iTRAQ8plex); K13(iTRAQ8plex)	CID	1.0000	0.0000	1	1	2965890	Unique	Used	0.984	0.579	0.923	0.880	0	0	4.66	0	19	200	3	660.01147	1978.01987	-2.02
aAEGISSDSLLSVPGQk	Unambiguous	5	1	Q8BPN8-2	N-Term(iTRAQ8plex); K17(iTRAQ8plex)	CID	1.0000	0.0000	1	1	3256830	Unique	Used	0.254	1.175	1.086	1.041	0	0	4.66	0	0	22	2	1134.13611	2267.26494	-2.39
gSETLGGGGGGAAGTR	Unambiguous	1	1	P31650	N-Term(iTRAQ8plex)	CID	1.0000	0.0000	1	1	2858781	Unique	Used	0.526	0.308	0.713	1.397	0	0	4.65	0	4	176	2	804.91321	1608.81914	-3.26
aHPPMIAVGSDDSSPNSMAk	Unambiguous	2	1	Q8R2U0-2	N-Term(iTRAQ8plex); K20(iTRAQ8plex)	CID	1.0000	0.0000	1	1	3067812	Unique	Used	1.577	1.156	0.859	0.610	0	0	4.65	0	0	200	3	874.11493	2620.33023	-0.68
gSQSVEGAGVMAGEPPSHSSGGR	Unambiguous	2	1	Q6PGL7	N-Term(iTRAQ8plex)	CID	1.0000	0.0000	1	1	2911692	Unique	Used	1.674	0.544	1.441	0.884	0	0	4.65	0	13	88	3	815.72809	2445.16971	-1.39

IIEESAIDEVVVTNTIPHEIQk	Unambiguous	1	1	Q8R574	N-Term(iTRAQ8plex); K22(iTRAQ8plex)	CID	1.0000	0.0000	1	1	3075176	Unique	Used	0.622	2.154	1.187	1.473	0	0	4.65	0	16	200	3	1029.24695	3085.72629	0.68
dELHIVEAEAmNYEGSPIk	Unambiguous	5	1	Q61937	N-Term(iTRAQ8plex); M11(Oxidation); K19(iTRAQ8plex)	CID	1.0000	0.0000	1	1	2813446	Unique	Used	2.858	1.158	1.175	0.629	0	0	4.65	0	2	65	3	923.81049	2769.41690	-2.06
IGSASSSHTSIQENHk	Unambiguous	2	1	P08414	N-Term(iTRAQ8plex); K16(iTRAQ8plex)	CID	1.0000	0.0000	1	1	3202770	Unique	Used	0.553	1.070	1.810	0.787	0	0	4.65	0	0	200	3	764.40985	2291.21500	-2.19
eIPGHSGEIGAYIAk	Unambiguous	2	1	Q9QYX7	N-Term(iTRAQ8plex); K15(iTRAQ8plex)	HCD	1.0000	0.0000	1	1	3186727	Unique	Used	0.467	0.969	1.550	0.887	0	0	4.64	0	0	82	2	1075.60669	2150.20610	-0.24
qIPGGGSGGGGSGSGGGGGGSGGGR	Unambiguous	2	1	Q8BMA6	N-Term(iTRAQ8plex)	CID	1.0000	0.0000	1	1	3290909	Unique	Used			0.509	0.485	0	0	4.64	0	0	200	2	1068.00684	2135.00640	-3.02
gcGGVITLNRPk	Unambiguous	2	1	Q8QZS1	N-Term(iTRAQ8plex); C2(Methylthio); K12(iTRAQ8plex)	HCD	1.0000	0.0000	1	1	3929030	Unique	Used	0.357	0.900	0.911	1.387	0	0	4.64	0	4	74	3	623.68994	1869.05527	-5.69
dVQTPFLVELEVLDGHEPDGGQR	Unambiguous	1	1	Q9QYR9	N-Term(iTRAQ8plex)	CID	1.0000	0.0000	1	1	2922185	Unique	Used	0.572	1.015	0.854	1.750	0	0	4.64	0	3	18	3	952.15472	2854.44962	-1.03
tGEPcVAELTEENFQR	Unambiguous	4	1	Q99MI1	N-Term(iTRAQ8plex); C5(Methylthio)	CID	1.0000	0.0000	1	1	2780850	Unique	Used	1.008	0.895	0.578	0.591	0	0	4.64	0	10	200	2	1087.01233	2173.01738	-1.56
aGQTQPNPGILPIQPALTPR	Unambiguous	2	1	Q3UHJ0-2	N-Term(iTRAQ8plex)	HCD	1.0000	0.0000	1	1	3915040	Unique	Used	0.568	1.487	1.685	1.194	0	0	4.64	0	3	61	2	1187.17590	2373.34453	-4.69
vIGNQSLVNELTFSAR	Unambiguous	2	1	O35459	N-Term(iTRAQ8plex)	HCD	1.0000	0.0000	1	1	3805093	Unique	Used	2.807	1.311	0.735	0.727	0	0	4.64	0	7	34	2	1026.56921	2052.13115	-3.90
gVMGGQSAGPQHTEAETIQk	Unambiguous	3	1	Q9Z1Z0	N-Term(iTRAQ8plex); K20(iTRAQ8plex)	CID	1.0000	0.0000	1	1	3006441	Unique	Used	0.611	1.285	0.741	0.810	0	0	4.63	0	0	80	3	878.79803	2634.37955	1.10
vTDANYGELQEHk	Unambiguous	2	1	Q8R0A6	N-Term(iTRAQ8plex); K13(iTRAQ8plex)	CID	1.0000	0.0000	1	1	2665682	Unique	Used	1.217	0.909	0.761	0.570	0	0	4.63	0	27	43	3	704.70923	2112.11313	-2.39
vPGEnGSPEMFLSHALAER	Unambiguous	1	1	Q8C0Q2	N-Term(iTRAQ8plex); N5(Deamidated)	CID	1.0000	0.0000	1	1	3171771	Unique	Used	1.062	0.738	1.040	0.891	0	0	4.63	0	21	200	3	782.72845	2346.17081	0.24

eSGAGGGcLQGLQDSLQQR	Unambiguous	1	1	O35544	N-Term(iTRAQ8plex); C8(Methylthio)	HCD	1.0000	0.0000	1	1	3414872	Unique	Used	0.548	0.952	1.364	1.008	0	0	4.62	0	18	73	2	1127.54150	2254.07573	-4.49
sVFDIPIFTEEFLNHSk	Unambiguous	2	1	Q9DC33	N-Term(iTRAQ8plex); K17(iTRAQ8plex)	CID	1.0000	0.0000	1	1	3500324	Unique	Used	1.443	0.873	1.836	1.240	0	0	4.62	0	23	75	3	877.81354	2631.42606	-0.71
ePGLFDLVIINDDLDk	Unambiguous	3	1	Q564G0	N-Term(iTRAQ8plex); K16(iTRAQ8plex)	CID	1.0000	0.0000	1	1	2960904	Unique	Used	1.153	0.904	1.669	1.181	0	0	4.62	0	10	128	2	1212.67639	2424.34551	-1.15
gSETLGGGGGGAAGTR	Unambiguous	1	1	P31650	N-Term(iTRAQ8plex)	CID	1.0000	0.0000	1	1	2841243	Unique	Used	0.379	0.311	0.655	1.492	0	0	4.61	0	2	28	2	804.91284	1608.81841	-3.71
sVTSAAPPSPATTHQHSLEEPSnGIR	Unambiguous	2	1	D3YVF0	N-Term(iTRAQ8plex); N23(Deamidated)	CID	1.0000	0.0000	1	1	2969639	Unique	Used	4.251	1.787	2.584	0.950	0	0	4.61	0	0	200	3	992.83466	2976.48941	-2.39
vNNSSLIGLGYTqTLkPGIk	Unambiguous	2	1	Q60932-2	N-Term(iTRAQ8plex); Q13(Deamidated); K16(iTRAQ8plex); K20(iTRAQ8plex)	HCD	1.0000	0.0000	1	1	3856883	Unique	Used	0.373	1.032	1.035	1.073	0	0	4.61	0	0	200	3	1006.26495	3016.78031	-0.23
aELGALPDDFIDSLEk	Unambiguous	1	1	Q91YP2	N-Term(iTRAQ8plex); K16(iTRAQ8plex)	CID	1.0000	0.0000	1	1	2942201	Unique	Used	1.102	0.662	0.622	0.655	0	0	4.61	0	12	31	2	1171.13892	2341.27056	-1.81
iGGHGAEYGAEALER	Unambiguous	2	1	Q91VB8	N-Term(iTRAQ8plex)	CID	1.0000	0.0000	1	1	2800520	Unique	Used	2.581	1.935		1.082	0	0	4.60	0	9	200	3	611.98407	1833.93766	-1.13
aSSATDGQLASPGEnGEVRPGQAk	Unambiguous	1	1	P16054	N-Term(iTRAQ8plex); N15(Deamidated); K24(iTRAQ8plex)	CID	1.0000	0.0000	1	1	2681407	Unique	Used	0.291	2.488	1.764	1.046	0	0	4.60	0	0	85	3	979.50824	2936.51017	-2.35
lVEVnGVNVEGETHHQVVQR	Unambiguous	1	1	Q9JHL1	N-Term(iTRAQ8plex); N5(Deamidated)	CID	0.0022	0.0000	1	1	2897516	Unique	Used	1.300	0.751	0.724	0.867	0	0	4.60	0	24	200	3	850.11340	2548.32566	-6.49
dHIIEVVGSSMPLIGDHQDEDk	Unambiguous	3	1	O88935	N-Term(iTRAQ8plex); K22(iTRAQ8plex)	CID	1.0000	0.0000	1	1	2995560	Unique	Used	0.539	1.687	1.966	1.233	0	0	4.60	0	6	28	3	1014.85693	3042.55625	-3.31
aGQTQPNPGILPIQPALTPR	Unambiguous	2	1	Q3UHJ0-2	N-Term(iTRAQ8plex)	HCD	1.0000	0.0000	1	1	3933689	Unique	Used	0.927	1.068	1.690	1.166	0	0	4.60	0	3	80	2	1187.17651	2373.34575	-4.18

dEDITEYQSILAAAVk	Unambiguous	1	1	Q9DCM2	N-Term(iTRAQ8plex); K16(iTRAQ8plex)	CID	1.0000	0.0000	1	1	3017560	Unique	Used		1.997	0.722	0.756	0	0	4.60	0	25	54	2	1187.64941	2374.29155	-1.97
dGSGYIDENELDALLk	Unambiguous	1	1	Q08331	N-Term(iTRAQ8plex); K16(iTRAQ8plex)	CID	1.0000	0.0000	1	1	2720897	Unique	Used		2.577		0.252	0	0	4.59	0	0	200	2	1180.62268	2360.23808	-2.59
qVGYENAGTVEFLVDk	Unambiguous	3	1	G5E8R3	N-Term(iTRAQ8plex); K16(iTRAQ8plex)	CID	1.0000	0.0000	1	1	2995215	Unique	Used	0.828	0.759	0.534	0.503	0	0	4.59	0	5	95	2	1189.14685	2377.28642	0.18
qLTEHAVEGDcDFHILk	Unambiguous	1	1	P29699	N-Term(iTRAQ8plex); C11(Methylthio); K17(iTRAQ8plex)	CID	1.0000	0.0000	1	1	3089900	Unique	Used	2.666	1.536	0.386	0.552	0	0	4.59	0	5	86	3	870.45001	2609.33548	1.62
aETLASSLQEDLAHTR	Unambiguous	1	1	Q2KN98	N-Term(iTRAQ8plex)	CID	1.0000	0.0000	1	1	3053953	Unique	Used	1.706	1.066	1.059	0.740	0	0	4.59	0	22	158	3	682.69434	2046.06845	-4.15
fEDGDLTLYQSNAILR	Unambiguous	2	1	P19157	N-Term(iTRAQ8plex)	HCD	1.0000	0.0000	1	1	3283823	Unique	Used	1.074	1.387	1.191	1.694	0	0	4.59	0	0	200	2	1080.06604	2159.12480	-1.78
INITPNSGATGNNAGPk	Unambiguous	1	1	Q62433	N-Term(iTRAQ8plex); K17(iTRAQ8plex)	CID	1.0000	0.0000	1	1	3407954	Unique	Used	0.908	0.429	0.964	1.397	0	0	4.58	0	0	73	2	1117.62000	2234.23271	-1.01
lGcVAAQnGEDAlk	Unambiguous	1	1	P16054	N-Term(iTRAQ8plex); C3(Methylthio); N8(Deamidated); K14(iTRAQ8plex)	CID	1.0000	0.0000	1	1	2884722	Unique	Used		0.822	2.205	1.111	0	0	4.58	0	22	74	3	682.02539	2044.06162	-2.33
gTGGVDTAAVGGVFDVSNADR	Unambiguous	1	1	Q04447	N-Term(iTRAQ8plex)	CID	1.0000	0.0000	1	1	3154602	Unique	Used	1.065	1.631	1.420	1.349	0	0	4.58	0	19	200	2	1135.07190	2269.13652	0.10
lEEGHSSAVAAHYNELQEVGLAk	Unambiguous	5	1	Q9D0L8	N-Term(iTRAQ8plex); K23(iTRAQ8plex)	CID	1.0000	0.0000	1	1	3087976	Unique	Used		0.727	0.966	1.058	0	0	4.58	0	0	200	3	1020.87781	3060.61887	-0.73
gATELTHEDYMEGILEVQAk	Unambiguous	2	1	O88685	N-Term(iTRAQ8plex); K20(iTRAQ8plex)	CID	1.0000	0.0000	1	1	2919440	Unique	Used	1.863	1.157	0.975	0.923	0	0	4.58	0	11	29	3	948.16016	2842.46592	-3.32
vAVLGASGGIGQPLSLLLk	Unambiguous	1	1	P08249	N-Term(iTRAQ8plex); K19(iTRAQ8plex)	CID	1.0000	0.0000	1	1	3936902	Unique	Used	1.294	0.973	1.313	1.557	0	0	4.58	0	9	92	3	801.16821	2401.49009	-4.26

tIGTGLVTDVPAmTEEDk	Unambiguous	2	1	Q8BFR5	N-Term(iTRAQ8plex); M13(Oxidation); K18(iTRAQ8plex)	CID	1.0000	0.0000	1	1	2900269	Unique	Used	0.533	0.840	1.045	1.068	0	0	4.58	0	0	177	2	1251.16309	2501.31890	-3.07
qFIAGLTELnDSPVPLELER	Unambiguous	3	1	Q5DQR4-3	N-Term(iTRAQ8plex); N10(Deamidated)	HCD	1.0000	0.0000	1	1	3217367	Unique	Used	0.760	0.656	1.197	1.249	0	0	4.58	0	0	49	2	1273.68762	2546.36797	0.92
sESNSSITATQPTLAk	Unambiguous	4	1	Q91ZU6	N-Term(iTRAQ8plex); K16(iTRAQ8plex)	CID	1.0000	0.0000	1	1	3122423	Unique	Used	0.477	0.448	1.038	1.142	0	0	4.57	0	0	81	2	1122.11658	2243.22588	-3.60
eHHLSEVQNmASEEk	Unambiguous	1	1	Q91VA7	N-Term(iTRAQ8plex); M10(Oxidation); K15(iTRAQ8plex)	CID	1.0000	0.0000	1	1	2646338	Unique	Used	1.010	0.717	1.377	1.502	0	0	4.57	0	20	200	3	798.06976	2392.19474	-3.18
eYAESIGAIVVETSAk	Unambiguous	2	1	Q921E2	N-Term(iTRAQ8plex); K16(iTRAQ8plex)	CID	1.0000	0.0000	1	1	3151482	Unique	Used	0.475	0.900	1.477	1.271	0	0	4.57	0	0	200	2	1138.13159	2275.25591	-3.65
eANHDGDFGITLTELR	Unambiguous	1	1	G5E829	N-Term(iTRAQ8plex)	CID	1.0000	0.0000	1	1	2901061	Unique	Used	0.479	0.831	1.045	0.664	0	0	4.55	0	5	200	3	698.02362	2092.05631	-2.39
vAVLGASGGIGQPLSLLLk	Unambiguous	1	1	P08249	N-Term(iTRAQ8plex); K19(iTRAQ8plex)	CID	1.0000	0.0000	1	1	3939315	Unique	Used	1.160	1.547	0.639	0.961	0	0	4.55	0	10	200	2	1201.24841	2401.48955	-4.49
vAVLGASGGIGQPLSLLLk	Unambiguous	1	1	P08249	N-Term(iTRAQ8plex); K19(iTRAQ8plex)	CID	1.0000	0.0000	1	1	3939207	Unique	Used	1.797	1.195	0.895	1.510	0	0	4.55	0	9	200	2	1201.24622	2401.48516	-6.32
nWETDVVLTDTLHLk	Unambiguous	5	1	Q9Z0J4	N-Term(iTRAQ8plex); K15(iTRAQ8plex)	CID	1.0000	0.0000	1	1	3260277	Unique	Used		1.865	0.874	1.167	0	0	4.55	0	30	52	3	798.11560	2392.33225	-0.43
fQEYHIQQNEALAAk	Unambiguous	1	1	Q9WV98	N-Term(iTRAQ8plex); K15(iTRAQ8plex)	CID	1.0000	0.0000	1	1	3087133	Unique	Used	1.168	1.322	1.193	0.916	0	0	4.55	0	25	122	3	800.10364	2398.29636	-0.50
dHIIEVVGSSMPLIGDHQDEDk	Unambiguous	3	1	O88935	N-Term(iTRAQ8plex); K22(iTRAQ8plex)	CID	1.0000	0.0000	1	1	2977035	Unique	Used	1.394	1.354	1.100	1.095	0	0	4.55	0	25	200	3	1014.85730	3042.55735	-2.95

gcIVDANLSVLNLVIVk	Unambiguous	1	1	P62754	N-Term(iTRAQ8plex); C2(Methylthio); K17(iTRAQ8plex)	CID	1.0000	0.0000	1	1	3879994	Unique	Used	0.599	0.658	1.133	1.053	0	0	4.55	0	28	37	2	1212.70813	2424.40898	-3.68
iPNIYAIGDVVAGPmLAHk	Unambiguous	1	1	O08749	N-Term(iTRAQ8plex); M15(Oxidation); K19(iTRAQ8plex)	CID	1.0000	0.0000	1	1	3764934	Unique	Used	1.648	2.156	1.269	1.293	0	0	4.55	0	18	35	3	868.49463	2603.46933	-5.64
vQRPEDASGGSSPSGTSk	Unambiguous	4	1	P70429-2	N-Term(iTRAQ8plex); K18(iTRAQ8plex)	CID	1.0000	0.0000	1	1	2822012	Unique	Used	0.949	1.002	1.059	0.918	0	0	4.55	0	0	93	3	785.74847	2355.23087	-2.22
lWSLDSDEPVADIEGHTVR	Unambiguous	1	1	Q9DAW6	N-Term(iTRAQ8plex)	CID	1.0000	0.0000	1	1	2920508	Unique	Used	1.363	1.214	0.655	0.829	0	0	4.55	0	23	200	3	815.08423	2443.23813	-1.07
gTAEGAVDAMLVHGQDAITVSnGGR	Unambiguous	2	1	Q8C7X2-2	N-Term(iTRAQ8plex); N22(Deamidated)	CID	1.0000	0.0000	1	1	3091275	Unique	Used	1.070	0.669	0.774	1.399	0	0	4.54	0	0	200	3	911.12628	2731.36429	0.70
iTVVDDADTVELcGALk	Unambiguous	2	1	Q3ULJ0-2	N-Term(iTRAQ8plex); C13(Methylthio); K17(iTRAQ8plex)	CID	1.0000	0.0000	1	1	3196088	Unique	Used	0.219	1.638	1.074	1.229	0	0	4.54	0	3	5	2	1208.64917	2416.29106	-0.57
rGPPPPPTASEPTR	Unambiguous	2	1	Q8CGF7-2	N-Term(iTRAQ8plex)	HCD	1.0000	0.0000	1	1	3269412	Unique	Used	1.239	0.877	2.019	0.908	0	0	4.54	1	16	200	3	588.66040	1763.96665	-2.26
dDGGIVIcEAQNQALPSGHSk	Unambiguous	1	1	Q8R464	N-Term(iTRAQ8plex); C8(Methylthio); K21(iTRAQ8plex)	CID	1.0000	0.0000	1	1	3011694	Unique	Used	0.833	0.466	1.082	0.930	0	0	4.53	0	21	50	3	931.80945	2793.41379	0.62
qAEIENPLEDPVTGDYVHk	Unambiguous	4	1	Q9Z1G4-3	N-Term(iTRAQ8plex); K19(iTRAQ8plex)	CID	1.0000	0.0000	1	1	2760642	Unique	Used	0.380	1.309	0.954	0.688	0	0	4.53	0	5	121	3	921.48560	2762.44223	-1.28
lATPTYGDLNHLVSATMSGVTTSLR	Unambiguous	1	1	Q9ERD7	N-Term(iTRAQ8plex)	CID	1.0000	0.0000	1	1	3940044	Unique	Used			2.617	1.578	0	0	4.53	0	4	200	3	970.51233	2909.52243	-4.14
lGNTISSLFGGGTSSDAk	Unambiguous	1	1	Q9JKR6	N-Term(iTRAQ8plex); K18(iTRAQ8plex)	CID	1.0000	0.0000	1	1	3765325	Unique	Used	2.208	1.422	1.014	1.068	0	0	4.53	0	12	56	3	774.08777	2320.24875	-5.07

dSVSAAEVGTGQYATTk	Unambiguous	6	1	Q9WV92-2	N-Term(iTRAQ8plex); K17(iTRAQ8plex)	CID	1.0000	0.0000	1	1	2845743	Unique	Used	0.568	0.905	1.436	1.203	0	0	4.53	0	0	200	2	1147.10535	2293.20342	-4.28
gTGGVDTAAVGAVFDISnADR	Unambiguous	1	1	P07310	N-Term(iTRAQ8plex); N18(Deamidated)	CID	1.0000	0.0000	1	1	3036177	Unique	Used	5.393	5.428	1.025	1.104	0	0	4.52	0	19	200	2	1149.57776	2298.14824	-1.47
gTGGVDTAAVGGVFDVSNADR	Unambiguous	1	1	Q04447	N-Term(iTRAQ8plex)	CID	1.0000	0.0000	1	1	3155047	Unique	Used		1.210	1.193		0	0	4.52	0	0	200	2	1135.06958	2269.13188	-1.95
gSLEADDLVSLDALSTVR	Unambiguous	1	1	P70261	N-Term(iTRAQ8plex)	CID	1.0000	0.0000	1	1	3153150	Unique	Used	0.922	0.823	0.815	0.840	0	0	4.52	0	0	200	2	1083.08044	2165.15361	-3.12
eGGSPALSTLFPPPR	Unambiguous	1	1	Q99MR3	N-Term(iTRAQ8plex)	HCD	1.0000	0.0000	1	1	3703301	Unique	Used	0.791	0.698	0.858	1.038	0	0	4.52	0	30	106	2	915.49982	1829.99236	-7.65
mILIQDGSQNTNVDkPLR	Unambiguous	1	1	Q3U0V1	N-Term(iTRAQ8plex); M1(Oxidation); K15(iTRAQ8plex)	CID	1.0000	0.0000	1	1	3452263	Unique	Used	1.316	1.427	1.183	0.940	0	0	4.52	0	7	43	3	889.49359	2666.46622	-3.53
qLTEHAVEGDcDFHILk	Unambiguous	1	1	P29699	N-Term(iTRAQ8plex); C11(Methylthio); K17(iTRAQ8plex)	CID	1.0000	0.0000	1	1	3071908	Unique	Used	2.722	1.391	0.476	0.501	0	0	4.51	0	6	151	3	870.44702	2609.32651	-1.82
vQDDEVGDGTTSVTVLAAELLR	Unambiguous	1	1	P80314	N-Term(iTRAQ8plex)	HCD	1.0000	0.0000	1	1	3133534	Unique	Used	1.406	1.109	1.067	1.349	0	0	4.51	0	26	176	2	1296.68555	2592.36382	-1.26
vGDENDnAPIFTkPVYEVSVR	Unambiguous	3	1	Q7TSK3-3	N-Term(iTRAQ8plex); N7(Deamidated); K13(iTRAQ8plex)	HCD	1.0000	0.0000	1	1	3149158	Unique	Used	0.733	1.980	0.977	0.877	0	0	4.51	0	0	200	3	986.86591	2958.58316	5.48
vPVASPSAHNISSSSGAPDR	Unambiguous	14	1	E9QMP6	N-Term(iTRAQ8plex)	HCD	1.0000	0.0000	1	1	3611651	Unique	Used	1.259	1.414	1.490	2.485	0	0	4.51	0	0	200	3	747.38727	2240.14725	-4.50
hLQLEQGVENYEESIAQLSR	Unambiguous	3	1	E9PX29	N-Term(iTRAQ8plex)	CID	1.0000	0.0000	1	1	3171206	Unique	Used	2.861	1.743	1.491	0.850	0	0	4.50	0	30	194	3	883.12573	2647.36264	-0.12
vADGQISTEVSEAPVASDkPk	Unambiguous	1	1	P31648	N-Term(iTRAQ8plex); K19(iTRAQ8plex); K21(iTRAQ8plex)	CID	1.0000	0.0000	1	1	2827919	Unique	Used	0.468	0.615	0.863	0.880	0	0	4.50	0	7	71	3	1014.23566	3040.69242	-0.20
rPASVSSAAAEHEAR	Unambiguous	1	1	E9Q1P8	N-Term(iTRAQ8plex)	CID	1.0000	0.0000	1	1	2908944	Unique	Used	1.391	0.945	1.206	0.932	0	0	4.50	0	0	200	3	614.99396	1842.96732	-2.77

rHEAAVPPLAVSSSRPEk	Unambiguous	3	1	Q8BJ05-2	N-Term(iTRAQ8plex); K18(iTRAQ8plex)	CID	1.0000	0.0000	1	1	3404603	Unique	Used		0.913	1.089	0.742	0	0	4.50	1	0	200	3	847.15192	2539.44120	-6.05
gLGTDEDSILNLLTSR	Unambiguous	1	1	P48036	N-Term(iTRAQ8plex)	HCD	1.0000	0.0000	1	1	3396946	Unique	Used	1.932	1.417	0.835	1.339	0	0	4.50	0	21	200	2	1004.54437	2008.08147	-2.49
vSAADSTQVDGGTPMVk	Unambiguous	6	1	A2AUK8	N-Term(iTRAQ8plex); K17(iTRAQ8plex)	CID	1.0000	0.0000	1	1	2933778	Unique	Used			1.266	0.908	0	0	4.50	0	7	123	2	1136.10779	2271.20830	-1.25
gPAGPGPGPGSGAAPEAR	Unambiguous	2	1	B1AS06	N-Term(iTRAQ8plex)	CID	1.0000	0.0000	1	1	2928516	Unique	Used	0.551	1.466	1.753	0.993	0	0	4.49	0	0	200	2	903.97144	1806.93559	-2.49
dVnGTTSGIGTGSSVEk	Unambiguous	3	1	A2APX7	N-Term(iTRAQ8plex); N3(Deamidated); K17(iTRAQ8plex)	CID	1.0000	0.0000	1	1	2713797	Unique	Used	0.555		1.552	1.890	0	0	4.49	0	0	200	2	1109.58594	2218.16460	-0.61
eSIQMAVGPFLHILEGnLLk	Unambiguous	1	1	Q8BP40	N-Term(iTRAQ8plex); N17(Deamidated); K20(iTRAQ8plex)	CID	1.0000	0.0000	1	1	3860997	Unique	Used	4.399	3.364			0	0	4.49	0	15	27	3	940.20929	2818.61332	4.80
gDGAPAPSGPPPPGTGR	Unambiguous	1	1	Q8BGH4	N-Term(iTRAQ8plex)	CID	1.0000	0.0000	1	1	2910863	Unique	Used	0.897	0.515	1.051	0.941	0	0	4.49	0	0	200	2	896.46490	1791.92253	-3.72
eVESIGAHLNAySTR	Unambiguous	1	1	Q9CZ13	N-Term(iTRAQ8plex); Y12(iTRAQ8plex)	HCD	1.0000	0.0000	1	1	3408828	Unique	Used	0.958	1.150	0.992	1.103	0	0	4.49	0	13	73	3	752.40918	2255.21299	-4.91
fkGEETVVIGqGkPYVFDR	Unambiguous	1	1	P28738	N-Term(iTRAQ8plex); K2(iTRAQ8plex); Q11(Deamidated); K13(iTRAQ8plex)	HCD	1.0000	0.0000	1	1	3367953	Unique	Used	0.688	1.127	1.161	1.087	0	0	4.49	1	0	174	3	1028.24829	3082.73032	-1.22
vLVTHQTGPDEDNPk	Unambiguous	1	1	Q80UG2	N-Term(iTRAQ8plex); K15(iTRAQ8plex)	CID	1.0000	0.0000	1	1	2681234	Unique	Used	0.833	0.603	0.939	1.053	0	0	4.48	0	0	200	3	753.41095	2258.21830	-2.41
gFEFVTASALISESk	Unambiguous	7	1	Q9QX47-3	N-Term(iTRAQ8plex); K15(iTRAQ8plex)	HCD	1.0000	0.0000	1	1	3418106	Unique	Used	0.952	0.918	0.926	0.686	0	0	4.48	0	0	79	2	1097.60840	2194.20952	-5.51
lSADMATVLQLLQR	Unambiguous	4	1	O35219	N-Term(iTRAQ8plex)	HCD	1.0000	0.0000	1	1	3918175	Unique	Used	1.584	1.065	0.624	1.058	0	0	4.48	0	30	48	2	932.03302	1863.05876	-4.73

tPYLGDVAIVVHPGk	Unambiguous	1	1	Q9EQF6	N-Term(iTRAQ8plex); K15(iTRAQ8plex)	HCD	1.0000	0.0000	1	1	3495817	Unique	Used	0.516	0.437	0.449	0.624	0	0	4.47	0	23	36	3	725.43024	2174.27616	-1.49
lSQVSDSVSGQTVVDPk	Unambiguous	2	1	Q91YR7	N-Term(iTRAQ8plex); K17(iTRAQ8plex)	CID	1.0000	0.0000	1	1	3147394	Unique	Used	1.086	0.946	1.000	0.780	0	0	4.46	0	0	190	2	1177.65198	2354.29668	-2.42
dSVSAAEVGTGQYATTk	Unambiguous	6	1	Q9WV92-2	N-Term(iTRAQ8plex); K17(iTRAQ8plex)	CID	1.0000	0.0000	1	1	2863558	Unique	Used	0.333	1.211	1.638	1.331	0	0	4.46	0	9	88	2	1147.10510	2293.20293	-4.49
lLHDLVQPPTITQQSPk	Unambiguous	1	1	Q810U4-5	N-Term(iTRAQ8plex); K17(iTRAQ8plex)	CID	1.0000	0.0000	1	1	3683069	Unique	Used	0.669	1.127	1.121	0.783	0	0	4.46	0	1	5	3	841.82440	2523.45865	-6.69
kAAAQLLQSQAQQSGAQQTk	Unambiguous	3	1	Q8C1B7-3	N-Term(iTRAQ8plex); K1(iTRAQ8plex); K20(iTRAQ8plex)	CID	1.0000	0.0000	1	1	3757009	Unique	Used	1.382	1.450	1.280	0.993	0	0	4.45	1	15	84	3	999.90863	2997.71134	-3.18
vAVLGASGGIGQPLSLLLk	Unambiguous	1	1	P08249	N-Term(iTRAQ8plex); K19(iTRAQ8plex)	CID	1.0000	0.0000	1	1	3937684	Unique	Used	1.701	1.186	0.808	0.892	0	0	4.45	0	28	200	2	1201.24890	2401.49053	-4.08
qGFNVVVESGAGEASk	Unambiguous	4	1	Q61941	N-Term(iTRAQ8plex); K16(iTRAQ8plex)	CID	1.0000	0.0000	1	1	2991306	Unique	Used	0.287	1.138	0.046	0.066	0	0	4.44	0	11	82	2	1094.09497	2187.18266	-1.81
iAPALISSGISVVEQEk	Unambiguous	4	1	P17183	N-Term(iTRAQ8plex); K17(iTRAQ8plex)	CID	1.0000	0.0000	1	1	3398955	Unique	Used	1.140	1.078	1.725	1.766	0	0	4.43	0	0	200	2	1175.19202	2349.37676	-3.51
vSASSPSVAAASQADSk	Unambiguous	8	1	P06537-3	N-Term(iTRAQ8plex); K17(iTRAQ8plex)	CID	1.0000	0.0000	1	1	2988047	Unique	Used	2.948	1.036	1.005	0.765	0	0	4.43	0	0	200	2	1086.08972	2171.17217	-1.97
gHTDSVQDISFDHSGk	Unambiguous	2	1	P63005	N-Term(iTRAQ8plex); K16(iTRAQ8plex)	CID	1.0000	0.0000	1	1	2824878	Unique	Used	2.287	0.964	1.051	1.011	0	0	4.43	0	20	200	3	780.06494	2338.18027	-3.48
eAEAQAAmEANSEGSLTRPk	Unambiguous	1	1	P63040	N-Term(iTRAQ8plex); M8(Oxidation); K20(iTRAQ8plex)	CID	1.0000	0.0000	1	1	2807592	Unique	Used	2.271	0.766	1.623	1.074	0	0	4.42	0	16	81	3	905.46460	2714.37925	-3.09

aLPSSEDLASLAPDLDk	Unambiguous	5	1	P58281-2	N-Term(iTRAQ8plex); K17(iTRAQ8plex)	CID	1.0000	0.0000	1	1	3152027	Unique	Used	0.713	1.128	1.575	0.897	0	0	4.42	0	20	200	2	1175.64880	2350.29033	-2.52
aVSPAATQPPPPSGAGk	Unambiguous	3	1	E9QQ25	N-Term(iTRAQ8plex); K17(iTRAQ8plex)	CID	1.0000	0.0000	1	1	3249116	Unique	Used	0.391	0.856	2.170	0.852	0	0	4.42	0	30	200	2	1071.11157	2141.21587	-0.78
tVAIHSDVDASSVHVk	Unambiguous	1	1	Q91ZA3	N-Term(iTRAQ8plex); K16(iTRAQ8plex)	CID	1.0000	0.0000	1	1	3121654	Unique	Used	1.819	0.844	1.249	0.942	0	0	4.42	0	29	110	3	758.42755	2273.26810	-1.28
qTPQAPSGLMEPPTPVTPVVQRPR	Unambiguous	2	1	Q8JZP2	N-Term(iTRAQ8plex)	HCD	1.0000	0.0000	1	1	3855440	Unique	Used	0.524	1.151	1.325	1.241	0	0	4.41	0	0	200	3	963.19281	2887.56388	-4.43
vVNVELPVEANLVWQLGR	Unambiguous	3	1	Q61699-2	N-Term(iTRAQ8plex)	HCD	1.0000	0.0000	1	1	3806203	Unique	Used	1.601	1.063	1.013	1.073	0	0	4.41	0	23	5	2	1170.16968	2339.33208	-2.94
gTVISPAELEAPILVPHTAQR	Unambiguous	3	1	Q8C754	N-Term(iTRAQ8plex)	CID	1.0000	0.0000	1	1	3538223	Unique	Used	0.626	0.750	0.959	0.994	0	0	4.41	0	9	19	3	835.14148	2503.40989	-3.51
lIEESAIDEVVVTNTIPHEIQk	Unambiguous	1	1	Q8R574	N-Term(iTRAQ8plex); K22(iTRAQ8plex)	CID	1.0000	0.0000	1	1	3075288	Unique	Used	0.520	1.532	1.447	1.480	0	0	4.40	0	3	89	3	1029.24573	3085.72263	-0.51
vTEGLVDVILYHQPDDk	Unambiguous	1	1	Q8VHM5	N-Term(iTRAQ8plex); K17(iTRAQ8plex)	CID	1.0000	0.0000	1	1	3132297	Unique	Used	0.471	0.605	0.827	0.866	0	0	4.40	0	4	67	3	850.47278	2549.40378	-1.33
mDGWGGPEELGLAPAPNPR	Unambiguous	3	1	Q8C3Q5-2	N-Term(iTRAQ8plex)	HCD	1.0000	0.0000	1	1	3259763	Unique	Used	1.440	3.040	2.366	0.879	0	0	4.40	0	17	97	2	1134.57312	2268.13896	0.18
yITGDQLGALYQDFVR	Unambiguous	2	1	P17183	N-Term(iTRAQ8plex)	CID	1.0000	0.0000	1	1	3670764	Unique	Used		2.690			0	0	4.40	0	0	200	2	1082.06885	2163.13042	-3.88
sEALSSVLDQEEAmDTk	Unambiguous	5	1	A2AU91	N-Term(iTRAQ8plex); M14(Oxidation); K17(iTRAQ8plex)	CID	1.0000	0.0000	1	1	2637049	Unique	Used	1.884	2.115	1.288	1.361	0	0	4.40	0	15	200	3	826.42188	2477.25107	-1.10
eQASHLGGAVFSGAGNIAAATGLVk	Unambiguous	1	1	Q91ZZ3	N-Term(iTRAQ8plex); K25(iTRAQ8plex)	CID	1.0000	0.0000	1	1	3783089	Unique	Used	1.231	1.984	1.202	1.251	0	0	4.39	0	0	93	3	978.87799	2934.61942	-2.19
gTGGVDTAATGSVFDISnLDR	Unambiguous	1	1	P30275	N-Term(iTRAQ8plex); N18(Deamidated)	CID	1.0000	0.0000	1	1	2996080	Unique	Used	1.324	1.094	1.157	1.300	0	0	4.39	0	11	200	2	1179.58801	2358.16875	-1.69
gTGGVDTAAVGGVFDVSNADR	Unambiguous	1	1	Q04447	N-Term(iTRAQ8plex)	CID	1.0000	0.0000	1	1	3089699	Unique	Used	2.411	1.468	1.009	1.031	0	0	4.39	0	0	200	2	1135.07190	2269.13652	0.10

eSLLEDIQANNALGDEVEAIVk	Unambiguous	3	1	A2ALU4	N-Term(iTRAQ8plex); K22(iTRAQ8plex)	CID	1.0000	0.0000	1	1	2942351	Unique	Used	3.887	3.555	2.787	1.591	0	0	4.39	0	7	40	3	993.54083	2978.60794	-2.14
aIEPPPLDAVIEAEHTLR	Unambiguous	1	1	E9QNN1	N-Term(iTRAQ8plex)	CID	1.0000	0.0000	1	1	3092855	Unique	Used		1.089	0.807	1.005	0	0	4.38	0	13	87	3	759.09259	2275.26322	1.40
kLFGQESGPSAEk	Unambiguous	1	1	Q9D6K8	N-Term(iTRAQ8plex); K1(iTRAQ8plex); K13(iTRAQ8plex)	HCD	1.0000	0.0000	1	1	3101959	Unique	Used	0.719	0.869	1.047	1.111	0	0	4.38	1	26	200	3	764.11035	2290.31650	-0.23
iAPALISSGISVVEQEk	Unambiguous	4	1	P17183	N-Term(iTRAQ8plex); K17(iTRAQ8plex)	CID	1.0000	0.0000	1	1	3399063	Unique	Used	2.586	2.349	2.114	1.957	0	0	4.38	0	0	200	2	1175.19153	2349.37578	-3.92
nEEDATELVGLAQAVnAR	Unambiguous	1	1	Q02053	N-Term(iTRAQ8plex); N16(Deamidated)	CID	1.0000	0.0000	1	1	2703656	Unique	Used		1.275			0	0	4.37	0	0	200	2	1103.06616	2205.12505	-2.31
gVHAEEGWPNSAYGVTk	Unambiguous	1	1	P48758	N-Term(iTRAQ8plex); K17(iTRAQ8plex)	CID	1.0000	0.0000	1	1	3048889	Unique	Used	0.317	1.571	0.981	1.362	0	0	4.37	0	19	200	2	1205.63074	2410.25420	-2.90
dGFTSEGEILEISDIQR	Unambiguous	1	1	Q8HW98	N-Term(iTRAQ8plex)	CID	1.0000	0.0000	1	1	2672891	Unique	Used	1.803	0.420	0.261	0.541	0	0	4.37	0	0	200	2	1107.06506	2213.12285	-0.51
aSGNYATVISHNPETk	Unambiguous	1	1	P62918	N-Term(iTRAQ8plex); K16(iTRAQ8plex)	CID	1.0000	0.0000	1	1	3066366	Unique	Used	0.450	0.561	0.861	0.966	0	0	4.36	0	3	27	3	766.41602	2297.23349	-0.49
vNNSSLIGLGYTQTLkPGIk	Unambiguous	2	1	Q60932-2	N-Term(iTRAQ8plex); K16(iTRAQ8plex); K20(iTRAQ8plex)	CID	1.0000	0.0000	1	1	3821598	Unique	Used		0.856	1.045	1.025	0	0	4.36	0	0	186	3	1005.93250	3015.78293	-4.66
gSETLGGGGGGAAGTR	Unambiguous	1	1	P31650	N-Term(iTRAQ8plex)	CID	1.0000	0.0000	1	1	2841601	Unique	Used	0.571	0.587	0.786	1.334	0	0	4.36	0	0	200	2	804.91351	1608.81975	-2.88
vAVLGASGGIGQPLSLLLk	Unambiguous	1	1	P08249	N-Term(iTRAQ8plex); K19(iTRAQ8plex)	CID	1.0000	0.0000	1	1	3936781	Unique	Used	1.119	0.684	1.086	1.087	0	0	4.34	0	2	84	3	801.16772	2401.48862	-4.87
lADAQSEAAmEEIEATVR	Unambiguous	1	1	Q8K3A0	N-Term(iTRAQ8plex); M10(Oxidation)	CID	1.0000	0.0000	1	1	2739272	Unique	Used	2.094		1.274	0.501	0	0	4.33	0	0	200	2	1127.56018	2254.11308	-1.97

gPAAAPGAASPPAEPk	Unambiguous	1	1	Q9QZM0	N-Term(iTRAQ8plex); K16(iTRAQ8plex)	CID	1.0000	0.0000	1	1	2912299	Unique	Used	1.915	2.023	0.920	0.675	0	0	4.33	0	0	164	2	999.06537	1997.12346	-2.11
sYLGGGAGAAGGGGADPGNk	Unambiguous	1	1	Q00899	N-Term(iTRAQ8plex); K20(iTRAQ8plex)	CID	1.0000	0.0000	1	1	2969677	Unique	Used	0.954	0.619	1.141	0.539	0	0	4.33	0	0	200	2	1121.58789	2242.16850	0.53
lFVGNLPADITEDEFk	Unambiguous	1	1	Q8VIJ6	N-Term(iTRAQ8plex); K16(iTRAQ8plex)	CID	1.0000	0.0000	1	1	3057144	Unique	Used	0.758	0.628	0.855	0.849	0	0	4.33	0	0	24	2	1208.66248	2416.31767	-1.82
lGEHNInVLEGNEQFIDAAk	Unambiguous	1	1	Q9Z1R9	N-Term(iTRAQ8plex); N7(Deamidated); K20(iTRAQ8plex)	CID	0.0278	0.0000	1	1	3014224	Unique	Used	1.632	1.645	0.782	0.852	0	0	4.32	0	4	32	3	940.83777	2820.49875	-0.04
tPTQTnGSNVPFkPR	Unambiguous	8	1	Q9ESZ8-5	N-Term(iTRAQ8plex); N6(Deamidated); K13(iTRAQ8plex)	HCD	1.0000	0.0000	1	1	3384106	Unique	Used	0.525	0.952	0.626	0.749	0	0	4.31	0	0	82	3	751.75122	2253.23911	-2.52
nAQAIEDMVGYAQETQHEk	Unambiguous	1	1	Q3TXS7	N-Term(iTRAQ8plex); K19(iTRAQ8plex)	CID	1.0000	0.0000	1	1	2938081	Unique	Used				0.966	0	0	4.31	0	8	147	3	924.13361	2770.38626	-2.32
gSETLGGGGGGAAGTR	Unambiguous	1	1	P31650	N-Term(iTRAQ8plex)	CID	1.0000	0.0000	1	1	2821787	Unique	Used	0.617	0.483	0.899	1.557	0	0	4.31	0	0	200	2	804.91583	1608.82439	0.00
mDENEFVAVTSTNAAk	Unambiguous	3	1	O35098	N-Term(iTRAQ8plex); M1(Oxidation); K16(iTRAQ8plex)	CID	1.0000	0.0000	1	1	2915425	Unique	Used	1.129	1.781	0.941	0.954	0	0	4.31	0	11	200	2	1176.09998	2351.19267	-3.53
lGSSEVEQVQLVVDGVk	Unambiguous	1	1	P07310	N-Term(iTRAQ8plex); K17(iTRAQ8plex)	CID	1.0000	0.0000	1	1	3172444	Unique	Used	12.433	15.900	0.372	0.849	0	0	4.31	0	9	156	2	1197.68396	2394.36064	-3.94
qGQETAVAPSLVAPALSkPk	Unambiguous	1	1	Q61166	N-Term(iTRAQ8plex); K18(iTRAQ8plex); K20(iTRAQ8plex)	CID	1.0000	0.0000	1	1	3472775	Unique	Used	0.604	0.632	0.668	0.560	0	0	4.30	0	0	71	3	968.91144	2904.71976	-3.04
aFMEEFGAEPELAVSAPGR	Unambiguous	1	1	Q9JIA6	N-Term(iTRAQ8plex)	CID	1.0000	0.0000	1	1	3093036	Unique	Used	0.418	1.075	0.860	1.195	0	0	4.30	0	2	127	2	1156.58118	2312.15508	0.66

iPIFSAAGLPHNEIAAqIcR	Unambiguous	2	1	P62814	N-Term(iTRAQ8plex); Q17(Deamidated); C19(Methylthio)	CID	0.0047	0.0000	1	1	3919799	Unique	Used	1.035	0.870	1.091	1.167	0	0	4.30	0	20	200	3	824.77649	2472.31491	4.19
vPSAGGGIGDGGQEPTTGNWR	Unambiguous	2	1	E9PUD2	N-Term(iTRAQ8plex)	CID	1.0000	0.0000	1	1	3124668	Unique	Used	1.744	0.812	0.451	0.541	0	0	4.30	0	0	72	2	1159.07422	2317.14116	-2.75
eAAEAIEAEGATAPLTELLHSR	Unambiguous	2	1	Q02248	N-Term(iTRAQ8plex)	CID	1.0000	0.0000	1	1	3036823	Unique	Used	0.459	1.116	0.820	0.778	0	0	4.30	0	19	32	3	861.79034	2583.35648	-0.15
eLEGLFHTIDSDNTNHVDTk	Unambiguous	1	1	Q91ZP9	N-Term(iTRAQ8plex); K20(iTRAQ8plex)	CID	1.0000	0.0000	1	1	2901589	Unique	Used	2.543	1.902	1.299	1.078	0	0	4.29	0	23	176	3	965.16010	2893.46573	-4.53
eTLGTGAFSEVVLAEEk	Unambiguous	2	1	Q8BW96	N-Term(iTRAQ8plex); K17(iTRAQ8plex)	CID	1.0000	0.0000	1	1	2886584	Unique	Used	1.635	2.525	2.312		0	0	4.29	0	0	200	2	1194.65698	2388.30669	-2.18
eYAESIGAIVVETSAk	Unambiguous	2	1	Q921E2	N-Term(iTRAQ8plex); K16(iTRAQ8plex)	CID	1.0000	0.0000	1	1	3151617	Unique	Used	0.535	0.824	0.969	1.052	0	0	4.29	0	0	179	2	1138.13550	2275.26372	-0.21
fEDGDLTLYQSNAILR	Unambiguous	2	1	P19157	N-Term(iTRAQ8plex)	HCD	1.0000	0.0000	1	1	3478565	Unique	Used	0.836	1.504	1.264	1.766	0	0	4.29	0	0	142	2	1080.06238	2159.11748	-5.17
gTGGVDTAAVGGVFDVSnADR	Unambiguous	1	1	Q04447	N-Term(iTRAQ8plex); N18(Deamidated)	CID	1.0000	0.0000	1	1	2938390	Unique	Used	1.531	1.047	1.293	1.411	0	0	4.29	0	0	4	2	1135.56274	2270.11821	-0.93
eSQISAEIELLPTNDk	Unambiguous	2	1	Q3TWV4	N-Term(iTRAQ8plex); K16(iTRAQ8plex)	CID	1.0000	0.0000	1	1	2832975	Unique	Used		1.187	1.475	1.157	0	0	4.28	0	15	200	2	1198.16162	2395.31597	-0.72
hGVYNPNkIFGVTTLDIVR	Unambiguous	1	1	P08249	N-Term(iTRAQ8plex); K8(iTRAQ8plex)	HCD	1.0000	0.0000	1	1	3914301	Unique	Used	1.097	0.355	1.283	1.441	0	0	4.27	1	15	146	3	917.85876	2751.56174	-5.42
lDHALSSPSSPcEEIk	Unambiguous	2	1	Q8BGT8	N-Term(iTRAQ8plex); C12(Methylthio); K16(iTRAQ8plex)	CID	1.0000	0.0000	1	1	3089138	Unique	Used	1.430	1.066	1.407	0.999	0	0	4.27	0	11	80	3	789.74335	2367.21549	0.42
hSPPELSQEEFGESFADPGSSR	Unambiguous	3	1	E9QAT4	N-Term(iTRAQ8plex)	CID	1.0000	0.0000	1	1	2684745	Unique	Used	3.492	2.738	1.138	0.741	0	0	4.26	0	0	200	3	898.75745	2694.25779	-0.30
qIPGGGSGGGGSGSGGGGGSGGGR	Unambiguous	2	1	Q8BMA6	N-Term(iTRAQ8plex)	CID	1.0000	0.0000	1	1	3311626	Unique	Used		1.228		1.141	0	0	4.26	0	0	200	2	1068.00757	2135.00786	-2.34

gDTPASAAGDTLSVPAPAVqHEDTIk	Unambiguous	3	1	Q8CAQ8-3	N-Term(iTRAQ8plex); Q20(Deamidated); K26(iTRAQ8plex)	CID	1.0000	0.0000	1	1	2899495	Unique	Used	1.923	1.706	1.444	1.054	0	0	4.26	0	15	200	3	1053.22192	3157.65122	1.20
eGSSGFGGPVVSSLYHEETIR	Unambiguous	1	1	Q9D061	N-Term(iTRAQ8plex)	CID	1.0000	0.0000	1	1	3089601	Unique	Used	1.146	2.104	1.340	1.366	0	0	4.26	0	21	88	3	838.09344	2512.26578	1.42
nEEDATELVGLAQAVNAR	Unambiguous	1	1	Q02053	N-Term(iTRAQ8plex); N1(Deamidated)	CID	1.0000	0.0000	1	1	2742052	Unique	Used	0.684	1.013	0.897	0.967	0	0	4.25	0	0	200	2	1103.06653	2205.12578	-1.98
gTGGVDTAAVGGVFDVSNADR	Unambiguous	1	1	Q04447	N-Term(iTRAQ8plex)	CID	1.0000	0.0000	1	1	3154940	Unique	Used			2.179	1.324	0	0	4.25	0	13	200	2	1135.06958	2269.13188	-1.95
sQGPALTASQSVHEQPTk	Unambiguous	9	1	B9EKN8	N-Term(iTRAQ8plex); K18(iTRAQ8plex)	CID	1.0000	0.0000	1	1	3045686	Unique	Used	0.898	0.720	1.597	0.966	0	0	4.25	0	19	200	3	825.45325	2474.34519	-0.32
vFEAnEEALGVVLHk	Unambiguous	1	1	O35857	N-Term(iTRAQ8plex); N5(Deamidated); K15(iTRAQ8plex)	CID	1.0000	0.0000	1	1	3017256	Unique	Used	0.590	1.210	1.586	1.782	0	0	4.24	0	26	195	3	755.42792	2264.26920	-2.44
nLDYVATSIHEAVTk	Unambiguous	1	1	P05201	N-Term(iTRAQ8plex); K15(iTRAQ8plex)	HCD	1.0000	0.0000	1	1	3369637	Unique	Used	2.017	0.981	0.900	1.014	0	0	4.24	0	0	200	2	1135.12976	2269.25224	-5.56
lSTLGSGGESGGDGSPGGAGATAAR	Unambiguous	1	1	Q80UU9	N-Term(iTRAQ8plex)	CID	1.0000	0.0000	1	1	3029188	Unique	Used	0.582	1.344	0.660	0.819	0	0	4.23	0	0	200	2	1197.58838	2394.16948	-4.38
vEPHQLAIDRPSPk	Unambiguous	7	1	Q8K341-4	N-Term(iTRAQ8plex); K14(iTRAQ8plex)	HCD	1.0000	0.0000	1	1	3272629	Unique	Used	0.763	0.618	0.928	0.876	0	0	4.22	0	0	174	3	732.42920	2195.27304	-1.21
gTGGVDTAAVGGVFDVSNADR	Unambiguous	1	1	Q04447	N-Term(iTRAQ8plex)	CID	1.0000	0.0000	1	1	3137579	Unique	Used			0.700		0	0	4.22	0	0	200	2	1135.07043	2269.13359	-1.19
gVSAIVYmVDAADQEk	Unambiguous	2	1	F6QKK2	N-Term(iTRAQ8plex); M8(Oxidation); K16(iTRAQ8plex)	CID	1.0000	0.0000	1	1	3053704	Unique	Used	0.623	0.707	0.880	0.963	0	0	4.21	0	20	200	2	1160.61865	2320.23003	-0.65
aVGFVSEDEYLEIQGITR	Unambiguous	3	1	D3Z396	N-Term(iTRAQ8plex)	CID	1.0000	0.0000	1	1	3076567	Unique	Used		2.387	0.822	0.882	0	0	4.21	0	0	200	2	1165.61267	2330.21807	-0.06
vEAGDVIYIEANSGAVk	Unambiguous	1	1	P60122	N-Term(iTRAQ8plex); K17(iTRAQ8plex)	CID	1.0000	0.0000	1	1	2957851	Unique	Used	0.749	0.764	0.650	0.945	0	0	4.21	0	21	200	2	1172.15576	2343.30425	1.10

aSLQSGSRPGQEGEGGLk	Unambiguous	1	1	Q8R409	N-Term(iTRAQ8plex); K18(iTRAQ8plex)	CID	1.0000	0.0000	1	1	2986401	Unique	Used	0.795	0.812	0.936	0.539	0	0	4.21	0	30	200	3	789.43005	2366.27561	-5.44
gSETLGGGGGGAAGTR	Unambiguous	1	1	P31650	N-Term(iTRAQ8plex)	CID	1.0000	0.0000	1	1	2821895	Unique	Used	0.809	0.401	0.791	1.608	0	0	4.19	0	12	6	2	804.91455	1608.82182	-1.59
iAPALISSGISVVEQEk	Unambiguous	4	1	P17183	N-Term(iTRAQ8plex); K17(iTRAQ8plex)	CID	1.0000	0.0000	1	1	3397748	Unique	Used	1.706	1.309	1.038	0.965	0	0	4.19	0	0	200	2	1175.18823	2349.36919	-6.73
mLIDAQAEAAEQLGk	Unambiguous	2	1	E9Q912	N-Term(iTRAQ8plex); K15(iTRAQ8plex)	CID	1.0000	0.0000	1	1	3109751	Unique	Used	1.118	0.817	1.086	0.663	0	0	4.18	0	0	58	3	732.74268	2196.21347	-0.92
ykPAPLATLPSTLDPAEYDVSPETR	Unambiguous	1	1	Q9CQC7	N-Term(iTRAQ8plex); K2(iTRAQ8plex)	HCD	1.0000	0.0000	1	1	3324408	Unique	Used	0.673	0.823	0.829	1.062	0	0	4.18	0	0	17	3	1113.93237	3339.78257	-3.22
eIGLLTEEVELYGETk	Unambiguous	1	1	Q922D8	N-Term(iTRAQ8plex); K16(iTRAQ8plex)	CID	1.0000	0.0000	1	1	2816163	Unique	Used	0.511	0.712	0.695	1.086	0	0	4.18	0	8	51	2	1216.17236	2431.33745	-2.22
aVVHAVDGIAEnGIQPLSSSEVDELIHk	Unambiguous	1	1	Q9Z0P4-2	N-Term(iTRAQ8plex); N12(Deamidated); K28(iTRAQ8plex)	CID	0.0120	0.0000	1	1	3056259	Unique	Used	1.050	0.629	0.955	0.663	0	0	4.18	0	9	200	3	1179.63831	3536.90036	-1.53
sALGEAAGAAGFNVLR	Unambiguous	2	1	Q99M31	N-Term(iTRAQ8plex)	HCD	1.0000	0.0000	1	1	3762753	Unique	Used	2.882	0.977	1.049	1.062	0	0	4.17	0	27	146	2	904.49573	1807.98418	-7.02
lVTEmGTYATQSALSSSRPTk	Unambiguous	1	1	Q9JIF7	N-Term(iTRAQ8plex); M5(Oxidation); K21(iTRAQ8plex)	CID	1.0000	0.0000	1	1	3797984	Unique	Used	0.399	0.995	0.551	0.699	0	0	4.17	0	0	106	3	951.51111	2852.51877	-3.39
aGDIITVLEQHPDGR	Unambiguous	4	1	Q6P9K8	N-Term(iTRAQ8plex)	CID	1.0000	0.0000	1	1	3090872	Unique	Used	0.918	0.769	1.204	0.889	0	0	4.16	0	21	200	3	642.35126	1925.03922	-0.12
tFEHVTSEIGAEEAEEVGVEHLLR	Unambiguous	1	1	P26516	N-Term(iTRAQ8plex)	CID	1.0000	0.0000	1	1	2870021	Unique	Used		1.853	0.996	0.924	0	0	4.16	0	7	200	3	995.83734	2985.49747	-4.47
vADGQISTEVSEAPVASDkPk	Unambiguous	1	1	P31648	N-Term(iTRAQ8plex); K19(iTRAQ8plex); K21(iTRAQ8plex)	CID	1.0000	0.0000	1	1	2847066	Unique	Used	0.690	0.650	1.391	1.401	0	0	4.16	0	0	200	3	1014.23627	3040.69425	0.41

qISQAYEVLADSk	Unambiguous	4	1	P63037	N-Term(iTRAQ8plex); K13(iTRAQ8plex)	HCD	1.0000	0.0000	1	1	3191721	Unique	Used	0.967	0.538	0.929	0.850	0	0	4.15	0	0	65	2	1030.57703	2060.14678	-0.80
sNLPATAGSEIPGGSTk	Unambiguous	1	1	Q9Z0U1	N-Term(iTRAQ8plex); K17(iTRAQ8plex)	CID	1.0000	0.0000	1	1	3144443	Unique	Used	1.328	0.692	0.962	0.832	0	0	4.15	0	0	116	2	1098.10974	2195.21221	-0.29
iGIVELAHVLPTEENFLLLFR	Unambiguous	1	1	P12658	N-Term(iTRAQ8plex)	HCD	1.0000	0.0000	1	1	3861082	Unique	Used	5.028	3.849	1.661	0.951	0	0	4.15	0	2	6	3	909.86035	2727.56650	-3.18
gTGGVDTAAVGGVFDVSNADR	Unambiguous	1	1	Q04447	N-Term(iTRAQ8plex)	CID	1.0000	0.0000	1	1	3136474	Unique	Used			1.093	2.353	0	0	4.15	0	0	200	2	1135.07007	2269.13286	-1.52
vLHEAEGHIVTcETNTGEVYR	Unambiguous	1	1	P62320	N-Term(iTRAQ8plex); C12(Methylthio)	CID	1.0000	0.0000	1	1	3070946	Unique	Used	1.443	0.879	0.939	0.848	0	0	4.15	0	27	200	3	903.10962	2707.31430	0.78
hLAIDSSNYEQESk	Unambiguous	1	1	Q9QYS2	N-Term(iTRAQ8plex); K14(iTRAQ8plex)	CID	1.0000	0.0000	1	1	2713064	Unique	Used		1.636	1.099	0.826	0	0	4.13	0	0	200	2	1115.08240	2229.15752	-1.46
lIASVADDEAAVPnNk	Unambiguous	2	1	P16125	N-Term(iTRAQ8plex); N14(Deamidated); K16(iTRAQ8plex)	CID	1.0000	0.0000	1	1	2654375	Unique	Used	1.189	0.799	0.961	1.492	0	0	4.13	0	23	200	2	1118.61462	2236.22197	-2.77
dASDDLDDLNFFNQk	Unambiguous	3	1	Q99L45	N-Term(iTRAQ8plex); K15(iTRAQ8plex)	CID	1.0000	0.0000	1	1	2688510	Unique	Used			0.374	0.515	0	0	4.11	0	5	200	2	1183.09009	2365.17290	-1.67
aAEGISSDSLLSVPGQk	Unambiguous	5	1	Q8BPN8-2	N-Term(iTRAQ8plex); K17(iTRAQ8plex)	CID	1.0000	0.0000	1	1	3234068	Unique	Used	0.308	1.294	1.039	0.818	0	0	4.11	0	5	27	2	1134.13672	2267.26616	-1.85
vGTLDVLVGLSDELAk	Unambiguous	1	1	Q9Z1G3	N-Term(iTRAQ8plex); K16(iTRAQ8plex)	CID	1.0000	0.0000	1	1	3352720	Unique	Used		1.712	1.051	1.368	0	0	4.10	0	11	19	2	1119.16113	2237.31499	-2.84
iQAAASPPAnATAASDTNAGDR	Unambiguous	2	1	E9QAQ5	N-Term(iTRAQ8plex); N10(Deamidated)	CID	1.0000	0.0000	1	1	2754753	Unique	Used	1.289	1.091	0.607	0.868	0	0	4.09	0	0	116	2	1188.08643	2375.16557	-3.60
sQGPALTASQSVHEQPTk	Unambiguous	9	1	B9EKN8	N-Term(iTRAQ8plex); K18(iTRAQ8plex)	CID	1.0000	0.0000	1	1	3045728	Unique	Used	0.843	0.701	1.353	0.776	0	0	4.09	0	0	200	3	825.45294	2474.34427	-0.69

sSGNSSSSLGEmVSGTFR	Unambiguous	4	1	Q8R0S4	N-Term(iTRAQ8plex); M12(Oxidation)	CID	1.0000	0.0000	1	1	3614152	Unique	Used	0.568	0.717	1.448	0.878	0	0	4.09	0	0	169	2	1055.50061	2109.99394	-4.04
gAGGAGTGPGGGGSGGVAk	Unambiguous	2	1	A2AQ87	N-Term(iTRAQ8plex); K19(iTRAQ8plex)	CID	1.0000	0.0000	1	1	3003785	Unique	Used	0.401	1.018	1.438	0.817	0	0	4.08	0	0	200	2	990.53870	1980.07012	-0.93
lQVSNVLSQPLAQAAVk	Unambiguous	2	1	Q9DBG6	N-Term(iTRAQ8plex); K17(iTRAQ8plex)	CID	1.0000	0.0000	1	1	3858813	Unique	Used	0.266	0.889	0.581	0.689	0	0	4.08	0	13	78	2	1187.71130	2374.41533	-5.28
aQSTGGSSDPVLHEk	Unambiguous	3	1	E9QP99	N-Term(iTRAQ8plex); K15(iTRAQ8plex)	CID	1.0000	0.0000	1	1	2646646	Unique	Used	1.763	0.724	1.572	0.835	0	0	4.08	0	16	200	3	707.71765	2121.13840	-0.59
dASEAPAGkPALQVPSSR	Unambiguous	2	1	Q6PEV3	N-Term(iTRAQ8plex); K9(iTRAQ8plex)	HCD	1.0000	0.0000	1	1	3206894	Unique	Used	1.162	0.723	1.382	0.954	0	0	4.08	0	0	23	3	797.11334	2389.32547	-1.74
nEEDATELVGLAQAVNAR	Unambiguous	1	1	Q02053	N-Term(iTRAQ8plex); N1(Deamidated)	CID	1.0000	0.0000	1	1	2742278	Unique	Used			1.217		0	0	4.08	0	0	200	2	1103.06714	2205.12700	-1.42
gEATAERPGEAAVASSPSk	Unambiguous	1	1	P26645	N-Term(iTRAQ8plex); K19(iTRAQ8plex)	CID	1.0000	0.0000	1	1	2728184	Unique	Used	0.550	0.402	0.428	0.687	0	0	4.07	0	0	200	2	1212.15100	2423.29473	-1.65
mDENEFVAVTSTNAAk	Unambiguous	3	1	O35098	N-Term(iTRAQ8plex); M1(Oxidation); K16(iTRAQ8plex)	CID	1.0000	0.0000	1	1	2898768	Unique	Used	0.459	0.511	0.589	0.504	0	0	4.06	0	0	200	2	1176.10010	2351.19292	-3.42
fDGGVVIAADmLGSYGSLAR	Unambiguous	1	1	P99026	N-Term(iTRAQ8plex); M11(Oxidation)	CID	1.0000	0.0000	1	1	3745340	Unique	Used	1.802	1.083	1.018	1.138	0	0	4.06	0	0	28	2	1160.09558	2319.18389	-5.11
qLEADImDINEIFk	Unambiguous	2	1	O70439	N-Term(iTRAQ8plex); M7(Oxidation); K14(iTRAQ8plex)	CID	1.0000	0.0000	1	1	3093838	Unique	Used	0.981	1.649	1.145	1.307	0	0	4.05	0	7	154	3	768.41840	2303.24063	-0.32
anATEFGLASGVFTR	Unambiguous	1	1	Q8R0Y6	N-Term(iTRAQ8plex); N2(Deamidated)	HCD	1.0000	0.0000	1	1	3393052	Unique	Used	1.295	1.215	1.186	1.354	0	0	4.05	0	30	112	2	923.48212	1845.95696	-4.30
tPEEEPLNLEGLVAHR	Unambiguous	1	1	E9Q0S6	N-Term(iTRAQ8plex)	CID	1.0000	0.0000	1	1	2901419	Unique	Used	1.582	1.596			0	0	4.05	0	13	96	3	703.37787	2108.11905	-4.72

vAVLGASGGIGQPLSLLLk	Unambiguous	1	1	P08249	N-Term(iTRAQ8plex); K19(iTRAQ8plex)	CID	1.0000	0.0000	1	1	3938009	Unique	Used	2.129	0.871	0.865	1.033	0	0	4.05	0	16	200	2	1201.25073	2401.49419	-2.55
vEGSSTASSGSQLAEGk	Unambiguous	1	1	Q6A0A9	N-Term(iTRAQ8plex); K17(iTRAQ8plex)	CID	1.0000	0.0000	1	1	2752433	Unique	Used	0.461	1.340	1.088	0.687	0	0	4.04	0	16	200	2	1102.08276	2203.15825	-3.65
nEEDATELVGLAQAVnAR	Unambiguous	1	1	Q02053	N-Term(iTRAQ8plex); N16(Deamidated)	CID	1.0000	0.0000	1	1	2740271	Unique	Used	1.435	1.270	1.484	1.606	0	0	4.03	0	6	200	2	1103.06836	2205.12944	-0.32
lYSGPGIVGNVLVDPSAR	Unambiguous	1	1	Q8BTZ7	N-Term(iTRAQ8plex)	CID	1.0000	0.0000	1	1	3805155	Unique	Used	0.508	0.732	0.789	0.995	0	0	4.02	0	6	26	2	1059.59473	2118.18218	-1.86
sIQGSSTSSSASSTLSHGEVk	Unambiguous	1	1	Q3UH60	N-Term(iTRAQ8plex); K21(iTRAQ8plex)	CID	1.0000	0.0000	1	1	3046060	Unique	Used	1.307	0.562	1.566	1.009	0	0	4.02	0	6	97	3	882.46344	2645.37577	-3.06
lIASVADDEAAVPNNk	Unambiguous	2	1	P16125	N-Term(iTRAQ8plex); K16(iTRAQ8plex)	CID	1.0000	0.0000	1	1	2884918	Unique	Used	0.853	0.744	1.351	0.850	0	0	4.02	0	5	200	2	1118.12231	2235.23735	-3.04
kYNTDcVqGLTHSk	Unambiguous	2	1	Q6PIC6	N-Term(iTRAQ8plex); K1(iTRAQ8plex); C6(Methylthio); Q8(Deamidated); K14(iTRAQ8plex)	CID	1.0000	0.0000	1	1	3066512	Unique	Used	0.575	0.673	1.152	1.121	0	0	4.01	1	0	200	3	851.78931	2553.35337	-1.36
tPIGSFLGSLASQPATk	Unambiguous	1	1	Q8QZT1	N-Term(iTRAQ8plex); K17(iTRAQ8plex)	CID	1.0000	0.0000	1	1	3898023	Unique	Used	0.756	0.848	0.817	0.826	0	0	4.01	0	27	10	2	1142.15564	2283.30400	-5.65
eEGVTFPSAGSqTVAAAAk	Unambiguous	2	1	O88811	N-Term(iTRAQ8plex); Q12(Deamidated); K19(iTRAQ8plex)	CID	1.0000	0.0000	1	1	3050554	Unique	Used	0.807	0.988	0.767	0.667	0	0	4.00	0	0	200	2	1215.65991	2430.31255	6.27
vFQPPPPPPAPSGDGATEk	Unambiguous	1	1	Q6R891	N-Term(iTRAQ8plex); K19(iTRAQ8plex)	CID	1.0000	0.0000	1	1	3088000	Unique	Used	0.513	1.198	1.252	1.056	0	0	4.00	0	26	26	3	833.12323	2497.35514	0.14

eAEAQAAMEANSEGSLTRPk	Unambiguous	1	1	P63040	N-Term(iTRAQ8plex); K20(iTRAQ8plex)	CID	1.0000	0.0000	1	1	2846408	Unique	Used	1.997	0.765	1.433	0.926	0	0	4.00	0	0	200	3	900.13232	2698.38242	-3.81
kGVAINMVTEEDkR	Unambiguous	1	1	P60843	N-Term(iTRAQ8plex); K1(iTRAQ8plex); K13(iTRAQ8plex)	HCD	1.0000	0.0000	1	1	3122721	Unique	Used	0.724	0.947	0.893	0.892	0	0	3.99	2	24	92	3	834.82196	2502.45133	1.43
rSEPFPAPPEDDAATVk	Unambiguous	1	1	Q8BG40	N-Term(iTRAQ8plex); K17(iTRAQ8plex)	CID	1.0000	0.0000	1	1	2714336	Unique	Used	1.738	1.047	1.208	1.172	0	0	3.99	1	0	200	3	812.44012	2435.30582	1.26
eAEAQAAMEANSEGSLTRPk	Unambiguous	1	1	P63040	N-Term(iTRAQ8plex); K20(iTRAQ8plex)	CID	1.0000	0.0000	1	1	2809397	Unique	Used	2.494	0.656	1.834	0.945	0	0	3.99	0	15	19	3	900.13141	2698.37967	-4.83
mSESLDTADPAVTGAk	Unambiguous	1	1	P63011	N-Term(iTRAQ8plex); M1(Oxidation); K16(iTRAQ8plex)	CID	1.0000	0.0000	1	1	2775536	Unique	Used	0.654	1.918	1.601	1.280	0	0	3.98	0	0	200	2	1109.07568	2217.14409	-4.00
aAAGELQEDSGLHVLAR	Unambiguous	1	1	Q8C845	N-Term(iTRAQ8plex)	CID	1.0000	0.0000	1	1	2991241	Unique	Used	2.002	0.873	1.355	0.987	0	0	3.97	0	0	33	3	681.03552	2041.09201	-2.95
vAVLGASGGIGQPLSLLLk	Unambiguous	1	1	P08249	N-Term(iTRAQ8plex); K19(iTRAQ8plex)	CID	1.0000	0.0000	1	1	3939636	Unique	Used	1.854	1.155	1.187	0.464	0	0	3.96	0	4	200	2	1201.24902	2401.49077	-3.98
mSESLDTADPAVTGAk	Unambiguous	1	1	P63011	N-Term(iTRAQ8plex); M1(Oxidation); K16(iTRAQ8plex)	CID	1.0000	0.0000	1	1	2775708	Unique	Used	0.347	1.006	1.192	0.901	0	0	3.96	0	0	200	2	1109.07739	2217.14751	-2.46
dAGEGLLAVQITDQEGkPQR	Unambiguous	1	1	Q80X90	N-Term(iTRAQ8plex); K17(iTRAQ8plex)	CID	1.0000	0.0000	1	1	2973670	Unique	Used	1.475	2.259	1.417	0.886	0	0	3.95	0	0	103	3	911.83765	2733.49839	-0.31
hSQFIGYPITLFVEk	Unambiguous	1	1	P07901	N-Term(iTRAQ8plex); K15(iTRAQ8plex)	CID	1.0000	0.0000	1	1	3744691	Unique	Used	0.698	0.784	1.002	1.083	0	0	3.95	0	0	27	3	796.45599	2387.35343	-2.07
eQGGQAPITPQQGQALAk	Unambiguous	1	1	P84096	N-Term(iTRAQ8plex); K17(iTRAQ8plex)	CID	1.0000	0.0000	1	1	3048065	Unique	Used	0.517	0.281	1.496	1.500	0	0	3.94	0	0	27	2	1187.16406	2373.32085	-5.83

eTSPETSLIQDEVALk	Unambiguous	1	1	P20357	N-Term(iTRAQ8plex); K16(iTRAQ8plex)	CID	1.0000	0.0000	1	1	2762174	Unique	Used	0.993	1.784	2.069	0.869	0	0	3.94	0	6	193	2	1184.65405	2368.30083	-2.52
sNLPATAGSEIPGGSTk	Unambiguous	1	1	Q9Z0U1	N-Term(iTRAQ8plex); K17(iTRAQ8plex)	HCD	1.0000	0.0000	1	1	3164558	Unique	Used	1.016	0.448	1.079	0.947	0	0	3.94	0	9	39	2	1098.10938	2195.21147	-0.62
eEPPSLRPAPPPISGGGYR	Unambiguous	1	1	Q8K0E8	N-Term(iTRAQ8plex)	HCD	1.0000	0.0000	1	1	3317719	Unique	Used	2.437	1.500	1.465	2.629	0	0	3.92	0	19	73	3	761.07574	2281.21268	-5.10
eQASHLGGAVFSGAGNIAAATGLVk	Unambiguous	1	1	Q91ZZ3	N-Term(iTRAQ8plex); K25(iTRAQ8plex)	CID	1.0000	0.0000	1	1	3807763	Unique	Used	1.696	2.215	1.534	0.937	0	0	3.90	0	20	200	3	978.87616	2934.61393	-4.06
lSTLGSGGESGGDGSPGGAGATAAR	Unambiguous	1	1	Q80UU9	N-Term(iTRAQ8plex)	CID	1.0000	0.0000	1	1	3028967	Unique	Used	0.646	1.362	0.761	1.045	0	0	3.89	0	0	75	2	1197.59021	2394.17314	-2.85
eADFQAVDNEVGLTk	Unambiguous	2	1	O54988-2	N-Term(iTRAQ8plex); K15(iTRAQ8plex)	CID	1.0000	0.0000	1	1	2702317	Unique	Used			3.076	1.754	0	0	3.89	0	0	200	2	1122.60144	2244.19560	-0.56
sSGLPITSAVDLEDAAk	Unambiguous	2	1	Q9Z2I8-2	N-Term(iTRAQ8plex); K17(iTRAQ8plex)	CID	1.0000	0.0000	1	1	2976159	Unique	Used	0.808	1.278	0.783	0.991	0	0	3.86	0	9	155	2	1141.63647	2282.26567	-1.91
iAPALISSGISVVEQEk	Unambiguous	4	1	P17183	N-Term(iTRAQ8plex); K17(iTRAQ8plex)	CID	1.0000	0.0000	1	1	3396146	Unique	Used	1.352	1.305	1.092	1.289	0	0	3.86	0	0	24	2	1175.19116	2349.37505	-4.24
iISGGEPQGALTSTMTHNEDLDR	Unambiguous	2	1	E9QAD4	N-Term(iTRAQ8plex)	CID	1.0000	0.0000	1	1	2992984	Unique	Used	1.571	1.192	1.010	0.977	0	0	3.83	0	12	151	3	916.12463	2746.35935	-0.97
vAPEEHPVLLTEAPLNPk	Unambiguous	6	1	P60710	N-Term(iTRAQ8plex); K18(iTRAQ8plex)	CID	0.0419	0.0000	1	1	3095619	Unique	Used	1.147	1.732	2.841	1.511	0	0	3.82	0	3	200	3	854.83258	2562.48319	3.11
lGAESPSIASTSSTEk	Unambiguous	4	1	Q68FH0	N-Term(iTRAQ8plex); K16(iTRAQ8plex)	CID	1.0000	0.0000	1	1	2932780	Unique	Used	1.552	0.611	1.332	0.915	0	0	3.81	0	0	200	2	1087.09131	2173.17534	-2.54
iGIIGGTGLDDPEILEGR	Unambiguous	1	1	Q9CQ65	N-Term(iTRAQ8plex)	HCD	1.0000	0.0000	1	1	3153327	Unique	Used	3.064	0.577	0.804	1.310	0	0	3.81	0	8	101	2	1065.09045	2129.17363	-0.94
vNHEPEPASGASPGATIPk	Unambiguous	3	1	O08539	N-Term(iTRAQ8plex); K19(iTRAQ8plex)	CID	1.0000	0.0000	1	1	2895817	Unique	Used	1.322	0.993	1.233	0.807	0	0	3.81	0	6	124	3	823.11548	2467.33188	-3.36

lDNLMLELDGTENk	Unambiguous	4	1	P17183	N-Term(iTRAQ8plex); K14(iTRAQ8plex)	CID	1.0000	0.0000	1	1	2942671	Unique	Used	2.039				0	0	3.80	0	26	200	3	738.40161	2213.19028	-1.87
dLGLSESGEDVNAAILDESGkk	Unambiguous	1	1	P08003	N-Term(iTRAQ8plex); K21(iTRAQ8plex); K22(iTRAQ8plex)	CID	1.0000	0.0000	1	1	2760979	Unique	Used	0.820	0.586	0.766	0.681	0	0	3.78	1	14	200	3	1053.91040	3159.71665	0.56
vLDLGSPQLAmHSIR	Unambiguous	1	1	Q9Z2W0	N-Term(iTRAQ8plex); M11(Oxidation)	CID	1.0000	0.0000	1	1	3819949	Unique	Used	2.226	0.892	0.662	1.217	0	0	3.76	0	25	113	3	653.03107	1957.07865	-2.89
sDTSDIPLLLSQVESk	Unambiguous	1	1	Q80VD1	N-Term(iTRAQ8plex); K16(iTRAQ8plex)	CID	1.0000	0.0000	1	1	3075278	Unique	Used	0.634	1.060	0.853	0.798	0	0	3.76	0	14	200	2	1170.65991	2340.31255	0.28
vVYEVHSGGTVVEnGVHk	Unambiguous	1	1	Q8BR92	N-Term(iTRAQ8plex); N14(Deamidated); K18(iTRAQ8plex)	CID	1.0000	0.0000	1	1	2932692	Unique	Used	1.100	0.909	1.147	0.916	0	0	3.76	0	9	80	3	840.45917	2519.36295	-3.38
vEAGDVIYIEANSGAVk	Unambiguous	1	1	P60122	N-Term(iTRAQ8plex); K17(iTRAQ8plex)	CID	1.0000	0.0000	1	1	2957734	Unique	Used	1.132	1.003	0.866	0.862	0	0	3.75	0	0	200	2	1172.15308	2343.29888	-1.19
lLDDNGNIAEELSILk	Unambiguous	1	1	Q9ERR7	N-Term(iTRAQ8plex); K16(iTRAQ8plex)	CID	1.0000	0.0000	1	1	3093803	Unique	Used	0.614	0.737	0.637	0.628	0	0	3.75	0	0	133	2	1183.17590	2365.34453	0.42
gTGGVDTAATGSVFDISnLDR	Unambiguous	1	1	P30275	N-Term(iTRAQ8plex); N18(Deamidated)	CID	1.0000	0.0000	1	1	2996463	Unique	Used	1.002	1.458	1.089	1.239	0	0	3.75	0	18	188	2	1179.58813	2358.16899	-1.59
lDNLMLELDGTENk	Unambiguous	4	1	P17183	N-Term(iTRAQ8plex); K14(iTRAQ8plex)	CID	1.0000	0.0000	1	1	2943579	Unique	Used	2.340	1.985	1.468	1.398	0	0	3.74	0	5	200	3	738.40283	2213.19394	-0.22
aPPAPPAAGEVTGGHPGER	Unambiguous	4	1	E9QP59	N-Term(iTRAQ8plex)	CID	1.0000	0.0000	1	1	2950660	Unique	Used	1.171	0.874	1.094	1.251	0	0	3.72	0	27	62	3	691.36401	2072.07749	-2.54
gEATAERPGEAAVASSPSk	Unambiguous	1	1	P26645	N-Term(iTRAQ8plex); K19(iTRAQ8plex)	CID	1.0000	0.0000	1	1	2750798	Unique	Used		0.402	0.769	0.587	0	0	3.72	0	0	200	2	1212.15027	2423.29326	-2.25

rSEALAGAPLDNAPk	Unambiguous	1	1	Q9DB15	N-Term(iTRAQ8plex); K15(iTRAQ8plex)	CID	1.0000	0.0000	1	1	3066307	Unique	Used	1.630	0.902	1.196	1.035	0	0	3.71	1	6	44	3	706.74054	2118.20707	-2.70
eDVQLVLLQADELHQGSk	Unambiguous	1	1	Q3UJU9	N-Term(iTRAQ8plex); K18(iTRAQ8plex)	CID	1.0000	0.0000	1	1	3014643	Unique	Used	0.629	1.020	0.980	1.173	0	0	3.70	0	3	20	3	877.49091	2630.45816	-1.09
hMVFLGGAVLADIMk	Unambiguous	1	1	P61161	N-Term(iTRAQ8plex); K15(iTRAQ8plex)	CID	1.0000	0.0000	1	1	3860305	Unique	Used	0.733	0.869	0.775	0.992	0	0	3.70	0	5	17	3	737.42407	2210.25766	-3.34
vGEPPPLGGVGGGGEGGGAGGGPGDR	Unambiguous	1	1	Q6NZL0	N-Term(iTRAQ8plex)	CID	1.0000	0.0000	1	1	2864665	Unique	Used	1.376	0.228	1.071	0.462	0	0	3.68	0	0	200	2	1211.10205	2421.19682	-3.86
nVPSAGGGIGDGGQEPTTGNWR	Unambiguous	3	1	Q8K1M6	N-Term(iTRAQ8plex)	CID	1.0000	0.0000	1	1	3049403	Unique	Used	0.223	1.525	1.033	1.144	0	0	3.67	0	0	25	2	1216.09656	2431.18584	-1.90
dHIIEVVGSSMPLIGDHQDEDk	Unambiguous	3	1	O88935	N-Term(iTRAQ8plex); K22(iTRAQ8plex)	CID	1.0000	0.0000	1	1	3034728	Unique	Used	0.896	1.771	1.586	1.408	0	0	3.67	0	0	200	3	1014.85895	3042.56229	-1.32
eRPPEEGAAAGLQGFGVDk	Unambiguous	2	1	Q9D6G9-2	N-Term(iTRAQ8plex); K19(iTRAQ8plex)	CID	1.0000	0.0000	1	1	2915787	Unique	Used	1.106	0.405	1.193	0.471	0	0	3.66	0	6	192	3	846.12500	2536.36045	-0.48
eDVQLVLLQADELHQGSk	Unambiguous	1	1	Q3UJU9	N-Term(iTRAQ8plex); K18(iTRAQ8plex)	CID	1.0000	0.0000	1	1	2995202	Unique	Used	0.895	1.172	0.990	1.149	0	0	3.64	0	5	124	3	877.49078	2630.45780	-1.23
gPSPSSPTPPPAAAPAEQAPR	Unambiguous	2	1	Q8K3I9	N-Term(iTRAQ8plex)	CID	1.0000	0.0000	1	1	3046754	Unique	Used	0.800	0.521	1.930	0.772	0	0	3.64	0	0	200	2	1144.10095	2287.19463	-1.69
IIAnDDMAELLLGESk	Unambiguous	1	1	E9Q6P5	N-Term(iTRAQ8plex); N4(Deamidated); K16(iTRAQ8plex)	CID	1.0000	0.0000	1	1	2922402	Unique	Used			1.235	0.798	0	0	3.63	0	21	200	2	1171.14148	2341.27568	-1.06
kEPQPSGPQPSGGAGGGGGk	Unambiguous	1	1	Q8JZQ2	N-Term(iTRAQ8plex); K1(iTRAQ8plex); K20(iTRAQ8plex)	CID	1.0000	0.0000	1	1	2927427	Unique	Used	0.413	1.023	1.011	1.134	0	0	3.62	1	0	200	3	888.15717	2662.45694	-4.02
vPSAGGGIGDGGQEPTTGNWR	Unambiguous	2	1	E9PUD2	N-Term(iTRAQ8plex)	CID	1.0000	0.0000	1	1	3087264	Unique	Used	0.695	0.287	0.517	0.676	0	0	3.62	0	0	122	2	1159.07642	2317.14556	-0.85
gSETLGGGGGGAAGTR	Unambiguous	1	1	P31650	N-Term(iTRAQ8plex)	CID	1.0000	0.0000	1	1	2822278	Unique	Used	1.778	0.492	0.714	1.377	0	0	3.62	0	6	200	2	804.91235	1608.81743	-4.32

eAEAQAAMEANSEGSLTRPk	Unambiguous	1	1	P63040	N-Term(iTRAQ8plex); K20(iTRAQ8plex)	CID	1.0000	0.0000	1	1	2809534	Unique	Used	2.644	1.370	1.721	1.133	0	0	3.62	0	12	89	3	900.13287	2698.38407	-3.20
gPAAAPGAASPPAEPk	Unambiguous	1	1	Q9QZM0	N-Term(iTRAQ8plex); K16(iTRAQ8plex)	CID	1.0000	0.0000	1	1	2895328	Unique	Used	1.477	2.226	0.967	0.781	0	0	3.61	0	0	58	2	999.06323	1997.11919	-4.25
nEEDATELVGLAQAVnAR	Unambiguous	1	1	Q02053	N-Term(iTRAQ8plex); N16(Deamidated)	CID	1.0000	0.0000	1	1	2741231	Unique	Used			0.790		0	0	3.59	0	20	200	2	1103.06677	2205.12627	-1.75
vSAADSTQVDGGTPmVk	Unambiguous	6	1	A2AUK8	N-Term(iTRAQ8plex); M15(Oxidation); K17(iTRAQ8plex)	CID	1.0000	0.0000	1	1	2896900	Unique	Used	1.089	1.629	0.827	0.651	0	0	3.59	0	0	200	2	1144.10120	2287.19512	-4.78
eGHEVVGVFTIPDkDGk	Unambiguous	1	1	Q8R0Y6	N-Term(iTRAQ8plex); K14(iTRAQ8plex); K17(iTRAQ8plex)	CID	1.0000	0.0000	1	1	3010183	Unique	Used	0.604	0.910	0.855	1.096	0	0	3.58	1	0	108	3	913.85217	2739.54197	-0.92
aDASSLTVDVTSPASk	Unambiguous	3	1	Q99JX3	N-Term(iTRAQ8plex); K16(iTRAQ8plex)	CID	1.0000	0.0000	1	1	3050426	Unique	Used	1.166	0.968	1.028	0.889	0	0	3.55	0	6	58	2	1079.09424	2157.18120	-2.20
eQASHLGGAVFSGAGNIAAATGLVk	Unambiguous	1	1	Q91ZZ3	N-Term(iTRAQ8plex); K25(iTRAQ8plex)	CID	1.0000	0.0000	1	1	3803331	Unique	Used	1.001	1.474	1.469	1.166	0	0	3.52	0	21	34	3	978.87408	2934.60770	-6.18
gTGGVDTAATGSVFDISnLDR	Unambiguous	1	1	P30275	N-Term(iTRAQ8plex); N18(Deamidated)	CID	1.0000	0.0000	1	1	2996208	Unique	Used	1.238	1.376	1.090	1.241	0	0	3.51	0	17	93	2	1179.58704	2358.16680	-2.52
eVIAVQGPPDGTVLVSIk	Unambiguous	4	1	Q8CHC4	N-Term(iTRAQ8plex); K18(iTRAQ8plex)	CID	1.0000	0.0000	1	1	3349069	Unique	Used	0.328	1.014	1.291	1.290	0	0	3.48	0	3	6	2	1215.72034	2430.43340	-3.89
vGLEAIELDDGAAVPk	Unambiguous	2	1	Q8BFT9	N-Term(iTRAQ8plex); K16(iTRAQ8plex)	CID	1.0000	0.0000	1	1	2886074	Unique	Used	0.490	1.229	0.879	0.714	0	0	3.47	0	13	200	2	1103.13025	2205.25322	-2.51
qGEQGPPGGPGNIATRPk	Unambiguous	2	1	Q5FW52-2	N-Term(iTRAQ8plex); K18(iTRAQ8plex)	CID	1.0000	0.0000	1	1	3225619	Unique	Used	1.039	2.020	4.082	0.883	0	0	3.47	0	0	200	3	790.44080	2369.30783	-2.87

iAPALISSGISVVEQEk	Unambiguous	4	1	P17183	N-Term(iTRAQ8plex); K17(iTRAQ8plex)	CID	1.0000	0.0000	1	1	3397427	Unique	Used	1.372	1.118	0.744	0.797	0	0	3.43	0	14	200	2	1175.19360	2349.37993	-2.16
vSAADSTQVDGGTPmVk	Unambiguous	6	1	A2AUK8	N-Term(iTRAQ8plex); M15(Oxidation); K17(iTRAQ8plex)	CID	1.0000	0.0000	1	1	2932289	Unique	Used	0.973	0.924	1.186	0.757	0	0	3.42	0	6	53	2	1144.10327	2287.19927	-2.97
vnHEPEPASGASPGATIPk	Unambiguous	3	1	O08539	N-Term(iTRAQ8plex); N2(Deamidated); K19(iTRAQ8plex)	CID	1.0000	0.0000	1	1	2774847	Unique	Used	1.922	2.011	2.435	1.116	0	0	3.42	0	0	200	2	1234.66272	2468.31816	-2.44
dHIIEVVGSSMPLIGDHQDEDk	Unambiguous	3	1	O88935	N-Term(iTRAQ8plex); K22(iTRAQ8plex)	CID	1.0000	0.0000	1	1	3073009	Unique	Used	1.742	2.218	1.872	1.393	0	0	3.42	0	25	200	3	1014.86133	3042.56943	1.02
gLLSLLNDFHSGk	Unambiguous	4	1	Q3UFK1	N-Term(iTRAQ8plex); K13(iTRAQ8plex)	CID	1.0000	0.0000	1	1	3805540	Unique	Used	1.009	0.758	1.606	1.257	0	0	3.42	0	0	39	3	670.39136	2009.15952	-2.24
fQDLGAAYEVLSDSEk	Unambiguous	1	1	Q99KV1	N-Term(iTRAQ8plex); K16(iTRAQ8plex)	CID	1.0000	0.0000	1	1	2998025	Unique	Used	0.805	0.607	0.599	0.674	0	0	3.39	0	7	51	2	1190.62634	2380.24541	-1.63
vVLGDSVDGGGADQLGGVAEVR	Unambiguous	1	1	P49070	N-Term(iTRAQ8plex)	CID	1.0000	0.0000	1	1	2938860	Unique	Used	1.666	1.328	1.366	0.951	0	0	3.38	0	12	109	2	1187.62585	2374.24443	-3.05
aSAELALGENNEVLk	Unambiguous	1	1	P05202	N-Term(iTRAQ8plex); K15(iTRAQ8plex)	CID	1.0000	0.0000	1	1	2849018	Unique	Used	1.474	1.014	0.788	1.254	0	0	3.36	0	8	45	2	1083.61023	2166.21318	-4.39
lDNVMLDAEGHIk	Unambiguous	2	1	P63318	N-Term(iTRAQ8plex); K13(iTRAQ8plex)	CID	1.0000	0.0000	1	1	3013314	Unique	Used	0.369	1.488	1.694	0.827	0	0	3.35	0	0	63	3	688.38556	2063.14212	0.26
fQLAVEALEQEk	Unambiguous	2	1	Q6P5H2	N-Term(iTRAQ8plex); K12(iTRAQ8plex)	CID	1.0000	0.0000	1	1	3093723	Unique	Used	1.475	1.474	0.553	0.959	0	0	3.33	0	0	200	3	671.72137	2013.14957	0.92
eVDGLLTSDPMGSPVSSk	Unambiguous	5	1	Q7TQH0-3	N-Term(iTRAQ8plex); K18(iTRAQ8plex)	CID	1.0000	0.0000	1	1	3034512	Unique	Used	1.990	1.117	1.481	1.255	0	0	3.32	0	15	200	2	1214.14465	2427.28203	-3.20

kTEAPAAAGPEAk	Unambiguous	1	1	Q91XV3	N-Term(iTRAQ8plex); K1(iTRAQ8plex); K13(iTRAQ8plex)	CID	1.0000	0.0000	1	1	2803707	Unique	Used	0.842	1.593	0.641	0.512	0	0	3.32	1	27	94	3	718.42621	2153.26407	-2.45
aHFGLPSVEAEDk	Unambiguous	1	1	P35585	N-Term(iTRAQ8plex); K13(iTRAQ8plex)	CID	1.0000	0.0000	1	1	2933772	Unique	Used	0.685	0.945	0.807	0.968	0	0	3.29	0	12	33	3	670.03644	2008.09476	-0.62
eVIAVQGPPDGTVLVSIk	Unambiguous	4	1	Q8CHC4	N-Term(iTRAQ8plex); K18(iTRAQ8plex)	CID	1.0000	0.0000	1	1	3348900	Unique	Used	1.367	1.407	0.974	1.099	0	0	3.22	0	0	60	2	1215.71985	2430.43242	-4.30
vVYEVHSGGTVVEnGVHk	Unambiguous	1	1	Q8BR92	N-Term(iTRAQ8plex); N14(Deamidated); K18(iTRAQ8plex)	CID	1.0000	0.0000	1	1	2932468	Unique	Used	1.126	0.715	0.787	0.800	0	0	3.22	0	0	134	3	840.45850	2519.36094	-4.18
nmLGLLGNVAEVk	Unambiguous	2	1	Q80ZJ6-2	N-Term(iTRAQ8plex); M2(Oxidation); K13(iTRAQ8plex)	CID	1.0000	0.0000	1	1	3662310	Unique	Used	2.151	1.598	1.051	1.091	0	0	3.21	0	22	46	3	661.38654	1982.14505	-5.78
vAEEHAPSIVFIDEIDAIGTk	Unambiguous	1	1	P62192	N-Term(iTRAQ8plex); K21(iTRAQ8plex)	CID	1.0000	0.0000	1	1	3017329	Unique	Used		0.833	0.747		0	0	3.21	0	10	34	3	954.86139	2862.56961	-0.48
IIASVADDEAAVPNNk	Unambiguous	2	1	P16125	N-Term(iTRAQ8plex); K16(iTRAQ8plex)	CID	1.0000	0.0000	1	1	2868060	Unique	Used	0.907	1.071	1.351	1.215	0	0	3.18	0	3	43	2	1118.12048	2235.23369	-4.68
nVQAEEMVEFSSGLk	Unambiguous	2	1	Q03265	N-Term(iTRAQ8plex); K15(iTRAQ8plex)	CID	1.0000	0.0000	1	1	3019968	Unique	Used	0.961	1.293	1.934	2.533	0	0	3.09	0	12	200	2	1138.60608	2276.20488	-0.19
vAVLGASGGIGQPLSLLLk	Unambiguous	1	1	P08249	N-Term(iTRAQ8plex); K19(iTRAQ8plex)	CID	1.0000	0.0000	1	1	3936379	Unique	Used	1.486	1.165	0.873	0.958	0	0	3.09	0	25	61	2	1201.24414	2401.48100	-8.04
fQDVPSAGGESVAGGGTVATALDk	Unambiguous	6	1	P51125-3	N-Term(iTRAQ8plex); K24(iTRAQ8plex)	CID	1.0000	0.0000	1	1	3090285	Unique	Used	3.384	1.358			0	0	3.08	0	0	148	2	1421.75562	2842.50395	-0.15

vGQAVDVVGQAGkPk	Unambiguous	2	1	Q8VDM4	N-Term(iTRAQ8plex); K13(iTRAQ8plex); K15(iTRAQ8plex)	CID	1.0000	0.0000	1	1	3364830	Unique	Used	0.598	0.936	0.925	0.971	0	0	3.02	0	29	80	3	789.14563	2365.42234	-4.54
vAVLGASGGIGQPLSLLLk	Unambiguous	1	1	P08249	N-Term(iTRAQ8plex); K19(iTRAQ8plex)	CID	1.0000	0.0000	1	1	3938217	Unique	Used	1.880	0.879	0.710	0.923	0	0	3.01	0	19	200	2	1201.24841	2401.48955	-4.49
IIEESPIDEVVVTNTVPHELQk	Unambiguous	2	1	Q9D0M1	N-Term(iTRAQ8plex); K22(iTRAQ8plex)	CID	1.0000	0.0000	1	1	3036119	Unique	Used	0.457	1.069	0.886	1.021	0	0	2.99	0	2	23	3	1033.24634	3097.72446	0.09
eTLGTGAFSEVVLAEEk	Unambiguous	2	1	Q8BW96	N-Term(iTRAQ8plex); K17(iTRAQ8plex)	CID	1.0000	0.0000	1	1	2886691	Unique	Used	1.361	1.476	1.139	0.718	0	0	2.94	0	0	200	2	1194.65662	2388.30596	-2.49
eQGQAPITPQQGQALAk	Unambiguous	1	1	P84096	N-Term(iTRAQ8plex); K17(iTRAQ8plex)	CID	1.0000	0.0000	1	1	3067343	Unique	Used	0.701	0.235	1.315	1.837	0	0	2.93	0	0	200	2	1187.17078	2373.33428	-0.17
vTVAGLAGkDPVQcSR	Unambiguous	5	1	Q99LX0	N-Term(iTRAQ8plex); K9(iTRAQ8plex); C14(Methylthio)	HCD	1.0000	0.0000	1	1	3721048	Unique	Used	0.936	1.169	1.088	1.208	0	0	2.86	1	5	15	3	752.41467	2255.22947	-7.37
aQSTDSLGTSSSLQSk	Unambiguous	6	1	O35551	N-Term(iTRAQ8plex); K16(iTRAQ8plex)	CID	1.0000	0.0000	1	1	3027535	Unique	Used	1.905	1.424	2.028	0.807	0	0	2.77	0	8	32	2	1103.09094	2205.17461	-3.31
tNTEVSEAMAEMSLGPk	Unambiguous	3	1	Q6P1H6-2	N-Term(iTRAQ8plex); K17(iTRAQ8plex)	CID	1.0000	0.0000	1	1	3014820	Unique	Used	1.351	1.158	1.918	2.572	0	0	2.55	0	0	145	2	1202.11829	2403.22930	-2.64
eEAAPPTPAPDDLAQLk	Unambiguous	2	1	Q80WJ7	N-Term(iTRAQ8plex); K17(iTRAQ8plex)	CID	1.0000	0.0000	1	1	2811916	Unique	Used	0.362	0.430	0.637	0.652	0	0	2.52	0	0	151	2	1186.14954	2371.29180	-2.02
qDGTHVVEAVDATHIGk	Unambiguous	1	1	Q8BH61	N-Term(iTRAQ8plex); K17(iTRAQ8plex)	CID	1.0000	0.0000	1	1	2880675	Unique	Used	1.486	2.028	1.232	1.528	0	0	2.49	0	24	200	3	795.76984	2385.29496	-1.40
mSINAEDVVVGDLVEVk	Unambiguous	1	1	Q8VDN2	N-Term(iTRAQ8plex); K17(iTRAQ8plex)	CID	1.0000	0.0000	1	1	3037571	Unique	Used	0.382	1.648	1.809	1.370	0	0	2.48	0	7	48	2	1213.17517	2425.34307	-1.59

dVLGTGAFSEVILAEDk	Unambiguous	1	1	Q91YS8	N-Term(iTRAQ8plex); K17(iTRAQ8plex)	CID	1.0000	0.0000	1	1	3017466	Unique	Used	1.297	1.686	0.871	0.900	0	0	2.38	0	19	39	2	1186.66113	2372.31499	-0.84
vAVLGASGGIGQPLSLLLk	Unambiguous	1	1	P08249	N-Term(iTRAQ8plex); K19(iTRAQ8plex)	CID	1.0000	0.0000	1	1	3939106	Unique	Used	1.319	1.209	0.571	0.597	0	0	2.34	0	24	200	2	1201.24768	2401.48808	-5.10
lPSIVVEPTEAGAVESGELR	Unambiguous	1	1	G3UW55	N-Term(iTRAQ8plex)	CID	1.0000	0.0000	1	1	2996610	Unique	Used	1.134	2.086	0.996	0.892	0	0	2.17	0	0	20	2	1179.14417	2357.28105	-2.37
sLLGVFEEDATAISNYMNQLYQAMHR	Unambiguous	1	1	Q8K3H0	N-Term(iTRAQ8plex)	CID	1.0000	0.0000	1	1	3861588	Unique	Used	1.252	1.243	1.127	1.084	1E-05	0	9.19	0	19	21	3	1102.54175	3305.61069	-3.94
sETEISTcGPALnQSTESSPSIATVcHSEEVR	Unambiguous	2	1	Q80U04-2	N-Term(iTRAQ8plex); C8(Methylthio); N13(Deamidated); C26(Methylthio)	CID	0.0011	0.0000	1	1	3109874	Unique	Used	1.918		0.976	0.530	1E-05	0	8.79	0	0	166	3	1249.57080	3746.69785	4.55
sGYQqAASEHGLVVIAPDTSPR	Unambiguous	8	1	Q9R0P3	N-Term(iTRAQ8plex); Q5(Deamidated)	CID	0.0138	0.0000	1	1	3276510	Unique	Used	0.641	0.463	0.773	0.649	1E-05	0	8.68	0	0	83	2	1294.66833	2588.32939	1.36
fQPALLDLSTVEDHnAQYFNFVELPAAALR	Unambiguous	2	1	Q8BW41-2	N-Term(iTRAQ8plex); N15(Deamidated)	CID	0.0279	0.0000	1	1	3706913	Unique	Used	1.240	0.816	0.586	1.223	1E-05	0	8.61	0	7	29	3	1232.30579	3694.90281	-0.86
eIAESSMFNLLEmDIEQVSQLSALVQAQLEYHk	Unambiguous	4	1	Q62420	N-Term(iTRAQ8plex); M13(Oxidation); K33(iTRAQ8plex)	CID	1.0000	0.0000	1	1	3646135	Unique	Used		2.255	2.642	1.091	1E-05	0	8.47	0	0	68	3	1473.42542	4418.26169	-2.36
gnDISSGTVLSDYVGSGPPSGTGLHR	Unambiguous	1	1	P70296	N-Term(iTRAQ8plex); N2(Deamidated)	CID	1.0000	0.0000	1	1	3279361	Unique	Used	1.965	1.307	1.113	1.033	1E-05	0	8.43	0	25	93	3	945.80438	2835.39859	-2.72
qLIYNYPEQLFGAAGVMAIEHADFAGVER	Unambiguous	1	1	P80314	N-Term(iTRAQ8plex)	CID	1.0000	0.0000	1	1	3623367	Unique	Used	0.957	0.765	0.882	1.392	1E-05	0	8.41	0	8	4	3	1171.92529	3513.76133	-4.73
eMQENDASMQNVVFDYLHATAFqGTPLAQAVEGPSENVR	Unambiguous	1	1	Q9CZ13	N-Term(iTRAQ8plex); Q23(Deamidated)	CID	0.0131	0.0000	1	1	3396167	Unique	Used			0.496		1E-05	0	8.40	0	0	17	3	1533.72900	4599.17246	0.29
vNAQAAAYEYMAAYIENAk	Unambiguous	1	1	O35864	N-Term(iTRAQ8plex); K19(iTRAQ8plex)	CID	1.0000	0.0000	1	1	3479591	Unique	Used	0.468	0.875	1.010	1.125	1E-05	0	8.31	0	0	20	2	1350.19763	2699.38799	-2.95

fEAPEALFQPHLINVEGVGVAELLFNTIQAADIDTR	Unambiguous	1	1	P61161	N-Term(iTRAQ8plex)	CID	1.0000	0.0000	1	1	3727675	Unique	Used		1.365	1.133	1.073	1E-05	0	8.30	0	0	4	3	1414.74231	4242.21238	-6.28
sLIDMYSEVLDVLSDYDASYNTQDHLPR	Unambiguous	5	1	P58281-2	N-Term(iTRAQ8plex)	CID	1.0000	0.0000	1	1	3419682	Unique	Used	2.220	1.887	1.239	1.616	1E-05	0	8.30	0	7	90	3	1188.56860	3563.69126	-6.78
vIcVGLNYADHcQEQNVR	Unambiguous	1	1	Q3TC72	N-Term(iTRAQ8plex); C3(Methylthio); C12(Methylthio)	CID	1.0000	0.0000	1	1	3665140	Unique	Used	1.689	1.157	1.022	1.355	1E-05	0	8.29	0	0	4	3	819.71552	2457.13199	-5.27
dMAIATGGAVFGEEGLNLNLEDVQAHDLGk	Unambiguous	1	1	P63038	N-Term(iTRAQ8plex); K30(iTRAQ8plex)	CID	1.0000	0.0000	1	1	3265589	Unique	Used	1.530	0.657	0.765	0.652	1E-05	0	8.28	0	0	200	3	1231.64124	3692.90915	1.08
tGDFQLHTNVNnGTEFGGSIYQk	Unambiguous	2	1	G3UX26	N-Term(iTRAQ8plex); N12(Deamidated); K23(iTRAQ8plex)	CID	1.0000	0.0000	1	1	3234194	Unique	Used	1.141	1.235	1.089	1.355	1E-05	0	8.27	0	13	89	3	1046.19556	3136.57212	-2.39
lIGQHLnGLGLNQTVDLLMQESGcR	Unambiguous	3	1	E0CYH4	N-Term(iTRAQ8plex); N7(Deamidated); C24(Methylthio)	CID	1.0000	0.0000	1	1	3824904	Unique	Used	0.954	1.134	1.425	1.447	1E-05	0	8.26	0	17	177	3	1020.85419	3060.54801	-3.37
dFTSEQIDEILQNHTEIVFAR	Unambiguous	2	1	Q6PIC6	N-Term(iTRAQ8plex)	CID	1.0000	0.0000	1	1	3357460	Unique	Used	0.608	0.401	0.768	1.346	1E-05	0	8.26	0	4	200	3	937.14606	2809.42362	-2.65
lYNPEnlYLSEHGGGAGNNWASGFSQGEk	Unambiguous	1	1	P83887	N-Term(iTRAQ8plex); N6(Deamidated); K29(iTRAQ8plex)	CID	1.0000	0.0000	1	1	3347290	Unique	Used			1.080	1.047	1E-05	0	8.23	0	0	30	3	1235.94214	3705.81186	2.37
iTQHFSTEEFEDEYVTDYTR	Unambiguous	2	1	Q9QYX7	N-Term(iTRAQ8plex)	CID	1.0000	0.0000	1	1	2813356	Unique	Used	0.914	0.882	1.815	1.175	1E-05	0	8.22	0	0	46	3	938.77289	2814.30411	-0.26
aENNPWVTPIADqFQLGVSHVFEYIR	Unambiguous	3	1	Q9D031	N-Term(iTRAQ8plex); Q13(Deamidated)	CID	0.0221	0.0000	1	1	3842683	Unique	Used	1.347	1.782	0.802	1.152	1E-05	0	8.16	0	21	75	3	1112.57544	3335.71177	3.43
eAVTPLPASSGHTEVLAATSLSPVPPR	Unambiguous	1	1	E9PV14	N-Term(iTRAQ8plex)	CID	1.0000	0.0000	1	1	3576851	Unique	Used	2.958	0.878	1.221	1.226	1E-05	0	8.13	0	4	51	3	996.87708	2988.61667	-4.75
gNDISSGTVLSDYVGSGPPSGTGLHR	Unambiguous	1	1	P70296	N-Term(iTRAQ8plex)	CID	1.0000	0.0000	1	1	3639485	Unique	Used	1.006	1.234	1.140	1.084	1E-05	0	8.12	0	0	30	3	945.47388	2834.40708	-5.37

tVFEALQAPAcHENMVk	Unambiguous	2	1	P17426-2	N-Term(iTRAQ8plex); C11(Methylthio); K17(iTRAQ8plex)	CID	1.0000	0.0000	1	1	3456485	Unique	Used	0.239	0.870	1.123	1.160	1E-05	0	8.11	0	14	32	3	848.10364	2542.29636	-4.46
gNDISSGTVLSDYVGSGPPSGTGLHR	Unambiguous	1	1	P70296	N-Term(iTRAQ8plex)	CID	1.0000	0.0000	1	1	3617788	Unique	Used	2.467	1.085	1.168	1.317	1E-05	0	8.10	0	0	53	3	945.47296	2834.40433	-6.34
sGYQQAASEHGLVVIAPDTSPR	Unambiguous	8	1	Q9R0P3	N-Term(iTRAQ8plex)	CID	1.0000	0.0000	1	1	3253736	Unique	Used	1.274	0.386	0.867	0.756	1E-05	0	8.09	0	0	74	2	1294.17554	2587.34380	0.75
nLHQSGFSLSGAqIDDNIPR	Unambiguous	1	1	O08553	N-Term(iTRAQ8plex); Q13(Deamidated)	CID	0.0297	0.0000	1	1	3367028	Unique	Used	2.297	1.286	1.432	1.151	1E-05	0	8.08	0	0	200	2	1237.63660	2474.26592	3.29
vEYPVMYSTDPEnGHMFNcIQR	Unambiguous	1	1	Q9CPU4	N-Term(iTRAQ8plex); N13(Deamidated); C19(Methylthio)	CID	1.0000	0.0000	1	1	3304239	Unique	Used	0.923	0.781	0.781	0.930	1E-05	0	8.08	0	4	45	3	994.44574	2981.32267	-0.59
vEYPVMYSTDPEnGHMFNcIQR	Unambiguous	1	1	Q9CPU4	N-Term(iTRAQ8plex); N13(Deamidated); C19(Methylthio)	CID	1.0000	0.0000	1	1	3282754	Unique	Used	1.071	0.813	1.071	1.109	1E-05	0	8.07	0	14	78	3	994.44635	2981.32450	0.02
qQLGTAVEMEIAQMLEENSR	Unambiguous	2	1	Q9JKK7	N-Term(iTRAQ8plex)	CID	1.0000	0.0000	1	1	3305735	Unique	Used	1.681	1.410	1.492	0.898	1E-05	0	8.07	0	0	16	2	1291.14636	2581.28545	-1.93
iVGLDQVTGMTETAFGSAYk	Unambiguous	3	1	Q5SV64	N-Term(iTRAQ8plex); K20(iTRAQ8plex)	CID	1.0000	0.0000	1	1	3601814	Unique	Used	0.304	0.988	1.129	0.976	1E-05	0	8.07	0	0	17	2	1348.71997	2696.43266	-3.69
qTIGNScGTIGLIHAVANNQDk	Unambiguous	1	1	Q9R0P9	N-Term(iTRAQ8plex); C7(Methylthio); K22(iTRAQ8plex)	CID	1.0000	0.0000	1	1	3703086	Unique	Used	0.816	0.717	1.537	1.554	1E-05	0	8.06	0	9	28	3	970.17194	2908.50125	-7.47
sNmHFTSSSTGGLSSSQSSYSPSSR	Unambiguous	2	1	Q80UY2	N-Term(iTRAQ8plex); M3(Oxidation)	CID	1.0000	0.0000	1	1	3754308	Unique	Used	0.703		1.170	0.788	1E-05	0	8.04	0	0	200	3	962.77057	2886.29715	-4.93
dFTSEQIDEILQNHTEIVFAR	Unambiguous	2	1	Q6PIC6	N-Term(iTRAQ8plex)	CID	1.0000	0.0000	1	1	3353582	Unique	Used	0.619	0.756	1.229	1.413	1E-05	0	8.04	0	2	10	3	937.14502	2809.42051	-3.76
qQLGTAVEMEIAQMLEENSR	Unambiguous	2	1	Q9JKK7	N-Term(iTRAQ8plex)	CID	1.0000	0.0000	1	1	3305724	Unique	Used	1.630	1.440	1.503	1.119	1E-05	0	8.03	0	0	137	2	1291.14526	2581.28325	-2.79

gTLSDEHAGVISVLAqQAAR	Unambiguous	2	1	Q9D1L9	N-Term(iTRAQ8plex); Q16(Deamidated)	CID	0.0137	0.0000	1	1	3576372	Unique	Used	2.340	0.927	1.538	0.868	1E-05	0	8.01	0	0	169	2	1164.63062	2328.25395	3.34
sINPDEAVAYGAAVQAAILSGDk	Unambiguous	2	1	P63017	N-Term(iTRAQ8plex); K23(iTRAQ8plex)	CID	1.0000	0.0000	1	1	3418834	Unique	Used	1.168	1.228	0.622	0.714	1E-05	0	8.00	0	0	26	2	1434.77441	2868.54155	-5.18
eAVTPLPASSGHTEVLAATSLSPVPPR	Unambiguous	1	1	E9PV14	N-Term(iTRAQ8plex)	CID	1.0000	0.0000	1	1	3597756	Unique	Used	2.767	0.627	0.851	0.767	1E-05	0	7.99	0	0	51	3	996.87781	2988.61887	-4.01
sMFPScEGTAQPLDNSMGDSDcLHk	Unambiguous	1	1	P47746	N-Term(iTRAQ8plex); C6(Methylthio); C22(Methylthio); K25(iTRAQ8plex)	CID	1.0000	0.0000	1	1	3260063	Unique	Used	0.626	0.771	1.420	1.239	1E-05	0	7.99	0	7	62	3	1124.16504	3370.48056	0.11
gTLSDEHAGVISVLAqQAAR	Unambiguous	2	1	Q9D1L9	N-Term(iTRAQ8plex); Q16(Deamidated)	CID	0.0213	0.0000	1	1	3576540	Unique	Used	2.709	1.030	1.272	0.894	1E-05	0	7.98	0	0	104	2	1164.63147	2328.25566	4.08
kADPQEAINcLNAAIDIYTDmGR	Unambiguous	1	1	P28663	K1(iTRAQ8plex); N-Term(iTRAQ8plex); C10(Methylthio); M21(Oxidation)	CID	1.0000	0.0000	1	1	3645169	Unique	Used	0.866	1.017	1.833	1.518	1E-05	0	7.97	1	4	7	3	1064.86426	3192.57822	-5.21
nILcFPIknENQEVIGVAELVNk	Unambiguous	3	1	Q5PR72	N-Term(iTRAQ8plex); C4(Methylthio); K8(iTRAQ8plex); N9(Deamidated); K23(iTRAQ8plex)	CID	0.0427	0.0000	1	1	3580852	Unique	Used	0.197	1.846	1.731	1.416	1E-05	0	7.97	1	25	18	3	1181.99609	3543.97373	1.54
aADFQLHTHVNDGTEFGGSIYQk	Unambiguous	3	1	J3QPE8	N-Term(iTRAQ8plex); K23(iTRAQ8plex)	CID	1.0000	0.0000	1	1	3255164	Unique	Used	0.518	0.823	1.183	1.143	1E-05	0	7.95	0	0	37	3	1048.53455	3143.58908	-3.70
icDPGLTSFEPEALGNLVEGMDFHR	Unambiguous	9	1	Q5SVJ0	N-Term(iTRAQ8plex); C2(Methylthio)	CID	1.0000	0.0000	1	1	3480012	Unique	Used		0.819	1.774	0.950	1E-05	0	7.94	0	0	0	3	1033.16028	3097.46628	-2.36

kYESAYGTQFTPcqLLLDHANNSSk	Unambiguous	1	1	Q8BMS1	N-Term(iTRAQ8plex); K1(iTRAQ8plex); C13(Methylthio); Q14(Deamidated); K25(iTRAQ8plex)	CID	1.0000	0.0000	1	1	3663784	Unique	Used	0.338	0.564	0.989	1.227	1E-05	0	7.94	1	0	67	3	1258.97693	3774.91623	-1.92
eAVTPLPASSGHTEVLAATSLSPVPPR	Unambiguous	1	1	E9PV14	N-Term(iTRAQ8plex)	CID	1.0000	0.0000	1	1	3577135	Unique	Used	2.637	0.973	2.200	2.194	1E-05	0	7.94	0	11	97	3	996.87769	2988.61850	-4.13
qQLGTAVEMEIAQMLEENSR	Unambiguous	2	1	Q9JKK7	N-Term(iTRAQ8plex)	CID	1.0000	0.0000	1	1	3284791	Unique	Used	1.230	1.358	1.284	0.971	1E-05	0	7.92	0	8	34	2	1291.14575	2581.28423	-2.41
wGEAGAEYVVESTGVFTTMEk	Unambiguous	3	1	P16858	N-Term(iTRAQ8plex); K21(iTRAQ8plex)	CID	1.0000	0.0000	1	1	3217212	Unique	Used		1.108	1.271	1.799	1E-05	0	7.92	0	0	20	2	1450.23108	2899.45488	-3.31
mGYAEEAPYDAIHVGAAAPVVPQALIDQLkPGGR	Unambiguous	5	1	P23506	N-Term(iTRAQ8plex); K30(iTRAQ8plex)	CID	1.0000	0.0000	1	1	3622757	Unique	Used	0.519	0.383	1.357	1.069	1E-05	0	7.91	0	0	15	3	1371.72961	4113.17429	-7.54
lSSVGGETSLAEMIATLSDAcER	Unambiguous	1	1	E9Q9B7	N-Term(iTRAQ8plex); C21(Methylthio)	CID	1.0000	0.0000	1	1	3624062	Unique	Used	0.554	0.703	0.606	0.866	1E-05	0	7.90	0	0	60	2	1345.64600	2690.28472	-5.30
lLGDASVSFTDncVVGIQANTER	Unambiguous	3	1	P97807-2	N-Term(iTRAQ8plex); N12(Deamidated); C13(Methylthio)	CID	0.0266	0.0000	1	1	3582921	Unique	Used	1.812	1.055	0.940	2.155	1E-05	0	7.90	0	0	200	2	1380.68091	2760.35454	2.12
vEYPVMYSTDPEnGHMFNcIQR	Unambiguous	1	1	Q9CPU4	N-Term(iTRAQ8plex); N13(Deamidated); C19(Methylthio)	CID	1.0000	0.0000	1	1	3304103	Unique	Used	0.810	0.748	0.814	0.735	1E-05	0	7.90	0	7	43	3	994.44324	2981.31516	-3.11
tGDFQLHTNVNnGTEFGGSIYQk	Unambiguous	2	1	G3UX26	N-Term(iTRAQ8plex); N12(Deamidated); K23(iTRAQ8plex)	CID	0.0330	0.0000	1	1	3233625	Unique	Used	0.726	1.053	1.323	1.477	1E-05	0	7.89	0	0	83	3	1046.19385	3136.56699	-4.03
aTLQEVVGLHSDmAVEAGAPYAER	Unambiguous	1	1	G3UYZ1	N-Term(iTRAQ8plex); M13(Oxidation)	CID	1.0000	0.0000	1	1	3300852	Unique	Used	0.914	0.113	1.146	0.163	1E-05	0	7.88	0	4	12	3	945.47937	2834.42356	-2.17

lVFFEVSPVSFHQVSEVLSEETSR	Unambiguous	3	1	D3YTU2	N-Term(iTRAQ8plex)	CID	1.0000	0.0000	1	1	3623432	Unique	Used	0.487	1.291	0.657	0.980	1E-05	0	7.88	0	26	18	3	1019.52911	3056.57279	-5.08
dYSLqIQNVDVTDDGPYTcSVQTQHTPR	Unambiguous	4	1	A0A4W9	N-Term(iTRAQ8plex); Q5(Deamidated); C19(Methylthio)	CID	0.0102	0.0000	1	1	3302061	Unique	Used	1.160	1.293	0.939	0.817	1E-05	0	7.87	0	0	124	3	1177.88513	3531.64084	2.78
gSQSSGSDSDSHFEQLMVNMLDER	Unambiguous	2	1	B8QI34	N-Term(iTRAQ8plex)	CID	1.0000	0.0000	1	1	2921661	Unique	Used	1.037	1.086	1.951	0.965	1E-05	0	7.87	0	17	191	3	987.44867	2960.33146	0.33
fQMPDQGMTSADDFFQGTk	Unambiguous	1	1	O08553	N-Term(iTRAQ8plex); K19(iTRAQ8plex)	CID	1.0000	0.0000	1	1	3283065	Unique	Used		1.410	0.801	0.900	1E-05	0	7.87	0	0	1	3	920.44458	2759.31919	-2.69
nTSNQAISqNTVFTIENNPGHR	Unambiguous	2	1	Q80XP9-2	N-Term(iTRAQ8plex); Q9(Deamidated)	CID	0.0140	0.0000	1	1	3431663	Unique	Used	1.044	1.024	0.955	0.995	1E-05	0	7.87	0	0	193	3	916.46265	2747.37339	3.02
fQMPDQGMTSADDFFQGTk	Unambiguous	1	1	O08553	N-Term(iTRAQ8plex); K19(iTRAQ8plex)	CID	1.0000	0.0000	1	1	3283424	Unique	Used	1.340	1.495	1.063	0.936	1E-05	0	7.86	0	5	17	3	920.44403	2759.31754	-3.28
aVQLTQALDTNTVGALLAEk	Unambiguous	2	1	Q3UPL0	N-Term(iTRAQ8plex); K20(iTRAQ8plex)	CID	1.0000	0.0000	1	1	3581065	Unique	Used	0.389	1.091	0.654	0.750	1E-05	0	7.86	0	0	21	2	1332.76770	2664.52812	-4.19
eLSSAVSPPnLHSDTPTFSYASLAGPTIPPR	Unambiguous	3	1	Q9QYR6	N-Term(iTRAQ8plex); N10(Deamidated)	CID	1.0000	0.0000	1	1	3684944	Unique	Used	5.402	1.473	2.091	0.923	1E-05	0	7.86	0	0	177	3	1172.26965	3514.79441	-1.83
vGFYVNTFQSIAGLEENFHk	Unambiguous	3	1	O08539	N-Term(iTRAQ8plex); K20(iTRAQ8plex)	CID	1.0000	0.0000	1	1	3686577	Unique	Used	3.794	1.545	1.367	1.021	1E-05	0	7.85	0	7	1	3	970.17993	2908.52524	-6.93
eATWESVSMMLQLGGTVIGSAR	Unambiguous	1	1	P47857	N-Term(iTRAQ8plex)	CID	1.0000	0.0000	1	1	3899120	Unique	Used	2.931	1.181	1.033	1.018	1E-05	0	7.84	0	26	36	2	1314.16992	2627.33257	-5.71
nIFLQYASTEVDGEHYMTPEDFVQR	Unambiguous	1	1	Q8BH59	N-Term(iTRAQ8plex)	CID	1.0000	0.0000	1	1	3262160	Unique	Used	0.743	1.452	1.230	1.235	1E-05	0	7.84	0	0	5	3	1098.52795	3293.56931	-1.04
qGLYELSANnFELHVSQGNHFIk	Unambiguous	2	1	E9PXX7	N-Term(iTRAQ8plex); N10(Deamidated); K23(iTRAQ8plex)	CID	0.0026	0.0000	1	1	3577579	Unique	Used	1.319	1.106	0.814	0.693	1E-05	0	7.83	0	21	200	3	1085.57471	3254.70957	1.25
sINPDEAVAYGAAVQAAILSGDk	Unambiguous	2	1	P63017	N-Term(iTRAQ8plex); K23(iTRAQ8plex)	CID	1.0000	0.0000	1	1	3459282	Unique	Used	0.760	0.893	0.701	0.795	1E-05	0	7.83	0	10	47	2	1434.77612	2868.54497	-3.99

tLALLAFDSPEESPFGDLLHMmQR	Unambiguous	1	1	Q9D7M1	N-Term(iTRAQ8plex); M22(Oxidation)	CID	0.0332	0.0000	1	1	3745851	Unique	Used	2.405	1.325	0.943	1.162	1E-05	0	7.83	0	7	60	3	1013.51001	3038.51548	-3.79
vEYPVMYSTDPENGHMFncIQR	Unambiguous	1	1	Q9CPU4	N-Term(iTRAQ8plex); N18(Deamidated); C19(Methylthio)	CID	0.0179	0.0000	1	1	3282747	Unique	Used	1.652	1.236	1.044	1.075	1E-05	0	7.83	0	0	200	3	994.44531	2981.32138	-1.02
sLSTYSAAALQSDLEDSLYk	Unambiguous	3	1	B1AQX6	N-Term(iTRAQ8plex); K20(iTRAQ8plex)	CID	1.0000	0.0000	1	1	3262192	Unique	Used	0.480	0.730	1.036	0.736	1E-05	0	7.82	0	10	8	2	1385.73401	2770.46074	0.01
qTTVSNSQQAYqEAFEISk	Unambiguous	3	1	Q9CQV8-2	N-Term(iTRAQ8plex); Q12(Deamidated); K19(iTRAQ8plex)	CID	0.0013	0.0000	1	1	3107119	Unique	Used	0.526	0.869	1.002	1.029	1E-05	0	7.82	0	0	200	2	1384.72107	2768.43486	5.40
cVEELPEWNFDGSSTFQSEGSnSDMYLHPVAMFR	Unambiguous	3	1	P15105	N-Term(iTRAQ8plex); C1(Methylthio); N22(Deamidated)	CID	0.0115	0.0000	1	1	3418847	Unique	Used	1.126	1.118	1.171	2.341	1E-05	0	7.82	0	0	22	3	1420.96289	4260.87412	3.26
aVANETGAFFFLINGPEIMSk	Unambiguous	1	1	Q01853	N-Term(iTRAQ8plex); K21(iTRAQ8plex)	CID	1.0000	0.0000	1	1	3686926	Unique	Used	0.453	0.733	0.788	0.783	1E-05	0	7.81	0	0	37	2	1432.76514	2864.52300	-8.65
nYPEGHqAAAVLPVLDLAQR	Unambiguous	1	1	Q9D6J6	N-Term(iTRAQ8plex); Q7(Deamidated)	CID	1.0000	0.0000	1	1	3537329	Unique	Used	2.068	0.823	1.352	1.109	1E-05	0	7.81	0	0	87	2	1234.16919	2467.33110	2.58
qSIAIDDcTFHQcVR	Unambiguous	2	1	Q3TWV4	N-Term(iTRAQ8plex); C8(Methylthio); C13(Methylthio)	CID	1.0000	0.0000	1	1	3537116	Unique	Used	0.218	0.799	1.455	1.399	1E-05	0	7.81	0	0	1	3	711.32513	2131.96085	-4.26
lLGDASVSFTDNcVVGIQANTER	Unambiguous	3	1	P97807-2	N-Term(iTRAQ8plex); C13(Methylthio)	CID	1.0000	0.0000	1	1	3582304	Unique	Used	2.061	1.015	1.028	1.376	1E-05	0	7.80	0	12	200	2	1380.17712	2759.34697	-6.41
gTLSDEHAGVISVLAqQAAR	Unambiguous	2	1	Q9D1L9	N-Term(iTRAQ8plex); Q16(Deamidated)	CID	0.0103	0.0000	1	1	3597228	Unique	Used			1.181		1E-05	0	7.80	0	24	200	2	1164.63037	2328.25347	3.13

aAFTTPDHAPLSPQSSVASSGSEQTEEqGSSR	Unambiguous	2	1	G5E8A7	N-Term(iTRAQ8plex); Q28(Deamidated)	CID	0.0013	0.0000	1	1	2933450	Unique	Used	1.248	0.633	0.852	1.256	1E-05	0	7.80	0	0	107	3	1184.56433	3551.67844	3.04
gAGQTVTIIAqYQPEDYAR	Unambiguous	9	1	Q91XM9-7	N-Term(iTRAQ8plex); Q11(Deamidated)	CID	0.0103	0.0000	1	1	3301453	Unique	Used	0.826	1.783	2.362	1.268	1E-05	0	7.80	0	27	200	2	1193.62097	2386.23467	6.46
dFTSEQIDEILQNHTEIVFAR	Unambiguous	2	1	Q6PIC6	N-Term(iTRAQ8plex)	CID	1.0000	0.0000	1	1	3374771	Unique	Used	1.113	0.959	1.076	1.133	1E-05	0	7.79	0	24	200	3	937.14471	2809.41959	-4.09
eAVTPLPASSGHTEVLAATSLSPVPPR	Unambiguous	1	1	E9PV14	N-Term(iTRAQ8plex)	CID	1.0000	0.0000	1	1	3576986	Unique	Used	3.004	0.822	1.146	0.975	1E-05	0	7.79	0	10	34	3	996.87628	2988.61429	-5.54
aLQSGQcAGAALDVFTEEPPR	Unambiguous	1	1	Q61753	N-Term(iTRAQ8plex); C7(Methylthio)	CID	1.0000	0.0000	1	1	3351617	Unique	Used	0.561	0.469	0.885	1.399	1E-05	0	7.79	0	0	8	2	1255.61389	2510.22051	-4.66
tGDFQLHTNVNnGTEFGGSIYQk	Unambiguous	2	1	G3UX26	N-Term(iTRAQ8plex); N12(Deamidated); K23(iTRAQ8plex)	CID	1.0000	0.0000	1	1	3233785	Unique	Used	0.670	1.148	1.106	1.512	1E-05	0	7.78	0	0	23	3	1046.19531	3136.57138	-2.63
dFTSEQIDEILQNHTEIVFAR	Unambiguous	2	1	Q6PIC6	N-Term(iTRAQ8plex)	CID	1.0000	0.0000	1	1	3395703	Unique	Used	2.205	1.163	1.447	1.185	1E-05	0	7.77	0	14	20	3	937.14215	2809.41190	-6.82
aAAQLLQSQAqQSGAQQTk	Unambiguous	3	1	Q8C1B7-3	N-Term(iTRAQ8plex); Q11(Deamidated); K19(iTRAQ8plex)	CID	0.0026	0.0000	1	1	3658060	Unique	Used	0.671	1.559	1.258	1.050	1E-05	0	7.77	0	0	200	2	1283.70789	2566.40849	1.53
lTPPVQEEMGHcDSVYLSEVGDTQVVVFk	Unambiguous	3	1	P42932	N-Term(iTRAQ8plex); C12(Methylthio); K29(iTRAQ8plex)	CID	1.0000	0.0000	1	1	3305059	Unique	Used	0.548	0.647	0.834	0.628	1E-05	0	7.76	0	0	41	3	1287.64661	3860.92527	-3.04
nISANEFLEFGSYQGNmFGTk	Unambiguous	3	1	B7ZCL8	N-Term(iTRAQ8plex); M17(Oxidation); K21(iTRAQ8plex)	CID	1.0000	0.0000	1	1	3560115	Unique	Used	1.207	1.324	0.937	1.012	1E-05	0	7.76	0	0	108	3	993.49371	2978.46659	-5.01
fQMPDQGMTSADDFFQGTk	Unambiguous	1	1	O08553	N-Term(iTRAQ8plex); K19(iTRAQ8plex)	CID	1.0000	0.0000	1	1	3304448	Unique	Used	1.773	1.426	1.125	1.267	1E-05	0	7.76	0	11	23	3	920.44519	2759.32102	-2.02

dMAIATGGAVFGEEGLNLNLEDVQAHDLGk	Unambiguous	1	1	P63038	N-Term(iTRAQ8plex); K30(iTRAQ8plex)	CID	1.0000	0.0000	1	1	3265125	Unique	Used	1.283	0.971	0.995	0.929	1E-05	0	7.75	0	0	200	3	1231.63831	3692.90036	-1.30
vETLSQVEVILQQSAADIAR	Unambiguous	2	1	P02469	N-Term(iTRAQ8plex)	CID	1.0000	0.0000	1	1	3645667	Unique	Used	1.566	0.548	0.451	0.383	1E-05	0	7.74	0	20	21	2	1237.68555	2474.36382	-5.27
sINPDEAVAYGAAVQAAILIGDk	Unambiguous	1	1	P17156	N-Term(iTRAQ8plex); K23(iTRAQ8plex)	CID	1.0000	0.0000	1	1	3440120	Unique	Used	0.787	4.672	1.230	1.037	1E-05	0	7.74	0	0	38	2	1447.80017	2894.59307	-5.31
aEcFETLSAmELAEQITLLDHIVFR	Unambiguous	3	1	P70392	N-Term(iTRAQ8plex); C3(Methylthio); M10(Oxidation)	CID	1.0000	0.0000	1	1	3727662	Unique	Used	0.417	1.630	0.841	1.221	1E-05	0	7.73	0	0	80	3	1082.53711	3245.59677	-7.13
lqLVGSGLHEAEAAGEAQQAVLEGLQGLLSR	Unambiguous	1	1	Q99K41	N-Term(iTRAQ8plex); Q2(Deamidated)	CID	1.0000	0.0000	1	1	3766533	Unique	Used	1.400	3.125	0.863	0.965	1E-05	0	7.73	0	0	30	3	1150.62622	3449.86411	2.84
vLGAHILGPGAGEMVnEAALALEYGAScEDIAR	Unambiguous	1	1	O08749	N-Term(iTRAQ8plex); N16(Deamidated); C28(Methylthio)	CID	1.0000	0.0000	1	1	3520665	Unique	Used	2.102	1.301	1.669	1.053	1E-05	0	7.73	0	10	97	3	1216.94006	3648.80564	1.13
sYIEGYVPSQADVAVFEAVSGPPPADLcHALR	Unambiguous	3	1	O70251	N-Term(iTRAQ8plex); C28(Methylthio)	CID	1.0000	0.0000	1	1	3623276	Unique	Used	1.586	0.680	1.100	0.538	1E-05	0	7.73	0	3	1	3	1236.94348	3708.81589	-5.01
dnVAGVTLPVFEHYHEGTDSYELTGLAR	Unambiguous	1	1	P57746	N-Term(iTRAQ8plex); N2(Deamidated)	CID	1.0000	0.0000	1	1	3324517	Unique	Used	0.845	1.692	1.163	1.077	1E-05	0	7.72	0	10	80	3	1132.56458	3395.67917	2.76
vSqIkPVSQPIGAEQQATLQNPDFVR	Unambiguous	2	1	Q80XP9-2	N-Term(iTRAQ8plex); Q3(Deamidated); K5(iTRAQ8plex)	CID	0.0117	0.0000	1	1	3535717	Unique	Used	1.051	0.946	1.131	1.120	1E-05	0	7.72	0	0	136	3	1153.97424	3459.90818	0.72
dMAIATGGAVFGEEGLNLNLEDVQAHDLGk	Unambiguous	1	1	P63038	N-Term(iTRAQ8plex); K30(iTRAQ8plex)	CID	1.0000	0.0000	1	1	3262220	Unique	Used	1.052	1.081	0.873	0.963	1E-05	0	7.71	0	2	3	3	1231.63916	3692.90293	-0.60
sVLPISQTQAAQEGALLYk	Unambiguous	2	1	Q3UV95	N-Term(iTRAQ8plex); K19(iTRAQ8plex)	CID	1.0000	0.0000	1	1	3497541	Unique	Used	0.860	2.140	0.469	1.430	1E-05	0	7.71	0	0	91	2	1313.25183	2625.49639	-4.13

IGSQATGVQGQAGqLLDTTESTLGR	Unambiguous	1	1	Q61001	N-Term(iTRAQ8plex); Q14(Deamidated)	CID	0.0078	0.0000	1	1	3457735	Unique	Used	1.295	2.804	0.911	1.449	1E-05	0	7.71	0	28	200	2	1397.23022	2793.45317	-0.04
eMEENFALEAANYQDTIGR	Unambiguous	1	1	P20152	N-Term(iTRAQ8plex)	CID	1.0000	0.0000	1	1	2833537	Unique	Used	0.766	2.566	0.774	1.293	1E-05	0	7.71	0	0	200	2	1253.09705	2505.18682	-0.07
sGGYTVLQVVEALGSSLENAEPR	Unambiguous	5	1	Q9D071-3	N-Term(iTRAQ8plex)	CID	1.0000	0.0000	1	1	3645875	Unique	Used	0.459	0.908	0.729	0.855	1E-05	0	7.70	0	8	23	2	1340.70105	2680.39482	-5.52
aSLDSAGASGNSPVLLPTPAVGGPR	Unambiguous	3	1	Q8C6B2-2	N-Term(iTRAQ8plex)	CID	1.0000	0.0000	1	1	3683203	Unique	Used	0.908	0.399	0.913	0.975	1E-05	0	7.70	0	0	128	2	1298.20288	2595.39849	-2.31
iGVGAMTWSPLAcGIVSGk	Unambiguous	3	1	P62482	N-Term(iTRAQ8plex); C13(Methylthio); K19(iTRAQ8plex)	CID	1.0000	0.0000	1	1	3936003	Unique	Used	0.471	0.518	0.941	1.286	1E-05	0	7.70	0	0	13	2	1251.17456	2501.34184	-4.84
sLEMLELGLSEAQVMmALSNHLNAVESEk	Unambiguous	5	1	Q5UE59	N-Term(iTRAQ8plex); M16(Oxidation); K29(iTRAQ8plex)	CID	0.0312	0.0000	1	1	3500650	Unique	Used	0.762	0.643	0.879	0.851	1E-05	0	7.69	0	18	58	3	1266.65503	3797.95053	-2.10
tGASFQQAQEEFSQGIFSSR	Unambiguous	1	1	Q9ERN0	N-Term(iTRAQ8plex)	CID	1.0000	0.0000	1	1	3518889	Unique	Used	0.571	0.726	0.628	0.766	1E-05	0	7.68	0	0	60	2	1255.11230	2509.21733	-3.51
eAVPLEDLVLPEPVGFLLVLLGPEAPHVDYTqLGR	Unambiguous	2	1	P04919-2	N-Term(iTRAQ8plex); Q32(Deamidated)	CID	1.0000	0.0000	1	1	3727861	Unique	Used		2.601		1.156	1E-05	0	7.68	0	30	186	3	1367.75220	4101.24204	-1.14
eGIcAIGGTSEQSSVGTQHSYSEEEk	Unambiguous	1	1	Q61233	N-Term(iTRAQ8plex); C4(Methylthio); K26(iTRAQ8plex)	CID	1.0000	0.0000	1	1	2757916	Unique	Used	1.843	0.583	0.797	0.829	1E-05	0	7.68	0	0	89	3	1123.19885	3367.58200	-1.75
aDTNSGDWNTEScQTLETQAAHTR	Unambiguous	7	1	H7BX92	N-Term(iTRAQ8plex); C13(Methylthio)	CID	1.0000	0.0000	1	1	2883890	Unique	Used	2.155	1.083	0.866	0.884	1E-05	0	7.68	0	0	200	3	996.11041	2986.31668	-1.43
tGDFQLHTNVNnGTEFGGSIYQk	Unambiguous	2	1	G3UX26	N-Term(iTRAQ8plex); N12(Deamidated); K23(iTRAQ8plex)	CID	0.0299	0.0000	1	1	3233799	Unique	Used	0.819	1.167	1.250	1.232	1E-05	0	7.68	0	0	42	3	1046.19434	3136.56845	-3.56

gPAVGIDLGTTYScVGVFQHGk	Unambiguous	4	1	P63017	N-Term(iTRAQ8plex); C14(Methylthio); K22(iTRAQ8plex)	CID	1.0000	0.0000	1	1	3805532	Unique	Used	1.541	1.369	0.985	0.896	1E-05	0	7.67	0	24	16	3	954.17041	2860.49668	0.71
ILLEAMDELEVAFNNTNVR	Unambiguous	2	1	Q5SWU9	N-Term(iTRAQ8plex)	CID	1.0000	0.0000	1	1	3521046	Unique	Used	0.567	0.679	0.673	1.003	1E-05	0	7.67	0	0	200	2	1248.15771	2495.30815	-1.47
sQMEFSISSLSIqEPSAATVTSDAR	Unambiguous	1	1	Q8C3W1	N-Term(iTRAQ8plex); Q13(Deamidated)	CID	1.0000	0.0000	1	1	3350521	Unique	Used	1.045	0.616	0.841	0.832	1E-05	0	7.67	0	0	35	2	1474.23352	2947.45976	3.01
IGVEPSHDDcVSVQHVcTIVSFR	Unambiguous	5	1	G3UVV4	N-Term(iTRAQ8plex); C10(Methylthio); C17(Methylthio)	CID	1.0000	0.0000	1	1	3726298	Unique	Used	0.612	0.960	1.335	1.403	1E-05	0	7.67	0	19	23	3	975.12805	2923.36960	-6.20
sIDDLEDQLYHQLEqNR	Unambiguous	3	1	E9Q452	N-Term(iTRAQ8plex); Q15(Deamidated)	CID	0.0078	0.0000	1	1	2765862	Unique	Used	2.202	1.234	1.574	0.818	1E-05	0	7.67	0	0	43	3	807.73230	2421.18235	-0.52
ecDEELQDTMLLLSQMGPDAHMR	Unambiguous	3	1	Q9EQZ6-3	N-Term(iTRAQ8plex); C2(Methylthio)	CID	1.0000	0.0000	1	1	3217653	Unique	Used	1.990	1.661	0.836	0.880	1E-05	0	7.66	0	24	134	3	1004.79169	3012.36051	1.89
yTPSGQSGAAASESLFISNHAy	Unambiguous	2	1	A6ZI44	N-Term(iTRAQ8plex); Y22(iTRAQ8plex)	CID	1.0000	0.0000	1	1	3411404	Unique	Used	0.351	1.233	0.835	0.985	1E-05	0	7.66	0	5	5	3	956.14941	2866.43369	-4.57
hTGPGILSMANAGPnTNGSQFFIcTAk	Unambiguous	1	1	P17742	N-Term(iTRAQ8plex); N15(Deamidated); C24(Methylthio); K27(iTRAQ8plex)	CID	0.0300	0.0000	1	1	3805158	Unique	Used	2.479	0.778	1.287	1.012	1E-05	0	7.66	0	15	77	3	1130.56396	3389.67734	-3.78
fQmPDQGMTSADDFFQGTk	Unambiguous	1	1	O08553	N-Term(iTRAQ8plex); M3(Oxidation); K19(iTRAQ8plex)	CID	0.0418	0.0000	1	1	3303414	Unique	Used	2.209	1.837	1.117	1.041	1E-05	0	7.66	0	3	32	3	925.77710	2775.31675	-1.72
dSEIFYQFTSFLSPGVIYHcDLTk	Unambiguous	1	1	Q9QUR6	N-Term(iTRAQ8plex); C20(Methylthio); K24(iTRAQ8plex)	CID	1.0000	0.0000	1	1	3766405	Unique	Used	0.767	0.483	0.768	0.618	1E-05	0	7.65	0	20	39	3	1155.57397	3464.70737	-8.47

fQmPDQGMTSADDFFQGTk	Unambiguous	1	1	O08553	N-Term(iTRAQ8plex); M3(Oxidation); K19(iTRAQ8plex)	CID	1.0000	0.0000	1	1	3307219	Unique	Used	1.331	0.923	0.857	0.688	1E-05	0	7.65	0	9	200	3	925.77667	2775.31546	-2.18
eSTFLDEGPNEQEITPLQHTPR	Unambiguous	3	1	Q9QYR6	N-Term(iTRAQ8plex)	CID	1.0000	0.0000	1	1	2778779	Unique	Used	3.222	2.185	2.272	1.370	1E-05	0	7.65	0	15	200	3	948.13995	2842.40531	-3.81
rLPASPVISNmPAQVDQGVATEDR	Unambiguous	2	1	A2A5V4	N-Term(iTRAQ8plex); M11(Oxidation)	CID	1.0000	0.0000	1	1	3386956	Unique	Used	1.123	1.491	1.442	1.541	1E-05	0	7.65	1	0	67	3	957.83307	2871.48465	-3.16
dMAIATGGAVFGEEGLNLNLEDVQAHDLGk	Unambiguous	1	1	P63038	N-Term(iTRAQ8plex); K30(iTRAQ8plex)	CID	1.0000	0.0000	1	1	3263421	Unique	Used	0.778	0.856	1.135	1.216	1E-05	0	7.65	0	0	140	3	1231.63879	3692.90183	-0.90
wGEAGAEYVVESTGVFTTMEk	Unambiguous	3	1	P16858	N-Term(iTRAQ8plex); K21(iTRAQ8plex)	CID	1.0000	0.0000	1	1	3218047	Unique	Used	0.515	1.117	1.395	1.171	1E-05	0	7.65	0	0	200	2	1450.23157	2899.45586	-2.97
kSQSVDITAPGFNPLGGAGk	Unambiguous	2	1	Q4KMM3	N-Term(iTRAQ8plex); K1(iTRAQ8plex); K20(iTRAQ8plex)	CID	1.0000	0.0000	1	1	3575258	Unique	Used	0.776	0.940	2.096	1.706	1E-05	0	7.64	1	9	61	3	952.87769	2856.61850	-5.67
aAAQLLQSQAQQSGAQQTk	Unambiguous	3	1	Q8C1B7-3	N-Term(iTRAQ8plex); K19(iTRAQ8plex)	CID	1.0000	0.0000	1	1	3679077	Unique	Used	0.587	0.980	1.184	1.096	1E-05	0	7.64	0	0	90	2	1283.20508	2565.40288	-6.88
qEEPVNIPQLQQQVETLYHSLLDR	Unambiguous	1	1	P08032	N-Term(iTRAQ8plex)	CID	1.0000	0.0000	1	1	3645470	Unique	Used	3.061		0.200	0.180	1E-05	0	7.64	0	8	13	3	1061.22461	3181.65927	-6.38
iIGLDQVAGMSETALPGAFk	Unambiguous	1	1	Q8VDD5	N-Term(iTRAQ8plex); K20(iTRAQ8plex)	CID	1.0000	0.0000	1	1	3645039	Unique	Used	0.387	1.075	0.683	0.914	1E-05	0	7.63	0	7	15	2	1313.73254	2626.45781	-5.99
gNDISSGTVLSDYVGSGPPSGTGLHR	Unambiguous	1	1	P70296	N-Term(iTRAQ8plex)	CID	1.0000	0.0000	1	1	3434604	Unique	Used	2.448	1.605	1.545	1.162	1E-05	0	7.63	0	6	53	3	945.47437	2834.40854	-4.85
sITIAnQTNcPLYVTk	Unambiguous	1	1	O08553	N-Term(iTRAQ8plex); N6(Deamidated); C10(Methylthio); K16(iTRAQ8plex)	CID	0.0079	0.0000	1	1	3599943	Unique	Used	1.004	1.332	1.066	1.371	1E-05	0	7.63	0	0	7	2	1211.14490	2421.28252	-6.31

vDEFPLcGHMVSDEYEqLSSEALEAAR	Unambiguous	4	1	I7HLV2	N-Term(iTRAQ8plex); C7(Methylthio); Q17(Deamidated)	CID	1.0000	0.0000	1	1	3112276	Unique	Used	0.749	0.557	0.700	0.994	1E-05	0	7.62	0	0	200	3	1126.18726	3376.54721	4.32
nGFSSVQATQLQTTQSVEGATGSAVk	Unambiguous	5	1	Q80X50	N-Term(iTRAQ8plex); N1(Deamidated); K26(iTRAQ8plex)	CID	1.0000	0.0000	1	1	3278821	Unique	Used	4.949	4.349	1.929	1.992	1E-05	0	7.62	0	0	88	3	1069.22815	3205.66989	-3.08
hGcAFLVDEVQTGGGcTGk	Unambiguous	1	1	P61922	N-Term(iTRAQ8plex); C3(Methylthio); C16(Methylthio); K19(iTRAQ8plex)	CID	1.0000	0.0000	1	1	3392242	Unique	Used	1.491	0.441	0.781	1.122	1E-05	0	7.62	0	0	200	2	1290.11060	2579.21391	-7.61
iGQGASGTVFTATDVALGQEVAIk	Unambiguous	1	1	Q8CIN4	N-Term(iTRAQ8plex); K24(iTRAQ8plex)	CID	1.0000	0.0000	1	1	3417829	Unique	Used	0.638	0.437	0.896	0.820	1E-05	0	7.61	0	0	87	2	1471.32117	2941.63506	-3.57
aGFAALQLEYFFTAGPDEVR	Unambiguous	1	1	Q9CZ30	N-Term(iTRAQ8plex)	CID	1.0000	0.0000	1	1	3645516	Unique	Used	0.600	1.159	0.856	1.068	1E-05	0	7.61	0	11	15	2	1253.64563	2506.28398	-3.22
sNYLLNTTIAGVEEADVVLLVGTNPR	Unambiguous	1	1	Q91VD9	N-Term(iTRAQ8plex)	CID	1.0000	0.0000	1	1	3602621	Unique	Used	0.885	1.522	2.362	1.960	1E-05	0	7.61	0	12	60	2	1531.83240	3062.65752	-3.30
qGQYSPMAIEEqVAVIYAGVR	Unambiguous	2	1	Q03265	N-Term(iTRAQ8plex); Q12(Deamidated)	CID	1.0000	0.0000	1	1	3603677	Unique	Used	1.095	0.987	0.828	1.370	1E-05	0	7.61	0	0	200	2	1307.68140	2614.35552	2.52
qSPINIDEDLTQVNVNLk	Unambiguous	1	1	B9EKR1	N-Term(iTRAQ8plex); K18(iTRAQ8plex)	CID	1.0000	0.0000	1	1	3282547	Unique	Used	1.156	0.957	1.179	0.731	1E-05	0	7.60	0	0	77	2	1324.74023	2648.47319	0.61
eVITSDDGSDcGDATELDISHSFSGSLDELSER	Unambiguous	2	1	Q99K47	N-Term(iTRAQ8plex); C11(Methylthio)	CID	1.0000	0.0000	1	1	2766163	Unique	Used	5.030	2.252	3.923	6.073	1E-05	0	7.60	0	0	200	3	1279.56836	3836.69052	0.00
sNYLLNTTIAGVEEADVVLLVGTNPR	Unambiguous	1	1	Q91VD9	N-Term(iTRAQ8plex)	CID	1.0000	0.0000	1	1	3581755	Unique	Used	1.110	1.406	0.677	1.095	1E-05	0	7.59	0	0	44	2	1531.82788	3062.64849	-6.25
dYSLQIQNVDVTDDGPYTcSVQTQHTPR	Unambiguous	4	1	A0A4W9	N-Term(iTRAQ8plex); C19(Methylthio)	CID	1.0000	0.0000	1	1	3323407	Unique	Used	1.321	1.716	0.972	0.665	1E-05	0	7.59	0	12	114	3	1177.54919	3530.63303	-3.96

iSHQFPLNNNDEDPNcAGIEGVLESYFqSLR	Unambiguous	1	1	Q1RLL3	N-Term(iTRAQ8plex); C16(Methylthio); Q28(Deamidated)	CID	1.0000	0.0000	1	1	3602452	Unique	Used	0.969	1.061	0.860	1.167	1E-05	0	7.59	0	0	45	3	1286.60632	3857.80442	-0.24
hNHDLSSYQDTIQQLEnELR	Unambiguous	2	1	P08553	N-Term(iTRAQ8plex); N17(Deamidated)	CID	1.0000	0.0000	1	1	2811274	Unique	Used	3.288	1.142	1.223	0.677	1E-05	0	7.59	0	0	93	3	915.78351	2745.33597	-0.81
sASAcPTPTHTPQDSLTGVGGDVQEAFAQSSR	Unambiguous	3	1	Q8CFR5	N-Term(iTRAQ8plex); C5(Methylthio)	CID	1.0000	0.0000	1	1	3370027	Unique	Used	1.279	0.859	1.403	1.261	1E-05	0	7.58	0	0	189	3	1184.88574	3552.64267	-5.94
qAFTDVATGSLGqGLGAAcGMAYTGk	Unambiguous	1	1	P40142	N-Term(iTRAQ8plex); Q13(Deamidated); C19(Methylthio); K26(iTRAQ8plex)	CID	1.0000	0.0000	1	1	3860465	Unique	Used	1.860	0.250	0.700	1.060	1E-05	0	7.58	0	0	30	2	1565.78198	3130.55669	3.14
lDAWDEAQVEFMASHGNEAAR	Unambiguous	1	1	E9PY16	N-Term(iTRAQ8plex)	CID	1.0000	0.0000	1	1	2959148	Unique	Used	1.232	0.942	0.767	0.805	1E-05	0	7.57	0	7	146	3	884.41931	2651.24338	-1.09
yGSGSGQQSVTGVTSVDDSNSYWR	Unambiguous	1	1	Q9DCT5	N-Term(iTRAQ8plex)	CID	1.0000	0.0000	1	1	3147706	Unique	Used	2.457	0.839	0.746	0.488	1E-05	0	7.57	0	11	95	2	1421.16162	2841.31597	-2.46
gAALDVHESEPFSFSQGPLk	Unambiguous	3	1	O88712-2	N-Term(iTRAQ8plex); K20(iTRAQ8plex)	CID	1.0000	0.0000	1	1	3212688	Unique	Used	1.263	1.374	1.025	1.098	1E-05	0	7.57	0	0	46	3	908.82147	2724.44986	1.65
aVQLYQQTANVFENEER	Unambiguous	2	1	Q9CWZ7	N-Term(iTRAQ8plex)	CID	1.0000	0.0000	1	1	3261182	Unique	Used	2.503	1.430	1.294	1.458	1E-05	0	7.57	0	0	77	2	1172.09790	2343.18852	0.10
eEALQNISADDLPDSASqAAHPQDSAFSYR	Unambiguous	7	1	Q6PAR5-2	N-Term(iTRAQ8plex); Q18(Deamidated)	CID	0.0185	0.0000	1	1	2901937	Unique	Used	1.410		0.984	0.677	1E-05	0	7.57	0	10	200	3	1180.22424	3538.65818	1.95
rDTSSNDInGGVEPTPSTVSTPSPSADLLGLR	Unambiguous	2	1	P17426-2	N-Term(iTRAQ8plex); N9(Deamidated)	CID	0.0489	0.0000	1	1	3108487	Unique	Used	1.351	0.934	0.739	1.195	1E-05	0	7.57	1	0	74	3	1182.60120	3545.78904	0.46
nLHQSGFSLSGAQIDDNIPR	Unambiguous	1	1	O08553	N-Term(iTRAQ8plex)	CID	1.0000	0.0000	1	1	3616970	Unique	Used	5.087	1.312	1.670	1.747	1E-05	0	7.57	0	0	200	2	1237.13306	2473.25884	-6.04
sINPDEAVAYGAAVqAAILSGDk	Unambiguous	2	1	P63017	N-Term(iTRAQ8plex); Q15(Deamidated); K23(iTRAQ8plex)	CID	0.0066	0.0000	1	1	3441590	Unique	Used		1.654	0.685	0.765	1E-05	0	7.57	0	0	200	2	1435.28088	2869.55449	4.90

nEAIQAAHDSVAQEGQcR	Unambiguous	1	1	Q9R0P9	N-Term(iTRAQ8plex); N1(Deamidated); C17(Methylthio)	CID	1.0000	0.0000	1	1	2755203	Unique	Used	1.869	1.097	1.344	1.288	1E-05	0	7.56	0	19	47	3	760.01959	2278.04422	-2.29
dYSLQIQNVDVTDDGPYTcSVQTQHTPR	Unambiguous	4	1	A0A4W9	N-Term(iTRAQ8plex); C19(Methylthio)	CID	1.0000	0.0000	1	1	3213746	Unique	Used	2.094	1.438	1.211	0.784	1E-05	0	7.56	0	0	22	3	1177.55310	3530.64475	-0.64
dYSLQIQNVDVTDDGPYTcSVQTQHTPR	Unambiguous	4	1	A0A4W9	N-Term(iTRAQ8plex); C19(Methylthio)	CID	1.0000	0.0000	1	1	3280978	Unique	Used	1.043	1.628	0.826	0.981	1E-05	0	7.56	0	15	116	3	1177.55139	3530.63962	-2.10
tAFNTDQGSAcSYEEEk	Unambiguous	1	1	Q61735-2	N-Term(iTRAQ8plex); C11(Methylthio); K17(iTRAQ8plex)	CID	1.0000	0.0000	1	1	2634491	Unique	Used	0.353	0.671	1.025	1.084	1E-05	0	7.55	0	0	200	2	1267.57813	2534.14897	-5.76
aTGHSGGGcISQGQSYDTDk	Unambiguous	1	1	Q8K274	N-Term(iTRAQ8plex); C9(Methylthio); K20(iTRAQ8plex)	CID	1.0000	0.0000	1	1	2861298	Unique	Used	1.049	0.770	1.297	1.371	1E-05	0	7.55	0	0	200	3	875.08026	2623.22623	-2.86
sLQLVVLDADTINHPAQLAk	Unambiguous	2	1	D3Z3Z3	N-Term(iTRAQ8plex); K20(iTRAQ8plex)	CID	1.0000	0.0000	1	1	3498094	Unique	Used		1.972	1.539	1.196	1E-05	0	7.55	0	0	87	3	918.86627	2754.58426	-4.79
qLNTIQNAEAVHFFcNYEESR	Unambiguous	4	1	P61922	N-Term(iTRAQ8plex); C15(Methylthio)	CID	1.0000	0.0000	1	1	3439010	Unique	Used	1.418	0.552	0.893	1.367	1E-05	0	7.55	0	12	37	3	955.11743	2863.33774	-2.38
iSFEEFcAVVGGLDIHk	Unambiguous	2	1	Q63810-2	N-Term(iTRAQ8plex); C7(Methylthio); K17(iTRAQ8plex)	CID	1.0000	0.0000	1	1	3690910	Unique	Used		2.188	1.830	0.356	1E-05	0	7.54	0	0	200	3	840.10968	2518.31449	-5.95
iLFIFIDSDHTDNQR	Unambiguous	1	1	P09103	N-Term(iTRAQ8plex)	CID	1.0000	0.0000	1	1	3520067	Unique	Used	3.344	0.880	0.908	0.603	1E-05	0	7.54	0	8	5	3	713.37415	2138.10788	-4.93
eMEENFALEAANYQDTIGR	Unambiguous	1	1	P20152	N-Term(iTRAQ8plex)	CID	1.0000	0.0000	1	1	2833771	Unique	Used	1.043	2.243	1.015	1.239	1E-05	0	7.54	0	17	200	2	1253.09534	2505.18340	-1.44

IVEVANLAcSISnNEEGVk	Unambiguous	4	1	Q61301-2	N-Term(iTRAQ8plex); C9(Methylthio); N13(Deamidated); K19(iTRAQ8plex)	CID	0.0438	0.0000	1	1	3262788	Unique	Used	0.534	1.012	0.718	0.885	1E-05	0	7.54	0	0	61	2	1322.68982	2644.37236	-2.24
nVVSQFVSSMSASADVLAMSk	Unambiguous	1	1	Q9CR68	N-Term(iTRAQ8plex); K21(iTRAQ8plex)	CID	1.0000	0.0000	1	1	3860895	Unique	Used	0.673	0.793	1.125	0.969	1E-05	0	7.53	0	27	24	2	1383.72656	2766.44585	-6.09
sLIDMYSEVLDVLSDYDASYNTQDHLPR	Unambiguous	5	1	P58281-2	N-Term(iTRAQ8plex)	CID	1.0000	0.0000	1	1	3396780	Unique	Used	0.804	1.146	1.259	1.445	1E-05	0	7.53	0	0	44	3	1188.57202	3563.70151	-3.91
kGTITIQDTGIGMTQEELVSnLGTIAR	Unambiguous	1	1	Q9CQN1	N-Term(iTRAQ8plex); K1(iTRAQ8plex); N21(Deamidated)	CID	0.0093	0.0000	1	1	3644565	Unique	Used	2.705	1.329	0.727	0.869	1E-05	0	7.53	1	19	41	3	1152.63489	3455.89011	0.70
qLQEQHYQqYMQQLYQVQLAQQQAALQk	Unambiguous	1	1	Q8BMP6	N-Term(iTRAQ8plex); Q9(Deamidated); K28(iTRAQ8plex)	CID	0.0053	0.0000	1	1	3744029	Unique	Used	0.556	0.847	0.526	0.543	1E-05	0	7.53	0	0	200	3	1358.04895	4072.13230	3.73
dYPLPDVAHVTmLSASQk	Unambiguous	1	1	P19783	N-Term(iTRAQ8plex); M12(Oxidation); K18(iTRAQ8plex)	CID	1.0000	0.0000	1	1	3495326	Unique	Used	0.702	1.141	0.966	1.038	1E-05	0	7.52	0	14	39	3	866.13129	2596.37931	-4.18
nIFLqYASTEVDGEHYMTPEDFVQR	Unambiguous	1	1	Q8BH59	N-Term(iTRAQ8plex); Q5(Deamidated)	CID	1.0000	0.0000	1	1	3263621	Unique	Used	1.090	1.065	1.673	1.215	1E-05	0	7.52	0	27	200	3	1098.86243	3294.57273	4.85
kADPQEAINcLNAAIDIYTDMGR	Unambiguous	1	1	P28663	N-Term(iTRAQ8plex); K1(iTRAQ8plex); C10(Methylthio)	CID	1.0000	0.0000	1	1	3645385	Unique	Used	1.623	1.345	2.266	2.278	1E-05	0	7.52	1	0	32	3	1059.53479	3176.58982	-3.19
kQTESSFMAGDINSTPTLNR	Unambiguous	5	1	E9Q3V9	N-Term(iTRAQ8plex); K1(iTRAQ8plex)	CID	1.0000	0.0000	1	1	3341595	Unique	Used	0.779	1.061	1.077	0.891	1E-05	0	7.52	1	0	23	3	935.82312	2805.45481	-4.06

aLQSGqcAGAALDVFTEEPPR	Unambiguous	1	1	Q61753	N-Term(iTRAQ8plex); Q6(Deamidated); C7(Methylthio)	CID	0.0373	0.0000	1	1	3351764	Unique	Used	0.891	1.301	1.505	1.071	1E-05	0	7.51	0	13	48	2	1256.11499	2511.22270	2.59
sITIAnQTNcPLYVTk	Unambiguous	1	1	O08553	N-Term(iTRAQ8plex); N6(Deamidated); C10(Methylthio); K16(iTRAQ8plex)	CID	0.0240	0.0000	1	1	3621113	Unique	Used	1.597	1.319	1.163	1.477	1E-05	0	7.51	0	0	96	2	1211.14380	2421.28032	-7.22
sVDLNFLPSVDPETVLQTGHELLSELQQR	Unambiguous	1	1	Q80UK0	N-Term(iTRAQ8plex)	CID	1.0000	0.0000	1	1	3727479	Unique	Used	0.509	1.099	0.927	0.643	1E-05	0	7.51	0	13	121	3	1190.28979	3568.85483	-7.10
sIEEAcFTLQYLNk	Unambiguous	1	1	P14873	N-Term(iTRAQ8plex); C6(Methylthio); K14(iTRAQ8plex)	CID	1.0000	0.0000	1	1	3581110	Unique	Used	0.493	0.717	1.082	1.130	1E-05	0	7.51	0	23	30	2	1157.10278	2313.19829	-4.17
mADPQSIQESQNLSMFLANHNR	Unambiguous	1	1	Q5SQX6	N-Term(iTRAQ8plex); M1(Oxidation)	CID	1.0000	0.0000	1	1	3618386	Unique	Used	0.979	1.387	1.738	1.400	1E-05	0	7.51	0	0	25	3	951.12524	2851.36118	-5.53
qGQYSPMAIEEQVAVIYAGVR	Unambiguous	2	1	Q03265	N-Term(iTRAQ8plex)	CID	1.0000	0.0000	1	1	3601971	Unique	Used	0.642	1.119	1.079	1.249	1E-05	0	7.50	0	6	7	2	1307.18115	2613.35503	-3.78
qTIENSQGAYQEAFDISk	Unambiguous	4	1	P68254-2	N-Term(iTRAQ8plex); K18(iTRAQ8plex)	CID	1.0000	0.0000	1	1	2956772	Unique	Used	0.915	0.785	0.927	1.117	1E-05	0	7.50	0	0	200	2	1319.17957	2637.35185	-3.73
kQLGSVSTGSTLQQSLELTR	Unambiguous	1	1	Q921G8	N-Term(iTRAQ8plex); K1(iTRAQ8plex)	CID	1.0000	0.0000	1	1	3721088	Unique	Used	0.695	1.210	1.029	1.072	1E-05	0	7.50	1	0	118	3	914.51764	2741.53836	-8.55
qLLHNFPPDQLTSSGAPFWSGPk	Unambiguous	1	1	Q02053	N-Term(iTRAQ8plex); K23(iTRAQ8plex)	CID	1.0000	0.0000	1	1	3804722	Unique	Used	0.986	1.234	0.567	0.764	1E-05	0	7.50	0	21	34	3	1044.89185	3132.66098	-3.76
dYLGDFIEHYAQLGPSQPPDLAqAQDEPR	Unambiguous	1	1	P42669	N-Term(iTRAQ8plex); Q23(Deamidated)	CID	0.0040	0.0000	1	1	3264482	Unique	Used				1.142	1E-05	0	7.50	0	22	200	3	1192.58643	3575.74472	5.99
sLEAIELGLGEAQVILALSSHLGAVESEk	Unambiguous	4	1	D3YXZ3	N-Term(iTRAQ8plex); K29(iTRAQ8plex)	CID	1.0000	0.0000	1	1	3500990	Unique	Used	0.722	1.056	0.866	1.020	1E-05	0	7.49	0	5	112	3	1191.33813	3571.99985	-1.28

ILGDASVSFTDncVVGIQANTER	Unambiguous	3	1	P97807-2	N-Term(iTRAQ8plex); N12(Deamidated); C13(Methylthio)	CID	1.0000	0.0000	1	1	3583023	Unique	Used	1.535	1.304	1.195	2.340	1E-05	0	7.49	0	0	200	2	1380.68103	2760.35478	2.21
IQDGQTFSSHGPGNPEAPGMDSIVR	Unambiguous	1	1	Q3V3R1	N-Term(iTRAQ8plex)	CID	1.0000	0.0000	1	1	3232589	Unique	Used	1.398	0.830	1.215	1.460	1E-05	0	7.49	0	0	58	3	967.80621	2901.40409	-2.17
IYPWGIVEVENQAHcDFVk	Unambiguous	1	1	Q9Z2Q6	N-Term(iTRAQ8plex); C15(Methylthio); K19(iTRAQ8plex)	CID	1.0000	0.0000	1	1	3395744	Unique	Used	0.365	1.357	1.660	1.372	1E-05	0	7.49	0	2	2	3	967.83026	2901.47623	-4.34
aLNALNNLSVNVENQTk	Unambiguous	3	1	Q9D0L7	N-Term(iTRAQ8plex); K17(iTRAQ8plex)	CID	1.0000	0.0000	1	1	3599661	Unique	Used	0.405	1.398	1.027	1.193	1E-05	0	7.49	0	0	29	2	1225.68774	2450.36821	-5.77
tSRPENAIIYSNNEDFQVGQAk	Unambiguous	1	1	P40142	N-Term(iTRAQ8plex); K22(iTRAQ8plex)	CID	1.0000	0.0000	1	1	3124760	Unique	Used	2.995	0.782	0.891	1.235	1E-05	0	7.49	0	0	54	3	1030.53760	3089.59824	-4.22
qLLHNFPPDQLTSSGAPFWSGPk	Unambiguous	1	1	Q02053	N-Term(iTRAQ8plex); K23(iTRAQ8plex)	CID	1.0000	0.0000	1	1	3804525	Unique	Used	0.141	0.992	1.084	0.923	1E-05	0	7.49	0	9	26	3	1044.89160	3132.66025	-3.99
sncNVAVINVGAPAAGMNAAVR	Unambiguous	3	1	Q8C605	N-Term(iTRAQ8plex); N2(Deamidated); C3(Methylthio)	CID	0.0227	0.0000	1	1	3764237	Unique	Used	1.692	0.851	0.970	1.150	1E-05	0	7.48	0	0	134	2	1225.61194	2450.21660	-3.70
dTNYTLNTDSLDWALYDHLMDFLADR	Unambiguous	2	1	Q8R5L1	N-Term(iTRAQ8plex)	CID	1.0000	0.0000	1	1	3440588	Unique	Used	2.281	1.088	0.933	0.635	1E-05	0	7.48	0	25	98	3	1141.53967	3422.60447	-3.17
fVSQTNGNLYIANVESSDR	Unambiguous	1	1	P12960	N-Term(iTRAQ8plex)	CID	1.0000	0.0000	1	1	3346301	Unique	Used	1.390	0.694	1.061	1.275	1E-05	0	7.48	0	0	44	2	1209.60974	2418.21221	-3.36
vLTLESNVDSTSSSScQInR	Unambiguous	2	1	O55106	N-Term(iTRAQ8plex); C16(Methylthio); N19(Deamidated)	CID	0.0027	0.0000	1	1	3392594	Unique	Used	0.667	0.741	1.227	0.737	1E-05	0	7.47	0	13	182	2	1246.10510	2491.20293	2.85
sVIIDcSGPqPQFHNAGSNR	Unambiguous	2	1	Q9CYI0	N-Term(iTRAQ8plex); C6(Methylthio); Q10(Deamidated)	CID	0.0067	0.0000	1	1	3759816	Unique	Used	0.341	0.968	0.879	1.165	1E-05	0	7.47	0	0	200	2	1239.60205	2478.19682	6.47

dVDFVTALcSqVVYEGLVDDTFR	Unambiguous	1	1	P59016	N-Term(iTRAQ8plex); C9(Methylthio); Q11(Deamidated)	CID	1.0000	0.0000	1	1	3542341	Unique	Used	0.880	1.051	1.110	1.646	1E-05	0	7.47	0	0	200	2	1471.71033	2942.41338	0.94
mEPIAIIITDTEISEFDVk	Unambiguous	2	1	D3YVF0	N-Term(iTRAQ8plex); K19(iTRAQ8plex)	CID	1.0000	0.0000	1	1	3133587	Unique	Used			1.847		1E-05	0	7.47	0	0	35	3	924.84448	2772.51889	-0.47
gMGSTFIVQGVTLGAEGIISEFTPLPR	Unambiguous	1	1	Q9CQS4	N-Term(iTRAQ8plex)	CID	1.0000	0.0000	1	1	3880464	Unique	Used	0.509	0.677	0.985	2.531	1E-05	0	7.46	0	11	29	2	1541.32385	3081.64043	-6.27
eVAAFAQFGSDLDAATQQLLSR	Unambiguous	2	1	Q03265	N-Term(iTRAQ8plex)	CID	1.0000	0.0000	1	1	3667193	Unique	Used	2.293	1.352	1.073	0.914	1E-05	0	7.46	0	0	190	3	881.45819	2642.36002	-4.85
vSLDGLQEPTHSVLqALNDLQESLTGSR	Unambiguous	3	1	Q8BNU0	N-Term(iTRAQ8plex); Q15(Deamidated)	CID	0.0174	0.0000	1	1	3824977	Unique	Used	1.157	0.871	0.930	1.047	1E-05	0	7.46	0	20	166	3	1104.91650	3312.73496	3.73
yGFLGGSQYSGSLESSIPVDVAR	Unambiguous	1	1	Q8BHL3	N-Term(iTRAQ8plex)	CID	1.0000	0.0000	1	1	3540664	Unique	Used	0.599	0.637	1.487	1.662	1E-05	0	7.46	0	0	29	2	1347.18188	2693.35649	-5.94
mMQDDEMnIFINLTkDEFAELR	Unambiguous	1	1	Q01065	N-Term(iTRAQ8plex); N8(Deamidated); K15(iTRAQ8plex)	CID	1.0000	0.0000	1	1	3262830	Unique	Used	0.295	0.655	2.926	1.353	1E-05	0	7.46	1	0	40	3	1104.88794	3312.64927	2.44
wGEAGAEYVVESTGVFTTMEk	Unambiguous	3	1	P16858	N-Term(iTRAQ8plex); K21(iTRAQ8plex)	CID	1.0000	0.0000	1	1	3240381	Unique	Used	0.403	0.750	0.890	1.157	1E-05	0	7.46	0	0	200	2	1450.24048	2899.47368	3.18
gPVAPAFALWDGELLTHSGLEVPk	Unambiguous	1	1	Q91WK1	N-Term(iTRAQ8plex); K24(iTRAQ8plex)	CID	1.0000	0.0000	1	1	3560767	Unique	Used	0.768	0.625	0.773	0.699	1E-05	0	7.45	0	30	33	3	1038.24255	3112.71311	-5.18
gAQAAIVVYDITNTDTFAR	Unambiguous	2	1	P35278	N-Term(iTRAQ8plex)	CID	1.0000	0.0000	1	1	3499941	Unique	Used	0.687	1.050	0.953	1.129	1E-05	0	7.45	0	0	12	2	1165.61462	2330.22197	-3.21
gGNVGInSFGFGGSNVHVILQPNTR	Unambiguous	1	1	P19096	N-Term(iTRAQ8plex); N7(Deamidated)	CID	0.0336	0.0000	1	1	3858002	Unique	Used	0.833	0.506	0.849	1.207	1E-05	0	7.44	0	17	73	3	949.49860	2846.48124	-1.39
qQNLILSYAEDETDVEGTVnGVTHTSTGGR	Unambiguous	2	1	Q99M87	N-Term(iTRAQ8plex); N20(Deamidated)	CID	1.0000	0.0000	1	1	2852355	Unique	Used				0.735	1E-05	0	7.43	0	22	200	3	1166.23438	3496.68857	-2.76
gAQAAIVVYDITnQETFAR	Unambiguous	1	1	P61021	N-Term(iTRAQ8plex); N13(Deamidated)	CID	0.0027	0.0000	1	1	3458977	Unique	Used	0.641	1.029	0.931	1.094	1E-05	0	7.43	0	0	160	2	1186.62915	2372.25102	4.64

lYsSMSDTNLAEAGLNYHAQR	Unambiguous	2	1	O88737	N-Term(iTRAQ8plex); S3(Phospho)	CID	0.0013	0.0000	1	1	3014499	Unique	Used	2.509	1.684	1.221	1.223	1E-05	0	7.43	0	17	143	3	909.09015	2725.25589	-1.32
eQASHLGGAVFSGAGnIAAATGLVk	Unambiguous	1	1	Q91ZZ3	N-Term(iTRAQ8plex); N16(Deamidated); K25(iTRAQ8plex)	CID	1.0000	0.0000	1	1	3643259	Unique	Used	2.891	1.356	1.937	2.559	1E-05	0	7.43	0	30	72	3	979.20331	2935.59537	-4.93
aLQSGQcAGAALDVFTEEPPR	Unambiguous	1	1	Q61753	N-Term(iTRAQ8plex); C7(Methylthio)	CID	1.0000	0.0000	1	1	3351447	Unique	Used	0.692	0.595	1.381	1.516	1E-05	0	7.43	0	25	39	2	1255.61597	2510.22466	-3.00
tQAQHSSPGEMASSPQGLDNPALLR	Unambiguous	1	1	A2ADY9	N-Term(iTRAQ8plex)	CID	1.0000	0.0000	1	1	3297597	Unique	Used	2.403	1.626	1.703	1.070	1E-05	0	7.43	0	0	106	3	966.15479	2896.44980	-0.96
wGEAGAEYVVESTGVFTTMEk	Unambiguous	3	1	P16858	N-Term(iTRAQ8plex); K21(iTRAQ8plex)	CID	1.0000	0.0000	1	1	3241430	Unique	Used		1.740	1.527	1.837	1E-05	0	7.43	0	0	200	2	1450.23169	2899.45610	-2.89
aFEDWLNDDLGSYQGAQEnR	Unambiguous	3	1	Q9EPJ9	N-Term(iTRAQ8plex); N19(Deamidated)	CID	1.0000	0.0000	1	1	2816050	Unique	Used			1.012	0.784	1E-05	0	7.43	0	5	111	3	878.40619	2633.20401	-0.68
tQPTDEEMLFIYSHFk	Unambiguous	4	1	P31786	N-Term(iTRAQ8plex); K16(iTRAQ8plex)	CID	1.0000	0.0000	1	1	3285392	Unique	Used	1.722	0.563	0.782	0.744	1E-05	0	7.43	0	9	200	3	865.45038	2594.33658	-2.14
gDVQAQVGLGQLHLHGGR	Unambiguous	3	1	Q9Z2G6	N-Term(iTRAQ8plex)	CID	1.0000	0.0000	1	1	3635362	Unique	Used	0.889	0.996	1.092	1.287	1E-05	0	7.42	0	27	138	3	716.06116	2146.16892	-4.40
wGEAGAEYVVESTGVFTTMEk	Unambiguous	3	1	P16858	N-Term(iTRAQ8plex); K21(iTRAQ8plex)	CID	1.0000	0.0000	1	1	3240301	Unique	Used	0.704	1.365	1.637	1.616	1E-05	0	7.42	0	0	200	2	1450.23401	2899.46074	-1.29
dFITALPEQGLDHIAENILSYLDAR	Unambiguous	4	1	Q5SRY7-4	N-Term(iTRAQ8plex)	CID	1.0000	0.0000	1	1	3645877	Unique	Used	2.689	0.889	1.089	0.923	1E-05	0	7.42	0	18	32	3	1040.21240	3118.62265	-4.39
tLTGTAALTVQSqEENLR	Unambiguous	1	1	P24527	N-Term(iTRAQ8plex); Q13(Deamidated)	CID	0.0162	0.0000	1	1	3278743	Unique	Used	1.198	1.537	1.034	1.219	1E-05	0	7.42	0	24	180	2	1119.10815	2237.20903	7.29
lTSTSTSDTQnINNSAQR	Unambiguous	1	1	Q8C180	N-Term(iTRAQ8plex); N11(Deamidated)	CID	0.0054	0.0000	1	1	3100781	Unique	Used	1.003	1.039	1.027	0.912	1E-05	0	7.42	0	0	200	2	1122.06433	2243.12139	7.15

eLAcDDPEAEQVALLAVDYLNNHLLQGFk	Unambiguous	1	1	P29699	N-Term(iTRAQ8plex); C4(Methylthio); K29(iTRAQ8plex)	CID	1.0000	0.0000	1	1	3500900	Unique	Used	2.130	1.606	0.325	0.555	1E-05	0	7.42	0	30	42	3	1294.99524	3882.97116	-4.01
sIVnNGHSFNVEFDDSQDNAVLk	Unambiguous	1	1	P00920	N-Term(iTRAQ8plex); N4(Deamidated); K23(iTRAQ8plex)	CID	0.0135	0.0000	1	1	2957678	Unique	Used	1.181	1.119	1.891	2.268	1E-05	0	7.42	0	0	89	3	1053.52991	3158.57517	-3.15
aAAQLLQSQAQQSGAQQTk	Unambiguous	3	1	Q8C1B7-3	N-Term(iTRAQ8plex); K19(iTRAQ8plex)	CID	1.0000	0.0000	1	1	3678946	Unique	Used	0.579	1.186	1.354	1.052	1E-05	0	7.42	0	0	129	2	1283.20691	2565.40654	-5.46
yFHVVIAGPQDSPFEGGTFk	Unambiguous	1	1	P61089	N-Term(iTRAQ8plex); K20(iTRAQ8plex)	CID	1.0000	0.0000	1	1	3538688	Unique	Used	2.205	0.771	0.814	0.994	1E-05	0	7.41	0	0	26	3	935.49573	2804.47263	-5.07
yTPSGQSGAAASESLFISNHAY	Unambiguous	2	1	A6ZI44	N-Term(iTRAQ8plex)	CID	1.0000	0.0000	1	1	3413721	Unique	Used	6.092	1.789	1.348	1.657	1E-05	0	7.41	0	0	75	2	1281.61914	2562.23100	-4.07
aEcFETLSAmELAEQITLLDHIVFR	Unambiguous	3	1	P70392	N-Term(iTRAQ8plex); C3(Methylthio); M10(Oxidation)	CID	1.0000	0.0000	1	1	3707362	Unique	Used	0.581	1.805	0.862	1.291	1E-05	0	7.40	0	0	77	3	1082.53613	3245.59385	-8.03
qLFALScAAEEQGMLPEDLSGVIR	Unambiguous	1	1	P08752	N-Term(iTRAQ8plex); C7(Methylthio)	CID	1.0000	0.0000	1	1	3500570	Unique	Used	0.505	1.124	0.946	1.906	1E-05	0	7.40	0	0	9	2	1464.23169	2927.45610	-2.00
eTEAALEFAQTQLAEQGEESR	Unambiguous	1	1	Q9D7M1	N-Term(iTRAQ8plex)	CID	1.0000	0.0000	1	1	2741798	Unique	Used	3.364	2.460	1.109	1.009	1E-05	0	7.39	0	3	200	2	1321.14624	2641.28520	-1.65
aTLqEVVGLHSDMAVEAGAPYAER	Unambiguous	1	1	G3UYZ1	N-Term(iTRAQ8plex); Q4(Deamidated)	CID	1.0000	0.0000	1	1	3323659	Unique	Used	1.360		1.333	0.526	1E-05	0	7.39	0	25	125	3	940.47961	2819.42429	1.94
dFTSEQIDEILQNHTEIVFAR	Unambiguous	2	1	Q6PIC6	N-Term(iTRAQ8plex)	CID	1.0000	0.0000	1	1	3351983	Unique	Used			1.889		1E-05	0	7.39	0	29	20	3	937.14636	2809.42453	-2.33
vTSAYLQDIENAYk	Unambiguous	1	1	Q99LC3	N-Term(iTRAQ8plex); K14(iTRAQ8plex)	CID	1.0000	0.0000	1	1	3193673	Unique	Used	0.547	0.867	1.112	1.395	1E-05	0	7.39	0	2	15	2	1112.10571	2223.20415	-3.41

sNIGAEGGPPPFLPFGqk	Unambiguous	5	1	Q91W18-2	N-Term(iTRAQ8plex); Q17(Deamidated); K18(iTRAQ8plex)	CID	1.0000	0.0000	1	1	3578730	Unique	Used	0.632	0.737	1.019	0.912	1E-05	0	7.39	0	0	170	2	1211.66785	2422.32842	2.34
icDPGLTSFEPEALGNLVEGMDFHR	Unambiguous	9	1	Q5SVJ0	N-Term(iTRAQ8plex); C2(Methylthio)	CID	1.0000	0.0000	1	1	3480998	Unique	Used		0.965	1.281	1.144	1E-05	0	7.39	0	28	200	3	1033.15747	3097.45786	-5.08
sVNESLNNLFITEEDYQALR	Unambiguous	2	1	Q68FD5	N-Term(iTRAQ8plex)	CID	1.0000	0.0000	1	1	3262200	Unique	Used	0.367	1.557	1.288	1.291	1E-05	0	7.39	0	0	22	2	1330.17566	2659.34404	-2.90
aADFQLHTHVNDGTEFGGSIYQk	Unambiguous	3	1	J3QPE8	N-Term(iTRAQ8plex); K23(iTRAQ8plex)	CID	1.0000	0.0000	1	1	3254999	Unique	Used	0.528	1.017	1.123	1.307	1E-05	0	7.38	0	0	83	3	1048.53271	3143.58359	-5.44
nMkDEGFnITAGIDEETGFVYGGNR	Unambiguous	2	1	F8VPN4	N-Term(iTRAQ8plex); K3(iTRAQ8plex); N8(Deamidated)	CID	1.0000	0.0000	1	1	3130703	Unique	Used		0.940	0.778	1.229	1E-05	0	7.38	1	0	116	3	1115.22119	3343.64902	3.83
dAFEHIVTQFSSVPVSVVSDSYDIYnAcEk	Unambiguous	1	1	Q99KQ4	N-Term(iTRAQ8plex); N26(Deamidated); C28(Methylthio); K30(iTRAQ8plex)	CID	1.0000	0.0000	1	1	3561143	Unique	Used	1.137	0.891	0.846	0.823	1E-05	0	7.38	0	21	57	3	1335.64856	4004.93113	-2.09
dEAIYEcVASNNVGEISVSTR	Unambiguous	17	1	Q64487-7	N-Term(iTRAQ8plex); C7(Methylthio)	CID	1.0000	0.0000	1	1	3172899	Unique	Used	1.402	1.158	1.058	0.924	1E-05	0	7.38	0	0	200	2	1303.61987	2606.23247	-2.14
vRPcVVYGGAEIGQQIR	Unambiguous	1	1	Q62167	N-Term(iTRAQ8plex); C4(Methylthio)	CID	1.0000	0.0000	1	1	3893944	Unique	Used	0.529	0.600	0.655	0.723	1E-05	0	7.38	0	1	18	3	732.39325	2195.16520	-3.62
qTTVSNSQQAYQEAFEISk	Unambiguous	3	1	Q9CQV8-2	N-Term(iTRAQ8plex); K19(iTRAQ8plex)	CID	1.0000	0.0000	1	1	3107016	Unique	Used	0.566	1.022	0.819	0.956	1E-05	0	7.37	0	0	18	2	1384.21875	2767.43022	-2.05
gLVHAEQGQGSGSPTGSSTR	Unambiguous	2	1	Q3URS9-2	N-Term(iTRAQ8plex)	CID	1.0000	0.0000	1	1	3291524	Unique	Used	1.249	1.003	1.233	1.024	1E-05	0	7.37	0	0	200	3	739.71118	2217.11899	1.26
yGSGSGQQSVTGVTSVDDSNSYWR	Unambiguous	1	1	Q9DCT5	N-Term(iTRAQ8plex)	CID	1.0000	0.0000	1	1	3126598	Unique	Used	1.929	0.782	1.058	0.669	1E-05	0	7.37	0	0	97	2	1421.16577	2841.32427	0.46

sITIAnQTNcPLYVTk	Unambiguous	1	1	O08553	N-Term(iTRAQ8plex); N6(Deamidated); C10(Methylthio); K16(iTRAQ8plex)	CID	0.0231	0.0000	1	1	3621317	Unique	Used	1.100	1.170	1.310	1.441	1E-05	0	7.37	0	0	5	2	1211.14502	2421.28276	-6.21
cLEILEQMNEDDSEQLQR	Unambiguous	3	1	O88597	N-Term(iTRAQ8plex); C1(Methylthio)	CID	1.0000	0.0000	1	1	2816426	Unique	Used	0.999	1.071	1.167	0.978	1E-05	0	7.37	0	14	200	2	1272.09216	2543.17705	1.59
sSTSWVDITQDFEDAcR	Unambiguous	4	1	Q6PHQ8	N-Term(iTRAQ8plex); C16(Methylthio)	CID	1.0000	0.0000	1	1	3173981	Unique	Used	0.518	0.835	0.887	0.992	1E-05	0	7.37	0	0	87	2	1155.51721	2310.02715	-2.13
sITIAnQTNcPLYVTk	Unambiguous	1	1	O08553	N-Term(iTRAQ8plex); N6(Deamidated); C10(Methylthio); K16(iTRAQ8plex)	CID	0.0204	0.0000	1	1	3621027	Unique	Used			1.245	1.187	1E-05	0	7.37	0	0	56	2	1211.14502	2421.28276	-6.21
vHAAVQPGSLDSESGIFAcAFDR	Unambiguous	2	1	Q922V4	N-Term(iTRAQ8plex); C19(Methylthio)	CID	1.0000	0.0000	1	1	3499268	Unique	Used	1.043	1.095	0.964	1.022	1E-05	0	7.37	0	25	102	3	909.77448	2727.30887	-3.10
fWEVISDEHGIDPTGTYHGDSDLQLER	Unambiguous	2	1	Q9D6F9	N-Term(iTRAQ8plex)	CID	1.0000	0.0000	1	1	3132002	Unique	Used	0.814	0.979	0.913	1.124	1E-05	0	7.36	0	14	165	3	1140.87866	3420.62143	-2.12
dVDFVTALcSQVVYEGLVDDTFR	Unambiguous	1	1	P59016	N-Term(iTRAQ8plex); C9(Methylthio)	CID	1.0000	0.0000	1	1	3561828	Unique	Used	0.537	0.829	0.853	0.916	1E-05	0	7.36	0	0	200	2	1471.21082	2941.41435	-4.16
dILGGnGISDEYHVIR	Unambiguous	1	1	Q60759	N-Term(iTRAQ8plex); N6(Deamidated)	CID	1.0000	0.0000	1	1	2956988	Unique	Used	0.591	0.930	0.867	1.060	1E-05	0	7.36	0	1	20	3	688.36139	2063.06961	-0.74
eIELLcQEHGQENDDLVQR	Unambiguous	4	1	E9Q6X0	N-Term(iTRAQ8plex); C6(Methylthio)	CID	1.0000	0.0000	1	1	2637751	Unique	Used	1.480	0.829	0.958	0.984	1E-05	0	7.36	0	5	200	3	873.42065	2618.24741	-0.72
sNVYQPTEMAVVLnGGTIPTAPPSHTGR	Unambiguous	1	1	Q923Z0	N-Term(iTRAQ8plex); N14(Deamidated)	CID	1.0000	0.0000	1	1	3476074	Unique	Used	1.092	0.711	1.189	0.802	1E-05	0	7.36	0	19	56	3	1067.21289	3199.62412	-3.72
dITFVAEFLTEHFNEAPDLYNR	Unambiguous	1	1	Q91W96	N-Term(iTRAQ8plex)	CID	1.0000	0.0000	1	1	3440357	Unique	Used	0.616	1.122	0.667	0.891	1E-05	0	7.36	0	0	24	3	982.48834	2945.45047	-4.04

sYIEGYVPSqADVAVFEAVSGPPPADLcHALR	Unambiguous	3	1	O70251	N-Term(iTRAQ8plex); Q10(Deamidated); C28(Methylthio)	CID	1.0000	0.0000	1	1	3625119	Unique	Used			0.590		1E-05	0	7.36	0	0	200	3	1237.27905	3709.82260	1.11
eQQPSPGPAGSHMAISHQPR	Unambiguous	4	1	H3BIW6	N-Term(iTRAQ8plex)	CID	1.0000	0.0000	1	1	3224233	Unique	Used	1.074	0.897	2.023	1.125	1E-05	0	7.35	0	28	200	3	806.07281	2416.20389	-2.28
gAQIASTGQLYAAWnGGLDR	Unambiguous	1	1	Q61361	N-Term(iTRAQ8plex); N15(Deamidated)	CID	1.0000	0.0000	1	1	3392844	Unique	Used	1.520	1.031	1.055	1.403	1E-05	0	7.35	0	0	39	2	1177.60010	2354.19292	-4.83
sASSTSSLFSSSSQLFPPSR	Unambiguous	6	1	Q3TQ29	N-Term(iTRAQ8plex)	CID	1.0000	0.0000	1	1	3933888	Unique	Used	0.471	0.836	0.844	1.006	1E-05	0	7.35	0	0	200	2	1182.59839	2364.18950	-3.82
gALGcPQEAATATAPGSGTAVTLR	Unambiguous	1	1	Q3UVU3	N-Term(iTRAQ8plex); C5(Methylthio)	CID	1.0000	0.0000	1	1	3640047	Unique	Used	0.261	0.668	0.688	0.979	1E-05	0	7.35	0	0	85	2	1275.64465	2550.28203	-5.42
tETVcTFQDGALVQHQQWDGk	Unambiguous	1	1	Q05816	N-Term(iTRAQ8plex); C5(Methylthio); K21(iTRAQ8plex)	CID	1.0000	0.0000	1	1	3257906	Unique	Used	1.813	0.381	0.761	0.698	1E-05	0	7.35	0	19	3	3	1015.83783	3045.49894	-0.98
nTPIYVSSPTWENHnAVFSAAGFk	Unambiguous	2	1	P05201	N-Term(iTRAQ8plex); N15(Deamidated); K24(iTRAQ8plex)	CID	0.0095	0.0000	1	1	3763161	Unique	Used	1.484	0.937	0.818	1.200	1E-05	0	7.34	0	0	105	3	1082.89221	3246.66208	-1.84
sIDDLEDQLYHQLEQnR	Unambiguous	3	1	E9Q452	N-Term(iTRAQ8plex); N16(Deamidated)	CID	0.0450	0.0000	1	1	2765718	Unique	Used	2.025	1.298	1.499	0.866	1E-05	0	7.34	0	8	200	3	807.73175	2421.18070	-1.20
aVMEQSGAWVQLSqkPEGINLQER	Unambiguous	2	1	D3YVV7	N-Term(iTRAQ8plex); Q14(Deamidated); K15(iTRAQ8plex)	CID	0.0095	0.0000	1	1	3516969	Unique	Used	1.103	1.399	1.319	1.257	1E-05	0	7.34	0	0	200	3	1103.25891	3307.76218	1.69
vATLEQSYEALcELAATR	Unambiguous	1	1	Q68FG2	N-Term(iTRAQ8plex); C12(Methylthio)	CID	1.0000	0.0000	1	1	3645709	Unique	Used	0.704	3.086	2.937	1.660	1E-05	0	7.34	0	1	2	2	1159.58093	2318.15459	-5.54
dFTSEQIDEILQNHTEIVFAR	Unambiguous	2	1	Q6PIC6	N-Term(iTRAQ8plex)	CID	1.0000	0.0000	1	1	3353882	Unique	Used	0.702	0.687	1.178	1.281	1E-05	0	7.34	0	1	15	3	937.14551	2809.42197	-3.24

sAGRPDAVGLPcPGGSEDTASDVSEPSGSAVSSPGDR	Unambiguous	1	1	Q6P1J1	N-Term(iTRAQ8plex); C12(Methylthio)	CID	1.0000	0.0000	1	1	2866410	Unique	Used	1.791		1.273	1.473	1E-05	0	7.34	0	0	104	3	1274.58606	3821.74363	-1.60
sLLGVFEEDATAISNYMNQLYQAMHR	Unambiguous	1	1	Q8K3H0	N-Term(iTRAQ8plex)	CID	1.0000	0.0000	1	1	3880659	Unique	Used	0.866	1.208	1.356	1.187	1E-05	0	7.34	0	19	34	3	1102.53699	3305.59641	-8.27
lVSQDNFGLELAAVEAAVR	Unambiguous	1	1	Q68FG2	N-Term(iTRAQ8plex)	CID	1.0000	0.0000	1	1	3419093	Unique	Used	0.568	3.773	2.519	1.312	1E-05	0	7.34	0	0	7	2	1153.63159	2306.25591	-4.31
iFDFQGLQHQVAQVATQLEATR	Unambiguous	2	1	Q9DBL1	N-Term(iTRAQ8plex)	CID	1.0000	0.0000	1	1	3898789	Unique	Used	0.336	1.555	0.887	1.323	1E-05	0	7.34	0	2	6	3	935.50055	2804.48709	-4.52
lELcAVHALNNVLqEQLFSQEAADEIck	Unambiguous	4	1	D3YTZ7	N-Term(iTRAQ8plex); C4(Methylthio); Q14(Deamidated); C27(Methylthio); K28(iTRAQ8plex)	CID	0.0395	0.0000	1	1	3561347	Unique	Used	0.836	0.307	0.976	1.157	1E-05	0	7.34	0	10	152	3	1277.30615	3829.90390	-1.44
sNVYQPTEMAVVLnGGTIPTAPPSHTGR	Unambiguous	1	1	Q923Z0	N-Term(iTRAQ8plex); N14(Deamidated)	CID	1.0000	0.0000	1	1	3475791	Unique	Used	1.479	1.280	1.274	0.950	1E-05	0	7.34	0	12	94	3	1067.21362	3199.62632	-3.03
qTIAHQQQLTNLQMAAQR	Unambiguous	2	1	Q3UEB3-2	N-Term(iTRAQ8plex)	CID	1.0000	0.0000	1	1	3872117	Unique	Used	0.665	0.816	0.555	0.599	1E-05	0	7.33	0	0	41	3	838.11261	2512.32328	-4.93
qTFIGHESDINAVAFFPNGYAFTTGSDDATcR	Unambiguous	4	1	P62880	N-Term(iTRAQ8plex); C31(Methylthio)	CID	1.0000	0.0000	1	1	3439644	Unique	Used		3.206	0.966	1.140	1E-05	0	7.33	0	0	19	3	1268.24670	3802.72556	-4.33
nPVWYQALTHGLNEEQR	Unambiguous	1	1	Q9EPL8	N-Term(iTRAQ8plex)	CID	1.0000	0.0000	1	1	3516795	Unique	Used	0.559	0.943	0.824	0.902	1E-05	0	7.33	0	9	8	3	787.07166	2359.20041	-3.93
gLAILSETEFLScANDASIR	Unambiguous	1	1	P27612	N-Term(iTRAQ8plex); C13(Methylthio)	CID	1.0000	0.0000	1	1	3561121	Unique	Used	1.144	0.959	0.997	1.087	1E-05	0	7.33	0	18	56	2	1230.61694	2460.22661	-6.13
aAFTVTTPQQQPASQEYGAHLEEER	Unambiguous	5	1	Q5NBX1-2	N-Term(iTRAQ8plex)	CID	1.0000	0.0000	1	1	2916031	Unique	Used			3.869	1.836	1E-05	0	7.33	0	0	200	3	1031.51184	3092.52097	-0.57
fQMPDQGMTSADDFFQGTk	Unambiguous	1	1	O08553	N-Term(iTRAQ8plex); K19(iTRAQ8plex)	CID	1.0000	0.0000	1	1	3306351	Unique	Used	1.126	0.475	1.348	1.746	1E-05	0	7.33	0	4	200	3	920.44336	2759.31552	-4.01
eDRPVSFYQLGSSQFQSNAVSLAR	Unambiguous	4	1	E9PX52	N-Term(iTRAQ8plex)	CID	1.0000	0.0000	1	1	3723580	Unique	Used	0.917	0.798	0.934	1.176	1E-05	0	7.33	0	5	40	3	997.50720	2990.50705	-6.81

aAAELLQSQGSQAGGSQTLk	Unambiguous	3	1	Q9R1T4-2	N-Term(iTRAQ8plex); K20(iTRAQ8plex)	CID	1.0000	0.0000	1	1	3411435	Unique	Used	0.585	0.930	1.536	1.654	1E-05	0	7.33	0	0	10	2	1277.20276	2553.39824	-4.34
aAMFPETLDEGMQIPSTqFDAAHPTNVQR	Unambiguous	3	1	Q02248	N-Term(iTRAQ8plex); Q18(Deamidated)	CID	0.0260	0.0000	1	1	3394299	Unique	Used	0.792	1.377	0.728	0.874	1E-05	0	7.32	0	0	157	3	1169.89954	3507.68406	0.37
iSAEGVDIVLDcLcGDNTGk	Unambiguous	1	1	Q80TB8	N-Term(iTRAQ8plex); C12(Methylthio); C14(Methylthio); K20(iTRAQ8plex)	CID	1.0000	0.0000	1	1	3374596	Unique	Used	1.221	0.388	0.976	1.034	1E-05	0	7.32	0	7	60	3	908.11450	2722.32895	-3.36
gGDFLEGnIITGAQMSQVNSR	Unambiguous	3	1	Q9R0H0	N-Term(iTRAQ8plex); N8(Deamidated)	CID	0.0041	0.0000	1	1	3437498	Unique	Used	0.991	0.701	0.524	0.827	1E-05	0	7.32	0	26	156	2	1250.12988	2499.25249	2.92
gYGHGQGSYSSYSnSYNSPGGGGSDYSYDSk	Unambiguous	2	1	Q9Z1X4	N-Term(iTRAQ8plex); N14(Deamidated); K32(iTRAQ8plex)	CID	1.0000	0.0000	1	1	2775535	Unique	Used	1.170		1.038		1E-05	0	7.32	0	0	200	3	1284.56030	3851.66635	-3.28
eVAAFAQFGSDLDAATQQLLSR	Unambiguous	2	1	Q03265	N-Term(iTRAQ8plex)	CID	1.0000	0.0000	1	1	3667835	Unique	Used	0.756	0.718	1.206	1.181	1E-05	0	7.32	0	0	195	2	1321.68176	2642.35625	-6.28
aVLASMDNENMHTPDIGGQGTTSQAIQDIIR	Unambiguous	1	1	P70404	N-Term(iTRAQ8plex)	CID	1.0000	0.0000	1	1	3394461	Unique	Used	0.997	1.444	0.982	1.564	1E-05	0	7.32	0	8	57	3	1196.58960	3587.75425	-5.42
tVQTAAANAASTAATSAAQNAFk	Unambiguous	2	1	Q8K021	N-Term(iTRAQ8plex); K23(iTRAQ8plex)	CID	1.0000	0.0000	1	1	3762126	Unique	Used	0.787	1.246	0.826	0.877	1E-05	0	7.32	0	0	65	2	1387.74500	2774.48271	-2.40
eLQSqISDTSVVLSMDNSR	Unambiguous	1	1	P11679	N-Term(iTRAQ8plex); Q5(Deamidated)	CID	0.0150	0.0000	1	1	3281474	Unique	Used	0.712	0.849	0.965	1.435	1E-05	0	7.32	0	0	187	2	1207.61353	2414.21977	7.23
gSYNLQDLLAEDkLPTLLGAEAnLNNIGDTNPR	Unambiguous	1	1	Q3UTR7	N-Term(iTRAQ8plex); K13(iTRAQ8plex); N23(Deamidated)	CID	0.0273	0.0000	1	1	3479694	Unique	Used	1.404	0.553	0.565	1.219	1E-05	0	7.32	1	0	24	3	1383.73364	4149.18637	-1.48

sAGDLGIAVcNVPAASVEETADSTLcHILNLYR	Unambiguous	3	1	O88712-2	N-Term(iTRAQ8plex); C10(Methylthio); C26(Methylthio)	CID	1.0000	0.0000	1	1	3785973	Unique	Used	0.578	1.263	1.293	1.243	1E-05	0	7.32	0	4	63	3	1266.95239	3798.84262	-1.36
lTHFPTVSGSPASLADSmQQk	Unambiguous	2	1	A3KGR9	N-Term(iTRAQ8plex); M18(Oxidation); K21(iTRAQ8plex)	CID	1.0000	0.0000	1	1	3659588	Unique	Used	1.195	0.712	0.975	1.066	1E-05	0	7.32	0	17	48	3	942.82990	2826.47513	-5.85
aNLMHNLGGEEVSVAck	Unambiguous	1	1	P29699	N-Term(iTRAQ8plex); C16(Methylthio); K17(iTRAQ8plex)	CID	1.0000	0.0000	1	1	3345416	Unique	Used	2.261	1.252	0.361	0.540	1E-05	0	7.32	0	0	35	2	1213.62256	2426.23784	-2.99
dFTSEQIDEILQNHTEIVFAR	Unambiguous	2	1	Q6PIC6	N-Term(iTRAQ8plex)	CID	1.0000	0.0000	1	1	3377054	Unique	Used		0.564	1.507	3.718	1E-05	0	7.31	0	0	200	3	937.15015	2809.43589	1.71
fQMPDQGMTSADDFFQGTk	Unambiguous	1	1	O08553	N-Term(iTRAQ8plex); K19(iTRAQ8plex)	CID	1.0000	0.0000	1	1	3308320	Unique	Used			2.190	2.253	1E-05	0	7.31	0	0	200	3	920.44489	2759.32010	-2.36
nSYVAGqYDDAASYk	Unambiguous	2	1	Q00612	N-Term(iTRAQ8plex); Q7(Deamidated); K15(iTRAQ8plex)	CID	1.0000	0.0000	1	1	2827906	Unique	Used	0.710	0.502	0.849	1.159	1E-05	0	7.31	0	0	191	2	1131.06702	2261.12676	3.77
sIVNnGHSFNVEFDDSQDNAVLk	Unambiguous	1	1	P00920	N-Term(iTRAQ8plex); N5(Deamidated); K23(iTRAQ8plex)	CID	0.0123	0.0000	1	1	2938660	Unique	Used	1.284	0.943	1.993	2.291	1E-05	0	7.31	0	1	13	3	1053.53015	3158.57590	-2.92
gQTLPLDFIEHFLALDGLqEAR	Unambiguous	2	1	A6H687	N-Term(iTRAQ8plex); Q19(Deamidated)	CID	1.0000	0.0000	1	1	3666650	Unique	Used			2.251	1.964	1E-05	0	7.31	0	0	87	3	930.16681	2788.48587	1.25
lQENLQQLQHSTNQLAk	Unambiguous	1	1	Q9ER00	N-Term(iTRAQ8plex); K17(iTRAQ8plex)	CID	1.0000	0.0000	1	1	3660214	Unique	Used	0.951	1.203	1.064	1.155	1E-05	0	7.31	0	21	45	3	867.81671	2601.43558	-8.20
eSLNANIVDAINQAADcWGIR	Unambiguous	4	1	Q99JB2	N-Term(iTRAQ8plex); C17(Methylthio)	CID	1.0000	0.0000	1	1	3806666	Unique	Used	0.797	1.000	0.958	0.959	1E-05	0	7.31	0	11	74	2	1312.14392	2623.28057	-4.02

eNSTFSINDAASHTSTMSSR	Unambiguous	2	1	Q8C0T5	N-Term(iTRAQ8plex)	CID	1.0000	0.0000	1	1	3228729	Unique	Used	1.172	1.181	2.531	0.989	1E-05	0	7.31	0	11	55	3	816.38135	2447.12949	-4.74
nSLIELPEDYScLLNQASHFR	Unambiguous	1	1	O70481	N-Term(iTRAQ8plex); C12(Methylthio)	CID	1.0000	0.0000	1	1	3665972	Unique	Used	1.080	0.721			1E-05	0	7.31	0	0	21	3	933.79077	2799.35776	-6.09
eLSSAVSPPnLHSDTPTFSYASLAGPTIPPR	Unambiguous	3	1	Q9QYR6	N-Term(iTRAQ8plex); N10(Deamidated)	CID	1.0000	0.0000	1	1	3725264	Unique	Used	2.892	1.410	1.887	1.152	1E-05	0	7.31	0	0	94	3	1172.26819	3514.79001	-3.08
tIGGGDDSFNTFFSETGAGk	Unambiguous	4	1	P68373	N-Term(iTRAQ8plex); K20(iTRAQ8plex)	CID	1.0000	0.0000	1	1	3236865	Unique	Used	0.402	0.996	1.114	1.544	1E-05	0	7.31	0	0	112	2	1308.64917	2616.29106	-4.89
eYLDLTASNNFYVDDFAR	Unambiguous	2	1	Q6P5E4	N-Term(iTRAQ8plex)	CID	1.0000	0.0000	1	1	3217009	Unique	Used	0.785	0.844	0.883	0.855	1E-05	0	7.30	0	0	90	2	1229.09900	2457.19072	1.26
dIGAIAQVHAEnGDIIAEEQQR	Unambiguous	1	1	O08553	N-Term(iTRAQ8plex); N12(Deamidated)	CID	1.0000	0.0000	1	1	2636310	Unique	Used	1.532	1.177	0.873	1.017	1E-05	0	7.30	0	9	200	3	894.79108	2682.35868	-1.89
tISASTQVQGGDFNLYENMR	Unambiguous	1	1	Q9EQF6	N-Term(iTRAQ8plex)	CID	1.0000	0.0000	1	1	3369706	Unique	Used	0.704	0.368	0.402	0.597	1E-05	0	7.30	0	0	78	2	1268.12048	2535.23369	-4.53
fQSQADQDQQASGLQSPPSR	Unambiguous	1	1	Q99LJ0	N-Term(iTRAQ8plex)	CID	1.0000	0.0000	1	1	2931999	Unique	Used	1.685	0.794	1.712	1.206	1E-05	0	7.30	0	0	200	2	1240.10376	2479.20024	-4.57
dAAEAEAWMGEQELHMMGQEk	Unambiguous	1	1	Q68FG2	N-Term(iTRAQ8plex); K21(iTRAQ8plex)	CID	1.0000	0.0000	1	1	2764165	Unique	Used		1.601	5.012	2.011	1E-05	0	7.30	0	17	200	3	1000.47589	2999.41312	-0.91
aSTNIQDTEGNTPLHLAcDEER	Unambiguous	1	1	Q9Z2X2	N-Term(iTRAQ8plex); C18(Methylthio)	CID	1.0000	0.0000	1	1	2759567	Unique	Used	1.340	1.350	1.007	1.159	1E-05	0	7.30	0	13	200	3	922.09845	2764.28080	-0.45
tISASTQVQGGDFNLYENMR	Unambiguous	1	1	Q9EQF6	N-Term(iTRAQ8plex)	CID	1.0000	0.0000	1	1	3346507	Unique	Used	0.656	0.263	0.485	0.689	1E-05	0	7.30	0	0	16	2	1268.12134	2535.23540	-3.85
lEQQcQQQQQQVEGLTHSLk	Unambiguous	1	1	E9Q7G0	N-Term(iTRAQ8plex); C5(Methylthio); K20(iTRAQ8plex)	CID	1.0000	0.0000	1	1	3370153	Unique	Used	1.098	0.939	0.852	0.905	1E-05	0	7.30	0	0	79	3	1003.18573	3007.54264	-4.11
dNVAGVTLPVFEHYHEGTDSYELTGLAR	Unambiguous	1	1	P57746	N-Term(iTRAQ8plex)	CID	1.0000	0.0000	1	1	3324531	Unique	Used	0.952	1.760	0.931	0.872	1E-05	0	7.30	0	0	69	3	1132.22815	3394.66989	-4.68

gLVEPVNVVDNGDGTHTVTYTPSqEGPYIVSVk	Unambiguous	1	1	Q80X90	N-Term(iTRAQ8plex); Q24(Deamidated); K33(iTRAQ8plex)	CID	0.0055	0.0000	1	1	3281615	Unique	Used	1.616	2.432	1.055	1.421	1E-05	0	7.30	0	0	69	3	1361.04797	4081.12937	1.64
icDPGLTSFEPEALGNLVEGMDFHR	Unambiguous	9	1	Q5SVJ0	N-Term(iTRAQ8plex); C2(Methylthio)	CID	1.0000	0.0000	1	1	3481950	Unique	Used		0.623	1.088	0.399	1E-05	0	7.30	0	17	200	3	1033.16113	3097.46885	-1.53
eVAAFAQFGSDLDAATQQLLSR	Unambiguous	2	1	Q03265	N-Term(iTRAQ8plex)	CID	1.0000	0.0000	1	1	3667312	Unique	Used	0.872	0.910	1.004	1.232	1E-05	0	7.30	0	5	103	2	1321.68213	2642.35698	-6.00
tPMGIILDALEQQEDNINk	Unambiguous	1	1	Q8R5J9	N-Term(iTRAQ8plex); K19(iTRAQ8plex)	CID	1.0000	0.0000	1	1	3239820	Unique	Used	0.381	0.916	1.184	1.124	1E-05	0	7.30	0	0	59	2	1375.74353	2750.47978	-2.09
vTITVDEYSSNPTqAFTHYNINQSR	Unambiguous	3	1	B1APT1	N-Term(iTRAQ8plex); Q14(Deamidated)	CID	0.0206	0.0000	1	1	3537554	Unique	Used	1.348	0.990	0.968	1.047	1E-05	0	7.29	0	0	70	3	1064.19385	3190.56699	2.35
qAALSAALQQSLQNAESWINR	Unambiguous	1	1	Q3UH60	N-Term(iTRAQ8plex)	CID	1.0000	0.0000	1	1	3918002	Unique	Used	1.569	0.371	1.082	0.886	1E-05	0	7.29	0	16	45	2	1302.18970	2603.37212	-4.71
gnDISSGTVLSDYVGSGPPSGTGLHR	Unambiguous	1	1	P70296	N-Term(iTRAQ8plex); N2(Deamidated)	CID	1.0000	0.0000	1	1	3256849	Unique	Used	2.056	1.019	1.655	0.843	1E-05	0	7.29	0	0	26	2	1418.20447	2835.40166	-1.64
nGFSSVqATQLQTTQSVEGATGSAVk	Unambiguous	5	1	Q80X50	N-Term(iTRAQ8plex); Q7(Deamidated); K26(iTRAQ8plex)	CID	0.0302	0.0000	1	1	3256284	Unique	Used	2.645	2.256	1.315	1.485	1E-05	0	7.29	0	0	96	3	1069.23108	3205.67868	-0.33
vVNAAIATASSAPGESEEPVVPSASR	Unambiguous	1	1	Q68FG2	N-Term(iTRAQ8plex)	CID	1.0000	0.0000	1	1	3302378	Unique	Used	0.258	1.855	2.477	1.687	1E-05	0	7.29	0	0	100	2	1400.73462	2800.46196	-0.42
lSSGEHIAAFcLTEPASGSDAASIQTR	Unambiguous	1	1	Q8JZN5	N-Term(iTRAQ8plex); C11(Methylthio)	CID	1.0000	0.0000	1	1	3478188	Unique	Used	1.796	1.396	2.750	2.636	1E-05	0	7.29	0	0	38	3	1023.83392	3069.48722	-1.69
rFPSPTPTNNFALDGTAGTVYLAAVNR	Unambiguous	1	1	Q3UH93	N-Term(iTRAQ8plex)	CID	1.0000	0.0000	1	1	3934595	Unique	Used	1.189	2.546	1.265	1.441	1E-05	0	7.29	1	7	101	3	1052.22131	3154.64939	-2.98
sQVFSTAADGQTQVEIk	Unambiguous	1	1	P38647	N-Term(iTRAQ8plex); K17(iTRAQ8plex)	CID	1.0000	0.0000	1	1	3126481	Unique	Used	0.772	0.802	1.100	0.978	1E-05	0	7.29	0	0	18	2	1209.15649	2417.30571	-3.13
iSLTQAGLEALFESNLLDDLk	Unambiguous	1	1	Q8BJY1	N-Term(iTRAQ8plex); K21(iTRAQ8plex)	CID	1.0000	0.0000	1	1	3541889	Unique	Used	0.445	1.155	0.782	1.194	1E-05	0	7.29	0	0	53	2	1449.81384	2898.62041	-2.79

tQPTDEEMLFIYSHFk	Unambiguous	4	1	P31786	N-Term(iTRAQ8plex); K16(iTRAQ8plex)	CID	1.0000	0.0000	1	1	3286053	Unique	Used	2.058	0.610	0.876	0.794	1E-05	0	7.28	0	3	200	3	865.45105	2594.33860	-1.36
dMHGDSEYNIMFGPDIcGPGTk	Unambiguous	1	1	P14211	N-Term(iTRAQ8plex); C17(Methylthio); K22(iTRAQ8plex)	CID	1.0000	0.0000	1	1	3171872	Unique	Used	1.275	0.746	1.017	0.716	1E-05	0	7.28	0	6	78	3	1013.46997	3038.39536	-0.78
sITIANQTNcPLYVTk	Unambiguous	1	1	O08553	N-Term(iTRAQ8plex); C10(Methylthio); K16(iTRAQ8plex)	CID	1.0000	0.0000	1	1	3802955	Unique	Used	1.734	2.276	0.866	0.929	1E-05	0	7.28	0	0	141	2	1210.65674	2420.30620	-3.14
sLAEQNLEFQGPLEISR	Unambiguous	1	1	Q8R105	N-Term(iTRAQ8plex)	CID	1.0000	0.0000	1	1	3302719	Unique	Used	1.690	0.883	0.854	0.685	1E-05	0	7.28	0	0	103	2	1118.09802	2235.18877	-1.59
eQINQQAQLQEFGQSSR	Unambiguous	7	1	F7BSN3	N-Term(iTRAQ8plex)	CID	1.0000	0.0000	1	1	3104304	Unique	Used	0.673	1.041	0.794	1.076	1E-05	0	7.28	0	0	200	2	1148.08362	2295.15996	-1.39
eTAAAHQASSsPPIDAATAEPYGFR	Unambiguous	1	1	P14873	N-Term(iTRAQ8plex); S11(Phospho)	CID	0.0330	0.0000	1	1	2669069	Unique	Used	1.781	0.844	1.034	0.850	1E-05	0	7.28	0	10	175	3	977.12653	2929.36502	-0.73
tQkPGESGInIVTADFVELGDFISTVIk	Unambiguous	2	1	G3X9A7	N-Term(iTRAQ8plex); K3(iTRAQ8plex); N10(Deamidated); K28(iTRAQ8plex)	CID	0.0453	0.0000	1	1	3602732	Unique	Used	0.705	2.296	0.866	0.888	1E-05	0	7.28	0	24	138	3	1298.05957	3892.16416	-2.10
tVYFAEEVqcEGNSFHk	Unambiguous	1	1	P97315	N-Term(iTRAQ8plex); Q9(Deamidated); C10(Methylthio); K17(iTRAQ8plex)	CID	0.0440	0.0000	1	1	3129052	Unique	Used	2.215	0.743	1.625	0.963	1E-05	0	7.28	0	0	157	2	1322.14380	2643.28032	4.67
gLGPSLTEDqPGPYLAPGLLGSNIHQQGR	Unambiguous	4	1	D3YZR8	N-Term(iTRAQ8plex); Q10(Deamidated)	CID	0.0385	0.0000	1	1	3663859	Unique	Used	1.278	1.057	0.921	1.033	1E-05	0	7.28	0	0	107	3	1093.24036	3277.70652	-1.66
qTTVSNSQQAYQEAFEISk	Unambiguous	3	1	Q9CQV8-2	N-Term(iTRAQ8plex); K19(iTRAQ8plex)	CID	1.0000	0.0000	1	1	3107161	Unique	Used	0.624	1.051	1.008	1.052	1E-05	0	7.28	0	0	157	2	1384.22070	2767.43413	-0.64

eGMTkDEcLQFTANALALAMER	Unambiguous	1	1	Q60692	N-Term(iTRAQ8plex); K5(iTRAQ8plex); C8(Methylthio)	CID	1.0000	0.0000	1	1	3686895	Unique	Used	1.180	0.838	1.084	1.073	1E-05	0	7.28	1	16	30	3	1032.84387	3096.51706	-8.95
rEHANIDAQSGSQAPnPSTTISPGk	Unambiguous	3	1	Q8VDQ8-2	N-Term(iTRAQ8plex); N16(Deamidated); K25(iTRAQ8plex)	CID	1.0000	0.0000	1	1	3161464	Unique	Used	0.770	0.119	1.131	1.393	1E-05	0	7.28	1	18	123	3	1058.22644	3172.66477	6.43
wGEAGAEYVVESTGVFTTMEk	Unambiguous	3	1	P16858	N-Term(iTRAQ8plex); K21(iTRAQ8plex)	CID	1.0000	0.0000	1	1	3218870	Unique	Used		0.799	1.264	1.133	1E-05	0	7.27	0	0	200	2	1450.23645	2899.46562	0.40
yAqPAAATAAAYSDSYGR	Unambiguous	5	1	Q8BIF2-2	N-Term(iTRAQ8plex); Q3(Deamidated)	CID	1.0000	0.0000	1	1	3231442	Unique	Used	1.189	0.873	1.458	0.846	1E-05	0	7.27	0	0	93	2	1070.02832	2139.04936	9.22
gLENMGVAFQSMMTGGNVGk	Unambiguous	1	1	Q8VDQ1	N-Term(iTRAQ8plex); K20(iTRAQ8plex)	CID	1.0000	0.0000	1	1	3805823	Unique	Used	1.010	0.905	0.998	1.123	1E-05	0	7.27	0	0	46	2	1318.66602	2636.32475	-7.90
vVTQLTGTLPSGVTEDDVALHcNSR	Unambiguous	6	1	Q0VF62	N-Term(iTRAQ8plex); C22(Methylthio)	CID	1.0000	0.0000	1	1	3539622	Unique	Used	1.127	1.169	0.753	0.806	1E-05	0	7.27	0	10	56	3	988.16608	2962.48368	-2.69
fQMPDQGMTSADDFFQGTk	Unambiguous	1	1	O08553	N-Term(iTRAQ8plex); K19(iTRAQ8plex)	CID	1.0000	0.0000	1	1	3307528	Unique	Used				0.606	1E-05	0	7.27	0	8	200	3	920.44476	2759.31974	-2.49
eIGncTQITNLDLQHNDLLDLPDTIGNLSSLNR	Unambiguous	1	1	O88520	N-Term(iTRAQ8plex); N4(Deamidated); C5(Methylthio)	CID	0.0151	0.0000	1	1	3645282	Unique	Used	0.554	0.688	0.950	0.986	1E-05	0	7.27	0	0	18	3	1334.33545	4000.99179	0.48
eVAAFAQFGSDLDAATQQLLSR	Unambiguous	2	1	Q03265	N-Term(iTRAQ8plex)	CID	1.0000	0.0000	1	1	3668387	Unique	Used	0.416	0.858	1.244	1.326	1E-05	0	7.27	0	9	200	2	1321.68445	2642.36162	-4.25
eGSLSPASVSSDTLSDLGISGLqDGLAFHMR	Unambiguous	2	1	Q5SWU9	N-Term(iTRAQ8plex); Q23(Deamidated)	CID	1.0000	0.0000	1	1	3560732	Unique	Used	1.307	1.194	1.214	1.282	1E-05	0	7.27	0	0	76	3	1151.58020	3452.72605	2.96
fQmPDQGMTSADDFFQGTk	Unambiguous	1	1	O08553	N-Term(iTRAQ8plex); M3(Oxidation); K19(iTRAQ8plex)	CID	0.0440	0.0000	1	1	3306774	Unique	Used		1.449	0.983	1.516	1E-05	0	7.27	0	6	200	3	925.77679	2775.31583	-2.05

lADELHMPSLPEMMFGDNVLR	Unambiguous	1	1	Q8BH58	N-Term(iTRAQ8plex)	CID	1.0000	0.0000	1	1	3520232	Unique	Used	0.783	1.653	0.946	0.774	1E-05	0	7.27	0	7	16	3	907.12054	2719.34708	-3.30
dkAPGQLEcETAIAALNScLR	Unambiguous	1	1	P26039	N-Term(iTRAQ8plex); K2(iTRAQ8plex); C9(Methylthio); C19(Methylthio)	CID	1.0000	0.0000	1	1	3785504	Unique	Used	0.861	1.085	0.730	0.973	1E-05	0	7.27	1	2	17	3	968.49225	2903.46219	-2.98
gIVDQSQQAYQEAFEISk	Unambiguous	1	1	P63101	N-Term(iTRAQ8plex); K18(iTRAQ8plex)	CID	1.0000	0.0000	1	1	3110111	Unique	Used	1.234	1.559	1.412	1.048	1E-05	0	7.27	0	0	200	2	1325.19946	2649.39165	-2.42
lEGVLAEVAQHYQDTLIR	Unambiguous	1	1	Q9JKF1	N-Term(iTRAQ8plex)	CID	1.0000	0.0000	1	1	3666232	Unique	Used	1.022	1.367	0.830	1.075	1E-05	0	7.27	0	6	11	3	787.09937	2359.28354	-3.74
fPEEGISPqNPDSPGLSDLNcGHLR	Unambiguous	2	1	D3Z0D9	N-Term(iTRAQ8plex); Q9(Deamidated); C21(Methylthio)	CID	0.0096	0.0000	1	1	3302658	Unique	Used	1.553	0.906	1.521	3.350	1E-05	0	7.27	0	0	99	3	1010.81671	3030.43558	3.83
gSLNPENPVQVSMDHLTTAIYDLLR	Unambiguous	3	1	Q61194-2	N-Term(iTRAQ8plex)	CID	1.0000	0.0000	1	1	3861282	Unique	Used			0.556	1.075	1E-05	0	7.27	0	22	62	3	1029.87183	3087.60093	-2.53
mcDPGMTAFEPEALGNLVEGLDFHR	Unambiguous	4	1	P11798	N-Term(iTRAQ8plex); C2(Methylthio)	CID	1.0000	0.0000	1	1	3480057	Unique	Used		1.895	1.871	1.129	1E-05	0	7.26	0	0	0	3	1033.81665	3099.43540	0.09
gLIEIISNAAEYENIPIR	Unambiguous	1	1	Q6P4T2	N-Term(iTRAQ8plex)	CID	1.0000	0.0000	1	1	3581388	Unique	Used	0.524	0.851	0.823	1.066	1E-05	0	7.26	0	10	15	2	1160.14270	2319.27812	-3.50
dMAIATGGAVFGEEGLNLNLEDVQAHDLGk	Unambiguous	1	1	P63038	N-Term(iTRAQ8plex); K30(iTRAQ8plex)	CID	1.0000	0.0000	1	1	3326517	Unique	Used	1.103	0.898	1.160	1.565	1E-05	0	7.26	0	0	31	3	1231.63708	3692.89670	-2.29
vTITVDEYSSNPTQAFTHYNINQSR	Unambiguous	3	1	B1APT1	N-Term(iTRAQ8plex)	CID	1.0000	0.0000	1	1	3537434	Unique	Used	1.511	0.944	1.199	1.320	1E-05	0	7.26	0	12	77	3	1063.85803	3189.55954	-4.99
mELLHPLGSDSPITGFLQk	Unambiguous	1	1	Q9D1I5	N-Term(iTRAQ8plex); M1(Oxidation); K19(iTRAQ8plex)	CID	1.0000	0.0000	1	1	3744707	Unique	Used	1.634	1.083	1.650	0.867	1E-05	0	7.26	0	3	18	3	903.16730	2707.48734	-2.82
eSTVTLQQAEYEFLSFVR	Unambiguous	1	1	Q9D8S4	N-Term(iTRAQ8plex)	CID	1.0000	0.0000	1	1	3396449	Unique	Used	2.122	1.218	1.503	1.093	1E-05	0	7.26	0	12	10	2	1226.13220	2451.25713	-5.64
nVAQYNANHPDFPMQIEQLER	Unambiguous	1	1	Q9JHU4	N-Term(iTRAQ8plex)	CID	1.0000	0.0000	1	1	3279884	Unique	Used	0.984	1.287	0.981	1.032	1E-05	0	7.26	0	18	91	3	940.13043	2818.37674	-4.17

eLSSAVSPPnLHSDTPTFSYASLAGPTIPPR	Unambiguous	3	1	Q9QYR6	N-Term(iTRAQ8plex); N10(Deamidated)	CID	1.0000	0.0000	1	1	3705054	Unique	Used	2.054	1.144	1.634	0.868	1E-05	0	7.26	0	0	95	3	1172.26917	3514.79294	-2.25
fLVLDEADGLLSQGYSDFINR	Unambiguous	1	1	Q91VR5	N-Term(iTRAQ8plex)	CID	1.0000	0.0000	1	1	3500879	Unique	Used	1.574	0.881	0.869	1.096	1E-05	0	7.25	0	0	118	3	892.79462	2676.36930	-4.87
aTQWGSFYSPAQTANNPcSR	Unambiguous	3	1	Q68FD9	N-Term(iTRAQ8plex); C18(Methylthio)	CID	1.0000	0.0000	1	1	3915012	Unique	Used	0.282	0.948	1.519	0.673	1E-05	0	7.25	0	0	200	2	1268.58105	2536.15483	-4.06
aEALAQISAAFEDLEQALQQR	Unambiguous	2	1	Q3TDT0	N-Term(iTRAQ8plex)	CID	1.0000	0.0000	1	1	3667030	Unique	Used	0.719	1.383	0.628	0.881	1E-05	0	7.25	0	14	200	2	1303.68176	2606.35625	-6.37
sASSTSSLFSSSSQLFPPSR	Unambiguous	6	1	Q3TQ29	N-Term(iTRAQ8plex)	CID	1.0000	0.0000	1	1	3934020	Unique	Used	0.513	0.627	0.870	1.080	1E-05	0	7.25	0	0	200	2	1182.59814	2364.18901	-4.02
sMFLQMGTTAGPPGEAGGGAGMAEAPR	Unambiguous	1	1	Q6R891	N-Term(iTRAQ8plex)	CID	1.0000	0.0000	1	1	3476593	Unique	Used	0.905	0.763	1.554	0.682	1E-05	0	7.25	0	0	60	2	1427.18103	2853.35478	-3.12
tSRPENAIIYSnNEDFQVGQAk	Unambiguous	1	1	P40142	N-Term(iTRAQ8plex); N12(Deamidated); K22(iTRAQ8plex)	CID	1.0000	0.0000	1	1	3125218	Unique	Used	2.123	0.978	1.033	1.046	1E-05	0	7.24	0	0	200	3	1030.87634	3090.61448	6.21
qTIEnSQGAYQEAFDISk	Unambiguous	4	1	P68254-2	N-Term(iTRAQ8plex); N5(Deamidated); K18(iTRAQ8plex)	CID	0.0124	0.0000	1	1	2937453	Unique	Used	0.898	0.997	0.844	0.950	1E-05	0	7.24	0	0	197	2	1319.68176	2638.35625	4.00
mNLFQSITSALDNSLAk	Unambiguous	2	1	Q6P3A8	N-Term(iTRAQ8plex); K17(iTRAQ8plex)	CID	1.0000	0.0000	1	1	3880195	Unique	Used	0.495	0.736	0.619	0.969	1E-05	0	7.24	0	18	20	2	1231.17859	2461.34990	-3.34
vIcVGLNYADHcQEQNVR	Unambiguous	1	1	Q3TC72	N-Term(iTRAQ8plex); C3(Methylthio); C12(Methylthio)	CID	1.0000	0.0000	1	1	3664990	Unique	Used	1.741	1.115	1.045	1.411	1E-05	0	7.24	0	13	24	3	819.71405	2457.12760	-7.06
eGGPNPENNSSLANILELcR	Unambiguous	1	1	Q8K0Z7	N-Term(iTRAQ8plex); C19(Methylthio)	CID	1.0000	0.0000	1	1	3459467	Unique	Used	0.598	0.573	0.937	0.965	1E-05	0	7.24	0	12	108	2	1239.10364	2477.20000	-2.70
sQAYADYIGFILTLNEGVk	Unambiguous	2	1	P58389	N-Term(iTRAQ8plex); K19(iTRAQ8plex)	CID	1.0000	0.0000	1	1	3706917	Unique	Used	0.625	1.719	0.964	1.129	1E-05	0	7.24	0	0	29	2	1355.73853	2710.46977	-7.92

gVMLAVDAVIAELk	Unambiguous	2	1	P63038	N-Term(iTRAQ8plex); K14(iTRAQ8plex)	CID	1.0000	0.0000	1	1	3602630	Unique	Used	1.307	1.213	1.016	0.859	1E-05	0	7.24	0	5	14	2	1019.11023	2037.21318	-5.25
gEMMDLQHGSLFLQTPk	Unambiguous	2	1	P16125	N-Term(iTRAQ8plex); K17(iTRAQ8plex)	CID	1.0000	0.0000	1	1	3476066	Unique	Used	0.568	1.060	1.190	1.428	1E-05	0	7.24	0	0	14	3	847.45209	2540.34171	-1.77
lQELTGSEGQVFMENVTQLLQSSHk	Unambiguous	2	1	Q8WTY4	N-Term(iTRAQ8plex); K25(iTRAQ8plex)	CID	1.0000	0.0000	1	1	3686878	Unique	Used	1.891	0.731	1.023	0.992	1E-05	0	7.24	0	0	16	3	1137.93164	3411.78037	-6.90
dEcLQFTANALALAMER	Unambiguous	1	1	Q60692	N-Term(iTRAQ8plex); C3(Methylthio)	CID	1.0000	0.0000	1	1	3727460	Unique	Used	1.722	0.774	0.897	0.811	1E-05	0	7.24	0	24	94	2	1123.54041	2246.07353	-8.30
iTQSSLTIcFPEYTGANk	Unambiguous	1	1	P08752	N-Term(iTRAQ8plex); C9(Methylthio); K18(iTRAQ8plex)	CID	1.0000	0.0000	1	1	3726538	Unique	Used	0.371	1.088	0.841	1.502	1E-05	0	7.24	0	13	12	2	1314.18115	2627.35503	-4.54
iQVLQQQADDAEER	Unambiguous	4	1	D3Z6I8	N-Term(iTRAQ8plex)	CID	1.0000	0.0000	1	1	2653124	Unique	Used	1.729	1.400	1.434	0.827	1E-05	0	7.24	0	4	95	2	974.00916	1947.01103	1.28
tLSLDEVYLIDSGAQYk	Unambiguous	1	1	Q6P1B1	N-Term(iTRAQ8plex); K17(iTRAQ8plex)	CID	1.0000	0.0000	1	1	3238777	Unique	Used	0.402	1.269	0.957	0.851	1E-05	0	7.24	0	6	26	2	1262.19189	2523.37651	-1.49
vVLAEVIQAFSAPEnAVR	Unambiguous	1	1	O35127	N-Term(iTRAQ8plex); N15(Deamidated)	CID	1.0000	0.0000	1	1	3645668	Unique	Used	1.815	1.464	1.332	1.155	1E-05	0	7.24	0	0	22	2	1109.61707	2218.22685	-5.28
aSFSYGALESNDSISELYTPATLPVMqEEHSSR	Unambiguous	1	1	Q8BGN5	N-Term(iTRAQ8plex); Q27(Deamidated)	CID	1.0000	0.0000	1	1	3283954	Unique	Used	0.853	0.882	1.417	1.072	1E-05	0	7.23	0	13	52	3	1307.96387	3921.87705	3.25
sSVSQSPAPTYPSDSHYTSLEqNVPR	Unambiguous	2	1	O88737	N-Term(iTRAQ8plex); Q22(Deamidated)	CID	0.0041	0.0000	1	1	3186611	Unique	Used	1.649	1.072	3.182	2.139	1E-05	0	7.23	0	0	44	3	1047.17932	3139.52341	3.57
eLANDELILTFGADDVVcTR	Unambiguous	1	1	P62965	N-Term(iTRAQ8plex); C18(Methylthio)	CID	1.0000	0.0000	1	1	3133330	Unique	Used	1.714	0.773	0.993	2.013	1E-05	0	7.23	0	0	79	2	1272.63379	2544.26030	-0.99
eHVISIEDSFVNSQEWTLSR	Unambiguous	3	1	F8VQE9	N-Term(iTRAQ8plex)	CID	1.0000	0.0000	1	1	3303774	Unique	Used	1.528	1.040	1.148	0.842	1E-05	0	7.23	0	22	95	3	894.12256	2680.35312	0.39

iWcFGPDGTGPNILTDITk	Unambiguous	1	1	P58252	N-Term(iTRAQ8plex); C3(Methylthio); K19(iTRAQ8plex)	CID	1.0000	0.0000	1	1	3706883	Unique	Used	0.316	0.747	0.838	0.904	1E-05	0	7.23	0	0	32	2	1351.69958	2702.39189	-8.28
aTYNNTQIQVVSATNASLAR	Unambiguous	2	1	Q3U8Y1	N-Term(iTRAQ8plex)	CID	1.0000	0.0000	1	1	3779840	Unique	Used	0.671	0.324	1.048	0.751	1E-05	0	7.23	0	7	200	2	1213.64514	2426.28301	-4.59
lSTVASTDILATVLEEMPPFPER	Unambiguous	1	1	P17427	N-Term(iTRAQ8plex)	CID	1.0000	0.0000	1	1	3602716	Unique	Used		2.637	1.145	1.321	1E-05	0	7.23	0	0	67	2	1410.74585	2820.48442	-5.79
aSTNIQDTEGNTPLHLAcDEER	Unambiguous	1	1	Q9Z2X2	N-Term(iTRAQ8plex); C18(Methylthio)	CID	1.0000	0.0000	1	1	2759685	Unique	Used	1.214	1.057	0.958	0.966	1E-05	0	7.23	0	23	172	3	922.09656	2764.27512	-2.50
gNDISSGTVLSDYVGSGPPSGTGLHR	Unambiguous	1	1	P70296	N-Term(iTRAQ8plex)	CID	1.0000	0.0000	1	1	3639639	Unique	Used	1.085	1.167	1.038	1.204	1E-05	0	7.23	0	0	57	3	945.47156	2834.40012	-7.83
sGTAHEYSScPDDAIFQSLAR	Unambiguous	1	1	Q00493	N-Term(iTRAQ8plex); C10(Methylthio)	CID	1.0000	0.0000	1	1	3301833	Unique	Used	2.090	1.182	0.912	1.271	1E-05	0	7.22	0	14	43	3	869.06897	2605.19236	-1.59
eMQDAFQNAYMELGGLGER	Unambiguous	3	1	Q6PIE5	N-Term(iTRAQ8plex)	CID	1.0000	0.0000	1	1	3352321	Unique	Used	0.806	0.588	1.013	1.577	1E-05	0	7.22	0	26	23	2	1232.07629	2463.14531	-5.43
eQINQQAQLQEFGQSSR	Unambiguous	7	1	F7BSN3	N-Term(iTRAQ8plex)	CID	1.0000	0.0000	1	1	3104230	Unique	Used	0.587	0.804	0.692	0.861	1E-05	0	7.22	0	0	200	2	1148.08435	2295.16142	-0.75
dHFmDEFFEQVEEIR	Unambiguous	1	1	P61264	N-Term(iTRAQ8plex); M4(Oxidation)	CID	1.0000	0.0000	1	1	2814976	Unique	Used	0.600	0.656	1.001	1.000	1E-05	0	7.22	0	0	42	3	764.35742	2291.05771	-0.68
dFSALESQLQDTQELLQEENR	Unambiguous	1	1	Q8VDD5	N-Term(iTRAQ8plex)	CID	1.0000	0.0000	1	1	2942289	Unique	Used	2.162	1.198	0.936	0.813	1E-05	0	7.22	0	1	10	2	1399.19141	2797.37554	-1.39
eMEENFALEAANYQDTIGR	Unambiguous	1	1	P20152	N-Term(iTRAQ8plex)	CID	1.0000	0.0000	1	1	2815253	Unique	Used	1.443	4.259	0.884	1.762	1E-05	0	7.22	0	0	200	2	1253.09558	2505.18389	-1.24
rPDEISQILAQSQGSITLk	Unambiguous	2	1	Q6XE40	N-Term(iTRAQ8plex); K19(iTRAQ8plex)	CID	1.0000	0.0000	1	1	3704630	Unique	Used	1.411	1.033	1.665	1.761	1E-05	0	7.22	0	7	20	3	898.18042	2692.52671	-6.95
nNAASEGVLASFFNSLLSk	Unambiguous	1	1	Q6PDL0	N-Term(iTRAQ8plex); K19(iTRAQ8plex)	CID	1.0000	0.0000	1	1	3880122	Unique	Used	1.384	0.828	1.096	1.019	1E-05	0	7.22	0	30	16	2	1289.20422	2577.40117	-4.72
lTEDDETFLSANASAVLSQFEk	Unambiguous	1	1	Q8K3C3	N-Term(iTRAQ8plex); K22(iTRAQ8plex)	CID	1.0000	0.0000	1	1	3305519	Unique	Used	1.413	0.998	1.024	0.743	1E-05	0	7.22	0	13	50	2	1512.28003	3023.55278	-4.71

iAEnNDLLWMDYHQk	Unambiguous	3	1	O08539	N-Term(iTRAQ8plex); N4(Deamidated); K15(iTRAQ8plex)	CID	0.0499	0.0000	1	1	2938375	Unique	Used	0.923	0.961	1.442	0.872	1E-05	0	7.22	0	4	12	3	833.76337	2499.27555	-1.73
eTFGVNNTVYGIDAMNPSSR	Unambiguous	2	1	Q3UGC7	N-Term(iTRAQ8plex)	CID	1.0000	0.0000	1	1	3370165	Unique	Used	1.098	0.666	1.757	0.636	1E-05	0	7.21	0	15	20	2	1238.60095	2476.19463	-5.43
gAGQTVTIIAQYQPEDYAR	Unambiguous	9	1	Q91XM9-7	N-Term(iTRAQ8plex)	CID	1.0000	0.0000	1	1	3322633	Unique	Used	1.490	2.815	2.024	1.087	1E-05	0	7.21	0	0	105	2	1193.11938	2385.23149	-1.57
dQLQTFSEEHPVLLTEAPLNPR	Unambiguous	1	1	P61164	N-Term(iTRAQ8plex)	CID	1.0000	0.0000	1	1	3214195	Unique	Used	0.641	0.704	0.896	0.822	1E-05	0	7.21	0	0	3	3	946.83514	2838.49088	-1.10
eVAAFAQFGSDLDAATQQLLSR	Unambiguous	2	1	Q03265	N-Term(iTRAQ8plex)	CID	1.0000	0.0000	1	1	3668823	Unique	Used	0.433	0.897	1.003	1.265	1E-05	0	7.21	0	0	200	2	1321.68396	2642.36064	-4.62
eYQLNDSAAYYLnDLER	Unambiguous	1	1	P08752	N-Term(iTRAQ8plex); N13(Deamidated)	CID	1.0000	0.0000	1	1	2637975	Unique	Used	0.567	1.265	1.022	1.388	1E-05	0	7.21	0	0	200	2	1191.57129	2382.13530	-2.11
qqSAPVSTLYPSTSNLLTNHQHEGR	Unambiguous	2	1	Q3UGN9	N-Term(iTRAQ8plex); Q2(Deamidated)	CID	0.0042	0.0000	1	1	3636497	Unique	Used	2.189	1.065	1.412	0.929	1E-05	0	7.21	0	0	92	3	1024.19043	3070.55674	2.32
sEPMSELSGATTSLATVALDPSSDR	Unambiguous	4	1	Q924N4	N-Term(iTRAQ8plex)	CID	1.0000	0.0000	1	1	3284110	Unique	Used	0.848	0.825	1.216	0.959	1E-05	0	7.21	0	25	26	2	1413.70251	2826.39775	-0.14
sVNESLNNLFITEEDYQALR	Unambiguous	2	1	Q68FD5	N-Term(iTRAQ8plex)	CID	1.0000	0.0000	1	1	3239070	Unique	Used	0.509	1.550	1.167	1.172	1E-05	0	7.21	0	0	35	2	1330.17920	2659.35112	-0.23
fQMPDQGMTSADDFFQGTk	Unambiguous	1	1	O08553	N-Term(iTRAQ8plex); K19(iTRAQ8plex)	CID	1.0000	0.0000	1	1	3283229	Unique	Used	1.584	1.098	1.003	0.899	1E-05	0	7.21	0	0	16	3	920.44397	2759.31736	-3.35
sFcPGGTDSVSPPPSIITQENLGR	Unambiguous	1	1	Q6ZPJ3	N-Term(iTRAQ8plex); C3(Methylthio)	CID	1.0000	0.0000	1	1	3600750	Unique	Used	0.750	0.661	0.784	1.011	1E-05	0	7.21	0	0	46	2	1405.18616	2809.36504	-5.44
kPGMFFNPEESELDLTYGNR	Unambiguous	1	1	Q00612	N-Term(iTRAQ8plex); K1(iTRAQ8plex)	CID	1.0000	0.0000	1	1	3259149	Unique	Used	0.499	0.553	1.032	1.273	1E-05	0	7.21	0	1	5	3	984.83667	2952.49546	-2.30
tISASTQVQGGDFNLYENMR	Unambiguous	1	1	Q9EQF6	N-Term(iTRAQ8plex)	CID	1.0000	0.0000	1	1	3369862	Unique	Used	0.708	0.554	0.467	0.608	1E-05	0	7.21	0	0	191	2	1268.12158	2535.23589	-3.66
nEAIQAAHDSVAQEGQcR	Unambiguous	1	1	Q9R0P9	N-Term(iTRAQ8plex); C17(Methylthio)	CID	1.0000	0.0000	1	1	3229514	Unique	Used	1.544	0.915	1.283	1.156	1E-05	0	7.21	0	0	200	2	1139.03125	2277.05522	-4.48
gNDISSGTVLSDYVGSGPPSGTGLHR	Unambiguous	1	1	P70296	N-Term(iTRAQ8plex)	CID	1.0000	0.0000	1	1	3495190	Unique	Used	1.459	1.552	1.950	1.615	1E-05	0	7.20	0	0	56	2	1417.71143	2834.41557	-2.37

sGTVPTPLVVGLGAAcELAQQEMEYDHk	Unambiguous	3	1	Q8C6I5	N-Term(iTRAQ8plex); C16(Methylthio); K28(iTRAQ8plex)	CID	1.0000	0.0000	1	1	3374165	Unique	Used	0.826	0.829	0.747	1.234	1E-05	0	7.20	0	23	43	3	1199.93872	3597.80161	-5.46
fQmPDQGMTSADDFFQGTk	Unambiguous	1	1	O08553	N-Term(iTRAQ8plex); M3(Oxidation); K19(iTRAQ8plex)	CID	0.0444	0.0000	1	1	3303283	Unique	Used	1.559	1.074	1.406	1.244	1E-05	0	7.20	0	11	47	3	925.77612	2775.31382	-2.77
sGMAQQIAYEMHLEqQSEGELQk	Unambiguous	5	1	A2ACC6	N-Term(iTRAQ8plex); Q15(Deamidated); K23(iTRAQ8plex)	CID	0.0389	0.0000	1	1	2995404	Unique	Used	0.759	0.963	1.477	1.265	1E-05	0	7.20	0	0	200	3	1082.21143	3244.61972	3.76
vIPAFMcQAGDFTNHnGTGGR	Unambiguous	1	1	Q99KR7	N-Term(iTRAQ8plex); C7(Methylthio); N16(Deamidated)	CID	1.0000	0.0000	1	1	3726490	Unique	Used	1.400	0.661	1.351	1.270	1E-05	0	7.20	0	14	18	3	848.72296	2544.15433	-7.58
ecQAALEVLQESPLYDcR	Unambiguous	2	1	O08842	N-Term(iTRAQ8plex); C2(Methylthio); C17(Methylthio)	CID	1.0000	0.0000	1	1	3374229	Unique	Used	0.505	0.380	0.694	0.959	1E-05	0	7.20	0	15	74	2	1232.06335	2463.11943	-5.53
lLGDASVSFTDNcVVGIQANTER	Unambiguous	3	1	P97807-2	N-Term(iTRAQ8plex); C13(Methylthio)	CID	1.0000	0.0000	1	1	3583216	Unique	Used	1.536	1.218	2.018	2.194	1E-05	0	7.20	0	0	200	2	1380.17969	2759.35210	-4.55
nSLcPSGGNILTPLLQQDcHQk	Unambiguous	1	1	P35762	N-Term(iTRAQ8plex); C4(Methylthio); C19(Methylthio); K22(iTRAQ8plex)	CID	1.0000	0.0000	1	1	3805513	Unique	Used	0.264	0.311	0.900	1.101	1E-05	0	7.20	0	14	6	3	1022.84967	3066.53446	-3.56
tISASTQVQGGDFNLYENMR	Unambiguous	1	1	Q9EQF6	N-Term(iTRAQ8plex)	CID	1.0000	0.0000	1	1	3346698	Unique	Used	0.518	0.232	0.408	0.563	1E-05	0	7.20	0	0	6	2	1268.12134	2535.23540	-3.85
kSQVFSTAADGQTQVEIk	Unambiguous	1	1	P38647	N-Term(iTRAQ8plex); K1(iTRAQ8plex); K18(iTRAQ8plex)	CID	1.0000	0.0000	1	1	3253172	Unique	Used	1.616	0.931	1.237	1.187	1E-05	0	7.20	1	0	54	3	950.53937	2849.60355	-3.53

gPMTGGIQGPGPINMGAGGPQGPR	Unambiguous	1	1	Q8C7E9	N-Term(iTRAQ8plex)	CID	1.0000	0.0000	1	1	3893958	Unique	Used	1.920	0.979	1.044	0.548	1E-05	0	7.19	0	0	200	2	1254.63208	2508.25688	-7.39
qLVSSMDVAETNVFFYPR	Unambiguous	1	1	G3X972	N-Term(iTRAQ8plex)	CID	1.0000	0.0000	1	1	3644919	Unique	Used	0.716	0.916	0.848	1.161	1E-05	0	7.19	0	10	29	2	1204.11133	2407.21538	-4.83
gEDSSPENILTYVAHTSALTYPPATR	Unambiguous	1	1	P80560	N-Term(iTRAQ8plex)	CID	1.0000	0.0000	1	1	3540826	Unique	Used		1.473	0.889	0.729	1E-05	0	7.19	0	19	14	3	1032.19031	3094.55637	-2.31
dVDFVTALcSQVVYEGLVDDTFR	Unambiguous	1	1	P59016	N-Term(iTRAQ8plex); C9(Methylthio)	CID	1.0000	0.0000	1	1	3582214	Unique	Used		1.966	1.704	1.663	1E-05	0	7.19	0	0	200	2	1471.21265	2941.41802	-2.92
dQDLNTYSLLAVFAATDGGITR	Unambiguous	7	1	Q6PHS9-4	N-Term(iTRAQ8plex)	CID	1.0000	0.0000	1	1	3581539	Unique	Used	0.532	0.433	0.797	1.233	1E-05	0	7.19	0	22	16	2	1323.18274	2645.35820	-5.40
yTPSGqSGAAASESLFISNHAY	Unambiguous	2	1	A6ZI44	N-Term(iTRAQ8plex); Q6(Deamidated)	CID	0.0181	0.0000	1	1	3234139	Unique	Used	1.277	0.892	1.171	1.121	1E-05	0	7.19	0	0	176	2	1282.12512	2563.24297	6.83
dLLETPGqYLVYNGDLVEYDADHMAQLQR	Unambiguous	1	1	Q6PGF7	N-Term(iTRAQ8plex); Q8(Deamidated)	CID	0.0209	0.0000	1	1	3262412	Unique	Used	5.148	1.080			1E-05	0	7.19	0	0	21	3	1224.60498	3671.80039	4.41
gRDPSPGLSSQEAnGQPTk	Unambiguous	4	1	Q8CFT0	N-Term(iTRAQ8plex); N14(Deamidated); K19(iTRAQ8plex)	CID	0.0473	0.0000	1	1	2663071	Unique	Used	1.033	0.405	0.654	0.640	1E-05	0	7.19	1	0	200	3	845.77740	2535.31766	-3.28
lADAQSEAAMEEIEATVR	Unambiguous	1	1	Q8K3A0	N-Term(iTRAQ8plex)	CID	1.0000	0.0000	1	1	2742802	Unique	Used	1.931	1.541	2.099	1.499	1E-05	0	7.19	0	21	200	2	1119.56262	2238.11797	-2.08
aIELQPHFPDAYcNLANALk	Unambiguous	2	1	Q8CGY8	N-Term(iTRAQ8plex); C13(Methylthio); K20(iTRAQ8plex)	CID	1.0000	0.0000	1	1	3706267	Unique	Used	0.460	1.250	1.202	0.925	1E-05	0	7.19	0	2	6	3	961.50385	2882.49698	-6.38
kPVLGQAGTLAVGTEGGLR	Unambiguous	4	1	F8WIE1	N-Term(iTRAQ8plex); K1(iTRAQ8plex)	CID	1.0000	0.0000	1	1	3700530	Unique	Used	0.569	1.290	1.062	1.271	1E-05	0	7.18	0	24	92	3	811.48309	2432.43473	-4.06
vLSTSTDLEAAVADALLLGDAR	Unambiguous	2	1	J3QP81	N-Term(iTRAQ8plex)	CID	1.0000	0.0000	1	1	3396750	Unique	Used	0.379	0.364	0.893	0.970	1E-05	0	7.18	0	0	39	2	1253.18420	2505.36113	-4.12
aGLTMNDIDAFEFHEAFSGQILANFk	Unambiguous	1	1	Q99JY0	N-Term(iTRAQ8plex); K26(iTRAQ8plex)	CID	1.0000	0.0000	1	1	3601969	Unique	Used	0.800	0.874	0.615	1.211	1E-05	0	7.18	0	0	41	3	1165.59705	3494.77658	-3.14
eSTFGYVHGVSGPVVTAcDMAGAAMYELVR	Unambiguous	5	1	P50516	N-Term(iTRAQ8plex); C18(Methylthio)	CID	1.0000	0.0000	1	1	3861040	Unique	Used	20.158	1.842	1.032	1.512	1E-05	0	7.18	0	21	13	3	1156.54797	3467.62937	-3.38

eTLVQDNIqWPTGLAVDYHNER	Unambiguous	1	1	Q91ZX7	N-Term(iTRAQ8plex); Q9(Deamidated)	CID	1.0000	0.0000	1	1	3303346	Unique	Used	1.130	1.708	0.924	0.822	1E-05	0	7.18	0	30	141	3	968.49255	2903.46311	5.28
dVLLVAQGEMALEEFLk	Unambiguous	1	1	Q9JHU4	N-Term(iTRAQ8plex); K17(iTRAQ8plex)	CID	1.0000	0.0000	1	1	3305912	Unique	Used	1.032	0.665			1E-05	0	7.18	0	7	200	3	838.47449	2513.40891	-2.27
vLGAHILGPGAGEmVNEAALALEYGAScEDIAR	Unambiguous	1	1	O08749	N-Term(iTRAQ8plex); M14(Oxidation); C28(Methylthio)	CID	1.0000	0.0000	1	1	3521288	Unique	Used			1.355	0.967	1E-05	0	7.18	0	26	200	3	1221.93921	3663.80307	-2.55
aQAALDSVSGLEQGHTQYLASSEDASALVAALTR	Unambiguous	1	1	Q9JKY5	N-Term(iTRAQ8plex)	CID	1.0000	0.0000	1	1	3623156	Unique	Used	0.487	0.419	0.669	0.647	1E-05	0	7.18	0	6	7	3	1245.63611	3734.89377	-5.41
fnSVVTNPQGHYNPSTGk	Unambiguous	1	1	Q02105	N-Term(iTRAQ8plex); N2(Deamidated); K18(iTRAQ8plex)	CID	0.0279	0.0000	1	1	3635190	Unique	Used	0.956	1.828	0.983	1.074	1E-05	0	7.18	0	29	200	3	852.78369	2556.33652	2.43
qASAAENHIHELEEALAGER	Unambiguous	2	1	P21619	N-Term(iTRAQ8plex)	CID	1.0000	0.0000	1	1	2813028	Unique	Used	1.009	1.120	1.618	1.658	1E-05	0	7.18	0	6	50	3	827.08722	2479.24710	-0.35
aLEQFLQEYFDGNLk	Unambiguous	1	1	P27773	N-Term(iTRAQ8plex); K15(iTRAQ8plex)	CID	1.0000	0.0000	1	1	3480075	Unique	Used	0.879	0.672	0.746	0.662	1E-05	0	7.18	0	14	25	2	1212.15393	2423.30058	-2.54
yTPSGQSGAAASESLFISNHAY	Unambiguous	2	1	A6ZI44	N-Term(iTRAQ8plex)	CID	1.0000	0.0000	1	1	3235540	Unique	Used	1.219	0.973	1.356	1.291	1E-05	0	7.18	0	0	67	2	1281.62170	2562.23613	-2.07
vEcVGSEGASEGIVLPLVSSnSGPR	Unambiguous	1	1	Q60625	N-Term(iTRAQ8plex); C3(Methylthio); N21(Deamidated)	CID	1.0000	0.0000	1	1	3284239	Unique	Used		8.519	3.894	0.691	1E-05	0	7.17	0	0	13	2	1397.69727	2794.38725	-1.18
scLIVEDSLLNSLPLPVAER	Unambiguous	2	1	B2RSI6	N-Term(iTRAQ8plex); C2(Methylthio)	CID	1.0000	0.0000	1	1	3707170	Unique	Used	0.440	0.795	1.306	1.133	1E-05	0	7.17	0	20	59	2	1259.67505	2518.34282	-5.36
yDELYSSLTTLLAAGSQLDTTR	Unambiguous	3	1	A2AN08-3	N-Term(iTRAQ8plex)	CID	1.0000	0.0000	1	1	3806777	Unique	Used	0.349	0.669	0.878	1.109	1E-05	0	7.17	0	8	42	2	1361.70593	2722.40459	-1.59
dIGAIAQVHAEnGDIIAEEQQR	Unambiguous	1	1	O08553	N-Term(iTRAQ8plex); N12(Deamidated)	CID	0.0139	0.0000	1	1	2830296	Unique	Used				1.429	1E-05	0	7.17	0	20	200	2	1341.69067	2682.37407	3.85
iDVPQDLEDDLTATPLSHATVPQSPAR	Unambiguous	2	1	Q8VDU5	N-Term(iTRAQ8plex)	CID	1.0000	0.0000	1	1	3111313	Unique	Used	1.888	0.723	0.990	0.917	1E-05	0	7.17	0	0	200	3	1064.22412	3190.65781	1.37

sADAPMFVMGVNHEk	Unambiguous	1	1	D3Z0Z9	N-Term(iTRAQ8plex); K15(iTRAQ8plex)	CID	1.0000	0.0000	1	1	3187473	Unique	Used	2.790	3.697	1.838	1.377	1E-05	0	7.17	0	0	14	3	747.72461	2241.15927	-1.08
lLGDASVSFTDNcVVGIQANTER	Unambiguous	3	1	P97807-2	N-Term(iTRAQ8plex); C13(Methylthio)	CID	1.0000	0.0000	1	1	3582097	Unique	Used	1.653	0.772	0.779	1.305	1E-05	0	7.17	0	13	200	2	1380.18481	2759.36235	-0.84
lNQPDcGYGAGGASVSVTPAqR	Unambiguous	1	1	Q3TL44	N-Term(iTRAQ8plex); C6(Methylthio); Q21(Deamidated)	CID	0.0014	0.0000	1	1	3494529	Unique	Used	0.364	0.872	1.202	1.703	1E-05	0	7.17	0	0	200	2	1250.10620	2499.20513	5.64
aGYTTDESSSSSLHTTR	Unambiguous	2	1	Q6P5B5	N-Term(iTRAQ8plex)	CID	1.0000	0.0000	1	1	2694873	Unique	Used	1.825	0.924	1.072	1.015	1E-05	0	7.17	0	0	200	3	702.00708	2104.00669	-1.40
kADNPQcLLGDFVTEFLk	Unambiguous	1	1	Q80UJ7	N-Term(iTRAQ8plex); K1(iTRAQ8plex); C7(Methylthio); K18(iTRAQ8plex)	CID	1.0000	0.0000	1	1	3500432	Unique	Used		1.451	0.674	1.048	1E-05	0	7.17	1	25	15	3	999.54596	2996.62333	-3.99
sqTEEDcTEELFDFLHAR	Unambiguous	1	1	P99028	N-Term(iTRAQ8plex); Q2(Deamidated); C7(Methylthio)	CID	1.0000	0.0000	1	1	2639909	Unique	Used	2.979	2.078	1.297	0.899	1E-05	0	7.17	0	1	82	3	841.04877	2521.13175	6.04
tQPTDEEMLFIYSHFk	Unambiguous	4	1	P31786	N-Term(iTRAQ8plex); K16(iTRAQ8plex)	CID	1.0000	0.0000	1	1	3283785	Unique	Used	1.830	0.743	1.038	0.745	1E-05	0	7.17	0	5	34	3	865.45123	2594.33915	-1.15
iAPVASALETTFDVVAAFSGVDLEGGTcSHPLTPDk	Unambiguous	3	1	Q8BIJ6	N-Term(iTRAQ8plex); C28(Methylthio); K36(iTRAQ8plex)	CID	1.0000	0.0000	1	1	3687632	Unique	Used	0.920	1.142	1.007	1.222	1E-05	0	7.17	0	13	200	3	1424.05530	4270.15134	-8.43
nLEYTASLLEAVYIDETR	Unambiguous	1	1	Q01065	N-Term(iTRAQ8plex)	CID	1.0000	0.0000	1	1	3305676	Unique	Used	0.436	0.863	2.371	1.247	1E-05	0	7.17	0	0	32	2	1202.62781	2404.24834	-2.76
nLEYTASLLEAVYIDETR	Unambiguous	1	1	Q01065	N-Term(iTRAQ8plex)	CID	1.0000	0.0000	1	1	3327240	Unique	Used	0.253	0.735	2.645	1.226	1E-05	0	7.17	0	0	20	2	1202.62903	2404.25078	-1.74

gQEHTIDELkQDSFPISLEqAVTDAAMTSk	Unambiguous	2	1	P20357	N-Term(iTRAQ8plex); K10(iTRAQ8plex); Q20(Deamidated); K30(iTRAQ8plex)	CID	0.0447	0.0000	1	1	3195644	Unique	Used	1.362	2.125	1.657	0.804	1E-05	0	7.16	1	0	93	3	1401.73730	4203.19736	1.86
iEDVLPFFPDFVTIDHFk	Unambiguous	1	1	Q8R307	N-Term(iTRAQ8plex); K18(iTRAQ8plex)	CID	1.0000	0.0000	1	1	3541572	Unique	Used	0.284	0.716	0.654	1.573	1E-05	0	7.16	0	0	21	3	929.84137	2787.50956	-4.41
ILGDASVSFTDncVVGIQANTER	Unambiguous	3	1	P97807-2	N-Term(iTRAQ8plex); N12(Deamidated); C13(Methylthio)	CID	1.0000	0.0000	1	1	3581400	Unique	Used	1.719	1.126	0.594	0.781	1E-05	0	7.16	0	25	63	2	1380.68127	2760.35527	2.39
aYFTEESGQIAVFDATNTTR	Unambiguous	4	1	Q6GTL7	N-Term(iTRAQ8plex)	CID	1.0000	0.0000	1	1	3214735	Unique	Used	0.952	0.955	1.083	1.303	1E-05	0	7.16	0	15	93	2	1263.12488	2525.24248	-1.48
fQmPDQGMTSADDFFQGTk	Unambiguous	1	1	O08553	N-Term(iTRAQ8plex); M3(Oxidation); K19(iTRAQ8plex)	CID	1.0000	0.0000	1	1	3281085	Unique	Used	0.888	0.781	0.944	1.113	1E-05	0	7.16	0	5	10	3	925.77661	2775.31528	-2.25
vATSAASGGHGSPLR	Unambiguous	4	1	A2AG50	N-Term(iTRAQ8plex)	CID	1.0000	0.0000	1	1	3631055	Unique	Used	1.232	0.506	1.464	1.158	1E-05	0	7.16	0	25	57	3	557.97113	1671.89884	-5.50
lPSPTAAPQQSASQATPVTQGQGR	Unambiguous	3	1	O88935	N-Term(iTRAQ8plex)	CID	1.0000	0.0000	1	1	3678120	Unique	Used	0.626	0.578	1.317	1.251	1E-05	0	7.16	0	2	28	3	894.80353	2682.39603	-5.71
dMAIATGGAVFGEEGLNLNLEDVQAHDLGk	Unambiguous	1	1	P63038	N-Term(iTRAQ8plex); K30(iTRAQ8plex)	CID	1.0000	0.0000	1	1	3263572	Unique	Used	0.818	0.726	1.072	1.128	1E-05	0	7.16	0	0	200	3	1231.63489	3692.89011	-4.07
vDQDQNVVHPQLLSLTSQLk	Unambiguous	2	1	Q91V14	N-Term(iTRAQ8plex); K20(iTRAQ8plex)	CID	1.0000	0.0000	1	1	3476335	Unique	Used	0.450	0.763	1.003	0.947	1E-05	0	7.16	0	0	8	3	957.54156	2870.61014	-3.31
aVSDASAGDYGSAIETLVTAISLIk	Unambiguous	4	1	Q6NVF9	N-Term(iTRAQ8plex); K25(iTRAQ8plex)	CID	1.0000	0.0000	1	1	3727731	Unique	Used		0.824	0.552	0.686	1E-05	0	7.16	0	0	138	2	1530.83655	3060.66582	-8.74
tVPLAGHVGFDSLPDQLVNk	Unambiguous	4	1	Q9R1T4-2	N-Term(iTRAQ8plex); K20(iTRAQ8plex)	CID	1.0000	0.0000	1	1	3517607	Unique	Used	0.571	0.964	1.332	1.603	1E-05	0	7.16	0	0	14	3	905.84418	2715.51798	-4.08
iNHFPEDTDYDHDSAEYLLR	Unambiguous	2	1	F7CZ64	N-Term(iTRAQ8plex)	CID	1.0000	0.0000	1	1	2760070	Unique	Used	3.661	3.399	1.094	1.461	1E-05	0	7.15	0	0	200	3	918.77081	2754.29789	1.07

iVENSEAVTEILNNAELLk	Unambiguous	1	1	Q8BJY1	N-Term(iTRAQ8plex); K19(iTRAQ8plex)	CID	1.0000	0.0000	1	1	3327011	Unique	Used	0.399	1.034	0.732	0.974	1E-05	0	7.15	0	0	37	2	1354.26477	2707.52226	-4.28
tSISTASATVSPASSAESAcTR	Unambiguous	1	1	P35831	N-Term(iTRAQ8plex); C20(Methylthio)	CID	1.0000	0.0000	1	1	3411088	Unique	Used	1.267	0.970	1.426	0.964	1E-05	0	7.15	0	15	137	2	1218.08264	2435.15801	-4.77
sSVSQSPAPTYPSDSHYTSLEQNVPR	Unambiguous	2	1	O88737	N-Term(iTRAQ8plex)	CID	1.0000	0.0000	1	1	3208907	Unique	Used	3.803	0.828	2.414	1.507	1E-05	0	7.15	0	0	40	3	1046.85010	3138.53574	2.41
lPQQSHSAFGAASSSSGFGk	Unambiguous	1	1	Q810A7	N-Term(iTRAQ8plex); K20(iTRAQ8plex)	CID	1.0000	0.0000	1	1	3657312	Unique	Used	0.885	0.667	1.061	0.667	1E-05	0	7.15	0	0	57	3	853.78082	2559.32792	-5.20
qQLGTAVEMEIAQMLEENSR	Unambiguous	2	1	Q9JKK7	N-Term(iTRAQ8plex)	CID	1.0000	0.0000	1	1	3327302	Unique	Used	1.844	1.370	1.589	1.322	1E-05	0	7.15	0	0	33	2	1291.14429	2581.28130	-3.54
lINQVLELQHTLEDLSAR	Unambiguous	3	1	Q78YZ6-3	N-Term(iTRAQ8plex)	CID	1.0000	0.0000	1	1	3727256	Unique	Used	2.826	0.750	1.204	0.932	1E-05	0	7.15	0	0	11	3	799.44769	2396.32853	-6.93
dAFEHIVTQFSSVPVSVVSDSYDIYNAcEk	Unambiguous	1	1	Q99KQ4	N-Term(iTRAQ8plex); C28(Methylthio); K30(iTRAQ8plex)	CID	1.0000	0.0000	1	1	3541546	Unique	Used	1.238	0.945	1.748	0.942	1E-05	0	7.15	0	8	31	3	1335.31482	4003.92990	-6.39
eMFGSGTAcVVcPVSDILYk	Unambiguous	3	1	Q8CBC8	N-Term(iTRAQ8plex); C9(Methylthio); C12(Methylthio); K20(iTRAQ8plex)	CID	1.0000	0.0000	1	1	3727101	Unique	Used	1.488	0.678	0.999	0.990	1E-05	0	7.15	0	0	40	2	1410.17957	2819.35185	-8.96
sLGGSATSLcLLPSEETSGEESR	Unambiguous	1	1	Q2WF71	N-Term(iTRAQ8plex); C10(Methylthio)	CID	1.0000	0.0000	1	1	3194373	Unique	Used	0.471	0.921	1.172	1.275	1E-05	0	7.15	0	20	118	2	1330.63354	2660.25981	-3.74
sYIEGYVPSQADVAVFEAVSGPPPADLcHALR	Unambiguous	3	1	O70251	N-Term(iTRAQ8plex); C28(Methylthio)	CID	1.0000	0.0000	1	1	3625462	Unique	Used	4.017				1E-05	0	7.15	0	24	200	3	1236.94409	3708.81772	-4.52
lPENVVAVPNLSEAVQDADLLVFVIPHqFIHk	Unambiguous	2	1	Q3ULJ0-2	N-Term(iTRAQ8plex); Q28(Deamidated); K32(iTRAQ8plex)	CID	0.0182	0.0000	1	1	3727340	Unique	Used		0.865	1.133	1.095	1E-05	0	7.15	0	14	46	3	1387.77490	4161.31015	-2.60

sAQLQHAVQSQGFIGYcQk	Unambiguous	2	1	Q3UPL0	N-Term(iTRAQ8plex); C17(Methylthio); K19(iTRAQ8plex)	CID	1.0000	0.0000	1	1	3820862	Unique	Used	0.619	0.991	0.682	0.989	1E-05	0	7.15	0	8	189	3	916.47632	2747.41440	-2.69
lTVPDDMAANcIYLNIPSk	Unambiguous	2	1	Q9CWS0	N-Term(iTRAQ8plex); C11(Methylthio); K19(iTRAQ8plex)	CID	1.0000	0.0000	1	1	3645069	Unique	Used	0.929	0.883	1.502	0.895	1E-05	0	7.15	0	7	13	2	1366.70959	2732.41191	-5.97
eEGNPVASMGHSEAETPVDAYSNGqAALMGER	Unambiguous	1	1	Q9JKZ2	N-Term(iTRAQ8plex); Q25(Deamidated)	CID	0.0014	0.0000	1	1	2636919	Unique	Used		1.403	1.719		1E-05	0	7.15	0	8	200	3	1203.88123	3609.62912	-2.36
sGDTVLYDLEAGSALLTLESR	Unambiguous	2	1	P58404-2	N-Term(iTRAQ8plex)	CID	1.0000	0.0000	1	1	3284746	Unique	Used	0.921	1.495	1.332	0.715	1E-05	0	7.15	0	0	34	2	1257.66455	2514.32182	-0.92
dDEEMNTWIQAISSAISSDk	Unambiguous	1	1	Q62261	N-Term(iTRAQ8plex); K20(iTRAQ8plex)	CID	1.0000	0.0000	1	1	3284795	Unique	Used	0.930	0.779	1.873	1.075	1E-05	0	7.15	0	0	65	2	1424.70947	2848.41167	-0.52
gLPHMFQQGGVFYNIMk	Unambiguous	1	1	Q8CDG3	N-Term(iTRAQ8plex); K17(iTRAQ8plex)	CID	1.0000	0.0000	1	1	3897269	Unique	Used	0.714	1.208	1.073	0.988	1E-05	0	7.15	0	0	85	3	859.12726	2575.36722	-3.96
wGEAGAEYVVESTGVFTTMEk	Unambiguous	3	1	P16858	N-Term(iTRAQ8plex); K21(iTRAQ8plex)	CID	1.0000	0.0000	1	1	3219078	Unique	Used	1.177	1.064	1.034	1.692	1E-05	0	7.15	0	3	200	3	967.15894	2899.46225	-0.76
fQMPDQGMTSADDFFQGTk	Unambiguous	1	1	O08553	N-Term(iTRAQ8plex); K19(iTRAQ8plex)	CID	1.0000	0.0000	1	1	3307725	Unique	Used			3.287		1E-05	0	7.15	0	12	200	3	920.44647	2759.32486	-0.63
tIGGGDDSFnTFFSETGAGk	Unambiguous	4	1	P68373	N-Term(iTRAQ8plex); N10(Deamidated); K20(iTRAQ8plex)	CID	1.0000	0.0000	1	1	3150679	Unique	Used	0.530	1.159	1.281	1.488	1E-05	0	7.14	0	0	200	2	1309.15784	2617.30840	7.84
aSFSYGALESnDSISELYTPATLPVMQEEHSSR	Unambiguous	1	1	Q8BGN5	N-Term(iTRAQ8plex); N11(Deamidated)	CID	1.0000	0.0000	1	1	3304942	Unique	Used	0.773	0.567	1.393	1.113	1E-05	0	7.14	0	0	21	3	1307.96313	3921.87485	2.69

lHISPSNMTNQNTPEYmEk	Unambiguous	1	1	P70288	N-Term(iTRAQ8plex); M17(Oxidation); K19(iTRAQ8plex)	CID	1.0000	0.0000	1	1	3229817	Unique	Used	1.083	0.630	0.942	0.663	1E-05	0	7.14	0	0	73	3	953.47864	2858.42136	-2.09
yTPSGQSGAAASESLFISNHAY	Unambiguous	2	1	A6ZI44	N-Term(iTRAQ8plex)	CID	1.0000	0.0000	1	1	3236013	Unique	Used	0.986	0.899	1.367	1.218	1E-05	0	7.14	0	12	114	2	1281.62366	2562.24004	-0.55
dELFALEQScAQVVLQAANER	Unambiguous	1	1	Q61553	N-Term(iTRAQ8plex); C10(Methylthio)	CID	1.0000	0.0000	1	1	3419737	Unique	Used	0.627	0.749	0.630	0.809	1E-05	0	7.14	0	0	29	2	1342.66406	2684.32085	-4.39
lLGDASVSFTDNcVVGIQANTER	Unambiguous	3	1	P97807-2	N-Term(iTRAQ8plex); C13(Methylthio)	CID	1.0000	0.0000	1	1	3582066	Unique	Used	1.565	1.156	1.027	1.148	1E-05	0	7.14	0	10	200	2	1380.17896	2759.35063	-5.08
rLSLESEGANEGATAAPELSALEEAFR	Unambiguous	1	1	Q7TQD2	N-Term(iTRAQ8plex)	CID	1.0000	0.0000	1	1	3132342	Unique	Used	2.504	2.547	1.590	1.580	1E-05	0	7.14	1	13	39	3	1041.53308	3122.58469	-1.98
nLHQSGFSLSGAQIDDNIPR	Unambiguous	1	1	O08553	N-Term(iTRAQ8plex)	CID	1.0000	0.0000	1	1	3494073	Unique	Used	1.345	1.487	1.133	0.984	1E-05	0	7.14	0	18	127	3	825.09271	2473.26358	-4.12
acADATLSqITNNIDPVGR	Unambiguous	3	1	P62874	N-Term(iTRAQ8plex); C2(Methylthio); Q9(Deamidated)	CID	0.0084	0.0000	1	1	3603235	Unique	Used	0.567	0.718	0.828	1.271	1E-05	0	7.13	0	0	200	2	1155.57556	2310.14385	2.87
gTVGEPTYDAEFQHFLR	Unambiguous	1	1	Q9JHU4	N-Term(iTRAQ8plex)	CID	1.0000	0.0000	1	1	3212801	Unique	Used	1.893	0.895	1.022	1.174	1E-05	0	7.13	0	0	148	3	757.71600	2271.13346	-0.59
eSLNANIVDAINQAADcWGIR	Unambiguous	4	1	Q99JB2	N-Term(iTRAQ8plex); C17(Methylthio)	CID	1.0000	0.0000	1	1	3806624	Unique	Used	1.057	0.516	1.027	1.038	1E-05	0	7.13	0	26	41	2	1312.14417	2623.28105	-3.83
qLFVLAGAAEEGFMTAELAGVIk	Unambiguous	1	1	B2RSH2	N-Term(iTRAQ8plex); K23(iTRAQ8plex)	CID	1.0000	0.0000	1	1	3581841	Unique	Used		1.085	1.070	0.755	1E-05	0	7.13	0	6	44	3	991.88647	2973.64487	-4.44
gDITLLEVDAIVNAANASLLGGGGVDGcIHR	Unambiguous	2	1	Q3UYG8	N-Term(iTRAQ8plex); C28(Methylthio)	CID	1.0000	0.0000	1	1	3727533	Unique	Used	1.185	1.097	1.348	1.025	1E-05	0	7.13	0	0	112	3	1124.24268	3370.71347	-7.93

dAVNScDITEYcTGDSGqcPPNLHk	Unambiguous	6	1	Q9R1V7	N-Term(iTRAQ8plex); C6(Methylthio); C12(Methylthio); Q18(Deamidated); C19(Methylthio); K25(iTRAQ8plex)	CID	1.0000	0.0000	1	1	3325550	Unique	Used	0.747	0.616	1.261	1.005	1E-05	0	7.13	0	0	92	3	1138.82983	3414.47495	1.46
lQEcIAELSQQLGTSEQAQR	Unambiguous	1	1	Q5SWZ5	N-Term(iTRAQ8plex); C4(Methylthio)	CID	1.0000	0.0000	1	1	3666596	Unique	Used	0.984	0.931	1.338	1.027	1E-05	0	7.13	0	17	39	2	1291.63977	2582.27226	-5.19
aVLASMDNENMHTPDIGGQGTTSQAIQDIIR	Unambiguous	1	1	P70404	N-Term(iTRAQ8plex)	CID	1.0000	0.0000	1	1	3372393	Unique	Used	0.617	1.052	1.516	1.950	1E-05	0	7.13	0	0	6	3	1196.59119	3587.75901	-4.09
eLSSAVSPPNLHSDTPTFSYASLAGPTIPPR	Unambiguous	3	1	Q9QYR6	N-Term(iTRAQ8plex)	CID	1.0000	0.0000	1	1	3705515	Unique	Used	2.807	1.103	1.824	0.940	1E-05	0	7.13	0	0	16	3	1171.93640	3513.79465	-6.31
tIGGGDDSFnTFFSETGAGk	Unambiguous	4	1	P68373	N-Term(iTRAQ8plex); N10(Deamidated); K20(iTRAQ8plex)	CID	1.0000	0.0000	1	1	3175103	Unique	Used		2.516			1E-05	0	7.13	0	0	200	2	1309.15283	2617.29839	4.02
tGDFQLHTNVNnGTEFGGSIYQk	Unambiguous	2	1	G3UX26	N-Term(iTRAQ8plex); N12(Deamidated); K23(iTRAQ8plex)	CID	1.0000	0.0000	1	1	3256861	Unique	Used	1.609	1.042	1.017	1.360	1E-05	0	7.12	0	0	35	3	1046.19568	3136.57248	-2.28
qTTVSNSQQAYQEAFEISk	Unambiguous	3	1	Q9CQV8-2	N-Term(iTRAQ8plex); K19(iTRAQ8plex)	CID	1.0000	0.0000	1	1	3089383	Unique	Used	0.709	1.210	0.738	0.658	1E-05	0	7.12	0	0	170	2	1384.21851	2767.42974	-2.23
eVAAFAQFGSDLDAATQQLLSR	Unambiguous	2	1	Q03265	N-Term(iTRAQ8plex)	CID	1.0000	0.0000	1	1	3666938	Unique	Used	0.851	1.053	1.054	1.343	1E-05	0	7.12	0	9	33	2	1321.68030	2642.35332	-7.39
aGYTDQVVIGMDVAASEFYR	Unambiguous	2	1	P17182	N-Term(iTRAQ8plex)	CID	1.0000	0.0000	1	1	3330011	Unique	Used	0.871	1.523	1.031	1.300	1E-05	0	7.12	0	0	200	2	1248.62280	2496.23833	0.01
gIVDQSQQAYQEAFEISk	Unambiguous	1	1	P63101	N-Term(iTRAQ8plex); K18(iTRAQ8plex)	CID	1.0000	0.0000	1	1	3130461	Unique	Used	1.313	1.690	1.101	1.089	1E-05	0	7.12	0	20	47	2	1325.20386	2649.40044	0.89
eGAAHAFAQYNLDQFTPVk	Unambiguous	2	1	P47754	N-Term(iTRAQ8plex); K19(iTRAQ8plex)	CID	1.0000	0.0000	1	1	3413919	Unique	Used	0.755	1.357	1.010	0.810	1E-05	0	7.12	0	5	5	3	905.81256	2715.42313	-4.42

gSIYLAGQNLQDVSLESLR	Unambiguous	1	1	Q61102	N-Term(iTRAQ8plex)	CID	1.0000	0.0000	1	1	3580549	Unique	Used	1.083	1.330	1.698	2.071	1E-05	0	7.12	0	0	20	2	1184.13892	2367.27056	-4.92
sQTEEDcTEELFDFLHAR	Unambiguous	1	1	P99028	N-Term(iTRAQ8plex); C7(Methylthio)	CID	1.0000	0.0000	1	1	2854736	Unique	Used			3.123	2.343	1E-05	0	7.12	0	10	200	3	840.71454	2520.12906	-1.37
tSEIQGANQLLELFDLGk	Unambiguous	1	1	Q9D967	N-Term(iTRAQ8plex); K18(iTRAQ8plex)	CID	1.0000	0.0000	1	1	3419155	Unique	Used	0.578	1.170	0.978	1.120	1E-05	0	7.12	0	0	13	2	1292.72180	2584.43633	-3.09
aVQLYQQTAnVFENEER	Unambiguous	2	1	Q9CWZ7	N-Term(iTRAQ8plex); N10(Deamidated)	CID	0.0070	0.0000	1	1	3260806	Unique	Used	2.303	1.446	1.184	1.070	1E-05	0	7.11	0	0	125	2	1172.59912	2344.19097	7.96
lLGDASVSFTDNcVVGIQANTER	Unambiguous	3	1	P97807-2	N-Term(iTRAQ8plex); C13(Methylthio)	CID	1.0000	0.0000	1	1	3581970	Unique	Used	1.344	0.953	1.216	1.841	1E-05	0	7.11	0	0	200	2	1380.18018	2759.35307	-4.20
vVNQLAAAYEQDLLPGGcTLR	Unambiguous	2	1	Q60649	N-Term(iTRAQ8plex); C18(Methylthio)	CID	1.0000	0.0000	1	1	3785543	Unique	Used	1.928	1.302	1.079	1.318	1E-05	0	7.11	0	12	81	2	1291.17041	2581.33354	-3.30
eAGLSQSHDDLSnTTATPSVR	Unambiguous	1	1	Q80X80	N-Term(iTRAQ8plex); N13(Deamidated)	CID	0.0450	0.0000	1	1	2862506	Unique	Used		1.790	3.123	1.376	1E-05	0	7.11	0	0	200	2	1246.12097	2491.23467	5.30
fQMPDQGMTSADDFFQGTk	Unambiguous	1	1	O08553	N-Term(iTRAQ8plex); K19(iTRAQ8plex)	CID	1.0000	0.0000	1	1	3304718	Unique	Used	1.975	1.264	0.803	1.398	1E-05	0	7.11	0	19	61	3	920.44604	2759.32358	-1.09
vINAAHSFYNGTTTLPISDEDR	Unambiguous	1	1	Q3THK7	N-Term(iTRAQ8plex)	CID	1.0000	0.0000	1	1	3436448	Unique	Used	1.358	1.079	1.094	1.066	1E-05	0	7.11	0	0	142	3	909.12701	2725.36649	-2.59
vFTTQELVQAFTHAPAALEADR	Unambiguous	1	1	Q8BK64	N-Term(iTRAQ8plex)	CID	1.0000	0.0000	1	1	3646232	Unique	Used	1.120	1.329	0.866	0.809	1E-05	0	7.11	0	0	200	3	907.14532	2719.42142	-5.28
sEQSSMSIEFGQEsPEHSLAMDFSR	Unambiguous	1	1	P14873	N-Term(iTRAQ8plex); S14(Phospho)	CID	1.0000	0.0000	1	1	2764932	Unique	Used	3.259	1.004	1.480	0.889	1E-05	0	7.11	0	1	77	3	1067.46692	3200.38620	0.18
nELFLDVLESVNLLMSPQGQVLSAHVSGR	Unambiguous	2	1	Q3TWV4	N-Term(iTRAQ8plex)	CID	1.0000	0.0000	1	1	3880522	Unique	Used	0.946	0.967	1.711	1.871	1E-05	0	7.11	0	16	17	3	1152.94983	3456.83493	-3.31
eTIEDVEEMLNNLPGVTSVHSR	Unambiguous	3	1	Q9WVJ2	N-Term(iTRAQ8plex)	CID	1.0000	0.0000	1	1	3326929	Unique	Used	2.060		0.698	1.125	1E-05	0	7.11	0	4	24	3	925.13409	2773.38773	-3.73
aLDLGTFTGYSALALALALPEAGR	Unambiguous	2	1	Q8BIG7	N-Term(iTRAQ8plex)	CID	1.0000	0.0000	1	1	3899373	Unique	Used	0.317	0.948	0.898	1.396	1E-05	0	7.11	0	9	14	2	1348.24548	2695.48369	-5.06
vGNPWDPNILYGPLHTk	Unambiguous	2	1	Q9DBF1-2	N-Term(iTRAQ8plex); K17(iTRAQ8plex)	CID	1.0000	0.0000	1	1	3684693	Unique	Used	0.489	1.533	1.009	1.494	1E-05	0	7.11	0	12	31	3	843.80359	2529.39621	-4.44

eIELLcQEHGQENDDLVQR	Unambiguous	4	1	E9Q6X0	N-Term(iTRAQ8plex); C6(Methylthio)	CID	1.0000	0.0000	1	1	2671258	Unique	Used	1.426	1.185	0.971	0.891	1E-05	0	7.11	0	10	200	3	873.41962	2618.24430	-1.90
iDAQAIEEFYGLTSDISk	Unambiguous	4	1	D3YVU0	N-Term(iTRAQ8plex); K18(iTRAQ8plex)	CID	1.0000	0.0000	1	1	3262660	Unique	Used	0.444	1.808	1.279	1.297	1E-05	0	7.11	0	6	17	2	1304.69824	2608.38921	-2.87
rFPSPTPTnNFALDGTAGTVYLAAVNR	Unambiguous	1	1	Q3UH93	N-Term(iTRAQ8plex); N9(Deamidated)	CID	0.0197	0.0000	1	1	3823676	Unique	Used	0.916	1.492	1.044	0.915	1E-05	0	7.11	1	27	200	3	1052.54834	3155.63047	-3.91
sIVDYkPSLDLLEQqHQLIQEALIFDNk	Unambiguous	1	1	P57780	N-Term(iTRAQ8plex); K6(iTRAQ8plex); Q15(Deamidated); K28(iTRAQ8plex)	CID	0.0225	0.0000	1	1	3541024	Unique	Used	0.914	2.442	0.841	0.571	1E-05	0	7.10	0	30	35	3	1404.45325	4211.34519	2.00
sSSSQTLTQFDSnIAPADPDTAIVHPVPIR	Unambiguous	3	1	O54916	N-Term(iTRAQ8plex); N13(Deamidated)	CID	0.0437	0.0000	1	1	3347665	Unique	Used			1.160		1E-05	0	7.10	0	14	26	3	1157.26343	3469.77573	0.11
sInGELYAPLQHVSAVLPGGAADR	Unambiguous	2	1	Q3UHD6-2	N-Term(iTRAQ8plex); N3(Deamidated)	CID	1.0000	0.0000	1	1	3580070	Unique	Used	0.555	0.774	1.118	0.795	1E-05	0	7.10	0	4	6	3	914.15417	2740.44797	-3.38
dLcGQSTTQAMDQYTFELSR	Unambiguous	1	1	P21614	N-Term(iTRAQ8plex); C3(Methylthio)	CID	1.0000	0.0000	1	1	3351192	Unique	Used	4.831	2.507	0.642	1.249	1E-05	0	7.10	0	0	18	2	1322.59790	2644.18852	-4.17
aAFDDAIAELDTLSEESYk	Unambiguous	1	1	P62259	N-Term(iTRAQ8plex); K19(iTRAQ8plex)	CID	1.0000	0.0000	1	1	2816570	Unique	Used	1.344	0.894	0.385	0.442	1E-05	0	7.10	0	28	200	2	1348.68909	2696.37090	-2.02
nLHQSGFSLSGTQVDEGVR	Unambiguous	3	1	E9PWE8	N-Term(iTRAQ8plex)	CID	1.0000	0.0000	1	1	3318081	Unique	Used	1.258	1.110	1.048	0.850	1E-05	0	7.10	0	11	90	3	779.06799	2335.18943	-2.15
dLkPENLLIDHqGYIQVTDFGFAk	Unambiguous	4	1	P68181	N-Term(iTRAQ8plex); K3(iTRAQ8plex); Q12(Deamidated); K24(iTRAQ8plex)	CID	0.0183	0.0000	1	1	3417023	Unique	Used	0.409	0.618	0.726	0.781	1E-05	0	7.10	0	0	94	3	1225.67981	3675.02488	1.39
aVQLYQQTANVFENEER	Unambiguous	2	1	Q9CWZ7	N-Term(iTRAQ8plex)	CID	1.0000	0.0000	1	1	3260975	Unique	Used	1.244	1.581	1.323	1.155	1E-05	0	7.10	0	0	1	2	1172.09631	2343.18535	-1.26

gSPGSIQGTPcPGTQLGPQPAASPSqLPADQSPHTLR	Unambiguous	4	1	Q5SSM3	N-Term(iTRAQ8plex); C11(Methylthio); Q26(Deamidated)	CID	0.0127	0.0000	1	1	3681567	Unique	Used	4.059	2.358	1.688	1.627	1E-05	0	7.10	0	0	133	3	1329.65723	3986.95713	-1.81
yYDQAEDESHSFTMTSETnTATVTR	Unambiguous	2	1	O08644	N-Term(iTRAQ8plex); N19(Deamidated)	CID	1.0000	0.0000	1	1	2652151	Unique	Used		1.756	1.485	0.571	1E-05	0	7.10	0	19	200	3	1063.81226	3189.42221	3.56
sAQcTAQPLSTGqGPcTPALEASR	Unambiguous	14	1	E9Q6Q2	N-Term(iTRAQ8plex); C4(Methylthio); Q13(Deamidated); C16(Methylthio)	CID	0.0465	0.0000	1	1	3683080	Unique	Used	0.517	0.614	1.662	1.184	1E-05	0	7.10	0	21	120	2	1386.14270	2771.27812	0.23
aGYTDQVVIGMDVAASEFYR	Unambiguous	2	1	P17182	N-Term(iTRAQ8plex)	CID	1.0000	0.0000	1	1	3327557	Unique	Used	1.010	1.449	1.371	1.483	1E-05	0	7.10	0	7	101	2	1248.61914	2496.23100	-2.92
gVASSPAISFGqQSTATLTPSPR	Unambiguous	4	1	Q19AB2	N-Term(iTRAQ8plex); Q12(Deamidated)	CID	0.0042	0.0000	1	1	3894351	Unique	Used	0.912	1.494	2.605	0.706	1E-05	0	7.10	0	0	200	2	1283.18176	2565.35625	3.89
yAEAGEGPVSHLATTVLQR	Unambiguous	2	1	O08601	N-Term(iTRAQ8plex)	CID	1.0000	0.0000	1	1	3536398	Unique	Used	0.881	1.036	0.812	1.010	1E-05	0	7.10	0	17	57	3	768.41119	2303.21903	-4.66
vTLDPELEEALASASDTELYDLAAVLGVHNLLNNPk	Unambiguous	2	1	Q9JKK7	N-Term(iTRAQ8plex); K36(iTRAQ8plex)	CID	1.0000	0.0000	1	1	3602744	Unique	Used			0.923	1.200	1E-05	0	7.10	0	0	165	3	1481.79065	4443.35739	-4.11
nSLISYLEQIHR	Unambiguous	1	1	Q00493	N-Term(iTRAQ8plex)	CID	1.0000	0.0000	1	1	3822951	Unique	Used	0.667	0.885	0.663	0.756	1E-05	0	7.10	0	5	19	3	593.00018	1776.98600	-2.82
tVQDALSSVQESDIAVVAR	Unambiguous	2	1	P33622	N-Term(iTRAQ8plex)	CID	1.0000	0.0000	1	1	3349745	Unique	Used	4.701	4.373	0.465	0.936	1E-05	0	7.10	0	0	15	2	1146.61597	2292.22466	-4.48
aRnDEYENLFNMVVEIPR	Unambiguous	2	1	Q91VM9	N-Term(iTRAQ8plex); N3(Deamidated)	CID	1.0000	0.0000	1	1	3305254	Unique	Used		1.604	1.215	1.044	1E-05	0	7.10	1	9	54	3	838.75812	2514.25980	-0.12
sITIANQTncPLYVTk	Unambiguous	1	1	O08553	N-Term(iTRAQ8plex); N9(Deamidated); C10(Methylthio); K16(iTRAQ8plex)	CID	0.0339	0.0000	1	1	3579206	Unique	Used	1.251	1.114	1.323	1.492	1E-05	0	7.09	0	0	58	2	1211.14612	2421.28496	-5.31

vLEDVTGEEFVLFMk	Unambiguous	1	1	O35841	N-Term(iTRAQ8plex); K15(iTRAQ8plex)	CID	1.0000	0.0000	1	1	3262840	Unique	Used	0.432	1.298	0.686	0.679	1E-05	0	7.09	0	20	36	2	1182.64600	2364.28472	-5.69
kHGcAFLVDEVQTGGGcTGk	Unambiguous	1	1	P61922	N-Term(iTRAQ8plex); K1(iTRAQ8plex); C4(Methylthio); C17(Methylthio); K20(iTRAQ8plex)	CID	1.0000	0.0000	1	1	3474954	Unique	Used	0.912	0.748	1.384	1.634	1E-05	0	7.09	1	20	116	3	1004.51581	3011.53287	-0.34
mADPQSIQESQNLSmFLANHNR	Unambiguous	1	1	Q5SQX6	N-Term(iTRAQ8plex); M15(Oxidation)	CID	1.0000	0.0000	1	1	3619256	Unique	Used	1.893	2.141	1.125	1.471	1E-05	0	7.09	0	11	31	3	951.12616	2851.36393	-4.57
aDRDESSPYAAmLAAQDVAQR	Unambiguous	3	1	P62264	N-Term(iTRAQ8plex); M12(Oxidation)	CID	1.0000	0.0000	1	1	3009171	Unique	Used	1.335	0.980	0.893	0.718	1E-05	0	7.09	1	4	23	3	862.42218	2585.25199	-1.87
yFHVVIAGPQDSPFEGGTFk	Unambiguous	1	1	P61089	N-Term(iTRAQ8plex); K20(iTRAQ8plex)	CID	1.0000	0.0000	1	1	3518249	Unique	Used	0.949	0.764	0.780	0.751	1E-05	0	7.09	0	11	29	3	935.49872	2804.48160	-1.87
qQNLILSYAEDETDVEGTVnGVTHTSTGGR	Unambiguous	2	1	Q99M87	N-Term(iTRAQ8plex); N20(Deamidated)	CID	1.0000	0.0000	1	1	2763135	Unique	Used	0.914	0.709	0.699	1.178	1E-05	0	7.09	0	21	200	3	1166.23669	3496.69553	-0.77
gQIEEAGWSYVGGWTGQGGSk	Unambiguous	1	1	P47857	N-Term(iTRAQ8plex); K21(iTRAQ8plex)	CID	1.0000	0.0000	1	1	3348486	Unique	Used	1.177	0.665	1.045	1.209	1E-05	0	7.09	0	0	6	2	1381.69885	2762.39043	-3.28
vFDFEVSSEDMATLLSYNR	Unambiguous	1	1	P45376	N-Term(iTRAQ8plex)	CID	1.0000	0.0000	1	1	3396419	Unique	Used	0.911	1.160	0.801	0.930	1E-05	0	7.09	0	2	5	2	1264.11487	2527.22246	-4.12
nLFAFFDMAYQGFASGDGDk	Unambiguous	1	1	P05202	N-Term(iTRAQ8plex); K20(iTRAQ8plex)	CID	1.0000	0.0000	1	1	3645706	Unique	Used	2.258	1.553	0.995	0.990	1E-05	0	7.09	0	0	44	2	1405.18140	2809.35552	-7.03
aASGGAALASPGPGSGSGTPTGSGGk	Unambiguous	1	1	Q3U0S6	N-Term(iTRAQ8plex); K26(iTRAQ8plex)	CID	1.0000	0.0000	1	1	3315434	Unique	Used	1.420	0.729	0.882	0.896	1E-05	0	7.09	0	22	190	3	889.13617	2665.39396	-2.36
eAmEAQIQNFGQTPSQLLIEPHPPR	Unambiguous	4	1	Q9EPN1-4	N-Term(iTRAQ8plex); M3(Oxidation)	CID	1.0000	0.0000	1	1	3475595	Unique	Used	0.859	1.395	0.782	0.719	1E-05	0	7.09	0	7	52	3	1051.20630	3151.60434	-3.35

aRPSQLPEQSSSAQQNAR	Unambiguous	1	1	P28740-2	N-Term(iTRAQ8plex)	CID	1.0000	0.0000	1	1	3291124	Unique	Used	0.470	0.480	0.951	0.819	1E-05	0	7.09	0	2	32	3	753.72791	2259.16916	-2.31
fIEAGQYNSHLYGTSVQSVR	Unambiguous	4	1	Q62108-3	N-Term(iTRAQ8plex)	CID	1.0000	0.0000	1	1	3721168	Unique	Used	0.799	2.048	1.239	1.265	1E-05	0	7.09	0	15	58	3	854.10321	2560.29508	-5.75
sGASGPENFQVGSMPPAQQQITSGQMHR	Unambiguous	1	1	P26039	N-Term(iTRAQ8plex)	CID	1.0000	0.0000	1	1	3533453	Unique	Used	1.123	1.378	0.626	0.989	1E-05	0	7.08	0	0	114	3	1077.85315	3231.54489	-3.99
cNENYTTDFIFNLYSEEGk	Unambiguous	1	1	P47857	N-Term(iTRAQ8plex); C1(Methylthio); K19(iTRAQ8plex)	CID	1.0000	0.0000	1	1	3239334	Unique	Used	1.078	0.864	0.963	1.436	1E-05	0	7.08	0	7	12	2	1471.19263	2941.37798	-2.20
aGGSASAMLQPLLDNQVGFk	Unambiguous	1	1	O09061	N-Term(iTRAQ8plex); K20(iTRAQ8plex)	CID	1.0000	0.0000	1	1	3706184	Unique	Used	1.382	0.697	0.724	0.975	1E-05	0	7.08	0	0	22	2	1306.71179	2612.41631	-6.28
vSDAISTqYPVVDHEFDAVVVGAGGAGLR	Unambiguous	1	1	Q8K2B3	N-Term(iTRAQ8plex); Q8(Deamidated)	CID	1.0000	0.0000	1	1	3355556	Unique	Used	2.163	1.578	1.129	1.486	1E-05	0	7.08	0	15	200	3	1078.89465	3234.66941	3.36
eQTLTTVQTEMVEQER	Unambiguous	2	1	E9QAH1	N-Term(iTRAQ8plex)	CID	1.0000	0.0000	1	1	2670131	Unique	Used	1.879	0.569	0.789	0.563	1E-05	0	7.08	0	0	200	2	1113.56226	2226.11724	-2.41
dHFMDEFFEQVEEIR	Unambiguous	1	1	P61264	N-Term(iTRAQ8plex)	CID	1.0000	0.0000	1	1	2853337	Unique	Used	1.491	0.970	0.952	1.238	1E-05	0	7.08	0	15	200	3	759.02380	2275.05686	-3.30
nTSSQTVGVATVGTQTIR	Unambiguous	2	1	E1U8D0	N-Term(iTRAQ8plex)	CID	1.0000	0.0000	1	1	3657052	Unique	Used	0.921	0.261	0.648	0.717	1E-05	0	7.08	0	0	184	2	1062.57471	2124.14214	-6.65
vAGHPDVVINNAAGNFISPSER	Unambiguous	1	1	Q9CQ62	N-Term(iTRAQ8plex)	CID	1.0000	0.0000	1	1	3535266	Unique	Used	0.641	0.875	0.781	1.500	1E-05	0	7.08	0	0	136	2	1284.67224	2568.33721	-3.93
vTSAYLQDIENAYk	Unambiguous	1	1	Q99LC3	N-Term(iTRAQ8plex); K14(iTRAQ8plex)	CID	1.0000	0.0000	1	1	3193898	Unique	Used	0.607	0.725	1.037	1.299	1E-05	0	7.08	0	0	24	2	1112.10754	2223.20781	-1.77
vIAVYDLGGGTFDISILEIQk	Unambiguous	1	1	P38647	N-Term(iTRAQ8plex); K21(iTRAQ8plex)	CID	1.0000	0.0000	1	1	3623594	Unique	Used	1.126	1.385	0.942	0.850	1E-05	0	7.08	0	0	21	2	1430.30933	2859.61138	-7.51
vQQMIGQIDGLITQLETTQQDGk	Unambiguous	1	1	Q9Z2I0	N-Term(iTRAQ8plex); K23(iTRAQ8plex)	CID	1.0000	0.0000	1	1	3542004	Unique	Used	1.427	1.351	2.494	1.954	1E-05	0	7.08	0	0	73	3	1051.57166	3152.70041	-2.48
lVLPMDDSEPALNSLDDTRHsPA	Unambiguous	2	1	A1L3P4	N-Term(iTRAQ8plex); S21(Phospho)	CID	1.0000	0.0000	1	1	2639330	Unique	Used	0.937	0.776	0.538	1.331	1E-05	0	7.08	1	0	200	3	959.79291	2877.36417	-0.08
aAATFNPELITHILDGSPENTR	Unambiguous	2	1	Q9R0H0	N-Term(iTRAQ8plex)	CID	1.0000	0.0000	1	1	3540926	Unique	Used	1.849				1E-05	0	7.08	0	14	12	3	891.13550	2671.39194	-2.79

vSDAISTqYPVVDHEFDAVVVGAGGAGLR	Unambiguous	1	1	Q8K2B3	N-Term(iTRAQ8plex); Q8(Deamidated)	CID	1.0000	0.0000	1	1	3354714	Unique	Used	1.529	0.989	1.045	1.176	1E-05	0	7.08	0	18	200	3	1078.89539	3234.67160	4.04
tVIQAEIDAAAELIDFFR	Unambiguous	1	1	Q8CHT0	N-Term(iTRAQ8plex)	CID	1.0000	0.0000	1	1	3480765	Unique	Used	0.577	1.636	0.780	1.432	1E-05	0	7.08	0	0	200	2	1163.63135	2326.25542	-1.84
iSIEMnGTLEDQLSHLk	Unambiguous	1	1	P57780	N-Term(iTRAQ8plex); N6(Deamidated); K17(iTRAQ8plex)	CID	1.0000	0.0000	1	1	3261425	Unique	Used	0.831	2.694	1.352	0.814	1E-05	0	7.08	0	2	13	3	846.46191	2537.37119	-1.18
lPSPTAAPQQSASQATPVTQGQGR	Unambiguous	3	1	O88935	N-Term(iTRAQ8plex)	CID	1.0000	0.0000	1	1	3677986	Unique	Used		1.908	0.373	0.758	1E-05	0	7.08	0	0	26	3	894.80347	2682.39585	-5.78
knGAVQAASAVVLQGGEEMQR	Unambiguous	1	1	Q11136	N-Term(iTRAQ8plex); K1(iTRAQ8plex); N2(Deamidated)	CID	1.0000	0.0000	1	1	3256434	Unique	Used	4.334	1.722	1.283	1.181	1E-05	0	7.08	1	0	59	3	918.16492	2752.48020	-2.58
dFTSEQIDEILQNHTEIVFAR	Unambiguous	2	1	Q6PIC6	N-Term(iTRAQ8plex)	CID	1.0000	0.0000	1	1	3352172	Unique	Used	0.937	0.669	1.131	1.362	1E-05	0	7.08	0	27	2	3	937.14545	2809.42179	-3.30
tVEIINVEDELPAASAVSVAAAAAVAGGAGVGGDSHLALPALER	Unambiguous	1	1	P0C192	N-Term(iTRAQ8plex)	CID	1.0000	0.0000	1	1	3541557	Unique	Used		1.491			1E-05	0	7.08	0	11	21	3	1481.78735	4443.34751	-4.50
dMAIATGGAVFGEEGLNLNLEDVQAHDLGk	Unambiguous	1	1	P63038	N-Term(iTRAQ8plex); K30(iTRAQ8plex)	CID	1.0000	0.0000	1	1	3263230	Unique	Used	0.941	1.127	0.953	1.332	1E-05	0	7.07	0	0	105	3	1231.63953	3692.90403	-0.30
gDAcTPTSTTVELVGSSGQnGDEk	Unambiguous	1	1	Q9ERZ4	N-Term(iTRAQ8plex); C4(Methylthio); N20(Deamidated); K24(iTRAQ8plex)	CID	1.0000	0.0000	1	1	2669907	Unique	Used	1.435	0.669	0.797	0.649	1E-05	0	7.07	0	7	200	3	1003.47845	3008.42081	-2.58
gYIWNYGAIPQTWEDPGHSDk	Unambiguous	1	1	Q9D819	N-Term(iTRAQ8plex); K21(iTRAQ8plex)	CID	1.0000	0.0000	1	1	3259510	Unique	Used	0.680	0.721	0.626	0.729	1E-05	0	7.07	0	0	13	3	1014.84485	3042.51999	-0.22
sLAEQNLEFQGPLEISR	Unambiguous	1	1	Q8R105	N-Term(iTRAQ8plex)	CID	1.0000	0.0000	1	1	3281692	Unique	Used	1.893	0.902	0.986	0.662	1E-05	0	7.07	0	0	88	2	1118.09656	2235.18584	-2.90

kLDLDNSGSLSVEEFmSLPELQQNPLVQR	Unambiguous	2	1	Q63810-2	N-Term(iTRAQ8plex); K1(iTRAQ8plex); M16(Oxidation)	CID	1.0000	0.0000	1	1	3519712	Unique	Used	4.813	4.155	0.688	0.564	1E-05	0	7.07	1	0	22	3	1304.35706	3911.05661	-2.96
tDDLVYHNVmTLVEAVLELk	Unambiguous	4	1	Q9QVP9	N-Term(iTRAQ8plex); M10(Oxidation); K20(iTRAQ8plex)	CID	1.0000	0.0000	1	1	3645882	Unique	Used		2.670	1.533	1.069	1E-05	0	7.07	0	9	41	3	976.20111	2926.58878	-5.76
dLVILLYETALLSSGFSLEDPQTHANR	Unambiguous	1	1	P07901	N-Term(iTRAQ8plex)	CID	1.0000	0.0000	1	1	3806773	Unique	Used		0.508	0.390	0.852	1E-05	0	7.07	0	1	8	3	1102.91858	3306.74118	-3.40
vEYPVMYSTDPENGHmFNcIQR	Unambiguous	1	1	Q9CPU4	N-Term(iTRAQ8plex); M16(Oxidation); C19(Methylthio)	CID	1.0000	0.0000	1	1	3391934	Unique	Used	1.140	1.434	1.503	1.725	1E-05	0	7.07	0	10	72	3	999.44720	2996.32706	-2.76
tSEPSNLSISDFSGIqDELHHLR	Unambiguous	6	1	Q3TVA9	N-Term(iTRAQ8plex); Q16(Deamidated)	CID	1.0000	0.0000	1	1	3302751	Unique	Used	1.444	1.475	0.893	1.018	1E-05	0	7.07	0	30	200	3	963.15460	2887.44925	4.03
yDGATIVPGDQGADYQHFIQQcTDDVR	Unambiguous	1	1	Q9CQI6	N-Term(iTRAQ8plex); C22(Methylthio)	CID	1.0000	0.0000	1	1	3239029	Unique	Used	2.163	0.930	0.850	1.087	1E-05	0	7.07	0	0	45	3	1121.51709	3362.53672	0.20
iGQGASGTVYTALDIATGQEVAIk	Unambiguous	3	1	Q61036-2	N-Term(iTRAQ8plex); K24(iTRAQ8plex)	CID	1.0000	0.0000	1	1	3459114	Unique	Used		2.475	1.356	1.471	1E-05	0	7.07	0	9	7	3	991.21851	2971.64096	-5.10
vSDAISTQYPVVDHEFDAVVVGAGGAGLR	Unambiguous	1	1	Q8K2B3	N-Term(iTRAQ8plex)	CID	1.0000	0.0000	1	1	3355012	Unique	Used	1.344	1.028	0.747	0.875	1E-05	0	7.07	0	0	200	3	1078.55518	3233.65097	-7.29
dDEEMNTWIQAISSAISSDk	Unambiguous	1	1	Q62261	N-Term(iTRAQ8plex); K20(iTRAQ8plex)	CID	1.0000	0.0000	1	1	3305799	Unique	Used	1.263	0.682	1.350	0.715	1E-05	0	7.07	0	0	75	2	1424.70825	2848.40923	-1.38
lYSSMSDTNLAEAGLNYHAQR	Unambiguous	2	1	O88737	N-Term(iTRAQ8plex)	CID	1.0000	0.0000	1	1	3390747	Unique	Used	0.919	0.728	1.217	0.885	1E-05	0	7.07	0	19	64	3	882.43311	2645.28476	-3.18
vETLHDFEAAnSDELNLQR	Unambiguous	1	1	Q7TQF7	N-Term(iTRAQ8plex); N11(Deamidated)	CID	1.0000	0.0000	1	1	2637584	Unique	Used		1.465	2.584	0.669	1E-05	0	7.07	0	2	26	3	836.08234	2506.23246	-1.57
eEPFFPPPEEFVFIHAVPVEER	Unambiguous	1	1	Q00PI9	N-Term(iTRAQ8plex)	CID	1.0000	0.0000	1	1	3112019	Unique	Used	0.853	0.982	0.645	0.787	1E-05	0	7.07	0	4	103	3	982.50439	2945.49863	-1.42

gFQQILAGEYDHLPEqAFYMVGPIEEAVAk	Unambiguous	1	1	P56480	N-Term(iTRAQ8plex); Q16(Deamidated); K30(iTRAQ8plex)	CID	1.0000	0.0000	1	1	3420640	Unique	Used	0.634	0.978	0.968	0.935	1E-05	0	7.06	0	21	200	3	1320.68518	3960.04099	1.49
tGSEPSPPQGGGSLGPSAPPEEIWVLR	Unambiguous	3	1	Q6P9K8	N-Term(iTRAQ8plex)	CID	1.0000	0.0000	1	1	3325188	Unique	Used	0.979	0.382	0.808	1.100	1E-05	0	7.06	0	0	25	2	1503.77454	3006.54180	-1.91
eYLDLTASNNFYVDDFAR	Unambiguous	2	1	Q6P5E4	N-Term(iTRAQ8plex)	CID	1.0000	0.0000	1	1	3217019	Unique	Used	1.015	1.049	0.640	0.762	1E-05	0	7.06	0	0	38	2	1229.09631	2457.18535	-0.92
lVDQALLTMDTYLGqFPDlk	Unambiguous	3	1	O08539	N-Term(iTRAQ8plex); Q15(Deamidated); K20(iTRAQ8plex)	CID	0.0397	0.0000	1	1	3687495	Unique	Used	1.082	0.919	1.381	1.062	1E-05	0	7.06	0	14	124	2	1445.79285	2890.57842	1.77
aQQEITVQQLMAHLDSIR	Unambiguous	1	1	Q8C3X2	N-Term(iTRAQ8plex)	CID	1.0000	0.0000	1	1	3765592	Unique	Used		2.775	0.571	1.152	1E-05	0	7.06	0	0	27	3	795.76447	2385.27884	-3.11
mSSVFEDQNAATHLIR	Unambiguous	4	1	A8Y5Q0	N-Term(iTRAQ8plex)	CID	1.0000	0.0000	1	1	3413003	Unique	Used	1.242	1.073	0.856	0.931	1E-05	0	7.06	0	23	89	3	708.36377	2123.07676	-4.24
yTPSGQSGAAASESLFISNHAy	Unambiguous	2	1	A6ZI44	N-Term(iTRAQ8plex); Y22(iTRAQ8plex)	CID	1.0000	0.0000	1	1	3453906	Unique	Used	0.293	1.036	0.681	0.945	1E-05	0	7.06	0	0	44	3	956.14807	2866.42966	-5.98
yTPSGQSGAAASESLFISNHAY	Unambiguous	2	1	A6ZI44	N-Term(iTRAQ8plex)	CID	1.0000	0.0000	1	1	3536749	Unique	Used	1.744	1.086	1.629	1.831	1E-05	0	7.06	0	0	136	2	1281.62000	2562.23271	-3.41
ekPGQVSEVAQLISQTLEQER	Unambiguous	6	1	H3BL28	N-Term(iTRAQ8plex); K2(iTRAQ8plex)	CID	1.0000	0.0000	1	1	3373425	Unique	Used	0.803	0.906	2.224	0.838	1E-05	0	7.06	0	4	5	3	993.21503	2977.63053	-3.69
vDLNSnGFIcDYELHELFk	Unambiguous	2	1	B1AX58	N-Term(iTRAQ8plex); N6(Deamidated); C10(Methylthio); K19(iTRAQ8plex)	CID	0.0142	0.0000	1	1	3326971	Unique	Used	1.120	0.762	0.895	0.994	1E-05	0	7.06	0	2	10	3	971.15021	2911.43607	-3.65
aDcTITMADSDLLALMTGk	Unambiguous	2	1	P32020	N-Term(iTRAQ8plex); C3(Methylthio); K19(iTRAQ8plex)	CID	1.0000	0.0000	1	1	3459773	Unique	Used	1.928	0.700	0.946	1.015	1E-05	0	7.06	0	9	183	2	1312.66003	2624.31279	-5.21
eTEAALEFAQTQLAEQGEESR	Unambiguous	1	1	Q9D7M1	N-Term(iTRAQ8plex)	CID	1.0000	0.0000	1	1	2764983	Unique	Used			1.189		1E-05	0	7.06	0	5	200	2	1321.14551	2641.28374	-2.20
iTYHPDGPEGQAYEVDFTPPFR	Unambiguous	2	1	Q99MN1	N-Term(iTRAQ8plex)	CID	1.0000	0.0000	1	1	3236242	Unique	Used	0.820	0.895	0.783	1.100	1E-05	0	7.06	0	20	4	3	947.46484	2840.37998	-1.20

fEFQPHMGDEVcQVSAQQPVQTELLmR	Unambiguous	3	1	E9QN14	N-Term(iTRAQ8plex); C12(Methylthio); M26(Oxidation)	CID	1.0000	0.0000	1	1	3438989	Unique	Used	1.138	0.939	1.051	1.313	1E-05	0	7.06	0	16	171	3	1171.88831	3513.65036	-2.11
gFQQILAGEYDHLPEqAFYMVGPIEEAVAk	Unambiguous	1	1	P56480	N-Term(iTRAQ8plex); Q16(Deamidated); K30(iTRAQ8plex)	CID	1.0000	0.0000	1	1	3459499	Unique	Used	1.254	0.929	1.144	0.836	1E-05	0	7.05	0	0	179	3	1320.68091	3960.02817	-1.74
lLAITQEASDTSWQALIDEDR	Unambiguous	5	1	D3Z7V3	N-Term(iTRAQ8plex)	CID	1.0000	0.0000	1	1	3262927	Unique	Used	0.853	1.045	1.443	1.305	1E-05	0	7.05	0	8	17	2	1340.19080	2679.37432	-1.37
tAVIDHHNYDVSDLGQHTLIVADTENLLk	Unambiguous	1	1	O54734	N-Term(iTRAQ8plex); K29(iTRAQ8plex)	CID	1.0000	0.0000	1	1	3347856	Unique	Used	0.522	0.611	0.868	0.738	1E-05	0	7.05	0	0	21	3	1280.67603	3840.01352	-6.60
gNDISSGTVLSDYVGSGPPSGTGLHR	Unambiguous	1	1	P70296	N-Term(iTRAQ8plex)	CID	1.0000	0.0000	1	1	3412492	Unique	Used	2.478	1.088	1.615	0.979	1E-05	0	7.05	0	0	128	2	1417.70984	2834.41240	-3.49
qGQYSPMAIEEQVAVIYAGVR	Unambiguous	2	1	Q03265	N-Term(iTRAQ8plex)	CID	1.0000	0.0000	1	1	3603371	Unique	Used	0.643	0.755	1.106	1.497	1E-05	0	7.05	0	15	200	2	1307.18091	2613.35454	-3.96
gnDISSGTVLSDYVGSGPPSGTGLHR	Unambiguous	1	1	P70296	N-Term(iTRAQ8plex); N2(Deamidated)	CID	1.0000	0.0000	1	1	3257173	Unique	Used	2.461	1.401	1.740	0.788	1E-05	0	7.05	0	0	19	2	1418.20447	2835.40166	-1.64
gITNLcVIGGDGSLTGADTFR	Unambiguous	1	1	P47857	N-Term(iTRAQ8plex); C6(Methylthio)	CID	1.0000	0.0000	1	1	3726961	Unique	Used	1.590	1.018	1.125	1.501	1E-05	0	7.05	0	6	19	2	1209.09851	2417.18974	-8.68
vQAQHDYTATDTDELQLk	Unambiguous	3	1	O08539	N-Term(iTRAQ8plex); K18(iTRAQ8plex)	CID	1.0000	0.0000	1	1	2809414	Unique	Used	1.154	1.366	1.114	0.943	1E-05	0	7.05	0	28	118	3	895.46716	2684.38694	-4.42
sVNESLNNLFITEEDYQALR	Unambiguous	2	1	Q68FD5	N-Term(iTRAQ8plex)	CID	1.0000	0.0000	1	1	3241052	Unique	Used	0.785	1.278	0.948	1.409	1E-05	0	7.05	0	0	200	2	1330.17957	2659.35185	0.04
sGGASEPLLPHqPSLETQLYcGQAEGGSEGPSTSGTLk	Unambiguous	2	1	P04919-2	N-Term(iTRAQ8plex); Q12(Deamidated); C21(Methylthio); K38(iTRAQ8plex)	CID	0.0128	0.0000	1	1	3235812	Unique	Used	0.518	0.812	0.495	1.171	1E-05	0	7.05	0	0	117	3	1476.06445	4426.17881	3.34
dIGAIAQVHAENGDIIAEEQQR	Unambiguous	1	1	O08553	N-Term(iTRAQ8plex)	CID	1.0000	0.0000	1	1	2830374	Unique	Used	1.060	1.009	1.196	1.560	1E-05	0	7.05	0	20	40	3	894.46320	2681.37503	-1.75
eTFGVNNTVYGIDAMNPSSR	Unambiguous	2	1	Q3UGC7	N-Term(iTRAQ8plex)	CID	1.0000	0.0000	1	1	3370302	Unique	Used	1.121	0.370	1.236	0.684	1E-05	0	7.05	0	7	38	2	1238.60303	2476.19878	-3.75

lLGQTDVAVYGVAVPGVEGk	Unambiguous	1	1	Q60714	N-Term(iTRAQ8plex); K20(iTRAQ8plex)	CID	1.0000	0.0000	1	1	3439409	Unique	Used	0.921	1.023	0.783	1.108	1E-05	0	7.05	0	13	144	2	1290.74182	2580.47637	-3.65
sTGALYAPDVcGASGPGYGISqSSR	Unambiguous	1	1	F7DCH5	N-Term(iTRAQ8plex); C11(Methylthio); Q22(Deamidated)	CID	1.0000	0.0000	1	1	3663328	Unique	Used	0.993		0.651	0.683	1E-05	0	7.04	0	0	51	2	1376.65137	2752.29546	3.42
iPDFQDSIFEYFNTAPLAHDLTFR	Unambiguous	1	1	Q7TT50	N-Term(iTRAQ8plex)	CID	1.0000	0.0000	1	1	3727295	Unique	Used	0.879	0.967	1.127	1.095	1E-05	0	7.04	0	11	44	3	1054.52783	3161.56894	-6.23
gGLHTVAVMNVGAPAAGMNAAVR	Unambiguous	1	1	P47857	N-Term(iTRAQ8plex)	CID	1.0000	0.0000	1	1	3931227	Unique	Used	2.667	1.148	1.048	1.104	1E-05	0	7.04	0	0	191	2	1234.65405	2468.30083	-6.51
eMVEGMGGTQSEQYQEFR	Unambiguous	3	1	Q6PF93	N-Term(iTRAQ8plex)	CID	1.0000	0.0000	1	1	2758882	Unique	Used		2.155	0.613	1.073	1E-05	0	7.04	0	0	200	2	1205.54639	2410.08550	-4.25
yHTSQSGDEMTSLSEYVSR	Unambiguous	2	1	P11499	N-Term(iTRAQ8plex)	CID	1.0000	0.0000	1	1	2991690	Unique	Used	0.476	1.390	1.139	1.069	1E-05	0	7.04	0	8	1	3	827.72095	2481.14829	-0.92
fSGWYDADLSPAGHEEAk	Unambiguous	1	1	Q9DBJ1	N-Term(iTRAQ8plex); K18(iTRAQ8plex)	CID	1.0000	0.0000	1	1	2918261	Unique	Used	2.576	0.863	0.721	1.039	1E-05	0	7.04	0	0	200	2	1294.63965	2588.27202	-6.10
gASFEELcHNYAQDR	Unambiguous	12	1	B2RRE2	N-Term(iTRAQ8plex); C8(Methylthio)	CID	1.0000	0.0000	1	1	3234803	Unique	Used	0.463	0.619	0.793	0.708	1E-05	0	7.04	0	3	11	3	697.31744	2089.93778	0.20
dSGVVSLIEALMNYPSSR	Unambiguous	1	1	Q8BUY8	N-Term(iTRAQ8plex)	CID	1.0000	0.0000	1	1	3880492	Unique	Used	0.442	0.609	0.499	1.064	1E-05	0	7.04	0	9	20	2	1121.58398	2242.16069	-3.77
lAQEnGWGVMVSHR	Unambiguous	2	1	P17183	N-Term(iTRAQ8plex); N5(Deamidated)	CID	1.0000	0.0000	1	1	3343119	Unique	Used	1.109	1.090	1.086	1.206	1E-05	0	7.04	0	9	1	3	630.32385	1888.95700	-3.80
iQQETGGLVDVLPLcGETSQR	Unambiguous	2	1	Q68FM7	N-Term(iTRAQ8plex); C15(Methylthio)	CID	1.0000	0.0000	1	1	3440079	Unique	Used	0.782	0.948	0.991	1.286	1E-05	0	7.04	0	26	22	2	1297.16211	2593.31694	-3.81
aSSHSTDLMEAMAmGSVEASYk	Unambiguous	1	1	P52480-2	N-Term(iTRAQ8plex); M14(Oxidation); K22(iTRAQ8plex)	CID	1.0000	0.0000	1	1	3212373	Unique	Used	1.447	1.045	0.972	0.837	1E-05	0	7.04	0	12	29	3	976.47021	2927.39609	-2.91
aVLDSTNADGISALHQAcIDENLEVVR	Unambiguous	2	1	Q3UMT1	N-Term(iTRAQ8plex); C18(Methylthio)	CID	1.0000	0.0000	1	1	3351411	Unique	Used	2.017	0.447	1.305	1.704	1E-05	0	7.04	0	25	30	3	1068.53198	3203.58139	-5.16

lLAESVTEVEIFGk	Unambiguous	1	1	Q9JHU4	N-Term(iTRAQ8plex); K14(iTRAQ8plex)	CID	1.0000	0.0000	1	1	3305318	Unique	Used	0.406	0.677	0.903	0.998	1E-05	0	7.04	0	22	22	2	1072.12451	2143.24175	-2.50
iEQLHQEQANNDFFLLAELLSDYIR	Unambiguous	1	1	Q6NZD2	N-Term(iTRAQ8plex)	CID	1.0000	0.0000	1	1	3602660	Unique	Used	1.172	0.990	0.986	1.413	1E-05	0	7.04	0	2	17	3	1108.57324	3323.70517	-4.90
wGEAGAEYVVESTGVFTTMEk	Unambiguous	3	1	P16858	N-Term(iTRAQ8plex); K21(iTRAQ8plex)	CID	1.0000	0.0000	1	1	3218542	Unique	Used	0.770	0.834	0.572	1.054	1E-05	0	7.04	0	5	200	3	967.16040	2899.46665	0.75
qTTAAAAATFSEQVGGGSGGAGR	Unambiguous	3	1	O88935	N-Term(iTRAQ8plex)	CID	1.0000	0.0000	1	1	3513379	Unique	Used	1.384	2.149	1.663	1.744	1E-05	0	7.04	0	0	200	2	1178.58960	2356.17192	-3.24
eVAAFAQFGSDLDAATQQLLSR	Unambiguous	2	1	Q03265	N-Term(iTRAQ8plex)	CID	1.0000	0.0000	1	1	3669138	Unique	Used		1.111	1.727	2.432	1E-05	0	7.04	0	7	200	2	1321.68152	2642.35576	-6.46
aEIVLLDHGLYQVLTEEFR	Unambiguous	2	1	Q9D0L4	N-Term(iTRAQ8plex)	CID	1.0000	0.0000	1	1	3419184	Unique	Used	0.555	1.364	0.934	1.189	1E-05	0	7.04	0	9	16	3	850.46655	2549.38510	-2.61
gFGSLPALEVLDLTYNNLNEHSLPGnFFYLTTLR	Unambiguous	2	1	Q9D031	N-Term(iTRAQ8plex); N26(Deamidated)	CID	1.0000	0.0000	1	1	3880346	Unique	Used		1.036	0.672	1.341	1E-05	0	7.04	0	28	23	3	1377.71753	4131.13803	-0.03
lAQDFLDSQNLSAYNTR	Unambiguous	1	1	Q99KK7	N-Term(iTRAQ8plex)	CID	1.0000	0.0000	1	1	3539818	Unique	Used	0.678	0.878	0.744	0.756	1E-05	0	7.03	0	6	55	2	1130.57520	2260.14311	-3.56
icDPGLTSFEPEALGNLVEGMDFHR	Unambiguous	9	1	Q5SVJ0	N-Term(iTRAQ8plex); C2(Methylthio)	CID	1.0000	0.0000	1	1	3482079	Unique	Used		0.979	1.377	1.221	1E-05	0	7.03	0	0	200	3	1033.15955	3097.46408	-3.07
kVADALANAAGHLDDLPGALSALSDLHAHk	Unambiguous	1	1	Q91VB8	N-Term(iTRAQ8plex); K1(iTRAQ8plex); K30(iTRAQ8plex)	CID	1.0000	0.0000	1	1	3726540	Unique	Used	3.775	4.408	0.711	1.007	1E-05	0	7.03	1	7	14	3	1302.05566	3904.15244	-7.29
eHANIDAQSGSQAPNPSTTIsPGk	Unambiguous	3	1	Q8VDQ8-2	N-Term(iTRAQ8plex); S21(Phospho); K24(iTRAQ8plex)	CID	1.0000	0.0000	1	1	2754072	Unique	Used	0.408	0.317	0.691	0.324	1E-05	0	7.03	0	0	200	3	1032.51160	3095.52024	-1.72
sYIEGYVPSqADVAVFEAVSGPPPADLcHALR	Unambiguous	3	1	O70251	N-Term(iTRAQ8plex); Q10(Deamidated); C28(Methylthio)	CID	1.0000	0.0000	1	1	3624884	Unique	Used	1.569	0.764	1.294	0.363	1E-05	0	7.03	0	0	200	3	1237.27710	3709.81675	-0.47

vDYILSVMnVPNFDFPPEFYEHAk	Unambiguous	5	1	Q6R0H7-4	N-Term(iTRAQ8plex); N9(Deamidated); K24(iTRAQ8plex)	CID	0.0057	0.0000	1	1	3706997	Unique	Used	1.095	1.272	0.702	1.126	1E-05	0	7.03	0	11	67	3	1160.92139	3480.74961	-4.33
sAFEAEVSEIAISQAEVDLALR	Unambiguous	1	1	Q80VQ0	N-Term(iTRAQ8plex)	CID	1.0000	0.0000	1	1	3133491	Unique	Used	1.370	1.058	1.604	1.987	1E-05	0	7.03	0	12	147	2	1326.70862	2652.40996	2.44
sLTLGYMAEAFGVGVDFIDQELSR	Unambiguous	1	1	Q99JI4	N-Term(iTRAQ8plex)	CID	1.0000	0.0000	1	1	3646242	Unique	Used	0.940	0.764	0.606	0.943	1E-05	0	7.03	0	0	200	2	1461.74219	2922.47710	-3.10
tTTnVLGDSLGAGIVEHLSR	Unambiguous	1	1	P56564	N-Term(iTRAQ8plex); N4(Deamidated)	CID	1.0000	0.0000	1	1	3663902	Unique	Used	0.707	1.575	1.356	1.164	1E-05	0	7.03	0	13	104	2	1173.13586	2345.26445	1.27
aLSAIAELLTSEHER	Unambiguous	19	1	G3X9V2	N-Term(iTRAQ8plex)	CID	1.0000	0.0000	1	1	3601937	Unique	Used	0.725	1.079	0.751	1.179	1E-05	0	7.03	0	0	24	3	648.69171	1944.06058	-5.06
eLSSAVSPPnLHSDTPTFSYASLAGPTIPPR	Unambiguous	3	1	Q9QYR6	N-Term(iTRAQ8plex); N10(Deamidated)	CID	1.0000	0.0000	1	1	3744115	Unique	Used	2.894	1.364	1.593	0.978	1E-05	0	7.03	0	17	200	3	1172.27600	3514.81345	3.59
eVAAFAQFGSDLDAATQQLLSR	Unambiguous	2	1	Q03265	N-Term(iTRAQ8plex)	CID	1.0000	0.0000	1	1	3667072	Unique	Used	0.859	0.877	1.489	1.245	1E-05	0	7.03	0	6	62	2	1321.68188	2642.35649	-6.19
sGASGPENFQVGSMPPAQQQITSGQMHR	Unambiguous	1	1	P26039	N-Term(iTRAQ8plex)	CID	1.0000	0.0000	1	1	3533517	Unique	Used	1.250	1.539	0.912	0.980	1E-05	0	7.03	0	11	199	3	1077.85229	3231.54233	-4.79
eQSGEYEcSASNDVAAPVVR	Unambiguous	5	1	D3Z396	N-Term(iTRAQ8plex); C8(Methylthio)	CID	1.0000	0.0000	1	1	2700495	Unique	Used	0.987	0.506	1.155	0.734	1E-05	0	7.03	0	0	200	2	1231.06726	2461.12724	-0.21
nLEYTASLLEAVYIDETR	Unambiguous	1	1	Q01065	N-Term(iTRAQ8plex)	CID	1.0000	0.0000	1	1	3284756	Unique	Used	0.497	0.899	2.801	1.789	1E-05	0	7.03	0	9	80	2	1202.63037	2404.25347	-0.63
tTPYQIAcGISQGLADNTVVAk	Unambiguous	1	1	Q9D0R2	N-Term(iTRAQ8plex); C8(Methylthio); K22(iTRAQ8plex)	CID	1.0000	0.0000	1	1	3765859	Unique	Used	0.728	0.649	0.846	0.687	1E-05	0	7.03	0	0	19	2	1452.77100	2904.53472	-2.51
vGVQDFVLLEnFTSEAAFIENLR	Unambiguous	5	1	Q9WTI7-3	N-Term(iTRAQ8plex); N11(Deamidated)	CID	0.0271	0.0000	1	1	3561541	Unique	Used	0.192	1.424	0.550	0.802	1E-05	0	7.02	0	0	200	2	1458.76868	2916.53008	0.11
wGEAGAEYVVESTGVFTTMEk	Unambiguous	3	1	P16858	N-Term(iTRAQ8plex); K21(iTRAQ8plex)	CID	1.0000	0.0000	1	1	3218809	Unique	Used		1.039	1.365	0.881	1E-05	0	7.02	0	0	200	2	1450.23401	2899.46074	-1.29

cPTDELSLSNcAVVNEk	Unambiguous	2	1	P46460	N-Term(iTRAQ8plex); C1(Methylthio); C11(Methylthio); K17(iTRAQ8plex)	CID	1.0000	0.0000	1	1	3304298	Unique	Used	0.474	1.142	1.728	1.102	1E-05	0	7.02	0	0	1	3	841.41125	2522.21921	-1.10
eHGYLGNLSAVASTEGTIEETLNEASR	Unambiguous	2	1	Q99P72	N-Term(iTRAQ8plex)	CID	1.0000	0.0000	1	1	3111834	Unique	Used	3.586	1.032	0.895	0.679	1E-05	0	7.02	0	0	46	2	1576.78418	3152.56108	-1.25
lGSFcLSEAGAGSDSFAMk	Unambiguous	2	1	Q9DBL1	N-Term(iTRAQ8plex); C5(Methylthio); K19(iTRAQ8plex)	CID	1.0000	0.0000	1	1	3479520	Unique	Used	0.688	1.029	0.850	1.428	1E-05	0	7.02	0	0	16	2	1266.62244	2532.23760	-0.70
tYFPHFDVSHGSAQVk	Unambiguous	2	1	Q91VB8	N-Term(iTRAQ8plex); K16(iTRAQ8plex)	CID	1.0000	0.0000	1	1	3481773	Unique	Used	2.873	3.073	0.338	1.110	1E-05	0	7.02	0	13	200	3	810.09747	2428.27787	-3.75
sLMLENTEcHSIYEESPER	Unambiguous	1	1	Q8CGF6	N-Term(iTRAQ8plex); C9(Methylthio)	CID	1.0000	0.0000	1	1	2867880	Unique	Used	1.485	0.822	0.913	0.856	1E-05	0	7.02	0	9	72	3	873.06433	2617.17844	-3.90
sLAEQNLEFQGPLEISR	Unambiguous	1	1	Q8R105	N-Term(iTRAQ8plex)	CID	1.0000	0.0000	1	1	3302858	Unique	Used	2.187	0.789	0.895	0.629	1E-05	0	7.02	0	6	14	2	1118.09778	2235.18828	-1.81
icDPGLTSFEPEALGNLVEGMDFHR	Unambiguous	9	1	Q5SVJ0	N-Term(iTRAQ8plex); C2(Methylthio)	CID	1.0000	0.0000	1	1	3482362	Unique	Used			0.723	0.763	1E-05	0	7.02	0	0	200	3	1033.16052	3097.46701	-2.12
aSGAVGLSYGAHSNLcVNQIVR	Unambiguous	1	1	Q9JHI5	N-Term(iTRAQ8plex); C16(Methylthio)	CID	1.0000	0.0000	1	1	3913822	Unique	Used	1.392	1.057	0.759	1.136	1E-05	0	7.02	0	16	200	3	856.10687	2566.30606	-4.35
gTVGEPTYDAEFQHFLR	Unambiguous	1	1	Q9JHU4	N-Term(iTRAQ8plex)	CID	1.0000	0.0000	1	1	3190718	Unique	Used	0.955	0.778	0.829	0.920	1E-05	0	7.02	0	7	20	3	757.71594	2271.13327	-0.67
kAEGATLSnATGAVESTSQAGDR	Unambiguous	1	1	Q61644	N-Term(iTRAQ8plex); K1(iTRAQ8plex); N9(Deamidated)	CID	1.0000	0.0000	1	1	2826768	Unique	Used	1.049	0.925	1.909	1.034	1E-05	0	7.02	1	0	107	3	944.15564	2830.45237	-4.10
fQmPDQGMTSADDFFQGTk	Unambiguous	1	1	O08553	N-Term(iTRAQ8plex); M3(Oxidation); K19(iTRAQ8plex)	CID	1.0000	0.0000	1	1	3303714	Unique	Used	1.935	0.950	0.950	0.746	1E-05	0	7.02	0	15	74	3	925.77600	2775.31345	-2.91

inScFSANTVEQIIENLR	Unambiguous	2	1	Q8QZS1	N-Term(iTRAQ8plex); N2(Deamidated); C4(Methylthio)	CID	1.0000	0.0000	1	1	3707259	Unique	Used	0.662	0.923	1.101	1.994	1E-05	0	7.02	0	30	44	2	1201.59668	2402.18608	-5.71
qGQYSPMAIEEQVAVIYAGVR	Unambiguous	2	1	Q03265	N-Term(iTRAQ8plex)	CID	1.0000	0.0000	1	1	3603271	Unique	Used	1.125	0.680	0.899	0.915	1E-05	0	7.02	0	0	200	2	1307.17957	2613.35185	-4.99
gSVHDFPEFDAnQDAEALYTAMk	Unambiguous	2	1	P14824	N-Term(iTRAQ8plex); N12(Deamidated); K23(iTRAQ8plex)	CID	1.0000	0.0000	1	1	2940616	Unique	Used	0.879	0.976	0.961	0.891	1E-05	0	7.02	0	7	200	3	1055.84888	3165.53208	0.78
dLYANNVLSGGTTMYPGIADR	Unambiguous	3	1	P62737	N-Term(iTRAQ8plex)	CID	1.0000	0.0000	1	1	3437843	Unique	Used	0.321	6.530	0.800	2.071	1E-05	0	7.02	0	0	78	2	1266.63428	2532.26128	-3.71
lGHVVmGNNAVSPYQQVIEk	Unambiguous	1	1	P60229	N-Term(iTRAQ8plex); M6(Oxidation); K20(iTRAQ8plex)	CID	1.0000	0.0000	1	1	3574826	Unique	Used	1.194	1.099	0.760	0.897	1E-05	0	7.02	0	0	176	3	936.51105	2807.51859	-5.30
qQLSAEELDAQLDAYnAR	Unambiguous	3	1	O08583	N-Term(iTRAQ8plex); N16(Deamidated)	CID	1.0000	0.0000	1	1	2637633	Unique	Used	1.260	0.196	0.859	0.555	1E-05	0	7.02	0	9	200	2	1170.58276	2340.15825	-1.67
hMQAVAEAWAQLQGSSAAR	Unambiguous	1	1	Q68FG2	N-Term(iTRAQ8plex)	CID	1.0000	0.0000	1	1	3914719	Unique	Used	0.559	2.271	2.298	1.379	1E-05	0	7.02	0	0	200	2	1158.58801	2316.16875	-5.78
lFVGGLNFNTDEqALEDHFSSFGPISEVVVVk	Unambiguous	2	1	O89086	N-Term(iTRAQ8plex); Q13(Deamidated); K32(iTRAQ8plex)	CID	0.0199	0.0000	1	1	3520302	Unique	Used	2.197	0.465	1.670	0.654	1E-05	0	7.02	0	13	46	3	1368.71643	4104.13474	-1.95
vFGLNTLMEIVTEAGPGSGEGNR	Unambiguous	10	1	A2AGR0	N-Term(iTRAQ8plex)	CID	1.0000	0.0000	1	1	3707148	Unique	Used	0.720	1.110	0.698	0.998	1E-05	0	7.02	0	16	147	2	1326.67334	2652.33940	-7.98
dNVcSPGGATIHALHVLESGGFR	Unambiguous	1	1	Q922W5	N-Term(iTRAQ8plex); C4(Methylthio)	CID	1.0000	0.0000	1	1	3784492	Unique	Used	1.081	0.767	0.694	0.732	1E-05	0	7.02	0	14	200	3	896.44647	2687.32486	-3.26
aGLGHLsPEGPLSLSEGPSSVGPEGGPGGVGAGGGSSTFPR	Unambiguous	2	1	B1AS06	N-Term(iTRAQ8plex); S7(Phospho)	CID	0.0214	0.0000	1	1	3172074	Unique	Used		0.971	0.855	0.992	1E-05	0	7.02	0	19	200	3	1352.98962	4056.95432	-1.59
sAAGSSSGFHSWAR	Unambiguous	1	1	P19246	N-Term(iTRAQ8plex)	CID	1.0000	0.0000	1	1	3832731	Unique	Used	2.200	0.213	1.255	1.843	1E-05	0	7.02	0	5	9	3	571.28467	1711.83945	-3.49
dAFQNAYLELGGLGER	Unambiguous	1	1	Q8VDN2	N-Term(iTRAQ8plex)	CID	1.0000	0.0000	1	1	3325875	Unique	Used	0.458	1.508	1.622	1.040	1E-05	0	7.01	0	3	15	2	1029.03137	2057.05547	-2.49

aQQLAAELEVEMMADMYNR	Unambiguous	1	1	P62073	N-Term(iTRAQ8plex)	CID	1.0000	0.0000	1	1	3217695	Unique	Used	2.121	0.959	1.515	1.046	1E-05	0	7.01	0	4	65	2	1259.10718	2517.20708	-0.77
dNVcSPGGATIHALHVLESGGFR	Unambiguous	1	1	Q922W5	N-Term(iTRAQ8plex); C4(Methylthio)	CID	1.0000	0.0000	1	1	3784512	Unique	Used	1.098	0.738	0.686	0.919	1E-05	0	7.01	0	0	200	3	896.44720	2687.32706	-2.45
eYQVETIVDTLcTNMLSDk	Unambiguous	2	1	Q6ZQ38	N-Term(iTRAQ8plex); C12(Methylthio); K19(iTRAQ8plex)	CID	1.0000	0.0000	1	1	3396865	Unique	Used	1.764	1.909	1.300	1.440	1E-05	0	7.01	0	19	200	3	952.80914	2856.41288	-5.64
tFPTVNPTTGEVIGHVAEGDR	Unambiguous	2	1	Q9CZS1	N-Term(iTRAQ8plex)	CID	1.0000	0.0000	1	1	3233812	Unique	Used	2.600	1.103	1.203	1.100	1E-05	0	7.01	0	19	25	3	834.43500	2501.29044	-1.36
fNLTGLNEQVPHYR	Unambiguous	5	1	P67871	N-Term(iTRAQ8plex)	CID	1.0000	0.0000	1	1	3681526	Unique	Used	1.323	1.122	0.807	0.668	1E-05	0	7.01	0	3	1	3	664.68732	1992.04740	-6.57
qLnTIQNAEAVHFFcNYEESR	Unambiguous	4	1	P61922	N-Term(iTRAQ8plex); N3(Deamidated); C15(Methylthio)	CID	0.0185	0.0000	1	1	3304374	Unique	Used	2.340	1.090	1.042	1.368	1E-05	0	7.01	0	18	52	3	955.44562	2864.32230	-2.19
yTPSGQSGAAASESLFISNHAY	Unambiguous	2	1	A6ZI44	N-Term(iTRAQ8plex)	CID	1.0000	0.0000	1	1	3235591	Unique	Used	1.128	0.912	1.051	1.397	1E-05	0	7.01	0	0	163	2	1281.61853	2562.22978	-4.55
eQTVQYILTMVDDmLQENHQR	Unambiguous	1	1	Q8BVE3	N-Term(iTRAQ8plex); M14(Oxidation)	CID	1.0000	0.0000	1	1	3686966	Unique	Used	1.461	1.056	0.733	0.836	1E-05	0	7.01	0	29	76	3	971.13916	2911.40293	-6.97
tVYFAEEVQcEGNSFHk	Unambiguous	1	1	P97315	N-Term(iTRAQ8plex); C10(Methylthio); K17(iTRAQ8plex)	CID	1.0000	0.0000	1	1	3090653	Unique	Used	1.716	0.877	1.919	0.854	1E-05	0	7.01	0	3	15	3	881.43195	2642.28128	-1.01
wGEAGAEYVVESTGVFTTMEk	Unambiguous	3	1	P16858	N-Term(iTRAQ8plex); K21(iTRAQ8plex)	CID	1.0000	0.0000	1	1	3219287	Unique	Used		1.383	1.102	0.727	1E-05	0	7.01	0	4	200	3	967.15930	2899.46335	-0.39
qSPINIDEDLTQVNVNLk	Unambiguous	1	1	B9EKR1	N-Term(iTRAQ8plex); K18(iTRAQ8plex)	CID	1.0000	0.0000	1	1	3260601	Unique	Used	1.667	1.322	1.556	0.969	1E-05	0	7.01	0	0	19	2	1324.73584	2648.46440	-2.71
eMEENFALEAANYQDTIGR	Unambiguous	1	1	P20152	N-Term(iTRAQ8plex)	CID	1.0000	0.0000	1	1	2833643	Unique	Used	0.469	2.160	0.649	1.040	1E-05	0	7.01	0	1	40	2	1253.09570	2505.18413	-1.15
sGSTAVGVLISPQHTYFINcGDSR	Unambiguous	1	1	P49443	N-Term(iTRAQ8plex); C20(Methylthio)	CID	1.0000	0.0000	1	1	3878030	Unique	Used	0.652	0.848	0.851	0.944	1E-05	0	7.01	0	20	16	3	953.80457	2859.39914	-2.81

dYLGDFIEHYAQLGPSQPPDLAQAQDEPR	Unambiguous	1	1	P42669	N-Term(iTRAQ8plex)	CID	1.0000	0.0000	1	1	3284178	Unique	Used	1.780	0.663	0.948	0.913	1E-05	0	7.01	0	10	17	3	1192.25134	3574.73948	0.05
aFVDFFSQcLHEEYk	Unambiguous	2	1	O70503	N-Term(iTRAQ8plex); C9(Methylthio); K15(iTRAQ8plex)	CID	1.0000	0.0000	1	1	3520221	Unique	Used	0.476	0.490	0.590	0.834	1E-05	0	7.01	0	17	28	3	839.74695	2517.22629	-5.57
IGTEEFPLIDQTFYPNHk	Unambiguous	3	1	O88935	N-Term(iTRAQ8plex); K18(iTRAQ8plex)	CID	1.0000	0.0000	1	1	3329062	Unique	Used	1.132	1.304	1.183	1.501	1E-05	0	7.01	0	20	200	3	919.82611	2757.46378	-2.56
eAmEAQIQNFGQTPSQLLIEPHPPR	Unambiguous	4	1	Q9EPN1-4	N-Term(iTRAQ8plex); M3(Oxidation)	CID	1.0000	0.0000	1	1	3455650	Unique	Used	3.439	1.714	0.965	1.042	1E-05	0	7.01	0	0	200	3	1051.20667	3151.60544	-3.00
vRPYIAEGESDTDSDLcTPcGPPPR	Unambiguous	7	1	B7ZBW1	N-Term(iTRAQ8plex); C17(Methylthio); C20(Methylthio)	CID	1.0000	0.0000	1	1	3130079	Unique	Used	2.074	1.518	1.611	1.441	1E-05	0	7.01	0	22	69	3	1024.46826	3071.39023	0.56
tFPTVNPTTGEVIGHVAEGDR	Unambiguous	2	1	Q9CZS1	N-Term(iTRAQ8plex)	CID	1.0000	0.0000	1	1	3211863	Unique	Used	1.513	0.746	1.155	1.651	1E-05	0	7.01	0	14	81	3	834.43488	2501.29007	-1.51
nSIFQLMIHTLDPLAEELGSYGk	Unambiguous	3	1	Q8BUN9	N-Term(iTRAQ8plex); K23(iTRAQ8plex)	CID	1.0000	0.0000	1	1	3727459	Unique	Used	0.521	0.870	1.154	0.826	1E-05	0	7.00	0	29	72	3	1062.23584	3184.69297	-7.65
lnSDPQFVLAQNVGTTHDLLDIcLR	Unambiguous	3	1	Q8R016	N-Term(iTRAQ8plex); N2(Deamidated); C23(Methylthio)	CID	1.0000	0.0000	1	1	3645412	Unique	Used	0.821	1.201	0.801	1.433	1E-05	0	7.00	0	12	16	3	1045.19812	3133.57981	-5.31
fIQDSIFGLcPHMTEDNk	Unambiguous	1	1	P27773	N-Term(iTRAQ8plex); C10(Methylthio); K18(iTRAQ8plex)	CID	1.0000	0.0000	1	1	3560754	Unique	Used	0.943	0.486	0.672	0.703	1E-05	0	7.00	0	0	65	3	917.12219	2749.35202	-3.21
enEcHFYAGGQVYPGEASR	Unambiguous	1	1	O08807	N-Term(iTRAQ8plex); N2(Deamidated); C4(Methylthio)	CID	1.0000	0.0000	1	1	2758895	Unique	Used	1.591	0.470	0.977	0.850	1E-05	0	7.00	0	9	200	3	822.36487	2465.08005	-0.14
yTPSGQSGAAASESLFISNHAy	Unambiguous	2	1	A6ZI44	N-Term(iTRAQ8plex); Y22(iTRAQ8plex)	CID	1.0000	0.0000	1	1	3388882	Unique	Used	2.088	1.260	1.141	1.300	1E-05	0	7.00	0	0	120	3	956.14838	2866.43058	-5.66

dLLVTGAYEITDQSGGAGGLR	Unambiguous	1	1	Q9D1D4	N-Term(iTRAQ8plex)	CID	1.0000	0.0000	1	1	3260704	Unique	Used	0.935	1.187	0.575	0.709	1E-05	0	7.00	0	0	53	2	1199.13513	2397.26299	2.75
yYLEETGPnSISYLGSk	Unambiguous	2	1	Q99J39-2	N-Term(iTRAQ8plex); N9(Deamidated); K17(iTRAQ8plex)	CID	1.0000	0.0000	1	1	3235546	Unique	Used	0.548	0.893	0.817	0.935	1E-05	0	7.00	0	0	200	2	1265.66919	2530.33110	5.44
aTAPVSFNYYGMITGPPASk	Unambiguous	3	1	Q8K2Q7	N-Term(iTRAQ8plex); K20(iTRAQ8plex)	CID	1.0000	0.0000	1	1	3783694	Unique	Used	0.590	0.558	0.629	0.815	1E-05	0	7.00	0	0	200	2	1340.71411	2680.42095	-2.09
lLDGDEGPNTGGMGAYcPAPQVSk	Unambiguous	3	1	Q64737	N-Term(iTRAQ8plex); C17(Methylthio); K24(iTRAQ8plex)	CID	1.0000	0.0000	1	1	3303790	Unique	Used	2.607	3.733	1.028	2.409	1E-05	0	7.00	0	5	27	3	1011.16174	3031.47068	-2.56
aTVLQNQLDESQQER	Unambiguous	2	1	E9Q7U2	N-Term(iTRAQ8plex)	CID	1.0000	0.0000	1	1	2847031	Unique	Used	1.494	1.390	1.784	1.158	1E-05	0	7.00	0	0	200	2	1032.03381	2063.06035	-3.28
vDSILLTHIGDDNLPGINSmLQR	Unambiguous	1	1	P14873	N-Term(iTRAQ8plex); M20(Oxidation)	CID	1.0000	0.0000	1	1	3686192	Unique	Used	0.564	0.390	0.752	0.828	1E-05	0	7.00	0	16	16	3	947.83496	2841.49033	-6.32
gPTGPPSTLPLGTQTSSGSSTLSk	Unambiguous	7	1	H3BJY2	N-Term(iTRAQ8plex); K24(iTRAQ8plex)	CID	1.0000	0.0000	1	1	3595660	Unique	Used	0.760	1.177	0.958	0.802	1E-05	0	7.00	0	26	35	3	956.18756	2866.54813	-4.79
lTGFHETSNINDFSAGVANR	Unambiguous	1	1	P15105	N-Term(iTRAQ8plex)	CID	1.0000	0.0000	1	1	3575476	Unique	Used	1.370	1.215	1.257	1.540	1E-05	0	7.00	0	2	15	3	818.74426	2454.21823	-5.43
lGGLTQAPGnPVLAVQINQDk	Unambiguous	2	1	P26369	N-Term(iTRAQ8plex); N10(Deamidated); K21(iTRAQ8plex)	CID	0.0243	0.0000	1	1	3664194	Unique	Used	0.755	0.854	1.003	0.972	1E-05	0	7.00	0	0	193	2	1371.78735	2742.56743	2.31
nQDEcVIALHDcnGDVNR	Unambiguous	5	1	Q80X50	N-Term(iTRAQ8plex); C5(Methylthio); C12(Methylthio); N13(Deamidated)	CID	1.0000	0.0000	1	1	2718181	Unique	Used	1.693	0.843	1.363	1.012	1E-05	0	7.00	0	8	200	3	804.68195	2412.03128	-1.73
lLGDASVSFTDNcVVGIQANTER	Unambiguous	3	1	P97807-2	N-Term(iTRAQ8plex); C13(Methylthio)	CID	1.0000	0.0000	1	1	3583670	Unique	Used				1.082	1E-05	0	7.00	0	0	200	2	1380.18018	2759.35307	-4.20

aITVASQTncPLYVTk	Unambiguous	5	1	E9PWE8	N-Term(iTRAQ8plex); N9(Deamidated); C10(Methylthio); K16(iTRAQ8plex)	CID	1.0000	0.0000	1	1	3579196	Unique	Used	0.835	0.635	0.501	0.625	1E-05	0	7.00	0	25	182	2	1182.63733	2364.26738	-3.79
qAEIENPLEDPVTGDYVHk	Unambiguous	4	1	Q9Z1G4-3	N-Term(iTRAQ8plex); K19(iTRAQ8plex)	CID	1.0000	0.0000	1	1	2737417	Unique	Used	0.265	1.742	1.508	1.089	1E-05	0	7.00	0	2	85	3	921.48590	2762.44315	-0.95
qTFTGHESDINAIcFFPnGNAFATGSDDATcR	Unambiguous	2	1	P62874	N-Term(iTRAQ8plex); C14(Methylthio); N18(Deamidated); C31(Methylthio)	CID	0.0243	0.0000	1	1	3459095	Unique	Used			0.738	0.612	1E-05	0	7.00	0	14	64	3	1268.88379	3804.63681	0.70
fQMPDQGMTSADDFFQGTk	Unambiguous	1	1	O08553	N-Term(iTRAQ8plex); K19(iTRAQ8plex)	CID	1.0000	0.0000	1	1	3307203	Unique	Used		0.716	0.871	1.034	1E-05	0	7.00	0	0	200	3	920.44611	2759.32376	-1.03
mADPQSIQESQNLSmFLANHNk	Unambiguous	4	1	Q7TMB8	N-Term(iTRAQ8plex); M15(Oxidation); K22(iTRAQ8plex)	CID	1.0000	0.0000	1	1	3435818	Unique	Used	0.786	1.506	0.881	1.088	1E-05	0	7.00	0	0	47	3	1043.19336	3127.56552	-3.40
hQVLFIADEIQTGLAR	Unambiguous	1	1	P29758	N-Term(iTRAQ8plex)	CID	1.0000	0.0000	1	1	3664255	Unique	Used	2.031	1.798	1.163	1.064	1E-05	0	6.99	0	5	9	3	705.72931	2115.17337	-6.18
yHTSQSGDEmTSLSEYVSR	Unambiguous	2	1	P11499	N-Term(iTRAQ8plex); M10(Oxidation)	CID	1.0000	0.0000	1	1	2989321	Unique	Used	1.172	0.982	0.945	1.122	1E-05	0	6.99	0	9	35	3	833.05157	2497.14017	-2.13
qGQYSPMAIEEQVAVIYAGVR	Unambiguous	2	1	Q03265	N-Term(iTRAQ8plex)	CID	1.0000	0.0000	1	1	3603466	Unique	Used	0.351	0.755	1.420	1.242	1E-05	0	6.99	0	0	200	2	1307.18042	2613.35356	-4.34
iVSGAAETDQEYYFGQVVR	Unambiguous	4	1	P06909	N-Term(iTRAQ8plex)	CID	1.0000	0.0000	1	1	3214522	Unique	Used			0.739	1.743	1E-05	0	6.99	0	0	133	2	1218.61816	2436.22905	-2.41
dFTSEQIDEILQNHTEIVFAR	Unambiguous	2	1	Q6PIC6	N-Term(iTRAQ8plex)	CID	1.0000	0.0000	1	1	3354662	Unique	Used	0.671	0.665	1.219	1.731	1E-05	0	6.99	0	5	57	3	937.14551	2809.42197	-3.24
sVNESLNNLFITEEDYQALR	Unambiguous	2	1	Q68FD5	N-Term(iTRAQ8plex)	CID	1.0000	0.0000	1	1	3240265	Unique	Used	0.526	1.331	1.715	1.914	1E-05	0	6.99	0	20	200	2	1330.17810	2659.34892	-1.06
dINQEVYNFLATAGAk	Unambiguous	1	1	Q99KI0	N-Term(iTRAQ8plex); K16(iTRAQ8plex)	CID	1.0000	0.0000	1	1	3542656	Unique	Used	0.743	0.837	0.945	1.185	1E-05	0	6.99	0	18	200	2	1181.64563	2362.28398	-0.99

gGSDNDASLVITDLTLEDYGR	Unambiguous	1	1	Q9QUP5	N-Term(iTRAQ8plex)	CID	1.0000	0.0000	1	1	2816014	Unique	Used	1.509	1.789	2.712	1.451	1E-05	0	6.99	0	12	55	2	1258.12573	2515.24419	-0.97
fVSPLTLVADEGWFIAESR	Unambiguous	3	1	Q8BGN3	N-Term(iTRAQ8plex)	CID	1.0000	0.0000	1	1	3666702	Unique	Used	2.165	0.150	0.826	1.075	1E-05	0	6.99	0	4	33	2	1221.14673	2441.28618	-6.44
eALSLAQMQEQTLQLEQQSk	Unambiguous	4	1	Q925I1	N-Term(iTRAQ8plex); K20(iTRAQ8plex)	CID	1.0000	0.0000	1	1	3260463	Unique	Used	0.737	1.115	1.170	1.003	1E-05	0	6.99	0	0	111	2	1456.28259	2911.55791	-2.63
vEIVAINDPFIDLNYmVYMFQYDSTHGk	Unambiguous	4	1	P16858	N-Term(iTRAQ8plex); M16(Oxidation); K28(iTRAQ8plex)	CID	1.0000	0.0000	1	1	3645318	Unique	Used	0.981	1.182	1.754	1.472	1E-05	0	6.99	0	12	23	3	1316.32544	3946.96177	-6.05
vGNPWDPNILYGPLHTk	Unambiguous	2	1	Q9DBF1-2	N-Term(iTRAQ8plex); K17(iTRAQ8plex)	CID	1.0000	0.0000	1	1	3684542	Unique	Used	0.278	1.246	1.082	1.523	1E-05	0	6.99	0	16	11	3	843.80182	2529.39090	-6.54
tIHQEDELIEIQSDTGTWYQR	Unambiguous	1	1	Q9JIS5	N-Term(iTRAQ8plex)	CID	1.0000	0.0000	1	1	2831923	Unique	Used	0.421	1.142	1.219	1.293	1E-05	0	6.99	0	7	77	2	1433.70764	2866.40801	-2.83
fEVAQELTLSSEAPQEADSFMGVESGHIk	Unambiguous	1	1	P20357	N-Term(iTRAQ8plex); K29(iTRAQ8plex)	CID	1.0000	0.0000	1	1	3195781	Unique	Used	1.800	2.203	1.250	0.772	1E-05	0	6.99	0	8	41	3	1248.96411	3744.87778	-2.95
eTAAAHQASSsPPIDAATAEPYGFR	Unambiguous	1	1	P14873	N-Term(iTRAQ8plex); S11(Phospho)	CID	0.0186	0.0000	1	1	2716756	Unique	Used	2.562	0.392	1.524	0.700	1E-05	0	6.99	0	0	89	3	977.12585	2929.36301	-1.42
nAQNINPSSSQNSQNFATYk	Unambiguous	4	1	P23819-4	N-Term(iTRAQ8plex); K20(iTRAQ8plex)	CID	1.0000	0.0000	1	1	3339610	Unique	Used		1.613	1.790	0.748	1E-05	0	6.99	0	0	9	2	1411.21130	2821.41533	-6.10
eLSSAVSPPNLHSDTPTFSYASLAGPTIPPR	Unambiguous	3	1	Q9QYR6	N-Term(iTRAQ8plex)	CID	1.0000	0.0000	1	1	3685249	Unique	Used	1.843	1.005	2.672	1.135	1E-05	0	6.99	0	0	93	3	1171.93555	3513.79209	-7.04
nVLGHMQQGGAPSPFDR	Unambiguous	2	1	Q8C605	N-Term(iTRAQ8plex)	CID	1.0000	0.0000	1	1	3699355	Unique	Used	1.649	1.119	1.020	1.085	1E-05	0	6.99	0	0	89	2	1058.03235	2115.05742	-6.32
tPAPVQSSSASVLNVNHIk	Unambiguous	2	1	Q99NH0	N-Term(iTRAQ8plex); K19(iTRAQ8plex)	CID	1.0000	0.0000	1	1	3636357	Unique	Used	1.001	0.690	1.081	0.691	1E-05	0	6.99	0	20	93	3	853.14948	2557.43387	-8.60
tLASLSPETSLFIIASkTFTTQETITNAETAk	Unambiguous	1	1	P06745	N-Term(iTRAQ8plex); K17(iTRAQ8plex); K32(iTRAQ8plex)	CID	1.0000	0.0000	1	1	3727049	Unique	Used	1.410	0.940	1.509	1.255	1E-05	0	6.98	1	0	14	3	1443.13257	4327.38315	-5.10

kSQVFSTAADGQTQVEIk	Unambiguous	1	1	P38647	N-Term(iTRAQ8plex); K1(iTRAQ8plex); K18(iTRAQ8plex)	CID	1.0000	0.0000	1	1	3253344	Unique	Used	7.399	1.290	1.164	1.223	1E-05	0	6.98	1	0	45	3	950.54407	2849.61765	1.42
sINPDEAVAYGAAVQAAILSGDk	Unambiguous	2	1	P63017	N-Term(iTRAQ8plex); K23(iTRAQ8plex)	CID	1.0000	0.0000	1	1	3479723	Unique	Used	1.028	1.043	0.765	0.876	1E-05	0	6.98	0	0	18	2	1434.77722	2868.54717	-3.22
eAEEEFWQNQHPQPYIFPDSPGGTSFER	Unambiguous	1	1	Q9CQJ8	N-Term(iTRAQ8plex)	CID	1.0000	0.0000	1	1	2940682	Unique	Used	1.304	0.971	0.693	0.770	1E-05	0	6.98	0	0	200	3	1209.56201	3626.67148	-1.44
kTESIDVmDAVGSNIVVSTR	Unambiguous	1	1	Q91VD9	N-Term(iTRAQ8plex); K1(iTRAQ8plex); M8(Oxidation)	CID	1.0000	0.0000	1	1	3367408	Unique	Used	0.848	1.120	1.117	1.101	1E-05	0	6.98	1	0	12	3	915.83105	2745.47861	-4.64
dQEGQDVLLFIDNIFR	Unambiguous	1	1	P56480	N-Term(iTRAQ8plex)	CID	1.0000	0.0000	1	1	3356270	Unique	Used	0.771	1.149	1.129	1.315	1E-05	0	6.98	0	0	200	2	1113.58289	2226.15849	-5.56
aLNALNNLSVNVENQTk	Unambiguous	3	1	Q9D0L7	N-Term(iTRAQ8plex); K17(iTRAQ8plex)	CID	1.0000	0.0000	1	1	3599511	Unique	Used	0.807	1.481	1.017	1.095	1E-05	0	6.98	0	0	89	2	1225.68481	2450.36235	-8.17
dAGYEFDIcFTSVQk	Unambiguous	1	1	Q9DBJ1	N-Term(iTRAQ8plex); C9(Methylthio); K15(iTRAQ8plex)	CID	1.0000	0.0000	1	1	3264840	Unique	Used	2.064	1.275	0.791	1.116	1E-05	0	6.98	0	0	200	2	1189.08691	2377.16655	0.03
IVEVANLAcSISnNEEGVk	Unambiguous	4	1	Q61301-2	N-Term(iTRAQ8plex); C9(Methylthio); N13(Deamidated); K19(iTRAQ8plex)	CID	0.0458	0.0000	1	1	3262801	Unique	Used	0.399	0.865	0.714	0.922	1E-05	0	6.98	0	0	38	2	1322.69397	2644.38066	0.90
tQPTDEEMLFIYSHFk	Unambiguous	4	1	P31786	N-Term(iTRAQ8plex); K16(iTRAQ8plex)	CID	1.0000	0.0000	1	1	3287717	Unique	Used	1.518	0.604	0.724	0.640	1E-05	0	6.98	0	10	200	3	865.45691	2594.35617	5.41
qQLGTAVEmEIAQMLEENSR	Unambiguous	2	1	Q9JKK7	N-Term(iTRAQ8plex); M9(Oxidation)	CID	1.0000	0.0000	1	1	3284751	Unique	Used	1.968	1.504	1.326	1.094	1E-05	0	6.98	0	7	49	2	1299.14294	2597.27861	-2.60
iGEHTPSALAIMENANVLAR	Unambiguous	3	1	A6ZI44	N-Term(iTRAQ8plex)	CID	1.0000	0.0000	1	1	3705080	Unique	Used	0.837	1.350	1.861	1.419	1E-05	0	6.98	0	0	0	3	804.43457	2411.28916	-5.29

vIQDSNNELLEPVcHQLFELYR	Unambiguous	3	1	Q8C729-2	N-Term(iTRAQ8plex); C14(Methylthio)	CID	1.0000	0.0000	1	1	3645349	Unique	Used	1.170	0.866	1.987	1.177	1E-05	0	6.98	0	7	7	3	1003.83728	3009.49729	-4.77
gTVVYGEPITASLGTDGSHYWSk	Unambiguous	1	1	O08553	N-Term(iTRAQ8plex); K23(iTRAQ8plex)	CID	1.0000	0.0000	1	1	3414384	Unique	Used	1.564	1.885	1.103	1.029	1E-05	0	6.98	0	0	55	3	1011.85889	3033.56211	-5.18
mcDPGMTAFEPEALGNLVEGLDFHR	Unambiguous	4	1	P11798	N-Term(iTRAQ8plex); M1(Oxidation); C2(Methylthio)	CID	0.0229	0.0000	1	1	3479832	Unique	Used		2.631	1.811	1.027	1E-05	0	6.98	0	8	3	3	1039.14380	3115.41684	-4.23
fPEEGISPQNPDSPGLSDLNcGHLR	Unambiguous	2	1	D3Z0D9	N-Term(iTRAQ8plex); C21(Methylthio)	CID	1.0000	0.0000	1	1	3302689	Unique	Used	1.760	1.133	1.399	1.630	1E-05	0	6.98	0	0	91	3	1010.48431	3029.43839	-0.52
aVQLYQQTANVFENEER	Unambiguous	2	1	Q9CWZ7	N-Term(iTRAQ8plex)	CID	1.0000	0.0000	1	1	3237998	Unique	Used	0.835	1.204	1.255	1.196	1E-05	0	6.98	0	3	19	2	1172.09546	2343.18364	-1.98
qLLHNFPPDqLTSSGAPFWSGPk	Unambiguous	1	1	Q02053	N-Term(iTRAQ8plex); Q10(Deamidated); K23(iTRAQ8plex)	CID	0.0273	0.0000	1	1	3859095	Unique	Used	1.193	1.723	1.113	0.899	1E-05	0	6.97	0	0	150	3	1045.22437	3133.65854	0.56
qTTAAAAATFSEQVGGGSGGAGR	Unambiguous	3	1	O88935	N-Term(iTRAQ8plex)	CID	1.0000	0.0000	1	1	3501806	Unique	Used	1.272	2.665	2.888	2.484	1E-05	0	6.97	0	14	200	2	1178.58972	2356.17217	-3.14
lTPLEAcAHSFFDELR	Unambiguous	2	1	E9QAQ5	N-Term(iTRAQ8plex); C7(Methylthio)	CID	1.0000	0.0000	1	1	3686997	Unique	Used	0.364	0.769	1.014	1.194	1E-05	0	6.97	0	7	9	3	733.69501	2199.07047	-8.00
lALEQHNWnMEAAVQDR	Unambiguous	5	1	Q3TDN2-2	N-Term(iTRAQ8plex); N9(Deamidated)	CID	0.0057	0.0000	1	1	3256024	Unique	Used	0.680	0.860	0.874	0.890	1E-05	0	6.97	0	0	126	2	1165.58508	2330.16289	5.46
vDEFPLcGHMVSDEYEQLSSEALEAAR	Unambiguous	4	1	I7HLV2	N-Term(iTRAQ8plex); C7(Methylthio)	CID	1.0000	0.0000	1	1	3112262	Unique	Used	0.488	0.379	0.731	0.824	1E-05	0	6.97	0	0	200	3	1125.85486	3375.55002	0.42
nDEYENLFNMVVEIPR	Unambiguous	2	1	Q91VM9	N-Term(iTRAQ8plex)	CID	1.0000	0.0000	1	1	3305528	Unique	Used	1.266	0.788	1.086	1.191	1E-05	0	6.97	0	0	21	2	1143.56909	2286.13091	-3.04
qFMDIFSLPEMTLLScVNEHFLk	Unambiguous	1	1	Q3UHB1	N-Term(iTRAQ8plex); C16(Methylthio); K23(iTRAQ8plex)	CID	1.0000	0.0000	1	1	3861394	Unique	Used	0.225	0.690	1.102	1.225	1E-05	0	6.97	0	21	23	3	1132.91333	3396.72544	-2.09

gEMMDLQHGSLFLQTPk	Unambiguous	2	1	P16125	N-Term(iTRAQ8plex); K17(iTRAQ8plex)	CID	1.0000	0.0000	1	1	3475886	Unique	Used	0.879	1.116	1.100	1.302	1E-05	0	6.97	0	15	162	3	847.44910	2540.33274	-5.30
aGYTDQVVIGMDVAASEFYR	Unambiguous	2	1	P17182	N-Term(iTRAQ8plex)	CID	1.0000	0.0000	1	1	3328432	Unique	Used	0.801	1.219	1.322	1.457	1E-05	0	6.97	0	4	200	2	1248.61865	2496.23003	-3.31
qPSQPTTSQSLGqLQAHAASAPGSNPR	Unambiguous	1	1	E9PZA8	N-Term(iTRAQ8plex); Q13(Deamidated)	CID	0.0086	0.0000	1	1	3834614	Unique	Used		2.096	1.377	0.988	1E-05	0	6.97	0	15	200	3	1007.85114	3021.53885	3.19
vGTGEPccDWVGDEGAGHFVk	Unambiguous	1	1	Q9DCD0	N-Term(iTRAQ8plex); C7(Methylthio); C8(Methylthio); K21(iTRAQ8plex)	CID	1.0000	0.0000	1	1	3151612	Unique	Used	0.484	0.996	0.730	1.143	1E-05	0	6.97	0	11	38	3	955.10760	2863.30826	-1.75
wGEAGAEYVVESTGVFTTMEk	Unambiguous	3	1	P16858	N-Term(iTRAQ8plex); K21(iTRAQ8plex)	CID	1.0000	0.0000	1	1	3262393	Unique	Used	0.556	0.861	0.898	1.204	1E-05	0	6.97	0	0	17	2	1450.23022	2899.45317	-3.90
nLHQSGFSLSGAQIDDNIPR	Unambiguous	1	1	O08553	N-Term(iTRAQ8plex)	CID	1.0000	0.0000	1	1	3638668	Unique	Used	1.649	1.582	1.052	1.107	1E-05	0	6.97	0	12	38	3	825.09094	2473.25827	-6.27
sLSNATIINEEVLNAmLk	Unambiguous	3	1	P14576	N-Term(iTRAQ8plex); M16(Oxidation); K18(iTRAQ8plex)	CID	1.0000	0.0000	1	1	3579611	Unique	Used	0.423	0.720	0.616	0.699	1E-05	0	6.97	0	0	98	2	1292.72021	2584.43315	-5.62
eHINLGcDVDFDIAGPSIR	Unambiguous	3	1	Q60932-2	N-Term(iTRAQ8plex); C7(Methylthio)	CID	1.0000	0.0000	1	1	3374821	Unique	Used	1.148	1.262	1.465	1.295	1E-05	0	6.97	0	5	150	3	807.73004	2421.17557	-3.69
aGYTDQVVIGMDVAASEFYR	Unambiguous	2	1	P17182	N-Term(iTRAQ8plex)	CID	1.0000	0.0000	1	1	3329282	Unique	Used	1.066	1.666	1.689	1.573	1E-05	0	6.97	0	0	200	2	1248.61926	2496.23125	-2.83
tQPTDEEMLFIYSHFk	Unambiguous	4	1	P31786	N-Term(iTRAQ8plex); K16(iTRAQ8plex)	CID	1.0000	0.0000	1	1	3288103	Unique	Used	2.224	0.929	0.968	0.837	1E-05	0	6.97	0	0	200	3	865.45227	2594.34226	0.05
qGQYSPMAIEEqVAVIYAGVR	Unambiguous	2	1	Q03265	N-Term(iTRAQ8plex); Q12(Deamidated)	CID	1.0000	0.0000	1	1	3603894	Unique	Used		0.744	1.108	1.337	1E-05	0	6.97	0	24	200	2	1307.68091	2614.35454	2.15

aASVAENcINYQQGTPHk	Unambiguous	1	1	P70202	N-Term(iTRAQ8plex); C8(Methylthio); K18(iTRAQ8plex)	CID	1.0000	0.0000	1	1	3388935	Unique	Used	0.861	0.814	0.897	1.071	1E-05	0	6.96	0	0	35	2	1293.14941	2585.29155	-5.62
aLIYGGEMAAAVAEVSEQLGk	Unambiguous	1	1	Q60714	N-Term(iTRAQ8plex); K21(iTRAQ8plex)	CID	1.0000	0.0000	1	1	3459710	Unique	Used	0.959	1.400	0.712	0.912	1E-05	0	6.96	0	15	200	2	1358.24146	2715.47563	-3.38
aGYTDQVVIGMDVAASEFYR	Unambiguous	2	1	P17182	N-Term(iTRAQ8plex)	CID	1.0000	0.0000	1	1	3327676	Unique	Used	1.275	1.531	1.028	1.218	1E-05	0	6.96	0	0	102	2	1248.61987	2496.23247	-2.34
wGEAGAEYVVESTGVFTTMEk	Unambiguous	3	1	P16858	N-Term(iTRAQ8plex); K21(iTRAQ8plex)	CID	1.0000	0.0000	1	1	3217393	Unique	Used	0.384	0.909	1.278	1.263	1E-05	0	6.96	0	0	39	2	1450.23328	2899.45928	-1.79
yTPSGqSGAAASESLFISNHAY	Unambiguous	2	1	A6ZI44	N-Term(iTRAQ8plex); Q6(Deamidated)	CID	0.0029	0.0000	1	1	3280098	Unique	Used	1.239	1.072	1.422	1.294	1E-05	0	6.96	0	26	200	2	1282.12463	2563.24199	6.45
aASQSAADYNQVVPTR	Unambiguous	4	1	B7ZCJ1	N-Term(iTRAQ8plex)	CID	1.0000	0.0000	1	1	3184506	Unique	Used	0.740	1.048	1.505	1.142	1E-05	0	6.96	0	0	32	2	991.51282	1982.01836	-3.10
eGIcALGGTSELSSEGTQHSYSEEEk	Unambiguous	2	1	B1AX58	N-Term(iTRAQ8plex); C4(Methylthio); K26(iTRAQ8plex)	CID	1.0000	0.0000	1	1	2717230	Unique	Used	0.809	0.463	1.220	0.583	1E-05	0	6.96	0	9	200	3	1128.19788	3382.57907	-2.51
rGPFELEAFYSDPQGVPYPEAk	Unambiguous	3	1	Q61699-2	N-Term(iTRAQ8plex); K22(iTRAQ8plex)	CID	1.0000	0.0000	1	1	3324242	Unique	Used	1.072	1.697	1.253	1.121	1E-05	0	6.96	1	13	14	3	1035.87256	3105.60312	-3.58
iTQHFSTEEFEDEYVTDYTR	Unambiguous	2	1	Q9QYX7	N-Term(iTRAQ8plex)	CID	1.0000	0.0000	1	1	2813478	Unique	Used	1.307	0.998	1.278	0.704	1E-05	0	6.96	0	0	62	3	938.77307	2814.30466	-0.06
eScLEAYTGIVQGLk	Unambiguous	1	1	P70168	N-Term(iTRAQ8plex); C3(Methylthio); K15(iTRAQ8plex)	CID	1.0000	0.0000	1	1	3601877	Unique	Used	0.338	1.969	0.724	0.832	1E-05	0	6.96	0	0	46	2	1133.10437	2265.20146	-2.86
gYFEELITMLEAALGLER	Unambiguous	2	1	Q68FD5	N-Term(iTRAQ8plex)	CID	1.0000	0.0000	1	1	3843611	Unique	Used	0.666	2.702	1.654	1.599	1E-05	0	6.96	0	27	91	2	1180.12842	2359.24956	-1.11
vLTFPEYIASLSDSGTk	Unambiguous	2	1	Q61137-2	N-Term(iTRAQ8plex); K17(iTRAQ8plex)	CID	1.0000	0.0000	1	1	3581225	Unique	Used	0.658	1.453	1.099	0.775	1E-05	0	6.96	0	28	25	2	1218.66882	2436.33037	-7.34

tNTSSVTTTITQATATANTANTENSPQALHNSQFLLTR	Unambiguous	2	1	Q80UY2	N-Term(iTRAQ8plex)	CID	1.0000	0.0000	1	1	3822137	Unique	Used	1.957			0.947	1E-05	0	6.96	0	0	116	3	1437.39038	4310.15659	-5.50
eFTGSQNSVFPVFDNLLLLDR	Unambiguous	1	1	Q9D2N9	N-Term(iTRAQ8plex)	CID	1.0000	0.0000	1	1	3687117	Unique	Used	0.512	0.865	0.763	1.061	1E-05	0	6.96	0	5	22	2	1358.21057	2715.41387	-5.80
aVGPHQFLGNQEAIQAAIk	Unambiguous	1	1	P15327	N-Term(iTRAQ8plex); K19(iTRAQ8plex)	CID	1.0000	0.0000	1	1	3596085	Unique	Used	1.258	0.943	0.686	1.010	1E-05	0	6.96	0	0	33	3	867.49243	2600.46274	-5.46
sIEEAcLTLQHLNR	Unambiguous	3	1	Q9QYR6	N-Term(iTRAQ8plex); C6(Methylthio)	CID	1.0000	0.0000	1	1	3726355	Unique	Used	0.708	1.157	1.375	1.344	1E-05	0	6.96	0	0	1	3	659.67352	1977.00602	-7.05
dHFMDEFFEQVEEIR	Unambiguous	1	1	P61264	N-Term(iTRAQ8plex)	CID	1.0000	0.0000	1	1	2816162	Unique	Used	0.699	0.784	1.644	1.610	1E-05	0	6.96	0	9	33	3	759.02563	2275.06235	-0.88
eVAAFAQFGSDLDAATQQLLSR	Unambiguous	2	1	Q03265	N-Term(iTRAQ8plex)	CID	1.0000	0.0000	1	1	3666579	Unique	Used	0.789	1.127	1.168	1.464	1E-05	0	6.96	0	4	20	2	1321.68323	2642.35918	-5.17
eEVEGESLDLcLQQLYk	Unambiguous	1	1	Q80TL0	N-Term(iTRAQ8plex); C11(Methylthio); K17(iTRAQ8plex)	CID	1.0000	0.0000	1	1	2979118	Unique	Used	0.577	0.889	1.419	0.874	1E-05	0	6.96	0	20	151	2	1325.67480	2650.34233	-5.33
gVLTGPEYEALDALPDAER	Unambiguous	1	1	Q9D1X0	N-Term(iTRAQ8plex)	CID	1.0000	0.0000	1	1	2815613	Unique	Used	1.359	0.837	1.304	1.007	1E-05	0	6.96	0	0	200	2	1160.60046	2320.19365	-1.66
dFTSEQIDEILQNHTEIVFAR	Unambiguous	2	1	Q6PIC6	N-Term(iTRAQ8plex)	CID	1.0000	0.0000	1	1	3358963	Unique	Used			1.523		1E-05	0	6.96	0	0	200	3	937.14508	2809.42069	-3.70
dSDWPFcSDDDWNHk	Unambiguous	2	1	Q99K47	N-Term(iTRAQ8plex); C7(Methylthio); K15(iTRAQ8plex)	CID	1.0000	0.0000	1	1	2814030	Unique	Used	1.504	1.544	1.432	3.743	1E-05	0	6.96	0	0	15	3	841.03802	2521.09952	-0.56
aQDAGVYQcLASNPVGTVVSk	Unambiguous	1	1	Q61330	N-Term(iTRAQ8plex); C9(Methylthio); K21(iTRAQ8plex)	CID	1.0000	0.0000	1	1	3644259	Unique	Used	0.571	0.580	1.006	1.188	1E-05	0	6.96	0	0	49	2	1381.22058	2761.43389	-4.88
tLEVVSPSqSVTGSAGHTPYYQSPTDEk	Unambiguous	1	1	P14873	N-Term(iTRAQ8plex); Q9(Deamidated); K28(iTRAQ8plex)	CID	1.0000	0.0000	1	1	3105578	Unique	Used	1.886	0.757	2.274	1.341	1E-05	0	6.96	0	15	146	3	1192.27539	3574.81162	2.98

scAEFEELTVSQLLLcLR	Unambiguous	1	1	Q8K3K8	N-Term(iTRAQ8plex); C2(Methylthio); C16(Methylthio)	CID	1.0000	0.0000	1	1	3807076	Unique	Used	2.686	0.705	1.035	0.938	1E-05	0	6.96	0	22	155	2	1225.60559	2450.20391	-2.72
sALFAQINqGESITHALk	Unambiguous	1	1	P40124	N-Term(iTRAQ8plex); Q9(Deamidated); K18(iTRAQ8plex)	CID	0.0014	0.0000	1	1	3683947	Unique	Used	0.746	1.394	1.265	0.967	1E-05	0	6.96	0	0	200	2	1269.21130	2537.41533	-1.22
eILEQLLEAIVVATNPSGR	Unambiguous	13	1	D3Z1W6	N-Term(iTRAQ8plex)	CID	1.0000	0.0000	1	1	3880812	Unique	Used	0.436	0.845	0.660	0.804	1E-05	0	6.95	0	24	103	2	1178.67004	2356.33281	-2.63
wGEAGAEYVVESTGVFTTMEk	Unambiguous	3	1	P16858	N-Term(iTRAQ8plex); K21(iTRAQ8plex)	CID	1.0000	0.0000	1	1	3240636	Unique	Used	0.989	0.742	0.707	0.976	1E-05	0	6.95	0	19	200	3	967.15820	2899.46006	-1.52
iLAVnGQSIINMPHADIVk	Unambiguous	4	1	Q9WVQ1	N-Term(iTRAQ8plex); N5(Deamidated); K19(iTRAQ8plex)	CID	0.0273	0.0000	1	1	3559632	Unique	Used	0.851	0.695	0.911	0.758	1E-05	0	6.95	0	10	108	3	881.50891	2642.51218	-1.46
sYVYAEEPNIDIHNFLGTFTR	Unambiguous	4	1	Q8C4G3	N-Term(iTRAQ8plex)	CID	1.0000	0.0000	1	1	3644825	Unique	Used		1.227			1E-05	0	6.95	0	0	9	3	930.80084	2790.38797	-5.78
yTPSGQSGAAASESLFISnHAY	Unambiguous	2	1	A6ZI44	N-Term(iTRAQ8plex); N19(Deamidated)	CID	1.0000	0.0000	1	1	3170109	Unique	Used	1.038	1.896	1.248	1.311	1E-05	0	6.95	0	0	200	2	1282.11353	2563.21977	-2.22
kIENLEALTELEVLDISFNMLR	Unambiguous	1	1	Q3UM45	N-Term(iTRAQ8plex); K1(iTRAQ8plex)	CID	1.0000	0.0000	1	1	3541827	Unique	Used	3.852	2.578	0.741	1.199	1E-05	0	6.95	1	10	53	3	1066.93152	3198.78000	-3.29
eTFGVnNTVYGIDAMNPSSR	Unambiguous	2	1	Q3UGC7	N-Term(iTRAQ8plex); N6(Deamidated)	CID	0.0072	0.0000	1	1	3214238	Unique	Used	1.227	0.409	1.102	0.806	1E-05	0	6.95	0	12	182	2	1239.10095	2477.19463	1.03
dmHGDSEYNIMFGPDIcGPGTk	Unambiguous	1	1	P14211	N-Term(iTRAQ8plex); M2(Oxidation); C17(Methylthio); K22(iTRAQ8plex)	CID	1.0000	0.0000	1	1	3151119	Unique	Used	1.607	1.014	0.928	0.557	1E-05	0	6.95	0	0	17	3	1018.80139	3054.38962	-0.99
aFSSSVVHVPGVNDIqSSSSTGQNISQISR	Unambiguous	2	1	Q61324-2	N-Term(iTRAQ8plex); Q16(Deamidated)	CID	0.0158	0.0000	1	1	3821294	Unique	Used	0.716	0.857	0.817	0.950	1E-05	0	6.95	0	0	200	3	1131.91455	3393.72910	3.01

skPSPQLSAETPVAALPEFPR	Unambiguous	1	1	E9Q0S6	N-Term(iTRAQ8plex); K2(iTRAQ8plex)	CID	1.0000	0.0000	1	1	3556995	Unique	Used	2.205	1.427	1.338	1.089	1E-05	0	6.95	0	10	30	3	944.19745	2830.57779	-5.16
vLGAHILGPGAGEmVNEAALALEYGAScEDIAR	Unambiguous	1	1	O08749	N-Term(iTRAQ8plex); M14(Oxidation); C28(Methylthio)	CID	1.0000	0.0000	1	1	3520211	Unique	Used	1.081	2.017	0.875	1.570	1E-05	0	6.95	0	19	36	3	1221.93665	3663.79538	-4.65
nQLEESEFTcAAAVk	Unambiguous	12	1	B2RRE2	N-Term(iTRAQ8plex); C10(Methylthio); K15(iTRAQ8plex)	CID	1.0000	0.0000	1	1	2957717	Unique	Used	0.902	0.720	1.283	0.817	1E-05	0	6.95	0	0	200	2	1147.57861	2294.14995	-5.14
sNISSPVTPTAASSSSTTPTR	Unambiguous	6	1	A2AU91	N-Term(iTRAQ8plex)	CID	1.0000	0.0000	1	1	3591739	Unique	Used	0.369	0.379	0.508	0.807	1E-05	0	6.95	0	0	57	2	1177.10474	2353.20220	-5.40
vMVATQQEMMDAQLTLQQR	Unambiguous	1	1	Q9CR61	N-Term(iTRAQ8plex)	CID	1.0000	0.0000	1	1	3519637	Unique	Used	0.605	1.016	1.027	0.963	1E-05	0	6.95	0	15	53	2	1263.13953	2525.27178	-4.39
nPVSGLLEYAQFTSQTcDFNLIEQSGPSHEPR	Unambiguous	3	1	Q99MU3-5	N-Term(iTRAQ8plex); C17(Methylthio)	CID	1.0000	0.0000	1	1	3706920	Unique	Used	2.029	0.641	0.755	0.935	1E-05	0	6.95	0	0	45	3	1305.61658	3914.83518	-7.16
qkPAPLPPASNAPQPGSSQQIQQR	Unambiguous	1	1	Q62415	N-Term(iTRAQ8plex); K2(iTRAQ8plex)	CID	1.0000	0.0000	1	1	3676139	Unique	Used	0.821	0.908	1.276	0.805	1E-05	0	6.95	0	0	57	3	1045.24060	3133.70725	-8.14
eAEEEFWQNQHPQPYIFPDSPGGTSFER	Unambiguous	1	1	Q9CQJ8	N-Term(iTRAQ8plex)	CID	1.0000	0.0000	1	1	2959367	Unique	Used		0.859	0.620	1.491	1E-05	0	6.95	0	0	200	3	1209.56384	3626.67698	0.08
fQMPDQGMTSADDFFQGTk	Unambiguous	1	1	O08553	N-Term(iTRAQ8plex); K19(iTRAQ8plex)	CID	1.0000	0.0000	1	1	3306661	Unique	Used	0.636	0.976	0.789	1.299	1E-05	0	6.95	0	0	200	3	920.44446	2759.31882	-2.82
sNVYQPTEMAVVLnGGTIPTAPPSHTGR	Unambiguous	1	1	Q923Z0	N-Term(iTRAQ8plex); N14(Deamidated)	CID	1.0000	0.0000	1	1	3455869	Unique	Used	0.534	0.558	0.925	0.848	1E-05	0	6.95	0	24	30	3	1067.21301	3199.62448	-3.60
tYPGVMHSScPQEMAAVk	Unambiguous	1	1	Q9WTL7	N-Term(iTRAQ8plex); C10(Methylthio); K18(iTRAQ8plex)	CID	1.0000	0.0000	1	1	3367069	Unique	Used	1.348	1.469	1.105	0.960	1E-05	0	6.95	0	0	83	3	864.09003	2590.25553	-7.40
eVAAFAQFGSDLDAATQQLLSR	Unambiguous	2	1	Q03265	N-Term(iTRAQ8plex)	CID	1.0000	0.0000	1	1	3668492	Unique	Used	0.652	1.029	1.281	1.554	1E-05	0	6.95	0	13	200	2	1321.68091	2642.35454	-6.93

eALNVFGDDYATEDGTGVR	Unambiguous	1	1	Q8R059	N-Term(iTRAQ8plex)	CID	1.0000	0.0000	1	1	2654609	Unique	Used	0.497	1.359	0.675	0.922	1E-05	0	6.95	0	3	200	2	1167.06213	2333.11699	-1.27
tASEMVLADDNFSTIVAAVEEGR	Unambiguous	4	1	O55143	N-Term(iTRAQ8plex)	CID	1.0000	0.0000	1	1	3112344	Unique	Used	0.271	0.836	0.876	1.035	1E-05	0	6.95	0	4	123	2	1365.18359	2729.35991	-0.27
qETLAQQQLQLEQIQQLQQQLHQQLEEQk	Unambiguous	2	1	Q9QYX7	N-Term(iTRAQ8plex); K29(iTRAQ8plex)	CID	1.0000	0.0000	1	1	3417802	Unique	Used	0.641	0.684	1.627	0.786	1E-05	0	6.95	0	0	61	3	1389.08276	4165.23374	-3.00
wGEAGAEYVVESTGVFTTMEk	Unambiguous	3	1	P16858	N-Term(iTRAQ8plex); K21(iTRAQ8plex)	CID	1.0000	0.0000	1	1	3218974	Unique	Used	0.815	1.319	1.570	1.605	1E-05	0	6.95	0	0	200	2	1450.23547	2899.46367	-0.28
iYPEEMIQTGISAIDGMNSIAR	Unambiguous	1	1	P62814	N-Term(iTRAQ8plex)	CID	1.0000	0.0000	1	1	3687012	Unique	Used	0.429	1.107	1.299	1.148	1E-05	0	6.95	0	1	2	2	1357.18738	2713.36748	-6.21
iSGETIFVTAPHEATAGIIGVNR	Unambiguous	2	1	Q68FD5	N-Term(iTRAQ8plex)	CID	1.0000	0.0000	1	1	3704555	Unique	Used	0.397	1.051	1.179	1.111	1E-05	0	6.95	0	0	0	3	886.48474	2657.43967	-6.34
dEAIYEcVASNNVGEISVSTR	Unambiguous	17	1	Q64487-7	N-Term(iTRAQ8plex); C7(Methylthio)	CID	1.0000	0.0000	1	1	3172912	Unique	Used	1.348	1.053	1.012	0.819	1E-05	0	6.94	0	0	200	2	1303.62256	2606.23784	-0.08
eVAAFAQFGSDLDAATQQLLSR	Unambiguous	2	1	Q03265	N-Term(iTRAQ8plex)	CID	1.0000	0.0000	1	1	3667950	Unique	Used	0.662	0.639	1.639	1.814	1E-05	0	6.94	0	14	200	2	1321.68372	2642.36016	-4.80
aGYTDQVVIGMDVAASEFYR	Unambiguous	2	1	P17182	N-Term(iTRAQ8plex)	CID	1.0000	0.0000	1	1	3328327	Unique	Used	0.973	1.368	1.230	1.303	1E-05	0	6.94	0	0	200	2	1248.62061	2496.23393	-1.75
gDFANANNFDNTYGQVSEDLR	Unambiguous	2	1	Q99K47	N-Term(iTRAQ8plex)	CID	1.0000	0.0000	1	1	2919464	Unique	Used	2.443	2.591	1.319	3.279	1E-05	0	6.94	0	0	200	2	1326.11133	2651.21538	-4.61
rGDQVALANWqSHPSLR	Unambiguous	3	1	Q61749	N-Term(iTRAQ8plex); Q11(Deamidated)	CID	0.0360	0.0000	1	1	3852577	Unique	Used	2.115	1.115	1.626	1.038	1E-05	0	6.94	1	0	200	3	747.40186	2240.19101	3.21
eVAAFAQFGSDLDAATQQLLSR	Unambiguous	2	1	Q03265	N-Term(iTRAQ8plex)	CID	1.0000	0.0000	1	1	3667732	Unique	Used	0.723	0.895	1.341	1.570	1E-05	0	6.94	0	4	168	2	1321.68127	2642.35527	-6.65
fQMPDQGMTSADDFFQGTk	Unambiguous	1	1	O08553	N-Term(iTRAQ8plex); K19(iTRAQ8plex)	CID	1.0000	0.0000	1	1	3307875	Unique	Used		1.633			1E-05	0	6.94	0	0	200	3	920.44452	2759.31900	-2.75
sVNESLNNLFITEEDYQALR	Unambiguous	2	1	Q68FD5	N-Term(iTRAQ8plex)	CID	1.0000	0.0000	1	1	3216848	Unique	Used	0.704	1.923	1.132	1.050	1E-05	0	6.94	0	0	200	2	1330.17932	2659.35137	-0.14
mAIITTHLQYQQEAIQk	Unambiguous	1	1	Q9CRC6	N-Term(iTRAQ8plex); K17(iTRAQ8plex)	CID	1.0000	0.0000	1	1	3702970	Unique	Used	1.427	1.082	1.385	1.004	1E-05	0	6.94	0	14	123	3	875.49005	2624.45560	-5.13
gAQSTPIQHSFLTDVSDVqEMER	Unambiguous	4	1	Q3UFK1	N-Term(iTRAQ8plex); Q19(Deamidated)	CID	1.0000	0.0000	1	1	3170130	Unique	Used	2.527	1.229	1.247	1.069	1E-05	0	6.94	0	19	200	3	960.80914	2880.41288	4.89

qcNNFLLHNLILDTGITQQLVER	Unambiguous	7	1	Q6PHS9-4	N-Term(iTRAQ8plex); C2(Methylthio)	CID	1.0000	0.0000	1	1	3879728	Unique	Used	0.753	1.514	0.380	0.930	1E-05	0	6.94	0	28	14	3	1011.53308	3032.58469	-3.86
gVASSPAISFGQQSTATLTPSPR	Unambiguous	4	1	Q19AB2	N-Term(iTRAQ8plex)	CID	1.0000	0.0000	1	1	3894372	Unique	Used	0.492	1.563	3.468	0.656	1E-05	0	6.94	0	0	126	2	1282.67896	2564.35063	-4.53
sNMHFTSSSTGGLSSSqSSYSPSSR	Unambiguous	2	1	Q80UY2	N-Term(iTRAQ8plex); Q17(Deamidated)	CID	1.0000	0.0000	1	1	3656080	Unique	Used	1.520	0.833	1.759	1.541	1E-05	0	6.94	0	27	200	3	957.77246	2871.30283	0.81
lSLESEGAnEGATAAPELSALEEAFR	Unambiguous	1	1	Q7TQD2	N-Term(iTRAQ8plex); N9(Deamidated)	CID	1.0000	0.0000	1	1	2871463	Unique	Used			7.968	6.355	1E-05	0	6.94	0	0	52	2	1484.23596	2967.46465	-3.07
eVAAFAQFGSDLDAATQQLLSR	Unambiguous	2	1	Q03265	N-Term(iTRAQ8plex)	CID	1.0000	0.0000	1	1	3669033	Unique	Used	0.855	0.689	1.972	2.656	1E-05	0	6.94	0	0	200	2	1321.68420	2642.36113	-4.43
aITVASQTNcPLYVTk	Unambiguous	5	1	E9PWE8	N-Term(iTRAQ8plex); C10(Methylthio); K16(iTRAQ8plex)	CID	1.0000	0.0000	1	1	3822793	Unique	Used	0.481	0.259	0.295	0.307	1E-05	0	6.94	0	4	72	2	1182.14575	2363.28423	-3.43
dmAIATGGAVFGEEGLNLNLEDVQAHDLGk	Unambiguous	1	1	P63038	N-Term(iTRAQ8plex); M2(Oxidation); K30(iTRAQ8plex)	CID	1.0000	0.0000	1	1	3152577	Unique	Used	2.538	1.678	1.322	1.281	1E-05	0	6.94	0	0	200	3	1236.96790	3708.88913	-2.95
kAcADATLSQITNNIDPVGR	Unambiguous	3	1	P62874	N-Term(iTRAQ8plex); K1(iTRAQ8plex); C3(Methylthio)	CID	1.0000	0.0000	1	1	3685158	Unique	Used	0.928	0.907	1.509	1.419	1E-05	0	6.94	1	6	20	3	914.48309	2741.43473	-6.86
aGYTDQVVIGMDVAASEFYR	Unambiguous	2	1	P17182	N-Term(iTRAQ8plex)	CID	1.0000	0.0000	1	1	3328850	Unique	Used	1.097	1.306	1.233	0.944	1E-05	0	6.94	0	0	200	2	1248.61902	2496.23076	-3.02
lVASGPSQTPVFETEGSAGTSR	Unambiguous	2	1	Q5SUQ9	N-Term(iTRAQ8plex)	CID	1.0000	0.0000	1	1	3278279	Unique	Used	0.501	0.989	0.630	1.001	1E-05	0	6.94	0	0	93	2	1241.63721	2482.26714	-2.27
dTFPSAFASGAAQQTQTQVk	Unambiguous	2	1	Q5SV85-2	N-Term(iTRAQ8plex); K20(iTRAQ8plex)	CID	1.0000	0.0000	1	1	3343682	Unique	Used	1.203	1.074	1.265	0.948	1E-05	0	6.94	0	0	37	2	1346.20544	2691.40361	-6.04
nASLASSNNDLQVAEEQYQR	Unambiguous	3	1	E9QP99	N-Term(iTRAQ8plex)	CID	1.0000	0.0000	1	1	2777781	Unique	Used	1.130	0.660	0.603	0.565	1E-05	0	6.94	0	0	200	2	1271.12451	2541.24175	-2.59
sSVELPPYSGTLcSIQAEDELPDGqEHQR	Unambiguous	1	1	P52479	N-Term(iTRAQ8plex); C13(Methylthio); Q25(Deamidated)	CID	0.0159	0.0000	1	1	2977828	Unique	Used	1.873	0.923	1.042	0.886	1E-05	0	6.94	0	0	200	3	1179.56030	3536.66635	5.65

lSGSNPYTTVTPQIINSk	Unambiguous	1	1	P57780	N-Term(iTRAQ8plex); K18(iTRAQ8plex)	CID	1.0000	0.0000	1	1	3682339	Unique	Used		1.679	1.293	0.772	1E-05	0	6.94	0	0	119	2	1264.70593	2528.40459	-5.33
eNPTTFMGHYLHEVAR	Unambiguous	1	1	P07724	N-Term(iTRAQ8plex)	CID	1.0000	0.0000	1	1	3411874	Unique	Used	6.473	1.966	0.324	0.591	1E-05	0	6.94	0	1	1	3	736.03534	2206.09146	-4.65
gGSAGPGEPEYGHDPASGGIFSSDYk	Unambiguous	2	1	Q9JME5	N-Term(iTRAQ8plex); K26(iTRAQ8plex)	CID	1.0000	0.0000	1	1	2810044	Unique	Used	1.276	1.840	1.717	1.016	1E-05	0	6.94	0	15	154	3	1049.83972	3147.50461	-2.23
gAVQFVTQYQHSSGQR	Unambiguous	2	1	E9Q1S3	N-Term(iTRAQ8plex)	CID	1.0000	0.0000	1	1	3890880	Unique	Used	1.063	1.007	0.892	0.948	1E-05	0	6.94	0	0	200	3	699.69415	2097.06791	-4.78
nLHQSGFSLSGAQIDDNIPR	Unambiguous	1	1	O08553	N-Term(iTRAQ8plex)	CID	1.0000	0.0000	1	1	3575341	Unique	Used	1.446	1.572	0.894	1.084	1E-05	0	6.93	0	10	18	3	825.09167	2473.26047	-5.38
tVIQAEIDAAAELIDFFR	Unambiguous	1	1	Q8CHT0	N-Term(iTRAQ8plex)	CID	1.0000	0.0000	1	1	3480785	Unique	Used	0.620	2.355	0.746	1.172	1E-05	0	6.93	0	17	200	2	1163.63147	2326.25566	-1.74
wGEAGAEYVVESTGVFTTMEk	Unambiguous	3	1	P16858	N-Term(iTRAQ8plex); K21(iTRAQ8plex)	CID	1.0000	0.0000	1	1	3239426	Unique	Used	0.419	1.086	1.036	1.245	1E-05	0	6.93	0	0	24	2	1450.23376	2899.46025	-1.45
yTPSGQSGAAASESLFISNHAY	Unambiguous	2	1	A6ZI44	N-Term(iTRAQ8plex)	CID	1.0000	0.0000	1	1	3413893	Unique	Used	4.893	1.813	1.293	1.861	1E-05	0	6.93	0	0	48	2	1281.61804	2562.22881	-4.93
sIHNLGTILTTnAVASETVATGEGLR	Unambiguous	3	1	Q9ESN6	N-Term(iTRAQ8plex); N12(Deamidated)	CID	1.0000	0.0000	1	1	3705199	Unique	Used	0.735	0.558	1.628	1.644	1E-05	0	6.93	0	0	200	2	1465.78992	2930.57256	-0.40
mTVNESEQLVScSMDDTVR	Unambiguous	1	1	O88342	N-Term(iTRAQ8plex); C12(Methylthio)	CID	1.0000	0.0000	1	1	2996363	Unique	Used	0.957	1.184	1.112	1.435	1E-05	0	6.93	0	0	200	2	1247.56323	2494.11919	-1.78
eTYEVLLSFIQAALGDQPR	Unambiguous	1	1	Q6P4T2	N-Term(iTRAQ8plex)	CID	1.0000	0.0000	1	1	3880701	Unique	Used	0.690	0.659	0.948	0.906	1E-05	0	6.93	0	12	32	2	1227.65930	2454.31133	-2.83
tTGLVGLAVcDTPHER	Unambiguous	1	1	Q9CPP6	N-Term(iTRAQ8plex); C10(Methylthio)	CID	1.0000	0.0000	1	1	3455488	Unique	Used	1.343	0.963	0.916	1.088	1E-05	0	6.93	0	1	16	3	673.67938	2019.02359	-3.44
qTIEnSQGAYQEAFDISk	Unambiguous	4	1	P68254-2	N-Term(iTRAQ8plex); N5(Deamidated); K18(iTRAQ8plex)	CID	0.0317	0.0000	1	1	2812252	Unique	Used	0.906	0.947	0.619	0.772	1E-05	0	6.93	0	0	200	2	1319.67554	2638.34380	-0.72
eHAPSIIFmDEIDSIGSSR	Unambiguous	2	1	P62196	N-Term(iTRAQ8plex); M9(Oxidation)	CID	1.0000	0.0000	1	1	3170438	Unique	Used	1.270	1.320	0.808	1.080	1E-05	0	6.93	0	13	144	3	808.73651	2424.19498	-2.87

nVQLDVTEAQADDEGGTLIAR	Unambiguous	1	1	Q810J8	N-Term(iTRAQ8plex)	CID	1.0000	0.0000	1	1	2636741	Unique	Used		0.723	1.011	1.187	1E-05	0	6.93	0	0	200	2	1260.14929	2519.29131	0.85
kQTESSFMAGDInSTPTLNR	Unambiguous	5	1	E9Q3V9	N-Term(iTRAQ8plex); K1(iTRAQ8plex); N13(Deamidated)	CID	1.0000	0.0000	1	1	3105131	Unique	Used	1.247	1.088	0.884	0.700	1E-05	0	6.93	1	0	189	3	936.15533	2806.45145	0.44
iSGETIFVTAPHEATAGIIGVnR	Unambiguous	2	1	Q68FD5	N-Term(iTRAQ8plex); N22(Deamidated)	CID	1.0000	0.0000	1	1	3707798	Unique	Used		1.171	1.164	1.214	1E-05	0	6.93	0	22	200	3	886.82184	2658.45096	3.93
hTGPGILSMANAGPNTNGSQFFIcTAk	Unambiguous	1	1	P17742	N-Term(iTRAQ8plex); C24(Methylthio); K27(iTRAQ8plex)	CID	1.0000	0.0000	1	1	3897310	Unique	Used	1.427	1.757	0.986	0.784	1E-05	0	6.93	0	13	40	3	1130.23303	3388.68454	-6.38
aLTPLQEEMAGLLQQIEVER	Unambiguous	1	1	Q9EQI8	N-Term(iTRAQ8plex)	CID	1.0000	0.0000	1	1	3459820	Unique	Used	1.191	0.776	0.768	0.694	1E-05	0	6.93	0	0	108	2	1286.69556	2572.38384	-4.69
wGEAGAEYVVESTGVFTTMEk	Unambiguous	3	1	P16858	N-Term(iTRAQ8plex); K21(iTRAQ8plex)	CID	1.0000	0.0000	1	1	3240992	Unique	Used	2.449	1.873	1.620	1.449	1E-05	0	6.93	0	12	199	3	967.15796	2899.45932	-1.78
kFVEVMSEYNATQSDYR	Unambiguous	2	1	O35526	N-Term(iTRAQ8plex); K1(iTRAQ8plex)	CID	1.0000	0.0000	1	1	3280021	Unique	Used	0.360	2.002	1.326	0.875	1E-05	0	6.93	1	2	17	3	892.45697	2675.35636	-1.20
fQMPDQGmTSADDFFQGTk	Unambiguous	1	1	O08553	N-Term(iTRAQ8plex); M8(Oxidation); K19(iTRAQ8plex)	CID	1.0000	0.0000	1	1	3304210	Unique	Used	2.121	1.099	1.019	1.064	1E-05	0	6.93	0	4	65	3	925.77576	2775.31272	-3.17
sIVnNGHSFNVEFDDSQDNAVLk	Unambiguous	1	1	P00920	N-Term(iTRAQ8plex); N4(Deamidated); K23(iTRAQ8plex)	CID	0.0260	0.0000	1	1	2957804	Unique	Used	1.894	0.871	0.881	0.956	1E-05	0	6.93	0	0	200	3	1053.53259	3158.58323	-0.60
nEAIQAAHDSVAQEGQcR	Unambiguous	1	1	Q9R0P9	N-Term(iTRAQ8plex); N1(Deamidated); C17(Methylthio)	CID	1.0000	0.0000	1	1	2755335	Unique	Used	2.407	1.297	1.770	1.105	1E-05	0	6.93	0	5	46	3	760.01996	2278.04532	-1.81
vLSQATPAMAGEAELATDQQER	Unambiguous	1	1	Q99P31	N-Term(iTRAQ8plex)	CID	1.0000	0.0000	1	1	2901751	Unique	Used	0.714	0.775	1.031	1.115	1E-05	0	6.93	0	0	200	2	1310.66235	2620.31743	-0.64

dYLGDFIEHYAQLGPSqPPDLAQAQDEPR	Unambiguous	1	1	P42669	N-Term(iTRAQ8plex); Q17(Deamidated)	CID	0.0418	0.0000	1	1	3263988	Unique	Used	1.542	0.601			1E-05	0	6.93	0	0	200	3	1192.58960	3575.75425	8.65
yTPSGQSGAAASESLFISNHAY	Unambiguous	2	1	A6ZI44	N-Term(iTRAQ8plex)	CID	1.0000	0.0000	1	1	3258147	Unique	Used	1.029	0.820	1.103	1.160	1E-05	0	6.93	0	0	33	2	1281.62317	2562.23906	-0.93
eHGYLGNLSAVASTEGTIEETLNEASR	Unambiguous	2	1	Q99P72	N-Term(iTRAQ8plex)	CID	1.0000	0.0000	1	1	3132762	Unique	Used	2.261	0.901	1.236	0.595	1E-05	0	6.92	0	0	36	2	1576.78601	3152.56474	-0.08
aGYTDQVVIGMDVAASEFYR	Unambiguous	2	1	P17182	N-Term(iTRAQ8plex)	CID	1.0000	0.0000	1	1	3327900	Unique	Used	0.996	1.162	1.265	1.113	1E-05	0	6.92	0	0	156	2	1248.61963	2496.23198	-2.53
acADATLSQITnNIDPVGR	Unambiguous	3	1	P62874	N-Term(iTRAQ8plex); C2(Methylthio); N12(Deamidated)	CID	0.0434	0.0000	1	1	3303715	Unique	Used	0.597	0.936	1.433	1.496	1E-05	0	6.92	0	6	68	2	1155.56995	2310.13262	-1.99
eYLQTHWEEEEcQQDVSLLR	Unambiguous	1	1	Q8BGB7	N-Term(iTRAQ8plex); C12(Methylthio)	CID	1.0000	0.0000	1	1	2852018	Unique	Used	2.234	0.761	1.478	0.902	1E-05	0	6.92	0	5	200	3	962.44965	2885.33438	-1.53
dFTSEQIDEILQnHTEIVFAR	Unambiguous	2	1	Q6PIC6	N-Term(iTRAQ8plex); N13(Deamidated)	CID	0.0029	0.0000	1	1	3374799	Unique	Used	0.769	0.701	1.513	1.502	1E-05	0	6.92	0	0	200	3	937.48157	2810.43015	5.36
vGIGAFPTEQINEIGDLLQNR	Unambiguous	3	1	P28650	N-Term(iTRAQ8plex)	CID	1.0000	0.0000	1	1	3645309	Unique	Used	1.026	0.309	1.351	1.956	1E-05	0	6.92	0	6	9	2	1294.69629	2588.38530	-5.16
vLGAHILGPGAGEmVNEAALALEYGAScEDIAR	Unambiguous	1	1	O08749	N-Term(iTRAQ8plex); M14(Oxidation); C28(Methylthio)	CID	1.0000	0.0000	1	1	3520420	Unique	Used	3.944	2.140	0.852	0.995	1E-05	0	6.92	0	22	51	3	1221.93994	3663.80527	-1.95
tQPTDEEMLFIYSHFk	Unambiguous	4	1	P31786	N-Term(iTRAQ8plex); K16(iTRAQ8plex)	CID	1.0000	0.0000	1	1	3282933	Unique	Used	2.062	0.733	0.930	0.762	1E-05	0	6.92	0	0	38	3	865.44794	2594.32926	-4.96
qQTPPGLcPLAGNSVHADk	Unambiguous	1	1	Q9D8S4	N-Term(iTRAQ8plex); C8(Methylthio); K19(iTRAQ8plex)	CID	1.0000	0.0000	1	1	3410960	Unique	Used	1.312	0.914	1.321	1.174	1E-05	0	6.92	0	0	92	3	863.11768	2587.33847	-7.61
iSVEDADEPPMFLAPSYIHEVQEnAAAGTVVGR	Unambiguous	1	1	P55288	N-Term(iTRAQ8plex); N24(Deamidated)	CID	0.0405	0.0000	1	1	3262255	Unique	Used	1.233	0.624	0.837	0.946	1E-05	0	6.92	0	8	21	3	1273.30627	3817.90427	3.80

iLQSSSEVGYDAMLGDFVnMVEk	Unambiguous	2	1	P63038	N-Term(iTRAQ8plex); N19(Deamidated); K23(iTRAQ8plex)	CID	0.0246	0.0000	1	1	3374125	Unique	Used	1.763	1.228	0.654	0.940	1E-05	0	6.92	0	0	50	2	1571.30408	3141.60088	2.02
gAGQTVTIIAQYQPEDYAR	Unambiguous	9	1	Q91XM9-7	N-Term(iTRAQ8plex)	CID	1.0000	0.0000	1	1	3301213	Unique	Used	0.712	2.292	2.444	1.157	1E-05	0	6.92	0	0	79	2	1193.11768	2385.22807	-3.00
eLSSAVSPPNLHSDTPTFSYASLAGPTIPPR	Unambiguous	3	1	Q9QYR6	N-Term(iTRAQ8plex)	CID	1.0000	0.0000	1	1	3685340	Unique	Used	2.517	1.476	1.430	0.685	1E-05	0	6.92	0	0	100	3	1171.93811	3513.79978	-4.85
qTTVSnSQQAYQEAFEISk	Unambiguous	3	1	Q9CQV8-2	N-Term(iTRAQ8plex); N6(Deamidated); K19(iTRAQ8plex)	CID	0.0246	0.0000	1	1	2956801	Unique	Used	0.929	1.466	0.897	1.081	1E-05	0	6.92	0	0	200	2	1384.71228	2768.41728	-0.95
iTQHFSTEEFEDEYVTDYTR	Unambiguous	2	1	Q9QYX7	N-Term(iTRAQ8plex)	CID	1.0000	0.0000	1	1	2813547	Unique	Used	1.995	0.821	4.320	3.369	1E-05	0	6.92	0	0	192	3	938.76996	2814.29532	-3.38
dIGAIAQVHAENGDIIAEEQQR	Unambiguous	1	1	O08553	N-Term(iTRAQ8plex)	CID	1.0000	0.0000	1	1	2811888	Unique	Used	1.768	0.995	1.340	1.397	1E-05	0	6.91	0	7	62	3	894.46301	2681.37448	-1.95
vMVATQQEMMDAQLTLQQR	Unambiguous	1	1	Q9CR61	N-Term(iTRAQ8plex)	CID	1.0000	0.0000	1	1	3519651	Unique	Used	0.654	1.139	0.838	1.187	1E-05	0	6.91	0	0	20	2	1263.14001	2525.27275	-4.00
lELNENPDTLHQDIQEVR	Unambiguous	2	1	Q8BG92	N-Term(iTRAQ8plex)	CID	1.0000	0.0000	1	1	2685435	Unique	Used	0.619	0.905	0.883	1.173	1E-05	0	6.91	0	4	98	3	823.09540	2467.27164	-0.59
wGEAGAEYVVESTGVFTTMEk	Unambiguous	3	1	P16858	N-Term(iTRAQ8plex); K21(iTRAQ8plex)	CID	1.0000	0.0000	1	1	3241533	Unique	Used		1.376	0.648	0.980	1E-05	0	6.91	0	0	200	2	1450.22803	2899.44878	-5.41
fIEAGQFNDNLYGTSIQSVR	Unambiguous	4	1	Q52KF7	N-Term(iTRAQ8plex)	CID	1.0000	0.0000	1	1	3539860	Unique	Used	0.749	1.334	1.496	0.923	1E-05	0	6.91	0	0	8	2	1282.15295	2563.29863	-4.23
sEVELVQLVIDGVNYLIDcER	Unambiguous	1	1	P30275	N-Term(iTRAQ8plex); C19(Methylthio)	CID	1.0000	0.0000	1	1	3624583	Unique	Used	0.604	0.707	0.811	1.039	1E-05	0	6.91	0	0	200	2	1378.70361	2756.39995	-5.57
sGFEPPGDFPFEDYSQHIYR	Unambiguous	4	1	E9PUI5	N-Term(iTRAQ8plex)	CID	1.0000	0.0000	1	1	3282124	Unique	Used	1.089	1.324	1.391	1.509	1E-05	0	6.91	0	30	83	3	898.09229	2692.26230	0.04
aVATEANNSTFFSVSSSDLMSk	Unambiguous	1	1	Q8VEJ9	N-Term(iTRAQ8plex); K22(iTRAQ8plex)	CID	1.0000	0.0000	1	1	3371442	Unique	Used	0.620	1.012	0.938	1.160	1E-05	0	6.91	0	0	165	2	1451.23108	2901.45488	-7.31
sASSTSSLFSSSSQLFPPSR	Unambiguous	6	1	Q3TQ29	N-Term(iTRAQ8plex)	CID	1.0000	0.0000	1	1	3934117	Unique	Used	0.670	0.643	0.907	0.813	1E-05	0	6.91	0	0	200	2	1182.59521	2364.18315	-6.50

cPTDELSLSNcAVVNEk	Unambiguous	2	1	P46460	N-Term(iTRAQ8plex); C1(Methylthio); C11(Methylthio); K17(iTRAQ8plex)	CID	1.0000	0.0000	1	1	3283130	Unique	Used	0.614	1.284	1.251	1.503	1E-05	0	6.91	0	22	90	2	1261.61365	2522.22002	-0.78
wGEAGAEYVVESTGVFTTMEk	Unambiguous	3	1	P16858	N-Term(iTRAQ8plex); K21(iTRAQ8plex)	CID	1.0000	0.0000	1	1	3218215	Unique	Used	1.464	1.591	1.925	1.285	1E-05	0	6.91	0	4	184	3	967.15875	2899.46170	-0.95
iAENNDLLWMDYHQk	Unambiguous	3	1	O08539	N-Term(iTRAQ8plex); K15(iTRAQ8plex)	CID	1.0000	0.0000	1	1	3128159	Unique	Used	0.881	0.959	1.684	1.347	1E-05	0	6.91	0	1	1	3	833.43542	2498.29172	-1.65
sSHTWVALSHIAGLcnR	Unambiguous	2	1	Q6PIC6	N-Term(iTRAQ8plex); C15(Methylthio); N16(Deamidated)	CID	1.0000	0.0000	1	1	3915427	Unique	Used	1.553	1.001	1.092	1.082	1E-05	0	6.91	0	0	200	3	735.03601	2203.09348	-5.42
kEQAQMLQTMQEQEQAAQGLR	Unambiguous	1	1	E9Q7G0	N-Term(iTRAQ8plex); K1(iTRAQ8plex)	CID	1.0000	0.0000	1	1	3189234	Unique	Used	1.586	0.592	0.864	0.642	1E-05	0	6.91	1	21	35	3	1018.86560	3054.58225	-3.25
sIEALLEAGQAQDTQASHAEANqQQTR	Unambiguous	2	1	Q99PL5	N-Term(iTRAQ8plex); Q23(Deamidated)	CID	0.0058	0.0000	1	1	2849877	Unique	Used	1.985	0.769	1.036	0.791	1E-05	0	6.91	0	0	200	3	1067.52539	3200.56162	-3.31
nVIIWGnHSSTQYPDVNHAk	Unambiguous	1	1	P14152	N-Term(iTRAQ8plex); N7(Deamidated); K20(iTRAQ8plex)	CID	0.0043	0.0000	1	1	3318748	Unique	Used	0.749	1.237	1.531	1.694	1E-05	0	6.91	0	11	147	3	963.83990	2889.50516	-1.82
vGEQAQVVIIDMNDPSNPIR	Unambiguous	2	1	Q68FD5	N-Term(iTRAQ8plex)	CID	1.0000	0.0000	1	1	3325531	Unique	Used	0.539	1.353	1.156	1.069	1E-05	0	6.91	0	0	13	2	1250.15759	2499.30791	-4.03
tcDISFSDPDDLLNFk	Unambiguous	4	1	P61082	N-Term(iTRAQ8plex); C2(Methylthio); K16(iTRAQ8plex)	CID	1.0000	0.0000	1	1	3284372	Unique	Used	0.351	0.770	0.919	0.942	1E-05	0	6.90	0	5	15	2	1242.61475	2484.22222	-1.01
aQMEQLnGVLLHLESELAQTR	Unambiguous	1	1	P05784	N-Term(iTRAQ8plex); N7(Deamidated)	CID	1.0000	0.0000	1	1	3305280	Unique	Used		2.039	0.975	0.974	1E-05	0	6.90	0	0	48	3	895.80951	2685.41397	-1.65

vIDIFDTDGnGEVDFkEFIEGVSQFSVk	Unambiguous	2	1	Q63810-2	N-Term(iTRAQ8plex); N10(Deamidated); K16(iTRAQ8plex); K28(iTRAQ8plex)	CID	1.0000	0.0000	1	1	3707097	Unique	Used	0.946	2.527	1.753	0.969	1E-05	0	6.90	1	0	107	3	1350.04260	4048.11325	-1.84
qAPQTVHLPSGETLDVFDAAER	Unambiguous	1	1	P28271	N-Term(iTRAQ8plex)	CID	1.0000	0.0000	1	1	3108824	Unique	Used	1.809	0.867	1.052	1.185	1E-05	0	6.90	0	0	37	3	895.79620	2685.37406	-1.71
vSDAISTqYPVVDHEFDAVVVGAGGAGLR	Unambiguous	1	1	Q8K2B3	N-Term(iTRAQ8plex); Q8(Deamidated)	CID	1.0000	0.0000	1	1	3355330	Unique	Used	2.507	1.233	1.104	1.146	1E-05	0	6.90	0	15	200	3	1078.89539	3234.67160	4.04
cTPSGFTLDDAIQTGVDnPGHPYIMTVGAVAGDEESYDVFk	Unambiguous	1	1	Q04447	N-Term(iTRAQ8plex); C1(Methylthio); N18(Deamidated); K41(iTRAQ8plex)	CID	0.0478	0.0000	1	1	3373661	Unique	Used	1.007	0.504	1.307	1.134	1E-05	0	6.90	0	0	18	3	1658.12158	4972.35019	0.74
gAQIASTGQLYAAWnGGLDR	Unambiguous	1	1	Q61361	N-Term(iTRAQ8plex); N15(Deamidated)	CID	1.0000	0.0000	1	1	3393307	Unique	Used	2.658	1.401	1.177	1.654	1E-05	0	6.90	0	0	57	2	1177.60461	2354.20195	-1.00
fIEAGQFNDNLYGTSIQSVR	Unambiguous	4	1	Q52KF7	N-Term(iTRAQ8plex)	CID	1.0000	0.0000	1	1	3559719	Unique	Used	0.536	1.879	1.887	1.080	1E-05	0	6.90	0	0	200	2	1282.15247	2563.29766	-4.61
tYFPHFDVSHGSAQVk	Unambiguous	2	1	Q91VB8	N-Term(iTRAQ8plex); K16(iTRAQ8plex)	CID	1.0000	0.0000	1	1	3658377	Unique	Used	12.762	4.705	1.082	3.817	1E-05	0	6.90	0	17	19	3	810.09381	2428.26688	-8.28
sIEEAcLTLQHLNR	Unambiguous	3	1	Q9QYR6	N-Term(iTRAQ8plex); C6(Methylthio)	CID	1.0000	0.0000	1	1	3726197	Unique	Used	0.583	1.094	0.984	0.965	1E-05	0	6.90	0	15	28	3	659.67401	1977.00748	-6.31
qLIYNYPEQLFGAAGVmAIEHADFAGVER	Unambiguous	1	1	P80314	N-Term(iTRAQ8plex); M17(Oxidation)	CID	1.0000	0.0000	1	1	3623188	Unique	Used	0.696	0.982	0.873	0.882	1E-05	0	6.90	0	4	5	3	1177.25708	3529.75669	-4.58
gQIHLDSQQDYQLLQAQR	Unambiguous	2	1	Q64237	N-Term(iTRAQ8plex)	CID	1.0000	0.0000	1	1	3298596	Unique	Used	2.335	1.320	1.102	1.295	1E-05	0	6.90	0	8	66	3	815.76331	2445.27536	-1.42
lVQDVANNTnEEAGDGTTTATVLAR	Unambiguous	3	1	P63038	N-Term(iTRAQ8plex); N10(Deamidated)	CID	1.0000	0.0000	1	1	2761650	Unique	Used	1.418	0.560	1.625	1.498	1E-05	0	6.90	0	2	200	2	1433.21985	2865.43242	-1.96

iAEnNDLLWMDYHQk	Unambiguous	3	1	O08539	N-Term(iTRAQ8plex); N4(Deamidated); K15(iTRAQ8plex)	CID	1.0000	0.0000	1	1	2957498	Unique	Used	1.060	1.073	1.690	1.364	1E-05	0	6.90	0	2	121	3	833.76477	2499.27976	-0.04
lPLTLAGASYEGVAVnDcIESGR	Unambiguous	1	1	P51175	N-Term(iTRAQ8plex); N16(Deamidated); C18(Methylthio)	CID	1.0000	0.0000	1	1	3500266	Unique	Used	0.913	1.177	0.936	1.413	1E-05	0	6.90	0	0	39	2	1343.67896	2686.35063	5.06
eMEENFALEAANYQDTIGR	Unambiguous	1	1	P20152	N-Term(iTRAQ8plex)	CID	1.0000	0.0000	1	1	2815005	Unique	Used	1.005	2.023	0.444	1.203	1E-05	0	6.90	0	4	65	2	1253.09607	2505.18486	-0.85
sEVELVQLVIDGVNYLIDcER	Unambiguous	1	1	P30275	N-Term(iTRAQ8plex); C19(Methylthio)	CID	1.0000	0.0000	1	1	3624569	Unique	Used		0.874	1.171	1.603	1E-05	0	6.90	0	0	200	2	1378.70447	2756.40166	-4.95
dTDAAVGDnIGYITFVLFPR	Unambiguous	2	1	Q9CVB6	N-Term(iTRAQ8plex); N9(Deamidated)	CID	1.0000	0.0000	1	1	3647231	Unique	Used	0.402	1.559	1.094	1.021	1E-05	0	6.90	0	0	200	2	1245.15320	2489.29912	5.01
eMEENFALEAANYQDTIGR	Unambiguous	1	1	P20152	N-Term(iTRAQ8plex)	CID	1.0000	0.0000	1	1	2814754	Unique	Used	1.359	2.417	0.687	1.197	1E-05	0	6.90	0	0	200	2	1253.09692	2505.18657	-0.17
tTEQLIEAVNnGDFEAYAk	Unambiguous	9	1	Q5SVJ0	N-Term(iTRAQ8plex); N11(Deamidated); K19(iTRAQ8plex)	CID	0.0275	0.0000	1	1	2834347	Unique	Used		0.709	1.578	0.913	1E-05	0	6.90	0	0	200	2	1361.70044	2722.39360	-3.54
dmHGDSEYNIMFGPDIcGPGTk	Unambiguous	1	1	P14211	N-Term(iTRAQ8plex); M2(Oxidation); C17(Methylthio); K22(iTRAQ8plex)	CID	1.0000	0.0000	1	1	3171147	Unique	Used	1.728	0.910	0.966	0.926	1E-05	0	6.90	0	9	152	3	1018.80011	3054.38578	-2.25
iGEHTPSALAImENANVLAR	Unambiguous	3	1	A6ZI44	N-Term(iTRAQ8plex); M12(Oxidation)	CID	1.0000	0.0000	1	1	3708315	Unique	Used	1.317	1.102	0.979	1.122	1E-05	0	6.90	0	11	200	3	809.76666	2427.28543	-4.70
kWEEVMANAQETGnLVMDVR	Unambiguous	4	1	E9PYI7	N-Term(iTRAQ8plex); K1(iTRAQ8plex); N14(Deamidated)	CID	1.0000	0.0000	1	1	3395139	Unique	Used	1.577	1.645	1.557	0.707	1E-05	0	6.90	1	20	111	3	977.17413	2929.50785	2.37

gEVPcTVTTASPLDDAVLSELk	Unambiguous	1	1	Q9DB20	N-Term(iTRAQ8plex); C5(Methylthio); K22(iTRAQ8plex)	CID	1.0000	0.0000	1	1	3262433	Unique	Used	0.619	0.829	1.128	1.260	1E-05	0	6.90	0	25	5	2	1450.26770	2899.52812	0.95
iLQSSSEVGYDAMLGDFVNMVEk	Unambiguous	2	1	P63038	N-Term(iTRAQ8plex); K23(iTRAQ8plex)	CID	1.0000	0.0000	1	1	3352791	Unique	Used	0.880	0.740	1.489	0.916	1E-05	0	6.90	0	0	8	2	1570.80127	3140.59526	-4.85
aTTSTNWILESQNINELk	Unambiguous	1	1	Q9R0A0	N-Term(iTRAQ8plex); K18(iTRAQ8plex)	CID	1.0000	0.0000	1	1	3348729	Unique	Used	0.760	0.806	0.749	0.842	1E-05	0	6.89	0	0	33	2	1335.72668	2670.44609	-3.68
wGEAGAEYVVESTGVFTTmEk	Unambiguous	3	1	P16858	N-Term(iTRAQ8plex); M19(Oxidation); K21(iTRAQ8plex)	CID	1.0000	0.0000	1	1	3216945	Unique	Used	0.617	0.896	0.941	1.018	1E-05	0	6.89	0	0	92	2	1458.23499	2915.46269	1.13
sLSLcNMFLDEMAk	Unambiguous	3	1	A2AS98	N-Term(iTRAQ8plex); C5(Methylthio); K14(iTRAQ8plex)	CID	1.0000	0.0000	1	1	3645562	Unique	Used	0.339	1.075	1.186	1.017	1E-05	0	6.89	0	0	32	2	1128.56580	2256.12432	-5.06
ePLAESAAHSVnQDQLDNQATVQR	Unambiguous	12	1	E9PXE2	N-Term(iTRAQ8plex); N12(Deamidated)	CID	0.0276	0.0000	1	1	2809304	Unique	Used	1.385	0.877	0.900	1.081	1E-05	0	6.89	0	0	200	3	976.15778	2926.45877	4.88
sTITIVAEEVSGTNDYVQLTFR	Unambiguous	2	1	Q9Z140	N-Term(iTRAQ8plex)	CID	1.0000	0.0000	1	1	3459412	Unique	Used	0.997	3.573	0.937	0.857	1E-05	0	6.89	0	0	146	2	1374.22278	2747.43828	-0.83
lLEAQPqVANLLPSAAQk	Unambiguous	2	1	Q69ZW3-2	N-Term(iTRAQ8plex); Q7(Deamidated); K18(iTRAQ8plex)	CID	0.0087	0.0000	1	1	3705962	Unique	Used	1.140	1.039	1.395	1.115	1E-05	0	6.89	0	29	127	2	1250.73059	2500.45391	-2.26
kVADALAnAAGHLDDLPGALSALSDLHAHk	Unambiguous	1	1	Q91VB8	N-Term(iTRAQ8plex); K1(iTRAQ8plex); N8(Deamidated); K30(iTRAQ8plex)	CID	1.0000	0.0000	1	1	3706254	Unique	Used	2.875	3.207	0.555	1.291	1E-05	0	6.89	1	9	13	3	1302.38989	3905.15512	-2.51
aLDPAPLAQPTPLGSVQTsPELEHR	Unambiguous	1	1	Q8BZB3	N-Term(iTRAQ8plex); S19(Phospho)	CID	0.0189	0.0000	1	1	2958419	Unique	Used	1.383	0.653	1.022	0.765	1E-05	0	6.89	0	29	200	3	1003.51898	3008.54239	0.90

sVNESLNNLFITEEDYQALR	Unambiguous	2	1	Q68FD5	N-Term(iTRAQ8plex)	CID	1.0000	0.0000	1	1	3263792	Unique	Used	0.671	1.364	1.064	1.102	1E-05	0	6.89	0	21	200	2	1330.18140	2659.35552	1.42
wGEAGAEYVVESTGVFTTMEk	Unambiguous	3	1	P16858	N-Term(iTRAQ8plex); K21(iTRAQ8plex)	CID	1.0000	0.0000	1	1	3241658	Unique	Used		0.845	1.094	1.261	1E-05	0	6.89	0	0	200	2	1450.23694	2899.46660	0.73
fGDEDITWQDEHSAPFSWETR	Unambiguous	1	1	Q8C3W1	N-Term(iTRAQ8plex)	CID	1.0000	0.0000	1	1	2886675	Unique	Used	1.560	1.101	0.867	0.637	1E-05	0	6.89	0	0	200	3	953.10339	2857.29563	-1.81
aGYTDQVVIGMDVAASEFYR	Unambiguous	2	1	P17182	N-Term(iTRAQ8plex)	CID	1.0000	0.0000	1	1	3328970	Unique	Used	1.206	1.372	1.179	1.079	1E-05	0	6.89	0	12	200	2	1248.62122	2496.23516	-1.26
dEVSMTAMSSSEEAScYR	Unambiguous	1	1	P0C8K7	N-Term(iTRAQ8plex); C16(Methylthio)	CID	1.0000	0.0000	1	1	2669666	Unique	Used	1.068	0.309	1.210	1.659	1E-05	0	6.89	0	0	134	2	1166.98621	2332.96514	-2.42
nYFESTDGLIWVVDSADR	Unambiguous	1	1	Q9D0J4	N-Term(iTRAQ8plex)	CID	1.0000	0.0000	1	1	3173779	Unique	Used	0.737	0.533	0.741	1.157	1E-05	0	6.89	0	0	37	2	1196.08875	2391.17021	-2.87
sLQDIIAILGMDELSEEDk	Unambiguous	1	1	P56480	N-Term(iTRAQ8plex); K19(iTRAQ8plex)	CID	1.0000	0.0000	1	1	3239979	Unique	Used	1.033	1.138	0.977	1.202	1E-05	0	6.89	0	8	63	2	1364.23145	2727.45561	-1.00
gDVEEVQGPGVVGEFPIISPGR	Unambiguous	3	1	Q9DC63	N-Term(iTRAQ8plex)	CID	1.0000	0.0000	1	1	3110912	Unique	Used	0.873	1.087	0.773	1.120	1E-05	0	6.89	0	0	200	2	1271.67297	2542.33867	-2.72
aQWGSNMQVTLIPTHDSEVMR	Unambiguous	1	1	P14873	N-Term(iTRAQ8plex)	CID	1.0000	0.0000	1	1	3641088	Unique	Used	2.035	1.178	1.186	0.952	1E-05	0	6.89	0	26	102	3	902.11823	2704.34012	-3.26
eVAAFAQFGSDLDAATQQLLSR	Unambiguous	2	1	Q03265	N-Term(iTRAQ8plex)	CID	1.0000	0.0000	1	1	3667627	Unique	Used	0.633	0.939	1.180	1.495	1E-05	0	6.88	0	0	151	2	1321.68298	2642.35869	-5.36
kGIVDQSQQAYQEAFEISk	Unambiguous	1	1	P63101	N-Term(iTRAQ8plex); K1(iTRAQ8plex); K19(iTRAQ8plex)	CID	1.0000	0.0000	1	1	3257356	Unique	Used	1.013	1.525	1.112	0.912	1E-05	0	6.88	1	10	19	3	1027.90247	3081.69284	-1.80
kLGGDVSPTQIDVSqFGSFk	Unambiguous	1	1	P14873	N-Term(iTRAQ8plex); K1(iTRAQ8plex); Q15(Deamidated); K20(iTRAQ8plex)	CID	1.0000	0.0000	1	1	3557518	Unique	Used	1.455	1.493	1.957	1.156	1E-05	0	6.88	1	18	149	3	1008.56732	3023.68741	1.89
rPPQDGSSAQNcSSSPAk	Unambiguous	4	1	E9PXG1	N-Term(iTRAQ8plex); C12(Methylthio); K18(iTRAQ8plex)	CID	1.0000	0.0000	1	1	3118246	Unique	Used	0.314	3.595	0.511	0.749	1E-05	0	6.88	0	0	80	3	824.41125	2471.21921	-1.44

nPEISHMLNNPDIMR	Unambiguous	2	1	Q8R317	N-Term(iTRAQ8plex)	CID	1.0000	0.0000	1	1	3389455	Unique	Used	1.399	2.263	0.471	0.868	1E-05	0	6.88	0	17	7	3	695.68561	2085.04227	-4.83
aVLASMDnENMHTPDIGGQGTTSQAIQDIIR	Unambiguous	1	1	P70404	N-Term(iTRAQ8plex); N8(Deamidated)	CID	0.0044	0.0000	1	1	3304456	Unique	Used	1.103	1.491	1.201	1.517	1E-05	0	6.88	0	0	166	3	1196.92029	3588.74631	-3.18
aLYDYEGQEHDELSFk	Unambiguous	1	1	Q9WVE8	N-Term(iTRAQ8plex); K16(iTRAQ8plex)	CID	1.0000	0.0000	1	1	2717282	Unique	Used	1.714	1.076	1.251	0.717	1E-05	0	6.88	0	8	200	3	851.42981	2552.27488	-0.65
sSVSQSPAPTYPSDSHYTSLEQNVPR	Unambiguous	2	1	O88737	N-Term(iTRAQ8plex)	CID	1.0000	0.0000	1	1	3297386	Unique	Used	2.475	1.522	2.598	1.281	1E-05	0	6.88	0	8	105	3	1046.84473	3138.51963	-2.73
gIVDQSQQAYQEAFEISk	Unambiguous	1	1	P63101	N-Term(iTRAQ8plex); K18(iTRAQ8plex)	CID	1.0000	0.0000	1	1	3110565	Unique	Used		1.256	1.116	0.960	1E-05	0	6.88	0	28	200	2	1325.20020	2649.39311	-1.87
nLHQSGFSLSGAQIDDNIPR	Unambiguous	1	1	O08553	N-Term(iTRAQ8plex)	CID	1.0000	0.0000	1	1	3514709	Unique	Used	1.459	1.195	1.462	1.769	1E-05	0	6.88	0	6	27	3	825.09131	2473.25937	-5.82
lHISPSNMTNQNTPEYMEk	Unambiguous	1	1	P70288	N-Term(iTRAQ8plex); K19(iTRAQ8plex)	CID	0.0349	0.0000	1	1	3231570	Unique	Used	1.067	1.186	1.164	0.965	1E-05	0	6.88	0	0	56	3	948.14728	2842.42728	-1.81
sYQAEHGPDFPWSVGEDMSADGR	Unambiguous	1	1	Q9Z1Q2	N-Term(iTRAQ8plex)	CID	1.0000	0.0000	1	1	2850204	Unique	Used	1.516	2.420	1.154	1.200	1E-05	0	6.88	0	6	51	3	948.09204	2842.26157	-2.31
gGDFLEGNIITGAQMSQVNSR	Unambiguous	3	1	Q9R0H0	N-Term(iTRAQ8plex)	CID	1.0000	0.0000	1	1	3457103	Unique	Used	1.013	0.809	0.650	1.076	1E-05	0	6.88	0	0	200	2	1249.63147	2498.25566	-2.21
hGLEVIYMIEPIDEYcVQQLk	Unambiguous	1	1	P07901	N-Term(iTRAQ8plex); C16(Methylthio); K21(iTRAQ8plex)	CID	1.0000	0.0000	1	1	3396195	Unique	Used		0.946		0.959	1E-05	0	6.88	0	2	2	3	1058.88428	3174.63828	-3.64
tQPTDEEMLFIYSHFk	Unambiguous	4	1	P31786	N-Term(iTRAQ8plex); K16(iTRAQ8plex)	CID	1.0000	0.0000	1	1	3284959	Unique	Used	1.650	0.648	0.867	0.771	1E-05	0	6.88	0	9	107	3	865.45105	2594.33860	-1.36
wGEAGAEYVVESTGVFTTMEk	Unambiguous	3	1	P16858	N-Term(iTRAQ8plex); K21(iTRAQ8plex)	CID	1.0000	0.0000	1	1	3219497	Unique	Used	1.850				1E-05	0	6.88	0	0	200	3	967.15997	2899.46537	0.31
dIGAIAQVHAEnGDIIAEEQQR	Unambiguous	1	1	O08553	N-Term(iTRAQ8plex); N12(Deamidated)	CID	1.0000	0.0000	1	1	2760677	Unique	Used	1.727	1.761	1.492	1.446	1E-05	0	6.88	0	26	200	3	894.79163	2682.36032	-1.27
yTPSGQSGAAASESLFISNHAY	Unambiguous	2	1	A6ZI44	N-Term(iTRAQ8plex)	CID	1.0000	0.0000	1	1	3234596	Unique	Used	0.983	0.807	1.474	1.167	1E-05	0	6.88	0	0	45	2	1281.62146	2562.23564	-2.26

gPGFNSQHQQGGSQASVTSVPR	Unambiguous	1	1	E9QQ02	N-Term(iTRAQ8plex)	CID	1.0000	0.0000	1	1	3550146	Unique	Used	1.417	0.585	1.482	0.766	1E-05	0	6.88	0	0	200	3	844.09088	2530.25809	-4.74
iGGGVGFGGLEAMNSMAGFGGVGR	Unambiguous	5	1	Q8C854	N-Term(iTRAQ8plex)	CID	1.0000	0.0000	1	1	3898492	Unique	Used	0.635	0.789	0.816	0.795	1E-05	0	6.88	0	0	19	2	1251.62390	2502.24053	-5.24
vEGAFPVTMLPGDGVGPELmHAVk	Unambiguous	1	1	Q91VA7	N-Term(iTRAQ8plex); M20(Oxidation); K24(iTRAQ8plex)	CID	1.0000	0.0000	1	1	3416897	Unique	Used	0.982	1.086	0.969	0.967	1E-05	0	6.88	0	8	59	3	1025.88184	3075.63095	-5.17
gSPGSIQGTPcPGTqLGPQPAASPSQLPADQSPHTLR	Unambiguous	4	1	Q5SSM3	N-Term(iTRAQ8plex); C11(Methylthio); Q15(Deamidated)	CID	0.0145	0.0000	1	1	3660815	Unique	Used	1.240		0.801	0.826	1E-05	0	6.88	0	0	49	3	1329.65942	3986.96372	-0.16
vIRPLDQPSSFDATPYIk	Unambiguous	1	1	Q6ZQ38	N-Term(iTRAQ8plex); K18(iTRAQ8plex)	CID	1.0000	0.0000	1	1	3557094	Unique	Used	0.292	1.429	0.927	1.199	1E-05	0	6.88	0	10	16	3	885.83331	2655.48539	-4.25
eVAAFAQFGSDLDAATQQLLSR	Unambiguous	2	1	Q03265	N-Term(iTRAQ8plex)	CID	1.0000	0.0000	1	1	3667522	Unique	Used	0.534	1.143	1.328	1.726	1E-05	0	6.88	0	4	174	2	1321.68140	2642.35552	-6.56
eLSSAVSPPNLHSDTPTFSYASLAGPTIPPR	Unambiguous	3	1	Q9QYR6	N-Term(iTRAQ8plex)	CID	1.0000	0.0000	1	1	3685085	Unique	Used	3.539	1.098	2.040	0.878	1E-05	0	6.88	0	12	90	3	1171.93628	3513.79428	-6.41
wGEAGAEYVVESTGVFTTMEk	Unambiguous	3	1	P16858	N-Term(iTRAQ8plex); K21(iTRAQ8plex)	CID	1.0000	0.0000	1	1	3241215	Unique	Used	0.999	1.062	0.704	0.976	1E-05	0	6.88	0	17	200	3	967.15796	2899.45932	-1.78
kFAEVEDVVNSILFLLSDR	Unambiguous	2	1	P08074	N-Term(iTRAQ8plex); K1(iTRAQ8plex)	CID	1.0000	0.0000	1	1	3861613	Unique	Used	0.822	2.735	0.688	1.031	1E-05	0	6.88	1	0	142	3	934.85852	2802.56101	-9.00
tGILMLNMGGPETLGEVQDFLQR	Unambiguous	2	1	P22315	N-Term(iTRAQ8plex)	CID	1.0000	0.0000	1	1	3727221	Unique	Used	0.736	1.084	1.026	0.922	1E-05	0	6.88	0	9	35	2	1412.23376	2823.46025	-3.01
eSTFLDEGPNEQEITPLQHTPR	Unambiguous	3	1	Q9QYR6	N-Term(iTRAQ8plex)	CID	1.0000	0.0000	1	1	2795247	Unique	Used	2.577	1.132	1.623	0.920	1E-05	0	6.88	0	0	200	3	948.14288	2842.41410	-0.72
aAAQLLQSQAQQSGAQQTk	Unambiguous	3	1	Q8C1B7-3	N-Term(iTRAQ8plex); K19(iTRAQ8plex)	CID	1.0000	0.0000	1	1	3658136	Unique	Used	0.810	1.301	1.132	0.777	1E-05	0	6.87	0	0	19	2	1283.20508	2565.40288	-6.88
dAGYEFDIcFTSVQk	Unambiguous	1	1	Q9DBJ1	N-Term(iTRAQ8plex); C9(Methylthio); K15(iTRAQ8plex)	CID	1.0000	0.0000	1	1	3265171	Unique	Used	1.979	0.991	1.373	1.465	1E-05	0	6.87	0	0	200	2	1189.08447	2377.16167	-2.02

sQVFSTAADGQTQVEIk	Unambiguous	1	1	P38647	N-Term(iTRAQ8plex); K17(iTRAQ8plex)	CID	1.0000	0.0000	1	1	3126656	Unique	Used	1.291	0.923	1.175	1.287	1E-05	0	6.87	0	0	76	2	1209.15674	2417.30620	-2.93
eMEENFALEAANYQDTIGR	Unambiguous	1	1	P20152	N-Term(iTRAQ8plex)	CID	1.0000	0.0000	1	1	2815127	Unique	Used	1.432	3.558	0.672	1.182	1E-05	0	6.87	0	0	190	2	1253.09619	2505.18511	-0.76
yVLQQLSNWQDAHSk	Unambiguous	2	1	O35668	N-Term(iTRAQ8plex); K15(iTRAQ8plex)	CID	1.0000	0.0000	1	1	3683129	Unique	Used	1.038	1.227	0.600	0.844	1E-05	0	6.87	0	8	11	3	809.10278	2425.29380	-6.04
sLSNATIINEEVLNAMLk	Unambiguous	3	1	P14576	N-Term(iTRAQ8plex); K18(iTRAQ8plex)	CID	1.0000	0.0000	1	1	3581125	Unique	Used	0.400	0.884	0.746	0.853	1E-05	0	6.87	0	29	24	2	1284.72498	2568.44267	-3.93
yTPSGQSGAAASESLFISNHAY	Unambiguous	2	1	A6ZI44	N-Term(iTRAQ8plex)	CID	1.0000	0.0000	1	1	3475499	Unique	Used	0.926	0.568	0.846	1.101	1E-05	0	6.87	0	0	150	2	1281.62085	2562.23442	-2.74
aGYTDQVVIGMDVAASEFYR	Unambiguous	2	1	P17182	N-Term(iTRAQ8plex)	CID	1.0000	0.0000	1	1	3354146	Unique	Used	1.025	1.455	1.231	1.305	1E-05	0	6.87	0	13	200	2	1248.61780	2496.22832	-4.00
lETYYNATEPVISFYDk	Unambiguous	2	1	Q9R0Y5	N-Term(iTRAQ8plex); K17(iTRAQ8plex)	CID	1.0000	0.0000	1	1	3262077	Unique	Used	1.438	0.950	1.390	0.876	1E-05	0	6.87	0	10	21	2	1331.19714	2661.38701	-1.44
iTSLAQLNAAnHDAAIFPGGFGAAk	Unambiguous	1	1	Q9D172	N-Term(iTRAQ8plex); N11(Deamidated); K25(iTRAQ8plex)	CID	1.0000	0.0000	1	1	3644509	Unique	Used	1.183	0.761	0.967	0.982	1E-05	0	6.87	0	19	4	3	1022.22168	3064.65049	-5.62
lVLASIDQADFQGFTYVNPDFVHPDAR	Unambiguous	2	1	P63318	N-Term(iTRAQ8plex)	CID	1.0000	0.0000	1	1	3581096	Unique	Used			1.802	2.472	1E-05	0	6.87	0	0	26	3	1113.90100	3339.68845	-2.04
dFTSEQIDEILQNHTEIVFAR	Unambiguous	2	1	Q6PIC6	N-Term(iTRAQ8plex)	CID	1.0000	0.0000	1	1	3376846	Unique	Used		0.624	1.223	1.225	1E-05	0	6.87	0	13	200	3	937.14581	2809.42289	-2.91
nASEVPMVLVGTqDAISAANPR	Unambiguous	3	1	F8VQE9	N-Term(iTRAQ8plex); Q13(Deamidated)	CID	0.0437	0.0000	1	1	3498918	Unique	Used	0.485	1.107	0.986	1.025	1E-05	0	6.87	0	0	117	2	1273.16663	2545.32598	0.99
yTPSGQSGAAASESLFISNHAY	Unambiguous	2	1	A6ZI44	N-Term(iTRAQ8plex)	CID	1.0000	0.0000	1	1	3496066	Unique	Used	0.441	0.776	0.971	1.175	1E-05	0	6.86	0	0	200	2	1281.62061	2562.23393	-2.93
aANLYASSPHSEFLDYVNAPIGk	Unambiguous	2	1	Q8R0N6	N-Term(iTRAQ8plex); K23(iTRAQ8plex)	CID	1.0000	0.0000	1	1	3517382	Unique	Used	2.057	0.680	0.758	1.256	1E-05	0	6.86	0	8	33	3	1024.87476	3072.60971	-5.01
rFTAQGLPDLNHSQVYAVk	Unambiguous	2	1	Q9EPU0-2	N-Term(iTRAQ8plex); K19(iTRAQ8plex)	CID	1.0000	0.0000	1	1	3759003	Unique	Used	0.457	0.648	0.750	0.909	1E-05	0	6.86	1	11	68	3	918.17902	2752.52250	-4.73

ILGDASVSFTDNcVVGIQANTER	Unambiguous	3	1	P97807-2	N-Term(iTRAQ8plex); C13(Methylthio)	CID	1.0000	0.0000	1	1	3583318	Unique	Used	1.878	0.785	0.879	1.083	1E-05	0	6.86	0	16	100	2	1380.17834	2759.34941	-5.53
aGGAGVPAFYTSTGYGTLVQEGGSPIk	Unambiguous	2	1	Q9D0K2	N-Term(iTRAQ8plex); K27(iTRAQ8plex)	CID	1.0000	0.0000	1	1	3518352	Unique	Used	1.515	1.097	0.676	1.574	1E-05	0	6.86	0	0	45	3	1065.23376	3193.68674	-3.85
vEGYSGTAGDSMNYHnGR	Unambiguous	5	1	Q80YX1	N-Term(iTRAQ8plex); N16(Deamidated)	CID	1.0000	0.0000	1	1	2665578	Unique	Used	0.711	0.757	1.056	1.318	1E-05	0	6.86	0	29	96	3	740.66736	2219.98752	-2.45
qFYDQALQQAVMDDDAnNAk	Unambiguous	1	1	P35762	N-Term(iTRAQ8plex); N17(Deamidated); K20(iTRAQ8plex)	CID	0.0102	0.0000	1	1	2739610	Unique	Used		0.606	1.297	1.732	1E-05	0	6.86	0	0	200	2	1447.70691	2894.40654	-0.76
nEAIQAAHDSVAQEGQcR	Unambiguous	1	1	Q9R0P9	N-Term(iTRAQ8plex); C17(Methylthio)	CID	1.0000	0.0000	1	1	3029341	Unique	Used	2.033	0.875	1.308	1.001	1E-05	0	6.86	0	0	3	3	759.69238	2277.06260	-1.25
rSsDSWDVWGSGSASNNk	Unambiguous	3	1	Q9EPJ9	N-Term(iTRAQ8plex); S3(Phospho); K18(iTRAQ8plex)	CID	1.0000	0.0000	1	1	2829010	Unique	Used	1.003	0.834	0.547	0.709	1E-05	0	6.85	1	0	37	3	876.74908	2628.23270	1.01
lQQTQAQVDEVVDIMR	Unambiguous	2	1	P63044	N-Term(iTRAQ8plex)	CID	1.0000	0.0000	1	1	3286476	Unique	Used	0.472	1.832	1.199	1.148	1E-05	0	6.85	0	0	200	2	1089.07898	2177.15068	-1.45
tIGGGDDSFNTFFSETGAGk	Unambiguous	4	1	P68373	N-Term(iTRAQ8plex); K20(iTRAQ8plex)	CID	1.0000	0.0000	1	1	3154676	Unique	Used		1.518	0.988	1.342	1E-05	0	6.85	0	0	200	2	1308.65686	2616.30644	0.99
cLEVLQASDNVLENLDGVANLPR	Unambiguous	1	1	Q9JHK4	N-Term(iTRAQ8plex); C1(Methylthio)	CID	1.0000	0.0000	1	1	3581922	Unique	Used	0.560	0.832	0.858	1.596	1E-05	0	6.85	0	0	139	2	1416.72961	2832.45195	-0.66
eAEEEFWQNQHPQPYIFPDSPGGTSFER	Unambiguous	1	1	Q9CQJ8	N-Term(iTRAQ8plex)	CID	1.0000	0.0000	1	1	2940493	Unique	Used	0.967	0.909	0.886	1.266	1E-05	0	6.85	0	25	148	3	1209.56189	3626.67112	-1.54
dFTSEQIDEILQNHTEIVFAR	Unambiguous	2	1	Q6PIC6	N-Term(iTRAQ8plex)	CID	1.0000	0.0000	1	1	3375103	Unique	Used	0.700	0.554	1.276	1.411	1E-05	0	6.85	0	7	200	3	937.14709	2809.42673	-1.54
aSTNIQDTEGnTPLHLAcDEER	Unambiguous	1	1	Q9Z2X2	N-Term(iTRAQ8plex); N11(Deamidated); C18(Methylthio)	CID	0.0088	0.0000	1	1	2759553	Unique	Used	1.304	0.925	1.046	0.937	1E-05	0	6.85	0	23	200	3	922.43030	2765.27634	3.72

iSPTFADAYSNMGNTLk	Unambiguous	2	1	Q8CGY8	N-Term(iTRAQ8plex); K17(iTRAQ8plex)	CID	1.0000	0.0000	1	1	3558497	Unique	Used	0.460	1.648	1.065	1.019	1E-05	0	6.85	0	0	200	2	1219.64001	2438.27275	-4.87
aFTHTAQYDEAISDYFR	Unambiguous	1	1	Q9CWJ9	N-Term(iTRAQ8plex)	CID	1.0000	0.0000	1	1	3323145	Unique	Used	0.925	1.510	0.998	1.611	1E-05	0	6.85	0	2	15	3	780.37732	2339.11740	-3.08
tQPTDEEMLFIYSHFk	Unambiguous	4	1	P31786	N-Term(iTRAQ8plex); K16(iTRAQ8plex)	CID	1.0000	0.0000	1	1	3285091	Unique	Used	1.993	0.662	0.937	0.795	1E-05	0	6.85	0	5	109	3	865.45044	2594.33677	-2.07
yGYIHLYDLETGTcIYmNR	Unambiguous	2	1	Q68FD5	N-Term(iTRAQ8plex); C14(Methylthio); M17(Oxidation)	CID	1.0000	0.0000	1	1	3665274	Unique	Used	1.333	1.094	0.818	1.098	1E-05	0	6.85	0	9	17	3	897.75104	2691.23856	-6.44
gAHGIIVVYDVTDQESFNNVk	Unambiguous	2	1	Q5SW88	N-Term(iTRAQ8plex); K21(iTRAQ8plex)	CID	1.0000	0.0000	1	1	3234744	Unique	Used	1.112	1.462	1.266	1.238	1E-05	0	6.85	0	6	8	3	971.85516	2913.55094	-1.98
kGTVVYGEPITASLGTDGSHYWSk	Unambiguous	1	1	O08553	N-Term(iTRAQ8plex); K1(iTRAQ8plex); K24(iTRAQ8plex)	CID	1.0000	0.0000	1	1	3434238	Unique	Used	0.919	1.237	1.440	1.299	1E-05	0	6.85	1	0	100	3	1155.96191	3465.87119	-2.01
sQAQTPVLSPSLPDMSLSTHPSVQAGGWR	Unambiguous	6	1	B2RUG9	N-Term(iTRAQ8plex)	CID	1.0000	0.0000	1	1	3840562	Unique	Used	1.485	0.886	1.196	0.700	1E-05	0	6.85	0	0	200	3	1113.57031	3338.69638	-4.26
tTTYQALLGSVDTEQDSTR	Unambiguous	3	1	Q8BVW0	N-Term(iTRAQ8plex)	CID	1.0000	0.0000	1	1	3108819	Unique	Used	1.820	1.896	0.850	1.147	1E-05	0	6.85	0	0	200	2	1195.60474	2390.20220	1.37
aMVALIDVFHQYSGR	Unambiguous	1	1	P50114	N-Term(iTRAQ8plex)	CID	1.0000	0.0000	1	1	3935955	Unique	Used	2.860	0.637	1.518	1.525	1E-05	0	6.85	0	6	3	3	671.02661	2011.06528	-4.20
kTVTYEDPQAVGGLASALDNR	Unambiguous	8	1	Q5SRX1-2	N-Term(iTRAQ8plex); K1(iTRAQ8plex)	CID	1.0000	0.0000	1	1	3256267	Unique	Used	1.838	1.411	1.059	1.091	1E-05	0	6.85	1	0	56	3	938.51080	2813.51786	-2.69
vHAAVQPGSLDSESGIFAcAFDR	Unambiguous	2	1	Q922V4	N-Term(iTRAQ8plex); C19(Methylthio)	CID	1.0000	0.0000	1	1	3499401	Unique	Used	1.905	1.692	1.235	1.562	1E-05	0	6.85	0	6	99	3	909.77594	2727.31327	-1.49
vEQLSSGLEHDNLEAHSPEQPPR	Unambiguous	3	1	Q99K01	N-Term(iTRAQ8plex)	CID	1.0000	0.0000	1	1	2810180	Unique	Used	1.466	1.112	0.881	0.984	1E-05	0	6.85	0	0	194	3	958.48083	2873.42795	-1.83
eTLAQTVLAEVPTQMVSYFR	Unambiguous	2	1	Q8C166	N-Term(iTRAQ8plex)	CID	1.0000	0.0000	1	1	3861045	Unique	Used	0.766	1.323	0.835	1.135	1E-05	0	6.85	0	30	18	2	1294.18677	2587.36626	-3.15

nAFAcFDEEATGTIQEDYLR	Unambiguous	1	1	Q3THE2	N-Term(iTRAQ8plex); C5(Methylthio)	CID	1.0000	0.0000	1	1	3017726	Unique	Used	0.961	0.361	1.283	0.832	1E-05	0	6.85	0	0	64	2	1322.10156	2643.19585	-1.92
iSAEGVDIVLDcLcGDNTGk	Unambiguous	1	1	Q80TB8	N-Term(iTRAQ8plex); C12(Methylthio); C14(Methylthio); K20(iTRAQ8plex)	CID	1.0000	0.0000	1	1	3353361	Unique	Used	0.629	0.656	0.620	1.063	1E-05	0	6.85	0	8	13	2	1361.66650	2722.32573	-4.54
sAAGSSSGFHSWAR	Unambiguous	1	1	P19246	N-Term(iTRAQ8plex)	CID	1.0000	0.0000	1	1	3753961	Unique	Used	3.304	0.234	1.855	1.264	1E-05	0	6.85	0	2	6	3	571.28387	1711.83707	-4.88
nTEINFQLGIEFDEVTADR	Unambiguous	2	1	P11404	N-Term(iTRAQ8plex)	CID	1.0000	0.0000	1	1	2942121	Unique	Used	4.401	1.487	1.775	0.978	1E-05	0	6.85	0	0	76	2	1315.64075	2630.27422	-5.56
aGLVVAPGHQGPQYPAGDEPLEEIk	Unambiguous	1	1	Q9JJG9	N-Term(iTRAQ8plex); K25(iTRAQ8plex)	CID	1.0000	0.0000	1	1	2956806	Unique	Used	0.413	0.627	0.961	1.870	1E-05	0	6.85	0	0	200	3	1060.91296	3180.72434	2.92
aVEASNAYSSILqAVQAAEDAAGQALR	Unambiguous	1	1	Q61001	N-Term(iTRAQ8plex); Q13(Deamidated)	CID	0.0044	0.0000	1	1	3746276	Unique	Used	1.536	3.185	0.600	1.463	1E-05	0	6.85	0	0	200	2	1505.27905	3009.55083	2.55
yAVDDVQYTDEIASVLTSR	Unambiguous	1	1	Q11136	N-Term(iTRAQ8plex)	CID	1.0000	0.0000	1	1	3173686	Unique	Used	2.247	0.723	0.577	0.930	1E-05	0	6.85	0	0	16	2	1225.11975	2449.23223	-3.20
lESTVLSILEAQASTLVGEk	Unambiguous	2	1	Q9QYX7	N-Term(iTRAQ8plex); K20(iTRAQ8plex)	CID	1.0000	0.0000	1	1	3624042	Unique	Used	1.719	1.244	2.990	1.198	1E-05	0	6.85	0	7	42	2	1348.77356	2696.53984	-5.35
lQELEScSGLGSTSDDTDVR	Unambiguous	9	1	Q6PAR5-2	N-Term(iTRAQ8plex); C7(Methylthio)	CID	1.0000	0.0000	1	1	2834771	Unique	Used	0.743	1.014	1.359	0.626	1E-05	0	6.84	0	19	143	2	1231.56824	2462.12920	-1.51
fADLAASHLEALR	Unambiguous	1	1	Q673H1	N-Term(iTRAQ8plex)	CID	1.0000	0.0000	1	1	3663291	Unique	Used	1.190	0.804	0.765	0.680	1E-05	0	6.84	0	0	133	3	573.32043	1717.94675	-4.17
gGQTGLQGPGLSVSGPqGHLESESGk	Unambiguous	1	1	E9Q616	N-Term(iTRAQ8plex); Q17(Deamidated); K26(iTRAQ8plex)	CID	1.0000	0.0000	1	1	3145075	Unique	Used	1.479	1.502	0.926	0.720	1E-05	0	6.84	0	0	88	3	1025.21021	3073.61606	4.86
yHTSQSGDEMTSLSEYVSR	Unambiguous	2	1	P11499	N-Term(iTRAQ8plex)	CID	1.0000	0.0000	1	1	3209966	Unique	Used	1.091	1.063	0.786	0.968	1E-05	0	6.84	0	16	86	3	827.72089	2481.14811	-0.99
wGEAGAEYVVESTGVFTTMEk	Unambiguous	3	1	P16858	N-Term(iTRAQ8plex); K21(iTRAQ8plex)	CID	1.0000	0.0000	1	1	3241958	Unique	Used		1.360	1.620	1.582	1E-05	0	6.84	0	18	200	3	967.15991	2899.46518	0.25

eALQGSAWQVSSEDVR	Unambiguous	2	1	A2BE28-2	N-Term(iTRAQ8plex)	CID	1.0000	0.0000	1	1	2955361	Unique	Used	0.831	0.840	0.892	0.711	1E-05	0	6.84	0	0	200	2	1033.52661	2066.04595	0.14
nLHQSGFSLSGAQIDDNIPR	Unambiguous	1	1	O08553	N-Term(iTRAQ8plex); N1(Deamidated)	CID	1.0000	0.0000	1	1	3300830	Unique	Used	1.895	1.128	1.191	1.039	1E-05	0	6.84	0	26	103	3	825.42047	2474.24686	-4.42
aVANETGAFFFLInGPEIMSk	Unambiguous	1	1	Q01853	N-Term(iTRAQ8plex); N14(Deamidated); K21(iTRAQ8plex)	CID	1.0000	0.0000	1	1	3440389	Unique	Used	2.351		1.548	1.501	1E-05	0	6.84	0	7	29	3	955.84497	2865.52036	-3.98
nLHQSGFSLSGAQIDDNIPR	Unambiguous	1	1	O08553	N-Term(iTRAQ8plex)	CID	1.0000	0.0000	1	1	3494103	Unique	Used	1.347	1.530	1.095	1.325	1E-05	0	6.84	0	0	123	3	825.09229	2473.26230	-4.64
iNEAFIEMATTEDAqAAVDYYTTTPALVFGkPVR	Unambiguous	1	1	Q8K310	N-Term(iTRAQ8plex); Q15(Deamidated); K31(iTRAQ8plex)	CID	1.0000	0.0000	1	1	3581395	Unique	Used	0.979	0.623	0.995	0.278	1E-05	0	6.84	0	21	45	3	1448.08203	4342.23154	-2.28
wGEAGAEYVVESTGVFTTMEk	Unambiguous	3	1	P16858	N-Term(iTRAQ8plex); K21(iTRAQ8plex)	CID	1.0000	0.0000	1	1	3240415	Unique	Used	0.540	1.062	1.515	1.158	1E-05	0	6.84	0	26	200	2	1450.23230	2899.45732	-2.47
eLSSAVSPPNLHSDTPTFSYASLAGPTIPPR	Unambiguous	3	1	Q9QYR6	N-Term(iTRAQ8plex)	CID	1.0000	0.0000	1	1	3725846	Unique	Used	1.933	1.130	1.062	0.926	1E-05	0	6.84	0	9	97	3	1171.93396	3513.78733	-8.39
vASVAHSAPSEAPScSPFGk	Unambiguous	5	1	B7ZCL8	N-Term(iTRAQ8plex); C15(Methylthio); K20(iTRAQ8plex)	CID	1.0000	0.0000	1	1	3595919	Unique	Used	1.248	1.042	1.068	1.288	1E-05	0	6.84	0	0	107	3	861.77014	2583.29587	-7.65
iGGGVGFGGLEAMNSMAGFGGVGR	Unambiguous	5	1	Q8C854	N-Term(iTRAQ8plex)	CID	1.0000	0.0000	1	1	3898478	Unique	Used	0.457	0.717	0.850	0.956	1E-05	0	6.84	0	13	22	2	1251.62415	2502.24101	-5.05
gITNLcVIGGDGSLTGADTFR	Unambiguous	1	1	P47857	N-Term(iTRAQ8plex); C6(Methylthio)	CID	1.0000	0.0000	1	1	3729360	Unique	Used	1.691	1.508	1.054	0.499	1E-05	0	6.84	0	0	200	2	1209.10010	2417.19292	-7.37
qGQYSPMAIEEQVAVIYAGVR	Unambiguous	2	1	Q03265	N-Term(iTRAQ8plex)	CID	1.0000	0.0000	1	1	3603959	Unique	Used	1.340		0.395	0.707	1E-05	0	6.84	0	22	200	2	1307.18250	2613.35771	-2.75
lLGDASVSFTDNcVVGIQANTER	Unambiguous	3	1	P97807-2	N-Term(iTRAQ8plex); C13(Methylthio)	CID	1.0000	0.0000	1	1	3581811	Unique	Used	1.725	1.234	0.780	0.833	1E-05	0	6.84	0	25	142	2	1380.17529	2759.34331	-7.74
aTLQEVVGLHSDMAVEAGAPYAER	Unambiguous	1	1	G3UYZ1	N-Term(iTRAQ8plex)	CID	1.0000	0.0000	1	1	3302233	Unique	Used	1.013	0.281	1.006	0.224	1E-05	0	6.84	0	8	35	3	940.14777	2818.42875	-2.15

gITNLcVIGGDGSLTGADTFR	Unambiguous	1	1	P47857	N-Term(iTRAQ8plex); C6(Methylthio)	CID	1.0000	0.0000	1	1	3730114	Unique	Used	2.489	1.551			1E-05	0	6.84	0	0	200	2	1209.09937	2417.19145	-7.97
iLTEAEIDAHLVALAER	Unambiguous	2	1	Q9QUM9	N-Term(iTRAQ8plex)	CID	1.0000	0.0000	1	1	3305138	Unique	Used	1.745	0.776	0.728	0.555	1E-05	0	6.84	0	0	36	3	723.41266	2168.22342	0.24
aESTSSISSTNSLSAATTPTVGSSR	Unambiguous	3	1	Q8VD37	N-Term(iTRAQ8plex)	CID	1.0000	0.0000	1	1	3386879	Unique	Used	0.877	0.812	1.710	1.506	1E-05	0	6.84	0	0	57	2	1352.17578	2703.34429	-5.33
vFQSSTSQEQVYNDcAk	Unambiguous	2	1	Q61768	N-Term(iTRAQ8plex); C15(Methylthio); K17(iTRAQ8plex)	CID	1.0000	0.0000	1	1	3107062	Unique	Used	0.626	0.395	0.827	0.967	1E-05	0	6.84	0	0	79	2	1294.63196	2588.25664	-0.58
vADVSISEVTTLLGNVHSPVVEGYVGENISGEVk	Unambiguous	3	1	P20357	N-Term(iTRAQ8plex); K34(iTRAQ8plex)	CID	1.0000	0.0000	1	1	3420802	Unique	Used	1.299	1.454	1.413	0.758	1E-05	0	6.84	0	0	200	3	1369.39856	4106.18113	-7.49
scSIDTSPGTSSLASPASQR	Unambiguous	3	1	E9PVB6	N-Term(iTRAQ8plex); C2(Methylthio)	CID	1.0000	0.0000	1	1	3493341	Unique	Used	0.864	0.240	0.739	1.261	1E-05	0	6.84	0	0	200	2	1151.54651	2302.08574	-4.34
yFDSFGDLSSASAIMGNPk	Unambiguous	1	1	P02089	N-Term(iTRAQ8plex); K19(iTRAQ8plex)	CID	1.0000	0.0000	1	1	3540881	Unique	Used	1.944	######	1.077	0.671	1E-05	0	6.84	0	0	7	2	1308.16028	2615.31328	-5.33
lcnGIQDcMDGSDEGAHcR	Unambiguous	3	1	Q91ZX7	N-Term(iTRAQ8plex); C2(Methylthio); N3(Deamidated); C8(Methylthio); C18(Methylthio)	CID	1.0000	0.0000	1	1	2886829	Unique	Used	1.830	1.755	1.017	0.927	1E-05	0	6.84	0	26	200	3	822.97797	2466.91935	-2.35
eQFGNNPFSSLAGNSDNSSSQPLR	Unambiguous	1	1	Q99NB8	N-Term(iTRAQ8plex)	CID	1.0000	0.0000	1	1	3414659	Unique	Used	1.692	1.114	1.958	1.631	1E-05	0	6.84	0	0	67	2	1429.17822	2857.34917	-5.72
eLQELVQAQAASGEELALAVAER	Unambiguous	1	1	Q9Z275	N-Term(iTRAQ8plex)	CID	1.0000	0.0000	1	1	3262851	Unique	Used	1.378	2.066	1.766	1.043	1E-05	0	6.84	0	5	15	2	1365.23474	2729.46221	-0.07
nENTFLDLTVQQIEHLNk	Unambiguous	2	1	Q91ZJ5	N-Term(iTRAQ8plex); K18(iTRAQ8plex)	CID	1.0000	0.0000	1	1	3351903	Unique	Used	0.631	0.879	1.048	1.516	1E-05	0	6.84	0	13	7	3	922.17175	2764.50071	-3.01
lVTFESVAVPLQQGAEQQR	Unambiguous	2	1	Q3UPL0	N-Term(iTRAQ8plex)	CID	1.0000	0.0000	1	1	3622698	Unique	Used	0.733	0.733	1.067	0.923	1E-05	0	6.84	0	30	17	2	1202.65393	2404.30058	-5.52
fLAEDYTLVDVLYYVTR	Unambiguous	2	1	E9PWG9	N-Term(iTRAQ8plex)	CID	1.0000	0.0000	1	1	3766888	Unique	Used	0.407	2.810	1.033	1.114	1E-05	0	6.84	0	0	200	2	1192.63611	2384.26494	-1.77

lQQQAALSPTTAPAVSSVSk	Unambiguous	1	1	Q8VHR5	N-Term(iTRAQ8plex); K20(iTRAQ8plex)	CID	1.0000	0.0000	1	1	3595459	Unique	Used	0.777	0.885	0.930	0.715	1E-05	0	6.83	0	0	74	2	1296.73450	2592.46172	-7.73
sGLGLGAASASTSGSGPADSVmR	Unambiguous	3	1	Q7TSJ2	N-Term(iTRAQ8plex); M22(Oxidation)	CID	1.0000	0.0000	1	1	3451653	Unique	Used	1.251	0.772	2.306	1.950	1E-05	0	6.83	0	0	200	2	1178.58240	2356.15752	-6.02
qFYDQALQQAVMDDDANNAk	Unambiguous	1	1	P35762	N-Term(iTRAQ8plex); K20(iTRAQ8plex)	CID	1.0000	0.0000	1	1	2850492	Unique	Used		1.703	5.486	6.151	1E-05	0	6.83	0	0	158	2	1447.21045	2893.41362	-3.84
iQEGVESLAGYADIFLR	Unambiguous	2	1	Q99MN9	N-Term(iTRAQ8plex)	CID	1.0000	0.0000	1	1	3666547	Unique	Used	0.471	1.213	1.166	1.366	1E-05	0	6.83	0	0	5	2	1093.08728	2185.16728	-6.14
vSDAISTqYPVVDHEFDAVVVGAGGAGLR	Unambiguous	1	1	Q8K2B3	N-Term(iTRAQ8plex); Q8(Deamidated)	CID	1.0000	0.0000	1	1	3353850	Unique	Used	1.211	0.988	1.254	1.277	1E-05	0	6.83	0	29	200	3	1078.89221	3234.66208	1.09
qTIGNScGTIGLIHAVANNQDk	Unambiguous	1	1	Q9R0P9	N-Term(iTRAQ8plex); C7(Methylthio); K22(iTRAQ8plex)	CID	1.0000	0.0000	1	1	3723007	Unique	Used	1.484	1.048	1.519	1.216	1E-05	0	6.83	0	0	116	3	970.16998	2908.49540	-9.49
eQDALDLYQHSLGELLVLLAAEAPGR	Unambiguous	2	1	Q3U3Q1-2	N-Term(iTRAQ8plex)	CID	1.0000	0.0000	1	1	3581915	Unique	Used	2.726	0.544	1.238	1.204	1E-05	0	6.83	0	21	116	3	1042.55896	3125.66233	-5.19
eQTVQDDLQWYQALVR	Unambiguous	1	1	Q8C419	N-Term(iTRAQ8plex)	CID	1.0000	0.0000	1	1	3262360	Unique	Used	0.623	1.221	1.510	1.109	1E-05	0	6.83	0	0	9	2	1148.59961	2296.19194	1.91
vGDSMSMGNDFDVFAHIGNDTSETR	Unambiguous	1	1	P21981	N-Term(iTRAQ8plex)	CID	1.0000	0.0000	1	1	3152201	Unique	Used	0.922	2.301	1.187	1.715	1E-05	0	6.83	0	0	151	3	1002.78894	3006.35227	0.35
mcDPGmTAFEPEALGNLVEGLDFHR	Unambiguous	4	1	P11798	N-Term(iTRAQ8plex); C2(Methylthio); M6(Oxidation)	CID	1.0000	0.0000	1	1	3459621	Unique	Used	1.152	1.267	1.007	0.674	1E-05	0	6.83	0	22	189	3	1039.14331	3115.41538	-4.70
fEGEPATHTQPGVQLQSNTYDLQESNVGLk	Unambiguous	4	1	Q9R1T4-2	N-Term(iTRAQ8plex); K30(iTRAQ8plex)	CID	1.0000	0.0000	1	1	3127560	Unique	Used	0.490	0.727	1.436	1.281	1E-05	0	6.83	0	0	18	3	1299.33325	3895.98520	-1.83
sFPNPFPDYEAAASAAGLAAGSAEETGR	Unambiguous	2	1	F6RJV6	N-Term(iTRAQ8plex)	CID	1.0000	0.0000	1	1	3352146	Unique	Used	0.972	2.393	0.609	1.055	1E-05	0	6.83	0	6	10	2	1529.73560	3058.46391	-1.89
qYDGVAELDQLLHYR	Unambiguous	1	1	P16014	N-Term(iTRAQ8plex)	CID	1.0000	0.0000	1	1	3261350	Unique	Used	0.846	0.377	0.206	0.247	1E-05	0	6.83	0	4	7	3	708.70526	2124.10123	-0.72
qGTFHSQQALEYGTk	Unambiguous	1	1	Q9WUM5	N-Term(iTRAQ8plex); K15(iTRAQ8plex)	CID	1.0000	0.0000	1	1	3183483	Unique	Used	1.395	1.251	1.557	1.620	1E-05	0	6.83	0	0	28	3	768.41016	2303.21592	-3.53

sNASLTnNQNLIQSLR	Unambiguous	2	1	Q7M739	N-Term(iTRAQ8plex); N7(Deamidated)	CID	0.0307	0.0000	1	1	3535780	Unique	Used	1.527	0.900	1.131	0.947	1E-05	0	6.83	0	23	101	2	1039.55774	2078.10820	-2.98
nAFAcFDEEAIGTIQEDYLR	Unambiguous	1	1	Q6ZWQ9	N-Term(iTRAQ8plex); C5(Methylthio)	CID	1.0000	0.0000	1	1	3217557	Unique	Used	1.432	0.490	0.464	0.568	1E-05	0	6.83	0	0	46	2	1328.11926	2655.23125	-2.29
qVLEVVSQDDPTWWQAk	Unambiguous	2	1	Q6XE40	N-Term(iTRAQ8plex); K17(iTRAQ8plex)	CID	1.0000	0.0000	1	1	3283659	Unique	Used	0.710	1.566	1.906	1.334	1E-05	0	6.83	0	0	200	2	1319.20435	2637.40141	-4.52
gVLTGPEYEALDALPDAER	Unambiguous	1	1	Q9D1X0	N-Term(iTRAQ8plex)	CID	1.0000	0.0000	1	1	2815365	Unique	Used	1.727	1.298	1.250	1.116	1E-05	0	6.83	0	0	13	2	1160.60156	2320.19585	-0.71
iAENnDLLWMDYHQk	Unambiguous	3	1	O08539	N-Term(iTRAQ8plex); N5(Deamidated); K15(iTRAQ8plex)	CID	0.0029	0.0000	1	1	2918855	Unique	Used	1.021	1.052	1.450	1.034	1E-05	0	6.82	0	1	24	3	833.76172	2499.27060	-3.71
iSEYLQTISDLETNcLDLR	Unambiguous	6	1	Q8C0J2-2	N-Term(iTRAQ8plex); C15(Methylthio)	CID	1.0000	0.0000	1	1	3542145	Unique	Used	1.151	0.749	1.030	0.813	1E-05	0	6.82	0	24	195	2	1288.64417	2576.28105	-3.09
vQAQHDYTATDTDELQLk	Unambiguous	3	1	O08539	N-Term(iTRAQ8plex); K18(iTRAQ8plex)	CID	1.0000	0.0000	1	1	2792791	Unique	Used	1.490	1.073	2.537	1.361	1E-05	0	6.82	0	0	200	2	1342.69958	2684.39189	-2.57
mcDPGMTAFEPEALGnLVEGLDFHR	Unambiguous	4	1	P11798	N-Term(iTRAQ8plex); C2(Methylthio); N16(Deamidated)	CID	1.0000	0.0000	1	1	3482067	Unique	Used		1.239	1.393	0.435	1E-05	0	6.82	0	9	200	3	1034.14417	3100.41794	-0.38
tGSAELcAEITGPDGVR	Unambiguous	2	1	Q3TDT0	N-Term(iTRAQ8plex); C7(Methylthio)	CID	1.0000	0.0000	1	1	3073428	Unique	Used	0.569	0.612	0.999	1.074	1E-05	0	6.82	0	9	37	2	1013.49750	2025.98772	-0.51
aNcGPPGLPEPAFLPDAER	Unambiguous	2	1	Q80U49	N-Term(iTRAQ8plex); C3(Methylthio)	CID	1.0000	0.0000	1	1	3325577	Unique	Used	0.957	0.477	1.186	1.073	1E-05	0	6.82	0	0	87	2	1151.06396	2301.12065	-4.51
lLGDASVSFTDNcVVGIQANTER	Unambiguous	3	1	P97807-2	N-Term(iTRAQ8plex); C13(Methylthio)	CID	1.0000	0.0000	1	1	3582758	Unique	Used	1.352		0.531	1.037	1E-05	0	6.82	0	0	200	2	1380.18164	2759.35600	-3.14
tFYGLHQDFPSVVVVGLGk	Unambiguous	2	1	Q9CPY7	N-Term(iTRAQ8plex); K19(iTRAQ8plex)	CID	1.0000	0.0000	1	1	3805329	Unique	Used	1.816	0.804	0.904	0.935	1E-05	0	6.82	0	0	69	2	1336.25012	2671.49297	-5.19

nLHQSGFSLSGAQIDDNIPR	Unambiguous	1	1	O08553	N-Term(iTRAQ8plex); N1(Deamidated)	CID	0.0176	0.0000	1	1	3344672	Unique	Used	2.450	2.863	3.919	3.691	1E-05	0	6.82	0	15	27	2	1237.62805	2474.24883	-3.62
sIATTLIDDTSSEVLDELYR	Unambiguous	8	1	Q921Z5	N-Term(iTRAQ8plex)	CID	1.0000	0.0000	1	1	3153925	Unique	Used	0.854	1.970	0.677	0.883	1E-05	0	6.82	0	9	176	2	1273.16101	2545.31474	-1.56
nLHQSGFSLSGAQIDDNIPR	Unambiguous	1	1	O08553	N-Term(iTRAQ8plex)	CID	1.0000	0.0000	1	1	3575203	Unique	Used	0.773	0.952	1.199	1.544	1E-05	0	6.82	0	28	16	3	825.09155	2473.26010	-5.53
sNVYQPTEMAVVLnGGTIPTAPPSHTGR	Unambiguous	1	1	Q923Z0	N-Term(iTRAQ8plex); N14(Deamidated)	CID	1.0000	0.0000	1	1	3476383	Unique	Used	1.527	0.991	1.309	1.206	1E-05	0	6.82	0	25	50	3	1067.21289	3199.62412	-3.72
sVNESLNNLFITEEDYQALR	Unambiguous	2	1	Q68FD5	N-Term(iTRAQ8plex)	CID	1.0000	0.0000	1	1	3240689	Unique	Used		1.469	1.032	1.164	1E-05	0	6.82	0	0	200	2	1330.17944	2659.35161	-0.05
vLGAHILGPGAGEmVNEAALALEYGAScEDIAR	Unambiguous	1	1	O08749	N-Term(iTRAQ8plex); M14(Oxidation); C28(Methylthio)	CID	1.0000	0.0000	1	1	3521121	Unique	Used		1.466			1E-05	0	6.82	0	18	200	3	1221.93665	3663.79538	-4.65
scSIDTSPGTSSLASPASQR	Unambiguous	3	1	E9PVB6	N-Term(iTRAQ8plex); C2(Methylthio)	CID	1.0000	0.0000	1	1	3493368	Unique	Used	1.180	0.363	0.866	1.208	1E-05	0	6.82	0	0	145	2	1151.54504	2302.08281	-5.61
nESSHGGEFGcTMEELR	Unambiguous	2	1	Q9R0K7	N-Term(iTRAQ8plex); C11(Methylthio)	CID	1.0000	0.0000	1	1	2757706	Unique	Used	0.750	0.424	1.694	1.063	1E-05	0	6.82	0	5	11	3	744.99109	2232.95871	-1.75
iQEFLSESEnGQHk	Unambiguous	2	1	Q6ZWR6-4	N-Term(iTRAQ8plex); N10(Deamidated); K14(iTRAQ8plex)	CID	0.0484	0.0000	1	1	2669922	Unique	Used	0.542	1.231	0.992	1.085	1E-05	0	6.82	0	11	200	3	752.39484	2255.16996	-2.87
dLcGQSTTQAMDQYTFELSR	Unambiguous	1	1	P21614	N-Term(iTRAQ8plex); C3(Methylthio)	CID	1.0000	0.0000	1	1	3351172	Unique	Used	5.294	2.591	0.648	1.046	1E-05	0	6.82	0	18	35	2	1322.59937	2644.19145	-3.06
sEMEETLTAIAEELAASAk	Unambiguous	1	1	P08553	N-Term(iTRAQ8plex); K19(iTRAQ8plex)	CID	1.0000	0.0000	1	1	3239961	Unique	Used	2.502		1.423	1.400	1E-05	0	6.82	0	0	11	3	868.12781	2602.36887	-2.07
yEDSNNLGTSHLLR	Unambiguous	1	1	Q9DB77	N-Term(iTRAQ8plex)	CID	1.0000	0.0000	1	1	3103347	Unique	Used	0.508	0.717	0.984	1.257	1E-05	0	6.82	0	3	0	3	641.66638	1922.98459	-1.45
dQEGqDVLLFIDNIFR	Unambiguous	1	1	P56480	N-Term(iTRAQ8plex); Q5(Deamidated)	CID	1.0000	0.0000	1	1	3263379	Unique	Used	1.142	2.144	1.140	1.358	1E-05	0	6.82	0	0	200	2	1114.07971	2227.15215	-1.23

sGFEPPGDFPFEDYSQHIYR	Unambiguous	4	1	E9PUI5	N-Term(iTRAQ8plex)	CID	1.0000	0.0000	1	1	3303189	Unique	Used	0.732	0.627	0.955	0.890	1E-05	0	6.81	0	9	35	3	898.08905	2692.25260	-3.56
lGIPMSVLMGANIASEVAEEk	Unambiguous	2	1	P13707	N-Term(iTRAQ8plex); K21(iTRAQ8plex)	CID	1.0000	0.0000	1	1	3480000	Unique	Used	0.666	0.488	1.719	1.337	1E-05	0	6.81	0	14	16	2	1384.25623	2767.50517	-5.18
tGSPGGPGVGGSPGGGAAGASPSLPPSAk	Unambiguous	1	1	Q8R1Q8	N-Term(iTRAQ8plex); K29(iTRAQ8plex)	CID	1.0000	0.0000	1	1	3408410	Unique	Used	1.549	1.234	0.689	1.009	1E-05	0	6.81	0	0	59	2	1470.78223	2940.55718	-2.20
qGQYSPMAIEEQVAVIYAGVR	Unambiguous	2	1	Q03265	N-Term(iTRAQ8plex)	CID	1.0000	0.0000	1	1	3623219	Unique	Used	0.721	1.170	1.172	1.455	1E-05	0	6.81	0	17	8	2	1307.17847	2613.34966	-5.83
lTPIPSTSPNLQAYHQGNTQVSR	Unambiguous	1	1	Q8BIW1	N-Term(iTRAQ8plex)	CID	1.0000	0.0000	1	1	3873176	Unique	Used			0.880		1E-05	0	6.81	0	0	100	3	938.49567	2813.47245	-4.39
vQGVAELAQALAAEGYYDAR	Unambiguous	2	1	E9PX29	N-Term(iTRAQ8plex)	CID	1.0000	0.0000	1	1	3541517	Unique	Used	0.466	1.010	1.051	1.278	1E-05	0	6.81	0	0	22	2	1200.12451	2399.24175	-3.82
sITIAnQTNcPLYVTk	Unambiguous	1	1	O08553	N-Term(iTRAQ8plex); N6(Deamidated); C10(Methylthio); K16(iTRAQ8plex)	CID	0.0176	0.0000	1	1	3599758	Unique	Used	1.354	1.501	1.211	1.294	1E-05	0	6.81	0	0	111	2	1211.14734	2421.28740	-4.30
fIEAGQYnDNLYGTSVQSVR	Unambiguous	10	1	Q91XM9-7	N-Term(iTRAQ8plex); N8(Deamidated)	CID	0.0396	0.0000	1	1	3214288	Unique	Used	1.563	2.025	1.675	0.886	1E-05	0	6.81	0	0	39	2	1283.63965	2566.27202	-0.28
fQMPDQGMTSADDFFQGTk	Unambiguous	1	1	O08553	N-Term(iTRAQ8plex); K19(iTRAQ8plex)	CID	1.0000	0.0000	1	1	3307306	Unique	Used			1.155	1.108	1E-05	0	6.81	0	5	200	3	920.44659	2759.32523	-0.50
vSEGcLTSSQDGVENDGLHR	Unambiguous	3	1	Q91VU7	N-Term(iTRAQ8plex); C5(Methylthio)	CID	1.0000	0.0000	1	1	2699781	Unique	Used	1.591	0.889	0.912	0.983	1E-05	0	6.81	0	26	200	3	818.38324	2453.13517	0.51
vYLASLETLDnGkPFQESYVLDLDEVIk	Unambiguous	1	1	Q9CZS1	N-Term(iTRAQ8plex); N11(Deamidated); K13(iTRAQ8plex); K28(iTRAQ8plex)	CID	1.0000	0.0000	1	1	3262598	Unique	Used	1.895	0.465	0.858	0.803	1E-05	0	6.81	0	0	30	3	1371.42004	4112.24558	-0.07
eQSGEYEcSASNDVAAPVVR	Unambiguous	5	1	D3Z396	N-Term(iTRAQ8plex); C8(Methylthio)	CID	1.0000	0.0000	1	1	2668979	Unique	Used	0.714	1.046	0.819	1.053	1E-05	0	6.81	0	0	200	2	1231.06396	2461.12065	-2.89

wAQPDPMAQAGFYHQPASSGDDR	Unambiguous	4	1	H9KUX8	N-Term(iTRAQ8plex)	CID	1.0000	0.0000	1	1	3233339	Unique	Used	1.194	0.900	1.071	0.998	1E-05	0	6.81	0	24	65	3	946.10522	2836.30112	-1.43
dIGAIAQVHAENGDIIAEEQQR	Unambiguous	1	1	O08553	N-Term(iTRAQ8plex)	CID	1.0000	0.0000	1	1	2811557	Unique	Used	1.633	1.477	1.201	1.587	1E-05	0	6.81	0	0	200	2	1341.19690	2681.38652	2.54
eTEGEEQAQYLQVTScPk	Unambiguous	1	1	P63141	N-Term(iTRAQ8plex); C16(Methylthio); K18(iTRAQ8plex)	CID	1.0000	0.0000	1	1	2811943	Unique	Used	0.566	0.455	1.286	1.443	1E-05	0	6.81	0	0	200	2	1347.65906	2694.31084	-3.82
vLMEDDDLYScVVENPISQVR	Unambiguous	1	1	Q640R3	N-Term(iTRAQ8plex); C11(Methylthio)	CID	1.0000	0.0000	1	1	3284495	Unique	Used	0.847	0.445	0.962	1.683	1E-05	0	6.81	0	0	25	2	1387.66797	2774.32866	-2.40
eAFQNAYLELGGLGER	Unambiguous	2	1	Q6PIC6	N-Term(iTRAQ8plex)	CID	1.0000	0.0000	1	1	3354482	Unique	Used	0.964	0.953	0.904	1.104	1E-05	0	6.81	0	0	200	2	1036.03711	2071.06694	-4.49
sIQDLTVTGTEPGQVSSR	Unambiguous	4	1	Q62073	N-Term(iTRAQ8plex)	CID	1.0000	0.0000	1	1	3232232	Unique	Used	1.544	0.843	1.587	1.067	1E-05	0	6.81	0	0	142	2	1090.07788	2179.14849	-1.09
yGSGSGQQSVTGVEESDDANSYWR	Unambiguous	1	1	Q9ESP1	N-Term(iTRAQ8plex)	CID	1.0000	0.0000	1	1	2652244	Unique	Used	1.767				1E-05	0	6.81	0	0	200	3	961.76996	2883.29532	-0.63
vAGPQPAQTGAPqGSLGEYLFER	Unambiguous	2	1	P29391	N-Term(iTRAQ8plex); Q13(Deamidated)	CID	1.0000	0.0000	1	1	3392818	Unique	Used	1.230	2.543	1.543	1.647	1E-05	0	6.81	0	0	137	2	1339.69043	2678.37358	0.28
sLDGLSGAcGGGGSSSGETGAGGGR	Unambiguous	1	1	Q9D6W8	N-Term(iTRAQ8plex); C9(Methylthio)	CID	1.0000	0.0000	1	1	3319675	Unique	Used	0.995	0.769	1.537	1.219	1E-05	0	6.81	0	0	90	2	1246.05554	2491.10381	-2.16
rPLGnFTPPPTYAETLSTAPVASR	Unambiguous	4	1	Q8CC35	N-Term(iTRAQ8plex); N5(Deamidated)	CID	1.0000	0.0000	1	1	3703209	Unique	Used	1.314	1.436	2.036	1.134	1E-05	0	6.81	0	5	62	3	950.17035	2848.49649	-6.41
qAATHISLDQEYDSESSQQWR	Unambiguous	2	1	E9PWG2	N-Term(iTRAQ8plex)	CID	1.0000	0.0000	1	1	2847177	Unique	Used	1.365	0.944	0.974	0.884	1E-05	0	6.81	0	0	140	3	928.44342	2783.31571	-0.63
vETLHDFEAAnSDELNLQR	Unambiguous	1	1	Q7TQF7	N-Term(iTRAQ8plex); N11(Deamidated)	CID	1.0000	0.0000	1	1	2637420	Unique	Used	2.279	1.499	1.622	0.826	1E-05	0	6.81	0	0	196	2	1253.61804	2506.22881	-3.02
lNDHFPLVVWQTGSGTQTNmNVNEVISNR	Unambiguous	2	1	P97807-2	N-Term(iTRAQ8plex); M20(Oxidation)	CID	1.0000	0.0000	1	1	3804352	Unique	Used	4.286	1.498	2.551	1.594	1E-05	0	6.81	0	0	64	3	1197.60022	3590.78611	-2.88
fQmPDQGMTSADDFFQGTk	Unambiguous	1	1	O08553	N-Term(iTRAQ8plex); M3(Oxidation); K19(iTRAQ8plex)	CID	1.0000	0.0000	1	1	3307115	Unique	Used	1.966	1.302	1.261	0.490	1E-05	0	6.80	0	0	200	3	925.77875	2775.32169	0.06

fLSQPFQVAEVFTGHMGk	Unambiguous	1	1	P56480	N-Term(iTRAQ8plex); K18(iTRAQ8plex)	CID	1.0000	0.0000	1	1	3864874	Unique	Used	1.160	0.812	0.895	1.731	1E-05	0	6.80	0	0	200	3	877.80988	2631.41507	-2.41
sIVSESDVSSFEIQHSGFVk	Unambiguous	1	1	G5E870	N-Term(iTRAQ8plex); K20(iTRAQ8plex)	CID	1.0000	0.0000	1	1	3280016	Unique	Used	1.013	1.355	0.887	1.011	1E-05	0	6.80	0	23	151	3	930.82916	2790.47293	-1.48
nIVAVGAGFcDGLGFGDNTk	Unambiguous	2	1	P13707	N-Term(iTRAQ8plex); C10(Methylthio); K20(iTRAQ8plex)	CID	1.0000	0.0000	1	1	3686739	Unique	Used	0.711	0.633	1.412	1.346	1E-05	0	6.80	0	10	25	2	1305.16113	2609.31499	-6.25
nATNVEQSFMTMAAEIk	Unambiguous	4	1	Q5SW88	N-Term(iTRAQ8plex); K17(iTRAQ8plex)	CID	1.0000	0.0000	1	1	3352202	Unique	Used	0.573	0.626	0.841	1.004	1E-05	0	6.80	0	0	5	2	1247.14490	2493.28252	-4.54
nTPIYVSSPTWENHnAVFSAAGFk	Unambiguous	2	1	P05201	N-Term(iTRAQ8plex); N15(Deamidated); K24(iTRAQ8plex)	CID	1.0000	0.0000	1	1	3538305	Unique	Used	1.904	0.717	0.762	1.079	1E-05	0	6.80	0	0	71	3	1082.88721	3246.64707	-6.46
gDMSEAELLILDEFTTLAR	Unambiguous	2	1	E9Q401	N-Term(iTRAQ8plex)	CID	1.0000	0.0000	1	1	3133781	Unique	Used	0.255	2.217	1.574	1.284	1E-05	0	6.80	0	0	77	2	1214.63110	2428.25493	-1.43
gTVLAEDQLAQMSk	Unambiguous	2	1	Q9CZ28	N-Term(iTRAQ8plex); K14(iTRAQ8plex)	CID	1.0000	0.0000	1	1	3070956	Unique	Used	1.160	1.359	0.851	0.607	1E-05	0	6.80	0	0	142	2	1050.08411	2099.16094	-0.85
hQGVNITDTGLEGLLFGMLDR	Unambiguous	1	1	Q8C547	N-Term(iTRAQ8plex)	CID	1.0000	0.0000	1	1	3727098	Unique	Used	2.083	1.231	0.703	1.162	1E-05	0	6.80	0	0	35	3	864.11829	2590.34031	-7.67
vGSDASSqQEFTScLk	Unambiguous	1	1	Q8VCI5	N-Term(iTRAQ8plex); Q8(Deamidated); C14(Methylthio); K16(iTRAQ8plex)	CID	0.0015	0.0000	1	1	3190400	Unique	Used	1.323	0.541	1.021	0.704	1E-05	0	6.80	0	0	151	2	1171.58081	2342.15434	3.36
lFVQLAGEDAEISAFELQTILR	Unambiguous	1	1	O08529	N-Term(iTRAQ8plex)	CID	1.0000	0.0000	1	1	3602825	Unique	Used	0.627	1.000	0.802	1.223	1E-05	0	6.80	0	0	40	2	1384.25598	2767.50469	-4.97
gFVYnVSPYMEYHPGGEDELMR	Unambiguous	3	1	Q3TDX8-5	N-Term(iTRAQ8plex); N5(Deamidated)	CID	1.0000	0.0000	1	1	3193019	Unique	Used	1.369	0.938	1.347	1.406	1E-05	0	6.80	0	15	89	3	965.78693	2895.34623	6.57
yTPSGQSGAAASESLFISNHAY	Unambiguous	2	1	A6ZI44	N-Term(iTRAQ8plex)	CID	1.0000	0.0000	1	1	3236070	Unique	Used	1.042	0.792	1.042	1.113	1E-05	0	6.80	0	0	193	2	1281.61987	2562.23247	-3.50

qQLSAEELDAQLDAYnAR	Unambiguous	3	1	O08583	N-Term(iTRAQ8plex); N16(Deamidated)	CID	1.0000	0.0000	1	1	2637523	Unique	Used	1.302	0.384	1.435	0.922	1E-05	0	6.80	0	0	200	2	1170.58325	2340.15923	-1.25
lLGDASVSFTDncVVGIQANTER	Unambiguous	3	1	P97807-2	N-Term(iTRAQ8plex); N12(Deamidated); C13(Methylthio)	CID	0.0279	0.0000	1	1	3582158	Unique	Used	1.439	0.966	1.068	1.042	1E-05	0	6.80	0	0	200	2	1380.67932	2760.35137	0.97
fQmPDQGMTSADDFFQGTk	Unambiguous	1	1	O08553	N-Term(iTRAQ8plex); M3(Oxidation); K19(iTRAQ8plex)	CID	1.0000	0.0000	1	1	3323445	Unique	Used	1.819	2.158	1.045	1.261	1E-05	0	6.80	0	0	20	3	925.77478	2775.30979	-4.23
eTAAAHQASSsPPIDAATAEPYGFR	Unambiguous	1	1	P14873	N-Term(iTRAQ8plex); S11(Phospho)	CID	0.0426	0.0000	1	1	2848283	Unique	Used	1.581	0.462	0.872	0.664	1E-05	0	6.80	0	2	48	3	977.12518	2929.36100	-2.11
dSLGDFIEHYAQLGPSSPEQLAAGAEEGGGPR	Unambiguous	1	1	O35295	N-Term(iTRAQ8plex)	CID	1.0000	0.0000	1	1	3217090	Unique	Used	0.966	0.813	0.723	0.608	1E-05	0	6.80	0	0	16	3	1187.24475	3559.71970	-1.32
fVSPLTLVADEGWFIAESR	Unambiguous	3	1	Q8BGN3	N-Term(iTRAQ8plex)	CID	1.0000	0.0000	1	1	3645788	Unique	Used	2.146	0.132	0.773	1.087	1E-05	0	6.80	0	18	38	2	1221.14734	2441.28740	-5.94
lQQADIEATLSPNLQPLGNYR	Unambiguous	1	1	G3X9N3	N-Term(iTRAQ8plex)	CID	1.0000	0.0000	1	1	3622686	Unique	Used	0.554	1.880	0.830	0.767	1E-05	0	6.80	0	14	20	2	1323.20715	2645.40703	-4.94
qFYDQALQQAVMDDDAnNAk	Unambiguous	1	1	P35762	N-Term(iTRAQ8plex); N17(Deamidated); K20(iTRAQ8plex)	CID	0.0029	0.0000	1	1	2739713	Unique	Used		0.309	1.134	1.789	1E-05	0	6.80	0	0	200	2	1447.70679	2894.40630	-0.84
eALNVFGDDYATEDGTGVR	Unambiguous	1	1	Q8R059	N-Term(iTRAQ8plex)	CID	1.0000	0.0000	1	1	2654506	Unique	Used		1.410	1.076	1.299	1E-05	0	6.80	0	9	200	2	1167.05994	2333.11260	-3.16
aFTHTAQYDEAISDYFR	Unambiguous	1	1	Q9CWJ9	N-Term(iTRAQ8plex)	CID	1.0000	0.0000	1	1	3323292	Unique	Used	0.707	0.987	0.894	1.137	1E-05	0	6.79	0	8	3	3	780.37659	2339.11521	-4.02
eYSDLLNTQDMYNDDLIHFSk	Unambiguous	2	1	B2RUJ5	N-Term(iTRAQ8plex); K21(iTRAQ8plex)	CID	1.0000	0.0000	1	1	3152482	Unique	Used	0.889	0.728	0.948	0.902	1E-05	0	6.79	0	0	158	3	1057.18970	3169.55454	-1.99
sTLINSLFLTDLYPER	Unambiguous	9	1	P42208	N-Term(iTRAQ8plex)	CID	1.0000	0.0000	1	1	3806279	Unique	Used	1.761	0.709	0.762	0.737	1E-05	0	6.79	0	23	6	2	1093.60217	2186.19707	-1.84
vAGTGEGGLEELVEELNSGk	Unambiguous	3	1	Q62418-3	N-Term(iTRAQ8plex); K20(iTRAQ8plex)	CID	1.0000	0.0000	1	1	2853327	Unique	Used	0.948	0.998	1.196	0.827	1E-05	0	6.79	0	21	200	2	1298.69800	2596.38872	-1.53

tTEQLIEAVNNGDFEAYAk	Unambiguous	9	1	Q5SVJ0	N-Term(iTRAQ8plex); K19(iTRAQ8plex)	CID	1.0000	0.0000	1	1	3075267	Unique	Used		1.068	1.424	1.609	1E-05	0	6.79	0	14	200	2	1361.21301	2721.41875	-0.17
iSFEEFcAVVGGLDIHk	Unambiguous	2	1	Q63810-2	N-Term(iTRAQ8plex); C7(Methylthio); K17(iTRAQ8plex)	CID	1.0000	0.0000	1	1	3686922	Unique	Used	0.652	1.650	1.998	0.728	1E-05	0	6.79	0	3	4	2	1259.65942	2518.31157	-7.11
vFQSSTSQEQVYNDcAk	Unambiguous	2	1	Q61768	N-Term(iTRAQ8plex); C15(Methylthio); K17(iTRAQ8plex)	CID	1.0000	0.0000	1	1	3127283	Unique	Used	0.814	0.538	0.668	1.084	1E-05	0	6.79	0	0	114	2	1294.63110	2588.25493	-1.24
tNELGDGGVGLVLQGLQNPTck	Unambiguous	1	1	Q91VI7	N-Term(iTRAQ8plex); C21(Methylthio); K22(iTRAQ8plex)	CID	1.0000	0.0000	1	1	3601777	Unique	Used	2.293	0.761	1.021	1.505	1E-05	0	6.79	0	14	13	2	1434.25195	2867.49663	-8.71
wGEAGAEYVVESTGVFTTMEk	Unambiguous	3	1	P16858	N-Term(iTRAQ8plex); K21(iTRAQ8plex)	CID	1.0000	0.0000	1	1	3241205	Unique	Used		0.880	0.656	1.121	1E-05	0	6.79	0	14	200	2	1450.23120	2899.45513	-3.22
nSAGGTSVGWDSPPASPLQR	Unambiguous	2	1	A2AKD7	N-Term(iTRAQ8plex)	CID	1.0000	0.0000	1	1	3432975	Unique	Used	0.984	0.672	0.894	0.879	1E-05	0	6.79	0	0	167	2	1144.57727	2288.14726	-4.41
sAAGSSSGFHSWAR	Unambiguous	1	1	P19246	N-Term(iTRAQ8plex)	CID	1.0000	0.0000	1	1	3850393	Unique	Used	2.464	0.502	1.312	1.929	1E-05	0	6.79	0	12	64	3	571.28314	1711.83487	-6.16
sQQDEGIVTVPqESPTASSLTLQSSHPTSDQER	Unambiguous	1	1	Q5SWP3	N-Term(iTRAQ8plex); Q12(Deamidated)	CID	1.0000	0.0000	1	1	2866038	Unique	Used	2.860	1.129	1.034	1.079	1E-05	0	6.79	0	0	200	3	1282.29675	3844.87571	3.38
eAFQNAYLELGGLGER	Unambiguous	2	1	Q6PIC6	N-Term(iTRAQ8plex)	CID	1.0000	0.0000	1	1	3356946	Unique	Used	0.796	0.421	1.468	1.047	1E-05	0	6.79	0	5	200	2	1036.03857	2071.06987	-3.07
fQMPDQGmTSADDFFQGTk	Unambiguous	1	1	O08553	N-Term(iTRAQ8plex); M8(Oxidation); K19(iTRAQ8plex)	CID	1.0000	0.0000	1	1	3281445	Unique	Used	1.372		1.348	0.794	1E-05	0	6.79	0	0	1	3	925.77679	2775.31583	-2.05
aAAQLLQSQAQQSGAQQTk	Unambiguous	3	1	Q8C1B7-3	N-Term(iTRAQ8plex); K19(iTRAQ8plex)	CID	1.0000	0.0000	1	1	3658331	Unique	Used	0.554	1.604	1.560	1.159	1E-05	0	6.79	0	4	23	2	1283.20618	2565.40508	-6.03
lQQTQNQVDEVVDIMR	Unambiguous	1	1	P63024	N-Term(iTRAQ8plex)	CID	1.0000	0.0000	1	1	3238237	Unique	Used	1.667	0.636	0.785	1.079	1E-05	0	6.79	0	5	49	2	1110.58179	2220.15630	-1.51

nMVHPNVIcDGcNGPVVGTR	Unambiguous	3	1	Q64337	N-Term(iTRAQ8plex); C9(Methylthio); C12(Methylthio)	CID	1.0000	0.0000	1	1	3839375	Unique	Used	0.827	1.351	1.045	1.275	1E-05	0	6.79	0	0	97	3	826.71802	2478.13950	-3.70
sPSSTLSGPDLAGHSSSATSTPEELR	Unambiguous	1	1	E9PV26	N-Term(iTRAQ8plex)	CID	1.0000	0.0000	1	1	3029575	Unique	Used	0.965	1.288	1.726	0.849	1E-05	0	6.79	0	0	200	3	959.14392	2875.41721	-1.79
sNSAPLIHGLSDSSPVFQAEAPSAR	Unambiguous	1	1	Q9DB52	N-Term(iTRAQ8plex)	CID	1.0000	0.0000	1	1	3474911	Unique	Used	3.217	1.673	1.628	1.356	1E-05	0	6.79	0	0	112	3	948.15765	2842.45841	-1.89
sNTSPEELGPLANQLTSDYGR	Unambiguous	1	1	P26039	N-Term(iTRAQ8plex)	CID	1.0000	0.0000	1	1	3192640	Unique	Used	0.881	2.035	0.606	0.969	1E-05	0	6.79	0	0	24	2	1277.13879	2553.27031	-1.25
lTcNDTSAALLISHIVQSEIGDFDEALDR	Unambiguous	2	1	F8VPU2	N-Term(iTRAQ8plex); C3(Methylthio)	CID	1.0000	0.0000	1	1	3374520	Unique	Used	0.232	0.950	0.755	0.861	1E-05	0	6.79	0	8	46	3	1166.24255	3496.71311	-3.19
vSSSPAPAVSWLYHNEEVTTIPDNR	Unambiguous	2	1	O35136	N-Term(iTRAQ8plex)	CID	1.0000	0.0000	1	1	3370918	Unique	Used	1.083	1.400	1.189	1.113	1E-05	0	6.79	0	0	145	3	1025.18579	3073.54282	-3.41
eAFQNAYLELGGLGER	Unambiguous	2	1	Q6PIC6	N-Term(iTRAQ8plex)	CID	1.0000	0.0000	1	1	3355910	Unique	Used	0.945	0.826	1.227	1.301	1E-05	0	6.79	0	20	200	2	1036.03735	2071.06743	-4.25
vEYPVMYSTDPENGHMFNcIqR	Unambiguous	1	1	Q9CPU4	N-Term(iTRAQ8plex); C19(Methylthio); Q21(Deamidated)	CID	0.0015	0.0000	1	1	3282904	Unique	Used	0.882	0.687	1.280	0.969	1E-05	0	6.79	0	0	82	3	994.44366	2981.31644	-2.68
wGEAGAEYVVESTGVFTTMEk	Unambiguous	3	1	P16858	N-Term(iTRAQ8plex); K21(iTRAQ8plex)	CID	1.0000	0.0000	1	1	3240758	Unique	Used	1.970		2.465	2.026	1E-05	0	6.79	0	19	195	3	967.15967	2899.46445	-0.01
sEQSSMSIEFGQEsPEHSLAMDFSR	Unambiguous	1	1	P14873	N-Term(iTRAQ8plex); S14(Phospho)	CID	1.0000	0.0000	1	1	2741589	Unique	Used	1.932	0.950	1.686	1.266	1E-05	0	6.79	0	5	200	3	1067.46582	3200.38291	-0.85
skEPSSPGTDDVFTPGSSDSPSSQR	Unambiguous	1	1	Q61165	N-Term(iTRAQ8plex); K2(iTRAQ8plex)	CID	1.0000	0.0000	1	1	2791023	Unique	Used	1.721	1.160	2.454	2.034	1E-05	0	6.79	1	5	200	3	1054.18591	3160.54319	-1.88
gITNLcVIGGDGSLTGADTFR	Unambiguous	1	1	P47857	N-Term(iTRAQ8plex); C6(Methylthio)	CID	1.0000	0.0000	1	1	3727551	Unique	Used	2.294	1.245	0.924	1.335	1E-05	0	6.79	0	13	81	2	1209.10437	2417.20146	-3.83
lDQEDALLGSYPVDDGcR	Unambiguous	1	1	Q9D1E6	N-Term(iTRAQ8plex); C17(Methylthio)	CID	1.0000	0.0000	1	1	2764799	Unique	Used	0.885	0.538	0.611	0.957	1E-05	0	6.79	0	11	200	2	1158.53967	2316.07207	-3.00

lkDLEEGIQALMQELEDGSPR	Unambiguous	1	1	P06880	N-Term(iTRAQ8plex); K2(iTRAQ8plex)	CID	1.0000	0.0000	1	1	3353178	Unique	Used		0.171	0.052	0.054	1E-05	0	6.78	1	0	9	3	993.86633	2979.58445	-2.48
sTPVTSFTNEDLLHGR	Unambiguous	2	1	Q8VHY0	N-Term(iTRAQ8plex)	CID	1.0000	0.0000	1	1	3298800	Unique	Used	1.233	0.813	0.868	0.962	1E-05	0	6.78	0	11	28	3	693.36365	2078.07639	-2.72
sLEQLDELITDLVIDSR	Unambiguous	2	1	A2AJA9	N-Term(iTRAQ8plex)	CID	1.0000	0.0000	1	1	3263480	Unique	Used	0.504	1.595	1.627	1.235	1E-05	0	6.78	0	10	136	2	1132.11951	2263.23174	-0.79
aLQSGQcAGAALDVFTEEPPR	Unambiguous	1	1	Q61753	N-Term(iTRAQ8plex); C7(Methylthio)	CID	1.0000	0.0000	1	1	3351431	Unique	Used	0.624	0.809	1.355	1.421	1E-05	0	6.78	0	15	34	2	1255.61536	2510.22344	-3.49
aVGFVSEDEYLEIQGITR	Unambiguous	3	1	D3Z396	N-Term(iTRAQ8plex)	CID	1.0000	0.0000	1	1	3111588	Unique	Used	1.256	1.213	1.069	0.870	1E-05	0	6.78	0	13	200	2	1165.61292	2330.21855	0.15
tQPTDEEMLFIYSHFk	Unambiguous	4	1	P31786	N-Term(iTRAQ8plex); K16(iTRAQ8plex)	CID	1.0000	0.0000	1	1	3286276	Unique	Used	2.151	0.585	0.783	0.665	1E-05	0	6.78	0	2	200	3	865.45154	2594.34006	-0.80
eYQLNDSAAYYLnDLDR	Unambiguous	1	1	B2RSH2	N-Term(iTRAQ8plex); N13(Deamidated)	CID	1.0000	0.0000	1	1	2638169	Unique	Used	0.563	1.282	1.292	1.375	1E-05	0	6.78	0	3	200	2	1184.56433	2368.12139	-1.39
tAIHTAAMDMLGGSGVENQcR	Unambiguous	3	1	Q2TPA8	N-Term(iTRAQ8plex); C20(Methylthio)	CID	1.0000	0.0000	1	1	3682980	Unique	Used	1.159	1.048	0.868	1.071	1E-05	0	6.78	0	0	31	3	838.05670	2512.15555	-6.52
tQPTDEEMLFIYSHFk	Unambiguous	4	1	P31786	N-Term(iTRAQ8plex); K16(iTRAQ8plex)	CID	1.0000	0.0000	1	1	3286506	Unique	Used	1.718	0.694	0.844	0.826	1E-05	0	6.78	0	2	200	3	865.45074	2594.33768	-1.72
iSGETIFVTAPHEATAGIIGVnR	Unambiguous	2	1	Q68FD5	N-Term(iTRAQ8plex); N22(Deamidated)	CID	1.0000	0.0000	1	1	3709206	Unique	Used		1.459	1.297	1.442	1E-05	0	6.78	0	9	200	3	886.81909	2658.44272	0.83
lTGFHETSNINDFSAGVANR	Unambiguous	1	1	P15105	N-Term(iTRAQ8plex)	CID	1.0000	0.0000	1	1	3555726	Unique	Used	1.626	1.595	0.948	1.279	1E-05	0	6.78	0	14	200	3	818.74286	2454.21402	-7.15
tDALEASSLLSSGVDcGLEEASLHSSFNLGPR	Unambiguous	3	1	Q80U23	N-Term(iTRAQ8plex); C16(Methylthio)	CID	1.0000	0.0000	1	1	3440064	Unique	Used	1.293	0.729	1.223	1.149	1E-05	0	6.78	0	27	37	3	1204.91553	3612.73203	-4.00
fTLNPNTTGVqNPHIER	Unambiguous	2	1	P47809	N-Term(iTRAQ8plex); Q11(Deamidated)	CID	0.0265	0.0000	1	1	3319134	Unique	Used	0.848	1.114	0.835	0.922	1E-05	0	6.78	0	21	23	3	748.39392	2243.16721	-2.25
sInGELYAPLQHVSAVLPGGAADR	Unambiguous	2	1	Q3UHD6-2	N-Term(iTRAQ8plex); N3(Deamidated)	CID	1.0000	0.0000	1	1	3643867	Unique	Used	0.998	1.248	1.003	0.973	1E-05	0	6.78	0	7	25	3	914.15295	2740.44431	-4.72

mTSHPVHQYYLTGAQDGSVR	Unambiguous	4	1	Q8BPN8-2	N-Term(iTRAQ8plex)	CID	1.0000	0.0000	1	1	3530077	Unique	Used	0.929	1.064	1.212	1.462	1E-05	0	6.78	0	0	200	3	851.08844	2551.25077	-6.19
qFYDQALQQAVMDDDANNAk	Unambiguous	1	1	P35762	N-Term(iTRAQ8plex); K20(iTRAQ8plex)	CID	1.0000	0.0000	1	1	2851181	Unique	Used	1.263		0.879	1.802	1E-05	0	6.78	0	9	200	2	1447.21094	2893.41460	-3.50
eVAAFAQFGSDLDAATQQLLSR	Unambiguous	2	1	Q03265	N-Term(iTRAQ8plex)	CID	1.0000	0.0000	1	1	3668271	Unique	Used	0.851	1.064	1.042	1.354	1E-05	0	6.78	0	5	200	2	1321.68286	2642.35845	-5.45
lGTEEFPLIDQTFYPNHk	Unambiguous	3	1	O88935	N-Term(iTRAQ8plex); K18(iTRAQ8plex)	CID	1.0000	0.0000	1	1	3329239	Unique	Used		0.790	1.268	0.841	1E-05	0	6.78	0	19	200	3	919.82422	2757.45810	-4.62
yTPSGQSGAAASESLFISNHAY	Unambiguous	2	1	A6ZI44	N-Term(iTRAQ8plex)	CID	1.0000	0.0000	1	1	3496200	Unique	Used	1.186	0.860	1.158	1.375	1E-05	0	6.78	0	0	200	2	1281.62280	2562.23833	-1.21
aEAGGGWEGSASYk	Unambiguous	2	1	P97765	N-Term(iTRAQ8plex); K14(iTRAQ8plex)	CID	1.0000	0.0000	1	1	2807924	Unique	Used	1.409	1.157	1.193	1.192	1E-05	0	6.78	0	0	76	2	989.50702	1978.00676	-2.99
sAIDLEEMASGLNk	Unambiguous	3	1	P14576	N-Term(iTRAQ8plex); K14(iTRAQ8plex)	CID	1.0000	0.0000	1	1	3150852	Unique	Used	0.485	0.951	0.654	0.663	1E-05	0	6.78	0	0	108	2	1043.56787	2086.12847	-1.26
tPMGIILDALEQQEDNINk	Unambiguous	1	1	Q8R5J9	N-Term(iTRAQ8plex); K19(iTRAQ8plex)	CID	1.0000	0.0000	1	1	3263001	Unique	Used	0.277	1.120	0.812	1.133	1E-05	0	6.78	0	11	21	2	1375.74585	2750.48442	-0.41
sSEYHLEDNAAYYLNDLER	Unambiguous	1	1	O70443	N-Term(iTRAQ8plex)	CID	1.0000	0.0000	1	1	2796391	Unique	Used	1.123	1.362	1.257	0.859	1E-05	0	6.78	0	29	200	3	869.41492	2606.23020	-0.41
qAPYENLnDQELGEYLAR	Unambiguous	1	1	Q03517	N-Term(iTRAQ8plex); N8(Deamidated)	CID	1.0000	0.0000	1	1	2763763	Unique	Used	1.658	0.647	0.685	0.812	1E-05	0	6.78	0	0	187	2	1214.59998	2428.19267	-0.31
yPVGIEVGPQPHGVLR	Unambiguous	1	1	Q8R3P0	N-Term(iTRAQ8plex)	CID	1.0000	0.0000	1	1	3616423	Unique	Used	3.507	0.259	0.835	1.667	1E-05	0	6.78	0	1	1	3	674.71503	2022.13053	-6.59
eGSSFEGLESSTASSMELEELR	Unambiguous	1	1	Q9QYE6	N-Term(iTRAQ8plex)	CID	1.0000	0.0000	1	1	2638895	Unique	Used			0.545		1E-05	0	6.78	0	0	200	2	1340.13403	2679.26079	-0.07
dFMAALEAEPYDDIVGETVEk	Unambiguous	5	1	P27546-2	N-Term(iTRAQ8plex); K21(iTRAQ8plex)	CID	1.0000	0.0000	1	1	2816136	Unique	Used	1.136	0.707	1.041	0.753	1E-05	0	6.78	0	8	106	2	1475.74548	2950.48369	-0.54
eHINLGcDVDFDIAGPSIR	Unambiguous	3	1	Q60932-2	N-Term(iTRAQ8plex); C7(Methylthio)	CID	1.0000	0.0000	1	1	3357798	Unique	Used	0.439	0.944	1.940	1.599	1E-05	0	6.77	0	1	200	3	807.73083	2421.17795	-2.71

tVYFAEEVqcEGNSFHk	Unambiguous	1	1	P97315	N-Term(iTRAQ8plex); Q9(Deamidated); C10(Methylthio); K17(iTRAQ8plex)	CID	1.0000	0.0000	1	1	3129195	Unique	Used	1.688	0.634	1.850	1.335	1E-05	0	6.77	0	0	105	2	1322.14514	2643.28301	5.69
qGQYSPMAIEEQVAVIYAGVR	Unambiguous	2	1	Q03265	N-Term(iTRAQ8plex)	CID	1.0000	0.0000	1	1	3603740	Unique	Used	1.184	1.106	5.559	5.395	1E-05	0	6.77	0	0	200	2	1307.18604	2613.36479	-0.04
eQLQDMGLIDLFSPEk	Unambiguous	1	1	P32261	N-Term(iTRAQ8plex); K16(iTRAQ8plex)	CID	1.0000	0.0000	1	1	3217358	Unique	Used	3.383	3.417	0.404	0.958	1E-05	0	6.77	0	0	119	2	1236.16858	2471.32988	-0.56
lDLLSDTNMVDFAMDVYk	Unambiguous	1	1	P60229	N-Term(iTRAQ8plex); K18(iTRAQ8plex)	CID	1.0000	0.0000	1	1	3352885	Unique	Used	0.538	1.064	0.849	0.931	1E-05	0	6.77	0	0	17	2	1349.69617	2698.38506	-2.90
lEGSEVQLVEYEASAAGLIR	Unambiguous	1	1	Q99KK7	N-Term(iTRAQ8plex)	CID	1.0000	0.0000	1	1	3196285	Unique	Used	0.661	0.711	0.585	0.813	1E-05	0	6.77	0	0	18	2	1219.65857	2438.30986	0.72
gSQSSGSDSDSHFEQLMVNMLDER	Unambiguous	2	1	B8QI34	N-Term(iTRAQ8plex)	CID	1.0000	0.0000	1	1	2921810	Unique	Used	1.493	1.034	1.398	0.747	1E-05	0	6.77	0	27	200	3	987.44751	2960.32798	-0.84
vETLHDFEAANSDELnLQR	Unambiguous	1	1	Q7TQF7	N-Term(iTRAQ8plex); N16(Deamidated)	CID	1.0000	0.0000	1	1	2671169	Unique	Used	1.133	1.021	1.618	1.115	1E-05	0	6.77	0	30	200	3	836.08295	2506.23429	-0.84
iISLSQLLQEDSLNVADLSSLR	Unambiguous	3	1	P97372	N-Term(iTRAQ8plex)	CID	1.0000	0.0000	1	1	3785909	Unique	Used	0.939	2.444	0.672	1.263	1E-05	0	6.77	0	2	26	2	1359.76050	2718.51372	-2.00
vAIQLNDTHPALSIPELMR	Unambiguous	1	1	Q8CI94	N-Term(iTRAQ8plex)	CID	1.0000	0.0000	1	1	3765082	Unique	Used	0.568	1.212	1.172	1.567	1E-05	0	6.77	0	0	3	3	808.11670	2422.33554	-3.10
aSADLMSYcEEHAR	Unambiguous	1	1	Q9DAS9	N-Term(iTRAQ8plex); C9(Methylthio)	CID	1.0000	0.0000	1	1	3191375	Unique	Used	1.070	0.601	0.978	1.047	1E-05	0	6.77	0	5	4	3	644.95557	1932.85215	-1.79
fIEAGQYNDNLYGTSVQSVR	Unambiguous	10	1	Q91XM9-7	N-Term(iTRAQ8plex)	CID	1.0000	0.0000	1	1	3347064	Unique	Used	1.013	1.208	1.449	1.024	1E-05	0	6.77	0	8	17	2	1283.14551	2565.28374	-1.95
fQMPDQGMTSADDFFQGTk	Unambiguous	1	1	O08553	N-Term(iTRAQ8plex); K19(iTRAQ8plex)	CID	1.0000	0.0000	1	1	3307979	Unique	Used				1.291	1E-05	0	6.77	0	4	200	3	920.44543	2759.32175	-1.76
hNHDLSSYQDTIQQLENELR	Unambiguous	2	1	P08553	N-Term(iTRAQ8plex)	CID	1.0000	0.0000	1	1	3236782	Unique	Used	15.801	2.349	1.307	0.385	1E-05	0	6.77	0	14	17	3	915.45477	2744.34977	-1.61
sADEPMTTFVVcNEcGNR	Unambiguous	3	1	P10711	N-Term(iTRAQ8plex); C12(Methylthio); C15(Methylthio)	CID	1.0000	0.0000	1	1	3283770	Unique	Used	1.982	0.692	1.152	0.784	1E-05	0	6.77	0	4	44	2	1185.00269	2368.99809	-1.08

yTPSGQSGAAASESLFISNHAY	Unambiguous	2	1	A6ZI44	N-Term(iTRAQ8plex)	CID	1.0000	0.0000	1	1	3235374	Unique	Used	1.417	1.186	1.210	1.381	1E-05	0	6.77	0	0	55	2	1281.62415	2562.24101	-0.17
lDGSMETTSEIVDSASHDcPLATQTYGTAAAk	Unambiguous	2	1	Q80W47	N-Term(iTRAQ8plex); C19(Methylthio); K32(iTRAQ8plex)	CID	1.0000	0.0000	1	1	3195805	Unique	Used	1.284	0.875	1.393	1.489	1E-05	0	6.77	0	15	29	3	1308.96045	3924.86679	-2.41
tSGELFAQAPVDQFPGTAVESVTDSSR	Unambiguous	1	1	Q9D1J1	N-Term(iTRAQ8plex)	CID	1.0000	0.0000	1	1	3261760	Unique	Used	1.382	0.649	1.097	0.885	1E-05	0	6.77	0	0	89	2	1550.77258	3100.53789	0.04
lVDGSLLTLDTYLGQFPDlk	Unambiguous	1	1	Q7TQF7	N-Term(iTRAQ8plex); K20(iTRAQ8plex)	CID	1.0000	0.0000	1	1	3645696	Unique	Used	0.666	2.259	1.634	1.075	1E-05	0	6.77	0	8	12	2	1408.79028	2816.57329	-6.16
nVAQYNANHPDFPMQIEQLER	Unambiguous	1	1	Q9JHU4	N-Term(iTRAQ8plex)	CID	1.0000	0.0000	1	1	3257368	Unique	Used	0.863	0.949	1.049	0.943	1E-05	0	6.77	0	9	27	3	940.13293	2818.38425	-1.50
gGYGGGMPANVQMQLVDTk	Unambiguous	1	1	Q8BGH7	N-Term(iTRAQ8plex); K19(iTRAQ8plex)	CID	1.0000	0.0000	1	1	3345382	Unique	Used	0.712	0.780	1.097	0.778	1E-05	0	6.77	0	0	15	2	1266.16064	2531.31401	-2.65
qkEVNENFAIDLIAQQPVNEVEHR	Unambiguous	2	1	P52503	N-Term(iTRAQ8plex); Q1(Deamidated); K2(iTRAQ8plex)	CID	0.0103	0.0000	1	1	3108687	Unique	Used	1.401	1.067	2.043	1.594	1E-05	0	6.77	1	0	200	3	1143.94727	3429.82724	1.42
gILSGTSDLLLTFDEAEVR	Unambiguous	1	1	Q64727	N-Term(iTRAQ8plex)	CID	1.0000	0.0000	1	1	3352675	Unique	Used	0.828	2.039	1.010	1.095	1E-05	0	6.77	0	25	18	2	1170.63013	2340.25298	-3.04
aVEYFQDNSPDSPELNk	Unambiguous	4	1	O35250	N-Term(iTRAQ8plex); K17(iTRAQ8plex)	CID	1.0000	0.0000	1	1	2830953	Unique	Used	0.519	1.615	0.887	0.953	1E-05	0	6.77	0	0	200	2	1281.15430	2561.30132	1.29
sWHNVYcVINNQEMGFYk	Unambiguous	1	1	Q62261	N-Term(iTRAQ8plex); C7(Methylthio); K18(iTRAQ8plex)	CID	1.0000	0.0000	1	1	3764088	Unique	Used	1.019	0.568	1.048	0.948	1E-05	0	6.77	0	21	69	3	962.80084	2886.38797	-3.69
sFESTVGQGSDTYSYIFR	Unambiguous	1	1	P24668	N-Term(iTRAQ8plex)	CID	1.0000	0.0000	1	1	3457413	Unique	Used	0.699	1.037	0.935	0.922	1E-05	0	6.77	0	0	73	2	1174.56677	2348.12627	-3.65
wGEAGAEYVVESTGVFTTmEk	Unambiguous	3	1	P16858	N-Term(iTRAQ8plex); M19(Oxidation); K21(iTRAQ8plex)	CID	1.0000	0.0000	1	1	3218232	Unique	Used			1.263	1.321	1E-05	0	6.76	0	0	200	3	972.48804	2915.44956	-3.37

qASISGPAPTkASGAPPGGQQR	Unambiguous	2	1	O88935	N-Term(iTRAQ8plex); K11(iTRAQ8plex)	CID	1.0000	0.0000	1	1	3631947	Unique	Used	12.415	1.893	7.069	1.796	1E-05	0	6.76	1	0	200	3	891.15564	2671.45237	-7.98
gAVDDDVAEADIISTVEFNHSGELLATGDk	Unambiguous	1	1	Q6P1F6	N-Term(iTRAQ8plex); K30(iTRAQ8plex)	CID	1.0000	0.0000	1	1	2834740	Unique	Used	1.051	1.162	1.577	1.026	1E-05	0	6.76	0	0	118	3	1232.95984	3696.86496	-1.41
qTTVSNSQQAYQEAFEISk	Unambiguous	3	1	Q9CQV8-2	N-Term(iTRAQ8plex); K19(iTRAQ8plex)	CID	1.0000	0.0000	1	1	3106768	Unique	Used		1.198	0.922	0.979	1E-05	0	6.76	0	6	81	2	1384.21960	2767.43193	-1.43
vPRPEDTLTYADLDmVHLSR	Unambiguous	7	1	Q6P6I8	N-Term(iTRAQ8plex); M15(Oxidation)	CID	1.0000	0.0000	1	1	3390478	Unique	Used	1.222	2.090	1.106	1.161	1E-05	0	6.76	0	25	69	3	883.45624	2648.35416	-4.33
dVPGTLLNIALLNLGSSDPSLR	Unambiguous	2	1	Q04690-2	N-Term(iTRAQ8plex)	CID	1.0000	0.0000	1	1	3899136	Unique	Used	0.555	1.128	0.919	1.169	1E-05	0	6.76	0	13	36	2	1285.22437	2569.44145	-3.47
qNLFQEADDFLcTFLPR	Unambiguous	3	1	P97372	N-Term(iTRAQ8plex); C12(Methylthio)	CID	1.0000	0.0000	1	1	3766955	Unique	Used	0.526	1.905	0.685	1.083	1E-05	0	6.76	0	18	98	2	1204.08557	2407.16387	-3.74
tIGGGDDSFNTFFSETGAGk	Unambiguous	4	1	P68373	N-Term(iTRAQ8plex); K20(iTRAQ8plex)	CID	1.0000	0.0000	1	1	3193328	Unique	Used	0.592	1.225	1.272	1.353	1E-05	0	6.76	0	2	17	2	1308.65271	2616.29814	-2.19
tSVTSLLDGLSQAFGEASSQSGGTDR	Unambiguous	2	1	Q6ZWR6-4	N-Term(iTRAQ8plex)	CID	1.0000	0.0000	1	1	3727228	Unique	Used	0.950	1.060	1.083	1.136	1E-05	0	6.76	0	7	19	2	1438.20300	2875.39873	-8.22
aVLASMDnENMHTPDIGGQGTTSQAIQDIIR	Unambiguous	1	1	P70404	N-Term(iTRAQ8plex); N8(Deamidated)	CID	1.0000	0.0000	1	1	3304599	Unique	Used	1.271	1.613	1.213	1.229	1E-05	0	6.76	0	0	103	3	1196.92346	3588.75583	-0.52
lVDQALLTMDTYLGQFPDIk	Unambiguous	3	1	O08539	N-Term(iTRAQ8plex); K20(iTRAQ8plex)	CID	1.0000	0.0000	1	1	3687405	Unique	Used	0.870	1.225	1.254	1.218	1E-05	0	6.76	0	0	56	2	1445.28540	2889.56352	-8.91
gFQQILAGEYDHLPEQAFYMVGPIEEAVAk	Unambiguous	1	1	P56480	N-Term(iTRAQ8plex); K30(iTRAQ8plex)	CID	1.0000	0.0000	1	1	3398526	Unique	Used	1.178	3.041	0.835	1.124	1E-05	0	6.76	0	0	200	3	1320.35144	3959.03977	-2.85
yHTSQSGDEmTSLSEYVSR	Unambiguous	2	1	P11499	N-Term(iTRAQ8plex); M10(Oxidation)	CID	1.0000	0.0000	1	1	2989185	Unique	Used	1.486	1.006	0.826	1.230	1E-05	0	6.76	0	18	18	3	833.05103	2497.13852	-2.79

iAEnNDLLWMDYHQk	Unambiguous	3	1	O08539	N-Term(iTRAQ8plex); N4(Deamidated); K15(iTRAQ8plex)	CID	0.0074	0.0000	1	1	2957620	Unique	Used	1.148	0.945	1.598	1.114	1E-05	0	6.76	0	27	113	3	833.76294	2499.27427	-2.24
lNQPDcGYGAGGASVSVTPAQR	Unambiguous	1	1	Q3TL44	N-Term(iTRAQ8plex); C6(Methylthio)	CID	1.0000	0.0000	1	1	3494511	Unique	Used	0.509	1.068	1.245	1.407	1E-05	0	6.76	0	0	200	2	1249.60364	2498.20000	-2.81
eQSGEYEcSASNDVAAPVVR	Unambiguous	5	1	D3Z396	N-Term(iTRAQ8plex); C8(Methylthio)	CID	1.0000	0.0000	1	1	2669104	Unique	Used	1.130	1.100	0.741	1.072	1E-05	0	6.76	0	0	200	2	1231.06641	2461.12554	-0.90
iEWLESHQDADIEDFk	Unambiguous	1	1	P20029	N-Term(iTRAQ8plex); K16(iTRAQ8plex)	CID	1.0000	0.0000	1	1	2833490	Unique	Used	2.209	0.864	0.839	0.741	1E-05	0	6.76	0	18	200	3	861.77747	2583.31784	-0.36
knGVQAMVEFDSVQSAQR	Unambiguous	5	1	G5E924	N-Term(iTRAQ8plex); K1(iTRAQ8plex); N2(Deamidated)	CID	1.0000	0.0000	1	1	3211322	Unique	Used	1.255	1.254	0.902	0.891	1E-05	0	6.76	1	0	83	3	868.45801	2603.35947	-4.37
aVAGEFSEPVTLETPAFMFR	Unambiguous	2	1	Q7TPM6	N-Term(iTRAQ8plex)	CID	1.0000	0.0000	1	1	3479643	Unique	Used	1.075	0.575	0.860	0.995	1E-05	0	6.76	0	7	13	2	1252.14417	2503.28105	-1.40
icDPGLTSFEPEALGNLVEGmDFHR	Unambiguous	9	1	Q5SVJ0	N-Term(iTRAQ8plex); C2(Methylthio); M21(Oxidation)	CID	1.0000	0.0000	1	1	3459356	Unique	Used	0.485	0.907	1.081	1.066	1E-05	0	6.76	0	11	26	3	1038.49036	3113.45652	-3.85
nIFLqYASTEVDGEHYMTPEDFVQR	Unambiguous	1	1	Q8BH59	N-Term(iTRAQ8plex); Q5(Deamidated)	CID	0.0251	0.0000	1	1	3263958	Unique	Used	1.844	0.858	1.224	1.718	1E-05	0	6.76	0	0	200	3	1098.86206	3294.57163	4.52
lNDHFPLVVWQTGSGTQTNmNVNEVISNR	Unambiguous	2	1	P97807-2	N-Term(iTRAQ8plex); M20(Oxidation)	CID	1.0000	0.0000	1	1	3804540	Unique	Used	3.118		0.794	1.049	1E-05	0	6.76	0	7	11	3	1197.59790	3590.77915	-4.82
aFALDVINSAHER	Unambiguous	4	1	Q91VM9	N-Term(iTRAQ8plex)	CID	1.0000	0.0000	1	1	3454329	Unique	Used	1.235	1.046	1.348	1.928	1E-05	0	6.76	0	25	155	3	582.98340	1746.93564	-4.83
qIETGPFLEAVAHLPPFFDcLGSPVFTPIk	Unambiguous	3	1	Q9JL62	N-Term(iTRAQ8plex); Q1(Deamidated); C20(Methylthio); K30(iTRAQ8plex)	CID	1.0000	0.0000	1	1	3880303	Unique	Used	0.974		0.941	1.068	1E-05	0	6.76	0	24	21	3	1309.36499	3926.08042	1.79

eIcSLFGEAPQNLSQSQR	Unambiguous	3	1	Q4FE56	N-Term(iTRAQ8plex); C3(Methylthio)	CID	1.0000	0.0000	1	1	3726667	Unique	Used	0.908	0.824	0.792	1.129	1E-05	0	6.75	0	15	24	2	1179.07141	2357.13555	-7.48
sNEYQLIDcAQYFLDk	Unambiguous	5	1	Q6R0H7-4	N-Term(iTRAQ8plex); C9(Methylthio); K16(iTRAQ8plex)	CID	1.0000	0.0000	1	1	3374045	Unique	Used	0.359	1.040	0.934	1.289	1E-05	0	6.75	0	11	28	2	1302.64478	2604.28227	-4.29
gFQqILAGEYDHLPEQAFYMVGPIEEAVAk	Unambiguous	1	1	P56480	N-Term(iTRAQ8plex); Q4(Deamidated); K30(iTRAQ8plex)	CID	0.0059	0.0000	1	1	3421447	Unique	Used	1.227		1.126	0.893	1E-05	0	6.75	0	0	200	3	1320.68713	3960.04685	2.97
aDVVMSMATDDFVk	Unambiguous	2	1	Q2TPA8	N-Term(iTRAQ8plex); K14(iTRAQ8plex)	CID	1.0000	0.0000	1	1	3132322	Unique	Used	1.007	0.874	0.735	0.899	1E-05	0	6.75	0	14	200	2	1069.05884	2137.11040	-1.22
eLEGVILELQEQLTGLIPGDHAPLAQGSk	Unambiguous	3	1	O08576-2	N-Term(iTRAQ8plex); K29(iTRAQ8plex)	CID	1.0000	0.0000	1	1	3581794	Unique	Used	0.676	0.994	0.849	0.999	1E-05	0	6.75	0	0	29	3	1222.01270	3664.02353	-5.01
gTALGSATSGSITk	Unambiguous	4	1	F8VQL9	N-Term(iTRAQ8plex); K14(iTRAQ8plex)	CID	1.0000	0.0000	1	1	3385026	Unique	Used	0.793	0.731	0.934	0.636	1E-05	0	6.75	0	0	135	2	930.03461	1859.06194	-4.04
vLGAHILGPGAGEMVNEAALALEYGAScEDIAR	Unambiguous	1	1	O08749	N-Term(iTRAQ8plex); C28(Methylthio)	CID	1.0000	0.0000	1	1	3521531	Unique	Used	1.142			0.805	1E-05	0	6.75	0	8	200	3	1216.60767	3647.80844	-2.48
eVITSDDGSDcGDATELDISHSFSGSLDELSER	Unambiguous	2	1	Q99K47	N-Term(iTRAQ8plex); C11(Methylthio)	CID	1.0000	0.0000	1	1	2639817	Unique	Used	0.898		0.692	1.279	1E-05	0	6.75	0	14	200	3	1279.56665	3836.68540	-1.34
eYEAELEAQLQQIETR	Unambiguous	4	1	Q9CZA6-2	N-Term(iTRAQ8plex)	CID	1.0000	0.0000	1	1	2815924	Unique	Used	3.300	0.672	0.941	0.807	1E-05	0	6.75	0	11	81	2	1127.57739	2254.14751	-1.33
ySSIDTLAEDLTHLLIR	Unambiguous	2	1	Q4QQM5	N-Term(iTRAQ8plex)	CID	1.0000	0.0000	1	1	3645673	Unique	Used	0.533	1.031	1.092	0.957	1E-05	0	6.75	0	18	68	3	755.41663	2264.23532	-3.84
tITNLQQQIQQHQR	Unambiguous	3	1	B1ATC3	N-Term(iTRAQ8plex)	CID	1.0000	0.0000	1	1	3872015	Unique	Used		1.142	0.883	0.805	1E-05	0	6.75	0	0	200	3	680.70972	2040.11460	-5.20
iPQSHIQQIcENILTSGENLSR	Unambiguous	1	1	O89112	N-Term(iTRAQ8plex); C10(Methylthio)	CID	1.0000	0.0000	1	1	3879638	Unique	Used	0.722	1.035	1.198	1.066	1E-05	0	6.75	0	0	13	3	944.14948	2830.43387	-5.48

lLGDASVSFTDNcVVGIQANTER	Unambiguous	3	1	P97807-2	N-Term(iTRAQ8plex); C13(Methylthio)	CID	1.0000	0.0000	1	1	3583433	Unique	Used	3.504	0.839			1E-05	0	6.75	0	0	200	2	1380.18213	2759.35698	-2.78
aGITPSYPQGSSWEHVSNNVR	Unambiguous	2	1	Q80VP0-2	N-Term(iTRAQ8plex)	CID	1.0000	0.0000	1	1	3533026	Unique	Used	1.221	1.646	1.261	1.075	1E-05	0	6.75	0	0	60	3	864.09991	2590.28519	-3.87
kSQIFSTASDNQPTVTIk	Unambiguous	1	1	P20029	N-Term(iTRAQ8plex); K1(iTRAQ8plex); K18(iTRAQ8plex)	CID	1.0000	0.0000	1	1	3493317	Unique	Used	0.768	0.852	0.790	0.539	1E-05	0	6.75	1	5	46	3	959.88361	2877.63626	-3.00
gVIINTASVAAFEGQVGQAAYSASk	Unambiguous	3	1	Q99N15	N-Term(iTRAQ8plex); K25(iTRAQ8plex)	CID	1.0000	0.0000	1	1	3644761	Unique	Used		0.474	1.308	0.674	1E-05	0	6.75	0	0	16	2	1524.32471	3047.64214	-6.60
wASLADSYSDAGLPEDGPGR	Unambiguous	1	1	Q80U49	N-Term(iTRAQ8plex)	CID	1.0000	0.0000	1	1	2996541	Unique	Used	1.340	0.412	1.274	1.048	1E-05	0	6.75	0	0	200	2	1184.56958	2368.13188	-1.72
lTSTSTSDTQNINNSAQR	Unambiguous	1	1	Q8C180	N-Term(iTRAQ8plex)	CID	1.0000	0.0000	1	1	3100830	Unique	Used	1.043	1.163	0.968	0.974	1E-05	0	6.75	0	0	129	2	1121.56128	2242.11528	-2.69
sAMNqVMEELENER	Unambiguous	3	1	Q3USJ8	N-Term(iTRAQ8plex); Q5(Deamidated)	CID	0.0030	0.0000	1	1	2797591	Unique	Used	1.321	1.145	1.027	1.234	1E-05	0	6.75	0	12	200	2	992.97565	1984.94402	9.17
gPPDSDFDGGVYHGR	Unambiguous	2	1	F6SPK0	N-Term(iTRAQ8plex)	CID	1.0000	0.0000	1	1	2631488	Unique	Used	1.539	0.573	0.858	0.999	1E-05	0	6.75	0	9	95	3	627.29919	1879.88303	-2.48
vQAQHDYTATDTDELQLk	Unambiguous	3	1	O08539	N-Term(iTRAQ8plex); K18(iTRAQ8plex)	CID	1.0000	0.0000	1	1	2777159	Unique	Used	0.999	1.198	1.315	1.456	1E-05	0	6.75	0	0	200	3	895.47058	2684.39719	-0.60
anGTAGPTEDHTDDFLGcLNIPIR	Unambiguous	2	1	E9Q350	N-Term(iTRAQ8plex); N2(Deamidated); C18(Methylthio)	CID	1.0000	0.0000	1	1	3239197	Unique	Used	0.475	0.473	0.574	1.184	1E-05	0	6.75	0	0	13	3	960.12512	2878.36081	-1.59
vLNNMEIGTSLYDEEGAk	Unambiguous	1	1	P09411	N-Term(iTRAQ8plex); K18(iTRAQ8plex)	CID	1.0000	0.0000	1	1	2958954	Unique	Used	0.605	1.320	0.818	1.026	1E-05	0	6.75	0	0	87	2	1296.17700	2591.34673	-0.63
mIVMASHMQEQEVGDGTNFVLVFAGALLELAEELLR	Unambiguous	4	1	P42932	N-Term(iTRAQ8plex)	CID	1.0000	0.0000	1	1	3881448	Unique	Used	1.599	1.614	1.486	1.232	1E-05	0	6.75	0	8	178	3	1422.39832	4265.18039	-3.75
gGDFLEGNIITGAQMSQVNSR	Unambiguous	3	1	Q9R0H0	N-Term(iTRAQ8plex)	CID	1.0000	0.0000	1	1	3437378	Unique	Used	1.045	0.837	0.633	0.922	1E-05	0	6.75	0	0	125	2	1249.63025	2498.25322	-3.19
gNDISSGTVLSDYVGSGPPSGTGLHR	Unambiguous	1	1	P70296	N-Term(iTRAQ8plex)	CID	1.0000	0.0000	1	1	3391944	Unique	Used	2.122	1.980	1.309	2.681	1E-05	0	6.75	0	0	138	3	945.47345	2834.40580	-5.82

eVAAFAQFGSDLDAATQQLLSR	Unambiguous	2	1	Q03265	N-Term(iTRAQ8plex)	CID	1.0000	0.0000	1	1	3669887	Unique	Used			1.265	1.737	1E-05	0	6.75	0	14	200	2	1321.68323	2642.35918	-5.17
dQEGQDVLLFIDNIFR	Unambiguous	1	1	P56480	N-Term(iTRAQ8plex)	CID	1.0000	0.0000	1	1	3374490	Unique	Used	1.322	1.498	1.068	1.265	1E-05	0	6.75	0	3	38	2	1113.58484	2226.16240	-3.80
nDVITVQTPAFAESVTEGDVR	Unambiguous	1	1	Q9D2G2	N-Term(iTRAQ8plex); N1(Deamidated)	CID	1.0000	0.0000	1	1	2833826	Unique	Used	1.766	1.235	1.749	1.689	1E-05	0	6.75	0	7	155	2	1277.15076	2553.29424	-1.74
lTGFHETSNINDFSAGVANR	Unambiguous	1	1	P15105	N-Term(iTRAQ8plex)	CID	1.0000	0.0000	1	1	3454040	Unique	Used	1.192	0.945	1.222	1.253	1E-05	0	6.75	0	14	30	3	818.74567	2454.22245	-3.72
fSGWYDADLSPAGHEEAk	Unambiguous	1	1	Q9DBJ1	N-Term(iTRAQ8plex); K18(iTRAQ8plex)	CID	1.0000	0.0000	1	1	2918138	Unique	Used	2.668	1.023	0.875	1.087	1E-05	0	6.75	0	7	70	2	1294.64075	2588.27422	-5.25
lPSPTAAPQQSASQATPVTQGQGR	Unambiguous	3	1	O88935	N-Term(iTRAQ8plex)	CID	1.0000	0.0000	1	1	3678153	Unique	Used	0.805	0.940	1.092	0.767	1E-05	0	6.75	0	0	66	3	894.80267	2682.39347	-6.66
tSDLTSTPSQPNFNTcTQcHNk	Unambiguous	2	1	O88737	N-Term(iTRAQ8plex); C16(Methylthio); C19(Methylthio); K22(iTRAQ8plex)	CID	1.0000	0.0000	1	1	3494079	Unique	Used	0.904	1.553	2.811	1.173	1E-05	0	6.75	0	2	15	3	1042.14819	3124.43003	-3.73
qASEQNWANYSAEQNR	Unambiguous	1	1	P23242	N-Term(iTRAQ8plex)	CID	1.0000	0.0000	1	1	2807076	Unique	Used	1.243	0.721	1.012	1.197	1E-05	0	6.75	0	0	200	2	1100.51563	2200.02397	-3.70
eAMEAQIQNFGQTPSQLLIEPHPPR	Unambiguous	4	1	Q9EPN1-4	N-Term(iTRAQ8plex)	CID	1.0000	0.0000	1	1	3457581	Unique	Used	0.778	1.262	1.388	1.014	1E-05	0	6.75	0	15	90	3	1045.87329	3135.60532	-4.68
sMVTAGFEPVVIENVLEGDELR	Unambiguous	2	1	Q6P6M7	N-Term(iTRAQ8plex)	CID	1.0000	0.0000	1	1	3174022	Unique	Used	1.707	0.886	0.724	1.100	1E-05	0	6.74	0	0	200	2	1354.71057	2708.41387	0.71
tQPTDEEMLFIYSHFk	Unambiguous	4	1	P31786	N-Term(iTRAQ8plex); K16(iTRAQ8plex)	CID	1.0000	0.0000	1	1	3283650	Unique	Used	1.780	0.603	0.909	0.846	1E-05	0	6.74	0	5	28	3	865.45074	2594.33768	-1.72
sSYLNIVGLVGSIDnDFcGTDMTIGTDSALHR	Unambiguous	1	1	P47857	N-Term(iTRAQ8plex); N15(Deamidated); C18(Methylthio)	CID	1.0000	0.0000	1	1	3666601	Unique	Used	1.003	0.260	1.234	1.493	1E-05	0	6.74	0	26	89	3	1241.59155	3722.76010	-1.44
fQMPDQGmTSADDFFQGTk	Unambiguous	1	1	O08553	N-Term(iTRAQ8plex); M8(Oxidation); K19(iTRAQ8plex)	CID	1.0000	0.0000	1	1	3307658	Unique	Used				0.506	1E-05	0	6.74	0	9	200	3	925.77917	2775.32297	0.52
lDEIFQTGLVAYVDLDER	Unambiguous	3	1	Q8VHM5	N-Term(iTRAQ8plex)	CID	1.0000	0.0000	1	1	3327453	Unique	Used	1.355	0.659	0.987	0.907	1E-05	0	6.74	0	18	88	2	1200.63049	2400.25371	-2.65

lQQGYNAIGFSQGGQFLR	Unambiguous	3	1	O88531	N-Term(iTRAQ8plex)	CID	1.0000	0.0000	1	1	3915847	Unique	Used	0.702	2.080	1.243	1.681	1E-05	0	6.74	0	2	9	2	1144.60364	2288.20000	-3.92
lEcQQALSHWLSNQIPR	Unambiguous	3	1	Q69ZH9	N-Term(iTRAQ8plex); C3(Methylthio)	CID	1.0000	0.0000	1	1	3935738	Unique	Used	0.771	0.724	0.982	1.239	1E-05	0	6.74	0	22	43	3	791.73907	2373.20267	-3.49
tIGGGDDSFNTFFSETGAGk	Unambiguous	4	1	P68373	N-Term(iTRAQ8plex); K20(iTRAQ8plex)	CID	1.0000	0.0000	1	1	3151012	Unique	Used	0.539	1.568	1.150	1.432	1E-05	0	6.74	0	0	25	2	1308.65344	2616.29961	-1.63
tFVQENVYDEFVER	Unambiguous	1	1	P47738	N-Term(iTRAQ8plex)	CID	1.0000	0.0000	1	1	2833343	Unique	Used	0.514	1.211	0.967	1.514	1E-05	0	6.74	0	0	4	2	1040.01819	2079.02910	-2.20
gITNLcVIGGDGSLTGADTFR	Unambiguous	1	1	P47857	N-Term(iTRAQ8plex); C6(Methylthio)	CID	1.0000	0.0000	1	1	3727217	Unique	Used	2.044		0.970	1.306	1E-05	0	6.74	0	21	57	2	1209.10083	2417.19438	-6.76
iPNIYAIGDVVAGPMLAHk	Unambiguous	1	1	O08749	N-Term(iTRAQ8plex); K19(iTRAQ8plex)	CID	1.0000	0.0000	1	1	3805776	Unique	Used	1.724	1.387	1.358	0.883	1E-05	0	6.74	0	20	24	3	863.16882	2587.49192	1.09
aDDPNSIFLIDHAWTcR	Unambiguous	1	1	Q3UDE2	N-Term(iTRAQ8plex); C16(Methylthio)	CID	1.0000	0.0000	1	1	3395099	Unique	Used	1.703	1.460	0.892	1.102	1E-05	0	6.74	0	11	154	3	775.37097	2324.09836	-5.26
nLHQSGFSLSGAQIDDNIPR	Unambiguous	1	1	O08553	N-Term(iTRAQ8plex)	CID	1.0000	0.0000	1	1	3411429	Unique	Used	1.668	1.426	1.879	1.994	1E-05	0	6.74	0	0	95	2	1237.13440	2473.26152	-4.95
aSEAGPLNDAGMELSSPk	Unambiguous	2	1	E9QAH1	N-Term(iTRAQ8plex); K18(iTRAQ8plex)	CID	1.0000	0.0000	1	1	2917173	Unique	Used	1.613	0.804	0.940	0.642	1E-05	0	6.74	0	0	200	2	1191.62073	2382.23418	-3.78
sYFSQYGEVVDcVIMk	Unambiguous	4	1	Q9JII5-2	N-Term(iTRAQ8plex); C12(Methylthio); K16(iTRAQ8plex)	CID	1.0000	0.0000	1	1	3439922	Unique	Used	1.777	0.729	0.886	0.624	1E-05	0	6.74	0	0	22	2	1261.62341	2522.23955	-7.71
fEGEPATHTQPGVQLQSNTYDLQESNVGLk	Unambiguous	4	1	Q9R1T4-2	N-Term(iTRAQ8plex); K30(iTRAQ8plex)	CID	1.0000	0.0000	1	1	3127537	Unique	Used	0.606	1.114	1.758	1.487	1E-05	0	6.74	0	0	48	3	1299.33887	3896.00205	2.49
vAIQLnDTHPALSIPELMR	Unambiguous	1	1	Q8CI94	N-Term(iTRAQ8plex); N6(Deamidated)	CID	1.0000	0.0000	1	1	3580761	Unique	Used	0.669	1.186	1.189	1.559	1E-05	0	6.74	0	0	11	3	808.44379	2423.31681	-4.23
aEQLGFAEVTPVTGASLR	Unambiguous	3	1	Q99MU3-5	N-Term(iTRAQ8plex)	CID	1.0000	0.0000	1	1	3415436	Unique	Used	0.599	0.723	0.833	1.095	1E-05	0	6.74	0	11	28	2	1075.58691	2150.16655	-4.38
lGDAVEQGVINNSVLGYFIGR	Unambiguous	1	1	Q9CZD3	N-Term(iTRAQ8plex)	CID	1.0000	0.0000	1	1	3842719	Unique	Used	0.758	0.686	0.778	1.018	1E-05	0	6.74	0	9	52	2	1263.18298	2525.35869	-3.14

sWIEEQEMGSFLSVAk	Unambiguous	2	1	Q9CPY7	N-Term(iTRAQ8plex); K16(iTRAQ8plex)	CID	1.0000	0.0000	1	1	3351655	Unique	Used	1.466	0.975	1.055	1.356	1E-05	0	6.74	0	15	41	2	1225.14380	2449.28032	-3.70
vFGLNTLmEIVTEAGPGSGEGNR	Unambiguous	10	1	A2AGR0	N-Term(iTRAQ8plex); M8(Oxidation)	CID	1.0000	0.0000	1	1	3706715	Unique	Used	0.804	1.056	1.186	1.276	1E-05	0	6.73	0	13	46	2	1334.67163	2668.33599	-7.31
qLAELNkNPVEGFSAGLIDDnDLYR	Unambiguous	1	1	P62254	N-Term(iTRAQ8plex); K7(iTRAQ8plex); N21(Deamidated)	CID	1.0000	0.0000	1	1	3326151	Unique	Used	1.643	1.058	0.818	0.827	1E-05	0	6.73	1	0	200	3	1134.26624	3400.78415	-0.12
vAGPQPAQTGAPQGSLGEYLFER	Unambiguous	2	1	P29391	N-Term(iTRAQ8plex)	CID	1.0000	0.0000	1	1	3393002	Unique	Used	2.152	2.501	1.328	1.817	1E-05	0	6.73	0	0	118	2	1339.18994	2677.37261	-6.06
gnDISSGTVLSDYVGSGPPSGTGLHR	Unambiguous	1	1	P70296	N-Term(iTRAQ8plex); N2(Deamidated)	CID	1.0000	0.0000	1	1	3257271	Unique	Used	2.368	1.183	1.892	1.093	1E-05	0	6.73	0	0	106	2	1418.20728	2835.40727	0.34
sNASLTNNQNLIQSLR	Unambiguous	2	1	Q7M739	N-Term(iTRAQ8plex)	CID	1.0000	0.0000	1	1	3855725	Unique	Used	1.406	0.677	1.308	0.778	1E-05	0	6.73	0	0	23	2	1039.06433	2077.12139	-4.33
vDVYDEGSYTcSVQTQHEPk	Unambiguous	2	1	Q3TYE5	N-Term(iTRAQ8plex); C11(Methylthio); K20(iTRAQ8plex)	CID	1.0000	0.0000	1	1	2955290	Unique	Used	0.727	0.971	0.901	0.862	1E-05	0	6.73	0	6	82	3	980.46991	2939.39518	-2.04
qLPQLTSLELQYVSPk	Unambiguous	1	1	Q9JLN9	N-Term(iTRAQ8plex); K16(iTRAQ8plex)	CID	1.0000	0.0000	1	1	3665512	Unique	Used	0.311	0.681	0.712	0.939	1E-05	0	6.73	0	17	14	2	1226.70776	2452.40825	-7.72
qFNLIPPVcEQAENHFFQR	Unambiguous	2	1	Q8VD73	N-Term(iTRAQ8plex); C9(Methylthio)	CID	1.0000	0.0000	1	1	3824545	Unique	Used	0.845	0.180	0.909	2.254	1E-05	0	6.73	0	25	52	3	889.77155	2667.30008	-4.25
aGYTDQVVIGMDVAASEFYR	Unambiguous	2	1	P17182	N-Term(iTRAQ8plex)	CID	1.0000	0.0000	1	1	3329075	Unique	Used	0.702	1.499	1.353	1.277	1E-05	0	6.73	0	0	200	2	1248.62073	2496.23418	-1.65
gVNIGGAGSYIYEkPQTEAPqVTGPIEVPVVR	Unambiguous	1	1	Q9DCT8	N-Term(iTRAQ8plex); K14(iTRAQ8plex); Q21(Deamidated)	CID	0.0238	0.0000	1	1	3518174	Unique	Used	0.942	1.094	1.223	0.686	1E-05	0	6.73	0	13	94	3	1312.38293	3935.13425	-0.84
mqSTGSAMPASSSFk	Unambiguous	3	1	H7BX26	N-Term(iTRAQ8plex); Q2(Deamidated); K15(iTRAQ8plex)	CID	1.0000	0.0000	1	1	3451703	Unique	Used	0.645	0.306	1.100	0.750	1E-05	0	6.73	0	0	200	2	1063.54285	2126.07842	3.09

tASGVTQLADIYEk	Unambiguous	6	1	O08532-5	N-Term(iTRAQ8plex); K14(iTRAQ8plex)	CID	1.0000	0.0000	1	1	3127964	Unique	Used	0.468	1.991	0.930	1.031	1E-05	0	6.73	0	0	5	2	1052.58923	2104.17119	-1.64
sLYGDLDAALSAEELADAPHSR	Unambiguous	2	1	E1U8D0	N-Term(iTRAQ8plex)	CID	1.0000	0.0000	1	1	2941394	Unique	Used	1.253	1.065	1.150	0.934	1E-05	0	6.73	0	10	200	3	869.10706	2605.30661	0.69
lkPYFLTDGTGTVTPANASGMNDGAAAVVLmk	Unambiguous	2	1	Q8CAY6	N-Term(iTRAQ8plex); K2(iTRAQ8plex); M31(Oxidation); K32(iTRAQ8plex)	CID	1.0000	0.0000	1	1	3664725	Unique	Used	2.414	0.788	0.468	0.578	1E-05	0	6.73	0	0	99	3	1380.40393	4139.19724	-7.56
qQLGTAVEMEIAQmLEENSR	Unambiguous	2	1	Q9JKK7	N-Term(iTRAQ8plex); M14(Oxidation)	CID	1.0000	0.0000	1	1	3305304	Unique	Used	1.486	1.409	1.398	1.139	1E-05	0	6.73	0	0	37	2	1299.14331	2597.27934	-2.31
fVDGLMIHSGDPVNYYVDTAVR	Unambiguous	1	1	P62908	N-Term(iTRAQ8plex)	CID	1.0000	0.0000	1	1	3458932	Unique	Used	0.514	0.575	0.987	0.899	1E-05	0	6.73	0	23	56	3	924.80084	2772.38797	-3.23
nIFLQYASTEVDGEHYMTPEDFVQR	Unambiguous	1	1	Q8BH59	N-Term(iTRAQ8plex); N1(Deamidated)	CID	0.0431	0.0000	1	1	3264111	Unique	Used		1.205	1.763	1.145	1E-05	0	6.73	0	17	200	3	1098.86462	3294.57932	6.85
acNDATLVQITSNMDSVGR	Unambiguous	1	1	P29387	N-Term(iTRAQ8plex); C2(Methylthio)	CID	1.0000	0.0000	1	1	3560395	Unique	Used	0.741	0.655	0.888	0.648	1E-05	0	6.73	0	0	62	2	1173.05652	2345.10576	-6.15
lYVTYFGGDEAAGLEPDLEcR	Unambiguous	1	1	Q8BGQ7	N-Term(iTRAQ8plex); C20(Methylthio)	CID	1.0000	0.0000	1	1	3093794	Unique	Used	0.371	0.505	0.793	0.889	1E-05	0	6.73	0	0	200	2	1334.63049	2668.25371	-1.50
lSEMTEAEQQqLIDDHFLFDkPVSPLLTAAGMAR	Unambiguous	2	1	P30275	N-Term(iTRAQ8plex); Q11(Deamidated); K21(iTRAQ8plex)	CID	0.0208	0.0000	1	1	3418694	Unique	Used	0.457	1.117	1.233	0.997	1E-05	0	6.73	0	0	13	3	1471.09985	4411.28501	1.69
nPVWYqALTHGLNEEQR	Unambiguous	1	1	Q9EPL8	N-Term(iTRAQ8plex); Q6(Deamidated)	CID	1.0000	0.0000	1	1	3516765	Unique	Used	0.781	1.313	0.946	1.153	1E-05	0	6.73	0	2	19	3	787.40576	2360.20273	3.82
lLLEAMDELEVAFNNTNVR	Unambiguous	2	1	Q5SWU9	N-Term(iTRAQ8plex)	CID	1.0000	0.0000	1	1	3542216	Unique	Used	0.926	0.869	0.660	0.955	1E-05	0	6.73	0	0	186	2	1248.15393	2495.30058	-4.50
fTFHLEPQVQAAQVADVPATSqR	Unambiguous	1	1	Q9JIG7	N-Term(iTRAQ8plex); Q22(Deamidated)	CID	1.0000	0.0000	1	1	3598529	Unique	Used	1.384	1.255	1.114	0.905	1E-05	0	6.73	0	20	104	3	949.16620	2845.48404	1.88

iGEHTPSALAImENANVLAR	Unambiguous	3	1	A6ZI44	N-Term(iTRAQ8plex); M12(Oxidation)	CID	1.0000	0.0000	1	1	3701904	Unique	Used	2.718	0.708	1.205	1.213	1E-05	0	6.73	0	9	12	3	809.76526	2427.28122	-6.43
fTFHLEPQVQAAQVADVPATSQR	Unambiguous	1	1	Q9JIG7	N-Term(iTRAQ8plex)	CID	1.0000	0.0000	1	1	3598449	Unique	Used	1.293	0.989	1.557	1.345	1E-05	0	6.73	0	0	13	3	948.83118	2844.47898	-5.52
fFPYYVYNIIGGLDEEGk	Unambiguous	1	1	O09061	N-Term(iTRAQ8plex); K18(iTRAQ8plex)	CID	1.0000	0.0000	1	1	3541675	Unique	Used	1.281	0.700	0.648	0.662	1E-05	0	6.72	0	9	29	2	1366.71558	2732.42388	-7.08
qkPAPLPPASNAPQPGSSQQIQQR	Unambiguous	1	1	Q62415	N-Term(iTRAQ8plex); K2(iTRAQ8plex)	CID	1.0000	0.0000	1	1	3655230	Unique	Used	0.860	0.897	1.277	0.702	1E-05	0	6.72	0	0	93	3	1045.24365	3133.71640	-5.21
hNHDLSSYQDTIQQLENELR	Unambiguous	2	1	P08553	N-Term(iTRAQ8plex)	CID	1.0000	0.0000	1	1	3215206	Unique	Used	1.341	1.072	1.137	1.288	1E-05	0	6.72	0	18	108	3	915.45563	2744.35233	-0.67
aGcEcLNESDEHGFDNcLR	Unambiguous	1	1	Q8CDN6	N-Term(iTRAQ8plex); C3(Methylthio); C5(Methylthio); C17(Methylthio)	CID	1.0000	0.0000	1	1	3015783	Unique	Used	0.724	0.595	0.987	1.214	1E-05	0	6.72	0	10	23	3	852.00146	2553.98984	-0.13
lHISPSNmTNQNTPEYMEk	Unambiguous	1	1	P70288	N-Term(iTRAQ8plex); M8(Oxidation); K19(iTRAQ8plex)	CID	1.0000	0.0000	1	1	3229596	Unique	Used	1.265	1.682	1.080	0.889	1E-05	0	6.72	0	0	78	3	953.47906	2858.42264	-1.65
iSGETIFVTAPHEATAGIIGVNR	Unambiguous	2	1	Q68FD5	N-Term(iTRAQ8plex)	CID	1.0000	0.0000	1	1	3684407	Unique	Used	0.512	1.286	1.455	1.181	1E-05	0	6.72	0	1	4	3	886.48468	2657.43949	-6.41
eAGFPPGVVNIVPGFGPTAGAAIASHEGVDk	Unambiguous	1	1	P47738	N-Term(iTRAQ8plex); K31(iTRAQ8plex)	CID	1.0000	0.0000	1	1	3458869	Unique	Used	0.882	1.715	0.901	1.347	1E-05	0	6.72	0	0	100	3	1190.64294	3569.91428	-1.99
vNDSTMLGASGDYADFQYLk	Unambiguous	1	1	P99026	N-Term(iTRAQ8plex); K20(iTRAQ8plex)	CID	1.0000	0.0000	1	1	3194153	Unique	Used	1.254	0.442	0.484	0.745	1E-05	0	6.72	0	4	7	2	1402.20264	2803.39800	-3.19
aDDPNSIFLIDHAWTcR	Unambiguous	1	1	Q3UDE2	N-Term(iTRAQ8plex); C16(Methylthio)	CID	1.0000	0.0000	1	1	3395031	Unique	Used	1.564	1.337	0.869	0.949	1E-05	0	6.72	0	19	56	3	775.37164	2324.10038	-4.40
lIDDAYGLVVDAVLGPGVR	Unambiguous	1	1	F6W8I0	N-Term(iTRAQ8plex)	CID	1.0000	0.0000	1	1	3786136	Unique	Used	0.209	4.528	6.090	1.011	1E-05	0	6.72	0	10	195	2	1123.63843	2246.26958	-0.13

sFSTADGAIYAMEInVEk	Unambiguous	1	1	Q8BR92	N-Term(iTRAQ8plex); N15(Deamidated); K18(iTRAQ8plex)	CID	1.0000	0.0000	1	1	3238165	Unique	Used	0.783	0.609	0.805	0.735	1E-05	0	6.72	0	0	137	2	1278.16724	2555.32720	4.38
gGYFLVDFYAPTSAVENILEHLAR	Unambiguous	1	1	P58064	N-Term(iTRAQ8plex)	CID	1.0000	0.0000	1	1	3861302	Unique	Used	0.889			1.346	1E-05	0	6.72	0	24	71	3	996.18921	2986.55307	-2.89
yDATMVEINPMVEDSDGk	Unambiguous	1	1	Q9Z2I9	N-Term(iTRAQ8plex); K18(iTRAQ8plex)	CID	1.0000	0.0000	1	1	2654909	Unique	Used	0.368	1.332	1.148	1.774	1E-05	0	6.72	0	0	200	2	1311.64563	2622.28398	-1.84
sLPGGSLQHEGVSGLGSSPFLSEANAER	Unambiguous	2	1	E9QLB8	N-Term(iTRAQ8plex)	CID	1.0000	0.0000	1	1	3369451	Unique	Used	1.241	1.050	0.939	1.191	1E-05	0	6.72	0	0	85	3	1029.85730	3087.55735	-2.47
fVSDDSIcVLGIck	Unambiguous	3	1	Q8C605	N-Term(iTRAQ8plex); C8(Methylthio); C13(Methylthio); K14(iTRAQ8plex)	CID	1.0000	0.0000	1	1	3623427	Unique	Used	1.289	1.122	0.961	1.114	1E-05	0	6.72	0	0	11	2	1100.05554	2199.10381	-4.75
dMAIATGGAVFGEEGLNLNLEDVQAHDLGk	Unambiguous	1	1	P63038	N-Term(iTRAQ8plex); K30(iTRAQ8plex)	CID	1.0000	0.0000	1	1	3264390	Unique	Used	1.553	0.795	1.098	1.051	1E-05	0	6.72	0	9	200	3	1231.64014	3692.90586	0.19
cLAVHPDmVTIATGQVAGTTk	Unambiguous	5	1	E9QK48	N-Term(iTRAQ8plex); C1(Methylthio); M8(Oxidation); K21(iTRAQ8plex)	CID	1.0000	0.0000	1	1	3780895	Unique	Used	0.339	0.585	0.902	0.991	1E-05	0	6.72	0	0	35	3	928.49261	2783.46329	-2.94
fQmPDQGMTSADDFFQGTk	Unambiguous	1	1	O08553	N-Term(iTRAQ8plex); M3(Oxidation); K19(iTRAQ8plex)	CID	0.0312	0.0000	1	1	3303011	Unique	Used	1.569	1.178	1.244	0.979	1E-05	0	6.72	0	20	80	3	925.77734	2775.31748	-1.46
iVEIVDAITTTAQSHQR	Unambiguous	1	1	P47857	N-Term(iTRAQ8plex)	CID	1.0000	0.0000	1	1	3843705	Unique	Used	2.470	1.461	0.688	0.644	1E-05	0	6.72	0	14	200	3	729.40546	2186.20182	-2.97
yFAGTMAEETAPAVLER	Unambiguous	1	1	Q64374	N-Term(iTRAQ8plex)	CID	1.0000	0.0000	1	1	3109632	Unique	Used	1.353	1.199	2.045	3.547	1E-05	0	6.72	0	30	124	2	1080.54919	2160.09111	-1.77
rAPAFLSAEEVQDHLR	Unambiguous	1	1	O54983	N-Term(iTRAQ8plex)	CID	1.0000	0.0000	1	1	3366357	Unique	Used	1.511	1.373	0.983	0.975	1E-05	0	6.72	1	30	83	3	715.05353	2143.14603	-4.76

eLASqPDVDGFLVGGASLkPEFVDIINAk	Unambiguous	2	1	P17751	N-Term(iTRAQ8plex); Q5(Deamidated); K19(iTRAQ8plex); K29(iTRAQ8plex)	CID	1.0000	0.0000	1	1	3396978	Unique	Used	1.148	1.757	1.473	1.204	1E-05	0	6.72	0	5	162	3	1315.06836	3943.19052	1.84
eVAAFAQFGSDLDAATQQLLSR	Unambiguous	2	1	Q03265	N-Term(iTRAQ8plex)	CID	1.0000	0.0000	1	1	3669564	Unique	Used		0.803	0.909	1.465	1E-05	0	6.72	0	4	200	2	1321.68298	2642.35869	-5.36
sQLTEDLGGTLDYcHSR	Unambiguous	12	1	E9PXE2	N-Term(iTRAQ8plex); C14(Methylthio)	CID	1.0000	0.0000	1	1	3302176	Unique	Used	1.047	1.313	1.109	1.202	1E-05	0	6.71	0	29	72	3	749.02081	2245.04789	-2.33
eSTFLDEGPNEQEITPLQHTPR	Unambiguous	3	1	Q9QYR6	N-Term(iTRAQ8plex)	CID	1.0000	0.0000	1	1	2812247	Unique	Used	2.751	1.174	1.666	0.886	1E-05	0	6.71	0	0	105	3	948.14288	2842.41410	-0.72
lTmQVTTLNENLGTVk	Unambiguous	1	1	Q8BL66	N-Term(iTRAQ8plex); M3(Oxidation); K16(iTRAQ8plex)	CID	1.0000	0.0000	1	1	3597790	Unique	Used	1.540	0.819	1.100	0.891	1E-05	0	6.71	0	0	139	2	1193.67139	2386.33550	-4.92
eALLESLGSHTPR	Unambiguous	1	1	Q3TYD6	N-Term(iTRAQ8plex)	CID	1.0000	0.0000	1	1	3455008	Unique	Used	1.678	0.933	1.534	0.852	1E-05	0	6.71	0	0	82	3	571.98346	1713.93583	-4.62
tIIDMSSANGVDYVIMGmPHR	Unambiguous	4	1	Q60597	N-Term(iTRAQ8plex); M18(Oxidation)	CID	1.0000	0.0000	1	1	3704982	Unique	Used				1.061	1E-05	0	6.71	0	0	173	3	876.43048	2627.27689	-6.29
gITNLcVIGGDGSLTGADTFR	Unambiguous	1	1	P47857	N-Term(iTRAQ8plex); C6(Methylthio)	CID	1.0000	0.0000	1	1	3729472	Unique	Used	2.162	0.968	0.748	1.144	1E-05	0	6.71	0	0	200	2	1209.10986	2417.21245	0.71
vAGHPDVVINNAAGnFISPSER	Unambiguous	1	1	Q9CQ62	N-Term(iTRAQ8plex); N15(Deamidated)	CID	1.0000	0.0000	1	1	3278427	Unique	Used	2.216	1.262	0.866	0.498	1E-05	0	6.71	0	18	163	3	857.11206	2569.32163	-3.77
eGGAGGGFGSPMDIFDMFFGGGGR	Unambiguous	4	1	P63037	N-Term(iTRAQ8plex)	CID	1.0000	0.0000	1	1	3843176	Unique	Used	0.769	0.458	0.991	0.954	1E-05	0	6.71	0	19	130	2	1314.09998	2627.19267	-1.34
qLVSSMDVAETNVFFYPR	Unambiguous	1	1	G3X972	N-Term(iTRAQ8plex)	CID	1.0000	0.0000	1	1	3644931	Unique	Used	0.543	0.945	0.882	1.197	1E-05	0	6.71	0	0	16	2	1204.11023	2407.21318	-5.74
lASVEPqGSEFLGTVHSLGR	Unambiguous	2	1	Q9R069	N-Term(iTRAQ8plex); Q7(Deamidated)	CID	1.0000	0.0000	1	1	3642041	Unique	Used	1.990	2.455	1.090	1.697	1E-05	0	6.71	0	0	92	2	1195.13904	2389.27080	1.77
dHFmDEFFEQVEEIR	Unambiguous	1	1	P61264	N-Term(iTRAQ8plex); M4(Oxidation)	CID	1.0000	0.0000	1	1	2814855	Unique	Used	0.725	1.093	1.465	1.408	1E-05	0	6.71	0	0	10	3	764.35870	2291.06156	1.00

lPNLVDMAAQVAAGMAYIER	Unambiguous	3	1	P39688	N-Term(iTRAQ8plex)	CID	1.0000	0.0000	1	1	3899538	Unique	Used	0.320	0.594	0.766	0.867	1E-05	0	6.71	0	4	13	2	1219.14197	2437.27666	-4.90
lSEVLQAVTDHDIPQQLVER	Unambiguous	5	1	E9PXY8	N-Term(iTRAQ8plex)	CID	1.0000	0.0000	1	1	3396044	Unique	Used	1.021	1.692	1.209	1.196	1E-05	0	6.71	0	6	3	3	865.47137	2594.39957	-3.72
aALETDENLLLcAPTGAGk	Unambiguous	1	1	Q6P4T2	N-Term(iTRAQ8plex); C12(Methylthio); K19(iTRAQ8plex)	CID	1.0000	0.0000	1	1	3216365	Unique	Used	0.651	0.733	0.739	0.821	1E-05	0	6.71	0	17	200	2	1271.17920	2541.35112	-0.09
mQTAGVTGHQHGSILSTLR	Unambiguous	1	1	Q8R0Y8	N-Term(iTRAQ8plex)	CID	1.0000	0.0000	1	1	3909585	Unique	Used	1.008	1.295	1.395	1.527	1E-05	0	6.71	0	0	200	3	766.74463	2298.21933	-4.23
aEAGGGWEGSASYk	Unambiguous	2	1	P97765	N-Term(iTRAQ8plex); K14(iTRAQ8plex)	CID	1.0000	0.0000	1	1	2826355	Unique	Used	1.977	1.412	1.345	1.218	1E-05	0	6.71	0	0	200	2	989.50891	1978.01055	-1.07
iLLLcVGEAGDTVQFAEYIQk	Unambiguous	1	1	Q9R1P3	N-Term(iTRAQ8plex); C5(Methylthio); K21(iTRAQ8plex)	CID	1.0000	0.0000	1	1	3666758	Unique	Used	1.587	0.779	0.718	0.724	1E-05	0	6.71	0	0	23	3	988.86633	2964.58445	-6.44
tYFPHFDVSHGSAQVk	Unambiguous	2	1	Q91VB8	N-Term(iTRAQ8plex); K16(iTRAQ8plex)	CID	1.0000	0.0000	1	1	3485500	Unique	Used	1.969	2.543	0.313	1.195	1E-05	0	6.71	0	0	200	3	810.09955	2428.28409	-1.19
gTGGVDTAATGSVFDISNLDR	Unambiguous	1	1	P30275	N-Term(iTRAQ8plex)	CID	1.0000	0.0000	1	1	3260551	Unique	Used	1.080	1.121	1.101	1.376	1E-05	0	6.71	0	0	90	2	1179.09521	2357.18315	-2.36
kEQAQMLQTMQEQEQAAQGLR	Unambiguous	1	1	E9Q7G0	N-Term(iTRAQ8plex); K1(iTRAQ8plex)	CID	1.0000	0.0000	1	1	3189342	Unique	Used	0.941	0.825	1.111	1.051	1E-05	0	6.71	1	25	63	3	1018.86688	3054.58609	-1.99
sFESTVGQGSDTYSYIFR	Unambiguous	1	1	P24668	N-Term(iTRAQ8plex)	CID	1.0000	0.0000	1	1	3477520	Unique	Used	0.772	1.457	0.911	1.000	1E-05	0	6.71	0	3	22	2	1174.56689	2348.12651	-3.55
aLVTGYFMQVAHLER	Unambiguous	1	1	O35286	N-Term(iTRAQ8plex)	CID	1.0000	0.0000	1	1	3898105	Unique	Used				0.889	1E-05	0	6.71	0	22	22	3	680.37067	2039.09745	-3.71
eVAAFAQFGSDLDAATQQLLSR	Unambiguous	2	1	Q03265	N-Term(iTRAQ8plex)	CID	1.0000	0.0000	1	1	3668057	Unique	Used	0.917	1.427	1.104	1.363	1E-05	0	6.71	0	14	200	2	1321.68115	2642.35503	-6.74
qIGVEHVVVYVNk	Unambiguous	3	1	Q8BFR5	N-Term(iTRAQ8plex); K13(iTRAQ8plex)	CID	1.0000	0.0000	1	1	3493232	Unique	Used	0.965	1.136	1.422	1.068	1E-05	0	6.71	0	4	53	2	1046.61914	2092.23100	-3.11

ekEGESSGSQEDQLcTALVNQLNR	Unambiguous	4	1	A2AN08-3	N-Term(iTRAQ8plex); K2(iTRAQ8plex); C15(Methylthio)	CID	1.0000	0.0000	1	1	3152216	Unique	Used	0.715	0.682	0.989	1.299	1E-05	0	6.71	1	0	200	3	1097.21130	3289.61936	-1.71
vFTFSVGQHNYDR	Unambiguous	6	1	O08532-5	N-Term(iTRAQ8plex)	CID	1.0000	0.0000	1	1	3720657	Unique	Used	1.335	1.975	1.029	1.046	1E-05	0	6.71	0	10	10	3	625.31720	1873.93705	-6.84
iYPEEMIQTGISAIDGMnSIAR	Unambiguous	1	1	P62814	N-Term(iTRAQ8plex); N18(Deamidated)	CID	1.0000	0.0000	1	1	3396318	Unique	Used	1.023	1.041	1.553	1.155	1E-05	0	6.71	0	8	9	2	1357.68103	2714.35478	-5.00
aITVASQTNcPLYVTk	Unambiguous	5	1	E9PWE8	N-Term(iTRAQ8plex); C10(Methylthio); K16(iTRAQ8plex)	CID	1.0000	0.0000	1	1	3823039	Unique	Used	0.711	0.455	0.473	0.572	1E-05	0	6.71	0	0	200	2	1182.14319	2363.27910	-5.60
fTLNPNTTGVQNPHIER	Unambiguous	2	1	P47809	N-Term(iTRAQ8plex)	CID	1.0000	0.0000	1	1	3615009	Unique	Used	1.372	1.054	1.172	1.201	1E-05	0	6.71	0	0	75	3	748.06329	2242.17533	-5.76
nASLASSNNDLQVAEEQYQR	Unambiguous	3	1	E9QP99	N-Term(iTRAQ8plex)	CID	1.0000	0.0000	1	1	2777665	Unique	Used	1.288	0.875	1.413	0.956	1E-05	0	6.71	0	0	200	2	1271.12830	2541.24932	0.39
aLAESWNAAFLESSAk	Unambiguous	1	1	Q921J2	N-Term(iTRAQ8plex); K16(iTRAQ8plex)	CID	1.0000	0.0000	1	1	3394202	Unique	Used	0.323	1.029	0.780	1.003	1E-05	0	6.71	0	6	26	2	1152.12341	2303.23955	-4.20
sTNGDTFLGGEDFDQALLR	Unambiguous	1	1	P38647	N-Term(iTRAQ8plex)	CID	1.0000	0.0000	1	1	2978171	Unique	Used	1.107	0.775	1.073	1.390	1E-05	0	6.71	0	0	200	2	1180.58582	2360.16435	-1.23
eFTDNINSAFEqGDFEk	Unambiguous	1	1	Q8K3A0	N-Term(iTRAQ8plex); Q12(Deamidated); K17(iTRAQ8plex)	CID	0.0119	0.0000	1	1	2851099	Unique	Used	1.056	1.389	3.510	2.181	1E-05	0	6.70	0	0	200	2	1300.64001	2600.27275	4.40
lGTQPDSASSTYQENYEQMk	Unambiguous	1	1	Q3ULD5	N-Term(iTRAQ8plex); K20(iTRAQ8plex)	CID	1.0000	0.0000	1	1	2828838	Unique	Used		1.533	0.757	1.529	1E-05	0	6.70	0	0	200	2	1443.20667	2885.40605	-0.80
kYPYVEVNSILGIDSAYDQNR	Unambiguous	1	1	Q6P5E4	N-Term(iTRAQ8plex); K1(iTRAQ8plex)	CID	1.0000	0.0000	1	1	3416289	Unique	Used	0.484	1.011	0.981	0.914	1E-05	0	6.70	1	9	42	3	1018.21027	3052.61625	-1.24
aVGFVSEDEYLEIQGITR	Unambiguous	3	1	D3Z396	N-Term(iTRAQ8plex)	CID	1.0000	0.0000	1	1	3132583	Unique	Used	0.971	1.340	1.238	0.945	1E-05	0	6.70	0	18	85	2	1165.61304	2330.21880	0.25
fHQLLDDESDPFDILR	Unambiguous	2	1	Q9JKS5	N-Term(iTRAQ8plex)	CID	1.0000	0.0000	1	1	3305195	Unique	Used	1.343	0.209	1.549	1.057	1E-05	0	6.70	0	13	8	3	755.38617	2264.14396	-2.73

sSLQQDGTVEQSVSSGLVnGR	Unambiguous	1	1	Q8BGT6	N-Term(iTRAQ8plex); N19(Deamidated)	CID	1.0000	0.0000	1	1	2916688	Unique	Used	3.325	0.288	0.894	0.571	1E-05	0	6.70	0	0	200	2	1227.12146	2453.23564	-2.67
tHnSALEYNIFEGMEcR	Unambiguous	1	1	O08553	N-Term(iTRAQ8plex); N3(Deamidated); C16(Methylthio)	CID	1.0000	0.0000	1	1	3151477	Unique	Used	0.736	1.157	0.951	0.946	1E-05	0	6.70	0	11	35	3	789.02240	2365.05265	-1.63
iVEIVDAITTTAQSHQR	Unambiguous	1	1	P47857	N-Term(iTRAQ8plex)	CID	1.0000	0.0000	1	1	3842468	Unique	Used	2.353	1.251	0.895	1.318	1E-05	0	6.70	0	0	3	3	729.40472	2186.19962	-3.97
gFQQILAGEYDHLPEqAFYMVGPIEEAVAk	Unambiguous	1	1	P56480	N-Term(iTRAQ8plex); Q16(Deamidated); K30(iTRAQ8plex)	CID	1.0000	0.0000	1	1	3398499	Unique	Used	1.313	0.656	1.141	1.449	1E-05	0	6.70	0	0	200	3	1320.68616	3960.04392	2.23
lkPFFEGMSqSSSQTEIGSLNSk	Unambiguous	1	1	Q8K212	N-Term(iTRAQ8plex); K2(iTRAQ8plex); Q10(Deamidated); K23(iTRAQ8plex)	CID	0.0418	0.0000	1	1	3578348	Unique	Used	0.724	1.022	1.145	0.996	1E-05	0	6.70	0	0	86	3	1139.28088	3415.82810	2.87
nQNEIIFGLNDGYYGAPcEHk	Unambiguous	1	1	Q8C0K8	N-Term(iTRAQ8plex); C18(Methylthio); K21(iTRAQ8plex)	CID	1.0000	0.0000	1	1	3236204	Unique	Used	0.787	1.017	1.269	1.565	1E-05	0	6.70	0	0	92	3	1012.83221	3036.48209	0.54
lTVPDDMAANcIYLNIPSk	Unambiguous	2	1	Q9CWS0	N-Term(iTRAQ8plex); C11(Methylthio); K19(iTRAQ8plex)	CID	1.0000	0.0000	1	1	3666024	Unique	Used	0.779	0.980	1.254	1.370	1E-05	0	6.70	0	21	12	2	1366.71179	2732.41631	-4.36
acADATLSQITNnIDPVGR	Unambiguous	3	1	P62874	N-Term(iTRAQ8plex); C2(Methylthio); N13(Deamidated)	CID	0.0030	0.0000	1	1	3603669	Unique	Used	0.600	1.270	1.144	1.112	1E-05	0	6.70	0	15	200	2	1155.57666	2310.14604	3.82
fPLYNLGFGNNLnYNFLETLALENHGLAR	Unambiguous	2	1	Q61704	N-Term(iTRAQ8plex); N13(Deamidated)	CID	0.0328	0.0000	1	1	3917908	Unique	Used	1.150	1.292	0.679	1.258	1E-05	0	6.70	0	11	31	3	1210.62463	3629.85935	-2.80

vTLDPELEEALASASDTELYDLAAVLGVHNLLNNPk	Unambiguous	2	1	Q9JKK7	N-Term(iTRAQ8plex); K36(iTRAQ8plex)	CID	1.0000	0.0000	1	1	3520846	Unique	Used			0.671	0.551	1E-05	0	6.70	0	4	10	3	1481.79199	4443.36142	-3.20
tIGGGDDSFNTFFSETGAGk	Unambiguous	4	1	P68373	N-Term(iTRAQ8plex); K20(iTRAQ8plex)	CID	1.0000	0.0000	1	1	3155437	Unique	Used		1.577	1.528	1.619	1E-05	0	6.70	0	0	200	2	1308.65698	2616.30669	1.08
eVLQQQHSVqVDQLR	Unambiguous	8	1	O88522	N-Term(iTRAQ8plex); Q10(Deamidated)	CID	0.0104	0.0000	1	1	3251533	Unique	Used	1.592	1.161	1.502	0.869	1E-05	0	6.70	0	30	200	2	1056.57825	2112.14922	6.67
qLNTIQnAEAVHFFcNYEESR	Unambiguous	4	1	P61922	N-Term(iTRAQ8plex); N7(Deamidated); C15(Methylthio)	CID	0.0104	0.0000	1	1	3283817	Unique	Used	1.142	0.540	1.307	1.375	1E-05	0	6.70	0	23	69	3	955.44690	2864.32614	-0.85
tAVTAFSSTEPGTSEqGFk	Unambiguous	3	1	F7AYW2	N-Term(iTRAQ8plex); Q16(Deamidated); K19(iTRAQ8plex)	CID	1.0000	0.0000	1	1	3105989	Unique	Used	0.963	0.429	1.420	0.866	1E-05	0	6.70	0	22	200	2	1277.66882	2554.33037	6.66
qSILELAQQWGEFk	Unambiguous	3	1	Q8CBC8	N-Term(iTRAQ8plex); K14(iTRAQ8plex)	CID	1.0000	0.0000	1	1	3623195	Unique	Used	1.762	0.625	0.630	1.001	1E-05	0	6.70	0	18	17	2	1143.13440	2285.26152	-5.92
vAGHPDVVINNAAGNFISPSER	Unambiguous	1	1	Q9CQ62	N-Term(iTRAQ8plex)	CID	1.0000	0.0000	1	1	3535166	Unique	Used	1.050	1.390	0.890	0.992	1E-05	0	6.70	0	0	24	3	856.78290	2568.33414	-5.12
dDGLADLLFVSSGPTNASAFTER	Unambiguous	4	1	P27546-2	N-Term(iTRAQ8plex)	CID	1.0000	0.0000	1	1	3326954	Unique	Used	2.569	0.413	0.831	0.835	1E-05	0	6.70	0	0	33	2	1344.17029	2687.33330	-4.98
aFSSSVVHVPGVNDIQSSSSTGQNISQISR	Unambiguous	2	1	Q61324-2	N-Term(iTRAQ8plex)	CID	1.0000	0.0000	1	1	3781731	Unique	Used	0.539	0.244	1.743	1.766	1E-05	0	6.70	0	11	200	3	1131.58154	3392.73008	-1.41
gFGFITFTNPEHASDAMR	Unambiguous	2	1	O89086	N-Term(iTRAQ8plex)	CID	1.0000	0.0000	1	1	3620309	Unique	Used	2.255	0.773	1.382	0.733	1E-05	0	6.70	0	19	152	2	1151.55811	2302.10893	-6.06
cAWGQQPDLAANEAQLLR	Unambiguous	3	1	Q9WVJ2	N-Term(iTRAQ8plex); C1(Methylthio)	CID	1.0000	0.0000	1	1	3499133	Unique	Used	0.674	0.730	0.808	0.884	1E-05	0	6.70	0	8	69	2	1167.58044	2334.15361	-4.32
eVAAFAQFGSDLDAATQQLLSR	Unambiguous	2	1	Q03265	N-Term(iTRAQ8plex)	CID	1.0000	0.0000	1	1	3668169	Unique	Used	0.612	0.988	1.153	1.510	1E-05	0	6.69	0	12	200	2	1321.68335	2642.35942	-5.08
nGVQAMVEFDSVQSAQR	Unambiguous	5	1	G5E924	N-Term(iTRAQ8plex)	CID	1.0000	0.0000	1	1	3256538	Unique	Used	0.688	0.710	0.963	0.971	1E-05	0	6.69	0	0	73	2	1085.54358	2170.07988	-3.05

dMAIATGGAVFGEEGLNLNLEDVQAHDLGk	Unambiguous	1	1	P63038	N-Term(iTRAQ8plex); K30(iTRAQ8plex)	CID	1.0000	0.0000	1	1	3265470	Unique	Used		0.344	0.838	0.794	1E-05	0	6.69	0	0	200	3	1231.63708	3692.89670	-2.29
cPTDELSLSNcAVVNEk	Unambiguous	2	1	P46460	N-Term(iTRAQ8plex); C1(Methylthio); C11(Methylthio); K17(iTRAQ8plex)	CID	1.0000	0.0000	1	1	3283351	Unique	Used	0.291	0.890	1.108	1.205	1E-05	0	6.69	0	0	19	2	1261.61108	2522.21489	-2.81
nLFAFFDMAYQGFASGDGDk	Unambiguous	1	1	P05202	N-Term(iTRAQ8plex); K20(iTRAQ8plex)	CID	1.0000	0.0000	1	1	3645736	Unique	Used	1.545	1.565	0.888	1.032	1E-05	0	6.69	0	0	89	2	1405.18311	2809.35893	-5.81
qSDPLNDDVLLAMEDMGLDk	Unambiguous	6	1	E9PU87	N-Term(iTRAQ8plex); K20(iTRAQ8plex)	CID	1.0000	0.0000	1	1	2816408	Unique	Used			0.662	0.718	1E-05	0	6.69	0	29	200	3	943.14655	2827.42508	-2.26
aGYTDQVVIGMDVAASEFYR	Unambiguous	2	1	P17182	N-Term(iTRAQ8plex)	CID	1.0000	0.0000	1	1	3329909	Unique	Used	0.602	0.575	1.036	1.313	1E-05	0	6.69	0	5	200	2	1248.62134	2496.23540	-1.16
sSVSQSPAPTYPSDSHYTSLEQnVPR	Unambiguous	2	1	O88737	N-Term(iTRAQ8plex); N23(Deamidated)	CID	0.0179	0.0000	1	1	3297373	Unique	Used	2.897	1.607	2.416	0.918	1E-05	0	6.69	0	0	149	3	1047.18066	3139.52744	4.85
lEEIAELVASSLPSPLR	Unambiguous	2	1	Q6P2K6	N-Term(iTRAQ8plex)	CID	1.0000	0.0000	1	1	3825492	Unique	Used	0.556	0.843	0.759	0.916	1E-05	0	6.69	0	3	200	2	1064.60815	2128.20903	-3.63
nADMSEDmQQDAVDcATQAMEk	Unambiguous	2	1	Q9D0M5	N-Term(iTRAQ8plex); M8(Oxidation); C15(Methylthio); K22(iTRAQ8plex)	CID	1.0000	0.0000	1	1	2813177	Unique	Used	1.216	1.514	0.836		1E-05	0	6.69	0	2	63	3	1034.45068	3101.33750	-2.46
tTSSANNPNLMYQDEcDR	Unambiguous	2	1	Q501J6	N-Term(iTRAQ8plex); C16(Methylthio)	CID	1.0000	0.0000	1	1	2757140	Unique	Used	0.492	0.360	0.637	0.611	1E-05	0	6.69	0	0	200	2	1205.02222	2409.03716	-2.14
kNAPcILFIDEIDAVGR	Unambiguous	1	1	Q8JZQ2	N-Term(iTRAQ8plex); K1(iTRAQ8plex); C5(Methylthio)	CID	1.0000	0.0000	1	1	3479708	Unique	Used	0.435	0.792	1.182	1.654	1E-05	0	6.69	1	13	36	3	843.46539	2528.38163	-0.38

aITVASQTNcPLYVTk	Unambiguous	5	1	E9PWE8	N-Term(iTRAQ8plex); C10(Methylthio); K16(iTRAQ8plex)	CID	1.0000	0.0000	1	1	3822651	Unique	Used	0.434	0.201	0.264	0.222	1E-05	0	6.69	0	0	1	3	788.43329	2363.28531	-2.97
sLEAEILQLQEELASSER	Unambiguous	3	1	Q5SV64	N-Term(iTRAQ8plex)	CID	1.0000	0.0000	1	1	3018074	Unique	Used	1.796	1.049	1.732	0.833	1E-05	0	6.69	0	16	164	2	1175.12329	2349.23931	-2.49
aTQWGSFYSPAQTANNPcSR	Unambiguous	3	1	Q68FD9	N-Term(iTRAQ8plex); C18(Methylthio)	CID	1.0000	0.0000	1	1	3915027	Unique	Used	0.369	1.113	1.428	0.910	1E-05	0	6.69	0	0	200	2	1268.58179	2536.15630	-3.48
aGYTDQVVIGMDVAASEFYR	Unambiguous	2	1	P17182	N-Term(iTRAQ8plex)	CID	1.0000	0.0000	1	1	3329596	Unique	Used		1.517	1.294	1.263	1E-05	0	6.69	0	5	200	2	1248.61951	2496.23174	-2.63
gLGPSLTEDQPGPYLAPGLLGSNIHQQGR	Unambiguous	4	1	D3YZR8	N-Term(iTRAQ8plex)	CID	1.0000	0.0000	1	1	3642777	Unique	Used	1.168	0.734	0.923	0.870	1E-05	0	6.69	0	4	9	3	1092.90930	3276.71335	-4.46
fVQLINTVLnGDQHFMSALDk	Unambiguous	3	1	Q9D4H8	N-Term(iTRAQ8plex); N10(Deamidated); K21(iTRAQ8plex)	CID	1.0000	0.0000	1	1	3687038	Unique	Used	0.697	0.912	1.014	1.513	1E-05	0	6.69	0	9	86	3	1000.53607	2999.59366	-6.16
sENGLEFTSSGSANTETTk	Unambiguous	3	1	Q60932-2	N-Term(iTRAQ8plex); K19(iTRAQ8plex)	CID	1.0000	0.0000	1	1	2698506	Unique	Used	0.477	0.775	1.313	1.355	1E-05	0	6.69	0	0	183	2	1284.64465	2568.28203	-2.55
eLHDMFVDMAMLVESQGEmIDR	Unambiguous	1	1	P61264	N-Term(iTRAQ8plex); M19(Oxidation)	CID	1.0000	0.0000	1	1	3305760	Unique	Used			0.874	0.712	1E-05	0	6.69	0	12	88	3	972.78644	2916.34476	-3.67
fAPPNMQAAEAVSHIqSSGPR	Unambiguous	1	1	Q9JME9	N-Term(iTRAQ8plex); Q16(Deamidated)	CID	0.0060	0.0000	1	1	3741000	Unique	Used	1.405	1.555	1.375	1.559	1E-05	0	6.69	0	0	200	3	834.09283	2500.26395	3.30
aVVHTHLLNPEWLVNYFGSLSVEDSLEcLR	Unambiguous	2	1	Q68FD5	N-Term(iTRAQ8plex); C28(Methylthio)	CID	1.0000	0.0000	1	1	3860982	Unique	Used			0.876	1.417	1E-05	0	6.69	0	19	16	3	1264.31030	3790.91635	-2.00
aGYTDQVVIGMDVAASEFYR	Unambiguous	2	1	P17182	N-Term(iTRAQ8plex)	CID	1.0000	0.0000	1	1	3354281	Unique	Used	0.990	1.227	1.203	1.302	1E-05	0	6.69	0	11	200	2	1248.61426	2496.22124	-6.84
gTGGVDTAAVGAVFDISNADR	Unambiguous	1	1	P07310	N-Term(iTRAQ8plex)	CID	1.0000	0.0000	1	1	3282658	Unique	Used	6.126	7.842	0.713	0.827	1E-05	0	6.69	0	13	200	2	1149.09277	2297.17827	4.64
lAEDAPNFDGPAAEGQPGQk	Unambiguous	5	1	G3XA17	N-Term(iTRAQ8plex); K20(iTRAQ8plex)	CID	1.0000	0.0000	1	1	2684873	Unique	Used		2.708			1E-05	0	6.69	0	0	200	3	874.11981	2620.34488	-0.58

eNLPqDFTNEGLGVSTDNAHVGGMLR	Unambiguous	3	1	D3YTZ9	N-Term(iTRAQ8plex); Q5(Deamidated)	CID	0.0015	0.0000	1	1	3258532	Unique	Used	1.955	1.476	1.847	1.923	1E-05	0	6.69	0	12	86	3	1026.17371	3076.50656	3.81
yVQQVqVScLDELEIcIHPDGVIPTLTFLR	Unambiguous	1	1	Q9DCT2	N-Term(iTRAQ8plex); Q6(Deamidated); C9(Methylthio); C16(Methylthio)	CID	0.0463	0.0000	1	1	3843141	Unique	Used	2.149	1.096	2.827	2.437	1E-05	0	6.69	0	17	200	3	1275.97803	3825.91953	-1.23
tYFPHFDVSHGSAQVk	Unambiguous	2	1	Q91VB8	N-Term(iTRAQ8plex); K16(iTRAQ8plex)	CID	1.0000	0.0000	1	1	3341400	Unique	Used	5.047	1.974	0.617	1.664	1E-05	0	6.69	0	6	17	3	810.09344	2428.26578	-8.73
wGEAGAEYVVESTGVFTTmEk	Unambiguous	3	1	P16858	N-Term(iTRAQ8plex); M19(Oxidation); K21(iTRAQ8plex)	CID	1.0000	0.0000	1	1	3240950	Unique	Used	1.931	2.167			1E-05	0	6.69	0	0	200	3	972.49194	2915.46128	0.65
mVVNEGADGGQSVYHIHLHVLGGR	Unambiguous	1	1	P70349	N-Term(iTRAQ8plex)	CID	1.0000	0.0000	1	1	3680393	Unique	Used	2.178	1.321	1.384	1.276	1E-05	0	6.69	0	0	92	3	950.49054	2849.45707	-7.46
sNTSPEELGPLANQLTSDYGR	Unambiguous	1	1	P26039	N-Term(iTRAQ8plex)	CID	1.0000	0.0000	1	1	3192483	Unique	Used	1.368	1.396	1.116	1.480	1E-05	0	6.69	0	19	130	2	1277.13965	2553.27202	-0.58
nNDGNLVIDSLLQYINQR	Unambiguous	2	1	Q07646-2	N-Term(iTRAQ8plex)	CID	1.0000	0.0000	1	1	3806527	Unique	Used	0.834	0.688	0.401	0.722	1E-05	0	6.68	0	0	42	2	1197.13538	2393.26348	-3.85
vYHPDYNNELTQFLPR	Unambiguous	1	1	Q9CZG9	N-Term(iTRAQ8plex)	CID	1.0000	0.0000	1	1	3558324	Unique	Used	1.222	1.172	1.123	0.927	1E-05	0	6.68	0	22	32	3	770.72852	2310.17099	-4.79
tQPTDEEMLFIYSHFk	Unambiguous	4	1	P31786	N-Term(iTRAQ8plex); K16(iTRAQ8plex)	CID	1.0000	0.0000	1	1	3304330	Unique	Used	2.245	0.904	1.059	0.964	1E-05	0	6.68	0	14	54	3	865.44977	2594.33475	-2.84
lTLYDIAHTPGVAADLSHIETR	Unambiguous	1	1	P08249	N-Term(iTRAQ8plex)	CID	1.0000	0.0000	1	1	3460584	Unique	Used	2.238	0.510	0.760	0.957	1E-05	0	6.68	0	20	200	3	899.81958	2697.44419	-2.67
mTSHPVHQYYLTGAQDGSVR	Unambiguous	4	1	Q8BPN8-2	N-Term(iTRAQ8plex); M1(Oxidation)	CID	1.0000	0.0000	1	1	3528789	Unique	Used	0.974	1.339	1.201	1.046	1E-05	0	6.68	0	0	151	3	856.42023	2567.24613	-5.97
eQSGEYEcSASNDVAAPVVR	Unambiguous	5	1	D3Z396	N-Term(iTRAQ8plex); C8(Methylthio)	CID	1.0000	0.0000	1	1	2668501	Unique	Used	0.975	0.984	1.260	0.900	1E-05	0	6.68	0	26	200	2	1231.06555	2461.12383	-1.60

tETGQELEcLQDHLDDLAVEcR	Unambiguous	3	1	Q61543	N-Term(iTRAQ8plex); C9(Methylthio); C21(Methylthio)	CID	1.0000	0.0000	1	1	2904979	Unique	Used	1.719	0.825	0.740	0.599	1E-05	0	6.68	0	12	200	3	971.77106	2913.29862	-1.89
eTLVQDNIQWPTGLAVDYHNER	Unambiguous	1	1	Q91ZX7	N-Term(iTRAQ8plex)	CID	1.0000	0.0000	1	1	3303204	Unique	Used	0.725	2.699	0.859	0.639	1E-05	0	6.68	0	30	34	3	968.15790	2902.45914	-1.59
vSVVEPGNFIAATSLYSPER	Unambiguous	1	1	Q80XN0	N-Term(iTRAQ8plex)	CID	1.0000	0.0000	1	1	3665664	Unique	Used	0.511	0.459	0.649	0.769	1E-05	0	6.68	0	0	14	2	1220.65039	2440.29350	-3.73
qSTSEPASAPFVPFSGGGqR	Unambiguous	4	1	Q8VBT9	N-Term(iTRAQ8plex); Q19(Deamidated)	CID	1.0000	0.0000	1	1	3514498	Unique	Used	1.935	1.202	0.830	0.891	1E-05	0	6.68	0	0	200	2	1156.58167	2312.15605	4.30
qLFALScAAEEQGmLPEDLSGVIR	Unambiguous	1	1	P08752	N-Term(iTRAQ8plex); C7(Methylthio); M14(Oxidation)	CID	1.0000	0.0000	1	1	3520287	Unique	Used	0.881	1.377	0.771	1.174	1E-05	0	6.68	0	0	53	2	1472.22961	2943.45195	-1.67
IVYQHDDSETIEDDIPFVATR	Unambiguous	2	1	Q8VHY0	N-Term(iTRAQ8plex)	CID	1.0000	0.0000	1	1	2781280	Unique	Used	1.016	0.812	1.661	1.251	1E-05	0	6.68	0	4	200	3	923.12958	2767.37418	0.47
dENPFASLTATSqPIATAAR	Unambiguous	3	1	Q9ES00	N-Term(iTRAQ8plex); Q13(Deamidated)	CID	1.0000	0.0000	1	1	3415732	Unique	Used	0.946	1.071	1.028	0.971	1E-05	0	6.68	0	0	173	2	1183.61438	2366.22148	3.08
aSMMYLENGTPDTAAMALER	Unambiguous	2	1	Q9CWZ7	N-Term(iTRAQ8plex)	CID	1.0000	0.0000	1	1	3236977	Unique	Used	2.061	1.781	1.146	0.907	1E-05	0	6.68	0	0	44	2	1238.59424	2476.18120	-0.51
dMAIATGGAVFGEEGLNLNLEDVQAHDLGk	Unambiguous	1	1	P63038	N-Term(iTRAQ8plex); K30(iTRAQ8plex)	CID	1.0000	0.0000	1	1	3284069	Unique	Used	0.737	0.758	0.931	0.730	1E-05	0	6.68	0	0	4	3	1231.63770	3692.89853	-1.79
aGGASPAASSTTQPPAQHR	Unambiguous	2	1	Q8R3Y8-2	N-Term(iTRAQ8plex)	CID	1.0000	0.0000	1	1	3245364	Unique	Used	1.294	1.713	0.786	0.815	1E-05	0	6.68	0	0	200	3	699.36407	2096.07767	-0.49
dmAIATGGAVFGEEGLNLNLEDVQAHDLGk	Unambiguous	1	1	P63038	N-Term(iTRAQ8plex); M2(Oxidation); K30(iTRAQ8plex)	CID	1.0000	0.0000	1	1	3283727	Unique	Used	0.824	0.741	1.135	1.054	1E-05	0	6.68	0	9	47	3	1236.96692	3708.88620	-3.74
gFVQDSPTVIHQVDETFR	Unambiguous	1	1	Q8BHW2	N-Term(iTRAQ8plex)	CID	1.0000	0.0000	1	1	3257337	Unique	Used	1.311	1.213	1.257	1.316	1E-05	0	6.68	0	5	11	3	793.74585	2379.22300	-0.71
dQEGQDVLLFIDNIFR	Unambiguous	1	1	P56480	N-Term(iTRAQ8plex)	CID	1.0000	0.0000	1	1	3354722	Unique	Used	1.171	1.468	0.976	1.101	1E-05	0	6.68	0	0	200	2	1113.58191	2226.15654	-6.44
lTEAYQVPDASVSSMHTALTR	Unambiguous	1	1	P58242	N-Term(iTRAQ8plex)	CID	1.0000	0.0000	1	1	3536548	Unique	Used	2.539	3.191	1.601	1.417	1E-05	0	6.68	0	0	76	3	861.10901	2581.31247	-4.24

gENLEAVVcEEPQVTYSk	Unambiguous	4	1	Q8BIF0-2	N-Term(iTRAQ8plex); C9(Methylthio); K18(iTRAQ8plex)	CID	1.0000	0.0000	1	1	2869595	Unique	Used		0.757	2.298	2.348	1E-05	0	6.68	0	0	200	2	1325.16504	2649.32280	-5.01
eVNEnFAIDLIAQQPVNEVEHR	Unambiguous	2	1	P52503	N-Term(iTRAQ8plex); N5(Deamidated)	CID	0.0060	0.0000	1	1	2887557	Unique	Used	1.517		1.242		1E-05	0	6.68	0	20	200	3	957.15765	2869.45841	-1.76
qAPYENLnDQELGEYLAR	Unambiguous	1	1	Q03517	N-Term(iTRAQ8plex); N8(Deamidated)	CID	0.0150	0.0000	1	1	2763197	Unique	Used	1.288	0.714	0.647	0.818	1E-05	0	6.67	0	7	200	2	1214.59583	2428.18437	-3.73
sAIRDNnPVVMLENELMYGVAFELPAEAQSk	Unambiguous	1	1	Q9D051	N-Term(iTRAQ8plex); N7(Deamidated); K31(iTRAQ8plex)	CID	0.0120	0.0000	1	1	3396503	Unique	Used	1.162	0.620	1.657	1.106	1E-05	0	6.67	1	0	42	3	1349.03577	4045.09275	1.37
mTTLFcINVLSEVcGQDITTk	Unambiguous	1	1	Q76MZ3	N-Term(iTRAQ8plex); C6(Methylthio); C14(Methylthio); K21(iTRAQ8plex)	CID	1.0000	0.0000	1	1	3899615	Unique	Used		1.121	0.786	1.624	1E-05	0	6.67	0	12	83	2	1508.75793	3016.50859	-2.02
vGAEGDSGFVSSEDVFETGPAMASR	Unambiguous	2	1	Q810U4	N-Term(iTRAQ8plex)	CID	1.0000	0.0000	1	1	2833133	Unique	Used	0.845	0.760	0.760	0.529	1E-05	0	6.67	0	0	68	2	1403.66040	2806.31352	-0.32
ITLQDQVDTLTFQSQSLR	Unambiguous	3	1	Q3UHD3-2	N-Term(iTRAQ8plex)	CID	1.0000	0.0000	1	1	3601273	Unique	Used	0.738	1.061	1.001	0.824	1E-05	0	6.67	0	29	41	2	1199.14539	2397.28349	-3.87
ILGDASVSFTDNcVVGIQANTER	Unambiguous	3	1	P97807-2	N-Term(iTRAQ8plex); C13(Methylthio)	CID	1.0000	0.0000	1	1	3582655	Unique	Used	1.962	1.253	1.404	1.185	1E-05	0	6.67	0	0	200	2	1380.18042	2759.35356	-4.02
mTEEEVEMLVAGHEDSNGcInYEELVR	Unambiguous	1	1	Q60605-2	N-Term(iTRAQ8plex); C19(Methylthio); N21(Deamidated)	CID	1.0000	0.0000	1	1	2816112	Unique	Used	1.900	0.986	1.040	0.506	1E-05	0	6.67	0	8	142	3	1149.85107	3447.53867	0.56
IPQQSHSAFGAASSSSGFGk	Unambiguous	1	1	Q810A7	N-Term(iTRAQ8plex); K20(iTRAQ8plex)	CID	1.0000	0.0000	1	1	3657300	Unique	Used	0.908	0.712	1.210	0.912	1E-05	0	6.67	0	0	99	3	853.78259	2559.33323	-3.13

qETLAQQQLQLEQIQQLQQQLHQQLEEQk	Unambiguous	2	1	Q9QYX7	N-Term(iTRAQ8plex); K29(iTRAQ8plex)	CID	1.0000	0.0000	1	1	3417881	Unique	Used	0.488	0.655	1.518	0.858	1E-05	0	6.67	0	0	48	3	1389.08069	4165.22751	-4.50
sELcEnLEAPALATk	Unambiguous	2	1	G3XA00	N-Term(iTRAQ8plex); C4(Methylthio); N6(Deamidated); K15(iTRAQ8plex)	CID	1.0000	0.0000	1	1	3237444	Unique	Used	0.740	0.508	1.117	0.874	1E-05	0	6.67	0	27	184	2	1122.59766	2244.18804	7.48
tQPTDEEMLFIYSHFk	Unambiguous	4	1	P31786	N-Term(iTRAQ8plex); K16(iTRAQ8plex)	CID	1.0000	0.0000	1	1	3304034	Unique	Used	2.012	0.802	0.986	0.896	1E-05	0	6.67	0	7	50	3	865.44940	2594.33365	-3.27
aGYTDQVVIGMDVAASEFYR	Unambiguous	2	1	P17182	N-Term(iTRAQ8plex)	CID	1.0000	0.0000	1	1	3326986	Unique	Used	0.851	1.465	1.227	1.421	1E-05	0	6.67	0	3	13	2	1248.61987	2496.23247	-2.34
yGGMFAAVEGAYEnk	Unambiguous	1	1	Q6P1J1	N-Term(iTRAQ8plex); N14(Deamidated); K15(iTRAQ8plex)	CID	1.0000	0.0000	1	1	3215721	Unique	Used	0.815	0.715	0.730	0.559	1E-05	0	6.67	0	0	172	2	1108.56909	2216.13091	6.98
aISFVGSnQAGEYIFER	Unambiguous	1	1	Q9EQ20	N-Term(iTRAQ8plex); N8(Deamidated)	CID	0.0195	0.0000	1	1	3261478	Unique	Used	0.945	1.798	1.176	1.623	1E-05	0	6.67	0	0	100	2	1097.05896	2193.11064	-1.08
iLNENPEEnLFVVHQAIk	Unambiguous	1	1	Q5EBJ4	N-Term(iTRAQ8plex); N9(Deamidated); K18(iTRAQ8plex)	CID	0.0390	0.0000	1	1	3193163	Unique	Used	2.366	0.401	1.713	1.195	1E-05	0	6.67	0	16	28	3	906.17328	2716.50528	-2.86
nIEYAASVLEAVYIDETR	Unambiguous	4	1	Q8BRR9	N-Term(iTRAQ8plex)	CID	1.0000	0.0000	1	1	3284790	Unique	Used	0.351	2.687	1.510	1.289	1E-05	0	6.67	0	0	41	2	1180.61658	2360.22588	-1.22
vHSPSGALEEcYVTEIDQDk	Unambiguous	3	1	B7FAU9	N-Term(iTRAQ8plex); C11(Methylthio); K20(iTRAQ8plex)	CID	1.0000	0.0000	1	1	2832035	Unique	Used	2.610	1.601	1.853	1.657	1E-05	0	6.67	0	0	200	3	958.80573	2874.40262	-2.93
ySTIGLAQQWDQLHQLGMR	Unambiguous	1	1	P08032	N-Term(iTRAQ8plex)	CID	1.0000	0.0000	1	1	3878202	Unique	Used	2.768	2.037	0.662	1.124	1E-05	0	6.67	0	0	67	3	850.44312	2549.31479	-3.49
aAFDDAIAELDTLSEESYk	Unambiguous	1	1	P62259	N-Term(iTRAQ8plex); K19(iTRAQ8plex)	CID	1.0000	0.0000	1	1	2816791	Unique	Used		3.038			1E-05	0	6.67	0	0	200	2	1348.68591	2696.36455	-4.38

eYQLNDSAAYYLnDLDR	Unambiguous	1	1	B2RSH2	N-Term(iTRAQ8plex); N13(Deamidated)	CID	1.0000	0.0000	1	1	2720253	Unique	Used		1.253	1.044	1.664	1E-05	0	6.67	0	0	200	2	1184.56360	2368.11992	-2.00
eVAAFAQFGSDLDAATQQLLSR	Unambiguous	2	1	Q03265	N-Term(iTRAQ8plex)	CID	1.0000	0.0000	1	1	3669346	Unique	Used	0.848	1.322	0.732	1.727	1E-05	0	6.67	0	6	200	2	1321.68396	2642.36064	-4.62
sSLFPFEDAFLDDSHGDqALSSGLSSPTR	Unambiguous	7	1	F6T0J8	N-Term(iTRAQ8plex); Q18(Deamidated)	CID	1.0000	0.0000	1	1	3305173	Unique	Used	1.044	0.593	1.487	2.197	1E-05	0	6.67	0	0	50	3	1130.21313	3388.62485	3.68
dFTSEQIDEILQnHTEIVFAR	Unambiguous	2	1	Q6PIC6	N-Term(iTRAQ8plex); N13(Deamidated)	CID	1.0000	0.0000	1	1	3017508	Unique	Used	0.975	0.748	0.904	1.602	1E-05	0	6.67	0	2	11	3	937.47485	2810.41001	-1.81
sLGGSATSLcLLPSEETSGEESR	Unambiguous	1	1	Q2WF71	N-Term(iTRAQ8plex); C10(Methylthio)	CID	1.0000	0.0000	1	1	3194342	Unique	Used	0.274	0.619	0.961	1.120	1E-05	0	6.67	0	0	55	2	1330.63477	2660.26225	-2.82
vADVSISEVTTLLGNVHSPVVEGYVGEnISGEVk	Unambiguous	3	1	P20357	N-Term(iTRAQ8plex); N28(Deamidated); K34(iTRAQ8plex)	CID	0.0015	0.0000	1	1	3397201	Unique	Used	0.967	1.194	1.748	0.850	1E-05	0	6.67	0	0	200	3	1369.73792	4107.19919	0.81
kFVEVMSEYNATQSDYR	Unambiguous	2	1	O35526	N-Term(iTRAQ8plex); K1(iTRAQ8plex)	CID	1.0000	0.0000	1	1	3279874	Unique	Used	0.484	2.788	1.671	1.067	1E-05	0	6.67	1	3	14	3	892.45581	2675.35288	-2.50
iATIQQTTEVFTSTLDDLEER	Unambiguous	3	1	Q9WV18	N-Term(iTRAQ8plex)	CID	1.0000	0.0000	1	1	3284895	Unique	Used	0.310	1.174	0.860	1.000	1E-05	0	6.67	0	0	43	2	1357.70496	2714.40264	-0.45
sScSLPPSLTTSTLASPSPPSSGHSTPR	Unambiguous	3	1	B8QI35	N-Term(iTRAQ8plex); C3(Methylthio)	CID	1.0000	0.0000	1	1	3931957	Unique	Used	1.610	4.020	1.512	0.976	1E-05	0	6.67	0	26	200	3	1030.50537	3089.50156	-5.51
iSLGTQEPSQPLPLGDLPqASVQGSELNYHR	Unambiguous	1	1	Q6P9J5	N-Term(iTRAQ8plex); Q19(Deamidated)	CID	0.0120	0.0000	1	1	3477972	Unique	Used	1.909	0.696	1.983	1.426	1E-05	0	6.67	0	0	110	3	1212.96875	3636.89170	2.88
fIEAGQYnDNLYGTSVQSVR	Unambiguous	10	1	Q91XM9-7	N-Term(iTRAQ8plex); N8(Deamidated)	CID	1.0000	0.0000	1	1	3192478	Unique	Used	1.237	1.046	1.048	1.063	1E-05	0	6.66	0	0	101	2	1283.63477	2566.26225	-4.09
sGAFGHLFRPDnFIFGQSGAGNNWAk	Unambiguous	1	1	Q9ERD7	N-Term(iTRAQ8plex); N12(Deamidated); K26(iTRAQ8plex)	CID	1.0000	0.0000	1	1	3916176	Unique	Used	0.600	0.878	1.102	1.085	1E-05	0	6.66	0	0	200	3	1135.58777	3404.74875	2.99

sYQAEHGPDFPWSVGEDMSADGR	Unambiguous	1	1	Q9Z1Q2	N-Term(iTRAQ8plex)	CID	1.0000	0.0000	1	1	2868093	Unique	Used	1.382	0.950	0.957	0.730	1E-05	0	6.66	0	9	200	3	948.09052	2842.25699	-3.92
qASISGPAPTkASGAPPGGQQR	Unambiguous	2	1	O88935	N-Term(iTRAQ8plex); K11(iTRAQ8plex)	CID	1.0000	0.0000	1	1	3610559	Unique	Used	1.929	2.509	2.919	1.640	1E-05	0	6.66	1	0	200	3	891.15710	2671.45676	-6.33
wGEAGAEYVVESTGVFTTMEk	Unambiguous	3	1	P16858	N-Term(iTRAQ8plex); K21(iTRAQ8plex)	CID	1.0000	0.0000	1	1	3241963	Unique	Used			1.127	0.826	1E-05	0	6.66	0	0	200	2	1450.23535	2899.46343	-0.36
tLmELLNQMDGFDTLHR	Unambiguous	1	1	P62334	N-Term(iTRAQ8plex); M3(Oxidation)	CID	1.0000	0.0000	1	1	3706596	Unique	Used	1.499	1.978	2.110	1.504	1E-05	0	6.66	0	21	8	3	785.39343	2354.16574	-5.50
sAYALGGLGSGIcPNk	Unambiguous	1	1	Q8C196	N-Term(iTRAQ8plex); C13(Methylthio); K16(iTRAQ8plex)	CID	1.0000	0.0000	1	1	3803374	Unique	Used	2.048	0.849	4.690	8.471	1E-05	0	6.66	0	0	124	2	1081.57996	2162.15264	-1.49
sSAAqAIHPGYGFLSENMEFAELck	Unambiguous	2	1	Q99MR8	N-Term(iTRAQ8plex); Q5(Deamidated); C24(Methylthio); K25(iTRAQ8plex)	CID	0.0315	0.0000	1	1	3459056	Unique	Used	0.762	0.843	1.398	1.773	1E-05	0	6.66	0	0	49	3	1119.21655	3355.63510	2.76
vNQSALEAVTPSPSFQqR	Unambiguous	1	1	Q8K3H0	N-Term(iTRAQ8plex); Q17(Deamidated)	CID	0.0180	0.0000	1	1	3596631	Unique	Used	1.678	1.422	1.326	1.358	1E-05	0	6.66	0	0	166	2	1132.59814	2264.18901	2.88
eLFLLGQHYVcEAk	Unambiguous	2	1	Q6ZPU9	N-Term(iTRAQ8plex); C11(Methylthio); K14(iTRAQ8plex)	CID	1.0000	0.0000	1	1	3539952	Unique	Used	0.427	0.936	0.725	0.895	1E-05	0	6.66	0	3	29	3	768.74750	2304.22794	-2.67
nEEQHFYWGGSQPGIQR	Unambiguous	1	1	O54991	N-Term(iTRAQ8plex)	CID	1.0000	0.0000	1	1	3123780	Unique	Used	0.760	0.737	1.706	1.631	1E-05	0	6.66	0	0	110	2	1169.06641	2337.12554	-2.53
sSLATmAHAQSLVEAQPNVDk	Unambiguous	5	1	Q60597	N-Term(iTRAQ8plex); M6(Oxidation); K21(iTRAQ8plex)	CID	1.0000	0.0000	1	1	3340641	Unique	Used		1.183	0.978	1.015	1E-05	0	6.66	0	8	19	3	941.16675	2821.48569	-4.18
fIEAGQFNDNLYGTSIQSVR	Unambiguous	4	1	Q52KF7	N-Term(iTRAQ8plex)	CID	1.0000	0.0000	1	1	3559811	Unique	Used	0.680	1.862	1.737	1.227	1E-05	0	6.66	0	0	98	2	1282.15344	2563.29961	-3.85
gQQYTDSFAQVNPLR	Unambiguous	3	1	Q99L20	N-Term(iTRAQ8plex)	CID	1.0000	0.0000	1	1	3576344	Unique	Used	0.344	0.864	0.646	1.668	1E-05	0	6.66	0	0	16	2	1014.52179	2028.03630	-4.41

aFLYLGQNNFAEAHk	Unambiguous	1	1	Q8K2L8	N-Term(iTRAQ8plex); K15(iTRAQ8plex)	CID	1.0000	0.0000	1	1	3618633	Unique	Used	0.744	1.535	0.903	0.960	1E-05	0	6.66	0	8	52	3	777.75800	2331.25943	-4.79
qATTIIADNIIFLSDQTk	Unambiguous	1	1	Q8R2K3	N-Term(iTRAQ8plex); K18(iTRAQ8plex)	CID	1.0000	0.0000	1	1	3479625	Unique	Used	1.754	0.822	1.268	0.907	1E-05	0	6.66	0	14	9	2	1300.73816	2600.46904	-2.52
sEEITTGSAWFSFLESHNk	Unambiguous	2	1	D3Z375	N-Term(iTRAQ8plex); K19(iTRAQ8plex)	CID	1.0000	0.0000	1	1	3350328	Unique	Used	1.813	1.519	1.234	0.732	1E-05	0	6.66	0	23	5	2	1389.70789	2778.40849	-3.98
yGFQEQLLYSVLEDLVSTGR	Unambiguous	2	1	Q8CCJ3	N-Term(iTRAQ8plex)	CID	1.0000	0.0000	1	1	3766899	Unique	Used	0.443	0.678	0.730	0.848	1E-05	0	6.66	0	6	143	2	1311.18774	2621.36821	-3.16
fPEQAGTLQNATTSSSLTAHLR	Unambiguous	2	1	P53995	N-Term(iTRAQ8plex)	CID	1.0000	0.0000	1	1	3740695	Unique	Used	0.976	0.867	0.900	1.064	1E-05	0	6.66	0	15	200	3	878.79407	2634.36765	-4.29
dQGScGScWAFGAVEAISDR	Unambiguous	1	1	P10605	N-Term(iTRAQ8plex); C5(Methylthio); C8(Methylthio)	CID	1.0000	0.0000	1	1	3419003	Unique	Used	1.185	1.051	1.721	1.029	1E-05	0	6.66	0	15	19	2	1228.02063	2455.03398	-4.62
rGDTSGETLHSLIQQGqQIASk	Unambiguous	1	1	Q91YM2	N-Term(iTRAQ8plex); Q17(Deamidated); K22(iTRAQ8plex)	CID	0.0090	0.0000	1	1	3639135	Unique	Used	0.590	1.132	0.838	1.100	1E-05	0	6.66	1	30	115	3	988.54041	2963.60666	2.01
wGEAGAEYVVESTGVFTTMEk	Unambiguous	3	1	P16858	N-Term(iTRAQ8plex); K21(iTRAQ8plex)	CID	1.0000	0.0000	1	1	3219995	Unique	Used			0.640	0.593	1E-05	0	6.66	0	7	200	3	967.16016	2899.46592	0.50
hQADIASYQDAIQQLDSELR	Unambiguous	1	1	P19246	N-Term(iTRAQ8plex)	CID	1.0000	0.0000	1	1	3172248	Unique	Used	1.914	1.007	1.004	1.040	1E-05	0	6.66	0	0	141	3	869.10535	2605.30149	-5.58
aMLSGPGQFAENETNEVNFR	Unambiguous	1	1	P83940	N-Term(iTRAQ8plex)	CID	1.0000	0.0000	1	1	3192042	Unique	Used	0.718	0.634	1.098	1.305	1E-05	0	6.66	0	0	4	2	1258.11169	2515.21611	-1.14
nGHnEMDEPMFTQPLMYk	Unambiguous	4	1	Q60597	N-Term(iTRAQ8plex); N4(Deamidated); K18(iTRAQ8plex)	CID	1.0000	0.0000	1	1	2919223	Unique	Used	1.676	1.253	0.963	0.910	1E-05	0	6.66	0	11	200	3	931.11560	2791.33225	-1.00
lEQVSSDEGIGTLAENLLEALR	Unambiguous	4	1	A2AN08-3	N-Term(iTRAQ8plex)	CID	1.0000	0.0000	1	1	3374521	Unique	Used	0.509	1.028	0.946	1.074	1E-05	0	6.66	0	14	63	2	1331.21057	2661.41387	-4.16
iVSGAAETDQEYYFGQVVR	Unambiguous	4	1	P06909	N-Term(iTRAQ8plex)	CID	1.0000	0.0000	1	1	3214650	Unique	Used	5.962	2.054	0.639	0.921	1E-05	0	6.65	0	0	26	2	1218.62000	2436.23271	-0.90

aEAGGGWEGSASYk	Unambiguous	2	1	P97765	N-Term(iTRAQ8plex); K14(iTRAQ8plex)	CID	1.0000	0.0000	1	1	2826491	Unique	Used	1.853	1.548	1.240	1.154	1E-05	0	6.65	0	0	65	2	989.50848	1978.00969	-1.51
eGnDLYHEMIESGVINLk	Unambiguous	1	1	P56480	N-Term(iTRAQ8plex); N3(Deamidated); K18(iTRAQ8plex)	CID	1.0000	0.0000	1	1	2941512	Unique	Used	1.002	1.100	1.223	1.217	1E-05	0	6.65	0	17	161	2	1335.69702	2670.38677	-1.42
dLYANNVLSGGTTMYPGIADR	Unambiguous	3	1	P62737	N-Term(iTRAQ8plex)	CID	1.0000	0.0000	1	1	3457552	Unique	Used	0.600	4.626	1.029	1.899	1E-05	0	6.65	0	15	189	2	1266.63550	2532.26372	-2.74
nVTDVVnTcHDAGISk	Unambiguous	1	1	Q99LF4	N-Term(iTRAQ8plex); N7(Deamidated); C9(Methylthio); K16(iTRAQ8plex)	CID	0.0286	0.0000	1	1	3344433	Unique	Used	1.070	0.623	0.783	1.036	1E-05	0	6.65	0	0	61	2	1164.60144	2328.19560	7.37
iDINMSGFNETDDLk	Unambiguous	2	1	B1AX58	N-Term(iTRAQ8plex); K15(iTRAQ8plex)	CID	1.0000	0.0000	1	1	2939617	Unique	Used	0.748	0.807	0.956	1.050	1E-05	0	6.65	0	0	160	2	1160.59460	2320.18193	-5.70
gHAPSTAAPAPSPAPMSSSVQSDEER	Unambiguous	1	1	Q8C996	N-Term(iTRAQ8plex)	CID	1.0000	0.0000	1	1	2843889	Unique	Used	1.700	2.644	0.766	0.785	1E-05	0	6.65	0	0	188	3	956.79230	2868.36234	-3.95
lQQTQAQVDEVVDIMR	Unambiguous	2	1	P63044	N-Term(iTRAQ8plex)	CID	1.0000	0.0000	1	1	3286140	Unique	Used	0.668	1.742	1.365	1.237	1E-05	0	6.65	0	4	200	2	1089.07971	2177.15215	-0.78
gnDISSGTVLSDYVGSGPPSGTGLHR	Unambiguous	1	1	P70296	N-Term(iTRAQ8plex); N2(Deamidated)	CID	1.0000	0.0000	1	1	3345394	Unique	Used	3.060	2.139	1.249	0.926	1E-05	0	6.65	0	21	4	3	945.80353	2835.39603	-3.63
eAEEEFWQNQHPQPYIFPDSPGGTSFER	Unambiguous	1	1	Q9CQJ8	N-Term(iTRAQ8plex)	CID	1.0000	0.0000	1	1	2939912	Unique	Used		0.695			1E-05	0	6.65	0	0	200	3	1209.56665	3626.68540	2.40
aVQLYQQTANVFENEER	Unambiguous	2	1	Q9CWZ7	N-Term(iTRAQ8plex)	CID	1.0000	0.0000	1	1	3261165	Unique	Used	1.933	1.632	1.295	1.158	1E-05	0	6.65	0	0	107	2	1172.09802	2343.18877	0.20
acADATLSqITNNIDPVGR	Unambiguous	3	1	P62874	N-Term(iTRAQ8plex); C2(Methylthio); Q9(Deamidated)	CID	0.0346	0.0000	1	1	3604073	Unique	Used	0.549	1.444	1.109	1.184	1E-05	0	6.65	0	17	200	2	1155.57727	2310.14726	4.35
nIEVMNSFELLSHTVEEk	Unambiguous	2	1	D3Z0F5	N-Term(iTRAQ8plex); K18(iTRAQ8plex)	CID	1.0000	0.0000	1	1	3239005	Unique	Used	0.858	0.862	0.832	1.116	1E-05	0	6.65	0	26	24	3	909.81915	2727.44291	-2.02

sMTEAEQQqLIDDHFLFDkPVSPLLLASGMAR	Unambiguous	1	1	Q04447	N-Term(iTRAQ8plex); Q9(Deamidated); K19(iTRAQ8plex)	CID	0.0090	0.0000	1	1	3519815	Unique	Used	1.172	0.730	1.231	1.196	1E-05	0	6.65	0	0	14	3	1399.73120	4197.17905	-0.75
lFESEFETSSTYISILNQk	Unambiguous	4	1	Q8BPN8-2	N-Term(iTRAQ8plex); K19(iTRAQ8plex)	CID	1.0000	0.0000	1	1	3418934	Unique	Used	0.512	1.295	1.424	2.214	1E-05	0	6.65	0	15	20	2	1422.75366	2844.50005	-4.47
qASISGPAPTkASGAPPGGQQR	Unambiguous	2	1	O88935	N-Term(iTRAQ8plex); K11(iTRAQ8plex)	CID	1.0000	0.0000	1	1	3610765	Unique	Used	7.543	1.493	6.088	1.409	1E-05	0	6.65	1	4	78	3	891.15729	2671.45731	-6.12
sEVELVQLVIDGVNYLIDcER	Unambiguous	1	1	P30275	N-Term(iTRAQ8plex); C19(Methylthio)	CID	1.0000	0.0000	1	1	3603283	Unique	Used	0.201	0.652	1.352	1.583	1E-05	0	6.65	0	0	200	2	1378.70337	2756.39946	-5.74
gQcGEnLAWASYDQTGk	Unambiguous	1	1	Q9CYL5	N-Term(iTRAQ8plex); C3(Methylthio); N6(Deamidated); K17(iTRAQ8plex)	CID	0.0165	0.0000	1	1	3190276	Unique	Used	0.585	1.040	0.936	1.358	1E-05	0	6.65	0	0	53	2	1242.10034	2483.19341	5.73
eLGGAIDSGAAYVLEQASSHIGNSTQAAVR	Unambiguous	3	1	Q91YH5	N-Term(iTRAQ8plex)	CID	1.0000	0.0000	1	1	3706335	Unique	Used	1.205	1.306	0.844	1.088	1E-05	0	6.65	0	24	31	3	1092.88757	3276.64817	-8.59
nQDEcVIALHDcnGDVNR	Unambiguous	5	1	Q80X50	N-Term(iTRAQ8plex); C5(Methylthio); C12(Methylthio); N13(Deamidated)	CID	1.0000	0.0000	1	1	2739066	Unique	Used	2.166	1.576	0.928	0.456	1E-05	0	6.65	0	0	200	3	804.68207	2412.03165	-1.58
eAGFPPGVVNIITGYGPTAGAAIAQHMDVDk	Unambiguous	1	1	Q9CZS1	N-Term(iTRAQ8plex); K31(iTRAQ8plex)	CID	1.0000	0.0000	1	1	3601776	Unique	Used	2.870	1.194	0.905	1.103	1E-05	0	6.65	0	21	18	3	1235.65308	3704.94468	-3.27
lGGDVSPTQIDVSQFGSFk	Unambiguous	1	1	P14873	N-Term(iTRAQ8plex); K19(iTRAQ8plex)	CID	1.0000	0.0000	1	1	3499177	Unique	Used	1.864	0.973	1.626	0.832	1E-05	0	6.65	0	0	9	2	1295.69617	2590.38506	-4.75
aGYTDqVVIGMDVAASEFYR	Unambiguous	2	1	P17182	N-Term(iTRAQ8plex); Q6(Deamidated)	CID	1.0000	0.0000	1	1	3354534	Unique	Used		1.553	1.186	0.899	1E-05	0	6.65	0	0	200	2	1249.11902	2497.23076	3.38

aLYDYDGQEQDELSFk	Unambiguous	1	1	Q61644	N-Term(iTRAQ8plex); K16(iTRAQ8plex)	CID	1.0000	0.0000	1	1	2654598	Unique	Used	0.886	1.332	1.769	1.012	1E-05	0	6.65	0	2	200	2	1265.13257	2529.25786	-1.07
sEMEETLTAIAEELAASAk	Unambiguous	1	1	P08553	N-Term(iTRAQ8plex); K19(iTRAQ8plex)	CID	1.0000	0.0000	1	1	3196706	Unique	Used	3.328	0.283	1.449	0.764	1E-05	0	6.65	0	0	62	2	1301.68823	2602.36919	-1.95
gNNTTSLLSQSVAk	Unambiguous	1	1	Q9DB77	N-Term(iTRAQ8plex); K14(iTRAQ8plex)	CID	1.0000	0.0000	1	1	3451640	Unique	Used	0.898	1.344	0.796	0.920	1E-05	0	6.65	0	0	200	2	1014.57758	2028.14787	-3.30
sNAQQIVAEMLSYLETADYSIR	Unambiguous	1	1	P17427	N-Term(iTRAQ8plex)	CID	1.0000	0.0000	1	1	3806904	Unique	Used		2.115	1.743	1.961	1E-05	0	6.65	0	0	103	2	1403.71277	2806.41826	-1.88
nEAIQAAHDSVAQEGqcR	Unambiguous	1	1	Q9R0P9	N-Term(iTRAQ8plex); Q16(Deamidated); C17(Methylthio)	CID	0.0421	0.0000	1	1	2896147	Unique	Used	2.769	1.877	1.366	1.001	1E-05	0	6.65	0	10	200	3	760.02692	2278.06620	7.35
gISNQPFPELHcAALk	Unambiguous	1	1	Q8BJY1	N-Term(iTRAQ8plex); C12(Methylthio); K16(iTRAQ8plex)	CID	1.0000	0.0000	1	1	3683625	Unique	Used	0.352	0.764	0.734	1.022	1E-05	0	6.64	0	7	11	3	793.75916	2379.26291	-6.08
qAAFQLISQVILEATEEFR	Unambiguous	6	1	O08715-2	N-Term(iTRAQ8plex)	CID	1.0000	0.0000	1	1	3861815	Unique	Used	1.250	0.966	1.168	1.004	1E-05	0	6.64	0	9	59	2	1249.18018	2497.35307	-2.96
qFYHLGWVTGTGGGISLk	Unambiguous	1	1	Q9WVQ5	N-Term(iTRAQ8plex); K18(iTRAQ8plex)	CID	1.0000	0.0000	1	1	3896131	Unique	Used	1.172	1.086	0.780	0.831	1E-05	0	6.64	0	10	103	3	843.80493	2529.40024	-2.85
dQEGQDVLLFIDNIFR	Unambiguous	1	1	P56480	N-Term(iTRAQ8plex)	CID	1.0000	0.0000	1	1	3353359	Unique	Used	1.433	1.639	1.110	1.278	1E-05	0	6.64	0	0	17	2	1113.58582	2226.16435	-2.93
qGFPDREsPVSDLTSTGLYQDk	Unambiguous	1	1	P14873	N-Term(iTRAQ8plex); S8(Phospho); K22(iTRAQ8plex)	CID	1.0000	0.0000	1	1	2812915	Unique	Used	1.547	0.450	0.923	0.617	1E-05	0	6.64	1	9	200	3	1043.51880	3128.54184	0.65
icDPGLTSFEPEALGnLVEGMDFHR	Unambiguous	9	1	Q5SVJ0	N-Term(iTRAQ8plex); C2(Methylthio); N16(Deamidated)	CID	1.0000	0.0000	1	1	3481899	Unique	Used		1.495	0.792	0.782	1E-05	0	6.64	0	0	200	3	1033.49255	3098.46311	1.77

iGEHTPSALAImENANVLAR	Unambiguous	3	1	A6ZI44	N-Term(iTRAQ8plex); M12(Oxidation)	CID	1.0000	0.0000	1	1	3709608	Unique	Used	1.252	0.969	0.581	1.208	1E-05	0	6.64	0	3	200	3	809.76550	2427.28196	-6.13
aGYTDQVVIGMDVAASEFYR	Unambiguous	2	1	P17182	N-Term(iTRAQ8plex)	CID	1.0000	0.0000	1	1	3326795	Unique	Used	1.203	1.436	1.449	1.385	1E-05	0	6.64	0	21	25	2	1248.62219	2496.23711	-0.48
lGSQATGVQGQAGQLLDTTESTLGR	Unambiguous	1	1	Q61001	N-Term(iTRAQ8plex)	CID	1.0000	0.0000	1	1	3457755	Unique	Used	1.763	3.215	1.198	1.450	1E-05	0	6.64	0	0	91	2	1396.72998	2792.45268	-5.94
sSGTGASVGPPQPSDQDTLVQR	Unambiguous	1	1	Q8CI51	N-Term(iTRAQ8plex)	CID	1.0000	0.0000	1	1	2952749	Unique	Used	1.547	1.200	0.836	0.718	1E-05	0	6.64	0	0	200	2	1244.62817	2488.24907	-3.67
aLEEALSIqASPSSPPAAFGSPEGVGGHLR	Unambiguous	5	1	Q9WUU8-2	N-Term(iTRAQ8plex); Q9(Deamidated)	CID	1.0000	0.0000	1	1	3518549	Unique	Used	1.547	0.988	1.306	1.411	1E-05	0	6.64	0	0	135	3	1079.89380	3237.66684	-0.81
qTIEnSQGAYQEAFDISk	Unambiguous	4	1	P68254-2	N-Term(iTRAQ8plex); N5(Deamidated); K18(iTRAQ8plex)	CID	0.0467	0.0000	1	1	2831370	Unique	Used			0.611	0.767	1E-05	0	6.64	0	10	200	2	1319.67346	2638.33965	-2.29
qTFIGHESDINAVAFFPnGYAFTTGSDDATcR	Unambiguous	4	1	P62880	N-Term(iTRAQ8plex); N18(Deamidated); C31(Methylthio)	CID	1.0000	0.0000	1	1	3439626	Unique	Used	1.425	2.839	1.308	1.546	1E-05	0	6.64	0	0	21	3	1268.58044	3803.72678	0.19
qLNTIQnAEAVHFFcNYEESR	Unambiguous	4	1	P61922	N-Term(iTRAQ8plex); N7(Deamidated); C15(Methylthio)	CID	0.0256	0.0000	1	1	3326401	Unique	Used	2.337	1.098	0.548	1.481	1E-05	0	6.64	0	21	9	3	955.44434	2864.31845	-3.53
wGEAGAEYVVESTGVFTTMEk	Unambiguous	3	1	P16858	N-Term(iTRAQ8plex); K21(iTRAQ8plex)	CID	1.0000	0.0000	1	1	3241068	Unique	Used		0.899	1.805	1.918	1E-05	0	6.64	0	0	200	2	1450.23608	2899.46489	0.14
eAFQNAYLELGGLGER	Unambiguous	2	1	Q6PIC6	N-Term(iTRAQ8plex)	CID	1.0000	0.0000	1	1	3357201	Unique	Used	1.024	0.332	1.630	1.424	1E-05	0	6.64	0	30	200	2	1036.03992	2071.07256	-1.77
fWEVISDEHGIDPTGTYHGDSDLqLDR	Unambiguous	1	1	P99024	N-Term(iTRAQ8plex); Q24(Deamidated)	CID	1.0000	0.0000	1	1	3132109	Unique	Used	0.932	1.274	1.255	1.383	1E-05	0	6.64	0	0	200	3	1136.54468	3407.61948	6.58
fQEYHIqQNEALAAk	Unambiguous	1	1	Q9WV98	N-Term(iTRAQ8plex); Q7(Deamidated); K15(iTRAQ8plex)	CID	0.0015	0.0000	1	1	3124634	Unique	Used	1.634	1.117	1.007	0.853	1E-05	0	6.64	0	0	168	2	1200.14978	2399.29228	4.47

dYSLQIQNVDVTDDGPYTcSVQTQHTPR	Unambiguous	4	1	A0A4W9	N-Term(iTRAQ8plex); C19(Methylthio)	CID	1.0000	0.0000	1	1	3214004	Unique	Used	3.625	1.203	1.273	1.191	1E-05	0	6.64	0	0	117	3	1177.55237	3530.64255	-1.27
tIVAINkDPEAPIFQVADYGIVADLFk	Unambiguous	1	1	Q99LC5	N-Term(iTRAQ8plex); K7(iTRAQ8plex); K27(iTRAQ8plex)	CID	1.0000	0.0000	1	1	3602151	Unique	Used	0.744	1.302	0.859	1.116	1E-05	0	6.64	1	17	12	3	1287.39917	3860.18296	-3.84
vLDGLTAGSSSASQQQQQQHPPGNR	Unambiguous	3	1	D3Z2Q2	N-Term(iTRAQ8plex)	CID	1.0000	0.0000	1	1	3531639	Unique	Used	0.836	1.054	1.406	1.193	1E-05	0	6.64	0	0	127	3	1008.50916	3023.51291	-2.25
qVQEAIAGISSAAQATSPTDEAk	Unambiguous	5	1	Q61301-2	N-Term(iTRAQ8plex); K23(iTRAQ8plex)	CID	1.0000	0.0000	1	1	3324020	Unique	Used	1.383	1.818	1.189	1.320	1E-05	0	6.64	0	0	22	3	961.18024	2881.52616	-3.55
rLEEDTEETSGLLEQLR	Unambiguous	3	1	E9QP99	N-Term(iTRAQ8plex)	CID	1.0000	0.0000	1	1	2761978	Unique	Used	2.098	0.876	1.292	0.988	1E-05	0	6.64	1	10	200	3	774.74103	2322.20853	-0.25
lQQTQAQVDEVVDIMR	Unambiguous	2	1	P63044	N-Term(iTRAQ8plex)	CID	1.0000	0.0000	1	1	3286245	Unique	Used	0.510	2.217	1.504	1.373	1E-05	0	6.64	0	0	200	2	1089.07959	2177.15190	-0.89
aISEAGAPPGSAGPLLLQPQQHR	Unambiguous	1	1	Q99MX7	N-Term(iTRAQ8plex)	CID	1.0000	0.0000	1	1	3680789	Unique	Used	1.079	1.390	1.228	1.064	1E-05	0	6.64	0	0	147	3	867.14355	2599.41611	-3.76
tGPQVGLcLDAVVFGGEDFR	Unambiguous	1	1	Q8R4N0	N-Term(iTRAQ8plex); C8(Methylthio)	CID	1.0000	0.0000	1	1	3500674	Unique	Used	0.848	0.709	0.700	1.149	1E-05	0	6.64	0	0	32	2	1215.60327	2430.19927	-4.42
eTVAYLQAHALTTEGIFR	Unambiguous	2	1	A2AH25	N-Term(iTRAQ8plex)	CID	1.0000	0.0000	1	1	3725019	Unique	Used	0.573	1.358	1.115	1.208	1E-05	0	6.64	0	0	174	2	1162.62292	2324.23857	-7.18
scVDVNEcATGFHR	Unambiguous	1	1	O88322	N-Term(iTRAQ8plex); C2(Methylthio); C8(Methylthio)	CID	1.0000	0.0000	1	1	3412156	Unique	Used	1.612	1.538	1.136	1.079	1E-05	0	6.64	0	10	30	3	645.28046	1933.82682	-3.25
gGYMDDGGYSMNFNMSSSR	Unambiguous	1	1	Q91VM5	N-Term(iTRAQ8plex)	CID	1.0000	0.0000	1	1	3257058	Unique	Used	0.937	0.389	1.255	0.643	1E-05	0	6.64	0	0	77	2	1190.49866	2379.99004	-1.93
eAFQnAYLELGGLGER	Unambiguous	2	1	Q6PIC6	N-Term(iTRAQ8plex); N5(Deamidated)	CID	0.0226	0.0000	1	1	3325091	Unique	Used	0.829	0.741	1.105	1.088	1E-05	0	6.64	0	0	187	2	1036.53784	2072.06841	3.94
aMLSGPGQFAENETNEVNFR	Unambiguous	1	1	P83940	N-Term(iTRAQ8plex)	CID	1.0000	0.0000	1	1	3170270	Unique	Used	1.011	1.126	1.067	1.034	1E-05	0	6.64	0	0	111	2	1258.10791	2515.20854	-4.15
vENQENVSNLVIDDTELk	Unambiguous	1	1	P40124	N-Term(iTRAQ8plex); K18(iTRAQ8plex)	CID	1.0000	0.0000	1	1	2637764	Unique	Used	0.675	0.974	1.001	0.842	1E-05	0	6.64	0	0	168	2	1334.21448	2667.42168	-3.04

sLLVIPNTLAVNAAQDSTDLVAk	Unambiguous	2	1	P11983	N-Term(iTRAQ8plex); K23(iTRAQ8plex)	CID	1.0000	0.0000	1	1	3601860	Unique	Used	0.513	0.998	0.781	1.049	1E-05	0	6.64	0	0	19	2	1481.34937	2961.69145	-5.64
dFSLILDDVAITELSFSR	Unambiguous	1	1	O35129	N-Term(iTRAQ8plex)	CID	1.0000	0.0000	1	1	3396814	Unique	Used	0.713	0.774	0.854	1.034	1E-05	0	6.64	0	29	120	2	1173.12683	2345.24639	-3.36
fIEAGQYnDNLYGTSVQSVR	Unambiguous	10	1	Q91XM9-7	N-Term(iTRAQ8plex); N8(Deamidated)	CID	0.0302	0.0000	1	1	3214159	Unique	Used	2.215	2.053	1.222	0.852	1E-05	0	6.63	0	0	200	2	1283.64026	2566.27324	0.19
sqAYADYIGFILTLNEGVk	Unambiguous	2	1	P58389	N-Term(iTRAQ8plex); Q2(Deamidated); K19(iTRAQ8plex)	CID	0.0030	0.0000	1	1	3706946	Unique	Used	0.527	1.239	0.913	1.114	1E-05	0	6.63	0	14	58	2	1356.24133	2711.47539	0.05
iHEGcEEPATHnALAk	Unambiguous	2	1	Q68FD5	N-Term(iTRAQ8plex); C5(Methylthio); N12(Deamidated); K16(iTRAQ8plex)	CID	1.0000	0.0000	1	1	2730855	Unique	Used	1.034	1.177	1.026	1.145	1E-05	0	6.63	0	17	200	3	792.40039	2375.18662	-3.29
sVNESLNNLFITEEDYQALR	Unambiguous	2	1	Q68FD5	N-Term(iTRAQ8plex)	CID	1.0000	0.0000	1	1	3240807	Unique	Used	0.718	2.089	1.502	1.853	1E-05	0	6.63	0	0	200	2	1330.17761	2659.34795	-1.43
eATDTPIVIqPASATETTQLTADSHPSYHTDGFN	Unambiguous	1	1	P60904	N-Term(iTRAQ8plex); Q10(Deamidated)	CID	0.0287	0.0000	1	1	2975599	Unique	Used			1.836	1.199	1E-05	0	6.63	0	0	200	3	1307.62793	3920.86924	1.93
sGDTVLYDLEAGSALLTLESR	Unambiguous	2	1	P58404-2	N-Term(iTRAQ8plex)	CID	1.0000	0.0000	1	1	3262880	Unique	Used	0.758	1.512	1.251	0.718	1E-05	0	6.63	0	7	14	2	1257.66516	2514.32305	-0.44
sSVPSGGSLFGDDEDDDLFSSAk	Unambiguous	1	1	Q6PGL7	N-Term(iTRAQ8plex); K23(iTRAQ8plex)	CID	1.0000	0.0000	1	1	2815066	Unique	Used	1.892	2.815	1.110	0.777	1E-05	0	6.63	0	0	200	3	980.81055	2940.41709	-1.25
vETLHDFEAANSDELNLqR	Unambiguous	1	1	Q7TQF7	N-Term(iTRAQ8plex); Q18(Deamidated)	CID	0.0226	0.0000	1	1	2654684	Unique	Used	2.445	2.322	2.308	0.847	1E-05	0	6.63	0	11	200	3	836.08319	2506.23502	-0.55
lTGFHETSNInDFSAGVANR	Unambiguous	1	1	P15105	N-Term(iTRAQ8plex); N11(Deamidated)	CID	1.0000	0.0000	1	1	3255727	Unique	Used	1.600	1.027	1.911	1.960	1E-05	0	6.63	0	0	69	2	1228.10913	2455.21099	-1.87
qEDSIWGFSGNANADSVVYYR	Unambiguous	2	1	D3YWB9	N-Term(iTRAQ8plex)	CID	1.0000	0.0000	1	1	3215362	Unique	Used	0.607	0.546	1.134	1.379	1E-05	0	6.63	0	0	122	2	1341.63928	2682.27129	-0.94
tVSYNILADTYAQTEFSR	Unambiguous	1	1	Q3TIU4	N-Term(iTRAQ8plex)	CID	1.0000	0.0000	1	1	3478667	Unique	Used	0.951	0.947	0.939	1.158	1E-05	0	6.63	0	0	200	2	1192.10278	2383.19829	-4.22

vYEIQDIYENSWTk	Unambiguous	1	1	Q8QZY1	N-Term(iTRAQ8plex); K14(iTRAQ8plex)	CID	1.0000	0.0000	1	1	3173248	Unique	Used	0.339	1.025	0.727	0.950	1E-05	0	6.63	0	0	200	2	1198.63013	2396.25298	-2.69
nmGGPYGGGNYGPGGSGGSGGYGGR	Unambiguous	3	1	O88569	N-Term(iTRAQ8plex); M2(Oxidation)	CID	1.0000	0.0000	1	1	3511053	Unique	Used	1.299	1.867	1.411	1.077	1E-05	0	6.63	0	0	200	3	837.37732	2510.11740	4.65
dMAIATGGAVFGEEGLNLNLEDVQAHDLGk	Unambiguous	1	1	P63038	N-Term(iTRAQ8plex); K30(iTRAQ8plex)	CID	1.0000	0.0000	1	1	3264227	Unique	Used	0.826	0.964	1.346	1.820	1E-05	0	6.63	0	0	200	3	1231.64331	3692.91538	2.77
kTESIDVmDAVGSNIVVSTR	Unambiguous	1	1	Q91VD9	N-Term(iTRAQ8plex); K1(iTRAQ8plex); M8(Oxidation)	CID	1.0000	0.0000	1	1	3367271	Unique	Used	1.035	1.337	1.107	1.109	1E-05	0	6.63	1	9	99	3	915.83228	2745.48227	-3.31
iVGLDQVTGmTETAFGSAYk	Unambiguous	3	1	Q5SV64	N-Term(iTRAQ8plex); M10(Oxidation); K20(iTRAQ8plex)	CID	1.0000	0.0000	1	1	3600830	Unique	Used	0.507	1.156	1.068	0.958	1E-05	0	6.63	0	21	138	2	1356.71619	2712.42510	-4.58
aLSEIAGITLPYDTLDQVR	Unambiguous	1	1	Q91VD9	N-Term(iTRAQ8plex)	CID	1.0000	0.0000	1	1	3582889	Unique	Used	1.341	0.959	1.557	1.354	1E-05	0	6.63	0	13	200	2	1190.14954	2379.29180	-6.54
qFGNILILSGDVNLAESDLAYTR	Unambiguous	2	1	P97355	N-Term(iTRAQ8plex)	CID	1.0000	0.0000	1	1	3561060	Unique	Used	0.820	1.055	1.464	1.819	1E-05	0	6.63	0	0	70	2	1407.24646	2813.48564	-4.66
eAmEAQIQNFGQTPSQLLIEPHPPR	Unambiguous	4	1	Q9EPN1-4	N-Term(iTRAQ8plex); M3(Oxidation)	CID	1.0000	0.0000	1	1	3475729	Unique	Used	1.133	1.410	1.386	1.097	1E-05	0	6.63	0	0	41	3	1051.20618	3151.60398	-3.46
fSITHISDSDAQSVGGPAAGAGGR	Unambiguous	5	1	B1AZF9	N-Term(iTRAQ8plex)	CID	1.0000	0.0000	1	1	3342171	Unique	Used	1.525	1.244	0.993	0.913	1E-05	0	6.63	0	12	45	3	854.76392	2562.27719	-3.08
dANAALLSNYEVFQLLTDLk	Unambiguous	1	1	O35427	N-Term(iTRAQ8plex); K20(iTRAQ8plex)	CID	1.0000	0.0000	1	1	3561351	Unique	Used	2.434	2.580	0.990	1.296	1E-05	0	6.63	0	29	63	3	949.52594	2846.56327	-4.49
tQPTDEEMLFIYSHFk	Unambiguous	4	1	P31786	N-Term(iTRAQ8plex); K16(iTRAQ8plex)	CID	1.0000	0.0000	1	1	3283500	Unique	Used	1.989	0.644	0.858	0.818	1E-05	0	6.63	0	2	14	3	865.44934	2594.33347	-3.34
iGEHTPSALAIMEnANVLAR	Unambiguous	3	1	A6ZI44	N-Term(iTRAQ8plex); N14(Deamidated)	CID	0.0211	0.0000	1	1	3725306	Unique	Used	0.913	1.058	0.898	1.120	1E-05	0	6.63	0	28	200	2	1206.64587	2412.28447	-0.61

lGLALNYSVFYYEIQNAPEQAcHLAk	Unambiguous	1	1	P61982	N-Term(iTRAQ8plex); C22(Methylthio); K26(iTRAQ8plex)	CID	1.0000	0.0000	1	1	3785534	Unique	Used	1.426	0.938	0.880	1.109	1E-05	0	6.63	0	0	1	3	1203.95728	3609.85727	-3.38
hQVLFIADEIQTGLAR	Unambiguous	1	1	P29758	N-Term(iTRAQ8plex)	CID	1.0000	0.0000	1	1	3643124	Unique	Used	2.726	2.011	0.569	1.184	1E-05	0	6.63	0	8	22	3	705.72888	2115.17209	-6.79
wGEAGAEYVVESTGVFTTMEk	Unambiguous	3	1	P16858	N-Term(iTRAQ8plex); K21(iTRAQ8plex)	CID	1.0000	0.0000	1	1	3240872	Unique	Used			0.837	0.644	1E-05	0	6.63	0	19	200	3	967.15790	2899.45914	-1.84
rEEEEFNTGPLSVLTQSVk	Unambiguous	1	1	P62317	N-Term(iTRAQ8plex); K19(iTRAQ8plex)	CID	1.0000	0.0000	1	1	3214066	Unique	Used	1.369	0.958	0.843	0.799	1E-05	0	6.63	1	0	110	3	924.50372	2771.49662	-2.53
gHLDNISNNLLIGAINIEnGk	Unambiguous	1	1	Q99KI0	N-Term(iTRAQ8plex); N19(Deamidated); K21(iTRAQ8plex)	CID	1.0000	0.0000	1	1	3416309	Unique	Used	1.492	0.969	1.151	0.819	1E-05	0	6.63	0	0	62	3	943.52301	2828.55448	-6.44
vIGVSSFHAmAEQcITDQk	Unambiguous	2	1	Q3UNZ8	N-Term(iTRAQ8plex); M10(Oxidation); C14(Methylthio); K19(iTRAQ8plex)	CID	1.0000	0.0000	1	1	3597744	Unique	Used	0.829	0.876	1.080	1.481	1E-05	0	6.63	0	0	53	3	912.12720	2734.36704	-5.59
fVTDQSAcAGSPSLVAAAR	Unambiguous	2	1	Q8VCY8-2	N-Term(iTRAQ8plex); C8(Methylthio)	CID	1.0000	0.0000	1	1	3723704	Unique	Used	0.268	1.574	1.195	0.891	1E-05	0	6.63	0	0	77	2	1101.04626	2201.08525	-6.56
gTGGVDTAATGSVFDISNLDR	Unambiguous	1	1	P30275	N-Term(iTRAQ8plex)	CID	1.0000	0.0000	1	1	3264738	Unique	Used	0.851	1.249	0.780	0.674	1E-05	0	6.63	0	0	200	2	1179.09741	2357.18755	-0.50
tSRPENAIIYSnNEDFQVGQAk	Unambiguous	1	1	P40142	N-Term(iTRAQ8plex); N12(Deamidated); K22(iTRAQ8plex)	CID	1.0000	0.0000	1	1	2954978	Unique	Used	2.693		1.152	0.872	1E-05	0	6.63	0	0	200	3	1030.86584	3090.58298	-3.98
aGYTDQVVIGMDVAASEFYR	Unambiguous	2	1	P17182	N-Term(iTRAQ8plex)	CID	1.0000	0.0000	1	1	3327426	Unique	Used	0.895	1.391	1.288	1.264	1E-05	0	6.63	0	6	74	2	1248.61914	2496.23100	-2.92
tGPAGGFQAAPHTAAPYSQAAPAR	Unambiguous	1	1	P47708	N-Term(iTRAQ8plex)	CID	1.0000	0.0000	1	1	3717122	Unique	Used	0.903	0.714	0.861	0.752	1E-05	0	6.62	0	0	177	3	867.10944	2599.31375	-7.01
vFPGFPHSQPEDEDPSAGLGEEGSSGSLSR	Unambiguous	1	1	Q5SWZ5	N-Term(iTRAQ8plex)	CID	1.0000	0.0000	1	1	2812671	Unique	Used	1.003	0.956	1.013	0.938	1E-05	0	6.62	0	12	161	3	1126.19934	3376.58347	-1.12

eAGGIDLFVGGIGPDGHIAFNEPGSSLVSR	Unambiguous	2	1	Q9CRC9	N-Term(iTRAQ8plex)	CID	1.0000	0.0000	1	1	3439691	Unique	Used	1.518	0.931	1.131	1.178	1E-05	0	6.62	0	9	26	3	1091.56519	3272.68100	-1.36
gSQYGGPGWPVYGEEEAGR	Unambiguous	2	1	Q68FD9	N-Term(iTRAQ8plex)	CID	1.0000	0.0000	1	1	2866544	Unique	Used	0.561	0.964	1.295	0.562	1E-05	0	6.62	0	0	91	2	1150.54395	2300.08061	-3.47
fTNASDDDVAMDEPSLQFR	Unambiguous	2	1	Q3UFK8	N-Term(iTRAQ8plex)	CID	1.0000	0.0000	1	1	2762984	Unique	Used	1.375	0.290	0.480	0.919	1E-05	0	6.62	0	11	200	2	1231.57593	2462.14458	-0.09
vGFYVNTFQSIAGLEENFHk	Unambiguous	3	1	O08539	N-Term(iTRAQ8plex); K20(iTRAQ8plex)	CID	1.0000	0.0000	1	1	3687948	Unique	Used	2.972	1.288	2.177	1.330	1E-05	0	6.62	0	12	200	3	970.18353	2908.53604	-3.22
eIELLcQEHGQENDDLVQR	Unambiguous	4	1	E9Q6X0	N-Term(iTRAQ8plex); C6(Methylthio)	CID	1.0000	0.0000	1	1	2671363	Unique	Used	1.157	0.989	1.463	1.176	1E-05	0	6.62	0	30	200	3	873.41943	2618.24375	-2.11
eLGDDcNFGPALGEVGEAmR	Unambiguous	5	1	Q62420	N-Term(iTRAQ8plex); C6(Methylthio); M19(Oxidation)	CID	1.0000	0.0000	1	1	3056213	Unique	Used			1.729	0.434	1E-05	0	6.62	0	6	71	3	816.03809	2446.09970	0.23
eAEEEFWQnQHPQPYIFPDSPGGTSFER	Unambiguous	1	1	Q9CQJ8	N-Term(iTRAQ8plex); N9(Deamidated)	CID	0.0045	0.0000	1	1	2814782	Unique	Used	0.904	0.948	0.847	0.764	1E-05	0	6.62	0	2	99	3	1209.89331	3627.66538	1.28
eSTSFQDVGPQAPVGSVYQk	Unambiguous	3	1	Q62418-3	N-Term(iTRAQ8plex); K20(iTRAQ8plex)	CID	1.0000	0.0000	1	1	3126435	Unique	Used	1.147	0.883	1.093	0.644	1E-05	0	6.62	0	0	9	2	1366.71912	2732.43096	-1.55
lLVDVDESTLSPEEQkER	Unambiguous	2	1	G5E866	N-Term(iTRAQ8plex); K16(iTRAQ8plex)	CID	1.0000	0.0000	1	1	2670138	Unique	Used	1.107	1.213	1.568	1.266	1E-05	0	6.62	1	23	200	3	899.15710	2695.45676	-1.61
aGATSEGVLANFFnSLLSk	Unambiguous	1	1	Q8R1Q8	N-Term(iTRAQ8plex); N14(Deamidated); K19(iTRAQ8plex)	CID	0.0166	0.0000	1	1	3541226	Unique	Used	1.271	1.076	1.417	0.762	1E-05	0	6.62	0	8	8	2	1268.19299	2535.37871	-5.06
gcTGDLLEIQQPVDLAALR	Unambiguous	2	1	Q8K010	N-Term(iTRAQ8plex); C2(Methylthio)	CID	1.0000	0.0000	1	1	3479611	Unique	Used	0.735	1.152	0.850	1.209	1E-05	0	6.62	0	0	12	2	1181.61914	2362.23100	-4.36
fASEIVDTVYEDGDSIR	Unambiguous	3	1	J3QQ16	N-Term(iTRAQ8plex)	CID	1.0000	0.0000	1	1	2816167	Unique	Used		6.004	0.705	1.550	1E-05	0	6.62	0	3	85	2	1110.55090	2220.09453	-1.30
gIQFLQEQGmLGAAVEDIAQFLHQEER	Unambiguous	1	1	A2A5R2	N-Term(iTRAQ8plex); M10(Oxidation)	CID	1.0000	0.0000	1	1	3561259	Unique	Used	1.070	1.063	0.875	1.132	1E-05	0	6.62	0	8	37	3	1126.56885	3377.69199	-5.41

vVTQLTGTLPSGVTEDDVALHcNSR	Unambiguous	6	1	Q0VF62	N-Term(iTRAQ8plex); C22(Methylthio)	CID	1.0000	0.0000	1	1	3539600	Unique	Used	1.405	1.259	1.150	0.972	1E-05	0	6.62	0	0	67	3	988.16394	2962.47727	-4.86
vYSPYQDYYEVVPPNAHEATYVR	Unambiguous	6	1	Q9QYE9-4	N-Term(iTRAQ8plex)	CID	1.0000	0.0000	1	1	3212912	Unique	Used	3.130	0.602	0.776	0.732	1E-05	0	6.62	0	4	32	3	1022.17090	3064.49814	-0.42
vAVVQYSGqGQQQPGR	Unambiguous	1	1	Q04857	N-Term(iTRAQ8plex); Q9(Deamidated)	CID	0.0060	0.0000	1	1	3677400	Unique	Used	0.573	7.600	0.652	1.515	1E-05	0	6.62	0	0	200	2	1004.03687	2007.06645	5.14
sINPDEAVAYGAAVqAAILSGDk	Unambiguous	2	1	P63017	N-Term(iTRAQ8plex); Q15(Deamidated); K23(iTRAQ8plex)	CID	1.0000	0.0000	1	1	3441166	Unique	Used	0.862	1.255	1.547	1.274	1E-05	0	6.62	0	19	200	2	1435.28101	2869.55474	4.99
nRDEVQALAFDEQR	Unambiguous	1	1	Q8R066	N-Term(iTRAQ8plex)	CID	1.0000	0.0000	1	1	2732438	Unique	Used	0.947	1.339	1.413	1.400	1E-05	0	6.62	1	8	85	3	665.67737	1995.01755	-1.12
aQQEFAAGVFSNPAVR	Unambiguous	3	1	E9Q855	N-Term(iTRAQ8plex)	CID	1.0000	0.0000	1	1	3640150	Unique	Used	0.349	0.604	1.081	1.004	1E-05	0	6.62	0	27	31	2	998.52478	1996.04228	-6.59
aDVGSIGGTMSHEFQLPVDIGEDR	Unambiguous	2	1	Q8CFI5	N-Term(iTRAQ8plex)	CID	1.0000	0.0000	1	1	3215847	Unique	Used	1.825	1.343	1.597	1.391	1E-05	0	6.62	0	7	147	3	945.46967	2834.39444	0.39
hVnGQDQIVPGLYAcGEAAcASVHGANR	Unambiguous	1	1	Q8K2B3	N-Term(iTRAQ8plex); N3(Deamidated); C15(Methylthio); C20(Methylthio)	CID	0.0211	0.0000	1	1	3644498	Unique	Used	1.347	0.865	0.824	1.035	1E-05	0	6.62	0	27	25	3	1078.82751	3234.46799	-5.43
gGNVGInSFGFGGSNVHVILQPNTR	Unambiguous	1	1	P19096	N-Term(iTRAQ8plex); N7(Deamidated)	CID	0.0015	0.0000	1	1	3858147	Unique	Used	0.801	0.698	0.719	0.988	1E-05	0	6.62	0	8	58	3	949.49384	2846.46695	-6.41
qLFVLAGAAEEGFMTAELAGVIk	Unambiguous	1	1	B2RSH2	N-Term(iTRAQ8plex); K23(iTRAQ8plex)	CID	1.0000	0.0000	1	1	3602675	Unique	Used	1.239	1.598	1.131	0.835	1E-05	0	6.62	0	11	56	3	991.88556	2973.64212	-5.36
gQVFDQSGAPLPNVIVEVQDR	Unambiguous	2	1	Q80V42	N-Term(iTRAQ8plex)	CID	1.0000	0.0000	1	1	3350561	Unique	Used	2.482	0.300	1.388	1.399	1E-05	0	6.62	0	0	23	2	1286.68018	2572.35307	-5.55
eYTEGLLDnVTVLIGIAYEGk	Unambiguous	2	1	Q9Z0S1	N-Term(iTRAQ8plex); N9(Deamidated); K21(iTRAQ8plex)	CID	1.0000	0.0000	1	1	3500880	Unique	Used	0.396	0.947	1.459	1.942	1E-05	0	6.62	0	0	119	2	1453.80042	2906.59355	2.62
aMLSGPGQFAENETNEVNFR	Unambiguous	1	1	P83940	N-Term(iTRAQ8plex)	CID	1.0000	0.0000	1	1	3191896	Unique	Used	1.168	1.221	1.026	0.969	1E-05	0	6.61	0	0	48	2	1258.10901	2515.21074	-3.28

qAHLcVLASNcDEPMYVk	Unambiguous	2	1	Q6ZWZ6	N-Term(iTRAQ8plex); C5(Methylthio); C11(Methylthio); K18(iTRAQ8plex)	CID	1.0000	0.0000	1	1	3437149	Unique	Used	1.240	1.698	1.374	0.900	1E-05	0	6.61	0	10	48	3	907.77295	2721.30429	-3.99
gNDISSGTVLSDYVGSGPPSGTGLHR	Unambiguous	1	1	P70296	N-Term(iTRAQ8plex)	CID	1.0000	0.0000	1	1	3389934	Unique	Used	2.651	1.945	1.802	1.240	1E-05	0	6.61	0	8	3	2	1417.70825	2834.40923	-4.61
gGEATWSGSEFEISFPDSPGAqAQADHLPR	Unambiguous	1	1	Q91X51	N-Term(iTRAQ8plex); Q22(Deamidated)	CID	0.0242	0.0000	1	1	3151261	Unique	Used	3.348	1.006	1.448	1.248	1E-05	0	6.61	0	0	148	3	1150.54919	3449.63303	4.10
nVmSAFGLTDDQVSGPPSAPTEDR	Unambiguous	3	1	Q9Z1A1	N-Term(iTRAQ8plex); M3(Oxidation)	CID	1.0000	0.0000	1	1	3212414	Unique	Used	1.446	0.529	1.074	0.839	1E-05	0	6.61	0	0	183	2	1406.16870	2811.33013	-3.86
aGYTDQVVIGMDVAASEFYR	Unambiguous	2	1	P17182	N-Term(iTRAQ8plex)	CID	1.0000	0.0000	1	1	3352352	Unique	Used	1.077	1.485	1.061	1.058	1E-05	0	6.61	0	0	14	2	1248.61768	2496.22807	-4.10
sITIANQTNcPLYVTk	Unambiguous	1	1	O08553	N-Term(iTRAQ8plex); C10(Methylthio); K16(iTRAQ8plex)	CID	1.0000	0.0000	1	1	3803257	Unique	Used	1.271	1.202	1.354	1.556	1E-05	0	6.61	0	21	55	2	1210.65393	2420.30058	-5.46
tGPAGGFQAAPHTAAPYSQAAPAR	Unambiguous	1	1	P47708	N-Term(iTRAQ8plex)	CID	1.0000	0.0000	1	1	3717128	Unique	Used	0.893	0.664	1.204	0.828	1E-05	0	6.61	0	17	165	3	867.11066	2599.31742	-5.60
aVTLHDQGTTQWADLSSQFYLR	Unambiguous	1	1	Q02053	N-Term(iTRAQ8plex)	CID	1.0000	0.0000	1	1	3705190	Unique	Used	1.144	1.184	0.857	1.171	1E-05	0	6.61	0	28	126	3	947.81458	2841.42917	-6.40
aAGAEEYAQQEVVkR	Unambiguous	2	1	P16330	N-Term(iTRAQ8plex); K14(iTRAQ8plex)	CID	1.0000	0.0000	1	1	3890615	Unique	Used	0.865	0.142	1.509	1.596	1E-05	0	6.61	1	4	143	3	753.08032	2257.22641	-5.90
aEAMQEASEAVPSGMLSVLGQR	Unambiguous	1	1	Q8R3F5	N-Term(iTRAQ8plex)	CID	1.0000	0.0000	1	1	3500113	Unique	Used	1.005	1.295	1.086	0.829	1E-05	0	6.61	0	0	24	2	1283.14783	2565.28838	-2.79
aNLMHnLGGEEVSVAck	Unambiguous	1	1	P29699	N-Term(iTRAQ8plex); N6(Deamidated); C16(Methylthio); K17(iTRAQ8plex)	CID	1.0000	0.0000	1	1	3345525	Unique	Used	3.374	1.714	0.430	0.594	1E-05	0	6.61	0	17	52	2	1214.12073	2427.23418	2.08
yFDSFGDLSSASAIMGNPk	Unambiguous	1	1	P02089	N-Term(iTRAQ8plex); K19(iTRAQ8plex)	CID	1.0000	0.0000	1	1	3560564	Unique	Used			3.648	0.737	1E-05	0	6.61	0	11	33	2	1308.16089	2615.31450	-4.87

aVGLISHVLEQNQEGDAAYR	Unambiguous	3	1	Q9JLZ3	N-Term(iTRAQ8plex)	CID	1.0000	0.0000	1	1	3393928	Unique	Used	1.095	0.831	1.426	1.749	1E-05	0	6.61	0	0	90	2	1237.64709	2474.28691	-2.93
tGSEPSPPqGGGSLGPSAPPEEIWVLR	Unambiguous	3	1	Q6P9K8	N-Term(iTRAQ8plex); Q9(Deamidated)	CID	1.0000	0.0000	1	1	3303779	Unique	Used	1.375	0.412	1.266	0.852	1E-05	0	6.61	0	28	157	2	1504.27698	3007.54668	5.03
gASQQDLPSGLAnScQQDR	Unambiguous	3	1	Q3UX37	N-Term(iTRAQ8plex); N13(Deamidated); C15(Methylthio)	CID	0.0257	0.0000	1	1	3209929	Unique	Used	0.379	0.169	0.542	0.501	1E-05	0	6.61	0	0	178	2	1163.54346	2326.07964	3.89
dILGGnGISDEYHVIR	Unambiguous	1	1	Q60759	N-Term(iTRAQ8plex); N6(Deamidated)	CID	1.0000	0.0000	1	1	2957110	Unique	Used	0.778	0.944	1.279	1.313	1E-05	0	6.61	0	4	117	3	688.36096	2063.06833	-1.36
sFESTVGQGSDTYSYIFR	Unambiguous	1	1	P24668	N-Term(iTRAQ8plex)	CID	1.0000	0.0000	1	1	3457177	Unique	Used	0.915	1.248	0.783	1.014	1E-05	0	6.61	0	0	200	2	1174.56848	2348.12969	-2.20
aGYTDQVVIGMDVAASEFYR	Unambiguous	2	1	P17182	N-Term(iTRAQ8plex)	CID	1.0000	0.0000	1	1	3355326	Unique	Used	1.008	1.183	1.088	1.516	1E-05	0	6.61	0	21	200	2	1248.61877	2496.23027	-3.22
vLVMTNDYYYTDIk	Unambiguous	1	1	Q9Z1L5	N-Term(iTRAQ8plex); K14(iTRAQ8plex)	CID	1.0000	0.0000	1	1	3325273	Unique	Used	0.589	0.683	1.093	0.772	1E-05	0	6.61	0	0	170	2	1173.62561	2346.24394	-3.08
yTPSGQSGAAASESLFISNHAY	Unambiguous	2	1	A6ZI44	N-Term(iTRAQ8plex)	CID	1.0000	0.0000	1	1	3258133	Unique	Used	0.979	0.705	1.570	1.556	1E-05	0	6.61	0	0	26	2	1281.61902	2562.23076	-4.17
dQAGEYEcSAEnDVSFPDVk	Unambiguous	3	1	A0A4W9	N-Term(iTRAQ8plex); C8(Methylthio); N12(Deamidated); K20(iTRAQ8plex)	CID	0.0454	0.0000	1	1	2637312	Unique	Used		1.336	1.078	0.525	1E-05	0	6.61	0	18	200	2	1429.65491	2858.30254	2.38
mQAqASIEGAPEVTMSSLFk	Unambiguous	5	1	A2ASZ8-5	N-Term(iTRAQ8plex); Q4(Deamidated); K20(iTRAQ8plex)	CID	0.0166	0.0000	1	1	3439208	Unique	Used	0.593	0.470	0.702	0.971	1E-05	0	6.61	0	0	200	2	1367.71887	2734.43047	1.91
nTSNQAISQNTVFTIENNPGHR	Unambiguous	2	1	Q80XP9-2	N-Term(iTRAQ8plex)	CID	1.0000	0.0000	1	1	3431685	Unique	Used	1.638	1.005	1.056	1.033	1E-05	0	6.61	0	0	61	3	916.12665	2746.36539	-5.71
lYVTYFGGDEAAGLEPDLEcR	Unambiguous	1	1	Q8BGQ7	N-Term(iTRAQ8plex); C20(Methylthio)	CID	1.0000	0.0000	1	1	3112109	Unique	Used	1.194	0.638	0.856	1.002	1E-05	0	6.61	0	0	63	2	1334.63135	2668.25542	-0.86

kYGGPPPDSVYSGqQPSVGTEIFVGk	Unambiguous	7	1	G3V018	N-Term(iTRAQ8plex); K1(iTRAQ8plex); Q14(Deamidated); K26(iTRAQ8plex)	CID	0.0136	0.0000	1	1	3368623	Unique	Used	1.098	0.983	0.989	0.770	1E-05	0	6.61	1	0	119	3	1203.31934	3607.94345	0.63
qLnTIQNAEAVHFFcNYEESR	Unambiguous	4	1	P61922	N-Term(iTRAQ8plex); N3(Deamidated); C15(Methylthio)	CID	0.0469	0.0000	1	1	3283430	Unique	Used	1.601	0.789	0.960	1.381	1E-05	0	6.61	0	30	128	3	955.44501	2864.32047	-2.83
sITIANQTncPLYVTk	Unambiguous	1	1	O08553	N-Term(iTRAQ8plex); N9(Deamidated); C10(Methylthio); K16(iTRAQ8plex)	CID	0.0242	0.0000	1	1	3579242	Unique	Used	1.291	0.972	1.148	1.155	1E-05	0	6.61	0	0	140	2	1211.14941	2421.29155	-2.58
kIISLSQLLQEDSLNVADLSSLR	Unambiguous	3	1	P97372	N-Term(iTRAQ8plex); K1(iTRAQ8plex)	CID	1.0000	0.0000	1	1	3806103	Unique	Used				1.147	1E-05	0	6.61	1	20	18	3	1050.94189	3150.81113	-2.65
mTVNESEQLVScSMDDTVR	Unambiguous	1	1	O88342	N-Term(iTRAQ8plex); C12(Methylthio)	CID	1.0000	0.0000	1	1	2977708	Unique	Used	1.045	0.982	0.995	1.147	1E-05	0	6.61	0	9	200	2	1247.56152	2494.11577	-3.15
skPYLTVDQMmDFINLk	Unambiguous	3	1	Q9Z1B3-3	N-Term(iTRAQ8plex); K2(iTRAQ8plex); M11(Oxidation); K17(iTRAQ8plex)	CID	1.0000	0.0000	1	1	3705500	Unique	Used	0.284	1.524	2.124	1.087	1E-05	0	6.61	0	13	27	3	991.21265	2971.62339	-5.60
sTVDGLQVTqLNMDHTTENEDELFR	Unambiguous	1	1	Q8CF89	N-Term(iTRAQ8plex); Q10(Deamidated)	CID	1.0000	0.0000	1	1	2902668	Unique	Used	1.175	1.230	1.374	0.748	1E-05	0	6.61	0	12	200	3	1066.51538	3197.53159	3.28
kTESIDVMDAVGSNIVVSTR	Unambiguous	1	1	Q91VD9	N-Term(iTRAQ8plex); K1(iTRAQ8plex)	CID	1.0000	0.0000	1	1	3392116	Unique	Used	1.295	1.181	1.088	1.152	1E-05	0	6.61	1	14	70	3	910.50018	2729.48600	-3.83

aAQLcGAGMAAVVEk	Unambiguous	5	1	G3UVV4	N-Term(iTRAQ8plex); C5(Methylthio); K15(iTRAQ8plex)	CID	1.0000	0.0000	1	1	3600578	Unique	Used	0.769	1.644	0.995	1.185	1E-05	0	6.61	0	24	108	2	1037.05151	2073.09575	-7.63
fQMPDQGMTSADDFFQGTk	Unambiguous	1	1	O08553	N-Term(iTRAQ8plex); K19(iTRAQ8plex)	CID	1.0000	0.0000	1	1	3304583	Unique	Used	2.181	1.343	1.218	1.833	1E-05	0	6.61	0	7	39	3	920.44366	2759.31644	-3.68
vYFQSPPGAAGEGPGGADDDGPVR	Unambiguous	1	1	Q62084	N-Term(iTRAQ8plex)	CID	1.0000	0.0000	1	1	2758926	Unique	Used	1.812	0.639	1.291	0.680	1E-05	0	6.60	0	0	200	2	1310.63367	2620.26006	0.70
qQDAQEFFLHLINMVER	Unambiguous	2	1	P56399	N-Term(iTRAQ8plex)	CID	1.0000	0.0000	1	1	3706926	Unique	Used	0.879	1.321	1.142	1.276	1E-05	0	6.60	0	26	15	3	808.08270	2422.23355	-6.43
nTSNQAISQNTVFTIENNPGHR	Unambiguous	2	1	Q80XP9-2	N-Term(iTRAQ8plex)	CID	1.0000	0.0000	1	1	3452135	Unique	Used	0.916	0.901	1.048	0.901	1E-05	0	6.60	0	24	163	3	916.12854	2746.37107	-3.65
qLGSLGVLGITAPVQYGGSGLGYLEHVLVMEEISR	Unambiguous	1	1	Q9JHI5	N-Term(iTRAQ8plex)	CID	1.0000	0.0000	1	1	3842773	Unique	Used	1.648	1.065	0.995	1.035	1E-05	0	6.60	0	15	37	3	1316.37500	3947.11045	-3.82
rEQEPGAGTPGIAAAEqQSVEFDSPDSGLPSSR	Unambiguous	2	1	Q80U12	N-Term(iTRAQ8plex); Q17(Deamidated)	CID	0.0258	0.0000	1	1	2811419	Unique	Used		1.925	0.847	0.528	1E-05	0	6.60	1	28	200	3	1225.93274	3675.78366	4.33
aESTSSISSTnSLSAATTPTVGSSR	Unambiguous	3	1	Q8VD37	N-Term(iTRAQ8plex); N11(Deamidated)	CID	1.0000	0.0000	1	1	3187076	Unique	Used	1.257	1.667	2.668	1.485	1E-05	0	6.60	0	0	72	2	1352.67261	2704.33794	-1.76
aLYLIATnGTPELQNPER	Unambiguous	2	1	Q61036-2	N-Term(iTRAQ8plex); N8(Deamidated)	CID	0.0061	0.0000	1	1	3622102	Unique	Used	0.863	1.142	0.826	1.262	1E-05	0	6.60	0	0	130	2	1153.12415	2305.24101	2.96
gYTVNVFDIHQGFDNPR	Unambiguous	1	1	Q9R1J0	N-Term(iTRAQ8plex)	CID	1.0000	0.0000	1	1	3496173	Unique	Used	0.959	0.591	0.865	0.975	1E-05	0	6.60	0	7	27	3	761.71863	2283.14133	-2.06
eAFQNAYLELGGLGER	Unambiguous	2	1	Q6PIC6	N-Term(iTRAQ8plex)	CID	1.0000	0.0000	1	1	3350135	Unique	Used	0.638	0.712	1.004	1.147	1E-05	0	6.60	0	8	15	2	1036.03796	2071.06865	-3.66
aGYTDQVVIGMDVAASEFYR	Unambiguous	2	1	P17182	N-Term(iTRAQ8plex)	CID	1.0000	0.0000	1	1	3354021	Unique	Used	0.874	1.231	1.151	1.220	1E-05	0	6.60	0	0	197	2	1248.62244	2496.23760	-0.28
mcDPGMTAFEPEALGNLVEGLDFHR	Unambiguous	4	1	P11798	N-Term(iTRAQ8plex); C2(Methylthio)	CID	1.0000	0.0000	1	1	3459609	Unique	Used		2.094	2.197	1.254	1E-05	0	6.60	0	0	6	3	1033.81140	3099.41965	-4.99
gITNLcVIGGDGSLTGADTFR	Unambiguous	1	1	P47857	N-Term(iTRAQ8plex); C6(Methylthio)	CID	1.0000	0.0000	1	1	3729257	Unique	Used	2.209	0.620	0.620	1.242	1E-05	0	6.60	0	0	200	2	1209.10046	2417.19365	-7.06
fGDEDITWQDEHSAPFSWETR	Unambiguous	1	1	Q8C3W1	N-Term(iTRAQ8plex)	CID	1.0000	0.0000	1	1	2903083	Unique	Used	1.537	0.640	0.916	0.650	1E-05	0	6.60	0	0	112	3	953.10175	2857.29068	-3.54

eGSYSISVLYGEEEVPR	Unambiguous	3	1	B7FAU9	N-Term(iTRAQ8plex)	CID	1.0000	0.0000	1	1	2813749	Unique	Used	0.971	4.714	0.769	1.046	1E-05	0	6.60	0	0	200	2	1109.55933	2218.11138	-3.05
gVPVAEEVSALFAGELNPVAPk	Unambiguous	1	1	O54774	N-Term(iTRAQ8plex); K22(iTRAQ8plex)	CID	1.0000	0.0000	1	1	3419191	Unique	Used	1.366	0.420	0.535	0.733	1E-05	0	6.60	0	14	31	2	1401.79028	2802.57329	-4.63
yTPSGQSGAAASESLFISNHAY	Unambiguous	2	1	A6ZI44	N-Term(iTRAQ8plex)	CID	1.0000	0.0000	1	1	3241326	Unique	Used				1.442	1E-05	0	6.60	0	0	200	2	1281.62622	2562.24516	1.45
vLAGDVETHAEMVHGAFQAQR	Unambiguous	2	1	Q9CYT6	N-Term(iTRAQ8plex)	CID	1.0000	0.0000	1	1	3411405	Unique	Used	1.039	1.179	1.338	0.864	1E-05	0	6.60	0	29	7	3	857.43860	2570.30124	-2.94
dSEIFYQFTSFLSPGVIYHcDLTk	Unambiguous	1	1	Q9QUR6	N-Term(iTRAQ8plex); C20(Methylthio); K24(iTRAQ8plex)	CID	1.0000	0.0000	1	1	3785722	Unique	Used	0.608	0.663	0.804	0.816	1E-05	0	6.60	0	0	42	3	1155.58191	3464.73117	-1.60
vGSHITGGDIYGIVNEnSLIk	Unambiguous	4	1	P50516	N-Term(iTRAQ8plex); N17(Deamidated); K21(iTRAQ8plex)	CID	1.0000	0.0000	1	1	3301908	Unique	Used	1.871	1.365	1.102	1.105	1E-05	0	6.60	0	0	72	3	932.51416	2795.52793	-4.32
dIILATDLAHHLR	Unambiguous	3	1	Q5PR72	N-Term(iTRAQ8plex)	CID	1.0000	0.0000	1	1	3576361	Unique	Used	0.756	1.373	1.262	1.242	1E-05	0	6.60	0	18	62	3	598.01501	1792.03049	-4.36
iGEHTPSALAIMENANVLAR	Unambiguous	3	1	A6ZI44	N-Term(iTRAQ8plex)	CID	1.0000	0.0000	1	1	3707633	Unique	Used	1.336	1.151	1.045	1.190	1E-05	0	6.60	0	18	200	3	804.43414	2411.28788	-5.82
iEESDQGPYAIILAPTR	Unambiguous	1	1	D3Z0M9	N-Term(iTRAQ8plex)	CID	1.0000	0.0000	1	1	3215198	Unique	Used	0.896	1.005	0.880	1.062	1E-05	0	6.60	0	17	200	2	1089.09534	2177.18340	3.58
aGYTDQVVIGMDVAASEFYR	Unambiguous	2	1	P17182	N-Term(iTRAQ8plex)	CID	1.0000	0.0000	1	1	3328745	Unique	Used	0.745	1.208	1.384	1.278	1E-05	0	6.60	0	4	200	2	1248.62000	2496.23271	-2.24
iGTPAMVAEASLPVHSGAAEnPGTSGSSk	Unambiguous	1	1	Q6A058	N-Term(iTRAQ8plex); N21(Deamidated); K29(iTRAQ8plex)	CID	1.0000	0.0000	1	1	3344676	Unique	Used	1.980	0.923	0.950	1.026	1E-05	0	6.60	0	0	59	3	1111.58167	3332.73044	1.52
qLQNISQAAASGGAk	Unambiguous	1	1	Q9JHU4	N-Term(iTRAQ8plex); K15(iTRAQ8plex)	CID	1.0000	0.0000	1	1	3531928	Unique	Used	0.362	0.630	0.825	1.066	1E-05	0	6.60	0	0	188	2	1026.58130	2052.15532	-5.12
nIFLqYASTEVDGEHYMTPEDFVQR	Unambiguous	1	1	Q8BH59	N-Term(iTRAQ8plex); Q5(Deamidated)	CID	1.0000	0.0000	1	1	3263830	Unique	Used	1.347	1.499	0.886	1.535	1E-05	0	6.60	0	15	200	3	1098.86316	3294.57492	5.52
eATWESVSMMLQLGGTVIGSAR	Unambiguous	1	1	P47857	N-Term(iTRAQ8plex)	CID	1.0000	0.0000	1	1	3918149	Unique	Used	3.170	1.205	1.017	1.028	1E-05	0	6.59	0	15	62	2	1314.17224	2627.33721	-3.95

sISMTTFTEPEVLFLQSR	Unambiguous	4	1	Q80WC7-2	N-Term(iTRAQ8plex)	CID	1.0000	0.0000	1	1	3706614	Unique	Used	1.142	1.778	1.485	1.292	1E-05	0	6.59	0	0	17	2	1195.62341	2390.23955	-7.71
eEIFGPVMSILTFGTEAEVLER	Unambiguous	2	1	Q9JLJ2	N-Term(iTRAQ8plex)	CID	1.0000	0.0000	1	1	3501140	Unique	Used	0.673	1.049	0.984	1.731	1E-05	0	6.59	0	8	198	2	1386.22070	2771.43413	-5.01
eVAAFAQFGSDLDAATQQLLSR	Unambiguous	2	1	Q03265	N-Term(iTRAQ8plex)	CID	1.0000	0.0000	1	1	3668928	Unique	Used	0.947	1.072	0.790	1.512	1E-05	0	6.59	0	5	200	2	1321.68262	2642.35796	-5.63
aQESVGIFEVTHQFVk	Unambiguous	3	1	Q8BHG2	N-Term(iTRAQ8plex); K16(iTRAQ8plex)	CID	1.0000	0.0000	1	1	3346908	Unique	Used	1.655	1.066	1.208	0.984	1E-05	0	6.59	0	7	3	3	809.78442	2427.33872	-4.35
eATDTPIVIQPASATETTqLTADSHPSYHTDGFN	Unambiguous	1	1	P60904	N-Term(iTRAQ8plex); Q19(Deamidated)	CID	0.0182	0.0000	1	1	2994642	Unique	Used	1.612	2.102	1.979	0.898	1E-05	0	6.59	0	0	200	3	1307.62793	3920.86924	1.93
aGYTDQVVIGMDVAASEFYR	Unambiguous	2	1	P17182	N-Term(iTRAQ8plex)	CID	1.0000	0.0000	1	1	3328640	Unique	Used	0.861	1.290	1.450	1.211	1E-05	0	6.59	0	0	200	2	1248.61975	2496.23223	-2.43
vQENLLASGVDLVTYITR	Unambiguous	1	1	Q9Z1G3	N-Term(iTRAQ8plex)	CID	1.0000	0.0000	1	1	3825001	Unique	Used	1.033	0.997	0.608	0.628	1E-05	0	6.59	0	5	52	2	1148.14331	2295.27934	-3.00
eSTFLDEGPNEQEITPLQHTPR	Unambiguous	3	1	Q9QYR6	N-Term(iTRAQ8plex)	CID	1.0000	0.0000	1	1	2811975	Unique	Used	2.260	1.082	2.057	1.233	1E-05	0	6.59	0	2	15	3	948.14484	2842.41996	1.34
fQmPDQGMTSADDFFQGTk	Unambiguous	1	1	O08553	N-Term(iTRAQ8plex); M3(Oxidation); K19(iTRAQ8plex)	CID	1.0000	0.0000	1	1	3306622	Unique	Used		1.211	1.275	1.359	1E-05	0	6.59	0	0	200	3	925.77521	2775.31107	-3.76
aVEASNAYSSILQAVQAAEDAAGQALR	Unambiguous	1	1	Q61001	N-Term(iTRAQ8plex)	CID	1.0000	0.0000	1	1	3766737	Unique	Used		1.774	1.181	1.545	1E-05	0	6.59	0	0	53	2	1504.78003	3008.55278	-2.12
icDPGLTSFEPEALGNLVEGMDFHR	Unambiguous	9	1	Q5SVJ0	N-Term(iTRAQ8plex); C2(Methylthio)	CID	1.0000	0.0000	1	1	3480618	Unique	Used	0.740	0.903	1.385	0.798	1E-05	0	6.59	0	9	200	3	1033.15759	3097.45823	-4.96
gTQPTVEAGVAHmIEALGVLEPR	Unambiguous	3	1	Q3U1Y4	N-Term(iTRAQ8plex); M13(Oxidation)	CID	1.0000	0.0000	1	1	3685519	Unique	Used	1.126	0.866	1.100	1.268	1E-05	0	6.59	0	17	121	3	899.14557	2695.42215	-6.31
iGEHTPSALAImENANVLAR	Unambiguous	3	1	A6ZI44	N-Term(iTRAQ8plex); M12(Oxidation)	CID	1.0000	0.0000	1	1	3701355	Unique	Used	3.114	0.948	0.686	0.612	1E-05	0	6.59	0	0	71	3	809.76593	2427.28324	-5.60
qLGLAEDEEVLFTVFAQGQk	Unambiguous	1	1	P70206	N-Term(iTRAQ8plex); K20(iTRAQ8plex)	CID	1.0000	0.0000	1	1	3262833	Unique	Used	0.208	0.999	0.526	0.876	1E-05	0	6.59	0	11	39	2	1415.77539	2830.54350	-0.45
iAFIMDESNVLDSGFLER	Unambiguous	1	1	Q9JHU4	N-Term(iTRAQ8plex)	CID	1.0000	0.0000	1	1	3352922	Unique	Used	0.533	0.833	1.058	1.064	1E-05	0	6.59	0	2	3	2	1180.60559	2360.20391	-3.02

eSTDGSINSYSSEGNLIFPGVR	Unambiguous	11	1	Q99NE5-5	N-Term(iTRAQ8plex)	CID	1.0000	0.0000	1	1	3150839	Unique	Used	0.726	1.097	1.523	1.012	1E-05	0	6.59	0	0	144	2	1317.14966	2633.29204	-2.91
tDEFQLHTNVnDGTEFGGSIYQk	Unambiguous	3	1	Q60932-2	N-Term(iTRAQ8plex); N11(Deamidated); K23(iTRAQ8plex)	CID	1.0000	0.0000	1	1	2957449	Unique	Used	1.139	1.412	1.089	1.212	1E-05	0	6.59	0	0	200	3	1070.53455	3209.58908	1.34
vASIQSEPGQQNVLLQQPLGR	Unambiguous	3	1	Q8BLN6	N-Term(iTRAQ8plex)	CID	1.0000	0.0000	1	1	3703305	Unique	Used	0.595	0.733	0.887	1.179	1E-05	0	6.59	0	0	163	2	1283.70801	2566.40874	-6.54
dINQEVYnFLATAGAk	Unambiguous	1	1	Q99KI0	N-Term(iTRAQ8plex); N8(Deamidated); K16(iTRAQ8plex)	CID	1.0000	0.0000	1	1	3544715	Unique	Used				1.973	1E-05	0	6.59	0	14	200	2	1182.14221	2363.27715	2.88
yEGSGDGGAAAQSLYIAnHAY	Unambiguous	1	1	P05063	N-Term(iTRAQ8plex); N18(Deamidated)	CID	1.0000	0.0000	1	1	2936773	Unique	Used	3.204	0.669	0.908	1.820	1E-05	0	6.59	0	10	74	2	1210.56970	2420.13213	0.54
aITHLNnNFMFGQk	Unambiguous	3	1	G5E924	N-Term(iTRAQ8plex); N7(Deamidated); K14(iTRAQ8plex)	CID	0.0228	0.0000	1	1	3555949	Unique	Used	0.859	1.110	0.973	1.044	1E-05	0	6.59	0	18	111	3	748.73529	2244.19132	-6.35
gPQAAQEQDFYTVELER	Unambiguous	4	1	Q6RHR9-3	N-Term(iTRAQ8plex)	CID	1.0000	0.0000	1	1	2701717	Unique	Used	2.609	0.668	0.795	0.995	1E-05	0	6.59	0	5	200	2	1143.06824	2285.12920	-2.63
aFLQGGQEATDIALLLR	Unambiguous	5	1	E9Q3M3	N-Term(iTRAQ8plex)	CID	1.0000	0.0000	1	1	3686667	Unique	Used	0.437	0.827	0.889	1.268	1E-05	0	6.59	0	1	2	2	1060.59778	2120.18828	-6.37
dNLTLWTSDMQGDGEEQNk	Unambiguous	1	1	P62259	N-Term(iTRAQ8plex); K19(iTRAQ8plex)	CID	1.0000	0.0000	1	1	2654237	Unique	Used	2.825				1E-05	0	6.59	0	23	200	3	930.45496	2789.35031	-0.21
sVPGALDkDLnSLEDTEDGVDLVSSR	Unambiguous	2	1	Q80T23-2	N-Term(iTRAQ8plex); K8(iTRAQ8plex); N11(Deamidated)	CID	1.0000	0.0000	1	1	2833995	Unique	Used			1.662	1.352	1E-05	0	6.59	1	8	200	3	1114.24658	3340.72519	1.04
vEGFNYAGMGnSTNk	Unambiguous	3	1	E9QNN1	N-Term(iTRAQ8plex); N11(Deamidated); K15(iTRAQ8plex)	CID	0.0076	0.0000	1	1	3298457	Unique	Used	1.031	0.979	0.875	0.898	1E-05	0	6.59	0	0	200	2	1099.56091	2198.11455	6.24

gENLEAVVcEEPQVTYSk	Unambiguous	4	1	Q8BIF0-2	N-Term(iTRAQ8plex); C9(Methylthio); K18(iTRAQ8plex)	CID	1.0000	0.0000	1	1	2886468	Unique	Used	1.139	0.956	2.343	3.020	1E-05	0	6.59	0	0	200	2	1325.16809	2649.32891	-2.71
tIGGGDDSFNTFFSETGAGk	Unambiguous	4	1	P68373	N-Term(iTRAQ8plex); K20(iTRAQ8plex)	CID	1.0000	0.0000	1	1	3152184	Unique	Used	0.464	1.588	0.968	1.294	1E-05	0	6.59	0	0	172	2	1308.65161	2616.29595	-3.03
ITLYDIAHTPGVAADLSHIETR	Unambiguous	1	1	P08249	N-Term(iTRAQ8plex)	CID	1.0000	0.0000	1	1	3477804	Unique	Used	2.053	0.958	0.807	0.856	1E-05	0	6.58	0	27	29	3	899.81775	2697.43869	-4.71
qAFTDVATGSLGQGLGAAcGMAYTGk	Unambiguous	1	1	P40142	N-Term(iTRAQ8plex); C19(Methylthio); K26(iTRAQ8plex)	CID	1.0000	0.0000	1	1	3842213	Unique	Used			0.653		1E-05	0	6.58	0	20	14	3	1043.85364	3129.54636	-5.27
qGTFHSQQALEYGTk	Unambiguous	1	1	Q9WUM5	N-Term(iTRAQ8plex); K15(iTRAQ8plex)	CID	1.0000	0.0000	1	1	3162833	Unique	Used	1.268	0.865	1.491	1.397	1E-05	0	6.58	0	6	72	2	1152.11047	2303.21367	-4.50
eAFQNAYLELGGLGER	Unambiguous	2	1	Q6PIC6	N-Term(iTRAQ8plex)	CID	1.0000	0.0000	1	1	3355809	Unique	Used	0.592	0.568	0.911	1.207	1E-05	0	6.58	0	18	200	2	1036.03772	2071.06816	-3.90
gLTESNMAqAAWESGcER	Unambiguous	2	1	Q8K284-2	N-Term(iTRAQ8plex); Q9(Deamidated); C16(Methylthio)	CID	0.0061	0.0000	1	1	3170340	Unique	Used	0.903	0.823	0.806	0.774	1E-05	0	6.58	0	0	200	2	1146.01331	2291.01933	6.48
gHYVLcDFGSATNk	Unambiguous	2	1	Q3UHJ0-2	N-Term(iTRAQ8plex); C6(Methylthio); K14(iTRAQ8plex)	CID	1.0000	0.0000	1	1	3514783	Unique	Used	0.738	1.185	1.080	1.256	1E-05	0	6.58	0	2	13	3	722.69989	2166.08512	-3.75
sSEYHLEDNAAYYLNDLER	Unambiguous	1	1	O70443	N-Term(iTRAQ8plex)	CID	1.0000	0.0000	1	1	2796491	Unique	Used	0.647	1.248	1.001	0.974	1E-05	0	6.58	0	13	192	3	869.41467	2606.22947	-0.69
qMNPYIQVTSHQNR	Unambiguous	1	1	Q02053	N-Term(iTRAQ8plex)	CID	1.0000	0.0000	1	1	3736738	Unique	Used	0.665	0.759	1.116	1.181	1E-05	0	6.58	0	1	5	3	674.01385	2020.02701	-3.27
ekTPAPAEGQcADFVSFYGGLAEASQR	Unambiguous	2	1	Q99J39-2	N-Term(iTRAQ8plex); K2(iTRAQ8plex); C11(Methylthio)	CID	1.0000	0.0000	1	1	3372997	Unique	Used	0.821	0.846	0.851	1.135	1E-05	0	6.58	1	11	94	3	1161.90356	3483.69614	-4.96
lEGLSENTDYTVLLQAAQEATR	Unambiguous	2	1	Q8BYI9	N-Term(iTRAQ8plex)	CID	1.0000	0.0000	1	1	3418988	Unique	Used	1.817	1.319	1.402	1.227	1E-05	0	6.58	0	0	10	2	1363.70557	2726.40386	-4.12

eLGSPPGISLETIDAAFScPGSSR	Unambiguous	1	1	Q91X72	N-Term(iTRAQ8plex); C19(Methylthio)	CID	1.0000	0.0000	1	1	3623386	Unique	Used	3.823	2.130	1.220	2.252	1E-05	0	6.58	0	0	20	2	1371.16724	2741.32720	-5.72
eGPNFITMEGTVSDHIER	Unambiguous	2	1	Q7TSH2	N-Term(iTRAQ8plex)	CID	1.0000	0.0000	1	1	2937298	Unique	Used	1.773	1.095	1.370	1.221	1E-05	0	6.58	0	29	51	3	779.38666	2336.14542	-1.74
fAPQFSGYQQQDSQELLAFILDGLHEDLNR	Unambiguous	1	1	P35123	N-Term(iTRAQ8plex)	CID	1.0000	0.0000	1	1	3581692	Unique	Used	0.645	0.841	1.158	1.207	1E-05	0	6.58	0	9	44	3	1261.96240	3783.87265	-5.14
sINPDEAVAYGAAVqAAILSGDk	Unambiguous	2	1	P63017	N-Term(iTRAQ8plex); Q15(Deamidated); K23(iTRAQ8plex)	CID	1.0000	0.0000	1	1	3441288	Unique	Used	1.025	0.543	1.155	1.219	1E-05	0	6.58	0	0	200	2	1435.27979	2869.55229	4.14
tGPAGGFQAAPHTAAPYSQAAPAR	Unambiguous	1	1	P47708	N-Term(iTRAQ8plex)	CID	1.0000	0.0000	1	1	3697215	Unique	Used	2.471	1.119	1.107	1.081	1E-05	0	6.58	0	0	200	3	867.10925	2599.31321	-7.22
yHTSQSGDEMTSLSEYVSR	Unambiguous	2	1	P11499	N-Term(iTRAQ8plex)	CID	1.0000	0.0000	1	1	3010660	Unique	Used	1.791	1.170	0.940	0.845	1E-05	0	6.58	0	8	85	3	827.72107	2481.14865	-0.77
lQQELDDLTVDLDHQR	Unambiguous	3	1	Q5SV64	N-Term(iTRAQ8plex)	CID	1.0000	0.0000	1	1	2817365	Unique	Used		1.151	2.021	1.704	1E-05	0	6.58	0	9	200	3	748.05786	2242.15903	-1.21
vASSVPVENFTIHGGLSR	Unambiguous	4	1	Q60597	N-Term(iTRAQ8plex)	CID	1.0000	0.0000	1	1	3800604	Unique	Used	1.032	0.808	1.014	1.250	1E-05	0	6.58	0	5	36	3	725.39795	2174.17929	-3.63
qASEQNWANYSAEQnR	Unambiguous	1	1	P23242	N-Term(iTRAQ8plex); N15(Deamidated)	CID	0.0015	0.0000	1	1	2632545	Unique	Used	0.868	0.852	1.723	1.973	1E-05	0	6.58	0	0	200	2	1101.00891	2201.01055	-2.54
eEQSQITSQVTGQIGWR	Unambiguous	2	1	Q3TWV4	N-Term(iTRAQ8plex)	CID	1.0000	0.0000	1	1	3368703	Unique	Used	0.318	1.235	1.151	1.142	1E-05	0	6.58	0	10	35	2	1126.07959	2251.15190	-4.52
qLAnSLSGVQNQLQSFNSYR	Unambiguous	1	1	Q68FG2	N-Term(iTRAQ8plex); N4(Deamidated)	CID	1.0000	0.0000	1	1	3804188	Unique	Used	0.937	2.964	1.603	1.217	1E-05	0	6.58	0	0	131	2	1280.15503	2559.30278	-3.03
aVGFVSEDEYLEIQGITR	Unambiguous	3	1	D3Z396	N-Term(iTRAQ8plex)	CID	1.0000	0.0000	1	1	3132569	Unique	Used	1.145	1.130	1.002	0.943	1E-05	0	6.58	0	11	181	2	1165.61133	2330.21538	-1.22
kGPPLGGGEGETESADTMPFVMLTR	Unambiguous	1	1	A2AT37	N-Term(iTRAQ8plex); K1(iTRAQ8plex)	CID	1.0000	0.0000	1	1	3302632	Unique	Used	1.077	1.170	1.256	1.012	1E-05	0	6.58	1	0	144	3	1062.55164	3185.64035	-0.90
gEMMDLQHGSLFLQTPk	Unambiguous	2	1	P16125	N-Term(iTRAQ8plex); K17(iTRAQ8plex)	CID	1.0000	0.0000	1	1	3414434	Unique	Used	0.544	0.776	1.246	1.180	1E-05	0	6.58	0	0	49	2	1270.67078	2540.33428	-4.69
nEAIQAAHDSVAQEGQcR	Unambiguous	1	1	Q9R0P9	N-Term(iTRAQ8plex); C17(Methylthio)	CID	1.0000	0.0000	1	1	3229590	Unique	Used	1.673	0.965	1.363	1.080	1E-05	0	6.58	0	0	2	3	759.69202	2277.06150	-1.73

sITIANQTNcPLYVTk	Unambiguous	1	1	O08553	N-Term(iTRAQ8plex); C10(Methylthio); K16(iTRAQ8plex)	CID	1.0000	0.0000	1	1	3840399	Unique	Used	0.798	1.032	1.013	1.452	1E-05	0	6.58	0	0	200	2	1210.65601	2420.30474	-3.74
lLGDASVSFTDNcVVGIQANTER	Unambiguous	3	1	P97807-2	N-Term(iTRAQ8plex); C13(Methylthio)	CID	1.0000	0.0000	1	1	3582537	Unique	Used	1.151	1.005	0.759	1.096	1E-05	0	6.57	0	0	200	2	1380.18115	2759.35503	-3.49
vNQIGSVTEAIQAck	Unambiguous	2	1	P17183	N-Term(iTRAQ8plex); C14(Methylthio); K15(iTRAQ8plex)	CID	1.0000	0.0000	1	1	3644495	Unique	Used	1.950	1.209	0.938	1.298	1E-05	0	6.57	0	4	7	2	1108.09875	2215.19023	-6.00
iEQGSLILSNVQPSDTMVTQcEAR	Unambiguous	3	1	A2AFG8	N-Term(iTRAQ8plex); C21(Methylthio)	CID	1.0000	0.0000	1	1	3439577	Unique	Used	0.371	0.685	0.973	0.730	1E-05	0	6.57	0	0	14	2	1485.23315	2969.45903	-3.18
lQQTQAQVDEVVDIMR	Unambiguous	2	1	P63044	N-Term(iTRAQ8plex)	CID	1.0000	0.0000	1	1	3285495	Unique	Used	0.506	1.989	1.390	1.243	1E-05	0	6.57	0	0	200	2	1089.07922	2177.15117	-1.23
gLGPSLTEDQPGPYLAPGLLGSNIHQQGR	Unambiguous	4	1	D3YZR8	N-Term(iTRAQ8plex)	CID	1.0000	0.0000	1	1	3704671	Unique	Used	0.704	0.638	1.025	0.883	1E-05	0	6.57	0	0	161	3	1092.90796	3276.70932	-5.68
lTGFHETSNlnDFSAGVANR	Unambiguous	1	1	P15105	N-Term(iTRAQ8plex); N11(Deamidated)	CID	1.0000	0.0000	1	1	3278414	Unique	Used	2.180	1.089	1.064	1.681	1E-05	0	6.57	0	10	30	3	819.07489	2455.21012	-2.23
nDITGAPSnPDVFSYGIETHEAYk	Unambiguous	1	1	Q9JI75	N-Term(iTRAQ8plex); N9(Deamidated); K24(iTRAQ8plex)	CID	1.0000	0.0000	1	1	3013048	Unique	Used	1.655	1.321	0.692	0.894	1E-05	0	6.57	0	0	164	3	1078.87646	3234.61484	2.99
vEIVAInDPFIDLNYMVYMFQYDSTHGk	Unambiguous	4	1	P16858	N-Term(iTRAQ8plex); N7(Deamidated); K28(iTRAQ8plex)	CID	0.0426	0.0000	1	1	3645857	Unique	Used	0.835	0.588	1.426	1.446	1E-05	0	6.57	0	10	70	3	1311.33337	3931.98557	2.75
sLGVAAEGLPDQYADGEAAR	Unambiguous	1	1	Q9QXF8	N-Term(iTRAQ8plex)	CID	1.0000	0.0000	1	1	2760059	Unique	Used	2.070	0.499	8.884	21.722	1E-05	0	6.57	0	0	142	2	1147.57800	2294.14873	-3.47
lAELEEFINGPnNAHIQQVGDR	Unambiguous	2	1	Q68FD5	N-Term(iTRAQ8plex); N12(Deamidated)	CID	1.0000	0.0000	1	1	3153660	Unique	Used	0.893	0.887	1.248	1.226	1E-05	0	6.57	0	20	172	3	923.80731	2769.40738	-1.31

aLAESWNAAFLESSAk	Unambiguous	1	1	Q921J2	N-Term(iTRAQ8plex); K16(iTRAQ8plex)	CID	1.0000	0.0000	1	1	3394043	Unique	Used		1.081	0.948	1.094	1E-05	0	6.57	0	13	109	2	1152.12158	2303.23589	-5.79
sFADENNVFHDPVDGWqNETPSGSLESPAQAR	Unambiguous	3	1	G5E8K5-2	N-Term(iTRAQ8plex); Q17(Deamidated)	CID	0.0396	0.0000	1	1	2939265	Unique	Used	1.317	0.849	1.442	1.439	1E-05	0	6.57	0	0	135	3	1269.58899	3806.75241	1.34
dSSTSPGDYVLSVSENSR	Unambiguous	5	1	Q64010	N-Term(iTRAQ8plex)	CID	1.0000	0.0000	1	1	2917370	Unique	Used	2.090	1.283	0.974	0.926	1E-05	0	6.57	0	0	36	2	1102.53442	2204.06157	-0.23
aGGGPGSQALAGDSLIPSPSLEESHk	Unambiguous	1	1	Q9ERE9	N-Term(iTRAQ8plex); K26(iTRAQ8plex)	CID	1.0000	0.0000	1	1	3070809	Unique	Used	1.737	0.966	0.871	0.679	1E-05	0	6.57	0	0	169	3	1024.21289	3070.62412	-0.82
gLFGGGAQSLDREELFGESSSGk	Unambiguous	4	1	D3Z2Q2	N-Term(iTRAQ8plex); K23(iTRAQ8plex)	CID	1.0000	0.0000	1	1	3212987	Unique	Used	0.516	1.170	1.060	1.073	1E-05	0	6.57	1	4	19	3	979.51105	2936.51859	-0.85
qkPAPLPPASNAPqPGSSQQIQQR	Unambiguous	1	1	Q62415	N-Term(iTRAQ8plex); K2(iTRAQ8plex); Q14(Deamidated)	CID	1.0000	0.0000	1	1	3676119	Unique	Used	0.755	0.888	1.565	0.934	1E-05	0	6.57	0	0	94	3	1045.57434	3134.70847	-2.64
rHHQEEVGELLGQIQGcGAAQAQAQAEAR	Unambiguous	1	1	P19246	N-Term(iTRAQ8plex); C17(Methylthio)	CID	1.0000	0.0000	1	1	3454611	Unique	Used	2.349			2.096	1E-05	0	6.57	1	0	107	3	1145.89868	3435.68149	-2.85
nLHQSGFSLSGTQVDEGVR	Unambiguous	3	1	E9PWE8	N-Term(iTRAQ8plex)	CID	1.0000	0.0000	1	1	3340887	Unique	Used	1.653	1.119	1.128	0.868	1E-05	0	6.57	0	9	7	3	779.06647	2335.18485	-4.11
nHLDQNPQLQWNTTEPSR	Unambiguous	1	1	Q80T41	N-Term(iTRAQ8plex)	CID	1.0000	0.0000	1	1	3207451	Unique	Used	1.335	0.948	1.177	1.095	1E-05	0	6.57	0	5	78	3	828.08472	2482.23960	0.76
iEESDQGPYAIILAPTR	Unambiguous	1	1	D3Z0M9	N-Term(iTRAQ8plex)	CID	1.0000	0.0000	1	1	3215213	Unique	Used	0.608	0.880	0.734	1.096	1E-05	0	6.57	0	0	148	2	1089.09204	2177.17681	0.55
vLTLESNVDSTSSSScQInR	Unambiguous	2	1	O55106	N-Term(iTRAQ8plex); C16(Methylthio); N19(Deamidated)	CID	0.0030	0.0000	1	1	3370619	Unique	Used			1.078	0.833	1E-05	0	6.57	0	0	200	2	1246.10632	2491.20537	3.83
qFLSGELEVELTPQGTLAER	Unambiguous	2	1	Q9D0K2	N-Term(iTRAQ8plex)	CID	1.0000	0.0000	1	1	3239032	Unique	Used	0.692	0.687	1.134	1.278	1E-05	0	6.57	0	17	8	2	1261.17456	2521.34184	-1.34

aASVAEnclNYQQGTPHk	Unambiguous	1	1	P70202	N-Term(iTRAQ8plex); N7(Deamidated); C8(Methylthio); K18(iTRAQ8plex)	CID	1.0000	0.0000	1	1	3068765	Unique	Used		1.184	0.783	1.195	1E-05	0	6.57	0	0	200	2	1293.64539	2586.28349	-2.56
gAQIASTGQLYAAWnGGLDR	Unambiguous	1	1	Q61361	N-Term(iTRAQ8plex); N15(Deamidated)	CID	1.0000	0.0000	1	1	3392693	Unique	Used	1.993	1.167	1.190	1.452	1E-05	0	6.57	0	0	70	2	1177.60010	2354.19292	-4.83
qTTAAAAATFSEqVGGGSGGAGR	Unambiguous	3	1	O88935	N-Term(iTRAQ8plex); Q13(Deamidated)	CID	1.0000	0.0000	1	1	3574081	Unique	Used	0.812	1.231	1.051	1.445	1E-05	0	6.57	0	0	200	2	1179.08972	2357.17217	3.64
scGPGTQVQEVVcINSDGEEVDR	Unambiguous	4	1	E9QNR5	N-Term(iTRAQ8plex); C2(Methylthio); C13(Methylthio)	CID	1.0000	0.0000	1	1	2832652	Unique	Used	0.791	1.241			1E-05	0	6.57	0	0	200	2	1409.12524	2817.24321	-1.20
dIPLQSAATLGVNPcTAYR	Unambiguous	2	1	Q9DCS3	N-Term(iTRAQ8plex); C15(Methylthio)	CID	1.0000	0.0000	1	1	3824225	Unique	Used	0.658	0.781	1.092	1.102	1E-05	0	6.57	0	12	32	2	1170.59558	2340.18389	-6.63
tTSkPLISVTAqSSTESTQDAYQDSR	Unambiguous	3	1	Q8BJ42	N-Term(iTRAQ8plex); K4(iTRAQ8plex); Q12(Deamidated)	CID	0.0030	0.0000	1	1	3104248	Unique	Used	0.869	3.427	6.208	1.358	1E-05	0	6.56	0	0	197	3	1137.58984	3410.75498	4.87
tIGGGDDSFNTFFSETGAGk	Unambiguous	4	1	P68373	N-Term(iTRAQ8plex); K20(iTRAQ8plex)	CID	1.0000	0.0000	1	1	3154262	Unique	Used	0.280	1.272	0.851	1.595	1E-05	0	6.56	0	0	200	2	1308.65210	2616.29692	-2.65
tYFPHFDVSHGSAQVk	Unambiguous	2	1	Q91VB8	N-Term(iTRAQ8plex); K16(iTRAQ8plex)	CID	1.0000	0.0000	1	1	3387295	Unique	Used	7.327	3.022	0.963	2.041	1E-05	0	6.56	0	5	41	3	810.09448	2428.26889	-7.45
vLGAHILGPGAGEmVNEAALALEYGAScEDIAR	Unambiguous	1	1	O08749	N-Term(iTRAQ8plex); M14(Oxidation); C28(Methylthio)	CID	1.0000	0.0000	1	1	3500147	Unique	Used	3.210	1.681	1.299	1.635	1E-05	0	6.56	0	2	4	3	1221.93945	3663.80381	-2.35
lMSQESYSDMSSQSFNETR	Unambiguous	2	1	Q80Y24	N-Term(iTRAQ8plex)	CID	1.0000	0.0000	1	1	2993142	Unique	Used	0.817	1.658	1.897	0.955	1E-05	0	6.56	0	0	180	2	1266.06934	2531.13140	-0.73

dITFVAEFLTEHFNEAPDLYNR	Unambiguous	1	1	Q91W96	N-Term(iTRAQ8plex)	CID	1.0000	0.0000	1	1	3419297	Unique	Used	1.459	1.456	1.037	1.262	1E-05	0	6.56	0	28	38	3	982.49243	2945.46274	0.13
tLGLGSLYGENDLLDLDGDPLR	Unambiguous	3	1	Q68FM7	N-Term(iTRAQ8plex)	CID	1.0000	0.0000	1	1	3239569	Unique	Used	0.787	0.622	1.195	1.070	1E-05	0	6.56	0	0	45	2	1325.69470	2650.38213	-2.15
aVVFETGEAGAASGAGSLPGEVR	Unambiguous	3	1	Q9QYR6	N-Term(iTRAQ8plex)	CID	1.0000	0.0000	1	1	3129653	Unique	Used	2.474	1.459	2.160	1.491	1E-05	0	6.56	0	0	99	2	1218.63794	2436.26860	0.52
icDPGLTSFEPEALGNLVEGMDFHR	Unambiguous	9	1	Q5SVJ0	N-Term(iTRAQ8plex); C2(Methylthio)	CID	1.0000	0.0000	1	1	3481477	Unique	Used		1.402	0.806	1.083	1E-05	0	6.56	0	0	200	3	1033.15918	3097.46299	-3.42
sFcSMAGPNLIAIGSSESAQk	Unambiguous	2	1	Q9CWS0	N-Term(iTRAQ8plex); C3(Methylthio); K21(iTRAQ8plex)	CID	1.0000	0.0000	1	1	3784983	Unique	Used	0.869	0.767	1.233	0.992	1E-05	0	6.56	0	0	28	2	1376.69287	2752.37847	-5.25
nHLDQNPQLQWNTTEPSR	Unambiguous	1	1	Q80T41	N-Term(iTRAQ8plex)	CID	1.0000	0.0000	1	1	3207601	Unique	Used	1.239	1.173	1.084	0.984	1E-05	0	6.56	0	8	32	3	828.08337	2482.23557	-0.86
sLLAAMEGYIGVDFEPLSSk	Unambiguous	1	1	Q80T41	N-Term(iTRAQ8plex); K20(iTRAQ8plex)	CID	1.0000	0.0000	1	1	3561219	Unique	Used			0.907	1.042	1E-05	0	6.56	0	14	54	3	912.49481	2735.46988	-3.21
sLYEGLVMTEVqIQk	Unambiguous	1	1	Q99LP6	N-Term(iTRAQ8plex); Q12(Deamidated); K15(iTRAQ8plex)	CID	0.0351	0.0000	1	1	3372775	Unique	Used	1.840	1.355	1.305	0.934	1E-05	0	6.56	0	0	200	2	1174.16101	2347.31474	4.60
mQAQMQMQMQGGDSDSGALGQHV	Unambiguous	3	1	P42208	N-Term(iTRAQ8plex)	CID	1.0000	0.0000	1	1	3071013	Unique	Used	5.130			0.969	1E-05	0	6.56	0	0	29	2	1370.11633	2739.22539	-0.28
gFGFITFTNPEHASDAMR	Unambiguous	2	1	O89086	N-Term(iTRAQ8plex)	CID	1.0000	0.0000	1	1	3620341	Unique	Used	2.422	1.149	1.628	0.845	1E-05	0	6.56	0	2	6	3	768.04169	2302.11051	-5.37
sYSPELIVHPVLDSSnAVEEVEk	Unambiguous	6	1	J3QMM7	N-Term(iTRAQ8plex); N16(Deamidated); K23(iTRAQ8plex)	CID	1.0000	0.0000	1	1	2813804	Unique	Used	2.224	1.129	0.532	0.465	1E-05	0	6.56	0	0	200	3	1050.89233	3150.66245	-1.36
tVAGIIVEPIQSEGGDNHASDDFFR	Unambiguous	2	1	P61922	N-Term(iTRAQ8plex)	CID	1.0000	0.0000	1	1	3016237	Unique	Used	2.591	0.752	1.122	1.407	1E-05	0	6.56	0	14	63	3	993.49646	2978.47483	-1.68
aAELIANSLATAGDGLIELR	Unambiguous	1	1	P67778	N-Term(iTRAQ8plex)	CID	1.0000	0.0000	1	1	3667772	Unique	Used	0.889	0.985	1.179	1.166	1E-05	0	6.56	0	0	200	2	1151.64233	2302.27739	-6.38
dVTEQLNLVTTWLQLQIPR	Unambiguous	4	1	G3UXZ5	N-Term(iTRAQ8plex)	CID	1.0000	0.0000	1	1	3918298	Unique	Used	0.664	1.894	0.586	0.892	1E-05	0	6.56	0	13	28	2	1286.22095	2571.43462	-3.98
vAGPQPAQTGAPQGSLGEYLFER	Unambiguous	2	1	P29391	N-Term(iTRAQ8plex)	CID	1.0000	0.0000	1	1	3392834	Unique	Used	1.269	1.988	1.918	1.834	1E-05	0	6.56	0	0	91	2	1339.19019	2677.37309	-5.87

aTAEFSTQTPSLTLSSDIPR	Unambiguous	2	1	O88737	N-Term(iTRAQ8plex)	CID	1.0000	0.0000	1	1	3415393	Unique	Used	0.971	1.124	2.340	0.990	1E-05	0	6.56	0	0	50	2	1213.63403	2426.26079	-4.50
IAASYSSDSEnQGSYSGVIPPPPGR	Unambiguous	7	1	O54916	N-Term(iTRAQ8plex); N11(Deamidated)	CID	0.0061	0.0000	1	1	3279253	Unique	Used		0.732	1.299	1.554	1E-05	0	6.56	0	0	59	2	1421.20227	2841.39726	4.49
eHGGPEGMDPDGVIESNWNEIVDNFDDMNLk	Unambiguous	5	1	P10630	N-Term(iTRAQ8plex); K31(iTRAQ8plex)	CID	1.0000	0.0000	1	1	2942391	Unique	Used				0.974	1E-05	0	6.56	0	8	84	3	1361.30493	4081.90024	-0.08
vLHMDQnPYYGGESASITPLEDLYk	Unambiguous	1	1	Q61598	N-Term(iTRAQ8plex); N7(Deamidated); K25(iTRAQ8plex)	CID	0.0168	0.0000	1	1	3260828	Unique	Used	2.871	1.282	1.026	0.710	1E-05	0	6.56	0	0	57	3	1150.58862	3449.75132	3.41
nLDkEYLPIGGLAEFck	Unambiguous	1	1	P05202	N-Term(iTRAQ8plex); K4(iTRAQ8plex); C16(Methylthio); K17(iTRAQ8plex)	CID	1.0000	0.0000	1	1	3479311	Unique	Used	1.204	0.671	1.483	1.760	1E-05	0	6.56	1	6	9	3	956.86108	2868.56870	-2.79
acADATLSQITNNIDPVGR	Unambiguous	3	1	P62874	N-Term(iTRAQ8plex); C2(Methylthio)	CID	1.0000	0.0000	1	1	3603087	Unique	Used		1.335	0.928	1.090	1E-05	0	6.56	0	11	200	2	1155.07861	2309.14995	-1.40
ITLYDIAHTPGVAADLSHIETR	Unambiguous	1	1	P08249	N-Term(iTRAQ8plex)	CID	1.0000	0.0000	1	1	3461146	Unique	Used	2.001	0.985	1.268	1.145	1E-05	0	6.56	0	7	200	3	899.81995	2697.44529	-2.27
IQQTQAQVDEVVDIMR	Unambiguous	2	1	P63044	N-Term(iTRAQ8plex)	CID	1.0000	0.0000	1	1	3285705	Unique	Used	0.832	2.440	1.078	1.203	1E-05	0	6.56	0	15	200	2	1089.07971	2177.15215	-0.78
vSLPTANPDVSSGITQIk	Unambiguous	1	1	Q8C419	N-Term(iTRAQ8plex); K18(iTRAQ8plex)	CID	1.0000	0.0000	1	1	3496075	Unique	Used	0.692	0.810	1.706	0.888	1E-05	0	6.56	0	0	200	2	1218.19763	2435.38799	-3.55
gDSALFAVnGFNILVDGGSDR	Unambiguous	3	1	Q9QYR6	N-Term(iTRAQ8plex); N9(Deamidated)	CID	0.0335	0.0000	1	1	3305451	Unique	Used	0.971	1.255	1.155	1.098	1E-05	0	6.56	0	0	14	2	1215.11328	2429.21929	-2.40
gFESPSDNSSAVLLQWHEk	Unambiguous	1	1	Q9CPW4	N-Term(iTRAQ8plex); K19(iTRAQ8plex)	CID	1.0000	0.0000	1	1	3212192	Unique	Used	1.047	1.874	0.722	0.592	1E-05	0	6.56	0	0	55	3	913.81244	2739.42276	1.05
dALRDEYDDLSDLNAVQMESVR	Unambiguous	1	1	Q80UU9	N-Term(iTRAQ8plex)	CID	1.0000	0.0000	1	1	2764079	Unique	Used	1.408	0.866	1.308	1.441	1E-05	0	6.56	1	24	200	3	953.46375	2858.37668	-0.49

lSLESEGAnEGATAAPELSALEEAFR	Unambiguous	1	1	Q7TQD2	N-Term(iTRAQ8plex); N9(Deamidated)	CID	1.0000	0.0000	1	1	2888283	Unique	Used			6.459	4.464	1E-05	0	6.56	0	0	200	2	1484.23682	2967.46636	-2.50
gFLGVmPAYSAAEDALTTk	Unambiguous	1	1	O54983	N-Term(iTRAQ8plex); M6(Oxidation); K19(iTRAQ8plex)	CID	1.0000	0.0000	1	1	3438578	Unique	Used	0.733	3.207	1.028	0.681	1E-05	0	6.55	0	0	168	2	1283.68274	2566.35820	-3.96
sLGVAAEGLPDQYADGEAAR	Unambiguous	1	1	Q9QXF8	N-Term(iTRAQ8plex)	CID	1.0000	0.0000	1	1	2759940	Unique	Used	7.471	4.172	4.073	6.319	1E-05	0	6.55	0	13	200	2	1147.57727	2294.14726	-4.11
fAEEADVVIVGAGPAGLSAAIR	Unambiguous	2	1	Q921G7	N-Term(iTRAQ8plex)	CID	1.0000	0.0000	1	1	3373767	Unique	Used	1.588	2.359	1.055	1.045	1E-05	0	6.55	0	10	28	2	1209.16797	2417.32866	-2.33
lTAVSVGVQGSGWGWLGFNk	Unambiguous	1	1	P09671	N-Term(iTRAQ8plex); K20(iTRAQ8plex)	CID	1.0000	0.0000	1	1	3935892	Unique	Used	2.402	1.895	0.661	0.800	1E-05	0	6.55	0	19	44	2	1336.23523	2671.46318	-6.94
aSANLGQGSGSQLVPTVcnGR	Unambiguous	1	1	Q14BI2	N-Term(iTRAQ8plex); C18(Methylthio); N19(Deamidated)	CID	1.0000	0.0000	1	1	3662378	Unique	Used	0.242	0.991	2.200	1.493	1E-05	0	6.55	0	0	69	2	1184.08276	2367.15825	-4.92
qLTVQMMQNPQILAALQER	Unambiguous	3	1	J3QP68	N-Term(iTRAQ8plex)	CID	1.0000	0.0000	1	1	3879325	Unique	Used	1.279	0.908	1.277	0.792	1E-05	0	6.55	0	7	12	2	1258.68103	2516.35478	-3.32
vNDEHPAYLASDEITTVR	Unambiguous	3	1	P58281-2	N-Term(iTRAQ8plex)	CID	1.0000	0.0000	1	1	2757309	Unique	Used	0.673	0.987	0.841	0.961	1E-05	0	6.55	0	0	200	2	1167.59583	2334.18437	-1.54
lNDYLQVETIQALEELAVR	Unambiguous	5	1	Q8BGW1	N-Term(iTRAQ8plex)	CID	1.0000	0.0000	1	1	3374358	Unique	Used	0.908	1.110	0.814	1.399	1E-05	0	6.55	0	0	49	2	1261.18872	2521.37016	-4.53
iQVLQQQADDAEER	Unambiguous	4	1	D3Z6I8	N-Term(iTRAQ8plex)	CID	1.0000	0.0000	1	1	2636069	Unique	Used	2.508	1.647	1.519	0.762	1E-05	0	6.55	0	0	40	2	974.00677	1947.00627	-1.16
gDAVGFQEASLAQYSATTAR	Unambiguous	2	1	O88737	N-Term(iTRAQ8plex)	CID	1.0000	0.0000	1	1	3257033	Unique	Used	1.281	1.352	1.875	0.762	1E-05	0	6.55	0	20	130	2	1174.09717	2347.18706	1.64
iSFYMTNYGEEGTHVGSAAALER	Unambiguous	2	1	Q3U3J1	N-Term(iTRAQ8plex)	CID	1.0000	0.0000	1	1	3301808	Unique	Used	1.278	1.127	0.943	0.971	1E-05	0	6.55	0	30	88	3	936.45667	2807.35544	-2.08
vEYPVMYSTDPENGHMFncIQR	Unambiguous	1	1	Q9CPU4	N-Term(iTRAQ8plex); N18(Deamidated); C19(Methylthio)	CID	1.0000	0.0000	1	1	3215197	Unique	Used	1.172	0.729	0.990	0.985	1E-05	0	6.55	0	13	200	3	994.44592	2981.32322	-0.41
lVQDVAnNTNEEAGDGTTTATVLAR	Unambiguous	3	1	P63038	N-Term(iTRAQ8plex); N7(Deamidated)	CID	0.0260	0.0000	1	1	2780137	Unique	Used	1.817	1.555	1.637	1.390	1E-05	0	6.55	0	13	200	2	1433.22253	2865.43779	-0.08

yNVYPTYDFAcPIVDSIEGVTHALR	Unambiguous	1	1	Q8CGC7	N-Term(iTRAQ8plex); C11(Methylthio)	CID	1.0000	0.0000	1	1	3766382	Unique	Used	0.798	0.424	0.952	0.895	1E-05	0	6.55	0	4	14	3	1065.18945	3193.55381	-3.21
qSFVLTTDEIIHqFGLPQTAFISIER	Unambiguous	3	1	E9QP54	N-Term(iTRAQ8plex); Q13(Deamidated)	CID	0.0412	0.0000	1	1	3824987	Unique	Used	1.413	0.735	0.874	1.151	1E-05	0	6.55	0	0	86	3	1099.25977	3295.76474	3.96
ILDLSSNPSIDESQLSLIADLPR	Unambiguous	2	1	Q8CIV8	N-Term(iTRAQ8plex)	CID	1.0000	0.0000	1	1	3480055	Unique	Used	1.975		0.823	2.099	1E-05	0	6.55	0	0	81	2	1400.76355	2800.51982	-1.72
eQSQPcAENTVLSSGLTAAR	Unambiguous	1	1	O54833	N-Term(iTRAQ8plex); C6(Methylthio)	CID	1.0000	0.0000	1	1	3300561	Unique	Used	0.560	0.514	1.234	1.096	1E-05	0	6.55	0	0	76	2	1206.58948	2412.17168	-3.50
tSISTASATVSPASSAESAcTR	Unambiguous	1	1	P35831	N-Term(iTRAQ8plex); C20(Methylthio)	CID	1.0000	0.0000	1	1	3411039	Unique	Used	1.126	1.032	1.339	1.098	1E-05	0	6.55	0	0	67	2	1218.08276	2435.15825	-4.67
gPAVGIDLGTTYScVGVFqHGk	Unambiguous	4	1	P63017	N-Term(iTRAQ8plex); C14(Methylthio); Q19(Deamidated); K22(iTRAQ8plex)	CID	1.0000	0.0000	1	1	3641541	Unique	Used	2.084	1.914	1.211	0.961	1E-05	0	6.55	0	27	39	3	954.50244	2861.49277	4.93
wGEAGAEYVVESTGVFTTMEk	Unambiguous	3	1	P16858	N-Term(iTRAQ8plex); K21(iTRAQ8plex)	CID	1.0000	0.0000	1	1	3241095	Unique	Used		0.923	2.329	1.877	1E-05	0	6.55	0	14	185	3	967.15979	2899.46482	0.12
lQDGQTFSSHGPGNPEAPGMDSIVR	Unambiguous	1	1	Q3V3R1	N-Term(iTRAQ8plex)	CID	1.0000	0.0000	1	1	3320278	Unique	Used	1.152	0.970	0.722	1.261	1E-05	0	6.55	0	0	62	3	967.80658	2901.40519	-1.79
vVNAPIFHVnSDDPEAVMYVck	Unambiguous	4	1	Q60597	N-Term(iTRAQ8plex); N10(Deamidated); C21(Methylthio); K22(iTRAQ8plex)	CID	1.0000	0.0000	1	1	3326624	Unique	Used	1.303	0.778	1.345	0.889	1E-05	0	6.55	0	24	29	3	1034.85413	3102.54782	-2.62
cQEILSVDcSTNNPAQANLR	Unambiguous	1	1	Q06890	N-Term(iTRAQ8plex); C1(Methylthio); C9(Methylthio)	CID	1.0000	0.0000	1	1	3644805	Unique	Used	0.844	1.152	1.118	1.038	1E-05	0	6.55	0	0	20	2	1286.59167	2572.17607	-6.59

knGVQAMV EFDSVQSA QR	Unambiguous	5	1	G5E924	N-Term(iTRAQ8plex); K1(iTRAQ8plex); N2(Deamidated)	CID	1.0000	0.0000	1	1	3232650	Unique	Used	1.095	1.086	1.068	0.853	1E-05	0	6.55	1	9	59	3	868.45941	2603.36368	-2.75
lVAGEYLSF FDFSGLTLD R	Unambiguous	4	1	Q6P1I6	N-Term(iTRAQ8plex)	CID	1.0000	0.0000	1	1	3806728	Unique	Used	0.658	0.651	0.665	1.039	1E-05	0	6.55	0	8	27	2	1227.64233	2454.27739	-3.47
sEQSSmSIE FGQESPEH SLAMDFSR	Unambiguous	1	1	P14873	N-Term(iTRAQ8plex); M6(Oxidation)	CID	1.0000	0.0000	1	1	2901192	Unique	Used	3.857	2.254	1.184	1.014	1E-05	0	6.55	0	0	200	3	1046.13867	3136.40146	-4.06
eVnENFAID LIAQQPVNE VEHR	Unambiguous	2	1	P52503	N-Term(iTRAQ8plex); N3(Deamidated)	CID	0.0076	0.0000	1	1	2852529	Unique	Used	2.540	0.695	2.507	1.384	1E-05	0	6.55	0	21	185	3	957.15808	2869.45969	-1.31
fQMPDQGM TSADDFFQ GTk	Unambiguous	1	1	O08553	N-Term(iTRAQ8plex); K19(iTRAQ8plex)	CID	1.0000	0.0000	1	1	3283489	Unique	Used	2.770	1.461	0.800	1.006	1E-05	0	6.55	0	16	71	3	920.44312	2759.31479	-4.28
eTEAALEFA QTQLAEQG EESR	Unambiguous	1	1	Q9D7M1	N-Term(iTRAQ8plex)	CID	1.0000	0.0000	1	1	2764881	Unique	Used	1.121				1E-05	0	6.55	0	16	200	2	1321.14575	2641.28423	-2.02
dYEIENQnP LASPTNTLL GSAk	Unambiguous	2	1	Q5F2E7	N-Term(iTRAQ8plex); N8(Deamidated); K22(iTRAQ8plex)	CID	0.0214	0.0000	1	1	3236715	Unique	Used	1.354	0.623	1.679	0.919	1E-05	0	6.55	0	0	75	2	1492.79272	2984.57817	3.63
cFSIDNPGY EPEVVAVH PGGDTVAV GGTDGNVR	Unambiguous	1	1	O88342	N-Term(iTRAQ8plex); C1(Methylthio)	CID	1.0000	0.0000	1	1	3193717	Unique	Used	1.458	1.193	1.060	1.444	1E-05	0	6.54	0	15	3	3	1226.91846	3678.74082	-1.72
iMDPNIVGn EHYDVAR	Unambiguous	1	1	P56480	N-Term(iTRAQ8plex); N9(Deamidated)	CID	1.0000	0.0000	1	1	2830868	Unique	Used	1.671	1.035	1.128	1.250	1E-05	0	6.54	0	4	8	3	716.69312	2148.06479	-2.32
dLVILLYETA LLSSGFSLE DPQTHANR	Unambiguous	1	1	P07901	N-Term(iTRAQ8plex)	CID	1.0000	0.0000	1	1	3787135	Unique	Used		1.665	1.538	2.438	1E-05	0	6.54	0	22	200	3	1102.91992	3306.74521	-2.18
hSPGEITGP GGHFMTSD SSLTk	Unambiguous	3	1	Q9QYR6	N-Term(iTRAQ8plex); K22(iTRAQ8plex)	CID	1.0000	0.0000	1	1	3251781	Unique	Used	2.020	1.059	2.016	0.799	1E-05	0	6.54	0	0	77	3	951.15363	2851.44632	-1.48
qLSPTEAFP VLVGDMDN SGSLnAQVI HQLSPGLR	Unambiguous	1	1	Q9QYA2	N-Term(iTRAQ8plex); N22(Deamidated)	CID	0.0153	0.0000	1	1	3765963	Unique	Used	0.987	1.166	1.363	1.172	1E-05	0	6.54	0	6	13	3	1299.34607	3896.02365	1.78
sLSLAVNAN VGDTAVQE LAR	Unambiguous	1	1	Q91W61	N-Term(iTRAQ8plex)	CID	1.0000	0.0000	1	1	3478076	Unique	Used	1.013	1.118	0.876	1.586	1E-05	0	6.54	0	0	200	2	1166.63818	2332.26909	-3.59

wGEAGAEYVVESTGVFTTMEk	Unambiguous	3	1	P16858	N-Term(iTRAQ8plex); K21(iTRAQ8plex)	CID	1.0000	0.0000	1	1	3219182	Unique	Used			0.999	0.827	1E-05	0	6.54	0	0	200	3	967.15955	2899.46408	-0.13
vTGGQTIQVETSSESPFPAk	Unambiguous	1	1	P20357	N-Term(iTRAQ8plex); K20(iTRAQ8plex)	CID	1.0000	0.0000	1	1	3233338	Unique	Used	1.806	2.033	1.562	0.905	1E-05	0	6.54	0	0	54	2	1336.22070	2671.43413	-2.18
kLGcEVLGVSVDSQFTHLAWINTPR	Unambiguous	2	1	Q61171	N-Term(iTRAQ8plex); K1(iTRAQ8plex); C4(Methylthio)	CID	1.0000	0.0000	1	1	3879282	Unique	Used	1.277	0.980	0.994	0.871	1E-05	0	6.54	1	7	19	3	1142.27588	3424.81308	-5.83
iDDLQMVLnQTEDHR	Unambiguous	4	1	Q9Z1G4-3	N-Term(iTRAQ8plex); N9(Deamidated)	CID	1.0000	0.0000	1	1	2834355	Unique	Used	0.855	1.183	1.160	1.110	1E-05	0	6.54	0	21	200	3	711.35687	2132.05606	-1.66
tVMAHGcYLSEEELnIFSER	Unambiguous	2	1	Q9R111	N-Term(iTRAQ8plex); C7(Methylthio); N15(Deamidated)	CID	1.0000	0.0000	1	1	3354076	Unique	Used	1.584	1.881	1.296	1.169	1E-05	0	6.54	0	22	200	3	893.75720	2679.25705	6.11
sEHSQTMcQLTSQNEALR	Unambiguous	4	1	E9PWI6	N-Term(iTRAQ8plex); C8(Methylthio)	CID	1.0000	0.0000	1	1	3296821	Unique	Used	1.663	1.507	1.065	1.070	1E-05	0	6.54	0	8	193	3	805.04327	2413.11527	-2.47
lSLAAAAGDPFAYPGAGGLYk	Unambiguous	3	1	B1AQX6	N-Term(iTRAQ8plex); K21(iTRAQ8plex)	CID	1.0000	0.0000	1	1	3706349	Unique	Used	3.058	0.710	0.754	0.616	1E-05	0	6.54	0	21	17	2	1309.71826	2618.42925	-5.60
qASGGPVDIGPEYQQDLDR	Unambiguous	2	1	P97450	N-Term(iTRAQ8plex)	CID	1.0000	0.0000	1	1	2635753	Unique	Used	3.079		0.930	0.795	1E-05	0	6.54	0	0	200	2	1175.08459	2349.16191	-0.25
fVHSENQHLVSPEALDFLDk	Unambiguous	1	1	Q60737	N-Term(iTRAQ8plex); K20(iTRAQ8plex)	CID	1.0000	0.0000	1	1	3280704	Unique	Used	1.581	1.170	1.237	0.990	1E-05	0	6.54	0	0	64	3	978.52325	2933.55521	-2.25
wGEAGAEYVVESTGVFTTmEk	Unambiguous	3	1	P16858	N-Term(iTRAQ8plex); M19(Oxidation); K21(iTRAQ8plex)	CID	1.0000	0.0000	1	1	3240829	Unique	Used		1.249			1E-05	0	6.54	0	9	200	3	972.48798	2915.44937	-3.43
vLSDDEEDVDFDIIHNANDTFTVk	Unambiguous	1	1	Q80X90	N-Term(iTRAQ8plex); K24(iTRAQ8plex)	CID	1.0000	0.0000	1	1	2815834	Unique	Used	1.053	1.954	0.925	0.781	1E-05	0	6.54	0	0	200	3	1120.56055	3359.66709	-2.06

sHSNQLVTDcISAMNPDTVLR	Unambiguous	2	1	Q9Z0S1	N-Term(iTRAQ8plex); C10(Methylthio)	CID	1.0000	0.0000	1	1	3784996	Unique	Used				1.277	1E-05	0	6.54	0	25	25	3	884.43243	2651.28275	-2.50
tGEmSGPVFTDSGIHIILR	Unambiguous	1	1	Q9QUR7	N-Term(iTRAQ8plex); M4(Oxidation)	CID	1.0000	0.0000	1	1	3537134	Unique	Used	2.601	1.493	1.264	1.104	1E-05	0	6.54	0	14	85	3	784.08020	2350.22605	-5.05
ePITVSSEQMSHFR	Unambiguous	1	1	P00920	N-Term(iTRAQ8plex)	CID	1.0000	0.0000	1	1	3252775	Unique	Used	1.694	1.325	1.456	1.616	1E-05	0	6.54	0	5	3	3	651.33185	1951.98099	-2.03
iREDLPNLESSEETEQINk	Unambiguous	1	1	Q9EQH3	N-Term(iTRAQ8plex); K19(iTRAQ8plex)	CID	1.0000	0.0000	1	1	2635277	Unique	Used	0.473	1.475	0.938	0.974	1E-05	0	6.54	1	0	186	3	951.50714	2852.50687	-1.04
fVSQTnGNLYIANVESSDR	Unambiguous	1	1	P12960	N-Term(iTRAQ8plex); N6(Deamidated)	CID	0.0306	0.0000	1	1	3108633	Unique	Used	1.036	0.939	1.236	0.967	1E-05	0	6.54	0	0	120	2	1210.10522	2419.20317	-0.48
eRVPEAQTGQASDYGLFLSDEDPR	Unambiguous	2	1	E9PUM4	N-Term(iTRAQ8plex)	CID	1.0000	0.0000	1	1	2778972	Unique	Used	1.166	0.577	1.867	1.130	1E-05	0	6.54	1	0	200	3	995.48895	2984.45230	-0.57
aASQQEIEQSIETLnMLMLDLEPASAAAPLHk	Unambiguous	1	1	E9Q0S6	N-Term(iTRAQ8plex); N15(Deamidated); K32(iTRAQ8plex)	CID	1.0000	0.0000	1	1	3419466	Unique	Used				0.640	1E-05	0	6.54	0	10	47	3	1353.71436	4059.12851	1.11
vNDEHPAYLASDEITTVR	Unambiguous	3	1	P58281-2	N-Term(iTRAQ8plex)	CID	1.0000	0.0000	1	1	2777142	Unique	Used	4.150	1.573	1.164	1.482	1E-05	0	6.54	0	0	200	2	1167.59216	2334.17705	-4.67
tYFPHFDVSHGSAQVk	Unambiguous	2	1	Q91VB8	N-Term(iTRAQ8plex); K16(iTRAQ8plex)	CID	1.0000	0.0000	1	1	3481294	Unique	Used	2.535	1.976	0.534	1.217	1E-05	0	6.54	0	15	200	3	810.09833	2428.28043	-2.70
qVLYQQSSGHNNR	Unambiguous	2	1	Q99M71	N-Term(iTRAQ8plex)	CID	1.0000	0.0000	1	1	3693944	Unique	Used	1.463	0.752	1.192	0.862	1E-05	0	6.54	0	6	48	3	612.31653	1834.93503	-6.07
mLQDDEDTVHLPFER	Unambiguous	2	1	Q8BTY2	N-Term(iTRAQ8plex)	CID	1.0000	0.0000	1	1	2739131	Unique	Used	0.415	0.801	1.245	1.049	1E-05	0	6.54	0	17	200	3	717.02142	2149.04972	-1.90
fQQQLEQLSAMGFLNR	Unambiguous	2	1	Q8R317	N-Term(iTRAQ8plex)	CID	1.0000	0.0000	1	1	3917675	Unique	Used	1.847	0.992	0.868	0.937	1E-05	0	6.54	0	23	52	2	1107.57971	2214.15215	-5.51
tIGGGDDSFNTFFSETGAGk	Unambiguous	4	1	P68373	N-Term(iTRAQ8plex); K20(iTRAQ8plex)	CID	1.0000	0.0000	1	1	3174443	Unique	Used		1.548	1.177	1.566	1E-05	0	6.54	0	17	200	2	1308.65198	2616.29668	-2.75
qLGSVSTGSTLQQSLELTR	Unambiguous	1	1	Q921G8	N-Term(iTRAQ8plex)	CID	1.0000	0.0000	1	1	3683151	Unique	Used		1.036	0.812	1.142	1E-05	0	6.54	0	0	80	2	1155.12634	2309.24541	-6.95
aQSFSGDFTHR	Unambiguous	6	1	Q8BXN9	N-Term(iTRAQ8plex)	CID	1.0000	0.0000	1	1	3294625	Unique	Used	0.580	0.607	0.797	0.823	1E-05	0	6.54	0	12	24	3	519.59503	1556.77054	-3.46

kTGSPGSPSAGGVqSTAk	Unambiguous	1	1	Q6PDL0	N-Term(iTRAQ8plex); K1(iTRAQ8plex); Q14(Deamidated); K18(iTRAQ8plex)	CID	1.0000	0.0000	1	1	3270229	Unique	Used	0.831	0.728	1.249	0.825	1E-05	0	6.54	1	16	200	3	844.14960	2530.43424	4.04
gAVPYVQAFDSLLANPVAEYLk	Unambiguous	2	1	P40124	N-Term(iTRAQ8plex); K22(iTRAQ8plex)	CID	1.0000	0.0000	1	1	3707085	Unique	Used	1.028	0.866	1.033	1.113	1E-05	0	6.54	0	0	77	3	991.88702	2973.64652	-2.73
aMSMASILTNTMEELEESR	Unambiguous	1	1	B1AWN6	N-Term(iTRAQ8plex)	CID	1.0000	0.0000	1	1	3353118	Unique	Used	0.430	1.628	1.533	1.584	1E-05	0	6.54	0	15	11	2	1224.08752	2447.16777	-3.79
fATASADGQIFIYDGk	Unambiguous	1	1	O88342	N-Term(iTRAQ8plex); K16(iTRAQ8plex)	CID	1.0000	0.0000	1	1	3302834	Unique	Used	1.180	1.394	0.928	1.043	1E-05	0	6.54	0	0	185	2	1156.61987	2312.23247	-2.53
qQMLHAQTFHSDSLLSQEEAAAAGDR	Unambiguous	3	1	Q8BHF7	N-Term(iTRAQ8plex)	CID	1.0000	0.0000	1	1	3070040	Unique	Used	1.085	0.763	0.873	1.021	1E-05	0	6.54	0	0	200	3	1049.17871	3145.52158	-1.89
fAPqFSGYQQQDSQELLAFILDGLHEDLNR	Unambiguous	1	1	P35123	N-Term(iTRAQ8plex); Q4(Deamidated)	CID	0.0291	0.0000	1	1	3602576	Unique	Used	1.130	0.755	0.892	0.779	1E-05	0	6.54	0	0	82	3	1262.29492	3784.87021	-1.57
eVLTGNDEVIGQVLSTLk	Unambiguous	2	1	Q3TKX1	N-Term(iTRAQ8plex); K18(iTRAQ8plex)	CID	1.0000	0.0000	1	1	3418903	Unique	Used	0.631	1.009	0.968	0.930	1E-05	0	6.53	0	25	18	2	1262.22449	2523.44170	-2.92
eHINLGcDVDFDIAGPSIR	Unambiguous	3	1	Q60932-2	N-Term(iTRAQ8plex); C7(Methylthio)	CID	1.0000	0.0000	1	1	3357357	Unique	Used	0.892	1.256	0.976	0.925	1E-05	0	6.53	0	7	200	3	807.73065	2421.17740	-2.93
eEVEDVSSYLcTELDk	Unambiguous	9	1	Q9ESN9-4	N-Term(iTRAQ8plex); C11(Methylthio); K16(iTRAQ8plex)	CID	1.0000	0.0000	1	1	2766363	Unique	Used	0.411	0.517	0.829	0.836	1E-05	0	6.53	0	6	200	2	1257.11536	2513.22344	-0.54
tcPSISSAISSAVTSPPVGnQSR	Unambiguous	1	1	E9Q828	N-Term(iTRAQ8plex); C2(Methylthio); N20(Deamidated)	CID	0.0138	0.0000	1	1	3859806	Unique	Used	0.545	3.157	2.413	2.029	1E-05	0	6.53	0	0	126	2	1299.14233	2597.27739	-3.06
aDLQNHLDTAQHALQDk	Unambiguous	1	1	Q8BL66	N-Term(iTRAQ8plex); K17(iTRAQ8plex)	CID	1.0000	0.0000	1	1	3165189	Unique	Used	1.673	0.984	1.053	0.734	1E-05	0	6.53	0	0	61	3	842.78717	2526.34696	-2.04

eMQDAFQnAYMELGGLGER	Unambiguous	3	1	Q6PIE5	N-Term(iTRAQ8plex); N8(Deamidated)	CID	0.0260	0.0000	1	1	2941417	Unique	Used	1.065	0.829	1.011	1.446	1E-05	0	6.53	0	0	200	2	1232.57410	2464.14092	-0.73
aGYTDQVVIGMDVAASEFYR	Unambiguous	2	1	P17182	N-Term(iTRAQ8plex)	CID	1.0000	0.0000	1	1	3328535	Unique	Used	0.881	1.398	1.298	1.502	1E-05	0	6.53	0	6	200	2	1248.61975	2496.23223	-2.43
yGGMFAAVEGAYENk	Unambiguous	1	1	Q6P1J1	N-Term(iTRAQ8plex); K15(iTRAQ8plex)	CID	1.0000	0.0000	1	1	3194215	Unique	Used	0.820	0.708	0.698	0.520	1E-05	0	6.53	0	5	7	3	739.04761	2215.12827	-1.42
aIIQSGSALSSWAVnYQPVk	Unambiguous	4	1	Q8BYM5	N-Term(iTRAQ8plex); N15(Deamidated); K20(iTRAQ8plex)	CID	0.0306	0.0000	1	1	3879043	Unique	Used	0.778	1.517	0.931	0.759	1E-05	0	6.53	0	0	54	2	1364.76636	2728.52544	4.54
dFPEYTFAIADEEDYATEVk	Unambiguous	1	1	P08003	N-Term(iTRAQ8plex); K20(iTRAQ8plex)	CID	1.0000	0.0000	1	1	2656787	Unique	Used	1.724		0.823	0.974	1E-05	0	6.53	0	18	200	3	987.82068	2961.44748	-0.93
gnAGQTNYGFANSTMER	Unambiguous	1	1	P19096	N-Term(iTRAQ8plex); N2(Deamidated)	CID	0.0368	0.0000	1	1	2897768	Unique	Used	0.739	0.374	0.970	1.110	1E-05	0	6.53	0	15	200	2	1061.98792	2122.96855	-3.78
aESADYSVTGHYPLNLPSVTVTNLDTFGR	Unambiguous	8	1	H3BL28	N-Term(iTRAQ8plex)	CID	1.0000	0.0000	1	1	3601550	Unique	Used	1.431	0.463	1.980	1.061	1E-05	0	6.53	0	11	4	3	1143.57654	3428.71506	-3.67
tVTSSGSSEPSPPEVDVSR	Unambiguous	1	1	Q8BMI0	N-Term(iTRAQ8plex)	CID	1.0000	0.0000	1	1	2754839	Unique	Used	0.784	0.583	1.138	1.054	1E-05	0	6.53	0	0	200	2	1111.55469	2222.10210	-3.13
aNNLHSGDNFQLNDSEIER	Unambiguous	2	1	B9EJ86	N-Term(iTRAQ8plex)	CID	1.0000	0.0000	1	1	2699089	Unique	Used	0.767	0.688	1.079	0.907	1E-05	0	6.53	0	0	73	3	826.40247	2477.19284	-1.24
sMSINLPMScLDSIALGFnFGR	Unambiguous	2	1	P20357	N-Term(iTRAQ8plex); C10(Methylthio); N19(Deamidated)	CID	1.0000	0.0000	1	1	3899524	Unique	Used	1.226	1.111	1.490	0.985	1E-05	0	6.53	0	16	125	2	1362.65649	2724.30571	-4.22
eNPETEEDVGPIVQHIYELR	Unambiguous	2	1	A2AKI5	N-Term(iTRAQ8plex)	CID	1.0000	0.0000	1	1	2672082	Unique	Used		1.997	1.147	1.374	1E-05	0	6.53	0	7	200	3	891.12091	2671.34818	-1.34
dLVILLYETALLSSGFSLEDPQTHANR	Unambiguous	1	1	P07901	N-Term(iTRAQ8plex)	CID	1.0000	0.0000	1	1	3843076	Unique	Used	1.230	0.853	1.158	1.221	1E-05	0	6.53	0	27	200	3	1102.92126	3306.74924	-0.96

vVETDPSPYcIVAPDTVIHcEGEPIk	Unambiguous	1	1	Q01853	N-Term(iTRAQ8plex); C10(Methylthio); C20(Methylthio); K26(iTRAQ8plex)	CID	1.0000	0.0000	1	1	3239004	Unique	Used	0.792	0.721	0.809	0.964	1E-05	0	6.53	0	0	27	3	1171.25415	3511.74790	1.04
nQDLALSnLESIPGGYNALR	Unambiguous	1	1	Q9QZM0	N-Term(iTRAQ8plex); N8(Deamidated)	CID	1.0000	0.0000	1	1	3325574	Unique	Used	2.204	1.143	1.075	1.132	1E-05	0	6.53	0	18	200	2	1225.64099	2450.27471	-3.36
sINPDEAVAYGAAVQAAILSGDk	Unambiguous	2	1	P63017	N-Term(iTRAQ8plex); K23(iTRAQ8plex)	CID	1.0000	0.0000	1	1	3441583	Unique	Used	2.062	1.820	0.410	1.283	1E-05	0	6.53	0	5	200	3	956.85474	2868.54966	-2.35
sLEGFFIYEEEGSGVPGSNR	Unambiguous	1	1	Q8CHX7	N-Term(iTRAQ8plex)	CID	1.0000	0.0000	1	1	3037221	Unique	Used	0.509	0.742	1.028	1.386	1E-05	0	6.53	0	7	188	2	1239.60864	2478.21001	0.36
eSYVETELIFALAk	Unambiguous	2	1	Q68FD5	N-Term(iTRAQ8plex); K14(iTRAQ8plex)	CID	1.0000	0.0000	1	1	3262583	Unique	Used	0.330	1.526	1.223	1.272	1E-05	0	6.53	0	8	17	2	1111.12988	2221.25249	-2.32
dDSGQYYcIASNDAGAAR	Unambiguous	1	1	Q9D8B7	N-Term(iTRAQ8plex); C8(Methylthio)	CID	1.0000	0.0000	1	1	2735214	Unique	Used	1.043	0.230	1.278	1.517	1E-05	0	6.53	0	0	87	2	1113.98499	2226.96269	-3.18
rPLGNFTPPPTYAETLSTAPVASR	Unambiguous	4	1	Q8CC35	N-Term(iTRAQ8plex)	CID	1.0000	0.0000	1	1	3894713	Unique	Used		2.824	4.608		1E-05	0	6.53	0	0	15	3	949.84344	2847.51578	-5.25
sALFAQINQGESITHALk	Unambiguous	1	1	P40124	N-Term(iTRAQ8plex); K18(iTRAQ8plex)	CID	1.0000	0.0000	1	1	3683968	Unique	Used	1.107	1.197	1.918	1.319	1E-05	0	6.53	0	15	113	2	1268.70911	2536.41094	-9.25
nPLSDPLYDcIFTVEGAGLTk	Unambiguous	1	1	P21981	N-Term(iTRAQ8plex); C10(Methylthio); K21(iTRAQ8plex)	CID	1.0000	0.0000	1	1	3480307	Unique	Used	0.569	2.107	0.844	1.235	1E-05	0	6.53	0	14	85	2	1454.25183	2907.49639	-4.44
fNSLNELVDYHR	Unambiguous	3	1	B1AT92	N-Term(iTRAQ8plex)	CID	1.0000	0.0000	1	1	3455709	Unique	Used	1.652	1.242	1.087	0.917	1E-05	0	6.53	0	5	74	3	604.31555	1810.93210	-3.79
aLYDYEGQEHDELSFk	Unambiguous	1	1	Q9WVE8	N-Term(iTRAQ8plex); K16(iTRAQ8plex)	CID	1.0000	0.0000	1	1	2717392	Unique	Used	1.511	1.190	1.379	0.806	1E-05	0	6.53	0	12	200	3	851.42761	2552.26828	-3.24
aTSSFSQSVSSLFGEDNVR	Unambiguous	1	1	Q8BXV2	N-Term(iTRAQ8plex)	CID	1.0000	0.0000	1	1	3685761	Unique	Used	0.752	1.008	0.720	0.999	1E-05	0	6.53	0	0	187	2	1161.57129	2322.13530	-7.01
aHDQNLANLALQALR	Unambiguous	1	1	Q8BQU6	N-Term(iTRAQ8plex)	CID	1.0000	0.0000	1	1	3802646	Unique	Used	1.424	0.916	0.713	0.767	1E-05	0	6.53	0	2	19	3	651.36798	1952.08939	-4.39

mSEEDEQGWcQGQLQSGR	Unambiguous	1	1	Q99JB8	N-Term(iTRAQ8plex); C10(Methylthio)	CID	1.0000	0.0000	1	1	2812106	Unique	Used	1.806	0.828	2.071	1.404	1E-05	0	6.53	0	0	200	2	1209.52100	2418.03472	-3.29
tTTNVLGDSLGAGIVEHLSR	Unambiguous	1	1	P56564	N-Term(iTRAQ8plex)	CID	1.0000	0.0000	1	1	3642546	Unique	Used	1.764	3.464	0.614	1.143	1E-05	0	6.53	0	23	32	3	782.09204	2344.26157	-6.78
lLEVSDDPQVLAVAAHDVGEYVR	Unambiguous	1	1	Q8BVE3	N-Term(iTRAQ8plex)	CID	1.0000	0.0000	1	1	3265443	Unique	Used		0.667			1E-05	0	6.53	0	20	200	3	933.83459	2799.48923	2.18
wGEAGAEYVVESTGVFTTMEk	Unambiguous	3	1	P16858	N-Term(iTRAQ8plex); K21(iTRAQ8plex)	CID	1.0000	0.0000	1	1	3240505	Unique	Used		1.385	0.892	1.119	1E-05	0	6.53	0	0	200	2	1450.23621	2899.46514	0.23
vINAAHSFYNGTTTLPISDEDR	Unambiguous	1	1	Q3THK7	N-Term(iTRAQ8plex)	CID	1.0000	0.0000	1	1	3436604	Unique	Used	2.027	1.011	1.016	1.116	1E-05	0	6.53	0	19	194	3	909.12677	2725.36576	-2.86
gPQAAQEQDFYTVELER	Unambiguous	4	1	Q6RHR9-3	N-Term(iTRAQ8plex)	CID	1.0000	0.0000	1	1	2717278	Unique	Used	2.926	1.381	0.684	0.901	1E-05	0	6.53	0	0	200	2	1143.06885	2285.13042	-2.09
qIFDEYENETFLcHR	Unambiguous	4	1	E0CYJ0	N-Term(iTRAQ8plex); C13(Methylthio)	CID	1.0000	0.0000	1	1	3055208	Unique	Used	0.718	1.283	0.697	0.685	1E-05	0	6.53	0	10	77	3	765.35321	2294.04508	-3.18
gTGGVDTAATGSVFDISNLDR	Unambiguous	1	1	P30275	N-Term(iTRAQ8plex)	CID	1.0000	0.0000	1	1	3264354	Unique	Used	1.526	1.556	0.851	0.945	1E-05	0	6.52	0	0	200	2	1179.09863	2357.18999	0.54
qSTqVMAASMSAFDPLk	Unambiguous	3	1	Q9Z1A1	N-Term(iTRAQ8plex); Q4(Deamidated); K17(iTRAQ8plex)	CID	1.0000	0.0000	1	1	3539453	Unique	Used	1.226	0.833	0.678	0.657	1E-05	0	6.52	0	12	45	2	1211.13904	2421.27080	3.85
eMQEVETNLQEVVFDYLHATAYQNTALGR	Unambiguous	1	1	Q9CXT8	N-Term(iTRAQ8plex)	CID	1.0000	0.0000	1	1	3440558	Unique	Used	0.921	1.036	1.070	0.968	1E-05	0	6.52	0	19	93	3	1225.26965	3673.79441	-4.54
qNPDNTGPALEWDNYDLR	Unambiguous	1	1	Q9ERU9	N-Term(iTRAQ8plex)	CID	1.0000	0.0000	1	1	2831957	Unique	Used	0.837	0.931	0.747	0.857	1E-05	0	6.52	0	0	200	2	1211.57886	2422.15044	-3.01
eYQLNDSAAYYLnDLDR	Unambiguous	1	1	B2RSH2	N-Term(iTRAQ8plex); N13(Deamidated)	CID	1.0000	0.0000	1	1	2919908	Unique	Used	0.743	1.466	1.351	1.537	1E-05	0	6.52	0	9	200	2	1184.56299	2368.11870	-2.52
nLFAFFDMAYQGFASGDGDkDAWAVR	Unambiguous	1	1	P05202	N-Term(iTRAQ8plex); K20(iTRAQ8plex)	CID	1.0000	0.0000	1	1	3727225	Unique	Used	1.579	1.516	1.127	1.172	1E-05	0	6.52	1	19	15	3	1169.90479	3507.69980	-7.27
vALITGITGQDGSYLAEFLLEk	Unambiguous	2	1	Q8K0C9	N-Term(iTRAQ8plex); K22(iTRAQ8plex)	CID	1.0000	0.0000	1	1	3687322	Unique	Used	1.225	0.935	0.796	1.166	1E-05	0	6.52	0	30	111	3	982.88690	2946.64615	-6.36

cPTDELSLSNcAVVNEk	Unambiguous	2	1	P46460	N-Term(iTRAQ8plex); C1(Methylthio); C11(Methylthio); K17(iTRAQ8plex)	CID	1.0000	0.0000	1	1	3304433	Unique	Used	0.732	1.263	1.585	1.952	1E-05	0	6.52	0	7	18	3	841.41003	2522.21555	-2.55
tDLNPDNLQGGDDLDPnYVLSSR	Unambiguous	1	1	Q04447	N-Term(iTRAQ8plex); N17(Deamidated)	CID	1.0000	0.0000	1	1	2814960	Unique	Used	1.615	0.800	1.414	0.923	1E-05	0	6.52	0	0	18	2	1412.17993	2823.35259	-2.16
vEGAFPVTMLPGDGVGPELmHAVk	Unambiguous	1	1	Q91VA7	N-Term(iTRAQ8plex); M20(Oxidation); K24(iTRAQ8plex)	CID	1.0000	0.0000	1	1	3458141	Unique	Used	0.644	0.872	0.986	1.293	1E-05	0	6.52	0	8	200	3	1025.88367	3075.63645	-3.38
tcLLNETGDEPFQYkN	Unambiguous	1	1	P15105	N-Term(iTRAQ8plex); C2(Methylthio); K15(iTRAQ8plex)	CID	1.0000	0.0000	1	1	3054442	Unique	Used	0.417	1.049	1.334	1.559	1E-05	0	6.52	1	0	23	2	1263.62476	2526.24224	-1.69
nATNVEQAFMTmAAEIk	Unambiguous	1	1	Q9D1G1	N-Term(iTRAQ8plex); M12(Oxidation); K17(iTRAQ8plex)	CID	1.0000	0.0000	1	1	3394091	Unique	Used	0.694	0.991	1.081	1.098	1E-05	0	6.52	0	0	62	2	1247.14575	2493.28423	-3.85
aVGFVSEDEYLEIqGITR	Unambiguous	3	1	D3Z396	N-Term(iTRAQ8plex); Q14(Deamidated)	CID	1.0000	0.0000	1	1	3075342	Unique	Used	0.705	0.985	1.089	1.100	1E-05	0	6.52	0	17	200	2	1166.11255	2331.21782	6.69
aILQQLGLNSTcDDSILVk	Unambiguous	5	1	G3UVV4	N-Term(iTRAQ8plex); C12(Methylthio); K19(iTRAQ8plex)	CID	1.0000	0.0000	1	1	3706554	Unique	Used	0.248	1.835	0.971	1.210	1E-05	0	6.52	0	5	10	2	1343.23108	2685.45488	-8.46
aGYTDQVVIGMDVAASEFYR	Unambiguous	2	1	P17182	N-Term(iTRAQ8plex)	CID	1.0000	0.0000	1	1	3329701	Unique	Used	1.522	1.245	1.696	1.745	1E-05	0	6.52	0	3	200	2	1248.62036	2496.23345	-1.95
aDcIEVQVQSSDTPVTQTTEAVk	Unambiguous	2	1	Q9WTQ5	N-Term(iTRAQ8plex); C3(Methylthio); K23(iTRAQ8plex)	CID	1.0000	0.0000	1	1	2957863	Unique	Used	1.533	1.314	1.015	1.102	1E-05	0	6.52	0	19	106	3	1035.19507	3103.57065	-1.34
ISISGNYNLETLMSPLGITR	Unambiguous	1	1	Q00898	N-Term(iTRAQ8plex)	CID	1.0000	0.0000	1	1	3917719	Unique	Used	18.183	11.315	7.503	0.599	1E-05	0	6.52	0	15	19	2	1242.17456	2483.34184	-2.55

wGEAGAEYVVESTGVFTTMEk	Unambiguous	3	1	P16858	N-Term(iTRAQ8plex); K21(iTRAQ8plex)	CID	1.0000	0.0000	1	1	3218433	Unique	Used	2.274	1.338			1E-05	0	6.52	0	0	200	3	967.16199	2899.47141	2.39
mPFPVNHGASSEDSLLQDAAk	Unambiguous	1	1	Q9R0P9	N-Term(iTRAQ8plex); M1(Oxidation); K21(iTRAQ8plex)	CID	1.0000	0.0000	1	1	3147134	Unique	Used	2.014	1.334	1.651	1.248	1E-05	0	6.52	0	0	22	3	946.82104	2838.44858	-2.37
tPVVQNAASIVQPSPAHVGQQGLSk	Unambiguous	2	1	Q8VHR5	N-Term(iTRAQ8plex); K25(iTRAQ8plex)	CID	1.0000	0.0000	1	1	3720534	Unique	Used	1.513	0.692	1.346	0.922	1E-05	0	6.52	0	0	113	3	1041.25208	3121.74167	-5.20
mQAQMQMQMQGGDSDSGALGQHV	Unambiguous	3	1	P42208	N-Term(iTRAQ8plex)	CID	1.0000	0.0000	1	1	3070875	Unique	Used	3.952	0.480	0.828	0.683	1E-05	0	6.52	0	0	137	2	1370.11499	2739.22270	-1.26
vGAGAPVYMAAVLEYLTAEILELAGNAAR	Unambiguous	4	1	Q64522	N-Term(iTRAQ8plex)	CID	1.0000	0.0000	1	1	3919198	Unique	Used		1.570	1.244	1.488	1E-05	0	6.52	0	14	59	2	1619.37268	3237.73808	-3.56
nIFLQYASTEVDGEHYmTPEDFVQR	Unambiguous	1	1	Q8BH59	N-Term(iTRAQ8plex); M17(Oxidation)	CID	1.0000	0.0000	1	1	3261467	Unique	Used	1.469	1.559	1.881	2.010	1E-05	0	6.52	0	0	62	3	1103.85803	3309.55954	-2.45
tLEVVSPSQSVTGSAGHTPYYQSPTDEk	Unambiguous	1	1	P14873	N-Term(iTRAQ8plex); K28(iTRAQ8plex)	CID	1.0000	0.0000	1	1	3210202	Unique	Used	0.972	0.980	1.351	0.695	1E-05	0	6.52	0	0	69	3	1191.94263	3573.81333	-1.02
lLDLYGWAQLSDTYVTLR	Unambiguous	3	1	Q9QYB8	N-Term(iTRAQ8plex)	CID	1.0000	0.0000	1	1	3861101	Unique	Used	1.802	0.874	1.653	1.428	1E-05	0	6.52	0	27	39	2	1216.15918	2431.31108	-2.64
qLcDNAGFDATNILNk	Unambiguous	1	1	P80313	N-Term(iTRAQ8plex); C3(Methylthio); K16(iTRAQ8plex)	CID	1.0000	0.0000	1	1	3518962	Unique	Used	0.441	0.837	0.896	1.099	1E-05	0	6.52	0	25	200	2	1196.10986	2391.21245	-5.55
gIVDQSQQAYQEAFEISk	Unambiguous	1	1	P63101	N-Term(iTRAQ8plex); K18(iTRAQ8plex)	CID	1.0000	0.0000	1	1	3110476	Unique	Used	0.972	1.405	1.168	0.690	1E-05	0	6.52	0	0	200	2	1325.20032	2649.39336	-1.78
vFTTQELVQAFTHAPAALEADR	Unambiguous	1	1	Q8BK64	N-Term(iTRAQ8plex)	CID	1.0000	0.0000	1	1	3648186	Unique	Used	2.075	1.045	1.821	1.737	1E-05	0	6.52	0	26	200	3	907.14557	2719.42215	-5.01
fQMPDQGMTSADDFFQGTk	Unambiguous	1	1	O08553	N-Term(iTRAQ8plex); K19(iTRAQ8plex)	CID	1.0000	0.0000	1	1	3307640	Unique	Used	1.303	2.244	1.559	1.241	1E-05	0	6.52	0	11	200	3	920.44537	2759.32157	-1.82
mAVAAQEGDAHPLGALPVGTLINNVESEPGR	Unambiguous	2	1	B1B1D8	N-Term(iTRAQ8plex); M1(Oxidation)	CID	1.0000	0.0000	1	1	3371616	Unique	Used	1.067	0.615	0.828	0.768	1E-05	0	6.52	0	10	71	3	1145.25610	3433.75376	-4.40

aAMTEHYVTQLELQDLQER	Unambiguous	2	1	Q6ZWR6-4	N-Term(iTRAQ8plex)	CID	1.0000	0.0000	1	1	3109318	Unique	Used	0.901	1.232	1.251	1.344	1E-05	0	6.52	0	9	99	3	860.43903	2579.30252	-2.03
iSGETIFVTAPHEATAGIIGVNR	Unambiguous	2	1	Q68FD5	N-Term(iTRAQ8plex)	CID	1.0000	0.0000	1	1	3684543	Unique	Used	0.493	1.190	1.281	1.296	1E-05	0	6.52	0	2	11	3	886.48431	2657.43839	-6.82
vNSMQTVFVGYGPTFk	Unambiguous	4	1	Q9R1E6	N-Term(iTRAQ8plex);K16(iTRAQ8plex)	CID	1.0000	0.0000	1	1	3841313	Unique	Used	2.248			0.606	1E-05	0	6.52	0	0	200	2	1192.14441	2383.28154	-5.25
aTQWGSFYSPAQTANNPcSR	Unambiguous	3	1	Q68FD9	N-Term(iTRAQ8plex);C18(Methylthio)	CID	1.0000	0.0000	1	1	3915156	Unique	Used	0.269	1.264	1.473	0.770	1E-05	0	6.52	0	0	200	2	1268.58044	2536.15361	-4.54
sVNESLNNLFITEEDYQALR	Unambiguous	2	1	Q68FD5	N-Term(iTRAQ8plex)	CID	1.0000	0.0000	1	1	3264303	Unique	Used	0.402	1.216	1.339	1.158	1E-05	0	6.52	0	13	200	2	1330.18018	2659.35307	0.50
gFQQILAGEYDHLPEqAFYMVGPIEEAVAk	Unambiguous	1	1	P56480	N-Term(iTRAQ8plex);Q16(Deamidated);K30(iTRAQ8plex)	CID	1.0000	0.0000	1	1	3398603	Unique	Used	2.442	1.987	0.897	0.743	1E-05	0	6.52	0	0	200	3	1320.68433	3960.03843	0.85
icEQFEAETPTDSEATQR	Unambiguous	2	1	Q8VCI5	N-Term(iTRAQ8plex);C2(Methylthio)	CID	1.0000	0.0000	1	1	2638227	Unique	Used	1.457		1.235	1.410	1E-05	0	6.51	0	0	200	2	1203.04309	2405.07891	-4.74
eGIcAIGGTSEQSSVGTQHSYSEEEk	Unambiguous	1	1	Q61233	N-Term(iTRAQ8plex);C4(Methylthio);K26(iTRAQ8plex)	CID	1.0000	0.0000	1	1	2757804	Unique	Used	2.359	0.727	0.877	1.041	1E-05	0	6.51	0	0	200	3	1123.19873	3367.58164	-1.86
fGDEDITWQDEHSAPFSWETR	Unambiguous	1	1	Q8C3W1	N-Term(iTRAQ8plex)	CID	1.0000	0.0000	1	1	2869500	Unique	Used	1.512	0.621	0.950	0.587	1E-05	0	6.51	0	2	133	3	953.10199	2857.29142	-3.28
sAAGSSSGFHSWAR	Unambiguous	1	1	P19246	N-Term(iTRAQ8plex)	CID	1.0000	0.0000	1	1	3888217	Unique	Used	1.946	0.744	0.854	1.217	1E-05	0	6.51	0	13	200	3	571.28387	1711.83707	-4.88
gPQAAQEQDFYTVELER	Unambiguous	4	1	Q6RHR9-3	N-Term(iTRAQ8plex)	CID	1.0000	0.0000	1	1	2717477	Unique	Used	1.679	0.724	0.722	1.196	1E-05	0	6.51	0	0	200	2	1143.07007	2285.13286	-1.02
gEMMDLQHGSLFLQTPk	Unambiguous	2	1	P16125	N-Term(iTRAQ8plex);K17(iTRAQ8plex)	CID	1.0000	0.0000	1	1	3391811	Unique	Used	0.557	0.866	1.280	1.176	1E-05	0	6.51	0	0	2	3	847.44965	2540.33438	-4.65
qSFLSYnGAEQFLEPAR	Unambiguous	1	1	Q9JI39	N-Term(iTRAQ8plex);N7(Deamidated)	CID	1.0000	0.0000	1	1	3283465	Unique	Used	0.809	1.049	1.028	1.556	1E-05	0	6.51	0	0	113	2	1131.56995	2262.13262	-0.82
eANAHEFISSFPDGYSTVVGER	Unambiguous	1	1	Q9CXJ4	N-Term(iTRAQ8plex)	CID	1.0000	0.0000	1	1	3235223	Unique	Used	1.450	0.891	1.319	1.263	1E-05	0	6.51	0	4	23	3	906.10718	2716.30698	-3.21

yAQPAAATAAAYSDSYGR	Unambiguous	5	1	Q8BIF2-2	N-Term(iTRAQ8plex)	CID	1.0000	0.0000	1	1	3231427	Unique	Used	1.077	0.947	1.361	0.838	1E-05	0	6.51	0	0	58	2	1069.52429	2138.04131	-2.02
lIAVIGDEDTVTGFLLGGIGELNk	Unambiguous	1	1	Q9D1K2	N-Term(iTRAQ8plex); K24(iTRAQ8plex)	CID	1.0000	0.0000	1	1	3602901	Unique	Used	1.143	0.760	0.917	0.737	1E-05	0	6.51	0	23	117	3	1018.24615	3052.72391	-4.99
eLNSYSIPFLGSDISVVk	Unambiguous	1	1	Q8R127	N-Term(iTRAQ8plex); K18(iTRAQ8plex)	CID	1.0000	0.0000	1	1	3560705	Unique	Used	0.468	0.563	0.730	1.014	1E-05	0	6.51	0	8	10	2	1288.71814	2576.42900	-5.52
dQEGQDVLLFIDNIFR	Unambiguous	1	1	P56480	N-Term(iTRAQ8plex)	CID	1.0000	0.0000	1	1	3355953	Unique	Used	1.209	1.616	0.868	1.068	1E-05	0	6.51	0	7	200	2	1113.58325	2226.15923	-5.23
tGAScPSGGHVADIYLANINk	Unambiguous	1	1	Q91Z67	N-Term(iTRAQ8plex); C5(Methylthio); K21(iTRAQ8plex)	CID	1.0000	0.0000	1	1	3576407	Unique	Used	0.627	0.862	1.025	0.987	1E-05	0	6.51	0	0	66	3	914.80359	2742.39621	-7.36
fQMPDQGMTSADDFFQGTk	Unambiguous	1	1	O08553	N-Term(iTRAQ8plex); K19(iTRAQ8plex)	CID	1.0000	0.0000	1	1	3286897	Unique	Used	1.629			2.100	1E-05	0	6.51	0	18	200	3	920.44635	2759.32450	-0.76
aNDTQEFNLSAYFER	Unambiguous	4	1	Q9D7X3	N-Term(iTRAQ8plex)	CID	1.0000	0.0000	1	1	3193973	Unique	Used	1.202	0.921	1.341	1.128	1E-05	0	6.51	0	3	10	2	1055.01160	2109.01592	-1.51
tYFPHFDVSHGSAQVk	Unambiguous	2	1	Q91VB8	N-Term(iTRAQ8plex); K16(iTRAQ8plex)	CID	1.0000	0.0000	1	1	3719472	Unique	Used	2.789	2.611	0.467	1.271	1E-05	0	6.51	0	14	14	3	810.09296	2428.26432	-9.33
dLAGNSEVVAGTGEQcLPFDEAR	Unambiguous	1	1	Q9WTS5	N-Term(iTRAQ8plex); C16(Methylthio)	CID	1.0000	0.0000	1	1	2920813	Unique	Used	0.727	0.530	0.379	1.317	1E-05	0	6.51	0	11	200	2	1364.64429	2728.28130	-1.76
eENAQQILNTAIPFR	Unambiguous	1	1	O35450	N-Term(iTRAQ8plex)	CID	1.0000	0.0000	1	1	3539781	Unique	Used	1.355	1.273	1.136	0.771	1E-05	0	6.51	0	0	95	2	1024.55322	2048.09917	-4.24
sAAEMYGSSFDLDYDFQR	Unambiguous	3	1	Q9Z204-3	N-Term(iTRAQ8plex)	CID	1.0000	0.0000	1	1	3055645	Unique	Used	0.791	0.775	0.756	0.952	1E-05	0	6.51	0	8	145	2	1203.54651	2406.08574	-0.19
gPPPSGIATLVSGIAGEEPQR	Unambiguous	2	1	E9Q3M3	N-Term(iTRAQ8plex)	CID	1.0000	0.0000	1	1	3519250	Unique	Used	0.411	0.705	1.085	1.099	1E-05	0	6.51	0	2	19	2	1169.13501	2337.26274	-3.82
tVMAHGcYLSEEELNIFSER	Unambiguous	2	1	Q9R111	N-Term(iTRAQ8plex); C7(Methylthio)	CID	1.0000	0.0000	1	1	3373088	Unique	Used	1.057	1.165	2.088	1.352	1E-05	0	6.51	0	20	94	3	893.42023	2678.24613	-3.94
aSLDSAGASGNSPVLLPTPAVGGPR	Unambiguous	3	1	Q8C6B2-2	N-Term(iTRAQ8plex)	CID	1.0000	0.0000	1	1	3683210	Unique	Used	0.554	0.331	1.042	0.864	1E-05	0	6.51	0	0	83	2	1298.19727	2595.38725	-6.64
nLALcPANHAPLQEAAVIPR	Unambiguous	1	1	Q02257	N-Term(iTRAQ8plex); C5(Methylthio)	CID	1.0000	0.0000	1	1	3821848	Unique	Used	0.787	0.914	0.827	0.911	1E-05	0	6.51	0	19	72	3	816.77319	2448.30503	-4.40

iFPSLELNITDLLEDTPR	Unambiguous	2	1	Q80TQ2	N-Term(iTRAQ8plex)	CID	1.0000	0.0000	1	1	3520804	Unique	Used	0.395	2.186	1.853	1.119	1E-05	0	6.51	0	0	77	2	1195.65637	2390.30547	-2.78
vIDIFDTDGnGEVDFkEFIEGVSQFSVk	Unambiguous	2	1	Q63810-2	N-Term(iTRAQ8plex); N10(Deamidated); K16(iTRAQ8plex); K28(iTRAQ8plex)	CID	0.0215	0.0000	1	1	3623751	Unique	Used	1.303	3.885	1.919	0.804	1E-05	0	6.51	1	25	67	3	1350.04370	4048.11655	-1.03
kTLNPAFNETFTFk	Unambiguous	1	1	P46097	N-Term(iTRAQ8plex); K1(iTRAQ8plex); K14(iTRAQ8plex)	CID	1.0000	0.0000	1	1	3617819	Unique	Used	0.604	0.345	1.127	1.175	1E-05	0	6.51	1	17	75	3	857.48816	2570.44992	-9.59
tIGGVISSPELAEDLTGTEILLER	Unambiguous	1	1	P08032	N-Term(iTRAQ8plex)	CID	1.0000	0.0000	1	1	3217580	Unique	Used	4.009	2.360	0.788	1.395	1E-05	0	6.51	0	0	48	2	1409.26990	2817.53252	-2.65
sITIANQTncPLYVTk	Unambiguous	1	1	O08553	N-Term(iTRAQ8plex); N9(Deamidated); C10(Methylthio); K16(iTRAQ8plex)	CID	0.0323	0.0000	1	1	3579071	Unique	Used	0.953	1.247	1.382	1.521	1E-05	0	6.51	0	8	71	2	1211.14380	2421.28032	-7.22
dMHGDSEYNImFGPDIcGPGTk	Unambiguous	1	1	P14211	N-Term(iTRAQ8plex); M11(Oxidation); C17(Methylthio); K22(iTRAQ8plex)	CID	1.0000	0.0000	1	1	3072910	Unique	Used	1.839	1.118	1.090	0.783	1E-05	0	6.51	0	19	200	3	1018.80243	3054.39273	0.03
yDDIEETnYFQQASELAk	Unambiguous	1	1	Q91VD9	N-Term(iTRAQ8plex); N8(Deamidated); K18(iTRAQ8plex)	CID	1.0000	0.0000	1	1	2764604	Unique	Used	0.845	0.800	1.268	0.808	1E-05	0	6.51	0	0	200	2	1387.18481	2773.36235	-1.49
iGcFALSEPGnGSDAGAASTTAR	Unambiguous	2	1	Q07417	N-Term(iTRAQ8plex); C3(Methylthio); N11(Deamidated)	CID	1.0000	0.0000	1	1	3283277	Unique	Used	0.873	1.239	1.175	1.496	1E-05	0	6.51	0	11	62	2	1252.58789	2504.16850	-0.59

acADATLSQITnNIDPVGR	Unambiguous	3	1	P62874	N-Term(iTRAQ8plex); C2(Methylthio); N12(Deamidated)	CID	0.0430	0.0000	1	1	3260033	Unique	Used	0.404	1.202	1.595	1.529	1E-05	0	6.51	0	1	2	2	1155.57031	2310.13335	-1.67
aVGPHQFLGNQEAIQAAlk	Unambiguous	1	1	P15327	N-Term(iTRAQ8plex); K19(iTRAQ8plex)	CID	1.0000	0.0000	1	1	3596122	Unique	Used	1.668	1.168	0.364	1.028	1E-05	0	6.51	0	0	140	2	1300.73413	2600.46099	-6.14
lAQIHIQQQDQcVAITEESk	Unambiguous	1	1	Q9Z0Y1	N-Term(iTRAQ8plex); C12(Methylthio); K20(iTRAQ8plex)	CID	1.0000	0.0000	1	1	3212795	Unique	Used	1.457	0.983	1.273	0.936	1E-05	0	6.51	0	30	43	3	979.51727	2936.53727	-1.97
sFESTVGQGSDTYSYIFR	Unambiguous	1	1	P24668	N-Term(iTRAQ8plex)	CID	1.0000	0.0000	1	1	3457280	Unique	Used	0.472	1.092	0.733	0.939	1E-05	0	6.50	0	0	7	2	1174.56628	2348.12529	-4.07
sNASLTNNQNLIQSLR	Unambiguous	2	1	Q7M739	N-Term(iTRAQ8plex)	CID	1.0000	0.0000	1	1	3855618	Unique	Used	1.667	0.908	1.159	0.788	1E-05	0	6.50	0	0	200	2	1039.06409	2077.12090	-4.56
qPSQPTTSQSLGQLQAHAASAPGSNPR	Unambiguous	1	1	E9PZA8	N-Term(iTRAQ8plex)	CID	1.0000	0.0000	1	1	3737193	Unique	Used		1.091	1.816	0.891	1E-05	0	6.50	0	0	200	3	1007.51874	3020.54166	-1.18
acADATLSQITnNIDPVGR	Unambiguous	3	1	P62874	N-Term(iTRAQ8plex); C2(Methylthio); N12(Deamidated)	CID	0.0446	0.0000	1	1	3259862	Unique	Used	0.563	1.088	1.042	1.181	1E-05	0	6.50	0	0	42	2	1155.56982	2310.13237	-2.09
fQMPDQGMTSADDFFQGTk	Unambiguous	1	1	O08553	N-Term(iTRAQ8plex); K19(iTRAQ8plex)	CID	1.0000	0.0000	1	1	3325590	Unique	Used	1.171	1.344	1.111	1.247	1E-05	0	6.50	0	0	17	2	1380.15967	2759.31206	-5.27
qDAGSEPVTPASLAALQSDVQPVGHDYVEEVR	Unambiguous	1	1	O88532	N-Term(iTRAQ8plex)	CID	1.0000	0.0000	1	1	2920734	Unique	Used			1.132	0.891	1E-05	0	6.50	0	0	200	3	1223.61890	3668.84214	2.03
qTTVSnSQQAYQEAFEISk	Unambiguous	3	1	Q9CQV8-2	N-Term(iTRAQ8plex); N6(Deamidated); K19(iTRAQ8plex)	CID	1.0000	0.0000	1	1	2918264	Unique	Used		1.698	1.311	0.892	1E-05	0	6.50	0	0	200	2	1384.71240	2768.41753	-0.86
tGAMLGQSEELQPLSqR	Unambiguous	4	1	Q5NCM5	N-Term(iTRAQ8plex); Q16(Deamidated)	CID	0.0046	0.0000	1	1	3278528	Unique	Used	1.094	1.203	1.949	1.169	1E-05	0	6.50	0	27	200	2	1075.56226	2150.11724	4.97
fQLQATTQQGPGEAIVR	Unambiguous	3	1	A2AFG8	N-Term(iTRAQ8plex)	CID	1.0000	0.0000	1	1	3555843	Unique	Used	0.596	0.857	0.721	0.738	1E-05	0	6.50	0	0	1	2	1074.58447	2148.16167	-4.59

sASQDcIETTPGAQEGk	Unambiguous	2	1	P98084	N-Term(iTRAQ8plex); C6(Methylthio); K17(iTRAQ8plex)	CID	1.0000	0.0000	1	1	2632732	Unique	Used		0.695	1.017	1.213	1E-05	0	6.50	0	0	200	2	1188.58325	2376.15923	-1.67
eGIcALGGTSELSSEGTQHSYSEEEk	Unambiguous	2	1	B1AX58	N-Term(iTRAQ8plex); C4(Methylthio); K26(iTRAQ8plex)	CID	1.0000	0.0000	1	1	2717211	Unique	Used	1.467	0.928	1.522	1.463	1E-05	0	6.50	0	0	200	3	1128.19739	3382.57761	-2.95
eTAAAHQASSsPPIDAATAEPYGFR	Unambiguous	1	1	P14873	N-Term(iTRAQ8plex); S11(Phospho)	CID	1.0000	0.0000	1	1	2736058	Unique	Used	7.018	1.378	0.833	0.969	1E-05	0	6.50	0	0	200	3	977.12677	2929.36576	-0.48
lNEDMAcSVAGITSDANVLTNELR	Unambiguous	3	1	Q9R1P0	N-Term(iTRAQ8plex); C7(Methylthio)	CID	1.0000	0.0000	1	1	3352685	Unique	Used	1.763	0.887	0.853	1.013	1E-05	0	6.50	0	0	10	2	1443.69580	2886.38432	-3.70
sYIEGYVPSqADVAVFEAVSGPPPADLcHALR	Unambiguous	3	1	O70251	N-Term(iTRAQ8plex); Q10(Deamidated); C28(Methylthio)	CID	1.0000	0.0000	1	1	3624424	Unique	Used	1.687	1.956	1.551	1.236	1E-05	0	6.50	0	24	200	3	1237.27820	3709.82004	0.42
sLPGGSLQHEGVSGLGSSPFLSEAnAER	Unambiguous	2	1	E9QLB8	N-Term(iTRAQ8plex); N25(Deamidated)	CID	0.0277	0.0000	1	1	3369484	Unique	Used	1.361	0.881	0.915	1.369	1E-05	0	6.50	0	0	97	3	1030.19153	3088.56003	3.58
lASEFFVDGLDSDESTLEHFk	Unambiguous	1	1	Q3UHY7	N-Term(iTRAQ8plex); K21(iTRAQ8plex)	CID	1.0000	0.0000	1	1	2978946	Unique	Used	1.297	0.612	1.190	0.853	1E-05	0	6.50	0	0	32	3	998.84216	2994.51194	-2.47
lEVSSLAQTSSAVASSTDGSIHTESVDGIPDPQR	Unambiguous	3	1	Q69ZZ6	N-Term(iTRAQ8plex)	CID	1.0000	0.0000	1	1	3192867	Unique	Used	1.930	1.114	1.277	0.997	1E-05	0	6.50	0	0	78	3	1249.29297	3745.86435	-0.73
sNcNVAVINVGAPAAGMNAAVR	Unambiguous	3	1	Q8C605	N-Term(iTRAQ8plex); C3(Methylthio)	CID	1.0000	0.0000	1	1	3933966	Unique	Used	2.285		0.956	1.068	1E-05	0	6.50	0	0	200	2	1225.11816	2449.22905	-5.15
qESGYLIEEIGDVLLAR	Unambiguous	7	1	E9PUF7	N-Term(iTRAQ8plex)	CID	1.0000	0.0000	1	1	3263062	Unique	Used	0.392	1.184	0.569	0.834	1E-05	0	6.50	0	0	37	2	1105.10205	2209.19682	-2.27
lFVGNLPTDITEEDFkR	Unambiguous	3	1	Q8R326	N-Term(iTRAQ8plex); K16(iTRAQ8plex)	CID	1.0000	0.0000	1	1	3324983	Unique	Used	0.696	0.811	0.892	0.869	1E-05	0	6.50	1	0	108	3	868.14508	2602.42069	-5.02
yGELEEGIDVLDADGNLVGSSk	Unambiguous	2	1	Q925N0	N-Term(iTRAQ8plex); K22(iTRAQ8plex)	CID	1.0000	0.0000	1	1	2766273	Unique	Used	0.549	0.852	0.889	1.022	1E-05	0	6.50	0	22	200	2	1444.75452	2888.50176	1.09

aGYTDQVVIGMDVAASEFYR	Unambiguous	2	1	P17182	N-Term(iTRAQ8plex)	CID	1.0000	0.0000	1	1	3354833	Unique	Used	0.990	1.108	1.644	1.361	1E-05	0	6.50	0	6	200	2	1248.61963	2496.23198	-2.53
enGTITAANASTLNDGAAALVLMTAEAAQR	Unambiguous	1	1	Q8QZT1	N-Term(iTRAQ8plex); N2(Deamidated)	CID	1.0000	0.0000	1	1	3396259	Unique	Used	0.624	0.501	0.636	1.009	1E-05	0	6.50	0	6	10	2	1625.82397	3250.64067	-3.74
lGTEEFPLIDqTFYPNHk	Unambiguous	3	1	O88935	N-Term(iTRAQ8plex); Q11(Deamidated); K18(iTRAQ8plex)	CID	0.0215	0.0000	1	1	3325835	Unique	Used	0.642	1.226	1.197	1.156	1E-05	0	6.50	0	23	82	3	920.15552	2758.45200	-1.04
lQNLDALTNLTVLSVQSNR	Unambiguous	1	1	Q3UM45	N-Term(iTRAQ8plex)	CID	1.0000	0.0000	1	1	3917650	Unique	Used	1.046	3.420	1.205	1.016	1E-05	0	6.50	0	4	8	2	1202.17310	2403.33891	-5.01
vETETVLSFVDDIISGAk	Unambiguous	2	1	Q61137-2	N-Term(iTRAQ8plex); K18(iTRAQ8plex)	CID	1.0000	0.0000	1	1	3374494	Unique	Used		1.833	0.630	0.440	1E-05	0	6.50	0	10	119	2	1266.20166	2531.39604	-4.14
vETLHDFEAANSDELnLQR	Unambiguous	1	1	Q7TQF7	N-Term(iTRAQ8plex); N16(Deamidated)	CID	0.0462	0.0000	1	1	2654792	Unique	Used	2.042	0.824	1.013	0.436	1E-05	0	6.50	0	5	200	3	836.08258	2506.23319	-1.28
kPPGAGAGAGAGAGAGPVPR	Unambiguous	1	1	P97793	N-Term(iTRAQ8plex); K1(iTRAQ8plex)	CID	1.0000	0.0000	1	1	3589586	Unique	Used	0.394	0.674	0.816	0.788	1E-05	0	6.50	0	0	148	3	742.09357	2224.26615	-4.95
tIGGGDDSFNTFFSETGAGk	Unambiguous	4	1	P68373	N-Term(iTRAQ8plex); K20(iTRAQ8plex)	CID	1.0000	0.0000	1	1	3155118	Unique	Used	0.930	1.723	1.176	1.284	1E-05	0	6.50	0	7	200	2	1308.65198	2616.29668	-2.75
aQQVFVDEGHTVQFVcR	Unambiguous	2	1	Q9D1T0	N-Term(iTRAQ8plex); C16(Methylthio)	CID	1.0000	0.0000	1	1	3476545	Unique	Used	0.926	1.375	1.046	0.823	1E-05	0	6.50	0	17	33	3	771.71753	2313.13803	-1.81
mTDSFTEQADQVTADVGk	Unambiguous	3	1	Q8BVI4	N-Term(iTRAQ8plex); K18(iTRAQ8plex)	CID	1.0000	0.0000	1	1	2832083	Unique	Used	0.595	0.564	1.724	2.523	1E-05	0	6.49	0	0	73	2	1276.14148	2551.27568	-1.96
sGETVINTAnYHDTSPYR	Unambiguous	5	1	E9Q3V9	N-Term(iTRAQ8plex); N10(Deamidated)	CID	0.0431	0.0000	1	1	3007873	Unique	Used	0.903	1.121	0.969	0.876	1E-05	0	6.49	0	0	200	2	1165.57153	2330.13579	6.67
sLQEHLPDVVVYNAGTDVLEGDR	Unambiguous	1	1	Q91WA3	N-Term(iTRAQ8plex)	CID	1.0000	0.0000	1	1	3130653	Unique	Used	1.078	1.352	1.544	1.772	1E-05	0	6.49	0	7	73	3	944.15411	2830.44779	-1.68
lTGFHETSNINDFSAGVANR	Unambiguous	1	1	P15105	N-Term(iTRAQ8plex)	CID	1.0000	0.0000	1	1	3473837	Unique	Used	1.315	0.941	1.723	1.957	1E-05	0	6.49	0	0	6	3	818.74634	2454.22446	-2.90

icDPGLTSFEPEALGNLVEGMDFHR	Unambiguous	9	1	Q5SVJ0	N-Term(iTRAQ8plex); C2(Methylthio)	CID	1.0000	0.0000	1	1	3480850	Unique	Used	0.880	0.860	1.295	0.946	1E-05	0	6.49	0	20	200	3	1033.16003	3097.46555	-2.60
tFVQENVYDEFVER	Unambiguous	1	1	P47738	N-Term(iTRAQ8plex)	CID	1.0000	0.0000	1	1	2833463	Unique	Used	0.712	1.529	0.957	1.657	1E-05	0	6.49	0	1	29	2	1040.01794	2079.02861	-2.44
nLHQSGFSLSGTQVDEGVR	Unambiguous	3	1	E9PWE8	N-Term(iTRAQ8plex)	CID	1.0000	0.0000	1	1	3340713	Unique	Used	1.182	0.215	0.334	0.370	1E-05	0	6.49	0	11	15	2	1168.09607	2335.18486	-4.11
kQSESSFITGDINSSASLNR	Unambiguous	22	1	Q80TS3-5	N-Term(iTRAQ8plex); K1(iTRAQ8plex)	CID	1.0000	0.0000	1	1	3318707	Unique	Used	0.971	1.529	1.355	1.250	1E-05	0	6.49	1	0	119	3	917.15472	2749.44962	-2.95
fQMPDQGMTSADDFFQGTk	Unambiguous	1	1	O08553	N-Term(iTRAQ8plex); K19(iTRAQ8plex)	CID	1.0000	0.0000	1	1	3307763	Unique	Used		1.043	1.281		1E-05	0	6.49	0	0	200	3	920.44458	2759.31919	-2.69
vIRPLDQPSSFDATPYIk	Unambiguous	1	1	Q6ZQ38	N-Term(iTRAQ8plex); K18(iTRAQ8plex)	CID	1.0000	0.0000	1	1	3577058	Unique	Used	0.367	1.537	0.833	1.141	1E-05	0	6.49	0	5	8	3	885.83209	2655.48172	-5.63
yEGSGDGGAAAQSLYIANHAY	Unambiguous	1	1	P05063	N-Term(iTRAQ8plex)	CID	1.0000	0.0000	1	1	2974870	Unique	Used	2.452	0.482	0.729	1.079	1E-05	0	6.49	0	25	189	2	1210.07446	2419.14165	-2.13
tQPTDEEMLFIYSHFk	Unambiguous	4	1	P31786	N-Term(iTRAQ8plex); K16(iTRAQ8plex)	CID	1.0000	0.0000	1	1	3260571	Unique	Used	1.990	0.733	0.931	0.951	1E-05	0	6.49	0	9	41	3	865.45111	2594.33878	-1.29
cAPGYYGNPSQGQPcHR	Unambiguous	3	1	E9PZ16	N-Term(iTRAQ8plex); C1(Methylthio); C15(Methylthio)	CID	1.0000	0.0000	1	1	3677900	Unique	Used	1.458	1.553	0.876	0.950	1E-05	0	6.49	0	5	82	3	744.31927	2230.94327	-5.55
qNYVDLVSSLSSSSEAPSQGk	Unambiguous	9	1	Q9WUR2-2	N-Term(iTRAQ8plex); K21(iTRAQ8plex)	CID	1.0000	0.0000	1	1	3347745	Unique	Used	0.762	1.025	0.791	0.989	1E-05	0	6.49	0	13	23	2	1396.22473	2791.44219	-5.32
iGEHTPSALAImENANVLAR	Unambiguous	3	1	A6ZI44	N-Term(iTRAQ8plex); M12(Oxidation)	CID	1.0000	0.0000	1	1	3701490	Unique	Used	1.263	1.086	0.958	1.000	1E-05	0	6.49	0	0	0	3	809.76605	2427.28360	-5.45
aQQEFAAGVFSNPAVR	Unambiguous	3	1	E9Q855	N-Term(iTRAQ8plex)	CID	1.0000	0.0000	1	1	3640318	Unique	Used	0.526	1.083	0.864	0.973	1E-05	0	6.49	0	0	3	2	998.52557	1996.04387	-5.80
gAGVTLNVLEmTADDLENALk	Unambiguous	12	1	Q63886	N-Term(iTRAQ8plex); M11(Oxidation); K21(iTRAQ8plex)	CID	1.0000	0.0000	1	1	3327399	Unique	Used	3.004	0.347	1.320	1.897	1E-05	0	6.49	0	26	127	3	933.50226	2798.49222	-5.17

eEGcTHVIVTTAcGSLR	Unambiguous	1	1	Q9CQ65	N-Term(iTRAQ8plex); C4(Methylthio); C13(Methylthio)	CID	1.0000	0.0000	1	1	3744273	Unique	Used	3.386	0.731	0.722	0.893	1E-05	0	6.49	0	0	200	2	1086.51172	2172.01616	-2.86
tIGGGDDSFNTFFSETGAGk	Unambiguous	4	1	P68373	N-Term(iTRAQ8plex); K20(iTRAQ8plex)	CID	1.0000	0.0000	1	1	3152925	Unique	Used	0.519	1.381	1.017	1.059	1E-05	0	6.49	0	0	200	2	1308.65405	2616.30083	-1.16
eIPQAMTkPEVVNILqPQTTMNPDR	Unambiguous	2	1	D3YVV7	N-Term(iTRAQ8plex); K8(iTRAQ8plex); Q16(Deamidated)	CID	0.0493	0.0000	1	1	3538564	Unique	Used	1.326	1.820	0.658	1.186	1E-05	0	6.49	0	18	91	3	1153.95374	3459.84665	0.85
fVTDqSAcAGSPSLVAAAR	Unambiguous	2	1	Q8VCY8-2	N-Term(iTRAQ8plex); Q5(Deamidated); C8(Methylthio)	CID	1.0000	0.0000	1	1	3703657	Unique	Used	0.433	1.862	1.350	0.804	1E-05	0	6.49	0	0	200	2	1101.54614	2202.08501	0.59
qPTELDALVFGHLYTILTTQLTSDELSEk	Unambiguous	1	1	O88441	N-Term(iTRAQ8plex); K29(iTRAQ8plex)	CID	1.0000	0.0000	1	1	3623802	Unique	Used	1.242	3.085	1.516	1.278	1E-05	0	6.49	0	3	14	3	1291.02368	3871.05649	-7.05
iMDPNIVGNEHYDVAR	Unambiguous	1	1	P56480	N-Term(iTRAQ8plex)	CID	1.0000	0.0000	1	1	3032401	Unique	Used	1.491	0.863	1.192	1.317	1E-05	0	6.49	0	11	31	3	716.36554	2147.08207	-1.72
fGTLnGFQILHDR	Unambiguous	3	1	Q4FE56	N-Term(iTRAQ8plex); N5(Deamidated)	CID	1.0000	0.0000	1	1	3558417	Unique	Used	1.019	1.140	1.028	1.136	1E-05	0	6.49	0	0	186	3	608.32648	1822.96488	-5.76
dTFPSAFASGAAQQTQTQVk	Unambiguous	2	1	Q5SV85-2	N-Term(iTRAQ8plex); K20(iTRAQ8plex)	CID	1.0000	0.0000	1	1	3343701	Unique	Used	0.983	1.115	1.638	1.027	1E-05	0	6.49	0	0	43	2	1346.20520	2691.40312	-6.23
eNENVINEYSSILEDQR	Unambiguous	1	1	Q8K0E8	N-Term(iTRAQ8plex)	CID	1.0000	0.0000	1	1	2655914	Unique	Used	2.298	2.224	1.535	3.885	1E-05	0	6.49	0	0	200	2	1178.57996	2356.15264	-1.87
gLETIASDVVSLASk	Unambiguous	1	1	Q8BMF4	N-Term(iTRAQ8plex); K15(iTRAQ8plex)	CID	1.0000	0.0000	1	1	3479965	Unique	Used	0.734	1.318	1.329	1.786	1E-05	0	6.49	0	19	80	2	1049.60815	2098.20903	-6.00
sQEVTDFLAPLQTSSR	Unambiguous	2	1	Q9QYX7	N-Term(iTRAQ8plex)	CID	1.0000	0.0000	1	1	3499070	Unique	Used	0.475	0.905	1.887	0.931	1E-05	0	6.49	0	0	28	2	1042.04932	2083.09136	-2.88
eVEEPSSGPYQEMQELPTDLLR	Unambiguous	1	1	Q7M732	N-Term(iTRAQ8plex)	CID	1.0000	0.0000	1	1	2815973	Unique	Used	2.811	0.636	0.402	0.696	1E-05	0	6.49	0	7	171	2	1426.20178	2851.39629	-0.39

fVLcPEcENPETDLHVNPk	Unambiguous	1	1	P59325	N-Term(iTRAQ8plex); C4(Methylthio); C7(Methylthio); K19(iTRAQ8plex)	CID	1.0000	0.0000	1	1	3261581	Unique	Used	1.114	0.698	1.023	1.051	1E-05	0	6.49	0	0	48	3	962.13531	2884.39139	-1.69
nLHqSGFSLSGAQIDDNIPR	Unambiguous	1	1	O08553	N-Term(iTRAQ8plex); Q4(Deamidated)	CID	0.0092	0.0000	1	1	3279767	Unique	Used	1.401	0.772	1.274	1.250	1E-05	0	6.49	0	8	17	3	825.42224	2474.25217	-2.27
sESNLSSVcYIFESNNEGEk	Unambiguous	1	1	Q8K3H0	N-Term(iTRAQ8plex); C9(Methylthio); K20(iTRAQ8plex)	CID	1.0000	0.0000	1	1	3111894	Unique	Used	1.666	1.085	1.203	0.886	1E-05	0	6.49	0	12	69	3	964.12671	2890.36557	-1.38
acADATLSQITNNIDPVGR	Unambiguous	3	1	P62874	N-Term(iTRAQ8plex); C2(Methylthio)	CID	1.0000	0.0000	1	1	3622781	Unique	Used	0.403	0.892	1.107	1.220	1E-05	0	6.49	0	0	5	2	1155.07239	2309.13750	-6.80
vADVSISEVTTLLGNVHSPVVEGYVGENISGEVk	Unambiguous	3	1	P20357	N-Term(iTRAQ8plex); K34(iTRAQ8plex)	CID	1.0000	0.0000	1	1	3421828	Unique	Used			1.070		1E-05	0	6.49	0	0	200	3	1369.39880	4106.18186	-7.31
sAQLQHAVQSQGFIGYcqk	Unambiguous	2	1	Q3UPL0	N-Term(iTRAQ8plex); C17(Methylthio); Q18(Deamidated); K19(iTRAQ8plex)	CID	1.0000	0.0000	1	1	3801326	Unique	Used	0.801	0.952	0.919	0.944	1E-05	0	6.49	0	0	132	3	916.80853	2748.41104	1.91
nLSNAEIcSVFLLDqNELVAk	Unambiguous	3	1	Q5PR72	N-Term(iTRAQ8plex); C8(Methylthio); Q15(Deamidated); K21(iTRAQ8plex)	CID	0.0062	0.0000	1	1	3520364	Unique	Used		2.768	1.724	1.260	1E-05	0	6.49	0	0	59	2	1488.29016	2975.57305	1.73
mVLAAAGGVEHQQLLDLAQk	Unambiguous	1	1	Q9CZ13	N-Term(iTRAQ8plex); K20(iTRAQ8plex)	CID	1.0000	0.0000	1	1	3579246	Unique	Used	0.740	1.045	1.176	1.128	1E-05	0	6.49	0	0	7	3	900.84595	2700.52329	-3.51
tGPAGGFQAAPHTAAPYSQAAPAR	Unambiguous	1	1	P47708	N-Term(iTRAQ8plex)	CID	1.0000	0.0000	1	1	3656020	Unique	Used	1.476	0.602	1.338	0.964	1E-05	0	6.49	0	0	200	3	867.11084	2599.31797	-5.39
qQMTALQSQLQQVQLER	Unambiguous	3	1	E9QP99	N-Term(iTRAQ8plex)	CID	1.0000	0.0000	1	1	3762764	Unique	Used	2.149	0.816	1.232	0.891	1E-05	0	6.49	0	0	131	2	1167.12646	2333.24565	-3.98

fNSLNELVDYHR	Unambiguous	3	1	B1AT92	N-Term(iTRAQ8plex)	CID	1.0000	0.0000	1	1	3435810	Unique	Used	2.887	1.344	1.009	0.612	1E-05	0	6.49	0	4	5	3	604.31549	1810.93192	-3.89
gDFIHVMDNSDPNWWk	Unambiguous	3	1	B1AT92	N-Term(iTRAQ8plex); K16(iTRAQ8plex)	CID	1.0000	0.0000	1	1	3303415	Unique	Used	1.394	1.199	1.046	0.804	1E-05	0	6.49	0	19	40	3	857.09387	2569.26706	-3.27
kNVLGHMQQGGSPTPFDR	Unambiguous	1	1	P47857	N-Term(iTRAQ8plex); K1(iTRAQ8plex)	CID	1.0000	0.0000	1	1	3756831	Unique	Used	1.254	0.845	0.987	1.299	1E-05	0	6.49	1	0	86	3	859.79565	2577.37241	-3.59
vADVSISEVTTLLGNVHSPVVEGYVGENISGEVk	Unambiguous	3	1	P20357	N-Term(iTRAQ8plex); K34(iTRAQ8plex)	CID	1.0000	0.0000	1	1	3397073	Unique	Used	1.836	2.791	1.799	0.943	1E-05	0	6.49	0	0	200	3	1369.40039	4106.18662	-6.15
sDIAAAQQSGmSLSNSk	Unambiguous	1	1	B1AQZ0	N-Term(iTRAQ8plex); M11(Oxidation); K17(iTRAQ8plex)	CID	1.0000	0.0000	1	1	3250920	Unique	Used	0.950	0.838	0.963	1.010	1E-05	0	6.49	0	15	90	3	773.73877	2319.20176	-2.31
eVAAFAQFGSDLDAATQQLLSR	Unambiguous	2	1	Q03265	N-Term(iTRAQ8plex)	CID	1.0000	0.0000	1	1	3669674	Unique	Used		1.562			1E-05	0	6.49	0	4	200	2	1321.68201	2642.35674	-6.09
qASEQNWANYSAEQNR	Unambiguous	1	1	P23242	N-Term(iTRAQ8plex)	CID	1.0000	0.0000	1	1	2826046	Unique	Used	0.924	0.905	1.650	2.332	1E-05	0	6.48	0	0	41	2	1100.51709	2200.02690	-2.37
aSLDSAGASGNSPVLLPTPAVGGPR	Unambiguous	3	1	Q8C6B2-2	N-Term(iTRAQ8plex)	CID	1.0000	0.0000	1	1	3683363	Unique	Used	1.152	0.369	0.732	0.762	1E-05	0	6.48	0	0	111	2	1298.19775	2595.38823	-6.27
aLQILMEGGTHMVcTGR	Unambiguous	2	1	Q8BW41-2	N-Term(iTRAQ8plex); C14(Methylthio)	CID	1.0000	0.0000	1	1	3935712	Unique	Used	0.957	1.508	1.188	1.550	1E-05	0	6.48	0	7	49	3	723.02881	2167.07187	-3.68
qVLYQQSSGHNNR	Unambiguous	2	1	Q99M71	N-Term(iTRAQ8plex)	CID	1.0000	0.0000	1	1	3673390	Unique	Used	1.445	0.929	1.204	0.808	1E-05	0	6.48	0	4	23	3	612.31671	1834.93558	-5.77
vTTLEEQLAGAHQQVSALQQGAGIR	Unambiguous	1	1	B8QI36	N-Term(iTRAQ8plex)	CID	1.0000	0.0000	1	1	3804979	Unique	Used	1.099	1.165	0.863	0.710	1E-05	0	6.48	0	14	49	3	970.52673	2909.56565	-3.12
lSEENIDSSAGnGVLTk	Unambiguous	5	1	G3X9J4	N-Term(iTRAQ8plex); N12(Deamidated); K17(iTRAQ8plex)	CID	1.0000	0.0000	1	1	2652443	Unique	Used	0.352	0.948	0.661	0.672	1E-05	0	6.48	0	0	200	2	1172.12585	2343.24443	-2.38
tVFEALQAPAcHENMVk	Unambiguous	2	1	P17426-2	N-Term(iTRAQ8plex); C11(Methylthio); K17(iTRAQ8plex)	CID	1.0000	0.0000	1	1	3456336	Unique	Used	0.353	0.899	1.024	1.091	1E-05	0	6.48	0	19	46	3	848.10498	2542.30039	-2.88

sGVSSSYVEEmYFAWLENPQSVHk	Unambiguous	1	1	E9Q7L0	N-Term(iTRAQ8plex); M11(Oxidation); K24(iTRAQ8plex)	CID	1.0000	0.0000	1	1	3417696	Unique	Used	0.887	0.884	1.340	1.236	1E-05	0	6.48	0	0	145	3	1133.56348	3398.67588	-1.92
gFGFITFTNPEHASDAMR	Unambiguous	2	1	O89086	N-Term(iTRAQ8plex)	CID	1.0000	0.0000	1	1	3620193	Unique	Used	1.882	0.698	1.143	0.816	1E-05	0	6.48	0	9	10	3	768.04163	2302.11032	-5.45
gAQIASTGQLYAAWnGGLDR	Unambiguous	1	1	Q61361	N-Term(iTRAQ8plex); N15(Deamidated)	CID	1.0000	0.0000	1	1	3371054	Unique	Used	1.816	1.239	0.876	0.951	1E-05	0	6.48	0	0	162	2	1177.60229	2354.19731	-2.97
aLSEIAGITLPYDTLDQVR	Unambiguous	1	1	Q91VD9	N-Term(iTRAQ8plex)	CID	1.0000	0.0000	1	1	3582255	Unique	Used	1.046	1.092	1.142	1.147	1E-05	0	6.48	0	18	200	2	1190.15271	2379.29814	-3.87
eYQLNDSAAYYLNDLDR	Unambiguous	1	1	B2RSH2	N-Term(iTRAQ8plex)	CID	1.0000	0.0000	1	1	2921122	Unique	Used	0.625	1.683	1.448	1.303	1E-05	0	6.48	0	2	98	2	1184.07239	2367.13750	-1.33
eGIcAIGGTSEQSSVGTQHSYSEEEk	Unambiguous	1	1	Q61233	N-Term(iTRAQ8plex); C4(Methylthio); K26(iTRAQ8plex)	CID	1.0000	0.0000	1	1	2757789	Unique	Used	2.121	0.977	0.564	0.769	1E-05	0	6.48	0	0	200	3	1123.20239	3367.59262	1.40
iTAPTHVPLQYIGPNQPQkPR	Unambiguous	1	1	Q9JJL8	N-Term(iTRAQ8plex); K19(iTRAQ8plex)	CID	1.0000	0.0000	1	1	3911243	Unique	Used	0.423	0.762	0.798	0.832	1E-05	0	6.48	0	0	200	3	988.56482	2963.67990	-8.12
mLDYLQGSGETPQTDIR	Unambiguous	3	1	Q8BHN3	N-Term(iTRAQ8plex)	CID	1.0000	0.0000	1	1	3214665	Unique	Used	0.428	0.981	0.699	0.827	1E-05	0	6.48	0	0	7	2	1114.55994	2228.11260	-2.03
sLQEHLPDVVVYNAGTDVLEGDR	Unambiguous	1	1	Q91WA3	N-Term(iTRAQ8plex)	CID	1.0000	0.0000	1	1	3109854	Unique	Used	1.615	1.205	1.376	1.157	1E-05	0	6.48	0	11	67	3	944.15704	2830.45658	1.43
fLcTPLAAAcPSGAEQGDAAGER	Unambiguous	2	1	F7BX42	N-Term(iTRAQ8plex); C3(Methylthio); C10(Methylthio)	CID	1.0000	0.0000	1	1	3623278	Unique	Used	1.288	5.181	1.904	0.753	1E-05	0	6.48	0	12	9	2	1316.09729	2631.18730	-3.99
fVQLINTVLnGDQHFMSALDk	Unambiguous	3	1	Q9D4H8	N-Term(iTRAQ8plex); N10(Deamidated); K21(iTRAQ8plex)	CID	1.0000	0.0000	1	1	3687054	Unique	Used	0.731	0.776	0.947	1.285	1E-05	0	6.48	0	7	53	3	1000.53546	2999.59183	-6.77
dLYANNVLSGGTTMYPGIADR	Unambiguous	3	1	P62737	N-Term(iTRAQ8plex)	CID	1.0000	0.0000	1	1	3437705	Unique	Used	1.694	5.166	0.617	1.441	1E-05	0	6.48	0	0	187	2	1266.63342	2532.25957	-4.38
aTENDIANFFSPLNPIR	Unambiguous	2	1	D3Z3N4	N-Term(iTRAQ8plex)	CID	1.0000	0.0000	1	1	3745623	Unique	Used	0.928	0.923	1.062	0.944	1E-05	0	6.48	0	7	6	2	1112.08557	2223.16387	-3.30

iYFAGTETATQWSGYMEGAVEAGER	Unambiguous	1	1	Q64133	N-Term(iTRAQ8plex)	CID	1.0000	0.0000	1	1	3326780	Unique	Used	1.153	1.147	0.701	1.105	1E-05	0	6.48	0	0	27	2	1514.71497	3028.42266	-2.46
eNSGVYTSQNQSPELQEYR	Unambiguous	4	1	E9QQ10	N-Term(iTRAQ8plex)	CID	1.0000	0.0000	1	1	2650194	Unique	Used		0.912	0.894	0.721	1E-05	0	6.48	0	0	200	2	1267.10742	2533.20757	-1.30
dFTSEQIDEILQNHTEIVFAR	Unambiguous	2	1	Q6PIC6	N-Term(iTRAQ8plex)	CID	1.0000	0.0000	1	1	3377495	Unique	Used				1.735	1E-05	0	6.48	0	0	200	3	937.14569	2809.42252	-3.04
tIGGGDDSFTTFFcETGAGk	Unambiguous	1	1	P68368	N-Term(iTRAQ8plex); C14(Methylthio); K20(iTRAQ8plex)	CID	1.0000	0.0000	1	1	3328163	Unique	Used		2.073	2.952	3.163	1E-05	0	6.48	0	0	200	2	1333.13708	2665.26689	-2.48
qGQYSPMAIEEQVAVIYAGVR	Unambiguous	2	1	Q03265	N-Term(iTRAQ8plex)	CID	1.0000	0.0000	1	1	3603016	Unique	Used	0.646	0.934	1.050	1.329	1E-05	0	6.48	0	15	200	2	1307.18079	2613.35430	-4.06
lAQPPSLVVDSHDAGVcQQSLk	Unambiguous	4	1	Q924T7-2	N-Term(iTRAQ8plex); C17(Methylthio); K22(iTRAQ8plex)	CID	1.0000	0.0000	1	1	3414677	Unique	Used	1.226	1.045	1.039	1.108	1E-05	0	6.48	0	0	94	3	982.85419	2946.54801	-5.36
aDIGVAMGIAGSDVSk	Unambiguous	2	1	Q6PIC6	N-Term(iTRAQ8plex); K16(iTRAQ8plex)	CID	1.0000	0.0000	1	1	3257878	Unique	Used		0.431	0.991	1.001	1E-05	0	6.48	0	0	14	3	700.39154	2099.16007	-1.27
dLTAVSnNAGVDNFGLGLLLR	Unambiguous	2	1	Q9D0K2	N-Term(iTRAQ8plex); N7(Deamidated)	CID	0.0278	0.0000	1	1	3581404	Unique	Used	0.579	0.709	0.531	0.610	1E-05	0	6.48	0	7	21	2	1232.66602	2464.32475	-4.16
eLHEVcHLLEQER	Unambiguous	7	1	O35551	N-Term(iTRAQ8plex); C6(Methylthio)	CID	1.0000	0.0000	1	1	3213895	Unique	Used	1.242	0.614	1.490	0.780	1E-05	0	6.48	0	4	12	3	662.33386	1984.98703	-0.82
fTLScTETLmSELGNIAk	Unambiguous	2	1	Q9R111	N-Term(iTRAQ8plex); C5(Methylthio); M10(Oxidation); K18(iTRAQ8plex)	CID	1.0000	0.0000	1	1	3766150	Unique	Used	1.251	3.729	1.119	1.005	1E-05	0	6.48	0	25	43	2	1314.67554	2628.34380	-4.03
qLTQDLLNSYIENEGk	Unambiguous	2	1	P48722	N-Term(iTRAQ8plex); K16(iTRAQ8plex)	CID	1.0000	0.0000	1	1	3215980	Unique	Used	0.759	0.607	1.043	1.292	1E-05	0	6.48	0	0	48	2	1237.17249	2473.33769	-0.72
iQAAASPPAnATAASDTNAGDR	Unambiguous	2	1	E9QAQ5	N-Term(iTRAQ8plex); N10(Deamidated)	CID	0.0448	0.0000	1	1	2731840	Unique	Used	0.376	0.820	1.086	1.166	1E-05	0	6.48	0	0	128	2	1188.08655	2375.16582	-3.50

mPSESAAQSLAVALPSQTR	Unambiguous	3	1	Q922V4	N-Term(iTRAQ8plex)	CID	1.0000	0.0000	1	1	3682912	Unique	Used	1.567	1.026	0.928	0.807	1E-05	0	6.48	0	0	66	2	1124.58997	2248.17266	-8.14
lALTTAEFLAYQcEk	Unambiguous	2	1	P62814	N-Term(iTRAQ8plex); C13(Methylthio); K15(iTRAQ8plex)	CID	1.0000	0.0000	1	1	3602240	Unique	Used	0.671	0.798	1.136	1.282	1E-05	0	6.48	0	22	24	2	1178.12488	2355.24248	-5.27
sITIAnQTNcPLYVTk	Unambiguous	1	1	O08553	N-Term(iTRAQ8plex); N6(Deamidated); C10(Methylthio); K16(iTRAQ8plex)	CID	0.0448	0.0000	1	1	3600265	Unique	Used	1.857	1.728	0.917	1.234	1E-05	0	6.48	0	0	125	2	1211.14526	2421.28325	-6.01
tkSEnGLEFTSSGSANTETTk	Unambiguous	3	1	Q60932-2	N-Term(iTRAQ8plex); K2(iTRAQ8plex); N5(Deamidated); K21(iTRAQ8plex)	CID	1.0000	0.0000	1	1	2754371	Unique	Used	0.666	1.226	1.520	1.265	1E-05	0	6.48	1	0	181	3	1034.87390	3102.60715	-4.34
fIEAGQYNSHLYGTSVQSVR	Unambiguous	4	1	Q62108-3	N-Term(iTRAQ8plex)	CID	1.0000	0.0000	1	1	3701184	Unique	Used	0.830	1.659	1.225	1.145	1E-05	0	6.48	0	0	140	3	854.10388	2560.29709	-4.96
gIVDQSQQAYQEAFEISk	Unambiguous	1	1	P63101	N-Term(iTRAQ8plex); K18(iTRAQ8plex)	CID	1.0000	0.0000	1	1	3109737	Unique	Used	1.037	1.265	1.102	0.840	1E-05	0	6.48	0	2	19	2	1325.20007	2649.39287	-1.96
qLPVQQDGFHTLPYQR	Unambiguous	2	1	B1AS06	N-Term(iTRAQ8plex)	CID	1.0000	0.0000	1	1	3615954	Unique	Used	0.568	0.967	1.280	0.923	1E-05	0	6.48	0	9	24	3	744.39832	2231.18039	-3.18
gAQIASTGQLYAAWnGGLDR	Unambiguous	1	1	Q61361	N-Term(iTRAQ8plex); N15(Deamidated)	CID	1.0000	0.0000	1	1	3392998	Unique	Used	1.819	1.115	1.194	1.259	1E-05	0	6.47	0	0	87	2	1177.59998	2354.19267	-4.94
iISAASEGGANVFTVSYFk	Unambiguous	1	1	Q922B2	N-Term(iTRAQ8plex); K19(iTRAQ8plex)	CID	1.0000	0.0000	1	1	3765721	Unique	Used	0.395	0.787	0.727	0.808	1E-05	0	6.47	0	4	8	2	1285.20569	2569.40410	-3.18
mATQASTLYSNNITk	Unambiguous	1	1	Q61941	N-Term(iTRAQ8plex); K15(iTRAQ8plex)	CID	1.0000	0.0000	1	1	3574524	Unique	Used	0.310	1.037	0.063	0.051	1E-05	0	6.47	0	0	169	2	1126.10742	2251.20757	-6.09
fATASADGQIFIYDGk	Unambiguous	1	1	O88342	N-Term(iTRAQ8plex); K16(iTRAQ8plex)	CID	1.0000	0.0000	1	1	3281761	Unique	Used	0.776	1.272	0.809	0.968	1E-05	0	6.47	0	0	12	2	1156.62061	2312.23393	-1.90

tLLTGLLDLQAQYPQFISR	Unambiguous	2	1	P61922	N-Term(iTRAQ8plex)	CID	1.0000	0.0000	1	1	3936248	Unique	Used	2.227	0.685	0.786	1.523	1E-05	0	6.47	0	15	78	2	1241.19971	2481.39214	-3.94
lQQTQAQVDEVVDIMR	Unambiguous	2	1	P63044	N-Term(iTRAQ8plex)	CID	1.0000	0.0000	1	1	3284587	Unique	Used	0.719	1.489	1.172	1.156	1E-05	0	6.47	0	17	51	2	1089.07690	2177.14653	-3.36
gLPHMFQQGGVFYNIMk	Unambiguous	1	1	Q8CDG3	N-Term(iTRAQ8plex); K17(iTRAQ8plex)	CID	1.0000	0.0000	1	1	3897415	Unique	Used	0.918	0.695	0.943	0.991	1E-05	0	6.47	0	10	127	3	859.12872	2575.37162	-2.26
eAGFPPGVVNIVPGFGPTAGAAIASHEGVDk	Unambiguous	1	1	P47738	N-Term(iTRAQ8plex); K31(iTRAQ8plex)	CID	1.0000	0.0000	1	1	3458677	Unique	Used	0.742	1.327	0.845	1.073	1E-05	0	6.47	0	0	200	3	1190.64026	3569.90622	-4.25
gDAcTPTSTTVELVGSSGQnGDEk	Unambiguous	1	1	Q9ERZ4	N-Term(iTRAQ8plex); C4(Methylthio); N20(Deamidated); K24(iTRAQ8plex)	CID	0.0077	0.0000	1	1	2653238	Unique	Used	1.642		1.422	1.305	1E-05	0	6.47	0	14	200	3	1003.47913	3008.42282	-1.91
dANSTEFEPNTPVPLVIDMPEHnPGDLGGTMR	Unambiguous	3	1	P70303	N-Term(iTRAQ8plex); N23(Deamidated)	CID	0.0077	0.0000	1	1	3131839	Unique	Used	1.130	1.735	1.339	1.256	1E-05	0	6.47	0	16	200	3	1252.60217	3755.79197	2.23
sTQDLSDVSTDEVGIPLR	Unambiguous	4	1	D3Z5J3	N-Term(iTRAQ8plex)	CID	1.0000	0.0000	1	1	2938524	Unique	Used	1.931	0.268	1.433	0.838	1E-05	0	6.47	0	0	18	2	1118.58228	2236.15727	-1.71
gAVVGIDLGTTNScVAVmEGk	Unambiguous	1	1	P38647	N-Term(iTRAQ8plex); C14(Methylthio); M18(Oxidation); K21(iTRAQ8plex)	CID	1.0000	0.0000	1	1	3518170	Unique	Used	0.922	1.009	1.094	0.917	1E-05	0	6.47	0	0	200	2	1346.19836	2691.38945	-3.05
aVEASNAYSSILQAVQAAEDAAGQALR	Unambiguous	1	1	Q61001	N-Term(iTRAQ8plex)	CID	1.0000	0.0000	1	1	3746295	Unique	Used		3.441	1.177	2.459	1E-05	0	6.47	0	0	200	2	1504.77576	3008.54424	-4.96
eLVNYGANVNAQSQk	Unambiguous	15	1	Q02357-5	N-Term(iTRAQ8plex); K15(iTRAQ8plex)	CID	1.0000	0.0000	1	1	3229796	Unique	Used	0.776	0.931	0.461	1.191	1E-05	0	6.47	0	0	140	2	1122.10852	2243.20976	-6.37
yDATMVEINPMVEDSDGk	Unambiguous	1	1	Q9Z2I9	N-Term(iTRAQ8plex); K18(iTRAQ8plex)	CID	1.0000	0.0000	1	1	2654815	Unique	Used		0.743			1E-05	0	6.47	0	11	200	2	1311.64551	2622.28374	-1.93
lPDFQDSIFEYFnTAPLAHDLTFR	Unambiguous	1	1	Q7TT50	N-Term(iTRAQ8plex); N13(Deamidated)	CID	1.0000	0.0000	1	1	3746092	Unique	Used	0.767	1.317	1.180	1.117	1E-05	0	6.47	0	0	144	3	1054.86401	3162.57749	1.53

aLQSGQcAGAALDVFTEEPPR	Unambiguous	1	1	Q61753	N-Term(iTRAQ8plex); C7(Methylthio)	CID	1.0000	0.0000	1	1	3351711	Unique	Used	0.951	0.771	1.539	1.596	1E-05	0	6.47	0	19	24	2	1255.61572	2510.22417	-3.20
iTSLAQLNAANHDAAIFPGGFGAAk	Unambiguous	1	1	Q9D172	N-Term(iTRAQ8plex); K25(iTRAQ8plex)	CID	1.0000	0.0000	1	1	3804918	Unique	Used	1.943	1.329	1.316	1.749	1E-05	0	6.47	0	14	39	3	1021.89484	3063.66996	-4.48
tALINADELPTDVASGEALLAR	Unambiguous	1	1	P08032	N-Term(iTRAQ8plex)	CID	1.0000	0.0000	1	1	3326715	Unique	Used	5.927	2.566	0.546	1.367	1E-05	0	6.47	0	14	11	2	1272.69043	2544.37358	-3.45
aTDLLLDDSLVSLFGNR	Unambiguous	4	1	P99029-2	N-Term(iTRAQ8plex)	CID	1.0000	0.0000	1	1	3605279	Unique	Used	0.948	1.032	1.108	1.509	1E-05	0	6.47	0	0	200	2	1077.08630	2153.16533	-4.78
sGGASHSELIHNLR	Unambiguous	6	1	P23506	N-Term(iTRAQ8plex)	CID	1.0000	0.0000	1	1	3360706	Unique	Used	1.024	1.272	1.493	1.487	1E-05	0	6.47	0	3	3	3	594.65405	1781.94760	-4.73
iVSGAAETDQEYYFGQVVR	Unambiguous	4	1	P06909	N-Term(iTRAQ8plex)	CID	1.0000	0.0000	1	1	3193024	Unique	Used	5.328	1.511	1.003	2.894	1E-05	0	6.47	0	0	148	2	1218.62085	2436.23442	-0.20
sAAGSSSGFHSWAR	Unambiguous	1	1	P19246	N-Term(iTRAQ8plex)	CID	1.0000	0.0000	1	1	3832624	Unique	Used	2.293	0.644	1.270	1.421	1E-05	0	6.47	0	11	200	3	571.28442	1711.83872	-3.92
yNNPAMLHNIDQEMLk	Unambiguous	1	1	O88543	N-Term(iTRAQ8plex); K16(iTRAQ8plex)	CID	1.0000	0.0000	1	1	3211715	Unique	Used	0.979	1.264	1.116	1.332	1E-05	0	6.47	0	12	103	3	847.11243	2539.32273	-1.20
tLEVVSPSQSVTGSAGHTPYYqSPTDEk	Unambiguous	1	1	P14873	N-Term(iTRAQ8plex); Q22(Deamidated); K28(iTRAQ8plex)	CID	1.0000	0.0000	1	1	3210182	Unique	Used	1.636	1.237	1.314	0.671	1E-05	0	6.47	0	0	200	3	1192.27454	3574.80906	2.26
aQESQQMIDFLNSVIVDLQR	Unambiguous	2	1	D3Z3M7	N-Term(iTRAQ8plex)	CID	1.0000	0.0000	1	1	3786065	Unique	Used	1.656	1.492	1.430	0.701	1E-05	0	6.47	0	7	165	2	1319.68933	2638.37139	-3.75
sVAALLAGTTEAILTPFER	Unambiguous	4	1	Q5HZI9	N-Term(iTRAQ8plex)	CID	1.0000	0.0000	1	1	3880431	Unique	Used	0.792	1.113	1.362	1.592	1E-05	0	6.47	0	22	4	2	1132.63818	2264.26909	-5.01
eVAAFAQFGSDLDAATQQLLSR	Unambiguous	2	1	Q03265	N-Term(iTRAQ8plex)	CID	1.0000	0.0000	1	1	3666345	Unique	Used	1.132	1.182	1.792	2.362	1E-05	0	6.47	0	8	28	2	1321.68115	2642.35503	-6.74
sQEPVTLDFLDAELENEIk	Unambiguous	4	1	O08532-5	N-Term(iTRAQ8plex); K19(iTRAQ8plex)	CID	1.0000	0.0000	1	1	2816209	Unique	Used	0.415	2.204	0.703	0.942	1E-05	0	6.47	0	9	111	2	1399.74951	2798.49175	-0.11
nTLIQFEDFGNHNAFR	Unambiguous	1	1	Q99KE1	N-Term(iTRAQ8plex)	CID	1.0000	0.0000	1	1	3577591	Unique	Used	1.570	1.219	0.999	1.330	1E-05	0	6.47	0	0	116	3	743.04218	2227.11197	-3.52
aFVDnYGVAMETAEk	Unambiguous	1	1	Q6PAJ1	N-Term(iTRAQ8plex); N5(Deamidated); K15(iTRAQ8plex)	CID	1.0000	0.0000	1	1	2937501	Unique	Used	0.607	0.990	1.364	1.230	1E-05	0	6.47	0	0	194	2	1127.58533	2254.16338	4.95

mDHFEGPPPNYVNTYPTDLSVGAGPAILR	Unambiguous	4	1	Q8BPN8-2	N-Term(iTRAQ8plex)	CID	1.0000	0.0000	1	1	3498836	Unique	Used	1.887	1.287	0.958	0.887	1E-05	0	6.47	0	11	120	3	1144.91003	3432.71555	-1.32
dQDFPAHENQPLLLTSGcQENPQEWVDR	Unambiguous	5	1	Q921T2-2	N-Term(iTRAQ8plex); C18(Methylthio)	CID	1.0000	0.0000	1	1	2996717	Unique	Used	1.467	1.105	0.873	1.322	1E-05	0	6.47	0	0	181	3	1206.22766	3616.66843	-1.53
nSLISYLEQIHR	Unambiguous	1	1	Q00493	N-Term(iTRAQ8plex); N1(Deamidated)	CID	1.0000	0.0000	1	1	3539601	Unique	Used	0.521	1.294	0.740	0.830	1E-05	0	6.47	0	18	61	3	593.32648	1777.96488	-5.70
aTENDIYNFFSPLNPMR	Unambiguous	1	1	P70333	N-Term(iTRAQ8plex)	CID	1.0000	0.0000	1	1	3766050	Unique	Used	0.724	0.792	1.052	1.069	1E-05	0	6.47	0	11	9	2	1167.07629	2333.14531	-3.65
fADVLGLEEETLGSVPSPAcALLLLFPLTAqHENFR	Unambiguous	1	1	Q9R0P9	N-Term(iTRAQ8plex); C20(Methylthio); Q31(Deamidated)	CID	0.0124	0.0000	1	1	3862753	Unique	Used				0.974	1E-05	0	6.47	0	0	200	3	1417.07349	4249.20591	4.49
gVIALcIEDGSIHR	Unambiguous	1	1	Q8K2B3	N-Term(iTRAQ8plex); C6(Methylthio)	CID	1.0000	0.0000	1	1	3621452	Unique	Used	0.606	0.830	0.845	0.855	1E-05	0	6.47	0	0	52	3	611.65857	1832.96115	-2.91
sALQAIGVEAHQAIMIGDDIVGDVGGAqQcGMR	Unambiguous	2	1	Q9D7I5-2	N-Term(iTRAQ8plex); Q28(Deamidated); C30(Methylthio)	CID	0.0015	0.0000	1	1	3541577	Unique	Used	2.028		0.774	1.237	1E-05	0	6.47	0	26	45	3	1221.26526	3661.78122	1.70
gDAVGFQEASLAQYSATTAR	Unambiguous	2	1	O88737	N-Term(iTRAQ8plex)	CID	1.0000	0.0000	1	1	3257013	Unique	Used	1.022	1.294	1.786	0.786	1E-05	0	6.47	0	0	80	2	1174.09595	2347.18462	0.60
eqGSSGLGSGSSGGGGSSSGLGSGYIGR	Unambiguous	2	1	Q3UHJ0-2	N-Term(iTRAQ8plex); Q2(Deamidated)	CID	1.0000	0.0000	1	1	3208027	Unique	Used		1.166	1.443	1.326	1E-05	0	6.46	0	0	198	2	1354.14807	2707.28887	6.61
tMSIVSYNHLGnNDGQNLSAPLQFR	Unambiguous	1	1	Q9JHU9	N-Term(iTRAQ8plex); N12(Deamidated)	CID	0.0046	0.0000	1	1	3857953	Unique	Used	1.456	2.000	0.717	0.817	1E-05	0	6.46	0	0	112	3	1027.84937	3081.53354	-1.00
tGLDSPTGFDSSDITANSFTVHWVAPR	Unambiguous	1	1	P11276	N-Term(iTRAQ8plex)	CID	1.0000	0.0000	1	1	3540108	Unique	Used	3.580	2.581	0.619	1.013	1E-05	0	6.46	0	14	74	3	1061.52576	3182.56272	-2.20
sEVELVQLVIDGVNYLIDcER	Unambiguous	1	1	P30275	N-Term(iTRAQ8plex); C19(Methylthio)	CID	1.0000	0.0000	1	1	3646506	Unique	Used	1.642	1.242	0.912	1.179	1E-05	0	6.46	0	0	200	2	1378.70325	2756.39922	-5.83
dPDNELYFAHGLFSAk	Unambiguous	2	1	G3X9A7	N-Term(iTRAQ8plex); K16(iTRAQ8plex)	CID	1.0000	0.0000	1	1	3236024	Unique	Used	0.874	1.813	1.120	1.102	1E-05	0	6.46	0	9	125	3	811.42468	2432.25949	-4.60

gAQGFGFTIADSPTGQR	Unambiguous	4	1	Q9WVQ1	N-Term(iTRAQ8plex)	CID	1.0000	0.0000	1	1	3596906	Unique	Used	1.041	1.139	1.214	0.878	1E-05	0	6.46	0	0	21	2	1007.51282	2014.01836	-5.59
tYFPHFDVSHGSAqVk	Unambiguous	2	1	Q91VB8	N-Term(iTRAQ8plex); Q14(Deamidated); K16(iTRAQ8plex)	CID	1.0000	0.0000	1	1	3658424	Unique	Used	25.641	7.213	0.979	5.481	1E-05	0	6.46	0	28	200	2	1215.13660	2429.26592	-2.09
lQQGYNAIGFSQGGQFLR	Unambiguous	3	1	O88531	N-Term(iTRAQ8plex)	CID	1.0000	0.0000	1	1	3915725	Unique	Used	0.807	2.056	1.300	1.655	1E-05	0	6.46	0	13	82	2	1144.60193	2288.19658	-5.41
lQQGYNAIGFSQGGQFLR	Unambiguous	3	1	O88531	N-Term(iTRAQ8plex)	CID	1.0000	0.0000	1	1	3934358	Unique	Used	0.699	1.733	0.741	1.028	1E-05	0	6.46	0	30	200	2	1144.60278	2288.19829	-4.67
vVLHDGDPNEPVSDYINANIImPEFETk	Unambiguous	2	1	P35235	N-Term(iTRAQ8plex); M22(Oxidation); K28(iTRAQ8plex)	CID	1.0000	0.0000	1	1	3216288	Unique	Used	0.868	0.703	1.038	2.177	1E-05	0	6.46	0	0	133	3	1260.97839	3780.92063	-1.21
sFLGSPAAMSPADQPPSTQPLAEGSHR	Unambiguous	3	1	A2AHC3	N-Term(iTRAQ8plex)	CID	1.0000	0.0000	1	1	3367710	Unique	Used	1.164	0.928	0.724	0.866	1E-05	0	6.46	0	0	36	3	1014.17102	3040.49851	-3.81
kTGQAAGFSYTDANk	Unambiguous	2	1	G3UWG1	N-Term(iTRAQ8plex); K1(iTRAQ8plex); K15(iTRAQ8plex)	CID	1.0000	0.0000	1	1	3294213	Unique	Used	0.960	0.658	1.119	0.780	1E-05	0	6.46	1	23	200	3	824.45654	2471.35508	-4.32
gSITDAmQPQQLHASEVLSSGFR	Unambiguous	1	1	Q8R2Z5	N-Term(iTRAQ8plex); M7(Oxidation)	CID	1.0000	0.0000	1	1	3680923	Unique	Used	0.960	1.001	1.105	1.138	1E-05	0	6.46	0	0	92	3	927.13483	2779.38993	-3.17
sPELQEEFGYNAETQk	Unambiguous	2	1	Q8K221	N-Term(iTRAQ8plex); K16(iTRAQ8plex)	CID	1.0000	0.0000	1	1	2635185	Unique	Used	0.402	1.429	0.982	0.866	1E-05	0	6.46	0	0	100	2	1239.62952	2478.25176	-3.69
lYNNITFEELGALLEIPAAk	Unambiguous	2	1	O88544	N-Term(iTRAQ8plex); K20(iTRAQ8plex)	CID	1.0000	0.0000	1	1	3623780	Unique	Used	0.582	1.154	1.406	1.480	1E-05	0	6.46	0	0	47	2	1414.29846	2827.58965	-6.01
qSSVSSTASVNLGDPTR	Unambiguous	4	1	B7ZNX6	N-Term(iTRAQ8plex)	CID	1.0000	0.0000	1	1	3295815	Unique	Used	1.570	0.765	1.442	1.129	1E-05	0	6.46	0	0	200	2	1005.51917	2010.03105	-4.73
lQQTQAQVDEVVDIMR	Unambiguous	2	1	P63044	N-Term(iTRAQ8plex)	CID	1.0000	0.0000	1	1	3285286	Unique	Used	0.635	2.145	1.157	1.314	1E-05	0	6.46	0	6	200	2	1089.07898	2177.15068	-1.45
aVGLISHVLEQNQEGDAAYR	Unambiguous	3	1	Q9JLZ3	N-Term(iTRAQ8plex)	CID	1.0000	0.0000	1	1	3371776	Unique	Used	1.048	0.912	1.479	1.920	1E-05	0	6.46	0	0	192	2	1237.64575	2474.28423	-4.02

aTVqVVILADPDGHEIcFVGDEAFR	Unambiguous	4	1	Q9CPV4	N-Term(iTRAQ8plex); Q4(Deamidated); C17(Methylthio)	CID	1.0000	0.0000	1	1	3354790	Unique	Used	1.162	1.041	0.747	1.184	1E-05	0	6.46	0	0	200	3	1018.17462	3052.50931	1.00
yTPSGQSGAAASESLFISNHAY	Unambiguous	2	1	A6ZI44	N-Term(iTRAQ8plex)	CID	1.0000	0.0000	1	1	3516147	Unique	Used	1.303	1.042	1.382	1.520	1E-05	0	6.46	0	0	200	2	1281.62268	2562.23808	-1.31
fcQEFQHYPAMGGVAPQALAVAASGPGSSFR	Unambiguous	2	1	D3YW52	N-Term(iTRAQ8plex); C2(Methylthio)	CID	1.0000	0.0000	1	1	3917284	Unique	Used	1.892	1.067	1.040	1.327	1E-05	0	6.46	0	0	29	3	1177.90051	3531.68698	-1.27
fSGWYDADLSPAGHEEAk	Unambiguous	1	1	Q9DBJ1	N-Term(iTRAQ8plex); K18(iTRAQ8plex)	CID	1.0000	0.0000	1	1	2900828	Unique	Used	2.398	1.002	0.842	0.942	1E-05	0	6.46	0	0	200	2	1294.64172	2588.27617	-4.49
mEMINVSGYVAQEk	Unambiguous	1	1	Q8CGK3	N-Term(iTRAQ8plex); K14(iTRAQ8plex)	CID	1.0000	0.0000	1	1	3347624	Unique	Used	0.552	0.864	1.028	1.013	1E-05	0	6.46	0	19	21	2	1104.08496	2207.16265	-1.56
gAANLQLIQALSDSEEEHDSAWDGR	Unambiguous	5	1	Q91VU6	N-Term(iTRAQ8plex)	CID	1.0000	0.0000	1	1	2852218	Unique	Used	1.793		2.405	2.071	1E-05	0	6.46	0	0	200	3	1006.15155	3016.44010	-4.97
aFTDEETEAQAGQASWPR	Unambiguous	1	1	Q08274	N-Term(iTRAQ8plex)	CID	1.0000	0.0000	1	1	2652454	Unique	Used	0.411	1.925	1.520	1.568	1E-05	0	6.46	0	0	200	2	1149.54773	2298.08818	-2.57
aGYTDQVVIGMDVAASEFYR	Unambiguous	2	1	P17182	N-Term(iTRAQ8plex)	CID	1.0000	0.0000	1	1	3329806	Unique	Used	0.741	1.977	0.710	0.874	1E-05	0	6.46	0	0	200	2	1248.62024	2496.23320	-2.04
gSLVDNIQQHFLLSDR	Unambiguous	2	1	Q9JI19	N-Term(iTRAQ8plex)	CID	1.0000	0.0000	1	1	3724943	Unique	Used	0.813	1.166	1.050	1.274	1E-05	0	6.46	0	15	45	3	716.05139	2146.13962	-7.57
iVEIVDAITTTAQSHQR	Unambiguous	1	1	P47857	N-Term(iTRAQ8plex)	CID	1.0000	0.0000	1	1	3844625	Unique	Used	2.548		0.939	1.354	1E-05	0	6.46	0	0	200	3	729.40515	2186.20090	-3.39
aGYTDQVVIGMDVAASEFYR	Unambiguous	2	1	P17182	N-Term(iTRAQ8plex)	CID	1.0000	0.0000	1	1	3328119	Unique	Used	1.194	1.310	1.658	1.686	1E-05	0	6.46	0	10	200	2	1248.62085	2496.23442	-1.55
lYNPENIYLSEHGGGAGNNWASGFSQGEk	Unambiguous	1	1	P83887	N-Term(iTRAQ8plex); K29(iTRAQ8plex)	CID	1.0000	0.0000	1	1	3347274	Unique	Used	1.167	1.099	0.817	0.979	1E-05	0	6.46	0	0	22	3	1235.60876	3704.81174	-1.97
qLETQNNNLQAQILALQR	Unambiguous	4	1	Q5SNZ0-2	N-Term(iTRAQ8plex)	CID	1.0000	0.0000	1	1	3763160	Unique	Used	1.618	0.966	1.439	0.793	1E-05	0	6.46	0	0	84	2	1200.16345	2399.31963	-4.69
sLYAEnLAGNVGEFTAYR	Unambiguous	3	1	D3YXV8	N-Term(iTRAQ8plex); N6(Deamidated)	CID	0.0294	0.0000	1	1	3438108	Unique	Used	0.850	0.797	0.928	1.139	1E-05	0	6.46	0	28	200	2	1140.58313	2280.15898	6.12
gQNLLLTNLQTIQGILER	Unambiguous	2	1	Q7M739	N-Term(iTRAQ8plex)	CID	1.0000	0.0000	1	1	3936373	Unique	Used			0.783	0.853	1E-05	0	6.46	0	0	125	2	1164.67383	2328.34038	-6.41
qTTAAAAATFSEQVGGGSGGAGR	Unambiguous	3	1	O88935	N-Term(iTRAQ8plex)	CID	1.0000	0.0000	1	1	3502526	Unique	Used			1.325	1.385	1E-05	0	6.46	0	0	200	2	1178.58728	2356.16728	-5.21

qIDHFPEDADYEADTAEYFLR	Unambiguous	1	1	O88602	N-Term(iTRAQ8plex)	CID	1.0000	0.0000	1	1	2672591	Unique	Used			0.879	0.568	1E-05	0	6.46	0	13	200	3	950.44397	2849.31736	-1.22
eScLEAYTGIVQGLk	Unambiguous	1	1	P70168	N-Term(iTRAQ8plex); C3(Methylthio); K15(iTRAQ8plex)	CID	1.0000	0.0000	1	1	3601881	Unique	Used	0.182	1.238	0.655	0.909	1E-05	0	6.46	0	5	7	2	1133.10291	2265.19853	-4.15
eGNDLYHEMIESGVINLk	Unambiguous	1	1	P56480	N-Term(iTRAQ8plex); K18(iTRAQ8plex)	CID	1.0000	0.0000	1	1	3110749	Unique	Used	1.191	1.334	0.929	0.922	1E-05	0	6.46	0	0	200	2	1335.21179	2669.41631	3.66
dHFmDEFFEQVEEIR	Unambiguous	1	1	P61264	N-Term(iTRAQ8plex); M4(Oxidation)	CID	1.0000	0.0000	1	1	2815101	Unique	Used	1.071	0.882	1.140	1.306	1E-05	0	6.46	0	9	200	3	764.35590	2291.05313	-2.68
nSSLAEFVQSLSQTMGFPQDQIR	Unambiguous	5	1	E9PXY8	N-Term(iTRAQ8plex)	CID	1.0000	0.0000	1	1	3899171	Unique	Used	1.207	0.776	1.628	1.068	1E-05	0	6.46	0	19	51	2	1444.22412	2887.44097	-5.29
rLTGFHETSNINDFSAGVANR	Unambiguous	1	1	P15105	N-Term(iTRAQ8plex)	CID	1.0000	0.0000	1	1	3698865	Unique	Used	1.387	1.050	1.961	1.742	1E-05	0	6.46	1	0	63	3	870.77594	2610.31327	-7.44
lQQPEAASGVLEYAMk	Unambiguous	1	1	Q9JLN9	N-Term(iTRAQ8plex); K16(iTRAQ8plex)	CID	1.0000	0.0000	1	1	3323795	Unique	Used	0.582	0.947	0.781	0.964	1E-05	0	6.46	0	0	186	2	1172.14124	2343.27519	-3.72
gGSAGPGEPEYGHDPASGGIFSSDYk	Unambiguous	2	1	Q9JME5	N-Term(iTRAQ8plex); K26(iTRAQ8plex)	CID	1.0000	0.0000	1	1	2810235	Unique	Used	0.424	0.841	1.056	1.116	1E-05	0	6.46	0	3	35	3	1049.84033	3147.50644	-1.65
dHFmDEFFEQVEEIR	Unambiguous	1	1	P61264	N-Term(iTRAQ8plex); M4(Oxidation)	CID	1.0000	0.0000	1	1	2833523	Unique	Used	0.718	0.584	1.337	1.285	1E-05	0	6.46	0	0	26	3	764.35760	2291.05826	-0.44
aDYAQLLEDMQnAFR	Unambiguous	1	1	P50516	N-Term(iTRAQ8plex); N12(Deamidated)	CID	0.0155	0.0000	1	1	3075676	Unique	Used	0.931	1.228	1.334	1.335	1E-05	0	6.45	0	2	22	2	1045.51221	2090.01714	0.22
tFYGLHQDFPSVVVVGLGk	Unambiguous	2	1	Q9CPY7	N-Term(iTRAQ8plex); K19(iTRAQ8plex)	CID	1.0000	0.0000	1	1	3805338	Unique	Used	1.744	0.688	1.213	1.464	1E-05	0	6.45	0	0	46	2	1336.25061	2671.49394	-4.83
eVSSGSEETFSYSDFLR	Unambiguous	1	1	O70200	N-Term(iTRAQ8plex)	CID	1.0000	0.0000	1	1	2939860	Unique	Used	2.261	1.148	1.136	0.695	1E-05	0	6.45	0	22	59	2	1122.53113	2244.05498	-2.69
gFEkPTENSSAVLLQWHEk	Unambiguous	2	1	A3KGQ6	N-Term(iTRAQ8plex); K4(iTRAQ8plex); K19(iTRAQ8plex)	CID	1.0000	0.0000	1	1	3343240	Unique	Used	0.400	2.908	1.031	0.783	1E-05	0	6.45	0	0	13	3	1038.24084	3112.70798	-3.69

aSANLGQGSGSQLVPTVcnGR	Unambiguous	1	1	Q14BI2	N-Term(iTRAQ8plex); C18(Methylthio); N19(Deamidated)	CID	1.0000	0.0000	1	1	3640841	Unique	Used	0.220	0.680	0.910	1.056	1E-05	0	6.45	0	0	115	2	1184.07837	2367.14946	-8.64
nLHQSGFSLSGAQIDDNIPR	Unambiguous	1	1	O08553	N-Term(iTRAQ8plex)	CID	1.0000	0.0000	1	1	3388892	Unique	Used	2.660	1.244	1.322	1.055	1E-05	0	6.45	0	24	44	3	825.09222	2473.26212	-4.71
qLLHnFPPDQLTSSGAPFWSGPk	Unambiguous	1	1	Q02053	N-Term(iTRAQ8plex); N5(Deamidated); K23(iTRAQ8plex)	CID	0.0078	0.0000	1	1	3804782	Unique	Used	0.880	1.323	0.774	0.843	1E-05	0	6.45	0	0	65	3	1045.22559	3133.66220	1.73
aLSEIAGITLPYDTLDQVR	Unambiguous	1	1	Q91VD9	N-Term(iTRAQ8plex)	CID	1.0000	0.0000	1	1	3582552	Unique	Used	1.486	1.338	0.793	1.060	1E-05	0	6.45	0	0	200	2	1190.15112	2379.29497	-5.21
vQQMIGQIDGLITQLETTQQDGk	Unambiguous	1	1	Q9Z2I0	N-Term(iTRAQ8plex); K23(iTRAQ8plex)	CID	1.0000	0.0000	1	1	3561574	Unique	Used	3.580	2.016	1.915	1.610	1E-05	0	6.45	0	28	200	3	1051.56982	3152.69492	-4.22
rDLELTPTSGTLcGSLSGk	Unambiguous	2	1	Q9WV34	N-Term(iTRAQ8plex); C13(Methylthio); K19(iTRAQ8plex)	CID	1.0000	0.0000	1	1	3619409	Unique	Used	1.944	1.524	2.192	2.479	1E-05	0	6.45	1	15	44	3	863.79468	2589.36948	-5.49
gITNLcVIGGDGSLTGADTFR	Unambiguous	1	1	P47857	N-Term(iTRAQ8plex); C6(Methylthio)	CID	1.0000	0.0000	1	1	3729898	Unique	Used	2.982		1.037	2.351	1E-05	0	6.45	0	10	200	2	1209.10168	2417.19609	-6.05
eVnENFAIDLIAQQPVNEVEHR	Unambiguous	2	1	P52503	N-Term(iTRAQ8plex); N3(Deamidated)	CID	1.0000	0.0000	1	1	2904240	Unique	Used	2.414	1.193	1.207		1E-05	0	6.45	0	27	200	3	957.15826	2869.46024	-1.12
gITNLcVIGGDGSLTGADTFR	Unambiguous	1	1	P47857	N-Term(iTRAQ8plex); C6(Methylthio)	CID	1.0000	0.0000	1	1	3729575	Unique	Used	2.117	0.808	1.157	0.786	1E-05	0	6.45	0	0	200	2	1209.10376	2417.20024	-4.34
dALRDEYDDLSDLNAVQMESVR	Unambiguous	1	1	Q80UU9	N-Term(iTRAQ8plex)	CID	1.0000	0.0000	1	1	2763957	Unique	Used	0.802	0.949	0.698	1.063	1E-05	0	6.45	1	2	60	3	953.46484	2858.37998	0.67
vLnNMEIGTSLYDEEGAk	Unambiguous	1	1	P09411	N-Term(iTRAQ8plex); N3(Deamidated); K18(iTRAQ8plex)	CID	0.0264	0.0000	1	1	2833798	Unique	Used	1.441	0.896	0.926	0.862	1E-05	0	6.45	0	9	200	2	1296.66882	2592.33037	-0.77
nTNSSMGPATPLEVPLR	Unambiguous	1	1	Q80VL1	N-Term(iTRAQ8plex)	CID	1.0000	0.0000	1	1	3535809	Unique	Used	1.160	0.865	0.648	0.795	1E-05	0	6.45	0	0	200	2	1044.55188	2088.09648	-4.63

tTQVPQYILDDFIQNDR	Unambiguous	4	1	E9QNN1	N-Term(iTRAQ8plex)	CID	1.0000	0.0000	1	1	3326697	Unique	Used	0.514	0.918	0.841	0.936	1E-05	0	6.45	0	0	20	2	1185.61462	2370.22197	-1.00
dFTSEQIDEILQNHTEIVFAR	Unambiguous	2	1	Q6PIC6	N-Term(iTRAQ8plex)	CID	1.0000	0.0000	1	1	3376381	Unique	Used		1.270	0.636	0.810	1E-05	0	6.45	0	7	200	3	937.14502	2809.42051	-3.76
aLYEGYAAVTDAPPEcFLTLR	Unambiguous	1	1	Q99KK7	N-Term(iTRAQ8plex); C16(Methylthio)	CID	1.0000	0.0000	1	1	3581331	Unique	Used	0.403	0.644	0.608	0.589	1E-05	0	6.45	0	0	31	2	1325.66174	2650.31621	-1.40
lSFGDTLQVQNVHGAFnALGGADR	Unambiguous	1	1	Q9DC16	N-Term(iTRAQ8plex); N17(Deamidated)	CID	1.0000	0.0000	1	1	3498475	Unique	Used	1.262	1.330	1.017	0.870	1E-05	0	6.45	0	22	49	3	931.47589	2792.41312	-4.97
sLGNVIDPLDVIHGVSLQGLYDQLLnSNLDPSEVEk	Unambiguous	2	1	Q9Z1Q9	N-Term(iTRAQ8plex); N26(Deamidated); K36(iTRAQ8plex)	CID	0.0047	0.0000	1	1	3645691	Unique	Used			1.978		1E-05	0	6.45	0	19	48	3	1500.80225	4500.39219	-1.09
aGYTDQVVIGMDVAASEFYR	Unambiguous	2	1	P17182	N-Term(iTRAQ8plex)	CID	1.0000	0.0000	1	1	3329180	Unique	Used	0.798	1.653	0.948	0.873	1E-05	0	6.45	0	27	200	2	1248.62012	2496.23296	-2.14
sLMLENTEcHSIYEESPER	Unambiguous	1	1	Q8CGF6	N-Term(iTRAQ8plex); C9(Methylthio)	CID	1.0000	0.0000	1	1	2849873	Unique	Used	0.857	0.929	1.408	1.154	1E-05	0	6.45	0	1	15	3	873.06549	2617.18192	-2.57
hPEGIVSEVEmLSSQER	Unambiguous	2	1	Q9WTQ5	N-Term(iTRAQ8plex); M11(Oxidation)	CID	1.0000	0.0000	1	1	2937986	Unique	Used	1.013	0.821	1.001	1.242	1E-05	0	6.45	0	6	33	3	749.71027	2247.11625	-2.98
aLSEIAGITLPYDTLDQVR	Unambiguous	1	1	Q91VD9	N-Term(iTRAQ8plex)	CID	1.0000	0.0000	1	1	3583480	Unique	Used	1.757	1.087	1.480	1.836	1E-05	0	6.45	0	0	200	2	1190.15076	2379.29424	-5.52
lEGAGSATIAEVEMPFYEDk	Unambiguous	2	1	P20357	N-Term(iTRAQ8plex); K20(iTRAQ8plex)	CID	1.0000	0.0000	1	1	2941900	Unique	Used	1.642	1.496	1.768	0.966	1E-05	0	6.45	0	3	17	2	1383.20898	2765.41069	-2.09
dLGATWVVLGHSER	Unambiguous	2	1	P17751	N-Term(iTRAQ8plex)	CID	1.0000	0.0000	1	1	3474642	Unique	Used	1.692	1.011	1.622	1.391	1E-05	0	6.45	0	5	4	3	615.33539	1843.99161	-2.84
fkPFTVEIVDSVEAYATMLR	Unambiguous	2	1	Q9D0F9	N-Term(iTRAQ8plex); K2(iTRAQ8plex)	CID	1.0000	0.0000	1	1	3766604	Unique	Used	0.299	0.821	0.773	1.417	1E-05	0	6.45	0	4	10	3	975.53619	2924.59403	-3.84
fQMPDQGMTSADDFFQGTk	Unambiguous	1	1	O08553	N-Term(iTRAQ8plex); K19(iTRAQ8plex)	CID	1.0000	0.0000	1	1	3306876	Unique	Used	1.039	0.898	2.691	2.303	1E-05	0	6.45	0	0	200	3	920.44513	2759.32083	-2.09
mTVNESEQLVScSMDDTVR	Unambiguous	1	1	O88342	N-Term(iTRAQ8plex); C12(Methylthio)	CID	1.0000	0.0000	1	1	2977583	Unique	Used	1.357	1.228	0.995	0.918	1E-05	0	6.45	0	0	82	2	1247.56372	2494.12016	-1.39

sENGLEFTSSGSANTETTk	Unambiguous	3	1	Q60932-2	N-Term(iTRAQ8plex); K19(iTRAQ8plex)	CID	1.0000	0.0000	1	1	2682607	Unique	Used			1.467	1.753	1E-05	0	6.45	0	0	200	2	1284.64722	2568.28716	-0.55
lGTEEFPLIDQTFYPNHk	Unambiguous	3	1	O88935	N-Term(iTRAQ8plex); K18(iTRAQ8plex)	CID	1.0000	0.0000	1	1	3303228	Unique	Used	0.962	0.838	2.310	2.051	1E-05	0	6.45	0	0	200	3	919.82800	2757.46946	-0.50
vGLIGScTNSSYEDMGR	Unambiguous	1	1	Q99KI0	N-Term(iTRAQ8plex); C7(Methylthio)	CID	1.0000	0.0000	1	1	3477773	Unique	Used	0.656	0.566	1.217	1.344	1E-05	0	6.45	0	0	1	2	1069.99133	2138.97539	-3.22
kDLYANNVLSGGTTmYPGIADR	Unambiguous	3	1	P62737	N-Term(iTRAQ8plex); K1(iTRAQ8plex); M15(Oxidation)	CID	1.0000	0.0000	1	1	3535449	Unique	Used	0.993	2.234	1.335	1.375	1E-05	0	6.44	1	21	58	3	994.18915	2980.55289	-4.37
nQGGYGGSSSSSSYGSGR	Unambiguous	3	1	Q5EBP8	N-Term(iTRAQ8plex)	CID	1.0000	0.0000	1	1	2771032	Unique	Used	1.913	0.436	0.986	0.485	1E-05	0	6.44	0	0	200	2	999.95435	1998.90141	-2.04
acLISMGYDLGEAEFAR	Unambiguous	1	1	Q9JI91	N-Term(iTRAQ8plex); C2(Methylthio)	CID	1.0000	0.0000	1	1	3419020	Unique	Used	0.636	5.621	6.015	1.131	1E-05	0	6.44	0	0	16	2	1098.52014	2196.03301	-5.08
sVNESLNNLFITEEDYQALR	Unambiguous	2	1	Q68FD5	N-Term(iTRAQ8plex)	CID	1.0000	0.0000	1	1	3264366	Unique	Used	1.158	1.628	1.060	1.400	1E-05	0	6.44	0	0	200	2	1330.18005	2659.35283	0.41
sSEYHLEDNAAYYLNDLER	Unambiguous	1	1	O70443	N-Term(iTRAQ8plex)	CID	1.0000	0.0000	1	1	2762431	Unique	Used	1.540	0.913	1.180	0.636	1E-05	0	6.44	0	14	200	3	869.41241	2606.22269	-3.29
sLQDIIAILGMDELSEEDkLTVSR	Unambiguous	1	1	P56480	N-Term(iTRAQ8plex); K19(iTRAQ8plex)	CID	1.0000	0.0000	1	1	3353597	Unique	Used	0.900	1.050	1.015	1.133	1E-05	0	6.44	1	5	31	3	1095.26355	3283.77610	-4.74
eLQSQISDTSVVLSMDNSR	Unambiguous	1	1	P11679	N-Term(iTRAQ8plex)	CID	1.0000	0.0000	1	1	3281454	Unique	Used	0.914	1.158	0.916	1.114	1E-05	0	6.44	0	19	172	2	1207.11157	2413.21587	-1.00
dkPVYYALEGSVAIAGAVIR	Unambiguous	4	1	Q64516	N-Term(iTRAQ8plex); K2(iTRAQ8plex)	CID	1.0000	0.0000	1	1	3785174	Unique	Used	0.853	0.756	1.445	1.710	1E-05	0	6.44	0	12	12	3	900.85431	2700.54837	-2.28
gHMLADVVAIIGTqDIVFGEIDR	Unambiguous	2	1	Q91WD5	N-Term(iTRAQ8plex); Q14(Deamidated)	CID	1.0000	0.0000	1	1	3645556	Unique	Used	1.499	1.503	1.442	1.504	1E-05	0	6.44	0	0	72	2	1387.73547	2774.46367	-2.33
qVLYQQSSGHNNR	Unambiguous	2	1	Q99M71	N-Term(iTRAQ8plex)	CID	1.0000	0.0000	1	1	3527193	Unique	Used	0.939	0.556	1.631	1.390	1E-05	0	6.44	0	26	126	3	612.31696	1834.93631	-5.37
eVAAFAQFGSDLDAATQQLLSR	Unambiguous	2	1	Q03265	N-Term(iTRAQ8plex)	CID	1.0000	0.0000	1	1	3669241	Unique	Used	0.547	1.365	1.213	1.327	1E-05	0	6.44	0	3	200	2	1321.68347	2642.35967	-4.99
gSFPLAASGPAQAAPAPPPEDAR	Unambiguous	1	1	Q9D4F2	N-Term(iTRAQ8plex)	CID	1.0000	0.0000	1	1	3255246	Unique	Used	0.288	0.865	1.117	0.998	1E-05	0	6.44	0	11	61	2	1240.14832	2479.28935	0.38

acADATLSQITnNIDPVGR	Unambiguous	3	1	P62874	N-Term(iTRAQ8plex); C2(Methylthio); N12(Deamidated)	CID	0.0342	0.0000	1	1	3457426	Unique	Used	0.494	1.239	1.303	1.319	1E-05	0	6.44	0	0	200	2	1155.56641	2310.12554	-5.05
aISFVGSnQAGEYIFER	Unambiguous	1	1	Q9EQ20	N-Term(iTRAQ8plex); N8(Deamidated)	CID	0.0171	0.0000	1	1	3261523	Unique	Used	1.103	1.439	1.204	1.214	1E-05	0	6.44	0	0	74	2	1097.06018	2193.11308	0.04
eLGDDcnFGPALGEVGEAMR	Unambiguous	5	1	Q62420	N-Term(iTRAQ8plex); C6(Methylthio); N7(Deamidated)	CID	1.0000	0.0000	1	1	2870360	Unique	Used	2.071	2.126	1.004	0.730	1E-05	0	6.44	0	0	200	2	1216.05835	2431.10942	8.71
vVWDSQAAEHFFEYk	Unambiguous	2	1	Q922Q9	N-Term(iTRAQ8plex); K15(iTRAQ8plex)	CID	1.0000	0.0000	1	1	3260435	Unique	Used	0.397	0.983	0.801	0.946	1E-05	0	6.44	0	24	15	3	822.09546	2464.27182	-1.60
vcIVGSGNWGSAIAk	Unambiguous	2	1	P13707	N-Term(iTRAQ8plex); C2(Methylthio); K15(iTRAQ8plex)	CID	1.0000	0.0000	1	1	3878045	Unique	Used	0.585	0.472	1.139	1.204	1E-05	0	6.44	0	5	17	2	1058.57495	2116.14263	-3.67
aAMFPLQVLGFEVDAVnSVQFSNHTGYAHWk	Unambiguous	2	1	Q8K183	N-Term(iTRAQ8plex); N17(Deamidated); K31(iTRAQ8plex)	CID	1.0000	0.0000	1	1	3860660	Unique	Used	1.306	0.804	1.047	1.308	1E-05	0	6.44	0	0	24	3	1358.36633	4073.08445	0.09
kLPEAPPSEEEEQEAWVNALLGR	Unambiguous	1	1	Q6ZPJ0	N-Term(iTRAQ8plex); K1(iTRAQ8plex)	CID	1.0000	0.0000	1	1	3132552	Unique	Used	0.503	0.893	0.926	1.163	1E-05	0	6.44	1	0	86	3	1067.57507	3200.71067	1.81
eFWEIVHSFTDEQk	Unambiguous	2	1	E9QKT1	N-Term(iTRAQ8plex); K14(iTRAQ8plex)	CID	1.0000	0.0000	1	1	3262269	Unique	Used	0.156	0.697	0.921	0.935	1E-05	0	6.44	0	6	12	3	801.75250	2403.24295	-0.49
lTGFHETSNINDFSAGVAnR	Unambiguous	1	1	P15105	N-Term(iTRAQ8plex); N19(Deamidated)	CID	1.0000	0.0000	1	1	3278633	Unique	Used	1.995	1.203	1.853	3.013	1E-05	0	6.44	0	0	200	2	1228.10901	2455.21074	-1.97
iQAAASPPANATAASDTnAGDR	Unambiguous	2	1	E9QAQ5	N-Term(iTRAQ8plex); N18(Deamidated)	CID	1.0000	0.0000	1	1	2731487	Unique	Used	0.931	1.068	1.105	1.173	1E-05	0	6.44	0	0	200	2	1188.08875	2375.17021	-1.65

aELEQHLEQEQEFQVNk	Unambiguous	1	1	D3YZP9	N-Term(iTRAQ8plex); K17(iTRAQ8plex)	CID	1.0000	0.0000	1	1	2700905	Unique	Used	1.570	0.718	0.918	0.629	1E-05	0	6.44	0	0	146	3	903.14063	2707.40732	-2.76
aMSIASILTNTMEELEESR	Unambiguous	1	1	A2ASI5	N-Term(iTRAQ8plex)	CID	1.0000	0.0000	1	1	3353400	Unique	Used	1.278	1.788	1.821	2.073	1E-05	0	6.44	0	0	76	2	1215.10742	2429.20757	-5.37
sPALASVASAVSEPEPQcELR	Unambiguous	1	1	Q3URQ4	N-Term(iTRAQ8plex); C18(Methylthio)	CID	1.0000	0.0000	1	1	3325360	Unique	Used	1.021	0.892	0.896	0.760	1E-05	0	6.44	0	0	134	2	1246.12024	2491.23320	-5.74
qLNTIQNAEAVHFFcnYEESR	Unambiguous	4	1	P61922	N-Term(iTRAQ8plex); C15(Methylthio); N16(Deamidated)	CID	1.0000	0.0000	1	1	3283163	Unique	Used	1.729	0.893	0.857	1.153	1E-05	0	6.44	0	25	57	3	955.44666	2864.32541	-1.11
yESSALPAGQLTSLPDYASR	Unambiguous	1	1	Q9CQN1	N-Term(iTRAQ8plex)	CID	1.0000	0.0000	1	1	3347736	Unique	Used	0.662	0.726	1.187	1.170	1E-05	0	6.44	0	0	9	2	1215.62146	2430.23564	-4.04
aGYTDQVVIGMDVAASEFYR	Unambiguous	2	1	P17182	N-Term(iTRAQ8plex)	CID	1.0000	0.0000	1	1	3353870	Unique	Used	1.354	1.681	1.064	1.263	1E-05	0	6.44	0	0	174	2	1248.62109	2496.23491	-1.36
gSDWLGDQDAIHYMTEQAPASVVELENYGMPFSR	Unambiguous	1	1	Q8K2B3	N-Term(iTRAQ8plex)	CID	1.0000	0.0000	1	1	3352500	Unique	Used	1.622	0.627	0.838	0.991	1E-05	0	6.44	0	12	29	3	1373.30432	4117.89841	-5.60
qSLQQcGEEGQSAHR	Unambiguous	1	1	Q68FE6	N-Term(iTRAQ8plex); C6(Methylthio)	CID	1.0000	0.0000	1	1	2823797	Unique	Used	1.445	0.820	1.103	1.090	1E-05	0	6.44	0	8	92	3	669.97876	2007.92173	-3.05
nTSSEPPEYQLMTGEQIHQER	Unambiguous	1	1	Q9CZD5	N-Term(iTRAQ8plex)	CID	1.0000	0.0000	1	1	2756683	Unique	Used	1.965	0.859	1.084	1.007	1E-05	0	6.44	0	13	200	3	926.78131	2778.32938	-0.47
dIFHDLIAcVTmDNLQER	Unambiguous	1	1	Q91Z67	N-Term(iTRAQ8plex); C9(Methylthio); M12(Oxidation)	CID	1.0000	0.0000	1	1	3602409	Unique	Used	1.189	1.554	0.715	1.107	1E-05	0	6.44	0	9	29	3	833.73444	2499.18875	-3.87
tSSPAIQNSVEDELEMATVR	Unambiguous	1	1	Q6PHZ8	N-Term(iTRAQ8plex)	CID	1.0000	0.0000	1	1	2833584	Unique	Used	0.630	0.576	0.876	0.582	1E-05	0	6.44	0	2	78	2	1241.12256	2481.23784	-2.69
vDLQQQImTIVDELGk	Unambiguous	5	1	Q8K341-4	N-Term(iTRAQ8plex); M8(Oxidation); K16(iTRAQ8plex)	CID	1.0000	0.0000	1	1	3459685	Unique	Used	0.372	0.428	0.681	0.577	1E-05	0	6.44	0	5	19	2	1227.68640	2454.36553	-3.23
iHFSTAPIQVFSTYSNEDYDR	Unambiguous	1	1	Q6R891	N-Term(iTRAQ8plex)	CID	1.0000	0.0000	1	1	3559037	Unique	Used	1.143	1.461	1.217	1.754	1E-05	0	6.44	0	11	108	3	932.12323	2794.35514	-2.68

eAAAEAISGAEEGRPDLLVnLPGELQPTk	Unambiguous	4	1	Q8BLK9-3	N-Term(iTRAQ8plex); N20(Deamidated); K29(iTRAQ8plex)	CID	1.0000	0.0000	1	1	3195087	Unique	Used	1.609	1.262	0.826	0.734	1E-05	0	6.44	0	0	67	3	1195.65247	3584.94284	4.44
gQGAAAAQQGGYEIPAR	Unambiguous	1	1	Q9DBS5	N-Term(iTRAQ8plex)	CID	1.0000	0.0000	1	1	3027132	Unique	Used	0.979	0.932	1.019	1.059	1E-05	0	6.44	0	0	200	2	975.00848	1949.00969	-2.37
vSQAASDLLAYcEAHVR	Unambiguous	1	1	P50153	N-Term(iTRAQ8plex); C12(Methylthio)	CID	1.0000	0.0000	1	1	3879855	Unique	Used	0.902	1.239	0.681	1.176	1E-05	0	6.44	0	0	37	3	728.36511	2183.08078	-3.82
sTTTTDMITEVGTAFSR	Unambiguous	5	1	J3QMM7	N-Term(iTRAQ8plex)	CID	1.0000	0.0000	1	1	3478321	Unique	Used	1.658	1.323	0.934	0.963	1E-05	0	6.44	0	6	42	2	1061.53003	2122.05278	-5.29
qETAASLVTSAVGQQnGNVVR	Unambiguous	2	1	Q61191	N-Term(iTRAQ8plex); N16(Deamidated)	CID	0.0171	0.0000	1	1	3391138	Unique	Used			0.951	0.931	1E-05	0	6.44	0	17	76	2	1217.64063	2434.27397	-4.12
sNISSPVTPTAASSSTTPTR	Unambiguous	6	1	A2AU91	N-Term(iTRAQ8plex)	CID	1.0000	0.0000	1	1	3612682	Unique	Used	0.536	0.426	0.705	1.087	1E-05	0	6.44	0	0	200	2	1177.10376	2353.20024	-6.23
gPMTGGIQGPGPINMGAGGPQGPR	Unambiguous	1	1	Q8C7E9	N-Term(iTRAQ8plex)	CID	1.0000	0.0000	1	1	3874853	Unique	Used	1.836	1.080	1.211	0.527	1E-05	0	6.44	0	0	199	2	1254.63708	2508.26689	-3.40
lQQTQAQVDEVVDIMR	Unambiguous	2	1	P63044	N-Term(iTRAQ8plex)	CID	1.0000	0.0000	1	1	3287103	Unique	Used	0.230	1.620	1.536	1.346	1E-05	0	6.44	0	0	200	2	1089.07922	2177.15117	-1.23
tASEMVLADDNFSTIVAAVEEGR	Unambiguous	4	1	O55143	N-Term(iTRAQ8plex)	CID	1.0000	0.0000	1	1	3076092	Unique	Used		0.974			1E-05	0	6.44	0	22	200	2	1365.18787	2729.36846	2.86
dVPWGVDSLITLAFQDQR	Unambiguous	1	1	Q61553	N-Term(iTRAQ8plex)	CID	1.0000	0.0000	1	1	3687147	Unique	Used	0.801	1.009	0.885	0.735	1E-05	0	6.44	0	2	13	2	1182.62195	2364.23662	-5.74
sAGPQSQLLASVIAEk	Unambiguous	2	1	Q9QYB8	N-Term(iTRAQ8plex); K16(iTRAQ8plex)	CID	1.0000	0.0000	1	1	3477264	Unique	Used	0.785	1.031	2.225	1.115	1E-05	0	6.44	0	0	15	2	1104.14282	2207.27837	-3.28
gIAPASPMLGnAGNPNk	Unambiguous	3	1	Q03141-3	N-Term(iTRAQ8plex); N11(Deamidated); K17(iTRAQ8plex)	CID	0.0062	0.0000	1	1	3553784	Unique	Used	0.446	0.640	0.974	0.743	1E-05	0	6.44	0	28	200	2	1109.61536	2218.22344	5.57
qLGSVSTGSTLQQSLELTR	Unambiguous	1	1	Q921G8	N-Term(iTRAQ8plex)	CID	1.0000	0.0000	1	1	3703362	Unique	Used	0.555	1.209	0.955	1.283	1E-05	0	6.44	0	20	82	2	1155.12634	2309.24541	-6.95
iMDPNIVGnEHYDVAR	Unambiguous	1	1	P56480	N-Term(iTRAQ8plex); N9(Deamidated)	CID	1.0000	0.0000	1	1	2830990	Unique	Used	5.184	1.218	1.156	0.920	1E-05	0	6.44	0	18	45	3	716.69348	2148.06589	-1.80

aLNEAcESVIQTAck	Unambiguous	2	1	Q61543	N-Term(iTRAQ8plex); C6(Methylthio); C14(Methylthio); K15(iTRAQ8plex)	CID	1.0000	0.0000	1	1	3645307	Unique	Used	0.712	0.649	0.775	0.684	1E-05	0	6.43	0	29	12	2	1140.56311	2280.11894	-5.59
aLYQTEAFTADFQQPTEAk	Unambiguous	1	1	P07759	N-Term(iTRAQ8plex); K19(iTRAQ8plex)	CID	1.0000	0.0000	1	1	3073378	Unique	Used	2.642	2.199	0.817	1.624	1E-05	0	6.43	0	14	200	2	1384.22058	2767.43389	-2.19
hTVTMIPGDGIGPELMLHVk	Unambiguous	1	1	P70404	N-Term(iTRAQ8plex); K20(iTRAQ8plex)	CID	1.0000	0.0000	1	1	3516636	Unique	Used	0.837	0.974	1.362	1.337	1E-05	0	6.43	0	0	200	3	918.50867	2753.51145	-6.86
iPATEDTPEVEGDVPELYHQR	Unambiguous	4	1	Q6ZPU9	N-Term(iTRAQ8plex)	CID	1.0000	0.0000	1	1	2636190	Unique	Used	0.887	1.052	0.604	0.918	1E-05	0	6.43	0	4	108	3	900.45233	2699.34244	-1.57
vSEGcLTSSqDGVENDGLHR	Unambiguous	3	1	Q91VU7	N-Term(iTRAQ8plex); C5(Methylthio); Q10(Deamidated)	CID	1.0000	0.0000	1	1	2715463	Unique	Used	2.434	1.354	1.222	1.269	1E-05	0	6.43	0	21	200	3	818.71710	2454.13675	7.67
vETLHDFEAANSDELNLqR	Unambiguous	1	1	Q7TQF7	N-Term(iTRAQ8plex); Q18(Deamidated)	CID	0.0047	0.0000	1	1	2654578	Unique	Used	1.638	1.328	1.487	1.145	1E-05	0	6.43	0	12	200	3	836.08386	2506.23703	0.26
eLGAFGLQVPSELGGLGLSNTQYAR	Unambiguous	2	1	P50544	N-Term(iTRAQ8plex)	CID	1.0000	0.0000	1	1	3785309	Unique	Used	1.049	0.994	0.472	0.985	1E-05	0	6.43	0	0	14	2	1441.26208	2881.51689	-6.71
lGTDQPLDqATISLQMGTNk	Unambiguous	2	1	Q08091	N-Term(iTRAQ8plex); Q9(Deamidated); K20(iTRAQ8plex)	CID	0.0078	0.0000	1	1	3348212	Unique	Used	0.467	14.401	1.815	3.555	1E-05	0	6.43	0	0	51	2	1370.74084	2740.47441	3.51
mTVAHMWFDnQIHEADTTEDQSGATFDk	Unambiguous	3	1	Q6PIE5	N-Term(iTRAQ8plex); N10(Deamidated); K28(iTRAQ8plex)	CID	0.0202	0.0000	1	1	2976676	Unique	Used	0.665	0.509	1.094	1.478	1E-05	0	6.43	0	10	200	3	1278.93604	3834.79355	2.57
iLTDYGFEGHPFR	Unambiguous	1	1	Q9DCT2	N-Term(iTRAQ8plex)	CID	1.0000	0.0000	1	1	3598479	Unique	Used	0.732	1.105	1.001	1.214	1E-05	0	6.43	0	12	25	3	619.32294	1855.95426	-5.50

aITVASQTncPLYVTk	Unambiguous	5	1	E9PWE8	N-Term(iTRAQ8plex); N9(Deamidated); C10(Methylthio); K16(iTRAQ8plex)	CID	1.0000	0.0000	1	1	3682012	Unique	Used	1.121	0.519	0.347	0.400	1E-05	0	6.43	0	0	200	2	1182.64795	2364.28862	5.19
wGEAGAEYVVESTGVFTTMEk	Unambiguous	3	1	P16858	N-Term(iTRAQ8plex); K21(iTRAQ8plex)	CID	1.0000	0.0000	1	1	3242066	Unique	Used			1.793	1.927	1E-05	0	6.43	0	0	200	2	1450.23499	2899.46269	-0.61
tSGVVQGVASVAEk	Unambiguous	1	1	Q91ZZ3	N-Term(iTRAQ8plex); K14(iTRAQ8plex)	CID	1.0000	0.0000	1	1	3277363	Unique	Used	1.220	1.231	1.680	0.989	1E-05	0	6.43	0	0	30	2	970.56531	1940.12334	-2.05
eLLESnFTLVGDDGTNk	Unambiguous	2	1	Q9D024	N-Term(iTRAQ8plex); N6(Deamidated); K17(iTRAQ8plex)	CID	1.0000	0.0000	1	1	2939765	Unique	Used	0.509	0.566	0.948	0.794	1E-05	0	6.43	0	0	145	2	1231.15649	2461.30571	5.62
eNNAVYAFLGLTAPPGSk	Unambiguous	1	1	Q9JJU8	N-Term(iTRAQ8plex); K18(iTRAQ8plex)	CID	1.0000	0.0000	1	1	3726597	Unique	Used	1.557	1.820	1.206	0.990	1E-05	0	6.43	0	19	13	2	1229.17505	2457.34282	-6.93
yTPSGQSGAAASESLFISNHAY	Unambiguous	2	1	A6ZI44	N-Term(iTRAQ8plex)	CID	1.0000	0.0000	1	1	3241311	Unique	Used		1.949			1E-05	0	6.43	0	18	200	2	1281.62524	2562.24321	0.69
tVPLAGHVGFDSLPDQLVNk	Unambiguous	4	1	Q9R1T4-2	N-Term(iTRAQ8plex); K20(iTRAQ8plex)	CID	1.0000	0.0000	1	1	3517396	Unique	Used	0.570	0.851	1.613	1.672	1E-05	0	6.43	0	0	14	3	905.84381	2715.51688	-4.48
mAEDQAETIANLIEEcGGLEk	Unambiguous	1	1	O35343	N-Term(iTRAQ8plex); C16(Methylthio); K21(iTRAQ8plex)	CID	1.0000	0.0000	1	1	3133637	Unique	Used	2.243	1.328	1.351	2.172	1E-05	0	6.43	0	7	150	3	973.48578	2918.44278	0.73
sLAESIDEALNcHPSGPMSEEPGSAQLEk	Unambiguous	5	1	E9QAD8	N-Term(iTRAQ8plex); C12(Methylthio); K29(iTRAQ8plex)	CID	1.0000	0.0000	1	1	3037178	Unique	Used	0.211	1.726	2.328	0.885	1E-05	0	6.43	0	9	200	3	1227.59521	3680.77109	0.20
aGYTDQVVIGMDVAASEFYR	Unambiguous	2	1	P17182	N-Term(iTRAQ8plex)	CID	1.0000	0.0000	1	1	3355718	Unique	Used	0.798	1.321	0.809	1.055	1E-05	0	6.43	0	22	200	2	1248.61670	2496.22612	-4.88

qQDAQEFFLHLINmVER	Unambiguous	2	1	P56399	N-Term(iTRAQ8plex); M14(Oxidation)	CID	1.0000	0.0000	1	1	3706671	Unique	Used	0.590	1.407	1.180	1.150	1E-05	0	6.43	0	3	1	3	813.41412	2438.22782	-6.66
ISGPPDASGAVFVLGGEGLR	Unambiguous	1	1	P60487	N-Term(iTRAQ8plex)	CID	1.0000	0.0000	1	1	3601545	Unique	Used	2.175	2.105	1.472	1.611	1E-05	0	6.43	0	0	23	2	1102.10364	2203.20000	-1.15
vSDAISTqYPVVDHEFDAVVVGAGGAGLR	Unambiguous	1	1	Q8K2B3	N-Term(iTRAQ8plex); Q8(Deamidated)	CID	1.0000	0.0000	1	1	3354068	Unique	Used	1.313	1.052	1.082	0.998	1E-05	0	6.43	0	12	200	3	1078.89319	3234.66501	2.00
aELFESPQEQqGALPVPGPSR	Unambiguous	2	1	Q3U1Y4	N-Term(iTRAQ8plex); Q11(Deamidated)	CID	0.0280	0.0000	1	1	3214134	Unique	Used	1.268	0.721	1.003	1.119	1E-05	0	6.43	0	0	200	2	1271.66345	2542.31963	4.11
nSLISYLEQIHR	Unambiguous	1	1	Q00493	N-Term(iTRAQ8plex)	CID	1.0000	0.0000	1	1	3822831	Unique	Used	0.690	1.451	0.640	0.825	1E-05	0	6.43	0	6	35	3	592.99988	1776.98508	-3.33
sQTEEDcTEELFDFLHAR	Unambiguous	1	1	P99028	N-Term(iTRAQ8plex); C7(Methylthio)	CID	1.0000	0.0000	1	1	2836013	Unique	Used	2.567	1.389	0.602	0.842	1E-05	0	6.43	0	0	200	3	840.71466	2520.12943	-1.22
vIQcFAETGQVQk	Unambiguous	2	1	Q68FD5	N-Term(iTRAQ8plex); C4(Methylthio); K13(iTRAQ8plex)	CID	1.0000	0.0000	1	1	3599898	Unique	Used	0.271	1.087	1.187	1.154	1E-05	0	6.43	0	0	2	2	1053.06458	2105.12187	-5.94
iATIQQTTEVFTSTLDDLEER	Unambiguous	3	1	Q9WV18	N-Term(iTRAQ8plex)	CID	1.0000	0.0000	1	1	3305830	Unique	Used	0.465	1.268	0.906	1.433	1E-05	0	6.43	0	0	123	2	1357.70496	2714.40264	-0.45
nmAEQIIQEIYSQVQSk	Unambiguous	1	1	P40142	N-Term(iTRAQ8plex); M2(Oxidation); K17(iTRAQ8plex)	CID	1.0000	0.0000	1	1	3706576	Unique	Used	2.004	0.738	0.570	1.086	1E-05	0	6.43	0	0	46	2	1317.19678	2633.38628	-7.69
eVNENFAIDLIAQQPVNEVEHR	Unambiguous	2	1	P52503	N-Term(iTRAQ8plex)	CID	1.0000	0.0000	1	1	3036209	Unique	Used	3.420	0.561	2.223	1.124	1E-05	0	6.43	0	4	9	3	956.83008	2868.47568	-1.31
tQPTDEEMLFIYSHFk	Unambiguous	4	1	P31786	N-Term(iTRAQ8plex); K16(iTRAQ8plex)	CID	1.0000	0.0000	1	1	3325519	Unique	Used	2.314	1.027	1.139	0.859	1E-05	0	6.43	0	6	51	3	865.44788	2594.32907	-5.03
IVQDVANNTnEEAGDGTTTATVLAR	Unambiguous	3	1	P63038	N-Term(iTRAQ8plex); N10(Deamidated)	CID	0.0156	0.0000	1	1	2762177	Unique	Used	4.228		0.892	1.311	1E-05	0	6.43	0	0	200	2	1433.22131	2865.43535	-0.93
qVLYQQSSGHNNR	Unambiguous	2	1	Q99M71	N-Term(iTRAQ8plex)	CID	1.0000	0.0000	1	1	3446557	Unique	Used	1.837	1.280	1.446	0.961	1E-05	0	6.43	0	5	200	3	612.31769	1834.93851	-4.17

vEYPVMYSTDPEnGHMFNcIQR	Unambiguous	1	1	Q9CPU4	N-Term(iTRAQ8plex); N13(Deamidated); C19(Methylthio)	CID	1.0000	0.0000	1	1	3282940	Unique	Used	1.134	1.119	1.432	1.120	1E-05	0	6.43	0	9	73	3	994.44598	2981.32340	-0.35
iVEIVDAITTTAQSHQR	Unambiguous	1	1	P47857	N-Term(iTRAQ8plex)	CID	1.0000	0.0000	1	1	3844416	Unique	Used	1.882	1.381	0.318	1.845	1E-05	0	6.43	0	15	200	3	729.40680	2186.20584	-1.12
sIELDGTFVGTEVPGELGALGTGPAEAR	Unambiguous	1	1	Q8K596	N-Term(iTRAQ8plex)	CID	1.0000	0.0000	1	1	3239157	Unique	Used			3.154	2.393	1E-05	0	6.43	0	0	18	2	1524.29468	3047.58208	-0.63
sEQSSmSIEFGQESPEHSLAMDFSR	Unambiguous	1	1	P14873	N-Term(iTRAQ8plex); M6(Oxidation)	CID	1.0000	0.0000	1	1	2918484	Unique	Used	1.510	0.763	1.505	1.089	1E-05	0	6.43	0	20	200	3	1046.14209	3136.41172	-0.79
iYLTADNLVLNLQDESFTR	Unambiguous	2	1	O35129	N-Term(iTRAQ8plex)	CID	1.0000	0.0000	1	1	3500478	Unique	Used	0.673	1.119	0.973	1.046	1E-05	0	6.43	0	11	17	2	1265.17627	2529.34526	-1.98
eMEENFALEAANYQDTIGR	Unambiguous	1	1	P20152	N-Term(iTRAQ8plex)	CID	1.0000	0.0000	1	1	2815328	Unique	Used	1.159	1.737	1.038	1.391	1E-05	0	6.43	0	0	200	2	1253.09802	2505.18877	0.71
dLTAVSnNAGVDNFGLGLLLR	Unambiguous	2	1	Q9D0K2	N-Term(iTRAQ8plex); N7(Deamidated)	CID	0.0156	0.0000	1	1	3623488	Unique	Used	0.552	0.691	0.533	0.497	1E-05	0	6.43	0	5	9	2	1232.66565	2464.32402	-4.45
qLNDLWDQIEQAHLELR	Unambiguous	1	1	Q6A068	N-Term(iTRAQ8plex)	CID	1.0000	0.0000	1	1	3418713	Unique	Used			1.268		1E-05	0	6.43	0	0	31	3	809.09625	2425.27420	-1.48
gNDISSGTVLSDYVGSGPPSGTGLHR	Unambiguous	1	1	P70296	N-Term(iTRAQ8plex)	CID	1.0000	0.0000	1	1	3576198	Unique	Used	1.344	1.139	0.915	0.711	1E-05	0	6.43	0	24	42	3	945.47394	2834.40726	-5.31
mVVNEGADGGQSVYHIHLHVLGGR	Unambiguous	1	1	P70349	N-Term(iTRAQ8plex); M1(Oxidation)	CID	1.0000	0.0000	1	1	3679265	Unique	Used	2.132	1.257	1.148	1.194	1E-05	0	6.43	0	0	88	3	955.82416	2865.45792	-5.35
lEENEAIDQDLQAYILHR	Unambiguous	2	1	A2A690	N-Term(iTRAQ8plex)	CID	1.0000	0.0000	1	1	2941584	Unique	Used	0.467	1.368	1.092	0.991	1E-05	0	6.42	0	15	76	3	825.43146	2474.27982	-1.26
vLNADTQEPMMDHPLR	Unambiguous	2	1	B2RUJ5	N-Term(iTRAQ8plex)	CID	1.0000	0.0000	1	1	3125402	Unique	Used	1.302	1.099	1.159	1.025	1E-05	0	6.42	0	28	23	3	724.36761	2171.08829	-0.39
gAVQFVTQYQHSSGQR	Unambiguous	2	1	E9Q1S3	N-Term(iTRAQ8plex)	CID	1.0000	0.0000	1	1	3872023	Unique	Used	0.787	0.666	0.725	0.636	1E-05	0	6.42	0	3	17	3	699.69489	2097.07010	-3.73
gFQQILAGEYDHLPEqAFYMVGPIEEAVAk	Unambiguous	1	1	P56480	N-Term(iTRAQ8plex); Q16(Deamidated); K30(iTRAQ8plex)	CID	1.0000	0.0000	1	1	3421674	Unique	Used		1.275		1.972	1E-05	0	6.42	0	25	200	3	1320.68164	3960.03037	-1.19

nFYGGnGIVGAQVPLGAGIALAck	Unambiguous	1	1	P35486	N-Term(iTRAQ8plex); N6(Deamidated); C23(Methylthio); K24(iTRAQ8plex)	CID	1.0000	0.0000	1	1	3806106	Unique	Used	0.397	0.726	1.005	1.162	1E-05	0	6.42	0	0	19	2	1473.28906	2945.57085	-4.41
vDAPVSLATGIPcSHPSIPDSFPEQPAFLSk	Unambiguous	2	1	Q9ES97-2	N-Term(iTRAQ8plex); C13(Methylthio); K31(iTRAQ8plex)	CID	1.0000	0.0000	1	1	3665743	Unique	Used	1.060	0.619	1.707	0.691	1E-05	0	6.42	0	0	13	3	1288.00012	3861.98581	-4.11
sTSSSPAPTqTLPNSITQR	Unambiguous	2	1	O08784	N-Term(iTRAQ8plex); Q10(Deamidated)	CID	0.0093	0.0000	1	1	3657104	Unique	Used	0.572	0.745	0.946	0.959	1E-05	0	6.42	0	0	200	2	1139.59717	2278.18706	1.84
aQTYYETLEQNDVVPEENWHTR	Unambiguous	1	1	Q6PAL8	N-Term(iTRAQ8plex)	CID	1.0000	0.0000	1	1	2831765	Unique	Used	1.904	1.227	1.138	1.009	1E-05	0	6.42	0	13	200	3	1009.48431	3026.43839	-1.66
lLEGSLGEPAPLPLPTPQQPPAGVPHR	Unambiguous	2	1	O88737	N-Term(iTRAQ8plex)	CID	1.0000	0.0000	1	1	3726223	Unique	Used	1.403	1.078	1.240	0.835	1E-05	0	6.42	0	13	30	3	1024.90442	3072.69870	-5.26
eTAAAHQASSsPPIDAATAEPYGFR	Unambiguous	1	1	P14873	N-Term(iTRAQ8plex); S11(Phospho)	CID	0.0358	0.0000	1	1	2701129	Unique	Used	2.149	0.966	1.382	1.116	1E-05	0	6.42	0	4	200	3	977.12598	2929.36338	-1.30
lEATLQEEAAIQQEHLEELk	Unambiguous	1	1	Q9Z2I0	N-Term(iTRAQ8plex); K20(iTRAQ8plex)	CID	1.0000	0.0000	1	1	2765525	Unique	Used	1.446	1.167	0.754	1.252	1E-05	0	6.42	0	19	200	3	977.53375	2930.58670	-2.21
eLVQPFEILFEGVEYIAR	Unambiguous	1	1	Q80YA7	N-Term(iTRAQ8plex)	CID	1.0000	0.0000	1	1	3542181	Unique	Used	0.843	0.843	0.952	1.071	1E-05	0	6.42	0	12	200	2	1228.66650	2456.32573	-4.97
vTFSATSQAQELQDPSqHSYYDSGLEESETPSSk	Unambiguous	2	1	F7BJK1	N-Term(iTRAQ8plex); Q17(Deamidated); K34(iTRAQ8plex)	CID	0.0140	0.0000	1	1	2830797	Unique	Used		1.669	1.945	0.928	1E-05	0	6.42	0	0	200	3	1448.35986	4343.06504	1.80
iNcMDSLEAIDQELSNVNAQADR	Unambiguous	1	1	Q8VD63	N-Term(iTRAQ8plex); C3(Methylthio)	CID	1.0000	0.0000	1	1	3262980	Unique	Used	0.496	0.835	0.628	0.869	1E-05	0	6.42	0	25	47	2	1450.18030	2899.35332	-0.18
qLANSLSGVQnQLQSFNSYR	Unambiguous	1	1	Q68FG2	N-Term(iTRAQ8plex); N11(Deamidated)	CID	0.0358	0.0000	1	1	3703479	Unique	Used	0.586	1.904	2.602	2.303	1E-05	0	6.42	0	0	193	2	1280.15186	2559.29643	-5.51

vGESTESGTVIFcDVLPGk	Unambiguous	3	1	Q80TQ2	N-Term(iTRAQ8plex); C13(Methylthio); K19(iTRAQ8plex)	CID	1.0000	0.0000	1	1	3305143	Unique	Used	1.017	2.026	1.997	1.342	1E-05	0	6.42	0	0	31	2	1296.67371	2592.34014	-4.20
qVLASAPTDNNLcHFR	Unambiguous	1	1	Q9DCC4	N-Term(iTRAQ8plex); C13(Methylthio)	CID	1.0000	0.0000	1	1	3761232	Unique	Used	0.955	0.616	0.624	0.856	1E-05	0	6.42	0	4	8	3	712.68970	2136.05454	-4.07
ckLEEMGFqDSDGDSQPFSLQETYEAk	Unambiguous	5	1	B1AQZ0	C1(Methylthio); N-Term(iTRAQ8plex); K2(iTRAQ8plex); Q9(Deamidated); K27(iTRAQ8plex)	CID	0.0016	0.0000	1	1	2959250	Unique	Used		1.119	0.579	1.355	1E-05	0	6.42	1	0	200	3	1347.97510	4041.91074	-1.59
qQNLILSYAEDETDVEGTVnGVTHTSTGGR	Unambiguous	2	1	Q99M87	N-Term(iTRAQ8plex); N20(Deamidated)	CID	1.0000	0.0000	1	1	2763272	Unique	Used	1.650	0.499	0.896	0.817	1E-05	0	6.42	0	10	200	3	1166.23608	3496.69370	-1.29
wGEAGAEYVVESTGVFTTMEk	Unambiguous	3	1	P16858	N-Term(iTRAQ8plex); K21(iTRAQ8plex)	CID	1.0000	0.0000	1	1	3242078	Unique	Used		2.242		1.134	1E-05	0	6.42	0	19	200	3	967.15753	2899.45804	-2.22
lEQVSSDEGIGTLAENLLEALR	Unambiguous	4	1	A2AN08-3	N-Term(iTRAQ8plex)	CID	1.0000	0.0000	1	1	3396811	Unique	Used	0.527	0.990	1.222	0.850	1E-05	0	6.42	0	12	66	2	1331.20984	2661.41240	-4.71
vAVLLMDTQGTFDSQSTLR	Unambiguous	1	1	Q8BH66	N-Term(iTRAQ8plex)	CID	1.0000	0.0000	1	1	3706659	Unique	Used	0.384	0.891	1.007	1.040	1E-05	0	6.42	0	5	16	2	1193.62537	2386.24346	-6.53
eEFVQEGAQDAYTYk	Unambiguous	1	1	Q8BLE7	N-Term(iTRAQ8plex); K15(iTRAQ8plex)	CID	1.0000	0.0000	1	1	2636518	Unique	Used	0.233	0.343	0.856	1.134	1E-05	0	6.42	0	0	200	2	1193.60486	2386.20244	0.05
lFVEDSIHDQFVQk	Unambiguous	1	1	Q8R0Y6	N-Term(iTRAQ8plex); K14(iTRAQ8plex)	CID	1.0000	0.0000	1	1	3149172	Unique	Used	0.670	0.722	0.953	1.213	1E-05	0	6.42	0	0	196	2	1157.13525	2313.26323	-2.91
aLEDVcIETIEAGFMTk	Unambiguous	1	1	O88844	N-Term(iTRAQ8plex); C6(Methylthio); K17(iTRAQ8plex)	CID	1.0000	0.0000	1	1	3327450	Unique	Used	0.591	1.171	0.841	0.779	1E-05	0	6.42	0	1	9	3	842.10022	2524.28611	-3.85
gSYYAGAPSGQTELSQER	Unambiguous	1	1	Q6PAL7	N-Term(iTRAQ8plex)	CID	1.0000	0.0000	1	1	2807707	Unique	Used	0.358	0.688	0.883	0.896	1E-05	0	6.42	0	0	200	2	1103.03857	2205.06987	-1.23

yHTSQSGDEMTSLSEYVSR	Unambiguous	2	1	P11499	N-Term(iTRAQ8plex)	CID	1.0000	0.0000	1	1	2973035	Unique	Used	0.667	1.301	1.666	1.126	1E-05	0	6.42	0	16	7	3	827.71863	2481.14133	-3.72
dDIGIILnQYIAEMVR	Unambiguous	2	1	Q9D1K2	N-Term(iTRAQ8plex); N9(Deamidated)	CID	0.0312	0.0000	1	1	3396908	Unique	Used	1.733	1.288	1.329	1.207	1E-05	0	6.42	0	0	200	2	1141.12000	2281.23271	-3.89
dQYLLTALELHTELLESGR	Unambiguous	4	1	G3X9J4	N-Term(iTRAQ8plex)	CID	1.0000	0.0000	1	1	3418746	Unique	Used	0.918	1.561	1.220	0.952	1E-05	0	6.42	0	5	9	3	835.78662	2505.34531	-1.98
iDVPQDLEDDLTATPLSHATVPQSPAR	Unambiguous	2	1	Q8VDU5	N-Term(iTRAQ8plex)	CID	1.0000	0.0000	1	1	3111269	Unique	Used	1.754	1.006	1.262	1.194	1E-05	0	6.42	0	28	200	3	1064.22180	3190.65085	-0.81
gTWESHPETAELVLSQGR	Unambiguous	2	1	P36916	N-Term(iTRAQ8plex)	CID	1.0000	0.0000	1	1	3146942	Unique	Used	0.909	0.991	1.219	1.138	1E-05	0	6.42	0	10	92	3	767.72919	2301.17301	-2.06
yTPSGQSGAAASESLFISNHAY	Unambiguous	2	1	A6ZI44	N-Term(iTRAQ8plex)	CID	1.0000	0.0000	1	1	3258770	Unique	Used	1.508	1.203	0.836	0.791	1E-05	0	6.42	0	0	134	2	1281.61865	2562.23003	-4.45
eNVVGQMGWSDSLGLNnGDLAR	Unambiguous	1	1	P58871	N-Term(iTRAQ8plex); N17(Deamidated)	CID	0.0078	0.0000	1	1	3238415	Unique	Used	1.396	1.108	1.706	1.313	1E-05	0	6.41	0	26	193	2	1319.14465	2637.28203	-2.32
wGEAGAEYVVESTGVFTTMEk	Unambiguous	3	1	P16858	N-Term(iTRAQ8plex); K21(iTRAQ8plex)	CID	1.0000	0.0000	1	1	3241473	Unique	Used		0.990	0.867	0.620	1E-05	0	6.41	0	0	200	2	1450.23352	2899.45976	-1.62
lMcPQEIVDYIADkk	Unambiguous	3	1	F6ZFT1	N-Term(iTRAQ8plex); C3(Methylthio); K14(iTRAQ8plex); K15(iTRAQ8plex)	CID	1.0000	0.0000	1	1	3459236	Unique	Used	1.131	0.951	1.546	0.729	1E-05	0	6.41	1	4	15	3	908.83295	2724.48429	-2.17
lVPLYAVVSEEPIYIVTEFMSk	Unambiguous	1	1	Q04736	N-Term(iTRAQ8plex); K22(iTRAQ8plex)	CID	1.0000	0.0000	1	1	3687628	Unique	Used	1.223	0.952	1.544	1.551	1E-05	0	6.41	0	20	175	3	1045.91394	3135.72727	-7.65
sSPQFGVTLVTYELLQR	Unambiguous	1	1	Q8BH59	N-Term(iTRAQ8plex)	CID	1.0000	0.0000	1	1	3860590	Unique	Used	0.729	1.071	1.286	1.834	1E-05	0	6.41	0	15	8	2	1121.61902	2242.23076	-3.47
aSGVcIDSESFLTTASIVSVLPQNR	Unambiguous	1	1	Q9CZC8	N-Term(iTRAQ8plex); C5(Methylthio)	CID	1.0000	0.0000	1	1	3860842	Unique	Used	1.296	0.790	0.913	0.936	1E-05	0	6.41	0	5	4	2	1472.74878	2944.49028	-5.42
vcPLPTTEDPGLPFHPnGk	Unambiguous	2	1	F6RJV6	N-Term(iTRAQ8plex); C2(Methylthio); N17(Deamidated); K19(iTRAQ8plex)	CID	1.0000	0.0000	1	1	3324753	Unique	Used	0.283	1.056	0.881	0.717	1E-05	0	6.41	0	2	7	3	892.12970	2674.37455	-3.15

eVAAFAqFGSDLDAATQQLLSR	Unambiguous	2	1	Q03265	N-Term(iTRAQ8plex); Q7(Deamidated)	CID	1.0000	0.0000	1	1	3666928	Unique	Used	0.838	0.851	1.070	1.222	1E-05	0	6.41	0	25	186	3	881.79034	2643.35648	-0.14
dFSALESQLQDTQELLQEENR	Unambiguous	1	1	Q8VDD5	N-Term(iTRAQ8plex)	CID	1.0000	0.0000	1	1	2960793	Unique	Used	1.901	1.993	1.488	0.855	1E-05	0	6.41	0	8	134	2	1399.19348	2797.37969	0.09
dMLYQVLAAEEPSVR	Unambiguous	3	1	Q91XH5	N-Term(iTRAQ8plex)	CID	1.0000	0.0000	1	1	3284054	Unique	Used	0.897	1.547	1.588	1.814	1E-05	0	6.41	0	9	8	2	1013.03320	2025.05913	-1.86
aEVEEFSFITqLSGLTDHLPVAMR	Unambiguous	1	1	Q9JJY3	N-Term(iTRAQ8plex); Q11(Deamidated)	CID	1.0000	0.0000	1	1	3687005	Unique	Used	0.579	1.025	1.213	1.549	1E-05	0	6.41	0	13	38	3	999.18469	2995.53952	0.20
yEAAGTLVTLSSAPTAIk	Unambiguous	1	1	Q9JIF7	N-Term(iTRAQ8plex); K18(iTRAQ8plex)	CID	1.0000	0.0000	1	1	3621070	Unique	Used	0.616	1.469	0.710	0.857	1E-05	0	6.41	0	0	80	2	1201.18640	2401.36553	-5.99
aVGQSVGGAFSSAk	Unambiguous	1	1	Q80U56	N-Term(iTRAQ8plex); K14(iTRAQ8plex)	CID	1.0000	0.0000	1	1	3511944	Unique	Used	0.536	1.183	1.067	0.946	1E-05	0	6.41	0	0	129	2	937.52863	1874.04997	-4.94
gTGGVDTAATGSVFDISNLDR	Unambiguous	1	1	P30275	N-Term(iTRAQ8plex)	CID	1.0000	0.0000	1	1	3264235	Unique	Used	0.818	1.488	1.055	1.032	1E-05	0	6.41	0	0	200	2	1179.10364	2357.20000	4.78
nIFLQYASTEVDGEHYMTPEDFVQR	Unambiguous	1	1	Q8BH59	N-Term(iTRAQ8plex)	CID	1.0000	0.0000	1	1	3264246	Unique	Used	0.938	1.032	1.685	1.571	1E-05	0	6.41	0	0	200	3	1098.52979	3293.57480	0.63
aVGPHQFLGNQEAIQAAIk	Unambiguous	1	1	P15327	N-Term(iTRAQ8plex); K19(iTRAQ8plex)	CID	1.0000	0.0000	1	1	3596254	Unique	Used	1.426	0.930	0.395	1.125	1E-05	0	6.41	0	0	104	2	1300.73206	2600.45683	-7.74
rGTGGVDTAATGSVFDISNLDR	Unambiguous	1	1	P30275	N-Term(iTRAQ8plex)	CID	1.0000	0.0000	1	1	3368689	Unique	Used	0.923	1.088	0.790	0.884	1E-05	0	6.41	1	18	58	3	838.43347	2513.28586	-1.58
vYEGSILEADcDILIPAASEk	Unambiguous	2	1	P26443	N-Term(iTRAQ8plex); C11(Methylthio); K21(iTRAQ8plex)	CID	1.0000	0.0000	1	1	3112296	Unique	Used	0.544	0.848	0.846	1.295	1E-05	0	6.41	0	0	200	2	1445.75330	2890.49932	-1.58
eALVDTLTGILSPVQEVR	Unambiguous	5	1	E9QKZ2	N-Term(iTRAQ8plex)	CID	1.0000	0.0000	1	1	3686998	Unique	Used	0.409	1.061	0.933	1.026	1E-05	0	6.41	0	17	30	2	1122.63513	2244.26299	-5.50
iYPEEMIQTGISAIDGMnSIAR	Unambiguous	1	1	P62814	N-Term(iTRAQ8plex); N18(Deamidated)	CID	1.0000	0.0000	1	1	3374078	Unique	Used	0.499	1.138	1.050	0.873	1E-05	0	6.41	0	0	35	2	1357.68127	2714.35527	-4.82

tAFDEAIAELDTLSEESYkDSTLIMqLLR	Unambiguous	1	1	P63101	N-Term(iTRAQ8plex); K19(iTRAQ8plex); Q26(Deamidated)	CID	1.0000	0.0000	1	1	3397030	Unique	Used	1.480	1.743	1.811	1.361	1E-05	0	6.41	1	18	200	3	1304.68384	3912.03696	1.85
ekPDDPLNYFIGGcAGGLTLGAR	Unambiguous	2	1	Q9D8B4	N-Term(iTRAQ8plex); K2(iTRAQ8plex); C14(Methylthio)	CID	1.0000	0.0000	1	1	3726776	Unique	Used	1.618	1.134	1.008	1.159	1E-05	0	6.41	0	11	6	3	1006.85303	3018.54453	-6.39
dQLQTFSEEHPVLLTEAPLNPR	Unambiguous	1	1	P61164	N-Term(iTRAQ8plex)	CID	1.0000	0.0000	1	1	3323661	Unique	Used	0.788	1.082	0.928	1.032	1E-05	0	6.41	0	19	101	3	946.83423	2838.48813	-2.07
eNIcTEVMALVPPADcR	Unambiguous	2	1	O35969	N-Term(iTRAQ8plex); C4(Methylthio); C16(Methylthio)	CID	1.0000	0.0000	1	1	3645657	Unique	Used	0.736	0.303	0.681	0.789	1E-05	0	6.41	0	24	22	2	1129.02185	2257.03642	-4.31
eELIQGVLAQVAEQFSR	Unambiguous	2	1	D3YTQ4	N-Term(iTRAQ8plex)	CID	1.0000	0.0000	1	1	3843180	Unique	Used	0.923	0.685	0.819	0.858	1E-05	0	6.41	0	18	200	2	1111.10669	2221.20610	-3.14
vFDFEVSSEDMATLLSYNR	Unambiguous	1	1	P45376	N-Term(iTRAQ8plex)	CID	1.0000	0.0000	1	1	3419274	Unique	Used	1.483	1.242	0.891	0.965	1E-05	0	6.41	0	14	18	2	1264.11316	2527.21904	-5.48
gTGGVDTAAVGGVFDVSNADR	Unambiguous	1	1	Q04447	N-Term(iTRAQ8plex)	CID	1.0000	0.0000	1	1	3190735	Unique	Used	1.496	1.159	1.315	1.297	1E-05	0	6.41	0	0	21	2	1135.06824	2269.12920	-3.13
ykVEYPVMYSTDPEnGHMFNcIQR	Unambiguous	1	1	Q9CPU4	N-Term(iTRAQ8plex); K2(iTRAQ8plex); N15(Deamidated); C21(Methylthio)	CID	0.0218	0.0000	1	1	3347819	Unique	Used	0.677	0.927	0.789	0.938	1E-05	0	6.41	1	11	18	3	1192.89575	3576.67270	-4.30
gAQAAIVVYDITNQETFAR	Unambiguous	1	1	P61021	N-Term(iTRAQ8plex)	CID	1.0000	0.0000	1	1	3479353	Unique	Used	0.531	0.920	1.073	1.163	1E-05	0	6.41	0	8	6	2	1186.12781	2371.24834	-3.23
qLEESVDSLGEELVQLR	Unambiguous	1	1	Q61768	N-Term(iTRAQ8plex)	CID	1.0000	0.0000	1	1	2942337	Unique	Used	1.423	0.631	1.139	0.895	1E-05	0	6.41	0	9	45	2	1124.60095	2248.19463	-1.27
iEQGSLILSNVQPSDTmVTQcEAR	Unambiguous	3	1	A2AFG8	N-Term(iTRAQ8plex); M17(Oxidation); C21(Methylthio)	CID	1.0000	0.0000	1	1	3438289	Unique	Used	0.893	1.273	0.896	1.113	1E-05	0	6.41	0	8	28	3	995.82227	2985.45224	-3.74
tNTGQALQYLSEHSFSPSQGDR	Unambiguous	2	1	Q8CIZ8	N-Term(iTRAQ8plex)	CID	1.0000	0.0000	1	1	3455064	Unique	Used	1.175	1.864	1.302	1.283	1E-05	0	6.41	0	30	200	3	909.77710	2727.31675	-4.00
iSGETIFVTAPHEATAGIIGVNR	Unambiguous	2	1	Q68FD5	N-Term(iTRAQ8plex)	CID	1.0000	0.0000	1	1	3708703	Unique	Used	0.735	1.449	1.710	1.569	1E-05	0	6.41	0	6	200	3	886.48615	2657.44388	-4.75

nSDGAVTWDEYNIQMYDR	Unambiguous	1	1	Q8BP92	N-Term(iTRAQ8plex)	CID	1.0000	0.0000	1	1	2868543	Unique	Used	2.473	0.675	1.089	0.834	1E-05	0	6.41	0	0	200	2	1241.06433	2481.12139	-3.25
gSDALLSNTVGTPAFMAPESLSETR	Unambiguous	5	1	Q8C078	N-Term(iTRAQ8plex)	CID	1.0000	0.0000	1	1	3372927	Unique	Used		1.873	1.370	1.208	1E-05	0	6.41	0	0	175	2	1428.21680	2855.42632	-4.77
yTPSGQSGAAASESLFISnHAY	Unambiguous	2	1	A6ZI44	N-Term(iTRAQ8plex); N19(Deamidated)	CID	1.0000	0.0000	1	1	3149902	Unique	Used	0.553	1.259	1.426	1.383	1E-05	0	6.41	0	2	19	2	1282.11401	2563.22075	-1.84
eIGINEDQFQEAcTSPLAk	Unambiguous	2	1	Q8C6E0	N-Term(iTRAQ8plex); C13(Methylthio); K19(iTRAQ8plex)	CID	1.0000	0.0000	1	1	3131626	Unique	Used	1.405	0.724	1.306	1.084	1E-05	0	6.40	0	0	96	2	1374.19275	2747.37822	-2.14
sGFEPPGDFPFEDYSqHIYR	Unambiguous	4	1	E9PUI5	N-Term(iTRAQ8plex); Q16(Deamidated)	CID	1.0000	0.0000	1	1	3282109	Unique	Used	0.964	0.989	1.157	1.041	1E-05	0	6.40	0	15	66	3	898.42328	2693.25528	3.37
gLSDFLGVISDTFAPSPDk	Unambiguous	2	1	Q80Y55	N-Term(iTRAQ8plex); K19(iTRAQ8plex)	CID	1.0000	0.0000	1	1	3602410	Unique	Used	0.695	1.475	1.407	1.017	1E-05	0	6.40	0	19	34	2	1287.69812	2574.38896	-0.88
qGHIYMEMNFTNk	Unambiguous	4	1	Q9DBG3	N-Term(iTRAQ8plex); K13(iTRAQ8plex)	CID	1.0000	0.0000	1	1	3366236	Unique	Used	1.126	1.299	1.172	1.239	1E-05	0	6.40	0	15	27	3	741.04657	2221.12516	-4.63
vADPVVTFcETVVETSSLk	Unambiguous	3	1	O08810	N-Term(iTRAQ8plex); C9(Methylthio); K19(iTRAQ8plex)	CID	1.0000	0.0000	1	1	3520885	Unique	Used	0.749	1.097	0.692	0.816	1E-05	0	6.40	0	13	200	2	1339.71143	2678.41557	-3.21
mTVVQAYVGDVHYk	Unambiguous	2	1	Q9R0K7	N-Term(iTRAQ8plex); K14(iTRAQ8plex)	CID	1.0000	0.0000	1	1	3577573	Unique	Used	0.800	0.699	1.114	0.893	1E-05	0	6.40	0	21	92	3	740.07373	2218.20664	-3.80
eTAAAHQASSsPPIDAATAEPYGFR	Unambiguous	1	1	P14873	N-Term(iTRAQ8plex); S11(Phospho)	CID	0.0328	0.0000	1	1	2700742	Unique	Used	7.145	0.805	1.507	0.491	1E-05	0	6.40	0	0	200	3	977.12494	2929.36026	-2.36
tVnDLEDSYGQQWTYEQR	Unambiguous	2	1	Q6PIC6	N-Term(iTRAQ8plex); N3(Deamidated)	CID	1.0000	0.0000	1	1	2637243	Unique	Used	0.477	0.964	1.178	1.239	1E-05	0	6.40	0	0	200	2	1269.08850	2537.16973	-1.46
nmQLSNTPDSGISLLHk	Unambiguous	3	1	E9QNG1	N-Term(iTRAQ8plex); M2(Oxidation); K17(iTRAQ8plex)	CID	1.0000	0.0000	1	1	3493031	Unique	Used	1.302	0.832	1.278	1.131	1E-05	0	6.40	0	0	200	3	827.11383	2479.32694	-6.69

lPDLSLDNMIGqSAIAGEEHPk	Unambiguous	2	1	G5E8J3	N-Term(iTRAQ8plex);<br>Q12(Deamidated);<br>K22(iTRAQ8plex)	CID	1.0000	0.0000	1	1	3304041	Unique	Used	1.723	1.085	1.043	0.957	1E-05	0	6.40	0	28	200	3	982.19373	2944.56662	4.05
tVEEVLGHFGVnESTGLSLEQVk	Unambiguous	3	1	O55143	N-Term(iTRAQ8plex);<br>N12(Deamidated);<br>K23(iTRAQ8plex)	CID	1.0000	0.0000	1	1	3326598	Unique	Used	0.967	1.079	1.238	0.810	1E-05	0	6.40	0	27	14	3	1027.88647	3081.64487	-3.78
eAFQNAYLELGGLGER	Unambiguous	2	1	Q6PIC6	N-Term(iTRAQ8plex)	CID	1.0000	0.0000	1	1	3356361	Unique	Used	0.891	0.908	0.989	0.995	1E-05	0	6.40	0	24	200	2	1036.03918	2071.07109	-2.48
eVAAFAQFGSDLDAATQQLLSR	Unambiguous	2	1	Q03265	N-Term(iTRAQ8plex)	CID	1.0000	0.0000	1	1	3669451	Unique	Used	1.092	1.182	1.354	1.257	1E-05	0	6.40	0	15	200	2	1321.68567	2642.36406	-3.32
tAcPGPFLQYNFDVTk	Unambiguous	4	1	Q9QXG4	N-Term(iTRAQ8plex);<br>C3(Methylthio);<br>K16(iTRAQ8plex)	CID	1.0000	0.0000	1	1	3686238	Unique	Used	0.550	0.400	0.813	0.968	1E-05	0	6.40	0	6	12	2	1228.12720	2455.24712	-5.67
sVSGASTGLSSSPLSSPR	Unambiguous	2	1	Q5RJI5	N-Term(iTRAQ8plex)	CID	1.0000	0.0000	1	1	3737976	Unique	Used	1.099	0.651	1.408	1.118	1E-05	0	6.40	0	0	200	2	991.02515	1981.04302	-3.73
fGDEDITWQDEHSAPFSWETR	Unambiguous	1	1	Q8C3W1	N-Term(iTRAQ8plex)	CID	1.0000	0.0000	1	1	2886569	Unique	Used	1.215	0.779	0.845	0.993	1E-05	0	6.40	0	2	200	3	953.10406	2857.29764	-1.10
lNATNIELATVQPGQNFHmFTk	Unambiguous	1	1	Q9Z2U1	N-Term(iTRAQ8plex);<br>M19(Oxidation);<br>K22(iTRAQ8plex)	CID	1.0000	0.0000	1	1	3683298	Unique	Used	1.946	1.175	0.817	1.162	1E-05	0	6.40	0	0	67	3	1033.54846	3098.63083	-7.93
lNITPNSGATGnNAGPk	Unambiguous	1	1	Q62433	N-Term(iTRAQ8plex);<br>N12(Deamidated);<br>K17(iTRAQ8plex)	CID	0.0312	0.0000	1	1	3185165	Unique	Used	0.877	0.610	1.647	2.009	1E-05	0	6.40	0	0	47	2	1118.11145	2235.21562	-1.50
fVSQTnGNLYIANVESSDR	Unambiguous	1	1	P12960	N-Term(iTRAQ8plex);<br>N6(Deamidated)	CID	0.0250	0.0000	1	1	3108764	Unique	Used	1.075	1.019	1.251	1.318	1E-05	0	6.40	0	0	18	2	1210.10449	2419.20171	-1.09
tGPAGGFQAAPHTAAPYSQAAPAR	Unambiguous	1	1	P47708	N-Term(iTRAQ8plex)	CID	1.0000	0.0000	1	1	3676982	Unique	Used	1.948	0.755	1.025	1.026	1E-05	0	6.40	0	0	200	3	867.11029	2599.31632	-6.02
qQEAPSNLSVNELLTPQGPR	Unambiguous	1	1	Q8CAD1	N-Term(iTRAQ8plex)	CID	1.0000	0.0000	1	1	3346793	Unique	Used	0.824	0.734	0.710	0.821	1E-05	0	6.40	0	9	18	2	1241.65942	2482.31157	-3.55

vAGHPDVVInNAAGNFISPSER	Unambiguous	1	1	Q9CQ62	N-Term(iTRAQ8plex); N10(Deamidated)	CID	0.0422	0.0000	1	1	3232962	Unique	Used	1.966	1.077	1.079	1.297	1E-05	0	6.40	0	24	72	3	857.11542	2569.33170	0.15
lVLPMDDSEPALNSLDDTRHsPA	Unambiguous	2	1	A1L3P4	N-Term(iTRAQ8plex); S21(Phospho)	CID	1.0000	0.0000	1	1	2639429	Unique	Used	0.276	0.292	0.661	1.111	1E-05	0	6.40	1	0	80	3	959.79272	2877.36362	-0.28
tGAAGDSLARPEEELPEIEnGQGSAAEISTIAk	Unambiguous	1	1	Q68FM6	N-Term(iTRAQ8plex); N20(Deamidated); K33(iTRAQ8plex)	CID	1.0000	0.0000	1	1	2813449	Unique	Used	1.158	1.209	1.395	0.992	1E-05	0	6.40	0	0	200	3	1307.67627	3921.01426	-1.13
dQEGQDVLLFIDNIFR	Unambiguous	1	1	P56480	N-Term(iTRAQ8plex)	CID	1.0000	0.0000	1	1	3355086	Unique	Used	1.177	1.019	1.067	1.315	1E-05	0	6.40	0	12	200	2	1113.58154	2226.15581	-6.77
gAGAFGYFEVTHDITR	Unambiguous	2	1	P24270	N-Term(iTRAQ8plex)	CID	1.0000	0.0000	1	1	3390983	Unique	Used	1.473	0.821	0.560	1.118	1E-05	0	6.40	0	7	5	3	682.34821	2045.03006	-4.59
qIQDMTPLSEQEMNAHLAEESR	Unambiguous	1	1	Q3UH93	N-Term(iTRAQ8plex)	CID	1.0000	0.0000	1	1	2848825	Unique	Used	1.103	4.412	2.307	0.895	1E-05	0	6.40	0	8	55	3	954.46094	2861.36826	-1.03
eSNVEEEVWHFLR	Unambiguous	2	1	Q91VM9	N-Term(iTRAQ8plex)	CID	1.0000	0.0000	1	1	3016728	Unique	Used	0.938	0.840	0.837	1.056	1E-05	0	6.40	0	2	5	3	660.00323	1977.99515	-1.06
vLRPPGGGSNFSLGFDEPAEQPVR	Unambiguous	1	1	P97825	N-Term(iTRAQ8plex)	CID	1.0000	0.0000	1	1	3557237	Unique	Used	2.265	1.681	1.278	1.257	1E-05	0	6.40	0	0	200	3	944.16022	2830.46610	-4.58
aMDQEITVNPQFVQk	Unambiguous	1	1	O88543	N-Term(iTRAQ8plex); K15(iTRAQ8plex)	CID	1.0000	0.0000	1	1	3256574	Unique	Used	0.759	1.225	0.958	0.982	1E-05	0	6.40	0	0	79	2	1178.63843	2356.26958	-4.06
vcIDSEHSSDTLLATLnk	Unambiguous	1	1	O08997	N-Term(iTRAQ8plex); C2(Methylthio); N17(Deamidated); K18(iTRAQ8plex)	CID	1.0000	0.0000	1	1	3347910	Unique	Used	1.975	0.684	1.380	0.876	1E-05	0	6.40	0	0	48	2	1301.17590	2601.34453	3.25
ySEVFEAINITNNEk	Unambiguous	3	1	Q60737	N-Term(iTRAQ8plex); K15(iTRAQ8plex)	CID	1.0000	0.0000	1	1	3215923	Unique	Used	0.810	1.206	1.107	0.875	1E-05	0	6.40	0	0	200	2	1190.13367	2379.26006	-2.18
aLEVFPEFAAAHSnLASVLQQQGk	Unambiguous	2	1	Q8CGY8	N-Term(iTRAQ8plex); N14(Deamidated); K24(iTRAQ8plex)	CID	1.0000	0.0000	1	1	3785079	Unique	Used	0.807	1.482	1.280	1.110	1E-05	0	6.40	0	0	41	3	1055.57922	3164.72312	0.95

aDTNSGDWnTEScQTLETQAAHTR	Unambiguous	7	1	H7BX92	N-Term(iTRAQ8plex); N9(Deamidated); C13(Methylthio)	CID	0.0125	0.0000	1	1	2900391	Unique	Used	1.900	1.391	1.416	1.251	1E-05	0	6.40	0	16	196	3	996.44183	2987.31095	2.00
eYQLNDSAAYYLNDLER	Unambiguous	1	1	P08752	N-Term(iTRAQ8plex)	CID	1.0000	0.0000	1	1	2940868	Unique	Used	0.954	1.235	0.957	1.348	1E-05	0	6.40	0	0	74	2	1191.07959	2381.15190	-1.85
dQLGGWFQSSLLTSVATR	Unambiguous	2	1	Q8BIJ6	N-Term(iTRAQ8plex)	CID	1.0000	0.0000	1	1	3898538	Unique	Used	0.921	0.979	1.038	1.237	1E-05	0	6.40	0	8	19	2	1135.60413	2270.20098	-3.23
eAFQNAYLELGGLGER	Unambiguous	2	1	Q6PIC6	N-Term(iTRAQ8plex)	CID	1.0000	0.0000	1	1	3356466	Unique	Used	1.102	0.520	0.791	0.733	1E-05	0	6.40	0	17	200	2	1036.03882	2071.07036	-2.83
iQDLEATLYNALQQEPGR	Unambiguous	2	1	D3Z4U5	N-Term(iTRAQ8plex)	CID	1.0000	0.0000	1	1	3520547	Unique	Used	0.462	0.814	0.885	0.801	1E-05	0	6.39	0	7	34	2	1182.12598	2363.24468	-2.63
eTEcTYFSTPLLLGk	Unambiguous	1	1	P08249	N-Term(iTRAQ8plex); C4(Methylthio); K15(iTRAQ8plex)	CID	1.0000	0.0000	1	1	3439762	Unique	Used	1.854	1.173	0.940	1.086	1E-05	0	6.39	0	17	42	2	1178.61450	2356.22173	-7.29
gIVDQSQQAYQEAFEISk	Unambiguous	1	1	P63101	N-Term(iTRAQ8plex); K18(iTRAQ8plex)	CID	1.0000	0.0000	1	1	3110400	Unique	Used	0.973	1.496	0.989	0.806	1E-05	0	6.39	0	0	200	2	1325.19751	2649.38774	-3.90
gSMAGSTGVHDTVVNQLLSk	Unambiguous	1	1	P46460	N-Term(iTRAQ8plex); K20(iTRAQ8plex)	CID	1.0000	0.0000	1	1	3639271	Unique	Used	0.429	1.219	1.895	1.555	1E-05	0	6.39	0	0	115	2	1305.20166	2609.39604	-8.33
yTPSGQSGAAASESLFISNHAY	Unambiguous	2	1	A6ZI44	N-Term(iTRAQ8plex)	CID	1.0000	0.0000	1	1	3234454	Unique	Used	1.355	1.262	1.367	1.201	1E-05	0	6.39	0	0	78	2	1281.62158	2562.23589	-2.17
hIPDEIFLLTAEQIAqEVSEQHLSQGR	Unambiguous	2	1	Q8BMF3	N-Term(iTRAQ8plex); Q16(Deamidated)	CID	0.0078	0.0000	1	1	3459441	Unique	Used	2.150	2.553	1.497	1.073	1E-05	0	6.39	0	0	158	3	1131.92773	3393.76865	2.75
sEGQATVIQQLEQTIEDLR	Unambiguous	1	1	Q9JL04	N-Term(iTRAQ8plex)	CID	1.0000	0.0000	1	1	3263045	Unique	Used	0.519	1.042	2.167	1.337	1E-05	0	6.39	0	8	22	2	1231.65625	2462.30522	0.47
eYHYGNNFGYIVAFkPFDGEEWk	Unambiguous	1	1	P12960	N-Term(iTRAQ8plex); K15(iTRAQ8plex); K23(iTRAQ8plex)	CID	1.0000	0.0000	1	1	3349218	Unique	Used	0.456	0.970	0.810	0.987	1E-05	0	6.39	0	6	10	3	1241.63513	3722.89084	-3.73
dEVQEVVYFPAVDHDAESk	Unambiguous	2	1	Q9CZC8	N-Term(iTRAQ8plex); K19(iTRAQ8plex)	CID	1.0000	0.0000	1	1	2636482	Unique	Used	1.873	0.881	0.986	0.991	1E-05	0	6.39	0	0	200	3	929.14319	2785.41501	0.32

mDYYDSEHHEDFEFISGTR	Unambiguous	1	1	Q60967	N-Term(iTRAQ8plex)	CID	1.0000	0.0000	1	1	2669491	Unique	Used	1.121	0.920	0.695	0.584	1E-05	0	6.39	0	0	200	3	894.72980	2682.17484	1.04
eLNQTSSSQLYLLSSYnALPPPHEALLER	Unambiguous	2	1	Q8BH55	N-Term(iTRAQ8plex); N17(Deamidated)	CID	0.0407	0.0000	1	1	3580483	Unique	Used	1.130	1.089	1.326	1.095	1E-05	0	6.39	0	0	51	3	1192.62268	3575.85349	-0.02
aSPTGTPGTTPPPSPGGGVGGAAWR	Unambiguous	2	1	Q5RJI5	N-Term(iTRAQ8plex)	CID	1.0000	0.0000	1	1	3699239	Unique	Used	0.794	0.604	1.586	1.705	1E-05	0	6.39	0	0	83	2	1269.15051	2537.29375	-4.48
sITIANQTNcPLYVTk	Unambiguous	1	1	O08553	N-Term(iTRAQ8plex); C10(Methylthio); K16(iTRAQ8plex)	CID	1.0000	0.0000	1	1	3934115	Unique	Used	0.865	0.938	1.506	1.562	1E-05	0	6.39	0	0	200	2	1210.65466	2420.30205	-4.85
rVTLDGDSEEMGVIPSk	Unambiguous	10	1	Q91XM9-7	N-Term(iTRAQ8plex); K17(iTRAQ8plex)	CID	1.0000	0.0000	1	1	2954792	Unique	Used	0.554	1.013	1.233	0.795	1E-05	0	6.39	1	4	25	3	814.44287	2441.31406	-1.07
iSGETIFVTAPHEATAGIIGVnR	Unambiguous	2	1	Q68FD5	N-Term(iTRAQ8plex); N22(Deamidated)	CID	1.0000	0.0000	1	1	3457504	Unique	Used	0.858	1.555	1.238	1.202	1E-05	0	6.39	0	17	47	3	886.81293	2658.42423	-6.13
aVQLYQQTANVFENEER	Unambiguous	2	1	Q9CWZ7	N-Term(iTRAQ8plex)	CID	1.0000	0.0000	1	1	3237846	Unique	Used	0.995	1.453	1.209	1.205	1E-05	0	6.39	0	0	1	2	1172.09607	2343.18486	-1.46
qTESSFMAGDINSTPTLNR	Unambiguous	5	1	E9Q3V9	N-Term(iTRAQ8plex)	CID	1.0000	0.0000	1	1	3255778	Unique	Used	0.583	0.816	0.960	0.786	1E-05	0	6.39	0	11	58	2	1187.08545	2373.16362	-0.94
[truncated: 623,627 more chars]
